# Uncovering transcriptional interactions via an adaptive fuzzy logic approach

**DOI:** 10.1186/1471-2105-10-400

**Published:** 2009-12-06

**Authors:** Cheng-Long Chuang, Kenneth Hung, Chung-Ming Chen, Grace S Shieh

**Affiliations:** 1 Institute of Biomedical Engineering, National Taiwan University, Taipei, Taiwan; 2 Institute of Statistical Science, Academia Sinica, Taipei, Taiwan

## Abstract

**Background:**

To date, only a limited number of transcriptional regulatory interactions have been uncovered. In a pilot study integrating sequence data with microarray data, a position weight matrix (PWM) performed poorly in inferring transcriptional interactions (TIs), which represent physical interactions between transcription factors (TF) and upstream sequences of target genes. Inferring a TI means that the promoter sequence of a target is inferred to match the consensus sequence motifs of a potential TF, and their interaction type such as AT or RT is also predicted. Thus, a robust PWM (rPWM) was developed to search for consensus sequence motifs. In addition to rPWM, one feature extracted from ChIP-chip data was incorporated to identify potential TIs under specific conditions. An interaction type classifier was assembled to predict activation/repression of potential TIs using microarray data. This approach, combining an adaptive (learning) fuzzy inference system and an interaction type classifier to predict transcriptional regulatory networks, was named AdaFuzzy.

**Results:**

AdaFuzzy was applied to predict TIs using real genomics data from *Saccharomyces cerevisiae*. Following one of the latest advances in predicting TIs, constrained probabilistic sparse matrix factorization (cPSMF), and using 19 transcription factors (TFs), we compared AdaFuzzy to four well-known approaches using over-representation analysis and gene set enrichment analysis. AdaFuzzy outperformed these four algorithms. Furthermore, AdaFuzzy was shown to perform comparably to 'ChIP-experimental method' in inferring TIs identified by two sets of large scale ChIP-chip data, respectively. AdaFuzzy was also able to classify all predicted TIs into one or more of the four promoter architectures. The results coincided with known promoter architectures in yeast and provided insights into transcriptional regulatory mechanisms.

**Conclusion:**

AdaFuzzy successfully integrates multiple types of data (sequence, ChIP, and microarray) to predict transcriptional regulatory networks. The validated success in the prediction results implies that AdaFuzzy can be applied to uncover TIs in yeast.

## Background

Identifying transcriptional interactions (TIs) is one of the central challenges in the post-genome era. When transcription factors (TFs) bind to cis-regulatory modules in the upstream DNA sequence of a target gene, its mRNA transcribes (expresses). In general, a cis-regulatory module consists of multiple TF binding sites, which may require several cooperating TFs to transcribe a given target gene. Therefore, predicting TIs for a whole genome can be computationally intensive. Nevertheless, integrating whole-genome DNA sequence, ChIP-chip, and microarray data may assist in the uncovering of gene regulatory networks.

Many types of resources have been exploited to predict gene regulatory networks; most of them use sequence data, localization data, gene expression data, protein structure data, or orthologs across different species. Sequence-based approaches focus on a group of genes and predict TIs within the group. Position weight matrices (PWMs) were incorporated to infer TF binding sites in [1]. Other approaches have been developed to find target genes with potential TIs such as, text mining [2], and support vector machines [3]. Besides sequence data, microarray data are also frequently used to reveal gene regulatory networks. The latest advance in Gaussian graphical models employed an empirical Bayes approach (EB-GGMs), and it can infer a large network of 3000+ genes [4]. The dynamics of pairwise TIs were studied using a nonlinear differential equation (NLDE) [5], which was shown to capture the behavior of transcriptional regulation with good accuracy. Other models proposed include Bayesian networks [6-9], state-space models [10], deterministic differential systems [11], linear differential systems [12], a linear dynamic model with latent factors [13], co-expression analysis [14], and machine learning [15,16]. In particular, [17] proposed a statistical approach (PAP) that incorporated sequence and microarray data to infer transcriptional regulators for co-regulated genes. PAP first gathers a set of co-expressed genes, then analyzes the regulatory sequence of these genes to identify potential TF binding sites.

Recently, integrating multiple types of data to infer TI has been proposed. Several approaches, including GRAM [18], COGRIM [19] and ReMoDiscovery [20], have been proposed to predict transcriptional regulatory networks using both TF binding information and microarray data. A two-stage constrained matrix decomposition model, called cPSMF [21], is the latest advanced algorithm proposed to predict TIs using ChIP-chip, sequence and microarray data. cPSMF considered the nonlinear structure in gene expression data of TIs, and used a linear combination of weighted TF activities to predict TIs and transcriptional modules. These approaches allow the prediction of TIs with more biological significance than models that use microarray or sequence data alone.

In our preliminary study [22], a conventional fuzzy-logic approach (FuzzyTRN) was proposed to integrate both DNA sequence and microarray data to infer TIs. Here, we present a further enhanced machine-learning (adaptive fuzzy) approach, called AdaFuzzy, to infer TIs, which incorporates DNA sequence, ChIP-chip and microarray data. A robust position weight matrix and a feature vector are proposed in AdaFuzzy. Furthermore, potential TF binding sites in upstream sequences of a specific target gene are identified by an adaptive neuro-fuzzy inference system (ANFIS) using sequence data. ChIP-chip data confirms that TIs do indeed occur under specific experimental conditions. In addition, microarray data is used to classify predicted TIs into activator-target or repressor-target relations via a weighted regression. After potential TIs are identified, AdaFuzzy also classifies their types of promoter architectures to provide insights into the organization of transcriptional regulatory interactions.

## Methods

**Figure 1:**
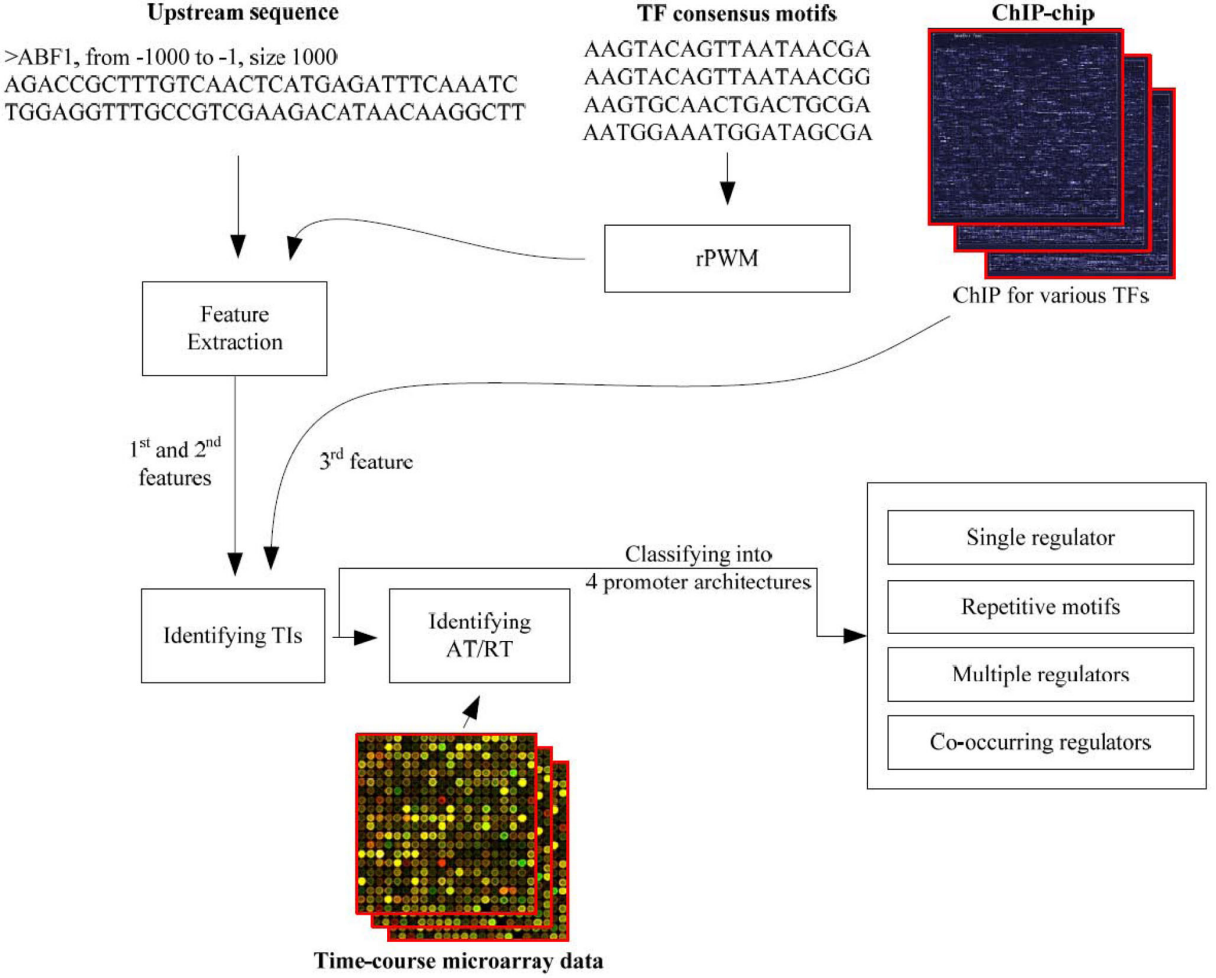
**Conceptual diagram of the AdaFuzzy algorithm**. First, consensus sequence motifs of a given TF are identified using a robust PWM (rPWM), which the rPWM is then used to search for potential TF binding sites in the upstream sequence of a given target gene via a vector of three features. Then this feature vector is incorporated to predict potential TIs by an adaptive fuzzy system (ANFIS), and a classifier is developed to infer the interaction types (AT or RT). Finally, all inferred TIs are categorized into one or more of the four major types of promoter architectures.

The proposed method (AdaFuzzy) consists of three parts. (1) Identifying consensus sequence motifs of a given TF using a robust PWM (rPWM). A rPWM is different from a PWM as it allows adjustment for gaps in the aligned sequence motifs. The rPWM of a TF is used to search for potential TF binding sites in the upstream sequence of a given target gene. For each pair of TF-target genes, a feature vector consisting of three indices to identify possible TF binding sites is constructed. The novelty of the feature vector lies in the adjustment for gaps of the aligned sequence motifs to make the score robust. (2) The feature vector and ChIP-chip data are then incorporated to predict potential TIs by an adaptive neuro-fuzzy inference system (ANFIS), which is a learning fuzzy approach. (3) Finally, a classifier is developed to infer the interaction types of predicted TIs (activator-target (AT) or repressor-target (RT) interaction) using microarray data. Figure 1 shows a conceptual schematic diagram of AdaFuzzy. The TI discussed here is the physical interaction between a TF and its target gene. After all TIs have been identified, they can be categorized into one or more of the four major types of promoter architectures defined in [23] to provide insights into the organization of transcriptional regulatory interactions. The details of the proposed method for inferring TIs are stated in the following sections.

### Identifying consensus sequence motif

For a group of genes of interest, suppose that some of them encode known TFs, for instance, a regulating gene that encodes a TF with *n *candidate sequence motifs of different length *k*_*i*_, which can be denoted by *B *= (*b*_*ij*_: *i *= 1, ..., *n*; *j *= 1, ..., *k*_*i*_), and *b*_*ij *_∈ {degenerate characters}. Each degenerate character is represented by two or more capital symbols with uniform frequencies and possibilities of occurrences. The details of these degenerate characters are summarized in the IUPAC website http://www.bioinformatics.org/sms/iupac.html.

ClustalW [24] is used to align the motifs of a regulating gene. To eliminate the length differences between motifs, the empty symbol '-' is inserted to fill in gaps introduced by the alignment. The aligned candidate sequence motifs of the regulating gene are denoted by ℜ = (*r*_*ij*_: *i *= 1, ..., *n*; *j *= 1, ..., *k*), *k *is the length of the aligned candidate sequence motifs of each TF, and *r*_*ij *_∈ {degenerate characters or '-'}. Then, the alignment result is summarized into a position-specific frequency matrix, denoted by *F*, as

where *ρ*_*x*, *j *_is the count of occurrences of nucleotide *x *at column *j *of ℜ calculated based on the occurrence probability of IUPAC nucleotide codes in additional file 1. Here, when computing *ρ*_*x*, *j *_the proportions of {A, C, G, T} in degenerate characters were also summed together with the frequencies of non-degenerate nucleotides. For instance, at the first column of three aligned sequences, there are A, C and Y, where Y assumes C and T with equal probability. Then *ρ*_*A*,1 _= 1, *ρ*_*C*,1 _= 1.5, *ρ*_*G*,1 _= 0 and *ρ*_*T*,1 _= 0.5. Next, in PWM the probability of observing a nucleotide *b *∈ {A, C, G, T} in the whole genome of a given organism is equal to *p*_*b*_. However, a large number of gaps in the aligned sequences would inflate the values in the PWM. To correct this, we propose to multiply the probability of a nucleotide in PWM by its proportion of valid nucleotides (non-gaps) in *n *sequences. For a given TF, the robust position weight, denoted by *S*, is proposed as

where *S*(*r*._*j *_= *b*) denotes the robust position-specific score of nucleotide symbol *b *occurring at column *j *of ℜ, *n *represents the number of candidate sequence motifs in ℜ, and *ω*_*j *_is the number of valid nucleotides (non-degenerate characters) at column *j *of ℜ. With small *ω*_*j*_, the value of *S*(*r*._*j *_= *b*) will be reduced to reflect that information of all aligned jth elements of candidate motif sequences (column *j *of ℜ) is less representative. Hence, a rPWM, denoted by *M*, can be derived as *M *= (*M*_*bj *_= *S*(*r*._*j *_= *b*); *b *∈ {A, C, G, T}; *j *= 1, ..., *k*), which is a 4 × *k *matrix. The rPWM can be used as a matching template to identify potential TF binding sites in the upstream sequence of a given target gene.

### Uncovering TF binding sites

For a given target gene, suppose that we have attained a fragment of its upstream sequence of length *l*, which can be denoted by *U *= (*u*_(-*x*)_: *x *= *l*, *l *- 1, ..., 1), where *u*_(-*x*) _∈ {A, C, G, T}, and *u*_(0) _is the transcription start site. The rPWM of a given TF is used to identify possible TF binding sites in the upstream sequence *U *of a given target gene. The first feature, a function that captures the probability that a candidate TF is indeed the regulator, is formulated as

where -*l *≤ *υ *≤ -*k*, and *k *is the length of the aligned candidate sequence motifs of each TF. A large score of  indicates a higher similarity between ℜ and the sequence fragment in the range [*υ*, *υ *+ *k - 1*] of the upstream sequence *U*, and hence the sequence fragment might be a potential TF binding site for the TF. The maximum score is obtained by matching the rPWM with sequence fragment *U' *in the range

The nucleotide fragment in the range [*υ*, *υ *+ *k - 1*] is used to determine the proportion of matched nucleotides which is the second feature. For a given pair of TF and target, the overall proportion of matched nucleotides adjusted for the effect of gaps is

where the indicator function *I*(*E*) = 1 if the event *E *holds; otherwise, 0. The weight (*ω*_*j*_/*n*) is to adjust for gaps in ℜ. The more valid nucleotides exist at column *j *of ℜ, the more important the matching event is. Large values of *O*(*υ*) indicate that ℜ and *U' *match well.

In addition, ChIP-chip data is also used to uncover TF-target gene interactions. For a given TF, the p-values of ChIP signals of all genes can be obtained after preprocessing all ChIP-chip data, and the p-value represents the significance level of a binding strength. Thus, the p-value of a TF associated with a given target gene, denoted by *κ*, is utilized as the third feature of AdaFuzzy.

Then, for any possible combination of TF and target gene, feature vectors [, *O*(*υ*), *κ*] can be calculated for predictions of TF binding sites via an ANFIS. The feature vector consists of information gathered by some similar but not completely overlapping features, and AdaFuzzy yields better results than using any subset of them. This concept is known as data fusion, a process of combining information gathered from multiple measurements into a single output to result in higher accuracy [25]. Data fusion has been proven effective in various applications. Thus, we use the feature vector to infer potential TIs by the ANFIS, introduced in the next subsection. Note that feature vectors [, *O*(*υ*), *κ*] with -*l *≤ *υ *≤ -*k *can be used to predict TF binding sites in the upstream sequence of a target gene for a given TF, and this additional information can be further used to identify the promoter architectures of target gene, which is stated in the next subsection.

### Identifying TIs using ANFIS

The three features above capture information about how a TF matches the promoter sequence of a target. However, whether a linear or nonlinear function of these features and what appropriate weights should be used to best summarize the information are unknown. Nevertheless, information on TIs, e.g. a few hundred pairs of TIs in the repository YEASTRACT already exists. Therefore, it is reasonable to take a learning approach. Here, a learning version of the fuzzy logic approach, called adaptive neuro-fuzzy inference system (ANFIS) is proposed to identify potential TIs from the three features. The three quantitative inputs of the ANFIS, , *O*(*υ*_max_), and *κ*, which are converted into qualitative descriptions by using some membership functions (e.g. large, medium, and small) for fuzzy reasoning, the parameters for which can be estimated by existing TIs. Then, the reasoning process (fuzzy rules) maps all combinations of the qualitative descriptions onto a decision score. For instance, a trained ANFIS may contain rules such as "if  is large, *O*(*υ*_max_) is large (the match of the sequences of a potential TF-target pair is good), and *κ *is small (ChIP signal intensity of a potential TF-target pair is significantly high), then the decision score is large (the chance that the potential TF-target pair is a TI is high)"; another extreme example would be, "if  is small, *O*(*υ*_max_) is small (the match of the sequences of a potential TF-target pair is bad), and *κ *is large (ChIP signal intensity of a potential TF-target pair is insignificant), then the decision score is small (the chance that the potential TF-target pair is a TI is small)". Finally, an overall decision score (denoted by *λ*) summarizes the reasoning results of the if-then rules for predicting TIs. By applying known TIs to train ANFIS, the parameters of membership functions for the fuzzy qualitative transformation and fuzzy rules can be automatically tweaked. When the number of membership functions or fuzzy rules of an ANFIS is large, an over-fitting problem will occur (i.e., the number of parameters is larger than the number of observations), but this disadvantage can be circumvented by setting a limit on the numbers of membership functions or fuzzy rules when initiating ANFIS.

Here, a Sugeno type-3 reasoning ANFIS [26,27] is used, which is the simplest model of ANFIS with a five-layer feed-forward architecture. A detailed description of this ANFIS can be found in additional file 2. Note that the cut-off for the decision score is also trained by existing TIs (say *c*), which is different from conventional ANFIS; the gradient descent method is used to train all parameters of the ANFIS. A decision score *λ *can be computed from the corresponding feature vectors [, *O*(*υ*), and *κ*] for any given *υ*. If the score *λ *of a given TF-target pair is greater than *c*, then this pair is predicted to be a TI. Furthermore, for all those *λ *scores greater than *c*, their associated *υ*'s are used to identify the positions of predicted TF binding sites.

### Classification of Promoter Architectures

After potential TIs are identified, insight into their transcriptional regulatory mechanism can be obtained if the prediction results include promoter architecture type. We thus used information from both sequence and ChIP-chip data to identify TF binding sites, and were able to predict some promoter architectures that ChIP experiments alone could not predict (see the experimental results section for details). In this subsection, we show how AdaFuzzy can classify the promoter architecture of identified TIs into at least one of the four types defined in [23], namely single regulator, repetitive motifs, multiple regulators and co-occurring regulators; see Figure 2 in [23] for an illustration of these architectures.

The first type of promoter architecture is single regulator architecture. This is the simplest type of architecture. For the upstream sequence of a given target gene, if there is only one TF with a *υ *that forms a feature vector [, *O*(*υ*_max_), *κ*] resulting in a *λ *> *c*, the predicted TI is classified as having single regulator architecture. The second type of promoter architecture is repetitive motif architecture. To identify this architecture, the feature vector [, *O*(*υ*), *κ*] was fed into the ANFIS for -*l *≤ *υ *≤ -*k *to identify all possible TF binding sites in the range [*υ*, *υ *+ *k - 1*] of *U*. If the upstream sequence of a given target gene is identified to contain multiple TF binding sites for a TF (multiple number of *υ *that results in *λ *> *c*), then the predicted TI is classified as having repetitive motifs architecture. The third type of promoter architecture is multiple regulator architecture. If the upstream sequence of a given target gene contains multiple binding sites for multiple TFs, all related TIs are classified as having multiple regulator architecture. The final type of promoter architecture is co-occurring regulators architecture. Such architecture is formed by a pair of TFs on the same target, where in general the distance between the two TFs is significantly closer than expected by chance, and the distance between two TF binding sites is the length of inter-sequence between them [23]. Furthermore, by plotting the distribution of inter-sequence lengths of all predicted TIs, any TF pair whose inter-sequence length has a p-value < 0.005 is classified as having co-occurring regulators architecture. Please note that the co-occurring regulators discussed here do not include the regulatory mechanism of heterodimers. In addition, these four types of promoter architecture are not necessarily mutually exclusive. An identified TI can be classified into one or more types of promoter architectures.

Due to the lack of a benchmark, it is hard to evaluate the classification of a TF-target pairs to types of promoter architectures. However, as long as the overall advantages of the classification outweigh the disadvantages, it is still worthwhile performing.

### Classification of AT/RT interactions

In our previous works [15,16], patterns in expression curves of paired genes were shown to be associated with the types of interactions, such as activator-target (AT) interaction and repressor-target (RT) interaction. The causal relation is inferred based on the observation of gene expression data taken with time lags to uncover the expression behavior of one gene that led to a delayed pattern of altered expression of its partner [28].

The patterns of paired gene expression curves can be used to identify the type of interaction between them. For example, a similar (anti-similar) pattern in a gene expression pattern (gradients with the same (different) signs) implies an AT (RT) interaction, and these patterns can be captured by the time-lagged gradients. To determine the type of interaction between a pair of genes, denoted by {*G*_1 _and *G*_2_}, we fitted a weighted least square regression to time-lagged gradients of expression levels of *G*_1 _and *G*_2_. Weighted least square regression was used since it can dampen the effect of noise in the microarray data. The slope of the regression line, denoted by *β*_1_, can be obtained by the command '*robustfit' *in MATLAB. The value of *β*_1 _can be used to infer the association between paired curves. A positive (negative) *β*_1 _indicates that overall the gradient signs of paired expression curves are of the same (opposite) sign, and this leads to a prediction of an AT (RT) interaction. The value of *β*_1 _can be mapped linearly to a decision score ranging from -1 to 1 to infer the interaction type of the gene pair. If the decision score is positive (negative), then the paired expression curves of *G*_1 _and *G*_2 _has a similar (anti-similar) pattern. The magnitude of the decision score indicates the strength of a pairwise interaction. For example, the decision score equals to 1 (- 1) indicates that the gene pair genes has a perfect positive (negative) association, and 0 means that there is no significant interaction between them. Furthermore, the p-value < 0.0001 of the decision score is used as the cutoff to predict an interaction type. Using such a stringent criterion can circumvent the noise in microarray data. Detailed description is in additional file 3. Note that a training version of this classifier can be used. However, this would be too complicated; for simplicity, the current version is used.

## Results and discussion

In this section, AdaFuzzy, consisting of an ANFIS and an interaction type classifier, is applied to identify condition-specific TIs by integrated analysis of sequence, ChIP-chip and microarray data. The demonstration of the experimental results is divided into two parts: (1) inferring TIs using data in the public domain, (2) classifying all predicted TIs into one of the four architectures.

In this subsection, upstream sequence data of genes (-1000 bp to -1 bp) in different species are gathered from EMBL-EBI database [29]. Sequence data of candidate sequence motifs are collected from YEASTRACT [30] and TRANSFAC [31]. By scanning the complete genome sequences of *S. cerevisiae*, the probabilities of observing nucleotides *p*_*b*_, *b *∈ {A, C, G, T} were calculated to be {0.3098, 0.1909, 0.1906, 0.3087}. The yeast ChIP-chip data set used was from [23], in which the genome-wide analysis contains 203 TFs in rich media condition, and 84 of them were also examined in at least one of 12 environmental conditions that may induce a stress response. A total of 19 TFs involved in the cell cycle and stress response were chosen to evaluate the performance of the proposed algorithm. The p-values of TF-gene pairs from [23] therein were used as the input (*κ*) of the ANFIS. Two publicly available yeast time-course microarray data sets were used, in which a cell cycle data set measured under normal growth conditions has 18 time points from [32], and the second set is related to yeast stress response to different experimental conditions, such as heat shock, amino acid starvation, nitrogen source deletion and progression into stationary phase [33]. There are 173 time points available in the second data set. The normalization process and missing-data imputation were conducted using zero transformation [34] and KNNimpute approaches [35], respectively.

Because ANFIS requires a complete training before it can produce any useful prediction, we collected 9609 positive TIs as training data set from YEASTRACT database. In addition, to control false negative rate of AdaFuzzy, 5260 negative TIs were formed by pairing up a TF with the other 18 TFs' target genes annotated in TRANSFAC and YEASTRACT.

### Inferring TIs using cell cycle/stress condition data in yeast

To see how AdaFuzzy performs, we compare AdaFuzzy with four well-known methods, cPSMF [21], GRAM [18], COGRIM [19] and ReMoDiscovery [20]. Similar to AdaFuzzy, these approaches predict TIs by performing an integrated analysis of sequence, ChIP-chip and microarray data. cPSMF unravels TIs and combinatorial gene regulation of TFs based on a two-stage constrained matrix decomposition model. GRAM utilizes an iterative search method to identify common TF binding sites of genes, then it relaxes its cutoff for co-expressed genes to extend the original gene set. COGRIM uses a Bayesian hierarchical model to represent expression level as a function of TF expression and binding strength. ReMoDiscovery is an intuitive method that concurrently analyzes all three types of data. Among these methods, AdaFuzzy, cPSMF and COGRIM are able to predict the interaction types (AT/RT) of the predicted TIs, while GRAM and ReMoDiscovery cannot. The comparison of these approaches was based on the same set of data (sequence, ChIP-chip and microarray data). In addition, since AdaFuzzy is a machine learning-based approach, a training set with 14491 gene pairs (9231 positives (TIs) and 5260 negatives (non-TIs)) was used to evaluate the performance of AdaFuzzy by 3-fold cross validation (CV) with 500 repeats.

Following one of the latest advances in predicting TIs [21], we compare these five algorithms using over-representation analysis [36] and gene set enrichment analysis (GSEA) [37] using the 19 TFs in [21], in which the target genes of all predicted TIs were clustered by GO terms. The over-representation analysis examines the predicted target genes and determines if there are gene sets which are statistically over-represented. GSEA attempts to determine whether members of a gene set (a set of predicted targets for a given TF) tend occur at the top (or bottom) of all genes considered; this gene set is expected to correlate with the phenotypic class distinction (targets or non-targets of the TF). The enrichment scores of GSEA can be calculated by the free software GSEA-P and its key steps are in p. 15546 of [38].

Using these two analyses, the performances of the four algorithms applied to the 19 TFs, and the average result of AdaFuzzy conducting 500 repeated 3-fold cross-validation experiments (CVs) are summarized in Table 1. All gene pairs were grouped into 19 subgroups by TFs, e.g. all gene pairs with TF_1 _being grouped into Subgroup 1, and CVs were performed on the subgroups. Therefore, the training set was formed from 13 randomly-selected subgroups, and the test set was constructed from the remaining subgroups. The proposed AdaFuzzy outperformed the other methods in both analyses. The averaged enrichment level of AdaFuzzy over all TIs associated with the 19 TFs in over-representation analysis was 6.00, better than those of cPSMF (5.81), ReMoDiscovery (5.40), COGRIM (5.22) and GRAM (4.96). In GSEA, the averaged enrichment level of AdaFuzzy was the highest (4.03), followed by cPSMF (3.65), COGRIM (3.42), GRAM (2.90) and ReMoDiscovery (2.64). These results imply that the target genes of all TIs identified by AdaFuzzy and cPSMF are more functionally relevant than the others. The average number of TIs identified by AdaFuzzy was 364, while those of cPSMF, COGRIM, ReMoDiscovery and GRAM were 91, 85, 74 and 32, respectively. This suggests that the machine learning-based AdaFuzzy is able to produce functionally coherent information on transcriptional regulatory mechanisms.

**Table 1:** Comparison of AdaFuzzy to other methods using over-representation analysis and gene set enrichment analysis.

TF	# TIs	Over-representation analysis					Gene set enrichment analysis				
		
		AdaFuzzy*	cPSMF	GRAM	COGRIM	ReMoDis	AdaFuzzy*	cPSMF	GRAM	COGRIM	ReMoDis
ABF1	598	5.48	5.93	6.12	5.74	4.55	4.57	4.56	4.60	5.05	3.76
ACE2	125	4.32	4.43	1.60	3.59	4.47	3.47	2.06	5.23	3.43	1.53
FKH1	164	4.53	4.62	4.79	1.76	4.05	4.26	4.51	1.91	2.99	2.04
FKH2	227	6.53	6.10	1.28	5.82	6.09	3.96	2.86	3.60	4.02	2.41
GCN4	306	6.96	7.23	6.64	7.40	7.61	4.36	5.71	2.09	1.48	4.72
LEU3	267	6.95	7.74	7.29	6.44	5.20	2.19	2.70	1.32	1.05	1.83
MBP1	315	6.48	6.08	6.15	4.93	6.17	4.78	4.17	4.21	4.91	3.40
MCM1	378	6.81	6.13	6.87	5.97	6.74	3.36	2.71	1.19	2.93	1.40
NDD1	60	4.33	1.64	1.82	1.66	4.49	3.09	2.67	2.40	3.09	3.47
RAP1	993	9.05	8.39	7.37	8.92	6.60	5.45	6.49	0.85	2.17	2.04
REB1	216	5.30	5.52	5.03	5.53	5.10	5.12	4.13	5.09	4.92	3.46
STB1	143	5.51	4.72	4.43	3.91	6.51	4.92	2.11	0.50	2.89	5.09
SWI4	346	6.73	7.06	5.61	5.76	6.42	4.52	5.22	4.25	4.73	1.56
SWI5	255	5.36	4.41	2.36	5.70	4.78	5.51	6.01	5.79	5.54	0.94
SWI6	237	5.93	5.66	4.58	4.79	4.90	3.64	2.81	4.08	4.23	4.23
HSF1	335	5.61	6.53	4.42	4.40	4.38	3.74	3.47	1.64	2.92	3.52
MSN4	310	5.93	6.31	5.51	5.67	5.72	4.01	2.77	0.86	4.58	1.93
SKN7	403	5.78	5.86	6.75	4.55	2.91	2.57	1.29	2.88	1.27	0.85
YAP1	1246	6.46	6.08	5.69	6.64	5.92	3.03	3.10	2.62	2.81	1.90

Averaged		6.00	5.81	4.96	5.22	5.40	4.03	3.65	2.90	3.42	2.64

* The performances of AdaFuzzy are average results of 500 repeated 3-fold CVs.

The performances of AdaFuzzy, 'ChIP-experimental method' and the other four methods applied to infer TIs of the 19 TFs are summarized in Table 2, and they were checked against the experimentally validated interactions from YEASTRACT as follows. Among 109130 possible TIs (19 TFs × the number of target genes), 6924 links were predicted to be TIs, in which the modified true-positive rate (mTPR) was 73% (6736/9231), and the modified false negative rate (mFNR) was 27% (2495/9231). In terms of sensitivity and specificity, AdaFuzzy outperformed the others (followed by 'ChIP only method' and cPSMF). If we manually relax the thresholds of some parameters in AdaFuzzy, e.g., the significance level of *β*_1 _or the cut-off *c *of the output of ANFIS, more TIs will be predicted, but this will also lead to higher false positive rates. The parameters of AdaFuzzy can be further tuned by users to meet their preference.

**Table 2:** The performances of AdaFuzzy and the other approaches, checked against validated TIs of the 19 TFs from YEASTRACT.

Methods	True positives	False positives	False negatives	True negatives	Sensitivity	Specificity	False Positive rate	False Negative rate
AdaFuzzy	6736	188	2495	108942	73.0%	99.8%	0.2%	27.0%
ChIP*	2799	108	6432	109022	30.7%	99.9%	0.1%	69.3%
cPSMF	1729	0	7502	109130	18.7%	100.0%	0.0%	81.3%
GRAM	1615	0	7616	109130	17.5%	100.0%	0.0%	82.5%
COGRIM	1406	0	7825	109130	15.2%	100.0%	0.0%	84.8%
ReMoDis.	608	0	8623	109130	6.6%	100.0%	0.0%	93.4%

2907 TIs identified by 'ChIP' were obtained by ChIP-chip experiment presented in Harbison *et al*. (2004) using criterion *p-value *< 0.001.

^† ^False-positives are assumed zero to estimate the best (upper bound of the) performance yielded by the other competitive methods.

We further compared AdaFuzzy and 'ChIP-experimental method', which applied the criterion of p-values < 0.001 to ChIP-chip experiment to identify TIs, using experimentally validated TFBSs in [23] and [38] intersecting with YEASTRACT. By definition, mFNR = 1-mTPR, so we only report mTPRs in the following. Under the rich media (stress) condition in [23], the mTPR of 'ChIP-experimental method' and AdaFuzzy, applied to 1955 and 1220 TIs, are 100% (100%) and 90% (82%), respectively. While under the normal (methyl-methanesulfonate exposure) condition, the mTPR of 'ChIP-experimental method' and AdaFuzzy, applied to 2529 and 1021 TIs, are 45% (18%) and 81% (59%), respectively. Detailed prediction results are summarized in additional file 4. The MATLAB code of AdaFuzzy is available at http://www.stat.sinica.edu.tw/~gshieh/AdaFuzzy.rar.

### Classifying Promoter Architectures

In the following, AdaFuzzy is applied to classify these 6865 TIs associated with the 19 aforementioned TFs to one or more types of promoter architectures in [23]. The predicted results are checked with biological information from the Saccharomyces Genome Database (SGD, http://www.yeastgenome.org).

For the results predicted by AdaFuzzy using cell cycle ChIP-chip and microarray data under the rich medium condition, ABF1 was predicted to be the TF regulating genes CDC24, GCN1 and IME4. These target genes share some common functions such as cellular developmental process and macromolecule metabolic process, and their promoters have been classified to single regulator architecture regulated solely by ABF1. Published literature shows that the ABF1 gene product binds to the upstream sequences of genes CDC24 [23,38,39], GCN1 [23] and IME4 [23,38,39]. Using the stress response data set in yeast, AdaFuzzy predicted that the gene product of REB1 interacted with the promoter sites of ABC1, MNP1 and NUT1. These target genes were found to be involved in cellular metabolic processes. Existing literature also confirms that protein *Reb1p *binds to the upstream sequences of ABC1 [24,38,40], MNP1 [38] and NUT1 [24,38,40], and these TIs were identified to be AT interactions. Figure 3 illustrates that the binding sites and their types of promoter architectures predicted by AdaFuzzy coincide with experimental results annotated in SGD.

**Figure 2:**
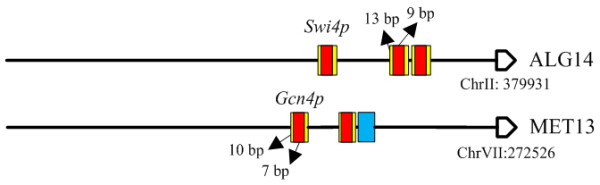
**Repetitive motifs promoter architecture identified by AdaFuzzy**. Yellow boxes represent binding sites predicted by AdaFuzzy, red boxes denote the results annotated in SGD or [23], and blue boxes show the results that are not annotated. Please note that the scale of the box (binding site) is not realistic.

**Figure 3:**
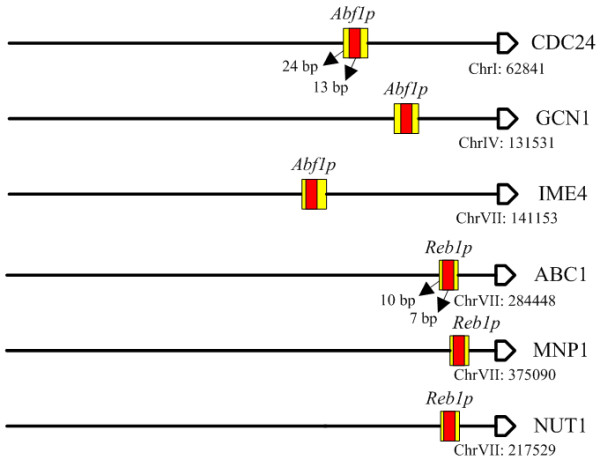
**Single regulator promoter architecture identified by AdaFuzzy**. Yellow box represents binding sites predicted by AdaFuzzy, and red box denotes the results in SGD. Please note that the scale of the box (binding site) is not realistic.

Several genes were predicted to have the repetitive motifs promoter architecture. Target genes with such promoter architecture are regulated in a graded manner by specific TFs. Among all TIs predicted by AdaFuzzy using the cell cycle yeast data set, ALG14 was classified to the repetitive motifs promoter architecture. The upstream sequence of ALG14 contains binding sites for the proteins of SWI4 and MBP1, while protein *Swi4p *was shown to regulate ALG14 [23,38,41,42]. When using the stress response yeast data set, MET13 was predicted to have the repetitive motifs architecture which is bound by protein *Gcn4p*, while the regulation of *Gcn4p *on MET13 under stress conditions has been demonstrated [23,43-45]. In addition, Gcn4p was identified to be an activator for MET13. Figure 2 shows that the predicted binding sites are in agreement with experimental results from SGD. Note that a potential binding site for *Gcn4p *was found in the upstream sequence of MET13 that was not annotated in SGD or [23].

**Figure 4:**
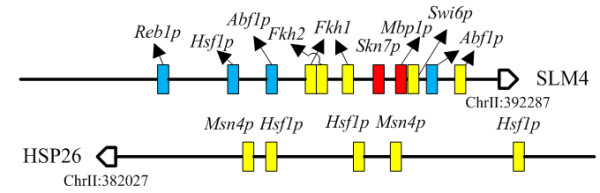
**Multiple regulators promoter architecture identified by AdaFuzzy**. Yellow box represents binding sites predicted by AdaFuzzy, red box denotes false negative, and blue boxes show the results that are not annotated in SGD or [23]. Please note that the scale of the box (binding site) is not realistic.

The multiple regulators promoter architecture type is commonly seen in the yeast genome. For instance, SLM4 has been classified to be co-regulated by multiple regulators using the yeast cell cycle data set, such as *Abf1p *[23,43], *Fkh1p *[23], *Fkh2p *[23,38,40,42] and *Swi6p *[38,40]. Applying AdaFuzzy to the stress response data set in yeast resulted in that HSP26 had multiple TF binding sites, while HSP26 was shown to be regulated by TFs *Msn4p *and *Hsf1p*, in [23,46-48] and [23,46-50], respectively. Msn4p was predicted to be an activator for HSP26. These prediction results are illustrated in Figure 4, which shows that the TF binding sites architectures classified by AdaFuzzy are consistent with experimental results in SGD. The binding sites of MBP1 and SKN7 were presented in the upstream sequence of SLM4, but AdaFuzzy applied to the rich medium data set failed to identify these as expected because the regulation of MBP1 and SKN7 on SLM4 occur only under stress conditions [23]. In addition, four binding sites were identified for Reb1p, Hsf1p and Abf1p, while these binding sites were not annotated in SGD or [23]. This shows that AdaFuzzy can predict novel results for biologists to test transcriptional regulatory interactions.

**Figure 5:**
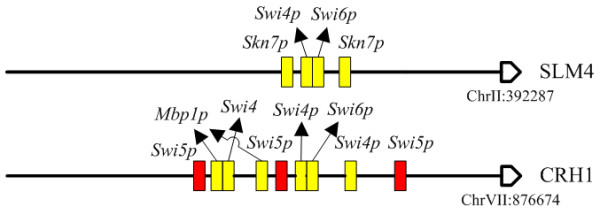
**Co-occurring regulators promoter architecture identified by AdaFuzzy**. Yellow boxes represent binding sites predicted by AdaFuzzy, and red boxes denotes false negative. Please note that the scale of the box (binding site) is not realistic.

The fourth type of promoter architecture consists of binding site sequences which are closer than expected by chance. This implies that two independent regulators may interact with each other before regulating their target gene. In Figure 5, *Fkh1p*-*Fkh2p *and *Mbp1p*-*Swi6p *are predicted to be co-occurring regulators that interact with the promoter of SLM4 under rich medium condition. AdaFuzzy predicted that *Swi4p*-*Swi6p *is likely to co-active OCH1, which is supported by [23,38,40-42]. Under stress conditions, *Swi4p*-*Swi6p *and *Swi4p*-*Mbp1p *were classified to be co-occurring repression and activation regulators of CRH1, and these were confirmed by previous publications [23,38,40-42,51]; these results are illustrated in Figure 5. In addition, 13 regulatory gene pairs, some validated and the others novel, identified by AdaFuzzy to be co-occurring regulators are summarized in Table 3. Among these results, MCM1-FKH1 and SWI4-MBP1 were not reported by ChIP-chip experiments in [23] but were validated by other biological experiments.

**Table 3:** The list of co-occurring regulators identified by AdaFuzzy.

Co-occurring Regulators	Literature support
ACE2-FKH2	Harbison *et al*. (2004) [23]
ACE2-SWI5	Harbison *et al*. (2004) [23]
FKH1-FKH2	Harbison *et al*. (2004) [23]
FKH2-NDD1	Harbison *et al*. (2004) [23]
MBP1-SWI6	Harbison *et al*. (2004) [23]
MCM1-NDD1	Harbison *et al*. (2004) [23]
SWI4-SWI6	Harbison *et al*. (2004) [23]
ACE2-FKH1	Tsai *et al*. (2005) [52] (Predicted with confident)
ACE2-SWI6	Tsai *et al*. (2005) [52] (Predicted with confident)
MCM1-FKH1	Kumar *et al*. (2000) [53]
NDD1-STB1	Tsai *et al*. (2005) [52] (Predicted with confident in Banerjee and Zhang (2003) [55])
ACE2-HSF1	Das *et al*. (2004) [54]) (Predicted with literature support in Banerjee and Zhang (2003) [55])
SWI4-MBP1	Mai and Breeden (1997) [50]

Taken together, AdaFuzzy performs well in identification of TIs and is able to classify promoter architecture types using genomics data in yeast to provide insights into the organization of transcriptional regulatory interactions.

## Conclusion

A novel algorithm AdaFuzzy is introduced for identifying TIs using sequence, ChIP-chip and microarray data. AdaFuzzy, cPSMF, GRAM, COGRIM and ReMoDiscovery were applied to TIs in yeast using genomic data from cell cycle and stress condition. AdaFuzzy performed better than the other methods in terms of over-representation analysis and GSEA, which were used in one of the latest advances [21]. Checked against known TIs of the preselected 19 TFs in [21] as annotated in databases and published literature, the mTPR and mFNR of AdaFuzzy were 72% and 28%, respectively. Furthermore, AdaFuzzy performed compatibly to 'ChIP-experimental method' in inferring TIs identified by two sets of large scale ChIP-chip experiments in [21] and [38]. This suggests that AdaFuzzy is useful for uncovering transcriptional regulatory interactions in yeast. AdaFuzzy can also classify the predicted TIs into one or more of the four promoter architectures in [23] to provide insights into the organization of transcriptional regulatory interactions. The classification results also coincide with known promoter architectures annotated in SGD and [23]. Some predicted TIs are not annotated in SGD and [23], and these can be tested further by biologists. However, AdaFuzzy is not able to predict TIs involved with heterodimers, which is an important regulatory mechanism in developmental and physiological processes in humans. We leave this for future research.

## Authors' contributions

CLC and GSS devised the method. CLC implemented the method and drafted the manuscript. KH helped implement the method. CMC and GSS supervised the methodology and implementation. GSS wrote the manuscript. All of the authors read and approved the final manuscript.

## Supplementary Material

Additional file 1 **IUPAC code**. The frequency table of degenerate characters defined in IUPAC. Click here for file

Additional file 2 **Adaptive neuro-fuzzy inference system (ANFIS)**. Description of the architecture and general theory of ANFIS. Click here for file

Additional file 3 **Classifier for ATRT**. Detailed descriptions of the classifier for prediction of AT/RT interactions. Click here for file

Additional file 4 **Prediction results**. The results predicted by the proposed AdaFuzzy. Click here for file
