# Supplementary material for: Uncovering transcriptional interactions via an adaptive fuzzy logic approach
Source: BMC Bioinformatics. 2009 Dec 6;10:400. doi: 10.1186/1471-2105-10-400 (PMC2797023; doi:10.1186/1471-2105-10-400)
Supplement: Additional file 1 — IUPAC code. The frequency table of degenerate characters defined in IUPAC. [file 1471-2105-10-400-S1.PDF]

Supplementary Material 1

Symbols of expanded version of IUPAC one letter codes

Symbol	Frequency (Probabilities)	Description
A	A: 12 (100%)	Adenine
C	C: 12 (100%)	Cytosine
G	G: 12 (100%)	Guanine
T	T: 12 (100%)	Thymine
R	A: 6 (50%) G: 6 (50%)	Purine
Y	C: 6 (50%) T: 6 (50%)	Pyrimidine
K	G: 6 (50%) T: 6 (50%)	Keto group
M	A: 6 (50%) C: 6 (50%)	Amino group
S	G: 6 (50%) C: 6 (50%)	Strong hydrogen binding
W	A: 6 (50%) T: 6 (50%)	Weak hydrogen binding
B	G: 4 (33%) T: 4 (33%) C: 4 (33%)	Not adenine
D	G: 4 (33%) A: 4 (33%) T: 4 (33%)	Not cytosine
H	A: 4 (33%) C: 4 (33%) T: 4 (33%)	Not guanine
V	G: 4 (33%) C: 4 (33%) A: 4 (33%)	Not thymine
N	A: 3 (25%) C: 3 (25%) G: 3 (25%) T: 3 (25%)	Any nucleotide
–	A: 0 (0%) C: 0 (0%) G: 0 (0%) T: 0 (0%)	No nucleotide exists
