# Supplementary material for: Uncovering transcriptional interactions via an adaptive fuzzy logic approach
Source: BMC Bioinformatics. 2009 Dec 6;10:400. doi: 10.1186/1471-2105-10-400 (PMC2797023; doi:10.1186/1471-2105-10-400)
Supplement: Additional file 2 — Adaptive neuro-fuzzy inference system (ANFIS). Description of the architecture and general theory of ANFIS. [file 1471-2105-10-400-S2.PDF]

Identifying TIs using ANFIS

The three features mentioned in the main text were to capture the information of how a TF matches the promoter sequence of a target. The three quantitative inputs of the ANFIS are $\hat{S}(\upsilon_{\max})$, $O(\upsilon_{\max})$, and κ, which are converted into qualitative descriptions (e.g. large, medium, and small) by some membership functions for fuzzy reasoning, in which the parameters can be tuned by existing TIs. Next, the reasoning process (fuzzy rules) maps all combinations of qualitative descriptions to a decision score. For instance, a trained ANFIS may contain rules such as "if $\hat{S}(\upsilon_{\max})$ is large, $O(\upsilon_{\max})$ is large (the match of a potential TF-target pair is good), and κ is small (ChIP signal intensity of a potential TF-target pair is significantly high), then the decision score is large (the chance that the TF-target pair is highly to be a TI)"; for another extreme example, "if $\hat{S}(\upsilon_{\max})$ is small, $O(\upsilon_{\max})$ is small (bad match of a potential TF-target pair), and κ is large (ChIP signal intensity of a potential TF-target pair is insignificant), then the decision score is small (the chance that the TF-target pair is not a TI)". Finally, an overall decision score (denoted by λ) summarizes the reasoning results of the if-then rules to predict TIs. By applying known TIs to train ANFIS, the parameters of membership functions for the fuzzy qualitative transformation and fuzzy rules can be automatically tweaked.

A Sugeno-type ANFIS [1, 2] is adopted in FuzzyTRN, in which network structure, fuzzy rules and output node can be trained by comparing the actual network output (the chance of a potential YF-target being a TI) to its target output (a known TI).
The schematic of a three-input one-output ANFIS is depicted in Figure 1, in which the inputs are $\hat{S}(\upsilon_{\max})$, $O(\upsilon_{\max})$, and κ, and the output is denoted by λ. The nodes denoted by a square, called adaptive nodes, have parameters, while the circle-shaped are fixed nodes (no parameters therein). In Figure 1, only nodes of layers 1, 4 and 5 are adaptive. Note that the cut-off for the decision score is also trained by existing TIs here (say c), which is different from conventional ANFIS. If a computed decision score λ is greater than c, then the given TF-target pair is predicted to be a TI. The gradient descent method is used to train all parameters of the ANFIS.

Layer 1 consists of three nodes, which are membership functions to transform quantitative inputs ($\hat{S}(\upsilon_{\max})$, $O(\upsilon_{\max})$, and κ,) to qualitative description (e.g. large, medium or small) for fuzzy reasoning. In general, the membership functions are bell-shaped ranging from 0 to 1, defined as

$$\mu_{A_n}(x,a,b,c)=\frac{1}{1+\left|\frac{x-c}{a}\right|^{2b}},$$

where μ_x denotes the bell-shaped function, A_n represents the qualitative label for such membership function (e.g. large, medium or small), and three parameters a, b and c control the width of the plateau, the slope, and the location of the center of the function.

The number of fuzzy rules in the rule base is n^m, where m is the number of inputs, and n is the number of membership function corresponding to each input. Therefore, there are 27 (3^3) rules (nodes) on layer 2. The fuzzy rule defines the logic of fuzzy inference, such as "if $\hat{S}(\upsilon_{\max})$ is large, $O(\upsilon_{\max})$ is large (the match of a potential TF-target pair is good), and κ is small (ChIP signal intensity of a potential TF-target pair is significantly high), then the decision score λ is large (the chance that the TF-target pair is highly to be a TI)". In evaluating the rules, we choose the product operation *and* for logic reasoning, defined as

$$w_i=\mu_{A_j}\left(\hat{S}(\upsilon_{\max})\right)\mu_{B_k}\left(O(\upsilon_{\max})\right)\mu_{C_l}\left(\kappa\right).$$

Normalized weights of all rules are generated by nodes on layer 3 by dividing weights of all rules by the sum of all weights, which can be formulated as

$$\tilde{w}_i=\frac{w_i}{\sum_{j=1}^{n^m}w_j},$$

where $\tilde{w}_i$ represents the normalized weights generated on layer 3.

Layer 4 is a fuzzy reasoning process that performs a simple linear combination of inputs $\hat{S}(\upsilon_{\max})$, $O(\upsilon_{\max})$, and κ by consequent parameters, which can be determined using least square estimator after the ANFIS is fed by the training set data. The aforementioned four layers are equivalent to "If-then" rules that transform qualitative descriptions characterized by a membership function to the domain that captures the imprecise means of reasoning. Layer 5 simply computes the sum of outputs from layer 4 to result in λ (the decision score), which can be defined as

$$\lambda = \frac{\sum_{j=1}^{n^m} \tilde{w}_j f_j \left(\hat{S}(\upsilon_{\max}), O(\upsilon_{\max}), \kappa\right)}{\sum_{j=1}^{n^m} \tilde{w}_j}.$$

To predict whether the regulatory gene encodes a potential TF for a given target gene, a tunable threshold with cut-off value c is used for the decision score. If $\lambda > c$, then the potential TF is predicted to a regulator (activator or repressor) of a target gene. After training set is inputted to the ANFIS (called forward pass), the consequent parameters of layer 4 can be obtained, while the error rates propagate backward to tweak the rest of parameters by the gradient descent method.

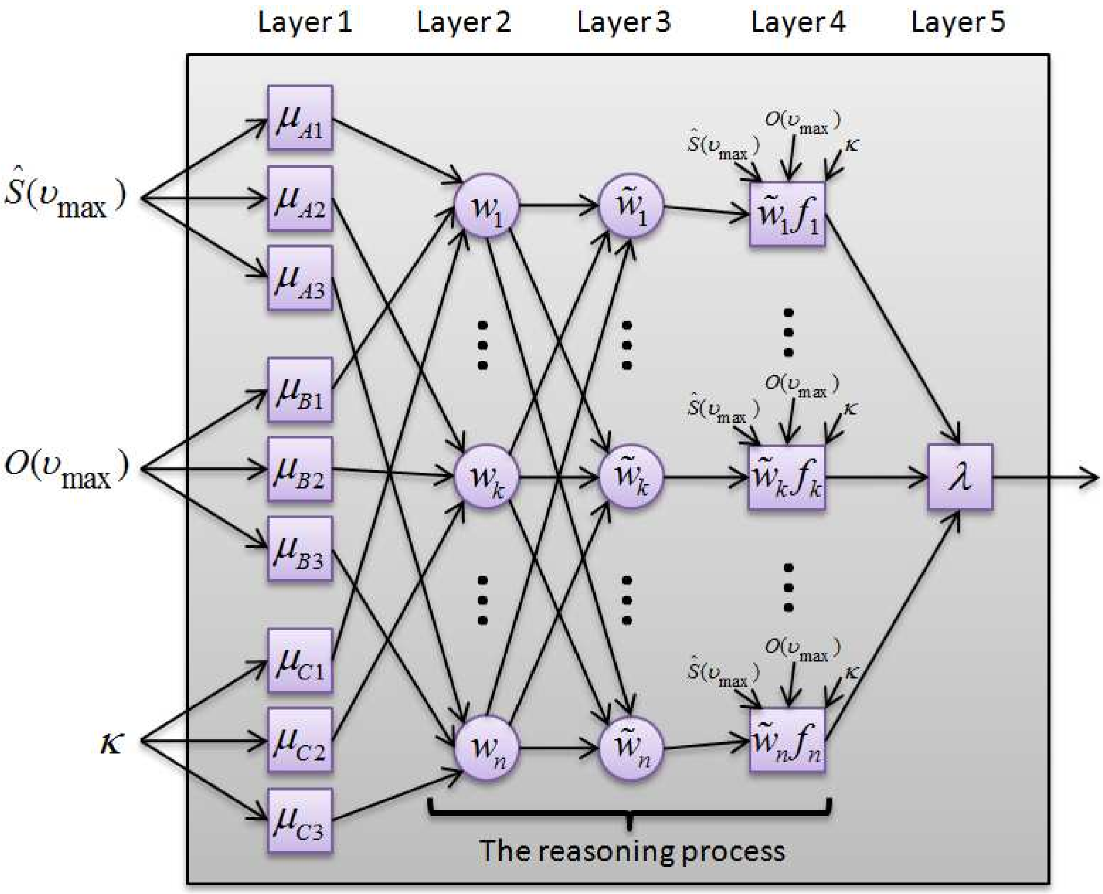

Figure 1. The architecture of the ANFIS in FuzzyTRN.

Reference

[1] Sugeno, M: *Industrial applications of fuzzy control*, Elsevier Science Pub. Co., 1985.

[2] Jang, JSR: **ANFIS: Adaptive-Network-based Fuzzy Inference Systems**, *IEEE Transactions on Systems, Man, and Cybernetics 1993*, **23**:665-685.
