# Supplementary material for: Uncovering transcriptional interactions via an adaptive fuzzy logic approach
Source: BMC Bioinformatics. 2009 Dec 6;10:400. doi: 10.1186/1471-2105-10-400 (PMC2797023; doi:10.1186/1471-2105-10-400)
Supplement: Additional file 3 — Classifier for ATRT. Detailed descriptions of the classifier for prediction of AT/RT interactions. [file 1471-2105-10-400-S3.PDF]

Classification of AT/RT interactions

In our previous works [1, 2], patterns in expression curves of TF-target pair (TI) were shown to be associated with the types of interactions, such as activator-target (AT) interaction and repressor-target (RT) interaction. The causal relation is inferred based on the observation of gene expression data taken with time lags to uncover the expression behavior of one gene that led to a delayed pattern of altered expression of its partner [3]. Specifically, let $G = (G_i(t) : i = 1, \ldots, I\,; t = 1, \ldots, T)$ be a I by T matrix, where $G_i(t)$ is the gene expression level of the i-th gene at time t. Suppose that G_1 is predicted to regulate a target gene G_2, the expression levels of G_1 ($G_1(t)$, $t = 1, \ldots, T$ - 1) and the lagged expression levels of the target gene ($G_2(t)$, $t = 2, \ldots, T$) are used to to infer the types of interactions as follows.

The patterns of paired gene expression curves can be used to identify the type of interaction between them. For example, a similar (anti-similar) pattern in a gene expression pattern, which corresponds to gradients with the same (different) signs, implies an AT (RT) interaction, and these patterns can be captured by the time-lagged gradients. The gradients of gene expression curves using their 1st-order derivatives with respect to the experiment time t, is formulated by

$$G_i'(t) = \frac{G_i(t+\Delta t) - G_i(t)}{\Delta t},$$

where $G_i(t + \Delta t)$ denotes the expression level of gene G_i at $(t + \Delta t)$th time point, and Δt is the time interval between two experiments. To identify the type of interaction between a pair of TF-target genes {G_1 and G_2}, we regressed G_1' on G_2' (that is, fitting a straight line to ($G_1'(t)$, $G_2'(t)$), and G_1' and G_2' are independent and dependent variable, respectively), via

$$G_1'(t) = \beta_0 + \beta_1 G_2'(t) + \varepsilon(t),$$

where β_0 is the intercept, β_1 is the slope of the regression line, and $\varepsilon(t)$ denotes the error at time t. Here, a weighted least square estimation on β_0 and β_1 was implemented to circumvent influence of outliers. A positive (negative) β_1 indicates that overall the gradient signs of paired expression curves are of the same (opposite) sign, and this leads to a prediction of an AT (RT) interaction. The value of β_1 can be obtained by the command '*robustfit*' in MATLAB. Furthermore, β_1 was mapped linearly to the interval of [-1, 1], denoted by $\tilde{\beta}_1$, to infer the interaction type of the

gene pair. If $\tilde{\beta}_1$ is positive (negative), then the paired expression curves of G_1 and G_2 has a similar (anti-similar) pattern. The magnitude $|\tilde{\beta}_1|$ indicates the strength of a pairwise interaction. For example, $\tilde{\beta}_1$ = 1 (–1) indicates that the predicted TI has a strong positive (negative) interaction, and 0 indicates that there is no significant interaction between them.

After $\tilde{\beta}_1$ of all predicted TIs have been calculated, we can assess the significance of $\tilde{\beta}_1$ by its p-value. The significance level of 0.0001 is used as a threshold to identify the AT/RT interactions of the predicted TIs under a specific condition of the microarray experiment. Note that a learning approach to determine the threshold can be implemented. However, this will be complicated, so a fixed threshold was used.

Reference

[1] Chuang CL *et al.*: GeneCFE-ANFIS: **A neuro-fuzzy inference system to infer gene-gene interactions based on recognition of microarray gene expression patterns.** *Biomed. Eng. Appl. Basis Comm.* 2007, **19**:71–78.

[2] Chuang CL *et al.*: **A pattern recognition approach to infer time-lagged genetic interactions.** *Bioinformatics* 2008, **24**:1183–1190.

[3] Reis BY *et al.*: **Approaching causality: discovering time-lag correlationships in genetic expression data with static and dynamic relevance networks.** *Proc. of RECOMB* 2000, 5.
