# Supplementary material for: Uncovering transcriptional interactions via an adaptive fuzzy logic approach
Source: BMC Bioinformatics. 2009 Dec 6;10:400. doi: 10.1186/1471-2105-10-400 (PMC2797023; doi:10.1186/1471-2105-10-400)
Supplement: Additional file 4 — Prediction results. The results predicted by the proposed AdaFuzzy. [file 1471-2105-10-400-S4.PDF]

True-positives TIs for training of ANFIS (TF-target)

ABF1	ABF1
ABF1	ACS1
ABF1	ADE3
ABF1	ADE5,7
ABF1	ADE8
ABF1	ADH1
ABF1	ADH5
ABF1	ADY2
ABF1	AGA1
ABF1	AHA1
ABF1	AHC2
ABF1	AIP1
ABF1	ALD3
ABF1	ALG1
ABF1	AME1
ABF1	AMS1
ABF1	ANT1
ABF1	APE3
ABF1	APL1
ABF1	APL2
ABF1	APS2
ABF1	ARC18
ABF1	ARC19
ABF1	ARC35
ABF1	ARN2
ABF1	ARO10
ABF1	ARO3
ABF1	ARO9
ABF1	ARP5
ABF1	ARP8
ABF1	ARP9
ABF1	ATO3
ABF1	ATP18
ABF1	ATP23
ABF1	ATP7
ABF1	AUR1
ABF1	BAP3
ABF1	BCP1
ABF1	BDF1
ABF1	BET2
ABF1	BET3
ABF1	BGL2
ABF1	BNA2
ABF1	BNA4
ABF1	BOP2
ABF1	BRE1
ABF1	BRX1
ABF1	BSD2
ABF1	BTN2
ABF1	BUD31
ABF1	BUD9
ABF1	BYE1
ABF1	CAD1
ABF1	CAF130
ABF1	CAF40
ABF1	CAP1
ABF1	CAR1
ABF1	CAR2
ABF1	CAT8
ABF1	CCT7
ABF1	CCT8
ABF1	CDA1
ABF1	CDC10
ABF1	CDC19
ABF1	CDC24
ABF1	CDC3
ABF1	CDC42
ABF1	CDS1
ABF1	CET1
ABF1	CFD1
ABF1	CHD1
ABF1	CHO1
ABF1	CHS5
ABF1	CIK1
ABF1	CKA1
ABF1	CLF1
ABF1	CLN1
ABF1	CMD1
ABF1	CMS1
ABF1	CNB1
ABF1	COP1
ABF1	COX23
ABF1	COX6
ABF1	COY1
ABF1	CPR1
ABF1	CST6
ABF1	CTF18
ABF1	CTF4
ABF1	CTS1
ABF1	CWC2
ABF1	CYB5
ABF1	CYK3
ABF1	DAL81
ABF1	DBP2
ABF1	DBP6
ABF1	DBP9
ABF1	DCC1
ABF1	DCP2
ABF1	DDR2
ABF1	DED1
ABF1	DFG10
ABF1	DFR1
ABF1	DHH1
ABF1	DHR2
ABF1	DIT1
ABF1	DIT2
ABF1	DMA1
ABF1	DMA2
ABF1	DOG1
ABF1	DPH2
ABF1	DPP1
ABF1	DPS1
ABF1	DSD1
ABF1	DSE1
ABF1	DSL1
ABF1	DSN1
ABF1	DSS4
ABF1	DUG1
ABF1	EBP2
ABF1	ECM16
ABF1	ECM17
ABF1	ECM22
ABF1	ECM25
ABF1	ECM33
ABF1	ECM40
ABF1	EDC1
ABF1	EFT2
ABF1	EGD1
ABF1	ELF1
ABF1	EMP47
ABF1	END3
ABF1	ERG28
ABF1	ERG3
ABF1	ERV25
ABF1	ERV46

ABF1	ESF1
ABF1	FAS1
ABF1	FBA1
ABF1	FCF1
ABF1	FES1
ABF1	FHL1
ABF1	FIN1
ABF1	FIP1
ABF1	FLC2
ABF1	FMP16
ABF1	FMP34
ABF1	FMP38
ABF1	FMP40
ABF1	FOX2
ABF1	FRE7
ABF1	FUI1
ABF1	FUR4
ABF1	FZF1
ABF1	GBP2
ABF1	GCN1
ABF1	GCN2
ABF1	GCN20
ABF1	GIC1
ABF1	GIM3
ABF1	GIS2
ABF1	GLE2
ABF1	GLG1
ABF1	GLT1
ABF1	GMH1
ABF1	GON7
ABF1	GPG1
ABF1	GPI17
ABF1	GPR1
ABF1	GPX1
ABF1	GRS1
ABF1	GRX6
ABF1	GSP1
ABF1	GTT3
ABF1	GUA1
ABF1	GUK1
ABF1	GUT2
ABF1	GZF3
ABF1	HBS1
ABF1	HCR1
ABF1	HDA2
ABF1	HIS4
ABF1	HIS7
ABF1	HMG2
ABF1	HNM1
ABF1	HOF1
ABF1	HOP1
ABF1	HOS2
ABF1	HSC82
ABF1	HSP104
ABF1	HSP12
ABF1	HSP150
ABF1	HSP26
ABF1	HSP30
ABF1	HSP42
ABF1	HSP60
ABF1	HSP78
ABF1	HSP82
ABF1	HST1
ABF1	HUA1
ABF1	IDH1
ABF1	IDP2
ABF1	IKI3
ABF1	IMD1
ABF1	IMD2
ABF1	IME4
ABF1	IMG2
ABF1	IML1
ABF1	INO1
ABF1	IOC2
ABF1	IPP1
ABF1	IQG1
ABF1	IRC19
ABF1	IRC23
ABF1	IRR1
ABF1	JLP1
ABF1	KAP95
ABF1	KAR2
ABF1	KEM1
ABF1	KIN2
ABF1	KRE1
ABF1	KRE9
ABF1	LAC1
ABF1	LAP2
ABF1	LAS21
ABF1	LCB1
ABF1	LDB16
ABF1	LDB19
ABF1	LOT5
ABF1	LPD1
ABF1	LRP1
ABF1	LTV1
ABF1	LYP1
ABF1	LYS21
ABF1	LYS4
ABF1	MAE1
ABF1	MAL12
ABF1	MAL32
ABF1	MAS1
ABF1	MBF1
ABF1	MCH2
ABF1	MCK1
ABF1	MCM16
ABF1	MDM20
ABF1	MDV1
ABF1	MEP1
ABF1	MER1
ABF1	MET3
ABF1	MFA1
ABF1	MGA1
ABF1	MGE1
ABF1	MHR1
ABF1	MIA40
ABF1	MIM1
ABF1	MIP6
ABF1	MKC7
ABF1	MKT1
ABF1	MLC1
ABF1	MLS1
ABF1	MMS1
ABF1	MNN10
ABF1	MNN11
ABF1	MOH1
ABF1	MRP20
ABF1	MRP49
ABF1	MRP7
ABF1	MRPL16
ABF1	MRPL28
ABF1	MRPL32
ABF1	MRPL36
ABF1	MRPL8
ABF1	MRPS12
ABF1	MRPS16
ABF1	MRPS18

ABF1	MRPS9
ABF1	MSC1
ABF1	MSH3
ABF1	MSL1
ABF1	MSN1
ABF1	MSS116
ABF1	MUP1
ABF1	MVP1
ABF1	NAB2
ABF1	NAS6
ABF1	NCB2
ABF1	NCE103
ABF1	NCS2
ABF1	NDE2
ABF1	NDJ1
ABF1	NDL1
ABF1	NEO1
ABF1	NFS1
ABF1	NFU1
ABF1	NGR1
ABF1	NHP2
ABF1	NHX1
ABF1	NIP7
ABF1	NIT3
ABF1	NKP1
ABF1	NMD2
ABF1	NMT1
ABF1	NOB1
ABF1	NOC2
ABF1	NOG2
ABF1	NOP1
ABF1	NOP12
ABF1	NOP14
ABF1	NOP53
ABF1	NOP58
ABF1	NPC2
ABF1	NPR1
ABF1	NPT1
ABF1	NQM1
ABF1	NRD1
ABF1	NSE4
ABF1	NSL1
ABF1	NTA1
ABF1	NTH1
ABF1	NUP1
ABF1	NUP133
ABF1	NUP170
ABF1	NUP57
ABF1	NUP84
ABF1	NUS1
ABF1	OCA5
ABF1	OPY1
ABF1	PAM16
ABF1	PAN6
ABF1	PAU1
ABF1	PDC1
ABF1	PEF1
ABF1	PEP12
ABF1	PES4
ABF1	PET10
ABF1	PET112
ABF1	PET20
ABF1	PET494
ABF1	PEX3
ABF1	PEX5
ABF1	PGA2
ABF1	PGS1
ABF1	PHB2
ABF1	PHO2
ABF1	PHO8
ABF1	PIK1
ABF1	PMA2
ABF1	PMP2
ABF1	PMT2
ABF1	POM152
ABF1	POP2
ABF1	POP8
ABF1	PPH21
ABF1	PRC1
ABF1	PRE6
ABF1	PRM2
ABF1	PRO3
ABF1	PRP4
ABF1	PRP8
ABF1	PRY1
ABF1	PSK1
ABF1	PSY4
ABF1	PTA1
ABF1	PUF3
ABF1	PUP1
ABF1	PUP2
ABF1	PUT3
ABF1	PUT4
ABF1	PWP2
ABF1	PZF1
ABF1	QCR8
ABF1	QCR9
ABF1	QNS1
ABF1	RAD23
ABF1	RAX2
ABF1	RBS1
ABF1	REC102
ABF1	RED1
ABF1	REF2
ABF1	RER1
ABF1	RET1
ABF1	RFA2
ABF1	RGD2
ABF1	RHO4
ABF1	RIB1
ABF1	RIF2
ABF1	RIM101
ABF1	RIX7
ABF1	RKM3
ABF1	RLF2
ABF1	RMD9
ABF1	RMR1
ABF1	RNA1
ABF1	RNA14
ABF1	RNH202
ABF1	RNH70
ABF1	RNP1
ABF1	ROT1
ABF1	RPA12
ABF1	RPB7
ABF1	RPB8
ABF1	RPC25
ABF1	RPC82
ABF1	RPF2
ABF1	RPL18B
ABF1	RPL2A
ABF1	RPL2B
ABF1	RPL3
ABF1	RPL36B
ABF1	RPL4A
ABF1	RPL5
ABF1	RPL7B
ABF1	RPN10

ABF1	RPN4	ABF1	SMK1	ABF1	SUP54
ABF1	RPN5	ABF1	SMX2	ABF1	SWI3
ABF1	RPN8	ABF1	SMY1	ABF1	SWI4
ABF1	RPO21	ABF1	SNA2	ABF1	SWS2
ABF1	RPO26	ABF1	SNA4	ABF1	SYF2
ABF1	RPO31	ABF1	SNF3	ABF1	SYS1
ABF1	RPP1A	ABF1	SNF7	ABF1	TAF4
ABF1	RPS0A	ABF1	SNF8	ABF1	TAF5
ABF1	RPS28A	ABF1	SNR14	ABF1	TAF9
ABF1	RPS28B	ABF1	SNR47	ABF1	TAO3
ABF1	RPT5	ABF1	SNR57	ABF1	TCB1
ABF1	RRI2	ABF1	SNU114	ABF1	TCO89
ABF1	RRP12	ABF1	SNX3	ABF1	TDH3
ABF1	RRP45	ABF1	SOD1	ABF1	TFA1
ABF1	RRP7	ABF1	SOF1	ABF1	TFB1
ABF1	RSC1	ABF1	SPC42	ABF1	TFG1
ABF1	RSC6	ABF1	SPO19	ABF1	TFS1
ABF1	RSF2	ABF1	SPP41	ABF1	THI12
ABF1	RSM19	ABF1	SPR3	ABF1	THI13
ABF1	RSM27	ABF1	SPS1	ABF1	THI5
ABF1	RTC3	ABF1	SPT15	ABF1	THO2
ABF1	RTN1	ABF1	SPT23	ABF1	THR4
ABF1	SAM37	ABF1	SPT8	ABF1	TIF1
ABF1	SAP4	ABF1	SRB2	ABF1	TIF11
ABF1	SCD6	ABF1	SRB8	ABF1	TK(CUU)D1
ABF1	SCL1	ABF1	SRP1	ABF1	TOK1
ABF1	SCS22	ABF1	SSA1	ABF1	TOM6
ABF1	SEC11	ABF1	SSA4	ABF1	TOM7
ABF1	SEC14	ABF1	SSC1	ABF1	TOM70
ABF1	SEC18	ABF1	SSE1	ABF1	TPM2
ABF1	SEC21	ABF1	SSE2	ABF1	TPN1
ABF1	SEC53	ABF1	SSF2	ABF1	TRM112
ABF1	SEC72	ABF1	SSH1	ABF1	TRM2
ABF1	SET3	ABF1	SSM4	ABF1	TRP3
ABF1	SET4	ABF1	SSO1	ABF1	TSC3
ABF1	SFC1	ABF1	SSZ1	ABF1	TUB2
ABF1	SGT1	ABF1	STE14	ABF1	TYS1
ABF1	SHC1	ABF1	STE7	ABF1	UBP1
ABF1	SHE10	ABF1	STI1	ABF1	UBP14
ABF1	SIP1	ABF1	STP1	ABF1	UBP6
ABF1	SKY1	ABF1	STP3	ABF1	UBR2
ABF1	SLA1	ABF1	SUF4	ABF1	UBX5
ABF1	SLA2	ABF1	SUI1	ABF1	UFD2
ABF1	SLC1	ABF1	SUN4	ABF1	UGA3
ABF1	SLM4	ABF1	SUP35	ABF1	ULI1
ABF1	SMF2	ABF1	SUP45	ABF1	ULP2

ABF1	UME6	ABF1	YFR018C	ABF1	YNL213C
ABF1	URA7	ABF1	YGR053C	ABF1	YNL313C
ABF1	URB1	ABF1	YGR066C	ABF1	YNR064C
ABF1	UTP11	ABF1	YGR203W	ABF1	YOL014W
ABF1	UTP13	ABF1	YGR251W	ABF1	YOL036W
ABF1	UTP15	ABF1	YGR269W	ABF1	YOL075C
ABF1	UTP18	ABF1	YHR050W-A	ABF1	YOL157C
ABF1	UTP8	ABF1	YHR078W	ABF1	YOR146W
ABF1	VBA2	ABF1	YHR080C	ABF1	YOR214C
ABF1	VHS2	ABF1	YHR162W	ABF1	YOR262W
ABF1	VID22	ABF1	YIL032C	ABF1	YOR289W
ABF1	VID27	ABF1	YIP3	ABF1	YOR309C
ABF1	VMA6	ABF1	YIR042C	ABF1	YPL229W
ABF1	VNX1	ABF1	YJL055W	ABF1	YPR045C
ABF1	VPS33	ABF1	YJL175W	ABF1	YPR063C
ABF1	VPS54	ABF1	YJL202C	ABF1	YPR077C
ABF1	VTH1	ABF1	YJR096W	ABF1	YPR148C
ABF1	VTH2	ABF1	YJR115W	ABF1	YPR153W
ABF1	VTI1	ABF1	YJR116W	ABF1	YPR157W
ABF1	WSC4	ABF1	YJR128W	ABF1	YPR172W
ABF1	YAP1801	ABF1	YJR162C	ABF1	YPT1
ABF1	YBR284W	ABF1	YKL069W	ABF1	YPT52
ABF1	YCG1	ABF1	YKL111C	ABF1	YRO2
ABF1	YCR001W	ABF1	YKL207W	ABF1	YSP2
ABF1	YDJ1	ABF1	YKR015C	ABF1	YSR3
ABF1	YDL012C	ABF1	YKT6	ABF1	YTA7
ABF1	YDL032W	ABF1	YLL053C	ABF1	ZAP1
ABF1	YDL034W	ABF1	YLR040C	ABF1	ZIP1
ABF1	YDL062W	ABF1	YLR041W	ACE2	AAD16
ABF1	YDL063C	ABF1	YLR173W	ACE2	AAD6
ABF1	YDR029W	ABF1	YLR179C	ACE2	AFG2
ABF1	YDR034W-B	ABF1	YLR230W	ACE2	AIM9
ABF1	YDR266C	ABF1	YLR400W	ACE2	AMN1
ABF1	YDR327W	ABF1	YML081W	ACE2	API2
ABF1	YDR340W	ABF1	YMR090W	ACE2	ASH1
ABF1	YDR360W	ABF1	YMR118C	ACE2	ASN2
ABF1	YDR391C	ABF1	YMR124W	ACE2	BAT2
ABF1	YDR455C	ABF1	YMR325W	ACE2	BEM2
ABF1	YDR476C	ABF1	YNG2	ACE2	BIO2
ABF1	YEH1	ABF1	YNL019C	ACE2	BMH1
ABF1	YEN1	ABF1	YNL043C	ACE2	BUD9
ABF1	YER071C	ABF1	YNL057W	ACE2	CDC26
ABF1	YER158C	ABF1	YNL086W	ACE2	CDC6
ABF1	YER187W	ABF1	YNL115C	ACE2	CHS7
ABF1	YER188W	ABF1	YNL190W	ACE2	CPR1
ABF1	YET3	ABF1	YNL208W	ACE2	CTS1

ACE2	CUE4	ACE2	OST5	ACE2	VPS55
ACE2	CUP1-1	ACE2	OTU2	ACE2	WSC4
ACE2	CUP1-2	ACE2	OXA1	ACE2	WTM1
ACE2	DSE1	ACE2	PAH1	ACE2	YAP1
ACE2	DSE2	ACE2	PCL10	ACE2	YCK2
ACE2	DSE3	ACE2	PCL2	ACE2	YDL041W
ACE2	DSE4	ACE2	PCL8	ACE2	YDR010C
ACE2	ECM32	ACE2	PCL9	ACE2	YDR154C
ACE2	EGD2	ACE2	PET122	ACE2	YDR157W
ACE2	EGT2	ACE2	PIR1	ACE2	YEA6
ACE2	ERG6	ACE2	PIR3	ACE2	YEL007W
ACE2	FRS2	ACE2	PMA1	ACE2	YEL008W
ACE2	GAT1	ACE2	PRY3	ACE2	YER077C
ACE2	GDH3	ACE2	PSA1	ACE2	YER078C
ACE2	GLO1	ACE2	PST1	ACE2	YER078W-A
ACE2	GTR1	ACE2	RAS2	ACE2	YER079W
ACE2	GUF1	ACE2	REE1	ACE2	YER152C
ACE2	HMS2	ACE2	REG2	ACE2	YER189W
ACE2	HO	ACE2	RME1	ACE2	YFR017C
ACE2	HOM2	ACE2	RMI1	ACE2	YGL006W-A
ACE2	HOT13	ACE2	ROT2	ACE2	YGL007C-A
ACE2	HSP150	ACE2	RPA14	ACE2	YGL007W
ACE2	ICS2	ACE2	RPA34	ACE2	YGR042W
ACE2	IFA38	ACE2	RPS30A	ACE2	YGR125W
ACE2	ISR1	ACE2	RPS4A	ACE2	YGR283C
ACE2	ITC1	ACE2	RSP5	ACE2	YHB1
ACE2	KSS1	ACE2	RUP1	ACE2	YHL012W
ACE2	LCP5	ACE2	SAP4	ACE2	YJL075C
ACE2	LSM3	ACE2	SBH1	ACE2	YJL077W-B
ACE2	MCM2	ACE2	SCP160	ACE2	YJL160C
ACE2	MCR1	ACE2	SCW11	ACE2	YJR146W
ACE2	MDH1	ACE2	SER3	ACE2	YJR149W
ACE2	MDJ2	ACE2	SFL1	ACE2	YKL151C
ACE2	MDM31	ACE2	SHE10	ACE2	YKR040C
ACE2	MGM101	ACE2	SIC1	ACE2	YKR041W
ACE2	MRP8	ACE2	SNA2	ACE2	YML003W
ACE2	MRPL4	ACE2	SNQ2	ACE2	YML100W-A
ACE2	MSR1	ACE2	SNR60	ACE2	YMR134W
ACE2	MTC3	ACE2	TAH11	ACE2	YMR135W-A
ACE2	MUM2	ACE2	TIM23	ACE2	YMR262W
ACE2	NIS1	ACE2	TPM1	ACE2	YNR018W
ACE2	NSA2	ACE2	TPS3	ACE2	YOR314W
ACE2	NUP100	ACE2	TSL1	ACE2	YPL025C
ACE2	NVJ1	ACE2	UBP6	ACE2	YPL158C
ACE2	OCA5	ACE2	UTH1	ACE2	YRF1-2
ACE2	OM14	ACE2	VID30	ACE2	YRF1-7

ACE2	ZWF1
FKH1	AAD15
FKH1	ACE2
FKH1	ADD37
FKH1	ADH4
FKH1	AFR1
FKH1	ALG1
FKH1	ALG14
FKH1	ALG3
FKH1	ALG5
FKH1	ALK1
FKH1	AMN1
FKH1	APE2
FKH1	APM3
FKH1	ARP7
FKH1	AVT4
FKH1	BAR1
FKH1	BDF1
FKH1	BNR1
FKH1	BRN1
FKH1	BUB1
FKH1	BUD3
FKH1	BUD4
FKH1	BUD8
FKH1	BUD9
FKH1	CDC20
FKH1	CDC39
FKH1	CDC5
FKH1	CDC7
FKH1	CDS1
FKH1	CHA1
FKH1	CHS2
FKH1	CHS7
FKH1	CIK1
FKH1	CLB1
FKH1	CLB2
FKH1	CLB4
FKH1	CLN1
FKH1	CMD1
FKH1	CSN9
FKH1	CTF18
FKH1	CTS1
FKH1	CUE4
FKH1	CWP2
FKH1	DBF2
FKH1	DEM1
FKH1	DIN7
FKH1	DPP1
FKH1	DSE1
FKH1	DSE2
FKH1	DUG1
FKH1	DYN1
FKH1	EAP1
FKH1	ECM33
FKH1	EDC3
FKH1	ERP3
FKH1	ERS1
FKH1	ESP1
FKH1	EXG1
FKH1	FAB1
FKH1	FHL1
FKH1	FIR1
FKH1	FKH2
FKH1	FMP34
FKH1	FMP48
FKH1	GAS3
FKH1	GAS5
FKH1	GLG1
FKH1	GLN1
FKH1	GLO3
FKH1	GND1
FKH1	GSG1
FKH1	GSH1
FKH1	GSM1
FKH1	HAP5
FKH1	HOF1
FKH1	HOG1
FKH1	HOS3
FKH1	HSP104
FKH1	HST3
FKH1	HUA1
FKH1	HXT1
FKH1	ICS2
FKH1	IDI1
FKH1	IFM1
FKH1	IME1
FKH1	IPI1
FKH1	IQG1
FKH1	IRC8
FKH1	JSN1
FKH1	KAR4
FKH1	KIP2
FKH1	KSS1
FKH1	LEU4
FKH1	LSM6
FKH1	MATA1
FKH1	MDM1
FKH1	MFA2
FKH1	MKK2
FKH1	MMR1
FKH1	MNT2
FKH1	MOB1
FKH1	MSH3
FKH1	MSM1
FKH1	MYO1
FKH1	NDE2
FKH1	NEW1
FKH1	NNF1
FKH1	NPP2
FKH1	NST1
FKH1	NUP145
FKH1	OPT1
FKH1	OPT2
FKH1	OPY2
FKH1	ORC6
FKH1	PBN1
FKH1	PDS1
FKH1	PDS5
FKH1	PES4
FKH1	PHO11
FKH1	PNP1
FKH1	PPN1
FKH1	PRP31
FKH1	PRY3
FKH1	PTC3
FKH1	PTH2
FKH1	RCR2
FKH1	REG2
FKH1	RET2
FKH1	RHO4
FKH1	RKM3
FKH1	RNR1
FKH1	RPL20B
FKH1	RPL31B
FKH1	RPL37B
FKH1	RPL39
FKH1	RPN10
FKH1	RPN11

FKH1	RPN12
FKH1	RPS22A
FKH1	RSP5
FKH1	RTC3
FKH1	SAM35
FKH1	SAP30
FKH1	SCJ1
FKH1	SCW11
FKH1	SEC14
FKH1	SEC28
FKH1	SET1
FKH1	SFG1
FKH1	SIF2
FKH1	SIL1
FKH1	SKM1
FKH1	SLM4
FKH1	SNF5
FKH1	SNX3
FKH1	SPC105
FKH1	SPC24
FKH1	SPS4
FKH1	SRC1
FKH1	SSO2
FKH1	STE12
FKH1	SUB2
FKH1	SUR7
FKH1	SVL3
FKH1	SWI5
FKH1	TEL2
FKH1	TEM1
FKH1	THO1
FKH1	TIF1
FKH1	TIM18
FKH1	TMN2
FKH1	TOM71
FKH1	TPO3
FKH1	TRM112
FKH1	TRS120
FKH1	TSL1
FKH1	TUB2
FKH1	TVP23
FKH1	UME1
FKH1	UTP4
FKH1	UTR2
FKH1	VAC17
FKH1	VBA3
FKH1	VPS54
FKH1	VTI1
FKH1	YAR070C
FKH1	YBL081W
FKH1	YBL083C
FKH1	YBR071W
FKH1	YBR138C
FKH1	YBR139W
FKH1	YCG1
FKH1	YCK3
FKH1	YCR075W-A
FKH1	YDR115W
FKH1	YDR179W-A
FKH1	YDR370C
FKH1	YFR018C
FKH1	YGL006W-A
FKH1	YGL007C-A
FKH1	YGL007W
FKH1	YGR050C
FKH1	YHK8
FKH1	YHP1
FKH1	YHR095W
FKH1	YHR182W
FKH1	YIL077C
FKH1	YIL158W
FKH1	YJR111C
FKH1	YKL069W
FKH1	YKL097C
FKH1	YKL171W
FKH1	YLL007C
FKH1	YLR352W
FKH1	YLR400W
FKH1	YLR407W
FKH1	YMC2
FKH1	YML119W
FKH1	YMR085W
FKH1	YMR086W
FKH1	YMR144W
FKH1	YNG2
FKH1	YNL058C
FKH1	YNL092W
FKH1	YNL174W
FKH1	YNL176C
FKH1	YNL213C
FKH1	YOL024W
FKH1	YOL114C
FKH1	YPI1
FKH1	YPL141C
FKH1	YPR013C
FKH1	YPR195C
FKH1	YPR196W
FKH1	YPT1
FKH1	YPT31
FKH1	YPT53
FKH1	YRB1
FKH1	YSY6
FKH1	YTA7
FKH1	ZRT1
FKH2	ABP140
FKH2	ACE2
FKH2	ACS1
FKH2	ADD37
FKH2	ADH4
FKH2	AHA1
FKH2	ALK1
FKH2	AMD1
FKH2	AMN1
FKH2	APC1
FKH2	APE2
FKH2	ARG5,6
FKH2	ARP7
FKH2	ASE1
FKH2	ASG1
FKH2	ATG26
FKH2	ATG8
FKH2	ATR1
FKH2	AYR1
FKH2	BDF1
FKH2	BNA4
FKH2	BNR1
FKH2	BNS1
FKH2	BRN1
FKH2	BUD3
FKH2	BUD4
FKH2	BUD7
FKH2	BUD9
FKH2	CDC15
FKH2	CDC20
FKH2	CDC5
FKH2	CDC6
FKH2	CDC7
FKH2	CDC8

FKH2	CHS2
FKH2	CHS7
FKH2	CIK1
FKH2	CIS3
FKH2	CKS1
FKH2	CLB1
FKH2	CLB2
FKH2	CLB6
FKH2	CLN1
FKH2	COG6
FKH2	COT1
FKH2	CPA2
FKH2	CPR6
FKH2	CTS1
FKH2	CUP9
FKH2	CUR1
FKH2	CWP1
FKH2	CWP2
FKH2	CYC7
FKH2	DBF2
FKH2	DCS2
FKH2	DIF1
FKH2	DIN7
FKH2	DIT2
FKH2	DSE1
FKH2	DSE2
FKH2	DUN1
FKH2	DUS3
FKH2	ECM19
FKH2	ECM2
FKH2	ECM33
FKH2	ECM38
FKH2	EFB1
FKH2	EKI1
FKH2	ELO1
FKH2	ERG26
FKH2	ERP3
FKH2	EXG1
FKH2	EXG2
FKH2	FIR1
FKH2	FKH1
FKH2	FKH2
FKH2	FLC2
FKH2	FRS2
FKH2	FRT2
FKH2	GAL80
FKH2	GAS3
FKH2	GAT1
FKH2	GDH2
FKH2	GIC1
FKH2	GIC2
FKH2	GLG1
FKH2	GLN1
FKH2	HDA3
FKH2	HEK2
FKH2	HHF1
FKH2	HHT1
FKH2	HOF1
FKH2	HOS3
FKH2	HPF1
FKH2	HSL7
FKH2	HSP10
FKH2	HSP30
FKH2	HSP60
FKH2	HST3
FKH2	HXT1
FKH2	HXT12
FKH2	ICS2
FKH2	IDI1
FKH2	IFM1
FKH2	IQG1
FKH2	IRC8
FKH2	JJJ3
FKH2	JSN1
FKH2	KAR4
FKH2	KGD2
FKH2	KIN3
FKH2	KIN4
FKH2	KIP2
FKH2	KRR1
FKH2	LCB5
FKH2	LSM3
FKH2	LST8
FKH2	LTE1
FKH2	MDG1
FKH2	MMR1
FKH2	MND1
FKH2	MNT2
FKH2	MOB1
FKH2	MRH1
FKH2	MRPL4
FKH2	MSB1
FKH2	MTC6
FKH2	MTR4
FKH2	MYO1
FKH2	MYO4
FKH2	NCE102
FKH2	NDD1
FKH2	NOP7
FKH2	NSR1
FKH2	NUM1
FKH2	OCA2
FKH2	OCA5
FKH2	OLE1
FKH2	OPT2
FKH2	OSH7
FKH2	PAI3
FKH2	PAU17
FKH2	PBY1
FKH2	PCL1
FKH2	PDS1
FKH2	PDS5
FKH2	PES4
FKH2	PEX13
FKH2	PGM2
FKH2	PHO11
FKH2	PHO3
FKH2	PHO5
FKH2	PIC2
FKH2	PLM2
FKH2	PMA1
FKH2	PMP1
FKH2	PMR1
FKH2	PPN1
FKH2	PRP31
FKH2	PRY1
FKH2	PRY3
FKH2	PST2
FKH2	PTR2
FKH2	PUP3
FKH2	RAD3
FKH2	RAD51
FKH2	RAX2
FKH2	RIB1
FKH2	RIM4
FKH2	RKI1
FKH2	RMD11
FKH2	RMI1

FKH2	RMT2	FKH2	TAH11	FKH2	YGL114W
FKH2	RNR1	FKH2	TDH1	FKH2	YGL117W
FKH2	ROG3	FKH2	TEM1	FKH2	YGR050C
FKH2	RPD3	FKH2	TFB1	FKH2	YGR168C
FKH2	RPL20B	FKH2	TIF1	FKH2	YHP1
FKH2	RPL37B	FKH2	TIM9	FKH2	YHR032W
FKH2	RPS16A	FKH2	TMA17	FKH2	YIL158W
FKH2	RPS1B	FKH2	TPO3	FKH2	YIL169C
FKH2	RPS30A	FKH2	TSA1	FKH2	YJR111C
FKH2	RRM3	FKH2	TT(AGU)N1	FKH2	YJR128W
FKH2	RSF2	FKH2	TUB3	FKH2	YKL066W
FKH2	RSP5	FKH2	TV(AAC)J	FKH2	YKL096C-B
FKH2	SCJ1	FKH2	TW(CCA)G1	FKH2	YKL097C
FKH2	SCO1	FKH2	UBI4	FKH2	YKR040C
FKH2	SCW11	FKH2	UBR1	FKH2	YKR041W
FKH2	SDC25	FKH2	UME1	FKH2	YLR042C
FKH2	SED1	FKH2	UTH1	FKH2	YLR301W
FKH2	SFG1	FKH2	UTP4	FKH2	YLR400W
FKH2	SFP1	FKH2	UTR2	FKH2	YMC2
FKH2	SGO1	FKH2	VPS73	FKH2	YML050W
FKH2	SHE1	FKH2	VTI1	FKH2	YML053C
FKH2	SIF2	FKH2	WHI4	FKH2	YML119W
FKH2	SIM1	FKH2	WSC4	FKH2	YMR031C
FKH2	SIS1	FKH2	WTM1	FKH2	YMR1
FKH2	SKM1	FKH2	YAP6	FKH2	YMR144W
FKH2	SLM2	FKH2	YAR070C	FKH2	YMR258C
FKH2	SLM4	FKH2	YBL029C-A	FKH2	YNL040W
FKH2	SMI1	FKH2	YBL029W	FKH2	YNL046W
FKH2	SNF4	FKH2	YBR134W	FKH2	YNL057W
FKH2	SNF6	FKH2	YBR138C	FKH2	YNL058C
FKH2	SNR66	FKH2	YBR139W	FKH2	YNL170W
FKH2	SPB1	FKH2	YBR241C	FKH2	YNL174W
FKH2	SPC24	FKH2	YCG1	FKH2	YNL176C
FKH2	SPO12	FKH2	YCR025C	FKH2	YNR014W
FKH2	SPS4	FKH2	YDR115W	FKH2	YOL024W
FKH2	SRC1	FKH2	YDR220C	FKH2	YOL114C
FKH2	SRL1	FKH2	YDR222W	FKH2	YOR020W-A
FKH2	SSA1	FKH2	YER067W	FKH2	YOR246C
FKH2	SSF2	FKH2	YER078C	FKH2	YOR248W
FKH2	SSO2	FKH2	YER079W	FKH2	YOR314W
FKH2	STT3	FKH2	YER189W	FKH2	YPL025C
FKH2	SUF11	FKH2	YFR017C	FKH2	YPL141C
FKH2	SUR7	FKH2	YGL006W-A	FKH2	YPL142C
FKH2	SUT1	FKH2	YGL007C-A	FKH2	YPR148C
FKH2	SVL3	FKH2	YGL007W	FKH2	YPR195C
FKH2	SWI5	FKH2	YGL102C	FKH2	YPR196W

FKH2	YPT31
FKH2	YRF1-2
FKH2	YRF1-3
FKH2	YRO2
FKH2	ZWF1
GCN4	AAD10
GCN4	AAT2
GCN4	ACO2
GCN4	ADE1
GCN4	ADE12
GCN4	ADE13
GCN4	ADE17
GCN4	ADE2
GCN4	ADE3
GCN4	ADE4
GCN4	ADE5,7
GCN4	ADE6
GCN4	ADE8
GCN4	ADH1
GCN4	ADH5
GCN4	ADO1
GCN4	AEP1
GCN4	AFG2
GCN4	AGC1
GCN4	AGP1
GCN4	AGX1
GCN4	ALB1
GCN4	ALD5
GCN4	ALD6
GCN4	ALT1
GCN4	AMD2
GCN4	ANB1
GCN4	ANT1
GCN4	APC5
GCN4	APN1
GCN4	AQR1
GCN4	ARG1
GCN4	ARG2
GCN4	ARG3
GCN4	ARG4
GCN4	ARG5,6
GCN4	ARG8
GCN4	ARG80
GCN4	ARO1
GCN4	ARO10
GCN4	ARO2
GCN4	ARO3
GCN4	ARO4
GCN4	ARO8
GCN4	ARO9
GCN4	ARP10
GCN4	ARP2
GCN4	ARV1
GCN4	ASC1
GCN4	ASF2
GCN4	ASG1
GCN4	ASI1
GCN4	ASN1
GCN4	ASN2
GCN4	ATG1
GCN4	ATG13
GCN4	ATH1
GCN4	ATO3
GCN4	ATP7
GCN4	ATP8
GCN4	ATR1
GCN4	BAP2
GCN4	BAS1
GCN4	BAT1
GCN4	BAT2
GCN4	BEM2
GCN4	BIO2
GCN4	BIO3
GCN4	BIO4
GCN4	BIO5
GCN4	BMH1
GCN4	BNA1
GCN4	BNA3
GCN4	BNA4
GCN4	BNA6
GCN4	BOP2
GCN4	BSC5
GCN4	BUB1
GCN4	BUD16
GCN4	CAF16
GCN4	CAN1
GCN4	CAR1
GCN4	CCT5
GCN4	CCT6
GCN4	CCT8
GCN4	CDC36
GCN4	CFD1
GCN4	CGR1
GCN4	CHA1
GCN4	CIN4
GCN4	CIN8
GCN4	CIS3
GCN4	CIT2
GCN4	CIT3
GCN4	CLN1
GCN4	CLN2
GCN4	CLN3
GCN4	COQ5
GCN4	COR1
GCN4	COX17
GCN4	COX9
GCN4	COY1
GCN4	CPA1
GCN4	CPA2
GCN4	CPT1
GCN4	CRC1
GCN4	CRN1
GCN4	CSN9
GCN4	CTK1
GCN4	CTS1
GCN4	CWP1
GCN4	CWP2
GCN4	CYC8
GCN4	CYS3
GCN4	CYS4
GCN4	DAL1
GCN4	DAL5
GCN4	DBF20
GCN4	DDR48
GCN4	DED1
GCN4	DED81
GCN4	DLD2
GCN4	DOA1
GCN4	DRS1
GCN4	DRS2
GCN4	DSE3
GCN4	DSK2
GCN4	DSS1
GCN4	DTD1
GCN4	ECM13
GCN4	ECM17
GCN4	ECM18
GCN4	ECM32

GCN4	ECM4
GCN4	ECM40
GCN4	EMI1
GCN4	ENA1
GCN4	ENA2
GCN4	ENO2
GCN4	ERG13
GCN4	ERG9
GCN4	ESBP6
GCN4	EXO70
GCN4	FAA3
GCN4	FBA1
GCN4	FLO1
GCN4	FMP21
GCN4	FMP23
GCN4	FMP34
GCN4	FOL2
GCN4	FRE8
GCN4	FRM2
GCN4	FRS2
GCN4	GAD1
GCN4	GAL1
GCN4	GAP1
GCN4	GAS4
GCN4	GAT1
GCN4	GAT2
GCN4	GCN20
GCN4	GCN4
GCN4	GCV1
GCN4	GCV2
GCN4	GCV3
GCN4	GDB1
GCN4	GDH1
GCN4	GGC1
GCN4	GIC2
GCN4	GID8
GCN4	GIP1
GCN4	GIP3
GCN4	GLC3
GCN4	GLG1
GCN4	GLK1
GCN4	GLN1
GCN4	GLN3
GCN4	GLT1
GCN4	GLY1
GCN4	GNP1
GCN4	GPD1
GCN4	GPD2
GCN4	GPG1
GCN4	GPH1
GCN4	GPM2
GCN4	GPM3
GCN4	GRE3
GCN4	GRX2
GCN4	GSY1
GCN4	GSY2
GCN4	HAL1
GCN4	HFA1
GCN4	HFD1
GCN4	HHF1
GCN4	HHF2
GCN4	HIS1
GCN4	HIS2
GCN4	HIS3
GCN4	HIS4
GCN4	HIS5
GCN4	HIS7
GCN4	HMS2
GCN4	HMT1
GCN4	HOM2
GCN4	HOM3
GCN4	HOR2
GCN4	HOS2
GCN4	HRB1
GCN4	HSH155
GCN4	HSP12
GCN4	HSP26
GCN4	HSP30
GCN4	HSP33
GCN4	HSP78
GCN4	HTA2
GCN4	HTB2
GCN4	HUA1
GCN4	HVG1
GCN4	HXK2
GCN4	HXT5
GCN4	HYP2
GCN4	ICL1
GCN4	ICY1
GCN4	ICY2
GCN4	IDH1
GCN4	IDP1
GCN4	IES6
GCN4	ILS1
GCN4	ILV1
GCN4	ILV2
GCN4	ILV3
GCN4	ILV5
GCN4	ILV6
GCN4	IMT2
GCN4	INP53
GCN4	IRC7
GCN4	ISA1
GCN4	ISU1
GCN4	ISW2
GCN4	JEN1
GCN4	JJJ3
GCN4	KAR1
GCN4	KAR5
GCN4	KAR9
GCN4	KRE5
GCN4	KRS1
GCN4	LAP3
GCN4	LAP4
GCN4	LEU1
GCN4	LEU2
GCN4	LEU3
GCN4	LEU4
GCN4	LEU9
GCN4	LPD1
GCN4	LSM12
GCN4	LSP1
GCN4	LST8
GCN4	LYP1
GCN4	LYS1
GCN4	LYS12
GCN4	LYS14
GCN4	LYS2
GCN4	LYS20
GCN4	LYS21
GCN4	LYS4
GCN4	LYS5
GCN4	LYS9
GCN4	MAE1
GCN4	MAS2
GCN4	MCH1
GCN4	MCH4
GCN4	MCT1

GCN4	MET1
GCN4	MET10
GCN4	MET13
GCN4	MET14
GCN4	MET16
GCN4	MET17
GCN4	MET2
GCN4	MET22
GCN4	MET28
GCN4	MET3
GCN4	MET4
GCN4	MLS1
GCN4	MOT2
GCN4	MRM1
GCN4	MRP7
GCN4	MRPS16
GCN4	MRPS17
GCN4	MRS4
GCN4	MSL1
GCN4	MTC4
GCN4	MTD1
GCN4	MTH1
GCN4	MTO1
GCN4	MUC1
GCN4	MUD1
GCN4	MUP3
GCN4	MYO1
GCN4	NAF1
GCN4	NAR1
GCN4	NCE103
GCN4	NDE2
GCN4	NIT1
GCN4	NNF1
GCN4	NOG1
GCN4	NOP13
GCN4	NPR1
GCN4	NPR2
GCN4	NQM1
GCN4	NRK1
GCN4	NRM1
GCN4	NRP1
GCN4	NTG1
GCN4	NTH1
GCN4	NUT2
GCN4	ODC2
GCN4	OPT1
GCN4	ORC3
GCN4	ORT1
GCN4	PAA1
GCN4	PCK1
GCN4	PCL5
GCN4	PDC1
GCN4	PDR8
GCN4	PDX3
GCN4	PEX14
GCN4	PEX2
GCN4	PEX21
GCN4	PEX5
GCN4	PFK2
GCN4	PHD1
GCN4	PHO11
GCN4	PHO5
GCN4	PHO8
GCN4	PIC2
GCN4	PIP2
GCN4	PIR1
GCN4	PIR3
GCN4	PKP1
GCN4	PKP2
GCN4	PLB1
GCN4	PNC1
GCN4	POB3
GCN4	POP4
GCN4	PRM5
GCN4	PRO1
GCN4	PRO2
GCN4	PRP28
GCN4	PSA1
GCN4	PSF2
GCN4	PSO2
GCN4	PTC2
GCN4	PTP1
GCN4	PUT3
GCN4	PYC2
GCN4	QDR2
GCN4	QDR3
GCN4	RAD14
GCN4	RAD16
GCN4	RAD26
GCN4	RAD5
GCN4	RAD55
GCN4	RAM2
GCN4	RAX1
GCN4	RBA50
GCN4	RGS2
GCN4	RIB1
GCN4	RIB3
GCN4	RIB5
GCN4	RIM101
GCN4	RIM9
GCN4	RNY1
GCN4	RPA34
GCN4	RPC17
GCN4	RPC37
GCN4	RPC40
GCN4	RPI1
GCN4	RPL11A
GCN4	RPL29
GCN4	RPL43B
GCN4	RPS0B
GCN4	RPS10A
GCN4	RPS12
GCN4	RPS24A
GCN4	RPS30A
GCN4	RPS30B
GCN4	RPS4A
GCN4	RRM3
GCN4	RRN11
GCN4	RRN7
GCN4	RRP12
GCN4	RRP5
GCN4	RSA4
GCN4	RTC2
GCN4	RTG3
GCN4	RTS3
GCN4	SAM4
GCN4	SAP4
GCN4	SCM3
GCN4	SCS3
GCN4	SDS22
GCN4	SDS24
GCN4	SEC4
GCN4	SER1
GCN4	SER3
GCN4	SER33
GCN4	SFT2
GCN4	SIP4
GCN4	SKP2

GCN4	SMF1	GCN4	TRI1	GCN4	YCR023C
GCN4	SMT3	GCN4	TRL1	GCN4	YCT1
GCN4	SNO1	GCN4	TRM1	GCN4	YDL025C
GCN4	SNQ2	GCN4	TRM11	GCN4	YDL183C
GCN4	SNR45	GCN4	TRM5	GCN4	YDL196W
GCN4	SNU13	GCN4	TRP1	GCN4	YDR010C
GCN4	SNZ1	GCN4	TRP2	GCN4	YDR034W-B
GCN4	SNZ2	GCN4	TRP3	GCN4	YDR341C
GCN4	SNZ3	GCN4	TRP4	GCN4	YDR509W
GCN4	SOD1	GCN4	TRP5	GCN4	YDR531W
GCN4	SOL3	GCN4	TRS20	GCN4	YEL045C
GCN4	SOV1	GCN4	TVP23	GCN4	YER085C
GCN4	SPE3	GCN4	UBC11	GCN4	YGL117W
GCN4	SPG4	GCN4	UBC13	GCN4	YGL118C
GCN4	SPO23	GCN4	UGA2	GCN4	YGL231C
GCN4	SPO75	GCN4	UGA3	GCN4	YGR066C
GCN4	SRX1	GCN4	UGX2	GCN4	YGR067C
GCN4	SRY1	GCN4	URA10	GCN4	YGR069W
GCN4	SSU1	GCN4	URA2	GCN4	YGR079W
GCN4	STB1	GCN4	URE2	GCN4	YGR269W
GCN4	STB4	GCN4	UTP9	GCN4	YHB1
GCN4	STE11	GCN4	UTR5	GCN4	YHI9
GCN4	STE12	GCN4	VAS1	GCN4	YHR020W
GCN4	STR3	GCN4	VHR1	GCN4	YHR022C
GCN4	SUL2	GCN4	VHT1	GCN4	YHR122W
GCN4	SWF1	GCN4	VMA13	GCN4	YHR162W
GCN4	TDH3	GCN4	VMA8	GCN4	YIL067C
GCN4	TEA1	GCN4	VPS41	GCN4	YIL077C
GCN4	TEF2	GCN4	VPS60	GCN4	YIL087C
GCN4	TFC7	GCN4	VTC1	GCN4	YIL092W
GCN4	THI20	GCN4	VTS1	GCN4	YIL152W
GCN4	THI7	GCN4	WAR1	GCN4	YIL165C
GCN4	THI73	GCN4	WTM1	GCN4	YIR035C
GCN4	THR1	GCN4	YAH1	GCN4	YJL086C
GCN4	THR4	GCN4	YAP1801	GCN4	YJL160C
GCN4	TIM22	GCN4	YAP5	GCN4	YJR008W
GCN4	TIP41	GCN4	YAR047C	GCN4	YJR018W
GCN4	TMA17	GCN4	YAR053W	GCN4	YJR026W
GCN4	TMT1	GCN4	YAT2	GCN4	YJR027W
GCN4	TOS1	GCN4	YBL044W	GCN4	YJR028W
GCN4	TPI1	GCN4	YBL081W	GCN4	YJR029W
GCN4	TPK1	GCN4	YBP2	GCN4	YJR096W
GCN4	TPK2	GCN4	YBR113W	GCN4	YJR111C
GCN4	TPN1	GCN4	YBR144C	GCN4	YJR146W
GCN4	TPS1	GCN4	YBT1	GCN4	YKE2
GCN4	TPT1	GCN4	YCL049C	GCN4	YKE4

GCN4	YLR049C
GCN4	YLR152C
GCN4	YLR194C
GCN4	YLR356W
GCN4	YLR358C
GCN4	YMC1
GCN4	YMC2
GCN4	YML087C
GCN4	YMR1
GCN4	YMR194C-A
GCN4	YMR196W
GCN4	YOL050C
GCN4	YOL057W
GCN4	YOR111W
GCN4	YOR203W
GCN4	YOR302W
GCN4	YPL150W
GCN4	YPL264C
GCN4	YPR038W
GCN4	YPR039W
GCN4	YPS6
GCN4	YRO2
GCN4	YSC84
GCN4	YSY6
GCN4	ZTA1
GCN4	ZUO1
GCN4	ZWF1
LEU3	AAT2
LEU3	ACO1
LEU3	ACS2
LEU3	ADD37
LEU3	ADE3
LEU3	ADE8
LEU3	ADH1
LEU3	ADH2
LEU3	ADH6
LEU3	ADR1
LEU3	AFG3
LEU3	AFI1
LEU3	AFR1
LEU3	AFT1
LEU3	AGA1
LEU3	AGP1
LEU3	AHA1
LEU3	AHP1
LEU3	ALD6
LEU3	AMN1
LEU3	ANB1
LEU3	APC1
LEU3	APE2
LEU3	AQY1
LEU3	ARG2
LEU3	ARG4
LEU3	ARG5,6
LEU3	ARG8
LEU3	ARG80
LEU3	ARO1
LEU3	ARO10
LEU3	ARO3
LEU3	ARO4
LEU3	ARO8
LEU3	ARO9
LEU3	ARR2
LEU3	ARR3
LEU3	ASN1
LEU3	ASN2
LEU3	ATH1
LEU3	ATO2
LEU3	ATP1
LEU3	AVO1
LEU3	AVO2
LEU3	AXL1
LEU3	BAT2
LEU3	BCK1
LEU3	BEM2
LEU3	BGL2
LEU3	BIO2
LEU3	BIO3
LEU3	BIR1
LEU3	BNA1
LEU3	BNA4
LEU3	BNA5
LEU3	BNI1
LEU3	BOI2
LEU3	BRF1
LEU3	BUD22
LEU3	BUD31
LEU3	CAM1
LEU3	CAR1
LEU3	CAR2
LEU3	CBP1
LEU3	CCS1
LEU3	CDA1
LEU3	CDC19
LEU3	CDC31
LEU3	CHL4
LEU3	CIT3
LEU3	COX19
LEU3	CPA2
LEU3	CPR1
LEU3	CRF1
LEU3	CRG1
LEU3	CSL4
LEU3	CTR2
LEU3	CUP5
LEU3	CUS1
LEU3	CWC27
LEU3	CWP1
LEU3	CYR1
LEU3	DAL1
LEU3	DAL2
LEU3	DAL3
LEU3	DAL7
LEU3	DCC1
LEU3	DEM1
LEU3	DFR1
LEU3	DGR2
LEU3	DIE2
LEU3	DIF1
LEU3	DIN7
LEU3	DOM34
LEU3	DON1
LEU3	DOT6
LEU3	DRE2
LEU3	DST1
LEU3	DUR3
LEU3	EAF7
LEU3	ECM17
LEU3	ECM32
LEU3	ECM33
LEU3	ECM40
LEU3	EDS1
LEU3	EFB1
LEU3	ELA1
LEU3	ELF1
LEU3	ENT5
LEU3	ERG26
LEU3	ERG3

LEU3	ESS1
LEU3	FBP1
LEU3	FET3
LEU3	FIT2
LEU3	FMP41
LEU3	FMP48
LEU3	FMP52
LEU3	FMT1
LEU3	FOL2
LEU3	FPR1
LEU3	FPS1
LEU3	FRS2
LEU3	FSF1
LEU3	FSH3
LEU3	FYV6
LEU3	GAC1
LEU3	GAS2
LEU3	GCD10
LEU3	GCV1
LEU3	GCV2
LEU3	GDB1
LEU3	GDH1
LEU3	GDH3
LEU3	GDS1
LEU3	GID8
LEU3	GIS1
LEU3	GIS4
LEU3	GLC3
LEU3	GLG1
LEU3	GLY1
LEU3	GNA1
LEU3	GPG1
LEU3	GPH1
LEU3	GPR1
LEU3	GRX6
LEU3	GSY1
LEU3	GSY2
LEU3	HAP1
LEU3	HAP4
LEU3	HAS1
LEU3	HER1
LEU3	HIS1
LEU3	HIS2
LEU3	HIS3
LEU3	HIS5
LEU3	HIS7
LEU3	HMO1
LEU3	HMS2
LEU3	HMX1
LEU3	HNT2
LEU3	HOG1
LEU3	HOM3
LEU3	HOR7
LEU3	HOT1
LEU3	HST4
LEU3	HTZ1
LEU3	HVG1
LEU3	HXT8
LEU3	ICL2
LEU3	ICS2
LEU3	ICY2
LEU3	IDP1
LEU3	IES1
LEU3	IES6
LEU3	ILV6
LEU3	IMD1
LEU3	IMD2
LEU3	INH1
LEU3	IRA1
LEU3	IRA2
LEU3	ISN1
LEU3	ISU1
LEU3	KRS1
LEU3	KTI12
LEU3	LEU9
LEU3	LPX1
LEU3	LRG1
LEU3	LYS1
LEU3	LYS20
LEU3	LYS21
LEU3	LYS5
LEU3	MAC1
LEU3	MAK32
LEU3	MCM1
LEU3	MCM3
LEU3	MDG1
LEU3	MDH3
LEU3	MDJ1
LEU3	MEP2
LEU3	MET1
LEU3	MET10
LEU3	MET13
LEU3	MET14
LEU3	MET16
LEU3	MET17
LEU3	MET22
LEU3	MET3
LEU3	MET32
LEU3	MET8
LEU3	MGA1
LEU3	MGR2
LEU3	MHP1
LEU3	MHT1
LEU3	MIC17
LEU3	MLH2
LEU3	MMF1
LEU3	MNN1
LEU3	MNN4
LEU3	MOT3
LEU3	MPS2
LEU3	MRM1
LEU3	MRPL11
LEU3	MRPL15
LEU3	MRPL24
LEU3	MSN4
LEU3	MSS1
LEU3	MTH1
LEU3	MTQ1
LEU3	MUM3
LEU3	MXR1
LEU3	NAR1
LEU3	NAT4
LEU3	NFS1
LEU3	NGG1
LEU3	NIF3
LEU3	NPP2
LEU3	NRT1
LEU3	NTH1
LEU3	NTH2
LEU3	ODC2
LEU3	OPY2
LEU3	ORC3
LEU3	ORM2
LEU3	ORT1
LEU3	OSH7
LEU3	OYE2
LEU3	PAC1
LEU3	PAU5

LEU3	PCL6	LEU3	RIM1	LEU3	SCT1
LEU3	PCL7	LEU3	RMD11	LEU3	SCW4
LEU3	PDC1	LEU3	RMD6	LEU3	SDP1
LEU3	PDC5	LEU3	RML2	LEU3	SDT1
LEU3	PDC6	LEU3	RNH203	LEU3	SEC23
LEU3	PDH1	LEU3	RNR4	LEU3	SED1
LEU3	PDR16	LEU3	ROM1	LEU3	SEN15
LEU3	PEA2	LEU3	ROM2	LEU3	SER1
LEU3	PER1	LEU3	RPA34	LEU3	SER33
LEU3	PET18	LEU3	RPB8	LEU3	SFL1
LEU3	PEX1	LEU3	RPB9	LEU3	SGO1
LEU3	PFK1	LEU3	RPI1	LEU3	SHM2
LEU3	PGM2	LEU3	RPL12A	LEU3	SHR5
LEU3	PHD1	LEU3	RPL13A	LEU3	SIL1
LEU3	PHO23	LEU3	RPL18B	LEU3	SIS2
LEU3	PHO4	LEU3	RPL29	LEU3	SLN1
LEU3	PHO80	LEU3	RPL33B	LEU3	SLX8
LEU3	PHO88	LEU3	RPL36A	LEU3	SMC1
LEU3	PKH2	LEU3	RPL41A	LEU3	SMC5
LEU3	PLC1	LEU3	RPL7A	LEU3	SMX2
LEU3	PMA1	LEU3	RPL7B	LEU3	SNA2
LEU3	PMA2	LEU3	RPL8B	LEU3	SNQ2
LEU3	PMP1	LEU3	RPM2	LEU3	SOK1
LEU3	PMP2	LEU3	RPP1	LEU3	SOK2
LEU3	POL1	LEU3	RPP1A	LEU3	SPA2
LEU3	POR1	LEU3	RPP2B	LEU3	SPE1
LEU3	PPA1	LEU3	RPR2	LEU3	SPO20
LEU3	PRO1	LEU3	RPS0B	LEU3	SPO75
LEU3	PRO2	LEU3	RPS10A	LEU3	SPS18
LEU3	PRP31	LEU3	RPS11B	LEU3	SRP40
LEU3	PRR1	LEU3	RPS14B	LEU3	SSC1
LEU3	PRR2	LEU3	RPS1B	LEU3	SSK1
LEU3	PSH1	LEU3	RPS26B	LEU3	SSK2
LEU3	PUP1	LEU3	RPS3	LEU3	SSK22
LEU3	PUT4	LEU3	RPS7B	LEU3	SSS1
LEU3	QCR6	LEU3	RRM3	LEU3	STE3
LEU3	RAD24	LEU3	RRP40	LEU3	STL1
LEU3	RAD54	LEU3	RRP6	LEU3	STP4
LEU3	RAD59	LEU3	RTS2	LEU3	STR2
LEU3	RBA50	LEU3	SAC7	LEU3	STR3
LEU3	REC102	LEU3	SAM3	LEU3	STU1
LEU3	RHO3	LEU3	SAM37	LEU3	SUL2
LEU3	RHO5	LEU3	SAM4	LEU3	SUR7
LEU3	RHR2	LEU3	SAR1	LEU3	SUT1
LEU3	RIB3	LEU3	SAS4	LEU3	TAH11
LEU3	RIB5	LEU3	SCS2	LEU3	TAP42

LEU3	TAT1
LEU3	TEC1
LEU3	TEF4
LEU3	THI2
LEU3	THI20
LEU3	THI21
LEU3	THI3
LEU3	THI4
LEU3	THO1
LEU3	THR1
LEU3	THR4
LEU3	TOP1
LEU3	TOR1
LEU3	TPC1
LEU3	TRP2
LEU3	TRP3
LEU3	TRP4
LEU3	TRP5
LEU3	TSL1
LEU3	TUB1
LEU3	TVP18
LEU3	TYE7
LEU3	TYW3
LEU3	UBC4
LEU3	UBX4
LEU3	UGP1
LEU3	UME6
LEU3	URA1
LEU3	URA2
LEU3	UTH1
LEU3	UTR1
LEU3	UTR5
LEU3	VBA2
LEU3	VHS2
LEU3	VMA7
LEU3	VOA1
LEU3	VTC1
LEU3	WBP1
LEU3	WHI5
LEU3	WSC3
LEU3	WSC4
LEU3	YAH1
LEU3	YAP5
LEU3	YAT1
LEU3	YBL029W
LEU3	YBL044W
LEU3	YCF1
LEU3	YCL056C
LEU3	YCR018C-A
LEU3	YDL144C
LEU3	YDL228C
LEU3	YDR042C
LEU3	YDR341C
LEU3	YEL007W
LEU3	YEL047C
LEU3	YER093C-A
LEU3	YER137C
LEU3	YFH7
LEU3	YFR017C
LEU3	YFR035C
LEU3	YGL101W
LEU3	YGL157W
LEU3	YGR021W
LEU3	YGR067C
LEU3	YGR146C
LEU3	YGR190C
LEU3	YHR033W
LEU3	YHR192W
LEU3	YIA6
LEU3	YIR016W
LEU3	YIR035C
LEU3	YJL043W
LEU3	YJL045W
LEU3	YJL216C
LEU3	YJR003C
LEU3	YJR018W
LEU3	YJR100C
LEU3	YJU2
LEU3	YKL037W
LEU3	YKL118W
LEU3	YKR041W
LEU3	YLR108C
LEU3	YLR162W
LEU3	YLR312C
LEU3	YLR356W
LEU3	YMR003W
LEU3	YMR099C
LEU3	YMR111C
LEU3	YMR147W
LEU3	YMR226C
LEU3	YMR291W
LEU3	YNL040W
LEU3	YNL050C
LEU3	YNL190W
LEU3	YNL195C
LEU3	YNR004W
LEU3	YOR062C
LEU3	YOR186W
LEU3	YOR223W
LEU3	YPL056C
LEU3	YPR071W
LEU3	YSA1
LEU3	YTM1
LEU3	YUR1
LEU3	ZAP1
LEU3	ZEO1
LEU3	ZWF1
MBP1	ABF1
MBP1	ACA1
MBP1	ACM1
MBP1	ACO1
MBP1	ACT1
MBP1	AEP3
MBP1	AFG2
MBP1	AGA1
MBP1	ALD3
MBP1	ALG14
MBP1	ALG9
MBP1	AOS1
MBP1	API2
MBP1	APJ1
MBP1	APN1
MBP1	APT2
MBP1	AQR1
MBP1	AQY2
MBP1	ARG1
MBP1	ARG5,6
MBP1	ASA1
MBP1	ASF1
MBP1	ASN2
MBP1	ATP16
MBP1	AXL2
MBP1	BTN2
MBP1	BUB1
MBP1	BUD31
MBP1	BUD9
MBP1	CAF120
MBP1	CAP1

MBP1	CDC13
MBP1	CDC21
MBP1	CDC45
MBP1	CDC55
MBP1	CDC6
MBP1	CDC7
MBP1	CDC9
MBP1	CHS1
MBP1	CHS2
MBP1	CHS7
MBP1	CIK1
MBP1	CIN2
MBP1	CIN8
MBP1	CLA4
MBP1	CLB1
MBP1	CLB2
MBP1	CLB5
MBP1	CLB6
MBP1	CLD1
MBP1	CLN1
MBP1	CLN2
MBP1	CLN3
MBP1	COP1
MBP1	COS1
MBP1	COS3
MBP1	COS4
MBP1	COS6
MBP1	COS8
MBP1	COX10
MBP1	COX17
MBP1	CRH1
MBP1	CTF18
MBP1	CUE4
MBP1	CWP1
MBP1	CWP2
MBP1	CYT1
MBP1	DCS2
MBP1	DDR2
MBP1	DDR48
MBP1	DIF1
MBP1	DIN7
MBP1	DNF2
MBP1	DPB2
MBP1	DSE1
MBP1	DSE2
MBP1	DSF2
MBP1	DUN1
MBP1	ECM13
MBP1	ECM33
MBP1	ECM38
MBP1	EGD2
MBP1	ELO1
MBP1	ENA1
MBP1	ERG26
MBP1	ERG4
MBP1	ERG9
MBP1	ERP3
MBP1	ERP5
MBP1	ERV25
MBP1	ESC8
MBP1	EST1
MBP1	EXG1
MBP1	FIG1
MBP1	FIT2
MBP1	FKS1
MBP1	FKS3
MBP1	FLO9
MBP1	FMP30
MBP1	FRE5
MBP1	FRE8
MBP1	GAD1
MBP1	GAL4
MBP1	GAS1
MBP1	GAS3
MBP1	GCD6
MBP1	GDH3
MBP1	GIC1
MBP1	GIC2
MBP1	GIN4
MBP1	GLO1
MBP1	GOR1
MBP1	GRC3
MBP1	GTR1
MBP1	GUP2
MBP1	HCM1
MBP1	HHF2
MBP1	HHT2
MBP1	HLR1
MBP1	HMG2
MBP1	HO
MBP1	HOT13
MBP1	HPR1
MBP1	HSL1
MBP1	HSP104
MBP1	HSP12
MBP1	HSP150
MBP1	HSP26
MBP1	HSP30
MBP1	HSP31
MBP1	HSP32
MBP1	HSP33
MBP1	HSP42
MBP1	HSP82
MBP1	HST2
MBP1	HTA2
MBP1	HTB2
MBP1	HTS1
MBP1	HXT10
MBP1	HXT17
MBP1	HXT5
MBP1	IDP2
MBP1	IQG1
MBP1	IRC15
MBP1	IRC23
MBP1	IRR1
MBP1	ISN1
MBP1	IVY1
MBP1	JEM1
MBP1	KAR4
MBP1	KAR9
MBP1	KRE6
MBP1	LAC1
MBP1	LAP4
MBP1	LRO1
MBP1	LSM3
MBP1	LSM4
MBP1	LTV1
MBP1	MAK32
MBP1	MAL33
MBP1	MBP1
MBP1	MCD1
MBP1	MCM2
MBP1	MCR1
MBP1	MDH1
MBP1	MDM31
MBP1	MDM35
MBP1	MDS3
MBP1	MEP3

MBP1	MF(ALPHA)1	MBP1	ORC4	MBP1	RAD54
MBP1	MGA1	MBP1	OSM1	MBP1	RBA50
MBP1	MGS1	MBP1	OTU2	MBP1	RBD2
MBP1	MKC7	MBP1	PAC10	MBP1	RCL1
MBP1	MLH3	MBP1	PAH1	MBP1	RDN5-6
MBP1	MNN1	MBP1	PAU2	MBP1	REG2
MBP1	MNN5	MBP1	PAU22	MBP1	REV3
MBP1	MNN9	MBP1	PCF11	MBP1	REV7
MBP1	MOT1	MBP1	PCL1	MBP1	RFA1
MBP1	MOT3	MBP1	PCL2	MBP1	RFA2
MBP1	MRC1	MBP1	PDR1	MBP1	RFC3
MBP1	MRF1	MBP1	PDR15	MBP1	RGT1
MBP1	MRL1	MBP1	PDR16	MBP1	RIF1
MBP1	MRP2	MBP1	PDR5	MBP1	RML2
MBP1	MRP8	MBP1	PDS1	MBP1	RNH202
MBP1	MRPL24	MBP1	PDS5	MBP1	RNH203
MBP1	MRPL4	MBP1	PET54	MBP1	RNR1
MBP1	MRPL40	MBP1	PEX10	MBP1	RNR4
MBP1	MRPS35	MBP1	PEX27	MBP1	RPA34
MBP1	MSA1	MBP1	PEX28	MBP1	RPL21A
MBP1	MSA2	MBP1	PGK1	MBP1	RPL31A
MBP1	MSB2	MBP1	PHO3	MBP1	RPL37B
MBP1	MSC1	MBP1	PIM1	MBP1	RPL7B
MBP1	MSH6	MBP1	PIR3	MBP1	RPL9A
MBP1	MSY1	MBP1	PLB2	MBP1	RPS0B
MBP1	MTC6	MBP1	PLC1	MBP1	RPS16A
MBP1	MTF2	MBP1	PLM2	MBP1	RPS22B
MBP1	MVD1	MBP1	PMA1	MBP1	RPS9A
MBP1	NAM2	MBP1	POL1	MBP1	RRD2
MBP1	NCA2	MBP1	POL12	MBP1	RSM10
MBP1	NCA3	MBP1	POL30	MBP1	RSP5
MBP1	NDD1	MBP1	POL32	MBP1	RTC2
MBP1	NIP100	MBP1	POP3	MBP1	RTT107
MBP1	NIS1	MBP1	POT1	MBP1	SAE2
MBP1	NKP1	MBP1	PPN1	MBP1	SBH1
MBP1	NQM1	MBP1	PRE1	MBP1	SCJ1
MBP1	NRG1	MBP1	PRE7	MBP1	SCP160
MBP1	NRG2	MBP1	PRM5	MBP1	SCS2
MBP1	NRM1	MBP1	PSA1	MBP1	SCW10
MBP1	NUD1	MBP1	PTK1	MBP1	SEC14
MBP1	NUP42	MBP1	PUP3	MBP1	SEC16
MBP1	NUT2	MBP1	QCR6	MBP1	SEC20
MBP1	NVJ1	MBP1	RAD27	MBP1	SEN1
MBP1	OCH1	MBP1	RAD3	MBP1	SEN2
MBP1	OM45	MBP1	RAD51	MBP1	SEN34
MBP1	OPY2	MBP1	RAD53	MBP1	SHO1

MBP1	SIM1
MBP1	SIP4
MBP1	SIT1
MBP1	SKG6
MBP1	SKI7
MBP1	SLM4
MBP1	SLY1
MBP1	SMC1
MBP1	SMC3
MBP1	SMF1
MBP1	SNA2
MBP1	SNO4
MBP1	SNQ2
MBP1	SNR190
MBP1	SOL4
MBP1	SPE1
MBP1	SPI1
MBP1	SPO12
MBP1	SPO16
MBP1	SPO73
MBP1	SPT21
MBP1	SRL1
MBP1	SRL3
MBP1	SSA1
MBP1	SSE2
MBP1	STI1
MBP1	STL1
MBP1	SUA7
MBP1	SUR2
MBP1	SVS1
MBP1	SWC7
MBP1	SWE1
MBP1	SWI4
MBP1	TAF12
MBP1	TAL1
MBP1	TD(GUC)J2
MBP1	TDH1
MBP1	TDH3
MBP1	TFC8
MBP1	TFS1
MBP1	TGS1
MBP1	THI22
MBP1	THI80
MBP1	TIM23
MBP1	TIR3
MBP1	TLG2
MBP1	TMA17
MBP1	TOF1
MBP1	TOS1
MBP1	TOS2
MBP1	TOS4
MBP1	TOS8
MBP1	TPM1
MBP1	TRF5
MBP1	TSA1
MBP1	TSL1
MBP1	TUB4
MBP1	TVP18
MBP1	TVP23
MBP1	TW(CCA)G2
MBP1	UBP13
MBP1	UFE1
MBP1	UIP3
MBP1	USA1
MBP1	UTP4
MBP1	VAC14
MBP1	VPS55
MBP1	VPS71
MBP1	VPS8
MBP1	VTC1
MBP1	VTC3
MBP1	WBP1
MBP1	WSC2
MBP1	WTM1
MBP1	YAR023C
MBP1	YAR068W
MBP1	YAT1
MBP1	YBL101W-B
MBP1	YBL108W
MBP1	YBL109W
MBP1	YBL111C
MBP1	YBR051W
MBP1	YBR071W
MBP1	YBR190W
MBP1	YCK2
MBP1	YCR018C-A
MBP1	YCR064C
MBP1	YDR034W-B
MBP1	YDR048C
MBP1	YDR094W
MBP1	YDR115W
MBP1	YDR210C-D
MBP1	YDR220C
MBP1	YDR222W
MBP1	YDR262W
MBP1	YDR391C
MBP1	YDR442W
MBP1	YDR509W
MBP1	YDR524C-B
MBP1	YDR526C
MBP1	YDR543C
MBP1	YDR544C
MBP1	YEL074W
MBP1	YEL077C
MBP1	YER066W
MBP1	YER071C
MBP1	YER078C
MBP1	YER078W-A
MBP1	YER079W
MBP1	YER097W
MBP1	YER189W
MBP1	YFL012W
MBP1	YFR017C
MBP1	YGL007W
MBP1	YGP1
MBP1	YGR079W
MBP1	YGR109W-A
MBP1	YGR109W-B
MBP1	YGR125W
MBP1	YGR153W
MBP1	YHL012W
MBP1	YHL049C
MBP1	YHP1
MBP1	YHR138C
MBP1	YHR159W
MBP1	YHR217C
MBP1	YHR218W
MBP1	YHR219W
MBP1	YIL141W
MBP1	YIL177C
MBP1	YJL045W
MBP1	YJL107C
MBP1	YJL185C
MBP1	YJL225C
MBP1	YJR011C
MBP1	YJR030C
MBP1	YKL097C
MBP1	YKL102C

MBP1	YKL151C	MBP1	YRF1-2	MCM1CCP1
MBP1	YLL066C	MBP1	YRF1-3	MCM1CCW12
MBP1	YLL067C	MBP1	YRF1-5	MCM1CDC19
MBP1	YLR035C-A	MBP1	YRF1-6	MCM1CDC20
MBP1	YLR049C	MBP1	YRF1-7	MCM1CDC28
MBP1	YLR104W	MBP1	YUR1	MCM1CDC5
MBP1	YLR297W	MBP1	ZWF1	MCM1CDC6
MBP1	YLR301W	MCM1ABF2		MCM1CHL4
MBP1	YLR366W	MCM1ACE2		MCM1CHS2
MBP1	YLR428C	MCM1AFR1		MCM1CIS3
MBP1	YLR462W	MCM1AGA1		MCM1CLA4
MBP1	YML003W	MCM1AGA2		MCM1CLB1
MBP1	YML133C	MCM1AGP1		MCM1CLB2
MBP1	YMR007W	MCM1AHC1		MCM1CLN1
MBP1	YMR144W	MCM1ALK1		MCM1CLN2
MBP1	YMR178W	MCM1AMN1		MCM1CLN3
MBP1	YMR258C	MCM1ARF3		MCM1CMP2
MBP1	YMR279C	MCM1ARG1		MCM1COQ5
MBP1	YMR304C-A	MCM1ARG3		MCM1COS9
MBP1	YMR306C-A	MCM1ARG8		MCM1CPR8
MBP1	YNL089C	MCM1ARO8		MCM1CRH1
MBP1	YNL296W	MCM1ARP5		MCM1CSG2
MBP1	YNL313C	MCM1ASE1		MCM1CSL4
MBP1	YNL338W	MCM1ASF1		MCM1CSN9
MBP1	YNR018W	MCM1ASG7		MCM1CTS2
MBP1	YOR052C	MCM1ASH1		MCM1CYC3
MBP1	YOR246C	MCM1ATG19		MCM1DAK1
MBP1	YOR248W	MCM1ATG26		MCM1DBF2
MBP1	YOR342C	MCM1AVT4		MCM1DGA1
MBP1	YOR390W	MCM1BAR1		MCM1DIT1
MBP1	YOX1	MCM1BBP1		MCM1DIT2
MBP1	YPK9	MCM1BET3		MCM1DPL1
MBP1	YPL014W	MCM1BNA3		MCM1DPS1
MBP1	YPL056C	MCM1BNS1		MCM1ECM15
MBP1	YPL158C	MCM1BPH1		MCM1ECM18
MBP1	YPL191C	MCM1BSC1		MCM1ECM25
MBP1	YPL257W	MCM1BST1		MCM1ECM33
MBP1	YPL264C	MCM1BUB1		MCM1EGT2
MBP1	YPL279C	MCM1BUD27		MCM1EKI1
MBP1	YPR027C	MCM1BUD4		MCM1ELO1
MBP1	YPR076W	MCM1BUD9		MCM1EMP70
MBP1	YPR078C	MCM1BUR6		MCM1EPL1
MBP1	YPR158C-C	MCM1CAF16		MCM1ERG28
MBP1	YPR158C-D	MCM1CAR1		MCM1ERG3
MBP1	YPR174C	MCM1CAR2		MCM1FAR1
MBP1	YRF1-1	MCM1CBT1		MCM1FAT1

MCM1FDH1
MCM1FDH2
MCM1FEN2
MCM1FKS1
MCM1FKS3
MCM1FRE2
MCM1FRQ1
MCM1FUN26
MCM1GDS1
MCM1GFA1
MCM1GFD1
MCM1GIC2
MCM1GIN4
MCM1GLC7
MCM1GLY1
MCM1GPA1
MCM1GPT2
MCM1GRR1
MCM1GYP8
MCM1HAP1
MCM1HCM1
MCM1HIP1
MCM1HLR1
MCM1HO
MCM1HOF1
MCM1HOG1
MCM1HSH49
MCM1HSK3
MCM1HSP12
MCM1HSP150
MCM1HST3
MCM1HST4
MCM1HTZ1
MCM1HUG1
MCM1ICS2
MCM1IES6
MCM1IME1
MCM1IQG1
MCM1IRC22
MCM1IRC8
MCM1IST2
MCM1ITC1
MCM1IXR1
MCM1JSN1
MCM1KAP123
MCM1KAR4
MCM1KCC4
MCM1KGD2
MCM1KIN3
MCM1KIN4
MCM1LSM2
MCM1LSM4
MCM1MAM33
MCM1MCM3
MCM1MCM5
MCM1MCM6
MCM1MCM7
MCM1MDJ1
MCM1MDJ2
MCM1MET1
MCM1MET2
MCM1MET32
MCM1MF(ALPHA)1
MCM1MF(ALPHA)2
MCM1MFA1
MCM1MFA2
MCM1MIC14
MCM1MIC17
MCM1MID2
MCM1MMR1
MCM1MNN1
MCM1MOB1
MCM1MON2
MCM1MPE1
MCM1MRD1
MCM1MRH1
MCM1MRPL28
MCM1MSA1
MCM1MSB2
MCM1MSC6
MCM1MSG5
MCM1MTC6
MCM1MTF2
MCM1MTR2
MCM1MUK1
MCM1MYO1
MCM1NCA3
MCM1NDI1
MCM1NDL1
MCM1NEM1
MCM1NEO1
MCM1NFU1
MCM1NRG2
MCM1NTE1
MCM1NUM1
MCM1NUR1
MCM1OCA2
MCM1OCA5
MCM1OPT2
MCM1PAC10
MCM1PAM17
MCM1PCK1
MCM1PCL1
MCM1PCL10
MCM1PDE2
MCM1PEA2
MCM1PEP7
MCM1PET130
MCM1PEX28
MCM1PEX32
MCM1PEX6
MCM1PEX8
MCM1PFK26
MCM1PHO3
MCM1PIG1
MCM1PIL1
MCM1PIR1
MCM1PIR3
MCM1PIS1
MCM1PKR1
MCM1PLB3
MCM1PLP1
MCM1PMA1
MCM1PMP1
MCM1PMT2
MCM1PNC1
MCM1POG1
MCM1POP7
MCM1PPN1
MCM1PPT2
MCM1PRY1
MCM1PST2
MCM1PTM1
MCM1PXA1
MCM1RAD28
MCM1RAM1
MCM1RAX2
MCM1REG1

MCM1REV1
MCM1REV7
MCM1RFC5
MCM1RNR1
MCM1ROX3
MCM1RPA190
MCM1RPA34
MCM1RPA43
MCM1RPC17
MCM1RPL11B
MCM1RPL15A
MCM1RPL15B
MCM1RPL32
MCM1RPL36A
MCM1RPL43B
MCM1RPN7
MCM1RPS22B
MCM1RPS24B
MCM1RSM26
MCM1RTC3
MCM1RVB1
MCM1RVB2
MCM1SAC7
MCM1SAG1
MCM1SAM3
MCM1SCO1
MCM1SCW10
MCM1SCW4
MCM1SEC28
MCM1SED1
MCM1SEN2
MCM1SFB3
MCM1SFG1
MCM1SHE9
MCM1SIM1
MCM1SIT1
MCM1SKG6
MCM1SKN1
MCM1SLM4
MCM1SMF3
MCM1SMI1
MCM1SML1
MCM1SNA2
MCM1SNF6
MCM1SPB1
MCM1SPI1
MCM1SPO12
MCM1SPS100
MCM1SPS4
MCM1SPT8
MCM1SRB7
MCM1SRL1
MCM1SST2
MCM1STB6
MCM1STE12
MCM1STE2
MCM1STE3
MCM1STE6
MCM1STP2
MCM1SUI2
MCM1SUR7
MCM1SWF1
MCM1SWI4
MCM1SWI5
MCM1TCM62
MCM1TFB2
MCM1TFC4
MCM1TIM10
MCM1TIM12
MCM1TIP20
MCM1TMA20
MCM1TOA2
MCM1TPA1
MCM1TPO3
MCM1TRA1
MCM1TRM12
MCM1TRS31
MCM1TVP18
MCM1TVP23
MCM1TYE7
MCM1UFD1
MCM1URA1
MCM1UTH1
MCM1UTR2
MCM1VAB2
MCM1VOA1
MCM1VPS63
MCM1VPS74
MCM1WSC4
MCM1YAP3
MCM1YAR064W
MCM1YBL059W
MCM1YBR071W
MCM1YBR138C
MCM1YBR139W
MCM1YCL056C
MCM1YCR024C-B
MCM1YCS4
MCM1YDR042C
MCM1YDR132C
MCM1YDR179W-A
MCM1YDR182W-A
MCM1YDR210C-D
MCM1YDR278C
MCM1YDR370C
MCM1YDR506C
MCM1YDR509W
MCM1YDR524C-B
MCM1YEA4
MCM1YEL045C
MCM1YEL047C
MCM1YER158C
MCM1YFH7
MCM1YGL006W-A
MCM1YGL007C-A
MCM1YGL114W
MCM1YGP1
MCM1YGR079W
MCM1YGR126W
MCM1YGR168C
MCM1YHL008C
MCM1YHL026C
MCM1YHP1
MCM1YHR022C
MCM1YHR022C-A
MCM1YIL077C
MCM1YIL158W
MCM1YIL169C
MCM1YJL049W
MCM1YJL127C-B
MCM1YJL160C
MCM1YJL171C
MCM1YJR054W
MCM1YJR100C
MCM1YKL105C
MCM1YKR040C
MCM1YKR041W
MCM1YLR030W

MCM1YLR040C		RAP1	ACO2	RAP1	ATG15
MCM1YLR154C-G		RAP1	ACS1	RAP1	ATG19
MCM1YLR287C		RAP1	ADA2	RAP1	ATG26
MCM1YLR302C		RAP1	ADE3	RAP1	ATG27
MCM1YML050W		RAP1	ADE5,7	RAP1	ATP7
MCM1YML053C		RAP1	ADE8	RAP1	AVT4
MCM1YML054C-A		RAP1	ADH1	RAP1	AZF1
MCM1YML119W		RAP1	ADH3	RAP1	BAT2
MCM1YMR001C-A		RAP1	ADI1	RAP1	BBC1
MCM1YMR031C		RAP1	ADP1	RAP1	BCK1
MCM1YMR122W-A		RAP1	AFG2	RAP1	BCK2
MCM1YMR252C		RAP1	AFT1	RAP1	BCY1
MCM1YMR253C		RAP1	AGP1	RAP1	BDF1
MCM1YNL058C		RAP1	AHC1	RAP1	BDS1
MCM1YNL146C-A		RAP1	AIM9	RAP1	BEM2
MCM1YNL146W		RAP1	AIP1	RAP1	BEM4
MCM1YNL190W		RAP1	AKR1	RAP1	BIK1
MCM1YNL295W		RAP1	ALD4	RAP1	BIO3
MCM1YNR061C		RAP1	ALD5	RAP1	BIO4
MCM1YNR062C		RAP1	ALF1	RAP1	BMH2
MCM1YNR063W		RAP1	ALG3	RAP1	BNA1
MCM1YOL013W-A		RAP1	ALG5	RAP1	BNI5
MCM1YOR022C		RAP1	ALP1	RAP1	BRR1
MCM1YOR093C		RAP1	ALT2	RAP1	BSC2
MCM1YOR246C		RAP1	ANP1	RAP1	BSC4
MCM1YOR342C		RAP1	APC11	RAP1	BSC5
MCM1YOR389W		RAP1	APE2	RAP1	BUD20
MCM1YOX1		RAP1	APJ1	RAP1	BUD22
MCM1YPL044C		RAP1	APL4	RAP1	BUD27
MCM1YPL071C		RAP1	APM3	RAP1	BUD3
MCM1YPL236C		RAP1	APS2	RAP1	BUD4
MCM1YPL276W		RAP1	APT2	RAP1	CAF120
MCM1YPL277C		RAP1	ARE2	RAP1	CAF40
MCM1YPL278C		RAP1	ARF1	RAP1	CAJ1
MCM1YPR091C		RAP1	ARF2	RAP1	CAR1
MCM1YPR157W		RAP1	ARG1	RAP1	CAR2
MCM1YPR196W		RAP1	ARG3	RAP1	CAT5
MCM1YPT6		RAP1	ARO10	RAP1	CBP2
MCM1YRF1-6		RAP1	ARR3	RAP1	CCA1
MCM1YTH1		RAP1	ASC1	RAP1	CCH1
MCM1ZDS2		RAP1	ASF1	RAP1	CCT6
MCM1ZRT2		RAP1	ASM4	RAP1	CCW12
NDD1	CLB2	RAP1	AST2	RAP1	CCW14
RAP1	AAC3	RAP1	ATF1	RAP1	CDC19
RAP1	ACA1	RAP1	ATF2	RAP1	CDC20
RAP1	ACE2	RAP1	ATG1	RAP1	CDC21

RAP1	CDC25	RAP1	CYC8	RAP1	ENB1
RAP1	CDC28	RAP1	DAL1	RAP1	ENO1
RAP1	CDC45	RAP1	DAN4	RAP1	ENO2
RAP1	CDC50	RAP1	DAP1	RAP1	ENT4
RAP1	CEX1	RAP1	DBF2	RAP1	EOS1
RAP1	CFT1	RAP1	DBF20	RAP1	EPL1
RAP1	CHO2	RAP1	DBP10	RAP1	EPT1
RAP1	CHS7	RAP1	DBP7	RAP1	ERG1
RAP1	CHZ1	RAP1	DDR2	RAP1	ERG13
RAP1	CLB1	RAP1	DEF1	RAP1	ERG5
RAP1	CLB2	RAP1	DET1	RAP1	ERV2
RAP1	CLN1	RAP1	DFR1	RAP1	ERV29
RAP1	CLN3	RAP1	DIF1	RAP1	FAA1
RAP1	CMS1	RAP1	DIP5	RAP1	FAB1
RAP1	COA2	RAP1	DIT1	RAP1	FAF1
RAP1	COF1	RAP1	DLD1	RAP1	FAS1
RAP1	COG4	RAP1	DLD3	RAP1	FAS2
RAP1	COQ2	RAP1	DMC1	RAP1	FBA1
RAP1	COS10	RAP1	DNF1	RAP1	FIG4
RAP1	COS111	RAP1	DNM1	RAP1	FIR1
RAP1	COS4	RAP1	DOT6	RAP1	FIT2
RAP1	COS6	RAP1	DPB11	RAP1	FKH2
RAP1	COT1	RAP1	DTR1	RAP1	FLC1
RAP1	COX11	RAP1	DUG3	RAP1	FLC2
RAP1	COX19	RAP1	DUR3	RAP1	FLO10
RAP1	COX4	RAP1	DUS3	RAP1	FMP16
RAP1	CPA1	RAP1	EAP1	RAP1	FMP23
RAP1	CPR4	RAP1	ECM12	RAP1	FMP40
RAP1	CRC1	RAP1	ECM13	RAP1	FMP48
RAP1	CRG1	RAP1	ECM16	RAP1	FMT1
RAP1	CRR1	RAP1	ECM17	RAP1	FOB1
RAP1	CRZ1	RAP1	ECM19	RAP1	FPK1
RAP1	CSI2	RAP1	ECM30	RAP1	FPS1
RAP1	CSM2	RAP1	ECM38	RAP1	FRE1
RAP1	CSR2	RAP1	EDC2	RAP1	FRE2
RAP1	CTA1	RAP1	EDS1	RAP1	FRE4
RAP1	CTR1	RAP1	EEB1	RAP1	FRE6
RAP1	CTS1	RAP1	EFB1	RAP1	FRE7
RAP1	CTT1	RAP1	EGT2	RAP1	FRE8
RAP1	CUE4	RAP1	EHD3	RAP1	FRS1
RAP1	CUE5	RAP1	EKI1	RAP1	FRS2
RAP1	CUP2	RAP1	ELP6	RAP1	FRT2
RAP1	CWC23	RAP1	EMI2	RAP1	FTR1
RAP1	CWP1	RAP1	EMT5	RAP1	FUI1
RAP1	CWP2	RAP1	ENA1	RAP1	FUN19
RAP1	CYC3	RAP1	ENA2	RAP1	FUR1

RAP1	FUR4
RAP1	FYV1
RAP1	FYV12
RAP1	FZO1
RAP1	GAC1
RAP1	GAD1
RAP1	GAS3
RAP1	GAT1
RAP1	GAT2
RAP1	GAT3
RAP1	GAT4
RAP1	GCN4
RAP1	GCR1
RAP1	GDS1
RAP1	GEA2
RAP1	GFD2
RAP1	GID8
RAP1	GIM3
RAP1	GIS3
RAP1	GIT1
RAP1	GLK1
RAP1	GLN1
RAP1	GLN3
RAP1	GLY1
RAP1	GPD1
RAP1	GPD2
RAP1	GPG1
RAP1	GPI1
RAP1	GPI10
RAP1	GPI17
RAP1	GPI2
RAP1	GPM1
RAP1	GPM2
RAP1	GPR1
RAP1	GRH1
RAP1	GRS1
RAP1	GSC2
RAP1	GTO3
RAP1	GTS1
RAP1	GUF1
RAP1	GUP1
RAP1	GUP2
RAP1	GUT1
RAP1	GYP5
RAP1	GYP6
RAP1	HAA1
RAP1	HAP1
RAP1	HAP3
RAP1	HAP5
RAP1	HAT2
RAP1	HCS1
RAP1	HEM13
RAP1	HHO1
RAP1	HIR3
RAP1	HIS4
RAP1	HIS7
RAP1	HKR1
RAP1	HMF1
RAP1	HMLALPHA1
RAP1	HMLALPHA2
RAP1	HMRA1
RAP1	HMRA2
RAP1	HMS1
RAP1	HMS2
RAP1	HOC1
RAP1	HOG1
RAP1	HOM6
RAP1	HOT13
RAP1	HRB1
RAP1	HRK1
RAP1	HRT1
RAP1	HSE1
RAP1	HSF1
RAP1	HSP104
RAP1	HSP12
RAP1	HSP150
RAP1	HSP26
RAP1	HSP30
RAP1	HSP33
RAP1	HSP78
RAP1	HST3
RAP1	HTB2
RAP1	HUG1
RAP1	HUL5
RAP1	HVG1
RAP1	HXT1
RAP1	HXT2
RAP1	HXT5
RAP1	HYP2
RAP1	IBA57
RAP1	ICS3
RAP1	ICY2
RAP1	IDP2
RAP1	IES1
RAP1	IES5
RAP1	IES6
RAP1	IFH1
RAP1	IGO1
RAP1	IMD1
RAP1	IMD4
RAP1	IME1
RAP1	INN1
RAP1	INO2
RAP1	IPP1
RAP1	IRC15
RAP1	IRC22
RAP1	IRC8
RAP1	ISR1
RAP1	IXR1
RAP1	IZH3
RAP1	JLP1
RAP1	JSN1
RAP1	KAP114
RAP1	KCC4
RAP1	KES1
RAP1	KIN4
RAP1	KNS1
RAP1	KRE29
RAP1	KRS1
RAP1	KTI11
RAP1	KTR5
RAP1	LAA1
RAP1	LAS17
RAP1	LCB2
RAP1	LCB3
RAP1	LDB18
RAP1	LEE1
RAP1	LGE1
RAP1	LOT5
RAP1	LSM12
RAP1	LSM5
RAP1	LSR1
RAP1	LST4
RAP1	LYP1
RAP1	MAE1
RAP1	MAM3

RAP1	MATALPHA1
RAP1	MATALPHA2
RAP1	MBP1
RAP1	MCH4
RAP1	MCH5
RAP1	MCM22
RAP1	MCM7
RAP1	MCR1
RAP1	MDJ1
RAP1	MDJ2
RAP1	MDY2
RAP1	MEC3
RAP1	MED7
RAP1	MEP2
RAP1	MET17
RAP1	MET2
RAP1	MET30
RAP1	MET6
RAP1	MGA1
RAP1	MIG3
RAP1	MIP6
RAP1	MLP2
RAP1	MMF1
RAP1	MMR1
RAP1	MNL1
RAP1	MNN1
RAP1	MNN4
RAP1	MNS1
RAP1	MOB2
RAP1	MOG1
RAP1	MOH1
RAP1	MON1
RAP1	MOT1
RAP1	MPC54
RAP1	MPS2
RAP1	MRE11
RAP1	MRL1
RAP1	MRM2
RAP1	MRP13
RAP1	MRP20
RAP1	MRP7
RAP1	MRPL15
RAP1	MRPL20
RAP1	MRPL24
RAP1	MRPL4
RAP1	MRPL44
RAP1	MRPL49
RAP1	MRS1
RAP1	MRS3
RAP1	MRT4
RAP1	MSB3
RAP1	MSC6
RAP1	MSN4
RAP1	MSR1
RAP1	MSW1
RAP1	MTC2
RAP1	MTC6
RAP1	MTH1
RAP1	MTR4
RAP1	MUB1
RAP1	MUC1
RAP1	MUP3
RAP1	MVD1
RAP1	NAB3
RAP1	NAT3
RAP1	NCA3
RAP1	NCE102
RAP1	NDL1
RAP1	NEO1
RAP1	NET1
RAP1	NEW1
RAP1	NFS1
RAP1	NFU1
RAP1	NIS1
RAP1	NMA1
RAP1	NMD5
RAP1	NOB1
RAP1	NOP12
RAP1	NPP1
RAP1	NQM1
RAP1	NRG1
RAP1	NSR1
RAP1	NTC20
RAP1	NUP2
RAP1	NUP42
RAP1	OAF1
RAP1	OM45
RAP1	OMA1
RAP1	OPI3
RAP1	ORC2
RAP1	ORC4
RAP1	OST5
RAP1	OYE3
RAP1	PAC1
RAP1	PAC11
RAP1	PAU21
RAP1	PAU3
RAP1	PAU4
RAP1	PBP2
RAP1	PCA1
RAP1	PCL1
RAP1	PCL5
RAP1	PCL7
RAP1	PCT1
RAP1	PDC1
RAP1	PDI1
RAP1	PDR11
RAP1	PDR12
RAP1	PDR15
RAP1	PDR5
RAP1	PDS1
RAP1	PDX3
RAP1	PEP5
RAP1	PET9
RAP1	PEX10
RAP1	PEX12
RAP1	PEX6
RAP1	PFA3
RAP1	PFK1
RAP1	PFK2
RAP1	PFK27
RAP1	PGA1
RAP1	PGI1
RAP1	PGK1
RAP1	PHM6
RAP1	PHM7
RAP1	PHO11
RAP1	PHO4
RAP1	PHO5
RAP1	PHO81
RAP1	PHO88
RAP1	PHO89
RAP1	PHO91
RAP1	PHS1
RAP1	PIL1
RAP1	PIM1
RAP1	PLB1
RAP1	PLM2

RAP1	PMA1	RAP1	RBA50	RAP1	RPL19B
RAP1	PMP1	RAP1	RDI1	RAP1	RPL1A
RAP1	PMP3	RAP1	REG2	RAP1	RPL1B
RAP1	PMR1	RAP1	REH1	RAP1	RPL20A
RAP1	PNS1	RAP1	RFT1	RAP1	RPL20B
RAP1	POC4	RAP1	RGP1	RAP1	RPL21A
RAP1	POG1	RAP1	RGS2	RAP1	RPL21B
RAP1	POL1	RAP1	RHO3	RAP1	RPL22A
RAP1	POL3	RAP1	RHO5	RAP1	RPL22B
RAP1	POP5	RAP1	RHR2	RAP1	RPL23A
RAP1	PPH22	RAP1	RIA1	RAP1	RPL23B
RAP1	PPN1	RAP1	RIB3	RAP1	RPL24A
RAP1	PRE2	RAP1	RKI1	RAP1	RPL24B
RAP1	PRE7	RAP1	RLM1	RAP1	RPL25
RAP1	PRM2	RAP1	RMD6	RAP1	RPL26A
RAP1	PRO1	RAP1	RNQ1	RAP1	RPL26B
RAP1	PRP16	RAP1	RNR2	RAP1	RPL27A
RAP1	PRP31	RAP1	RNR3	RAP1	RPL27B
RAP1	PRS1	RAP1	RNR4	RAP1	RPL28
RAP1	PRT1	RAP1	ROX3	RAP1	RPL29
RAP1	PRY1	RAP1	RPA190	RAP1	RPL2A
RAP1	PRY3	RAP1	RPA34	RAP1	RPL2B
RAP1	PSA1	RAP1	RPA43	RAP1	RPL3
RAP1	PSY2	RAP1	RPC31	RAP1	RPL30
RAP1	PTC1	RAP1	RPC37	RAP1	RPL31A
RAP1	PTC2	RAP1	RPC40	RAP1	RPL31B
RAP1	PTI1	RAP1	RPH1	RAP1	RPL32
RAP1	PTK1	RAP1	RPI1	RAP1	RPL33A
RAP1	PTK2	RAP1	RPL10	RAP1	RPL33B
RAP1	PTM1	RAP1	RPL11A	RAP1	RPL34A
RAP1	PTP1	RAP1	RPL11B	RAP1	RPL34B
RAP1	PUB1	RAP1	RPL12A	RAP1	RPL35A
RAP1	PUF6	RAP1	RPL12B	RAP1	RPL35B
RAP1	PUP2	RAP1	RPL13A	RAP1	RPL36A
RAP1	PUT1	RAP1	RPL13B	RAP1	RPL36B
RAP1	PUT2	RAP1	RPL14A	RAP1	RPL37A
RAP1	PUT3	RAP1	RPL14B	RAP1	RPL37B
RAP1	PUT4	RAP1	RPL15A	RAP1	RPL38
RAP1	PYK2	RAP1	RPL15B	RAP1	RPL39
RAP1	QCR6	RAP1	RPL16A	RAP1	RPL40A
RAP1	QDR2	RAP1	RPL16B	RAP1	RPL40B
RAP1	RAD2	RAP1	RPL17A	RAP1	RPL41A
RAP1	RAP1	RAP1	RPL17B	RAP1	RPL41B
RAP1	RAS1	RAP1	RPL18A	RAP1	RPL42A
RAP1	RAX1	RAP1	RPL18B	RAP1	RPL42B
RAP1	RAX2	RAP1	RPL19A	RAP1	RPL43A

RAP1	RPL43B	RAP1	RPS21B	RAP1	RVS161
RAP1	RPL4A	RAP1	RPS22A	RAP1	RVS167
RAP1	RPL4B	RAP1	RPS22B	RAP1	RXT3
RAP1	RPL5	RAP1	RPS23A	RAP1	SAM1
RAP1	RPL6A	RAP1	RPS23B	RAP1	SAM2
RAP1	RPL6B	RAP1	RPS24A	RAP1	SAP1
RAP1	RPL7A	RAP1	RPS24B	RAP1	SAS2
RAP1	RPL7B	RAP1	RPS25A	RAP1	SAS3
RAP1	RPL8A	RAP1	RPS25B	RAP1	SCP1
RAP1	RPL8B	RAP1	RPS26A	RAP1	SCT1
RAP1	RPL9A	RAP1	RPS26B	RAP1	SCY1
RAP1	RPL9B	RAP1	RPS27A	RAP1	SEC10
RAP1	RPN11	RAP1	RPS27B	RAP1	SEC13
RAP1	RPN2	RAP1	RPS28A	RAP1	SEC17
RAP1	RPO21	RAP1	RPS28B	RAP1	SEC31
RAP1	RPP0	RAP1	RPS29A	RAP1	SEC59
RAP1	RPP1A	RAP1	RPS29B	RAP1	SED1
RAP1	RPP1B	RAP1	RPS3	RAP1	SEO1
RAP1	RPP2A	RAP1	RPS30A	RAP1	SER1
RAP1	RPP2B	RAP1	RPS30B	RAP1	SER3
RAP1	RPR1	RAP1	RPS31	RAP1	SES1
RAP1	RPR2	RAP1	RPS4A	RAP1	SFA1
RAP1	RPS0A	RAP1	RPS4B	RAP1	SFG1
RAP1	RPS0B	RAP1	RPS5	RAP1	SFL1
RAP1	RPS10A	RAP1	RPS6A	RAP1	SFP1
RAP1	RPS10B	RAP1	RPS6B	RAP1	SFT1
RAP1	RPS11A	RAP1	RPS7A	RAP1	SGA1
RAP1	RPS11B	RAP1	RPS7B	RAP1	SGM1
RAP1	RPS12	RAP1	RPS8A	RAP1	SGT1
RAP1	RPS13	RAP1	RPS8B	RAP1	SGV1
RAP1	RPS14A	RAP1	RPS9A	RAP1	SHE9
RAP1	RPS14B	RAP1	RPS9B	RAP1	SHR5
RAP1	RPS15	RAP1	RRN5	RAP1	SHY1
RAP1	RPS16A	RAP1	RRN6	RAP1	SIM1
RAP1	RPS16B	RAP1	RRN7	RAP1	SIP2
RAP1	RPS17A	RAP1	RRP14	RAP1	SIP4
RAP1	RPS17B	RAP1	RRP5	RAP1	SIR1
RAP1	RPS18A	RAP1	RSM10	RAP1	SIR3
RAP1	RPS18B	RAP1	RSM7	RAP1	SIZ1
RAP1	RPS19A	RAP1	RSN1	RAP1	SKN1
RAP1	RPS19B	RAP1	RSP5	RAP1	SKP2
RAP1	RPS1A	RAP1	RTA1	RAP1	SKT5
RAP1	RPS1B	RAP1	RTC4	RAP1	SLD5
RAP1	RPS2	RAP1	RTS3	RAP1	SLG1
RAP1	RPS20	RAP1	RTT109	RAP1	SLM3
RAP1	RPS21A	RAP1	RUP1	RAP1	SLS1

RAP1	SLX8	RAP1	STB5	RAP1	TIR4
RAP1	SLY1	RAP1	STE23	RAP1	TKL2
RAP1	SMP1	RAP1	STE3	RAP1	TL(CAA)G3
RAP1	SNF4	RAP1	STF1	RAP1	TMA10
RAP1	SNF5	RAP1	STR3	RAP1	TNA1
RAP1	SNO4	RAP1	SUE1	RAP1	TOD6
RAP1	SNR17A	RAP1	SUN4	RAP1	TOS1
RAP1	SNR17B	RAP1	SUP17	RAP1	TOS4
RAP1	SNR72	RAP1	SUR1	RAP1	TOS6
RAP1	SNR73	RAP1	SUR7	RAP1	TOS8
RAP1	SNR74	RAP1	SUV3	RAP1	TPI1
RAP1	SNR75	RAP1	SVS1	RAP1	TPM1
RAP1	SNR76	RAP1	SWC3	RAP1	TPO1
RAP1	SNR77	RAP1	SWC5	RAP1	TRM12
RAP1	SNR78	RAP1	SWI1	RAP1	TRM2
RAP1	SNR87	RAP1	SWI5	RAP1	TRM5
RAP1	SNX3	RAP1	SWM1	RAP1	TRM9
RAP1	SOK2	RAP1	SYC1	RAP1	TRP3
RAP1	SOL4	RAP1	SYF1	RAP1	TRR4
RAP1	SOP4	RAP1	SYG1	RAP1	TRS23
RAP1	SOV1	RAP1	TA(AGC)D	RAP1	TS(GCU)L
RAP1	SPO12	RAP1	TA(AGC)G	RAP1	TSR1
RAP1	SPO19	RAP1	TA(AGC)K2	RAP1	TTI1
RAP1	SPO20	RAP1	TDH2	RAP1	TUB4
RAP1	SPO73	RAP1	TDH3	RAP1	TVP15
RAP1	SPS100	RAP1	TEA1	RAP1	TYE7
RAP1	SPS4	RAP1	TEF1	RAP1	UBA1
RAP1	SPT16	RAP1	TEF2	RAP1	UBC11
RAP1	SPT20	RAP1	TEF4	RAP1	UBI4
RAP1	SPT3	RAP1	TEL1	RAP1	UBP2
RAP1	SPT5	RAP1	TEM1	RAP1	UBP5
RAP1	SRL1	RAP1	TEX1	RAP1	UBX2
RAP1	SRO9	RAP1	TFA1	RAP1	UFD4
RAP1	SRP1	RAP1	TFB1	RAP1	UFO1
RAP1	SRP40	RAP1	TFB4	RAP1	UGA1
RAP1	SRT1	RAP1	TGL2	RAP1	UGO1
RAP1	SSA1	RAP1	THI73	RAP1	UGX2
RAP1	SSA3	RAP1	TIM17	RAP1	ULS1
RAP1	SSB1	RAP1	TIM21	RAP1	USV1
RAP1	SSD1	RAP1	TIM23	RAP1	UTH1
RAP1	SSF2	RAP1	TIM8	RAP1	UTP15
RAP1	SSL1	RAP1	TIM9	RAP1	UTP4
RAP1	SSN8	RAP1	TIP20	RAP1	UTP6
RAP1	SSS1	RAP1	TIP41	RAP1	UTR2
RAP1	SST2	RAP1	TIR1	RAP1	UTR5
RAP1	STB1	RAP1	TIR3	RAP1	VAC17

RAP1	VBA5
RAP1	VEL1
RAP1	VHR1
RAP1	VHS1
RAP1	VID24
RAP1	VMA6
RAP1	VPS3
RAP1	VPS36
RAP1	VPS53
RAP1	VPS61
RAP1	VPS73
RAP1	VRP1
RAP1	VTS1
RAP1	WHI2
RAP1	WSC4
RAP1	XBP1
RAP1	YAL018C
RAP1	YAL046C
RAP1	YAL064C-A
RAP1	YAL064W
RAP1	YAP1802
RAP1	YAP6
RAP1	YAT1
RAP1	YBL028C
RAP1	YBL029C-A
RAP1	YBL029W
RAP1	YBL053W
RAP1	YBL071C
RAP1	YBL071C-B
RAP1	YBL081W
RAP1	YBL086C
RAP1	YBL111C
RAP1	YBR056W-A
RAP1	YBR138C
RAP1	YBR139W
RAP1	YBR190W
RAP1	YBR196C-A
RAP1	YBR196C-B
RAP1	YBR197C
RAP1	YBR219C
RAP1	YCF1
RAP1	YCG1
RAP1	YCK2
RAP1	YCL041C
RAP1	YCL042W
RAP1	YCL065W
RAP1	YCR006C
RAP1	YCR024C-B
RAP1	YCR025C
RAP1	YCT1
RAP1	YDL012C
RAP1	YDL032W
RAP1	YDL034W
RAP1	YDL129W
RAP1	YDL133W
RAP1	YDL173W
RAP1	YDL186W
RAP1	YDL187C
RAP1	YDR042C
RAP1	YDR063W
RAP1	YDR065W
RAP1	YDR089W
RAP1	YDR109C
RAP1	YDR112W
RAP1	YDR132C
RAP1	YDR186C
RAP1	YDR187C
RAP1	YDR193W
RAP1	YDR246W-A
RAP1	YDR274C
RAP1	YDR387C
RAP1	YDR391C
RAP1	YDR524C-B
RAP1	YDR543C
RAP1	YDR544C
RAP1	YEL007W
RAP1	YEL008W
RAP1	YEL023C
RAP1	YEL043W
RAP1	YEL045C
RAP1	YEL057C
RAP1	YEL074W
RAP1	YEL075C
RAP1	YEL076C
RAP1	YEL076C-A
RAP1	YEL1
RAP1	YER034W
RAP1	YER053C-A
RAP1	YER078C
RAP1	YER091C-A
RAP1	YER130C
RAP1	YER158C
RAP1	YER188C-A
RAP1	YER189W
RAP1	YFL015C
RAP1	YFL034W
RAP1	YFL051C
RAP1	YFL064C
RAP1	YFR017C
RAP1	YFR018C
RAP1	YFR032C
RAP1	YFR032C-B
RAP1	YFR035C
RAP1	YGK3
RAP1	YGL006W-A
RAP1	YGL007C-A
RAP1	YGL007W
RAP1	YGL072C
RAP1	YGL074C
RAP1	YGL114W
RAP1	YGL146C
RAP1	YGL177W
RAP1	YGL188C
RAP1	YGL188C-A
RAP1	YGL242C
RAP1	YGL261C
RAP1	YGR012W
RAP1	YGR031W
RAP1	YGR050C
RAP1	YGR051C
RAP1	YGR117C
RAP1	YGR149W
RAP1	YGR176W
RAP1	YGR203W
RAP1	YGR283C
RAP1	YHB1
RAP1	YHL015W-A
RAP1	YHL037C
RAP1	YHL045W
RAP1	YHL046C
RAP1	YHL049C
RAP1	YHP1
RAP1	YHR009C
RAP1	YHR032W
RAP1	YHR033W
RAP1	YHR095W
RAP1	YHR097C
RAP1	YHR122W

RAP1	YHR177W	RAP1	YLR137W	RAP1	YNL011C
RAP1	YIL002W-A	RAP1	YLR140W	RAP1	YNL024C
RAP1	YIL012W	RAP1	YLR154C-G	RAP1	YNL024C-A
RAP1	YIL046W-A	RAP1	YLR162W-A	RAP1	YNL028W
RAP1	YIL054W	RAP1	YLR164W	RAP1	YNL067W-A
RAP1	YIL055C	RAP1	YLR173W	RAP1	YNL067W-B
RAP1	YIL057C	RAP1	YLR184W	RAP1	YNL144C
RAP1	YIL158W	RAP1	YLR224W	RAP1	YNL150W
RAP1	YIL177C	RAP1	YLR255C	RAP1	YNL162W-A
RAP1	YIR014W	RAP1	YLR264C-A	RAP1	YNL165W
RAP1	YIR016W	RAP1	YLR287C	RAP1	YNL181W
RAP1	YIR042C	RAP1	YLR312C	RAP1	YNL190W
RAP1	YJL027C	RAP1	YLR326W	RAP1	YNL217W
RAP1	YJL028W	RAP1	YLR338W	RAP1	YNL277W-A
RAP1	YJL045W	RAP1	YLR339C	RAP1	YNL337W
RAP1	YJL049W	RAP1	YLR400W	RAP1	YNL338W
RAP1	YJL135W	RAP1	YLR402W	RAP1	YNR014W
RAP1	YJL206C	RAP1	YLR406C-A	RAP1	YNR018W
RAP1	YJL213W	RAP1	YLR407W	RAP1	YNR042W
RAP1	YJL218W	RAP1	YLR415C	RAP1	YNR070W
RAP1	YJL225C	RAP1	YLR463C	RAP1	YOL019W
RAP1	YJR114W	RAP1	YLR465C	RAP1	YOL029C
RAP1	YJR115W	RAP1	YMC1	RAP1	YOL075C
RAP1	YJR146W	RAP1	YML050W	RAP1	YOL083W
RAP1	YKL030W	RAP1	YML053C	RAP1	YOL085C
RAP1	YKL031W	RAP1	YML089C	RAP1	YOL161C
RAP1	YKL063C	RAP1	YML100W-A	RAP1	YOR008C-A
RAP1	YKL083W	RAP1	YML101C-A	RAP1	YOR008W-B
RAP1	YKL096C-B	RAP1	YML133C	RAP1	YOR032W-A
RAP1	YKL097C	RAP1	YML6	RAP1	YOR041C
RAP1	YKL151C	RAP1	YMR013W-A	RAP1	YOR152C
RAP1	YKL161C	RAP1	YMR027W	RAP1	YOR161C-C
RAP1	YKL177W	RAP1	YMR086C-A	RAP1	YOR186W
RAP1	YKL183C-A	RAP1	YMR086W	RAP1	YOR203W
RAP1	YKL225W	RAP1	YMR087W	RAP1	YOR268C
RAP1	YKR012C	RAP1	YMR099C	RAP1	YOR292C
RAP1	YKR040C	RAP1	YMR135W-A	RAP1	YOR302W
RAP1	YKR041W	RAP1	YMR144W	RAP1	YOR316C-A
RAP1	YLL037W	RAP1	YMR193C-A	RAP1	YOR338W
RAP1	YLL067C	RAP1	YMR230W-A	RAP1	YOR342C
RAP1	YLR012C	RAP1	YMR242W-A	RAP1	YOR343C
RAP1	YLR073C	RAP1	YMR244W	RAP1	YOR365C
RAP1	YLR076C	RAP1	YMR265C	RAP1	YOR366W
RAP1	YLR104W	RAP1	YMR295C	RAP1	YOR378W
RAP1	YLR111W	RAP1	YMR326C	RAP1	YOR387C
RAP1	YLR112W	RAP1	YNL010W	RAP1	YOR389W

RAP1	YOS1
RAP1	YPI1
RAP1	YPL056C
RAP1	YPL080C
RAP1	YPL113C
RAP1	YPL162C
RAP1	YPL191C
RAP1	YPL197C
RAP1	YPL229W
RAP1	YPL277C
RAP1	YPL278C
RAP1	YPR148C
RAP1	YPT31
RAP1	YRF1-1
RAP1	YRF1-2
RAP1	YRF1-4
RAP1	YRF1-5
RAP1	YRF1-6
RAP1	YRF1-7
RAP1	YSP3
RAP1	YSR3
RAP1	YTP1
RAP1	ZEO1
RAP1	ZIM17
RAP1	ZRC1
RAP1	ZRG8
RAP1	ZRT1
RAP1	ZRT3
RAP1	ZTA1
REB1	ABC1
REB1	ABD1
REB1	ACC1
REB1	ACT1
REB1	ADE2
REB1	ALA1
REB1	APL4
REB1	APS1
REB1	ARF2
REB1	ARF3
REB1	ARG5,6
REB1	ARL1
REB1	ARP7
REB1	ASI3
REB1	ASK1
REB1	ATH1
REB1	ATP4
REB1	AVT3
REB1	BCD1
REB1	BET3
REB1	BFR1
REB1	BLM10
REB1	BPH1
REB1	BUD27
REB1	CAT8
REB1	CBK1
REB1	CCL1
REB1	CCW12
REB1	CDC21
REB1	CDC39
REB1	CDC5
REB1	CDC50
REB1	CDC9
REB1	CEF1
REB1	CIS3
REB1	CLB2
REB1	CLB3
REB1	COG3
REB1	COX13
REB1	COX14
REB1	CPR1
REB1	CRP1
REB1	CSE1
REB1	CSM3
REB1	CSR1
REB1	CTI6
REB1	CTR9
REB1	CYB5
REB1	DCP2
REB1	DOC1
REB1	DOM34
REB1	EFG1
REB1	EHD3
REB1	ELO1
REB1	EMP24
REB1	EMP70
REB1	ENO1
REB1	ENT1
REB1	EPL1
REB1	FAS1
REB1	FAS2
REB1	FMP13
REB1	FMP42
REB1	FMP43
REB1	FMT1
REB1	FRE2
REB1	GAL1
REB1	GAL10
REB1	GCD11
REB1	GCS1
REB1	GCV2
REB1	GCY1
REB1	GEA2
REB1	GFA1
REB1	GIP4
REB1	GLC8
REB1	GLN3
REB1	GOS1
REB1	GPI12
REB1	GRR1
REB1	GTS1
REB1	GUS1
REB1	HAP2
REB1	HOG1
REB1	HOM2
REB1	HOS4
REB1	HRR25
REB1	HSC82
REB1	HSH49
REB1	HVG1
REB1	IBA57
REB1	IFH1
REB1	IGO1
REB1	ILV1
REB1	IME2
REB1	IME4
REB1	IMT4
REB1	IRC19
REB1	IRC23
REB1	ISA2
REB1	ISD11
REB1	JJJ3
REB1	KRE5
REB1	KRS1
REB1	LHS1
REB1	LIP5
REB1	MAM1
REB1	MCM5
REB1	MET1

REB1	MF(ALPHA)2	REB1	PRP2	REB1	SHP1
REB1	MLP2	REB1	PRP22	REB1	SHR3
REB1	MLS1	REB1	PRP43	REB1	SHS1
REB1	MMS1	REB1	PRP5	REB1	SIN3
REB1	MND1	REB1	PSD1	REB1	SKI7
REB1	MNP1	REB1	PUS1	REB1	SKP1
REB1	MOT2	REB1	RAD28	REB1	SMK1
REB1	MPE1	REB1	RAD5	REB1	SMP3
REB1	MPH1	REB1	RAP1	REB1	SMX3
REB1	MRP10	REB1	RAT1	REB1	SNC1
REB1	MRPL10	REB1	RCR2	REB1	SNR189
REB1	MRPL23	REB1	REB1	REB1	SNR190
REB1	MRPL51	REB1	REG1	REB1	SNR70
REB1	MRPS17	REB1	RGM1	REB1	SNR8
REB1	NAS6	REB1	RGT2	REB1	SNT309
REB1	NAT2	REB1	RHO2	REB1	SOR1
REB1	NDI1	REB1	RIX7	REB1	SPC24
REB1	NHP6A	REB1	RKM4	REB1	SPP2
REB1	NOP6	REB1	RMT2	REB1	SRP21
REB1	NOP8	REB1	RPA14	REB1	STB4
REB1	NPC2	REB1	RPA34	REB1	STB6
REB1	NPL4	REB1	RPB11	REB1	STE18
REB1	NST1	REB1	RPB9	REB1	STH1
REB1	NUP116	REB1	RPC19	REB1	SUB1
REB1	NUP157	REB1	RPL15B	REB1	SUI2
REB1	NUT1	REB1	RPL40A	REB1	SWI5
REB1	OKP1	REB1	RPL42A	REB1	TAF2
REB1	OST1	REB1	RPN2	REB1	TCO89
REB1	PAC10	REB1	RPO21	REB1	TDH3
REB1	PAN3	REB1	RPO31	REB1	TEF4
REB1	PCS60	REB1	RPS29A	REB1	THR1
REB1	PEP7	REB1	RPT5	REB1	TIF3
REB1	PFY1	REB1	RRN6	REB1	TIM11
REB1	PGC1	REB1	RRP14	REB1	TIM44
REB1	PGK1	REB1	RTF1	REB1	TKL1
REB1	PHB2	REB1	RXT2	REB1	TOA2
REB1	PHO91	REB1	SCJ1	REB1	TOM1
REB1	PIB1	REB1	SDH1	REB1	TOP1
REB1	PKH3	REB1	SDP1	REB1	TOR2
REB1	POL2	REB1	SEC23	REB1	TPA1
REB1	POL3	REB1	SEC24	REB1	TPI1
REB1	PPA1	REB1	SEC4	REB1	TQ(UUG)E1
REB1	PPH22	REB1	SEC61	REB1	TQ(UUG)E2
REB1	PRE1	REB1	SEC66	REB1	TRP1
REB1	PRE3	REB1	SEC8	REB1	TRP5
REB1	PRE4	REB1	SGN1	REB1	TS(AGA)D1

REB1	TUF1
REB1	TW(CCA)G2
REB1	UBA3
REB1	UBC8
REB1	UBR1
REB1	UFE1
REB1	URK1
REB1	VAC8
REB1	VMA10
REB1	VPS13
REB1	YAP1802
REB1	YBL059C-A
REB1	YBL059W
REB1	YBR096W
REB1	YDL186W
REB1	YDL233W
REB1	YDR029W
REB1	YDR154C
REB1	YDR157W
REB1	YDR455C
REB1	YEL023C
REB1	YER039C-A
REB1	YER066C-A
REB1	YER156C
REB1	YET2
REB1	YGL088W
REB1	YGL152C
REB1	YGL159W
REB1	YGL182C
REB1	YGL193C
REB1	YGR011W
REB1	YGR012W
REB1	YGR079W
REB1	YGR235C
REB1	YGR242W
REB1	YIF1
REB1	YIL108W
REB1	YIP4
REB1	YIR003W
REB1	YJR085C
REB1	YJR087W
REB1	YJR098C
REB1	YJR100C
REB1	YJR128W
REB1	YKE4
REB1	YKR015C
REB1	YLR171W
REB1	YLR224W
REB1	YLR366W
REB1	YLR379W
REB1	YML002W
REB1	YML119W
REB1	YMR122W-A
REB1	YMR185W
REB1	YNL092W
REB1	YNL115C
REB1	YNL171C
REB1	YNR040W
REB1	YOR093C
REB1	YOR186W
REB1	YOR287C
REB1	YOR318C
REB1	YOR325W
REB1	YPL182C
REB1	YPR053C
REB1	YPR076W
REB1	YPR099C
REB1	YPT7
REB1	YTM1
REB1	YUH1
REB1	ZRG17
STB1	API2
STB1	BUD31
STB1	CLB6
STB1	CLN2
STB1	CSI2
STB1	ELC1
STB1	FUN26
STB1	GIC1
STB1	GIC2
STB1	GVP36
STB1	IRC6
STB1	LTV1
STB1	MCM16
STB1	MGM101
STB1	MGR2
STB1	MRPS9
STB1	NDD1
STB1	NSE3
STB1	NTR2
STB1	OAZ1
STB1	PEX15
STB1	RFC3
STB1	RPL37B
STB1	RPS10A
STB1	RPS11B
STB1	RUP1
STB1	SFB3
STB1	SNO3
STB1	SPO16
STB1	SPO77
STB1	TOS2
STB1	VTC2
STB1	YAL064C-A
STB1	YDR367W
STB1	YER084W
STB1	YFR011C
STB1	YGR277C
STB1	YKL097C
STB1	YMR306C-A
STB1	YOR246C
STB1	YPL056C
SWI4	ABF2
SWI4	ACM1
SWI4	ACS2
SWI4	ADK1
SWI4	ADR1
SWI4	ADY2
SWI4	AEP3
SWI4	AGA1
SWI4	AGE1
SWI4	AGP1
SWI4	AHT1
SWI4	ALD3
SWI4	ALG14
SWI4	AMS1
SWI4	APC2
SWI4	API2
SWI4	APT2
SWI4	AQR1
SWI4	ARG5,6
SWI4	ASG7
SWI4	ATF1
SWI4	ATG11
SWI4	AUR1
SWI4	AXL2
SWI4	AYR1
SWI4	BAP3

SWI4	BAR1
SWI4	BAT2
SWI4	BBP1
SWI4	BFA1
SWI4	BMH1
SWI4	BNA5
SWI4	BTN2
SWI4	BUB3
SWI4	BUD31
SWI4	BUD9
SWI4	CAF120
SWI4	CAF40
SWI4	CAP1
SWI4	CBF2
SWI4	CCT2
SWI4	CCT6
SWI4	CCW12
SWI4	CDC21
SWI4	CDC48
SWI4	CDC6
SWI4	CHS1
SWI4	CHS3
SWI4	CHS5
SWI4	CIK1
SWI4	CIN1
SWI4	CIS3
SWI4	CLA4
SWI4	CLB1
SWI4	CLB2
SWI4	CLB5
SWI4	CLB6
SWI4	CLD1
SWI4	CLN1
SWI4	CLN2
SWI4	CLN3
SWI4	COQ10
SWI4	COS3
SWI4	COS6
SWI4	COS8
SWI4	CPR8
SWI4	CRH1
SWI4	CSH1
SWI4	CSI2
SWI4	CTF18
SWI4	CUE4
SWI4	CUS2
SWI4	CWP1
SWI4	CWP2
SWI4	CYC8
SWI4	DAL5
SWI4	DAM1
SWI4	DBF2
SWI4	DDR48
SWI4	DEM1
SWI4	DGA1
SWI4	DIF1
SWI4	DIN7
SWI4	DIP2
SWI4	DOT6
SWI4	DSF2
SWI4	DUN1
SWI4	DUO1
SWI4	ECM32
SWI4	ECM33
SWI4	ECM38
SWI4	ECM8
SWI4	ELO1
SWI4	EMG1
SWI4	ENP1
SWI4	ERG11
SWI4	ERG3
SWI4	ERG4
SWI4	ERP3
SWI4	EST1
SWI4	EXG1
SWI4	EXG2
SWI4	FIT2
SWI4	FKS1
SWI4	FKS3
SWI4	FLC1
SWI4	FMP27
SWI4	FRE5
SWI4	FTR1
SWI4	FUS3
SWI4	FZF1
SWI4	GAC1
SWI4	GAS1
SWI4	GAS2
SWI4	GAT2
SWI4	GCD6
SWI4	GFA1
SWI4	GIC1
SWI4	GIC2
SWI4	GID8
SWI4	GIM4
SWI4	GIN4
SWI4	GIT1
SWI4	GNP1
SWI4	GON7
SWI4	GPM2
SWI4	GSC2
SWI4	GSY2
SWI4	GTR1
SWI4	GUT2
SWI4	HAA1
SWI4	HAL1
SWI4	HAP1
SWI4	HAP4
SWI4	HAP5
SWI4	HBT1
SWI4	HCH1
SWI4	HCM1
SWI4	HCR1
SWI4	HEH2
SWI4	HEM12
SWI4	HEM13
SWI4	HHF2
SWI4	HHO1
SWI4	HHT2
SWI4	HIP1
SWI4	HMS2
SWI4	HO
SWI4	HOG1
SWI4	HPF1
SWI4	HSL1
SWI4	HSP12
SWI4	HSP26
SWI4	HSP30
SWI4	HSP33
SWI4	HSP42
SWI4	HTA1
SWI4	HTA2
SWI4	HTB1
SWI4	HTB2
SWI4	HTZ1
SWI4	HXK2
SWI4	HXT11
SWI4	HXT12

SWI4	HXT2	SWI4	MPT5	SWI4	PMC1
SWI4	HXT3	SWI4	MRPL4	SWI4	PMS1
SWI4	ICY1	SWI4	MRS1	SWI4	PNC1
SWI4	ICY2	SWI4	MSB2	SWI4	PNS1
SWI4	IPT1	SWI4	MSC1	SWI4	POG1
SWI4	IRC15	SWI4	MTC2	SWI4	POL30
SWI4	IRC22	SWI4	MUC1	SWI4	POP3
SWI4	IRS4	SWI4	MVD1	SWI4	PPN1
SWI4	ITC1	SWI4	NAN1	SWI4	PRI2
SWI4	KAP120	SWI4	NAT4	SWI4	PRM5
SWI4	KAP95	SWI4	NCE102	SWI4	PRY1
SWI4	KAR4	SWI4	NDD1	SWI4	PRY2
SWI4	KRE6	SWI4	NDL1	SWI4	PSA1
SWI4	KSS1	SWI4	NOP16	SWI4	PSE1
SWI4	KTI12	SWI4	NRM1	SWI4	PSO2
SWI4	LAC1	SWI4	NRT1	SWI4	PST1
SWI4	LAP4	SWI4	NUD1	SWI4	PTR2
SWI4	LEE1	SWI4	OCH1	SWI4	PUP3
SWI4	LOH1	SWI4	OPT2	SWI4	PUT4
SWI4	LSM3	SWI4	OXA1	SWI4	PYC1
SWI4	LSM4	SWI4	PAN5	SWI4	QDR2
SWI4	LSM5	SWI4	PAU21	SWI4	RAD16
SWI4	LTE1	SWI4	PCL1	SWI4	RAD18
SWI4	LYS2	SWI4	PCL2	SWI4	RAD27
SWI4	LYS20	SWI4	PCL5	SWI4	RAX2
SWI4	MAE1	SWI4	PDC1	SWI4	RBA50
SWI4	MAL11	SWI4	PDR1	SWI4	RCL1
SWI4	MAL12	SWI4	PDR16	SWI4	RDN5-6
SWI4	MAL32	SWI4	PDR5	SWI4	RDR1
SWI4	MCD1	SWI4	PET122	SWI4	REF2
SWI4	MCD4	SWI4	PET54	SWI4	REG2
SWI4	MCH2	SWI4	PET9	SWI4	REV7
SWI4	MCM2	SWI4	PEX28	SWI4	RFC3
SWI4	MDJ1	SWI4	PFA5	SWI4	RHO5
SWI4	MDM12	SWI4	PGM2	SWI4	RIM101
SWI4	MFA1	SWI4	PHD1	SWI4	RIM4
SWI4	MFA2	SWI4	PHO3	SWI4	RKM4
SWI4	MGA1	SWI4	PHO5	SWI4	RMI1
SWI4	MGR3	SWI4	PHO81	SWI4	RNR1
SWI4	MHR1	SWI4	PIL1	SWI4	RNR2
SWI4	MID2	SWI4	PKR1	SWI4	RNR3
SWI4	MLH2	SWI4	PLB2	SWI4	RNR4
SWI4	MNN1	SWI4	PLB3	SWI4	ROT1
SWI4	MNN5	SWI4	PLC1	SWI4	RPA34
SWI4	MON2	SWI4	PLM2	SWI4	RPE1
SWI4	MOT3	SWI4	PMA1	SWI4	RPG1

SWI4	RPL18B	SWI4	SPS4	SWI4	TOS1
SWI4	RPL34B	SWI4	SPT21	SWI4	TOS2
SWI4	RPL37A	SWI4	SPT8	SWI4	TOS3
SWI4	RPL37B	SWI4	SRD1	SWI4	TOS4
SWI4	RPL9A	SWI4	SRL1	SWI4	TOS6
SWI4	RPR2	SWI4	SRM1	SWI4	TOS8
SWI4	RPS16A	SWI4	SRP40	SWI4	TPO2
SWI4	RPS3	SWI4	SRP68	SWI4	TPO3
SWI4	RPT1	SWI4	SRS2	SWI4	TRF5
SWI4	RRD1	SWI4	SSA4	SWI4	TRM2
SWI4	RRP5	SWI4	SSE2	SWI4	TRS23
SWI4	RSR1	SWI4	SSK22	SWI4	TSA1
SWI4	RTC3	SWI4	SSL2	SWI4	TSA2
SWI4	RTC4	SWI4	SSU1	SWI4	TSL1
SWI4	RUP1	SWI4	STE6	SWI4	TVP18
SWI4	SCW10	SWI4	STP4	SWI4	TYE7
SWI4	SCW4	SWI4	STR3	SWI4	ULA1
SWI4	SEC16	SWI4	SUE1	SWI4	URE2
SWI4	SEC53	SWI4	SUP3	SWI4	USV1
SWI4	SED1	SWI4	SUR1	SWI4	UTH1
SWI4	SFG1	SWI4	SUR2	SWI4	UTR2
SWI4	SFL1	SWI4	SUT2	SWI4	VAC17
SWI4	SGA1	SWI4	SVS1	SWI4	VCX1
SWI4	SHC1	SWI4	SWE1	SWI4	VHR1
SWI4	SHE10	SWI4	SWI4	SWI4	VID30
SWI4	SIM1	SWI4	SYC1	SWI4	VPS20
SWI4	SIR4	SWI4	TA(AGC)P	SWI4	VPS71
SWI4	SIT4	SWI4	TAT2	SWI4	WSC2
SWI4	SKG6	SWI4	TCP1	SWI4	YAL064C-A
SWI4	SKM1	SWI4	TFP1	SWI4	YAL064W
SWI4	SKS1	SWI4	TFS1	SWI4	YAP5
SWI4	SLM4	SWI4	TGL5	SWI4	YAR068W
SWI4	SLX5	SWI4	TGS1	SWI4	YAR075W
SWI4	SMF2	SWI4	THI22	SWI4	YAT1
SWI4	SMT3	SWI4	THI7	SWI4	YBL029C-A
SWI4	SNA2	SWI4	THI72	SWI4	YBL029W
SWI4	SNF11	SWI4	THP2	SWI4	YBL104C
SWI4	SNQ2	SWI4	TIP1	SWI4	YBL108W
SWI4	SNU71	SWI4	TIR1	SWI4	YBL109W
SWI4	SOK2	SWI4	TIR3	SWI4	YBL111C
SWI4	SOL4	SWI4	TK(UUU)D	SWI4	YBR071W
SWI4	SPB1	SWI4	TLG2	SWI4	YBR113W
SWI4	SPC29	SWI4	TOK1	SWI4	YCR064C
SWI4	SPE4	SWI4	TOM1	SWI4	YDL183C
SWI4	SPO20	SWI4	TOP1	SWI4	YDL186W
SWI4	SPO77	SWI4	TOP3	SWI4	YDL187C

SWI4	YDR010C
SWI4	YDR132C
SWI4	YDR134C
SWI4	YDR186C
SWI4	YDR222W
SWI4	YDR442W
SWI4	YDR509W
SWI4	YDR524C-B
SWI4	YDR526C
SWI4	YDR541C
SWI4	YDR542W
SWI4	YDR543C
SWI4	YDR544C
SWI4	YEF1
SWI4	YEL007W
SWI4	YEL074W
SWI4	YEL075C
SWI4	YER010C
SWI4	YER078C
SWI4	YER079W
SWI4	YER138W-A
SWI4	YER189W
SWI4	YFL063W
SWI4	YFL064C
SWI4	YFR017C
SWI4	YGL007W
SWI4	YGL036W
SWI4	YGL177W
SWI4	YGR012W
SWI4	YGR045C
SWI4	YGR050C
SWI4	YGR109W-A
SWI4	YGR109W-B
SWI4	YGR111W
SWI4	YGR151C
SWI4	YGR153W
SWI4	YGR190C
SWI4	YGR250C
SWI4	YGR251W
SWI4	YHB1
SWI4	YHL026C
SWI4	YHL049C
SWI4	YHP1
SWI4	YHR078W
SWI4	YHR213W
SWI4	YHR214W
SWI4	YHR217C
SWI4	YHR218W
SWI4	YHR219W
SWI4	YIL141W
SWI4	YIL169C
SWI4	YIL177C
SWI4	YIR018C-A
SWI4	YIR020C
SWI4	YJL107C
SWI4	YJL118W
SWI4	YJL160C
SWI4	YJL185C
SWI4	YJL225C
SWI4	YJR003C
SWI4	YJR030C
SWI4	YJR054W
SWI4	YJR116W
SWI4	YJU2
SWI4	YKL044W
SWI4	YKL096C-B
SWI4	YKL097C
SWI4	YKL102C
SWI4	YKL151C
SWI4	YKR011C
SWI4	YKR040C
SWI4	YKR041W
SWI4	YLL066C
SWI4	YLL067C
SWI4	YLR035C-A
SWI4	YLR042C
SWI4	YLR111W
SWI4	YLR112W
SWI4	YLR125W
SWI4	YLR159W
SWI4	YLR162W
SWI4	YLR184W
SWI4	YLR255C
SWI4	YLR256W-A
SWI4	YLR301W
SWI4	YLR462W
SWI4	YML133C
SWI4	YMR135W-A
SWI4	YMR144W
SWI4	YMR178W
SWI4	YMR194C-A
SWI4	YMR194C-B
SWI4	YMR244W
SWI4	YMR279C
SWI4	YMR304C-A
SWI4	YMR306C-A
SWI4	YNL179C
SWI4	YNL295W
SWI4	YNL296W
SWI4	YNL337W
SWI4	YNL338W
SWI4	YNR062C
SWI4	YNR070W
SWI4	YOL019W
SWI4	YOL019W-A
SWI4	YOL114C
SWI4	YOR114W
SWI4	YOR152C
SWI4	YOR246C
SWI4	YOR248W
SWI4	YOR302W
SWI4	YOR314W
SWI4	YOR338W
SWI4	YOR342C
SWI4	YOR343C
SWI4	YOR378W
SWI4	YOR390W
SWI4	YOX1
SWI4	YPL009C
SWI4	YPL014W
SWI4	YPL025C
SWI4	YPL056C
SWI4	YPL068C
SWI4	YPL088W
SWI4	YPL162C
SWI4	YPR013C
SWI4	YPR015C
SWI4	YPR148C
SWI4	YPR158C-C
SWI4	YPR158C-D
SWI4	YPR202W
SWI4	YPR204W
SWI4	YPS1
SWI4	YPS3
SWI4	YRF1-1
SWI4	YRF1-2
SWI4	YRF1-3
SWI4	YRF1-5

SWI4	YRF1-6
SWI4	YRF1-7
SWI4	YRO2
SWI4	YSY6
SWI4	ZEO1
SWI5	ALG14
SWI5	AMN1
SWI5	ASH1
SWI5	AST1
SWI5	AYT1
SWI5	BNI1
SWI5	BOS1
SWI5	BSC4
SWI5	BUD9
SWI5	BUL2
SWI5	CBK1
SWI5	CDC34
SWI5	CDC6
SWI5	CHS1
SWI5	CLB4
SWI5	CLN3
SWI5	COS1
SWI5	COS3
SWI5	COS4
SWI5	COS6
SWI5	CPR1
SWI5	CRH1
SWI5	CTK3
SWI5	CTS1
SWI5	CUE4
SWI5	CYK3
SWI5	DBP9
SWI5	DDI3
SWI5	DDR48
SWI5	DFR1
SWI5	DJP1
SWI5	DLD1
SWI5	DPM1
SWI5	DSE1
SWI5	DSE2
SWI5	DSE3
SWI5	DSE4
SWI5	ECM32
SWI5	EGT2
SWI5	ENO1
SWI5	ENT2
SWI5	ERG25
SWI5	EXG1
SWI5	FAA3
SWI5	FLC3
SWI5	FLO9
SWI5	FOB1
SWI5	FRS2
SWI5	FUN26
SWI5	FUR1
SWI5	GAS5
SWI5	GAT1
SWI5	GAT2
SWI5	GAT3
SWI5	GCS1
SWI5	GDH3
SWI5	GPR1
SWI5	GTO3
SWI5	GTR1
SWI5	HBT1
SWI5	HO
SWI5	HOR7
SWI5	HRD3
SWI5	HSP12
SWI5	HSP150
SWI5	HXT3
SWI5	ICS2
SWI5	ISR1
SWI5	IWR1
SWI5	KEX2
SWI5	KSS1
SWI5	LAA1
SWI5	LAP3
SWI5	LOT6
SWI5	LSM3
SWI5	MDJ2
SWI5	MET18
SWI5	MFA2
SWI5	MGM101
SWI5	MMF1
SWI5	MNN9
SWI5	MRPL4
SWI5	MSR1
SWI5	MTC3
SWI5	MTR2
SWI5	NCB2
SWI5	NIS1
SWI5	OCA5
SWI5	OST5
SWI5	OTU2
SWI5	PAH1
SWI5	PCL2
SWI5	PCL7
SWI5	PCL9
SWI5	PFA3
SWI5	PGA3
SWI5	PHO81
SWI5	PIL1
SWI5	PIR1
SWI5	PIR3
SWI5	PRI2
SWI5	PRP9
SWI5	PRY3
SWI5	PSK1
SWI5	PST1
SWI5	PTH1
SWI5	PUP2
SWI5	PUT4
SWI5	REE1
SWI5	RME1
SWI5	RPL2A
SWI5	RPL31B
SWI5	RUP1
SWI5	RVB2
SWI5	SCP160
SWI5	SCW11
SWI5	SEN2
SWI5	SFL1
SWI5	SHE10
SWI5	SHS1
SWI5	SIC1
SWI5	SMX3
SWI5	TAF13
SWI5	TAH11
SWI5	TAO3
SWI5	TEC1
SWI5	TGS1
SWI5	TIM23
SWI5	TM(CAU)C
SWI5	TPM1
SWI5	TPS3
SWI5	TSL1
SWI5	TSR1

SWI5	UBC4	SWI5	YLL066C	SWI6	AYR1
SWI5	URM1	SWI5	YLL067C	SWI6	BAR1
SWI5	UTP5	SWI5	YLR012C	SWI6	BBP1
SWI5	VBA3	SWI5	YLR035C-A	SWI6	BFA1
SWI5	VCX1	SWI5	YLR049C	SWI6	BUB1
SWI5	VID30	SWI5	YLR162W	SWI6	CCW12
SWI5	VPS55	SWI5	YLR194C	SWI6	CDC21
SWI5	VTH1	SWI5	YLR346C	SWI6	CDC45
SWI5	WHI4	SWI5	YLR407W	SWI6	CDC5
SWI5	YAL018C	SWI5	YLR462W	SWI6	CDC6
SWI5	YAR009C	SWI5	YLR463C	SWI6	CHS5
SWI5	YBL108W	SWI5	YLR464W	SWI6	CIK1
SWI5	YBL109W	SWI5	YLR465C	SWI6	CLB5
SWI5	YBL111C	SWI5	YML131W	SWI6	CLB6
SWI5	YBL112C	SWI5	YML133C	SWI6	CLD1
SWI5	YBL113C	SWI5	YMR134W	SWI6	CLN1
SWI5	YBR071W	SWI5	YMR135W-A	SWI6	CLN2
SWI5	YDL032W	SWI5	YMR172C-A	SWI6	COX7
SWI5	YDL034W	SWI5	YMR173W-A	SWI6	CRD1
SWI5	YDL114W	SWI5	YMR262W	SWI6	CRH1
SWI5	YDL118W	SWI5	YNL033W	SWI6	CSE1
SWI5	YDL119C	SWI5	YNL046W	SWI6	CSI2
SWI5	YDL173W	SWI5	YNL146W	SWI6	CSN9
SWI5	YDL180W	SWI5	YNL193W	SWI6	CWP2
SWI5	YDR112W	SWI5	YNL324W	SWI6	CYT1
SWI5	YDR543C	SWI5	YNL337W	SWI6	DIN7
SWI5	YEL077C	SWI5	YNL338W	SWI6	DSF2
SWI5	YER078C	SWI5	YNR018W	SWI6	DUN1
SWI5	YER078W-A	SWI5	YOR262W	SWI6	ECM33
SWI5	YER079W	SWI5	YOS9	SWI6	ECM38
SWI5	YER138C	SWI5	YPL158C	SWI6	ELO1
SWI5	YER189W	SWI5	YRF1-1	SWI6	ERP3
SWI5	YFL063W	SWI5	YRF1-2	SWI6	ESF2
SWI5	YFL064C	SWI5	YRF1-3	SWI6	FAR1
SWI5	YFL065C	SWI5	YRF1-4	SWI6	FAR7
SWI5	YFL067W	SWI5	YRF1-5	SWI6	FKS1
SWI5	YFR017C	SWI5	YRF1-6	SWI6	FKS3
SWI5	YGL108C	SWI5	YRF1-7	SWI6	FLO10
SWI5	YGL140C	SWI5	ZWF1	SWI6	GAS1
SWI5	YHB1	SWI6	AAD6	SWI6	GAS3
SWI5	YHR138C	SWI6	AAP1	SWI6	GAT2
SWI5	YIL174W	SWI6	API2	SWI6	GDB1
SWI5	YIL177C	SWI6	ARG5,6	SWI6	GIC1
SWI5	YIR003W	SWI6	ASN2	SWI6	GIC2
SWI5	YJL160C	SWI6	ATO3	SWI6	GID8
SWI5	YJL225C	SWI6	ATP16	SWI6	GIM4

SWI6	GIN4	SWI6	PUP3	SWI6	YDR524C-B
SWI6	GOR1	SWI6	QCR10	SWI6	YEL010W
SWI6	GTR1	SWI6	RAD27	SWI6	YER078C
SWI6	GTT1	SWI6	RBA50	SWI6	YGL140C
SWI6	HCM1	SWI6	REX3	SWI6	YGR012W
SWI6	HHO1	SWI6	RFC3	SWI6	YGR125W
SWI6	HO	SWI6	RIX7	SWI6	YHP1
SWI6	HOG1	SWI6	RNR1	SWI6	YHR003C
SWI6	HSL1	SWI6	RNR4	SWI6	YIL169C
SWI6	HXK1	SWI6	ROT1	SWI6	YJL185C
SWI6	IMP2	SWI6	RPL18B	SWI6	YJR030C
SWI6	IRC22	SWI6	RPL37A	SWI6	YJR054W
SWI6	IRR1	SWI6	RPL37B	SWI6	YKL066W
SWI6	KSS1	SWI6	RTR1	SWI6	YKL096C-B
SWI6	LAC1	SWI6	SCJ1	SWI6	YKL161C
SWI6	LAP4	SWI6	SCW10	SWI6	YKR011C
SWI6	LCP5	SWI6	SER3	SWI6	YLR297W
SWI6	LIN1	SWI6	SIM1	SWI6	YLR301W
SWI6	LPE10	SWI6	SKG6	SWI6	YML083C
SWI6	MCH2	SWI6	SLM4	SWI6	YMR178W
SWI6	MKC7	SWI6	SMC3	SWI6	YMR306C-A
SWI6	MNN1	SWI6	SNU71	SWI6	YNL089C
SWI6	MNN5	SWI6	SOK2	SWI6	YNL296W
SWI6	MRPL4	SWI6	SPO16	SWI6	YNL313C
SWI6	MSH6	SWI6	SRL1	SWI6	YOL114C
SWI6	MTF2	SWI6	SUT2	SWI6	YOL161C
SWI6	MTR4	SWI6	SVS1	SWI6	YOR105W
SWI6	NDD1	SWI6	SWE1	SWI6	YOR246C
SWI6	NTE1	SWI6	SWI4	SWI6	YOR314W
SWI6	OCH1	SWI6	TE(CUC)D	SWI6	YOX1
SWI6	OPY2	SWI6	TOK1	SWI6	YPT52
SWI6	OTU2	SWI6	TOS2	SWI6	YRA2
SWI6	PCL1	SWI6	TOS3	HSF1	AAC3
SWI6	PCL2	SWI6	TOS4	HSF1	AAD6
SWI6	PDS1	SWI6	TOS6	HSF1	ACA1
SWI6	PDS5	SWI6	TPK1	HSF1	ACC1
SWI6	PET54	SWI6	TPS2	HSF1	ACT1
SWI6	PEX28	SWI6	TRF5	HSF1	AEP3
SWI6	PFK26	SWI6	TRM2	HSF1	AFG3
SWI6	PHD1	SWI6	TSA1	HSF1	AHA1
SWI6	PLC1	SWI6	TYE7	HSF1	AHP1
SWI6	PLM2	SWI6	URA1	HSF1	ALD1
SWI6	PPN1	SWI6	UTR2	HSF1	ALD2
SWI6	PRE1	SWI6	VCX1	HSF1	ALD3
SWI6	PRE8	SWI6	VPS74	HSF1	ALD4
SWI6	PRY2	SWI6	YDL211C	HSF1	ALD5

HSF1	ALG13
HSF1	APA1
HSF1	APJ1
HSF1	APP1
HSF1	ARB1
HSF1	ARO1
HSF1	ARP7
HSF1	ASK1
HSF1	ATC1
HSF1	ATG1
HSF1	ATG19
HSF1	ATG8
HSF1	ATH1
HSF1	ATO2
HSF1	ATP17
HSF1	AVT6
HSF1	BAG7
HSF1	BBC1
HSF1	BDS1
HSF1	BET4
HSF1	BOP2
HSF1	BSC2
HSF1	BSC5
HSF1	BSC6
HSF1	BTN2
HSF1	BUB3
HSF1	BUD7
HSF1	BUL1
HSF1	CAK1
HSF1	CAT2
HSF1	CEM1
HSF1	CHO2
HSF1	CIN1
HSF1	CIT3
HSF1	CMP2
HSF1	CNS1
HSF1	COQ4
HSF1	COQ6
HSF1	CPR1
HSF1	CPR6
HSF1	CPS1
HSF1	CRC1
HSF1	CRG1
HSF1	CSE2
HSF1	CSM4
HSF1	CSR2
HSF1	CTF13
HSF1	CTH1
HSF1	CTL1
HSF1	CTT1
HSF1	CUE4
HSF1	CUP1-1
HSF1	CUP1-2
HSF1	CUR1
HSF1	CWC23
HSF1	CWP1
HSF1	CYB2
HSF1	CYC7
HSF1	DAK2
HSF1	DAL4
HSF1	DAL7
HSF1	DAP1
HSF1	DBP1
HSF1	DDI1
HSF1	DDI3
HSF1	DDR2
HSF1	DEP1
HSF1	DIT1
HSF1	DNM1
HSF1	DOC1
HSF1	DPB2
HSF1	DRE2
HSF1	ECI1
HSF1	ECM21
HSF1	ECM23
HSF1	ECM29
HSF1	ECM4
HSF1	ECM8
HSF1	EDC2
HSF1	EFB1
HSF1	EMI2
HSF1	EMI5
HSF1	ENO2
HSF1	ENP1
HSF1	ENT2
HSF1	ERO1
HSF1	ERV29
HSF1	ESBP6
HSF1	EST1
HSF1	EXG2
HSF1	FAA1
HSF1	FAL1
HSF1	FAR3
HSF1	FES1
HSF1	FIT2
HSF1	FMP12
HSF1	FMP16
HSF1	FMP23
HSF1	FMP33
HSF1	FMP40
HSF1	FMP45
HSF1	FMP48
HSF1	FMP52
HSF1	FMS1
HSF1	FOX2
HSF1	FSH1
HSF1	FSP2
HSF1	FTH1
HSF1	GCY1
HSF1	GDE1
HSF1	GFD1
HSF1	GGA1
HSF1	GIC2
HSF1	GIP2
HSF1	GIR2
HSF1	GLC3
HSF1	GLO4
HSF1	GOR1
HSF1	GPH1
HSF1	GPM1
HSF1	GRC3
HSF1	GRE1
HSF1	GRE2
HSF1	GRE3
HSF1	GRS2
HSF1	GSP1
HSF1	GTB1
HSF1	GTO1
HSF1	GTO3
HSF1	HAC1
HSF1	HBT1
HSF1	HCH1
HSF1	HEK2
HSF1	HFD1
HSF1	HHF1
HSF1	HIF1
HSF1	HLR1
HSF1	HMS1

HSF1	HMX1
HSF1	HOM2
HSF1	HOM3
HSF1	HOR7
HSF1	HOS4
HSF1	HPR1
HSF1	HRD1
HSF1	HRD3
HSF1	HRK1
HSF1	HSC82
HSF1	HSP10
HSF1	HSP104
HSF1	HSP12
HSF1	HSP26
HSF1	HSP30
HSF1	HSP42
HSF1	HSP60
HSF1	HSP78
HSF1	HSP82
HSF1	HUA1
HSF1	HXK1
HSF1	HXT16
HSF1	HXT3
HSF1	HXT5
HSF1	HXT9
HSF1	ICY2
HSF1	ILS1
HSF1	ILV2
HSF1	IML2
HSF1	INO2
HSF1	IRA2
HSF1	IRC18
HSF1	ISA1
HSF1	ISF1
HSF1	JEM1
HSF1	JIP4
HSF1	JLP1
HSF1	JSN1
HSF1	KAP95
HSF1	KAR2
HSF1	KNS1
HSF1	KRR1
HSF1	KSP1
HSF1	KTI12
HSF1	LCB5
HSF1	LEE1
HSF1	LIN1
HSF1	LSM8
HSF1	LST8
HSF1	MAG1
HSF1	MAK21
HSF1	MAM3
HSF1	MBF1
HSF1	MCH2
HSF1	MDJ1
HSF1	MDL2
HSF1	MEF2
HSF1	MET2
HSF1	MET28
HSF1	MET31
HSF1	MET8
HSF1	MEX67
HSF1	MGA1
HSF1	MHP1
HSF1	MLS1
HSF1	MMT1
HSF1	MOH1
HSF1	MPA43
HSF1	MPM1
HSF1	MRD1
HSF1	MRT4
HSF1	MTQ1
HSF1	MUP3
HSF1	NAF1
HSF1	NAT4
HSF1	NBP1
HSF1	NBP35
HSF1	NCA3
HSF1	NIS1
HSF1	NPL4
HSF1	NQM1
HSF1	NUP1
HSF1	OLA1
HSF1	OPI10
HSF1	ORC2
HSF1	OSW2
HSF1	OTU1
HSF1	OYE2
HSF1	PAA1
HSF1	PAI3
HSF1	PAU17
HSF1	PCK1
HSF1	PDC6
HSF1	PDE1
HSF1	PDH1
HSF1	PDR11
HSF1	PDR12
HSF1	PDR15
HSF1	PDR3
HSF1	PEP8
HSF1	PES4
HSF1	PET10
HSF1	PEX18
HSF1	PEX30
HSF1	PGK1
HSF1	PGM2
HSF1	PHM8
HSF1	PHO2
HSF1	PHR1
HSF1	PIB1
HSF1	PIN3
HSF1	PIR3
HSF1	PMA1
HSF1	PMC1
HSF1	PMR1
HSF1	PNC1
HSF1	PNS1
HSF1	POP3
HSF1	POX1
HSF1	PPM2
HSF1	PRD1
HSF1	PRE6
HSF1	PRK1
HSF1	PRM5
HSF1	PRM6
HSF1	PRM8
HSF1	PRM9
HSF1	PRP46
HSF1	PRR2
HSF1	PRX1
HSF1	PSF1
HSF1	PUF3
HSF1	PUT3
HSF1	PYC1
HSF1	RAD14
HSF1	RAD28
HSF1	RAX1
HSF1	REB1

HSF1	REC104
HSF1	REC114
HSF1	REG2
HSF1	REH1
HSF1	RFS1
HSF1	RIB1
HSF1	RIF1
HSF1	RIM11
HSF1	RIM13
HSF1	RIM4
HSF1	RLF2
HSF1	RNH202
HSF1	RNH203
HSF1	RNR3
HSF1	ROD1
HSF1	ROG1
HSF1	ROT1
HSF1	RPA14
HSF1	RPA34
HSF1	RPN12
HSF1	RPN4
HSF1	RPS8B
HSF1	RPT2
HSF1	RPT3
HSF1	RSC58
HSF1	RTC1
HSF1	RTC3
HSF1	RTS3
HSF1	SAE3
HSF1	SAM35
HSF1	SBP1
HSF1	SCS22
HSF1	SCT1
HSF1	SDH3
HSF1	SDP1
HSF1	SDS23
HSF1	SDS24
HSF1	SEC1
HSF1	SEM1
HSF1	SEO1
HSF1	SET2
HSF1	SFK1
HSF1	SGT2
HSF1	SIL1
HSF1	SIP4
HSF1	SIS1
HSF1	SKI6
HSF1	SKO1
HSF1	SKS1
HSF1	SLF1
HSF1	SLZ1
HSF1	SMC2
HSF1	SMC3
HSF1	SNG1
HSF1	SNQ2
HSF1	SNZ2
HSF1	SOD2
HSF1	SOL1
HSF1	SOL4
HSF1	SOR1
HSF1	SPE2
HSF1	SPG4
HSF1	SPI1
HSF1	SPO22
HSF1	SPO71
HSF1	SPO75
HSF1	SPR1
HSF1	SPT7
HSF1	SRL4
HSF1	SSA1
HSF1	SSA2
HSF1	SSA3
HSF1	SSA4
HSF1	SSC1
HSF1	SSE1
HSF1	SSE2
HSF1	SSH4
HSF1	STB5
HSF1	STE24
HSF1	STI1
HSF1	SUL1
HSF1	SVL3
HSF1	SWM1
HSF1	SYM1
HSF1	TA(AGC)G
HSF1	TAD2
HSF1	TAH11
HSF1	TDH3
HSF1	TEC1
HSF1	TEF2
HSF1	TGL2
HSF1	THI2
HSF1	THI72
HSF1	TIP1
HSF1	TIP41
HSF1	TKL2
HSF1	TMA10
HSF1	TOP3
HSF1	TPK1
HSF1	TPO1
HSF1	TRM82
HSF1	TSL1
HSF1	TYE7
HSF1	UBC13
HSF1	UBC4
HSF1	UBC5
HSF1	UBC8
HSF1	UBI4
HSF1	UBP15
HSF1	UBX3
HSF1	UBX4
HSF1	UBX5
HSF1	UFD1
HSF1	UFD4
HSF1	UGA1
HSF1	UGA2
HSF1	UGP1
HSF1	UGX2
HSF1	UMP1
HSF1	UPC2
HSF1	URA10
HSF1	USV1
HSF1	UTR5
HSF1	VEL1
HSF1	VIK1
HSF1	VPS29
HSF1	VPS61
HSF1	VPS62
HSF1	VPS73
HSF1	WSC4
HSF1	XKS1
HSF1	XYL2
HSF1	YAL004W
HSF1	YAP1801
HSF1	YAP6
HSF1	YAR1
HSF1	YBL073W
HSF1	YBR051W

HSF1	YBR053C	HSF1	YGL036W	HSF1	YMR196W
HSF1	YBR062C	HSF1	YGL230C	HSF1	YMR244W
HSF1	YBR071W	HSF1	YGP1	HSF1	YMR245W
HSF1	YBR085C-A	HSF1	YGR050C	HSF1	YMR252C
HSF1	YBR090C	HSF1	YGR146C	HSF1	YMR253C
HSF1	YBR116C	HSF1	YGR210C	HSF1	YMR258C
HSF1	YBR139W	HSF1	YGR250C	HSF1	YMR262W
HSF1	YBR219C	HSF1	YGR251W	HSF1	YNL134C
HSF1	YBR241C	HSF1	YHR138C	HSF1	YNL146W
HSF1	YBR284W	HSF1	YHR173C	HSF1	YNL155W
HSF1	YBR285W	HSF1	YJL045W	HSF1	YNL234W
HSF1	YCR061W	HSF1	YJL068C	HSF1	YNL305C
HSF1	YCR076C	HSF1	YJL144W	HSF1	YNR068C
HSF1	YCR099C	HSF1	YJR008W	HSF1	YOL038C-A
HSF1	YCR102C	HSF1	YJR107W	HSF1	YOL048C
HSF1	YDJ1	HSF1	YKE2	HSF1	YOL131W
HSF1	YDL057W	HSF1	YKE4	HSF1	YOL162W
HSF1	YDL119C	HSF1	YKL027W	HSF1	YOL163W
HSF1	YDL176W	HSF1	YKL037W	HSF1	YOP1
HSF1	YDL177C	HSF1	YKL070W	HSF1	YOR008C-A
HSF1	YDL180W	HSF1	YKL091C	HSF1	YOR020W-A
HSF1	YDL199C	HSF1	YKL123W	HSF1	YOR021C
HSF1	YDR010C	HSF1	YKR041W	HSF1	YOR052C
HSF1	YDR018C	HSF1	YKR075C	HSF1	YOR059C
HSF1	YDR034W-B	HSF1	YLL023C	HSF1	YOR300W
HSF1	YDR061W	HSF1	YLL032C	HSF1	YOR387C
HSF1	YDR065W	HSF1	YLL037W	HSF1	YPI1
HSF1	YDR124W	HSF1	YLL056C	HSF1	YPL113C
HSF1	YDR154C	HSF1	YLL058W	HSF1	YPL150W
HSF1	YDR157W	HSF1	YLR042C	HSF1	YPL168W
HSF1	YDR170W-A	HSF1	YLR050C	HSF1	YPL247C
HSF1	YDR222W	HSF1	YLR064W	HSF1	YPL276W
HSF1	YDR248C	HSF1	YLR108C	HSF1	YPP1
HSF1	YDR374C	HSF1	YLR118C	HSF1	YPR015C
HSF1	YDR476C	HSF1	YLR125W	HSF1	YPR036W-A
HSF1	YDR506C	HSF1	YLR126C	HSF1	YPR038W
HSF1	YEL045C	HSF1	YLR164W	HSF1	YPR039W
HSF1	YER067W	HSF1	YLR217W	HSF1	YPR153W
HSF1	YER079W	HSF1	YLR345W	HSF1	YPR157W
HSF1	YFL012W	HSF1	YLR349W	HSF1	YPR174C
HSF1	YFL040W	HSF1	YLR356W	HSF1	YPR196W
HSF1	YFL042C	HSF1	YLR445W	HSF1	YPS5
HSF1	YFL067W	HSF1	YML053C	HSF1	YPS6
HSF1	YGL006W-A	HSF1	YMR085W	HSF1	YRO2
HSF1	YGL007C-A	HSF1	YMR185W	HSF1	YSC84
HSF1	YGL007W	HSF1	YMR187C	HSF1	YTH1

HSF1	YTP1	MSN4	CIT3	MSN4	EXG2
HSF1	ZEO1	MSN4	CMP2	MSN4	FHL1
HSF1	ZIP1	MSN4	COP1	MSN4	FIS1
HSF1	ZPR1	MSN4	COQ1	MSN4	FIT2
HSF1	ZRG8	MSN4	COQ6	MSN4	FLR1
MSN4	AAC3	MSN4	COS1	MSN4	FMP12
MSN4	ACH1	MSN4	COS3	MSN4	FMP45
MSN4	ADR1	MSN4	COS6	MSN4	FPR1
MSN4	AFG1	MSN4	COX14	MSN4	FPS1
MSN4	AFR1	MSN4	COX17	MSN4	FUM1
MSN4	AGP1	MSN4	COX20	MSN4	GAC1
MSN4	AGX1	MSN4	COX5A	MSN4	GAD1
MSN4	AHP1	MSN4	CPA2	MSN4	GAL1
MSN4	ALD2	MSN4	CPR3	MSN4	GAT1
MSN4	ALD3	MSN4	CPR6	MSN4	GDB1
MSN4	ALD4	MSN4	CRM1	MSN4	GDH3
MSN4	ALD5	MSN4	CRS5	MSN4	GIP2
MSN4	ALG7	MSN4	CTR1	MSN4	GIP3
MSN4	ANB1	MSN4	CTT1	MSN4	GLC3
MSN4	APC9	MSN4	CUE4	MSN4	GLK1
MSN4	APE2	MSN4	CWC27	MSN4	GLO1
MSN4	APE3	MSN4	CYB2	MSN4	GLO2
MSN4	APJ1	MSN4	CYC7	MSN4	GOR1
MSN4	ARA1	MSN4	DAK1	MSN4	GPD1
MSN4	ARF1	MSN4	DAP1	MSN4	GPG1
MSN4	ARN1	MSN4	DCS2	MSN4	GPH1
MSN4	ARN2	MSN4	DDI3	MSN4	GPI1
MSN4	ARR3	MSN4	DDR2	MSN4	GPM2
MSN4	ASI1	MSN4	DDR48	MSN4	GPT2
MSN4	ATG13	MSN4	DFR1	MSN4	GPX1
MSN4	ATG8	MSN4	DLD1	MSN4	GPX2
MSN4	ATP25	MSN4	DOG2	MSN4	GRE1
MSN4	ATR1	MSN4	DOM34	MSN4	GRE2
MSN4	AYT1	MSN4	DOS2	MSN4	GRE3
MSN4	BCY1	MSN4	DSF1	MSN4	GRX1
MSN4	BDH2	MSN4	DYN1	MSN4	GRX2
MSN4	BEM2	MSN4	ECM3	MSN4	GRX6
MSN4	BGL2	MSN4	ECM38	MSN4	GRX7
MSN4	BTN2	MSN4	ECM4	MSN4	GSC2
MSN4	BUD19	MSN4	EFB1	MSN4	GSH1
MSN4	CAF20	MSN4	EMI1	MSN4	GSY1
MSN4	CBP2	MSN4	EMI2	MSN4	GSY2
MSN4	CBP6	MSN4	ERG6	MSN4	GTO1
MSN4	CCP1	MSN4	ERP2	MSN4	GTO3
MSN4	CDC48	MSN4	ERR1	MSN4	GTT1
MSN4	CHA1	MSN4	ESA1	MSN4	GTT2

MSN4	GTT3
MSN4	GUD1
MSN4	GUT2
MSN4	HBT1
MSN4	HER1
MSN4	HIS5
MSN4	HMRA2
MSN4	HOR2
MSN4	HOR7
MSN4	HPF1
MSN4	HRD1
MSN4	HSP104
MSN4	HSP12
MSN4	HSP150
MSN4	HSP26
MSN4	HSP30
MSN4	HSP31
MSN4	HSP42
MSN4	HSP78
MSN4	HSP82
MSN4	HUG1
MSN4	HXK1
MSN4	HXT11
MSN4	HXT13
MSN4	HXT15
MSN4	HXT17
MSN4	HXT3
MSN4	HXT4
MSN4	HXT6
MSN4	HXT7
MSN4	IBA57
MSN4	IDH1
MSN4	ILM1
MSN4	IME1
MSN4	IMP2
MSN4	IPT1
MSN4	IRC24
MSN4	IRE1
MSN4	ISA1
MSN4	IST2
MSN4	ISU2
MSN4	IVY1
MSN4	IZH1
MSN4	KHA1
MSN4	KSS1
MSN4	KTR2
MSN4	LAA1
MSN4	LAP4
MSN4	LEU2
MSN4	LIN1
MSN4	LSM3
MSN4	LSM4
MSN4	LSP1
MSN4	LST7
MSN4	LST8
MSN4	LYS2
MSN4	LYS20
MSN4	MAL31
MSN4	MAL32
MSN4	MBA1
MSN4	MBF1
MSN4	MCR1
MSN4	MDH1
MSN4	MDH2
MSN4	MDH3
MSN4	MEI4
MSN4	MKC7
MSN4	MMF1
MSN4	MNN10
MSN4	MOT2
MSN4	MRK1
MSN4	MRPL4
MSN4	MRS4
MSN4	MSC1
MSN4	MSK1
MSN4	MSN4
MSN4	MSR1
MSN4	MSS11
MSN4	MTD1
MSN4	MTF1
MSN4	NAM8
MSN4	NCE102
MSN4	NCE103
MSN4	NEO1
MSN4	NIS1
MSN4	NNF2
MSN4	NOP53
MSN4	NQM1
MSN4	NTH1
MSN4	OCA6
MSN4	OCH1
MSN4	OLA1
MSN4	OLE1
MSN4	OM45
MSN4	OPI10
MSN4	OPI3
MSN4	OSH2
MSN4	OSW1
MSN4	OYE3
MSN4	PAH1
MSN4	PAI3
MSN4	PAU4
MSN4	PBA1
MSN4	PCF11
MSN4	PCL7
MSN4	PDE2
MSN4	PDR15
MSN4	PDX1
MSN4	PEP4
MSN4	PEX32
MSN4	PGA3
MSN4	PGM2
MSN4	PHM7
MSN4	PHM8
MSN4	PIB1
MSN4	PIC2
MSN4	PIH1
MSN4	PIL1
MSN4	PKC1
MSN4	PMC1
MSN4	PNC1
MSN4	POP7
MSN4	POT1
MSN4	PRE2
MSN4	PRE5
MSN4	PRP22
MSN4	PRP46
MSN4	PRX1
MSN4	PST2
MSN4	PTK2
MSN4	PUP2
MSN4	PYC1
MSN4	PYK2
MSN4	RAD53
MSN4	RAD57
MSN4	RDH54
MSN4	RDS3
MSN4	REC104

MSN4	RFS1	MSN4	SSP2	MSN4	WTM2
MSN4	RGD1	MSN4	STB4	MSN4	YAK1
MSN4	RHO4	MSN4	STE5	MSN4	YAP1
MSN4	RIM4	MSN4	STF2	MSN4	YAT1
MSN4	RIO1	MSN4	SUC2	MSN4	YBL029C-A
MSN4	RLP7	MSN4	SUR1	MSN4	YBL059W
MSN4	RNR3	MSN4	SYM1	MSN4	YBL104C
MSN4	RNY1	MSN4	TAF12	MSN4	YBL108W
MSN4	ROD1	MSN4	TAF7	MSN4	YBL109W
MSN4	ROX1	MSN4	TCM62	MSN4	YBR012C
MSN4	RPA135	MSN4	TDH3	MSN4	YBR013C
MSN4	RPF1	MSN4	TES1	MSN4	YBR056W
MSN4	RPL11A	MSN4	TFC8	MSN4	YBR071W
MSN4	RPL2A	MSN4	TFS1	MSN4	YBR074W
MSN4	RPL7B	MSN4	TKL2	MSN4	YBR134W
MSN4	RPN3	MSN4	TOM1	MSN4	YBR241C
MSN4	RPN4	MSN4	TPK2	MSN4	YBR284W
MSN4	RPS8B	MSN4	TPS1	MSN4	YCL042W
MSN4	RTC3	MSN4	TPS2	MSN4	YCL065W
MSN4	RTN2	MSN4	TPS3	MSN4	YCR022C
MSN4	SAP185	MSN4	TRP4	MSN4	YDL124W
MSN4	SBA1	MSN4	TRR1	MSN4	YDL180W
MSN4	SBP1	MSN4	TRS120	MSN4	YDL237W
MSN4	SCC2	MSN4	TRX2	MSN4	YDR010C
MSN4	SEC7	MSN4	TRX3	MSN4	YDR042C
MSN4	SGE1	MSN4	TSA1	MSN4	YDR061W
MSN4	SIP18	MSN4	TSA2	MSN4	YDR089W
MSN4	SIS1	MSN4	TSL1	MSN4	YDR098C-A
MSN4	SLH1	MSN4	TVP23	MSN4	YDR098C-B
MSN4	SML1	MSN4	TYR1	MSN4	YDR102C
MSN4	SNF11	MSN4	UBA1	MSN4	YDR179W-A
MSN4	SNQ2	MSN4	UBI4	MSN4	YDR210C-D
MSN4	SOD2	MSN4	UBP9	MSN4	YDR455C
MSN4	SOL4	MSN4	UGA2	MSN4	YDR544C
MSN4	SPG3	MSN4	UGP1	MSN4	YEL010W
MSN4	SPG4	MSN4	UPF3	MSN4	YEL047C
MSN4	SPI1	MSN4	URC2	MSN4	YEL073C
MSN4	SPO7	MSN4	USA1	MSN4	YEL077C
MSN4	SPS100	MSN4	VAN1	MSN4	YER066C-A
MSN4	SQS1	MSN4	VBA3	MSN4	YER067W
MSN4	SSA1	MSN4	VPS52	MSN4	YER188C-A
MSN4	SSA2	MSN4	VPS62	MSN4	YER189W
MSN4	SSA3	MSN4	VPS73	MSN4	YFR017C
MSN4	SSA4	MSN4	VPS74	MSN4	YGL159W
MSN4	SSE1	MSN4	VTH1	MSN4	YGP1
MSN4	SSE2	MSN4	WTM1	MSN4	YGR031W

MSN4	YGR050C	MSN4	YMR090W	MSN4	ZRT1
MSN4	YGR071C	MSN4	YMR119W-A	MSN4	ZWF1
MSN4	YGR127W	MSN4	YMR172C-A	SKN7	AAC1
MSN4	YGR210C	MSN4	YMR173W-A	SKN7	ABF2
MSN4	YGR250C	MSN4	YMR178W	SKN7	ACO1
MSN4	YGR272C	MSN4	YMR315W	SKN7	AFG2
MSN4	YGR283C	MSN4	YNL011C	SKN7	AFT2
MSN4	YHK8	MSN4	YNL134C	SKN7	AGA2
MSN4	YHR032W	MSN4	YNL144C	SKN7	AHP1
MSN4	YHR033W	MSN4	YNL146W	SKN7	ALD5
MSN4	YHR035W	MSN4	YNL194C	SKN7	ALG7
MSN4	YIL169C	MSN4	YNL195C	SKN7	AMN1
MSN4	YIL174W	MSN4	YNL200C	SKN7	APC1
MSN4	YIL175W	MSN4	YNL337W	SKN7	API2
MSN4	YIL176C	MSN4	YNL338W	SKN7	APQ12
MSN4	YIL177C	MSN4	YNR014W	SKN7	APT2
MSN4	YJL225C	MSN4	YNR034W-A	SKN7	ARF2
MSN4	YJR011C	MSN4	YNR064C	SKN7	ARG1
MSN4	YJR054W	MSN4	YNR073C	SKN7	ASF1
MSN4	YJR096W	MSN4	YOL057W	SKN7	ATP1
MSN4	YKL071W	MSN4	YOL150C	SKN7	ATP14
MSN4	YKL151C	MSN4	YOL157C	SKN7	BAT2
MSN4	YKL223W	MSN4	YOL159C	SKN7	BER1
MSN4	YKL225W	MSN4	YOR021C	SKN7	BIM1
MSN4	YKR005C	MSN4	YOR228C	SKN7	BNA2
MSN4	YLL023C	MSN4	YOR235W	SKN7	CAF20
MSN4	YLL056C	MSN4	YOR262W	SKN7	CBF2
MSN4	YLL067C	MSN4	YOR292C	SKN7	CCP1
MSN4	YLR053C	MSN4	YPL109C	SKN7	CCW12
MSN4	YLR108C	MSN4	YPL150W	SKN7	CDC48
MSN4	YLR126C	MSN4	YPL272C	SKN7	CET1
MSN4	YLR162W	MSN4	YPR013C	SKN7	CHC1
MSN4	YLR164W	MSN4	YPR153W	SKN7	CHS1
MSN4	YLR177W	MSN4	YPR202W	SKN7	CIN1
MSN4	YLR281C	MSN4	YPR203W	SKN7	CIN4
MSN4	YLR294C	MSN4	YPS6	SKN7	CIS3
MSN4	YLR302C	MSN4	YRF1-1	SKN7	CLB1
MSN4	YLR345W	MSN4	YRF1-2	SKN7	CLN1
MSN4	YLR346C	MSN4	YRF1-3	SKN7	CLN2
MSN4	YLR462W	MSN4	YRF1-4	SKN7	COX19
MSN4	YLR463C	MSN4	YRF1-5	SKN7	CPR1
MSN4	YLR464W	MSN4	YRF1-7	SKN7	CRR1
MSN4	YLR465C	MSN4	YRO2	SKN7	CTT1
MSN4	YML131W	MSN4	YSH1	SKN7	CUE4
MSN4	YMR086C-A	MSN4	YSN1	SKN7	CUP9
MSN4	YMR087W	MSN4	YTH1	SKN7	CWP2

SKN7	CYT2
SKN7	DAK1
SKN7	DAK2
SKN7	DAL7
SKN7	DAN2
SKN7	DBP10
SKN7	DBP5
SKN7	DDR2
SKN7	DDR48
SKN7	DED1
SKN7	DIA3
SKN7	DNM1
SKN7	DOT6
SKN7	DPS1
SKN7	EAR1
SKN7	ECM33
SKN7	ERG6
SKN7	ERG9
SKN7	ERP6
SKN7	EXG1
SKN7	FKS3
SKN7	FLO1
SKN7	FLO9
SKN7	FMC1
SKN7	FMP48
SKN7	FRE1
SKN7	FRS2
SKN7	GAS1
SKN7	GAT1
SKN7	GAT2
SKN7	GDH3
SKN7	GGA1
SKN7	GIC2
SKN7	GID8
SKN7	GIP3
SKN7	GIT1
SKN7	GLY1
SKN7	GPH1
SKN7	GPX2
SKN7	GRE2
SKN7	GSC2
SKN7	GSH1
SKN7	GSH2
SKN7	GSY2
SKN7	GTO3
SKN7	GTR1
SKN7	GTT3
SKN7	GUT2
SKN7	GVP36
SKN7	HAA1
SKN7	HAP4
SKN7	HEF3
SKN7	HER1
SKN7	HLR1
SKN7	HMI1
SKN7	HMS1
SKN7	HMS2
SKN7	HMX1
SKN7	HOM2
SKN7	HOR7
SKN7	HOT1
SKN7	HPF1
SKN7	HRT3
SKN7	HSP104
SKN7	HSP12
SKN7	HSP26
SKN7	HSP30
SKN7	HSP42
SKN7	HSP78
SKN7	HSP82
SKN7	HXT11
SKN7	HXT12
SKN7	ICS2
SKN7	ICY1
SKN7	IDH2
SKN7	IES6
SKN7	INO1
SKN7	IRC25
SKN7	ISA1
SKN7	ISU1
SKN7	ISU2
SKN7	ISW2
SKN7	JSN1
SKN7	KNH1
SKN7	KNS1
SKN7	KOG1
SKN7	KRE6
SKN7	KTI12
SKN7	LAT1
SKN7	LEU5
SKN7	LSB3
SKN7	LSM2
SKN7	LSM3
SKN7	LTP1
SKN7	LTV1
SKN7	LYS20
SKN7	MAK32
SKN7	MDG1
SKN7	MDH2
SKN7	MET12
SKN7	MET13
SKN7	MET16
SKN7	MET2
SKN7	MET6
SKN7	MFA1
SKN7	MFA2
SKN7	MGA1
SKN7	MGM101
SKN7	MIR1
SKN7	MMF1
SKN7	MMP1
SKN7	MNN1
SKN7	MOD5
SKN7	MOT3
SKN7	MPR1
SKN7	MRP8
SKN7	MRPL4
SKN7	MRS4
SKN7	MSB2
SKN7	MSN2
SKN7	MSN4
SKN7	MSS11
SKN7	MTC3
SKN7	MUC1
SKN7	NCA3
SKN7	NCE102
SKN7	NDE1
SKN7	NEM1
SKN7	NQM1
SKN7	NRG1
SKN7	NRG2
SKN7	OCH1
SKN7	OLA1
SKN7	OLE1
SKN7	OST5
SKN7	OTU2
SKN7	OXA1
SKN7	OYE3

SKN7	PAC11	SKN7	RPM2	SKN7	TA(AGC)P
SKN7	PAH1	SKN7	RPP2A	SKN7	TAH1
SKN7	PAI3	SKN7	RPS15	SKN7	TAL1
SKN7	PBI2	SKN7	RPS3	SKN7	TCB2
SKN7	PCK1	SKN7	RSC6	SKN7	TFG2
SKN7	PCL7	SKN7	RUP1	SKN7	THI7
SKN7	PDS5	SKN7	SCW10	SKN7	THI72
SKN7	PEA2	SKN7	SCW4	SKN7	TIM23
SKN7	PET122	SKN7	SDC25	SKN7	TIR1
SKN7	PET9	SKN7	SDP1	SKN7	TL(GAG)G
SKN7	PHO91	SKN7	SDS23	SKN7	TOS8
SKN7	PIL1	SKN7	SEC39	SKN7	TPO4
SKN7	PMA1	SKN7	SED1	SKN7	TPS3
SKN7	PMC1	SKN7	SET4	SKN7	TRM13
SKN7	PMP2	SKN7	SFG1	SKN7	TRR1
SKN7	POL32	SKN7	SFL1	SKN7	TRS20
SKN7	POR2	SKN7	SGA1	SKN7	TRX2
SKN7	PRI1	SKN7	SGV1	SKN7	TRX3
SKN7	PRI2	SKN7	SHE10	SKN7	TSA1
SKN7	PRM5	SKN7	SHM2	SKN7	TSA2
SKN7	PRP9	SKN7	SHQ1	SKN7	TSL1
SKN7	PRY1	SKN7	SKN7	SKN7	TVP18
SKN7	PSO2	SKN7	SLM4	SKN7	UBA1
SKN7	PTC1	SKN7	SNA2	SKN7	UBX6
SKN7	PTP2	SKN7	SNZ2	SKN7	UFO1
SKN7	PTR2	SKN7	SNZ3	SKN7	UGP1
SKN7	PUS9	SKN7	SOD1	SKN7	UIP5
SKN7	PUT4	SKN7	SOD2	SKN7	URA3
SKN7	PYC1	SKN7	SOK1	SKN7	USA1
SKN7	QCR10	SKN7	SOK2	SKN7	USV1
SKN7	RAS2	SKN7	SOL4	SKN7	UTH1
SKN7	RAX2	SKN7	SPI1	SKN7	VAM3
SKN7	RBA50	SKN7	SPO1	SKN7	VTC3
SKN7	RDI1	SKN7	SPO20	SKN7	YAP1
SKN7	RET1	SKN7	SRP40	SKN7	YAP5
SKN7	RGS2	SKN7	SRP68	SKN7	YAR047C
SKN7	RHO3	SKN7	SRX1	SKN7	YAR062W
SKN7	RIM20	SKN7	SSA1	SKN7	YBL029W
SKN7	RME1	SKN7	SSA4	SKN7	YBR032W
SKN7	RMI1	SKN7	SSE2	SKN7	YBR051W
SKN7	ROX1	SKN7	SSK22	SKN7	YBR053C
SKN7	RPA14	SKN7	SSY5	SKN7	YBR085C-A
SKN7	RPA34	SKN7	STB4	SKN7	YCK3
SKN7	RPI1	SKN7	SUE1	SKN7	YCR018C-A
SKN7	RPL35B	SKN7	SUR7	SKN7	YCR022C
SKN7	RPL6A	SKN7	SUT2	SKN7	YCR102C

SKN7	YCT1
SKN7	YDL038C
SKN7	YDL129W
SKN7	YDR154C
SKN7	YDR157W
SKN7	YDR357C
SKN7	YDR442W
SKN7	YDR526C
SKN7	YEH2
SKN7	YEL045C
SKN7	YER010C
SKN7	YER078C
SKN7	YER079W
SKN7	YER152C
SKN7	YET2
SKN7	YFL054C
SKN7	YFR017C
SKN7	YGL007W
SKN7	YGL039W
SKN7	YGR015C
SKN7	YGR016W
SKN7	YGR045C
SKN7	YGR050C
SKN7	YGR107W
SKN7	YGR130C
SKN7	YGR210C
SKN7	YHL012W
SKN7	YHR126C
SKN7	YHR138C
SKN7	YHR213W
SKN7	YIL100W
SKN7	YIR020C
SKN7	YJL107C
SKN7	YJL113W
SKN7	YJR079W
SKN7	YJR096W
SKN7	YKL037W
SKN7	YKL044W
SKN7	YKL063C
SKN7	YKL066W
SKN7	YKL097C
SKN7	YKR040C
SKN7	YKR041W
SKN7	YKR043C
SKN7	YKR045C
SKN7	YKR075C
SKN7	YKR096W
SKN7	YLL017W
SKN7	YLL020C
SKN7	YLR042C
SKN7	YLR108C
SKN7	YLR111W
SKN7	YLR112W
SKN7	YLR253W
SKN7	YLR257W
SKN7	YLR294C
SKN7	YLR296W
SKN7	YLR301W
SKN7	YLR414C
SKN7	YML053C
SKN7	YML089C
SKN7	YMR018W
SKN7	YMR086W
SKN7	YMR134W
SKN7	YMR135W-A
SKN7	YMR172C-A
SKN7	YMR173W-A
SKN7	YMR252C
SKN7	YMR304C-A
SKN7	YNL092W
SKN7	YNL143C
SKN7	YNL146W
SKN7	YNL170W
SKN7	YNL234W
SKN7	YNL235C
SKN7	YNR014W
SKN7	YNR018W
SKN7	YOL048C
SKN7	YOL050C
SKN7	YOR029W
SKN7	YOR314W
SKN7	YOR343W-A
SKN7	YOR343W-B
SKN7	YPL025C
SKN7	YPL041C
SKN7	YPR013C
SKN7	YPR063C
SKN7	YPR064W
SKN7	YPR148C
SKN7	YPR158C-C
SKN7	YPR158C-D
SKN7	YPS1
SKN7	YPS3
SKN7	YPT11
SKN7	YRO2
SKN7	ZEO1
SKN7	ZPR1
SKN7	ZPS1
SKN7	ZWF1
YAP1	AAC3
YAP1	AAD10
YAP1	AAD14
YAP1	AAD15
YAP1	AAD16
YAP1	AAD3
YAP1	AAD4
YAP1	AAD6
YAP1	AAH1
YAP1	AAP1
YAP1	ABP1
YAP1	ACA1
YAP1	ACH1
YAP1	ACO1
YAP1	ACS2
YAP1	ACT1
YAP1	ADE1
YAP1	ADE12
YAP1	ADE17
YAP1	ADE2
YAP1	ADE3
YAP1	ADE8
YAP1	ADH1
YAP1	ADH2
YAP1	ADH3
YAP1	ADH5
YAP1	ADH6
YAP1	ADH7
YAP1	ADI1
YAP1	ADO1
YAP1	ADY3
YAP1	AEP1
YAP1	AFG1
YAP1	AFT1
YAP1	AFT2
YAP1	AGA1
YAP1	AGP1
YAP1	AGP3
YAP1	AHA1

YAP1	AHC2	YAP1	ARO9	YAP1	BEM2
YAP1	AHP1	YAP1	ARP3	YAP1	BET1
YAP1	AI1	YAP1	ARP5	YAP1	BET3
YAP1	AI2	YAP1	ARR2	YAP1	BET4
YAP1	AI3	YAP1	ARR3	YAP1	BET5
YAP1	AI4	YAP1	ASC1	YAP1	BFR1
YAP1	AI5_ALPHA	YAP1	ASE1	YAP1	BI3
YAP1	AIF1	YAP1	ASG7	YAP1	BIK1
YAP1	AIM9	YAP1	ASN1	YAP1	BIO2
YAP1	ALD3	YAP1	ASP1	YAP1	BIT61
YAP1	ALD4	YAP1	ASP3-1	YAP1	BLM10
YAP1	ALD5	YAP1	ASP3-3	YAP1	BNA1
YAP1	ALD6	YAP1	ASR1	YAP1	BNA3
YAP1	ALG1	YAP1	AST2	YAP1	BNI1
YAP1	ALG13	YAP1	ATG1	YAP1	BOI1
YAP1	ALG7	YAP1	ATG10	YAP1	BOP2
YAP1	ALG8	YAP1	ATG19	YAP1	BPL1
YAP1	ALO1	YAP1	ATG26	YAP1	BRO1
YAP1	ALR1	YAP1	ATG8	YAP1	BSC5
YAP1	ALR2	YAP1	ATH1	YAP1	BSP1
YAP1	ALT1	YAP1	ATM1	YAP1	BTN2
YAP1	AMD1	YAP1	ATO3	YAP1	BUD20
YAP1	AMN1	YAP1	ATP1	YAP1	BUD28
YAP1	AMS1	YAP1	ATP12	YAP1	BUD7
YAP1	ANB1	YAP1	ATP14	YAP1	CAF130
YAP1	APA1	YAP1	ATP15	YAP1	CAF20
YAP1	APJ1	YAP1	ATP2	YAP1	CAK1
YAP1	APL6	YAP1	ATP3	YAP1	CAN1
YAP1	APP1	YAP1	ATP5	YAP1	CAP1
YAP1	ARA2	YAP1	ATP6	YAP1	CAR1
YAP1	ARC15	YAP1	ATP8	YAP1	CBF2
YAP1	ARC35	YAP1	ATR1	YAP1	CBF5
YAP1	ARD1	YAP1	AVT6	YAP1	CBP2
YAP1	ARE2	YAP1	AVT7	YAP1	CBP3
YAP1	ARF1	YAP1	AYR1	YAP1	CBP4
YAP1	ARG1	YAP1	AYT1	YAP1	CCC1
YAP1	ARG3	YAP1	AZR1	YAP1	CCP1
YAP1	ARG4	YAP1	BAP3	YAP1	CCS1
YAP1	ARG5,6	YAP1	BAT1	YAP1	CCT2
YAP1	ARN1	YAP1	BAT2	YAP1	CCT3
YAP1	ARO1	YAP1	BCH1	YAP1	CCT4
YAP1	ARO10	YAP1	BCH2	YAP1	CCT5
YAP1	ARO3	YAP1	BCP1	YAP1	CCT7
YAP1	ARO4	YAP1	BCY1	YAP1	CCT8
YAP1	ARO8	YAP1	BDH1	YAP1	CCW12
YAP1	ARO80	YAP1	BDS1	YAP1	CDC1

YAP1	CDC19	YAP1	CTA1	YAP1	DIM1
YAP1	CDC33	YAP1	CTF8	YAP1	DIT1
YAP1	CDC34	YAP1	CTM1	YAP1	DJP1
YAP1	CDC43	YAP1	CTR2	YAP1	DLD3
YAP1	CDC48	YAP1	CTS1	YAP1	DOA1
YAP1	CDC60	YAP1	CTT1	YAP1	DOA4
YAP1	CDC7	YAP1	CUP1-1	YAP1	DON1
YAP1	CDH1	YAP1	CUP1-2	YAP1	DPH5
YAP1	CEM1	YAP1	CUR1	YAP1	DPL1
YAP1	CFD1	YAP1	CWC15	YAP1	DPM1
YAP1	CHC1	YAP1	CWC23	YAP1	DPS1
YAP1	CHO2	YAP1	CWC25	YAP1	DRE2
YAP1	CHS6	YAP1	CWP1	YAP1	DSD1
YAP1	CHS7	YAP1	CYC1	YAP1	DSE3
YAP1	CIN5	YAP1	CYM1	YAP1	DSE4
YAP1	CIS3	YAP1	CYS3	YAP1	DSF2
YAP1	CIT2	YAP1	CYS4	YAP1	DSK2
YAP1	CKA2	YAP1	CYT2	YAP1	DSL1
YAP1	CLB1	YAP1	DAD3	YAP1	DST1
YAP1	CLB3	YAP1	DAK1	YAP1	DTR1
YAP1	CNS1	YAP1	DAK2	YAP1	DUG1
YAP1	COA1	YAP1	DAL2	YAP1	DUG2
YAP1	COB	YAP1	DAL3	YAP1	DUG3
YAP1	COF1	YAP1	DAL4	YAP1	DUR1,2
YAP1	COQ1	YAP1	DAL5	YAP1	DYN1
YAP1	COR1	YAP1	DAL7	YAP1	ECM13
YAP1	COS10	YAP1	DAN3	YAP1	ECM16
YAP1	COS12	YAP1	DAN4	YAP1	ECM17
YAP1	COS2	YAP1	DAP1	YAP1	ECM21
YAP1	COS3	YAP1	DBP2	YAP1	ECM23
YAP1	COS7	YAP1	DBP9	YAP1	ECM38
YAP1	COX1	YAP1	DCD1	YAP1	ECM4
YAP1	COX10	YAP1	DCG1	YAP1	ECM40
YAP1	COX15	YAP1	DCP1	YAP1	EDC2
YAP1	COX6	YAP1	DCS1	YAP1	EFB1
YAP1	CPA1	YAP1	DCS2	YAP1	EFT1
YAP1	CPA2	YAP1	DDI1	YAP1	EFT2
YAP1	CPD1	YAP1	DDP1	YAP1	EGD1
YAP1	CPR6	YAP1	DDR48	YAP1	EGT2
YAP1	CPT1	YAP1	DED81	YAP1	EHD3
YAP1	CRD1	YAP1	DEF1	YAP1	EHT1
YAP1	CRG1	YAP1	DEP1	YAP1	ELP6
YAP1	CRH1	YAP1	DFG16	YAP1	EMI2
YAP1	CRR1	YAP1	DFR1	YAP1	EMI5
YAP1	CSE1	YAP1	DIA2	YAP1	EMP70
YAP1	CSI2	YAP1	DIG2	YAP1	ENA1

YAP1	ENA5
YAP1	END3
YAP1	ENO2
YAP1	ENT2
YAP1	ERB1
YAP1	ERG1
YAP1	ERG11
YAP1	ERG13
YAP1	ERG24
YAP1	ERG25
YAP1	ERG26
YAP1	ERG28
YAP1	ERG3
YAP1	ERG5
YAP1	ERG6
YAP1	ERG7
YAP1	ERG9
YAP1	ERO1
YAP1	ERP5
YAP1	ERV14
YAP1	ESC1
YAP1	EUG1
YAP1	EXG2
YAP1	FAA3
YAP1	FAA4
YAP1	FAF1
YAP1	FAR10
YAP1	FAR3
YAP1	FAR8
YAP1	FAS1
YAP1	FBA1
YAP1	FCF2
YAP1	FCY2
YAP1	FEN2
YAP1	FES1
YAP1	FET3
YAP1	FET4
YAP1	FIT1
YAP1	FIT3
YAP1	FKS1
YAP1	FKS3
YAP1	FLO8
YAP1	FLR1
YAP1	FMP12
YAP1	FMP22
YAP1	FMP23
YAP1	FMP25
YAP1	FMP27
YAP1	FMP34
YAP1	FMP41
YAP1	FMP42
YAP1	FMP45
YAP1	FMP46
YAP1	FMP48
YAP1	FMP52
YAP1	FMS1
YAP1	FPR1
YAP1	FPR4
YAP1	FRE1
YAP1	FRE2
YAP1	FRE4
YAP1	FRE6
YAP1	FRE7
YAP1	FRE8
YAP1	FRM2
YAP1	FRQ1
YAP1	FRS2
YAP1	FSP2
YAP1	FTH1
YAP1	FUI1
YAP1	FUM1
YAP1	FUR1
YAP1	FUS1
YAP1	FYV1
YAP1	FZO1
YAP1	GAB1
YAP1	GAD1
YAP1	GAP1
YAP1	GAR1
YAP1	GAT1
YAP1	GCN1
YAP1	GCN3
YAP1	GCN4
YAP1	GCV1
YAP1	GCV2
YAP1	GCY1
YAP1	GDB1
YAP1	GDH1
YAP1	GDH2
YAP1	GDH3
YAP1	GDI1
YAP1	GDT1
YAP1	GET3
YAP1	GIC2
YAP1	GID7
YAP1	GIP1
YAP1	GIS2
YAP1	GIT1
YAP1	GLG1
YAP1	GLK1
YAP1	GLN1
YAP1	GLN4
YAP1	GLO1
YAP1	GLO2
YAP1	GLO4
YAP1	GLR1
YAP1	GLT1
YAP1	GLY1
YAP1	GND1
YAP1	GOR1
YAP1	GPD1
YAP1	GPH1
YAP1	GPI14
YAP1	GPI18
YAP1	GPM2
YAP1	GPT2
YAP1	GPX2
YAP1	GRE1
YAP1	GRE2
YAP1	GRE3
YAP1	GRS1
YAP1	GRX1
YAP1	GRX2
YAP1	GRX3
YAP1	GRX5
YAP1	GSH1
YAP1	GSH2
YAP1	GSM1
YAP1	GSY1
YAP1	GSY2
YAP1	GTO1
YAP1	GTR1
YAP1	GTT1
YAP1	GTT2
YAP1	GUD1
YAP1	GUK1
YAP1	GUP1
YAP1	GUS1

YAP1	GUT2	YAP1	HSP60	YAP1	IRA2
YAP1	GYP8	YAP1	HSP78	YAP1	IRC24
YAP1	GZF3	YAP1	HSP82	YAP1	IRC6
YAP1	HAC1	YAP1	HST2	YAP1	IRC7
YAP1	HAP1	YAP1	HSV2	YAP1	ISA2
YAP1	HAP3	YAP1	HTS1	YAP1	IST1
YAP1	HAP4	YAP1	HTZ1	YAP1	IST2
YAP1	HBN1	YAP1	HUA2	YAP1	ISU2
YAP1	HEF3	YAP1	HUG1	YAP1	ITC1
YAP1	HEK2	YAP1	HUL5	YAP1	ITR1
YAP1	HEM15	YAP1	HXK1	YAP1	IVY1
YAP1	HER1	YAP1	HXT11	YAP1	JID1
YAP1	HFM1	YAP1	HXT12	YAP1	JJJ1
YAP1	HHF1	YAP1	HXT2	YAP1	KAP123
YAP1	HHF2	YAP1	HXT3	YAP1	KAR2
YAP1	HHT1	YAP1	HXT4	YAP1	KEL2
YAP1	HHT2	YAP1	HXT5	YAP1	KEL3
YAP1	HIP1	YAP1	HXT6	YAP1	KES1
YAP1	HIS1	YAP1	HXT9	YAP1	KIN1
YAP1	HIS4	YAP1	HYP2	YAP1	KIN28
YAP1	HIS5	YAP1	HYR1	YAP1	KIP1
YAP1	HMRA1	YAP1	IBA57	YAP1	KNS1
YAP1	HMS2	YAP1	ICE2	YAP1	KOG1
YAP1	HMX1	YAP1	ICY1	YAP1	KRE2
YAP1	HNM1	YAP1	ICY2	YAP1	KRE29
YAP1	HNT1	YAP1	IDH2	YAP1	KRE33
YAP1	HNT3	YAP1	IDP1	YAP1	KRS1
YAP1	HO	YAP1	IES1	YAP1	KSS1
YAP1	HOG1	YAP1	IES4	YAP1	KTI12
YAP1	HOM6	YAP1	IES6	YAP1	KTR2
YAP1	HOR2	YAP1	IFH1	YAP1	KTR4
YAP1	HOR7	YAP1	IKS1	YAP1	LAP3
YAP1	HOT13	YAP1	ILS1	YAP1	LAP4
YAP1	HPF1	YAP1	ILV3	YAP1	LAS1
YAP1	HPT1	YAP1	ILV5	YAP1	LCB3
YAP1	HSC82	YAP1	ILV6	YAP1	LCB5
YAP1	HSH49	YAP1	IMD1	YAP1	LDB18
YAP1	HSL1	YAP1	IMD2	YAP1	LDB19
YAP1	HSP10	YAP1	IMD3	YAP1	LEU1
YAP1	HSP104	YAP1	IMD4	YAP1	LEU4
YAP1	HSP12	YAP1	IME2	YAP1	LEU9
YAP1	HSP150	YAP1	IMP3	YAP1	LHS1
YAP1	HSP26	YAP1	INO1	YAP1	LIA1
YAP1	HSP30	YAP1	IOC2	YAP1	LOH1
YAP1	HSP31	YAP1	IPI3	YAP1	LOS1
YAP1	HSP42	YAP1	IPP1	YAP1	LRS4

YAP1	LSB1
YAP1	LSB6
YAP1	LSM3
YAP1	LST4
YAP1	LYP1
YAP1	LYS1
YAP1	LYS20
YAP1	LYS21
YAP1	MAD2
YAP1	MAE1
YAP1	MAG1
YAP1	MAK10
YAP1	MAK21
YAP1	MAK32
YAP1	MAL32
YAP1	MAS1
YAP1	MBF1
YAP1	MBR1
YAP1	MCH1
YAP1	MCH2
YAP1	MCH4
YAP1	MCH5
YAP1	MCM4
YAP1	MDG1
YAP1	MDH1
YAP1	MDH2
YAP1	MDJ1
YAP1	MDJ2
YAP1	MDM1
YAP1	MDM10
YAP1	MDN1
YAP1	MDV1
YAP1	MEC1
YAP1	MES1
YAP1	MET10
YAP1	MET13
YAP1	MET14
YAP1	MET16
YAP1	MET17
YAP1	MET22
YAP1	MET3
YAP1	MET30
YAP1	MET4
YAP1	MET6
YAP1	MET7
YAP1	MF(ALPHA)1
YAP1	MF(ALPHA)2
YAP1	MGA1
YAP1	MGR3
YAP1	MIH1
YAP1	MKC7
YAP1	MKT1
YAP1	MMP1
YAP1	MMS2
YAP1	MMT1
YAP1	MND2
YAP1	MNN10
YAP1	MNN2
YAP1	MOH1
YAP1	MOT1
YAP1	MOT2
YAP1	MPD1
YAP1	MPE1
YAP1	MPH1
YAP1	MPR1
YAP1	MPT5
YAP1	MRD1
YAP1	MRH1
YAP1	MRI1
YAP1	MRL1
YAP1	MRM2
YAP1	MRP8
YAP1	MRPL11
YAP1	MRPL15
YAP1	MRPL19
YAP1	MRPL24
YAP1	MRPL39
YAP1	MRPL4
YAP1	MRPL40
YAP1	MRPL49
YAP1	MRPL51
YAP1	MRPS35
YAP1	MRS1
YAP1	MRS4
YAP1	MSA2
YAP1	MSC1
YAP1	MSF1
YAP1	MSI1
YAP1	MSN1
YAP1	MSN2
YAP1	MSN4
YAP1	MSN5
YAP1	MSS4
YAP1	MSW1
YAP1	MTC5
YAP1	MUD1
YAP1	MUP1
YAP1	MUP3
YAP1	MXR1
YAP1	MYO1
YAP1	NAB3
YAP1	NAM7
YAP1	NAN1
YAP1	NAR1
YAP1	NAT1
YAP1	NBP1
YAP1	NBP35
YAP1	NCA3
YAP1	NCE101
YAP1	NCE103
YAP1	NDE2
YAP1	NDL1
YAP1	NDT80
YAP1	NET1
YAP1	NEW1
YAP1	NFS1
YAP1	NFU1
YAP1	NGL2
YAP1	NIP1
YAP1	NIP7
YAP1	NIS1
YAP1	NIT1
YAP1	NMA2
YAP1	NNT1
YAP1	NOG1
YAP1	NOG2
YAP1	NOP1
YAP1	NOP16
YAP1	NOP56
YAP1	NOP58
YAP1	NOP6
YAP1	NOP8
YAP1	NPA3
YAP1	NPL4
YAP1	NPT1
YAP1	NRG2
YAP1	NRM1
YAP1	NRP1

YAP1	NSR1	YAP1	PDX3	YAP1	PPT2
YAP1	NST1	YAP1	PEP12	YAP1	PRB1
YAP1	NTC20	YAP1	PEP4	YAP1	PRC1
YAP1	NTG1	YAP1	PEP8	YAP1	PRD1
YAP1	NUG1	YAP1	PER1	YAP1	PRE1
YAP1	NUP100	YAP1	PET127	YAP1	PRE10
YAP1	NUP157	YAP1	PEX2	YAP1	PRE4
YAP1	NUP53	YAP1	PEX6	YAP1	PRE5
YAP1	NUT2	YAP1	PFK1	YAP1	PRE6
YAP1	OAC1	YAP1	PFK2	YAP1	PRE7
YAP1	OCH1	YAP1	PFY1	YAP1	PRE8
YAP1	ODC2	YAP1	PGK1	YAP1	PRE9
YAP1	OLA1	YAP1	PGM2	YAP1	PRM4
YAP1	OLE1	YAP1	PHD1	YAP1	PRM5
YAP1	OM45	YAP1	PHM7	YAP1	PRM7
YAP1	OPI10	YAP1	PHM8	YAP1	PRM8
YAP1	OPT2	YAP1	PHO11	YAP1	PRO2
YAP1	ORC3	YAP1	PHO12	YAP1	PRP21
YAP1	OSH2	YAP1	PHO23	YAP1	PRP3
YAP1	OSM1	YAP1	PHO3	YAP1	PRP8
YAP1	OYE2	YAP1	PHO5	YAP1	PRS1
YAP1	OYE3	YAP1	PHO8	YAP1	PRX1
YAP1	PAM17	YAP1	PHO81	YAP1	PRY2
YAP1	PAN1	YAP1	PHO84	YAP1	PSA1
YAP1	PAN2	YAP1	PHS1	YAP1	PSD1
YAP1	PAN3	YAP1	PIG2	YAP1	PSD2
YAP1	PAP2	YAP1	PIR1	YAP1	PSE1
YAP1	PAT1	YAP1	PIR3	YAP1	PSF1
YAP1	PAU15	YAP1	PKC1	YAP1	PSK1
YAP1	PAU16	YAP1	PKH2	YAP1	PST1
YAP1	PAU21	YAP1	PLB1	YAP1	PST2
YAP1	PAU3	YAP1	PLB2	YAP1	PTC2
YAP1	PAU4	YAP1	PLC1	YAP1	PTC4
YAP1	PAU6	YAP1	PLM2	YAP1	PTM1
YAP1	PBY1	YAP1	PMD1	YAP1	PTR3
YAP1	PCD1	YAP1	PMI40	YAP1	PUB1
YAP1	PCL6	YAP1	PMP1	YAP1	PUF3
YAP1	PDA1	YAP1	PMT6	YAP1	PUF6
YAP1	PDB1	YAP1	PNC1	YAP1	PUP2
YAP1	PDC1	YAP1	PNO1	YAP1	PUT4
YAP1	PDC6	YAP1	POL1	YAP1	PXR1
YAP1	PDE1	YAP1	POL12	YAP1	PYC2
YAP1	PDI1	YAP1	POL3	YAP1	QCR10
YAP1	PDR16	YAP1	POL4	YAP1	QCR2
YAP1	PDR5	YAP1	POP8	YAP1	QCR6
YAP1	PDX1	YAP1	PPG1	YAP1	RAD1

YAP1	RAD16
YAP1	RAD23
YAP1	RAD4
YAP1	RAM2
YAP1	RAP1
YAP1	RBA50
YAP1	RCK1
YAP1	RCL1
YAP1	RCN1
YAP1	RCR2
YAP1	RCY1
YAP1	RDH54
YAP1	RDS1
YAP1	RDS3
YAP1	REB1
YAP1	REC102
YAP1	RED1
YAP1	REG1
YAP1	REH1
YAP1	RER1
YAP1	RET3
YAP1	REX3
YAP1	RFA1
YAP1	RFA2
YAP1	RGD1
YAP1	RGP1
YAP1	RHO3
YAP1	RHO4
YAP1	RHO5
YAP1	RIB1
YAP1	RIB3
YAP1	RIB4
YAP1	RIB5
YAP1	RIF1
YAP1	RIM11
YAP1	RIM15
YAP1	RIM21
YAP1	RIO2
YAP1	RIP1
YAP1	RKI1
YAP1	RKM1
YAP1	RKM2
YAP1	RKM4
YAP1	RKR1
YAP1	RLM1
YAP1	RLP24
YAP1	RMD6
YAP1	RML2
YAP1	RNA1
YAP1	RNH70
YAP1	RNR1
YAP1	RNR2
YAP1	RNR3
YAP1	RNR4
YAP1	RNT1
YAP1	ROD1
YAP1	ROG3
YAP1	ROM1
YAP1	ROM2
YAP1	ROT2
YAP1	ROX1
YAP1	ROX3
YAP1	RPA135
YAP1	RPA14
YAP1	RPA190
YAP1	RPA34
YAP1	RPB5
YAP1	RPB8
YAP1	RPC40
YAP1	RPF2
YAP1	RPI1
YAP1	RPL10
YAP1	RPL11A
YAP1	RPL11B
YAP1	RPL12A
YAP1	RPL12B
YAP1	RPL13A
YAP1	RPL13B
YAP1	RPL14A
YAP1	RPL14B
YAP1	RPL15A
YAP1	RPL15B
YAP1	RPL16B
YAP1	RPL17A
YAP1	RPL17B
YAP1	RPL18A
YAP1	RPL18B
YAP1	RPL19B
YAP1	RPL1A
YAP1	RPL1B
YAP1	RPL20A
YAP1	RPL20B
YAP1	RPL21A
YAP1	RPL21B
YAP1	RPL22A
YAP1	RPL22B
YAP1	RPL23A
YAP1	RPL23B
YAP1	RPL24A
YAP1	RPL24B
YAP1	RPL25
YAP1	RPL26A
YAP1	RPL26B
YAP1	RPL27A
YAP1	RPL27B
YAP1	RPL28
YAP1	RPL2A
YAP1	RPL3
YAP1	RPL30
YAP1	RPL31A
YAP1	RPL31B
YAP1	RPL32
YAP1	RPL33A
YAP1	RPL33B
YAP1	RPL34A
YAP1	RPL35A
YAP1	RPL35B
YAP1	RPL37A
YAP1	RPL37B
YAP1	RPL38
YAP1	RPL39
YAP1	RPL40B
YAP1	RPL41A
YAP1	RPL42A
YAP1	RPL43A
YAP1	RPL4A
YAP1	RPL4B
YAP1	RPL5
YAP1	RPL6A
YAP1	RPL6B
YAP1	RPL7A
YAP1	RPL7B
YAP1	RPL8A
YAP1	RPL8B
YAP1	RPL9A
YAP1	RPL9B
YAP1	RPM2
YAP1	RPN1

YAP1	RPN10
YAP1	RPN11
YAP1	RPN12
YAP1	RPN2
YAP1	RPN3
YAP1	RPN4
YAP1	RPN5
YAP1	RPN6
YAP1	RPN7
YAP1	RPN8
YAP1	RPN9
YAP1	RPP0
YAP1	RPP1A
YAP1	RPP1B
YAP1	RPP2B
YAP1	RPS0A
YAP1	RPS0B
YAP1	RPS10A
YAP1	RPS10B
YAP1	RPS11B
YAP1	RPS12
YAP1	RPS13
YAP1	RPS14A
YAP1	RPS14B
YAP1	RPS15
YAP1	RPS16A
YAP1	RPS16B
YAP1	RPS17A
YAP1	RPS17B
YAP1	RPS18A
YAP1	RPS18B
YAP1	RPS19B
YAP1	RPS1A
YAP1	RPS1B
YAP1	RPS2
YAP1	RPS20
YAP1	RPS21A
YAP1	RPS22A
YAP1	RPS22B
YAP1	RPS23A
YAP1	RPS23B
YAP1	RPS24A
YAP1	RPS24B
YAP1	RPS25A
YAP1	RPS25B
YAP1	RPS26A
YAP1	RPS26B
YAP1	RPS27A
YAP1	RPS27B
YAP1	RPS28A
YAP1	RPS28B
YAP1	RPS29A
YAP1	RPS29B
YAP1	RPS3
YAP1	RPS30B
YAP1	RPS31
YAP1	RPS4A
YAP1	RPS4B
YAP1	RPS5
YAP1	RPS6A
YAP1	RPS7A
YAP1	RPS7B
YAP1	RPS8A
YAP1	RPS8B
YAP1	RPS9A
YAP1	RPT1
YAP1	RPT2
YAP1	RPT3
YAP1	RPT4
YAP1	RPT6
YAP1	RRI2
YAP1	RRP14
YAP1	RRP3
YAP1	RRP9
YAP1	RSC2
YAP1	RSC30
YAP1	RSC58
YAP1	RSF2
YAP1	RSM10
YAP1	RSM22
YAP1	RSN1
YAP1	RSP5
YAP1	RTC3
YAP1	RTG2
YAP1	RTN2
YAP1	RTS3
YAP1	RTT105
YAP1	RTT109
YAP1	RVB2
YAP1	RVS167
YAP1	RXT2
YAP1	SAE2
YAP1	SAH1
YAP1	SAM1
YAP1	SAM2
YAP1	SAM3
YAP1	SAM35
YAP1	SAM4
YAP1	SAP155
YAP1	SAP185
YAP1	SAS4
YAP1	SAT4
YAP1	SBA1
YAP1	SBE22
YAP1	SBP1
YAP1	SCC4
YAP1	SCEI
YAP1	SCP160
YAP1	SCS3
YAP1	SCS7
YAP1	SCT1
YAP1	SCW10
YAP1	SDH2
YAP1	SDL1
YAP1	SDO1
YAP1	SEC18
YAP1	SEC21
YAP1	SEC53
YAP1	SEC62
YAP1	SEC65
YAP1	SEC66
YAP1	SEC9
YAP1	SED1
YAP1	SEM1
YAP1	SEN2
YAP1	SEO1
YAP1	SER1
YAP1	SER2
YAP1	SER3
YAP1	SER33
YAP1	SFA1
YAP1	SFG1
YAP1	SFP1
YAP1	SGT2
YAP1	SHC1
YAP1	SHM1
YAP1	SHM2
YAP1	SHP1

YAP1	SHR5
YAP1	SIM1
YAP1	SIP18
YAP1	SIR4
YAP1	SIS1
YAP1	SIS2
YAP1	SIW14
YAP1	SKG6
YAP1	SKM1
YAP1	SLA1
YAP1	SLG1
YAP1	SLM5
YAP1	SLS1
YAP1	SLU7
YAP1	SMB1
YAP1	SMF1
YAP1	SMY1
YAP1	SNF4
YAP1	SNG1
YAP1	SNO4
YAP1	SNQ2
YAP1	SNR18
YAP1	SNR57
YAP1	SNR6
YAP1	SNR60
YAP1	SNR66
YAP1	SNT309
YAP1	SNU13
YAP1	SNX4
YAP1	SNZ1
YAP1	SNZ2
YAP1	SNZ3
YAP1	SOD1
YAP1	SOD2
YAP1	SOL1
YAP1	SOL2
YAP1	SOL4
YAP1	SOY1
YAP1	SPC110
YAP1	SPC19
YAP1	SPC34
YAP1	SPC42
YAP1	SPC97
YAP1	SPE1
YAP1	SPE3
YAP1	SPG1
YAP1	SPG5
YAP1	SPI1
YAP1	SPL2
YAP1	SPO1
YAP1	SPO77
YAP1	SPR3
YAP1	SPS1
YAP1	SPS2
YAP1	SPS4
YAP1	SPT15
YAP1	SPT20
YAP1	SPT23
YAP1	SPT5
YAP1	SPT7
YAP1	SRB7
YAP1	SRL1
YAP1	SRO9
YAP1	SRP1
YAP1	SRP21
YAP1	SRP40
YAP1	SRV2
YAP1	SRX1
YAP1	SRY1
YAP1	SSA1
YAP1	SSA2
YAP1	SSA3
YAP1	SSA4
YAP1	SSB1
YAP1	SSB2
YAP1	SSC1
YAP1	SSE1
YAP1	SSE2
YAP1	SSF1
YAP1	SSK2
YAP1	SSL1
YAP1	SSL2
YAP1	SSN2
YAP1	SSO2
YAP1	SSP1
YAP1	SSP120
YAP1	SSS1
YAP1	SSU1
YAP1	STB5
YAP1	STD1
YAP1	STE2
YAP1	STE7
YAP1	STF2
YAP1	STI1
YAP1	STM1
YAP1	STP1
YAP1	STP4
YAP1	STR3
YAP1	STU2
YAP1	SUA7
YAP1	SUB2
YAP1	SUC2
YAP1	SUI1
YAP1	SUL1
YAP1	SUL2
YAP1	SUP17
YAP1	SUP35
YAP1	SUR2
YAP1	SUR4
YAP1	SUR7
YAP1	SUT1
YAP1	SVL3
YAP1	SVS1
YAP1	SWH1
YAP1	SWI3
YAP1	SWI5
YAP1	SWP82
YAP1	SWR1
YAP1	SYC1
YAP1	TA(AGC)K2
YAP1	TAF13
YAP1	TAH18
YAP1	TAL1
YAP1	TAT1
YAP1	TBF1
YAP1	TBS1
YAP1	TCB2
YAP1	TD(GUC)K
YAP1	TDH1
YAP1	TDH2
YAP1	TDH3
YAP1	TEF4
YAP1	TEM1
YAP1	TFC7
YAP1	TFP1
YAP1	TFS1
YAP1	TH(GUG)K
YAP1	THI11

YAP1	THI12	YAP1	TSC10	YAP1	UTP22
YAP1	THI13	YAP1	TSL1	YAP1	UTP9
YAP1	THI2	YAP1	TSR1	YAP1	UTR1
YAP1	THI5	YAP1	TUB2	YAP1	UTR2
YAP1	THI74	YAP1	TUL1	YAP1	VAM10
YAP1	THI80	YAP1	TUM1	YAP1	VAN1
YAP1	THR1	YAP1	TUS1	YAP1	VAS1
YAP1	THR4	YAP1	TV(CAC)D	YAP1	VBA4
YAP1	TIF1	YAP1	TVP23	YAP1	VBA5
YAP1	TIF11	YAP1	TW(CCA)G1	YAP1	VEL1
YAP1	TIF3	YAP1	TYE7	YAP1	VHR1
YAP1	TIF4631	YAP1	UBA1	YAP1	VHT1
YAP1	TIF6	YAP1	UBA3	YAP1	VID24
YAP1	TIP1	YAP1	UBA4	YAP1	VID27
YAP1	TIP20	YAP1	UBC12	YAP1	VID30
YAP1	TIR4	YAP1	UBC13	YAP1	VMA13
YAP1	TKL1	YAP1	UBC6	YAP1	VMA2
YAP1	TKL2	YAP1	UBI4	YAP1	VMA22
YAP1	TMA10	YAP1	UBP13	YAP1	VMA5
YAP1	TMA19	YAP1	UBP2	YAP1	VMA7
YAP1	TMA46	YAP1	UBP5	YAP1	VMR1
YAP1	TMT1	YAP1	UBP6	YAP1	VPS15
YAP1	TOF1	YAP1	UBP7	YAP1	VPS29
YAP1	TOM20	YAP1	UBR1	YAP1	VPS45
YAP1	TOM40	YAP1	UBX4	YAP1	VPS51
YAP1	TOP2	YAP1	UBX7	YAP1	VPS55
YAP1	TOS2	YAP1	UFD2	YAP1	VPS61
YAP1	TOS6	YAP1	UFO1	YAP1	VPS71
YAP1	TP(UGG)L	YAP1	UGA1	YAP1	VPS72
YAP1	TP(UGG)O3	YAP1	UGA3	YAP1	VPS8
YAP1	TPO1	YAP1	UGX2	YAP1	VTC1
YAP1	TPO2	YAP1	UMP1	YAP1	VTC2
YAP1	TPO4	YAP1	URA1	YAP1	VTC3
YAP1	TPS1	YAP1	URA10	YAP1	VTC4
YAP1	TPS2	YAP1	URA2	YAP1	WHI2
YAP1	TRM11	YAP1	URA3	YAP1	WSC4
YAP1	TRM3	YAP1	URA4	YAP1	WSP1
YAP1	TRP2	YAP1	URA5	YAP1	WSS1
YAP1	TRP4	YAP1	URA7	YAP1	WTM1
YAP1	TRR1	YAP1	URA8	YAP1	YAF9
YAP1	TRR2	YAP1	URN1	YAP1	YAL037W
YAP1	TRX1	YAP1	USA1	YAP1	YAL044W-A
YAP1	TRX2	YAP1	USO1	YAP1	YAL045C
YAP1	TRX3	YAP1	UTP15	YAP1	YAP1
YAP1	TSA1	YAP1	UTP20	YAP1	YAP1801
YAP1	TSA2	YAP1	UTP21	YAP1	YAP5

YAP1	YAP6	YAP1	YCR024C-B	YAP1	YDR433W
YAP1	YAP7	YAP1	YCR050C	YAP1	YDR474C
YAP1	YAR009C	YAP1	YCR087W	YAP1	YDR514C
YAP1	YAR010C	YAP1	YCR099C	YAP1	YDR524C-B
YAP1	YAR030C	YAP1	YCR100C	YAP1	YDR531W
YAP1	YAR075W	YAP1	YCR102C	YAP1	YDR532C
YAP1	YBL005W-A	YAP1	YCR102W-A	YAP1	YDR544C
YAP1	YBL028C	YAP1	YCT1	YAP1	YEA4
YAP1	YBL044W	YAP1	YDC1	YAP1	YEF3
YAP1	YBL073W	YAP1	YDJ1	YAP1	YEL007W
YAP1	YBL086C	YAP1	YDL025C	YAP1	YEL008W
YAP1	YBL095W	YAP1	YDL026W	YAP1	YEL020C
YAP1	YBL101W-A	YAP1	YDL068W	YAP1	YEL045C
YAP1	YBL101W-B	YAP1	YDL071C	YAP1	YEL047C
YAP1	YBL107C	YAP1	YDL121C	YAP1	YEL073C
YAP1	YBL109W	YAP1	YDL124W	YAP1	YEL074W
YAP1	YBR012W-A	YAP1	YDL144C	YAP1	YER053C-A
YAP1	YBR012W-B	YAP1	YDL159W-A	YAP1	YER067W
YAP1	YBR013C	YAP1	YDL180W	YAP1	YER078C
YAP1	YBR016W	YAP1	YDL183C	YAP1	YER079W
YAP1	YBR053C	YAP1	YDL211C	YAP1	YER130C
YAP1	YBR056W	YAP1	YDL218W	YAP1	YER138C
YAP1	YBR071W	YAP1	YDL233W	YAP1	YER156C
YAP1	YBR074W	YAP1	YDL237W	YAP1	YER160C
YAP1	YBR075W	YAP1	YDL241W	YAP1	YER189W
YAP1	YBR085C-A	YAP1	YDL242W	YAP1	YFH1
YAP1	YBR089W	YAP1	YDR010C	YAP1	YFL034W
YAP1	YBR099C	YAP1	YDR042C	YAP1	YFL042C
YAP1	YBR134W	YAP1	YDR089W	YAP1	YFL052W
YAP1	YBR137W	YAP1	YDR124W	YAP1	YFL063W
YAP1	YBR138C	YAP1	YDR131C	YAP1	YFL065C
YAP1	YBR139W	YAP1	YDR132C	YAP1	YFR017C
YAP1	YBR144C	YAP1	YDR154C	YAP1	YFR018C
YAP1	YBR184W	YAP1	YDR157W	YAP1	YFR039C
YAP1	YBR190W	YAP1	YDR185C	YAP1	YFR057W
YAP1	YBR197C	YAP1	YDR186C	YAP1	YGL036W
YAP1	YBR224W	YAP1	YDR210W	YAP1	YGL052W
YAP1	YBR239C	YAP1	YDR250C	YAP1	YGL101W
YAP1	YBR285W	YAP1	YDR262W	YAP1	YGL114W
YAP1	YCF1	YAP1	YDR266C	YAP1	YGL117W
YAP1	YCL042W	YAP1	YDR278C	YAP1	YGL140C
YAP1	YCL049C	YAP1	YDR307W	YAP1	YGL188C
YAP1	YCL056C	YAP1	YDR355C	YAP1	YGL242C
YAP1	YCP4	YAP1	YDR381C-A	YAP1	YGL260W
YAP1	YCR006C	YAP1	YDR391C	YAP1	YGP1
YAP1	YCR018C-A	YAP1	YDR417C	YAP1	YGR011W

YAP1	YGR038C-A	YAP1	YIL165C	YAP1	YKL107W
YAP1	YGR038C-B	YAP1	YIL168W	YAP1	YKL118W
YAP1	YGR067C	YAP1	YIL169C	YAP1	YKL151C
YAP1	YGR069W	YAP1	YIL171W	YAP1	YKL153W
YAP1	YGR117C	YAP1	YIL172C	YAP1	YKL187C
YAP1	YGR125W	YAP1	YIL174W	YAP1	YKL223W
YAP1	YGR127W	YAP1	YIP1	YAP1	YKR011C
YAP1	YGR130C	YAP1	YIR014W	YAP1	YKR041W
YAP1	YGR146C	YAP1	YIR040C	YAP1	YKR070W
YAP1	YGR161W-A	YAP1	YJL012C-A	YAP1	YKR075C
YAP1	YGR161W-B	YAP1	YJL028W	YAP1	YKR078W
YAP1	YGR210C	YAP1	YJL045W	YAP1	YKR096W
YAP1	YGR266W	YAP1	YJL047C-A	YAP1	YLL030C
YAP1	YGR272C	YAP1	YJL052C-A	YAP1	YLL032C
YAP1	YGR287C	YAP1	YJL064W	YAP1	YLL054C
YAP1	YHB1	YAP1	YJL070C	YAP1	YLL056C
YAP1	YHC1	YAP1	YJL144W	YAP1	YLL058W
YAP1	YHI9	YAP1	YJL160C	YAP1	YLL059C
YAP1	YHK8	YAP1	YJL175W	YAP1	YLL066C
YAP1	YHL044W	YAP1	YJL213W	YAP1	YLL067C
YAP1	YHM2	YAP1	YJL218W	YAP1	YLR041W
YAP1	YHR007C-A	YAP1	YJL220W	YAP1	YLR042C
YAP1	YHR020W	YAP1	YJR011C	YAP1	YLR053C
YAP1	YHR022C	YAP1	YJR023C	YAP1	YLR073C
YAP1	YHR022C-A	YAP1	YJR026W	YAP1	YLR076C
YAP1	YHR033W	YAP1	YJR027W	YAP1	YLR108C
YAP1	YHR054C	YAP1	YJR028W	YAP1	YLR149C
YAP1	YHR095W	YAP1	YJR029W	YAP1	YLR159W
YAP1	YHR097C	YAP1	YJR071W	YAP1	YLR164W
YAP1	YHR138C	YAP1	YJR096W	YAP1	YLR169W
YAP1	YHR145C	YAP1	YJR115W	YAP1	YLR179C
YAP1	YHR180W	YAP1	YJR120W	YAP1	YLR198C
YAP1	YHR202W	YAP1	YJR146W	YAP1	YLR241W
YAP1	YHR214C-B	YAP1	YJR151W-A	YAP1	YLR257W
YAP1	YHR214C-C	YAP1	YJR157W	YAP1	YLR281C
YAP1	YHR214W-A	YAP1	YKL023W	YAP1	YLR297W
YAP1	YIL057C	YAP1	YKL030W	YAP1	YLR301W
YAP1	YIL060W	YAP1	YKL044W	YAP1	YLR302C
YAP1	YIL082W-A	YAP1	YKL066W	YAP1	YLR339C
YAP1	YIL086C	YAP1	YKL069W	YAP1	YLR345W
YAP1	YIL087C	YAP1	YKL070W	YAP1	YLR346C
YAP1	YIL091C	YAP1	YKL071W	YAP1	YLR352W
YAP1	YIL092W	YAP1	YKL075C	YAP1	YLR358C
YAP1	YIL096C	YAP1	YKL077W	YAP1	YLR366W
YAP1	YIL102C	YAP1	YKL083W	YAP1	YLR412C-A
YAP1	YIL108W	YAP1	YKL105C	YAP1	YLR413W

YAP1	YLR414C	YAP1	YNL213C	YAP1	YPL088W
YAP1	YLR426W	YAP1	YNL234W	YAP1	YPL199C
YAP1	YLR428C	YAP1	YNL247W	YAP1	YPL238C
YAP1	YLR458W	YAP1	YNL260C	YAP1	YPL272C
YAP1	YLR460C	YAP1	YNL285W	YAP1	YPL277C
YAP1	YMC1	YAP1	YNL324W	YAP1	YPP1
YAP1	YME1	YAP1	YNR014W	YAP1	YPR002C-A
YAP1	YML007C-A	YAP1	YNR018W	YAP1	YPR013C
YAP1	YML039W	YAP1	YNR034W-A	YAP1	YPR015C
YAP1	YML040W	YAP1	YNR062C	YAP1	YPR036W-A
YAP1	YML045W	YAP1	YNR066C	YAP1	YPR063C
YAP1	YML050W	YAP1	YNR068C	YAP1	YPR064W
YAP1	YML082W	YAP1	YNR070W	YAP1	YPR078C
YAP1	YML083C	YAP1	YNR073C	YAP1	YPR099C
YAP1	YML087C	YAP1	YNR075C-A	YAP1	YPR1
YAP1	YML096W	YAP1	YOL019W	YAP1	YPR114W
YAP1	YML108W	YAP1	YOL048C	YAP1	YPR202W
YAP1	YML131W	YAP1	YOL057W	YAP1	YPT1
YAP1	YMR045C	YAP1	YOL098C	YAP1	YRA1
YAP1	YMR046C	YAP1	YOL114C	YAP1	YRB1
YAP1	YMR051C	YAP1	YOL118C	YAP1	YRB2
YAP1	YMR084W	YAP1	YOL150C	YAP1	YRO2
YAP1	YMR090W	YAP1	YOL157C	YAP1	YRR1
YAP1	YMR1	YAP1	YOL164W-A	YAP1	YSA1
YAP1	YMR144W	YAP1	YOR008C-A	YAP1	YSC84
YAP1	YMR147W	YAP1	YOR052C	YAP1	YSP1
YAP1	YMR172C-A	YAP1	YOR059C	YAP1	YSR3
YAP1	YMR173W-A	YAP1	YOR093C	YAP1	YTA12
YAP1	YMR196W	YAP1	YOR139C	YAP1	YTM1
YAP1	YMR244W	YAP1	YOR152C	YAP1	YTP1
YAP1	YMR258C	YAP1	YOR225W	YAP1	ZEO1
YAP1	YMR295C	YAP1	YOR248W	YAP1	ZIM17
YAP1	YMR315W	YAP1	YOR285W	YAP1	ZPR1
YAP1	YNK1	YAP1	YOR292C	YAP1	ZRG8
YAP1	YNL022C	YAP1	YOR302W	YAP1	ZRT1
YAP1	YNL057W	YAP1	YOR309C	YAP1	ZTA1
YAP1	YNL086W	YAP1	YOR314W	YAP1	ZWF1
YAP1	YNL092W	YAP1	YOR385W	ADR1	ACS1
YAP1	YNL134C	YAP1	YOS1	ARO80	ARO10
YAP1	YNL143C	YAP1	YOS9	ARO80	ARO9
YAP1	YNL155W	YAP1	YOX1	CAT8	ACS1
YAP1	YNL174W	YAP1	YPC1	CAT8	FBP1
YAP1	YNL176C	YAP1	YPI1	CAT8	FBP1
YAP1	YNL179C	YAP1	YPL062W	CAT8	ICL1
YAP1	YNL208W	YAP1	YPL067C	CAT8	IDP2
YAP1	YNL211C	YAP1	YPL080C	CAT8	JEN1

CAT8	JEN1
CAT8	MDH2
CAT8	MDH2
CAT8	MDH2
CAT8	MLS1
CAT8	MLS1
CAT8	PCK1
CAT8	SFC1
CAT8	SFC1
CAT8	SFC1
CAT8	SIP4
CBF1	CENIX
CBF1	CENVI
CBF1	CENXII
DAL80	UGA4
DAL81	UGA1
DAL81	UGA4
DSC1	cdc9
GAL4	GAL2
GIS1	SSA3
LEU3	BAP2
LEU3	ILV2
MAC1	CTR3
MAC1	CTR3
MAC1	FRE7
MAC1	FRE7
MAC1	FRE7
MIG1	ENA1
MIG1	FBP1
MIG1	MEL1
MIG1	PDC1
RAP1	PYK1
RCS1	FET4
RCS1	FET4
REB1	ACS1
ROX1	AAC3
ROX1	ANB1
ROX1	ANB1
ROX1	ANB1
ROX1	ANB1
ROX1	COX5b
ROX1	COX5b
ROX1	CPR1
ROX1	CYC7
ROX1	ERG11
ROX1	FET4

ROX1	FET4
ROX1	FET4
ROX1	FET4
ROX1	FET4
ROX1	FET4
ROX1	HEM13
ROX1	HEM13
ROX1	HEM13
ROX1	HMG1
ROX1	HMG2
ROX1	OLE1
ROX1	OLE1
ROX1	OLE1
ROX1	ROX1
ROX1	ROX1
RPN4	RFT1
RPN4	RPT4
RPN4	RPT6
SIN1	HO
SIP4FBP1	
SIP4MDH2	
SIP4MDH2	
SIP4MDH2	
SIP4SIP4	
SKO1	SUC2
SUT1	DAN1
SUT1	RRP12
SWI4	CLN2
SWI4	CLN2
SWI4	HO
SWI5	CTS1
SWI6	CLN2
SWI6	CLN2
SWI6	HO
TOA1	LEU2
TOA2	LEU2
UGA3	UGA1
UGA3	UGA4
UME6	ACS1
UME6	PHR1
UME6	spo13
UPC2	ERG2
ZAP1	FET4
ZAP1	ZRT1
ZAP1	ZRT2
ZAP1	ZRT3

CUP2	CUP1
GZF3	UGA4
HSF1	SSA4
RAP1	OPI3
SUT1	DAN1
ABF2	ARS1
ABF2	ARS1
ABF2	ARS1
CHA4	CHA1
CHA4	CHA1
CUP2	CUP1
CUP2	SOD1
DAL82	dal7
GAL4	MEL1
GCR1	GLK1
HAP1	CTT1
HAP1	CYB2
HSF1	hsp70
MAL63	MAL61-62
MAL63	MAL61-62
MCM1	CLB2
MCM1	CLB2
MCM1	CLB2
MCM1	MFalpha1
MCM1	MFalpha2
MCM1	SWI5
MET31	MET28
MET32	MET28
MSN2	GLK1
MSN4	GLK1
RAP1	FAS1
RAP1	HMLsilencer
RAP1	HMRsilencer
RAP1	PDC1
RAP1	RNR2
REB1	FAS1
REB1	FAS2
STE12	STE2
STE12	Ty917
MCM1	CDC46
MCM1	CDC47
MCM1	CDC47
MCM1	CDC6
MCM1	CDC6
MCM1	CLN3
MCM1	CLN3

MCM1	STE3
MCM1	SWI4
STE12	MFA1
STE12	MFA2
SWI4	HO
SWI6	HO
ACE2	CTS1
ACE2	SIC1
ADR1	ADH2
AZF1	CLN3
CRZ1	FKS2
CUP2	CUP1
CUP2	CUP1
CUP2	CUP1
DAL80	CAN1
DAL80	CAN1
DAL80	DAL3
DAL80	DAL3
DAL80	GAT1
DAL80	GAT1
DAL82	dal4
GAL4	GAL2
GAL4	GAL7
GAL4	GAL7
GAL4	GAL80
GAL80	GAL7
GCN4	HIS3
GCN4	HIS3
GCN4	HIS3
GCN4	HIS3
GCN4	HIS3
GCN4	HIS3
GCN4	HIS4
GCN4	HIS4
GCN4	HIS4
GCN4	HIS4
GCN4	ILV1
GCN4	ILV1
GCN4	ILV1
GCN4	ILV2
GCN4	TRP4
GCN4	TRP4
GCN4	Ty1
GCR1	Adh1
GCR1	ENO1
GCR1	ENO2
GCR1	PGK
GCR1	PYK1
GCR1	TPI
GCR1	TPI
GCR1	Ty2-917
GCR1	Ty2-917
GCR1	Ty2-917
GLN3	GAT1
GLN3	GAT1
HAC1	EUG1
HAC1	FPR2
HAC1	KAR2
HAC1	LHS1
HAC1	PDI1
HAC1	PDI1
HAP2	CYC1
HAP2	CYC1
HAP3	CYC1
HAP3	CYC1
HSF1	CUP1
HSF1	CUP1
INO2	ACS2
INO2	INO1
INO2	INO1
INO4	ACS2
LEU3	GDH1
LYS14	LYS1
LYS14	LYS9
LYS14	LYS9
MAC1	CTR1
MAC1	CTR1
MAC1	FRE1
MAC1	FRE1
MCM1	MFalpha1
MCM1	STE2
MCM1	STE3
MCM1	STE3
MCM1	STE6
MCM1	Ty1
MCM1	Ty1
MED8	GLK1
MED8	HXK1
MED8	HXT1
MED8	SUC2
MED8	SUC2
MIG1	FPS1
MIG1	GAL1-10
MIG1	GAL3
MIG1	GAL4
MIG1	HAP4
MIG1	MAL2R
MIG1	MAL61-62
MIG1	MAL61-62
MIG1	MAL63
MIG1	SUC2
MIG1	SUC2
MOT3	ANB1
MOT3	CYC1
MSN4	Ty2-917
MSN4	Ty2-917
NDT80	SPS4
NRG1	STA1
NRG1	STA1
PDR1	IPT1
PDR1	PDR10
PDR1	PDR10
PDR1	PDR15
PDR1	PDR15
PDR1	PDR5
PDR1	PDR5
PDR1	PDR5
PDR1	YOR1
PDR3	HXT11
PDR3	IPT1
PDR3	PDR3
PDR3	PDR3
PDR3	PDR5
PDR3	PDR5
PDR3	PDR5
PDR3	SNQ2
PDR3	SNQ2
PDR3	SNQ2
PHO2	HO
PHO2	HO
PHO2	PHO5
PHO4	PHO5
PHO4	PHO5
PHO4	PHO8
PPR1	URA3
RAP1	ENO1
RAP1	ENO2
RAP1	MAT-alpha

RAP1	PYK1
RAP1	rp51A
RAP1	rp51A
RAP1	rp51A
RAP1	TEF1
RAP1	TEF2
RAP1	TPI
RCS1	CCC2
RCS1	FET3
RCS1	FRE1
RCS1	FRE2
RCS1	FTH1
RCS1	FTR1
REB1	35SrRNA
REB1	35SrRNA
RFX1	RFX1
RFX1	RFX1
RFX1	RNR2
RFX1	RNR2
RFX1	RNR3
RFX1	RNR3
RFX1	RNR3
RFX1	RNR4
RFX1	RNR4
RPH1	PHR1
RPN4	RPT2
RPN4	RPT5
RTG1	CIT2
RTG1	CIT2
RTG3	CIT2
RTG3	CIT2
SKO1	ENA1
SKO1	HIS3
STE12	MFA2
SUM1	SMK1
SWI5	HO
SWI5	HO
SWI5	SIC1
TEA1	TY61
YHP1	IME1
ZAP1	ZAP1
ZAP1	ZRT1
ZAP1	ZRT1
ZAP1	ZRT2
ADR1	ADH2
ADR1	CTA1
BAS1	HIS4
BAS1	HIS4
DAL82	dal7
GAL4	GAL1-10
GAL4	GAL1-10
GAL4	GAL1-10
GAL4	GAL1-10
GAL4	GAL1-10
GAL4	GAL1-10
GAL4	GAL1-10
GAL4	GAL1-10
GAL4	GAL1-10
GAL80	GAL1-10
GCN4	ADE4
GCN4	ADE4
GCN4	ADE4
HAP1	CYC1
HAP1	CYC7
HAP1	CYT1
LEU3	LEU2
MCM1	cdc28
MCM1	MFalpha1
MCM1	MFalpha1
MED8	HXK2
MED8	HXK2
PHO2	HIS4
PHO2	HIS4
PHO4	PHO8
RAP1	BCY
RAP1	ENO1
RAP1	LSR1
RAP1	PGK
RAP1	PHO5
RAP1	PYK1
RAP1	rpL16
REB1	ACT1
REB1	cdc9
REB1	GAL1-10
REB1	RAP1
REB1	TRP1
REB1	TRP5
REB1	X40
REB1	Y30
RIM101	PHR1
RIM101	PHR1
RME1	IME1

False-negatives TIs for training of ANFIS (false TF-target)

ABF1	ACS1
ABF1	ADE3
ABF1	ADE5,7
ABF1	ADE8
ABF1	ADH1
ABF1	ADH5
ABF1	ADY2
ABF1	AGA1
ABF1	AHA1
ABF1	AHC2
ABF1	AIP1
ABF1	ALD3
ABF1	ALG1
ABF1	AMS1
ABF1	APS2
ABF1	ARC35
ABF1	ARO10
ABF1	ARO3
ABF1	ARO9
ABF1	ARP5
ABF1	ATO3
ABF1	ATP7
ABF1	AUR1
ABF1	BAP3
ABF1	BCP1
ABF1	BDF1
ABF1	BET3
ABF1	BGL2
ABF1	BNA2
ABF1	BNA4
ABF1	BOP2
ABF1	BTN2
ABF1	BUD31
ABF1	BUD9
ABF1	CAF130
ABF1	CAF40
ABF1	CAP1
ABF1	CAR1
ABF1	CAR2
ABF1	CAT8
ABF1	CCT7
ABF1	CCT8
ABF1	CDA1
ABF1	CDC19
ABF1	CDS1
ABF1	CET1
ABF1	CFD1
ABF1	CHS5
ABF1	CIK1
ABF1	CLN1
ABF1	CMD1
ABF1	CMS1
ABF1	COX6
ABF1	CPR1
ABF1	CTF18
ABF1	CTS1
ABF1	CYB5
ABF1	CYK3
ABF1	DBP2
ABF1	DBP9
ABF1	DCC1
ABF1	DCP2
ABF1	DDR2
ABF1	DED1
ABF1	DFR1
ABF1	DIT1
ABF1	DIT2
ABF1	DPP1
ABF1	DPS1
ABF1	DSD1
ABF1	DSE1
ABF1	DSL1
ABF1	DUG1
ABF1	ECM16
ABF1	ECM17
ABF1	ECM33
ABF1	ECM40
ABF1	EFT2
ABF1	EGD1
ABF1	ELF1
ABF1	END3
ABF1	ERG28
ABF1	ERG3
ABF1	FAS1
ABF1	FBA1
ABF1	FES1
ABF1	FHL1
ABF1	FLC2
ABF1	FMP16
ABF1	FMP34
ABF1	FMP40
ABF1	FOX2
ABF1	FRE7
ABF1	FUI1
ABF1	FUR4
ABF1	FZF1
ABF1	GCN1
ABF1	GIC1
ABF1	GIM3
ABF1	GIS2
ABF1	GLG1
ABF1	GLT1
ABF1	GON7
ABF1	GPG1
ABF1	GPI17
ABF1	GPR1
ABF1	GRS1
ABF1	GRX6
ABF1	GSP1
ABF1	GTT3
ABF1	GUK1
ABF1	GUT2
ABF1	GZF3
ABF1	HCR1
ABF1	HIS4
ABF1	HIS7
ABF1	HNM1
ABF1	HOF1
ABF1	HSC82
ABF1	HSP104
ABF1	HSP12
ABF1	HSP150
ABF1	HSP26
ABF1	HSP30
ABF1	HSP42
ABF1	HSP60
ABF1	HSP78
ABF1	HSP82
ABF1	HUA1
ABF1	IDP2
ABF1	IMD1
ABF1	IMD2
ABF1	IME4
ABF1	INO1
ABF1	IOC2

ABF1	IPP1
ABF1	IQG1
ABF1	IRC19
ABF1	IRC23
ABF1	IRR1
ABF1	JLP1
ABF1	KAP95
ABF1	KAR2
ABF1	LAC1
ABF1	LDB19
ABF1	LOT5
ABF1	LTV1
ABF1	LYP1
ABF1	LYS21
ABF1	MAE1
ABF1	MAL12
ABF1	MAL32
ABF1	MAS1
ABF1	MBF1
ABF1	MCH2
ABF1	MCM16
ABF1	MDV1
ABF1	MET3
ABF1	MFA1
ABF1	MGA1
ABF1	MHR1
ABF1	MIP6
ABF1	MKC7
ABF1	MKT1
ABF1	MLS1
ABF1	MMS1
ABF1	MNN10
ABF1	MOH1
ABF1	MRP20
ABF1	MRP7
ABF1	MRPS9
ABF1	MSC1
ABF1	MSH3
ABF1	MSN1
ABF1	MUP1
ABF1	NAS6
ABF1	NCB2
ABF1	NCE103
ABF1	NDE2
ABF1	NDL1
ABF1	NEO1
ABF1	NFS1
ABF1	NFU1
ABF1	NIP7
ABF1	NOB1
ABF1	NOG2
ABF1	NOP1
ABF1	NOP12
ABF1	NOP58
ABF1	NPC2
ABF1	NPT1
ABF1	NQM1
ABF1	NTH1
ABF1	NUP1
ABF1	OCA5
ABF1	PDC1
ABF1	PEP12
ABF1	PES4
ABF1	PET10
ABF1	PHB2
ABF1	PHO2
ABF1	PHO8
ABF1	PMA2
ABF1	PMP2
ABF1	POP8
ABF1	PRC1
ABF1	PRE6
ABF1	PRM2
ABF1	PRP8
ABF1	PRY1
ABF1	PSK1
ABF1	PUF3
ABF1	PUP1
ABF1	PUP2
ABF1	PUT3
ABF1	PUT4
ABF1	RAD23
ABF1	RAX2
ABF1	REC102
ABF1	RED1
ABF1	REF2
ABF1	RER1
ABF1	RET1
ABF1	RFA2
ABF1	RHO4
ABF1	RIB1
ABF1	RIM101
ABF1	RIX7
ABF1	RKM3
ABF1	RLF2
ABF1	RNA1
ABF1	RNH202
ABF1	RNH70
ABF1	ROT1
ABF1	RPB8
ABF1	RPF2
ABF1	RPL18B
ABF1	RPL2A
ABF1	RPL2B
ABF1	RPL3
ABF1	RPL36B
ABF1	RPL4A
ABF1	RPL5
ABF1	RPL7B
ABF1	RPN10
ABF1	RPN4
ABF1	RPN5
ABF1	RPN8
ABF1	RPO21
ABF1	RPO31
ABF1	RPP1A
ABF1	RPS0A
ABF1	RPS28A
ABF1	RPS28B
ABF1	RPT5
ABF1	RRI2
ABF1	RSC6
ABF1	RSF2
ABF1	RTC3
ABF1	SAM37
ABF1	SAP4
ABF1	SCS22
ABF1	SEC14
ABF1	SEC18
ABF1	SEC21
ABF1	SEC53
ABF1	SET4
ABF1	SGT1
ABF1	SHC1
ABF1	SHE10
ABF1	SLA1
ABF1	SLM4
ABF1	SMF2

ABF1	SMK1
ABF1	SMX2
ABF1	SMY1
ABF1	SNA2
ABF1	SNR57
ABF1	SNX3
ABF1	SOD1
ABF1	SPC42
ABF1	SPO19
ABF1	SPR3
ABF1	SPS1
ABF1	SPT15
ABF1	SPT23
ABF1	SPT8
ABF1	SRP1
ABF1	SSA1
ABF1	SSA4
ABF1	SSC1
ABF1	SSE1
ABF1	SSE2
ABF1	SSF2
ABF1	STE7
ABF1	STI1
ABF1	STP1
ABF1	SUI1
ABF1	SUN4
ABF1	SUP35
ABF1	SWI3
ABF1	SWI4
ABF1	TAO3
ABF1	TCO89
ABF1	TDH3
ABF1	TFA1
ABF1	TFB1
ABF1	TFS1
ABF1	THI12
ABF1	THI13
ABF1	THI5
ABF1	THR4
ABF1	TIF1
ABF1	TIF11
ABF1	TOK1
ABF1	TRM112
ABF1	TRM2
ABF1	TRP3
ABF1	TUB2
ABF1	UBP6
ABF1	UBX5
ABF1	UFD2
ABF1	UGA3
ABF1	UME6
ABF1	URA7
ABF1	UTP15
ABF1	VBA2
ABF1	VHS2
ABF1	VID27
ABF1	VMA6
ABF1	VPS54
ABF1	VTH1
ABF1	VTI1
ABF1	WSC4
ABF1	YAP1801
ABF1	YBR284W
ABF1	YCG1
ABF1	YDJ1
ABF1	YDL012C
ABF1	YDL032W
ABF1	YDL034W
ABF1	YDR029W
ABF1	YDR034W-B
ABF1	YDR266C
ABF1	YDR391C
ABF1	YDR455C
ABF1	YDR476C
ABF1	YER158C
ABF1	YFR018C
ABF1	YGR203W
ABF1	YGR251W
ABF1	YHR078W
ABF1	YIR042C
ABF1	YJL175W
ABF1	YJR096W
ABF1	YJR115W
ABF1	YJR116W
ABF1	YJR128W
ABF1	YKL069W
ABF1	YKR015C
ABF1	YLR041W
ABF1	YLR173W
ABF1	YLR179C
ABF1	YLR400W
ABF1	YMR090W
ABF1	YNG2
ABF1	YNL057W
ABF1	YNL086W
ABF1	YNL115C
ABF1	YNL190W
ABF1	YNL208W
ABF1	YNL213C
ABF1	YNL313C
ABF1	YOL075C
ABF1	YOL157C
ABF1	YOR262W
ABF1	YOR309C
ABF1	YPL229W
ABF1	YPR063C
ABF1	YPR148C
ABF1	YPR153W
ABF1	YPR157W
ABF1	YPT1
ABF1	YPT52
ABF1	YRO2
ABF1	YSR3
ABF1	YTA7
ABF1	ZAP1
ABF1	ZIP1
ACE2	AAD16
ACE2	AAD6
ACE2	AFG2
ACE2	AIM9
ACE2	AMN1
ACE2	API2
ACE2	ASH1
ACE2	ASN2
ACE2	BAT2
ACE2	BEM2
ACE2	BIO2
ACE2	BMH1
ACE2	BUD9
ACE2	CDC6
ACE2	CHS7
ACE2	CPR1
ACE2	CTS1
ACE2	CUE4
ACE2	CUP1-1
ACE2	CUP1-2
ACE2	DSE1
ACE2	DSE2

ACE2	DSE3
ACE2	DSE4
ACE2	ECM32
ACE2	EGT2
ACE2	ERG6
ACE2	FRS2
ACE2	GAT1
ACE2	GDH3
ACE2	GLO1
ACE2	GTR1
ACE2	GUF1
ACE2	HMS2
ACE2	HO
ACE2	HOM2
ACE2	HOT13
ACE2	HSP150
ACE2	ICS2
ACE2	ISR1
ACE2	ITC1
ACE2	KSS1
ACE2	LCP5
ACE2	LSM3
ACE2	MCM2
ACE2	MCR1
ACE2	MDH1
ACE2	MDJ2
ACE2	MGM101
ACE2	MRP8
ACE2	MRPL4
ACE2	MSR1
ACE2	MTC3
ACE2	NIS1
ACE2	NUP100
ACE2	OCA5
ACE2	OST5
ACE2	OTU2
ACE2	OXA1
ACE2	PAH1
ACE2	PCL2
ACE2	PCL9
ACE2	PET122
ACE2	PIR1
ACE2	PIR3
ACE2	PMA1
ACE2	PRY3
ACE2	PSA1
ACE2	PST1
ACE2	RAS2
ACE2	REE1
ACE2	REG2
ACE2	RME1
ACE2	RMI1
ACE2	ROT2
ACE2	RPA14
ACE2	RPA34
ACE2	RPS30A
ACE2	RPS4A
ACE2	RSP5
ACE2	RUP1
ACE2	SAP4
ACE2	SCP160
ACE2	SCW11
ACE2	SER3
ACE2	SFL1
ACE2	SHE10
ACE2	SIC1
ACE2	SNA2
ACE2	SNQ2
ACE2	SNR60
ACE2	TAH11
ACE2	TIM23
ACE2	TPM1
ACE2	TPS3
ACE2	TSL1
ACE2	UBP6
ACE2	UTH1
ACE2	VID30
ACE2	VPS55
ACE2	WSC4
ACE2	WTM1
ACE2	YAP1
ACE2	YCK2
ACE2	YDR010C
ACE2	YDR154C
ACE2	YDR157W
ACE2	YEL007W
ACE2	YEL008W
ACE2	YER078C
ACE2	YER078W-A
ACE2	YER079W
ACE2	YER152C
ACE2	YER189W
ACE2	YFR017C
ACE2	YGL006W-A
ACE2	YGL007C-A
ACE2	YGL007W
ACE2	YGR125W
ACE2	YGR283C
ACE2	YHB1
ACE2	YHL012W
ACE2	YJL160C
ACE2	YJR146W
ACE2	YKL151C
ACE2	YKR040C
ACE2	YKR041W
ACE2	YML100W-A
ACE2	YMR134W
ACE2	YMR135W-A
ACE2	YMR262W
ACE2	YNR018W
ACE2	YOR314W
ACE2	YPL025C
ACE2	YPL158C
ACE2	YRF1-2
ACE2	YRF1-7
ACE2	ZWF1
ADR1	ACS1
ADR1	ADH2
ADR1	CTA1
ARO80	ARO10
ARO80	ARO9
AZF1	CLN3
BAS1	HIS4
CAT8	ACS1
CAT8	FBP1
CAT8	IDP2
CAT8	MDH2
CAT8	MLS1
CAT8	PCK1
CAT8	SFC1
CAT8	SIP4
CHA4	CHA1
CUP2	CUP1
CUP2	SOD1
DAL80	CAN1
DAL80	DAL3
DAL80	GAT1
DAL81	UGA1

DSC1	cdc9	FKH1	DSE1	FKH1	MYO1
FKH1	AAD15	FKH1	DSE2	FKH1	NDE2
FKH1	ACE2	FKH1	DUG1	FKH1	NEW1
FKH1	ADD37	FKH1	DYN1	FKH1	NPP2
FKH1	ADH4	FKH1	EAP1	FKH1	NST1
FKH1	AFR1	FKH1	ECM33	FKH1	OPT2
FKH1	ALG1	FKH1	ERP3	FKH1	OPY2
FKH1	ALG14	FKH1	EXG1	FKH1	PDS1
FKH1	ALG3	FKH1	FAB1	FKH1	PDS5
FKH1	ALG5	FKH1	FHL1	FKH1	PES4
FKH1	ALK1	FKH1	FIR1	FKH1	PHO11
FKH1	AMN1	FKH1	FKH2	FKH1	PPN1
FKH1	APE2	FKH1	FMP34	FKH1	PRP31
FKH1	APM3	FKH1	FMP48	FKH1	PRY3
FKH1	ARP7	FKH1	GAS3	FKH1	RCR2
FKH1	AVT4	FKH1	GAS5	FKH1	REG2
FKH1	BAR1	FKH1	GLG1	FKH1	RHO4
FKH1	BDF1	FKH1	GLN1	FKH1	RKM3
FKH1	BNR1	FKH1	GND1	FKH1	RNR1
FKH1	BRN1	FKH1	GSH1	FKH1	RPL20B
FKH1	BUB1	FKH1	GSM1	FKH1	RPL31B
FKH1	BUD3	FKH1	HAP5	FKH1	RPL37B
FKH1	BUD4	FKH1	HOF1	FKH1	RPL39
FKH1	BUD9	FKH1	HOG1	FKH1	RPN10
FKH1	CDC20	FKH1	HOS3	FKH1	RPN11
FKH1	CDC39	FKH1	HSP104	FKH1	RPN12
FKH1	CDC5	FKH1	HST3	FKH1	RPS22A
FKH1	CDC7	FKH1	HUA1	FKH1	RSP5
FKH1	CDS1	FKH1	HXT1	FKH1	RTC3
FKH1	CHS2	FKH1	ICS2	FKH1	SAM35
FKH1	CHS7	FKH1	IDI1	FKH1	SCJ1
FKH1	CIK1	FKH1	IFM1	FKH1	SCW11
FKH1	CLB1	FKH1	IME1	FKH1	SEC14
FKH1	CLB2	FKH1	IQG1	FKH1	SFG1
FKH1	CLB4	FKH1	IRC8	FKH1	SIF2
FKH1	CLN1	FKH1	JSN1	FKH1	SIL1
FKH1	CMD1	FKH1	KAR4	FKH1	SKM1
FKH1	CSN9	FKH1	KIP2	FKH1	SLM4
FKH1	CTF18	FKH1	KSS1	FKH1	SNF5
FKH1	CTS1	FKH1	LEU4	FKH1	SNX3
FKH1	CUE4	FKH1	MDM1	FKH1	SPC24
FKH1	CWP2	FKH1	MFA2	FKH1	SPS4
FKH1	DBF2	FKH1	MMR1	FKH1	SRC1
FKH1	DEM1	FKH1	MNT2	FKH1	SSO2
FKH1	DIN7	FKH1	MOB1	FKH1	SUB2
FKH1	DPP1	FKH1	MSH3	FKH1	SUR7

FKH1	SVL3
FKH1	SWI5
FKH1	TEM1
FKH1	THO1
FKH1	TIF1
FKH1	TPO3
FKH1	TRM112
FKH1	TSL1
FKH1	TUB2
FKH1	TVP23
FKH1	UME1
FKH1	UTP4
FKH1	UTR2
FKH1	VAC17
FKH1	VBA3
FKH1	VPS54
FKH1	VTI1
FKH1	YAR070C
FKH1	YBL081W
FKH1	YBR071W
FKH1	YBR138C
FKH1	YBR139W
FKH1	YCG1
FKH1	YCK3
FKH1	YDR115W
FKH1	YFR018C
FKH1	YGL006W-A
FKH1	YGL007C-A
FKH1	YGL007W
FKH1	YGR050C
FKH1	YHK8
FKH1	YHP1
FKH1	YHR095W
FKH1	YIL158W
FKH1	YJR111C
FKH1	YKL069W
FKH1	YKL097C
FKH1	YLR352W
FKH1	YLR400W
FKH1	YLR407W
FKH1	YMC2
FKH1	YML119W
FKH1	YMR085W
FKH1	YMR086W
FKH1	YMR144W
FKH1	YNG2
FKH1	YNL058C
FKH1	YNL092W
FKH1	YNL174W
FKH1	YNL176C
FKH1	YNL213C
FKH1	YOL024W
FKH1	YOL114C
FKH1	YPI1
FKH1	YPL141C
FKH1	YPR013C
FKH1	YPR195C
FKH1	YPR196W
FKH1	YPT1
FKH1	YPT31
FKH1	YRB1
FKH1	YSY6
FKH1	YTA7
FKH1	ZRT1
FKH2	ACE2
FKH2	ACS1
FKH2	ADD37
FKH2	ADH4
FKH2	AHA1
FKH2	ALK1
FKH2	AMD1
FKH2	AMN1
FKH2	APC1
FKH2	APE2
FKH2	ARG5,6
FKH2	ARP7
FKH2	ASE1
FKH2	ATG26
FKH2	ATG8
FKH2	ATR1
FKH2	AYR1
FKH2	BDF1
FKH2	BNA4
FKH2	BNR1
FKH2	BRN1
FKH2	BUD3
FKH2	BUD4
FKH2	BUD7
FKH2	BUD9
FKH2	CDC20
FKH2	CDC5
FKH2	CDC6
FKH2	CDC7
FKH2	CHS2
FKH2	CHS7
FKH2	CIK1
FKH2	CIS3
FKH2	CLB1
FKH2	CLB2
FKH2	CLB6
FKH2	CLN1
FKH2	COT1
FKH2	CPA2
FKH2	CPR6
FKH2	CTS1
FKH2	CUP9
FKH2	CUR1
FKH2	CWP1
FKH2	CWP2
FKH2	CYC7
FKH2	DBF2
FKH2	DCS2
FKH2	DIF1
FKH2	DIN7
FKH2	DIT2
FKH2	DSE1
FKH2	DSE2
FKH2	DUN1
FKH2	DUS3
FKH2	ECM19
FKH2	ECM33
FKH2	ECM38
FKH2	EFB1
FKH2	EKI1
FKH2	ELO1
FKH2	ERG26
FKH2	ERP3
FKH2	EXG1
FKH2	EXG2
FKH2	FIR1
FKH2	FKH2
FKH2	FLC2
FKH2	FRS2
FKH2	FRT2
FKH2	GAS3
FKH2	GAT1
FKH2	GDH2
FKH2	GIC1

FKH2	GIC2	FKH2	OSH7	FKH2	SGO1
FKH2	GLG1	FKH2	PAI3	FKH2	SIF2
FKH2	GLN1	FKH2	PAU17	FKH2	SIM1
FKH2	HEK2	FKH2	PBY1	FKH2	SIS1
FKH2	HHF1	FKH2	PCL1	FKH2	SKM1
FKH2	HHT1	FKH2	PDS1	FKH2	SLM4
FKH2	HOF1	FKH2	PDS5	FKH2	SNF4
FKH2	HOS3	FKH2	PES4	FKH2	SNR66
FKH2	HPF1	FKH2	PGM2	FKH2	SPB1
FKH2	HSP10	FKH2	PHO11	FKH2	SPC24
FKH2	HSP30	FKH2	PHO3	FKH2	SPO12
FKH2	HSP60	FKH2	PHO5	FKH2	SPS4
FKH2	HST3	FKH2	PLM2	FKH2	SRC1
FKH2	HXT1	FKH2	PMA1	FKH2	SRL1
FKH2	HXT12	FKH2	PMP1	FKH2	SSA1
FKH2	ICS2	FKH2	PMR1	FKH2	SSF2
FKH2	IDI1	FKH2	PPN1	FKH2	SSO2
FKH2	IFM1	FKH2	PRP31	FKH2	SUR7
FKH2	IQG1	FKH2	PRY1	FKH2	SUT1
FKH2	IRC8	FKH2	PRY3	FKH2	SVL3
FKH2	JJJ3	FKH2	PST2	FKH2	SWI5
FKH2	JSN1	FKH2	PTR2	FKH2	TAH11
FKH2	KAR4	FKH2	PUP3	FKH2	TDH1
FKH2	KIN4	FKH2	RAX2	FKH2	TEM1
FKH2	KIP2	FKH2	RIB1	FKH2	TFB1
FKH2	KRR1	FKH2	RIM4	FKH2	TIF1
FKH2	LCB5	FKH2	RKI1	FKH2	TIM9
FKH2	LSM3	FKH2	RMD11	FKH2	TPO3
FKH2	LST8	FKH2	RMI1	FKH2	TSA1
FKH2	LTE1	FKH2	RMT2	FKH2	TW(CCA)G1
FKH2	MDG1	FKH2	RNR1	FKH2	UBI4
FKH2	MMR1	FKH2	ROG3	FKH2	UBR1
FKH2	MND1	FKH2	RPL20B	FKH2	UME1
FKH2	MNT2	FKH2	RPL37B	FKH2	UTH1
FKH2	MOB1	FKH2	RPS16A	FKH2	UTP4
FKH2	MRH1	FKH2	RPS1B	FKH2	UTR2
FKH2	MRPL4	FKH2	RPS30A	FKH2	VPS73
FKH2	MTC6	FKH2	RRM3	FKH2	VTI1
FKH2	MTR4	FKH2	RSF2	FKH2	WHI4
FKH2	MYO1	FKH2	RSP5	FKH2	WSC4
FKH2	NCE102	FKH2	SCJ1	FKH2	WTM1
FKH2	NDD1	FKH2	SCW11	FKH2	YAP6
FKH2	NSR1	FKH2	SDC25	FKH2	YAR070C
FKH2	OCA5	FKH2	SED1	FKH2	YBL029C-A
FKH2	OLE1	FKH2	SFG1	FKH2	YBL029W
FKH2	OPT2	FKH2	SFP1	FKH2	YBR134W

FKH2	YBR138C	FKH2	YNR014W	HSF1	AAC3
FKH2	YBR139W	FKH2	YOL024W	HSF1	AAD6
FKH2	YBR241C	FKH2	YOL114C	HSF1	ACA1
FKH2	YCG1	FKH2	YOR020W-A	HSF1	ACC1
FKH2	YCR025C	FKH2	YOR246C	HSF1	ACT1
FKH2	YDR115W	FKH2	YOR248W	HSF1	AEP3
FKH2	YDR222W	FKH2	YOR314W	HSF1	AFG3
FKH2	YER067W	FKH2	YPL025C	HSF1	AHA1
FKH2	YER078C	FKH2	YPL141C	HSF1	AHP1
FKH2	YER079W	FKH2	YPR148C	HSF1	ALD3
FKH2	YER189W	FKH2	YPR195C	HSF1	ALD4
FKH2	YFR017C	FKH2	YPR196W	HSF1	ALD5
FKH2	YGL006W-A	FKH2	YPT31	HSF1	ALG13
FKH2	YGL007C-A	FKH2	YRF1-2	HSF1	APA1
FKH2	YGL007W	FKH2	YRF1-3	HSF1	APJ1
FKH2	YGL114W	FKH2	YRO2	HSF1	APP1
FKH2	YGL117W	FKH2	ZWF1	HSF1	ARO1
FKH2	YGR050C	GAL4	GAL1-10	HSF1	ARP7
FKH2	YHP1	GAL4	GAL80	HSF1	ASK1
FKH2	YHR032W	GAL80	GAL1-10	HSF1	ATG1
FKH2	YIL158W	GCN4	HIS3	HSF1	ATG19
FKH2	YIL169C	GCN4	HIS4	HSF1	ATG8
FKH2	YJR111C	GCN4	ILV1	HSF1	ATH1
FKH2	YJR128W	GCN4	ILV2	HSF1	ATO2
FKH2	YKL066W	GCN4	TRP4	HSF1	AVT6
FKH2	YKL096C-B	GCN4	Ty1	HSF1	BBC1
FKH2	YKL097C	GCR1	ENO1	HSF1	BDS1
FKH2	YKR040C	GCR1	ENO2	HSF1	BET4
FKH2	YKR041W	GCR1	GLK1	HSF1	BOP2
FKH2	YLR042C	GCR1	PGK	HSF1	BSC2
FKH2	YLR301W	GCR1	PYK1	HSF1	BSC5
FKH2	YLR400W	GCR1	TPI	HSF1	BTN2
FKH2	YMC2	GCR1	Ty2-917	HSF1	BUB3
FKH2	YML050W	GIS1	SSA3	HSF1	BUD7
FKH2	YML053C	GLN3	GAT1	HSF1	CAK1
FKH2	YML119W	HAC1	EUG1	HSF1	CEM1
FKH2	YMR1	HAC1	KAR2	HSF1	CHO2
FKH2	YMR144W	HAC1	LHS1	HSF1	CIN1
FKH2	YMR258C	HAC1	PDI1	HSF1	CIT3
FKH2	YNL040W	HAP1	CTT1	HSF1	CNS1
FKH2	YNL046W	HAP1	CYB2	HSF1	CPR1
FKH2	YNL057W	HAP1	CYC1	HSF1	CPR6
FKH2	YNL058C	HAP1	CYC7	HSF1	CRC1
FKH2	YNL170W	HAP1	CYT1	HSF1	CRG1
FKH2	YNL174W	HAP2	CYC1	HSF1	CSR2
FKH2	YNL176C	HAP3	CYC1	HSF1	CTT1

HSF1	CUE4	HSF1	FOX2	HSF1	ICY2
HSF1	CUP1-1	HSF1	FSP2	HSF1	ILS1
HSF1	CUP1-2	HSF1	FTH1	HSF1	ILV2
HSF1	CUR1	HSF1	GCY1	HSF1	INO2
HSF1	CWC23	HSF1	GGA1	HSF1	IRA2
HSF1	CWP1	HSF1	GIC2	HSF1	ISA1
HSF1	CYC7	HSF1	GLC3	HSF1	JLP1
HSF1	DAK2	HSF1	GLO4	HSF1	JSN1
HSF1	DAL4	HSF1	GOR1	HSF1	KAP95
HSF1	DAL7	HSF1	GPH1	HSF1	KAR2
HSF1	DAP1	HSF1	GPM1	HSF1	KNS1
HSF1	DDI1	HSF1	GRE1	HSF1	KRR1
HSF1	DDI3	HSF1	GRE2	HSF1	KTI12
HSF1	DDR2	HSF1	GRE3	HSF1	LCB5
HSF1	DEP1	HSF1	GSP1	HSF1	LEE1
HSF1	DIT1	HSF1	GTO1	HSF1	LIN1
HSF1	DNM1	HSF1	GTO3	HSF1	LST8
HSF1	DOC1	HSF1	HAC1	HSF1	MAG1
HSF1	DRE2	HSF1	HBT1	HSF1	MAK21
HSF1	ECM21	HSF1	HCH1	HSF1	MAM3
HSF1	ECM23	HSF1	HEK2	HSF1	MBF1
HSF1	ECM4	HSF1	HHF1	HSF1	MCH2
HSF1	ECM8	HSF1	HLR1	HSF1	MDJ1
HSF1	EDC2	HSF1	HMS1	HSF1	MET2
HSF1	EFB1	HSF1	HMX1	HSF1	MET8
HSF1	EMI2	HSF1	HOM2	HSF1	MGA1
HSF1	EMI5	HSF1	HOM3	HSF1	MHP1
HSF1	ENO2	HSF1	HOR7	HSF1	MLS1
HSF1	ENP1	HSF1	HOS4	HSF1	MMT1
HSF1	ENT2	HSF1	HRD3	HSF1	MOH1
HSF1	ERO1	HSF1	HRK1	HSF1	MRD1
HSF1	ERV29	HSF1	HSC82	HSF1	MRT4
HSF1	EST1	HSF1	HSP10	HSF1	MTQ1
HSF1	EXG2	HSF1	HSP104	HSF1	MUP3
HSF1	FAA1	HSF1	HSP12	HSF1	NAT4
HSF1	FAR3	HSF1	HSP26	HSF1	NBP1
HSF1	FES1	HSF1	HSP30	HSF1	NBP35
HSF1	FIT2	HSF1	HSP42	HSF1	NCA3
HSF1	FMP12	HSF1	HSP60	HSF1	NIS1
HSF1	FMP16	HSF1	HSP78	HSF1	NPL4
HSF1	FMP23	HSF1	HSP82	HSF1	NQM1
HSF1	FMP40	HSF1	HUA1	HSF1	NUP1
HSF1	FMP45	HSF1	HXK1	HSF1	OLA1
HSF1	FMP48	HSF1	HXT3	HSF1	OPI10
HSF1	FMP52	HSF1	HXT5	HSF1	ORC2
HSF1	FMS1	HSF1	HXT9	HSF1	OYE2

HSF1	PAI3
HSF1	PAU17
HSF1	PCK1
HSF1	PDC6
HSF1	PDE1
HSF1	PDH1
HSF1	PDR11
HSF1	PDR12
HSF1	PDR15
HSF1	PEP8
HSF1	PES4
HSF1	PET10
HSF1	PGK1
HSF1	PGM2
HSF1	PHM8
HSF1	PHO2
HSF1	PIB1
HSF1	PIR3
HSF1	PMA1
HSF1	PMC1
HSF1	PMR1
HSF1	PNC1
HSF1	PNS1
HSF1	POP3
HSF1	PRD1
HSF1	PRE6
HSF1	PRM5
HSF1	PRM8
HSF1	PRR2
HSF1	PRX1
HSF1	PSF1
HSF1	PUF3
HSF1	PUT3
HSF1	PYC1
HSF1	RAD28
HSF1	RAX1
HSF1	REB1
HSF1	REG2
HSF1	REH1
HSF1	RIB1
HSF1	RIF1
HSF1	RIM11
HSF1	RIM4
HSF1	RLF2
HSF1	RNH202
HSF1	RNH203
HSF1	RNR3
HSF1	ROD1
HSF1	ROT1
HSF1	RPA14
HSF1	RPA34
HSF1	RPN12
HSF1	RPN4
HSF1	RPS8B
HSF1	RPT2
HSF1	RPT3
HSF1	RSC58
HSF1	RTC3
HSF1	RTS3
HSF1	SAM35
HSF1	SBP1
HSF1	SCS22
HSF1	SCT1
HSF1	SDP1
HSF1	SDS23
HSF1	SEM1
HSF1	SEO1
HSF1	SGT2
HSF1	SIL1
HSF1	SIP4
HSF1	SIS1
HSF1	SKS1
HSF1	SMC3
HSF1	SNG1
HSF1	SNQ2
HSF1	SNZ2
HSF1	SOD2
HSF1	SOL1
HSF1	SOL4
HSF1	SOR1
HSF1	SPI1
HSF1	SPO75
HSF1	SPT7
HSF1	SSA1
HSF1	SSA2
HSF1	SSA3
HSF1	SSA4
HSF1	SSC1
HSF1	SSE1
HSF1	SSE2
HSF1	STB5
HSF1	STI1
HSF1	SUL1
HSF1	SVL3
HSF1	SWM1
HSF1	TA(AGC)G
HSF1	TAH11
HSF1	TDH3
HSF1	TEC1
HSF1	TEF2
HSF1	TGL2
HSF1	THI2
HSF1	THI72
HSF1	TIP1
HSF1	TIP41
HSF1	TKL2
HSF1	TMA10
HSF1	TOP3
HSF1	TPK1
HSF1	TPO1
HSF1	TSL1
HSF1	TYE7
HSF1	UBC13
HSF1	UBC4
HSF1	UBC8
HSF1	UBI4
HSF1	UBX4
HSF1	UBX5
HSF1	UFD4
HSF1	UGA1
HSF1	UGP1
HSF1	UGX2
HSF1	UMP1
HSF1	URA10
HSF1	USV1
HSF1	UTR5
HSF1	VEL1
HSF1	VPS29
HSF1	VPS61
HSF1	VPS73
HSF1	WSC4
HSF1	YAP1801
HSF1	YAP6
HSF1	YBL073W
HSF1	YBR051W
HSF1	YBR053C
HSF1	YBR071W
HSF1	YBR085C-A

HSF1	YBR139W	HSF1	YLR108C	LEU3	ADE8
HSF1	YBR219C	HSF1	YLR125W	LEU3	ADH1
HSF1	YBR241C	HSF1	YLR164W	LEU3	ADH2
HSF1	YBR284W	HSF1	YLR345W	LEU3	ADH6
HSF1	YBR285W	HSF1	YLR356W	LEU3	ADR1
HSF1	YCR099C	HSF1	YML053C	LEU3	AFG3
HSF1	YCR102C	HSF1	YMR085W	LEU3	AFR1
HSF1	YDJ1	HSF1	YMR185W	LEU3	AFT1
HSF1	YDL119C	HSF1	YMR196W	LEU3	AGA1
HSF1	YDL180W	HSF1	YMR244W	LEU3	AGP1
HSF1	YDR010C	HSF1	YMR252C	LEU3	AHA1
HSF1	YDR034W-B	HSF1	YMR258C	LEU3	AHP1
HSF1	YDR065W	HSF1	YMR262W	LEU3	ALD6
HSF1	YDR124W	HSF1	YNL134C	LEU3	AMN1
HSF1	YDR154C	HSF1	YNL146W	LEU3	ANB1
HSF1	YDR157W	HSF1	YNL155W	LEU3	APC1
HSF1	YDR222W	HSF1	YNL234W	LEU3	APE2
HSF1	YDR476C	HSF1	YNR068C	LEU3	ARG4
HSF1	YEL045C	HSF1	YOL048C	LEU3	ARG5,6
HSF1	YER067W	HSF1	YOR008C-A	LEU3	ARO1
HSF1	YER079W	HSF1	YOR020W-A	LEU3	ARO10
HSF1	YFL042C	HSF1	YOR052C	LEU3	ARO3
HSF1	YFL067W	HSF1	YOR059C	LEU3	ARO4
HSF1	YGL006W-A	HSF1	YOR387C	LEU3	ARO8
HSF1	YGL007C-A	HSF1	YPI1	LEU3	ARO9
HSF1	YGL007W	HSF1	YPL113C	LEU3	ARR2
HSF1	YGL036W	HSF1	YPP1	LEU3	ARR3
HSF1	YGP1	HSF1	YPR015C	LEU3	ASN1
HSF1	YGR050C	HSF1	YPR036W-A	LEU3	ASN2
HSF1	YGR146C	HSF1	YPR153W	LEU3	ATH1
HSF1	YGR210C	HSF1	YPR157W	LEU3	ATO2
HSF1	YGR250C	HSF1	YPR196W	LEU3	ATP1
HSF1	YGR251W	HSF1	YRO2	LEU3	BAT2
HSF1	YHR138C	HSF1	YSC84	LEU3	BCK1
HSF1	YJL045W	HSF1	YTP1	LEU3	BEM2
HSF1	YJL144W	HSF1	ZEO1	LEU3	BGL2
HSF1	YKE4	HSF1	ZIP1	LEU3	BIO2
HSF1	YKL037W	HSF1	ZPR1	LEU3	BIO3
HSF1	YKL070W	HSF1	ZRG8	LEU3	BNA1
HSF1	YKR041W	INO2	ACS2	LEU3	BNA4
HSF1	YKR075C	INO2	INO1	LEU3	BNA5
HSF1	YLL032C	INO4	ACS2	LEU3	BNI1
HSF1	YLL037W	LEU3	ACO1	LEU3	BUD22
HSF1	YLL056C	LEU3	ACS2	LEU3	BUD31
HSF1	YLL058W	LEU3	ADD37	LEU3	CAR1
HSF1	YLR042C	LEU3	ADE3	LEU3	CAR2

LEU3	CCS1	LEU3	GCV2	LEU3	LYS21
LEU3	CDA1	LEU3	GDB1	LEU3	MAK32
LEU3	CDC19	LEU3	GDH1	LEU3	MDG1
LEU3	CIT3	LEU3	GDH3	LEU3	MDJ1
LEU3	COX19	LEU3	GDS1	LEU3	MEP2
LEU3	CPA2	LEU3	GID8	LEU3	MET1
LEU3	CPR1	LEU3	GLC3	LEU3	MET10
LEU3	CRG1	LEU3	GLG1	LEU3	MET13
LEU3	CTR2	LEU3	GLY1	LEU3	MET14
LEU3	CWP1	LEU3	GPG1	LEU3	MET16
LEU3	DAL1	LEU3	GPH1	LEU3	MET17
LEU3	DAL2	LEU3	GPR1	LEU3	MET22
LEU3	DAL3	LEU3	GRX6	LEU3	MET3
LEU3	DAL7	LEU3	GSY1	LEU3	MET8
LEU3	DCC1	LEU3	GSY2	LEU3	MGA1
LEU3	DEM1	LEU3	HAP1	LEU3	MGR2
LEU3	DFR1	LEU3	HAP4	LEU3	MHP1
LEU3	DIF1	LEU3	HER1	LEU3	MLH2
LEU3	DIN7	LEU3	HIS1	LEU3	MMF1
LEU3	DOM34	LEU3	HIS3	LEU3	MNN1
LEU3	DON1	LEU3	HIS5	LEU3	MNN4
LEU3	DOT6	LEU3	HIS7	LEU3	MOT3
LEU3	DRE2	LEU3	HMS2	LEU3	MPS2
LEU3	DST1	LEU3	HMX1	LEU3	MRPL11
LEU3	DUR3	LEU3	HOG1	LEU3	MRPL15
LEU3	ECM17	LEU3	HOM3	LEU3	MRPL24
LEU3	ECM32	LEU3	HOR7	LEU3	MSN4
LEU3	ECM33	LEU3	HOT1	LEU3	MTH1
LEU3	ECM40	LEU3	HTZ1	LEU3	MTQ1
LEU3	EDS1	LEU3	HVG1	LEU3	MXR1
LEU3	EFB1	LEU3	ICS2	LEU3	NAR1
LEU3	ELF1	LEU3	ICY2	LEU3	NAT4
LEU3	ERG26	LEU3	IDP1	LEU3	NFS1
LEU3	ERG3	LEU3	IES1	LEU3	NPP2
LEU3	FET3	LEU3	IES6	LEU3	NRT1
LEU3	FIT2	LEU3	ILV2	LEU3	NTH1
LEU3	FMP41	LEU3	ILV6	LEU3	ODC2
LEU3	FMP48	LEU3	IMD1	LEU3	OPY2
LEU3	FMP52	LEU3	IMD2	LEU3	ORC3
LEU3	FMT1	LEU3	IRA2	LEU3	OSH7
LEU3	FPR1	LEU3	ISU1	LEU3	OYE2
LEU3	FPS1	LEU3	KRS1	LEU3	PAC1
LEU3	FRS2	LEU3	KTI12	LEU3	PCL6
LEU3	GAC1	LEU3	LEU9	LEU3	PCL7
LEU3	GAS2	LEU3	LYS1	LEU3	PDC1
LEU3	GCV1	LEU3	LYS20	LEU3	PDC6

LEU3	PDH1	LEU3	RPL29	LEU3	SRP40
LEU3	PDR16	LEU3	RPL33B	LEU3	SSC1
LEU3	PEA2	LEU3	RPL36A	LEU3	SSK2
LEU3	PER1	LEU3	RPL41A	LEU3	SSK22
LEU3	PFK1	LEU3	RPL7A	LEU3	SSS1
LEU3	PGM2	LEU3	RPL7B	LEU3	STE3
LEU3	PHD1	LEU3	RPL8B	LEU3	STP4
LEU3	PHO23	LEU3	RPM2	LEU3	STR3
LEU3	PHO4	LEU3	RPP1A	LEU3	SUL2
LEU3	PHO88	LEU3	RPP2B	LEU3	SUR7
LEU3	PKH2	LEU3	RPR2	LEU3	SUT1
LEU3	PLC1	LEU3	RPS0B	LEU3	TAH11
LEU3	PMA1	LEU3	RPS10A	LEU3	TAT1
LEU3	PMA2	LEU3	RPS11B	LEU3	TEC1
LEU3	PMP1	LEU3	RPS14B	LEU3	TEF4
LEU3	PMP2	LEU3	RPS1B	LEU3	THI2
LEU3	POL1	LEU3	RPS26B	LEU3	THO1
LEU3	PPA1	LEU3	RPS3	LEU3	THR1
LEU3	PRO1	LEU3	RPS7B	LEU3	THR4
LEU3	PRO2	LEU3	RRM3	LEU3	TOP1
LEU3	PRP31	LEU3	SAM3	LEU3	TRP2
LEU3	PRR2	LEU3	SAM37	LEU3	TRP3
LEU3	PUP1	LEU3	SAM4	LEU3	TRP4
LEU3	PUT4	LEU3	SAS4	LEU3	TRP5
LEU3	QCR6	LEU3	SCT1	LEU3	TSL1
LEU3	RBA50	LEU3	SCW4	LEU3	TVP18
LEU3	REC102	LEU3	SDP1	LEU3	TYE7
LEU3	RHO3	LEU3	SEC23	LEU3	UBC4
LEU3	RHO5	LEU3	SED1	LEU3	UBX4
LEU3	RHR2	LEU3	SER1	LEU3	UGP1
LEU3	RIB3	LEU3	SER33	LEU3	UME6
LEU3	RIB5	LEU3	SFL1	LEU3	URA1
LEU3	RMD11	LEU3	SGO1	LEU3	URA2
LEU3	RMD6	LEU3	SHM2	LEU3	UTH1
LEU3	RML2	LEU3	SHR5	LEU3	UTR1
LEU3	RNH203	LEU3	SIL1	LEU3	UTR5
LEU3	RNR4	LEU3	SIS2	LEU3	VBA2
LEU3	ROM1	LEU3	SLX8	LEU3	VHS2
LEU3	ROM2	LEU3	SMX2	LEU3	VMA7
LEU3	RPA34	LEU3	SNA2	LEU3	VTC1
LEU3	RPB8	LEU3	SNQ2	LEU3	WSC4
LEU3	RPB9	LEU3	SOK1	LEU3	YAP5
LEU3	RPI1	LEU3	SOK2	LEU3	YAT1
LEU3	RPL12A	LEU3	SPE1	LEU3	YBL029W
LEU3	RPL13A	LEU3	SPO20	LEU3	YBL044W
LEU3	RPL18B	LEU3	SPO75	LEU3	YCF1

LEU3	YCL056C	MCM1	SWI4	RAP1	AFG2
LEU3	YCR018C-A	MCM1	SWI5	RAP1	AFT1
LEU3	YDL144C	MCM1	Ty1	RAP1	AGP1
LEU3	YDR042C	MED8	GLK1	RAP1	AIM9
LEU3	YEL007W	MED8	HXK1	RAP1	AIP1
LEU3	YEL047C	MED8	HXK2	RAP1	ALD4
LEU3	YFR017C	MED8	HXT1	RAP1	ALD5
LEU3	YFR035C	MED8	SUC2	RAP1	ALG3
LEU3	YGL101W	MET31	MET28	RAP1	ALG5
LEU3	YGR067C	MET32	MET28	RAP1	APE2
LEU3	YGR146C	MIG1	ENA1	RAP1	APJ1
LEU3	YGR190C	MIG1	FBP1	RAP1	APL4
LEU3	YHR033W	MIG1	FPS1	RAP1	APM3
LEU3	YIR016W	MIG1	GAL1-10	RAP1	APS2
LEU3	YJL045W	MIG1	HAP4	RAP1	APT2
LEU3	YJR003C	MIG1	PDC1	RAP1	ARE2
LEU3	YJR100C	MIG1	SUC2	RAP1	ARF1
LEU3	YJU2	MOT3	ANB1	RAP1	ARF2
LEU3	YKL037W	MOT3	CYC1	RAP1	ARG1
LEU3	YKL118W	MSN2	GLK1	RAP1	ARG3
LEU3	YKR041W	MSN4	GLK1	RAP1	ARO10
LEU3	YLR108C	NDT80	SPS4	RAP1	ARR3
LEU3	YLR162W	PDR1	IPT1	RAP1	ASC1
LEU3	YLR312C	PDR1	PDR15	RAP1	ASF1
LEU3	YLR356W	PDR1	PDR5	RAP1	AST2
LEU3	YMR099C	PDR3	HXT11	RAP1	ATF1
LEU3	YMR147W	PDR3	IPT1	RAP1	ATG1
LEU3	YNL040W	PDR3	PDR3	RAP1	ATG19
LEU3	YNL190W	PDR3	PDR5	RAP1	ATG26
LEU3	YOR186W	PDR3	SNQ2	RAP1	ATP7
LEU3	YPL056C	PHO2	HIS4	RAP1	AVT4
LEU3	YSA1	PHO2	HO	RAP1	BAT2
LEU3	YTM1	PHO2	PHO5	RAP1	BBC1
LEU3	ZAP1	PHO4	PHO5	RAP1	BCK1
LEU3	ZEO1	PHO4	PHO8	RAP1	BCY1
LEU3	ZWF1	PPR1	URA3	RAP1	BDF1
LYS14	LYS1	RAP1	AAC3	RAP1	BDS1
MAC1	CTR1	RAP1	ACA1	RAP1	BEM2
MAC1	FRE1	RAP1	ACE2	RAP1	BIK1
MAC1	FRE7	RAP1	ACS1	RAP1	BIO3
MCM1	CDC6	RAP1	ADE3	RAP1	BNA1
MCM1	CLB2	RAP1	ADE5,7	RAP1	BSC2
MCM1	CLN3	RAP1	ADE8	RAP1	BSC4
MCM1	STE2	RAP1	ADH1	RAP1	BSC5
MCM1	STE3	RAP1	ADH3	RAP1	BUD20
MCM1	STE6	RAP1	ADI1	RAP1	BUD22

RAP1	BUD27
RAP1	BUD3
RAP1	BUD4
RAP1	CAF120
RAP1	CAF40
RAP1	CAR1
RAP1	CAR2
RAP1	CBP2
RAP1	CCT6
RAP1	CCW12
RAP1	CDC19
RAP1	CDC20
RAP1	CDC21
RAP1	CDC45
RAP1	CDC50
RAP1	CHO2
RAP1	CHS7
RAP1	CLB1
RAP1	CLB2
RAP1	CLN1
RAP1	CLN3
RAP1	CMS1
RAP1	COF1
RAP1	COS10
RAP1	COS4
RAP1	COS6
RAP1	COT1
RAP1	COX19
RAP1	CPA1
RAP1	CRC1
RAP1	CRG1
RAP1	CRR1
RAP1	CSI2
RAP1	CSR2
RAP1	CTA1
RAP1	CTS1
RAP1	CTT1
RAP1	CUE4
RAP1	CWC23
RAP1	CWP1
RAP1	CWP2
RAP1	CYC8
RAP1	DAL1
RAP1	DAN4
RAP1	DAP1
RAP1	DBF2
RAP1	DBP10
RAP1	DDR2
RAP1	DEF1
RAP1	DFR1
RAP1	DIF1
RAP1	DIT1
RAP1	DLD1
RAP1	DLD3
RAP1	DNM1
RAP1	DOT6
RAP1	DTR1
RAP1	DUG3
RAP1	DUR3
RAP1	DUS3
RAP1	EAP1
RAP1	ECM13
RAP1	ECM16
RAP1	ECM17
RAP1	ECM19
RAP1	ECM38
RAP1	EDC2
RAP1	EDS1
RAP1	EFB1
RAP1	EGT2
RAP1	EHD3
RAP1	EKI1
RAP1	ELP6
RAP1	EMI2
RAP1	ENA1
RAP1	ENO1
RAP1	ENO2
RAP1	EPL1
RAP1	ERG1
RAP1	ERG13
RAP1	ERG5
RAP1	ERV29
RAP1	FAA1
RAP1	FAB1
RAP1	FAF1
RAP1	FAS1
RAP1	FAS2
RAP1	FBA1
RAP1	FIR1
RAP1	FIT2
RAP1	FKH2
RAP1	FLC1
RAP1	FLC2
RAP1	FLO10
RAP1	FMP16
RAP1	FMP23
RAP1	FMP40
RAP1	FMP48
RAP1	FMT1
RAP1	FOB1
RAP1	FPS1
RAP1	FRE1
RAP1	FRE2
RAP1	FRE4
RAP1	FRE6
RAP1	FRE7
RAP1	FRE8
RAP1	FRS2
RAP1	FRT2
RAP1	FTR1
RAP1	FUI1
RAP1	FUR1
RAP1	FUR4
RAP1	FYV1
RAP1	FZO1
RAP1	GAC1
RAP1	GAD1
RAP1	GAS3
RAP1	GAT1
RAP1	GAT2
RAP1	GAT3
RAP1	GCN4
RAP1	GDS1
RAP1	GEA2
RAP1	GID8
RAP1	GIM3
RAP1	GIT1
RAP1	GLK1
RAP1	GLN1
RAP1	GLN3
RAP1	GLY1
RAP1	GPD1
RAP1	GPG1
RAP1	GPI17
RAP1	GPM1
RAP1	GPM2
RAP1	GPR1
RAP1	GRS1

RAP1	GSC2	RAP1	INO2	RAP1	MRP7
RAP1	GTO3	RAP1	IPP1	RAP1	MRPL15
RAP1	GTS1	RAP1	IRC15	RAP1	MRPL24
RAP1	GUF1	RAP1	IRC22	RAP1	MRPL4
RAP1	GUP1	RAP1	IRC8	RAP1	MRPL49
RAP1	HAA1	RAP1	ISR1	RAP1	MRS1
RAP1	HAP1	RAP1	JLP1	RAP1	MRT4
RAP1	HAP3	RAP1	JSN1	RAP1	MSN4
RAP1	HAP5	RAP1	KES1	RAP1	MSR1
RAP1	HEM13	RAP1	KIN4	RAP1	MSW1
RAP1	HHO1	RAP1	KNS1	RAP1	MTC2
RAP1	HIS4	RAP1	KRE29	RAP1	MTC6
RAP1	HIS7	RAP1	KRS1	RAP1	MTH1
RAP1	HMRA1	RAP1	LAA1	RAP1	MTR4
RAP1	HMS1	RAP1	LCB3	RAP1	MUC1
RAP1	HMS2	RAP1	LDB18	RAP1	MUP3
RAP1	HOG1	RAP1	LEE1	RAP1	MVD1
RAP1	HOM6	RAP1	LOT5	RAP1	NAB3
RAP1	HOT13	RAP1	LSM5	RAP1	NCA3
RAP1	HRK1	RAP1	LST4	RAP1	NCE102
RAP1	HSP104	RAP1	LYP1	RAP1	NDL1
RAP1	HSP12	RAP1	MAE1	RAP1	NEO1
RAP1	HSP150	RAP1	MAM3	RAP1	NET1
RAP1	HSP26	RAP1	MCH4	RAP1	NEW1
RAP1	HSP30	RAP1	MCH5	RAP1	NFS1
RAP1	HSP33	RAP1	MCR1	RAP1	NFU1
RAP1	HSP78	RAP1	MDJ1	RAP1	NIS1
RAP1	HST3	RAP1	MDJ2	RAP1	NOB1
RAP1	HTB2	RAP1	MEP2	RAP1	NOP12
RAP1	HUG1	RAP1	MET17	RAP1	NQM1
RAP1	HUL5	RAP1	MET2	RAP1	NRG1
RAP1	HVG1	RAP1	MET30	RAP1	NSR1
RAP1	HXT1	RAP1	MET6	RAP1	NTC20
RAP1	HXT2	RAP1	MGA1	RAP1	OM45
RAP1	HXT5	RAP1	MIP6	RAP1	ORC2
RAP1	HYP2	RAP1	MLP2	RAP1	OST5
RAP1	IBA57	RAP1	MMF1	RAP1	OYE3
RAP1	ICY2	RAP1	MMR1	RAP1	PAC1
RAP1	IDP2	RAP1	MNN1	RAP1	PAC11
RAP1	IES1	RAP1	MNN4	RAP1	PAU21
RAP1	IES6	RAP1	MOH1	RAP1	PAU3
RAP1	IFH1	RAP1	MOT1	RAP1	PAU4
RAP1	IGO1	RAP1	MPS2	RAP1	PCL1
RAP1	IMD1	RAP1	MRL1	RAP1	PCL5
RAP1	IMD4	RAP1	MRM2	RAP1	PCL7
RAP1	IME1	RAP1	MRP20	RAP1	PDC1

RAP1	PDI1
RAP1	PDR11
RAP1	PDR12
RAP1	PDR15
RAP1	PDR5
RAP1	PDS1
RAP1	PDX3
RAP1	PET9
RAP1	PEX6
RAP1	PFA3
RAP1	PFK1
RAP1	PFK2
RAP1	PGK1
RAP1	PHM7
RAP1	PHO11
RAP1	PHO4
RAP1	PHO5
RAP1	PHO81
RAP1	PHO88
RAP1	PHO91
RAP1	PHS1
RAP1	PIL1
RAP1	PLB1
RAP1	PLM2
RAP1	PMA1
RAP1	PMP1
RAP1	PMR1
RAP1	PNS1
RAP1	POG1
RAP1	POL1
RAP1	POL3
RAP1	PPH22
RAP1	PPN1
RAP1	PRE7
RAP1	PRM2
RAP1	PRO1
RAP1	PRP31
RAP1	PRS1
RAP1	PRY1
RAP1	PRY3
RAP1	PSA1
RAP1	PTC1
RAP1	PTC2
RAP1	PTM1
RAP1	PUB1
RAP1	PUF6
RAP1	PUP2
RAP1	PUT3
RAP1	PUT4
RAP1	QCR6
RAP1	QDR2
RAP1	RAP1
RAP1	RAX1
RAP1	RAX2
RAP1	RBA50
RAP1	RDI1
RAP1	REG2
RAP1	REH1
RAP1	RGP1
RAP1	RGS2
RAP1	RHO3
RAP1	RHO5
RAP1	RHR2
RAP1	RIB3
RAP1	RKI1
RAP1	RLM1
RAP1	RMD6
RAP1	RNR2
RAP1	RNR3
RAP1	RNR4
RAP1	ROX3
RAP1	RPA190
RAP1	RPA34
RAP1	RPC40
RAP1	RPI1
RAP1	RPL10
RAP1	RPL11A
RAP1	RPL11B
RAP1	RPL12A
RAP1	RPL12B
RAP1	RPL13A
RAP1	RPL13B
RAP1	RPL14A
RAP1	RPL14B
RAP1	RPL15A
RAP1	RPL15B
RAP1	RPL16B
RAP1	RPL17A
RAP1	RPL17B
RAP1	RPL18A
RAP1	RPL18B
RAP1	RPL19B
RAP1	RPL1A
RAP1	RPL1B
RAP1	RPL20A
RAP1	RPL20B
RAP1	RPL21A
RAP1	RPL21B
RAP1	RPL22A
RAP1	RPL22B
RAP1	RPL23A
RAP1	RPL23B
RAP1	RPL24A
RAP1	RPL24B
RAP1	RPL25
RAP1	RPL26A
RAP1	RPL26B
RAP1	RPL27A
RAP1	RPL27B
RAP1	RPL28
RAP1	RPL29
RAP1	RPL2A
RAP1	RPL2B
RAP1	RPL3
RAP1	RPL30
RAP1	RPL31A
RAP1	RPL31B
RAP1	RPL32
RAP1	RPL33A
RAP1	RPL33B
RAP1	RPL34A
RAP1	RPL34B
RAP1	RPL35A
RAP1	RPL35B
RAP1	RPL36A
RAP1	RPL36B
RAP1	RPL37A
RAP1	RPL37B
RAP1	RPL38
RAP1	RPL39
RAP1	RPL40A
RAP1	RPL40B
RAP1	RPL41A
RAP1	RPL42A
RAP1	RPL43A
RAP1	RPL4A
RAP1	RPL4B
RAP1	RPL5

RAP1	RPL6A
RAP1	RPL6B
RAP1	RPL7A
RAP1	RPL7B
RAP1	RPL8A
RAP1	RPL8B
RAP1	RPL9A
RAP1	RPL9B
RAP1	RPN11
RAP1	RPN2
RAP1	RPO21
RAP1	RPP0
RAP1	RPP1A
RAP1	RPP1B
RAP1	RPP2A
RAP1	RPP2B
RAP1	RPR2
RAP1	RPS0A
RAP1	RPS0B
RAP1	RPS10A
RAP1	RPS10B
RAP1	RPS11B
RAP1	RPS12
RAP1	RPS13
RAP1	RPS14A
RAP1	RPS14B
RAP1	RPS15
RAP1	RPS16A
RAP1	RPS16B
RAP1	RPS17A
RAP1	RPS17B
RAP1	RPS18A
RAP1	RPS18B
RAP1	RPS19B
RAP1	RPS1A
RAP1	RPS1B
RAP1	RPS2
RAP1	RPS20
RAP1	RPS21A
RAP1	RPS22A
RAP1	RPS22B
RAP1	RPS23A
RAP1	RPS23B
RAP1	RPS24A
RAP1	RPS24B
RAP1	RPS25A
RAP1	RPS25B
RAP1	RPS26A
RAP1	RPS26B
RAP1	RPS27A
RAP1	RPS27B
RAP1	RPS28A
RAP1	RPS28B
RAP1	RPS29A
RAP1	RPS29B
RAP1	RPS3
RAP1	RPS30A
RAP1	RPS30B
RAP1	RPS31
RAP1	RPS4A
RAP1	RPS4B
RAP1	RPS5
RAP1	RPS6A
RAP1	RPS7A
RAP1	RPS7B
RAP1	RPS8A
RAP1	RPS8B
RAP1	RPS9A
RAP1	RRN6
RAP1	RRP14
RAP1	RRP5
RAP1	RSM10
RAP1	RSN1
RAP1	RSP5
RAP1	RTC4
RAP1	RTS3
RAP1	RTT109
RAP1	RUP1
RAP1	RVS167
RAP1	SAM1
RAP1	SAM2
RAP1	SCT1
RAP1	SED1
RAP1	SEO1
RAP1	SER1
RAP1	SER3
RAP1	SFA1
RAP1	SFG1
RAP1	SFL1
RAP1	SFP1
RAP1	SGA1
RAP1	SGT1
RAP1	SGV1
RAP1	SHR5
RAP1	SIM1
RAP1	SIP4
RAP1	SLG1
RAP1	SLS1
RAP1	SLX8
RAP1	SNF4
RAP1	SNF5
RAP1	SNO4
RAP1	SNX3
RAP1	SOK2
RAP1	SOL4
RAP1	SPO12
RAP1	SPO19
RAP1	SPO20
RAP1	SPS4
RAP1	SPT20
RAP1	SPT5
RAP1	SRL1
RAP1	SRO9
RAP1	SRP1
RAP1	SRP40
RAP1	SSA1
RAP1	SSA3
RAP1	SSB1
RAP1	SSF2
RAP1	SSL1
RAP1	SSS1
RAP1	STB5
RAP1	STE3
RAP1	STR3
RAP1	SUE1
RAP1	SUN4
RAP1	SUP17
RAP1	SUR1
RAP1	SUR7
RAP1	SVS1
RAP1	SWI5
RAP1	SWM1
RAP1	SYC1
RAP1	TA(AGC)G
RAP1	TA(AGC)K2
RAP1	TDH2
RAP1	TDH3
RAP1	TEF2

RAP1	TEF4	RAP1	VPS61	RAP1	YEL007W
RAP1	TEM1	RAP1	VPS73	RAP1	YEL008W
RAP1	TFA1	RAP1	WHI2	RAP1	YEL023C
RAP1	TFB1	RAP1	WSC4	RAP1	YEL045C
RAP1	TGL2	RAP1	YAL018C	RAP1	YEL074W
RAP1	TIM23	RAP1	YAL064C-A	RAP1	YEL075C
RAP1	TIM9	RAP1	YAL064W	RAP1	YER053C-A
RAP1	TIP20	RAP1	YAP1802	RAP1	YER078C
RAP1	TIP41	RAP1	YAP6	RAP1	YER130C
RAP1	TIR1	RAP1	YAT1	RAP1	YER158C
RAP1	TIR3	RAP1	YBL028C	RAP1	YER189W
RAP1	TIR4	RAP1	YBL029C-A	RAP1	YFL034W
RAP1	TKL2	RAP1	YBL029W	RAP1	YFL064C
RAP1	TMA10	RAP1	YBL081W	RAP1	YFR017C
RAP1	TOS1	RAP1	YBL086C	RAP1	YFR018C
RAP1	TOS4	RAP1	YBL111C	RAP1	YFR035C
RAP1	TOS6	RAP1	YBR138C	RAP1	YGL006W-A
RAP1	TOS8	RAP1	YBR139W	RAP1	YGL007C-A
RAP1	TPI1	RAP1	YBR190W	RAP1	YGL007W
RAP1	TPM1	RAP1	YBR197C	RAP1	YGL114W
RAP1	TPO1	RAP1	YBR219C	RAP1	YGL177W
RAP1	TRM2	RAP1	YCF1	RAP1	YGL188C
RAP1	TRP3	RAP1	YCG1	RAP1	YGL242C
RAP1	TRS23	RAP1	YCK2	RAP1	YGR012W
RAP1	TSR1	RAP1	YCL042W	RAP1	YGR050C
RAP1	TYE7	RAP1	YCR006C	RAP1	YGR117C
RAP1	UBA1	RAP1	YCR024C-B	RAP1	YGR203W
RAP1	UBI4	RAP1	YCR025C	RAP1	YGR283C
RAP1	UBP2	RAP1	YCT1	RAP1	YHB1
RAP1	UBP5	RAP1	YDL012C	RAP1	YHL049C
RAP1	UFD4	RAP1	YDL032W	RAP1	YHP1
RAP1	UFO1	RAP1	YDL034W	RAP1	YHR032W
RAP1	UGA1	RAP1	YDL129W	RAP1	YHR033W
RAP1	UGX2	RAP1	YDL173W	RAP1	YHR095W
RAP1	USV1	RAP1	YDL186W	RAP1	YHR097C
RAP1	UTH1	RAP1	YDL187C	RAP1	YIL057C
RAP1	UTP15	RAP1	YDR042C	RAP1	YIL158W
RAP1	UTP4	RAP1	YDR065W	RAP1	YIL177C
RAP1	UTR2	RAP1	YDR089W	RAP1	YIR014W
RAP1	UTR5	RAP1	YDR112W	RAP1	YIR016W
RAP1	VAC17	RAP1	YDR132C	RAP1	YIR042C
RAP1	VBA5	RAP1	YDR186C	RAP1	YJL028W
RAP1	VEL1	RAP1	YDR391C	RAP1	YJL045W
RAP1	VHR1	RAP1	YDR524C-B	RAP1	YJL213W
RAP1	VID24	RAP1	YDR543C	RAP1	YJL218W
RAP1	VMA6	RAP1	YDR544C	RAP1	YJL225C

RAP1	YJR115W
RAP1	YJR146W
RAP1	YKL030W
RAP1	YKL063C
RAP1	YKL083W
RAP1	YKL096C-B
RAP1	YKL097C
RAP1	YKL151C
RAP1	YKL161C
RAP1	YKR040C
RAP1	YKR041W
RAP1	YLL037W
RAP1	YLL067C
RAP1	YLR012C
RAP1	YLR073C
RAP1	YLR076C
RAP1	YLR111W
RAP1	YLR112W
RAP1	YLR164W
RAP1	YLR173W
RAP1	YLR184W
RAP1	YLR224W
RAP1	YLR255C
RAP1	YLR312C
RAP1	YLR339C
RAP1	YLR400W
RAP1	YLR407W
RAP1	YLR463C
RAP1	YLR465C
RAP1	YMC1
RAP1	YML050W
RAP1	YML053C
RAP1	YML089C
RAP1	YML100W-A
RAP1	YML133C
RAP1	YMR086W
RAP1	YMR099C
RAP1	YMR135W-A
RAP1	YMR144W
RAP1	YMR244W
RAP1	YMR295C
RAP1	YNL190W
RAP1	YNL337W
RAP1	YNL338W
RAP1	YNR014W
RAP1	YNR018W
RAP1	YNR070W
RAP1	YOL019W
RAP1	YOL075C
RAP1	YOL161C
RAP1	YOR008C-A
RAP1	YOR152C
RAP1	YOR186W
RAP1	YOR292C
RAP1	YOR302W
RAP1	YOR338W
RAP1	YOR342C
RAP1	YOR343C
RAP1	YOR378W
RAP1	YOR387C
RAP1	YOS1
RAP1	YPI1
RAP1	YPL056C
RAP1	YPL080C
RAP1	YPL113C
RAP1	YPL162C
RAP1	YPL229W
RAP1	YPL277C
RAP1	YPR148C
RAP1	YPT31
RAP1	YRF1-1
RAP1	YRF1-2
RAP1	YRF1-4
RAP1	YRF1-5
RAP1	YRF1-6
RAP1	YRF1-7
RAP1	YSR3
RAP1	YTP1
RAP1	ZEO1
RAP1	ZIM17
RAP1	ZRG8
RAP1	ZRT1
RAP1	ZTA1
RCS1	FET3
RCS1	FET4
RCS1	FRE1
RCS1	FRE2
RCS1	FTH1
RCS1	FTR1
REB1	ACC1
REB1	ACS1
REB1	ACT1
REB1	ADE2
REB1	APL4
REB1	ARF2
REB1	ARG5,6
REB1	ARP7
REB1	ASK1
REB1	ATH1
REB1	BET3
REB1	BFR1
REB1	BLM10
REB1	BUD27
REB1	CAT8
REB1	CBK1
REB1	CCW12
REB1	CDC21
REB1	CDC39
REB1	CDC5
REB1	CDC50
REB1	CIS3
REB1	CLB2
REB1	CLB3
REB1	CPR1
REB1	CSE1
REB1	CYB5
REB1	DCP2
REB1	DOC1
REB1	DOM34
REB1	EHD3
REB1	ELO1
REB1	EMP70
REB1	ENO1
REB1	EPL1
REB1	FAS1
REB1	FAS2
REB1	FMP42
REB1	FMT1
REB1	FRE2
REB1	GCS1
REB1	GCV2
REB1	GCY1
REB1	GEA2
REB1	GFA1
REB1	GLN3
REB1	GTS1
REB1	GUS1
REB1	HOG1

REB1	HOM2
REB1	HOS4
REB1	HSC82
REB1	HSH49
REB1	HVG1
REB1	IBA57
REB1	IFH1
REB1	IGO1
REB1	ILV1
REB1	IME2
REB1	IME4
REB1	IRC19
REB1	IRC23
REB1	ISA2
REB1	JJJ3
REB1	KRS1
REB1	LHS1
REB1	MET1
REB1	MF(ALPHA)2
REB1	MLP2
REB1	MLS1
REB1	MMS1
REB1	MND1
REB1	MOT2
REB1	MPE1
REB1	MPH1
REB1	MRPL51
REB1	NAS6
REB1	NOP6
REB1	NOP8
REB1	NPC2
REB1	NPL4
REB1	NST1
REB1	NUP157
REB1	PAN3
REB1	PFY1
REB1	PGK1
REB1	PHB2
REB1	PHO91
REB1	PIB1
REB1	POL3
REB1	PPA1
REB1	PPH22
REB1	PRE1
REB1	PRE4
REB1	PSD1
REB1	RAD28
REB1	RAP1
REB1	RCR2
REB1	REB1
REB1	REG1
REB1	RIX7
REB1	RKM4
REB1	RMT2
REB1	RPA14
REB1	RPA34
REB1	RPB9
REB1	RPL15B
REB1	RPL40A
REB1	RPL42A
REB1	RPN2
REB1	RPO21
REB1	RPO31
REB1	RPS29A
REB1	RPT5
REB1	RRN6
REB1	RRP14
REB1	RXT2
REB1	SCJ1
REB1	SDP1
REB1	SEC23
REB1	SEC66
REB1	SHP1
REB1	SHS1
REB1	SMK1
REB1	SMX3
REB1	SNT309
REB1	SOR1
REB1	SPC24
REB1	SRP21
REB1	STB4
REB1	SWI5
REB1	TCO89
REB1	TDH3
REB1	TEF4
REB1	THR1
REB1	TIF3
REB1	TKL1
REB1	TOM1
REB1	TOP1
REB1	TPI1
REB1	TRP5
REB1	UBA3
REB1	UBC8
REB1	UBR1
REB1	YAP1802
REB1	YDL186W
REB1	YDL233W
REB1	YDR029W
REB1	YDR154C
REB1	YDR157W
REB1	YDR455C
REB1	YEL023C
REB1	YER156C
REB1	YET2
REB1	YGR011W
REB1	YGR012W
REB1	YIL108W
REB1	YIR003W
REB1	YJR100C
REB1	YJR128W
REB1	YKE4
REB1	YKR015C
REB1	YLR224W
REB1	YLR366W
REB1	YML119W
REB1	YMR185W
REB1	YNL092W
REB1	YNL115C
REB1	YOR093C
REB1	YOR186W
REB1	YPR099C
REB1	YTM1
RFX1	RNR2
RFX1	RNR3
RFX1	RNR4
RIM101	PHR1
RME1	IME1
ROX1	AAC3
ROX1	ANB1
ROX1	CPR1
ROX1	CYC7
ROX1	ERG11
ROX1	FET4
ROX1	HEM13
ROX1	HMG2
ROX1	OLE1
ROX1	ROX1

RPH1	PHR1
RPN4	RFT1
RPN4	RPT2
RPN4	RPT4
RPN4	RPT5
RPN4	RPT6
RTG1	CIT2
RTG3	CIT2
SIN1	HO
SIP4FBP1	
SIP4MDH2	
SIP4SIP4	
SKN7	ABF2
SKN7	ACO1
SKN7	AFG2
SKN7	AFT2
SKN7	AHP1
SKN7	ALD5
SKN7	ALG7
SKN7	AMN1
SKN7	APC1
SKN7	API2
SKN7	APT2
SKN7	ARF2
SKN7	ARG1
SKN7	ASF1
SKN7	ATP1
SKN7	ATP14
SKN7	BAT2
SKN7	BNA2
SKN7	CAF20
SKN7	CBF2
SKN7	CCP1
SKN7	CCW12
SKN7	CDC48
SKN7	CET1
SKN7	CHC1
SKN7	CHS1
SKN7	CIN1
SKN7	CIS3
SKN7	CLB1
SKN7	CLN1
SKN7	CLN2
SKN7	COX19
SKN7	CPR1
SKN7	CRR1
SKN7	CTT1
SKN7	CUE4
SKN7	CUP9
SKN7	CWP2
SKN7	CYT2
SKN7	DAK1
SKN7	DAK2
SKN7	DAL7
SKN7	DBP10
SKN7	DDR2
SKN7	DDR48
SKN7	DED1
SKN7	DNM1
SKN7	DOT6
SKN7	DPS1
SKN7	ECM33
SKN7	ERG6
SKN7	ERG9
SKN7	EXG1
SKN7	FKS3
SKN7	FLO9
SKN7	FMP48
SKN7	FRE1
SKN7	FRS2
SKN7	GAS1
SKN7	GAT1
SKN7	GAT2
SKN7	GDH3
SKN7	GGA1
SKN7	GIC2
SKN7	GID8
SKN7	GIT1
SKN7	GLY1
SKN7	GPH1
SKN7	GPX2
SKN7	GRE2
SKN7	GSC2
SKN7	GSH1
SKN7	GSH2
SKN7	GSY2
SKN7	GTO3
SKN7	GTR1
SKN7	GTT3
SKN7	GUT2
SKN7	GVP36
SKN7	HAA1
SKN7	HAP4
SKN7	HEF3
SKN7	HER1
SKN7	HLR1
SKN7	HMS1
SKN7	HMS2
SKN7	HMX1
SKN7	HOM2
SKN7	HOR7
SKN7	HOT1
SKN7	HPF1
SKN7	HSP104
SKN7	HSP12
SKN7	HSP26
SKN7	HSP30
SKN7	HSP42
SKN7	HSP78
SKN7	HSP82
SKN7	HXT11
SKN7	HXT12
SKN7	ICS2
SKN7	ICY1
SKN7	IDH2
SKN7	IES6
SKN7	INO1
SKN7	ISA1
SKN7	ISU1
SKN7	ISU2
SKN7	JSN1
SKN7	KNS1
SKN7	KOG1
SKN7	KRE6
SKN7	KTI12
SKN7	LSM3
SKN7	LTV1
SKN7	LYS20
SKN7	MAK32
SKN7	MDG1
SKN7	MDH2
SKN7	MET13
SKN7	MET16
SKN7	MET2
SKN7	MET6
SKN7	MFA1
SKN7	MFA2
SKN7	MGA1

SKN7	MGM101	SKN7	PTR2	SKN7	SOL4
SKN7	MMF1	SKN7	PUT4	SKN7	SPI1
SKN7	MMP1	SKN7	PYC1	SKN7	SPO1
SKN7	MNN1	SKN7	QCR10	SKN7	SPO20
SKN7	MOT3	SKN7	RAS2	SKN7	SRP40
SKN7	MPR1	SKN7	RAX2	SKN7	SRP68
SKN7	MRP8	SKN7	RBA50	SKN7	SRX1
SKN7	MRPL4	SKN7	RDI1	SKN7	SSA1
SKN7	MRS4	SKN7	RET1	SKN7	SSA4
SKN7	MSB2	SKN7	RGS2	SKN7	SSE2
SKN7	MSN2	SKN7	RHO3	SKN7	SSK22
SKN7	MSN4	SKN7	RME1	SKN7	STB4
SKN7	MTC3	SKN7	RMI1	SKN7	SUE1
SKN7	MUC1	SKN7	ROX1	SKN7	SUR7
SKN7	NCA3	SKN7	RPA14	SKN7	SUT2
SKN7	NCE102	SKN7	RPA34	SKN7	TA(AGC)P
SKN7	NQM1	SKN7	RPI1	SKN7	TAL1
SKN7	NRG1	SKN7	RPL35B	SKN7	TCB2
SKN7	NRG2	SKN7	RPL6A	SKN7	THI7
SKN7	OCH1	SKN7	RPM2	SKN7	THI72
SKN7	OLA1	SKN7	RPP2A	SKN7	TIM23
SKN7	OLE1	SKN7	RPS15	SKN7	TIR1
SKN7	OST5	SKN7	RPS3	SKN7	TOS8
SKN7	OTU2	SKN7	RSC6	SKN7	TPO4
SKN7	OXA1	SKN7	RUP1	SKN7	TPS3
SKN7	OYE3	SKN7	SCW10	SKN7	TRR1
SKN7	PAC11	SKN7	SCW4	SKN7	TRX2
SKN7	PAH1	SKN7	SDC25	SKN7	TRX3
SKN7	PAI3	SKN7	SDP1	SKN7	TSA1
SKN7	PCK1	SKN7	SDS23	SKN7	TSA2
SKN7	PCL7	SKN7	SED1	SKN7	TSL1
SKN7	PDS5	SKN7	SET4	SKN7	TVP18
SKN7	PEA2	SKN7	SFG1	SKN7	UBA1
SKN7	PET122	SKN7	SFL1	SKN7	UFO1
SKN7	PET9	SKN7	SGA1	SKN7	UGP1
SKN7	PHO91	SKN7	SGV1	SKN7	URA3
SKN7	PIL1	SKN7	SHE10	SKN7	USA1
SKN7	PMA1	SKN7	SHM2	SKN7	USV1
SKN7	PMC1	SKN7	SLM4	SKN7	UTH1
SKN7	PMP2	SKN7	SNA2	SKN7	VTC3
SKN7	PRI2	SKN7	SNZ2	SKN7	YAP1
SKN7	PRM5	SKN7	SNZ3	SKN7	YAP5
SKN7	PRP9	SKN7	SOD1	SKN7	YBL029W
SKN7	PRY1	SKN7	SOD2	SKN7	YBR051W
SKN7	PSO2	SKN7	SOK1	SKN7	YBR053C
SKN7	PTC1	SKN7	SOK2	SKN7	YBR085C-A

SKN7	YCK3
SKN7	YCR018C-A
SKN7	YCR102C
SKN7	YCT1
SKN7	YDL129W
SKN7	YDR154C
SKN7	YDR157W
SKN7	YDR442W
SKN7	YDR526C
SKN7	YEL045C
SKN7	YER010C
SKN7	YER078C
SKN7	YER079W
SKN7	YER152C
SKN7	YET2
SKN7	YFR017C
SKN7	YGL007W
SKN7	YGR045C
SKN7	YGR050C
SKN7	YGR130C
SKN7	YGR210C
SKN7	YHL012W
SKN7	YHR138C
SKN7	YHR213W
SKN7	YIR020C
SKN7	YJL107C
SKN7	YJR096W
SKN7	YKL037W
SKN7	YKL044W
SKN7	YKL063C
SKN7	YKL066W
SKN7	YKL097C
SKN7	YKR040C
SKN7	YKR041W
SKN7	YKR075C
SKN7	YKR096W
SKN7	YLR042C
SKN7	YLR108C
SKN7	YLR111W
SKN7	YLR112W
SKN7	YLR257W
SKN7	YLR301W
SKN7	YLR414C
SKN7	YML053C
SKN7	YML089C
SKN7	YMR086W
SKN7	YMR134W
SKN7	YMR135W-A
SKN7	YMR172C-A
SKN7	YMR173W-A
SKN7	YMR252C
SKN7	YMR304C-A
SKN7	YNL092W
SKN7	YNL143C
SKN7	YNL146W
SKN7	YNL170W
SKN7	YNL234W
SKN7	YNR014W
SKN7	YNR018W
SKN7	YOL048C
SKN7	YOR314W
SKN7	YPL025C
SKN7	YPR013C
SKN7	YPR063C
SKN7	YPR064W
SKN7	YPR148C
SKN7	YPR158C-C
SKN7	YPR158C-D
SKN7	YPS1
SKN7	YPS3
SKN7	YRO2
SKN7	ZEO1
SKN7	ZPR1
SKN7	ZWF1
SKO1	ENA1
SKO1	HIS3
SKO1	SUC2
STB1	API2
STB1	BUD31
STB1	CLB6
STB1	CLN2
STB1	CSI2
STB1	FUN26
STB1	GIC1
STB1	GIC2
STB1	GVP36
STB1	IRC6
STB1	LTV1
STB1	MCM16
STB1	MGM101
STB1	MGR2
STB1	MRPS9
STB1	NDD1
STB1	RFC3
STB1	RPL37B
STB1	RPS10A
STB1	RPS11B
STB1	RUP1
STB1	SPO16
STB1	SPO77
STB1	TOS2
STB1	VTC2
STB1	YAL064C-A
STB1	YKL097C
STB1	YMR306C-A
STB1	YOR246C
STB1	YPL056C
STE12	MFA1
STE12	MFA2
STE12	STE2
SUM1	SMK1
SUT1	RRP12
SWI4	ABF2
SWI4	ACS2
SWI4	ADR1
SWI4	ADY2
SWI4	AEP3
SWI4	AGA1
SWI4	AGP1
SWI4	ALD3
SWI4	ALG14
SWI4	AMS1
SWI4	API2
SWI4	APT2
SWI4	ARG5,6
SWI4	ASG7
SWI4	ATF1
SWI4	AUR1
SWI4	AYR1
SWI4	BAP3
SWI4	BAR1
SWI4	BAT2
SWI4	BBP1
SWI4	BFA1
SWI4	BMH1
SWI4	BNA5
SWI4	BTN2
SWI4	BUB3

SWI4	BUD31
SWI4	BUD9
SWI4	CAF120
SWI4	CAF40
SWI4	CAP1
SWI4	CBF2
SWI4	CCT2
SWI4	CCT6
SWI4	CCW12
SWI4	CDC21
SWI4	CDC48
SWI4	CDC6
SWI4	CHS1
SWI4	CHS5
SWI4	CIK1
SWI4	CIN1
SWI4	CIS3
SWI4	CLB1
SWI4	CLB2
SWI4	CLB5
SWI4	CLB6
SWI4	CLD1
SWI4	CLN1
SWI4	CLN2
SWI4	CLN3
SWI4	COS3
SWI4	COS6
SWI4	CRH1
SWI4	CSI2
SWI4	CTF18
SWI4	CUE4
SWI4	CWP1
SWI4	CWP2
SWI4	CYC8
SWI4	DAL5
SWI4	DBF2
SWI4	DDR48
SWI4	DEM1
SWI4	DIF1
SWI4	DIN7
SWI4	DOT6
SWI4	DSF2
SWI4	DUN1
SWI4	ECM32
SWI4	ECM33
SWI4	ECM38
SWI4	ECM8
SWI4	ELO1
SWI4	ENP1
SWI4	ERG11
SWI4	ERG3
SWI4	ERP3
SWI4	EST1
SWI4	EXG1
SWI4	EXG2
SWI4	FIT2
SWI4	FKS1
SWI4	FKS3
SWI4	FLC1
SWI4	FMP27
SWI4	FTR1
SWI4	FZF1
SWI4	GAC1
SWI4	GAS1
SWI4	GAS2
SWI4	GAT2
SWI4	GFA1
SWI4	GIC1
SWI4	GIC2
SWI4	GID8
SWI4	GIM4
SWI4	GIN4
SWI4	GIT1
SWI4	GON7
SWI4	GPM2
SWI4	GSC2
SWI4	GSY2
SWI4	GTR1
SWI4	GUT2
SWI4	HAA1
SWI4	HAP1
SWI4	HAP4
SWI4	HAP5
SWI4	HBT1
SWI4	HCH1
SWI4	HCM1
SWI4	HCR1
SWI4	HEM13
SWI4	HHF2
SWI4	HHO1
SWI4	HHT2
SWI4	HIP1
SWI4	HMS2
SWI4	HO
SWI4	HOG1
SWI4	HPF1
SWI4	HSL1
SWI4	HSP12
SWI4	HSP26
SWI4	HSP30
SWI4	HSP33
SWI4	HSP42
SWI4	HTB2
SWI4	HTZ1
SWI4	HXT11
SWI4	HXT12
SWI4	HXT2
SWI4	HXT3
SWI4	ICY1
SWI4	ICY2
SWI4	IRC15
SWI4	IRC22
SWI4	ITC1
SWI4	KAP95
SWI4	KAR4
SWI4	KRE6
SWI4	KSS1
SWI4	KTI12
SWI4	LAC1
SWI4	LAP4
SWI4	LEE1
SWI4	LOH1
SWI4	LSM3
SWI4	LSM5
SWI4	LTE1
SWI4	LYS20
SWI4	MAE1
SWI4	MAL12
SWI4	MAL32
SWI4	MCH2
SWI4	MCM2
SWI4	MDJ1
SWI4	MFA1
SWI4	MFA2
SWI4	MGA1
SWI4	MGR3
SWI4	MHR1
SWI4	MLH2

SWI4	MNN1
SWI4	MNN5
SWI4	MOT3
SWI4	MPT5
SWI4	MRPL4
SWI4	MRS1
SWI4	MSB2
SWI4	MSC1
SWI4	MTC2
SWI4	MUC1
SWI4	MVD1
SWI4	NAN1
SWI4	NAT4
SWI4	NCE102
SWI4	NDD1
SWI4	NDL1
SWI4	NOP16
SWI4	NRM1
SWI4	NRT1
SWI4	OCH1
SWI4	OPT2
SWI4	OXA1
SWI4	PAU21
SWI4	PCL1
SWI4	PCL2
SWI4	PCL5
SWI4	PDC1
SWI4	PDR16
SWI4	PDR5
SWI4	PET122
SWI4	PET54
SWI4	PET9
SWI4	PEX28
SWI4	PGM2
SWI4	PHD1
SWI4	PHO3
SWI4	PHO5
SWI4	PHO81
SWI4	PIL1
SWI4	PLB2
SWI4	PLC1
SWI4	PLM2
SWI4	PMA1
SWI4	PMC1
SWI4	PNC1
SWI4	PNS1
SWI4	POG1
SWI4	POP3
SWI4	PPN1
SWI4	PRI2
SWI4	PRM5
SWI4	PRY1
SWI4	PRY2
SWI4	PSA1
SWI4	PSE1
SWI4	PSO2
SWI4	PST1
SWI4	PTR2
SWI4	PUP3
SWI4	PUT4
SWI4	PYC1
SWI4	QDR2
SWI4	RAD16
SWI4	RAD27
SWI4	RAX2
SWI4	RBA50
SWI4	RCL1
SWI4	REF2
SWI4	REG2
SWI4	RFC3
SWI4	RHO5
SWI4	RIM101
SWI4	RIM4
SWI4	RKM4
SWI4	RMI1
SWI4	RNR1
SWI4	RNR2
SWI4	RNR3
SWI4	RNR4
SWI4	ROT1
SWI4	RPA34
SWI4	RPL18B
SWI4	RPL34B
SWI4	RPL37A
SWI4	RPL37B
SWI4	RPL9A
SWI4	RPR2
SWI4	RPS16A
SWI4	RPS3
SWI4	RPT1
SWI4	RRP5
SWI4	RTC3
SWI4	RTC4
SWI4	RUP1
SWI4	SCW10
SWI4	SCW4
SWI4	SEC53
SWI4	SED1
SWI4	SFG1
SWI4	SFL1
SWI4	SGA1
SWI4	SHC1
SWI4	SHE10
SWI4	SIM1
SWI4	SIR4
SWI4	SKG6
SWI4	SKM1
SWI4	SKS1
SWI4	SLM4
SWI4	SMF2
SWI4	SNA2
SWI4	SNQ2
SWI4	SNU71
SWI4	SOK2
SWI4	SOL4
SWI4	SPB1
SWI4	SPO20
SWI4	SPO77
SWI4	SPS4
SWI4	SPT8
SWI4	SRL1
SWI4	SRP40
SWI4	SRP68
SWI4	SSA4
SWI4	SSE2
SWI4	SSK22
SWI4	SSL2
SWI4	SSU1
SWI4	STE6
SWI4	STP4
SWI4	STR3
SWI4	SUE1
SWI4	SUR1
SWI4	SUR2
SWI4	SUT2
SWI4	SVS1
SWI4	SWE1
SWI4	SWI4

SWI4	SYC1	SWI4	YBL109W	SWI4	YJR003C
SWI4	TA(AGC)P	SWI4	YBL111C	SWI4	YJR030C
SWI4	TFP1	SWI4	YBR071W	SWI4	YJR054W
SWI4	TFS1	SWI4	YDL183C	SWI4	YJR116W
SWI4	TGS1	SWI4	YDL186W	SWI4	YJU2
SWI4	THI7	SWI4	YDL187C	SWI4	YKL044W
SWI4	THI72	SWI4	YDR010C	SWI4	YKL096C-B
SWI4	TIP1	SWI4	YDR132C	SWI4	YKL097C
SWI4	TIR1	SWI4	YDR186C	SWI4	YKL151C
SWI4	TIR3	SWI4	YDR222W	SWI4	YKR011C
SWI4	TOK1	SWI4	YDR442W	SWI4	YKR040C
SWI4	TOM1	SWI4	YDR524C-B	SWI4	YKR041W
SWI4	TOP1	SWI4	YDR526C	SWI4	YLL066C
SWI4	TOP3	SWI4	YDR543C	SWI4	YLL067C
SWI4	TOS1	SWI4	YDR544C	SWI4	YLR035C-A
SWI4	TOS2	SWI4	YEL007W	SWI4	YLR042C
SWI4	TOS3	SWI4	YEL074W	SWI4	YLR111W
SWI4	TOS4	SWI4	YEL075C	SWI4	YLR112W
SWI4	TOS6	SWI4	YER010C	SWI4	YLR125W
SWI4	TOS8	SWI4	YER078C	SWI4	YLR159W
SWI4	TPO2	SWI4	YER079W	SWI4	YLR162W
SWI4	TPO3	SWI4	YER189W	SWI4	YLR184W
SWI4	TRF5	SWI4	YFL063W	SWI4	YLR255C
SWI4	TRM2	SWI4	YFL064C	SWI4	YLR301W
SWI4	TRS23	SWI4	YFR017C	SWI4	YLR462W
SWI4	TSA1	SWI4	YGL007W	SWI4	YML133C
SWI4	TSA2	SWI4	YGL036W	SWI4	YMR135W-A
SWI4	TSL1	SWI4	YGL177W	SWI4	YMR144W
SWI4	TVP18	SWI4	YGR012W	SWI4	YMR178W
SWI4	TYE7	SWI4	YGR045C	SWI4	YMR244W
SWI4	USV1	SWI4	YGR050C	SWI4	YMR304C-A
SWI4	UTH1	SWI4	YGR190C	SWI4	YMR306C-A
SWI4	UTR2	SWI4	YGR250C	SWI4	YNL179C
SWI4	VAC17	SWI4	YGR251W	SWI4	YNL296W
SWI4	VCX1	SWI4	YHB1	SWI4	YNL337W
SWI4	VHR1	SWI4	YHL049C	SWI4	YNL338W
SWI4	VID30	SWI4	YHP1	SWI4	YNR062C
SWI4	VPS71	SWI4	YHR078W	SWI4	YNR070W
SWI4	YAL064C-A	SWI4	YHR213W	SWI4	YOL019W
SWI4	YAL064W	SWI4	YIL169C	SWI4	YOL114C
SWI4	YAP5	SWI4	YIL177C	SWI4	YOR152C
SWI4	YAR075W	SWI4	YIR020C	SWI4	YOR246C
SWI4	YAT1	SWI4	YJL107C	SWI4	YOR248W
SWI4	YBL029C-A	SWI4	YJL160C	SWI4	YOR302W
SWI4	YBL029W	SWI4	YJL185C	SWI4	YOR314W
SWI4	YBL108W	SWI4	YJL225C	SWI4	YOR338W

SWI4	YOR342C	SWI5	DBP9	SWI5	MFA2
SWI4	YOR343C	SWI5	DDI3	SWI5	MGM101
SWI4	YOR378W	SWI5	DDR48	SWI5	MMF1
SWI4	YOX1	SWI5	DFR1	SWI5	MRPL4
SWI4	YPL025C	SWI5	DJP1	SWI5	MSR1
SWI4	YPL056C	SWI5	DLD1	SWI5	MTC3
SWI4	YPL088W	SWI5	DPM1	SWI5	NCB2
SWI4	YPL162C	SWI5	DSE1	SWI5	NIS1
SWI4	YPR013C	SWI5	DSE2	SWI5	OCA5
SWI4	YPR015C	SWI5	DSE3	SWI5	OST5
SWI4	YPR148C	SWI5	DSE4	SWI5	OTU2
SWI4	YPR158C-C	SWI5	ECM32	SWI5	PAH1
SWI4	YPR158C-D	SWI5	EGT2	SWI5	PCL2
SWI4	YPR202W	SWI5	ENO1	SWI5	PCL7
SWI4	YPS1	SWI5	ENT2	SWI5	PCL9
SWI4	YPS3	SWI5	ERG25	SWI5	PFA3
SWI4	YRF1-1	SWI5	EXG1	SWI5	PHO81
SWI4	YRF1-2	SWI5	FAA3	SWI5	PIL1
SWI4	YRF1-3	SWI5	FLO9	SWI5	PIR1
SWI4	YRF1-5	SWI5	FOB1	SWI5	PIR3
SWI4	YRF1-6	SWI5	FRS2	SWI5	PRI2
SWI4	YRF1-7	SWI5	FUN26	SWI5	PRP9
SWI4	YRO2	SWI5	FUR1	SWI5	PRY3
SWI4	YSY6	SWI5	GAS5	SWI5	PSK1
SWI4	ZEO1	SWI5	GAT1	SWI5	PST1
SWI5	ALG14	SWI5	GAT2	SWI5	PUP2
SWI5	AMN1	SWI5	GAT3	SWI5	PUT4
SWI5	ASH1	SWI5	GCS1	SWI5	REE1
SWI5	AYT1	SWI5	GDH3	SWI5	RME1
SWI5	BNI1	SWI5	GPR1	SWI5	RPL2A
SWI5	BSC4	SWI5	GTO3	SWI5	RPL31B
SWI5	BUD9	SWI5	GTR1	SWI5	RUP1
SWI5	CBK1	SWI5	HBT1	SWI5	RVB2
SWI5	CDC34	SWI5	HO	SWI5	SCP160
SWI5	CDC6	SWI5	HOR7	SWI5	SCW11
SWI5	CHS1	SWI5	HRD3	SWI5	SEN2
SWI5	CLB4	SWI5	HSP12	SWI5	SFL1
SWI5	CLN3	SWI5	HSP150	SWI5	SHE10
SWI5	COS3	SWI5	HXT3	SWI5	SHS1
SWI5	COS4	SWI5	ICS2	SWI5	SIC1
SWI5	COS6	SWI5	ISR1	SWI5	SMX3
SWI5	CPR1	SWI5	KSS1	SWI5	TAF13
SWI5	CRH1	SWI5	LAA1	SWI5	TAH11
SWI5	CTS1	SWI5	LAP3	SWI5	TAO3
SWI5	CUE4	SWI5	LSM3	SWI5	TEC1
SWI5	CYK3	SWI5	MDJ2	SWI5	TGS1

SWI5	TIM23	SWI5	YLR035C-A	SWI6	CDC6
SWI5	TPM1	SWI5	YLR162W	SWI6	CHS5
SWI5	TPS3	SWI5	YLR346C	SWI6	CIK1
SWI5	TSL1	SWI5	YLR407W	SWI6	CLB5
SWI5	TSR1	SWI5	YLR462W	SWI6	CLB6
SWI5	UBC4	SWI5	YLR463C	SWI6	CLD1
SWI5	VBA3	SWI5	YLR465C	SWI6	CLN1
SWI5	VCX1	SWI5	YML131W	SWI6	CLN2
SWI5	VID30	SWI5	YML133C	SWI6	CRD1
SWI5	VPS55	SWI5	YMR134W	SWI6	CRH1
SWI5	VTH1	SWI5	YMR135W-A	SWI6	CSE1
SWI5	WHI4	SWI5	YMR172C-A	SWI6	CSI2
SWI5	YAL018C	SWI5	YMR173W-A	SWI6	CSN9
SWI5	YAR009C	SWI5	YMR262W	SWI6	CWP2
SWI5	YBL108W	SWI5	YNL046W	SWI6	DIN7
SWI5	YBL109W	SWI5	YNL146W	SWI6	DSF2
SWI5	YBL111C	SWI5	YNL324W	SWI6	DUN1
SWI5	YBR071W	SWI5	YNL337W	SWI6	ECM33
SWI5	YDL032W	SWI5	YNL338W	SWI6	ECM38
SWI5	YDL034W	SWI5	YNR018W	SWI6	ELO1
SWI5	YDL119C	SWI5	YOR262W	SWI6	ERP3
SWI5	YDL173W	SWI5	YOS9	SWI6	FKS1
SWI5	YDL180W	SWI5	YPL158C	SWI6	FKS3
SWI5	YDR112W	SWI5	YRF1-1	SWI6	FLO10
SWI5	YDR543C	SWI5	YRF1-2	SWI6	GAS1
SWI5	YER078C	SWI5	YRF1-3	SWI6	GAS3
SWI5	YER078W-A	SWI5	YRF1-4	SWI6	GAT2
SWI5	YER079W	SWI5	YRF1-5	SWI6	GDB1
SWI5	YER138C	SWI5	YRF1-6	SWI6	GIC1
SWI5	YER189W	SWI5	YRF1-7	SWI6	GIC2
SWI5	YFL063W	SWI5	ZWF1	SWI6	GID8
SWI5	YFL064C	SWI6	AAD6	SWI6	GIM4
SWI5	YFL065C	SWI6	AAP1	SWI6	GIN4
SWI5	YFL067W	SWI6	API2	SWI6	GOR1
SWI5	YFR017C	SWI6	ARG5,6	SWI6	GTR1
SWI5	YGL140C	SWI6	ASN2	SWI6	GTT1
SWI5	YHB1	SWI6	ATO3	SWI6	HCM1
SWI5	YHR138C	SWI6	AYR1	SWI6	HHO1
SWI5	YIL174W	SWI6	BAR1	SWI6	HO
SWI5	YIL177C	SWI6	BBP1	SWI6	HOG1
SWI5	YIR003W	SWI6	BFA1	SWI6	HSL1
SWI5	YJL160C	SWI6	BUB1	SWI6	HXK1
SWI5	YJL225C	SWI6	CCW12	SWI6	IRC22
SWI5	YLL066C	SWI6	CDC21	SWI6	IRR1
SWI5	YLL067C	SWI6	CDC45	SWI6	KSS1
SWI5	YLR012C	SWI6	CDC5	SWI6	LAC1

SWI6	LAP4	SWI6	SNU71	SWI6	YOR246C
SWI6	LCP5	SWI6	SOK2	SWI6	YOR314W
SWI6	LIN1	SWI6	SPO16	SWI6	YOX1
SWI6	MCH2	SWI6	SRL1	SWI6	YPT52
SWI6	MKC7	SWI6	SUT2	TOA1	LEU2
SWI6	MNN1	SWI6	SVS1	TOA2	LEU2
SWI6	MNN5	SWI6	SWE1	UGA3	UGA1
SWI6	MRPL4	SWI6	SWI4	UME6	ACS1
SWI6	MTR4	SWI6	TOK1	UME6	PHR1
SWI6	NDD1	SWI6	TOS2	YAP1	AAC3
SWI6	OCH1	SWI6	TOS3	YAP1	AAD15
SWI6	OPY2	SWI6	TOS4	YAP1	AAD16
SWI6	OTU2	SWI6	TOS6	YAP1	AAD6
SWI6	PCL1	SWI6	TPK1	YAP1	AAP1
SWI6	PCL2	SWI6	TPS2	YAP1	ACA1
SWI6	PDS1	SWI6	TRF5	YAP1	ACO1
SWI6	PDS5	SWI6	TRM2	YAP1	ACS2
SWI6	PET54	SWI6	TSA1	YAP1	ACT1
SWI6	PEX28	SWI6	TYE7	YAP1	ADE2
SWI6	PHD1	SWI6	URA1	YAP1	ADE3
SWI6	PLC1	SWI6	UTR2	YAP1	ADE8
SWI6	PLM2	SWI6	VCX1	YAP1	ADH1
SWI6	PPN1	SWI6	YDL211C	YAP1	ADH2
SWI6	PRE1	SWI6	YDR524C-B	YAP1	ADH3
SWI6	PRE8	SWI6	YER078C	YAP1	ADH5
SWI6	PRY2	SWI6	YGL140C	YAP1	ADH6
SWI6	PUP3	SWI6	YGR012W	YAP1	ADI1
SWI6	QCR10	SWI6	YGR125W	YAP1	AFT1
SWI6	RAD27	SWI6	YHP1	YAP1	AFT2
SWI6	RBA50	SWI6	YIL169C	YAP1	AGA1
SWI6	REX3	SWI6	YJL185C	YAP1	AGP1
SWI6	RFC3	SWI6	YJR030C	YAP1	AHA1
SWI6	RIX7	SWI6	YJR054W	YAP1	AHC2
SWI6	RNR1	SWI6	YKL066W	YAP1	AHP1
SWI6	RNR4	SWI6	YKL096C-B	YAP1	AIM9
SWI6	ROT1	SWI6	YKL161C	YAP1	ALD3
SWI6	RPL18B	SWI6	YKR011C	YAP1	ALD4
SWI6	RPL37A	SWI6	YLR297W	YAP1	ALD5
SWI6	RPL37B	SWI6	YLR301W	YAP1	ALD6
SWI6	SCJ1	SWI6	YML083C	YAP1	ALG1
SWI6	SCW10	SWI6	YMR178W	YAP1	ALG13
SWI6	SER3	SWI6	YMR306C-A	YAP1	ALG7
SWI6	SIM1	SWI6	YNL296W	YAP1	AMD1
SWI6	SKG6	SWI6	YNL313C	YAP1	AMN1
SWI6	SLM4	SWI6	YOL114C	YAP1	AMS1
SWI6	SMC3	SWI6	YOL161C	YAP1	ANB1

YAP1	APA1
YAP1	APJ1
YAP1	APP1
YAP1	ARC35
YAP1	ARE2
YAP1	ARF1
YAP1	ARG1
YAP1	ARG3
YAP1	ARG4
YAP1	ARG5,6
YAP1	ARO1
YAP1	ARO10
YAP1	ARO3
YAP1	ARO4
YAP1	ARO8
YAP1	ARO9
YAP1	ARP5
YAP1	ARR2
YAP1	ARR3
YAP1	ASC1
YAP1	ASE1
YAP1	ASG7
YAP1	ASN1
YAP1	AST2
YAP1	ATG1
YAP1	ATG19
YAP1	ATG26
YAP1	ATG8
YAP1	ATH1
YAP1	ATO3
YAP1	ATP1
YAP1	ATP14
YAP1	ATR1
YAP1	AVT6
YAP1	AYR1
YAP1	AYT1
YAP1	BAP3
YAP1	BAT2
YAP1	BCP1
YAP1	BCY1
YAP1	BDS1
YAP1	BEM2
YAP1	BET3
YAP1	BET4
YAP1	BFR1
YAP1	BIK1
YAP1	BIO2
YAP1	BLM10
YAP1	BNA1
YAP1	BNI1
YAP1	BOP2
YAP1	BSC5
YAP1	BTN2
YAP1	BUD20
YAP1	BUD7
YAP1	CAF130
YAP1	CAF20
YAP1	CAK1
YAP1	CAP1
YAP1	CAR1
YAP1	CBF2
YAP1	CBP2
YAP1	CCP1
YAP1	CCS1
YAP1	CCT2
YAP1	CCT7
YAP1	CCT8
YAP1	CCW12
YAP1	CDC19
YAP1	CDC34
YAP1	CDC48
YAP1	CDC7
YAP1	CEM1
YAP1	CFD1
YAP1	CHC1
YAP1	CHO2
YAP1	CHS7
YAP1	CIS3
YAP1	CLB1
YAP1	CLB3
YAP1	CNS1
YAP1	COF1
YAP1	COS10
YAP1	COS3
YAP1	COX6
YAP1	CPA1
YAP1	CPA2
YAP1	CPR6
YAP1	CRD1
YAP1	CRG1
YAP1	CRH1
YAP1	CRR1
YAP1	CSE1
YAP1	CSI2
YAP1	CTA1
YAP1	CTR2
YAP1	CTS1
YAP1	CTT1
YAP1	CUP1-1
YAP1	CUP1-2
YAP1	CUR1
YAP1	CWC23
YAP1	CWP1
YAP1	CYT2
YAP1	DAK1
YAP1	DAK2
YAP1	DAL2
YAP1	DAL3
YAP1	DAL4
YAP1	DAL5
YAP1	DAL7
YAP1	DAN4
YAP1	DAP1
YAP1	DBP2
YAP1	DBP9
YAP1	DCS2
YAP1	DDI1
YAP1	DDR48
YAP1	DEF1
YAP1	DEP1
YAP1	DFR1
YAP1	DIT1
YAP1	DJP1
YAP1	DLD3
YAP1	DON1
YAP1	DPM1
YAP1	DPS1
YAP1	DRE2
YAP1	DSD1
YAP1	DSE3
YAP1	DSE4
YAP1	DSF2
YAP1	DSL1
YAP1	DST1
YAP1	DTR1
YAP1	DUG1
YAP1	DUG3
YAP1	DYN1

YAP1	ECM13	YAP1	FMP34	YAP1	GPM2
YAP1	ECM16	YAP1	FMP41	YAP1	GPX2
YAP1	ECM17	YAP1	FMP42	YAP1	GRE1
YAP1	ECM21	YAP1	FMP45	YAP1	GRE2
YAP1	ECM23	YAP1	FMP48	YAP1	GRE3
YAP1	ECM38	YAP1	FMP52	YAP1	GRS1
YAP1	ECM4	YAP1	FMS1	YAP1	GSH1
YAP1	ECM40	YAP1	FPR1	YAP1	GSH2
YAP1	EDC2	YAP1	FRE1	YAP1	GSM1
YAP1	EFB1	YAP1	FRE2	YAP1	GSY1
YAP1	EFT2	YAP1	FRE4	YAP1	GSY2
YAP1	EGD1	YAP1	FRE6	YAP1	GTO1
YAP1	EGT2	YAP1	FRE7	YAP1	GTR1
YAP1	EHD3	YAP1	FRE8	YAP1	GTT1
YAP1	ELP6	YAP1	FRS2	YAP1	GUK1
YAP1	EMI2	YAP1	FSP2	YAP1	GUP1
YAP1	EMI5	YAP1	FTH1	YAP1	GUS1
YAP1	EMP70	YAP1	FUI1	YAP1	GUT2
YAP1	ENA1	YAP1	FUR1	YAP1	GZF3
YAP1	END3	YAP1	FYV1	YAP1	HAC1
YAP1	ENO2	YAP1	FZO1	YAP1	HAP1
YAP1	ENT2	YAP1	GAD1	YAP1	HAP3
YAP1	ERG1	YAP1	GAT1	YAP1	HAP4
YAP1	ERG11	YAP1	GCN1	YAP1	HEF3
YAP1	ERG13	YAP1	GCN4	YAP1	HEK2
YAP1	ERG25	YAP1	GCV1	YAP1	HER1
YAP1	ERG26	YAP1	GCV2	YAP1	HHF1
YAP1	ERG28	YAP1	GCY1	YAP1	HHF2
YAP1	ERG3	YAP1	GDB1	YAP1	HHT1
YAP1	ERG5	YAP1	GDH1	YAP1	HHT2
YAP1	ERG6	YAP1	GDH2	YAP1	HIP1
YAP1	ERG9	YAP1	GDH3	YAP1	HIS1
YAP1	ERO1	YAP1	GIC2	YAP1	HIS4
YAP1	EXG2	YAP1	GIS2	YAP1	HIS5
YAP1	FAA3	YAP1	GIT1	YAP1	HMRA1
YAP1	FAF1	YAP1	GLG1	YAP1	HMS2
YAP1	FAR3	YAP1	GLK1	YAP1	HMX1
YAP1	FAS1	YAP1	GLN1	YAP1	HNM1
YAP1	FBA1	YAP1	GLO1	YAP1	HO
YAP1	FES1	YAP1	GLO4	YAP1	HOG1
YAP1	FET3	YAP1	GLT1	YAP1	HOM6
YAP1	FKS1	YAP1	GLY1	YAP1	HOR7
YAP1	FKS3	YAP1	GND1	YAP1	HOT13
YAP1	FMP12	YAP1	GOR1	YAP1	HPF1
YAP1	FMP23	YAP1	GPD1	YAP1	HSC82
YAP1	FMP27	YAP1	GPH1	YAP1	HSH49

YAP1	HSL1	YAP1	KNS1	YAP1	MET30
YAP1	HSP10	YAP1	KOG1	YAP1	MET6
YAP1	HSP104	YAP1	KRE29	YAP1	MF(ALPHA)2
YAP1	HSP12	YAP1	KRS1	YAP1	MGA1
YAP1	HSP150	YAP1	KSS1	YAP1	MGR3
YAP1	HSP26	YAP1	KTI12	YAP1	MKC7
YAP1	HSP30	YAP1	LAP3	YAP1	MKT1
YAP1	HSP42	YAP1	LAP4	YAP1	MMP1
YAP1	HSP60	YAP1	LCB3	YAP1	MMT1
YAP1	HSP78	YAP1	LCB5	YAP1	MNN10
YAP1	HSP82	YAP1	LDB18	YAP1	MOH1
YAP1	HTZ1	YAP1	LDB19	YAP1	MOT1
YAP1	HUG1	YAP1	LEU4	YAP1	MOT2
YAP1	HUL5	YAP1	LEU9	YAP1	MPE1
YAP1	HXK1	YAP1	LHS1	YAP1	MPH1
YAP1	HXT11	YAP1	LOH1	YAP1	MPR1
YAP1	HXT12	YAP1	LSM3	YAP1	MPT5
YAP1	HXT2	YAP1	LST4	YAP1	MRD1
YAP1	HXT3	YAP1	LYP1	YAP1	MRH1
YAP1	HXT5	YAP1	LYS1	YAP1	MRL1
YAP1	HXT9	YAP1	LYS20	YAP1	MRM2
YAP1	HYP2	YAP1	LYS21	YAP1	MRP8
YAP1	IBA57	YAP1	MAE1	YAP1	MRPL11
YAP1	ICY1	YAP1	MAG1	YAP1	MRPL15
YAP1	ICY2	YAP1	MAK21	YAP1	MRPL24
YAP1	IDH2	YAP1	MAK32	YAP1	MRPL4
YAP1	IDP1	YAP1	MAL32	YAP1	MRPL49
YAP1	IES1	YAP1	MAS1	YAP1	MRPL51
YAP1	IES6	YAP1	MBF1	YAP1	MRS1
YAP1	IFH1	YAP1	MCH2	YAP1	MRS4
YAP1	ILS1	YAP1	MCH4	YAP1	MSC1
YAP1	ILV6	YAP1	MCH5	YAP1	MSN1
YAP1	IMD1	YAP1	MDG1	YAP1	MSN2
YAP1	IMD2	YAP1	MDH1	YAP1	MSN4
YAP1	IMD4	YAP1	MDH2	YAP1	MSW1
YAP1	IME2	YAP1	MDJ1	YAP1	MUP1
YAP1	INO1	YAP1	MDJ2	YAP1	MUP3
YAP1	IOC2	YAP1	MDM1	YAP1	MXR1
YAP1	IPP1	YAP1	MDV1	YAP1	MYO1
YAP1	IRA2	YAP1	MET10	YAP1	NAB3
YAP1	IRC6	YAP1	MET13	YAP1	NAN1
YAP1	ISA2	YAP1	MET14	YAP1	NAR1
YAP1	ISU2	YAP1	MET16	YAP1	NBP1
YAP1	ITC1	YAP1	MET17	YAP1	NBP35
YAP1	KAR2	YAP1	MET22	YAP1	NCA3
YAP1	KES1	YAP1	MET3	YAP1	NCE103

YAP1	NDE2
YAP1	NDL1
YAP1	NET1
YAP1	NEW1
YAP1	NFS1
YAP1	NFU1
YAP1	NIP7
YAP1	NIS1
YAP1	NOG2
YAP1	NOP1
YAP1	NOP16
YAP1	NOP58
YAP1	NOP6
YAP1	NOP8
YAP1	NPL4
YAP1	NPT1
YAP1	NRG2
YAP1	NRM1
YAP1	NSR1
YAP1	NST1
YAP1	NTC20
YAP1	NUP100
YAP1	NUP157
YAP1	OCH1
YAP1	ODC2
YAP1	OLA1
YAP1	OLE1
YAP1	OM45
YAP1	OPI10
YAP1	OPT2
YAP1	ORC3
YAP1	OYE2
YAP1	OYE3
YAP1	PAN3
YAP1	PAU21
YAP1	PAU3
YAP1	PAU4
YAP1	PBY1
YAP1	PCL6
YAP1	PDC1
YAP1	PDC6
YAP1	PDE1
YAP1	PDI1
YAP1	PDR16
YAP1	PDR5
YAP1	PDX3
YAP1	PEP12
YAP1	PEP8
YAP1	PER1
YAP1	PEX6
YAP1	PFK1
YAP1	PFK2
YAP1	PFY1
YAP1	PGK1
YAP1	PGM2
YAP1	PHD1
YAP1	PHM7
YAP1	PHM8
YAP1	PHO11
YAP1	PHO23
YAP1	PHO3
YAP1	PHO5
YAP1	PHO8
YAP1	PHO81
YAP1	PHS1
YAP1	PIR1
YAP1	PIR3
YAP1	PKH2
YAP1	PLB1
YAP1	PLB2
YAP1	PLC1
YAP1	PLM2
YAP1	PMP1
YAP1	PNC1
YAP1	POL1
YAP1	POL3
YAP1	POP8
YAP1	PRC1
YAP1	PRD1
YAP1	PRE1
YAP1	PRE4
YAP1	PRE6
YAP1	PRE7
YAP1	PRE8
YAP1	PRM5
YAP1	PRM8
YAP1	PRO2
YAP1	PRP8
YAP1	PRS1
YAP1	PRX1
YAP1	PRY2
YAP1	PSA1
YAP1	PSD1
YAP1	PSE1
YAP1	PSF1
YAP1	PSK1
YAP1	PST1
YAP1	PST2
YAP1	PTC2
YAP1	PTM1
YAP1	PUB1
YAP1	PUF3
YAP1	PUF6
YAP1	PUP2
YAP1	PUT4
YAP1	QCR10
YAP1	QCR6
YAP1	RAD16
YAP1	RAD23
YAP1	RAP1
YAP1	RBA50
YAP1	RCL1
YAP1	RCR2
YAP1	REB1
YAP1	REC102
YAP1	RED1
YAP1	REG1
YAP1	REH1
YAP1	RER1
YAP1	REX3
YAP1	RFA2
YAP1	RGP1
YAP1	RHO3
YAP1	RHO4
YAP1	RHO5
YAP1	RIB1
YAP1	RIB3
YAP1	RIB5
YAP1	RIF1
YAP1	RIM11
YAP1	RKI1
YAP1	RKM4
YAP1	RLM1
YAP1	RMD6
YAP1	RML2
YAP1	RNA1
YAP1	RNH70
YAP1	RNR1

YAP1	RNR2
YAP1	RNR3
YAP1	RNR4
YAP1	ROD1
YAP1	ROG3
YAP1	ROM1
YAP1	ROM2
YAP1	ROT2
YAP1	ROX1
YAP1	ROX3
YAP1	RPA14
YAP1	RPA190
YAP1	RPA34
YAP1	RPB8
YAP1	RPC40
YAP1	RPF2
YAP1	RPI1
YAP1	RPL10
YAP1	RPL11A
YAP1	RPL11B
YAP1	RPL12A
YAP1	RPL12B
YAP1	RPL13A
YAP1	RPL13B
YAP1	RPL14A
YAP1	RPL14B
YAP1	RPL15A
YAP1	RPL15B
YAP1	RPL16B
YAP1	RPL17A
YAP1	RPL17B
YAP1	RPL18A
YAP1	RPL18B
YAP1	RPL19B
YAP1	RPL1A
YAP1	RPL1B
YAP1	RPL20A
YAP1	RPL20B
YAP1	RPL21A
YAP1	RPL21B
YAP1	RPL22A
YAP1	RPL22B
YAP1	RPL23A
YAP1	RPL23B
YAP1	RPL24A
YAP1	RPL24B
YAP1	RPL25
YAP1	RPL26A
YAP1	RPL26B
YAP1	RPL27A
YAP1	RPL27B
YAP1	RPL28
YAP1	RPL2A
YAP1	RPL3
YAP1	RPL30
YAP1	RPL31A
YAP1	RPL31B
YAP1	RPL32
YAP1	RPL33A
YAP1	RPL33B
YAP1	RPL34A
YAP1	RPL35A
YAP1	RPL35B
YAP1	RPL37A
YAP1	RPL37B
YAP1	RPL38
YAP1	RPL39
YAP1	RPL40B
YAP1	RPL41A
YAP1	RPL42A
YAP1	RPL43A
YAP1	RPL4A
YAP1	RPL4B
YAP1	RPL5
YAP1	RPL6A
YAP1	RPL6B
YAP1	RPL7A
YAP1	RPL7B
YAP1	RPL8A
YAP1	RPL8B
YAP1	RPL9A
YAP1	RPL9B
YAP1	RPM2
YAP1	RPN10
YAP1	RPN11
YAP1	RPN12
YAP1	RPN2
YAP1	RPN4
YAP1	RPN5
YAP1	RPN8
YAP1	RPP0
YAP1	RPP1A
YAP1	RPP1B
YAP1	RPP2B
YAP1	RPS0A
YAP1	RPS0B
YAP1	RPS10A
YAP1	RPS10B
YAP1	RPS11B
YAP1	RPS12
YAP1	RPS13
YAP1	RPS14A
YAP1	RPS14B
YAP1	RPS15
YAP1	RPS16A
YAP1	RPS16B
YAP1	RPS17A
YAP1	RPS17B
YAP1	RPS18A
YAP1	RPS18B
YAP1	RPS19B
YAP1	RPS1A
YAP1	RPS1B
YAP1	RPS2
YAP1	RPS20
YAP1	RPS21A
YAP1	RPS22A
YAP1	RPS22B
YAP1	RPS23A
YAP1	RPS23B
YAP1	RPS24A
YAP1	RPS24B
YAP1	RPS25A
YAP1	RPS25B
YAP1	RPS26A
YAP1	RPS26B
YAP1	RPS27A
YAP1	RPS27B
YAP1	RPS28A
YAP1	RPS28B
YAP1	RPS29A
YAP1	RPS29B
YAP1	RPS3
YAP1	RPS30B
YAP1	RPS31
YAP1	RPS4A
YAP1	RPS4B
YAP1	RPS5

YAP1	RPS6A	YAP1	SGT2	YAP1	SRP40
YAP1	RPS7A	YAP1	SHC1	YAP1	SRX1
YAP1	RPS7B	YAP1	SHM2	YAP1	SSA1
YAP1	RPS8A	YAP1	SHP1	YAP1	SSA2
YAP1	RPS8B	YAP1	SHR5	YAP1	SSA3
YAP1	RPS9A	YAP1	SIM1	YAP1	SSA4
YAP1	RPT1	YAP1	SIR4	YAP1	SSB1
YAP1	RPT2	YAP1	SIS1	YAP1	SSC1
YAP1	RPT3	YAP1	SIS2	YAP1	SSE1
YAP1	RRI2	YAP1	SKG6	YAP1	SSE2
YAP1	RRP14	YAP1	SKM1	YAP1	SSK2
YAP1	RSC58	YAP1	SLA1	YAP1	SSL1
YAP1	RSF2	YAP1	SLG1	YAP1	SSL2
YAP1	RSM10	YAP1	SLS1	YAP1	SSO2
YAP1	RSN1	YAP1	SMY1	YAP1	SSS1
YAP1	RSP5	YAP1	SNF4	YAP1	SSU1
YAP1	RTC3	YAP1	SNG1	YAP1	STB5
YAP1	RTS3	YAP1	SNO4	YAP1	STE2
YAP1	RTT109	YAP1	SNQ2	YAP1	STE7
YAP1	RVB2	YAP1	SNR57	YAP1	STI1
YAP1	RVS167	YAP1	SNR60	YAP1	STP1
YAP1	RXT2	YAP1	SNR66	YAP1	STP4
YAP1	SAM1	YAP1	SNT309	YAP1	STR3
YAP1	SAM2	YAP1	SNZ2	YAP1	SUB2
YAP1	SAM3	YAP1	SNZ3	YAP1	SUI1
YAP1	SAM35	YAP1	SOD1	YAP1	SUL1
YAP1	SAM4	YAP1	SOD2	YAP1	SUL2
YAP1	SAS4	YAP1	SOL1	YAP1	SUP17
YAP1	SBP1	YAP1	SOL4	YAP1	SUP35
YAP1	SCP160	YAP1	SPC42	YAP1	SUR2
YAP1	SCT1	YAP1	SPE1	YAP1	SUR7
YAP1	SCW10	YAP1	SPI1	YAP1	SUT1
YAP1	SEC18	YAP1	SPO1	YAP1	SVL3
YAP1	SEC21	YAP1	SPO77	YAP1	SVS1
YAP1	SEC53	YAP1	SPR3	YAP1	SWI3
YAP1	SEC66	YAP1	SPS1	YAP1	SWI5
YAP1	SED1	YAP1	SPS4	YAP1	SYC1
YAP1	SEM1	YAP1	SPT15	YAP1	TA(AGC)K2
YAP1	SEN2	YAP1	SPT20	YAP1	TAF13
YAP1	SEO1	YAP1	SPT23	YAP1	TAL1
YAP1	SER1	YAP1	SPT5	YAP1	TAT1
YAP1	SER3	YAP1	SPT7	YAP1	TCB2
YAP1	SER33	YAP1	SRL1	YAP1	TDH1
YAP1	SFA1	YAP1	SRO9	YAP1	TDH2
YAP1	SFG1	YAP1	SRP1	YAP1	TDH3
YAP1	SFP1	YAP1	SRP21	YAP1	TEF4

YAP1	TEM1
YAP1	TFP1
YAP1	TFS1
YAP1	THI12
YAP1	THI13
YAP1	THI2
YAP1	THI5
YAP1	THR1
YAP1	THR4
YAP1	TIF1
YAP1	TIF11
YAP1	TIF3
YAP1	TIP1
YAP1	TIP20
YAP1	TIR4
YAP1	TKL1
YAP1	TKL2
YAP1	TMA10
YAP1	TOS2
YAP1	TOS6
YAP1	TPO1
YAP1	TPO2
YAP1	TPO4
YAP1	TPS2
YAP1	TRP2
YAP1	TRP4
YAP1	TRR1
YAP1	TRX2
YAP1	TRX3
YAP1	TSA1
YAP1	TSA2
YAP1	TSL1
YAP1	TSR1
YAP1	TUB2
YAP1	TVP23
YAP1	TW(CCA)G1
YAP1	TYE7
YAP1	UBA1
YAP1	UBA3
YAP1	UBC13
YAP1	UBI4
YAP1	UBP2
YAP1	UBP5
YAP1	UBP6
YAP1	UBR1
YAP1	UBX4
YAP1	UFD2
YAP1	UFO1
YAP1	UGA1
YAP1	UGA3
YAP1	UGX2
YAP1	UMP1
YAP1	URA1
YAP1	URA10
YAP1	URA2
YAP1	URA3
YAP1	URA7
YAP1	USA1
YAP1	UTP15
YAP1	UTR1
YAP1	UTR2
YAP1	VBA5
YAP1	VEL1
YAP1	VHR1
YAP1	VID24
YAP1	VID27
YAP1	VID30
YAP1	VMA7
YAP1	VPS29
YAP1	VPS55
YAP1	VPS61
YAP1	VPS71
YAP1	VTC1
YAP1	VTC2
YAP1	VTC3
YAP1	WHI2
YAP1	WSC4
YAP1	WTM1
YAP1	YAP1
YAP1	YAP1801
YAP1	YAP5
YAP1	YAP6
YAP1	YAR009C
YAP1	YAR075W
YAP1	YBL028C
YAP1	YBL044W
YAP1	YBL073W
YAP1	YBL086C
YAP1	YBL109W
YAP1	YBR053C
YAP1	YBR071W
YAP1	YBR085C-A
YAP1	YBR134W
YAP1	YBR138C
YAP1	YBR139W
YAP1	YBR190W
YAP1	YBR197C
YAP1	YBR285W
YAP1	YCF1
YAP1	YCL042W
YAP1	YCL056C
YAP1	YCR006C
YAP1	YCR018C-A
YAP1	YCR024C-B
YAP1	YCR099C
YAP1	YCR102C
YAP1	YCT1
YAP1	YDJ1
YAP1	YDL144C
YAP1	YDL180W
YAP1	YDL183C
YAP1	YDL211C
YAP1	YDL233W
YAP1	YDR010C
YAP1	YDR042C
YAP1	YDR089W
YAP1	YDR124W
YAP1	YDR132C
YAP1	YDR154C
YAP1	YDR157W
YAP1	YDR186C
YAP1	YDR266C
YAP1	YDR391C
YAP1	YDR524C-B
YAP1	YDR544C
YAP1	YEL007W
YAP1	YEL008W
YAP1	YEL045C
YAP1	YEL047C
YAP1	YEL074W
YAP1	YER053C-A
YAP1	YER067W
YAP1	YER078C
YAP1	YER079W
YAP1	YER130C
YAP1	YER138C
YAP1	YER156C
YAP1	YER189W

YAP1	YFL034W	YAP1	YKL070W	YAP1	YNL092W
YAP1	YFL042C	YAP1	YKL083W	YAP1	YNL134C
YAP1	YFL063W	YAP1	YKL118W	YAP1	YNL143C
YAP1	YFL065C	YAP1	YKL151C	YAP1	YNL155W
YAP1	YFR017C	YAP1	YKR011C	YAP1	YNL174W
YAP1	YFR018C	YAP1	YKR041W	YAP1	YNL176C
YAP1	YGL036W	YAP1	YKR075C	YAP1	YNL179C
YAP1	YGL101W	YAP1	YKR096W	YAP1	YNL208W
YAP1	YGL114W	YAP1	YLL032C	YAP1	YNL213C
YAP1	YGL117W	YAP1	YLL056C	YAP1	YNL234W
YAP1	YGL140C	YAP1	YLL058W	YAP1	YNL324W
YAP1	YGL188C	YAP1	YLL066C	YAP1	YNR014W
YAP1	YGL242C	YAP1	YLL067C	YAP1	YNR018W
YAP1	YGP1	YAP1	YLR041W	YAP1	YNR062C
YAP1	YGR011W	YAP1	YLR042C	YAP1	YNR068C
YAP1	YGR067C	YAP1	YLR073C	YAP1	YNR070W
YAP1	YGR117C	YAP1	YLR076C	YAP1	YOL019W
YAP1	YGR125W	YAP1	YLR108C	YAP1	YOL048C
YAP1	YGR130C	YAP1	YLR159W	YAP1	YOL114C
YAP1	YGR146C	YAP1	YLR164W	YAP1	YOL157C
YAP1	YGR210C	YAP1	YLR179C	YAP1	YOR008C-A
YAP1	YHB1	YAP1	YLR257W	YAP1	YOR052C
YAP1	YHK8	YAP1	YLR297W	YAP1	YOR059C
YAP1	YHR033W	YAP1	YLR301W	YAP1	YOR093C
YAP1	YHR095W	YAP1	YLR339C	YAP1	YOR152C
YAP1	YHR097C	YAP1	YLR345W	YAP1	YOR248W
YAP1	YHR138C	YAP1	YLR346C	YAP1	YOR292C
YAP1	YIL057C	YAP1	YLR352W	YAP1	YOR302W
YAP1	YIL108W	YAP1	YLR366W	YAP1	YOR309C
YAP1	YIL169C	YAP1	YLR414C	YAP1	YOR314W
YAP1	YIL174W	YAP1	YMC1	YAP1	YOS1
YAP1	YIR014W	YAP1	YML050W	YAP1	YOS9
YAP1	YJL028W	YAP1	YML083C	YAP1	YOX1
YAP1	YJL045W	YAP1	YML131W	YAP1	YPI1
YAP1	YJL144W	YAP1	YMR090W	YAP1	YPL080C
YAP1	YJL160C	YAP1	YMR1	YAP1	YPL088W
YAP1	YJL175W	YAP1	YMR144W	YAP1	YPL277C
YAP1	YJL213W	YAP1	YMR147W	YAP1	YPP1
YAP1	YJL218W	YAP1	YMR172C-A	YAP1	YPR013C
YAP1	YJR096W	YAP1	YMR173W-A	YAP1	YPR015C
YAP1	YJR115W	YAP1	YMR196W	YAP1	YPR036W-A
YAP1	YJR146W	YAP1	YMR244W	YAP1	YPR063C
YAP1	YKL030W	YAP1	YMR258C	YAP1	YPR064W
YAP1	YKL044W	YAP1	YMR295C	YAP1	YPR099C
YAP1	YKL066W	YAP1	YNL057W	YAP1	YPR202W
YAP1	YKL069W	YAP1	YNL086W	YAP1	YPT1

YAP1	YRB1	YAP1	ZEO1	YHP1	IME1
YAP1	YRO2	YAP1	ZIM17	ZAP1	FET4
YAP1	YSA1	YAP1	ZPR1	ZAP1	ZAP1
YAP1	YSC84	YAP1	ZRG8	ZAP1	ZRT1
YAP1	YSR3	YAP1	ZRT1	ZAP1	ZRT3
YAP1	YTM1	YAP1	ZTA1		
YAP1	YTP1	YAP1	ZWF1		

Candidate sequence motifs

TF	Candidate sequence motif
ABF1	AGCCGTAAATAGTTATCTTCCAAG
ABF1	RTCRYYYN{3}ACG
ABF1	RTCRYBN{4}ACG
ABF1	TCN{7}ACG
ABF1	RTCRN{6}ACGNR
ABF1	TCRTN{5}AYGA
ABF1	TNNCGTNNNNNNTGAT
ACA1	TGACGTCA
ACA1	TTACGTAA
ACE2	ACCAGC
ADR1	TTGGRGN{6,38}CYCCAA
ADR1	TTGGRG
AFT1	YRCACCCR
AFT1	TGCACCC
AFT1	GGCACCC
AFT1	TGCACCCA
AFT2	YRCACCCR
AFT2	TGCACCC
AFT2	GGCACCC
AFT2	CGCACCC
ARG80	CCTCTAAAGG
ARG81	WWN{3}YARNHN{3}VVCGR
ARG81	AAGTACAGTTAATAACGA
ARG81	AAGTACAGTTAATAACGG
ARG81	AAGTGCAACTGACTGCGA
ARG81	AATGGAAATGGATAGCGA
ARR1	TTAATAA
ASH1	YTGAT
AZF1	AAGAAAAA
AZF1	AAAAGAAA
BAS1	TGACTC
CAD1	TTACTAA
CAT8	NCCDTYNVNCCGN
CAT8	YCCNYTNRRCCGN
CBF1	RTCACGTG
CBF1	RTCACRTG
CHA4	CGGN{10}CCG
CHA4	CGGN{10}CCA
CIN5	TTACTAA
CRZ1	CACCAGTCGGTGGCTGTGCGCTTG
CRZ1	GAATGGCTG
CRZ1	GGGTGGCTG
CRZ1	GNGGCKCA
CST6	TGACGTCA
CST6	TTACGTAA
CUP2	HTHNNGCTGD
CUP2	GCGTCTTTTCCGCTGA
CUP2	TCTTTTGCTG
CUP2	TCTTTTTGCTG
CUP2	TCTTTTTTGCTG
DAL80	GATAAGN{15,20}GATAAG
DAL81	SAAAWNTGCGBT
DAL81	GAAAATTGCGTT
DAL81	CGGCN{6}GCCG
DAL82	SAAAWNTGCGBT
DAL82	GAAAATTGCGTT
ECM22	TCGTATA
FKH1	GTMAACAA
FKH1	RTAAAYAA
FKH1	RYMAAYA
FKH1	RYAAACAWW
FKH2	GTMAACAA
FKH2	RTAAAYAA
FKH2	RYMAAYA
FKH2	RYAAACAWW
FLO8	TTTGCN{97}GCAAA
FZF1	CGTATCGTATAAGGCAACAATAG
FZF1	YGSMNMCTATCAYTTYY
GAL4	CGGN{11}CCG
GAT1	GATAAG
GCN4	TTACGTAA
GCN4	TGATTCA
GCN4	TGACTGA
GCN4	TGACTMT
GCN4	RRTGACTC
GCN4	TGACTC
GCN4	TGASTCA
GCN4	TTGCGCAA
GCN4	TTGCGTGA
GCN4	GCACGTAG
GCN4	CACGTG
GCR1	CTTCC

GCR1	CWTCC
GIS1	TWAGGGAT
GIS1	AGGGG
GLN3	GATAAG
GLN3	GATTAG
GSM1	CGGNNNNNNNNCGG
GSM1	CGGNNNNNNNNNCGG
GZF3	GATAAG
HAC1	CAGCGTG
HAC1	ATGGTATCAT
HAC1	TGACGTCA
HAC1	CCAGC
HAP1	CGGN{3}TANCGG
HAP1	CGGN{6}CGG
HAP1	CGGN{3}TANCGGN{3}TA
HAP2	TNATTGGT
HAP3	TNATTGGT
HAP4	TNATTGGT
HAP5	TNATTGGT
HCM1	WAAYAAACAAW
HSF1	NGAANNTTCN
HSF1	NTTCNNGAAN
HSF1	NGAANNTTCNNGAAN
HSF1	NGAANNNNNNNGAANNNNNN NGAAN
IME1	TTTTCHHCG
INO2	ATGTGAAAT
INO2	CATGTGAAAT
INO2	WYTTCAYRTGS
INO4	ATGTGAAAT
INO4	CATGTGAAAT
INO4	WYTTCAYRTGS
INO4	TYTTCACATGY
KAR4	YAANNCAAANNCNGNYT
KAR4	CTAANNCAAANNCNGNYT
KAR4	TTAANNCAAANNCNGNYT
LEU3	CCGN{4}CGG
LEU3	CCGGNNCCGG
LEU3	CCGTTAACGG
LYS14	TCCRNYGGA
MAC1	TTTGCKCR
MAC1	WWWTTTGCTCR
MBP1	ACGCGT
MCM1	TTACCNAATTNGGTAA
MCM1	TTWCCYAAWNNGGWAAWW
MCM1	DCCYWWWNNRG
MCM1	CCYWWWNNRG
MCM1	CCTAATTAGG
MET31	AAACTGTGG
MET31	AAACTGTG
MET32	AAACTGTGG
MET32	AAACTGTG
MET4	TCACGTG
MIG1	AATTRTCCGGGG
MIG1	TATAGTGCGGGG
MIG1	ATTTTTGTGGGG
MIG1	GAATAYCTGGGG
MIG1	TTATTTCTGGGG
MIG1	TAAAAGCCGGGG
MIG1	TTAAAAGCGGGG
MIG1	TTAAACGTGGGG
MIG1	ATAAAACTGGGG
MIG1	ATAAACGCGGGG
MIG1	ATTAATGTGGGG
MIG1	AAAAATGCGGGG
MIG1	ATTTTGCGGGG
MIG2	AATTRTCCGGGG
MIG2	TATAGTGCGGGG
MIG2	ATTTTTGTGGGG
MIG2	GAATAYCTGGGG
MIG2	TTATTTCTGGGG
MIG2	TAAAAGCCGGGG
MIG2	TTAAAAGCGGGG
MIG2	TTAAACGTGGGG
MIG2	ATAAAACTGGGG
MIG2	ATAAACGCGGGG
MIG2	ATTAATGTGGGG
MIG2	AAAAATGCGGGG
MIG2	ATTTTGCGGGG
MIG3	AATTRTCCGGGG
MIG3	TATAGTGCGGGG
MIG3	ATTTTTGTGGGG
MIG3	GAATAYCTGGGG
MIG3	TTATTTCTGGGG
MIG3	TAAAAGCCGGGG
MIG3	TTAAAAGCGGGG
MIG3	TTAAACGTGGGG
MIG3	ATAAAACTGGGG
MIG3	ATAAACGCGGGG

MIG3 ATTAATGTGGGG
MIG3 AAAAATGCGGGG
MIG3 ATTTTGCGGGG
MOT3 WAGGTA
MOT3 CAGGYA
MOT3 AAGAGG
MOT3 ATGGAT
MOT3 TAGGTA
MOT3 TAGGAT
MOT3 TMGGAA
MOT3 AAGGKA
MOT3 AAGGWT
MSN2 CCCCT
MSN4 CCCCT
MSS11 TTTGCN{97}GCAAA
NDT80 GNCRCAAAW
NRG1 CCCCT
NRG1 CCCTC
OAF1 CGGN{3}TNRN{8,12}CCG
PDR1 TCCGCGGA
PDR1 TCCGTGGA
PDR1 TCCACGGA
PDR1 TCCGCGCA
PDR1 TCCGCGGG
PDR3 TCCGCGGA
PDR3 TCCGTGGA
PDR3 TCCACGGA
PDR3 TCCGCGCA
PDR3 TCCGCGGG
PDR8 TCCGHGGA
PHO4 CACGTK
PHO4 CACGTGGG
PIP2CGGN{3}TNRN{8,12}CCG
PPR1 CGGN{6}CCG
PUT3 CGGN{10}CCG
RAP1 ACACCCRYACAY
RAP1 ACACCCAYACAYYY
REB1 CCGGGTAA
REB1 TGTTACCCGT
REB1 CCGGGTGGAT
RFX1 TCGCCATGGCAAC
RFX1 TTGCCATGGCAAC
RFX1 TCGCCATGACAAC
RFX1 CTATTGCTGCAAC
RFX1 TCTCTGTGGCAAC
RFX1 TTGTCACAGCAAC
RFX1 TTGTCGCAGCAAC
RFX1 TTGTTGTGGCAAC
RFX1 TTTCCACAGCAAC
RFX1 TYGCCATGCCAAC
RGT1 CGGANNA
RLM1 CTAWWWWTAG
RLM1 TAWWWWTAGM
RME1 GTACCACAAAA
RME1 GTACCTCAAAA
RME1 GAACCTCAARA
ROX1 TCTATTGTTTCCC
ROX1 YYYATTGTTCTC
RPH1 CCCCT
RPN4 GGTGGCAAA
RTG1 GTCAC
RTG1 GGTAC
RTG3 GTCAC
RTG3 GGTAC
SFL1 AGAANTTCC
SIP4TCCATTSRTCCGR
SIP4NCCDTYNVNCCGN
SIP4YCCNYTNRRCCGN
SKN7 GGCYGGC
SKN7 AGAACGTTC
SKN7 ATTTGGYTGGGCC
SKN7 GGCGAGATCT
SKN7 GGCCCAGA
SKN7 GGCCGGC
SKN7 GGCCAGA
SKN7 GGCTGGC
SKO1 TGACGTTT
SKO1 TGACGTCA
SKO1 TTACGTAA
SMP1 ACTACTAWWWWTAG
STB5 TCTCCGCGAAC
STB5 CGGNS
STE12 TGAAACA
STP1 CGGCTC
STP1 CGGCN{6}CGGC
STP2 CGGCTC
STP2
CGGGGTGNNNNNNNCGCACC
G
STP2 CNCACCNG
SUM1 GNCRCAAAW
SWI4 CACGAAA

SWI4	TTTTCGCT	XBP1	CTCGA
SWI5	ACCAGC	YAP1	TTACTAA
TEC1	TTCTCACATTCTTC	YAP1	TGACTCA
TEC1	CATTCT	YAP1	TGACTAA
TEC1	CATTCC	YAP1	TKACAAA
UGA3	SGCGGNWTTT	YAP3	TTACTAA
UME6	TAGCCGCCGA	YAP3	TGACTCA
UPC2	TCGTATA	YRR1	WCCGYKKWW
UPC2	TCGTTYAG	ZAP1	ACCYYNAAGGT
WAR1	CCGN{23}CGG		

Upstream sequence of target genes

Target	Upstream sequence
ACADM	CCACTACTGGGACTGTGGACTACAGAATTTCTACTTGAGAGGCATT<br>TCTGCCTTGGAATGGGACATTAACAGAAGCTATCCCTATGACTGGA<br>GGACATAAAATGATCTTGACACCTAAAATACACATGCTGTCTGGAG<br>TGATGTCAGAGAAACACTCTAACGGGGATAGCAGTGCCCAGAAGA<br>GTACATAATAAAATGGGAATGGATCTTGCTACCTGAGGAGTGCAAA<br>GAGGATATACTCATGAGCTGCGAGCCTCTTTCTCTCCTAGGACTGAT<br>CCTGGAACTGGTGAGGAGTTGCTGGATACCATAGTACCTGACAAAC<br>AGCTCTAACCTAAGAGTTGCTTGGCTTGTGGATGGCAGTTCCAAGG<br>TCATCAGGCAACATCCTGTTTGGAAGGCATTACTCTGATTGAAGAA<br>GAGTCAAGAAAACTTTTTTGTTTTGAGCTCTTTATAACTTATAATGA<br>TTAAGTGTATTTTTGTAAGCAAAATTTGTCCTTCTCTCTATATGAGTT<br>CTCCCAAATTTGAAAACTATTTGTGTTATTTTATGGCAATATAGTTAT<br>GTACATATGTTCAATATGAATGTTTTCTTTTGTAATAGGACACAATTG<br>GAGACACATTATTTTACCAAGGCTTTGACTGGAATGGTCTATTTTCA<br>GACATGATCAATCTGCTTGGGGAATTGAAGTTGACTGCAGAGAGCC<br>AATAAAAAGCCCATGGAAAGACTGGTTCCCTAAGAAGTTTCCTGAC<br>CCGTGGTAAGTAATGAATGTCACTTTCTAACAGGATCAGAAATCTC<br>AAGATATTTTGAGACCTCAAGAAGAGAGAAATTTGTACAGGTATTA<br>TAAATGCAGTCTAATGGTGAATCCTTGGCTTTGCTTCTGGCGTTGAG<br>GCTTTAAAACACTGGAATAAAAAATTCTGATAGTTCTAGCAAAGCC<br>AACTTAAAAGGAGCCTATATGGCTGATCACTATTCCTGCTACAATTT<br>ATTCAAATAACCAGGCCAATATAATGAGACAAATTTATTTTGCAAAT<br>AAATTGGTCCTAGTATAATTTATCCTTGGTAGAAATAAGAAAACTGG<br>AGAAAAAAAAATCTTAGATTCTAGCCCTGACCACTGCTTTTGAGTT<br>TTTATAATTTGCCTATAATTTGGACTAAATCCTGAATTATTTCCTGGCT<br>ACAAGAAGTCTCTAAAAAGGAACTTGGTTTTAATTTTCTTCATGAT<br>GCTTTTAGTTGGCTGCTTACTCGAATAGGCTCCTTTCATTTTTGTTCT<br>GGCATACAAATTCTCTTCTGATTGTAATCCTTGTGTGGATTGTATTTC<br>TACTATTCAAATTATTGATGCTATGTATCTTTTGTTGTTTTACTTCCGA<br>GAAAACTAAAGTCATAGTATTCTGAAGACTAGAGAAATTCAACGAA<br>GCCTGTCAATCTCCCTTGTTTGAAATCCCAAGGAGTATGTTGGCAAT<br>GAGAAACCAATATGGCCCAGTGGGATTTCAAAGGCTCTGGGACCAT<br>TGCAAAAAAGAAGGGTGCGTGAGACTCTACACATGAATTAGTGGA<br>ATGTGGAGGACAAAGCAACGCCACCTAGGATGTTAATCCACCATGT<br>GGACTTCCCCTGTTCTGGGAAGGCTTCTAAGATTTCCCCTCTCTCTA<br>TTGTTTCTTGTGTAAAAGCATGTACTTACTGTAAATCCTGCCCTTAG<br>ATCAAGACAACTTTGATGTTATTGTATTTCAATTGTCCTACATGTCCC<br>TTCTGAGTCACCCTTTCCCTGTGGTATACAACCCCTGGGTCTGAGG<br>GGTAATGGCACTGGGATCCACCATCTTGTCTCACTGCCGCCTGAGA<br>TGCAGACAGGGCTTCTGTTTTTAAGTCTCCATTAAATGTTTCTTTTT<br>GAGAAACGTTAATGTGTCAGCCTCTTTCTTTAGCCTCTCAGCTTCCT<br>TAGACTTTGGAGCGTAGGTCTGTATAGTCCTGCTTACCTTGAAATAG<br>ATGGGTTAGGGAGAAGGAACACCAGGGAGACGTTCGAATTATTTC<br>ACCTAAAAATGTAGAGAAGTTTTGTGAAACAGTGCTTAATTAATAA

	AAAGGTATTTCTTGAGAGATGGTTCTATTCCTACTACTGGCAAAAT<br>GTAGATGAAAGATTGTTTGGCATGATGCCAAATATTACCCTATAAATT<br>ACTTCCTAATTGCAAAGGGAAAACTATAACTTTACCATAGAAAGAA<br>CTGTCTGTCAATAATCAAATGATCAAATAGCATTGTCAACAAAGAG<br>TCAAACTTTGTAAAATATTTGAAGAGATTTATTCCGAGCCAAATATG<br>AGTGATCATGGCCCACGACACAGTCCTTGGGAGATCCTGAGAACAT<br>GTGCTCAGGGTGGTTGGGGTGCAGGTTGGTTTTATATATTTTAGGGA<br>AATATGAGACTTCAATCAAATACATTTAAGAAATACATTGGTTCAGT<br>TCAGAAAGACAGGACAACTTGAAGCAGGGGTGGAGCTGGGGGGG<br>TAGGCGGGGGGAGGTTCCAGGTTATAGGCAGATTTAAACATTTTCT<br>GGCTGACACTTGGTTAAGTTTATCTAAAGACCTGGAACCAACAGAA<br>AGGAAATGTCTGGGTTAAGATTAAAGGATTGTGGAGACCAAAGTTC<br>TTATTGTGCAAAGGAAGCCTTCAGGTGAGAACAGAAAAGCATTCT<br>GTTATCTCTCTCCTTCAGAGAGAAGAGATTGTAAATGTTTCTTATCA<br>GACTTAAGGTCTGTGTTGATGTTAACATCACTGAGGTATAAAGAGG<br>CATGTCCAACCCCCACTTCCCATCATGGCCTGAAGTAGTCTTTCAG<br>GTTAAATTTAAATTTTAGAGTGCCCTAGCCTACGAGAAAGTCCATTC<br>AGATAGTTGGGGGGAATTTAGAATTTTGTTTTTGGTTTACAGCAAG<br>CTTGTCCAACCTGCTGCCCACAGACCACATGCAACCCACAGGCCA<br>CATGCAGCTCAGGACAGCTTTGCATGCAGCCCAACATAAATTTGTA<br>AGCTTTCTTAAAACGTTATGAAATTTTCTTGCGTTTTTGTTTTGGTTT<br>GTAGCTTATCAGTTAACATTAGTGTTAGTGTATTTTATGTATAGCCCA<br>AGACAATTCTTTTTCTTCCAGTGTGGCCCAGGGAAGCCAAAAGATT<br>GGACACTGCTGGCTTACACCATCACCAATAGTGGGACAAATCTGAT<br>ATTAAATGCCTCCTAATGTAATACAATATTAACTACACAGTATGACCT<br>TTGAAGTATCCTTATCCAATGTTTAATCTGAACTCCTCAAGCCTCTG<br>GTAACTTGCAACTGATAGGAAATACAAAATCTAGAGGTACAAAATA<br>AATAAAATCGGACAATCCAGCATTAGGGCATTCTTCATGACAACTG<br>CTTGGTATCTTAAATTTGTTTAAAAAGACAGTTATTATTGGGAAAAA<br>TGAATTAGATTAAAAGCTTTAGATTAAAAGAAGCCAAAGAAAGTTA<br>AAGCCCAACTTCAATGGGCAATCATTCATAGGATCCTGGTTCAAAA<br>ACAAACTATTTACAAAAGGTATTCTGGAGACAACTGGACAACTGG<br>AGAAATGTAAATATTCTCTGGACCTTAAATAATAGTAGCGAATAATT<br>GTTAATTTTCATTGATGTGATAAAGGTTTTATAGGAAATGGTTATATA<br>GGAAAATGTCCTTGTAGGAGACATATAGCTAAAGTATTCTGAACTG<br>AAGTGTCTTATCTACAACCTACAGATCTGAGAAAAATAAAACCAAC<br>AACCAAAACACACACACATAGGTACACATATAAATATATAAATAAAT<br>ATGGAAAAATATTTGAATCTAGGTGATAATGATATTGAGAAGAAATT<br>ACTCAGGCCGATAGTACGAGTATGGGAGTCCTCAGTGTAAAAAGTG<br>AAGTAGAGGTTCCTCTTCAAATGGACTTTCCTTTCCGTCTAATTAGG<br>AATAAACAGTAACTTCTCTTAAAAGCAAAATTTATTCAAAGAACTG<br>TGCTAACATATTGGAGGCCGAAACAATGAGGTTCGTGATCAACTCA<br>GTATATCACTGGAGGCTATATGAGTAAGCAGCAAACTGTTTCTCATA<br>AATGCAGAACGTTGGCGAACTGACAAACTGCGTATGCCACCCAGG<br>AGGACTGCTGAGGGTAGTTACGATCTAGGCACAAATGTTTCTTATG<br>ATTAGGCATAATTGAAGCCTGTCAGTAACAATATGAACCTGTGATCA

	ATTAAGCAGCTGACCAATCGTTACCACCTCCTCCCTGCTCCTGTTAC<br>CCAAAAAATAGGAAGGGCTGTGGAAGCTCTGTGGCTGCCTTTGCT<br>CACGAGAAGCAGGGAGCTCTCTTCTTCTTCCCCTGGCCCTTTCCTT<br>AAAACAGTTCCTTTTGTCTTAAGTTATCATTTCTACGTTCGTCCCTT<br>CATTTGATCTCACAATGATGGTCTCAAGCAGTAACAGTAGTAACTG<br>CTGTAACGATGGTCTCAAGTAGTAACCGTGGCAGTTGGCCACACTA<br>ACATTCTTTGATATCTGTTGGCCGTAATAAAGAAATCAATGTACTTT<br>GTGCTCTTAGCTGCCACATTTTAGCCTAGATATTTGCCCTGGCATGC<br>TTATACTGGTCCAAGGAAGCATTAGGCCATAGCCTGTTTTCTTCCTT<br>ATCTGGAGGTGTTTTTACCTTTCTCAGCATTCCACAAGTTGCTTCCT<br>CCTTCCTTTGTTCTCCTCTGTCTTTGCCTCTTTTGGAAAGTTCTAAG<br>TTGCTAGCCAATCAGGACAAGTACAGAGGTCCCCTTCCAGCCAATG<br>GAAACCGGACACGGTAGTAGGGTGCACGTGTCAGGTTATAAATGA<br>CCCTGTCTCCTTTATGCGTGTGTGCTCTCGTGGCAAGACTACTAGCG<br>AGCGGCAACCTTTCTGCAGAAAGTAAACTAGCCTTGCTAAGAGCT<br>CCTTTGTCTCAGTGTTGATTTTTGCAACACTGAGCGCCCGTTCCCA<br>ACATCAGTAAGGCTTTTCTTTTTAATGAAAAGCAGCCCCAAATCCTT<br>TTCTAAGAGCAGCCTGTGAAGTCGAGCTGCAGGCACAGACAAACA<br>AGGGGG
ACAT2	TAAGGGGTGTTAGAATGTAGGTAGGAGAAAATGTTTTTAAGGTGGA<br>AGAATGTCAAAATTTGCTCCATTTTCTCAGAGGTAATTAAGTATAAA<br>AGTATGATATTGAATGGCCTTCAAATGTGTAGCTTTCATATATGGTTG<br>AAATGAAATTCCATTTCTGTTAAAAGCAAAGTATTGTTTTAGACATA<br>ACTTGTAATACAATCAAGGAACCTTGGGAGTGGAGTTATAATACTC<br>GAAAACTGAGGTACAACTTATTTGAAAGTAATTGCTGCTTTTGCTT<br>GTTTAGCTGATACAGGTATATCCGGTCTTCAGCAGTATGTTAAATAC<br>TGCACTGTCTCTTGATTAACCTTTTCTTTTTTTTCTTTTTCTCTTTTTT<br>TTTTTTGCCGAGAGTCTCGCTCTGCCACCCAGGCTGGAGTGCAGTG<br>GCACCATCGTGGCTCACTGCAACCTCCACCTCCCGGATTCAAGTAA<br>TTCTCCTGCCTCAGACTCCTGAGTAGCTGGGATTACAGGCGTGCGC<br>CACCATGCCCGGCTTATTTTTGTATTTTTAGAAGAGATGAGGTTTCA<br>CTGTGTTGGCCAGGCTGGTCTCGAACTCCTGACCTTGTGATCCACC<br>CACCTCGGCCTCCCAAAGTGCTGGGATTACAGGTGTGAGCCACCG<br>CGCCCAACCTTGATTAACCTTTTCTACAGTCTGCTCAAGTGAGTAA<br>AAAATCCTATACGATTTTTAATTTGCATGTGATTAAACATTTTGGTGT<br>TTGATATATCAGATACTGTGTTTGGCTTTTGTTATGTACATCCTTCAA<br>CTTGACCAATTTAAACAAAAATGTAATTTCTCTGTCCTGTTGGTGTA<br>ATGTAATCAAGAGTTTGTGATAGGAAATTAATTTTTCAACCAGTGAT<br>ACAATTTTATGACTGAATTTAATATGGACCTGTTAATGTGACATTTTC<br>ATGTTTCTGTTGAGTCTGCCTTGATGAATAACTCAGAGCACTTATTC<br>TGTGGTACACAGAGTCAGTATTTCTCATTTCTGGAATTGGATGCTTT<br>ATTTTCTGGTATGCCTGTGATAAGAGAGGTGATAGAGTGGCTTCTGC<br>ATCTAATTGGTTTGAATCCTGGTATTACACTTAACTGATTTGGGCAA<br>ATGTATCCTCGAGGCTCGATATTTTGTTTTGTTTTTCTCCCCTGTAAG<br>AGCACTCACTGCTGTCTTAGCTTGGGCTGCTGTGAAAAAAAAAAA<br>AAACCACAGACTGGGTGGCTTTACACGCGCATTCCTCACTGTTTTT

	GGAGGCTGGGAAGTCTAAAGATCACAGTGCCCCCTGATTGGTGCC<br>CCAGTTTGGTTCCTGGTAGTCCTCATACAGAGAGCGCAATAGATGT<br>CAGTTTCCTTGGCTCCTCAGGTGTATTGGATACAACTGACTACTCCA<br>GTTTTTGAGACACTTTTGCAAAACAATGTAAAGCCATTCCTTCTGA<br>CAACTTACTTTCTCAGATGTTCCCTCTTTCATGAGTAGTTATGCCCT<br>GTACTCAAATCATTGCCTGCTGCTCTACGTCACTGTCTTGGGAGTTC<br>ACCTATCCCTGTGGTTATAGTGAACACTTTCATAATCCCAGGTAATG<br>TCATTAACTGATCTGGACCCATCATGGGGTGGCTGTGCTGAGGGAG<br>TGTTGAGCTAGCCCCTAGTGGTAAGGAGTACACTGCTGACCTTCAT<br>CCTATGGCAATTCCACTTTGGTGATTCACTGTTCCCCTCATGGAGAC<br>CAAGGAGGAATTTGCTGCATGGTAGGTTGGTGAATCCAGCCTGTAG<br>CAAAGACCTGGCTTGGACTCCTGCAACTCTTTATGAAACTTAACCA<br>AGAATGATTGCATCTTCCAGCGGGGGAAGATGCTGAGAAGGCCCC<br>TTCAGGATAAACTGGCTCCATAGCACTGCTGTAATGATGGCTCCTCA<br>TGAGTAAGCACTTGGAGATTTTTGTGGCTTCCTGCCCACTCACCCT<br>GCTCACCAGTCCAGGTAGTTTATGATTTGAGATCTAGATGTAATTTT<br>ATTTATTTCTTTTTTTTTTTTTTGAGATGGAGTTTCGCTCTTGTTGCC<br>CAGGCTGGAGTACAATGGTGCAATCTCGGCCCACCACAACCTCCG<br>CCTCCCAGGTTCAAGCAATTCTCCTGCCTCAGCCTCCCTAGTAGCT<br>GGGATTTACAGGCATGTGGCACCACGCCCAGCTAATTTTGTATTTTA<br>GTAGAGACAGAGTTTCTGCATGTTGGTCAGGCAGGTCTTGAACTCC<br>CGACCTCAGGTGATCTGCCCGCCTCGGCCTCCCAAAGTGCTGGGAT<br>TACAGGCATGAGCCACCGCGGCCGGCCAAGACATAATTTTAAAAAT<br>TATCTTCCAGCTGGCCGGGCGCGGTGGCTCACGCCTGTAATCCCAG<br>CACTTTGGGAGGCCGAGGTGGGCGGATCACAAGGTCAGGAGTTCG<br>AGATCAGCCTGACTAACATGGTGAAACCCCGTCTTTACTAAAAATAT<br>AAAAATTAGCTGGGCATGATGGCACGGGCCTGTAATCCCAGCTACT<br>CAGGACTCTGAGGCAGAAGAATCGCTTGAACCCGGGAGGTGGAG<br>GTTGTGGTGAGCCGAGATAGCGCCACTGCACTCCAGCCTGGGTGA<br>CAGAGCGAGCTCCGTCTCAAAAAAAAAAAAAAAAATTCCTCTTCTT<br>TCCTTCATTCATTATTCTTAGTCTCTGTCTGTCTCATATGAGTAGATA<br>ATCATATGAGTAGCTATAATGAGGACTCCTCAGGGGACAGATTCTGT<br>TTGGCCACTCAGCAGAAGCATGCAATTACCTTCTCAAGATTGGCTC<br>CTAAACTTACTCTAGATAGAGCCTTCAGGCAGGAAACTCTATATCCC<br>TTGGCTCCAGAAGAATATATTCATACTCATTCAAATGTGCTAGGCAC<br>TGGCCATAAAGAATATTGGTGTTCATTCATAACTGCCTATAATGTGCT<br>AGGAGCTGTGCATAAAGCAGTGTAACAGGATCATCTTGGAAAGCTT<br>ATGTAGTCAGTAGAGGAGACAGATAAACTAGGAATTACATGGTAAG<br>TTGAAAGAGGAAGTTAGGGGTAAGATGCTGCACCAAATGCAGGAG<br>ATTGGAAGGTTTTTCTGGAAGAAGTGATGTTTAGGGGGAGTTTCAG<br>AGGTAAATTTCTGGTTTGGTTAACAGAAGATAGTTTCACTGCTCAC<br>TGAGATTACAGAAGAAACAGGCTGGAAGAGTTTGGTTTCAATATGT<br>CAAATTTGATGTGCTTATAGGAAATTTTATTTATTTGAGACAGTTTCT<br>CTCTGTCGCTCAGGCTGGAGTGCAGTGGTGTGATCTCGGCTCACTG<br>AAACCTCTGCCTCCTAGGTTCAAGCGATTTTCCCATGTCAGCCTCC<br>CCAGTAGCTGGGATTACAGGCGCACGGCACCACGCCCGGCTAATTT

	TTCTATTTTATGGTAGAAACGGGATATCACCATGTTGGCCAGGCTGG TGTCAAACTGCTGACCTCAAATGATCCGCCCTCCTCGGCCTCCCAA AGTGCTGGGATTACAGGTGTGAGCCAAGGTGCCCGACCTGATGTG CTTGTAAGAATTTAAATGAAGAGGGTCAGTCAGCTTAGGAGACGA GGTTAGATGTAAGGATTTCAGACTTGTCAGTATACAGGTGGTGATTG ATGCCGTAGGAATGACTGGTATTACCCAGGGAGCTGGTGCAAAGTA GGAGCCTTGGGGAACATGTTAAGGGGATGTGAGAGTAAGCCCACA AAGACTCAGAAGTGGCCAGGGAGTAAGGGGAAAATGGGATAGAAT AGAGCAAAAGCCACGGGAGGCATTCCTTGAATTCCTTGGACTCAG ATATGTTAGGGGACAGGAAACACATTGAAGACAACTGAAGAGACT TCAGTGTGCTGCTGAGTTCTTAGCAGAGTAGACAGCTGATTTCACC ACTATTCCAGACCCTAAAGATTTCTCCTGAAATTGCACTGTTATGCA ATTTTTTTCCTTCTCTATTGCCCCACCTCTGGGGCCTACACTCTTTAC CTTCTCTGATTTATCTGACCCATCCGCATATCTCTGCAAAGTCTTAAT GTAGGTTTCCAGGATTTAATGATTCTTTCAATCAACTTTAAAGTTCT TCAGGAAGATTCTCCAGGTCCGTCAACAGTTTTCCCCTTCATGACT TTGTGACAAAGCTTTCTTGTTCCACTATCCAGGTGTTCCCAACAAG TTGCTGTAACGGTGGCCCCTCATAAGCTTGCCTGGAAACCCTAGAA AGCTCAGAAAAATTATGAACTTTGCCCAGTAAGAGGCTGGGAAGT CAGAAAGGGTTTAAACAAAACTGACCCCACTTGTTACATGCTCCT TTCACTGTCCAAACGTCAACCAAAGGAACTTTTGTGACGCTAAGA CGCTCCCGGCGTCCTCACCCGTTCACAGCCAGAGTTGATCTTAGAA GGCAGGGGGCTCGCACCGAGACGCTCCTAGCAAGCCCCTCACGCG AGCGAATGACTGGCGCCAGGAGAAACGCATAGGACCTCCCGAAGC CGCCGAGAGCAAAAGGACCCATCAGAGCTGTGCTGAGACCCGCCG CGGGCCGGGCTCGCACTCGAGATTCTCGCCCGTGGGATTCCAGTCC TGAGCTGAACGAGAGATAAGTCTTTGTCACCCCCCAGTCTTTACTG TTTCGAGAAACCCTAAGATTTTTAAAAAAAAGCCCAAGACGTCA AGTCGGGGAACTCCAACATTGGGGTGTCCAGAAACACTGGGGTTT CAGGCGCTCTGTAAATGCGTCCGAGGTCCGCGGTTCTGCCCGCAA GCCCAGACCCCGGCCTCACTTCCGCCCCGCGCCCCGCGCCCCGGC CTCACTTCCGCCCCGCGCCCCGCGCCCCGCGCCCCGCGCGCCTCCG CCCGCCCCTGCTCCGGCCTTGCGCCTGCGCACAGTGGGATGCGCG GGGAGGTGGTGCGCGGGGAGGTGGAGGGCGAGGGGCGGGGCTAC CTCAGGTCCCGCCCGCGGCAGGCCTGTGGGCTGCGAGGAGGAGCT TTGCCTAGCTTGCAGGCAGCGCAGGGCAGACGGCGG
ACE2	ATTTTAATTTTGCTTATGGGGACCTGCTTATTCTCAGAATATGACAGA TTGATGAGGACTTATTAGACATGCATGAAGTTAGCAGATAACTGAA CCCATGACTCTGTGGAGAGAAAAGGTCTCTTAAGCAATCTGGGATC AAAATGTAGCCCCATTGCCTATCAGTTGTGGAAGTTTGAATTAATCA TGGGAACAGTTGGTTTCCTTAATTATAAAATGGATCTCACTATTTCT ACCTGTAAAGTTGTTGTGAAGAGTTTATATACAGGAGCAGGTACAA AGCATATGCAACCAACAAATACAACATCTGTAAATATTGAGAGGGA GTCATTTACCTTTATGACTTTCCCCTTCTTCCTCAAGATAGCGTGAC AGACTCAAGTTTTCTCTTCTCTGTATGTAGGAAGTTTTATACCAGGA CCTGACCATGACTGGGTGATGAAAGCACGCTGAGAGGTTCTCTATG

	GCTGGGCTCATGTAGACTACTGGCTGACTCCCCTTTCCTTTCATATA<br>TGACTCTACTCATTCCATTGGTGAGCTCCTCCATCCTGCTGTTTCAG<br>CACAAACTAGCAGACTTCTCCTACAATGCAGGATCCTATAGTCCAA<br>ACAGACCTTTGAGTCAAACTCCCAGCCAGTCTCTGTCTGCTGCAGT<br>GTCTCTGAAAGATGACCTTGCAGATGGTTTGATTAAACATTCCAGT<br>GTGGAAGTTCTCTAATTATTACACAGCTCCTTTCATTTTCTGTGGCT<br>TTTATTACTAGGCTTACAACATTACTCTCTGTGTTCTTATGTATTAATT<br>CAAGTAATATGACATCCCTATGAAATGGATATAAAACTATCATTGGC<br>CTTGAAATAAATTAGGTGCAGGGAGGATATTACCTGCCTAAGGTCA<br>CACAGCTAACAAGTGGCAGAGTAAATCCACTATGCTAAGTCTTCTT<br>CTAAATGGCAGTACCATGATGGAAGAAAGCTGTCCACCCTGTCTTC<br>TCTTCCATATCTAAACCATTCTCCAAATTATATTATTTTAGATCCATCT<br>GCTCTCCTATTTATTTTCCTCTGGATGCTCTCTTGTGCGTCTCTAAAA<br>TTGTGGTTCACAGAAATGAATGTTATAGTCTAGATTATTTTGTATCTA<br>ATGCTTATATTAGCAAAGTCTAATATAGTATAAGATGTAATTATAGGC<br>CCAGTAATTTTTACTGGGCCTCCTAGTTGGATCTTGAAGCTCATACT<br>AAGCTTTTATTCAACCAGCTTGACTGGGCTGCTGCAGTAGCTCATT<br>AACTAGCCTCCCTGCTTCCATCCTTACCCCTTTAGTGTCCATTCTCTA<br>AGCAGCAGCCAAAGTGACCCTGCTAAAGCTTCTACTAGATCACTTC<br>TCTGCTTGAAGCCTTCCAGTGTCTTCCCATGTTGCTGAGAGTAAAG<br>CTGAAGTCTCACAGTTAATTGTCTTTAAGACCTCCCTGGTGTGACC<br>CTAGCTTCTTCCTTGACTTCACCTCCTGCCACTTTCCCCTGCACACT<br>GAGCTCCAGTCACAAAGACCTCTGATGTTCTTGAAATCTGCCTACA<br>TCCTTTACACTTGCTGCTCCCTCTCCCTAGGAAAACTTCTTCACCCA<br>GAGATCCGCACAACTTTCATCACTTCTTTCCGGTCTTTGCTCAAAC<br>GTCACCTTCTCAGAGAGGCCCTCCTTATAGAAAATCCCTCTCCCCA<br>CCCCATAATTCTCTATCCTCCATTTCTTGCTTATTATAAGTCATGACAT<br>TTATCACTATTTTTGTCTTCCTTCTATTGTCACCTCATATAATACAATA<br>TTTATTAGCTTACATTCAAAATCTAGAATAGTGTTTGGCATATAGTAG<br>GTATTCAATAAACAATGATTGAAGCAAGATTCTTGTTGTTTTGGACA<br>TGAATTGTTGTTTACCAGAGGACTGCCTAATCCTGTACTGTGTAATT<br>GTTTCAGGGGACATTTGGGTCTAGTCATAGCATTTTACATTCTGTTA<br>AATCCCTTATTGAAAAGGAAGATACACTACGTCTTTGGATATATTTT<br>TTTACTATTGTATTCTATCAGAGAGGAGATAGGGCAGGCAGCATCTG<br>ACTTATTCCTAGTGAATTCCTAATGGACCAGAGATATATTTTTCCAA<br>GTGCAAATCACATTCTAAAAATTTTACTTTAGGCCGGGTGCGGTGG<br>CTCATGCCTGTAATCCCAGCACTTTGGGAGGCCGAGGCGGGCAGAT<br>CACGAGGTCAGAAGATCGAGACCATCCTGGCTAACACAGTGAAAC<br>CCCGTCTCTATTAAAAATACAAAAATATTAGCTGCGCATGGTGGCGG<br>GCGCCTGTAGTCCCAGCTACTCGGGAGGCCGAGGCAGGAGAATGG<br>CGTGAACCTGGGAGGCGGAGCTTGCAGTGAGCGGAGATCGCGCCG<br>CTGCACTCCAGCCTGGGTGACAAAGCGAGACTCCGTCTCAAAAAA<br>AAAAAAATTTACTTTATAGCTTTACTAGGTGTTAAGGTCAAACTTCC<br>CTTTACCATTATGAAATCCACCCCCTCCTCCAATCTTTTTGAAACAT<br>AGAACATCTTTCCTGCATCTTTGGTCTTCTAATTTTTTCTGATTCTCC<br>AATTTTTCTCAAAGACAATACATTCCTTTAGACCAATTGTTTTTCTG

	GGTCTCATCTCCTGAGTCTTCAGTGAATTCTTTCCATTCTCGGAATG<br>CGCTCAGAGAGAACGCTTCTGGGGAACTATCAGAACATTGATATCT<br>TCTCAAAAAAGTGTGAACAAAAATTCCAATAATTCAGTCAGTGCTT<br>GCAGTTGGCCATTCTGGGCTAGTTTTTCTACAGAATCACTGTAGACT<br>ATTCTCCCTTTTCCCTTGCTTCTCAAAGTTTCTTGGCTTCTAGCATTT<br>TCCTGCCTGCCTCACTGCTGTCCCAGGCTCCTTGTTCATTTATTCTT<br>AGCTTTCAGATTTTATATCTTGCTTTCTCCATTTCAGTTTGATCAAGT<br>TAATCGTATTTTCTGCCTCAGCGAATTGAATTTGGACTACTCCCATTT<br>TTTCCACTAAACCCACGTGTTTCATTTAGGAACTTACCTGTGTAGAA<br>ATTTATTTCCGCTTTTATTACAGTAACATTTCAACCTTTTCTTCAGAT<br>ACTCGTGTGCTGTCCTCTTTCTTGCCTGTTTTTTCTCCTATTGTGTGC<br>AAGGAGTGAGTGGATCTAGGTTTCCTGGAATGTGGGAGGAGCTTTT<br>CTGAAATTTCTGCACTGATTTTTTTTTTTTACCCTCATCTCACTTTCA<br>TTTAAAATTCTTTATTATTATTACTTATATTTGTGAGCTGCTTTATTTTT<br>TTAAAAGGAGAGTATATTTTGATTCACATCAAGGGAAGTAAGAGTC<br>CACCTCTTTTTGATGCAGTGTAACTAAATCTGTCATCTTCACTGAGG<br>CACAAGGCCCTCTGTCACATTTTATCTATGCCTCCACTATTGCTTTTA<br>GACTGTTGACTACAAGTTAGTTAAGAGCCTATACTCTAGAATCAGA<br>ATACATAGATCCATGTTCTGATTCCATCATTTGTTAGCTGAGTGAGAT<br>ATAGCAATTTACTTAAACTTAGTTTCATAATTCATAAAATGGTGATAA<br>AATATTTGCCTATAGGTTTCTTGTGATGCTCAAATGAGATATTATAAC<br>ACAAAGCACTTTGAATAGTTACTTATAATTGTTTTTTTCCTATTCTAC<br>AATAATATACACAACTTTGGAATAAGGAAAAGCAGTGGACATTTTT<br>TTAAAGGCTTGATTATTGCAATGTCACCTGAACCTGGAAGACTTGT<br>TTTTCTGGGTGAAGAAATATTTTCTCTGTGTCAGAGTTTCACAATCA<br>TCGTCAGGTAGGCCCTTGAACCCTGCCATTTAAAGTGCTCCTCTCTT<br>TGATCTGTGGCACTCATACATACACTCTGGCAATGAGGACACTGAG<br>CTCGCTTCTGAAATTTGACAAGATAACCACTAAAATCTCTTTGAATT<br>CTATGTTGTTGTGATCCCATGGCTACAGAGGATCAGGAGTTGACATA<br>GATACTCTTTGGATTTCATACCATGTGGAGGCTTTCTTACTTCCACG<br>TGACCTTGACTGAGTTTTGAATAGGTAAGTGAAGGAGAAGGAGGC<br>ACTCAAAGAAGTCAGCCACAGAACCAGTGTGAGAAATAGGAAATG<br>AGCTTTTTTAAGTTTTGCAAAGGCAGATCAGGAGAGTTGACCTGTG<br>GAGTGGAGAGTAGTCATAATTTTAAAAAATGGCCATGGAAATTAAA<br>ACTGATCAGAAATGGCTGGGCACAGTGGCTCACGCCTGTAATCCTA<br>GCACTTTGGGAGGCCGAGTTGGGCAGATCACAAGGTCAGGAGATA<br>GAGACCGTCCTGACTAACACGGTGAAACCCCGTCTCTATTAAAAAT<br>ACAAAAACTTAGCTGGGCGTGGTGGTGGGCACCTGTAGTCCCAGC<br>TACTCGGGAGGCTGAGGCAGGAGAATGGCGTGAACCTGGGAGGC<br>AGAGCTTGCAGTGAGCCGAGATCGCGCCACTGTGCTCCAGCCTGG<br>GCGACAGACCGAGACTCAGTCTCAAAAAAAAAAAAAAAAAAAAA<br>ACTGATCAGAAATATGAATTCCATAAAGACAAGGGCAAAGTCATGT<br>ATTTGGAAGGGAAAATGTTGCCCAAGTAGAGAGTTTCTGGGAATAT<br>GATCTTGAAATAAAAATAAATGTGAGATAACCTATTAATGAAATTGT<br>CTGAAAACCATACAAACACCAACATTATCTTCATGATCCCTAGTTCT<br>AGACCTCTTTGGTCACTGTAAAATTATAACATTTTCCGTGTATCTTTA

	ACAGCTTTCTAGGAAAATATTAACCAAAAGTACCGGTTTTGATTTG<br>GCCATAAAGTGACAGGAGAGGTAAGGTTCTCTAGGATTAAAGAATA<br>ACGTATTCTTATTTGATTCACTTTAAAAAATTATTCTAAAATCTGTTA<br>CATATCTGTCCTCTCCAGGATGAACTTTATATTGGCTCAGCAGATTG<br>TTTACTGTGTTCTTCTTCTTTTTCTTTTTTTGGTCTTTCCTGCTCAGC<br>GCCCAACCCAAGTTCAAAGGCTGA
ACLY	GCTACTCAGGAGGCTGAGGCAGGAGAATCGTTTGAACCTGGGAGG<br>TGGAGGTTGAAGTGAGCCCAGATCACACCATTGCACTCCAGCCTG<br>GGTGACAGGGTGAGACTCCCTCTCAAAACAAACAAACAAACAAA<br>CAAAAAAATAGTAGAACTGGACACTTAGGTAGACCCTTCTCCAGGC<br>TGCATCTCCTCCCTCCTCTCCCTGCCCTTCCCCTACAATTGCTTTCTT<br>TCATTCACATATTATAAGAGCCTTTTTTTAGTCATGTTTACAGTGAAT<br>GCTTAATATGTGTCCAGTCCCATGCTAGGCACTGGAGATGCCAAAA<br>TGAACAAGGCACTCTCCTAGGCCTTGGGTTGCTTACAGTCAGATAA<br>AGGAGATCCACTGGATAGGATCTTTCCCTCTTACTCACCTGCAGCA<br>GGTTGCAGGGTACAGGCCCAAGGACTTGGAGTCTGAAGATCATTA<br>GGTTAGTTGATCATAGGCAAAATGATGAAAACACTTAGTTAGCTCA<br>AGTGGCTGCTGTGAGTAGTATGCCTTTCACATAGTAGGTGCTCAATA<br>AATATTAACTAAAATTGGTCCAAATCTAGGAGGAAGTCCTTAGCCA<br>GTTTTAGGCTTCTGGAACCCAGGTGGAATGCCCAAACCTGGGCTTG<br>GAGATGGTGCCTATAACCCTCTGTATTCTGATAGGGACCTGTCCTCT<br>AGTCCAGTCTAGAAGAGGAGGGAGGTGATTCAAATGGGAAAATCA<br>AAAGAGTTTAGAGCTTTTATTTTATTTTTTATTTTTTGTAGAGATGGG<br>GGTTTTGCCATGTTAGCCAGACTGGTTTTGAACTCCTGAGCTCAGG<br>CAGTCCACCCGCCTTGGCCTCCCAAAGTGTTGGGATTACAGGCGTG<br>AGCCACTGCACCCAGCCTGGTTTAGAGCTTTTAAGTTAGAAAATAG<br>AATGATGAGAAGATGGGTTCTAGATTGATTAATTCAAGGCAGATGA<br>ACCTCTGTGGGTGCATGGTGGGGTAATGAGAGAGTCTTACTTGAGG<br>AGGCTGGGCGTTGGGGGCAGAAACTTGGTATGAGACCACTGAGTG<br>AACTGGAAAACCTCAGTGGCTGGCTCATGGGCCCTCTGGCCAGCA<br>CCTCTTCCCCCTCACTCTCTGGACAGTTATTTTTTGTTGTTCCTGCC<br>CAGGAATAGTCAAACTTAGGCCTAGCAACACCTAAATGGAGGACA<br>ATCATTCAGCAAACATTTATTGAGTACTTACTGTGTAACCAGTCCCT<br>ATTTAAACTGCTAGAAATACAGCAATGAAGAAAATAGACAAAGATC<br>CCTATCTTCGTCATGCACATACGCAAGTATATCTGAAGGATAAATTAT<br>TAGAAGTGGAATTACTGGACCAAAGGGCAAGTGAATTTAAAATGTT<br>TACTGATATTACCAAATTGACCTTCAAAGAGTTTATTATATTTTATGT<br>CCCGCCTGGATGACCATTTCCCCAGGAGTGTATTTGTCTTTCTTTTT<br>TTTTTTCTCATACAGGGTCTCACTCTGTCACCCAGGCTGATGTTCAG<br>TGGTGCAGTCACGGCTGACTGCATCTTTTTTTTTTTTTTTTTTTTTG<br>GCTCGCTCTGTCACCCAGCCTGGAGTGCAGTGGCGTGATCTCGGCT<br>CACTGCAACCTCCGCCTCCTGGGTTCAAACGATTCTCCTGCCTCAG<br>CCTCCTGAGTAGCTGGGACTACAGGCACGTGCCACCATGCCCGGCT<br>ATTTTTTTTGTATTTTTAGTAGAGCTTGGGTTTCACTTTGTTAGCCAG<br>GATAGTCTCAATCTCCTGACCTTGTGATCCGCCCGCCTCGGCCTCCC<br>CAAGTGTTGGGATTATAGGCGTGAGCCACTGCACCCAGCCAGCCTC

	CCGAGTAACTGGGATTACAGGCATGTGCCACCACATCCAGCTAATT TTTTGTATTTTTAGTAGAGATGGGTTTTGCCATGTTGGCCAGGCTGG TCTCGAACTCCTGGCCTCAAGTGATCTGCCTGCCTCAGCCTCCCAA AGTGCTGGGATTACAGGCTCGAGCCACCGTGCCCACCCAGCTCAC TGCGTCTTTGACCTCCTGGGCTCAAGTGATTCTCCCACCTCTGCCTC CTGAGTAGCTTGGACTACAGGTGTGTGCCACCATGCCTAGCTAATT TTTTTTTTTTGAGACAGTGACTCACTATGTTGCCCAGGCTGGTTTTG AACTCCTGGACGCAAGTGATTTTCCCACCTTGGCCTACCAAAGTGC TCGGATTATAGGCGTGAGCCAGCATGCCCAGCTGCCCAGCTAATTT TTTATTTTTTGTAGTGACATAGACTCACTGTATTGCCAAGGCTGGGT TGGAACTCCTGGGCTCAAGCCCTCCTTGGCCTCTCAAAGTGATGGG ATTACAGGCCTGAGCCTATGCACCTGGCCTCCCAGGAGTATTTCCTT CCTTCCTTCTTTCTTTTCTTTTCCTTTTTTTCTTTTTTGTTTTTTTTTT TTTTTTCTGAGACAGGGTCACACCCTGTCGCCCAGGCTGGAGTGCA CAATCTCAGCTCACTGCAGCCTTGATCTCCTGGACTCAAGTGATCC TTCCACCTCGGTCTCCCAAGTAGCTGGGACTACAGGTGTGCACCAC CATGCCCTGCTAATTTTTGTTTATTTTTTTGTTTTGTTTTGTTTTGTTT TGAGACGGAGTCTCACTCTGTGGCCCAGGCTGGAGTGCAATGGCA CGATCTCTGCTCACTGCAACCTCTGCCTCCTGGGTTCAAGCAATTC TCCTGCCTCAGCCTCCCAGGTAGCTGAGATTACAGGCACCCGATAC AGTGCCCAGCTAAATTTTTTTTTTTTTTTTGCTTTGAGATGGAGTTT TGGTCTTGTTGCCCCCGCTAGAGTGCACTGGTGCGATCTCGGCTCA CTGCAACCTCCGCCTCCTGGGTTCAAGCGATTCTCCTGCCTCCGCC TCCTGGGTTCAAGCGATTCTCCTGCCTCCGCCTCCTGGGTTCAAGC GATTCTCCTGACTCAGCCTCCTGAGTAGCTGGGATTACAGGCACCT GCCACCACGCCCAGCTAATTTTTTGTATTTTTAGTAGAAACAGGGTT TCACCTGTTGGCCAGGCTGGTCTGGAACGCTTGACCTCGAACTCA GGTGATCTACCCACCTAGGCCTCCCAAATTGCTGGGATTACAGGCA TGTGAATCACCACGCCCGGTCTTTTTATTTTTTGTAGAGATTTGATG GTGGTGGTGGGGGCTCTTACCATGTTACCCAGGCTGTACTTGAACT TCTGGGCTCTAGCAATCTGCCTGACTTGGCCTTCCAAAATTCTGGG ATTACAGGCATGAGACAGCATGCCTTGCCTGGAGTGTATCTTTTTTT TTTTTTTTTTTTTTTTGAGATAGAATCCTTCTCTGTCACCCAGGTTGG AGTGCAGTGGTGCAATCTCACCTCACTGCAACCTCCACCTCCCGGG TTTAAGCGATTCTCATGCCTCAACCTCAAGAGTAGCTGGGATTTATA GGCGTGTGCCACCATGCCTGGCTAATTTTTGTATTTTTTGTAGAGAT GGGGTTTTGCCTTGTTGGTCTTGAACTCCTGACCTCAAGTGATCCC CCACCTTGGCCTTCCAAAGTGCTGGGATTACAGGTGTGAGCCACCA AGACTGGCCACCCCCATTTAGGACAAGAGAAGAGAGGTGATAAG GAACTGCTATTTCAAAAAGATGAATGGGAGGGAAGGGACCAGAAA GTGACATCATCAGGGAACACTTGATTCAGGCATGGGTAGCAAGTTC AGTTACCTTACTCCTGGCAGCAACAGGATTGGGGGAGGGGGTCTT GGAGAAGATTTTGTCTGCAAATGTCGCAACACTGTTGCAGTGTGAG TATGACCCAAAGTGCTTGGCAGGGGGACAGGCCTGCTGCTTCCCT GCATTCTGGGCTGACCTCTTCACCTGAGTCGGTCAGTGGACGCTTT TGGCCTCCTTCCTGGAGCTAGACAGCTGTTAGGCTTAGGGAGCAGC

	TCCGGACCTACAGGGTTGGGGTGCAGGTAGGGAGGGAGGCTGCAG<br>CATGTCAGAGTGGTAAATACATGTCCTGATTTCCAGGACTGATTTCA<br>CATATTCTGCCCCACAAGTTCACCAGCACCTGTCAGATTGGATGTC<br>CCAGTTTTTGGTTTGGAAAATTCGATCTGTCTCAGGTAATGGAGAA<br>GGGGGAGACAGCAGTTCATTCATTCCAGTGTCTAAGCCTGTGGTTG<br>GTGGGAACAAGAGACTGTCTGGGATGGCTCCAGCCCTTCGCTGGA<br>ATCTCGCATTGAATTCCTAGCTCCGCCCTGATCCGTGCTGAGGCGA<br>GCAGGTGAGTGGGACGGACAGCGCTGTGCCTGCACCCTCCTAGCC<br>CCACTTGCTTACCGCGGCCCCTGCCTTATCCCACACTGGGAAGTAT<br>GCTCTGTTAGCCTCGGCTCAGCCTTGCCTCATCGGCCCAGTGGGGA<br>GAGTCCAGGGAGGAGAGAGGCTCCACGTGCTAAAGGAAAGCGGC<br>CTGCCAGACACTGCTGCTTAAGGAGACTGTCTAAGCAATCGGATTC<br>GGTGCGGCCGAGATGCAGACGTCCGTTTCTCCCAAGGGAAGGAGC<br>AAGGGTACTCCAGGTCCCAAAGCTGCGTTACAGCCAGTTGGGCTA<br>ATCCTACCCGCCCCTCCCCCAACTCCCCGAGTGGCAATCTGTCCAG<br>CCCCTCAAGCGATCAGGCCACAACCCCCAGCACCGCGCGTTCTCCT<br>CCCAGCCTAGCCCGGCCCATCTCCCCACCCGGGGCCCCGCCCCGGC<br>CCCGCAGGCTCCCAAGAGGCGGTGGATGGGCCAGCGGGACTACAA<br>GTCCCAGCAGGCCCCGGGGCCCGCCCTCGTGGGGCGGAGCCAAGC<br>CAGCAGCGAATTGGGAGGAGCCCTGGCGCTCAGGCTAGGGAACGC<br>GTGTGGCCAATCGCGGGGCCGTTCTCGCCGCGAGCCGATGGGGGC<br>GGGGAAAAGTCCGGCTGGGCCGGGACAAAAGCCGGATCCCGGGA<br>AGCTACCGGCTGCTGGGGTGCTCCGGATTTTGCGGGGTTCGTCGG
ACTB	GGTTGCAGTGAGCTGAGATTGTGCCACTGCAGTCCAGCCTGGGCG<br>ACAGAGCAAGACTCCATCTCAAAACAACAACAACTAAAAACGAAC<br>AACAACAACAACAAAAAACTGAGGCCAGGTGCAGTGGCTCACAC<br>CTCTAATCCCAGCAATTTGGGAGGCTGAGGTGAGAGGATCACTTCA<br>GCTCAGCAGTTCGAGACCAGCCCAGGCAACACAGGGAGACAGAC<br>CCTGTTATATTGCAGAGAGACCCCATCTCCACAAAATATAAAAATAT<br>TAGTCAGATGTGGTGGCATCCCTGCCGTCCCAGCTACTCAGGAGGC<br>TGAGACAGGAGGATCGCTTGAGCCCAGGAGGTCAAGGCTGCAGTG<br>AGCTGTGATCATGCCACTGCACTCCAGCCTGGGCCACAGAGCTAG<br>ACCCTGTCTCTAAAATTTTTAGAGACCTTATCTCTAAAAATAAATTA<br>AATAAATAAACCGGGAGCACCTACTTTTTCTTTTTCTTTTACTTTTTT<br>TTTTTTTTTTTGGAGACAGGGTCTCTATCACCCAGGCTGGAGTGCG<br>GTGGCATGATCTTGGTTCACTGCAGCCTCGACCCCTCAGGCTCAGG<br>CAGTTCTCCCACCTTAGCCTCCCCAGTAGCTGGGACTACAGGCACA<br>TGCCACCATGCCCGGCTAATTTTGTCTTTTTTTTTTTTTTTGGTAGAG<br>ACAGGGTCTCACCATGCTCCTCAAAACTCCTGGACTCGAGAGATCC<br>TCCTGCCTCGGCCTCCCAAAATGCTGGGATTACGGTGTGAGCCGCT<br>GTGCCCGGCTATTTTATTTTTAAATGAATAAAAGCTGGAGCACCCAA<br>CTTTTTTGTTGTTGTTTTTCTGAGACAGAGTTTTGCTCGTCACCCAG<br>GCTGGAGTGCAGCGGCGCAATCTCGGCCCACTGCAACCTCTGCCT<br>CCCGGGTTCAAGCGATTCTCCTGCCTCAGCCTCCTGAGTAGCTGGG<br>ATTACAGGCACGCGCCACTATGCCCAGCTAATATTTTGTATTTTTAGT<br>AGAGACAAGGTTTCATCATGTTTGCCAGGCTGATCTTGAATTTTTG

	ACCTCAGGTGATCCACCCGCCTTGACCTCCCAAAGTGCAGGGATTA<br>CAGGCGTGAGCCACCACGCCCAGCCTGGAGCACCTAATTTTTAAAT<br>TTAATTTTTCTTTTCTTTTTTTTTTTTTTTTGAGACAGGGTCTTCCTC<br>TGTCATCCAAGCTGGAATGCAGTGACTTGATCATAGCTCACTATAAC<br>CTTGACCTTCCACGCTGAAGCAATGCTCCTGCCCCAGCCTCCCAAT<br>TAGCTGGGAGTACAGGCACGTGCCACAACACCCAGCTGATTTTGTA<br>GAGATAGGATCTCCCGTGTTGCCCAAGCTTGTCTCCAACTCCTGGG<br>CTCGAGCGATCTTCCCTCCTCGGCCTCCCAAAATGCTGGGATTACA<br>GGTACAAGCCATCACACCCCAGTGGGAGAGACCCCACTTGCTGCC<br>ACGTGACCATGGGCTGATGGTGTCCTCTCTGGGCCTCAGGTGATAA<br>ATTCTGAGGAGGGAGGTAGGGCCCAGGAATTCCGACTTTCAAACA<br>GCCTTTGGACTCAGGCCTTGGGGACATTCCAGGGGACCCTTTACCA<br>GGGAGGGGCAGTGTGAGGCAGGAGGCAGGGCTGCCGCCTCAGGG<br>ACCCTGAGGCTAGAGTCCTTCCCACCGCATTTGGGGACTTGAGCAC<br>TTCACCAGTCCTGACTCAGTTTCCTAGTCTGTGAATGGGGGTGTGT<br>CTCCTGGAATGTTGCCCCCCCTCATTCATTCATTCTCCTCTCACTGA<br>GCACCTACCGTGTTCAGGGTCCCTGTCCTCCTAGAGCTAACTCAAG<br>TCTTGGAGGGAAGCTCTGTCTCAGTTCCTTCCTCTACAAAATGGAG<br>CTTGAGGACGGGCACGGTGGCTCATGCCTGTAATCCCAGGACTTTG<br>GGAGGCTGAGGTGGGAGGATCGCTTGAGCCCCCCGGAGCTGGAGA<br>CCAGCCTGGGCAACATGGTGAGAAGCCATACCTCTACAGATAATTT<br>AAAAATTAGCCAGGCTTGGAAGTGTGTGCCTATAGTCCCAGCTACT<br>CGTGAAGGCTTTGAGCCCAGGAGGTCGAGGCTGCAGTGAGCTGTG<br>ATCGCACTCCAGCCTGGGCATCACAGCAAGACCTTGTATCAAAAAT<br>AGTAATAATAATAAAAAGGAGGTTGGATTCCCTCCTGGCAGGATAG<br>GGAGGGCGCTGCAGTGCCCAGGGCAAGGTGGCTGGGTGGTTGTTT<br>TGCGGGAGGGCCAAGGAGTGGTCCCTGGGTCTGCGCTGTAAGAGT<br>TGGTTGCCTGGAGGCCTTGCAGGGTGGGGGTGTCACCAAAGCAAA<br>GGCTTGGAGGGGAAAAAACAAAAGTCCCAGCCAGGCAGGTGGAG<br>TCCCCTTGGCTGAGTTCAACCGAGGGTTCTCCGGGGGCTGCGTGC<br>GTGCCCCAGTGACAGCTCCGAAAGCTCCCTTACAGGGCAAAGTTC<br>CCAAGCACAGAAGAGAACCTGTTCACTTCTCCCCTGCTCGGCCCG<br>CCCCCTGGCCAGGCACCTCTACTTCCTCTTTTCCTGCTCCGCTGCTT<br>GCTTTCTCTCTTCAGCTCCTCCCTGCCCCTCACCCCAGGCTGCTCG<br>GCCACCTCCAACCTGCCACCTGAGGACACCCAGGCAGTCACTCAT<br>TCAACAGCGAGGAGCCCTGGGGTGGGTGTAGTGGGAAGGAGTGG<br>GGGTGACGGAGACCCTGGGAGGGCTCGCAGCCTGGTGGCTGAGG<br>CCCAGTTCTAAATGCCAGCTGCAAGCCTTGGTCTGAGGTAGGGAG<br>GAAGGCGTGGCTGCAGAGGCTAAAACGCTTCCCCAAAGAGGGGCT<br>TTCTGGGATGGGACTTGAAGGGTGCATAGGAGAGCACTAGGAAGT<br>GGCCGCTGCAGACAGAGGGAACCACAAGCCAGGAGGACAGGCCA<br>GGAATGCTGCAGCCCGGGGCGGGGTGGGGCTGGAGCTCCTGTCTC<br>TTGGCCAGCTGAATGGAGGCCCAGTGGCAACACAGGTCCTGCCTG<br>GGGATCAGGTCTGCTCTGCACCCCACCTTGCTGCCTGGAGCCGCCC<br>ACCTGACAACCTCTCATCCCTGCTCTGCAGATCCGGTCCCATCCCC<br>ACTGCCCACCCCACCCCCCCAGCACTCCACCCAGTTCAACGTTCCA

	CGAACCCCCAGAACCAGCCCTCATCAACAGGCAGCAAGAAGGGCC CCCCGCCCATCGCCCCACAACGCCAGCCGGGTGAACGTTGGCAGG TCCTGAGGCAGCTGGCAAGACGCCTGCAGCTGAAAGATACAAGGC CAGGGACAGGACAGTCCCATCCCCAGGAGGCAGGGAGTATACAGG CTGGGGAAGTTTGCCCTTGCGTGGGGTGGTGATGGAGGAGGCTCA GCAAGTCTTCTGGACTGTGAACCTGTGTCTGCCACTGTGTGCTGGG TGGTGGTCATCTTTCCCACCAGGCTGTGGCCTCTGCAACCTTCAAG GGAGGAGCAGGTCCCATTGGCTGAGCACAGCCTTGTACCGTGAAC TGGAACAAGCAGCCTCCTTCCTGGCCACAGGTTCCATGTCCTTATAT GGACTCATCTTTGCCTATTGCGACACACACTCAGTGAACACCTACT ACGCGCTGCAAAGAGCCCCGCAGGCCTGAGGTGCCCCCACCTCAC CACTCTTCCTATTTTTGTGTAAAAATCCAGCTTCTTGTCACCACCTC CAAGGAGGGGGAGGAGGAGGAAGGCAGGTTCCTCTAGGCTGAGC CGAATGCCCCTCTGTGGTCCCACGCCACTGATCGCTGCATGCCCAC CACCTGGGTACACACAGTCTGTGATTCCCGGAGCAGAACGGACCC TGCCCACCCGGTCTTGTGTGCTACTCAGTGGACAGACCCAAGGCA AGAAAGGGTGACAAGGACAGGGTCTTCCCAGGCTGGCTTTGAGTT CCTAGCACCGCCCCGCCCCCAATCCTCTGTGGCACATGGAGTCTTG GTCCCCAGAGTCCCCCAGCGGCCTCCAGATGGTCTGGGAGGGCAG TTCAGCTGTGGCTGCGCATAGCAGACATACAACGGACGGTGGGCC CAGACCCAGGCTGTGTAGACCCAGCCCCCCCGCCCCGCAGTGCCT AGGTCACCCACTAACGCCCCAGGCCTTGTCTTGGCTGGGCGTGACT GTTACCCTCAAAAGCAGGCAGCTCCAGGGTAAAAGGTGCCCTGCC CTGTAGAGCCCACCTTCCTTCCCAGGGCTGCGGCTGGGTAGGTTTG TAGCCTTCATCACGGGCCACCTCCAGCCACTGGACCGCTGGCCCCT GCCCTGTCCTGGGGAGTGTGGTCCTGCGACTTCTAAGTGGCCGCA AGCCACCTGACTCCCCCAACACCACACTCTACCTCTCAAGCCCAG GTCTCTCCCTAGTGACCCACCCAGCACATTTAGCTAGCTGAGCCCC ACAGCCAGAGGTCCTCAGGCCCTGCTTTCAGGGCAGTTGCTCTGA AGTCGGCAAGGGGGGAGTGACTGCCTGGCCACTCCATGCCCTCCAA GAGCTCCTTCTGCAGGAGCGTACAGAACCCAGGGCCCTGGCACCC GTGCAGACCCTGGCCCACCCCACCTGGGCGCTCAGTGCCCAAGAG ATGTCCACACCTAGGATGTCCCGCGGTGGGTGGGGGGCCCGAGAG ACGGGCAGGCCGGGGGCAGGCCTGGCCATGCGGGGCCGAACCGG GCACTGCCCAGCGTGGGGCGCGGGGGCCACGGCGCGCGCCCCCA GCCCCCGGGCCCAGCACCCCAAGGCGGCCAACGCCAAAACTCTCC CTCCTCCTCTTCCTCAATCTCGCTCTCGCTCTTTTTTTTTTTCGCAAA AGGAGGGGAGAGGGGGTAAAAAAATGCTGCACTGTGCGGCGAAG CCGGTGAGTGAGCGGCGCGGGGCCAATCAGCGTGCGCCGTTCCGA AAGTTGCCTTTTATGGCTCGAGCGGCCGCGGCGGCGCCCTATAAAA CCCAGCGGCGCGACGCGCCACCACCGCCGAGACCGCGTCCGCCCC GCGAGCACAGAGC
ADA	GAGTGCCAGGAAGTTAATGTCCTGGAAGCATTGCCAGAGGGGAGG GGGCAGTTAGATTCCCCAGCTTCCTCGCTCCTCTGCTGGGTTAAGC CTGCAACATATTCATCATCTCCCAGAGTGCTGCCCAGGGATAGAGC TCCAGGTGCTCAGTGTGGTGACCTGCTCATGTCAGCTTCTTCCCCT

	TTCCTGTCTTGTTTCGCACCCACATCCTTCCCATGTTTCCTCTTCCC<br>AAATAAACTACTTGCATTGAAATCTTTGTCTCAGGCTCTGCTTCTGG<br>GGAAACCTGAACCTAGCGAGGAGAGTTTAAGGAAAGCAACAACG<br>GGTGGCGTGGGACTCTGGTAACAAAGGGAACCACTGCCTTTCAAG<br>AAAACGGCAGGAGGCCAGGCACGGTAGCTCATGCCTGTAACGCCA<br>ACACTTTGGGAGGCCAAGGTGGGTGGATTATTTGAGGTCAGGAGT<br>TCAAGACCAGCCTGGCCAACATGGTAAAACCAAGTTTCTACTAAA<br>AATACAAAAATTAGCCGGGCATAGTGGTGGGTGCCTGTAATTCCAG<br>CTACTCCAGAGGCTGAGGCATGAGAATTGCTTGAGCCCAGGAGGT<br>GGAGGTTGCAGTGAGCTGGGATTGCACCACTGCACTCCACCCTGG<br>GCGACAGAGTGAGACTCTGTCTCAAAATCAATCAATCAATCAATAG<br>GCAAGAGGGAATCTGGAATGGGGAGGGTATGGAGAGGTGGGCCA<br>GGAGGACTGACAGGAGATGTGGCCTTGGTAGAGAGACACAGTCAG<br>CCCAGTGACTGTGCACCGAGGGAAAGGGGGAATGAATATCCTGAC<br>CTCTCTCCTCTCATCTGCTGTTCTCCTGCTGGTGCTCCCTGTTGACA<br>AAACCCAACCAGAGCCAGAGGGCAGGAGAGCCCATTGGTCTCACC<br>CACAGCAGCATGGCTGCCGGACACAGAGGAGCAGAGCAGCGTGC<br>AGAAGGGTGGAGAGTGCACCTGCAGGGGCAAACAGAAGAACTGC<br>ATCACGCAGCCTGGCCCTTTGGATCTGACCCATTTGGAATGCAGAA<br>TTTTGATAGTCTAGGATCTGGGTAAAGGGTTTTCCAGGTGTCAGGA<br>TGGAAGTGACTAAGGTGCAGAGGCTGGAGGGCTGGGGCAGGTAG<br>AAGCAAGCATTCCTGTTACCTACTGCTGTGTGACAATCTCCCCCTA<br>AAACACAATGGCTTAAAATAACATCCATTTCATTACATATCTCAATA<br>CTATAGGTCAGGAATTTGGGCTGGGCTTACTTGGGTAATTCTTCTGT<br>CCCACATGGCATTGACCAAAGCCTGGTTTTCAGTGGGCAGCTGGGC<br>TGGATGGCCCAACACAGCTTCGCTAACATGATTGCTGTCTTCGTAG<br>GGATGGTGGAAGCCTGGGCTCAGTGGGACTGTCAACTGGAATGGC<br>CATATGTGGACTCTCTTAGCATGATGGTCTCTTCTAGAAGCTTGGGT<br>TCCCAGAGAGAATGTTCAAGAGGCCCCAAAGGACACCACAAAGCT<br>TCTTTATGACCAAGGCTCGGAAATCCAGGAAGCTTGCTCCCATCAC<br>GCTCTATTACTCCAACAAGTCACTCAGGCCAGCCCAGGTCCAAGAG<br>GAGGAAACCTAGACTCCATCTTGCAATGTGAAGAATTGCAAATAAT<br>TTGTGTCACCCTTAAGCAACCAGCAACTCATCTAGGTTGATTGGCA<br>TTTCAGCAATGTGGTGGGAAGTGGTGGGACTGATGTTGAAGAGGG<br>ACTTGAATGTCATGAGAGGCTGGGGAGGCAATAAGGTGGGGAGTG<br>AAGTTTCTCGAGTCAGATTCAAATTTAAACCCCAGTTTTGCCACTTA<br>CAACCCATGAGCCAAGCAGGCTGTCTCTCTATCTGAACCTCAGTGT<br>CCTCATCTGTAAAATGAGGAGAACACCTCCTACATCTGAGGATGAC<br>TGTAAAGATGAAATGGGATGGGTGCTTATAAAGTGCTTCCCAGTGT<br>ACCTGGCTCCAAACCTGTCTCAGTAAATGGCAGCCCCTATTATTGA<br>ACCCGAGTAACACAGAGAGCCAAGAAAGGATCTTACAAAAAACTC<br>CCCTGGCTTTGACAATGTATGAGACCCACTGATAGGGTTTGGCTTT<br>GTGTCCTCACCCAAATCTCATCTAGTAGCTCCCATAATTCCTACATG<br>TTGTGGGAGAGACTCGGCGGGAGATAATTGAATCATGGGGGATGGT<br>CTTTCCCATGCTGTTCTTGTGATAGTAAATAAGTCTCACAAGATCTG<br>ATGGTTTTAAAAATGGGAGTTTCCCTGCAGGCGCTCTCTCTTTGTCT

	ACTGCCATCCATGTAAGACGTGACTTGCTCCTCCTTTGCCTTCTGCC<br>ATGATTGCAAGGCCTCCCCACCATTGTGGAACTGTAAGTCTATTAA<br>AGCCTCTTTCTTTTGTAAATTACCCAGTCTCAGGTATGTCTTTTTTT<br>TTTTTTCATGAGATGGAGTTTCGCTCTTGTTGCCCAGGCTGGAATGC<br>AATGGTGTAATCTTGGCTCACCACAACCTCCACCTCCCAGGTTCAA<br>GCGATTCTCCTGCCTCAGCCTCCCGAGTAGCTGGGATTACAGTCAT<br>ACACCACCACGCCTGGCTAATTTTGTATTTTTTTTTTTTTTTTAGTA<br>GAGACGGGGTTTCACCATGTTGGTCAGGCTGGTCTCAAACTCCCG<br>ACCTCAGGTGATCCTCCTGCCTTGGCCTCCCAAAGTCCTGGGATTA<br>CAGGCATGAACCACTGCGCCCAGGCTCGGGTATGTCTTCATCAGTA<br>GCATGAAAATAATGGACTAATACAGCCACCCTCTCCCTCACTCCCA<br>CATACAACCAAACCCCAAATCCAGCTGATTTTACACCCTAAATGCA<br>GCTTGAATATGAGTTTCTCCACTTCCCCCACTGACATCACTATGCCC<br>TACCCAGACCATGGCAGTTGCCTCCTTCCTGGTATCCTGTCCTCCCT<br>CACCCCCGCTGGCCCCCTGTAATGCCCTCCCCTCACAGCAGGGAGC<br>CCAGGCTTCTCAAAGTGCCCTGTGGGTGCGAACCACCTGGGGGTC<br>CTGTTTGTATAAAATACAGATTCTACTTCAGTAGGTCTGGGATGGGG<br>TCTGAAAGTCTGCATTTGTAGTCAGCTCCCAGGTGATGTGGGTGCT<br>GATGATCCCTGGATCACACTTTCAGTAGCTGGAGAATATTTTTTCCA<br>AATAAAAGGGTGATTTTGTCTCGCCTCCACTTAAAACACTCCACTG<br>ACTTCCTAGGAATCCCACACCATCGCTGGGTCCCACATCCCTGGCA<br>GGATTCAGCTCCCATCAGACCTTCTAGCCCCTTGCTCTCCACTCTCC<br>CACTCTCTCTTTCCCCCTTGTTTATGGGTTTGTTAATTTATTTATGATG<br>AAATGAAATGAAGCTACCATCCACCCCAGTACTGGAACATTATCAAT<br>AACCTGTGTGTGGCCAGGCGTGGTGGCTCATGCCTGTAATCACGCC<br>TTGGGAAGCCGAGGTGGGTGGATCATGTGAGGTCAGGTGTTCGAG<br>ACCAGCCTGGCCAACATGGTGAAACCCCGTCTCTACTACAAATCCA<br>AAACTTAGCAGGGCACGGTGCCACGCGCCTGTAATCCCAGCTACTC<br>GGGACGCTGAGGCCGAGAACTGCTTAAAATCCAGGAGGTGGAGGT<br>TGCAGTGAGCCGAGATTTCGCCACTGCACTCCAGCCTGGGCGACA<br>GAGCAAGAGTCCATCTCAAAAAAACAAAAACAAAAACAAAAAAA<br>CAAAAAACAAAAATTAGCCAGGCGTGGTTGTGGGCGCCTATAATCC<br>CAGCTACTCGGGAGGCTGAGACAGGAAAATCGCTTGAAACGCTGG<br>GGGTGCGGGGGAGCGGTGGGGAGGAGGCGGGCCAGAGGGGCAG<br>AGGTTGCAGTGAGCCCAGATCGCGCCACTTCACTGCAGCCTCCGC<br>GAAAGAGCGAAACTCCGTCTCAGTAAATAAATAAATAAATAAATAA<br>ATAAATAAATAAATAAATAAATAACCTGTACCCGCGTGTTATTTCCCT<br>CCGTCCTTACCTCCTCCCGGCTCCTTCCCTTTCACCTGAGATAACCA<br>CTCTTCTCGTATCTATGCTCATCTTTCCCTTGCTTTACATTTTTTCCAC<br>CGATGCATGTGTCTAAACATACATACTTTTGGTTTTGCTTTTACACAT<br>TCTAAAAGTTGCACCATTGTATGCAGTTTTCCGCAACTTAGTTTTTT<br>TCACTCAACATTGTTTCTGAGACATTGTTTCTGTTGTTGTCTGGCTG<br>AAGTTCATTCCGTTTCACTGCTGTCTAACGTTTCATGGTGTGAATAT<br>TCCGGTTTATTTGCCCACTCGCCCGTGGAGGGGCATTTGAGGGTGT<br>TTCCAATGTTCCTGTTATTCGGAATAGCGCTGGTGTGAACATTCTGC<br>ACAGGTCTCTGGCTGCGCCTGGGCGGGTTTCTTAAAGGTGAATGCC

	CAGGAGGGGACTGTCTGTGTTCTCCCTCCCTCCGAGCTCCAGCCTTCCTCGCCTCCTTTCACTCCCAGCTCCCTGGAGTCTCTCACGTAGAATGTCCTCTCCACCCCCACCCACCCCTGATGAACTCCTGCAGGTTCTGCAGGCCACGGCTGGCCCCCCTCGAAAGTTCCTTAACTATACAATTATGGTGTGTGTTTCTGCGACGAGCGTCCGTCTATCCGGTGGAAGGCACGCCGCTCGAGGCTTGCGATGCTCCCGGGGTCCCCGCTTCTAGCTTGGGCCTGGCGCACAGCAGCGCCCAGACTGCAGGGGGACGCTTGAAAGTTGCTGGAGGAGCCGGGGGGAAGGCAGCGCCCAGCGAGGCGGCTGGAGCGCGCGCCCACAGGTGGGTCCGGTCGGGCGCCGCGGGGCCGTAGTTTTCGGGTCGGCGGGCGAGGACGCCGGGTCCAGAATTCCAGGAAATGCGCGATCCAGGCCGGCGGGCGGGGCGGGGGCTCCGGCGAGAGGGCGGGCCCCGGGAACGGCGGCGGGCGGGGCGGGAGGCGGGGCCCGGCCCGTTAAGAAGAGCGTGGCCGGCCGCGGCCACCGCTGGCCCCAGGGAAAGCCGAG
ADM	TTCCTCCATTTCGAATATTAGATCCCCCTAGTCTTGCTTTTTCTTCAGAATACTTAACACAGTTTATAATCATGTAAATACATAATTCTGATAAGGATTTGTTTATATCTGGCTACGTCACTAGGCTGTAAGCTCGTTGAGGGTAAGTTTGTTGATGACTGCATTGGTGATTACTGTATGTTGAGAACTTAGCACAGTGCTGGGTATATAATATATAATCAGTAAATATTTGTTGAAAGAATGAAAAAACTATCAATTCATTCAGGCTTATAAATCAGAATTGCTTCTTGGCTGAGCACAGTGGCTCATACCTGTAATCCTAGCACTTTGGTAGGCCAAGGCGAGTGGATCTCTTGAGATCAGGAGTTCAAGACCAGCGTGGGCAACATGATGAAACCACATCTCTATAAAAAGAAAAAAATATATATAAATATATATACATATATAAAATAATTGCTTCTTTCCAGGTGCTTCTAATCTGATATCACAGGATCCCTATCTGGAGACAAGTAGCATGATGGTTGCTTTCATTAACACCATTCTCTTTGGCTATGGATGATTCAAATGGCCTAATCATCTCTTTTATCATCCATTCATTAATGCCATTTATTGTGCCCTCTGTAGCAAATTTCTCTTGAAGTAATTTCATTTTGTCCTGTTACAGGTATTATTGTAACAAGCTTCTTTAGGTTTACTACTTTCTCTTCTCTCTCTCTCTTTTTTTTTTTTTTTTCTTTGTAGGGATGGATTTTTGCCATGTTGCCAGGCTGGTCTTGAACTCCTGAGCTCAAGAGATCCACTTGCCTCAGCCTCCCAAAGTGCTAGGATTACAGGCACGAGCCACTGTACTGGACCATCTCTTCTCTTTCATTTGTTCTGCAGTAATTTACCTTATTTAAGCTTCAGGGAGGTTTGCCTTCTGGCTTTACCATGTACAAGAACTGACTGTTACTCTCTGCCTGTTTTGTACAGGAGCACAAAAATGAGGCAGCAAACGCATTACTTTTCTTTATGCTGATGCTCTGGAAAATATTATGCCCTAAAGAGGGAGGTTAACTGTCCTCCTTGCCCTTACCACTATAAACCAGACAGAAATTTAAAATATGGAAACAGTGTTTTTTATTCTGTGTCAGGGTTTTAAACTTGGTCAACCTCCCGGAACTGTATGCCTATTTTATACTTTAATCGGTTTATGTACATGTTCATTTTTCTGGAAAGGATCTATATTTTTATTAGGTCCTTAGAGAAGTCAGAGATCCCAAAAAAGTGCCCTGGGCTTTGTATCCTGCTTTACTTCTCCTTGTAAGTGATCAAATCCACAAGGCTGATTGTGTTCTTTCTCTAAATCACTTTGTGTTATGACTTCAGGATGCATCTTGAACTTTGTTTTCAAGTGAACTTTGTAAACAAGTCCTCAGCTTAAAAGACATTTTGAACTATATTAATAATAAATGCATCATAAAATTTCCTGTTTGATAAAGGAGGCATCTGAGATAATTATGCAATGC

	ACTTAGCTACTCACATTTTAGTTATTCTTATTAATATCATTGATATTTA<br>TTATTTTTCAATGCTTCTCTGAAAGACCTGAGTATGGCCACTCTGGC<br>TTTAAGAACTGTGTCCACAGTGCTAGCTGAGAAACCACTACCACAT<br>ATTTTTTTAAACTCCAAACTTTACTAAGAATCAGGCTTGGATATAGG<br>GCCTAAAAATCATTCAATTGGAATGTGAAGAAGTTGAAACACCATT<br>ACGCAAAAACCCTTTAGCATATGTGTATGTGTGTCTCCCTAATGTCA<br>GGATGTACACATTTATTACAGTGGTTTGGAGGTTTATTTGTAATCTG<br>ACCTTTTGAAATTCTGGAGCACACAAAAAAAGTAGATCTTCATTAA<br>CAAGGATTTTTTTTCTTTTTCTTTTCTCTTTTCTTGCCAGAAATTTA<br>AAGATGTTTCTTCCTTCACTTCCTTCCTTCCTTGTACTGTCCTTGCTC<br>AACGTGAATCTACAATCACTGCCACTTGAATGGATGCAACTTATGTT<br>AATAAATTGCCAGGATCAGAACTCCCACTGCAGCATTTCCTTCTCCA<br>TAGAACCACTGAACCAGAAATGCATTCTAGTGATTTTTTTTTGTCGT<br>TAACGATGATTAATTAAACAAACTCGAGATTTCACGAGAAAAAACA<br>TAGATGCCCAGATTTAACAAACTCCTTATCTTTTTTCGACTTAAACC<br>CACGAAGCTTTGGGGAGCACTCTAGCCCCTGCTACTCACCCATGCA<br>AGCGGGGTGCGCGCTCGCGCACACACTCACTCACTCCAAGATAGG<br>GGCTTTCTAGGAAAATACTTTGAGCTGCCAATCCAAGATTTTACTTA<br>AAGAATTGACTTGGTTCTCAAGTGACAGATCTCTAAGTGCCAGAGC<br>GGCAAAGTGCACCAGGCTGATAAAGTGCATGCCAAGACTCTCAGG<br>TGGGAATTCTTGTCACAGCCAAGAGGTTGCTGGCATAGGCACCCG<br>AGGCTGGCAGAGCCTAGCCTCTGAGTCTCCCTACACTCCTGGGCTT<br>GCTTTCCTTTTTGAAGCTGCCTGCAGCTCAAAGAGGAGTTTGAACC<br>AAGGGCACTTGCTTCGCGATCCTCCTGGCAGAGCCACAGCTCAAG<br>TTTCCCCCACGGAATCACGAGCTTTTGTAAAGGGCAGCGAGGAGG<br>CAGCGAGGTGGCAGCGAGGTACAGTCGCAGAGTTCTCTGGAGAAC<br>GAACACCCCCGCCGCGCCCCACCCCCCAGGACTGGTGGAGACTGG<br>AAGGAACGAGTCCAGACCACGGGACCTTCCCTGGCCGTGGGCCGA<br>CGCCTAGCTGTCCTTGACCTCCCCAGCAGTCGGAGGCGCGCGCGG<br>GAGAAATGAAAGTAAAACCTGTCCTGGCGCGGAACCAAGTGGAA<br>GCTGGCGACAAGTAGGTCGCATCCTCAGAGCGACTGATAGCCAGT<br>CCTGGATCTGCGCGAAGAGCTTGACTCGCAGAGGATAAAGTGAAA<br>GGAAAAGGAAATGGGAGACAAGGGAGAAGAACAATAGTTAGGCT<br>GGGGAGGCTCCTGGTCACCGAGCAGGCGTTCAGTAATAGCTGCTG<br>TCTGCGGTGCAGACGGACTAGTCGCCCGGCCAGGCCACTTCGGTG<br>ACTGCCCTGCGCCCAGCCGGGCGGCCGGATCCAGACCAGTGCGCC<br>GCGCTGCACTCCTCTAACCCTCCGCGTCCCAACCCCTTACCAGGGC<br>CAGGGTCAGGGCCCGGAATCTGTCCTCCGTCCTCAGATGGAATTCA<br>GGTCCGCTCAGGTGACTCCTTCCAGGAAGAGTCCTTAAATAACTTC<br>CGCGCGGCCATTTCTTCCCCAGGTGCAATGTCCACTGTTGAGTCCC<br>GAGAGTGAAAAGGCAGCTAAGCCAGCAGTGCGCGGCCCAGACAG<br>ACCACTCTGGGCAAGGTCCGCGCGACCCACTGCCCAGCCGCCTTT<br>GCACCGGAGCTCAGAGCCATATGGCAGAGTAGGAAGGAAGCTTAA<br>AGGTCTGCTGGGGACCGGCTCTAAGATGGGGACTCGAGAGATGGG<br>ACATGACTCGCCCACCTTCACACAGCGAATTGCCGGGAGGTGAGG<br>ACTAGAAGCCTGTTTGGCTAGTGGTCTCGCACCTTTGGCCTCTCCG

	TCGAGTCGCTGGGCTTCAGGACATTCGATTAGAGGAGTGGTTAGTA<br>GGACCCCAGAAACCCACAAGCACGAAGCAGGAAGCCCCGTGGCT<br>TAGCCTTTCCTTCCCAGTTTGGCCCCCAGGAGAGAAGTAAGAAAG<br>AGAAGAAGCTGTGATGAAAGAGCACAAACGGGTGACAAACGTGT<br>CTAGCGTGATTCATCATGAACAGGCACAAATTCTTTGGGCGGGGGC<br>TAGGACTCTCCTTTGCCCCTTGAGAAGTTGGTGACCCCAGCCTAGA<br>GGAATCCACGCCGCGCCCCAGTCGGCCGTGCTAGCGTGTCGGGCC<br>GAGTAGGGACTCTGCTGCTTCCTACCTGCAGGGTGACTTCCCCTCT<br>CTGCAGATACCTCCCCTCTCTGAGCTTGAATTTTCTACCTGCTGAAA<br>TGGGAAAAGGAATGTTACCTTCCTTGCCTGACTCAAGGGTGGCTGT<br>GAAGCTCAAAGTAGACTGGGATGTGGCCGTGCTTGGTAAACTGTA<br>AAATGATTAGCATACGTGAAGCGTTAGTGTGCTCCCTGGCAGTCAG<br>TCCTCACGTTTACGATGGATTAATGAAGGCAGCCAGGCACAATCTC<br>AGGTTATGACCTTATAAGGCATAATAGGATTACAAGGAGGCAAAAT<br>GAAACAGTATATTTCCTCCTAATTTTGGTTATTCCTATGTTTCCAAAT<br>AGAAATGAAAATTCTGAAATTTCAGATAATTCCCCCCTCCAAATCCC<br>AATTCTCAATTTAGCTTTTCTGAGCCTTGTCCCTACCGTGAAATCTT<br>ACTAGCTCACCCCGAAACGCCGGGGTCTTGGTCCTCTGGGGGACT<br>AGTGAGTCCTGCTCCTCTCGGCTTTGCTCCGTCCCTGCCCCGGCCC<br>CTCCGCGGGAGTCTGGGGTCGCGGCCCGGTCAGGCGCCAAGCCGG<br>CAGGGGGGCGCGGTCCACTTGAGGCCACAGCTCCCCAGGTCCAGG<br>GCTCGGGCCATCCGCGCTGTCCCTTCCGCGGGCTCTTGCTGTTCTT<br>CGCCAGGAGGCTTTGCACTCCGGGCCGCATGGGTCCTGGATAAGG<br>ACCTCAAGAGGTTGTAGTGGCGCCCCTTCGCTCCGGCGTACTGTCT<br>GAACCCTGTGCCCAAAGAGGGCTGTGGCAGTTCCTGCACCAACCG<br>CCTGGAGCCCATACCTAAGCCCTCTGGGCACGAGGGACCTCCTCTC<br>CCCCGCTCCCCCCTCCCCCCATCCCAACTCCAGCCCCAAAGGAAGC<br>AATGCGCGCGTCCGAGAGCAGGAGCGCGCGTGGCTGAGGAAAGA<br>AAGGGAAGGCAACCGGGCAGCCCAGGCCCCGCCCCGCCGCTCCCC<br>CACCCGTGCGCTTATAAAGCACAGGAACCAGAGCTGGCCACTCAG<br>TGGTTTCTTGGTGACACTGGATAGAACAGCTCAAGCCTTGC
AFP	TTCTATGGGGCTTGTTTTAAGCTTGGCAACTTGCAACAGGGTTCAC<br>TGACTTTCTCCCCAGGCCCAAGGTACTGTCCTCTTTTCATATCTGTT<br>TTGGGGCCTCTGGGGCTTGAATATCTGAGAAAATATAAACATTTCAA<br>TAATGTTCTGTGGTGAGATGAGTATGAGAGATGTGTCATTCATTTGT<br>ATCAATGAATGAATGAGGACAATTAGTGTATAAATCCTTAGTACAAC<br>AATCTGAGGGTAGGGGTGGTACTATTCAATTTCTATTTATAAAGATA<br>CTTATTTCTATTTATTTATGCTTGTGACAAATGTTTTGTTCGGGACCA<br>CAGGAATCACAAAGATGAGTCTTTGAATTTAAGAAGTTAATGGTCC<br>AGGAATAATTACATAGCTTACAAATGACTATGATATACCATCAAACA<br>AGAGGTTCCATGAGAAAATAATCTGAAAGGTTTAATAAGTTGTCAA<br>AGGTGAGAGGGCTCTTCTCTAGCTAGAGACTAATCAGAAATACATT<br>CAGGGATAATTATTTGAATAGACCTTAAGGGTTGGGTACATTTTGTT<br>CAAGCATTGATGGAGAAGGAGAGTGAATATTTGAAAACATTTTCAA<br>CTAACCAACCACCCAATCCAACAAACAAAAAATGAAAAGAATCTC<br>AGAAACAGTGAGATAAGAGAAGGAATTTCTCACAACCCACACGT

	ATAGCTCAACTGCTCTGAAGAAGTATATATCTAATATTTAACACTAA<br>CATCATGCTAATAATGATAATAATTACTGTCATTTTTAATGTCTATAA<br>GTACCAGGCATTTAGAAGATATTATTCCATTTATATATCAAAATAAAC<br>TTGAGGGGATAGATCATTTTCATGATATATGAGAAAAATTAAAAATC<br>AGATTGAATTATTTGCCTGTCATACAGCTAATAATTGACCATAAGAC<br>AATTAGATTTAAATTAGTTTTGAATCTTTCTAATACCAAAGTTCAGTT<br>TACTGTTCCATGTTGCTTCTGAGTGGCTTCACAGACTTATGAAAAA<br>GTAAACGGAATCAGAATTACATCAATGCAAAAGCATTGCTGTGAAC<br>TCTGTACTTAGGACTAAACTTTGAGCAATAACACATATAGATTGAGG<br>ATTGTTTGCTGTTAGTATACAAACTCTGGTTCAAAGCTCCTCTTTATT<br>GCTTGTCTTGGAAAATTTGCTGTTCTTCATGGTTTCTCTTTTCACTG<br>CTATCTATTTTTCTCAACCACTCACATGGCTACAATAACTGTCTGCA<br>AGCTTATGATTCCCAAATATCTATCTCTAGCCTCAATCTTGTTCCAGA<br>AGATAAAAAGTAGTATTCAAATGCACATCAACGTCTCCACTTGGAG<br>GGCTTAAAGACGTTTCAACATACAAACCGGGGAGTTTTGCCTGGAA<br>TGTTTCCTAAAATGTGTCCTGTAGCACATAGGGTCCTCTTGTTCCTT<br>AAAATCTAATTACTTTTAGCCCAGTGCTCATCCCACCTATGGGGAGA<br>TGAGAGTGAAAAGGGAGCCTGATTAATAATTACACTAAGTCAATAG<br>GCATAGAGCCAGGACTGTTTGGGTAAACTGGTCACTTTATCTTAAA<br>CTAAATATATCCAAAACTGAACATGTACTTAGTTACTAAGTCTTTGA<br>CTTTATCTCATTCATACCACTCAGCTTTATCCAGGCCACTTATTTGAC<br>AGTATTATTGCGAAAACTTCCTAACTGGTCTCCTTATCATAGTCTTAT<br>CCCCTTTTGAAACAAAAGAGACAGTTTCAAAATACAAATATGATTT<br>TTATTAGCTCCCTTTTGTTGTCTATAATAGTCCCAGAAGGAGTTATAA<br>ACTCCATTTAAAAAGTCTTTGAGATGTGGCCCTTGCCAACTTTGCC<br>AGGAATTCCCAATATCTAGTATTTTCTACTATTAAACTTTGTGCCTCT<br>TCAAAACTGCATTTTCTCTCATTCCCTAAGTGTGCATTGTTTTCCCTT<br>ACCGGTTGGTTTTTCCACCACCTTTTACATTTTCCTGGAACACTATA<br>CCCTCCCTCTTCATTTGGCCCACCTCTAATTTTCTTTCAGATCTCCAT<br>GAAGATGTTACTTCCTCCAGGAAGCCTTATCTGACCCCTCCAAAGA<br>TGTCATGAGTTCCTCTTTTCATTCTACTAATCACAGCATCCATCACA<br>CCATGTTGTGATTACTGATACTATTGTCTGTTTCTCTGATTAGGCAGT<br>AAGCTCAACAAGAGCTACATGGTGCCTGTCTCTTGTTGCTGATTATT<br>CCCATCCAAAAACAGTGCCTGGAATGCAGACTTAACATTTTATTGA<br>ATGAATAAATAAAACCCCATCTATCGAGTGCTACTTTGTGCAAGACC<br>CGGTTCTGAGGCATTTATATTTATTGATTTATTTAATTCTCATTTAACC<br>ATGAAGGAGGTACTATCACTATCCTTATTTTATAGTTGATAAAGATAA<br>AGCCCAGAGAAATGAATTAACTCACCCAAAGTCATGTAGCTAAGTG<br>ACAGGGCAAAAATTCAAACCAGTTCCCCAACTTTACGTGATTAATA<br>CTGTGCTATACTGCCTCTCTGATCATATGGCATGGAATGCAGACATC<br>TGCTCCGTAAGGCAGAATATGGAAGGAGATTGGAGGATGACACAA<br>AACCAGCATAATATCAGAGGAAAAGTCCAAACAGGACCTGAACTG<br>ATAGAAAAGTTGTTACTCCTGGTGTAGTCGCATCGACATCTTGATGA<br>ACTGGTGGCTGACACAACATACATTGGCTTGATGTGTACATATTATT<br>TGTAGTTGTGTGTGTATTTTTATATATATATTTGTAATATTGAAATAGT<br>CATAATTTACTAAAGGCCTACCATTTGCCAGGCATTTTTACATTTGTC

	CCCTCTAATCTTTTGATGAGATGATCAGATTGGATTACTTGGCCTTG<br>AAGATGATATATCTATATCTACATCTATATCTATATCTATATCTATATCTA<br>TATCTATATATGTATATCAGAAAAGCTGAAATATGTTTGTAAAGTTA<br>TAAAGATTTCAGACTTTATAGAATCTGGGATTTGCCAAATGTAACCC<br>CTTTCTCTACATTAAACCCATGTTGGAACAAATACATTTATTATTCAT<br>TCATCAAATGTTGCTGAGTCCTGGCTATGAACCAGACACTGTGAAA<br>GCCTTTGGGATATTTTGCCCATGCTTGGGCAAGCTTATATAGTTTGCT<br>TCATAAAACTCTATTTCAGTTCTTCATAACTAATACTTCATGACTATT<br>GCTTTTCAGGTATTCCTTCATAACAAATACTTTGGCTTTCATATATTT<br>GAGTAAAGTCCCCCTTGAGGAAGAGTAGAAGAACTGCACTTTGTA<br>AATACTATCCTGGAATCCAAACGGATAGACAAGGATGGTGCTACCT<br>CTTTCTGGAGAGTACGTGAGCAAGGCCTGTTTTGTTAACATGTTCC<br>TTAGGAGACAAAACTTAGGAGAGACACGCATAGCAGAAAATGGAC<br>AAAAACTAACAAATGAATGGGAATTGTACTTGATTAGCATTGAAGA<br>CCTTGTTTATACTATGATAAATGTTTGTATTTGCTGGAAGTGCTACTG<br>ACGGTAAACCCTTTTTGTTTAAATGTGTGCCCTAGTAGCTTGCAGTA<br>TGATCTATTTTTTAAGTACTGTACTTAGCTTATTTAAAAATTTTATGTT<br>TAAAATTGCATACTGCTCTTTCATTGAAGAAGTTTTGAGAGAGAGA<br>TAGAATTAAATTCACTTATCTTACCATCTAGAGAAACCCAATGTTAA<br>AACTTTGTTGTCCATTATTTCTGTCTTTTATTCAACATTTTTTTAGA<br>GGGTGGGAGGAATACAGAGGAGGTACAATGATACACAAATGAGAG<br>CACTCTCCATGTATTGTTTTGTCCTGTTTTTCAGTTAACAATATATTAT<br>GAGCATATTTCCATTTCATTAAATATTCTTCCACAAAGTTATTTTGAT<br>GGCTGTATATCACCCTACTTTATGAATGTACCATATTAATTTATTTCCT<br>GGTGTGGGTTATTTGATTTTATAATCTTACCTTTAGAATAATGAAACA<br>CCTGTGAAGCTTTAGAAAATACTGGTGCCTGGGTCTCAACTCCACA<br>GATTCTGATTTAACTGGTCTGGGTTACAGACTAGGCATTGGGAATTC<br>AAAAAGTTCCCCCAGTGATTCTAATGTGTAGCCAAGATCGGGAACC<br>CTTGTAGACAGGGATGATAGGAGGTGAGCCACTCTTAGCATCCATC<br>ATTTAGTATTAACATCATCATCTTGAGTTGCTAAGTGAATGATGCAC<br>CTGACCCACTTTATAAAGACACATGTGCAAATAAAATTATTATAGGA<br>CTTGGTTTATTAGGGCTTGTGCTCTAAGTTTTCTATGTTAAGCCATAC<br>ATCGCATACTAAATACTTTAAAATGTACCTTATTGACATACATATTAA<br>GTGAAAAGTGTTTCTGAGCTAAACAATGACAGCATAATTATCAAGC<br>AATGATAATTTGAAATGAATTTATTATTCTGCAACTTAGGGACAAGT<br>CATCTCTCTGAATTTTTTGTACTTTGAGAGTATTTGTTATATTTGCAA<br>GATGAAGAGTCTGAATTGGTCAGACAATGTCTTGTGTGCCTGGCAT<br>ATGATAGGCATTTAATAGTTTAAAGAATTAATGTATTTAGATGAATT<br>GCATACCAAATCTGCTGTCTTTTCTTTATGGCTTCATTAACTTAATTT<br>GAGAGAAATTAATTATTCTGCAACTTAGGGACAAGTCATCTCTTTG<br>AATATTCTGTAGTTTGAGGAGAATATTTGTTATATTTGCAAAATAAAA<br>TAAGTTTGCAAGTTTTTTTTTTCTGCCCCAAAGAGCTCTGTGTCCTT<br>GAACATAAAATACAAATAACCGCTATGCTGTTAATTATTGGCAAATG<br>TCCCATTTTCAACCTAAGGAAATACCATAAAGTAACAGATATACCAA<br>CAAAAGGTTACTAGTTAACAGGCATTGCCTGAAAAGAGTATAAAAG<br>AATTTCAGCATGATTTTCCATATTGTGCTTCCACCACTGCC

AGT	AAACGACTTCTGTCTGTCAACAGATGAATAGATAAAGATTTGGCAT<br>ACTGTATACACACAATGGGATACTATTCAGCCTTCAAAAAAGCATA<br>AAGGCCAGGCATGGTGGCTCATGTCTTAATCCCAGCACTCCGGGAG<br>GCTGAGGTGGGCAGATCACTTGAGCTCAGGAGTTTGAGACCAGCC<br>TGGGCACCATGGTGAAACCCTGTCTCTACAAAAAAGTACAAAAAA<br>GTTGGCCAGGTGTCATGGCATGCATCTGTAGTCCCAACTACCAGGG<br>AGGCAGAGGTGGGTGGATCACTTAAGCCAGGGAGGTCAAGACTGC<br>AGTGAGCCATGATTGTGCCACTGCACTCCAGCCTGGGTGACAAAG<br>TGAGACCCTGTCTCACAAAAAAAAAAAAAAAAAAAAGCGGTGTGG<br>AACTGTGCCGCTTGCTACAACATGGATGACTGGAAGACATTATGCT<br>AAATGAAACAAGGCAGATACAGAAAGGAAAATACTGCATGACCTC<br>ATTTATATGTGGAATCTTTTTTTTTTTAAAGTGAATATACAGAGATA<br>GAGAATACAACAGTGGTTACCAGGAGTTGAGGAGGAGAGCAGGG<br>AGGAGATGTAGGTCAAATGATACAAAGCAGCAGATATATACGATGA<br>ACAAGTCTAGAGATCCAGCGTACAACATGAGGACTATACTTAAAAA<br>TGTCACATTGCACTTGGGATTTTTGCTAAAAGAGTAGACTTTACGT<br>GTCCTTGCCACCTAAATGAGTAACTGTGAGATGATGGATATGTTAAT<br>TTTCTTGACTACCATAATCATTTTACTATCTTCATATCAAAATATCATG<br>CTGTACACCTTATATAAATACAGTAAAAATTATTATTAAAAAAGAAA<br>GAACAGGCTGAGAATGGTGGCTTATGCCTGTAATCCCAACACTTTG<br>GGAAGTTGAGGTAGGAGGATCACTTGAGACCAGTAGTTCAAGATC<br>AGCCTGGGCAACATAGCAAGACCCTATCTCTACAAAAAATAAAAAT<br>AAAAAAATTAGCCAGATATGGTAGCATGCACCTGTAGTCCTAGTTAC<br>TCTGGAGACTGAGGCAGGAGGATTCTTTGAGACATGATCATGCCAC<br>TGCACTCCAGTTTGGAGGACAGAGCAAGACCCCAACTCTAAAACA<br>ACACACAAAAAATACAGCCATTTCTCCTGGGTGAAATGGGGGAGCT<br>CAGACTATTTTGTTTTATTTGTCAGGAGTCTAAAGCCCATGTGAGCC<br>AACTTAAGAAAATTCTGTTTGTATTTGAGTGTTCTTTATTGGAAAGA<br>AATGGACTTTTCCCTAATGAGCCCATTTTACAAATCGAGAGCTCCA<br>AATGCCCGGTTCCAGCCCCCATGATATAGATGGGCAGCAATGAGGA<br>CCAGAAATGCTGGGAAGTGTCCTTGTCATGCAAGGAAAACTTGGA<br>ACTTCAAGGATCCATCATGGGATGCAGGACAGTAGGCTCCACCCCT<br>CTTCCTGCTTTCCAGTAAATGATGTTCAGGGCTCACATGCCTCTTCC<br>AAGGCATGATGTGGGGTTGCAGTTCTGATCCCAGCAGAGAACAAG<br>GCTCCTGTGAAACAATTAGTTTTGGCTCAGAGGCAAAAAATGGAA<br>ACCCCATTCCTGTATTTACCCTTCATTCTTTCCTTACCTCATACAGCT<br>GGTTCCAGGTTTGATTGCATCATATACATAAATAAATAATCTGCTTTC<br>CTGGTTTGGTTTAGTTTTGCTGGAGAGAGGAGTTTATGTGTTCATCC<br>CATGACTAGCTGGGTGGTCAAGAGATTGGAAAGTAGGAGTTCTAGT<br>TTAGACCAAGTCTCATCTCAGAAACCACAGAGTAGAACTGATCCCA<br>AACGTCATCATCCCTGTAGGGAAAAGAAAGAGAGATCAGACTGTT<br>ACTGTGTCTATATAGAAAGGGAAGACATAAGAGACTCCATTTTGAA<br>AAAGAGCTATACTTTAAACAATTGCTTTGCTGAGATGTTGTTAATTT<br>GTAGCTTTGCCCCAGCCACTTTGACCCAACCTGGAGCTCACAAAA<br>ACATGTGTTGTATAAAATCAAGGTTTAAGGGATCTAGGGCTGTGTC<br>AAAGTGGCTGGGGCAAAGCTACAAATTAACAACATCTCAGCAAAG

CAATTGTTTAAAGTACAGCTCTTTTTCAAAATGGAGTCTCTTATGTC
TTCCCTTTCTATATAGACACAGTAACAGTCTGATCTCTCTTTTTTTC
CCTACATATCCCAATAGCACCTGCCTGACTAATACATCATGCTCTGC
TGACTCCATATGTGGCTGGTTTCCTGGATCCGATTCTGACTGATGGT
ATCTGTTCTCACGCTGCTAATAAAGACATACCAGAGACTGGGTAATT
TATAAAGAAAAAGAGGTTTAATGGACTTATGTGTTCCACGTGGCTG
GGGAGGCCTCACAATCATGGTGGAAGGTGAAGGAGGAGCAAAAG
CACATCTTACATGGTGGCTGGCAAAAGAGAGAAAGCATGTTCAGG
GGAACTCCCCTTTATAAAACCATCAGATCTCATGAGACTTATGCACT
ATCACAAGAACAGCATCAGAAAGATCCACCCTCATGATTCAATTAC
CTCCCACTGGGTCCCTCCCATGACACATGGGAATTATGGGAGCTAC
AATTGGAGATTTGGGTGGGGACACAGCCAAACCATATCAGATGGCT
TATTTGGTTTCTATGTAGAACCTCTGCTTTTCATTCAACAGTCTTCAT
TTAGCCACAGATAAGCTCTGTCCCTAACTTCCACTGATGGAATGTAC
ACATAAGAAACTTCCACTGATGGAATGAACACAGAAGGTGCCTACT
GGGAAGAAAACTGGCCTGAATCTGAGCTGGGTCAAATGTCTGCAG
TCAGTTTGAATGGCTGCTCCTTATGGGAATAATTTACATTCTCAATA
AAATTCTCTAGCAATTTTCTGATTGATTTTAATGAGCTTTAAAGCCTT
ACGTAGAAGATCCCCCAGCTGATAGTCAGCCTTGGGCATGGATTAA
GGGCTTTTAACCAATCTTGCAACAAGTTTAAGCAGATATTCTTTATT
GGGTCCAATCTAACCAAAATTATTTCTTATGTTCTCCCCAGTAACG
TGTCATTATTAAGAGAAGTTTGGCTTGCTTAGAGGCCAAATTTAGA
GGGTCCTGAAATTTTATTTTCTTTTACACCACTTTCCAGCATGTTAC
CTGATCAGTTGTTTATTATCTTTGCTGTTGAATGGAGTGATCATTCCA
AGGGCCCGAGGCAGGAGGCCCAGGCACAGTGGAAACTCTCCCAA
AGACCAGGATCTTTGTTTTGTTCCCTGACATATGCTGAGCACCAGG
AATAGTGAGTGAATGAAACAAATTGTGAGGCTTTAAAGAGCCGAA
ATATTTAAACACTGGGCACAAGGTTGTTGCTTAATCAGTGCTAGATC
CTTACCTCCCCCTTGTGTCCAGGTGGACTTGTTACTGCAGTTAAAC
CACTTGCTGATCCTCAAACAACTAGTTAGTGGCACAGCCAGGCCTA
GGACCCCAGTCTCTACTGTTCCAACTAACCCATTCGCAGGCAGGAG
CACTTTGAATGGTCTCTTATTTTAAAAAAATTAAATTAAAATTGTCTA
TTTATTTAGAGACAGAGTCTTACTCTGTAGCCCAGGCTCGAGTGCA
GTGGTGCAATCATAGCTCACTGTAACCTCCATCTCCTGGCCTCAAA
AAGTGTTTGAATTACAGATGCGAGGCACTGTACCTGGCCCGAATGT
TCTGTTCAGACAAAGCCACCTCTAAGTCGCTGTGGGGCCCCAGAC
AAGTGATTTTTGAGGAGTCCCTATCTATAGGAACAAAGTAATTAAA
AAAATGTATTTCAGAATTTACAGGCCCATGTGAGATATGATTTTTTA
AATGAAGATTTAGAGTAATGGGTAAAAAAGAGGTATTTGTGTGTTT
GTTGATTGTTCAGTCAGTGAATGTACAGCTTCTGCCTCATATCCAGG
CACCATCTCTTCCTGCTCTTTGTTGTTAAATGTTCCATTCCTGGGTAA
TTTCATGTCTGCCATCGTGGATATGCCGTGGCTCCTTGAACCTGCTT
GTGTTGAAGCAGGATCTTCCTTCCTGTCCCTTCAGTGCCCTAATACC
ATGTATTTAAGGCTGGACACATCACCACTCCCAACCTGCCTCACCC
ACTGCGTCACTTGTGATCACTGGCTTCTGGCGACTCTCACCAAGGT
CTCTGTCATGCCCTGTTATAATGACTACAAAAGCAAGTCTTACCTAT

	AGGAAAATAAGAATTATAACCCTTTTACTGGTCATGTGAAACTTACC<br>ATTTGCAATTTGTACAGCATAAACACAGAACAGCACATCTTTCAAT<br>GCCTGCATCCTGAAGGCATTTTGTTTGTGTCTTTCAATCTGGCTGTG<br>CTATTGTTGGTGTTTAACAGTCTCCCCAGCTACACTGGAAACTTCC<br>AGAAGGCACTTTTCACTTGCTTGTGTGTTTTCCCCAGTGTCTATTAG<br>AGGCCTTTGCACAGGGTAGGCTCTTTGGAGCAGCTGAAGGTCACA<br>CATCCCATGAGCGGGCAGCAGGGTCAGAAGTGGCCCCCGTGTTGC<br>CTAAGCAAGACTCTCCCCTGCCCTCTGCCCTCTGCACCTCCGGCCT<br>GCATGTCCCTGTGGCCTCTTGGGGGTACATCTCCCGGGGCTGGGTC<br>AGAAGGCCTGGGTGGTTGGCCTCAGGCTGTCACACACCTAGGGAG<br>ATGCTCCCGTTTCTGGGAACCTTGGCCCCGACTCCTGCAAACTTCG<br>GTAAATGTGTAACTCGACCCTGCACCGGCTCACTCTGTTCAGCAGT<br>GAAACTCTGCATCGATCACTAAGACTTCCTGGAAGAGGTCCCAGC<br>GTGAGTGTCGCTTCTGGCATCTGTCCTTCTGGCCAGCCTGTGGTCT<br>GGCCAAGTGATGTAACCCTCCTCTCCAGCCTGTGCACAGGCAGCCT<br>GGGAACAGCTCCATCCCCACCCCTCAGCTATAAATAGGGCATCGTG<br>ACCCGGCCGGGGGAAGAAGCTGCCGTTGTTCTGGGTAC
AKR1C4	GGAACGAGAATCACTTGAACCCAGGAGGCAGAGTGTGCAGTGAG<br>CCGAGATCGTGCCACTGCACTGTAGCCTGGGCAATAGAGCAAGAC<br>TCCATTTCAAAAAAAAATAGAAACATCTCAAATTAACCACCTAAT<br>GTCACACCCCTAAGAACTAGAAAAACAAGAACAAATCAAACCCAA<br>AGCTAGCAGAAAAAAAATAAGAAATATTAGAGGAGAACCAAATG<br>AGATTGAGACTAAAACAATGATGCCAGAATCAAAAATATTAAAAAT<br>ATGTTATTTGAAAGGTTAAACAAAATTATAGACTGCTAGTTATATTA<br>ACCAAGAAAGACAGAGAAAATTCAAATGAGCAAAATCAGAAATGA<br>CAAAGGCAACATTGCAACTGTTAGAACAGAACACAAAAACTAACA<br>AATATTGCTATGAACATCTCTGTGCACACAAAATAGAAAACCTGAA<br>GAAAATGATTGAATTTTTGGAAATACACAAGTTCTCAAAATTGAAC<br>CAGAAATAAATAGAAATTCTAAACACACCAATAATAAGCAAGCACA<br>TTTAATCAGTAAAAGAGCAAGAACAAAAAAATCCCAGGACGAGAT<br>GAATTCACAGCCAAATTTTATGAGATGTACAAAGAAGATCCGAAAC<br>AAACCTTCCTGAAGCTATTTCATAGTATCCCTCATTATAGGAAACAA<br>GTGTCACCTTGATACCAAAATCAGGCAACAACACGACTAAAAATG<br>AAATCTAAAGGCCGATATCCCTGATGAACATGGATGCAAAAATTCT<br>CAACAAAATACTACCAAACTGAAACAAAGAGCACATCGAAGAGAT<br>AATTTATCATGATCAAGTGGGTTTTCTTGCAGGAATAAAGGATGTTT<br>CAACATTCACAAATTAATAAATTTGATTCACCATATAACTAAGATTTA<br>AACAAAAACCATATGATCATCTCAAAAGAGACATAAAGCATTGCAT<br>AAAATCCAACATTTCTTTCTCATAAAACCCTTAATAAACTATGTATTG<br>AAGACATATCTCAAAATAATAAGTCTTACATAACAAACCTGCAGCC<br>AACGTCTTCCTGAATAGGAAAATGTTGAAAGCATTCACCCTACGAA<br>CTGGCAAAAGACAAGAATGTCCATTGTCACCACTCTTACTTAACAT<br>AGAACTGGAGGTCCTCACCAGAAAACCCAGGCAGTAGAAAAAAT<br>AAAAGCCATCCAAGTTGGAAAAGAGAAATTCAAATTATCAATTTTT<br>TTCTGATGACATGATTTTATACCTATCAAACTATAATGACTCCTTCAA<br>AGGACTGCTTGATTGAAAAAAAAAACTTCAATAAAATTTGAAGAT

	ACAAAATCAATGTAGAAAAATCAGTAGCATTTCTATACACCAATAAT<br>GTTGAAGCTAAAACCCAATTAATAATTCAGTCCCATTTGCAATAGAC<br>AAAAACAGAATAAAATATCTAGATATAAATAAAATTAAGCAGGTGA<br>AAGATCTCTACAAGGAGAAATACAAAATACTGATGAAAAAAATCAT<br>GGATGAAACAAGCAAATAAAAAGATTACCATGCTTATGGATTGGGA<br>GAATCAATATCATGAAAATGATCATACTGCCCAAAGCAAACTACTGA<br>TTCAATGCAGTTCCTAACAAAGTACCAACATTATTCTTCATATAATTA<br>CAAAAAAATCTAAATTTCATAAAAAGGCAATCCTAAGCAAAAGAA<br>CAAAGTCACAGGTATCACTTTACCTGACTTCAAATTATACTAACAGA<br>CTATAGTAACTAAAATAACATGGTACTGATACAACAGTAGACACGT<br>GGATAAATAAGAATAGAGAACCTAGAAATAAAGCTACATACATACA<br>ACAAACTGATCTTTGACAAAGTTAAAGAAAAACAAACAATGAGAA<br>AAGGACACTGTAACCATTAAATGGTATTGAGAAAATTGGCTAGCCA<br>AAGCTGAATAATGAAACTAAACCCCTATCTCTTACCATTGGCAAAG<br>TTAAGTCAACGTGGATTAAGGACCTACAAGGAAAATCTGAAACTAT<br>GAATGTCCTAGGAGAAAACCTTGGAAAAACTCTTCTGGACATTGA<br>GCTAGGCCAAGATATAATGATGAAGATGCCAAAAAGGAAATGTAAC<br>AAAGACAGATAAATGGGAATTAAACTAAAAAACTTATGGGCAGCA<br>AGAGAAATAATCAACAGAATATCTACAGAATGGGAAAAATATTTTC<br>AAATTTAGGCTCCAACAAAGGACTTTTATTTAGATTCTATAAGGAAC<br>TCAAACCAACTCAACAAGTAAAACACAAACAATCCCATTAAAAAC<br>TAGGCAGAAGACATGAACAGACATTCATCAAATGAAGACATACAA<br>GCAGCTGACAAATATACAGGAAACTGTTTTTAATAGTCATCAGAAA<br>AATGCAAATTAAAAGCACATTGAGATGTCATCTTGCACCAGTCAGA<br>ATGACTATTATTAAAATGTCAAAAAACAACAAATATCAGTGTGGATG<br>TGGAAAGAAGGCAAAATGTGCATATACATACTAATATGCATATATATA<br>TATACTAAGTGTACATACGTATACTAAGCATATATGCTTATGGAATACT<br>AAGCATCCACTAAAAAGAGTGAAATTAAGTCCTTTTCAGCAGCATG<br>GATGGAACTAAAGGATATTATTCTAAGTAAACTATCGTACAAGCACA<br>ACAAAATACCACATATTCTCATTTATAAGGGGAAGCTAAACAATGTG<br>TACACACGGACATAAACATGGAAATAATAGACATCAAGTACTTCAA<br>AAGGGAGAAAGGTGGAGACAGGGGCAAAGATGACAAAACTTCCT<br>ATTGGGTACAATGTTCACTATTTAAGTGATGTGTACACCAGAGGCCC<br>AAACCTCACCAGTATGTAATGTGTCTATATAACAAACCTACACATGT<br>GCCTATTTAATCTAAAATAATTTTTAAGACTTTAATAAGAAATAAAAA<br>TCCGAAAGTCTTTCCTTACTAATATCAATGCCTGTAATTCTTCACAG<br>TTTGTCAGTTTATTTTCTTCCTCATAATGAACGCTGTTTTCCTGTTTC<br>TTTGTAGCTTTGTTATTGTTTTTATCGCACATTTGAAAAACAGCCAC<br>ATTTCATAATCTATATATATTCTGTGTCATGACAATGATCAGCTAATAT<br>GCTTGGCATATTCTTAGTCTCGACATCAGCCCTACATGAAAACTTAA<br>GGTCTTCTCAGTTCTTTTCTGAACGTGCATCTGCCTAGACTCTGTGT<br>GCCCTATTTGATTTCTCCAAATACCCAATGGTTTTGAATACCTTATCA<br>TGTCAAAAATTCACAGCTCAGCTTCTCCTAAGTGCCATAGATGGCC<br>TATTGTATGTCTCTTCCCCCTAATCTCTTGCCAGTGGCATCTGTGCAT<br>CTATAGTCACCCTGCATCTCTACTGAGCCACTCTATAAATAAATGAG<br>TTTATTTTAGTTCATTTCAGATTGTTTTTTGATAATTCCATGGGAGAT

	TTGAGAGCTTTCTAATCTACCATCCTGCTGATGACACTCTGTGTGAA<br>AATTTTTAAATGGCTCTATTATATTCCATTGCTTCAATGTATAGTAATT<br>TACCTAATTATTTCTTTTATTGTTCCTTTTAGGAAGAAGTGAATAGA<br>AGAAAAGAGGGTGTTGCAAATCAAAAGCACATAGAGATATCATCTT<br>ACACCAGTCAGAATGGCTATTCTTAAAATGTCAGACAACAACAGAT<br>ATTGGTATGGATTCTTCACAGATGTTAATCATCTTCGTTTTTTGTTTT<br>TTGTTTTTTTTTTTTTTTTTGAGACAGTCTTGCTCTGTCGCCCAGGC<br>TGGAGTGCAGTGACCCAATCTTGGCTCACTGCAACCTCTGCCTCCT<br>GAGTTTAAGCAATTGTCTTGCCTCAGCCTCCTGAGTAACTGGGATT<br>ACAGGTATCCACTACCACTCCAGACTAATTTTTGTATTTTTCGTAGA<br>GACAAGATTTCACTTGATTGGTCTCAAACTCCTGACCTCAAGTAAA<br>CCTACCGCCTTGGCCTTCCAAAGTGCTGGGGTTACAGGCGTGAGCC<br>ACTGCCCCCGGCCTAATCATCGTGTTCTAGTCTGCCCTCCAATGGGA<br>GAAACAAGTAAAATTATGCCATGTGAGGATTATTCACCAATTTATTT<br>TAATTACTTTTTTCTTTATAACATTTAATAAGATCACAAATTATATAAA<br>ATAGTTATCAGCTTTTTGGGAAGTTACTTTTGCTGGTTTCTTATAAA<br>ACTGATGGAAGATACAAACACTATTAAAGAACTGTTTGCATGTTGC<br>AAATGATGTCCAAAGTCCAAACATTGTTAATAATTAATACTCCAATA<br>AACATCATGTCAGAATTTCTGTTTTCTTTTCCCTTTGAACCTTTGCA<br>GGATTGCCACATCATCAGGACCACACCTTCATCAGGAATGAATATTC<br>CTACTACAATTAAAGAAGAAACAAAATTAATTTGTTGGTGAAAAAC<br>GTAAAAAGAGAAATTTTCTTTGGTTTTGTTTAATTTCTTTATTGAGG<br>GTCACCACTAAAAAAAAAATGCTCACTGGTCATTCTTTTGAATACTG<br>TCTGAGAGAAAGATGTAAGATGGTTGATTATTTCAAATGACAGAAG<br>ATAAAGATGGGATATCATCATGGCATGAACAAAAACAAGATTTGTA<br>GCTGGAGGTATTTTATAGTCTAACATGATCACCAATCATTCTATAAAC<br>CTGTTGGATGAGTTTATCAGACAGACAGAGAGAGAGATTGATTGAT<br>TCTGAATAGAAAATTTCACTTTAGAAAAAAATATTTTGACTATATAAT<br>AATGTATGTAAAAATTCTCTTTGATAAGAAACGAGTGAACTGGATT<br>CAATTTTCCTCACAGCCTGTGTAATACACCATCACTTGCTTCCTCCT<br>ACATGCCATTGATTAGCCCCAGGGAGCAGTGCAGCACTGCCTGCCC<br>ATGTTTTACATAACCCCTGAATATAAATGCCAGATGTTGCTGAAGGA<br>AACAGGATCTGCTTAGTGAAAGAAGTGGCAAGCAATGGATCCCAA<br>ATATCAG
AKT2	CTTTTAAATATTTTATTTATTTATTTATTTATTTATTTATTTATTTTTTGA<br>GCTAGAGTCTCGCTCTGTCACCCAGGCTGGAGTGCAGTGGCACAG<br>TCTCGGCTCACTGCAACCTCTGCCTCCTGGGTTCAAGTGATTCTCAT<br>GCCTCCGCCTCCCAGTAGCTGGGATTACAGGTGCCTGCCACCTCCC<br>GGGCTAATTTTTGTATTTTTAGTAGAGACGGGGTTTCGCCATGTTGG<br>CCAGGCTGGTCTCTAACTCCTGGCCTCAAGTGATCCTCCTGCCTCG<br>GCCTCCCAAAGTGCTGGGATTACAGGTGTGAGACACTGTGTCCAG<br>CCTAATATTTTAAATAAAAAATAAAAATGGGGCCGGGTACGGTGGC<br>TCACGCCTATAATCCCAGCACTTTGGGAGGCTGAGGTGGGCGGATC<br>ACGAGGTCAGGAGATTGAGACCATCCTGGCTAACATGGTGAAACC<br>TTGTCTCTACTAAAAATACAAAAATCAGCCGGGCATGGTTGTGGGT<br>GCCAGTAATCCCAGCTACTCGGGAGGCTGAGGCAGAAGAATCACT

	TGAACCCAGGAGGTGGAGGTTGCAGTGAGCCGAGATCGCACCACT<br>GCACTCCAGCCTGGTGACTGAGAGAGACTCCATCTCAAAAAAAAA<br>AATTATTTTGTGACTAGTCCCCACAGCTTGTCCAGTTGCCATCGATA<br>AGGAGACTCCTCTGTGAGAAGCCTTGGAATGGACTCCTGGGCCTG<br>ATTTCTACTCCCTGGGCACAGAAGGGGGCTTTGAGGAGATGTCCTC<br>TCCAACCCTTGGGGCAAGATCCCTCATCGTTTTCTTTTTTCTGAATC<br>AAGAGACCTTTGAACAGCTTTCAATAAGTTACCGGCCCCCTCTACC<br>CAGCAAAGTGCATGTGCAGTCCATACTTATAGTTATGGGGAGAGAG<br>TTTATAGACTAGATTAAGATGGAAGAAAATGGGGTGAGGTTGTAGG<br>GAATTTTCTTCTGGTTCACACTTTTTTTCTTTCTTTTTTTTTTTTTTT<br>GAGATAGAGTTTTGCTCTTGTTGACAAGGCTGGAGTGCAATGGTGC<br>CACCTCCGCTCACTGCAACCTCCACCTCCCGGGTTCAAGGAATTCT<br>CCTGCCTCAGTCTCCCAAGTGGCTGGGATTATAGGCGCCTGCCACC<br>ACGCCCGGCTAAATTTTTGTATTTTTAGTAGAAATGGGGTCTCACCA<br>TGTTGGCCAGGCTGTTCTCGAACTCCTGACCTCAGGTGATCCACCT<br>GCCTCTGCCTCCCAAAGTGCTGGGATTACAGGTGTGAGCCACCATG<br>CCCAGTCGCCTCGATCGATCACCCTTGATGCTGAAATGGAAAGGGT<br>CTCGCTGTGTTGCCCTGCCACAAGATTCAAAGTGGCCCTCAAGTTT<br>CACCCTTGATGCTGAAAATAGAGAAAGAGTCTCGCTATGTTGCCCA<br>GGATGGCCTTTGAGGCCCCCCAAAGATAACTAAGCCATTTGCAGAA<br>GTAACATAGGGCACAAAAGTAGGGCCAATACTTTTCCATTTTCTAG<br>CATGAGTTGAGTTAAGAGCACAGGCTCTGGTATCAAACTGTCTGGG<br>TTCAAATACTGTCTTTGTCCTTAACTTGTCCTGGGAACTCAAGCAA<br>GGCCCTTAATGTGCTGTTACATTTAACCTCCAGGGTTACTGTGTAGA<br>TAGCAACAGGATAGCATCAGTGGGAGTGTGTTTACACAGAACTTGG<br>CCTGTAACAATTGCTAAATAAATTATATTATTACTGTCTTAATAGAAT<br>AAAGAAAGGCAAGCTCAGAGAAGTGAAGTGACTTTGACAAGGAC<br>ACAGAAAAGATACGATTAGAACCTGGGCCTACTCATCATCAATGCT<br>AAAAAGTGTTGTTTACCTCCTAGACTAATGTTTTACAAACTACAAG<br>GCACAACCAGAAAATCAGTTCAGCAGGGTTGCGGAATTGAAATAA<br>ATAGTATATTAAGGGCTGGGCGTGGTGGCTCATGTCTGTAATCCCAG<br>CACTTTGAGAGGCCAAGGTGGGCAGATCACCTGAGGTCAGGAGTT<br>CGAGACCAGCCTGACCAACATGGAGAAACACCATCTCTACTAAAA<br>ATACAAAATTAGCCAGGCGTGGTGGTGCATGCCTGTAATACCAGCT<br>ACTTGGGAGGCTGAGGCAGGAGAATCGCTTGAACCCAGGAGGCG<br>GAGGTTGTGGTGAGCCGAGATCGTGCTATTGTACTCCAGCCTGGGC<br>AACAAGAACAAAACTCAGACTCAAAAATAAATAAATAAATAAAAAT<br>ACAAAAATTAGCCAGGTGTGGTGGTGCACGCCTGTAATCCCTCTAC<br>TCAGGAGGCTGAGGCAGGAGAATCACTTGAACCTAGGAGGCGGA<br>GGTTTCAGTGAGCCGAGATGGCGGATGGCACCACTGCACTCCAGC<br>CTGGGAGACAGAGTGAGACTGTGTCAAAAAAAAAAAAAAAAAAA<br>AAGAATATTAGGCCAAGTGCGGTGGCTCACATCTCTAATCCCAGTT<br>CTTTGGGAGGCTAAGGTGGGTGGATCGCCTCAGCTCAGGAGTCTG<br>AGACCAGTCTGGGCAACATGGCGAAACTCCGTCTCTACCAAAAAT<br>ACAAAAAAATTAGCTGGGCATAGTGGCGCACATCTGTGGTCCCAGT<br>ACTTGGGAGGCCGAAGTGGGAGGATCGCTTGAGCATGGGAGGTGT

	CGGTTGCAGTGAACTGAGATTGTGCCACTGCATTTCAACCTCGGTG<br>ACGGAGGGGGTGAAACCCTGTCTCAAAAAAACAATAAAAAACCA<br>AAACCAGAAAGAGCCGGGTGCAGTGGCTCACACCTGTTATCCCGG<br>CACTTTGGGAGGCCAAGGCAGGTGGATCACGAGGTCAAGAGATGG<br>AGACTATCCTGGCCAACATGGTGAAACCCCCTCTCTACTAAAAATA<br>CAAAAAAATTAGCTGGGCGTGTTGGCGCGTGCCTGTAATCCCAGCT<br>ACTCAGGAGGCCGAGGCAGGAGAATCGCTTGAACCTGGGAGGCA<br>GAGGGCAGAGGTTGCAGTGAGCCAAGATCTCGCCACTGCACTCCA<br>GCCTGGCGATGCAGTGAGTCTCAAAAAAAAAAAAAAAAAAAAAG<br>AGAAAGCCACTGCCCTTGACTACCAGTGCTGCAAGGAGTTTATACA<br>CAAGCACATGGGTGGTTTCAGTGAGATCTCAGTGTGTCATAATTAA<br>GTGCCCTAGGAGGGGCTAGGATTCAAGTTGGCCCAGGGGAGACTT<br>AGACGTAGGGGAATGGCCTGTGAGCTGTTCAGTGTGGACAGGAGT<br>GAAGAGGGTTGGTCACAGTAACCTTGAGTCTAGATTAGGGCAGAA<br>GGGCTAATGCTTGAAACAAACATTCTGCAAGAGCCCACTCTGGGC<br>AATGTTGGATACACACTGATCTCTGAGACAGTCTTGGTCTCTTCCTT<br>TACTGTGTTTCCTGTGCAGTGGGGGAAACAGACCTGTCACCAGAC<br>AGTAATAGATGAAAATGGTTAGGGCTGTGAAGGGGGAGGCATTGG<br>TCAGAGTTATTAACACCGACTGATGGGGAAGACCGGGAATTAGGAA<br>TACCAAGCAGCAAGTTCCAAGCTAGAAACCAAATAAGAAAGGGCT<br>AGTTCCAGCATTAGGTTTACGTCTCAGAAAACGAAAGCCGAACCCT<br>CAGCAGGATTTGCCTGAAACACTCTCACAGACTGAGGTGGACCCA<br>GGATTCAACTCTCAGTCAGGCATTTTTCTCACTTACCAATGCATACA<br>ATTGCCAAGTTATATGGCATACTGGAACCCAAATTCAGGAGGCAGC<br>ACAATCCCCTATGAGAGAGGAGGGGCTGGGAGCCAGATGTCTCAC<br>AGCTGTCCAGTATACTAGCTAATATACGGATTAGGATCTGTGCAAAG<br>CTCTTTTCTTGCAGGGTCATTTTTTTTCTTTTTTCTTTCTTTTCTTTTT<br>TTTTTTTTTTTTTGAGACGGAGACTTGCACTGTCGCCCGGGCCGGA<br>GTGCAATGGCATGATCTCGGCTCACTGAAACCTCTGCCTCCCGGGT<br>TCACGCGATTCTCCTGCCTCAAGCCTCCCAAGTAGTTGGGATTACA<br>GGCACACACCGACATGCCCGGCTTTTTTTTTTTTTTTTGTATTTTGA<br>GTAGAGACGGGGTTTCACTATGTTGGCCAGGCTGTCTCGAACTCCT<br>GACCTCATGATCCGTCTGCCTTGGCCTCCCCAAGTGCTAGGATTAC<br>AGGAGTGAGCCACCGCGCCCGGCCGCAGGGTGATTTTAAGGCCTT<br>TATGGGCCCAAGTTGCATTATAAAATGTTATATCCCAGATTCACTGG<br>AATATGTACGATGAGAGCAGGTGGCATTAATATCTTGCAGTAATGAA<br>CTAATTGGTAAAAACCGTTTTGTTTACATTAAAATGGTTCAAAAGCC<br>AGCGACACAGCGATGATTGTTTCCATTTAGCAGATGTGGATACTGA<br>GGCACAGAAAACGGTCAAGCCTTCTGCCCACAGTCACCAGAAAGG<br>AGGAAATTCAAGTCTGGGGTTGTGTGGCTTCAGAGCCTGAGCTCA<br>ACCATGACGAGCACACTGAAAAGGAAACCCCTCCTAACTAACTCC<br>CGCTCCGGGGTCCTTTAAGAGCGTCAGAACCCAGAACTGAGTAAA<br>ACAAGATCAAAGCGTATGTAAATACGGACTAGGGGCTGCCGGGAA<br>ATAATCACTACGGCTAGGCCCTAGGGAAAGCAGACGTCGGAGTCCT<br>AGTGCGCCTGCGTAGCCCCATCTCGCCTTTCCGCCCCACGGCCTAG<br>CGAGGCCCATCTCTAACCCACACAGGCCCAAAGTTGTCTAATGTGC

	GCAGGCGCCCTGCTGTGAGCCCGCCTCCTTCTCTGCCCTGCTCTTG<br>CGCTCTCGCTCTCTCAGAGTTCGTCTCTGGCTCTTTGTACGTCACTT<br>CCTGTTTGTGGCGCGAGAGGGCGGTATTTCCGTCCAGAGGGTGGG<br>AGGGGGCGGTAAGCGGGGGCTGGGGGGAGGGGGCGGGGGGGGCC<br>GCGCCGTGCTAGCCGTTGGGCCTGCCTCGGAGGAGGCGTCGCCGC<br>CGCCGCTGCCGCTGCCGGCGCCGTTGCCGCTGC
ALB	AATACTGAACAATTCAATGCTTATATACCCAAAAATATTTTACAATTA<br>TTTTTGAAAGCATTTATATTATGTAGTTTATTATAATTTGGCTAGAGAT<br>GAATTTCTCTCAAAAAAGTGAACATCTGCCAATTATTTATATGATATA<br>TTTCTTAAAACTTACACTTTTTTGGATCAAATAATTCATTTATTGATG<br>TCAGCGCACACATACCTTCTTGGAAAACTCCTGAGAATTTCAAGGC<br>TTAAATTCCAAGCTTCAGCTGGGTTACGGGTTGAGAAAGTTTTAGC<br>TAAGAACTGGCACTAAAAGATTACAAAACATCTCGCAAAGCTTAAA<br>GCTATAGAAGACATCAAGAAGAAAAAATACCTTTTAATAAAAACAT<br>TCTAAACTCTATCCACTAAAATTTCAACATGAGACTATTTTAGCTATA<br>CAATCTTAGTTGTTTTATAGAAGATTGATAAAGTTCAATTTTATAACT<br>TTAAACTTAAGTAACAAGTTTCTTGAATTTCATGTTTAAAATATAGA<br>ATATGAATAGATTCAAAGTGCATTCATTTCTGTTTAATTTCCATCTGT<br>GGCTATCCTTATGAGACAGGATTAGTATTAATTTCAATTTATATGTTTA<br>TAGATTCTATTAGTTGTTAATTTATTAAGAATGGATAAACATGGATTG<br>CAGACATTAACTATATTTTTCTTGCACTTGAAAAACTTGATAAAAAA<br>ATATGAGAAAGTCTCATTAATATAGTTTAAATAAGTTTAAATGAACTT<br>TTTATTCTTTAAAAATATTAAGCTGAACTAGCTAGAGCGTAATACTT<br>GAGGAGCAATTGTGAGGCTTAGGAATGAACAAGCTCTCAAACTTG<br>AATCCTAATTGCCTAACTTGATATATTCTTTCCTACCACTCAGGTCCT<br>TTTCTCATTTGATAAGGCTGTCACATTGAAAACATGGAATTGCAGAA<br>TGTTCAAGGTAGAAGGATCGTTAAAGATAATTTAGTCCAACTTGAC<br>CCATATGGCATAGAGATATATGCAGATCTGCATGTCCAGTTCTGCAG<br>CTAGAAACTGACACTGAGCTGAATTAAACCACACGGAGAAGAACT<br>GAGAAATTTAAAGTAAAAGGTTAGGAAATTCGAATGTATATATATTC<br>CAGAGGCCCAGTGTAACATGAAGAAAGTCAAGGAGAGGGACTCTT<br>TCATCATGGAAGATCTGGGGGCAGAGGGACTTAAAAGGTGGGACA<br>AGCCAAAGATGGTGACCTAGGGTGGTTTTTATGATATAACCTTTTTT<br>CTCCTCATTCCCTATGAAATCTTCAGCCAATTCCATATTTCTCACTAA<br>TTCAAGTGAATATCTGGGGGGATGGCTTATGGAGGGAAATAAGGGA<br>GCAATTGTTTGGATTATATTCCAATTTTTTATTTGCATTAGAATACCTA<br>TATGTTTATACATTTTTATTTGCATTAGAAATACTTATATAAGTTTATA<br>CACTTTCATTCTAACTTTTTTTGAGGGAAATGTATCTCCATAAGTGA<br>ATAAGAAGTTAAATTACTGATTATGTTTCAATTGGTTAGTTTGATGCT<br>TAACAGTCTTTCACTTTTCCACTCTTGCATATTTTGTAGAGAGTAAT<br>ACAAGCTCTTCATGATAGGAAGGATCTGCATATGTTTATTTTATAGTA<br>GAAAAATGGTAAGATTTTCCTGTCCCCAATTTTCTCTCCAAACAAAT<br>CCAAATTTCTTGTATTTTGGAATGACTCTGGGTAAAGTGGTTGTTTA<br>TAGAAAGTCTCTTATAAAATATTGAGGATATTATTAGATATTATTACAT<br>TTCCATGAATATGTTTCCCTTTATCTTTAAGAGAAGAAGAGTCAGAA<br>ATATAACCTATCAGAGTGGGAGATGGCATAAAAGCTGGACTAAATG

	GATTGCTAGATGGAGGGCAAACCTGGTGTGAATGACTCAGTGAGA<br>AGCTTCGGGAGTCCTGAGGGTAGCAGAAGGGTGCGGATTTAAAGT<br>TACTGTTAGAGTGGCTGGAAAATGGGAGACCGGTTCAGAGACATTT<br>TATCTACTTAAAAACTGTGCCTTTTGTATCACGTCAAAGTGAATGCA<br>AAACAAAGAACAAAAGGGTTAAAGGCTCAGGTTTAAATCCCAGGT<br>ATATGTACATTTCAATTGAGGTATTTTTTTTTTCTTTTCTAAATGATCA<br>GTACACTTATTCTTTCTAAAGAAAATACTTTTCTTAACTACTCTCTAT<br>TTTTAAACTTCTCCCACAAAGATGAGAAAACATTTAAAAATCATTG<br>GGGCTATTTTTCTGTTTACCGAGTAAAGAGAATCTCTAAACCATATT<br>TATAACTCTTACTCTAAATATTTGCATTTACCCTCATGCCAGAGCCCG<br>TTGATGACTGACTAAACAGAGTTTCAAAGTTTGAAGAACAGGAAA<br>TTTAGAAATGACTAACAATTATGTAGGTTTATTTCTCTCAGTATAGAA<br>TGTTCATATAGAATTAATGCCAGAGGTTTTCAGAGAAAAATGCAGA<br>AATTTTTACTTTGCAAATCCAGAAGATGCAATTGTTCAAGTATTTCT<br>TAAGAAACATTAATTTTAAGTATGCAGATATCATTGAGAATTAAATAT<br>TTTAATTTCTAAACTATTAATCTTTTAGTAGGATGCACATATGCAAAA<br>TGCCTCATTAGTACTGTAAGAAAAGATTCTTGGCCGGGCGCGGTGG<br>CTCATGACTGTAATCCCAGCACTTTGGGAGGCCGAGGTGGGCGGAT<br>GACGAGGTCAGGAGATCGAGACCACCCTGGCACACGGTGAAACCC<br>CGTCTCTACTAAAGATACAAAAAATTAGCCGGGCGTGATGGCGGGC<br>GCCTGTAGTCCCAGCTACTCGGGAGGCTGAGGCAGAAGAATGGCG<br>TGAACTCGGGAGGCGGAGCTTGCAAGTGAGCCGAGATAGTGCCAC<br>TGCACTCCAGTCTGGGCGAAAGAGCGAGACTCCATCTCAAAAAAA<br>AAAAAAAAAAAAGAAAAGATTCTTTTAGGTTTCATCAATTTTGTTT<br>TAAAGCTAGGGCTCTTCATTAGATATAGGAAAATCAATTCAAAGTTT<br>CTATTCAGTCATGATGAATTTGAGATTTTTTTAGGTTTCTTTGTATTT<br>AACAATATATTACATTATAATGTTGTGGTGAAAACTAAATGGACTAAT<br>ATTATTCTTTTCATTTGTTAAATGAAAAAGTATGCACAAAGTATATGT<br>GAGAGTGACAAAGGCCTGAATTTGTCAATTAGTAACAATTGTATTC<br>AACAGTAAGGATTTTATGTTTGGGTAGGCCTTTCCCAGGGACTTCTA<br>CAAGGAAAAAGCTAGAGTTGGTTACTGACTTCTAATAAATAATGCC<br>TACAATTTCTAGGAAGTTAAAAGTTGACATAATTTATCCAAGAAAG<br>AATTATTTTCTTAACTTAGAATAGTTTCTTTTTTCTTTTCAGATGTAG<br>GTTTTTCTGGCTTTAGAAAAAATGCTTGTTTTTCTTCAATGGAAAAT<br>AGGCACACTTGTTTTATGTCTGTTCATCTGTAGTCAGAAAGACAAG<br>TCTGGTATTTCCTTTCAGGACTCCCTTGAGTCATTAAAAAAAATCTT<br>CCTATCTATCTATGTATCTATCATCCATCTAGCTTTGATTTTTTCCTCT<br>TCTGTGCTTTATTAGTTAATTAGTACCCATTTCTGAAGAAGAAATAA<br>CATAAGATTATAGAAAATAATTTCTTTCATTGTAAGACTGAATAGAA<br>AAAATTTTCTTTCATTATAAGACTGAGTAGAAAAAATAATACTTTGT<br>TAGTCTCTGTGCCTCTATGTGCCATGAGGAAATTTGACTACTGGTTT<br>TGACTGACTGAGTTATTTAATTAAGTAAAATAACTGGCTTAGTACTA<br>ATTATTGTTCTGTAGTATCAGAGAAAGTTGTTCTTCCTACTGGTTGA<br>GCTCAGTAGTTCTTCATATTCTGAGCAAAAGGGCAGAGGTAGGATA<br>GCTTTTCTGAGGTAGAGATAAGAACCTTGGGTAGGGAAGGAAGAT<br>TTATGAAATATTTAAAAAATTATTCTTCCTTCGCTTTGTTTTTAGACA

	TAATGTTAAATTTATTTTGAAATTTAAAGCAACATAAAAGAACATGT<br>GATTTTTCTACTTATTGAAAGAGAGAAAGGAAAAAAATATGAAACA<br>GGGATGGAAAGAATCCTATGCCTGGTGAAGGTCAAGGGTTCTCATA<br>ACCTACAGAGAATTTGGGGTCAGCCTGTCCTATTGTATATTATGGCA<br>AAGATAATCATCATCTCATTTGGGTCCATTTTCCTCTCCATCTCTGCT<br>TAACTGAAGATCCCATGAGATATACTCACACTGAATCTAAATAGCCT<br>ATCTCAGGGCTTGAATCACATGTGGGCCACAGCAGGAATGGGAAC<br>ATGGAATTTCTAAGTCCTATCTTACTTGTTATTGTTGCTATGTCTTTTT<br>CTTAGTTTGCATCTGAGGCAACATCAGCTTTTTCAGACAGAATGGC<br>TTTGGAATAGTAAAAAAGACACAGAAGCCCTAAAATATGTATGTAT<br>GTATATGTGTGTGTGCATGCGTGAGTACTTGTGTGTAAATTTTTCATT<br>ATCTATAGGTAAAAGCACACTTGGAATTAGCAATAGATGCAATTTGG<br>GACTTAACTCTTTCAGTATGTCTTATTTCTAAGCAAAGTATTTAGTTT<br>GGTTAGTAATTACTAAACACTGAGAACTAAATTGCAAACACCAAGA<br>ACTAAAATGTTCAAGTGGGAAATTACAGTTAAATACCATGGTAATG<br>AATAAAAGGTACAAATCGTTTAAACTCTTATGTAAAATTTGATAAGA<br>TGTTTTACACAACTTTAATACATTGACAAGGTCTTGTGGAGAAAAC<br>AGTTCCAGATGGTAAATATACACAAGGGATTTAGTCAAACAATTTTT<br>TGGCAAGAATATTATGAATTTTGTAATCGGTTGGCAGCCAATGAAAT<br>ACAAAGATGAGTCTAGTTAATAATCTACAATTATTGGTTAAAGAAGT<br>ATATTAGTGCTAATTTCCCTCCGTTTGTCCTAGCTTTTCTCTTCTGTC<br>AACCCCAC
AMBP	CTGGAGTGCAATGGAGTGATCTTGGCTCACTGCAACCTCCGCCTCC<br>TGGGTTGAAATGATTCTTCTGCCTCAGGCTCCCGAGTAGCTGGGAT<br>TACAGTCGTGCGCGACCACGCCCAGCTAATTTTGTATTTTTAGTAGA<br>GATGGGAGTTTCACTATGTTGGCCAGGCTGGTCTCCAACTCCTGAC<br>CTCAGGTGATCCGCCCCCTTCAGCCTCCCAAAGTGCTGGGATTACA<br>GGCCTGAGCCACTCCACTCCAAAAAGATTCTTAATAGAGGATTTAG<br>AACGACATGCTCTTGTAGTGCTCTGCGTGGACTTGGCAGACAGTAA<br>GAACTTAGTACAGTTATAGTAGCCTTCGTGGTCTGCAACAACTGCA<br>CCCAATTCTGGTTCCCCAGCAAAACTATTTTTTTAAGGGCCCCAAC<br>CAAGCATCAAGCTCTCTAGCAGGGTAGGGATGGGCAAGGTAGACG<br>CTAAATGTTTTACACTCACCAGGGGATTTTAAGTAGGCTTGGGGCA<br>AGGAAGCCTGGACACGAGCCCGCATTGTAGCTCTTTGAGCCTTTGC<br>TCCTGGCCTCTGTCTCCATCTGTACAGAACAATCTGTGAGCAAACT<br>CCGGTGGTTCTTTCGGGAAGTTCTCTGCTGCCCCCTGGTGGACAAG<br>GGAAGTTCACACCTTAGTGCAGTTGCAGCTATGTATGGAGTCCTTA<br>GCTGGAGCACATGCGGTACCAAGCGCCCATGATGACAGGGGTCCT<br>ACGTATCTACACACTTGGGGTGCATTTTCACAAGTGACCCTCATTCA<br>GTTATTATGTCAGCTCTGAAGCCATGGAGGGAAATGACTGCCCCAT<br>TGTCCCGCAGCAAGCCACAAGGACTAGGACTCAAGTCTCTGGGTG<br>GACTTGCTCTTTGGGTTGGAGGTGTAGGGATGAGTAACAATGCCTA<br>GAAATCACAGGGCAGTGAGAACACCCCTCCACATCACCAGTCATG<br>GAGTTATGGGTGCTGTGCACCTCCAGCTATGCCATGAGGACCCTGA<br>GTGGACAGGCTGTGGCACGAGGCCATGGAAATTGAGCAAGGTCAG<br>ACATTGGAGGCAAGGCCTCCTGTCACGGAGAAAGTCACCTTATCAT

	TCCACCATTGGCAGAGGAGGACTCAAAGGTTGACCACACCCTCTG<br>GTCTGACTCCCAGTCAATGTCCCTGATGTCCCTGTGGGTCCAGTCA<br>CTGGGTCCTAGTGCCCTGGCCCGGCCCCTTCCTTCTTCCTGGAAAA<br>TCTCCTAAGGAGTTGCTTTCCTGGGTCTCCAGCTTACGTTACCTCCT<br>GATTGATCTCTCCCGATTACTAATCTGGTTGACCTTGACTTCTGAAA<br>ACCCACCAGTGCCCCTGTGCACTCCCAAGCAACACCCCTTGGGAC<br>GGCAACACGATCCCACCTACTGGACCTCAGCCACCTCCCCAGCCTC<br>ACTCTCACCACTCCCTTCTGTCCCCAAGCTCCAGACCTGTGGATTTA<br>TTTGTATTTTTTGGATAAAAATAAAAATAATTTTCTATTTTCTTCCCTA<br>AGCCTTTGCAATTGCTGTTCCCTGTGCATGGAACCCACTCCCCTCC<br>CCCATGCACCCATCCCACTGCACTGTTGTCTAGCTCACTCCTACTCT<br>TGGAGTCTGACTCCCCTGTCCCGGCCTCTGCTTCCCTGATCACTGC<br>AGCAACCATCCTGTCTTATCACCTTCTCACCTCGTCTGTTCCCCAAC<br>CCCAGGCCTCAAGGGAGCTCTGGGAGGCCTGTCATATGCCCATCCT<br>GTCCTCAGCACCAAGCATGGAGTGTGTATTTGGCAAAAGAACAAG<br>TAGACACAAGAATAAATGATGAGGGGAGAGCCTGGGTGCCACAGT<br>CAGGCCTATCTGGCCCCGACTCGGCCACACACTGGGTGACCTTGAC<br>CAAGTCCCATCCCCTCCCTGAGCCCCAGTATGTGCATCTGAAAATC<br>GAGGCCTCTATTCCACATTGCACTGGGTACCCCTGAGGCTCTGAGG<br>CCTCCTGTGAGGGGCAAGACCCTCCCCCACAACCAAATGGTTTCA<br>GAACCTAGCAATTCCCATGGACTCTTAGACCCCTGGGCCCCCTAGG<br>AGGGCAGGGACCCCAGAGCCTCCATCCTCCCACCCCAGCAGCCAG<br>AAGGGGAGGAGGGGGCAGCAGCTGTCTGACCACTGTTGGTCTTGC<br>AACTTGTGTCCCCAGGTTAATTTTTAAAAAGCAGTCAAAAGTCCAA<br>GTGGCCCTTGGCAGCATTTACTCTCTCTGTTTGCTCTGGTTAATAAT<br>CTCAGGAGCACAAACATTCCTGGAGGCAGGAGAAGAAATCAACAT<br>CCTGGACTTATCCTCTGGGCCTCTCCCCACCCCCAGGAGAGGCTGT<br>GCAACTGAATGAACAGCCCTGTCCAAGAGAGGCTAGGACTGGGGC<br>TTCTCCACCCCTGCGCGTCTCAGGCTTCCCATCTGGACCATGGGGT<br>TGATAACTGTAGTCACGCAGGCCCAACATGAAGGTCAGCGAGCCAT<br>CTTGGTGCACTGTAAGCGGGCTTAGTAGGATGATTTTATTCCTATTA<br>ATGATTCATTGTTATTTATTTATTCTTATACCAGGCCTGCCTCCTCCAA<br>TGGCAGACACCCCTGAGCCACCACACAGGTAAGGCAGTGGACAGT<br>TTACAGTTCACTTCCTCACACATTGTCTCTCTTGGCCTCTTGACAGA<br>CAGAACAAGAACTGTTTTCTTCCGTTCACAGATACGGAAAGTGGTC<br>TTTAGCGAGGGAAAGCCACCTCCAATATCATGCAGAACAAGGAGA<br>AGTTGGGGGCTACCACCTCCTCATTACCATCATTAACTAAAACATCA<br>AAGATTGAGAAACTTTGTGAACACAAGGAACACGAAGATAACTGT<br>CCCAGAAAGCTAAACCAAGACAGTAACTGTGCTTAAACTTACATCA<br>TGGCTTTGAGCTTCCTAGCAGCCAAAGCAAGAAGGGAATATAATCA<br>CTGGCCAAGGTTAGGGTGACGGAGCAAAGGAAACACATTTATGAA<br>TAGAAGCACACTTGTTCTGGATACCAGTGAAAGAGGGATGTACTGA<br>CTGAATCTTACAGAAGGGACATGATACACCATTTGTTTCTGCACTG<br>ACTACATGGGGGAATCCTTTGATTCTCACATAAGCAGGGGTCTTAAT<br>CTTGGGACTGCCTGCCCGAAGGGGTCATTCCCAGCTCTAACAGAG<br>AGGTGGCTGAGAGTATGGAATGTTCCATGCCAAGAGCAGGGAGGA

	AGTAAGGACAGTAGATTTAACCACTGGAGAAGGGAAATTCATAGA GAGGCCTTTGGTATTGCTGGCAAGAGTGTGAGCTCACAGACAACG GACCTGGGTTTTAAAAAATTCTCATTTAAAATCTTAAATTTAGGCTG AGCGCAGTGGCTTACGCCTGTAATCCCAGCACTTTGGGAGGCTGAG GTGAGAGGATCACCTGAGGTCAGGAGTTAGAGACCAGTCTGGCCA ATATGGTGAAACGCTGTCTCTACTAAAAATACAAAAATTAGCCAGG CATGGTGGTGGGCACCTGTAATCTCAGCTACTCGGGAGGCTGAGGC ACAAGAATCACTTCAGCCCAGAATGTGGAGGTTGCAGTGAGCCGA GATCGCACCACTGCACTCCAGCCTGGGCGACAAGAGTGAAACTCC GTCTCAAAATAATAAATAAATAAATAAAACCTTAAGTTTAATAACTA CTGTATTAGTTTCCTAGGGCTGCTAGGAAATGTGTCTTCTCTCAGCA CTGGAGGTCAGACACCTGAGACCAAATTTTCAGCAGAGCCATGTT CCCTTTGAAGATTCTGGGGAAAAGTCCTTCCTTGCCTTTTGCTAGC TTTTGGTGGCTGCCATCAATCCTTGGTGATTTTTGACGTGTCACTGC ATCCCTCCCATCTCTGCCTTCGGCGTCACGCCACATCTTCCCCGTGC TTCTCTCTGTGTCCCTGTGTCTGAGTCTCCCTCACCATTCTCTTATAA AGATACCGGTCATTGGATTTAAAACCCCTGTCATCCAGTTCAACCTC ATCTAACTAATCACATCTGCAAATCAGGTTCATTCTGCGGTTCGGGG TGCACATGGATTCTGGAGGGGGCACTGTTCAACCCACCACAAACA CTCAGCTCTTACTCTATGCTGGGTACTGTCCTAGCGCATTGCAGGTG CTCACTCAACAATGCTAGGAGCTAGGAACTATTATTATCCCCATTTA CAGATGAGGAAACCAAGGCACTGAAAGGTAAAGTGACTTGCCCAA GGTCACACAGCAGTGAGTGCCACTGTAGGCTGCCTGGCTCCCACG TCCATGGTCTCTACCACGCCTTTCCCAGCTACTCCACTCCCGGCTGA GGGATTCTGGGCAAGTCACTTCACTTCTTGGAGACTCAACTCCTCT GCCTGTAAAGGGGACAAATAGCACCTACTTCGTAAGCTTGTTGTGG GGATTTACTGGACACCTGTGGCACGTGTAAAGTGCCTGGTACACAG CCAGCACATAGCAGGTGCTCAGTGAACGGAAGCTCGTAACAGTCA TCATTGTTAATGTCTCTATTATTTGCAAAGGCTAGGGCAAGCCTGGG GGCTGGAGAAGGAACTCTGGAGACAGGATACCTGGGTTCTGGTCT TGGCTCTGCCCATAGACTTCCCTGAGGTCTCTGGCAAGTCACTTCC TCTCCTTGGGCCTCAGTTTTTTCCTGTGAGAAATGGGCCCAATCAG GCTTGACCTGCTGGCTCACAGAGCTGTGAGGTCCAGATGGCATGA CTCAGCAGAAAGGCCAGGTCGCTCCCCATCCTCGCATGCCCTCTGT GGGGGACTTTTGGGGGGCGGAGCCCAATCCTGGTGCTCCAGGGCC CTATTGTGCTTGGCCAATGCCTCTTCTGAAGCAGCCATCCCGGCCTC TTGGTACTGCTGACCCCAGCCAGGCTACAGGGATCGATTGGAGCTG TCCTTGGGGCTGTAATTGGCCCCAGCTGAGCAGGGCAAACACTGA GGTCAACTACAAGCCACAGGCCCCTTCCCCAGCCTCAGTTCACAG CTGCCCTGTTGCA
AMHR2	GTGTGGCACATTTCTATATTTTTTAGTCTCCTTCATAGCCACTTCTTC CTGTGTCCCTTTAGTATTTGTGTTCCTGTCTCGTGTCCTTTCCTTTCC CTACCCTTTTCCCTCTTAAACTCTAACATAAGACTCTAACATAAGAC TGTGTCCATGTTGATGACTCCCAAATCTTGAGCCCTAACTTGTCTCT TGACCTGGAAGTCTGCTTGCTAGACATTTTCATCTGGATGTCCTCCA GATAAATTCAACACAGTAGAAGGAGAAATAATTCTTTGTGTTTCAC

	ATCTCCCTTAATAATATCACCCACTGCTCCTTGATCACTCATACTTGA<br>AACCTCAGAATCATCTTTTAGTTCTCCCTTTCACCTACTAACACTAA<br>TTTGATTGCCAATCCTGTCAACATTACCTATACAATATATATCAAATT<br>TTTCCTCTTTTTTTTTTTTGAGATGGAGTCTCGCTCTGTCGCCAGGC<br>TGGAGTGCAGTGGCGTGATCTCGGCTCACTGCAACCTCCGCCTCCC<br>AGGTTCAAGCAATTCTCCTGCGTCAGCCTCCTGAGTGGCTGGGATT<br>ACAGGCGCGCGCCACCACGCCCAGTTAATTTTTGTATTTTTAGTAGA<br>GACGGGTTTTCACCATGTTGGCCAGGGTGGTCTCCATCTCTTGACC<br>TCGTGATCTGCCCGCCTCGGCCTCCCAAAGTGCTGGGATTACAGGC<br>GTGAGCCACCACGCCCGGCCTCCTCTTTTATTTTCATTTCCACTGTC<br>TCAGTACTCACTTTGTTTACTGCTTAGGTCTTGCAACTGATCTCTCC<br>AAACCCCAGTAGTCATCCATCTTCCAATTGCCACCAGGCCCCTGCC<br>TAAAATTCTTTGCTTTTAGGGAAAGCCTAAGCTCCTTGATACAGAAT<br>TGAAAACCTGTCCAATCTGGTCCGTCTCTCTCTAAGGTGATTTTCTG<br>TCTCATATCTCATACTGCTTCCTCCTCACTCCTTCCACCTTTAACACA<br>CAAGCCTTTGTGTTTATATCTCCATGCCTTTACTCTTGCCATTCCCTT<br>TGCTTAGAAGGACTGTTCTCCTCTTACCATTGTGTTTACCTTTGGGA<br>CTATTTAAATATCCCCTTCTCTGTGAAACTCTCTGGGCACCTTTCTTC<br>TATGCCCTAGGCAGAATTAGCTTACTTGATCTATTTGTATAGCTCTTT<br>CCTCATGCCCCTAGTAGGAAACACAGTTTTCCGGTTGCTTGTCAATA<br>TCCACCTGCATTTCTACACTATGAGTTCCCAGAGGGCAGTAATCAC<br>ACCTCTATCTTTGTAGGCCCAGCACCTAATGAAATACATTTTTTTTG<br>GCCCCGCGCAGTGGCTCACGCCTGTAATCCCAGCACTTTGGAAGG<br>CCTAGGCGGTGGATCACCTGAGATCAGGAGTTTGAAACCAGCCTG<br>GCCAACATAGTGAAACCCCATCTCTACTAAAAATACAAAAAATTAG<br>CCAGGCATGGTGACAGGCATCTGTAATCCTAGCTACTTGGGAGGCC<br>GAGGCAGGAGAATTGCTTGAACCCAGGAGGCGGAGGTTGCAGTG<br>AGCGGAGATTGCACCACTTTACTCCAGCCTGAATGACAGAGTGAG<br>ACTCCGTCTCAAAAAAAAAGGAAATTTTTTTTTAAAAATAATATAG<br>GTGGGGGTCTCGCTATGTTACCCAGGGGGACTCCTGGCCTCCCAAA<br>GTACTGGGATTACAGACATGAACCACAGCGCCCAGCCTCTATCACC<br>ATCTTAACTCTAAAACCTAACAGCCCAGAGCAGGTATAGTTCCCATA<br>AACCTGACTGGAATGTTTCTATCTTGGAAAGAATTAGTTATCCGAA<br>GCTTTGGGTGAGGAGACTGCCTTTCTGCCTGATCCTTACGCTGCTC<br>CCACTCTGGAGGAGATAGTCTCCTGAGACATTCCAGACAAAAGAA<br>GCTATTTCACCTTCAAATCTCCCCTCACACCCCACCACACACACCA<br>CCCCAGACTTATCTGATCTGGGGCTAGTGAAGGAAAGTAGCTGGAA<br>GCACAACATTCCATCTCACTGGCAAGGCCCTGCAGACTCCTGGAA<br>GGGTCCAAAGAAGGATGGGCGGCTATCTCGAGCCCTGGTCATTCCT<br>CCCGCTGCTGTTTCACAACTACTGGAACTTTTACTATCTCTTCCTTT<br>CATGCAGGTTTCCCAGAAGAAAGACCCCAGAATCAAAAAGTCAGT<br>ACTCAAGCTGGGCGCAGTGGCTCCCGTCTGTAATCCCGGCACTTTG<br>GGAGGCCGAGGGGGGCGGATTACCTGAGGTCAGGAGTTTGAGACC<br>AGCCTGGCCAACATGGCGAAACCCCGTCTCTACTAAAAATACAAA<br>AATTAGCCGGGCGTGGTGGTATGCGCCTGTAGTCCCAGCTACTCAG<br>GAAGCTGAGGCCGGAGAATCGCTTGAACCCGGTAGGCAGAGGTTG

	CAGTGAGCCGAGATCGCACCACTGCACTCCAGCCTGGGTGACAGA<br>GCGAGACTCCGTCTCAAAAAAAAAAAAAAAAATGTCGGTACTCAG<br>ACTGTCCTCCCTGGTCTAAGCCAGATCATTCCAAGAAAGGGTTTCC<br>AGGAAGCCACTAGGGGGACAGAAAGCTAGGGAAACAGAGAACAA<br>GTCCCCTCTGACTCTTAATTCTCAAGGAGCCAGTGGCTCACAGGTG<br>TGGAAGTGATTACCTATTTCTGTTGTTTCTTACTCTGGGAAAGAGTC<br>TGGTGGAGTTGGACTCCTCTGTCTCTACTGCAGAACAAGCCATAGC<br>CCTCTCCTCCCAACTCCATGCTGAAACTCTCCACATCCTGTGCCCG<br>CCCTTCACATTTTATAGTCCCTGAAGCTTAGTAAATGTAGGAGGTAT<br>ACTATATGTATTAGTCATCAGAGGAGGTACTTTCTGCCCTCTCACCG<br>CAGCAGGGAATTTCTCCTCTGCTGACCTCTGGTGGTGATAGTTTGA<br>ACTGTACCCTCCCTTTTTAGTTAGTGGCCTGTTGGCAGGTAATGATG<br>CATATTTTCTCAACAATTTAAATTCAAATGTCCAAGTATAGTATTAAG<br>TGGTAATTTTTTTCATTGTTGAATTTTGTACGTCTTTCAGGCAGTAT<br>AGTGTAATACAAAGAATAGAGCCGGCCGGGTGCGGTGGCTCACGC<br>CTGCAATCCCAGCACTTTGGGCGGCCGAGGCGGAGGGATCACGAG<br>GTTAAGAGATCGACCCCTTCGGCCGGGCGCGGTGGCTCACGCCTGT<br>AATCTCAGCACTTTGAGAGGCCGAGGCGGGCGGATCACGAGGTCA<br>GGAGATCAAGACCATCCTGGCTAACACGGTGAAAACCCGTCTCTAC<br>TAAAAATACAAAAAATTAGCCGGGCGTGGTGGCGGCCGCCTGTAGT<br>CCCAGCTGCTCGGGAGGCTGAGGCAGGAGAATGGCGTTAACCCGG<br>GAGGCAGAGCTTGCAGTGAGCCGAGATCGCGCTACTGCACTCCAG<br>CCTGGGCGACAGAGCAAGACTCCATCTCAAAAAAAAAAAAAAAA<br>AAAAAAAAGATCGACCCCATCCTGGCCAACATGGTGAAACTCCGT<br>CTCTACTAAAAGTACAAAAATAATAATAAAAATAAGACTAGAACCT<br>GGACTTTGGAGTCAGACCTGCCTAAGTTCAAATTCCAGCTCTGCTA<br>TTTCCTAGCTGAATGACTTTGGGCGACTTACGTAAGTTCAGTTTTCT<br>TACCTATAAAATGGAAATAATAAAAATAATAGGCCAGGCACAGTGG<br>CTCACACTTGTAATCCCAGCACTTTGGGAGGCCAAGGCGGGCAGA<br>TCACGAGGTCACGAGATCGAGACTATCCTGGCCAACATGGTGAAA<br>CCCTGTCTCTACTAAAAATACAAAAATTAGCTGGACATGGTGGTGC<br>GTGCCTGTAATCCCAGCTACTCGGGAGGCTGAGGCAGGAGAATCG<br>CTTGAACCAGGGAGTCAGAGGTTGCAGTGAGCCAAGATGGCACCA<br>CTGCACTCTAGCCTGGTGACAGAGCGACACTCGGTCTCAAAAAAT<br>AAATAAATAAAATAATAATAATAAATATTTCAGGGGAGAATTAGATG<br>AGATGATTGTAAAATGCATAGCCCTAGCTGTGCCCCAGCACATAGTA<br>GGTGCTTGATAAGAAATTTGTTACCTCCTTAATAATGGGTATTTGTTC<br>AGCCTGTGAACCAATGTGTTTTTTTTTTTTAAGTAAAATCCACGGCT<br>AGGCATGGTGGCTCACGCCTATAATCCCAGCACTTTGGGAGGCCGA<br>GGCGGGCAAATCACCTGAGGTAAGGAGTTCGAGACTAGCCTGGCT<br>GACATGGTGAAACCCCGTCTCTACTAAAAATACAAAAATTAGCCAG<br>GTGTGGTGGCAGGCGCCTGTAATCCCAGCTACTCAGGAGGCTGAG<br>GCAGGAGAATAGCTTGAACCCAGGAGGCGGAGGTTGCAGTGAGCT<br>GCGATCGTGCCACTGCACTCCAGCCTGGGGGAGAAGAGCGAGACT<br>CCGTCTCAAAAAAAAAAAAAAAAAAATCCACATAGCAAAATTTAC<br>CATTGTTACTGTTTTCAATGTGTTTTTTAAAAATTCAGGTAAGTTTT

	GTATACTTTCTGATTCTTAAGTTGGAAAACAAGGTAACCTCTAATAT<br>GGGCTGTGAGGCCTTCCTCTGCCCAAGCAGTTGCAGATATGAACCC<br>TGAATATAAAGAGATCTTAGGCTAGAAAGGATCTTGGGCAGAGCTG<br>AATGGCTCTAAGCATTTGACCTCACATTGGTTTCCTCCTGAGAAGA<br>GTCAACAGAGTCCAGCATCTTCTTCCAAGGTCAGGGAAGGGCAAA<br>GATTTGAAATTAGGCATCACTGGGTTCTCAGCTGGGCCTCCAAGTT<br>CCCCTCCTCTCACTATGCAGAGCAAAGAGGAGGTTGCAGAAGAGA<br>AGAGGATATGAGATTGGTTCTCTGCTCCTCCCTTTCTTTCCCTGCTT<br>TCCCCACACCCACCCTCTCCCCCACAGCAGCCTTCTCCCCCACAGA<br>GGCTGGGATAGGATGAGGGGGGCGGAGTTGGGGACTGAGGGATCA<br>GAAGCCCCAGGATGCCCTGTATCTGAAGAAAGATTTGGCCAGGGG
ANPEP	TAGGAGCTACCTAGAGGAACAATTTTTTCTTTTTTTCTTTTTTTTTTT<br>TTTTTTACTTGAGATGGAGTCTCACTCTGTCGCCCAGGCTGGAGTG<br>CAGTGGCACGATTTCGGCCCACTGCAACCTCCGCCTCCCGGGTTCA<br>TGCCATTCTCCTGCCTCAGCCTCCCGAGTAGTTGGGATTACAGGCG<br>CCCACCACCACGCCCGGCTAATTTTTTGTATTTTTAGTAGAGATGAG<br>GTTTCACCGTGTTAGCCAGGATGGTCTCGATCTGCTGACCTCGTGA<br>TCCGTCCGCCTCAGCCTCCCGAAGTGCAGGGATTACAAGCATGAGC<br>CAACACGCCCGGCCCAGGAACAATTTTTAAACCAATAGTCATGAAT<br>TGTTAATATAAATTAACACATCCAACCATGATTTTATCGCTCAGAAG<br>GAGGCTAAGAAAGTGAATTGATTTAAAGAAAAATACGAAGTAAATA<br>ATCACAAAGGTGGATCACGAAGGTGGTTTGTGCACAGCCCAGAAG<br>CCCAGGAAGGAATGTCGGGTAGAGAGGGTGGAACCTGTCCCGAGG<br>ATCAAGGCTCTGAAGTTCCATCCTTAACTGCTGTCTCTCATTCTTGC<br>ACTGATGCCCCATTCCACAAGCACGTGTTGCCCTCCCACTGCACGC<br>CAGGTGTAGAGGATACGGCACACCTGGTGCTACCCTCATGGAGGC<br>ACTTGGAGGGCTGCCTGGGGGCAGCACCACTGACTTGTGTCTCCA<br>GGGCAAGTGGGGAGTGGATGAAGCAAAGGCAGGAAGCTTTCCAG<br>GCCCAAGGATCAGCTTGTGTGTAGGGAGGAAGGGAGAATGAAGCG<br>GGGAAAAGCAGGTTGGATTTGAACTGAAAGAGGCCACTGGGGTG<br>GAGCACAGAAGTGACAGGGGAGAGCGTTTGAGATGAGGCCAGAG<br>AATCACTGGGGGCTGGATCACACAGGCCCTGTAGGTCACGGCAAG<br>GACTGTGGGTGTTGTCATTCATTTGGCCAACATGTATTGGGCTCCTG<br>CCTTGTGCTGGGCATGGAGGGGCATACGATGCCACCAGGCCTTCCT<br>TTGAGGTGCTTGGGTGCAGCAGCAGGCTTTTGAACACACTGGTAG<br>CATTTTTCTTTTTCTTTTTTTTTTTTCTTTTTTGAAACAGAGTCTCGT<br>TCTGTTGTCGTCCAGACTGGAGTGTAGTGGCACGATCTCCGCCCAC<br>TGCAACCTCCACCTCTTGGGTTCGAGCGATTCTTCCGCCTCAGCCC<br>CCCAAGTAGCTGGGACTACAGGCGTGCGCCACCACGTCCAGCTAA<br>TTTTGTATTTTTATTTATTTATTTATTTATTTATTTTTTGAGACAG<br>AGTCTTACACCGTCCCCCAGGCTGGAGTACAGTGGCACAATCTCGG<br>CTCACTGCAACCTCTGCCTCCCGAGTTCAAGCGATTCTCCTGCCTC<br>AGCCTCCTGAGTAGCTGGGATTACGGGCACCTGCTACCATGCCCAG<br>CTAATTTTTTGTATTTTTATTAGAGACGAGGTTTCACCATGTTGGCCA<br>GGCTGGTCTCCAACTCCTGACCTCGTGATCTGCCCACCTCAGCCTC<br>CTAAAGTGCTGGGATTACAGGCATGAGCCACCGCGCTCAGCCTCAG

	TGGCATTTTCCTAAGTGCTGTGATAGAGGTGGGAACTGGAGACTGG<br>GGAAGCGCAGAGGGGAGTGGGGGCTGGTGTGCATGATAAGGCAGT<br>GACATTGAGTTGGGTTTTGAAGGATGAGTAGGAGTTTGACAAGAG<br>GGAGAGGAAAGGCATTCCTGGCAGTGGGAACAGAATGAGCAAAG<br>TCACAGAGGTATGAAAAAGCCCCGATGATCTGGGGAGACGCTGGC<br>AGCTGGAGCCACAGTGGGCAGTAGAAAGAGGACAGGCCAGAGGG<br>TCAAGTTCACAGAAGGTATTAAGGCCCAAGCCAGAGTTAAGGTTTC<br>ACTCTGCAGGTTGGGCACCAGGTTTGAATGGGAGAGTCCCACCCA<br>CACTCTTAGGCAGAGCCCTCTGCTGACGTGTGCTGGTGGGGTTGG<br>GTGACAGGGGAGGGTAGCATAGCCGCCACTGCCCGATGGGGTGTG<br>GAGGTGGCCAGGCCTGGGCTGTGGCAGGGGACTGGACGAGGGAA<br>GCCCTTTTGTGTGTTCCTGAAGGTGGGGGCTGCAGAGCATGGCTGT<br>CTCTGTGCCCCCAGCCTGGGTTGGCCTGGTGCCTAGAGCAGGGAC<br>CTCTCATTATAGAGCCAACTTTGGGCTACCTGCTGCACCAACTTGG<br>AAGACCAGAGGCCTTGTCCAGAAGAGTAAAGATTCCAGCCAGCTG<br>GGCCTATGGTCTGAATCCAAAGCAAACAGGAAGAGAGTATGGTGG<br>GCCAGGCAGGGCCTCTTTCTTTGTTTAGAAAAGGGATTAGTCCAGG<br>CCGACTTGTGGGTCTTTCTGCTGCAAAGGTGGGAGGGGGCTGCAG<br>GCTCCGTCAGGGACATATCAAAAGGCTTTTCAGGGGCAGGCATGGT<br>GCCCCAGTGCCAGCCTGGGGGGTGCTGTAAAGATGGGCTCCCTTG<br>GCGAATTCCATCCACCCTGATGGGAGGAGCCTGGCAGCATGTGCTG<br>GGAGCCACTGCTCCTTCTCTCACCACGCCCAAGCTGGAGCCCTTCC<br>TGACACCTGATTTCGCACGATTGCCCCTTGGGGTCATCACCCAAGC<br>CTAATGGGGTGTCTGCTAGTCACTGCAGTGGGAGGTCCATCCTGGT<br>CCCAGACCAAGGCCCCTTGGGGAAATGCCAAAGGCTCAGAACACC<br>CTCAGTCTCAAAGGTGGGCACCCAGGACTCCCCTAAAGACCCTGG<br>GCTCAGACTCGGCCTCCATACACCAGACGAGAGGCGGCAGGTATTT<br>GTGTTTTGCTTATGCCTGATTGAACTAATCCACGTGTCCCTTCAGTT<br>CACACATTGCATTTATTTAAGAAGCCTTCCTGCAAGCTCAGGGGTG<br>GCAGGGGTGGCAGGGGTGGCAGACGAGTGAGGCCCTCAGGGAGC<br>AGAGCTGAGGGAGCAGGAAGGCATGTGGGGCCCTGACATGCTGGG<br>GCCTGGCACGAGTCCGGCCTGCCTGCCATGCTTGGAGAAGGGCTC<br>AGAAGAGGCAGGCACTTCTTCTGGGGGCAGGTGTGGGGTGGGGA<br>CAAGCTGGGAGACAGAGGCCAGAAAGACTTTGGTAAGGAGTTTCT<br>CCTGGACTGACTCATTCATTCATTTACTCAATCCTTCGTGCATGTGC<br>GCATCCACTCAACATCTCAGTAATGAGGACCTACTGTGTGCCAGGC<br>ACTGTGCTAAGTGCTGGTGAGGCCCTGCTGTCATGGGGCCTGCAGT<br>CCAGGACTCAAGTTATCACCACACAGATCAAAGGGAAAACCACGC<br>TGAGTTCTGGGGCTTTGCAGAACAGGTCTGTGGAGCTAATGGAGC<br>TCATAGGAGCTTGACTTGGTCAGGGAGGTCAGGGCGGCTTCGCTG<br>CGGAAGACTTGTAGGATGGAAAGGAATTAACCAAGGCAATAGGGA<br>AGAGAAGATCCAGGCAGAGGGAACAGGTTGTGCCAAGGCCCTGG<br>GGCTTGATGAAGGCCAGAGTGGCTGGGGTACTGCAACAGGGGGGA<br>AGAGGCCAGGAAATGGGCAGGCTGACTGTACCCGGGCTTGTGGGT<br>GCCTTTAGAAGGTCTGGTCTGGACTCTACAAGCAAAAGGAAGGTT<br>TTTTTTAGCAGTGGCATAAAATGATCCAATTTGTGTTGAGCAGGTTC

	AAGCAGGCCCGAATGGAGGCAGGGAGTCCATAGGGGCGCTGCTGT<br>GTCCACATAAATGATGAGAATGGTTTTGACCAAGTGCATCTTGCGA<br>TGGGGGAATGTGGGTGAATTGGATGTGGAGTGAGGTCAGGCGGTG<br>TCGAGGTCTGAGAAACTGGGCTGGTGGATGGGGAAAGTGACAATG<br>AACATAATTTTCCTGTAAGGTGTGTATTTTATTTTGTATGGTAAGTAA<br>TATCTTCATTTCTACCAAGGATAGAAAGAGGCAATTGTTGAGCTTTG<br>GGTAAGAAAGAAGGGAACGATTTTTTTCTTTTTTCTTTTGAGTCTC<br>ACTCTGTCACCCAGGCTGGAGTGCAGTGGTGCGATCTCAGCTCACT<br>GCAATCTCTGCCTCCCGGATTCAAGCAATTCTCTGCCTCAGCCTCCC<br>AAGTAGCTGGGATTACAGACGCCCGCCACCACGCCCAACTAATTTT<br>TTTTGTATTTTTAGTAGAGACGGGGTTTCGCCATGTTGGCCAGGCTG<br>GTCTTGAACTCCTAACCTCGTGATCCACCCACCTCGGCCTTCCAAA<br>GTGCTAGGATTACAGGCGTGAGCCACCACGCCCAGCCGGGAATGA<br>TTTTTAATCATTCTACTATTTGGTGTTTAATATGTTTTCTCACTAAATC<br>TGTTCAACAAACCTGAGAGGCAAATACTGTTATTTATTTATGAGGAG<br>AGAGGTTCAGAAGGGTAAAGTTACCTGCTCAAGGTCACAGCTGGA<br>AAGAGGTGAAGCCTTCAGCAAAAAAAGCAAAACAACTCATTTCAT<br>TTTATTCCGTGAGCTGATAATGGAGGAAAAAGTATTTGGCAAGAAG<br>CGGCAGGGAGGGGAACCTGGAGGAACTCCCTGCTCTTGAAGAATG<br>CAAGGGAGGCTGGGACCTTGTCACTGAGGAGCCCCTGGCATCCTT<br>CCAGAGATGCAACCAAAGGGGTGACCCCAGTGTGTTCTTAATAGG<br>AGCCGGGAACCCCTTCTCTGTGCACCTCCCACCTCTTGCCCCTGGG<br>AAGTCCTTCCCTGACTCTAACCTCAGTTCTGATGCTGTTCCCACCCT<br>CTCAGCTCTCAAGCAGATCAATGCAATGCCACCTGGCCGCTTGCTT<br>TGCCCACTGGGCCTACAGCCGGAAGCCTGCCCTTCAGCCCTCGGG<br>CCTGATCCCAGGCCGCCTGCAGCCTGTAACCAGACACTGTTTGCTT<br>CCAGCAGGCACCCCCCGAGCCCAGCTCCACACACCGTTCCTGGAT<br>CTCCTCTCCCCAGGCGGAGCGTGCCCCTGCCCAGTCCAGTGACCTT<br>CGCCTGTTGGAGCCCTGGTTAATTTTGCCCAGTCTGCCTGTTG
APC	TGGAGGTTGTTTTCTTTTTCCTGTCTCTAATCATACTTAGTTGTTTTT<br>TTTTTTTTAAATCCAACTTTTGGATGGTTTTCTTTTTCTCTTTTTTTTT<br>TTGTTTTTTTTTTTTGAGATGGAGTCTTGCTCTGTCTCCCTGGCTGG<br>AGTGCAGTGGCGCGATCTCGGCTCACTGCATCCTCCACCTCCCAGG<br>TTCAAGCTAGTCTCTCACCTCAGCCTCCCAAGTAGCTGAGATTACA<br>GGCATATGCCACCACGCCTGGCTAATTTTTGTATTTTTAGTAGAGAT<br>CGGGTTTCACCATGTTGGCCAGACTGGTCTTGAACTCCTGACCTCA<br>AGTGATCCGCCCGCTTCAGCCTCCCAAAGTGCTGGGATTACAAGCA<br>TGAGCCACCGCCACCTGGCCAATGCTTTGCTTTTCAAACTCTTCAA<br>AGGGTATGTAATAAAAAGGAAGGCTTACCACCAGACACCATACCTT<br>GATTCTATAAGCAGTGACTGTATTTTTGTATGTTCTTTAAGTGATATT<br>TTTTATCCTTATGCACTCCTTTATTTTTACTGGAAGAGGAGGGACCA<br>CATGAGGCATTAGTGATGAGCATTCTTTTTTTTTTTCTTGGCTTTTGC<br>CTGTTTTTTATGTTTGTTTGTTTTGTTTTTCATCAACCTCATTTTTACA<br>TCAGCCTCATTCTTCTTAATACCAATGTAGTATTTCACTGTACAGATG<br>TGCATTATTTATTTTAACGTAGTTGTGCTTTTATAGCAAATAGAAACT<br>TTCCCATTTGTTTGAAGTACCCTAAGAATCTCCTTTATTACTCCTAGT

	CTAGTAACGGACATCATTACTTTAGAGAACTCTACTTGGCCCTCTGT<br>GTGTCTTACACCCATTAAACAAATTAAAATTAAAATGTTCCATACCA<br>TCCTTTGAAGAGGGTGTACACCATTTAAATATCTTTACTTAAAAAAA<br>TAAAAGTCTGCTCTCATAAGCAATGAAATGTTTTTCAAACATTTAAA<br>TCTCTGTTCCTTTTTGCAAGTTATCCTAGTGTGTTGTATGAGGAATA<br>GATGCAGTTTTTTCTTTTTCCTCATGGCTGTCCTATTTCCCAGCACC<br>ACTTTAAATTCCATCTTTTTACAAGTATTGAGATGCTACTTTAATCAT<br>ATACTGAATTTATATATATTTACTTCTGGATTTTAAACATTGTGTTCTC<br>GTGGTTGATCTATTCATGTACTGGTATTGTAGCTTTTTAAAAAACTTT<br>TAATTTTAAATAACTTTGGACTTACAGAAGAGTTGGAAAAATAGTA<br>CAGAGAGTTTCTATATACCTTTTACAGATTCCCCAGATGATTAACAT<br>CTTATATATCTGTACTACAATTATCAAAACAAAAAAACTAATCTATTG<br>GTACAATATAGTTAACTACATTTATATGGTTTTAATTATAAAGGCTTT<br>GTACAATATTTTAATATGTTGTTGGTTTACTCCCTGCTCTTCTTTCATA<br>TTTTCAATTTTTTAAATGTCTAGCTGCAGAAAATAAACAAAAGCTTT<br>ATATAGTATTTTTACTGGGACTGTAAAATTTATAAGTTAGCTTAGGGA<br>GAAATGGCATTTTTATGTTGCTGAATCTTGGTATACCAGAATAAGAA<br>ACGTCTTTCCATTTGTTGAAGTCTACTTTTCTATCTTTCTCATATATGT<br>TTTGGACTCTTTTTGTAGTTTATGCCCAGATATTTGCATTTTTTATTGT<br>AAATGGGATCTTTTCTTTCATTTATCTTCTGTCTGGTTTCTCTCCCCT<br>ATAAATGAATGATTTCTGTATATTTTGTACTATACTGTATTGCCAAATT<br>ATATTATTATAATAATATTTCATCGATGCTTTTGTATATTCCAGGTATAT<br>AATTGTGTTATCTGTGAATAATGTTCCCTTTCTAATTTTTTTCTAATTT<br>TATTCATTAATTTGTGTTGACTAATATTATGACTAATAGAATGGTACC<br>CTGACTGTAGGACTATATCCAGTGTTTCCACATTAAGTATGCTGTTG<br>GCCTTGGGGTTGATAGATCATGTTAAAGAAATGTTCTTTTTTATTCA<br>GTATTTTCAACAAGAATGGGTATTAAATTTGTAAAGTGTCTTTTCCT<br>CATCTTTGGCATGAACATATGATTTTTTCCCATATCTATTATGTTAAAT<br>GATATAATTAATTTTCTAATAGTGACTTTTTCATTGTAAATTATATTTT<br>TCATCTCTTTTGGGATCAGTTCTGATAGATTATATCTTCTAGTAAATTA<br>TTTTGGGTTTTCAAATTTGTTTGCATAGAGTTGAGCAGAGTCATCTT<br>TTATGATTCTTTTGCTTTCCTGCCTTCTTATTCATATTTTCCTCATTTC<br>ATCTAAAAATGCTGGAAAATATATCTTTGTTTTTCCATGAAAACAAA<br>ACAATAAAAGCAGCTCCATTAATGTAAAAAAGAAGTGATCTCAAAC<br>TTCTGAAAATAATAATCCTCCACATTTAATTCTGATTATCTTTTGCCA<br>GCCTGAAATTTTTTGCTTCCTTGCTTTAATTTCTTACATTACTTGTAA<br>ATTTATTTTCTAATGAACATTTGAATAAAAGTCTAGATAATAGTCCTT<br>GTGCTTTCTTCTCCCCTACCCCACACTTACAAGGTGCAGCAGTGTC<br>TTTAGTGGTCATCTTAACTGCAGAAAAACAAATTGAGAGGTTTGCA<br>TGCTCTCAGTAAAGTGAAAGATTGGGAGGTTTTCCCCATTTCATGG<br>TGTGTGGCAGCTGTAATGTGATCACACAAGTTTCAAATTTGAAAAA<br>TGTTTTAAAGATATATTTGGTATGCAATTCGTGTAATTGCAAAAAAG<br>GCATTCCAAAGTAAATTTATGACTTCACAAATTTTGTATGACTTCAC<br>AAGTTTTGGATTAGGGAGGTATGGCTGCAGCATCCTAAAACAATAT<br>AAGGTTGGGTATTCTTCCATGAAACTTGTTATTTTTTCCATAAAGTT<br>AGATTCTTTCTAGAGCAGAAAGAACTTTAATGATTGGGAATCTGGA

	GTTAAGGGAAAAAAAGATGATTAAAAGTTTAATTGTTCATCTGAAG<br>AGTTGATTTTTTATTCCTGTAATAAAGGGTACTTTTAGCAGTCTCTG<br>CTCATCTTGCCCATCCGGCTCTTTTTGTGGTTGTGTAAGGTTATAAC<br>TTCTGTGTCTCAGTAAACTTGTGCATGCCCATTTTTTTCTCTGTTACT<br>ACCTTTTCTCTTATTTTGTTTTATTATTTTGATGTAAAATTACCTGTTA<br>ATTTTATTTGAAATGAGAAATTTTAAGGTTCACATTATTCAAATTCTG<br>TCAGATCCCTACCTCTGTCATATGGTTTATAATGTGCTGGGTATTTTC<br>AGACCTGCTTATTAAAAAGATGTAAAACAAAATAATGATCACTCCT<br>GTGGATTTTTCCTTTATTTTTGAGATGTCTCCTTTGGCTGCATTACTT<br>CTTCACCCCTTGCCCATTGATCAGAGGAGGGGTCTTAACTATGGGT<br>GAACCCTATATCTTACTGAAGAGGTTATGTTACATGTATATTTTCATA<br>ATATAACTTACATTTACATAGTACTTTTATTTTTAGCATACCTTTTTTT<br>ATTAATCCTAATAATATCACTGTAAGTTATGTTGAAGCAGATTGTAAG<br>TGTTCATTTACAAATTGTGAAATGAATTAAAATGAAAGGGCAAAGA<br>TTAAATCATGACCAGGCCTGAAATTAACACACAAGACTCAATTTTT<br>TTCAACCAAAGACTTTTGTAGGTGATCCCTGCCTGCAGGACTCCCC<br>TTCCTCCTCAGATGTCATTGGATTGTACCAGGTTTACTGTAGATTCT<br>AGCCGTTGTAGAACTAACTAGATCTAAGATGAGTCCCCTGATTTCCT<br>TTGGTAGAGTCTTCCAATTGCTGAACTCCAATATTGTCGTGACTAGC<br>CAGTGTTACAACCTGTCTGCCTTATTTTGTGTAATGGATTTCATATTA<br>CAGAGGCATTTTTTTAATGTCAAGATGTTTAAGTATTGCTTAAGTGC<br>AAACTACTTAATACTTTTTAGCTATTAAGTAATTAAGATAGGCAGGA<br>TTTTATTTGTTCCAAAATGATTTGACCTAAACTAAAAAGAGAATGTG<br>GATCTCCTGAATCTTACTTGGTTAATCTTAATATAACTCCTAGCATTC<br>TATAATTCTTCCTAAAGTCCTCTTACCTGGCTATCTTTTGTATCTTCTT<br>TGTCTCTCCTCTTCTTTCCCAGTCATAATAACTGCCAGACTCTGCTT<br>CATTTCTCTTTGACAGTCTCTACTCCTAAGGTCATCCATTCTCTTTAG<br>GTATCTTTTGGCCTCAGTTTGAGCACAGCAGATCCCAAGACCACAT<br>ATGCCATAGCATAGGCTATTATAGTCAACCTTTTGAATAAATGTGATT<br>GAACTTTATGTTAGTAATTCTTATTTACCATCTTCCTATCAAAAAGGC<br>TTAAAGTCTTCATTTAATGCTCTCCTTCATGTCCATTTTGTTAAATGA<br>TTGCCTTTTAATGACATCTTAGAACTTCAGAACTATTTCACCATGGA<br>GGATGTGTAAGATTAGCCTTTTATCAAATAAAAAGTGTGAAATGGA<br>ATATGTAATCTCATTAATCCATTCTGGCTCTAAAATTCTGTGACTATC<br>AGATAAAATTCAGAAATAAAATAGTATTACTAATATAAATAAATTTTT<br>ATCATAATTATATTTCCTAAGTTTTGCCTGTAAGAATGGGTAAAATAT<br>CTTTAAAACCTTGAAGAAATTATTACTTGATAGAAAGTTTAATCCAT<br>CTGTGAGAAGGCAAATGTATTCAGACACAACTAAAGTTCTCTCTTC<br>TATTTTAATTTCATTTATCTTGAACTAAGACTCCACTGTTTCATCCTC<br>TTAGATGCTGCTACTTGAACAATATTGTTTTGAGACCAAAAACTAGC<br>ATATTAACACAATTCTTCTTAAACGTCTTAAGAGTTTTGTTTCCTTTA<br>CCCCTTTCTTTAAAAACAAGCAGCCACTAAATTTTTTAGTAGTGAAT<br>TTCAAAATCCTTTTTAACCTTATAGGTCCAAGGGTAGCCAAGGATG
APOB	ACCATCCCCCTGTCTCCCTTCTCCCCAGAGACTCCAGTAGCCTGGC<br>GTCATCACAGGGGCCAGACATATCCAACATGTTCCCAGCTTCCTGC<br>CACTGCACTTTCAGTGTGCCTCCCTCTTCAGTTACCCAAATCCTGCC

	CACCATTCCAGAGCCAGTTCAATCTCACCCATCCAGGACCCCCGAG ACCCCCATCGTACCACTATAGTCTAACTGTGGTGTAGACCCCACACT GGGCACATTGCGTACGCTCATTATTGGCTGTGACGTCTGATTATGCC CTTCTCCTGGTCTGGAAGCTCTCGGAGGTGCTCCATAATACATGAA GAGAAGTAGTGCTGGTGTGGGAATAGTGAGGTGTGTTTATCCATCC AGCTATCCGGCACCAGCACTGGTCTCAGCTTTCTGAGGTAACACGT TCTGAGCCTTAGTCTTGAGAGAACATAAAGAAAACTTTTTTTAAAA GTAGTAAAAAGTGGCTGACAAAAGCTGACCAAAAGCCTTTCAAAA GAAATGCTAAGTTATATCTAAGAAAGTTTACCCAAGGTCAGGCAAA TATGAAACCTAAAGCTAGACGTGGGGAAGAACTTCCGGAGAGTTG CAATTCCCTGTGCCCCAGCATCCTCAGGAGGGCATGCCCACATCTG ATTTAGAAATCTGTGTAAAATGAGTGAAGGGTTCTATTTCTTGGGCA GTGTGGGCACAGGTCTTTGGAGAGGTCGATGGCCTCCCATAAAATC CTTCCTGCTTGATGGTTCTGGATCCTCAGCCACAGCTCCTAATAGCC ATGAGGTTTGAGCCCAAAATAATTTATGTGTTTGTTTTTCAGCCCC AAAATTTCCATAGAATCAAAGTAGTCAGAGCTGAATGGGGCTAAGA GACCGTCCATTCCTGTCTTCTCATCACACAGATGAGGGACTGCCAC CCAGAGCCGTAGAAACTGTCCCATGGCCCCAGTTCCCAGACCCTTC CTCTCTCCTACAGCTCCAAGTTCACTGTGCATTCTAAATGAAGATGT AAACATAAGCAGCAACACTCAAGAGTAAAAATGAAGTGTGCATAT GAAAGAAACCTATTCACATGGACCATATTACATTATAATCACAGTGT TTACTGCTTGACTACCATCTGCCTGGCCTAGCAAGGGTGTCAGTGA GGAAGAGAGGACAAGGGGTACCAATCTGTGAACTACACATGGTTC TTGCTCTCCCAGCTTCTCTCTCCCATTGGCAAGGCAACAGGTAAAC ACATGAAAAATCAAATAATGCTATAAGAGAAAAATGTATTCAGGAC AACAACAGGTTTGTATGAAGGCCTTTCATCATCGTTGTCCTACCTAG AAACTGAATGACAGGGAATCAGAGTCACAAGCTATGAAGTCTAAC TGGGCTGTTCCCAGAGAAAGATTCAGTGCAGTAGGTGGGGCTGCA GCCAGCCCTGGGTGGGTGGAAGGATGACATCCACATAGGCAAGAG GGTGATAATTCACTTACGCAGCTCCTCACTGCACATTGAACCCTGCT GACTTCTGGCTTCTCTCCCGGGAGGAACTGCGACTCAACATTCTGA CCTTATCTCTTGGGTAGCAGAATGATGGAGAAGGAAAGTTTCTTTT TGCTTCTCGCAGGGGTTAATCATCCATCTGGAATGCCTACATTTGGT TGACAATGGCTCACCCTATCATCTTCCTCCTGAACCATTCACCTAAA TGTGCCATTTCTTTCCTGATAGTTCTCATTTGTGTGTGTGTGTGTGT GTGTGTGTGTGTGCACGTGCTCACACATGCATGCTGTCACTGGGTA AACAGGCCACCCTGGGCACAGTTCCATCTACAATGTTTGAAGTTTA CTTTCCAGCTTCTGGGCATCATTTGCAATTATAATGCTGTCATAGGC AGAAACGAGATAGGCTAATTAATCGTTGTCAATACTGATCCCTATTT GCCAGATGAGATTTTGGAGCAGCATGGCTGGGAATAATTGGTATAG ACTGTATTTCCTTGCTTTATGTCACTGGAAATATTTATTTAAGCATCA CGGTCGCTATGCATAAATATCCTGGAAAATGGGGTATAGCTGAATGG TGCAGATTCATTCATTCATATTCAGCAAATTATGTTCTAAGCACCTAC TTCAGTATGTGAACAGCACTAAACTCAGAATATTGGTCTGCTGGGG TCCTTTATTAGCTTCCATGATTCCCTGAACTTGGCCAAGACCCTTCT GGTCGGCTGCAGATAGGCACAATGGATAGTTTTGCTTCTAGATAATG

	TAACTGGGACATTCAGCATTATCTATCGCCTTGAAATTCCTCTAGTC<br>AGGTGGCTTTCTAATGGGTACCCAGAGCCCTATGACTACCCAGATT<br>GATGGTGCACCCAACAGGACTTTGCATTTATGAGCTGATAAGTCAC<br>AGTCACTAGCTGAGATTAATCTGTGTGACACCAGAATGTGTCTCTAT<br>CTAAAGGAAAAGGGATGAAGGGTGATATCTTTGGTCACAAGTAATG<br>TATTTCCATGTAGTCTTTGACAAAGGATCTAAGTGGATTTTGTAATT<br>GAAGAAAAATCTATGCACTAATCTTTACAGCATTTCCGTGAGTGTAC<br>GCAAGTCAGCTCAACAATTCAACATTTGCTCTGTGGGGTTGTGCTA<br>GACCCTGTCAGGGGATAACTACTGCTGGCTGGGGCCCAGTTCAGG<br>GAAGACTTGCCAAAGACCATCAGGAAAAGAGGGAAGCTGAGTCTT<br>AGGTTTCTTCCTTTAGAGATGGTGACAGTCCTCTCACCACCTCCAA<br>GCATCTCACAATGTTTCCCTGCCTCCAAGTCATCAAATTCATTTTTG<br>ATTCCTACTTCATAAAAATTACATTCTCCCAGCACTTTGGGAGGCCA<br>AGGCGGGCAGATCATGAGGTCAGGAGTTCAAGACCAGCCTGATCA<br>ACATGGTGAAACACCGTCTCTACTAAAAATACAAAAATTAGCTGGG<br>CATAGTGGCACTCACCTGTTATCTCAGTTACTTGGGAGGCTAAGGC<br>AGGAGAATCGCTTAAACCCGGGAGGCAGAGGTTGCAGTGAGCCGA<br>GATTGTACCACTACACTCCAGCCTGGGTGACAGAGGGAGACTCCAT<br>CTCAAATAAATAAATTAAAAAAAAAAATATATATATATATGTATATATT<br>CTCTATGGATGCTGACCATTGGACCCTGGTTTCATCTGCACGTAACA<br>GAGTAAGCTTGGACTTGTGCTTGTAAATTAAAGCTCGACACCTCCT<br>TTTGGCTTCTCTATACCTGAATATTCTTACTCACTCTCCTTAATGTGA<br>ATATGCATGGAAGCAGGACCATTTCCTCAAACACTAGCAGCAGCGA<br>ACCCTGTGGAAAGTCAGTCCACATAGAATAATTCAAATAAAGTGTT<br>CAGAGAAATGGGGTTTCAGAGCAATTACTTTTTCCAGACCTTTCAC<br>AAATCAGTGGTGTAGGTATGACCAGCCTTGAGTTGAGACCTCTGTA<br>ATATCCATCTTTAATAACATTAATATGCTGTGGATGAGCAACTGATCA<br>CTGGAGGGAGTTTAGCTGCCCATAGGAGTTCATGGCTAATGACAAT<br>ATCTGAATAAGGACAGGTGTGGAGCCCAGGTGCAGGAAGCAGGCG<br>AAGGTCTTTCTGTGAGTCTCCTCTGAGGGAACTGGGTCTTTATACAT<br>AGTTACTGTTTCAGAATTGATCCTTCTGGAATCATCAGTCTTCACCA<br>GTAGCTTGTTACATCTGGGGTTATCTCATAATTCAAACAAAGCTGAC<br>AAGTTGTAACAATGAGCACACACTGACTTCTGCAACAGGCGCTGT<br>CCACTTCCCATCCGCACTCTACCGGCTTGCTCCTGGCCGCCTCCCA<br>CTCGCCTTCCTGGGTGGTCCCCCAGCAGTTATACCTACCTGGTTGTC<br>GCCCCCTCTATCCTACCACAATTGCTCACTAGCGGTTTCCTGCGTAC<br>ACAGCTTGTCTCCCTAACCAGAGTGGAGGTGCCTTGGGGACACAG<br>CCAGGCTCAGACATTCACTCAGCTCATCATAGTGCCATCCCATCAAT<br>AACCCCTTCTGAGTGATCCTGGGTTAGTAAACCGAGTGTCCCTGAA<br>ATTCCACTACCGCTGATTCCCTCCAGCTGGGCAGAGGCAGCGAGCG<br>CTGGCTGAAGCTTCCGGTGGGAAATGGGCAGTGCCTAGAAGAGAA<br>GGAAACGATGCATGAGAAGGTTCCAGATGTCTATGAGGAACATGA<br>CGTGTCCTGTCCACTACTCTGCTTTTCCTCGTCCGCCTCCCCACCAC<br>TGGAGGAAACCTAGAAGCTGGTGCAGGAAATCCTCCTCTCAACAA<br>CCCAAGAACACTTTGCACAAGAGGGGTGCGCCCTCGGAGGTTGCT<br>CTTCCCCAGAGGCCTCTCCTCGCTGGGGTTTCTTGAAGACAGATAC

	TTGGACTCCTGCTGGGACCAGGCAGGCCACCCATCCTCAGGGGCA<br>GTGACTGGTCACTCACCAGACCTCCCTGCATCCCCCTTCTCTCTCCT<br>CCCCCAGCACGGGCTGAACCCCGCAGCCACAGATTCTGATCAGGA<br>TTAGGGTGTGGGTGCAAATCCAAGGTCCACCAAAATGGAAAAGAA<br>GTAACCGATGGGAACACGTCTCCACCAAGACAGCGCTCAGGACTG<br>GTTCTCCTCGTGGCTCCCAATTCAGTCCAGGAGAAGCAGAGATTTT<br>GTCCCCATGGTGGGTCATCTGAAGAAGGCACCCCTGGTCAGGGCA<br>GGCTTCTCAGACCCTGAGGCGCTGGCCATGGCCCCACTGAGACAC<br>AGGAAGGGCCGCGCCAGAGCACTGAAGACGCTTGGGGAAGGGAA<br>CCCACCTGGGACCCAGCCCCTGGTGGCTGCGGCTGCATCCCAGGT<br>GGGCCCCCTCCCCGAGGCTCTTCAAGGCTCAAAGAGAAGCCAGTG<br>TAGAAAAGCAAACAGGTCAGGCCCGGGAGGCGCCCTTTGGACCTT<br>TTGCAATCCTGGCGCTCTTGCAGCCTGGGCTTCCTATAAATGGGGT<br>GCGGGCGCCGGCCGCGCATTCCCACCGGGACCTGCGGGGCTG
APOE	GGCCTGGAGAGGAGGGCACTGTCATGTCTCTAGCTGGGAAATACA<br>CATGTGAGCCTGGCGCCTGGGTCCGAGGGTGGAGGGGCTGGGCCC<br>CTGGACTCCTCCTGGGTCTGAGGGAGGACGGGCTAGGGCCCTGGA<br>CACTCAGGTCTGAGGGAGGAGGCCTGGGTTCCCAGATGCCCAAAT<br>CCCCTTGGTAATGAGACCCCGCCTCCACCCCACTCTCTGACAGTGA<br>ACAACTGGTTGGCAACGGTAACGTTGGGCCAGGCGGGCATGCACG<br>CAACATACTACCACAAAGCCAGTGACCAGGTGAGTGGGTGCAGGG<br>ACTAGCTGGTGCTGCCAGGGGCTGCTGGGCCTGGAAGTCCAGGTG<br>GGGCCACTTGCTAATTCTCATGTGTTGCTCCGGCCCCTCCAGCTGC<br>AGGTGGGTGTGGAGTTTGAGGCCAGCACAAGGATGCAGGACACCA<br>GCGTCTCCTTCGGGTACCAGCTGGACCTGCCCAAGGCCAACCTCCT<br>CTTCAAAGGTAAAGGTCTCGGTTCCCCTACGCGGGAAACAGGCAG<br>GAGGTGACTCAACTCTGAGTGGATGTGTGGGCCACCACAGGTGCT<br>GGAGGACAGTGTGCTGCCACCCTGTGGGCCTCCACATTACCAGGG<br>AACACTTGTTAAAAGGTAGGTGGGGCCGGGTGCGGTGGCTCACGC<br>CTGTAATCCCAGCACTTTGGGAGGCCAAGGCGGGCCGAGGTAAGG<br>AGATTGAGACCATCCTGGCTAACACGGTGAAACTCCGTCTCTACTA<br>AAAATACAAAAACAAAATTAGCCGGGTGTGGTTGCGGGTGCCTATA<br>GTCCCAACTACTGAGGCTGAGGCGGGAAAATGGTATGAACCCAGG<br>AGGCGGAGCTTGCGGTGAGCCGAGATCGTGCCACCGCACTCCAGC<br>CTGGGTGACAGAGCAAGACTCCATCTCAAAAAAAAAAAAGTAGGT<br>GGACAACCCTCTACTATGTTTTATGCTTGGAAAAAAAAAGTAGGTA<br>GAGCAGCCAGGCGTGGTGACTCACGCCTGTAATCCCAGCATTTTGG<br>GAGGCCAAGCCAGGTAGAATACTTGAGGCCAGGAGTTGGAGACCA<br>GCCTGGCCAACGTGGTGAAATCCCCTCTCTACTAAAAGTACAAAAA<br>TTAGCCAGGTGTGGTAGCGTGCTGCAACTGTAGTCCCCGCTACTTA<br>GGAGGCTGAGGCACAAGAATCACTTGAACCTGGGAGGCGGAGGTT<br>GCAGGGAGTTGAGACTGCACCACTGCACTCCAGCCTGGGTGACAG<br>AGTGAGACTCCATCTCCAAAAAAAATAAAATGAAATAAATAAATAA<br>ATGTTAAAAAAAATCTGGTGGAGCATCTGATGGGTGTTTGGGCCAA<br>GCTGGAGCTTTGTCCATCCCCTCTTATTTTTCTGCACTTGACTCTCTT<br>ATTTTTCTGAGACTGGTCTCCCTCTGTCGCCCAGGCTAGAGTGCAG

	CAGTGCAACTGCGGCTCACTGCAGCCTCCACCTCCCGGGCTCAAG CAGCCTTCCCACCTCAGCCTCCTGAGTAGCTAGGACCACAGGTGTA TGCCACCAGGCCCAGCTAATTTTTTTGATAGTTTTGGGAGACATGG GGGTTTCACCATGTTGCCCAGGCTGGTCTCGAACTCCTGGACTCAA GCCTTGGCCTCCCAAAGTGCTGGGATTATAGGTGTGAGCCACCACA CCCAGCCAGGGTAGAAGGCACTTTGGAAGCCTCGAGCCTGCCCCA TTCATCTTACGTTAGTGGAAACTGAGGCTTCCAGAGGTTTCAAGGT CACAACTAAATCCAGAACCTCATCTCAGGCACACTGGTCGTAGTCC CAATGTCCAGTCTTAAGTCTTCTTGGATATCTGTGGCTCACAGATTT TGGGTGTTTGAGCCTCCTGCTGAGCACTGCTGGGGCCACAGCGGT GACCAGCCCTGTCTTCACGGGACTCAGTGAGAGGAACAGATTCAT CCGCAGAGTGGGCAGGACTAGGTTGGGGGAACCCAGGGGTCTAGA GGGCTTTTCAGAGGGCAGGGGTCACTGAGCGGAGAGCAGAGGAG GAGTGAGCCATTTGCTCCAGCGTGAAGTTGTTGGTGTGATGGGGTT TCAGGGTGGCAGGAGCAGTGTGGTTAAAGGTCTGGAAGCTGTCGG CATGTGGCTGGTATCCAAGGTGGCCAGGAACTCTGCATGGATATGG TGGGAAGCTGGCACGCCTCTCACCTCAGCTCTTCCCTGCAGGCTCT GTGGATAGCAACTGGATCGTGGGTGCCACGCTGGAGAAGAAGCTC CCACCCCTGCCCCTGACACTGGCCCTTGGGGCCTTCCTGAATCACC GCAAGAACAAGTTTCAGTGTGGCTTTGGCCTCACCATCGGCTGAG CCCTCCTGGCCCCCGCCTTCCACGCCCTTCCGATTCCACCTCCACCT CCACCTCCCCCTGCCACAGAGGGGAGACCTGAGCCCCCCTCCCTT CCCTCCCCCCTTGGGGGTCGGGGGGGACATTGGAAAGGAGGGACC CCGCCACCCCAGCAGCTGAGGAGGGGATTCTGGAACTGAATGGCG CTTCGGGATTCTGAGTAGCAGGGGCAGCATGCCCAGTGGGCCTGG GGTCCCGGGAGGGATTCCGGAATTGAGGGGCACGCAGGATTCTGA GCACCAGGGGCAGAGGCGGCCAGACAACCTCAGGGAGGAGTGTC CTGGCGTCCCCATCCTCCAAAGGGCCTGGGCCCGCCCCGAGGGGG CAGCGAGAGGAGCTTCCCCATCCCCGGTCAGTCCACCCTGCCCCGT CCACTTTCCCATCTCCTCGGTATAAATCATGTTTATAAGTTATGGAAG AACCGGGACATTTTACAGAAAAAAAACAAAAAACAACAAAAAATA TACGTGGGAAAAAAAACGATGGGAGGCCTCCGTTTTCTCAAGTGT GTCTGGCCTGTTTTGAGCATTTCATCCGGAGTCTGGCCGCCCTGAC CTTCCCCCAGCCGCCTGCAGGGGGCGCCAGAGGGCCGGAGCACGG AAAGCAGCGGATCCTTGATGCTGCCTTAAGTCCGGCTCAGAGGGG CGCAGCGTGGCCTGGGGTCGCTATCTTCCCATCCGGAACATCTGCC CTGCTGGGGGACACTACGGGCCTTCCCTTGCCTGAGGGTAGGGTCT CAAGGTCACTTGCCCCCAGCTTGACCTGGCCGGAGTGGCTATAGAG GACTTTGTCCCTGCAGACTGCAGCAGCAGAGATGACACTGTCTCTG AGTGCAGAGATGGGGGCAGGGAGCTGGGAGAGGGTTCAAGCTAC TGGAACAGCTTCAGAACAACTAGGGTACTAGGAACTGCTGTGTCA GGGAGAAGGGGCTCAAGGACTCGCAGGCCTGGGAGGAGGGGCCT AGGCCAGCCATGGGAGTTGGGTCACCTGTGTCTGAGGACTTGGTG CTGTCTGGATTTTGCCAACCTAGGGCTGGGGTCAGCTGATGCCCAC CACGACTCCCGAGCCTCCAGGAACTGAAACCCTGTCTGCCCCCAG GGTCTGGGGAAGGAGGCTGCTGAGTAGAACCAACCCCAGGTTACC

	AACCCCACCTCAGCCACCCCTTGCCAGCCAAAGCAAACAGGCCCG GCCCGGCACTGGGGGTTCCTTCTCGAACCAGGAGTTCAGCCTCCC CTGACCCGCAGAATCTTCTGATCCCACCCGCTCCAGGAGCCAGGAA TGAGTCCCAGTCTCTCCCAGTTCTCACTGTGTGGTTTTGCCATTCGT CTTGCTGCTGAACCACGGGTTTCTCCTCTGAAACATCTGGGATTTAT AACAGGGCTTAGGAAAGTGACAGCGTCTGAGCGTTCACTGTGGCC TGTCCATTGCTAGCCCTAACATAGGACCGCTGTGTGCCAGGGCTGT CCTCCATGCTCAATACACGTTAGCTTGTCACCAAACATACCCGTGCC GCTGCTTTCCCAGTCTGATGAGCAAAGGAACTTGATGCTCAGAGA GGACAAGTCATTTGCCCAAGGTCACACAGCTGGCAACTGGCAGAG CCAGGATTCACGCCCTGGCAATTTGACTCCAGAATCCTAACCTTAA CCCAGAAGCACGGCTTCAAGCCCCTGGAAACCACAATACCTGTGG CAGCCAGGGGGAGGTGCTGGAATCTCATTTCACATGTGGGGAGGG GGCTCCCCTGTGCTCAAGGTCACAACCAAAGAGGAAGCTGTGATT AAAACCCAGGTCCCATTTGCAAAGCCTCGACTTTTAGCAGGTGCAT CATACTGTTCCCACCCCTCCCATCCCACTTCTGTCCAGCCGCCTAGC CCCACTTTCTTTTTTTTCTTTTTTTGAGACAGTCTCCCTCTTGCTGA GGCTGGAGTGCAGTGGCGAGATCTCGGCTCACTGTAACCTCCGCCT CCCGGGTTCAAGCGATTCTCCTGCCTCAGCCTCCCAAGTAGCTAGG ATTACAGGCGCCCGCCACCACGCCTGGCTAACTTTTGTATTTTTAGT AGAGATGGGGTTTCACCATGTTGGCCAGGCTGGTCTCAAACTCCTG ACCTTAAGTGATTCGCCCACTGTGGCCTCCCAAAGTGCTGGGATTA CAGGCGTGAGCTACCGCCCCCAGCCCCTCCCATCCCACTTCTGTCC AGCCCCCTAGCCCTACTTTCTTTCTGGGATCCAGGAGTCCAGATCC CCAGCCCCCTCTCCAGATTACATTCATCCAGGCACAGGAAAGGACA GGGTCAGGAAAGGAGGACTCTGGGCGGCAGCCTCCACATTCCCCT TCCACGCTTGGCCCCCAGAATGGAGGAGGGTGTCTGTATTACTGGG CGAGGTGTCCTCCCTTCCTGGGGACTGTGGGGGGTGGTCAAAAGA CCTCTATGCCCCACCTCCTTCCTCCCTCTGCCCTGCTGTGCCTGGGG CAGGGGGAGAACAGCCCACCTCGTGACTGGGGGCTGGCCCAGCCC GCCCTATCCCTGGGGGAGGGGGCGGGACAGGGGGAGCCCTATAAT TGGACAAGTCTGGGATCCTTGAGTCCTACTCAGCCCCAGCGGAGG TGAAGGACGTCCTT
APP	AACGCTATTTACAGTTTAGACTTTTGTAGCTATTGAAGGCTGACATT GAGATAAAGAAGTTAATCATGTCCTTCTGTCTTGGAGGAGGTAGAA AGAGATGAGAATGAATACAATTCAGGATCTACTTCTGGTCTTTGATG AGGAGTTAGCACACGGTTCTGGGAGGAAAGACAGGTTAAGAGGCA TGTGAAACTCTCAAATACGTCACTGCGTCTGCCAACGTACATGATA CCCAGCAAGCTCACATCTTCATGGAAAGCATGGTAATTCCCAACAC TACCGGAAGTCTGGAGTGGCTAAGTAATCCATATATTCAACCAGGA AGCAGCTAAAGAAATATTCTAATTACCTAGGAAGGTTTCTGATTTCA AAAGGACATGAATAAAAAGTAGAAGGAATCCACTCCCAAGGACGG ACATCAGAGTAGCTTAAAATGTGAGAATAATTTTAGGGGAATTTTAG AGGTTTGGTTATAGACTTATGTTCCCCCAAAATTCATATGTTGAAGC CCTAACCCCCAGTACCTTAGAACATGACTGTATTTGGGTAGGGCCTT TGAAGAGCTAATTAAATTAAGGCCACTGGCGTGGGCCCTAATATAAT

	CTGGCTGGTATTCTTGTAAGAGGAGGAGATTAGGACACACAGAAAT<br>ACCAGAGGTACCTGTGCAGAGGAAAGAACGTGTGAGGACTTAGCA<br>AGGGTGCAGCCATCTGCAAGCCAAGGAGACCTCTGAGGATTCCAA<br>TCCTATCTGCATCTTGATCTTAGACTTTTCTGGAACTGTGAGAAAAT<br>AAATTTCTTGGTTTAAGCCACCCAGTCTGTGATATTTTGTTATGGCA<br>GCTCTAGTAAACTAATACAGATTTTAAATGTCATTAAATGTCAATGTT<br>TAAGCTTTGACAAAATTTTCTAAAGGAAAGTATAAAAGGTCATTTT<br>CTTTCTTTTCAGAGCCTGATGATTGCGGGAGGGGTAAGCCAGCTGC<br>ATGGGGATCATGATGCAATGCTGATGCAGGACAGACAGAAAGTAGA<br>TCTCTTCCATTTCTATTTTTTTTTTTTCTGTTGAGTTGAATGATCTTC<br>AGACTGAAAATGAAAGAAAGGTCACTGGAAATAAAGGCCAAAGAT<br>GAGTGACAGGATTATAGAATAAGTCTTAGCTGTTCTAAAGAAGGAC<br>ATATTATGTACCCCCACCCCCAAATTCATATGTTGAAGTCCTAACCC<br>GACAGTGTCTCAAAATGTGACCATATTTGGAGATAGGGTCAAAGAT<br>GTAATTAAGGTTAAATGAGGTCATTAGCATGGATCCTAACCCAATAT<br>CTGCTGTCCTTATAACAAGAGGAGATTAGGGCACAGTAAGACACAG<br>AGGGAAGACCATGTGAGAATACAGGGAGAAGGTGGCCATCTGCAA<br>GCCAAGGAGAGAGGCCTCAGAAGTAACCAACTCAGCCAACACCTC<br>GATTTCAGACTTCCAGCCTCCTGAAATGTGAGGAAATACATTTCTG<br>GTGTTTGATCCATCCAGTCTATGGTAAGTTATGGCACCCCTGCAGGG<br>TTCATCTGGCTCAGACTTAACGATTGCTTTTGGTGATATTTATAGGG<br>CACAGATAACAGCCTAAACACAAGACGACAGAAACGCGGCCCAGC<br>AGACTATGCATAAAATAGAAATGGGGTATCTGGACCAATTGGAGTC<br>TGCAGTGGGATGCGGTTACTAAAACAGTCAAATGCAACATGAGGCT<br>CCAGGCAGAGTAGTGGGCAACATCTCCCATGTTGCAGCAGTCAGA<br>GCACACTTCGAGTACTGTAAAAAGACACAGACAAGCCAGAACACA<br>TTTAGAGAATGGCCAAGGTGTGGAAGGAACCAGAAACCATGCCAT<br>TATGCAACTGTTGAAGGAAGTGCCTGTTTTACCTTGTGAAGAGAAG<br>ACTCTAGAGGAAGAAGTAGCATGAAAACCGCTGGCAAATTTGTAA<br>AGATCTGAAGTGTGGAAAAGAATTATTCTGCTTGGTCACTGGGGAT<br>ACAAGGATATCTGAGTGGGAGTTTAAAGGCGGGGGATGTGAGCTTT<br>AAATGGGATAAGAACATTCTAGTAACCAGAAATGCCCAAAGATAGA<br>ATGCACAGTCTGGAGAGCCAGTGAATATCTCACAAATGGAGACACT<br>TGAAACTAGGATGGGGATGCTGTTGTAGGAATTCCAGCAGACAAGT<br>GGTTGTTGGTTCCTTCCCCAACTTTGTAGGGTTATAACTAGGGATGT<br>TCCTGCGTTTTCTGCTTGGAGGATCTGCAAGACACCTCAGGGCAGG<br>AAATGGCATTAAATGCAGAACAGAGCTAGTGGCTGAAAAGCAAAA<br>AGCCATCAGGATCTCTGAGTAGTGAAGGAACCAGAGAACATGCAG<br>GCAATGTCCATCATTCTGACGCAATCAGCAGCATAATCATCTTCCCC<br>CAGGAACATCTTGACCAGGGAATGTGTCAGTGTGGTGAATTTCAAC<br>AGTGGAAAGAGAAACTGCTAAATCTAAGAACTTTAATTTTTATAGA<br>TTATGATCTCATCTCTACAATTTTGAATTTCATGCTCAATAAAAGTTC<br>CTTACTCTCTTTTTTTTTTTTTTGAGACGGAGTCTCGCTCTGTCGCCC<br>AGGCTGGAGTGCAGTGGCGCGATCTCGGCTCACTTCAAGCTCAGC<br>CTCCCGGGTTCACGCCATTCTCCTGCCTCAGCCTCCCCAGTAGCTG<br>GGACTACAGGCGCCCGCCACGACGCCCGGCTAATTTTTTGTATTTTT

	AGTAGAGACGGGGTTTCACCGTGTTAGCCAGGATGGTGTTGATCTC<br>CTGACCTCGTGATCCGCCCGCCTCAGCCTCCCAAAGAAAAGTCCCT<br>CACTCTTAAAGTTGCCTCCTCCTTCCCAGGGCTGGCTTCATGGGCA<br>TGCAACCCTGGAGAGTCTCACAGGCCCTGCGGTGGGAGGAGCCCC<br>ATGCTTGGTTTAACGCTCTGCCATTGCCATCTTAAAATTCTTAATTTA<br>ATTTTTTTTCTTTTTTTTGAGGTGGAGTCTCGCTCTGTCGCCCAGGC<br>TGGAGTGCAATGGCACAATCTTGGCTCACTGCAACCTCCGCCTCCC<br>AGGTTCAAGCGATTCTCCTGCCTCAGCCTCTGGAGTAGCTGGGATT<br>ACAGGCAGGAGTAACCACGCTCGGCTAATTTTTGCATTTTTAGTAG<br>AGATGGGGGTTTCACCATGTTGGCCAGGCTGGTCTAGAACTCCTGA<br>CCTCAGGTGATCTGCCCACCTGGGCCTCCTAAAGTGCTGGGATTAC<br>AGGCATGAGCCACCAGGCCCGGCCTTAAAATTCTTAATAATGTAAC<br>AAAGGGTCTCACGTTTGCATTTTGCAGTGGACTCTGCAAGATTTGT<br>AGCTTTGGACCACGTTTCTCTTTGCATTCAGATACCTTCTTTTTTGC<br>CTTATTTGCTCATGCAGACCCGGAACAAATACGGAATTGCGGTGGG<br>TAAATGTGGTGCAGAAAGTGAACAACTGGGTTTGTCCTGTCACTTT<br>AGGCTTTTCCCTGCTGTCCCAGCTTCATGTCACTTACTTGCTATTAG<br>ATTTGGGAGTTCATTAGCTTCATTTTCCTGATGTATAAATAGGAATAA<br>TAGTAACAGCCTCTTTGGCTTTTGTAGGAAGTAAATGACATGAAGC<br>GTATAAACAAATACTGCATGACAATAAATATTTGTCCTTATTTGTTGA<br>GGACATCCAAAGGACATTCAGGGGCAAAAGTAATCCAAGAGTCAA<br>GACTGAATGCCTAGTGCGGGAAAAGACACACAAGACAACATTTAG<br>GGGAGCTGGTACAGAAATGACTTCCCAGGAAGGAAGTCTGTACCC<br>CGCTGGCTGAGCCATCCTTCCCGGGCCTAGGCACCCTTGTCAGCGC<br>AATGAGCAAGGGAGAGAAGGCAGGCTGCAGTGCAGCCCTCAGAA<br>GGGCCAGAGCACTCCCTGGCTTCAGTCCTTCGCTCCAAGCCCTGTG<br>TGGAGTGGGCTGTGGCTTGGTAACTAAATGCTACTTCAGGTCAAGA<br>GCAGGGGATATATCTGGGCAGTTCTAGAGCATTCTAAACTATCTGGA<br>CACTAACTGGACAGTGGACGGTTTGTGTTTAATCCAGGAGAAAGT<br>GGCATGGCAGAAGGTTCATTTCTATAATTCAGGACAGACACAATGA<br>AGAACAAGGGCAGCGTTTGAGGTCAGAAGTCCTCATTTACGGGGG<br>TCGAATACGAATGATCTCTCCTAATTTTTCCTTCTTCCCCAACTCAG<br>ATGGATGTTACATCCCTGCTTAACAACAAAAAAAGACCCCCCGCCC<br>CGCAAAATCCACACTGACCACCCCCTTTAACAAAACAAAACCAAA<br>AACAAACAAAAATATAAGAAAGAAACAAAACCCAAGCCCAGAAC<br>CCTGCTTTCAAGAAGAAGTAAATGGGTTGGCCGCTTCTTTGCCAGG<br>TCCTGCGCCTTGCTCCTTTGGTTCGTTCTAAAGATAGAAATTCCAGG<br>TTGCTCGTGCCTGCTTTTGACGTTGGGGGTTAAAAAATGAGGTTTT<br>GCTGTCTCAACAAGCAAAGAAAATCCTATTTCCTTTAAGCTTCACT<br>CGTTCTCATTCTCTTCCAGAAACGCCTGCCCCACCTCTCCAAACCG<br>AGAGAAAAAACGAAATGCGGATAAAAACGCACCCTAGCAGCAGTC<br>CTTTATACGACACCCCCGGGAGGCCTGCGGGGTCGGATGATTCAAG<br>CTCACGGGGACGAGCAGGAGCGCTCTCGACTTTTCTAGAGCCTCA<br>GCGTCCTAGGACTCACCTTTCCCTGATCCTGCACCGTCCCTCTCCTG<br>GCCCCAGACTCTCCCTCCCACTGTTCACGAAGCCCAGGTGGGCCG<br>TCGGCCGGGGAGCGGAGGGGGCGCGTGGGGTGCAGGCGGCGCCA

	AGGGCGCGTGCACCTGTGGGCGCGGGGCGCGAGGGCCCCTCCCGG<br>CGCGAGCGGGCGCAGTTCCCCGGCGGCGCCGCTAGGGGTCTCTCT<br>CGGGTGCCGAGCGGGGTGGGCCGGATCAGCTGACTCGCCTGGCTC<br>TGAGCCCCGCCGCCGCGCTCGGGCTCCGTCAGTTTCCTCGGCAGC<br>GGTAGGCGAG
AR	TAGCCTGGAAGAGGGCAGCCAGGGGAGAAGTTAGGGCTGGAGCTA<br>TGAGAAAGGATAAGATGAGATGATGGCTTCAACATTGAGGACAGA<br>AAGAATATTGAGATGAGAAAGTAGTCCATTATAAGCATCTATGCAAA<br>GGAAATAGCAGATGTCCACAAATCAGCAGAGGCAACAACTCTGAA<br>AGTTTATTCATAAGCCCCTCTTTTCATCTCCAATCCAGTTCAAATGTA<br>ATTATTTAAATTGTTCTTCACTCTCCTTCCTGGATCATGAATGAGCTC<br>CTTAAATGCAGGGTCCACAGTGTCCTATTCATCAGTGAATTCCAAGT<br>GCCTAGCACAGAGCCTGGCAAATAGTAAATGCTTAACAAATATTCG<br>TTCAGTGCATGAATTGGAGTGATTCTCTACTTTGCTCATAAGTTGAA<br>AAAAGGTTTATTACATACCTAAATATGCTGAAATCACAGGGCATTTG<br>GCAACCCCCCAAAACCAAAACTCCCAGTTTGGAAACAGAATTTTA<br>ATTCTGTGAAAATAAAATCCATTCATTTATTCAAAAAATATTTATTAA<br>ACAATGACCATGTCCCACACCAGGCTGAGTCCTAAGGATTCAATGA<br>TGAACAAAAACCAACATGATTCCTGCTCTTAGGAAACATACAGTTC<br>AGTGAGGAAAACAGATTGTGAGAAGTCCTCCAACAAATACTGGGT<br>GCTATTAAAATATATTAAAAGGTGAGTGGGTGAGGGACTTGAGCTA<br>GCCTAGGTGGTTCAGGAAGTCTTCCTGGATGTGCTGATATGCATAG<br>GCATTAACTAGATAAATAGAGAGAAGGATGAACCAACATTGCAGGT<br>AGAGGGAACAGAATATGCAAAGGCAGGAAGGATTATGGAGTCGTT<br>GGAGGACCTGAATAAAGGCCAGTGTAAGTGGATCTCAGAAAACAG<br>GAGGAAAGGTGTATGAGATGAGATCAGAGAGGCAGATCATGTGGG<br>GTATGGTTAATGTTTTGGACTTTTCTATTAAGAGCAATGGGGAGACA<br>GTGACAGGACTTAAACGGGGAAATAATATGACCAGATTAAACTTTC<br>TAAAAAACCCTCTATGCAAATATATATTGAGAGTTAATTATTGACAA<br>AGATTCAAAGGCAACAAAGTGGAGAGAGAATAGTATTTTCAAAAA<br>ATGGTGCCAAAACAATAGGACATCTATATTAAAAGTTGGGTATCTGT<br>CTACAAAACTTAATTCAAAATGGATCACAGACCTAAATGTAAAACT<br>GAAAGCTATACAACTTCTGGAAGAAAACACAGATGGGAATCTGTG<br>TGATCTTGAGTTTGAAAATGATTTATTATATCTGACACCATAATCCGT<br>AAGTTAACATAATTCATAAGTGAACAAAGTGATGAACTGGACTTCA<br>TCAGAATTTAAAATGTTTGTGCTTCAAAAGACACTGGTATGATAATG<br>AAGACAAACTACAGATAAGATATTGTTGAATCATATTTCTGATAAAG<br>GAATTGTGCTCAGAATACATAACTCTAAAACCCCCATAATAAATTAC<br>AAGTAGCCCAATTAAAAAAAAAAAAAAGAGAAAAAATTTACAGTCT<br>TCATCAAAGAAAGTATACAATTGTAAAATAAGCACATGAAAAATGC<br>TCTGCATCTTTATTCATGGGAGAAATAAAAATTAAATGGGAAAGAC<br>ACCTCTAATTAGAATACTAAAATTAAAAAGACTGACCATACCAAGTA<br>TTGGTGAAGTGGAAATGTAAAATGATACAATCACTTAGGAAGATGA<br>TTTGGAAGTTTCTTACAAAAGTAGGTGTATACCTACCCTGTGACTCA<br>CCCATTCCATGGCTAAGTATTTACCTGAGAGAAATGAAAGAATACAT<br>CCATACAAAGATGTTTATACAAATATTTATAGCAGTTTTATTTGTAGT

	AGCCCCAAACTGAAAAGAACCCAAATGTCCATCAAAAGTGAATGG<br>ATAAACAAAGCGTGGTACAGCAATGCAATAGAATACTACTTAGCAA<br>TAAAGAAGAATGAGCTAGTGATATACATAACAGCTTAAATGTACATC<br>AAAGGCATTGTGCTCAGTGAAAGATGCAAGTAAAAAAAAAAAAAG<br>AGTACATGCTGTATAGTTCCATTGACATAAAACTCTGGAAAGTGAA<br>AAACAGTCTATACTGACAGAAAGCAGATCATTGGTTGCCTGAGGAG<br>GAGGAGTATAGGAGAGGTGGAGGGAAAATGTACAAAGTGGCACAA<br>TAAAAACTTTTGGAATCATAGATATATTCACTATCTTGATTGAGTGAT<br>GATTTCATGAGTGCACGCGTGTGTCAAAAATGATCAATTTATGCAA<br>CTTTAAATATGTGCAGTTTATTGTATATATCAATTATACCTCAGTACGG<br>CTATTAAAAAGAAACCCTCTGGCTGCACAATGCAGAACTGATTCTA<br>GGAAAGAGTGGAGGGAGGATGACCATTTACAGTGCTCCAGGTGGA<br>AGAGAACGGTGCCTTCTGGAAGTGAACTAGGTTGGCAACAACAGA<br>GATGAAATAAATGGGCAGATGTGTGAGATACTTAGGAAATAAAACC<br>CGATGGTCACCATTTTCCAAAGGTCAGCTCATCCTGGCTTTCCAGA<br>GCAAAGAGCTAGGGAAGACTTTATTAATAAATCCCTCTTGAAGTTG<br>CAGAGGAAGCTTATAGCAGAAACTTACTCTCAACCTGACTAATCTG<br>AGAGAACACCTCTGGTTCCATTTGATTACTAAAAAACTGCAAAGAA<br>CAGGAGGAGAAAGAAGAAGAAAGCTGGTACAAACAGTGAACTTA<br>TATAATATTAATCAATAATTGTCTCTTGTTCTTAAAAGCAATGGGAAG<br>AAAATGAGATTTGAGCTGGAAGATCAGAGTTCAAAATCCAAATAA<br>AGTATATGGCCCTAATATGCTTATAGTAGTTAACCTTTCCTGATAATG<br>ATATAATTGTTGACAGCACCATCTTTAAAAATAAAAATAACATAGTA<br>ATCCTTCAGATTTGTAGAATGCTTTCCTGTTTACAAGTTTGTTCTATA<br>CACATTATGTCTTTTAAATGACACACTAGCCTTCTGAGGGTAACTTA<br>TATTGGCAACAGTTTTCAGATGTGGAAACTGTGAAGACAATGTTGG<br>TGATGTGGAAGCAACATAAACTTTGGAGTCTTTCAGACCCAGGTTT<br>GAATGTCAGACTGCTTTTTATTCAGAGTAACTTCAGAGCATTATTTC<br>TCACCTTAATTTTTTTTCAGGCCTCTTTGTGTCTATGTGTCCTCTTCA<br>CTCCTGTCCATTGTTCATTCAGTGATTTTTGCACCTTCCTTCACTGTT<br>AGTGTGTAGACACATAGTTCTCCTGGCTCTGAGACCTATGTTAATTC<br>CATTCTACCATCCTGCCAGCCCACTCAATTCCTATTGAGCAATGCTA<br>GTTGAAAGTTGTGGTGGGATTAAATGTTGCAATGAGTATTCAAATG<br>AGGTTGAAGTATCTACGCATTCTACTTACATATGGTGAGGTATATTCA<br>AGGAAGGCTGTAGCCATTAAAATCTCAGGAAATAATTTTTCACCTC<br>CTCAGGTGAAAGGGTCTTCAGGCCTTTGTGTTCTGGAAGGTTCATT<br>TATAGCCATTTCCCAAATGACAATGCGATTGATGAGTCTAGAGTCTA<br>GCTCAAATAGCAATGGACTGGAAGACTAGTTTAGGTTTTACTAATG<br>TGGAACATAGAACAAATTATGTCCTTGTTTCAGCCTGTTCATCTGTG<br>AAATAGAGCCTATCATATCCAGTCTTCCTTGCCTTTAGGTTTGAGTT<br>ACCTTCTTTGGTCAAGGTAAGTAAATGCCTATGATGTTTGGCTGTGC<br>ACAAGATAAAGCTACAACAAAGCTACAACCCATCTTTTCTCTGTAG<br>AAGACTGCAAAAAGCAAAAGAGACCCAGGCAAAAATCTCGGAAT<br>GACTTTTGGAACAGAGAGCCTCCCCAGAATCAGAAGTCAAAGGAA<br>TTTAAAACATAGGGAGGCCCAGGGTCTCTACTGACATAAAGGAAA<br>GATGTTTTCCTTATAGGTTTACGTTTACATTTTCTCTCTCTTTCCATTC

	CCACTTGCATCTCCACCTTTACACAGGGCTTATGGGACCTCCTCCACAAAAGAGCAGTTGCAGTAACCCACATCATCCTCTACGCCTGGCTGTCCATCAAGAGGCGAAAAGCAGCCCTATATAGGTTCTATCCTTGGATAGTTCCAGTTGTAAAGTTTAAAATATGCGAAGGCAACTTGGAAAAGCAAGCGGCTGCATACAAAGCAAACGTTTACAGAGCTCTGGACAAAATTGAGCGCCTATGTGTACATGGCAAGTGTTTTTAGTGTTTGTGTGTTTACCTGCTTGTCTGGGTGATTTTGCCTTTGAGAGTCTGGATGAGAAATGCATGGTTAAAGGCAATTCCAGACAGGAAGAAAGGCAGAGAAGAGGGTAGAAATGACCTCTGATTCTTGGGGCTGAGGGTTCCTAGAGCAAATGGCACAATGCCACGAGGCCCGATCTATCCCTATGACGGAATCTAAGGTTTCAGCAAGTATCTGCTGGCTTGGTCATGGCTTGCTCCTCAGTTTGTAGGAGACTCTCCCACTCTCCCATCTGCGCGCTCTTATCAGTCCTGAAAAGAACCCCTGGCAGCCAGGAGCAGGTATTCCTATCGTCCTTTTCCTCCCTCCCTCGCCTCCACCCTGTTGGTTTTTTAGATTGGGCTTTGGAACCAAATTTGGTGAGTGCTGGCCTCCAGGAAATCTGGAGCCCTGGCGCCTAAACCTTGGTTTAGGAAAGCAGGAGCTATTCAGGAAGCAGGGGTCCTCCAGGGCTAGAGCTAGCCTCTCCTGCCCTCGCCCACGCTGCGCCAGCACTTGTTTCTCCAAAGCCACTAGGCAGGCGTTAGCGCGCGGTGAGGGGAGGGGAGAAAAGGAAAGGGGAGGGGAGGGAAAAGGAGGTGGGAAGGCAAGGAGGCCGGCCCGGTGGGGGCGGGACCCGACTCGCAAACTGTTGCATTTGCTCTCCACCTCCCAGCGCCCCCTCCGAGATCCCGGGGAGCCAGCTTGCT
AVP	ACACAAAAAATGAATTGTGAATTGCAGTAGGTGCTATGAAATTCATCAACATAGTCCCAGACAGAAGATAACAGAGATGGGGGATGCTCTAGATAGGGCAGTCAGGGGAGCCTCCTCTTAAGAGATGCCATTTAAACCAATCAAATGAATCCTGGGGAGGGTGCGGGTAGAGGAATCTTCCAGGACACGGAAACAGCAGGATCTCAGATCCCAAAGCAGCAGTTGGAGTTGAGACTGATTTTAGAGGTAACATAAGCAACAGTGAGGGAAAGAGAAGACCCAGGGTGAGTCTCAGGTTCTGGGAGGAGCAGCTGGTTGGATGGTGGTAAGATCACACAGGTGGGAACCCTGGAAGAGCAGGGAAACAGCTGAGAATCTGGAGTTGCGTGTTGACTTTGTTAAGTTGGAGAAGCCTATTGGATATCCAATGTGAGGTGTCACTGGGCAGTTGAAACTATAAGTCTGGCTCTTTGGGGAGAGGCCCAGGATGGAAATACCAGTTTGGGAATTATTAGCTTGTAGTAAGTATATAAAATTGTGGGATCACCCAGGAAGAGAAGGGACACAGGACTGATTCCTGGGATGCTCCAACGTTTAGAAGTCAAGGTCAAAGAGAAGGAGCCAGAAACGGAGACAGAGAGAACACCCGGTGTAACAGAAGGAAAACCAAGAGAGTGTAGCATCGGTGGAACCAAACAAAGTGTGTTTCAGAAAAGGATAAGTGTGGCCGGGCGCGGTGGCTCACGCCTATAATCCCAGCCCTTTGGGAGTCCGAGGCAGGCAGATCATGAGGTCAGGAGATCGAGACCATCCTGGCTAACACGGTGAAACCCCGTCTCTACTAAAAATACAAAAAATTAGCCGGGCGCGGTGGCGGGCACCTGTAGTCCCAGCTACTCGGGAGGCTGAGGCAGGAGAATGGCATGAACCCAGGAGGCGGAGCTTGCAGTGAGCGGAGATCACACCACTGCACTCCAGCCTGGGCGACAGAGCAAGACTCCGTCTCAGAAAAAAAAAAAAAAAAGAAAAGGATAAGTGTTCACCCATGTCAAATGCTGCTAGATCATGTGAAATAAGGACATAGACA

	TCACCACCACATTTTCGTTTTTTTTTTTTTTTTTTTTTTTTTTGAGAC<br>GGAGTCTTGCTCTGTTGCCCAGGCTGGAGTGCAGTGGCACGATCTT<br>GGCTCACTGCAACCTCCGCCTCCTGGGTTCAGGCGATTTTCCTGCC<br>TCAGCCTCCCGAGTAGCTGGGATTACAGGCAGGTGCCACCACGCC<br>CGGCTAATTTTTCTATTTTTAGTAGAGACGAGGTTTTACTATGCTGG<br>CCAGGCTGGTCTCGAACACCTGACCTCGTGATCCACCGGCCTCGGC<br>CTCCCAAAGTGCTGGGATTACAGGTGTAAGCCATTGCGCCCAGCCG<br>ACCACATTTTTCAATATGGAAGTAGTTTAACACCTTGACAACAGGA<br>GTTTCAATGGGCTGGTTTGAGCAGAAGCCCAGGTGGAGGGAGTTG<br>AAGAATAAATGTTGACTTCTGGATAAATGGCAGGTGGAGTGAGACC<br>CCATCTCTAAAAACAAAAACAAAAACAAAAACAAAAACAAAGGG<br>CAGATGGACCACAAATGAAAAACCAAGATGACCACTAACTCCAAG<br>GAATACAAAAAGTTGTTCACAGATAGAAAGATCATCATAATATACTA<br>CGTGGCTTAGCTCAGAGGGCAGAATTGCAATCTAACTTGTAATACT<br>GATCTATCATAACGTCATTGTATTTAGAAAATGGTGGATGAAGGGTA<br>TACATCAGGGGAGGGGGAAGGAACTGGTGAAGGAGCTGACTCCTT<br>TCTTTTGGAAGGATGAATGAATCAGGATCATGAGCTGAGATGTAGG<br>AGATAAAAGGACCGAGAAGATGTAAAAAGTAGATTGGGAAAGGAA<br>ACCTGTAAGGGACGTGTAGAAGGGTGTGCAGTGTCTGGGGGGTCC<br>ATGTGACATATGGGGTGATGTGTTGGAAATGCAGCCAGTCAGCACA<br>GGATAGAGACTTTTCCCAGTCAGCCAAGGTTCCGAGAAGGTAGGT<br>CCTGGGAGAAAGGGGCCTCGGTGTGGGAGGCCGGTCTTAGAATGA<br>AGATCCTGGTAGACAGATCTGGTCAGCACAGAGGTCCTGGCTGTCC<br>CATGGCTTGGGCCCTTCAGTCAGTCTGGCCTCATCCGTTTTCAGTTG<br>TTGACTCAACCCCCTGCAGTGAAGAAGGGGTAAGGTGCTGGGCCA<br>AGGCGCCTTAGCCAGGCCTCCAGCCTCTCTCCTCAGCTAGCCTCAC<br>AACCCACAGAGTCTCAGCGGGGCAACGGCCTCAGACCAAGCTTGT<br>TTCCAGGCCAGGAGTAGAATGAAAATGTTCCTCCTAGACTAATCCA<br>GGAAGCTGCTCTCTCTGGAAAAATCTCCTAGCTCTTAACAAGAATA<br>ATGGCATTGGCCAGGCATGGTGGTTCATGCCTGTAATCCCAGCACT<br>TTGGGAGGCTGAGTGGGGTGGATCACCTGAGGCCAGGAGTTTGAG<br>ACCAGCCTGGCCAACATGGTGAAACCCTGTCTCTACTAAAAATACA<br>AAAATTAGCTGGACGTGGAGATGGGCGCCTGTAATCCCAGCTTCTC<br>AGGAGGCTGAGGCAGAAGAATCGCTTGAACCTGGGAGGCAGAGG<br>TTGCAGTGAGCCAAGATCGCGTCACTGCACTCCAGCCTGGGCAAC<br>AAGAGCAAAACTCCATCTCAAACAAAACAAAACAAAGAATAATGG<br>CATTACTAATGCAATACTACTGTTCATTAACTTTACAATGCTGCAGCC<br>CCCTTTTTCTTTTTTTTGAGACGGAGTCTCGCTCTGTCGCCCAGGCT<br>GGAGTGCAGTGGCGCGATCTTGGCTCACTGCAAGCTCTGCCTCCCA<br>GGTCCATGCCATTCTCCTGCCTCAGCCTCCTGAGTAGCTGGGACTA<br>CAGGCGCCCGCCATCACACCCGGCTAATTTTTTTTTTTTTTTGGATT<br>TTTAGTAGAGACGGGGTTTCACCATGTTAGCCAGGATGGTCTAGAT<br>CTCCTGACCTCATGATCCGCCTGCCTCGGCCTCCCAAAGTGCTGGG<br>ATTACAGGCATGAGCCACCGCACCTGGCCACATGCAGGCACTTTTC<br>TAAAGCTACTTTTTCTTTTGAAACAGTGTCTCACTCTCACCCAAGCT<br>GGAGTGCAGTGGCTCAGGTATGGCTCACTGCAGCCTGGGCTCAAG

	CAATTCTTCCACCTCAGCCTCCCGAGTAGCTGGGACCACAGGCATG<br>CACCACCACACCTGCCCAATTTTTAAATTATCTGTAGTGATAAGGTC<br>TATGTTGTCCAGGCTGGTCTCGAACTCCTGGGCTCAAGTGATCCTT<br>CTGCCTTTGCCTCTCAAAGTGCTGGGATTACAGGTGTGAGCCACCG<br>TGCCCAGCCTTAAAGCTGTTGTTTTTTTTTTTCAATTTAAAATGTATT<br>TATAGGCCGGGCGCGGCGGCTCACACTTGTAATCCCAGCACTTTGG<br>GAGGCCAAGGCAGGAGGATCACAAGGTCAGGTTCAAGACCAGCC<br>TGGCCAACATGGCAAAACCCCATCTTTAGTAAAAATACAAAAAAAT<br>TAGCCAGGCGTGGTGGTAGGCGCCTGTAATCCCAGCTACTTGGGAG<br>GCCGAGGCAAGAGAATCACTTGAACCCCGGAGTTGGAAGCTGCAG<br>TGAGCTGAGATCACGCCACTGCACTCCAGTCTGGGTGACAGAACG<br>GGACTCTGTCTCAAAAAAAAAGTATTTATTTATTTATTTAAAATTAG<br>AGATGGGGCCGAGCACGGTGGCTCATGCCTGTAATCCCAGCACTTT<br>GGGAGGCCGAGGCGGGTGGATCACTTGAAGCCAGGAGTTCAAGA<br>CCAGACTGGCCAATATGGTGAAACCCCATTTCTACTAAAAATACAA<br>AAATTAACCTGGCGTGGTGACGCGTGCCTGTAATCCCAGCTACTTG<br>GGAGGCTAAGGCAGGAGGATCGCTTGAACCCGGGAGGTGGAGGTT<br>GTACTGAGCCGAGATTGTGCCACTGCACTCCAGCCTGGGTTGACA<br>GAGTGAGACTCTGTCTCAAAAAAAAAAAAAAAAAAAATAGAGATGG<br>GGTCTTGCTATGTTGCCGAGACTGGTCTCAACCTCCTGTCTGGGTC<br>TCCCAAGTAGCTGGGATTACAGGCATAAGCTGCCATGCCTGGCTCT<br>AAAGCTTTTGTATAATAAATTATTTATTTCTGTCCTCACATTGACCCT<br>AGGAGGTGGGTACCATGATTTTCCTTACTTCACACTGGCAGAAAGT<br>AAGGCACAGAGAAGTCCAGTGAGCTGCCCAAGCCATGAAGTGTCG<br>AGCAACAGAGGCCAGACCCTGAGCCAAGCTATTGGGAGCCAGTGT<br>TCCTGCAATAACAACTGCCCAGTGTTGCCTCTCAAAGGGTCCTTGC<br>AGTCCCTGAGGGGTCATTCCTGAAAAGACCGACGCCATGGCCTGA<br>GTATAGGAGGCCCCTCCTGGGTTGCTGGCCCCCTCACTGCCCTTGC<br>TGGAGATGGGGCTGCTGTGTCCCCTGGAATTTCCTCGCAGCACCTC<br>TATGCACCCTGGAGAGGAGGCCAAGAGCCTGTGGGAACTGCGAGA<br>GCTGGGGACTGCGCACAGCTTGAGAGGCAGGGGGTACCGGGCCTG<br>AACCCCAGACCAGCGGGGCATTCTGGTGGCCCAGGGAGAGAGGCC<br>ATGTCCTCTTTAGACCTGCCACCTTGGGATTAGGGACCAAAAGTGG<br>CTTTCTCAGGCTGGGTCCATTTAGGTATGGCTCCTCCTGCACCTTCC<br>CCCAGGCCCAGACACCCCCCACCCCCGGCTCTCCCACCCCAGGCC<br>TGAGATGTACCTGCCCTGGTTGCCACATACTAAGGTCCTGGTGGGG<br>GTAGGAATTGGGACAAACTGTTGTCAGGTTTCCTGGGGCTCCCCCC<br>GCCTTTGAACCTTACTTGGCATGGAGTTCTTTCTCCCCATAGTAGCT<br>CTCCACACTCATGTTTCTACCTGGGAGGGGGTGACGGGTCATGTGC<br>CAATGTGTCCCCAAGGCCCCCAGTTTCAGGGCCTGAGTCCCCATG
B4GALT5	CAGGCTGGAGTCCAGTGGCACGATCTCAGCTCACTACAACCTCCG<br>CCTCCTGGGTTCAAGTGATTCTCCTGTCTCAGTCTCCCGAGTAGCT<br>GGGATTACAGGTGCCCACCACCACACCTGGCTAATTTTTGTATTTTT<br>AGTAGAGACGGGGTTTCACCATGTTGGCCAGGCTGGTCTCAAACT<br>CTTGACCTCAGGTGACCTGCCCATCTCAGCCTTCCAAAGTGCTGGG<br>ATTACAGGCATGAGCCACCATGCCCTGCCGGATTGTTTTCTTTATGT

	GTGTGATTTCAGCTAGCCTGCAATTGGGTGTTGACAAGCACAAGAG<br>GCCCAACCTGTGCTCTCCTGAGATTTCACCAAGGACCTCCCTTTCT<br>TTAGGCCACTCCCCTGCGCTAAAACCTTCAAGGGCTCTCTATGGCT<br>CAGAGGAAAAAGTTAAGAATAGTGATGGCTCCCATGGATTGAGCA<br>CTTCCTACATATCAGGCCCTTTATAGAGATACTATTTGTAATCTGTGC<br>ACAATCCTAGAAAGAAGGAACTGTCTGATCTCCATTTTACAGATGC<br>GGAAATGGAGGTTCCGAGAGGTGAAGTGACTTGCCAAGATGACAG<br>AGTGAGGGCAGGGAGGAAGGAAGACTGGCAGCCAGATCCATCTAA<br>TTCCAGGGCCCATGCTGAGCCACAGGGTCAGGCCTCACTCCTCCA<br>GCCTCACCCCTCACCTCTTTCTTTTCCTTGGCAGGACTAGCTGCTCC<br>AACCAGGCAGGCCTCTTCTCACTGCCCCTTGAGCAAACCTGTCTCA<br>ATGTCCCTTCAAAGACCATCTAGAATCTCACAGAACACCTTTTCCTA<br>CTGCCAGCCATGCTGAGTCCTGCCCTTATGACAGTGTCCCTCAGTT<br>AGTCACTTTCCTGTAGCTCATATGTCACTAGGCTGCAAGAATGTCTT<br>AGTCATCTTTGCATCTGCCATGCCCAGCACACAGTCACTGCACACC<br>TGTCGTCCTTCAACAATTATTCAGCGCCTACTGGCTGCTGAGCACT<br>GTGCTAGGTGCAAGGATACAGCAGAGAGTGAAACCAAATCCCTGC<br>TCTCGTGAAGTTTACATTCTGATCTGGGAGAAGAAAATGAACAAGT<br>AAACGCGTATATGTATAGTAGAATGTCAGGGGATAAAAAGTGCTATG<br>GAGAAAAATCAAGCTGAGGAAGGCTATGGATAGAGAGTGCTGGGA<br>TGGGGAGAAGAAAGCTACTTTAGAGGAAAGGTCTTTTTGAGACGG<br>TAACATTTGAGCAGAGATTAGAATACAGTGAGTTTGTCTGAGGAGC<br>TGAATAGTCAAATGAATATATAATGAATACATGCTTTAGTGAGTTGG<br>AAACCTAACTTGGAGATGAAAAAGTAGTGTGGAGGAAGGAGTTTG<br>AGGTAGGGATCCCTGGATACTCCTGACATACTTCTTTTTCTTCCATTT<br>AAAAACATAAACTGCCGGAGGCGGTGGCTCATGCCTGTAATCCCAG<br>CACTTTGGGAGGTCGAGGCAGGCAGATTACCTGAGGTCAGGAGTT<br>CGAGACCAGTCTGACCAACATGGTGAAACCCTGTCTCTACTAAAAA<br>TGCAAAATTAGCCGGGCATGGTGGCGTATGCCTGTAATCCCAGCTA<br>CTTGGGAGGCTGAGGCAGGAGAATCTCTTGAACCTGGAAGGCGAA<br>GGTTGCGGTGAGCAGACAATGTGCCATTGCACTGCAGCCTGGGCA<br>ACAAGAGCGAAACTCCACTGAAACAAACAAACAGACAAAAACCA<br>TAACCAGTTTATTTATTTATTTTTGAGACGGAGTCTTGCTCTGTCGCC<br>CAGGCTGGAGTGCAGTGGTATGATCTCAGCTCATTGCAACCTCCAC<br>CTCCAGAGTTCAAGCGATTCTTGTGCCTCAGCCTCCCAAATAGCTG<br>GGATTACAGGCTGCCTGACACCGTGCCTGGCTAATTTTTGTATTTTG<br>CATTTTTTTTTTGAGGTGGAGTCTCACTCTGTTGTCCAGGCTGGAGT<br>GCAATGGCACGATTTTGGCTCACTGCAACCTCTGCCTCTGCCTCCC<br>AGGTTCAAGCAATCCTCCTGCCTCAGCCTCCCGAGTAGCTGGGATT<br>ACAGGCACGTGCCACCACGCCCAGCTAATTTTTGTATGTTTTAGTAG<br>AGATGGGGTTTCATGTTGTTGGCCAGGCTGGTCTCAAACTCTTCAC<br>CTCAAGTGATCTGCCTGCCTCGGCCTCCCAAAGTGGTGGGGTTACA<br>GGCGTGAGCCACTGTACCCGGCCTAAAACATAACCAATTTATTTTGA<br>TGCCTTGTTATTTCAGATAGGTCAGAAATAGAGACTGATCATTGGTA<br>CATAGTTTAAATGTAGGAAGGTTTCTCTTTCTCTGTCTATCCAGAAC<br>AGACTGGATGTTACAGAATGTTATGGTATAATTGTTAATTCTCTTAGT

	ATTACTGTGGTTTTGAAGGATAATGTAATTTTCCTTAGGATTTGCTAC TGAAGTATTAAGGCATGAAGTACTAGTAAGGCAACAACTTACTTTT TTTTATTGTTTTTTTGAGTCAGGGTCTCACTCTGTCTCCCAGGCTGG AGTGCAGTGGCGCTATCTTGGCTCACTGCAACCTCCACCTCCTGGG CTTAAGAGATCCTCCCACCTCAGCCTCCCAAGTAGCTGGGACTATA GGCACACGTCATCACGCCCAGCTAATTCTTTGTATTTTTTGTGGAA ATGGAGTTTTGCCATGTTGGCCAGACTGATCTCAAACTCCTGAGCT CAAGCGATCCATCTGCCTTTGCCTCCCAAAGTGCTGGGATTACAGG TGTAATCGCCCAGCCTACCTTTAAATATTTAGCAAAAAATAAAAATT TTCAACATATGAATATAGATACATACATGTGGAAAAATGATAACAAT GATCAAATCCAGGTGGGGAGTGTACAGATGTTTCTTGTACTATTCTT TCAACTTTTCTGTATGTTTGAAAATGTTTCATAGATACGAGGGTTGG GAGGGGCCATTGTGTGGGCCAACAAAAACTATTTGCAAGTTAGATG ATTCCGGCTAGTCTGGGATTTCTGGTCCAGGGCCTTACCTGCACTAT TTCATCGAATCCACATCACATCCCCGTGGGGTGGGCAGCACTGGGT CCGATTCACAGGTGATGAATAGAGAAGCTCAGAGAGGTAAGTGAC TTTCCCAGAGTCACACAGCCAGGAGATGCCAAGCCGAGATTTGAA CTCACATCTATCTGAGTCAAAATTCTCTGCTTTCCTTTTTGATCGTTT AAGCCTCTTCTCTTCCACCATACCATTCTCCAGGGGTATTGAATTAG GTGACAGCTAAGCACTCTTACTATGTGCACTTTGCATGTATTACTTA AATCCTCGCAACAACCTGATGAGGTTGCTAATAACCTCCTCCATTTT ACCGAGGCACAGAGAGTTTAACTTGCGTGAGGTCACACAGCTAAC AATGGAAAAGCTGGGAGTCAGAGAACCTGGGCAGTGTGGCTCTAG ACTCCCCACTTAAAGGATCAAAGAAACACTGTAAAAAGTAGAAAT CACTTTAGGAATATAAATCAGAATTATTGTTGATTCTCACAGAATCC CTGCAAAGTCCTCTCCCCTCCCCACTGCTTCTGGCATTCCAAGACA GTACCCTAGTTTCAGATATGTCTACACAGGGACACTGGCCTCCTCA GTCCAGCTCCTTGAGGCTACTGCTTCTTGCCATAAATTTCTAGGACT GTTCATTGCCTTCTGAGGTCCTTTGAGCTGATGCTGATCTGAAAGCA TTTTTTTGGATATGGTCCCATACCCAGGTGATGGGGATAGGATACCG ATTTGCCTGCAGGATAAGAATTAGAGGTCATACAGACTGCTGAGAC AGTTACAACCTAGAGGTCAAGGGCCTGACATGGGAGACAGGCCAG TTTTGATTTAAGCCCTTACTGCAAGGCTTGGGTGAATTGCCTTTCCA TCTAGAAACCTCAGTTTCCTCATTTGTATGATAGTAATAATAACAGC ACACATCTCAAGACTGGTGGGGGAATTTCATGGCATGAGTGAAGTC CATGTTTCTCAGCAGAAGTCTAGAGACATTGACAATTGTTATCATTA TGCTATTTCTGATACAGTAGGATCCCTGACTACAGCTCCAAGCTCAT CCCTATTTCCCCCCCTACTTCACTGGATTTTATCCATGTTGGCCTTGG ATTCAACTCGGGGGCAGATCAGCCCTTTTCCTACCTCAGGGCCTTT ACACCTGCTGTTCCCCCTGGTATGACTGCTTTTTCCCCAGCTTATTC AAAAAAGCTGGCGCTTTCTCACCCTTTTATATCTCAGTTCGTGTCAC TGCTCAGTGAGGCCTTCCTTGACCACTCCAGCCTGCCTGCAATACA TCCTCCTGTTTTATCGTATTCATATGTGTTACCAGTACCTGATATTACT AGCTGATTTGTTTCTTTTATCTCTCACATAATGTAAGTTTCAAGTAGA CAGAGATTTTTATCGTTTTTTTTCTCTGTAGCATGCCCAGCGCCAAC AACTGTGTCTGGCATTCAATAAATAAATGGTGAATTGAATGAATGAA

	TGAATGAATGAATGAGGGTGTATACCAGGCCGAACCCCCAAGCCTT<br>GCAGTCCCCACTCTTCTGCTGCAGTGGGCAACGAGATGAGTCAGA<br>CTTTGTAGATGGCCCCTTGGCCCCCGAGATGGGGGACGAGCTAGCG<br>CGGAGCACTTGGGAGACATTCCCCGAAAGCCTGGGCGGTGGCCGC<br>ACTGGGAGAGGGTCTGTCGCCATTTGGCGATTACTCCATGCGGGCT<br>GCTGTGGGCGCCGAGCGAGGGTCGGCGGCGAGCGCAAGCGCGCG<br>GGTCTTTTGCGACGGTGCCCGGCGGCACTGGCCCCGCCTCCCGCG<br>CGTGCGCCCCGCCTCCGCCCCCGCCGCTGCGCCCGCTCGGGGCCG<br>CTCTGGCCCCCGCCCGCTTCATGTGGCCCGGCCCGCGACGGCCGGC<br>GGCTGGGAGCGGCGAGGCGGCGGCGGCGGCGAGTGGCGGCCCGC<br>GAGGCCCGGGAGGCGGTGGCCGAGGCCCAGGC
BAX	CTGCTTGAGTGTCCTCACTGTCACACGTGTCCGTGCAAAGAGACC<br>ACCTAACAGACTTTGTGTGAGCAACAAGGCTGTTTATTTCACCTGG<br>GTGCAGGTGGGCTGAGTCTGAAAAGGGAGTCAGCAAAGGGTGGT<br>GGGATTATCATTAGTTCTTATAGGTTTGGGATAGGTGTACAAATTACA<br>TTCTCAAGAGTGGGGAGAATATTGCAAAGTACCTTCTTAAGTGGGG<br>TGGAGGGGAGAATATATCATATCAGTTAGAGTGGGGCAGGAACAAA<br>TCACCATGGTGGAATGTCATCAGTTAAGGCTATTTTCACTTCTTTTG<br>TGGATCTTCAGTTGCTTCCGGCCATCTGGATGTGTACGTGCAGGTC<br>ACAGGGGATATGACGGCTTAGCTTGGGCTCAGAGGCCTGACACTC<br>ACCACATGGCAGCTGGCTTTCCTTAAAGGAAGTGATCTGAAAGGG<br>AACAAGGTGGAAGCTGTAGTGGCTTTTCTGACCTGTCCTGTGAAAT<br>CACACTCTGTCATTTCCACAATATCCTCTTGGTCACACTGTTTAGCC<br>CTATTGAGTGTGGGCAGAGACCACACAGCAGCATGAATACCTGGA<br>GGTGTGACTCCTCAGGTGCCATCTTGGATGTGGGCTTAATAGTTTTG<br>ATTCAGCATCTTTATTATTATAAAGAGTTTGGCTGGGTGCGGTGGCT<br>CACGCCTGTAATCCCAGCACTTTGGGAGGCCAAGGCGGGCGGATC<br>ATGAGGGCAAGAGATCGAGACCATCCTGGCCAACATGGCAAAAGC<br>CTATCTCTAATAAAAATATCAAAATTAGCAGGGCGTTGTCACACGCC<br>TATAGTCCCAGCTACTCAGGAGGCTGAGGCAGGAGAATCACTTGA<br>ACCCGGAGGCGGAGGTTGCAGTGAACTGAGATCACACCACTGCAC<br>TCCAGCCTGGCGACAGAGCAAGACTCCGTCTCAAAAAACAAAACA<br>AAGCAAAACAAAACAAAACACACAAAAAAGAGTTTTAGTCAGGC<br>ACAGTGACGCATGCCTGTAGTCCCAGCACTGTGGGAAACTGAGGC<br>AGGTGGCTCACTTGAGCCCAGGGGTTTGAGACCAGCCTGGGCAAC<br>ATGGCAAAACCCTCTCCCTACAAAACATACAAAATTAGCAGTGCAT<br>AGTGGCACACACGTGTAGTCCCAGCTACTCCAGAAGCTGAGGTGG<br>GAGGATCCCTTGAGCCCAGGAGGTCGAGGTGGCAGTGAGCCACAG<br>TTGTGTCATTGCACTCCAGCCTGGACGACAGAGGGAGATCCTGTCT<br>CAAAATAAATAAATAAAAATAAAAATAAACAGTTTTGACTTCACAA<br>CTAGCTAAAAAGTGTATTCCCTCACTCAGTGACTGTACTGTTCAGA<br>GGTGTATACCTGCATTAAAAGCCCTTTCCTTCCTTCTCTGTAACTGG<br>AGTAGGGAAGGGCTATCTCATTGGACTGGAGTAACACACACAGATA<br>AAGCCGGATGCAAAGTTAACAGGAAACACTATTTCTCTCAAGGATA<br>CGCTTTGTTTGTTTTTTTTTTGAGATGAAGTCTCGCTCTGTCACCCG<br>AGGTGGAGTGCAATGGCACGATCTCTGCTCACTGCAGCCTCTGCCT

	CCTGGGTTCAAGTGATTCTCTGGCCTCAGCCTCCCAAGTAGCTGGG<br>ATTACAGGAGCACACCAGTACGCCCAGCTAATTTTTGTATTTTTAGT<br>AGAGACAGGGTTTCACCATGTTGGCCAGGCTGGTCTCAAACTCCT<br>GACCTCAGGTGATCTGCCTGTCTTGGCCTCCCAAAGTGGTGGGATT<br>TCAGGTGTGAGCCACCACGCTGGCCAAGGACATGGTTTCTTACAG<br>AGACTTTGTTCTCTAAATTCATAAATTGTTGGAAATTCTATCAGTAA<br>AAATGAAACATCCGAGTCTTGCTGACAGGATCTAATCCACTTGATA<br>CAGAGTAGCAGCCTTGATTTCCAAAGCAGGTGCACAGCTTCAGATA<br>AAGGGTTTCTGGATGCAACATTTCACATGTACCTTCTTGTTTCCAGC<br>GATTCAGGACACTGGTTTCACTTCACAGTCCTGATCCAATGTTGAC<br>CTTGCTTTGCTCTAAGCTATCATTTGGTTGTCACCTAAGCTCTACCC<br>TCCCCCTTTATCTTGGCTTTTTCTTTTCTTTTTTCTTTTTGAGACAGG<br>CTCTTCCTCTGTCACCCAGTCTGATTGCAGTGATGCAATTGATCACA<br>GCTCACGGCAGCCGGGACCTCCCAAGCTCAAACAATCTTCCCACC<br>TCAGCCTCCCAAGTAGCTGGGACTACAGGCACGCACCACCACATC<br>CAGCTAATTTTCTTTTTTTTCTGCTTCCTTTTCTTTTTGTTTTTTTTTA<br>GATAGAGGCTTGCTCTGTTGCCCAGGCTGGGGTGCAGTGGCACGAT<br>CTTGGCTCACTGCAACCTCTGCCTCTTGGGTTCAAGCGATTCTCCT<br>GCCTCAGCCTCCCAAGCAGCTGGGACTGCAGGCACGCGCCACCAC<br>TCCCAGCTAATTTTTTTGTATTTTTAGTGGAGACGGGGTTTCGCCAT<br>GTTGGCTAGGCTGGTCACAAACTCCTGACCTCAGATGATACACCCA<br>CTTCGGCCTCCCACACAGCTGGTATTACAGGTGTGAGCTACCACGC<br>CCGGCCCCCCCTCCTTTCTTTTGTTTTTAGTTGACACAGGGTCTCA<br>CCATGGTACAGCCCAGGCTGGTCCTGAACTCCTGGCTTCAGGTGAT<br>CCTCCTGCCTTGGCCTCCCAAAGTGCTGGGACTATAGGAATGAGCC<br>ATCACACCTGGCCCCTTTCTTCAATTTTCAAAACAAACTGATCCTTC<br>AAGGTCAAGAGGAAATACCTCCTCTGAGAAGTCTTCTCTGAATGTC<br>AGAGGCAGACAATGTCTGATTTCTGCATGCTCCCCAACATTCAATC<br>ATACAGTTATTGAATAACACATTTTGAGAGATAACTATGAATCAAGT<br>AACATGCTGGTTTCTGGGAGAATTGAGGACAAATTAACCTTGTGGA<br>AATTTTGGGTGGATGAAAAAAACCAACATGAAATTAAAACACTGC<br>ACACATTTACAGCTGTGAGAAGCATTACACATCCTGGGTGCTATGC<br>GAGCTTTTTTTTTTTTTTTTTTTTTTTTTGAGTTGGAGTTTCCCTCTT<br>GTTACTGAGGCTGGAGTGCAAGGTCACGATCTCAGCTCACTGCAA<br>CCTCTGCCTCCAGATTCAAACGATTCCCCTGCCTCAGCTCCCGAGT<br>AGCTGGGACTACAGGTGCCTGCCACCACACCTGGCTAATTTAGAAT<br>TTTTAGTAGGGATAGGGTTTCACCGTGTTGGCCAGGCTGGTCTCAA<br>ACTCCTGACCTCAGGTGATCTACCCATCTCGGCCTCCCAAAGTGCT<br>GGGATTACAGAAGTGAGCCACTGAGCCCAACCAGGAGCTTTTTCG<br>AGAAAGAAGGAAGTCCAAGAGATCTTCCTGACACCCTAGTCTGAC<br>TCTGCCCTTTGCCTGCTCAAAATTTCCCCATGCTTCCCAGCGGCCTT<br>CTGGACATAGATCAAGTCCCTTCTCTGACAGGCCCAAACCCTTTAT<br>CATCTGATCCTAGCTCATTTTTCTGAGTTTTCCTTAGTTGCTATTATTT<br>TCTGTCTAAAGTGACATGTCATAATATTCATAAAGCACACAAGTCTT<br>ATGTGTACAGCTCAATGAATTGTAAATATGTGTATACCCGGCCGGGC<br>ACAGTGGCTCACGCCTGTAATCCCAGCACTTTGGGAGGCCGAGGC

	AGGTGGATCACTTGAGGTCAGGAGCTTGAGACCAGCCTGACCAAC<br>ATAGTGAAACCCCATCTTTACTAAAAATACAAAATTAGCTGGGCGT<br>GGTGTCGCATGCCTGCAATTCCAGCTACTTGGGAGGCTGAGGCAG<br>GAGAATTGCTTGAACCCGGAGGCAGAGGTTGCAGTAAGCCGAGAT<br>CGTGCCATTGCACTCCATCCTGGGCAACAAGAGCAAAACTCCGTCT<br>CAAAATAATAATAATAATAATAATAATAATAATAATAATGTGTATA<br>CCCATGTAAACACCATTCAGATAAAAATATGGCATATTTGGGGCACC<br>CGGGGAGTGTCTCTTGTGGCCCCTCCCCTCCATACCCTGCTGATCTA<br>TCAGCACAGATTAGTTTCTGCCACTTTTTAAACTTCATATTCCTTTTC<br>TTTTTACACAAACACAAACATTCGAGTCATGACTGGGTGGGGTGGC<br>TCAAGCCTGTAATCTCAGCACTTTGGGAGGCCAAGGTGCGAGGAT<br>CGCTTGAGTCTGGGAGTTCAGAGACCAGCCTGGGCAACATAGAGA<br>GACCTCATCTCCACATAAAAAGTTTTAAAAATTAACCAGGGGCGGT<br>GTAGTCCCAGCTACTCAGGAGGCTGAGGTGGGAGGCTTCAGCCCG<br>GGAATTCCAGACTGCAGTGAGCCATGATTGGGCCACTGCACTCCAG<br>CCTGGGCAACACAGTGAGACCCTGTCTCAAAAAAAAAAAAAAAA<br>AAAACAGGAAAAAACAAACAAACAGAAAAGCAGGCCTGGCGCGG<br>TAGCTCATGCCTGTAATCCCAGCGCTTTGGAAGGCTGAGACGGGGT<br>TATCTCTTGGGCTCACAAGTTAGAGACAAGCCTGGGCGTGGGCTAT<br>ATTGCTAGATCCAGGTCTCTGCAAAAAACAAAACCACTCAGTTTTT<br>AGTCATCTATAACGTCCTGCCTGGAAGCATGCTATTTTGGGCCTCTG<br>AGCTTTTGCACTTGCTAATTCCTTCTGCGCTGGGGAGAGCTCAAAC<br>CCTGCCCGAAACTTCTAAAAATGGTGCCTGGATAAATGAAGGCATT<br>AGAGCTGCGATTGGACGGACGGCTGTTGGACGGCGCCACTGCTGG<br>CACTTATCGGGAGATGCTCATTGGACAGTCACGTGACGGGACCAA<br>ACCTCCCGAGGGAGCGAGGCAGGTGCGGTCACGTGACCCGGCGG<br>CGCTGCGGGGCAGCGGCCATTTTGCGGGGCGGCCACGTGAAGGAC<br>GCACGTTCAGCGGGGCTCTCACGTGACCCGGGCGCGCTGCGGCCG<br>CCCGCGCGGACCCGG
BCL2	GAAGATTTGATACACACACATGATTTTTTTTTTCTTTTCTTTTCTTTT<br>TTTTTTTGAGACAGGGTATCAGTCTGTCACCCAGGCTGCAGTGCAG<br>TGGTGTGATCACAGCTCACTACAGCCTCCACCTCCTGGGCTCAAGT<br>GATCGTCCTATCTCAGCCTTCAGAGTAGATGGGACTACAGGCACAC<br>ACCACCATGCCCCGCTAATTTTTGTATTTTTTGTGGAGATAGAGTTT<br>TGTCATGTTGTGCAGGCTGGTCTCAAACTCTTGGCCTCAAGCAATA<br>CACCCACCGCAGCCTCCCAAAGTGCTGAGATTACAGGCATGAGCA<br>ACCACACCTGGCCGATACATACCTATATTAAACATTAGTATGTTCATG<br>TTAGAATAATGTACCTTTTGAATTTCATAAACTTGGAGAATATTTATA<br>TTGATGATGGATGAAAGAACTTTCTTGGATGGATGAAGAGAGTAAC<br>GCTGTGAAACAACCAGCAGGTGGCGAAAACTGGCAATCAAAAGCT<br>TTTTGTTTGGTGGCCTGGGGAATGAAGACGGAAAGAAAACACAGG<br>CCATTCAGACTCTTGATACAATCTCCATTCCCTGCATCTTGTTTTTTT<br>TCTCTTCCTGGTGCCACGCTACTTGTAGAATCCAACCAGGTAAAGC<br>TGCCAAAAGGGTCATCCATTGGCTCTTACAAGTAGAAAACATCTTG<br>GAAAGTGAAAAGTCCACTGTGCATATGTTTGTAAGGTTGTTGGAAG<br>GTCTCAGGCATAGATCTAGGATTCAAATCCAGTTACTCCTGCCTCAG

	GCTGGTGTTCCCTCCCCCACCTCAGCATTGCCCAAAGACGAGTAGT<br>CAATGTCACATACTTCCTGGGAATACTTGCCCTTATGCTTTAAAATG<br>GAATCTAATAAACATGGAATCTGAACGCAGGAATGGTTTCACTCTT<br>TCATTTGAAAGAGTATCTAGAACATTCCCAGGGAAAATATAACCCCT<br>AGCCAAAGACTGCAATACAGACCTGTCTCAAGACTGATTATAGCCA<br>AGATGCCACATAAGGAATCAGTCTGGGAAAATCCATAGAGTGAGGC<br>TCTGTGGGAGCAAAGGAAGACGAAAATCAGTCAGCTTTTCTTTCTC<br>TGGAAGTAGGGGATCCGTTTCCTTCTGGCTGCCCCCTTTGCAGAAG<br>TACAGTTTCTTTTGCAGGTTTGTCCTATCATTTCCTCACTCATATGCT<br>GAGTATTAGGAGCTTGAAGCCTTTCAATTCCTCTTAGGTAATTTTGG<br>GGCTTTAAAATACGCTTTCAAGATTTCTAAACCATACTGTTGTGCAA<br>TTGGTATGAATTTATGTGAGAACATTTATTCTAGGTCAATCTATACCC<br>AGTGTCTATCCAGACCAAAACACCTCCCACGCGCATAAAAGGGAC<br>TCTGTCCCAACCATCAGAAGGGCAAGAAGGAGGATCTCCTTTCATC<br>CCCTCTTGCCTGGATAAGAAATTTGTACCCAGGCCCCCATTCCTATG<br>TGAGAGAAGTTGGCTTGTTGGGCTGATGGGATACAATAAATGAAGA<br>AATAAAATAAAAACACCCAAGAGAGATGGCAGTGCGTATAGTCCCA<br>GCTATTCATGAGGCTGAGGTGGGAGAATCCTTCGAGCCCAGAAGTT<br>CGAGTCCAGCCTGGGCAACATAGCAAAAGCCATCTCTTAAAAAAA<br>AAAAAAAAAGGCCAACTAAGTAAAAATTAAAAAAATCATAATTTG<br>GTGTGCTTTTCTGGCTTTTTAAAGAATGTTTTGATTTTAGAGTAGGA<br>ATGAGACAAAATAAAGATGTCAGGCAGGGCACAGTGGCTCATGTC<br>TGTAATCCCAGCACTTTGGGAGGCTGAGGCAGGCGGATAACGAGG<br>TCAGGAGTTCGAGACCAGCCTGGCCAGTATGGCGAAACCCCGTCT<br>CTACTAAAAATACAAAAATTAGCAGAGCGTAGTGGCGTGCACGTGT<br>AATCCCAGCTACTCAGGAGGCTGAGGCAGAATTGCTTGAGCCTGG<br>GAGGCAGAGGTTGCAGTGAGCTGAGATCGCACCACTGCACACCAG<br>CCTCCAAGATACAACAGAGCAAGACTCTGTCTCAAAAAAAAAAAA<br>AAAGTCATAGCATATTTGTACACATTGTAGTACTCATTTGTCATCTTT<br>CTTGACCCCAATAATCCAGTGTCCCTATATATTTGCACTCGAGCCCTA<br>TTAAGTAAGCCGCTGTGCTTCTAGAAGACCTTTTTCTTTTCTTGGTG<br>CTTTGTCAAAGACTCTTGGAGATAAAAATACACACGTGCAACTTGT<br>TTGTCCTCTTGTCCTTTTTTGCTAGGGGCTATTCATGCTGATTAATTT<br>AAAACTGTCTGCTTGCGCGTACACACGTCTGCGAGTGTGAATGTGT<br>ATGTGTGTATCTATGTACCTCATTTGAGAAAGTGCGGCCAACTAGGA<br>TTGGCTACGAGGCAAAGGTGGAGACCTTTAGGAGCCCACCCACCC<br>CAGCGTTAGGACGGTGGGCCTGAAAGTTACTATATGGAAGTCCTCA<br>TCGTGTAGCACTAAACCAGTGTAAAAGGTGTTAGGGACAGAGGGA<br>AAACATTGACTTAAACTGTCGTAAAGCCCTTGATAAACCCCTTCCC<br>TGGAGCGCTGAGTTCTGCATGGCCTGGGCCACGGACTAGGTGTTC<br>AGGTGGACACGGGCGGGGATGCGCGTGCGTGTGTAGTGCGCGGAC<br>ACCTAGGAAGCTACTTGAAAGTAAACACCACGCTCGGGGCGTCCC<br>TAGACATTGCTTAAAACGTGCAGAGTCACCTGTCTTCACAGCAGGG<br>CAGCGCTGAGGTCCCACTGCTGGGGGCGGTGGGGGGCGGCATTGG<br>CCTGGGTCTTCCCCCGGCGGCCGAGCGCCGGTAACACAACGTGTG<br>TGTGTGTAGGCGCGTGTACACACTCTCATACACGGCTAGAAAGGGT

	CCAGGCGACACACACACTCCCACATACACGGCTAGAAAAGGTCCA<br>GGCGAGACACACACACACACACACACACACACACACACTCCACA<br>CACACTCACACGGCCAGAAAGGGTCCAGGCGGTTCCCGGCGCTTT<br>TCCAGCCCTTGTTTTCATGGCGCACCCTCCCGCCAGCCGCCCCCCT<br>CCGCACTCCGTCGTCCGCCCGGCCCGGCCGCGTGCGGTTCCCCGG<br>GAGCCCCCACCCCGTCGCGGACCCCAGCGACCACCAAGTCCGCAC<br>GCGGCCTGCCGCAGGCCTGAGCAGAAGGCCCCGCGCACACCCACC<br>GCGCCGCGGCCGCGCGGGAGGCCTGTGCCGCCCGCGCCACCCACT<br>GGCCGGGCCCCGCGGGCGCAGCGGAGCGGGCGGGTGGCCGGCCC<br>GGACGCGCCCTCCCCGGCCGCGGCCCCGCGCGCCATGTGCCCCCG<br>GCGGGACGCGCCACTCCCGGGCCTGCCGCGGCGCCTTTAACCCGG<br>GCCAGGGAGCGGGGCGGAGGGGGCGGTCGGGTGGCTCAGAGGAG<br>GGCTCTTTCTTTCTTCTTTTTTTGAATGAACCGTGTGACGTTACGCA<br>CAGGAAACCGGTCGGGCTGTGCAGAGAATGAAGTAAGAGGACAG<br>GCACCACAGCCCCGCTCCCGCCCCCTTCCTCCCGCGCCCGCCCCTC<br>CGCGCCGCCTGCCCGCCCGCCCGCCGCGCTCCCGCCCGCCGCTCTC<br>CGTGGCCCCGCCGCGCTGCCGCCGCCGCCGCTGCCAGCGAAGGTG<br>CCGGGGCTCCGGGCCCTCCCTGCCGGCGGCCGTCAGCGCTCGGAG<br>CGGGCTGCGCGGCGGGAGCTCCGGGAGGCGGCCGTAGCCAGCGC<br>CGCCGCGCAGGACCAGGAGGAGGAGAAAGGGTGCGCAGCCCGGA<br>GGCGGGGTGCGCCGGTGGGGTGCAGCGGAAGAGGGGGTCCAGGG<br>GGGAGAACTTCGTAGCAGTCATCCTTTTTAGGAAAAGAGGGAAAA<br>AATAAAACCCTCCCCCACCACCTCCTTCTCCCCACCCCTCGCCGCA<br>CCACACACAGCGCGGGCTTCTAGCGCTCGGCACCGGCGGGCCAGG<br>CGCGTCCTGCCTTCATTTATCCAGCAGCTTTTCGGAAAATGCATTTG<br>CTGTTCGGAGTTTAATCAGAAGAGGATTCCTGCCTCCGTCCCCGGC<br>TCCTTCATCGTCCCCTCTCCCCTGTCTCTCTCCTGGGGAGGCGTGA<br>AGCGGTCCCGTGGATAGAGATTCATGCCTGTGCCCGCGCGTGTGTG<br>CGCGCGTGTAAATTGCCGAGAAGGGGAAAACATCACAGGACTTCT<br>GCGAATACCGGACTGAAAATTGTAATTCATCTGCCGCCGCCGCTGC<br>CTTTTTTTTTTCTCGAGCTCTTGAGATCTCCGGTTGGGATTCCTGCG<br>GATTGACATTTCTGTGAAGCAGAAGTCTGGGAATCGATCTGGAAAT<br>CCTCCTAATTTTTACTCCCTCTCCCCGCGACTCCTGATTCATTGGGA<br>AGTTTCAAATCAGCTATAACTGGAGAGTGCTGAAGATTGATGGGAT<br>CGTTGCCTTATGCATTTGTTTTGGTTTTACAAAAAGGAAACTTGACA<br>GAGGATCATGCTGTACTTAAAAAATACAAGTAAGTTCTCTGCACAG<br>GAAATTGGTTTAATGTAACTTTCAATGGAAACCTTTGAGATTTTTTA<br>CTTAAAGTGCATTCGAGTAAATTTAATTTCCAGGCAGCTTAATACAT<br>TCTTTTTAGCCGTGTTACTTGTAGTGTGTATGCCCTGCTTTCACTCA<br>GTGTGTACAGGGAAACGCACCTGATTTTTTACTTATTAGTTTGTTTT<br>TTCTTTAACCTTTCAGCATCACAGAGGAAGTAGACTGATATTAACAA<br>TACTTACTAATAATAACGTGCCTCATGAAATAAAGATCCGAAAGGAA<br>TTGGAATAAAAATTTCCTGCATCTCATGCCAAGGGGGAAACACCAG<br>AATCAAGTGTTCCGCGTGATTGAAGACACCCCCTCGTCCAAGAATG<br>CAAAGCACATCCAATAAAATAGCTGGATTATAACTCCTCTTCTTTCT<br>CTGGGGGCCGTGGGGTGGGAGCTGGGGCGAGAGGTGCCGTTGGC

	CCCCGTTGCTTTTCCTCTG
BCL2L1	AAGTCTTGAAAGTGACATAACCCCAGCTTGGGCAATATGGCGAAAC ACCGTCTCTACAAAAAAATACAAAAATTAGCTGGGTGTGGTGGCAC ATGCCTGTGGTCCCAGCTACTAGGGAGACTGAGGTGGGAGGATTG CTTGGGCCTGGGAGGTCAAGGCTGCAGTGAGCCGTGATTGTGTCA CTGCACTACAGCCTGGGCAACAGCGAGACCCTGTCAAGAAAAGAA AGAAAGAAAGAGAGAGAGAGAGAGAAGAAGGGAGGGAAAGAAA CATTCCATCGCTGTAATTTGTCATTAGTCACACAGAACAACTGTGGT TCAGTGTGGGAGGGAAACACACAAAGGCATGAATACGAAGAGGC AGGGATATTTAGGTGCCATCTTGGAGGAAGCTAGATGCTACAAACT CTAAATTGTATATTGCCTATTTCTAAACTGTATATAAGTAGAATCTTC CTACATTATTCTTTTGCCATTTTTTCCATTCAATATGTTTCTGAGATTC CCTTTTTTTTTTTTTTTGAGAGGGTGTCTTACTCTGTCACCCAGGCT GGAGTACAGTGGTATGATCACAGCTCACTGTAGCCTTGACCTCCCA GGCTTAAGCGATTCTTTTGCCTCAATCTACTCAGTTGCTAGGGACA ACAGGTGTATGCCACCAGGCCTGGCTAATTGTTCTTTTTCTGTTTTT GTTTTTGTTTTATTTTGTAGAGAGGGGGTCTCACTATATTGCCTAGG CTGGTATTGAACTCCTGGGCTCAAGCATCTTTCCGCCTTGGACTCC CAAAGTGGTGGGATTACAGGTGTGAGCCACCATGGCTGGTCTGTTT CTGGGATTCTTCTTTTTTTTTTGAGGCAGAGTCTCACTCTGTTGCCC AGGCTGGAGTGTGGTGGCATGCTCTCAGCTCATTGCAAACTCTGTC TCCTGGGTTCAAGTGATTCTCCTGCCTCAGCCTCCTGAGTAGCTGG GATTACAGGCACCTGCCACCATGCCTAGCTAATTTTTGTATTTGTAG TAGAGACGGACTTTCACCATGTTGGCCAGGCTGGTCTTGAACCCCT GACCTCGTGATCCACCCGCCTTGGCCTCCCAAAGTGTTGGTACTAC AGACGTGAGCCACTGTGCCCGGCCAAAGTTAGCATGTTTTTAGATA GTGTTATATAATTAAAAATAATAGCTGGACGCAATGGCTCACGCCTG TAATCCCAGCACTTTGAGAGGCCGAGGCCGGCGGATCACAAAGTC AGGAGTTCGAGACCAGCCTGACCAACATGGTGAGACAGGAGAATC ACTTGAAGCCGGGAGGCACAGGTTGCAGTAAGCCGAGGTTGTGCC ACTGCACTCCGGCCTTGGCGATAGAACAAGACTCTGTATCAAAAAA AAAAAAAAAAAAAAGAGGGAGGCTGAGGCCAGAGAATCGCTTGA ACCCAGGAGGCAGAGGTTGCAGTGAGCCAAGATCATGCCACTGCA CTCCAGCCTGGGCAACAGAGCAAGACTCCATCTCAAAAAAAAAAA AAAAAGAAAAAGAAAAAAGAAAAAAAATACAAAGTTATGTGTAG ACAACATAGAGAAGTATAAAAAAGAAAACTTATTTTCTTTAGATTTA AATGTAACTTATTTTCTTTAAATTAGATTCCAGTCAAAGATCATTTGA GAGCCATTTTCAAGACTTGCAAAATTCTTTTTCCAAAATATGTATCC ATCTTTCCAAAATATAAATTTCTTCATGTTCTTTCTGGTTTAAAAAAA TCTTTGCTGGCTCCTCTGGTGCTATTTGATGTGCTGCTCGCATGGAT ACACATTTATATTCCATCCCCTCTCCACCACTCCCCTCAGCCATCCC AATTTATTTCCAGGACACCATGGGCTCTCACGCTGGGCTGCCAGGC CTTCGCTCAAGCAATATCTTCTGCTTAAAATGCTCCTACATCCTTTCT CACCCAAGCAAAATTCTATTCTTTTGAGGGCCAGTTCAGTAAAGCC CCTATCTGTGGACAGAGTTTATCTGCCCTCTGCTGGGCTCCCAAAG CACACTGAGGCTTCCATAATGATAGTTTTCACATTGAGATAATTGTG

	GCACCATAGGTTCAAAAATGTAAATCAGACCGGGCGCGCTGGCTCA<br>CACCTGTAATCCCAGCACTTTGGGAGGCCAAGGTGGGCGGATCAC<br>GAGGTCAGGAGTTCGAGACTAGCCTGACCAACTGGTGAAACCCCG<br>TCTCTACTAAAAATACAAAAATTAGCCGGGCGTGGTGGCAGGTGCC<br>TGTAATCCCAGCTACTCAGGAGGCTGAGGCAGGAGAATCACTTGA<br>ACCCAGGAGGCGAAGGCTGCAGTGAGCCGAGATCGCACCACCGC<br>ACTCCAGCTTGGGTGACAGAGCAAGACTCCATCTCGAAAAACAGC<br>AAAAAAAATGTAAATCAGAGCCAGGTGTGGTGGCTAATGCCTGTAA<br>TTCCAGGACTTTGGGAGGCCGACATAGACAGATCACTTGAGCCCA<br>GGAGTTTGAGACCAGCCTGGGCAACATGGGGAAACTCCATTCTCT<br>ACAAAAAATACAAAAATTAGCTGGGTGTGGAGGTGCGAGCCTGTA<br>TTCACAACTACTGGGGAGGCTGAGGTGGGAGGATCACCTGGGATG<br>GGGAGGTCGAGGCTGCTGTGAGCTGAGATCACACCATTGCACTGC<br>AGGCTGGGTGACATACCCTGTCTCAAAAAAAAAAAAAAAAAAAA<br>GGCCGGGCACGGTGGCTCATGTCTGTAATCCCAGCACTTTGGGAGG<br>CTGAGGCAGTCGGATCACCTGAGGTCAGCAGTTTGAGACCAGCCT<br>GGCCAAAATGGTGAAACCCTGTCTCTACTAAAAATACAAAAATTAG<br>CCGGGCGTGGTGGTGTGTACCTGTAGTCCCAGATACTAGGGAGGCT<br>GAGGCAGGACAATCACTTGAACCTGGCGGGCAGAGTTTACAGTGA<br>GCCAAGATAGCACCACTGCACTCCAGCCTGTGCAACAGAGCAAGA<br>CTCCATCTCGAACAAACAACAATAACAAAAAAAAAAAAAAAAATG<br>AGGAAAGAGTTCAACAGAAAGACCACCTGAAAAAGGGGTAAACA<br>ATATGAAGAAAATTCACCAATACAAGATCCACCACCCTCCAAAGAA<br>AGACTAAAAAATATGACAAATTGCTCAACTTCATTCATAATAAAAA<br>AAGAAATATGAAAGTGCCTTCGGTTTTTACCCATCTAATTGGCAAG<br>GATAAAACATTTGATAACACACTGTGTGGTTCAGGGTGTAAGGAAA<br>TTGGCGGGGCGCGGTGGCTCACGCCTGTAAACGATTCTCCTGTAAG<br>CGAGTCTGCTGCCTCAGCCTCCCGAGTAGCTGGGATTACAGGCATG<br>CGCCACCACGCCCAGCTAATTTTTTGTATTTTTAGTAGAGACGGGGT<br>TTCACCACATTGGCCACGCTGGTCTTGAACTCCTAACCTCAGGTGA<br>TCCGCCCGCCTCGGCCTCCCAAAGTGCTGGGATTACGGGCGTGAG<br>CCACCGCGCCCGGCCTGTCCTATGGAGTTTACAATTAATAACTCTCT<br>TAATCATCACAATAGCTCAACGAGAGAGGTTGGTACTATTACCCATT<br>TTACAATTGAGGACATTGAAGCACAGAGCAGTTGAGAAACTTGCCT<br>AACTAGCAGAGTTAGAATCCAGACAAAGTGCTTAACCACAAGGCG<br>ATGCTTCTTCTATATATAAACTGACGGACGGATGAAATAGGCTGATT<br>GACTGATGAGTGACTGACTGACTGACTGAATGAATAAATGGAAATG<br>TGATTATAAATGTTGATTTTTTGCGCCTCCCGATTCCCCAGATCTACC<br>GCGAGGGGGCGATCTCTCCCCGGTCCTGATGCCAGTCACTCAAGG<br>CGCGCACTCCCTTTGCGTCTCGGGCTCGCGCGCGTTGCCGCGGCAC<br>CGGAAGTGACTGAGCTTGCAAGTTCCCCTGTCTCTTCAGGGGAAA<br>CTGAGGCCGGCTTGTTCGGGAGAGACGGCGCGAGCAGTCAGCCAG<br>GTAGGCCGGCAGCCAGGTAGGCCGGCCCGGGTCCGCGGCGCGGAA<br>CTCGGCCGCGAAGAGCTCTTGCGTCTGGAAGCTACCGGGCCGATG<br>AAGGGGGATGTGGCCCCCCACGGCTCGCGGGGCTCGCAGGTGAGA<br>GCGCCGCCTCCCCTGTGCGTGACAGCCGTTGCGCCCAATAGGAGC

	CTTCGCCGTCCCGCTTCCGTGCCGGCTGGGCTCGCTGATTGGCTGC GCTGGGGCGGACCGCCGGGGGCGAGGCCCCTCCTCTCAGGAAGGT CTAGGGGGCGCCCCCGGGGGAGGGCGGTTGCCTAGCAACGGGGCG GTGGCTCGGCGGGTACCCGGCCTAGTGAGCTCGAGAAGCCGCTAG GCCGCAGCCGGCCCACCCTGGGCTCCGGAGAGGTGCAGCCCCCGG CCCCGGAGCCCTCCCGGGGGCGCCAGAAAGCCACTTTGGGCCTGT CCCCAGGAAAACGTGGTCTCAGCTGTGGGGCCGTCGAGCAGGCCT CGACCCCTCTTCCTGACCTGAGGAGAGGCCCAGCGCCTCACCCTC ACCCAGTCTTTGTGCAGGGCGCCGGCGGCCATTGTGTCCGGGCGG GGAATGGAGGACCCGGCAATCCCCAACTGCCACGTTCTGGGCCTTT GGGGAATTCAGAGCAAACCAGCGGCATTTGTTGGGGGTCTCCGGC CTTCAACATCACAGACAGGCCTGGGGGTGGCCTTCCCAAAGTCAG ATTGCAGATCTGAGGCAGTTTCCCCCTCCCTGCGTCCCTCACTGAA ACCTTGAACCCCATTGAGAAGTCCCTTTAGGGTTTCGGACGCCTCC ACCTCACCCTGGGCTGGTGCTTAAATAGAAAAAAGAAAAACAAAA ACCAACTAAATCCATACCAGCCACCTCCGGGAGAGTACTCCTGGCT CCCAGTAGGAGGCGGAGAGCCAAGGGGCGTGCAAGAGAGAGGGG GCTGGGCTCCCGGGTGGCAGGAGGCCGCGGCTGCGGAGCGGCCG CCCTCGATCCGGGCGATGGAGGAGGAAGCAAGCGAGGGGGCT
BCL3	CTCAAAAAAAAAAAAATCTGTAGAGACAGGGTGTCCCCTTGTTGC CCAGACTGGTCTTGAACTCCTGGTTTCAAGGGATTCTTCCTTCTAC CTTGGCCTCCCAAAGTGCTGGGACTACAGGAGCACACTCCATGCC CGGCCCCATTCGCCACTATTAAAGTCACAAGGGGCTGGGCATGGTG GTTTATACCTGTAATCTCAGCATTTTGGGAGGCCAAGGCAGGTGGA TCACTTGAGGTCAGGAGGTTGAGAACAGCTTGGCCAACATGGTGA AACCCAGTCTCCACTAAACATACAAAAATTAGCCGGGCATGGTGGC GGACGCCTGTAATCCCAGCTACTTGGGAAGCTGAGGCAGGAGAAT TGCTTGAACCAGGGATTGGAGATTGCAGTGAGCCGAGATCATGGC ACTGCACTCCAGCCTGAGCGACAGAGCGAGACTCCATCTCAAAAA ATAAAAATTAAAAAATAATAAAGTCACAAGGAAGGCACAGAGAGG GAAAGCCACCTGCCTAAGGTCACATAACTAGCACATGGACGAGCC AGGGCTCAAAACTATGTCTCTAAGCCCAGCATGTTGAATTCTATTTT CCCCAATGAAGCAGGTCCTGCTATTGAAAACACAGGATATTTGATG AGGACAATAATAATATATAATGTTATATTATTATTATTATTATTATTA TTATTATTATTATAAAACTAATAGCAGCTAAATTGCATCAAGAGCCTT CTATACATCAGACCCCATGGGACAGAGTCATATTTTCTTGATGTGAT CCACTCTACAGATGAGGAAGCTGAGGCTCCACGAGGTTAAGTAAC TGCCCGGAAGTCACAAAGCTAACGATCAGGAGACAGGACTTGGAC CCAGGCCTCAATTCAAAACAAAGTAGGACAAGGAAGCCAGATGCG GTGGCTCACGCCTGTAATCCCAGCACTTTGGGAGACCGAGGAGGG AGGTTCGCTTGAGCCCAGGAGTTCGAGAACACCCTGGGCAACACA GTGAGACCCCCTGAGTCTACAAATTGTTTTTTTATTAGCCAGTCAT TGCGGCACATGCCTGTAGTCCCACCTACTTGGGAGGTTGAGGCAGG AGGATCATTTGAGTCCAGGAGTTCAAGGCTACAGTGAGCTATGATC TCACCACTGCACTTCAGCCTGGGAGACAGAGCCAGATCTTGTCTCA AAAAAAAAGCAGGACAAGACCCAGTAATTGAGCCAGATGGGAAC

	TTAGGCCCTGACCAGCCCTACTCTCTCCTCTTAGAAAGGGAAATTG<br>AGGCCCCTGAGAGCAAGAAAGCTGCCTCAAGCCTACAGACTTCCA<br>GATGGAATTTCGTTTCCCTCTCAGGACTTTGAGCAGGTAAATTCCT<br>GGCCTTTCCTCCCCCAGGATGGGTTGGGGAAAAAGAAACAGCAGC<br>TGGGGAGGAGATCTGAACAGGTACAAAGGGCGGCCTGCATGAGGG<br>GAAAGTGGTTAGACACCAAGCAGAACTTCCTGGCCTCCTCTGGGA<br>AGTTTCTAAGACTCATCCAGGGGCTGCCCCTGCGGGTGGGGGCCCT<br>CCAGAGGCCAAGGCCCAGAGAAAGGAGACCCTAGTCCTTTCCGCC<br>TGGGGTGCAAGGAGCCGGGGGAGGGTGGGGGTGGCCATCCTGGTT<br>CCGCATCCTCCGTTCCCGACATTGGAGGGAGGCTGCTGTGGGAGCT<br>GGGACTTTCCTGAGAGCAGAAGATTCTGGGAAATTAGAGACAGCT<br>GCAGGGCGGGGCTGCCTCCCAGCCTCCCTTCCCTTCCCTCCCCTTG<br>GTCCCTGCTGGCTCCGGAGGCACGCCTCTCCACCCCTCCACCCCAC<br>CAGCTCCTCCTCCCCTAGTCCATGCCTTCCACCCTCGGACCCTCAG<br>ATCTTTTCCCCTGAGCCTCTTTCTTCTCCCTCAGATCTGGCCTCTAC<br>CCTCAGACTCTCTCCAAATCTTCTTTCCTCAGACCCTTCTTTCCCCT<br>CCAACTCTCTCCTCCTCCCTCAGAGCTGCGTCCCCTCACTTAGTGC<br>TTCTCAGACTTCCTCAGACTGTGCCCTCTCCCTCAGATACTCTCCCC<br>TCCCCTCAGACCCTCACCTCCTCACTCAGACCCTCACTTCCTCCTTC<br>AGACCCTCACCTCCTCCCTCAGACCCTTACCTCCTCCTTCAAACCC<br>TCACCTATTCTCTCAAACCCTCACCTCCTTCCTCAGATGCTTATCTC<br>CTCCTTCAGACCCTCACCTCCTCCCTCAGACCCCCACCTCCTCCTTC<br>AGAGCCTCACCTCCTCTCAGACCCTCACCTCCTCCCTCCAAACCTC<br>ACCTCCACCTTCAGACCCTTACCTCCTCCTTTAGACCCTCACCTCCT<br>CTCTCAAACCCTCACCTCCTTCCTCACATGCTTATCTCCTCCTTCAG<br>ACTCTCACCTCCTCCCTCAGACCCCCACCTCCTTCAGACCCTCGTC<br>TCCTCTCTCAGACCCTCACCTCCTCCCTTAGCCCCTCATCTCCTCCC<br>CCCGAACCTCACCTCCACCTTCAGACCCTCACCTCCTCCTTCAGAC<br>CCTCACCTCCTCTCTCAAACCCTCGCCTCCTTCCTCAGACTCTTATC<br>TCCTCCTTCAGACCCTTGTCTCCTCCCTCAGACCTTCACCTCCTCCC<br>TCCAAACCTCGCCTCCACCCTCAGACCCTTATTTCCTCCATCAGACC<br>CTTACCTCCTCCAACAGACCCTGTCCTCCTCCTTCAGATCCTCTCCC<br>CCTCCTCCCACTAAACCATCTACTTCTCTCTCAGACCTATCTTCCTT<br>CTTCAGATCCTCCTCCTTCTGACCCTTTTATCCCTCTTCCAACCTCA<br>GAGAACCATCAGCACCCTCTCTTCCAGCAGCACCGTCTCTCCCAGC<br>CCTTCGAAGCTCCCACCGGACCCATCACATCTTCTCCCCTCTACAG<br>TCCTGCTAGGTTGGAACCACTGCACCAGCTGCCCTGGGATGCCAG<br>GAGTGGGACAGGCTTGGGTGTCCTTCCCACCCTACCTCTGAGGGTC<br>CCCACCCTAAGCCTCGCCCCTCCAAACCTCAGCTTTCCTTTCTGTG<br>CATTGGGATAGTGCTCCACACCCCTCTCCTGTGTCTGAGGGTCCCC<br>AGGGGTGGGGTTGGATGGGCAGGGAACACAGCCCCCAGCCTGTCC<br>AACCGAATTCCTCCCCTCTTCCCTTGCTGTGGCCCCCACAACACCA<br>GCAGGCAGGGGTTGCCATTGGTCCCATTTTCCAGAAGAACAAACT<br>GAGTCTCAAGGCAGGGGATGGCTTGTCTGAGGTCCCACCGGGAGC<br>AAGGGGTGGAGGTGGAATTAACACAGGTCTGTGCTCGGGATGTTT<br>CGCCATGAAGGGGGATAAGGGAGATCCAGAGAGAGGTCGAAAGG

	AAGGGAAGGGGTGAGGCCTCTCCACACAGCCACTTCTCACATCCT<br>GGGTTAACGATCCACTCGGGTGGGGCCCGAGTCCGGCCAAAGTCC<br>CTTCCCGGGGCCCCAATACCCACTTTCAAGGTCCCAGGGGACACA<br>GAGACAGCAACAGAGATGGGGAGACAGAGACAGAGGGCGGGGCA<br>GGAGACAACCAGAGGGAGGTCAGGCAAGTTTCGAAGACAGAAGG<br>AGACCGGGGGAGAGAACCAGAGAGACGCCGCGTGCCCCCTGGGC<br>AAGTCCGGGGTGCAGCCCCCATGCCCCGCCCCCTGTGTCCCCAAA<br>GCCTCCCTCTCCGCCTCCTGTGACTCAGTGACCCGGACTCAACCCC<br>AGCGGCTGCCCCGCCCCCAGGGAAATCCGGGAAAGCCCCGGACCT<br>TCGGGGCCCCACCCCGGGGCGGGGAGAGGGAGAGGAGGGGAGGA<br>GACGGGGAAGAGTCCCGCCTCCCTCCTCCCTCCCTCCCATCTCCAG<br>CCTGAGTCATGCCCCCAACCCGCCTAGCCTGTTCCCCACCCATCAA<br>CCCCTCCTCGAGGTGGGAGGGGAAGTCGGGGGGAAGCCAAACTG<br>CTCCCCGCTCCTGCAGCACCGGCCTCGGTCGCGCTGACTCTGGCCT<br>GGTGTCCGTGTCTCTTGCTATCTCTCTTTCTCTCAAGATCTCTGCGC<br>CTGTCTCCATGTCTCTTTCTCTCTGTGCACCTAGGATTTTCCGAGCA<br>CCCACCCCGGTGCCCCGCGGGCCCCGGCTGGGGGCAGGGCCCCCA<br>ACGAGTGCAGAGACACAATCAGTGGAGCGCTCCCCACCCTCACCC<br>CACCCCCAGCCCCTTTAGACCCACAGCTGATGAGGCACGTGGAGT<br>GGCAGAGATGGAAGAGGGGAGGGAGAGGAATCGTTCCTGGCCGC<br>CTGCCGAGTGCCAGTCCCTGCCCCGGGTGCGTCTCTTCACTCCCAC<br>TAAGGAGGAAACGGCTCAGAGAGGGGAAGTGTTTGGCCAAGGTC<br>ACCCAGCGAGTAAGTGGGAACCGAGATTCCAAACCCCGTCCCAGA<br>GATGCCAAGGCCTCCTGAGAGACAGAGAAGCAGACAGCAACTGA<br>GAGGCAGAGAGATGGTGACAGACACAAAGAGACAAAAGAGAGAG<br>ACAGAGACGGAACAGAGCACACAGAGACAAACGCGGGGTTGCGG<br>AGAGAAACACCTACTCAGACAGGAGAACCAGAGAGACAGTTACA<br>GACTCAGAGATAGAGATGTTGAGAGATAGGGCCAGAAAGACAAAA<br>ACAGAGGCAGAGAGAGCGGCCCTTGGCAGCAGGGGTGGGGACAC<br>CCCCCCACCCCCCGACCCCGCCTCCTCTCCCCCCACCCCTCCTTTC<br>CTCTCCCTCCCCCGCCGAGGCCTGGCTGCCCCAGGCGCCGCGGGC<br>CGGGAGGGGGCAAGCGGGGCGCGGCGCGGGCGGGGCGCAGGGCA<br>GGCTGCACCTCAGAGCGGCGGGAGCAGCGGCGGGTCCAGGAAAC<br>CCCTGGGGCGTACGGGTGGCCCCGGGGGGGCCGGGGGCGGGGAG<br>GCGGGCGGCCGGCACCGCCCCGGCCGACAAAAGTCCCTTCAGTTC<br>AGCCGGCTGCAGGGGAAGTCCCGGCGCCCGGCGAAACCACCCTCC<br>CGTGCAGCCGAGCCCAGCCGCTCTCCGGCCGCCGTCCCCGGCGGC<br>CCCATGCCCCG
BCL6	AGCTGTAATTAGAAACCAAGGGTTTGTGTGTGTGTGCGCGCGTGTG<br>TGTGTGTGTGTGTGTGTGTGTGTTTGCAACAAATACAGTGTTTTTTT<br>TTTTCTCCCTACACTGTGCCCCCGTGGAGTCACATTTGTGTGTCTGT<br>GTCTCTGTACGTACATAAGTTACACAGACACTGACATGTAGGAAAC<br>GTGCACCAAAGTGTCTGTCTTCTGACCTCAGGTAACAGTATAATGA<br>CTTGAATTTCAGGCAGCTGAAAGGTTTCTGCCGGTGGAGGTTGAAA<br>TAAACAAGAAAAGCCACTGTGGAGATGTGAATGGAAAAGTACCGA<br>GCCCTCCCTCCCTCCGCACATTCTTCCGCTCTCCAGCTCTCCCTGCC

	ATCGAGCTGGCTTCAGATAGGCTTCTGCATGGTCAGGTGTACAAGA<br>GGGCGGTGGGGAGAAGAAAAAAAAAATGCAGGCACAACACGCAA<br>ATCAAGTTTTTCCACTTCTAGCCTTAGGTAGTAGAGACAGCTAAGTA<br>CAGCAGCCAGCAGCCCGGCAACGGCAGCGGGTGGACCAGCCACC<br>CTGAGTTTACAAACACTCAAGTGCTTTCCTTCCCTCATCCCTCTCAG<br>AGTCCAGCTGCTGCTTTCCTTCATGCTAAGGTTTCATAGGAAGTGA<br>AAACTCTGCTATTCAAAACAGCGATCGAACGCAATAAACAAATCAT<br>TACACACCCCCTAACCCCCATCACTTCTCTATTTTAAGCTTCTGATAT<br>TTATTCCCATTTTAAATAAGTGAGAAAAGTGTGGAAAATTAGTGTTT<br>GGGGGTAAACTCTGAGCCAGGCTGAAAAGGTTTCTAAAGGAAAAA<br>AAAATCTCAGAACAATAAAGGCTAAAAGCAGGCAGCATATGGATG<br>AAAATTAAACACTGATACTTCCTTTTCAGAAGGCAGTAGCTGGAAA<br>TTATACACTTTTTTAATGTCTCAAAACTTTCTGCTCATCTTGCTATGT<br>TAAAAACGCCTTTCTTTCTCCAAGGATACTACAAAAAGCTTGTTTA<br>CAACAGTTCTAAATGAAGGATTTGAAATAAAACGAACAGGTAAAAT<br>TTAACAAGTCTGATAGATAGTGTCTCCCAAATCTATCAAAAGCAGT<br>GCCAAGTACTTCAATGTAGCTGAGAGGCATAAATAAACCCAAATGA<br>CCATCAAAACTCATCATGACTTGGAGTTCGCTCTGAGTTTTGCAGT<br>TTACAAAGAGACCATGGCAGCCTTGCTTCCCTCAGTTCTACAAGGA<br>CACAAGATATACACCTACAGACTCAAGTTGTCAGATTACACTGATC<br>CTCTAAAATGACAGAGGGCCAGCAAATCATGCAGACCCATTTTCAG<br>TTGTGTTCCTGGGGTCACACATGCTCCTAGTGAAGACCCAGCCTAT<br>AATCCTGAAAGAAGAAAGCCTAGAGAAGGTGATTGATTTGAAAAA<br>GTCTTCCCAGTTTTAAAATCTTTAGTCCTATGATGTGGTATCTTAAAG<br>ACCTACCAAGGTGCCAGAGGTTCCTGACAGGTGAAACCAACTTCC<br>TCTTGTGAGCCCCCTTAGAAAGAGGACAGAACCGTGTTTATTCCAA<br>GGATAGGTTCTTTTTCAGCTATGACTGAATTGTGGGGAAGGTTTTGC<br>AAGGGGGAATTGGATGTGAAGTCTGTTCTTTTCCTCAAATAGATGT<br>AATATTAGGACCAGGCTATTTTATTTTGTAATAAAGCTTATATTTACC<br>CAGCAGCAATGATCAGGGACCTATTCTTATGCCCAGTCCATGAGGC<br>AAAGAGGTTGGCCTGGTCCTCTACTGAGTATTAGCAGCCAGTAACC<br>ATTAAATCATGGGACTAGTTGAAATGTAGTGCCCCGAAGTCTGCAG<br>GAATTATTCATACGACCCCAGACATGGAATCACTCTTTAGAGCTTCT<br>TAAGGATGATTTAAAAGAATCAGAATACGTTCAAGTCAGCCCTTTC<br>TTTAATCCTGTAACACGGCACTGCGGGAGTGAGGGAGGCCCACATA<br>GTGATGCCAACTGGATACTGAGGAGAGGTCAAGAATGAAAGAAGA<br>AATGACATTCTGGAAGAAATTCAACTGGTATAATATTTGACAAAGTT<br>ACTTTCCTAGGAATTGAAAAGAGATTGAGAGGCGGGTGCACAATTT<br>TCCTCACCATTCATTCAGTTCAAAGTAAAAGAGACTCACCGAAAAG<br>TAAGTGCCTATCTTTAGAAAATTTTCAATAATGATTTTCTCTTTCTTT<br>CTAACTGGTCTTCTGTTCTGGTCAATTTCTTTCAGTGTAAACACATT<br>GATTTGGCAAAAAGCAGTAGGAAAATGTGGCACTCTGGCACTTGG<br>TCCCAGAAATAATATGCTGGGAAGATTTGAGGTCCCTGGTGATGAG<br>GTTAATTATATATGAACCAGCCCTGGTGGGTTCTCCCTCTAGGGGCT<br>CACTGCAGAGAATTAAAGAGGGCTGAGGTATGAGAAGGGTGAATT<br>CCTTCCCAGCCCCCACTCTGGCTGGTTCTACTACTGCCTTAGAGAG

	CAGATTTCCCTTTGCTCTGCAGCGCCCCCATGGGGCTAAGAGTGGA<br>GTGGCAAAGGGAACACAGGAGGGACAAGCTGTGTTTCAGGTTGA<br>GGGGGGCGGTGGATGAGGCTGAATGGCAGTTTTGACAAAGAAAAA<br>AGTGACCAAAAATCATAAAAATAATCTTTTGAGGGCCCAATAGTAA<br>GGCAGAGCCATACAATTCACATTCCAAACCATATAGATACGTCTGAG<br>AAATCCTAAAGTGCTAATTGCTCATAAAAGAAAAAATTACACATATA<br>AACACACAAAGAAAATCCCTTCCACAAAATCGGGGTGTCATTTTGC<br>ATCCAGCGGGATTCATTTTAATTTCTTTGAAAATGAGAAGGAAGGG<br>GACTCAAATGAAAAAGCAGATAGTCTGCCTTCTGGCAGAATAAATC<br>TGAACTTGACAATATCATGTGTCTTTGGGGGTAAAACGTACATTTCA<br>ACAACAGTGACAGGATTAGGCCTATGTATATTTTTCAAAAACCGTTC<br>ACAAGACAGGCTTTTCTGCAGAGGCTGCAGTAATCCATCTGTCAAT<br>AAGTATTAAAATATTCAGATTTCACAGGGACAGACACTTTAACGCAT<br>ATTTCCTAAGCTCCAGCCCTTGTGGAAAATAATCAACCTCTTTGCAC<br>CTTTCTGGGTTTTAAAACCTAAAATACAGCCTTTAAAAATGTGTGTG<br>TGTTGTGGGGTAGGGGGGTGCATTGCCAACAACATTTTCGGTGATA<br>GATGGAACTTCTTACGGGACTGTCAATGAAAGAGATTTTCCAAATA<br>TCCCAGCAAACAGCAATCTTTCACAGCTCTGATCACTCCTCCATTAT<br>AAACCCAAATTTTGGGTTGAGATAGGTAGATTATTTTAGACATATCT<br>TTATTAGAAATTAACAAGTGACGAGATTTTGTGGAAGCTTTAAGAA<br>TTCATCTGTAATTTAATAAGTCGCTTGAAGGACTCTCATAGCCAAGG<br>CTCAGAACAGCCTGACCTTTGAAAGCTGCTTCTGGTCCAAACATTT<br>TGGGCTAATTCTTGAGGAATCTGAAATATTATTTTCCCCTCACACCC<br>TTCTTTTAAGAGAGAGACATAAAAGAAACAAGAGTCTCCCTTATTC<br>AGGGATGAGTAGGAGGGGAAAAAACCCGAACCAACATTTAAATAA<br>GGAAACTAGCAGCTCTGAACAAACAAACTAGGACCCACAATGAAA<br>TGATTCTGCACTGCAATTGCCTTTAAAAAGAAAGTAATAGAGAAAA<br>AGAGAAGGAAAGAATTTCTCCTTCTTCTCTACCCCCCCCCCACCCC<br>ACCCCCCAACTCAGCTTCAAAGCTAAGAAGACTGTGCTGCGTGTA<br>GTGCATTGTAGTTGTGGCAGTCTGTTCTAAATACAGGCAGTATCTGT<br>GATACTGGCACGGCAGGCCTTTAGAATTCCCTCCGGCTGATCTCTTA<br>AACACAGACTGAAGAGATTTTTTTACAACGACCTTGAAACGAGCC<br>TCGAAAACAAAAATCTCAAGACCTTAAGAGAAAACAAAACACAA<br>ACAGGTATTTGGCTCACAGAATTTTGTAGAAAACACACACATACCA<br>CCCCGCCACCCCCACCCTCCCCCCCACACACACGTTTCTTGCAACA<br>AGAAATTTCCCAAGAGTCAACAATAACAGATTAAACCCACCACTTG<br>CTGTCCTGGAAAGAAACAAACCAAACCAAAACAAATCCTTTGAAC<br>ATTTCTCTGAAGTGCAGGAGAGACACACTTCAGCAAAAGTCCAAG<br>GGGGAAAAAGAAAATTGCACCAAAGGAAAAAAAAAAAAAAAAA<br>GTGGGGGCTGGGATTGTTACATATGGCCAAAAATTTAAGCTTCTTTC<br>AATAGTATTAGTATTGAAATAATACATCTTTAAAACGCTTGAGGGATT<br>AGATAGGGAAAGAAAAGGCACGTACAAAAAAATCCAACCGATGCC<br>GATCCTGTGATTTACGTAACACCACAAACTTGCAAAAGGCAAAAAA<br>TCAGAAGCAAAAATCCATAAACCATCAAAATACAGAAACCAAAAA<br>TCCCAAGCCACCACACCAGAAAGAAAAAAACCCAGAACAACAGC<br>AAAAACCCCTGTCCTAAATAAAAATAAAGCAAATGAACCCACCGA

	AAACTGCTTGGCAAATATTTTTCTCGTGGTGCCTAATATTCTAGTTG<br>GAAAGAGCTGTGATGTTTATTTTATTTTATTTTTCTCTTACTCGCCTC<br>TCTAACCCTACTATATATATAACATACTTTTCCCAGTGGTTCAAACCT<br>CTCGCTCCCTTTTGTGCATTTAGCTCGATCTGCTGAGTTTATGGGTA<br>AGAAAGAAGGAATTAGCCCCAGACCCCGGGAAAGCAAAGCGCAC<br>TCCCCCTCTTATGTCACCGAATAGCAAATTAGTTCTCAGAATTCCAG<br>AGGCCGAGCTTTGCTACAGCGAAGGCGCCGACGTCACAGAGGAG<br>GAGCCCACGTGATGGTGGCGGAGCAGGCCATACCATCGTCTTGGG<br>CCCGGGGAGGGAGAGCCACCTTCAGGCCCCTCGAGCCTCGAACCG<br>GAAC
BDNF	GTAGAAAATCTCTCACTCCAAACATCACACAGCCTAAATAGGTGAG<br>TCTCAAAAATAAGCTAATGTTCATCTTTCATCTGATTCAATGTCCTG<br>AAACCCTTTGGTTTAAATTTGTTAATTCTTCTCATGGCTTTTCTCCTA<br>GCAAAACCAACTAATACCACAGCTATTTATTACTGTCAGCTCTAACT<br>TATGCCCACAATCTCACATCCCTTTTGACCACGCTTATAGAACTATT<br>ACAACAAGTAAACCAAATTTATTCTTCATTATTAATTTTTAAATGTTC<br>TCAGCACAAATCTGGTAACTTGGAGGGCTACAAGTTGATATTTCTC<br>ATATGTTTGGGGGTTTAGTCTCAACAGTTTCTTAATGGTTTCTATGCC<br>GTTTTTCTTGATCCAACTAAATATTATTCCCAGATGGGATCAGCTTTT<br>GACCCTCTTGTTCTACTCTCCTAGTCTTGGCCCTTCTAAAAGTTTCT<br>TGCTGTGGTTCCTTTCTTTTGTCTGCCACTAATGGCTATGCCTGGTT<br>ACATAACTCCTGTAACAGGTGTTGACCAATTTGAACACATTTTGGTA<br>TGGTATTGAGCTATTCTTATGGTTCATAAAAAGCTTAGTGAGAACGT<br>AACATCTCATGAATAGGGAAATTACTTCTCCCTTAAGGTTTTTCTCA<br>GGACAGGCCTCATACAAGAATTTCAAGGATTGCGAGTGACATAGTT<br>TAACATTGGACCAGGCCTTTCAAATTATCCAGGATGAGTTTGAAAA<br>CACCTGTGCCACTCTGCTCAACAGCAGAGTTTTCTGTTTACTAAGT<br>ATTTTCCCTATGCTAATTACGGAAAGTTTCAACAGTTTTTTAGGCC<br>AACTTATTTGATGCTAGACTAGACAACTTATTTTTTTTTCTTGCAAG<br>GAATACTGAAGGTAGGAGTAACTAGGAAGCTTAAATAAACATAAAT<br>ATAAAATGCTTATAGTGATAGAATTGACCTCAGCCAATTAAAATTATT<br>AATAGAAAAAACATGTCAATGTCAAGCCTACTACCTCTGTTCTCAC<br>TTGAGTAATGAGGATTAGTTTATATTTCCCGACAAGAATAGATGGGA<br>ATTCAAATTTCTTCCTGACCTTTGTTCCCCCTGGAACATTGGGTTAG<br>GATCATATTAGAACATAACCAAAAAGAAATAAAGATTCAGACGAAT<br>TCACAATTAATTTTTAAGCCCCACAAAAGTGAAATAGGTAGCATTAT<br>TTTTTCAAGCTGTGAAACTTTCCCTCATTTTAGTAATAGAGAAAATG<br>TTCAGATTATAAACTTGGAAACTTTGCTCCTAACATATCAATTATGCC<br>AGAGGCCAATTTTTAAGAAGAAGAGAAATGCATGCTCTATATTCTC<br>AGCATCATCCTTGCCCACAATAGGGAAATAATTTTGTAAAATGTTTG<br>ATTTTAGACCTCCAAAATTATCTCTATATGCTACCTGAATTAAGCAAA<br>TAAAAAATAATATTTAGAATTCCATGCAAGGCACTGGTACAATTTTG<br>TTTATCTTGGCTTCATTGTTTTTGAATGTAAGATGTACTTTTAAGGCA<br>AATAAGTACATGTTTTAAGCTGGTCGCATACAGTATTGGCAATGCTA<br>TAATCACAAATCAGAAAGTTTGGAAATGCTTACAAGTGTTAAGAGG<br>TGTGATTCATCATGGTTATCTGAATTGGCATCTGATCTTCTTTTCTTT

	CTAAATATCCCTGACATTTCTGACTCCTCTGTCTTTTCCTCAGTAAA<br>ACTGCACCACACACTGGAAAGCGAAGATACACACATTTATTTATATA<br>ATGTCAAGGGAGAGTAGGAATAAGAAGATTGGCCATAGACCCACC<br>CAATCAGAGTCTGGGAAATGAGAACACTTTTTCCTTCAGCAGAAAT<br>GCTGACGTGCCAATGTGAATTTAGCAGAAAAAAGATTTGCCATAAC<br>TTCTAAGTGAGCAGCCTTCAGAATGCTAGCTTAGATTCCTGGCATTA<br>ACTTGCCAGGTATTTTTTCAGGAAGGAAATAAATTACAATTGAGCTT<br>AAAAACCTGAGGGTAGAACTCATTTTCAAGCAAATGTGAAGCATC<br>AGTTTGAAGTTAACAAAGTTAAAGTTTGGAGTAGGGTTCCTCCAGT<br>CCTTTATAATGTAGTACAAGTATTTTTTTAAATGTATAACACTAGCC<br>TTTTAAATTGTATTGTGCTACTAAAAGAAATTGTGCCTGCATTCATCT<br>TACAACCTGGGAACCAACGCAGAGGGTCTGTGGGGTAGCGGTATC<br>CAGCTTCATGCCCTCTGTCCTTTATTGCTTTCTGGTTAGCCTGCGTAT<br>TTCACATACATTAAATATTCCACAATAAACTCTGCCATCTGTGCTGTA<br>GGGTAGTTTGTATTGGTCATGTGCTCTGTCAAGTTGACAGAGGTGC<br>AAAGCTAAATGTGTGACACTCGAAGAATATGCATATATTTGAATAAT<br>TTGACTATTTAGTCCAACAATTTGCAAAGGCGCTCTGAATGATCAC<br>ACATTCTGATAACACTTCCAAGGAACAGATAGCTTCACTTAGGGGG<br>TGGGGGAGATGGAAGCAGGGTTATTTCTAGCAGGAATTCTTGAGTT<br>CACTGAAGTCTTGTCCCTGGTACTTCACTGTGTGAACGTGGGTAAA<br>TTATTTCCTGGCAGAGGATCGGATTCTTCTTTTATAAAACGGGTAAA<br>TAATTTCTGTCACTAGTCTTTAGAAGTTCTAAAATAGCTAATGTTAG<br>TGAATTCATTTTGCTAACTGTAAACCCTTAGGTAAATTGAACTGAGT<br>ATGTAATAATATTATATATTCAGTTCAACAGCACATTCTTGGTAACCA<br>CAAGAGGGTCCAGGAAAGGAAACTGTTTATAAATCTTTCCCTTTAG<br>CAAAATTAATGTTGGAGTCTTTAGGGAAATTCTTACAGCAATAGTCT<br>TCGCAATTATTAGGTCAAACCCCTTTGAGATTACAGAAAAACGCAC<br>ACACACAGAAAGCTGCCTGCAGAATTTGGGTGTGGGCTTGGTGGG<br>AGATTCCTCTGATACCCAGTGTTGTACCCCCAAGAGAGTGTTTCTC<br>AAAGTGTGACTTCAGATTGTCTGCATTCGAATTGCTTGTGGTATTTA<br>TTAAAATTATAACTCCTGGGCCCTGCCCCACCCCTACTAAATCACAA<br>TTTCAGGAGGAGGGACCTTCATTTAACACTCACCCAGGTGATTTT<br>TATGCTCCGAGGAGGTCCAGGGACTCCAAGTTAAGTACGGTACTGC<br>TGTCTTATTCTTTATTCTAAATTTTAAGGTCTGCACAAATTGGTTGAA<br>CTAATGAGAAGAAAATTCAGCTTTAAAGCAGAAACACAGGTAGAC<br>GGTTGACAGAGTTCATCAAATGGATAATTGAAAATGTCCTCTGGAC<br>CCTAGCCATATAAGTTCTCTTCAAGGGTCTTGGCTACAGGCAAATG<br>AGAACCCGAAAGGCTATTTGCTCTTTTGCTGCGGGCAGTGGTGGG<br>GGTGGAGGGCGGGGGAGGATTAACTGAGCCAGTTCTGCCCCCACC<br>CTNCGAATCACCTACCCCCACTCTGGTTAAAGCAGAAGACTTTTTA<br>TTTATCTTGGCTGCCCTGGTTCGTTATTAAAAGGGTTAGCTTATACGT<br>GTGTTTGCTGGGGCTGGAAGTGAAAACATCTGCAAAAGCATGCAA<br>TGCCCTGGAACGGAACTCTTCTAATAAAAGATGTATCATTTTAAATG<br>CGCTGAATTTTGATTCTGGTAATTCGTGCACTAGAGTGTCTATTTCG<br>AGGCAGCGGAGGTATCATATGACAGCGCACGTCAAGGCACCGTGG<br>AGCCCTCTCGTGGACTCCCACCCACTTTCCCATTCACCGCGGAGAG

	GGCTGCTCTCGCTGCCGCTCCCCCCGGCGAACTAGCATGAAATCTC<br>CCTGCCTCTGCCGAGATCAAATGGAGCTTCTCGCTGATGGGGTGCG<br>AGTATTACCTCCGCCATGCAATTTCCACTATCAATAATTTAACTTCTT<br>TGCTGCAGAACAGAAGGAGTACATACCGGGCACCAAAGACTCGCG<br>CCCCCTCCCCCCTTTAATTAAGCGAAGGAACGTGAAAAAATAATA<br>GAGTGTGGGAGTTTTGGGGCCGAAGTCTTTCCCGGAGCAGCTGCC<br>TTGATGGTTACTTTGACAAGTAGTGACTGAAAAGGTGGGTTTGTTT<br>TCTTTCTTTCTCTTTCCGTTTTTCTGTTTGGTCGGCTAGAAAGCGTG<br>TGGCTTTAGCGAGGTCTGTCATTGCCTGGGCTTCCTGGCTGGAACA<br>AGTAACTTGGTGTAACGTTATCTGGGGGCGTTCATCAATAAAAAAT<br>GCTGTTATTATCTTGATTGAATTCCTATTAGGCAAACTCTAGAGAGG<br>TCAGTGCGCGAACTCTGTTTAAGCCGGCGTGTTTAAGGCAGCAGA<br>GTAAACCAATAGCCCCCATGCTCTGTGCGATTTCATTGTGTGCTCGC<br>GTTCGCAAGCTCCGTAGTGCAGGAAGGTGCGGGAAGGTGTGTCTG<br>TGGCCCGGGAAACGCACGCCCTCTCCCAGAGAACTTGGGTGCTGG<br>GATGGGGAGGAAGGGGAGAGTTGAAAGCTAGGGGAGCGAGACCT<br>CGGGGCGTGCGATTCTCACTCGCTCCCTCCCGCCCCAGCGCCCACA<br>GCCGGGGTTTCTGCAGAGGGCGCGGGACGCGGGGTTCCCCGGGGC<br>TGAGGCTGGGGCTGGAACACCCCTCGAAGCCGCGGGCGTCCTGTC<br>CAAGGCGCCCCAGGAGGGCGCAGGACTCGCAGGGCGATGTCGCG<br>GGGCCCTAGGGGAGGAGGTGAGGACAGGCCCCGGGGGAGCGGGG<br>AGTTCCGGGCGCCCCTCGGTTCCCCGCGCGAGGAAAAGACGCGGC<br>GTTCCCTTTAAGCGGCCGCCTCGAACGGGTATCGGTAGCGCGGGCG<br>AGCGGGGAGCGGGGGGCGGGGTGCGGGGGGGGGGGGCGGCGC<br>CGTTTGACCAATCGAAGCTCAACCGAAGAGCTAAATAATGTCTGAC<br>CCGGGCGCAAGGCGCAGCCTGGAGCTCCGGGTCCCCGACGCTGCC<br>GCCGCCGCGCCCGGGC
BHLHB3	GAGCACACGTGAAATGCAGAACAGCCTGCATGAATGGTTTTGATCA<br>TCACATAAATATGAAAATACAAATGATTTCAGCAGAAATAAAATTTA<br>TTTCTAATACTCAATATTATCTGCTCCTTGAAGCTATGCAAAGACTAA<br>TAAGTGCATAAACTTCTTGAGCAGTGTTACATAAATGTAAAACATG<br>GTTCATAGGTATGTTTATGGCTACAGACACATTAATTGATAGTCTTCA<br>GAAAGACTTAAGGTCCTTTACAAATTAAACGGGGACACGTGTGCT<br>GTTTCTGCTCATTTGCTGAGTATGCAACTGGAAAACCCAATCTTCTA<br>TTCTGGCATTTAAAATCCGGTACTTTCAGCAAATAACATCATCAATA<br>TTTTAAAGTCATACTGGCTTCTGGTGAGTAGCAGTGGAATTGCCGT<br>CCCTCCTTAAATCCTTCTCTGAGGCACCCTTCTTTCTGTGGTGTGCC<br>TGACAACATTAACTCTTCTCCCTTGAGAGTAGGTGTGCATGCCAAG<br>AGCCGAAATTCCTAGGGAAGCTTTTGTAAGTCCAGATCCCACCCCG<br>CTATCCCCTCCAGGCCTTGATTTTAGCATCAACCCAAAACTTTTTTA<br>GATTGTTTTGGACTCTGCTTAACTTTGATGTAAAACGTCTGCAGATT<br>CTTGTCCGCTCTGTAAAACAGAGGTGATCAAGAATGGCTTCTGCCC<br>TTCAGATGGGCCATTTTCACAGTATCACAGGCAGGAGACTCTACGC<br>CCCTAAGCAGATTTTGGGGCCATCAGAGATGTACAGTTAGTTTGGA<br>TCACACTTGGGTTCCTAAAACACCCTGGAGTTAATTCTCCAGAAGG<br>GCAGTAGGGCTTGCAGAACCCTAAAGAGGCAGAAAACCTCTATTC

	CCCATGGAAACTTTAATCCTTTCAGGCAAGTTCTGTTTCTGTTGGA<br>AATCCAAGACTTTGCAAACAGGATAAGGCCTTCTAAAGTCACAGG<br>GTGAATATAATGAGTGCAGGACTTAGGATATTTAGGCAGGGGGGAG<br>AATTGGCCTAAGTATTTCCTTTCATTAGTGTCTCTATAGGTAATGATT<br>TCCTTACTTAACCAAAGAAAAGCAATGGAAGCAGCTACCCCTCGCC<br>TATTAAAAGGAGAAAGATGGATATCTTTAATTGAAAGCAAAGCATC<br>ACAAAATTCTTTAATTATAAGCTCAAAGGGGCTTTGAGCTCATCTTG<br>TATCTTCTTGGCTTTCAGCAGGATAGCATTAAGCTTTCCTAAAAAGA<br>TAAGACTCAACCTATTTTAAAACATATCCAAAGATGGCATTCTAGTT<br>TCTGTTCCTTTGTTAGCGTACTTTTTTTCCCTTTCTAACCTCACCACA<br>GGTGAACTTTTCCTATTACTAACCTGAAGTCTTCACATTAGAGCTTG<br>GCTCAGTTTGCTCAGCAGAGCTGGTGAACAGCTGGTCACCATCAG<br>CCTTTTCTAAACTGTACTTCCATCTGTCCATCTGTACAAAATTATAAG<br>AGTTAATTAGGATATTTGGATTTAAATCAATAAATTTGAAGCTAGATG<br>CCAACTGAATGACTCACTGACATGGCAAAAGACTGCCTCTCTTCTT<br>GCCATAGCTTTTCTTATCTCTGTCATCAGCTCCAATGCTAACACCTC<br>TCCCTTTTCCATCTCTCTTGCAAAACTCCCCACACAATTTCATTCAC<br>ACACACACACAAATGTGCACACTTATACTGGTAAATAGAAAAACAT<br>CCCTGAAGCAGCTAGTTAACACTTATACCTTACCACTCACTATTCAC<br>ATTGAGGTGTAAGAGTCGTCCAGGGTCCAGACTCTCGAACCCACT<br>GTCTGGGCTTAAATCCTGGCTCTGGGACCTTGGAAATATTTTGCAAT<br>CTCACTTTGTAAGCATTTTCTCATCTGTAGAATGGAAACAATAATAG<br>TATCTACCTTAGAGGGTTGTGGAAATGATTAATGACTTAATACATAA<br>AAAGCTCTTTGAATGCAGTCTGGTACTCTAATCTTAAAGGCTATATA<br>GTTGTTAGCTATTGTTACACCCTTCTTGCGCAAAGGGATATTCAGAG<br>GCATCAAACTATGCCCAATATTCCAGAAGCCAAGAGTTTGAGAAAG<br>AACAGGAATGGGATTCAAGTTTAAGGGAAGTGGGTAACCAAGAAG<br>AGTCACCAGGGTGAAAGATCAAAGAAAAAAAAGGGCAAAAAGCA<br>AACAGGAGATTGACAGAACTACAGAGAAATGGCAAAAGCTGGCAT<br>TTAACAGGGAAAAGGTAGACAGAGAACATAAGGGAAAGGAAGAT<br>AAAGAAGAAATGAAAAAGGGAAACATGAGTTAAAAGGTGAATATG<br>TAAAGGAAAGACAGAAAAGAAGAGGAAAAGGGGATCAAGAACA<br>ACAAAGGAGATAAAAATTGGGAAAAGACCCCATTTGCAGTTTGAG<br>TAGGTATCACGGGAAAAGGGTACGGAAGTGGGGAAGCTTGGGGCA<br>GAAAGGATGGGGGGAGAGAGGAGCCTCAAAGAGATAACAGAAAA<br>AAGCTTCATGTGTCATTTGCAGCTTTTTGTCCCTTTAAATTATGGGG<br>TGCTGCAAGCTTATTTAACCCACCTTTGAGCCTCAAGGCTCCTGATC<br>GTGTTCCTCCTATTTCAACTTGCATTTAATCTCTGCCCCAGACTTAG<br>CTTCTAAGGTTTGCCCTGGTCAGATGTCTGCCTTGTGTTTTAGCCC<br>GACGAGGCCAAAATGCAATGATCTTGTCTTTATTTCATTTTCCCTAA<br>CCAGTCATCCTTTTCAAGTGAAATTTAGCTCTGGAGCAAATATTTAC<br>ATCCCCTTTCTACTCCAAACATTCAGAAATCTAGAAGGGGACCTGG<br>TGTTTTAATTTTTAAATTATTCACCTAACGGCATTCCCATTTTTAGTC<br>AATTAAAAAGAGGTAAAAATGATACAGTACTGCAGGAATAGGTCGC<br>TTTTAGGGCTCCAACGTGCCCTCCCCATCGAAGGGTCTTAATTACCT<br>AACCTTTTGGAAATAGGAGAGAGGTCGGACCGAGATTAAAATAA

	AGTCAGCATTTTAAAAATACTTTTTAAATTAAGAACCATCGTGATTA<br>AATTGCCTGTTTGGTGCAAAATACGTTCCTCTATTTGAGGAAGTCG<br>AGAGACCTTAAAAGGCCCTCTAAACAGATGAACTGAACGGACCGC<br>GAGCGTCTCCTCACGCCGGGGTGATCCTGGGCCGCCGCCGCCGCC<br>CGAGTCACGAAGCCAGACGCAGTGCGCGTCTCCGCCCAGGACTCC<br>GGAGACTGCAGCAGCGCGAGCGGCCGCCGCGGCCCGGGCTGCTCC<br>TGGAGTCGGGGAGGAGACGCGACTCCCGCAGACACGTGACCTCCT<br>CCCAGCCGCCGCTTCCCGCGACCCAGATCCGTCCCCGGAACCCGT<br>GCCCGGCGGGGCGGGCCGGGGGACACGTAGCGGGCGCCCCAGTC<br>CCTGCTGCGCCCTGGTGCATGTACCTTTGGCGTTCTTCTCCCCTCGG<br>TTCACTTATCACCGGCAATAATCAACGTTTCAGAGATCACCCCGGG<br>GAGAATGTAAATATCCCTCTCTGTGCAGGAAAAACCTTGGTTTTTT<br>CTCTATGTCTTTATGTATATCTAGAGAAATATATATATAGCCTCCATCC<br>CACCCCTGGGAGTCCCTCAGGACATGCCTTCCCCTGGGCTGGCAG<br>GGCGGCGTGGGCAGTGCGCATCCCCGGGCACCTCCCGGTAACTCT<br>CACTAGCAGTGAGTCATGCTCCCAGGGTGGCTCTGGGGAAAGGTA<br>ACCCGGCTCTTGCTGGCCTGTGATTCCCGGCGCCGAGCGCCGCGGC<br>TGCAGGGCGAGCGCGCGCGCGCACGGGTTCCCCGAGCAGTGATGT<br>GGCAGCAGCGGCAGGTCGGATCACGTTGCTGGCCCTGTGCTGGTA<br>CCCGTTTGAGACCGGATTAAGGAGGGAGGCTTTGGAGGTGCCAGC<br>GGTAGTAAACGTGGGCTCTGCACACTCCGGGCGTCTCTTATCGCGC<br>GGGGGGCGGGGGGATGGGGAAAGCACGTAGCGTCACCGGAGGC<br>TCAGAACGCGGCAGAGTTGAGCGGGCGGGTGATAAATGCTGCATA<br>GCTTTTCTGTGGTAGCCCCTCTCGTGCCACCCCCTCTCGTGCCACC<br>CCCTTCTCCCTTCCCAGCCCCCACTCAACCCCGGCTCGCTCCCCGC<br>CGCGTTCCTCCAGGAGCACACGCGGCCCCAAACGTCGTCATCTGCT<br>GCTGTGATTAGTGGCACCGCAATTCCGGCTGCTGCGGGGAAGTTTA<br>GTTGGCCCAGGGACGCCGTGTCACCGAAAGACACTTGGATTGCGA<br>CATTCGGACCCAGTGACAACGTTTTCATGTATCTTAAATCCTTCAAG<br>GAGCGGATTGCAAGTTGCTTCTTCTCGAGGCAACCTCTCCACCCAG<br>CGCCAAGGAGTCCCTCCAGGCCACTGATCAGTGAGCAAACAAGTC<br>TCCAAAATTTCCTTTGGGACTCAAGTTTACCCACCGCGAATTCTCTT<br>TTCACTTCCTTTCTATGGTTCAAACCAACAGCACAATCGGTGGAAA<br>AGCAAAAAGCCACGTTCACATTCCTGCACCGTAACAACGGACGAA<br>CCCAGGGAGTCGCATTTTGGAGCCAAGGAAGCTCAGGCTGCCCCG<br>CACCCCTGCACCCCCACTCCCCCCTCCTCCTGCCGACTGCATCGCC<br>TTTGGGAAGATGAATAGCTTGGCCGAAGTTGTTTTTCCTTTCCGGT<br>GGCCCTGATCAGGTGTCCCCGCCCCCTCCTGCCGTTCGCCTTCTCT<br>CGCCGCCTTCTCCCGACGCCCCCGCCCGGCCCGGCCCCTCCTCCCC<br>CGCCCCCCTCCGGGCTGGACCGCGGATGGTACGTTCCGCACGTGA<br>GCTGGGTGCTGGTCTGGCCGGCGACGCGCGTGCCCTGTGGCCAAA<br>CACTGCCTGGAGTGAGAGCAAACTACCAGCGCAGTGGGGCCGGCG<br>CGAGTGTGCGTGTGTGTGCGTGTGTGTGTGCGAGCGCGGTGGAGG<br>GGGGGGACCAACTGCTTCACACTTTCAACACTGCACTGAAGAGGG<br>AGAGCGAGAG
BRCA1	CGGGGTCTCGAAAAAAGGAGAATGGGATGAGAAGGATATATGGGT

	AGTGTCATTTTTTAACTTGCAGATTTCATCCTAGTCTTCCAGTTATCG<br>TTTCCTAGCACTCCATGTTCCCAAGATAGTGTCACCACCCCAAGGA<br>CTCTCTCTCATTTTCTTTGCCTGGGCCCTCTTTCTACTGAGGAGTCG<br>TGGCCTTCCATCAGTAGAAGCCGGATGTTCTTGTGTCCGAAATTGG<br>TGGGTTCTTGGTCTCACTGACTTCAAGAATGAAGTTGCGGACCCTC<br>ACGGTGAGTGGTACAGTTCTTAAAGATGATGTGTCCAGAGTTTGTT<br>CCTTCTGATGTTCGGACGTGTTCAGAGTTACCTCCTTCTGGTGGATT<br>CGTGGTCTCGCTGGCTTCAGGAGTGAAGCTGCAGACCTTTGCGGT<br>GAGTGTTACAGCTCTTAAGGCGGCATGTCTGGAGTTTGTTCGTTCC<br>TCCCGTCTGGAGTTGTTCATTCCTCCTGGTGGGTTCGTGGTCTCGCT<br>GGCTTCAGGAGTGAAGCTGCAGACCTCTGCGGTCGGTGTTACCAG<br>CAGATAAATGCTATGCGGACCCAAAGAGTGAGCAGCAGCAAGATTT<br>ATTGCAAAGAGCACAAGAACAAAGCTTCCACAGCGTGGAAGGAG<br>ACCAGAGCGGGTTGCTGCTGCTGGCTCAGGCAGCCTGCATTTTTTT<br>TTTTTTTTTTTTTTTTTTGAGATGGAGTCTCCCTCTGTCACCCAGG<br>CTGGAATGCAGTGGTGCAATCTGGGCTCACTGCAAGCTCCGCCTCC<br>CGGGTTCACGCCATTCTCCTGCCTCAACCTCCCCAGTAGAGGGGAT<br>TACAGGCACCCACCACCGCACCCAGCTAATATTTTGTCTTTTTAGTA<br>GAGTCGGGGTTTCACTGTGTTAGCCAGGATGGTCTCGATCTCTTGA<br>CCTCGTGATCCACCCCTCTAGGCCTCCCAAATTGCTGGGATTACAG<br>GTGTGAGCCACTGGCACCCAGCGGGGCAGCCTGCTTTTATTCCCTT<br>ATCTGACCCCACCCACATCCTGTTGATTGGTCCATTTTACAGAGAGC<br>TAATTGGTCCGTTTTGACAGGGTGCTGATTGGTGCATTTACAATCCC<br>TGAGCTAGATATACACAGAGTGCTGATTGGTGCATTTACAATCCTCT<br>AGCTAGACATAAAAATTCTCCAAGTCCCCACTACATTTGCTAGACA<br>CAGAGCACTGATTGGTGCGTTTACAAACCTTTAGCTAGACACAGAG<br>TGCTGATTGGTGCATTTGCAAACCTTGAGCTAGACACAGAGCACTG<br>ATTGGTGCATTTACAATCCTTTAGCTAGACACAGAAGTTCTCCAAGT<br>GCCCACCAGATTAGCTAGATACAGAGTGCTGATTGGTGCATCCCCA<br>AACCCCAAGCTAGACACAGAGTGCTGACTGGTGCATATAAAATCCT<br>CAGGCTAGACATAAAAGTTTTCCAAGTCCCCATCTGACTCAGGAGC<br>CCAGCTGGCTTCACCTAGTGGATCCTGCGCAGGGCTGTGCCGGGC<br>GCCTGCACTCCTCTCAGCCCTTGGGCAGTCGATGGGACCGGGCGCT<br>GAGGAGCAGGGGGCGGTGCCCGTCGGGGAGGCTCAGGCCACGCT<br>GGAGCTCACAGGGGTTGGGAGGGGGCTCGGGCATGGCGGGCTGC<br>AGGTCCTGAGCCTTGCCCTGTGCAGGGCGGCTGGGGCCCGGTGAG<br>AATTCAAGCGGGGTGCAGGCGGGCCGGCAGTGCTGGGGGACCCG<br>GCGCACCCTCTGCAGCTGCTGGCCCGGGTGCTAGGCCCCTGACTG<br>CCCGGGGCCGGGGGTGCGGGGCCCGCTGAGCCCGCGCCCACCTGG<br>AACTCGCGCTGGCTGGCGAGCGCTGCGCGCAGCCCCAGTTCCCAC<br>ACCCGCCTCTCCCTCCACACTTCCCCGCAAGCAGAGGGAGCCGGC<br>TCTGGCTTCGGCCAGCCCAGAGAGGGGCCCCCACAGCGCAGTGGC<br>GGGCTGAAGGGCTCCTCCAGCACGGCCAGAATGGACGCCAAGGCC<br>GAGGAGGCGCCGAGAGCGAGCGAGGGCTGCTAGCACGTTGTCAC<br>CTCGCATTCTGAACCACAGACTCTCCAACTCTCCGGCGCTTTTCGC<br>CCACTCGGTCCCTCAGAACACGAAGGGCTCTCTCATCCTGTCACTA

	AAACGATTAGCTGTCCGGAGACACGGAAAAAGTCGCCCCTCTTCT<br>TTGCAGGATTCCTCCCTTGAACTTCTCCAAACCCTCTTAGTGTGAC<br>GTGACCCCACCCCTAGCTAACCCAGGCTGCTTCCTTACCAGCTTCC<br>CGCCCCCTGGGGAGGCGGCAATGCAAAGACCGTCCGCTGCCAGCT<br>CTGCCGCTATCTCTGTGGGGTGAATCTAACATGGCGGACAAAGACA<br>GTAACTAGTCCCGTTTCTCCGCGTTTTCGCCAAGAAGATTGGCTCTT<br>ACCACTTGTCCCTCAAAACGACCACCCCATTGACTGGTGGCGATTG<br>CGTCGACGGAGACGGGGCAAAAGCAAGCTGAACCCGAAAAATAA<br>CAAACACTGGGGCTGAGGGGTGGAACTACGAGTGCGCAGACATGG<br>GCCAGAGCGCATTTCCCCTGCCCCAGGCAAATTCGGCGCTCACTGC<br>GTCCCCGCAGGCCACTGACCTTACAAGACTACTTGCCCCAGACTCC<br>TGGGGCTGGATGGGAATTGTAGTCTCCCTAAAGAGTTGTACGTATC<br>TTTTTAAGGCCTAGTTTCTGCTTTCAAAATACGAAAACATAACACTC<br>CAGTCCATAACTGTTGACAAGTACAAGCGCGCACAGGTCTCCAATC<br>TATCCACTGGATTTCCGTGAGAATTGTGCCCGCTCTGGTATTGGATG<br>TTCCTCTCCATAAGACTACAGTTTCTAAGGAACACTGTGGCGAAGA<br>CCTTTCATTCCGCAACGCATGCTGGAAATAATTATTTCCCTCCACCC<br>CCCCAACAATCCTTATTACTTATATTTACCGAAACTGGAGACCTCCA<br>TTAGGGCGGAAAGAGTGGGGGATTGGGACCTCTTCTTACGACTGCT<br>TTGGACAATAGGTAGCGATTCTGACCTTCGTACAGCAATTACTGTGA<br>TGCAATAAGCCGCAACTGGAAGAGTAGAGGCTAGAGGGCAGGCAC<br>TTTATGGCAAACTCAGGTAGAATTCTTCCTCTTCCGTCTCTTTCCTT<br>TTACGTCATCCGGGGGCAGACTGGGTGGCCAATCCAGAGCCCCGA<br>GAGACGCTTGGCTCTTTCTGTCCCTCCCATCCTCTGATTGTACCTTG<br>ATTTCGTATTCTGAGAGGCTGCTGCTTAGCGGTAGCCCCTTGGTTTC<br>CGTGGCAACGGAAAAGCGCGGGAATTACAGATAAATTAAAACTGC<br>GACTGCGCGGCGTGAGC
C3	AGATAGATTGATTCAGTCAGTCAGGTCAAGGTTAACTTGAATTAATC<br>AGTAATAGGGTGGAAGAAGGGGATGGCCTTGCTGTGGGTTCTGGA<br>GAAAAATTCTAGGAAAGCAGCCACCTCAGCCTGGAATTAGACGAT<br>GGGATAGGGGTTTCCCAGCTGCTCCCAGGCCTGGCTGCCCCTTTGT<br>TGGGGAAGGGGAGGGATGGGATATAGGGGACAGTGAGTGAACTCA<br>GGCAGGTGTGAGCCGGGGGCATCTGGGTCCCCCACCCAGAAATCA<br>TTCCCACTTCCTTCCTCTTATTTCTTTCTTTTTCCTGTCTTGCTCTG<br>TCATTCAGGCTGGGGGGCAGTGGTGCAGTCATAGCTCAGTGCAGC<br>CTCTAACTCCTCCTGCCTCAGCCTCCCGAGGAGCTGGGACTGCAGG<br>CACGCCACCATGCCCTGCTAATTTTTTTTTTTTTTTCAATTGTAGAG<br>ACGAAGTCTCACTGTATTTCTCAGGCTGGTCTCGAACTCCTGGACT<br>CAAACAATGCTCTCACCTCGGCCTCCCGAAAGTGCTGGGATTACAA<br>GCACGAGCCACCGCACCCTGGCCCCTTCTCATTTTCCCCTTGCACC<br>CCAGCTAGGATTGCCAAACAGAATACAGGACGCTCAGTTACATTTG<br>AATTTCAGATAAATAACAACTACTTTTTCAGTATATGTAGCTTCCAGA<br>TAACCCACGAATGGTCAGCCCGGTTGGCCACACTCTCCCTCCTTGA<br>TTCCGGGAATGCTGGGCTGGGTGGGCCTCAAAATGGAAAGTACCC<br>CAACACACACCCAGACCTCCTTCTCTCCCTCCCCTGCTGGCTCATC<br>CTTGTGCACTATCCCCCTCCCAAACCTCTGGACACCAATGCACATC

	TCCCAGAAAAAAGTCACGAGGTTCTGAAGAATTCCCGGTCTCATCT<br>CCCTCCCTCCTTCCCTCCCAGTAGGCTACCATCTGCTCCAGCCTCCA<br>ACCCCCTCACTTCTCATCCTGCCCCTCCCCTCTGGTCACTTCTTGGA<br>GGTCAGGGTAGGGCCAGACCCTTTCCAGGTTCAAGTGATTCTCCTG<br>CTTCAGCCTCCCGAGTAGCTGGGATTATAGGCACCTGCCACCATGC<br>TCAGCTAATTCTTTGTTGTTGTTGTTTGTTTTGTTTTGTTTTGAGACA<br>GAGTCTCGCTCTTGTCGCCCAGGCTAGAGTGCAGTGGCACGATCTT<br>GGCTCACTGCAACCTCCGCCTCCCAGGTTCAAGTAATTCTCCTGCC<br>TCGGCCTCCCCAGTAGCTGGGATTACAGGTGCCCGCCACCAATCCT<br>AGTTAATTTTTGTATTTTTAGTAGAGATGGGGTTTCACCATGTTGGC<br>CAGGCTGGTCTTGAACTCCTGACCTCAGGTGATCCACCCATCTCGG<br>CCTTCCAAAGTGCTGGGATGACAGGTGTGAGCCACCATGCCTAGCC<br>AGCTAATTTTTGTATTTTTTAGTAGAAACAGGGTTTCACCATGTTAG<br>CCAGGCTGGTCTCGAACCCCCGACCTCCAGCGATCCCCCAGCCTCA<br>GCCTCCCAAAGTGCTGGGATGACAGGCGTGAGCCACCACACCTGG<br>CCCCTCTGAGCCTGGTGGCTTCTAGGCATCCTGGTTTCTTTAATTGT<br>CACAACAACCAGAACTATCTTCAGTCGCATTGTTTAGTTGGATTAA<br>CCGAGGCTCAGAGAAAAGAGGAACCCAGGCTTGCCGCTAGACAG<br>AGGCCAGACAGGAATTCCTTCTCAAGGTTGTCAAACCACAGTGCC<br>GAATGCTTGAGTCTAGAATGAAACCAGGAAATGGGGTGGCTTGAG<br>GAGAAAGTGGGGGATAGAAGATGGAATGGGGCAATTGGGAGATCC<br>AGTTTCTTTCCTTTTTTTAATTTTTTTTTTTTTTTTTGGCAACAGGGT<br>CTCTCTCTGTCACCCAGGCTGGAGTGCAGTGGTGCAATCTCAGCTC<br>ACTGCAACCTCTGCCTCCCGGCTTCAAGCGATTCTCCTGCCTCAGC<br>CTCCTGAGTAGCTGGGATTACAGGCACCCACCACCACGCCTGGCTA<br>ATTTTTGTACTTTCAGTAAAGACGGGGTTTCACCATGTTGGCCAGG<br>CTGGTCTCCAACTCCTGGCCTCAAGTGATCTGCCTGCCTCGGCCTC<br>CCAAAGTGCTGGGATTACAGACGTGAGCCACTGCGCCTGGCAAGG<br>GGATGCAGTTTCAAAAGCTGAACCCCAATTCTGGAGAGCAAGCAG<br>GTATTTTCATTCTCTCTCCTCCTCCTCCTCCTCTTCCAAAGAGTGTG<br>TCGCAATCAGTGCAGACAGACGCCAGGTTTGTTCTCATGCTCCACG<br>CCTCCCCCTACCCCTGGCACGGAAAAGAATGTGGTTTACAGGAAAT<br>CAGAGAAAACTCCCCATTAACCCCTTCAGTGGGGTTTCAGAAACC<br>GCCTCTCCAGGGATAAGGGGGCCCCACCCACAGACCCTTCTCCTGC<br>CCTCACCATCCACCTCGTATGCCTGGGCAGCAATGCTGCAGAACGT<br>CAGAGGAATGCCAGTTAAAATGACACCGGCTGCCGGGGTGTGGTG<br>GCTCACTTCTATAATCCCAGCACTCTGGGAGGCCGAGGTGGGCGGA<br>TCACCTGAGGTCAGGAGTTTGAGACCAGCCTGGCCAACATGGCGA<br>AACCCTTTCTCTACTAAAAATACAAAAAATAAAAAATAAAAGAAAA<br>AAAAAATTAGCCAGGTGTAGTGGCGCATGCCTGTGATCCCAGCTCT<br>TTGGGAGGCTGAGGCAGGAGAATCACTTGAACCCAGGAGGCAGA<br>GGTTGCAGTGAGCTGAGATGGCGCCACTGCACTCCACCCTGGGTG<br>ACAGCACAAGACTCCATTTAAAAAAACAAAACAAAACAAAAAAA<br>ATGACACCAGGGTACCAGTTTTCACCCATAAGGCTGGCAAAAATCT<br>TCAAGTTCATCAACATGCCCTTGTGATGAGGCTGTGGAAGAAACTG<br>ACAATTCATTTCATGCAGGGCTCATAAGTGTGTAAATCAATACAACT

	TCTGTGCAGGGGAATTTGGCAATATCTAGCAAGATTACCAGTGCATTCAGAGATTGACCCAACATATTTCCTTTCATTGCAACGACAACTCTATGAAGCAGGTGGTAAGGGTTTCCTTTTCCATGAACAAACTGAGGCTCAGGGCGGTAATCAGTAGCTTACCCAAAGATCACAGCTAGTTTCAGAGCTAGAAAATAACGCAGGTTCAAGCTTATTCACTGCAGAGAGCCTGGTGTGAAGCCACAGATGTCAGTCTCTCCATCAAGAAGAGGCTGGTGGCTGGACACAGCGGCTCACGCCTGTAATTCCAACACTTTGGGAGGCCAAGGTAGGTGGGTCACTTGAAGTCAGGAGTTCAAGACCAGCCTGGCCAACATGGTGAAACCCCTTGTCCACTAAAAATACAAAAATTGCCAGACGTGGTGGTGCTCACCTATAATTTCAGCCATTCCGGAGGCTGAGGCAGGAGAATTGTTTGAACCCAGGAGGTGGAGGGTGCAGTGAGCTGAGATAGCGCCACTGCCCTCCAGCCTGGGTGACAGGGCAAGACTCTAAAAAAAAAACACTCAAACAAACAAAATATCCCCCAAAAAGTAGGAGGCTGGTTACTTTCTCACAATATAACAAGAGGCCTGTAACCTGTAAGAATGAGGCAGTTCTTTGCTCACTGAGGTGAAATAGCCTCTGAGGTATATTGTTCATGAAAAAACGAAACAAAACGAAACCCAAGATTTAACTGAAGAGACCAGGAAGAATAGTATGTGCTATGTGCTGTCCACAGGGCACAGTAGTTCACACCAGCACTTTGTGAGGCTGCTGCGGGAGGATCACTTGAGCCCAGGAGTTCAAGACTGGACTGGGCAACATCGTGGGACCCCCATCTCCACAAAAATAAAAAAATTATCCGGGCATGGTGGCGGCCACCCGTAGTCCCGGCTACTTGGGTGGTTGAGCCAGGATGATCACTTGACCCCAGGAGGTTGAGGCTGCAGTGAGCTGTGATTGCACCACTGCAATTTAGCCTGAGTGACAGAATGAAAAAAAAATTTTTTTAAAGGAAAACACAAAAAGAATATGCTGTCAACAGGGATGGGAGGAAGACCACCTTTACTGCTATACACATTTGTACCTTTTAGATGTTGATCAATATGAATATATTATACACACAGACACACACACACAGACACACACACACACACACAAACAATACAATTTAATATCCTAAGAGGATATTGACATTAGACAGGTACAAAAGCTCTAGAAATGAGGACTTTCCTCAGTGATGACTTTTTTCACCACCAAAGTCACTCAGGCATCCTGACAAGGGTAAGTGAGGGGAGCCTCCTTGGAAAATAAACTCACTTGGATAGTGAACTCCTGCACATACCTCAAAGCCCATCTGAAATGTCCCCTCCTACAGGAAGTTTTCCCTGACCCTCCAAGAAGCAGAGTTCTATTTCACTGGGGAAAACATTTCTTCTTCTTCTTTTTTTTCCCTGCCCTGCACATGAGCTAGAAAACATTTCATGAAACTGGGAGTTTCTGTGCTGGGCTCTGTCCCTCCCCCATTCTACTTCCCCTCCCTCAGCATGGAAGCCTCTGGAAGTGGGGCTCTGACTCCCAGCCTACAGAGAGATTCCTAGGAAGTGTTCGACTGATAAACGCATGGCCAAAAGTGAACTGGGGATGAGGTCCAAGACATCTGCGGTGGGGGGTTCTCCAGACCTTAGTGTTCTTCCACTACAAAGTGGGTCCAACAGAGAAAGGTCTGTGTTCACCAGGTGGCCCTGACCCTGGGAGAGTCCAGGGCAGGGTGCAGCTGCATTCATGCTGCTGGGGAACATGCCCTCAGGTTACTCACCCCATGGACATGTTGGCCCCAGGGACTGAAAAGCTTAGGAAATGGTATTGAGAAATCTGGGGCAGCCCCAAAAGGGGAGAGGCCATGGGGAGAAGGGGGGGCTGAGTGGGGGAAAGGCAGGAGCCAGATAAAAAGCCAGCTCCAGCAGGCGCTGCTCACTCCTCCCCATCCTCTCCCTCTGTC
C4BPA	AGCAATGTGAGCAAAGCAGAGTGGCTATAAATACAGCTGAAGCTTT

	GCTTGCCTGCCCGCTGCTCACTTTCTGCTGTGTGGCCCAGTTCCTAA<br>TAGTCCGTGGACCGGTACCGGTCTGTGGCCCTGGGGTTGGGGATCC<br>CTGTTATAGAGCAAAGCACATAAGCACAATGCGTGAGAATTGGAAA<br>AGTCTCTAAAATTCTTCTAGGCTCCAAAACTAAACAAACAACACTG<br>AACAGATCCATTTTCATTGAGATTCTTCTTTTAGTTCATATTTATTACT<br>GGGATAATATTAATAAGTATATTTAAGCCAGAAAATATAAATGATTTG<br>CCATGTTGAAAACCCAAATCTTTACTTAAGCTAGCCTGGTTCTGAG<br>CCCCCATTCAGAGAGCTAACTGCTGCTCACTGGCTTCAGAGTATATA<br>GCTGGGTCTCAGTCCTTATGTTGTAACTTGTGAAAAATTTTCTTTCT<br>TCAGCTTGCCTTTCAGGAGAGTAAGAACCTCTGCGAAGCCATGGA<br>GAACTTTATGCAACAATTAAAGGAAAGTGGCATGACAATGGAGGA<br>GCTAAAATATTCTCTGGAGCTGAAGAAAGCTGAGTTGAAGGCAAA<br>ATTGTTGTAACACTACAGCTGAGCAGATGTAATAGAAATAAACCTAT<br>GAATAAATTTTCTTCTTGGTTCTGAAATTGGTTTCAGATTTACCTCTT<br>ATTGGGCTGAAATTGCCAACAGCAGTTGTATAATGCACCATGTGTG<br>TGTGTGTGTGTGTGTGTGTGTGTGTGTGTGTGTATGTGTGTGTGTGT<br>GTGTGTGTGTGTTGTTCTCAGAATGTGAAAAGGCCAAATGTAGTGG<br>TTGTCTAAGGCCAGAATTAGCAACATATTATTGGAGAATAGAGGGC<br>TTAATTTCCAGTCTGTAAGGAGAATGGAAAACTCTGATCTCAGCCG<br>AGAGAGTAACTCGTATTTTAACATGAGACCATGAATTATGAAACCA<br>AAACTAACCGCAACTTATGAGGCAAAAATTAACCTTCCATTTTGGA<br>AAAGATGCTTAGAATAGTTACATCCTTAAAAATGACCCACTGGGAC<br>AGAAGGAGAGCTAGGACCCAAATTCTCCTCCCAGTCTTGCCCACAT<br>CTGGCTTACGGAAACAGGTTTCCAAAGCTGCAGGAAAATTGCTTAC<br>TGCCTCTTGAATGAAACCTGGTCCTGCATGATAAATTAGAGATGGT<br>GCAACACAAGTGAATATATTTCTTCTCTTTTTTAGAAACAAAAATTT<br>TATATTATTTTTTACTTACAATTCTCCTTTTTTAGAGGGTAGAAGAAA<br>TAAGAATCCTAAGGCTTTCAATCAAAATGTCATACTGAGCTGGCAT<br>GACAAGCTTCTCCCACCTCTCGTCCTGACTGCTACCTGAAACACGT<br>AGGATATCTAGATAAAGTGCAACCAAAATATATAAGCCAGCTTGAAA<br>TAATGGAAAATCGCTAGGTAAAGTTATGAAGGGGAATAAGAGATGG<br>ACTCTTGAGTACCCCAGAGTCTACTAATAGGCACAAGACATGTACA<br>CACGTAGCATTATTTTAGAGTGGTTAGAATGAACTAAACATTTTCAG<br>ACAACAGTGGGCTATGAGAGCTCAGATGAGAGGAGGTTCACTTCT<br>GTCTGGGAGGAGGAGGCAGGAATATTGTCAAGGAAGAAGTCACAG<br>TTATTTGGGCCTTCAAAAGTAAGTGAGAATTAGAGACAAAGATTAA<br>AGAGCTCATTCCAGTTGGAAAGAATATTTGAGAAATGGAGTAAGAC<br>AATAATAAGATTTAAAACCAATTTTTTTTTTTGAGATGGAGTTTCGC<br>TCTTGTTGCCCAGGCTAGAGTGCAATGGCGTGATCTCAGCTCACCA<br>CAACCTCTGCCTCCTGGGTTCAAGCGATTCTCCTGCCTCTGCCTCCT<br>GAGTAGCTGGGATTACAGGCATGCGCCACCATGCCCGGCTAATTTT<br>GTATTTTTAGTAGAGACGGGGTTTCCCTATGTTGGTCAGGCTGTCTC<br>GAACTCCTGACCTCAGGTGATCTGCCCGCCTCGGCCTCCCAAAGTG<br>CTGGGATTACAAGCATGAGCTACCACGCCTGGCCAATAATAAGATT<br>TTTAACCAAGTAAATTCATTCCTAGGATTAATCTAACTGGAGGAAAA<br>TGATTTGAAATTGAAGCTACTTCTTATTTGTACATATTATCTATCTAG

	GAGGATCTTATGAGCATTTGAAATGTCTACTCTTAGGAAAATTTTGA<br>ATAAATTATGGTACTTTAAATGTAATGCAGACCTATATGGTGATAAAT<br>ATGAGGAAGTAGAAAATCTTAAAAATACAATCTTAAGTAAAAATAG<br>AATATTTAATAACAAGGGCTTATGATTGCATTTATGTAAAACCTTTAT<br>TCTTGTGGGAAAGGATTAGACAATAATAGGCACAGATAAAAACATT<br>TGTCTTAGAATATGAAATTATAAGTGTTTATTCTCTTAAAACTTTTTA<br>ATGTTGTAAAATGAATTTCTAATAATAATTTTAGAAGGCATTGCACA<br>GAGGCAGAAAAAATGGGATATGTTTAGGAACTTGCACATAGCCTGG<br>ACTGGCCTAGATGAAGCATGGAAATTGGTGAGAGAGAAGGTGGCT<br>GGAAAGATAAGTCAAAGCCAGTTTATGGAGGCTTTTGAATGACGTG<br>CTAGAGAATATACACTTGTTTTTGGTAGGCAGAGAATATACATTTGT<br>TTTTTGGCAGTCAGACCATGAAGACATGGAAGCCTTGCAAACAATG<br>AAGTAATAAGGACAGAACATAAAGAAAGATTTCTCTCACAATGTTG<br>GTTGTAGTATTGGTTGATGAGGGAAAAGAACTAAGAGAAACCAAC<br>GAAAGGACTCCTACAATAGTCCATATGGGAGGCATTATCAACCTGA<br>ACTGGTTTGGTGGCAGCAGAAATGAAAAAGCAGCAGCAGACACG<br>AGAGATATTGAAAACTTGTATCAAAACAACCTGTCAACTAATCAAA<br>CATTGAAGGGTGAGGGAGAGGAAGAGTTCAAAAGTGAATCTGAAA<br>TTTTATTCCCGAGAACCAGAGGTCAGAAAAAGCTGGGGTCAGAAA<br>GTGAAGCTTGTTGGCCGGAAATGAGAAATTCCTCTTTGGATGTGCT<br>GAGGGTGAAGTGCTTGCAAGATGTCCAGTGGGGGGAAAAAAAAA<br>ACAAAACCTAAGAAAGTAAGTCTTGAGTTTCGGAGAGTAATCAGA<br>AATAATAATGTAGTGCTGGCTAGTCTTCTTACTGGTAATAGTTAAAG<br>CTGTGACAGAAGGCAAGATCATTAAAATTGCGAAGATAATTTTTTT<br>AAAAAAAGAAGAGCCTGAGATATATCTATATTTAGGAATGAGAAGG<br>AGGAAGAGAAGGTAACAGTAACTAAGAAAGAGAAATTAGTTAAGT<br>TAAGGGAATCAGGTAAGTTTAGTGTCATGGAAGCCAAAGGAGAAG<br>AGAGTTTCAGAAAGGAAATGATCAGCAGTGACAAAGGCTGCAGAC<br>AGATCCTAAGAAATGAATTAAAGATATTTTGGACTTGGACCTAAGG<br>GCGTGCCTCAGAGAGACATGTCAGTAGAAGAGTGGTAGAAGCCAG<br>ATGGGAAAGCAACAGGTGGAGTGATGAATGAGAAAGAACAGGCA<br>GGTAGAACAAGTCACACCTTTGAGAAGTTCAGCATCACACTAAAG<br>AAGAAAGGTGGAATTTGAGGTATTGGCAAAATTGAAGGGACATTT<br>CAGGATGAGAGATGCATTGAAAATGAGGCAGGAAAAGAAGAAAC<br>AATTCTCCTCGGAATGAGGAGTGAATAGGAGGGAAAATTTGACATT<br>GATGAAGCTAAGGTAGAATGATTTTTAGTAAAGTAGGAGGTAAGAG<br>CATCTGCTGAGAGGGAATGTGACAATTTGGTGACCTGGAGAGAAA<br>AATCTGCAAGAGTCACTGTGGAAAGTAAACGAAAGGACTTCAATA<br>CATTGGAAAACGGTACTTTAAAGAAATAGCAGCTTTAAAGGTGATA<br>TTTCTTCCAATTTAGTCTCAAGACTGTGCAATCTGGAATCCTCCTCT<br>ATCTTCATTCTGGTCAAATCAGTGTCTCACCTTGGTGACCTAGCGCA<br>TGGTTTCTTTACATCTAATATCCTTACCACTTTTATCTAATAATTTCTA<br>CCTTTTATCCAAATCTCACATCTTCTGTAGAAACTTCCTTGATTAACT<br>CAACCTGTTGTAGTTTAGTTCCTTTTCTGAAACTCTTCTGGGACTTT<br>CAGTATTTATTACAGAAAAAGTGAAGTTCTCAGAAAGAATTGCCTT<br>TTAATAGCAATCTTCTGAGCACACAAATCTTAACCTTGAGGATTAGT

	AGCTTAATTGGACTATGTAAGTGCTTGTGGTCTTTTTAGATCTATGC<br>GAGAGGAAACATGGCCGGTCCTTCATTGCGTTCTTCTGAGCGCACT<br>GGATAGATCAATTTTTGGAGGCCCTCTGGTGCCTGACATGTAGTAG<br>CAGTTAACATTTGCTGAACTCCAAATACGTTCTAGATAACTCAGCAC<br>ATAGTGGAAACCCAATGCATGTTTGATAAATTCCCCACCTTTTGTAA<br>TTCCTTGGGGTGGGATAGAGACGAGCTCTGGGCAAGAATATCAGTT<br>TTCAAACTCCAGGAAAGCATACTGGACCTAGTGTCATGGAAAGTG<br>GATCATTTTTGCGGAGGCTCTCTGAACCTCGCAGTATCCAAGGTTT<br>CCAGGCTAGCTGGACTGTGTCTTGCCATAACCCTTATGTGTTTATGA<br>AAAGAAAAAAGAATTCTGGCTTCAAATTCAAATTACCTTTCCACTT<br>AGGGGAAATGGTTGGCAGAGGAGAAAATAAACGATTGCGACATGT<br>TACGAAGAATGAGGACTAGCAAAGAGGAAGGAGCTTAGGTAAACA<br>GTGCTGCTTTATTTCTGCTGTTAATCATTCATTGGGCCCGTCAAAAG<br>TTTCTGCCCATCTATTTCCATCAACCGTCCTTGACCAGCCAACCACA<br>TGGCTGAAATTCAGGGACTCTTTGGTGGAGCAATTACCAGTCAACT<br>TCAGGGTATTATGATAAACTCTGATCTGGGGAGGAACC
CASP2	CTATCGTACTCTCCTTCCGGCAACCAAGAGACTTAAGCTACAAAGG<br>TCCGCCTCTCCCAGACTCTCCCCTTACACCTCAGGCCCATACCTTTT<br>GGCCCCGTGCCTCTTAGCCTACACCTTCCCATTCCCACAACTCACTC<br>CTTAACAAACCCCCTTTCCTATCACACACACCAATGACCCTTGGCC<br>AATTTCCTTCTATTCATATGTTAACTGTAGACTGCAGTCCTGATTCCA<br>GCTGTACTTTCCCCTTATGAGCAGAACAGTGAGGAAATTGGCTGAA<br>ACAGAACAACAATCAGCCCCCCTCACTCCTGTGCCTACTTTGCATA<br>GAGACACACCTCATTTAGACTCCCACAGCCTTCCTGTTAATGTCTCC<br>TTCCTCTCTCTGCTATCGATCTGGGGCTCTGGGACAGAGAATCTTCA<br>ATATCCCACCAGTGCCCACACTTATTACCACTACAGACACAGTTATT<br>CTCATTCCCATACTCTCTCTCTCTCTCTCTCACACACACACACACAC<br>ACACACCCTCTCCTATGCTTCCCCACCCCAGCTGTCCCTGGGGAGC<br>CTGGATTGGATTTGTGCCCACGGTCTGCAGCTTCCCTCGGTCTGTCT<br>GCTGGTCACCCTCTGAAGGAACGGGGCCCAGTTTTGTTCACCCTCT<br>GGCTCAAGGACCAAGTATGCTTCTACTCACTCTCTACCTCCAAGCA<br>CCTACCCTACTCCGACTTAACTGCCTGACCCACAACTAAAAAGAAC<br>AGCTCTTTGATCTTCACAGGTGTGGTGAGGCCTGGTGAGAGGGGA<br>GCCTTCTAGTTAGTGACCTACCACGTTTCAGGAACCATGTTAGTTTC<br>CAGGATTCAGAGATGATTAAAACTAATCACGCACCTTGAGAAACTC<br>ACAGTGGAAGGAAAATCAGTAAGCAGAAAAATGCTACATAACCAG<br>GTAAATGCTGGGACAAATTTAGGAAGGAACGATGTGGATACACTGG<br>AGCAGGCTCCAAAGCAGCATCACCTCGGAGGAGGATCTTGAAGAC<br>TCAAGAGTTAACTGGGTCAAGAAATATACACACTAACACTTCTGAC<br>AGGTCTGCAGAATACAGGGAGTCCATAAAAATAGCACTTGGGTAAT<br>GGAAGCAGGACACACCATTCCATGCCCACAGCATTACTTTCTGTGT<br>GCAGTGCACTGTGTGATGATCTACAGAGATTAAATAAGCCGAGGTC<br>TCCGCTTCCAGTCACTTGGGAGAGACGCACAAGGTAAATTACCCA<br>AATAGAAGGCAAACTGCCCAGGGCTGTAATTAACATACAATATGCG<br>CCGAGAGGGAAAGGTTAATGCTAACTCCGGTCCTAAGTACAAAGAT<br>CATTGGGAGTCAGGGGTGCACTCTCATTAGGGTACAGCATGGGTGG

	GTGGAGAGAGTAAAAACCAGAACAAATCAAGGTTCAGCAGTTGG<br>AAGTCAGCCTTAGGGAGAAAAGGGGCGGAGACTGACTGGGCCAG<br>AACAGGGATTGGGACAGAAAAAGAGGGTGGGGCCAGTGGGCTCC<br>ACCTACCCGCTCCGGCCCTTCCCACCACCCCCCACCCCATCTACTTT<br>CTACAGTCTGTGGAGACAGGTGAGCGACGAACTTCTGAGACAGGT<br>GTGGGTGCGAGGGTCGGGAGGGTCATGGGATTGGGACCGAGGTGT<br>GAGGAGGGAATCTGCAATTCCTTGCTACACAGAGCGCTGGCAACT<br>TCTGACAGGCTGTTTCTGGGGTATGGGCTGCCTCGGGTTGTTGCTG<br>TTACAAGGAAAGAAAAGAGTTCCCCTGCCCACCGCCTCCCAGCCA<br>CTGGGCTACCTCCTGGCAGGAAATTTGCAAACTGAGTTTAACAAGT<br>TAGGATCAGCAGAGGGTAGAGGAGGGCCCTGGCAGATGTGGGGTC<br>TAGAAGAGGACAGGAGTTATCAGGGCCTCCGGCCATTGTGCTGGG<br>CCTTTGCCTGTACAATTGTTTCTCAAGCAGTTGTGTCCCTGTGGCTT<br>TGGTGCGCCTGTGTGCACTTTCTCCCTCCACCTGGAGCATGGGCTA<br>ACACCGGAGGAAAGGAAAAGACAGAGTCAGACAGGGTAAGTGGG<br>GCTCCCTCCCCTCTTCTCTCTAACGGGGCTGGATTGAGGGCCTCTG<br>GCTGGGGAGGTGGGGGTGGAGATCCAGTAGGAGCAATAACAGAGG<br>AAGGGCAGGGCCTGCCCCATCACCTGAATTCCAGAGATGCCAGTG<br>TGCACTGAAGCCCCAGGCAGGGCGTGCCCAGGACCGGATCCTGGA<br>TGGTGGTAAGGGACAAAGCTGGAAGGGAGACTACAGGGAAGGAG<br>AAAGGAAAGAAGGCAGAGCCATGACACAGTCAACTTACAGAATG<br>GCTGGGAGTCAAAGCTCCTGGGCTGGTTTCCTGGCTATTCCACACA<br>CTCCAAGACATAGAGAGAATTTCGGAACTGGAGGGGACTCTACAG<br>ATGATTTATTTGAGATTGAAGAACCTAGAAACCAAACAAAGAAATA<br>TCATATGGTTACTTTGGTATCTGACACAGCCATGACACCAATTGCTA<br>GTGTAGACACAATAGCTGTGTGTCTTTTTGCAGGAGCCTGGGGAGG<br>GGCCATGGTGCCAATGCACTTACTGGGGAGACTGGAGAAGCCGCT<br>TCTCCTCCTGTGCTGCGCCTCCTTCCTACTGGGGCTGGCTTTGCTGG<br>GCATAAAGACGGACATCACCCCCGTTGCTTATTTCTTTCTCACATTG<br>GGTGGCTTCTTCTTGTTTGCCTATCTCCTGGTCCGGTTTCTGGAATG<br>GGGGCTTCGGTCCCAGCTCCAATCAATGCAGACTGAGAGCCCAGG<br>GCCCTCAGGCAATGCACGGTGAGTTAAGGGTGGGCATCGTAAGAG<br>GAATACACCTTGGGCCCATCCTCCCTCTCTCCCTGTAGTCTCTGAGG<br>CAGAGAGTCTCTGGTTTCCTGTCATTGGCATCAGCGGGAGGAGGG<br>GTCAGAGGCCAACAGAGGATCTTTTGCCCACAGCAGACAAAAAAA<br>AGGGAAAAAGGGCTTCTTTCATGGAAGCCACCAATGGCCTTATTTC<br>TCTTCCCTCAGGGACAATGAAGCCTTTGAAGTGCCAGTCTATGAAG<br>AGGCCGTGGTGGGACTAGAATCCCAGTGCCGCCCCCAAGAGTTGG<br>ACCAACCACCCCCCTACAGCACTGTTGTGATACCCCCAGCACCTGA<br>GGAGGAACAACCTAGCCATCCAGAGGGGTCCAGGAGAGCCAAAC<br>TGGAACAGAGGCGAATGGCCTCAGAGGGGTCCATGGCCCAGGAAG<br>GAAGCCCTGGAAGAGCTCCAATCAACCTTCGGCTTCGGGGACCAC<br>GGGCTGTGTCCACTGCTCCTGATCTGCAGAGCTTGGCGGCAGTCCC<br>CACATTAGAGCCTCTGACTCCACCCCCTGCCTATGATGTCTGCTTTG<br>GTCACCCTGATGATGATAGTGTTTTTATGAGGACAACTGGGCACC<br>CCCTTAAATGACTCTCCCAAGATTTCTCTTCTCTCCACACCAGACCT

	CGTTCATTTGACTAACATTTTCCAGCGCCTACTATGTGTCAGAAACA<br>AGTGTTTCTGCCTGGACATCATAAATGGGGACTTGGACCCTGAGGA<br>GAGTCAGGCCACGGTAAGCCCTTCCCAGCTGAGATATGGGTGGCAT<br>AATTTGAGTCTTCTGGCAACATTTGGTGACCTACCCCATATCCAATA<br>TTTCCAGCGTTAGATTGAGGATGAGGTAGGGAGGTGATCCAGAGA<br>AGGCGGAGAAGGAAGAAGTAACCTCTGAGTGGCGGCTATTGCTTC<br>TGTTCCAGGTGCTGTTCGAGCTGTTAGAACCCTTAGGCTTGACAGC<br>TTTGTGAGTTATTATTGAAAAATGAGGATTCCAAGAGTCAGAGGAG<br>TTTGATAATGTGCACGAGGGCACACTGCTAGTAAATAACATTAAAAT<br>AACTGGAATGAACTCCTGATCCCAAAGCCTATTGTGTTTTCAACAC<br>AAGGTAATAGGAAGCCAAGCAACTTGCATGTCTCCCAATCCCTGTC<br>TAGGGACAGCTGCTGGTGCAGGATGGAAGACTTCCTATAAACAGA<br>ATGGGCGACCAATTGTGTCTACAGAGGGGGTGGGCTGAGCATGGG<br>CACGCATGTCCCCAGCCTCCTGGACGAGCAAAGTCAGTCAAAGCG<br>CTGGTGATCCCTGCTCCGCGTGCGTAGCAGTGTCTGTGCCTCTCCT<br>GCCCAGGGGCTAGAGAGCAGTCTCCAGTGCAGGGTCCCCATCCTAT<br>CTGAAAACAGTGGAGTCAGTGACCCCAGGTGGAGGGACTTCCAGT<br>TTTAACAATGGGTTGGTATGAAGGCCAGAAGGAGTGAAACATGTG<br>AACTTTCTGGCGAGGAGACTCCCTCTTGAATCATCCATATGTAGCCC<br>CGGGGTCACTTGCGAAGAGTCTCTAACAGCTTCTGCCTCACTCCTA<br>AGCTCTGACCGCTAGGCTTTCACCCCAGCCGCCGGTCCTGATTCCT<br>GAGATCCCAATATTGAGCACCAGGTTTCCTGGAATTGTGTGCTGCG<br>GCTGTGATGTAGGTTTCGGTCGCTGGAAGCTGCTAAACCATAGCTG<br>ACGCCCCCTCCTAAGCCAGCCTTCCTTCCCCGCGCGGGCATCTGTC<br>CAGGGCCTTGTCGCGCTCTCAGTCTCCTTCGCAGCCTGGCCCCAAC<br>CGTTCAACTTCAATAAAGCAAAACTCCAGCCACGCCCGTCTCCGTG<br>AAGTTATCGCCATAGGCCGGCCAGGGGGCGCGAGAGGCACCGGGG<br>TGATTTCCGCGGGAATCGATAACCAATCGGATTCCCAGGCCGAACG<br>GAGCACACCCGCCCGCCCTCGCTCTTTCCCCGCCCTTTGCCCCGCC<br>CCGCTCCTTCCCCTCCTTTTGTCTGTCCGCCGAGCACCCCACTTCAC<br>CCCATTGGACCGCGCGGCCGCCGCTAGAGCTCTGCGCCTGCGCAC<br>GCACCGGGCCGGGGACTGGGTGGCCTGGTGTGTGGGCGCGGCAG<br>GGCGCAGGCGCAGGCGCAGTGTGCGTCCGCGTCTGAGGGGAGGG<br>ATGTGGGGGAAGCGACGGCCCCCGGTTTGTTTGGGC
CCNA2	ATTTTATAGAAAAGGAGAGGGAGTTTTTAAATCTGACAACATTTCTA<br>CTATCTCCATCCTAAAAGATGTGCTTTCTAAAGAAGCTACAAAAAG<br>GAAAATTAACCTCAACATATCATACGGTAAACAAACAAAATTAATAA<br>TTCTTTCTGATGTTTATTTTGTGTTGTTGTTACATTATTAATGTTTCTG<br>ATCAAAGTGATTTATCTAACTGCATCTGGTCAACTCCTTCTAGAGAT<br>AAATGAAGTATCAGTCAAACACACTTTAAAGCTAATCCACCCAAAG<br>CTGGAGTACCAGTTGCTTTTGGCTAAGAAAGTGCAGTTAATTGATG<br>CTTTAAAAGTAAGTACATGTGTCGTCGTGGACTGGTGATTATTATTT<br>GAAGTCATAGGATTTCTAAAATTATCAATACTTTAACATTACATTGGA<br>TTGAATTTTGAATATTAAGGTGCTTCTGTTTTTCATTTTAAATAGGAA<br>TTACAGATTCATGAGGGAAATACGAACTTTCTGATACCAGAATATCA<br>CTGTATTCTAGAAGAGGCAGATCACCTACAGGAAGAATACAAAAA

	GCAACCTGCACATCTTGAAAGACTCTATGGTTAGTGGACCATTCTA
	GAATACCTTGCCATATTTCTTTTCAACTGGCTATTTCCCAAGGATCAT
	GTAAGAGAAGCTGGGCAGAAAGATAAAAAGATTACATCAGTCATG
	ATTCATGAACCAGTCATGAATCAGTTGACAACAAATTTATTGCACA
	GAATATTTCTGTATTTTGTCAAGCTACTTTTAATATTTAATTCTTTTAC
	TTGATAAAATGCAAGTATATTAAGAAATAAGTATACTGTGATGAATT
	AATCTATATATATGAACAAACCTGGTATAAAATGAATGTAATCTATGA
	ACCTTTAGAGCTTAGACTGTATTTCACACAAATAATTGTCATGTTTT
	GTTGCTATTGTGAATTATAAAAATGCAGCATTTAAAATTTTTAAGGC
	AAAATTTAATAAAGGATATACAGTATTTGTGTTGTGTCAATTTATAGT
	TCAAAACTTTGGTGGTGAGAAGCTGAATGTTTCACAAATTGTATTG
	AAACAGAGTAAGGGAGGTGAGGAGAGGGAAAGAAGGAGTGAGG
	AAGGAAGAAACAGCTTAAGAAAAACAATTTTGTTAGCAATCTTTAC
	ATAAGACCTGTTGTATATAGCACACTATTTACACTGTCTTCATAATCA
	GGTGAGATATTTAATCCTTACAATAGCTCTGCAAGGTAGGTGGTATT
	ATCTTCATTTTTCATATGCTTTAACTGAAATCTGAGTCTAAGGAATTT
	GTTGTCACCTAGCAAGTTAGTGATCATGCCTGCATTAGAATCTTCTC
	ACCCCAAAATGCATGCCTTTTACACTATGCCAAGCCTTCCATAGTCA
	CTGTAGAGCCAATTGGTATCACTAGTACACATGGATCCTTAAGCCTG
	CCTACTGAATTTTTTATTACATATCCAGAGACTTTCTCACAAACTGT
	AGCTATATTTAATGTCTTAACCTTTATTACATGACAAGCACATGATCT
	CTTTTATCCTTATAATAACTTATGAGATTGGCTGTACATAATATCACT
	GATTGCAAAATCCAGACAGCTTTGTAAACCAAAGTTTTTTTCATAA
	ATAATTTGACAGCAAAATCTTGACTTAATCTGAATTAATTTAATAAC
	AAAATCTGACTTAAACTGACATGAGGTTTTATATATATTTTTTGTTTG
	TTTGTTTGTTTGTTTTTTGAGACAGAGTCTCGCTTGGTCTCCCAGGC
	TGGAGTCAGTGGCGCGATCTCGGCTTACTGCAGCCTCTGCCCCCTG
	GGTTCAAGCAATTCTCCTGCCTCAGCCTCCTGAGTAGCTGCGATTA
	CAGGTGTGCGCCACCATGCCCAGCTAATTTTTTTGTACTTTTAGTAG
	AGACAGGGTTTCACCATATTGGCCAGGCTGGTCTCCAACCCCTGAC
	TTCGTGATCTGCCCACCTCAGCCTCCCAAAGTGCTGGGATTACAGG
	CATGAGCCACCGTGCCCAGCCCAAGGTTATATCTTTTTTATTCCACT
	TAGTGCAAATATTCATCCGTATATTTTGTGCCAGAAATAGTACTATGT
	TTTGCTATTGGATGCTGCTACTCAGTCCCCTTGGGGTTAATACCATA
	GTATATTCCTTTCATTATCTTCCTAAAATAGGAAAAATTCTGAAATAC
	ATCTTGCATCAAGCTTTTTGGATAAAGGATTGACAGCTTGAATTATT
	CCTGTTTTACATATGAGAAAACTCAAGGTGAAATGATTTGCCCAGA
	TCTTTGAACCTAGGACTATTTGCCTTCAAGTCAAATCCCAAGAATG
	GTCAACATATAGGTGGAATATTTTAATTCAGATAAGCTGTTAAAAAT
	AGAATCTGCCTAGACTATGTCAAAATGGTCAGCTTGGTGGAAAAAA
	TATACCACTTGTTGTGATGTTATATATACTGATAGCAGTTCAAAAATG
	GCCAGTAGTTGATTGTCTGAATTAATAGCTTGTAATCAATTGTTAAG
	AGGGTTATTAGGCAGATGATTTATATCTTGAAACTTGTTTTAAAATC
	CCCCCAAAATGACTATTTTTAAAAACATGGCTTTTAGGTTTGTGTAT
	GTTAAATCATGGAATTACAGGTGCTTAAAAATAGATTATCAAGCAAT
	TACTTTAGTGAAATACTGTGTCATTAAATATAACTAATCACTGACAG

	GAAATTTATGTTTTTTCTTTAAAGGCATGATCACTGATCTTTTCATAG<br>ATAAATTTAAGTTTAAAGGCACCAATGTAAAAACTAAAGTACCCCT<br>TCTATTGGAAATTCTGGACAGTTATGACCAAAATGCATTGATTTCAT<br>TCTTCGATGCAGCATGACATACAAATAAGGTAAAGTCCCAAAGAAG<br>TGGTCTGTTTAAATGAAAAATGTTAAAAGAGAATTAGAAGTTAACT<br>GTGTATGTACTTTAATTTCAAATGCTTTTTTATATCTAAAAATATTTGA<br>GATATAATGGCTTTTTAAAGTAATGCTTCTATTTCTTTTGACAAATTG<br>AACTTTCTAAAACTAAAAGAGTCTTTTTATTTTTAAAACACAAGTA<br>GAATGATTTAAATAGGATTTTAATGAATTTTTGGCAAGTGGCTGTTT<br>TAATTTTTAAATTGAGAAGTAATATGTGGAAAATTAAAATATATTCAA<br>ATGTATTACAAGGTACATAAACACATAAACATGTAGACCGCTTTATA<br>GGCTTCAAAATGTATTTTTATATATTACCTTATTTGATCTTCACAACT<br>GTTGTAAAATTTGTCAAATCATATAAGAAAACTGATTCTCAGTGAAA<br>TTTTGTGATTAAGAGGACATGCTAATATTTTGTAAAGCTGGGACCAG<br>AACCCAAGTACTGGTACTTGCTTTGTGTCCTGGTTACTATGTATGTT<br>GCCAGCTAGTGGAAATAACCTACCAATTGTTTTTTCTAGGAGATAG<br>GATTGATTTGCAACTGATATTCCTTTTGCTTGGTAGTGTTTTATGACT<br>CTGCTGCAGTTTGTGGAAATGCTGGACTTGGCTCAGTGCTATAAAT<br>TGTAGCCAGGAAATAAGAAGAGCTTACACAGAGTCGGGGGATCTT<br>AATTAGACATGCTGTAACCAGTGGAGCTATTCAGCGTGCTTCAAGT<br>GAAGAGTGCAGAAATAGTGATGCCATCTTAGATTTTCCTACACTAGT<br>ATCTTCTAGATCTCTTTTTATCATATATGACATTAAGGCTACAGAAGT<br>TTTGTGCATTAATCTTAATTCATATTTATTAATTTCAAGGGAATATTGA<br>AAGGTTTTAAAATGGCATACAGATAATGGAAAATAAGCTTTATGTTT<br>CAAGAAGGGCAGACTCACGAATTTTTTAGAAAACAGTGCTTCTTAA<br>TGATATGCTATTATACTTATTAGTTATGTGCAAGCTTTGTATATTCTTAT<br>ATTTATATATAAATATAAAAATTTGTTAAAGGCACGTATAGTTAAGAG<br>AGTTTTATTTTAATAAGGTCATATTGTTTTTACTATGTTTAAAAAACT<br>TTACTTCTGAAAGGAACATAATTATATCTAGGTCACTAGAACGTCAT<br>TGTGTTTTTTGTTGGTTGCCACAGCTTGGGGAAAAATAGAAAAAAA<br>TTAATGACTGTATTTGAATATTTTGTAATGCACTGCTATTTATTATATA<br>TATCAACAGTAGTTCAAGGTGCCATCTTAAATTAATTGCATCTTCATT<br>AGGAAAAATAAAAAGCATAAAACACAATTTCTGGTTACTATGAATA<br>AACGCCTAAATGTTAAGATGACATTACAGTCTTGACACTTGAGTAC<br>TGTATTACTATGTGAGCTCCGTGTTAAATAATTTATGCACATTATTTA<br>ATCCTAACAACCATATGACTGTAGTTATTAGTCCCTATTAACACATAA<br>GAAAACGGAGAATCGGAGATACTGAAAAACGTGCCCCAGATTTTA<br>GACCTTTGGAAAAAGTCACTTAAGCTAACTAGACGTCCCAGAGCTA<br>AAGGCTGGGCAACCCAAATGATAGTCGCCAAAGTTTAATTCCGTTT<br>AATTCCCTAAAAGGCTTAGAGTCAGCCTTCGGACAGCCTCGCTCAC<br>TAGGTGGCTCAGCTTAAAATAATCGGAAGCGTCGGGCCCTAAATCC<br>TACCTCTCCCCGCCCCGCGCAGGCGTTTTCTCCCGCCCCAGCCAGT<br>TTGTTTCTCCCTCCTGCCCCGCCCCTGCTCAGTTTCCTTTGGTTTAC<br>CCTTCACTCGCCTGCGACCCTGTCGCCTTGAATGACGTCAAGGCCG<br>CGAGCGCTTTCATTGGTCCATTTCAATAGTCGCGGGATACTTGAACT<br>GCAAGAACAGCCGCCGCTCCGGCGGGCTGCTCGCTGCATCTCTGG

	GCGTCTTTGGCTCGCCACGCTGGGCAGTGCCTGCCTGCGCCTTTCG CAACCTCCTCGGCCCTGCGTGGTCTCGAGCTGGGTGAGCGAGCGG GCGGGCTGGTAGGCTGGCCTGGGCTGCGACCGGCGGCTACGACTA TTCTTTGGCC
CCND3	CCGCGCCCAGCCGAAGAGTGCTCAATTTTTACTGATAGGAATGGAC AAGCAAAGAGTTTAAAGATTAATGTGAAATATTGTACAAATATTAC AGATGCTCATAATAGTGACCTGGAATACTGTAAGCACCGAGATAGC TCCTTGATTCTCAAAGAAAAAAGCTAAATTATATGTAGTTTGGTTTA CTGCTTTTTTTTTTTTTTTTTTTTTTTTTTTTGAGACAGGTCTCTGT TGCCCAGGGTGGAGTGCAGTAGTGCGATCTGGGCTCACTGCAATCT CCATCTCCCAGGCTCAAACAATTCTCCTGCCTCAGCCTCCTGAGTA GCTGGGACTACAGGCGTGCGCCACTGCGTTCGGCTAATTTTTATATT TTTTTTTTGGTAGAGATGGGGTTTTACTGTGTTGGCCAGGCGGTCTC AAACTCCTGGCCTCAAGTGATCTGCCTGCCCCTGCCTCCCAACGTG CTAGGATTACAGGTGTGAGCCACCGTGCCCGGCCAACTTTGTGCTT TTAAGTCCCGCAATCACAACTAACTTTTAAAAAAGGGTTCACCAC GTGCCAGCCACTCTTCTATGTATTTTAAACAAATCAACCCATTTCCT CCTCACAACCACTCTGTGGGGGGGTACTACTGTTATCACCATTTTTA GAAAAGGAAACTGAGGCACAGAGAGGTGGTTTAACTTAGCCAAAT TCACACAGTGAGGGAGTGGAGTGGCAGAGGGGTGTGGCTCCAGG CCCTGGAGGGTCCTCTCCATACGCTAGACCATGCGTTTACCGTTAG AAGCCAGGTCTTCAGGATTTGCTAGAGATGCAATTCTAGGAGTAGG GCTCTTGCTGCTCAAACTTTTGCTTTTCTTAGAGGGGTTATGGATGC ATGTTCATAACAGAAAGGGTACTCCTTGAGAAAGGGAATAATAATA ATCATTTCAGAAACTACTATCTCTGAATGCTTGCTTTGTGCCAGGCA CAGGTAAGACATTTTCTGCTTGTTTAATTTCAGAACATCATTATAG GGCTATTATATTATCCCCACTGGATAAAAGAGGAAGCAGAGAGGTT CCAGTAATTCTCTCAAAGTCATAAGCTGTGGAGCCAGGTTTGAACT GAAAGCAGTCTCTGACTCCAGAACCCTGTTTAGCACCCCTGTTCTC AGCCCCATGGCTCGCTTGGCTCTCCCCAGCATCTCAATGGGTCTGA AGGTAACTAAGCCATGGCATGTGGACTGTGGGGTGATGGGCCTCAC CCATCACCTTGTTCAGCCTCTCACTTCTCAGATGCGGACTTTTTTTT TTGGAGACAGAGTTTCGCTCTTGTTGCCCTGGCTAGAGTGCAATGG CACGATCTCGGCTCACTGCAGCCTCTGGCTCCAGGGTTCAAGTGAT TCTCCTGCTCAGCCTCCGGAGTAGCTGGGATTACAGGCATCCACCA CCACACCTGGCTAATTTTTTGTATTTTTAGTAGAGACAAGGTTTTAC CATGTTGGCCAGGCTGGTCTCGAACTCCTGACTTCAGGTGATCCAC CCGTCTTGGCATCCCAAAGTGCTGGGATTACAGGCGTGAGCCACCG TGCCTGGCCCAGATGGGGGTATTTTGATGATGAAGAGGAAGAGAA AGAAAATGTATCTTTCCTCCTTGGGGTTTCTTATTTGGCCTGTGACA TTCCATTCCCACCCCTCCTCCAATTTGCCATTTTATTGACTTTTTCTA ACTGGATGTACCTGGGTAGAAACTCCTCATACTATTGGCCCTGGTCA TTGCTTTGTCTTTAGTTTTCCTTTTAGAAAAACAAAAACAAAAACA AGAAAAACAAGAGGAGCCTCTATTTCTTTGAGGTGGAGGTAACTG GTGAACTTGTGCTGGTCCTGCATTGAGCTCACCTGCCTCCCCAGTG GCCAGTCCTTGGCCCTGAAAGTCAGGGATGATCTAGCTCTAGGCAT

	GGCTATTCCATCCAGGGGTGAGGCAGAAGTATCTCTCCTAAAGTCC<br>TGCTCACCAGGAGCTCCCGTCCCCATACTACAGGTTCACATCCAGC<br>TTTCAGGACTAGTCAGTCTATGTGGCCCTCCCTCAATTAATAAATCA<br>GCAACTAATTTGCCAGGTGCGGTGGTTTGTGCCTGTAATCCCAGCA<br>CTTTAGGAAGCTGAGGCAGGCAGATCACTTGAGGTCAGGAGTTCG<br>AGACCAGCCTGGCCAACATGGTGAAATCCCGTATCTACTGAAAATA<br>CAAAAATTAGCCGGGCATGGTGGTATGCACCCGTAATCCCAGCTAC<br>TCAGGAAGCTGAGGCAGGAGAATCACTTGAAACCAGGAGGCAGA<br>GGTTGCAGTAAGCTGCACTCCAGCCTGGTGACAAGAGCAAAACTT<br>TGTGTCAAAAAAACAAAGAAAACCAAAAAACAAAGAAAAACACA<br>AAAAACCCTTCTATTTGTTAAAAAAAAAAAAATCCACCGTGAACCA<br>AAAATTAGTAAAAACAATGAACTAAAATTTTGTTTTTGCAAAATGTA<br>TGATAACAAAATGTTAAGGAAGGTCATGTGCCGTTATGGTTCACTG<br>CAGCCTTGAACTCCTGGGCTCAAGCGATCCTCCTGCTTCGGTCTCC<br>CTAGTAGCTGGGACTACAGGCTTGTGCCACCGCACCCAGCTTATTT<br>TTTTTTTTTATTTTTTGTAGAGATAGGAGTCTTGCTTTGTTGTCCAGG<br>CTGGTCTTCAACTCCTAGCTTCCAGTGATCCTCCTGCCTCAGCCTCC<br>CAAAGTGCTGGGCTGATGGGACATTTTTATACATAGTGCCATGTTAC<br>TATAAATGAGAAGTTTTAAAAATACTGATTTTAAAAATTAATTTATGT<br>CAAGAATTTTTATAACCAAAGTTAAAAAACCAAACAAAAAATATGA<br>AAAGGGTTAATATCTTTGAGAGGTGATGAGAACTTATAAGTCAATA<br>AGAGAAAACAAACATCCCTATAAATGAATAAGCTAAGGACATGAAT<br>GGGTAATGTACATAAGAAATGTAAATGTCTAGTAATATGCCAAAATA<br>GATTTATTATTACTAATAAGCCACTTTCACTCTCTAGTTGGCAGAGTT<br>GTTTTGAAAAATAGATATGTAATGATGGTGGAAAAGATTGGTTTAAC<br>TATTCAGCAGGAAAATTTGGCAATTAGAAGTGTATCAAAAGCCTTA<br>GAATGTTTCATAACCTTAGATTGGGAAATTCCACTTCTAGAAATTAA<br>TTCACTTCTAGAAATAATCATGAGTGTGCACAAAGATATTACCACAA<br>AAATATTTTACAGTATTATGTCTAATAGAGAAGAACTAGAAATAATTT<br>AAATTTCCACCAATACAGGTTTGCCAAAATACATTTTGTACATTCAC<br>CTAATGGTATATTATGTCCCTATTACAAATTATGTCCTAGAATATTTAA<br>TAGCATGGAAAAGTGTTAACAGTATTTTTTTAATGAAAAAAGCTTA<br>CAAAACAGTTTGTGATGATTCCATTTAAAATGTGTGTTTATTCATAG<br>AACAAAGATTAGAAAAATAAACATTGATATATTAAAGGGTTATTTCA<br>TGGCAAATTGCAAATGATTATTTCCTTTTTTTGTGGCTTATTTGTATT<br>TTTGAAGTTTTCTACAATGTAAAAGAATATTTTATGATATGAAAACT<br>ACAATACAATTTATAATATAAGAAAGAATAATTCGGCCGGGAACGGT<br>GGCTCACGCCTGTAATCCCAGCACTTTGGAGGCCGAGACCGGCG<br>GATCACGAGGTCAGGGGTTCAAGACTAGCCTGGCCAACATAGTGA<br>AACCCCATCTCTACGAAAAATACAAAAATTAGTCAGGCATGGTGGT<br>GCGTGCCTGTAGTCCCAGCTACTCGGGAATTGCTTGAACCCGGGAG<br>GTGGAGGTTGCAGTGAGCCCAGATCGCACCACTGCACTCCAGCTT<br>GAGCAACAGAGTGAGACTTCGTCTCAAAAAAAAAAAAAAAAAAA<br>AAAAAGAATAATTAACAGAAAATGGTTAGACACTTCCTTAGTGTCT<br>CCTAAGTCAGGAGGACCCCAGTAGGGCAGGGATCCTCATGGCCTC<br>CTCCCATTTGGAGCATTATTGGAGGTCTTTTTCGGCCTCTTCGTCAA

	GTGGAATCTAGCTTCCGGTAAAACTACAAAGTAACCAAAAGTTTGG GAGGTGGAAGAAATGCAACCGGTAGATCTCACAGAGTCTGTGCAA GAAACTGATTCAATGAGAATCTAGTTTCTCCGTCCACAGTTTCTCC AAACAGAAACTAAGGCCGACTTTAGGGGCTTGTCCAAACCTAGGC AAGCAACTTAACAAGGTGAGGCCATGACTCCATGGCCTTTCCGTTC TGTTATATGCTGACTTAGACTAAAGCTCTCATACTTTAAAGTGCACA GAAATCTAGTTAAAATGCAGATTCTGATTCAGGTTAGGGGTGGGCC TGAGAGTCTGCATTTCTAACCAGCTCCCAGGCGATGACCACGCACG GGACAGGTCTGGGATCACAGTTTAACTAGCAATGGTGTAGAACAC AGAATCTGCAGCAAGAAGGCCAGCTTCCCAATCCTAGCTCTGCCAC GGACCAACTGAATGACAGTTGCCTCGGTTTCCGAGTTTTCGTGAAG ATGTAGTGAGTCATTACATCGTGAGGCTTTCGAGCAGCGTTCACTA AGAACTAGCTCTGACATTATTTATCGCATTCCTTAGAGCAAGCAGCC GGTGAAGTAGGGTTTGACGAATGAATAAGTGAATGAATGACCTTTG GAGAAAAATTGTTTCCTGGGTGACTAGAGTCCGAGAAGCAAAATG GGAGGGCCCGTGGGTGGGTAGGAGGCCCACCTCCTAGAAAGTTCT CTGCACCCGGTGGTCCAGAGGGCCTGGAGTGCCCGGAAGCCGGCC GGCTTGCGCTCAGCGGCCCAATGGGGCCGCGGGAGGGAGGGGAG AGCGCTCAGCCAACCCTTTCCGTCCGGGGCGCCGCAGCCCCGCCC CTCGGAGCGTTGCGACGTCCGAGCATTCCACGGTTGCTACATCGTC GCGAGGGGCGGGGCGCCTGTCAGGGAAGCGGCGCGCG
CD40	TAGGAATCAGCCACATGGCTGGCCCACAGCCAAAGCCAACAGGTC CTCTGTACCCTGTATTTAGGGAGCCAGATAGTTTCCCTCCAGGTTT ACTAAAGGAGGCAGAACTCGCTTTACACCCACAGCCCAAAATTCC AATTTCCTCTTCCTTGCCCATACAGCAATCTTGGGGAGGAAGGAGG TCCTTGGTTTTACTGAGTAGTCAGGTGTTCATAGGAGAAGGCCATT TCCTCCCTCCCCGTTAAGGGTGGGGTAGTGGAAATTCAAAGGAAGT CCCTCATGGAGACTGAGAGGTGACTGCAAGCATGGGGTGCTGGGA TGCTGTTCCCTGATGGGTGGTGGACTGTGGGCAGTGGACTGTGCCT ATCTGAGCAGGGTAGAGGAGAGGTGAGAAGAGGGGCATTGTGGTG TAGCTGACTGGATGCGTGAGCTTCTCTAGGTCAAGTGGACGTTGCA GTAATCAGCCTGGCTGGAGCACTTTTCTGGTGGCCTACTCAAGCAG GTTGACTGCTGGGCAAGAGGGTCAGGACACTGGTAGGCCCTAGGT GGTGGGGTGGGGTAGGCGAGGTCAAGCCTGGGCTGGATGACCCTG TGAGAGAATTGTGCAGTGGAGAAGCCGCTAAGGAACTTACTCATTT GCCCATGAGAAACCATATATGGACACGGGGTTGGGGTGGGGGTGGT AGGGTATGTCTCCCAATCAGACCATCCGCCCCACCTCCACCAAGTC CTTTTCCTCCTAGCAGGCAGGGTCCTGGCTCAAGTTAACTCTTGAA TGAGTAAGAGAGAAGATAAAATGAGATTTAAATTGAGTTTGAGAAT AAAGGTCTAAAGGATGGAGGTTTAACAACAGAAAGTGATCCAAAA GTAATGCAATCTGCCCAAAATGTCATTAAGGGATGGAAAAGGGAGA TTCAACACAGCATGGCTGAAGGCAGAGATTAGGGGAGAAAATCTT CATCATTATACCCCACAGAGTTGAATCGCCTCAATACACTGGCTCCA CAAGTAAACAGAAAAATTGAAATACAGTGTGGTCGGGCTAGAGTC TTGCTTGGGTAAAGCTGCCCCAGGTTCTGAGAGAGCACAGGGGGA TAGGTTGGTTGTTAGAGAGAGATTTCCAGAGGAGGGTTGCCTGAGA

	TGGACCTTGAAGGGTGAGAAAGAGTTGGCCAGGTGAAAGGAGAG TGGCAGTGGGAAGGGAGCTCTCCAGGCAGAGGTGACAGCACGGG ACATCTGGGGAGTGGGGCTGGAGCATGAAGGGGAAGGCAGGAAG GATGCTTAGAGATGTACACCAGGAGGCCCATGGCCTTGCAGCTCTG CAAGCTTAAGGGGTGAGGGCCCTGGAGAGCCACTTTAAACCTTGG AGCAACACAATTGGATTTGCATATTAGACAGAACACTCTGGATGCC AGATTCTGGCAGCGCTGTGGACACTGGACCTCTAGGCATGCTCCTC ACCCATTCACCTGGCACTGTGAACCCACCTCCCTACAAAGGCCATT TCATCTCTGAGTTTCCAAAATCCTGGCTCCCTACTCTTTTGAATGTC CTTTGCCCCATTCCTATTGCAGAACCCTTCATTGTTCCCTTTTTGATT GGCCTCCGAATATTGCCACTTTCCCTTAAATCATCTTCTCGCTTTCCT CCTCCAGCACACTCTTCTCAGCTCAACAAGATTCTAGGCCTTCTCT GGGCCTGTGCCCACCAGCACATGCCTCCTCAGGATATGTATATACTA AAATATCTTCCCTTTATCTTATCTGGTCTTCGCCCCATGGGGCAACC CGAAGGCTAACATTTATTCCTTCACACATTAATTTAAGCTGCTTTAAT TGCTGCTATGATATGTTTTATATAATATAATTAATGTTTTAATTCTGTGT ACTTGCTTCCCCAGGCAGTTTTGTGAAAGGCCCAATTTTGGAATCA AGCAAATAAAGAATAAGTAATGGCTGAAATCATTATAGTAACACCC CTCTCTCAAAGGATTTAAAACCGTGCTTGTTTTTTTCAAAGTATGTT AAAAAGGGAAAAGATATTTTAATCTTCTCTCCATCCTGGGCATGTTT GGGTGGCTTTAATGAAGTTCCCTGGACCCTGATTTCAAGAGTTTCT GGCCTTTTGGTATCTGACCACCATTCTCAAGAGGCCACTGCAGCTT GAGGTCTGTGTTGAGATTACCAGCTTCGCACCCCCTGCCACCAACT CTTTGTCATGATTGGAGGCTGTACTTAGGATGAGAAGTTGCTGAAC CCACTCATTCATTTATTCATTCATTCAGCACCCATATATTGGTGTCCTA TTATGTACCTAGGACTGGGATAGATCCAGTCCTGCCCTCAGGGAGC TCACAGTCAAATGGGACCAGGCAGAAGGAGTTATGAGCTGGCATG CTAAATGCTGTGGCAACACAGGGGAGGAGGCAGGGTGGGATAGAG TACCTACCTCTGCCTCAGGGAGTTGGGGAAGGTGTAAGAGGAGGC AACATTTAAACTGTTTAAGAAAGGAGAAGGAAAGGAAGGAAACAT CAAGAGCAATGAGATAAACCGTAAGGAAAAAAGATTGTGTGCCTG GGAAGTGGGGAGAGGCTGAAGCCAGTGTGCATGGGAGGACGTGG CAGGAAGTAAGCATAGAAAGAGAGGTTGGGGCTGAGTGAGGAAG GGTGTGCTTTGCCAGGATAAGAAATTTGGATCATCCTGAGGGTGCA GGGAAACCCAAAGAGGCTTTTATGCAGGAAAGTGTCCCAGTCGAT CAGATCTGGAGTTTATCAAGAGAATCGCAGCAGTGTAAAGAGTTGC CTGGCACATAGTAGGTGCTCAATAAATCCTGTGGAATGAGAGAGGC TGTTGGGACAGTCCAGATGAAAGATGGCACCCTCTGAATTAGGGC AGTGGCAGAGGTGACAGAAAATAAGGGATGGATCCTAGAGGCAGA ATCCACAAGACTTGCAAATGTGCCCTTAACAGCAGAAGCCATGAG AATGGGGTTAGAAGTGTCCTCCTCTTCCATCCTCCCTGATCTGTGAG GTCTGGCCTCTTGTCCTCTTCCAGAAATTGAGAGCTTGGAGGGTCA TCACCTCAACCGTTGTCTAGTTCAAGCTCCTAAGTTTTCCTTGGAG AAAAATTTAGGCCTAAGGGAACACAGTCCTTCAGAGGCAGAGGCT GCACCAGAACCCAGGTGTTCCATGCTGCAAAGCAGAGTGTTAACA CTGGTTCAAGAACATCTTGAGGGCCAGGTGCGGTGGCTCATGCCTG

	TAATCTCAGCACTTTGGGAGGCCAAGGCAGGCGGATCACCTGAGG<br>TCAGGAGTTTGAGACCAGCCTGGCCAACGTGGAGAAACCCCGTCT<br>CTACTAAAAATACAAAAATTAGCTGGGCGTGGTGGCGCCAGCCTGT<br>AATCCCAGCTACTCGGGAGGCTGAGGCAGGAGAATTGCTTGAACC<br>CAGGAGGCAGAGGTTGCAGTGAGCCGAGATCATGCCACTGCACTC<br>CAGCCTGGGCAACAGAATGAGACTCTGTCAAAAAAAACCCAAAA<br>ACCAAAAACCAAACAAACACCCAAAAAACAAAACGAAACAAAAA<br>ACAAAAAAACAAAAGCACGTCTTCAGAGTTCATGAACCCAGGAAA<br>TGTAGGCACAAGTGTGTGTGTTTCTGCAAAATGAGAGGGTCCCGA<br>GTTTCCTAACATACTTAAAGTGTTTTATGATGCCACAAAAGGCTGGC<br>CCACTCTTTTTTTTTTTTTGAGATGGAGTTTTGCACTGTCGCCCAGG<br>CTGAAGTGCAGTGATGTAATCATTGCAATCATGGCTCACTGCAACC<br>TTGACTTCTTGAGCTCAAGCGATCCTCCTGCCTCAGCCTCCTGAGT<br>AGCTGGGACTACAGGTGTTTGCCACCATGCCTGGCTAACTTAAAAA<br>TTTCTTTATTTTGTAGAGATGGGGGTCTTGCCATGTTGCCAGGCTGG<br>TCTTGAACTCCTGGCCTCAAGCAATCTCCCTTTTTGGCCTTCCAAA<br>GTGTTAGATTACAGGCGTAAGCCACCGCGCCTGGCCCCCACCTTTA<br>TTTTTATTTTTATTTATTATTTATTTTTTTTTTTTTTGAGACTGAGTCTT<br>GCTCTGCCTTCGAGGCTGGAGTGCAGTGGCACGATCTCGGCTCATT<br>GCAATCTCTGCCTCTGCGGTTCAAGCGATTCTCCTGCCTCAGCCTC<br>CCGAGTAGCTGGGATTACAGGCGAACGCCACTACATCCGGTTAATT<br>TTTGTATTTTTAGTAGAGACGGAGTTTCACTATGTTGGCCAGGTTGG<br>TCTCGAACTCCTGACCTCAAGTGATCTGCCCGCCTCGGCCTCCTAA<br>AGTGCTGGATTACAGGCGTGAGCCACCGCGCCCGGCCCCACTCTTA<br>ATAAATGCCTGTCTCCAGGTGCTGGGTGGGAGGTGGGATGGAATGG<br>AATGAGGTGAGGACGCATGGATGCATGGATGAATGGATGGGAAGTT<br>GAGACGACGCGCCCACACGAGGGAATTTCCTTTGAAAGAGAGCGA<br>AATTCTGAGTTGGGAAACTCTTCCTTGAAACGCCTCCCCATACCCC<br>AGCTGTGGCCTTCCCGTTTTCTGCGTGGTGGTGTGGGGGGAACTTC<br>CTCAGGCCTCTCCGCAGTGGAGCCTCTTTCGGTTCTGCCAGGATAC<br>CTAGAGGCAGCGGAGAGCGGGGCAGGGAGGGGAAAACCGTGAGG<br>GTCCCTGTGGCAGGCCCCAGCACCCATGGGATCTCTCTCCGGTCGC<br>AGGAAGCAGGCTAGCTCCTAGCCCGCCTCGGCTTGGCCTTTGTGGG<br>ACCTGGGGGCAAAGAAGAAGAGCTGTCTCTGGGACCATGCCTCCT<br>CCCGTACACAGCAAGATGCGTCCCTAAACTCCCGGGGGAATTAGAC<br>TTGTGGGAATGTTCTGGGGAAACTCCTGCGCGGTGAATTGCTGGGG<br>GCTCCGCCCCCCCGATAGGTGGACCGCGATTGGTCTTTGAAGACCC<br>CGCCCCTTTCCTGGGCGGGGCCAAGGCTGGGGCAGGGGAGTCAGC<br>AGAGGCCTCGCTCGGGCGCCCAGTGGTCC
CD53	CCTATCAAACTACCAACACTATTCTTCACAGGACTAGAAAAAAACT<br>ATTTTAAAATTCATATGGAGCTAAAAACAAATAGCCAAGATAATCCT<br>AAGCAAAAAGAACAAAGCTGGAAGCATCATGTTACCTGACTTTGA<br>ACTATACTACAAGGCTATCATAACCAAAACAGCATGGTACTGATACA<br>GAAACAGATGCATAGACCAATGGAACAGAATAGAGAGCCCAGAAA<br>TAATGCTGCACACCTACAACCATCTGATCTTCAACAAAGCTGACAA<br>AAACAAGCAATATGGAAAGGACTCCATACTCAATAAATGGTACTGG

	GCTAACTGGCTATCCATATGCAGAAGATTGAAACTGGACCCCTTCC<br>TTATACCATATACCAAAATCAACTCAAGATGGATTAAATTTAAATCT<br>GAAACCTAAAACTTTAAAAACCCTGGAAATAACCTAGGAAATACC<br>ATTCTGGACAAAGGCCCTGGTAAAGACTTGATGATGAAGATGCCAA<br>AAGCAATTGTGACAAAAACAAGAATTGATAAATAAGGCCTAATTAA<br>ACCAAAGAGCTTCTGAACAGAAAGGGAAACTATCAACAGAGTAAA<br>TAGACAGCCTGGAGCATGGGAGGAAATATCTGCAAACTATGTATCT<br>GACAAAGGTCTAATATCCAAATCTATAAGAAGGTTAAACTAATTAAC<br>AAGCAAAAACCAAACAACTCCATTAAAAAGTGAGCAAAGGACATG<br>AATAGACATTTGTCAAAGGAAGTCACACATGTAGCCAACAGGCATA<br>TGAAAAAATACTCAGCATCGCTAATTATTAGAAAAATGCAAATCAA<br>AACCACAATGAGATACCATCTCACACCAGTCAAAATGCCTATTATTA<br>AAAAGCCAAAAAATAACAGGTGTTGGCGAGGTTTCAGAGTAAAGA<br>GAATGCTTATACGCTGTTGGTGGGAATGTAAATTAGATTAGCCAGTG<br>TGGAAAACAGTTTGGAAATTTCTCAAAGAACATAAAACAGAACTG<br>CCTTTTGACCCAGCAATCCCATTACTGGGTACATATCTAAAGGAATA<br>CAAATTGTTCTATCATAAAGACACACGCATGTGTATGTTCATCATAG<br>CACTCTTTACAATAACAAAGACACGGAATCAATCTAAATGCCCATC<br>AATGATAGACTGGATAAAGAAAATGTGGTACATATACACCATGGAAT<br>ACTATGCAGTCATAAGAAAGAATGAGATGATGTTCTTTGCAGCAAC<br>ATGGATGCAGGTGGAGGGCCTTATCCTAAGCAATTTAATGCAGGAA<br>GAGAAAACCAAATATTGCATATTCTCACTTATAAGTGGGAACTCAA<br>CACTGAGTACACATGGACGCAAAGAAGGGAAAAACAGACACTAG<br>GGCCTACTTGAGGGTGGAGCGGGGAAGGAGGGAGAGGATTAAAA<br>AACTACCTGTTGGTCACTATACTTACCCCCTGGGTGATGAAATAATC<br>TGTACACCAAAACCCTGTCACATGCAATTTACCTATATAATAAGCCT<br>GCATATGTACGTTTGAACCTAAAATGAAAGTTAAGAATGATGTTCTA<br>CAGCATTTTCTTCAAAACTTTACTCTTAGAGCCCATAAGCAAAATCC<br>AATGTACTACTAGTTAACTTTAATCTCCCATTTGAAGTTTCCAATCTT<br>ATAATTCTCACAAAATCTATGCCTCTTAAACTCTAATATCCTTATATA<br>CTTAAATAACTCTGAAATAAACTTGGAAGTTCTATCTGTATTCTCATA<br>TTTCAACAGTGATTATCTTCCAATAATAAAACAGAACTACTCACTTA<br>GAAGGCTTTTGAAAAAGGCCTAAATGGGGGCTAAATACAAAATGT<br>CTATGTGAGTATTTGTCTATTCAGAATGTATTTTGGCAAATACGACTG<br>CACAGTAGTAGAGTACTCATAATTTGTATTCCCGCTATTCGTCTGGC<br>CAAGTTCAAGTTTCCTAAACTCTCAGATTCTATTATCATCTATGATGG<br>TGATCAATATACCCCGTATTGAGAAGACCAAGCCATGAGTCTGAGC<br>TTCTGCTAAATGCCTTTTCTAATTTAGTTTTTCAAAGTTTTTGAGCG<br>TTCTTTTGGGTTATTCAATAACATGGGGTTTCCTGATATTGTCTTATT<br>CTCTATTTGTTTTTGTTCTTGTCCCTCCAAATGAATTCTAAATATCTG<br>ATGGTCAGAAACAGAATTAATTCTCAATAAAATTTTATAGGTATGGG<br>TATAAGTGGGCACGTTGTTCTCTACCAAACCCCTAAACCCAGGAAA<br>CAGCCACCTCTAGTTGTCCGGCACTTGGAACAACCCTTCCCAGTGA<br>AATGATGGAAAGAGGCACATTCAAGGAAAAATGTTCTGCATTAAAA<br>TGGTCACCTATTTTGCTGTGAACAATTTGTCTTGAGATAGTCTTCCC<br>GATATTCCTAAGATTCCCCCTTATTTATCACTTCTCTCTCTGCAGATG

	TTCAGTCTACCTATCTCTTTCTTCTCCTTACTCTCTCTGCCTGTCCCA<br>AATTTCTGGTTCCCCTAACTTGCAGGCCTCACATTACCTGAAACTAT<br>TCAAATATTTATAGAACACCTCTTATGTGCCAGACCCAATACCAGGC<br>ACTAAAAATACAGTCATGAGCAAGATCACCCAACATACACAGAGTA<br>ACACAGAACCTACAGAGCTTTCAGTCTAGTGGGGAAAACAATGAA<br>AAACAAGGAATGACAGAGGGTGGAGGAATGCCTGTGGGCAATGG<br>GAGCAGAGAGCAGGAGTAACTGTTCCTGCCTGGAGGGCTTTCTGG<br>TGCTATGATGGGGGATGAGTAAGGAATTAAGTGAACAGGTGAGAG<br>GATATTCTAGAGAGAGCTCGTGAGACAGAACTAGATTGATTTGTTT<br>AAGCAACTAAAAGGAATTCCATATCCAGTGTTACCCAGTGTGAGGA<br>GATCTTGGGGGTGGGGCCGGGGAAGAAATAGCCGGCTGAGAGTTT<br>TAAGCTGGGGAGTCATGTGATTAAATTTGCATTTTAGAAGGCTCTCC<br>TCCCTTCTTCCTGAGGACAGACTGAAGAAACATCCAAGGTGGTCTT<br>GAAGGACACTGGGATCCTGTAACACAGGTAAAAGATAAAGACTAA<br>TGTAATTTCTATGGGGTTGGAGAGAGGTGGTCTGGTTTGAGAAATA<br>TTTAGGCAAAAGCATAACTGACAGGACTTGGTGGCTGATAATAGGT<br>GGTGGTGTGGGAGAGAGAGTAGCTTAAGAGGACACCTGGTTTCTC<br>ATGAACGTGGCTGAAAGGCCAGTCAGGCCTTTCCTGGATGGTGGA<br>CATGGTGGGAGGAGCAGGCTTGAGTGTGGGGATGCCAAGTTAATC<br>AAACTGAAGTTGGATATTGGATTTGAGGTGCCTGGGAGAGAGTGG<br>AGCTCAAAGGAGAGATCATGGCATCTATATAGATTTGTGAATCTTTG<br>GAACAGACAGATAGTAATTAGAGCCATCAGAATGGGTGAGATAATT<br>AGGAAAATATGAGTTCAGCCAGAAGCCTCTCCTCAGCCCCACCACT<br>TCAGGGACCTGTAAGCATGGCAGCAGCTCTGAGTGAGGGTCAGTG<br>ATTCCAGCCGAGGTGCAGAGACTTTTTAATAGCATTCCCTAAAGTGT<br>AGTTACACTGAAAAATTGTTCCCCAAGAGAAACTACACACACACA<br>CACACACACACACACACACACACACCACACAGTTCCATGCTCAGATA<br>AGTTTGGAGAAATCTATATATTATTTCCCCTGCTTAGAGAATATTAAG<br>GACTGACAAGTTCTGCAGTAAAGACCTTTTTACCTTCATTTAATTCA<br>GTTTTCCCCACAAGACAGCCCTGGCTGGAGATTACTCTGGATCTGA<br>ATGTCTCAGGGTGAAGGAAGCGGGAGACAGCTAGCTTTCCCAGAG<br>GCATGCCCGCAAGTCAGCACCGCAGAGTGCATCTATCTGTTCATTTA<br>TAAGATGAAAACATTGGGGCCCAGTGATTGCTTCCAAGTTTCATAG<br>TTAGCTAGTGATACATTTGGGTTGTCACATACTCCTTTGACCCAATC<br>TAAGAAAATTCCAGTGATGCAGCATAGTGAAACCAGAAACACATTT<br>TTCAAAATATATTGATCACAGTGTTTCTCAAAAGGCCTTCTCTGTGA<br>CCTTGAATTTTAATCCTACCTCTGACATGTCTTAGTTGTATGAGCTTG<br>GGCAAATTTTCTAACCTCTTTGAGCGCCTGTTTTCTTGTATATAAATA<br>AGGATGTAACAGTAACCAGTAGGATTATTATTAGGTGTTACCATTAA<br>TAAACTGAGGCTGACAGATTGAGACTTGCTCAAGATCTCACAGCTA<br>AAAGAGATAAAGCTGGAACTGAAACTCAGATCTTTGGCTCCAAAT<br>GTGGTGTTTCCTGTACTAACCAAATCATTTCTACCAGTGCCCCTGGG<br>ATTATGCCTTGGGGACTCACCTTAAGACAGTCTTGCTCTCGAGACA<br>GAGAAGTCTGCAAATGGTGTCCAATTCAAGTCTAGAGACTGGCTG<br>AAGCCAGACAGAGGGAGTATGAGGAGGGGGCAGAGAATCTTCAG<br>AAAGACATTCCAAAGTCTCGGGCCTAGAAAATGTTTAGAAATATTG

	GTTCCTGTGTTCCCCTCCAGTCTGTCTACCTGAATGGGAAACTGGTGGTGGGGCCAGGCATACTTCTTTTTGAACAAATACACAGTTAATTCTGAGATGCCACCTTGATTGAGAAGCACTGTCCTTTTCTAAGCAAAAATCATTGCCAAATTACCCTAGAATCCACCACTGCTCACAGATCTGTGCCTGAACCAACTTGGTTTTGAAGTTTTCTTTTGTTTCACAAAACTAGTTTAGGGTGGGCTGTGAGAGGAAGCTTCTAAAAAGCCACTGGGAAAGTACTAAATCTCTGAGACTAAAGAGGGCGGACTCAGCCTCACTTCCTCCTTCTTCTCATCTGGGCTTCCTGTCGTCACAGCATGATCATATTTTTTCACCCTTCACTTCTCCTTTTACACAAATAGCCCCGGATATCTGTGTTACCAGCCTTGTCTCGGCCACCTCAA
CD79A	CCGCTGTGACCAGATGTCCCTCCCAGTTGGGAAGACTAAACTGGTTTGGCCAATATCTCCCAGGATTCCCCTGTCCAAATTATTCCTGGGATCTGACCCATTTCCTGGAAAGGGGCGAGCCTGGGTTTTGAAGTTCAAACTAGAGTTTAAATCACAACTCTGCCCCTAATCCCACGTAACCCTGTGCACCTTATCCTCTGAGCCTTCGTCTCCTCGTGTGTAAGCCTGCTAACTCCTGAGAGACTGGACAAGATGCCGTGGAGAGCCTTGGCTCTGAGTAGCTCTGCTGCTGGACAGTTCAGTTGGCAGTCCCCAAACGTAAGGCGTAAGAGTTTAAGAAGTATCGGCCAGGCACAGTGGCTCACACCTGTAATCTCAACACTTTGGGAGGCCGAGACGGGCTGATCACCTGAGGTTGGGAGCTTGAGAACAGCCTGACCAACATGGAGAAACCCTGTCTCTAAAAATACCAAAATTAGTTGGGCATGGTGGCGCGCACCTGTAATCCCAGCTACTCAGGAGGCTGAGGCAGAAGAATCGCTCGAACCCCGGAGGCGGAGGTTGCAGTGGGTTGAGATCGAGCAATTGCAATCCAGCCTGGGCAACAGAGCGAGACTCCATTTCAAAAAAAGAATCAATAAAAATGTTAAAAGCACCGTTGTTTACACTCAGCAACTCTGTGAAGTAAGTGCCGAAAAGTGCTTTGGAGGTAGGTAAAGTAAGCCATAGGCTGGGTAACTTGCCAGATATCCCACAGCTGGAGCCATCATATGAACCCAAGCAACCCAGCTGCATCCTGGACCTAGCCACTCCAAGGGCTCTGTGCCTCAGAAAAGAGGCCCAAAGGGTTGCACTCTAGCTGCAGGCCAGTCACTCCCCATTCTGGGCTCTAGTTTCCTCTTTTGGCCATTAATGAAAACATTTCCCAGAGCTGACATGAGAACTCAATGCCAAGCGTCCGGTTTGCACAGCAGCAGGCATTGCGCTTCCCAGGTAGGAAGCAAGGTCTAGGTGTCCCGAGCAGGCCTGCAATCCCGCAGCCGCGCCGCTCCAGCCACTGGGGAGCAGTAGAGGGCCTAGAAAGAAGCTCAGCCACCGAAGGCTCTTGAAGGTGAATTCCTAGGGCTTCCAGGTCTGGAGCCAGGCTTATTCCTGGGAGGGGGGTTGTAACGGGGAATTGAAGTGCAGAGGGGCTAGTGACCCCAGCAGAACACCTGGGGTCCTGGTTCCCAGGGCTACAGAACTGTCCTAGATACTGTGCCAAATGCACTGCAGAATATTCTTGCAGTCTTATCAAGTGGGGGGTTTGTACCCATGTTATAGGAGAAAAAACTGAGTTTCAGATGACCAGTCAGTTGGCAGAGCTGAGTTGTAAAAGAATCCTGGACTCTTGAGGCCCAGGCTCTTCTGCGTGGATGGGAATTGCCCTTTTCCCATCTCCTGGGTTCACGCCATTCTGCCTCAGCCTCCCCAGCAGCTGGGACTACAGGCACACGCCGCCATGCCCGGCTAATTTTTGTATTTTTAGTAGAGACGGGGTTTCACTGTGTTAGCCAGGATGGTTTTGATCTCCTGACCTTCTGATCCGCCCGCCTCAGCCTCCCAAAGTGTTGGGATTACAGGCGTGAGCCACCGCGCCCAGC

	TGGCAATTGCACTTTTTTAAAATTCTGCACTCTAGGAGGCAGAGGT<br>GGGCAGATCACTTGAGGTCAAGAGTTTGAGACCTTGGCCTGGAGT<br>GGTGGCTCACACCTGTAATCCCAACACTTGGAGAGGCCGAGGCAG<br>GTGGATCACCTGAGGTCAGAAGTTCGAGACCAGCCTGGCCAACAT<br>GATGGAACCCTGTCTCTACTAAAAAATACAAAAATTAGCTGGACGT<br>GGAGGCAGGAGAATCGCTTGAATGCAGGAAGTGGAGGTCACAGTG<br>AGCCAAGATTGCACCATTGTACTCCAGTCTGGGCGACAAGAGTGA<br>AACACCGTCTCAAAAAAAAAAAAAAAAAAAGAGTTTGAGACCAGC<br>CTGGCCAACATGGTGAAACCCTGTGTCTATGAAAAAAAAAATTGG<br>CTGGGCATGGTGGTGCACACCTGTAATTCCAGCTACTTGGGAGGCT<br>GAGACAGGAGAACAGCTTGAACCCAGGAGGTGGAGGCTGCAGTG<br>AGCCAAGATTGTGCCGCCACTGCACTTCAGCCTGGGCAACAGAGC<br>AAGACTCCATCTCAAAAAACAAAATCTGAAATACTTCAGGCATGCA<br>AGTATTTAGCATTCAGTATTCATAATGTAAGAAAAACCTATGAAGCA<br>CAACCCAGTTTAAGTAACACAGGCAGGGCAGGGTGCAGTGCCTAT<br>AATCCCAGCACTTCGGGAGGCCAAGCTGGGAGAATCACTTGAGGT<br>CAGGACTTCCAGGCCAGCCTGGGCAACACAGTGAGACCCTGTCTT<br>TACAAAAAATTTAAAAATAGGCCCAGTGCGGTGGATCATGCCTGT<br>AATCCCAGCACTTTGGGAGGCCGAGGCGAGTGGATCATCAGGTCA<br>GGAGATTGAGACCATCCTGGCTAACACGGTGAAACCCCATCTCTAC<br>TAAAAAAATACAAAAAATTAGCCGGTCATGGTGGCATCTGCTTGTA<br>GTCCCAGCTACTCAGGAGGCTGAGGCAGGAGAATCACTTGAACCC<br>GGGAGGCGGAGGTTGCAGTGAGCCAAGATCACACCATCGCACTCC<br>AGCCTGGGCTACACAGCGAGACTCTGTCTCAAAAGAAAAAAAAAA<br>TTAAAAATAGCCAGGCATGGTGTGTGCACCTGTAATCCCAGCTACT<br>TGGGAAGCTGAGGCAAGAGAATCACTTGAGCCCAGGAGTTTGACG<br>TTATGATTATGCCACTGGACACCAGCCTTGGTGACAGAGAGAGGCC<br>CTATCTCTAAAAAAGAAAAAAAAAAAAAAGTGGCCAGCCACGGTGGC<br>TCCCGCCTGTAATCCCAGCACTTTGGGAGGCTGAGGTGGGTGGATT<br>ACCTGAGGTCAGGAGTGTGAGACCAGCCTGCCCAACATGGTGAAA<br>CCTGGTCTCTACTAAAAATACAAAAATATAGCCAGGCGTGGTGGTG<br>GCCGCCTGTAATCCCAGCTACTGAGGAGGCTGAGGCAGGAGAATC<br>ACTTGAACCTGGGAGGCAGAGGTTGCAGTGAGCCAAGATCACGCC<br>ACTGCACTTCAGCCTGGGTGACAAAGCGAGACTCCGTCTCAAAAA<br>AAAAAAAAAAAAAAAAAGTAACACAGGAAATAAAACCAAGACAAG<br>ATGAGCTCTCTGTGTCTCTGTGTGTCCCTCCCCAGACCCAACCCTG<br>GCGTCCCCCAGAGGTAACCCCTCCCAGGAACTGGGCGTTTGCCAC<br>TCCCCTGCACGGGTCTACACCTCTGCTACAAACGTGTGTCCCTAAG<br>CAGCGGACGCCTTTTGAAAACAAGTCAACTGTATCATGCTACGATA<br>TTTTTTCTGATGTTTGTGCTGTTTCTGAGACGGCCTTATGACACCTT<br>CCCCTATTCTGTAGCATTCTGTTGGCTCACTTTGCCACAGTGTATGC<br>ACCCATTCTCCCACAAATGGACTTTTCATTTGCTTCTCAATGTTTGC<br>TACCAAAAATCTCCCTGCAATAAATTTATTTTATATATCCCCTTGTAT<br>GTGTGTGAGGGTCCCCTAACTGCTCACAAATATCTCAGCAACTCCG<br>CCCTCCCCACTCAGACCTCATTTCCCTCCTACCAGACATGCTCAAA<br>GGAAACTGGGAGCAACACAAAGATGACTTTGAATCTCTGTCCACC

	ACCCCTAGACTGTATGACACCCGCAAGTGACAACCTCCCCTCTCTG<br>GCCTCGGTTTCCCCCTCTGTAAAATGGGGGATGGCCTACCTTGCCA<br>GTCATGGCAGGTCAATATAAAACAACTTCTGGCTGTGCACAGTGGC<br>ACATGCCTGTAATCCCAGCACTTTGGGAAGCTGAGGCAGGAGGATT<br>GCTTGAGGCCAGGAGTTCAAGACCAGCCTGGGCAATATAGCAAGA<br>TCCCATCCCTCTTAAAAAAAAAAAAAGAAGAAAAATATATGTTGTTG<br>TTGTTATTATTGTTATTATTAGAGACAGAGTTTCGCTTTTGTCACCCA<br>GGCTGGAGTGCAATGGTGTGATCTCAGCTCACTGCAACCTCCACCT<br>CCTGGGTTCAACTGATTCTCCTGCCTCAGCCTCCTGAGTAGCTGGA<br>ATAATAGGCATGTGCCACAACATCTGGCTAATTTTGTATTTTTAGTAG<br>AGATGGGGTTTCGCCATGTTGGCCAGGCTGGTCTCAAACTCGACCT<br>CAGGTGATTCTCCTGTCTCAGCCTCCCAAAGGGCTGGGATTACAGG<br>CATAAGCCACCATAGCTGGCCTGTTTTTTTAAAAAAAGAATTCCAGT<br>AAAGAGCTGATCATGGTTCTCACTCCTTGAATACCAGGAACACCAT<br>CTCGTATCACATAATGAGACAGGGAGACATTCTGGTCCTCATCTCAC<br>AGATGAAAAATGTCAAGCTTCGAAGGATCAAAGTGCCCACCTAGT<br>CACACGGGTAGTCAGCCACAGGTCAGCCTGCCTTATTTATTCTTCAT<br>GAGTATTTATAGTGACTAACATTTACTGGGCGCCTACTGTGGGCCAT<br>TTCTGTGCATGTGACAACCCCTTTAAGTCCTTGTTTCTAATCCCAAG<br>AAGCAAGGAAATGGGGTCAGGGAAGGGACAAGGTTTGCCCAAGT<br>CCAGGCAGGGGGAGAGGTCAAGCTCAGAACCATCACCTGCCCATG<br>ACACATGCCCAGGACTCAGGTTCCCTAGGCTTCCTTCCAAAGGCTC<br>AGCAGTGACGAGCCAGCCCTTGAACCAGCCTCTTCCCCCACCCAA<br>GCAGCCACCTCTCAGGGGAATTGTGGCCACCACAGGTGCAGGGAG<br>CAGTTTCTCTCCACTCACAGCCTGAAGCATACCCGGCAGGGGCTGT<br>CCCCAGGCCCAACAAGCAAAGGGCCCAGTAGCGAGGGCCACTGG<br>AGCCCATCTCCGGGGGGCTGGGCAGGAAGTAGGGTGGGGTTTGGG<br>GTAGGGATCTGGTACCCTGGGACTGCTGCAACTCAAACTAACCAAC<br>CC
CD8A	GTGCAGGGTTATCTTGAATAGAAATTTTTGAAAATCTCTTCCTCTCT<br>TTACTCTCTCTTTTCCTGAGTTTTTTAAAGTAGCCTCTGACTTTCCA<br>GGGCCCACAGTCAGAACTAGGCCTTACATGGGTGGCTGTGGGCTCT<br>CTTTCAATCAAGAGAAAATCAGATCCAAATCCCTGTTTCAAGCAGA<br>GAGCCTGGCTCCAGCCTTCCTCTATAGGCAAAGCACTGTGAAACTC<br>TGTTAGCTGAAGGAGTCAAACTCTTGGTGCCTCTACACTAAGAACA<br>GACCCTCAAGACCCAGGCCCAAGAATGGTTTGCTAAGAACAGATC<br>CCAGCAAGCCAGCCCCTGATTCCAGCCATTGTCCTGCAACTACATC<br>CTTGAAGACGTTTATCTTGCTTTTGAGTACATCTGCCTTCTCCCCTT<br>CCTCCTCTTCTTTTGTTTTTAACTATACCATTGCTATATGTTAAAGCAT<br>GGTATCAGCTGATCTTGATCAGACATATCAAATGCCTCATTCTAAAC<br>AACCTCTCTCCTAATTCTTCCTTTATCTTCAAACCCAACTATATGTGA<br>AGCCTTTCCTGACCACTCTTACTTGGAATGAACTATCTAAGCCTCCT<br>CTGGTCTGCTGTACCCTACTTCTACCTTCCTTTAGCTTTTACTGAGC<br>AAGAATTTCTTGAGTGTGTGCCACTCAAGTGTGTGCCAAGCCCTCT<br>GTTTGGCACCCTGGTGTAAACATGAGAGGGACAAGGCTCCTTCCTC<br>AAGGGACCAAGGGTTTGGTTGTCAAGCTGGACAGGCAAACAAAC

	CATTGTGCTCCAGTCTGCAGATACCCAAGGTCCGGGTGACACAGA<br>GAAGAGAAGGATGTGGTCTGTACTGTCTGGGAGTGGGAGGGGGAA<br>GGAGCTAAACCCCACAGGAAGGAGATGCTTGAGCTGGGTCTTAAG<br>GGAGAGGAGTTTGGTAAGCAGAGAGCAGAGGTGAGAACATTCTTG<br>GCTGAGGGAATAGCATGTTTAATGGCTCTGAAGTGGAAAGGACTAG<br>GGTCCGCTTGAGAAATGAGAGATGCGGAACCTAGGAGGAGTGAAG<br>AAGGTGACAGACAAGAGGACAGAGACTATGAAACCAAGTGTGCA<br>GGGCCTCACAGGCAACATTAGGGATCCCCGACATTCTACTAGGGAC<br>ACCAGGAAGTCCTTTCAGGTTGAAGCAGGGGGTTGGCATGATGAG<br>ATTTTCTCTAGAAGGGACCAGAGCAGAAATGGGGTTGGCCAGTTA<br>AGAGGCCATTGCAAAAGTCCAGGAAAGAAGGACAGCAGCTAGAT<br>GAACTTGGGGAACTCGGGGTGGAGGAAAAGAAAGGTGACAGAAG<br>TGAGGCCAAATTGATTGGTATTTCCAATTAAGATAAAACAAGCCCT<br>GATAACTCTAGAGTTTCTAGCCCTGGACAAGCTAACTCCTTCCAAG<br>AACAGTAACATCTCCATAGACCTGAATTGGCGATGGGGACACAGCA<br>GGCAATTTCCCAGCCATGATTCCAAGTTGTCCCTGCAGTTCATGTCT<br>TAGGAGCAGTGGCACAGAACCCTGATGGATTTCCTACCACTGCTCT<br>GCCCGGTTTTGCATTTTGAATATGTCTTTAATCTACATTAAAGTTTTC<br>CATTTCCATGGACCAGTTTAAATCAGCATAGAAACTGGTTACTATCT<br>TACTCTATCTACTTCTGGATATTTTTTAAATAAGGGGCTTGCTTAAA<br>GGAGTGCAGTCATTCACATTTCTTCATGCCATCCCAAGTCAGTGCTA<br>CAGTGTGGGGTTTGGGAACTTACCTGGCAGTGATGGACAGGCCCA<br>ACCCAGCCCTCTGCACACCTGGTCTACAGAAAGTTTCTCCAGAACT<br>TGTGCAATTGCATCTGTTCTGCCACTGCAAGGCGGATGTCATTGTG<br>GTCACATCGAAAATCCACCACGTTCTGCAAAAAATATCACTGGGGT<br>CACAAAGCCTCCTTGATGGGTTTGACATGTTGCCTTATCCTCCCCAC<br>CCCAACCCCCATGGTGCTCTGTACAAAGGGGCTGCCACCCCACTG<br>GCTGACACTTTTCTGAGCTGCAGGGCTAGAAGGCCCCCAAGCTGA<br>TTTCCGTGAGACTAAGATGGAACTAGTCTATTTTAAAGCAGTACAC<br>CCCCAAATCTGGCCATCATCACACTCAGGGGCCTGATTCTCAAGAT<br>CCAAATAGGTGGAGGTTGACTCTGGCCCCCTCAGTGCTTCCACCAC<br>TGGGCAAGTGCAGTTTCGTGTCTAGCCATCTTCCTTACCACCCTGTT<br>GATTGTAGCCTTTGGCCAGCAGGGCCATTTGACACTCCTGATACAG<br>TGCTGGTGGTGCTGAGCAAAATGGCAAACTACCATCTACAGCAAG<br>AATTGATGTGTTGGTGGCCTGTCTGGACTTGATTGCTTCCTTGTCTC<br>CCTCATTCATGTGAGTTGAGGGACAGCACTGTGTCTACACAGCGAG<br>GGACCCTCATACCATGCACACACACACACACATGCATACATCGATA<br>GGCCAGTTATAACCATCATTGTAAAGGTGTTTTCTTCCCCAACTCCC<br>TCATCTCCCATTTGAAAAAGCTGCAGGTACTGGTGCAAGCCCTCAG<br>TGATACAGATACATTATATCTACCACAGCCTTGCTAGTGTTGCAATAA<br>TGAGAAATCGCCTAAATGTCCACTAACAGCAGAATTAAATTATGGA<br>ACACCCATATATAATGCACCCATTTATAAGAATGGGGTGGATCTTCAT<br>CTGCTGAACGCTCTCCTTCTACTGTGAAACAAGATAGGTTGCAAAA<br>AAATATTTATCTCATACAATCCCATGTGTCTAAGATGATGTATAAACA<br>TCTGTTTATGTACAGATAATATCTAAGGGACACATTCAACTGTAACA<br>GTGGTTTTCTGTGGGGAGCAGGTGACAGTGAGGAGAGTTGAGACA

	GGGAACTTTGTGTTTGTTTTCGTACAAGAATATATTGCACTGTTGTC<br>ACTTAAAAGACATTTTTAAAAATTTCTGAATATAAAATGTTACATGT<br>TCCATGCTAGAATAAGCATCTTGGATGCTTTGAGGGAGCTGGAAGG<br>AGGTACTGAAATCAAAATAATTAAAAAAAAAAAAAAAAAGCCCATC<br>CAGGCCCTGATATGGTGGCTCACACCTGTAATCTCAACACTTTGGA<br>GGCCAAGGCGGGGCAAAGCGCCTGACCTCGGGCATTGGAGACCA<br>GCCTGGGCAACATGGTGAAACCCCATCTCTACCAAAAATACAAAA<br>AATTAGCTGGGCATGGTGGTGTGCTCCTGTGGTCCCAGCTACTTGG<br>GAGGCTGAGGTGGAAGGATCACTTGAGCCTGGGAGGTAGAGGCTG<br>CAGTGAGCCGAGGTGGTGCCACACTGCACTCCAGCCTGGGCAACA<br>CAGCCAGACCCCATCTCAAAAAAAAAAAATAGCAACAACGACAAAG<br>AACCCATCCAACACTTAAGTTTGTACTTCACAAGGACAAGGAGAAT<br>GCAGTTCAACAAGCACAGCTCAGCCCATGCTGTGTTTCTGGGGATG<br>GAGGACACATCATGAAGTCTCGGCATGGTCCTCGCCCAGCAGAACT<br>AATGGGTGGGGCAGACACATGGAACACAGAATGCCAGGTGTCTTG<br>TCTCTAGGATCAAGCAGTAGCAATGTGAGGTGCAGCCACTATGGGA<br>TTCAAAGTGGAAAGACTCAGAAACCCTGAAGAAATGGGAAGAGG<br>GCATGCCACTGGAGCTGGAAGGTTTCAGGACAGCAACAGTCAAAA<br>AGGAAAGATGAGCCTGTAGTCCCAGCTACTCAGGGGGCTGGGGTG<br>GGAGGATCACTGGAGCTCAGGAGTCCCAAGGCCAGCCTGAGCAAA<br>ACAGCGAGACTCCAGTCTTTTTTATTTTATTTTATTTTTTAAAGAAAC<br>AAAAAGGAAGGGGACACACATGTGTTAGGGACAGAAAAGAGAAA<br>ACCGCCTCTACCCAAGCATTCACCCACATCACCCACACCTCCCTGC<br>AGAGCACCCAGAGCTGGGGGTGAAAGAAATGAGGTCCAAATGAG<br>ACAGCACAGGAGCTGCCTCCAGGGCTTAAACAGACCAGCATTCCA<br>GGCCGAGGGACCGCAAGTGCAAGGGCGTGAAAGCACAGAGCGCA<br>GGGGTTGAATGACTTCAAGCCTGTGAAGCTGCAGCTGCAGGTGTAT<br>GGGAAAGGCAGGGCAGGGGGCTGTGCGGAGGCTGGGAGGAGCCA<br>GCACCCAAGGGCTGGTCAACCAAGCTGGGGGTTGAATTTCCATCC<br>AGCAATGCAGGCCATGGGAGGCTGCAGCAGTGACGCTGTCAGATC<br>CCCTTTGTGAGAATAATAATTTTTATAACAACGTGGCTGGAGGACTG<br>ATCAGGAGAGAGACTGGTGTGAATTGAAGGCTGTTGCAATGGCTC<br>CAAGAAGAGATGAGGCTGTGTGGTGAGTTTAGCCGCTGGATGAAA<br>GGCCGGAAGAATGAGGTCAGCAGCGCACTGACACCGACACCCAA<br>AGCTTCGGCTGCTGCCGCTCTCATGGAAATCTCCTGGGGGAAGGG<br>AGAGGGTCCTTCCTCGGTGAAAACTGGGGCTGCTCTAGCGAGTTC<br>CTCAGAAGCGGGCAGGTCGCTAGTTCCTCTTCCTTTTCAGCCCTCA<br>GTGCCCATTTTGCCAATAAAAAGTCCCAAGGTGACAGTACAAGAG<br>ACGCCTTTAGTGAAGGCAAAGGAAGGGACACTCCCCTCCTTTGCT<br>GCCTACTCTCGCCCTCACTTCTTGAAATCTTTGGTCTCCCTTCACCC<br>ACTCTGTCACTCTCACAAGACAACCATTTCCAAGGACTATTTCCAA<br>GCCCTTTTCCTCATCCCCAAACCCGCAGTTTTCAGCTGCCCCCAGT<br>TGCCTGGCCAGGCTGCCTCGACGGCCCTATTCACGGGCCCCAGCCT<br>CCTCGCCGGGCTGGAAGGCGACAACCGCGAAAAGGAGGGTGACT<br>CTCCTCGGCGGGGGCTTCGGGTGACATCACATCCTCCAAATGCGAA<br>ATCAGGCTCCGGGCCGGCCGAAGGGCGCAACTTTCCCCCCTCGGC

	GCCCCACCGGCTCCCGCGCGCCTCCCCTCGCG
CDC2	TATTATAAGGGCATTAATCCCATTAATGAGGGAAAAGCCCTCGAGGT TTAATAATCACCTCCCGAAGGCCCTACCTCTTAATACCATCACCTTG GGGTTTAAGTTCCAACATATGCATTTAGGAGGGGCACATACCTTCA AATTATAGCAATCCGCAAGTGATATACACTTCAGGCCCTATGGAACC TACACTTACCCTTGGCCATATCCAGCCACGTGGATGTCCCACAGTAC TTCACTATCAATATGCCCAAAGTGATGCTCCATGCCCCATAGCTGCC CCTTAGCATCTCTCTAGAACAGCAGGACCCTCAGCCTGTTGCAAAG CCACACACCCAAGTCATCCCTGATGACTCCTTCTCCCTTTTTAACC CAGTCCCAGCTCCTAAAAATATCTCATTGTCATTTCTTCCTCTTGAA ACTGACATCTCCCCAAGTTCAGCACCTGCTGGGACTGTTGCAGAGC CCTCCATTGTCTGCTGGCTCTGATGCTGCATCACCAGCCCTCTTCAT GACTTTTCCCCCCTAACTCCCACTTATGACCATGTCACTGCCTTGTT CACATTTTTTGTTCAAGTCCAACGTCTAATCCAAAACTTTTAGCAT GACCTTGAACATTACCTTCAAGGTATAGATATTATTTAACTACCTAGC CTAATGTTTATGACCCTTGCTGAGCACCCAGATTCTGTTTATGTTCA GCGTACTGAATTAATTTCAGTCTACAAACTTGCATTGTTCTTTCTTG TCAATGGATCAAGCTCTCCACCTTCATTTGTCTTCTTGGCAAACTCT TTTTATTCCTTAAGACTCAGTTCAGTCAACTTTGCTATGAAGCCTTG CCTGATGCCCTCTCCACTATGTTCTGCACACAGCAGTCTTCACCATA TTGCATTGTAAACATTTGCACCCCACCTAGGTGGAAGATCCACAGC TACTCACCTGTTATCCTTGATCCTCATTAGTCCCAAGGTTGACATATG AATCTCTTCTCCCTACACCATGCCCAGTTTCTAAAATTCCAATGAGA GATTAAGGTGGGTTGACATGTATGCATTTAAAGTACAACTACCAAC AGGTTTAAAGTGATCTCTTTCTCTTTTTGTGGGGCGAGAATGAAGAT AGGGGGCATGGAGGTTGTATATGACAAACTTTTATTATCTGTATTTTA CACTTTGGATTATTTCTTAAAATTTACAGAAAAATCACATTACTTTTA TAATCAGAAAAACACCCAACTCTGTCATAGCAACGATAGTTCAGTA TTTTCACTTGTGGTGAAAAGACCTGAATCTATATATCTTGTATTTTCT GTCACTTTCCTTTCTGAATTAACATGAAAAAGGATAAATCTTCTTCT TTTGCATACATTTTCCGGCAACTTTTAAATTTTTTTTTCTTTTTATAT ATTTGTCTGTATAGAGACAGGGTCTTACTATGTTGCCAAGCTGGCCT CAAACTCCTGGCCTCAAGGGATCCTCCACCTTGGCCTCCCAAAGTG CTGGGATTACAGGTGTGAGCCACTGTGCCTGGCCAATTTTTACCATT TTTATCCAGTAGCAATGGAGGCCTTGGCAAGACTGATTCATTCATTC ATTTATTTATTCGTTCATTTTTGGGCTAGGGTAGTACATTCACGTGGC TCAATATTCAAAAGATACAAAACTCTCCTTTCCACCTCTGTACCAGG TCACTCTTGTATTCCACAGAGGGCACATTTTTATTAGTTTCTTCCAC CTCCTTCCCGAAGTGTATTTTCTTTTCTTTTCTTTTTTTTTTAGAAGG AGTCTCGCTCTATCACTAGGCTGGAGTGCAGTGGCGCCATCTCGGC TCACTGCAACATTCGCCTCCAGGGTTCAAGCGATTCTCCTGACTCA GCCTCCCGAGTAGCTGGGATTACAGGCATGTGCCACCACGCCCAGC TAATTTTTGTATTTTTAGTAGAGATGGGTTTCACCACGTTGGCCAG GATGGTCTCGATCTCCTGACAACGTGATCCACCCGCCTTGGCCTCC CAGAGTGCTGAGATTATAGGCGTGAGACACCGCGCCCGGCCCGGA GGTGTATTTTCAAGCAAATGTTCTCACATTTTTCTTATCTAAAAAAA

	ACACTTGTCTTGACCTGGGCTGCCATAACAAAATGCCATAGACTAC<br>GTGCCTTAAACAACAAAATTTTATTTTCACTCAGTTCTGGAGGCTTG<br>GTGTGCAAGTTCAGAGGGCGGGCAACATCAGGTTCTGGCAAGGGC<br>TGACTTCCTCACTGACAGGTGCCTTCTCATTCTAACCTCACATGAG<br>AGCAACCAAGCTCTTCAGCGTCTCTTCTTATAAGAGCACTAATTCCA<br>TCACGAGGGCCCCACCCTCATGTCCTCATCTAAACCTAATTACCTCC<br>CAAAGCCTCATCTCCAAACACCATCCCAGTGGGGTGAGGCCTTCA<br>ACGTATGAATTAGCGGGGTGGGGGACAACAAACATTCAGACCAT<br>AGAAATACGTAAATATAAGAAACAAAACCAAAAAGACTTATGTGCA<br>ATACCAACTGATCTTTTCTTTTCCTTCCAAAATGGAGTTTACCAAAA<br>TACAAATATGAAGTTTTTCAGAGCACATACCTTCCCCTGCTCTCCTC<br>TGCAGTTTTTTTTTTTTTAATACCACCTCTAGGGTAGTTTCCTAAAA<br>TATGGTTCCAAGGCTGTGTGTACCTCTCCTGCTCAGATCCTTTGGAA<br>CAGTGTTCAGCGCTCATCAGGATTCTGACTGTAAACTCCCTTCTCT<br>GACTTTAACATCACAGGATGCAGTACTCCCTACATTCTACCATGGGG<br>GGCACCCTGAATTACCTGGGTTGACTTTCTGGCCTCTGTGCTCTACT<br>TATACAATTTCCTCTGTATGGAAGGCCCACTCCAGAATCCTTTCCCC<br>AGCCTTTGTTCAAATGTCACTTCTGCTAGCATTAGCAGTTTCCTCCT<br>CTGGGTTCTCACATTGCCCTGTAACATAAGTTACAAGGTAAGTACC<br>AGAAGAGAATTCCCAATTATCATCCTGCTCCCCTACCCTCAACACCT<br>CCAGCGGTAACTGTATCTTATTCATTTTTGTGTCTATGAAGCACAGT<br>GTTGCACTTTGGAGATACTACATAGAAGTTGGCCCAAAGGGTAATA<br>AACTGTATTTGCCAAGATGATAGCATGGATTTACAATAACCTCTGAA<br>ACTTTATAAAGAAAGATGATTGAGTTAAATTGATACACCTAGCATCA<br>CAGCTGTGGAAGCTTGTGGTCTTCTTTATGTTAAAGGTAAAATGGAT<br>ATAATGAATTAGTTGCCATTTTTAGATGATATAGAAGTTGAATTCCTC<br>TTAAGCCTCTCAAAGAGAAAGACATATTAAAATTCATTAAAACCAG<br>TACCATAAACTGCACAGAACAGCCTGGTTCCCTAGAGCACTTCTCT<br>GGGGCAGTGAGTTTTCATTTAAAAAATGAAGTTTGGATTTTAAACA<br>TAAGTTTTAAAAGTGATTAGGCTCCAGCAGGAGTTCTTAAATTCCTA<br>CCTACACCATGACTCCCAAATGCTCTGCCTCTGTTGCCTAGGAACA<br>AAAATTGTCAAGGTTGAGCCAGATGCAATGTATAACACCAGATGAT<br>AGATGCAGCTCATTGTGTTAAAAGGTGTTAAACACTTAAGTGCATG<br>TGATATACTCTATATCACAAGGGTAAGGAAGTTCCATTCCATATCCTG<br>ATTTGGGTCTTCTAATACATACTTGTAGTGGATGTGGTAATCAGTCTT<br>TGTTCTATGTCCCATTAGGATCAATGCAACCAGAAATCCTTAAAATG<br>GATTTTTATTATGTCTAGCTCTGTTTTACTGTTCTATTTGTTTATTTTA<br>AAAAGATTTTAGAATGGTGTTTAGGTTGGGCTGGTGGCTCACGCCT<br>GTAATCTCAGCACTTTGGGAGGCCAAGGCAGGAGGATTGCTGGAG<br>CCTAGGAGTTGGAGACCAGCTAGGGCAACATAAGGAGACATTGTC<br>TCCGCAAACAAACAGAAAAAAAAAAAGTAAGAAAGAAAGAGAG<br>AAAGAAGGAAAGAACAAGAAAAAGGAAAAAAAAATTAACTGCAG<br>TAAGTGCAGAAATCTCTCTCAAGGATATGATGGATGGCAATGAAGA<br>CAGAGTTTTTGACACTGGATTATTTAAAGCATAATTTAAGCCTCTTT<br>CTCTCCCCTCATAGAATTGTGAATTTTTGTGTTTTAATTATATTTAAG<br>CCAGGCGTGGTGGCGAGTGCCTGTAGTCGCAGCTGCGCTGGAGGC

	TGAGGCCGATTGCTTGAGCCCAGGATTTGGAGGCCAGCATGCGCA<br>ACATAATGAGACCCAGTCTCTAAATGCATGCCTCTCTCTATATATTTA<br>AAATTCTGATGTGAAAATATTTTAAAATTTAATACATTTCAAATGTTT<br>TTAATTGTATAATAAACAAAATGTAAATAATAAAATAATTTAATATTA<br>AATTCAAAAATGAGGTAGAAACAAAGCACAGCGATATAAATAATAA<br>ATTTTCCTTTACATTTTTGAGGCGGTCTTTTGAGTTTTCCATTTCCTT<br>CTTAAGGTCACTGAAATGTGCTCCTTGGAGCCAGCCCGCAAATCAC<br>GCATTTAGAAAAACATAACTATACACTCCTAACCCTAAGTATTAGAA<br>GTGAAAGTAATGGAATCTCGATGTAAACACAATATCACTTTTTTGAT<br>GAGCTATTTTGAGTATAATAAATTTGAACTGTGCCAATGCTGGGAGA<br>AAAAATTTAAAAGAAGAACGGAGCGAACAGTAGCTTCCTGCTCCG<br>CTGACTAGAAACAGTAGGACGACACTCTCCCGACTGGAGGAGAGC<br>GCTTGCGCTCGCACTCAGTTGGCGCCCGCCCTCCTGCTTTTTCTCTA<br>GCCGCCCTTTCCTCTTTCTTTCGCGCTCTAGCCACCCGGGAAGGCC<br>TGCCCAGCGTAGCTGGGCTCTGATTGGCTGCTTTGAAAGTCTACGG<br>GCTACCCGATTGGTGAATCCGGGGCCCTTTAGCGCGGTGAGTTTGA<br>AACTGCTCGCACTTGGCTTCAAAGCTGGCTCTT
CDC25A	TTGTATTTTTAGTAGAGATGGGGCTTTCTTTTCTTTTTTTTTTTTTTT<br>TTGAGAGGGAGTCTCGCACTGTCACCCAGGCTGGAGTGCAGTAGT<br>GCCATCTCCACTCACTTCAACCACCGCTTCCCAGGTTCAAGCAATT<br>CTCTTGCCTCAGCCTCCCAAGTAGCTGGGATTACAGGTGCCCACCA<br>CCACGCACCACTAATTTTTTGTATTTTTAGTAGAGATGGGGTTTCAC<br>TATGTTAGCCAGGCTGGTCTTGAACTCCTGACCTCATGATCCACCCA<br>TCTCGGTTTCCCAAAGTGCTGGGATTACAGGCGTGAGCCACCATGC<br>CCGGCCAAGATGGTGTTTCACCGTGTTGGCCAGGCTGGTTTTGAAC<br>TCCTGACCTCGGCCTCCCAAAGTGCCGGGATTACAGGTGTGAGTCA<br>CTGTGCCCGGCCAGGAGTTTTACTGTGAAGGGAAGGAATGAATATA<br>GTTGGAGGGGAAAGTGGCATCAAGGAAAGGCTTTTTATCACTAATA<br>ATAAACATTTCATTGAAATACACCATTCATACAGAACAGCTAACAAA<br>TCTTGAGTGTGTAGCTCAATGAATTTCCACAAGGTGAACACACTCA<br>GGTAACCAGCACCCAGATCAAGAAATGGAAAGTGGCCAGGTGCAG<br>TGGCTCATGCATATAATGCTAACACTTTGGGAGTCCAAGACGGGAG<br>GATCGCTTGAGCTCGAGAGTTTGAGACCAGCCTGGGCATCATAGTT<br>AGACTCCCGTCTCTAAAATTATACATATATTTTTTTCTGAGACAGAG<br>TCTTGCTCTGTTGCCCAGGCTGGAGTGCAATGGCATGATCTCAGTT<br>CACTGTAACCTCCGCCTCCTGGGTTCAAGTGATTCTACTGCCTCAG<br>TCTCCCAAGTAGCTGGGATTACAGGCGTGTGCCACCACACCCGGCT<br>AATTTTTTGTATTTTTAGTAGAGACGGAGTTTTGCCATGTTGGCCAC<br>ACTGGTCTCGAACTCCTGACCTCATGATCCACCCACCTCGGTTTCC<br>CAAGGTGCTGGGATTACAGGCGTGAGCCACCATGCCCAGCCAAAA<br>TAAAAAATAAAATTTTTAATTAGCTAGGTTTATGGGTGCATGCCTGT<br>AGTTCAGGCTACTCAGGAAGCTGAGGTGGGAGGATCACTTGAACC<br>CAGAAGTTCGAGGCTATAGTGAGCCATGATCATGTCACTGCACTCC<br>AACCTGGGCCACAGAGCAAGACTCTGTCTCAAAAAGTGAAAAATA<br>AATAAGAAACAGAATATGACCAGTTGCCAATATCCCCTTCATGGCC<br>CCTTGCAGTCACCACCATCTTTCTGAAAGGTAACTAGTACCATAACT

	AACTTTTTTTTTTTTTTGAGACGGAGTCTCGCCCTGTCACCCAGGCT<br>GGAGTGCAGTGGCTCAATCTCGGTTCACTGCAACCTCCACCTCCTG<br>GGTTCAAGCAATTCTCATGCCTCAGCCTCCATAGTAGCTGGGATTAC<br>AGTTGTGCACCACCATACATGGCTAATTTTTGTGTATGTATATATGTG<br>TGTATATATATGTTTGTGTATATTACATGTGTGTATATATGTATATTACA<br>TATGTGTGTATATATGTATATATACATATATATGTATATGTATATATACAT<br>ATATATGTATATGTATATATACATATATATGTATATGTATGTACACATATA<br>TATGTATATTATGTATATATACACATGTATGTATATTATGTATATATACAC<br>ACATATATGTGTGTGTGTGTATATATATATATATTTTTTTTTAGTAGAG<br>ATGAGGTTTCACTATGTTGGCCAGGCTGGTCTCGAACTCCTGGCCT<br>CAAGTGATTCGCCTGCCTCAGCCTCCCAAAGTGCTGGGATTACAGG<br>TGTGAGCCAGACCTAAACAGCATGTTCTCTTTTGTGTCTTCCTTCTT<br>ATGTTTGACATTGATGTGAGATTCACTCCTACTGTGGGTAGCGCTTC<br>TGTCCTTTTCATCACTGTATAAAATAACTTTGTTCTATTTACCCATTTT<br>ATTGTTGATGGGCATTAGGGTTGCTTTCTGTTTGGAGCTAAAATGAT<br>TAACATGGCCATGAATATTTGTGTACATGCATTTTGGCACACTTGAA<br>CTGTATTGTAGCTTCTGTGCCAACTTTTCCCTTCCCATGGCCTTCTG<br>TGGCCACCTCCTCGCCTTCCTACATGATTTCCATAGTTGCCTGTGAC<br>CTCATTCTGGCCACCTTCAGTGACTCTCCTGGGTGCACCTCTGAAC<br>TATGGACTCGGCTACTCCCTTGTGGTTTCTATCTCAGCCCCAGCTTT<br>GCGGTCCTGTCTCACTACATCAAGTCCTCAATGCTGGTGCTCCCCA<br>GATCCAGGGTCCTAAAAACCCTTTGTACATCCTCAATGGGCATTACC<br>ATCTTCATGCTAATCAACACCAAATCTGCCTCCTGACTACACCCCCC<br>AGCCAGTCCACCAGCACAAAACTGAGCTCATCAAATTCCTCCAAG<br>AAGTATGCCTCCAATTGTTGAAATGTTACATCCACCCAGGTACTTGA<br>ATCATTACCTAAGAATCATTCTAGCCTCCTCCCTTCCCTCTAGCCCCC<br>TACAATCTTAACTCTCAATTATCAAAATCTGATGAGATCTACCTCCTT<br>CAGGGCTCACAGAATCATTCCCTTCCTTTGTCTCCAAGGCCCCCAC<br>CTTGCTTCAGAATCTCCCAGACTTTTGAGACAGCCACCTCCTTTTTC<br>CTCAGGCCTCCCCACTCTCCTCCACTGATCCACCCTAAAGCAAATC<br>TGATCCGGTTATCACACACCCTCCACTGTCCACAGGATAAGGCCAA<br>ACTCTTCAGCAAACTCCAATCCTTTCAAGAACTGTCCCTCACAGCC<br>AGTCCCATGTGCCCATTCTAGAACCGACTGGATCAACTCAGGAGTA<br>GCTCCCGGCAAGTACGGCGTTCCTCCTCACCTCCTGGCTGCCGAGG<br>GCCGTTTCCTTAGAAAGCACTTCCCACCAGCCCCAAGCTCCCCTTC<br>TCCAGCAACCTTGACCTTTAGTTTTAATCTCCTTTAGGACGTGCCCC<br>CCACCGTACTGCCTTCCCCAGTCCCCGCCCCTCCTCTAGGTGCCCA<br>CCATCCCTCCACAAACCACAGCTATTGTCATGGTAATAATTCCATTAT<br>TAGAAGGAGCTTTATGCTCAGCGGACCAGAGCTCCCTGAGAGCAG<br>GGATTGTGGTATTATTTCAAGCACTGAATTTGTTCTAGTAATCCTTAA<br>ATAAACATATGTTGAATGAATGAATGCAGTAACAGGAATGTTGAGC<br>CAAGTTTTGTTTTTGTTTTTTGAGACGGAGTCTCGCTCTGTTGCCCA<br>GGCTGGAGTGCAGTGGCTCGGTCTTGGCTCACTGCAAGCTCCGCC<br>TCCCGAGTTCAGCCATTCTTCTGCCTCAGCCTCCCGAGTAGCTGGG<br>ACTACAGGCGCCCGCCACCACGCCCGGTAATTTTTTGTATTTTTAGT<br>AGAGCCGGGGTTTCACCATGTTAGCCAGGATGGTCTCGATCTCCTG

	ACCCCGTGATCCGCCCGCCTCGGTCTCCCAAAGTGCTGGGATTACA<br>GGCGTGAGCCACCGCTCCCGGCCTGTTGAGCCAAGTATTAAACCAC<br>ATAAAACACAGGAGGAGAGGAAAGTATTCAGAGATACGACAGGGA<br>GAACTATTTGAAACAGTGGGACCACAGACCATGTGAACTGTGCAG<br>AGAGGCCTAAATAGCCTGGGGTTTCTGGAGGGAAGGGAGGTTCCT<br>GGGTGGTGGAGCACTGGTGCACAGAAAAGGAGATGAATCCTGAGA<br>GAGAGGTTCCTCACTGTGAAGGGTCTGGTGTTGGGGCCAAAGGTT<br>TGGGGTTTCCACCTGTCCAGCATGGCTTTTCAAACTGTAGGGTTTT<br>TTTTTTTTGAGTGGGGGGCAGGGGGTGTCTCACTTTGTTGCCCAGG<br>CTGGTCTCAAATTCGTGGGCTCAGGCAATCCTCCCACCTCGCCCTC<br>CCAAAGTGCTGGGATGATAGGCGTGAGCCACTAAGCCCAGCAGTA<br>GGTTGCAATTAAATCATGCACTTATGGCCCTCAAAATCCGTCTAGGA<br>GCTGCCACAGGTTCGGCGCTGTAAAAGTAAATATGCCCACTTAGAA<br>AATGGGGATAATTCCTATGTCACAGAGTTGTAAAGATTAAATGAATT<br>AATACAGATTAACACACTGGGAACAGTGTCAAGTGCATAAGCATTA<br>TGTAAACTTTAGCTATTATTTGCTATTATTGTGTTTGCTGTTATTTCTC<br>TCTAGGAGCTCCCAGGGGGCTAAGAAGTGGTGGGAAAGAAAGAA<br>ATGATTCTAAGAGCATCCAATAAGGGCTAGAATGGAAGTGAGCAAA<br>AAATGCTGAGGCCCACAGCACAGGTTGTACTGCGGGGTTCAAATC<br>CCATGAGGCCAGCAGCACCCAGGGTCTGTGAGCCCTCCAGAGTTG<br>GGCCCTGGTGGTCGAGTCCAGTCCTGGGGGTCATTGCATTCCCTCC<br>CTCATTATAAAATGGGGCCTGGAGGCCCGGGGCGGAAGAAAGGGG<br>TCCACAATACTGCACGGTTAGAGGCCGAGCCAAGGCTGGATCCGG<br>CCAGACCTCCACAGGTCTTCCTTAGCCTCCACATTGCCTCAGAGTG<br>TGGGGCGCCCGGCTGGGGGCGAGGTAGCGGAGGCCCAAAGGGGG<br>CCGAAGCTAACTGGACGGCAGCTCGCGATGGGAACTACGCTTCCC<br>AGCATGCGACGGGGCAAAGGGGCCTTTCAGCCGCGAGCAGCGCCT<br>CGCAGGTTCTGCTGGGAGTTTTCATTGACCTCTGCTCCCCCTCTCAT<br>TTTGATCCCCGCTCTTCTGCTCTGGGCTCCGCCCCCTTCTGAGAGCC<br>GATGACCTGGCAGAGTCCCGCGAGCCGCTTTCTTCTTCCCCTCTCA<br>TTGGCCCAGCCTAGCTGCCATTCGGTTGAGAGGAGGAGAAGTTGC<br>TTACTGATTGGTGGATTCCGTTTGGCGCCAACTAGGAAAGGGGGGC<br>GGGGCAGCAGCTGGCCCCACTGAGCCGCTATTACCGCGAAAGGCC<br>GGCCTGGCTGCGACAG
CDK2	CAGAGGCTGATTTCTCAGGAAGTGCTTGTTCCCTCCATCCAAGGCC<br>CGCAACTGGGACACGCTCACGGAGAAAGGCACCTGGTCTGGGATT<br>GGAGCCAGAAAAGGTGAGAACCAGGCCTGAGCCACAGCTTCTCCT<br>TTCACAGCCACACCCTCCAACTCCAAGCTGACTGGCTGGCCCTTCA<br>CTGGCACTAAGCCTCACATTTCTCTGCCTAGGTCTTCCTGGCCCTTC<br>TACTTTAAGTTTGCACAGCATTTCACCTTTCTCCTTTCCAGTCCATC<br>CAAGCCTCCCTGCCTTCTCTTGCCCTACAACTGGCCTTGCCCCTTCC<br>TAAACCCTTACCAGTAATGGTGAAGGCTGAGCTGGAATGAGCAAG<br>AGGCACATAGCTCCGGGATCCCCGGCGATGGTAGACAGTCACTTCC<br>ATGGTGTGTGTGCCCAGCATTGCCCTGCCTGTCCCAATGCTCAGCC<br>CAGACACTGGGCCCCCTAGAACTTGCCAGTATTGGCCTGAAGTTTT<br>TGGAATGAAAAGCAAGGAAGAAGAGATTACTGGTCAAAGGGGAC

	TGAGGGACAGGCTTCGAAACGGCACTGGAGATGGGAGGTCAGGA<br>AGTATGATTACTTCTGAGGGTGTTTGGAAGGCTGTGATATGGGGGG<br>ACAGGGAAGGGGTCCTAATGAGGCATTCCATGGGGTAGGACTACA<br>ATTGAGGAAGAGTTGCCAGAAAGAATTATGATAGAGTTGAAGGGG<br>GCTAGGTGCTAAGAAAGAGAAAAGATCTAAGAGGAAAATGAAGAT<br>TAGAGAAAATCACAGAAGTTAAGGTGGAAGGGGCGGCCAAAAGA<br>AAGGTGATCAGGTAGAAAGAAGTAAACACAAGTGTGGATGATAGG<br>CTGAGAAGGGAGTCCCTCACCCCAGGTCTTCCAGACATAAACAAA<br>GCTTCTCTTCTGAGACCAAGAGCCAGATGGGCAAGGTCCACCATC<br>AGGGAAGATGCAGGCATCGTCAGTTTCCTGGGGATACACTGGCTGT<br>CCTCCCCACACCTGGCTCCCTGAAAGATAAATACAGAGTCACTCTC<br>AAACCCTGGCATTCTTTTTTGTATATCAAAACCCCTAAAATTGCTCC<br>CAGGGTATCAGAGAGGGGCTCTGGGAATATTCCCTAGCTAGAAAGT<br>ACATTCTCCATGATATAACCATTTGGGGTGGTAGTTGGGGGTTGAGG<br>GTGTGGAAACTACATTTTTTTTTAGAGACAGGGTCTTAGTCTGATG<br>CCCAGGCTGGAGTGCAGTGGTGTGATCACAGCTCACTGGAGTGTC<br>AACCTCCCAAGGTCAAGCAATCCTCCCACCCGAGCTTCCCGAGTA<br>GCCGGGAACGACGTGTTATTNNNNNNNNNNNNNNNNNNNNNNNNNNNNNN<br>NNNNNNNNNNNNNNNNNNNNNNNNNNNNNNNNNNNNNNNNNNNNNNNNNN<br>NNNNNNNNNNNNNNNNNNNNNNNNNNNNNNNNNNNNNNNNTGATTATTTG<br>CAGCGGGTTTTGATACCCGCTGGCGAGATGGTGTTTTAAACAGGGC<br>CCACAAAATTATGTAACTGACCAAGAGAGGTCTGAGTATTTATAAG<br>GATATGCTGGACCTCCATCTGCCCCACCCAGGTCCAACAACTATCA<br>ACACCTTGCCACATTTGCTTTGTTTATCCTTACTAGTTGTTGTTTTTG<br>TTTTTGTTTTGGCTAAAGTATGTAAAAGCAAATCCCAGACATCGTGT<br>TATTTTAACCCTACAAATTTAAAATGTATGAGTATTTTCTTGCATAAG<br>CACAATGTCATTTCATGCTAGACAAAATTATCAAACTCAGTCCATAT<br>AAAATTTCATGAATATCTGGAAAAATGTCTTATGATTTTTAAAATCA<br>GGATCCAAAATATAAGTACAGTCTTTTTGTTAAGAGCCTTAGGTGG<br>GTGGGTGTGAACAGACTCTTTCCAGGATACATTAAGGAATATAGTA<br>AAAGAAAAAGACTTGAAACTGCAGCACAGCGAGGAAAGTCACAT<br>CAAGGAGAACTTCCTAGAAATCTGTGACACTAAACATCTAGGGAG<br>AAAGCAGGGCTGGGATATAATGTATGTATTTCAGCACAGCAGAAAC<br>TACCCTCACCCCATTTTTCCCTTTCCTGCAGAAACCTCTCTCCCCTC<br>CCCTTCCCTCCCCTCTCCTCTCCTTTCTGTATATAGTTCAGCACAGC<br>AAAAACTACCCTCACCCCATTTTTCCCTTTCCTGCAGAAACTTCCTT<br>CCCTTTCCTTTCCCTTTCCCCTTTCCTTTCCTTTTGATACAGGGTTTT<br>GCTCTCTCACCCTGCGTGCAGTGGCACGTTCATCACTCACTGCAGC<br>CTCAAACTCCTAGGCTGAAGCCATCTTCCTCATTAGCCTCCCAAGT<br>GGCTAGGACCACAGGTGCTCACCACCAAGCCTGGCTAATTTTTTTT<br>TTTTTTTTTTTTGAGACAGAGTTTCGCTCTTGTTGCCCAGGCTGGAA<br>GGCTGGAGTACAATGGCACGATCTCAGCTTACTACAACCTCCGCCT<br>CCCGGGTTCAAGCGATTCTCCTGCCTCAGCCTCCTGAGTGGCTGGG<br>ATTACAGGCATGCACCACCACGCCCAGCTAATTTTGTATTTTTAGTA<br>GAGACTGGGTTTCTCTATGTTGGTCAGGCTGGTCTCAAACTCCCAA<br>CTTCAGCTGATCCACCTGCCTTGGTCTCGCAAAGTGCTGGGATTAC

	AGGCGTGAGCCACCAAGCCTGGCCTAGGCCTGGCTAATTTTTTTAC<br>GTTTCGTAGAGACAAGGTCTCACTATGTTGCTCAGGCTGGTCTTGA<br>ACTCCTGGGCTCAAGCAATCCTATGACCACGGCCTCCCAAAGTGCT<br>GGGATTACAGGTGTGAGCCACTACACCCGGCCTCCTTATCTCTTTTC<br>TTTTCTTTTCTTTTTTTATTTTTTTGAGACAGAGTCTTGCTGTTGCCC<br>AGGCTGGAATGCAATGGTGTGATCTCGGCTCACTGCAACCTCTGCC<br>TCCCAGGTTCAAGCGATTCTCATGCCTCGGCCTCCCAAGTAGTTGG<br>GATTACAGGTGCGTACCACCATGCCCAGCTAATTTTTGTATTTTTAGT<br>AGAGATGGAGTTTCACCATATTGGCCAGGCTGGTCTGACCTCAAGT<br>GATCCACCCGTCTCAGCCTCTCAAAGTGCTAGGATTACAGGCATGA<br>GTTACTGTGCCGGGCCTCATCCCCTTTCTTTTTTTTTTTTTTTTTGAG<br>ACGGAGACTCGCTCTGTCACCCAGGCTGGAGTGCATTGGTGTGATC<br>TTGGCTCACTGCAACCTCTGCCTTCTGGGTTCAAGTGATTCTCCAG<br>CCTCAGCCTTCTGAGTAGCTGGGACTACAGGCATGTGCCACCATGC<br>CCAGCTAATTTTTGTACTTTTAGTAGAGACGGGGGTTTCACCATATT<br>GGCCAGGCTGGTCTCGAACTCCTGACCTCGTGATCCACCTGCTTCA<br>GCCTCCCAAAGTGCTGGGATTACAGGCGTGAGCCACGGCGCCCGG<br>CCTCATTCCCTTTCTAATGTGTGCTGTAACCCTAGTTGATTCTGCTG<br>GGGGAGGATCTGGAATCATCTATTGAGCATCCCACCACTAGAACAA<br>GAGAAGTCATAAGATACCCCATTCTTTCTCCACTTCCAACTCTCCTC<br>AGAGCAGCACGGGACATTCCAGACCCTCTCACCACCCCACCCCCG<br>CCCCTTGCTCCTGTCACGAGGCCCAATCCCCCAGAGCCCTTTCATG<br>TGATGCTCAGCTGAGACCCCTCTGCCTATCTCTCCAGCCCCAGAAC<br>AAAAGGTCTGGGCTGTGTTCACCCGTACCCATCCCAAGAGGAGAG<br>GATTGTATCTCCTTAGTGTTCTTACCCAATACCACATTCTCTGACTCC<br>CCACTTTAAATCTCCTACCCCAAGAGACGTTTCCTAAAAGAGTCTC<br>CTGCACATTTCACCCCTTTGGAGCCCGTGCTCCAGCTCACTGCCAA<br>GGAAGCCTCAGAATCAGTGCCACAGAGACCTTCTATCCTTCTGCCC<br>CTCCAAGCATATGAGATTATTTCAGGCAAAATCCCAAATCTCCATG<br>TAGGGAGGGAGCCCAGGTGCCCACATCCTCACTAAATCAAATGAGT<br>GGGAGACGCCTGAAATATTGCCGCTATCTCCCATTAGGAGGACAGA<br>AACCAGAGCAGGGGGAATTCATCCCCAAGTTCACACCTGCTCATGT<br>CCACATATCCACACATACCTTTTGTAGCCCCCACAGCCAGCAAAGC<br>ACCTATCACAGCCAAATGAAGAAGGCATCTTTTTAGCACCAGATCC<br>ATTGTGTTCTTCCCTCCAGCAACCAAAGGCACTGGGGGGACTGGG<br>ATAGGCCCCTATATAAGAAAAGGGTGCTCATTTGCATAGCCCTTCCT<br>CTTCTCCCTCAGAGAAGGCCTGGGAGGGGCCGGAGGAGAGGAAA<br>AAGGAAAAACTGACAAAGGGATCCTGGTCCCTAGACATTGCTTTC<br>CCATCCTGCTACTCAATGACAGTTTCTGGTTTCACTGGGTCACTCTC<br>ATCTTGATGCACTCCCGGGCAAGAGCTAACTGAAAGGCAGCTGCGT<br>AACACATACCAGACACAACAGTTTATCATGGGAGAGTGAATTAAAC<br>CAGGAACTTCTCAAAAAGACAGAAACAGAACCCTGCATCCCAAAA<br>CAGAGACAGTGTCGGGGTATGCTATGAGGCAGAGGCAACGACTCC<br>TCTGAGGCCCAGGATTCCTTTGCAACGAGATTCCCGGCTTCCTGGT<br>TTCCAAAGGAGGCTCCTGGGCCAGGTGGAATTTAACCTTAGGTCGA<br>TACATATTTTCCCCAGGATTGGTGAAAATAGTGAGTTAGAAATCAAG

	AAATCCCTACAGACTAGAGACATAGGTAGGAAACTTGGACCCAAA<br>GCAGGTACTTGGGAAGAGTGTTCAGCCGCCCCACCGGGGAAGAAA<br>CTTGTCTAGCCCCCAAGAACCAAACTTGCCTGGGGCGGGATCATTT<br>GCGGGGAGGAGCGCTGGGCTCGCCCCCCGGCTCTGACGTTGACCA<br>ATAGAAAGGCCTGGGGGCGGAGCCAGGGAAACGCGGGAAGCAGG<br>GGCGGGGCCTCTGGTG
CDK4	CGCGCCCAGCATTTTTTTTTTCTTTTCTTTCTTTTTTCTTTTTTTTTGT<br>GGCGGGTGGGGAGTGGTGATTGTCCCAGTGCCTAGAACAGTGCCT<br>GGAATATAAGTGGCACCCAATAAATATCTCTGTGAATAAATTAAGGG<br>CTTAGGAAGGATATACAGACACACGGGAGACTGTGGTAACTGGCG<br>GGGAAGGGTGGCTTTGGGAAAAGAGTAAATGAAATAGGCTTCCTG<br>GAGGGTCCCTGAGTCCAATAGCTAAAAGGTCCTCGCCCAATCTCCG<br>AGTCCTCTCCTTGCACCTCACCTGCAGTGGGTTCTTGGTGCTGATC<br>GCCAGGACCTGGTACTTGAAGTAGCAGAGCTCACAGCTCCAGGAG<br>CCCCTCTCGCTGATCCAGCGGATGAGGCAGGGCTGATGCGTGCAGC<br>GCACTGAGCCGTCGCAGCGGCAGGGGCTTAAGAGCTCCCCCTGGA<br>GGAAGAGTGAGGAGGGGCCCTGTCAGCCATCACCACCCCGACCCC<br>ACTCAGCCGCGCTCCCCGACAGGGAAACCTCCTCTTCAGGTCACTT<br>TCCCACCTTAAATAACTCAGGCCTTTCACCCAAGACAGGGCAAGA<br>GGCTAAGTAAGGCTGCAACAGAAAAGAAGCTTCCCTTGGAGATCA<br>AGGGAAACTTCCCATACCAAAGAGATGAAAGATGGAAAGGGATGG<br>GGGGATTAGAGGGGAGAAGGGCTGGGTCTTCTCTTTTCCTAGAATT<br>TGGGATGGGTAGTCTTTAAGACTTCTCAAAAGAAAGGCTTCCTCTT<br>AGGATCTGGAGGGAGGGGAGTGTTAGAGCTTCTGAGCCATGCTTT<br>CCTTCCCCCACCCCATCATTGTAAAGTTACTTCACAGATCTGGCGCA<br>AACTGGGATGGGTTATGGTCCTCGGTTTCCACGTACTAAAAAGGGG<br>AGAAATCGCGGAGGTGGGAGTGAAGGGTGTTTCCCTTTAATCATGG<br>AATCAATTCTCACTACACAGAGACACCCCCTGGCACTATTCTGGGA<br>CAACTTTGCCCATAAAATTTTAACAAAGAATACTCTTTAGAATGAGA<br>TAGAAAGTTGCAGAATGGGAAGTGAAAAAAAGTGCCCCACGGGG<br>AGAAATACTGAGCTAAGCAAGGGCGCTGGCTGGGCCCCTCCCTTC<br>AAGGGGGGCACAGAAGCACTCTCCTTTCTGCCCACCTGCAAGAGA<br>ACAGGTCCCCTTAGCTTACGGAGACCCTGGGGCGAGGGAAATAGG<br>AGAGAACAGCAGTCCCTGTTAATGGGTGAGAGGGATTTTGCGCTCT<br>CCGGGGAGAAAGCCCCACCCCTTCCTGGGGACCCTTGACCCTCCG<br>ACACAAACAAGCGCTCCAGGAGTCCCCTGCCAGCCCCTCGGCCTC<br>ACCCCATTCCCACTCCGTGTCCTGGGGCCCGCCCAGCCTCCTTAGG<br>CGCCGCGCCTGCTCGCGGTCTCCGCTGACACCCCAGGTGCGCGCC<br>TCCACCCTCCGCGCCCGGCCAGGTGCCCAGGCCTGACCTGCTCCG<br>GGCCCTGGAAGCAGATCCGGCACTGAGGGGTTCGGAGTCCGCTGT<br>CCAGGCTACTGCTGAGCGACAGGGCGTCCAGCGCGCCCGGGGGCG<br>GCAGCGGCGGCGCGGGCGGCGGCAGGGGTCCGGCCCGCGGCTCC<br>TTGTCGCCGGCCAGGCCCCGGGCCCGCGGCTCCGACCCGTAGTACT<br>CCTCCTCGTCGCCGTCGCCGTCCCGGGTGGAGCAGCCAGCGAAGG<br>GCGCCCAGCCGCAGCCGCCTCCCCGGCCCCCCCGGGGTTGCGGCT<br>CGGCCCGGGGCCGCCCCCCGCCTGTCAGCACCAGCAGCTTCAGCT

	CGTTCAGAAACATGCGGAGCCGAGACTTGAGCATCGTCCGGACAC<br>CGGGGGCGGGGGGCAGCGTGCAAGGGGGGCTCGCTAGCGGCGGG<br>GGGAGGGGAGAGGACAAGGCCGGGAAGGGGGCGCGCGCTAGAG<br>AGCCGAGCCGGGCCGGGGTCTTCGGGGCCTGCGACAGCCGTTCAC<br>TGCTGCCACCGCCGGGGCTCCGGTGCTTGCGGGGCCGAGCGGGCT<br>GGCGGCCCGGGGGCGGCGGGGTGGGCGGCCATGGCCAGGGCAGG<br>GGGCGGGGAGGGGGGCTCAGGGTGCCGCACCCCGCGGGTCTGGG<br>TCTGGAGCGGGCCGGGCCCCCGGGGTTACGCGCCCGGGTCCTCCT<br>GCGGCGGGGCCGCCCCCCATCGCCGTTCTCTCGGTCCCAGTCCCCC<br>TCCGGCTCCGGGATCAGGCTTGGCTCGGCCCGTGCGCTCGGCGGT<br>GGCAAATGGCGGCTCCTTCCGGCGGCGGCGGCGGCGGAGACCCCC<br>CCGGAGTGGCGGGCGGGCGGGGCGGGCGCCGGGGAGCGGGTGAA<br>GGATGGAGGGAGGGGCGGGGCGGGGAGGGGGAGAGGAGAAGGG<br>GCCGCGGCTGCGCATCCCCGCCCTGCCCCGCACGAGCAGGTGTCC<br>CAGGGGCGGGGGCAAGTGTGGGATCCCGAGGAGGGTGGAGGAAG<br>GGGAGTGGCCAAGGAAGGGGAGTGTGCACACAAGTTGACTAGGT<br>GTGTGTCTGGGAGTGGGGGATGAGCGGGTGAGCATGGAAGGTGAG<br>TGGTTAAATGACGCAGGTGTACCAGGGAGGGTGAGGCTTGTGCAA<br>AATGGGAATGTGCAAAGAAAGAGCTGTGCAAGTGTCGGAGGTGTG<br>AGGGAGGTACATGGGCAGGGGTGGGACTCAAGCAATATACACTTTA<br>CCCAAAGGGCTGAAAAGTGTGATCGATGGGGACTGGGAAAGAGA<br>GGTGTGTAAGGAATGGAATCGGATTGTCTGAGAAACAGAAGCCTG<br>TGATTGTAATGTGTGAAGGAGGTGAGGGAGGGAGCAGGGGAGGTA<br>GGCGTGCTAAAGGGATGGGAGTGTCTTTCCTGGTACTCTCCTGCCC<br>TCACCTCCACTACACTGAAGTGAACAATACACACACTTACCCCTTT<br>CCTTTTATGGAGACTTCCCTCTGAGGCCAAGTTCTACCCCCATCACA<br>TTTTTTTCTTTCTTACACTTACATTCTCCCTCTCCTCCCCTTTGCAAA<br>GCTGAGGGAAAAGGGATTCCAGGGGAGCTCATTGGGTCAAACATA<br>CTGGGAAAAAATTTAATTAGTGAAAGAGTAATTAACAGAAGAAGG<br>GAGGGGACAGAGCCTGGGACAGACTGCCAAATTAAGGTTCCTCAA<br>TAGAAAGTGATGGAGAGACAGTAATGTTCCCATGAAGTATTCAAAT<br>GTTTTGGATGGACTAATGAATGAATCAGAAAAAGGGGCTGGGACC<br>CAAGCAGACAGAGAGGACGTCTTCTGTTGCAAAGACAGTAAGCTG<br>TGAAGGTCTTATGTGGAAGGTGAGGCTGAAGTGGGGAATGCTGGT<br>GTGGGAAGGGAGTTTGAAAAACTCACAGACAATTATCTTAGCCTTC<br>CAGAAGGCTTACAAAGCACCCACCCCTAGCAAGGTAGCCAATAAA<br>ACATTCACCTCTTGAGCCCCTTAGTCAGTTCAGTCCTTGAGGTTTTT<br>CTTACTAGTGTTCCAGCTGCCCTTGCCAGGAGGGGCCCAGGGCTAC<br>AAGTATCAGGATGGAAAGAGGAGGCATCTGTATTAATATGTATTCTA<br>TTTTGCTCTAAGTCATGGTTTATATTCATCTTCTAGTTTGAACTCTTAT<br>ATTCTCAGCCAGATGGTTAAGGAAAACAGAAGTTAGAGTGGAAGG<br>AGTGGCAAATAGGGTAATAAAATGGCTTTAGATTTTAGACAAATTCT<br>CACAAATGAGACACTAACCGCTTTCCTCCTTTACTCCCATACCCCCA<br>AAGGGCTGGGAGATAAAGACCTTGGAGCCAATAAAACAAAGTAGG<br>AGAAATCTACTTGTGTCTACCTAGGTGTCTGGGCAGAGGCCATTTG<br>GGAACAAGTGTGTTCTGGACAGTGCTAAGTGCTAAGAAAGCGGCA

	CTAGGGAAAAAGAGGGAAGCACAATAACTCCAAATGAGTAGTCAC<br>AAGGTTTGCAGAAGGGGGCATGTCATTTTCATTCATTGTGGTGGTC<br>TGGAGGGTGCAGGGGGTGTGGTGGTAACTTTCTTGCACATGTGCAA<br>TCCTGTATCCGTCTTATATATGATGTGATAAATAATATCATATACATAC<br>ATATATACATAGAAGCATATATGCACACATACAAATATCACATAATCA<br>TAGAACCTTCCTACTGGAAAAAAAAAAAAAAACTTAAAAGGTCAAG<br>TATTCCTGTCTCCTGCTCATCAATAACTTCTCTGCCAGATGGCCTCA<br>GGTCTTTGCATTAACACTCCCAGGGAGGTTTGGAAGTTGGAAAAG<br>TGAGAGGATTACCTTGTCACTGAGCCTGTTGGATTACTGTATTAATC<br>TGCCTTCCGTCATCCTTTAATCGTAGTTTTGCCCTCAAATGGGCATT<br>GTTTACTCCCTATTGTAGAAGACCCCATTGTTGGCTACATCTTTATAG<br>ATAGTCGTACCACTACTTTAAAGGGCCAGTGACAGACACCAAAAA<br>CAAAATAAAAACCCACATACACACTGGAAGCAAGCACTCAGTAAA<br>ATGTTGTAGAATTAATGAATGAAACAATGACACAAATGAGAGTATG<br>TTTTCTCATGGTGGAGCGAAAAGGTGACAGCATCACCTCTGGTACC<br>CCAACTCCCACCCCCTCCCCAATGCAGACAGGCTGAAAGACCGGT<br>AGTGAGACTGGAGTTCAGCCTTCAGACCGGTAGTGAGACAATCCT<br>TCAGCCGGGAGTTGGGCTCTGGGTGGCCTAGGTTGCCATGGCACC<br>GCCTCGGGCTCCACCCTCTCTTGTCCCCCTCACCGCTCCCCCCCTG<br>CAGCGGGGGTTGTGGCAGCCAGTCACGTGCCCGCCGCGTAGCCAC<br>ACCTCTGCTCCTCAGAGCAATGTCAAGCGGTCACGTGTGATAGCAA<br>CAGATCACGTGGCTGCCATCGCCCCTCCGCCCCCTTACACTCTTCG<br>CCCTCCTCCCAGTCGAAGCACCTCCTGTCCGCCCCTCAGCGCATGG<br>GTGGCGGTCACGTGCCCAGAACGTCCGGCGTTCGCCCCGCCCTCC<br>CAGTTTCCGCGCGCCTC
CDKN1A	ATAGAGAAACCCTCTCTCTACTAAAATACAAACATTGGGTGGGGCG<br>AGTCATCGTCTGACGTCTGGCCGTGAGATGTTTCGGGAGCCGGGGT<br>CTCTCCGCTGCAGACATGACGAAGGGCCTTGTTTTAGGAATCTATT<br>CCAAAGAAAAAGAAGATGATGTGCCACAGTTCACAAGTGCAGGAG<br>AGAATCTTGATAAATTGATAGCTGGAAAGCTGAGAGAGACTTTGAA<br>CATATCTGGACCACCTCTGAAGGCAGGCAAGACTCGAAACTTTTAT<br>GGTCTGCATCAGGACTTCCCCAGCGTGGTGCTAGTTGGCCTCGGCA<br>AAAAGGCAGCCAGAATCGACGAACAGGAAAACTGGCAGGAAGGC<br>AAAGAAAACATCAGAGCTGCTGTTGCAGCAGGATGCAGGCAGATT<br>CAAGACCTGGAGCTCTCTTCCGTGGAGGTGGATCCCTGTAGAGATG<br>CTCAGGCTGCTGAGGAGGGCGCGGTGCTTGGTCTCTATGAATACGA<br>TGACCTAAAGCAAAAAAAGAAGATGGCTATGTCGGTGAAGCTCTAT<br>GGAACTGGGGATCAGGAGGCCTGGCAGAAAGGAGTCCTGTTTGCT<br>TCTGGGCAGAACTTGGCATGATGGAGACGCCAGCCAGCGAGATGA<br>TGCCAACCAGATTTGCCGAAATTATTGAGAAGAATCTCAAAAGCGC<br>TAGTAGTAAACCGAGTTTCATATCAGACCCAGGTCTTGGATTGAGG<br>AACAGGCAATGGGATCATTCCTCAGTGTGGCCAAAGGATCTGACG<br>AGCCCTCAGTCTTCTTGGAAATTCACTACATAGGCAGCCCCAATGC<br>AGACAAACCACCCCTTGTTTGTTGGGAAAGGAATTACCTTTGACAG<br>TGGTGGTATCTCCATCAAGGCTTCTGCAAATATGGACCTCATGAGG<br>GCCGACATGGGAGGAGCTACAACTATATGCTCAGCCATTGTGTCTG

	CTGCAAATCTCAGTTTGCCCATTAATATTATAGGTCTGGCCCCTCTGT<br>GAAAACATGCCCAGCGGCAAGGCCAACAAGCTGGGGGATGTTGTT<br>AGAGCCAGGAACAGGAAGACCATCCAGGTTGGTAACACTGATGCT<br>GAGGGAGGCTCATACTGGCTGATGCGCTCTGTTACGTGCACACAT<br>TTAACCCGAAGGTCATCCTCAATGCCACCACCTTAACAGGTGTCAT<br>AGATGTAGCTTTGGGGTCAGGTGCCACTGGGGTCTTTACCAATTCA<br>TCCTGGCTCTGGAACAAGCTCTTCGAGGCCAGCATTGAAACAGGG<br>GACCGTGTCTGGAGGATGCCTCTCTTCAAACATTGTACAAGACAGG<br>TTGTAGATTGCCAGCTGGCTGATGTTAACAACATTGGAAAATATAGA<br>TCTGCGGGAGCATGTACATCTGCGGCATTCCTGAAAGAATTCGTGA<br>CTCATCCTAAGTGGGCACATTTAGACATAGCAGGTGTGATGACCAA<br>CAAAGATGAGGTTCCCTATCTATGGAAAGGCATGACCGGGAGGCCC<br>ACAAGGACTCTCATAGAGTTCTTACTTCGTTTCAGTCAAGACAATG<br>CTTAGTTCAGATACTCAAAAATGTCTTCACTCTATCTTAAATTGGAC<br>AGTTGAAGTTAAAAGGTTTTTGAATGAATGGATGAAAATATTTTAA<br>AGGAGGCAATTTATATTTAAAAATGTAGAACACAATGAAATTTTTAT<br>GCCTTGATTTTTTTTTCATTTTACACAAAGATTTATATATTTTTTTTT<br>GAGACGGAGTCTCACTCTGTCACCCAGGCTGGAGTGCAGGTGGCA<br>TGATCTCAGCTCACTGCAACCTCCGCCTCCTAGGTTCAAGCGATTC<br>TCCCACCTCAGCCACCTGAATACCTGGGACTACAGGTGCCCACCAC<br>CATGCCCGGCTGATTTTTGTATTTTTAATGGAGACGGGGTTTCACCA<br>TATTGGCCAGGCTGGTCTCAAAACTCCTGACCCTGTGATCTGCCCG<br>CCTCGGCCTCCCAAAGTGCTGGGATTACAGGCGTAAGCCACCACG<br>CCCGGCCAGTATATATTTTTAATTGAGAAGCAAAATTGTACTTCAGA<br>TTTGTGATGCTAGGAACATGAGCAAACTGAAAATTACTAACCACTT<br>GTCAGAAACAATAAATCCAACTTTTTGTGCAAAAAAAAAAAATACA<br>AATATTAGCTGGGCATGGTGGTGCATGCCTGTAATCCCAGCTACTCG<br>GGAGGCTGAGGCAGAATTGCTTGAACCTGGGAGGCGGAGACTGCA<br>GTGAGCTGAGATTGTGCCACTGCTGACTTTGTCTCAAAAAACAAA<br>ACAAAACAAAAAAACAAAATGAAAACAAAAAGCCAGGGCTGCCT<br>CTGCTCAATAATGTTCTATCTTTGTTCCGCCTCTTCTCTGGGGTCTCA<br>CTTCTTGGGAGCCTGTGTGAAGGTGAATTCCTCTGAAAGCTGACTG<br>CCCCTATTTGGGACTCCCCAGTCTCTTTCTGAGAAATGGTGACATTG<br>TTCCCAGCACTTCCTCTCCCTTCCTAGGCAGCTTCTGCAGCCACCA<br>CTGAGCCTTCCTCACATCCTCCTTCTTCAGGCTTGGGCTTTCCACCT<br>TTCACCATTCCCCTACCCCATGCTGCTCCACCGCACTCTGGGGAGG<br>GGGCTGGACTGGGCACTCTTGTCCCCCAGGCTGAGCCTCCCTCCAT<br>CCCTATGCTGCCTGCTTCCCAGGAACATGCTTGGGCAGCAGGCTGT<br>GGCTCTGATTGGCTTTCTGGCCGTCAGGAACATGTCCCAACATGTT<br>GAGCTCTGGCATAGAAGAGGCTGGTGGCTATTTTGTCCTTGGGCTG<br>CCTGTTTTCAGGTGAGGAAGGGGATGGTAGGAGACAGGAGACCTC<br>TAAAGACCCCAGGTAAACCTTAGCCTGTTACTCTGAACAGGGTATG<br>TGATCTGCCAGCAGATCCTTGCGACAGGGCTGGGATCTGATGCATG<br>TGTGCTTGTGTGAGTGTGTGCTGGGAGTCAGATTCTGTGTGTGACT<br>TTTAACAGCCTGCTCCCTTGCCTTTTTCAGGGCAGAAGTCCTCCCTT<br>AGAGTGTGTCTGGGTACACATTCAAGTGCATGGTTGCAAACTTTTT

	TTTTTAAAGCACTGAATAGTACTAGACACTTAGTAGGTACTTAAGA AATATTGAATGTCGTGGTGGTGGTGAGCTAGAAGTTATAAAAAAAA TTCTTTCCCAAAAACAACAACAAAAAGAATTATTTCATTGTGAAGC TCAGTACCACAAAAATTTAAATAATTCATTACAAGCCTTTATTAAAA AAAATTTTCTCCCCAAAGTAAACAGACAGACAATGTCTAGTCTATT TGAAATGCCTGAAAGCAGAGGGGCTTCAAGGCAGTGGGAGAAGG TGCCTGTCCTCTGCTGGACATTTGACAACCAGCCCTTTGGATGGTT TGGATGTATAGGAGCGAAGGTGCAGACAGCAGTGGGGCTTAGAGT GGGGTCCTGAGGCTGTGCCGTGGCCTTTCTGGGGTTTAGCCACAAT CCTGGCCTGACTCCAGGGCGAGGCAGGCCAAGGGGGTCTGCTACT GTGTCCTCCCACCCCTACCTGGGCTCCCATCCCCACAGCAGAGGAG AAAGAAGCCTGTCCTCCCCGAGGTCAGCTGCGTTAGAGGAAGAAG ACTGGGCATGTCTGGGCAGAGATTTCCAGACTCTGAGCAGCCTGA GATGTCAGTAATTGTAGCTGCTCCAAGCCTGGGTTCTGTTTTTTAGT GGGATTTCTGTTCAGATGAACAATCCATCCTCTGCAATTTTTTAAAA GCAAAACTGCAAATGTTTCAGGCACAGAAAGGAGGCAAAGGTGA AGTCCAGGGGAGGTCAGGGGTGTGAGGTAGATGGGAGCGGATAGA CACATCACTCATTTCTGTGTCTGTCAGAAGAACCAGTAGACACTTC CAGAATTGTCCTTTATTTATGTCATCTCCATAAACCATCTGCAAATGA GGGTTATTTGGCATTTTTGTCATTTTGGAGCCACAGAAATAAAGGAT GACAAGCAGAGAGCCCCGGGCAGGAGGCAAAAGTCCTGTGTTCC AACTATAGTCATTTCTTTGCTGCATGATCTGAGTTAGGTCACCAGAC TTCTCTGAGCCCCAGTTTCCCCAGCAGTGTATACGGGCTATGTGGG GAGTATTCAGGAGACAGACAACTCACTCGTCAAATCCTCCCCTTCC TGGCCAACAAAGCTGCTGCAACCACAGGGATTTCTTCTGTTCAGGT GAGTGTAGGGTGTAGGGAGATTGGTTCAATGTCCAATTCTTCTGTTT CCCTGGAGATCAGGTTGCCCTTTTTTGGTAGTCTCTCCAATTCCCTC CTTCCCGGAAGCATGTGACAATCAACAACTTTGTATACTTAAGTTC AGTGGACCTCAATTTCCTCATCTGTGAAATAAACGGGACTGAAAAA TCATTCTGGCCTCAAGATGCTTTGTTGGGGTGTCTAGGTGCTCCAG GTGCTTCTGGGAGAGGTGACCTAGTGAGGGATCAGTGGGAATAGA GGTGATATTGTGGGGCTTTTCTGGAAATTGCAGAGAGGTGCATCGT TTTTATAATTTATGAATTTTTATGTATTAATGTCATCCTCCTGATCTTTT CAGCTGCATTGGGTAAATCCTTGCCTGCCAGAGTGGGTCAGCGGTG AGCCAGAAAGGGGGCTCATTCTAACAGTGCTGTGTCCTCCTGGAG AGTGCCAACTCATTCTCCAAGTAAAAAAAGCCAGATTTGTGGCTCA CTTCGTGGGGAAATGTGTCCAGCGCACCAACGCAGGCGAGGGACT GGGGGAGGAGGGAAGTGCCCTCCTGCAGCACGCGAGGTTCCGGG ACCGGCTGGCCTGCTGGAACTCGGCCAGGCTCAGCTGGCTCGGCG CTGGGCAGCCAGGAGCCTGGGCCCCGGGGAGGGCGGTCCCGGGC GGCGCGGTGGGCCGAGCGCGGGTCCCGCCTCCTTGAGGCGGGCCC GGGCGGGGCGGTTGTATATCAGGGCCGCGCTGAGCTGCGCCAGCT GAGGTGTGAGCAGCTGCCGAA
CETP	AGGTGGAAGTGTACTGAAAAGCTCGTGTTTTATCTTTATACGTTTAC ATGGGGCCCCAAAAAGGGGGAAAACCCACTTTGATAGGGATATGA CACAGGGACGCAGGAGCCAACCGAAAGAGCCCCCAGTGGCCAAA

	GATGGAGCAACTTGAGCAACAAAATAAATAAAGTAAGCATCAAATT<br>ATAGCCCAAAGTATAAAATAAGTGTCTATGAGTCTATACTCACATAG<br>ATGATTTAATACATTCATATTAATAAATGGGAGAGCACTTTGAGAGG<br>CCAAGGTGGGCAGATCACTTGAGCCCAGGAGTCTGAGACCAGATT<br>AGGCACTGTGATGAAACCCTGTCTCTACCAAAAACACAAAAATTA<br>GCTGGGCATGGTGGGGCATGCCTATAGTTCCAGTTACTTGGGAGGC<br>TGGGGAGGGAGGATTGCTTGAGCCTGGGAGACGGAGGTTGCAGTG<br>AGCTGTGATCTCACCACTGCACTCCAGCCTGGGCAACAGAGTGAG<br>GCCCTGTCTCAAAATAAATAAATATATAAATAAGATAAATATATAAAT<br>AAAAATAAAAATAAACGGAAAGAAGAGACAAATCTTCTGTGCAG<br>GAGAATCCCAAATGAATTCTGTAGATTCTCCACCCTACAGGAGGGC<br>ACACACAACTCCAGGCTGCCTTAGTGACCTTCTTCCAAAGAGTACA<br>GTACGGGAAGGGGGAGCGGGGAGAATTAACTTCACAGTGGAGAAA<br>TCTGACAAATACGACCTCCGCCAAGTGATCGAGGTCACATCAGCTG<br>TCATGAATTATGTTGATAGTGGGCATCCTGGATATGATGGGATGAAA<br>TGGCACTTTACCTCCACGACCCTTTTCACAACAGCCCGTAATGCCA<br>GTATAAGGAAAACATCAGACCAGGAGTGGTGGCTCACGCTTGTAAT<br>CCGAGCACTTTGGGAGGCCAAGGTGGGTGGATCACAGGTCAGGAG<br>TTTGAGACCAGCCTGGACAACATGGTGAAATCCTGTCTCTACTAAA<br>AATACACAAACTAGCCAGGCACGGTGGCGGGTGCCTGTAATCCCA<br>GCTACTTGGGAGGCTGAGGCAGAAGAACCGCTTGAACCCGGGAG<br>GCGGAGGTTGCAGTGAGCTGAGATCGCGCCACTGCACTCCAGCCA<br>GGTTGACAGAGTGAGACTCCATCTCAAAAAAAAAAAAAAAAAAA<br>GAAAAGAAAAGAAAAAGAGAAAAGAAAAACATCAGACAGATCCC<br>AACAGAAGGGCATCCTACAGTATACGTGACCACTGCTCCTCAAACC<br>TGTCAAGACTATCAGAAACAAGAGAAACTGTCACAGCTACAAGGA<br>GATGTGACAATTAATTGTGATTTTTTTTTTTGAGATGTGGTCTCGCTT<br>TGTCACCCAGGCTGCAGTACAGTGGCACAATCACAGCTCACTGCA<br>GCCTCGAACTCTGGGGCTCAAGTGATCCTCCCATCCATCTCAGCCT<br>CCCAAGTAGCTGGGGCTATATGCGTGCACCACCACACCCAGCTAAT<br>TTTTGTATTTTTTTAGAGACGGTATTTCGCCATGTTGCCCAGGCTGG<br>TCTCGAACTCCTGGATTCAAGCAATCCACCTAACTTGGCCTCCCAA<br>AGTGCTGGGATTACAGGCATGAGCCACCATGCCTTGCCAAATGTGA<br>TGTATTCTTGATGGGATCCTGGAAGAGGAAAAAGATATTAGGTAAA<br>AACTAAGGACATCTGAATAACCATGGATTTCAGTAATGTATCCACAC<br>TGATTCATTAATTGTAACAAATATACCGTATGAATGCAAGATGTTCAT<br>AATGAGGCCGGGGCGGTGGCTCATGCCTGTAATTCCAGCACTTTGG<br>GAGGCTGAGGCGGAAGGATCTCTTGAGCTCAGGAGTTCGAGACCA<br>GCCTGGGTAACACAGTGAGATCCCCATCTCTACAAAAAATTTTTG<br>AAATTAGCCAGGTGTGGTGGTGGCACATGCCTGTAGCCCCAGCTAC<br>TCAGAAGACTGAGACAGGAGGATTGCTTGAGCCGGGAGGTTGAGG<br>CTGCAGTAAGCCAGGATCACGCCACTACACTGCAGTCTGAGCAAC<br>AGAGCGACACCCTGTCTCAATGATAATAATAACAATAATAATAATAA<br>TGGAAACTCAGTATGGGTTGTATATGGGAAACTTGCTCAGTTTCTCT<br>GAAAATCTAAAATTCTTCTGAAAATCAAAGTCTGCTTAAAAATTCA<br>CATGGGGTTTGCATTCTCCTCCATGACAAGGTTTTGCAGGTTATGAT

	TACAGAGGCTGGAGAGGGTGCAGGTTATCCCGCCCCTCCCTGTCCC<br>AGGGATTCCCTTCCCCAGGAGCTGTGTCTCCCCTGTGAGAGAGGGT<br>GAGCTTCCATGACCCCAAGCCTCTTGCCCTCTGACTCCGGTATTCTT<br>AGAAGCTGGGACCAGCACTGAGCCCAAATTCCCGAAGCGCTCAAA<br>TACTGGCTTTCTGTCCCTATGTGACCTGGAGCTTGTAGTTTAACTTC<br>TCTCTGCCTCACTGTTTGACCTATAAAGCAGGGCAATCAAGGCATC<br>CCGGGGGTGGCTATGAAGAGTGAATGAGATAGCAGACAACCCAGA<br>TGCCTACCGACAGGTGAAGGGACCAACACAGTGCGGTATAGGCGT<br>ATAAGGGAATGGAGTATGGACACAGCCTACAACACAGACAAACCT<br>TGAAGACATTCCTCTAAGGGACATAGGCCAGGCATGGTGGCTCACA<br>CCTGTAATCCTAGCATTCTGGGAGGCCGAGGCGGGCAGATCACTTG<br>AGGTCAGGAGTTGAGACCAGCCTGGCCAACATGGCAAAATCCCGC<br>CTCTACTAAAAATACAAACATTAGCTGGGTGTGGTGGTAGGTACCT<br>GTAATCCTGGATACTCGGGAGACTGAGGCAAGAGAGTTGCTGGAA<br>CCCGGGAGGTGGAGGTTGCAGTGAGCCGAGATTGTGCCACTGCAC<br>TTCAGCCTGGGCGACAGAGCGAGACTCTGTCGAAAGAAAGAAAG<br>GGAGGAAGGAAGGAAGAAAGAAAGGGAAGGGAAGGGAAGGGAG<br>GGGAGGGGAGGGGAGGGGAGGGAAGGGAGAGAGAAAGAAGAAA<br>GAGAAAGAAAGGAAGAAAGAAAAAGAAGGAAAGAAAGAAAGAA<br>AGAAAGAAAGAAAGAAAGAAAGAAAGAAAGAAAGAAAGAAAGA<br>AAGAAAGAAAAGAGTGAACCCAGGAACGAAAGATCACACACACT<br>GTATGACTCTATTTACATGAAATGTTCAGAGTAGGCAAATCCATAGA<br>GACAGAAAGCACATTTATGGTTGCCAGGAGCTGGGAAAGGGCAGG<br>ATGGGGAATGACTGTTTATTGGATGTGGGGCTCTATTTTGGGGTGAT<br>GAGAATGTTCTGGAATTAAATTCATGGCTGCATAACACTGTGAACAT<br>ACTAAATGCCCCTGAATTGTACACTTTAAAATGGTTAAAGTGGCAA<br>GTTTTCACTAAGCAGTAAATTAAATTCTACTACAATTTTAAAAAGAC<br>TAAAAAATAATTTAAAAAAGATTAAATGAGATAACGCAAAAAAGCA<br>TTATCTCGAAAATACAGCTGATATTAGTATAATTCTTACTAAGTTTTA<br>AGAGTCTAAGGTGCAGGATTCTAAGTTTAAAGGGATAGGCTCTTTT<br>GGTTTTTTGGTTTAGTTATTTGGTTTTTTTTTTTAATCCATTATCCCCA<br>CCCTTGGGAGGCCCCCAGCACCCAGTCTGCACTAGAGGATGGGGC<br>CCACCTCCCTTTTCTCTCCAGGCCCAGCCACTGACCACCAGTACCC<br>TGGCCAGGGGCACCCTCGGTCATTGCCCTCCGTGGCCCAAGGAAG<br>GGAACAGAAACAACAGCCAAGAAGACAATAGCCGCCGGGAAGTC<br>CTCACATTTCTGGAGAAATAGAGCCCATTAATGAATGAAGTTCCTCC<br>AGCCTGATCGGAGGACGGGGTGCTGGGGAGGCCTGGGCTAAAGG<br>GCTCACCTCCAGCCCCCACCCTGGCAGGGCCGATGGTACATGCTCA<br>CTCAGTGAGGGGGCTCCAGAGGTCTGTGGGTACGAACCCAAGGGC<br>TGGTGCCCAGGGGCAATCAGCTTATGTCTCTGAGCCTTGGGAAACA<br>GTGAGGGTCAGCCCGGCTCCCCACGTGCTTCTGGGCAGCTTTGGTA<br>TTGGAGCAGGTGCAAACTCGGGACTAGGGCAGGACCCCCTGAGAG<br>GCGACTGAGCAAGGCCATCCCGACTCATGTTTCCTTGGCCCTGCCC<br>GGGGCACAGCATCCTGCCCACATCCCTGCAGCCCTGGCTCCTTCCT<br>AGGGGCTCTGAGGAGGCAGCACTTGGTCATCTGGTCACAGTTGCT<br>GCAGGGCAGTTCTTGGCCCCAGCTGTAGGTAAAGTACTGTATGTTG

	TAATTTTTTGAAAGATAACACGTTCACACAACTCAGAATTGAAATGCCACAGACATTCCCCCTGCTCCGCCCCTTTCCCCCGGATACCCAGTTTCTCCCGGAGGCAGCCAATGATCTCAGAGGCTGTATACCCCCCCAGAGTTATTTTATGCATATCAAGGAAAGTCTACATAGAGGACTGTTTCTGGGGTACCCAGATGCAGCGTCAAATGCCATGGAATACTACAGTGAGGACATTATCCTTTCAAGCTTTCAAATCAGAGCAAGGAAAGGTCGATGCTAGAGTTTCTCTAGCACCCATGAAGCCCTCTCCCTTTTTCTACTGAGTTTTACTTTACAGGCAACAGCAGGCTTCAAGCTTGGGGTCATTGTCGGGCAACAGTATCTGGCAAGAATTCAATGTCTTTTTCTCATAGTCATTGTATTTTGGCCTCTTTCTATTTATGGCAACTGAGAGAGAAAGCTTATTCCTAGATATATGTATTTAAGTAAAAAATAAATGAATTCATGGAAACATATTAAGCAATTATCCAGATAACATAAGGGATGGCAAAAATGGTGCAGATGGTGGAGGGGAGACAAGTAGAAGTTGGGGTGCTCTTGTTGAATGTCTGGCTCTGAACTCTAGAGGAGGCCGCAGGGGCTGGGCAGGAAGGAGGTGAATCTCTGGGGCCAGGAAGACC
CFTR	AAATGATTTTACAATTGCAAGAGTACTTGATTTACCCCTTTACATTTAGTTCAAATACCAAAAATTTCTTAAGGAATGAGAAATTCCAATGTTCCTGAGAATTCTGATAGCTTTTAGAGAGTTCAGTTTTCTGTAGCATTCCATTTTGCAATCCTATACAAATTTCTAATTTATAACCAGTGGTATGTAATGATAATTTCTAATATTTATTAAGTGTTTATTGGGTTCTAAGTGCTTTACGTCTGATATATGTATCACATTTAATTTATTTCATCCAGTGGTTCTTAACTGGGGACAACTTTGTACCTCTCTCCCCAACATATTTGGCAATCTCTGGAGATAGTCCTGGATCTCCAGATCTATCTGTCACAACCTAGGATGTATGTGGTCCTACGAGCATCCAGTGAATAGAAGCTAGAGATACTGCTGAACATTCCACAGTACAAGGGCAACCCCCACATCAAAGAATTATCCACACCCAAATGTCAGTAGTACTGAGGTAGAGAGACCCTAACTTAATCTGTTCAACAATCCTATGAGGTGATTTTTTTTTTTTTTTTGAGATAAGGTCTTACTCTGTCACCTAAACTGGAGTGCAGTGGCATGATCACAGCTCACTGCAGCCTCGATCTCCCAGGCTCAAGCCATCCACCTGCCTCAGCCTCCCAAGTAGCTGAGATCAGAAGCATGCACCACCACACCTGGCTATTTTTTTATTTTTTTGTAGAGACAAGGTCTTACTGTGTTGCCCAGGCTGATCTCAAACTCCTGAGCTCAAGCAATCCTCCTGCCTCAGCTTCTCAAAGTTCTGGGATTACAGGCATGAGCCATGGCACCTGACCAAGGTGAGTGTATTTAACCTCATTTTCAGGCAAGGAAACAAAAGACAGAAAAGTTAAGTAGCTTACTTAAGGTCACAGAGCTAAGTGTGGTGCCAGGATTGAAAACCTAGTTCTTTATTGCTTTAGCACAAGCTATTTCCACTATACTCTGTCATGTTCAGAGAATGTTGATGTCCATCAGTGGATTCTAAATTTTGAAGGATGGAGATACTGCCTTATTCTGTACATCTGCTTTAGCACCCAAGCTCTTGCTTGGTGAAAAATTAATAGTAAACATTCATCTTTTGAGCATCTTCAAATATCCCCTTTAGAATGACATTCAATTATTAGGTCAGTAACCCCAAGAGAAAACGGTTGTTTGAGTGTATATACTGTATTACAAAATAAGGGGTGAATTCAAAGGAAAACATAAGATGCAATTCGTGCCTCCAAGGAGGTTGTAGGGAAGAGGGGTTATGAATGTATGTAAATAGAAGTTGGTGTGCGTGTGTGTTTATAAACAGAATTGTCAGACCAAACATTATTTTGGAAGCAGTAAAAGTAAACTAGAATCTGGCCTAGTCATGTCCCAGGACACCTCTTTCAAGTCCTGAAACATCTTTGT

	AAGACTGTAATGTGTGTTTACATCCTAGGTAATCACTGTGGCCCACT<br>GTTGAAGAGCTGTGGCTGTTCTTACCCTTCTAGCTTAGATAAACTTA<br>TAAGCACAACCAGACTACATATATGAAGCTGAAGAGACCTTGTCTT<br>TTTTTAACGAGCTTTTCTTCCCGATAGGAGTGACTATTTCTTTTCTTC<br>TTCCACATTTTCAGGTTTTAGTGTACTTGTGATTGCTACCCACTTATC<br>ACTATTAAAGTCTACTCAGGAGAGAATCTGAGAAACACTCTCAAAT<br>TAAGTTGAACATGATGGATAAGTAAAGTATTGTGAAAGTTCACTCT<br>CATGATTTCTAATGGTGAAACCTGGCAGGGTGACTAATCTTTGACG<br>AGAAGGTTATCACTTATAATCTTTCATATATTGAGATCATTTGTAAGA<br>AGCACCCAGCACATTGCTGAACACAAAGTAGGTATTAAATAAATGT<br>TGGCTTCCTTTTCTCCTACTCATCCTCGCTCTTCTTTTTAATATACCTT<br>TAAAATGATGCCACAGAAATGGCCACCCAATCTTCTATATTTAAGGT<br>CAGTTCTTGCATTAGGAAATTCTATAGGGGAAGTATGTGAAGTATGT<br>GTAGTCAGTCATTAAATGCTTGGGCTCTGGCCACAGATTGTTTAGGT<br>TTAAATCCCAGTTTCCTCTTTTATTATTAATTGTGCAACTTGCTTGGG<br>AAAACATGAAACTTGTTTTTCCTCAGGTTCATTATCTGTAATATATAG<br>TGAATGAAGAAGTTTCCTGTCCCATGAAGGTGTTGTAAAGATTAAA<br>AAAGGCAAATTAGGCTGTGTATTTGTCATAATAATTGGCATATATGGT<br>AAGTGACCAACAACCATAAGGTATTATAAAATTGTTATAAAATGATA<br>TGAGCTATCATTGAGCAGCATGAAAGAAGAGCTTCACTGTTTCACC<br>TACTATCACCCTGGCCCATTAATCTCTTTCCTGTTCCTGACATTTCAG<br>AGATACGTTTAGGATTTCAATCATGACCTTAAGCCACATTTGAACAA<br>TTTTCTGGTGGATAAGTCCTCATTCCCACATTATGTATGTACCTAGAT<br>GCAAATCCTGAATATCATGTCGCAATTAGTGCATCTGGACATGCTTG<br>CTAACTGTGTTAAAGCTCTGAATAATGGTAAAGTTTTATTTCTACCA<br>AAACAAATTTGGGCTGTAATGTTTTATGATAAAAATCTGTGGTCTTC<br>CTATGTACATGTGTGTGTACATGCTTAAAATGCAATGTTATAGTTAAA<br>TGTAATTCATTAAAAGTATGTAACTCCAGTGGCTACTTAGTTTGGCT<br>ACTTGGTTTGTAGATTTCTGCTTTCCTGTTTCATTGTTAAACAGGTC<br>TAGAAGTTATTATTTCATGAAACTAATGTGAGGAAAAAGACTATGTT<br>GATATATAAGTGACATTATATAAATACATGAGGGATGATTTGATTAGA<br>AGCAGTATTACACAGTGATAGGAGTAATGGTTTAGAACTAGACTCA<br>GGTTTGAATCTTAGCTCTATCATTATAGGCATTTACTTAACTTTTCTT<br>GTTTGCTTAACTGAAAACTGAAGATAATAACACCTATTTACATGGTT<br>GTTATAAGGGTTATATGAATAATGTCTGGCAAATAGTAAGAACTCAA<br>GTAACTGTTTCACTCTTTCCAGAAGGAGATTGGCTGAAAAATATTT<br>GGAGTCTCCTCCAGCCATATTCCTTGGTCAGCTTCTATGATCCTCTT<br>TGGAGCTTAATTCTTAATCCCTTTATTTTCACTTGCTTGTTGATAACA<br>AAGAAGAACTAATTATTAATTTATTTCAAAATGCATGTATTATATTTG<br>ATGGGCCACACTAACAGTTATAAACCAAACAACAGATTGGGAATGG<br>GGAAGTGGATGTGGTGAGTTCAATCACATGTCTGGGAAAAGTCAAT<br>AGTGAAGACAGAGTCTCACAATTTTTTGTCATAATGGAGAGATGAA<br>AACACAGGTAGAGGATTTCAAACAACAGAGTGGATGGTGAGTTAA<br>AAATGCTGAAATTCTTTCCTGGTGTCTAACTTAATGCAATGTGGTTT<br>ATCTCTTTGCTCTTTTCTCTACTATTCAAATTTAGGATAATAAAGATT<br>AAATGTTTCTAAATCTTACTTTACAATATCAAGAAAAAAAGGTATGC

	TTTTGCCCACGGAAGGGCAAAGCAGAGCTATGAAAACCTGCTGAA<br>CACATTCTTTATTTTCAACACAGGTTCTTGTCTTTCCATCATGAAAT<br>GCACATTTTATTTGTACTGTATTTGGGTGACCACAAGTCAACAACA<br>AGATAATTCACAAGACCCTTGCCTTAGATGTGTCGGCAATAAAGTA<br>ATCAGGCCAAAATTTTTACTTTCCTTTGAATTTTTCAATTCAAACAC<br>AATGTATGCTTGCTTTTACACAGTAGGGTTCAGGGATTAGAGGGTT<br>GGCTCTTTAAAAACCGTCAGAGACACAGGCAATCCTACACAAAATT<br>CTCAGAAGGAAGGCGCCTACGCCTGGGAATGCCCAGATGCCCCTC<br>AGAGAGTTGAAGATGGCGTTTCTCTGAGTCAGGTCAAAGTTAACA<br>CATTACCTTCGCTTCAAAGACTGCTTGGCTTCCTTTCGGTGGATTAG<br>TCAAGATGTTTTGCTGACTGAGACTAGGAAATCTATAGGAGGGCGG<br>GTTAGTTTACATTGTTCCTTGTCATTATCGCTAAAACACTCCAAAGC<br>CTTCCTTAAAAATGCGCACTGGGCTAAAAAGGATAGACAAGGAAC<br>ACATCCTGGGCCGGTAATTACGCAAAGCATTATCTCCTCTTACCTCC<br>TTGCAGATTTTTTTTTCTCTTTCAGTACGTGTCCTAAGATTTCTGTGC<br>CACCCTTGGAGTTCACTCACCTAAACCTGAAACTAATAAAGCTTGG<br>TTCTTTTCTCCGACACGCAAAGGAAGCGCTAAGGTAAATGCATCAG<br>ACCCACACTGCCGCGGAACTTTTCGGCTCTCTAAGGCTGTATTTTG<br>ATATACGAAAGGCACATTTTCCTTCCCTTTTCAAAATGCACCTTGCA<br>AACGTAACAGGAACCCGACTAGGATCATCGGGAAAAGGAGGAGG<br>AGGAGGAAGGCAGGCTCCGGGGAAGCTGGTGGCAGCGGGTCCTG<br>GGTCTGGCGGACCCTGACGCGAAGGAGGGTCTAGGAAGCTCTCCG<br>GGGAGCCGGTTCTCCCGCCGGTGGCTTCTTCTGTCCTCCAGCGTTG<br>CCAACTGGACCTAAAGAGAGGCCGCGACTGTCGCCCACCTGCGGG<br>ATGGGCCTGGTGCTGGGCGGTAAGGACACGGACCTGGAAGGAGCG<br>CGCGCGAGGGAGGGAGGCTGGGAGTCAGAATCGGGAAAGGGAGG<br>TGCGGGGCGGCGAGGGAGCGAAGGAGGAGAGGAGGAAGGAGCG<br>GGAGGGGTGCTGGCGGGGGTGCGTAGTGGGTGGAGAAAGCCGCT<br>AGAGCAAATTTGGGGCCGGACCAGGCAGCACTCGGCTTTTAACCT<br>GGGCAGTGAAGGCGGGGGAAAGAGCAAAAGGAAGGGGTGGTGTG<br>CGGAGTAGGGGTGGGTGGGGGGAATTGGAAGCAAATGACATCACA<br>GC
CGA	CTCATAAGACTTCCCCTGCAAAATTAGGGTAGGAAGTTACTTCATCA<br>TTTACTCTTTCACCTCCCCATCCTGCAATATGCATAAAACTGAACAA<br>TTCTATCAATACAATTGCTTCTCTTTCCTTCTTGTACAAAACCTGTTT<br>CTCTCATGAATCCTTTCATAGTTGATTACTGAAAGCTTTCTGAAATG<br>GAAGTGGGAGGAGATATGTGCAAAGCAAAAAGAAATGGAAAAAT<br>GTTAAAAAAATTAGTTTTCACCTGTGAGATTTTTCTCCCTTTCTTGG<br>CAGCATTATTTGCCTAGTCTATTGACATTTCAGACTAGAGAAAGATT<br>AGCTATAATTGTACCAGCAGTTTCTTTATCTCTCTGAGGGTTCCAGT<br>GACAGAGGTTGTTAGCTTGAGAAACAGGATGTTTGCATTATTGATT<br>AGGCCACACTGTTTTGACTGAAATAGTAGAGAATATTCTTGGCTGC<br>TTTACATGTGTCAAGTCAGTTTGCTTCTTGTCCATCAGTTGCAGGGG<br>GATTTTGTTGCTGCCACTGTGTTAGATATCACATCTCAGGTGATTCT<br>TTCTGACTGTCAGTCACCTTATCTGTTGTTGTTGATTAAAAACCTCA<br>TCTCAATCAAGAAATTTTCTGCTGCCTGTTTTGGTGTTTGTGACCAG

	GAGATAGTTGTTTTGGGTCTTTGAGCTGTTTCTCATAGATTCTTAATA<br>GCAGGATGTTTCAGCTGTTTGTACAGAGGGGAGGCTCACAGAACT<br>GAACAAATAACTTTACCATTTTTGATTTAGTTGTGCTACAGGGACAG<br>TTGAAGATGAAAACAAACCATATTGCTCCTTACTTACATTCCTGTAT<br>TGCTTAATAAAACAATGCTATGCAGTCACGGTTCTCAAGCTTTGACT<br>GATGAAATGCAAATTTCTGGGCTCAACTGAGAGAGATTTTGGTCCA<br>GCAGGACCAGGATAGGATGGGACCCACGAACCTGTATTTTTAATCC<br>ACACCCTAGTAGTTCTGATTTTAGACAAAACATGAGATATACTTCAA<br>GAAACACAGCCACACAGCATTAAGGAGTCAAGTGAAGATTAGAAA<br>ATTAGGGTGTTAGGAGCCTCATGACAAGTCAAAGAGAATGGTGAG<br>GGAAAATCCCAATGCTTCTAATTTATAGTAAACAGACTGTTAGCAA<br>GTTAGTGATATGATTTATGTCTAATTTCTCTTTGATAATGCACTTTCG<br>TTGATACACTTGGTAAAGAGACACTCGATAATTATATCTAATAATAAT<br>CTAAACAAATTTAAAATATTTAATTATCATTGATTGAATATTTTTATTA<br>ATATAGGTCACAACAGTAGAGAACCCAAAAATGGACCTCAAAATAT<br>TTAGGAAGTAAGTAGAATTTCAAATTAGTGGAGAAAGAAATGTTGA<br>TTATTTCATACATGTTATTACAACACCTGGCTTGTCATTTAGGTTTAA<br>AAAGAAGATAGATTTCTACTTCATTTCCAAAATAAATTACAGATGAA<br>TTAAAGGGCTAAACCTAAAATAAATAACACTGTATACAGTTGAGAA<br>TAAAATATGGGCACTTCTTTTTAGAATCTTGGCATTACATAAACTCT<br>GCAAGTCTAAAAGTAAAAGATACATAAATTTGACCTCATAAAAACA<br>ACTGTAAAACTACCATAAAAGAGTCAAAAGTCAAACTTAAAATTAA<br>AGACAGAATTAAGAACTTGAGAAAGATTTTTGAACAGGCAGTTCA<br>CAGAAAAAAAATTCTAAATGACCACAACTTATGAAATTATGTTCAG<br>TCCTGCTCAAAATTATTTACATGCAAATAAAAATAAAATATTGTTTTT<br>CATCTGTTAGTTCAACAAAAATTAACATATTTCATAGCATTAACTTTG<br>AGGAGGGGTGGAGAAGAGTGAGTACCTGAGCAACTTCTACTCAAG<br>TTTAAAATACTCATATTCTTTGATCCAACAATTCCACTTCTAAAAACT<br>TATTCTAGAACTATAATTATATATGTAACCAGAGCAAGATGATAATTA<br>TAATATTGTTTATAACATTTAAAAACTGGAAATGATGCAAATGGTCA<br>TTAATAGAATGCTGGTTGAATAATTCATGGTCCTTTATATCTTGCAAT<br>ATTCAAAAGTATGGGTTAGATCCACACATTTGGATATGAAAGAAAC<br>CACCAAGATACATTGTTATGTTTTTAAAAACAGAAAGAAGGCAAGG<br>CACTAAACAATGTGTATCTTGTGTTTCTATTTGTATACAGCAAAACC<br>CCCGAAAATGTATGTATATACATAGTAAATTTCTGGAAACATTAAGA<br>AATTTTAAAAAATAAATACCTTTGGGAATGGAATTAAGGATTTTGGA<br>GTAAGAGGAAGACTTATTTTTCATTGTATATACTTTTCTACTACTTGA<br>AAATTTAAAATTGTGTAATGTATCGTTAAAATTACCAAAGAAAAAG<br>CCACTTGGAAATGCAAGGATTTTACATACCTGAGCTGTTCTGATTAC<br>AGCATAATGTTTGTGATAATCATGTTTTGCTATTAGAAAGTTCAAATT<br>ATGTTGAATAAAACAATCAAGTAAGCATATGAAACATTTACAAAGA<br>TGGTTATTTAAAAAAATTCAGCGTGTAAATGCAAAACTTCATTAGG<br>AAAAAAACTCATGCATTTAACATAGTGTGTACATACTTAACTGTGTC<br>ATACTGCTTTGCAAAGAAGTTCCTGACCTGTATACTCTATAGGGCTC<br>TCAGGACCTTGCCTAGTAAAGGTGGCGAGTTGCCCTGTGTCATCCC<br>AGTTCTTCAGCCAGCCTATCTTTTTGGTGACAGATGCTTCAAACTTT

	AGCAGCTGGGAGGTAACTGTCAATGACTGAGGAGTGGTATTTTTAT<br>CCTTCACCCACCAAACCTGCTCTTACCCAGAGTAAAGAAAATAGAA<br>AATAGAACTATCAGATATGGGATAAAACTGTCAATAATAAATGGAAG<br>CAGCCACATGAAATAGTGAAACTTAGAAACATGTTTTTGTTAACAG<br>TACTTTCCCCAATTATAAAAGTAATATATGTTTGTTGTAGAAAGTACA<br>GAGAAGTATAATTTATACCATACCAACAGAGATTCTCTAAGCACAAA<br>GGCAGGGGTTCCTCTAATTTTTGTATTCTGTTTGTTATTTTAAGGAG<br>AATTTCAACAGGGATGGAGACTTAAATATAATTTGTTTGTATATACAT<br>AGAGTATCTGCAGAAACACAAGTAAGAAACTGGAAACATTGCTTC<br>TGAGTAGAATAATTGAGGAAATGGGGAAGATAAGTCAGGTGTTAAA<br>GGCACTGACTTTTGTATTATTTATGTAGCCACATCACTAAAAAAATT<br>AAAAAACAGTATGTAAACTTTAAAAACACAATAAACATTTTCATAA<br>GAATTCCCTAATATTCATAATTCTTATATAGAAATGTGAAATAAAAAT<br>AAAATCCACTATGAAATCATAAGCCAAACAAAAATAATTTAATAAG<br>AGCATCCTAGAAACATTGTTTCTATGTAATCATTCAGATAAAAAAGA<br>AATAAAAGGATGTAAAGAAGGCACAAATAAAAGAGAAAGAATAGA<br>ACAAAGAAGAGTGAAATGCCTAGATGGAAAGATTTTCAAAATAAG<br>TCTGCTACATTGAAAATATACTGCCAGTTGGTTGATATTTAAATGTAG<br>AAATTCTTGAATAATGGTGGCATCAATCATCACTAGGAAGGCCCGG<br>CACGGTGGCTCATGCCTGTGATCCCAGCACTTTTGGAAGCCGAGGT<br>GGGTGGATCACTTGAGGTCAGGGGTTAAACCAGCCTGGCCAACAT<br>GGTGAAACTCCTGTCTCTACCAAAAATACAAAAATTAGCCAGGCAT<br>TATGGCGCACACCTGTAGTCCCAGCTACTCGGAGGCTGAGGCAGG<br>AGAAGTGCTTGAACCCGGGAGGTGTAGACTGCAGTGAGATTGTGC<br>CACTGCACTCCAGCTTGGGAGACAGAGCGAGATTCCGTCTCCCCC<br>ACTCGTCCCCCAAAAAGGAGCATCACTAAGAAAAGGTGAATGGTT<br>GGGATGCATACTGGAAGGAAACAACGGAAATCTGAAAAGGTGTAA<br>GAACCTAAACAAATTTGTTTATCACAGAAAATAAATCACAAAACAA<br>CTTTGCGTTCTTTGGCAAGTTTCTTTATGTTAAACAAGAATTGCTTT<br>TTGCATCACATAGATCTTCTAAACTCTTTGTTGAAGAGGTCCTTGGT<br>AGTCTGTATCTAAGCCAGTTCCTTACGGAAGTGGCACTGAGCGGAG<br>TAGATAAAGATAGGAACTTTTGAAGGGTCATAATCTCTGTGTGCAA<br>AAAAGAAGCCACAGTAGTCTGAAGAGCTGTGCAGGTTTTAGGGTG<br>ACACTGGGTTGGGAACCTTGGAGCTAAGTGTCCCACACCTGGCAA<br>GCCATGACATACATATTTTCTGTTCAGGCAGAAACTGAGCTTTACAA<br>AAGTGAAATGAGAAAAAAAAAAAAAACCAAAAACCAGGCACGTAT<br>ATTGAGAACCATTCAGTCCTTCTTAGAATTGCCTCATACCTTTCTCA<br>TGCATCTTTATTAAATTCAGATGCAAATTAATTTTAGAAAAGTCTAA<br>ATAGGTGTGTGTTTTATTTTTCTGTTTCCTAATTAAATAGTGGTATAA<br>GCCTGGAAATGCTCTATATCTATTTTCGGAAATCTATAGCTCTTGTTT<br>AGGTAAATATCAGGTACTTAGCTAATTAAATGTCTCTTGTTTATAGG<br>AAAGTGTCAGCTTTCAGGATGTTATGTGTATGGCTCAATAAAATTAC<br>GTACAAAGTGACAGCGTACTCTCTTTTCATGGGCTGACCTTGTCGT<br>CACCATCACCTGAAAATGGCTCCAAACAAAAATGACCTAAGGGTT<br>GAAACAAGATAAGATCAAATTGACGTCATGGTAAAAATTGACGTCA<br>TGGTAATTACACCAAGTACCCTTCAATCATTGGATGGAATTTCCTGT

	TGATCCCAGGGCTTAGATGCAGGTGGAAACACTCTGCTGGTATAAAAGCAGGTGAGGACTTCATTAACTGCAGTTACTGAGAACTCATAAGACG
CHRNB2	CCCACTAGAATTCCTGCAATTACTGTAGGCAGTTGAAACCCAATAATATGTATTCATGTATCTATCTAAACACAGGAAAGGTACAGCAAAAATACAGTGTTATAATTTTATGGGGGCCACCTTTGTATATTGCAATCTTGTTGACTGAGAAGTCATTACATGGTACATGACTGTATTTGTATACCAAAAGTATGAACAAGAGTGTTCACAGCAGTATTATTTTTTAATAGTCCAAATAGGAAACAACCAAAATATCCATCAATAGTGAAACGGATAAATGATAGTGTATTCATACAATGGAATAGTCCACAGCAATGAAGATGAATGAACTACAACTATATTCAGCAGTGTTCACGATTCTCACTAACATGTTTTTTAAAAAAACAGACACAGGCCGGGCATGGTAGCTCACACATGTAATCCCAGCACTTTGGAAGGCCGAGGCAGGTGGATCACCTGAGGTCAGGAGTTCAAGACCAGCCTGACCAACATGGTGAAACCCCATCTCTACTAAAAATGCAAAAAAATTAGCCGGGTGTGGTGGCACATGCCTGTAATCCAAGCTACTCAGGAGGCTGAGGCAGGAGAATTGCTTGAACCCGGGAGGCGGAGGTTGCAGTGAGCCAAGATCATGCCATTGCACTCCAGCCTGGGCAACAAGAGCAAAACTCCCATCTCAAAACAAACAAACAAACAAAAAAAACCAGACACACACTGAAGATGATGATTCCATTTTTCCATTTATAAAAAGTTCCACAGGCAAAGTCGATCTATGCTATTAGAAGACAGGACAGTAGTTATTTATTTTACCTTCAGTCATTTTTTTAAAATTGTGGTAAAATTACACATAAAATCTGCCACTTTAACTAATTTAAAGTGTATGACTCAGTGGCATTCAGTGCATTCACAAGTTGTATAACCATTACTACTGTCTAGTTCCAGGTAAGTAGTCACCACCTCCCATTCCTCCCTCCTCCCAAGGTGTTTTTTTTTTGAGATGGAGTCTCGCTCTGTCGCCCAGGCTAGAGTGTGCAGTGGTGCGATCTCGGCTCACTGCAAGCTCCACCTCCCGGGTTCACGCCATTCTCCTGCCTCAGCCTCCCGAGTAGCTGGAGCTACAGGTGCCCGCCACGATGCCCGGCTAATTTTTTTATATTTTTAGTAGAGACGGGGTTTCACTGTGTTAGGATGGTCTCGATCTCCTGACCTTGTGATCCACCCGCCTCAGCCTCCCAAAGTGCTGGGATTACAGGTGTGAGCCATCACGCCTGGCCCCAAGGTGTTACTTTTGAAGAAGAGAGAGTAACTGCTGGAAGGGTCATCATGGGGACTTCTGGATATTAGTTATGTGTGTTTCTTTAAAAAAAAAAATCTAAATTGCGGCTATATGGCTGTGTTTATGTTGTGAAAATTTAGCTGTACACTTGTATATACTTTGCTGTATGAATGTTATATCCGAGTATATATAGATAAATTTGGAGGGGTATGTGTGTGTGTGAAATATATTATTGCTAACACTGATTTTTTTCCTGGGAGGTAGGAGTATGAATCCATTTTGTACTTACTATGTTTTGCTGACTTGTTTTCCAATTGGTATTCATGACGAATACATGTATTACTTTTGTAATAAAAATTTTAAAATTTAAAACATCTTTTTTTTTTTTTTTGAGATGGCGTCTCATTCTGTTGCCCAGGCTGGGAGTGAAGAAGTGGTGCAATCTCGGCTCACTGCAACCTCTGCCTCCCAGGTTCAAGCAATTCTTCCTGCCTCAGTCTCCTGAGTAGCTGGGATTACAGGCATGCGCCACCACACCAGGCTAATTTTTGTATTTTTCAGTAGAGACCGGGTTTCACCACGTTGGCCAGGCTGGTCTTGAATTCCTGATCTCAGGTGATCTGCCTTGGCCTCCCTAAGTGCCAGGATTACAGGTGCGAGCCACCACACTCAGCCTAAAACGCCTTAAATAAAGGACAATAACATTGATTTTAAGCCTTTCCC

	CCCAACCTTTTTTTTTTTTTTTTTTTTTGAGACAGGGTCTGGCTCTG<br>TTGCCCAGGCTGGAGTTCAGTGACATAGTCTCGGCTCACTGCAACC<br>ACCTCCACGGCTCACGATATCCTCCCTCCTCAGCCTCCCAAGTACT<br>GGGAGGTGTTGCCCAGGCTGGGAGCACGTGCCACCACACCTGGCT<br>AATATTTTTAAATTTTTTGTAGAAACAGGGTTTCACCATGTTGCCCA<br>GGCTGATCTTGAACTCTTGAGCTCAGTCCATCCACCTGCCCCAGCC<br>TCCCAAAGTGCTTGGATTACGGACGTGAGCCACCGCTTTCCACCAA<br>TTTTAAGCCTTTCAAGAGAAAACATTCCTTATCATCAGTGGCAATGG<br>GAGTGTCTCACCAAAACTTTGCCCAATTACTCTTTAGCCTCAGGATT<br>GATACTTCTGCATCTGTTCATCACCCTCTGTGGTTCTGGACCTGTCC<br>ACTCTTCCTGGCTGCCTTTCTTCTTGGTGGGTATGGAAGGAGTATGT<br>GTGGGAGAGATGAGGAAGGAGGTGTTCCCGCCCTGCTTGGACACT<br>GGGTGGTTGTGCTCAGAAGACTCACTCCTTGCCAGGCTGGAAGTA<br>AACTTGAAGCAAAAGCTGGGGGATTTGAGAAGAGAAGGTAGACT<br>GGCCTGGAAGTTGCATCAGGAGAAGCCAGAGGCTTTAAGCAAGCA<br>AATGACCCCCTAGTTTCTCATCCCATAAACAAGCAACCATTACAGA<br>AAGAGATTTTTGTTAAGAGAAGACTTTCACAGCCTTCCTCAATCAT<br>TCTGCATCCACCAAAACTCCCTGTATTTCTTAGGAGTTTGGAATACC<br>TTTAAGTCATCCTTGTCTTAATGACTTTCTCACCTTTCAAATCTCAAT<br>TTTCATCTTCATCCCTTCTAGGTTCTTAGACACTATTAACCTCTTCTT<br>CCTCTGTGCTTCCATCCATAGCACTTCCTTTATATGTCTGTTAGAACA<br>GCCATCACATTCTATAATAATTTGCTGCCAAACTGTGATCTCCTTAAT<br>AGCTGGGACTAAGTCTTTTTGATCACTGCATCTTCAACACATATGGT<br>CCACAACACACATGCAGTTTCCTGGCAAACCACATATACCTAACAA<br>ATGTTGATCTAAATTGTTGAAGGAAGATCTTCCCACCCTCTATCTTT<br>TCCTTAAGCAGAGGGACTACACACATTCCTTCTCTTTTCCTTAAGGC<br>TTTTCTTCACTACTTTTCTTTGTTACAAGGTTTCTTTAATTCTTAAGA<br>GATTATTGTGCGCTTACACATGCGCATGCCCCGGAACAAGTCGTGC<br>AGATTTACGCCCATTTCCATGTGCATCATGGGGTCTGTAGTCCTTCT<br>ACTTCTCCAGTCCTCTTCTAGAACTTTTCTGCTTCTTCCCTTAATTCC<br>ACGACCTGGGGACATGGTCAATAAGTTGCTTTTAGCTGGGCTTTTAT<br>TTATTTATTTGTTTAGTTTTTGAAATGGGGTCTTGCTTCATTGCCCAG<br>GCTGGAGTGCAGTTGCGCCATCATGCCCCAAACTGCAGCCCCGAC<br>CTCCCTGGCTTAAGTGATCCTCCCACCTCAGCCTCTCGAGTAGCTG<br>GGACTATAGGCACACACCATCATGCCCGACTAATTTTTAAACTTTTT<br>GTACACACGTGATCTCATTATGTTGCCCAGGCTGGTTTCAAACGCCT<br>GGGCTCAAGTGATCCTTCTGCCTCAGCCTTTGAAAGTGCTGAGATT<br>ACGGGCTTAAGTCACCTCTCCTGGTCCCTAGCTGAGCTTTTAGAGA<br>GAAATTTTCCCTACACATTAAAAAGTGTCAGTCCAGTACTAGCCCTT<br>TTCCTTACCTGTTGAGCCCTTATAATAATATGCCAGGCACTGTGTTG<br>ATTCATAATCTAGAGAGGCAGCCACTGCCTCAGGTGGATTCATTTAT<br>TCATTGGACATGCTCTGGAAACATATAACAAAGTATCTGATCTAGTT<br>CTTGGTCAGGCGCAGCTTCCTTAAGGAATTGACATTCCTGCCAAAT<br>GCTCAAAGTTAAGTAGAAGGTGATGAGGGGAACATGGGTTATAAA<br>AGAGCCCAAGAACCATGCATTTAAGTCCCTGAAGTGTGGGGGA<br>TGATGGGACGGATAAGATGTTCAAGGATCCTGAAGACCTTGCCAAT

	AGTTGGAATGTGGAGCTGGGGGTGGAGTGACCCAGATGAGGTTGA<br>AGTAAGCAGGGACAAAACAGGACAGAGCCTTGCTGTATGCTAAGT<br>CTGCTTTAGTCAGGAATCTTTTGGTTGAAAGTGACAACGAACCCAC<br>TGAAGGAAAAAAAGGGGCTAAGAGTTCAGGGTTCTCATCTTTGGT<br>CTCCTTTGTGTTAGCTTCTCTCTTGATCAGGCCTCTGTGAGTGGTCT<br>CTGATCTCCCTAGTCACACAGAAGCCAACAGCTGGAGTCAAGAGA<br>AGGGGGTGAGGAAGGCCGATGAATAGGGGCTACTTGTCTTTCCACT<br>ATTACCCTCAAGTCTACCTTCTTTGCAGGCAAGTCTAGACCTCCTCA<br>GGTAGACCCTCAGTGGGAGGGCAGGAAGCTTAGCCTTTTAGACTC<br>CAAAGCCGTTTGCTTTCTTAAAAATAGCAAACCCACAGCCCCCTAT<br>CTCTTTAAGGGGCTAAACACATTTTCTCTGCTGTATCCTCAGCCTAC<br>CTGGGGAGCAGGACCATTTGGGTTTGGTAAGAGAAAAAAGGAGG<br>GCGAAAACCCCGGACACTCCTCCTGTCTCCCGACGCTCGTTCTGGC<br>CGTCTGGCCCTCAGTACTAAGCAGGCCCTGACCTACTTGTCTTACT<br>CTTGCTGCTTCTTCCCGCCGGGCCTCTTGCCCCTTCTCCGACCATGC<br>TGCAGGTCCTGCCCCAACTTTAGGACTTCAGAACTCCTCCACCGTG<br>CCGTGGAGGAACAGCCCTTCTTCCACCTCACGCACACCCTACCAC<br>GGCAGAACCAATCGAAGACTATCCCAGCCGGGGACCCCCCTTTTTT<br>TTCCTGGGACCCAGCGTTTCCGCCTCCGGGGCGCAGACTCCTCCCC<br>CTCACCGTCCCAATTG
CLDN2	TCCCAAAGTGCTGGGATTATAGGCGTAAGCCACCGCACCCGGCTGA<br>GCTCCTGAAGATTTTTAAGCATGGCAAAGCGTGAGGTGAAAGTCA<br>CAATTTGATTTCAACTTTACAAAGATCTTTGACTACTGGGTAGGTAA<br>TTCATTATTGCAGGATTGGGGCAGAAGTGGAATTAGAGAGATCAAT<br>TTGAGGTGTCTATGAGATTTTCAAATAGACAAGAAACGATGGTTGC<br>ATAGTCTGAACTATGGTTTAGGGCAGTGGCGACAGAAAGAAGTAGA<br>TGGATTCACAGTCTATTTTTGAAGGCAGACCTGATGGTACATTATAA<br>TTAATTATATGTCAACAAGAGGTGAAAAAGCAAGGATGACTGGCTT<br>GGGTTATTAACTCAGTGACAGCCCCACTGAGGTAAGGAACACTGCA<br>TGAGGAGCAGTTTGGGCATGGGGAGCAAAAAGGGCAGATGATTCC<br>AGCTTTGCAAATGTTGAATTTGAGGCCATTAAAACATTCAGGTAGG<br>GCCATTCCAAGTAATAGTTAACTACACAAATCTTGAGTTCAGGAGA<br>GAAATCTGTGGCAGAGATATGAATTCAGGAGTCACTGGCATGGAGA<br>CAGAGAGGATGAGATAACCCATGAAGAATGTGAAGGATAAGAAGA<br>GCCATCTAGAAGTCGAGATGAAACCCTGAGGAGGCTAGAGGGGCA<br>GAGATGGATAGTTAGACTTAAAGAGTGAGCAGAAGAAGAGGAACT<br>TATAAGGGAAAACTAGAAGAATCATTTTGAGAAGTAGTTAAAATGG<br>AGTGGTGTTACAGAAAACAAGGGAGAAGATAGTTTTATGAAGGAG<br>AGAGACAAGGTGAGATGACAGAGATATGATCATTGAATTTTGGATC<br>AAGAAAAGAGTTGATAACTTTGGTGAGAATATTTTCAGTGGAGGGC<br>TAATGGTGGAAGCCAGACTGCAGAGGGCTGAGCAGTGAGTGGGA<br>GGGTGAAGAAGTGGAGACATCAATAATGAATGGCTTGTTTAAGGAT<br>CTTGGCTGCAAAGAGAATGATCAAGATAGGAGTAATAGCTGCACAG<br>GGAGGAGGTAGAGTAGGGAGCTCAAAAAGTAGTAGTAAATGTTTG<br>AAGCAGTCATCAGAAGGGTGCTTACCAGGGAGAAGTAGAAGGATT<br>TCCAAGCAGCACTGAAGGCCTCAATGAGATACATAGTCATGATCAG

	GACAGGTACAATTGTGCCAAGGTTAGCAATAAGAAACCAATGTCA<br>AGAAAGACTTGCTAATGAGTGACAGAGGTGAGATTCAAACTATGTT<br>TCTCTCCCTTCATCAAATGTTTATCGGCTCCTGCTCTGCACTGCTTT<br>GCATCAACACTGGCATTGGTGTTTCCAATGCTGATGAATCGTATTGA<br>CTATCAATCATGTGCCCAATGCTGTGGAAGCATGATAGACATTTAAT<br>ATATATTTTTTCACCCTTAAATTGTTTAGAATTTGGTTGAGGAGACA<br>AAATATTCCACAAATGCATATATATTATAAATATATATAATATACATTAT<br>ATATATACATAGTGAAGCTCTGCTGTCTCTCTCTCCCCCCTTACCTCT<br>TTCTTCCTCTGATCCCCCATCTCTCTCTTTCTTCTTTCCCAACGTTGG<br>GCAGCACTTCAATGACCCTTACACTGAAGCGTTATTGACTCATTTG<br>GAAAGTCCCTATATTTTAGTCATATGGAGCCCCTCAAACCTCTTTGC<br>CAACTTCATTCATTTATTAAGAAGACAGTTTTTGAATTGCTGCTCTG<br>GATAAAGCAGTATGTAAGGCACATGAGATACAGAGATACATAAGAT<br>ATAGTCTTGCCCTCAAGTAGCTCACAGGCTAATAGGGAAGAATGAG<br>GGTTTAAACAGGAAATTGCAACATAATGCAAAATGTACTATCTCTGC<br>CTCAGTTTCTTCATCTATACAATAGGGATTATTAGTGCCTACCACAA<br>AAAAACCTGTGACGACTAAATAAGTTAGCATGTAAAAAGTGCTTAT<br>AATGGTCCCATAAGAAACACTACATAAGTGTTGCTATTATTATTATTA<br>CTATGATGGAAGTAAACACAAGATACTGTAAGAATAGACAGGAGG<br>GGTACTTGACCCAGGCTAGAGTAAGAGACATCTGTGCTGAATCCTA<br>AAAGATGAGAAGGCACTAAACAGAGGAAGAAGAATACATAACACT<br>CTGCATAGAATATTCTGTTCTATTTCTATTTGGGAGCAAAAGACCAC<br>ATGGCATGATTGAAGAACTGGAAGTAATTTAATTCCACAATAGTACA<br>GACTATGAGGAGGGAAATGGCAAGAGATGAAGCTTGAGAGGTCAT<br>GGGGTAAACCAAGCAAGTTTGGACTTTACTTTTTAGCAAATGGGGA<br>GCCACTGGAATGCTTTATGTAGAGTAGTGACTCGATCAGATTAATGT<br>GTTGAAAGATCACCTTGGCAATGATGTGGAGAATGAATTGGGAGA<br>AAGACAAGTTAGAAAGCTGTTGCTATAATCCAGGTGAAAGAGAAT<br>GATGAGGTCGTGCATGGTGGCTCACACCTGTAATCCCAGCATTTTG<br>GGAGGCTGAGGTGGGCGGATCACTTGCAGTCAGGAGTTGGAGATC<br>AGCCTGGCCAGCATGGCAAAACCCTGTCTCTACTAAAAATACAAAA<br>TTAGCTGGGCGTGGTGGCGTGCACCTGTAGTCCCAGCTACTCAGGA<br>AGCTGAGGCACAAGAATCACTTGAACCTGGGAGGTGGAGATTGCA<br>GTGAGCTAAGATCACGCCACCCCACTCCAGGCTGGGCAACAAAAG<br>CAAAATTCGGTCTCAAAAAAAGAATGGTGAAACTGAACTAGGGAA<br>GTAGCAGTGGAAATGGAGAGAAAGGAACCATGGATTTGAGGTATAT<br>TCATAAGGTAGAACTGGTGGGACATAGGGACTATAGGATGCAGGAG<br>GTAAGTAGAAGAAAGGTGCTAAAGATCACTTTCCGGATTTTGGTCT<br>GGCACAAGTGGATGGATAGAGGTATCATTCAGTGAACACAGGGAA<br>CACAGGGAAGAACTGTTTGGGGAAAGCTGCATGGGGGATGTGATG<br>AGTTCACTTTTGGACATGTCAAATTCGAGATGTCTATGAGATTTCCC<br>TGTGGAGATTCTGACTAGGTGATTGCTCTGCCAGTCTTGAAGCTCA<br>GAAGGGTCTATCCTGGAAATTCAGATTTGGGAGGGTCAGTGGATGG<br>GTAGTAGATTAAGTTATGGGGGCAAGATGAGTTTGCTCAGGGACAG<br>AGTGTTAATTGAGAAAAGGATGGACTTCTGAGGAATACTCACATTT<br>AAGAGATGAGTAGGGAAAGAGAAATCTTGAAGAAGGCTAAAATGG

	AATAGCCAGATTGTGGTGGGTTGAAAAATTAGTGGGTTAGTTCGGG<br>CACGGTGGCTCATGCCTGTAATCCTAGCACTTTGGGAGGCTGAGGC<br>AGGTGGATCACTTGAGCTTAGGAGTTCAAGACCAGTGTGGGCAAC<br>ATGGCAAAACCTGGTCTCTACAAAAAATACACACACACACACACA<br>ACTAGCCAGGTGTGGTGATGCGCTCCTGTAGTCCCAGCTACTTGTG<br>GGGCTGAGGCAGGAGGATCACACACACACACACACACACACACA<br>CACACACACACACAACTAGCCAGGTGTGGTGATGCGCTCCTGTAGT<br>CCCAGCTACTTGTGGGGCTGAGGCAGGAGGATCACACACACACAC<br>ACACACACACACACACACACACACACAAACTAGCCAGGTGTGGTG<br>ATGCGCTCCTGTAGTCCCAGCTACTTGTGGGGCTGAGGCAGGAGGA<br>TCACTTGAGCCCAGGAGGTCGAGGCTGCAATGAGCCGAGATCATG<br>CCACTGCACTCCAGCCTGGGTGCCAAAGTGAGAACCTGTCTCAAA<br>AAGAAAGAAAGAAAAATTAGTGGATCAGAGGGTCTTGTAGAGAAT<br>GGGGAGGTGTGCCCTTGACCCTTAGTGTCCTGAATCTTGGCAACAC<br>CGAGGGCTCCTTGAACACGGCAAAATCTTATATGGCTCTGAGATTC<br>CAAAGCATTGACTCAGATACCTGCCTCATGCAAAGCCCTATATTCTA<br>GAGCAGTTTCCCTTTCCTCTGTGGCAGACTCTTGTCCCCCCTAACA<br>GATGGCCCAGGGAATTTCAGGGCCCCCTCTCAGTCCTGGAACCCTT<br>GTTCCAGAGTGCTCCCTCATCATCCAAGAGGCTGATGATGGGAGCA<br>TCTATTAGGAGACTGGACAGGAAATGTCTGGGCATGTTATACATGC<br>AGGAGGCCTTAGACTAGGCTGCAGAGGGGGATTTGGGCATGGCTG<br>GGAGGATCTGAACTCTCAGAGTATGGACAGAAGGCTTTGCTGCCC<br>ACCCCCATCTACCCTGGAGTAGATTTTCACCATGGGCAGAATGATCC<br>AGGGCTAGGCCACTACTCTCTAGGCCCCTGGAGATTCAAGAGGCCT<br>CTAACAAACTGGAGTCCAAGACTACATTCTAGGATCTGTTCCTCCT<br>GATGTAGTCTGCAGTTTGGCCTCAGTCTGCAATTGAGGGGCCCTAT<br>GGCACTGTTGCTTGGCAATGTATTAAACAGCAGGCCTTGGAGACTA<br>GCACTTGAGTTAACACAGCCACCACAACCACCACTGCCATCATCAC<br>CTTCCCGGAAAGCAGCCACCTGTCTGGCTCCTGGCTTTGTCCAGCT<br>GCCAACCTAAGGCATGTGCCTACGCAGGAGGCGATGACATTTTGGC<br>TCCACGTTCAAAGTTGTTTTTTTTTTCCTTTCTCATGTGTTATTTCTA<br>AAGATAACAAAGGTCAAAAGGCATCCAGCGTTTTCTGGTTTCTCAT<br>AAGCTTCTGGTCAATATTTAATCTGGTTTATGGATTTTTTTTAGGTCT<br>TCTAGATGCCTTCTTGAGGCTGCTTGTGGCCACCCACAGACACTTG<br>TAAGGAGGAGAGAAGTCAGCCTGGCAGAGAGACTCTGAAATGAG<br>GGATTAGAGGTGTTCAAGGAGCAAGAGCTTCAGCCTGAAGACAAG<br>GGAGCAGTCCCTGAAGACGCTTCTACTGAGAGGTCTGCCATGGCC<br>TCTCTTGGCCTCCAACTTG
COL1A1	AGGCTGGAGTGCAATGGTGTGATCTCGGTTCACTGCAACCCCCGCC<br>TCCTGGGTTCAAGTGATTCTCCTGCCTCAGCCTCCCAAGTAGCTGG<br>TACTACAGGCCCATGCCGCCATGCCGGGCTAATTTTTGTATTTTTAGT<br>AGAGATGGAGTTTCACCATGTTGGCTAGGCTGGGGTCTCAAACTCT<br>CGACCTCAGGTGATCCGACTGCCTCAGCCTCCCAAAATGTTGGGAT<br>TACAGGTGTGAGCCACCGCGCCCTGCCAAGCCAACTTTTAAGAAG<br>AACTACACTGCCTCCAGACATCAGGAGCAGGGAGGGATATGACAG<br>AAAAGCTGCCAGCTACCCAGCTGGGAGCTCACTCCAGCAGGAAAG

	ATGGGGCTGACCCCTCACTTCTGACTACCAGAGAGCAGCAGTGAG<br>AACTGGGGTCCCCTAACTCAACAACATCCCCCATCCAGTGCATTATT<br>CTCAGCTGGGGCCTGGCTGGGACCTGGTGGGGTGGCTGGAGAGGG<br>CTTGGGAGCTGGGGCCATGACAGACCTCGTGTCACCTGGGGCCAT<br>ACCCCCAGCCCTTCTCTCTCCAGCTCCCAAGTTCTCCTCCTAGGAC<br>TGGGGTGGGGGTGGTGGAGGCAGCGGTCAGAGAATGGGGTGGGA<br>TCCGTCCAGGACTAGAAAACAAAGTTCATCTTCAAGAGCCTGGAA<br>TGAGGGCTTGTTCTAGGGGAGAATGAGGGGGAGGCTGGGCCTGGC<br>TATGGACATTTGAGGGCTCGGGTCACCTGGAAAGAAAAGGAAAGG<br>GGGAAGGAGAGCAAAAGAGGCAAATGAGATCCAGAAAAGGAGCT<br>CTGGAGACAGAGGGGGATGGAGAAAGCAGCGAAGAAACTTGGAA<br>GACAGAGGAGGGATGCAGGGGCCTGAAAGGAACGCAGGATCCGG<br>GCTGGGAAGAAAGAGAAAGAGCAGGGGACAGATGCCTCTCTAGA<br>CTCCTGCCACAGCCAGACTCCTCCTCCCAGTCGCTGCCCTCTGACC<br>TTGCTTCCTGGCCTCCCACACACTCCTTAGCCCACTCCAGTCCGGC<br>TTCCTTGTCCATCTCTCCACCACAGCAGCTCACGCTGCGGGGATCA<br>GTCACCACCATTCTGCTAAGTCCAGGGAACATTTTACTCCTTACCAT<br>ACTAGTTCTCTCTGCAGCATATAACTCTCCTTCCTTCTAGTACCATCT<br>CTTTCCTCTGCACTCATGACCCCCCAATCCCTCTCACCCAGCCCTCC<br>TTCTCTGGCCACTCTTGCCCTATCTCCTCCTCCTCCACCCAGCCCTT<br>ACACCCTGAGGGTCCCCGAGGCTCAGTGCCAGCCCCTCCACACTC<br>CCTCCCTCCATGCTTTCTCTGAGACAACACCATCCACAACCCCAGC<br>TTCAGTTACCATCATCCTGGGACCCACATTTGTCTCTCCTCTGAACT<br>CCTGACCCAAGTGCCCAAGGGTTTCCTCCGTATTTCTAATTGGATGT<br>CTCACAGGCTTCAAACACTCAGCATGGACAAAACCTACTTCATCAT<br>CTTCCCCCTAACCCTGGAAAGGAAGGAAGGAGGGAGGAAGGGAG<br>AGAGGGAAATCCTAGGAGGCTGGAGGACTTGGGTTTGGGGCTAAG<br>GCATAGAAGAGCAGAGCTGTACCTGGAAGCTGAGGAACCTAGCAA<br>AGGGCTCCGACTGTGCAATGCTAAAGCGAGGGAAGAAGAAGGGC<br>CTGCACACACCCGGGGTCTATCCCCAGACTCTGCTCCAGGCTCCCT<br>CAACAGGAACTGTGCAGGGCTCCATGATAGAGACTCATGGAGAGC<br>TTCATCCTGGGGTCTTCACTGCACCCCCACTCTTAGACCCCGCCCTT<br>CTGTCTCCTCAGACCCTCTCCTAGGAACCCCCTCTTTATCATCAGTC<br>TAGGGCTAGAGGTGGGAGCAGAGAAGCGTTCTGGAGAATTGGAGT<br>TGGGGGAAGAGCAACAGAACCTCAACTGGGGGCTGCAGCAGCTG<br>GAAAGGGTGACCCCGATGTGCAGGGCAGGGGTGCATGCCCCCCAC<br>CAGGTGCACAACACCTGGGCTACCTCTTCCAGGTGTGTTCTCCTCC<br>TTTGTCCCATACCATGGAGTCCAGATGGGGCACCTGAGGAGCTCTG<br>AGGGTGGGGGCCTTTTCCCTCCTTCTTTGGTGTTGGAGGGGGGCAC<br>TTGGGGAAATTTGGAGAAGGAGAGAACTCAAGCTCAGCACTTTCC<br>TCTTTCTGTTTTTCTTCTTAAGGAATTTTTTTTCCCTAACTGCTGATG<br>ACTTTACCATTTCTTGGGGGTGTGGGGAGGAGATTTTGGCTTTTGC<br>TCCCCCCCACTTTAAGTGCCGGACAAAGTCTTACATTCCACAAGAA<br>GCCAGAGCTTCAGAGTTTCCTAAAGATGAGGTGGCGTCTCCTCCTC<br>TCACAGGCACAAGCTCCCTCCTCCTCCCACCCCCCATCCCCCCAGT<br>CTGCAGCCCTCAATCCTGGCCAGGAAGGCCCAGCCAGGCTGGGAG

	GAGACCCCAAGCACATTCTTCCTCTCACTGTCATACTGCAGAAATT<br>AAAGACACATCTTCAGCCTGGGCACCCGCCAAGCGTTTTAAGTCG<br>AAGAGTGGCAGGGGAGGCCTTGAGCCTCAGCTCCATGCCACGTGT<br>AAAGGATGCTTGGAAACTGTCTGCCTCGGCCCCTGGGAGGAAGGC<br>CTGGAACTGGACATTGGGGTGGTGGCTGTCACACGCCAGGCACAC<br>AAAACTCCAAAGCCAGGGATCCCCAAATATCCTTCAGAACCCCAG<br>GCCCATGATGTAGCAACCCCCAATTCACACCTTGGAGGTTTCAACT<br>CTTCTTTAAGATGGGCGTGGGAAAGCCTGGATGGGAAACATATGGG<br>GAGGGGCGGGGAGCTGCAGGCAGGAGCCCTTCTTACTACGAAAAC<br>CCAAGAAGCAAGGAAGTGGACAGGTCACTAACCCTCATACTACCA<br>AGCCCTGCGGCACCCTGCCCTAGACCACCACTCTAAATGTCTGTTC<br>CCTCCAAAAACAGGACCCCTGTCGCCTATTAGGGAGGGGTTCTCTT<br>GGAACTGACCCACAGTAGGGGGCAGGACTTTGGTGGGTTCAAGAA<br>CTGCCATCTCAGCACCTCAGCCCCCTAGTCCTGCCCTGCAGTCGCT<br>GGCACTAGGCGGGGGCAGACCCTGGGCCACAAGTTGCTGCCACAT<br>GGTCGGGATAATTGATGAAGGTCCATCCCTCCATTGCTGTCTCCAGC<br>CCTGCCTCTCTGGAAACTCTATATTTTCCCTTTAATTATAGCCCCTGC<br>AGTCTCCCTCTGCTGCCCCACCCGCACCGCTCATCCTGGCTGCCCA<br>CGGCCAGCCGGCCAGCCGACGTGGCTCCCTCCCCTTCTGTTCCTTT<br>TTTTTCCCCTTTGCCTTCGTTGCACAAAACCAGCTGGGGGAGGGCG<br>TGGAGAGGGGCGGGGGGAGGCAATGGAATCTTGGATGGTTTGGGG<br>GAGGCGGGACTCCCCGCTTCCACGTTTGCAGCTCTGGAGCACCCG<br>GGGTGGGGAGCTGCACAGGAGGGAGAGAAATGAACAGGGCACTG<br>CAAGGAGACCCCCAGGCCTTCTCTCAGCCCTACAGAGTTTCTCAG<br>GACGAGGTAGATTGGGGTTGAGGCAGAGCCTTGTTGGGGGAATGG<br>GACATGGAGGAAGAAAGGACGTGGAGTTCTAGAGCCATCTTCCTT<br>AGATATAGCCTGCTGTCCTTCGGGTCCCCAGACCCTTTCAGAGTGT<br>ACAGATGATTCTCTCTGGTTCCTAAGGCATAGAGCAATGACCGGGA<br>TTTTCAAGAAAGAGATGAGGCAGTGGGAAGTAGCCCCTAAAACAA<br>AGTCAATCATCCTCTGCAGCCCATCCCACACCCCCAAAGGAAAGTT<br>TCACCCAGACACCCAAAATATCCCATACATCCCCAACACTGAGTCC<br>AGGTACAACTGGAGAAGGGGCTTTATGCAGCTCCCAGAAAGACAC<br>CCCTTTAGCTAAGTGCCCTCCCTCCACCCAGGTTCTCTCTGGTTTGA<br>CTGTGCTGGGAAGGAGGGTCTCTAAGCAGCCCCTGGCCACAGCCA<br>TGGCAAACAAAACTCTTCTCTAAGTCACCAATGATCACAGGCCTCC<br>CACTAAAAATACTTCCCAACTCTGGGGTGGAAGAGTTTGGGGGATG<br>AATTTTTAGGGGATTGCAAGCCCCAATCCCCACCTCTGTGTCCCTAG<br>AATCCCCCACCCCTACCTTGGCTGCTCCATCACCCAACCACCAAAG<br>CTTTCTTCTGCAGAGGCCACCTAGTCATGTTTCTCACCCTGCACCTC<br>AGCCTCCCCACTCCATCTCTCAATCATGCCTAGGGTTTGGAGGAAG<br>GCATTTGATTCTGTTCTGGAGCACAGCAGAAGAATTGACATCCTCA<br>AAATTAAAACTCCCTTGCCTGCACCCCTCCCTCAGATATCTGATTCT<br>TAATGTCTAGAAAGGAATCTGTAAATTGTTCCCCAAATATTCCTAAG<br>CTCCATCCCCTAGCCACACCAGAAGACACCCCCAAACAGGCACAT<br>CTTTTTAATTCCCAGCTTCCTCTGTTTTGGAGAGGTCCTCAGCATGC<br>CTCTTTATGCCCCTCCCTTAGCTCTTGCCAGGATATCAGAGGGTGAC

	TGGGGCACAGCCAGGAGGACCCCCTCCCCAACACCCCCAACCCTT<br>CCACCTTTGGAAGTCTCCCCACCCAGCTCCCCAGTTCCCCAGTTCC<br>ACTTCTTCTAGATTGGAGGTCCCAGGAAGAGAGCAGAGGGGCACC<br>CCTACCCACTGGTTAGCCCACGCCATTCTGAGGACCCAGCTGCACC<br>CCTACCACAGCACCTCTGGCCCAGGCTGGGCTGGGGGGCTGGGGA<br>GGCAGAGCTGCGAAGAGGGGAGATGTGGGGTGGACTCCCTTCCCT<br>CCTCCTCCCCCTCTCCATTCCAACTCCCAAATTGGGGGCCGGGCCA<br>GGCAGCTCTGATTGGCTGGGGCACGGGCGGCCGGCTCCCCCTCTC<br>CGAGGGGCAGGGTTCCTCCCTGCTCTCCATCAGGACAGTATAAAAG<br>GGGCCCGGGCCAGTCGTCGGAGCAGACGGGAGTTTCTCCTCGGGG
COL1A2	ACTTCTGAAACATTTCTATAGCTCATCAACTTCTCTTGAGCTCCACC<br>ACATCTATCCTAGTTCAGGCCACCATCATTTCCCCTCAGAATTCCTG<br>CTGCAGTCTCCTTACTGTTCTCCCTGTCTCTACTGTGCTCCCCTTCT<br>AATCGTTTCTCCACACTGCAGCCCCAGTCATCTTATTCAAAACACTC<br>TCTGGTTTCCTACCCTCTCTGTTTAAAATAATCTAAACACTCCCATA<br>GCACACTTGAGAAAACACAAACACCAGAATACAGCCTAAAAGGTC<br>CTTTTTGATCTGGTCCCTGCCAAAACCACCAATCTCATCTCTAGCTC<br>CTTCCTCTATCCTCCCTCTTTTCCACCACAATCCCATCGTACTGAAAT<br>GCTCCATTCCTCAGATTGCCAAATTTTTCAATGCCTCTGTGCCCACA<br>CCACTCTCACACCTCTTTTTAAAATTTTGTTTGTATTTAAGGGTTGA<br>ACAAGGATGTTGCATCCTCCAGGAAGCCCTCCTTGATTCCACTTATC<br>TGAATCTAGATGAGGTGTCCATCCTAAATCCTCCCATAAACATGTAA<br>GCATTCTATTCCTCCATTACAGTATTCATCACACTCTTCTCTATCCTC<br>CAACAGACTATAAGCCCCCTGAGGGCAATGACCTTGGCATTCTGTT<br>TCATAATTATATACCCACCAACCCAGTAAGTGCCTGGCATAACACTA<br>AATATTGCTTGAGTAAACATACATCTTACCAGCTGGGCGAGGTGGC<br>TCATGCCTGTAATCCTAGCACTTTGGGAGGCCGAGGTGGGTGGATC<br>ACCTGAGGTCAAGAGTTCCAGACTAGCCTGGCAAACATGGTGAAG<br>CCTCCTCTCTACTGAAAAATAGAAAAATTAGCCAGGGGTGGTTGTG<br>GACACCTGTAATCCCAGCTACTTGGGAGGCTGAGGCAGGAGAATC<br>GCTTGAACCCAAGAGGCAGAGGTTGCAGTGAGCTGAGATCGTGTC<br>ATTGCACTCCAGCCTGAGCAACAGAGCAAAAACTCTGTCTCAAAA<br>AAAAAAAAATATATATATATATATCTTGACTCATTTAAATAATCAATG<br>GGAACTGAATATCCTAATTGATATGACACAGAGCAGGAATCTCCAT<br>GCCTTTCAAACAAATATCACAGAAAGAAATCAGCCCTTGCTGGGCA<br>CAGTGGCTCACACCTGTAATCCAAGCACTTTAAGAGGCAGAGGCA<br>GGTGGATCACTTGAGCTCAGTTGGAGTTTAAGAAAAGCCTAGGCA<br>ACATGGTGAAACCTCATCTCTACAAAGAATACAAAAAATTAGCCTG<br>GCATGGTGGCATGCACCTGTAGTCCCATCCACTAGGGAGGCTGGGG<br>CAAGAGGATCACGTGAATCCAGGAATTCGAGGCTGCAGTGCCCCC<br>AGATCATACTACTGCACTCCAGCCTGGGTGATAAAGTGAGACCTTC<br>TCAATAAATAAATAAATACAAAATTAAAAACCAGCACTTAACTCTTC<br>AGCTACCACATTAATTCTCAGCAAATACCTTCTTAGCAGTCGTTTGA<br>CAATCAAGTCAACTAGAACTGGAACTAGAATGAGATAACATGGTAT<br>TGCTTCTTCTCACAGAGTAGATCCTCAATGAATGTTTGAAAGGAGA<br>TGTCAGTCCGATGTTTACCTTTGATCAAAGAAAGGAATATAAGATTA

	AGACATCAGGGCCTCGTCAGGCTGTTCTGCATTTCTGTATTAGCCA GACTCTCAACCATTTTCTTGCCATATATAAACATGTGGCCCAGTTTG TAATCAGTGGGTTATAGGAAGCTAAAGAAATTATCTTTTCTTTCATA GAAAATAATTTGGGCATAGGAACCCAGGTATTAACCGTCTAGCACC AAAGTTGTAGGAATCTTTAGTCTCCTCTGCCTCCCTTCTTCCATCCC TCTACTCTCAGTCTTGACCCCACCCCACCCCTTACTTGTTTTTCCTT CTACTCATTTTCTCCTCCCTCCCTCTCCCCTCTCCCCACACTAGAAC CATCAGGGCCCTCACCAGGGCATTCAGTTCAGGTTCTGGGTCAGCT CTCACAGCTCTACCAGTCCCACCCCCATCCCCAGGAAAAACTGCTC CTTATTTGGAGTCACAAAAATATTTAACAGAGATCTAACTGACCACT AAAAATTCCTCCTTTAAAACAAACACCTAATCAACTATTTTCCCCCA AGTTATATGGAAAAACAGCTGCAATTAGAACTTGATTCTCACTTTAA GAAAGAAAGATTCTTGTTTGGTTTTCTCCACTTTCATTTTTTGTTTC TAGGTCCAGGGCCTCCCACCAAATGCTGACGGCTGCCTGCTTCAAA CCCTGCCACATCAGCAGGGAGGGAGCACAGCGGCATTTGGTATTTG CATCAGTTTCCAGGAATGCTTTTCAAGTTATCAGTTCACTCTGCTGC CTTTAGCAGAGACGTTTTCCCTCTAAGTTTATAGATGTCTGCTTCAA TTTACAGTCCTCTAATTCTCAAAAACTTGCTGAGGATTTTTCTTTTT TGGCTAAAAGAGAAATTATAATCACACTTCTAAAAACATTCTATGTT TTAAACTATTTTGACAACAACAACAAAACCTACAATGAAATGTTTA AAACTTGGGATATCTATTGCAAATCCAATGATTGAGCCAACTTGACC CCAGAAGCGTTATCAGATAACCTCACTGAAGGGTATTTGGAGGGTT TCTTTCATAAATCTGTTGCATTACCACCCTGAGTCATTTTGCTCAGA ATTAGTCTCTGACTCTCAGCAACACAGGACAAATACACACATATGC CCTGCAAAGGTAATTCAGCACAGTGGTAACAATGATTCTTAGAAAT CATTTCTCACTCTTCTGATATGCAGAAAAAAATTTGTTATGATGTAGT ATTGAAGTTTTTCTTTCCTGATAAAAATGATTTCCACTTTAAAAGTT TTTTGTTAGTTCTGTAACGGTGATATTTCAGGGAAATGTTAAAAATG TTCTTGGAATATACAATTCAACCTCAGGTCTTTTGTTGTTGTTGTTC CTAGAACCTAGAAAACTTCAAACATTGTTGCCTAGTTAGAAAAAAA TTTGAATGTGGATTGCTCCCTGTAAACCCCCTTCTAGGAATGACCA GTAACCCTTTCAAATTCTTTCACTCCCAGTTACTTCAAAAAATCATC CAAAGTGGTCTCCCAAGTGAGTGCCTTTAATTAGAATAAAACAAGA GTTTATTATAGTTTTTGGTTATCCACTTTTACTTGCATTAACCTTTTTT TCTTCTTTTACATTTAGAAAGAGTAACCTGCTTTAGAATAGTCCCTT TTATTTACAGAAGCTGCTGATGGAGTTAACTTCTGCAGAAATTCTTC CTTAAGGCAAAGCAAAAAAAGCGGGGAGGGGGTGGGGGGAAGGA AGGGAAAAAGATTCTCAGGGAACTACAGCCCACTTGCTTCTGTTTC TTAGAGACAGAACTGACCTAAAGATGCCCCCTTTGCGATGACTTCT GGGATAGAGCAGCACTCTAACTAGGCCCCCGCTGCCTCATGGGGAC CTTAGGCAAGTAGAGGAGAGGCCTGACACACACACACACACACAC ACACACACGCACACGCGCGCGCGCGCACACACACACACACAGCCT TTCAAACCTAGGGCCTGGAATGCCATCCCAAGAGGCTTTAGAAAA AGGCACAGGACCTTTGGCCTCCCACCTCAGGGTCAAAGTACCAGT TCCTCCTCTCCCTAGTAGGGAGTGGAGGGTTGGATGGAGGCGGCC AGAGAAGAGGGAAGTTGGGTGCTGGGGAGAGAGTTAACATCCAC

	GTTGGTGGGCGCACTGCTTGGGGTGTTACCAGCGAAGACTACGAA GACCCCAAGCTCGAATCAGAAGGGCCTCTGGATGTGCTAGGGGAG GTGCTTGGGTGTGGCTGTAAGAGATGGGACAGAGAGTAAGCAGCA AGGTCAAGAGGGACCGGGGGGCTCACGGGAGGGTTGAAGGGTCC AGGCTCAGGGTAGAACTGGTAAATCCAGACAAGGAGCCCATGGAG AAGGGGAGGGGAGACTGGAAACCATGAAAGATCCCCCACCGCAG CCTCAGAAAGGAGAGACTGAGAAATAAGTTCTCGGTCTCCAGGTC GGTTGGAGTCGTGTCGGAGTGCCAGACCATCCCCCAAAAGACCCT CTTTGGAATGAGCCTCAGCAAAGGCAAGCTAGGAGGTCGAAGGAC TTCCCCAGGTGACTCGGTCTAGTCTAGAGTTCGCAAAGCCTATCCT CCCTGTAGCCGGGTGCCAAGCAGCCTCGAGCCTGCTCCCCAGCCC ACCTGCCAACAAAAGGCGCCCTCCGACTGCAACCCAGCCCTCCAC AGACAGGACCCGCCCTTTCCCGAAGTCATAAGACAAAGAGAGTGC ATCACTGCTGAAACAGTGGGCGCACACGAGCCCCAAAGCTAGAGA AAAGCTGGACGGGGCTGGGGGCGGGGTGCAGGGGTGGAGGGGCG GGGAGGCGGGCTCCGGCTGCGCCACGCTATCGAGTCTTCCCTCCCT CCTTCTCTGCCCCCTCCGCTCCCGCTGGAGCCCTCCACCCTACAAG TGGCCTACAGGGCACAGGTGAGGCGGGACTGGACAGCTCCTGCTT TGATCGCCGGAGATCTGCAAATTCTGCCCATGTCGGGGCTGCAGAG CACTCCGACGTGTCCCATAGTGTTTCCAAACTTGGAAAGGGCGGG GGAGGGCGGGAGGATGCGGAGGGCGGAGGTATGCAGACAACGAG TCAGAGTTTCCCCTTGAAAGCCTCAAAAGTGTCCACGTCCTCAAA AAGAATGGAACCAATTTAAGAAGCCAGCCCCGTGGCCACGTCCCT TCCCCCATTCGCTCCCTCCTCTGCGCCCCCGCAGGCTCCTCCCAGC TGTGGCTGCCCGGGCCCCCAGCCCCAGCCCTCCCATTGGTGGAGG CCCTTTTGGAGGCACCCTAGGGCCAGGGAAACTTTTGCCGTATAAA TAGGGCAGATCCGGGCTTTATTATTTTAGCACCACGGCAGCAGGAG GTTTCG
COL4A2	AAATGAGTTCAGTCCTGAACATATTTGCTCAGTTTGAGATCAAAGC AAGACAGCCAAGGGGAGGCGTCGCGTGGAAATGTGAAGACGTTG AACTGGAGTTCAAGAAGGAGTTCTGGAGTCCCCTTCAGGGAGTAA TGGTGCATCATGGAGCAGACCCCCAGTGGAAGTTAGAGAGTGCAA AGAACAAAGAGAACCACGCAGAGCTGCCCCTGCAGCTCACCCAC ACTGAGGGGCATGGGAAGAGGAAGAAGACAGAAGCAGAGACACG GAAAGAGCGCTAGGAGGGACCCATGTCCCAGAGTTAGGACCAGGA GAACAATGGAGGGAAACGAGGCTGGAACCAGCTCACTGTGTACCA GACTATCAAAGTCTTCACCCCAAAGTCATTCCTACAGTGTCAAAAG ACTCAGAAGAAATTCACCATTAAAACATGCTCTGGTTGACATCCCT TTTCATTGTATGCATAAGAATGGTAGGAATATGTCTTTTGTATTGTTC TATAGTTGACATTTTTTCATGGTTGCTTAATAATAGCAGGAAGAACA ATGCTCTAACATTATTTTAGGCATTCTATTGTTGCTTGAGCTGACTCT AGCCCAAGAACTCAAAATACCAAACCTCCTTGTGAGACATTTTAAT GAGGTTAATCCTTCTTCAATTTGGGGAACAATTATTATAACAAATAT CATAGAATTCATCAAAACCACTGTTGTCATTTAGCCACTTCTTGATG TTCTTCCACTGTCTACAAAGGTTTGTCTTAGATCATCACTGGTGGGT TCCATTTGTCTGCAAAATCTTTACCAGCTCTTAACTAAATAACTGAA

	CTTGTTATGACCATCCTTGCCAGTTCTCAGAAATTTGCCAACAGCCT<br>TGTTAATGAATAATGTAGGGTTTTGTGAATTCCCTCATGGCCTGGCC<br>CAAGTAAGGGTAGGTTTTCTGGGTCAGTTCCTAAAGGCACTCCTGA<br>CTAGAATCAGTCATCCATGCCGCAACTTTCCAAACATCCCCTGCCT<br>GCCTACCTGCCACCTCCGAGAAAAGGGACAGGACCACATACTGGG<br>GCCTCAGTTGGAGCTGGGAGCTCTTTTTTAACACTGGTCTCCTAAA<br>CCCTGCCCTTCCAATTCTTTCCACCCCTCTGAGAGCAAAGTTCAGC<br>CACCAAGATTGTTAATCACAGAAACAATGACATTTTGGTATCTCAA<br>AGGAGCAAACTTGAAGGTCATCTTTCAGTGCAAAGACCCTTAGAC<br>TTGAGAGCAGACAGGCAGAATGACCATGTCTTCTGTGTATAGTACA<br>CCATCACAGTGCACATGGAAATGACATGCTGGTGTATAGTTCCTATT<br>TCTCCCTCATATTTCAGAGGCGGAAGCCCCTCCCAGAAGTTTCCTC<br>TGAGTTCCCCAAAGCTGGATTCAGTGTTCCTATAGCCCTTCGATTCC<br>CCTGATTGTGACATTTACCAGCCTGACTTGGATTCGTCTGTTTCTTT<br>TCCAATATTTCTCACCTGACCTTCCTGAGGACAGAGAGTGGGGGTG<br>AGTCCCTGCCGCATGGCCAGCACCTGGCAAGGTCATGGCAGGAAG<br>GAGGTGTTCCCGATGTGTGTGTGGAATGAAGGAAGGCACAAACGG<br>GTCCGTGTGAGGAAGGGACAGACCTGCCAGGGAAACTGAGGTATA<br>GAGTTTGATGAGTACCCCCAAATCATCTGCTTAAAAGCAGGTATAA<br>AATGTAAGGTCCTCTGGTGCCTAAAGCACACTCATCCTGTGTACAC<br>GTGACTATTAATAATATATAGCATTGATAATTCTGTGAATCTAGTCAG<br>AGTACATGAACATTGTGTCTTCTGGTTGGTCTTAAAGTTATGTCCCT<br>TCACCCTAGTCCAATTCCATCTATTAATATAAAGAGCTTGCAGATATA<br>AGGAATAGCTTCACCAATATCACTCCTGAGATCCTGGGCGATGCTG<br>CTCTTGTTGGGACTGATTCAGAAACCAGCCTTCTCTTCTAAACTACA<br>TTTCCAGTAGAACATGGTCCATACTTATATGGGAATGAAATCTCACT<br>TAGTTTGAACTCTAGGTCATTCAGTCCCAGAGGTGGTGACTAATGG<br>TCAGGAGATGACAGTATTGCTGGACAAGCATCACCACACTGAGGG<br>GAAAGTTCACGTTTCTAATCCTGAGAAATAATCTGTTAAGAAGCAT<br>CACACAGAACCAGCTAGAGGGGAGTGTGAAAGCTGCTGCAGGAA<br>GGGTTCATTCCGCCCAGTGCAACTGAGTGACTAAGAGTAGAGGGT<br>GCTCACAATCAGAACACTGCTGGCACGTGTTTGTCTGGAGCCTGCA<br>AGCTATTCCTCCTTTCTTGCTGAAGAAAAGTGGAGGAAGGGAGGA<br>GCGGGGAGTAGAGTGGTAGGTTCAGCGTGGGATAAAATCAATGGT<br>TTAGAAAATCCACCCATGAGTACGTAAACAATATGACCATAGCAGAT<br>ACAGCAAATACAAAATTAAACAGCCTTTCATGAAGAAAAAACAAT<br>AAGGGCAATTCATGCCTGGCACACAGCAGGTGATGTCTGGATGTAC<br>AAATGCAGGTGAAGTGTGATTCAGACAGTGCAAAGGAGAGGTAAG<br>CCAACAGTCACTTCCACTGGCTTCTGAATGAGGATCTGATCAAATC<br>CACAGCATCTTATCATTCAAAATGTGCTTTTAAATTATGTTTTGCTTG<br>GATCAGTATTGAAGCTATTTCAGTGTTATGGTACATCTAACAGCTGA<br>TTTTGGAAGGAGGTAAGCCAAAATGTTATTGATGGCATGAAAAGTG<br>AGACAAATTTTAAAATTAGGCAGATTCAGTTTTTAAGTGATTTTCTT<br>GTATACCCATACATCATCTGCACATTAAGCACTTTTCTCTAATTAAGC<br>CAGGGAAAATACAGAGTAATGATAATATCTGTGAGGTCAGCTGTGA<br>TCAGAAGTGCCCACAGGCTGCATCTCTTAAAAACAGTCCATGGCAC

	CTCAAGGGACACTGCAAAGACAGTGGCCGGGAATGTAATTGAGCT<br>TTAGGAAATGATTAAAGGGAAGTAAAATCATCTCCAACTCAACATT<br>AATGCGTTTGATTTCCCGGGTGAGAACAGGATTTGCGTAACTGAGT<br>GGTTCTTTCTTTCTACAGTCCTGGATGTTTGGAAAGCCAGCTCTTAC<br>AGTTAGCTTTCTTTGCTTTGCTAACATTATCCATCAGAAAATCGAAA<br>TGCAAACAGCTCCTATTTCCCAATGAAGAACTTTCTCTGATTTACCT<br>TACCATCATCAGGCTTCACCATCTCACCTTGGAAGCGTTCAATCTTT<br>TGGTCAATATTTCTAGACCAAAATAAAGTGCTCTCAAGCCTGTTCTT<br>TGGAAAGTATTTTGTCATCATTACCAAAGAGTGTTTAAATAACTGGC<br>TATGAAGTTTGGGGAGACTGGGCTGGGAGGTACAATCTGAAGTTAA<br>TTGCTGATGTAATCATTAGTCCTTTCAATACCCAGTGGAGAAGCTGC<br>CTCCAGGGAGATAAGCTCTCAATTAGGAGACGTGTGTGTATATCAC<br>CAGTTCTCTCTGGCATGTAACAGTTAACAATTTCTATACAGTTAAGT<br>GGATTTAGTGAGTATCATTCTTCCAGGAGAGAAGTAAAATTTTTTTT<br>TAAAGAGGAAAACAAAGAACAAGATTAAGAACAGTAAAAGAATA<br>AGTATGGAAAATCTGGTAAGTAGCAAAAATCATTTTTAAGCATTTGA<br>GGGAGAAACTGCAAAATCTGTTCAGAAGCCACACCTTCCGGCCTG<br>TGGGACTGCATGGCTGTGACACTGAGCAACAGCTGAAGTCTTTATT<br>CTCTCGGAGGGGAAGGAAAAAAGGAAACAACAGTCAGTCCAGGC<br>TTGTCCACGGCTGGAAGCCTCGACTCCTGCAAACCTCCTGCAAAC<br>CGATCTCCCAGATCCTTTCTGGGCAAGCACATAACCAAAGGAAATG<br>AAAACTAGAAAAACAATTTGTGCATGGAACAATGACATGGAGTTTC<br>ACTTTGGTTTCATGCTCAAATATGAATGTGGTTATAATTTTTTAAAAT<br>ATATATTAATAGAAGGCAACGAGTTGAGGATGGTGGCTTCTTCAAG<br>CCGGGCGTCCAGAAGAGCCCCACTTCTGCCTTGGAGCGCGCCTTA<br>GGCAGCAGTCGTGGGAAAGCTCAGATACAGCTGTTTCCAGGCAAA<br>GGGCCCAGAGCTTTTCAGCCTCAGCCACCAGGGTCTCCAGACAAG<br>GAGAAATACGCCCAAAGCTGCTCCCAGGGGCTAGGAGCTCGGAGC<br>TCCCAGTGTGGGTCTCTGAGCAGCGCGGGGTGGGCGCTCCCGGCC<br>CTCCAGCCCCCAACTCCCTCGCGCACCCGGGACGGGTGAAGGCGC<br>CCAGCTGCCGAGCATACCCGCGACGCGGGTTCAAATCCCGCCGGC<br>TCCCAGGCACCCTCACCCCGCCACAGCGCGGGCTGTTTCTCCTCCC<br>TTCCCCGGAGGAATTCCTTAACTCTCCAGTACCCATACCATAACAAA<br>GGAGGCTCGGTCCACCAATGCGCGATCGTAGCCTACAGGGACCCC<br>CAACGAACCCCGGCCCAGAGAATGCACCTGGCCGTGCCACGCGCG<br>ACCCCCGAGGGGCAGGCGGACGGGTCCAGGCGCGGACAAAGGGG<br>CCTCTCGGGGCGCCCAAGCTCGGGGCGGGACGCCGGGAGCGGAG<br>CTGGCCGGGAACTCACCTTCGCAGCGGCCCGGCTGTGCTCCTCGT<br>GGAGCAGAAGGGCGGCGGGCAGCAGCAGCAGCCAGACGCTGAGC<br>CGGGGCCCCATGGTGGCGCGCCCGAGGCGGCGAGGGACGGCTGCC<br>CGGCGTGCGGGGGCCGCGGCGGACAGCTAGCTCTCGGAAGGCCG<br>GACTTCCAGCGCTACGCACCGTCCCGGGTGCGGCGGCTCCAAGCG<br>GAGACCTGAGCGCGGCGGGCCGAGCTCCCCAATTTGTTGGCGCTG<br>CCCCCTCCCCCCCGGCGGTGCGCGGGCGGCGCCTCAAAGGGGAGG<br>ACCCTGCGGCGCGGGTAAGAGGCGGCGGGAGCGCGCGGCCCGGG<br>AGTGTGGCTGCAGTGCGCCGGGAC

COL7A1	GTCAGGGAGGTCCTTGTCAACCTGAGGCTGGAGATTGTTTGTGTAC<br>CTGTGGGTCCAGGATTGGGCTGGGTGGACCTCTGAGCCTTCTCTCA<br>CCCTTAAAGGCACTACTCCTGTGTGTGAGGACATCGGACGCAGCCT<br>CCTGACCTATGGCCGCCGCATCCCCCTGGCTGAATGGGAAAGCCGG<br>ATTGCGGTAACATGGACCCCTGGGAGAGGTTCTGGAGTCTTGGCCT<br>GGCAGACACCTGGGAGGTGTAGGGTTGGGGCCTGGCATAGCGCAG<br>CCTGCTCCTATAGGCATCCCTGGTGTCCTAGGCAGCTGTCACCTGC<br>AGGGGTAGCATAGTGGGAGGGCAGGGCATAGGTGCTGATGGCTGT<br>GGAGATGGGCGGGGCCCATGGAGGTCTATAGCAGGGATCCCTTCTG<br>CCTTTCCTCTTTTGGGCTGGGGACAGCTGCATGCTGCCTGTGGGAG<br>ATCTCTGCTAGTTGGATGATGCTGACTGGCTGAGGTGGTGGCCAGG<br>TGCCCGTTAGCCAGTATGCCCTCTGCCTCACCCTGCAGGAGGTGGA<br>TGCCAGTGTGGTACGTGAGATCTGCTCCAAGTACATCTATGACCAG<br>TGCCCAGCAGTGGCTGGATATGGTAAGTGGCCTAGGGGGTCTGCTC<br>TTTGTTTATTTCCTCAGGCCGTTCTCTTGGCCTCCCCTTCCACCTCT<br>GCTCCCGCACTGCTGCTCAGCTTACAGAGCAGCCAGTTAAGGCCCC<br>AAGCAGCTCTCCCCAGTCCCCTGGCTGCCAGGGAGTTCTTGCTCAG<br>AAAACCTGTCCCAGCAGTTCCTTGACTGCCTGCCAAGGTGCCTTCA<br>TCAATACCCTGCACCCGTCAGGTAACGGGCACCTCCATGTGGGGTG<br>GGGCTTCAAGGGCCAGAACATGAAGGGACAGGCTCAGCACCCCTG<br>GGGGAATGTTCTCTCCTCTCTTTCCACTCAAAGCCTGTCTTGTGCTG<br>CTCTGGGCAGCATCTCCGAGCTGGGTGCGGCACAGGGCCAGGTGT<br>CCCTGTGCAGGCCAGGTACCCCACCCTGACGCTCCACCCTATCCCC<br>ACAGGCCCCATTGAGCAGCTCCCAGACTACAACCGGATCCGTAGC<br>GGCATGTTCTGGCTGCGCTTCTAGGCGGGAAGCCTATGTAAGCAAG<br>AGGGCAGGGCCGGGGTTTGTGGTCCCCCCCCCACCACAAACACAG<br>CACTTCGGCTCCTCTAACCTGTGCCACAGGTGACCACCAATAAAAT<br>CCTCTGCTGAGAAGTGTGTCATCCCTCTTTTGGCATTGGCATGGAG<br>TCCATGGGGCAGTAGATGGGGAGCAAGGACTTCCCTCTAGTTCTGT<br>GGCTCCTGGATCCTCCTGCCTTCCCACAGGCTGATGTGTCCTCAGC<br>CAGGGGCAAGCGTGGGGGTTGCAGCTGAGGCCGGAAGTCTTTGAT<br>GCCTACAGGGAGCTGAGCCCAGATGTTTCCACTCAGCCACTTAGGG<br>GAGTTCCTCAGCCAGCTGGTCCCAAGATTGGGCTGTGGGAGTCCAT<br>AGGAAGTGTAAACCCAGCCTTTGCCCCTGTAGGAGTCCCAGCCTG<br>GAATCCCTGCAGGCTCAGGTGGGAAACCCCTCACTGTGGACCTTG<br>TGCCAAGTCACCCCCTGGTGGTCCCTGGTGGACCAGAAGGGGCCA<br>AGGGGTGTCTCCAAGGATGCCTCTTGTGTGTGTTTACTCTGCATTCT<br>GGGAGGAGGATAGCCTGGGGCATAGTGTTCTCATTTGGTTTCTAAC<br>TGCTGCCTGGACAAGCAGGGCAGGAATCCTTTGCCTCCGCTGACTC<br>AGGCTCCAAGAATAGCCCTGAGCTCAGTGGGGCCCGAGCCCCCTC<br>CCTCTACTACATTTGTCCCAGGCAAGTCCAGAGCTGCTGGCCTGAC<br>AATCCCCTCCCCATTTTCCTGAGGCAGCCAGGACCTGGTACAGCTC<br>TTAGCTACCTAGTCCCTCTGTTATCTCTTTTGGCTCTGGTCCCCATGG<br>TGGGTGCGCAGGTAGGCTGCTCCTCCTGCACCCCCCAGTGCTTCCA<br>CAGTCAGGTTCTCTGGGATCCCAGGGGGAAGTTGAAAGCGAGGGG<br>CCCAATTTTCCTTTGAGGAAGTCGCTGCCTGGCAGTCACAAGCAGG

	GTGCTGGGACAAGGTCTTTTAGAAGATGGCTGTTCAGAAGTCCCCC<br>CAGCATCGTAGCTTGGAAGTAGCCCAAGGAACCCTTGTATCACTAT<br>CACCCTGCCCCACTGCCTTTCCCTCACCATTGGTTGCCATACGTTGC<br>CAATTCGGAGACATTTGTTAAGCACCTTCTACGGCCCGGGACTGGG<br>GCATTGCGCAGATCATTCTACTTTGTGAACGGCTGGAGAGAAGATG<br>CTGGGCAGCGCAGAACCCTGACAGGCCCAACCCTGGGTGCTCATG<br>GAGGTGCAACACCCAGGCAGGGGAACAAGTGCCAAGTCTGTTGTC<br>AAGTTTGAGCTCCTGGAGGATGGGGCTTCATTTCTAAGCCCAGCCT<br>GGCCCTTAGAGGCTACAGGTGTGCTGGACACGAGAGCTTGAGTTG<br>CTGGGAGTGGTCTGGTTCTAAGGGACCTTGCGTACCAAGCCAGGG<br>ACTCTGGTCTTTACCCTGGAGCCATGCTGGGGAGGTGTGTTGGAGG<br>TTACTGTGAAGAGAAGTGACTTGGTCAGAGTTGAGATTCAGCATG<br>GGGACTCTCAGGCCATATTTCTGTATCAGTCTTGGTGAGCTGTGCTG<br>TGGCAGAACAGAGGTCATGGCCACACGAACTGGAGCCAGCTGTAG<br>GGGACACAAAGAGGACTACATTCCCAGCAGGGCTGGGAGACGCG<br>GAGGCACGAGTGGGTTGGAGGAGGGTAATCTTCCATAGTCCAGTG<br>AGGAGTTTGGTGGTGTCCAGGGTACAGTGGGATGTGTGGGGCTGG<br>GACCTGATAAAGAGGCCCTGGTTGGAAACAGATGGGGTGAGGTAG<br>TGACTGAGGCCAGGGAAGGAAATGTTCAGGGAAAGCTTGGGAGG<br>GAGAAGGATGTGCTCTGGGGGCTCCACCGCCCTGATGGGGTTGCA<br>GGTAGGCACTGGCCTCGGGCTGTGCCCTAGGCTGTGTTCCTCCCAG<br>GAGGCCAGAGCTGGGGCTTCAGAGGGCCACACTCCTGCTCGTGAG<br>TCTCATAGAAGCTCCAGACGTGGAGTCATTCCAACCTTACTCCTAC<br>CTTCTCAACAGCCTCTCCCAGCACCCTTTGAGAGACCCTCTCACAG<br>CAGCCTGTCCTTTCTTTTGGAGGCACCTCAATTCTGATGAGGTTTGC<br>CTGAGCACCCACCCTCCCAGCTGGGCCTCCTGTCTCCCCTAAGACT<br>GGCCCACGTGTGCACATATGTCTGTACCGTACTGTCCATGCCACTG<br>GCCTGCTCTTGCCCGCCCTGGCTGCACGGGAGGAGGTGACAGTCT<br>TAGGGGGAAGGGGCTGCCTGTGTGATAACATACCTGGTTCTCTGCA<br>GCCCCCCACCTGGAGCACACCTTGCAGGGATGGGGGGTCCTGGGA<br>CATGGCTGAGGAGACCCTGACCTTGGAATAAGGTCATAGCACTATG<br>CCCCAGGATAGTTTGGGGGGTCAGTAATAAGGGATAGAGCCCCAGC<br>CTTCCTTGGGCAATAGGAAAGCAGGCAGCATGGATGGTCTTCACTG<br>CCTCCAGCTCTGGCCACACCAGAGTCCTCGGGCTGTGGAGGAGTC<br>CAGCCAGTGCTGTCCCCATATCTCCCTGTGGATCCCAGGGGAGGCT<br>GTGGGGCCGGGCATATTAGAAGGGGCCCAGCAGCCTTGGTGAGTAT<br>AGATGTGCTTTCTGGGCTGCCCAGAGTTATTCAGGATTAGCAGGAA<br>AAAAAGGGGGAGCAGGACCCAGATGGGGGCCTGCCCTTCTTAGCA<br>GTGCCTCTCTGCCTTAGTGTCACCCATCTAATGGAGAGATATGCATA<br>TACAGAGCCCCACCTCCTTCGGACAGTGGCCCTGGCGTGGTGTGT<br>GTTGGAGGACTGTCCACAGCCTCAGAAGCGAAGCTGGGGGAGGG<br>TGTGAAGTCAGCCTGGGTAAGGGGACAGGAGTAAGGTAGGGGGC<br>AGTTATGAAGCTGGAAGCTACAGGTACCATGAGGGCAGTGAGGAA<br>AGGAGGAAGGGGCCCAAGGGGACCTCGGCCTGGTAGGGGAGATG<br>GGTGGCAGGGACCTACAAGAAAAGTAGATGGAAGGAGAGGATGC<br>CTGTGGGTGAGGACATGTAGGTAGGGGGACCTGGCCTGGGGCATG

	GAGGGCTCGATTCCTGAGAGATGAGCTGTCACTGTGGGTGGTCCC GCCCTCCACAGATGGGCGGCCCCCTCCCTGCAGGCACAGCTGGATT GCATCTGCCTGTGACATGGTAGGGGATGTGGGACACTGGGATGTAT GCATAAGCATGACACAGGTTCTCTCCTCCTTCCGGCTTATCCCCTAC AAAGAGGGGGTGAGTTACTTGGATCCAGGCCAAGGGGACCTTGGT TTCCCTAAGACCGGCCCAGAGTCACTCATTTGCCAGGGCTTCTTGC CTGTCAAGGAGATCCGGGTGGGGCCCAGGAGGCCCACCAGACAGA TGGCTGAATCACAGGAGTGGCCGGCGGGACCCATGGCCTGAGGGC TTGTCTGGGCACCCCCACTGGATTGGGGGTGAGTCATCCCCAACTG CAGCCCCACCCCCCACGGCGCTGCTGCCTTGTGGCTCTGCAGGAA CCTGTCCACTCCTCAGCCTGGTCACTGTGATTGACCTAAAGCAGCC AAGACCTGTGACCTTAGATGGAGTTAGGGGTACTCCCTCAGCATCT GCCCATGCAGAACCTTCTGGGAAATTCCCAGAAGCCACGGGGGGT CGGGGGGTTTATAGTTAAGTGCGTCATATCGTTTGTCTGGGGGAGG GGTGGGGGGGGCGGCGACCTCTCAGGGATATGGGTGAGGGCGGGT GCCTGGGTTCCCGCCTGCCGCTCCGCCCCCCGAGATCAGGGACTTT TCTCTGCTCTGCCCGAGAGACTGCAGCGGCGGCGGCGGGAGCGGG CGGACGCGCAGGCAAGACCAGGACTCGGGCTGGAGGGGCGCTGG GCTCGGA
COX7A2L	CTCAAGTGATCCGCTGGTCTTGGCCTCCGAAAGTGCTGGGATTACA GGTGTGAGCCACCACACCCAGCCCGCCCCTTGTTTTGTTACTGAAT AGATTCAGGTAGCTTTTCTATAGACAAAAAGTTCTACTACCTCAAA AGAAAATGATCCATTGACATTAGCCTCCTTAAGACCCAGGTGTATG GTTCTTAATCTTTTCCACTTGATCACTAATTTCCATGGCTGTCTGGGC ACTAATGTAAGTAACTACCCAGAGTGGTTGCTCATTTCTCCCAGCAA TGTGTGGAGGTGATATGTTTGTTCTTATTCATTTGTTCATTCATTCAT TCTTTTCTCCAACAGCGCTAGGAAGAAGAACCACTATAGTGTTAAC TGTAATGGGCAGAATGGCCCTTTAGTCACCCTTCCTGTGGCCTAAC ACTTGGACACTGTCACTTAAGAGGCCCCTCACATCTACGCAGGAGG TGATTAAAGAACATTCTAAATTACCTGGTAATTTGGGGGTGTAAGTA CATGAAAGCATTTTTAAAATTCTAATTTGTATGTTTTTGGTGGACTTT GTGTAATTTCTTTCTTTTTTTTTTTCTTCGGGACGGAGTTTTGTTCTT GTCGCCCAGGCTGGAGTGCAGTGGCATGATCTGGGCTCACTGCAA CCTCTGCCTCTTGGGTTCAAGCAATTCTCCTGACTCAGCCTCACCT GTAGCTGGGATTACAGATGCATGCCACCATGCCCAACTAATTTTTGT ATTTGTAGTAGAGATGGGGTTTCACCATGTTTGTAAGGCTGGTCCC GAACTCCTGATGTCAGGTGATCCACCTGCCTTGGCCTCCCAGAGTG CTGGGATTACAGGTGTGAGCCACCGTGCCCAACCGACTTTGTATTT CTGTGTGTGTATGTGGGCTTACAGAACTCATGTATGCTCACTGTGAA ATATTTCAACACTGCAGATGTATGTGAGGTAGAGAGTGGGAATCCT CAATGATGAGAAACCACCACCCGTCCCCCAAACTGGAGGCCACTG CTAATTCTTTCATTCTATGCATAAGACAACTGTGTGTACTGTAATTTT ACAGATGATGTCAATGTAAAATAGCATGACTCTGGTTTTGGATCTCA ACTTTTTTCTATGCATAATTAAGTGTATTTAAAATGATTTAAAATAAA TTATGAGTCATACCTCAGCCTTTCTTAACCAGTGAGATGAAATGTGA TGTGTCAAAATTGAGTTAAATCCATATTCTCTGTGGATGTGCTTTTG

	ACTTGCACATAAGGAAAAGCAGGCAGGCAAAAGTGAGGCAGTTTC<br>ACACACTGTATTGACATTCAACAACGAGAAGGTCAGAGTAGGGAG<br>TTGATGTCAATATGTTCATCCTGTACCGCAATAAAGCTGTCCCGGCA<br>ACTGCAATCCCAGCTGCCATTTCCCCTCCTTTGGGTGAGAAATGAG<br>CCATTCAGACCCTCAGCTCTTTAGCTATGAAAACACCATGCTTTAAG<br>AAAGATTTGGTTTTCAATATCAATATAATCTAGGTTTCAGGCTTGGA<br>AGATAAAATTTCCACACCAGATAGGGCTTTATTTGACAAAAACAGT<br>AAGAGATGACAGCTGAGATTTCTGCCACATGAAAATTACCGAATAA<br>TTGCATGGCAGGTGTCATCATTCTGAATATCATAAAGTGAGTTTAGC<br>ACTCCATTTTGCTTTCCAGTATTTTACTGGGGATGCTTTTCTTGGGG<br>TTATCAAGCAGTTTTAAATGTTGGGAAAATGTAAAAAGAGAGAGTA<br>GACATAAACAACAATGGAAAAGGTAACACTTCACTATAACATTGCA<br>CTATGTATGTTCCATTTTCCCTCAGGGAAACTACCGTAGAATGGGCA<br>AAATCCTTCAGAAACGGGAGCCCACAATGACCTCAACTCCTGCAG<br>GCCTACATGTTTTTTTATCTCTGCCAGACCCACTGTCAATTTTTTTTT<br>TTTTTTTTTTTGAGACGGAGTCTCGCTCTGTCGCCCAGGCTGGAG<br>TGCAGTGGCGCGATCTCGGCTCACTGCAAGCTCCGCCTCCCGGGTT<br>CACGCCATTCTCCTGCCTCAGCCTCCCGAGTAGCTGGGACTACAGG<br>CGCCCGCTACCACGCCCGGCTAATTTTTATATCACTGTCAATTTTTT<br>AAAAGTCAGCTTGGAAAAAATGAGAGAATTTAAAAAATTAACAAC<br>TATTTCACAGTTGGTTCCTATCTCTACTCAGTATTACCCAATTTAAAT<br>TTTATGTTGGTTTTCCATCCATTCCCCAGGCGCTTTCACAGAGCGGC<br>CTGCCAGAGGCCATTAGCACACACCCCTCACAAATGGAGAAGCAG<br>CGGCATTCAGATTCACCAGGAGCTGGACTGGCATTTGCACTCACTC<br>AGTTCACCTGGTGATTGGCAAGCTTCTCTACTCTGAGTCGTGGTCA<br>CTTCCCTTCAGAATAGAATGTGGGGAACAACCTGAAGGGATCGTGA<br>TAAAAAAAGATTGTCTTTGATGTTGGATGGGATATGCAAATACGAAT<br>GAGAATAGGGTGCCTTAGTACTGTCAGAGTGTGCTGTGTTTTATTTG<br>GGAAATGAGACAAAATCTTCCCATGATCAGCCATTCCATGTGACAT<br>CCCAACCCACACTCTGCTACTGACATTTTATCTCCCACTGAACAGC<br>CTGGCTGTTGTAACATTTTCAGGATTCTTAATTTATGTCTTGCAGGAT<br>CTAAAATCAGATGGCAGGAACTTTAAGAAAATAACCAGTGTTGACA<br>GGCACCTTATAAAAGAAGCTGGTACACCTTAGTGCTGCTAAAAGTT<br>AGAGAATATTCTTTCGTTTCTTCTTCCAAAATATGTCCAGAAGTCAG<br>TGGGATGGAGACAGTAGCTGTCCCAAATCATTTAATTTCTCTTTTCA<br>ATCTAAACTCTATGAAAGGCTTTTTCTTTCACTCAATATTTGCTAGC<br>GCCTGGGACTCAAGAAGGCTAATGTCAATGGATCATTTTCTTTTGA<br>GGTAGTCGAACTTTTTGTCGTAGAAAAATCTTCCTGAATCTATTCAG<br>TATCAAAACATGGGGTGGGCCTGGCACGGTGGCTCATGCCTGTAAT<br>CCCAGCATTTTGGGAGTCTTGAGTTTGGCAGATCACTTGAGCCCAG<br>GAGTTTGAGACTAGCCTGGGAAACATGGTGAAACCCCATCTCTACT<br>AAAAATACAAAAAATTAGCCAGGCGTGGTGGCGCGCATCTGTGGTT<br>CCAGCTACCCGGGAGACTGAGGTAGGAGAATCACCTCAGCCTGGG<br>AAGTCAAGGCTGCAATGAGCCGAGATCACACCACTGTACTCCAGC<br>CTGGGACAGAGTGAGACCCTGTTTCAAAAAGAATTTAAAAAAAAA<br>AAAAAAAAAAAGGAATCACTAGTCCCAAACCACTTATCCTTGGA

	GAAATATGGACATAAACCCAAATTGCATGTTCTTTCCATTATATCAC<br>AGTGTCTCTTTGTAAAAGTGGCAACTTTGGGCTGGCTTACATGAAT<br>TCGCTGAAACCTTTACAACATATTGGCGGTGCTGAATACTTGGGGC<br>TCTTGAACGTAAGCCTGCAACCAGGATGCTGGTGTACACCCTCACC<br>CTGGAATGCTCAGCCTTGATCCTCCTCTTTTCAGAATGGCTTCATAT<br>CTATTTATTTGTAACATAGTGCTTAACAGTAGGAAGTCTTGAGCCAA<br>ATGCCTAGCTCTGCCACTTATCCCTGACCTGAGCAAGTTACTTAGCT<br>TCTCCAGGCGTCATTTTCCTGTATTCTAAAATGGAGATGATAATAGC<br>ATTTACCTTATAGGATTGTTGTGAAGATTAAATGAGTTTAGGGCAAT<br>GCTATATATAGGTTAGTATCATCCATCAATTTGCAACATGATTTCATA<br>AGTGCATCTAGAATATGCTTGTGTAAACCTATGGTTAGATGATGAAG<br>CTAAGAAAAGCCTGAGTCCATCCCTGGAGAGTAAGAAACTAGTATA<br>CTCCTTTTACACAGTACCTTGCACACAGCACAGGCTTAACAGACAT<br>ATTTAAATTGAACTGAAGCGAATACTACCAGAACTGAAAGTGGTAG<br>TTCCAGATCTTTCAGGCTCAAGGTCCAGATTTTACAAGTGTTTCTGA<br>TCACCCTCCCCCTACCTCATTTATCGCAAAGAGCAGAGGCCAGGAA<br>AAGGATGAATAATCAATGACCTTAAAACTCAGGTGAGAATAAAAAC<br>CAGTTCTGAGAAACCGTGTGACATTTACATCCTGGTACCTGAGCTT<br>TAGATCACTCTGCATGTGTATTTCCTCTGCACAAAGCCCAGGAGGC<br>TACTTTTATATTATTGGGGCAAATCTCCCAACAGCCCTGGGTACTAA<br>ACAGCCTTCAGCAGGTGCTTCCAAATTTTCTTATTTGCTTCAACTCC<br>CAGGTTGCCAGGGGAAGCTTGGCCGACAAGTCTATACTCCTGGGC<br>AGACTGAGCAACAGCAGGTGGTGGCTTCTGGATAAGAGGTGCTCT<br>AGGTCGGAAGGAAATAAATGAGACTACGATCAGTTAAAGTTCGGTA<br>AAGCAAGAGACGCCTTCAACCCGACGACCAGACGGTGGCTCCGCA<br>GCACAGACTCTCTCGGATGGTGGAATTCCCAACCGGCTTCGATGTA<br>GCCCCGCCCCTACCCAGACCACGCCCACCAATCACCGCCCTCTCCC<br>CCCCAGGCCCCGCCTGGAGCCCGCCCCTCCTAAAGCATGCTCCACC<br>TGACTCCGACACTGCCGGGCTTAGTCCGCCGGGCTTAGTCCGTGGA<br>GCGCTTGCGCATTTCTTGAGTTGTCGTGGCACTGCCCAAATCTGAA<br>TGGGGGAAAGAGGGAACTTCATATTTCCTACAGACTCCGCCACAGC<br>GACTTTGCGTGCTCTCTTAGAGCTGGTTTTGCCTTTCGTGGTCCTGA<br>GAACCCTGGCTTTTGGTTCCCCAATGCGGCTTAAGTGTGCAGGTTT<br>TAAGTTAGGCGATCTTCGGGCAGCCCTAGTCCCAGCACCGGGTCCT<br>TGCGCTGAGTCTCGGGACCACAGCCGGGGAGGCGGGGTCCTTCTC<br>TGGGGCGGTCGCGTTGGCA
CPT1B	AAATGTTTACTGCGTACCCACCGGGTGCCCGCATCCCGCACTCTGC<br>CCGCGCTGTGCGGCCCTGCCTAGCGCGCGCGCCTCCTCCAGGCCTC<br>ACCCACGCTCAGGACGGACGCGCGCTGGACGGCTCTTCCTTGTCG<br>GAGCGCCCCAGGGGTCGGGGAAGAGGGCCCGGCAAGGGAGCCCT<br>CGCGCCGGAGCTGCAGCTGCAGCCGCCGCCCCGCCGCCCCGCCGG<br>CTCCCACGGGGCAGAGACGCAGCTCCTCTCCGGTCTTCCCGTACGC<br>TACCGCGCCCGGGCAGTTCCTCGCCCGCGCACGCGCCGCTCCGCC<br>AACTGATTGGCCTCCGGCGCCTCGGATTGGCCCAGGCCGTCCAACA<br>GCAGCCCCGCCCAGAGAGAGACCATTGGTCCTTGCCCATAGGGGC<br>GGGGCCCGGCAGAGATCTGCGGAATTCGGCCTTCGGAAAGAGCCC

	CCGGGCCGGGGCACGGAGAGAGCCGAGCGCCGCAGCCGTGAGCC<br>GAATAGAGCCGGAGAGACCCGAGTATGACCGGAGAAGCCCAGGCC<br>GGCCGGAAGAGGAGCCGAGCGCGGCCGGAAGGAACCGAGCCCGT<br>CCGAAGGGAGCGGAGCGCAGCCTGGCCTGGGGCCCGGTCGAGCC<br>CGCGCCATGGCGGCCGAGGCGACAGCTGTGGCCGGAAGCGGGGCT<br>GTTGGCGGCTGCCTGGCCAAAGACGGCTTGCAGCAGTCTAAGTGC<br>CCGGACACTACCCCAAAACGGCGGCGCGCCTCGTCGCTGTCGCGT<br>GACGCCGAGCGCCGAGCCTACCAATGGTGCCGGGAGTACTTGGGC<br>GGGGCCTGGCGCCGAGTGCAGCCCGAGGAGCTGAGGGTTTACCCC<br>GTGAGGTGGGAGGTCAGGGGTCAGCCTCTCCGGTGCGCGGATCGG<br>GGTCAGGGGTCAGCCGCGGGGCCCTCAGGATGCTCCATGTTTTCGC<br>CCCCCTCTTGCGCCCGCGCCTGGGGCGGGGCGGGGCCGGCCTGGC<br>CGGGAGGGGGCCGGGGCCGCGGCAGGTAGGGCCGGCCGCGGGCT<br>GAGCGCGCCTGGTGTGGGTCTGCAGCGGAGGCCTCAGCAACCTGC<br>TCTTCCGCTGCTCGCTCCCGGACCACCTGCCCAGCGTTGGCGAGGA<br>GCCCCGGGAGGTGCTTCTGCGGCTGTACGGAGCCATCTTGCAGGT<br>GAGGGGGGTGTGAGCGCCGCAGCACCAGTGGCTTTAGGGCCTGTC<br>GCTTACGCGATGCGGGTAGTATTGTTCCCGTTGCGCAGTTGAGGAC<br>ACCGAGGTTCACGGTCTGAGTAACACCTCATTACACCGAAGCCTGG<br>GCCTGTATTCCCAGAGCTTTGGGAGGCTGAGGCGAGAGGATCACTT<br>GAGCACAGGAGTTCGAGACCAGCCTGGACAACATAGTGAGACCCC<br>CATCTCTAAATAAAAATAGACCAACGCTAAAGCCTGTGCTCCAGAG<br>CCTCCAGGCAATTGGATCAGAAGTCGCAGCTCTGGTGGGAGGAAG<br>GCGAGCCCTCATGTGTGTCCCTGTGCCACTTTGCCTTGGCCCCTTT<br>GCTGTCCATCCTTTTTCAGGGCGTGGACTCCCTGGTGCTAGAAAGC<br>GTGATGTTCGCCATACTTGCGGAGCGGTCGCTGGGGCCCCAGCTGT<br>ACGGAGTCTTCCCAGAGGGCCGGCTGGAACAGTACATCCCAGTAC<br>GGGCCCAGTCCTACCCTCTCCTCCCCAAGGCACCTCCACCCCCTAA<br>CCCTACCCCAGTGCCCAATGTCTGCCTCCACATCCCTCACCCCAAG<br>TAGGGAATTTCCCCCAAAACCCTGACTTCCCCTCATTGCCCCAGCC<br>CTTCCCACTCCTGCCCCAGCCCCATCACCACCCTGATAGCTTCCTG<br>GGTGCAGAGTCGGCCATTGAAAACTCAAGAGCTTCGAGAGCCAGT<br>GTTGTCAGCAGCCATTGCCACGAAGATGGCGCAATTTCATGGCATG<br>GAGATGCCTTTCACCAAGGAGCCCCACTGGCTGTTTGGGACCATGG<br>AGCGGTGAGTCAGGAGCCTCCTCAGGGCTCCTGTACTCCTGAGCT<br>GAACCTCCATGCCAGCGGATGTGTCGGGGGCTCTATCCAGCCCTCC<br>ACTTGAGTGGATCTGATGTCATGGGCTGTCACAGAACAGGCTTTTA<br>AATGCTTGCCAGAGGCCACATGCAGTGCCTCATGCCTGTAATCCCA<br>GAACTTTGGGAGCCGAGGTGGGTGAGGTGGGAGGATCACTTGAGC<br>CCAGGAGGCGGAGGTTGCAGTGAGCTGAGCTCATGCCATTGCACT<br>TCAGCCTGGGTGAGAGCAAGACTCTGTCTCAAAACACCAAACAAA<br>AAAAGAAAAAAGAAATGATTGCCAAGGCCAGGCACGGTGGCTCAT<br>GCCTGTAATCCTAGCACTTCGGGAGGCTGAGGTGAGCAGATTGCCT<br>GAGCTCAGGAGTTCAAAACCAGCCTGGACAACACAGTGAAACCCT<br>GTCTCTATTAAAATACAAAAAAATTAGCCAGGCATGGCAGCATGCA<br>CCTGTAGTCCCAGCTACTCGGGAGGCTGAGGCAGGAGAATTGCTT

	GAACCCGGGAGGCAGAGGTTGCAGTGAGCCAAGATCGCGCCACTG<br>CACTCCAGCCTGGGTGACAGAGCAAGACTCCATTTCAAAAGAAAA<br>AAAGAAATGATTGCCGGACAAGTCACACAGTGATAGGAGCAGGGG<br>TGCAGTAGTCTGGCCTGAGGGGTAGATAGGCGAGGGGTGGGAAAG<br>AATAGCAGAGCCAGTGTATCCTATTCTTTGGGCTTCAGGTACCTAAA<br>ACAGATCCAGGACCTGCCCCCAACTGGCCTCCCTGAGATGAACCT<br>GCTGGAGATGTACAGCCTGAAGGATGAGATGGGCAACCTCAGGTG<br>AGGGCAGGCAGGACAAGGCTAATGGTAATGGTGTCCGCCCTTCCA<br>GTAGTTGCTGAGGGCTGGTGTCAGGGCCTGGCCTGTTAGGGTGGCT<br>CTGATCCTCCTCTAGTCACTCCTGCTCAGGACCCATGCTCCCATACC<br>CCTGTAGGAAGTTACTAGAGTCTACCCCATCGCCAGTCGTCTTCTG<br>CCACAATGACATCCAGGAAGGTAGGAGAAGGCATCTGAGTCTCCT<br>AACCCAAGATGGAAGAGCCAGAGGGCTCTGGAGTGAGCAGAACC<br>TCACCCCATTCCCCCAGGGAACATCTTGCTGCTCTCAGAGCCAGAA<br>AATGCTGACAGCCTCATGCTGGTGGACTTCGAGTACAGCAGTTATA<br>ACTATAGGTGAGGCTGGAAAGATGGCTTCCCATAGATCTGTTCCCAT<br>AGGGCTCTTGAAAACAGGCCAGCTGCCCAGGGCATTTGGGGACTG<br>AATGTCCACCTTATTCTCCCAGGGGCTTTGACATTGGGAACCATTTT<br>TGTGAGTGGGTTTATGATTATACTCACGAGGAATGGCCTTTCTACAA<br>AGCAAGGCCCACAGACTACCCCACTCAAGAACAGCAGGTATGTGG<br>GCCAGAGGCTGGGGAGCAGGACCCATCCTGTGAGGAAGGAGGGA<br>GGTGGAGTCTGGAAGGAATGGCCGGAAAGGATGTTACCTGGGAAA<br>TACTCCACAGTCTCCCCAATTCCTGACTCTTGGCCATTGATCGTAGT<br>TGCATTTTATTCGTCATTACCTGGCAGAGGCAAAGAAAGGTGAGAC<br>CCTCTCCCAAGAGGAGCAGAGAAAACTGGAAGAAGATTTGCTGGT<br>AGAAGTCAGTCGGTGAGGAAGGAGGGGCAGGGTGGGGTAGGGCA<br>GAGCAGAGGAAAGAGGGATTGGGGAAGAGGCAGATTTATCAAGCT<br>GCAGGGAAGGTGGCTGTGGAGAGTGGAGTTAGGACAGTGGGGGA<br>GAAGTTAAAACTGTAGGGATTGGCCAACCTGGGTGAGGGGATCCA<br>AGCAGTGCTAGACATGCTCTTGAATGCCCCTCCTTTTCCTGCCCTCC<br>CCCCAGGTATGCTCTGGCATCCCATTTCTTCTGGGGTCTGTGGTCCA<br>TCCTCCAGGCATCCATGTCCACCATAGAATTTGGTTACTTGGTAAGT<br>GACCCTGGGGATGGGAATGCTAGCTGGGGGGCTGGGGAGCAGCAG<br>CAGCCACACTCTTCCAGGAGGCCTGGGGAGTCCCGGGTGGCTGTG<br>GGCAGCCTGAGGTGGATGTAGAATGCTGGTCCCACGTCTTCTCACC<br>ACTGTGTGGGGTGGGTTTCCTTCCCTAGGACTATGCCCAGTCTCGG<br>TTCCAGTTCTACTTCCAGCAGAAGGGGCAGCTGACCAGTGTCCACT<br>CCTCATCCTGACTCCACCCTCCCACTCCTTGGATTTCTCCTGGAGCC<br>TCCAGGGCAGGACCTTGGAGGGAGGAACAACGAGCAGAAGGCCC<br>TGGCGACTGGGCTGAGCCCCCAAGTGAAACTGAGGTTCAGGAGAC<br>CGGCCTGTTCCTGAGTTTGAGTAGGTCCCCATGGCTGGCAGGCCAG<br>AGCCCCGTGCTGTGTATGTAACACAATAAACAAGCTTCTTCTTCCC<br>ACCCTGTCCTGGCCCTGCTGAGCAGCAGCAGAAAGTACCAAACCG<br>AGCAGTACACACAAAGGGACTCTTCAGTGCTCTGGGATTGAAAGT<br>GGTTAGCGTTCATGCTGCCAGTTGGGGTCCCCCATCCCTCCCCAGT<br>CCCCTGGCTGCAGCTTAGAATAATAAATACTAGGACTTGGGGAGGA

	GGAGAGTGATGGGGGTATGAAGACGACCCTGAGGTGGGGATGCCG CCCGGAGCACCAGCGATCCCAGAACAGGCAGCAGCTGACACATCG GTGACCTTTTCCCTACATTTGGCTATTTTTAGCTCTAAAGCCACCAT CCTCACGAGACTCTGGGGCCCCCCAGGCTCCCAGACCTTTGAGCA ACCTTCACCGCACAGAAACCCAGCCGCGCCCTGCAATTCCCACCG CGGAAGGTGGGTGGGTTCTGGTTCTCGCCCCACGTTTTCCCCGAC CCCGATTTGGGAGTAGGTGTCAGGTTCCTGGTGAGGGCGGGGCGG GGGTGGCTAGGCCTGAAGGACGTGGGGACACGGGCCAGAGTGGC TGGCCCCACGCACGGACAGGAGTGAAC
CRH	AAAAATAATTAACGGACAATCTAGAAGTAGAAGTATAGATGAAAGA GACTGAGAGATCAAGGTGCAGGAGGGTGTCTGAAAAGTTGAAGG GATAAATAATCTCATCTTACAATCTGGGAGTTAAGAGATATTGTTTAT ATTTGAAGACTCAAGAAATAATTCTGTGGGTATATTACTTAAAAGTG CAAGGGTAAGATGAAAAAGAATAAAAAGCAGGTCATTAGGGCAGT GATCAACAATGAAGAATGAATAGAAAATTTGGCACATATTGATGCA ATACCATGCAATAAAAAAAGGATTTCACTTGGATATAGGAAAATGCT TGAGATATAATTTTAAATGGAAAAAAGTAGATTATAAAATTTTAACT ATAAAATGTTATTTAGTTATGTCTGGATAATATGTACAGTTTATGGGT AATTTTTATACTTCATTCATTCAACAGATTTTCTGCATTTCATTCATTC AACAGGCTTACTGGAAATCTACCATGTTTGGGGCAAGAGAAATGGA TATGGCAACTTACAAAGATGATCAAAGCCCTGCTACACAGTTTACT GTCCAGCAAAGGAGGTACATATTGTACCAGTAACTATAACAAAATG TGAGCTTTACAATGAAGGACATTCATGCTATTAGGAAAATATGAAGC ACAGGTGCTGAACCTAATATAGGAATCTGGGGAAGATTTCTTCAAC AAATTATTTTGCAATGAATTAATTAATTAATGTAAATTGACAAATAAA AATTGTATATGTTTATGGTGTATGACATGTTTTGATATATGCGTATATT GTGGAATGGCTAAATCAAGTTATCAACATATCCATTACCTCACTTAC TTACCAATTTCTGGTGATGAGAACATTTAAAATTTACTCTCTCAATTA TTTTAAATGTTAAAATGAGAGTTTTTTTTTAAGCAGAGATTCAGTA TTAAGAACATGGTGTTATCAAAATTATGATTATGAGCTCAGGAAAAA TGCTCAAGGGAAACAACAAAATATTAATTATCTGTGATGATCTTTAG ATTCAGATATTCTGAGGAATTTTATATTCTTTATGCTTTTCTGATTTTT CTAAGTTTTCTGCAAAGAGCACATATGGCTTTGATAATCAGAAGAG ACTAACAGAAGTTATTGTTGCCAGTGAAGCAGTTAGATATTTTGCTG TTTGAGGGCCTTTCCTCAGTCCTCCTGTGGAAGCAGAAGGCAGTTA CTTAATCTATCTAAAGAACAGTTACAAACAATTACCAAACCACCTG TCCACCCTAGGCATTTCTGAAGGCTCTCATTGGAAACAGTAGATTC ATTTCAACAAATGTTTCTGTTAGCCAACTTGGAGATGGTGTTTGTTC ACCACGAGCTGTGCTAATAGAAGCTTGTGAAGGTACAAGGTGATAC AAGTGACAAAAATATGCCCACTAGTTACCTATTATTTCTGATATTTCT TTATTAGACTGTTGTGTTGGCTCTGTTTTAATTTACCTATACCGTATAT TATGTAGCCATTGAAAAATATGATTTAAAACATGAAGTAATCCCTGT TTTTGTGCCCATGTTTCTTGGATTGTGTTTTTAGGTTCTAAAAGCATT GAAATTGATTCTCCAATGACTCCTTTCCAGAAGTTATTCTTACATGT AAGATCAGATCTGTCAGTGGTGATTTCTATGTATTTGGATATTTAGTT TATAGCACATTTAAGTTATCTGCATTATGTTATTTCTCAAATCATAGAT

	CTATATTATAATTTTAAGAATCCCTTTCACCCTCCATACTGTATCCAA<br>AGATCACTTTTTTCAAAGGTCACCTAGGCAGAATAATCAAATTAATG<br>CTTTTAATTTGGTAATACTGAAAAGTAAATTGCAATGTATGCACACA<br>CAGATTGAAAATCAGGTGCCACAGACATGAGCATGCACAGAGAAT<br>TTCTGCATTCTCATGCCTTAGTTTATCAAATAAGGAAAATGTATAAA<br>AAGCTACTCCACAATTGGTGTGTGAATATATTACTTTATCTAAATGCA<br>TCTTCTCAGGCCAGGCATGGTGATTGATGCCTATAATTCCAACTGCT<br>CAGGAGTCTGAGGATCGCTTGAGTCCTGGAGTTCTAGGCTGCAGT<br>GAGTATCACAGTGCCTTCAGCCTGGGCAAGAAAGTGAGATTCTAGC<br>TCTAAAATATTTTAAAATTCATCTTTTCACCTCAGTTTGTGTGCCTCT<br>GCTGGAAAAGAAAGTCCAAAGGTTATTGTTACATTATGCAAATAATA<br>TGGGCTTGCAATCAAAAGAGCTGGTTCCTAATTCTCACTTTACCACT<br>AACTTGCTGAGTGACTTCAGGTAAGTCACTTAACTTCTCTGGTTCT<br>CATTTAAACCAAGTGATCTCTTTAAGTCATTTCTAATGTGAAAACTG<br>CGTGATTTAATGAGATATACATTTTGGATAATGATATGGTTAGATTGT<br>GTCCCCACCCAAATCTCATCTTGAATTGTAGCCCCCATAATTCCCAC<br>GTGTTGTGGGAGAGACCTGGTGGGAGGTAACTGAATCATAAGGGT<br>GGGTTGTTCCCATGCTGTTCTTGTGATAGTAAATAAGTCTCATCAGA<br>TCTGATGGTTTTAGAAAGGGGAGTTCTCTTTCACATGCTCTCTCTTG<br>CCTGCCACCATGTAAGACGTGTCTTTGCTTCTCCATTGCCTTCTGCC<br>ATGATTGTGAGGCCTCCCCTACCATGTGAAAATGTGAGTCCATTAA<br>ACTGCTTTCCTTTATAAATTACCCAGTCTCAGGTATGTCTTTATTAGC<br>AGTGTGAGAACAGACAAATACAGATAAATTGTGGCTGTGCATAGCT<br>TCTCTTCCTCTGGGGTTCAGGAACATTTTTTTCCTTACTCCCTAGTT<br>GGAGCCACTTTGGCTCTATTAATGACTTACCCCAAGAAAACCTCAC<br>AGCAAGGACTCAACAGTGATGAGGGAGCTATACTAAAACAATATCC<br>CCCAGATAGCCCAATGGGAGAGAGATTTGTATGGGCATGGGTTTAG<br>TCAGAGCCCAAAACTTGTTGTGTCTTCTCTGGAAGAACCTCCCAGA<br>CCTCCCCAGCTAAACTCATAACCACATCCCTTTCCTAGTTTATCTTCT<br>GTGTCCATTTACTGCACTGAAACTTCCTATTCCAAGTCAACGTTTCA<br>CCTCCAGATTGACATCCAAGCTGAAATCTGCTAACAGGGTCAGAAA<br>ACAGTTCATAAAGGAAAGTAAGCAAAAATGTTGAACCCATTCCAG<br>CTTAATAAAAAAAAAAAAAAGCCCACAATAACAAAATCTAAGGTTCT<br>GTGTGCTTTCCTACCAATATAAAACACAGCTTTCTCCCAGAAACTC<br>AACCATTTTCCATAATCATGATCTTCTATTAACAACTCACATCTTTCC<br>AAGTAGGCACATTCTCTCTGTTCTCTTATGTCAAAAGTACATAACAT<br>GGTGCAGTGATCCAACTATGGGCTGGCTTCTCTGCAAACTGTCAAC<br>ATTTCTCTCCCTGCCTTCCCTTTGTTCCTAAGAGCAGTTGTGGATTG<br>GTATAGACCCAAAATGGAAGTTGAAATATGCATTGCATCATTTGTCA<br>CATTAGCTACAAAGGAAGCAGCAGAAGTAATCAGAAATGAGGAGC<br>AGGAAATTTCCGATAAACAACTCTGATGGTTATCTGGAAGCTGAAT<br>TGAATCACAGGCACAAGAACTTGGAATTGCATTTTGAATTGCAGAA<br>TTAGAGGTAACTGCCTCCCACCTATATGATAGTGTCAATTAATCAAA<br>AATTGAATATAGTTTTACAGGCTTAGCATTCCATTCCATTCTGCGACA<br>TGTCAGAGAGCATTTATTTATTTAACAGGCTGACATGAAGCACATTT<br>GGATTTGGAAAAAGAAATAATTAAAAAGAACCACCCTGGGTGGCC

	CAGATGTGGATACTATTATGGATGTTGATAGTATTTGGAGGTGAAAT<br>GAACGAGTTTTTTTTACAGACTCAATATTGACCAATGAAAACATGT<br>AACTAATGAATTTAGCCCTGTAGTCAAGCAGTTTAAGCCTTTTTCAA<br>CATAGATTAGGAAGTTAGAATGGATGCCTCTCATTGAGTTTGCATTC<br>AGTAGAATAAGTAAAGCTTGTTTAACTCATGCATCTCTCACTGGCCG<br>CCTCTCTCTTTAATTTGGGAATAGAAAAGGGAGTCCACAATTAATTT<br>TAGATTGTGAGAAGAGAGACAAAAAAAGGAGCAGAGAGTTTCTG<br>AGATAACCTAAAATCTTTGCCGCACCCCTTACTAACCCCGGTTTGTG<br>CCCCTTCACTATGGGAGTAGCTCTTGTCATCATCTAAAAAACTTGAA<br>CTGCATTTTGAGAGATTTATTGGCCTTGCTTCTGCAGGCTCATAACT<br>CCTTTATGTGCTTGCTCTTGGGAGGAAAAAGCAGATAGACGTTTAA<br>AAGCTGGATGTTCCTGCACACCCCTCTCCTGATGCTTCATTCTTTTC<br>CAGGCAGAAAGATGGTGGGACTCTGTCTCTAGCAAAGGGATATTTC<br>CAGATACTGAGGTGTTGTCAGAGACACCTGGTCAGGGAGGTTAGG<br>AGAAGGGGCATCCAGGTCCACCCCCTCCAACTGGCTGCTGCTTTCC<br>TGGCAGGGCTGCACTGGGACACCTCACTTCCTTCCCACTTCCCCTT<br>CCTCCTCCCATTCGCTGTCTCTTTGCACACCCCTAATATGGCCTTTCA<br>TAGTAAGAGGTCAATATGTTTTCACACTTGGGAAATCTCATTCAAG<br>AATTTTTGTCAATGGACAAGTCATAAGAAGCCCTTCCATTTTAGGGC<br>TCGTTGACGTCACCAAGAGGCGATAAATATCTGTTGATATAATTGGA<br>TGTGAGATTCAGTGTTGAGATAGCAAAATTCTGCCCCTCGTTCCTT<br>GGCAGGGCCCTATGATTTATGCAGGAGCAGAGGCAGCACGCAATC<br>GAGCTGTCAAGAGAGCGTCAGCTTATTAGGCAAATGCTGCGTGGTT<br>TTTGAAGAGGGTCGACACTATAAAATCCCACTCCAGGCTCTGGAGT<br>GGAGAAACTCAGAGACCAAGTCCATTG
CRP	CTCAAACAATGTTATTGAGGCATGGTCTATCTCTCAGCTCTACTCGT<br>GAGTCAAGGATGGTGTATTAGTTGGTTTTCACACTGCTGTAAAGAA<br>CTACCTGAGTATGGGTAATTTATAAACAAAAGAAATTTTAAATGAAC<br>TTACAGTTCCACATGTTTGGGGAGGACTCATGAAACTTACAATCAT<br>GGTGGAAGGTGAAGGGGAAGCAGGCATTTTCTTCACAAGGCAGCA<br>GGAGAGAGACAGTGTGAGTGGGGGACTGCCAAGCACTTTTATTTA<br>AATCATCAGACCTAGTGAGAACTCATTATCATGAGCACAGCATGGG<br>CAAAACTACCTCCACGATCCAATCTTCTCCCACCATGTCCCTCCCTC<br>AACTCATGGGGATTACAATTTGAGATGACATTTGGGTGGGAACACA<br>GAACCAAACCATATCATTCCACCTCTGGCTCCTCCAAAATATCATGT<br>TCTTTTCACATTTCAAAACCAATCATACCTTCCCAACAGTCACCCAA<br>ACTTAACTCATTTCAGCATTAACTCAAAAGTCCAAGTCTAAAGTTC<br>CATCTGAGAAAAGGCAAGTCACTTCTGCCTATTAGCCTAGTAAAAT<br>AAAAAACAAGTTAGTTACTTCCAAGATACAGTGGGGGTATAGGCAT<br>TGGGTAAATGGTCCTGTTTGAAATGGGAGAAATTGGCCAAAACAA<br>AGGGGCCACAGGCCCCATGTAAATCCAAAATCTGGCAGGACACTC<br>ATGAAATCTTAAAGCTCCAAAATAATCTCCTTTGATTCTTTGTCTCA<br>CATCCAGGGCATGCTGATGCAAGCGGTAGGCTTCCATGGCCTTGGG<br>TAGCTCCATACTTGTGGCTCTTCAGGGTACAGCCCCTGTGGCTGCTT<br>TCACAGGCTGGCATTGAACACTTGCAAGCTTTTCTAAGCACAAGGT<br>GCAAACTGTCAGTGGTTCTACCATTCTGGGATCTGGAGGACAGTGG

	CCCTCTTCTCACAGATCCACTAGGCAGTGCCCCAGTGGGGACTCTG<br>TGTGGAGACTCCAACCCCACATTTCCCTGCTGCATTGCCCTAGTAG<br>AGGTTTTCTGTGAGGGCTCCATGCCTGCAGAAGACTTCTGCCTGAA<br>CATCCAGGTGTTTCCATACATCTTCTGAAATCTAGACAGAAACTCCC<br>AAAGCTCAACTCTTGTCTTCTGTGCATCTGCACCCTCAACACTACT<br>TGGAAGCCACCAAGGCTTGGGGCTTGTGCCCTCTGAAGCAATGGC<br>CTGAGCTATATACATTGCCCCCTTTTAGCCATGGCTGGAGCCGCAGC<br>AGCTGGCACACAGGGTGCCATGTTCCTGGGCTGCACAGAGCAGCG<br>GGGCCCTGGGCCTGGCCCATGATACCATTTTTTCCTCCTAGGCTTTT<br>GGACCTCTGATGGGAGGGGCTGCCATGAAGATCTTCTGAAATGACC<br>TGAAGACATTTTCCTCATTGTTTTGGCTATCAACATTCATCTCCTCAT<br>TACTTATGCAAATTTCTGCAGCCAGCTTGAATTTTTCCCCAGAAAAT<br>GGGTTTTTCTTTTCTACCACATGGTCAGGCTGCACATTTTCCAAACT<br>TTTATGCTCCCTTTCCCTTTTAAACATAAGTTCCAATTTCAGATCATC<br>TCTTTGTGAACACATATGATTGTATGTTTTCAGAAAAAGCCAGGTCA<br>CTTCTTGAATGCTTTGGTGCTTAGAAATTTCTTAAGCACCAAAGCAT<br>TCAAGAAATCATGTCTCTTAAGTTAAAAGTTCCACAGATCTCTAGG<br>GCATGGGCAAAATGCCACCATTGTCTTTGCTAAAACATAGAAAGAG<br>TGACCTTTACTCCCGTTTCCAATAAGTTCCTCATCTCCATCTAAGGA<br>CACCTCTGCATGAACTTCATTTTCCATATCACTATCAGCATTTTGGTC<br>AAAACCATTCAACAAAACTCAGGAAGTTCCAAGCTTTTCCACATCT<br>TCCTGTCTTCTCCTGAGCCCTCCAAACTCTTCCAGCCTCTGCCCCTA<br>GTTGGTTCCAAAGTTGCTTCCACATTTTTAGGAATCTTTATAGCAGT<br>ATCCCACTATCCTGGTACCAATTTTCTGTATTAGTTTGTTTTTACACT<br>GCTATAAAGAGCTACCTGAAACCGGGTAATTTATAAACAAAAGAGG<br>TTCAATTGACTCACAGTTCTGCATGGCTCGGGAGGCCTCAGGAAAC<br>TTACAATTATGGCAGAAGGCAAAGGGGAAGCAGGTACCTTCTTCAC<br>AAGGTGGCAGGAGAGATAGAGGGTGGGGAGGAACTGCCAAACAC<br>TTTTAAACCATCAGATCTTGTGAGAACCCACTCACTAAAATGAGAA<br>CAGCATGGGGGAAACCGCTCCTATGATCCACCCACCTCCCACCAGG<br>TCCCTCCCTTGACATGTGGGGATTGCAATTCAAGATGAGATTTGGG<br>TGAGGACACAGAGCCAAACCATATCAGATAGAGAGTGGTGGGACA<br>GAGGACCACTTCTTTTAGGCATAAAGCCTTGAAATTATTTCACTTTT<br>AAACTAGGAGCAAGAATTCAGGAGGAAGTTGGCTGGGCATGATGG<br>CTCATGCCTGTAATCCCAGCACTTTGGGAGGCTGAGGTGGGCAGAT<br>CATGAGGTCAGGAGATTGAGACCATTCTGGCTAACATGGTGAAACC<br>CCGTCTCTACTAAAAATACAAAAAATTAGCTGGGTGTGGTGGCGTG<br>TGCCTATAATCCCAGCTACTCAGAAGGCTGAGGCAGGAGAATCACT<br>TGAACCCAGGAGGCAGAGGTTGCAGTGAGCTGAGATTATGTCACT<br>GCACTCCAGCCTGGGCGACAGAGCGAGATTCCATCTCACAAAAAA<br>AAAAAAAAAAGAAAAGAAAAGAAAGAATTCAGGAGGAAGTTATA<br>AACTGTGTTGAATATTGCCAAGAAGCTGGGTAAAATAAGAACAGA<br>AAATTCATTATTAGATTTAATGAGAGAGGTGTTTGTTTATGTTGGCTT<br>CACTAGAGTGGTGAAGGCCAATGGCTGATTTCCATGGATTGAGGAG<br>GGAACAGGAGATGGAAAATAGAGGTTGTCAACCCACGTAAACTTT<br>TCCTTCCTTCCTCCCTCTCTCCCTCCCATCCTTCCTTTTTCTCTTTT

	CTTTCTTCTTTCTCTAAAAGATTAAATATATTATAGTATGGTTTTGTG GAAATGATCAAGTAAAGATGAATAAATTAATAATACAGAAGATAGA GAGGATACCTATAGGAGCAAAATTCCTTGCATAGTCGACAGGGTCT GGGATCCACTGAAGAATTGCAAGGGTTGGCCTTAGATGTAACACAG GGAGAGCTCATCCCTTTTAATAGAGGAAGACAGAAAGGCTGGTAC ATTGGGTTGTAGGAGGATATATAAGGATATCAGTTAGGTGTTAAGAA CAGAAAGACTGAGTTAACAGTGATTTAACAAAAGAGGAGAGTATT TCTCCTTTAAGTAAATTTTCAGGTAGGCAGCCTAGAGCTGATGCAG TGGCTCCATACCCAATTATCCAGGCTCCATCTATCTTGTTGCCATGCC AACCTCAACATGCAGCTTACACTTGCTGGCCTGAAAGTGAACTATT CCATCTCCTGCTATCATGACTGAATACTAGTCAAAGGGAAAAAGGG GGAAGTGGGAGGGGAAAGCACATACCTTTCTTAAAGAGCACAACC TAGAGATGGTCCCTATTCTGTTCATATCTCGTTGGCCTTAATTTAATC ACATCATCACTGCAAGCTACAGTGGGGGCTGGGAAATTTGGTGTTT GAGGGGATGACCAAGCGCCCAACTGAAAATGTTATTCTTCTCTTAT GAGAAGTAGAGAATGGATGTTAGGGAATGGCTAGAAGACTCTACTA CAAAGGATACGGTGGTTCTTTTCTGATTGCTTCTAACTTCTCAATGA AAGGTGAGTTCATCAGCTGAATGAAAGAATAAATGAGATTTTGGGG ACTTGAGGAGCAAGGAGAAGGAGTATAATAGTCATTTTGAAGAGT GAGTTAAGTAGGGAACTGTAGTAAGATTGACAGACAGTGTGGAGG GATTACTTGAATCTTGTGAATAGAGGAAAGAGTAGAATCAGATTATC CTGACTCCTGCCTGAAGCTTTACATATTCAGAGAAAAATGTTGGAA GAAACTTTGATATAATGCTATGTCTGTGATCAGGCACACATTTTACT GGACTTTTACTGTCAGGGCCGTCATTTAGTGCCAAGATGTCTAGAG AGTTCTTAATAAGTGTACTCAATTGGCTGAGAAAATGTGTCCATGC AAAAAACCAAACACCGCGTGTTCTCACTCATAGATGGGAATTGAA CAATGAGAATACTTGGACACAGGAAGGGGAACATCACACTCTGGG GACTGTTGTGGGGTGGGGGGAGGGGGGAGGGATAGCATTAGAAGA TATACCTAATGCTAAATGATGAGTTAATGGGTGCAGCACACCAGCAT GGCACATGTATACATATGTAACTAACCTGCACATTGTGCACATGTAC CCTAAAACTTAAAGTATAATAATAATAAAAAAATGTGTCCATGGCTC TGGGAGGAGCATGTTTGTTTTCCTCATTTCCCAGTCTGTAAATAAGC AAATTGAAAGGGGTTAGTGATAATGTCCATCTCCAGAAGCTGTCAG ATTTCCTTTGTCAAACTCTATGATTTGGGCTGAAGTAGGTGTTGGAG AGGCAGCTACCACGTGCACCCAGATGGCCACTCGTTTAATATGTTA CCATTTCCCATTATTTTCGCAGGATAGATAGCCAAAGTGGAGCCCTG AGAGATTTCTTCATTTTTCCTGTCATAAAGAATTGGTAATTCAGTAG TCATAGGAGTTTGTAATAAATAACTCACATTGATTTCTCTGTTCTGA AATAATTTTGCTTCCCCTCTTCCCGAAGCTCTGACACCTGCCCCAAC AAGCAATGTTGGAAAATTATTTACATAGTGGCGCAAACTCCCTTACT GCTTTGGATATAAATCCAGGCAGGAGGAGGTAGCTCTAAGGCAAGA GATCTAGGACTTCTAGCCCCTGAACTTTCAG
CSNK2B	TGTAGTCAGGGAACTCCTCCTGGGGGAAGTGAGACTTGCACGAAG CTGGGCAATTCTTGGTCCAGGGGGGTTGAAAGAATAGGTGGGGGA CTCCCAGGAGGGTCTGGGACCTGAAAGTGAACCCAGATTGGCAGG GAGGTGACCTTATCATGCCACCTGGAGAGGCTGCCCCTTCTGACTC

	AGGTGGGACTTGCATGTGGCTCCCAGGCTTCTGTTTGGCTTCCTCA<br>AAATAGCTTCCAGAAAAGTGAATAAACCACAAATGGTTGATTTATT<br>TCTGACTCTCAGCCCGTCTCTCACGAAGACAGAGCCTATTGACCAA<br>AAACTTCAGGATCTGCATCTGGGCAGATCCCAGGAAGGGGAAGTC<br>AAAGGGCCCAGGTCAGAGGCCCAAGTTCAGACTTCAGCAGCAGA<br>CTAGGGTCAGACTTTACCAAAGTCAGAACTCGAGGTTCATGTAAGT<br>CCTTAGATCCCGCTCCCAAGCCCTGTCTTTCTCCTCCCTCCTTCTCT<br>CCTCCCTCCAGCTCAGTGTGGCCACCCGAGGGGGTCTCTCCCTCCC<br>AGCCACAGCTCGGGTATCCCAAGCTGGGAAATGTGTCACTCGGGG<br>CTGGGGTGCTGATCTGTAGCCTAGTCCTTCCTGGTCTCTCTTGAGG<br>ACAGTGGGGATGGGATTGGCACGGCCCTCACCCCGGGGTCCCAGC<br>CCCATTCCTGGCTCCCAGCCCCCCCTCAGCAGCAGTTTGAAGCCCG<br>GGCTGGAGATGGGCACCCCAAGTGGAAGGTTGGGAGGCTGAGGA<br>CCCTGCGACAGTGACAGCAGGTGAGCAGTGGATGTGCGGTGGTTG<br>GAATCTTGGAAGTGGGTGTCACAGTTCTCGCAGTACTGGAGGGAG<br>GGAGTAGGAGACCTGCAGAGAAAGAAGAAAAAGCATTAAGGGCA<br>GGGGAAGGAAAAGGGGAAGAGTTGAGGCCTCAGAGGGGGCTGGC<br>AGGGTAGAATAGGATCTTTTCAGCTTTTCTGCTAAGGAACAAATTG<br>CCAGCTAGGCATAGTGGCTCACGCCTGTAATCCCAACACTTTGGGA<br>GGCAGAGGCGGGCAGATGGCTTTGAGCTCAGGAGTTTGAGACCAG<br>CCTGGGCAAAATGGCAACGCCTGCTTTTTTTTTTTTTTTTTTGAG<br>ATGGAGTCTTGCTCTGCTGCCCAGGTTGGAGTGCAGTGCCATGATC<br>CTGGCTCACTGCAACTTCCACCTTAGCGATTCTCCTGCATCAGCCTC<br>CCAAGTAGCCGGGATTACAGGCACATGCCACCATGTCCCGGCAAA<br>GCCTGCTTTCTACAAAAAATATGCTTGAGCCCAGGAAGCGGAGGTT<br>GCAGTGAGCTGAAATCACACCATTGCACACCAGCCTGGGCGACAG<br>AGTGAGATGAGTGAGACTTTGTCTCAAAAAAAAAAAAAAAAAAA<br>AAAAAAGGGACAAATTGCCTTCCTTCCTACTTAACAGTGAGGGATC<br>CAGGCTGGTCCAAAGGTGGTGGTGAGTTATCTGAATTAATTGTTCA<br>CTCAGTTACAGATCAAACTCCTTACTCCACTTTTCCCCTCCTTCTCA<br>CTACTGCACTTGACTTGTCTTAAAAACAAATTTCTTTAAACCATTGT<br>GGGATCCAGAGCAGAATAGTTGAAAGAAAAAAATGGTAACCAGAC<br>CTAGCAAACTCTTGGGCAAGGGGAGGGACATTAGTCATAATGACTA<br>TAGCTAACATTCATGTATTGCATACTATGCGGCATGCACTATTCTAGC<br>ATTTTACATATATTAACCCATTGAATCCTAACAACGATTCTTACTACC<br>CCCATTTCTAAGATGAGAAAACTGGAACATGTAGACATTAGGTTGT<br>TTGCCCAAGTAAGTGGAATCAGGCTTTAAATCCAGGGAGCTCATGT<br>TTATAACCACTTGACTATACTACCCTGTCAACCTACACATGAGGATA<br>AGGAAAGAACTCTTCAGCACTGTGCTGGGGCGTCTGGTGTGGTGT<br>GGCTGGGAGAGGCAGAACACAATGAGACATGGGTCTGAGCTAAAG<br>TTTCCCCTTACCGGTTTTCCGGGCTCCTTGTCTCTCCATGGCTCTCC<br>CTGACCATGCGGGCTACCTCAGGGAAGCCAGCTTCTTCAGCGAGC<br>TGAGCCGCATCCCTGCCACTCAGCTCACAGACCCCCACCCAGGCA<br>GCCCCACGGCCCAGGAGATAGCTCACAGCTGCCCCCTGGCCCGCT<br>CGAGCAGCACACATCAGTGGGGTCCACCAGAAGGCATCCCGGGCG<br>TTGATATTCCCCCCAGCTCCTCCTGCCTCATGCGGTTCCAGCAGTCT

	CCTAAGTTCTGGCAGGTCCCCCTCCTGGGCTGCCCTCAGTATCCGG<br>TGAGTCATCTTATCCTCAGCCTCAAGGGATCTCCCTTGTCCATGTCT<br>TCCTGATGCTCCTTCTGCCACTGCTTCTGCTGCTGGTGCCTTCATTA<br>TTCTTCTTTTCTTTCTCTTTCTTTCTCTGGCAGGTTCAGTCTGAGATC<br>TCTGGGAGTCAGGAGCGCTGCTCTCATCCCCAATCAGGGCCTCATA<br>GAAAGCTCGGGCTGCAGCCCCATCCAGGGTGGACTCTGGCTTCTC<br>GGGCTGTGGCTGCTGCTGCCCATCCTTCCAGAGGTCGCTGGGGTCA<br>GTGGCTGGGGTGAAGGTGATGAGCAAGGGCCGGGACATGGCTTTT<br>GGGAGAACTGAGAAAATGATACCAGGCAAGGGAAGGATGAGACA<br>AGTAAGCCAAGCTCGTGGTGACCCTGTAGCAACCACAGCCTCAGA<br>GACCTGCTGGGATGAGAAAAAGTAGTCAAAAACACTTTCCTGCCA<br>CTAAAGTAACCCCACAACTTAGGACTCTGCAGGGCCTAAGGGAGA<br>GAGACTTTGCGTAAAAACATGGAACCCTACAATACCGACTTTGCTC<br>CTTAGTAAAGATTAATAAAACTCCATGAGACTGTTGTCCAGAGGTC<br>CTGCGTCCGGCCCCCACCCCCATCCTCACCAACAATAAACACCAGC<br>CTCTTTCTGAAACCACTTTCCCACCCCGTAAGACATACCAGTAGGA<br>AAAAAAAATCAGCCTGGCCCTTTAAGTCTTCCGCGATCCCATTTCG<br>GAGTTTCCTCTTCCCAAACAAAAATAGATGGGTCACTCCCTAGAAG<br>ATCTCGGGGAGAGTCTCCTATACGTGTTGCTGTGTAGCTTCCGTACC<br>GCAAAATGGCGCCATTCTAATCAGAAGAGTTGACACAATCAAATAG<br>CCACACGGCACGAAGACGCATGCGTGGCGACAACAACAACAAAA<br>ACCACAACCCACATTACTTGAGGGCTCGGGCGTGCGCAAAGCTCC<br>GGGTTCAGTTTCCCGCGCTGGAACTTTTTCAATAGTAAACGAGCAA<br>AGCTCCGCGCGCCCAGGTGGCGCGAGCACTAGGATCTGTCGGTTG<br>GGGTCCTACTTTTACATAACGCCCCCACAATGCCCTTCGCCTTCCTC<br>AACGTGGCCCCCGCTCCAAGCCCATTTTCTGGAGCCAGGAATCCAC<br>TCTGTGGGTTAGGAAAGGCCCTCAGGAGGCGGAGGGAAACCTGTG<br>GAATGCCGAGAAGCCGTGTAATGAAATAACGTCACGCCTGCCCCTC<br>ACCATTACTCTGACCAGGGTTCGAAGGTCACACTTAGAGCCTAAGG<br>GGAAATGGAGAAGTGCAAAGGGACGAGCAGAATGGCTGGCACCA<br>CCTCAGGTTAGCGCACTGGGACGTTCCAGTTCTCACACCGCCCACC<br>CCACCCCACCCAAGTCCCTACGCACGGAGCCAAGCCGCACCTCTC<br>CCCTCATGAGGCAGGAGCCCGGAGGAAACAGTATGCCCGTCAAGG<br>GTCTCTGGCGGGACTGATTCGCACTAGGGGCCCAACAGGCAATAA<br>GGACCCAGCGGATTGGCCGAGGATAGGCCAGTCCCCTGGGCAGCA<br>GCGCCGCGCCGGGACTAGAGGGGAACGTGAGGAGAGCTGCGGAA<br>AGAGATCCAGCCTGGCTTCCTCCTTTCCCCGCCCTAAGTCAGCCTC<br>TTCACCCAGTGAGCACAAAACTGTATTGCCCAGACTCCCGGGCCCC<br>GAACGCCATACCTGGCTTCCGCTTCCGGTGGCTTCTCGTTGTGCCC<br>CGCCCGCAAGCGCCCTCCTCCGGGCCTTCGTGACAGCCAGGTCGT<br>GCGCGGGTCATCCTGGGATTGGTAGTTCGCTTTCTCTCATTTAGCCA<br>GTTTCTTTCTCTACCGGGGACTCCGTGTCCCGGCATCCACCGCGGC<br>ACCTGACCCTTGGCGCTTGCGTGTTGCCCTCTTCCCCACCCTCCCT<br>AATTTCCACTCCCCCCACCCCACTTCGCCTGCCGCGGTCGGGTCCG<br>CGGCCTGCGCTGTAGCGGTCGCCGCCGTTCCCTGGAAGTAGCAACT<br>TCCCTACCCCACCCCAGTCCTGGTCCCCGTCCAGCCGGTGAGTCTG

	AAGTCGTCGCTGCTCCGAGTCCCTTGTCGCTGGGAGCGGCACATG<br>GGGTCTCCGGACTTTGATGTGGGGGCGGGGGAGGAAGCGACCAGG<br>TCCGGCACGAAGGAGGGAGAGGTGGCCTGAGGAGCGGAGGGGGG<br>ATGTGTGGATTCCGGTGAAAGGGACCTGACAATCGCCCCCAACCC<br>GTGAGAAAAGGAGGAGCCCGGTTCTTGCTTGAGAATGATAAACTT<br>GGAAACCCTTGGGAAAGGCGTGGGGGTCATGCAGAGACTTGTATT<br>GGTAGGGAGCCTGAGTCGAGGTCCCTGCCGGAGTTGACACAGAGG<br>AGAGAGGGCCCTGGCCTTCGGGAGCTCCAGGGATGTGGGTCGGGC<br>TGGTGGGTCAAAGTATCTGTTGGCTTCTTTCAAGTGGTGGGACCCC<br>AAAGAATGTTTAACTTCAAAGAAAAGGGGCTGAGATGTAAATTAG<br>AGGAGCTGGAGAGGAGTGCTTCAGAGTTTGGGTTGCTTTAAGAAA<br>GGGTGGTTCCGAATTCTCCCGTGGTTGGAGGGCCGAATGTGGGAG<br>GAGGGAGGATACCAGAGGCAGGGAAGGAGAACTTGAGCTTTACTG<br>ACACTGTTCTTTTTCTAGCTGACGTGAAGATGAGCAGCTCAGAGGA<br>GGTGTCCT
CYP11A1	CAATGTATAGAGGGGGACAAGGAGCCACAGTGTGCACCCAGGCAG<br>GGAGTGCCTGGGTACTCATTCACCCTTCAAGTTGGGGTGTGGGGGT<br>TCCTCTGGACTTACAGGAGTGATGAGGATTTAGAGGAGCAGGGAG<br>AGGCTGCCCTGGGTAGCTAAGATTAGGGCTCAGGTTCCAGTTCACC<br>ATGTGGAGTTAAGTTCTCATAGGAGGGCTGCAATGGGTGGGCCCCT<br>AGGAACAGCCCTCAGAGATGCCCTTTGCAGAGGCTCCCAACCTGG<br>CAACACTCCTTTGCCGGGGGCTCTGGGCCTTTGACTTCTGGGTCCA<br>GTCTTCTCCCCTCCTTTACCCCAATTACTCCTGCCCCTAAAGCTATG<br>TCAACTCTGTCCCGTATTTCCCTTCATTTCACTCTTGTTGGACAGAA<br>GGAATAGCCCAGGATCCATAAAAAGAAACAGTCCCCAAAGCAGGG<br>ACCCCTTTCCTCCAGGACACAGAACATTCAGTCTTCATAATCCCTG<br>ACTACATAAGCCCTGGCCCGGGCAGGAACAATGTGTTTCATTCCTT<br>CCTTGGGAATGTCTCTTTACCATATGGCCACATGACCCCTCCCAAGG<br>CCAAACAAACAAACAAACAGGAAAAACCCCAAACAGCAAAAGGA<br>ATCTATTTAATAATAGTTTTAACAGCCACATCTGATACTTAAGCTGAG<br>AAATGCCATAACCTTGAGTCCCATCTCTAGGGCTGATTTCTCTCCAA<br>ACATCTTAGGTCCTTGCCTCTCCCTCCTCCCTTTAAACTGTTAGAGT<br>GGGGTGACACCGAAACAGATGAAATGCCCTCAGAGAAAGAAATAG<br>GGTGGAGTCGGGGGGACCATCTGACTTAGTGAAATACCCTGAGCT<br>GTGCATGTGAAAACAAACAGGGAAAGAATCACCTAATTTTCATGTA<br>TGGTGTCCATACCCTAAGCTGAGCAAGAGGGTGATGTTTAAGGCAA<br>TGATATTAGGTGGGCTACTTTGAGAACCTCTGAATCTTTTAAGTGCT<br>TTTTTTTATCTTGCAACAGCTCTGAGAGGCAGGTCTGATTATCTCCA<br>TTTTAAATATGAAAAAATTAAGTCTCAAGTAGCTCAAGTGTCTTGCC<br>CTATGCCACGCGACCACTCAGTGGTTGAACTAGGATTTGAACCTGA<br>GTCCATGTGGCTCCAAAACCGTTGCTTGTTTCATGATACAAAACTGT<br>ATTATTATGTCTATGGTCTGGCCAGCACCTCTGCTAGCGGATGTATTG<br>CCATCCAGAGCAAAACGACATTGTCCAGCCTCCCTGTACCTACCTC<br>CCTGTACCTAGGTACAGTCATGTGGTTACTGAATGGAAGGTCTTGA<br>CTGAGTTGTCCAGGTTCTTGGCATGTTAAAGAATTGAACAAAACGC<br>ACAAAGCAACAAAAGACAAAGCAACGAAAGAATGGAGTAATGAA

	GGCACAGATTTATTGAAGATGTGCAAGTACAATTCACAGAGCAAGA<br>GTGGGCTTGAGCAAGTGGCTCAGGAGCCCCTTCAATTAGAGATTTT<br>ATTAAGCTAAAGAACATGAACACCCCCGGTGCCCTCCAGAGGCCTC<br>CAATCAGTTACATCCAATGAAGGATTGATCCGTGACCAATTAGAGG<br>CTTAAGTGGCGACTTGGCCTGTGGTTATCACAGAAGTGAAGATGTG<br>GCCTGTATGTTGCCTAATCCTGCCTAGAATTGGCTGTACCTGCTGTG<br>CCTTTTGCTTATGTGAACTGGCTGCACCTGCTCTTCTTTTGTTCCTA<br>CCTTAATCCTTGGTTGCCCTAATTCCCTATTCTCCTGCCTCAATGTGG<br>TTAAGTTCCCACCAATCAGATATGGTTGGACTCGTGTGGGACTTTG<br>AGGAAGGGGCTTAAATGAAGCTGGCTCTTCAGGGAGGGCCCCTTT<br>TGGTCTTCTTGAGTTCTTCCGCCTTCTGGCCAGCAACACGGTTAAT<br>GCTCCAGCAGCAGCTTGGACCATCGGGTGGCCTTAGAAATGCCAA<br>CCAAATGTCTTCGTGGTGAAGCAGGAAGATGGAGCCTGGCATTTTG<br>CTGGGACTGACACACTCACCCTGGACTGCCCAGCCTTGGACATCCT<br>TTATGGGAGAGAGAATACACTTCTATCTTCTTTAAGTCTGTGATTTG<br>GATTTTCTGTTTATCCATCCAAGCTGCCTGCAATGAATAAAATCATG<br>TTCCCCGTCCTAGGCAACGTTTAGATGGGTCTAAATGTGTACACCCT<br>CTACTCTGAGAATGATAGGATAGTTACAGAATGTGGGAGGCAGGAG<br>TGGGAGGAGAAAGCACTATAAGTATGGGCCTGTCTTGAGAGAGCC<br>AAGAAGGACTTTGGCATAGCCGAGCACTAGGCTAACCTCACCTAGC<br>AGAGGATTCACTTGCCCATCGTAAGGGGCTTGGTGAGGGTTGGAC<br>CCTAACTTCCCATCACTTTTGAGAGGGTGAAAATTTCAGTTTGGGC<br>ACTGGCAAGAACAGAGAGAGGGAGGGAAAAATTATGTAATATACA<br>CATGTATATGTGTAATATATATATTTTGAAATGTATGTATCATTCATTAA<br>TCTTCTCAATAGTCCTGAAACAGCCACTGAAAAGTTTTGAAGCAGG<br>GCCAGTTTAATCCACTGAGCTTGGAAGCTGATCCTACACCCTGGTC<br>AAGCTTCAGGGATGGCAGCCGCTTGTGAGAAACCCTGAGCATGAG<br>CCACTCAGCCACCCAGCCACCCAGTCACCCAGCCACCCAGCCACC<br>CAGTCACCCAGAAAAGCTGCTCCTGGGTGCTGCACCCTCGGAAGC<br>TGTGAGATAATAAACATTTATTGTTTTAAGCCACTAAATTTTGGGATA<br>ATTTGTTAAGCAGCAGTAAACAGCTAATACATTCAGCCTTGTTTGGA<br>GTGAGTGATGTGTTTCTGGAAGCTCTTTCAGAGAAGTGAGGGAGC<br>TATTCTCCCAGAAGCCACAGCAAACCTTTCCCTGTGTTTCATTGGC<br>CCAAACTGAATCGCTGGCCTATGCTGTGATGTGACCATGGCCATTG<br>GAGAGGATGAGGCAATAACCTCCAGCCCGGGCCACTTCTGGGGAG<br>GCGGTCAGTGCCCCACAACACTGGGGGAGGTGCGGAGGCCTGAA<br>CGGAAGTTGGGGTGGCTGCCAAGAGGACCACAAGTTCTTCCATGC<br>CACATCGATTAGGGCTCCTTCTGAGGGAGGAATGTGGGGCTGCGTA<br>GAACAATGGGATTGACTTTAAGTCAGAAAGTTATAAATGTCACCTC<br>AGTGCTGAGACCCTTGGAGGAAAAACTAGTCCTTGGAAGACTGCT<br>TTTCTTTGTGGAAGCTCATCACCCTGCCGCTGCTCGTGAGACACTG<br>CCTTCCTTGGCTGATGTCATTCCAGGCTCAAGGTCATCAGTGAGGC<br>AAAACAGGCTTTCTCCATACTCTCTTTATCAGAAGGTTCATGACTGA<br>TGAGGTAGTGGTCACTCCAGCGGGAAGAGCAACAACCACTCTTGA<br>TAAGTACTTTTTTTTTTTCTTCTAAAAACTGTTGCTCTAAATTTGTTG<br>AAAGTGGTTCAACAGTATTGGAGTCTGGGGTCAAGTGGCTGTGTA

	AGGACAACTTTTGCCATTGTGGGTGATCTATGGGCTGACACATACA ACAGAAGAGGCCAGGAGGATGTCACTCGTGTGTGTGTGTGTGTGT GTGTGTTTGGTAACAGCTCTATTGAGATATAATTCACACACCATACA ATTCACCCATTTAAAGTATGCAATTCAATGGCTTTTAGTGTATCAGA GAGTTGTTCAATAATTACCACAATATACTTTAGAATATTTTCATTTTC ATCATCCTAAGAATCCCTACACACTTTATGTCCCATCCTCTAATTCCT CTATCCCCCTAGCTCTAAGCAACCACCAGTCTATTTTCTGTCTCTTA GATGTGTGTTTTAAATGCCTTATTTGCCAGAAAATAAGATTTTGGGG AAAAAAAGCAAGAAGTTGTACCTTCCAATGATAAGTATAAGGTATA GTGTAGATTGCTGTCATGAAATGCTCTACATGGCACATGTATGTTTT CTGACCTACACATTTTTACCAAGTGGGCTGTAATTTTGTTGAAATGA CTTGAAAACTCTACAGGTGACTGACCCTTGTTGTTTGGGAAAGTGG TAGGTGCAGGGTGATGGGGGGTGGGTGTGGGGGCTGACCTGGGCT GGAAGGATGGGTCTGGGGATATGATATGATGGGAGGGGGTATGGAA GCAGCTCTGAGGGGAAAGCAGCACAGATCAAGTGTGTGGACAGG GAAGAGCTGACATCCTGAGTTCCATGGCAACCAGATTTGCCAAGGT CTTAGAGTGTGTCCAGAGTGGAGCCTGACCACAGACCTCAGCTCA AGGGACCCAGAGCCCCTCTGAGTCAGCTGTACTGAATTACAGCCCC AAATCTGGGTCAACTGGGGAGAGACGACGAGGATTAGGGTTCCAA GGTGAAACTGTGCCATTGCGCTCCAGCCTGGGCAACAAGAATGAA ACTCTCTTAAAATAAAATAAAATAAAATAAAATAAAATAAAATAAAA TAGCCTAAGGATGCATTTCTCAGAACTTATCCCTGTTGTTCAATGAT GTGTGTCTATACAGTGGGGCCATAACTAAGACGTATGTTGCCCAAG CTGGCAAGATAGCTCTGACCTTCTCTTGGGCCCCTCATTTCCCCCA AACACAGGTTGTCTGCAGTCTTGACCAATGGCTGCCAGGGCATGG ACTCCGCTGCAGGGGCCAGTGGGAGGCCCCAGCTCAGGCAAAAG CACAGGCAGATATTTCAGGAGTCTGCTAGGGCTGGCACTGAGGGC AGAGACAGAGGGGTCTCCCTGTCCTTTGGAGAACCTCACGCTGCA GAAATTCCAGACTGAACCTTCATACCGAGTAGGGGAGGAGCTGTCT GCGGGTTTGAGCCTGCAGCAGGAGGAAGGACGTGAACATTTTATC AGCTTCTGGTATGGCCTTGAGCTGGTAGTTATAATCTTGGCCCTGGT GGCCCAGGGCTACAGTCATCCTAGCAGTCCCCGCTGAAGTGGAGC AGGTACAG
CYP11B1	AATCACTTGAACCCGGGAGGTGGAGGTTGTGGTGAGCTGAGATTG CGCCACTGCACTCCAGCCTGGGCAACAGAGTGAGACTCTGTCTGA AAAAAAAAAAAAAAAAAAGAAGTATCACCTGACACTGGTTAGAATG GCCTCTATAAAAAAGGGGGAAGATGACAGTGTTGTGAGAATGCAG ACAAAGGGGATTCTAGTACCCTGTTGATGGGAATGTAAATTATCAC AGCCATTATGGCAAACAGAATGAAAGTTTCTTAAAAATTAAAAATA AAATTACCATATGACCCACCCATGTCATGGTGAGTCTACATAAAGGA AATGAAATCAGTATGCTGAAGATACCTGCACTCCCATGTTGATTGCA GCATTATTCACAATAATGGAATCAACCTAAGTGTCCATCAACAGATG AATAAAGAAAATGTGGAATATAGACACAAAGGCTTTAATTCAGCCT TAAAAAAATAAGGAAATCTATTATTTGTGACAACATGGATGAAGCT GGAAAATGGTTATGTGAAATAAGCCAGGCACGGCCAGGCGCGGTG GCTCACGCCTGTAATCCCAGCACTTTGGGAGGCCGAGGGGAGTGG

	ATCACGAGGTCAGGAGATCGAGACCATCCTGGCTAACACGGTGAA<br>ACCCTGTCTCTACTAAAAATACAAAAAAATTAGCCAGGCATGGTGG<br>TGGGCGCCTATAGTCCCAGCTACTGGGGAGGCTGAGGCAGGAGAA<br>TGGCATAAACCCGGGAGGTGGAGCTTGCAGTGAGCCAAGATCGCG<br>CCACTGCACTCTAGCCTGGGTAACAGTGAGACTCTGTCTCAAAAAT<br>AAATAAATAAATAAATAAAAAATAAGCCAGGCACAAAAAAAGCAA<br>ACACCAACACATAATCTCACTTATACTGTGGAATCTAAAAAAATAG<br>AACCCACAGAAACAGAGAGTGGAATGGTGGCTACCAGCAGTTGAG<br>GGCAGGGAGGCAGACGTACCTGGAGGGTTGCGGTGGGGAGACTG<br>TTGGTCAGCAGGTGAAAGGATACAAAGTTTCAGTTAGCCAGGGGG<br>AAAAAGGCCAAGAGATTGATGATGTGACATGGAACCGTGGTTAATA<br>ACCACGGACTTGAAAATCTCTAAGAGAGTAGACTTGATGTATTCTA<br>AACACACACAAAAAGTGTCTGAGCTAATAAACATATTATTTAGCTT<br>GATTGTGGCCAAAATTATATTTCAGATTTTATATACATATTTCAAAAC<br>ATCATGCTGCACAGGATAGATACATGTAATTTTAATTGTCAACTAAA<br>AGTTAATGAAATAAAATTATTTTTATTTTTATTTTTATTATTATTATACT<br>TTAAGTTTTAGGGTACATGTGCACAATGTGCAGGTTACATATGTATA<br>CATGTGCCATGTTGGTGCGCTGCACCCATTAACTCGTCATTTAGCAT<br>TAGGTATATCTCCTAATACTATCCCTCCCCCCTCCCCCCACCCCACA<br>ACAGTCCCCAGAGTGTGATGTTGTCCTTCCTGTGTCCATGTGTTCTC<br>ATTGTTCAATTCCCATCTATGAGTGAGAACATGTGGTGTTTGGTTTT<br>GTGTCATTGAGATAGTTTACTGAGAATGATGATTTCCAATTTCATCC<br>ATGTCCCTACAAAGGACATGAACTCATCATTTTTTTATGGCTGCATA<br>GTATTCCATGGTGTATATGTGCCACATTTTCTTAATCCAGTCTATCAT<br>TGTTGGACATTTGGGTTGGTTCCAAGTCTCTGCCATTGTGAATAGTG<br>CCACAATAAACATACGTGTGCATGTGTCTTTATAGCAGCATGATTTA<br>CAGTCCTTTGGGTATATACACAGTAATGGGATGGCTGGGTCAAATG<br>GTATTTCTAGTTCTAGATCCCTGAGGAATCGCCACACTGACTTCCAC<br>AATGGTTGAACTAGTTTACAGTCCCACCAACAGTGTAAAAGTGTTC<br>CTATTTCTCCACATCCTCTCCAGCACCTGTTGTTTCCTGACTTTTTAA<br>TGATTGCCATTCCAACTGGTGTGAGATGGTATCTCATTGTGGTTTTG<br>ATTTGCATTTCTCTGATGGCCAGTGATGATGAGCAATTTTTCATGTG<br>TGTTTTGGCTGCATAAATGTCTTCTTTTGAGAAGTGTCTGTTCATAT<br>CCTTTGCCCACTTTTTGATGGGGTTGTTTGTTTTTTTCTTGTAAATTT<br>GTTTGAGTTCATTGTAAATTCTGGATATTAGCCCTTTGTCAGATAGTA<br>GGTTGCAAAAATTTTCTCCCATTTTGTAGGTTGCCTGTTCACTCTGA<br>TGGTAGTTTCTTTTGCTGTGCAGAAGCTCTTTAGTTTAGTTAGATCC<br>CATTTGTCAATTTTGGCTTTTGTTGCCATTGCTTTTGGTGTTTTAGAC<br>ATGAAGTCCTTGCCCATGCCTATGTCCTGAATGGTGATGCCTAGGTT<br>TTCTTCTAGGGTTTTTATGGTTTTAGGTCTAACGTTTAAGTCTTTAAT<br>CCATCTTGAATTAATTTTTGTATAAGGTGTAAGGAAGGGATCCAGTT<br>TCAGCTTTCTACATATGGCTAGCCAGTTTTCCCAGCACCATTTATTA<br>AATAGGGAATCCTTTACCCATTGTTTGTTTTTCTCAGGTTTGTCAAA<br>GATCAGATAGTTGTAGATATGCGGCATTATTTCTGAGGGCTCTGTTC<br>TGTTCCATTGATCTATATCTCTGTTTTGGTACCAGTACCATGCTGTTT<br>TGGTTACTGTAGCCTTGTAGTATAGTTTGAAGTCAGGTAGCGTGATG

	CCTCCAGCTTTGTTCTTTTGGCTTAGGATTGACTTGGTGATGCAGGC<br>TCTTTTTTGGTTCCATATGAACTTTAAAGTAGTTTTTTCCAATTCTGT<br>GAAGAAAGTCATTGGTAGCTTGATGGGGATGGCATTGAATCTATAA<br>ATTACCTTGGGCAGTATGGCCATTTTCACGATATTGATTCTTCCTACC<br>CATGAGCATGGAATGTTCTTCCATTTGTTTGTATCCTCTTTTATTTCA<br>TTGAGCAGTGGTTTGTAGTTCTCCTTAAAGAGATCCTTCGCATCCCT<br>TGTAAGTTGGATTCCTAAGTATTTTATTCTCTTTGAAGCAATTGTGAA<br>TGGGAGTTCACTCATGATTTGGCTCTCTGTTTGTCTGTTAAGTGTGT<br>ATAAGAATGCTTGTGATTTTTGTACGTTGATTTTGTATCCTGAGACTT<br>TGCTGAAGTTGCTTATCAGCTTAAGGAGATTTTGGGCTGAGACAAT<br>GGGGTTTTCTAGATATACAATCATGTCGTCTGCAAACAGAGACAATT<br>TGACTTCCTCTTTTCCTAATTGAATACCCTTTATTTCCTTCTCCTGCC<br>TAATTGCCCTGGCCAGAACTTCCAACACTATGTTGAATAGGAGTGG<br>TGAGAGAGGGCATTCCTGTCTTGTACCAGTTTTCAAAGGGAATGCT<br>TCCAGTTTTTGACCATTCAGTATGATATTGGCTGTGGGTTTGCCATA<br>GATAGCTCTTATTATTTTGAGATACGTCCCATCAATACCTAATTTATT<br>GAGAGTTTTTAGCATGAAGGTTGTTGAATTTTGTCAAAGGCCTTTT<br>CTGCATCTATTGAGATAATCATGTGGTTTTTGTCTTTGGTTCTGTTTA<br>TATGCTGGATTACATTTATTGATTTGCATATATTGAACCAGCCTTGCA<br>TCCCAGGGATGAAGCCCACTTGATCATGGTGGATAAGCTTTTTGAT<br>GTGCTGCTGGATTCGGTTTGCCAGTATTTTATTGAGGATTTTTGCAT<br>CAATGTTCATCAAGGATATTGGTCTAAAATTCTCTTTTTTGGTTGTGT<br>CTCTGCCCGGCTTTGGTATCAGGATGATGCTGGCCTCATAAAATGAG<br>TTAGGGAGGATTCCCTCTCTTTCTATTGATTGGAATAGTTTCAGAAG<br>GAATGGTACCAGTTCCTCCTTGTACGTCTGGTATAATTCGGCTGTGA<br>ATCCATCTGGTCATGGACTCTTTTTGGTTGGTAATCTATTGATTATTG<br>CCACAATTTCAGATCCTGTTATTGGTCTATTCAGAGATTCAACTTCTT<br>ACTGGTTTAGTCTTGGGAGAGTGTATGTGTCGAGGAATTTATCCATT<br>TCTTCTAGATTTTCTAGTTTATTTGCGTAGAGGTGTTTGTAGTATTCT<br>CTGATGGTAGTTTGTATTTCTGTGGGATCGGTGGTGATATCCCCTTTA<br>TCATTTTTTATTGCATCTATTTGATTCTTCTCTCTTTTTTTTCTTTATTAG<br>TCTTGCTAGCGGTCTATCAATTTTGTTGATCCTTTCAAAAAACCAGC<br>TCCTGGATTCATTAATTTTTTGAAGGGTTTTTTGTGTCTCTATTTCCT<br>TCAGTTCTGCACTGATTTTAGTTATTTCTTGCCTTCTGCTAGTTTTGA<br>ATGTGTTTGCTCTTGCTTTTCTAGTTCTTTTAATTGTGATGTTAGGGT<br>GTCAGTTTTGGATCTTTCCTGCTTTCTCTTGTGGGCATTTAGTGCTAT<br>AAATTTCCCTCTACACACTGCTTTGAATGTGTTCCAGAGATTCTGGT<br>ATGCTGTGTCTTTGTTCTCGTTGGTTTCAAGAACATCTTTATTTCTG<br>CCTTCATTTTGTTACGTACCCAGTAGTCATTCAGGAGCAGGTTGCTC<br>AGTTTCCATGTAATTGAGCGGTTTTGAGTGAGTTTCTTAATCCTGAG<br>TTCTAGTTTGATTGCACTAAAATTTTTAAAAAGTAAAAAAAAATACAT<br>GTGGTTTAATACAATTCATGCCAACTCATTCCCTCGTTTTTTGCTATA<br>AACCTTGCAAGGAGATGAATAATCCAAGGCTCTTGGATAAGATAAG<br>GGCCCCATCCATCTTGCTCCTCTCAGCCCTGGAGGAGGAGGGAGA<br>GTCCTTTTCCCCTGTCTACGCTCATGCACCCCCAATGAGTCCCTGCC<br>TCCAGCCCTGACCTCTGCCCTCGGTCTCTCAGGCAGATCCAGGGCC

	AGTTCTCCCATGACGTGATCCCTCTCGAAGGCAAGGCACCAGGCA<br>AGATAAAAGGATTGCAGCTGAACAGGGTGGAGGGAGCATTGGAAT<br>GGCACTCAGGGCAAAG
CYP1A1	CTGCACCTCTACTTCCCAGGCAGAGAAGGGGACAGAGGCCCTGCT<br>CCACACGGCGCCACTCCGGTTCCTCATGGATGGCGTCCGCATCTTC<br>CTCCAGGCCCAGGATGGCGTCTGTGGACAAGGGCAGCTCAAGCCC<br>CAGGCTCCACTACACCCCATCTCCCCCTTCACTCTCTCACTTGGCCC<br>GGGCTGGGCTCCCGGAAGAGAAGGCGACAATGCCAAGGCCTACCA<br>TTAAATAAGCGGCCAGCCAGGGAAGCAGGAGTCAGAGCCCAGCAC<br>AGCTGGAGGGGCCTTTGCTGGGGAGGGCAATGAATGCCAGCACCA<br>TCCACGTCGTCAGTGAAGCCTCACCGCAGTGTGTGCTGTCTGTTTT<br>TATAAGACCCCATCTAACAGATGAGGAAGGTGAGGCTCATAAAAGT<br>TAAGGAACTAAGTGGATTGGATGGAATTCAGCTCCATATGACTCCA<br>AAGCACAAGCTTTTTCCACCACGAGAGAGATTTGTGTCTAACCCTT<br>CCCATTTCTCAGATGACGAAACTGAGGCCTTGTGACAAAAAAGGA<br>AGTGCCTGAGACCACAGAACACAGGAGAGCACTTTAGGAGGGAGT<br>AACTGCAAGAAGGCCCAAGACTCTAGGCTGTGGCCAAATGAGGCC<br>AGAGCTTCCTGGTGGACAGGACCTAGGGGGCATTCCAAGGTGAGG<br>AAAGCAGGAAAGCAAAGATGTGAAGGTGAGAACCGGCCCACAGG<br>ACTGCGATGAAAGAATGCAGGATGGAGTATGTGTGTGAGAATTGCC<br>ACTGGCGGTGGTGGGGCGGGGAGGAAGGAGCCCTGCATGGTAGG<br>AAAAAGGAATGGGAGAGAAACTGAGGCAGTCTTGGCAGGAACTG<br>GGGGCAGGGAACTCAGAGGCCCTACTCGAGCTCGCTCCCCACCCT<br>CCAACCTGCCAGCCTGGAACCTGGCCCAGGGCCCAGACTCTGCCA<br>GGACGTTCTGGAGGCAGCTAATCCAGCCTGTTTGCTCTATAAAGGC<br>CAGGGATGGGGGTAGGGAGGCTCAGAGCTGCAAGGCCACAAGTC<br>CCAGGGCCTCTGGAAGCTGTCACTGCCTCTGAAATCCTTGCAGAA<br>GGCTTTCAGCCTTACAAGGCCTGTGGGTGAGACCTTGCCCCAGAC<br>CATGGGCTCCTTCAGCCTCACTCTGCCTGGTATCTCTGGGCTGGGC<br>ACAGAGGAACATCAGGGTGAACGAATGAATGAACAGACAATGCAC<br>ACATCACTGGGTGATCTTGGAGAAGGTGCCTCTGCTCCCTCACATG<br>ATCTGTGACACCCAGATTTGGCAAGGCCCATAGCAGTGAGTGTTGT<br>ATAAGTTTTGCCATCCTGGTCCTGAAGCAGGGCCCAGCTTGGTGCT<br>GTGAGAAACAGCCCACCTTTTTGTTACCTCCTGTGTCTCTATGAAG<br>GAGGACTGGAAGAAACCCTACTGACAGGGCCTATCATCTTAAAGG<br>AATATTTTGGGCTGGGCGCGGTGGCTCATGCCTATAATCCCAGCACT<br>TTGGGAAGCCGAGGCAGGCGGATCATCTGAGGTCAGTAGTTCAAG<br>ACCAGCCTGGCCAACATGGTGACAACCCGTCTCTACTTAAAAAATA<br>CAAAAATTAGGCTGGGCGCGGTGGCTCGCCTGTAATCCCAGCACTT<br>TGGGAGGCCAAGGCGGGCGGATCACGAGGTCAGGAGATCGAAAC<br>CATCCTGGCTAACAAGGTGAAACCCCATCTCTACTAAAAGTACAAA<br>AAATTAGCTGGGGGTGGTGGCAGGCACCTGTAGTCCCAGCTACTCG<br>GGAGACTGAGGCAGGAGAATGGCATGAACCCAGGAGGTGGAGCT<br>TGCAGTGAGNNNNNNNNNNNNNNNNNNNNNNNNNNNNNNNNNN<br>NNNNNNNNNNNNNNNNNNNNNNNNNNNNNNNNNNNNNNNNNNN<br>NNNNNNNNNNNNNNNNNNNNNNNCTTTCTATATATTCTTTGGGTAG

	ATTCTACGTAGACAGTCATGTAATTTGTTAATAAAAACAGCTTTACT
	TTTTCCTTTCCAATATGTATGTCTCTTATTTTTCCTTCTTGTTTTATTG
	TACTAGTTAGGACTTCCACTACATTGTTGAATAGGAGTGGTGAAAG
	AGGACATTCTTGACTGGTTCCAATATTGGGGAAGTGTTTAATCTCTC
	ATTATTAAGTACCATTCTTCTTTGTTAAAAAATCTTTCTGAATCTGTG
	CCATAAGTATGTCTCTTATGTAAGCCACAGTGATTTGGCTTTGTTAA
	CCAATCTAAAAAGATTTTTTTTTTTTTTAAACAGGGTCTCACTCTGT
	ACCCCAGGCTGGAGTGCAGTGGCCAAACATGGCTCCCCAGGCTCA
	AGTCATCTTCCTGCCTCAGCCTCCTGAGTGGCTGGGGCTACAGGCA
	CATACCACCACATCTAGCTAATATATAATATATATAAGTGTGTATAAGT
	GGAGTCTTGCTCTGGTGCCCAGGCTGGAGTGCATGGAATACAATGG
	TGCAATCATGGCTCACTGCAACCTTGGCCTCCCAGGTTCAAGCAAT
	TCTCCTGCCTCAGCCTCCTGAGGAGCTGGGACTACAGGCGCACGC
	CACCACACCCAGCTAATTTTTGTATTTTTAGTAGAGTCAGGGTTTCA
	CCATTTTGGCCAGGATGGTTTTCAACCTCTTGACCTCGTGATTCATC
	CGCCTTGGCCTCCCAAAGTGCTGGGATTACAGGCGTGAGCCACTG
	CACCTGGCCAAGAACTCACATTTTATAGACAGAAATCCACAGCCGA
	TCTGCTTTCTGTCTTTGCTTTGCCTGCTAAGAGGCTCAGAATTAAAT
	CCATGGCCCAACTCTCAGGTGGACAGACCTCACAGCATATGTTTTG
	GAAGCCATGCTCCTAGACTATTTCACGCTCCACTCTAATCCTCATTG
	TTCTTGCTCACTCCCTCTATCGTGTAGTTCCATCATCACAAACCCTT
	TTTTGAACAAGATCTGGTAGAAGACACACTCTTTGTAGGTCTGGGT
	TTGGAAGTCCCTGAATACAAACCTATGGGCTAAGATGCTCTCATGC
	CTGCTTCCACCCACAACACACATACAGGGAAGGGCTGGTGCCCAC
	TCACCTGGGATGAGGATGCTTCCCTCCAGGTAGCCCCCAGTTCTCA
	TGGCCAGCCACAGGCCCGCTGGGATGGAGCCGGGAGAGTGGAGG
	TTTGTGCTGGTCCACATTGCCTTCTGTGTGCCTGGGACTTAGTGTGT
	GAGCATGTGTCTGTGTGTTCACAGGTCAGAGGTAGAGGAAGGGCA
	GGGGTCCAAGGAGAGGCAGAGGAGAAGAGAGAAGTCCTGGGGCA
	GGGAAGCAGAGAGGCAGAGGAGAAACAGCTTGGGCAGGGGTGGG
	GGGTGTTGAGGCGGTGGAGCAGGAGACAAGCCTGGAAGGGGTCC
	TGGGGTGCCTGGCACTGTCCCAGGCCTTGCCCCACCCTGGACAGA
	GGATGGCTGTGACAGACCCACTCCCCACCAAGTCACAGCCCCTCC
	AGCACTTTGAGGGACCCTGGGCATAGCTGGTCCCACCCCTGCCCTC
	ACCTCCATGATGGCTCAGAAGGGTATCCAGCCCCAGGCAATGCCAG
	CCTTGGGCCTTTGAAGCTGAGCTTGTACTCAACCTGCGTAGAGGAG
	GACACCCGNNNNNNNNNNNNNNNNNNNNNNNNNNNNNNNNNNNNNNN
	NNNNNNNNNNNNNNNNNNNNNNNNNNNNNNNNNNNNNNNNNNNNNNN
	NNNNNNNNNNNNNNNNNNNNNNNCATGCCAAATGGCACTGGGGCT
	TCGTGTCGTGCCACAGCGTGGACCGAAAATGCGGACACATGCAGG
	CTGCCTCTCCTCGCAGGCAGAAGCCACACGCAGACCTAGACCCTT
	TGCACCGCATCCCCTTATTCAATCGCGCACCCGCCACCCTTCGACA
	GTTCCTCTCCCTCCACCCCAACCCCACGCCGCGCGCGAGGCTGGCC
	CTTTAAGAGCCCCGCCCCGACTCCCTCCCCCCTCGCGTGACTGCGA
	GCCCCCGCGCCGGGCCGGGGAATGGGTCGGCTGGGTGGCTGCGCG
	GGCCTCCGGTCCTTCTCACGCAACGCCTGGGCACCGCGCCTCCGG

	GCCAGGTGGGGCGGGGACGGGCCGCCTGACCTCTGCCCCCTAGAG<br>GGATGTCGCCGGCGCACGCAAGCTAGCCGGGGGTAGGGTGGGGGC<br>TCCGCGCCAGGTGCCCCCTCCGTGGTCCCTGGGCCCGAGTCTTTCC<br>GTGGCCCCCCGCCGCCGGATTTCTGTGCTCTGCCAATCAAAGCACT<br>AGCCACCCCGGGAGCCAAGAGGGACCCTCAAGGGCCGGTGGGTC<br>CTGGCTGGAGGGACCGCGCGTTGCAATCAGCACTAAGGCGATCCT<br>AGAGGCTGCGAGGAGCCGCTAGTGAGCGCTCAGCGAGCCTGCCCC<br>TTCGCCATCCATTCCGATCCTTCAATCAAGAGGCGCGAACCTCAGC<br>TAGTCGCCCGGGCTCTGGGGGACAGGTCCAGCCCCGCGGCGCCTC<br>TGGCCTTCCGGCCCCCGTGACCTCAGGGCTGGGGTCGCAGCGCTT<br>CTCACGCGAGCCGGGACTCAGTAACCCCGGGAAGGAGGTCACCAC<br>GGGGCAGCCCCGCCCCCGCCTGCCGAGTCCTGGTAGGCTGTAGCG<br>CTGGGGAGGCATCTGCACGCCCAGCGTTCCAGTGGGTGCAAAAAT<br>GACGAAGAGGAGTCCCCGCGCCCCAGGATGGAGCTTCCCGTACCC<br>TCTCTTCGGGCTGTCCTGGGACTTCTCCCTCAAGCCCCCTCCTCGG<br>CTGGGTTCTGCACTGCCCTTGGGACGCCTTGGAATTGGGACTTCCA<br>GGTGTTCCCAGCCCTCACCCCTCTATGTACAGGCACCGAGATGTGT<br>CCCATAGTGGGTTCTTGCCCACCCGACCCCCCACCCCCGCCGCCCT<br>CCGCCACCTTTCTCTCCAATCCCAGAGAGACCAGCCCGGTTCAGGC<br>TGCTTCTCCCTCCATCTCAGCTCGCTCCAGGGAAGGAGGCGTGGCC<br>ACACGTACAAGCCCGCCTATAAAGGTGCAGTACTTCACCCTCACCC<br>TGAAGGTGACAGTTCTTG
CYP27A1	CACTCTGGGAGGCTGAGGCAGGTGGATCATCTATGGTCAGGAGTTT<br>AAGACCAGCCTGGCCAACATGGTGAAACCCTGTCTCTACTAAAAAT<br>GCAAAAATTAGCCAGATGTGGTGACGGGCACCTGTAATCCCACCTA<br>CGTGGGAGACTGAGACAGGAGAATAGCTTGAACCCAGTGTTATAA<br>ATAAATTTTCAGTGGTGCCAAAGAAATAGCACTCAAACATAAATTT<br>AATTTTCTCAGCAAGGCAATTTTACTTCTATAGAAGGGTGCGACTTG<br>TGGATGGAGCAATGGCAAGAGACACCTGAATAAAGGAGGGGAAG<br>GGGTTCTTATTCCTGATGCAGGTAGCCCCTACTGCTGTGTCATTCCC<br>CTATTGGCTAGGGTTGGACTGCACAGTCTAAGCTAATTCCGATTGGC<br>TACTTTAAAGAGAGTAGGGACACAAGTCAGAGTGGCGGGGTGAGT<br>AGTTTGGTGGGAACAGGTGACTCAAGATGACTCAGGTCAGAGTAG<br>GTGACCAGGAGTGACTCAGGACGGAGCAGGTGATAGAGGCTAGGA<br>GGTGGTTGTTTACTGGAACTAGGGGCAAGGAGACGAAAACTTTAA<br>AATGAAGAGCTGAACATAACTGATACATTGATTCTTTGGAGAGGAT<br>CTCAGAACTCATTGTACTTAACAATTTACAGGCTAAAACCTTTGAA<br>GAGGAATTTATTATATCCTACACCAGGAAGCAGAAGTTGTAGTGAC<br>CCGAGATCGTGCCACTGCACTCCACCTGTCAGGGAAACATCTCAA<br>GCATGAGGTCATGAAAGTCAAGGCCCCCAGCTGCTGCCCTTGGGC<br>AACATGGGAGAGGTGAAATTTAACGTTTGCTTCTCCTCGTAGTGAT<br>TCCCCTCTGAAAATGTCCTAGCTTTTTGGTAAAGGAACGTGCACTG<br>TCTGGTGTGTGGGTCTTGTGAACCCTGAGGAGGTGGAGTGGCAGG<br>AGATGGGGGCTTTCTTCTTTCCCATTTCCTCCTGGTGTCCAGGGAA<br>GATGCCCGTTCACCTTGGGAGGACTGAGACTCGGAGCTCCAGGTA<br>AAACTGGTGGGCTAGGGGAGGTCCCCAAATGTTGGTGAGACCTTG

	ACCCTGGCCAGTATCCAGGTTCTTGACACCACTGCAAGAAGGAATT<br>CAAGGATGAGTCAGAAAATAGTGAAAATATGGAGATTTATTGCAGT<br>GAAAAGTACACATTAAAGAAAGGGGAGTGTGGCCGGCTCAAGA<br>GAGGCAGTTGCACCAAGAGACAGTTGCACCATAGGGTTTAGAGTT<br>ACCATCTTTATGGGTTTCTTTAACCAAGGGGTGAATATTCATGAAGA<br>TTTCTGGAAAAAGGTAAAGATTTCTTGGAACTATGGTATCATCCTTT<br>TTTTTTTTTTTTTTTTTTTTTTTTTTTTTTTTTTTTTTTTTTTTGTGACA<br>GAGTCTCTCTCTGTAGCCCAGACTACAACCTCTGCCTCCCAGTCCC<br>GGTTCAAGCAATTCTCCTGCCTCAGCCTCCTGAGTAGCTGGAATTA<br>CAGGCACGTGCCACCATGCCCAGCTAATTTTTGTATTTTTAGTAGAG<br>ATGGGGTTTTACCATGTTGGCCAGGCTGGTCTTGAACTCCTGACCT<br>GGTGATCCGCCCACCTCGGCCTCCCAAAGTGCTGGGATCACAAGT<br>GTGAGCCACTGTGCCCAGCTGGTGCCACTCATGTTTGCACCAAATA<br>TGGGTGCTCCTAGAACTGTCACAGCGCTGGTGGGTGTGTAATTTAA<br>TATGTTAATGAGTGTATAATGAGGTCCTAGGTGAAACCTAGGTCAAA<br>TCCAGTGCTGTGTTGGTTCCCATCAGTCTTAGCCAGCTTGGCCCAC<br>ATCTTGATTTTCGGGGTCTTCTTGGCCCTTGCAGCTATTTTAACAGT<br>TTCCTTTTGCTAGTTGTGTGAAGCTGCTGCCTGGAATTGTTTATTTC<br>CCTGCAACCACCGTGTATTATTCCTGCCTAATTTGACTGCCCGCAGA<br>GATTTTTATTCTTTATTTTTAAGGGTTGATGAGGGAGGGAGGAGGTC<br>AGTGTTTTATATTTACTTTTTGTTGATTAGGGTCATTGGCCATGTCTA<br>GTTAGGGCATGGAAATTCCTAGCTGCCTGTTTTAAGGGTCCCAAGG<br>GTGGGTTATTAGGAGAGTCTGAATCCGAGGCAGGGATTGGTTGGAA<br>TTTTTGTATGACCATTATTTTGACATGGAACTGCCATAATCCAGAAG<br>ACACAAACTTTATAAGGAGGTTAAACAAGCAAGGACCAAAGATTA<br>GTAGAAATTAGAGTTATTAAAGGTTCCAGGAAAGGTAGGAACTGTG<br>TCACACTTAGTAGGTATTTTTTTGATGAGGTCCCAGATGGTTTGAGC<br>AGTGTGCTTATTGAAATTATGTAGCCAGGTAGCATGTTGGTAAATCT<br>TTTTGTTTGTTTGTTTGAGACGGAGTCTTGCTCTGTCACCCAGGCT<br>GGAGTGCAGTGGCGTGATCTCGGCTCACTGCAAGTTCCGCCTCCCA<br>GGTTCACGCCATTCTCCTGCCTCAGCCTCCCGAGTAGCTGGGACCA<br>CAGGCGCCCGCCACCACGCCCGGCTAATTTTTTGTATTTTTAATAGA<br>GACAGGGTTTCACCGTGTTAGCCAGGATGGTCTCGATCTCCCGACC<br>TCGTGATCCGCCCTCCTCGGCCTCCCAAAGTGCTGGGATTACAGGC<br>TTGAGCCACCATGCCCGGCCGCGTGTTGGTAAATCTTTTGAGCTGG<br>CAGTCTAATTAATCCTGAGTTGTTAATATAAAGAACAGGTGTGGTTT<br>ATTACTGTGTAAACCCCTTTGTTTAGCTAGAAGATAAAGCCTTCTGC<br>TTTTTGATCATTTGAATGGCTTATGTTTTGGCCCTGTTAGGGCCTCAT<br>ACAATGGCTTAGCAATTCTGCAAAATCCTGCTATACCCAGGAAAGT<br>TCTTAGGTTTTTTTTTTGTTGTTGTTGTTGTTTATAGAGGTTTTACTT<br>CTAAGTAAACTTTTGTTTGATGGACAAGGTCCAGTTTCCTGAGTTT<br>AGAATATACTACAGGTATTTCACTTTTGGTAGAGAAATTTGGGCTTG<br>GATGGAGAGAACTGATTTTTTTTAAGGAAGTTTAGGACTTGAATGG<br>CATTTTTATTTGAGTCTTCTTTAGTGGGGCTGTATACAAAGATGTTAT<br>TTACATACTGGAGTGTGGCTCTCCTTTTTAGCTCTGGCTTTCTTAAC<br>TTTTGTGTCAAGGCATTTTTGAACAAGCAAGGGCTATCCCTAAAGT

	CATGAGGCAGTATTGTCCTGGTGTACCATTGGGTAATAGTAGTTTCA GGATTAGTCTACTTGAAAGGAAATAAGTTCTGAGAGTCGAAGTGCA AAGGAATACAAAAGAAAGCATTCTTATGATCTAACACTGTGAACTA ATTGGTGTTTTCTGGTATTTGGTTAAGTATAGTATAAGGATTTGGCAC TATAGGATGTAAGAGAATTATGACCTCGTTAATGGCTCCTAAGTCTT GAACTAGATCATATATATATATATATATATATAATTTTTTTTTTTGAGA TGGAGTCTCACTCTGTTGTCCAGGTTGGAGTGCATGGCACAATCTC AGCTCACTGCAATCTCCGCCTCCCGGGTTCAAGCGATTCTCCTGCC TCAGTCTCCCCAAGTAGCTGGGATCACGTGTGCCACCACGTTTTTA AAGCATGGCCTCCTGTCCTTCCTGTCCAAAAACCAAATGAAAAATA TGGTCTAGGTTGGGTGCTGTGGCTCACACCTGTAATTCCAGCACTT TGGGAGGCCGAGGTAGGTAGATCATCTGAGGTCAGGAGTTTGAGA CCAGCCTGGCCAACATGGCAAAACCCTATCTCTACTAAAAATACAA AAATTAGCCGGGCGTGGTGGCGGGCACCTCTAATTCCAGCTACTCT GGAGGCTGAGACCGGAGAATTGCTTGAACCGGGAGGCGGAGGTT GCAGCGAGCCAGGATTGCACCACTGCACTCCAGCCTGGACGACAG GGTGAGACTCTGGGGAGTGGGGAGGAGAGAATTAACAAACTATAT GACACAAAGGAACAAAAATACACAAGACAGGATAAGAGAGAGGA AGGATACAGGGAGAGCTAACATACATATAGCAGTTTGAAGTCCCGG GAAAGGAGAAAAAAATGGGGAGGAGCAATATTTGAACAGATAATA TCTGAGAAACTCCCCAGACTGATCAAAGACATCCAGTCACAGAGT CAAGAAACTAGGAGTCCTAGAATAAATAAGTAGAAAGCCACACCT AGCAACTCAGCTGTCAGCCTAATTGAACTTAATATGCCCTTTCTTCT CTGGCTGCCTGTAAATCACCAATTTTTCAAATGCTGTTAGAACCAG CTGTACCATCCTGCTGGGTCCTTCATTCCTGGCTTGAATGTGATCTT TGACCCTGTATTTATTTTATACTTGCGTGAGTTATGTTTTTAAACTTT TTGACAACTATACTTTGAATATACATGAAACATAGGATTCATAAGAC AATACTTTTTTTTTCTTCTTGCTGTACGTTTTCCGTACTATGTTACTC TTTCCATTTTATTAAAAATAATTCTGGCCATCATCTACGAAATCTGGT CTCAACCTGCAGTTTGAAATATCCTGCCTTGGCAGACGCGGCAGGG AGGTGCGTGGAGGGAGAATTGAATTAAAAAAAAAAACAACAAAA CACGAATATTTAGATTTCTTTGTTCAGCGGCCCCCCTCCAGGGATCA GATGACTGGCCCCCCTCGCTCCGAACTGACTCCGGGATCAATCCGG AAGGCCATTGGGAGAAGCCGAGGGCAGCTTAGCCACGGCCGGTTC CCGTTCCCTCCAGGACGCGAGGGTCGCCTTGGGTGGGGAACCGCG ACCGGGCGAGGACCTATCCCGGTGTGGGGCTTCCCGATTTCGAAA GAATCTCGCTGCACCCCCGCCCAGAGTTCAGACCAAGCGAAAAGT TATTTGAGAGGCCTCGGGGGCGCGGGGTGAGGAGTCGTGGCGGAG GCCTTGGTCGGGGCGCCGTGGATATCCCCGAGTCACCGCGTC
CYP2A6	CTGAAATGTGAAACACAGACATTTCACTCTCTGACCACAACCACTA ATACCCCAACTCCCCCACTGCTTCATTGCCAATGGCATGAGGGCTAT ACTGGCCAACTAGGTCTTTGCTTAAGGTTCCAAAGAGTGCAAACA CTATAGAAGGTCAAGTCTCCCTCCTATTAAAAGACTTTTTTTTTCTA ACATTTTGAAATCTTTTGTTACATGTTTACACGGAAGTATTTGAAGA AATACAAGGGAGTACAAGCAGTTTTTCTAATGTTTCATAAAGAAAA ACAGGTGGGGCCTAGCAGTCCAAAGTGGTGATTAACAGCATGAAA

	CTTACATTCACAGCCAGGCGTGGTAGCTCATGCCTGTAATCCCAGC<br>ACTTTGGGAGGCCGAAGTGGGCAGATCACCTGAGGTCAGGAGTTT<br>GAAACCAGCCTGACAAATATGGTGAACCCCGTGTCTACTAAAAATA<br>GAAAAATGAGCTGTGTCTGGTGGCTATAATCCCAGCTACTCGGGAG<br>GCTGAGGCTGGAGAATCACTTGAACCCAGGAGGCAGAGGTTGCAG<br>TGAGCCAAGATCGCACCATTGCACTCGAGCCTGGGCAACAAGAGC<br>AAAAGAGCGAAACTCCATCTCAAAAAAAAAAAAATTTACCATCACA<br>CAAGACCATACTAAGACTCTACACTCATTAGCTGTGAAATGCAGAA<br>GCAAGTCACTTATGACCGGCTTTGGGGGAACTTATCTAACTCATTTG<br>CAAAATGGAGATGATAAAGCTAACCTCAGTCTTGCAATCAGGAGTT<br>CAATTAAGATTATGCTCATAAAAAGCTTCCTGCAGTTCTGGGGGTTT<br>TTTCAAGCAGATAAATGGAGCTGGGATAGGACTGTTTTAATCCAAA<br>CACAGATGAGCACAGGAAAATATCACATGTTCACGTGTATGCAACA<br>TGGCCCACAAGTCTGCACACACACACCAGAATGAACACATGAAAA<br>CATCCACGTACTGGCCGGGCACGGTGGCTCACACCTGTAATCCCAG<br>CACTTTGGGAGTCCGAGGCGGGCGGATCATGAGGTCAGGAGATCG<br>AGACCATCCTGGCTAACATGGTGAAACCCCATCTCTACTAAAAATA<br>CAAAAAATTAGCCGGGCATGGTGGCAGGCACCTGTAGCCCCAGCC<br>ACTCAGCAGGCTGAGGCAGATGAATGGTGTAAACCTGGGAGGCAG<br>AGGTTCCAGCCGAGACCATGCCACTGCCCCCCAGCGTGGGGACAG<br>AGCAAGACTCTGTCTCAAAAAAAAAAACAAAGAAAAAAACATCC<br>ACATACTTATATGTAGTCACCCAATTGTGAATGTATACACACAAATAT<br>GAATATGCAGCATTCATCTTGTTCTACACAGAAATACACATCGTAGA<br>CACCCCTAAATCATGTGTGCACACATGCTGTATGCAGAGACAGACA<br>CAAAGATTGGCAAGCATTGAGAAACAGTGCCACACCCTGTCAGGC<br>ACACACACACATGTGCATGTGCAGACATAAGTTGGAATGGATACCC<br>ATTATCAAGCACAAACATACACACCACAAAGCCTGGTGCACACATA<br>AATTCATGTATCAACATGAACATGCTGAAACATACATACACGTAGAC<br>ACTAAAAGCATGCACCCAGAATGACACACACACAGTTGTATAAATG<br>AGGATAAACATGGCAGAGGCATCCAGGAGAACCCACTGGACAAAA<br>TGGCACAAAACACAAAGATGCATGAGCACACAGGGTGATTGAGGG<br>GAACACTGACTTGCAGGGAAGGAGAAAGAGAAAGATAGAAAGAT<br>GGGATCCAAGACAAGCATAGACAGACAGGTGGGCAAAGTCCTGTG<br>CAGGGCTCCAGGGCCAACCAGAATAACCTGAGAGTCTAATGCTGG<br>CCTGTCCTTCCGCAGATACTGCCTAACTCAGCACCACACTCCAGGA<br>GGCCATGAAGGATTAATTTCCTATTCACAGCTCCATGGGGTAAAGCT<br>TGGCTGTGCTGAGGAAGGGATGGCGAAGGAGGTTCCCAAAGACTC<br>TGTCCCTTTCCTCCTTCCCAAAGGAAAAGATTTGTGAGCTTTAGAC<br>TAGAGAATGTAAAAGCAAGGAATGTGCTTAGGGAACATCTCATCCA<br>TCCGCTTCATCCTACAGATTAAAGAGAACAACTACCATTCATTGAG<br>CACTTACTGCCATGTACCTCATCTGCATTAACACTTATACTTCTCCCC<br>TAAATAGCCAAAGAGGTAGGAGCTGTTCTTGGCTCCATAAGACTCA<br>GAGAGATGAAGTCAGCTGTTCAAAGCCACACAACAAAGAAAGGAT<br>AAGGTTAGGATTCCAATCCAGGCCTTAATCATTGCACCATCCATTCA<br>GAAAGGGAAACTGAGGCCCAGCAAAGGAAAGGGATCTTCCTGGG<br>CTCGCCACTAACTCACTCTGAAAGCCTGTTCCCAAGACACTGCCCA

	CTGGGACAAGTCCCTCCCACATGATTTCAGACACCATTCTCTCACT<br>CCATGCTCTAGGGTAATCAACAAGTCCAAAATTCATTGACCTCTATG<br>TCCACAAATACTAGAGTTCTCCCAACCTGTCTCTCCCCATCCCCTCC<br>CTGGCCAGGTCATGGCAACCTGCTGATCACTCAGTGCACTTCCTAG<br>TGCCAACCCCAGTGAGAAACAAACTCCCCAGATTTCTGTCTCCCAC<br>CTTTTTAAGAAACACGAAGGAAAAGGAGGAGCCAGGCCCAGGTG<br>CAGACTGGTGAACAGGATTAGAGCGATTGATATCTGCCAAGCTGGT<br>GCTAATCTTCCTTCCCACCTCTTCCCCTCCAAAATCAACAAACAAC<br>TTTCCTCTCTCTGCCTTGTTCTCTGACCTTTGTTCCCTCCACACAGG<br>TCTCAGAGCTCAGCATCCTAGAACAGTTTGAGCAAGATGTCAGTCC<br>TGGGGGTGCCAACCTCACCCACCTCCACTTGTCCAAAGTTGGTGA<br>CCCAGAGGACAGGTGCAACCCTAAATGGTCCAACCACCACAGCCT<br>GTCCTGTGGCCATCTGGGGCCCCTCTCAGGGTCTGGCTGAGGCTCA<br>TCTCAGACCCACATAGGACCAATATCAGCACCTGCCTCATGCTCATG<br>TCACAGCCCACTGGGACCAACCTCAGACCCTATATGGTGCCACCCT<br>TACGCCCTCCTGAAGCAGGACACTCTTATGCCCTCAATGACACCTG<br>AGACAACACACAGAGCCCTCTTGGCACCAAACATGACTTCAATATT<br>CTCCTGGTGTCAAGTTCAGTGCCAACTTGTTTCCCATCCTCGCATAC<br>ACCTGGGGGCAGTCCCAAAGCCAACACGATGCCTTTCCCTCCCTG<br>GCCATACCTGATGCTGATATCAGAACCCACCAATGTCATCAGTGTGT<br>CCACTGCTGATGCCAGTCCCAGCACTCACCTCCTGCTAAGGCTGGC<br>CTGCACTTGACTCCCAGGGTGATCTAGGCCAGTCAATGAAGGGGG<br>GTGAGGAGAAAGTTCACATCCCAGCTCCCTCACTTACTCCCTGTGT<br>GACCTCACTCAAGAGGATTTATGGAGTCTAGATCTGCTCATCTTCAA<br>AATGAGGATGATCATGATGAACCAACCTCATTGTTACAAAGATTCA<br>ACCAGATCATACACACCTAGACTTAATCTTCCCGTATACGACCATGC<br>TCACCAAAGGGTAACTGTCCTGTTTACTAAGGAAGAATTTCCCTGA<br>GGGAAGGACAGGAGGGCTACTCATCCCTTCTCCACTCACACCCAC<br>CCCAGGATCTGCCTCTGTGCCTAATACTGGAGTTTACCCCAATCTCT<br>TCTGCCACTGTTCCCTCTGCCTGCTAATAGTAGTAGCCCCTGACAAA<br>GCAGGAATCATTCTTAAAGGAGACTTAACTCACCCTCGAATGTGAT<br>CTTCTCTTCCCAAACACTCCCTTTCCACTGGCAGGAAAACCAAATC<br>CAGAAAGGGGAACTAAGTACACAGAGCAGGAGAGATGGGAGTTC<br>AGGGCCACTCACACATGTCCCCTGCCCACTGTCTGTTTTCTGTCCT<br>CTGTAGATCTTTATATAAAATGAGAAACATAAACAACAATCATAATA<br>TTAATAGGGATGATACAATCAATCTAGTGGGTTTCCCTGAGGATCTG<br>GGTTGGAAACCAGCGGACAACCCTTGGGACACTTGAGATTTTTCC<br>ACCATTTGGGGGTTGTTATTACTATTTCTCCAGACCTAGCCAATCCC<br>TCTGCCAACCGCTAACTCCAGGTACTCTTCTCCAGGTGTGGGGAAA<br>GTTCCCCTGAAATATGGCTCTGGTCTTCCTCCCCTTCCCAATCAGAG<br>ATGGGCAGTGGAGGTTCTATGGCACCCATCCTGGCCTCACTCTGAG<br>GTTCCAATGAGGATTCTGGGCATCAAGAGACAGCTCTGGGCAAAA<br>GCAAAATCAAGTCAGCCCCTGGACCCAGTGCTGGGCTGCTGGGCT<br>TTCTGGGAGAACCCGCTGGGCTTGCTACACACTCCTCCTCCCAGAA<br>ACTCCACACCCACAGCCCTGGGTCTTCCTAGCCCCGAGACTTTCAA<br>GTCCATATGCCTGGAATCCCCCTTCCTGAGACCCTTAACCCTGCATC

	CTCCACAACAGAAGACCCCTAAATGCACAGCCACACTTTGTCTTAC<br>CCTAATAAAACCCAGACCTTTGGATTCCTCTCCCCTGGAACCCCCA<br>GATCCACAACTTTGGGGTGCATTCTCACTCTCAGACCCCAAATCCA<br>AAGCCCAAGTGCTCCCCTATGCAAATATTCCAAACTCCTCAGTTCTA<br>CAGCTTATCTGTTGCCCCCTCCTAAATCCACAGCCCTGCGGCACCC<br>CTCCTGAAGTACCACAGATTTAGTCTGGAGGCCCCCTCTCTGTTCA<br>GCTGCCCTGGGGTCCCCTTATCCTCCCTTGCTGGCTGTGTCCCAAG<br>CTAGGCAGGATTCATGGTGGGGCATGTAGTTGGGAGGTGAAATGAG<br>GTAATTATGTAATCAGCCAAAGTCCATCCCTCTTTTTCAGGCAGTAT<br>AAAGGCAAACCACCCCAGCCGTCACCATCTATCATCCCACTACCAC<br>CATGCTGGCCTCAGGGA
CYP2C8	AAGAAATTTAACTCATTTCATACCCCCCATTCTCCCACTACCCTTCC<br>CATCCTCTGGTAACTCTACTGTCTATTAGAGAAGTTACTCTCTCATA<br>GTTACAAAAGGCTGGGAAGGGTAGTGGGAGGGTGGGGGTATCTGG<br>GGTGCTTAATGAATGCAAAAAAATAGTTAAAATAAAAGACCTACTA<br>TTTGATAGAAAACCAGAGTGATTATAGTCATTAATAATTGAATTGTA<br>CCTTTTAAAATAACTAAAAGAGCATAATTATATTGTAACTCAAAGGA<br>TAAATGCTTGAGGGGATGGATATTCTATTTTTTGTGATGTGATTTTTA<br>CACATTACATGCCTGTATCAACACATCTCATATACTCCATAAATAAGT<br>ACACTGACTATGTACCCACAGAAATTAAAAATTAAAGAAAAAAATT<br>AAATGAATTCAAAAACAAAGAGAGTCTCTCTACTGTAAACCTTACA<br>AAAAATAATGTGAAATCCTTACTCCTATATTAGAACAGAATAGAAAA<br>CCCATAGATAAAGCCACATACCTATGGCCAACAGATCTTTGGCAAA<br>GTTAACAAAGATATACACTGTGGAAAGGACACCCTATTCAATAAAT<br>GGTGCTGGGAAAATTAGCTCGCCATAAACAGAAGAATGAAATTGG<br>GCCCCTAATTCTTACTACATATCAAAATTAACTTAAGATGGATTAAA<br>GACAAATATGAGACCTAAAACCATAAAAATCATAGAAGAAAACCTA<br>GGAAAAATTCTGACATTGGCCTAGGCAAATAATTCATAACTAAGAC<br>CTCTAAAACAAATGCAACAATATCCTGTGACAATTGGAACTTTGCC<br>CAGTTTTTAATGGGGTTATTTGTTTTGTCTTATTGACTTGTTTGAGTT<br>CCTTATAAATTCTGGATATTAGCTCTTTTTTGAATGCACAGTTTGCAA<br>ATATTTTACTCTATTTTGTAGGTTGTCTGTTTACTCTGTTAATTATTTC<br>TTTGCTGTGCAGAAGCTTTTTAGTTTAATTAAGTCCCATTTGTCTTA<br>AAATGTTTTAGCAAAAATAAGAATCTAGATGTAAATCTTACACTTTT<br>CACAAAAGTTAACTCCAAATTGTTTATAGAACTAAATGTGAAACAC<br>AAAACTATAAAACTCTGAGAAGATAACATAGGAGAAAATATAGAAG<br>ACCTTGGCAATGGTTTTTCAAATACAACAACAAGTGCACAATCCAT<br>GAAAGAACAAATCAATAATCCGAACTTTGTTAAAACAAAAAAAAA<br>GTTTTACTCTGTAAAAGACACTATCAAGGGAGGGAGAAGTCCAGCT<br>AAAAGAGGAATAATAGTTCCAAAAGATACAAGAATATATCTTTATAA<br>GAATAAAACACTGTCATCCAAAATACCCAAAGAACTCTTAAAGCTC<br>AGCAACAAAAAAGTGGACACCCTCAATAGAAACATGGGCAAAAGA<br>TTTGAACAGACACCCCACCCATGAAGATAAACAGATGTCAAATAAG<br>CATGAAAACTGTTCAACATTACACATAATTAGGGGCCTGCAAACGA<br>AGACAACAATGACACATACCTTTATAATAATGAACATCTAAGGCACT<br>GATAAAACCAAATGTTGGCAAGGATTTGAAGCAATGGGAACTATTA

	GTTATTGCTGATATGAATGCAAAATGTCACAGACACTCTGGAAGAG<br>AGTTTGGTGGCTTTTACAAAACTAAAATATGCTTACCATATGATCCA<br>GCAGTTGTGCTCCTTGATATTTGCCAAATGAGTTGAAAACATATGTC<br>CACACAAAACTTGCACAAGGACATTTATAGCATCTTTATTCATTATT<br>GCCAAAATTGGAAGCAACCAAGATATCCTTCACTAGATGAACAGAT<br>AAACTGTGGTACATCAATGCAAGGGAATATTATTATATTACTATTTGG<br>TGGTAAGAAGAAATGAGCTTTCAAGCCATGAGACGACATGGAGAA<br>ACCTTCTGCATATTAATAAGTGATGGAAACCTGCCTGAAAAGGCTA<br>CATACTGTGTGATTCCAACTATATAACAATCTCTTATAGTAAAGGCA<br>AAACCATGGCAACACTAAAGTGAACTGTGGTTCACTTGTTACCAAG<br>GACTTGCATGGAAGGGAGAGAATACGCAGAGCATGGGGGGTTTTT<br>AGGACAGCAAATCTATTCTGTATGATGCTACGATGGTGGATATATGG<br>CATTATACATCAGTCAAATTTATAGAATGTACAACACAAACAGTAAA<br>CCCTATGTGAACTTCGAACTTTGGTTGATGATTATGTCTTATTGTTGG<br>CTCACTGACTGTAACTAATGAACCCCACTGATGGGGGTTGTTGATT<br>GTGGGAAAGACTATTTGTAGGTGTGGTGAAGGGGTTTGTGGGAAC<br>TGTTGCTCGGAGCCTAAAATTGCTATAAAAATAAATTCTATTAATTA<br>AAAAAAAACCCCGCTCTAATTCACAACCCTATCCAAGCTGAGGTGG<br>GTGTCAATCTGTGACTGTAGAGGGCTTAATTAATGCTAATACACTCT<br>TGATCATTTGGACTTAGGTGGGAATTCTATAGGATAGGGTATTTCAC<br>TGAACCACCAAGCAGGAAAACTGGGATTCTAATACAAAATCTTTTA<br>CTGGTGCATTGATAATGCTCTAACTCACTGAGTCACCAATTGCTCAT<br>TCCTGAAAAACAAAGCAAAATTAAATTAGTAGATCTCAGAGATCCC<br>GTCTGTCTTTAAATTATCTATGTTCCTTTTATTCTATAAAAAGAAAGG<br>TCAAGGCAGGAGCCTCAGCTCAGGAGAAGAAACAAGGAGCAGAG<br>CAAGGGCAACTGTTTCTCAAGGAATAAAATTATTGCTCTAAAGAGA<br>GAAAGTGAACTTATTTTATCCAAATAAACTAGCTTATACCTACGTGA<br>GTGAGGCAGCAAATTACTACTTCCCTTTGCCCTGGATAAAGGGTTC<br>ACCAGGACCTGGACTCACTCACCTTTTAAAGGTTATAAAACCAAAC<br>ACGTCTGACCCACATTTTACTCAACTGGTGCTAGAATTATTAACTAA<br>ATTAATGTTTATTTTGAAAGTCACTGATTAGATTAATCCACAAGTATT<br>GAATTTTAGTCAATCTTGGTGGCCCGGTTTAACTGGATGTTTTGCTT<br>AAAAGGAAGGCAGCAAGATGCAGGGGTTATGGTTTCCAGCCCCAG<br>CTTGGTCACTTGCATTCTGTGTGTCCTTAGCTAAAGTACTGAATCTC<br>CATGGTCTAACTTTCTCCTCTCTAAACTGGGAATAATTTTACAGTGG<br>GCAAAGATAATTGAGAGAATAAAAAGAGATGTGATGAGTGTGAAA<br>ATTCTCTGTAAATTTGTCATAATGTCTATAAACATAATCGATAAAACA<br>TTGTATAACTGGGTCTTAATATTTTCTTAATGAAAGAGCTGGAAATA<br>ACTGTACTGGTCAATTTAGAATAAAGGTAATCTTTTCAGAGCATGCC<br>TTTGTATACACACTTTGTTATTAGTGATCTAGTAATGTTCATAAATCC<br>AGTTGTATTTAGATCTTCATGACCATTGACTATCAGTTCCCATTTCAG<br>GTCTGCACATTGCAGTGGTTCTGTGCCCTGGGTCCATTCAGTGATTT<br>CCCTGTGTTCCATCTTCTGTTGAATCCACAACTGTTGTTCTGTGTAT<br>AATTTCTCTTCCTTGCTGTGTATGATTACATTCTATTATTTGTAACAAT<br>AACAGACCAAAAACAATAGAAGCAGCCATGTCTGGAGGTGACTGG<br>AAGGTGGAGAAGCCATAGATTTTCAAGCCCTGTGCCATAAATTATG

	TGAGATTGGCCCTTTCCTTAATAGTGCTGAACAACTTTCACTTGTGA GGTGATGCAGAGGGGAGAACTCTAATTTTTATTTCTTCTTTTGAGCG TCTCCGGTCCTCTTATCCTTATAAACAAATAACGGACTTCTATTTAAT GTGAAGCCTGTTGCTTTCTGAACAGAGTCAAGGTGGCGTATCTTCA GAGTAACTAATGTCTGGGGTTTGTTTTGTTTTTCTAAAATTGTTCTT GAGCCAGCTGTGGTGTAAGTGGTAATGAACCCCAATGGGTATCAGA AGATCTCTGCTCAAATCCCGGTTTTACCGGCAATGAGCTGTGTGGC ACTGACAGGTGTCCTGTTCTCCCAGAGTTTCTTTCCCAATTTGAAA AATAAAAAATGATAATCTTTATACTCCAGTCTCTTTTAATGATGAATA TACATTTATATATATACTTTTATATATTTAATATAATATTTAATAGTATAA ATATGTATTTATGTTATTATTATGTAATAATGTATGTAACACTCCCTGC TAATTCAGTTTGTCTCTTTGACATGTAAAGTAAATAATCACCTATTAT TATAATAATGTAATAATAACACAAATATTATTATGTAATAACATATATAT TTATGTATATTGTTTATATACATTTAAATATATATAAATATACATTTATTA GCTAATAATTTGATATATGTATGGTAATTCAACATGTATGAGTTATATT CACTATTTCATGTTTAGGCAGCTGTATTTTAAGTGAACTATACTAAAT ATTTGAAAGGCTTTTGTTATCAAGGGCTAAGTCTCCTATTTTTTGATA TAGCATTACAATGTACATTTTTTATACACAAAATATAGAATACACTGA TTTCCCTCAAGGTCATAAATTCCCAACTGGTCATTAATCTGAGAATA TTGAATTTTGAGTATATTCTAACATAGAATCATTTACTTCAGTGTTTC TCCATCATCACAGCACATTGGAACAACCAGGGACTTTTAATTAAAA ATACCTGGGCTCCAATCCAATACAATTAAACCAGAATCTCCTAGATT GGCACTGGAAAGAAGGAGTAGGACAAAAGAACATTTTATTTCTATC CATGGGCCAAAGTCCACTCAGAAAAAAAGTATAAATTGGATCTAGG TGATTGTTTACTTTACATGTCAAAGAGACACACACTAAATTAGCAG GGAGTGTTATAAAAACTTTGGAGTGCAAGCTCACAGCTGTCTTAAT AAGAAGAGAAGGCTTCAAT
CYP2C9	CCAAAAATATGAAATCCCTAGGTGGAAGTCTGTCAAAATACATATAT CACTTGAAGAAAACTACAAAACTCTGATGAAAGAAAACAAAGATA TAAATAAATACACAAATATTTCATGTTCATGGATATGAAGACCGTTG TTAGTCTTTTTGATTTGATCTATAGATCAACACAATAAGAATCAAAC TCCAGGAAAGTTTTATTATTTTTATTATTTTTTATCTTTAGATGCTAAC AATCTGATTGTAAAGTTTATGTGGAGAGGCCAAAAAACCCAGAATA GCCAACGTTAAGGAGAACAAAGTCAAATGACTGACCTTACCCAAT TCAACTTCAAGACTTACTATGAAGCTAATCAAGACAGTGTGCAATT GGTGAAAAAAGTCAAATAGATTAATGCAAGTGAGTAAAGAGTCTG AAGTAGATCATCAGAAATATAGTCAACTGATCTTTGAAAAAAAAGG AAAGACAATTCAATGGGGGAAAAATGGTCTTTTCAACGAAGACTA ATGGAGTAACTGGAGGTTCACATACGGAAAAATGAATCTGGAAATA GACCTTACACCTTTAATGAAAGTTAACTAAAAATATATTATAGATCTA AGCCGGGTGCAGTGGCTTATGCCAGTATTCCCAGTACTTTGGGAGG CTGAGGTAGGCAGATCACAGGTCAGGAGATCGAGACCATCCTGGC TAACACGGTGAAACCCCCATCTCTACTAAAAATACAAAAAATTAGC TGGGCATGGTGGCACGCACCTGTAGTCCCAGGTTGTCAAGAGGCT GAGGCAGGAGAATCGCTTGAACTCAGGAGGCAGAGGTTGAAGTG AGCCGAGTTGAGAGCCACTGCACTCCAGCCTAGGTGACAGAGTGA

	GACTCAGTCTCAGGAAAAAAAAAAAAAAAAAAAAAAAAATATATATAT<br>ATATATATGTGTATATATATATATATGTGTATATATATATATATGTGTATAT<br>ATATATATATATATATATACGTGTGTGTATATATATATATACATATATATAT<br>TAGATCCAAATATAAAATGCAAAACTATGAATCTCTGATAGTATAAG<br>ATAGGAGAAAATAAAGATGACCTTGATCAGCAATGGTTTTTAAAAT<br>ACTACAATGAAGGTATAATCCATGAAATAAAGAATGACTAAGCTGG<br>ACCTTATTAAAATTTAAACTTCTACTCTGCAAAAGACACTATCAAGG<br>GAATGAGATGAAAAGACAGAGACTGGGAGAAATACTTTCAAAAGA<br>TATATCTGATAAACGACTTCTCTCCAAAAATTTTTAAAACTCCACAA<br>TTAGAAATCAGACACCTTGATTTAAAAAATGGGCAACAGATCTGAA<br>CAGACACCTCACCCAAGAAGAAATACAGATGGCAAATAAATATATG<br>AAAAATGCTCATCATTAAACGTCACTACGGACCTGCAAACTAAAAC<br>CCCAATGAGATACAACTACACACTTATACAATGGCTAAAATCCAAA<br>ACACTGATAAAACCAAATGTTGACAAGGATGTGCAGCAATGAGAA<br>CTAAGTCATTGCTGGTGGAAATGCAAAATGATAAAGACACTTCAGG<br>AGACAGATGGGCAGTGCCTAAAAAAACTAAAATATGTTTACCACAT<br>TATCTGGTAGTTGTGCTCTTTGGTATTTGCCAAATAAGTTGAAAATA<br>CATTTCCACACAAAAATCTGCACATGGATATTTATAGCAGCTTTATTC<br>ATCATTGTCAAAAATTAGAAATCACCAAGATGTTCTTCAATAGATAA<br>ATGAATAGACAACGTGATAAACCTATACAATGAATGTTATTTAGTGT<br>TAACAAGGAATGAACTATCAAGCCATGAAAAGACATGCAGAAACT<br>TTATACATATGAGTAAGTTAAATCTTAAAAGGCTACATACTGTATGAT<br>TCCAACCGTATTACATTTTGGAAAAGGCAAAACTATGCCTACACTG<br>CAAAGATTATTTGTTGCTAGGGCTTGCAAGGAAGGGAGAGAGAAC<br>ACGTAAAGCACAGAGGTTCTTAGGGCAGTAAAGCTATTTTATGTGA<br>TATTACAATGGCACATGTCATTATGCATTAGTCAAATCCCATAGAATG<br>TACAACACAAAGAATGAACCCTACATAAACTATGAGCTTTGGTTGA<br>TGATGATGCGTCAATGTTGGTTCATTGATTGTAACTAATGAGAGTTC<br>TTGATTGTGAGAGAGACTGTGTGTGGGTGCAGGGAAAGAGGCATG<br>TGGTAATTATATACTTTCCACTTAGTGTTGCCAGAAACCCCAAACTG<br>TTTCAAAAGCCTACTCTAATCCACCATTCTAGTCAAGCTGAGGTTTG<br>TATTACTCAGTGACTGTGGAGGGCTTAATGTTGATACTCCCCTGATC<br>ATTTGGACTGAGATGTGAATTCTATGAGATGGGATGTGACATGTCAC<br>TGAATTGGGAGTTGAAAAACTGGGATTCTAAGAAAAGTCTTGGCT<br>GGGCGTTGTGGCTCATGCCTGTAATCCCAGCACTTTGGGAGACCTA<br>GGTGGGTGGATCACAAGGTCGGGAGATCAAGATCATCCTGCCCAA<br>CATGGTGAAACCCTGTCTGTACTAAAAATACAAAAAAATTAGCTGG<br>GCGTGGTGGTGGGCACCTGTAGTCCCAGCTACTTGGGAGGCTGAG<br>GCAGGAGAATGGCATGAACCCAGGAGCTGGAAATTGCAGTGAGCC<br>GAGATCGCACCACTGCACTCCAGCCTGGGTGACAGAGCGAGACTC<br>TGTCTAAAAAAAAAAAAAGAAAAGAAAAAGTCTTATACTGAGGCA<br>TTGTGATTGTGATACTTTGTCTCACTGAGTCAATAATTGCTCATTTCT<br>TAAAAAAAAAAAAAGCAAAGTTTAGAGTAGTTGATCTCAGATATCCC<br>TTCTATCTACACATTATCTATAATTCTTTCTTTCTGTAAACTGAAAGG<br>TCTAGGAAGGAGCCGCAGCTCAGCAGGAGAGAGGAGGAGCTGAG<br>CTGGGACCCCTACCTCCTGAGGAATGAAATGATTATTATAAAGACA

	GCAACCGAGCTTATTTTACCCAAAATAAGGTAGTATATTTCTGTTAG<br>AGTTTAGAGTTTCATGAGTCAGGGACCAAGTTATTGCTTTTCTTTGC<br>CCTGTATAAAGGCTTCTCCAAGGCCTTTGACTTACCTAAGTACTAAA<br>TGTTATAAAACCAAACTCTTCTGACCTCTCAATCTAGTCAACTGGG<br>GCTGTAATTATTAATGAAATTAATGTTTATTTTGAAAATAATTTACTA<br>GACTGAATTACGAAATCCTGAATCATTGTACACTATCAGTAAATATT<br>GGTGGACCCAACTGAACTGAATGTTTTGCTTGAAATGAAACCTTTG<br>AGATGCAGGGCTTATGGGTTCTAGTCCCAGCTCTAGCACTAGCAGA<br>CAGCATGTTCTTGGCTAAGATACTGAATCTTCAAGGCTCAGCTTCCT<br>CATTCCGGAAATGGGTCAATTTTATTGTAAGCAGAGGTAATTGAGA<br>GATTCAAAAGGGACATGAGGTGTAACAATTCTCTGTAAATTGTTAG<br>AATCCCTGTTAAAAATGACCAGTAAAGCTTTGTGCAACTGTGTCTT<br>GACATAACTTTATTTTTCTTAATAAAAGAAATGGAAATAACCTCACT<br>AGGGAATTTAGAACAAATATGATGATATCTTTAAAGAAAATGGCTTT<br>GCACAAGTATTGACATTAATGATCTAGTAAAGTGTATCTTTCTAGTT<br>GTATTTAGATCCTCAACTCAGTATGTCAGCTCCTGTTAAGGTCTATA<br>CATTGTGGTGGTTCTGTGCTGTGGGTCCATTTAGTGATTTCCCTACC<br>TCCCATCTTTTATTGCATCCACAACTGTGGTTCTGTCCATAATTTCCT<br>TTGCTTTCTGTGCATTATTACATCATATCTGAAAATGAGAAACCAAA<br>AACAATAGAAAGCAGCCATGTCTGGAGGTGACTGGGGGGTCGAGA<br>AGCCCTAGTTTCTCAAACCCTTAGCACCAAATTTTTCCCTCAGTTAC<br>ACTGAGCGTTTCACTTCTGCAGTGATGGAGAAGGGAGATCCCTTAT<br>TTCTTCTCATGAGCATCTCTGGTGCTGTTTCCCTTAGAGACAAATAA<br>GGGGTTCTATTTAATGTGAAGCCTGTTTTATGAACAGAATAAATGTG<br>GTGTATATTCAGAATAACTAATGTTTGGAAGTTTGTTTTATTTTGCTA<br>AAATTGTTCTCAAGGCAGCTCTGGTGTAAGAGATAATACACCACGA<br>TGGGCATCAGAAGACCTCAGCTCAAATCCCAGTTCTGCCAGCTATG<br>AGCTGTGTGGCACCAACAGGTGTCCTGTTCTCCCAGGGTCTCCCTT<br>TTCCCATTTGAAAAATAAAAAATAACAATTCCTGCCTTCAGGAATTT<br>TTTTTAGGGGGTTTAATGGTAAAGGTGTTTATATCTGCTAAGGTAAT<br>TTACTTGATATATGTTTGGTTATTTAAGATATATGAGTTATGTTAGCTA<br>TTTCATGTTTAGGCTGCTGTATTTTTAGTAGGCTATATTAAATATTTG<br>AAAGGATTTCATTATAAAGAACAAAGTCTCCTAATCTTTGATATAGC<br>ATTGACATACTTTTTAAATATACAAGGCATAGAATATGGCCATTTCTG<br>TTAAATCATATATTCCCAACTGGTTATTAATCTAAGAATTCAGAATTT<br>TGAGTAATTGCTTTTGCATCAGATTATTTACTTCAGTGCTCTCAATTA<br>TGATGGTGCATTAGAACCATCTGGGTTAACATTTGTTTTTTATTACCA<br>ATACCTAGGCTCCAACCAAGTACAGTGAAACTGGAATGTACAGAGT<br>GGACAATGGAACGAAGGAGAACAAGACCAAAGGACATTTTATTTT<br>TATCTGTATCAGTGGGTCAAAGTCCTTTCAGAAGGAGCATATAGTG<br>GACCTAGGTGATTGGTCAATTTATCCATCAAAGAGGCACACACCGA<br>ATTAGCATGGAGTGTTATAAAAGGCTTGGAGTGCAAGCTCATGGTT<br>GTCTTAACAAGAAGAGAAGGCTTCAATGGATTCTCTTGTGGTCCTT<br>GTGC
CYP2D6	GCTCATTTTTGTCTTTTTAGTAGTGATGGGTTTCGCCATGTTGGCCA<br>GTCTGGTTTCAAACTCCTGACTTCACGTGACCACCAGCCTCAGCCT

	CCCAAAGTGCTGGGATTACAGGCGTGAGCCACCGAGACCAGCCTC<br>ACCTCATTCACTCTTACCTGGACGCCTGACTTTACTTGAGATACAGG<br>CATAGTGATTCTCAGCAGGAAACAGCCTGCCCCCACGTCACGCCCA<br>GAGACCCATCACTGGCTGCCTGGCTTGGTGACAAAGTCCATGCGTA<br>AGTCTTGGCTGGGGTGGATATGAATAGGCATATGCCAAGAATCAAC<br>CCATTCCCTGGCTAGGGTGGGAGACTGTGTTGTGCTCCCCCAGACC<br>ACCCTCAGGTTCAGTGATTTCTAGAAGGTCTCACAGCCCTAGAAAA<br>GCTGTTATTCTCCCTGTTAACAGTTTATTACAGAGAAGGGTACAGAT<br>TAAAGTCAGCAAAGATGAAAGGCACAGGGACCAGAGTCCAGAAT<br>GACCAGGCCAAGGCTGCAGCTCTCTTTTCTGGTGGACTCCTACAGG<br>CAGTGCTTAATTCTCCCCCAACAGTAAGTGAGGCAGCAGAGAGCC<br>CTGCCAGCCACGGAAGCTCACCTGGGCCTTGGTGTCCATGGTTTTT<br>GTTGGGAGTTGGTCATCCTAGGCTTGAGCCCCCGCAGCATGGCTGA<br>CCTCAGTTACTCAGTCTCCAGCCCCTCCTGAAGTCAGATGGATACA<br>GGCCTGACGGCCCCACCCTCGATCACATTGTTGGCATAAACTGTGT<br>TGTACGGTCCAAGGCCCTAGCTATGTACAAAGACACTATTTCAGGC<br>AGGACATTCCAAGGCCTTAGCAGATATCTCCCAGCCTCCTGTCAAG<br>AGTCAGTTTGGACTCTTGGTCCAGTGGCTTGCATTGTGCAAGGAAT<br>GACTTCCCCACTTTTTACTACACAGGCCACCCCTCTTGGCTCTAAC<br>AGCAAAATGATATTAGTTTGAGCATCTGTGTGTGTGTGTGTGTGTGT<br>GTGTGTGTGTGTGTTTTCTTGAGACAGGGTCTTGCTCTGTCACCGA<br>GGCTGGAGTGCAGTGATGCCATCAGGGCTCACTGCAGCCTTGACTT<br>CCTGGGTTCAAGCAATCCTCCCATCTCAGCCTCCCTAGTAGCTGGG<br>ACTGCAGGCACATGCCACCATGCTTTGCTAATTTTTGTATTTTTTGT<br>AGAGACGGAGTTTCACCATGTTGGCCAGGCTGCTTTCGAACTCCCT<br>ATCTCAGGTCATCTGACTGCCTCAGCCCCCCAGAGTGCTGGGATTA<br>CAGGTGTAAGCTACTGTGCCCAGCCAAATTTCCTTCCTAATTTCTTC<br>ATTGAACCACTGGCCATTCCGGACCATATTGTTTAATTTTCACGTGT<br>ATGTATAGTTTCCAGAATTCCTCTTGTTGTTGATTTCCACTTTTATTC<br>TGTTGTGGTCAGAGAAGATGCTTGATATTATTTTAACATTTGTAATGT<br>TTTAAGACTTGCTTTGTGACCTAACATATGGTGTATCCTTGAGAATG<br>ATCCATGTGCTGAGGAGAAGAATGTGTATTCTGCAGACTTTAGACG<br>AAGTGTTCTGTAAGTATCTAGTAGGTCCATTTCTTTTGTAGTGCAGA<br>TTAAGTCTAATGTTTTCTTATTGGGTTTCCATCTGGGACACCCGTCC<br>AATGCTGAATGTGGGGTGTTGACGTCTTTAGCTGTTATTGCGTTAAC<br>GTCTCTCTTGGGCTCCAATAACATTTGCTTTACGTGCTCCAGTGTTG<br>TGTGCATATGTATTTACAATTGTTATATTCTGTTGCTGGATGACCTTC<br>TTTGTCTCCTCTTACAGTTTTTTTGGTTGTTGTTGTTTGTTTGTTTTG<br>TTTTGGAGACGGAGTCTCGCTCTGTCACCCAGGCTGGAGTGCAGT<br>GGCGCGATCTTGGCTCACTGCAAGCTTCGCCTCCCAGGTTGACGCC<br>ATTCTCCTGCCTCAGCCTCCTGAGTAGCTGGGACTACAGGCGCCCG<br>CCACCACGCCTGGCTAATTTTTTGTATTTTTAGTAGAGACGGGGTTT<br>CACCATGTTAGCCAGGATAGTCTCAATCTCCTGACCTCGTGATCCGC<br>CCGTCTTGGCCTCCCAAAGTGCTGGGATTACAGGCGTGAGCCACC<br>ACACCCGGCCTCCTCTTACAGTTTTTGTTTTAAAATCTGTTCTGTCT<br>AAGTATTGCTACTCCTGCTCTTTTTTGTTTTCCATTGGCATGGAGTAT

	CTTTTTCCATCCCTTTATTTTCAGTCCATGTGTATCTTTACAGGTGAA<br>GTGTGTTTCTTCTAGACAAAAGAGCATTGAGCTTTGCTTTTTCATCC<br>ATTCAGCCACTCTGTGTCTTTGTATTGGAGAGTTTAGTCCATTTACA<br>TTCAATGTTATTATTGCTAAGCAGGGACTTACTCCTGCTATTTTGTTA<br>TTTCTTTTCTCACTGTTTTGTGGTCTTCTCTTTTTTTTTTTTTTTTTT<br>TTTTTTTCCTTGTCTTCCTTTTAATGAAGGTGATTTTCTCTGGTGGTA<br>TGATTTAATTTCTTGCTTTTTTGTGTGTGTGTATCCATTGTGTGTTTT<br>TTCTTCTTTTCTTTTTGAGACACAGTCTCACTTATTGTGTGCTTTTTG<br>ATTTGAGGTTGCCGTGAGGCTTGCAAATATTATCTTATAACTCATTAT<br>TTTAAACGGATGACAACACTGATTGTGTAAACAAACATAAAGCAAA<br>AGGAAGACTAATAAAAACTCTACACTTTAAGTTCATCTTAGTGCTTT<br>TTAACTTTTTGTTGTTTCTCTTTTTTTGTTTTTGAGATAAAGTCTTGC<br>TCTGTTGCCCAGGCTAGAGTGCAGTGGCACGATCTCAGCTCACTGT<br>AACCTCCACTTCCCAGGTTCAACCGATTCTCCTGCCTCAGCCTCCT<br>GGGTAGCAGGCGCCCACCACCATGCCCAGCTAAATTTTTTGTATTTT<br>TAGTAGAGATGGGGTTTCACCATGTTGGCCAGGCTTGTCTCGAACT<br>CCTGCCCTCAGGTGATCCACCCACCTCAGCCTTACAAAGTGCTGGG<br>ATTACCTGCGTGAGCCACCGGGTCCGGCCTCTTTATGTCTTACTGTA<br>CTGTCTGTCTTGAAAAGTACTTATTATTTTTGATTGGTTCATCATTTA<br>GTCTAATTAAAATAAGAGTAGTTTACACACCACAATTACAGTATTAT<br>AATACTCTGTTTTTCTGTGTGCTTACTATTACCAGTGAGTTTTGTACC<br>TTTAGATGATTTCTTCTTGCTCATTAATATCCTTTTTTTTTTCAGATTG<br>AAAAACTCCCTTTAGCATTTCTTGTGGGATATAGGTCTGGTGTTGAT<br>GAAATCTCGCAGCTTTTGTTTGTCTGGGAAGGTCTTTATTTCTCCTT<br>CCTGTTGGAAGGATATTTTTGCCAGATACGTTATTCTAGGCTAAAAG<br>TTTTTTTTCCTTCAGCACTTTAAATATGTCATGCCACTCCCCCCTGGC<br>CTGTAAGGTTTCCACTGGAAAGGTGGCTGCCCCATGTCATGTATTG<br>GAGCTCTACTGCATGTTATTTGTTTCTTTTCTCTTGCTGCTTTTAGGA<br>TCCTTTCTTTATCCTTGACCTTTCGGAGTTTAATTATCAGATGCCTTG<br>AGGTCGTCTTCTTTGGGTTAAATCTGCTTGGTGTTCTATAAACTTCT<br>TGTACAAAAAATCAGCCAGGCATGGTGGTGGGCACCTGTAATCCCA<br>GCTACTTGGGAGGCTGAGGCAGGAGAATCGCTTGAACCCTGGAGG<br>TGGAGGTTGCAGTGAGCCGAGATCGCATCATTGCACTCCCACCTGG<br>GCGACAGAGCAAAACTCCGTCTCAAAAAAAAAATTATTTGGGCTC<br>GGTGGTGCCTGTAGTCCCAGCTACTTGGGAGGCAGGAGGTCCACT<br>TGATGTTGAGATTGCAGTGAGCCATGATCCTGCCACTGCACTCCGG<br>CCCGGGCAACAGAGTGAGACCCTGTCTAAAGAAAAAATAAAAATA<br>AAAAAGCAACATATCCTAAATAAAGGATCCTCCATAATGTTTCCACC<br>AGATTTCTAATCAGAAACATGGAGGCCAGGAAGCAGTGGAGAATG<br>ACGACCCTCAGGCAGCCCTGGAGGATGCTGTCACAGGCTGGGGCA<br>AGGGCCTTCAGGCTACCAACTGGGAGCTCTGGGAACAGCCCTGTT<br>GCAAACAGGAAGTCATGGCCCGGCCAGAGCCCAGAATGTGGGCTG<br>AGCTGGGATCCATGTGACAGCTTTGAGGCTCACCGGGAGCAGCCT<br>CTGGACAGGAGAGGTCCCATCCAGGAAACCTCGGGCATGGCTGGG<br>AAGTGGGGTACTTGGTGCCGGGTCTGTATGTGTGTGTGACTGGTGT<br>GTGTGAGAGAGAATGTGTGCCCTGAGTGTCAGTGTGAGTCTGTGTA

	TGTGTGAATATTGTCTTTGTGTGGGTGATTTTCTGCATGTGTAATCGT<br>GTCCCTGCAAGTGTGAACAAGTGGACAAGTGTCTGGGAGTGGACA<br>AGAGATCTGTGCACCATCAGGTGTGTGCATAGCGTCTGTGCATGTC<br>AAGAGTGCAAGGTGAAGTGAAGGGACCAGGCCCATGATGCCACTC<br>ATCATCAGGAGCTCTAAGGCCCCAGGTAAGTGCCAGTGACAGATAA<br>GGGTGCTGAAGGTCACTCTGGAGTGGGCAGGTGGGGGTAGGGAA<br>AGGGCAAGGTCATGTTCTGGAGGAGGGGTTGTGACTACATTAGGG<br>TGTATGAGCCTAGCTGGGAGGTGGATGGCCGGGTCCACTGAGACC<br>CTGGTTATCCCAGAAGCCTGTGTGGGCTTGGGGAGCTTGGAGTGG<br>GGAGAGGGGGTGACTTCTCCGACCAGGCCTTTCTACCACCCTACCC<br>TGGGTAAGGGCCTGGAGCAGGAAGCAGCGGCAAGGACCTCTGGA<br>GCAGCCCATACCTGCCCTGGCCTGACTCTGCCACTGGCAGCACAGT<br>CAACACAGCAGGTTCACTCACAGCAGAGGGCGAAGGCCATCATCA<br>GCTCCCTTTATAAGGGAAGGGTCACGCGCTCGGTGTGCCGAGAGT<br>GTCCTGCCTGGTCCTC
CYP3A4	AGGACAAGGGCAGGCAGAGAGAAGGAAGGACATTGCTTCACCCC<br>AGCCCTCACTGACGAGTTTGCTAGGGGACCTCACTTTGTCCCAGAG<br>TAGGGGCAGAACTCTGGCCACTACCCATTCAGAAGGCCTGGGCTG<br>CACTGCTAGTTCCTCACTAACTCTGTGTGGCCTTGGGCAAGGTTGG<br>GCCTGTGTTAACAGATTATGACCCTGGGCTCTCAAGCTAGAGGATC<br>TAAATTTGAATCCTGGCTCTGCTAAAGCAATTAGTGATGTAAACTTT<br>AATGGGTCAGTTAACCTTCCTGTGGCTTAGTTTGCTCATCTGTAAAA<br>TAGGGATCATAACAGTATCAATACCACATGATTGTTGGACAGATTGA<br>ATCAGTTAATGCAGGGGAAGTACTTAGCATGACACGTATTCACTATC<br>ATTTCCTGGAGTAAGAGCTGTGTGTGAGTGGGTGTGAGCATGTGTG<br>AAACCTTTTCTCTGCAATCTCAGTTAAGAAACCAATCCAGAATTTA<br>AAGTTCAGGGCCTAAATGGGTGGTTATCTTCTCCCAGTTCCATCCTA<br>TCCCACCTTTGCTCTTCCTCCCGCCCACAGGAGCTGTTGGTCCTTG<br>ATTGGGCTGGAAGACCTGGTGGACCCTAAGTGATCTATAAGAGGAG<br>AATAGAGAACAGGGAATGTCTTCAAAAATCCTAGAGGGACACAGA<br>GGCTGAGAGGCAGGCAGTCCTGCAGGGGTCTTCTGATTGGGACAA<br>GGAGAACCTTGGTCTTCACAGGCCAATTCTGGTCAGTTTCCCCCAT<br>GGACAGATGAGGAAACAGGCCCAGGAATATCCAAGGTCTCACACT<br>TCCCATCTGTCAAGTCTTGTTGATGCTGTTGTATTCATGTCTCTCAA<br>AGGGAGATAGAGTTTAGGGAAGAAAGAAGGATCAACTGTGTCTGA<br>TACCACTGGGAGCTTAAGTAAAGGGTTCTTTTACTTCATAGCATTTA<br>TCCCAATTTGTAATTCAGTATTATTTGTGTGGCTGTTTGGTGTCTCTT<br>TCTCCTATATGAGTGCTAGCTTCATAAGGGCAAGGATTTTGATTCTTT<br>AATATTTAGTGCTTGCCACATGCCCTGAACACAGCAGGCATACAGG<br>CTAACCAACATACAGTGGCATGAAAGTCATGAAAGTGAGACACCT<br>ACCTCCTCCAGTGCCAAGAGAGCATAACCATGCACCTGTCACTCTC<br>CTCAACACCACCCCCAAGCATGAGGCCCAAAAGCATTAGCTAATCC<br>CCTCCTCCAGCCACTAAAACTTAAAGGCCAGGTGTGGTGGCTCCCA<br>TCTGAAATCCCAGAACTTCAGGAGACAGCAGCAGGAGGATCACTT<br>GAGGCCAGGAGTTTGAGATCAGCCTGGGCAACATAGCTAGGTCCC<br>ATCTGTACTAAAAATTAGCTGGGCGTTGTTGCATGCCTGTAGTCCCA

	GCTACTAAGGAGGCTGAGGTGGGAGGATCACTTGAGCCCAGGAGG<br>TGGAAACAACAGTAAGCTATAATCACAGCACTGAACTCTAGCCTGG<br>GCAACAGAGTGACACCCTGCCTCAAAACAATTTTAAAAATAAATAA<br>GAGCAAAACTTAGATACCACGTGGTCACCCCAACATGCAAAATCA<br>AGTTTTCCCCTACTGAGAAGAATGGGGACTTGACAGCTGAGTTACA<br>GAGAGATAATCTTCTTCTTCTTTTTTTTTTTGGTTTACATCCTCAAG<br>ATCATGACTTGTGAAATTTGAATCGAATACACATGTAATTCCAGAGC<br>AATGTTGCCTCCGCATACCATCAGCAATTCACTTGGCTACTGGAAGT<br>CAGGATAAGCTTCCCAGAAGAGAGGTACCACTTGGGCTACCAGTAT<br>AAAAGGATGAAAATATCAGAGTGATGGTGTTCTTTACAACGTTGAG<br>TCCCTGGACAGCCTGTCCACTGATGCTGATATCTGAGCCTAATGCTT<br>CTCTGAATGTTGAGATTGAACTTTGATCCAATGAAACTAGAACGAG<br>AAAGAAGATAAGTCTTTCATTGTTGATAAGGACATTATGTTTCTCAT<br>ACTTGTATGATTATTTTTCCTTAGCTGTACTATAATTATCTGCTTATTT<br>GTCTCTGCTCTATGTGCTTAGGGTACAAAGTTGACCAAGACCAACT<br>TTGGTTGGAAGCATAGTACTAAGAGCACAGTACTGAGAGCACAGC<br>TTTAAAAAACATGATGAAGGCTTTAATACAGGAAATGAGCAGGGG<br>AGAGGCATGTGGTGGTTGGATGTATCTTCCTTGACACAGTCAGTGC<br>AGCTCTCAGTAGTCAAGTCCCTACATGTTAGAAGATGTTACCTTCTG<br>TGGAATTAAGTGGCAGAACTTGCCTTCAATTATTTCCTTTGCAGAA<br>CAACACCAACTGCATTAGTTAGGACACAGTGCTGGCTGCATTTAAG<br>TCCCAAGCGATGATTAGTCTCTCACTGTTGGTATAGATTCAAACCAA<br>TCAGACCACCTCCTAAAGTTTGTAGGGCAGGTAAATCCTCATCTTA<br>GAATAAAAATCATCTTACCAAGTATGTGTTTTAGAGGCAAGAAGAA<br>AACATATTTGTTTCTGTAAGAGTTTTGTTTAAAAAAAATATAAGAAA<br>GGCTCTCGGTTTAGGTGAGGTAATGAAGTTGTTGATAGTTATCAGAT<br>GACACTGGAATCTTTACTTCTCTGAATGTGTTCTGTGCATCTCTCAG<br>TGTGGGAACATAGAGAGGGAGATCCTCCAGCAATGCCACTGATATG<br>GTCAGAAACTGCATCTTTCTTTCTCCCTGCTGAGATGAGATGGAGT<br>CCTTTGTTCTAGAAGACCCATGGTGGTGCCGCTGGGAGTAACCCTT<br>GAGACAGGAACACAAATCCCAACCAATTTGTGGTTGCAGCCTTGA<br>GTCTCACTATTTCCCATAGTGATGCGTAGCAGGGAATGGCAGGTGC<br>ACCAGAGCAGGAGAGGACCTAATATCTCCCTTCCTGTTAGCTTTTA<br>TAAAGTTTTATTGTGATCAGTAGCAGTTGGGAAGCTACTTGCAGTC<br>ACTGAGCCTCAGTTTCTACATCTGTAAACTGGGGATAGTAGCATGG<br>CCCCTACTTAATGTGCTCAGCAAAGCCACTGAAAGGAGACAGAAA<br>TGTATCTAAATTCCCTGGACTTTTATCCTACCTCTCTTGGGGATTGTC<br>ACCACCTTCCCATGTTTGTCCTTTTTGGTTTGATGCTTGCTGTCACT<br>TCTTTCCTTAGGTGCCTCTCTGTACGGCTCTTTTATCCCAGGGATTC<br>CAGAGTTACAGCACATGCATACCACCATCCAAGCATGTTTATTTGTC<br>TCCTGCTTCACTAGGCTGTCCCCAAGGAACATGTGGCTCCCGGCAC<br>ACACCTGGCACAACACTGCACATGACATTCACCCACTTGGCCTTGA<br>ATCTGACAAGGAATCTGGCATGATGTTCACCCACTCAGGCCAGGTG<br>CCGAGCAGCCCTGGAGGCTTAGGGGCCAGAGGGATGGGAAAAGG<br>TGTCTTTCTGGGGTGAGTATCAGTTTCTGCAGGAGGGCTGAATGTG<br>AGAAAGAATAAAGAGAGAAGGAAGCGAACAAGCACAGCTTAAAC

	ATCGCCTATTTCTATTGAGTTTTAAGAACGCTGTGATTTTGTTTGTCATGCAATCCATTCATCAGGCCAGGCAGACACAGAACTTGGGTGTGAGTGACGATAATGAGCTGATATAATTTCACACCCTCATCACTGAGATCTCTCCCATCAGGAATGGGTCAGGGAGCTCACAGGTGGCAGCAACTGCTATTACAGGCCTCATCTCTACCAGCTCCTGGGGCCTGCCCTCCTCCCATTAGAAAATCCTCCACTTGTCAAAAAGGAAGCCATTTGCTTTGAACTCCAATTCCACCCCCAAGAGGCTGGGACCATCTTATTGGAGTCCTTGATGCTGTGTGACCTGCAGTGACCACTGCCCCATCATTGCTGGCTGAGGTGGTTGGGGTCCATCTGGCTATCTGGGCAGCTGTTCTCTTCTCTCCTTTCTCTCCTGTTTCCAGACATGCAGTATTTCCAGAGAGAAGGGGCCACTCTTTGGCAAAGAACCTGTCTAACTTGCTATCTATGGCAGGACCTTTGAAGGGTTCACAGGAAGCAGCACAAATTGATACTATTCCACCAAGCCATCAGCTCCATCTCATCCATGCCCTGTCTCTCCTTTAGGGGTCCCCTTGCCAACAGAATCACAGAGGACCAGCCTGAAAGTGCAGAGACAGCAGCTGAGGCACAGCCAAGAGCTCTGGCTGTATTAATGACCTAAGAAGTCACCAGAAAGTCAGAAGGGATGACATGCAGAGGCCCAGCAATCTCAGCTAAGTCAACTCCACCAGCCTTTCTAGTTGCCCACTGTGTGTACAGCACCCTGGTAGGGACCAGAGCCATGACAGGGAATAAGACTAGACTATGCCCTTGAGGAGCTCACCTCTGTTCAGGGAAACAGGCGTGGAAACACAATGGTGGTAAAGAGGAAAGAGGACAATAGGATTGCATGAAGGGGATGGAAAGTGCCCAGGGGAGGAAATGGTTACATCTGTGTGAGGAGTTTGGTGAGGAAAGACTCTAAGAGAAGGCTCTGTCTGTCTGGGTTTGGAAGGATGTGTAGGAGTCTTCTAGGGGGCACAGGCACACTCCAGGCATAGGTAAAGATCTGTAGGTGTGGCTTGTTGGGATGAATTTCAAGTATTTTGGAATGAGGACAGCCATAGAGACAAGGGCAGGAGAGAGGCGATTTAATAGATTTTATGCCAATGGCTCCACTTGAGTTTCTGATAAGAACCCAGAACCCTTGGACTCCCCAGTAACATTGATTGAGTTGTTTATGATACCTCATAGAATATGAACTCAAAGGAGGTCAGTGAGTGGTGTGTGTGTGATTCTTTGCCAACTTCCAAGGTGGAGAAGCCTCTTCCAACTGCAGGCAGAGCACAGGTGGCCCTGCTACTGGCTGCAGCTCCAGCCCTGCCTCCTTCTCTAGCATATAAACAATCCAACAGCCTCACTGAATCACTGCTGTGCAGGGCAGGAAAGCT
CYP4B1	GCAGCATGATTTATAATCCTTTGGGTATACACCCAGTAATGGGATGGCTGGGTCAAATGGTATTTCTAGTTCTAGATCCCTGAGGAATCACCACACTGACTTACACAATGGTTGAACTAGTTTACAGTCCCACCAACAGTGTAAAAGTGTTCCTATTTCTCCACATCTTCTCCAACACCTGTTGTTTCCTGACTTTTTAATGATTGCCATTCTAACTGGTGTGAGATGGTATCTCATTGTGGTTTAGATTTGCATTTCTCTGATGGCCAGTGATGATGAGCATTTTCTCATGGGTCTGTTGGCTGCATAAATGTCTTCTTTCGAGAAGTGTCTGTTCATATCCTTCACCCACTTTTTGATGGGGTTATTTGATTTTTTCTTGTAAATTTGTTTAAGTTCTTTGTAGTTTCTGGATATTAGCCCTTTGCCAGATGGGTAGATTGTAAAAATTTTCACCCATTCTGTAGGTTGCCTGTTCACTCTGATGGTAGTTTCTTTTGGTGTGCAGAAGCTCTTTAGTTTAATTAGATCCCATTTGTCAATTTTGGCTTTTGTTGCCATTGCTTTTGGTGTTTTAGTCATGAAGTCCTTGCCCATGCCTATGTCCTGAATG

	GTATTGCCTAGGTTTTCTTCTAGGGTTTTTATGGTTTTAGGTCTAACA<br>TTTAAGTCTTTAATCCATCTTGAATTAATTTTTGTATAAGGTGTAAGG<br>AAGGGATCCAGTTTCAGCTTTCTACATATGGTTAGCCAGCTTTCCCA<br>GCACCATTTATTAAATAGGGAATCGTTTCCCCATTTCTTGTTTTTGTC<br>AGGTTTGTCAAAGCTTAGATGGTTGTAGATGTGTGATATTATTTCTG<br>AGGGCTCTGTTCTGTTCCATTGGTCTATATCTCTGTTTTAGCACCAGT<br>ACCATGTTGTTTTGGTTACTGTAGCCTTGTAGTGTAGTTTGAAGTCA<br>GGTAGCATGATGCCTCCAGCTTTGTTCTTTTGGCTTAGGATTGACTT<br>GGCAATGTGGGCTCTTTTTTGGTTCCATATGAACTTTAAAGTAGTTT<br>TTTCCAATTCTGTGAAAAAAAGTCATTGGTAGCTTGATGGGGATGG<br>CATTGAATCTATAAATTACCTTGGGCAGTATGGCCATTTTCATGATAT<br>TGATTCTTCCTATCCATGAGCATGGAATGTTCTTCCATTTGTTTGTAT<br>CCTCTTTTATTTCATTGAGCAGTGGTTTGTAGCTCTCCTTGAAGAGG<br>TCCTTCCCATCCCTTGTAAGTTGGATTCCTAGGTATTTTATTCTCTTT<br>GAAGCAATTGTGAATGGGAGTTCACTCATGATTTTGCTCTGTGTTTG<br>TCTGTTATTGGTGTATAGGAATGCTTGTGATTTTTGCACATTGATTTT<br>GTATCCTGAGACTTTGCTGAATTTGCTTATTAGCTTAAGGAGATTTT<br>AGATTTTGGGTTGAGATGATAGAGTTTTCTAAATATGCAATCATGTC<br>ATCTGCAAACAGGGACAATTTGACTTCCTCTTTTCCTAATTGAATGC<br>CCTTTATTTCTTTCTCCTGCCTGATTGCCCTGGCCAGAACTTCCAAC<br>ACTATGTTGAATAGGAGTGGTGAGAGAGGGCATCCCTGTCTTGTGC<br>CAGTTTTCAAAGGGAATGCTTCCAGTTTTTGCCCATTCAGTATGATA<br>TTGGCTGTGGGTTTGTCATAAATAGCTCTTATTATTTTGAGATACATC<br>CCATCAATACCTAATTTATTCAGAATTTTTAGCATGAAGGGCTGTTG<br>AATTTTGTTGAAGGCCTTTTCTGTATCTATTGAGATAATCATGTGGTT<br>TTTGTCTTTGGTTCTGTTTATATGATGGATTACATTTATTGATTTGCAT<br>ATGTTGAACCAGTCTTGCATCCCAGGGATGAAGCCAACTTGATCAT<br>GGTGGATAAGCTTTTTGATGTGCTGCTGGATTCCGTTTGCCAGTATT<br>TTATTGAGGATTTTTGCATCGATGTTCATCAGGGATATTGGTCTAAAA<br>TTCTCTTTTTTTGTTGTGTCTCTGCCAGATTTTGGTATCAGGATGATG<br>CTGGTCTCATAAAATGAGTTAGGGAGGATTCCCTCTTTTTCTATCGA<br>TTGGAATAGTTTCAGAAGGAATGGTACCACCTCCTCTTTGTACCTCC<br>GGTAGAATTCGGCTGTGAATCCGTCTGGTCCTGGATTTTTTTGATT<br>GGTAGGCTATTAATTATTGCCTCAATTTCAGAGCCTGTTATTGGTCTA<br>TTCAGGGATTCACCTTCTTCCTGGTTTATTCTTGGGAGGGTGTATGT<br>GTCCAGGAATTTATCCATTTCTTCTAGATTTTCTAGTTTATTTGCATA<br>GAGGTGTTTATAGTATTCTCTCATGGTAGTTTGTATTTCTGTGGGATT<br>GGTGGTGATACCCCCCTTCATTTTTTATTGCGTCTATTTGATTCTTCT<br>CTCTTTTCTTCATTAGTCTTGCTAGCGGTCTATCAATTTTGTTGATCC<br>TTTCAAAAAACCAGCTCCTCGATTCATTGATTTTTTTGAAGGGTTTT<br>CTGTGTCTCTGTCTCCTTCAGTTCTGCTCTGATCTTAGTTATTTCTTG<br>CCTTCTGCTAGCTTTTGAATGTGTTTGATCTTGCTTCTCTAGTTCTTT<br>TAATTGTGATATTAGGGTGTCAATTTTAGATATTTCCTCCTTTCTCTT<br>GTGGGCATTTAGTGCTATAAATTTCCTCCACACATTGCTTTAAATGC<br>ATCCCAGAGTCAAGACCCATCAGTGTGCTATATTCAGGAGACCCAT<br>CTCATGTGCAGAGACACACATAGGCTCAAAATAAAGGATGGGGG

	AAGATCTACCAAGCAAATGGAAAACAAAAAAAGGCAGGGGTTGC<br>AATCCTAGTCTCTGATAAAACAGACTTTAAACCAACAAAGATCAGA<br>AGAGACAAAGAAGGCCATTACATAATGGTAAAGGGATCAATTCATT<br>AAGAAGAGCTAAGTATCCTAAATATATATGCACCCAATACAGGAGC<br>ACCCACATTCATAAAACAAGTCCTTAGAGACCTACAAAGAGACTTA<br>GACTTCCACACAATAATAATGGGAGACTTTAACACCCCACTGTCAA<br>CATTAGACAGATCAACAAGACAGAAAGTTAAACAAGGATATCCAG<br>GAATTGAACTCAGCTCTGCACCAAGCGGACCTAATAGACATCTACA<br>GAACTCTTCACCCCAAATCAACAGAATATACATTCTTCTCAGCACC<br>ACATCGCACTTATGCCAAAATTGACCACATAGTTGGAAGTAAAGCA<br>CTCCTCAGCAAATGTAAAAGAACAGAAATTATAACAAACTGTCTCT<br>CAGACCACAGTGCAATCAAACTAGAACTCAGGATTAATGAACTCAC<br>TCAAAACCGCTCAACTACATGGAAACTGAACAACCTGCTCCTGAAT<br>GACTACTGGGTACATAATGAAATGAAGGCAGAAATAAAGATGTTGT<br>TTGAAACCAATAAGAACAAAGATACAACATGACTTGCTTTTTAAAT<br>GGTGCACTCAATGTCTCGATCCTATAAAATGAAGCACAAATGTGAA<br>ATTTCAGGAAGTGTGACAGACCAATGGAGAAGACACTTTTGGAGT<br>TCTTTCATTGTACTCTCTTCTTGCTTTCTCTGCCTATGGGTAAGATTG<br>GACATTATGTCTCCCCAGCAGAGGAGGAGGCTTGGTCTCTTGTGCC<br>AGAGGGGATAAGGCAGCCATGTTGATGGCTTATCATTCTCTGAATTA<br>GAACCTGTGATCTGAGTTCTGAGATTTGAGATCCAAGTCCACCTGA<br>TTGCTGGTTTTTTTCCTGCCACCTCCCTGCCAGACTGTCCTTGCTCT<br>TATCTTGCTGGGAGGCTGACTTCTGCAGACTGACTCCCCAGGCTCC<br>TTGCCATTTCACTTCTGATTGGCTTGGCTGATTGGAGACACTAGTAG<br>GAGATGGGAGATGGCAGATGAAAGAGTTTAGGACATTTTTGCTCTG<br>TTCTTCCACTGTTCCAGCATCACTTCTCTCGCACTAGCTGTGTTCCC<br>CATAAATACAACCCCCACCAAGCTACAGGATCATGATTTTCTTCCCT<br>TCTTCCTTTAGTCCTGGGGTATTGATGCATTTCTTGTTGTTTCAAGTC<br>TCTGCATGCCACATCATATCTTGTTTGATTTCTTTAATCTGCCTGCCT<br>CTGCAAATAACATACCCTCCCCAGGCAAGGCAGTGGTCTGAGCCA<br>AACTTGTTCTTCCACAGAAGCCTGGGCTGTGGCACTGGGAGCTGC<br>CAATTTCTTGCTGTGCAACTTTGGATATTTCCCTACCTCTCTTGAGC<br>TTAAGTGGCTCCATCCAGCCAGGAGGGATTGAATTTGATGACCTCT<br>CATCACCCTGAAAGTTCTATGTTCCTAAGTGTCAAAATGGCACTATT<br>GTTTTAGGGGTGGAGTTGGCCCCTTTCTGACCTGGTATTTCCCTTTT<br>GGGGCTAGAACCTTGTCTCTGAAGGTGCCTCACAAAAAAGAACTC<br>AAAGCATTGCGAGCCTAGCATAAATGCCAGCTCTCTTAAGGGTTCA<br>CATTGAAGGGGACACTGGAAGCTTTCTTGAGAGAAAGCATAGGCA<br>AGCTGAGCCTCAGCTTCATTATCTGGGAAATGGGTATAAAATGTGA<br>GTGCCTCTGACCTCCTTGCTGTTTGGGTTAAATGAGATAGTATGTAA<br>AAAGTGCCTGAAACATTCACAGTGCTTGTATGAGAAGACAGTGGTT<br>ATTATGCTGATGATTACACCAAGAATTGCCATCTGTTTGTTCTAAGG<br>AATCAAGGTGGGATACCCATTGCCTGTTTTTTTTGGAGTCCACTATT<br>AGGTTTCTCTTTAGCTGAATTTTGGGAGAAGGCCACTAGGCTCTCT<br>CCTCTGTTGTTTGCAGTTTTGGAGGTTGCTGTCTCCGCCTTATGGCA<br>CTCAGCCCAGCCTTGGCTGCTGCTGGCTGCAGGGAGTGGCTAGGG

	CTGGACGCTGACAAGATGCATGGGTCCAGAGTATAAAGGAACCCA<br>GGAGCAGCTGAAGGCAGGTCAGATGAAGGCTAGGTGGCTGGAACT<br>GCAACCATGGTGCCCAGCT
CYP8B1	GCCTACTCCCCGCCGCGCCTCCCGGAGACTCCCTCGCTGAGGCTTC<br>GGGGGCCGGAAGTGCACCCGCCCCTTTCCCGAGCGCCCGCGGGCC<br>CTCTGGCTGTCCCGGAGGCCGCCTCCGTGTGTCTTTGGTCCTACCT<br>GGCCCCCTAGCCCACCATCGTAACCCCGCCCCGGCCCCTGACAGTT<br>CCCGGACCGAAGGGGCGAGGAGGGGACGTGCCGCCCGCTCCCGG<br>TGTTGGCTTGAGTTGCTGGTCCCCAGCCCCTCGGGAGGGAGAGGA<br>GGGCAGTCCCGTGGGCTGACTTCCCAGGGGCAAGCCAGTCTCCTC<br>CCGAGGAAGAGCCCGTATTCCCTGCCTGTCCATCCGGCAGCCCTGC<br>GTGGCCCCCGTGGGCTGGCCCCGGACACAAGCGTGGCTGGGCCGG<br>CTGGCGTGAGAGGGGTTCAGTGCCACCGATGTGTCAAAGTCCTAA<br>CCCGCACAGAGCCTGCCGGTTCTGCACCCTGTGGAGATTCTTCCCC<br>CTTCTCCGAGTGCCCTGGGCTCATTGGCCCCAGGGTGGATATGCTC<br>TCGGTACCTTGGGACCACCCTGCTGCATCCCCTGACCAGCACCCCT<br>CTTCGGCCGCTAGCACCTCCACCACACACAACACACATTGACTGG<br>GTCTTGTGGTCCTCCAGGCCTCCGCTGAATCAGTGTGGCCCAATCA<br>GTGTGGCCTTCCTGACTGCCACCTCCCTTCATGCTCTGCTCTTCTTT<br>GCAGCATTTGTCACAATTACAGCCATACCGCGAAGATATTGCGGGT<br>TGGGTTTCAAACCTCCATAGTAAAATGAGTCGCACGAATTTTGTTTC<br>CGGGGAATATAAAAATTATGTTTAGACTATACACCATTAAGTGTGCA<br>AAAGCATTATATCTAAAATACATATATATACCTTAATTTTTAAAAAAA<br>ACTTTATTGCTAAAAAATGCTAATGATCATCTGAGCCTTCAGTGATT<br>CCTGTCTTCACCCTGGACTGATCAGGGTGGTGGTTGCTGAGGGCTG<br>GGGTGGCTGTGACAATTTCTTAAAATAAGACAACAATCAAGGTTGC<br>CGCACCAGTTGACTCTTCCTTTTAGGAAAGATTTCTCTGTAGCATGG<br>AATGCTGTTTGATAGCATTTTACCCACAATAGAACTTTTTTTCAAAA<br>TTGGAGTCAATGCTCTCAAACTCTACTGCTGCTTTACTAACTTTATG<br>TAATATTCTAAATTCTTTATTGTCATTTCAACAGTGTTCCCAGCATCC<br>TCGCCAGGATTAGTTTCCATCTCAAGAAACCACTTTCTTAGCTCATC<br>CGTAAGAAGTTACTCCTCATCCATTCAAGTTCAATCAGGCGATTGC<br>AGCAATTCAGTCACATCCTCTGGCACTACTTCTAATTCTAATTCTCTA<br>TTTCCACCCATCTGCAGCGACTTCCTCCACTGAAGCCTTGAACCCC<br>TCAAAGTCATTCATGAGGGTTGGAATCAACTTCTTTCAAACTCTTG<br>TTCATGTTGATATGTTAACCTCCTCCCATGAATCACAATTGTTCTTAG<br>TGGCTCTAAAATGGTGAGTTCTTTCCATAAGGTTTTCAATTTACTTT<br>GCCCAGATCCATTAGAGGAATCGCCATCTACAGCAGCTATAGCCTTA<br>CGAAATGTATTTCTGAATACTAAGACTGAAAAGTTGAAATTACTCCT<br>TGATCCATGGGTTGCAGAATGGATGTTGTGTTAGCAGGCATGAAAA<br>CAACATTCATCTCTTCGAACATCTTCCTCAGAGATCTCAGGTGCATT<br>GTTAATGAGTAGTAATACATTGAAAAGAATCTTTTTTGTGAGCAGTA<br>AGTCTCAGCAGTGGGCCTAAGTAAACCATGCTGCAAACAGATGAG<br>CTGCCATCCAGGCTTTGTTTTGTTTGTTTTGAGACAGGGTCTCACTC<br>TGTCGTCCAGGCTGAAGTACAGTGACATGATCTTGGCTCACTGCAG<br>CCTCAACCTCCCAGGCTCAGGTGATCCTCCCACCTCAGCCTCCCAA

	GTAGCTGGGACCACAGGCATGCACCACCATACCTGGCTAATTTTTG TAGAGACAGGGTTTCACCATGTTACCCAGGCTGGGCTCCAAGCTCT GGGCTCAAGCAATCCACTTGCCTTAGCCTTCAAAATTGCTGGGATT GCAGGCGCGAGCCACCACACCTAGCCTTGCTACTTATAGAGTACAG GCAGAGTAGATTTAGCATAATTCTGAAGGGCCCTAGGATTTTCAGA ATGGTCGTGAGCTTTGGCTTCAACTGAAAGTCACCAGCTACCTTAG CTCCTAAAAAGAGAGTCATCCTACCCTTAGAGGCTTTGAAGCAGGG CATTGACTTTGCTCTAGGAAAGTCCTTAGATGGTCTCTTCTTCCAAT ATAAGGCCGTTTCATCTACATTAAAAATCTGTTGCTTAGTGTAGCCA TCTTCATCAATTATCTTAGCTACATCTTTTAGATAACTTGCTGCAACT TCTTCATCAACACTTGCTGCTTCACGTTGCACTTCTATGTTATAGAG ATGGCTTCTTTCCTTAAACCTAATGAACCAACCTATGCTACCTTCAA ACTTTTCTCCAACTGCTTCCTCATCACTTTCAGCCTTCATAGAATTG AAGAGAGTTAAAACATTGCTCTGGATTAGGCTTTGGCTAAGGGAAT GTTGTAGCTGGTTTGATCTATGCAGGCCACTAAAACTTTCTTCATAT CAGCAATAAAGCTGTTTTTCTTATTATTCATGTATTCACTAGAGTAGC ACTTCTAATTTCCTTCAAGAAATTTTTTCTTTTGCATTTACAACTTGG ATAACTGTGTAGTGCAACAAACCTAGATTGCAGCCTATCTTGGCCTT CCTCACTAAGCTTAATCATTTCTGGCTTGTGATTTAAAGTGAAATTT AAAGTGATTTAAAGCGCAGCTCTTCCTTACACTTGAACACTTAGAT GCCATTGTAGGGTTATTAATTGGCCTAATTTCAAAATAGCTGTGTCT TGGGAAACAGGAAAGCCCAAGGAGAGATAGAAAGAGGGGAGAAC AGCTGGTCAGTGGAACAGTCAGAATACACACAACATTTACCAATTA AGTTTGCATCATATATGGGCCCAGTTCATGGTGCCCCAAAACAATTA CAGTACTAACATATCACAGATCGCCATAACAGCTGTAATGATAATAA TGAAAAAATTTGAAATATTGTGAGAATTACAAAATGTGACACAGAG ACACAAAGTTAGCACACACTGTTGGAAAAATGGTGCCAATAGTTTT CCTCAATGCAGGGTTGCCACAAACCTTCAATTTGTAAAAAACATAG TATCTGTGAAGTGCAATAAAGCAAAGCACATTAAATGAGAAATATG TGTATAATTACTTTGGGCAGTGATTTAATATCTTTCTCCCTCCATTAG ACCATAAACTGCAAGAGAGCAGGGACTGTGTCTATCTCATTCATGA TTATAATCCCAGATCGTAGCAACATTTCTGCCAGTAAGTGCCCAATA AATATCTAGTGAATGGATAGATGGGGCTTCTCCAAGGAAGGAGGGA ATGAATAGATGAGTGTGGGTTAATGAATGAGGTAAGAATGAATGTG AAATAATGGTTCATCCATTAAAAACTAGGGGACACGGTTCAGTTCA TTGGATTTGGCCCAGTTTCAGACCGAATTCTAAGGTTGAGGAGAGT TGATGATGCCAAGTACTGTGGTGGTCCTGAACAATTAAAGAGGGAT TCTGGGAAGCAGAGTGTGGACAGTTCCAACTCCCTGCCAAGGGGA AGCTCATAGGCAAAGGAAGCTCACTCCAGAGGGGATATGGAAGTT CCATACCCTCTTTTGTCTGAAGAGCCGAAGTCCCTGTTCTCAGGTC GTTAGGAAGTTAAAAAGTAATTTGGAGGTTATCAGAACTGATTGAA TTGAGTTTGAACCTCACCTATAGCAACAATGGGCCAGGCTGCTTGA CTAATGCCTTGGCGTCAATGGTACAGTTTTCTCCCTCTTGAGCTGCT GGCAGGGACCTGGGCTGACATGTCTCAGAAGGCCCTTAGTCAATG ATTAGCTTATCTCAAGGCCCCAAGCCAGGGCAGCTGTCAAAGAGG GTCCCCACTGCGTTCTGCACCTAGATCCTCATTGTGAAATGAAGTAT

	GAAGTGATTGAGTGAGGTCTCTATTGTCTCTGACATTTTACAATTCCAGGATTCTGCCTTCTTGTCAGAGAAGTGTATAGGCAAGCAGTTGGGCAGGTGAGAGGGCTCCGGGTGAGAGGCTCAGAGACTGAGGGCTCAGCCTCTGCTTGAAGAGTCATCATCTGGGAGGCTCTGTGGCCTCCTCAGATGAGTTCATTCACACTCATACCCCAAGATGGAGTCAACACCCCCTCCACCATCTCAGCCTTCTCAGCATCTAAAGCCCCAGCATCAATGCCTCTTTTTTTGGGTTAGGGGTCAGAGCTGTTGTGGAAGGGCATACAGTCATTCTTCACTTGCCTTTGACTGTGTACTCTGTGCACATGGAGGTAGGAGCAGACATGACTTCAACAAGGTCATGCCCCCTTGGCAAGCATCTTTGAGACCAGAGAGGAAGACAGACTAGGGAAAGAATGAGGAGATAAGCACGGGCTGCTGTGAGGTCCAGGGGAGCAGGCAAAGGTAAGAGAAAAGGCTTTAGGATACTAACTAACATATATGGAGCACTAGCATGAGCCAGGCACTATTCTAAGTGCTTTTCAGGTGTTATCTCTTTTTGCCTCACAACAGCACCTACAAGGCACTGTAATTATCCCTACTTCACAGATGAGGGAGTGGAGCCACAGTGAGGTTAACTTACTTGACCAAGGGGGCCAAGTAGGAATGGAGGCATTTGTTGAGTCTTCTAAAGATGAGGAAAGAGTGGAAGTGAGATTTTGTAAGTGCTTGATTCATTTCTACCAACTGAACTGGCAAATAAATAAAAGCATGAGTAAATGGGGGTATAAATAGTCTGTCAGCTATGGGGGTGGGAGTGGGCTCAAGGCAGGCTTAGAGAGAAGGTGCAAGAGCTGTCTGAAAAG
DBH	CAGTGCCGGTCCTGAGCAAGATGGCCATTTCACAGATGAGGAAAGAAGGCTCAGGGCTGTGGGGACTGTCCGGGTGGGTCACATGGCCGGCAGGTTTCAGGGCCTCTCTTTCCATCTGGTGCCCACAGTTACGCTGTGGCTTGGGTGTGGTCTGGAGCTACTGCCTCAGGACCCACCACCATCCTGGCAGTGTGGTCCCTTCAGAAAAGCTGAAAATGCAAAAATCAGGCACATGCACCTCCCCCCATGAGCTGCTCAAGAGAGAGGAGCAGTCACGCATCCTTATGGAGAAAAGGAGAAGCAGGACCCAGAAAGGGTTTCCCTGTAAGATTCCTCAGGGCCCAGCAGGCCTCAGCCCCAAGATTGAGGCCAAATTATTGGAGGGGTGTGTGTGTGTGTGTGTGTGTGTAAAACTGAGTATGTGAGCATGAGCCCAGAGCTGGGGGTGCAGGCCACGCAGGTGTGAGAGTCCACCTCCCCATCTCGCTGACCCCTGTGTATGGAGGAGAGATGGAGGCGGGGGAGGCTGTGTCCTGCCCAGGCCAGCCGCCTGCCAGTGACAGTGGAGGACTTGCTCACAGTGGGGCTGCTTGGGGTCACACAGGAGTGGCCTGGCAGGCCTGGCAGCCCCAGCCCTGGAGCCAGCCACAGACTCCTGGGTTCTGCTTCCCTGGGAGCTTCCAGGGGAGTGTCACTGGGGCTCACGATACCGACCCCTTCTGGGATCAGTGGCCCCTTGCTTTTGGCACAGAGCTTCAGGGCCAGAGGGTGCCTCTAATCCAGTAGCTGAGGAAAGCTTTCAGTGGTGCTCTGGGCACCTCTGTGACCTGCCCTGGGCCCAGTCCTCATGCTGATCCCACTGTAGCCACCCTACTCAGTGCCCCAGGCCCTGGACACCTCAGGCCATGGGATATCCCAAGCATTCAAACTCTCACGGCCACCTACGCTTATGAGCAGAGGCAGAGGCATGGTGCCACCGCTGCCTCCTCAGGCAGGATATGCTTTTTGTTTGTTTGTTTTGAGACAGGGTTGTTCGCTGTCATTCAGGCTGGAGTGCAGTGGTGTAGTCAGGGCTCATTGCAGCCTCAAACTCCTGGGCTCAAGCGATCCTCCCGCCTCAGCCTCCCGAGTAGCTGGGACTACAGTCTTGTGCCACTATGCCTGGCTAATTTGTTTTTATTTTA

	TTTTGGTAGAGACTGGGTCATGCTATGTTCCCCAGGCTGGTCTCGA<br>ACTCCTGGGTTCAAGCAATCCTCCCGTCTTTGCCTCCTAAAGTCCT<br>GGGATTATAGAGGTATGAGTCACTGTGCCCAGCCCAGAATGTGCCT<br>CTTAACTTTCATACCGCAGCCCCTATGCTCAGGACGAGGAGGGACA<br>TAGGCCAGCTGCCCCAAGTTCCCCAAGGGGACTGTGCCACCCTGG<br>AGTGGGCTCTGTGATGGACAGAGTCACCGTCTCCCAGCAGCTCCC<br>AATCCATCCCATCACAAGTCAGGTCTAGGCACTGCCATCAGACTGA<br>TAGTTGAATTTTTCCTGCCAAGAGGAGGTCCCTAAGAGCCTGAGCC<br>ACAGGCGAGGGACAGTGCTGTCCTGTGGCCAGGCCCCGTTGTCAC<br>TGGGGAGTGGCCTGGCCTGTTCACTCACCCAGCTCCAGTCTGCACC<br>ACCGTCGCTCCACTTGGGGTTGTCACTGCCTGAGGTTAGGACGTCA<br>GCAGAAGCCAGCCTCATGTGGCAGAGGCGATGGGGAATTACAGCC<br>TGCAAAGGCTGATGGCATCAGAGAGATTCAGGAAAGCAAAGGGCT<br>TATTAAAAAGCTGAAATTGGTTCTAAACAAGGTCAGCCGTCGATGC<br>GCCTTGGAGGGAAGCCAGGCGCTGAGCAGCAGGAATGGGGCTTAT<br>TTCCTGACACCGTGGCCGTGACACCCTGCACCTCCCCGGCCTACCC<br>CACCCCCAACCCCACTCCCACCCCCATTCCCATCCCCACCCCCACC<br>GCCACCTCTGGAGGGCAACGTGCACCCAGACACTGGAGCAGAGG<br>CAGCAGGGGTGGGAGCAGGGAGGAAAGCCCCCCGCGGGGCACCA<br>CAGGGAGGCATGTGGCCAAGAGAGCACCTGGCAAGCAGGTGTCA<br>CCTCTGAGGAGGGTGAGGGCAGGAGGAGAGCTGACTGTTGCTTCC<br>TTACCCTGGGGTCGGATTGCTCCCTTTTAAAATCCATTAATGCAAGG<br>AAGGGCGCAGCCACGGGGCCATCCGATTGGATTCGCTCCCTCCCGC<br>CCGGCCCCAGTCTCCGCAGATTTTCTCTGCGGTTGCATTTTTCATCT<br>GAGCGTCCCTTCCTCTTGACTCCAGTGGAATTTTTCTGACCTTGGCT<br>GACAAGGTGTGCAGCCCCCTCCAGCTCAGGGCACCCTCTTTGGGG<br>TGCTTGCCCCAGGCACTGGCCCACCCTGTGAGCTCAGGCGCATTGT<br>CTCGCCTCTCCGAGGCTGACTCTCCTGTGTCTGAGACAGGGATGAT<br>CGCACACCCAGCCACCCAAGGCTGGCCAGGATGGAGGCAGGGCA<br>GGGGAAGCCCTGGTGCGGCCTGACTTGTGTCTCCTGTCCCTCCCTA<br>GGACTTAGTTATCAGTATTTTGGAATCAGCTGAATCATTTCTGTCAA<br>ATGCTAGAGGAAAGCCAGTTTTGAGTTGGATAAAGAAAATGTCTTT<br>GATTCCTGTTAAAAATAAACATGTGTGCATAACACTGCTGAGCACG<br>AGACCGCACCTGCCGTCCCTCCTTAGAGCTCCCAGCAGCTGTTGGT<br>CTTTGGCGTATCCGGGAGAGGAGCGAGGCCTGGAGGTGTGTTGAG<br>CCGTCCAAGGGCTGGTAGCATCAGGGGCTCTTCACTGCCTCTCGCT<br>CCACAGAACCGCTGGGTTGAAGGAAGCTGGAGGGATCAATTGTCC<br>TTGTCATCCAACTTCCTGTGTCTGTAGATGGGGAGGAGGCGCTGGG<br>TTGGAAAGTGCTTCTCTGAGGACGCCAGCTGCGCATTCAGGAGGG<br>CGGAGCAGGATATGACCCCAGAGCTCTGACCCCGGAGGAGCAGGA<br>GGGGTGCCTGGGGCCTCAATACCCCAGAGGGGAGAGAGGTGCCCC<br>CCTTCCCACAACGCGGGCCCCAGCTGGTGCTCGTTTAGCCTGTTGT<br>CTAGCTGGAGCCACCTGGGTGGCCACCAGGCTGCTGGGAGGGCTC<br>ACCCCTGAGCTGGGTGTGCTGTGGAGCCCGTGACCCTCACCTCTGC<br>CTCTGCTGGCTTTTGTTATCCCGTGCTGGGAACACCAGTGCCTGCC<br>CACCCCGGAGGCTCTTCGTATCACCGGCTCGCTGTGGCCGCTCCCC

	AGTCCCGAGAGTGAATGTCCGGGATGCAGCAGACACCTGCTTACA<br>AGGCACAGGTGAGGAGGTTTTGGAACTAATCTGAGGCATGAAGGT<br>GAGGTGCTCTCAGGCGACGGAGGTGGCATGTCCTGATGGGAAAGC<br>CAGGCCTTCGGAGGCTGAGACCCTTCCCAAGGCTCACCAGAGAAC<br>TCCCAAGAGTGTCTTTCCCCTGGCCTGGGAGACAATTTCACACCAA<br>TGCCACACTCTATTGCACAGGTGATTATGCACTTGGTCCTCACCTGC<br>CTGGGCTGTGGGTGGGGGCGAACGGTGCCCCTCTGGGTGCAATCA<br>GTGGGGAGGGGCTCATGGCCTGAGCTTGGCTTTGCAGGATGGCCT<br>GGCCACTGTGGGGCAGAGAGCACTGCCTGCGAGGGCCAGGGGAA<br>GGCTGGCACTTTCTCCAAATTTCACGTTCGTGCAAAGACACAGTCA<br>TTCCTTTCTACAGCGTAGAGCTCAGAGCTGAAGCAGCCTCCAGACC<br>TGCTGTCATGGGTATTTAAGGACCTAAATGTTGCTGTCAGCCTATGA<br>CATGAATGTGCCCCTAAGGCTAGATTCTGGGTTTCTCCAGAGAGAC<br>GAGAAACAGGAGGGAAAAGGAAGGAAGGGAGGGAGGAGGCTGG<br>GGAGGAGGGACAGCTTCTAGTCCAGCTGGAGAGATCTGTCAACCC<br>AGCCTGGGGGGTGGAGCTGGAGGGATCAAGCAGAATGTCCTGAAG<br>GCAGCTGCCCTCAGTCTACTTGCGGGAGAGGACAGGAGGGAGAG<br>GTGCCGTGGTGAGACTGACCCTCGGGCCCACGGGTGGATGATGGC<br>AAAGGTCCTGTGGCAGGTGTGGGAGAAGCAGGGCTGCGGCCAGC<br>TGCCCTGAGAGGCTTCAAATTCAGGCCAGATCTGCTCTGGGCAAG<br>AGGAAGCTGTGGGTTTGGGAGCTTCAGAGACAGGGGAGCAGGCC<br>TGTCACTCACCCTCTTTTCCTTAAAGGCTGTCACCCCCAGCAGTGC<br>ACCCTGCAGCCTGCCTATCCCCTGTGCAGCTCCAGCTCCGTCTGTC<br>CCCAGCAATGCACCCCACGCCCATGTCCCCCACTCCCTATGATGCT<br>CTCGCGCCTTCTGGAGCAGCAGTGGTACCAGCTGGAGGCTGTGGA<br>AAAATTGAGAGGAGACAAAAGTGGGAAGAGCAGGCCGTGGAGAG<br>AGTGTCTAAACACAGGGACTATTTGGAGATTGTCTTATTTTTGGTTT<br>GAAATGGGCCCACCTGCTAGTATTGTACAAACGGCTCTGCTCGCTA<br>GGCCAGGCCTGCAGTGGCTACGGGCCTATCCTTCACTGGTCAGCCT<br>GGAGCAGCTCCTCGGACCTCAGTCTGCTCATCCGTGGGTAGAGGC<br>GACGCAGACCCCATCCCACTGGGGACTGGGCCAGACAGCCTGTGA<br>GGCAGTCAGCTGGTGCCTGGCCAGAGGGTGTCTGAAAGGCCTCTT<br>GGCTGCAGGGTGCATCTGCTTTTGGGACAGCTCTTCAGAGCCATCT<br>CAGAAGGGACAGCATCCGCCTGTCTACTTCAACTCCCACTGATGAC<br>GTCCATGTGTCATTAGTGCCAATTAGAGGAGGGCAGCAGGCTGAGT<br>GCTTGGCCTGGGGCGCAAGCTTGTGGGAGGGAAAATTGGATTCCC<br>CGCTAGACAAATGTGATTACCCGTGCTGCCTGGACCCACCCCATTC<br>AGGACCAGGGCATAAATGGCCAGGTGGGACCAGAGAGCTCACCCC<br>AGCCATGCCCGCCCTCAGTC
DDB2	CACCTGAGGTCAGGAGTTCGAGACCAGCCTGGCCAACATGGTGAA<br>ACCCCGTCTCTACTAAAAATACAAAATTAGCTGGGCATGGTGGCGC<br>ATGCCTGTAATCCCAGCTACTCTGGGTGCTGAGGCAGAATTCCCTG<br>AGCCCAGGAGGTGGAGACCGCAGTGAGCTGAGATTGCGCCACCAC<br>ACTCCAGCCTAGGTGGCAGAGCGAGACTCCGTCTCAGAACAAAAC<br>AAAACAAAAAACCAAAACACCACCACCAACAACAAAAACTCTGC<br>TCCCCCAATGCTTGGGGAAACTGACTTGAGTAATAATAAAACTCCG

	GTATCCTGCACAGCTGGCTCTGCGTGAATTACTCTTTCTCTGTTGCA<br>ATTCCCCTGTCTTGATGAGTTGGCTCAGGCAAAGTGAACCCCTTGG<br>GTCGTTACAACAGTACTACTGTCTCCATAGAATTAGCTTTCCTGAGT<br>GCATCCAAAAATCTTAATTGGTGACCAGGCATGGTAGTTCATGCCT<br>GTAATCAAAGCAATTTGGGAAGCTGAGGCTAGAGGATGGCTTGAG<br>CCCAGGAGTTCAAGGCCAGTCTGGGCAACATAGTGAGACCTTGTC<br>TCTATAAAACCAAAAACCAAAAACCTTGGTGGGCATAGCATACTTA<br>GTCTTCAAATGTGTACCTACCTTGAAGTATTTTACTTGTTTCACATAT<br>ATTTTTATTCAGTTCCCAGTAGGTTGTAAATCTTTTAGAGGCAAAGT<br>CCATTTCTTACACTTCAATATCCCCTGCAACTGAGCACCCTGCTGAA<br>GGACAGGTTGGTCTTAGTTGGCATGTTGGTTGGTCTGAATAAGGGG<br>AGGGGGCGAACAGTTTGCTTCTGCAACACAGCTCTGAAAACACAG<br>CTCTAGGCTAGCAGGGGAGAAGCTCCCAGCTTGCCCCTTTCCCAGG<br>CAAAACCATTTCTTTTTTTTAATTTTTAATTTTTTATTTTTTGAGACA<br>GAGTCTTACTCTGTCGTCCAGGCTGGAGTGCAGTGATGTGATCTCA<br>GCCTTTTTGTGTTTTTTTTTCTTTTTTTTGAGACGGAGTCTCACTCT<br>GTCACCCAGGCTGGAGTGCAGTGGCGCGATCTCGGCTCACTGTAA<br>GCTCTGCCTCCCAAGTTCACGCCATTCTCCTGCCTCAGCCTCCCGA<br>GTAGCTGGGACTACAGGCGCCCGCCACCACGCCTGGCTAATTTTTT<br>TTGTATTTTTAGTAGAGACAGGGTTTCACCGTGTTAGCCAGGATGGT<br>CTCAATCTCCTGACCTCGTGATCTGCCCGCCTGGGCCTCCCAAAGT<br>GCTGGGATTACAGGCGTGAGCCACCACGCCTGGCCGTGATCTCAG<br>CTTACTGCAACCTCTGCCTTCCCGGTTCAAGCGATTCTCATGCCTCA<br>GCCTCCTGAGTAGCTGGGATGACCGGCACGTGCCCCATGCCTGGCT<br>AATTTTTGTATTTTTAATAGAGAGGGGTTTCACCATGTTGGCCAGGT<br>GGGTCTCAAACTCCTGGCCTCAGGTGATCTGCCCACCTCGGTCTCC<br>TATTGGGATTATAGGCGTGAGCCACCGCAACCGGCCTTCTAATTTCC<br>CTTTTGGAGCATTCATTCCGTTGCAAGAGCTTATTTAATTTTTATTTA<br>TTTATTTATTTATTTATTTTTGAGACAGAGTTTTGCTCTTGTTGCCCA<br>GGCTGGAGGGCTATCACGCCATCTTGGCTCACCGCAACCTTCGCCT<br>CCTGGGTCCAAGTGATTGTCCTGCCTCAGGCTCCTGAGTAGCTGGG<br>ATTACAGGCATGTGCCACCATGCCCTGCTAATTTTGTATTTTCACTA<br>GAGATGGGTTTTCTCCATGTTGGTCAGGCAGGTCTCGAACTCCCGA<br>CCTCAGGTGATCCGGCCGCCTCGGCCTCCCAAAGTGCTGGAATTAC<br>AGGCGTGAGCCACTGCGCCTGGCCAATTTTTATTTATTTATTTAGAA<br>ACAGGGTAAACCTCTGTCATCCGGGCTGGAGTGCAGTGGCACAAT<br>TGTAGCTCACTGCAGTCCCAAACTCCTGGGCTTAAGCGATCCTCCT<br>GCCTTGGCCTCCCAAGTAGCTGGGACTACAGGCGTGTGCTACCGCA<br>CCTAGCTAAGTTGTGTATTTTCTAATTTTTTAGACAGGGGTCTCAGT<br>ATTGCTGAGGCTGATCTCCAACTCCTGGGCTGAAGCGATCCTATCC<br>TCTTGCCCTCTCGAAGTGCTGAGATTACCAGCATGAGCCACCACAC<br>TGGACCAAGAGTTGATTTTGGTAACATCTTCTGTCCCTATCTGTCTG<br>AAAAATCCTTGAGAGTAGGATCTACCATTAATGCATTAAACAAATTA<br>TTATTTTTTGTTTGTTTATTTTGAGATGGAGTCTTACTCTGTAACCCA<br>GGCTAGAGTGCAGTGGCGCTATCTCAGCTCACTGAAACCTCTGCCT<br>CCCAGGTTCAAGTGATTCTCCTGCCTCAGCCTCCTGAATAGCTGGG

	ATTACAGGTGCGTGCCATGATGCCCAGCTAATTTTTATATTTATTTA<br>TTTTTATTTTTATTTTTTTGAGTTGGAGTCTTGCTCTGTCGCCTAGGC<br>TGGAATGCTGTGGCATGATCTTGGCTCACTGCAGCCTCCCTCTCCTC<br>GGTTCAAGCGATTCTCCTACCTCAGCCTCCTGAGTAGCTGGGACCA<br>CAGGCAAGTGCCACCACGCCCCAGCTAATTTTTGTATTTTTAGTAGA<br>GACGGGGTTTCACCATGTTGGTCAGGCTAGTCTCGAACTCCTGACC<br>TCAAGTGATCTGCCTGCTTCATCCTCCCAAAGCGCTGGGATTACAG<br>GCATGAGCCACTGCGCCTGATGGTATTTTTAGTAGAGAAAGGGTTT<br>CACCATGTTGGCCCGGCTGGTCTTGAACTCCTGGCCTCAAGTGATT<br>CACCTGCCTTAGGCTCCCAAAGTGCTGGGATTACAGGCTTGAGCCA<br>ATGCGATGGCCTACCATTAATGCATTTTTGTATATCTCCTCCCACCCC<br>CACAAACTGTCAGGCACGGGGCTTAGTGGGTGGAGGAAAGGCCC<br>AGCAATGCCTGAGTAAGCCCAACTCCTGCCTGCCTCTTCGCAGAAA<br>CTAGGGTGCATCCAAACTGTCTCTTCTCAGGCTAGGCAGGACAGCG<br>CGGCCTCACCTGTGAACCTTCAAGACTTGGACTTTGGCTGAAGCG<br>AGGGCGTAGGAGTTCTAAGTACCGCTGGCTGCACGCCCTCTGGCCC<br>ATCACGTCCCAGGCTGCTCCTAGGTGGACACCCAACAGGTTAGGC<br>AAGTCTTAGTTAAAAGCAGTGAGAGGCCAAGAGGCTGGGCATGGT<br>GGCTCACGCCTATAATCCCAGCATTTTGGGAGGCCGAGGCAGGCAG<br>ATCACAAGGTCAGAAGATCGAGACCATCCTGGCCAACATGGTGAA<br>ACCCTGTCTCTACTAAAATACAAAAAATTAGTCAGGCGTGGTGGTG<br>TGTGCCTCTAGTCCCAGCTGCTCCGGAGACTGAGGCAGGGGAATC<br>GCTTGAACCCGGGAGGCGGAGATTGCAGTGAGCCGAGATTGTGCC<br>ACTGCACTCCGGCCTGGGCAACAGAGTGAGACTCCGTCTCAAAAA<br>AAAAAAAAAAAAAAAAGCAGTGAGGAGGCTGGGCACAGTGGCTC<br>ACACCTGTAATCTCAGCACTTTGGCAGACCGAGGTGGATGGATGCC<br>AGGAGTTCTAGACCAGCCTAGGCAACTTAGGGAGACTCTGTCTCTA<br>CAAAAAATGCAAAAATTAGCTGAGTGTTGCGGCGCGCACCTGTAG<br>TCCCAGCTACTCGGGAGGCTGAGGTGGGAGGATCGATGTCCAGGA<br>AGTCCAGCCTGCATTGAGCCCACATCCTGCCACTGCACTCCAGCCT<br>GGGTGACAGAGAGAGGCCCTGTCTCAAAAAAAAAAAAAAAAAAAA<br>AGAAAGAGAGAAAGAAAGAAAGAAAAAATAAAAACAGCCAACG<br>ACCAAACAAATAAAAGAGCAGTGAGAACCAGACACGGTGGCTCAT<br>GCCTGTAATCCCAGCACTTTGGGAGGCAGAGGCAGGAGGATCACT<br>TGAGCCCAGGAGGTCCAGACCAACGTGGGAAACATAGCAAGACCT<br>TGTCTCTACAAAAATAAAAATAAAAAAATTAGGCTTGGGGATACAG<br>GTCTGCAAGCCTACTTGGGAGGCTGAGGTGGGAGGATCAGTGAAA<br>CTTGAGGTCAAGGCTGCAGTGAGCTGTGATTACATCACTGCACTCC<br>AGCCTGGGTAACGGAAGAGAGACACTGCCTCTAGGAAAAAAAAA<br>AAAAAAAAAAAAAAAAAGGCAAGGAGTTCCAATCATTCTCCCTTCA<br>CAGAGAAGCAGCTTGGATTCGATGGGACTGTAGTTTCTGGAATCCG<br>ACTGCAAAGCTGACCGCTGGTAGAGCCCAGCTTCTAACTTAGGAA<br>AGGCACTAGCTCTCTACAAAGCCGCCACTCCCCAACTACACCCTGT<br>AGGGACCAGCCAATCCCAAAGCCCGAGCGGGCGTCATCACCAATT<br>GCAAGGCGGGAGACAGAGCCTTGGCTGGCACCGGGCCTCCTCCCC<br>GGACCCGCAGAGGCCTGGCAGCGCCGCGTTTGAGGAGGGTTCGTG

	TCAGGAAGTCAAGGCTGGATGATCGCTTAAGCCCAGGAGTTAAAG ACCGGCCCGGACAACAAAGCGAGACCCCGTCTCTCCAGAAAAAG AAAAAAAGAGAGAGAGAGAGAGAAATTAGCCGGGCGTGGTGGCG TACGCTTGTAGTCCCATCTACTTAGGAGGCTGAGGTGGGAGGATCG CTTGAGCCCAGGAGGTGGAGGCTGCACTGAGCTATGATCGCCCCA CTGCCCTCCAGAGTGAGCGACAGAGCCAGACCCTGTTGCTAAAAA AAAAAAAAAAATCCATAAAGCCGGGGACCATCTTTGCTCCAGGGA GGTCAGCTGACCCGGGCTGGCACTGGCCCTGGCGCAGTTCCCGCC CCTCCCGGGAGCGCTGGCACCGCCCCTTGGCACCACCCCCTCCCC GCGCCCCCGCCTTCCAGGAAGGGGCGGGGTCTCCGAGACGGGTGG GGCCGGAGCTCCAAGCTGGTTTGAACAAGC
DFFB	ACAAATCCAATCTTGTGGGGCATTCTGCACGTTTGGGCTGTTTATAA GAGCAGGAAAAACTTCTTTAAAACAAAGGAATTCACTAACACATG GACATTTAAAGGGTTGGTTCATGCCTTGCTAACACACACAATAATG CACTAAGTATTCAGAATTTTCCCATCTCTTTGAAAAGAGTCTATTTT TTTTTGTTAAACACTTTTATAGATGCTTCTGGTTCTGCTTAAGATATC ATTTAAGTTATCTTGACTTTTCCCAAATATGTGTCCAAAAAGTTTGA CCAGTGCTGTGGATTGTGCTCCCCAAAAGGATACGCTGAAGTTCCA CGTGTGGTACCCGTGAAGGAGACCTCATTTGAAAATAGGGTCTCTG TGGGTGTGATCTAGTTAAAATGAGGTCCTCGGGGTGGGCCCAATGC AACGTGACTTCTAAGAAGGGAAGAGACACAGCCACACAAAGGGA GAGCGCCATGTGGTGACAGAGGCAGAGACCGGAGCAACGCATCTC TAAGCGGGCCGGCCAAGGACGGCTGAGGATGGCCAGCAGGCGCC CAAGGTTAGGAAGAAGCCAGGAGGGATCCTGCCCTACAGCCTCCA GAGCGAGAGTGCAGCCCTGTCCATGCAGCCATCTGACTTCTGCCTG CAGAATGAATTTCTGGTGTTTTAAGCCATCTAGTTTATGATATTTTGC TATGTCTAGAATATAAACACAAACTTCTTTCTATTAGTTAGGAAACT AATACAACCAGTATGTTAATTAGTTATACAATACCAAGGGGAAAAA GGCAACGTAAGCGCTAGCATTAACAACTGCTAAATGAATGTGTATG ACCAGAAGGAAGAGCAGGAATGACAGGCAGGCATATTAAGTACAA AAAGGTTCATGCAGAGTTAGGTTGAGGGACTGACTCACAGGACAT TGAGCCCAATTTGCTCAGGGACTCTGGGTTACTTTTCAGTTACTAA GCCAGTCAGATTCCTCCTAAGTAAGCCAATTTGGGTTAGGTCTAAAT CATCGGGGGAAAAAAAAAAAGGCTCCTCACTAAACCAGGATAAAG AAGACTCCATCAGAAAGTTCTCTGGGATAAGATATACTATTAATTGA AATTCCCAATAGGAGGTAACATATAAACATACCCCCACATAAAACTG TACGTATTTTGACATGCTTGATGCATGATCTAGATCTTTCTTTTACTT TCATTGAGAATTCGAAGAGGACCACTTCAATATATTAATTATTGGCT GGCATTATTGCTAATGTTTCTTGGAATGTATGAACAAAAGCACTAAA ATAGAATGATAAACCATCGAGATTCTAGTTATTTTTTGCACAAAGTT CACTGCAAGATGTTACTGTATATTAAACAAAAACAAAACTAAACCA ACTCAGAAAATAATTTTTAAAAAGCCCCCCAGAACAGGATGAGGCT GGGTGTGGTGGTTCCTGCCTATAATCTCAGCACTTTGGGAGACCGA GGTGGAAGGATCACTTGAGCCCAGGAGTTCGAGACCAGCCTGGGT AAGAGAGTGAGACCCCGTTTCTAAAAAACCAAACAAAAAAAGAA TTTTGAAAGGATGAGTGTGATTTAACCCACTGTTAGCTGCACGGCC

	CCACCTGCACTCTGGGCAACCTATGCCAGAAACGCACAGCCCATG<br>GCTCCCACTCCTTCGTGACCTGGCACTTCTGGGTCGCATGGGCTCT<br>GAGCCTAGAGGTACTTTTCCCTTGGCCACAGCATCTCTCTTCACCC<br>ACTCCCCATCCAACAGTGTGCCAGGTCTTATCAGGCCTGCCCTTCC<br>TTCTGATCCCACTGTCTTTTCTAGACAGCAACCACCTGGTGTCCCC<br>ACCTGAAGCGCCTCCCTCAGACAAGTCCTAAGTTCCTATTGTGTGA<br>ACGTGCCTCAGGCCCAGCTCCAGTCATCTCCATCCATGTCCTAACT<br>CCAGAGCTCTTGGTGGGCTCCCCATCTAGGGATCAATGCGCCAACT<br>CTCCCCTGACCCTCACTTCTTGTCCTGAATTCTGAAGTCCAGCCAA<br>ACAATCAAAGCTCAAAGTTAATACCCTTCAGCTCTGAAGCCCTCCA<br>ATCCTTTCATCAGCTTTTTTTCCTTTTTTGAGACAAGAGTCTCAATC<br>TATCACCCAGGCTGGAGTGCAGTGGCGTGATCTGGGCTCATTGTAA<br>CCTCTGTCTCCCTGGTTCATGTGATTCTTGTGGTTCAGCCTCTCAAG<br>TAGCTGGGATTACAGGCATGCACCACCATGCCTGGCTAATTTTTTTT<br>TTTTTTTTTTTTTGGTATTTTAGTAGAGACGGGGTTTTGCCATGTTGG<br>CCAGGCTGGTCTCGAACTCCTGGCCAATCTGCCCGCCTTGGCCTCC<br>CAAAGTGCTGGGATTACAGGCATAAGCCACCATGCCAGGCCCCTTT<br>CATCATCTTTCAGGGCAGGGCTTGTCACTCCCTATTCCACCTTGAAA<br>ATACCTCTTGTGCCCAGGCTGTTGGGTTCCAGTCTCCAAATTTTTTT<br>TTTTTTTTTTTTGAGAGAGGGTCTCACTCTGTCACCCAGGCTGGAG<br>TGCAGTGGCGTGATCGCGGCTCACTGCGACCTCTGCCTCCTGGGTT<br>CAAGTGATTCTCCCGCCTCAGCCTCCCAAGTAGCTAGTATTACAGG<br>TGCGCACCACCACACCCAGCTAATTTTTGTATTTTTAGTAGAGATGG<br>GGTTTCACCATGTTGGCCAGGCTGGTCTCGAACTCCTGACCTTAGG<br>TGATCCACCCGCCTCAGCCTCCCAAAGTGTTGGGATTACAGGCGTG<br>GCCAATAAGCCCAGCCCCGATTTTTTTTTTTTTTTTTTTTTTAATACA<br>GGTTCTCGCTCTGTTGCCCAGGCTGTACTGCTGCAGTGGCGTGATC<br>ATAGCTCACTGTAACCATGAACTCTCGGGCTCAAATGATCCTCTTGT<br>CTCAGCCTCCTGAGCAGCTAGGACTACAGGCACATGCCACCATGCA<br>CAGCTACTTTTTTTTTTTTTTTTGTAGGGATGGGGTTTCACTGTGTT<br>GTCTAGGCTGGTCTTGAACTCCTCCCACCTTAGTGTCCCAAAGTGT<br>TGGGATTACAGGCGTGAGCCGCCGAGCCTGGCTAGTCTCTGAACCT<br>AACCAACACTCTCTGTTACGGCTCGAAGACATAAATATAGGACCTT<br>TTCAGTCAAACAGAAGGGTGTTTCCAGTATGCTATACTGCCCCCAA<br>CAAATCTAAAACAAAGTACTGGCATTTAAGAACCAGCTAAGAGAA<br>ATGCACAGGGACTGTGGAGGCAATGCTATTACCTCAGTCTGGTATT<br>TGAAAAGGATTCCTTTTTTAGTCTACCCCCTCCCAAAGACCTTCCA<br>GTTGTACCCTAGGGTCCCCTCTGTGTAAACCTTCAAGTGTTACATAA<br>GATACTTGGAATGTCACTGAAGGATGCCACTTTTAGGCTCTATAAAA<br>ATCACCATTAACAATTTGGGAAGAAGCAAAGCTCTGTTGAACATCC<br>AGTTATTGGCTGGTCTTCCTCACCAGAGGCATGAGGGATCCCTGTT<br>GTCCCAGGGGCAGCCATCGACATCCCGACAAATGCCAGAGTACATC<br>TCTCCCCAGGCAGCGATGACTCCATGGCGGTCTCATCCCTCACTAC<br>ACTTTTCCTAGTGCTGGCATGGTAAAAGGCAATTAACTATAAATTAA<br>ACGGAAACTCAGTGTAGCAACTTCCATTTTGTTACAAGACTAGGAA<br>ACAAAACTTGTCTCTGATTTTCTTTTTAAGAAGACTTTTAAGTGTGA

	CTGCGCGCGGTGGCTCATGCCTGTAATCCCAGCACTTCGGGAGGCC<br>GAGGAGGGCGTATTACTTGAGGTCAGGAGTTCGAGACCAGCCTGG<br>CCAACATGGTGAAACCCCGTCTCTACTAAAAATAAAAAAATTAGTC<br>GGGCTACTCGGGAGGCTGAGGCAGGAGAATCGCTTGAACCCGGGA<br>GGCGGAGGCTGCAGTGAGCTGAGATCGTGCCATTGCACTCCAGCC<br>TGGGCGACAGAGCGAGACCGTCTCAAAGGCTTATGGGTTTCTGATC<br>AAGTCATCTTAGCGACTGAAAGGGCTTCAGAGAAATCTGAACTTAA<br>GTGCAAACAAAACAAAACAAACTCCAAAACAAAAAACAACCAAC<br>CATCAGACTTTAATCTCTATAGGGACAGAGCAATTTTCCTCAAACTT<br>TTGCTCAGCAGAACACATCTGAAACTGGCCCCCTATGTGCTACGCA<br>TCCAGAAGAGGAAAAGCCGGATCTGAAAACAATATTCCACGCTAA<br>ATGGGCCGTCCCGGCCGGGGCAGGCGGTCCCAGCACCCGTGCTGA<br>GCTCCCCGGCATACGGCACGACGGGGCCGCGCCGGCTCTTCGGCG<br>ACAGCAAGGCCGGCATCTAACTCCACACCGTCCGCGCTTGCACCG<br>CGCACCCAGGCTTCGTACCCATCTCCCATGTGGGGACCGCGCCCCC<br>GCGGTGCCCGCGCCCCGCGCCCCGCGCCCCGGTCCCCACTCCGGC<br>CCTGGGCCGGCGCCGCATCGGCCCGGAAGGGACCTGCNNNNNNN<br>NNNNNNNNNNNNNNNNNNNNNNNNNNNNNNNNNNNNNNNNNNN<br>NNNNNNNNNNNNNNNNNNNNNNNNNNNNNNNNNNNNNNNNNNN<br>NNNNNNNCCCCTTCCGCCGCCCCAGGCCGCCTTGCACCCCAAGCT<br>GCTGCGGCAGTGACAGGGAAGAAGCAGACCCGGCCACCTCTGCCC<br>ATAGCCCCGGCCGCAGCCTCCACTCTCATGACAACCAAGCCCAGCC<br>CTGGGCCAGGGCCTCTGCGCGTGCGCGACCCCGCCCCACCCCATG<br>CCGCGCCTGCGCACAAGGGGAGGGCTCCGCTACTCTGGCCCTCAG<br>GGTTGTGTAGTTTCCGGGGCCACGGCACCTGGGCACACCAGGTGG<br>CGTCGCGCCTTTGCTTTCCTGAGCCTTCTGAGTAAGGTAATGTGGT<br>GTCCGTGGGGCGACGCCTGCGCACAAGACCGCGTCGGGCCTCACT<br>TTTCCCAGGCCTTCTGGGTAATGTAGTTTCCGGGATCGGCACCCGG<br>CCTGTGCCAGCTTGCA
DIO2	ACAGAAATTTTTCATGCTACAGTTCATTCTATTAGCTTCCTCATTTCC<br>ACTGAGGTTAGAATAGAATATCTAGTTTATTTTCTTTGAGCCTTACTT<br>AGTGTACTGATTCTTGAGGGAGGAGGTGTGGCATCCTTTGCTCTCA<br>TTGGGAACAAACTACACCATTACCTCTGAAATGGCATGAGTCAGCA<br>TGTTTCTTAGTATATTTTGTCATGCTTGGTGTTCACATTTTCAGCATA<br>TGAGGGAGGTGATGGATAAGTGACTTTTTAAAAAAGCTGCTGTAAA<br>GCCATTTATAAAGAATGACCTATTTTATTTTAAAACTTAATATCATCC<br>GGTGGTTTCCTACGAGCCGAGGAATGAAACAAAGTAAAGGAAATG<br>TTGTTCCAGAGATGATGACTTTTTTTGACCATTTTCCTTTGTTTCTTT<br>TGGTCTACTGCATTGGGTCAAGTATTTAGGGAGAAAGAAGAGATTG<br>AGTCACAGTTTCAGAAAAGAAAGGGTGAGTTCAATTTCCTCATTTT<br>CAGCTTTTTATTTATTTATTTATTTATTTTTGGTCATGAATGAATTCAC<br>CATGTATAACTTTACAAAGTCTTCTTCTCAATTCCCCTACCACCCCT<br>CTTCCCACAGCATCCCAAACTCTGGGTGTCTCTAGAGGCTGATAAA<br>CTGTTCTCAAAACCAAATAAGAGTCAAGGCACCTGGAGAGGTTTG<br>GGCAGTGGGGAGAGGGGATTGGCTATCAATGTGGATCCAGACTAG<br>GCCAATCCTAAGTGAAGTATATTGTTGTCTCCTAACAGCTGTTCTGT

	GGCCTCTGTGAAATGCGGTGGTGGCTTTCATCCAGATACCGGACAG CTGTTGCCTCACAAAGAGCATCGCCACAATTAGCATTAACTGGCTC TTTGTTGGTGGCCTCCACTGGCAACTCATTGAATCTTTTCTTTCCCT GCCTATCTCCTACCATCTTTATCTCCCTTTTTCTCTTCATTTTTGCTTT CCCTCTCCTTTTTATTTCCTTTGTCTTTTTCTCCCCCTCTGTCTTACC AGTAGGTAGCCTGGAAAAAAAAGAGAAGCAACAGTTCCTGAGGG AAGAATGCATTAACAACTCGGATGGGTGCTGTCTGCCAGCACTGCA GAGGAACCTACGGAGAGGGCTGCTGGGAGAGAATGCTCATACCTC CCAAGGCGTCAGCCTGCCCTCCCGCCCCCAAGTTGCTTTGCTCAAG GTGTGTCAGAGTTTACTCTGGGATTGTGTATTTTATCCATTGAGCTT CTCCTTTCATCACAAGGCAGAGAACACGGCTGTGTTTGTGGAATGG ATGGGCCCACAGGGAATTTTGAGTGATTCAGTAGTTGTGGTGTAAG CCGTTCGTCATAACTCCCTGTCAGGCAGAGTCACTGGAGGCAGTAG GTTATACAGTTTGTGATTCCTTGCTCTGATAAGAGCGGCGCTGTTTT AGGCGTTATGTATGGCAACCATATTCTCAAATCCAAAATCAGCATGC CTGCTGTTTCCAGACATTTTTGGATTTATAATGTGTTTATTTGACATG TTTTTAAGAAAGTAAAAATGAAATGCCATTTTTTAAATTATGCACTT GACAAATCACTGGAATTAGAAAGAATAAAATTCCACGAAACAAAG GCAGCTTGAAAGTCCATGGTATGAGTTGTTAACATGACAGAACTGT CTGGATGCTCTTCTGCTCTTTCAAATAAGAATAAGGAAATAGTAATG GCAACTGTTGTGCTTAAAATTTCCAAGTCTGCTTTCAGATTATCCAA CTTTATTTTCTCAATCTCAATTTTAGAGTCAAGAAGAGGACTAAAG AAAGTCTTGTGACTCATATGCTTGTTATTTTCCAACCAGAAAGAGG CAACAGTAGGACATGATGCTCTTTTACTATGGGAATATAAAAAAGTT AAACAATTTTGCAAAGGTTTTTTCATAAAAAGCCCAAAGCATTTTG CCCTAGGCTGCTTCTCTTTGGAATTTAAAACCCGTGTGGAATATCGT ATGGGGAAATGCAACCGACATGCCCCTCGTTGGTTTGCATTTAACT CATTACATGTTGATTGCTAAGAACACATACATGACCAAGAAGATAG CCAAGCCCTTTGAAAGAACATATGAAATCTAAACAAGGAGCCTGA GTCGGGACAGTCTTAAACAGATGTGAAGTCCTCACAGCCTTTGCCA TTTTGAAGATTAAATTACTAGCAAACCACTTTCAACAAGGGTTTTG GTTCTCATAGTAATGACCAGGACCCATAACAGACATTAAAGAAAAA AAGTGAGGCTTATTTAGAGTTAGGGAAAATATCCCAACACCCGCTT GGACTCCCTAATTTTGGTAATCTGCTTTCTCTCATCTATATGATGGGA AAAGTCATACTCTGTCACCTTAGGATTATTCTGTTACTTAATGAGTAT AAAATTACTTGAAAATCAATGTGGTAAATATTCTTAATAACTGGAAT GAGCAATTGTGCACCATGCATAAAAAAAAGAAATAGCTTTGGAGAAA TGTAATTTCTTGGATGGACAGATTGTCTATCTGCTAGAGGAATGGCC CTCAGTTTTGTGATGTGTTTATCGTTCATTTAGAAAAATGATTGAAG AAATCTGAGAAATGACATAACCAGATTGGCTGTGTATCTTTTTGTTC TCCTATTTAAAAAAAAATTCCCTTCAAACACTTCTAACCTCAATAAA TAAATAAATAAAAAGAAACAAAAGCCCCCAACAGAGTCCTAATTCT AACTTATAACCACTTAAAGTCTGGATATGCTTTGAACTTCACATTTA AAAGAAAGGTTTTTCTGATTTTCTATCAATCCCTCTCCTTAGCTTGC CATTTACAAACATACTTTCCTCCTTCAGAGCAAAAGCAAGTGAGAG AGAATTGGTTTAAAGCTGCTGAAGTCTCCACTGCAGGGGAGCACT

	GGTTTAATTCAATCAAGGTAGAATGAAGGTCTGAGAAGGGGAGAG ATTCTAAGGCCTGGGCAATTGAGTGGAAGCAGCATTTCCTGAGTCT ACACCTCCTTCTGCCATTGTTGGTGGGAACTATGGCAATATAGCTTT CACATCTCCACCAGTTAAAATACTGTGCTATCAACTCCCTGCCTTTG TTCTTTCTTTTTTCCCATAAGGACATTGAGTAGTTTGAGAAGATACA TGCAGTAAATGTACATTGCATATATTAGTAATATTATTTTGCTAACATT GTATGCTAGTCATAGGGAAGGTGTAATTTTTCTATGTGGTTTATATTA TTTTTCTAAGCTTTGAAGTCTTTTAACAATTTCGTCCATGAGATGCA AATTCAAAACTCATTAGGAGCCAAACTAATGAAAAAAATGAAAGA ATTTGTAGGGAATGCTGAGGGTTGTGTCACAAGAGAGGGCATTGC ACAAGGCCACCACGTTTTACAATTCCAGGGCACCCCCATTCACATC ATATTGTATGTGAATGGTGCCCCCTAGTGTAGAGCAATGCAGAGGC CCTCTACATGCCCTGTTCATTCATTCATTCAGGACGTCCTCAATGAG CCAACTGTGCGCCAAGCATGGTTCTAGGTGTTGGGGAGGTAGCAG TGAACAAAGTGGACAAGAATCCCTGCTCTCACGGAACCTAAAGGC ATTCTTTTTTGATTATTGTTTTTTTAAAGGCAAAAAAGCAACCTGCC AACCAAACAAAATGCATTAATATGCAGAATTCAGCTAACAGGACGA CAAATTAATGACTCTTGTCTAGGCTTTTAAATTATTTCTGTGATCATG AAGGTTTTAGCCACATGGAAAAAATACGCAATACGCTAACCACAAG CTTGCACCTACTGCTTAGCTTATGCCTAAGCTCTGGCTACATAGAAA ACTCAGCTATCTATTACCCAGTATCTAAAACTCAGTATCTATCACTGA GAAAACTCAGTGATCTATTAGGTAACACAGGATGAAAAAGTAGAAT AAAGTGGGGGTCATGCCATTCTGCCAAAGCATTGTCTGCCTAAATA CCTGTCCTAAAGACTCATGGGAGAGGCAAGCCAATGAACAATGGT CAATGTGGCACCTTGAACCAATTCAGTCGGCTCTTAACTTCCCATAC ATTTAATTAAAATTTTTTTCAAACAAAAAGGAAGATTCCTTAAAGTA TCAGGTCTCTTAGGGGTTATGCTAACAGAAGCAGGTGAAACACCA AATTTAAAGAGCTTTCATTAATAATTTCTCAGCCTTATGGCTTCTTAA TGCTATTCAATGGAAAGTTTTAATTTTATCTGCAAAATGAAGCTAAA AGAATGTCGCCTAGCTCCTTCCCTGTCTGGAGGAACTTGGATTTTTT TTTTTTTTTTCATTAGAAGCTTGGGTAAAACTAAAAAGAAACCTGTA TTCAAGTTTCTGCAAGAAGCTTTAAAGAAATGCTGGGATGGTACTC AAATAGTTTACAAGTTTTCCACCTACAACTCATTTTGCATTTTCTTA AATAAGATAATGACTCAAAAAAATTTAAAGGACCTAGGTCACAGAT CTTACAAAGGGTTGGAGTCATGGTATAAAGGTATAAAGATTGTATAT AAATGTTCATATTTTCCAAGAAACAGAGCAAAGACCGCCTGTGATA ACTCTTCCTTTTTTCTACCTGCCTTCCAGGATCCTTTCTTTCCAAAA CCCCTCATAAATTAATGATAATGATAATCAGAAGAGAGAGTGTTGGC CATATCAATCTGCACATTATATTAAATTACTTTTCCCAAAGTTTGGAA GCATTTCCTGAAGGCTGTCAAGGGTATTAGTTTTTTTCTCTCTTTAA ATCACAAAGCAAAATTCCACTGTCTCTCCCCAACCCCTTCTAAAAA AAAAAAGTTGTCGAGTTTTGCTTTTCTATTCACTGCAATCCTGGCC AAAGTAAAGCCCTCTTTCTCAATGACGTCAAGATCTTTACCAAGAT TAGGCTTTCACTTCTCTATTGCAGCAATTAGCCAGGGAATGTATAAA AGGCGTCAGGGAGACTCACTGGGCTGCAGAGAGAGGCACTTTGCA C

DKK1	CATACACACACACACACACACACACTTTTTAAAAAGTCGCCTGGTCCA<br>AGTAATTCACCTTATTTCCAGGCACTTAATACTTACATGCTAGTCTCT<br>TCAAAATCGACATGCTCAGTATCAGTGTCAATGATTATTACTTGATC<br>TTTAGGCTGCATAAAAGAACAGACTCCTTGCAGGATGTTCTTATTTA<br>ACCTGAGTACAAAAGGCCTTCTCTTGGCAGTGCTGAAAGAAAAAG<br>AAAAGAAAGAGAGAGAGAAAGAAAGGGAGACTTTTGTAGTGAAA<br>AGGAACCTCTCCATCTCCCCTGCATCCCATTTTATAAAGAAAAGCC<br>AGTACCACACCAAGAGTTAAATGGCTTTCCCATATTTAGGAGCCAG<br>ATTATGGCTGACACCCTATACAGAGTACCATCCCTAGCCTCTTAACT<br>CCTTGGCACGATGCCTTTCTTGGCCATACCCTGGGAGAAGATCTGT<br>CTTATAAAGGAGGTAATAATAGGAACCCAGTGACATGGAAGAAAAT<br>TGTTTTTCTGTAGATGCGGAGACTGTGTCATGGGATCCTACATCTTG<br>CTTTCTGCAGACACTGGCCTATCCTTAGGGTTGGAGATGTTAAATCC<br>TTTGGGGACTCAAAGAGGACATTGCACTCCCTATACCCACCACCAC<br>AGACCTTTCTGAGTGTCAGGACCTCAGAAAGGATGCAGGCAGATA<br>TCTATCTGGTTAGGCACTATCTTCACTGGGTTTAGAGGCTGGAACCC<br>AGCCTCTACTCTCAGATTAGCTTTTAATGTCTTACTCCAGTAAAACA<br>CATCTTCTGAGCTTGGTTAGAAAGCTACCACGGAGCCTGATTTATCT<br>GGGCTGGCCATCTCCACCGCCCAAATAAATGTAATCACGGTCAAAT<br>CTTGCCCTCTGTTTGTACTGGGGAGTGCAGAAAATCCAGCTCTCAG<br>GGGAGTCATAACATGGACCAGGTCTAAAAGTTCTTTTAACATCATG<br>AAGAACATTAAGACCAAACTGTTTTAAAAGTCAATGAGAAAGTCAT<br>ATCATTTTTTACAAAACATCAGGAATGAGGAATCCCTGCTATACTCA<br>AGATTGACACTAACAAAAATGTTGGCAACACCCACCGTAAGGAAT<br>GATATGTATGCGGTATTTTCCTAGCAAAGTAAAATTATATGGCAACA<br>AATCTGCTTCTTTTGATTTAATGAAGAGAAAGAAGGAAAGAAGGG<br>AAGGAGGGAGAGAAAAAAGAAAAGAGGAAAGAAGGGAATTTAA<br>AATACAGCAGTAGACTGGGCGCGGTGTCTCACTCCTGTAATCCCAG<br>CACTTTGGGAGGCCAAGGTGGGAGGATCACTTGAGCCTAAGAGTT<br>TAAGACCAGCTCTGGCTAGCAACATAGTGAGACCCTGTTTCTACAA<br>AAAAATTAAAAATTAGCCAGGTGTGGTGGCATGCAGCTGTAGTCCC<br>AGCTACTTGGGGGTGAGGTGGAAGAACCGCTTGAGCCTGGGAGGT<br>CAAGGCTGCAGTTAACCACGATTGCACCACTGTGCTCCAGCCTGA<br>GCGACAGAATGAGATCCTGTCTCAAAAAATATATAAAAATATATAAA<br>ATAAAAAAAATCAGCAGTCACAAATAATAGAGATGGAAAAATAGTT<br>GGTGAATAGCATGGAGTGTTTTGGAGTATATTTGTCTGTCTCCCTCA<br>TGGATGAAAAGGTTGAGAAATATTCTATGCATGGCATCCAGCACTT<br>AAAATATAAAGAAGACATGTGTTAGCATGCTGTTCCCGAGTTATGA<br>GGTTCAATTCCACGCTGATAAGCATATTTCACCTATTCAAAAATATAA<br>TACAAAATATTAAATTTGTTACATTAATTATGATCATATTTATACCAGA<br>CTGAATAGGAGGCAGGGTTCCCTACAGGACTAAATAAACTCAGTTT<br>AATAAACTAAATTCATCTACTCTGGCTCCCACCTCCTCTGTCACCCA<br>GCTGTAAGAGAGACTCAGACAATCACCAGAACCCTCTCTCCTTCCT<br>AAGACATGTTGGTCACAGTTCCTGCACATTTTTCTTGCTCTCCATGA<br>CCTAGTATCTGCCAAAATATAATCGATGTTGGAAAAATTTTTACCCC<br>CAGCCCCATGTAAGTATGTTGTATTTCCCCAACTAGTCACAGACTGA

	AAGCTGAGGACAAGAAGCCTGCCTTTCATATTGTGAGATATGCTCC
	CAATAATGGATACTCACCGAATATTAACTGGCTGCTGATGGAACAAT
	GAGTTTTACTATACCTCTTTATCTACCTTGCACTCTTGTTGCCAATGA
	ACAAACCACATCCTGGAAAGGATTTTCAAATGGAGAAAAAGTACT
	GAAAAAATAAATTTCCTCATTTGGAACAAAGGTTTGATTTGCTATGT
	ACTTAGCCCTCTCTGAGGACCACCCATGTAATTTCTCCCCTGAATAA
	TTTGTGAGGTTACCTCTCCTCTGCCCACTAAATGATTTGAAGGTAGA
	TTATATGTGAACTCATCTTTTATCACACAATATCATGTATAATGCTTAG
	CACAAAGTAGGAACTCAGATATTGACCAACTCATTATTAGTTTTAAA
	CTCATGACAACTTCAGGAGCAATGTGAAAGCTGAGGAAGGAGGTA
	GAAGCTAGCCCATTCTTGAAACTCTATGCTTGGTTTAGCTCTCCTAG
	CTCAATACCACAGATTCTGTCCAGACTCAGTGAATATTGAAACATAA
	TAGACAAATGATATGTGTTCAGAGATCAAGGTTGAATTTATGCAAC
	AAGCTTCTTTGTTATAACAGAATTTTCCTTTTGTTGATTAAATTGTTC
	CACTTTCTCTTCTTTTGCCCCAGGCAATGGATTTTTTTTGTGAAGCC
	AAGCTTTTAATGAACCAAGTTCATTAAATGTATTAATCATATACTATC
	TCTTCAGAAAAAAAAGGCAAGAGATTGGCCTTTAGTGTGGCACAAA
	TATATTTCTAGATTCAAAATATTTAAACAGGTTAATAGGACTAAAAA
	GAAACATTTTCAACCTTCTGATTGGCAGGCGGATTTTTCTCTACGTT
	TCAAAGTCATTCAAACTTAACACAGCTTGCAGATTTCCTAGTACAC
	TGAAGGTGCTTAACGCATTGATTCCTGACAGGATAATTTCCTGGAC
	AGATGGAATTTGGGATGGGAAGGACACTAATATAACTAGGGTACCA
	TTCAAGAATTGTTTAGATGATCTTCTTAATATTACCTTTGAAACTGA
	AGGAACTAGAACACAGACCATAGTAAATTGTGTTTTAATTACTCCTT
	CTCCCAGTAAACTTTAGGATTTTTTTACTTTAAGATTGTCACTGGCT
	TTACTTGGTGGTGGCCCCAGTTCTGACTATAGTAGGTCAGCATGTG
	GAGTCATTATAGACAGCCTAAATCTATACAGCTTAGAGGAAGACAA
	AATAAATATATCTTACTCTTTTTTAATTCAATAGTGATTATTCAATCAT
	CTCTCCCTTTTCCAAACTCACAAAACAGTTGCTTATTTCTGACACT
	TATAATATCAACCTGTTTTTAAAAGAATTATGTATGCTTTTATATTTAT
	TGCTATAATTTTATTGTTACTACATCTTTTATTATTACTGTAATTAGAAT
	AAAGAGAACTTTATTATTTAAAATGAGAGCTGTGTCTCATAAATATT
	TGTATTCACTGTGCCCCTCCCCCCACCACCCATCTCACCAAAAAATT
	TAAAAAATAAAAATAAGGTAACAACCCAGAGGGCAGCCCAATGCC
	AGGGCTCTAGGCTTCCAATAAGTTTTTGTTGAATTAAATAGATCATT
	TCCCATTTATAAGCATGACATCATTCTTATACACCAGCCTTACTTTAT
	TATATCCACACACCAATTTCAATGACGTCAAATTCACTTTCTAATTC
	ACTTTCAGCTCTTCTTCTCCTCAAGGTTGTTTCTTTTCTCCCTAGAA
	AGGGTATTGCGTGGTCCCGTGCAAGGTAAGACTTGGGAGCAAACC
	CTGCATTCGGAAGCGTTGCGATGTGATATACTAATGAGGAAGTCAG
	GCGCTAAGCCCTAAGGGTTTCTTATTTCTCCACATTAGCCCACCACT
	GAGAAGGGAGAACCGGGGAAAGGCGACTGACCTGGCACGTTGGA
	CCCTGTCCGCTTTGGTCCCGGCCACTTTGATCTCACGCGTCTGCCTA
	ATCAAGTTCATCTACCGCCGCGATTGCCCTGATTCAAAGAACAACA
	TTAAATGGTTTGATTATCGGATGGTCATTTCCCTCTTCCCTCCACCCC
	TGATCTAATTTCTAAACGCCAGTCTCTCGCCTCCCTCTCTAAACTTC

	CCATCAAAAGAGAATTAGCTCTTCTTCCTTCTTACCCTCGTTTGATG<br>TGCAAATTTCCTTCTCTTTGCACATTTTTCTCGCAGTGGCGGGGGG<br>AGGTGGGGGAGGAGGGCAACTGAAGGACCTCAAAGCCGGGGATC<br>TAACTCAGATCTGCAAACTGCGACTCTAAAGGGTTAATGAGCAACT<br>TGCACCCGCCACCTGGCCCTCTCAGAGGCGTCTTGCTAGAGCCATT<br>GAATTACAGACCGGTTTGTATTTAATACTGAATATGACTTGTGTGCA<br>CAGTCAGCGAGTATTGGCATAAAGGAGAGGGGCAAAAAGTGCTTT<br>TGCAGAGCCTATCACCCCTCGGCTCTGTAAAGTATTTCAGATCCGGA<br>TAATTCAACCCTTACTGCCAGGCAAGGGCACCCAAGTTCCCAGAGT<br>TCCTGCTGCCGCCTGCATTTATTAAAGTCGTCTGCTATAACGCTCGC<br>TGGTAGCCTTCACCCCGAAGGTGAGCCGGGCCAGCCGAGCGACTA<br>AGCAAGGGAGGGGCGGGGTGAAGAGTGTCAAAGGCCCCCTTCAT<br>GTACACAAACACACCCCCTCCCAGCCCCTCCCAGCGCTTTGAAATC<br>CCATCCCGGCTTTGTTGTCTCCCTCCCAAGGGGCCGGAATGCTCCG<br>GGCCCGCGGTATAAAGGCAGCCGCGGTGGCGGTGGCGGCGCAGAG<br>CTCTGTGCTCCCTGCAGTCAGGACTCTGGGACCGCAGGGGGCTCC<br>CGGACCCTGAC
DUSP1	AGGTTTGGTTCACAGGCTCTAGTGTGCCAATCACTGTCTTATATAAC<br>CTTCCTATCAGCCCTACAAGATCGATATGATTATTATTCCCGCTTTAA<br>AGATGAGAAAACTGAGTCTCACAGAGGTAAAGTAACTTGCCCAAA<br>TACACACAGCTGGTGAGTAAGCAAGCTGGGATTCTAATCCAGGCA<br>GTCTAACTCTGTAACTTGGTTTTAACAACAATGCTGTTACCTACATA<br>TGATTGCCTTCTAATCTTCTTTTATTCTTCGTTACAAAAGTTACACAT<br>TTAAGTTTAAAAAGTTCAAATCATTCAGAGTCATAAGGAAGAAAAT<br>GAGAGGAACTCCACCTTCCAAAGATAAACACTGTTAAGGGTTTGG<br>ACACACAGCTCACACAATGCTGACTACGGTTAGAATGTTCTGAACA<br>CTGTGTGACTTTGGACAGATTGCCTAACCTCTCTGGGCTCTTGCCT<br>CTCAAATGAGGAGGTTAGACAGGATGACACAGCTCTGTGGGCCCT<br>GCCAGGTCTGAGCATGGAGATCTGTTTGTAAAGATTGTGCAATGGC<br>AGCTAGCTCTGTCCCAACTGCCTGGGTGTAGAGGGTGAAGTCAGC<br>CCCTTGCCAGTCCCTCAAGGTAGGCCTGAGTGGCTACAGAGTCTCT<br>TCAGCAGCGGCACTAGGGCCTGAGTTGGGAGTCTTAGAAGGCTCC<br>AGGCTCCCTGCTGGGCTGACACCAGGGACCACTCCGTCCAGCGAG<br>AAGAACTCCAGGAAGCTGGGAGGCAGAGGCTTCAGCCAGTGCTG<br>CCAGACATGGGGCTGGCAGACGCGGAGGCCTCAACAGACCCAGG<br>CCCAGCTCCCTCTCTCAAGTCAGACAGGCGCTGGGTTTGGATCTGT<br>GTCTGATGATTACCAGCTTTATAACCTTAGGCATGTGACCTTTGGTA<br>TGTCTCCTCTCTGAGCCTCTTGCCTTGCCTATAAAATGGGGACAAC<br>AAGCACCTCACAGGATCACTGATAGATTCATGGAATGATACCTGTAA<br>TGCACCTGGCCCAGAGGTGAGGCCTAATGAGTGTCAGTGACCACC<br>CTCCTCTCTTCTGACACTTTTCAACAGTACCCTTTGAAACAATTCTA<br>CCGTTTCCCAAATTGTGAACCTACTATGTGCCAGGCACTGCATCAA<br>AGGTAGTAGGCTGGGGAGTGCAATGTTGAGAAAAACACACCCCTT<br>GCCCTCAAGCTGACCAACTTAGAACCTGGGGAGAGTACGACAAAG<br>ATCTAGGTGACTTGTACACAATCAGAATATACGGACAATGTCCTCCA<br>GACGCAGGAGAGGGAGAAGTTGGAGAAACCAGAGGAGTCTTCAC

	CTGGAAAGACTATGGGGGAGCCCAGAGGGGCCACTGTCTGGCAGG<br>GGGAGGGGGAGGAATGTGAGCAAAGATGTGGGAGAGGACAAGAA<br>CATTCTAGGAACACAGGCCCACAGAATTTTCCTGGAGCACAAGAG<br>AGAAAGCAAAGAGAGTGGGAAGCTGAGCCATATAGATGGCCGAGG<br>CCCAGTGGCAGTGAGTTTTGGATTTGCTAAGAAATGGGGAGTTATA<br>GAAGGCTATTGAGGGGAGGAACCCCCATACAGCACAGGGGAGACA<br>GGAAAAACGCCGTGGTTTTTGAAAGAGGGAACATGAATGTGGGGC<br>CCTGCTCCCTCTCTTGAGAGCACCCTCTTTGGGTGTCATCCACATCA<br>GGTTCTTATACACTGTTTTACTTGGTCCTAAATGGATGTTATTCCCAG<br>TTTACAAATGGGGAAACAGAGGCTCAGAAACGGTAAGCTGCTTGC<br>CCAAGGTCACCAAGCAGACAGTGAGCGGGGGCCTCTTTCTGACTG<br>TCATCAGCGGGAGCTCAACTCTTTTCTTTTCCTGTGATCCCAAGAG<br>AGATCCAAAGTCGCCTTTGAGAGCAGGATTAGGACAGGGATTGTG<br>AGCTAGGGCCAACCCTGGCTACACTGAGTCTCCCCAGAGTCCCTG<br>GAGGTGAATGTTATTATTCTTCTCTCCATCATGGGAAGGAGGGGGT<br>GGAGCTCAGCACCATGGGGGCACTTACTCAAGGCCACACATTAAA<br>GGTAGAAAATCCAGGCCAGCTTTTTGGGGAGTGTGGAACCCTGAA<br>CCCTTCTTTCCGCTACTCCTCACTGTCTCCAGGTTTATTCCTAAGCA<br>GGACTAGGTAGAATTGGGAACAGCCCAGGTCAGAAAGGCCCAATT<br>CTCTCCCCTGAGTCAACATCCACTGATTTATCTGCTTCACTCCCTCT<br>GGCAGCCAGGCCCACAAGGCCAGTCCCTCACTCCTTCTGTGGTTAC<br>AGAGACCAGATTATGCTGGGCTCTCTTTGGCTCCATCCTCAGTCAG<br>AAGCAGCATCTCCACTGCCTGGATTGGGGGCCTAGGTGGCTACACG<br>TTTACCACTTGTCGCCGGGCAGGTGACCCACTGGCCCGAGCACTGT<br>GTGTTAGTGGGCCTTAAAGAGGAGCTCTGCCTGAGTTGGGCCCCTG<br>TCCACTGATGTATCTCATAAGCAGATGGCTTGCAGTGACCTTCTCCC<br>TGGTTCTGAGGCAAGTCTCTGAAAGGCACAGGGGCATGATGATATA<br>TAAAGAACCATTCTGCTGGGAAGGGGAAACCCTAGAGATACAGTG<br>AATCATAAAGAGCAGGGGTGAGGTAGGGCCTGAGTTCCCTAAACC<br>CCCCATCTCTCACTTGGGCCTCAAACCATCTACAAAGTGGGCCAGT<br>GTAGACGCACTGTACAGACGTGGGTTTGGAGGCTCAGTAGGGGTC<br>GAGACTCACCTCTGACCATCCAGCAGCAAAATGACAGGGTTCAAG<br>TCTACTCTCTGCACTCTTTCCAGCAAGGGAGGAGAGAGAAAAGCA<br>GGAGAGGCCAGCAGACACCTGGCTCTAGGGCCAGACTGCGCCACT<br>ACTACTATGATAACGGCATTAAAAAGTTTTCTTTTCTTTTTTTTTTTT<br>GAGACAGAGTCTTGCTCTGTCACCAGGCTGGAGTGCAGTGGTGTG<br>ATCTTGGCTCACTGCAACCTCTGCCTCCCGGGTTCAGCCTCAGGCT<br>CTCGAGTAACTGGGATTACAGGCGCCCACCACCACACCCAACTAAT<br>TTTTGTATTTTTAGTAGAGACAGGGTTTCACCATGTTGGCCAGGATG<br>GTCTCGATCTCCTGACCTCATGATCCGCCCCCCCACGGCCTCCCGA<br>AGTGCTGGGATTACAGGTGTGAGCCACAGCGCCCAGCTCTTAAAA<br>AGTTTTCTTTTTGAAAAATTTCGGTAGGGGAAGGTATAATAAATGCT<br>CTTGGTGAGAAAAAAAATGGGACAAATCAAGTCGGTATGCAGTGA<br>AAAGTCAGCAGCTGTAACATTCTATGATTCAACGCAAAAACTATGT<br>TGAGTTCCTCAGCTGAATAAGTAGTATAAACAATTTCTTTTTCTTCTA<br>TCTCTCCATACAGGGAGGGCGTTGCCCTTATCTGAGGACCTCTTTG

	CTGTCCTCGACCAATGTTCAAATGAAGGTAGAAGACGCCCTTTTAA AAAAAAAAGCACTCCATGCCCTGAACTTTCTAGAAGAATCCAGG CAGAACATTTGCGCAGGCGAAAACACACAAGCTAAGCGAGGCAAA TGCAGAAGTTGCCACTGGTGATACAGCTCGCACAGCGACGACACA GGGTGGCCAGCGAAATCCCCTCCCCCAGGAGGGGAGGAAACCGC AGAATGTTCCTGACTCGGCACCCGGGCGGGTGGCGCAATGTTTATG TTTGTGTACCCAGCGCGTCGCGTCGCTGCAGCAGGCTCCGCTGTCC AGGGGGCCGTCACTGGGACTCAGGGCACGGAGATCGCTGGGCGG GGCGGGGGTCTTCCCAAGTGTTGCGATCCAGGTCCTGGCAATCCGC CCACAATGGCCCGGGATTGGATTTTGCTTTCGGCCTATAACGGCCG CGACGACAGGGAGCGAGGGTTGTGGCCGGCTTCTGTTCCGGGTTG GAGGCCCCCAGCCCAGCGCTTAGTGGGCGCTCACTGTGTATACTGC CTATTTGTATAATAAAGAAAGGGATGGAGAAGCTCAGTCTGGAGCC AAGGTGACAGAAACGTGTCGGGCGGCCCCTGGGGGGCGCCGTCG AGTCGCGCTGCGGGTCGCCCCTCACCCCTCCAAACGCCAAACCCT GGAATCCACTGCGGGGCCTTGCGACCCCTCACCGGGATTTCAGCCC CCTGACCTGCCTCCCTTGGCTCCAAGTCTTCCGGGGGCCACAAGAC TAGGAATAGCATTATTTCCCGGTGGGAGTTTGCTTGCTCACACTTTC ATGGCAAATGTAGATATTTTAAAGCCACCTTAAAGAAAAGTCTGGG AAACAGGAAAGCATTGCCCTGAACCTCTCCGCCCCAACTCGCTCG AGTCGGTCTTGGTAGGGACGACTTTTCTATCGGCAGAGTTGTTCAT TTTTCTCTAAACTTCATCTTAGGCGCCTATTTGCTGCCCCCACCCCA GTAGTGTGGTTCTGGGCAAGTCAGTCGCTTCGCCTGGGGAACCTC AGTTTCCCCGCCTGTTAGTGATGAGGAAATGAGGAAAAGGGGCAC AAGAGTATGCAAAAGCACAGGAAGCCCCTTTCGGTTCAGGTCGGC GGCCGAGGGGCACGGGGAAGGGGACTCGGGGAGGGAGAGAGGG AGGAGCGGCGAGGCCAAGGCAGGTGGCACGTCGCCCGTTCTCCCG GCCGCTCGCATCCCGGAGGTCAGCCTCAGCTGGCAGCGAGCCCTC CTCCTCCCCGCTGGGCCTGGAGCGCGGCGCGGGTCCGGTCCCTGG GGACGCGCCAAGAGCAGGCCGGACAGCAGGGCGGGGGCCGGGCC GCGAGCCAGCGCGCGGCGAGCGGACCCAGCTCCGAGGCTGATGAC GTCTCCCCCTCTGGCTCGGCGGCGCCTGGCCTGGCAGGGCGGGTG ACGTCACCGCCCCGTCACGTGATCACCATTCAAACAAACACCCCCC CTCCCCCTGCGCGCGGGTCTGGCCCGCCCCGTCCCCCAGAGGCCG CATATAAACGCGCTCCCCGGGCCAGGCTCGCTGCGAAGGACATTTG GGCTGT
E2F1	GTCCATATGTTATCTTTGGTTACTTGCAAGGCAGAGAAGCTGTGCTG GTACCAGTCAGTTCCTTGGCGGTAATAAGACTAACAGCAGCTCACA TTTTTTGGGTGCTCACTCTGTGCCAGGCACTGTACATGAGGGGTGG CTGTTCATCCCTGGAATGGCAGGGCAAGAACTGAGTGAGGCATGC CAGGGAAGGACTCTGGTGGCCTGGAGGGTAAGGCCCTCGCTAGCT GGGGGACAACAACTGTTTAGCACCGTGATGGCCTGGCCAGTATAGC CAGATACTGCTGTTTGAAAAGAAAGCCAGAGGCCAAGCGTGGTGG CTCACGCCTGTAATCCCAGCACTTTGGGAGGCCGAGGCGGGTGGA TCATCTGAGGTCAGGAGTTCCAGACCAGCCTGGCCAACATGGTGA AACCCCGTCTCTACTAAAAATACAAAAATTAGCCTGGTATGGTGGC

	ACACACCTGTAATCCCAGCTACTTGGGAGGCTGAAGCAGGAGAAT<br>TGCCAGAACCTGGGAGACGGAGGTTGCAGTGAGCTGAGATCACGC<br>CACGGCACTTCAGCCTGGGTGACAGGGAGACTCCCATTTCAAAAG<br>AAAAAAAAAAAGAGGCCAGGCACGGTGGCTTACGCTTATAATCCC<br>AGCACTTTGGGAACCCGAGGCGGGCGGATCACCTGAGGTCAGGAG<br>TTCGAGAACAGCCTGACCAACATGGAGAAACCCCGTCTTTACTAA<br>AGATACAAAATTAGCCGGGCTTGGTGGCAGGCGCCTGTAATCCCAG<br>CTACTGGGGAGGCTGAGGCAGGAGAATCACTTGAACCCGGGAGGC<br>GGAGGTTGTGGTGAGCCGAGATCACACCACTGCACTCCAGCCTGG<br>GCAACAATAGTGAAACTCCGTCTCAAAAAAAAAAAAAGAAAAGAG<br>AAAGAAAAGCCAGACATGTTAATTTTTATATGGTATTTTCCAATTTC<br>CTCAAATCTTAAAACAAATTCATAATTCAAGAAAAATCTTCTATGGG<br>CAAATTGAGTTATATCTGGGGGCCAGTTTGCAAACTCTGCTTGTTTT<br>GTTTTCTTAAACTTTGACACGCATCGTGTAAAGAAGCTGTTATCTGT<br>TGTTCTGCAGATGAGGCAAGCAAAGCTCTTCAAGAGGGTGAATCA<br>CTTCCCCAAGATCACAGAGGCGGGAAGCCACAGGGTTGGGGGCCA<br>CCCGACTCCAAAGGCTTGTTTCTATGTTAGACTGCCTTTTGGATGGG<br>CAAGAGGTCTGTTCAGATATAGCCAGAACTGGCACAGTGACAGAAT<br>ATCTCCAGGGACAGTAAACTGTCAGTCAGGGTAGCATTGAGGGATT<br>GTTGCAGATGCAAAACTCCCCACCTCCCTCTGCCACAACTCAACAA<br>TGGACATCTCTGTGCCCTTCATAAAATTAAGTCATCACAGAATCTTT<br>TCTCCCAATTATGCATTCCTTATCATGACTGGATATTCATGTTTGGTG<br>AATTTTTGTTTTTTTGTAAGCTAAGAATGTATGCTTAGGGGAGGTGC<br>TGTTTGTGGAATAAATAGGTTTAAATTATTTGTGTCACTTGTCAAAC<br>TTCTTAACAAGCATACTGTGCAGTGACAGAGAAATAATTCCTAACT<br>TTCCCACTGGACTGTGAGTTCCAGGGGTCTGTTTTACTTATAACTGT<br>GTCCCCAGCACCTAGCACAATGTCTGACACATAATAGGTGCTCAGT<br>AAATATCTGTGGATTGAATGAAGTGAATTGACTTGTCCAAGGTCAT<br>GTTGACCAAGATTTCTGATTACACCCACCTGATTTAGAAGCCCATGC<br>TATCACCTCTACACCACTCTGCTTCTTGGAAAGAACCAAGGGAAAG<br>AGGTCTCCAGGCACAGGATTGTGGGCCCAACAGATTGCATCCCCTC<br>GAACTCCTCTAGTTTGGTTGGTGAAGGTGGTGATGGAAATAAGCAC<br>CTATGAACCAAGCCACTATCCACCATAATACCCTTTAGAAACTACAC<br>TTTATAATCTTGACAGCCCTGAGCCTGGCTTTTGGGGTATTTACAGA<br>GGGTGGCAAGGGCAGGTCCTGGGTTTTGCAGTGGGACAAGTGAAG<br>TTCTCATCTCAGTTCTGCTGTGTGACCTTGAGCTATGAATGCCCCTC<br>TCTGAGCCTCTTCCTTCCTGCACCATGTGGATAAACAAGCTTACTTA<br>CGGGATTATGGGGTTGAAAGGAGATCATCAATGGAAGTGGCTGCCC<br>AGTGACTGTGGATTTTTTGGAAACTGTTTTTTGAAAACTGTTGACA<br>TTGAGAACAGCCTTATCAGGAGCTGCCAGGCTTAGGGCCAAGGGG<br>CCTCATCTATCAGGCTTTTCTGCTTTCAAGTAGGTATTAATAGGTACA<br>GAAGGCCCTTATCTCTTCCTCAGTTTCATGCTAGGAATGCTGGTCTT<br>CACTCCTTTCTGCTTCAGCAGGACCTGAAGGATTGTGGGACGGAG<br>GGCGCCTACTTCTACCTGGATGTGTACTTGAATACTCTGGGGCGGG<br>CAGGACTTTGGAAAACCCCCAACCTCAGCCTCACCAAGCCTGAAA<br>AATAACCTTTTCATCTACTTGGTAAACTCAGGCCCTTTTGGCCTCAA

	ATTTCCTCATCATAAAATGGAGCAATAATAATAGACATCTCTCTTTGC<br>CAGCCACCACAGTTAAACCAGCACAGTGCTTGGTATATAATAGGTG<br>CTTAATAAGTGTTTGTTGAATGACTTGGGTTTGAGAGGGCTTCACA<br>CAAATGAATGTTAGTGTTACTGGGACCCTGTGGGAAGGTGCAGGG<br>AAAGGACACTCTTTCCATCTGGAATGGGAATGTGCTGGTTTCTCTT<br>GCTCTCTGCCAGCACCTGCGTTGGTTGTTTAGAATGGGACCTACCC<br>CTCAGCTTCTTTATGGAGAGGGGATGGGGAAGGGGGACAGGCAGA<br>CCTCAGGGCTGGGAATGCAGTGTAGGCCCAATTTGGTCACTGGTTG<br>GCTTCACAGTTACTTTCTCCTGAAAGGGCCTCAGTTTTCCTATCCAT<br>ACAGCAAGAGGCTTATGTGGTCTCTGTGGTCCCTTCTAGCTATGATA<br>CATTCTAGGATTTGCAATGAGTAATTTCAGAAACTACTCATTGGAGG<br>GGAAAACAACTCCTGATTTCCTAAACCACCTCATAGTTTGTAAAAC<br>ACTTTCCAATTCCTTATGCTTTTCATTCTCCTGGCAACCCTGGAAGG<br>GGCCAGGGCAAGGTCTGTGTGCCCATTTGAAACTAAGGGTGAGAA<br>GCATCCTCAGGAGCTGTGATTTGAGGCTGCACCACTCATTTTGATTA<br>TGCTGGTACACCAGTTTGCTTTCAAATGTGAGTATGCATAAAAATAA<br>CCTGGAAGCTTGGTGAGGTGGCAGTCACCTGGAGTACCAGCTATG<br>CTGGAGGCTGAGGCGGGAGGATCTCTTGAGCCCAGGGGTTTGAGA<br>CTACCCTGGGCAACATAGTGAGACCTCGTCTATATAAAATAAATAGG<br>AAAAAAAAAATAAGATACTGGCTTTTCTAATTCCCTAAGTGTCAAA<br>AAAAAAAAAAGAAAAAGAATAACAAATATCACCTGGAAAGGGGA<br>CATGGCATTGTTAAAATGCAGATTCTTGGGCACCTAGAGATTTTAAA<br>TTGGCAGGGCCAGGGTGGGCCCAAAATTAGCAAGTGACCACGTGG<br>TTCTGAAGCCAGTGGCCTAAGGACCACCCTTGCAGAACCGTGGTC<br>TCCTTGTCACAGTCTAGGCAGCCTCTGGCTTAGCCTCTGTTTCTTTC<br>ATAACCTTTCTCAGCGCCTGCTCTGGGCCAGACCAGTGTTGGGAGG<br>AGTCGCTACTGAGCTCCTAGATTGGCAGGGGAGGCAGATGGAGAA<br>AAGGAGTGTGTGTGGTCAGCATTGGAGCAGAGGCAGCAGTGGGCA<br>ATAGAGGAAGTGAGTAAATCCTTGGGAGGGCTCCCTAGAAGTGAT<br>GTGTTTTCTTTTTTTGTTTTAGAGACAGGATCTCGCTCTGTCGCCCA<br>GGCTGGTGTGCAGTGGCATGATCATAGCTCACTGCAGCCTCGACTT<br>CTCGGGCTCAAGCAATCCTCCCACCTCAGCCTCCCAAGTAGCTGGG<br>ACTACGGGCACACGCCACCATGCCTGGCTAATTTTTGTATTTTTTGT<br>AGAGATGGGTCTTCACCATGTTGATCAGGCTGGTCTCGAACTCCTG<br>GGCTCATGCGATCCACCCCGCCAGCTGATTACAGGGATTCCGGTGG<br>TGAGCCACCGCGCCCAGACGCCACTTCATCGTATTGTAAACGTCTG<br>TTACCTTTCTGTTCCCCTGTCTACTGGACTGTGAGCTCCTTAGGGCC<br>ACGAATTGAGGATGGGGCACAGAGCAAGCTCTCCAAACGTTTGTT<br>GAATGAGTGAGGGAATGAATGAGTTCAAGCAGATGCTATACGTTGG<br>CTGTTGGAGATTTTGGCTAAAATGGGACTTGCAGGAAAGCCCGAC<br>GTCCCCCTCGCCATTTCCAGGCACCGCTCTTCAGCTTGGGCTCTGG<br>GTGAGCGGGATAGGGCTGGGTGCAGGATTAGGATAATGTCATGGGT<br>GAGGCAAGTTGAGGATGGAAGAGGTGGCTGATGGCTGGGCTGTGG<br>AACTGATGATCCTGAAAAGAAGAGGGGACAGTCTCTGGAAATCTA<br>AGCTGAGGCTGTTGGGGGCTACAGGTTGAGGGTCACGTGCAGAAG<br>AGAGGCTCTGTTCTGAACCTGCACTATAGAAAGGTCAGTGGGATGC

	GGGAGCGTCGGGGCGGGGCGGGGCCTATGTTCCGGTGTCCCCACG CCTCCAGCAGGGGACGCCCGGGCTGGGGGCGGGGAGTCACACCG CGCCTGGTACCATCCGGACAAAGCCTGCGCGCGCCCCGCCCCGCC ATTGGCCGTACCGCCCCGCGCCGCCGCCCCATCCCGCCCCTCGCCG CCGGGTCCGGCGCGTTAAAGCCAATAGGAACCGCCGCCGTTGTTCC CGTCACGGCCGGGGCAGCCAATTGTGGCGGCGCTCGGCGGCTCGT GGCTCTTTCGCGGCAAAAAGGATTTGGCGCGTAAAAGTGGCCGGG ACTTTGCAGGCAGCGGCGGC
EBAG9	TGTTTGAAGTAGCAGTGTATAAAATTCACACCCAGTTATAGACTGA GTAATTATAGTTGCTATGGTAATGGGCTCTGTGCCTATGAGCAAAAA AAAAAAATTCCTTTTGTGCAAGATATTTATGACTGTGCCTGGCTACA TGGCAATAGTATTTTCCTTTGGTTTTTCATTACAGTAACTTATGCATT TAGGCAGATGATCCTTTATATATGTAAACGCTATAAATTCTTAGGAAG AAAATAAATTTATTAAGTTTTTCACATCAAAAATTTCTAGTATGATTA TGAGTTCAATTTTCAAGTGAAAGTAATCCTTAAAGGATTTAATTTCA AATAAGAACTACGTACAAAATATAGATTTAAATTAATTTTCACTGTAT GAAATGTACAGTATACATAGACCAACTCCTTTAACAAGAATTTCTGG TATGTCTTAAAGGGCATGGGTGGTTAGAGGGAGATCCCCAAACAAA CAATTCAAAATTAAGAGTTTAAAAAGCAACAATCAAGACACATATC CTCCTAAGTGAATTTGAATTAGACACTGGAAAATTTAAAAAGGTCA GTTACTATGAAGAAGGTGTAACAATATTTATATTAATCAGACAGCAA AGTAACAAACTGACTTAGAGGAAGGAAAATAGTAAAATACAAAAG ACTAAGTTAGCAGATCATTTTGATATCCAGATGTGCTGAAATGATGG ACACTGAGAAGAAAGAATAGACAAAACTTGGCAACAGATCTTGGT ATGAAATAAATATCCCATGAGTAGTACATAAAAGCCTGGAGAGAGA GGGGAAGGATTGTAAGTGTCATAGTTCTGACAGCTGCTCTTTCATC CTATGCCTTTAAAACAAAGGGAACAAAGAAGGGAACAAAGAAGGT AGAAGCGGGGGTATGGAGGGTTGACTTCTGGCTGTCCCTCCCTGTT TCCCTTTGTTAATATATTGCTAGTAGACATGTCTACTTCTGGTTGCTG ATGACATAAAATTCACCTCTATTTCTTGGAAGCACTATTCCATGTTG TGAGCTAAAGGGGATTGTACTGATCTTGAAGATAACAAATCTGCCT GCTTCAAAGGTTTCACCGATTTTATTGCTGTGTTTCCAACACATTTC CACATATGGTATGATATTTAATTTGCAAAATAAATCCGTAAAGCAGG AATTACTAACCTAATTTTGCAGATGAGAAAACTGAGGCTTAAAGTT GTTCAAGGTCAAAGTCACGATGTCCATCATGCTGTAGCTCAATGAA TGAGTCTCACATTGTTTCATGTTGTTTTATTTCTACTTTCTCAATCCA TGTACAAGATAGATTTACAAACTACTGTTTTATGTTGGTTGTATGAAT TTCAATACATTTACTGTTAGAAATAACAAAAGGTCTTAGACTGCCA GATGCTGTTCCCATCAACTCTACTTAATTCATAATATAGCTGAACTCT GTCATAAATCTAATAACGATGTGGACATTAGCCAGAAGTATGATGTG GCTGAACCTCACCACTGAACACAAAATGAGAATTCACCTCATCTTT TCATTTGAATTTATTTCTACTAAAATATATGTATTATTTTTCTTGATTTC TAACCAGGCCCCTTCTGAAGAAAACTGGTTAACTCTAAATTGTTAC TAAATTTTGAATCTTCCTGTATTTAGCAATAAAAGTTACTGCTAATTG TAATAACAATTATGAGGAAAAATTTATTTAAATGCTTCATAGAAAGT TAATTTTCATTTAATAGAAAAGAAATAAAAAATCTGCTTCAGACTGT

	TTGATTTCTCTATTAGGCAATTGACCCTAACTAGTATATGGTGATTTT<br>GAGTATTAAAAAAGAAGTTATAATTTTCCAGAATATTAATGTTTATTA<br>TTTTGAAAGCAAGTAAAATTCTCCAGTAGGAAGATTAATAAAACAA<br>TTCATTAAAATTCTCATCAGAAAATCCTATGCTTTCAAATGTATCTGC<br>TCAAAGATGTCACTCTATGTGCGGCAAAGCTTTGTTTTACTTGATCA<br>AGAAATGAATGTGACTTTATGATCTTGTATTCAACTGTGAACAGCTG<br>CTCATTAATAAAAAGCCATTAAAGTCCAAGTCAGAACAGCAGGTCT<br>AAAATTGTGTTTGAGGGAAAAAAAACCCACCCTTTGATGAAAAGC<br>CAGGGAACTGGGTTGGGAAAGATCTGGAAGCAGAGGGCTGTGTTT<br>GCAGTCTGCTCCTACCTCCTGGGCCAGGTCAGGTGACAAGGCCAG<br>AGTTAGCCCCAGCTGGAGAAGTCAGAACAAAGCCTATGTAGGCTT<br>TGAAAGGGGAGAGTAAGTGGTGGGCTGGAGGTTGATAGTGTGAAT<br>AAATCAAGCTGGTAATTGGGCAGAGGCTGATCAGAAGCGTTAAAG<br>TGAGCAAAATGGTTTGGACACAGAGCAGACAGAATTTACTATATTC<br>CATTGAGTAAAAGAAATTCATATGCCAGGTTCTATCCCTTATGTTCC<br>TCCCATTTGACATCTGGTGGTTTCCTACCCCCGTCCCACACAAGCTT<br>TGGGGAGAGGACAGGACTTAAAGTTCCCTGTTCCCAAGAGGTGGT<br>CCCTAAACCCAATCCCAAATTATTGACTTGTGATCCCAATTATGGGC<br>TTACTATTTAACAAGTAAAGATGATTTTAAAATATTATTTTATAGGTAT<br>TACTAAAGTAAATTTTAAATTTGCAGTCTTTAGCGAGCTTTGTTTTC<br>TGTTTTGCTATTGTGTTGGGAAACAAGTACTCAGTAACTAGCTGTTT<br>CTGGAAAAGTATATACATTTCGTTTTGTTTCATTTGGAAATGGCAAA<br>TTTAATAAAGAAAACTGAAGAATCAGGAAAAAGAAGAAAATATCC<br>TTGGGCATAATAAATTGTCTAAAGCAATCAACTCTTTAAGAAAAAA<br>AATTTAAACACGTTTATATCCGAATATAGGTTATTTTGGATTTTTTTTT<br>TTTTTTTTTTTGAGTCAGGGTCCTGCTCTGTCTCCCAGGCTGGAGTG<br>CAGGGCCCAATCATGGCTCACTGTAGTCTCGACCTCCCAGGCTCAG<br>GTGATTCTTCCCACCTCAGCCTTCTGAGTAGCTGGGACTACAGGCA<br>TGCGCCACCAAACCTGGCTAATTTTTAAATTATTTGTAGAAACGGA<br>GTTTCGCCCGTTGCCTGGGCTGGTCTTAAATTCCTGGGCTCAAGCG<br>TTCCTCCTGCCTCGGCCTCCCAAAGTGCTGAGATTACAGGCATGAG<br>TTACCGTGCCCAGCTAGATAACTCGATTTTTTAAATCCAAAAAAAAT<br>TTAGAAATTTTATATTGAAATTTAGGCATTAGAATTCTAGAGATTAAA<br>GTTAGAGCAGGTGTTTAAGGGACTGAAAGGGGTTGCATGGCCTTT<br>GGGAAATGCAATTACCGGAAATGATACTGTAGTAATGCAATAGTAAT<br>GCAGTAGTAGTAGTAGCTGCAGCAACAACTGTTATCTATGAATGTTT<br>GCTATGTGCCAGGCTCTGTGCTAAGAGAGCTTTGCAAGCATTACTA<br>CTGCATGAAGTAGGCACTATTATGGCCATTTTACTGATGAGGAAATT<br>GAGGTGAACAGATTAAATAATTTTACCCAACTTTACACAGAACCAT<br>TCCAGAGCACGAGCCATAAACACATCTTTCCCGTCTTGGAAATTAA<br>ATTATTAATAAAAAACGTGGTGCAAATTGGAAAGAGGACGGTTGTG<br>GTAGATTATCACAAAGTTAAGTGGGTAGGTGGTTGCTGGGTCAGGG<br>TGGGAGAACAAAGGCAGCTGTAATAAAAAAAAAAATTAAAGGCAT<br>ATAAAGAAAAAGGCTTGTTGAGAACAATTTGCATAAACTGATTTTG<br>GCCTTAGACCCTATTCACTTCTCCCTCACCAGTCACTTTGCAACTGT<br>GGAGCATAGTATTCTGAGGGTCCCCTGCACATAACAACCCCTTTCAT

	AGCATACAAGATAAAAAGAAGAATCAGGCTGCAAAGAGAGAAGC<br>ACTGAAGGAAAGAACATCTCAGGTTGAAAGAAGGGCAAGTCCAG<br>GGTGCATGTGAGGAACAAATGCATGGACACGGTGAAGTGCCACTT<br>CTTCATTTCTAGTTCAACACAATTCAATTAAAAATTACTGGGAGGAG<br>GCTACGTCTTGAATTAAACTCAGCATTCCACATCTTTGAGGTGGCG<br>GAATGACTGAGGGGAGGGGGTCTGTCTAGCCACATCCAAATCTTCC<br>TGAGACAGAGGGAGATCCCTCATTTGTTGCAGTGTATTTTCCTTGG<br>TCGCATCTGACACAGGGGTTCTGTGGGTTGAACAGAAAGGGACTT<br>TGCTGGAGGGGAGGCACTCTGCATAGAAGGAAAGTAGGGACATCT<br>GGCAGAAGCAGGCGCTCCGAGAATAAACAGTCAAAGCAGCTCGC<br>AATAGTCCCAAAAAGCTACCCCTCACCGAACCTGGCGTGCCTGTCG<br>CCGCTTGCCCCGCTCCCAGGGCTCATTGGCTCCCAGTCTCGACGTC<br>AGACCCGTAGCCTCTCACCTATTGGGCCAGCCAGGACCTGAGGCG<br>GAGTGCAGAAGCGAGGGCCTGGAGACAGCCGAGAGCCGCCTTCA<br>AGGAGGGCTGGGGGCGGGGCTAGAGGAGGGGCTGGGGGCGGGTT<br>TCCCGATGAAGGGGCGGCCATGGCAGCTGCGCAGAGGCAACGCAG<br>GCTGCTACGGAGCGCGCGCCCGGCTTTGAATGAGCGGGGCTGGGA<br>GTGAGCGGGCGGAGCGCGAGCTCGAGGAAGAGACAGGCAGCGCG<br>CGTGAGCGCGCCTTGTGTGCGCGCGCGGCCCGCGGCAGCTCGGAG<br>CCTCCGCCGGGCGGGCGGGGAGGGGGAGGGGCAGGTGAGTGTGT<br>GCGGTTCGCGCGTGCCTGGGAGGGGCTTTCCTCTCCCTACCCCCAT<br>CCCGACCCCAGCCCTAGCCTCTGGGGCATTGTCTGCCCTTCGCCGT<br>CGGCCCTCCGCCTAGCCGCGCACTTCCCGCCCTCCCACCTTCCTTT<br>CGCCCTTCCACCAGACCTCCCTCGACGCCCGACAGCTGCTCTGGGT<br>ACTGTTTCCGGGTCAGGGTGACCTCTG
EGFR	GGATCACCTGAGCCTGGGAAGTTGAGGTTGCAGTGAGCCAAGGTC<br>ACGCCACTGCACTCTGGATTGGGCAACAGAGCCAGACCCTGTCTC<br>AAAAAAAAGAAAAATTCCATGGCTCTGCTTACATTATCCATCTGATC<br>TTACATGTTGCCTATTTTTTCCATTAAAACTCCTAGCCTATTAATCAT<br>AGTTTTTTTATAATTAATACTCCGATGTGATAATGTCTTAGTCCAATT<br>ACTGTGGTTATAACAGAATGCCACAAACTGGGTGATTTATAAACAA<br>AAGAAGCTGATTTAGGCTGATTTAGAGGCTGGGGAGTCCAAGAGC<br>TTGGTGCTAGCATCTGATGAGTGTCTTCTTGCTTCATCATAACATGG<br>GAGAGGGCATCACGTGTGAAGAGAGCTTACTCTTATAACATAGCCA<br>CTCCCACAAGAATTAACCCACCGCCATGAGAGCCATGTGAATTCAT<br>TCATGAGGACAGCGGGTTAAGTTTCCAATATATGGACTTTTCGGGG<br>ACACATTCAAACCACAGCAGTTAGTTGTAACGTTCGTGTCATGTCT<br>CATTCTGGTTCTGATGCTTGTGCAGTCTCTTCAAACTGCGTCTTTGC<br>CTTTTAGTGTGCCTTGCAATGTGGAAATGATATACTGGGTAAGAGG<br>AGCTGTAGTAAAGAGGCTTCTAGTGACGTAGTGACAAGCTGTGGG<br>GAGAGGGAGTGTTGCACAGTCCTGCCGCATGTCACAGTCTTCCAG<br>TGAGCCTGTGTCCCTGGACTGTGAACTTCATGCTTGCTTCTCAGCT<br>TCCCCAGCCCCTTAGATGGTACAGAACTGTTGGAGGGGGGTGGAG<br>TTGTATATTTCCCTTGCTCTGGGTAGGTCACCCTCTGATAAAACACC<br>AGGTTAGGCCTCTGGTGAAATAATTTCTCCTGAGGGCAGACCTTCT<br>ATTAATAATAGAATGTTCCAACCTATTTCAAAATGGTTCCTCTTCTCC

	TTCCACTGCCAGAAGCATAATGAGATTTTCCCCCTAATATTCGTGGTAAGGACCTAGCAGAGCTCCAGGAGGTAACACTCTCAAGTGTCTCATACTACCCTGCACCATGACTGGGCTCTGCTGGAGTTCTTAATTTGCAGAACTGCCCACACTGAGCCTCCCGCAATTTCTCAATTACAGGGCAAACTTTCCCAGCCGGCACTGGGTCCTTGGAGGTTTCTGTCTGCTGGTTTCTTCCTCTGGAGGTTGTGCTTCTGTGTTTGCCTGTCTCTCCAATTTGGGGGGCAGTGGTTTGCCCAATGACCTCAATTCTCTGAAAGAGCTAAGAAGAGGTGTTAATTTTTCGGTTTGCTCAGCTTTCTACTTGTTGCTAGAATGGAGCGCCAATAGTGCCTCCTATAGTGACATGTAACCCTCAACTCTAGAGATGATGAAGCATACTAATGACAAAGGAGAAATGCTTCAGCAGTTTTCTGTCAGCACATTACCCCTTGAAAAAGCTGCTTCTTCCACATTCTGCAAGAGATGGGTCTCAACTCAGAGCTCAAGGCAAATGACTTCCTTCAAGGAGAAGGAATAAACAGTCTCAGAAACCATGAAAGCCTGCCCCCAGGAGTGTCCCTGAACCTCAGCAGGGGCCACACTTACCTTGCAGAAATAGGTGAGGCATGCTCCTGGTACAAAATCCCAATGGTACAGAAGGACAAATTGAAAAACAAGTCTCCCTCTAAACCCCTGACCCCGAGCTACCTAGTTCTCCTCCCTAGAGGCAAAGCTGTTACCAGATTCTTGTATCTCCTTAACATATATCCTTAGAAGAGCTGTCAAGTGAACACATGTTTAAGTGAAAACCTATTTTAGAAGTGCATTTTCTTAAGGAACTTTAGGGTTGGAAGGAACCTGTGTCAGTCCTTAATTCACAACCTCCATTAGTACTTATTGTTCTTGCACAAAAATCTTTCTCAAAAAAGCCCTTTCCACTCTGACATAGCTTATTCTACTTTTACTTAGCTCCAATAACTTATAAAACATATTTTTGAAAGTCTAAAATCTGCCACTATGTTTTTTTTTCCTAATCAATCTTTACTTTGACCTCTAAGCCAGAGAAAACAGGTGGTCAAATGCCTTTTGCCTAAGATGGAACTTAGAATATTTGAAGACCTCAGATCTTCACCCTGCCAAATAACGTGTTTCTCCTCCCCTTTCACAGAGCATTTGGTTTTAGGAAATTCAGAGCCACATTCCTTATAGACAAGACTAAACTCTTATTCAACATACTCAGAAACTTCTTCTAAGAGGATAACCACTCATCAGAGGAAAAAAGTTTCTCATGTACAGCTGGCAAAGGGATGGAACCATCTGTGTTATTAAAATTGACAGACGCTTATGAGATTTATTAAGGGAAATACTAGAGTCTTAGTACATACTTGCTAATATAGCATACATGAAGGCTTTATCTATAATTTTTTTGGCCAAGCAGAAATTTTGGTATTACTCACCCTAACAAATTTCCAAGACATTATGAAATAGAATTTTAGGTCCTGACATCACCATTTGTCTCAGGTTTTGAAGCGTTGCTGGACAAGAGGGGTAAAACACGGCTCTGCCTTGGATTCAAAGTTGGCCTCTCATACTAGCAAGTATACCTTGGTATCCTGGTCACTTCTCCCGGCCACAGCATCACATTGCTATAAAAGGCAGATACAAGTATTAACCAGCTCACAGGTTATCAGATAAGCTTAGTCTGACCAATGCTTAACACAGCAACTGGGCCACTATTGTCATTCCTGTGGTGGTGGCACACACACCCAGCCTCTGTCCGGGCCATGGTCTAGGACCACCCTCCACAGAGGCTGTGAGCTAGAGCCCTAACTGTGCAGGGCCCTAACTATGCCAGGCTACTTATCTCTCTTAAGAGGACTTCATTAGTGCCTGCTCGGCCATACAGTTTTTTACTTACCAAGTAACACAGTTATCAGCACACTCCAGGTACTAGCCAAGGACTACAAAATCAACGTGAATGTCAGCTTTTGTATCAAAAGCTCAAAGGAGAAACTCAAACTTTACATAGATGTCCCATGAAGATGTTCAGCAAACCCATTCTTCTCTGTTCCCTGGAATCCATCCCA

	GTATTGTGCTATGTGTGTGTCTAGTAATTCTTTACAAAAAGCTCTGT<br>TTCTTGTGATGCTATCAGATCACATTGAAGAATATACAAGCCGTACT<br>ATGAAGGCTGTTGTCTCATATAGTCCTAACGTAGTGAGAACTGATGT<br>TCTTACATGCTGTCTTTTTGGGCACTCAAAGAAATTCCTGTACAGTC<br>TTACAAATCAGTTGTAGCTTAAATTGATTTGTGTTGTGACTTGTACA<br>CACAGGTCACATTCCCTTGACAGAAAATATAGTTTAAAACCAAATT<br>TGCAGCCCTTGTTAAGTGAATGCACAGGACTTTATTGTATTCAGGTC<br>TTTTATTGTAAGACTCACTCCTGTCTTCATTTTATGTTCCACTGTTGT<br>GCTTCCCATTTGCCTTTCTCTAGTTTTGTTTTCTGTGTTTCTACGGAC<br>TGCTCTCAGCCCAGGTGTGCAGGAAGCACACACATGCCTGCAGAG<br>CCTTCATGGCCTCTGCATTCAGGGCATGACTTCAACGCACAGTGGC<br>TGTACTGATTTGTTAAAACAAAGGAACAGATTACTTCTCCTAATTCA<br>CAGGGAAGTTCCAGGTTGTGCGGGCAGTGAGCAGACCTGTGTCTG<br>TCTGCGCTTGCCCTGGTGAAAAACCCCACCGTTCAGGCTGCAGGG<br>TGCGAGACCCAGGCACAAACATTTTGCTGGATGAGGAGGAAAGAT<br>GTAAGGTTGCTCCCCTTCAGAGACAGCAAAGGGCAGGTCTGTAGC<br>TTCACTTACTTCAGGATTGTGATTTTTGACAGAGCCGAGAGATCAG<br>GGTTGTTGAACCAGGCCTGAAGGTCCTAGTGAATCTCGTGAAGAG<br>AGGAGGGGTCTGGCTGTAACATGGACCTAGAGGACATTTTTACTGC<br>AGGAGAAGGAACAGTGGGGATGGGGTGGACTTGCCAAAGGAATAT<br>AGCTCAAGTTCCTGCAGCCCAAAAAAGCTCAGTTTCTTTTGGCCAA<br>AGCTTCCGCGAGTTTCCCTGGCATTTCTCCTGCGGGAGCTACAGGG<br>GCAGTGGGACACTTAGCCTCTCTAAAAGCACCTCCACGGCTGTTTG<br>TGTCAAGCCTTTATTCCAAGAGCTTCACTTTTGCGAAGTAATGTGCT<br>TCACACATTGGCTTCAAAGTACCCATGGCTGGTTGCAATAAACATT<br>AAGGAGGCCTGTCTCTGCACCCGGAGTTGGGTGCCCTCATTTCAGA<br>TGATTTCGAGGGTGCTTGACAAGATCTGAAGGACCCTCGGACTTTA<br>GAGCACCACCTCGGACGCCTGGCACCCCTGCCGCGCGGGCACGGC<br>GACCTCCTCAGCTGCCAGGCCAGCCTCTGATCCCCGAGAGGGTCC<br>CGTAGTGCTGCAGGGGAGGTGGGGACCCGAATAAAGGAGCAGTTT<br>CCCCGTCGGTGCCATTATCCGACGCTGGCTCTAAGGCTCGGCCAGT<br>CTGTCTAAAGCTGGTACAAGTTTGCTTTGTAAAACAAAAGAAGGG<br>AAAGGGGGAAGGGGACCCTGGCACAGATTTGGCTCGACCTGGACA<br>TAGGCTGGGCCTGCAAGTCCGCGGGGACCGGGTCCAGAGGGGCA<br>GTGCTGGGAACGCCCCTCTCGGAAATTAACTCCTCAGGGCACCCG<br>CTCCCCTCCCATGCGCCGCCCCACTCCCGCCGGAGACTAGGTCCCG<br>CGGGGGCCACCGCTGTCCACCGCCTCCGGCGGCCGCTGGCCTTGG<br>GTCCCCGCTGCTGGTTCTCCTCCCTCCTCCTCGCATTCTCCTCCTCC<br>TCTGCTCCTCCCGATCCCTCCTCCGCCGCCTGGTCCCTCCTCCTCCC<br>GCCCTGCCTCCCCGCGCCTCGGCCCGCGCGAGCTAGACGTCCGGG<br>CAGCCCCCGGCGCAGCGCGGCCGCAGCAGCCTCCGCCCCCCGCAC<br>GGTGTGAGCGCCCGACGCGGCCGAGGCGGCCGGAGTCCCGAGCT
EGR1	CCCTCTATGCAGTCAGTTTCCTCTCCTCTTTTTTATCTTTTATCGCTG<br>CATAAATGCCACCTCCTCCAATATGCCTTTCCCTCACCTTCCTAGTAT<br>ATCAGCCACACTCACCATGATCTGAAATTATCCTTTTTATTTGCATAA<br>CTGTTTATTTCTGTCTATCCCACTAAATGGTATTTTCCATGAGAGCAA

	AGCCTCACTGCAGAATCTCTAAGACTCAGCACTGCACCCAATACAC<br>ACAGTGGGCAATAAACCTTTTCAAAAGAATGAATGTTTTATTTAACT<br>TTACCTTCTGCTACTTCCCAAGAGGAAATCTCTCTACTCTAGCCCAT<br>TTCGTCCCCTTCCAGCCTCATTTTCCCCAGACAGACCACACATGGG<br>CGCTCCTGGCCAACCCACCCCCAAGCCTGGCCTGAAACAGCTTCT<br>CACTTCCCCACCACAAAGTTTGGGCCTTCTCACGTTCCTTCTCCTC<br>CAAAATCTCTTCATTGTCCCTGACCGTCTCCCAGCCTCCTGGAATCT<br>CTCCCTTCTCTTATCTCCTATGTCCCTTAGTGTCACTGAGTTTTGCTG<br>GCTAACAGGGCTGCTTCCTTTCTATTCTTTATTCTCTTGTAACATAGA<br>ATCCAGGCTCTCTTCTACCCAGCTGTAAGATCCTTAGAGGCAAAGG<br>CCTATGCCATGGATCTTATTCCCAAACCTTGACTGCAGTTAGTCCTC<br>AGGGAAGTTTTATTGTTGTTGATAATGGGTGTTCAACTGGTTAGGT<br>TGTGAGTGAGAGAAACTAACTGAGGATGTTTTATTTTATTCAATAAA<br>AAAGGAATTCATTGGCCTGCATAACTAAAAAGCTCAGGCTGAGTCC<br>AGGGACCTTCTGTACGTTTGTCTCTCTCTTCTGTATGTCTCTTCTCTC<br>TGACTTCCCCTGCATTGGCTTCATTCTCAGGCAGGCTCTCCAGGCC<br>TGTGGGCGAAACGGCCTCCAGGCTTACATCCTATTGGCTTAGTACC<br>ATCAGGGAAAGGGAATTTCCCTTTCCCAAGAGTTCTGGAAAAAGT<br>GATTCTCATTGGCCTGGTTTGGGTCATGTGCCCATCCTGACTCAATC<br>ACTACAGCTAGGGAAATGAACCACTCCTAGGCTAGGCCACCCCTGG<br>GTGGATGGCCAGGCCCAGGGAACATACCTACTTATAGATGTAGAGT<br>AGGGGCTATCTGCCCCCAACCACATGGATTAAGGTGGCTCCCCAAA<br>GGAAACTGCTGGCTGGGGAATAGTAATAAAAAGAAAGGGGAATTG<br>ATGTTGGTCATGCAAAACAAAAGATGTAGACGTAGACTATGAGAAT<br>TTCCCATGCTGAGCTTCAGCAAGAACCTGACAAATACGTTAGGTGA<br>GCTGCACAGGAGAATCATCGGGGAACTGGTTTGCCCTGGCTGGGG<br>CAGAGGATGTTCTCTCCCAGTCTGGGTGGCTCTCCAGTGACAGCAC<br>AAGAATCCCAGGCCGATACCTTGGCTCAGTGCTGCCACTCGCAAGC<br>TCGGGAAGATTGTGATGCAGCTGGTCCAGGGTAAAAGCTGTCTTTT<br>TCAGACTTCCACAGGCGATTCTGCTGCACAGTCAAGGTTGAAAAAT<br>GCTGCTCTAGTAAACGCTCTACTGCAGCAAGATGGGTTCAGATCAA<br>CTCTTAGGGATTACGTCCCCTGTGATAGGATTGGTATGTTTTTCCCCA<br>TCTGCACAGCTTTCCAGAAAAGGGTCACTGGAGTTTTCTTGGAGG<br>CTGAGGATGGGTGGGTGAACTGTGGATGACTCTATGGATCCAAAGC<br>CTCTGAAAAGAACAATAGCCACTGTTTGTTGAATGCCAATTGCAAG<br>GCAGGTACTGTGTGTTGGGACTTGACATATGGCAGCCCGCATAAGT<br>TTCAGTCCTCACAACAATACTCCCATGTGGGTATTATTACTATTATTT<br>ATTTATTTGAGACAGAGTCTCACTCTGTCGCCCAGGCTTGAGTGCA<br>GTGTTGTGATCTCGGCTCACTGCAAGCTCCACCTCCCGGGTTCACG<br>CCACTCTCCTGCCTCAGCCTCCGGAGTAGCTGGGACTACAGGTGGC<br>CACCACCACGCCCGGCTAATTTTTTTGTATTTTTAGTAGAGATGGGG<br>TTTCACCGTGTTAGCCAGGATGGTCTCGATCTCCTGATCTCATGATC<br>CGCCTGCCTCGGCCTCCCAAAGTGCTGGGATTACAGGCATGAGCCA<br>CTGTGCCCGGCCCCATGTGGGTATTATTAACATTAGCCCCATTTTAC<br>AGTTGAAGAAAGTGAGACACAAAAAGCATGGCTGGCTGGGCACG<br>GTGGCTCACGCCTGTAATACCAGCACTTTGGGAGGCCAAGGTAGGT

	GGATCACCTGAGATCAGGAGTTCAAGATCAGGCTGGCCAACATGG<br>TGAAACCTGGTCTCTACTAAAAATACAAAAATTAGCCATGTGTGGT<br>GGGGTGCACCTGTGGTCCCAGCTACTAGGGAGGCTGAGGCAGGAA<br>AATAGCTTGAACCCAGGAGGCGGAGGTCGCAGTGAGCCAAAATTG<br>CCCCATTGCACTCCAGCCTGGGTGACACAGGGAGACTCTGTCTTAA<br>AAAAAGCAAAAACAAACAAGTAAACAAAAAGCTTGGCTGGCTGG<br>GTGCTGTGGCTCACACCTGGAATGCCAGCACTTTGGGAGGCCGAA<br>GTGGGTGAATCGCTTGAGCTCAAGAGTTCAAGACCAGCCTGGGCA<br>ACACAGCGAAACCCCTCTCTACGAAAATACAAAAAAAAAAAAAAA<br>AAAAAAAAGTAAAAGCCAGGCGTGGTGGCAGGCACCTGTAGTCCA<br>AGCTACTCGAGAGGAGGAGGCTGGAGGATCACTTGAGCCTGGGAG<br>GCGGAGGTTGCAGTGAGCTCGCGCCACTGCACTCCAACCTGGGTG<br>CCAGCGTGAGACCCCGTCTCAGAAAGAATAAAAACATTAAAAAAA<br>AAATTTGGCTAAGGTACCCTACCAGGGAGTGGCAAAATGGACATTC<br>AGACACAAGGCCATCTGCGCTGCAACAGCCTGGCCTTCCTGCCCTT<br>GCGGCAGGAGTCCTCTGAGAGGCGCATCACTCCTGCCCCAATGGA<br>CAACTCGGTAGACAGTGGGAGTGAGCCCCCCACCTCCCCAGCGGA<br>CTTGAGACGGCAGGCTCCGAGACGAGGGAGTCCTGGTTCATTAAG<br>TTGGTTTTTATAAGAAAACATGTTTGGAGGGGGGACAGCCACAGA<br>GGGATTAAGTCCAAGAAAGTTACACCCTCCCCCACCTAATCCCCCT<br>GACCCCGACCTCCAGAGGCTGTTGGGGTTCACAGAGGCCCTCACC<br>TCCTCCCTTCCCTCTCGGTGTCGTCAAGCACCCTCCTTCCCCACATT<br>CTCTTTCTGCTTTCTTTTTAAATCCAGAAAAAACAGCACCTCCTCTG<br>GATTCAGAGCTAGAGCAGGAGGAGCCTTCCCTTCCCGGAATCCCTG<br>TTCCCTTTGGGGTGAGCAACTGACTGCGTCGTGGGGGCGGGGAGG<br>GCTTCCCTGTTCGCGTTCGGCCCCAGGGAGACCTGCGGGAATCGTT<br>CTCCCTCGCCACCACCCACCCCCTGCTTCCTTCTCCCCCTCGCCTTG<br>GCCAGGCTCGGGGTGAGGAGTGTTATCCCGGAGTCTGGGCGCCTC<br>GGCAGTGACGGCTCCCCAGGGACTGCAGGGGGAGCCCGGGCTGC<br>AGCGCCTGCTCAGTTCGTGCTCACTGCGTCGAAGGCTCCCCCGGCC<br>TGGCTCCGCGCCCAGCGCCGCATCCGGGAGGAGGAGCGAGGAGG<br>CGGCGGAAGAGCCCGCGCGGCCGGAGTCCGGGGCTGGGAGTGGA<br>GAGGGAACCTCCAGGGGGCAGCACCGAGCCGCCAAGCCGGTCCT<br>CTCTTCGCGCCCAGCCCGGGGTCCCCAGACAGCCCATAGGGAAGC<br>CCCTCTTTCGGATTCCCGCAGTGTGGGCCGGCCCTCCACCTGGACT<br>GGATAAAGGGGGGAAAGTGACCCCTCACCACAAGGACCATTATCT<br>CCTGGTGAGAACAAGAATCAGGCCTCTCTTGGGGCAATCAGCTTCC<br>CCACTTCGGTCCCCCAAAGGTGGGCTCTTTGCCGGCGGGGACTAG<br>GGAACAGCCTTTCGGTTCCGGGGGAGCACAGGGGACCCCAGGCAC<br>CAGCAGCCCCATCCCACCGACAGGTGGCAGAGGCAAGGCAGCTCA<br>CTGCTATACAGTGTCCCAAGAACCAAGTGGCCGTGACTTCCTATCC<br>TCAATTTCCCAGCGACACCCGGAAAGACACCGTGCCATAGATCGA<br>GGCCCGGGGTCAAGGCCCCGCCTCTCCTGGGCGGCCCCTGCCCAG<br>GCGGGCCCAGCCGCTCCTCCCCCGCACTCCCGGTTCGCTCTCACGG<br>TCCCTGAGGTGGGCGGGCGGGCCCTGGATGACAGCGATAGAACCC<br>CGGCCCGACTCGCCCTCGCCCCCGCTCTGGGTCTGGGCTTCCCCAG

	CCTAGTTCACGCCTAGGAGCCGCCTGAGCAGCCGCGCGCCCAGCG CCACACGCCACGAGCCCTCCCCGCCTGGGCGTCCCCGGATCCCGC GAGCGCTCGGGCTCCCGGCTTGGAACCAGGGAGGAGGGAGGGAG CGAGGGAGCAACCAGCTGCGACCCGGAAATGCCATATAAGGAGCA GGAAGGATCCCCCGCCGGAACAACCCTTATTTGGGCAGCACCTTAT TTGGAGTGGCCCGATATGGCCCGGCCGCTTCCGGCTCTGGGAGGA GGGAAGAAGGCGGAGGGAGGGGCAACGCGGGAACTCCGGAGCTG CGCGGGTCCCGGAGGCCCCGGCGGCGGCTAGAGCTCTAGGCTTCC CCGAAGCCTGGGCGCCTGGGATGCGGGCGCGGGCGCGGGCCCTAG GGTGCAGGATGGAGGTGCCGGGCGCTGTCGGATGGGGGGCTTCAC GTCACTCCGGGTCCTCCCGGCCGGTCCTGCCATATTAGGGCTTCCT GCTTCCCATATATGGCCATGTACGTCACGACGGAGGCGGACCCGTG CCGTTCCAGACCCTTCAAATAGAGGCGGATCCGGGGAGTCGCGAG AGATCCCAGCGCGCAGAACTTGGGGAGCCGCCGC
EGR2	GGAGACACTCCAAGGAGGATGCCTGTATTGGCTACCTTCAGCCCCA GTGCACTAGACATGGAGATGGCCTCAGAAGTTTCCACCAACCATGG ACTTTCTCCATTCTTCAAATGAACTTTGCTTCCCTCCACCTTGGCAC CTTTCAACACCTGTTCTTTCTCCCTGGAATGCTGTCTCCACTGACCT TCCCCCAATCCTTGCACATCCTTCAGATCTCTGCTTAGAGAAGTCTT CCTCAACCTCCTAGATTCAACTGGGTCCTTTGTCATTTTCTTTCATT GGGATCAAACACTGTCATTGTTCATAGTCATGTATTTGTGTGATGCA CAATGCTAATTCCATGGGGTGGGAACCAGGCCTAATTTAGGCACC ATTATATCCCTGGGGTTTGGTGTAGTGATGGACACCTGGTAGAGAG GCACACGAAATATCTAAAGGGAACCTGAGCACCCACTGCTTGGGC CAGTGCTGCTGCTTGTGCTGCTGCTTCTACAAAATTAGATCATAGAG CTCTTATCTCCTCCTGTCCAGATTATTTGTGCCAATGACACGTGACT TAAGTTGTACATTGGACTGGTGCAAAGCAATGCTTTCCTCAAGCTT CACAACAGTTCTGATTGGCGTAGCCTGGGCTTGGTTTATAGCCTCT GGGATTACCAGATTAGTAGTGAGATGCTGAAACGTAGAGAACCATT TATCACTTTTTTTCAGCATGCAGGGGCACTTTAGGTGGATGGCAGA GTTGGCAATTGATCTGATTTTCACCGAAGTGCACATGTTCTATGCCA TTCAAGTTTGAGGTCCCCACCCTCATGAGTGTCCACAGTTAAGGCA AATTCTGACCTTCTTTTGTCTTCCACTCCAGTAACAGTATTAGTATG GAAGGGAACAAAAATGACATTACAATAGGTAGAGCCTATAATGATG TGCAAGAATGTTAATAAATCAAAACACAAATATGAACAAATTATTTG TGACATCCCTCTTGGCTGATGGTGATCCTGTCACCACACTGTAAATG AGAATTTCAAATCTGGGTTAAAAAAAAAAACCTGAAAATCACTCA GGAGACTCTGGGGTCCAGCTAGGACCCTCAGGTTGCCACCCCTGC TTGTCACCCTAAACACTCTCCCGCAACCCCAGGAGCAAGCCTACTA AAGGGTTTCTCTGCATCGTTGTTATTTTACTTTCTGGCTCTGGAATC TGGACAAAGCAAATTGGAATCTGGCATGAGTTTGCTACTCTTTTAAT AGCCCTGATACATCTTGGAGTGCCCCATTTAGTCGGGCATTTTTACA GGTCTATTTTAAAGAAAGTTGTTTCTTTGTTGGATTTTTAATCAAAA CAAAAAAGTTCAAATGCTTAATAGTAGGGACCATGGTACATGTTTG TATTCAACTCTCACATTCTCCATCCCCACACCCCTCCACCTCCAGCA CAGTATCTTGCTCAGTAAACCCTTTGGAGTTTTTACTTGTCCGCACT

	TCTTTCTTTTCTCACCTCTTCTTGTCTCCAGGAAGGATACTGTGGGG<br>CTAGCTTCTCTTATGACAGCGCCTATTAGTATGCTGGCTTTGTGCCA<br>GCCTGGCTATTCCATCCACAGTCTAGGTCTGGAGAGCCCAAGAACT<br>CAAATTCTCCCTTTACAGTGGGCAGGGGGCGTGTCCATGACAGTCC<br>CTTCCTCCTTGCCTGTAGAAAGCCCTGACCTCAGCTTTGCTCTCCTC<br>AGAAATGGCAAAGAAAGGAGATGAAATTGGCGGAGGCGGCCTGAT<br>TCTTCCCTAGTGGGCTGAGTCAGGCTCCCAATCCTCAAAGAAGTGT<br>TCACTTTTCAGACCTGGGTTTTCCTGAGGGCACGATGTAGCGACCA<br>CTGTGCGCTGTACTTGGATTCAGAAGACCCAGCTGCAAGTTTGGAC<br>ACGACCACTTACCAGCTACGTGACTTTAGGCAAGCCACTTTAGCTT<br>TCTGAGCTTATCCACAGGGTGGCTGGCTTGAAAGGGTGCGCCTTGA<br>TTCTGTTCCATTATCTGCACTACCCATATTTAAGGGTTGGTACTTCAA<br>GAGACACTCAAAAAAACAAAGGTTACGAGAGTTATCTACTGACTT<br>GTTTCAGCGGAGGTTCAATAAAGGCCTCCCAGGACTGGCCCCAGG<br>CATTCCCTGGCCTCCCCGCAGCTGCCGCCCGTCCCCGCCCCCACTC<br>CATTCCCTGCGCACCCCAGGCTGATGCAGCGTAGCTCTTAGGGGGA<br>GGTGTGAGTCCTTGGAAAGCTCACAAGAATTTCAGAGGGTCAGTC<br>ACTCTCATTTACTCTAATGTGCTTTCAAGACTCCTTGATGGGGCCCC<br>CTCCTCCACCCGCCTTCTACTGGCATCCTCCTCCGGGAAAGGTGGC<br>AGCCGAAGACCTCGCGGTTTTCCCTCGCTCAGGCACCCCGCCCCG<br>CCCCCAGCCCCGGTCCTGCCCTCCGCGCCCTGCTGGCCGGGCACTC<br>GGGCGTCCAGGAGCTCCCCCTCGGGGTGGCGCGCGGCGACCGCCG<br>GCGGGTTTGGCAGCAGCGCCAGTCGCGGGGCGGCCCGGGCAACTC<br>GCCCGCCTGCCGGTCCGCCCACGTGCGCAGAGAGGAGCGAAGCGC<br>GAGCAGTCGCCTCGCCCGCGCTTGCTGCCGGTGGGTGCCTCACCTA<br>AGGGGCCGGCGGGGGCCGTAACCCGAGCCCCATCGCAGGGATCCG<br>CATTTGCATAATTCGGCTTTCCCCCGTTCTTTCGGACTCAGGGCCCG<br>AGATGCGAGTCGGACTCCCTTCCGAGGCGTCGTCTTGCCCATGGTC<br>AGCGCGGGGGCCGCGGGACCCGCCAGAGCGCAGGCGGAGTGGGC<br>TGGCGCACGTGGTAAGAACGCAGCTATCTTTTCTTGCTTTTCTTTTC<br>TTTCTTTCTTTCTTGAGAAATAGGACTTTGCACATTTTTGCTGGGGT<br>GGGGTATCTACTAGGGTGCTAACCTTGCTGCGATTTCTTTCCATGTC<br>CTTTTCATCCCCAATTGGAAAAAAGCGCACAGCGCGAGCGAGAAA<br>CTTTCCCAAATGTGCTCTGGGTGACTTAAGTCAGAGAGATGGCCGG<br>CCGCTGGATACCGGCTGTTTTGGAGGGAGGGGGAGAAGCAGCGCT<br>TCAGTGGAAGAGCAGAGGGAGAGACCGCTCCCGGCAGCCTCAGG<br>GCCCTGACCGCGGTGCGCTGGGCCCGGCGAGAGCTGGCGCACGGC<br>TGCAGCGGTCGAGGCAGGTGAGCGGCTGAAGACCCGCCTGCAGC<br>GCCGCGCTTCCTCCACGGCGTGGCGCCGTGGCTCCCGCCCGCCCC<br>GGCCCACACTAAGCTAGGGCCGCTCCGGGCCCGGGGTGAAAAAGG<br>GACAGGACAGGCCTGCGAGGCTGACCCTGCGCGTTATCCCTGGCC<br>TCCGCGGCTTTGGAGCGGGAAAGGGCTCTGGGGCAGTTCGCAACC<br>TGCACAAACGACCATGAATGAGTCATGCAGGCGGCGGTGCGCTGG<br>ACTTGGGCACCCGGGACCAGCCTCCTGTGGGGCCCCGGACAGGCC<br>CGGCTATCCATTGCGGGCTGGGGCGCACTGGGAGGTAGAAGCTTTG<br>CGCCCAGCAAGCCAGAACCTGCTGCTATTTGGACTTTGGAAAAGTT

	TTTAAAAATTGCTTTGGGAACGAAACAGAAGGGTTTCTACCGGGGT<br>CTCGCAAAGTAAAACGAGTGGGTTGGGGGATGGGGATGGCGTCTT<br>GAAAGTGATTAGAGATGCTTTCGGCCGTCACTGAGGACCGCGCTG<br>GGCTCACCGAGGTGTTTTTTGGGGAGGGAGAGAAAATATTTATTCT<br>AGTACGTGTATTCTGGCTCGGGTGCGGGCAGGGCGCGGTCCTGGAT<br>CCTGGGCTATGGGGGTCAGGGGGATTCTGTGGCTGCAGGGACCGG<br>TAGTGAAGGTTAGGCCAGTGTCTTCATAGAACCTCCTGTCCAGCGA<br>GCCCGGAGCCCGCTCTGCCGCCGGGACACTGCTGGCAACGACGGC<br>GCGCGGACGCAGTCAACAGGGAGGTGTGTAGTGTAAGGTGTGTGA<br>GCGGGTGTGTGAAGAGGGCAGCGGGGGCGGGAGTGTGTAGCGGA<br>AGTGGAGCGAGTGTGAGTGTGTGTGTATGTGTGTCTGGAGGGGCA<br>GGGTGAGTGTCCAAGCTTCCATCTGGACGCCCCCACGCAGCCGAC<br>CGCCCAGACGCGCCGGGAAGGGCGCCCGCTCCGCGCAGCAGCTG<br>AAGGGGGGCGGGGAGCGCGCGGGCAGAGGAAGGAGGGTGTCTCC<br>GGCTGAGGATTTCTCTCGAAGCTCCCCACAGTAATTCTGCTCCTCCT<br>AGGGTTTACCCTCCCAGTCCCAAGGTGCCCAAAATACTTAAACAAA<br>CAAACAACCCAAACCTGTTCCGTTTTCGCTCCTGTATAAATAGAAC<br>AGACTTTCCAAAAAAGCAATACGCATTCACTCTTATCACCAGCCAC<br>TTCTTTCCACCAAGTAATTCAGAAAAAAGCAGTCAGCTTCCGTGAA<br>TGCATGTAGCTTTTTTTTTCTTCGCTGCCTCCTTTTGCTTGCGGTTTT<br>GAGCTGCCAAGAAAGTGAGTAGGGGTTTGACTGTAGTGTCTCGGC<br>TCCGCTCGGTTTCTTTCCGAAGTTTAATTTTCCGGAATGGCTCCCAA<br>ACAAGGGCTAGGGAGGCGGAGCCGCCGGCACCGGATCTTCGCCTT<br>TTTTGGAAAGTCCCAGAGAACCGGAATTCCTCCCCGCCCCGGGAG<br>GCTGAGCCGCAGCCTGGAGCAGCTCGCTCTCCTGCTGTGGCTGTG<br>ACTGGGTCCATGGCGCGCCCTCTCCCCCGCGATCCCTGCGCCCCGG<br>ACCCAGGCTGCAGCTAGGGCCGCGCGCCCGGGACCCCAGCGATCC<br>CCGCCGCCTGCGAGCTGGAAGTCGCGAGCCCGGCGGAGTCCGCGC<br>GGCCGAGCCCGTGTATGCAAATCAGCCATGTGACGGCAAAAGCCG<br>CCTGGCCCAGCCCTGTTCCTCAGTCCATATATGGGCAGCGACGTCA<br>CGGGTATTGAAGACCTGCCCATAAATACTTAGAGCAACACTTTCCG<br>TCTAACTGAGCGAGGAGCAATTGATTA
EGR3	TTTTTGTTTGTTTGTTTTTGTTTTTGTTTTTTTGTGTTTTTTTTGAGA<br>CAGGAGTCTCACTCTGTTGCCCAGGCTAGAGTGCAGTGGCGCGAT<br>CTCAGCTCACTGCAACCTCCGCCTCCCGGGTTCAAGCAATTCTCCA<br>GTCTCAGCCTCCTGAATAGCTGGGACTACAGGCGCCTGCTACCATG<br>CCAGGCTAATTTTTGTATTTTTAGTAGAGACGGGGTTTCACCAATTG<br>GGACGCTCACTAAACCTATAATAGCNCTCAACTNNNNNCCTCTCTC<br>NNNNNNNNNNNNNNNNNNNNNNNNNNNNNNNNNNNNNNNNNNNNNN<br>NNNNNNNNNNNNNNNNNNNNNNNNNNNNNNNNNNNNNNNNNNNNNN<br>NNNNNNNNNNNNNNAAACTCGTTTGTTGTTTGAAGAGGTTTAACT<br>TGGTAACCAGAATCTTTAAATTGGGCACAGAAAGATATACTGGATG<br>GCTGGACAAGATAATGTATTTCTTTGATGATCATTACTAGATTTACTA<br>ATTGCTAACAGTTCATGAAGGTTTTTTTGCAGTGTCTAGACAATGTC<br>AGCCCAAGAGACTGAAATTCTAAATGAACAAACACTGAGAAATATT<br>TGAAGAATTCATTCAGTAATTACCTATGTAACATTCTTGCTAAAGGT

	GTTTAATTTAAGCTGAGCTTAGTTATAGTAGACAATCAGACAAATCC<br>AGTTTGTGGGACTTTCTACAAGACAGTTGGCCTGAACTCTAAAAAT<br>TTCAAAGTGGTGAAAACGAAACGAAACAAAAAGGGCAGGAAGAC<br>TGTTCTAGATGAAAGAAGTTGAAAGAGAAATGACAGTCAAGTACA<br>GTGTCAAGCCTGGATTAAGAAAACAAAAAACCATAGAGGACATTT<br>TGGGGATAACTGGGAAAATTTGAATATGGTGTATATATTAGACGGTA<br>TTATACCTTTTTTTTGTTTTTTTTTTTTTTTGAGATGGAGTTTCGCTCT<br>TGTTGCCCAGGCTGGAATGCAATAGCGTGATCTCGGCTCACCGCAA<br>CCTCTGCCTGCCGGGTTCAAGCGATTCTCCTGCCTCAGCCTCCCGA<br>GTAGCTGGGATTACAGGCATGCACCACCGCACTGGGCTAATTTTATA<br>TTTTTAGTAGAGACGAGGTTTCTCCGTATTGGCCAGGCTGGTCTCG<br>AACTCCTGACCTCAGGTGATCTGTCGACCTTGGCCTCTCAAAGTGC<br>TGGGATTACAGGTGTCAGCCACCGCGCCCGCCAGAGTTGTTTTTTT<br>TTTTTTTAAAACATAAAGTGAGGTGGGGTGGGGGACACAAAGGCA<br>GGCAGTGGGAGAGAGGCCCGGGCGGGCAGGCAGCCTCCCTTTCCA<br>CATCCCTGAGAAGGTCAACCAGCTGATAATCCCACCAAAAATAACA<br>TTCCCACTCTTCCCTGTGGCAGAGGAAGAAAAAGGAAAGTCCACA<br>AGTACAGGCTGTTTCTAGAATTTCTCAGTGGCTCATGGAAATTTTGA<br>GTTTCCCTGCCATCACACACATCCTGAAATGCTGGGGGAGGGGGCC<br>CAGTGCTGCCAAGAGGGAGTGATGCCAGAGTCACCATGGCCACAG<br>CCGGGAGCTGGCCAGCAGAGCCACTGCACACACCAGTTCACCCAC<br>GGCCAAGGCTTCTGCCGTGGGAGGCACCACAGAGCGTCTGCTGGG<br>TGTTAGGGGGCGGCCGAGGAGCTGGGAACAGGAACTGAGACATG<br>GAATTTCTGTTCTGAGAGCTGGCACTTGGTCAGCAGCCCGGGCCTG<br>GGGGAGTGGGTGACTTTGTACTTTTTGCATCTTTCTGGGAAGGGAG<br>ATGGGAACAGCGTGGCTTGCTGCCCAGGGACCTGGTATCCTGAGA<br>ATTACCCAGCCCATCAGATGGAAAACAGCCAGCCCTGGAGAAACG<br>GTCAGCATTGGAATTGGAATACAGATACGCCAATTGGAGTCACATG<br>GGCCTGGGATGACAGGGCACAGGACGCGCCCTCAGTGAGCAGGA<br>GAGAAGGGCGGAGGCAGGGAGNNNNNNNNNNNNNNNNNNNNN<br>NNNNNNNNNNNNNNNNNNNNNNNNNNNNNNNNNNNNNNNNNN<br>NNNNNNNNNNNNNNNNNNNNNNNNNNNNNNNNNNGCCCTGAG<br>TAGTTCTAACGCACTCAGCTGAGTGAACCATGCATCCACCACCACA<br>CTACAGCCTGTGTGGCAGAGCAAGACCCTGTTCTATAAGAAAGAAT<br>GAGAGAAAAAGAGAGAGAGAGAGAGAAAAAGGAAGGAGGGAGA<br>AGAGAGAGAGAAGGGAGGGAAGGAGCAGAGAGAAAGAGAGACA<br>GAGTGAAAGAAAAAGAAGGAAGCACTTTTCTTTATCTTCACCTATT<br>TATTTATATGTTTATTTATTGAGACAGGGTCTTGCTCTGTCGCCCAAG<br>CTGGAGTGCAGTGGCATGATCATAGCTCACTGCAGCCTCTATCTCCC<br>TGGGCTCAAGTGATCCTCTAACCTCAGCCTCCCAAGTAGCTGGGAC<br>CACAGGCATGCCACTGCCATGTCCAGCTATTTTTTTCTTTTTTTAGA<br>GATGGGGGGTCTTCCTATGTTGCCCACTCTGGTCTGGAACTCCTGG<br>ACTAAAGCGATCCTCCCACCTTGGCCTCACAAAGTGTTGGGATTAG<br>AGGCATGAGGCACCGTGCCGGCTGCTCTTTTCTTTTAGTGGCCTAG<br>TTCCAATAGAGTTAAACATTCATTGCTTTCTCAATGTCTTTTCTGATT<br>AAAACAAAAACAAACAAAACATCTAGAGCCACTCCATGAAAATTT

	AAAAAGAAGAAAGCTCTAATTGAGCTCCCTTCCCAAACAATTGTTT<br>TCCATAACAAATTAGGTTTAAGTTCATTGCTTGTGTGGTGGTGTTTT<br>TAGACATTAACAATTCTCAAGAATCATTGGAGAGCCCAATTTCATTC<br>AGCTGGTGGATCTCTCCACCTGGCCAGATGTCCACTTGGTGAATTT<br>TGTATCCCCCCACTTTTTTTTATTTTCTGCATTGGTCAGTCAGAAGA<br>CATAAATATTTGAAATAACCAAGATTCATGTTTAACTATCATTTCTAC<br>ACATTTCTTCAAATCTGGGTTTCCTTCTTACAGGGCTTGTGAGCTTT<br>GGTGTATTTCCTCATGAAACTCCCTAAGCGCTTCCTAAACCTTGATT<br>AATGACCAGCTCCTCTGAGACATAATTATGAACACTTTTAATTTAAC<br>AAAAGCACAGGGAACCCTTGTTTCCACTGTTGTGTGAAAGTAACC<br>TGGCGTCAGTTTCTATTTATATGAAGCCATTAAATGTTCAACTTCTGT<br>GTTAAATGCAGGTAAGAGAACTGTAGAATATTTTAAAAACCATAGT<br>AAAATGAGCATTTCTAAAATAGCTACACAAAACAAAAGGTATATTAT<br>AATGTATAATCTGCAATTTTATATGAAAAGCACTATATAGGCCAGGC<br>ACGGTGGCTCACGTCTGTAATCCCAGCACTTTGGGAGGCTGAGGC<br>AGGTGGATCATGAGGTCAGGCGATCGAGACTATCCTGGCTAACACA<br>GTGAAACCCTGTCTCTACTAAAAATACAAAAAATTAGCCAGGTGTG<br>GTGGCACGTGCCTGTAGTCCCAGCTACTCAGGAGGCTGAGGCAGG<br>AGAATTGTTTGAACCCAGGAGGCAGAGGTTGCAATGAGCTGAGAT<br>CACGCCACTGCACTCCAGCCTGGGCAACAGAGGGAGACTCCATCA<br>AAAAAAAAAAAAAAAGAGGGAATTGGGAGTCCCAGACCGGCTGC<br>GGCAGCTCGCCCCTATAATCCCAGCACTTTGGGAGGCCGAGATGGG<br>CGGATTACTTGAGGCCAGGAGTTAAAGACCAGCCTGGCCAACATG<br>GTAAAACCTTGTTTCTACTAAAAACACAACGATTAGCTGGGCATGA<br>TGGCACAGGCTTGTAATCCCAACTACACAGGAGGCTGAAACAAGA<br>GAATGGCTTGAACCCAGGAGGTGGAGTTTGCAGCGAGCCGAGATC<br>ACACCACTGCACTCCAGCCTGGACGACAGAGCAAGACCCTGTCTC<br>CAAAACGAAAAAAAAAGAATTGGGAGCCCCAAAGCTGCCGTCAC<br>TTCCTGCTTGTGACTGTTGTTGTTTGCATCATTGCAGAACAGGAGA<br>AAATGGTGGCAACCAGTACATAACACATTTGGTGGTTCCACAGGTA<br>AGATACCAAGGTAAACAGCACTGGCATTCAAGTTGCTTGGACCAA<br>AAATTTAGAATCAGGGACGCCTTTCCACACACTGTATCCTGTCAAC<br>TTCCTCCCCCTACCGTCTATTTTCCTATACAGCAGATCTTTCTAAACT<br>TAAAGCATAAAACTGATTTGTCATTCCTCTGGTTAAAACTCTCTTGT<br>GGTCTAAGGGCGATGGCCCACGCCTGTAATCCCAGCACTCTGGGAG<br>GCTGAGGCAGCAGGATTACTTGAGCCCCAGGACTTGGAGACCAGA<br>CTGGGCAACTTAGTGAGACCCCGTCTCTAAAAAAAAATTAAATTAG<br>CCAGGCTGGTTGAGGTAGGTGGGAGGATCTCTTGAGTCCAGGAGT<br>TTGAAGCTGCAGTGACCTCTGACCCCATCTCTAAAACAAACAAAC<br>AAAAACAAATGTCTTCTCCTTTCCCGAAGTGGTCTGACTCCTGTCA<br>ACTTTGAGCTCCCACCAACCTTCTTCCCTCATGTCTGCTGCAGTCC<br>AGACAGACTGGCCTTGTTTCTGCTGTTTACACATACTGGCCTCAAAT<br>AGATGAAGCTTATTTCTGTCTCAGCATTCTTGCGTTGGCCGGCCCTT<br>TGGCTTAGAACATTCTTCTACAGTCACAAAGCTCAACTCAGATGTC<br>ATCTGGTTAGAGCCCTTTCCTGATCACCTCTATCCCTGTCTGCCCAT<br>TCCCTACCTCACTCCCCCGTGTTAATTTATCTGTAGCACTTGGTTTTT

	GTTTATTTGCCCCTCATCCTTAAGGGAAAGTGCGGATGNNNNNNNN<br>NNNNNNNNNNNNNNNNNNNNNNNNNNNNNNNNNNNNNNNNNNNN<br>NNNNNNNNNNNNNNNNNNNNNNNNNNNNNNNNNNNNNNNNNNNN<br>NNNNNNATCCGCCCCACCCCCCCCCACGACCCAGC
EIF2S1	ATTTCTGTTCTTGCTGCCATATGCCAGATACAATTTAATAATGAAAA<br>AAGTGGAGCCCAGTTCGAGGATGTAGAAGTAAGACCCTGTCTCAA<br>AAAAAAAAAAAAAAAAAAAAAAAAAAAAAAGACTAGAAATAAAAT<br>GTCATACCATGCAGTAGTACATGCTACAATAAAATTAAAATAAGGAA<br>TTATGACAGAAGCAAACATGGTGTTCACTTTAGATTGGGTGATCAG<br>GAACAGCCTCTCTTAAGAGACTAGAACAGCCAAAGGAGCCAGTTA<br>TAAGTTCCGGGGAAAGAGTATTTCAGGCAGGAAAAAGAGCTAATG<br>AAAAGTACCTGGAGGACCTTAGGAAGAATGCTCTAAATAAGGTAA<br>GTGGTACAGTGGCTTATGTCTATAATCAGCACTTTGGGAGGCTAAG<br>GTGGGTAGATTGCTTGAGTTCAAGACCAGCCTGGGAAACAAGGTG<br>AAACCCTGTCTCTACAAAAAAAAAAAAATATCAAAATTACCTGGGC<br>ATGGTAGGGCACACCGGTAATCCCATATACAAAGAAGGCTAAGGTG<br>AGAGGACTGATTGAGCCCAGGAGGTTGAGGCAGTGAGCTGTGATC<br>ATGCCACTGCACTGCAGCCTGGACAACAGAGCAAGACTCGGTCTC<br>AAAAAAACAAGTGTTTGGGTAATAAAATTCAACAATAAGGTTGTAT<br>AACCTAGACACAGTATAACCTAGACACAGTGATTTAAGGGGATATA<br>ATTTCATATGACTGGTGTTTTAAAATCGCATGTACTAATAAACTTTTT<br>TGGCCAGGTGCAGTGGCTCACACCTGTAATACCAACACTTTGGGAG<br>GCCGAGGCAGGTGGATAACTTGAGGTCAGGAGTTCGAGACCACCC<br>TGGCCAACATAGTGAAACCCCATCTCTACCAAAAAATACAAAAATT<br>AGCTGGGTATGGTGCTGCATCCCTGTGGTCCCAGCTACTCAGGAGT<br>CTGAGGCACAAAAATCACTTGAACCTGGCAGGCAGAGGTTGCAGT<br>GAGCCAAGATCGGGTCACTGCACTCCAGCCTGGGCAACAGAGGGA<br>AAACCTGCCTCAAAAAAACAAAAACAAAAAAAAAAAAACAAAAA<br>CAAACAAACACCATTTGGCCAAATTCTTAAATTATACTGGAATCTTT<br>TACCATAGGGCTAAGATCACTAAGACAATTCTATAGCTGAAAGGGA<br>CTTTAGACATTAACTTAAAGATTAGAAAAATGAGGCTTAGAAAAGT<br>AAAACAACTTTCTCAAAGTTACTCAGTGAAGAAAAGGCAGGGCAG<br>GGACTTGAACCAGACTTTTGATTCCACTGACTTTCCCAATGCTCAA<br>ACTAAGGCTCCAACCTACGTTCACTGTACCCCAAACATGTGCCTCC<br>CCTTAAAGAGAACAATTATCACTGGCTGACTCTGCACTGACTGGTC<br>TCACAAGAGGTCTTATCAAGTGAAGGTCGTTTAGTTTCCAGCCTGA<br>GAAGGAGCTATCCATCTCAATGTGTGCATTTAACATAATGTATTGTT<br>GATAGTAAATACTAGAACTTGGGCTCAAAGAGCTGGATCAAATTTA<br>AGTCAAATTGTAGTACTGCATATTCCAGCCAGTTTATAGTAAAAGGT<br>AAACTGGTTGATAAAATTGAATTTACCATGTTAATTTTATTTTCCTCC<br>TAGGGAATGGCATCTTTGTGAAATGGGCAATTTTAATTCCTGAAACT<br>CTAAGAGCAGCGTGCACACTTACAGCTAATATGTAGCAGAACCTGG<br>ATTTAGTGGACTTCTGTTGACTCCAAATTTTTCATGCGTCTTTTACTT<br>CCACTCTACAATTGTTTTTGCTTTCTCTGGTACGAAAGTGGGTGAA<br>AGTAACATCACAGAATAATACTGTTGAATGGAAATGTCTGTGAAAC<br>AAGCTGCCAGTATATTTTCTAGCCTATGGCTTTTAAAACTTGCTCCT

	TTACATGTTTCAGAGCTGCTCAGTCCTACTCAAAAAGTTTGACCTTT<br>AGCTAGGATGATGACTTTTCTTCCAGCTGAATCAAAGACATCTGAC<br>TACATGACAAAAAGCCTGCTGCCAGCCATGGGGAAAATATTTAGAG<br>AAGAGATTATTTCCTGCACCTGATTTTCCTCTCAATCAGCAAGCTGA<br>GAGCTTTCAAATAGTAGTTTCACCTTCATGAGCCTATCATTTTCTTAC<br>CTAAGCCCAAAGATGTCTATTTTTGCCTCTTAGTCTCAAAGTAAAAG<br>TTTCAAAGTAAAAACAAAAAAGTTATTCTGACTGCTGTCAAAGATA<br>AACCCAGCCAGACATTCGTTAAAGTAGTGAAAACAGATTTTATTCA<br>GTAAACCACTGATAGTAGGGGAAAGGGCTGAGCTCAATTCTCATTT<br>GTGCAGAGGTGACTGGCCATTTTAAAAAAGAGAGTAAGGGAGTGG<br>CAGGGAGCCAGCCGCAAGAAGAGTCAGAGAAGAGAAACCTTAAA<br>GTGTTGGTCAGTGTAAACGCCATACTACCTGGCAATTAGTAAGTTAC<br>TATTCTATCCTCTCACACAGACTGGGAGACAAAGCCCCTATCCCTCC<br>TGATGATTACGTTTCAAAGAAATGGCTCTCAAAGATACTTGAAGGG<br>ACACTTCAGAGTTGGAAGAGATACATATTAACAATTGTAAGCCTGT<br>TTCGTAAATGCTCTAAAAAGGGAACGAGAGGGGTCTATCAATTAGG<br>TGTTGACTAGAACAAACAGTAAATTCTCCCCCTAGCTTTGAGCTTT<br>TTCAGGCAGGCATTTTAATGGCGGGGGCGGCGGGGGGCGGCCTGG<br>GGTCACCCTAGGGGCAGGCCTTACATTGCTAGAAGATAAACTAGTG<br>TCGATGTCTTTCAGTGCAGAAGTTTTGACTGAGTCATCACGTCCTA<br>GGAGTTCTGCAGTTGTCACTACCTCGGGATAGTCCTCATCTTTATTT<br>CCCCACAACTAGCTTGCTGCCTACTTAGTGCAAACAGCTATTAGAA<br>AGTCAACAGGTGAAGGTAAAAAATTTAAAAACTTCATTTCATTAAG<br>AGCTATTTAGTCACCGGGCAGAAAGAAATCTGGGCTTCAGGTTTGA<br>ATATAACTTTTATTTTAAGCATCCTCAGATTCTCTCAAAGGACAGAA<br>TGAGGAAAAACAACAAAACACCCCCACAGAGATAGGACCACAATA<br>AAAGGGTCTAAATGTCAGGGTCAAAACTGATTTTCTGCTTCCTCAC<br>TGAAAAGAAAAAGGGAAAGCCAGGCGCGGTGGCTCCCATCTATAA<br>TCTCAGCCGCCAGGGAGGCTGAGGCCGGAGGATCACTTGAGCCCA<br>GGGGTTTGAGGCTGCTGTGAGCTCTGATCATACCACTGCACCCCAG<br>CCTGGGAGACCCTGTCTCTTATTAAAACAAACAAACAACAAACTA<br>AATAAAAATAAACAGGATGAGGAGGAGAAAGGGAGCAAGTTGTTA<br>GGCACATTTGAGAAAACGTCAAAATTTTCCTTACAAACTAGCCAAA<br>CACCTACAGTGATATGCCATTTTCCTGACAAAGAATTCAGGCCCCA<br>AAAGAAAGGCAGCCCACAACTGAGCGTATTCCCAAGCATGAGTTC<br>TTTCAAGAAATATTTTTTTGTGGACCTTCTATGGATTAAGACACTCT<br>TTCATCCTGGAGAGAGCCCACAGTCTAGTGGGAGAGAAAGACACA<br>TAAACAAATATCTACTTTACAGCGTGATGACCGGTATTAACACAGGT<br>CTGAACAAGGTGCAGCGGTGAGAGAGCAAAAGCAGTGCCTCAGA<br>AACACAGGGATATCCTCTCACAGCAGGTGACATTTGGCGGGATCTT<br>GGAAAATGAGTAAGGGGATCCAAGATCGAATCCGAGGGCTATGCTA<br>GGGCGATTCGATTCCATCCCCACTTTGGCTTAGGGACCTCTTCTATA<br>TCCTCAGTAAACTTCCCTTCTCAGAGCATTTATTAAAACCTCCTTGG<br>CCTTTCCAGACGAATGTCTGGCTACGTAAACTGTGAGCTCTGTAGG<br>GCAGAGGTGGCGACGGTTCAGTTATATAAATCCAGTGCCCGGCTCA<br>ATAAGGGTATGTGGAATGGATGAATCCATTAATGAATAAAGACAGC

	AGACCTCCACAAAAGCCCGCCGGCCGGGCGCCTTTTCCTACCGGC<br>TGGGATCGCTTCGTCCAGCCTGATTCACTCAGGAGGAATGCGCGAA<br>GAGGCCGTCAGGAAGCCTCGCCCTAGACTCAAGAAAAGAACCTGG<br>GAAAAGCTCAGGATCCTCCCAGGAAACAAGGACCCTCACTGTGGC<br>CCAGGGTCTGAAGGGACCGCGCTCCGGGTTCCTAAACCTGTGAAA<br>GCGGCTTATCCCATTCCTTTACTTACATTCGCGAGGGAAAGATTTCA<br>ATTCGGTCTTTGCCCGACATTCTGACGATAACTTTTCGGCTCGGGTC<br>CCCGGCCGGGCAACCGAGGCTGCAATAGCTCCAGAACTGGCCTCC<br>ACAGTGTCTTCCTCTACGGGAGTCAGCTGGTTGTGTCACTTGACCC<br>CTCTTGTTGCTAAGAGGCAGCAGGGACACCCATTTTCACTTCCGCT<br>TCCGGTTGTCACAGGCGACGAAGGGGGAGGACAGAACAGAGGCG<br>TTGCCCGGATGTCGAGGCGCGCGCACCGCGGAACCACCCCTCAAT<br>TCCGGTTCGCCTCCCAACCCCTCCCCATCCAGCTTCCCTGCGGCTG<br>CGCAATACGCGGCACGCGCCAGGCCAAAGACTAGGAGCGAAGCGT<br>GCACGCCTTGTCTTTCTCCACCTTTCGTCTTTGGTCACAGCGCCATC<br>TCATGGTTAGCTGAAACTTTGAAAGAGGATTCATTCTTCAGAGGCA<br>AGGAATCGGAGTCCTATGTCTTTCTCGCGCCTTGAAGGTTACTTAG<br>CTAAAGCTAAGATAGCGAGAAACAGTACTTTTCAGGTCCTTCCATG<br>ATCTACATTCGTCCTTGTTCTCGGAGGACCCAGACTCTATGGTTAAT<br>GATGAAGAGGGGCCTCTATTCGTTCAGCGCTTGCGCATGCGAGGAG<br>GTTCCGCATGCGCGGTGGAGTGAGCGAAGCGCACGCTGAGGAGGA<br>TCGGCGGCCGGTGAGGGGG
ENG	TCAAACTCCTGACCTCATGTGATCCACCCACCTTGGCCTCCCAAAG<br>TGCTGGGATTACAGGCGTGAGCCACCACACCCGGTCCTTCATTTTT<br>TATTTTTTAGAGACAAGGTCTTGCTCTGTCACCCAGGCTGGAACAC<br>AGTGACGTGATCACAGCTTACTGCAGCCTTGAACTCCTGGGCTCAA<br>GCAATCCTCCTGCCTCAGCCTCCTGAGTAGCCGGGACTGCAGGCTT<br>TTACCACTAAGCCTGGCTCAAATCTGCATTTATAAGAAGCTTCCAGA<br>AGAAACAAGACCACACTTTGAGTAGCAACAGTCTAGGGCATGACA<br>TTTTATGGCCGAGACTCGTTGGTGGGTAACAAAACCAACAGATGAG<br>CTTGTGACGAGTAGTGAAAGAAAATGCAACAGGTTGGGGGTTACT<br>GGAGGGCATCACAGCCGCAAATCTATCTACAGGACCACAAAATGC<br>GTTTCCATTATTTGAGTCTGAGTCCCAGGGTCTGTGTTGGGACGTG<br>ACACCAGGGTCGCAAAGGTTTGCGAAAACCTGGCTTGGGAGTGAG<br>GGTAAACAAAGAAGGAAGGGGCCAGCAACCAAGCCTGGGGGAAC<br>AAGGAGGAACCAGGGAGGAGACAGATAAGGTGACCAGGGAGGTG<br>GGAGGAAAGCCAGGACAGAATGTTCTGGAAGCCAAGGGAAAAAG<br>GCCTGGATTTCAATTATCCTCTGCCTCTTCCCAGCCCTGTGGCCTTG<br>CAAACCTGAGCCTCAGTTTCTTCCATCGTAAAATGCTGACATGACA<br>GCCTCAGCCACTGAAGTGGCTGCAAAGTGGCACTTGGCACAGGGC<br>CAGGCAACCTCATGGATGGTGGTGCAATTCCAATTCTTGTCTTGCC<br>CTTTGAACTCCTCCAGACCAGCAGGCTGCCCTCCCCTTGTGCAGAT<br>GAAGAAACTGAGGCTCAGAAAGTGGAAAGATCTGGCTGGGTGCG<br>GTGGCTCACGCCTGTAATCCCAGCACTTTGGGAGGGTAAGGCGGG<br>TGGATCACTTGAGGTCAGGAGTTCAAGACCAGCCTGGGCAACATG<br>GTGAAATCCCGTCTCTACTAAAAGTACAAAAATTAGCCGGGTGTGA

	TGGTGCATGTTCCCAGCTACTCGGGAGGCTGAGGCAGGAGAATTG<br>CTTGAACCCAGGAAGCGGAAGTTGCAGTGAGCCAAGATCATGCCA<br>CTGCACTCCAGCCTGGGTGACAGAGTGAGACTCTGTCTCAAACAA<br>ACAAAGATCCTGCCTGATGCCACCTGGCCAGTGTAGGGCAGAGCC<br>TGGGCACCCTGCTCCGCCCTGTGGGTCTGGCCCTGCTGTTATCAAT<br>GGCCCCTGGCTCCAGGCCAGTGCTGGAGACAGTCAGCCGCCTGGT<br>GGGTCCCTGGGGGCCGCCTGAAATTCCTTCAGTGGCCAGTGGCCA<br>GGGTGGACGTGCTCTGTCTTTTCCTGCAGCCCTGGCCTTCCTGGCC<br>AGCCAGGAGGAAGAAAGAGCAGAAAGTGTGCATCTGCCCTCTGTG<br>GGGTCCAGGCACAGGGGCCTGGCCATCAGCCACGCGTCTCTCGGG<br>CGTGTGGACAGACGGCACCTGAACACATCCGTATCAGTCAAAGCC<br>CCGGCAGGAAACAGATGGCACGTTCCAGTAGGATCATTAGAGGAG<br>AGTTTGGTGAAGGGTCTGTTTACACAGGTGTGGTCGGGGTGTAGG<br>GAAGCCCCAAGGGACGGTGCAGAACCCCAGGGCTGGCAGCGGCG<br>TGGGCTGTGACCACCCTCAGCCTGAAGAGGCCAGGGGAGGAGCTG<br>AGTCCACAGCTGGACAGAGCTGGGTGGAGGGGGTCCCCAACAGG<br>ACCGTGGCCTTCAGTGGAGGGAGGCCACCAGTATGCAGTGACCCT<br>GCAGGGAAGGGGCTGGAAGACGGACCCTCCTCCTCCTGCCTCCTC<br>TGACCTCTGCAGGGGTGGGGAAGTGGATGTGTCCCACAAGAGGGA<br>GAGTGCAGCTGGGGGATGTAGAAGACAGCTACCCCAAGGCCTGTG<br>CCCAGCAGGGAGGCCATGTGGCCACCAGGCTTCCTCGGGCAGGAG<br>ACCCAGTCCAGGACTGGCCTTTTCTCCTGGGAACCAATGACAAGG<br>CCCACTTCTCTGGGCCTCCATGACCCCCACCCCTGCCTTGACTTCTA<br>GGGTCCCTCATGCTTTCAGCAAATCCATCTCAAGAGCTGAAAGGCC<br>CTGGGCTCTGTGCTGGGGACACAGCAGTGCAAAGGGTGTCAAAGT<br>CTCTGCCTTCATGGGGCTTCTAATCAAGTAGAGAGATACATGCAGAT<br>GGCTGCACACAGGCTTAGGTGTGACAGGGAAGGTCAGGATGCTGT<br>GGGAGCCAGAGGTGACTTCCGCTTCACCCCCCACCCGCGGTGGTC<br>CCAATCTCTTCCTTCCTCCATGAGGTGTCTGGGGTCGGGGCCCCAG<br>TTCTTCCTGGAGTCCATCTGGAGTCTCTCCTACTTTCTAGAGAATCC<br>ATTGGGTCTTGTTTACAACGTGGATGGGGACAGACTGTGCAGCGTG<br>GAGGGAAGGGGAGGGAGGGAGTTTTGGGAAGAGCCTCCTGTGGG<br>GTCCCTGTCACTGCCCCAGATGCCCCCAACACCCTGTGATACCTGC<br>AGCCCCTGCCACATCTGTCCCTCACTCCAAACTCAGCTCAGGGGAT<br>GGTGGCAGGGAAGGAGGTGAGCTTGGACCCAGGCAGCCCTGGGG<br>TCACCACCCTCCAGCTGGGGTTCCTCCTCTGTAAAGTGGAGGTATA<br>ACGGTACCCACCTCCTGGGGTGGCTGTGAGGATTCAGAGCTGATAA<br>GGTGAACGCCTAGGGCGGGCCCTGGTGCAGAGAGAGCGCTCAGCT<br>CCTAGGGCTGGATTAACTGTCCCTGGGGCACAGATCTCGGTCTGGG<br>GCCTGTGGAAACCTCAGAGCCACCCCTGAACCCCCACCGAGCCAC<br>CCTTTGCCTCGCAGTGCCCATGGCCTTGTCTCCGAGGTTACAGGAA<br>AAGGCAGAGGAGATGCCCTTCTCAGGGTGGCCCTCTGGGAGAGGA<br>CACTCTCCCTTGACCTCAAAGCCACGCTTGGCTGCAAACTGGCCA<br>GGCAGCCACAAGGCTGGGCAAGCAAAACTATCCCTAATCCCCACC<br>CAAAGAGCCACACCGACCCTCCCAGCCGCTGTGACAGCTCCTGCA<br>GAGACAAACACACGGCCTACTCTTGTCACCCGGGCCGGCCAATAA

	GCACGGAGAGGCAAGGCCTCAGACCCTGGACAGACATCCTCCCTC CAGAGGCACCCAGGGCCTCAGCCTTCTCCTCCCTCCCTGGGCCTCA ATTTCTCCACCTGTGACCCAGGGCAGGTGGATCCAGGGAGAAGAA CCTTCTGGCTCCATCTCACCGTGGGTCCTGCCAGCACACACAAAGA TTTGGCCTCTCAAAGCCTAGCTCTGCCAGCGTCCTTCTGCTCAAGA ACTCTCCATGACTCCCAGTGGCCCTAAGGACAAAGTCCTGGCATTT GAGGCCCTCCCAATGCAGGGCCAGACTCTGCCTCTCCAGCTTCCTG TCCCCACCACACCCCTGCTGGTCTCACGGTGGTCCGACTGTTTCCT GCTTCTGTGCCTTTGCTTAGTCTGGCACCCCTGCCTGGCATGCTTTC CTCACCCCTTCTTCTCCCCAATCCCAACTCACCCAGTCTTTCAAAG GGCAGGCCTAAATACCAGGCCCTCCAGGTGGCCCAGGATTCCTTCT CTGAGCTTTCATGGGCCTGGCCCTGGGTGCTACCTGTGAGTAGTCC CACGGTGGGTACATAGTAGGTGCGCTTACTGTTTGCAGAATGAACA TGGGACAGTTTGGGGACTGTCACCCAGCTCAGGGAGCACTGATGG GGAAGCATCTCCTGTATGTCCCAGGGCTCAGTGCTGTAGTGTCCTG ACCCTCAGAAATCTCATAATGGCTTGGTCAGGAAGGCATCGTGCCC CACTTTGCAAACAGGGGGTGCTGAGAATTGAGGGGCCTTGTCCAA GGTCTCATGGCTAGGAGCAAGCAGAATCGGATTTGAACCCAGGGC CACGTGACTTCAGAAGTGCCATTAAAGTCCCCATAATTTGGAGCTG TCTTCTTTTTTTTTTTCTTTTCTTTTTTTTGAGACCGAGCCTCACTCT GTCACCTAGGCCAGGAGTGCAGTGGTCTGATCTCAGCTCACTGCA ACCTCCGCCTCCTAGGTTCAAGTGATTCTCTAGCCTCAGCCTCCCA AGTAGCTGGGACTACAGGCGCACGTCATCATGCCCAGCTAACTTTT GTATTTTTAGTAGAGATGGGTTTTCACCATGTTGGTCAGGCTGGTCT CGAACTCCTGACCTCAAGTGATCCGTCTGCCTCGGCCTCTCAAAGT GCTGGGATTATAGGCTTGAGCCACTACACTCGGCCTGGAGCTGTGT TTTGTCGGTGAAGGATTTTCCACCCATGAAGGGGTCAGACGTGAA GTGTGTGGCCCTGGGCAGCTCCTCTGAGCCCAGAGACGCCAGCCC TAGCCGCCTTGCTGTGCCACTTTGGGACTTCCCTCCCTAGCCTGAG CTTCAGTTTTCCTGCCTGTTAGGCAGCCCCATGTCAACTGCACTTAG TAGGCCGGGTTTGATGCCCGACAAGACGTGAAGTGGTGGAGGTGG GCAGGATCCCAGCGCTACCATCTTCTTGAACCAGTGATCTCAACAC ATCGGATTTCTGTTTCCTCATCTGCAAAATGGGATCAGTGAGCTCAG GTGGGTCACAAATTCTACAGGAACTACTTTAGCCAAGACCGGCCCC CTGAAAGTTCCCCTCGGTGGGCTGTTAGGGTGATTGTTTTCATCTGT GGGGCTCCCTGATGCGTCCCACCCACCAGCCTTGGAGAGGGTGGG ATGGGAGGGTGGGGTGCTTGGGGAGACAAGCCTAGAGCCTGGGCC CTCCCACCCCACTGCCTCCCCCCATCCCAGGGCCCCCCACCCAGTG ACAAAGCCCGTGGCACTTCCTCTACCCGGTTGGCAGGCGGCCTGG CCCAGCCCCTTCTCTAAGGAAGCGCATTTCCTGCCTCCCTGGGCCG GCCGGGCTGGATGAGC
ENO3	CCCCCCCACCACACACAGGCAGGCTGTCAGTCACCCTGAAACAAT AAGGCTTTTCAAAAGAGGAAAATCAAGCTTAAACGCTGGAGAGGA ACAGACTAAAAACTTAGGGGTCAAAGGCTCATAGACTGTTGATTCA TGTGTTCAGGCTAGAAAGGGCTGTGGATGTGCATGCCACCTCCAGG TTCTAACAAAAGAACTCAAACGATGAACTCGATGATTCAAGACCCC

	AACAATCTACCTATACTCCTAGAAAACAGTTTCTAATCCGAGCTCAA<br>GGCAGTAAGTAATTTAAGAGGTAATGCAGTTCAGCAACTTCGAATT<br>ACAAGATGAGGAACTGAGACAGAATGGAGGGACTATCCCGTGTTC<br>CAGCATCCAGCAGACAAGGAACACAATACTGGTCTGACTCCCTTCA<br>TGTTGGGGAATCACACAAAAAAGCACCCTCAAGATTACCAGAAGG<br>CGGTACATTAGAGATCTTGGAGCTAAAGGAAGGGTAAGACTCAGG<br>AACTCACTCTTGTCAGTCTTGGTGACAGTGACATTGAAGGTGGGG<br>GCCCCACCGGTGCTCTTGGTACGAAGATCCATGCTAAATTCCCCATC<br>CTGCAGCAGTGAGTCCCGGATCACCGAACATTTCTGGCCCCCAAGT<br>GTCAGCCCATTCACGTAAAAACTTGACCGGTCTTTGCCAACCAGGA<br>CACCCACCTCAGCTGGCTGGAAGAGGAACCAAGCGCCCATCAAGT<br>TAGTCAGTGCACCAAGATTCCCACCGTGTTCTCCAAAGACCCACCT<br>GTCTTCCACTTCTGAGAACCACCCCGCCGTGGGGGCTAAGTATAAA<br>TTATATACTCAGTACACATCAATGAACTGTGAGAAACTCTGAGGAC<br>TGTGGGACGTTAGTGCAGAAAAAGATCATGATTCTGAAACTTAGAA<br>TATGGAACCAGGGAGGAAGGGATGACATACTCCTCTAGAGATTTAG<br>ATGTCAGTGTTAATGGGGAAAGTAAACCCAGGAAATAAAGGGAGT<br>CAGTGAGGTAAGTGAGGAGCCTTTGGATGCTCAGAACTCTCCTTCA<br>GGAAAAGCAGAAGCGGAGAGGGGAAGGGAGGGGGGCGGGCGAG<br>GCAAGAGAAGCAACTTTGCCCTTATTTGGTCAAAGGTTCTGCAGG<br>AGCTGTTAGGGCCCGGACGCCTGGGTCTCCAAGTAACCTAGAGTTT<br>AGCTCCAGGTATCTCTGCTCTGAGATGAGGAAGCAGACCCCTGGG<br>GGCTTTCCGGGAAAGTTGGAAAACTTTTGAAGGTGGACAGGGGCC<br>AGACCCGGCAGGCCAGCTCTCGGGTCTAGGTACCCCGGGAACCTT<br>TCCCCCCTCCCCCTTTCACCCCAAGCCCCCACATCCGGTCCCCTCC<br>CGCCCGGAAACCCCCTTCCCCCCCCTTTTGAACTTCCGCTCCCCCT<br>CCCCACTTCCGGGCAGTGTGGACAGGGAGATGGTGGTGGGAGCAG<br>CGGTAGTAAACCAATATACTTTCGCTTATGCTGTCTCAGAACTTCTC<br>ACAAAGTTCCCCTGCTCCAGGCCCCGCCGGATGGCGGGAAGGGAG<br>GGCGAGGGGACTTCCGGGATTGGCCTCGCAGGAATGTTGAATCCA<br>ACGTGCTGAGCTGGGGGGGCGTGTGGTGGCCTCGCCCTCTCTAAC<br>GGAGTTAGGAGGGGCCAGAAGCCCACAGAGGGTGGGCAAGGGAC<br>CAAGACCACGCGCCTGGGGCTCCCTTCCGCACCAGGAGAAACAAT<br>GGTAGAGGGACGCGGGCTGGCAGCCGGACGGGGAGCTGGGGGTC<br>CAAGGATCCCCGGGTCCCCTCTCAATCCCAATCCCCAGTAAAAACT<br>GAGGCGCGTCCCCGCCCCGCCCTGGGAGAGGCGGAAGTGGGCCG<br>CCGCACCGGGCGCCGCGCCCCTCCCCGCCCTGTGCCCCGGATGTAA<br>CGCCCCGTCGCGGAAAGCGGGGTAGCGGGCGGGATGGGCGCCGCC<br>GCCTGGGGCATAGGACCTACGGGCAACTGAGGGACCCACTCACGT<br>GCAGCCGCCATTCGCGCCGCTTCCAGGGCAAGCACCCAGTCAGGG<br>CCTCTCGCTGCGCGCCCTAGACCGCGCCCGCCCCCACCCAAGTCCC<br>TCCCTCAGGGTCCAAACCGGAGCAGTGCCCCGGACCGCGCAGGCC<br>TCGCAGTACCGTGATGTTGACGAACGTTTTCCCGGGGACGGCGGC<br>CCAGACGGAGGGCGAGTCCTTGTAGCCCACGATGGCCGCGTCCTG<br>ACAGGTCCCGTCCGCCATGAGGTTGTCGATGTAGGCGTTCCACCCG<br>GCCATGGCGCTGCTACTGGGGCTGCTCTCGGCGCTGCTGCTGGGGC

	CGCGGACTGGGCTCGAGCTGCCTCGGCTGGCGGGCGGGGGGAGG<br>CGGAGAGCTCGGGGCACGCGCTGCCGTCCGGACCGCGGCTCCGCT<br>CGCTGTGCAGCAGCCCTCGCACCGCCACTTCCTGCTCCTCCCCCCC<br>CCCGCCCCGCTAGATTTTTATTTGCAAAGGACGGTAGGGGCGGGGC<br>CTACTCCCCATTCCCTCCCTCCCCCACTCGCCAGCCCAGACACCGA<br>ACTTTGCAAATGCAATTTTAAAAATCCCTCCCCCTATTGCAAAATTT<br>GCAGAGTTCGATGGAAAGCAATGCAGAAGGATGGGGGTGGCGG<br>GAGAGAGGTGAAGGGTAGGGTTTGCTCCAATTTGCACTGAATATAA<br>ACCAGACTTTGGTATCGGGGCAGGGAGTGAGCCAGAAGAAGGAA<br>GGAAGGAGAAAGCAGGAGGAATTAGCAAGCTGCAAATTTAGCAAA<br>TTAAGGAGAGTTCGGGGAGCCCAGGGTTAAACGGGATGACTCCTC<br>CTTCCCCCACCTCCGCCGAAGGGAAGGACCGGCGGGCGGCGGCGC<br>GTGTGCCCGCGCCTGCGCCTGCGCCGGGGCCGGGAGTGCATGGGG<br>CGGTCGGGGCCGGAGCCGGCTGCACGGGAGCGCGCCCGGCCCCGA<br>CCCGTGTGGACCCTGCGGGAAAGGAAGGCGGGCTGGGGACGTGT<br>CTCCCGCCTGGCGGAAGGGGCGGGGGCGAAGCCGTGCGCGCTGCC<br>GCCTCACCACCCTTGATTTTACTTATTCAAGTAATAAGAAAGCAAA<br>GAAGAGTTTGGCCAAATCCTCTAGTCGCTCTCATAGCTGAAGGCAC<br>CCACCGGGGCCACTTCTGTCCCCAGCGTCTTCTCGGGTCGTGCAGG<br>TTGGGTTCACCAGGGCCCATTCTCAGTCTAGAGGATTGTCCCTTCT<br>CTGGATCGGCTCTAGCCCAGGGCCTCACCCCACATCCCAGCCCCGC<br>CCGCCCCAGGCAGGCAATGTCTGGATCACCGGCCGTGCCCCTTGGC<br>CTGTGTCTGCAGAGGTCACAGAGCGCGGACACCCGCGGGGCTTAA<br>CAGGCGGGGCTATTTTTCGAGGCGGAAGAATACTCAAGTTGCAAG<br>AGTGGCAACTTTTTCAAGAAGGGGAAAGTCCCACTTCGGGCCCTT<br>TGGGTTTCTTCGCAGTCAGATGTGCGCTCACAGCCACCTGGGGAG<br>GGCGGGACGGGCGGGACGGCCGTGCGGCGGCCGCGGTGAAACCG<br>CCTCCTCCGACCCGCCGCCGAGGGCTGGGCGGCTTCCGGAGCCTG<br>GGCTAGGGGCAAGGGGTCACCGAGCGCGGCCCGGGCGGAAAGGG<br>GGTTGGTTTTCTCTCTCCTCCCGAGCGCGGCGCGCATCCCTGGCGT<br>CCACGCCGAATCCCACAGTCCCCGACGCCCCTCGAGTCCGTGTTCC<br>TCGACAGCCGCGCGGCTGAGTCACTGGCGGGCTCGGGCGGGGCCG<br>CACCCGGGCACGTGGCGGCGCTCCCCGCCCGCCATCTCCTGACCCC<br>TGGCCCACCGCACCCCTACCCCAGGGTGGAAAAATCCCGGGAGGA<br>GCGGCCTGAGATGAGGGGGCGGGGCGAGAGGGGAGACTGGACGG<br>GTGGCGGGGCAGGTGGCCTGGGGTGGGGGCTGGGAGGCCGCGCG<br>GGCCGGCGGGGCGGGGAGCAGGGGTGGGGAGAGGGCGGCGGGG<br>GTGAGTCACCGGGCGCGCGCTGCCCCGGCGCCGACGGGAAGGGC<br>CTCCGAGTCGGGCCGAGGCGCCGTGTGTCCCCCGCCGGTCGGCTG<br>GTATGGCTGCAGAGGAGCTCGGGGCAGACCCCGCACTGGCCCCAG<br>AGCCGGCAAAGTGGAGATCTCTACCGAGGGCAGAGACCTACCTCC<br>CCGCAGTGCTACAAGTGGGGCGCCGGAAGAGCCCCAGGCGTGCA<br>GAAGCTCACAAAAGGCCACCCGTCCTCGGTCCATTCATTTTTGTTC<br>ACTGTTGATTCAGCCCCATTCATTGATGGGCTGGGGCCGTGCGCTG<br>AGCGCCCACAGTCGATGGGGAAAGGGGCTCTGACCGACAGTCCCC<br>ACGCCGGGCGACAAGTGCTGTCCCAGCGTTATCAGTCGGGCGCCT

	TGCCAGCCGAAAGGGCCTGTCTAAATTCGTTTCCTGTCCCCTAACTCATCCCGGCGCTGGCTGGCCTGGAGAGGGTAGGATGGGGCGGCGCCGAGAATGGCCGTTATGAGGACCCTAAGAGGTGAGACCCTCTCGCCTTCTGGGGTGGGGGGTCCCGTCCTTTCCCCCACTGAGGACAGAGGCCCGCCCAGCGATCTGAGCATGTGTGGACGTCAATCTTGCAGCCCCTCTTCCAGGCCCCCTCCCCAGCCTTGCAGGGCTCAGGTTACCCCTGGCCTTTCCTAAAGGTCACTCATTCCTCTTGACGTTTGCAAAAGGGGAATGTAATCCTGGGGTGGGGGGAGACCCCTCATCTGTAGCCCCTCCCTTGCTCCTCCCAAAGGGTGGAATTAGAACAGGGACTGTTATTGGGAGACAGAAAGTGGGGGATAGTAGTTGACCTTTGGTAAGGGGGCAGGTGCCCAGGGCCAGAGGCTTCTGCTTCAGGCTGTAGTGGGCACTTGGCTGCCAGCCCAGTGTGAAGGGGGGAGGATGGAGAGAAAGAGAGGCGGGGCTGGCTGGGGACCGAGTGGCTCAGGGATAAATGCGCAGCCTGAGAGGGGGTGAGCTGACACTGTCCCAGCTGCCACCTAGA
EPHX1	GACGGAGTTTCACCATGTTGGCCAGGCTGGTCTTGAACTCCTGACCTCAGGTGATCTGCCCACCTTGGCCTCCAAAGTGCTGGGATTACAAGCATGAGCCACCACGCCTGTCCCCAAGTATATTTCTTAGCTCACTCTTTTCAGCATGTAATTTACAGAAGGGTAATCTGAATTGTAAACACGATTTCTAAGTTAAAACTCCTGGGTCAGCTCGTGATGAAGTAGCAATTTATAAAAGAGATAGTATAGTAATAAATGAGACAACTACACTCGGAAATGTGGAATCATCAACTCTTATGCGAAAATAGCCTATGAGACTCTTGTGTTTTTTCATTCATGCCATGCGCTTCCTCACTTCCATCCCATTCCATTCCACATGCTCTTCTTTCTGCCTGGATTATTCCCATTCCACCATTATTTTTCTCCTGGTAAACTCCTATTCATACTTAAAAACCCTGCTCAATGACCCCTTCTCTGTGAAAACTTTGTGGCAAAACCTAACCACTTCTGCTTGTGCACACTTCTCTTCCATCTGTGTGCATATAGTATGTGTCTATAACTGGAAGGGTGTTTATCTGTTATCCGTTTGTCTGCCCTGCTAAAGTGTGAGCTCCTACAGTTACAGGGTTGTGTGTTAGTCCTGGTGCCTGCACAGTACCCAGCCAGTAGTTACACTCAGTGTTGCTGAACCAACCTCTGGTCCAAAAACATGTTTATACTTGCTTTTCCAAGGCGCCAATCCAAATCTCTTATTTACTTTAAAAAACAAATAACTTCTTGGCACTTTAAAAACCACATTCAAATAACAGAAAAAGAGTTCATTTGAACTTTAAGTATCAATCTTAAAATGAATTGTATGTTTTAAAACTTGACTTAGTGACATCACTTAAGAGAATTTTTAAAAAGCAAACTTTTATTTTTACCCCAGTGTAACAAATATTAAGTTCCATTAAATTATTAAACTGATCTACCTTTTCTTTTTTTTTTGAGATGGAGTCTTGCTCTGTTGCCCAGGCTGGAGTCTAGTGGGGCAATCTCGGCTCACTGCAACCTCCGCCTACCCAGGTTCAAGCGATTCTTCTGCCTCAGCCTCCCGAGTAGCTGGGACTACAAGTGCACACTACCACACCCAGCTAATTTTTGTGTTTTTAGTAGAGACGGGGTTTCACCATGTTGGCCAGGCTGGTCACGAACTCCTGACCTAAAGTGATCTGCTCGCCTTGGCCTCCCAAAGTGCTGGGATTACAGGTGTGAGCCACCATACCCAGCCTGTTTTTCATTTTTTGGTTCTTTTTTTTTTTTTTTTTTTTTTTGAGACAGGTTCTTCCTCTCAGGCTGGAGTGCAGTGATCATAGCTCACTGCAGCCTCAACCACCTGGGCTCAAGCAATCTTCCCACTTCAGCCTCCCGAGTAGCTGGGACCTCAGGCATGCACCACCATGCCTGGCTAATTTTTTTTTATTATTTGTGGAGATGGGGTTTTGCCATGTTGCC

	CAGGCTGGTCTCAAACTCCTGGGCTCAAGTAATCTACCTGCGTTGG<br>CCTCCCAAAGTGCTGGGATTACAGGTGATTTACCTTTTAACTGTGCC<br>AAAGAACAAAGCACCTACTTTATTTGCTCTCCTGTATTTAAAATGGT<br>CCTTTATACCTCAGATCCAGAAAGATAAACACAAGTAAACTAACAA<br>GTACTCTACTTGTATCATTTGTTGTTTTCTAACAGTTTACATTTAGTC<br>CATCTTTGAAGGGTAAAGACCATTGATTGCTCATCTAACTTCTTCAC<br>CTTGAACTTAGTGAGATTTTCTTCCACCTTTCACAAATGCATGCCCA<br>AAATTTGACTTCAGTGTAGAATTGGAATTAAAATGTATGCAATCCCA<br>TTTCTTACAGTAGGAGCCAAATGTTCATCAAGTTCCCCTCTGAGCTC<br>AGTATCTTGTAGCTAAATGACCACTATCTTCCAGTCACGTTTTTAAA<br>AAAAATCATGCTGGTTTGCAACAAAAATTGTATTTTCTTTACTAGGC<br>AAGTGAACCATTTTGTTGGGAAGAGTACCTTGGCTTCGAGAAAAA<br>AATGATTTCATTCTGAAAGGCTTCCCCTCCAACCCCCTCCTGCACA<br>AACTCTCAGCCCAGTAACTTGTCAGTATTCTGGAAAAGTGCATTAC<br>AAAACAAAAACTCGAATCAGATAAAATATATCTCTGGAACTTTTGA<br>GGCAGATCAATGAAGCTCTGATTTCAGGTGCTAGCAAAATAAAGCA<br>GGCACTCTCATTTTGTATTGTTTTACTTCTCAGTTTTTCATGGTGTCT<br>CCTAAACTTTGATGTTTTGAAGAAGTTCTAATGTTTGCTACATCAGC<br>AGGAGCTGACCACAGGGAAGAGATAAATATAGCTCTCCTTGGAAA<br>GGTGAAGGCTTAGCCACCAGAATTCTGCCCCTTGATGCTGAGAATG<br>CACCTTCTCCGAACAGATGGAAAAGAGGGGTGGAGATTACCCCAG<br>GGACATATCTTGCAAAATGCTGATACCCGAAGATGTACATAGCCTTG<br>TGCTTTGGTCATCCTCCCAAATCATGTCCTTGGTGAAGGCACATGG<br>AGAGGAGAGCAGATGCTGCCAGACCACGGTGCCTGACGCCATGCA<br>CTCCCTCACGTCCAGCCTGTTTATGTGCACACGGCTGCAGCGGGAC<br>CTTGGGGTGTGGGCTAAATAACAAGAACAGGGGCTCCTCTGGGGT<br>CTGTTACAAGGCAGAGATGCTCCACGGTAATTAGGGATTTAGGGGA<br>AGAGGAAGGAACACACATGTTGATAATAAGTAGTTCAACATTATTAT<br>CCCCATTTTACAGATGCAGCTACTGAGCTTAGAGAGGTTTCATAACT<br>TGCCCAGAGCCCCTCAACTAGTAAACAGTGGAGCCGAGATTTGAA<br>CGACCCAAAGAGGAAAGCAAACAAGCAGCCCCTTTATTACTTTTTT<br>TTTTTTTTAAGATGAGGGTCTTGCTATGTTGCCCAGGCTGATATTGA<br>ATTCCTGGCCACAAGTGATTCACCACCTTGGCCCCACGAAGTACTG<br>GGATTACAGTCATGAGCTACCACACTGGGCCAAAGCCCTCGTATTG<br>TAACCTCCAAGATGTTTTCATTTCTAAAGTCAAAGGTTGTCAGAGT<br>TGCTGTTTTGCAGGGAGCCATGGGATGGGGAGTCTCCGGGAAACC<br>CAGGCTGGCTCCTTAAATGGCATCACCCAAGTTGGCCATCAAGCTT<br>CCCCAGACTCAGTCACTCGGCCCCCTCTTTCCCTGGCTGTAGAGCA<br>GGGGTTTGGACTCGAGGCTGCTGAGTTCCTCTCCAGCTTCCATGCC<br>CAGTGGCAGTGACTTATCAACTGGCCCACCCGGTGGATGTAGGGGT<br>GATGCAGCCCTGCTCTGTCCTCTTGAGTGCTGGGTGGCCATGAGAG<br>AGGGAGGCCATGAGAGGGAGGGGAGGGTTCTCATCTGCTTAGTGC<br>CACCCCCTCCCCAGCCCACCCCAGGTCCTCTGCCGCCTTGGGTTCC<br>TGCGTGAGCAGTCCTTCTCACTCTGGGCGATGTTCGCTTTCACCAG<br>CCCAACCCCAAGAAGTCCACCCTGGGGAGGACAGAGGGAGTCTCC<br>ACGGCTCCACACCCCCATGCTTTGTCGGAGATCTGGCCAGGCAGA

	GTGGTGCCCTCTTGACTGAGCCTCGAGCCTTTGCTTTCCCCTTACTC CACATAAAGCCGCAGCCCTGGCGAGGGAGCCCAGAGGCCTGAGG ACAGCCTGCTGGGTGCCTGGCACTCTCTAGATCCTCCCTGCCTGGG CGGTGGGCATCTGCAATTCTCCCTGTCTCGAACTGCAGCAGAATGT GTGGGCAGGGTTAGGCAGATAGAGAGTGGGTGCCTGGACTCATTG GTCAGACTCTAAGACCGCCCACCCCCGCCGCCCCCGCAGTCCTTCC CAGGACTGGCTGACCCACCAGATAGGGGAGGGTGTTGTTTCTTATT ATTACATTTTTTGAGAAAAGCTATGCAGGCCTGGGAGACGTCTGTG CCCTAGGAGTCCCTTCTGGCCTGAAGACTCAAGGCTGCAAACAGG TGCTGAGAGGGAACAAGAAGGGGAGTTGGGGTGCAACACACCCA GTGACAGACCAGGAGTGAGTGCTGTGGCCACAGATTCTAATCCTC ACAACTGCAGTGTGATTTAGGCTAAGCCCCTTGCCTACTCCGATTCT TGCCTTAAATAAGTGAAAAGGAAAGTCAAGTTTGATGAGTTGTGGC TCTTTAGTGGGTGGATTTGCATGTACCATGGATTACATTTACCAGGG TCTAGGAATTGTCAAGCGCCCAGCAGGCTGCCATCAGGCCTCCGA GCTCCTTCGCCAGGCGGCTTTATTTGGAGTAAGAGGAAAGCAAAG GTTTGAGGCTCAGTCAAGAGGGCGCCACTCTGCCTGGCCAGCTCT CCGACAAAGCAGACGGGGGGGTGTGGAGCCGTGGTGACCCCCTCC GTCCTCCCCAGGGTGGGCTCCTTGGGCTTGGGCTGGTGGAAGTGA ATATTGCCTGCAGCAAGAAGAAGGACTGTTCATGTGGGAGCGCAA GGCGGCAGCGGGGTTGGAAACCCACCCCCACACAGTCAACACCTG CTGTCACACCAGGCTAATCAAGAACACTTTTCTTTGGTGCATTAAC CACCTGCTATTAAATAAAGGGCTGTTACTAGGTGGCAAAGTAACAC ACTGCAGTAGAGCCTATTTGTAATCTGAGATCAGTGGCTGTAAGGC ACCATCCTTGAGCCAAGCAAGTACTAATATTCTTGTTATCTTAATTAG AATGCAAGAGATCGTGTTTTTAAAATTCTCTTTGAATCTGTCACTTT GCCCTCCTCTTCCTGGGTGATACACCTGGTAGTGCTGGTGGGGGCC ATCATAATGCCCCTTGTCCCAGATTCCCTTCTTTTAGATGGGACTCG AGCACTGATCATTTCAGCCCTGTATCTCTCAGGTCAGCGTGGTTCA GTTTGCTGTGCAGAGTCCAGGGGAGATAACCACGCTGTGCACACA TGAGATTGGCTGACTTGGCAGGACTGTGCAATTG
EPO	GGTTCCTGCTCAGGGAATTAGCACAAATGAGGCTGTTCAGTGGGA AAGTTAATTGAGCAGCAGGGTTAGGGATTAACTGGAGGGGTCAAT GAGGAGATGCTCTGCAGTGGTCCTGGGCCTGTGGGTCAGCATCTGC TAGAAGATGAAGAGCAGGCAGATGACGGGGGTGGGGGGCACTGG GCAGGGGGATGGTGGGCATTGGTCGGGGAGCCCCATGGAGCTTCT TGTTTTCTTCCTGTTCCTCAAGGACAGATGAATGGACCCTGCATTGC TCAGTATCCAAGAGGCTCTGGGCTCCAAACCAGCCTCTCCCCTCTT AGCAGAATTACTATGGCTTCAAGCCGGGTGTGGTGGCTCACGCCTG TAATCCCAGCACTTTGGGAGGCCGAGGCGGGCGGATCACTTGAGG TTGGGAGTTCGAGACCAGCCTGGCCAACATGGCGAAACCCCATCT CTACTGAAAATACAAAAATTACCGGGCGTGGTGGCACATGCCTGTA ATTCCAGCTACTCGGGAGGCTGAGGCAGGAGAATTGCTTGAACCC GGGAGGTGGAGGTTGCAGTGAGCTGAGATAATGGCACCACTGCAC TCCAGCCTGGGCAATAGAGTGAGACTCCATCTCAAAAAGAGAATTA CTATGGCTTCGGATTGGGTATGGGGTGGACCAAGATCCTCTCCTCCC

	TCCCAAGCTGCAATCACAGCTCTCTCTCCAGAAGCCCCAGGGGAC<br>AAGGCAGTCACTACTCCTATCCAGCAGGCTACAGCATTTAGAGGCT<br>GGTGCCCCTGGCCCATCTCTCACTCCTCTTCAACTGCAGACAAGGA<br>TGCAGGGACCCCCGAGAAGCTGTGTGAGGGGAGGGGCAGGTCCC<br>ACAGCCCAAAAGGCTCCTCCTCCTCCAGCTGTGTCCACCCCACCCA<br>TCTGTCTCCGGCCCCCTCTCCATGGTTAGGTCAGGGCATTGACGCA<br>GGAGTCAGGGTCCTGGGAGAGCAGAGGCTGGGGAAGGGGAGCAG<br>CCAAGACAGAACGCACGTGCGGCCATGTGTGGATGTGAGTGTGAT<br>GGTGCACGTCCCAGGACTCTGGAAGGGATCAGAACCAGGAGCCGA<br>GTGGGGCTGGGGGAGCCTGGACAGCAGTTTTGGGAAGGCTCAAG<br>GTGAGCAGAGGATGCAGGGGAGCAGGGTCTGAGGGAATTAGGCC<br>AGCCAGAAGCATACCAGCCTCTGTCTCCTGACCCCCACCCAACCAT<br>CTTGCCAAACCACTCATTTTTTCTTTTTTTTTTTTGAGACAGGGTCT<br>CCCTTTGTTGCCCAGTCTAGAGTGCAGTGGCGTGATCATAGCTCAC<br>TGCAGCCTCAACCTCCCGGGCTCAAGAGATCCTCTGACCTCAGCCT<br>CCTGAGTAGCTGGGACTACAGATGTGCACCACCATGCCCAGCTAGC<br>TAAACAAAAATAAAAATTATAGACATGGGATCTCACTATGTTGCCCA<br>GGCTGGTCTCAAACTCCTGGCCTCAAGCAATCCTCCTGCCTCAGCC<br>TCCCAAAATGTTGGGATTACAGGTGTGAGCCACTACACTTGGCCCA<br>AATCCCCCTCCCATCAAGCTCTTCACCTCACCACACCCTCATGGTCC<br>CTCACCCCCAACAGGTCTTTATTCAAGTCAGTTTCTCAGCCTAGTGT<br>CTCTGACTACCTTATTTAGATAACCCTGCCTGTGCCTTTTCTCCTTA<br>GTTCTGGCTATTTGAAATCCTCTATCTTTTCTTTTCTTTTCTTTCTTTT<br>TTTTTTTTTTTTTGAGACAGGGTCTCATTCTGTTGCCCAGGGTGGA<br>GTGCAGTAGCAAAACCACAGCTCACTGCAGCCTCAACTTCCTGGG<br>CTGAAGTGATCCTCCCACCTCAGCCTCCCAAGTAGCTGGGACTATA<br>GGCACATGCCATCATGCCCAGCTAATTTTTGTATTTTTTTGTAAAGAT<br>GGGGTTTCACCATGTTGCCCAGGCTGGTCAAGCCAACTCCTGGGCT<br>CAAGTGATCCTCTTGCCTTGGCCTCCCGAAGTGCTGGGATTACAGG<br>CATGAGCCACCTCACCCAGCCAAAATCCTCTGTCTTGTACCCATTTT<br>TGTGTTCATCATTGGCAACCCCACTAGAACATCAGCTCCACCAGGG<br>CAGGCATTCCTGGCTGTATCATTCACTGCTCTATCCCCAGGTCCTTG<br>GCCAGGGGTAGGTAGGAAATCCATAAATATCTTTTGAATGAATGAG<br>TGAATGTTTCCCACTCTTTCCAAACAAAATCCAAATTCTTCAGAGA<br>GGCATTCAAGGCCATAGACAGCCTGACTCCTCCCTAAGCAGCACCC<br>TGGCCTCATCTTCCACCTCTCTCTTCTCCATCTTCCCTTCTGCTCACC<br>TGTAGGGAGACCCCCTGAAACTACTGCTACGGAATAAAAGATGAAA<br>TGCTCCTGATTATTGTAAATACAAAATTGCATGCAGGATTGTGTACA<br>AACAATGCCAGGTTGGACTGCCAGAATGAGCCAACAGCCTGTGAT<br>GTGCTTCCGCCTGCGGAGAGCCTATGAACGGACCTGCAGTCAGGG<br>AGGTTTCACATCACCAAGATTTCTATCCCAGAAAAGCAGATGTTCAT<br>AGCTCTGGGAATGGAATGCGACCCTTGTGGAGAGCCTATAAACGG<br>ACGCATGGGGGCGGGGGGCACCTGTCCATATGGATAAGATAGGGCT<br>ATAAACGCCCTCATCTTGCCATGGCTCTTCTAGGCCTCTTTAGGGTT<br>AAGGCATACTCCCTTCTGAGAATTTCTGGTCTAACTGGTTGTCTAGC<br>TTCATGTCCTGTTTCCATGGATTGTCTGTAACCAGCTTTTGTTGCAA

	TTGTTACTGCTGATTAGTATCTTGCTAATCATAGGTTATGGAAAGATT<br>GTGTTTCTGTTTTAAGGCTCTGTTAGAAATTACTGATGCACACACTA<br>TATTGTAAATTCTTATCTCTGTATACTGTACTTCTGCATACAAATGTA<br>CTGTACTTCTACATATAAATGTTATGTTAAAGAATTAATTCATCCCCA<br>TGTGACCATCTCACCTCATAATCAAATGACCCTAAATCCCTCACTAA<br>CCTACCCCCGCCCTCACTAAACTTAATAATAAATGATGGTACATCTG<br>GTGCATTGTTGACACCACGGGACCAGAAGGCGGTGACCCCCCGGA<br>CCAGCTTTCACTATCTTGTGTGTGTCTATTATTTCTCAACCTGCTGAT<br>CCGCCTGGGAACAAAGAGAGAGCCCTGTTGCATTGCGGGCTGCTG<br>GCCAGATCCCGCAATACTCACCTGACTCTCCATTCACTTTGCTGAAT<br>CGACAATCCCCCACCCACCTCATACCTCTTCCCATTGATGTCCTACC<br>CCCCTTCAAGGCTGAGGTCATTGTCACCTCCTCCCTCAAATCTTTCT<br>TGATTTCTCCCGGCTAACTCTTACAGCTCAGATCTACACATTCCAGT<br>CTTTTTTTGTTTTTGAGACAGGCTCTCACTCTGTCGCCCAGGCTGC<br>AGTGCAGTGGCACGATCATGGCTCACTACGGCCTCGAACTCCTGGG<br>CTCAGGTGATCCTCCTGCCTTAGCCTCCTGCATATTTGGGACTACAG<br>GCGTGAGCCACCACACCTGACTAATTATTTTTTATTTATTTATTTATTT<br>TAGAGACAAGGTCTTGCCATGTTGTCCGGGCTGGTCTCGAACTCCT<br>GGGCTCAAAGGATCTTCCTGCCTTGGTCTCCCAAAGTGCTGGGATT<br>ATAGGTGTCAGCTGCGGCGCCTGGACCTTTCCTGTCTTTTATGAAA<br>CCTGAATGGGATAGGCTGGTAGTTTCACCACACCCATTTGACAGAT<br>GAGGACATTGAGGGCTCAAGGACGAGGCCACTTTCTAAGGTGTGA<br>GAGACCAGCTAGTCTTGGTCTCCTGCTCTGGGAATCTCACTCCTCT<br>GGCTCAGGGTTTCCAGAAGCCATAAAACCTTAGCTGTAAATCCCAG<br>CCCCCATCACTCTTGGTGTTAGCTGTATTTCAGTGTTCTTAAGAACT<br>CAGCAATGCAGCCTAGCTAACCTACACCACAGGTCAAATAAACAGA<br>TGTCAAGGTGCATGTGTGTCCTGCACAATGGACTGTGTGCTCTGTG<br>CACTAAAAGTTAAGTGTCTGGGGTGGGGGCTGGGTGCGGTGGCTC<br>ACGCCTGCAATCCCAGCACTTTGAGAGGCCGAGGAGGGTGGATCA<br>CCTGAGGTCAGGAGTTCAAGACCGGTATGGGCAATATGGCAACAC<br>CCCATCTCTACTAAAAATACAAAACATAGCCGGGTGTAAAGGTGTG<br>CCTGTAGTCCCAGCTGCTCCAGAGGCTGAAGCAGGAGAATTGCTT<br>GAACCCAGGAGGCAGAGGTTGTAGTGAACGGGAGATGGCACCACT<br>GCACTGCCTGGGCAACATAGGGAGGCTACATCTCCAGAAAAAAAA<br>AAAAAAGTTAAGTGTCTGGGTTTGCAGAGTACTAGACGGTGAGGA<br>GTGAGGTGGGGAGGAAGAACCCCAAATTTCTTGCCCTATTTGCCCC<br>CATCAAATTCCTCAACATGGTCAACATTGTTTCTAGAACATGTCCTG<br>GGATTGTGGGAAGGGAGACCACTCATTTGCCCCTCCCTAAAGCTTC<br>TGGGCTTCCAGACCCAGCTACTTTGCGGAACTCAGCAACCCAGGC<br>ATCTCTGAGTCTCCGCCCAAGACCGGGATGCCCCCCAGGGGAGGT<br>GTCCGGGAGCCCAGCCTTTCCCAGATAGCACGCTCCGCCAGTCCCA<br>AGGGTGCGCAACCGGCTGCACTCCCCTCCCGCGACCCAGGGCCCG<br>GGAGCAGCCCCCATGACCCACACGCACGTCTGCAGCAGCCCCGCT<br>CACGCCCCGGCGAGCCTCAACCCAGGCGTCCTGCCCCTGCTCTGA<br>CCCCGGGTGGCCCCTACCCCTGGCGACCCCTCACGCACACAGCCT<br>CTCCCCCACCCCCACCCGCGCACGCACACATGCAGATAACAGCCCC

	GACCCCCGGCCAGAGCCGCAGAGTCCCTGGGCCACCCCGGCCGCTCGCTGCGCTGCGCCGCACCGCGCTGTCCTCCCGGAGCCGGACCGGGGCCACCGC
EPOR	CTTGACACAGGGGCCAGGCGCGGTGGCTCACGCCTGTAATCCCAACACTTTGGGAGGCCGAGGCGGGCGGATCACCTGAGGTCAGGAGTTCGAGACCAGCCTCAACATGGAGAAACCCGTCTCTACTAAAAATACAAAATTAGCGGAGTGTGGTGGTACATGCCTGTAATCCCAGCTATTCGGGAGGCTGAGGCAGGAGAATCACTTGAACCTGGGAGGCGGAGGTTGCAGTGAGCCGAGATTGTGCCATTGCACTCCAGCCTGGGTGACAAGAGCGAAACTCCATCTCAAAAAAAAAGAAAAAGAAAAAGAACTTGACACAATCTAATTTAATCCTTACCATAGCTCTATGGAGTAGACATTATTTTCACTTTATACATTTTATTTATTTATTTACTTATTTATTTATTTTTAGAGACAGTGTCTTGCTCTGTAACCAGACTGGAGTGCAGTGGTGCGATCATAGCTCGTGGCAGCCTCGAACTCCTAGACTCAGGTGATCCTACTCAACCTCCTGAGTAGCTGGGACTACCGGCACATACCACCACACCAGGCTAATTTTTCTTCTTTTTTGTAGAGTCAGGGTCTTGTTATGTTGCCCAGGCTGGTCTCAGACTACTATCTTCAATTGATTCCCCTGCCTCGGCCTTCCAAAGTGCTGGAATTACAGGCAAGAGCCACCATGTCTGGGCCGGAAGAGACACTTATCGTCCACTATTTTTTTTTTTTTTGAGATGGGGTCTCACTCTGTCACCCAGGCTGGAGTGCCGTGGTGTGAACATGGTTCACTGTAGCCTCCACCTCCTGGGCTCAATTAATCGTCCCACTTCAGCCCCCTGAGTAGCTGAGACCACAGACGCATGCCACCTCGCCTGGCTAACTTAAAAACAAAGTTGGCCGGGTGCGATGGCTCACGCCTGTAATCCTGGCACTTTGGGAGGCTGAGGCGGGTGGATCACCTGAGGTCAGAAGTTCAAGACCAGCCTGGCCAACATGGTGAAACCCTATCTCTACTAAAATACAAAAATTAGCCATGGCGGGTGCCTATAATCCCAGCTACTCAGGATGCTGAGATGGGAGAATCACTTGAACCTGGGAGACGGTGGTTCCAGTGAGCTGAGGTCATACCACTGCACTCTAGCCTGGGCAGCTGAGCGAGACTCCGTCTCAAAAAAAAAAAAAAAAAAAAGTTGGCTGGGCGTGGTGGCTCACACCTGTAATCCCAACACTCCAACACTTTGGGAGGCCAAGGCAGGCGCATCACCTGAGGTCAGGAGTTCGAGACCAGCCTGGCCAACATATAGCGAAACCCCGTCTCTACTAAAAAAAATACAAAAATTAGCTGGGCATGGTGGCACACATCTGTAGTCCCAGCTACTCGGGAAGCTGAGGCAGAAGAATCGCTTGAACCCGGGAAACAGAGGTTGCAGTGAGCCGAGATGGCGCCATTGCACTCCAGCTTAGGCGACAGAAAAAGACTCCGTCTCTCAAACAAACAAACAATGTTTTGTAGAGACGGGATCTCACTCTGTCACCCAGGCTGGAATGCAGCGGCATGAACATGGTTCACTGCAGCCTCCACCTTCTGGGCTTAAGTGATCCTCCCACCTCAGCCTCCCGAGTAGCTAGGACCACAGACGGGTGCCACCTCATCTGGCTTACTTAAAAAAACAAAAAACAAAAAACAACAACAAAAAAAAAACGTGTTTTATAGAGACAAGGTCTCACCATGTCAGCCAGGCTTCAGGCAAGGGAGGGTTAAGTAACTTACTCAAGGTCCCATGGTAAGTGGGTGCTGTTCAAATATTGATCCAACTCCAGAAAACATGCATTTAACTCTGGGGCCCGTTTGCCATTCATAACAATTTATTGTGACCAAATTTTGAAACAGGTGTCCATCTTGTTTGTTCCACCTCCCTCTCACATACATGCACAGTCTAAATATCAATTTCCTGAGGAAGAGATTTG

	CGTTGACTTCCGTTTTTTAAAAGCGCAGGGTTACGCAGGCACAGTT<br>GTACCCAGTGGCTAACACGAGATCAGCTGTTTCTCTACCTCTCTCA<br>GCTTGATTTTCTCTACATCTCTAATCTTTTACTCCACAAAATATTTCT<br>TGAGCTTCTACTGTGCTCCAGGCCCTGTTCTATATGCTAGGGATACA<br>GCAAAGAACAAAAGGGTAAAAAAAAATCCCTGCCTGAGGCTGGG<br>CGCGGTGGCTCATGCCTGTAATCCCAGCACTTTGGGAGGCCAAGGC<br>GGGCGGGTCACAAAGTCAGGAGTTCGAGACCAGCCTGGCCAACAT<br>GGCGAAACCCCGTCTCTACTAAAAATACAAAAAAATAGCCGGGCAT<br>GGTGGCGGGTGCCTGTAATCCCAGCTACCCGGGAGGCAGAGGCAG<br>GAGAATCACTTGAACCCAGGAGGCGGAGGTTGCAGTAAACCAAGA<br>TCATGCCATTGTACTGCAGCCTGGGCAACAAGAGCAAGACTCCAC<br>AAATCAATCAATCAATCAATCAATCAATCCCTGGCCGGGCTCAGTG<br>ACTCATGCCAGTAATCCCAGCACTTTGGGAGGCTGAGGCGGGGGG<br>ATCACTTGAGGTCAGGAGCTCAAGACAAGCCTGGCCAACACAGCA<br>AAACCCCGTTTCAACTAAAACTACAAAAATTAGCTGGGTGTGGTGG<br>CACACACCTGTAGTCCCAACTACCCTGGAGTCTGAGGAAGGAGAA<br>TCGCTTGAATCCAGGAGGCGAAGGCTGCAGTGAGCCAAGATTGTG<br>CCATTGCACTCCAACATGGGCAACAGAGCAAGACACCATCTAAAA<br>AAAAAAAAAAAAACCTGCTTTCATGGAATAGTAAGGGCAGCGAAT<br>CAATAACAAAACATCAAATACATGTCAGGTGACAATAAGCACATTG<br>GCTACAAACAAAAACAAAAAACCCACCTGCTTAATAGAAACAGGG<br>TAGGAGTATTGCCATTTTATTTTATTTATATTTATTTATATTTATTTATTT<br>ATTTTATTTATTTATTTTTTTGAGACGGAGTCTCGCTCTGTTGCCCAG<br>GCTGGAGTGCAGTGGCACAATCTTGGCTCACTGCAAGCTCCACCC<br>ACTGGGTTCACACCATTCTCCTGCCTCAGCCTCCCGAGTAGCTGGG<br>ACTACAGGCACCCGCCACCACGCCCGGCTAATTTTTTCTATTTTTAG<br>TAAAGACAGGGTTTCACTGTGTTAGCCAGGATGGTCTCGATCTCCT<br>GACCTTGTGATCCACCCACCTCGGCCTCCCAAAGTGCTGGGATTAC<br>AGGCATGAGCCACTGTGCATGGACTATTTATTTATTTTTTTGAAACA<br>GAGTTTCAATCTTGTTGCACAGCCTGGAGTGCAATGGTGTGATCTC<br>AGCTCACTGCAACCTCTGCCTTCTGGTTTCAAGCAATTCTCCTGCC<br>TCAGCCTCCTGAGTAGCTGGGATTACAGGCACCCACCACCACGCTC<br>GAATATATATATATATTTTTTGAGACGGAGTCCCGCTCTGTCACCAGG<br>CTGGAGTGCAGTGGCACAATATCGGCTCACTGAAACCTCCGGCTCC<br>TGGGTTCAAGCGATTCTCCTGCAGCCTCCCAAGTAGCTGGGATTAC<br>AGGCATGCAGCACCACGCCCATCTAATTTTTGTATTTTTGGTAGAGA<br>TGGGGTTTTACCATGTTGGCCAGGATGGTCTTGATCTCTTGACCTCG<br>TGATCTGCCCACCTCGGCCTCCCAAAGTGCTGGGATTACAGGCGTG<br>AGCCACCGCGCCCGGCCTACGCCTGGCTAATTTTTGTATTTTTAGTA<br>GAGACGTGGTTTCGCCATGTTGCCCAGGCTGGTCTCGAACTCCTGA<br>CCTCATGATCCGCCTGTCTCGGCCTCCCAAAGTGTTGGGATTACAG<br>GTATGAGCCACCGCGCCCAGCCAATTATTATTATTTTTTGAGATGCA<br>GTCTCACTCTGTTGCCCAGGCTGGAGTTGCAGTGGCATGATCTTGG<br>CTCACTGCAATCTTCATCTCCCAGACTGAAGCAGTTCTCATGCCTC<br>AGCCTCCTGAGTAGCTGGGATTACAGGCACACGCCACCACACCTG<br>GCTAATTTTTGTATTTTTAGTAGAGATGGGATTTCACCATGTTGGCC

	AGGCTGGTCTCAAACTCCTGACCTCAAGTGATTTGCCCACGTCGGC<br>CTCCCAAAGTGCTGGGATTATAGGCGTGAGCCACCGGGCCCAGCCC<br>AAGAGAATAAAAATGTGGGTGGTAAAAATTTTTTTCCCAAAAATTC<br>GTAAATGAAAATCTCACATATTATGCATACTGCCCAGGAGCATGGCC<br>TAGCACTGTGCAAACACTCAACTGCTGGTCGTTGCAAGGATTATTA<br>TTGGCCGGCTTCAGTGGCTCGTGCTGGTATTCCCAGCACATTGGGA<br>GATGGAGGCTGGAGGATTGCTTAAGTCCGGGATTTCAAGACCAGC<br>CTGGACAACATAGTGGGATCCCATCTCTACAAAGAATTTTAAAAATT<br>AGCCAGGTGCAGTGGGAAGATTGCTTCAGTCCAGAGGCTGCAGTG<br>AGCTATGATTGTGCCACTGCACTCCAGCCTGGGTGACAGAGCAAC<br>ACCCTGAGACAGAGAGAGAGAGGGGGAAGGAGGGAAGGAGGGA<br>AGGAAGGAAGGAAGGAAGGAAGGAAGGAAGGAAGGAAGGAAGG<br>AAGGAAAGGAGAGAGAGAGAGAGAGAGAGAGAGAGAGAGAGAG<br>AAAATAATTTTTATTTATTTCCAGGCTGGGAAGAGATGCTGATTTCT<br>GCGATAAAATCAGTAGGTACATTTTTTGGAATGTTCGCTATGTGCCA<br>GGCTAGATTTTACAGATGAGAAGTCTGAAGCTCAGGTAAGGTAAGT<br>CACCTGTCCAGGGCCACAAAGAAAAAAAAAAACGTGTGTCTGAAG<br>CCAGAACGGGAGCTGTTGCGGCCCAACTCCCTCCCCTGCCCCCAA<br>GCGGCCTCTGGGCTCGGGAAGGGCCCCTGCCTCCTCCCGCCAGGC<br>ACTTATCTCTACCCAGGCTGAGTGCTGGCCCCGCCCCCTCGGGGAT<br>CTGCCACTTAGAGGCGCCTGGTCGGGAAGG
F11	CCTGCCAAGGCATGGGAACCCCCCACGTAAACTTGGAACTTTTCCT<br>TTCAATTGTTGCTTATGTCTGTCACTTTACTTGACTGTGAGCAGCTT<br>CTAGGGAGGCTCTCTCATTCATAGTGCATATCATGGGCATCCAGTAA<br>ACGGCTAATGATGATGATACACCTTTATTACAAAAATAAAGGCAATG<br>GAGACTGAAGAGGTGAAATGATTAGCTCAAGGTCACTGGCAAGGA<br>CAGAGCTGGAACAAAGCTCAGCATTCCTATGCCAGGCAATGTCCTC<br>TCACTGATATCCGGTTGCTTCTCACTAGGAGAAGGATGAAAGATGA<br>CAGAGCATTTATAACCACCATTTGTTATTTTCATTATCAACTGACACT<br>GGTGTTTCTCTGCCCGGAGCGATTTTGTCCTCCCTTCACCTCAGGA<br>AACATTCGGCAACGTCTAGAGACACTTTTGGTTGTCCTAATGGGAG<br>AGGGTATGCAACTGGCTGTAGTGGGTAGAGGCCAGGGATGCAGCT<br>AAACATCCTGCAGTGTGTGGGACAGCCCCTCGCAAGAGAGAGTTA<br>TCCTGCCCCAATATCAATAATGCCAGGGTTGGGAAAGCCTGATCTCA<br>TGTCAGCATTAAAACCTTGTAATGAAAAGAGGACATTTCGGCCGGG<br>CGCGGTGGCTCACGCCTGGAATCCCAGCACTTTGGGAGGCTGAGG<br>TGGGCGGATCGCGAGATCAGGAGATCGAGACTATCCTGGCTAACAC<br>GGTGAAACCCCGTCTCTACTTAAAATACAAAAAAAAAAAATTAGCC<br>GGGCGTGGTGGCAGGCACCTGCAGTCCCAGCTACTCGGGAGGTTG<br>AGGCAGGAGAATGGCGTGAACCCGGGAGGTGGAGCTTGCAGTGA<br>GCCGAGATCACACCACTGCACTCCAGCCTGGGCGACAGAGCGAGA<br>CTCCGTCTCAAAAAAAAAAAAAAAAAAAAAGAAAAAGAAAAACA<br>AAAGAGGACATTTCAGGAACAGCAAGAAAGGGGTTGTAGGACATG<br>GCGGGCTGTGCCCTTTCTCTTCCTTTATTTTTACTTTTTGATACATT<br>CTATTTGTACATATTTGTGGGGCACATGTGAAATTGTGTTATGTGCAT<br>AGAATGTGTAATGATCAAGTCAGGATGTTGGCATACCCATCACCCA

	ACCATTTCTATGTGTTGAGTACATTTCAAGTCCTTTTCTAGCTATTTA<br>GAAAACTATAATACCTTGTTGTTGACTGCAGTCACCCTACTCTTCTG<br>TCAAACATTAGAAAGCATTCCTTCTATCTAACTCTGTGGTTGTACCC<br>CTTCACCAACCTCTCTTCGTCCTCACCACCACCCCCCCACACACAC<br>CCTTCCCAGCCTCTCGTAACCACCATATACTCTCTACGGCCATGAAA<br>TCATCTTTCGTGGGATCATGCAATGTTTGTCTTTCTGTGCTTGGCTT<br>ACTTTACTTAACATAATGACCTCCAGTTCCATCCACATTGCTGCAAA<br>TGGAATGATTTCCTTCTTTTTTATTGTGTCCATTTTCAAAGCAAGTTC<br>TGAAAGACAAGAGAAGGAGCTCTGAGAGTTGATTATGGCCATGCC<br>TGAGAAGGGGTCTCAGTCAACTAATGCCGTTGTGGACATTCCTGTC<br>CCAAGCAGAAGGCTTTAGGTCACAATATGTCCAAATGTCCAATACA<br>TCTCCCACCTATGATCCATTGGAAAGAAAACAAACCTCGGGACCCC<br>CAAAATCACTAAGCCACATGGAAAAGTAAAGCTGGGAACTGCATT<br>GGGCAAACCTTCCTCCCATTCTATTCCTAAATAAAATAGCTTTCTTA<br>AGCTATAAAAAACCTTTTTTAAGCTTTAAAAAGGTTTTAAAAAGCT<br>ACATTCCTCACAATTTGCCCATGAGGAAATTCCTTGTGGAAAAAGG<br>ACAGGCAGAAGTCAGAGTCACCCCTCTGCTGACATGAGACAGGTG<br>CGTATCTGATTGCCTCGTTTGCCATATTGTTTCACTGGGCCACACTA<br>AGGCATGAGTGACTATTCTTGTAAATTGCGTATTTGGTGAAAGGCTA<br>ATTGGAAACTCGAAAAAATGCTTCTGTTTGTCTCTTATCTACCTATG<br>ACCTGGAAGCCCCCTTCCCTTCTTCGAGTTGTCCCACCTTTTAGGA<br>CCAAACCAATGTACATCTTGCATAGATTGATTGATGTCTTATATCTCC<br>CTAAAATGTACAAAATCAGCTTGGGCCCTGACCACCTTGGACACAT<br>GTCATCAGGACCTCATGAGGCTGTGTCATAGGTACATCCTTATCCTT<br>GGCAAAATAAACTTTCTAAATTGATTGGGACCTGTCTCAGATACTTT<br>TTGATTTAGAATCCTAACTTCTGACTTGTACTTGGGTCTCTGCAGAC<br>TAACTTCTATATTCTTCCTAAGACTTTGTCTAGTTATTCCTCATTTTCA<br>TCAGTCTTCGAATATTCAAAAACATTTCATTTCATTGAAAAAAAATT<br>GAAAAAAGTAGTTGATATCAGAATTTTAAACTGCAATGCATTAGTTG<br>AAAATAATTTTTTAAGGATATCTATGGTCAGTTTAAAAGAAAGGTCT<br>ATTTAATCTCAAAGAGTTTCTATGTAGTCAAGACTTTTATTCTAGGC<br>TGGGCACGGTGGCTCATGCCTGTAATTCCAGCACTTGGGAGGCCAA<br>GGCAGGTGGATCGCTTGTGCCCAGGCATTCCAGACCAGCTTGGGG<br>AACACAGCAAAACCCTGTCTCTACAAAAAATACAACAATTAGCCAT<br>GCGGAGTGACATGCGGCTGTATTCCTAGCTATTCAGGAGGCTGAGG<br>TGGGAGGATCACCTGGTGGCTGCGTTGAGTGGAGATCACACCACT<br>GTACTCTCGTCTGGGCGACAGGAGTGAGACCTTGTCTGAAAAAAA<br>ATTATTCTACTTTTATTTGAATAGACAAAATTTTTTTGAATCCTGATA<br>CTATTTCAAAGACCTTAAAGATTTCACTAACTAGCTAAATCAAACTT<br>AGGAAAATATTATTTGGTTTTCCAGTGAAGCATTTCTCTTATTTAGA<br>GCCTTTCTGTTTCTCTCAATAGGGTTGGAGAGTTATCCTTATCTTCTT<br>TTTATTGGGGCTTAAGAAGAGAGATGAGGTTCCATGGAGTAAACAA<br>TACAAGGATATAAGGACCTCATATAATCTCACGTATCCATTTTCCATG<br>AAAGCCATTCTTGGCACGAATTTGCCATTCTATGTTTGAGCCTCATA<br>AAAGGCAATGCATCATAAATATGCACTTTTTGGGTAGCTGGAATGTA<br>CTTTAAACGGGAAACTCAAGAAAACAGCTAGGAACTCTTTATGCC

	AGGATAAGTCAAATGCACTGGCAATAGATCAAATAAAAATATCTGA GTAAATGCCTTCCAGATAGAACATCACCCTAGTTCTTCAGCACTTTA CAAAAAGATCTGTTCTCAGTTCACATTCTCTTAATTCCCTGTCATTT CTCCCCGGTCTTCCAAATATGTTTTTAAATTATGATTCCATGTATAA ACACAAGATCGTATTTTGCTTTTCTATGAAAAATTGAGAGCCTTCTC TGAACCATAAACGTATCTTTCTCCAAAATGTATTCAAATCAGGCATA TTGAAAACTGTTTTGTGTTCGTGTTGTGAGGTTTTTAGGGGACCG CACAGAAACCCTCAGCACTAGTTAGTGCCTTGTGTTCTAAATGAGG AAACGGAAGCCAAGGACCTGCAGGTGGCTGGGGACTGTGCACAG GCTGGTTCCGCTCAAGTCACATGACTCGTCAGCCAGTGAGTAGGTC TGGGTAGCAGTTGGCATGGATGAACATGCCCTCCATAGGCTTTCAG ACCTTCTTCAAGGCCAAAGGGAAGGCCTTCATGGAAATATAATTAT GTGAAACACCCCAGAATTTTTTCACAAACTTTCCTGCCTATAAACG CCAGGTCCTACCACTGTCCAGTGTCCCACTTCCCACTGTCCAGTGG GAAGACTCCCTCCAGGACAAAACCACCCTTTCCTTCAGGGATGTG ACGTGCTGTGCTTTCGTTGACAGTCATGCAGCTGCTAGACATGTCA CTTCCTAGCTCTCCATTCGGGTCTGTGTCCCAGGACCTAAAGAAAG CTAAAAGAAGCCGGGCGTGGTGGCTCATGCCTGTCATCCCAGCATT TTGGGAGGCTGAGGCTGGCGGATCACTGGAGATCAGGAGTTTGAG ACCAGTCTGACCAGCATGGTGAAACCCTGTCTCTACTAAAAATACA AAAAAAGAAAAGAAAAAAGAAAAAAAAATAAGCTGGGCGTGGTG GCGCGGGCCTGTAGTCCCAGCTACTCCGGAGGCGGAGGCAGGACA ATCACTTGAACCTGGAAGGCGGAGGTTGCAGTGAGCTGAGATCGC GTCATTGCACTCCAGCCTGTGCGACGAGAGACTCTGTCTCAAAAG AAAGAAAGAAAGAGAGAAAGAAAGAAAGAAAGAAAAGAAAGAA AGAAAGAAGAAAGAAAGAAAAGAAAGAAAGTAAGAAGCAAGCA AGCTGAAATAGCTTATTTTTCTGTTTTAAAAAATAGACTTTTAGTGT TCACAAGTAAGAATAAAAAAAAAAAAAAGCTTGGCCTTAGGAGGAA TCCTATCTGCTTAGGCCTCTGAGAGGCAGCGTCACCTGAAGGGAAA CTGACTGGGCAAGAGCCAGGCTCTTAGGAGCTGTCTGTACTTTGCT TCCCATCCGTCCGCTCTCCCCCAGCAGCCAAAGTCTCCTCCCTCCA TTATATTGCAAATCAATGAATCCATTAGACTTTCCATGTTTTCTTTTT AGACTTTGAGATTCAAGGTCAGCTAGAATTAATGGTTAACAGCTGA CAGGTGAAAGTGTGGTTAAATGCAGCTTTGGCTAGCAGGCACACA GGCAAAATCAAGTTCTACATCTGTCCCTGTGTATGTCACTTGTTTGA ATACGAAATAAAATTAAAAAAATAAATTCAGTGTATTGAGAAAGCA AGCAATTCTCTCAAGGTATATTTCTGACATACTAAGATTTTAACGAC TTTCACAAATATGCTGTACTGAGAGAGAATGTTACA
F12	TAAATAGTGGAATGAACAGAACTAGGTGGCTAAGTAGGCTGGGAG CATGAGGAAGAGAGAGCTCTTGGTTGCAAGCAACAGAAAATGACT CTAGCTGCTTCAACAAAAGGGAATTTACTTTGTGAGAAGGTCTTCG GGAGTTCACGGCATCAGCTCGAAGGAATAGGCTTGGAAATGTGCA GGAATTAAGAGCCACTGGAAGACTAGGAAGCAGGAAGTACAGTGA TTTCCCTTGAGCAGACACGGAAGTTGTCCACCTAATATCCATGCCA CCGCTCTTCCCTGTTTGCAGAGTCCCAGTTCTGTTCCAGTGGCCAC TTTACCTCACATGCCTCTGACCCAGATCCAGGGTTAATAGGTTTAGG

	CCAGTGATGGGGATCCCCTTTTCCTTGCCAAACATGGCTTAGGGAT<br>GTCATGTGAGCCTGTTCTAGCTAAGAAAGCATGAGGAGAGGTCTGC<br>TTGAGATTTGAGGGAGGGTTCCTCATTCCTAAGAAGGAGCTAGTGG<br>AAGGGAAGGTGCCTCTTCTTTGCTTGAACATAATTGAGTTGAAGTG<br>TGATTCCTGGAGCTGTGACAGCCATCTTGTGACCATGAGGGCAGGC<br>AGCCTGAAGGTCTGCACACAGAAGACAAACAAAAAAAGAAAGAA<br>CTTGGGTTCTTGATGACATTGTTGAACCACTGAAAGCACTGTTCCT<br>GGAGTCCTGCTGCTCCAGACATCTGGTGACATGGGATGATGAATTC<br>GAATTGCCCTACTGCATATGCTTGGTTTTTGGAAGGCATCCTGACTT<br>AATGCCATGTAGCCGGGATGATTTGGTCAGGGCACAGGTGGTGCAA<br>TGCCAGACCTCCAGCTTTTCCTGCCATCCTTGTGTGGCTTGATCAG<br>GATTCAGAGCCCTGGGAGAAAACACTATTGGCTGAGTTTAGGTCCC<br>TCTGGCTATACTATGGTAGGGAGAGGTTGGTCTGGTCCATTAGGCTT<br>CCATAGTGGAGATGGTCTCTAGAAAGTACCCTTTCAGCCGGGTGTG<br>GTGGCTCACGCCTGTAATTCCAGCACTTCGGGAGGCCGAGGTGGG<br>CAGATCATGAGGTCAGGAGTTCGAGACCAACCTGGCCAACATGGT<br>GAAGCCCTGGTCTCTACTAAGGAGACAAAAAATTAGCCGGGCATG<br>GTGGCGTGGGCCTGTAATGGCAGCTACTTGGGAGGCTGAGGCAGG<br>AGAATCACTTGAACCTGGGAGGTGGAGGTTGTAGTGAGCCGAGAT<br>CGCACCATGGCAGTCCAGTTTGGGCGACAGCGCGAGACTCCATCT<br>CAAATAAATAACTAAATAAAAATAATAATAAAGTACCCTTTCACCAC<br>CACAGCTGGGGAGGGGTGATCACAAAATGAAACTGGGAGAGGAA<br>GAGGAAGGGAGATGGATGTTGAACATCCAAATGACCATTAATGTCC<br>ACCTGACACCTCATCTCACACTCCCTACAAGGCACCTTATGCTCAC<br>CACATTCCCCATTTGACACGCTGCATTCAGGCTTCTGTGCAGTTGTA<br>TTACTCACTCTGCCTGCAAACCCCTACTTGTGGCGAAGACCTGTTC<br>AAAAGGCCCCTTCCATGTAGAGGAACAGCTGCCTTCTCCCCAAGA<br>GGAAGGAGCCAATGTGGGAGTGGCCTTAGGGCCAGGGTCTATACT<br>CAGGAGCAATCAAGAACTGACAGAGCCAGGCATGATGGCATGAAC<br>CTGTAATCCCAGCTATTCAGGAGGATGAAGCGAGAGGATCACTTGC<br>GCCCAGAAGTTCCAGGCTAGCCTGGGCAACTTAATAAGACCGAGT<br>CTCTTTGAAACAACAAAAAAGGTGACAAAGGTAGTTTCGCTTTAAT<br>GACTCGTCAATATATCTAATAGTTTCACTTTCATCATGGAGTTAACA<br>GCTGTCACGTCAATGACTTTGAAACCAGTTACCTTTGAAACCAGTG<br>GTTGGAAAGGTGCCCCTCCTCTCCTGCCCTCCTCTTTCTGTGTTTCT<br>GTTCCAAGAAGTCTTCCACAGCCTGTCTCTCTTCCAGCAGAATTAA<br>TAAATTGCTACACTCAGTCTTCATTTCCCATAAATTACTTTGTATAAC<br>TTAGGACTTTGTATAACTGAAAGTGACAGAAGTCCACTCAAATTGG<br>TTTTATTGGCTCATATAACTGAGAAGTCTATTTTTTTTTTTTTTGGAG<br>ATACAGTCTCAGTCTGCTTCCCAGACTGGAGTGCAGTGGCACAATC<br>ACGGCTCACAGCATTTTGGACCTCTGGGCTCAAGCAATCCTCCTGC<br>CTCAGACTCACAAGTAGCCGGAACTACAGGCACGCGCCACCATGC<br>CTGGCTAATGTTAAATTTTTTTGGAGAGATGGGATCTCACTATGTTG<br>CCTACATCAGTCTGGAACTCCTGGGCTCAAGTGATCCTCAAGTGAT<br>CCCCAGCCTCCCAGAGTGTTGGGATTACAGGCGTGAGCCACTGTG<br>CCTAGCCATAACTGACCAAGTCTTAAGGATACTTCTAGTTTTAGGAT

	GTAGGCTCTCAAAGTCATGAGAAATCAATCCATCAGTACTAGTTCT<br>CAAATGTCGTGTAACAAATCACCCTAACTTGTGGCTTAAAGCAACA<br>ACATTTAATGATGATTTATCACAGTTTCTGATGGTCAGTTTTGCCTTG<br>AGGTCTTTCATAAGATTGCAAGCCAAATGTCAGCGAGGGCTGCAGT<br>ATCCACTTCCAAGGCAGCTTATTCATGTGGCTGTCCAATTGGCACTG<br>GCTGTCAGCAGGGTGCTTTCCTTTCTCCCTACATGGGCTTCTCTGCA<br>GGGCTGCATGTGTGTCCTCACAACACAGTGGCTGGCTTCCTCCAGA<br>GTGAGCAACCTAAGAGACCGAGGCAGAAACTGCAATGTCTTTTAT<br>GACCTGGGCTTGGAAGTCCAACACCCTCACTTCTGCCATATTTTTTT<br>TTTTTTTGAGACAGTCCCTGTCGCCCAGGCTGGAGTGCAATGGCAC<br>AATCTCTGCTCACTACAACCTCCACTTCCTGGGTTCAAGCGATTCTC<br>CTGCCTCAGCCTCACGGGTAGCTGGGATTACAGGCACACGCCACTA<br>CGCCCGGCTAATTTTTGTATTTTTCATAGAGATGGGGTTTGGCCAAC<br>ACGTTGGCCAGGCCGGTCTTGAACTCCTGACCTAGAGTGATCTGCC<br>TGCCTTGGCATCCCAAAGTGCTGGGATTACAGGTGTGAGCCACCAC<br>ACCCAGCCACTTCTGTCATATTCTGATGGACACACAGAACAGCCCT<br>AACTCAATGTGGGAAGACACCATACAAGGGCATGAATCCTAGGTG<br>GTGAGGATCAATGGGGGCTGTCTTTGAGGCTGTCTACCACAGCATC<br>TTTCCATCCTTCCTGCCCTGTTTGCTTTGCTTTTCCTATGTGTAGGCT<br>TCATTCTCAAACAGGCCCTCCCTAGAGAGTGACAAAGGTGATCATC<br>AATGTGTTCAGACCCACATGCTCTGTGCTTAGTAACCCCAGTGCAA<br>CTTTTTTGCTTTCCCAAAAGTTCTGGCAAAAGTCCCAAGCTAGCAC<br>TTTAATTGGCCTAAATTGTGTATATGCTTATCTCTGAACCAATCACTG<br>TGGATTAGAGATGTCATGCTCTGATTGACCAGACCTAGGCCACATCT<br>CTAGCCCTAGCTCTGAGGGTAGAGTTGGCAGCACTAGAGCCCATGG<br>AAGAAGTAAGAGAGGAGTCGTTGCTAAAGGAAAAATCAAAGTGTC<br>ATTACCGAACCAGGACAGATGCTGGGCAGCACATGTGCACCCCGT<br>CTTCTTCTCATGTTCCAGCTGCACATCTTAGTGCCCCTTGGTTTAGC<br>ACTTTTCTCATTAAATCATTTGCTTTCTTGCCTCACTTCCTGTGGTTG<br>GTAGAATGCTAAGATGGCCCCAAGATCTCTACCCCTGGTGTTTGCA<br>CACCTCCCAGTTATTCTGTCAAACATGAATGTAGATGCTTCTGTGAA<br>AGAATTTTGCACATGTAATTTAAGTCCCAAATTGTTTGACCTTAAAA<br>TAAGGAGAATGGCAGGGCCAGGCATGGTGGCTCATACCTGTAATCC<br>CAGCACTTTGGGAGGCCAAGGCGGGCAGATCACGAGGTCAGGAGA<br>TCGAGGCCATCCTGGCTAACACAGTGAAACCCCATCTCTACTAAAA<br>ATACAAAAAATTAGCTGGGCGTGGTGGCGGGTGCCTGTATTCCCAG<br>CTACCCAGGAGGCTGAGGCAGGAGAATGGCGTGAACCCGGGAGG<br>CGTAGCTTGCAGTGAGCCAAGATCGTGCCACTGCACTCCAGCCTG<br>GGTGACAGAGCCAGACTCTGTCTCAAAAAAAAAAAAAAAAAAGGA<br>GAATGGCTTTGGTGGGCCTGACCTAGTCAGGTGAGTTCTTAAAAGG<br>CGACACATGGCCCGGTGCAGTGGCTCAGGCCTGTAATCCCAGCACT<br>TTGGGAGGCCGAGGCGGGTGGATCACGAGGTCAGGAGATCGAGAC<br>CATCCTGGCTAACATGGTGAAACCCCGTCTCTACTAAAAAGACAAA<br>AAATTAGCTGGGCGTGGTGGTGGGCTCCTGTAGTCCCAGCTACTCG<br>GGAGGCTGAGGCAGGAGAATGGCGTGAACCCGGGAGGCGGAGCT<br>TGCAGTGAGCGGAGATTGCGCCACTGCACTCCAGCCTGGGCGACA

	GAGCGAGACTCCGTCTCAAAAAAAAAAAAAAAAAAAAAAGAAAAT<br>TAAAAGTGGGTATTGTTGTAAGATGCTGAGTTTATGGTAGTTTGTTA<br>CATGACAATAGAAAATGAACACACTTCACAGTGGACTCCAAGATCC<br>CCATGATCTTTGATCTCCTTAACCTCCTGATCTCCACAGGACCCAGA<br>GCATAAGAATGTCCCTTCTTCTGCTTCCAGTCCCACTATCTAGAAAA<br>GAGAGGAGGAGCCCAGCTCTTCATTTCACCCCCACCCACAAACTC<br>CCAACTTTCCGGCCCTCAAGGGGTGACCAAGGAAGTTGCTCCACT<br>TGGCTTTCCACAAACAGCCTGTGCCCCACCAGGCTCAGGAGGGCA<br>GCTTGACCAATCTCTATTTCCAAGACCTTTGGCCAGTCCTATTGATC<br>TGGACTCCTGGATAGG
F7	TGCCTCAGCCTCCCGAGGAGCTGGGACTACAGGCGCCCGCCACCA<br>CGCCCGGCTCATTTTTTGTATTTTTAGTAGAGACGGGGTTTCACTGC<br>GTTAGCCGGGATGGTCTCGATCTCCTGACCTCGTGATCTCCCACCTC<br>AGCCTCCCAAAGTGCTGGGATTACAGGCGTGAGCCACCGCGCCCG<br>GCTGCAGCAAAAATTTTAAGTACTGAAATCCTTTAGAACCATCCCC<br>ATTGGCAGCAGAACTGAACGGGGCCCCACTACTCTCCAGCGCCATT<br>TAGCCGCAGTACTAGTGACTACCACCGCAAAGGAAAAGGAAACAA<br>GGCCAGCGCGGCGGCTCAGGCCTGTCATCCCAGCACTTTGGGAGG<br>CCGAGGCGGCAGATCACGAGGTCAGGAGATCGAGACCATCCTGGC<br>TAACACGGTGAAACCCCGTCTCTATTAAAAATACAAAATTAGCCGG<br>GCGTGGTGGTGCATGCCTGTAATCCCAGCTACTCGGGAGGCTGAGA<br>CGGGAGAATCGCCTGATCCCAGGAGGCAGAGGTTGCCGTGAGCCG<br>AGATGTCGCCATTGCACTCCAGCCTGGGCAACAAGAGTGAAACTC<br>CATCTCAAAAAAAGAAAAAAAAGAAAAAGGAACCAAGGCATGCTA<br>ATTGAAAAATAACTTTTCATATCTAGAAAAACCAGGTGAATTCATAA<br>ACTATTGAAACTAAGAAGTCCACAAGATCAACCCACAAAAAGTTAT<br>TAACATTCCTAGCAATAACAATACTATTTTAACAGCAACCTAAATAC<br>TAAAATAAATGTAACAAAAGTTTTGGACAAAATTATGAAACATGAT<br>AGAAGGTCATAAAAGAAGGCCTAAATGGGAGATTTTTATGGATGGG<br>ACAATTCAGTATCACCAAAGTGTTAACTCTCCCCCAAGTTAATCTGT<br>GTATTTCATCAAAATTCCAACAGAATTCTTTGTGGAATTAGGAAGTT<br>GATTGTAATAAACATAGAAGATAAAGGTCTGAGTATAGCCGTGGTG<br>ATTCGGGGAGCAGAGGCAGGAGGAGGAGGGCTCACCTACCAGGC<br>ATCTGGATAGATCATAAACCTGTCATCAGTCGTGACAAGGGGGAGA<br>GAGGCTAAACTGGTCGAGCAGAATTCAGATTCCCAGGCAGGGATG<br>TGTGCAGGGACCTGGCTTGTCCTGGGGCTGGTCAATCTTTGGCACG<br>GAATGGCCTCTTCAGGAACGGGTGTTATTGGGACAGCTGCCTTTCC<br>ATGTGGATGATGGCATAAAAGTATAGCCCCCCCAAACTTAAGATTAA<br>AACATTATTATGAGGAAACAGAATATTCTTATTGCCTAGGGCAAGGA<br>TAGATTTCTTACAAAAGACAAGCTACACTTTAAGAGATGGACAAAA<br>AGATAAGATGGACAATCTGAGCACATCCCATGGTGAATTTCTCTCC<br>ACGCTAAGACCCCAGACAAGGGTGAAAAGCAGGACCAAGGCCCA<br>GAGAAGAAAAATTCTGCTCAAAATCATAACTGTTCAACCTTGCATA<br>CATTCTCTCTCTTAAACTGAAAGTTAGCATCCTACAGCACTGGTGTG<br>ACACCCGGCAGGCCCGAGGGCTCAGCTGCCTCCAGCTGTGGCCCC<br>AAGAAGGCAAAGCACCCGGGTGGGGTGGAGGGAGCAGCCCCGTG

	CTCTACTGGCTGACAGTGGTGCCCAGGCCCAGCTCCCTTCTCTGCA<br>CAGACAGCAGAGTTCCGCTTAGCACAGGCTTTTCCTCGCCAGTGC<br>ACACACTGTCAGCCATTGGAACTGGCCTGCAAGACAGCTTGCTGT<br>GGCGGCTTTGCAGGCACAGGGCACACAGGCATCCGTGCACGCAGC<br>TCCTCTGTCCTCCCATGCCCTGCACCCTCCGTCACAAGGAAACCCT<br>TGCTTCTAGGATTGCACCAAATAGGATTTGGAACCAATTGGTGCCC<br>AAGCTCAAATCCTGTTAGTGGCTGCCTGTTTCCCTGGCATACATGCG<br>TCCTGCCGAGATGCATTTGGCACCAGGTGGGATGTGCTCACTGGTT<br>CCCAAAACGAGAACACATAGCAGTTTCCCTGGATGTTTGTTCAATA<br>CTATTCCCGGGACCTGCCCGTAGATTCCAATTCCTTATGGGTGTGGA<br>AGAGCCTGGAAATCACGTGTGTGTAGGTGTGTGGCATGTGTATAGG<br>GGTGTGTTTATGTGGTGTGACTTTGGGGTATGAGTGTGGCATGTGTA<br>CGGTGTGTGTGAGTGTGGTATGTGTATGGCGTGAGTTTGGGTTGTG<br>CGAGCGTGGTTCTCATGTACGAGTGTGAGCATGGAGATTCTTTGGT<br>AGGGAAGGAGTCAGGGTCTCCCTCTGTCGCCCAGGCTTGAGTGCA<br>GTGGCACAATCTCAGCTCATTGCAACCTCTGCCTCCCGGGTTCTCT<br>GTCTCTGGGACCACAGGCATGAACCACCAAGCCCAGCTAATTTTTT<br>TATTTTTTGTCTAGCTTTGGTCTTGCTATGTTGCCCAGGTTGGTCTG<br>GAACTCCTTTCCTCAAGCGATCCTCAGTCCTCAGCCTCCCAAAGTG<br>CTGGGATTACAGGCATGAGCCACCACCCAAGAGATTCTGATGTCAC<br>AGACATCTGACTCACACACTCCCTGGACAGCTCACGTGTGCCCACC<br>ACCACAGTCAGGGATGGCTGCAGATGAACAAGGTGCTGACGTGGT<br>TTCTGTAGCCCCCAAACAAGCAACTTGCCTTGAGATGACAACCAA<br>AGTTTTCCTGTGTCCTCCACACTCAAGAGTGACTGTGAGGCGGAG<br>GGGCCCAGCCCTTCTTGCAGGCGGGAATGAGTGGATGGGTGGATC<br>AACAGAGGCTGCCACAGGAGAGAGGGAGGCCTGGCCTGGGAACA<br>GAGCTGTGACCGTGCCCTTCCCCAGGGTAGGGGCTGAAGGACCCT<br>CCCATCCTAGTGACAGGGCCACAGCATGTCCAAGGAGGCCCCAGA<br>GGAGGTCCCGGGAGTCCTGGGAGAGCCTGGTTAGCCTCCCTGAAG<br>GGAGGAAGTGGGGTTTTGTGAGAGGGATGGTGCAGCAGCCCCCAC<br>ACCTGCTACTCCGTGTGGCCGGGTCCAGCCCCAGGCAAGGTTCCA<br>GGCATGCCCCTGGGACAGACGTGGGAGGGAGACCAGCAGGCAGG<br>TCCCCCTCAGGGAGGTATGGAGCGGGTCCCCGGGGCCAGGTGGAG<br>CCCCTCACACCTGTGTCCTCAAGGACCCACACCAGGACAATGGCC<br>ATAGGCGGGCACCTGCAGCCGCCCTGGCCAGCAGTGAGACGGGCA<br>GAGAGCCCAGCCAGATAGCCCGGTTCCTGCCCCCTCCCGCCTGCCC<br>GCACAGAGAGGGCTGCATGCAGGTGAGGGTCCATCACCTGAAGAG<br>CTTCACCAGCCCCGAGGCCATGAGCCACTGAATCTCTCAGGGGGTT<br>TTGGCGCTTGAGGTTCCCCAGGTCATTCTGCTGCCCAGAGAGGGTT<br>GAGGACCTCTGACCTGGAGCCCCAAAGGGGAAGGGTACCCTAAGA<br>CCAGCCAGCCATCTACTCTCAGGTGGGAAAGGGGTAGGGTTTAGAT<br>ATGGCCCCCTCCCCTCCACGTCCTGAAATTACCTTGGGCTGTGCTG<br>GCCGAGAGCCGCGGGGACCAGATTTGAGCTGGACGTGAGCTTTAT<br>TCCAGACGGAGCAGCTACTTTGTAAGCGATCTCCCTGGGATCGATA<br>TCATGGAGGGGCACAGATGGGCTTCAGCTGTGCTGGGTGGAAGAG<br>AAAACATGCCCTGTCGGCCCCTCAGCAAGTGCTCTGCTCTCTGGAC

	TGAGATGCAGGTCAGCAGCAAGCAGGGAGGCTTTGGGGGTGCTGG<br>GGGCTGCTTTGAAAGGCACCCCTGAGCTAAGACTTGGCTGTTGGG<br>AAGGAATCACCTCCCTCCCTCCAGCCGTAAAAAGAACTGGACATC<br>AGCTTCCAAAGAAGTCATCAGCAATGCAACTGTTCACATGGAGGAT<br>ACTCCCTGCTTGAGGGGTCAGACAGGCCTGCTGGGCAACCCAGGA<br>GGCTTGGATGACCGTCTACCCCAGTGTTTTTGGGATGGAAAGTTCC<br>ACATTCTGAGAACCCTCAGTCCCTGGGCAACCTGGGGTGGTTAGTC<br>ACCACAGCTTGTGGCTTGGGCCCATGACAGCAGGTAGAAATGACG<br>TGGACTGCCGCCAGCCGGGCACAGTGGCTCACGCCTGTAATCCCA<br>GCACTTTGGGAGGCTGAGGCATGTGGATCACTTGAGGTCAGGAGT<br>TCGAAACCAGCCTGGTCAACACGGTGAAACCCCATCTCTGCTAAA<br>AAAAAAAAATATATATATATAAATTAGCCAGGCATGGTGACGTGCAC<br>CTGTGGTCCCAGCTACTCAGGAGGCTGAGGCACAAGAATCACTTG<br>AACCCGGGAGGTGGAGGTTGCAGTGAGATTGCACCAGTGCACTCT<br>CCAGCCTGGCAACAGAGCAAGACTCTGTCTCAAACAAACAAAACA<br>AAACAAACAAAAAGACGTAAGATGTGGACCGCTGGAGAATGGGG<br>GTGCTGCCTGCAGTCAAAACGGAGTGGGGGTGCCCAGCTCAGGGC<br>CAGAATGATCCTATTCCCGGCACTTCTCAGTGAGGCTCTGTGGCTC<br>ACCTAAGAAACCAGCCTCCCTTGCAGGCAACGGCCTAGCTGGCCT<br>GGTCTGGAGGCTCTCTTCAAATATTTACATCCACACCCAAGATACA<br>GTCTTGAGATTTGACTCGCATGATTGCTATGGGACAAGTTTTCATCT<br>GCAGTTTAAATCTGTTTCCCAACTTACATTAGGGGTTTGGAATTCTA<br>GATCGTATTTGAAGTGTTGGTGCCACACACACCTTAACACCTGCAC<br>GCTGGCAACAAAACCGTCCGCTCTGCAGCACAGCTGGGGTCACCT<br>GACCTTTCTCCTGTCCCCCCCACTTGAGCTCAGTGGCTGGGCAGCA<br>GGGGATGCATGGCCACTGGCCGGCCAGGTGCAGCTCTCAGCTGGG<br>GTGTTCAGAGGACGCCTGTGTCCTCCCCTCCCCCATCCCTCTGTCA<br>CCCTTGGAGGCAGAGAACTTTGCCCGTCAGTCCCATGGGGAATGTC<br>AACAGGC
FAAH	ATGCCCAGCTAATTTTTATATTTTTAGTAGAGATGGGGTTTCACTGTG<br>TTAGCTAGGATGGTCTCGATCTCCTGACCTTGTGATCCATCTGCCTT<br>GGCCTCCCAAAGTGCTGGGATTACAGGTGTGAGCCACCTCACCCG<br>GCCTACTTTGACAATCTTTTTAAAAGTGTCCTTGTAGACTGCACTGG<br>AAGCAAGCCCTATTAGGCATTCGATTTGCCCAGTTTTTCCCTTTTCC<br>AGAGCCTCCAAAGTCCACTTGCCTGAGGGCCATGACTAAAGTGGT<br>GGCCTCTTTTTTATCCTGTTTGTCCCATTCCACCTGCTCCTCCTGATC<br>TTTATTATAAAAAACCAAGGTTTCCAAGTTCAATAGGGTTTCTAAGT<br>TTTGCTCCAGGCCTAAGGCGGACTTTTGAAGTTTTTTTCTAATGTCT<br>GCAGCTGACTGAGTGATAAACTTATTCTTCAAGATTAGTTGGCCTTC<br>AATAGAGTTAGGTGACAGAGAGGTATGCTTTCTCAATGCCTCCCTT<br>AGTCTCTCCAGAAAGGCAGTAGGATTTTCTTCCTTTCCCTGTATTAT<br>AGTGGACATCATTGAATAATTCATAGGTTTCTTCCTAGTTTTCCTTAG<br>TCCTTCTAGCACGCAAGTTAGCAAATGTCTGCTGCACCAATCTCCAT<br>GTTCTGATTCTGTGTCCCAGTGAGGGTCTACACTGGGAACTGCCTG<br>CTGGCCTGTGGGGAATCGTTCTTTCCTCTGTTGTCATCTTGTCATTG<br>ACCTGACTGAGATACCAGAGATCACCAAACTCTCGGGCTGCAGTTA

	TGGCAGCACTTCTCTCATTTGGGTTTAGTGTCTGATTTAGCAATAAC<br>ATTATATCTTTCCATGTCAGATCAAAGGATTGTCCTAACCCTTGGAA<br>AACATCAATATAGCCATCAGGGTTATCTGAGAATTTACCTAGGCCTA<br>TTTTAATTTGCTTTAAGTCTGAGAGAGAAAAAGGTACATGCACTCT<br>GGCTGGGCCGAATTCTCCTCCTCCCATTGCTTGGAGAGGGGCATAA<br>TTGGGGAATATTAGCACTCTTTGGTTCATTGTTTACCCCTTTGTCTAT<br>CTCCTTTTGGACGTTTGGGTTGAAGGGGGGTCCTCATTAGTTGGGG<br>AAGGAGTCGGGGGGACACCAGGGTAGGGAGGTAGACTCTAGGGC<br>TTCCTGTAGGACATAAATCACACTTTTTACATAATTGCGAGTTGTCT<br>CTTAATGAAAAGAAAGTTTGTACATATGGCACTTCATTCCATTTGCC<br>TTCTTCTCTACAAAAGAGGTCTCACTGTAAGATGGTGTTATAATTTA<br>TATTTCCCTCAGGAGGCCAGGTTTCTCCCCCTTGAAGAGGATATCAT<br>GGCCAGGTGGTACTGCAGAAGAATATAAGTCATTTCTTTCTTAGCGT<br>CTGAGGGTCAAATTGGTCCCAAATCTCCAGAATACATCTTAGGGGC<br>GTTTTTGCCTTGTGGGGAACGTTTCCCATTGCTTTGGAGGTCCCTTC<br>GTGGTCGCCAAATGTTACTGGGGGGTCCTTGCTCCCAGAGCTCCCA<br>AGATGGTGGCGGGCCGCTTCCAAGATGGTGGCAAGCCTGGTGTTC<br>TCTGACCTGGGGTTCTTGGCCTCACGAATTCCAAGGAATGGAATCT<br>TGGGCCATGCGGTGAGTTTTATAGCTCTATTAGAAGCCGTGGGTCAT<br>GGAAGAGAACTGTGGAACCCAGTGACTAGTGTTCAGCTTGATTAG<br>GATGAACCTGGGCACTTAGCCGTGCAGGAACAATGGCAAACCTTT<br>AGCCTGATTGGGAGCGGCAATGGGCGCCTCGCTGGATCAGGAGCA<br>CAGTAGACACCCTGCCGGATCCAGAGGGATGGAAGTCGGCGGCGG<br>GTCTGCGATGGCAGCAAACAGCAGTGGATGTCGAGCGAAAGCTCA<br>GCTCCAGCCATAGCAAACACGGACCAGAAGAGAGTGCAGTTGCAA<br>GATTTAATAGAGTGAAAACAGAGCTCCCATACAAAGGGAGGGGAC<br>CCAAAGAGGGTAGCCATTTTTTCACTTTTTTTTTTTAACTTTTGTTG<br>AAAACATTTTAAGTTTGGAATTTCAACTATTCTTTGCTATTAATAAG<br>ACCTCATTCAGTCCATATTAACTTAGAATTGGTATAGATGGGTCCTTC<br>CTGATTCTGTAAGTGCTTTAAGGCTTGGCTGAGTGCAAACAGCTCT<br>CACATTTGAACAGGCCAATTATTAGGCAATTTTCCTAACTCTGCTTC<br>TACAAGAGTTTCCTTATCACTTACTGAATACCCATTGTGTCTTTTTCC<br>CTCAATTACCCAGGAGGAACCATCTATCATCCTGTCCTGAAGGGAG<br>TTCTTCCTAGGTCTGGTCGGACCTTTGTATGGTAATTAATTAAGATTT<br>AGATCCCATGTTAGGAAACCTGCTGGGTTAAGGGAATTTTCAGTGG<br>TTAATATTAAATCATTTTTTTCTAACAGAACAGCCTCATACTTTAAGG<br>TTCTTGAGTCAGTAAGCTACCTTTTTTTTTTTTTGACTTAGGATAGTT<br>CTGACCTGATGAGGTGTGCTCACAATGAGGTTTCCTCTAAAAGTTG<br>TTTTTCTACTTTCTTCTGTTAGCAAAGCAGTTGCTGCTACAGATTGA<br>ATGCATTTGGGCCATCTGCCGGTTACTGGGTTAAGGAGTTTTGATTA<br>GGGAGGTTACGGGTTGTCAGTGGCCTCAGTGCTTTTGGGCTACATC<br>CTTGTTTACACTGACAACAAGGTGGTATTGGAGTGTTATAGGGTCAT<br>GGAGAAGACCTTCAATTATCAATTACAGGTTTTGAATTTTATCCTGG<br>CTTTTAAAGGAATAGGGTACACTGTTTTCTCTTTACTACTTCTATCTC<br>TCTTTCTCTCTCTCTCTCTTTGACTCTCTCTCTCTTTCCCTGTCTCTG<br>ACTCCCTCTCTCTCTCTCTTTTTCTTTTGAGACAGAGTCTAGCTCTA

	TCACCCAGGCTGGAGTGCAGTGGCGCGATCTCGGCTCACTGCAAG<br>CTCCGCCTCCCAGGTTCACGCCATTCTCCTGCCTCAGCCTCCTGAG<br>TAGCTGGGACTACAGGCGCCCGCCACCACGCCTGGCTAATTTTTTT<br>GTATTTTTAGTAGAGATGGGGTTTCACGGTGTTAGCCAGGATGGTCT<br>TGATCTCCTGACCTCGTGATCCACCGGCCTTGGCCTCCCAAAGTGC<br>TGGGATTACAGGCATGAGCCACCACGCCTGGCTTCTGTCTTTCTGT<br>CTCTTTCTCTCTCTGCCTCTCTTTCTCTCTCTTTGCCTCTCTCTTTCT<br>CTCCAACTTCCTCTCCCAGTTTCTCTTTCCTCTCTGCTGGTCTTTCC<br>CTGCCTCTGCCAGCTGCTTATGCTGCTGTTCTCCCCTCTCCTTCCCC<br>TTCCCCTAGGGAAGGGACCGTCAGGAGTGCAGCTACTTTTTCTTCC<br>CCGAGAAGAAAGGAAAGGGGAATTCTGAATATTTTTCTTACTACCG<br>GAGGTTTGTGTGAGGTTTTTGATCCCCTGAAATTTGTGGAAGGCTC<br>AACACCTCAAACCAGGGGGTATCTTGCCTTGCCTGACCTGGAAGG<br>CTCAACCCCTCAAACCAGGGGGTGTTTTGCCTTGCTGCCCTGGAAG<br>GCTCAATCCCTCAAACTAGGAGGTGTCTTGCCTTGCTTCCCTGGAA<br>GGTTGACCTGTTTCCTCCCTTTCCCCCTCTGAAGGTCCTTTGCACAC<br>TTCCCACTCGTGTTGTCCTCTCTGGCTGCTCCCCCAAGGGAGAATT<br>AGGCCCCCCTCAGCATTGGCATTCCAGTAGAAATCCCACCGCAGGA<br>TCCTCCCTAAGCCATATGAGGTAGCTACGGAAACGTGGAGAGGACC<br>CACTCACTCCGTCCAGCAGTAGGACTTGTCATCATCCACACGAACA<br>ACACCACAGGTAGGGTTGTTTGTGATCATTCACGCACACACACATT<br>TAGCCCTCCAGAATTTGACCACCAAGGAAGTACCTTACCGGCTCCC<br>TCGGCTTCTTCCTTTGTCTGTGCACAGAGTTGTCTCTGCAGTGTGT<br>GAGGATGCTTTACCCTAGGTTGCCTGCTGGTTTCTTTTCACATTGCT<br>GAGAGCTCAGGTTATTTCTTGCACTGGGTGAGTCCTGATTTCTCAC<br>CCCTGAGGCCATCACAAGAGGGTGGGGCATACCTCCTCAGGAGAG<br>AGAACCAGAGACTGCCCCTGAAGGGGAATGTAATCACGAGCAAGC<br>CCCCAAATTGTTATAAATAAAGTTTCCCCCCAGTGCCGCAAAAGAA<br>ATAGCACTCGAATATAAAATTTTCTTTTTTTTCTTCTCAGCAAGGCA<br>ATTTACTTCTATAGAAGGGTGCGCCCTCACAGACGGAGCAAAGGTG<br>AGCGCACACCTGGACAAGGGAGGGGAAGGTGGTTCTTATGCCTGA<br>CCCACGTGATTCCTGATGCTGTGTCCTTCCCCTGTTGGCTAGGGTTA<br>GACCTATAGGCTAAACTAATTCCGATTGGCTAATTTAAAGACAGTGA<br>GGGGGTGAGTGGTTTGGCGGGAAAAATGGTTACCGCAGAGCAGGA<br>AATCGGAATGAACCACGGTGAAGAATGAGTGAGGGAGGAGCAGGT<br>AATCGAAAAATGTTGCTTTATGAGGAAGTTAAGTTTAAAAGTAGAA<br>GGCAAAGAATTGAAGATACTGACATATTGATTCTTTTAAGATAAATT<br>TAGAACTCATATCTAACAGCTGGCATGTCTGGCATGGGGCTCCGGC<br>TGGGGCGCAGTCTTCAGCATTTGGGGCTCTGGGGTGCAGGGGGCA<br>GAGGACGGCCGGGGCTGTGGTGCCCAACCGGTGCTAAACTCAAGC<br>TTGGGAGGTCGCCCACGAGAAACAGTCGCCCTTCCCCACCCGCCT<br>GCTCGGCTTGGCGGGGAGCGGGATGTCGCTGGGGGCGATGCCAGG<br>CGGGCCGCCTCTGATAGCCTCTAGGCTCTGGCGCGCTTCCCCGCCC<br>CTCCCGCGCAGGCGGGCGTGGGATCCCGGCTGATCCAGTCCGGGT<br>TTTGCGGCGGAGCGGGCGGGCTGCGCGTGCGGCGGCTTCAACTGT<br>CGCGGTAGGCAGCAGCAGGCTG

FGFR4	AAAAAAAAAAAAAAAGAACAAGACTGGGCCCAGAGTTAGGGCCAG<br>GCCCGGGCTGCAGTGGGGCCTGGGCAGGGGCCGGGCCGGCTTTAG<br>TGGCAGCCCTTCTGCACAAGAGCCCCAGTGTTCCCTGGGCCCGGC<br>TGCCATCCAAGAGCAACATCGTTGGCACCAGGCAACGTCTGCCCTC<br>TACCCCCCGATGACTCTGCCCCCAGGCAGGTGGGGGGTAGGACCT<br>GGCCTCAGGAGACCCTCCTGGCCCAGCCCAGCATTCCAGAGGCAC<br>AGAGTCTGCAAACCCCTGCCTGGGCCTGGTGTCTCCTCAGGACTG<br>CTGGCTAAAAGGTCAAGGAGTGCAGTGCTCAGTCCCAGCCCCCAC<br>ACCTCCCTCCCCAACACACTCACCTTCAGGTGAATCTGCACCTTCC<br>CGGGCCACAGTTAAGGATGGAGACAGAAAGCATCTGATGCAGTAG<br>CATCAATGATCCCACTCTGGAGAGTTATCCCGAGCTCTCCATCTTTG<br>CCCCAGAAGACAGGGGTTCGAGTTCTGCAATTCACTTGCTAGATGA<br>CCTCAAATAAGTAATTCCATCTCCCTGAGCTTGTTTCTTCACCTACA<br>AATCAGAGCTAAGGATTCCTATGCCATTTGGCTTGGTGGAAGAATT<br>AAAGGATATAACCCATGTACAACACCCAGCATGATGCCTGGCATGT<br>AACGGGAGCTGAAGAGTCACGAGCTCAGGAACAGGAACACGTTC<br>ATTTCCCCCAACACTCCTAGGAAGAAAGAAGCTGGAGCAGCCGGG<br>AGCCGTTGCTCACGCCTGTAATGCCAGCACTTTGGGAGGCCGAGA<br>CGGGTGGATCACCTGAGGTCAGGAGATCGAGACCAGCCTGGCCAA<br>CATGGTGAAACCCCGTCTCTACAAAAATACAAAAATTAGCTGGGTG<br>TGGTGGCAGGCGCCTGTAGTCCCGGCTACTAGGAAGGCTGAGGCA<br>GGAGAGGCAGGAGAATTGCTTGAGCCTGGGAGGCGGAGGTTGCA<br>GTGAGCCAAGATCACGCCACTGCACTCCAGCCTGGGCAATAGAGC<br>GAGGCTCCATCTCCACAAAAAAAAAAAAAAAAAAAAGGAAGAAGA<br>GGAAGCTGGAGCATGACGGCTTTCTGGGTGGGGAAACTGAGGCAG<br>AGGCAGGTTAACAGCAGGATCTTAGGGTGACAGGCTCTTGCCTCTG<br>CTAAGCGAGAGAGAGAGAGAGAGAGAGTGTGTGTGTGTGTGT<br>GTGTGTGTCTGAATGTATTTTTTTTTTTTTTGAGACAGGGTCTTGC<br>TCTGTTTCCCAGGCTAGAGTGCAGTGCGTGATCTTGGTTCAATGCA<br>ACATCTGCCTCCCAGGTTCAAGTGATTCTCCTGCCTCAGCCACCCC<br>AGTAGCTGGGATTACAAGCGTGTGCCACCATGCCTGGCCAATTTTT<br>GTATTTTTAGTAGAGATGGGGTTTTACCATGTTGGCCAGGGTGGTCT<br>CGAACTCCTGACCTCAAGTGATCCGCCCACCTCAGACTCCCAAAGT<br>GCTGGGATTACAGGAGTGAGCCACTGTGCCCAGTCGTGTCTGAATG<br>TCTTTGAGAAAGAGACTGCAAATGTGACTGGTGAAAGATTGCGTG<br>TCAGCAAGAGAGTGAGACAGTGTGAAAGGCTTAGGTGCTCTCCCT<br>GACTGGCTCAGCTTATCTCCCAGGCTGTAACTGATTACTTATCTGTC<br>TGTCCCCACTACAATGTCAGCAGCCCCTTGAAGGGTAGAAGTAGG<br>GTCCAGGCCAGGTGAGGTGGCTCACATCTGTAATCCCAGCATTTGA<br>GGAGGCTGACATGGGCAGATAACTTGAGGTCAGGAGTTTGAGACT<br>AGCCTGGTCAATATGGTGAAACCTTGTCTCTACTAAAAATATAAAAA<br>TTAGCCAGGTGTAGTAGTGCACATTTGTAATCCCAGCTACTCAGGA<br>GCCTGAGGCAGGAGAATCACTTGAACCCAGGAGGCAGAGCTTGCA<br>GTGAACTAAGATCACACCACTGCACTCCAGCCTGGACAACAGAGC<br>AAGACACTGTCTCCCAAAGAAAAGAAAAGAAAAAGAGTCCAATT<br>CGGGGCTTTATCCCCAGGATTTAAAACAGTGGATGCCACATAATGA

	ACCTTCAATAAATAATCATTCAGATGAATGAGTGAATGAGTGAATGA<br>CTTTGTGAGGGAGCATCAGCCTCTGTGGAGGGTCAGTGTGAAAGG<br>TTATGAGTGCGGGGAGAGGAGGCAGACAAGGGATATGGGGAACAT<br>ATCCTAGGGACATCAGAGGGTTGCAAATCTGAGGGATTTGCGCATC<br>TCAGGAGACAAGGAGGTTGCAAGACCTGAGCTATGAAAACTTTTA<br>GTTTGTCTTTGGCTCTGGTAAACTCAGGAAATGCCCGGGAAGGTGA<br>ATGAAAGCCATGTGGGGTCCAGCTCCAGCTGGCGGCTCACCCTGTC<br>TCCCCCCACAACCACATTCACAAGGGGAGCCAGCAGAGGGCCAGA<br>GGCAGGGAGCTGAACCCCTCCAGCAGTCCGGGAGGTCTGGCAAG<br>GTACCCTCTGGGCAGGCAGCTCCCAGGGCCAGGAGCCTCATCTGT<br>CCTGGGAGCTCCAGCAAGGCAGGACTCTAGAGAAGGGAACCAGG<br>ACAAATTGTTTTTAAAAGATAACCAATTAAAAAAAAATTTATAATAT<br>ACATATATATGTGTGGGTGTGTCTGTGTGTATGTATATACACACAGAC<br>ACACAGACACACACAGACACACACACACACACACACACACACATA<br>TATATATATATATATATTTTTTTTTTTTTTGGAGACAAAGTCTCACTC<br>TGTCACCCAGTCTGGAGTGCAATGGCACGATCTCGGCTCACTGCAA<br>CCTCCACCTCCCGGGTTCAAGCAATTCTCTGCCTCAGCCTCCCTAGT<br>AGCTGAGATTACAGGCACATGCCACCATGCCTGGCTAATTTTTTTGT<br>ATTTTTAGTAGAGCTGGGGTTTCACCATCTTGCCAGGTTGGTCTTGA<br>ACTCCTGACCTCGTGATCCACCCACCTAAGCCTCCCAAAGTGCTGA<br>GATTACAGGCGTGAGCCACCGTGCCCAGCCTAATATATATTTTTAAT<br>AGAGACGAGATCTCACTATGTTGCCCAGGCTGGCCTCGAACTCCTG<br>AGTTCAAGCGATCCTCCCGATTCAGCCTCCCACAGTGCTGGGATTAT<br>AGGCATGAGCCACCATGCCTGGCCTCAATTATTTTCTGATTTATCTTT<br>GGGAGGAGACCATTCTTTCGCTTAAGAAGAAAAAGAAAAAAACAA<br>TTTTAAGTCTCTTTAATGGGCCTTACAGTTATGCAAAGATATGCCAC<br>TATCCCTTTGGGAGTGAGGGTGGAAAGAACTAGACTTGCCTGCGTA<br>ACAATGAGAAAGGAAGTTGCTTCTTAAGACAGGGTCTACACTAGCT<br>AGAGGGGACAACCAGGACCTGAGCCTCAGACAGGGTCTCGCTCCA<br>TAGCCTGTTGCCCAGGCTACAGTGCAGTGGAGCGATCACGGCTCAC<br>TGCTGCCTCGACTTCCTGGGCTCAAGCAATCCTCTGGCCTCAGCCT<br>CCCAAGTAGCTGGGACCACAGGTGCACCCAGCCCATTTCCCTTCCT<br>CTCAAGGTGCCTTAAGAGAAGGAGAAGGCTTGAGATTCAGGTGAG<br>GGATGGGCAGAGATCAGAGGCTGCCTGGCCTCCTGGTTTGGCCAC<br>AGTCTAGATGCTCAGGAGAGAAGTGCTTACAGGTGAGGCAAACAT<br>GAGCCAGGTGGGCACGGAGCTATCTGGGAGCACCTACTGTATGCC<br>AGACACTTGAACTCTAATGGTGAGGTCTGAGGACACAGTTCCTCTA<br>CTGAAAAATAGAGACAGGGCAACTATGTTGCCAGGGCTGGCCTCA<br>CACTCCTGAATTCAAGCTGTCCTCCTACCTCAGTCTCCTAGTAACTA<br>GAACTACAGGCACCCGTCATTGTGCCCGGCCATTCCACAGCTATTT<br>ACATAATTAACTGTTCAGTTACAATTCTGGCAAGCGTGCTGAAGAA<br>ATAACAGGTCCTAAATAATTACAGCTAGTATTACAAAGCAAGCGCT<br>GCATGCCAGGCTTACACATACTGCTAACATGTCTACTTTTTGCAACA<br>ACCTCTTGTGGGTGAGACTATCATTTGCCTGTTTTACTGAAAGGAC<br>AGTGAAGACTGAGGCCTTGTGTGATGGTGCTGAGAGCTGGGCCTA<br>TGACGTCCAGCCCTGTAGTCTGTGCTCTGAACCACTTCACCCTGTT

	TCTTTCTCCATCCTCACAGTAGAGACGTCATCTAAGAGGAGCTTGC<br>AAGTCCAGAGGGCTTCCTGAAGGAAGTGAGCTGAGAATAATATGA<br>AATTAACCAGACCAAGGGCCATTCCAGGAAGAGAGAATGGTACGT<br>GAAACTGCCCAGGAGTAGCAGGGTGGGAGCCAGGAGGCGTGGGT<br>CATGGCAATCTATGTATAAAGAGCTATGGGAATCTATAGGAAAATTT<br>AAGGGAGTGGCTGACACAGTCAGATTTGTGTTGTAAGTATCACTCC<br>GCAGGTTGCGCAGGGAACAAATGAGGGAGCAGAAGGAAGGGGTT<br>CTCCTATCCGCTGCACGGCACTGCGCAGAAGACAGGGGAGCCAGG<br>CATTCCCTGAAGGGTGAAAAGCAAGGAGTAGAGCTGGGTAGTAGA<br>CTAGAATTTAGGAGCCTGGCCTGGGGCCTGGGTGGGGCGAAAGAG<br>GCGGAGCCTGAATGGGGTGTGTATAGGGGGGTTGCGTGTAGGGGT<br>GTGTGTATAGGCTGGGGCGGGGTCCCGGGAGTGGGCTGACTGGGT<br>CGGGGGCGGGGCTCTCCAGGTGGGCGGGGATCTTGGCCACCCCTG<br>GCCACACCTCTCTCCGGCTCGAGCTGGTCTAGGCGGGGCGGGCCC<br>GAGGGGGTGTGGCAGGAGGTGGGCGGGCCCGGGTGGGGGGGGGG<br>GGGGCGTGGAAGGAGGGGCGGGCCCGAGCAGGAGGGGGCGGGCC<br>CGAGGGGCGGGGTGGGACAGGAGGTGGGCCGCTCGCGGCCACGC<br>CGCCGTCGCGGGTACATTCCTCGCTCCCGGCCGAGGAGCGCTCGG<br>GCTGTCTGCG
G6PC	TGCAATTACAGGTGCCAGCCACCTCACTGGGCTATTTTTTTTTTTTT<br>TTTGAAATAGGGTCTCGTTCTGTCACCCAGGCTGGAGTGTAATGGT<br>GTGATCTCGGCTCGCTGCAACTTCCACCTCCTGGGTTCGAGGGATT<br>CTCTTGCCTCAGCCTCCTGAGTAGCTGGGATTACAGGCGCCCACCA<br>CTACACCTGGCTAATTTTTGTATTTTTAGTAGAGATGGGGTTTCACC<br>ATGTTGGTCAGGCTGGTCTTGAACTCTTGACCTTGGGTGATCCACC<br>CACCTCAGCCTCCCAAAATGCTTGGATTACAGGCGTGAGCCACTGC<br>ACCTGGCCAATTTTTGTATTTTTTGTAGAGATGGGGTTTCACCATGT<br>TGGCTAGGCTGGTCTCAAACTCCTAAGCTCAAGTGATCCACCAACC<br>TCAGCCTCTCAAAGTGCTGGGTTTACAGGTGTGAGCCATCGCACCA<br>GGCCGTGACTATTTTAAATAAAAAACCCTTGAGAGGTTTTTCACC<br>CTTGGGAGCCTGGAAGAGATTTTTCCCCTATCTTTCACCTATAAAG<br>ATAGGACTGGCGGGGGATGGTGGCTTTTGTTTGTCCTCTTTGCACT<br>TTGGGATGCCGAGTCGGTTGTATCACCTGAGGTCAGGAGTTCGAGA<br>CCAGCCTGGCCCAACATGGCAAAACCCCATCTCTACTAAAAATACA<br>AAAATTAATCGGGCCTGGTGGCACATGCCTGTAATCCCAGCTACTT<br>GGGGGGCTGAGGCACAGGAATCGGTGGATCTCAGGAGGCAGAGG<br>TTGCAGTGAGCCAAGATCATCGCATTGCACTCCCAGCCTGGGCAAC<br>AAGAGTGATACTCCGTCTCAAAAAAAAAAAAAAAAAAGGACTGAC<br>ACAAAAGAACAATTGTTCTTCTCCTTCCTTTATCCCATTATCCATCA<br>CAGAAAAGAAGACCAAAAATGTAACCATAAAAAAGTCTCAACAGA<br>CCCTTTTTCAAGACAATGACTGTCTCTGAGGACCATTTAAAATCCA<br>GGGAGAACTATTTACAAGTTAATTTCTGTTCCTCGATCCAATCATTC<br>TCCCTCTGCCCTTCGACAGAATTCCTCTTCTCCTCCTTCCCATAACC<br>TGTTTTTAACAGGATCCAAGCCACCCATTCTTTCTGCAACCTCAAG<br>ATGGTGGATGAGCTTCCATACCTCACTGGGAAGTTGGATCTTCATTC<br>TTAAGCCTCCCATGTATACATGTTAAATACATTTGTAATCTTTTTCTC

	ATATTAATTAATCTGCCTTATGTCAGTGACTTTTCAGCGAACTTGTAG<br>GTAGTCACTCACATAAGGATCCTCCAGTTTACATAGTTTTGTAAATG<br>TCAGGTCACCCTGTCCCACTAGACCACCAAGGTCCTGGAGGACAC<br>CTGGGATTTAACTTTTTTTTTTTTTTTTTTTTTGAGACAGTCTCGCTC<br>TGTCACTGAGGCTGGAGTGCAGTGTCAGGATCTCCACTCACTGCA<br>ACCTCCACCTCCCGGGTTTAAGTGATTCTCCTGCTTCAGCCTCCCG<br>AGTAGCTGAGACTACAGGTGCCCCTCATCATACCCGGCTAATTTTTA<br>TATTTTTAGTAGAGATGGGGTTTTGCTATATTGGCCAGGCTGGTCTT<br>AAACTCCTGACCTCAGGTGATCCACCCTCCTCAGCCTCCCAAAGTG<br>CTGGGATTACAGGCGTGAGCCACCATGCCCAGCCTGGCTTTTAACT<br>TTTAATTCCTCAGAGAGCCAGGCATGGTGCTTTGCTCAGGAAATGT<br>TGGTTGAATTAAACCGGAGCACTTCTTGAAAAGGGAAAATAACAA<br>AGAGTTAGAAGGAGATGGCGGGAACCCCTCTCTGGAGATAGTTCTT<br>TAAATTAGTGGATTCTGCAGGGCACTGTTGCTCATACCTGTAATCCC<br>AGCATTTTGGGAGGCCAAGGCAGGAGGACCACCTGATGCCGGGAG<br>TTTGAGACCAGTCTGGGCAATGTAGAGAGACCCCCATCTCCACAA<br>AAAATAAAAAGTTAGCTGGGTTTGGTGGTGTGCGCCTGTAGGCCCA<br>GCTACCCAGAGACTGATGCAAAACGATCCCTTAAGCCCAGGAGAC<br>TGAGGCTGCAGTGAGCTGTGATGGTGCCACCGCACTCCAGCCTGG<br>GTAACAGAGTGAGACCCCGTCTCAAATAAACAGATAAATGAGTGG<br>ATTCTCAGCAAAACTTCTAGCCACTCGCCTCATATATCCACAAGACC<br>TTTGAGAATCCACGGTGTCTCGATGCAGTCAGCTTTCTAACAAGCT<br>GGGGCCTCACCTGTTTTCCCACGGATAAAAACGTGCTGGAGGAAG<br>CAGAAAGGGGCTGGCAGGTGGAAAGATGAGGACCAGCTCATCGTC<br>TCATGACTATGAGGTTGCTCTGATCCAGAGGGTCCCCCTGCCTGGT<br>GGCCCACCGCCAGGAAGACTCCCACTGTCCCTGGATGCCCAGAGT<br>GGGATGTCAACTCCATCACTTATCAACTCCTTATCCATAGGGGTATT<br>CTTCCTGAGGCGTCTCAGAAAACAGGGCCCTCCCCATATGCTGACC<br>ACATAATAGAACCCCTCCCAACTCAGAGACCCTGGCTGCTAGCTGC<br>CCTGGCATGACCCAGACAGTGGCCTTTGTATATGTTTTTAGACTCAC<br>CTTGACTCACCTCTGACCATAGAAACTCTCATCCCAGAGGTCACTG<br>CAATAGTTACTCCACAACAGAGGCTTATCTGGGTAGAGGGAGGCTC<br>CCTACCTATGGCCCAGCAGCCCTGACAGTGCAGATCACATATACCC<br>CACGCCCCAGCACTGCCTGCCACGCATGGGCTTACTTTACACCCAC<br>CCACAGTCACCAACACATTACCTGCTCTCCAAGGTTAGGCGTGGCA<br>GGAGAAGTTTGCTTGGACCAGCAGAAACCATGCAGTCAAGGACAA<br>CTGGAGTCAGCATGGGCTGGGTGCGAGCCCTTGGTGGGGTGNGGG<br>NNAGGAGACTCCAGGTCATACCTCCTGGAGGATGTTTTAATCATTT<br>CCAGCATGGAATGCTGTCAACTTTTGCCACAGATTCATTAGCTCTG<br>AGTTTCTTTTTTCTGTCCCCAGCTACCCCTTACATGTCAATATGGAC<br>TTAATGATGGGAAATTCAGGCAAGTTTTTAAACATTTTATTCCCCCT<br>GGCTCTTATCCTCAAAAAATGCATGAATTTGGAGGCAGTGGCTCAT<br>GCCTGTAATCCCAATGCTTTGCTAGGTTGAGGCGGGAGGATCACTT<br>GAAGCCAGGAATTTGAGACCAGCCTGGGCCGCATAGTGAGACCCC<br>GTTTCTACAAAAATAAATAAATAAATAATAAATAATAGTGATATGAA<br>GCATGATTAAATAGCCCTATTTTTTAAAATGCATGAGTTCGTTACCTG

	ATTCATTCCCTGGTTCCTTTCACAGTCCTCCGTGACCCAAGTGTTAG<br>GGTTTTGGTCTCTCTACTATTTGTAGGCTGATATATAGTATACACACA<br>CACACACACACACATATACACACACACAGTGTATCTTGAGCTTTCT<br>TTTGTATATCTACACACATATGTATAAGAAAGCTCAAGATATAGAAG<br>CCCTTTTTCAAAAATAACTGAAAGTTTCAAACTCTTTAAGTCTCCA<br>GTTACCATTTTGCTGGTATTCTTATTTGGAACCATACATTCATCATATT<br>GTTGCACAGTAAGACTATACATTCATTATTTTGCTTAAACGTATGAG<br>TTAAAACACTTGGCCAGGCATGGTGGTTCACACCTGTAATCCCAGA<br>GCTTTGGGAAGCCAAGACTGGCAGATCTCTTGAGCTCAGGAATTC<br>AAGACCAGCCTGGGCAACATGGAAAAACCCCATCTCTACAAAAGA<br>TAGAAAAATTAGCCAGGCATGGTGGCGTGTGCCTGTGGTCCCAGCT<br>ACTCAGGAGGCTGAGGTGGGAGGATCACATTAGCCCAGGAGGTTG<br>AGGCTGCAGTGAGCCGTGATTATGCCACTGCACTCCAGCCTGGGA<br>GACAGAGTGAGACCCTGTTTCAAAAAAAAGAGAGAGAAAATTTAA<br>AAAAGAAAACAACACCAAGGGCTGTAACTTTAAGGTCATTAAATG<br>AATTAATCACTGCATTCAAAAACGATTACTTTCTGGCCCTAAGAGA<br>CATGAGGCCAATACCAGGAAGGGGGTTGATCTCCCAAACCAGAGG<br>CAGACCCTAGACTCTAATACAGTTAAGGAAAGACCAGCAAGATGAT<br>AGTCCCCAATACAATAGAAGTTACTATATTTTATTTGTTGTTTTTCTT<br>TTGTTTTGTTTTGTTTTGTTTTGTTTTGTTTTAGAGACTGGGGTCTTG<br>CTCGATTGCCCAGGGTGTAGTGCAGCGGTGGGACAATAGCTCACTG<br>CAGACTCCAACTCCTGGGCTCAAGCAATCCTCCTGCCTCAGCCTCC<br>TGAATAGCTGGGACTACAAGGGTACACCATCACACACACACCAAAAC<br>AATTTTTTAAATTTTTGTGTAGAAACGAGGGTCTTGCTTTGTTGCCC<br>AGGCTGGTCTCCAACTCCTGGCTTCAAGGGATCCTCCCACCTCAGC<br>CTCCCAAATTGCTGGGATTACAGGTGTGAGCCACCACAACCAGCC<br>AGAACTTTACTAATTTTAAAATTAAGAACTTAAAACTTGAATAGCTA<br>GAGCACCAAGATTTTTCTTTGTCCCCAAATAAGTGCAGTTGCAGGC<br>ATAGAAAATCTGACATCTTTGCAAGAATCATCGTGGATGTAGACTCT<br>GTCCTGTGTCTCTGGCCTGGTATCGGGGACCAGGAGGGCAGACCC<br>TTGCACTGCCAAGAAGCATGCCAAAGTTAATCATTGGCCCTGCTGA<br>GTACATGGCCGATCAGGCTGTTTTTGTGTGCCTGTTTTTCTATTTTAC<br>GTAAATCACCCTGAACATGTTTGCATCAACCTACTGGTGATGCACCT<br>TTGATCAATACATTTTAGACAAACGTGGTTTTTGAGTCCAAAGATCA<br>GGGCTGGGTTGACCTGAATACTGGATACAGGGCATATAAAACAGGG<br>GCAAGGCACAGACTCATAGCAGAGCAATCACCACCAAGCCT
GADD45A	ACTAGGGGTTTTTCTCTGAGTAAAAAGAAAAGCCATTGATAACTTT<br>GGGTTAAGGAATTGACTGGGATCTGACTTACTATTTACAGGATCACT<br>TCGGCTGCTGGAGGTGAAGGGAGGAAAGGCAAAAGCAGAGGGAG<br>ACCAGTTGTTACATTTTCCTAGGTGGGAGATGATGCTGGTGGTAGT<br>GGTGATGAGAAATGGTTGAATTATTTTACATTTTGAAATGGAGCCTA<br>TAGGATTTACTGATGGACTGGATGTGGTTGTGAGAAAAAGGAATCA<br>AGAATGACACCAAGGTGTGGGGGGCTGAACAGTGGGAAGAATGG<br>AGTTGCCATTGACCTATTGTAGGCACAGATTTGGCCAGACAATCAG<br>GAGAGCAACAATTGTCATTTTAAGTTTAAGATGCTTACTATATATTGA<br>TGCAGATATGATGAATAAGCGCTGAGGTTCAGAAGAGATGACTGAC

	CAGGTTTGAAATATATTTGGGAGTCATCAGCATATAAATAATTAAGG<br>CCATGGGACCATATGAGGTCACCAAGAGAGTGAGTACAGAGAAGA<br>GGTATAAGCATGAGCTGTTAAGAAGTTAGGAAGAAGAAAACTTTG<br>CAATGAAGTTGATGGGTGAATAGAATTGTTTCTTTTATTTCAGAGAG<br>AGAAAACTCACAAATTGGCTGGGCGCAGTGGCTCACACCTGTAAT<br>CCCAGCACTTTGCGAGGCCAAGGTGGGAGGACTGCTTAAGCCCAG<br>GCGTTTGAGACCAGCCTGGGCAACAAAGCAAGACCCCGTTTCTTT<br>GGAAAATTTTAAAATTAGGCTGGTGTGATGGCATGTGCCTATAGTCC<br>CAGGTACTCAGGAGGCTGAGGTAGGAAGATCGCTTGAGCCTGGGA<br>GTTCAAGACTGCAGTGAGCTATGACAGTGCCACTGCACTCTAGTCT<br>GGATGACAGAGGATGACCCTGTCTCAAAAAAGAAAAAAAAAAAA<br>GCAAACTCACAAATCTGTTCAAGGTGCAAAAACAACCTACCAGTG<br>GCCAATCACCAACTCAGACCACCTACTTCCCTCACCAGTGATTAGG<br>CTACAAAGCAGAGACTCTCAGAACATTCCTCCCATGATAGAAGCCT<br>TCTGATACGCACCGACAGAGAGGTGAGGGGTTAGGGGGTGTTGAT<br>CTTGAAGAATGTTCTAGTTAGTCTCTTCATCTGACTTCTCCACTGGC<br>TGGGCCTTGCTTGGCTGGTGAAATGTGAGCAGCTGATGGCAGCAC<br>AAGGTGGCCAGCCCAAAGCTCAAATTAACACTATGATTACTACCAT<br>GATTCTTAATAGCTGAAGAGAATTTCCACAGGATAAGGATTTATTGA<br>ACACACAAAAAAGATGGCATTAAGAATATTATACTAGTTTTTTGTTG<br>TTGTTTTTGTTTTTGTTTTGAGACGGAGTTTCAATCTTGTCACCCAG<br>GCTGGAGTGCAATGGCACCATCTCGGCTCACTGCAACCTCCACCTC<br>CAGATTCAAGTGATTCTCCTGCCTCAGCCTCCCGAGTAGCTAAGATT<br>ACAGGTGCCTGCCACCGCGCCTGGCTAATTTTTGTATTTTTAGTAGA<br>GCTGGGGTTTCACCATGTTAGCCAGGCTGGTCTCAAACTCCTTAGC<br>CAGGCTGGTCTCAAGCTCCTGACCTCAGGTGATCCACCCGCCTTGG<br>CCTCCCAAAGTGCTGGGATTACAGGTGTGAGCCACCACACCTGGC<br>CCCAACTAATTTTTGTATTTTTAGTAGAGGTGGGGTTTTACCATGTT<br>GGCCAGGCTGGTTTTGAACTCCTGACCTCAGGTGATCCGACCTCCT<br>CAGCCTCTCAAAGTGCTGGGATTACAGGCGTGAGCTACTGCACCTG<br>ACCAGGAACCTTATGATTTTTAACAAGAAAAAATAATACAATACAAT<br>TCTAGGTAGGTGGAAAATACATCCATCATAGTTGAACTGATCTTCTC<br>CTTTGATGTGGACAATGATCTAATACTGAAATAACAAGCATTATATTT<br>CTTTCAGATGTTAAAGCATTGATTGTTTTTTGAAAGAAAGAAAGAA<br>TAATTCTGCATTATGGAACCAGTAGTAGGAATGTATGTAGGATAAGC<br>ATGACTAAAATATCAAAAAGATCTTTAATATGCAAAGCAAGTAGCA<br>GTGCTAAAATAATTGAACAATTCTTGAGTATATATTTCCATATGATTT<br>CTAAATATAATCATTTGGTATCTTTACAAACATTACTACAAGAGCATT<br>CTACTCCTGTGTACTTGGCAGTTGTCAGAGTTACCTGTAGTGGTTTT<br>TAAAATGACATCACATATTGGAGTTATCACACTGATTATAAAGAAAT<br>ATATATATATATATAATATTGATATAGGAAAATTTCTCTGATTTACCATT<br>GACTATCAATGTCCTCCAGGACTAATAAACAGTTAATGATAAATCAA<br>CTATGTCCATTCTAATGTGTAACTACAATCTAACTGTGTAGCTAAATG<br>CAGTTTTTACTTCCAGTCATGAGGCCCAACTTTGAAGATAATCACTG<br>AGAAAACATTCTAGGTATACTCAGCAATCAGGCAAAATGATGTAAC<br>GTATCTGAGTCAGAAAGTATGACAAATGAATATTTTTGCATACTGGA

	AGCCCAAGCTTATCTCAGAGAAAATCCCCAAAATACTTGGGCAAAT<br>AACTTTCTGATGAAGTCAAATATAGCATGCAGTATAATTTTTGAGTG<br>GAGGACTAAGCCTATAATAAGAAGTAACCATTTGATATCACATTTTT<br>ACTGGATATAGTCGACTTGGGTGGGGCACTTTAGGACTGTGGTTCA<br>TTTGAATTGGTGTAAACAATACACCGGTTCTACTGTCCTACAGCCTC<br>CATTCAGATGACTGAAGTCATGGGACTTTCAGCATAGCTAGCTGAT<br>GACAGTGCATACTATTTTGTCCCAAAATCCAGTTCAAGCATGGACAT<br>ACCAATAAGAGCCTAAGCTCTTTAAAGGCAAAGGACCAGGAATTGT<br>ACAGTTCTTGGTATAGAAGAAGACAGGCAAAAGTGTTTTTGAACTA<br>ACGTTAAATGTGCAATATGTTAGAATTCATGCAATGCACAGGACTGC<br>AGGATTCTGATATCTTATTTAACTCTCAAATTCTATTCAACTCAATAA<br>ACCTTGACTGTGCTTCTACTAAATGCAGGTATTGTACTAGGAGCTGA<br>GGACACCAAACTGATGAAGTCCTTGCTGTCAAGAAACTCACATGAT<br>TCCCTAATTCTTTGTCAGCTTGCTGTGATCACATTTTCTTCCCAAGA<br>ACCTCTAAGAAATGCCTAGTGGATAGAACCTTGGAGTTCCACGGAA<br>CATATTAACAATCGCCAAATGATGACTCAGGCTAGATTGTGTAATTC<br>AGGTTTTGTCTGCAAAACTGAAAATGCTTCGGTAACCTACCTAAAT<br>TTCAATGTTGAGGAATTCTTTAAGAAAGACATCAAATGTTAAGATTT<br>AAGGCATAGATATGAGATACATAGTCATGCTTAGGTGAATTATGCAC<br>TGACCATGACCATTTCTTTACTCAAATGTTGTCCATGGCTGACAACA<br>CAGTGAAAAAATGAGTGCAAAATGACAACTCAAATAAATGAACCA<br>GAAAACCTATCACTTTTCTTTTCCACCAAATTAAGATCAAGAGAGC<br>TGGAGAATATTTTGTCTAGAGTGATAAAAACATAAGGGTGCAAAAC<br>TTCCAGGTACCTTTGCAGAAATTACTTCTGTGACCTTTGGCTGTACA<br>GCAACCTTAATAATGCAAGCACTGTTTTGAATGCAAGCATGTGGGA<br>GCCATTTTCACCACTTTTGATGACTTCAGTAGGTTTAAGAAATGTTT<br>TTGCTTTTATTGCATAAACCATAAAACAAAGGAAGGGACTTTTGAA<br>CTACTCAGTGAGAGTCTATATATTAAAGTTTGTTTTTCAAAAATGTG<br>TAACTACCATTTGCAGTTTTAAAGGTCTGCTTTCCACCTACAAGTTG<br>CCATTATCTCAAAGGTGAAATTTTAGCATATGACTAAAAACTTCCTA<br>TAGTTACAGCTTCATGATTCAGCATCTAACATCAATAATTCACAGTG<br>AGATCATAGGAGGCTCTCTGTGGAAGGTAACGACATACATACGTTA<br>GGAAAGGAAGCTTAGGGCATATCGAGAGCATTTTGAATTTAGACTT<br>GTGGGCTGTGTGGGTGTCAGATGGTTGTCTCTCAGCTGGTGGGCGT<br>CCAGAAGGATCCTTGTTTGGGCAAGGCTCTTTGAGAAAGGAGAAT<br>CTGGGTTGCCAGGGATTCCCACATGTGGTCACCAGCTCCCCACGCA<br>GACCAGCTCACGATTTCCCAGTTACACCGGGCAGGTGGGAAACCG<br>TTCTGCTTTCTGTGGAAAAGATTCTAACTTGGTTCCCTGCCATCCCT<br>GAATACAAACGGGTTGGTTTTTCTTTTTTCAGCTTCCAACCCTTGCA<br>GCTTTCCAAAAATAAATCAAACCAGCCATCAGGGCACCGAAATAAT<br>ACTACTGCTAATAAGCAGCTTCGCCTAGACTTAGATAAACAACACT<br>TCTGAGGTAAACTTTGCCCCGGAGGTCTGGAGACACTTTTTTAATG<br>TAACCTGCTTACTAATAATTACTAGACTTCAGTGCATTAACCCTGGA<br>AATAGATTTTAATAGCCACCCCTTAAAACAAAAGACATGAAAAGAT<br>AATAAGAAAAAAGTGCCGCAACTATTATAGAAAAACACTTGGCAG<br>CCTGCTTCAGCCCAAGCTGAGGCCACCTCTAGCCTCTGCTAAAGCC

	CCCCACTCCCAATGGTCCCCGCCAACCGGATAAGAGTGCGCGCGG<br>GACCCGCCTTCCCCTCTCGGCACCGCCCCCGCCCCCGCCCCCTCGG<br>CTCGCCTCCCGCGTGGCTCCTCCCTTTTCCGCTCCTCTCAACCTGAC<br>TCCAGGAGCTGGGGTCAAATTGCTGGAGCAGGCTGATTTGCATAGC<br>CCAATGGCCAAGCTGCATGCAAATGAGGCGGAAGGTGGTTGGCTG<br>AGGGTTGGCAGGATAACCCCGGAGAGCGGGGCCCTTTGTCCTCCA<br>GTGGCTGGTAGGCAGTGGCTGGG
GFAP	CTGATTTTATTCTTACAACTACCTGGGGAGCAGGCATGGTTGAATCC<br>CCATTTTACAGACGTGGAAATGAAGACTCAGGCAAGGTTCTTAAAG<br>TCATCAAAGACTGCACAGCTACTGAGTGGCCAAGGCTTTGCTCCCT<br>CTCCTTGAGGACAGTGCTATAGCAGTCAGAGTCAAGTCTAGAGAA<br>CAAAGCGGGTGATGCCCCTGGGTGAAACAAATTTGGGTTTTAGAA<br>AACTCCGCCTACAGCTTTCTCCCTGCCTCTAAACTGCGGTGGCTGT<br>GGCGGCTCACACCTGACCAGCTGAGCACCACCATCTAGTGGCCAC<br>CAGGCTCAGTTCCCTTCCTCGGGACAGGCCATGGATGAACAGACTT<br>GCTGGTCACCAAGGCTCAGCCAGAGAGACTGTGTTCTCTTCACCC<br>AGCTTGCTGACCACCACGCTGTGCCAGGCACTGGGCCAGGGCCAA<br>GGGCCCAAGCAGAACCAGCGGGCAGCAGTCATGTGGCAAAACTG<br>CCGTTTGAATTAGAGGCTGGCCTATTTGGCAAACACCAGGCACCCT<br>GTGTGTGGGTTGATGCCACATGTGGGGCTGGTCTGTCCCAGGAGGC<br>ATCTGGGCTTTACCCTGTAAGCAGAAGGTCATCTAGCAGATTCTGAT<br>GTGAGGGAGGGGCTCAGGAAGGTCATCTTGTCCTCACCTCAGCTT<br>GAAGCAGGCACAGCTTGGCTGTAGGCTCCTTGAAAGGGCATCCAG<br>ACTTCTCTCTCCATGGCTGGTATTATCCTGCTGTGCAGTCTAGAAGG<br>CAGTCTAGGGTTTGGCCAGAGGCTTTAGAAGTTGGGGCCTTCCATT<br>TGGGGAAGCAGGAGAGGAGGCACCCCAGAGAATGGGGCCTGAGC<br>CAGGCTAGGAGGATGGGGGGTTTGGGTGGAGGCTGGACGGGCTGA<br>GGGCCTACCCTTCTCTGTTTGCTGTGTCTGCCTGCAGCAGATGCAA<br>ATCAGAGTGTGGTTTAGAACAGAGAGAGGACAGTCACAGCGGCTC<br>ACATCTGTAATCCTAGCACTTTGGGAGGCTGAGGCCAGAGGATCAC<br>TTGAGGCCAGGAGTTCAAGACCAGCCTGGGCAACATAACAAGACT<br>TGGTCTCTGAAAAAAAAAATTATCTGGGCACACTGTCATGTGCCTG<br>TAGTCCCAGCTCCTTGGCAGGCTGTGGCAGGAGGATTGCTTGAGCT<br>CAGAAGTCTGAGGCTGCAGTGGGCTATGATGGTGCCACTGCACTCT<br>GGCCCTGGGTGGCAAAGTGAGACTTTGTCTCTAAAAAAAAAAAAA<br>GAGAGAGAGAGAACATACTGTCATACTGTCCACCACTAGGGCTTG<br>GTACATGAATGCATATAGTGGAAAATCATGCAGCTGTTAAATGATGA<br>TGCAGACAAATATTTATTGATGTGGAAAGATGTTCACAACATATTGT<br>TGAGTGAGAAAAGCAAATTACAAAATAATTTGTACGGCATGAGCCC<br>ATTTTGAAAAAAATGATTATATATGTGTGGAAAAAATCTGGTAGTGT<br>TTATCTCCAGGTGGCAAAATGATGGCTAATTACATTTTTTTGTATTGC<br>TTATCTATAGTTTCTGACCTTTCTACAATGAATATTTAATTAAAAAGA<br>TAATGAAAGTTTGGAACACAACCACGACCAAATCTGTCTGTATCTT<br>TGGTTCTCTGTGCAGACACCTTTAGATAGTTAAGGGTTGGCCAGGC<br>GTGATCTATATGTACCGGTTGTATAGGTCATGCCTGTAATCCCAGCA<br>CTTTGGGAGGAAGAGGTGGGAGGGTCACTTGAGCCCAACAGTTCG

	AGACCAGCCTGGGCAACATGGCGAAACCTTGTCTCTAGTAAAATAC<br>AAAAATGAGCTGGGCATGGTGGTGTATCTCTGTGGTCCCAGCTACT<br>AGAGAGGCTGAGGTGGGTGGATCGCTTGAGCCAGGGAGATTGAGG<br>CTGTAGTGAGCTGTGATAGCACCACTTCACTCTAGCCTGGGCAACA<br>GAGCAAGACCCTGTCTCAAAAAACAAAAACAAGATAGTTAAGGGT<br>TGAGTGTGAGGAAAGGTCTGAGGGTTGAGAAGGGGTGGAGGATG<br>CACCTGGGCCTATGACAGGGGTCCACGGAGGTGGCTGATGGCAAA<br>AGCTGGGGGACTCCAACTGCTGATGCTGAAACAAGCTTGTGTCTC<br>ACATACACAGGGACAGTTCACTGAGCTTCAATGACAGGCACCTCCT<br>GCTCATCACATCTTTTCTCTCTAGGACAGCTTTGCCCTTATTTTAACT<br>AGACTTCCCTTGAACCAAAAGGGAAGGCTACATGCTGTGACTTGC<br>TGGGCAGCCTGGAAAGGCGGGCCACTCCTAGCCACAGAGATGAGA<br>CAGAGTTCAGACAAGAGCTTATCCCCAGTCTTCCTTTTCTATTTTGT<br>TTATTTTATTTTATTTTTTTATTTATTGAGACAGAGTCTCTGTCACCCN<br>AGGCTNGGGGTGCAGTGATGCGACATTGGCTTACTGCAGTCTCCAC<br>CTCCTGGGCTCAGGTGATCCTCCCACCTCAGCCTCCCGAATAGCTG<br>GGATCACAGTAGTGCACCACCATACCTGGCTAATTTTTTTGTATTTT<br>TTGTACAGACAAAATTTCACCACATTNGNNCCCAGGCTGGTCTCGA<br>ACTCCTGGACTCAAGCGATCCGCCCACCTCAGCCTCCCAAAGTGCT<br>CGGATTACAGGCATGAGCCACTATGCCCAGCCTTGCTCTTCCTTTAA<br>AGCCTCCTGTCCTTCCCCAGGTCCCCAGTTCATAGCAGGATCAAAG<br>GTCACTGGGCGCTCACCCCGTCTTCAAGATGCTCTTTCCTATGTCAC<br>TGCTTACGCCCAGGTCAGATGTGACTAGAGCCTAAGGAGCTCCCAC<br>CTCCCTCTCTGTGCTGGGACTCACAGAGGGAGACCTCAGGAGGCA<br>GTCTGTCCATCACATGTCCAAATGCAGAGCATACCCTGGGCTGGGC<br>GCAGTGGCGCACAACTGTAATTCCAGCACTTTGGGAGGCTGATGTG<br>GAAGGATCACTTGAGCCCAGAAGTTCTAGACCAGCCTGGGCAACA<br>TGGCAAGACCCTATCTCTACAAAAAAAGTTAAAAAATCAGCCACGT<br>GTGGTGACACACACCTGTAGTCCCAGCTATTCAGGAGGCTGAGGT<br>GAGGGGATCACTTAAGGCTGGGAGGTTGAGGCTGCAGTGAGTCGT<br>GGTTGCGCCACTGCACTCCAGCCTGGGCAACAGTGAGACCCTGTC<br>TCAAAAGACAAAAAAAAAAAAAAAAAAAAAAAGAACATATCCTG<br>GTGTGGAGTAGGGGACGCTGCTCTGACAGAGGCTCGGGGGCCTGA<br>GCTGGCTCTGTGAGCTGGGGAGGAGGCAGACAGCCAGGCCTTGTC<br>TGCAAGCAGACCTGGCAGCATTGGGCTGGCCGCCCCCCAGGGCCT<br>CCTCTTCATGCCCAGTGAATGACTCACCTTGGCACAGACACAATGT<br>TCGGGGTGGGCACAGTGCCTGCTTCCCGCCGCACCCCAGCCCCCC<br>TCAAATGCCTTCCGAGAAGCCCATTGAGCAGGGGGCTTGCATTGCA<br>CCCCAGCCTGACAGCCTGGCATCTTGGGATAAAAGCAGCACAGCC<br>CCCTAGGGGCTGCCCTTGCTGTGTGGCGCCACCGGCGGTGGAGAA<br>CAAGGCTCTATTCAGCCTGTGCCCAGGAAAGGGGATCAGGGGATG<br>CCCAGGCATGGACAGTGGGTGGCAGGGGGGGAGNAGGAGGGCTG<br>TCTGCTTCCCAGAAGTCCAAGGACACAAATGGGTGAGGGGACTGG<br>GCAGGGTTCTGACCCTGTGGGACCAGAGTGGAGGGCGTAGATGGA<br>CCTGAAGTCTCCAGGGACAACAGGGCCCAGGTCTCAGGCTCCTAG<br>TTGGGCCCAGTGGCTCCAGCGTTTCCAAACCCATCCATCCCCAGAG

	GTTCTTCCCATCTCTCCAGGCTGATGTGTGGGAACTCGAGGAAATA<br>AATCTCCAGTGGGAGACGGAGGGGTGGCCAGGGAAACGGGGCGC<br>TGCAGGAATAAAGACGAGCCAGCACAGCCAGCTCATGTGTAACGG<br>CTTTGTGGAGCTGTCAAGGCCTGGTCTCTGGGAGAGAGGCACAGG<br>GAGGCCAGACAAGGAAGGGGTGACCTGGAGGGACAGATCCAGGG<br>GCTAAAGTCCTGATAAGGCAAGAGAGTGCCGGCCCCCTCTTGCCCT<br>ATCAGGACCTCCACTGCCACATAGAGGCCATGATTGACCCTTAGAC<br>AAAGGGCTGGTGTCCAATCCCAGCCCCCAGCCCCAGAACTCCAGG<br>GAATGAATGGGCAGAGAGCAGGAATGTGGGACATCTGTGTTCAAG<br>GGAAGGACTCCAGGAGTCTGCTGGGAATGAGGCCTAGTAGGAAAT<br>GAGGTGGCCCTTGAGGGTACAGAACAGGTTCATTCTTCGCCAAATT<br>CCCAGCACCTTGCAGGCACTTACAGCTGAGTGAGATAATGCCTGGG<br>TTATGAAATCAAAAAGTTGGAAAGCAGGTCAGAGGTCATCTGGTAC<br>AGCCCTTCCTTCCCTTTTTTTTTTTTTTTTTGTGAGACAAGGTCTCT<br>CTCTGTTGCCCAGGCTGGAGTGGCGCAAACACAGCTCACTGCAGC<br>CTCAACCTACTGGGCTCAAGCAATCCTCCAGCCTCAGCCTCCCAAA<br>GTGCTGGGATTACAAGCATGAGCCACCCCACTCAGCCCTTTCCTTC<br>CTTTTTAATTGATGCATAATAATTGTAAGTATTCATCATGGTCCAACC<br>AACCCTTTCTTGACCCACCTTCCTAGAGAGAGGGTCCTCTTGCTTC<br>AGCGGTCAGGGCCCCAGACCCATGGTCTGGCTCCAGGTACCACCT<br>GCCTCATGCAGGAGTTGGCGTGCCCAGGAAGCTCTGCCTCTGGGC<br>ACAGTGACCTCAGTGGGGTGAGGGGAGCTCTCCCCATAGCTGGGC<br>TGCGGCCCAACCCCACCCCCTCAGGCTATGCCAGGGGGTGTTGCC<br>AGGGGCACCCGGGCATCGCCAGTCTAGCCCACTCCTTCATAAAGCC<br>CTCGCATCCCAGGAGCGAGCAGAGCCAGAGCAGGATGGAGAGGA<br>G
GFI1B	TTTCTGCTGGGGCCCCTGGGCCTGACCTGGGCTCATTTTCCCCCGA<br>CTCCTCCACGGACAAGTCTTGTTTTCCCCTTGAATCTTTTTTTTTTTT<br>GACAGAGTCTCGCTGTGTCACCCAGGCTGGAGTGCAGTGGCGCCA<br>TCTGGGCTCACCGCAACCTCCGCCTTCTGGGTTCAAGTGATTCTCC<br>AGCCTCAGCCTCCCGAGTAGCTGGGATTACAGGTGCCTGCCACCAC<br>ACCCAGCTAAGTTTTGTATTTTTAGTAGAGACGGGGTTTCCCCATGT<br>TGGCCAGGCTGGTCTTGAACTCCTGACTTCAGGTGATCCGCCTGCC<br>TTGGCCTCCCAAAGTGCTGGGATTACAGGCGTGAGCCACCACGCC<br>CGGCCTCCCTTGAGTCTTTAAACATAAAGGAAGTCAAAAAGGTTCT<br>CTGTAGCTGACCCTGAGAACCAGCTGAGTCTTACCCAACCCTCCCA<br>AAAGGGGAACCCGAGTGACAAGTCTTTTAGTCAAGGTGGTTCAAT<br>CTCTCACTGAAGGTCAACGAATTCTACATGGGATTTTAGAGCAAAA<br>GGGAAGTTTAGAAATCTCTTAGCCCAACCGTCCCTTTTAAGAGATA<br>GGAAAACCAAGCCCCAGGTTGGTGGCTGATGCGGTCACAAGCTCC<br>AGGACCCCAAATCAGACTCAACTCCTTGGGCAACAAAGGGGCCTC<br>ATTACAGGGACATCACAGGCCTGTTGACCCAAGTCCAACTGGAAG<br>TTAGGGTGCTGGCTCCTGCCAGGGTGCAGGGTCGCCTGTCTTCAAG<br>AAGCACTGACTGAGATTCTGACTCAGGGTCAGGGGGACTCTATCA<br>GGATCGGGGGTTATGGAGTGATAACAGTGAGCCTCTTTCCGGAGGT<br>CCGATACCATCAAGTCCTCTGTTTTATGAAGAATTCCTGAGCTTCTT

	TCATAATCTCTGCTCTTAGCTCCTTCTCAGAACGGAGTTCATGTCAA<br>TCAAAACCAAGTGGTTGTTAATTAACTACAGTATAGGTGATTGGTTC<br>CTGTTTTTCTTTAAAAAAAAAACAACTCTGCTAACAGGGGCCAAA<br>AGGAAGTTCACCGGGTGCATTTGGCCCCTCTGTGAGCCAGAGGGC<br>TTGGCCGTGGCGTTGGCTGTGTCACTGTCACTTGCCAAGCTCAGGG<br>GAGAGAACACTGGGGGGAGGCAAGAAAGCGGGTGTGGGGCTCAG<br>GGGAGATCACTAGAAGAAGCATGAGAATCACCTTCCTGAGGCTGG<br>AGAACCCCAGAAGCCAGCCCCATCTTGCCCTGTGCCTAAGTGAAA<br>AGTGCTTGGCCAGGGCTCATGAGCGCTAGAAAGTGTCCTTGTTGTG<br>GGGCCCAGGGTTAGAGTCAGTTGACCTGGGTTCTCCTTAGGCCCTG<br>AGCCCAGGAGAATACCAATGCTTCCTGAAGGGAAGTCTGTCCCTG<br>GCCTGGCCCCACCAAGACTCTTCAGGAGCCATGTGGGTCTTTTAGG<br>AGCCTGGAAAAGACTTGAGGGTCAGAGGGTTTGATTTTCTCCCTC<br>CTTTTTTTTTTTTTTTTTTTTTGAGACAGAGTGTGGCTCTGTCACCC<br>AGGCTGAAGTGCAGTGGTGGTGTGATCTCGGCTCACTGCAACCTC<br>CGCCTCCCGGGTTCAAGCGATTCTCCTGCCTCAGCCTCCTGAGTAG<br>CTGGAACTGTAGGCAAGTGCCACCATGCCCGGCTAATTTTTGTATTT<br>TTTGTAGAGACGAGGTTTCACTGTGTTGGCTAGGCTGGTCTCGAAC<br>TCCTGATCTTGTGATCCGTCCGCCTCGGCCTCCCAAAATGTTGGGAT<br>TACAGGTGTGAGCCACTGCACCCGGCCTGATTTTTCTCCCCTCTTG<br>CCCCAGCACCCTGCATTTCCCTCTGTGCTGCAGGCGGTGTTCACCG<br>GGTGCTGTGAGGACCTCTGACAGTGCTGGGTGGCACAGTGGCTTG<br>AGAGTAAAATCCTAACAGGCCAGGAGCCCTGGGTCTGAGTCACTT<br>TCCTCTCTGCACCTCAGTTTTCCCACCTGCAACCTGGGAAGACACT<br>GCTTGACACGGGCACACGAGGGAGCTTCAAACTGCTGTTTTCATCC<br>CTAGCACGTCGGCTCCACTCCCTCTGCAGAGAGAAGCCCATGCCG<br>GGCACTGCCCAGCACTTGGCACCCATTCTGCACCGAGTAACTGCCC<br>ACCTGCTGGGCTGAAAGTCAAGAGCATGGAGGCCTGGGGCCAGAC<br>AGACCCCAGTCCTTCCCTGCTCACTCTGCCCCAGGGTCCTGAGCCC<br>TCCAGGCTTCAGCTCCTTCTTTATAAAATGGGACAGCAGCGGGACT<br>CAGCATCTGGATTAAGCATCAGAATTAAAGGTGTAATAAACAAGGC<br>AGGCTAAGCAGCCCCCTGCCAGAAGGTTTGACCAGCTCCTCTTCTA<br>TCACAGAAGCCTTGGCGAGTACCTTTGGTTCTAGTTGATTTCCTCAT<br>AAATATTATGCGCAAAAAAGGATCATCTTTTGATATTTTGATAGGCA<br>AACCATGACCCTCACAGGCCCCCTCAAAACAAAGTGCAAGGGGAA<br>CCCCACACCCCCAGTCCTCCTCCTGCTCACTGGGTCCCCTCCTCTC<br>CCCCTAGGGTCTGGCCTCCCCATGGGCTTTCTTGAAGCGAAGAAGA<br>GTTAAAAGCGGGAAGGAAGGGGGGTGTGGGGAGCCCTGGTGAGG<br>GAGGGGGAGGAGGCAAGAGGAGGAAGACGTGTCCCTGGGAGATA<br>AGGCCGTGGGCTGCCCCCGGCCCGAGCCTGTGTTCAGAGAGCCGA<br>GAATCTGACCGCGGGAAGGGACCCCAGAGGCCTTCAAGGTCCTTT<br>CTCCTAATTTTAGAGCTCAGAGAGTACAGGGCCTTGAGCGCGGCCC<br>TGGCTTCTGGGAGGCTGCTGCAGCCCTTGGCACAGGGCTTACCCC<br>GTCCTTTGAATGCACCTGCAGTGGGCTGGGTGCTGGGATGCGGGG<br>GAGCACATGCTGAGCACCTCTCACACTCGCTCCTGATGGATAAAGG<br>TCCTTTCTCGCTCCCCACCATCCCATGTGGTTGGATTGGGATCCCCG

	TTTAGCAGATATGAGCACTGAGGCTCAGTGATGAGCCTTACAGCAG GCAAGGGCTGGGCCTGAGCTTGGCCCCCTGTGCAGAGCCCCCAGT CCAAACTCTTGATGCCCCCAGGCTCTGGGCCAGGGGGAAGCAGGC CTGGGCATAGTCGGGTCCCTCTTCTGCCTGGCTGTGGTTCATCTCCT TGGGGAAGGGGTGAGTCCCTGGCCCTTTTCCAAGCTGCATTCTCTA ATCTCCCACCTCCCACCCTGCTCCTGGGCCAAGGCCTTATCACCCA GGCCCTCACTGCTGTTCTGAAGCAGCTGTGATCCCTCTCCCGCCTT CTCCGGACCCTATCACCCCTGCAGCCTTGCATGGCGGCTGGCCCAA GGGGTGGGGGGGTGGGACTCCTCTGACGCCTCAGGCATGGGCCTG GTGTCGGCCTGGGTGCAGCGTGGGGGCAGAAAGGATAGAGGACC ACCAGAAGATGCTCTCCAGAGTGCCCGGGGGAACCTCACAGGCTG AGTGCAGGGATGTGAACCATTCTGGGGGCCAGGCAGGGCTCTCAG CTCCAGAGGGCTCTGCAGGGGAAGCCAGGAGTCCTGGGGTGGTCA AGTTGGTCCAGCCCTGTCACTCCCTGACTCTGCCTGGGATGGTCCC TTGACTGATGTGGTCCTTGGTTTCTTCTCCCACTCCCTGACTGGTGG GGTTGGTAGTGATGCTGGGCTGCCCACGGAAGCTGGGGGCTGTTC GCCTCTTCTCCTGTGTCCATGAAGCCTCAGGCCTCCAGGTGCAGTG GAGTTGGGGGTCCTCTCAGGTCTTGAGTCCCCACACCCCACTCCTC AGAGAGAGAAAGCCCCTGTCTCTCTAGGGAACAGACTCTGGGCCA GGGCTGTGGACAAGGCGCAGCCGTGCTCTGGCGGCTGTGGCTTGC GGCTTGCAGGAGCACGTGGATTGGCAATGTCCCCTCGTTTGCTGCA GCTTCTCTGCCAGAGCCCCCAGTGATAGACACTGAACACCTGTGAT GCACCTGTCAGCACTGCAAGCCTGGACATCCCCACTTACAGCAAA GGGGATAGGCCCAGAGAGGTTAAGTGTGTGTCCCAAATTGCACAG CAAGTGAACTGAGATTCTAACCCACGAACCACTGATTCCAAAACCT AAGCTCAGACCACCCGTTTCTATGGCCTCTGAAGGTGAATCTCACT GGTACCCAAATGTAGCTTTCTAGAATTGGGGTCCACTGTGTGTCAC AGAGGGAGACCCCAGTCCCTGCCTTTCCTTTTCTGCTCCATTGCAG CCCAGGTCATGCCAATGGGAGCAGGCTTGTCTGAGCGTTTTCTAGG GGGCGATGCATTCATTTCCTGGTGAAGGAGCAGTGAGGGAAGTTA GGATTTGTAGCTGCCGTAACAAAATAGCACAAACAGGTGGCTTAAA ACCAACAGAAATTTATTCTTTCATGGTTCTGGGATCTAGAAATCCAA AATCGAGGTGTCCGCAAAGTGGGTTCTTTCTGCAGACGCCAGGGG AGAGGGTTTCATGCCCCTCTCTGAGTTCTGGCGGCTGTCGGCACTG CCTGGCATTCTCTGGCTGATAGAAGCTCCGAGTTCTGGCGGCTGTC GGCACTGCCTGGCATTCTCTGGCTGATAGAAGTGTCACTCTCTGCT CTGTTGTCGGCACACGGCGTTCTCCCTGGGTGTCTGTGTCTCTGGG TCCAAATTTCCATCTTCTTATAAGGACACGAGTCATCTTGGATTGAG GGCCCATCCTAACCCGGAATGACCTCATCATAACTTGATTACATCTG CAAAAACACCCTATTGCTAATAAGGTCACATTCACAGGTTCTGGGT GGACATGAACTTTGGGGGCCACTGTTCAACCCAGTATAGCTGGTGT TTTATCTCAGGAGGAGGTTCTGAGATATGGAGGGGTATTGTTCTCTT GTCCTTCCTAACCGCTCCCATCCCTCCCCCTTGCAGGTGTGGGGTG CACACTGAAAATGCCACGCTCCTTCCTGGTGAAGA
GJA1	TATTTATCCACTGGCTGGAATTCTTTCCCACTCAACAAAGCAAAAC AAATTCTTCCCTTTACAAATACCTTTTTGTGTAGTTTGGTTTACATAA

	GATTCGCTTGGAGGTAAATGTGGGCTACAAAAATGAGTTGAAATTT<br>TACACAGCTCAAATCAATCTTTGATCCTTAAACATTTTTCAAGGATG<br>ATAGAATGCTAAGTAAATGGGAAAGACGTTCTCAGTTAAGTTGAAT<br>ACTTCAGGGTTATGAAGCGAATTGACGGACCCCAGAAACTTATCTT<br>TTGAAAAAGGGATACCAGCACAAGTGGTAATACGATGAGAAATCTT<br>CACTGCTCTGAAAGATGTAATTGACCAACAGTCACATAAAGAACAC<br>ATTTTCTGGCAAGTTTTACCAATTTGGGTAGAGCACTTCTAAACTTG<br>CAAACTTATGACTTCCAAATTAACTTTTACTTCTTTCTTGCTGTCTG<br>GTAAACGTTAGTTAAAATGGAAATTTGGTGCATAGAAAGGATTATTC<br>TGATGCGAAGGAAAGGAAATCTGTAAGATAATGCCATCTTATCTCAT<br>TACAAAATCAACCTACACCCGCCTAGTCTCAGATAGTCTTTCTATTC<br>TTCTACAAATGCCTTGAATTGCCCCAGAGGGATTGCAAATTTTCTGA<br>TTCTTCTTCACTTACTTTCATCTCAGCCAGCTGATAACCTTAGCTACT<br>TTGAGAAAATACCTTCAAACCCTAGTTTCATGCGGCCTATACTGTCT<br>GAAAACATTTTCTTTATGTTGCACTCATTCTCTTTTTAACGGAGTAG<br>CTGTCCTTTCAAAGTTAACTACTCCTCCAGTGCACAAGTAATATTTT<br>CCCACAAATAAGCCCACTTGTAGCTATTTTAAATACCTTTCCCTTTC<br>CCAGATGTTACCTTGCCTCTTTTTTCATTCTTTTTGTCTGCATTAAAT<br>GAGGAAATCCACCAAGGCTGTGTGTGTGCTTTTTGGTACACTGCCT<br>ATATCCCATCTCTTAGTAATTTCATCTGTACCTGTGGCTTCTAATTAC<br>AAATCTTTTTTTTTTCTTTCTTTTTTTTTTCAGATCCTCTCACCTCAG<br>CCTCCTGAGTAGTTGGGACTACAGAGGCCTGCCACTATGTCTAAAT<br>AACCTTTTTATTTGTTGTAGACACAGGGTCTCCCTATGTTGCCCAGG<br>GTGGTCTTGAACTCTTGGGTTCAAGTTGTCATCCCACCTCAGCCTC<br>CCAAGTGCTGGGATTACAGAGTTGAACCACTGTGTCTTCCCAATCA<br>CAAATCTTATAACAATTTTTTTTTTTTTTGAGACTGAGTCTCACTCTG<br>TTGCCCAGTTGTAGTGCATTGGTGTTATCTTGGCTCACTGCAACCTC<br>CACCTTTCAGGTTCAGGCAACTCTCGTGCCTCAGCCTCCCGGGAGG<br>CTGGGACTACAGGTGTGTGCCACCACGCCCAGCTAATTTTTGTATTT<br>TTAGTAGAGACGGGATTTCTCCATGTTGGTCAGGCTGGTCTCGAAC<br>ACCTGACCTCAGGTGATCTGCCCGCCTTGGCCACCCAAAGTGCTGG<br>GATTACAAGCATGAGCCACTGTGCCCCGCTGTAACAATTTTAAAGT<br>GAACGCCACAGCTCCTGTTTTAAATTGTCAGTCATCGGTCTTTACCT<br>AGATATTTCAGTTCTTCATACCAAAATATTTCAAACAAATCTTTGTAT<br>CTCTTTCAGCAGGTCAGTCCCTCCCCGAGCTTCCTTTTTTTGTGTTA<br>GTAACATTTTCCCAATCAAAACTCCAAAAATCGGTTTCCACAGCCT<br>CCTCTTCCTTGTCTTCCCATTCATCCATCATCAAGTTCTGGTAATTCT<br>ACCTTCCTTTCCTTTCTTTCTGCTAGTATTGCCTTTCAAGTTCCTATA<br>GCTTACTGCTCTCCCATTACAATACTCTTCTTACAAGTCTAATCCGG<br>AGCCACCAATTATTTAACGTTTATGCTACTCAATGAGGTGAAAGTAA<br>TCCATCAGAGTTTATGTGGGATAGGCATTTAAAGGCCTTAAAAATCA<br>CTCCATTCATTTCTTAAAATTTCAGAAATTCCTATTTTGAAACAAAG<br>GTAAAAATCCAAAAAAAGGAGGTGGTTCTCATATTAAGAAATATTTC<br>TCACAATTCTGAAAAAAACTTACCCTCCATTGTGCCAACAATGTGC<br>ATTTTGCAAAATATATATAGCTACATGGAGAAAACAGAATCAAAGG<br>CCTGGCAGGTCATTTGGCCTAAAGAAGCCCTGCAGAAGACAGAAA

	CTGTAAATAACTGTTTCTAGTTCTCCAAAGAGCAGTGTTAATCAGG<br>CTGCATCCATTTCTTCTACATAGACAAGGTCTGGCAATCAGAGAAG<br>ACACAGTTCTAGGAAGTCAGTGTTTATATTAAAGAAATGGCATTGA<br>CATTTCTGAGAATATGAGAAAAACACATTTAAAAAAAAAAAACTAT<br>TCCACAATTTTTGCAAAACATTCCAATGGAGACAAGCTTCTAAGGT<br>CAGATAAGTGAGATACTTTTGAACAATTTTGCTATTGATAAAAATAT<br>AACTCACCAACACTGCTAGAGTATGAGTTATAATAGAAGTTGCTAC<br>CTGTCATTTAGAGACCCTGTTAGTTGTCCATGAGAGAAAACTGTGG<br>GGCCTTTTGCAAAACCTAGTTTGTCTTGCACATGATTACTCATAGCA<br>TTGCATTTCAGTGCCATTATCCCAAAAGAAGAGAAGCTTCTTCAGA<br>ATGTCTTATCAATGATATTTTCATGAAATCATGTTGAGATCTTGTATC<br>AATGATCATGGAAAAATGATTTAGAAAGAAAAGACATAGTACGTT<br>CATGTATTAAGAGAAGTAACTAATTACTGGCTCATGCTATGAGTAAT<br>TCCTTTGGCTCAGAAAATAGTTCAAGTGATATCCATGAATAATTTAA<br>CTTGTTATAAGGAAGTTCTTTAGCTCTATTTCCAATTGGTCACATAG<br>GTAATAGATTAAAAAGAAAAGAATACTTTACAAGAATATTTAAACAT<br>CAACATTCATCTATAGACCTAACAAAACATAACAGAAATAAACACA<br>ATTTTTTTTGCTTTAAAAATCCTATTATAAAACAGTCAATGACAACTT<br>TTCTTTCATGTAACTTATTCTGAATTAGCATCATGGTAATGTTTTTTAT<br>AATTGTAGTTGATAAATAATTTTACAAAAATAAATTGTTATGTAAATT<br>AAATGAAGCATACTTTTTTGAAAAGAAGACATGTATTTTCTTACGAT<br>AAAAAGAATAAATCATTTTCATGAATATGTTCTAAGTCCAATGAAAT<br>AATTTTTAGTTAAGACAAAACAATAGATTTATTTAAAGAACAATATA<br>AATACAAGACCAAAACAACATTTATAGGTTGCTGAACCTGAAGACT<br>CGAAAGTCAGGTTACAATTTCTACTTCCAGAAGATTGTTTTTCCCCT<br>TTCTTAATTTAAAGAAAAATGTACTCTTGGAAATAGGATAGCCAAC<br>AAGTAAAAGCAAAATTGTTCCTTAAAATTGCAACATTTGGGCCCAG<br>CGCGGCGGCTCACGCCTGTGATCCCAGCACTTTGGGAGGCGGAGG<br>CCTGTAATCCCAGCCACCCAGGAGGCTGAGGCAGGAGAATCACTT<br>GAAGCCGGAAGGCGGAGGTTGCGGTGAGCAGAGATGGCGCCACT<br>GCACTCTAGTTTGGGCAACAAGAGCGAAACTCCGTCTCAGAAAAG<br>AAAAAAAAAAACCTGCAACATTTCAAAAGATGTTATATTAGTCATT<br>TCAGTGAAAGTTTAATTAGGGAACGGTGTCTACCAGAATATATTTGG<br>CATCCTTCTTTTTTCTGGTATTTGCCTGAGTGAGAAAAGTGTTAGGA<br>ATAGGAAGGCCAGATGAGTTTGCTTTCCCTCCAGAGAATACTGGCC<br>AAGAAATTTCCTGTCCAACCTCAGGAGCATCACTGAAGCCTGTTTA<br>TGAGGAATTGTCCTACCACAACACTGTGTTATGGCATTTGGGAAGC<br>CCTCAGAGGAGCTGCCTGCTCTAAGATTGAGGCCACATGACTCAG<br>GCCATTATAAACCCTAATGCAGAGCCCTGAAGAGGGAGAGCATTTG<br>GAAGGGGATAGGGAAGCACATAAAGATGATATGCTCAGCTTTACTG<br>TCGTTGGGCAGAAAACTCTCTAGTGGGCTTGAGAGGGGGAACTGA<br>TTTTAAGGAGGGCTGAATAGTTTGGGAACCTACTTAATGCAACCAT<br>AGTCTGTATTTTTAAAATCCTCACATTTCACTACAGATTTGGGTTTTA<br>GTGTCTTGGCATAAATAGGGCCTTAATATAAATTCTTAGTTTTCTGGA<br>GACATGGTAGAGGTCAGATATCAAGATTTCATAGGCAAACAAAACA<br>TGCTATATATGTAAAAAATGTATATACACACACAGCCTTTTGCTAAA

	CTGAAAGAAAGTAAAAATTCAAAGCAGTCAAGGGGTTGAAAGATG GATTGGGTATGGACTTTTCTTCATCTATGTCAATTTTTGAAATCTCTC TCCCATCTCCACCATTCCCTTTGTTAATTGTCCTTGCTTACATTTAAA GGAAGGGAATTAGATCATCAAACAGGTCACTCACACCTACCTCTAA CTTCACACACCTTGGTCAATGTGAAACAAGCTTGAACTGGTTATAT GCTTCCCCACCATTTTTATTTATTGTAAGCTACTTTAAAAATTTTAGA CAATGTTGTAATAAAAATATACACCTTTTTCCCCCATTATCCCTGGTC TTCTGGTGAAGCAAGCAGTGTAATCAATGATTCTTATGTGTGAGGA GCAACTCCAAGTAGAGGCGTTCATGTCCCTAATGCTTTTTTATTTTT CCTCATCAGCAAATTTAAACTACAACTTTATCCTGATCCCACTGCTG CTCTTTGCCTCTTCTCCCCCCAGCCCTTCCACATTTACCAGCCTCCT CCCTACCTCCTCCCAGCCTTTCCCTTTGCCCTCCCCTTTCTTCTAGC CCCTCCTCCCAGTTGAGTCAGTGGCTTGAAACTTTTAAAAGCTCTG TGCTCCAAGTTACAAAAAAGCTTTTACGAGGTATCA
GK	GCAGAAAAGATACCCAGAAATGCGGAAGCGACTTTGGAACTGAGT AACAGGCAGAGGTTGGAACAGTTTGGAGAGCTCAGAAGAAGATG ACAGAAAAATGTGAGAAAGTTTAGAAGTCCCTAGAGATTTGTTGAA TGGCTTTGACAAAAATGCTGATAATGATATGGACAATGAAATCCAC GCCGAGATGGTGTCATATGGAGATGAGAAACTTGTTGGGAAATGAA GCAAAGGTGACTCTTGTTATGTTTTAGCAAAGAGACTGGCGGCATT TTGTCCCTGCCCTAGAGAACTGTGGAACTTTGAACTTGAGAGAGAT GATTTAGGGTATCTAGTGGAAGAAATTTCTAAGCAAAGCATTCAAG AGGTGACTTGGGTACTGTTAAAGGCATTCAGTTTCGAAAGGGAAA CAGAGCATAAAAGTTTGGAAAATTTGCAGCCTGACAATACAATAGA AAAGAAAATCCCATTTTCTCAGGCAAAATTCAAGACAGCTGCAGA AATTTGCATAAGTTACAAGGAACTGACTGTTAATCACCAAGACAAT GGGGAAAATGTCTCCAGGGCATGTCAGAGACCTTTGCAGCAGCCC CTTCCATCACAGGCCCAGAGGCCTAGGAGGAAAAAGTGGTTTCAT AGGCTGAGCCCAGGGTCCCTGTGCTGTGTGCAGCCTAAGGACACG GTGCCCTGTGTCCCAGCCACTCCAGCCGTGGCTGAAAGGGGCCAA CATAGAGCTCAGGCCGTGGCCTCCAAGGGTGCAAGCCTCAAGCCT TGGCAGCTTCCACGTGGTGTTGAGCCTGTGAGTACACAGAAGTCA AGAATTGAGGTTTGGTAACCTCCACCTCGATTTCAGATGTATGAAA ATGCCTGTATGCCCAGGCAGAAGTTTGCTGCAGGGGCGGGGTCCTC ATGGGGAACCTCTGCTAGGGCAGTGCAGAAGAGAAATGTGGGGTC AGAGTCCCCACACAGAATCCCTACTGGGGTACTGCCTAGTGGAGCT GTGAGAAGAGGGCCACCATCCTCCAGAACCCAGAATGGTATCACT GATAGCCTGCACCATGTGCCTGGAAAAGCCATAGACACTCAACACC AGCCCCTGAAGGCAGCCAGGAGGTAGGAGCTGCCCAAGACTGTG GGAACCTACATCTTGCATCAGTGTCACCTGGATGTGAGACATGCAG TCAAAGGAGATCATTCTGGAGCTTTAAGATTTGACTGCCCCACTGG ATTTCAGACTTGCATGGGGCCAGTAGCCCCTTTGTTTTGGCCAATTT CTCCCATTTGGAATGGCTGTATTTACCCAATGCTTGTATCCCCATTGT ATCTAAGAAGTAACTAACTTGCCTTTGATTTTACAGGCTCATAGGCG AAAGGGACTTGACTTGTCTGGGATGAGACTTTGGACTGTGCACCTC TGAGTTAATGCTGAAATAAGTTAAGACTTTGGGGGACTGTTGGGAA

	GGCATAATTGGTTTTGAAATGTGAGGATATGAGATTTGGGAGGGGC<br>CAGAGGTGGAATGATATAGTTTGGTTCTGTGTCTCCACAAAATCTCA<br>TCTTGCAGCTCCCATAATTCCCATGTGTTGTGGGAGGGACCCAGTG<br>GAAGACAACTGAATTATGGGGATGGGTCTTTCCCATGCTGTTCTCG<br>TGATAGTGAATAGGTATCATGAGATCTGATGGTTTTAAAAATGGGAG<br>CTTCCTTGTACAAGCTCTCTTTGCCTGCTGCCATCCACATAAGATAT<br>GACTTGCTCCTCCTTGCCTTTCACCATGATTGTGAGGTCTCCCCAGC<br>CATGTGGAACTGTTAAGTCCAATAAACCTCTTTCTTTTGTAAATTGC<br>CCAGTCTCGGATATGTCTTTATCAGCAGCATGGAAATGGACTAATAC<br>AAACACTTACTATTAGAAGTTCTTCTGTACGTTGCTCTAAAATCTTT<br>TTCACTCTTATTTTGCAACACTGATAATTCTGCCACCACAATAAACT<br>GAACAAGATTAATCTGTCTCTTATGAAATTCCTCAAAATCTTACAGA<br>GAGCATTATATTTTCCCAAATTATCCTTCAATGGTCTGTACAGTCCTA<br>TGTCCTTCAACCACTGCTTGCACAACATGATTTGGGGTCTTCATCAT<br>CACCCTGACTATAAATTCAGGGCTCTAGTGCATCAGTCTTTCTCAAA<br>ACACTGAACCGAAAAATAAACTCAATTTCACAGCTATGGTCTGATC<br>TGGACAGAACAGAACAGGATAATCACCTTACACCTTTCTGGACACG<br>AATACATAATTGGTTCCCACTGAATATACCATCAACTAAAAGCCTAA<br>CATCTTTGCATCAGTTTGGTTAATATAAGCCTAACACAACACAGCCA<br>AAGTGAAAAATTCTTCCGTTTCAGTGAATGAATTCTTTTTCATCCTA<br>CTGTATCTCAGTAAGGTACAGTAAAAATCAACAGTTACTGCACACT<br>TGCTGTAAATATGCACACACTAAAATGTACCTACAAAGAATTTACCT<br>AAAATGTAATGCTCAAAGGCAGGTCATCTCAGAGTTTAATCTGTTT<br>CCTCTACTTATACTCAGAGTTAGTTCCCATATCTATGATCTTGCTTCA<br>TTCACCTCCCCTCTGTGTTCATTGCCCTAGTTCTAACGCTATTACCA<br>ACCTCAATTATTGCTTCCAAACCTATCACCCACATACTGACAGGTCA<br>TTTTGTTTATGGATCATAGCTTGTGACATTAGTAATTTACCTTCAGTA<br>GTTCACCACTGGCCACTGAATAAAGCCCAACATAGTTATACTGACA<br>TTTAATCATCAGTTGCTTATCTCTAAACAAATACCATAACTGCAATAG<br>GGCACAATTCCAAGTAGTAAAGACTATGCCATCAATATGCTATATAC<br>GTTAAACACATAAACTACACACACATAATATATAATATACATATGAAT<br>CCTTCCATATATGTATGTGGAATATACATTCCATTCCTTCTATAATGAA<br>CATATATGCTCATTACTCTCTAAAGCAATTGAACTTTTCTGTTTCCCT<br>CTAGGAAATAAAATAAGCTAATCATGAAATGAAAGACAGTCCCTAG<br>AATTTGCCATACCAGGTCAAGAGAGAGCATCAGGGCTGGGCACGG<br>TGGCTCACGCCTGTAATCCTAGCACTTTGGGAGGCTGAGGCGGGTG<br>GATCACGAGGTCAGGAGATCGAGACCATCCTGGCTAACACAGTGA<br>ACCCCTATCTCTACTAAAAATACAAAAAATTAGCCAGGCGAGGTGG<br>CAGGCACCTGTAGTCCCAGCTACTAGGGAGGCTGAGGCAGGAGAA<br>TGGCGTGAACCCGGGAGGCAGTGCTCACAGTGAGCCGAGATCGCA<br>CCACTGCACTCCAGCCTGGGTGACAGAGCGAGACTCCATCTCAATA<br>AAAAATGAAAAAAAAAAAAAGAGAGAGAGAGAGAGCATCAGGTT<br>AGGACCCTCATCCCAAATAAGTGTAATAAAATCAACAATATAAGTCT<br>CAAGTGTTATTTGTTCTGTTTTGTATCCTGTAATAAGGTACAAAGTA<br>CCCTTTGTATTTGATTTCTAAACACCAAGCACCTTTTTGAACAAAAT<br>TAAAATGCCATGATGGGTCTCCACAATTCCTCTATACTCAATGCAAA

	GTCCCATTTGAATAGTGAGGGCAAAAGTATGAATAACAATAGTCTC<br>TGAGATCCACAAGCAGCTCATCCCTAACAGACAAAGTGTTTTTGGG<br>AAAACAGAAATATCTCTGGTCTTCATTTTCCTAATTAATAAAATGAA<br>AGTGTTAGACTAGATAACTGCTAAGATTTCCTATAACATTAATACTCT<br>GTGATTCTTTGACTTTCAACACTATTAATCTTTAGGAGAAAAAAACC<br>ATGTATGTCTCCAGAAGTTCTATTAACATAAGGCAGTTTTAATGAAA<br>CCTGAAAACAGTTAACTCATGTAAGCTAGAAAAAATTCCTGTCAAC<br>AAGGCATAGCAGAGACTGCATGTAGTTTACTAAGGAATAAAGAGG<br>ACCATAATGAATGCATGAATATACAAATAATTAACTCTTTTAAGAAG<br>CATCATTTGAGTTGGCATGACTTCAGCTAAAAAGTGCTGTGTATCA<br>AGTGCTTTCACAGTTCTCCAAAGGAAGAATCTTCTGTATCAGTAAA<br>TTAACACTGCAATAGACCCTTGAATAATCAGCATCTATTCTTTGGCA<br>TGCATATCCCATGGCTAAGGCTAGCAATACGATTATTAAAACCACTC<br>ACTCAACACAATTTCTTTCAAGAGGGTGGCATTCAAATCATGAGCT<br>TATTCTAACCGATGTTTATTCCAAGCAGTGATTCCTAGCCTAAATTAC<br>AGAAAGGTGGGCAAATGATTGAAGAAATTTTCTGTAGAAGAAACT<br>GTTATAAAAACTGGTCTTACAGAGAATAATGCATATTCCAAATATAC<br>TTAATTAGGGTGTGAGTTTATTGTTACTGGACTATAAAGTTAGATTAT<br>AAGCAAGGGTTAAATTGTACTGAGCCTATTATCTATCACCCACAACA<br>GTGTGGGCAATGCCAGGCACATAAAGAGGATAGCTTTGAAATGACT<br>GACTACTGGGTCTAGGCTGAACGGCCAAGAGTTGGCTGGAAGAAC<br>AAGGGCCAAAGAGCACAGATTTATGAAGAAATACAAGAGAATGAG<br>AAGGCACACATCTAGTCCGAACCAAGGTAGGGACCCCAGAGTTCT<br>AAATACCAGAAAGGTCCAACAGACCCTGGAACTGCAGTCTAAGGT<br>GATGAATGGGTTTGTGGGGGGAAGATGATAGTCACAGGGAGCTATG<br>AAGGAGTCTCACGTGAGCCAACAGTATAGATTCTGAAGGCGTTTAT<br>GGCAGGAGAAACAAAATACGCAGGGAAAGATAATCTCAGTGTTAA<br>AGACAGATGTATGCCTGGAGGCCAAGACAGAAAATGAGCTGCAGA<br>GAGAACGCAGTAGCCTGCTAGCCAGCCAGTAAAGAAGTGAGGCTA<br>GTTCAGCGGACTCGCGCGGCCTCGCTCTTTGGACTCGTCACCCTCC<br>CCTCCCCCTCCCGCCGCCGTCACCCAGGAAAC
GNRHR	TTATACCCAGTGTCCCATGTGGGATCAGGCTAAGGAAGGTTAGACT<br>TCAGCTGAACACACATTTGCTTCGATTCTTCCTCTGCCCTGTCTTGC<br>TTTCCTCACTCTCTTAGAGGTTTCACCTTAAAGCACACTCTCAAGTC<br>GCTTGCACAAGAATCCCCATCTCCAGCTCTGCTCTACGGAATTCAA<br>CTTAATACATGAGTTGATTTAGTTTTCCACTGACTTGAACTGTTAAG<br>GGAAACATTTTTCTTATACTTTCCCTAAAATACATGTGTGTATTTTTC<br>TAATTCAATAAATCCAATACAGTAAACCCAATTGAATCATTATTGTTC<br>CAGAATATCTTGTCTAAAGTTCATCTTTCCAATGAGGAAGTTTCCTT<br>GTATTGTATGGTAAAGTTAAATGCAGGTGGTTTAAGCATTTAAACAA<br>AAACAAAAAAGCATTTAAAAAAAAACACTATACTAAGTTTAGGACA<br>TATTCTTACATTTGACAAAACACTTGTTACACTTTTTTTTCCTTTTTA<br>TTTGTCTGTTTCTCACAGCAGAATGCAAGTGAATTTTCAGGTAACT<br>AACCATAACCCTGGTTAGTAGAACGCCAGTTAATCAAGTTTAACTAT<br>TCGAAGACTCTTGCTTTAAAAGATTTTTCTTTGTTTTTATGGATGTTT<br>GTAAAGAAAAAATTCTATGCAAGTAATACTATATCATAGATATACAC

	CCCTATCTACGAAATTACACCTTAAAACAAAGGCCTTGTGTATGTGA<br>TATCTGCATCATAGATAAAGAGAAATAAGCAGAAGAAAGGGGTTTT<br>TATTCTTCCCGTTCCTGTTATCATTTCTACCTTTGAAAAATAATGCTA<br>ATAAATTTCCAAATTATACAACCTAGCAAATCAATTAGTGGAAAAAT<br>TTCAGATAAAAGTAACTATAAAGGAAGTGAGTGATTTACCTGAATA<br>AAACTATTTTTCAAAGATTTTATTAAATAACTACAGCATCTCTAGGCT<br>TCAACGGTATTTTTTTGTTTTGTGAGACAGGATCTTGCTCTGTCTCA<br>GACTGGAGTGGCTCAATCTCAGCTCGCTGCAACATTCGCCTCCTGG<br>GTTCAAGGGATCCTCCCACCTTAGCCTCCAGAGTACCTGGACTACA<br>GGTGGGTGCCACCATGCTTGACTAATTTTTAAATAATTTTTTTGTAG<br>AGATAGGCTCTCCCTATATTGCCCAAGCTTCATCTTTGTTTTAATCTT<br>GATCCTTAAATATTTTAAAAATACAATATTTATTTTTATAAAATTCAAT<br>TTTTAAAAAGCTATTTTATTTTAAAAGTCCTAAAATAAGAAAAGAAG<br>AAATAATAATAAAAGTGAATATGTTATATAGAAATTAACAGGATGTC<br>AATTTCATGTGTGCCCAGTCTTACTGAAACTTAAAGAGAAACCAAT<br>ACTAATAATCATATTTTTCTAAATTGTATTTTAAAATCTTATTTATATTA<br>TTATGCATTTGCATCATAAATTCTGTTTTTGTTTGTTTTTGAGACAGA<br>GTCTCACTCTGTCACCCAGGATGGAGTGCAGTGGTGTGATCTCAAC<br>TCACTGCAACCTCCACCTCCTGGGTTCAAGTGATTCTCCTGCCTCA<br>GCCTCCTGAATAGCTGGGATTACAGGTGCATGCCACCATGCCCAGC<br>TAATTTTTATATTTTTAGTAGAGGCGGGGTTTCACCATGTTGGCCAG<br>GCTGATCTTGAACTGCTCACCCTGTGATCCACCCAGCTTGGCCTCC<br>CAAAGTGCTGGGATTACAGGTGCGAGCCACCGTGCCCGGCATAAAT<br>TCTTAATTAATACAATTATTGGGGATCTAGCTTTCAAATTTCCAATTC<br>TGAAGAAGGTGGAAATAAGATACATTGTATTTTAAAATTGAATTGCA<br>TCATGAAATGGAAATGCTGTGTGTCAACAGAAGATTATCAGCAAAA<br>GAACAAAATTGAAATTATACTCTTTTTAATACAAGCAAACTACATAA<br>AGTTTATGTAAATAAGAATATCTCGGCCTCACGCGGTGGCTCCTGCC<br>TGTAATCCCAGCACTTTGGGAGGCCGAGGTGGGTGGATCACGAGG<br>TCAGGAGTTCGAGCCAGCCTGGGCAATATGGTGAAACCCTGTCTCT<br>ACTAAAAATACAAAAATTAGCTGTGCATGGTGGTGCACACCTGTAG<br>TCCCAGCTACTTGGGAGGCTGAGGCCGAAGAACCACTTGAACCCA<br>GGAGGCGAAGGTTGCAGTGAGCCGAGATCGTGCCACTGCACTCCA<br>GCCTGGGCGACAGAGCAAGACTCTTGATTGAAAAAAAAAATTCAT<br>AACAATACAATATAATTATAAAATATGTTTGGAAATATCAAATACGAG<br>CTTTGGCTAGCCTTATTTTTGGTATCCACAAGTATAGTGTGTAGTACA<br>TAATGCTTAATAAGTGGTTAATTGAATTGTTGAAATAAATATAATTCT<br>TATATTCCTGGTTTAAATCTTTTTATATATTGTTAATTATACTTAAAAG<br>TAAAAGATTACTACTTTTAGTATTTAGTGAACATAAATTTTGATAAGT<br>AAACATACTTATTATGCTTCTGCTTTTCTGTTCATTTCATATGTTGGT<br>GATTTATATTATATATGCATTTCCTTAATCTGATCTCCAAAATCGTACA<br>TATTTAACCATAATTTCCCATCATTTACCTCTAACACATCTATACTGA<br>AATAAAAATTGCAACAATGTTTTACAGTGTGATTCTCCAAATAATGA<br>GCACTCTTGGAATAATGAAAGTGAATCTGCCATGTTAAATGCTATGC<br>ATAATTTAACATGGCTTAGAAAAATTTGTATGGCTTTTAGAATTTGTT<br>GCTCTCCTTCACAACAATTTTGTGTATATATTTCCTCATACTACTTCA

	TCCACAGTCCTTCCTCAAATGCTTCCATTTAAACTTAAATTTTAAAA
	GTTCCCCTTTTACTCATTTCACAGTTGTCGTAGACACTATCATTCTTT
	GCTCAACAAAGTAAGTCATGTTTTCAAAAACTAAAAAAAAAAAAA
	TTCTTTTTTTTCTGATATTGTGCTACTTCCATCTTCTCTCCTTACAGG
	AAACAAAATTTTTTCCCAAATACATTTTCATACACATTCTTCTTATTT
	GAAAGAGCTGCCTTCCTCTATTATTTTATTCTCATGAGTAAAGAGGT
	ATGAAAAATATTTCTTGCTCCCCTGGGTCACTTCTAAACCATCATCA
	CTCTTTCCCTTCAGAAGTTGGACTTGTAATTCAAAAAAAAAAAAAA
	AAAAACTGGCTAGTTAGGTTCAAGAAGATGATGGGAAAAACACTA
	TGAAGAAAAAAAAGATACTATAATTGAAAACCAACTCAGGTGATG
	AATCAGCAGGAGTTGCTCATCCTTAGAGCTGCCTGGGCTCCTGTTG
	GCTTTCTAGGTCTTGTGTATCATGAAAGATAACTCCATTATCTAAAA
	CAGTCTCCCTTTCCTGCAACTAAAAGGGCCTGTTAACACGGCCCTT
	CATTCACTAAGGACTATGGCACCTGGCTCATAGACCTTTCCACCCC
	AACTGCTACCAGAAAGTAGCATTGCATAGTAGTTAAAGACAGGAGC
	TCTGAAGGCAGACTGCATGAGATGTAACACAATTCTGTGGTGTCCT
	AATTAGGGAAAAGGAGTCAGGCTGGTGGGAGCAGGGGAAAGCAA
	AAAGAAAAAGCAGATAAGCTACAAGGACACATAGCCCTCCTGTGC
	AAATAACTCTCAATCTTCCTATGCCCAACTATCACCAGACACCTGCA
	AGTTAGCTCACTGCAACCTTGGCATTATCAGTACTGCACAAAGCCC
	TCTTCAGCATACAGCATAAACACTATCCTATAAAATCTCCAGCAAGC
	CTTTGTTTCTTTGCAGTCAGCTTCCCTTCCGCTGATCCTGCCCATTG
	TCTCCCTGGCAACGTATTTTCCTCCCTTCTCTAACAAATTTGCCTTT
	CTTTTACCTACAACGGTAAACCCTTTTGTCCCTGCACCTCGGGCCTA
	GATAGCCACCGCTCCCCCATGACAAACTCTAATATGGATCAGGTATG
	TAAATGTCTCTGCTTCACCTTCCTCAACCATAAGACGAAAATAATAG
	TACTGACTTCTAGGGTTGTTGTAAGGTCTAAATGTGTTAATACGTGT
	AACATGCTTAGAAAAGTGCTCGACACAAAGTAAGTACCCAATAAAT
	ATTAACTATGAGTAATATTATCAGTATTGACAGCACCTTCAAAATCTA
	TATAGTTGACTCATCCAACAACTTAGATTCAACAACTTAGAGCTCCT
	CAAATGCGTCAAACATCATTCGCCCTCTACTTCCTCTACCGACTTTC
	ATAGCCACACCCTGCACTTGAGGGCTTTATACCTTTCATGTTGTGTT
	GATAGCAAGGGTTTTCCAAATAGATTAACCTCCCTGAATTTTGATTG
	CCCACTATTTCCCAAATCCCTTCAAGGCATTCTCTGTATGCTACACA
	ATACACTAAGTATATATAAAGAAAGGAGTATATTACTCCATTCTTTAT
	ATAGGTAAAGCCTAAACTCCTTTTCTTGGAATATAGGCTCTCCAGAT
	CTGAAGTCTGCCTAATTATTTACACTTTTGCTTTCACATACCCTTTGA
	ACTTTCTCACATTGTCTTCGTGTTTGCATGTGCTGCTCCAGCTTCTA
	AGCATGCCCTCCCTGTCCTCATACCCCATTCCCCAGCCACTTATTAC
	GCTATATGCTGTAGTCCCATTCAGCTCGGTTGCAACCTCTTCCCTAA
	TGAATCAGTCCATCATTAACAAAGAAAGGGAGGGAGGGAGGGAGG
	AAGAGAGGAATGAAAGGAGGAAAAGGGAAGGAAGGGGAAGGGA
	AGGGGAAGGGAAGGGGAAGGGAAGGGAAGGGAAGGAAGGGGAA
	GGGAAGGAATGGGAGGAAAAGGGACAAATAATGAATGATATGCTC
	TAATCTTTTTCCCCTAGATATAGAAGACAAAGAGCAAAATATACTTC
	ACTAAAATTGGATTTTTACATAAATGTTCTTTCCTTTGTTTTTTGGTT

	GCTGGTCCACTTACAAACA
GPD2	TATTTGTTTGATTTGGTTTTAATACTATAAGCAAAAAAGAAGCAGTA<br>GCAAGTGTTTGTTGAGAAGGGAAGGTTTTTGTTTTTTGCTTTTTTTT<br>TTCAATATTGTCTGTGGAGCTGGCATAGTAATTACTGAGAGCAATTC<br>CTAAATTAATGATTATCTCCCACTTCTCCATGAAAGTATCTTAATAAT<br>GAAAAGAAAGTGCTTAGGCATATAGGAAATTGTGTTCCTGTTACAT<br>ACTCTAATCAGCAACCAAACCCTTTCAGTGGAATTCTTGTTATATTA<br>GGAGCAAGACACAGGGCTAGAGATAGGGAGGCAGAAAGCTACTTA<br>GATACTCATAAAAAGAATTGTCAAAAAAGGCTTTAAAGTGTTATGT<br>ATTAGGCTAGAAAGAAAAATTTGTTATTATATTTTATTCTCTTTAGAA<br>AAGATTCATGGAATAAATAGGGCATAGTGTTTTATTTTATACGTGTCT<br>GCGTGTCTGTGAAATCTTTTTTTTTTTTTTTTTTGAGATAGAGTCTC<br>GCCCTGTTGCCCAGGCTGGAGTGCAATGGCGCAATCTCAGCTCACT<br>GCAACCTCTGCCTCCCGGGTTCAAGCAATTCTCCTACCTCAGCCTC<br>CCGAGTAGCTGGGACTACAGGCGTGTGCCACCAGACCTGGCTAATT<br>TTTGTATTTTTTACTAGAGACAAGGTTCCACCATGTTGGTCAGGCTG<br>CTCTTGAACTCCTGACCTCAGGTGATCCTCCTGCCTTGGCCTCCCA<br>AAGTGCTGGGATTACAGGCATGAGCCAACGTGCCCGGCCAGTATCT<br>GTAAAATCTTAAAAGAATGATCATAAACCTCTACATTTTGAAATTAA<br>TATTCATTAAAAATTTTTTAATTTCCATTTTTATTTTAGATTCAGGGGC<br>TACATGTGTAGGTTTGTTTCAAGGGTAGATTGTGTGGTGCTGAGGT<br>TTGGACTTCTATTGATTCCATCACCCACATACTGAACATAGTACCAA<br>ATTGGAAGTTTTTCAGCCCCTGTCCCCCTCCCTCCTTTTGGATTCCC<br>CAGTGCCTATTCCCATCTTTATGTCTGAGTGAAACCATGATTTAGCT<br>CACACTTACAAGTGAGAACATGTGATACTTGGTTTTCCGTTCCTACA<br>TTAATTTGCTTAGGATAACGGCCTGCAGCTGCATCCATATGCTGCAG<br>CTGCATCCATGTTGCTACAAAGTGATTTTGTTCTTTTTTAGGGCTGT<br>GCATTTTTAAGAATTAACAACTTTGAAAATATGCTCTAAATAACTTAT<br>GCTCTAATTGCAAGTCAGCAATGTTTTATTGGAGAAAATTATCTACT<br>ATACAACAGTTTCCTATTTATTTTATTCAAGTCAATAGTTGAGATTAG<br>ATGTGTGTGTGAAGAGTGGGAGAGTGGGAACTATAGAGCAGAGGT<br>GCACGGTCTTGAAAAAGAAGATTCAGAGTAAATTGTATGCTGATCA<br>GGTGAAGCTTCTTTCCTTGTCTGTGCCAATTGACCCCACTCTCATGC<br>ACACCTTTTTCCTTCTGTGTCCTTCCTGAATCCCACCAGATGCCAAC<br>ATCTTTCTGCTGTCCTTTGGTTGTTCTGGGCCCCAAATTTGGCAAGA<br>GGAATCACGTCCAGTGCTCCTGACTTTTCTCAGGAAAAGGTGGTTA<br>GTGAAACCACCTTTGCAAAATTATGACCTAGACAGTGAAAGAGATC<br>TAACTTAACCGACTGCAACTTGTTTCTAACCTACAAGCTGTCCTTGT<br>TCATTCCTGGGCGTAGGCTGAACTAACTTTGGGAAAAACTTAGTTT<br>ACAGTTTATAGTTTTAACAAAGGTGGTAACAGCCCATTCCCAAAGC<br>AGACCTCCCTCTTGCCTGGGGACTAGATTGCCTTTGTAGGACTAAC<br>AGCCACAAAAGATTAGAAACTATGGTTAAGGAGTCATTCTGACGCT<br>CCCTAAACTGCTTCTAAGATCAGTGCTTGAGATATTTTGCAGACCCT<br>GCACTTGACGGATCAGCTGGTACCACCTAGATCAATAAACTGACTC<br>CTCTGACCTTGTGGCCCTCCACCCAGGAACTGACTTAGTGCAAGA<br>AGACAGCTCCAACTCCCTATGATTTCATCCCTGAGCAATCAGCACT

	CCTGGCTCACTGGCTTCCCCCAACCCACCAAGTTGTCCTTAAAAAA<br>TCCGCTCCACGAATGCTCCGGAGTCTGATTTGAGTAATAATAAAAC<br>TCTGGTCTCCCGCACAGCTGGCTTTGCGTGAATTACTCTTGCAATTC<br>CCTTGTCTTGATCAATCGACTCTGTCTAGGCAGCGGGCAAGGTGAA<br>CCCCTTGGGCGGTTACACTAGCTAGCAGTGTGACTAGGACTAGTGG<br>GGAAAATAATCAGCTGACATAGTATGGATTGGACCAGATCGCTCAC<br>CTAAGCATTCATTTCAAATCAAGAAACATTTAAACATCAGTTATATT<br>CAGGCACTATGCTAGGCACTAGAGTACAAAAGTGAGTAACTGATGG<br>TCCCATTTCCCAAGCACTGAACTGATCTGGGATAATCAGTGAGAAG<br>GAAATCCAGGACTGGCACCCAACTCAACCAGATTATTCCTTTTCTG<br>GGTAACATGTTGCATTTCTAGATAAATGATGAGGGTTTGAAAAAAA<br>TGATAAAAATATTGGTTTGATTTGTCAAACTAAAGAAAAAAGAACT<br>GAGGCATACTTAATATAACCAGTCTCATGATGTTGCCCAGTCACAAG<br>TTTTTATTTATTTATTTTTTGAGATGGAGTCTCACTCTGTTGCCCAGG<br>GTGGAGTTCAGTGGTGCCATCTCAGCTCACTGTAACCTCTGCCTCC<br>CAGGTTCAAGTGATTCTCCTGCCTCAGCTTCCTGAGTAGCTGGGAT<br>TACAGGTGTTTGCCACCATGCCCGGCTAATTTTTGTATTTTTAGTAG<br>AGATAGGGTTTCACCATGTTGGTTAGGCTGGTCTTGAACTCTTGAC<br>CTCGTGATCTGCCCACCTCAGCCTCCCAAAGTGCTGAGATTACAGG<br>CGTGACCCACTGCGCCTGCCCGGCCACAAGGAAGTTTTTAAAGGA<br>AAAGAAGAGGCAGTTTCTAAATTGTTTACCAATAATTTACATAAAA<br>AAACAAACTATTAATTGTCTATAGATTGTTCTTCGTATCACAAATTCC<br>AGGAACATGAAGATATGGTTGGGTAGTCAGTCATGAACAAAATGCC<br>TTTAAACAATCGCCTCTGAGCATGAGTGTGGGGGGTGCAACTTATG<br>TCCCATATTCAAGGTCTCTCTGGGCCTGATAAATTTTGCATACCTCA<br>CATTGCTCAAATGCTCTGAACTATTTTTCTTTTCTCAACTACAATTTC<br>CTTTTTCTGGTTGCTCATGATTCAACTGGGGGAGCTTTTAAACCGTG<br>GTTCTCAAACTTTAATGTGCATCAGAACTACCCAAAGGGCTTGTTA<br>AATCTGGGTAGCTCAGCCCCAGCCCCAGAGTCTATGAATCAGGTCA<br>CTGGAGTACAGTTTCTGTCAAGTTCTCAGGTGATGCTGATGCTGCG<br>GGCTCCAGTGACTACACTTTTAGAGCCAGTTTTAAAATACAAGGAC<br>TTGCAGATCCCACGTCCAGACTGACTCATTGGACTTGGGGATGGAA<br>ATCTGGCATTCCACCCCTCCCTGCCCCCTCCCCCCAATTTTAATGTG<br>TCCAAGGGTGAGAATCATTGCTCCTCTGTGGACTATGGATGGTTCC<br>CTGAAGTTACTGAGTTTGGGATTTAATAAATATATGCATTCCTCTGG<br>GTCAACTACAGGAATTCTGCGGTATTTAAGGACATTAAATGAGTGA<br>CTTAATACTTTATTTAAAAAAATACCACTGCTTTTTGGTTCACACTAA<br>TTTTCTTCCTTCTTTTGGAAAGGCATTTCCGTCCATCTCCTGGATATT<br>TTCCAATACCACGATAGAAAGGTTATTAGTACAGCACCCAGAGGTC<br>TTCGGAGCAAAGCTGTTAAAGAAGTGGGGTCCATTTGAGCCCCAA<br>CTTTGCCTTCCTTAGGTCCCCTTGGGCTTGTCTAGCCCTAGGAGTTC<br>CAACAAGTAAGCTGGGCATGCTCAGTGGGCAGGGTCGTTTTTTTTT<br>TTTGGGTGGCGGGGGGTTGCAAGTGGGAAGCCTGCTGTTCAGCTG<br>CCGGGGCTCTCCGCCTCCCCCCACCTGTATGAGGCTGGGTCTGGGG<br>AACCTGTGCTCAGCATTCCACCCCCTGGAGCTTGGGCTTGGTCTTC<br>CCTGCGGGTCCCTGCGCTGACATTCAGGCGGGGAGCCAGGAGGCC

	TGGCGCGCCTCCAGAGCCCGCCGGGGGAGCCGGGCGAGGGTTCTG<br>GGCTCTGACGGCGGGGTCGCAGGGTCGCCCGCCTCCTGGACACGT<br>CTGTAGGCCTAGGGAAGCCTGCCGGCCGGGAGGTACAGAGTAGGA<br>GAAGCCAGATCCCAGGGCGGACAACGAGAAGTCGTCAGGTAAGC<br>CAGCCCCGCCAGCGTGCCGGCGGACGAGTGTCTCCTCTCCCGCAG<br>GCAGATGGCCAGGGCGGCCCGCATGGGCGAGGGCACAGGAAATCG<br>GACACCGCCACCGTCTTCCCCAGCCTGGCCTAGCTCAGGCTTTCCA<br>GTCCGCCAGCCCGCTGGCAAGGCGGGGCGGAGGTCTGGAGTCCGG<br>CATCGCCGAGGCACAAAGCAGGAACTGCAGCCAGGGGCGAGGGC<br>CCTTGCGTGTGGCGCTGCGCGACCCCACCCGGCGGCAGGACCCGC<br>GTCCCCGTCTAGTCACGTTCATTGGGCCACGGAGACCACCTCGCTC<br>GCCGCCCGCTGTTGGCTTTCCCTCCCACACCCCCGCCAACCCCGG<br>CCCCGGTTCCCGCCCCCACTTCCGCGTTTCCCCGCTGGGCCGGAGG<br>AGCCTGGGCCCGAGGTATCCGGGCAGGAGGTGGCGGCCTCGCCGC<br>CCTGGTCAGGCCTCCGGGAGGGAGCAGGCGCGGGAGCGGGAGGA<br>GGAAGTCGGGAAGAGGGAAGGCGGGGGCGCGGCCCGCCCGGCGC<br>TCGAGTTACACCCGCCCAGCCCGCCTCGAGTGACAGCCCCGCGCG<br>CCTCGCTGGGAGCACCCGGGCCGAGGCTC
GSS	CTGGCCCAGGCCTGTGCTAGGGGCTGGAAGACAGAGGAATTCCAC<br>TCTGAATGGCCAAAGCAGTGGCCCCCAGCCAGGTGGGGAGATGGC<br>CTCATAAACAACAGTATGAGGCAGGGTGATATCACTGCTAAAAAGA<br>GACAGTAGCCATGTGCACAGATGCAGACCATGTGTGATGCAGACCC<br>TGGGGCTGACAGAGATGTTCCAATCAAAAGGCACAGTCCAGTGGC<br>TGGGTGCGGTGGCTCCCGCCTGTAATCCCAGCACTTTGGGAGGCTG<br>AGGCGGGTGGATCACAAGGTCAGGAGATCGAGACCATCCTGGCTA<br>ACACGGTGAAACCCCATCTCTACTAAAAATAGAAAAAATTAGGCGG<br>GCGTGGTGGCAGGCGCCTGTAGTCCCAGCTACTCGGGAGGCTGAG<br>GTAGGAGAATGGCATGAACACGGGAGGCGGAGCTTGCAGTGAGCC<br>AAGATGGCGCCACTGCACTCTAGCCTGGGCTACAAAGCAAGACTC<br>CATCTCAAAAAAACAAAAACAAAAAAAACCCACAGTCCAGACTTG<br>GAGAGAGGCCCTCGCAGGGAGAAAGGCAAGACACAAAATTAAGG<br>GATTGGCCTCCATTCACAGTGAAGTACATCCCTGCATACCTTTTTTC<br>ACCTAATTCAGACCCAAATGTTCCCACAAACCCCAAGAACAGAGG<br>ATGAAATTGATTCAACAGTTAGCTTTGGGAAGTCTGAGCCCATGGC<br>ATGTCCCAAGGTACATATGTGGGAACACCATGGCTGACACTGCTGC<br>TGTGATCTTTGGCAAGTCAGGATACCTCTTCAAGCCTTCCTTTCCCC<br>GGCTGTAAAATGGAGGCTATAAAGATACTGATGTGATAGGGAGGTA<br>AGGGAAAGTGAGATAACGACTGTAAAGTCATTAGCCAAGTACCTG<br>ACACTTAGCGACCAGATCAATAAAGGGTAGTTATTGTTAGGAAAAA<br>ACAGATTTCACTTTGAAAGTGAGGAAAGCCAGGCGCGGTGGCTCA<br>CACCTGTAATCCCAGCACTTTGGGAGGCTGAGGCAGGCAGATCAC<br>GAGGTCAGGAGTTCGAGACCAGCCTGGACAGCAAGGTGAAACCC<br>TGTCTCCACTAAAAATACAAAAAATTAGCTGGGCATGGTGGCATGT<br>GCCTGTAATCCCAGCTACTCGGGAGACTGAGGCAGGAGAATTGCCT<br>GAACCCGGGAGGCAGAGGTTGCAGTGAGCCAAGATCGTGCCACTG<br>CACTCCAGCCTGGGTGACAGAGAGAGACTCCATCTCAAAAAAAAA

	AAAAAGAAAGAAAGAAAAAAAAATAAAATGAGGAAAGTGGGATTC
	GGAGAGGTGAGATGACTTCTGCACAGTCAGGATTCAAACTCAGGT
	CTGAGACACCAGAGGCTCAGGGCAAATCATCTCCCTCTGCTCCGG
	AATCTTCAGCAGGTTCCCTACAGTCCACTACATGGACTCCATCCTTG
	CCAGGGAGTGAGGATCCAGCAAAGGCCTGGGGCCAAAAAGACCC
	TGGGCAAGTTCCCCAATCCTATGTGTCTTGATCTGAGATCCCCAAGT
	GAGCCTGAGAAGGTCTGTGAATTCCTCCACGTAGCTGATGCAAAAT
	TTTATACTAATGGATCTCAGACGAGTCTATAGCCTCAAAAGTAACAA
	ACCACAGACTTGACCTGACTTAGCATGGTTTCTTATTCACCCAATGG
	AGATACGATTTCTACCTTGAAAGGCTGTGAAGGGATTAAATGAGGT
	AATTTGTGAGAAGTACTTGAAACATCCTCAGCTTTCATTAAATGTTG
	GTTTCCTTCTCTTTCTCTCTCCCCAAACTATTTCCATCAGTTAGCAAA
	TATTATTGAGCATCTGCCACGTGGCAGGCACCAGTCAGGTCCTGGG
	GGTAAAGTGGTGAACAAGACAGACATGGCCCATTTTCATGGGGCT
	CACATTCTAGCAGAGGGAAACAGCAAACAAATAAAAGCAACAATT
	TCAGATATGAATAACTGCTATGAAGAAAATACAAGGCCGGGCACGG
	TGGCTCACGCCTGTAATCCCAGCACTTTGAGAGGCAGAGGCGGAC
	GGATCACAAGGTCAGGAGTTTGAGACCAGCCTGATCAACATGGTG
	AAACCCTGTCTCTCCTAAAAATACAAAAATTAGCCGGGCATGGTGG
	CACGTGCCTGTAATCCCAGCTACTCAGGAGACTGAGGCAGGAGAA
	TTGTTTGAACCCAGGAGACAGAGGTTGCAGTGAGCCAAGATCGCA
	CCACTGCACTCCAGCCTGGGCAACAGAGTGAGACTCCATCTCAAA
	AAAAAGAAAAGGATAACGTGATAGACTTATAGGGTGGGGCAGCCT
	CCAGGGATGAAACATCTGAATGACCAAAGGAGCCAGTCATGCCAG
	GATTTTGGAGGAAAGCACCCAGGCAGAGAGTGCAGAAAGGGCAA
	ACGCTCCCTGGAAAGATTCTTAGTCAAGAGTCCTTCACTCCCAGTC
	CTACCACAAACTGGGTCACCTTGAACAAGTCACGTAACTTCTGAG
	GCTCAGCTGCCACATCTACAAAATGGGAATAAAGACATCTTACCTG
	CCACATTGTGAGAGGTTTCAACCAAAGGGCTGTTAAGGTCTGGGAT
	CCTCCCCAAATCTCACCATAGACACCTGATACTCATCACTTGGCACC
	CGTCTTGGAAGAGGGGAACCTGCACAGAGAACCCTGGGTCATGCT
	TTTGATTTTTAATTTCATGCTGCACTAGAAATAGCTTCTTTTGTTCCT
	GGTTGACCCAGGAGCCTCTTCCTGCCACCTGGGGCCTATTCTAGTT
	AACAGCTGCTTATCCCCTCAGGTACAAAAGCCAACGAGGAAAGGA
	CATCAGGAAACATTGTTCTGGGAATAACCAGACACCTATCTGCCAC
	CATCTCCCCCCATCCCGTGACCACACACGGGAGACTGGAGGACTC
	AGCCTGTCCTGTAGTCAGATAATGTACATGGTTTATTTAAAGAGTCA
	AAAGGGGCCGGGCGCAGTGGCTAACGCCTGTAATCCTAGCACTCT
	GGGAGGCTGGGGCGGGTGGATCACCTGAGCTCAAGAGTTTCAGAC
	CAATCTGGCCAACATGGTGAAACCCTGTCTCTACCAAAAATACAAA
	AATTAGCCGGGTGTGGTGGTGGACGCCTGTAATCCCAGCTACTTGG
	GAGGCTGAGGCAGGAGAATTGCTTGAACCTGGGAAGTGGAGGTTG
	CAGTGAGCTGAGATCGTGCCACTGCACTCCAGCCTGGGCAACAAC
	AACGAAAACTCCGTCTCAAAAAAAAAAAAAAAAAAAAAAGAGTCA
	AAAGGATCTTGGTCCCTGGGTTGGGCCACTGATTTACGATCACTAG
	GAGTTCTCACTCCTAAATTTCTTTGATCTGTTCGCTTCGCTCCATCT

	CCACAGCTGCTGCACTGGCTACAGCCTCATGATCTCACATCTTAACT<br>CTCTCTGCTTTCTCTGGCCCATCTCCCCACTTCCAAACCATTTGTCA<br>CGCTGTAACCAGTGCCTAACACACAAAACTAACCATGTCCTTCCCC<br>TGCTTAAAGCCCTTAGCTCCTGTTGCTCAGTGGAACTGGCGTCTGA<br>GGCTACCTCTCCAAGCCTAAGCGCCTAAGTCCTGTTGCAACCCTCA<br>GGCCCTTCCTCATTATCCACACCAAACTCCTCAGTGTCTGTAAAAC<br>AAGCCGAGCAACCCTCAGAATTATATGCCTTCGCTGCTCGTTTGTTT<br>TGTTTTTAGGACAGGGTCTCACGCTGTCACCCAGGCAGGAGTGCT<br>GCAGCGTGATCTCAGCTCACAGCAGCCTCCGTCTCCGGGGCTCAA<br>GAATTCTCACGCCGCAGCCTCCCGAGTAGCTGGGATTACAGGCACG<br>GGCCAACACACCCGGCTAATTCTTGTATTTCTAGTAGAGACGGGGT<br>TTCGCCATGTTGCCCAGCCTGGTCTCCAACTCCTGAGCTCAAGTAA<br>TCCACCTGCCTCGGCCTCTCAAAGTGCTGGGATTACAGGTCTGAGC<br>CACTGCACCCAGCCAGCCTTTGCTGCTTTTGTTCCTGCAATTTGGA<br>ACACTGTCCCCATCCCAGCCTCTCACCTCTACCCCTACCTCCTTCAC<br>TACCTATACCTTCCTATCCATCCTTCAAGACCCCAAAAACCATCCCT<br>GATTCCTTCAGAAAGGCAGTTTATTGCCTATCTTATCAGACTGAAAG<br>CAGTGGCTGTGTCTTATTTATGGTTAATTCCCTAGAAGCTGGACTGA<br>TACATTCCATTTAACTAAAATTCGTATCAGGTGCTTCGGACTGCAGA<br>CAAGCCTATCACAACCCAGAAGGAAGAAACAGGGAAGGCACCTG<br>GGGGCTGCCAAGCAATGAGGTGGGGGGTAGGAATCATGAATCCGC<br>ATATTTTTAAAAACTGCCCCAGATCCTGATGTAAACGGTACAAGAG<br>AGTCTGAGAAACACAGGGCTCCCCTCAAACAGTCCTGACTTCAGC<br>ATTCCTGGAAAAATGAAAATCCTTTCCTTTTGCCTCTAATGCTTTCC<br>CTGCTGGTATCCCAGGTTAAAAAAAAATAGATAAAATCAGGGGGAT<br>TTTTCTGGGACTTGGCTGGGCTGGGAAACAAGCCTGGGTTCTAATA<br>CAGGCTCAGCCCCTGACGTACTATGGGCCCCTGCCCCTCCTTGGGG<br>CCTCCATTACCACGGCCACCCCCACCCTTATCAATTGTGTGCCCCTG<br>AGGTAGTGACTGTCCCGCTCTGAGCATTAGTTTCCCCATCTTCCACT<br>AGTCGTCGTCAGCTCTGACGCTCTATGAGCTATGCATACCCGTAGCT<br>CCCCGCCGACCCCGATGGTCCCCTCCCCTCCTTCCCAAGGTCCATC<br>CGCCAGGGTGCAGCCGACGCACTCCTAATGCTAAGGCCGCCCTCTC<br>ATCGACCGCCCCTTCCTGGCCTCGACTCAGCGCCAAAGGTATGGGT<br>CTCTGCCCCGCCTGCTCTTTAAGCCTAGCCGGGGCGGTCAGCGCAA<br>GCGCACTGGGTCGCATCGAGGCCCCGCCCCCTGAGCCTGGGTAGC<br>GGCGCGAGGGCCGGGAGAACCGTTCGCGGAGGAAAGG
HAND1	GGCAAGAACCTCCTGGCCAAAATGTAAAGAACGTCACTTCCTGCT<br>TGGACCCTTTCTGGCCTGACCACCCTCGTCTCAGGAAAGAAGGTAA<br>TAAGGGGAGACAGAGGCTGCTGTTCACCCCAGGTCCCCTCTGGAG<br>ATCTGGGCTTTAGCCGCGCGTCTGCCACTCATCGGCCGTGTGCCCTT<br>AGGAAAGTTCCTTCCCCTCTCTGGGCTAGTTTCCCGTCTGCAAACA<br>GAACCTTGGAGCTCCCAGGAGCAATCTACTCCGTGCCTATCTCTTC<br>CCTGCCCCGACTGTCCCCCATCCCTGGGAGCAGGCTGGGCCAGATT<br>GGAAAGTCCTAGGTCCTCTTCAGACATGGCAGAGAAATGTTTAAGG<br>GATTTGCGCGCTCAAGCTGCGGGGTAAATGGTCCGCGGGCTGATTT<br>ATGGCGCGTTACTGCGCGACGCGATTCCCCAAGACAGCGAGAATAT

	TTTACACCGCGCTAATATAAACAGGCGGCCGGCCGGGTCCTTTTTAT<br>GGAGTTCCTGTAATTACCCGGGCCCTGACAGGTCCTTGATTTACTG<br>CCCTATTTAGATAAGGGGTCCGGTGCGGGGATTGCCCGTGGCGTCA<br>AGGTCACGGGACTGCGGCGGGAGAAGGTTTTGCGGTAGGCGCCGC<br>GGCCTGGGCTTCCAGAGGCCTGCCAGTCCGGGGGGCGCGGATAGG<br>GGTGAGGGTAGGAGAGGGGCTAGGGCAAGAGCGAGACCCCACCA<br>CTGCAGAACGTCCAGGCTTGAGGGACCTTCGCATTGCCCGGGTAC<br>AGCTGTCCTACTGTTATGGTTGGGGGAACTGAGTCCCGTAGGTGAC<br>AAGACTGCACCCCCCCACCAAGTCATTTAGGGAGGGACATGGGGGT<br>GGAGGGTGGAATCTGGAACTGGAATTCGGGTCTCTGGCAACATAAT<br>TCCTACACCTGGCGATGTCTCTGTATCTCCAGTCTTATGATGGCTGG<br>GTTGTGTAGGGTCAGTAGGGAATGGGAAGGAACCTAAGGCAGTCA<br>GAAGCAGCTAGGTCAGGTCAGAACAGAGCCCTGCAAGGAGGATTC<br>AGTGTCCTGGTCCTTCTCCCCATCCAGAGCTTTGTAGTCATGTGGC<br>GGCATGTAATTCCATTTCCGCCCCTATGAAATTAATATTCCTAAAAAC<br>AGAGCCACCATTTATCCAGGTTTACTGTCACTGTGCTAAGAGCCTC<br>GCCTTGTGCTTACTCTTACGCCAGCCCTCCACAGAGGTTATTATTGT<br>CCTCCAATTTACAGAAGAGAAAACTGAGGTGTAGAAGCTTCACAC<br>AGGGAGGAATGATGTGCACAGTTTTAACTCAGATGGGCCCGATTCC<br>ATAGCAAGGTGGAATGTGTTTTATGTGCATAAGCCCTCTACTTTGTA<br>AAACAGGGTAAAGAAGAGTTCCTGCCGGGTCTCACCACTACAACT<br>GGGGGAAGAAACGAGAAGCCTGCATTTGAAAATACTAAGAAGGCC<br>AACCCCCCTGAAGGCACTCTTTAGGTGAGCAAGGTTCGTTAAGAG<br>GAGGCTGCAGGGGCAGTGTGGGCTAGGACCCTGGGAGTTCTAGTC<br>CTTGGTCTGTTGAGGTAGGCAAGAGGAAAGCGGAGTCCTTCTGTA<br>GGGATCCCTGAGCTGAGCACTAAGTCCCAGGAGGAACCAGGGCAG<br>GGTCCCTGCAATAGTGAGGAGCTGGGAATTCCAGAATTCCAGACTA<br>TCAATCCTATTCCCTTCTTCTGCAACTGGGGAAACTGAGGCTCGGA<br>CTGGAAAGGAACAAAGAGGTGCTAGATAGGGAAAGCCAGGACTCT<br>TGCCTGTCACATCTCTTGCCTGCCAGCCTAAAGCAATTTTAGCCAA<br>AATCCTAGGTCTCAGACCTCCTCCTACAATATAGCTTGGGCAGAGAT<br>AGAGAACAACCTTACAAGGTCCCCACCATCCCGGGAGTTCTGCTG<br>CTCTCCACGGACTACAGTAAAGCATTTTGGGGTCTCTGAGAAGGCA<br>GTCTAGAGCCTAGTTGTACTGCAGTCTCCCCCGACGTCATCCTGCT<br>GGGGGTTGCCCCAGCCCCAAGCCTCAGGAGATCCCTTAGGGCTGA<br>GTTTCTGGTGGGGGCACCGCTTGAAAAATGACACTGAATACCTTCT<br>CTAGGTTGGGGAACAGTGCTTTCCACATCACTGGCAGCTGGAGAC<br>ACCCTGCTCTTTAAGACCCATGGGGGGCTGCCAGGTAGTCTCAGGG<br>GCCCTCAAGGGGTGTCATAGGACTGGGACTCCTCACCCAGTACTGA<br>TAATGCATTCCCCACCACACTAACCAGGCCAGATATATGGGGGATGT<br>ATACAACCTTGAGTGATCTGTGTAACATCATAAAAGCAACTAGGAC<br>TCATATGGGCAGGCGCGTGGGCCACACATTTGTATGTGTGTTATGTG<br>TGAGCATGTGAGAACCTCTATTTGTGAGCACCTGTGCATATGTAGTA<br>GATATTGTGACTGTGTACTTGTGCACTGAAGGCTTAGCGGTGACAC<br>TGAGAATATACAAAATGTATGATGGGATTTTGGAAGTTTGTGTTGCG<br>AATACAATTGTGGAGAATCTTAGGAAGATACAAGCTGGTTTAAATT

	GTGGCAATGAGATTATGTGTGAAACCCTGCATTTGTGTAGTATTCAG AATTTATGTGTTTCTACCATATGACTTTGGTCTCTGTGCATGCCTGAA TGGAGGATTGGGAGCGGCTCCCAGGTGTAACACATCTGAAGCGTG ATGAGTATGTACCCTGTGGGTGACAGAGAGACAATGTGTAAAATCC TAGGAATGCATGAATGCTGTGTGTACCGTGGCTGTGCGTATTACTGT GAGAATACACATACTTTTGGGCGTCTTATGAAGATACGGGCTTTTGT GTACGGCGGCAAGGTTTTGTGAGCCCTTGTGCATGTACCTGTGAAT GTATAATCTGAGGGCCTAGTGTGCCGCTCTGAGAGGCACTGTTGCA GATTGTACTTGGATTAGGACAAAGACTGGGCGTGTGTTGAGGATTG TGTGTTGAGGTCCTTCTCTCCACAGCGTAATTACTCTCTACCTGCGC TCTTTCTAGAATGCCTCCAGGCCAGGGAGGCTTGGGACCCCGCAG AGCCGCCGCAGCAGCGGGCCCCCAGCGCTCCCTCCCCGCGTGCCT CCGGCTCCGCGCTCTGCCCTACCCCAGCGCTGCTGGCGCCAGGCG CCGGGGAGAGCGGGGGGTTTCCAGCCCGCCTCGGGCAGGAAACC AGGACCTCCCTGCACCACCCCCCCATGCACCTCCAGGGCCCACAG CCCTGCTGAGCTCCCACAGGTGGAACCAGCAGGCCCAGGCTTTTG TCTCCGTCACTCCTGTATGTAACCCCCCACCCTTCATTCACTCTCTT GTCCAGGAGTGGAATTCCACTCATCCACTCCCTACCGGACCTCGAG AAAATCGCTCTTCTTCTCTGGGCTTCACGTTCCATATCTGTTCAAGG AGAGAATGGGCCTTTCTCCCTCTGTCTTTGGACTAGTAACTATGATA GGCACATAGTAGGCATCAATAAATAATTGTTGAATGAATGGGTGAAT GATTTGCCTTCTAAAAAGAGCACTCACATTAACTTCTCCTTTCCCCC TCCCCTCGACTTTGCTTTGAAGAGAGGCACACTCCCTCTACCACAC CGAGGGAGGGGGCGTTGAGCTGAGAAAGGTTGAGAGAATGAGGG ACCCAGGTAGGTGGACATCGGCCAAGAAAGGAACCACAGCGGGA GGTAAGACCGAGAGTCCCCAGCTTGAAGCGTCACCACTCCGGGAT TCCCAGATTCCAACGCGAGCCTGGGGAAAGCCCACAGTGGAGAGA GTCCGGCTGGCAGGGAATGGCCCTACCCCCGGGGTGAAATCTCGG AGGGTCGTGCAGCCGAGTCGCGCCTCTGCGCTGATGCGTGAGAGA TGCCGGACGTCGCGTTTGCCTGTGCGAGCCTCGCGGATGCTGTGCA GTCTTGGTCCCCTCTGCGTGTGTCTAACGCCGAATGCTGGTGTCTC GAGGTGTGAGCTTCGGGGCCGGTGTCTTTAAAGAACCAAAGATTC TTAAGGAGTGATGATCTGGGTAGAGCGGCCCGACGTAGCCGCGCTC CCAGGTCTCGGTGCGAGTCCTGCGGACAGACCAGAGGAGACCTGC TGGCCAGATGCCCCGGGCCCAAGGCGGACGCCAGACTGTCTCTGC GCCAGCCGGGCTGGCCTTCGGAATGGATCAGGCACCCGGGAGGCC GGAGTGGATCTCAGACCCTCAAGCCGGGAACAAACCCGTCGATGC CCGTGGGCCTGGAGTCCGCCTCCTCCTTCCCGCCCCACCCCTACCC CTGCCTCCGAAAGGCTTCTTCGCTGGTCAGTAGCTGCGTGCCCGTC TGCCTGAGGCTGGGTCAGAATTGGCGGGCTGGTAACGACCCCGTG CACAAGCGGCTCCCAGTCTCTCCAGAAAGGGCCGATGACTAAGGG GTGGGGGTGGGGGCGGAGGGCTGGAAGGTGTTAGGGAAGAACGT TAGCGGCCTATCCTGTCTTCAGCAGCGCCCTCTCATCTTCTAGCTCT GACGCCGAGCAGAGCAGTTGGAGCTCGGGACTGGGAACTGCTGG AATTCCTATTTAGACTTCTAGACAGTCTAGAAACAAGAACCTTTCTT TCCCTGGGCCTCAGTTTCCTTGTCTGTAAAATCAAAAGGCGGGCTC

	TAGGTGTAGGCCTTCTTTTCGCTTGGTGATTCTGGATTCCTTTCCTTGGATCCGTGGGGAGGGGGTGGCAGCAACAGTCCAGGGCGTTGGCCGTCCTGTGCCTCAAGTACGTAGTCCCCGTGCCCGCCCCCTCAACACCCCCAGCAGCCCGCCCCCCTAAGCCCGCAGAGCAGGGAGCTGAGTGGGAGGGGCAGAGGCGGGGCCGGTTCCCAGTCCCTGCTGGCGGACTAGAGTGGCGCGGGCTGAGCGTAAAACCTGGGATAGCCACTCCCCCTTTTCCTTATCCCCGCCCCCCTGCCATTGGCTCCCGGGAGAGGTTGACATCAAAGCCGCGGTCTTATATAAGCCAGATCCGC
HIF1A	GGCTGTATCCAGTCTATTGATGAGCCCATTAAAGGCATTTTTCATTTGTTCTATTGTTTTTATTTCTAGCATTTCCTTTCTATTCTTTCTTAGAGCTTCCATATCTCTGCGTACATTACCCATGTGTTCTTGCATGTTGTCCACTGTTTCCTTAGAATCCTTGGCATATTAATCATATTTATTTAAAATTTTTGGTCTCAAATTCAAAAATCTCTGCCATATGTGAGTCTGATTCTGATGTTTGGTTTGTCTCTTCAGACTGTGTTTTCTGTCTTTTAGCATATTTTGTAATTTTTCTATCATTGTTGTCATTGTTGAAAGCCAGACATGCTGTATCAGTTAAAAAGAACTTAGGTAGATGCGCCTTTAAGTGTGAGGTTTCATGTTTATCTGGCAAAGAGGTAGGCTTTGTTACTGTTCGTGGTGTCAGAGGCTAAAATTTCCTACTGTGTCCTTGTTTTTGCTTCCTATGTTGTCTTTGGCTGTCCCCAGAGACTCTTAAAATAGGGTCTGAGGTTTGAGTTCTTTTAGCTGTAATGCCCCGTTATTACACAGGAGCTCTTCTGATGTGGTGATAAGGTGAGGGGGAGAGGAGGACTTTCTGCAGTCCTATGATTAGGGCTCAAGTCTTTTAGTGAGCTTGAGTTGAGTATTTCCCTTCTCCCCTGTGGAAGGCTAGAGCTGACCAGAGTTGGTAATTTCCCTTCCTCCAGATTGGTTAGCCTTTGGTAGATTCATCTGGTTAGGCTCTGATAGAGAGTTTCCCTTGAGGCCAGGTCTTGTTAAGAAGAACAGAGAGCCCTGAGAGTATTTCACGATGGTTACTTACCCCTTCTCCCTGGCAAAAGCAAAGCAGATTTTTCTCAGATCTTTACAGTGAGAATCTGACAGGATTCACAGAGGTAAAACTGAGGTAAGTATTGAGGCCCCTCTCAGACTGAGCCTCCTTGGAGTTTTTAACTCTCAAGCTAGTCTGCACTGAGCCTCCAGCAATTCCCCAATTACAGTTTAGTGTTCCTACTGATGTTGGCTCCAGCTGTGAAACTAGCTTCAGCTTCTGGCTTCTGTGCCTGGGCTCTGCTCCTGGTAAACTGTGATTCTCTGAAAAGCTGTGATTCTCTGTATCTATCTGTCTGTCTCTCTAGTTTTTAGGGCAGTGGTTTTTCCTGTGACCTCAATTCTCTGGTGGATCTAAGAAGAGTTACTGATTTCAGTTTGCTTAGCTTTTTTTTTTCTTGTTGTGAGGATGGGAGTGACAACTTCCAAGCTCTTTACATGTTGAACAGGAAACTGAAAGCCCCTTGGTGTTCCTTTGTAAATTCATCTTAAAAATATTTATCATAATTGAAAAGTGCTAATATCAAATTTTCAGTCTGTTTATATTCCCCCTAAACTCAGATAAATATACATTTTATTTTGTGTGTCTGTGTGTGTGGGTTTTGTTGTTGTTTGTTTTTTGTGTTTTTTTTTAAGATATAGGGTCTTGCTCTGTCAAGGCTGGAGTGCAGTGGCACAATCGTACATCTCTGCAGCCTCGAACTCCTGGGAGAAAGTGATCCTCCCGCTTTAGCCTTCAGAGTAGCTACGACTACAGGCACTAACCACCAAGCCCAGCTAATTTTTAAAATTTTTTGTAGAGATGGGAGTTTCACTTTGTTGCCCAGGCTGGTCTCAAACTCTTGGCCTCAAGTGATCCTCCTGCCTCAGCCTCCCAAAATGTTGGAATCACAGATACTTTGTGTCTTGATTCTTGAAAGGAAAAAACAAAGATTTTTAAT

	GCCTCTTATCTTGTACGCACTTTCCTTCCAAACAATACCCTTTTGCT<br>GCCATTGTTCTCGTTATGAATAGCTTAAAGAAAAAGAAACAACTAA<br>GGGTAGTAATAGGCCAGGAATCACTTACTGAATACTAGGTCTTCTTG<br>TATAGTTTGATACCCTATAAATTGTGTGCATCTGATGCATTTCACCTT<br>CAAAAGGCTCAATGCTCTGTATTATTTAGTAGTAATCAAAATTTCAA<br>GTTTTACTTAACCTCCTGATTCACTGCCCAATTTCCTAATAAATACG<br>GGCTAAGGGTCAATGGGGTCATTTGCAAGTAATCTTGTAGTCTACTC<br>AGAAAGTTCTGCAAAGTTAGAAAGTGATTAAATGACTGTTTGTTAA<br>GATATACTTACATAGTAATAACCTAAATGCATTTGTTAAGTGGTTGTA<br>GAGAGAGGGATTTAAAATTTTATCCTATATGAAATTTTCCTTTTTGGT<br>GTCTGTTATTTAATAGGATTGTTTGAATTAGGGGATACTATTTGGTGC<br>CTTTGTAACTATATGAAAATTAGTTGGTTGAATATTACTGCTTTCCAT<br>GTTCATATTTATATTTGTATAGACATATATATATATACACATATACTACT<br>TTCCTTTCCATTTTCATATTTATATTTGTGTATACACATATACATAAAC<br>ATATATTTTATACATTTTTGAAAAGGAAAATTAACTTAAGGGCATATT<br>TAATGAATATTCAAAAATTTTTTTGCTGATCAAATTATCATTCTGCTT<br>TAAACTTTTGAAATGATCCAAAAAAATTTTAAATGACTTAGATTTAC<br>TGTTACAAAATGCTTGTCTTTTGATGTCACAAACATTATATACTATAA<br>TCACTGGCCAGAGATAATTGCTATAAGTATAATGAAAAGGGAAATG<br>ATGGAAGATCTCTGCAGCTATCCTCATAAATGAGGGTGGGAACACG<br>ATGGGCAGTTCCAAAGTTGAAAATAGAGAATATATGTGGATTTATAT<br>TAACATAATTGGTATTCTTGGATAGTTAAAAATGGCTAAACTGTAGG<br>AGAAGCCCGAGTAATTACTGTTAACAGAGGAATAAATTTGAGGGCA<br>ATAATAATGATGATAGGCCAGGCACTGTGGCTCATGCCTGTAATCCC<br>AGCACTTTGGGAACCCGAGGCGAGCGGACCACCTGAGGTCAGGA<br>GTTCGAGAGCAGCCTGGCCAACATGGTGAAACCTCGTCTCTACTAA<br>AAATAGAAAAATTATCCGAGTGTGGTGGTGCGTGCCTGTAATCCCA<br>GCTACTTGGGAGGCTGAGGCAGGAGAATCACTTGTACCTGGGAGG<br>CGGAGTTGCAGTGAGCCGAAATCGCGCCACTGCGCTCCAGCCTGT<br>GGGCCAGAGCGAGACTCCGCCTCAGAATAATAATAATGATAATAATA<br>ATAACGCCACCAACAATACTAAGAGCTAACATTTACTGAGTGCTTA<br>CTATGCACCAGATATTGTTCTAAGTATACATTTATTATCTCATTTAACC<br>ATCCATAATACTGTGGTATAGACACTTTTATATCCATTTTATAAATAAG<br>TAAACTGAGTTATGGAGAGATTAAACGACTTGCCAGTAAGATTCAA<br>AGCCTGTGTACAAGCTCACGCTTGATTCTGGAGCCAGTGTTCTTAA<br>CACAGTATCTTGAGAATGTTAAACTAAAAAGTTTTTAATTTACAGTA<br>TTCTTTCCACAATTAAAAAAGAAATTATGAGTAATTATTTTTAGTTCT<br>TTCTTCTCTTCAGGCATTTCCCATGGTTCTTTTCAAGACATAATACAT<br>ATCATTTAGTGTTGTAGATCTGAAAAAACAAAAGTAGCGTGAAGAT<br>CAAAAATTTTCTAAAGAGACGGAGTCTCGCTACGTTCCCTAGGCTG<br>GAACACCCAGGCTTCTCCAGCCTCACACCTCTGAGTAGCTGGAAC<br>CACCCTGTCCGCTAAGGTCAATGTTTAATCGTATCTTTGTAGGTCTA<br>CTGACCAGTTAAAAAGAGGTGCTGTATACATTGGTTGTTGTCTTGT<br>CAGAGTTTGATGCTTCTATATAGACCATTGTTTTTACATGCTAATACA<br>ATTGAAAGCCACTACAGATATTTATATTTACAACCCAAAGCTAGGTT<br>TTAACAAGAAACTCATAAGGCAAAGGTGAGAAGTAAAATAATTTAG

	CGCCAAGTGGAGATATATGTGCAATGCTACTTTGTTGGGCTCAAAA<br>CATATTTTTCTTTTAGAAGACTGACAGGCTTGAAGTTTATGCCTCCA<br>AAGACAAAAGTGATTATGTTTTGTTTAGTAGCTTGCAAAGTTGCCA<br>AAGGCCATTTTTTCTACTCTTTCCCTGAAATTGGTTTATATGCTTATT<br>AAAGTCATTTATACCTATTTGCAAATGCTTAACATAGTTTCAGATTTT<br>AAGATTTCCCTGCAACTTTATTTCCCTTGAAGTTTACAGCAACAGG<br>AGTTCATTTTTATTTTTAATTGCATTTATTCAGTAAGTAAACTCCGCC<br>ACAGAAAAACTTAGTAGACAAGGTGAGTTCCCCTGTGCTCCGTGG<br>CAAAGAGTGCGGTGGGTGACATTGACCCATGGTTAGGTAATCTGGT<br>AAGGAAAGACCCCGTTGTAACACATCTGAGCAACGAGACCAAAGG<br>AAGGGCTTGCTGCCACGAGGCGAAGTCTGCTTTTTTGAACAGAGA<br>GCCCAGCAGAGTTGGGCGGCAATCGTGCCCAGCACTGAGGCCGAG<br>GAGAAAGAGAGCAGGAGCATTACATTACTGCACCAAGAGTAGGAA<br>AATATGATGCATGTTTGGGACCAGGCAACCGAAATCCCTTCTCAGC<br>AGCGCCTCCCAAAGCCGGGCACCGCCTTCCTTCGGAGAAGGCGCA<br>GAGTCCCCAGACTCGGGCTGAGCCGCACCCCCATCTCCTTTCTCTT<br>TCCTCCGCCGCTAAACACAGACGAGCACGTGAGCGTCGCAGCCCG<br>TCCCAGCTGTGCCTCAGCTGACCGCCTCCTGATTGGCTGAGAGCGG<br>CGTGGGCTGGGGTGGGGACTTGCCGCCTGCGTCGCTCGCCATTGGA<br>TCTCGAGGAACCCGCCTCCACCTCAGGTGAGGCGGGCTTGCGGGA<br>GCGCGCGCCGGCCTGGGCAGGCGAGCGGGCGCGCTCCCGCCCCCT<br>CTCCCCTCCCCGCGCGCCCGAGCGCGCCTCCGCCCTTGCCCGCCCC<br>CTGACGCTGCCTCAGCTCCTCAGTGCACAGTGCTGCCTCGTCTGAG<br>GGGACAGGAGGATCACCCTCTTCGTCGCT
HLA-DRA	GTGTTCCCTACACACCAGACACGGGCCATGAAGGAATCTGAAGAG<br>CCTACCAAACCTTGCACAAGAGAAAAGCTTTACTTGGAACATCATC<br>CAGGCTCAGAGAACACAAATATTTCATTTCCAGTAAGACGTTTCTG<br>GTCTTTTTCTCTTCCTCCCCTTCCCTGAACCTACCCTAGATGAGCTA<br>TGGCCTCAAAGTGCCAGTAGAACGTAAGAAGGAAGGAGAACCAC<br>ACTCATTCCTGCCTTCAACAATTTACACAGGGATAGAAAGAGATTTA<br>TATTAAATCAAGTTGGGACTTTCAATTATTATATAGTACCAAACAATC<br>TAATTGCTGAACTAAGATATACTTGTGCAATTTAAGGGAATTGTAGA<br>ATAGCATATTAATTAGAATCAAGAAAATAATTCATGAAGTATGCTATA<br>ATTCCTACCCAAGCGCAGGGGAATAGCATCTCTAATGAAATTCTCTA<br>AAGAGGCAAGAGCAGGCACAATGAGTTTTTGTTTGATTAAAGATTC<br>CATTTAGTGCTTATCCAACCTAGCAATTACATTTGTATGCTTCAGATG<br>TTTTTAAAAAAATAAACAAAAGAAAGTACCTTAAATAAAGAATAGG<br>ATCAAATAGTATTTAAACAATTGAGTAAATTAAAAAATTATATGAATT<br>AGATTGATTGAAATTGATACTTTCCTAATTCTCCTCCTTCAACACAC<br>AGACACACACACACACACACACACACACACACACGTATGCATACA<br>AACACATCTGAATTCTATAAAATCATTCTGACCTTGATGAGATTCCA<br>TAGTTTACTCATGCAACAGAACATAATGTCTAAATGAAGTTTCTGGT<br>CTCTGTTTTACATGGATGATTGAGTAAAATCATTCCCTATTCCTGGA<br>AGAATAGCTAAGAAAGGATTCACAGGTGAGGACATGCGTTTTTTCA<br>GAAGATGAGAACAAAGATGAGAAGATGAGAGCAACAGAATGTCCT<br>ATATCCTAATGCTCTGTGCTGACTTCGGAGTGGCCAATATGATAGAG

	ATGGAAGGAACTCTGAAAACAAATTGCCAGAATTTCTAAGGAACA<br>GGAGATGTTGAGTGAGTGAATCAAGCCATGGACTGGCTGTATGGG<br>GGCAGCTATTAGAGACAACTACCCTTAGACTTCTTTGGTGATTGGTC<br>AAGCTAATCTTTTCCTTCAGAGTCTCTCAATTATAAGACTTAGCTTG<br>TGCCATTTAGAACAGACAAGAACACAGAGAATTATAGAACAATCTG<br>ACTACAGGTTCTTAAGTTATAGCAATGAAACTTGTAGTTGGCCGGC<br>AGGAAAATATTCTGAGATGTGGATTCAAAGTTTCTAAGTGTGCACA<br>CGTACACACACACACCCCTACCTGCATGCGTTTTCTAATTTACAAAG<br>ACTACTCAAGTAAAGAGGGGTAATTTCACACCCCAGGAGGTCTGTA<br>TAAAGATAACTCTGGTCTTTAAAGCATCGGGTTTCAGGTAGAGGTG<br>AAGAGAGAATGAATCAAACTCAAACTGCCATCCTCCCAGGTTAAA<br>GATGAGTCCAGTCATTGTGGAGCCCTCTATTAACACAGGACATGCT<br>AGGAAGGCCCATTAACCCACTGCCCTAGCACATTTGTTAACGTCCT<br>AGTGCATTTGTTGATATCAACAGTTCACAGTTTTTATTCTGATAGGG<br>ATCTATTCCAGCAGACCAGCTTCTGTGACCTCTCAGGATGCGAAAA<br>AGTAACACAAGAAAAGCTTCTTATGTAGTGAATTGAGAAGGAAATA<br>CCTAGATCAATATTCCCTCAGCACCTCTGGTAGGAAGTCCTTAGTAG<br>GAGAAAAACACCATGAAGACCCTTAGTGCAGAAGGAAAAGGGGG<br>TAGGGGGTGGTGGAAGGGAAGCTAAAAGAAGGGGCTGGAGGTTC<br>TCAGAATTCAAACCACACAAACAAATGAAGTATTGAGGTCCCAGA<br>CTTGATCTGGGCCCAGTGTGAAAGCCCTAACTTATTTCTCCAGAAG<br>AATATGTCCTCTGGTTTTAGACTTGGCACTGTGGGGAGAACCAGAG<br>TGATCTATGGTGGATATACACACAAACATAGACACACATATTTGCAT<br>TTAGTAATTTTTGTAAAATTTCCATTTGCTTCTCTGATCCTGTCTGTA<br>TCTTTGGGAATAGATGTAAGAATATTACATCTCTCAGGCTTGCTCTG<br>CCCCAGGTTTCTGAACGTGGAATACATTTCTCCAGGGAAACTCAGT<br>ATTATGAGATTTGGGAGGTGGAAGTTAGGCCACAGCCATCTCAGGG<br>ACAGGTTTCACAGACATGAGTTTTGGCAGCAGCCTTGTGTTCTAAA<br>GACATTTACTCCTAGGGGCTCTAGAGGATCTGCAACATCAGCAGAG<br>GCTTCCTGTGGGTTCCTGATCTTTTAAAATTAGGGTTCTGCAGTGAC<br>TTCTGCTCCTCCAGACCCCCTAACAGTTTTAAGGGCTAATTCCCTGT<br>AATATATTCAGTTCTGCTTAGACTGATTACAGGGATTCCTATTTCTTG<br>ACTGAATTCTCATGGCTATAGTGGCTCGTCACCATTTGACATCACCA<br>AGAAGTCCTCATTCAGGTGCCTTTGGAAATTCCCTCAAACACACAG<br>GAAATTAGAGTTTGAAAGAAAACGGAGAACCATGAGCACTGTCCA<br>AATAGGAACTTCTCTCCTATCACAGAGAAAGGGAACTGAAAGTCAT<br>TTCTCAAGTCTCCCAAATTTAGTAATCTCACAAGAAGAACCAATCA<br>GTGTTCTAGGACTAAACAGTGTCATAAGTTGCTGAGCAACAACTTG<br>GATTGAAGATGCTATTATAATATATGAAATGTCTTTGAATTTACCATG<br>TTTTTCTCAAGCACCATTTAAGAACAAGGCATTATGGCAGCCAGCA<br>AAGGGCAGACATAGAAAATTATACATGGTTTTGCCTCTAAAAGAGG<br>AGATGACAAGCTTAAATCATAGGATCAGACTCTTAGCACAGACTGA<br>TACCATAGGCTCTCATCTGGCCCATTCTCCTGACTCTTTACCTTTCA<br>GGAAAGGTATTCCTGAAAAATTGCAGGAGAGACCATGCTGTAGGT<br>CTCTTTCTAGCGATCTAGGAGTTAATGCCACAGTGTGTTCAAAGCC<br>CTTTGATGCGATCAGATAATCAGTAATGTATGGAATATTTGTGTTCAT

	AACTTGTGAGAACGGCTGCATGGCAGGACAAGACCCCAGCACAAC<br>AGTATGGAAAATCCACCCTAAGCAGACATGTCATGACTGATGTTGA<br>ACAATGGACTCACCAGCCAGGCACGGTGGCTCATGCCTGTAATCCC<br>AGCACTTTGGGAGGCAGAAGCAGGCAGATCACGAGGTCAGGAGAT<br>CAAAACCATCCTGGTTAACATGGTGCAACCCCGTCTCTACTGAAAA<br>TACAAAAAAAAAAAAAAAAAAAATTGGCCGGGCATGGTGGCGGGT<br>GCCTGTAGTCCTAGCTACTCGGGAGGCTGAGGCAGGAGAATGGCG<br>TGAACCCAGGAGGCAGAGCTTTCAGTGAGCCGAGATCGTGCCACT<br>GCACTCCAGCCTGGGCGACAGAGCAAGACTTCCGTCTCGAAAACA<br>AAACAACAACAAAAAAAACAATGGATTCACCATCCGATGGGCTCC<br>CTCACTGCCAGGTCACTCTTCATGGAAGTATTTGTATTCCAGTCCTT<br>TCTGTGGAAAGAACTTAACATTCTCCTTTTCATAACACTGTATCTTC<br>AGAAACAAGAGAGTCGAAGTCTCCTAATTTTCAGGAGTGTCTATGT<br>TGAACATCAAAATATATTCTTTAGAGCAGATCTTTAATAATCATATGA<br>CAAGAGAAAAACTTTCATAATCTTATGACATGAGGGAAGGAATATT<br>AAAGCCGTTCTGTGGGTTATTATCTCTAACGTTCCCAATAGAATAGG<br>CTTTGCCAGCTGGGTGCGGTGGCTCATGCCTGTAATCCCAGCACTT<br>TGAGAGGCCAAGGCGGGCAAATCACGAGGTCAGGAGTCTGAGAC<br>CAGCCTGACCAACATGGTGAAACCCCGTCTCTACTAAAAATACAAA<br>AATTAGCCGGGCATGGTGGTGGGCGCCTGTAATCCCAGCTACTCAG<br>GAGGCTGAGGCAGGAGAATCGCTTGAACCCGGGAGGCGGAGATTA<br>CAATGAGCTGAGATCACGCCACCAACTCCAGCTTGGGCGACAGAG<br>CAAGACTCTGTCTAAAAAAAAAAAAAAAAAAAAAAAAAAAAAAAA<br>AAAAAAAAAAAATAGGCTTTGCCCATTATACTCTCTCATATTCATTG<br>ACCTGAATCCTCAAATGAGGTGTGTCCATTAGTCAACTCCAATCTCT<br>TGTCATATATAAGATGGTAGAGATGAGAAGAAGGTAGCTCCTTTACA<br>GCCCACTATTTCCACTAACTACTACCTGTGTTTCAAGATACAGCCTT<br>TCATCCTTCTCCAGTGTTGAGAGTGTTGAACCTCAGAGTTTCTCCT<br>CTCATTTTCTCTAAATGAGATACAATGCCAGCCATCCCAAGCTCTTG<br>GCCTGAGTTGTTCATCTTGAAGTCTAGGACTCCAAGAAGCATGAAA<br>GAGCTTCTTTAGTGAAGCTATGTCCTCAGTACTGCCAAAATTCAGA<br>CAATCTCCATGGCCTGACAATTTACCTTCTATTTGGGTAATTTATTGT<br>CCCTTACGCAAACTCTCCAGCTGTCATGGCACAGACATATGATCTGT<br>ATTTAGCTCTCACTTTAGGTGTTTCCATTGATTCTATTCTCACTAATG<br>TGCTTCAGGTATATCCCTGTCTAGAACTCAGATTGGGGTTAAAGAGT<br>CTGTCCGTCATTGACCAACAGTCTTAAATACTTGATTTGTTGTCGTT<br>GTTGTCCTGTTTGTTTAAGAACTTTACTTCTTTATCCAATGAACGGA<br>GTATCTTGTGTCCTGGACCCTTTGCAAGAACCCTTCCCCTAGCAAC<br>AGATGCGTCATCTCAAAATATTTTTCTGATTGGCCAAAGAGTAATTG<br>ATTTGCATTTTAATGGTCAGACTCTATTACACCCCACATTCTCTTTTC<br>TTTTATTCTTGTCTGTTCTGCCTCACTCC
HLA-G	AGCTAAACATCACTGATCATTAGAAAAATGCAAATCAATACTACAAT<br>GAGATACCATCTCATGCCAGTCAGAATGGCAATTATTAAAAGTCAA<br>GAAACAACAGATGCTGGCAAGGTTGCAGAGAAATAGGAAGGCTTT<br>TACACTGTTGGTGGAAATGTAAATTGGTTCAACCATTGTGGAAGAC<br>AGTGTGGCAATTCCTCAAAGATTTAGAACCAGAAATACCATTTGAC

	CCAGCAATCCCATTAAAGGTTATATACCCAAAGGAATATAAATCATT<br>CTATTATAAAGGTATATGCATGTGTATGTTCATTGCAGCACTATTCAC<br>AATAGCAAAGACATGGAATCAACCCAAATGCCCACAAATGAGGAA<br>CTGGATAAAGAAAATATGGTACATATACACCACGGAATATTATGCAA<br>CCATAAAAAGGAATGAGATCAAGTCCTTTGCAGAGATACGAATGAA<br>GCTGGAAGCCATTATCCTCAGCAAACTCACACAGGAACAGAAAAC<br>CAAACACCGCATGTTCTCACTTATAATTGGGAACTGAGCAATGAGA<br>ATACATGGAACCAGGGAGAGGAAAAACACACAATGGGGCCTGTTC<br>GGGGAGGGCAGTGATGGGGGGATCATTAGGAAAAATAGCTAATGA<br>ATGCCAGGGTTAACACCTAGGTGATGGGTTGATAGGTACAGCCAAC<br>CACCATGGCACACGATTACCTATGTAACAAACCTGCACATCCTGCA<br>CACGTACCCTGGAACTTAAAATTAAATTAAATTAAATTAAATTAAAA<br>GATAAGCTTAAAGCATTAAAGAAAAATAATTAGATAAAAGAAGTCT<br>TTGATTTACAAAATCCTGAAACAATAGTTTTAATTTTGCTTTTAACAT<br>ATACGTAAGTCCTTTAGTACAGCTCTCTTTCAGAGGTGCAGCTTAAT<br>TCCCTCTCTTAAGTGTGGCTTGGACTTAATGATGCACTTCTGATATG<br>GCCTGTCTCTGTGTTCCCACCCAAATCTCATTTTGAATTGTCATGCG<br>AATTCTAATCCCCACATATTGGCGGAGGGACTTCATGGGAGGTGAT<br>CGAATCATGGGGATGATTCCCCCAAGCTGTGGAAGTCAGCGGTTGA<br>ACCTATTTTTCCTAATGCTCCCCTCAGCACTGCCCTCCCATAATAGG<br>CTCCAGTGTGTGATGTTCCTCTCCCTGTGTCCATGTGTTCTCATTGC<br>TCAGCTCCCAGTTACAAGTGAGAACATGTGGTGTTTGGTTTCCTGT<br>TCCTGTGTTAGCTTGCTGAGGATAATGGCTTCCAGCTTCATCCATAT<br>CCCTGCAAAGGACTTGATCTCATTCCTTTTTATGGCTGCATAATATTC<br>CATGGTGTATATGTACCATATAAGGGGATTTTCCCCACTTCACTCTGC<br>ATTTTTCTCTCCTGCCACCATGTGAAGAATGACATGTTTGCTTCCCC<br>TTCTGCCATGATTGTAAGTTTCCTGGGGCAGCCTCCTCAGCCATGC<br>ACAACTGTGAGTCAACTAAACCTCTTGCCTTTATAAATTACCCAGTC<br>TCAGGTATTTCTTTATAGCAGTGTGAGAACAGACTAATACAACTTCT<br>AACTGATAGAGTAATGTTGACATAACAGTTTGTGACTCTGGGTGTA<br>GAATGTGAAACTCACTATGGCTTCCACCTTCTCTCTCTCTGTCTCTG<br>GGATCATGAGCTCTTGGGGACCCAGCTGCTGTGCCATAAGCAGCCC<br>TGCAGGAAGGTCCATGTGGCTAAGAACTGAGGCCCCCTGGGACCA<br>GACAGCAAGGAACTAGGCTTTTCCAACAGCCATGTGACTAAGCCAT<br>GTTTCACATGAATCCCTAGCCCCAGTGAAGCCCTCAGACGATGCAG<br>CCCTAGGCTGACAACTGGACTGCAACCTTGTGAGAGGCCCTGAGC<br>CAGAAGCACTCAGGAAAACCGCTCCTGGATTCCTGACCATTAGAA<br>ACTGTGGGAGATGATGAATATTTGTTGTTTTGAGCTGCTAAGTTTTA<br>CATAATTTGTTACACAATAGTAAATAACTAATACATTTTCACAAGAG<br>AGGATGTATTATTACACATTAATTTGCATTTGCTCTAAATTTATCATCA<br>TCATATTACTATTTTTGAGACAGGGTCTTGCTCTGTCACCCAGGCTG<br>GAGTGCAGTGGCATGATCACCATGCACTGCAGTGTCGACCTCCTGG<br>GCTCAAGGGATCCTCTGATCTCAGCCTCTTGAGTAGCTGGGACTAT<br>AGGCATGAATTAACATGCCTGGCTAATTTCTAATTTTTTTGTAGAG<br>ATGGGGGTTTCACCATGTTGCCCAGGCTGATCTTGAACTTCTGGAG<br>TCAAATCTGCCTTCCTCTGCCTTCAACAGTGCTAGGATTGCAGGCG

	TGAGCCACCACACCTGGTCTAAATTAACTATAAGATATTAAACATGT<br>AACTTAGTTTTAAAAGGAAAGGAGAATTTCCACGGCTGAAGAGGA<br>TGTATTTTATTACTATTCACAATGATCACTTTACTTGAACTTCAATTT<br>CCAACTGTGTCCAAATTAAACACAAAAGGAAGATCCAGCCCTTCCT<br>GGGCTGATTCTATCATGGCTCCCAACAACCAGCTCCTGGTCATTCA<br>CCTTCCCCCAGTTATTCAACCAACTCTAATGTAGGTGCTGCTGTGAA<br>GGGATTTAGCAGATATAATTAAGGGCCTCAATTAGTTGACTTTAGGC<br>TGAGTTTATCCTGCTTGGACTGTCCTAATAAGGAGAGTCCTTGAAA<br>GGACTGGGTTCTTCCTGAGCATAGAGATTCACAGTGTGAGAGGGAT<br>TCAGCATAAGGGGTTTCCTCCACTGTGGGCTTTGAAAATGAAGGGG<br>CTGTGTAGGAAAGGACGCTGGTGGGCACCATGCATTGAGTGCAGC<br>CCTCCCTGTTCTCTACAGTGACAGCCAGTGAGGAACAGGGACCTC<br>AGTCTTACAACTGCCAGAAACTGCATTCTGCCACCTCTGTATAAGC<br>CTGAAGGAGGATTCAAAATGAAAACACAGGTTTAGGAAGACCGGA<br>ACAGAGATTCCATCCACATCATGCCCAGATTTCTGATTAAGAAACTA<br>TAAACAACAAATGAGTGTTATTTGGCCAGGCGTGGTAGTGCACACC<br>TGTATCCTAACATTTGAGGAGCTGACACAGGAGGAACACTTGCAGC<br>CAGGACTTTGAGACCAGCTAGGATAATATAGTGAGACACTCGTCTC<br>TACATTTCTTTTTAATTAGCTGGGCATGGTGGCACTTGCCTGCAGTC<br>CTAGCTACTCTGAAGACTGAGGTAGGAGGGTCCCTTGAGCCCAGG<br>AATTTGAGGCTGCAGTGAGCCATGATCATGTGACTGCACTTCATCC<br>TGGATGACAGAGGGAGACTCTGTCTCTAAAAATAAATCAATGAATA<br>CAATAAATGGGTGCTGTTTAAAGCCAATGTTTGTGACAATTTGTTAC<br>CCAGTCTTATAAAATTCATACACAGACTCAAAAGACTCCTGGAATG<br>AACTGATGAATTGATACGCACACTAGTTACATAAAATAAAATCTTTT<br>TTAACTTTTTAGTGTTTTACATTTTATAATTTTCTGTGATGCAATTTA<br>ATACACTCATAATTCATTCATTCAGCCAAGAAAAAATAATTTAGTCC<br>CTACAATGAACCAGGTATGCCCTCATATGCTCAAGTGCCTGACATTC<br>TAGAAGCTTCACAAGAATGAGGTGGAGCCACTGGAGTGTTTTAGG<br>TGGAGAAATGACACACTCTGACTCATAGTAGCAGGACCACTATAGA<br>GAGAACACTCATGTAGCAGGTCATGGAACAGTGCTAGAGCCACAG<br>TTCAGGAGTGAGAGGGTGGTGGGGATTAAGGGGAGAAGAGGGCC<br>TGAGGGATGAGAGGGACGGAGGGAAGGGCTGGAGGAGCAGGAGG<br>TGAGGAAAAGGAGCAGAGGAAAGAATTCCAAAGCAGCAGAACTC<br>TTAGGTTTAAACACATTGTTTTATAGATTTTAATACATCCATCTACAG<br>AGCCTCGCTGGGTGTTCTTTGCAGTTGGCCTTTAATATCTTATGTGG<br>GTCTGCCTAGAAACTAATTGTTTTTTATGTTAATCAGGTTTAAAAAA<br>TACTAAGTATTCCTAAAAAATATACACTCCACTCACATGTGGATACT<br>TCCTAAAAACAGGCAGTGCGTGAGCACTAGTGAGGGGCATTGTGA<br>CTGCACTGAACACTTACAACTGTGAGGTGAATAAAGTTTGTGCTGG<br>CTCCTGGTTGCAACATATAGTAACATAGTGTGGTACTTTGTCTTGAG<br>GAGATGTCCTGGACTCACACGGAAACTTAGGGCTACGGAATGAAG<br>GTAAATTTAAAATAAAACAAGCGGGAGTCACAGATACACTGTCTGG<br>GAAAGTGAAACTTAAGAGCTTTGTGAGTCGTGTTGTAATGCTTTTA<br>GATGCATTTATATACCAACAGGCCAAAGTCACATTTTTTACCGATTA<br>GATTCCTGATCATTCAGGGGTTACCAAGGTTATGCTACCCACTATAG

	TTAATAAACAAAAAGCAAACTGGTCTCTATTCTATCTCATGCACTCA<br>GGCACAACTTTTCCAGATTTAAGGGGGAAAAAAAACCCTGTCTTTA<br>CACCTACAATCCCAGGGCGAGCTCACTCTCTGGCAACAAGCTCCCT<br>GGGGTGATTTTTCTTCTAGAAGAGTACAGGAGGACAGGCAAGGAG<br>TGGGAGGCAGGGAGTCCAGTTCAGGGACAGGGATTCCGGGATGAA<br>AAGTGAAGGGAGAGGGCCAGGGACCTTGCCGAGGGTTTCTCCCTG<br>GTTTCTCAGACAGCTCCTGGGCCAAGACTCAGGGAGACACTGAGA<br>CAGAACGCTTGGCACAAGAGTAGCGGGGTCAGGGCGAAGTCCCA<br>GGGCCTCAAGCGTGGCTCTCAGGGTCTCAGGCCCCACAGGCGGTG<br>TATGGGTTGGGGAGGCCCCGCGTTGGGGATTCTCTCCTCCTTCTCCT<br>AACCTGTGTCGGGTCCTTCTTCCTGGATACTCACCGGGCGGCCCCA<br>GTTCTCACTCCCATTAGGTGACAGGTTTTTAGAG
HMGB1	GGGGATTGTTTATTTCTGCATCATAATCTAATCTATCCCAACTGATAC<br>TACAGCTTAAACTAAATATGTACACTTTATTAGTTTGTACCTTTTGAA<br>ATTGTTGTTTGGTAGTAACAAACTATCTAATAGCAACAATCTCATAC<br>AATTTGACTTATTATAACTGAGAAAATAGGAATATAAGTTTCTACTCA<br>TGAATGCTTTTCCTCCCAGATGATAATTACTTATTAAGAGAAGAGTA<br>TATTTATTACTTGTATTTTATTTTATTTTGTTTTATTTTATTTTTTGAGA<br>CGGAGTTTCGCTTTTTTTGCCCAGGCTGGAGTGCAATGGCGCTATC<br>TGGGCTCACAGCAACCTCCGCCTCCTGGGTTCAAGCGATTCTCCTC<br>CCTCAGCCTCCCCAGTAGCTGGGATTACAGGCATGTGCCACCACGC<br>CTGGCTAATTTTGTATTTTCTGTCGAGACACGGTTTCTCCATGTTGA<br>TCAGGCTAGTCTTGAACTCCCAACCTCAGGTGATCTGCCCTCCTCG<br>GCCTCCTAAAGTGCTGGGATTACAGGTGTGAGCCACCGTGCCCGGC<br>CTGTATTTTATTTTTATAAGGAACATCATAGCAGAGTTAGAGAAAAT<br>GTTGCAAATATTTACGCATTCCTAAATCCACAGCAATGCTCATCATT<br>GATCGAATGTCCTCCATGTGTCACTTTCTAGGAATGTATTAGGTGGG<br>CAAGACAGACATGACCCATACCTTCATGGAGATTTCATTCATTCTAG<br>TGATGGAGTTCAACTATAAACAATAATTGCAGGCTAGGAAATGAAT<br>AGATTGTTTTAAGTCAAAATTACAGAGTAAAAGGAACTCATGGATA<br>TGGTGGTGGATGATGGCCTCTCTGAAGAGTTGATGGTTGAGGCCTG<br>AAGAATGAGAAAAGTGTTCTAGGCTGAGGGGACAGCATAATGCAA<br>ATGGCATCTCCATGTCCCGGAGGCAGATCAGATCTGCCCAGTGTAG<br>CTAGAATGTAGTGAGGAAGGGGATCATGATGTGAGGTAAAGCTGA<br>AGAAATGGAGTATGGACATGTCATACAGAGCTTTGATGGCCAGGGA<br>GGCAGTTTGAATTTTTTTTTTAAGTCAATGGGAAGCTATGGAAGGA<br>AAATGTGAAGAAAAAGACACGAAGCAGATTATTTCTTCAACGCTG<br>ATCACTGTCTCTGCTGGGAGTCCCATATACGTACTGACACAAAGGG<br>GAAGTGAGGGGACCAGTGAGGAGGCTACTACAGGTCAGAGGTGAT<br>GGTTATCTGGACCAGGGTTGTGGCAGTGAAGAGGAAGAGAAATGG<br>ACAAAATCAAGATGTATTCAGAAGGTCAAAACAGAACTTGTTGATG<br>CTCTGGATGTGGAAAATGAGGGAAAGGAGACCAGCAAGAATGGCT<br>TCCAGACTTCTGGCTTGGATCCTTGGGTGGACATTGGTGCCACTTC<br>TTTTTTTTTAGATGGAGTCTCACTCTCTCCCCCAGGCTAGAGTGCAG<br>TAGCATGATCTCAGCTCACTACAACCTCTGCCTCCTGGGTTCAAGC<br>AATTCTCCTGCCTCAACCTCCCGAGTAGCTGGGATTACAGGCGCCC

	GCCACCAAGCCTGGCTAATTTTTTTTGTATTTTTAGTAGAGATAGGG TTTTACCATGTTGTCCAGGCTGGTTTTGAACTCCTGACCTCAGGTGA TCCACCCACCTCGCCTCCCAAAGTGCTGGGATTGCAGGCGTGAGC CACCGTGCCCGGCCAAACATGATGCCACTTATTAAAACAGGAAATA CCAAGGGAGGACTAAGGTTTTCAAGACATGAGGGGATCAAGAGTT CAGTTCTGGACATGCTAAGCACAGGATACCCTGGAAACATCTGAGT GGAGATATCAAGTAGGAAGCTCTGTGTGTGTCTGAAATCCAGAGAA ACAATCTGGGCTGGACATATGAACTGGGGCATCATCAACACATCAC TGGAATTTCAGGCTTGGAGAATAAATGTAAAGGGAGAAGAAAAAA GGTTCTCTGATGCCTGAGGAACCCCTGGCATTTAGAGGTTTGGGAG GAAACAAAGAGCAGCCATGAGGCTGGGGGAAAGCCAGAAGGGTG AGTCTTCACAGAAGCTGAGAAGAGAGTGGTTTAAGAAAACCATGG GTCAGCCTTGTCAAATGCTGCTGAGAGGCGGAATGAGAAAAGGAC AGAGAAGATGCCCTGGATCTGGCAACCTGGAGGTCTTTGGCAACC CTGATAATGTGTTTCAGGAGAGCGGTAGGACCCAAAGGGGTTCTCT AGTGGGAAATACAGATAACAGGTACAGTAAAAAATGTTTTAAAATT TGGCTAAGAAGGGGATAAGAGAAGAAGGGTGAGGGAGGAGAAGC CTAAATTAACTGAGTGTTTATGTTAAAGAGAGACTGTGAAAAATCC TGGAAGTGACTGAGTTTACCTGGAAGCTACCAGATTGTTTTCTACC ATCAGCAAAAATGGAGACTTGAGAACTAGCTAACTTGCTATACATG TGCACAACTGCATTGTGTGGCCTCTTTCTGAAATACCATGTTCCTTA AGGGTCACCTTCTCTTCTTTCTTTTCCCACATTTCTGGAAGGAAATT GTTTGGGTTGCTTTAATTATTATAGCTTTTCTATCCCTACTTTGTCTTT TTTTGTTGCTTAAGTTCTGAATGATATGCAGTCACTGAAGAATGTTC TTTTATTTCAGAAAGAATTATTGTTCCTCTCTAACTCAGAAGTGCTG ACCTATTTGGCTTCTGTATCTGTTGATACAGTTCACTTCCCTCTTAGC TTAAGGGTCATTAAATGAGGGTAAAATTTCATTATAAATTTTATTTTT AAAATGTTTAATTAACAGTTTTAATTGAAAAAATTCACACACTTTAG GTAAAGAAAAAAAGTTAAATAGCACAAAAGATTATAGAATAAATAA GTCCTCCCCAGAACCCTAGCCCTTGCTCCAGAGGAAACTGTTGCCA TCACTACCTTGTGTGAACTATTCTATATACATACAATTCTAAGGCTAC ATCTCCTGCCTGTAAAACAAAAATAAAAATAAAAACAAAACCCTCA AATGTGAGCATATTTACACACTCTGTGCTTTGCTTTTTTCATATTTCA ATATAACATGGAGGCTTTTGCATAAAGTCAAGCATCGCTTAACAAC AAGGATATGTTCTGAGAAATGTGCCACTAGACAATTTTGTCTTCATT GGAATAACCTATAGTGTACTTACACAAACATAGATGGTATAGTCTAT CAGACACCTAAGCTATATGGTATAATGTGTTGTTTTTAGGCTACAAA CCTGTATAGCATGTTCCTGTACAAAATACTGTAGGCAATTATAACAC AGTGGTAAAAAATTGTGTATCTAAACATATGTAAACTTAGAAAAGG TACAGCAAAAATATGGTATTATAATCTTACGGGACCACCATCCTATAT GTGGTTCATTGTTGATCACAACATTGTTATGTGGTCCATGATGATAC AGCTATGTCATTCTTATTCATGGTTCCATAGTATTCCATTTTATGGATG TGCCATTATGTTAACAAGTTTCTACTGATGAATATTTATGTTTTCCTA AGCATTTTAAAACCAAACCAAAAATTTTCTTGAATGATTACACAAA ACCTTTGCCTAAAAACACACAAAACCTTTAACCAATATGAAGTAGA CAACTGTCATCCTAAATCCCAAAGACTGTTGGATTGGGAATAACTA

	CTGTTTTCCTGTTTTGTAGTAAAAAAAAGGTGGCTGATGTTTGTGT<br>GACTCCTTCATCTATGAAATCTGGGGATATCAGTGACACTAATCAAT<br>GCCACAGGAATAATGAGAAGAGAAAAAAATACGAAAGTTTTAAGA<br>CGTTTAGAAAAACAATCTTTTAGAATAAAATAAATATTTTATGGGCT<br>ATCAAATGGCTTATATAGCAAATGCATAGTATTTTGTATTTTCTTAGAT<br>AATATGCCACTAGATTTTTTTCAAAATAGCAATGTTTCCTAAAGATG<br>CTTTCCTTCACACTTATATGGACAATTAATATTTTCCACTTCAAACAA<br>TACTAAGACTTCTATGGGGTTGGGGGGGCGTGGATTTCATGGACCT<br>AAGTAACGTATTCTGAGGGTGCTTAAAGTGCCCACAGGCCTTCCCC<br>TAGTATCACCACTGATTTCAGGGGCAGGGAAGTGGAGATTTTAGGA<br>AAATAAAGTTTATGAAGGTTTAGAAGTTTTTTTTTGTTTTTTGTTTTT<br>CTTTTTTGTGAGACAGAGTTTCGCTCTTGTTGCCCAGGCTGGAGTG<br>CAATGGTGCGATCTTGGCTTACTGCAACCTCTGCCTCCCAGGTTCA<br>AGTGATTCTCCTGCCTCAGCCTCCTGAGTAGCTGGGACTACACGCA<br>CATGCACCACCATGCCTGGCTAATCTTATATTTTTAGTAGAGACAGG<br>GTTTCTCCATGTTGATCAGGCTGGTCTCAAACTCCCGACCTCAGGT<br>GATCTACCTGCCTTGGCCTCCCAAAGTGCTGGGACTACAGGTGTGA<br>GTCACTGCGCCTGGCAGAAGTCATTCTTAAAGATTTTGTCTTTTATC<br>CCATGGGCTCTGGGGAGACGCTGGAGCTTCTGATCAGGAGGATGA<br>CATATAGTAATTGGCAGTGGCATGCAGAATAATTTAGAGGAGAGAG<br>GGAAAGAAGAGAATCAAAAAGGACATTATAATAGTCCAACTGAGG<br>TGAGGAAAGCCTTGGCTGGAATGGGAACAGCAGAAGCAAAAAGT<br>AAAAAGGACCTAAGAGTGAATTTAACTTTTTTTCCTGCCCTAGCAC<br>ACTTGAGAAGACAAATCCTATTAACAGTGATTCACTCTGCATTCAA<br>AACTTTGTTATAAGTGCAGCAGAAAACCCAAATTAAAAGGCTAAGC<br>TATTAAAGTATATTGCTACAAATCATTAAACTATATAAAAACAAGGA<br>AGGTTAATGTTACAGAGTGGAGAGGGTGAGGAGGCTATGCCCGGC<br>TCCCACTCTCACAGCCACTGCAGTACATTGAGCTCCACAGAGACA<br>GTGCCGGGGCAAGTGAGAG
HMGN1	GGGTCAACTGTATTTGATGGGAAGGAAATCTAGCATATCCTTAATTT<br>TGGTTATAAAATGGTCCAGAAATTAAGAAAACTTTTTTATAACTTCT<br>GTACAAAGCAGTTTAGCAAAATATATCAAGAACTTTAAACTAGCAC<br>TTTATTTCTGGAAATGTCTTCTATGAAAATCGGAAATGTGGAAAAAC<br>ATTGCTTCTTGGCCTTTTGCCTAAGATCCAAGTGTAGAAATGCAGA<br>AAAAGATTTAGGTGTTAAGGTGATCATTGTGATTTTTTTTTTTTTTT<br>TTTTTTGAGACAGAGTCTCACTCTGTCACCCAGGCTGGAGTACCAT<br>GGCGCGATCTCGGCTCACTGCAATCTCCAGCTCCCAGGTTCAAGTG<br>ATTTTCCTGCCTCAGCCTCCTGAGTAGCTGGGATTATAGGCGCGAG<br>CCACCATGCCCGGCTAATTTTTGTATTTTTAGTAGAGACGAGGTTTC<br>ACCATGTTGCCGAGGCTGGTCTCGAACTCTTGACCTCAGGTGATCC<br>ACCTGCCTCAGCCTCCCAAAGTGCTGAGATTACAGGCGTAAGCCAC<br>CGTGCCCAGCCACCTGGGCTTTTCTTTGCACCATATATTCTAGATGG<br>GGTACTAGAAGAGCTAACAACCTGGAAACATCAACGTGTAAAACA<br>GCAACAACAAAAAGCAGAATGAAGACAGGACAATTATTGTGCTTG<br>AAGATAGAAAAATAGAAATTATCAAATCCTAACACCATAAAGAAAA<br>TAGACTTAAAAGGACAGAACCTCAGGAACATGTTGGACTATAACA

	AAAGACCTAGCATTCATGTCTTCAGAGTTCCAGAAGGAGAGAAGA<br>AAGAGAGAGGGCTAAAAAATATTCAAAGAATGGCAGAAAATTTAC<br>CAAATTTGGCAAAAGATATAAACCTACAAATTTAAGAAGCCAAATT<br>GAGGAGCATGGTTAAGGGTGGGAGTACTCCCATTGGGAAGAAGGG<br>AATTACATGAAAATTTGCTGGTACTGAATGTTGGGATCCTCTATCTT<br>CTACCTTCCAACTCTGCTTCTAGAACACAGGCAGCTAAGGCATATC<br>ACTCCAGGAATGAGATTTGAAATATTTTTGTATGATCATATGCTAAA<br>AAAAAAAAAAGACTTAATGATACTGATTTACCACAGAAAGAGCCT<br>GGGTGGATCAACCAGTGAAGAGGAGATGAGGAGGAGAAGAAAGG<br>AAGAGAGAAGAGAGATGGGGGAAGAGGTTGGTGGCAAGAACAGA<br>AGAAAGCAAATTGTCAAATTGTAAAGGTTTCTCAGAACCAAAGGT<br>CATAAATTTCTAGACTGAAAGGGCCCACTTAGTGTCCAACACATAA<br>TGAAATTTGGGAATACTGGGACCAAAGAGAAGGTCCAATAAGATTC<br>CAGAGAGGAAAAAGATAGGTTTATATAAAGAATCAAGAGGTAGAAT<br>GGCATCAGACATCAGACCTTTCCTTTTTTTTTTTTTTTTTAAGACG<br>GAGTCTTGCTCTGTTGCCCAGGCTGGAGTGCAGTGGTGCAGTCTTG<br>GCTCACTGCAACCTCCACTTCCCAGGCTCAAGCAATTCTCATGCCT<br>CAGCCTCCCGAGTAGCTGAGACTGCAGGCATGCGCCACCATACCCA<br>GCTAATTTTTGTATTTTTAGTAGAGATGGGGTTTCACTATGTTTACCA<br>GGCTGGTCTGGAACTCCCGGCCTCAAGACATCCTCCCACCTCGGCC<br>TCCCAAAGTGCTGGGATTACAGATGTAAGCCACCACACCCAATCCA<br>GACTTTTCAAAAGTAATTCTTAGTTTTAAAAAGCTATGAAAATGTAT<br>TTTCAAAAATTTTAGGGAAAGTGATATCCCACTGTATAATTTTATACC<br>TAGGCACACTATAAGTCAAGTGTAGAAAAAATACATTTTCAAATATG<br>TGAGGTCTCAAAAGTTATGTCCAATGCACCCTTTCTTAGGAAGTTA<br>ATGGATGTCATCTACAGTCAAGGGAGTAAATCAAGAAATACGAAGA<br>CATCAGATTCAAAATGTAGAGAAGCCACAACCTAAGAGAAAAACA<br>AAGGGAATTCCTGGGATGATGGCAAAGTGATAACAGCTATGTACAT<br>AGCAGGCCTAGAGAACCACTTCAGAATAGAACTGGTCAGAGAACT<br>CTGGAAGAGTGCTTTAAATAAAATGAAACCAGTATTTTATTTCAAAC<br>ACAATCTATTGGCTTTGAACATATTGAAAGGAGATTTTTACAACTTA<br>AGGAATTTGGGGCTAAATTAATGAAAGCCACGTAGAAAATGAGCA<br>AATGGCTGGGCGCAGTGGCTCACGCCTGTAATACCAGCACTTTGGG<br>AGGCCAAGACGGACGGATTGCCTGAGGTCAGGAGTTCAAGACCAG<br>CCTGGCTAACATGGTGAAACCCCATCTCTACTAAAAATACAAAAAA<br>TAGCCGGATGTGGTGGCGCACGCCTGTAGTCCCAGATACGTGGGAG<br>GCTGAGGCAGGAGAATTGCTTGAACCCGGGAGGCAGAGGAGTTGC<br>AGTGAGCCCGAGATCACACCACTGCACTCTAGCCTGGGTGACAAA<br>GAAAGGCTCCGTCTCAAAAAAAAAAAAAAAAAAAAAAGAAAGAAA<br>GAAAAAAGAAAATGAGCAAATGAAAAGCAATACAATTATTGACTG<br>CTGGGAAAACGTTTTTTGCAGGAATGAAAAATCAGCTCACACCATA<br>TGGCTCACCTGTGAATCACATTTTTCAAACATATTAAGATAAATGCT<br>GAATACTTAAGATATAACCATATTGGTGAATGAAAGGCCAGGAAAG<br>AGTGGTTGTATATTAAGGAAATGAGGCAAAATGAAAGAGATAACTA<br>AATTCTTATCTTTCAGAGTTGGAAGTCAATATCCAATGCCAAAACTG<br>GAAACAAAACTTTAAGTAGCAATATAAGCATATTATTTAAAGATAAA

	GATATAAATGTCAAAAAATAATCAGCTAAAAGAGTTAAAAATGGTT GCCTCTGAGGAGCAAGAATTAGAGGGTGATAGAAGACCATCAAGG AATTCTATTTTTCTTTCTTTTTTTTTTTTTTGAGACTGAGTATCATCTA CGGCCATACCACTCTGAACACGCCCCATCTTGTCTGATCTCGGAAG CTAAGCAGGGTCGGGCCTGGTTAGTGCTTGGATGGGAGATGGAGT CTCGCTCTGTCACTCAGGCTGGAGTGCAGTAGCATGATCTCGGCTC ACTGCAACCTCTGCCTCCTGGGTTCCAGCGATTCTCCTGCCCTAGC CTTCCACGTAGCTGGGACTATAGGTGCACGCCACCATGCCTGGCTA ATTTTGTATTTTTAGTAGAGACAGGGTTTCAGCACGTTGGCCAGGC TGGTCTCAAACTCCTGACCTCAGGTGATCTGCCTGCCTCGGCCTCC CGGCCTCTTTTATTTATTTATTTATTTATTTATTTATTTATTTTGAGAAG TAGTCTTGCTCTGTTGCCCAGGCTGGAGTGCAGTGGCGCGATCTCG GCTCACTGCAACCTCCACCTCCCAGGTTCAAGCTATTCTCCTGCCT CAGCCTCCTGAGTAGCTGGCATTACAGGCACGCACCACCACTCCAG GCTAATTTTTGTATTTTTTAGTAGAGATGGGGTTTCACCACGTTGGC CAGGCTTGTCTTGAACTCCTGACATCAGGTGATCCACCCGCCTCGG CTTCCCCAAGTGCTGGGATTACAGGCGTGAGCCACCGCACCCAGC CTGAACTCTATTTTTCATAACAAACCTGGTACAGCTATTTGATTCTA AACTATATGCATGTATAATACTGAAAAAAATCAAAACTAGGCCAGG CCAGGTGCAGTGGCTCACGCCTGTAATCCCAGTACTTTGGGAGGCT GAGGCGGGTGGATCATCTGAGGTCGAGAGTTTGAGACCAGCCTGG CCAATATGGTGAAACCCTATCTCTACTAAAAATACAATAATTAGCCA GGCTGTGGTGGTGCATGCCTGTAATCCCAGCTACTTGGGAGGCTGA GGCTGGAGAATCGCTTGAACCTGGGAGGCAGAGGTTGCAGTGAGC TGAGATTGCACCGCTGCACTCCAGCTGGGGCAACAGAGTGAGACC CTGTCTCAAAAAAAAAAACAAAAACAAACACAAACAAACAAAAA AAACTAGGCCAGGCATGGTGGCTCATTCCTGTAATCCCAGCGCTTT GAGAGGCTGAGGCAGCAGGATCACTGGAGGCCAGGAGTTCAAGA CCAGCCTAGGCAACATAGTGAGACCCTGTCTGTACCAAAAAAAAA AAAAAAAAAAAAAAAAATCAAGAATGGTGGCACAGGTAGTCCTAG CTAATTGGGAGGCTGAGCAGGGAGGATTGTTTGAGCCCAGGAGTT CGAGGCTGCAGTGAGCTATGATCGTGCCACTGCACTTTAGCCTGGG CAACAGAGTGAAACCCTGTTTCTTAAAAAAAAGAGAGAGAACTAA AATATTAAAATATAAAGCTCAGTATCATATTTCGTAATGACTCTTTGG AAATATATTTTTGTCCTGTCACAAATATGGATTGCAGATCATTTATTA TCTTCAAACACCCAGTTTTAAGTTGTTATTCTATTTTCTAAGTTTTCT TCTGTCCCATAACTGCTTTGAAGAACATAATTTATATTAAATTAGTTA TAAACTTTGTATTTCAAAGTTTGGTTAGTGATTTGAAGATATTTCCT GTATTTAAGTGATTATTGTTTTATGTGCTGATGATATAACACTTACTG ATATTTAAAATATTTTTTGTGACATAACTCTACCGAAAAATAAAGAA TTTCATTGGCTGTACTTGAAAATTTTTAGAACAGATTTTGGAAGTGT TTGTGTTAATACTAATTTTGTGCCAACATTTTAAAATTTGTATTAATG TGTAATTTATTAAATTTTTAAAAAACTATCAACTTTGGTTCTGAAGC ACTGATTTTCAGACAAGTGCAGGGAAGTATCACTATACTATGGTGA AATAAGATGAATAAACAATATAGCATGTTGTTCATGAAGCTAAATCA ACTTAAGAATTTTACTCTTGGTGGGTCTCAGTAGCTCGGGCGGC

HNF4A	AGCTCCCTTACCACCGTCCACAGCAGAGGCACAAGCTCCCTACACA<br>TGCCTCCCCAACTGCAGGTTGCCCAGGGCAATCCCTTCCCGTCTCA<br>ACTGGGTCTCTCTGTGGGCTCTGAGGTCCTCTGCTATCCCCTGGAA<br>TCTGCTGCCTCTTGAGCTGGGGCCCGACAGCAAGTGAGCAGAGGT<br>AAAGAACAGCTGCTAAATTGATGGCATTGAGGCTAGTGCTTGTTCA<br>TGGCTGGGTGTGTGGTACCTGGAGCTGTCCTGAGCAGGGGTCCTG<br>ATTCCAACTGATTATGTGGCCAGTGTGTCATGCAATCAATGACCAAT<br>GACAGTGATTGCTACTATTGTACTGTATGCCTACACGTGGTCATTTA<br>ATGCTTGAACCATCCCTACAAAGAAGGTATCAACCCAATTTTTCTGA<br>TGCTTAAACTAAGGCTGAGAAAAGTAGAAGTTGAGGTCACAGAGT<br>TTGGTACATTGGCACTGCCTGGATTTTAACCTAGATCTATCTGACCC<br>ACAAGGTCTGTGGTTTATTCCCCTAAAGAGAGGTCAGGGGAAGAG<br>AGGCTAAGAAGCTTATCTACTCTCTCATTTAATCTTCACGACAAACG<br>ACAAGTTAGACCCCTTGTACAGAAGGAGTAACCAAGGCTTCGAAA<br>TAAAAACTTGCCTTGTGCAGACAGCTGGCCCTGGGCTTGGAATATA<br>AATAGTCACTGGTATACAGGCACCAGAGACCAATACCCACAGGCTA<br>ACACCCACTCAGTCCACAGAAAGAAGCTTAATATTTTTTATAAGCAT<br>AGAAATAAAAACCAAACCAATACTGTGACATTTGCTCTGTGACTTC<br>CTGTATTTATTATGTGCTCATATGAAAGCACCTAAGCATTGTCAGGAT<br>AATGTTCTTCCTCACTGGAGGGCGTTATGAAGTTGCTTTCTTTTTTC<br>CACTAGTTAAAAAAAAAATCCCTTGGAGGAAGGGAATGAAACTTT<br>TTATAATTGATACATAATAATTGTACATATATATGGAGCACATGTATTT<br>TGATACATGCATACCATGCAAAGTATTTAGGATATCCATCACCTCAA<br>ACATTTATCATTTCTTTGTGTTGGGTACATTTCAGATCTTCTCTTCTG<br>GCTATTTTGAAATATAAAGTAAATGATTGTTAACTATAATCGCTCTAC<br>TGTGCTATAAGAACTAGATCTTATTCCTTCTATCTCACTTATTTTTGT<br>ACCCATGAACCAACGTCTCTTCATCCCCTGAAGAAAACATTCTTTT<br>TTTTTTTTTAGACAGAGTCTTGCTCTGTCACCCAGGTGCAGTGGAA<br>CAATCTCGGCTCACTGCAGCTTCCACCTCCCAGGTTCAAGTGATTC<br>TCCTGCCTCAGCCTCCCAAGTAGCCTGGACTGCAGGGGCATGCCAC<br>CATGCCTAGCTAATTTTTGATTTTTGTATTTTTAGTAGAGACAGGGTT<br>TCTCCATGTTGGCCAGGCTGGTCTTGAACTCCTGACCTCAGGTGAT<br>CTGCCTGCCTCGGCCTCCCAAGATGCTAGGATTACAGGTGTGAGCC<br>CCTGTGCCTGGCCTGAAACTTTCAAGGTCTCAAGACACTGTACATT<br>TAAAGTACCCCAATGTTTTCCTTTTCCACCTCCCGTTCTTTCTCCTTA<br>TCTAACATGAAGAACATTGTATTAAATCTGATTTTTAAGGAGTTTTT<br>GTCTGTTATGTGTTAAGGGTTTGTTTGGTACCCCAATGAAAACATTC<br>TGCATTGCAGACCAAAGTGTGCTTATTTCAGGGGCATGGGATCATT<br>GCGTTCTTAGCCATTGTCACAAAATACATGGAGTCATATTTAAAATG<br>TAAAGTTGTAACATACATATGTTTAAAATGGAAATACAAAGCAAAA<br>AGATGGATGTACAATGTCATGTGTATTCAGTTCTCTGTACTTAGTAA<br>ATGTAACAAACTTTCTGTCTATTACTGAACTATAGGTGCAAGGACAC<br>GTCTCCAGTTACTTTGCATCTGTAATCCACAAAGATTCTGGGCAGCT<br>GTATTATCATAGCTTGGTTTAAATAAACTATGTAGTAACAATGAACA<br>ACAAGAACAAAAGAAATAATAGCTTGCCTGAGGTCACAGAGCCAG<br>CAGATCTTATAGGTGGTGCGTCATTTATATGAGGAATGTGTATCTAG

	GAGATACTGTTTGGCGTTGGCTAAGGGTGGAATGAATCATGTATGTA ATAGATTATGCAATTTACAGTGATGAGTGGATAGATTTACTTATGGG GTAGGTTATAATGTGGTGGGATGTGAATCTAGAATGTGCCTCCTAGG CAGTCATGCAGATTGTGCATTTGGTAAGTCAGGTATGTCATGATTCA TGTATCTAATAGCTCTTCATTTGCAGTGTCCACCTAGTAGCTCTTGG ATATAACGGGTCATGGCTGTGGCATGGTGTGGTTGATCTTGGTTGAT CGTAGACCTTATTTAATTGAATCTAAGATGTCATCTATGGAAAAATG CATTGTTATTTTTGTTGCCAATGAAATTCTGACATGACATTGATCACA TTCCCCGTTAAGCAATGTGGAACTGTGCCTCAGAGTGGAAGAGGC CATGTATTCCTGGATTGTGTGGGGAGTAGCTGTGCCTGTGGCCTGC CATAGGTGCCAGGCAGGTGGCAGAGGCCTGGGCTCAGCCCTTGGA GGCTCCTCCCGGAGCTGCCCTCCACATCTCTTCTGTGTTTACCCCTT GGTCGGATCTTCTGTTTCTTCCCACCTCTGCACTGCCCCCAAGGCC GGTGGAGCCAGGTCAGATACTCCTGAGGTAGGCATGGGAGGAACC CGCCGGTATTTGGTCTCTTTCAGCCCCTTATCAAGTCTCATAGTGTG GGTTGCAGGGGGCTGCCTCTTGCTCCCTCCCTCAGAGTCTTGTCCT GGCTAACAGAAAACTGAGCCAGGATCATCTTTCTATTTTACAGAGG GAGAAATTGAAGCCCTGAGAAGAGACGATGTAAATAAAGTTATCG GAATAGTGACTGAGCTAGTTCCTAACCCCAGGTCTCCTGACATCAA ATCTAGCCTCTCTTGTCCTCCACAACAGTTGCTCCCTCTCCCCTTTC CCTACAGGCAATAGCACTCCCCAGTCATCTCAGCTTCAGAGGTCAG ATCAAGTGGACAGAATAAAGCTAAGCAGGGCAGAGAAAGGGCTTC TAGCAGTGGAAAGAACATGAAGATGCCTCCTCATACCACTCGAACA CACATGCCCTGACTCCCGATTTGCTCACTCATTAATCCACCCACACA TTCACTCATTCACTCACTCATTAATTCACCCACCCATTCACTCATTAA TTCACCCACCCATTTGCTCACTTACTCAGTAATTTACCCTCATTCGTT CATTCACTCACTCATTCATGCATTCACTCAACTCACTCATTAATTCTC CCTGTCTCTAATTCACTCACTCCTAATTCACCCACTCATCAATTCCC CCACACACTCACTCATTAATTGGTGCTCCCACTAATTCGCCCACTCT TGCTCACAGGTGTCACCGAGGCCCTCCAACCTGCCCTTCCAGCCA GCAGTGGAGGACAGGCTCCCAGGCCTCCCAAGTCTCAGGCTTTTC TTCCTCTGCCCTCACTCTCTGCCTTCTACATCAAGACTTTACTTCCC CAGATTGATTGGCAGCCCTTGAAAATGTCTGCACAGAAGGCAATGA GGGCTGGAGGGAGTGAGAAGAACAGAGTGCACCATGAGCTTCAG ACAGCCAGGACCAGCTGTGTAATTTTCAGGGCCCCGTCCAAAACA AAAATGCACAATCCCTTGTTCAAAAGTCATGATGAATTTTAGGACA ACCACAGCAGAGCACTGAACCAGGAACCAGGACTTTCTAAGGGTT GGGTTGCCTGTGACTGCACTGGCGATACCCCCACAAAGCCCACTCT GAAGGTAGGAGACGGGTGGAGAGAAACAGGGGGATGGCAAGGGG GATACGAAACAGGGAGAGGGAGGAGGGGGAAGAGGATGGACGTC TACCAGGCCCCACTTGGTGCTTGATTTATGCCATCTCATTTCCTTCTC AAACCACCCTTTGAAGTTGATTGTACATTTTACAGAAAAGGAAACT GAGGCTCGGAGAGGAGAATCATTTACCCAAGGTCCCAGTTAGTAG ACGGTAGGTGCCTGAATGTAAATCCAGGTCTCTGCCTGCTCCGGGA GGGGGTGGGGGTGAGGGAAACAGGAGAATGTGATGGGAAAATCC GAGATGGAGCCAGCCTGGGCCAGAAACACTGGGAGCTGTGGGAG

	ACGGAGAGGGGCAGGGTGGGATCACAGGGAGCAGGAGCGGGGAA<br>TTGGAGGTGAATCTGGCCCTCCCAAACTTCCAGTCCATTCTGCTCC<br>CAGGGGAACCGGGAAACTGCGGGGGAACTGGAAGGGAGCTCCCA<br>GAACAAGGATCCAGAAGATTGGCATCTGGGGCCTGGGATTTAGGTT<br>TCTAAATCGTGGGCCATGGGGCAGCCTTATCTCTGCAAAAGCATTG<br>AGGGTAGAAGTCAATGATTTGGGAAGTTATTGAATTAGGGGATCTC<br>GGAGGTAGGCTGTCAGTGCCTGATAGTATCAGTTAGAATGCCTGAC<br>TTGGGGTGACAATGGCTTGGAGGGGTGGGTGAGTCAAGGGTCAAA<br>TGAGTGCCCGTGAGTCATGATGCCTGCCTTGTACAATTGATAACTG<br>AACATCGGTGAGTTAGGGCCCCAGCAGTTGTAATTAGCACCCCGGG<br>TGTCAGCCAGAAACCAACAAACAGCCAAATCCCTGCAGCCCCGCC<br>CAGCCTATCCACCGGCGGGGGACCGATTAACCATTAACCCCCACCC<br>CTCCCCGGCAGAGCCTCCACCCCTTCACAGAGGCTAGGCCAAGAC<br>TCCCAGCAGATCTTCCCAGAGGACGGTTTGAAAGGAAGGCAGAGA<br>GGGCACTGGGAGGAGGCAGTGGGAGGGCGGAGGGCGGGGGCCTT<br>CGGGGTGGGCGCCCAGGGTAGGGCAGGTGGCCGCGGCGTGGAGG<br>CAGGGAGAATGCGACTCTCCAAAACCCTCGTCGACATGGAC
HOXA10	GGGCTCTCGGGAACGTGGCTGGGTTTGTGGGGTGACGCGGTAGGG<br>AGCCCCCACCCCATTGCTCCCTAAGGTTGGAAAGCAGGGGGAGGC<br>CTAAGTAGAAAAGTTGAGGATTTTAGCTCCTTGCGCCAGCATTTCA<br>CTCAAGGCGAATCTTTCGATTTTGTAGAAATACACAGAATAGAATG<br>GGCTAGGGATGAGAGGCATCTAGGCGAAAGTGTTCCAGCTGTGCT<br>GCGATAGGGGGTTGTGGGAGGGCAAAAAAAAAAAAAAAAGGTGAT<br>CGCGGGTTGAGGAAAACAAAGTTTCCATTCTAAACAATGGGGTGG<br>TAGAAGTCTGCGGCCGCTAAGGCGAGCTTATTCCCAGGGGCAGAA<br>GTCCTGGAGGTCACTGGTTTCCTACTAGGTGCATCTCTATGTCTGCC<br>CGGCAGCTTGGAGAAAACCTTTGTTCCGAAAAAGGCCAATTCGGA<br>GAAGTCGCATGTGATTTTTGAGAGTGCCAGACAAGTCTCTGTGTTG<br>CATACTTGGAGTACGCTGTTATAAACCCAGCTGCAATTTCGCAGCAA<br>TTTCTTACATATGAAAATTAGATCTTTTGTAGAAGATATTTTGCAAAT<br>ATGCAAAAATGTATTGGTAAATACTAATACTTTTTTAAAAAGCTGGA<br>ACCGAGGATAAATTTTGACGGAGCAATCACTCCATTGGGGTTGTCA<br>TCAAACACATTTTAAATGAAAAAATTCCATTACATTTTGAAGGATCG<br>ATTCAAATTCCCTCTTTCTCCTATATTTAATTTTTTCTACTATTTTTCC<br>ATTAGTTTCCCACATCTTTTTCTTTTCCCAAATATACAGTGCGTGGTG<br>AGTGGGGTGAATCCCAGAGAAAGTAGGCACTGCCTGTTTGTTGCGT<br>AAATTTTGCCTATCATAGTTACTAGTCTTTGTTGAAGGCTGGCTCCC<br>AAACTATGACGGAGCAAAGCAACAAGGCAAGAAATGAGCGCCCA<br>CCCAGTGAGCTGCTCTCTGCAGCGGGAGCCCTCCCTACAATGCCAG<br>AGCCGCCTCCAGTTCTAAGGCCAGAGGCTGTTTATTATGAACAAAT<br>CTAAGAATATTGCAATTCAAAGGGTGTGTGAAAAGGCCCCAACACT<br>TCCCTAACAGGGGAAGGGCCATCTTAATGGTTGGATTTAGAAAACT<br>TTAAAAGTATGTTGTTTAGTAATAGATTGCACCTGATAGATTCAAAA<br>CAGACACATCAAATATCCCTGAAAATGTTGACATTCTTAAAGATGAA<br>TGGGAAAAGAACATCATTGCTTGTGGAAAAATTTTCTAACTATGCAT<br>AAATGCATGGATATGAGAAGCTCTGATTTTTTTTTCTTTTTTTGCTAA

	AGCCAGATATGTAAAAGCAGCCTTTATTTTTCCTCTGTGGGACTGCA<br>GCCCAACTCCACCGCTGGATAATTCAGAGTCTTGAAAGCATTGAGA<br>GTGCCCTCCAGATATAAATTGTTTACTAGCAGTTTCGATCTCAGGAC<br>CTGGTATCTTTTCATTAGGAACTTGTTTTTCATAATAGTTTATTTCTC<br>ACTGCTTACAAGGGCCTACTCAGAAGGAGTACCTAGTGAACTGCA<br>GCCTAATCCTTGCCGAAGGCCTTAATCCATGGGCCTGGTGTTTACTT<br>TGGCCCCATAGGGCTGGCAGGTTATTACCCGGGTAGAAATGGGCAA<br>TATTTCCTCCTAAATAAAACATGGGCTCTGGAGGTGACTGTGTAACC<br>GCTGCATAAGGCCTGGTTGAGGCAAGCAGGCACTGAAGATTTAGG<br>TCAAAGTTACCCTGAGGGAGAGGGAGAAATTTAGTAGCTGGGAAG<br>AATGGCAAAAAAGAAAGAAGGCAAATGAGCACGAGGGAGGAAAA<br>GTGCTTGGAAGCCGACCCACACTACAACTGAATCGCTTTCTTCATC<br>TCTTGGGGTGGGGAATCTCAGGAAAGCCGAGGCGCTCAATAAATG<br>GAATATTTTATTGAGAAACCTAACACTCCAGTGTTAAAGTCTAGAG<br>AGCATGGGAGCCTTGGGGGTCGGTCAGGGATGCTGGGGGCTAGGA<br>GACCAACTGAGAGGGCCAAGGAGGCGTTTAACTTGAGCTTCTGCT<br>GCGTTAGTATCCTCTCATTTGAACCAGCAGCAGCCGGCCCGCCTGC<br>AGCCGGCCAGCTCACCGGCAGTGTCTCGCTGCGTTTCCCACTCAGC<br>AAGCCCTGGTGGGAGTTGGAAGGCAGTCCTGGGCCAGAGAGGGG<br>CTGCCATACATGAGCTTACACACTCTTTTTAAAAGCCAGAAGGAGC<br>TCAAGCCCAACATCCCGTTTGGTTTATTACAAGGGGGAAAAAGTCA<br>TACTAAAGAAACACATCTGGCTCACCACAGAAAGGTCACTGAAGA<br>CCCAAGTGCCAACGCCACGGCTTGCGAGGCCCATAGCAGGCCTCC<br>CAGCTCCTCTACTCTTCGGCTGGGGCAGCCTTTTGTAGCAGATGCG<br>ATACAAACTGACTTTTGTAACCTTGGTTGGCACGTGCAGGTCCCTG<br>GATTCTATCCAGCCTAGAAGCCCATTTGGCTGCCCTACGGGGCCCT<br>GACCTGAGCTGTCTGCTGCAGGGCACCCAGCTTGTCTTGCCAGCC<br>AAATTGGCAGGGCCCCCAAGTCCCAGGACACTGGGGGACTCTGGA<br>AGACCCAAGGAAAAGACTGAGGGGTGTGTGAACTCCTTCGAAATG<br>ATGGATCAAATTGTGGGAATATACAGATATGCATTTTTCACAGAATG<br>AGTCAAAACTTACTTGGATTTTCCAGAAGGGTCCTGACTCCCTAAA<br>AGGTTAGGAAAGATAGGTTAAGATCATTGTCTTTTACTTTATTTGTAT<br>AGCCTCCACTATCTCAAATATATACTGGACCTTCCCAGATGTCGGCC<br>TGGGAAGTTGCTTTCTGCATTTAGCCTGGAGAGGCATGACTCTAGG<br>CCCAGGTCCTGGCCCACTGACCTGCCACGGCTCATTATCCTTGAGG<br>AAACCTCGAAGGTCTTGGGACCCACTTGCCAAATGACACAGCTTTT<br>CTGAGAGGCATGGGCAGTGCTGAAGAGCAAAGCTTCCTTCCTAGG<br>CAGCCCCCAGCTCTGTGGCATTTCCAGGTGGCATTGCTAAGGGCAA<br>CAGTCCCAGCCAGTTCCACCCTTCTGGTGAGCAGGGTAAGGACCA<br>GGCAGATCCGGGAAGCTAAAGATAGGGCCACTGGCTGCGGTGCTC<br>TGAAGCTTTTCTGCACTCAGGTTCGTCCGTCTCATGGGCCTAGAGG<br>GTAGAGAATTTGCCTTGGCCTTGGGCGCAGGAGCATGCGAAGAGC<br>GAAGGAGAGTTAATGATCTCTTGGCACAGACCCTGTCTTGATCCCT<br>CTAAAGCCGCTGCTGCAGCTTCAAGACCCTGTCAGGCTGGAGAGA<br>ACTGGCTTTCTTATTCTCTCTCCCCTCCTGTTTCTCTCTCTTTTCTCT<br>CCCTCTGTCCGTCTCTCCTTCTATCTCTCTCTTTTTCTCTCCATCCTC

	CTCCTCCGCACCCTGATGCTCAGTCCTTACGGCCGGGTCACCGTGG GGCGCCGAGGTTGAAGTGGCTGGAGCGCCGCGCTAGCGGTGGAGC GGCCTTCTGGGCCCGGAGCTCGGAGCAGCCACTGCCCGCAGCGAC CGCAGCTGCTTGAATCTCAGAAGCCAGAGCTGGAGCTCTACCTAGC CAGCTCTCTCTGCGCTTTCAGGGTCCTCTCAGGCTCCTCTTCCACCT TGGGCTGGATTCACACTGGGAGGAAGGAGGCAAAGTGCCGCGTAT GACCCGGATGCAAGAGTCTTTCCCAGCTGAAGGCAGCGGCCTGAG CGGTCTATTTCCTGGGATGCCGGCGTTCAGTAATGCCAGCTCCGAC TGGGGCCGGAGGGGCGGGCAGGCCTAGAGTATCCTGGAGACCTGT AGCCCACGGGCCAGGGGCAAGGCAGCGACCTCCCGGGCTCCACTG GATCTGGCCGGAAAAGGTTGCATTTCCTGAGACTAGCATGAGGCTC ACACTTGGGGGCTAGCACCCCAGGCCGGCCATTTCCAGTTGGCTAA AACAGAGCCCCTGAGCCTGCTGAGCAAAGGGCCTGACCTCCACAT GCTCTTATAACAGGACCATGAGCCCAGCCCGGCCTTGCAGGGAAA GGATTTCCTCACCGAGGCCTCGTTAAAATGCACACACTGCAGGAGC AAGGACTGCCCTAGCTGAGGGTATGGGAGGGAGGGGAAGAGGGG AGGGGACAGAGAGGTAGGAGTGTAGTGACACTGGTTCTTGTTGTA GCCTCCAGGAGCATATGAGTGCCAAAGCTAACAGGAGAGCAGGAA CATAAAGCAGCGGAGGTGTACACCGGGCACACCCCTCCAAGTCTC TCACTTTGGGCCAGCCAAGGACCCCAAGGCCAGCATTTTACTTCCC CGAAATGACTGTGGGCTTAGAGTCTTCCCTAGGTGATGAGGGGATG CTTGGATCTGGCTCTGGCCTTGGGAGTGGGAGTGGGGCCGTCTTTC CATCAAGGCTGGCAGCCGCTTCCCACCCCCAGTCCCGCCAGCCCA CCAAAGTCCACTGACAGGAGTTGTAGAGAAGGCAAGCCGGGTGA GCACAACACCAGAGGAGAAGATCCTAACTGGATCAACGGACTAGG GGAGAAAAGTGTTGGGGGTTAGGGGCTAGGGAACAACCACTGCAT TTTGGGGAAAAATCTGTGGATACTGGAAGTAGAGGCAAGTCAGCT TCTAGTTCACTCTGGGCTCCTGACACTCTGAGGATCCTGACGGCCA TAGGGCCAGGTTCGCCACTTTTGAGGAGGCAGAATTCAGACAAGG GGCCAGTTCCAGATCCAGAGCTGAATGGGAAGCGCGTGAGTCCCC GACACGGCCGCTTCAGTGGTTAGAGTACACCGGGGACTAGTGGCA GCTTCTGCGACAGGGATGGTGAGCACCCAAGGCAGACTGGGCGCG CACCATAAACACCCCACTTGTTAACTGCGCGGGGCCCAAGGGCCCT TCTAAGGGACCCCGGTATGGGGCTCACGTCCGGGTAGGGGCGGGG CGAGCCCAATGGCCAGGCCCCCCACCAGCCACG
HOXB4	GTATTGATGAGAACGATTCTTCGGGGGGAGGGGGAAGCTATTGTTT TGTATTTAAAGGACAGTTAAAGCTCTGAAAGTTATGTCGCCGGCTT TGACCAGCCCAGCCCAGCACCTCTCTCAGGGTTCTATCTTCCCCAA ACCTGCTGGGTTGTGTGGACCAAAATCTTGGCCCCAAGAGGTGGA GTGGTCATTTCTAGGCCCTGGAGCTTCCAAAGCTTTTGTAGAAATG AGGATATCTCTGACACTTGGGGGAGCAAATAAAGCAGATGGAGTGT GCAATGGGGTGGGGATCCGGGCCCTGGCCATACAGGCATTTAGGGG TAAGGTTCAGGGAAGGGGCCTCTTGCTCAGGCCTGCTCTCTGGGG CCCAGGTGCCCAAAGCCATGCTTTCAGTTTTCCCCGTGCCAGCCTC ATTCTCAGGCCTCAAAAGGATCCCACATGAAACCCCACACTCTCTC TGGAACCCGAGCTGACAAAATATGGGAATGATGGAAAATCAAAAC

	AGGGGCGTGGCCGACTGACTCCTATCCGAATGGGACTTTTTTCGGG<br>TGGGGGTTGGATGAAGAGAAGAGAAGGCTCTGGCGAAAAGTCGA<br>GCTGGAGAGGATGAGCTGAATCAAATTTCTGCTCTGTCATTTTTTTC<br>CCAAGTAATGACCTACGCAAAATTCAATATGACCCAGCGAACTGCG<br>CGAGCATATTATAGTAACTGCCTGCTCGTGGGGGAGGCTGGGAGAG<br>AGGTGAACCGCAGGTCACGGCGTCTAAAAATTATTAAAATGTTTGA<br>GAGCCTCGTGACGCGCCTAGCTGTTTAACAAAGACTGCCAAAGTAT<br>GAGATTAACACGAAAACTTGCGCTTCCAAAACAAAAACACACCAA<br>AAAAAAAAGTCACGTTCCGGCTTCTGAGGGAGCCCGTGGCTGTGT<br>CCGCCGCCGCGGTCCGGTTTTCCGGACACACAATAGTGGACAGGA<br>GAGAGCTCAGCTGTTGCCCGGCGCGTCCGCGGGCGGCTCGGCGGC<br>CGGCGGGGCTGGGGAGCCTCCCGGTGAGTACCCTCGCCCGCGCCC<br>CCTCTGGGCCTTTCTCTCCTCCGCGCCGCCGCCGCCGGTGCGCAGG<br>CCGGCTGGGGACTCGCCTCCGCTCGGGCCTTCGGTTTCGCTCCCTC<br>GCTGAGTCCCTTCTCCCCGCCAACAAAAGGTGATGATTCCTAGCGG<br>ACTGGGCACGCCTGGACGGCGGAGGGTGGCTGGGAGGGGCGAGG<br>TCACGCTAGCAACCTGTGGCTGCCAGGAGAAGGCGGCTGGGAAGC<br>CACGGAAAGACCCGGGAGCGCAGGGGAGGTGGCTCAGCTCGCGC<br>GCAGCCCCTGCTCTTATTTACTATTATGCGTGTTGCTGTTCGTATCC<br>TTCTGGGCGGTGAAGGGGAGAGGCAGCCTCCCGCTCCGGTGAAAC<br>CCGTCCTGGGAGTCGCCCATAAACCCTGTTCTCCATCTCGGGCGGC<br>TGGAGGGGTGAGGGGGGTGCGGCGCCTTGGTGGGATTGGCAGGTG<br>GTGACTTCCAGGGCGCAGAGGAGAAAAATCTCTGCTCTTGGGCGC<br>CAGGATCAAGGATACCCAAACCCGCGGTGGCTCTGCCGCCTGTTCC<br>TCTCTCTTGGGGTGCTGGGGCACCTGGGCCGCATCCCTTGTGAAGT<br>GTAGAGCAGCAGAGAGGCCCGGCCGCCGGCCCAGCGGCCCTCCCA<br>AACTTCCGAAATGCCGACGGGTTGGACGGGTTTCCGAGAGCTGGC<br>GAGTTGAGAAGGACAGGAGGTATGGGGTGGGGGAGGGGCCTGGA<br>CCGTTGGAGTGGGTCCTGGAGACCCTAAGTGGCCTATGACAAGATT<br>TGGAAAGGTCCCTGGTTGACGCTAGGGAGCTGGTGGGGATCCTAG<br>CAGCGCATTTTGTCTGTGGGCCCTGGGCCAGACTCTAGTCTCCCTG<br>AGTGCCCAGATGGGGGTCTGAGATTACCTATTGGACCCTCTCTTGG<br>CAAAGACAACCATAGTGTGATAGTTTGCTTTTTTCTCCCAAATATGT<br>GTGAGTTCAGCCTGTCTGGGAGAATGTTGATGTTAGAAATCAGGCC<br>CAGAGGCCAACATTGGCAATTCCTAGATTTAAGATTAAAAGAAAAT<br>CAGAGGGTCTCTGGACCTAGTTGTGGGCAGGTGGGAACTCTCCTC<br>CTACCCACCTGCAAGGTCCCAGGACTGGGCATAGGCAGAGATCTCT<br>GGAGTGTTGCAGTTAGAGGGTTGTTTTTGTGTTGTTCCTGTATTTGT<br>TAATGAAATTGATATTATTTTGACTTCCTCACTAAAGATGCAAGTATT<br>TCTCAGACCTAGGGAGTTGAGTAGGGAGTCTGGGTGGGGTGGAGG<br>CTTTTTCTATGCACATTTTCCCTTACTCTGTCACAGCTTTCACAGTCT<br>CAGTTCCCTCCAGCCCAATCCCCAGACTCTCAGCTTAGTAAATTCA<br>CGGCTTCTGGGAGCTTCAGAAAGTTGGTTCGTAAACCCCAGATGCC<br>CATTTTGGCCCTACCACCAGCAACCCAAGCAGGTGGCTAGGCGAG<br>TTCATATGTGTTTAGAAGGAAAGGACCCGTCTGGGGCTAGGAAGGT<br>GGGGAAATGGGGTTCCTCCCCACACCTCCCCGGGATAGACCCTCTC

	TCCTGGCTCCCAGGATGTGTGAGAGGCTGGGAGGGGCCCTATATCA
	TCTGGCTGCATCTTTTATGGTTCCTCATTGCCCTTCATTCGGAAATG
	AAACGCCTTTGATCTTTTCTCAGGTTGTAAACGGAATTGCCCGTCAT
	TAAATATCTGGGGCCCCATCTGCTTGGTCTCCCAGCATTTTTCTTTA
	CAGCTGTTTCTCTCTCTTTTTACCACTGAATTTTTCCAAAAGGACTT
	TTATTACACCACCTTCACACCCAGCCGCCTCTTCCTCCTCCCTTCGC
	TGAGAGGCGCTTTGGAAAATATCTAGATATTCGTTTGATCACAAACT
	AAAGGCTTGGAGGGGCACGGAGGAGCCGATTGGGGTTTTGTTTTT
	TCTAAAAAAAAAAAAAAAAAAAAAAGTCCTGGGGGGAGGGGAGG
	GGCGCGAAGGCACCCAGAAAGGCCTGAATCTGACTTCGTGGCAGC
	CTCAGAATGACCCTTCCTCCTTTTGTGCTTAGCTAATGTTTACATCT
	CATAATTCATGCGCCACCGAGAGTTGCGCGGCGGCGGCGGAGGCA
	AGGTTCTCGTCCCTTTGCGAACTGGCTACTTGAAATTCTAATAGAA
	GAGGAGAATTGAAAACCTTGTAATCCCAAGAACAGACTCGCACTG
	CCTTTTTCTGTTCCCAGAGCTCAAAACTAGAACAAAACGAAATAAA
	ACCAAAGCACTCAAACCACACCCCAAACGAAGAAGGCGCGGAAA
	GTAGGAGAACTGGAAAATTTCTGGGCCAAGAAGATCTGTCTGTCTT
	CTGTATATACCCTGTAGATCCGAATTTGTGTAAGGAATTTTGTGGTC
	ACAAATTCGTATCTAGGGGAATATGTAGTTGACATAAACACTCCGCT
	CTTATTTTTCCAGAAGAAAAAAATATATATATATGTATATGTAGTATTT
	TTCTGAATGAGGACAGTCTGGTGACTGGCCACACGAAGACTCCTT
	CCTCTTTATTCCTCTATCTTTTCCTCCTTCAACTGGTTAGAGATGGAG
	AAATCATCTACCTGAACTCTCCCCACGCCTGCCCCTGGGAGCCTTG
	GTCCCTTTTCACATCTCTTTAAGAGGTTAATGTTATTGTTGTTGAAG
	TTTTAAATTTATCTTCTCGTCCCAAACGCACCCATTTTCTATTCTGGG
	CTCAGGGAGGCTTTATTGGAAATTGCGAAAAAAAATGACTTGTCTT
	CAGTGTGCGAAGAAGGCAAAGGAAATTTGTTTAACAAACGGATGC
	TGAGGCCGCCTTTCCGTTTCAGTTCAGAACAAGATGACCTGGAAAT
	ACTCGATTACCAAGGAGTGTTATTGTCTGTGTGGGTCACCTCTATGG
	CTGTATCCCCAGTATCAAAGGGATGTGAGATTTCTATTCGCCGCGGA
	GGCGGCGACAATTAGAACGTCTCGGACCCATGGTTAGTATTGCTCG
	GGTGGTTATGGAATTAGCAAATTGCTAATCAATTTTGCTCTCAAAAG
	TTGCAGGTTTTTTTACCCCAGCGCTCTGTCTATAGTGTGGGAAACC
	AAGTCGTTTCTCCAGGCTTGGGCCGTGTGGGTTAAAGGGAGGAGT
	GGATAGGAGAAGGGGGACAGACGGACTGACGGACACAGTCTAGCT
	AACCGTGCAGCTAAATGAGATTTGGAAGGCGAAGTCTCCCCGAATT
	AGTGCATGAATTTCCTATCGATCCGCCCGCGGTTCCATTCATCTCTG
	ACAAGTCCCTCTGCGCACCCGCCCTCTAGCTCTGGACGCCTCCTCT
	CTCACCCCTACCCTTCCCCATCCAGCTGCAGAGAAAAGCCCCAAGC
	TCTGTGCTCTGGCCCCCGTATGGGTTCCCCTTTCGAGACGGAGCCC
	CCAACCCGCCAGTAACCTAACTCCCCATAGAGACGCAGGGTGCTAG
	CGCTCCCTCCCGCCGAGGGGAGGAGACTGGGGCCTTTGGAAGGTA
	CAGAATGCTGGGCAGAGGTGAGGCAGAACAGGAGGGCTACACCG
	GGAGCCCCAACTTCTTAAGGGATCTAGGGTTTGCGCTCTGCCTAGC
	GCCCCAACAGGCCCTCCAGTCGCAACCTGCGCGCGCTCTCTCTCTC
	GCTCTCTCTCTGATCCCGGAGAGAGCGAGGCAAGGAGGGGGTCGC

	CCCGCAGGAGCCCTATGTAAATCCTGGTGTTGGGTGGGTGGGTGGG<br>GAGGGGGAAGGGAAGAAGGGGAAATAAACCTCTTTGGCTGGAGT<br>GGGGTCCGGGTGAGCAGATTTCCTTATCCGGGAATCGCAGGCGGG<br>GCGGCCATTGGCTCGGAGGATCACGTGGGCGCCTAACTTTGTTCAC<br>TTGACAGTAAGTAGGAGGGCTTTCGGAAACAGGAAAACGAGTCAG<br>GGGTCGGAATAAATTTTAGTATATTTTGTGGGCAATTCCCAGAAATT<br>AATGGCTATGAGTTCTTTTTTGATCA
HOXD9	CCGCCAGTCCTGGCCCCACGCTGATGGCGCCCGGGCAGAGGAAAA<br>GCTTGCCGGTTTTATTTTTCCTGAGCTAGACCTGAACACAACAAAA<br>GAGCGCAAAGGAGACCTGCGGCTCATAAACACGACCACAGAGCCT<br>CTTTTCTCCTGCTCAGATTTGCAGTTCCAGTTTTGCCTTGAGCCCAA<br>TGATCATGTTAAGGTGATCCAGGGCACCGTGTTCGTGTTCAAGTGT<br>ATGCACCCCGCATCCTGCGAGCTTGGGGGTGGTGAGGGGAAAGAG<br>ATGGCTGGGCTGGTTGGTGCTTGAGTTGGGAAACAGGGCTTACTG<br>CCTTTGCTGGGCTAGGTAACCTTGGCTTTGTTTAGGAAAAGTGCTG<br>CAGTCTTTGCAATCCGTCGGCAAAGAGGGCAAAGGCGGAGGGGG<br>AGAGTGGAACCCGCATTGCCCTCCCTGCAAGGCCAGCCTTAGGGC<br>TGGGCTAAGGCAAAGAGCCAGGGATCTGGCTTTTTGAGAAGGAAC<br>CCTCCTCCTCTCCCCCAGTGCTTAGAGGTGGGCCACAGTAGGGGGC<br>TCCCTTTCTGGGGGAATGCTTTAGTGTGGGGGCAAGAAGACATGA<br>AAATTAAGGAAATTCTGGGGAATGCAACAATACCCAGGCAAGGTG<br>GGGGAAGGTGTCTCGCTTCCCCATTTATCTTTTGAAAGAGAATGGG<br>CACCTATAAACCTGACTGTCAGGATTCCTGACTGCCTAGGAGAGGT<br>GGGGAAGAAGTGGCAGATTTGGGGACCTGAGGCAGCAGTGGGGT<br>TGGTAGGCTTGTCCAGGTCGTGGCGTATTCCCCTCCGTCCCTGTTAG<br>GAGCTGAACCCTTAGAATGTTGCTGGGGAGATCTGGAAAGTTTACT<br>ATTCTACTAATGTTTTGTACAAGTGAGAAAGTTGAAAGAGAGAGCG<br>AGAACCCAAATGCAGACTGTCCTGCCATCATGTCATTTAAGTAATGT<br>GGCATCAATGTAAGATTCCCTTCCAAGGCCCACTTCATGTGAGTAAT<br>GTTTAATACTAGCATTTTCCAAAGCGGCCTGGCTGCCAGCAGGGTC<br>ACGGCCAAGGGTACATTTGAACAGTCTGAAGAAAAAAACAAAAA<br>CGAAAACCAAAACCAAAACCAAAACAAAAACAAAAACAAAAACA<br>AACAAACAAAAAACCTCTTGATTTTTTTCTTCTTCTCCCTTTAATTT<br>TGTTAGAGGAAATCAAGTCTGATACACCAACCAGCAATTGGCTCAC<br>TGCAAAGAGTGGCAGAAAGAAGAGGTGCCCTTACACTAAGCACCA<br>AACGCTGGAATTAGAAAAAGAGTTCTTGTTCAATATGTACCTCACC<br>CGCGAGCGCCGCCTAGAGATCAGTAAGAGCGTTAACCTCACCGAC<br>AGGCAGGTCAAGATTTGGTTTCAAAACCGCCGAATGAAACTCAAG<br>AAGATGAGCCGAGAGAACCGGATCCGAGAACTGACCGCCAACCTC<br>ACGTTTTCTTAGGTCTGAGGCCGGTCTGAGGCCGGTCAGAGGCCA<br>GGATTGGAGAGGGGGCACCGCGTTCCAGGGCCCAGTGCTGGAGGA<br>CTGGGAAAGCGGAAACAAAACCTTCACCGCTCTTTGTTTGTTGTTT<br>TGTTGTATTTTGTTTTCCTGCTAGAATGTGACTTTGGGGTCATTATGT<br>TCGTGCTGCAAGTGATCTGTAATCCCTATGAGTATATATATATATATAT<br>ATATATATATATAAAAACTTAGCACGTGTAATTTATTATTTTTTCATCG<br>TAATGCAGGGTAACTATTATTGCGCATTTTCATTTGGGTCTTAACTTA

	TTGGAACTGTAGAGCATCCATCCATCCATCCATCCAGCAATGTGACT<br>TTTTCATGTCTTTCCTAACACAAAAGGTCTATGTGTGTGGTTAGTCC<br>ATGAACTCATGGCATTTTGAATACATCCAGTACTTTAAAAATGACAT<br>ATATATTTAAAAAAAAAAAGATTAAGAAAACCCACAAGTTGGAGGG<br>AGGGGGACTTAAAAAGCACATTACAATGTATCTTTTCACAAATGAA<br>TTTAGCAGTTGTCCTTGGTGAGATGGGATATTGGCGATTTATGCCTT<br>GTAGCCTTTCCCTTGTGGTGCATCTGTGGTTTGGTAGAAGTACAAC<br>AGCAACCTGTCCTTTCTGTGCATGTTCTGGTCGCATGTATAATGCAA<br>TAAACTCTGGAAATGAGTTCACTCCCTCTGCTTTCTGAAATGGAAA<br>TATGTTATGGTGGAAATGAAAGCCTATGGTGAGATTATCTTCTGGTT<br>ACACTCCCTGTTTGGGGCATTTGGGCAGGGGAGTGATAGACTAGTA<br>GGGGAAGGGAGATGGGGGAGAAAAGCTGGAGGAGGCCTAGGGTG<br>TTGGATTTTGGCAGTGGTTGGGGGAGAGGAATTATAAGCTAGCTTG<br>AGAGTGAAGTTTTCATAATTGGGAGGAAGGGGAGTCTCCTCTTTCC<br>TTTCCCAGTCCCCAGTGATAGTAACATAATTGCGCTCTCAATGGGTG<br>TGAGCTTTCCTCTGGCCTGAACCTGGTAAGTAAGCCTATACCCCAA<br>GCCACTTTCTCCTCAAAGCTTCCCATTTGTGTGTTTTCTCCTCTTTG<br>GTTTTGGTTGTGTTGTTTTTAATGCTTTCAGTGGCATCTTGGTGATTT<br>CTGGCTGGCGAGCAATCATCAGGGGCTAGGTTGAAGCTAGTCTTGC<br>CCACCTGGAAGTTGCCGGCCTCCATTACAGGAGCAAGGACAAACA<br>GCAGTGTAGCACTGCAGCGGATCCAATTCTGCCCCCTTTCCCTCAG<br>CCCTACCCCCATCCCAAGCGCAAGACAGCCAGACCCCAGAGAAGC<br>CGAGGATGGGTGAGTTTTCCCATCCCACTTCGCCTTGATCTCCTTGT<br>GGACGGGTTTTATGCTCAGTCATTACCTTTTAGTGGCCCACATGAAA<br>TTTTGTTAAAGGAAGAAATGAAAAGATTTTCCCCAGTCAGTCTTTC<br>CTCTATTTAATTACAAAATGCTGGTGGGAACTGCTGCATCTGGGATG<br>CAAGAAAATGCAGAAAGGGTGACTGAAAATTTTGCAAATGAACAT<br>GACTTCCCATGAAGTCTAATGTTCCATTCGCTGCCATGGTCCAGGG<br>GACTCCCACCAGCTTCCACCGGCTTCAACAGGATCTCCACTAGAGA<br>GCCCAGACTTATCTAGTCCTGTCGGGGAAAAGGGAGAAGAGGCCT<br>TGCAGGAGAAAGCTAACAGAAAATTCGTTACCTGAGGTCCTGCCT<br>GCAGTTTCAAATAGCTTCCAGCAGTTTTACAAAACACATCCTTTCCA<br>TTTCTTCCTTTTAAATGTTTCCCTAAGAACGATCCATTTAGGTGCTAT<br>AAGTCCTCAGCCAGGGAGTCTCTGGGACACTGGCATTCAAAATTTT<br>AAACTTCCGCCCCAAAACCAGGAACATTCCAAGACAGAACTCTTT<br>TAGGGGGCCATTTCCTGGGGGTGGGGGAGAGGGCTTGGAATCAAT<br>GCTAGATTGAAAACGTTGTAATAGCTTTGCCCCAGACTTAACACCG<br>GTTGGGCAGGAGGAGGGTAATTTTTATTTAGCCGTTTCTCCGATCAT<br>GTGGGGAATACCATTAGCTGTTGATAGCGGGCCATGTATCCGAGGA<br>AAGCCTGAGCTACAAGGCAAAGGCATCCCATCTGGAACAAAATCA<br>GAAAGCTATTGGCAAAGGTAATCAATCAGGCCATAAATAGCCATTT<br>ACCCGCTTCCTTTTCGGGGCTGGAGGTGGGCCGGGAGCCCTCCAA<br>GGGTGAGCTGGGCAACTTGTAGAGCAAGGAATATGCCCTCCGCTG<br>CCGGCGCCCCGGCCGCTTTTGTCTGGGCTCCCAGCCGGGCTTCCGA<br>GGCTTTGTACCATGGATTTGGGAGTGACAATGGGCATTTCCCTCAG<br>ATTCAAGGCTGCTCAACCTCACCTCTGTAGGGGGAAAAAAATCAG

	AAGGGAGTGTCCCAAGGACCTAGCCATTCGGCCGAATTTTTAGAC<br>ATTTTGGGAGTCTCCTCCGAGGCCTTTAAGTGCGAACCGCGCGAAG<br>CGGCCCTGCCCGGGGAGACTCGCTGAGGCAGGGCTGAGGCGGCG<br>GGCGGGAGCAAGCTGCTCTAGCATTTGGGTTCTGCCCTGTGGCGTG<br>TTCTCTTCCAGGGCCTTTCCAGCATCATCGGAGAAGACGAAGCACC<br>CTGGCCGCCACTGTCCGTGCTGCGCCAACTCGCCCGGCCGCCCGC<br>CCTTCCGAGGGCAGGCAGAAGCCCCTCTGTGTCCTCCACCGCCGC<br>GCCCCGGCTCGCCCCTCGGGCCGCGGCGTGTGCCCAGCCTCACGT<br>CGGGGTGTGTGTGGCCGCGCGGGCGTGTGTGAGTGTGGCAGGGGG<br>AGGGGGCCCTCCGATCTGCTCCATCCGTCCGTTTATTAGGGACACA<br>TTAATCTATAATCAAATACACCTCATAAAATTTTTATTGAAAGGCATA<br>ATATCATTACAGAGGTCTTCCACCTGTTTAAACAACACGACAAGC<br>TGTGAGCAAGCGTGTGTGTGGGGATGTGTGGGGAGGGGTGGGTGT<br>GAGTAGGGAGAGAGGCGAGGGGAGAACAGCTCCCCTCGGGCGCT<br>AGGGGCCGCCCCGAGGGCCCGCCTGCCTCGGGCGACACCGGCCTG<br>GCGCCCCCGCGGCCGCTCCGTGTGCCCTGGACTCGCCGCCCGCGG<br>CTCGGAAGCTGGAGAGTCAGCGACGGGGCCCGACTGCGGGACCG<br>AGGGCTGCAAGAAGAAGCGAACAAATAGTCCCCAGCGCCTCCTCT<br>GGATGCGGTCGCGTCTGTGGTCCTGGCAGCCGCTGGGCGGGCCAG<br>GCCAGGTCGGGCCGGGCCGAGCCGGGCACATGGACCTGGGCCTGC<br>GGGCTCTAATTGCGGCGCTTATGTTGATGATTTTTTTTTAATCACAG<br>CAGCCCCCAGTTTAGCGGACTGATTTACTCCCGGTATTGGTAAATAT<br>GATCACGTGGGCCGCGCGACCAATGGTGGAGGCTGCAGCCTGCGA<br>ACTAGTCGGTGGCTCGGGCGCCGGCGGGGAGCTGCTCGGCGGCGG<br>ACAGTGTAATGTTGGGTGGGAGTGCGG
HSD17B1	GGCTCTACTAAAAAATACAAAAAATTAGCCGGGAGTGGTGGTGGG<br>CGCCTGGAGTCCCAGCTACTCGGGAGGCTGAGGCAGGAGAATGGC<br>GTGAACCTGAGAGGCGGAGCTTGCAGTGAGCCGAGATCGCGCCAC<br>TGCACTCCAGCCTGGGCAACAGACAGAGCGAGACTCCGTCTCAAA<br>AAAAAAAAAAACAAAAGAAATCAGACGGGGAGATGCACAGTAAC<br>ACAACAAATATCTCTACTTAAATGGGCTCCCCATCTTCCATTTCTTG<br>GTTCTATTATTTTTTACTCAAATCCTAACCTTCTAACTCAAAATTGCT<br>GTATATCCTTAATCACTTTTTTGGGTCATTTTTATTTATTTATTTTTTTG<br>GGGGGGCGGACAAAAATAAAATTTTTACCTTTCATTTGGGAAATAA<br>CAAATTCTTTTATAACCTTCTAAAAATACAAGTTTGGGTCACTGAAA<br>CTAGTGACTGCAAAAAACANNNNNNNNNNNNNNNNNNNNNNNNNN<br>NNNNNNNNNNNNNNNNNNNNNNNNNNNNNNNNNNNNNNNNNNNNNN<br>NNNNNNNNNNNNNNNNNNNNNNNNNNNNNNNNNNAGCGCTCTAAG<br>GCAGGAAGATTGGTTGGTTAAGTCCAGGAGGTTGAGGCACCAGTG<br>AACGGGTTCACCCCCACTGCCCCCCAGGCTGGGAAACAGGCCAAG<br>GCCCTGTTTAAAAAAAAAAAAAAAATCTTTAAGAAAAAATAGTTTGT<br>GAGCCCCCAGGAGGGAGAAGGAGTAACAGGTTATCCCAGTCCCCA<br>AACTGAAAAATTCAGGGGGAATTTCCAAAGTTGACTAAATTTGCAG<br>TTGTTTTTGCCAAGGTGGAAAGTTCTCCCGCTCATTATGGTTGCCG<br>AAAAATGTTTTCAGCCGGGGGCAAGGGCTTACCCCTAAAATCCCAC<br>CACTTTGGGAGGTTAAGGGGGGAGGATGGCTTAACCCCCGGAGTT

	TGAGCCCAGCCTGGCCAATAAAATGAGACCCTGTTTTTATTTAAAA<br>AAAAAAAAAAAATTAGCCGGGTATGGTGATGCAACCCTGTAGTTTCA<br>GCTACTCAGGAGGCTTGCTTGAGCCTGAAAGGTTGAGGCCCCAGT<br>GACCCGTGGTTGTGCCCCTGCACTCCAGCCTGGTTGACAAATTTGA<br>GCCCCTTTTTCAAAAAAAAAAAAAAAAAAAAAAAAAAANAAAAAA<br>AAAAAAAAGAAATAAAAATGTTTTAGTTTTTTTCTTATTACACTGTT<br>TTATCTGCTTGTGAGTTTTTTGGCCCGCATGAATAGACATTGCAGGA<br>CTAAACGAATGCAGAGGAATCCTCCCTTTTTTTCCTCCATTCCTTAC<br>CCATACCATCTCTAAAGTACTTCATCTTCAATTACATCTTGTCCATCC<br>ACATAGTTGCAAAGCTTGTTTTACCGCTTGACTTCTAGGTTACATCT<br>CTAAAATGACAAGTATAATGTGACCTTATTATATACAAAATACATCAA<br>TCTGATGTTGATACTCCCTTTTATTTAGGAATTTTGTGTTGAGGGCT<br>GGAAGGAAAGGAGAAGCACACACTTATCTATACCATTTTATATTTGA<br>TAAACCTGTAAGAGAACATTAATATTAATTACCTGGTTACCTTGGAG<br>GGGTCAACTCTAGTTCTTTGAGGTGATCTGTCTATTAAGTTTTGCTG<br>TTTGGAAGAATTTTTTTTTTTTTTTTGAGATAAGAGTCTGGCTTTGT<br>CACCCAGGCTGAAGTGCAATGGCGCCATCTTGGCTCACTGCAACCT<br>CTGCCTCCTGGGTTCAAGCAATTCTCCTGTCCTAGTCTCCCAAGTA<br>ACTGAGATTACAGGTGCCCTCACCATGCCCAGCTAATTTTTAAATTT<br>TTTAAATAGAGACAAGGTTTCACCATGTTGGCTAGGCTGGTCTCAA<br>ACTCCTGATCTCAAGTGATCCGCCCGCTTCGACCTCCCAAAGTACT<br>GGGATTAGAGGCATGAGCCGCTGTACCTGGCCAGAAGAATCTATTT<br>TTTCTTTTTTTTTTTTTTTGAGACGGAGTCTTAGCTCTGTCGCCAGG<br>CTAGAGTGCAGTGGCGTGATCTTGGCTCACTGCAACCTCTGCCTCC<br>CAGGTTCAAGCAATTCTCCTGCCTCAGCCTCCTGAGTAGCTGGGAT<br>TACAGGCATGCACCGCTACGCCCAGCTAATTTTTGTATTTTTAGTAG<br>AGACGGGGTTGCACCATGTTAGCCAGGATGATCTCGATCTCCTGAC<br>CTTGTGATCCACCCACCTCGGCATCCCAAAGTGCTGGGATTACAGG<br>AATGAGCCACCGCGCCCAGCCAGAATCTATTTGTGTGTGTGTGTG<br>TGTGTGTGTGTGTATATATATATATATATTTCGTATATATATTTTTGG<br>GTATATAAATATTTTGTGGGTATATATATATATATATATATATATATA<br>TATATATTTTTTTTTTTTTTTTTTTTTTTTTTTTGAGACAGAGTCTCAC<br>TCTGTCACCCAGGCTAGAGTGCAGTGGCACAGATCTCCACTCACTT<br>CAACCTCCGCCTCCAGGGTTCAAGTAATACTCCTGCCTCAGCCTCC<br>TGAGTAGCTGGGGTTAAGGTGTGCGCCACTGCGCCCAGCTAATTTT<br>GTATTTTAGTAGAGACGGAGTTTCGTTTTGTTGACCAGGCTGGTTTC<br>GAATNCCTGGGGATNCTNTNAGAGCCGGACCGTGTTTTATTAAGAC<br>TAGTTTNNNNNNNNNNNNNNNNNNNNNNNNNNNNNNNNNNNNNN<br>NNNNNNNNNNNNNNNNNNNNNNNNNNNNNNNNNNNNNNNNNNNN<br>NNNNNNNNNNNNNNNNNNNNNCCACACCCGGCCAGAAAATGTGCT<br>TTAAATTCAAGGGCCCAGGCACGGTGGCTCGTGCCTGTAATCCCAG<br>CCTGCACTTTGGGAGGCCTAAGTGGATGGACTATTTGAGGTCAGGA<br>GTTTGAGACCAGCCTGGCCAACATGGTGAAACCCCATCTCTACTAA<br>AAATACAAAAATTAGCCAGGCATGGTGGTGCGCACCTGTAATCCCA<br>GCTACTTGAAGGCTGATGGAGGAGAATTGCTTGAGGTTGGGAGGC<br>AGAGGTGGCAGTGAGCTGAGTGAATGGCACTTGCACTCCAGCCTG

	GGCAACAGAGTGAGACTCTGTCTCAAAAAAATAAAATAAAATAAA AAAATTCAAGGGACCATTGGAACCTTTGGGAAGACTAGTGGCCCT GGGCCATGGAGTAAAAACTAGATTGCAAAAGGAAACTTATCCTTTT TATTTTTTTTAATTTTNTAATTTAATTTTGCAAATCTCTNGTTACATTC CAATTTTAGGAAAGGGGGCGCCCAAAACAATCACTGCTTAACCTTA AACCACAGTGGGCACAAAGGGATGTTTGAAAAAATCATAAAATTA AAATGTGTGGAAGATAAAGAAGTGGTCGGTGAGTATACACTTTTTT TTGGAGGGTTCAGGGTCTCGCTCTGTTGCCCAGGCTGAAGTACAGT GATATAATCACCATGCACTGGAACCTCAACCTCTCAGGCGCAAGCA ATCCNTCCCACCTCAGCCTACTGAGTAGCTGGAACTACAGGCACAT GCTGCCATGCTCAGCTAGTGAAAAAAAGTTTTTTGATGCAGCTGTA GCAAAATATTGCTTATAATTATCAATATTTTAAAAATACAGAAATTGC TGGGTGTGATGGCTCACACCTGTAATCCCAGCATTTTGGGATGCCA AGGTGAGAGGATCAATTGAGCCCAGGAGTTTGAGACCAGCCTGGG CAACATGCAGACCCCCTCTCTCTACAAAAAAATAAATAAATAAATA AAAATTAGCCAGGCATGGTGGCACATGCCTATAGTTCCAGCACTTT GGGAGGCTAAGGCAGGAGGATTGCTTGAGCCCAGGAGTTTGAAGT TACAGTAAGCTATGATCGTGCCATTGCACTCTAGCCTGGGCAACAG AGTGAGACCTTGTCTCTAAAAAAAAAAATTAAATACAGAAGTTATT AGGCCCACTTCAAGATCTCGTTATTGAATGCATTAATAAAGGACAC AAAATAAAAGAAAATGTATAACTATGTCAAAAATTATTCAGGCAAC TTAAAAATATTGTAACAACATATTGTATATAATTGGTTCCTGTGGTAA TTCTATGTGTATCATTTTATGTATTCAAAAAACACCGGGGCGGTGGC CCATGCCTATAATCCCAGCACTTTGGGAGGATGAGGCAGGCGGATC ACCTGAGGTCAGGAGTTTCGAGACCAGCCTGGCCAACATGGTATTT TTGTACTAAAAATACAAAAAAATTACCTGGCCGTGGTGGTGGGCAT CTGTAATCCCAGCTACTTGGGAGGCGGAGGCAGGAGGATTGCTTG AACCGGGAAGGCAGAGGTTGCGCCACTGCACTCCAGCATGGGTGA CAGAGCGAGACTCCGCCTCAAAAAACAAAAAAAAAAAACAAAAA AACCCCCACCAGACTGCCGAAGGAATCCACAGTTAAAAAAGGTTA GGAACCCTTGCTCCAAGAGAAAAGAGAGAAAGGGAGACTTGTAG TGTCTGGGGAACCTGGCTTTTTAGGAGTAGAAGAGAGGCTAACAT GTTAAAATACTGCAAGGGCAGAGAGGCGATTTTGAGAGCCAAGGT CCCGGAGGAGGCTGAAAGAGTGGAACCCAGGGCTCAGCAAGAGA AATGAAGTACAGGAAGGAAACCTTTCTCCTCAATAAAAGGGAGGA AAGAATTAGAGTGGATAACAATAAAGTTTCAGTGGGGTGGGAGTG AAAGGCAGGAATTGAGGGGGGATGTGTTTACTCTCACACCAAGCG CGCATACAGACCCCTAGTCCCNNNNNNNNNNNNNNNNNNNNNNN NNNNNNNNNNNNNNNNNNNNNNNNNNNNNNNNNNNNNNNNNNN NNNNNNNNNNNNNNNNNNNNNNNNNNNNNNNNNNNNCAATGTTGCT GAAACCAGGTTTCTCTANAGAACACCACGGGTCTACTCTGTNACCT GGCCTGGTNCTTGACTCCTGGGCTNNGGGGAGGGCCCTTTCCAGA GCCTCCCAAAGTGCTGGGATTAC
HSD3B1	CCAAGGTAATTTATAGATTCAATGCCATCCCCATCAAGCTACCAATG ACTTTTTTCACAGAATTGGAAAAAACTACTTTAAAGTTCATATGGA AACAAAAAAGAGCCTGCATCGCCAAGTCAATCCTAAGCCAAAAGA

	ACACAGCCAGAGGCAACACGCTACCTGACTTCAAACTATACTAAA
	AGGCTACAGTAACCAAAACAGCATGGTACTGGTACCAAAACAGAG
	ATATAGATCAATGGAACAGAACAGAGACCTCAGAAATAATGCCACA
	TATCTACAACTATCTGATCTTTGACAAACCTGACAAAAACAAGAAA
	TGGGGAAAGGATTCCCTATTTAATAAATGGTGCTGGGAAAACTGGC
	TAGCCATGTGTAGAAAGCTGAAACTGGATTCCTTCCTTACACCTTAT
	ATGAAAATTAATTCAAGATGGGTTAAAGACTTACATGTTAGACCTAA
	AACCATAAAAACCCTAGAAGAAAACTTAGGCAATACCATTCAGGAC
	ATAGGCATGGGCAAGGACTTCATGTCTAAAACACCAAAAGCAATG
	GCAACAAAAGCCAGAATTGACAAATGGGATCTACTGAAACTAAAG
	AGCTTCTGCACAGCAGGAGAAACTACCATCAGAGTGAACAGGCAA
	CCTACAAAATGGGAGAAAATTTTCACAACCTCCTCATCTGACAAAG
	GGCTAATATCCAGAATCTACAATGAACCCAGACAAATTTACAAGAA
	AAAAACAAACAACCCCATAAAAAAGTGGGCAAAGGATATGAACAG
	ACACTTCTCAAAAGAAGACATTTATGCAGCCAAAAAACACATGAA
	AAAATGCTCATCATCACTGGCCATCAGAGAAATGCAAATCAAAAGC
	ACAATGAGACACCATCTCACACCAGTTAGAATGGCAATCATTAAAA
	AGTCAGGAAACAACAAGTGCTGGAGAGGATGTGGAGAAATAGGA
	ACACTTTTACAATGTTGGTGGGACTGTAAACTAGTTCAACCATTGT
	GGAAGTCAGTGTGGTGATTTCTCAGGGATCTAGAACTGGAAATACC
	ATTTGACCCAGCCATCCCATTACTGGGTATATACCCAAAGGATTATA
	AATCATGCTGCTATAAAGACACATGCACACGTATGTTTATTGTGGCA
	CTATTCACAATAGCAAAGACTTGGAACCAACCCAAATGTCCAACAA
	TGATAGACTGGATTAAGAAAATGTGGCACATATACGCCATGGAATAC
	TATGCAGCCATAAAAAATGATGAGTTCATGTCCTTTGTAGGGACGT
	GGATGAAGCTGGAAACCATCATTCTCAGCAAACTATTGCAAGGACA
	AAAAACCAAACACCGCATGTTCTCACTCATAGGTGGGAATTGAAC
	AATGAGAACACATGGACACAGGAAGGGGAACATCACACTCCGGGG
	ACAGTTGTGGGGTGGGGGGAGGGGGGAGGGATAGCATTTGGAGAT
	ATACCTAATGCTAAATGCCGAATTAATGGGTGTAGCACATCAACATG
	TCACATGTACCTGCACATTGTGTACATGTACCCTAAAACTTAAAGTA
	TAATAATAATAAAATAAATAAATAAATAAACAATTGAAAGAAGTGAA
	AAACAATGATGTGAAAAATTGAACTACTCTTGCAGTGTTGATGGGA
	CATGCAGCCACATTGGAAAACAGATTGCCAGTTCCTTAAAATGTTA
	AATATACATTTACCTTATGATCTAGCACTTTCACTCCTAGCTGTCTAC
	CCCAAAGAAACAGAAACCTATGTCCACACAACACATGAATGTGAAT
	ATTCATAGCAGCTTTATCCATAATAGTCCATAAAGTGGAAACACATC
	AAATATCGATCAATTGATGAATGAATAAACAAATGGGAGCCATCCAT
	ACAATTTAATAGGGTTTGGCACCTAAAAGGAGTAAAATATTGATACG
	TGCTACAACATAGATGAACCACAAAAGAACATTAAGCTGGCCAGG
	TGTGGTGACTCACACCTGTAATCCCAGCACTTTGGGAGGCCAAGGT
	GGGTGGATCATTTGGGGTCAAAAGTTTAGATCAGCCTGGCCAACAT
	GATGAAATCCCGCCTCTACTAAAAATACAAAAATTACCCAGGCATG
	GTGGCAGGTGCCTGGAGGCTGAGGCAGGAGAATCACTTGAACCCG
	GAAGTGGAGGTTGAAGTGAGCCAACATCGCACCACTGCACTCCAG
	CCTGGGCAACAGAGCAAGACTCTGTCTCAAAAAAAAAAAAAAAGTA

	TATTAAGCTAAGTGAAAGAATAGAAATACAAAGACCAAATATTCTAT<br>GATTCCATTTATATAATGTCTAGAAAAGGCAAATCTGTAAAGTCAGT<br>GAGTCAATTAAAGGTTGCCTTGAGCTGTGAGTAAGAGTAAGGAAT<br>GGTGACTGATGAGCACGAGGGATTTCTTAGGAATGATAGAAATGTT<br>GGAAAACAGGATTACAGTGATGGTCCACACAAGTATAAAATTTTAC<br>TAAAAATAATTGAACTGTACACTTAAAACAAGCAAATGTGATGTCA<br>ATTATACCTCAGTAAACCTATTTAAAAATAAAAGAAAGTTGGTAGTA<br>TGGTTCCCAATCACTCTGGACCTTTGTTTTATTTCTCCCTTTCTTTAG<br>GGCTTATATTTATTGCTTCCACTCAGAGACTCCAAGACACATGAGCT<br>GAAATCTACGAGCCTCCAGAACGTTCAACAGCTGAAAGAAAGTCC<br>AGCCCCACACTGAAGGCGATGGGGGATCTTTAAGGTGCTCTCCAG<br>CACTGCTACAGCAGGCAAAGGCATGCGATGCACCTGGGTAGTTTCA<br>GATAGTACTCCACAAGTCTCAGCAAGACCCAGGGCCTGTAAATAGT<br>TCTGATAGTTTCAGAGAACAAGCTGCTACATCTTGTAGCACAAGGC<br>CAACCTTTCCTAGGCAAGTGGTTTTGAAGCATTGGTAGTGAGGTCC<br>AAAGTAGAAGATGAATGTCTACAACATGGAGGAGGGCAAGACCCA<br>GAGTGGGGTGTCAAACTAAATCATTTGTAGAGTATTACTTTCAACA<br>ACTGCAGAAAGCATTCTTTTCAAGTGCTTATGGCATGTTCACCAAT<br>GTAGACCATATGCTGGACCATAAAACAAATCTCACTAAATATTCAAG<br>AATCAAAATCATACAGTGCATATGCTCTGGCCACAGTAGTATTTAAT<br>TAGAAATCCATAACAATGAGATATCTAGAAAATCCTCACTTTTAGAA<br>AACAAGCAACACACTTCTGAATAACACTTGGGTCAGAGAAGAAAC<br>CACAAGACAAAACTTTAAATATTATTAACTGAATGATGATAACCCAA<br>TACATCAAAATGTTGGGTACAGCTCACACAGTGCTTAAAGAGAAAT<br>TTACAACTTTTAATGCCTATAGTGGAAATAGGAAAGGAGTAAAGAA<br>AAACAATTTTCTATGCTTTTATTATCAGGATCCTGAAAAACGGGGCT<br>GGGTGCAGTGGCTCACGCCTGTAATCCCAGCACTTTGGGAGGCTG<br>AGGCGTGCAGATCACAAAGTCAGGAGTTTGATACCAGCTGGTCAA<br>CATGATGAAACCCCACCTCTACTGAAAATACAAAAATTAGCCCGGT<br>GCAGTGGCGGGCACCTGTAATCCCAGCTACTTCGGAGGCTGAGGT<br>GGGAGAATTGCTTGAACCCGGGAGGCAGAGGTTGCAGTGAGCTGA<br>GATGGTGCCCCTGCACTTCAGCCTGGGTGACAGAGCAAGACTCCA<br>TCTCAAAAAAAAAAAAAAAAAAATGTGAAAAACGAGTGAAAAAAA<br>AAAACAAAATTAATAAATGTAAGCCAATAATAAAAATAATGAAAAT<br>CATGAAATAGAAAAATAAACAACAGAGACAATCAACGGTCAATTTT<br>TCTGTACTCCCTGCACAAAACTTCATTTCAAAATAATTTTATATTTAA<br>ATATATATCCAGAACCACAGAGATCTTAGAAGAAAAAATTAGAGAA<br>ATATAGAAATAAAATGAGAACTGTGGGAATAAAAATCAGGAAGTAG<br>TTGCCTATGAGGTAGATTGGGGGAAGGTCAGGGAATTAACAGGGA<br>ACAGGCAGAAAACAACTTTCTGGAGTGACGGAAATGGTCTATATCT<br>TTTTTGGGGTGCTACTTACATGGGCATATATAACTGTCAAGTTCCAC<br>TGAACTGAACACTGAAGACCTGGGCATTTTAATGCATGTAAATTATA<br>TTTCAATTTTTTTGAGTAAAGTGTCTTGGGAAGCTTATGGCGGTGAT<br>ATTTGGGCAGTTCTGGGGCATAGGCTTCAGAAGCATTCACAGTATG<br>TCCAGTTCAAGAATTTGCTGAGAATCGTTTTGCAGCCAAAAATATG<br>GATAAGTTCGAACACCTATGATAAGAGTCATTGGGGCATACAACCA

	CAAATCCCCACTCTTGTGATACAGGCCATATCATTAAGTGGTTATCA CCAGATAGCTTTCCTTCAATGATGTTGTTCATCTAGGAACCCAGGGC TCTCCAGGGGCAAATGACCATTGAGGTTTCAGATATATTGGGTAGG AAAGAAAGTCACCTCACATAAAACTAAGTGATTGGAGCTGTCACCA TTGCAAATTTCTAAATTTTGTAAGACAGCAGCAACATTTCAAATGAT AGTCTGAGAGAGGAGATAATGGGCTTACATGGATTTCCCTCCAGAT GGATTTACTGAACAAGGGCACAATTCAAATTGCACTTGCAGATTTC TCCTGGTCTAGTTTAAGTTCACAGATTGTGGATCCCAGACAGCTGG TATCAACTGACTAGTGTCCTGTTAAGGCTAAACCCAAGACTCTTTG CCACACTGCAGCATTAGGATGGGACTTCTCTTCCTGTTCCTGGGAA GAATTAGAGATGTAACCCAAAGGTCACTATTTTTCTGAGATAAGGAT CCCATAGGAGGAGAGAGCAATGAGTACATGGCCAGAGATCAAAGT GATAAGGGTTGGGCCAGAAGCCACAGTGCATAAAGCTTCAGACTT GCCACAGGAAATGAGGTGAGAAGTACGTCCACTCTTCTGTCCAGC TTTTAACAAT
HSD3B2	GTATGAGACATAGACATTTTATTTAGTCAAACACTCAACAATGTTTC AACATCTACTCTGTACACGGTGCTAAGGATCAATAAAGGAGTAGTC CAGTTTTAAGACAACACAACAAAAACAAACTCTCAACTCCACTGG CTGTAACACCTTTCTACTGAGATCTTCATGGCACAAATTATTGATGA ACAGGTATTTTTTTTTAGTGGTCACTCTCTTCACTTTTTCCTTCTACT CCTCAACGCACAGATGAATTGTGGGTTGTACTTTCCAGTTGAACTT TCAACCCAAATATTCGAGGAAATTGCTCTTACCAAATTACTTAGGAT GTTGCCAAAACCAATGGTCAATTCTCTTCCTATTGTATTTGATGCAC CAGCAGCATCCATCATAGTGGACCACGCTCCTCTTGAAACACTTTC TCCAGTTGACTTTTACAATGCCATCCCCTCCTAATTTCCTCCTGCC ACCAACTTCTACTCAGTATCTTTAGCTGGCCCCTCCTCCTCTTTTCA ACCTCTAAATGTTGATATATCTATTCCAGGATTCAGTCTGGAAACTTT TCCTTTTATCCATGAATATTATTACCCTAGCTTATTTCATCTAGTCCTG TGCCTTTAAAAATTATTATATGCTTCTCACATACAAATTGACCTCTCT AGGACTAGCACAGGGGGTGTAATTATCCTAAAGTAACCCAGAATTT TAATAAGCTGGCCAAGAAGAGGGATTTCCTTTTTCCAGCATGAGG GACTGTGCCTAATATAGCTCCCTGTAGAATGGGTGTCTTCTCCCCTA AGAAAAGGGAAACTCTTTGCATGAGTCCCAAGGACTCTAGGTGCT GGGTAGTTGATTTTCGCAGACAATCTTGTGAATAGCTGGCACCTAA ATCCAGACTGTTTCACGCCAACAGCCTGTATTCCTCCCTTGAGGAT CCTGTGTCCTTGTGCTTACATCATAAAAAGTTATAATAAGGGATGTA AGCCTGTGTCCTTGTGCTTACATCATAAAAAGTTATAATAAGGGAGA TTAGTGTTCCTACACTGGAATGAATACTCACATTGTTAGCCCAGCAA TTTCCTAACAATTTATGCATCAGCATAAGATAGGAGGATGGGGAAAT CATGCTGCCTATTCTTTTGCTGATCAGTAATCTTTGTGAGTTTTCCTA ATACAGCATATTATTTAACCCGTGATCATCATATTGAGAAATTCTTTA CAAACTCTGATGCCAATAGGTGGTAACTCAGATGAGTATCACTTTAC ACAGCATGCTGACCTTCTCTGAGCATGTGGAGGACAATTTCATTGT CTCAGGATTGCATGTATGAAAGACTATCATTTTTTCCATTGAATTTGC TTTTGTCAAAATCAATTGGTCGTACGAATTTAGGCCATTAACTCACA CATAAGCAAAAATTCTCTCAACATGGATTATAGGCCTAAATGTAAGG

	GCTAAACTATACACTTCTTGAAGAAATCAGAGATTAATACATACATG<br>ACACTGGATTAGGCAAAAGCCCAAATTTTCCTGGTAGAAGGAAAA<br>AAGTCGAAAAATGAGACATGATCAAAACTAAAAATGTTTACATGTT<br>AAAAGACATCAATAAGAAAATGAAAAGATAAGCCACAGACTGGAG<br>GAAAAAAATTTAAATCATATATTTGATAAAGGACTCATATCCAGAAT<br>GTAAAATGCACTCACAACTAAACAACAAAAAGAAAAATAACCCAA<br>TTTTAAAATGAGCAAGATATTTGAATAATCATTTCACCAAAAAGGTG<br>TAGGAATGGCCAATAAAGTACAGAAAAGATGGTGAGCATCATCAGT<br>CAGCAGGGAAATACAAATCAAAACTACAATGTGACACCACTTCTTA<br>CCCACTAGAATGCTATACTAAAAATAAAACAATAACAATTGGAGGA<br>AGTGAAAGCACAATGATGTGGGAAAATTGAAATACTCATGCATTGT<br>TGATGGGACAGGCAGCCATGTTCAAAAACATTTTGGCTGTTCCTTA<br>AAACATTAAAGATGCACTTACCATATGACCTAGCAGTTTTACTCCTG<br>TCAACCCTAAAGAAATAAAAATGTATGTCTACACAATACATGAATGT<br>GAATATTCATAGCAGCTTTATCCATAATGCACTTTGGGAGGCCAAGG<br>TGGGCAGATCATGAGGTCAAGAGATCGAGACCATCCTGGCCAACAT<br>GGTGAGACCTTGTCTCTCCTAAAAATACAAAAATTAGCTGGGCATG<br>GTGGCGTGCACCTGTAGTCCCAGCTACTCGGGAGGCTGAGGCAGG<br>AGAATCGCTTGAACCCAGGAGGCGGAGGTTGCAGTGAGCCGGGAT<br>TATGCTACTGCACTCCAGCCTGGTGACAGAGTGACACTCCATCTCA<br>AAAAAAAAAAAAAAAAAGGGGGGAAACACATAAATATCTATCAA<br>CTGATAAATGAATAAACTAATGGCAGCCATCCATACAATTTAATAGA<br>ATTTGCCACCTAAAAGGAATGAAATATTGATACATGCTACAACATAG<br>ATGAAACACATAAGTATATTAAGCTATGTGAAAGAAGGCAGATGCA<br>AAAGACCACATATTACATGATTCTAGTTATATGGTGTCCAGAAAATC<br>AAATCTGTGGAGTCAGGAAGTCATGAGGGCTGCCTAGAGCTGGGG<br>GCTGGGAATAAGAGGTGGCAAATGATAAGCATGAGGGATTTCTTAG<br>GAAAGATAGAAATGTTCCAAAACAGGATTGTGATGATGGTTACACA<br>AGTCTAAAATTTTACTAAAAATAATTGAACTGTACACTTAAAATGGG<br>AATGTTATAATATGTCAATTATACTTCAGTAAAGCTGTTTAAAAACA<br>GAAAGTTGGTAGTGTGGCCCCCAGCAGCCTTGCTCTTTGTCCTACT<br>GCTCACTTTCCTAAAGAAAGCAAAATATTTATTGGTTTAAAGAAAG<br>CTTACAGTTATTGCTTCCACTCAGACTCCAAGAGACACTGCCTGAA<br>CTCTAAGAGCCTCCAGAACTCTCAACAGCTGAAGGAACATTTGGT<br>CCCACCCTGAAGGTCATGGGGGTCTGTAAGGTGCACTCCAGCATCT<br>GATATGGCAGGCAAAGGTGTGTGATGCACCTGGGTGGTTTCAGATA<br>GTGCTCCACAAGTCCCAGGGCCCGTGTCTAGTTCTGAGAGCTTCAG<br>AAGACAAGCTGCTACACCTTGCATCACAGGACCAACCTTTCCTAGG<br>CAAGTGGGTTTGAAGCATTGGCAGCCAGGTTCCAAGTAGAAGGTG<br>AATGTCTACACACAGGAGAAAGGCAAGACCCAGAATGGGGAGTCA<br>AGCTAAATCATTTGTAGAGTATGACTTCCAACAACTTCAGAAAGCA<br>TTCTTTTGAAGTGCTTATGGCATTTCCACCAACATAGACCATAGGCT<br>GGACCATAAAACAAATCTCAATATATATCAAAGAATCAAAATCATAC<br>AGAGTATATGCTCTGGCCACAATAATATTTAATTAGAAATACATAAC<br>AATAAGAAAATCCCCAGTTTTAGAAAACATGCAACATGCTTCTCAA<br>TAACACTTGGGTCAAAGAAGACATCACAAGAAAAAATTCAAAACG

	TCAGTAACTGAATAATGATAACCCAATGCATCAAAATGTTGGATACA<br>GCTAACATAGTGTTTAGAGAGAAATTTACAACTTTAAATGCCTATGG<br>TGGAAATGAGCAAAGAAGTAAAGTCAATTATCTATGCTTTTATCATA<br>AGAACCCTGAAAAAGAAGAGTAAAATAAACCCAAAGTTAATAAAC<br>ATTTAAGCCAATAATAAAAATAATGAAAATCATGAAATAGAAAAATG<br>AACAAACAATAGAGACAATCAGTGGTCAATTTTTCTGTACTCCATG<br>AACAAAACTTCAGTTCAGAATGATTTTATATTTAAATATATATCCAGA<br>ATCACAAAGATCTTAGAAGAAAAAATTAGGAAAATATAGGAATAAA<br>GTGGGAACTCTGTGGGAATAAAAATCAGAAAGTAATTGCCTACCAG<br>GTAAACTCGGGGGAGGGCAGGCAATTGACTAAGAACAGGCAGATA<br>AAACTTTCTGGAGTGAAGGAAATGGTGGTCTTCTTTTCGTTGGTAC<br>TTACATGGACATATATAACTGTCAAGGTTCATTGACAATGAACACTT<br>AAGTGTACACTGAACGCTTAAGATCTGGGCCTTGTAATGCCCAGAT<br>TACATCTACTTAAAAAAACTACATCTCTATTTTTTTAAGTAGAGAAA<br>AGTGTCTTGGGGAGTTTATGGCAGTGATGTTTGGGCAGACTGGGGC<br>ATAGGTTTCAGAAGCATTCATAGCATCTTCAGTTCAACAATTTGCTA<br>AGAATGGTTTTGCAGCCAAAAATGTAAGATGAGTGCAAATATCTAT<br>GATTAGAGTCATTGGGCATACAACTACAAATCCCCACACTTGTATTA<br>CAGGCCATATGATTAATTCGTTATCACTAGATACCTTTCCTTCAATGA<br>GGTTCTTCCATTAGGAACCCAGAGCTCTCCAGGGAAAAATTGCAAT<br>TGAGGTTTTGGATATATTGGGTGGAAAAGAAAGTCACCTCACATAA<br>AACTTAGTGATTGGAGCTGTCACCATTGAAAATTTCTAAACTTTGC<br>AAGACAGCAGTGACGTTTCAAATGACACTCTCAGAGAGTTGATAAT<br>GGGCTAAAATAGATCTCCCTCCAGGTGGATTTACTGTACAAGGACA<br>ACATTTACATTGCACTTGGAGACTTCTCCCAGTTTGGTTTAAGTTCA<br>CAGATTGCAGATCCCAGACAGCTGGTATCAACTGACCAGTGTTCTG<br>TTAAGGCTAAAGCCAAGACTCTTTATCACACTGTGGCCTTAAGATT<br>GGATTTCTCTTCCTGTTCCTGGGAAGAATTAGAGATATAACCTAAAG<br>GTCACTATTATTCTGAGAAAAGGGATTCTGGAGGAGGAGGGAGCA<br>ATGAGTATGTGGCAGGAGTTCAAGGTAATAAGGGCTGAGACACAA<br>GCCACAGAGCATAAAGCTCCAGTCCTTCCTCCAGGGATGAGGCAGT<br>AAGGACTTGGACTCCTC
HTR1A	ACAAATTTCAAATACTTATTTTATGCTTTTGAACAAATGCATGAACT<br>TCCAGGGAAAGAGTAGGGTCGTTTTCAATACTGATCATTGAACTTT<br>CATTGGCATGTTCTTTTATTCCCTTCACTAACTTCTGATTATGTAAAG<br>AAGTCTCAGAATGCAGCAATCACATCTTGTTTTCTACTTGTCTTTTA<br>CTCAGAATTTCCCAGAGAACTGGAAGCTTACCAAATACTTTAAAGA<br>ATGGGGAGAATGTAACATCCACATGCAAATCTCTTTCCTAATATATC<br>CTCCCATGAAAGAGACTTTTTTTTCAGTCTTCACTCTCTAAGGCCAT<br>CCAGCTCTCTTTTTCCATACTTTAGAGCGATTATCAGTAGAGAGAGA<br>ATTAATATTTTTACTTTGCATCTGTATTATTACTATTATTGTTTAATCAT<br>TATTGTGTGTTTATTATGAGCCAGTGAAATGTGTTAAATGGTTTAATC<br>TTCACAAGTCTATTATTACACTCGTTTTATTACTGAGGAAATAAGAG<br>GGTTAATTAATTTGTTCAAGTCACACACATAGTGAACACATGTTATA<br>TTTTTCACTCTTTATGTACTGTGCTTTTAGTATGTGTACTCACTGCAC<br>TAAGTCAACCATACAATTCTCAAAAGCAGAGATTGAAGCTTCCAAT

	GTTTGTGTTTTATCATTGAAAACCCAGTGCAATTCCAGGCCTGCAA<br>GTGCCTGACAGGGATAAAAAGCAGTGAAGAAATGATATTATTAGAG<br>AAGGTAAATGGTGAGGATTAGATAAGAAAAAAGCAAAACACAACA<br>AAAAAGTCAAGAAAACAATACAAAAACAAACCGTGTTGTTTTAAA<br>GAAAAATTATTTTCTTGGCATTTTGGTTGCATTTCTTGAATCTTTTCA<br>GTGATAATCAGAAGACCACATTTCCCAAATTATGTAAGGAAAATTTC<br>CTATAAGTTGTTTCTCCTAGTCATTTCCAATCAAAATTCATAGTTCTG<br>AACAAAACACAATTGCCCTACTGCTACAGTATTTGGGGATTGGGAA<br>GAATTAAATTTAGAATAATATTTTTAACCTAACAAAATAATTGATTCT<br>AATTTCAATTACATGAAAAAAAAGTAAAAACTTTTTTTTCAGTAGG<br>AGCTTTTAGAAACAACTTTTATAATCTCATAAGCAAATATTCAACTC<br>TTTACTGTATAGATTCTTTCTTCAGTAATAAAAACAATTCAGCCATAG<br>TTTGGGGATAATGGACAAACATACCAATTACTGAGAAACTCCAACC<br>ATCAGCCTTCAGTAATGATAGGGCTTCAAGGTTCACAGCAAAACTT<br>CAGCTTAAATCTTTTTAGTTTGTGTCCCTGGTCAAGGAATTACATTG<br>AAATGCTGGGTGTGTATCAAATTTCCTGTGAAAATGTTAAAAATATT<br>TATCTTTTTAAAACTATTTTAGAGAAATTTCACATGATGGATGATATA<br>TCATACCCTAAATTCATAACCTGACAAGTTTATGCTGGCATCTAGTA<br>AATTCAAAAAGGAATAAAGGCAGAATGACTAGAATTTTTCTTTTCA<br>AATGATATCATCAATAATATCCGTTATAAAGCTTGCTTTTCTTTAGGT<br>TAACTTTAGAGGCCTTGAAGAATAAGAGCTCATCTCTTTATGCAGG<br>AGCTTTGGTTTGCAGCATTTACTTAAGAAATATTTGGTATTCTGTATC<br>TTTAAGAGTTAAACATAGAAGAATTGGCTAAGTGAAAATGAAGAA<br>ACGCAATATCATTCTGCATATATCATTTATTATATATCACAGTATTATTA<br>GTTTTAAAAAGTTAAACATAAATATCTATTAGTCACTTGCAGACTAT<br>GGAACCATAGTCAGTGCTGCATACTTTCATACTTCTGTTTCCCTCCT<br>AGTATTCATAAGTGTGCCTTTAAAAACTTTTAAATTGTAAGAAATAA<br>AATGTTTGATATATTATGTATATTATTACTAAGAAAAAACTTGAATTA<br>CTTTGGATTTTTAAAAACTTTGATAAATTCTACATCATAGCATATTGA<br>AGCAGAATAACAAATGCTATACCTCAGGAATATTAATTCCAGATTTT<br>ACAGCATTTTAACTTTCTTGATGAGAAAAAATAAATTTGTCAGTTAT<br>TAAACTATTTGGGATCCAACAGATGAAAGCAGAATTCTAACTAACA<br>TATTTATTGATTTATTTGTGATTTACATATTTACATGTGTTGTTTGACA<br>CAATTCTTAATTATGTTCTTGATATGCATATATTTGCTTCTTAAATTTT<br>AAGTTTCCTTTATTTTACTTTGTTTATAGTCTCAACTATAATTTCAAA<br>GTTTAATTTTAGATAATTCAGCTTTTAAATATTTTCCCATTATAATTTT<br>TGGACCTCTAACTCTATTTTAACTGTAAATACAGTTCTGTATTTGTGA<br>AGAGACTTTAGAAGTGGAAATAGATACCTTCACAAATCTTAAAAAG<br>ACTTCTTCAGAGTCTGTAAACAGCATTACCATGTATACTTATCTCTTT<br>CTTTTCAATGCCATGAATCATCACAAATGGCAGGCTCATTGTGGTGG<br>CATGCTGAAATGATTGAGTGGGAACTGTGCCCAGCACTGAACTATA<br>AAAAAAAAAAACAAACAAAACCTTATCCAAACACACTGTCCTGTA<br>TTGTAATGCATTGGCCCAACTGGATTCTTTTTGATGCTTTGGTGATT<br>GCTCTTTTGTTTGGGCTTGGAGAATTCAGAGCTATGAAATTCAGAG<br>CTCAGATTTGAACACAATATTAAGATTATTGCAATCTGTAGTGAATC<br>TGTTCATGTTATCCAGTGTCAACTGCTTTTGAGATTGCATTCCTTTC

	ACCTCAGGCATGCAATCAGGATGTATAAGTGAAATGTTGTGTGGTAT GTTTACTGTAGTTGCTTAGAAGTCCCATTCTTTACCAATGCTCAAAT GTGATTAAATTTGTTTTTCTTGTTAAAGGAAACAGCTTAGAACAAA CCCTTGTAAGTATCTTTATTTTCAGTGATTTAACATTTCCAAATGTTA AATCATTTGGAAAATGCAATACTATTCGTTTCTCCAACAAAAGGTAA ATTTATGTCAGTTCCAAAGTTCAGGTTATGACAGCACAAAACCAAC ACAGGTGAAAGTGTTAGCCTAGCTTTATTAAAATGGCATTCCCAGTT AGAACTTGTGAATGACAGATACTTCAGGCTTTCGAAGGAAGCTAA AACATATAATAGGCCTGATATATAAGGTTCAGAGCAAAAGAGGGCA CTAAAATAAATTTTTAAAGAAAATAGGAAGGAGACAAAACTCAATA CTACCTTGTCTTTTAATAACTGTCTTCCTCTTTCTAAAAGTTGTTGTA TTTCCTCAATACTTGCTTCATTTCTGCATAAGGGTTTCCAGATGGCA CTCTAAAACATTTGCCAGAAGGTGGCGACATAAAACCTCATTGCTT AGAACTGTCCCAGGTGCTGAACCCAGTTTCTGAGATTAAGAGAGG CTAGCCGGCTAGCGAACCGGGATTCCACCAAGTTTCCCCCAGAGGT TTGCAGGCTCTGGTAAGAAGTGCAAAAGGCCATGTGAAATGCCAG GCTTCACTTAGAACACATATGCAAAATATTTCCATCCCTGAATTTACT AGCCACAAAGCTATGGGAAGTGGCAGTGTCACTGAAATTACAAGT GTAGTAGTGATGGAAAAGTGTGTGTGTGTTTAGAATATATATCACAC TGAGTTTTGTTCTTCATTTCGAGATGCAGTTGTTTACCTCTCCTTGT CCTTTGACACGTCCTTTATAATTTCGTTCTCTCCCGGTTCCCCAACG TTAAAAAAAAAGTCACAGGCAATATTCTCCCTGAGGGAGTAAGGC TGGACTGTTAGATGATAACGGAGGTACCGTTTTGTTGTTGTTGTCGT CGTTGTTCGTTTGTTTTTGGAGACGGAGTCTCGCTCTGTCGCCCAG GCTGGAGTGCAATGGCGCGAGAACGGAGGTAGCTTTTTAAAAACG AAGACACACTCGGTCTTCTTCCATCAATTAGCAATAATTGGGAGAC TGACCCAGGACTGTTCACCTTCCCATTCAGGCTCCCTATGCTTCCTT TTCTCATCTCCTATTGCCACTCTGGGATGCTGACACGATTTAAGAAT TTGGCAGATAATATGAGGCAAGGAGTAGTTGGAATTCCCTCCCCCA AGTTTTTCCAACCCCAGTTTTGCTGGGTTGGAGGCGGAGTTTATTT GTTACAACCTTGGTCTGACCGGCAGGGATCTGGTGTGTGTAAGTGA GTTCTGAGTCTCTGTTGACAAAAAGAGACTCGAATGCAAAGACGC TGAGCTAGAGGGAGAGGAGGGCGGGGACCCAGAGGAAAGAGGCA CTCCTCGGGGTTGGGGAAGTATTAGGAGGGGAGGGTTAGAGTGGG AGGGAAGGAGCCTGGCTTTCGAAGCGACTCACAGAGGGATAAATA AAGGGAAGTGAGGAGGAAGAGGGAGACTGAAAGGGAAGGCAGG TGGGGAGAAGGGGGACGAAAGAGGCAGAAGAGAGAGAAGAGAG GAGGAGAGAGGGGGAGAGAGGGAAGGAAGGAAATAGGGAGAGG AGGGTCACAGAGTGACCGTGGAGGATGGGGCTTCTCGGTTCTAGAT ATTTCTGGGATTGGAGACTGTTTGCTAGTGGGGAGACTCCAGCTCC GGCAGCCAGTTCGGGAGCGGCAAAGTAAAATGGACAGCGACAGA CAGACGTTCCAGCCACCTCTCCGCCGCCGGGAGATCCTGGAGCTG CTTTCAGGCCAACTCCAGTTTCCCAGCTGGAGCTTCTGAACGCGCT GGACTGCGAGAGCCCAGGGAGCGCCTGAAAGCTGCTCCTCGGAGA TACCCTTCGCCGAAGCAGTAAGAACTTCCTGCTTGGGTCTCTGCAT TCCCTTCCTCCGAAACTTCCCAGGAGAAGGGCGGAAGACCCCAGG

	GGAAGGGGCGAGGCGAATCTTCGCGCTGCTTTTTCTTCCCTCCCCC TTCCCGCGCCGGGCGCGCAGGCATGGATGTGCTCAGCCCTGGTCA GG
ID3	TTTCCAACAAATTCCAATGCTTTCTGAGTCTCTCTGCACAGAAAAC AATGTTTTCCCCTAGTACCCCAACACTTCGGTGTCTGTCTCCCTACC TAATGGATTTACCTTTGCTTTTCCCTATCTGGTAGAATAAAACCCTCT CAAGTCCAAGCTACCTTCCCAGGAGGTCCTTTGTGAGGGCAGAAG GAGAAGCATAGGTGCAGGAGGATACAGGCTTTGGGAGACAGGCAG ACCTGGGTGCAAATCTTGGACCTGCCACTTATCTAGCTATGTGACCT TGGGCAAGCACATTCACCTTTCTGAGCCACTGTCTCTCTGTCTATAA AAGGGATCAGCGGGAAAATGTGTAAGAAGGCAAACAGGACTTGG CAAACAGAACTCAATGAATGATGATGGTGGCGGTGGCGCTGAGTG CTTCTGAGTTAGAGAGTAGCCCATCCTGGAGGCCTTTGAAATCCCC ACCCCTGTAAGGACTCATGCCTTAGATCCTGATGCTCCTCACATTCC TGACAACAGCCTCAGCAGGGACCACTCAACTCACTTGGTACCACC TATCCCACTCAACAGGGGCTACATCACATTACAGCACATAAAAATA ACTGTTTCCCACACCATTTTCCATCCCTTGTCCCCTTTGACTCTCCC AGCTGTGAGGTAGATGGATGGTATTTTCCTCCCAATTTAATAGAAGA GACAGCTGAGACAGAGTGAAGGTAGGAGAAGTCAGGGCAAGGAT TGAGCCTGGTCTTGATTCTGAATCCCCTGTGCTTTCCTTGTATCACC TGAATCTCAAGCAATACTTTGGGCTCCAGACTCTCTGAGGGGAGGA GAAGGAGAAGGGATGTTCTATGTGTGTGACTGCAAAGTCCTCACC ACACCTTCTAGGCACTTATGAGCAAGGAGATACATATTATGAAATGT GGCATTTGGGAATGTTCTAAGACCACCCTAATGCTATTAAGAGTGCC GGGCGCGGTGGCTCACACCTGTAATCCCAGCACTTTGGGAGACCA AGTCGGGCGGATCATGAGGTCAGGAGTTCGAGACCAGCCTGACCA ATATGGTGAAACCCTGTCTCTACTAAAAATACAAAAATTAGCCGGG TGTGGTGGCGTGCGCCTGTAGTCCCAGCTACTCAGGAGACTGAGA CAGAAGAATCGTTTGAACCCAGGAGGTGGAGGTTGCGGTGAGCCG AGATCACAACACTGCACTCCACCCTGGGCGACGGAGTGAGACTCC ATCCCAAAAAAAAAAAAGAGAGAGAGGGCAGAGTGGACAGAGAG TCAAATGAGAGGAGGTCTGGGTTCTGGCCATAGGCCAGCTACTGAC ATCCCAAGTAAACGTGAGCAAGTTTCTGAGGTCCCCATGATCCCCA TGTCTGGGACATAAATGGGAAAAAGATGATAGATAGATAATAAATA GAGGCTCAGCTTAGACAAAAAGGCATCTGCAATCCTTGTCATCATT ACTTTGACTTTGAAATCAACAATTTAGAGAGCCCACTACCACGAAA TTAAAAAATTCTAAAATTTTTTAAAAAACAAAACAGTATAACTTAA AGAGTTCTGCCAACTTCTCAAAAAAAAAAAAATCAGAGAGCACAA CATTGAATTTTTTTTTCTTTTTTTTATTTTGGCAGAGGAAGATTGAGT TCCTGGAAATGCTTACAAAATGATTCCTGACACTGGGAGGAGATAG TTAGAAAAAAGAAGAAGAAAAATTATACACATATATAGATAAAGGA AGAAGGTGGAAAGAAAGGGAATAGACAAAAGAAAATAAAGAACT AAAAGAGAAAGAAAAAAAAAGACTTTAAAGGGCCCCAATTGAAG CAACATTATCAAGTTCTCAGCATCTGCAATTCACAAGCCGCTCGCG GTTGGGGCGATGCAGACCCCGCCCCTTTCTCGGGTGTCCGGCGCTC ATTGGTGGGGGAGGCAGTCGCCGGCCGGTGACTGCCCGAGGAAAG

	GTTGCCTGGGACACGCATCCCTGTGTGAACGCATGACGTCCCACCC<br>TGGCGCCAGGCTGTCTGGGGCTGAGTCTTAGATCAACACAGCTGT<br>GGGACCGGGACCCACAGCTGGGCAAAGGAGCGGATTCCTCAACA<br>AAAAATAGGATTGTGAGAAAAGTTCTGAAACAAAAACAGCGCAGA<br>CAAAAGCCTTAATGACATCCTTTCATAAAAATAAAAAATGCAAGAG<br>GGGGGAAAAAGCTGGTAAAAGGAGCAGAACTGGGAACAGAAAGT<br>TCAGAGGCATTTGGAATAAGAGGCTGAGGCCCCACCACTGCTAAA<br>CAAGTTGCAGAAGGCCTGAGCTAGCCTGTCCAGTCTGCTGTGGGG<br>AAAAAGAAGTCTCTTGGGTTTAAACACTATTCAGTGTTCCTAACCA<br>TAACCTGCTTCTTTCAGAATTAGAATCTTAGGCTAGAGAGGACCATT<br>AAAGGGCACTTGCTTCCTCCCAACCTACCCACTATCTTTTTTTTTTT<br>TTTTTTTTTAAAGAGAAAATGAAGCCCAGGAAGGGAGGAAATTCG<br>TCCAAGGGCACACTGCAAATTATTGGCATGCACAGAACAGAATTTT<br>GCTTGCTAATTCCACAGCATCCTTCTGCCTTATGTCACACAGCCCAT<br>GGGCATTTCAGCCTGGAAGCATCCTGGGAAATAATAAATGCGAAGG<br>TGCTTTGCAAACCTAAATCACTATACAAGCCTGAGGCATCAGCTGC<br>AGTAGTAGAGGAAGGAAAAGAAGGGAGGAGAATCTGGGTAGACT<br>GAGCACCTTCCACACAACTGGCCCATGCTGGGTCCCCCAGGCAGT<br>AAGAAGACTAAGATCAGATGGAGTGAACACATCAAGTGCTTTGCA<br>GAGAAGAGGCATTCAATAATTATCGAATGAATGAATGAGTAGACCC<br>AATTAGTTCCATTTTATAGATGGGAAAATAGAAGCACCCAGGATAAC<br>TCAGCCAAAGTCTACACAATTAGTTTAAACAAAAAAAGAAGAAGA<br>AGAAGAAGAATGGGCATTTGCACAGAAATCTGCCTGGCCCGTGATT<br>TTTCTAGAACCCTCTGGACTTGACCGTTTTGCCACAGAAGTCTGCA<br>AGATAGGAAAAGAAGGATCTTCTCTTCGGTTATTCAGGAGTTCCTA<br>CAGCCATTATTTTGCCTTTCCTGACCAAATAGCTGGCACTTGACGGC<br>TGTAGCATGTAAAGACTGACTTGCCCACTCACAGGTTTTCTTCCTCT<br>TTTCTTACTGAAGAGCCTGTCCTTGAAAAAAACATTCCAGGAAGA<br>AATCTTTCTTGGGCCACAGAGCCCTGTGGTTTGATGGAAAAGAACG<br>AGGCTTCAAGACTGGCTCTGCTATTACTTGTAGTGTGACATTGGGC<br>ATGTCTTAAATGCCTTCCTTGGGCCCCTCAGCATCTTTCACCTACAT<br>ATCCAAATTTCACACAACCTTGGACTTCTACAATTGGAAAGGATTTT<br>AAATAATCACTTAGTACCATCGTCTATTTTACTCACAAGGCAATTAA<br>AGCCAGAGTACCTGGGCTTGTTCAAGATTACATGACAAATTATTGT<br>CAGGGCTAGCATGATGAACCCAGGGCTCTCAATTCCCAGTCAGTGT<br>CCTTTTCAACCGTTTTGCGACTCTCTTTGGCTCTGAGCCCCCTTGTC<br>CTGTTAATCAGTGCCACACATTATCGAACTCTTGTTTCCTGTGTTAA<br>TCTTGTCTCCCCAAATTGTGAGATTCTTGAAAGCAGGAGCCACTGT<br>TTTATGCGGAGGGACTCCATAAATATTCACCAATGATTCATTAGGAA<br>AGCAACTATTGGGGGAATCCCACTTGGTCATTTCCAAAATGGAAAG<br>CAGCATCTTGGAAGTGCAGGTCCCCAAATGTCTAGGGAGGTGACA<br>AGAAGGCAAGCAAAGAATCAAAGAATACTGAGCACAGTGTCCTGT<br>TGCAATACAGGACTTAGTTTGCCGGCCCTCTGGTTCCTTTCCTGGTC<br>TTTCAGATGGAGCTGGAGCTACTTCAGCAGGGCTAAGAATAATCAA<br>GTCCAGGTGGCCTGCCCTTCCATAGCAGAAAGGGACAGACAATAG<br>AGCAATGAAATTACCACTGTTTTCTACACAGAGGTGTGTGGAAAGT

	GAAGAGAGGGGAAGTTAAGAAATTTGCTGACCCAATTGGGTGGGG<br>GGGGGCTCTTAAAAAGGTTTATGTAGAAATTGTCCCATCTGTGCCT<br>CCATATTTAAAACCTTGTCCAAAATTCTAATTGAACTCACAAAGCAC<br>AACTGTGCATATATTTAGTTCGTTATGGAGTTTATGGGGCAGAGGGT<br>CCAGGCTCCTTTTTTCCCCATGCAAAAAGCATGGGCTCAAGCTTTC<br>TTCTTTTCCCCTGTTGCTCAAATAAATAGTGTTCTTTGCTCAAACCC<br>CCTTTCCCTCCTCCTTCTGCAATCTCAGCGCCTAGCGCAAATCTGTT<br>TTCTTCATTGTAACCTCAGCTTCACCGCAATTAATTTTTTTTCCCTCT<br>GGTCACAAGATAATTCCTGACGCCAGTGAGTCTGGAGGTCAGACG<br>AACAGCAAATTGGGGAACAAGGCGGCACTAATTCCTTACAAGTTTC<br>CCTTGAAAAATCTTTCGCTTAAAAAAAACGGGGGGTGGGGGGAGC<br>TTCTTTGCTGTTCAGGGATTTATGACCTCGGAGGAGCTGTGGCTCG<br>AACCAGTGTTGGGCTAAAGGCGGACTGGCAGGGGGCAGGGAAGC<br>TCAAAGATCTGGGGTGCTGCCAGGAAAAAGCAAATTCTGGAAGTT<br>AATGGTTTTGAGTGATTTTTAAATCCTTGCTGGCGGAGAGGCCCGC<br>CTCTCCCCGGTATCAGCGCTTCCTCATTCTTTGAATCCGCGGCTCCG<br>CGGTCTTCGGCGTCAGACCAGCCGGAGGAAGCCTGTTTGCAATTTA<br>AGCGGGCTGTGAACGCCCAGGGCCGGCGGGGGCAGGGCCGAGGC<br>GGGCCATTTTGAATAAAGAGGCGTGCCTTCCAGGCAGGCTCTATAA<br>GTGACCGCCGCGGCGAGCGTGCGCGCGTTGCAGGTCACTGTAGCG<br>GGACTTCTTTTGGTTTTCTTTCTCTTT
IFNA1	AATGCAAGAACTAATGCACCTATCATGATAGTTTTGACACCTGTTGC<br>AATACTTTTAAATAAAGCCTTCCTTACTGTTATACCAAGTGTCAGAA<br>TAAATTTTTCTTTACCAGATCCTTAAATTAAGGCAGTACAATCAACA<br>CTGCAGCATACTCTGGTGACCTACAACATAAAAGGCATGAACCAGC<br>TGGCAAGTCAGGTGACTCAGGGTCTTTTGTCATCTCAGTCCTCATC<br>CTCTAATGCCATGGACTCTCCTCATTTCTCCAGAAGTGTTTCCATTT<br>CCACACCTTTAATAATTCCTTTACTTTAGTTAACCTTTCCAGGTGTG<br>CTCTCCCTGAGCTCTGAGCCTTAGTTCATTGGTTTAGCTCTGATTTA<br>GTTCATGTCTGTAGCCTGACAATGACTTGTTCTTTCTTAAATTTGTG<br>CTACACAGGTGGAAATCTAAAGTATGGTGCATATTTGATTAAAAGAA<br>TAGTACTATGTAGAATTTTAAATGGATAGAAATATATAGGGTTTATGA<br>ATACATTTACTGGGGCAAACAACATGTTTTACAAATACACTCATGTT<br>TATGATAGTGTATCCTCAAGAAGACAAAACTAGAATATAACGTATAT<br>ACTCCTTTACAAGGAGCATAAGTAAAGCGGGGCCCAATTTCATTGT<br>AATGTTGCCATGAGCAAATGGGGCATTACAGTGAAGGAGACAAAT<br>GGAAACTGAAGAGAAAGACTCTGTCATTACATAGGCAGGACTCTA<br>CTAGGAAAGGATTGACTGGTCTATAAATCAGCTCTGGTTCTTATGGC<br>CCGAAATATTCCCAGAGAGCATGTAAGAGCTAAGACTTCAGAGTTG<br>AATTTTCTGCATCCAAACTGTAGCTCCAAAACTTAATAGCTTCCTGT<br>GCTTTCATTGTTTCTTTTGTAAATGGGGACAATAGTAAAATCTGTCA<br>CTGTGTGCCTTGGTCAGTGATATCTCCAGTGAATAAATGGGGTTAG<br>GGACATGGTAGATGTTCAGTAAATACGAAAAACAAAAGTATTCTTT<br>ATTGGCTAAGTGAGCATCGATTATGCAAGTGCTAAAAACAGTGTCT<br>GACTGAAAATAAAGACTGTCTAGAGTTTAGATATCTCATTCCTCTTA<br>TTAACATTATCTTTATTATATAACTCTATTAAGTTGGGGAAGTGTCCT

	TTGAAATTATCAAGTCTCTTGTTTCTTATTAGAAATATAACAAGAAA<br>AAAATTGATCTTTGGAGTGTACTTATCCACTAGTGGAGAGCTTGGT<br>GCAATGAACACTTAACTATTTTCCAGAGAGAATATTGCACTCTCTAA<br>AGTTTGCCTTTCTTGTGTATAAATGCTTGCAGTTGGTGTGTTTGCAA<br>GATGGGCTTCAGTAAATCTGAAAACACCCTGTGGACCAGGAGTAAT<br>TACTTTTCTTCCAGCCCATACTTATTTGCTACCTTTGACCTATATCCC<br>TTCTACCATTTTGCTTCTGTTCTTTCCCACCCCTTAACCTCCATCATG<br>GCTGGGCTGAATGCAGCTAAGGTACATTTTGTCATTGTTTTTCTTA<br>GTGGGATATTACTTCCATTTACATTTTAATCACATTTAGATATATTTTT<br>GTTTTATTTTTGTCCCCATTCATTAGGGTGCAAAAAGTGACAATTTT<br>TGCTTTTTATGAAAAGAGAAAGAAAGATATCATGTCTGGCATGCTAT<br>GGAAAACAACTGTACAAGTCATTTATGTTATAACATTTACATCTCATT<br>ATGAACATTTAGGACAAAGATGGTTTCCTTTAGTTGGATCAGAATC<br>AACAAAGAACCTCTGAAGTTTCTTTGGAAATTTGAATTTTCATGCA<br>AAAAAGTGTTTTTAATCAAATTGAGGATATTTTTTCAATTGTGTATTC<br>TCATTGAGTATGATGCTACTCCCAAGGGTGTAAAAGTGTAGTTCTTG<br>AGAGTGGGAACAAATCTTACAGATTTAAATGATTTGTGGCCCTTCA<br>AAGTTCAACCCTATCTAACAAAGTCTTATTCCTTAGTTTTTTGTTTTC<br>TCCCCATTGGGTTTTCTTGTGTATCACATGAGAAGCATTGACATTAA<br>GTTCATGGAAGATACACAAAATGTATATAAGTTCAGTGTTACAAACC<br>TATGGAAAATGAGTGGCTGTGATTGCAGTACTTTCCAAGGCTTACT<br>AGATCTCACAGTCATATCAAGAGATGTGTCCTCTGCATACATATGTT<br>TTGTCACTGGCCATCTTATAGTTGTTGCTTATTTTTGATCCTTAGCAT<br>TTCTAACTGTGTTGTGGGCTTTGAGGATAATTTATATTTTATTAATTCT<br>ACAAAAATTGTTCAGCACATCAATAATTAGGTCAAGCATCGAAGAA<br>AAAAGTCTTGACAATATTTAGGTGACTAGCAGCTAGTGAAGTTAAA<br>AAATTTCACACATGCAACAAAATTAAAAGTTAAGTACAACCCTGCC<br>ACAGAGGCCGGGAGAGGCGACTGGCCCTTTGTTGGTCTGTGAGAA<br>ATCAAACTTCCAACATGACAAGCAGGAAGCCTGCGGTTAAGGGAG<br>TCAGCGTGTCACAAATACAAGCACCTGGGGTCTTCCAAGAGCATCA<br>TACCTCCTCCAGCCCCTGGAGCCACCTGCTTTCCTAACTGGCTGTG<br>GGAAATGGCCTGAGGTCCCAGATGCTGCCACTGTGCTGCTGCCTTC<br>CACTCTCTCCAAGCAGAGCGCAAGTTCAGCCACCTTTGAAAGACA<br>CCCACCCCCTGGCCTGGGGATGCACAAGTTCAGAGCTTTGCAGGG<br>AGTAACCATGGGTTATGGCTTCATGAAAATGTCCTACTCACCAGTG<br>CCTTTTTCGCAGATGTGGATGTGGAGGCATGAGGGAGGGTAATTAT<br>TGGATTACCAAGGTGCTGCTAAGAGCAGAGGAGGAAACCCCTATT<br>CCCAGTCATATGTCTGGTGTGACATTTCACTAACCCATTTAAGTGCA<br>CAGGCCTCCAAATATCTACCTAATGAGTATGATGGTTTGGGCATTTT<br>ACACTTAAAATCACTGGCCTCATGTCAACTGAAGCCTGACTGGCCA<br>GTGTCTCAAAGTCACAGATGATGACCTGATCCCTCAGGAACAGATG<br>GTGTTTCAGCTTTGTGGGAGTGACTGTCAAGGTATGGAGCACTTAG<br>GTGGTCTTTGAAACCTGTCAGGTTTCACATCTCTGCTTTGAGTGAA<br>AAGCTCATCACCCACAGTAGTGAGGAATGTGCCTATACTTGCTGGG<br>GCTGATCTGTTGAAACTTTATGTGTAGACAATGGAAACATCCAGGA<br>GCATTTCTGCTTTCATGTAGCCTCTTAATAATTGATGTCCCTGAAGTC

	CTGTGTCCTCAGGGACAGTTCCTCTGATTCCTGGATTGTACCAGCTT<br>TCATGATGTTAAATCTAATCTGTAAAACTCTGAGCATTAATCTCCATG<br>AAAATAAGACCTTGTTCTTTCTCATTTAAATGCCCCTTTTTTTCTTG<br>TCTAATATTCTGAGTAGGATTTCCAGTACTGTGTTGAAAGAAGTAGT<br>GAGAGTGGGCATCCTTATCTTATAATAAATCTCAGAAAAAAGATTCC<br>AACATTTCACCACTGACTATAGTGTTAGCTAATGGCTTATCTTATAGC<br>TAATAAGGAATGTGTCTCTTTATTCTGAGGTATATTCTTTATATACCTA<br>ATTTGTTATAAATTGTATTAGAGATGGATTTTAAATTTTGTCAAAATA<br>ATTTTAGGCATGCATAAAAATAGTTATGATTTTTAATCTTTTATTGTGT<br>AAATAAGGAGTATGGCATTTATTGATTCCAACATATTAAAATATTCTT<br>GCATCCCAGGAATAAATCAAGCTTGATCATAATAAATGATCCTTTTAT<br>AGTGCTTTTGAATTTCATTTGCAACTACTTTGTGGATGATTTTGCAT<br>CTGTGTTCATCAGGAATATTGGCTGTAATTTTTTTTTTTCTTGTAATG<br>TCCATCTCTGGCTTAGGTATCAAGGTAATGCTGGCTTCATAAAATGA<br>GTTTGGGAGTATTCCTCCTTAATTTTTTCAAAGATTTGGTTCTGTTTA<br>AATGTTTGGTGAAATTCAGGAGTAATCAGATCTAAAAAAACGTAAG<br>AACACTAAAAATAAATGATAAAAATATTTTAAAGTTTTAAGTGTTTA<br>TCACTTTCAAATAATTTTTAAGTATTCATTACTTAGAAAATGTTTATC<br>TATAAACATTAGAAAATGTTTATAATTTGTTAATTTGCTTATGTAATTT<br>GATACACTACAATTTATTTGTTTGTTTGTTTTTTGAAACAGAGTCTC<br>ACTCTGACACCCAGGCTGGAGTGCAGTGGCACAATCTCGGCTCAC<br>TGCAACCTCCACCTCATGAGTTCAAGGAATTCCCTGCCTCGGCCTC<br>CTGAGTAGCTGGGATTACAGGTGCACACAACCATGCCTAGCTAATT<br>TTTTTTTTGTTTTTTTTTTGTTTTTTTCGTTGAGATGGGGTTTCACCG<br>TCTTGGCCAGGCTGGTCTTGAACTCCTGACCTCGTGATCCACCCAC<br>CTCGGACTCCCAAAGTGCTGGGATTACAGGCGTGAGCCACCTCGC<br>CAGGCCTACAGTACAATTTATATCAGTAAATAAATTACGTTGAAGTC<br>CATAAGCAAATTTAGTTATAAAGTTAAGAAAAATTTGAATAGATTTT<br>AAATTTAACTTTTAGCTTAAATTTTTCATTTGGTTATTTTTAAACCTG<br>CATTGAATACAAAGATTAAACTTTGTACTTTTTGATTATAGAGATATA<br>CATATAGTATATAAATAGATACATATTGTATCTGTGTTATTAAATTTTC<br>ACGGTGGGTTCAATTAGGAAAAAGATATCTAAAAAGTCTCTGGGAA<br>CAAGATGGGGAAGACAATAATGAAAAACAAAACATTTGAGAAACA<br>CGGCTCTAAACTCATGTAAAGAGTGCATGAAGGAAAGCAAAAACA<br>GAAATGGAAAGTGGCCCAGAAGCATTAAGAAAGTGGAAATCAGTA<br>TGTTCCCTATTTAAGGCATTTGCAGGAAGCAAGGCCTTCAGAGAAC<br>CTAGAGCCCAAGGTTCAGAG
IGF2R	ATATTTTATGAGCCCAGGATCTAGCACAGTTTTTGGCCAAAAGTAGT<br>TTTTCTTTATTCCCATGTGACTTTTGCTTAAGCAAAAGGCCTTGTAC<br>AGCAAATACAGCTTCTGTATTTTACCATGGCCTTGCACTTGCTGTTC<br>TTTGTGCCTACAATGTCCTTTCCCTAAATTTCCCCCATTCTTCCAGAT<br>TCACTCAAAAGCAATCTCTTTCATGATGAGATCTCTTTCCCAATTCT<br>CATTTTGAAAAATGTCAAACCTATAGAAAAGTTGAAAGAATAGTAC<br>AAGAGCACTGATATATTCTTCACTACATTTGTGACTGTTAATATTTTT<br>AACCTCTGCTTGCTATTCATACATATATTCTTTTTTTCCCCTGAAATC<br>ATTTGAAAGTTGCCAACATCATGACACTTCACTTTCAGCATGTATCA

	CATAAGAACAAGCACATCGTCCTATGTAATCACAGTAGCTTTATCTT<br>ATGAAATTTAACATTGATTCAATATTATTTACTGTACATTTTTTCTTGG<br>CAGTTTATGATCTTAGAAATCAAGAAGCCTAAAATGAATCACTGTG<br>TGTTTTGAGGGTCTTTTGGTAGAATCATGTTCCTTTCTGGGTGTTCT<br>AAAAAGTTCCTGGATCTTTGGGGTGAGATTCTGTTTGAGTTTCAGA<br>ACAATGTGTGCATGCCATCAGTTTGATAGCTCATGGTTTGTGTTCCA<br>AAGGGGCAGCTATTAGTGTTACCTGTCCCTGCTATTGCTCTTGACAT<br>CATGCAGGGACATCCTCAGTAGCTGCTCTTTCTGCCCTTGGTCTGTC<br>ATTTGAGCTTGTGTTTCCAAAGTCAGATGCAGGAAGATTGGGTCCT<br>CACAACAGGGCGGTGGTTGGAGCACCTGGATGCCAAGCCTGGATC<br>TGTCCTATCATTTGAGGGACATTTGTGTTCTTGGCTATTTGTGTTCTT<br>AGGCTGCCATAACAAAGTACCTCTGGGTGGCTTAAAATGACAGAAA<br>TTGTCTCACACAGTTCTGGAGGCCGGAAGTCCAAAACCAAGGCAT<br>TGGCAGGGTCACGCTCCCTCTGAAGGCTCTAGGGGAGAATCTGTTC<br>CATGCCTTTGTCCCAGCTTCTGGTGTTGCTGGCAATCCTTGGTGTTC<br>CTTGGCTTGTAGATGCATAGCACCTCCATTCTCATATGGCTGCCTTC<br>TCCTTGTGTGTTTCTTGCCCACTTCTTGAAAGTACACCAGTCATACT<br>GGATTAAGAACCCACCCTACTCTAGTATAACCTCATCATAATTTATCC<br>ATAAGGAATGACCCTATTTTCAAATAAGGTCACACTTGAAGTACTG<br>GGGGTTATGAGTTCAACGTATTCTTTTAGGGGACACAATTCAGCCTA<br>TAACAGGGGGTCTTAGGCTGATATCTGTGAGTCTCTGCTTCCTCAA<br>AGGTTTTTCAGAAAATTTAAATGAAATACAGGAGAATTATGCCCTG<br>CAGTATTTGACTTTACAGCAGGAGTCCCCCAACCCTCCGGGCCATG<br>GACTTGTACTGGTCTGTGGCCTGTTAGGAACCTGGCCGCACAGCAG<br>GAGAACGAGGGCATTACCGCTGAGCTCCGCCTCCTGTCAGATAAGT<br>GGTGGTGCAAACCCTATTGTGCATGAGAGGGGTCTAGGGTGGACT<br>GTCCTTGTGATGCCTGATGAAACATCCCCCACAACAGCCCTCCACC<br>ATCCATGGAAAAATTGTCTTCCACGAAATTGGTGCCTGGTGCCAAA<br>AGGTTGGGGACTGCTGCTTTAAAGTCCCTTATATAGGATTTACATTA<br>AAAATTTTCCAGTGGTCTCCAAATGTCCCTTATACCTTCCTATTTTTG<br>GGTCTAGGCTCCAAGTAAGAATCACGCATTGCATTTTGTCATGCCTT<br>TTTAGTCTCCTTTAATTTAGGACAATGGTTCTCAAACTTCCATGTGTA<br>TGAGAATCTCCTGGAGAGCTTGCTAGAGCAATCTCCTGGTGCCAAA<br>CTTCCACGTTTATGAGAATCTCCTGGAGAGCTTGCTAGAGCAATCT<br>CCTGGTGCCAAACTTCCACGTGTATGAGAATCTCCTGGAGAGCTTG<br>CTAGAGCAATCTCCCGGTGCCAAACTTCCATGTGTATGAGAATCTC<br>CTGGAGAGCTTGCTAGAGCAATCTCCTGGTGCCCATCTCCAGAGAT<br>TTTCATTCAATAGGTTGAGGCAGGACCCATGAAATGGCATTTCTAAC<br>AAGCTGGTAGGTGACACAAAAGTTGCTTGTCTAAGGACCGCACTT<br>TTGAAAACCACTGATCTAGAACAGAGGTTGGCAAGCTTTTTCTGCG<br>GAGGGCCATATGGTAAAATATGTTAGCATTTTCAGGTCATACAGTCT<br>CTGTTGCAACGACTCAACTGTGTCATTGCAGGCAAAAGCAGCCCT<br>AGATAATACATAAACACATGGGTGTGGCCGTATTCCCACAAAACTTT<br>ATTTACAAAACAGCTGCCTTACCACATTTGACCCACAGGTTATTGCT<br>TGCTACATGGATTTAGAGTAGCACTTCCACTTTTTTTCTCTTTTTTTT<br>TTTGGAGGAGAGTTGTTTTTTCAGCGCATTAATATTTTGCAATAGTC

	CAGTTCAGTTTTCTTGTCATATGTTTCACAGTTTGAATTTGATTGTTT TATCTCAATTAGATTAAGTTGAACTTCTTGGCAAGACTACTTGTGAT AGTGTGTACGGTAGGGAATAACTGTGAAAGACTTTGAAATGGACC AAAAATATTAGTGGTCAACTGTAATGGAATGCTAGCTGTTTAGTACA GTCTTTTGGGTTATCTGATTCTATGGGGTCTTGTGTCTGCCACTGTC TTTGAGGTATTTTACAACTCTGTAAATTGTAGACTACTGATGGTAATA TACACGATTTTTGTCTTTAGAAATGCAAATTGTGTCTATTGCGTACG CAGCTGTTTTTTGACCTATGGTTACTGACCAATTTTCCATCTATAGAC AACCTTATTTCAGCATTCCTAACTGCTTGAATATTTGTTGGATTCTCC TTGGATTTTCCTTTGAACCAGCTGTGACATGTTACCGGTTCTCACAT TAATGAATGAGCTAAGGAATACCTTTGATACATGTTCATTCTACATCT GAGTCATGATTATACCATCTTTTAAATATTACCCTTTAGCATTTTTTAT TGCATCACTTTAGAAAGCAGAAAATGCTAGTTTTTTAAAAATTGGT GATACTAGTTTTAATTACTTGATTAAGGTGGTTCTACCAGATCTTTCC ATTGTGAAGAGGTATTTTGTGTTTTTTAAGGAAGTATACTTTGAAGG TGATACTTTGAGACAAAGAGAATATTCCGCTTCACAATAATTTTTCA CCAGATGGTTTTAGTATTTACTGATGGTCTTTGCCTGTATCCATTATA ACACTGGAGTTGCAAAACACAAACCCTTTTTCTTTTTTTTTTTTTTT GAGATGGAATCTCGCTCTGTTTCCCAGGCTGGAGTGCAGTGGCACG ATCTCTGCTCACTGCAACCTCTGCCTCCTGGGTTCAAGCGATTCTCC TGCCTCAGCCTCCCGAGTAGCTGGGACTACAGGCGTGTGCCACCA CGCCCGGTTAATTTTTTGTATTTTTAGTAGAGATGGGATTTCACTGT GTTAGCCAGGATGGTCTCGATCTCCTGACCTTGTGATCCGCCCGCC TTGGCCTCCCAAAGTGCTGGGTTTACAGTCGCGAGCCACCGCGCC CGGTCACCACAAACCCTTTTTCGACGTCCACAGTTTACACCTGAGC TCTCCCTTTGACCCACTACAACACTTTCTTTTCTTGGGCAATATATTA ATAGCACGGTGGTTATGTCACAGGCCTGTGACTGGCTTGGGCTCAA ACCCGGGCTTTTTCACTTACTAGCTGCAAGACTAACTAGTTATTTAA TTTCTCCATTCCTCACTTTCCTTATCTGCAAAACGGATGCAATAAAG GCACCCCCTCTTACAGGACTGTTAGGATTAAATGAGCTAAATCACA GTGTCCGAACACTTCAGTACTTCGTACATAATTACTTGCTCAAGAAA TGTTAGCTATTATTGGGTCATGATTATTTATATGTGTTTGAATTCCAGC TTTGCCACATCCCAGAGTGTCTTTGAGTAAGTCACTGAGTATCCTTG TGCCTCAATTCCTCATCTATAAGTAGGTTCCCATCTCATAGGTTTTTA AAGATTCATGCTTACATGCTTAGCAGTGTCTGGTATGGAATAAGCAC TAAAAAAGATGAGCTTTTATGATGATGATGATTACTATTTTCAGTCAT GATTATTTTGTATATTTACTCTAAAAGAAAGTGAATTTTTTTAGTTCA AGACTTTTAGATTCATCGTTCTACTTTCATTGTGGCTAGCTCATGGC CATCCACGTAGTGAATTAATAAAGCTTTGTTGAGTGGCACTTGCTTG ACAGCGACACTTTTGTCCACTCTTTCACCTTGCTGGATTTGTGCTGC CTTCCTTGCTGTAGGAAGCCCACGCTTGGGAAATAACATTCCCACA GAAGGTCTGAGAGGCTCTGACGGGCCAAGGTTGTAAACCCCCATG ATTTGACTCCACGGGCAGTTAAAGTGTGTTCCCACCCCCCACGGAT CCGCTGCCACTAGGCTGTGCGAAGGCATTTACCTCCCTGGCACTTT GATCCAAGTTCCCTATTTTCCCCTACTGGTCGATCGAGGTTGGGAAC AGTTGCTCTAACCTGGGAAGCACCCGCCACGATCCAGCAGGGCGC

	GGTGGGAGGGGAAACTGAGGCCCGGCTAGGCACCCCCGCCGCGC<br>CCAGCCCGCCGAGAGGCTCAGGCCCCGGGAGGCACCTGGAGCCTG<br>CGCCCCGCCCGCGCCGCTTCCTGTCCCGCGCGCTGCGCTCACGTGA<br>CCCGGGCCTGGGAGGAGCCGGGGCGGGCGGGGGTCACCTGAACA<br>AGGGAGTCACGTGAGCGGGGGGCGGGGGTGGGGGGGCGGTGCCG<br>GGCGGCTGTCACGTGACGCGGTTCCGGGGCCGCCGCTGCCGCTGT<br>CGCTGTCGCCGAGCCCAGTCGAGCCGCGCTCACC
IGFBP6	TTGGTTTCACCAGGCTGGTTTCGAATTCCTGACCTCAGGTGATCTG<br>CCCACCTCGGCCTCCCAAAGTGCTGGGATTACAGGCGTGAGTAAG<br>CACACCTGGCCTAAAAAATTAAAAAATTAGCCAGATGTGGTGGTGC<br>GTGGCTGTGGTCCCAACTACTCTGGAGGCTGAAATGGAAGGATTAC<br>TTGAGCCCATGAGGTTGAGGCTGCAGTGAGCTGTGACTGAGCCAC<br>TGCACTCCAGCCTGGGTGACAGAGTGAGACCCTGTTTCAAAAAAA<br>AAAGAATGTGGCTAGTGATGAGGGGAGTGAAGGGAGGGTCCTTGT<br>AAGCTGAAAGCTTGGAGTTCAGGGGAAGAAATGATCTCTGAGCAC<br>CAATTATGCCTCATCCTCATTTCCCCTTATCCCATTTCAATCCCCAAG<br>ATGAGAAACTACCAGGGGGTTCTGGGGAAGGTGATTCAGCCAGCT<br>AACTGAAGCAGAGTGTGTTTCAGGGTCATTGTCTACGGGACCCTG<br>AATGTCAGGTGGAGCACAGGTTCTATGGTAGGGGAGGACATCGAG<br>GAATAACACACACCGGGACCTGTGGGGTCGGGGGGCAAGGGGAG<br>GGAGAGCATCAGATCAAATAGCTAAGGCATGTGGGGTTTAATACAT<br>AAATATATGGACTGGGCATGGTGGCTTATGCCTGTAATCCCAGCATT<br>TTGAGAGGCTGAGGCGGGCAGATCATGAGGTCAGGAGTTGGAGAC<br>CAGCATGATCAACATGGTGAAACCTCGTCTCTACTAAAAATACAAA<br>AATTAGCCGGGCACGGTGGCACGTGGCTGTAATCCCAGCTACTCAG<br>GAGGCTGAGGCAGGAGAATCACCTGAACCCAGGAGGCAGAGGTT<br>GCAGTGAGCCGAGTTCGCGTCACTGCACTCCAACCTGGGCGACAG<br>AGCCAGACTCCGTCTCAAAAAAAACGAAAAACAAAAAACATAAAT<br>ACATAAGTGATGGATTGATAGGTGCAGCAAACCACTGTGGCACATG<br>TTTACCTATGTAACAAACCTGCACATTCTGCACATACATCCCAGAAC<br>TTAAAGTAAAATTTAAAAACCAAGAAATAAAAAGTAAAGTGCCCC<br>CGAAATCCTTGAGGAGAAGAGAATTATGGGATCAGGAGCACATTTC<br>AGCAGGACCAGTTTGGATGGTATGTGTGGGAGAGATGGCAGAAAA<br>CAGAGGCTGCAGGTTAGCTGAGGTTTACACAGTAGTTCCCATCAGG<br>CCCACGTCAGCCTTAGGCCATGTCATGATGTGATGTGGTCATTGAA<br>CTTCCTCTCTGGGACTTTCTGTCTGTGGACTGTGCCACTGTTTCTCG<br>TCTCAAATTTAAAGCTGAATATGGTCCAGGCAGGCCCCTTCCTTTAT<br>CCCTTGTTCTCAAAAGGAAGTCAGCACTTGGGGCACCATGAATTTA<br>GAGGGACGGGTTGAGTGTGAGAGACACAGCATGAAGGATCAGAA<br>AACCAGGGTATAATGTAGATGCGGAGGAGGAAGTTCCAGTAGAGTT<br>TGTTTATGGAAAAGCTTAAGTGATAGCCCAAGCTATTTAGCCTTTCC<br>TCAGCCTGGAAGAAACTTCTCAGCAAACCCTTCTCACACAAAGGC<br>CAGACAGAATCCGACAGTTTTGTACCTTGGCCCACACTCTTGGTCT<br>GAGTGCTGAACATGTGCTTCAGATTTCTGCCCTGGGTACTAAGGCA<br>GAGGGCATCCCAATGGCAGGAAGGCAGAGTTCAAGCGTCTCAGTC<br>TGCAAAGAGACCGGTTAGACCCTCAGTGCTGATGGCCATACAACTG

	GGGATCATGCTAGAGGGAGGGGGAAACAGGGAAGTGTAATCCTGG GGTGGTGGGTGGAACTGGACTGTGCAATCGGGCTTAGAATCTAGA AAGTAACAGCTGGGCAGGTGGGAGCGGCAGGCAGGGAGAATCTG AGGCAGTGGTAGCAGCGTGGGAAGCGGGGGCTGGAAATTGAGGA GGGTTGAGGAGGGAGTGGTGGATGTTAGAAGCTGGCAAAGTGTTG GGCTTTTTCTGAACGATAACCTACAAAGGAGTAGCACAGCCTTTTT TTTTTTTTTTTTTTTTTTTTTTTTTTTTTCTNTGAGACAGGGTCTCACT CTGGTTGTTCCAGGCTGGAGTGCAGTGGTGTGATCTTGGCTCATCC CTTCCACCTTAGCCTCCTAGGTAGCTGGGACTAAAGGCATGCACCA CCACATCTGGCTGATATTTTGTATTTTTAGTAGAGATGGGGCTTGGT CATGTTGCCCAGGCTGGTCTGGAACTCCTGGGCTCAATTGATCCTC CCACCTTGGCCTCCCAGAGTGATGGGATTACAGGCATGAATCACTA TGACAAACATCTTTAAAGAGCCACATCAATCTGGGTGCAGTGGCTC ACACCTATAATTCCAGAACTCTGGGAGGCCAAGGCGGAAGGATCA CTTGAGCCCAGGAGTTCCAGACCAGCCTGGGCAACATGTCGAAAC CCCCTCTCTGCTAAAAATACAATTAGCCAGGCGTGGTGGTGTGTGC CTGTAGTCCCAGCTACCTGGGAGGCTAAGGTGGGAGGATCTTCTGA GCCCAGGAGGTCAAGGTTACAGTGAGCCGTGATGGAGTCACTGCA CTCCAGCCTGGGAAATAAAATGAGAACCTGCCTCAAAAAAAAAAA AAATCTAGAACCAGACCAGAAATACCATTTGACCCAACGATCCCAT TACTGGGTATATACCCAAAGGAATATAAATCATTCTATTATAAAAATA CATGCACATGTATGTTTATTGCAGCACTATTCACAATAGCAAAGACA TAGAACCAACCCAAATGCATGGAATACTATGCAGCCATAAAAAGGA ATGTGATAATGTCCTCTGCAGGGACATGGATGAAGCTGGAAGCCAT CATTCTCAGCAAACTAACACAGGAACAGAAAACCAAACACTACAT GTTCTCACTTATAAGTGGGGGCTGAAAACAATGAGAAAACATGGA CACAGGGAGGGGAACAACACACACTGGGGCCTGTGGCGGGGTAG GGGCTAGGGGAGGGAGAGCATCAGGTCAAATAGCTAATGTATGCG GGACTTAATACCTAGGTGTTGGGTTGACGGGCAGCAAACCACATGG CACATGTTTACCTATGTAACAAACCTGCACATTCTGCACATGTATCC CAGAACTTAAAATAAAATAAAATAAAATTTAAATTTAAAAAAGAGC CACACTGGGATCCTGCAAGAGCACAGCCATCTCTTTCCCCTTCCCA ACTTCCCACAGGCTTTCCATCTTATTCAGGAGCACCAACCCGAAGT CCAGGAGAAAGACCCTAAGACCCTAGCCAGAACCATGTGTAGGAC AAAATAAATAGTGATAAAGGGTGTGCAGCAGGACCCCAGTACTCTG GAACCCATCTCTGACAGCCTTGGTTCCAGGGAGCTTGGTCAGAGCT TGTGGCAGGGGGTTGAAGAGCCTTGGTGGAAGGGGTGCTTTCTCT CACCCCTTTCCCAATTTTGCCCTTCCTCTCCTCTGCCCTGGTCTACT TCTCCCATCAGCAGTAGCTTCTCCCATCAGCTGCCCCACAACCCTC TCCTCCCTGCCTCTCCAGTCTGCATCAGTATCTCAGGGAAACAGGA AAACACCATTCTCCAAACACATGTCCTAGGGTCCCAGGGTTGCATG GCGAAGCGGGCTGTGCTGACAATGAGGTTCGTATTATTTCTATTTAC CTAAAAGTTCAAGAAGGCCTTGCTGACATTGTTGCTTGGGGCCCAG CATTTAAACTTAGTCACTGCCCAGAGCCCTTGTTTTAGGAAAGAAT GAGATGGTCCCTGTTGCATAACCCAGAGTTGGGTGGGTGTGGGTTG CCGAAGGAAACAATCCCTCTTCCTCTCTCTTGTGAGGCCTGGCTAG

	GAGTGCCTGGGATGGGCGGGACACTGCCTGGGGAAGGCGGGTGG<br>GGAGCGGTGGAACCCGACCCTTGGAGCGAAGGCGGACAAGTAGG<br>TAGGCAAAGATGCTGAGTAGTCCCTCCCCAAAGTCCCCTGGGCAC<br>AAGACAGTCTGATGAATCTAGAAAACCCCATGGATGACTGGGAGG<br>GAAGGAAACGGATTCTGGGAGAGGAGAGGAATGGAGGAGCGAGC<br>TTACCTGAAGGACGGGGATGGGGAGGGGGGTGGGGTCTGCTCTCT<br>GGCCACTGTCCCCAAGAGCATCCCGAATACGAGTTCCTTTAATTCT<br>CTCCCTCTCTAGTTTCAACCTGCCAATTTCCCCCTTTCCCTGCCTCC<br>CAGCCAGTTCTCACCCTGCACACAGCCCTCTCCAATCCTTCCAGAC<br>TCCGCATGCGCCCACCACAATGCTAGGGTCTCCTACGGTTCATTCA<br>AGTTGTATCCCTCCCTGCCTCCCGGCCCTTCCCCCATTGGCTTACCC<br>CAGTCTGGTTCCCCTCGGGCCCCTTTTCTCCATAGTCTTACCTCAAG<br>GGTGCTTTCTCTTACTCCTTTCCCAATTTTGCCCTTCCTCTCCTCTGC<br>CCTGTTCTACTTCTCCCATCAGCAGTAGAATTTCCCAAGACCCCGTT<br>ATCCACCCTCTCCATCTTTTCTCTGTCCCTCCACATTTGCCCACTCCT<br>CTTGTGATCCACACACACCATCTCTTCTCAGTTCCCTTTCAGCCCCC<br>GTCTTCTCACTCCCCACCGGGTCCCTGCATGCCCCTCGTCGTCCTAC<br>ACATACACACTAAGTGGATTGCACTCCCGTCCTCCCCTTCTTCCGCT<br>CCTCCCCACCCTCCAGTCCCTCCAGCCAGCTGTACAGCTCCCAAAT<br>TCCGGGCCGAGATTCCCGGGAACAAAGCAAGAAAAATCAGAGCAA<br>TTTGGGAAGTAAACAGAAACTGGAAAGGGAGAGGGAAAGAAGAG<br>TGGGGAGGACCCAGGTTGGGGGGGCCCTCTCCAGTCCCACCCAGT<br>TTAGGGAATGCCCCTGCCCTCTCCCCCACCCCCCTTCACCTGGCTC<br>TTAAAGGGCCCGGCCCCTGGCCGGCGGCTACTTAAGACAGAGGGG<br>CGGCGGCGGGCAGCAGCTGCGCTGCGACTGCTCTGGA
IGLL1	GCCAGGTGTGGTGGCATGTTCCTCTAGTTCCAGCTACTTCGGAGGC<br>TGAAGTGGGAAGATCGCTTGAGCCTAAGAGTTGGAGGCTGCAGTC<br>AGCCCTGATTGCACCACTGCACTCCAGCTTGGCTGACACAGCAAG<br>ACCCTGTCTCTAAAACAGAAAGGAGGGAAGGAAGGAGAGAAAGA<br>AGGGAAAGGAAGGAGAGAGAGAGGAAAAAAAAGAAAGAGAGAA<br>AAGAAACTGCTGAATAAGCCAACAGTTGGGGAACTCAAGAAACA<br>AAATTAGGTAACATTTTAAACTGAACAATGATGAGACCACCACATA<br>AAAATTTGTAGGATACAGATAAATCAGCACATAGAGGGAAATTGGT<br>AACTTAAATCCCAGCCCTTTGGGAGGCTGAGGTGGGAGGGATTCCT<br>TGAGCCCTGGAGGTTGAGGCTGCAGTGAGCTATGATTGCACCATTA<br>CATTCCAGCCTGGGCAACAGAGTGAGACCCTGTCTCAAAAAAAAG<br>ACTGTTGTTTAATTTTCACAAGTTTGTGAAATGTAAAATTTAAATGT<br>TAATCCCACCCACAAAATAATGCCACAGAAACATCAGAATAATATTT<br>GACCAAATATCTGGCACTGTGGCCCTGCCAAGTTGACACATAAAAT<br>TAAACATCACCCTGGCCGTAAATAATGGTAGGTACATCATCATCCTC<br>ATGTCCAAGGAAACCTCAAGACTTCTGGGCTTTGAAGAATATTTTG<br>AAGCTGCCAAAATAACAGAGATCACTTCCAGTGACCCTGAAAACT<br>TGAGAGTTTCTAGAACCTTCACTTCTGGTTCCTGAGGAAAATGGTC<br>CTGTATTTCCTCTGGAGACAGCCTGAGAGAGAGGGATCCTTGGTGG<br>TGCAGGCGGAGTCTTTGAGGCCTGGGGCATAGAGAGTGGGCAGGG<br>GACTGACCAGCAGGAGGCCAGAATGGGGGACACCCACAAACACG

	GGAGCAGGGCAGAGGTGACTAGATGGGGCAGTCACCTGTGCTCTG AGAGAGGACAGCCAGGCAGGACCCAGAGGGCTCTGCAGAGGCAC CAGGGACCCTAAAAAGGGAGGGCACTGAGCAGGACAGCAGGGCC CAGCCTCCTCTGCCCAGGCCTGGACACATCTCAGTCCAGGAAGGG GTGAGGTGAGCCTGGGTGGTGAGAGCTGCAGCTGCACCTGCTGGA GAGGCCCGGCCCAGCCCAGCCCCGCAGGGTCCCCAGGATCCAGCC AGACCCAGATTTCCCGCCACAGAAAGATGTGGTCTGAGGCTGCCT CTGTTGAGAGGCCATGGCAGGTGCAGGGTCAGGCAGGGCCTGAAA GGGAGCGGCTGGGGGCACTATTTAAAACAGTCGCTTCCTCTCCCGA GACCTGGGGCTGTCCTTGGGGGAACCACAGTTTCTGAGTCCCAGG CAGTGATGAGTGTCACACTCAAGGGGGGTGCTGGGGAGGGAGCA GCTTCAGCCCAGACCCCCAGTGCAGGTGCTGCTCCAGCCCTGAAG GTGAGAGGAGCCAGGGTCGGGGGCAGCAGTGGACCAAGGCTCCC AACAGGGGAGAAGCTAGACTGGATTGGGGCCTCCCAGGGGCTTCC CCTCTACCTGCATCAGTGTTTCTTAGGGTGTCCACATCCCATTACTG CTCAGCACCTTGCCCATCTCCCCGAGGATCGGGAGCTGTCTGGCAT GCAGCCTTTGGCGCTGAGGGGACTCTCAGTGTTCGTGGGACACTG GGATCTGAGCATCAGTCCATGGAGCCTGAGCTCACTATGGCTCTCG TGATCTCTATGAGATAATGTGAAGTCTTCTGTTTGTCTCTCTGTCTGT CTGTTACAGCATCCCACAACCATGGCTGGCAGATGCCAAGGGGATG TGTGAGATGGCCCAGCTCCAGGATGAAACTCAACTTTAGGGAAGC TGGGGAGAGCCTCACAGCGCTGGACAAGCCATCAGATGGCACTGC CCCCAGGGACACACCCGGGGGCGTGGACAGGGATATGGAGGACCT AAGGCAAAGGGTGGCTTCAAACGTGACAGCAGATCAGCCGCTGGG GAAATACCAGAGGGGATCTGACAGCTGGGCCAGGTGTTCCAGGAC AGCACTTTCCAAAAGATTTTCCATCCAAACTTCCAGCAAGAAGTAG ATCTTCCATCGTTATCCAGAGCATATCTACGCATTCAGGTAACTGAA GCAACATTCCAGGAACCCTCACCATCCCTCCCGTTGAACAGTTCGC TCTCCAGCTGGAGTCAGCTCTGGGCTGCCTCACTTTAACAAAATGC TGGTTGCAGCCATTGGAATTAATTTTACACCCTCTGAGTTGCAGTGC AAAATTTGAAGACTGGCATTTGGAAGCTCAGTATCATCAAGGATTC ATTTATTTTATGATGGTTGACAACATTTTCCAGGCCACACTAACTGG GTCAGCTGGTAATAATAATAATATGATAATTGGCCGGGCACGATGGC TCACGCCTGTAATCCCAGCTCTTTGGGAGGCTGAGGTGGGCTGATC GCCTGAGATCAGGAGTTCGGGACCAGCCTGATCAACATGGTGAAA CCCCGTCTCTACTAGAAACACAAAAATTTAGCATGATGGCGGGCAC CTGTAGACCAGCTGCCTGGGAGGCTGAAGCAGGAGAATTGCTCGA ACCCGGGAGGCAGAGGCCACAGTGAGCCGAGATAGCACCATTGCA CTTCAGCCTGGGCAGCAGAGTGAAACTCCATCTCAAAATAATAATA ATAATAATAATAATAATAAGCCCCTGAAGGTATTACACATGCAGCAC TTTCCAGTTTAAAAAGGACTTTCAAAGGACAAGTAGTGTATGGTCC CATCCATAGGAGGTATCCAGAGTACCCAAATATAGAGATGGGAACT AGCATGGTGGGTGCCAGGGGCTGGGGGAGGGAGGAATGGGAAGT TGGTGTTGAATGGGGACAGAATTTGAGTTCTGCAAGATGAAAAAG ATCTAGACATGCCATTGCACTCCAGCCTGGGCAACAGAGTGAGACT CCGTCTCAAAAAAAAAAAAAGAAAAAGCCAGTTGCGGTGGCTCAC

	GCCTGTAAACCCAGCACTTTGGGAGGCCGAGGCAGGTGGATCATG AGGTCAGGAGTTTGAGACCAGCCTGACCAACATGCTGAAACCCCA TCTCTACTAATAATACAAAAATTAGCCGGCATGGTGGTGGGCGCCT GTAATCCCAGCTACTCAGGAGGCTGAGACAGGAGAATCGCTTGAA CCCGGGAGAAAGAGATTGCCATGAGCTAAGATTGCGCCACTGCAC TCCAGCCTGGGCAACAGAGCAAGACTCTGAAAAAAGAAAAATCTA GAAATGGATGGTGGTGATGGTTGCACGACATTGTGAATGTAGTTAA TGCCACTGAACTGTGTACTTAAAAATGGTCAAGATGGGCCAGGCGC GGTGGCTCACACCTGTGATCCCAGCACTTTGGGAGGCCCAGGCGG GTGGATCATGAGGTTAGGAGATCGAGACCAGCCTGGCCAACATGG TGAACCCCATCTCTACTAAAAATACAAAAATTAGCTTGTCGTGGTG GTGCATGCCTGTAATCCCAGCTACTCAGGAGGCTGAGGCAGGAGA ATCGCTTGAACCAGGGAGTTGGAGGTTTCAGTGAGCTGAGATCAC GCCACACTGCACTCCAGCCTGGCGACACAGCAAGACTCTCAAAAA AAAAAAAAAAAAGGTCAAGATGATAAATTTTAGGTTGTGTGTATGT TACTGCTTTTTCACAGATGAAGAAAACAACACTGACATGCATTATC TCCCAACACCAACCTCAACCCAGCCCTGGAGTGTGAGCAGAGCCA GCAGCATTTCCAGCCCCGCCTCTGGAGGAAGGAGAGGAACCTGAG ACTCTTGGGTGACCTGAGGCGTCTCTGGCAGCACCAGCAAAAGAG CCTGGCCCTGGCCGCCCAGCACAGGTGCTCAGTCCCTCCTGACTGC CACTGCTCCCTCAACACCAAAGCACAGGGTCGCGAGATGTTGGGA GAGGGTTGGGGATGCTGGGCTGGGAGAGGACCCGGGGGCCTGTCA GAAGCCATGACATGATGCACTCACCTCTCAGGAGGAAGCTACTGG CCTTTGAGTGAAATCTATAAGAATGAACTCTGGAGATGTATCGTACA ACATGGAGTCTACAGTTAAAAACTGTATTGTATACTGGAAAATTGCT AAGATTAGATCTTCCATGTTCTCACTACAAAAGCATAAGTATGTGAG ATGACGGACGTGTGTGTCAGCTTGATTTAATCATTTTGCAGTGTGGC AAAGCATCGCACTGTAGGTGGTAAATATATATACAGTTTTGTCAATT ATACCTTCAGGAAGCTGGGGTGGTTGGTGTGTGTGTGTGTGTCTGT GTGATGTGAGGTCAGTGAGGTGCTGAACATGGTGCATTAAACAGCA TTTTCATTCTTATCTCATGATTCCTGGACTTGAACCGTTGAGTGGCT TCTGGCTAAACCCAAACCTGTTTCCTGCTCTCTAACCACACCTCCC TCCAGGACCGGTTGGAGGAGCCTCAGCCCAGGCCCCTGAGCTCAA GCCCTGGGGGACTCCTGCTGGGCAGCTCTCTGGACCCCAGTGAGA TGCTCCACAGACCCAGGGCGCCCTCGGGGAAGTGGGGAAGGAGG GAAGTCAGGTGCATCACCTCTTGGGACCAGCCCCGAGGGGACTCA AGCCCATCCAGGGCGCAGGTGCCTCCAAAGGAAAGCCCCAAGGG AGGGTCTTGGGGGGCCATGGCGCATCCCACCTCCCTGTGTGACCTC AGCTCTTGCTGACCCTCTGCCTGTGGTGGGAGGTCCCCAAACAAA GCACCCAGGGCCCCAGGGGCAAGGCCAAGCTGACAGGTGTGGGA GGCAGTTGAGCCCTGGATCCTGACCACAGCAGGGCAGTTGGCAGA TGTGCCTCTGGGACAGAACCCCAGGGGTAACGGTCATGGACTCTG GAAGGGGCCTGGCTGGGGGCAGGGACCAGAGGATGAGGATGGGG CCACATGGACTGGGGTGCAATGG
IL10	GCCATTTGCTTAGCATGAGAGTAGCTGGGAGGCAGATTCTTTTTTT TTTTTTTTTTTTGAGACGGAGTCTCGCTCTGTCGCCCAGGCTGGAG

	TGCAGTGACGCGATCTCGGCTCACTGCAACCTCCGCCTCCCAGGTT<br>CACGCCATTCTTCTGCCTCAGCCTCCCAAGTAGCTGGGACTACAGG<br>CGCCAACCACCACACCTAGCTAAATTTTTTTTTTTTTTTTTTTTTT<br>TTGAGACGGAGTCTCGCTCTGTCGCCCAGGCTGGAGTGCAGTGAC<br>GCGATCTCGGCTCACTGCAAGCTCCGCCTCCCAGGTTCACGCCATT<br>CTCCTGCCTCAGCCTCCCGAGTAGCTGGGACTACAGGCACCTGCCA<br>CCACGCCCAGCTAATTTTTCTATTTTTAGTAGAGACAGGGTTTCAC<br>CGTGTTAGCCAGGGTGGTCTCAATCTCCTGACCTCGTGATCCGTCC<br>ACCTCGGCCTCCCAAAGTGTTGGGATTACAGGCGTGAGCCACCGT<br>GCCCGACAAGGGAGGCAGATTCTTTTTTTTTTTTTTTTTTTTTTT<br>GTAGTTGCAAGATTTAATACAGTGAAAACAGAGCTCCCATACAAAG<br>GGAAGGGACCCAAAGAGGGTAGCCATTGCCGGCTTGGATGCCCGG<br>GTTTATGTCCCGATCATTGTCCCTCCCGCTGTGCTCTCAGGCGATAG<br>ATGATTGGCTATTTCTTTACCTCCCGTTTTTGCCTAATTAGCATTTA<br>GTGAGCTCTCTTTACCACCTGATTGGTCGGGTGTGAGCTAAGTTGC<br>AAGCCCCGTGTTTAAAGGTATATGCGGTCACCTTCCCAGCTAGGCTT<br>AGGGATTCTTAGTTGGTCTAGGAAATCCAGCTAGTCCTGTCTCTCA<br>GTCCCCGCTCTCAACAGGAAAACCCAAGTGCTGTTGGGGAGGTTG<br>GCCGATGACTGCTCTAATGGGAGGCAGATTCTTTATAAGATCTTGTA<br>AACTGTAGAATGCACCCTCCAAAATCTATTTGCATAAGCACACACA<br>CACACACACACACACACACCCCAGCAGTTCTTGCCTGCCCAGATTC<br>CTCTGCAGCTAAAGTGATGAAACTTACTGGGCGGAGCTTCCTAAAA<br>AGATTATTAGGGTCTCCTGGGTTGGTGTGCCTTTAAACCTTTGGACT<br>TTACCACCTCCTATCTCTCCTATCTCCTTGCAACAAAGGTTAGGAGA<br>ACAAGAATGCAGAAAAAACGGGTCCTGGATGACATCTGAGTGCCT<br>GCTTTGGGCTTCTTGATGAGTGAGACAGAAAATAAAATACAACCCC<br>CTCTTTTAAAAGCCATGCTTACTCAGGTTTTCCTTCATTTGCAGCTA<br>AATACAGAAATGAGAGAATATTTTGGAGCAGGGATGGAAGAAGAG<br>AGGTATTCCCCTTCCCACAACCTTCTGATTTCCCAGTACATCCCCCA<br>CTGGAAAAATTCATTTAAAATCAGTATAATAAGCATTGATTAGATGC<br>CTACTATGCATCTGGGCTTGAGGGCAAACTGGACTCAGGCCTTTTG<br>GCCTCAAGAAGCTCACAGTGTGAGAGTGGCATTTGTGTCCTCTTGA<br>AATTCACAGGACTAAATTGTGCCCAGGCTGACATTCTATCCATCCAT<br>AGGTGCCTGCCTTCTCACTTCCCTCTCTTCATGGGCTCTTGCCTTGT<br>ACCAAAATCCAAACCCAAATCTCCTCACATGTGAGTGTTGGCATTC<br>ATGTCTCAGACATGACCTATGGGCTTGGGACTTTTCCCCGTGGACC<br>CCAGTGACTTTTCAGATGAACAGGTATCTTCAAAAACTTGAGAAAT<br>AGGAGTCCTGTTTGTTGTTCTTGTTGCTTTGTCAATATAAGGACACA<br>GGGTCTTTATTCAAATGTTCATATCTATCTCTTGACAGAAATACTATG<br>AGACATATTGATGGAGAAGCCGTTATCTCCATATGCTAAATGAGGAC<br>TTGCACCAGGGAACTTGCCCATGGTTCTCTCCAACCACTTAAATTC<br>TGAAATTTTGAAATGAGAGTGGACAGTAATTTCAAATCAATGGGGA<br>AAGAATCAAATCTTCAGCAAATGGCTTGAGATAATTAGCTACACATT<br>TCAGAACAAATAAAGAAGTCAGATCCGGGCCGGGCACAGTGGCTC<br>ATGCCTGTAATCTCAGCACTCTGGGAGGCCAAGGCGGGCGGATCAT<br>AAGGTCAGGAGATCGAGACCATCCTGGTTAACACAGTGAAACCCC

	GTCTCTAATAAAAATACAAAAAAAAATAAAAAAACTTAGCCGGGC<br>GTGGTGCCAGGCGCCTGTAGTCCCAGCTACTCGGGACGCTGAGGC<br>AGGAGAATGGCTTGAACTCGGGAGGCAGAGCTTGCAGTGAGCTGA<br>GATCATGCCACTGCACTCCAGCCTGGGCAACAGAGCGAGACTCTG<br>TCTCAAAAAAAAAAAAGAAGTCAGATCCTAACCTCAACCCTATTTA<br>ACAGATTATAGATGAAAGAAAGGTACAAATGGCTTTTACATACCTC<br>CCTTCTCCCTGACATTTTGTATGTGTGTGTGTGTGTATTTACACACA<br>CATCTCATATAAGGAAATTGAAGGGAGGCTGCCTGCATCCCTGAGT<br>CACTCTCCCTCTCCTTCTGAATGCTTACCTGTGCCCAGACCACCTCC<br>TTAGCCTCGCACCCTCCAGGCTTACAGGGCACTCTTCTATGCCCATC<br>CCAAGTATAGCTGATACCTTCCAAGGGCCAGACTTGGTGCTAAGTA<br>CCAAGTACGCAAAGATTAATAAAACAATGTCCTGTTTCAGGGAGCT<br>CAAAGCTGATTCGGCAGGGCATGGTGTGTACATGAATGATAACCAC<br>GTAGGGTTGCAGGTTTCCTAGTGAGGTAAGCACAAGGCAAGATGG<br>GAAACAAAGGAAGGAGGGGTTCACAGCCTCACCCAGAGTCCAGA<br>ACCCCTGGCCTGCCTGGTGCCCATGCTGAGTCCACTTCTGGAACAC<br>CCAGCTCAGAGAGGGGGTTAGACCTGCAGGCTAACACAGACACAG<br>CCCAGAAAACCCAGGAGCCGAGGGGGAAGGAGAAAGGTGCAAGA<br>AGGGGAAACCCAGGTCCTGGTCCCCTTCTCTCTGCTTCCTGGCAGC<br>AGAACTCAGACAGAACCCTTAAGCCAGTCTAAGTCTGGCAGGACC<br>AGTAAGTTCTGAGTTAGCTCCATACTAGTTTCTAGCAGGCTCTTTCT<br>CACTTCCTGATTCTTAGGTTTCTACATTGACACTCCCTGAAGAGTTG<br>GGAAGAGACACCACAGTCCCCTGACCCTGATCCATAGGTCACACA<br>GCAGGGACATCCACAGGGTGGGCGTGGGCCCTCTCATCCCTCCCTC<br>CCACTCACTTCACGCTGGCTGGGCCCCAAGGTGTTTGCACCCCTTG<br>CAGTGAGTGACCTTCTCTAGTGCAGCAAGCTCAGAACCTGCTGCC<br>ACTGGAGTTGTCCCATTGCTGATGCAGAAAGGTGAAGAACTAGCA<br>GAACACTGGAAATGCCCTCCATCTGGGTCCATGGCTACTTAAGCTC<br>AATGCTCCCTGGCAGGCAGGAGGACAGGTGCTATTGCCCTGTTGG<br>GACAGATGAAAAACAGACACAGGGAGGATGAGTGATTTGCCCTGA<br>CTATAGAGTGGCAGGGCCAAGGCAGAGCCCAGGCCTCCTGCACCT<br>AGGTCAGTGTTCCTCCCAGTTACAGTCTAAACTGGAATGGCAGGCA<br>AAGCCCCTGTGGAAGGGGAAGGTGAAGGCTCAATCAAAGGATCCC<br>CAGAGACTTTCCAGATATCTGAAGAAGTCCTGATGTCACTGCCCCG<br>GTCCTTCCCCAGGTAGAGCAACACTCCTCGCCGCAACCCAACTGG<br>CTCCCCTTACCTTCTACACACACACACACACACACACACACACACA<br>CACACACACACACAAATCCAAGACAACACTACTAAGGCTTCTTTG<br>GGAAGGGGAAGTAGGGATAGGTAAGAGGAAAGTAAGGGACCTCCT<br>ATCCAGCCTCCATGGAATCCTGACTTCTTTTCCTTGTTATTTCAACTT<br>CTTCCACCCCATCTTTTAAACTTTAGACTCCAGCCACAGAAGCTTA<br>CAACTAAAAGAAACTCTAAGGCCAATTTAATCCAAGGTTTCATTCTA<br>TGTGCTGGAGATGGTGTACAGTAGGGTGAGGAAACCAAATTCTCA<br>GTTGGCACTGGTGTACCCTTGTACAGGTGATGTAATATCTCTGTGCC<br>TCAGTTTGCTCACTATAAAATAGAGACGGTAGGGGTCATGGTGAGC<br>ACTACCTGACTAGCATATAAGAAGCTTTCAGCAAGTGCAGACTACT<br>CTTACCCACTTCCCCCAAGCACAGTTGGGGTGGGGGACAGCTGAA

	GAGGTGGAAACATGTGCCTGAGAATCCTAATGAAATCGGGGTAAA<br>GGAGCCTGGAACACATCCTGTGACCCCGCCTGTACTGTAGGAAGC<br>CAGTCTCTGGAAAGTAAAATGGAAGGGCTGCTTGGGAACTTTGAG<br>GATATTTAGCCCACCCCCTCATTTTTACTTGGGGAAACTAAGGCCCA<br>GAGACCTAAGGTGACTGCCTAAGTTAGCAAGGAGAAGTCTTGGGT<br>ATTCATCCCAGGTTGGGGGGACCCAATTATTTCTCAATCCCATTGTA<br>TTCTGGAATGGGCAATTTGTCCACGTCACTGTGACCTAGGAACACG<br>CGAATGAGAACCCACAGCTGAGGGCCTCTGCGCACAGAACAGCTG<br>TTCTCCCCAGGAAATCAACTTTTTTTAATTGAGAAGCTAAAAAATTA<br>TTCTAAGAGAGGTAGCCCATCCTAAAAATAGCTGTAATGCAGAAGT<br>TCATGTTCAACCAATCATTTTTGCTTACGATGCAAAAATTGAAAACT<br>AAGTTTATTAGAGAGGTTAGAGAAGGAGGAGCTCTAAGCAGAAAA<br>AATCCTGTGCCGGGAAACCTTGATTGTGGCTTTTTAATGAATGAAG<br>AGGCCTCCCTGAGCTTACAATATAAAAGGGGGACAGAGAGGTGAA<br>GGTCTACACATCAGGGGCTTGCTCTTGCAAAACCAAACCACAAGA<br>CAGACTTGCAAAAG
IL5RA	CGAACACGACTCACCAGAGCCTCAAACTCCTGGGCTCAAGCTATC<br>CTCCCACCTCAACCTCCCAAAGTGCTGGTATTACAGGCTAAAGTGG<br>GTTATTTTTCTTTTTATTGTTTGGTTTTAAGAGTTTTTGACATATTCTA<br>AATAAAAGTTCCTTGTCAGATATGTCTTTGCAAATATTTTTCCCATGC<br>TGTGGCTTGTCTTTTCACTTCCTTGTATTTTATTTTATTTTATTTCAGA<br>CAGAGTCTTGCTCTGTTGACCAGGCTGGAGTGCAGTGGCACGATCT<br>CAGCTCACTGCAACCTTCGCCTCCCGAGTTCAAGCCATTCTCCTGT<br>CTCAGCCTTTGAGTAGCTGGGATTACAGGCACCTGCCACCACACCT<br>GGCTAATTTTTGTGTTTTTTAGTAGAGACAGGGTTTCACCATGCTGG<br>TCAGGCTGGTCTTAAACTCCTGACCTCAGGTGATCTGCCTACCTTG<br>GCTTCCCAAAGTGCTGGGATTAGAGACGTGAGCCACTGCATCCGGT<br>CTTTTTTCACTTTCTTCATGGTATCCTTTGAAGCACAAAAGCTTTTA<br>ATTTTGGTGAAATCCAGTTTATCTATTTTTTTCTTTTGTTGTGTCTGC<br>TTTTGTATCTTATTGAAGAAACCATTGGGTAGGTGCGGCGGCTCATG<br>CCTGTAATCCCAGCTCTTTGGGAGGCCAAGGTGGGTGGATCACCTG<br>AGATCAGGAGTTCGAGACCAGCCTGGCCAACATGGCAAAACCCCA<br>TCTCTAGTAAAAATATAAAACTTAGCCGGGTGTGGTGGCACACGCC<br>TGTAATCCCAACTTCTCGGGAGGCTGAGGCAGGAGAATCGCATGAA<br>TCCAGGAGGCAGGGGAGTTGGCAGTGAGCTGAGATCATACCACTG<br>CACTCCAGCCTAGGCGACAGAGTAAGATTCTGTCTCAAAAAAAAA<br>AAAAAAAAAAAAGAAAGAAACAAAATGAAGGTCATGAAGATTTAT<br>GCCTATGTTTTCTTCTAAGAATTTTATAGTTTTGCCTCTTACTTTTAG<br>GTCTTTGATCCCATTGGAATTCATTTTTGTCTATGCTGCGAGGCATTC<br>TTTTGCATGTGGATATGCACCACTTATTGAAAGGCTGATCTTTTCCC<br>CGCTGAATTGTCTTGACACTCTTGTCAAAAATCAATTGACCCTAAAT<br>GCGAGAGTTTATTTTTGACTCTCAATTCTATTAAATTGATCTGTATGT<br>CTCTAATATTTTTTAAAATAACTTTTTCTGCCTGCATTTGTACAGAAT<br>AAAACGTTTGTAAATATAGAAACACAGAATAAAAGTAAGAGAAAC<br>ACTAACCCTGCCGTGGTAGAATGAACTGTTGGTCCCATTTCTTCACT<br>CCCCACTAGGAGTTGTCCATGTCCACATCCTTATCATGGCCTTATCA

	TGGTGGAATGTACTTTGGGCTTCGTCCATTTGACTCCGGGCTTGGC<br>CATGCAATTTGCTCAGCCTCTTGAGATGTCAGGGAATGTGACATGA<br>GCAGATGCTTGATGCGTGCTTGTGCAGTTGGTCCTGCTCTCTTGCA<br>CTTTTGCTGTCACCATAAAAAGATTATGCCCTGGGTAGCCACTGGTT<br>CCAAATGAATGGCAGATACTTGGAACAAATCTGAACTCACTTTGTG<br>ATCTGGATCCCAGCCCAAGATTAAGCAGACCCCAGTCAAAACTCA<br>GACACATGGGCTACAAATACATGCCTATCACAGTTTACCACTGAGG<br>GTTCTGTGGTTGCTTGTTCTGTAGCAATAGCTGACTAATACACCTAC<br>CACTTAGAGATAACTATTCCTGTTAGTCTGATCTTCTGACCAAAAAT<br>AAGACTGTAAAAAAAAATTACATGTGTGTATACCCTCCTGTTGTTTT<br>TACTTAACATTTTTTCATAATACCCTATGTTATTAAAAGTTCTTGATT<br>GGCTCATACCTGTAATCCCAGCACTTTGGGAGGCCAAGGTGGGAG<br>GATCACAAGCCAGGAGTTTGAGACCACCCTGGCCAACATGGTGAA<br>ACCCTCTCCCTACTAAAAATACAAAAATTATCTGGCATGGTGGCACA<br>TGCCTGCAATCCCAGCTACTCCGGGGGCTAAGGCACGAGAATCACT<br>TGAACCCAGGAGGCAGAGACTGCAGTGAGCCGAGATCCTGCCACT<br>GCACTCCAGCCTGGGTGACAGGGCACCACCCTGTCTCAAAAGAAA<br>AAAAAAAGTTATTGATAAACATTCCTTTTGACATCCACATCACATAT<br>TTCAACCTTGAGCAGTTACATATTTCTCTTCACCATTCCTATAGTACT<br>TTTACTATTTTACTATTGTAAAGGTACAATGAATTTTGCTCTCTTTTG<br>TATAAATCTTGATCTACATTTTTCCATTATTTCCATGGAATCAAGTTCT<br>AGAAGTTGATACCTTTAATAAGCTCCTTTAACCAAAGTTCATGGTAG<br>AGCCACTGAACCATGATTTTCGTTTTATTACTATTCATATTTCTATTTG<br>GCTAAACTCACATTGTTTTGCTTAAAGGATGCAGACACATTTAATTT<br>CACTTTTTAAAAAAACGACTTTTGTGAATTCTTCCAGCATGTTCTAT<br>CATACTGTCTTTGGACGTTTACACTTTATTATAAAGAATTAAATTATT<br>GCCATAAAAAACCAAACAGGCCTCGCTCAGTGGCTCACGTCTGTAA<br>TCCCAGCACTTTGGGAGGCCGAGGTGGGTGGATCACCTGTCAGGA<br>GTTCAAGACCAGACTGACCAGCGTGGTGAAACCCCATCTCTACTAA<br>AAATACAAAAAAATTAGCTGGGTGTGGTGGCGGGGGCCTGTAATCC<br>CAGCTACTCAGGAGGCTGAGGCAGGAGAATCGGTTGAACCCGGGA<br>GGTGGAGGTTGTGGTGAGCTGAGATCGCGCCATTGCACTCCAGCCT<br>AGGCAACAAGACTGAAATTCCATCTCAAAACAAACAAACAACCCC<br>CCCGCAACCCCCCGCCAGAAAAAAAAAAAGTCCCTGCCTCTCATT<br>TTTTCGTTTGTGCTTATACAGAAACATAGAGACAGTGTTTCTGTATG<br>AGCATGTGTTTGCTTTGTTTGTTTGTTTGTTTTTATTGGCGTAGTTAA<br>TTAAAGATCATATACCCAGGAAGGTGGTATGGAAGGAGCAGCCAGA<br>TCTAATTTTCTTTATACCCTTCACCGCGCCTAGCATAGTGCATAGGTG<br>CATTCAATGAATATTTGAGTAAATGAATGTAAGAAATATACAGACCA<br>TTGGCTGAGTGTGGTGGCTCACACCTGTAATCCCAGAACTTTAAGA<br>GGCCAAGGCGGGTGCATCACTTGAGGTCAGGAATTTGAGACAAGC<br>CTGGCCAACATGGTGAAACCCCATCTCTACCAAAAATACAAAAAAT<br>TAGCCAGGCGTGGTGGCGGGTGCCTGTAATCCCAACTACTCGGGA<br>GGCTGAGGCAAGAGAATCGCTTGAACCTGGGAGGCAGAGGTTGCA<br>GTGAGCAGAGATTGCGCCACTGCACTCCAGCCTGGGCGACAGAGT<br>GAGACTTCATCTCAAAAAATATACATATATATATACGGAGAGAGGCC

	ATTAAAGCAGGCTTTTAAGCCAGAAATTACTGTCTTCCCAATGATTA<br>ATAAGTATTTTTAAGGTTTCATTAGTGACAAATATGTTACATGTATAA<br>ACAGATGGGCAGGACTTAAAAGAGTAGGCCAGATAGTTGATACTAT<br>ATCTAAACTTTTCTGGGTGCCTCTTAGCAAATAAATGGATCTCTCTC<br>TGTTCCTTGTTTTACTTCCCCTTTCCCCGAGGAAAATCACATTTTTC<br>AGGTCTGACAACAATTTTCAACCTGATAACAATTGCTCCGGTTTCAT<br>TTTGGCAACTGCTAAAATGGTCACCTTGGGAACCCCAGGCACACA<br>CGCTGACTGTACTTGCACAGCTTCTACACTGAGCCTTTTGGAAATT<br>GCCTCCAGGAAGCCTGTCTCTCATCTCACAAACAGAACCACTGATC<br>CAACCTGGGCCACCATCTTTCTGCATCTGCTCTGTCTGAGACTCTCC<br>CAGTCAGCTCCTAAGGAGACAGGGAATCCTGCATCACTCATCGCTT<br>AGGCTAGACTGAAGGTCTCCTTCCAAAGGCAACTACTTACTACTCT<br>CTGCCGTGGGTCATACAATAAGCACTACTCTTATCTATTCACACGCT<br>AAGCTACTTTTTTAAAGCCAGCTTTGCTAGCTAGCCACCTCGAAATT<br>CCCCAGAAGGAATCAAACAGAAATGGAACAAAGGACAGGAAAAG<br>CCAAGGAGGTTTGCTGAAATTGAAGCATTCTGAAGACAGCTGGGT<br>GGTAAAATGGATGTGACGTGAGGAAGGTGGTCTTGGGTGCACCCA<br>CAGACCAACACTGCCATTGTTACAATTTGTGTAGGATGATCTCAGG<br>TGACAGACAGCAGCAATTTATCACACCCAGAAACGCATCCCCCAC<br>ACATATTTACTTGAGGGTACCAGACCTGCTCACAAAGCAGAGAAG<br>AGCTAAGGCGGTTCTCTAAGGGCAGAGAATTGCTGCTATTGCCTAG<br>TGAGTGGGGAGAGGGTACTCCTCAGGCCTTACTTCCTATCAAATCA<br>TGTGTCAGTGTTGCCTAGGAGACAGAGGCACAGTAACTACTGTAGC<br>CAAACAAGGCACATAAACAAAACAGAAATGCAACGCTTTAGAGTA<br>CCCACGGAAAACTTGTTTACCTTGTCACCATGAGTAAAAGTTAATT<br>CCCACTCCTGAAGAGAGCAAACCAACTCTGAAAGAGAGTGAAAAT<br>GCAGACAAGACAGTTATCAGATAATGGCTATCTGGACGAGAGATTC<br>TTTCGTTTGACAGCAGTTTGGTTGTTGGGAGTTCCAGTTCAGCTCC<br>TGCACAGTTGCTCTGTACAAATCCTCCTCCATATTTGCTTAGAGAAA<br>ACGTGTTGCCATCCCATCATGAAGGAAGCTGCCTGAGAGTTTTTAA<br>CCATTACAGCCGTGATGATGAAAGAGTGAAGAACCGCCTCTAAGTT<br>AAAAAGTGCACCCAGAGATAAGGTTCGTTCTCAATGCCCTGCCGCT<br>GCTTCTCATCGCATGGCCAC
IL8	TTTTGTTTTACTTTTTTTTTTTTTTCTTGCGACAGGCTCTCTGTCACC<br>CAGGCTGGAGTACAGTGGCATGATAACAGCTCCCTGCAGCCTCGAC<br>CTCCCTGGCTCAAGTGATCTTCCCACCTCAGCCTCCTGAGTAGCTG<br>GGACTACAGGCATGCACCACCACGCCCGGCTAATATTTGTATATTTT<br>TGTAGAGACAGAGTTTCATCATGTTGCCCAGGCTAGTCTTGAACTC<br>CTGGGCTCAAGTGCTCCACCTGAATTGGCCTTCCAAATTACAGGCA<br>TAGCCACCACAGTGAGCCCTTTTTTTTTTTTAATAAATTTAACAAAG<br>AAAAGAAATAGCTACAAGTAAATATTTACTTTTTTTTTTCATATTGAA<br>GATTGGCTGATCTTAGCCTTTCTAAGTCCTTAATAAATGCCTGTTTAC<br>ACAACTGGTGTCTTCCCCTAAACCTAAACTTCAAACTTCAAACTCC<br>AAACTCCACCGATTTGGATAGTATTAGAAATGGCATTCATATTAATG<br>GTTATAGTACATTTTTTTACATGTGAATGCAAATGGAATTGTTAGGCA<br>TTAAAAAAGAAAGCTTATATAGTGGAAGAAAATAAAGCATCTAGAC

	ATAAGCTTTAAGAGATCTATTGTGTTAATACAGCTTTACTTTTTGAGT<br>GGTAAGCTTTTAAAAAGAAATGTGGTGCTCTAACTCCAGGAAAAG<br>ATAAGGGTGACTGAAGTGATAGTCTAGAGGAAAAAGATGCAGACA<br>TTTACTGAGTACCTCCAATGTGCCAGGTGCCATTCTGGGCATTTTCA<br>TTATGTTTCCTCATTTAATTCTCATGGTGATCCTTTGGAACTGTGTTA<br>TTCTCATTTTTACAGATGAGGTAACTGAGAGACAGTCAGATTAAAG<br>AACTGCCTATGATTGTTTGGCTAATAATAAGTGGAGGGGTGAGGCT<br>TGAAGGCAGGTTTGTCTTATTCCAACACCCATACATACCCTTAAATT<br>TAAGTTATTCTGACTTGTGTTGCTCAAATCCAATGTGTTCAGCTGTT<br>TGCTTCTCCAATTACCAAGATTTTTCTTTAAAAGGTAGGACACTTTT<br>GGCAACACGAACCAACTTTGCTCAGTATTGTTATAAACTGTTAACT<br>GGAGACATTTGAATTTGGAGATGGAACTGAAATGGTCTTGCGGTAC<br>TAGAGAAGATCAAGTTATCACATAAACAAAGTACAGAGCTGAGAA<br>CATATTTTAAATCTTTCCACTAACTCTGACTTTTATTGACTAAAATTT<br>TAGTGGGCAGTATGTTTATGTTTATGACTCTTAACATTAACAACATC<br>GTAAGTCAAACTCACTAATATATGTTAAGCATTCTGTTTATGATTCTT<br>TTAACCTAGAGGATTGTTGAGCTGGGACTAATTTCCTCAAATGGGA<br>AAAAAACCCAGGTGAGAGCTGAGACTGCTCCTGAGACTGAGAAA<br>GGCAGCTCTGACGGGATCTCAGATTTTAGCAGCAGGAGTTGAACA<br>ATGGGCATAGAATCAGCTTGCCCAAGATCTCCTGATTAATAAACCAT<br>GGAACAAGATTTAAACCCAAGTTCATTTCATTTCAAAGCTCATACC<br>ACATTTTGCCCACCATATTTTGCTTTGTTATATGACTACAACTTAGTT<br>CAGGCTTACAAAAAAGTCCTAATTCTAAAATTCCTATGGCGTGGGT<br>GGGAGGGGATTTAGATGATTTTGCATAGGCAAGAAACACCCAGTTT<br>CATGGAGTTTGATGGAAGAGTTATGTACTAATATGGGAAAAGTAGA<br>GGCCATCTTTGTCTTTGTTCTTTCTTTTTTAGACGGGAGTCTCGCTC<br>TTTCACCCAGGCTGGAGTGCAGTGGCGCTATCTCGGCTCACTGCAA<br>GCTCCGCCTCCTGGGTTCACGCCATTCTCCTGCCTCAGCCTCCTGA<br>GTAGCTGGGACTACAGGCGCCCGCCACCGCTCCCGGCTAATTTTTT<br>ATATTTTCAGTAGAGACGGGGTTTCACCGTGTTAGCTAAGATGGTTT<br>GGATCTCCTGAACTCGTGATCCGCCCGCCTCGGACTCCCAAAGTGC<br>TGGGATTACAGGCTTGAGCCACCGCGCCCGGCCGTCTTTGTTCTTT<br>CTTGAACTCTTCCTTTTCTTGGGTGATAGACTTCGTCAACGTCTAAT<br>GAGGATATCTAGGTGCTAGTCTCTGCTCATCAAATGATTCTTATGGC<br>TCAGGAGCCGAATGGGACGTAAATAAACAGTTAAGTCTCATGAACT<br>CACTTTGCATTCATCTCTAGAAGATGACAAAACATTTGTATTTATGT<br>GTAGCGTGGCACTTTAGTTAAACTTTGTACCCCACTTTGCTCTATTT<br>TAAAGCAGAATATCCTTAAAAAGGATACTTAGTCCTGCTTTTTTTTT<br>TCCGCCTAAGCCCATTTAGTCCTTCTACTCATTATGCAAGGACTCAA<br>ATGGTTATCTTTACAGAAGTGAGACAAGATAGAATCAATGCTCTTGT<br>AGTCACTTCATCTTTGTCCATTCCCACTTCTGATGGAGAGGGTTCTA<br>GGACATAATGCACTGAAGGTTACATTGTGAGAGATGAACAACATTT<br>GCAAAAGAGGTCTTTTTGCCTTGGAAAGGCTTCATTCTTAAAAAAA<br>AATGTGAGCATCAAGGTTAAGTAGACCTCATTAGCTCAAACTTTAA<br>GGATGATATCAGGATAAAGTTGGGCCCATGAGAAGAGAATGAGAG<br>GGAGATATAGTGACATGAAAATAAGGAGGAAAACGAGGTGTCTATG

	TAAGTTGGGCTCACCATAAATACAAAGGCAACCGTTAGGGAAAAG
	CAAAGAAGTCTTTGCACATCCTCAGAACTCTGAATGTCTTAGTGAT
	GCTGTATGAGTGAGTCTTAATGATAGTGAACTGAATCAGTCAAGCC
	AGGTTGTGTCCATATGAGAATGTGTCTTTGCTAAACATGCCAACATC
	ACTGAAGCAAAGAAACTTGGAGTTTTCTTTAAGATATAGGTCTTTT
	TTACCTATCCGGCCCAAGCTTTCTCTTCTTGTCACTCCATGCACTGT
	GTTCCGTATGCTAAATAGTTTGAGAAACCCAAATGGGCCATGTTCG
	CCTACATTTCATTGTCCTGTACTTCCTGTCCTGTACTAGCAAAGCAG
	TCCCATTGGTCTTTCTTCTCCTCATTAACAATAAAGGTAACACTTTT
	GATGTTGTTTCTTCAGAAAACCTTCATTCATCAAAACTGCCTCAAA
	GATCATGTTTGTTTGATTCCAGAACTTCCTGTAATTACCTGTTATTGT
	AACACTCATCACTGTATTTTACTTACTTGTGTAACTAATTTTCCATAT
	TCTGCACTAGACAACAAAGTCCTTTAAGTCAGGTACTATATCTATTT
	ACATAGCATTCACATCTCCTACAATAAGGGACATTAGCAGATAAACA
	ACACATATTAAATGAATAATGAAGTTTCTGAAATACTACAGTTGAAA
	ACTATAGGAGCTACATTATATAGAATAAACATTTACTTTGCTATAGAA
	TTCAGTGTAACCCAGGCATTATTTTATCCTCAAGTCTTAGGTTGGTT
	GGAGAAAGATAACAAAAAGAAACATGATTGTGCAGAAACAGACA
	AACCTTTTTGGAAAGCATTTGAAAATGGCATTCCCCCTCCACAGTG
	TGTTCACAGTGTGGGCAAATTCACTGCTCTGTCGTACTTTCTGAAA
	ATGAAGAACTGTTACACCAAGGTGAATTATTTATAAATTATGTACTT
	GCCCAGAAGCGAACAGACTTTTACTATCATAAGAACCCTTCCTTGG
	TGCTCTTTATCTACAGAATCCAAGACCTTTCAAGAAAGGTCTTGGA
	TTCTTTTCTTCAGGACACTAGGACATAAAGCCACCTTTTTATGATTT
	GTTGAAATTTCTCACTCCATCCCTTTTGCTAGTGATCATGGGTCCTC
	AGAGGTCAGACTTGGTGTCCTTGGATAAAGAGCATGAAGCAACAG
	TGGCTGAACCAGAGTTGGAACCCAGATGCTCTTTCCACTAAGCATA
	CAACTTTCCATTAGATAACACCTCCCTCCCACCCCAACCAAGCAGC
	TCCAGTGCACCACTTTCTGGAGCATAAACATACCTTAACTTTACAAC
	TTGAGTGGCCTTGAATACTGTTCCTATCTGGAATGTGCTGTTCTCTT
	TCATCTTCCTCTATTGAAGCCCTCCTATTCCTCAATGCCTTGCTCCAA
	CTGCCTTTGGAAGATTCTGCTCTTATGCCTCCACTGGAATTAATGTC
	TTAGTACCACTTGTCTATTCTGCTATATAGTCAGTCCTTACATTGCTT
	TCTTCTTCTGATAGACCAAACTCTTTAAGGACAAGTACCTAGTCTTA
	TCTATTTCTAGATCCCCCACATTACTCAGAAAGTTACTCCATAAATG
	TTTGTGGAACTGATTTCTATGTGAAGCACATGTGCCCCTTCACTCTG
	TTAACATGCATTAGAAAACTAAATCTTTTGAAAAGTTGTAGTATGCC
	CCCTAAGAGCAGTAACAGTTCCTAGAAACTCTCTAAAATGCTTAGA
	AAAAGATTTATTTTAAATTACCTCCCCAATAAAATGATTGGCTGGCT
	TATCTTCACCATCATGATAGCATCTGTAATTAACTGAAAAAAAATAA
	TTATGCCATTAAAAGAAAATCATCCATGATCTTGTTCTAACACCTGC
	CACTCTAGTACTATATCTGTCACATGGTACTATGATAAAGTTATCTAG
	AAATAAAAAAGCATACAATTGATAATTCACCAAATTGTGGAGCTTC
	AGTATTTTAAATGTATATTAAAATTAAATTATTTTAAAGATCAAAGAA
	AACTTTCGTCATACTCCGTATTTGATAAGGAACAAATAGGAAGTGT
	GATGACTCAGGTTTGCCCTGAGGGGATGGGCCATCAGTTGCAAATC

	GTGGAATTTCCTCTGACATAATGAAAAGATGAGGGTGCATAAGTTC<br>TCTAGTAGGGTGATGATATAAAAAGCCACCGGAGCACTCCATAAGG<br>CACAAACTTTCAGAGACAGCAGAGCACACAAGCTTCTAGGAC
INS	ACACCTGGACCCCTGGCTTGTGGGCTCCCGGAGCTGGTGCAGAGT<br>CTGGCTCTCCCCATCACACACAGGGCCCTGCTGAGCCCTGATCCGT<br>GCACTGCATGGGTGGCTGGGCCCAGTCAGGAGGAGGCTGGACCTT<br>GCCACAGCTGTGTCTCTCATTACAGTCCCTGGAGACCCAAAGTCTC<br>TGGGTAGCTTCCACCAGTCCCTCAAATCCTGGCTTCTTTGTGGTGA<br>GCACAGGGTGTGGGGTGTCCTGCTCCGAGGCCATAGGAACTCAAG<br>AAGCAACTGGAGCCTGGGTCAGTGTGGGTGAGGGGCTGCCAGGTC<br>CCCCCCGATAGTGGCTTCTGCCCCCCAGCCCTTGGAAATCACCCCA<br>GATGGTCACTCCTAAGTCTCGGCCACGATACGTGTTTGGTTACCCTT<br>CAGGTGCCCCAAACCCAGGCGTCGTCTCTCCCATGCCCTGGATTCC<br>TGGTCTCAGGATGGATCAGCGTCCACCCTGGCTTCAAAACGCCCTC<br>CAGGCGACCAGCCTCGAGCCCTTCTGTCACCCAGCAGCCACACAC<br>CCTGCCCTGCCACACTGACCACGCCCGGGGGCTGGATCTCATCATC<br>CTTGGGTCTCTAGGCCTCCCCACCCCACTGCCACCCCCGATCCCCT<br>CTGGCCATCCTGTCATTGTCTGAACCCTTCATGAGATTTCTAGAGCC<br>CCTGGATGAAATTACATGGACTGCCCCGGACCAGAGGCACAGAGG<br>CCGGCACAGCTGCCCGCTCCACCCACTGCCTGGTCTAGGCCAGGC<br>CCCTCCTGGTGTCGTGACAGGGCCTTCCCACTCCCAGTTACTTCTT<br>GCCCATGGCCCACAGAAGCTTCCATCACAATGCATCTGGGGTGCAG<br>GCTGCTCAGCTTCCCCTGCCAGGCCTTGAAGACGCGGCCCACTCAT<br>TCAGCTGTGTCCTCGAGACCCTGACGCCCTCCTGGAGCGTTCACTG<br>AATGGAAAGGTTCCAGGTGGTCACGGTGGGAGCACCTCTGACTGG<br>CAGACCATGTGTCAGTCCTCCCCAAGGCCAAGGCAGGCTTGCCTC<br>CTCCCGGGCTCCTGATCAACATGGTTCCAAGGTCTGTTCTTTCTCCC<br>CTGGGTGTGAGTGTGTGATTTGAGTCTGCGTGTGTGTGTGTGTGGG<br>TGTGATCTGTGTGAGTGTGTGTGTGCATGGGTGTGATGTGTGTGAG<br>TATATGTGTGTGAGATGTGAGCGTGTGTGATGTGTGTGTGGTGGTGT<br>GATATGTGTGTGTGTGTGTGCATGGGTGTGTTGGATGGTATGGTACA<br>GGCTCATTCAGTCTCTGCACTCCGCCTCCTGCTCAGTCTGGTGGAC<br>GCTTTCCTACCACTGCGTGCGCTTTCTGAGCAGTGCCGACTGAGGA<br>GGCAGCGGCCCCTGCTCTGTGTTCTCCTGGGGTTGCCCACCTCTCC<br>AGCCTCCCCTCCTCAGGTCAGGCCTCTCCTGGGCTACAGGCTCACT<br>TGTGCATTTTCTTGGTGCACCCTGGAGTGGACTCACTCTCGCCGTG<br>AGTGTCGCTGAAACACTGGCCTCCTCCCAGTTCCCACCCCGGTGTA<br>TGTTGGGATGGGATGCCCCTCCCAGGAAGGTGCAGCAGGCTGCTG<br>CGCAGAGTGTGTTCAACCGTCTCCATTCTCTCACCGTCCCTGAGTG<br>TGCCTGCCTGGGGTCATCAAACAAATCATAAAGTAAAGCCTGAAAG<br>AATCAACCTGCTTCCAAGTAACTGAACTGCATGCAGCCCAGGACA<br>AAGTCCAAACAAAATGTATAAGAGTACAAAATGCTGAACACCCAA<br>AAAGGTACAATTCACAATGCACAACAACCAATCAAAGATGACTAA<br>GCACACGAAAAGGCAGGAAAATATGCCCCACAATGAGGAAGATCA<br>GCCAACTGAAACTGATGCAGACGGACACAAAATTATCAGAAAAAG<br>ACATTGAAACAGCTTCCTAACTGTGTTCCACAGCTGCAAGAAGTTA

	AAGACATGAAGTATAGTTTAAAAGACTCAAATACAACTTCTAAAGT<br>AAAAACTACCATGCCTGAGATGAAAAAATACACTGGATGAGATTAA<br>CAGCAAAAGAAAAGAATACTGAATTTGAAGACATAGAAATGGAAA<br>CCATTCAAAATAAAATACAGAGAGACAAAAGAAATGTTTTTAACTG<br>CATCGATGAGTTCTGGTGTAACTCCAGGTGGCCCAATATATGGATAA<br>TTGGCTTCCTTGAAGGAGAGATGTGTGTATGTTTGGGGCAGGAGGG<br>AGAAAGAAAAATGTTTGAAGAAATAATGACTGAAACATTTCCAAA<br>CTTAATGAAAACTATAAACCAAGAAACTCAATGACTCCCTAGGGCA<br>GGAAACAGGACTAAAACCAAGCCAAGGTACATCATAATCAAATTGT<br>TTAAACTAGTGATAAAATGAGAATCTTAAAAGCAACTAGAGGCAAA<br>AGGAACACGGTACGTGCAGAGGAACGGATGGCCGCAGATTTCCCA<br>CAAGACGCAATGCAAGAAGACGGGAAAGCAAGACCTTTAAATTGC<br>TGAAACAAAAAGAACCCATCAGCATCAGAATGCTATGCCTGGGTAA<br>AACATCCTTCACAAACGAAATTTAAATAAGAACATTTTGGAGATATA<br>AAAGCTGAAGCTCCATCGCCGGCAGAACCGTGCCGTGAATGACGT<br>TCAAGAAATTCACTCAGGCTGAAGCGGCGTGAAATCAGACAGAAA<br>TCTACATTCTACTGGAAATGGTAACTACATGGGCAAACACGTGAGA<br>TTTTAAAATCATTTAAATTTCTTTGGAGATGCAAATCAAAACCACAA<br>GGAGGCCAGGCGCGGTGGCTCTCGCCTGTAATCCCAGCACTTTGG<br>GAGGCCGAGGTGGGCGGATCACTTGAGGTCAGGAGTTTGAGAGCG<br>GCCTAACCAACGTGGTGAAACCCTGTCTCTGCTAAAAATACAAAAA<br>TTAGCCAGGTGTCGTGGCGCATGCCTGTAATCTCAGCTACTCAGGA<br>GGCTGAGGCAGGAGAATTGCTTGAACCCGGGAGTCGGAGCTTGCA<br>GTGAGCCAAGATCGCACCACTGCACTCCAGCCTGGGCAACAGAGC<br>GAGACTCTGTCTCAAAAAACAAAACAAAACAAAACAACCCACAA<br>GGATATACCATCTCACACCAGTCAGAATGCCTATTACTAAAAAGTCA<br>AAAAACAGATGCTGGGGAGGTTGTGGAAAAGAAAGAACGCTTTTC<br>TCTTGTTGGTGGAAGTGTAAATTAGTTCAGCCATCGTGGAAGACGA<br>TATGGTAACTCCTCAGACACCTAGAGACAGAACTACCATTCAACCC<br>AGCAATCCTTTTACTGGGTATATACCTAGAGGAATATAAGTCATTCTA<br>TCATAAAGACACATGCACGCCATTGCAGCACTATACAATAGCAAGG<br>ACATGGAATCAACCTAAATGCCCATCAGTGATAGACTGGATAGGCT<br>GGGCGCGGTGGCTCATGCCTGTAATCGCAGCAGTTTGGGAGGCCG<br>AGGTGGGTGGATCACGTGAAGCCAGGACTTCGAGACCAGCCTGAC<br>CAGCATAGCGAAACCCCGTCTCTACTGGAAATAGAAAATTAGCCAG<br>GTGTGGTGGTGTGCACCTGTGGTCCTAGCTACTTGGGAGGCTGAGG<br>CAGGAGAATGGCTTGAACCCGGGAGGCAGGAGTGGCAGTGAGCC<br>AGATTGCACCATTGCACTCTANNNNNNNNNNNNNNNNNNNNNNN<br>NNNNNNNNNNNNNNNNNNNNNNNNNNNNNNNNNNNNNNNNNNNNN<br>NNNNNNNNNNNNNNNNNNNNNNNNNNNNNNNNNNNNCTGCCCCTG<br>GTGCTAAGAGGCAGGTAGGGGCTGCAGGCAGCAGGGCTCGGAGC<br>CCATGCCCCCTCACCATGGGTCAGGCTGGACCTCCAGGTGCCTGTT<br>CTGGGGAGCTGGGAGGGCCGGAGGGGTGTACCCCAGGGGCTCAG<br>CCCAGATGACACTATGGGGGTGATGGTGTCATGGGACCTGGCCAGG<br>AGAGGGGAGATGGGCTCCCAGAAGAGGAGTGGGGGCTGAGAGGG<br>TGCCTGGGGGGCCAGGACGGAGCTGGGCCAGTGCACAGCTTCCCA

	CACCTGCCCACCCCCAGAGTCCTGCCGCCACCCCCAGATCACACG<br>GAAGATGAGGTCCGAGTGGCCTGCTGAGGACTTGCTGCTTGTCCC<br>CAGGTCCCCAGGTCATGCCCTCCTTCTGCCACCCTGGGGAGCTGAG<br>GGCCTCAGCTGGGGCTGCTGTCCTAAGGCAGGGTGGGAACTAGGC<br>AGCCAGCAGGGAGGGGACCCCTCCCTCACTCCCACTCTCCCACCC<br>CCACCACCTTGGCCCATCCATGGCGGCATCTTGGGCCATCCGGGAC<br>TGGGGACAGGGGTCCTGGGGACAGGGGTCCGGGGACAGGGTCCT<br>GGGGACAGGGGTGTGAGGACAGGGGTCCCGGGGACAGGGGTGTG<br>GGGACAGGGGTCCCGGGGACAGGGGTGTGGGGACAGGGGTGTGG<br>GGACAGGGGTGTGGGGACAGGGGTGTGGGGACAGGGGTGTGGGG<br>ACAGGGGTGTGGGGACAGGGGTCCTGGGGACAGGGGTGTGGGGA<br>CAGGGGTGTGGGGACAGGGGTGTGGGGACAGGGGTCCTGGGGAC<br>AGGGGTCCTGGGGACAGGGGTCTGGGGACAGCAGCGCAAAGAGC<br>CCCGCCCTGCAGCCTCCAGCTCTCCTGGTCTAATGTGGAAAGTGGC<br>CCAGGTGAGGGCTTTGCTCTCCTGGAGACATTTGCCCCCAGCTGTG<br>AGCAGGGACAGGTCTGGCCACCGGGCCCCTGGTTAAGACTCTAAT<br>GACCCGCTGGTCCTGAGGAAGAGGTGCTGACGACCAAGGAGATCT<br>TCCCACAGACCCAGCACCAGGGAAATGGTCCGGAAATTGCAGCCT<br>CAGCCCCCAGCCATCTGCCGACCCCCCCACCCCAGGCCCTAATGGG<br>CCAGGCGGCAGGGGTTGACAGGTAGGGGAGATGGGCTCTGAGACT<br>ATAAAGCCAGCGGGGGCCCAGCAGCCCTCAGCCCTCCAGGACAGG<br>CTGCATCAGAAGAGGCCATCAAGC
INSM1	AACACACGTTGGCCTGTGGGGAGTGGGGGGAGAGTAAGAGCAGG<br>GAGAAGAGTGGGGGGAGAGTAAAGGAGGGGGGAGAGAGAGAAG<br>GAAAGAGAGAAGGGGAGAGGAATGGGAGACTGGGAGAGAGGTG<br>GGGGGGAAGAGAGGGAGAGAAGACAGAAACAAGCGAGGGAAGA<br>GACAGGAGAGAGGGACGAGAGGAGAGAGGGGAGCAGAAAGACA<br>GCAAAGAGGGCTGGGGGAACTGGATGGGGAGGGAGGGACAGTGG<br>GAAGAAGGGGCTGGGAGAGAAGAAGGGGGCCAGTTATGGGGGGG<br>ACACCACAGGTCGGGGGGGGGATTAGGAGTGAAAGAGATGGGAG<br>AGAGAGGAGAAGGAGGGAGAGGTGACACCGTGGGGAGCAAGAA<br>AGGAGGGGAAAGAAAAGAAAGGAAGAGACAGAAGGAGAGAGAA<br>GGACAGGAGGAGGGAGAGGAGGCGGATGACACAAGGAGGGAAC<br>CAGAGGGCGAGGGAGGAGAGCAGGGACGGGAGAAGAGGGAAAG<br>GCGGAGGAGAGAGGAACGGCGGTGGGGGGGGAGGAGCACCCGCC<br>AACCCCATAGCAAGAACGCTCGCCAGGGTAGAAAGTATGCCCTTGT<br>GGACCCCAGGAGTCTGGCTGCCTCTTCGTCCTCCTGCGGCAGGCG<br>AGGCGGGGTGCAGGTGTGAGTGGGGTGGGAGGAAGGGGAAAGGA<br>TCGGGGTCGCCGCAGCCTTTCTGGTCAGTTTCGCTGGGTTTTGTCA<br>GTTCCGTTTGAGACCGAGGCGCACGGACTAGCGGCCGCCAGCACC<br>TCACAGGTGGAGGACACCTCTTGCGCCCCAAGGTCAATGTCATCCC<br>GATGGGTCCCGTGCCGTCCTGACTCTCCCTGACCTAATTCTTTGGTT<br>ATGACTTTCTCACCTTCTGGGCTTCGCCTCTGCCAGCTTTCGCCGAC<br>GTCGCCCTTCCCGCCGCCCGCCCGGGCCTTCCTACCCGCTCTCCTC<br>CCTCCCTGGCCCGACGGTCCCACTGAGGCAAAAACAAACAGCCCT<br>GAGGGCACTTCAAGGAGCGTGCAGTGGCCCCGACGGTACTCTCGG

	GGGGATAAAAGAGGTGGCCAGCCACGCCGTCGGCCCCCGGCCGAA<br>TAATGGGCGCGCAGCAGATTGCCAGGGGGCACTTGAAACTCGAGG<br>GGAGGGAAATACTTGAGCGCGGTCCCTCCCCGCGAGGACGCACGA<br>CGCAGCCTCGCGGATCGTCCCTTGTTCGCTCTCTTTCATGCACTCTC<br>CTTTTGTGTGGAATTATCTAAACGGGAGATCCCTGGCAGCTTTATGC<br>TAATGCGCCGAACAAAGGCAACGGCCCGCGGGCGGGAGGCCCGG<br>AAGGTGCCTGGGGCCGGGCCGGGCGGGGTGGCGGCCAGGCGGGC<br>TGCGCGTGTCTCTCGCGTGCCCCTGGGACTGACACAACAACCCCTC<br>ATTCCTGCAAATACAAAGCCTCTCACCGGCTCGGGTCGGGCGCACC<br>CGGCGCAGGGAGGCGCTGGTCCCTCCGGGCGGCGGGCACTGCAGC<br>GGTAACGCACGGTTCCTGCCGGTTGTGGCTCGGGGGACTGAGGGC<br>CGGGAGTGGGGGCTTCTGTGAAGCCAGAAGAGAAGTCTGCAGCA<br>GAGTGGTGATGAGGGGCGCTGACACCTCACCCCCAACGCCGACGT<br>CTCCAAACGCGATACCCTCTGCGGGGTGAGAATGCGGGCCCGCCC<br>GGCTCCTCCCGTGAGGCCAGGGCCTCCTGTTCTCCTAGACACCCCA<br>AGGAGCCAACTCCTCCGCAGAAGTTCCCCGCTTCTGCTCTTATTTC<br>CAAGCTTCGCGCTTTCTACAAACTCCCTGTTGCCTTGACTTTGATTT<br>CCAGCCGTGGTGAGGGTCAGAGTGAACCCCGGCGCGCTCCCCGAC<br>GGCATCCCCGCACACCAGGATAGGAGAAATTGGAGGGCCTGGGGC<br>CTCGGGCTCCGCAGTCGTCGGAGGAAGAACCCACCGCGGGGTCCG<br>CAAGGGAAAGTGAAGAGGCCCGGGATTTTTCCAAAGCGCTGGCCA<br>GGACCCCGAAGGAAGGGGAGGAGTCACCTGAAGCCGGGGAAGGC<br>CCCTTGGGTGCTCTGCCTTGGATCCTTATGTTCACTGACTTTCGCGA<br>AGCCCCCCTGGAGGGGGGCAAATCCGCGCTGTTTCCCCCAACTTA<br>ACTTCACGCGGCCCATTCTCCGAATCGCGTGCGCCAAGGCCGGCGT<br>CGGGGGCGCACTGTGATGGCGCTTTGCTCTTCCCAGGCGAAGACA<br>AATATCTCGACCCGATGTCCCCATGCATGCGGGTGCACAGGTTCTG<br>AGAAAATCACGCAGAAGGCGGCTGCGATCCACGGCCCGGGCTGGG<br>CTACACCTGCTTGGAGGTCGCTGGTGTGTGCGTGCGCGCGTGCGC<br>AGCCTAACTCCTTCCAGCCCCGGGTGGTGAAATTAGTTTCCAATACT<br>AGTACCTTTCAAGTTTCCATTTATTTTGACTGTACCCGTCTGAAAGC<br>AGATTTCATTTACGCTAGCAAGACTGGAAGCCACAAATAAAGAAA<br>AAGAAACGGATTCATTGAATCTCAGTATCTCAGTAGCGTTTAATAGA<br>TTCCCTTGGCTAAATTCAGAGCCCTGTTTGAATTCACTCACGCAGG<br>AGTTGTTTTGTTTTTTTTGTTGGTAGAGGTGCCGGTTTCCCATCCCC<br>ACGGCTTATTTACAATTATGGGTGTTGTTATTTTTAAAGGATTCACA<br>AGGTGACTAACTCTCTGTTATCTGTTGAATGGAAGGAGTGTTACATA<br>TGCAAGACCGAATGGGGCAGGCCGAGCCCCCGCCCTATGCTATTCT<br>ACAATCCAGATGGGATTCGTGATTAGAAAATAAAATTGGCCAAAGC<br>TGTCTCGGAATCCTTTTCCCACTTCTAATTTCATCAGCACTATTAATC<br>AGAGCTCAAAAGAGCAAGGAAATAACATCAGTTACTTTTAGATAGA<br>AATATGTTTATAAATTTTAGATAAACTCATTTTAAGATTTCTAACTTTT<br>GGGGAGACTAGCACTCACCACAGCATTTTTAAAACTAGGGTGAGA<br>CTTTTCAAAATATATTTCACTATATGCATTCTTCCCTTTTCAGTGTGT<br>GAGTTAAAATATTACAAACATAAAATTATCAAATATCTTTCCCTTGAT<br>TAACCAACCTGATTAACCCCTTAATTAATTAAGCCTAACAACTGTCC

	CCATCACAACTTATGACTCATGTGGACTCTATTTTTACATGTTTAATT<br>TCTACGCCAGGGTATTTTCAGAGGGAAAATCCAAAACAATTAAATA<br>CCTTCAGTGGAATTAAGCGTAAGTAAAACTCAGCAAAATCTGTCTG<br>GTATACAAAGCTTTGCAGAAAAATGTTTCCTTCTCTTTCAATAAAGT<br>GACATTTTCCTCACAGCATATGACATTTTATGTGGCTTGGCTATTTGT<br>TCCCCAGTGTTGGACGATCAGGGCCACTACGCACTCGTGGGGTTCT<br>CTGCCCTGTCCCCCGAAGATTCTGCCTTTTGTGTTTCCTCGGGGCTG<br>GGCTGGACAGAGGATGGAGGCAGGCGGCCCAGTCTGGGTCCAAA<br>CGGAACGGCAGCGGAGGTGGGGGTGGGGGTGGGGGCGGGGGGAA<br>TCTTCGCTGTTGTCTCTTCCTGGTGGCACAGGGGAGGCGGCTTGTC<br>CTCTCGGAGGCCTCAGCCTGCCTCAGAGTACAGATTGCCCCCCCTC<br>CCCCCGTCCAGCACGCGTCTCTTTTGCGTCCAGATTGGCCGCGACC<br>GGAGCTCAATAGCAGGAGGTTAATTTCCTTCACAAAGGTGAAGGG<br>GGCACGGCTCCGTGGGGGCTGCGCCCAGACAGGCAGCCCCTTTAT<br>TTCGCAGCGCCTTGATTGGAGCCCTTGATTTAGCATCTGATGTCAAC<br>CGGCAAACAAAATGCCCGCTCGGAATGAAAATGCATGAGGCCGCG<br>CGGGGAGGAGAAAGCCAACTTCACTGGGGCGCACGAGGCCGACC<br>GCGCGTTTCGGGCTTCGGCCAACTCGACCCGGATTAACCGAGCCTT<br>GGACCACGCGGAAGCTCCGGGCGGCCTGGGTCGGGCCCCGCCAA<br>AGATGCCATTCCCAAGCCTCCAAGAACCCAAAAGTTCAGAAATTC<br>ACGTTCCTCTGGGAACCCAGCCCAGCCCGCCCGCGCTCCAATCCCG<br>CGCTTTCGGGAGACTGAGAAGGCCGTGCCAATTCCTTCTCAAACTC<br>GAAAGAAACCTTCTCTAGCCCCGTGGGCGCGCGGAGGCTGCGAGC<br>ACAAACATCGCCCTCGGCCACTGCCAGAAGGCCGGGCCCCCTGTC<br>CACACTTGGAACCCCGGGGAACCCTTTTGCTTGGCCTCTTGGGTCC<br>AGCGGCCCATCCGTCCAAGGTCCGGGCGGAGGCCGTCCGGACCCT<br>GCTGCTCTCTCGGATTCTTGTTTATTTCCCAAACACCACGCGGAGC<br>CACTGCGCCTCCGCAACGATCTCCCCCGCACCGCCCCGGCGCGCCC<br>CCGCCCCCACCCAATCAGCGCGCACAACTTCCCCCTCGGCTCCGGC<br>TCGCGGATTGAACCCTCCTGACATATTTGGGGCCATTCTTCTCCTTT<br>GTTGCTATTTTGCTAGCGACCCGCGGGTAATCCCCGCGCGGGAGGG<br>GGGCGTGCATTGTCGCGCTGATGGACGGGCCCATTTGGCGGCTCCG<br>CGCCCCCCGGAGGAGAGACACAAAGCCCAGGCACGTGCGCCTCCC<br>CATAGAGAAGCAGCAGACCGTGAAGGGAGGCGGGGCCGGGCGTG<br>TGCCTGGACCGGGCGGGGCGGCGGCGCCGGGCGGGGCGACCAGG<br>GGCGCGCGCGGGGGCCCCGCGCCCTCAGGTACATCTGCCGCACCT<br>ACCGGGCGACCCCCGAGTCCCGGCCCCCTTTTGGCCGCCCCATCGC<br>CCTCCCACCCTGCCAGGCTGAGGAGCTGCGGACGCGCTGATTGGC<br>TCCAGGGGAAGCGGGAGGCGAGAACAATGGCCCCCTCCCCCCGTT<br>AAAAGGGAGCGGCTGCCGGGCCCGGGGACAGGGACGCGCGTGCA<br>GGGCGCAGAGCTGGGCCGAGCCGTC
IRF4	ACCCTCCAGCAACCCCTGACTCCCCGACCGCCCCACCCCCTGCCG<br>AGCACGTCTACTCAGCCCCATAACTGCTTGTCCTTCCCTCCTCTGCC<br>ACCCATGCACCTGCCCGTCTGAGAGCTCTCTACCTCACCCCGAGGC<br>CTCCCCGGCCTCCTGGCCATTGTTCTCTCTCGGGCGGTACCCACACT<br>ATGGCCAGATAATTCCTCCTTTACTGTAGTTCTTACCTTATTACGGGG

	GAATATGAGCCAAAGCCATGTAACTCGCTGCCGGGCAGGAACTAA CAGAGCCAGCACATGCTTCTCCCGTACCCTTCTCTCCATCAGGTGG CCAGGAATGTTCCGGATCCCCGTGGCTTTGCCATGGATATCTCATTT GAACAAGAAGTAACCCCCCCCCCAGAATCTCAGGGGGTCTCGTTA CCACAGCATAACCTAACCTTTCTAGAAAGATACACTCCTCTATCCAG CATGCCTAGAGTCTTATGTGTGACAAGTGACGGTATGCTGTTTCAC AAAATTTCGCTGAAAGAGCCGTTGATGAACTTTAGAATAACCCAAC ATTAGGCGCTGTTACAAATGGGTGGAATTAGACAAAGCCGTTTGCC TTTCACCTCTTGCTTCAGTTTTCAGAAAATTGCCCAAAGGTATTGCA AACCATTTGCGTTTTCATTGGCTTGGTTAAGGAGTTGGCAGTGTCA ACTGGCCCTGTGTAAAGCCACGTCACTGTTTCTGTAGAGAGCATGA CACGTCAGAGAGGTGGGAAGTGTTGCCAGGACTCTTCTTAGAATG CTGGCAAGAAGAGCAGGCGAAAAGGGAAGAGCTGTTTATAAACA GGGAAAGGAGACATCACCTCGTATCCTTGGCTTCTCATTACGTCATC GATTACAATCACAAGTCTCATTTCCTACAAGCCTGAAAGCGGGTTC TTCTCTGCATGTAGGAAAAAGCCCCGCTTACTAACTCTGCCCTCAA AGAGATCACAAGCTGGTCCTGAACGCCCTGTGCCCTGGGAATGCTT ACTGTCGAAGCTCTGCAAATTTTCTCATTGAAGGCACTCAGGGCAT TCTGCTAATTAAATTAGGAAAATGTGTACAGCTCTACTTGAAAAAT CCAGGTTTATGCTCTGTATTCTATTACCAAAGAATTTATCACTTTTCA AATAACATTCAATTATTTAAACTGAGTGTGCAGTGAGAGCATTTAT AATATAATCTCATTTCCAGAGTGTTCCATCCCAACTCCTCTCTGGTAT AAGTAGCTTAGAAGGTTAGATTTTTTTTTCCAGAGGATTAAACATT CGAGAAGAGTGGCTAAGAGGCAGCACCAGGTTTCTCTCATTTCCTC AAAGAGAATTTAGCCTTGTGAAGAAGAATAAAGTGAAGGTCAATG GTTAGATCCATGGCTTAAAAATAAAATGCAGCTTTCCTTACTCTGAA TTTACCATAACCAAAAAGGCCACGAGGCAGCCATTAATGATCCTAA GTTGCAACAGAAAGAAAAAAACACCTACTTAGGGTTAAACGAGGA TTCAGTGGTCACTTGCCGCCGGCCTCCACTTCACAGGGGCGGAGG ATGAGGCCAGGGAAGACTTTCTTACCCACACTGACCTGACCAGAG ATGAAACTCCCCCTCCTATGACAGCCTGCACTCAGAGGCTGATTCT GAAATAGTCATGCCAAATGCAGCCAATCCCTGTGTACAAGGAGAAA AAATAGTTCCTCAGAGCTGTGTCCTTGGGCCAGCCATCTGGACCTC TCACAATCTCTTTCGTTCTCCCTACACTTGGGCTGAATCCCGCCTTC CTTGCCTGCCAGGTTTGGTTTGGGGGCCAAATGAGTGAAAGTCCGT GAGATACCCCTAAGTATTGAAGTGCGTAGTAAATGATAATAGCCCTG ATTTGTTGAACATGATGATGTGCCAGGCATTGCGCAAAGCATCATTT CATCATCACAACAAACACTGCTGGGAGGCAGGGACACCCCCACCC TCCTTGTACACCCAGAAGTAGGACTTTGGTTAAATGGCTCTCCCAA GGCCTCACAGCCTTAAAAACTCAAGCACTTATTTAATCCCCAATGAT ACTACCTTTTGGCCATGCAATGTGTCATGGCCAGTAATTAGATTTGC AAACACATATAACCAAAGAGGATTAGAAGAGACATATATTCTATATA AATTACATGAAAATAGCACTTTCAGTATTTTCCTGATTTTTTCTGGTA ATTAAAAATGATTGCCCTTTATATTTCTTTATGGATACAGTTGCATTG TTTTTAGCCAATTGTAGCCCCCCCCTTTAAAGAGTGAATTAGTGGGA AAATAGTGTTATCCAAGATTTTAAGAATCTAGTGTGTGTGTTTGTGT

	GTGTAGGATGGGTTTTTCTGAAATATTTTACTTGCATGTGAATGTATA<br>ATTTTAAAGATTGGCTTTTAAAATACTGAATTGAGGTAAATTCAGCT<br>TTTCTTGCAGACTCTTTACGATAAATGTGAAATTGAAAATGGAAATG<br>CTCCATTCAGATGGTGTGCCGACTACAACCTCAACATACGGATAATT<br>CACTAAATAAGTAACTTCCTCGTGGAATATCACGGTCAGCCTTTGTT<br>TTGGGTACACATTCAATGAACTGGTTTCCTATTAGTTTCTGCTTGTC<br>AACAAGTTGTGTCATTTACTGATAAATATCCAGCCTTTTACTAAGGA<br>ATACCGATTTTCTCAAGTCTCAGTGTGTGGAGGGGGTGGGAGTTTG<br>GTGGGGAAGCTTTTCAATTTGAGAACCTTTGCCGTTTTTTGTTTCAA<br>TCGTGACTAAGATATAAAAGTCCTTGGTCACCACTGCCAGCTGCTA<br>CCTGTTCACTGCCTCCCTGACGGGTAATGAGGCCATCCGGAGTTTT<br>AGTGATTAAAATGTTTTATTACTTGTATTCACATCATCATTAAGAAAA<br>ATAACTTCTAGTGGATCAATATGGCAAATGAGTAAACCAGAAGATG<br>GCGTTGAAGATTAGCAATTACATTTAAAAACAAGGTAGTGGTGTGA<br>AGGCTCAGAGTTTCACACAACGACTGTTGTTCTGAACTAAAAGCA<br>GAGGATGTGGATGAGTGAGGGGAGGGTATGTTGAAAATGACTAAA<br>CCCGGTGCAGAGGACACGTATACGAAAGTGCAAAAAGCTCTCATT<br>TGCCATTTTTTGTCCTCAGCAAACTCCTCTCTGGTATGTTTGTGAAC<br>GTTTGAGGGAAAGGTAACACTGATTAAAATATAATGCAACAAGAAG<br>GCAACGATCTTTGCTTTGTTCACTGATGTGTTCCAAGTGCCTAGGA<br>CGGTTCCTGACAAGTAGCAGGTGCTCAAAATACATTTGTCAAGTGA<br>ATAAATCTGTGAAAGGAAAGGAAGAAAAACAGGTTGTGACCAGA<br>ACTTTTTGTGAACAAAACCCTGTTGTTTATTACATAGACTTTGGTAA<br>TGGAAAACTTACTTGGGTGAAATAAAAAATGGCATCCTGTTAATTT<br>CTGAGAACGTCTGTTGGTTTTAGGAAAAGCATACCTATCCATACCTC<br>ACTACCTGTTTTGCTCTAGCCTAAACATAGATTCTTCTTTGGTTAGG<br>AGACATTGACCCAATATATAAGATTAAAACAAAAACTGAGAAATAA<br>GTGAAATCAAAAGCTTTTCAAAAGTTGCAGGTTGACCTACGGTGG<br>TGGTGGTCTAAAATCATATAGAAACCAGAGATCTGATGGTTTACCTG<br>AAAATGCGAAGTAAATGCACATACTCAGCATCTCAGACGATCGAAA<br>GCTCAACGGGTGAAAGCTCAGGGGGTTAATTTTGCGATTAAGGACA<br>TCTTGGAAAAGTATGTAAAATCCCTGGTCCACTTAAGTATTATTCCT<br>ATTTTGGGCTTTTATTTATTTATTTTGAGATGGAGTCTTGCTCTGTCG<br>CCCACCTCGGCTCACTGCAACTTCAGCCTCCCGGGTTCAAGCGATT<br>CTCGTGGCTCGTCCTCTCAAGTAGCTGGGGCCACGCCCGGCTAATT<br>TTTGTATTTTTAGTAGAGATGGGGTTTCACCGTGTTGGCCAGGCTGG<br>TCTCGAACTCCTGACCTCAGGCGATCCACCGCCTCGGCCTCCCAAA<br>GTGCTGGGATTACAGGCTTGAGCCACAGCGCCTGGCCTATTTTGGG<br>CTTTTATACCCCACTGGTAAACTGCTTTCCTCCAGGTTGAGGTTAAA<br>ACGACATCATTTTAAGGTGAACTGAAGTCTGGAAGTGATTAAGCAC<br>TTGGATCCTTAGGGAGCCTCTCCCCGCCCCCATCTCTTTCATGCTAA<br>GATAATTAAAACTTCGGGGCCGGGGCATTGTCTGAGTCACTTCAAT<br>TCACCAGCCTAATAGATGCAAAAGGATGTAAGCATGTCAGACACGC<br>AGAGACAGTATTTGAATCAAGCTTAATAGCTCAAGGGAGCTGGGCC<br>ATTTCCTATTTTCTTTTTAGTGAGTGCGATGTTCTCTAAACACCGCG<br>GAGAGGCAGGGTTCCCGGTGATGGCCTTGCCGAGGGTGCTCCCGC

	AACCTCCACCTCCAGTTCTCTTTGGACCATTCCTCCGTCTTCCGTTA<br>CACGCTCTGCAAAGCGAAGTCCCCTTCGCACCAGATTCCCGCTACT<br>ACACGCCCCCCATTTCCCGCCCTGGCCACATCGCTGCAGTTTAGTG<br>ATTGACTGGCCTCCTGAGGTCCTGGCGCAAAGGCGAGATTCGCATT<br>TCGCACCTCGCCCTTCGCGGGAAACGGCCCCAGTGACAGTCCCCG<br>AAGCGGCGCGCGCCCGGCTGGAGGTGCGCTCTCCGGGCGCGGCGC<br>GCGGAGGGTCGCCAAGGGCGCGGGAACCCCACCCCGGCCGCGGC<br>AGCCCCCAGCCTTCACGCCGGCCCTGAGGCTCGCCCGCCCGGCCG<br>GCCCCGGCTCTCGGCTTGCAAAGTCCCTCTCCCCAGTCCAACCCCC<br>GGCCCCCACAGGCCTCGGCGCCCCGCCCCGCCCCAGGCCCCGCCC<br>CAGAGAGTTCTATAAAGTTCCTCTTTCCCACCTCGCACTCTCAGTTT<br>CACCGCTCGATCTTGGGACCCACCGCTG
IRS2	AAAAACGTTTTAATCGGCAGCTAGGATAGCAACATGAAGCAACAC<br>ACAGGACATCAATTTCATCATGCAAAATACCGGTGTATTATATTACA<br>GGGGCCAAGCATTCCAAGGAAAAGTAATGATCTAAGAAGATGCTCT<br>TGCATTTTAAAAATATCTACTAGTGTTATACAGTCATTAATGTATAATT<br>TCCATTTCAATAATAGGAAGCCTTCTGGGAAAAAAAGCTTGTGTTG<br>CTTCTTAAATCTAGAAACACATTCTCAAAATTCAAACAGAACAAAA<br>TCCAGAGGCATATTAATAAATATTTTCAACTCCTAACCGCAGTCACT<br>TTTTAATTGGGCATTTATGTATTTCTTCAACATAATTATATTTATAATG<br>AAAAAATGTTGAGAATACTGGATTAGCTGCATTTGTAACTACAACAT<br>ATTTTTCTAAACAGTATTAATTCAATTGATAAGACTTTAAAAAAGTTA<br>TAGTAGTTTGTTCTATCATATATGCACATTAAATAATCATTTATTTTAG<br>AAAATCCTCCCTCATAAAATGAACATTTAGGAAAGAGACTGATCCA<br>TATTTTTAACAAGAATAATAAACACACAAGTAGAAAGCCTTTATAAT<br>TTCCAAGGGGCTGATCAATTCCATTAACAAATCCTGATACTTTAAAT<br>GTTTGCAATGCCATACATAGCAAATGATCCAAACAGCAGTTATATTC<br>TCTCATGTAAGTCATTCTGTTGCCCATACACAGAGTTAGCATGTTAC<br>AAAACTCTTGTGAAATTTCAGTTGAAACATTAGCAAATAAATTTGC<br>TGGTAAAGTGCAAGAAGTTTCCTTATAAACCCTAGATGAGGAAAAG<br>TCTGTCCACAGTTCGTTCATCAGAGGAATGGAATTTCTCAAATGAG<br>CTACTGAACAACATTATCTAATAAAATGAAATATAATGAAATGGTGC<br>CACTCATTTTAAAGATTAAATATATCCTTTGACAGAAACCTGTGAAG<br>TTAAAAATCTTTCCCTCTTAAAATACCTTTTAAATTAAAGCTATTCTA<br>ACTAGTTATATAGCATTCCACTTAATCTGCTTAGTTTTTAGATTGAGC<br>TCACCTAAATACAAAATACTGTCAAACACCTACAGGGCAAAGAACT<br>AAAAAAAGAAAGAAAATTTCACAAGCAATAAAATGCTATTCACCTT<br>TCTCCTCCCATACTTGCCTTTCCTATATGAAACAAAGAAAATAATTA<br>GTGATGTTCATTATTGGTAGTGCTACTAGTATTTGTATTGTGAACCTG<br>TAACATATTTTTAAATCTCTACACTTTTATATAATTGATTCTTTCTTGA<br>AAAGTAATTATTTCAGAAGACTTCTAATAAAATAATCATTATTGTATA<br>TGATTCTGTTAAAAATAGAAAAAACAAACAAACAGAAAAAACTCC<br>CTGCTGAAAGATATGGCCATTTTATTAGCTGTGAAAGGAAAGAAAG<br>CTTCCAAGGAAAGATCTTGGACCATGAGAAAAAATTTCAACCAGG<br>CTGTTTGCTGGCAAGAGGCCATGCCAGCCCCAGCCAGTCCTGCGT<br>GCAGAGCATGTTGGCAGATGTTATTAGCTGACTTTTGTTGAAAAAG

	TATGGAAGAACAGTTGGAAAATCACAAGGTGTAGAACAAGCTTTA<br>AATAAATAAATAGCTTATCTGGCAATAGAAAGGGGGAAAGAAAACA<br>AGTTCCTCGGTTGAGGAAGAGGACTGGGATACTTCTAATTACAGAG<br>TTTCTTACTGTTGGTTTGTTTCATGGGCTGACTCTCAATGAGAATTG<br>AAATTGTATCATCCACATTTGTATTTGGAGGTTCTAACTGCAGAATT<br>GCAATTAATTTCCAGTTTGCTGTAGGAAATTTATACTCTCTATGACA<br>GAATACCACGGACATAGGTGATAATTCTTTAACCTTCTCTCTTTTGT<br>CCCCAGTCTGGTAACTGAGGCAGTACATGGGGAAAGTGCTAACTG<br>CCAATCCAGAGCTGCTGACACCTTAAAAACACTGGCCTTTAACACT<br>AGGATGGCCCCAAGAAACAAGCAAACAGGAAAAGATGACACCTG<br>CTAGCGTGTGACAGGCACAGCAAGTAAATGTTCAATACGGATATGA<br>AAATGCAACTGCATAATAAAAGTCAACATGAAAAAGAAAAAGATT<br>GCAAAATATACTTTCTCAGAAGAAACCATCACATGATGTTTAAAAG<br>TGGTGGTTCCTTTAGCATTGTCTACTGATGGCTGGCAACATACAGG<br>AACATAGAGGTGGCCCATTTACATCCTCATAAAAGTGGGATGTAAG<br>TGATCACAAAGCCAATAAAACTACACCACATTTTCTTCAAGCACCA<br>AGATAAGAATCCCTTCATCTTTCCTGACTAGACATATTGCTAGGCAA<br>CATTTGTGAACGTAAGAAAAGGTAACAAGATCTAAAAAGAACGCC<br>GAAGCATAATGTTCATAAAGGTGGCGCCCCCAACACACACAATCAT<br>ACACACATACCTATACACACACAATCACACAATCTATTATATAAACA<br>AACACGCACACATGCACACATAGGTGCACAGACTTAAAATGGCATT<br>TCTTGAGGTATTTTTTTTTAAGGTATGATTTAAAAATGGAAGATCAA<br>CTTTAAACACACCTTTTTGAGGCACACTGGGTGATATTTGCCACAAT<br>GATTATTCTTCCCAAATTCAGGCTACTGATCAATAATCCTAACTGTAT<br>ACATGCTAAAAATAAAAACGCTATAATCTTTGTACTAGTCAGGGCTC<br>TCTTGGCACATAAAATTAGCCAACACAATCTTCAAGTCACATGTTAC<br>AAAAACATATTAAAATATATATCACTCTTTCAAAAATTACACTTATTT<br>TCTCTTAGATTAAGATTTTATTACTTTTAAGATACTTCTATGAATTATA<br>GAGGACATCCAAAAGTAAACATCCGGGACTTAAAAACAATAACCA<br>ATATTATAAGATAAATTATTGCTGTAAATTATTTCATGAAGGCACAAT<br>TAAAAAAAAAATAAAATAAACAGCCAAAGCCTGTTTTGTTGAGTTT<br>TCTTCGTTGCGCAGGATCGGGAGCTTGCTGGGATCTGAACTCAGGA<br>AGCCCTCACCTCACCCTGACTTGGTCATTGAGGAGACGTAATTGCA<br>GGGGTGCTTATTTGCTATAGGCTTTGAAAGTCACAAAAGCCCAGTA<br>GATACATGAATTCGAAGCAGTTCTCCAAGGGCATCTCCAACTTGGC<br>AGTCACCGCCCGCACCGCGCACACAGGTAGTGGACGCCCGCCCAC<br>CTGCCCGTCTGCTGGGCGCAGGGCGCTCCTGGGGTGGGGAGGGCG<br>CTCCTGTGGGGGGAAAAGGGGCGCTCCTGCCTCGGAGGGGGAAA<br>GGACGCCCCTGTGGAAGGAAGGGGGGGCTCTTGAAGCGGTGTGGT<br>GAGAGGACGCTTCCGAGGAAGGAAGGGGGTGCTCCTGCAGCGGT<br>GGGGGGAGAGGACGCCCCTGCGGAAGGAAGGCGGCGCTCCTGCG<br>GGGGCTGGAGAGACGCCCGCGCGGTCTCCCGGCTCAGGGACCGCT<br>GGCTCGCGGGTCCGCCCTCTCCCCGGCGCTATGGAAACCGCACTTT<br>CTCCGCCTGCCGTCCCCGATCTCTTTCTCCTATTACCTCCCAAACGC<br>GATTTCAACTTCCCGACAAGCTGACAAGTGAATGGCGCAGAGGAC<br>GCCCCGTAAAGCGCCCGCGAGCGGGTGGGACACAGGCGCCCCCTC

	CGCGCCCCTCTGTCCTCCACGGAAGGGCGCGCCGCTCTCGATCTGGAGCGCGGTGCTCGCGAACCCGCGGCCGCGGGCCCTGGAACCTCCCCGCGTTTCGCGGCTGCGGGGGGACGGAGATGCGGCCGGGAGACGGGGACGGGAAACGGGGGACGGGGATGGGGCGCGCCCACCCCGAGCGCGGGAGCGGCCGCCCTCGCCCAAAATGGGGACCCCGCGCCCCGAGAGCACGGCCCGGCTCGCCGCCCATGCTTCCACTGCAAGGCCGCAGCCCCCGCCGCCGATCCCGATCCCCCGCCCCGCCCAGCCGCGGCCCCGCCCGGCGCCCCCTCCCCGGCGGAAGCTCAAGCCCAATTAATTGAGTCCGAGGCGGGAGGGAAGGGCCCTGCGCGCCGTGGCCCGCCCCGCCCCTCTTCCGCGCCCCTTTTCCCGCCCTGGGTGGCATCTCCTCCGCCGGCATCCACAACAAGCCGCTGATTAATGAGGCCGGGGCCGCCCCACCCCGCCCGGCCGGGCCGGGGCCTTCGCGGGAGGGGGAGGGGACGGCGGGAAACGCGGCCCGGGGAGAAAGGGGGGCGGGGCGGGGGCGCGCGGCCCGCCCCCGCGAGCGCCGCGCCGATTGGCCGAGCGCGCCGTCCGTCGGGGGGCGCGGCGCCAATGCGAGGCAGCGGGGCGGGGCGGCCGCGCTGTGTGTGCCTGCGTAACGCCGAGTCACATGTTGTTTTGCTCTTCTTAGTTCAGTCACTCGGTGCGCGATGTGTTACTCACTGTGCGGCGGGGACCGCGACGAGCCCGGGTCGCCGTTGGCAGCAGCAGCAGCAACACCAGCAGCAGCAGCAGCCCCGGCGGCGGCGCGGACCCCGAGCGCCCGGGCGCACCCCGGCTTCCCGGAGCGCGACGCGGCGGCAGCAGCCCCGGTGCGGCCGCGCGCGCCTTAGGCTCGGCCCCGCGGCTCGGGGACCCCGACTCCCGGCCCAGCGAGCGCGTCCCCCGGCGCCGCCCGAGAGCCCGAGGAGGCAGCGGCCGCAGGCAGCCGGGGAGGGGGGCGGCCACCGCCCGCGCCGGGCATCCTCAGGAGCCCCAGAGCGCGGAGGGCGCGGCGCCGCCGAGCGGTGCTGGCCCCCGCGGGCCTCCCCGGACCTTCCCCACCGCCTGGGCCCGAGGGACGCGTGATCGGGCGGGCGGCCGGGCGCAAGGGTGGGAGGGAGCCGCCCCCGCCCGCGCCCCCTCCGCCCCTCGCCCCAACCCCTGGGCGCCGGGCCCGGGCCGCGCGGCCTGAAGCGCCCGCGATGGCGAGCCCGCCGCGGCACGGGC
ISG15	TAGGAAGAGGAAGCTCCATCAGCACGTCCCCAGGAAGCTGGTTTGTTGGGAAAGGGGTGAGAGCAGCACTCCAGGAGGTGGCCAATGAACCTACTATGCGGGATTTCGGCTGGACTTTTCCTGACCAAGCCAGGGGCTTAATGTTACCAAGCAGGTTGCAGGCAGGAGGCTGTCACCTGCTTGACAGAAATCCTTGGGGCAAATGCCATCTCCATGTGGAGGCAGCAATGAAAGCATGAAGGATCTGAGCTGACGTTACAGTGAGAGTGTCCCCCCTTCCTGGCACTCAGGCAAGGATGCTCCCCAGATGATGGACACGGGGACCCTCCCCAGATGACAGACACGGGGACCCTCCCCAGATGACGGACACGGGGACCCTCCCCAGATGACGGACACGGGGACCCTCCCCAGATGACGGACACGGGGACCCTGCCAGATGACGGACACATGGACCCTCCTCAGATGACGGACACATGGACCCTCCCCAGATGACGGACACATGGACCCTCCCCAGATGACAGACACATGGACCCTCCCAGATGACGGACACATGAACCCTCCCCACATGGACAGGAAGATTCCAGCAATGTTAGAGATGATAGAATTCATAGACAAGGATATGAAAGCTGCTGCTACAACTTACTCCAAGAAGCTATTCCAGGTGTGGTTGTGTGTGCCTATAGTCCCAGCTACTCAAGATGCCAAGGCCAGCCTGGGCAACAAAGCG

	AGATCTCATCTCTAAACAAAAACAGAAATAAACTAGAAAAGTGGA
	AGCTAGAGGAAATATCGAATGTGTTAGGGACTTTGAAGTTACAAAA
	ATCCCAACGGGGAACTTCTAGAGATGAAAATTATGATGTGTGATTT
	GAGAAATGCACTGGGGGGAAGTACCAACAGGTTAGACACTGGAAA
	GGAAAACAGTAGTCAACTCGAAACTATAACAATAAAAACTATTTAA
	AATAAAACAGATTTTAAAAACAGNNNNNNNNNNNNNNNNNNNN
	NNNNNNNNNNNNNNNNNNNNNNNNNNNNNNNNNNNNNNNNNN
	NNNNNNNNNNNNNNNNNNNNNNNNNNNNNNNNNNNAGGCTGA
	GGCAGGAGAATTGCTTGAGCCCTGGAGGCGGAGCTTGCAGTGAGC
	TGAGATGGCACCACTGCACTCCAGCCTGGGCGACAGCAAGACTCC
	GTCTCAAAAAAAAAAAAAAAGAAAAGAAATCAGGAAACATATACCA
	TGTTTAACAGGGCATCTAAAATGATAAAAATAAATAGAAAATCCTG
	GGTTTTTGTTTGTTTTGCTTGGGTTTGGTTTGGTTTTGAGACAGGG
	TCTCGCTCTGTCACCCAGGCTGGAGTGCAGTGGTGCGATCACAGCT
	CACTGCAGCCTCCACCTCCCGGGTTCAAGCTATCCTCCCACCTCAG
	CCTCCAAAGTAGCCAGGACTACAGGTGTGCGCCACCACGCCCGGC
	CAATTTTTTTATGTATTGCAGGGACAGGGTTTCACCATGTTGCCCAG
	GCTGGTCTCAAACTCCTGGGCTCAAGCCATCTGCCTGCCTCGGCCT
	CCCAAAGTCCTGGGACTGTGGTGTTTGTTTGTTTGAGACAGGGTCT
	TGTTCTCGTCGCCCAGGCTGGAGGGCAGTGGCGCGATCTCAGCTC
	ACTGCAACCTCCACCTCCAGGGCTCAAGCAATTCTCCTGCCTCAGC
	CTCTCGAGTAGCTGGGACTACAGGTGTGAGCCACCACACCCAGCT
	AATTTTTGCATTTTTAGTAGAGATGGGGTTTCACCATGTTGGCTAGG
	CTGGTCTTGAACTCCTGACCTCAAGTGATTTGCCCGCCTCGTCCTC
	CCAAAGCGCTGGGATCACAGGCGTGAGCCGCCACCCCGCCTCCTC
	TTTGGTTTTCAGATGCTGACAATTTTGGTGACCCCTGAGATTTGCTG
	GGAGACGGGGAAGGGCCAGGATTCATATTCAGAATATGTTGATTTT
	TCACTCTTACACTTGTTTATTTAAAAATTATTTATTTATTTATTTGAAT
	CTTATTTAAATATTTTATTTATTTGTTTATTACTGAGTCTTAGCTATCCT
	TCTCTTGTTATGTGCTGTGTGTAACAACAACAATTTGGGAACAGCC
	TAAGTATTCAGTGATGTGGGATGGGTTTTAACTAAATTAACTCTGTA
	CTGTAACAGGCTCATTCTTCCATGGGAAACATGCAGTTGTTAAAAC
	AACCTGCACAGATGCGTCCACCAACACGGATAGATGCGTGTTTTTG
	TTTTTTGGGTTTTTTTTTTTTTTTTTTGAGACAGGGTCTCGCTCTCTC
	ACCCAGGCTGGAGTGCAGTGAAGCGATCTCGGCTCACTGCAGCCT
	CCGCCTCCCAGGCTCAAGTGATTCTCCTGCCTCAGCCTCCCAAGTA
	TCTGGGATTACAGGTGCCCACCACCACGCCCAGCTAATTTTTGTATT
	TTTAGTAGAGATGGGGTTTCACCATGTTGGCCAGGCTGGTCTCAAA
	CTGCTGACCTCAGGTGATCTACCCGCCTCGGTCTCTCAAAGTGCTG
	GGATTACAGGTGAGCCACTGCGCCCGGCCCATGCCCAGCTAATTTT
	TCGCATTTTTAGTAGGGACGGAGTTTCACCATATTGGCCAGGCTGG
	TCTTGAACTCCTGACCTCAGGTGATCTGCCTGCCTCGGCCTCCCAA
	AGTGCTGGGATTACAGGCGTGAGCCCCTGTACCTGGCCTTTTTTGT
	TGTTGTTGTATTTTTAGTAGAGATGGGGTTTCGCCATGTAGGTCAGG
	CTGGTCTCGAACTCCTGATCTCAAATGATCCTCCCGCCTCAGCCTCC
	CGAAATGCCGGCATTACAGGCATGAGCCGCCTGGCCTGGATAGATG

	TTTATGATACACTGTTGCATGAATGAAGCAGGTTACAAAACAGTGA<br>AAGTATGGTGAGGCCTTTTCACCTTTGCTTTTTATTGATGTGAAATT<br>CATATAACATAAAACTAACCATTTTAAAGAATGAAATCCAGTGGCGT<br>TTAGTTATTTACCACGTTGTCCAACCATCACCTCTGTGGAGTTCTGA<br>AACATTGTCACCCCCAAAGGGGCTTGTGGCTCTGAAGCAATCACCC<br>CCCACTCCCCCTCCCTGGCCCCCGGTAACGGGGTCGGCTTCCTGTG<br>CTGCGGCTCTACCAGTTCCGGTCGTTTCCCCTGCGGTGAATGGAAC<br>CAACACTCTGCGGCCTTCGTGTCTGGCTCTTTCCACTTAGCATAAC<br>GTTTTCAAGGTTCTTTCACATGAATCAGTGCCTCAGTCCTTTTCATG<br>GCTGCATAATATTCCGTTGTGTGGACATTCCACACTTTGTGTGTCCA<br>TCCATCACTGATGGACATGTGCTCCGTTCCTGCTACTTGTTTATTGT<br>AAACTGTGCTGCCATGGACATTTGTATGCAAGTATTTGAACACCTAT<br>TTTCAATTCTTTTGGACACATGCCTAGAAGTGGAACTGCTGGGTTC<br>CCAATAATTCTGTTGAACGTTTTGAGCATCGCGGCGGCCGCACTGT<br>TTTACATTCTCAACAGCAATGCATGTACCAGGATTCCAGTTCCTCTA<br>TGTATTCCCCAGTGCTTGTTACTGCCTTTATGTTTATTTTATATTATTT<br>TTTGAGACTGTCTTGCTCTGCTGCCCAGGCTGGAGTGCATTGGTGC<br>AATCTTGGCTCACCACAATCTCTGCCTCCTGGGTTCAAGGGATTCT<br>CCCGCCTCAGCCTCCCAAGTAGCTGGGATTACAGGCGTGCACCACC<br>ACGCCCAGCTAATTTTTGTATTTTTAGTAGAGATGGGGTTTCTACTA<br>AAAATGTTGGCCAGGCTGGTCTCAAACTCCTGACCTTAGGTGATCC<br>GCCCGCCTCAGCCTCCCAAAGTGTTGGGATTGCAGGCGTGAGCCA<br>CCGCACCCGGCCTGGCCTTTATTTTTATTATTACAGTCATACCAGCA<br>GGAAATAGCATCTCACTGGGGTTTTGATTTGCATTTCCCCAATTAAT<br>AATGATGTTGAACATCACTTTACAGCCGTTTCTATGTCATTGGAGAA<br>ATGTCTATTGAAGTCTTTTGGCCATTTGAAAATTGAGTTGCCTTTTT<br>TTTTTATTTTTATTTTTTATTGAGTTGTAAGAGTTCTCTATATGTCCTG<br>GATGCTATGCCCTCATCAGATAGATAATTTGCAAATATTTCTTCCCAT<br>TCTATGGATTGTCTTTTCACTTTCTCAATAGTGTCCCAGAGTTCATTT<br>TTGTAGAAAATAAAAGATAGGTCTCTTTTATTAAAAAACAATCTGA<br>GGCTCCGGGTGCAGTGGCTCACGCCTGTAATCCCAGCAGTTTCAGA<br>GGCCGAGGCAGGTGGATCACTTGAGCCCAGGAGTTCGAGATCAGC<br>CTGGGCGACATGGCGAGACCCCCATCTCTACTAAAAATACAAAAAA<br>TTAGCCGGGCCTGGTGGTGCACCCCTGTGGTCCCAGCTACGTGGGA<br>GGCTGAGGTGAGAGGATCGCTTTAGCCTGGCAGGCGGAGGTTGCA<br>ATGAGATGAGATCGTGCCTCTGCACTCCAGCCTGGGCGACAGAGT<br>GAGAGACCCTGTCTCAAAAACACAAAAACAACAACAAAAAAACA<br>CCAATCTGAGCAAATACTGCCCTAAACCGAGTGTTGTTATCTCTGG<br>GTAGTTTGGAGTTCTTGTTTCTCAATTAACCATGGGGATGTTTTCCA<br>AGTTTACTAATTTTGCAAGTTGGTAAATGGAAAATGAAACCATTAGT<br>CCATGTGATGACAGCTTTAGTGCATCCTGTGAAGGATCTGGAATGC<br>GCGATATTTAGGTGTTTCCAGGGTGTTGGGTGGGGGTGGGGATGCC<br>GTCCGCTGTCCGGAGTCCCCGCCACTTTTGCTTTTCCCTGTCTTTCG<br>GTCATTCGGTTTTGTTTCTTCCGCTCACTCTGGGGCATGCCTCGGGA<br>AAGGGAAACCGAAACTGAAGCCAAATTTGGCCACCAGCGCAGGCT<br>CGGCGGCACGCCCCCTGACGTGTGTGCCTCAGGCTTATAATAGGGC

	CGGTGCTGCCTGCCGAAGCCGGCGGCTGAGAGGCAGCGAACTCAT<br>CT
IVL	TAAATAGAGAAGCTCCTATGCACCAGGCGCAGTGTAAGGCATTTCC<br>CATGCATTCTCTGAGGACTCCTCCATCTCAGACATTCTGCTGCTGGA<br>CAGAGAATCAACTGTACAAAATGTGATTTCCCCAACAATGAGCCCA<br>AAGGGGAGGGAATTTCCTTTAGTTGGAGAAGGGGGAACTGAGTGT<br>CTCTTTCCTCTACTACTGGCAGCCTTTATGCCTCTGCTCCTCTTGCT<br>GCCACCAAGAGCAGAGCTGTGGCTGCCCTTCCACAGCTCTCAGAT<br>CCAAGAACAAAGGCCAGAACTGGTGACTGAGATCCAGTGATCCAA<br>CAAGCCCAGGAGAGAAAGCTATAAGAAGATGTAAGAGAGGAAAG<br>CAGGTCAGGACAGACAGATCCACCCAGAAGCCCAAAAAGGATGA<br>GTCAGCCCTCTTGGCCCTTTCTTGGTCTCATCTCCCTTCCTTGAATA<br>GCAGACGGGCACAAAAGGTGAGAGAAGAGCCAAGGTCAGGTCCA<br>GGAAGCAATGGAAAAAGATGAGGAGGATTCAAAGTTTGTGGGGAA<br>GGTCGGGCACCGTGGCTCACGCCTGTAATCTCAGCACTTTGGGAG<br>GCTGAGGCGGGCAGACCATGAGGTTAGGATTTCGAAACCAGCCTG<br>GCCAACATAGTGAAACCCCATCACTACTAAAAATACAAAAATTATC<br>CAGGCATGTTGGCACATGCCTGTAGTCCCAGCTACTCAGGAGGCTG<br>AGGCAGGAGAATCGCTTGAATTGGGGAGGCAGAGGTTGCAGTGAG<br>CCAAGATTGCACCACTGCACTCAAGCCTGAGAGACAGAGTGGACT<br>CTGTCTCAAAAAAAAAAAAAAAAAAAAGAAGTGTGTGGGGAGACA<br>ACTAGTGCAGGTTGGCAGGTTGAGGACTGAGAGCCGCTGATGTAG<br>GAGACAGTGCCCTGGGGAGAGGAGGAGGCTTGTGAGAGGGAGAG<br>GAGGCTAGCAGCCTCAGAGCATGGGGCAGTGCCATACCAGGAAGC<br>AGATGAGGCATGGCTATTCAAGATGAGAATGCATGATTTTCTGGCC<br>CAAGTCACCTTCCAGGACCATCTGCTATGACCCCTCTGGCAATCTG<br>TTAAGAGCTGTCCTCAGCATCCCTCCCCACAGGACATTGAATCACC<br>ATAACCAATTTCTTCTGCCCCCTACCACTAAGCAACAGCTCAAATAG<br>GTTGCCTCACTCAGGAGAGAGACAAACTAGCCAGCTTCTGCCTCC<br>AAATTTGAAATAGCCAGTGCATCCTTAGGCTGGGGCAGAGTTGGGG<br>CCAAAGCTTCTCCATGTGTCATGGGATATGAGCTCATCCTTATTATGT<br>TGGGTGGGGGTTGGACAGTTACCCAGACTTGTCATGTGGACCTGG<br>AGCTTATGAGGTCATTCACATAGGCAGTGAAAGAACCTCTCCCATAT<br>ACGTGAATGCCTGTCTCCCAAATGGGGCAACCTGTGGGCAGAATA<br>AGGGACTTCTCAGCCCTAGAATGTTGAGGTTTCCCCAACCCCTCCC<br>TTGCATACACACACACACAAACACTCCCTCAGCTGTATCCACTGCC<br>CTCTTTCCCACACCCTAGCTTTGCCCAGCAGTCAAAGGCTCACACA<br>TACCATCTTCTCCTTAAGGCTCTTATTATGCCGTGAGTCAGAGGGCG<br>GGAGGCAGATCTGGCAGATACTGAGCCCCTGCTAACCCATAAGACC<br>GGTGTGACTTCCTTGATCTGAGTCTGCTGCCCCAGACTGACTGTCA<br>CGGGCTGGGAAGAGGCAGATTCCCCCCAGATGAAGTCAGCAGCAG<br>AGCACAAGGGCATCAGCGCCAAAGTAAGGATGCTTGATTAGTTCTT<br>CAGGGCAGAGTGGGCTGTGCTTCCTCTGCCCCAGAAAATGGCACA<br>GTCCCTGTTCTATGGGAAAAAGAATGTGAGGTCCCTGGGTGGGCTC<br>AGGGAACAGAGAGGTCATGAGGAGGGGATAGCACTGCAGAAACC<br>AAGGGTGCCTTGTGAGTCCTCCCTCTGTCTTTTTAGGCATGATCCA

	GGAACATGACAAAATTAGTGCTTTAAATAGATTTACTTGGGGCTAA<br>GAGAAATGTGCCTGTCAGGAAAACTATGGGGAATCAGGACACTTC<br>TCAAAATTAGCCCCACTGAGTATTGTCTTTATAATTCCTTCTTTTGG<br>ATTAGATTGTAAAAAAGAGAGTGTAAATGAATGATGTCCATATAATA<br>AGTTATTAGCCAACCATTAAAAAGAAAGGGAAGAAATAAATCAGTT<br>TGGTTTTTACACACACATACAGACACACACATATAAACATTGATCAA<br>CACTGAAATGTTTAATAGTCATTATTTTCGGGTCGTAAAATTCACTG<br>TTCTTCAATGAATACTTGTAGAGCACATATTATATGCAGTAGTTTTGA<br>TAGGTTCTAGGGGTATAGTGGAAAACATACCAGGTATACGCTGCTCT<br>TAGCTTATTTTCCAGTGGGAAAGATAGACAATAAGCAAGTGAACAA<br>ATGCAAATAAATTACTCTAGATTGTTATAAGTGAAATTAAGTACCAA<br>TCCTTTAGATATGGTACACAGAGAAGGATCTCTGACAGACCCCAAC<br>ATTGACACTGAAGCTGAAAGGCATAAAAGAACCAGAGACCTGGGG<br>AGGGGCCGGTGGGCAGAAGGAGAGCAGGTGCCAAGCCCCCAGGT<br>GGAGAGCTCTGGGCTCATCTCAGGAACCGAAGGCCCTCAGTGAGG<br>TAAGAATATACCTCTCAGGGAGAGATTGACATGAATTGGGGCCCCA<br>GAAGAAGGCAGAAGCCAGGTACCCAGGGTCTTTTAAACCACGGCA<br>GTGAGTTTGAATGTTATTTCAAGTGTGCTGGTGCACTGTTGGCACG<br>GGGGAGAGATGTGCTCAAATCCCCACTCTGAAAGATTTCTTAAGCT<br>ATTTCTAGAGTATGATTTACAACAGGAAATGGATGATTTGATTCTGA<br>TCTTTATACCTTCATGCATTTAAAAAAGTACTTAAGAAAGTAGTTTG<br>GTTTGTCATTATAAAAAGCAATACTTATTTTTATATTGTGTAGATTCA<br>ATCTTGTTTCCTTGCCTAGAGTGGGCCGTGCTTTGGAGTTCTTATGA<br>GCATGGCATTCCTGAGAACTTCTCTAACTGCAGCCTCGGGCATAGA<br>GGCTGGGCAGCAAGTGGCAGCAGCAGAGGACTCCTAGAAGCCTTC<br>TACTTGACTCTACTTGGCCTAAAGTCAAACTCCCTCCACCAAAGAC<br>AGAGTTTATTTCCACATAGGATGGAGTTAAAAAATATATTCTGAGAG<br>AGGAAGGGCTTGTGGCCCAAGAGAACACCCCAGAAATACCACCCC<br>TTCATGGGAAGTGACTCTATCTTCAAACATATAACCCAGCCTGGACA<br>TCCCCGAAAGACACATAACTTTCCATTTCATGCCCTTGAAAGTGAA<br>TCTTTTGGCCTAATAATGAGAACAAACTCATTTTGAAAGTGGAAAA<br>ATTGAGATTCAGAGCAGAAGTTTGACTAAGGTCACAAAACAGTAG<br>GATGCCTCACTCAGCTCCCTGTGCCTAGGTCAGAAAAGCATCACAG<br>GAATAGTTGAGCTACCAGAATCCTCTGGCCAGGCAGGAGCTGTGTG<br>TCCCTGGGAAATGGGGCCCTAAAGGGTTTGCTGCTTAAGATGCCTG<br>TGGTGAGTCAGGAAGGGGTTAGAGGAAGTTGACCAACTAGAGTGG<br>TGAAACCTGTCCATCACCTTCAACCTGGAGGGAGGCCAGGCTGCA<br>GAATGATATAAAGAGTGCCCTGACTCCTGCTCAGCTCAGCACTCCA<br>CCAAAGCCTCTGCCTCAGCCTTACTGTGAGTCTGGTAAGTGTCGGA<br>TGGTAGAACCAGGGTTGGGACTCGGGACCTCCAACAGCATACGAT<br>GTGGTGGGGGTGGGCAGCCTGGGTGGGGGTGGGCATTACTCTGGG<br>GCTGGATTCAGCTGGACTTTCATTCTAGGGGGACTCGAGTCAGAGT<br>ACTGAGAGAAAAGTGCCTTGGCACAGAAGTGCAGAACAGAGAGT<br>AATCATCCTATGTCCCATCTTTTCTTGTGACCATATTTTTGGATTTGT<br>GTGTGAGAGAGAATTATGGAAGGGAGGAGGGGAATAGCATTCAAC<br>TTCTTTCCTAAACCTCTTGGGTTTTGACAGACCATCATTTTGCCTTC

	TTTATGGAGGGAGAGGTTCAGGGAAGAGCTTCTACCTTTTGGCTAT<br>GCTGCACAGAGGGATGGCAGAATGGGGAAACCTTTCTATTTGGAG<br>AAACCTAGGCAGAGCTGGGACAGGAAAACTCAACTTAGAAGTATA<br>AGACTTGGAAGAACAACCTCCAACTCTCAGCAACCTTCCAGCTCC<br>CGCAGCCCCACCCCAGACACAAGGACTGCAGCTAAACCTCAGAAG<br>GTCAGGAGAGAAAGCAGCCCTGGGGTTGAATAGGCCAACCTGCTG<br>GCTTTACAGGGGGGAAAACCAAATCCCAGGAGACTAAGTGACATG<br>CCCAGAAACACACAGCATTCCAATGGGAGATTCAGGCCTAGAGCA<br>TGTCCTGTGGCTCCAGTCTGGAGGTCACACCATGACCTCTTAGGTC<br>CTCTCTGGCACGGCCTATTGGTTTTCTAGGACTTGGTGTTCTCCAAG<br>AGACATTTCATTCCCTAAGGCCTTACTCCTCACTGTGACATAATCCC<br>AGAACGCATCTCTGCTCCTTGGTCAGTGAAGCGATGAGGGTGGAC<br>ACAAGGACTAGACAAGAGCAGACAGTGAGCTGGCACCTGACCCA<br>CCCTTGCAGAACAGCCCTGCAGACAGATCTCCTTGTTGGCTCTCAC<br>CTGGGAACAAGGAGGCTCCTAGGAGGACCTTTCTCTGCCCCTCCA<br>CATTTCCACCCTTCTCTCTCTGCTGCTTTTGGGAAATGATAGTCCAG<br>AGGTGGTAGAACAGTACCCTGCCCAAGGGAAGAGGGGATGCTAAA<br>AAACCAGATACTTCTGCAGATTCCCAAGGTTTCATCTATTTCCTTTG<br>CCTTCAGCCTGTGCATCAGACCTCTTCTGTCTTTCAGGTTGACAGT<br>AGCTTCTAAGATGTCCCAGCAACACACACTGCCAG
JAG1	CTGGATCTACTCAACTTATGCTTGAATGAGCAAACTTGTGGCCTGG<br>TTGTGGTTGCTACAACTATACCCTGATTTTCAGTGTCTGTTATCTATA<br>GGTTGGTGGTGGGTTTTTTTCCATGTAGAATATCTTGTGATGTGGGA<br>GAATGATGTTGTGCTTCCTTAGGGAGCTGGCTGGACAGGTTCATGG<br>GAGGCAGTAACCTGGCGGGACATGTGAGCTTCACTGGGCTCTGCT<br>GGAAAGGGGCAGAAAGATAACATATCCGTGGAACAGGATTTGTGA<br>ACTTGTGTTAACATCTCTGCAAGTTCAAACAAGGAATAAGGAAGAT<br>CTTAATGGATATTTTACATGTGCCTTCTCTACAATTTGAACCTAACTG<br>GAATGAAGAATGGGAAGAAAGAACTACTTTCCTTTCGTTCACTCTT<br>TTTTTTTTTTTTTTTTTTTTTTTTTGAGATGGAGTTTCGCTCTTGTTG<br>CCCAGGCTGGAGTGCAATGGTGCCATCTCATCTCGGCTCACCGCAA<br>CCTCTGCCTCCCAGGTTCAAGCAATTCTCCTGCCTCAGCCTCCTGA<br>GTAGCTGGGATTACAGGCATGCACCACCACGCCTGGCTAATTTTGTA<br>TTTTTAGTAGAGACGGGGTTTCTCCATGTTGAGGCTGTTCTCGAAC<br>TCCTGACCTCAGGTAATCCGCCCGCCTTGGCCTCCCAAAGTGCTGG<br>GATTAGAGGCGTCAGCCACCGCTCCCAGCCTCATTCACTCTTAAAA<br>TCTACAGTATCACCATTCCTTTGAAACACACTCTCCACTCAGTAGTG<br>CTGAAGACCAGTATTGTGGTGTTGCCCACCATATTCTTGGCACCCA<br>CAAAATACCTGGCAAATCAATAACTATCTTCCCCAAGATTTAAGAA<br>GGAAAATTTTCAAACATAGAAAAATGTCGAAGAATTTTGCAGTGAA<br>CACCACTGTAACACCTAAATTCTACCATTAAGACTTTACTGGCTGGG<br>CGCTGTGGCTTATGCCTGTAATCCCAGCACTTTGGGTGGCCAAGGC<br>AGGAGGATCATCTGAGGTCAGGAGTTTTAAAGCAGCCATGGTCAA<br>CATGGTGAAACCCCATCTCTACTAAAAAATACAAAAATTAGCCGTG<br>TATGATGGCATGCATCTAATCCCAGCTGCTCGGGAGGCTGAAACAG<br>GAGAATCACCTGAACCTGGGAGGCGGAGATTGCAGTGAGCCAAGA

	CTGTGCCACTGCGCTCCAGCCTGGACAACAGGCTCTGTCTCAAAA<br>AAACAAAAAAAGACTTTACTATTTGCTTCAGCGTGTAACCACCCA<br>CCCAGCCATCCTCTCTGATCCCTCTAATTTTGTATGCATTTCAGTGTC<br>AGTTGCAGATATCATTCCACTTCCCCGCTAAATGCTTCAGCACTCAT<br>ATAACTGGAGGTGAATATCTGTTCACAATTCTTTTTTCCTCTTTTGA<br>ACACCTAATGAAATACAATCTTAAATGTACTATTTAATAAATTTTGAC<br>AAATGCATACACCTGTGTAGCCCAAACACCTATCAAAAATATGAAA<br>CATTACCCCACAATGCCACCAGAAGAGCCTCAAGCCCATCCCAGTG<br>AAGCTCTGCTCCCACCCACCCACCCTCAAAATCACTCTTGTGATCT<br>TTTTTCACCAGTTTTTCCTGTTCTGGAACTCCGTATAAATAAATTCAT<br>ACAGGATGTAGTCTTTTGTGTCTGGCTTATTTCACTTAACATAATATT<br>TTTTAGATCATCGATGTTACTGCTTATAATAGCAGTTCTCAATAAATA<br>TTGATTAGCTTATTTTAACCACAGTGTTGATTAACCAAGGATTACAG<br>TTGATTAGATGTACTTAAATACAGCTAATATAGCTTGAATGTCTCTTA<br>TAAAGCTTTTGCAGGTTCAGAACTGCTATATGTTGTTGACTAGATAA<br>AAGTGGGGCAAGTTTTCTGGGAAAAGAAAAAATGAGGCATCCATC<br>ATCTTTTTAGAATTAAAGGAAAAGATATCCAAGGATGATGTTTGAGC<br>ACACGAGACAGGTCAGATAGTAACTGACTACAAGCCTCTCCCCTTC<br>TCTCAGGTGTAGGTATGACAGCTAAAGATGGCATTTTCCACAGGAA<br>GGTTGTTCTGGAATATTCATTTACACTTGGAGGCTATACCCCAAAGC<br>AGGACAAGCCCTGCCAAGGGGGAATCTCTGTATTTCAGTTCTCTCG<br>GCAGCAGTTCCTCATTCTCTAAGGAGGGGGCCTGACAACCGGCTTC<br>AAGGAAGCAGAGGATAGAGCTTCCACTCAGTGTCTGTTGTGGTAA<br>ACGTGACTGGCTTTTTGGGGGAGTCCCAATTATTTGGAAGAGCCCC<br>AACTGAAAATTTTCAAAAGAATTCCCTCTAGCCCACAACTGCCTTC<br>TCTAAAAGTGGGTTCCCAGGCTCAGGAAGGGTGTGGGACTCTGTG<br>CTATGTGACATAAAAATTTTGTGTTTATCAGTGATTTGACATTCTTGT<br>TAGGTGTAATCAGGTAGTTTTCACCTCTCAGAGTTTATATTTTCTTTC<br>CTATCAGTCAAATGGGCTAAATTCACTGGTGGACTGGAGGTGACAC<br>CAGCTTGCATGTGGCCCAGATTGCAACTGGTGGCACCTGCAATCTC<br>AGGTAGCCCTTCTAGGTGAAGCCAGGTGGAGATCTAAACACTTTAA<br>CTAAAGGTCTGGCTCCGCAGCCTCTTTCCTGAGAGCTGTGGGGGG<br>CACAAGGCACAGAATTAAAACAGGGTCCGACATAGGGAACTCCAA<br>GGAGTGTGCTCTTTAAGATCACACCAGCCTTTTTCAGCACCATTTT<br>GCCTTTTTTTTTTTTTTTTTTTTTTTTGAGATGGAGTCTCGCTCTGTC<br>GCCAGGCTGGAGTGCAGTGGCATGATCTCAGCTCACTGCAACCTCC<br>GCCTCCCAGGTTGAAGCTATTCTCCTCTCTCAGCCTCCCGAGTAGC<br>TGGGACTACAGGTATGTGCCACCACGCCCAGCTAATTTTTGTATTTG<br>TAGTAGAGACCGGGTTTCACCATGTTGACCAGGATGGTCTCAATCT<br>CTTGACCTCGTGATCCACCCGCCTCGGCCTCCCAGAGTGCTGGGAT<br>TGCAGGCGTGACCCACAGCTCGCGGCCCATTTTACTTTTAAACCGT<br>TATTTAGCACCCAAACTCAGGGATACTTGGGAGTAGAAATTAGAAC<br>GGGACAGATAAGTGGGGAGACTGGTGTTGATGGAAACCTCAGTGA<br>GAAAAGTGTAGATGGGATTACAGTTGACAACTGGTTGCCTTGATTT<br>AGGATAATTGGGGAAAGGGAGGCTGGGGCACATTGAAACACCCAG<br>GTTGTCGCTGCCTACCCCAGCTGCTAACATGACCAGCTGTAAACTT

	GCTCTGTTCTTAGAAACCTCCCTTCCAAAACATGGATTCTCCCGCC AGTGCTCAAGATGGGACACATTACCACTAAGTAAGCACTTTTCTTA GGAGCATTTCTTAGGCTGTTAAAATGCCAAGACTCCTAACCATCCTT GGTAGCTTTGAACCTATGTAAAGAGGTCAGTTAGCTGCCCTAGTTTA TTCCTCAGAATGATGAGATTTGGCACTGAAAATATTGTTCATACTGA GCCTCCCATCAAATGCAATAAGTAGATAACCACAAATACCCACCCTT TTATGCTCATAACTCAGCATGTTGGAAACTATTTTCCCTTGACCTATT GCCAGCCCATCACGCCCCACACCCAGGAGAACTCAAGCCCCAAAC CGGCCGCTGAATAGTCACGCTTTTCTGCAGGACATACCTACTATTAG GGCCAAAACTTTGTCCACCCTTCAAAGGAAGTCGATGTTTCCATAT AAAGGTCCCCTCAAATGCAACAGCAAGCCCGTGGGAGAGGGGTTA GCAGAGGCTACGGCCAGCCCACGGGCTTTCTTCGACCTTGATTATG ACCAGGAGTGTAGCTGTTAATTGCGAGGCTTGCCTCAAGGTGGAA AACAGTATCGGTTTCCACTGCCACCCCAGAGAAGGCAAGTTCCGC GGCTGGCGGTGCTGGGGACACGGTCCCTCCCAGGCCCATCTCTTGC CACCCAGAGAGCTGCTCGGAGGCCGCCTACAGGTGCAATCCCGGC ACTGCGGCCGGGGCGTCGGGCCGGGGAGGGCGTCCAAGCCCACC AGCATCTCCGCCGGCCCTTCCCAAAGCCTGAACAGGGCCCCGGCG TGCCCGCCGCCTTCTACCCCCGGTTTCCCCGCGCCTCTGCCCCGGC GCGGTTTGGATAGGAAGCTGGGAGCCCTCCCAGGCTCCGCAGACT CGGATTTGGGAGGGGGTGGGACGCGGCCGAGGCTTCCCCTCGAAT CTGCGGCAAGCCTGGCTCCAGGAAAGTTTTTCAAAGTTCCCAGCA GCGTCTGCCCAGGTCGCCTCCGCGGGGCGAGCAGACGGCGGCAAG CGCGCCAGCCTCGCCGCCGCCTCTGCCGCCAGCAGAGCGCTCTGG GCGGCTCGCTCGCGGGAAGCGGGCCGAACTCCCGGCGGGCAGGC AGGCCCTCCTCCCGGGGCGAAAGCCGCAGCTGACGCAGGCGGTTC GGAAGGCGGAAGCTGCCCCGCTCCGACCGCTCAGTCAGCGCCGCG GCGCCTACACCTGGGGCCCCGACGCGCGGGCAAAGGCGCACGGCC CGGGGCGCCCGAGGGGGCGGTCCCCGCTGGGGGCCTCCAGGCGTC CCTGAGCAACGATCCCTTCCAAGTACCTCCCCGCACTCTCCCTTCC CTCCTGGCCCGAAGCTCCCGAGGGCGGGGGTTGGTGTGGGGCCCT GGTTCTTCTACGCCGCCCTGAGCATCCCGCTGCCCCCAACCCCTTC CAAGTTCCTCCTCGCACTACCCCCTCCCCAGCAACGTGAAGGGGA GGGGCGTGCCCAGGGTGAGCACGCCCTCTCATGAATATTAATAAGC GCGCATGCGCCCTGCCCGGCGTGCTGGGTAGAGGTGGCCAGCCCC GGCCGCTGCTGCCAGACGGGCTCTCCGGGTCCTTCTCCGAGAGCC GGGCGGGCACGCGTCATTGTGTTACCTGCGGCCGGCCCGCGAGCT AGGCT
JUP	TCGAGACCATCCTGGCTAACACTGTGAAACCCCGTCTCTACTGAGA AAAAAAAAAACAAAAAATTAGCCAGGCATGGTGGCACGTGCCTGT AGTCCCGGCTACTCAGGAGGCTGAGGCAGAAGAATCACTTGAACC CGGGAGGCGGAGGTTGCAGTGAGCTGAGATCGCACCACTGCACTC CAGCCTGGGTGACAGAGTGAGACTCCAAAAACAAAAAACAAAAA ACAAAAAACAGGCCAGGACCTGGAGAGAAACTGGGTGTAAAGAT GGCTCTGGCCAGGTGTGGTGGCTCACACCTGTAATCCCCACACTTT GGGAGGCTGAGGTGGGAGGATCACTTGAAGCCAGGAGCTCCAGA

	CCAACCTGGGCAACATGGTGAGACCTCATCTCTACCAAAAAAAAG AAAAAAAATTAGCTGGGTGTGGTAGCGGCTGTGGTCAGCTACTCA GGAGGCTGAGGCTAGGGGATCCCTTGAGCCCAGGAGGTTGAGGCT GCAGTGAGCTGTGATTTCGCCACTGCACTCCAGTTCTGGCAGCAGA GTGAGACCCTGTCTCTGAGAAAAAAAAAAAAAAAAAAAAAAAGGC TGGCTTTGTCCCTGGGCTGAGAGATGGCTCATACCCAAGGTGCCTC TAGCTTTCTGCATTGCCTTTCTTTCAAGTCTGCCATCCGGGAAGAAC CTCCCGTTGTGTGATTTCCTTTTCTGCTGCTGCAAAGCGCGGGGAA GAGGCTACTGGGTGATGAAAATGTACCATCATCAGCCTGAAGTTTG GCAACCCAGCTGGTCACCCTGGGCTCCCCTTTATAGAAGTGGTTAT GTCTTAGGCTACCTGAGGCTGAAGAGGCAGGAAGAAGGGTGTGTG TGTGTGTGTGTGTGTGTGTGTGTGTGTGTGCGCGCGCGTGTATTAG AGAAAGCTGGTGTTTGTGTATCTGAATCTGCACCCGCAAGTGTATAT CCCTGAGGTGTGAGCATAGAAGGTTGTGTGTGTGTGTGTGTGTGTG GCTTGTGTACGTCTAAATTTCTACCTGTCTCTGGGGAAAAGGCCCC CATAGAATAAGGACTGTGGGTTTTTTTCTTTTTTTTTACTTTTGAGC TGGAGTTTCGCACTTGTTGCCCAGGCTGGAGTGCAATGGCACGATT TCTGCTCACTGCAACCTCTGCCTCCCGGGTTCAAGCGATTCTCCTG CCTCAGCCTCCTGAGTAGCTGGGATTACAGGTGTGTGCCACCACGC CCAGCTAATTTTTTGTATTTTTAGTAGAGATGGGGTTTCTCTGTGTTG GTCAGGCTGGTCTCGAACTTCCGACCTTGGATGATCCACCCGCCTC GGCCTCCCAAAGTGCTGGGATTACAGGCATGAGCCACTGTGCCCG GCCCTAAGGACTGTGTTTGAATGGAGAACAGAAACTCTTCCATCTC CTTGTATGGCCCAGAGGCCTCGTGGAAGATGAGGGGGGTTGTGAG ATAAGGAGAGGACGGTGGCCTGGTGGTCCTGGACACTGGCTGATG GTGTGCACCCTCCCCTAGAAAAGAGCCTCCCTCCCACCAGTCCCTC ACTGAACCTCTCTGCGCCAGTTACTTCTGTCTCTGCAGCAGCTTGA GATGGGCCATGTGGTACCCTTATATTCTCTACTCTGAAGCACTAGCA AGCTGCAAGGTTGAGTTACCCATATGTGTTTTTGGCAGCCCATGGA CTCTGCTTCCACTGAGCCCTGGGCAAGAGGAAGGAATTGAAACTG CAGGAATAGGAATTGAGGCTGGACATTAGAACAAGGAGACAGGGG CATTCACCATAAGGGAGGCTGCAGGATCTTCTTTCCTGGACATTTGT CAGAGAATTCTTGCCTTTAAAGGTTTGCAGGCAGGGGACTGGATAA GATGACCTTGCGGCCTTTTCTTCAAGTTCAAGCTACAGGGGAGACT GTGGTCACAGAAACCCCCATCCTATCCCTACATACCTCCTTTCACAA GAGACCAAATTGCCTGTGCAGACAGCTGAGTCTAGGAAACATCATT TCTGATTCTATTCTGTATTCTCAGATACAGATGAATCTCAGTGCCTGC CAGGCACATAGTAGGTGCTTAAAGAGAATCCCTTGATCTGGGAGGA GAGACTCAGCTCTAATCCCAGTCTTTGCCGCTGCTGTGGTGGTGCT TCCACCAGTCCCAGCCTGTGTGTGTTTAGCTCTGGGGCTGGTGTTATG CAACATGTTCCATGCTAAGACACGCAGGGGGCATTCTGGGGAGAC ACACACACACACACACACACACACACACACACACACGGTTTATTCTG AAACCTTATTTCTGCTCCTCCACCCACATGGGCTCCTCCACCCCTCT CTGCAGTCAACCAGCCCTACCCTGCCTCTTTGCTGAGGTCAGGAGA AATAGGAAGGAGTCAGGCCAGGCTTGGAGCTGGGACGTATGTGTG TGTGCATGTGTGGAGGTGGGAGGTGGGCAGAAGGGGGAAGAAAT

	GAAACCCTGGCAGCTGCAGCAGTCCAGGATTGTGAGCCCATTCCTT CCCCAGCTGTGGCCTGGCTACAGCTCTATGTGACAGGGTGACAAGC AGGTGGATGGGGCAGGGGTAAGGCAGGGATAAAGATAGGGAGGTA AGCTGGAGGATGGAGAGTTGAGGAAACAGGCAAAGGGCGGTGGC CCTGGTTATGGGATGGCCACTAGTTCAGGGATGTCCGTATCTCCCTG GCCCTGCTTTTGGATGGCGCAACTTCAGAAGGGAGAATGTTCAGCT ACCATTCTTTTCTCTGGTGGGGAAAGTGGTCAAGGTTGCTGCTAAG GTACCTTCATCCAGCCCATCCTGTGTGCTAGCTTTGGGGCTGTAGA GCTTTGTTGATGGGGGACCCTTTTCTAAACCACCACTCCTCCAAAA CCCACCCACACACGCTGCTTCTGCTTATCCTTCTATGAGCTCTTTGA AGGTTGGGATCATTTTTATTTCCTGTGTCACCAGGTTTTATTTTCTGT GTTCACCAGTGCCTAACATACAGTTGACAATGAATACTCAAGTGTT GAATGAAGGAATGTTTGTAGGGCCCTTGGCACACCATACAGCCACG AGCAGCATTCATGTCATCAACCACAGAAAGGAAGTGCCCTTGTCTT CTTAAGCTGCCTGTGAGAAGTCCTTTTTATTTTTTTGAGACAGAGTC TCACTCTGTCACCCAGGCTGGAGTGCAATGGCATGATCTCAGCTCA CTGCAACCTCTGCCTCCCGGGTTCACGCCATTCTCCTGCCTCAGCC TCCCGAGTAGCTGGCATTACAGGTGCCCGCTGCCATGCCTGGCTAA TTTCTGTATTTTCAGTAGAGATGGGGTTTCACCATGTCGGCCAGGCT GGTCTCGAACTCCTGACCTCAAGTGATCTGCCCACCTTGGCCTCCC AAATTGCTGGGATTACAGGCATGAGCCACCACGCCTGGCCGAGAA GTTCTCTCTGATCTCTACCCCAGGCTCTCCTATCGCAGAGGGGTGTG CTGCCCGGTTTATTTTTTTCTTTTCTTTTTTTTTTTTTTGTTTGAGATG GAGTCTCGCTCTGTCATGCCCAGGCTGGAGTGCAGTGACATGGTCT CGGCTCACTGCAACCTCCGCCTCCCAGGTTCAAGCAATTCTCCTGC CTCAGTCTCCCAGGCAGCTGGGATTATAGTTGCCCGCCACCACGCT CGGCTAACTTTTTGTATTTTTTGTAGAGATGGGGCTTCACCATATTG GCCAGGCTGGTGTTGAACTCCCGACCTCAGGTGATCCACCCGCCTT TGCCTCCCAAAATGCTGAGATTACAGGAGTGAGCCACTGCGCCTG GCCTGCCCCGTTTCTTTCATGCCAGGGCTGGATTTCCTTCAGATCTG CTGCTTGATGTTTCCAAGCCCACCAAAGCCTCCTGACACCAATACT GTATTTTTGTGCCAATTAATTGTTCTGTCTCTCCTCCAAGTGTTTGTG CCTTTTCTCTGCCCCTTCCCCATTGCCTTGACATTTTATGTCTATGCT CCTCTTCCATGAGGGGAACAGCTGGTTTGATGGTCCTTCAAATGAG AGGAAGGAGGTTCTAGCTTGGGGTGTGCCACTTTGTAGTTTTGTGA CTTCTCTGAACTTCGGTTTCTTCATCTTTACAATGAGGGTCGTTTTC TCATCCTAACTATTTTTCAGAGCTGTCACAATAATTAAAAAAAAACA TACCGTTAATTTAAATTTCTAACCTTTAGCAGTTTCTGAGTGCTTTC ACAAACCTGACCTCATTTAATCCCCACATACTCCAGTGAGGTAGATA TGGCTAATGTATCCAGTTTACAGAAGAGGAGAAACAGGGTGAAGT AACTTGCCTAAAATCATACCAAGTGGGAGAAGACCAAACCTGGCC TTCTAATTCTAAATCCCAGGAGTACTCCATCTTTCGGGAAAAAAATC CCACCCCAGCACAAAGCCTATGAAATGCTATACAAATATGTGATTAC TACTTTGGGGCAAGGATGAATAAGCCCAAAGAAGCATCATGCCTGA CGCTGAGTTGGCTGGCCGGTGAAGGCGTCTGTCCCTTCCCTTGGTA TCCCTATGACTTACCTGTTGGACAGGTAGGGGGAAGGGGAGAGTAA

	TGAGTCTCACCTGCTCAGAGCAAGGGTGGGGCAAGACACACCCCA TCCCTTCCATTGGTTTTTCCTTAGTCTTACTGACAGAGCCTTGTCC AATCAGGAGGAAGTAACTTTCTATCTGCCAATAGATGCAATGTTAG GATGAGACCTCAAGTTAGAGTCCATCCCTAGAGCCGACTGGCAGTC CCCGGGGCCAATGGCAAGCGGATAAACAGAGGCGGCCGTGGAAG AGGACTGGAGGCGAGGCTCCGCCCCTCCACGGGACAGTCAGGCG AGATAGCCAGTGAGCTCGCACCAGAGGGTGGGCGTCTCCCCCAGG GGCGGAGCTTCGAGGTGGCGAGGGGCGTGGCTTGGCTGTCAGGTC TCTTCGCCTTTTGTTCGGTTACTGAGTTGCTGCCTTGGCCAGAGTCC GGAGCAGCCGCCGC
KIR2DL3	GGGGGACTCTGTCTCAAAAAATAAAAATAAAACATACATAATTATG ACACACAGAAATTACAAAGGCAACTGGATACCAACCATCATTTTTC TATTTCTCTGTGTTTAATTCTTTGACCCTTTATCTTATCCATTAAACAA TCAGGTTAAACCTCTTCCTTATTTGGCTTTCTGTGAGCTTGGGATCA TATGGAAAATGTGAAAGCCTCCTGAACCCACCAGCACAGGTCCTG GAATAGAGAACGTGCTCTGTTCATGGCATAAAACTTGCCCCTTCAC CCAAATCCCCCAATTCATCTCTACTTCCAATCACCTATGGAGATACA GATAGATCATGGGGAGGTAAACACTAATACTCTTTGGAGTGAGCTC AGATCTTGGACTCAGAGACCAGTGCCAGCACTAGCCCCTGGTCAC ATTTCGTACTAACTCACAGAAGGACAGGCTGTATTGAAACAATAAA CGACGGAGAGGGCGGTCCTTCCCCGTGCTTCTCGGGTGGAATAGC AGCCTAATATATGTCTCAGCAGATCACAAAAAGTAGCATGTTGTTCC TGGGCTACATCATTATTTCATGGCTGTTTGATTTAAGTCAGTTCTACT TCACTTTTTTTATCTTGATTTCATTTTTTCTTTCTTTTCTTGGAGAATG TAATTTTTTTTGAGTCAAGAGGGTTGTGGTGGTAGAAACTGTAAAG CACATTCGCTGTGTATCAATCCCAATCCAGTCTTCCCAGAGAAGATT CTAAACACCTCCTGGAATGCACCTGGGCCTATACCAATTCCTATCAC TCACCGTCACTCCAGGGAGACAGAACACACAGAGAACACATTACA CAGGCAGGTTCATTACTAACAGATAAGCAGCGAGTGACAACAGAA ACCTACATTTCAATGTGAGCCAGTCCCTCAAGGCTCAGAAAAGCTG CTCGAGACATGTGGAGTCACCCCATATGCAGTGTATCTGGGGGAAA TCAAAAAGCAGCCCAGCCTGGGTTTTGTACCCTGGAGCCACAGGA AGCACTCAGCTAAAGCACTGCATGACGTCCTCCTCCAGGAAGAAC AGGAAGACAGCCCAGGCTGTTCTGGGATGTTCCTCCTGATCTCAGG ACGTTGCTGTCTTAGTCCATTTTTGTTGCTCTAAAGGAACACTTGAG CCTGGGTAACTTCTAAAGAAAAGAAATGTGTTTGCCTCACAGTTCT GCAGGCTGTACTGGAAGCATGGCACCAGCATCTATTTCTTGTGACG GCCTCAGGCTGCTCCCACTCTGGCAGAAGGGAAGGAGGGTCTGTC TGTGCAGAGACCACAGAGATCACACGGCAAGAGAGGGACCAAGG GGGAGGGGGAGCGATGGAGCTTCCAAGCTCTTTTAACAACCAGTT CTCCAGGAACTAATAGAGGGGGAACTTGCTAACCCCGTCTCCTTGG AACAGCATTGATCTGTTCATGATGGATCCACCTCCATGACCCAAAC AACTCCCAAGAGGCCCAACCTCCCACCCTGGGGGTTACATTTCAAT GTGAGGTTTGAAGGGGTCAAACATCTAAACTAAAGCAGTTGTATCC TCAGCACGTTCTATGGTTACTACAACTGAGAAAGCAGGAGGAAGCT AGGTCTCCCGCCATCTGGGTGCTTGTCCTAAAGAGACGTTGTATGT

	GGTTACCTGTCAATCAAGAAATGTGAGACAATTCATATAGAGGAAC<br>TGCTATGATTAGCTTCTTATTGGTGTCTTGTCTTCCTCCAGGTAACTC<br>CAGACACCTGCACGTTCTGATTGGGACCTCAGTGGTCATCATCCCC<br>TTTGCTATCCTCCTCTTCTTTCTCCTTCATCGCTGGTGTGCCAACAA<br>AAAGAGTAAGTCTCACGAAGCAGAAGCCAGAGAGCTCAGGGCCA<br>TGTGGGGAAGCAGGATGGGAGCACTCAGGTGTGTGTTCCTCACAG<br>GCAGGATGGTCCCTGGCCCAAGGCAGGAGCCACAGAGGCAGGAC<br>TTTCTAGAGAGAGCACCAGACTCCCTGCCTCTGCCTTCAGCTCACA<br>GACCATTGCCTGATTCTGAACCGTATCCTCACATCCCCTGCAGCCAC<br>TCACATCCAGGAGAAGGTTCCATGACAGGCAGAAAGTGGGACACA<br>GAATCAATAGGATGGGAACTCAGAGCTATACATGGGATGGATCCTT<br>GAGCTCAGAGAGATAGAATGTCTGAGTCTGCTGTTGGCAACTGAG<br>GGACCTCAGGCACCTATGGCCTCCCCCTGTATGTTGGTATCTGCTTA<br>TGAAATGAGGACCCAGAAGTGCCCTCCGAGCTGTTTTGACGACTT<br>CCGTCTTCTACAGATGCTGTTGTAATGGACCAAGAGCCTGCAGGGA<br>ACAGAACAGTGAACAGGGAGGTAGGTGCTCCTCCGCCCAGCCTCG<br>TGGCTAGTCTTATTCCCAAAGAGTCCTGGAAAATGTGAGCACCCTC<br>CCTCACTCAGCATTTCCCTCCCTCCAGGACTCTGATGAACAAGACC<br>CTCAGGAGGTGACATACGCACAGTTGAATCACTGCGTTTTCACACA<br>GAGAAAAATCACTCGCCCTTCTCAGAGGCCCAAGACACCCCCAAC<br>AGATACCAGCGTGTAACACGGAACTTCCAAATGCTGAGCGCAGATC<br>CAAAGTTGTCTTCTGTCCACTAGCACCACAGTCAGGCCTTGATGGG<br>ATCTTCTAGGGAGACAATAGCCCTGTCTCAAAACCGGGTTGCCAGC<br>TCCCATGTACCAGCAGCTGGACTCTGAAGGCGTGAGTCTGCATCTT<br>AGGGCATCGCTCTTCCTCACACCACGAATCTGAACATGCCTCTCTC<br>TTGCTTACAAATGTCTAAGGTCCCCACTGCCTGCTGGAGAGAAAAC<br>ACACTTGCTTAGCCCACAATTCTCCATTTCACTTGACCCCTGCCCAC<br>CTCTCCAACCTAACTGGCTTACTTCCTAGTCTACTTGAGGCTGCGAT<br>CACACTGAGGAACTCACAATTCCAAACATATAAGAGGCTCCCTCTT<br>AACACGGCACTTAGATACGTGCTATTCCACCTTTCCTCAGAGTATCT<br>TTCAGCCTTCTGTCAGCAGTAAAACTTATAAATTTTTTTTATAATTTC<br>AATGTAGTTTTCTCTTCTTCAAGTAAACATGTCTGCCCTCATGGTTT<br>CGTCAATGGGACTCTTTTCTTGCCTAAGGCTTCCGGTGTTATCATTA<br>CCACGTCCACATAACCCCATCTGTTCTCCGCTGGGTTCTCACCCCTG<br>GACTCTGAGCTTCTGGAAGCAGGGTGGAGCCTGAATTGTCTCTGA<br>GACTCCAATTTCCATCCAAAGATGCAGCACATAGGAGGTTCCAAGG<br>ATGGTGAATCAGATGAACAAGTGATATTCTTACTCTCTGCAGATCTG<br>GAAAGCTGGCAGAGTCATTCCACGATGAAACATTTGTAGAGTCATA<br>GGCCTTGTTAGTCTCATCTCCACAGGGACACGTATCAACACATCATC<br>TTTCATACTACTATAAATAGACAGTCACTCCTCCATATCTCTGGGGTT<br>TACACATGTTTATTGAATCAGCAATAAATCAAAAATATTTTGAGAAA<br>AAAAATCCCCGAAGTTTCAAAAAGCAAAAAACTATGTTGAATCGA<br>CACAAATTGAGTGGCGTGTAGGCTGTGTCAGGAATTATAAGTAATC<br>AAGAGATGATTTCATGTATACAGGAGGATGTGCATGGGTTCTATGCA<br>ATTGCTATGCTATTTTTTTTTTTTTTGAGACAGTCTCACTCTCTCACC<br>CAGGCTGGAGTGCAGTGGCGTGATCTCAACTCACTGCAACCTCCG

	CCTTCCAGGTTCAAGCGATTCTCTTCCCTCAGCCTCCCCAGTAGCC<br>TCCCCTAGGATTACAGGCACGTGCCACCCTGCACAGATAAATTTTTT<br>TGTGTGTATATTTTTAGTAGAGATGGGGTTTCAGAATGTTGGACCAG<br>CTGGTCTTGAACTCCTGACCTTGTGATCTACCCAGCTCAGCCTCCC<br>AAAGTGCTGGGATTACAGGCGTGAGCCACGGTGCCCAGCTTCACTA<br>TGCCATTTCATGCAAGGGGCTTGAGCATCTGCAGATTTTGGTATCTG<br>AATGGGGATCCTGGAACCAATCACCCAGGTATAGTGAAGGACCATG<br>GTATATAATTTTTATTTGTCAATCTTAAAAATAAAGCATAAAAAATTT<br>ACAACAACAAGATAAAAAATAAGAAGTGTTTTATAGTGTGAGGAT<br>AAGTTTAGATTTATTTTTTCCTACGTGTAACCCTATGGTCCTGTGTTA<br>TTTGTTGAGAAAATATTCTATTCCACCTTAAACTACATGGCAGCCTT<br>TGTCAACTATAAAGGGACTGTGTATCCACAGATGTATTTAGACACA<br>GTTTTCTGTCCAGTGGTTCTCTGTATCCCCTCTCATGAGGATGCTGC<br>ATTTTATATAAACTTATAGAACCCCTTAAAATTTGGTAACCTGAGTCC<br>TCTGATTTGTTATTATAGGTTATTTAGTTTGCTTTTTTTTTTTTTCTTG<br>AGACAGACTCTTCCTCTGTCACCCAAGCTGGAGTTCAGTGGCTTGA<br>GCTCAGCTCACTGCAACCTCCGTCTCCCAGGTTCAAGCTATTCTGA<br>TGCCTCTGGTTTAGTAGTAGAAACTCAAGCAGGAAAATTAGAATGG<br>CTTCTTGTCACAATTACTCTGATAATGTTAATAATACCTGTTAGACAT<br>TTTGCACATTACATATGAAGAAGAGTTTGAATCTCAGATAAAAACA<br>AAAATACATCAAAAATCTTTAATGTAAGCACAGAATTCAATCATCTC<br>GTGTATGAGAGGTTGGATCTGAGACGTCTTTTGAGTCTGGTCGTAG<br>TGAAGGACGCAAGGTGTCAATTCTAGTGAGAACAATTTCCAGGAA<br>GCCATGTTCCGCTCTTGAGCGAGCACCCACTGGGCCTCATGCAAGG<br>TAGAAAGAGCCTGCGTACGTCACCCTCCCATGATGTGGTCAACATG<br>TAAACTGCATGGGCAGGGCGCCAAATAACATCCTGTGCGCTGCTGA<br>GCTGAGCTGGGGCGCGGCCGCCTGTCTGC
KIR3DL1	TTCTCTATAATGACTTCTTTGATCCTTTATCCTATCCGTAAGAAAATC<br>AGGCGAAAACATCTTCCTTATTTGGCTTTCTGTGAGCATGAGATCAT<br>ATGGAAAATGTGAAACCCACCAGCACAGGTCCTGGAATAGAGAAC<br>GTGATCTGTTCATGGCACAAAACTTGCCCCTTCACCCAAATCCCCC<br>ACCTCACCCCTACTTCCAATCACATTAATGATACAGATAGATCATGG<br>GGAGGTAAAAACTAATATTCTTTGGAGTTCAGATCGTAGACTCAGA<br>GACCAGTGCCAGCACTATCTCCTGGTCACCTTTTGGAGTAATTCAC<br>AGAAAGACAGGCTGTATTGAAGCAACAGATGATGGAGGGGGTGGT<br>CTTTCCCCCAGACTCTCGGGTGGAACAGCAGCCTAATATCTGACTC<br>CCAAGATGACAAAAGTAGCATGTTGCCCACGAGCTTCATCATTATTT<br>CCTGGCTGTTTGATATAAGACAGCTCAACCTCACTTATGTTGATTTC<br>AATGTCACTGTTTTTTCCTTTTCTTGGAGAATGTAATTTGTTTGAGT<br>CAAGAGGGTTGTGGATGTAGAAACTGTAAAGCACATTCACTGTGTA<br>TCAATCCCAGTCCAGTCTTCCCAGAGAAGACTCTAAACACCTCCCA<br>TACTGCACCTGGGGCTGTGCCAATTTCTATCACTCACCATCACTCCA<br>GGGAGACAGAACACACAGGGAATACATTACATAGGCAGGTTCATTA<br>CTTATAGATAAGCAGCGAGTGACAACAGAAACCTTCCTTTCAGGGT<br>GAGCCAGTCCCTCAAGGCTCAGAAAAACTGCTCAGGACACATGGA<br>GTCACTTCATGTGCACTGTAGCTGGGGGAAGCCAGAAAGCAGCCC

	AGCCTGGGTTTTGTACCCTGGAGCCACAGGGAACACTCAGCTAAA<br>GCACTGCATGATGTTCTCCTCCAGGAAGAACAGGAAGACAGCCCA<br>GGCTGTTCTGAGACGTTCCTCCTGATCTCAGGATGTTGCTGTCTTAG<br>CCTATTTTTGTTGCTATAAAAGAACACTTGAGCCTGGGTATCTTCTA<br>AAGAAAAGAGATGTGTTTGGCTCACTGATCTGCACGCTGTACTAGA<br>AGCAGGACACTACCATCTATTTCTGGCTGCGGCCTCAGGCTGCTCC<br>CACACTGACAGAAGAGAAGGGGGTCCTGCGTGTGCAGAGACCAC<br>AGAGATCACATGGCAAGAGAGGGAGAAAGGGGGTGTGATGGAGC<br>TTCCAAGCTCTTTTTAAGAATCAACTCTCCAGGGTACTAATAGAGG<br>GAGAACTTGCTAACCCCGTCCTCTGGGGACAGCATTAATCTATTCAT<br>GATGGATCCACCCCCATGACCAAAACACCCCTCCCAATAGGCACAA<br>CTCCCACACTGGGGATTAAATTTCAAAGTGGGGTTTGGAGGGGTCA<br>AACATTGAAACAATAGCAGTTGTATCATCAGCACATTCTATTGTTATT<br>ATGAAAACTATAACGGAGAAAGCAGGAGAAAGCTGGGTCTCCCGC<br>CTCGTGGGTGCTTGTCTTAAAGAGGTGTTTATGTGGTTGCCTGGC<br>AACCAAGAAATGAGAGACAATCCACAAAGAGGAACTGCTATGGTT<br>AGCTTCTTATTGGATTCCCATCTTCCTCCAGGTATCGCCAGACACCT<br>GCATGCTGTGATTAGGTACTCAGTGGCCATCATCCTCTTTACCATCC<br>TTCCCTTCTTTCTCCTTCATCGCTGGTGCTCCAAAAAAAAAGTAAG<br>CCTCACGAAGCAGAGGCCAGAGAACTCAGGGCCCTGTGCGGAAG<br>CAGGATGGGAGCACGCAGGTGTGTGTTCCTCACTGGCAGGAAAGT<br>CTCTGGCCCAAGGCAGGAGCCAGAGGCAGAGCTTTCTAGAGAGAG<br>CACCAGACACCCTGCCCCTGCCTTCAGCTCACAGACCGTTGCCTGA<br>TTGTGAACTGTATCCTCACGTCCCCTGCAGCCACTCACATCCAGGA<br>GAAGATTCCATGACAGGCAGAAAGTGGGAGATAGAATCAATGGGA<br>TGGGAACTGACAGCTATTCATGGAATGGGGTCTTGCACTCAGAGAG<br>ATGGAATGTCTGAGTCTGGCTGTTGGCAGCTGAGGGACCTCAGGC<br>ACCTATGGCCTCCCCCTGTGTGTTGGTATCTGTTCATGAAATGAGGA<br>CCCAGAAGTGCCCTCCCAGCTGTTTTGATTGCTTCCGTCTCCTACA<br>GATGCTGCTGTAATGAACCAAGAGCCTGCGGACACAGAACAGTG<br>AACAGGGAGGTAGGTCCTCCTAGCCCAGCCTCATGGATACAGTCTT<br>ATTCCGAAATAGTCCTGAAAAATGTGAACACCCTCCCTCACTCAGG<br>ATTTCCCTCTCTCCAGGACTCTGATGAACAAGACCCTCAGGAGGTG<br>ACATACGCACAGTTGGATCACTGCATTTTCACACAGAGAAAAATCA<br>CTGGCCCTTCTCAGAGGAGCAAGAGACCCTCAACAGATACCAGCG<br>TGTGTATAGAACTTCCAAATGCTGAGCCCAGAGCGTTGTCTCCTGC<br>CCATGAGCACCACAGTCAGGCCTTGATGGGATCTTCTAGGGAGACA<br>ACAGCCCTGTCTCAAACCCAGCTTGCCAGCTCTAATGTACCAGCAG<br>CTGGAATCTGAAGGCGTGAGTCTCCATCTTAGAGCATCACTCTTCC<br>TCACACCACAAATCTGGTGCCTGTCTCTTGCTTACCAATGTCTAAG<br>GTCCCCACTGCCTGCTGCAGAGAAAACACACTCCTTTGCTTAGCCC<br>ACAATTCTCTATTTCACTTGACCCCTGCCCACCTCTCCAACCTAACT<br>GGCTTACTTCCTAGTCTACTTGAGGCTGCAATCACACTGAGGAACT<br>CACAATTCCAAACATACAAGAGGCTCTCTCTTAACACGGCACTTAG<br>ACACGTGCTGTTCCACCTTCCCTCGTGCTGTTCCACCTTTCCTCAG<br>ACTATTTTTCAGCCTTCTGGCATCAGCAAACCTTATAAAATTTTTTT

	GATTTCAGTGTAGTTCTCTCCTCTTCAAATAAACATGTCTGCCTTCA TTCTTTAGGTGACTCTTTTTTTGGCTGAAAGTTTCCAGTGTTATCATT ACCATGTCCAAATAACTCCAACTGTTCTCCACTGGGTTCTCACCCC TGGACTTGGAGCTTCTGGAAGCAGGGTGGAGCCTGATTTGTCTCTG AGACTCCAATTTCCATCCAAAGATGCAGCACATAAGAGGTTCCAAG GATCGTGAATCACATGAACAAGTGATATTCTTACTCTCTGCAGACCT GGAAAGCTGGCAGAGTCATTCCATGATGAAACATTTGTAGAGTCAT AGGCCTTGTTAGTCTCATCTCCACGGGGACACATATCAACACATCAT CTTTCATACTATAAATATACAGTCGGTCCTCTGTATCTGTGGGATTTA CAGGTGTTTATTGAACCAAATATAAATCAAAAATATTCAGAGAAAA AATCCACAAAGTTTCAAAAAGCAAAACTATGTTGAATGGACACAA ATGAAGCTGTGTGTAGGCTGTATCAGGAATTATAAATAATCAAGGGA TGATTTCATGTACACAGGAGGATGTGCATGGGTTATTTGCAAATGCT GTGCCATTTCATGTAAGAGGCTTGAGCGTCTGCAGATTGTGCTATCT GAGTGGAGATCCTGAAACCAATCACCCACGAATAGTGAGGGATGA CTGTATATAATTTTTATTTCTCAATTTTAAATATAAAACATAAAAAAA TTACAATAACAAGATAAAATAAACAAGTGTTTTATAGTGTGAGAATA CGTTTAGATATATTTTTCTCTATGTGTAACCCTTGGGCCCATGTTATTT ATTGAGAAGACATTCTATTCCACCTTAAACCACATGGCAGCCTTTGT CAACTATAAAGGGACTGTGTGTACACGGATGTATTTTAGACACTGTT TTCTGCTCAGTGGCTCTCTCTCTGTCCACTCTCTTGAGAATGCTGCA TTTTATGCAGCCTTATACAACCCCTAAAATTTGGTAGCTGGAGTCCT CTAGTTATTTATTATAGGCTATTTGCTATGCTTTTTTTATTTTTCTTGA GGCAGAGTCTCGCTCTGTTGCCCAGGCTGGAGTGCAGTGGCACGA TCTCGGCTCACTGCAACTTCCGCCTCCCAGGTTCAAGGGATTCCGT GCCTCAGCCTCTTGAATAGCTGGCATTACAAGTGCCTGCTACCAGG CATGGCTAATTTTTGTATTTTTAGCAGAGACATGGTTTCACTATATTG GCCAGGCTGGTCTCAAACTCCTGACCTCGGTTGATCACTCACCTCG GCTTCCAAAGTGCTGGGGAAATTGATTTTCTATAGCATTATGTTACT GGATATTTCTGTAAAATTTAAAATGAGGGAGGCAGAGAGACAGAG AGAGAGCAAACCATGAGTTGGAACTCTGGAATCTTGGGACATGAG ACAAATTCTAGATAAATCTACAAAAATCCAGAATTTACATGTTGTGA TTTTTGCTGATAAAGTACAATTCTAAGATTGTAAATAATTGCATAATC CTTCCCTGGGAGTTTAAATCATTTGAACTGGTTCTGCTGTAATACTA GAAATACAATCATGAAAAATTCTAATGGTTTATTGTCACAATTGCTC TGAAAACCTTAATAATACCTATTAGATATTTTGCATATTACACAGGAA GAAGAGTTTGAATCTCAGATAAAAACAATAAAAATACATGAAAAGT CTTTCATGTTAGCACAGATTTTAGGCATCTCGTGTTCGGGAGGTTGG ATCTGAGACGTGTTTTGAGTTGGTCATAGTGAAGGACGCGAGGTGT CAATTCTAGTGAGAGCAATTTCCAGGAAGCCATGTTCCGCTCTTGA GCGAGCACCCACTGGGCCTCATGCAAGGTAGAAAGAGCCTGCGTA CGTCACCCTCCCATGATGTGGTCAACATGTAAACTGCATGGGCAGG GCGCCAAATAACATCCTGTGCGCTGCTGAGCTGAGCTGGGGCGCA GCCGCCTGTCTGCACCGGCAGCACCATGTCGC
KIT	AAAAGTCGGCCGGACACAGTGGCTCATGCCTGTAATCCCAGCACTT CGGGAGGCTGAAGTCAGGAGACTGAGACCATCCTGGCTAACATGG

	TGAAACCCCATCTCTACTAAAAATACAAAAACAAAATTAGCCAGGC<br>ATGGTGGCGGGCGCCTGTAGTCCCAGCTACTCGGGAAGCTGAGGC<br>AGAGGAATGGCGTGAACCCGGGAGGCGGAGCTTGCAGTGAGCTG<br>AGATTGCACCACTGCACTCCAGCCTTGGCGACAGAGTGAGACTCC<br>GTCTCAAAAAAAAAAAAAAAAAAAAGTCCACGAGCATCTCGAAA<br>CAAACAGGCTCAACTCCAAATCCTTCACTGTCCACTAACAAGTACC<br>TCCATGTCTTGCTGGATGGGAGCACATGTAGCTCCACAATACTTTTG<br>GTCACACAGGCCTATTGAATCTTAACTTCCTTATTCATCCCCTCTCA<br>CTATTCTCACCTCTGTGGACTTAATTCAGTTCCCTGTTTCTCCTGTG<br>GATCACTGCATTAGGCTCCTTACCATTTTCTTCCTGCCTTAACTTTTG<br>CCCCCTTTCAAGTCACCCTTCACTGAGTTTCTTCACTATCTTTCCAA<br>AAATGTAAATCTTAGCACAACAGGCTGCAGCTTAAAGTCCTTTAGT<br>GACTCCCCGTAGCTCAGAGGATGAGGTTCTCATTTCGGAGTATTTA<br>CAGTTCTTGTCTATCTCTGTGGCCTCGACTCTCCCCACTCTCCTCCA<br>AGCCCCATTTCCTTGACTGGGCAGCACTCCTTGTTCTTCCTATTCCT<br>TATGCTGTTTCCTGCCTCTAGCCCCGTGCGTTTGTACTTCCCACTGC<br>TGGAACATTCTTCTCTCCTTTCCCTTTCCCCGCTCCTGATCCTTCAG<br>AGTCTAATACCCACCTCTCTGGGAGGCCACATGAGCTCACTGGACA<br>GGTGCTCCTCTGTGTGCAAACATCACTGTGCATGGCTCTGTTAGAG<br>TACTTCATGCCATGTAATTTTTGCCCCTTTATTCATCTCTCCCCTCATT<br>TGCTGGAAATCCTGTGAGGGCAGCATCTGTGTCTTGTCTAACTTGG<br>TATCCCTGACACCTAATACACTACATCTGACACAGAGATGTTCAATT<br>AATACTGAATGAAAGCTGGAACGAAGACCATACATTATCTCCTTTC<br>AGACTCACAAACTGTGAAGTGGTTATTGTTAGTATAGAATCTAACC<br>GGGGGATTGTATTAATCTATTCATTTCTTCAACTTTATGGACAAAAG<br>ACTTTCAGATGATTGCTGAAAACCAAGAAAAGCTATTAAAAAGCA<br>AACTTGAAAAGGATTATACTCTTGTTGTAGTAATTCCACCCCTGGTT<br>AGGAATGCATACTTAATAACTAATCAGAAGCAGGCAAGTTTTATTCA<br>CCAAGACATTAATCATCAAGTAATTTATGATAGTAAAAAAATTGGAA<br>AAAAAAGAGAGAAATGATTAAACACCCAGAATTATGGTACATCCAT<br>AAAATTGAACATTATACTGCCATTAAACAGTGTGTTTCCAAAGGATT<br>TTTAGTGATCTGTAATAATGCTCATGTTATTTGTGAAAAAGCTGGAT<br>AGAAAACTGAAAATATACCAAGATCTTAAATTATGTAAAGGGAAAA<br>AAACCTCACACATAAATAGAGAAAAAAGAAAGGAACATATTAAAA<br>CAAACTCTTCAGTGATTGTATCTTGAGTGCCTTTTTTGTTTTTTATAA<br>TTTTTGGTATTTTCCAAATTTTCTACAATAAGCATCTTTTACAGTCAG<br>AAATATAATAAAATATTTTCAAGTTCGAGATTTTAGCTGTGTTAGTTG<br>TATTTGTGTGGCATTATTACTCTTTCTTCTGTTGATGGTGCATCAGTT<br>TTTCTTTGGGGATGCACCCTTCCCCATCTCAGGCCTCTGGTGTGAA<br>GTGGATCCCTGACCCCGGCTTCAGGGGTGGGCACATGACTCAGAC<br>ATGGCCAATCAGCATACTCCATCCCTCTGGCTCCTTCCCAATGGGCA<br>CATGACTTAAGCCAGGTCAGTGAGACTCGGTTTCTCCCATAAACTT<br>TTACAGGAGATGTTTATGGGGGCAGGAAAACTCTACTTCCGTTGGT<br>ACTAGTGAGCTGTAAGCCTAGAGCTGCCCTGAGTGTATACAAGGTT<br>GGCAAAACAACACAGGAAAGTATTGAGGATAAAGGTGGAGGAAA<br>GTGTACGTGCACTTGTGCACACATGCATCCACACACACTCGCCCTT

	CAAGTCCTGATAACTTTGAGTCCTTGGATCTAGGAAAGTCTGAAAC<br>ACTATTGTCCCTGGACTTTCTGGTTATAAGAGCCAATACATTTAGGC<br>TTCTTTGTTTGCTTGCTTAATTTAAGCTGCTTCTATAAGTTGCAATCG<br>AGAGTCCTTGCTAAAAGAATTATGAAAACAATGAAATACATAAAGC<br>ACAATCCCTGAACATGCCCAGGACTATCACAAAATACAGTATAGAA<br>TTAATCCATACATCCTACCTGAGATATAAAACATTTCCATCATTGCCA<br>ATGTTCCATTGGACACTGCTGATCTATAATATAGTTCCTTATATATTAC<br>AGCATATATGTCCACTCATAGTGCCTTATATATATGCTTCCTTTCTTTA<br>TGTAACAGGCAGTGGGTATTATTAAAAACAGCTAACAACCCTAAAG<br>GTACAAGTACTGGAGGCTCTCTGTTGTGGCCAGCATTAAAATTTTAA<br>TTCCTGGATCTAAGGAGATTTAGCGTGTCTCAAGGTTGGGCTGGGA<br>GATATTTTAGGGTATGGTTTTTTTTGTAATCAGTACAGAAGGATTTC<br>AATATTGTGTCAACCAATAAGGCCAAATTGGTGTGTACTGGCTGAA<br>CATAAGCTCTGTTAGCAGATATTATAGTTTCTTACTTGAGTACCTATT<br>TTCCCTGCTGGACTGTGAATTCCCCTAGACAGAGACTTTGTCTTACT<br>CATTTATGTGGGTCTTATGCCTACCTAGGTGCTCAAAAGATATTTGC<br>TGAATGAAAAACAGGAATGACGGTCCAACATGGTATAACTCCCTAT<br>TCAGTTTTTTTTTTTCCTGATAGAAGTTCCGTGCCTAGAATTAAATT<br>GCAATATGGTCTCTTTTCACCCACTCTTTTGAAGATGTTATCCTCTCC<br>AATATCAGCTGATTGAAGAAGAAAATTGTAAATTTTCTCTCTCAGGC<br>ATAATGTTGATATTTTAACTCATAGTGATTTTTTCTCAATACAAAATG<br>AATTTTAGAAATAAATCAAGATCCCAGAAACTTATGATTGCTTTGAG<br>TGGTATCTTTTATCTGTCATCCTAAGAAAAACATGAGATCTGATGGC<br>CCAGAGCACAGAGACCTTTGGTATTTCAGTTCTCCTACAGCCGCAT<br>GACTCGAACCTTTTGCTGGGAGATTGGGATGTAGGTCCCCATACTT<br>GATTATAAGGACTGTGAGGGCAGGGGCCACATCTGCATATCTGTCTA<br>TTAATAATAGTACTCCTGGTACCTAGCTCCTCCTTGCTTCACAGCAG<br>ACACTAAACAAATGCTTGCTGAGTGAGTAAATACTTACATTTAAAG<br>AGAGGCAATTATACACATTGATCATTTGATCACCCTCTAGCACTGAT<br>AATTTTTAAGTGATTAGCTTTAAAAATCATTTTTCTTACATCAACCTG<br>GGTCCTCACACTATTAAACAACAGGAGAGCTTTCAATATTAAATGA<br>AGTGGGGCAGCTGGGCAACTGTAAATTTTGGATGATACTTCAGAGA<br>AGAGTCTAGGTGTATCTGTTCTATCCCTTTTTCTGATGGGCATCTGC<br>CATTTATTTTTCCATTCTTAAGCTCTCAGGAGGGATGTCTTATTAGTT<br>TATCAGAATGCATAGGGAAAGCTCATCTTTCTTGGAGTAGGTCTTCC<br>AATAAGTGTTCAAGCCCTGGGTCTTCCCTCACTACAATAATAAACAT<br>ACCTGCTCTACCCTCCTCACAAAGCTGTTATCGTGAGCATGAAAAG<br>ACGAGTCGCTGTGGGGAAGAACTATTAACCCCTATACAGATACTGT<br>TGTTGCTTTCCGTTCAAGGATCCACCATGAGACTGTGGGTAAGGCA<br>CTTTCTGGGCAGTGAAATCGGGCTTAAGAGAATCATTGTACTTTTAT<br>TTTGCTCCTGGGACCGGCAAGGGCAGTTAAGAGCCCTAGCCCTAA<br>AACTGTCGTCTGTTACAGAGGCAGAGAAATCATTACTGGGGTTGGC<br>AGCGTTGGGGGGAGGGGGGAAAACGTGTATGAAAACCTGGGCTCC<br>CAGAGCAAACTTCGCCACGCGCCCTCCATCGCCCCTGTCTCCACAC<br>AAGTCCCGGCAGGGGCTTCTTGGGCGGAGGCCACGCCGACCAATA<br>GGAACCCACTGTGTTCCTACAGGTTACGAAGCAGGTGGAGAAATT

	GAGCAGAAACAATTAGCGAAACCGGGCTCAGCCTTTACCGCGGTG<br>CCAGGAGCTCCTAACAGGCCTGGAGGGGAATGTCGGGGCTCAATT<br>TCCTAACGCTCCCCTCCCCATCCCCATGCCACCTCCACGAGCAGCG<br>GCGTCCAGCCTCCTCCCGCCCGAACGTGCTCGAGGGGCGGGCAGT<br>CGACCTTTATTGTCTGGGGAGCACCTGGCAGGTGGCGGGCCCGTG<br>CCCTAACGTGTGCGTGGTGCCCAGCTTCACAAAGCGAGCGGGCAG<br>CACCTCCTTGGTCCGGGAACGGCCTCAGCCTGGCCGTCCACATCCC<br>AGGGGTGGAAAGGTGGAGAGAGAAAGGGGCTCCGGAGTCAAGAG<br>CGGGGAGAGAGGGCGCGCGCGCCCTCCTCCTCCCGGCGGGCACGG<br>CCCCCCGGCATTAACACGTCGAAAGAGCAGGGGCCAGACGCCGCC<br>GGGAAGAAGCGAGACCCGGGCGGGCGCGAGGGAGGGGAGGCGA<br>GGAGGGGCGTGGCCGGCGCGCAGAGGGAGGGCGCTGGGAGGAGG<br>GGCTGCTGCTCGCCGCTCGCGGCTCTGGGGGCTCGGCTTTGCCGCG<br>CTCGCTGCACTTGGGCGAGAGCTGGAACGTGGACCAGAGCTCGGA<br>TCCCATCGCAGCTACCGCGATGA
KLF5	TTCTTTGACTATACATAGCATGAATGTTTTCCCATGTCACTAAATGTT<br>CCTTAATAATAAGAATTTTTCCCCTTTTTTTCCAACTACTCAACTTTT<br>TGGTGGCATAACAGTAGCCATGGTATGGATGTATTGCTTAACTACTC<br>CCCTAATGTTGGACACCTAACCTAGGTTATTTCCAATTTTATTTTAAT<br>TTAAATTAATTATTATTATTTGAGACAGGGTCTTGCTCTGTCACCCAG<br>GCTGGAGTGCAGTGGTGTGATCATAGCTCACAGTAACCTTGAACTC<br>CTGGGCTCAAGTAATCCTCCTGCCTCAGCCTTCCAAGTAGCTGGGA<br>CTACCGGCATACATCACCATGCCTGGTTAATATTAAACATTTTTGTAG<br>AGATAGGGTCTCACTATGTTGCTCAAGCTGGTCTCCAGCTCCTGGC<br>CTCAAGCAATCCTCCCATCTTAGTCTCCCAAAGGGCTGGGATTATAG<br>GTGTGAGCCACCATGCCTGGCCCAATTTTATATATAAAGATATATGTG<br>TGTGTGTATATATATATATATATGTGTATATATATGTGTGTGTGTGTATAT<br>ATATACACATACACCTTTCCTAGAAGAGGAATTATCTATCATTTTCAA<br>AGTTATTTACATTTTAGCCTTTTTCATACTTATTGTCAAATTGTCCTTC<br>CCCAAAGTTTTAAAGTTTACACAACCATCAACAGTGTATGGAAGTT<br>AAGGCATGAAGAACTCACATTGCTTTTAGCACTACTTTTACACACT<br>TCATCTATAAATCAACATCTAAAAGAGTATTTCCGTGTGACCCATGC<br>ACGGTTCATTTGAGCATTCACAGGGTGTTCCCCACAGCCTGGTACA<br>TTACAGCTTTTGTCATAACATGAACAGCAATCGGCTCAAAACACTG<br>CCCATTGTGATGCTGATGATGAGACCTCCCTAATGTTCTAAAAGATT<br>GTTTGGTGTAACCACTTTAGCTCAGACCTTTCTTTTAACCTTGCCTA<br>TCATGTTTCGAGTCAGAATTTAAATACTGTGCAGTTTAAGCTACAAT<br>ACGCTTGGCCTATAACTTGGTTCCAGGCATTTATATTTATGTCACTTT<br>TGTCTACTTATTATACTAACAAGGTGGAAAAAGCAATCCCAGTCTCT<br>CCAAAAGGTAAAACTCTCCTGTTTTTTTGGATATTATTAGAGTTTTA<br>AAATTAGGCTAGATAAATTATAGTACCCTTAGGAATAAGTGCATAAA<br>GGGAGTTTTATGAAATATTTTAAGAAGAACACATTTAAAAATCCAA<br>ACTGAAATATAAAATGTAATTTCTTAAGAACTGGAGGCAAGAGACA<br>TTACAAAAATAAAATATTAAATTGTTGTCTTAAGACATAATTTTAATT<br>AATATAAATAGAAGTATTTAGTCTTATTTAAACTGCATATTTTTTCATC<br>ACAAAAGCAATGAACAGAGCATCTTCTCTCTTGTCTCGTAATCTTTT

	GAGTCAAAATGCTTTAAATTCCAACTAATCTCAGTTGAAGCTTCTAT TTATCAGGCTATTTCCTTAATGACTTTTTTTCTGATGTGTGGCTTTAG GTGGTTGCCATTAATGAAAAAATACAAAGACTACAGATGTGAAATG TTGGAGAGGACAATAATTACAATAGTTTTAAGTTTTCACTGATACAG ATTCTAACAGGGGTGTGCAATCAATGACTACCAAAAAGTCACTTTA ATTAAATAATCTTACCCTAGAGAAGAGTGTGCTCCCGTATAAAGCTT TATTTTTCATTTTTATTCTTGTTCTTTTTTCTGTGGCCCAAGATCACTT GGCAATTTGATACTGATTTGGCAGAAATAAATATTCTATGAATATTTT TCTACTCAAAGTGAATACAGTTTTGTTGGCTGTGATACTCTATACCT CTTAATTTCAAGCTGTGACGTTTTTGCCAAACAACTGAATTTAGATT TGCCAAACAACTGAATTTAGATATTCCTTTTGCCGTGTCGTTGTGTG AACATTGCCCTAGTGATAGCTTCATATTTCCCTCATCTCATCATTCAG AAGCTATTTATCTATTTTTTTCCACTGCCTCCTTCAAACCCCCTCCCC CAACATTGAATGCCTGTTCAGCCCCCGCATATTCATCCGTATTTATTC CCGTAAAGATAGTGACACTTATGGTTTATTCTAAGACTAAATCTGTG TTGCCCAGTGACTAAGGACTTAAGCATGCACACTGCTTCTATGCTTA CCCTCCTACCTAACTTGACTACTCAACATTTTTATTTCATTCCCTCAG AATCCCCTGTTTTGGCACAGTTCAGTTAACACACCACTGCCAGGCC ACTAATTGATTTCTGAAACCTGACATTTGTTTTTTGTTTTCTCCTATC TTCCCTAATGGTACAGCACTACTAAGCGGGAAGGAATGCAAAGAA CTAGAAAGTTCAAAGTAATTGAGGATCTGTCCATTCACACTGTCTG GCTTCAAGCCAATGTAGAACCTGGAACATTTGAAAAATATTTATCA AGTTTTAACCCTTTTTACTAAGTCGGTGAAACAGGTTTGCAGCTGT AGCATTTATGTTAAAAGCAACAGCAAGGAAACTTGGTCGAACATGT TCTTTATATGTTCAGAATCACATGGCAAATGTCTCCGAAAAGGATTA CTTTCCAATTTCCGTACTATTGTACAGTGCTAAGCTAGTTATTTTCTG TGAGACTGCAGAGCAGGGCATTTCAGAGAAACTATTACTTAAAAGT GGCAGCCCTATTTCATGAATCTTTATGCCCAAAATGGAATTTTAAAA CCTGCTACTGTATTGGAGCAAAAAGGAATATATTCAGTTACTATAAG AACCCCAACGCTAGCTTCATATCTATATTATGAAAAGTGGAAATATG AAAAATTAACAAGACTAAAAATATTAAAAACTTAGTTGTATGGTTTT TTTTTTGCAATGAAAAAGCTTTTGTAAATGAAAGCATGTTAAAAAG TGGTGTGAAATTATAGAAGCATTTCCAGTAAAATACATGGATTTTAT TGCAGTGAAAACTTAGGTTGAAAGACCGGGCCACTGAAGCTCTCG TAAAAAGACAAAAGGAAAGGTCTAGATAGGTTTAAAAGCACATTA AGATACAGCTACCGAATGTATTAAATCTAAGTTAGATTTAGCTTAAA GTTACAAGAGCACAAAAGTTCTATTAAGAATTATCTACATCACATTT AGGTTTGATGTTATCAGAATTAAGTAACTAAAAATCTTTCAAAGCTC AATTTTTTAAAAGAGTTACATTAAATCCCATCCTAATTCTATAATCTG TTTCTAAGCTAATTAATTACATGATAGGTTTTACATTAGAGAAAAGC AGGACAGCTTTTTAAAGATGAAGATGGACAGCATGGGGCTGCTTG GTTAGAGAAAAACTTGCAAAAGCACTTATTAAATAATACTTTGATAA CATGAAAGTAGTCATCAGCCATTCTCTGAAGGCAGTGATCACAGCT ATAAAAATGAATCATGTGTCAACAGGAAGAGAAGGAAAAGGAGAA AAACCCAGTTTACAGCATAATTTTATATTTTCTAATAATAAATACATT TCTTTATGTGCATATTAATTTTCTACCCATTGAAAATGAGATCCATAC

	CTTTGATAGATACACTCTTTGAAGTGAACGTTTTTATCCAAGACTTT<br>TTTTTAAAGCTGTTTTACTCTGAAGTGATGAATAGGCTGTGGTATAT<br>GTAAAACTGTCTAATGGTATATTGTGTGTCAACATATGTACATTTGTA<br>GTAATATCACAGGCATTTTTATTGCAGTTTATTGTTCCGTCCTCCCAA<br>TAAGCCAGATAAAATCATGTACAAGAACATTAAGGATTAAGGGAAG<br>TGGTTTCCTTAGGTGATTGCTGGCAAAAATTCACCCTCCCATTATAA<br>CAACATAGTAAATGGATGGTAATATATTTGGCTATTACTTTCCAACAA<br>ATTTGGATTCCCAGTTTTCAGAGCTGCTCTGTGTCTATGTTTGCGGC<br>TTTTTGATTGTCTCTTCGTAGGAAAAGGTGCCACAATGTGAGGTAA<br>ATGTGCTAGGTTTCTGCTTTGTAAATATTAAAGTCCATTTTTTGAACA<br>TACAGTGCTAGCAAAAATAAGTCAAAGATGTAATGTCTATTTAAATA<br>CACGTTTGAGCTAGTCGTGCTTAAATTAGATTCCATGCTTCGGCTAG<br>AATCAACACGGCTTAAATATTAGAAACTTCAAAATTCATATGGCCCA<br>AGCCCTCCTCCCCACACACGGATGGGAAGTAGAAATGTGTGGGAA<br>AACGTCTAGCAATTAAAATCCTGAAGGAGTGGGAAAAACCCAGGC<br>TTTTATGCAAGGATCTGAAAAAACGCTTGCCATCAAATCTTTTATTC<br>AGGCATTCTGAATTTGCCTTGTTAAACGGATACACTCACTGCTAAA<br>GATACACAGGGACAAGAGCTGTTTTAAATTTGTTTATCACACCGCC<br>CCTAGTTTTCTGCTAAAGAGAAGTACATTTTGGATATTTAAAAGTAC<br>ACACTCATTAGACAAATCTCTAAAAGCCTCAGCGCCCCTACCTAGC<br>TGCCTTCTGGGTTTCAGCTTTTGAGTTTCAGGTCCTTGCACAACAC<br>CTAAGACGTGCAATACTCTTTTTCTTCCTAATCCTGGCAGAGAACA<br>GTGCGAAGAGATCTCCCTCCGCGTCTCCACCCTAATTCCCCCTCTTC<br>CCAGATTCCACCCGCTCCGCTACTTCCCTCCGCCCCCGGCTCCGCC<br>AGCCCAATCTGTCAGAGAAGTTGTGTACAAACTGCGCGGCCGCCC<br>TGCGCGCGAAGCTCGTGGCCCGAGAGGGGTGCGGTCGGGCCGAC<br>GGAGGCGGGGCCCTGGCTGCCTCTCTCCCTGCTCATAGGCTGGCCG<br>CTCAGGCCTGGCCGGCCTCGGGGCCTCGGGATTCGCGGCGGCGCT<br>GCCAATCAGGCGATCGGGCCCCGCCCCCCCGGAGTTGGGTGAAAT<br>AGAGGCGGGCGTCAAGTGTCAGTAGTCGCGGGGCAGGTACGTGCG<br>CTCGCGGTTCTCTCG
KRT16	AAGCTGCAGATCTGTGGTTGGATTGTGAAGGGCTATGACAGCCCCA<br>CCTTCAGAAGGGGGGCGTCAAACTTGCAGGAGGGGAGAGGACCC<br>CCGATGGTGGGCCTGGCACTCACAGATTTCCCCAGATCCCTGGAGC<br>CATCACAGGGGCAGAGCCTGGGCCTGGACATTCGTTCTCCCCCAG<br>GAACCCTGCTCCCTCTGTTTCTCTGCAGGGCCCCAGCACCCCATCG<br>TGGCCTGACTTTAGTGTATTAGGATTGTCTTTACCACCTTCTCCCCC<br>AGAAGACTATGTCTCCAGGGCTTCTCTGTCTTGTTCTTTGAGTCTG<br>AGTGCCCAGCACAGTGCCAGGCACACAGTAGGTGCTTTATAAACA<br>CCTGCTCATAAACATAACGCTAGCCACCAGCCTTTGCCAGGTGCTTT<br>ATTTATTTGGCCTCAAATCCCCTAATATGCCTGTGACACAGGGCTGT<br>TATTCCCATTTCCCAGACAAGGAAACTGAGGCTCAGAGAGGCTGA<br>ATATCCTGCCCAAGGTTTCGCCCCTGCTTGCCCCGATTCCAGGCTGC<br>TCTGTCCTGGCTCAGTGGGGCTCTACTTTTCCTTCCTCCTGCCCTGA<br>AGCCTGTGGCTCTGGCTTGTCCACCCAGGGCTGGGGGCCCTGCAG<br>CAGCCCCCTCTCTCCCCTCAGCTTCTCTGCCCCACTCCTCAGCCCC

	CTAAGCTGCCTCCTGCCTTCCCAGTCTGAGTTGGCTGGGGAGCTTG<br>TAAAACAGCCCCACCAGCAGGCTTGTCCTGAGTGGTGCTGATGCA<br>GGAGTTGGAGCAGGGCTGCGAATCTTCTTGGGGACTCTGATGATCT<br>GTCTGGTTGGAGAATCACTGCAAGCCAAGGCATGGCATTGGCCAAT<br>GGTGATTCCCGGCCTCCTCAGGTATGCTATGATCTCCTGCCTCATGC<br>TTGACATGGGCAGGACGAAGGAGGGAGGAAGGAGGCAGGGTGCG<br>GGGAGGAATCCTCTGCCTGGAGGATGCCCATTGGGCTTCTCTATGC<br>TCACCAGGCTTAGTGGTCCTGGCTCTTGGCCACCTTGGGGCAGGTG<br>TGCATCACTGATGCAGAGGAAACGCCTCCTCCTCCTCTGTCAACTC<br>AGATCCTCTGAGGCCCTGCTGGTGCCTCCCTCTGCTGGGACCCTTT<br>CAGGGATCAAGGCCAGGAGGGCAGCCCCCTCCTCTACTGCTGAGG<br>CTCAGCGGGGTAATGCTTACCCCAGCTGCCTCCTGTGGTGTGGGCG<br>TGGCAGGCAAGCCCAGTACCCTAGGGTTTCTAGCCAGGTTCTGCAT<br>TTTCCAGCGTTAGTTTTCCCTCCAGGGTCAAATGATAAGTTCTTCTA<br>AGGTCAGAGACTATGTCATCATTTCCCTCTTCACCCCCAGCCCCCG<br>AGAGCTGTGCACACGGCAGGTGCGTGATCTATGGAGTTGGCTGAG<br>CAGTTTGGCGCGTTGGTCAGCAACTTCCTGCAGCTTCTCAGCCCAC<br>GCAGATCCTCAGGTCTGCAGTGCTGGGCTTGTGTATAATCCCACCC<br>TGACACCTCCTGGAATGGGGAACTTGGCGGGGGGCAGTGAGCCAA<br>TGAGAAAGGGGTGTGTGTGCACGCACACACGGTGGGGGCAGAGA<br>GTGAAGAGTTCTCGGAGACTCCACTGTGAGAGTCGGTGTGAATCC<br>TGGCTTGGGAGTTGGAGCTGGCTCCGGTCTGTGCTCTCTCACTTTT<br>GTGCTGCTTAACCTGGATAAGGCTTTCGCCCTCTCCGGTTGGGGTAT<br>TGATAGGCTTTGGTGACCTTGAAGACCATTTTCAGTTCTGAGGGTG<br>ATGGGTCTGTGGTTGGACTGGAACTCCAGGCCCACCCTGACACCT<br>GAACGCTGAGGTTTTTGTCCAAGATTCCGGGGTCCCCTTTGTCCCT<br>CTTTGGGGTGGGGGTGGGTGTGGGGAAGAGAAAGAGAGATCAGT<br>GTCTCTTTCTATACGGACTGTGTCTATATAGAAAGAAGTAGACATGA<br>GAGACTCCATTTTGTTCTGTACTAAGAAAAATTCTTCTGCCTTGAGA<br>TGCTGTTAATCTGTAACCCTACCCCAACCCTGTGCTCGCAGAAACA<br>TGCCCTGTGTCGACTCAAGGTTTAATGGATTTAGGGCTATGCAGGAT<br>GTGCTTTGTTAAACAAATGCTTGAAGGCAGCTAGCTTGTTAAAAGT<br>CATCACCACTCCCTAATCTCAAGTACCCAGGGACACAAAACACTGC<br>GGAAGGAAGGCCGCAGGGACCTCTGCCTAGGAAAGCCAGGTTTCT<br>CCCCATGTGATAGTCTGAAATATGGCCTCATGGGAAGTGAAAGACC<br>TGACCGTCCCCCAGCCCGACACCCGTAAAGGGTCTGTGCTGAGGA<br>GGATTAGTAAAAGAGGAAGGCCTCTTTGCAGTTGAGATAAGAGGA<br>AGGCATCTGTCTCCTGCTCATCCCTGGGCAATGGAATGTCTCGGTGT<br>AAAACCCGATTGTATATTCCATCTACTGAGATAGGAGAAAACCGCC<br>TTAGGGCTGGAGGTGAGACATGCTGGCGGTAACACTGCTCTTTAAT<br>GCACCGAGATGTTTATGTATGTGCACATCAAAGCACAGCACCTTTT<br>CCTTAACCTTGTTTATGACACAGAGACATTTGTTCACATTTTCCTGC<br>TGGCCCTCTCCCCACTATTACCCTATTGTCCTGCCACATCCCCCTCT<br>GGGAGATGGTAGAGATGGTAGAGATAATGATCAATAAATACTGAGG<br>GAACTCGGAGACTGGGGCCAGCGTGGGTCCTCTGTATGCTGAGCG<br>CCAGTCCCCTGGGCCCATTTTTCTTTCTCTATACTTTGTCTCTGTGTC

	TCTTTCTTTTCTCAGTCTCTCATCCCACCCCACGAGAAACACCCAC<br>AGTTGTGGAGGGGCAGGCCACCCCTTCAGGTGGGGAGTGTCGGGT<br>TCCCTCTCTGAGGTTTGGCTCTGAATGCATGTGCATCCATTCTGCTC<br>TTACCTCTGATCCCAGAGTGACCAGGGGAGATGTAGCAGCGACAA<br>AACCCTGGTTCTGCTGAGACAAGGAATTGCCTGCCCCAGTGGAGC<br>CCAGGCAGAGGCAGGCTGTCTCTGGGCTCCTCACCTCAACAGAGG<br>CACTGCAGAGCAAGGCCAGTGGCCAGCACCAAGGGATGCCCCACG<br>GCACCCTGAGGAGGCCCCGTCTTGGTGCCCAGATCCCTCCTCCTCC<br>CTCCAGTCCTCGGTGCTGCACCAGCCTCTCAGGCCAAGGCCACGC<br>CCACCCTGGGCAGACCTCATGCCAGTGATCGTGGGGAGGTGGGTG<br>AGAGTTGGGTATACTGGCCTCACCTGCAGGCATGGTGGGAGCCTGG<br>AGAGGGCTGAGGTCTGGGTTCTAGTTGCAGCTCTGCAGCTTAAAG<br>CAGAGTGACTTTGAGTGAATCTCCACCTGTCTCTGGTTACCCCTCT<br>GTCCCCTCTGATCAAGAAAATGATATAAATTTGAGATGCACAGAGA<br>GAGGGCTCAGGATGACAGAACAGGCTTAGGCAAGGAGAACAGGC<br>AGGCAGGTTCACATCTCAGCTTCCAGTGCCTGGTGTCTTGGGAAGC<br>CCACTTATCCTCCCTGGATCCCAGCTCCCTCACCTGAGGACTGGTG<br>GCAGGAATAACGCCCCCGTCATCCCTGCTCACAGAACCGTACAACA<br>CACACCAGGCTCTCCATGCGTGCCTGAGTGGGCCCATGACCGGTGC<br>TCTCCCCCAGCTCAGCTGGATGAATGGAGCTGCCAGGCCCAGCGT<br>GGGATCTGTGGACACAGGAGAGAGAACTGCTGGGAACGGATCCCC<br>AGCCAGTCCTGTTTCTACAGGGTAGGGGGGAACTCTGAGGCCCAC<br>GGCGGGGTCAGGGGTCACACCTGGCTGCAGTGGCAGGACGGGGC<br>TCCCTCCCACCGGCCTGCAGGTAGGGTGCCTGTGATTAGGAGAGG<br>GCGGTAGAGGAGTGGAGGAGCTAGGTGTGGGGCTGTGGTGTTCTT<br>CAGCTGGGAGTGTGGGGGACGCAGGGCCCCGCGTAGCGGTTCGGG<br>AGGGTGCCAGTGTGTAGAGGTGAGTGTGAGGAGCAGGTGGCTGG<br>AGGGGGAGGATGAGGAGGATGAGGGGCAGGGCTGGCCCTTGGGG<br>AAGGGCCGGTGGGGTGCTTCGCGCATGTTTCTTTGTGGCTAGTGGT<br>GTGTCATCCCCTTTTACAGATGAGAAAACTGAGGACCAAGGGGCT<br>GTAAGACCAGCCTGTGCTGTACAGCTGGTAAGGGCAGAGCAGGGA<br>CTCAGTGCCGCCTGTCTGGCTCCATGGCCAGTGCTGTTTGTATGGT<br>GCACACACTTCTGGAGATCCCGGGGGCTCAAGGCAGCCTCCGGAA<br>CTGGAATTAGATCCAAGAGGGGAAAGAGAGTCATAGGATTTTTAGA<br>ACCAGAAGGGACTCTGCAATCACCCAGTCCAGCCTGGTCACCACT<br>GAGGGGAAACAGGCCCAGAGGTGGGAGGTGGGGAGCCTCAGGCC<br>ACACAGCAAGCAGAGTGAAGACACGAACTCTGCCCCTGCCCAGA<br>GTGGGACATGTAGGATCCCCGCAACTGCTCCCCAAGACAGCCCAG<br>GATGGCATCACTGAGGTCTCTTTCAGCCAAGGCTGTCACTGTGGGG<br>CAGGGAGTTCTTCTGAAGGGCTGACTCACTGCCTGGGGACGCAGT<br>TGCCACAAAGCCACCTGTGCCAAGGCCCCACTGGCCCCAAGTGCT<br>CCAGGAACGGGAGCCTGATTCCCGGCCACCTAGCCTGAGTCACCA<br>CCGACTCGCATTTGTGTGTTTTTCTCTCAGCTCCACACTCTCAAAGC<br>TGGGTGGGAACTCTGAGCCAGCACACAGCGGAGTTGATCCTGGGC<br>TGAATAATCCAGAGTGAGGAGTTGGACGGGACTGGGAGTGATGAA<br>ATCCAGAGGGGAACCTGGAGTCAGCAGTTAGGAGGGCCCCGCCTT

	CCCCAGGTGCATATAAAGGTCTCTGGGGTTGGAGGCAGCCACAGCATGCTCTTAGCCTTCCTGAGCACCTTCCCTTTTTTCAGCCAACTGCTCGCTCGCTCACCTCCCTCCTTGGCACGATGAGCACCTGCAGCCACCAGTTCA
L1CAM	GTGAGGTTGGGAGTGCAGGGACTAAGCAGCAGTCATAAAGGACTTCTGGGCCCCCAGGTACCTGTGGGGGCTCCCTGGAGCAGCCCCCTTTATGTGCTGTCTACATAACACATAACAAGGCTTTCCTCTGGCTTCCCCCACCTCTGGGACTTGGGCTGCATCACCATAGAAACCCGCCAGCCACGGCCTGGGGAGCGGGGCGGGGAGGGCCAGCGGGAGCCTTAAAGGGACACAGTGCACAGGGTGTAGGTGACACCCACACCATCGTATACACACCAACAGGCACAGAAGCCCCCCAGGCTCCCAGGCCTGCAAGCACATGGGCCCAGCACCACACTCTGACCCTTTCATGTGCCCTGTGCTGAGCCCCCAGCCCCACCCCCACTCTGGCCCACTGAGTCACCAGGCACCATGTCCCCAGGAGACAAGGGCAGGAGAAGCCGGATAGTCTCTGCTGTGCTGTTGAAGGGCTCTCTTGTTGCTTCTCCTTTGAGAGGAACTCCAGGCAGAACCTGAGAACTGACAGGCAGGTGTGCAGGGCACATGTGCAGGGCACCGCTGGAGGTTCCCTTCTCAAGGTGTGTGGTGGTGGTGGTGATGGGGTGTGTGTGTGTGTTTTCAGGGAGGAGGCAGTCACTCCAGGTCTAGCCAGGCTCCACAACCTCCCAGGTCCCTCCCTGGCAAGCTGCTTCCCATCAGACCAAAGCAGGCACTTTGGAGCAGAGGACAGAGGACTCCCTGAGGGACCTGCGGGTGTAGTGGGAGGCCAGGTGGGGTGGGATGGGGCAAGGCTCTTCTGGGGCTGAACCCCTCCCTGTAGGCTGCTGGGCGTGACCCTTGCCTCCCTGGCCCTTTCCCACCGGGGAGGATGGGTTTGGGACCGGACTTACTCAGGAATGGCCCCATCTTCAACTGCCCCAGCACAGCGGCCACTGTCCACTCAGTGCATGGTCACGCAGGCTCACTCTTGGCCTAACACATGTGCGTCTCCTCCAGCCAGGTGACCACTTACTCCCAGCCAGCAGCAATATTGGAAGGCTTCCCTGGGATTTTCTGGGTGCTTCCCCCTCCCCCCGTTCCTACCCGCAGCAGGAATCCCCCGACCCCCACCTGTTCCCTGGCATGGCATGGGTCAGCCAGCAGTGTGCCATTCATCCTGTCTCACGGCCGTCCCTTGCCCAGTGCCACAAGTGGGCAGGGCTTGATGCTCTGTTGCGTGGGGCTTTGTAGGCCTCCGGCCCCAGCTCACAGCTGCTCCACCCTCTGTCTGTCTCTCTACCCTGCTCCCAGGCCAGCGTGACGTCCACGCCGGGAAGCCCCTCCACTGACCCAGGCCCATCTTCCCTCCTCCCCGCCCCTTGCCTGGCACAGCCCCCAGGGACAAGGACGCTGTGCAGGCGGCCTGCGCGCACGCGCACGTGCACACGCCCACCCCCACCCCTCACTCTTCTCGCTCTTTCTGCCATTTCTCAGCGGCGCACTCTGCAGCTGCCTCTCACCCTTTGTGCTCCGGTTTCCTCTTCTCTCCCTTTCTCTGCTGCCTGGGTCTCCGTTTCGGAATTTCCCCACCCCCTAACCATCTCCATCGTGGTTAGCGCTGTCTTCCTACGTCTGTCTTTGTCTCCTGCTCCCCCCTGCCTCTTCTCACTCTGTCTGTCACCCTTGGCAGACCCGGCCTTGGCTCCATCTGTGGAACACTCCCCCCTCCCCGCTCCTTTGCCCTTAGTGGTGCTGGTGGGGGTAGAGGGCCTGCTGAGCTGACAACCCTAGCTGCTTGCTCTGTAATTGCCTGAGGAATGGTGAGAGATCAGAGACCCAGCAGCAGTGGGAGGAGAGGAGGGAGCGCCCCCTCCCAGCTGTTATATCTCGCAACCTTCTGGCTGCTTCCTGCCTGTCTCCGACTGCCCAGGGCGGT

	GGCAAGCTAGGTGGCAAATACTGGGAGACCAAAAAAGAAGTTGGA<br>TATGAGCCTGTGGGGAGATGGGATGGGGCTGGGATGGGGCAGGGA<br>GCGGGGACCAGGGGTTTGGGGATATTGTATTTCCCCTGAGCGTACA<br>TGCCAAACACCATTCCAGGATTCTAGATTTGGAGGACAAATAGAGG<br>AACTTGCGTGGCAGAAATTATCGACCTCCAGAGCACCCAAAAGCC<br>TCTGACATGCAGGAGGGTCCTTCTTTGAGCTTTTCCTTGCTCCGGTT<br>TCCTCCCCTCCCCCTCCTAAGACCAGTGCTCAGAGCTCCCAAGGCT<br>GCTGTTTGATCATCTGCTCTCTGATCCGAGTCTCCTACTGCTCCTAC<br>AGGCCTTGAAGTTGAGGCCAGTGCTGCCTGGGGCCTCCCCTGATCC<br>ACTGGCTTTGTAGGGAGAGGCCGGGGTGGAACAGATCCACTGTTG<br>CTCTTCCCTTCTGGGCCCAGGTCCAGCTCTTGCAGGCTGGGAATCA<br>CTCTTCTTACCCTCCAAGTCATCTCTTGGCCTCCAGGCCATGCACGC<br>TGACCCACCCTGCTGCTTCTCCACCACCCTCTGCAGCAAGCTCTTG<br>CCCAGAGTCCCCACGGGATCTGCCGCATTCACAGCTCTTCTTGGGT<br>CACTCTGTTACACAAAACTGACAAGGCCCAGAGCCTTAGCACAGC<br>ATGGCTGACTTCTCTACCATCCTTTCGGTGTTCCTGCTGAGGCTCGG<br>TGCCCGCTTTGATAAGGTCCTGGCTGGGCAGAGGGAGGATGCACA<br>GTATGGATTTAATGAAGGCGTCTAGTCTGGGACTTCAGCCTCCAAG<br>CTCTGTCACCTAACAACACCCTTTCTTGCCTTGCCTGCACTTTAACC<br>ACTTCCTGTTTGGAATTGAAGTGGTCTGGCCACTTGGGAGTCTGTT<br>TCCTCTTGTCATTGTCAGAGGAGCCCCCTCTTTCAGACCCTTAGTCT<br>TTGGCTCCCCTTACTTGTAGGTGAATATGGACATGGCCGGTGTGGCT<br>TGGGTTTGGGGTGGACACATGCAGCAGTTGAGCTCTGAAGACTCA<br>GACAAAGACCTAGAAATTGGGACTTTGAGACACACAAGAAGTGTT<br>AGAAACACTGTCACGGAAAATGTCCCAGAGCTTGTCCTGACCCCT<br>GTCCTGACCCTAACCTGGAGCATCTGGAAGGGAGGAGGCCTCAGT<br>GGCCTCTCTTGACCCCCAGGGGGCTGAGGAGCAGCAGGGGGTTGT<br>TTTCCTCCTTCCTCTCTTCTCCCACACCAGTTCTCAAGGGAAGGCTT<br>CCTTCCCCATTCTTTCTCTCTTTTTTTGAGATGGAGTTTCGCTCTTG<br>TTGCCCAGGCTGGAGTGCAATGGTGCGATCTTGGCTCACTGCAACT<br>TCCGCCTCCTGGGTTCAAGAGATTCTCCTGCCTCAGCCTCCCAAGT<br>AGCTGGGATTACAGGAATGCACCACCACGCCAGGCTAACTTTTTTT<br>TTTTTTTTGAGACAGAGTCTTGCTCTGTCACCCAGGCTGGAGTACA<br>ATGGCGCAATCTCCGCTCACCGCAACCTCTGCCTCCTGGGTTCAAG<br>TGATTCTCCTGCCTCAGCCTCCTGAGTAGCTGGGATTACAGGCATG<br>AGCCACCACGCCTGGCTTTTTTTTTTTTTTTTTTTTTTTTTTTTTGTAT<br>TTTTAGTAGAGACAGGATTTCACCATGTTGGCCAGGCTGGTCTCAA<br>ACTGCTGACCTCATGATCCGCCTGCCTCAGCCTCCCAAAGTGCTGG<br>GATTACAGGCGTGAGCCACCACGCCCAGCCCTAATTTTGTATTTTTA<br>CTAGAGACAGGGTTTCACCATGTTGGCCTGGCTGGTTTTGAACTCC<br>TGACTTCAGGTGATCCACCCACCTCGGCCTCCCAAAGTGCTGGGAT<br>TACAGGCATGAGCCACTGTGCCTGGCCTTTTATTTTTATTTTTATTTA<br>TTTTTTTAGACAGAGTCTCACTCCATCACCCAGGCTGGGGGGCAGT<br>GGCACGATCTTGGCTCACTGCAACCTCCGCCTCTCAGGCTCAAGTG<br>ATTCTCATGCCTCAGCCTCCCAAGTAGCTGGCATTACAGGCGCCCA<br>CCACCAAGGCTAATTTTTTTTCTTTTTGTATTTTTAGTAGAGACGGG

	GTTCCGCCATGTTGGCCAGGCTGGTCTCAAACTCCCAACCTCAAGA<br>GATCCACCCACCTCAGCCTCCCAAACTACTGGGATTACAGGCGTGA<br>GCCACCACGCCTGGCCCTTTCCCCATTCTTTCTTCATTTTCCTGAAA<br>CTATTTTGTGAAATGCTGCCATCTTGGGCCCCTTTCCTCCCTTCTCTA<br>TCCCACTTCCCAATCCCCCCATTAGCTTTTTAGGTCTGTACAGCACC<br>CAGGCATCAGTTGGCTCTGAAAGCTATAGCAATGTGACTGCAAGAA<br>GAATTCTTCTCTACATGTGTGTGCACACAGAGATAATAGATACAGGG<br>ATTCATCCCCACAGATACATAAAGACATACAAGAAGACACACGTAG<br>CTAGCAGACTTGTGTGTTGATTCCCTCAGGCACACACACTAACACA<br>CCCACTCATGAGTGCAACCCCTCCCAGAGAAGACACACCACAGAA<br>ATACCAGCTCACATACAGCTCACTCCCTGCTTGCAGATCCACAACC<br>ACACACATGCCCACGGACACCTGCACATGCAGACACATACGGGGA<br>TGTGCATGCCATGCACAGGGGAGCTGATGGGGCAACAAGTTGGTG<br>CTGGGAAGGCAGCTGGCAGGAAAGGTGCAGACAGAGAGTAGAGA<br>GGGAATAAAAGGCAACCTAGAGGCCCAGCCAGGGCTATCTCTTCC<br>AGATTTCTCCCTCCAAGGTGGGGAGGGGTGCGGATGGGGAGGGGA<br>GGGCTGGAGAAGCCGGCCAGCTGGGGGCGGGGCTGAGGGCGGGG<br>CCAAGAGCCCGGGCTTACCCAGATGTTAGTCACTAACGTCCTTCCG<br>TTCTCTCGTCTCTCTTCCCCCACCCTTCCCTTCCCTTCTCCCCTCTCC<br>CAGTGCCCCCACTCCCAACTCCCGCCCCAAGCCGCCCACCAGCCC<br>CCTTCCCCTCCGGCCGGAGCCTGAACCGAGCCCGGCTGGCTGTGC<br>TGCGCGGTGCCGCCGGGAAAGATGGT
LCAT	GCGTGCCAGTGCCTTGCAGGGAGTGGCCAGCATGTGGTGACTCCC<br>AGGGACCCCGAGGCAGCGCTTGTTTGGTTGACTCTGTCCAGGCTG<br>GGGTCCTCGCCCACCCTGCTCCGTGGCTGACGCTCCTTCTTGCTTG<br>TGCGGTTATGAAAGGCCTCCTCTTCCCTGCCCACTCAGCCTGCAGA<br>CTTGACAGGGGCCCTGTACCCCTGAGCAGGCTCCATCCCAGCTACA<br>GCCAGGCCTGCTGGAGCCAGCACCCTCTTGTCCACCCCCCCACAC<br>CCCTTCCTCTCACCCAAGAGCACACCTGAAACTGTTTGCCACAGC<br>CATCTTGGGTGGTGACCCCCCTTGTGCCCAAATCAGAGGCTACATC<br>TCAGCCTTTATCTCGGGCCTCCTGCTGGGAGCCCCTGCCCCCTGGC<br>ATGCCCTGTGTGGGTCCTCCTGCCTTCCTGCGCACCCGTCCTCGGG<br>TGCCTTCCTGGAGGGCTCATGCAGCTGGCCCTGCCCATGCGTGGGA<br>CTCCCACGCCCACCGCAGTGACTCTTAAGTATCTCTGACTGGTTGC<br>ATTCAAATGGTCCAGCTTATCTTCCATCCTCCCCTTCATGCATGGCA<br>GCCTCTCATCCAGCTGGTCACTCAGGCCAGAGGCCTGAATGTGGCC<br>TAGAGTCTCCAGGCACTGAGCTCTTCCTGCTGCCCCTGCACCACCC<br>TCTTCAGCTCTGCCCTGTTACTCCCCTTCCTACCTCCTCCAACAGCA<br>AACCCTTGCCCATCCTCTGCGATCTGGCTCCGAGGACTCCTCCACA<br>GTAGTCTCATTGCCTCAGGCCATGGAGGTGGCAGCAATGAGGTTGA<br>GAAGACCTTGCTGAGCCATGCAGGGCAGGGCCGGGCCACGCCCCG<br>TTTCTGGGCCAGGCCCAGCCTGACATCAGGTCCCCAGCTGCCGAG<br>GAGGCTGCTCTTCGCCAACCGCCCTCTGTGCCGCAGGTTCTTTCTG<br>ATGTGCTACCTGTTCGTGAACCTCGCCTGTGCGGTGCAGACACTCC<br>TGAGGACCCCCAACTGGCGGCCCCGGTTCAAGTACTATCACTGGTG<br>AGCCATGCCTCTGACACGGGAGACCCTGGGGAGGCCAGGAGCCTC

	ATTCTGGGTGAGGCTTGGGTTTCCTGGGTAGGGAGCTCTTGAAACA<br>CTGTGGGGAGGGAGATTATAGGGCTGCGGGGGACTGGACCTAGGT<br>CTTACACCCAGTGACCTGACCACCCACCTGGGACCTCCCACCCGG<br>GACCTGACAGGGCTACCAGGGCATGGGCCAGGCTGTGGCCAGGGG<br>TCCTCCTGGTCCTGCCCTGACTCTGCCCTCCCCTCTCGTCCAGGGC<br>GCTGTCCTTCCTGGGCATGAGTCTCTGCCTGGCCCTTATGTTTGTCT<br>CCTCCTGGTACTATGCCCTGGTGGCCATGCTCATCGCCGGCATGATC<br>TACAAATACATCGAGTACCAAGGGTGAGTGAGCACTGCCACCTGG<br>CCTCTGCCAGCTGAGGGGGCCCGGCCGAGCAGGCAGGGTGGTCTG<br>GCATTGGGGTGGGCAGGGAGCTGGGCCACCGTGGGTGCCAGGACT<br>GGGGACTGTAACTCAGGACCCCTGGGTGGTCTTGAGGGGCAGAAC<br>CCCTCCACCTCGCATCCACATAGACAGAACCCCCTGGGCGGCACCA<br>GGGGCTCACCAGCTGTGGTGGAATCTGCAGGGCTGAGAAGGAGTG<br>GGGTGACGGGATCCGAGGCCTGTCCCTGAGCGCTGCCCGCTACGC<br>GCTGTTGCGGCTGGAGGAGGGGCCTCCTCACACCAAGAACTGGCG<br>GTGAGTTCCCGCCAGACCCTGGCCAGGGCGCAGAGGTTCTTAGGG<br>GTCGCTGTCGGGGTCAGAGGGTCGAGAGGTGCCGGCTCCCCATCC<br>TGGGGCTGCCCGGGGCACCCTCCTGGGCCATGCTACCCAAACCTG<br>CAGGGACCACGACCCGAGAAGGGAAGAAGAGGAGGGGCTCCCTA<br>GAACGGCCGGCCCTGGTCTCAGGCTCCCCCCACCCGGCCACTGCA<br>GGCCGCAGCTGCTGGTGCTGCTGAAGCTGGACGAGGACCTCCACG<br>TGAAGTACCCGCGGCTCCTCACCTTCGCCTCCCAGCTCAAGGCTGG<br>CAAGGGCCTGACCATTGTTGGTTCTGTCATCCAGGGGAGCTTCTTG<br>GAGAGCTATGGCGAGGCTCAGGCCGCCGAGCAGGTACTTGCTTTG<br>GGGAGGGAGAGCTGAGCCAGACTACAGGGGTCTGGACTGCAGCT<br>GGCACGGAGGGGAGGGCCCAGTCTTGGCCAGGGTGGCCAGCCAG<br>GCAGCCAGCACTGCTGCCACCTGAGAGCTGGCACAAGGGGGAGC<br>CAGCAGTCCCATCTTGAAAACTTGGCCTCTGGGTAGGCAGCTTGTT<br>GCCCCCAGGCCCTCCTCCACCCAAAAGTCGTTTTGTTCCCGAGGGG<br>CCTTGGGAGGGAGCCCAGAGGCAGGAAGCTGATGGCCTCCCATTG<br>GCCCTGGGTGTGTTCCCCACAGACCATCAAGAACATGATGGAAATT<br>GAGAAGGTGAAGGGCTTCTGCCAGGTGGTGGTGGCCAGCAAGGT<br>GCGGGAGGGGCTGGCCCACCTCATCCAGTCCTGTGGCCTGGGAGG<br>CATGCGGCATAACTCCGTGGTGCTGGGCTGGCCCTACGGCTGGCGA<br>CAGAGCGAGGACCCCCGTGCCTGGAAGACCTTCATTGGTGTGTGA<br>AGGCCTGGGGCCTGGTGGGCAGGGGTGAAGTGAGGTGGCAGTGG<br>GCGGAGGGCCACAGACTGGTGGTCAGCCGTGCTCCTCCTCCCCAG<br>ACACCGTGCGCTGCACTACGGCTGCCCACCTGGCCCTGCTCGTGCC<br>CAAGAACATCGCCTTCTACCCCAGCAACCACGAGCGCTACCTGGA<br>GGGCCACATAGACGTGTGGTGGATCGTGCACGATGGTGGCATGCTC<br>ATGCTTCTGCCCTTCCTGCTGCGCCAGCATAAGGTAGACCAGGGGG<br>TGGGTGCAGATGAGAGGGGCTAGGCCCTTGGAGGAGCAGCTGTGA<br>CCAAACCGCCCACCTGGTCCCTTGCCTCAGGTCTGGAGGAAGTGC<br>CGGATGCGCATCTTCACAGTGGCCCAGATGGATGACAACAGCATCC<br>AGATGAAGAAGGACCTGGCTGTCTTTCTGTACCATCTGCGCCTTGA<br>GGCCGAGGTGGAGGTGGTGGAGATGGTGAGCCCTCTGCCCTGCCC

	TGGCCCCGTGGATACCATGTCCCGTGCAGCTGGGTCCTCACGGCCA ACCCTGTCCCCAGCATAACAGTGACATCTCTGCATACACCTACGAG CGGACGCTGATGATGGAGCAGCGGTCGCAGATGCTGCGGCAGATG AGACTGACCAAGACTGAGCGGGAGCGAGAAGTCAGTGCCCTTTGT GGTTCACGCCAGGGCGGGTGGCAGGGTCAGGCCTTTGGAGACCGC ATCGGTGGGTGCGGAGGAATCAGAATGACTCACCGCAGCCCTACC CAAGCTAGTGCTCATTTCCCGGAGGCAAGGTGGTCACATTGGGCCA GACAGGCCCCATCCCCCTTTTCCTATGGGACCAAGGCTATCTCCTGC AATTGTCCTCAGGCCCAGCTGGTCAAGGATCGGCACTCGGCCCTGC GGCTGGAGAGCCTGTACTCGGACGAGGAAGATGAGTCTGCAGTGG GGGCTGACAAGATCCAGATGACGTGGACCAGGGACAAGTACATGA CTGAGACCTGGGACCCCAGCCATGCCCCTGACAATTTCCGGGAGCT GGTGCACATTAAGCCGTGAGTGCAGCAAAAACAGCCCCAGCGGCA CTGGAATCTAGGGGTGGAGGTGGGGTGGGGCAGACAAGTAGTGTT AGATGGGCCAGGGGTGGGGACCCTCCTTGCAGGATGGCTCATGGC GACCTGTGACTGGCTACACTACGTGTAGGGACCAATCCAATGTGCG GCGCATGCACACTGCTGTGAAGCTCAATGAAGTCATTGTCACGCGC TCCCACGACGCCCGCCTGGTTCTCCTAAACATGCCTGGCCCACCCA GGAACAGTGAGGGCGACGAGAACTGTATCCCTTTGTGGAGAGGCA GGCAGTTGGGAGGTGGATAGCATGGGAGGTCAAGCCAGCGGCTTC TCTCCTGGGCACCCCTGGGTGGGCATGGCCCAGGTGGCCCGGGTT GCCCGTTGATTCTGTTGTTGCCTCCTTGACTTGAGGCCCTGCAGAC ATGGAGTTCCTCGAGGTGCTGACCGAGGGCCTTGAGCGGGTGCTG TTGGTGCGCGGTGGTGGCCGTGAAGTCATCACCATCTACTCCTGAG CCCAGTGTCATCTTGTGGCCTGGAGTCGAGGTCTTGGCCAGGACAT AACAAGCTGTGGTCTGGGGTAACAGCCTCTTCCCAGCACCCACCT GCCAGCCCTGCTTGCCTGGCCCTGTCCTGGACCCAGCTTTGCTAGG TCTCCTTGGAAACCAGGCCTGGGCCTCAAAATGGAGATGGATCCCA GGTCTTGTGGGACCCTGGGATGTTTGGGGACTTTACTATCTAGCAC CCCAGTAGGCCTGTCCTGGCCAGAGAAGACTGGTAGGGGCCGAGT GGGGTTTGAAGGCAGCCGGCCCGGCCCAGCCCAGGAGCGCTATTT ATTGCATATTTATTGTTTGGATGTCACCATCAGAGACGAAGGGAAG GGTAGCCAGGGAGGGAGTCCAGCCCAGCTGCCTGCAGGAAGATCT GGCTCAGTCTACTATGGGCAGGGCCCCCCACCAAGCTGAGCCGAAT GGAGACAGCTGAGCTGAGGCCTGACTTTTTCAATAAAACATTGTGT AGTTCTGGGCCTCCTGCTGCCCCGGCTCTGTTTCCCCTGGCGCCAA GAGAAGAAGGCGGAACTGAACCCAGGCCCAGAGCCGGCTCCCTG AGGCTGTGCCCCTTTCCGGCAATCTCTGGCCACAACCCCCACTGGC CAGGCCGTCCCTCCCACTGGCCCTAGGGCCCCTCCCACTCCCACAC CAGATAAGGACAGCCCAGTGCCGCTTTCTCTGGCAGTAGGCACCA GGGCTGGAATGGGGCCGCCCGG
LOR	GGTTTAATCTGTAGTTCCATGCTGAAACCCTCCACTCCTCATCATCT GACAAGATCATCTCTCAACCGCAGTCAGTCAATAGGTCTCAAGAGT TCCTTCAATTCCACTGGGATTTACTGAGCACCTATTAATGCCCAGCA CCATGCCAGGCACTGTGTAGCATCTGCTTTAAGGACCATCCCCTCTA AGGATACAAGTATAGGCAAGAACCATGTCCTAATCTGTCAAACTGC

	AGAAGTCAAATGTGCTATCAAGCACACCTTGCAGGGTTAGGTGTCT<br>AAAGGCTATAAACCTTAAAAATCTGAACTGCCAAGTCTTACAAAAC<br>CCCAAATACCTATGTGTCATTCTACAATTCTACAAAGTCCCATTATTT<br>CCTCACTGATATGATAAAAGGTGATAATCAGTTGCTGCCCACACAG<br>ATATCCCATTAGGACTACTGCTTCTGCTCCAGAAAGATGAAATGAG<br>AAACCAGGTTCATATATTCGTATATCAGGCAAGGTTAACTCAGGGCT<br>GAAAGCCACAAGATCTGGTACTCAGTATCATGTCACATCAACCTCT<br>TCTGCATGTTAACGAAATATTTATACTAGTTACAAGTTATATATATATA<br>TATATATCTCTACAGATATATCTCTATATATATGTAACTCTATATATATAT<br>AACTTGTAACTAGTACACATATTTAGTTAACATGCAGAAGAGGCTGA<br>TGTGACATAGTTATATATACATATATGACTATATATAGTCATATATGTAT<br>ATAAAACTATATATAGTCATATATGTATATAAAACTATATATAGTCATAT<br>ATGTATATAAAACTATAGTCACATATGTATATAAAACTATATACAGTTA<br>TGTATGTATGTAAAACTATATACAGTTATGTATGTATGTAAAACTATAT<br>ACAGTTATGTATGTATGTAAAACTATATACAGTTATGTATGTATGTAA<br>AACTATATACAGTTATGTATGTATGTAAAACTATATACAGTTATGTATG<br>TATGTAAAACTATATACAGTTATGTATGTATGTAAAACTATATACAGTT<br>ATGTATGTATGTAAAACTATATATAGTTAAATATGTATATCCTTTCTTAT<br>TAAATGCTATATAGGGTGGCAGAGGCAGGCATGGGGCACCATCCCC<br>ACCCGCGACTCTACCAAAAAGACCCATGCATAGCTTTGGAAAGCCT<br>TCTGGCTCTTTGATGCTTAACATGGTGCCCAGTACATTTGTTTTGGC<br>TGGATTAACAAATAAAGAATTAGCATCTTAAAATTAATAGAAAAAA<br>AAACCCTATATTTCTTAGAAGAAAATGAACCCCCCAAAGCCAACAT<br>GGACAATTTGAAGATTAGTCATTTTGGGTCAACTGTACTTTAGGTTC<br>CCGTTTTGTTTATTTTTTGAGACAGGGTCTCACTCTGTCACCCAGGC<br>TGGAGGGCAATGGTGCAATCTTGGCGCACTGCAGCCTTGACCTCCC<br>AGGCAGAAGCGATCCTCCCATCACAGCCTCCTGAGTAGCTGGGACT<br>ACGGGCACATGCCACCATGCCCAATTAATTTTGTTTATTTTTTTGTA<br>GAGATGAGGTCTCACTATGTTACCCAGGCTGGTCTCAAACTCTTGG<br>GCCCAAGCGATTCTCCCACCTCAGCCTCCAGAGTAGATGGCACTAC<br>AGGCGCATGGCACCACACCTCGTTAATTTTTGTATTTTTTGTAGAGA<br>CAGGGTCTTGCCATGTTGTGCAGGCTGGTCTCAAACTCCTGGGCTC<br>AAGCAATCCTCTCACCTTGGCCTCCCAAAGTGCTAGAATTACAGGC<br>ATGAGCCACTGTGCCCGGCCTAGTTCCCAAATTTTAGAATTCCATGG<br>AGAAGTAATATCTCCCTCAGAAAAAAGTGGTAATCATTACACCATA<br>GCCAAATTTTCATTTTGACATGGCAGGACATAAAAGAAACAACTAC<br>AGCCTCATGTCACCACATCTTTTAAAGGAAGAGGTAGGAAGGCCAT<br>GATTTCAGAATTGAAGACTTTCTTTCCCAAGAATACCCTGCTGACG<br>TCCTTTTACTTACACTTTTGCTAAGGACTGGTGAACACATAGCAATG<br>GGCCAACAAGGGACCACACATATCAGTACTGGGAACCGTCGTCCA<br>CACGTGCTCCTCTTTCCAATGTGGCCCAGCTAAATAAAACTGTTCTT<br>TCTTATACCCACAGCAATCACTAATCGCGGCCTGTTTCCCTCCAAAT<br>TCTCCACACTGCCTCAACTTCAAGCAGAGAAACTTGCCTTCTCCAA<br>CAGATCCTGTCTTCTCCCTACTGGGTCTCCTCTAAGGAGGAGAGCA<br>CCTGAGTTCCTCTTGGGTTCACCTGGGTTTATGCTGTCCAAACTGA<br>AACTAGTGCCACAAGTTTCTGATGGCCACAGTCTAATCAGGATCAT

	CTGGATCATTAAAAGTGCACTATAATTAAGAATTTATTATTGGAGCT<br>GTCATTTCAAAACATAACTGGGCAGAACAAAGTTTGACTGTGGTAT<br>CTTAAACAATACCAATTGACTTCCGGTTATGAAAGAAGCAGCATCC<br>TGTTCTGCATGCGCAGAGGTGATGCTACTGGTCCAGAGTCAACAAG<br>AAAGTTTTGAGTTATTCTCCTAGGAGAAGAATTGGACTTGCCCAAT<br>CCCTCCATGGACTCCTTTCTGGCCTTGGTATCCAAGGGAAGGCCAG<br>GTTGGAAGTAAGGATGTGCTGTTATATGAGAAAACAACAAAAAAG<br>ACAGCATCACCTTCTACACTTAACATAACTATTCTCCCTTCACATGC<br>CTGTGAAATAGGTACTACTATTTTTATTTGATAGACAAGGTAACTGA<br>GGGTCAAAAAGCTTGATCATTCCACAGTCAATAAGTACTTACTGAG<br>TGCCTCCCATGTGCAGGTACTGCACTAAGAGCTATGACTCTGCCCA<br>AGGTGACACAGCTGGCAAGTAGCAAAGGGAGCACCCAATCTGTCA<br>CCTAATCTCAAGTCTATCCCATTCAATGACTGCCCCTCAGGCTAAGG<br>TCAGTGTTTTGCCACACAACACCACAGGACAACAGAGCCAAAGAG<br>CCTTTGTGGGATCACATGGTCCATCTCTTTAGTTTGCTCTGCTGCAA<br>GGAAAGTCAGTATTGATTACCCTTCTCAGACTGTCTCAAATGCCTG<br>CTGTCTCTGGGGATGAATCAAAGTAGCTTCCCACGTCACATTAGTT<br>ACAGCACTGTCTATGGTGTGCAGTCCAGAGCTGGGTGCTGGGGAC<br>TCATGCCTCCAGTGGGCAGGACAGAAACACCCCAGCAAAACACAC<br>ATGAAATCCTGGGAGATAAACTTTTGCAAAGGAGTAACGGGTTCTA<br>AAGTTTTGAGGATTTTTAGAGTAGCAGGACCAGAAGTCAAACAGT<br>AGGATTTAGAAGGTCCTACTCCTTGGATAAAGCACTGAGATCTTCA<br>GTTTGACTCTCTTAGGGCACCTCAAGACTCTGGGACCTATTCCTCA<br>AGCACAGCCCTGTGGTCACCTGGTATGCATCCTGTGCCAGAGGTTT<br>TGGAAACAATGTCTGCCATCCACTCTGACTGGGTGACCCCACTGAT<br>GAGCCTGCCACACTGTTGCATCAGAGAAGGGGCCAGTCACACACC<br>AGGCTGCCCATCTCAAGAATGCCAAAACCTTCATGAATGGGCCATC<br>CTGTGCCTGCAATCACAGGGAGGTGGGGCCGACAGCCACGGGTCA<br>CGTAACTGAGGCCAAACACAAGAAGCTGGCCTGGATCAATGAGTC<br>AGGGAGAGCTCTATATATAACCTCAGGAGATCAGTGCTCCTCACATT<br>GCCAGCATCTTCTCTCCTCACTCACCCTTCCTGGTGCTTTGGGTAAG<br>TGTGGTTCTACTGACTCTCTCATTTTCCCAGCTGGTCTTGCCCAGGC<br>CTGACTAGATTAGATGGACCAGGGCCTCTTTCCCCTTTGGGAGCTAT<br>AGGACCTCTGCCTTCCCAAAAGCACTCACATTTAGAGGGCGGTCA<br>GGAAAGGAGCAGGGGATGAGCTGCTGCCATGCAGATGGTGTTTCT<br>GGTCTTCTGGCCAGAATGTAAACTCCACAAAGACAAGACTATCTCC<br>TGCCTCTCTGGCACCCGCATAGGGGCAGGCATGGTGCCGGGCACA<br>GAAGGACTCTGCAGAGGCTGTCCAAGGCAGCCTGTGCACAGGCTG<br>AGCAGACCTTGTGAACCTGTCAGGAGGAGAGGCTGAGCCACTCTC<br>AAGAGAGTAGGGAGAATGATCCAAAAAAGTTGCAGATGGGAGGGA<br>TTTCAGGTGCCACAGAAATCCAATTGCTGTTTTACAGAGTTAGAAG<br>TTCTGAGAGGGAAATACAACTTCCTACTAGTCAAAGTCCCTTCTAC<br>CAAACTTGGACTTGAGAAAATCATGGAAGAGATGGCTAATAGCTTC<br>TGGTCGGGGATGTTGGTCCAAAGGACCACCAGGGTCCTTCCCCTGT<br>TTCCAGGCAGGGCCACAGCAGAGCCTGTCTTTTCTAGTGACTAGCC<br>CTTGGTTCATGGCCTAGCTCAGTGGGACAGACCAAGAGTTTGAACT

	AAGAGCTTCTGCAGAGGATGGAGTGCAACAGCCCTCAATAGAATG<br>AAGTCCGACAAACCTCTCTCTGTTGTGCTGGGAATGGGTAAAATCT<br>CCTATCCTAGGTAGAGGTTTGTGGTAGTTTTCTATTTGCACTCAGAG<br>AATCAGTGTTGAGATTGGAAGAGGCCTGAAAGATGAAGTGTTCAA<br>ACCCTTTCATTTAACAGGAAATGAGAAAGAGGCGGGCGTGTGGGT<br>GATCTGCCCTCAAATCACACAACAGTGGTCAGAGGCAGAGCTGGG<br>AGAAAGAATGGGCACTGCTAACTGGGCTGTTTAAAGCATAATGAA<br>GGCTTTCTGCAGGGGAATGAGGAACTCAACTTTTCCAAAAGCAGC<br>AATCATAAGAAACCCCGCTGAGGGCTCTCCGGGCACCTGAAGGAG<br>CCCTGGGAGGATGGCGATGTTGCCTATGGATGCAGCTGCCTCCGTA<br>AGTATCCTCTTGCAGCTGGTCCAGCGATGCTCTCTTCTCCCCTTCCA<br>GGCTCTCCTTCCTTCTCAG
LPL	ATTCAACATAGTGTTGGAAGTTCTGGCCAGGGCAATCAGGCAGGA<br>GAAGGAAATAAAGGGTATTCAATTAGGAAAAGAGGAAGTCAAATT<br>GTCCCTGTTTGCAGATGACATGATTGTACATCTAGAAAACCCCATCA<br>TCTCTGCCCAAAATCTTCTCAAGCTGATAAGCAACTTCAGCAAAGT<br>CTCAGGATACAAAATCAATGTACAAAAATCACAAGCATTCTTATACA<br>CCAATAACAGACAAACAGAGAGCCAAATCATGAGTGAACTCCCAT<br>TCACAATTGCTTCAAAGAGAATACAATACCTAGGAATCCAACTTAC<br>AAGGATGTGAAGGACCTCTTCAAGGAGAACTACAAACCACTGCTC<br>AAGGAAATAAAAGAGGATACAAACAAATGGAAGAACATTCCACGC<br>TTATGGCTAGGAAGATTCAATATCGTGAAAATGGCCATACTGCCCAA<br>GGTAATTTATGGATTCAATGCCATCCCCATCAAGCTACCAATGACTT<br>TCTTCACAGAATTGGAAAAAACTACTTTAAAGTTCATATGGAACCA<br>AAAAAGAGACCGCATCGCCAAGTCAATCCTAAGCCAAAAGAACAA<br>AGCTGGAGGCATCACGCTACCTGACTTCAAACTATACTACAAGGCT<br>ACAGTCACCAAAACAGCATGGTAGTGGTACCAAAACAGAGATATA<br>GATCAATGGAACAGAACAGAGGCCTCAGAAATAATGTCCTATATCT<br>ACAACTATCTGATCTTTGACAAACCTGACAAAAACAAGAAATGGG<br>GAAAGTATTCCCTATTTAATAAATGGTGCTGGGAAAACTGGCTAGC<br>CATATGTAAAAAGCTGAAACTGGATCCCTTCCTTACACCTTATACAA<br>AAATTAATTTAAGATGGATTAAAGACTTAAATGTTAGATCTAACTGC<br>ATAAAAACCCTAGAAGAAAACCTAGGCAATACCATTCAGGACATAG<br>GCATGGTCAAGGACTTCATGACTAAAACACCAAAAGCAATGGCAA<br>CAGAAGCCAAAATTGACAAATGGTATCTAATTAAACTAAAGAGCTT<br>CTGCACAGCAAAAGAAACTACCATCAGAGTGAACAGGCAACCTAC<br>AGAATGGGAGAAAATTTTCGCAACCTACTCATCTGACAAAGGGCTA<br>ATATCCAGAATCTACAATGAACTCAAACAAATTTACAAGAAAAAAA<br>CAAACAAACCCATCAAAAAGTGGGCGAAGGATATGAACAGACACT<br>TATCAAAAGAAGACATTTATGCAGCCAAAAGACACATGAAAAAAT<br>GCTCACTGGCCATCAGAGAAATGCAAATCAAAACCACAATGAGAT<br>ACCATCTCACACCAGTTAGAATGGCGATCATTAAAAACTCAGGAAA<br>CAACAGGTGCTGGAGAGGATGTGGAGAGATAGGAACACTTTTACA<br>CTGTTGGTGGGACTGTAAACTAGTTCAACCATTGTGGAAGTCAGTG<br>TGGTGATTCCTCAGGGATCTAGAACTAGAAATACCATTTGACCCAG<br>CCGTCCCATTACTGGGTATATACCCGAAGGATTATAAATCATGCTGCT

	ATAAAGACACATGCACATGTATGTTTATTGTGGCACTATTCACAATA<br>GCAAAGACTTGGAACCAACCCAAATGTCCAACAACGATAGACTGG<br>ATTAAGAAAATGTGGCACATATACACCATGGAATACTATGCAGCCAT<br>AAAAAATGATGAGTTCATGTCCTTTGTAGGGACATGGATGAAACTG<br>GAAAACATCATTCTCAGCAAACTATCACAAGGACAAAAAACCAAA<br>CACCACATGTTCTCACTCATAGGTGGGAATTGAACAATGCAAACAC<br>ATGGACACAGGAAGGGGAACATCACACACTGGGGACTGTTGTGGG<br>GTGGGGGGAGGGGGAGGGATAGCATTAGGAGATATACCTAATGTAA<br>ATGACAAGTTAATAGGTGCAGCACACCAACATGGCACAGGTATACA<br>TATGTAACAAACCTGCACGTTGTGCACATTTACCCTAAAACTCAAA<br>CTATAATAATAATAAAATGTTTTAAAAATTCAATAGATTTTACAATATT<br>TTTAAATAATTATGACTTTGTATTTATACATTTTATTTGTATTAGTATGT<br>TTTCTTACATTAATTATACTTTTTATAAGGACAAGATCCGACTCAGTA<br>CTGATGTTCTTTAGAGCCTACTTACACATTTCATTGTATTTTACTTTA<br>GTAAGAAAGCCACAAAATAAAAATCTACATTAAAATATGTGTGTAC<br>AAAAAAAATCTGATTCCCAGCCTATTCCTGTTTGCAATTGCTGCAA<br>CATTAAAATATAGCACATCAAATACATATTTGCTTCTCGGCTAGGATC<br>GCCAAAGAAAAAACATCTTATGATTTGTGGTTGCTGTTATTTAATCT<br>TCCCTGAGCTTCATCTTCCCTCATCTGTAGAAGGGGAATATTATTGA<br>GTTTGGAGTGCTTTTGTAATGATTAAACACAGTAGAAGATGAAAAG<br>TGCCCAGTGCAATTAGCAGGCCTTCAATCAAAATAAGCAAGCTAGC<br>TAGCTAGCTGGCTGGCCAGCTAAATAATGCTTCCCTACATCCAGAAT<br>TTCCAGTTGTTTCATTAGATATGCACATTCAAGTTTTTACTTAGGTAA<br>TTTTAAATGTGTCCCAGTGGCAAGTGTCCTGCTAAAGGGGTACTGT<br>TAGCTTCTTCTCACCTAAAACTTAGTTTGTTACAAAGGAAATGCCAA<br>TTATGGAAAAAGTCAAGCCTTGAGCTACTGAAGGCCGAGTAACCT<br>CGCCAAAGTTTGGATGAGTCGCTTCACTAATGCACATGCAATCCTG<br>ACCTAGAGATCATGTGAGTTTCTGCGTTAGAAGGCGGGCACCTGAT<br>GGCTCCCAGTGCAGCCAATCAGGAGAGAACGTTGCCGCCACACCC<br>ACCTGCTCCGCGCTGGCCCTGGGAGAGGAGCCAACCTGTCTTGCT<br>TTTTTTTTTTTTTTAACCAGGCTAAGCTGCTGTAAATTAAGTCTTCCT<br>GGAAATCTCAGCAACTCTATTGGACGTGCTAATGGCACAGTTCACT<br>CCAAACTGGATTCATATTTAAATATTACATATCCTCCAACTCTTGAGT<br>GCTTTAATTTTGTTATCTGGTTAGCCTGGGAAATCATTTTAAAAATAC<br>GCTATAAGTATCAGACTTTCCCTTAGCAATTATATAAAATCTCCTTGC<br>AGTTTTCTGAAAGGCACAGAAGTGCAGTTTGATTTGGTTGTCTTAT<br>CAAAGGACATTTATGCATGAAAATAGGCTGCAGGAGTATTCTATATA<br>AGATAGCAGGCAGTTCTGCACGTCTGTTTATATGCCATTCACACCAT<br>GAACACATCCAATGCAATTGTATTCATCTTCTGTCATTTTTTAAAATT<br>AGAGTTTACTACATGATTTTTGGAGGACGGCCATATCATAGTTATTC<br>ATCCCTTGCATGGTCTGTTCTGCCACTTCTAAAGTTCTGCGAAGTTT<br>AATTTTTGGCAGGTTTCAAGATTCTCTACATACTTTGTAGGTTAGAG<br>TGAACGTGCACAGTATATTGTGTAATAATTGCAATACAAGACGGTGT<br>GTTGTGTTGTGGCACGGAATGCATCAACTTAAAGATAGAATTGTTAT<br>GCAGAATAGAGACCTTTGAAATATTTGGGTATTATCAACTTTTATTTC<br>ATTTTTTGAGAAAAACTTAAAAAGTGAGCCCAGTTTTTTAAAAAGC

	ATTTCATTGTTCTAACCTACAATTGTGCATTGTATGTTTGTGTGTGTG<br>TGACGGAGGGTGGGATGAATAAAAGGGGACATTAAAAATATTCAG<br>ACCATAAATATAGTAGATTGGAGGTTCTGATTTGATGAGCCAGTTTC<br>TCAGCCATAAACTGAGAGGGGGTTGGGGATACACTTCATTGTCCTT<br>CCTGGCTAATGTAAATCCCTTATATTTAAAAAGATATTTAAAAGTATT<br>CCAAGCATTTTGGCAGAAAAGCATAGTATCTAATGTTATTTTTTTCTT<br>ATTTTATGTGCATGCCTCTTATCCATTTAAAAATAGCTTTACTGACCT<br>ATAATTTACACACTATATAATTCTCCCATTGAAAGTGCATAATTCTGT<br>TGCTTTTAGTATATTTACAGAGTTGTGCAGCATCAGCATAATGTAATC<br>TAGAACATTGTCATCAACTACCCCCAAATCTCTATTCTTCCCTTCCC<br>CTATTAATTACCCAGCCCCAGGCAAGCACTGATCTACTTTTGGTCTC<br>TATGGATTTGTCTATTTGTGGACACTTTAAATGGAATCATACAATATG<br>TGTCTTTTGCGACTATCTTCTTTCACTTATCATAACTCAATACGGCTT<br>TAGATTATTTGACCTCGATGTTCTGCCTCTGAACATAAAATATTATCC<br>TTGCATTCCTTGATGAGTTTGAGGATTGAGAATAATTTGCATGAGAC<br>AAAAATTAGAAACTAGTTAGAGCAAGTAGGCTTTTCTCCATCACAT<br>AAGCTGATCCATCTTGCCAATGTTAAAACACCAGATTGTACAAGCA<br>CAAGCTGGGACGCAATGTGTGTCCCTCTATCCCTACATTGACTTTGC<br>GGGGGTGGGGATGGGGTGCGGGGTGAGTGAGGGAGGACTGCAAG<br>TGACAAACAGGATTCGTCAAAAGAGAGGTGTATTAAAGTGCCGAT<br>CAAATGTAATTTAACAGCTAAACTTTCCCTCCTTGGAAAACAGGTG<br>ATTGTTGAGTATTTAACGTGAATCGATGTAAACCTGTGTTTGGTGCT<br>TAGACAGGGGGCCCCCGGGTAGAGTGGAACCCCTTAAGCTAAGCG<br>AACAGGAGCCTAACAAAGCAAATTTTTCCGTCTGCCCTTTCCCCCT<br>CTTCTCGTTGGCAGGGTTGATCCTCATTACTGTTTGCTCAAACGTTT<br>AGAAGTGAATTTAGGTCCCTCCCCCCAACTTATGATTTTATAGCCAA<br>TAGGTGATGAGGTTTATTTGCATATTTCCAGTCACATAAGCAGCCTT<br>GGCGTGAAAACAGTGTCAGACTCGATTCCCCCTCTTCCTCCTCCTC<br>AAGGGAA
LTF	TGGGGTGCTATCCCGGCCAGGAGAATTGAAAACTCAGAAGTGGGG<br>TGGGACTGGAAGCATGAGCTCATTCCACTGCCCCTTGCCCACTGGG<br>GAGCAAGCTCAGCAGGTCTTTGGAGAAGCCAGCCCCAGGAGGGCT<br>GTCCCTGTGAGGAGAGGGCCAGAGGGCAGAGGGTCCTGCAGAGG<br>AGGAGGGTTAGGTGAGGGGAGGGAAGAGGAAGAGGAAGAAGAG<br>GGCTGATCCAAGCAGACAGAAGCTCCTTGTCTCTGTGGGGCCTTTG<br>TAAATTGGGCAACATTTGGCAACAATACGTGTTGGCTAATGAAGCC<br>ACTGTTTTCCTGCCTGCTGCCCTTCAGCAGATCTTCCCTCCTGATGG<br>AAGCTCCCAGGAGCCCAGAAACGTTCCCAGACTCTTAGGGCCTTT<br>GGCTGTCTTCAAAAGGGCCAATGTGGGGCCAAATGTGAGGGCCTT<br>TCTCTCAAGGTCATTGTCTGTGTTGGGATCTTGGGGGGACTTGGGT<br>GGGTAGAGACACAGGCAGCTCTCCATCCATCTCTTTCTCCTTCTCC<br>CCCATTCTCTGCCCCTCTCTTCAGGGTGATCAGCAAATAAATGATCA<br>ATACTAAGACACCAAGGCCCTAAGAAGTCACTGGATGGGGTCCCG<br>GGTGGATTTCTAGCTCTACTGTCCACTGGCTGTGAGACCATGAACA<br>GGGTGCATTGCCCAAGTCCACTCAGCTGGAAACAGGTATAACAAG<br>CACCTTTTGGTATTTCCAGAGCATCAGAAGCCAGCTGATGTGTGGA

	AGGGGCCAGGCAGGGCCTGCTTCACGGAAGGTTCAATTCTTGCCA<br>GGTTCCCTTTGTGCTGAGATCCCTGGGCCTCAGTCCCTGCACCCTG<br>AGCTCTCAGAGGGTTCTTGGCTCATGGAGGAGAAAGCCCCTGGCT<br>GTGGGCCTAATGGGAGACTCAGGGATAGCAGTTGTATCTCACTCAC<br>TGTGTGACCTCAGGCGACACTCAGAGTACCTGTTTTTAGAATGGAA<br>AGAGTGACTGTCTTAACTCACAGAGTCGTTGTTAAAGCCATGTGAA<br>ACAAAGTCACTGACAGCACTATTTGGATTTTAAAATGTCATCCAAAT<br>ATTAAAGATTATTATCCTATTTCTCTGCTAGGGATGGCACAGCCACC<br>CATACACAAACACACCCACACTAATGTGGACACTGCATGCCACCAA<br>TGATGAACGTAGGCAGCTGTGTGCTGGTCAACTGGCCCTCTGGGG<br>GTGTGGAAGGAGGAAGCCCTGATTTAGAGCATTTGCTAATTCCATG<br>GCATAAATACATCGACTATGCCTGATATCAAGCTGCCATCAGTTAAC<br>CCACCCCAAATCCCTAAATGTTTAACAAGTGGTTCTCAAGTCAGCA<br>ATCTCTCACCACTGGCAGGGAAAAGATACAGGGCCCAAGGTCTCC<br>CATCTGCCAAGCTATTGTCCAAGCGTGAGTTGATGTCTCAGGAAGG<br>CCTTTTCTGCCTTATCCCACCTGAAGCCAGTTCTACTCAGATCTCAC<br>ACTTCCTTCCCAACACTCTTCTTGGGGTTTGAAGCTAGAGGCTCAT<br>AGAGGGCAGGGTGTGAGGTTTACTGGCTTGCAGGGAGGACTTAGG<br>TTGGCCAGAGACTGTTGCCTGCTAATGGGGCTTGGGCCTGCAATTC<br>TGTAAAGGCTCCCTGGAGGAGTTTTGAGGGTGGGGGCAGAGCAGG<br>GTTAGGGGGAAGGGAGTCAGCCTCACACACCTGCCTGTTGAACAC<br>TAGCTCTTGCCACTGCATGGCAGTTTCCACAGCAGCTACCTTCTTTG<br>TGCTCCTTAAACTCATGTTACTGTCTCAGCATTTGGAAACCTGCATT<br>CTGCTTCCCTGCAGGCTCTTTGAGATCTGACCCAACTCCTGGCACG<br>TTGGAGATGTCCCATAGACCAGTGTTGAATGAATGGGTAAGTTAAT<br>GAGTCCATGTACACAGGCTTTGGCATGGCCTGGCTTAGGCAGCCCT<br>GACCCTCACTATCCTTCAGACCCAACACATTACAAACAGGATCCTT<br>TGTCCCTTTCTTAGCCTGTTCCTCTTCCTCATTTATTCATTCCTTCAA<br>CCGATATTGATCATCAAGTGCCTATAACATGCACAGAGCTAACTGGC<br>CAAAGGATTTGAGGGTGCCTCTGCGGCTCTTTGGTGGGTTTGGTGG<br>AAAGCGGTAGGAAATATGAGAAAAGGTTCAGGGTTGGAGGGACTG<br>GGTGAGTTGCATTTTCGATGCATTAAATTTAGGCGCCTTCAGGACCT<br>GCAAGAGTACCTTTCCAGAGGCAGATGAACACATGATGTGGAGCT<br>CAGGATAGAGGAGGAGGATTTTGTTAATCCTTCTTCCAACAAGAGT<br>TCCAAGCCATGGCTGGGTGCAATGGTTTATGCCTGTAATTCCAGCA<br>CTTTGGGAGGCCAAGGTGGGAGGATCGCTTGGGCCCAGGAGGTCA<br>AGGCTGCAGTGAGCTGTGATTGCACCACCACTGCATACCAGCCTAG<br>GTGACAGAGGGAGACCCTGTCGGTAAAAAAAAAAAAAAAAAAAAA<br>GAAAAGAAAAGAAAAGAAAAAAAAGAATTCCTGGCCAGAAAGCCA<br>GAGGGAACTTTTGTTGTCCCTAGTGCTCAGGTAATTGGTCACTGGG<br>TTCATGCTCCCAAGCCTTTACTTGTTTGGTTGTTTACTTTACAGAAT<br>GCTCTACCTGTAGAAATCCCACCCTTCCTTCAGGTACCCAGGCTAA<br>ATCTTCCTGGAGTTCTTCTGGAGCCCTCATGATGGGTGCTGGTCATT<br>CCTCCTCTGGGCCCTCACACTCACTTGCTTTATGCTGCTTGGCCTCT<br>TCTTAGGGAGCCTCTGTGATAGAAGTGCATTCCTGTGACTGTCCCT<br>CCCCCATAGTGCTGTGAGCTCCTGAGGGCAAGGACAGACCCCTCTT

	CTCTGTCTCTCATGGCATCTACCAGTTCTGGCATGAATGAATGAATG<br>AATGAATGAATGAATGCTGTTAATAGATCAGTGAAACTTCATTGTTT<br>TTTGGGGCAGACTCATCCCCTACATCAGCCTCCTCTGCAAATGGCC<br>TTGAGGGGCTGCTCTGCCTGTGTCCAGATGCTCACATCCCTGCCCT<br>GGGGCTGGGCTGTTCCAACAGGCACAGCAGGAAAACAGTCTGCCC<br>TGTTGCCTACCCATGCTGCCTTCAGGGCACTCCTTAAGCTGAGCCT<br>CTTGGTAGTGGCCCCAGGTTCTCTGTGTTCTTGCCAACACTGTAAC<br>ATACTTAAGAGGGCCCAGGGCCCTCTGACCTCCCAGTCATCCTTTT<br>TTAAAATTTGGACTCAGAAAAAGAGACACGGGTTATGATGTTTCTT<br>AATTCTTTTATAATGATGAAAAGGCAAAGTCTTGTTGCCAATTTAGG<br>TACAAAGATGCTTCAGCACTCCTGGGAATTAGTCAATTTTGTATTTC<br>TTCAGTATTTTTGAAAGAACTTATTGCAATTATTGATGATGGCAACT<br>TTAAATGGTGCAATATCATGTTTCCAAACAATGAGAGACCTTGGATC<br>TGTCACCCCAAAACCCAGCTGGTGATTCTAGCACCAAATCTCAGAC<br>CCCAGTCTCATGGCAGGCAAGCATGATCCCTGATTAGCCTCCACCC<br>TGTGCCTTGGCAGGATCATGCCCAGAAATGGAGGGGCTCCCCAGC<br>CTCCTTAATGGCCTCTCCACCTTGGGCTGAGCCTGCTTCTCCATGGC<br>TAGGTGCCCACTGCATGCTCACTCTTGGCAGAGCTGGCTCCCTGGG<br>GCATTGCCTATCTGCTCCAGGAAATGCTTTTTAGTACCAAGTAGTCT<br>AAGCAGAGTCAAGACCAGCTTTTCAGAATAAGCAGATTTCAGAGT<br>CACATATTGGTCAGACTCACCTGCCAAGAAAGGTGTTCAGGTGACC<br>TATTATTTCCCTCACTGTTAGTACCTTTTTTCTTCACTTAGTTACCCTT<br>TTTTTTTTTGGTGGGGGTTACAGAGTCTTACTCTATTGCCCAGGCTG<br>CAGTGCAGTGACATGATCATGGCTCACTGCCACCTTCATCTCCCAG<br>GCTCAAATGGTCCTCCCACTTTAGCCTCCCAAGTAGCTGGGACCAT<br>AGGCATACACCACCATGCTGGGCTAATTTTTGTATTTTTTGTAGAGA<br>TGGGGGTTTCCCTATGTTGCCCAGGCTAGTCTTGAACTCCTGGGCT<br>CAAGCGATCCTCCCATCTTGGCCTCCCAAAGTGCTGGGATTACAGG<br>CATGAGCCACTGTGCCCTGCCTAGTTACTCTTGGGCTAAGTTCACAT<br>CCATACACACAGGATATTCTTTCTGAGGCCCCCAATGTGTCCCACA<br>GGCACCATGCTGTATGTGACACTCCCCTAGAGATGGATGTTTAGTTT<br>GCTTCCAACTGATTAATGGCATGCAGTGGTGCCTGGAAACATTTGT<br>ACCTGGGGTGCTGTGTGTCATGGGAATGTATTTACGAGATGTATTCT<br>TAGAAGCAGTATTCTAGCTTTTGAATTTTAAAATCTGACATTTATGG<br>CGATTGTTAAAATGAGGTTACCATTTCCTACTGAATACTATCAACAC<br>CAAAAAAGAAGAAGGAGGAGATGGAGAAAAAAAAGACAAAAAA<br>AAAAAAAAGTGGTAGGGCATCTTAGCCATAGGGCATCTTTCTCATT<br>GGCAAATAAGAACATGGAACCAGCCTTGGGTGGTGGCCATTCCCCT<br>CTGAGGTCCCTGTCTGTTTTCTGGGAGCTGTATTGTGGGTCTCAGC<br>AGGGCAGGGAGATACCCCATGGGCAGCTTGCCTGAGACTCTGGGC<br>AGCCTCTCTTTTCTCTGTCAGCTGTCCCTAGGCTGCTGCTGGGGGT<br>GGTCGGGTCATCTTTTCAACTCTCAGCTCACTGCTGAGCCAAGGTG<br>AAAGCAAATCCACCTGCCCTAACTGGCTCCTAGGCACCTTCAAGGT<br>CATCTGCTGAAGAAGATAGCAGTCTCACAGGTCAAGGCGATCTTCA<br>AGTAAAGACCCTCTGCTCTGTGTCCTGCCCTCTAGAAGGCACTGAG<br>ACCAGAGCTGGGACAGGGCTCAGGGGGCTGCGACTCCTAGGGGCT

	TGCAGACTAGT
MAOB	AAGGCTACATAAATGTAACCAAATAATTGATTCTGCATCAGTGAGCT<br>TACACTATGAAGTTAGAATTATATAAAGAAGAAAAATTCATTCAAAA<br>TAACTCTGTTCAACAACCAATAATTAGTATTCGGCCCCTAATATTTAT<br>TTTTAATTTCCTTTTTACTAAAAGAATTGGTGAAAATCCTTGACCCA<br>ATGTTCTTAAGAAATGTTTGTTCTGATTTTCACTTCTTCTTGATCAG<br>AATTTTTGCTATTTGACCTTCCTCTGTGTGCCATACAGTTCTTTTCAT<br>GTCATCTGTTCTGATACATCATTCCTTGTTTAAGGCCCCATGTATGAG<br>TTATGAAGTATTCTTTTTTATTTTTTAAGTTATTATTTTCTTTTAGAGA<br>TGAGGTCTCACTATATTGCCCAGGCTTGTCTCAAACTCCTGAGCTCC<br>AGCAATCTGCCCACATCAGCCTCCCAAAGTGCTAGGATTACAGGCA<br>TAAGCCACCATACCCTGCCAAGTTGTAGAAATATTCTTAGTCTTGAC<br>ATCACTCATTGTTAAAAAAAATTCACTTTGGGTGCAGATTGCTTAGC<br>ACTTATAATGCAAATAAGGTCTTGTTTTTCTTCAACATGCTCTACAG<br>CATGTTTGGGGATGACCTCTATCTTTCCGAAGACCTGTGACTTCGTG<br>TCTCTGCCTGACTTTGTATGTTCTGGGGCCAACCCCTCTGTAATGTG<br>CCCCTATGTTTCCTGTCCTGCTCATACTATGACAATGGAACCATAAG<br>GAAAAAGGCCATATTCTGGTTCTTTGGAAAGTAAAAGTTATTTTTCT<br>TTCTGTCTCAATCTTGTCTCTATAAGCCAGGTCAGCACCTTCACATG<br>GCTGTCGGTCTATTGAGGTGAGTTGTGAATGCCCACCTGTCATTTG<br>ACTTGTTCCTTGCCAACTTTACTTTGATCAAATACCTGACCAATCCC<br>TGGCTCTGGGGTAGGTCTTTCCTTTGGCATCTTAAACCAGCCTGTG<br>GACTTTTGAGGTGGGCCTAGATTCTGTAGCCCATTTTCCTGTGAAA<br>GGCCTTGACTTGGATTGAAGCTGGGCTCCTCCAACTTCTGTGTTGC<br>CTGAGGATGTGTGATCCCCCCTAAACAAGTCCTTGGGTCCAAAAGC<br>AAGGTCTTGCCCCATTGAAGGGTCCCCTACCACCTTCTCATTGACC<br>TCAAGGGAAAGATTTGGATGCTTTCAGATTAGGATCCAAGCTGATG<br>ATGAACACTGCACTGGGCAGAACCAGGAGCCCTATAACACTTCCAA<br>TTCAACATTATTAAAAAGCTATCCTGGCTGTAACGGCTGACCCTTTC<br>ATCACTTTATCCCTCTATATGTTTTTAAAAAGATTAACAGAGTCATTG<br>GGGAAAAAATGGAAAGCTCTGAAGAATCTAATTTTAAAACAAATCT<br>TCTATCCCCTGTCAAGCTTCCTGCATTAAAAAAAAAAAGCGAAATGT<br>ACAACTTAGATAATATCTGGTTAACTCTGTTCTGCACAGCCTACTTC<br>TTTATTGAAACCGCCTGTGGCATCATGAGACATTCGCTGAAATATCC<br>GTTAATGAAAAGCTGGACAGTAAGAAAGAAGTAATAAGAACATTTT<br>ATCTCTTTGGGAATCTTCTAATAAAAAAATGAAAGGAAAGTAAGTT<br>CATTTTAATTGATGAAAGGGACATAGATTTCATCAAGTTGAAAAGG<br>CACACAAACACTGCTAGGGGCAAACTGCACTCCTCTGCGCCTTTTG<br>GTTAGAGCTGTTGAGTACTGCTCCATTGGAACGTGAATTAGGTTTAT<br>TGTAATGGTCTGTTTCCATATTTAGTTTTCCCAGAATTCACATTGCTT<br>CTTGTTTATAATGGCATATACCTAATGATGAGCACAATGTCTGACAC<br>ACAGTTGTTGCTAAATAAATTATTCTTGAATGGATGAATAAAAGATA<br>ATGTATTATCCCTTCCAGCAGGAATTGATTATCTAGTTGGGAGGGGA<br>AGACAGAAACATACACAAAAATAAGTGATGATTCCTATCATTGTATT<br>TATTGTTAATCGTCTACTGTTATCATCGTATTCTTCACTATTAATTTG<br>TGGTATTTGTGATAAGTCATAAAGGTAGCCAAAGATGTTCTTTCTAC

	CTTAGCCTGTGTATGTTTCTTCCATTATTCCAGCCTCAGGCTTGCCTC<br>TCAATTTAGCACTTGCTGATCAGCCTGCCTGGAGTGCTCATCTTCCT<br>CAGTAGCTAATTAACTCCTATTCATCCTCCAAGCCCCAGTAGAAATG<br>TCATTTCCTCCTGGAGGTCCTCTGTGGTTGCCTGGGCTGGGTTATCT<br>AGCTCTGCTTTATGTTCCAGGGCAATATTCAGAATTATGCTTGTGAT<br>CACTCTTCCTGTATGTCTTCCCTGACAGAGCATGAGCCCCATGAAG<br>GCAGGGACAGACTCCATCCTTTTCATCTCTTTCCCAGGGCTCAGCA<br>TAGCAGGTGTTTAATAAATGCTGGTATAATGAATGGACACTTTCTTA<br>CAAAGAGCACCTTGGAATAGGTAAGGGGCTCTAGAGCATGGAACT<br>GGTTCTCAAAATGATGGGGAGGCAGAGGCCTGGAATAGACAATGT<br>GATCAAAGTGTGTGGTCCATGGACCAGCAGCACTGACATCACCTG<br>GAGCTTGTTAGAAATGTGTCTTCTCAGACCCCACCCAAAATCTGGG<br>TGATTTGTATGCACGTTCAAGTTTGAAAAGTCCTGCTTTTCATTTGG<br>CCACACAATGCAGATGGAGAAGACAGACCTCAGAAATCATCCCAC<br>CTAAGAGACTGAGGGTCCTGAATGGATCCAGATGATTGCCTGTATG<br>TGGGGACAACAGGTGGCTGCTGTTTTTCCTATGACCAAGTCCTAGG<br>TTCTAATTCAATCCCCACCCCCATCCCTTTTAAAAATATACCTCATGG<br>AGCACAAGGTAACTGATTACCTTTGTTTGGGGAAAATGCTGTTTGC<br>TAATGAATTTTCTCCAGATACCATGGAACCGTGTGGATCCTCTCCTG<br>CCCTCATTGCCAGTTGGACATAGAGAAGGGTTTATCAGTCTGCCTTT<br>TATTCAAAGTCTTAGATCCCCAGGCTCCTGGCTGACTTGCACTGTTG<br>GAACTAAGCAAAAGAGATTCCAAGAAGATCCCACAAGGATCTCGT<br>GTTAGTCCATTTTGTGCTGCTAGTACAGAATACCTAAGGGGGGTAAT<br>TTACATTGAAGAGAAATTTATTGTATCATGGTTCTAGAGTCTGCAAA<br>TTCCAAGATGAGATGTTGGCATCTGGCAAAGGCCTTCCCAATATGT<br>CATCCCATGGCAGAAGGGCAATGAGGGTTAGAAGGAGCAAGAGGT<br>CAAATTTGCTGTCTCAGGCCCTTTTTATAGTGGCATTAATCCATTCAT<br>GAGGATGGAGCCCTTGGAACCTAAACATGCCCCAACATTGTTGCAT<br>TGAGGATTGAATTTCCAACACGTGAACTTTGGGGGACACATTCAAA<br>CCGTAGCAGATCCTCTCCATTCATCCTACCTCACCTGGCACGTAATT<br>CACTTGCAGGCTAGGTGGGGAGACAAGAGTATCCACAGCCAAGAA<br>GCCCACAGCTTCAGGGACCTGTGAACAGAAGTGGACCAGATAGGG<br>GATGGCAAGGAGGAGGTCAGCACAAACTGTCTGCAGGGTGAGGTA<br>GCTTCATTCTCTGCAGTGGGGACATGAGGTAGGGATCGGGGTTCAG<br>GCACAGGGTCTGACTGTTTTATACCAAAGCAGGACAGATGGAAGT<br>CAGAGGTGGACAAAGACTTTTCCTGTCACCTGTGACTCAGTCACA<br>GAAGTGCCCAAATTGTCTTCTGGTCTGAGGACAGCAGAAGCTCAG<br>GATACCTTTCTAGCAGGTAGATACAGGGAATGGTGTGGGATGAGGC<br>AGAGGTTTGCTTCAGGTTCTCATCAAATTCCCCAGATCTGAAGCCA<br>GCCCCTCTCAGGCCTGAACAGAAGCACTTCACCTGTTTATTTCTTG<br>AAAAGCCATTCTATGTTGCTGCTTCCCACTTTTAGTTCTCAGAGGTG<br>GCTTTCCTACTGTCCAGAAGACATAAACTTCTGTTTTCCCTGGAGTC<br>ACCTTAGCTTAAACTCCTATAATCTTGTTGAATCATCCCTGTGTTTGG<br>CTAAACCAGGCTCCTCATACCCACGTTCCAGGGTCACACAGGTAAC<br>TTGGCTTGCTGCTTCTGGGGGCTCTGAATCCCACAATGCCGATCCA<br>CCACCTCTTCCCTGCTGCCACTAGTCTCCTGGGGTCTAGCCTCAAC

	AGCTTCCACCAAGGACACACTGCATTGCTTAAGAGTGTATTTAAGG<br>TGCGGCATTTGTGATGCTAGGGAGGAGCCTTCCTGACTTAATCACG<br>TAGGGGGAGTGGGGAAAGAAGTGGGTATTTGGAAGGAGGGTGAG<br>GTGGGCTGGGAATGCCAAGAAATGGTAGTACTGTGTGGTGAATCA<br>AAGTGGGTCTTGGAATCTTCCTAGCTTTTCTTTTCTTTTCTTTTCTTT<br>TTTTTTCTTTCTGACTTTTTATCTTATGTTGTCACTCACCATGTTTTGT<br>TTTCCTCATCTGCAAAATGGATTGTTGTAAGCTTGAACAGGAATCAT<br>GCATAAAGTACTGAGCACAGTGCCTGGTACCTTGAAATCACTCAAT<br>GGAAGTTTCTTATTATTTGCCCTACACCCAAGGAGTAATTGGGGCCC<br>TGAAGGAAGACACCCCAATTTGCCGTCCTCCCAGCCGCCAAGCCT<br>GATAGTGCACTAGGGAGGCTGATGGGAAAATACCGTGCAGAAGGC<br>TGAGGAGTGGGGATTCTGGGGCGGGACTCTTTGAAGTCCTAGGTG<br>ACCTCTCCGCCCAGGCACCCGCCCTCCCCGGGCCTGGGCTGCAGA<br>GCTGCGGGCGGGGGCGGTGGCCCTGCGCTGCACCCGGCCTGCCTG<br>CGCGTAGGCGGGCGGGCGGGGCTGCGCGTCCGGGCTCCCGGGGCT<br>GGTAATATAGCGGCTCGCCGAGGCGCTGGTGCACGGGGGCAGCGC<br>GCAGCAGGCCGGCGGGCAGGCGGGCGGGCTGGCTGGCACGCAGG<br>ACTGGGATCGAGG
MB	GGCCTGGCGCTGGTCCAGGAGGGTAGACTCTGCCAGGGAGGCACA<br>AGCTAAGCTGGGGGTGGGGTGGGAGGGCCTGCTGGCAGGAGGGA<br>GGCACTGCCCAGACTGGCCTCCAAAGGCTGGTTCCCAGAAGGAGG<br>AGGAAGAGAGTAGTGAAGATCGTGGGGAGTCCTGGCTTTGAGGAG<br>CCAGAGTTCTCCTTCTTGCCACATCTTTGATCTCCCTTGGTGGTGTC<br>TTAAGCCCAGATTTACCAAAGGGAATTGTCAGCTGTCCAAGGGCTA<br>GCAAATTCCTAGGTCACCTAGATTGGATTTTCTGACCATAAAAACTG<br>TGGGCCAGGTGCACAGCTGCCTGAGGGGCTCAAACCTGTGCAGGT<br>GGGTGCTGGAATCCCCTTTGCTATAGCGTTCTCTTTGTGTGCATGTG<br>ACCAGAGGTGAGAGTGGCTGGAGACCGAAGATGGCAGAGCCCGT<br>CTTTCTCCACCAGACTGAGGCCCCCGACTTGCCTTGCCTGGGGAGG<br>GTGCTCAGTGGAAGTCCTGGGATGTTCAGTCTGGACAAGAAAAGT<br>CTTGGGTGAGCCTGCTCCTTCTCTTGTGAGGCAGACTCAGCTGGGC<br>CTCTATATCCCCAAGGAGCAGGCGGAGCTGCCGCAAGGAAGTGCA<br>CGTGACATGGAGGCATCTTTTGGTTCGACATAAAAAAGGATTTTTA<br>AAGCTTTAGGTAGTGAGCTCACCACTCCCAGGAGTATTCAAGCAGA<br>TGCCAGATGATGATCTGTAGGGAAAACTATGTTTAGTGGGAAATTG<br>GACAAAATACCGTTGAAGGCCTCTTTTAGTCACAAGATACTATGGC<br>CCTATAATTCTAGATTTCTCTGCTTCTAGCTCTCTATAAGTCTGGAAT<br>CTAGAACCCAAGTTTCTCCGGTGCTGAAAAGTTGAAGACTTTGAC<br>ACTCTAGAATTTTAATTCTGGGATCTCAAGATTCCAGAATTGGGAGC<br>CTTGAGGATGATTCTACAAGTCTCAGTTTTGGATTCAAAGGATCCT<br>GTGATATGAAAGGGAAAAAGAGGAGGTCTGACTTGGTGGGATCCA<br>GTTTTGCCCCACTGGGTATCAGACTAATGTGCCCTGCATTCGTGCGT<br>GTGTGTGCGTGTGTGTAGCATGAGCGTGTAAGGCTGTAGAAAGTTT<br>TGCACACAAAGGCAGCCTGGCTTTCCTATCATGTGTCTGCAGCTTC<br>TCTGCCCTGGCCATAATGAAGGCTGTCACAGGGATAATAATAGCCC<br>CCAAACAGAGCTGGTGACTCCAGGTAGCCATAGCAACTGTGAGGT

	AACGCTGCTCTGTTGTAGAGATGTGTGAGTACAAACAGATGTCGGT<br>GCCTTCCCATTAGCTTAAATGGTAAAACTTAAAAGGTCCATCAACTT<br>CAAAGCTACAGGTTTCCATGAGCATGCCAGTTAATGGGGGCGCTTG<br>CCAAGCGGGTGATAGCTTCATATTAAACATTTCAGCATCAGTAATGG<br>GTTGATGAGATTTTAAATGCTCAGGCCATCCCCAGACAACTCAGGA<br>GGCCAGAGACGGGAGAAGAGCTGGGATTCGGGTAGTAATCAGCCC<br>TGCCCTGCCCCGCACCCTCAGGGGTGTTCTGACACTCTCGGGAGG<br>GCACAGGCCAGAGAGCATTTGAAAACCGCACATTGCGTCTCAATCT<br>CCAGGAGTCTCAGATCTGCCTCTTGGCCTTCCCCAGCGCACCAGGC<br>AGGGGGCCCCAGGTCAGATGTGGCACAGCTCAATGCCATCTCCTTC<br>TTTCTGCCCTCCTCTCTCACACCCTGGACTCACCTCCAGCGAGTGA<br>CCAGCAGTAAGGGTCAAACTGCTGAGAGTCTGAGACCGGGGCAG<br>ACCCTCTCAGAGTTGTCTTTCCTTCTGAGGCTGGGCCTGGCACAGA<br>CTGGGGCCTCAGTAATGGGCTTTCAAATGAATGAATGAACAAAGG<br>AACAAACAGATGACTGACTGACTGAATGAATGAATGAGATCCCCTT<br>TGGCATCCCCATGGCTTCTTTTTTTCTTTTTGAGATGGAGGCTCGCT<br>CTGTTGCCAGGCTGGAGTACAGCGATCTCGGCTCACTGCAACCTCT<br>GCCTCCCGGGTTCAAGTGATTCTCCTGCCTCAGCCTCCCAAGTAGC<br>TGGGACTACAGGTTCGTGCCACCATGCCCAGCTAATTTTTGTATTTT<br>TAGTAGAGACAGGGCTTGAACATGTTAGCCAGGATAGTCTCGATCT<br>CTTGACCTTGTGATCCACCCACCTCAGCCTCCCAAATTGCTGGGATT<br>ACAGGCGTGAGCCACCACGCCCGGCCCCCCATGGCATTTTAGCTAC<br>TTATTTTGTTACTGCTTCTTCCACTGCTAAAGAGATGGGCCTTTGGA<br>GGTCGGCTGGCCTAGCATTTGGGGTTGACTTCTGGAATGGTCACTA<br>GGGAAGGGGCGCCAGAGGTGCAGCCGGTGCCTCTGACCCTTTTGG<br>TCGCTAGGAGTCAGCCGACTCAGTACACAGGACTCACTGAATGGA<br>GACACAAGGCTCCTCCAGGGAGTGGCGGCTCATGGCAATCCTAGA<br>ATGGTCACCAGCCAGGCTTTAGAGACCCACACAGAGGGCGTTCTG<br>ACCCAAAGTTGCACTGGGGAACTCCAAGTTTGGGGATTCTTTGAAT<br>TTAACTCTTTTTCTAGCTACATTTCCTATTATTTGTCCAATTCTTACCA<br>AACATCTCTGTTCACATTCTGAAGCTGGGATCTGACTGGCAGAGCT<br>AGTAGATGCTGACTATTCAGATGGAGCCCTGACATTGGCTTTCTCA<br>GCTTGGCTGTGACTGGCAGCAGGTTTGCGGGAGAACTGTGTGTCC<br>CAGAACATGACTGGCTACACCTGCACCTCAGCAAGATTGGGGCAG<br>GGCAGTTATCTTCAAAAAGCTGTGTAGGTGGGGCAGTCATTACTGA<br>CAAATCCAGTGCAGACCCAGGATGGCCCAAACACTGGCTTATCCTT<br>TCTGAATCTCATCTCCCACAGCTGTAAAGCGGGGTGGTGCTCGCTA<br>CCTCACAGAGGTGTTGTAAAGATTAGATGTAATCTTGCCAAGCAGC<br>CACTTTGTAAACTGTATAGTCTTATGCAGATGGAAGGAAGGGCCTG<br>TGCCTACCTTGATCATAGCACTAAACAAACTGTACTGTATTTTCATT<br>CCTCTTAGTTATCTCCCTAAAAAGACTCTGAGTTCCTTGAACACAG<br>GAAGGTGTTTTATTTGATTTTGTTATCCTCAGCATGTAGCAGTGTCT<br>GACACACAGTAGGTGCTCTATCACTGTGAGAGGGATGGATGGATGG<br>GTGGAGTTACAGATGGATAGAAGGATAGATGGAGGGATGGGTGGAT<br>GATGGATGGATAGATGGATGGAGGGGGGATGATGAATGGAGGGATA<br>ATGAGTGGATGAATGAGGGAATGGGTGGATGGATGGATGGAGGGA

	TGGAGGAACAGATAGATAGATGGAGGGATGGGTGGGTGATGGATG<br>GATAGATGGATGGAGGGAGGGATGATGAATGGAGGGATAATGAATG<br>GATGAATGAGGGGATGGGTGGATGGATGAATGGAGGGATGATGGG<br>TGGATGAATGAATTGAGGGATGGATGGATGAACACATGGATGGATG<br>GATAGATGGATAGATGGAGGAACTGGTGGATTTTGGATGGATGGGT<br>GGATGGATAGATGAATGAATGCCTGGATAGACAAAGAGATGATGGA<br>TAGATGAATAGATGAATTAAGGGATGTCGGATAGATGGAGGGATTG<br>ATAGATGTTGGATGGATGGGTGGTGGATGGATAGATGAGTGAATGC<br>ATGGATAGACAAAGAGATGATGGATGGATGAATTAAGGGATGACAG<br>ATGGATGGATGGATGAGTAACTGGATGGACAAGTGGATAAATGGAT<br>AGATGGTTGAATACCTGAATGGATTGAAGGAGGATGCATGGATGTA<br>AGATAAGGCTAATCATCCTCCACTCTCTTTCTTTGCAAAACCATCCA<br>CCCATTTACTCAATAAACATTTATTCAGTTCAAACTTGGCACAAAGC<br>ACCATGTGAGGCCCAAGAGATACGTGGGTTAATAAAACAGAGCTC<br>CTGCCCTCCTGAAAACTGCAAAGAAAGGGGCGTGGCTTCCTGAGT<br>TCAAATCCCAACTCTGCCAGCGACTAGTTGTACATCAGTGATGTTTC<br>CCTACTTTCTCTCAATTAAATAGGGATAATGTCAGTACCTATCACATT<br>GGGAGGTCTTGCGGGGATTAAATGAGTTACCAAATGCCAAGTGTTT<br>GGGACAGGGCCTGGCACCCAGCAAAGTCTCTTGTGAGTGCTGGCT<br>GCTATTATCCTAATGGAGAAGATGGCATGAAAACCAGGAAATAGGA<br>TGCCCTTTGGGAAGCAATGCAACAGGAACTTACACAAAGAAAGGA<br>AAGGAGGAAGCAATTAGTGGTGTCTCAAAGGAGTATGTCAAGAAA<br>AACTTTTCAGAGGGAAACCTTTGAGCAGGGTCATGAAAACAGGAG<br>TTCTCTAAGAGATTGTGGACTTGCCTGGGACCACCTGGCTATAAGC<br>ACAAAACCATCCGGTTCCTTTCTGTCACTTCTGGCTGGGTGAGGGG<br>TCTCTGGCAAAGGGGCAGAAGGTGCGTGAGAGGTTGCGAATGGCC<br>AGGACTGTCCTGGGGCCAGCCGGGGCACCTGGTGGCCAAGCTTAG<br>AAACATGACAGGTCCTCTTGGGAGGGCTGACCGCAGGGAGCGTTG<br>GGTTTCAGGCTGCTGGCGTCGGCTTCTGTGGTGCCCTTTCTGTCGG<br>CTATGAGAGTCCAGACAGTGCCCAACCTCCTCCCCTTCTTTCCACA<br>CGCACAACCACCCCACCCCCTGTGGCCTGAGCTGTCCTGCCTCGCC<br>ACAATGGCACCTGCCCTAAAATAGCTTCCCATGTGAGGGCTAGAGA<br>AAGGAAAAGATTAGACCCTCCCTGGATGAGAGAGAGAAAGTGAA<br>GGAGGGCAGGGGAGGGGGACAGCGAGCCATTGAGCGATCTTTGTC<br>AAGCATCCCAGAAGGTATAAAAACGCCCTTGGGACCAGGCAGCCT<br>CAAACCCCAGCTGTTGGGGCCAGGACAC
MC2R	GACAGCTCCCACCACCAGAACCCAGAGCTCAGGAGCACAGTCCTA<br>CACTGGCTCTCTCTAAAGCCTGGATTACAGGACTGCCCTCTAGAAA<br>AGCCTGAAAGCCCCTGGTAAACAGTACTCAGCCACTCAGTCCTCCT<br>TCCCCAGCTCTTCTCCAGAAGAGCAATCCGAGAAAACACCCAGCA<br>GCGCAGAGGAAAGCGGCACAGTGTTGCTAACTTATCTTCAGCAAG<br>ATCCACGGAACCCCAAAAACACAGCCCATGGGCCCCAGGACGCCT<br>GCAGCCCTGCTGCTCAAAGGCTTTCTGTAGCATTAACTCCCTTCCTA<br>AACCACAAGTGGTGTCTACAGGTCTTGCTGTTCTCTAAATTTCAGA<br>GAGATTTTTCTCTAAATGAAATTTAGAGAACAGAAAGATTAAAAC<br>CATCCTGATTTGGTCAGATACAGAGGTTTAAGATTGTTAAGTTATTT

	TGTGTTCATTTTTTTACTAAAGCCATTAAAACTTCCTTATCAAATGAA<br>TATTCATGGAAGCGCTAAGATTAATCATTTTTGGAGCCAAACAGATC<br>TTGATTTAAATCCCAGCTGTGCAACACAAAGCCTGTGGGACTTTAG<br>ACCGGTCTCTTGGTCTCTGGGCCTCAACTCCTCAGGGGTGAGTCAG<br>ATCAGGGGTGAGTCAGAGAGAATAGGACTCCCTTGTATTGTCAACA<br>AGAGGATTAAAATGAGACCCACCATGCACAGCACAGGACACAAGA<br>CCAGCTGGCACATAACAAGAATTTAACACATAATAGAGCCTTCTCC<br>TTACCCTTGGAGCCTTTAAAAAATATAAAAACTATATATATTTTTTTT<br>GGTCAAAGATGATGATCAATATAAAAATCTCACTTTAGGCCCATGCT<br>CCCTGGACTTTCCAAAGAGCATCTAACAGAGTGAGCCACATTTGGG<br>GCCACATTTGAGTTATCCGGGGCCATGTTCTTGTGCCCTGTGATGCT<br>GTTAACTCGATGATTTGTGCGCCCCTCCCAGCCAAAAAACAAAAGT<br>CGGTTTTGCTTTTCCTTGCAGTCCCTTGAACAGTTATAACTACCCCA<br>ATTGTCTGCTCTGCCTGCCAAGACCTACGCCTTCAGGCAAACGAAT<br>CAACCAAGTTTCTTTCTGGGTACAGATTATAAAGCTATGAAAAACTT<br>GTCTCTTTTCTCCTTTCTCATCCACCACCCCATCTCTGCCTCCTGGG<br>GAGCACTGCAGAGTTAGAGCCTTCTAAGACCTTGTTTACTGCAAGC<br>TCAAGGTGATGGAGCTTGGTCTTCGGGCCAGGAGTTAACTCCAGA<br>GGTTCCAATAACATAAGAAAGAATGAGATTTAAAGATCACTCTGCC<br>AGGTGGGATGGTTCATATCTGTAATCCCCAGTGCCTTGGGAAGCTA<br>AAGTGGGAGGATGCCTTGAGCCCAGGAGCTTGAGACAGGTTTCAA<br>TAGGGTATGGACAGAGGAAAGCAGCATGGAATTGAAGGGTGGACA<br>TTTATTGCCCAGCAAACCACAAACCCTGGAAGGAGGTGGAAGGGC<br>TCTAGACCTGTGTTGTTTGCAGTGAAGAAATCAAGACACAAAAAA<br>GCAAATGGGAGAGGACAGCAGACGGCTTTGAATGGATGAGAGCCA<br>GACATATGGCAGGTGCAGACCCCTATCCAAGTTCTTTAAGCCAAAG<br>ACATTGTTGAAATATTTGGATCATTGACGCCACCTCACAGCAGGTC<br>CTTGTGAAGTTTAAATGGAGTGAGTTTTGCTTCTTCTAATTGTCATT<br>TAGTGTGTTCTACAGGCAATCTCTAAGCAGGAACAACAGGAGCATA<br>GCAATGCAGCAGAATTTACAAATCCTACTTGGCACTGAGAAAGGCC<br>AAGTCGAGAGGAAAGTGACTGCTGTCAATTTTATTGCAGTTTGTGT<br>TGGAGTAGGGGGTGCTGTGTCTGGTCTCTTGCATCTGTTGTTATTAC<br>CCTTGCCACTTGTGAAAGGAGTGTGACATGGTGGTCACAAGGCGG<br>GCTCTGCTGTATCCTCTCCAGACTGTGGCTTTGGGCGTTTGCCCAA<br>CCTTTCTGGGGTAGCTTCCTCTGGGGGTTGAGTGAGTGAATGCATC<br>TGGAGAATTAGGTGGTGCCCATGGCATGCTGAGCACTCATAAAGGC<br>CACCAATTCGTGTCACTGGCCCTTCAACCAAGAATGGGGCCACAC<br>ACCACCTGCAGCCCCCACCACCAGCTTGGCAGCATCCACAAACGT<br>GCAGTAGCAATGCTTGATTGGTGGCCTTTTGAAGGGACCCCATGGG<br>CACACTGTGTATCTGTCCAAATTCAGGGCTCCCCATGGATTTATAGG<br>ATTGGCAGCTGCAGGTCAGCAATGCACAGCAACTGAAGCTGGGCT<br>CCCAAACCACCTGTTTCTAAAAAAATATTACAACCACTTTCAACAT<br>GTCTTTTTTTTTTTATGAATTTTACCATCAAATGCATCTGAAATTGGA<br>AATATATACTAATTCTGATTACATTTAATGGACTCATGAGATGGGAGT<br>ATCTGTCCCAGCAGAGTCTCCATACATGGCCCATGCTGGCCACAGG<br>TGTTGGCCCCTGTATTAACCAGGATTCTGAAGAGGAAAATAAACAG

	CAGGAGATGGGTAGAGACATAAAAGATTTACTATAAGGAATTGGCA<br>CACACAGTTATGGGGCTGCGCAGTCCCAAGCTCTACACTCAGCAA<br>GCTGGAGACCCAGGAGAGTAGATGATGTGTTCTGGTCTGAAAGCC<br>AGCAGACGTGAGACCTAAGAGGAGCTCACACTTCTGTGCTAGTCC<br>GAAGGCAGGAAGAGACCAGTGTCTCCATCAGGCAGTCAGGCAGG<br>AGGAGCCCCCCAGGTCGTCGAGGAGCTGGTCTGTTATCCTCAGGCT<br>TTCTGCTGATTGGACAAGGCCCTCCCCCATCCCAGAGGGCCTCTGT<br>TTTACTCAGTAACCTGTTCAAATGTGCATCTCACCCAGAAACACCC<br>TCAGACACACCTAGAACACTGTCTCACCACATACCTGGCACCCTGA<br>AACTCAGTCAGGCTGAAACATGAACACCAACCACCACAGGCCCCC<br>TCCCATCACCGATGCTTCCCCAGCAGGTGGGGCACAGCACACAGG<br>ACCCCTCCCATCCCTGAGGCTTCCCCAGCAGGTGGGACACAGCAC<br>ACAGGCTTCCCCTGCCATCCCCGAGGCTTCCCCAGCAGGTGGGAC<br>ACAGCACACAGGCTTCCCCTGCCATCCCTGAGGCTTTCCCAGCAGG<br>TGGGACACAGCACACAGGCCCCCTCCCATCCCTGAGGCTTCCCCA<br>GCAGGTGGGACACAGCACACAGGCTTCCCCTCCCATCCCCGAGGC<br>TTCCCCAGCAGGTGGGACACAGCACACAGGCTTCCCCTCCCATCCC<br>CGAGGCTTCCCCAGCAGGTGGGACACAGCACACAGGCTTCCCCGG<br>GCCTGGGCAGGAGGCACGAAGGTGACTCATCCCTGTCTGCCCAGG<br>ACTGAGGTGGTTCCTGAGATGCAGAACTTCTGGCTTTTAAACCCTG<br>TCCATGTTTCAGGCAAACAAGGTAAATTGGCCATTCCAGGCAGAAC<br>CCTCACAGCAGCTACGCCCTGTCAACCCCTTCCTTCCGCATCCACC<br>GGACCCACAAACCTCCTTCTGAACACTTGACCTCTGACCCAATCCT<br>TTCCAAGGTAATTAGTTCCTTTCCAGACAAGTCCCTAGAGTGAGGC<br>CGTGATGGGCTCCCCGTGGACTTCAGATCATAGGCTGTGCTCCCAA<br>ATTTGTCCTCCCTAGGAAGTGTCTTCCTCGCCACGTGGTGCTGGCT<br>TTCCCCACTGGTGCACTGCCGCAGTCCGCCTTCACCCCAGAGACAC<br>AGAAAATAAGAAGTCTCCAGAAATGCCAGTCCCAGGAGCTCCACC<br>TTTGAAACAGAAGTGCTGACGCTCATTGTATAGTCATCTTTGGGAAT<br>GATACATCAGCACCTTTACCGTTATCCCACAGGCTGTGGGGTCAGG<br>CAGTGACCCAGGGAGACGAGCAGTGAGAAGGAATGGACTAAACA<br>TCTCCCGAGTCCACCGAGCCCAGCCACCGCCTCCCAGTCAGAGAA<br>GGCAGCTGTGGGCTCTCCTGCTCCCACATGGACCTTTCCTTGCTTT<br>ACCTCTTTTTTCATCAAGGGCACCGCTGCTGTTCATCTGTTCATCCC<br>ACACTCACGTGTGTTCACGAGAAGGATGGGAGGGAAAGTGGGGC<br>GGCAAAGAATAATCTTTGCTATCATCTCTCGGCTCAAAATTCCACTA<br>AAGCTTTTTCTCACAAAGTGCTTAAGATGAGGGCCCAGTATATTATT<br>TTACCTAAATTACAACACTTTATATATATTTTTATAAATGTAAGGGGT<br>ACAAAAGTGCCATTTTGTTACATGGATATACCGTGTAGTGGTGAAG<br>CCTGGGCTTTTAGTGTATCTGTCATCAGAATAACATACGTGTTACCC<br>ATAGGGTAATTTCTCATCACCCGCCCCCTCCCACCCTTCCGAGTCTC<br>CAATGTCCATTCCACACTCTATATCCACGTGTATGCATTATTTAGCTC<br>CCACATATAAGTGAGAACATGTAGTATTTGACTTCCTCTTTCTGAGT<br>TATTTCACTTTGATAATGGCCTCCACTTCCATCCATGTTGCTGCAAA<br>AGACATGACCTTATTCTTTTTGATAGCTGGGGAGTACTCCATTGTGT<br>ATATGTACCACATTTTCTTTATCCATTCACCCATTGATGAACACTTTA

	GTTGATTCCATATCTTTGCTATTGTCACTAGTGCTGCAATAAACATAC ATGTGCAGGCTCCTTCTAATATACTGATTTCTTTATATTTATGGAGG GAATAGAGTTTCTTTAAGCGAGTGTGGCTGGTTTTATTTTCTAGTG TACTTTGCAACTAATATTTCTGTATACTCCCTTTTAGGTGATTGGGAG ATTTTAACTTAGATCTCCAGCAAGTGCTACAAGGAAGAAAAGATCC TGAAGAATCAATCAAGTTTTCCGTGAAGTCAAGTCCAAGTAACATC CCCGCCTTAACCACAAGCAGGAGAAATGAAGCACATTATCAACTCG TATG
MDM2	TTTACTTTTTTATTTTCCAGGACAGAGTCTTGCTCTGCCGCCCAGGC TCGAGTGCAGTGGCGCGATCTCGGCTCACTGCAACATCTGCCTCCC AGGTTCAAGAGATTCTGCTGCCTCAGCCTCCCGAGCAGCTGAGATT ACAGGCTTGCACCACCATGCCCGCCTAATTTTTGTATTTTTAGCAGG GACGGGGTTTTACATGCTGGTCTCAAACTCCTGACCTTCAGTGATC TGCCTGTCTTGGCCTCCCAAAGTACTGGGATTACAGGCGTGAGCCA CTGCGTCCGGCCCGGATTTTGCCTTTTAAATAACACTATTCAGTTCC AGATGTTTGGAAAGACATAAGGAATTTGAACTACTTCTGTAATTTTA GATGGTCTCATAAATATTGTGAAACTCAGCATTCTAAACACGGTTTC TGAGCTAAATCTTGTTTAGTTTTCACCATGCTTTAAAGATTAGTTGT GGCCAGGCACAGTGGCTCACGCCTGTAATCCCAGCATTTTGGGAG GGTGAGACGGGTGGATCACCTGAGGCCAGGAGTTCGAGACCAGCC TGGCCAACATAGTGAAATCCCGTCTCTACTAAAAATACAAAAATTA GCCGGGTATGGTGGCACAAGCTTGTAATCCCAGCTACTCGGGAGGC TGAGGCAGGAGAATCACTTGAACCCAGGAGGTGGAGGCTGCAATG AGCTGAGATCGTGCCACTGCACTCCAACCTGGGTGACAGAGTGAG ACTCCGTCTCAAAAAAAAAAAAAAAAAGAAAGACAAAAGATTAGTT ATGACTAATAAATACAAGTACGAAGTGGCCCTTGGGTCAAGATAAG TCTGTTGTAGACTTTTTTTTTTCCCCTTGAGACATGGTCTCACTCTG TCACATAGGCTGGAGTGCAGTGGCAGGATCACACCACAACCTATCC GGGCTCAAGCCATCCTCCCAGCTCTGGCTCCCAAGTAGCTAGGACT ATAGGTGAGTGCCACCACGCCTGGCTAATTTTTTTTTGCAATTTATA GAGACAGGGTTTCGCCATGTTGCTCAGGCTGGTCTCAAACTCTTGG CCTCAATAGATCCTCCAGCCTTGGCCTCCCACAGTGCTGGGATTAC AGGTGTGAGCCTTGTTTGCAGAGTTTAGGTCCCAGAGAATATAAAG ACACTCAAAGTATCTCAACCTCTTGGAACACCTCTTGGGCATCAAG AACAACGCTACTAGAAATAAGCAGTTTGCCTCTAGAGAAACTTCAA GTAAGCACAACAAAGGGCAAAGTGACACAAGATAACTGTGGAATG CAAGCAAACAGCAGCAGCTGGTCTGTATTTTTTTAAATGGCAAAGT CACAAACAACAGCAACTGCTTTTAGAGTGAGCAGGTAGTTTGCTTT CTTTTTTTTCAAATGGAACTCACTTTTTAAGTGATGACATGCACTTG GGGGTGGGTAGGTGTGAGTTCATTTGTGTTTTGAAATCCCAAATTA ATGAGCATGATAAACCGCTATTGCTGGCCCTGTCTGACTCTCTACTC TGAGTAATTGGTGGGTTCCCCCCAACCTTCTCCTAACCCTCACCCTT TTTCCCTTTCCATTGACTTGGGACAGTCCTTGTCAAAGTGCCAATC ACAGTGGGAAGGAGGAGAAGGGGGGAGGTCACAGTGCATGGGAT TCAAGGTCACAGTGGGCGGAGCAACGGGGATCACCAGGCAAGTA AGTCAAGTTCCCGCTGCTCAAGTCCAGCTGTCTGCCACGGTAGTGC

	AACCCTTGGCGGACGACCCAGGACGCGTTGACGCGGCAGTCGCGC<br>CATCTCTTCTTGCCTGCATTTATCTGCGCTGTTTGAAGCCGTGTGAC<br>CCATCAGGACCCAGAGGCTGTCTGATCACATTATTGGGCTGTCTGC<br>TGTCTTCGGGTTAGGAGATCCTGGTTGGCCTGAAGGGAAGAAGAT<br>CAGAGGAAGACCTATCGAGGATGGGTTTCTCCTGCTTCTCCTCGGG<br>TTGGCGCTGCCCAGGATCCGCTTCCTGGCCTCGGTGTACTCGGCTT<br>CCCACTGTGCCAGGGACTTGACTGGAAGGGCGGGCCTGCTAGCGG<br>ACTTGGGGCTGCTGACCACACCGTTGCTGGTGGGCCTCTTGAGGAT<br>GCGGACCTGTGGAGGGGGTCCCGGGGGAAAATTATCATCCTGAATC<br>ACAATGGGCACTTTGGGAGATTTGAATTTCCTGCTTTCTGTGTGATC<br>TTCAGTTTTTTTTCCAGACGTCTATTTCCCCGCTGTCTGCCGTCTCC<br>TCCCAGCTCTCAGCAACCTCCTCATATTCCATCTTACTCGCCACTTT<br>CCAGATCGCTTGCTTTCTTATTTCCACATTTTCTCTGACTTTTGCAGT<br>ATTCAATATTATTACCTGATTCTGATTTTTCAGACATCACTATATGGTC<br>CTTGGCTTTTTGGTCGTTCCTCTCATTCCTTTGCTGTTTCAAGCTCT<br>GTTCCCAGCCTAAGCTTTTTACCACCGAATTCTGTTCTGTGACCCAT<br>AACTTAAAACTAACCCAACTTTTCATTCCCTCCAGACTTTTTTTGGT<br>AAGCAGTATCATCATGCTTTAGGTGGACACTGAGTCATACTGCTGT<br>GGTAGTGCTGGTACACGTAGTTTTGAATACCAGGTTCTTGAGTGAA<br>TCCTTTTTACTTCTCTCTCTCTCCCCCACCCCCGCTTCTCCCTTCTGC<br>CTTCATTTCTGACCCTTCTTTTCTGGTTCCATTAGAAAAGGACTAAT<br>CTTATCAGTTCTGCCTTTTTTTTTCTGCTCTAATAATTTTGTCAAGAT<br>AATTTTTTCAAAGCACAAGGCCAATGAGAAGGGGGAAAGTCGGTC<br>CAGAAGGCAGCCGGCCCGACTATTCTGAGTTTGAATCTACTCTTGG<br>TGGTCCTGTTAAAATACAGTTTGAAGCAAATCACTTGTTTTTATGCT<br>TCTAATGTCTACTCTGTCGAAAGCCCAAATTTCCTTGCTTGGCATTT<br>AAGGAGACTCCAAGTTCTTAAAACTTCTTCTTGACTTTCATGCAAG<br>TCAAACGGATTTATTTATTGTATCTCAAACAGTCCTCTTATATTTTCC<br>TTTCTGTATTTTTTGATAATTTCTTGTGCCTGGAATATTCTCTGCTTT<br>TATTTAAGTCTCACCATCTTTCAAGACACAAAATATGTGTGATCTCT<br>GAGGCCTTTGTGAACTGCCTCCAGCATTTACTAAGCAATTAATTCTG<br>TCTCTGAACTTTTATAGCACAAACCCATTCGTTCACTTGATATTTAA<br>CAAATATCAACCTCTATCACTTATCTCTTTTTTTTTTTTTTTCTTCTGA<br>GATGGAGTCTTGCTCTGTCACCCAGGCTGGACTGCAGTGGCGCGAT<br>CTCCGCTCACTGCAACCTCTGCCTCCCGAGATCAAGCGATTCTTCT<br>GCCTCAGCCTCCCAAGTGGCTAGGATTACAGGCGCCCGCCACGGG<br>GCCCGGCTAATTTTTGTATTTTTAGTAGAGACAAGGTTTCACCATCT<br>TGGCCAGGCTGGTCTCGAACTCCTAACCTCGTGATCCACCCACCTC<br>AGCCTCCCAAAGTGCTGGGATTACAAGCGTGAGCCACCGTGCGCA<br>GCCTATCACTTATCTTTTCATGCTTACATTTTCTCACTGCTTGAGATT<br>TTCTTTCATCCAGGGTCAAGCACTGAGTCTATTAGAAACCCCACTG<br>TATATGATCTGATGAAGATGAGATTATCTTTTTAAAAGCCACATATTA<br>AATCTCTTAAGTTAATAATATTGGCAATCAACTTAAATCAACTGAGT<br>GACCACTATGTTTAAGGAAGTTTCCTTTCTGGTAGGCTGGGAAGCG<br>GGAGAAGGGAAGGATATAACTTTATAAAAAAAAAAAAGCTGCAGA<br>AGGGAAGGATATAACTTTATAAAAAAAGGTCCTTGTTCACAAGGAG

	CTAATCACTTACTGGCCAGAGAAGAAACGTGTTCCTTCTTTGCAAT<br>GGCTGTGTTTTTGGGTTTAGCTAACTCAATTCTTTATGTCCTCAATTT<br>ATGAGACCGCATCAAAAACGTTTTTGCCACATCTCTGTGGGAAAGT<br>AGGTGAGTCAGAATGAGTGCATAATATATAAGTATATATTTTAATGAT<br>AATGAATGTGTAGATTAACAAACCCAAACTTTGTTTTTGTTTTTTTG<br>AGAGTCTTGCCCTGTCGCCCAGGCTGGAGTGCAATGGCATGATCTT<br>GGCTCACTGCAGCCTCTGCCTCCCGGGTTCACAGGATTGTCCTGCC<br>TCAGCCTCCGGAGTAGAGTAGCTGGGATTACAGGTGACCGCCACC<br>ACGCCCGACTAATTTTTGTAGTTTTTAGTAGAGACGGCGTTTCCCAT<br>GTTGGCCAGGTTGGTCTTGAACTCCTGACCTCAAGTGATCCACCCG<br>CCTCGGCCTACCCAAAGTGATGGGATTACAAGCGTGAGCCACCGC<br>GCCCAGCCAAACCCAAACATTCTAAAAGCGCAGAGTAACCGCTCC<br>CCTCCCCGCCAACGGTAACCAGCATCTTCGTTCTCCAACCAGAACA<br>GCTGTATGCACCGGTGTATAAGCCTGCCTTAAGTGCTATTTTTAATCT<br>TTTTTAAAAACAGGCCTTAAAATAACTCATTTGGGTACAACTCCAG<br>CGGCTGGTGGAGTGGGCTTAATATATTAAACAGCTGTTAATTTTGGT<br>TTCTTTTTGGTAACAGCGACACGGAGATCCGCATTTGTCGCAGTTT<br>CCACCGCGGGCGGGAAGTGTAAACACAAGAAATACAAACATAGCG<br>CAACGGCTAAAGGAGTGTCACAGCGCCAAACCTTGCTGGCTCCGC<br>GCCAGGCCGAGCCCCACCCTCTCAGCTCGCGGCCAACACCCCCAC<br>CCCGCCTCACAGCCCGCCGCGCCCGCGGGGCGACACCCCCCACTC<br>CATCATCCCGGAGGTGGTGCGGCCGAGCCCCGGACCCAATTGGCG<br>GAAGCGGGGCCGGTTGTGTGCGCGCGCACAAATGCCCGGATGCGC<br>CGCGACGACCCGCGCGCTCCCCTCGGGCGGTAGGGGGCGCGCACC<br>GAGGCACCGCGGCGAGCTTGGCTGCTTC
MEFV	ACCACTGCACTCCAGCCTGGGTGACAGAGTGAGACTCTGTCTCTA<br>AATAAATAAATAAAAATAAAAAAATATGAAACCAATCCCCTGCACT<br>CACTAACCCTATTATTCTGCTGTTTTTTCCCCCAACTTATCACTCCCT<br>GATACATTGTGTATTTGTTTGTTTATATCAATTCACACTTTCCTATAAG<br>AATGTGGGCTCTTGGCTGGGCGCAGTGGCTCATGCCTGTAATCCCA<br>GCACTTTGGGAGGCCGAGGTGGGCAGATCACAAGGTCAGGAGATC<br>GAGACCATCCTGGCTAACACGGTGAAACCCCGTCTCTACTAAAAAT<br>ACAAAAATTAGCCGGGCGTGGTGGCAGGCGCCTGTAGTTCCAGCT<br>ACTCGGGAGGCTGAGGCAGGAGGATGGCGTGAACCCGGGAGGTG<br>GAGCTTGCAGTGAGCTGAGATAGCACCACTGCACTCCAGCCTGGG<br>CAATATAGCTAGACTCCGTCAAAAAAAAAAAAAATGCGGGATCTTCT<br>GAGCCAGGCGCAGCAGCTCACGCCTGTAATCCCAGCACTTTGGGA<br>GGCCGAGGTGGGTAAATCACTTGAGGCCGGGAGTTTAAGACCAGC<br>CTGGCCAACATGGTGAAACCCCGTCTCTACTAAAAATACAAAAATT<br>AGCCAGGCGTGGTTGTCGGTACCTATAATCCCAACTACTTGGGACG<br>CTGAGGCAGGAGGATCGCTCAAACACTGGAGGCAGAGGTTGCAGT<br>GGCGAGATTGCGCTACTGCCCTCCAGCCTGGGCGACTGAGTGAAA<br>TTCCATCTCGAAAAAAAAAAAAAGAATGTGGCCTCTTCTGAAGGGC<br>ATTTATATCAGAAAAAAAAAGAATGTGAGCACTGCAAGAAGTTTTTT<br>TCTGTTCAGTTCTGTATCAGTAGTACCTAGAATAGCATCTGGCATAC<br>AGCAGGTGCTCCAAGAGTAAATGAATGGCCCTGTGGTGCCTCTTCC

	TAGCTAGACATGTGTTCAGTATGTCAATTAACCCATCTAAACAGGGT<br>GGGCAATTGTCCCAATTTCCCCGGACTGTGGTCGTTCCCAGAACTA<br>GGCTTTCAGTGCTAAGACTGGGCAAATTGGGTTTTGCTCCTAGACA<br>AAATGTGTTGGTCACCCTACCTGTAAGCCTACATTTCATCATCTATG<br>AAAAAATAGTTGGCTGGTCACGGTGGCGCACGCCTGTAATCCCAG<br>AACTTTGGGAGGCTGAGGCGGGCGAATCACAAGGTCAGGAGTTCA<br>AGACCAGCCTGGCCAACATGGTGAAACCCCGTCTCTACTAAAAATA<br>CAAAAAATTAGCTGGGTGTAATGGCGGGCACCTGTAATGCCAGCTA<br>CTCGGGAGGCAGAGGCAGGAGAATCGCTTGAACCCGGGAGGCGG<br>AGGTTGCAGTGGGCCATGATCTTGCCACTGCACTCCAGCCCAGGTG<br>ATAAGAGTGAGACTCTGTCTCAAAATAAATCAAGTAAATAAATAAG<br>TAAATACTATATACCTCCTTTATCATGATGGTTTAGGGGCACGGGACT<br>GATACTCCCTTAGAAGTTAAGCTAATTGTGAAAGACTTTTTTTTTTT<br>TTAAATACGGAATCTTGCTCTGTTGCCCAACCTGGAGTGCAGTGAT<br>GCAATCTCGACTCACTGAAACCTCCACCTCCCAGGTTCAAGCAGTT<br>CTACCTCAGCCTCCGGAGTACCTGGGATTACAGGCGCGGGCCACCT<br>CACCTGGCTAATTTTTGTATTTTCATTAGAGACGAGGTTTTGCCGTG<br>TTGGCCAGGCTGGTCTGGAACTCCTGACCTCAAGTGATCCACCTGT<br>CTCGGCCTTCCAAAGTGTTGAAATTACAGGCGTGCGCCACCACACC<br>TGGTCTGAAAGACTTTTTAAAACTATCTTTTTTGCTTATTACGAAAG<br>TAATACAATCTTCATTTTTTTAAATCCCAAAATGTGCAGAAGTAAAT<br>GCCATAGAAAGTGAAACCCCTGGTGACCAGGAGTGGAGTGAAGGT<br>CGTGGTGTCTGAGCCGCACATTTCCCAGGAGTAATAGCCGAGAGTA<br>GACCCTTTGCAGCAGCTTTGTTGGGTTTAGATTTGCCATGTCACCCC<br>AAATAAGGTCACACATCCAATGGCAGGGGCACACAAGACAGCCCA<br>TGACCATTCCACTTTGATTATTAAACACTTAAATGACGTTAGAAGGG<br>CAATAATTCTATTTTCACTGTGAGAAATTACTGGAACCCCTCCAAAC<br>AAAAGCTTTGTGAGCCTGCCTTGCCCTAGAAAGTCCAAAGTGGCA<br>TCAAAACACTGCCTTCCAGAAGTCCTGCCTCAGAGCAGCCTCTGTT<br>GCAAAACGTGTCACCGCAATTGCAAAATATCCTGCTTTAGACCAGA<br>AAGTATCATTCCCTGCCCAATTCTGTCATGGGGTTTTGGTATCAGCT<br>CCGAGGAGTTTTCTGACCAGACGAGATGGGATCACACAGCACCCA<br>CAGCGTCACTGCACAGTCTCATCCTTTCTCCATCTGGTAGTGGGAG<br>ACTGCCTGGGAGGTGCTGGGTTGCCACCGTCCAGAGTCAGGGACG<br>GGTGCTGGCCCCAGTGATACCCCATTCAACACCCTTCTTCTGGCCC<br>AGCCTAAAAATTCCAAAAATCTGCTGTCTGCACCATCAATGTGCAT<br>TTCATCCACTTTTTTTTTTTTTTTTTTTTTTTTTTTTGAGACAGGGT<br>GTCTTGCTGTGTCACCCAGGCTGGAGAGCAGTGGTGCAATCACAG<br>CTCATTGCAGCCTGAACCTAGGCTCAAGCAATCTTCCCACCTCAGC<br>CTCCTGAGTAGCAGCAGGGACCACCAGTGTGTGCCACCACACCCA<br>GCTAATATTTTTTCTATTTTTTGTAGAGGCAGGGTCTCCTTATGTTGC<br>CCAGGCTAATCTCAAACTCTTGGGCTTAAGTAATCCTCCTGTCTCAG<br>CCTCCCAAGGGCTGGGATTGCAGGTGTGAGCCACTACACCCAGCC<br>CAATTCACCCTTTTTTTAGGGAAGATGTTGGGTTTGGGCTCAGAAA<br>GATCTAGCAAACTGCATGTTTGTTGTCAGCTATGAAAACAATGCAA<br>AAACACCAAATACTCTGTGAAATAGGAAACAGGTGGACCAGCTGG

	ACAGTAAGTTAAATTCAACCAACCAGAACTCATCAAAGCCTTGAAT<br>CCCAGGCTGGGCACACACAGAGGAGCTCACACCCAGAGCTGGGT<br>GTTCAGCTAGGCGTCGCAATGTTGTGACTGACCTGTGGAAGTGACC<br>CATCCTGGACCTCAGTGGTCAGCTGGAAATGTGACATCCATGGGTA<br>GTCATTAACAGTAAAATCACAAATTATTGTCATGCAGGAGACGATG<br>TTGTGAAGTGGTTTGACGTTTGGATCTTGGCTCTAGGCAAGTCACT<br>AAGCTACAGATTCTTGGCTGCACAGGGGGATGATAAACGCAGCCCT<br>GCAGGGTGCTGCAGAGATTCTAGGAGGAAGTGTTTATAAAGCTAGT<br>GGTGGCTGGTGCCGATGAAGTGCTGGTGAAGGGTAAATTGCATTAT<br>TAACAAAACTTCTGGCCGGGCGCAGTGGCTCACACCTGTAATCCCA<br>GCACTTTGGGAGGCCAAAGCGAGTGGATCGCTTGAGATCAGGAGT<br>TCAAGACCAGCCTGCGCAACATAGTGAGACCCTGTCTCTATAAAAA<br>ATACAAAAATTGGCCAGATGTGGTGGAATGCACCTGTAGTCTCAGC<br>TACTTGGGAGGCTGAGGTTGGAGGGTTGCTCAAGCCCAGGAGGCA<br>GAAGTTGCAGTGAGCCAAGATCCCACTACTGCACTCCAGCCTGAG<br>TGACTGAGCAAGACCCTATCTAAAAATAAAAAAATTAGGGTATGCA<br>ACGGTCAGAAGTAGGTAAGTGAGGAGTCAGTTCACCCTACATGTAA<br>TTTTTCATTTATTTATTTTTTTTGAGACAAAGTCCCGCTCTGTCGCCC<br>AAGGTGGAGTACAATGGCGCAACCTCGGCTCACTGCAACATCTGC<br>CTCCCATGTTCAAGTGATTCTCCTGCCTCAGCCTCCTGAGTAGCTG<br>GGAGTACAGGTGCGTGCCACCATGCCTGGCTAATTTTTGTATTTTTA<br>GTAGAGATGGGGTTTTACTGTGTTGGCCAGGCTGGTCTTGTACTCC<br>CAACCTGAGGTGATCCACCCACCTCGGCCTCCCAAAGTGCTGGGAT<br>TACAGGCGTTAGCCACTGTGCCCTGCCCCCAACATGTAACTTCTGTT<br>AGCTTCAAAGCCACCTCTGGGGCCCTGCACCACATATGAGCTGAA<br>GGACACCCGTGCCTTTTCACCCGTGTAGCTCCAGCATCTTGGCACA<br>CTGTCTAGAATGTTCAATGAATGTGCACGGAAGAGCATTCTGGCTC<br>CAGGGAGCGAGGACTGAGTCAGCTCTGGGAACAGATGAGTCAGG<br>CTGGTGGTCCAGGCATTGCTTTTCAAGTCCTTCATGTGGCTGGAAG<br>AACCAGTCAACTGGAACCGGATCAACAGGGGTGATGGCATGGCAA<br>GAGTTATCTCCTGGCAGTGCCCTTCTGGCCTCACTTGCCTTCTTGGG<br>CCAGGAAAGGCAAAGCTCACAGGACTGTATTCAGTGCCCACCCCT<br>TCCCCCGTCCTGTGCCATTGGCTCTGGAAGGTCCCTGAAACCCCGA<br>GTCTGGAGGAGAACAGTTGACCAGCAGGGCGGGCCCTCAGCATAG<br>TCCTCTCTGTTCCCACTCACCCGCTCTGCCAGCCCCAGATCCTGGC<br>AGGAAGGAAGATTGGAGGGGGTGTCTGGAATCCAATCCCAGACCT<br>TCCCTTGCAGACTTGCCCATCTGTCTGTGGTCTAGTGTGGAGGCGA<br>GGTCCAGGGTTTGGGAGGGGTGTGGGGGCACATGTCTGCCAAGGC<br>ATGGAGCCCTCCCAGCTGGAAAATCCTCTGAACCTGTAAGAAGAG<br>AACACAGCCGGCATGGACACACCCTTACCCTTAGTCTCAGTTCCCA<br>CCAAGACACAGAGCATTTCCTGTGCCTTTTCCGCTATTTCACAACC<br>TGCCTTTTCTTGCTCACCAAGGACAGAGGCTTCTTTTCCTACCAGA<br>AGCCAGACAGCTGGCTCGAGCC
MIP	CACCCTCTTGGACGGAGCTACAGCTACCCAAACTGCATCTGTGGAT<br>CCGAGCTTCCCTGTGCTCTTGAGGGAGAGCCGGGAGCAGGTAAGA<br>TCTGCCTTCCCAGGTGCCACTGTGGCTGCCCTTCCAGGTACAGGAG

	CTGGGTGTTTCTATAGCCTGCATGCTTGGGAGCCCCAGGAAGGACC<br>CCTGAACCCTGCAGTCATGGGGGTGTCTGGTGTCTGTCTGGCCTAC<br>CTCCAGTCCAGGCACCTGCTCTGATCTTGGAGCGGGGTTTGGGGCT<br>GAACCCAGGGACCATGAATGGCAGTGGGAGGCAGATTGATTCCAA<br>GGCAGAAGGGGGCAGATCCCCAGTAAGGCCCCACCTTTGGGCCAG<br>GGAGGACCTGAAGGCTGGGGGCTGGACTGACAGTCCCACGGACC<br>AGAGTGGGGACTTGTGGTATCTCTTCCAGGCCCACCCATGGCTTCC<br>CGTGGGCCAATTGGCACAAACTTTCTTCCCTCTGAGGTCCATAAAA<br>GCCCTGGGGTCAGCCAGAGCAGGGCAGAGGATGGCCAGAGGACA<br>AAGAAGGCAGAAAGATGTCAGTGCAACCAGCTGCAGAGATGCAG<br>AGAGGAGTATCCTCTGCTGAGAGCTGCAGAGATGACCTGTTGGCA<br>GAGAGGAGCTACTCTCTCTGCAGAGAGCTTCAGAGACCTGCAGAG<br>ATGTCTGAATGACCTGCCTGCAGAGAGGAGCCACCCTCTCCAGGG<br>CCTCCTCTCTGCTGAGAACTGAATACTCCATGGGATGACCTGCCTA<br>CAGAGAGGAGCTACCCACTGTGGGTCTCTTCTGAGCTGTTCTAACA<br>CTAAATAAAACTCTTCATCTTCACCCTTCACTTGTCTGTGACCCTTA<br>TTCTTCCTAGATGCAGGACAAGAACAAGAACTCGAGCACCATGGC<br>CACAGAGGTTTTTCTGTAAGAAAAATCGATCCCATAACACTAGGAT<br>TACAGACACAAGCCACTGGGCCTGGCCTTAATTTTTGTCTAATAGA<br>CTTTTTTTTTTTTTTTTTTTTGAGACAGTCTCACTCTGTCACCCAGG<br>CTGGAGTGCAGTGGTGCGATCTCGGTTCACTGCAACCTCCGCCTCC<br>TGGGTTCAAGCAACTCTCGTGCCTCAGCCTCCCAAGTNGAGGGATT<br>TCTGGTGCACACCACCACGCCTGGCTAATTTTTTGTATCTTTTTTAG<br>TAGAGATGGGCTTTCACCATGTTGCCCCGTCTGGTCTCGAGCTCCT<br>GAGCTCAGGCAATCTGCCTGTCTTGGCCTCCAAAAGTGCTGGGACT<br>GCAGCCGTGAGCTACCATGCCTGGCCTCAGCCTGATATATCTTGAA<br>GCTCTGTTATATAGAGAGCGTCTTTTGACCATGGTTTTTTTGAGACA<br>CACTGTTGCTCTGTTGCCTAGGCTGGAGTGCAATGGCGTGATCTCG<br>GCTCACTGCAGCCTCCGCCTCCTGGGTTCAAGCGATTCTCCTGCCT<br>CAGTCTCCCGAGTAGCTGGGATTACAGGCAGGTGCCACCACGCCT<br>GGCTAATTTTTTGTATTTTTAGTAGAGGTGGGGTTTCTCCACGTTGG<br>CCAGGCTGGTCTCAAACTCCTGGCTTCAGATGATCCACCCACCTCA<br>GCCTCCCAAAGTGCTGGGATTACATATGTGAGCCACCGCACCTGGC<br>CTATCCTTTTACATTCAACTTACCTATGTCATTATATTTACATGTACAT<br>GTACATATACATGTTGTTTTTTATTTTTCTACCAGTCTTTTAATTGGTA<br>TGCCTAGATCATTTACATTTAATGTAATTTTTGATATATTAGGGCTAA<br>GTCTGCCATTTTATGTTTCTTTTTGTTTGTTTCAGCTCTCATTTTCATT<br>ACTCTTTGACCTTTTTCCCCTTCCTGTGGATTACTTGAACATTTCTTT<br>AAATTCCTTTTTGATTTATCTGTTATAAATTTTTATTTATTATGAATGT<br>TTTTAGTGGTTGGTCTAGATATTGCATTATACATATGAAACCTTCCCC<br>ATTTACTGGTGTCAACATTTTATCAGTTTGAGTAAAGTATAGAAATC<br>TTACCTTCATTTAAGGTCCCTAACCATTCCCTTCCCCTTGTATACTAT<br>CATTATCTTAACATTTCCTAATCATATATGNNNNNNNNNNNNNNNNN<br>NNNNNNNNNNNNNNNNNNNNNNNNNNNNNNNNNNNNNNNNNNNNNNN<br>NNNNNNNNNNNNNNNNNNNNNNNNNNNNNNNNNNNNNNNNNNNTTA<br>GCGAATGAGNNCCNNTTTGCATNNNNCGTGGACTNCCNCNTTCCA

	AANNAGCCCCGTATATAAGTTAGAAGTGGAGTTGCTGAGTCAGAAA TTATGTAATTTGACCTGGCATGGTGGCTTACACCTGTAATCCCAGCA CTTTGGGAGGCCGAGAGGGGCAGATCACTTAAGGTCAGGAGTTTG AGACCAGAGTGGCCAACATGGTAAAACCCCGTTTCTACTAAAAATA CAAAAATTAGCTGGGCATGGTGGTGGGCACCTATAGTCCCAGCTAC TTGGGAGGCTGAGACATAAAAATTGCAGAGGTTGCAGTGAGCTGA TTGTGCCACTGCACTCCAGCCTGGGCAACAGAGTGAGACTCTGTC TCAAAGAAAAAAAAAAAGAAATTATGTAATTTGTTCATCTTTATAAGT AGGTGTTAAACTGTGTCCCAAAGTCATTATCTCAGGTGGGGAACAG TGCCTCACGCCTGTAATCTCAACACTTTGGGAGGCTGAGGTGACAG GATCACTTGAGCCCAGGAGTTTGAGGCCAGATTAGGCAACACAGT GAGACCCCATCTCTACAAAAAATAAAAAAGTTAGCTGAGCATGGTG GTAAACACCTGTAGTCCCAGCTATTTGGGAGGCTGGGGTAGGAGGA TCGCTTGAGCCCAGGAGGTTGAAAATGCAATGAGCTGTGATTGCAC CACTCGACTCCAGCATGGGTGACAGGGTGAAACCCTGTCTAAAAA AAAAAAAAAAAAAAAGTAATATCTCAGTTTATACTCCCACCAGCAG TTGTAACTGGTTCTTTTTTTTTTTTTTGAGACGGAGTTTCGCTCTTG TTGCCCAGGCTAGAGTACAATGGCGTGATCTCGGCTCACCACAACC TCTGCCTCCCGGGTTCCAACGATTCTCCTGCCTCAACCTCCCGAGT AGCTGGGATTACAGGTACCCACCACCATGGCCAGCTAATTTTTCGTA TTTTTAGTAGAGACGGGGTTTCATTATGTTGGTCAGGCTGGTCTCGA ACTCCTGACCTCAGGTGATCCACCCGCGTTGGCCTCCCAAAGTGCT GAGATTACAGGCGTGAGCCACCGCACCCAGCTGGTTCTGTCACTTT TTATCCTCACCTAATTGGACCAGAGTATCTGTGGCCAAAATCTCTGC TCTTAACACCATGATGTGCTACCCTCTCTCTTTATCCTGTCCAGGGG AACTTTTCTGGAGGCAGTGTGAACCCTGCATGCTTCCTGAGGCCAG CAGTGGTGACTGGGATCTGGGAGCATTACTGGGTGAGATGCACTG AGGGTTACAGGGCCTTAAGTCAGGAGACTGATGAGGCTTCTAGAAT ATAGGAGATTCCAATGACTTCTAGTGGACTGTTTCTCTTCTATGTAA TTGAGAGCTCCAAACCCTTTGGGTGCTCTCTGACCTGTTCTCAGCA ACTCTTCCTGCCAGTGGATAGGTTAGATGGACCCTCTACCTTCACTA TAACTCTCTTTCTGTCCATCCCCAAGCTGGGTATTATGAGGAGAAA GCACAGCCCCTGATCTGTGTTCCTTCTCTCTCCCCACAGGTGTACT GGGTAGGACCCATGCTGGGCACCATTCTTGCAGGCCTCACCTCGAA TTTCTGCTCTCTGGTGCTTCATGAGAAAAGTTTGTGGCCGGTATGA CTTGCCACTATGTGGAGATGGTAGAACCTATCAGTATGTCCCATTCT ACCTTCTCCATCACCACACAACCCCACGCCTAGGTCCCAAGTCCCT CACCAAGCCCGAGCACAACTGAACCTCTTTCCCCAAACTTTCCTTC CCCATATTATCCTTTTCCCTCTCTTTGATCGGCTGTTTCCCCAGATCT GATCCCAGCACACCCATACCTTTCCCTGGGGATCAAGGCTTAGGGT GTGCTGATCATGGCTGTATTAAAGGACATTAGACCTTCCCATTGCTT CATAGCCATGACTGTTTCCTTCCTTTCAGTACTTCTTGCTATGACCAT GTGAGTTAGGAAAGATGAAATACAAGCTGATTCTAAGGTTTAACTA CGAGTCCCTTTCTTATTCCTATCCCTCCAACCCCACTCCCAGCCCAT GCAGGGATGATAGTGTCTTCTCTCTGTCGGTCCCTGTAGTTTAAAA AGCCAGGCCTAGGGTTTGAAAAGGGAGGTGCTGTCATGGACTGGG

	GGTAGGGAACCCGGGCTCAGAGAAAGCCAGACGCAGCAGAACTAT<br>TTTGTCCCAGAGCTGCAGGGTCCACAGATTCCAGCCCAAACTAGCC<br>AATGGGAGAAAGGCTTCTAATCCCTGGGAACTAAAGAATTCTCTTT<br>AGTTATCCAACTTCCCTGCTGAACCCCACTCCTTACCACTCCCCCCA<br>ACCCTTACCCCTGGCAGACTCAGAGGAGGCTGAGGCCCCCAGGAG<br>CTCAGCCCCAAATGCCATGAAGTGAGCTCAGCTTGATGGACCTTCC<br>AAAGAGAGCTGAAGAGTATTCTAATGTTGATGTCAATCAGGACAAT<br>GAACCCCAATTTTGACACACACAGAAGGGCAGACTCTTCCAGTCC<br>TGCTGTTCTTCACCCCCACTTCTCGTAGTCTCTCTTGCTGTGACCCC<br>AATCCCACCCTCACTGCCATGGCTCTCTCGGCTCATCTCCCAGTTGA<br>GAAAGGCGGGAAAATCCAGCATTTTTACCATGTAGGGGAGGGGAC<br>TTAGCCCTCCACAGCTGTGAAGGGGTTAAGAGGCTGGGCCTGCTA<br>CCTCAGCCTGCCCCTCCCAGGGATTAAGAGTCCTCTATAAAGGGGA<br>CTGTCCACCCAGACAAGGCCATGGGGGTAGCAGGGACCCAGGCA
MITF	GGGCTGAAAACTTCAAAATCAGGGCCTGGCAGAGCCTAGCTTCGC<br>CAAGGTCAGCCCACCAGGAGCCCTGCCTTCGTCTCCATAGGAAGG<br>ACACATGTACAGCCCTTGCCCCCGGCCCTCTCATTCCCACTTCTGCT<br>TGGCAATGCTCTCCATCTCCCTTATGTGGACTCTTGTTCTTGTCTGA<br>TCTCTTGTCAAATTGTTATTTTGTAATGAACTGCGTCTCCTTATTAAA<br>GAAATGAGCTGAAAGAAACCTGGGGGTGGGGGGTGTCTCTGGTTG<br>TTTTTGCTGCGCTGAGGTGGGGGTGAGGGAGTGGGAGCTGCCTGT<br>GAGGGTCAGTATGGTGGGTGAGGGTGGTGAGATGGTCCTGAGGCT<br>CTGGCTTCTAACCCAGAATCTCCCTTCAGGGAAGCACCAAAAGGG<br>GCTGAGGTCCCCTCTCTCCTGGGGTTGGGGGAAGACCAGATTGGC<br>ACTCTGGTGGGTGCTGGGGCCCACACATCTCCTGAGAACACATGCC<br>AGGCTGTGGAACTGTGGGGAGTGTGGTGGTGCCGGGGAACATTGT<br>CTGGGAGGCTGGGCGGGGCGGGGTGGTGGTGGGGACTATATTCTT<br>GAATTCCCTTCCAGTAGAGCTGGGCTTAGGGAACAGCCACAGTCTG<br>AGCCCAATGACGAGAGGGAGGCTGGCCCTGGGAAGTGGGAATTTG<br>AAAAGTAACAATTTCTACAAAGTCCTCAGTTGACTCTTTGCAAATA<br>ACTTGAAATGTCTTTAACTTCTGAAGCAAAGATGTTAGGGACCAGG<br>GATAGGGCGTCAGAATTTTTAGGGAAAGGGAGAGTGGGACAGCAT<br>TGCCCCCAAAGACCAGGGAAGAGTAAAACTCCTTCTTCGGTTCCC<br>ACTTCCACCTGTCTCCTCTCCTTGAGGATAGACCAGCAGCTACCATT<br>TTTGTCAGTTTACACCAGCACCTGTCCTCCAGGAACACCGTTGGCT<br>AGATGTGTGCTTGTCCATCAGGTCCAAAGGCATGGGGGCAGTGAAT<br>GCAAGCAGGGATGGGAGCTGCCCATCTGAGGGAGCATGAGCCCAG<br>ATCCTTTAACATCTATCCTGTCTTGCTGCTTCAGTCCCTAAGGCTGG<br>GTAGGGTGAGGGGCATGAGCCTTTCAGCAGACGGACTGGGATTTAT<br>CACCTGAGGGTCTCCTGGCTAAGAATATGAGCTTTTTACTCTGCCAA<br>TAGGTTGTGGCCTTCTCCTTTCCCCCCAAGAGGGCTGGACCACGTG<br>ACGGCTAGGTGATACTGTATCTGCAGACCTTACTACAACCAGTGGG<br>GGTCAGAAAGAGAAATGACATTTGCAGTCTAGGAACCCTTTGCATC<br>GGTCATGCTTCTTTGCCTCTGTTTTGTGCCCCAGGACAGGACTGAA<br>TTCTTCAACCATGACATCAAGTGTGCTTGTTCTGCTGCTGCTTGGGT<br>TACAAACAGCTGGGTGACATCAGGGGAGAAGAATCTTNNNNNNNN

	NNNNNNNNNNNNNNNNNNNNNNNNNNNNNNNNNNNNNNNNNNNNNNNN
	NNNNNNNNNNNNNNNNNNNNNNNNNNNNNNNNNNNNNNNNNNNNNNNN
	NNNNNNGCCTGGGCAACATAGCAAGACCCTTATCTCTAAAAATATA
	ATAAATAAATAAATAAACTAAAAGGATTAAGAAAATCTACACTCGCT
	TCTACTTGAATTCTGGTTTACAAATAGTAGAGAGAATAGAAGAATA
	GTGGATATCTAGATAGGGATATTTCTTCCCCAGTAAATTCTGTTGTCA
	GCCAAGATGGTAGTATCTATAGCTACCATCTTAAACTGACATGTGAT
	GTGCTCCAGGATCTCTTTAGAATGAAACTGATGGAAAATAACCTGT
	CAGTTTTACCCAGGAAGTGGTAAAGCTGAAGTGTGACAGTTAGGA
	GGTGATCCATTCCGATTCTCTTGCTATCCATTCAAGATCTGAATGAA
	GAAAGGAAACAGGTGAAATTACAGGAGTGCTAACAATGAATAAAA
	GTTACAAAGCCTTTGGGTGTTTGCCCTTTAACTCAGATAGCTAGCAT
	AATTTATATGAAATATAAGTTATATCAATAAATAATATTTTATGATAAA
	GTATTCTAGCTTTATTTTGCTGTCTATACTTCAGGTGTTCAGCAGTAC
	TCAACTTTTGCTATCTGAATTTCTTAGTGTGGTTAGTCATCTGGAAA
	GTCAAAAACATGCAAACATTATCAGGAAATCATAAAGATGTTTCTG
	TCCTCTTTAATGCTACATATATGTTCTCATTTGCATACTTCTTTGGCTA
	TTTAAAAATTAATGAAAGCAAAGTTATTGACTAAAAAGCTATGAATT
	AGGGTGGCTGAAGACAGAAAATTATATTTATATGGCTTTTTAAGTTG
	TCAAAGGGCTATAGAGCCTCATAATAATGCTATGAGGGAGTTTGGA
	CAGGGTTAGTATCTTTCTCCAGCATCTTCTCAGGGAGGGAAGTAAA
	CATAGAGAGGCTATGTCACACACCACCTGTCCACAGTGGGGAGTG
	ACATTGCTGGTGCAGTGATGATGCTAGAATGCCAACCACATTCTAG
	ATCAGGGGTTCTCAAAGTGTGATCAGAGGGCCCTGGTTGTATCTAA
	GACCCTTGCAGGAGTTCTGCCAGATCTGGACTATTTCAGAATAACA
	CTAAGATATTGTTTTCCTTTTTCACTCTCATTTCACCAGTAAACAATA
	GAGTTTTCCGAAGGCTACATGTCGTGTTGAGATGTCATTGTTCTGA
	CATCCTGTGAGATATCTACTAATGTATAATCGTGTGTTTTCAAAAATT
	CTCAATTTTAATTTCTAATATGCTAAATATTGATAGATATAATCCATGT
	AAACACAATAAATTTAAAGAGTGTAAAAGGATCCTAAGACAAAAAT
	GTTTGAGAATTGCTGTTTTAGATATTGTGCTAAGGCCGTTAGAAACA
	TGATCTCATTTAATGCAGAAAACACACATATGAGGCAAGCTCTACT
	GTCATTCCCATTCAATATGCTAGGAGATGAGGGCCTGGAGAAGTTA
	ATGAATTGCAGAAGGTACACGGGTTGTTAAGTTGCAAAGATAGAAT
	CTGAACCCAGGTTTCAAGTTAAGAACTCCACTGTCTGTGTTGTCAG
	GCACATTGTCTTTCAAAGAAGCACCAGGAAATGCCTGGATTTGGAC
	TAATTTCTCAGAAGAGAAACTTCTTCCCATAACCTAGGCTATCTCCA
	AGGGAATGTAAATATGAAGATTATCCTTATAGACAGTGTAAAATAGA
	GATTATTTTCTAGTAGTTTGTGGGCCGAGGCTTGTACTCTGTGAAAC
	ACAAAACAGTTGAGCATCGGATCTTTAAACCTGGAGGATTTTACTT
	AAAAAAGAAGTTTGGATTGCCAGCTACCCTTGAAAAATTTGGAGAT
	TTTGAGCTAGGCTGGACTCATATTCCCACATGGCCACAATCAGCTG
	CAGTTTTTATCTGCTGTTCCTGCTAGATAGATGTGTGTCCTCTGGTTT
	GCCACAGACCTCACTACTCCTTATTACCTTCCTCGTAGCTCATGTTC
	CTCATTTATGTTGCCTGTTTGAGGTATTTGGGTTAGAAACAGCTGGA
	GATGTTCTGTCTACTGCATAGTGTTTATAGGCCATCTGCTTTCAACTT

	ACGTGAAAAATAAAAAGTTATTAACAGTAAAAGAGCCAAATATATC CCATTCTGCTTACCCAAACCTGGATGTAATAAAGACCTCACTGACA GTGCCACATAGAAAGCAAATTGCTTCTTTCTTTACTAAAGCATAATA TTCTGATTCATTAAAGAATTAGGATCTGCCAGTGAATTAACATTTATT TTTAAACATGAATCTCTTTTCTTTTTAAGTGTAATAGTGAAATAAGC ACATTCTACTCATTGCCCTCATTTTATGTTTATTTTAGTAATGTCTACT GTCATCATTATCATGTCTGGACCCTTTATAAAAAAAAAATCTCTGTG AACCTCTGTGTACATATTAAATCAGTCATTTGAAACAATATGAGATT GTCAACCTAACGGACTGCATTATCCTGGGCATTTAGACAACATTATT GAACTTCAAATTTTTAGCTTAACATAACATTTGTCAGCAGTAGATAT TAAGCATTTATCATAATTTATAAGTATATATTTGCTTGCCTCTTTTCTT TTTTTGCCTCACCCCAGAATATAAGTTTCATAAAGCTGGAAACTATA TCTGTTTGTTCACAATGTATATTCTATGCTTTGTACAGTGTTTAGCAC TTAACAGTCAATAATGAATGAATAAAGGAATAAATATGTAAATATAT CAGTGTTGTATATGATAACTCATTCTTCTGTAATCTTAAGAATAATAT GTAAAAATTCTCAGCAAAAATGCTCATGCTTTTTTCATTTGAGAATT AAATAGGGGAGATATTTATTTCTTATTATCCACATAAATATATATCCAC TTCTGTGTGTGCTATGTTCTATTTAGGATAGTGCCCGGTCTTCCTGAT GTGAGGTCAATAGGCAGGTTACACATCTATGTAGCTAAGAATAAGG TGCATATTAAGACCAGGATGCAAGAAGAGGCTGTTGAACTTGAACA TTCAGCACAGAGTCTCTTCTCTTCTATACTAAAAGATAGTCATCCTG CAGTCGGAAGTGGCAGTTATTCGGCCATTGGAAAAGTCTCATGTAA TAGTAGCTTTTAGATGATGTCTCCTCCAAAGGGGCATTCTGCTATTA ACCTATTGCTGAAAGAGAAATACCATTGTCTATTAATACTACTGGAA CTAAAGATGAATAGTGAATTGGCCTTGATCTGACAGTGAGTTTGAC TTTGATAGCTCGTCACTTAAAAAGGTTCTTTTATATTTATGAAAAAA AGCATGACGTCAAGCCAGGGGGAAAAATTGATATCAACATTTAAGA CCAAACTCGTAGGGCTTCCAAAAAAAAAGGCCCTTATGTGAACGTTT TTTTTTACATGCATAACTAATTAGCTTAGGTTATTATAAGCAGGGCTT CTGTGATAAAGTTTCCGGTGGTGTCTCGGGATACCTTGTTTATAGTA CCTTC
MLANA	CTCCTATGAAAGGGCAACAGGACTTTCTGGGATTTTCAGGCATAAC CCTGAAGTTCAGGTAGAAGTTGTTTTCCCCAAATCTTTTGAGCCAG GGGCTTGTAGCCTTCCCAAAAGGGAACACAGTGGACGCATGGCTA GCGGGGACCGAAAGCGGAAGCCCGAGCAGCCGGCTATCAACCCTC GTTTGTAGCAATACCTCTGCGGGGTGTGTGAGAGATCAAAAGCTCA CAATATTGTGGGGAGCACTGAAGAGGGCCTAAGCCCCAAGCAGGA TGAGTCAAGAGGATTCTGGCTCCTGAGCACTGTAAGCAGATGCCA AAGAGGCATCTCTGGCCCCTTCATGAGGTGGGCCTGTGATGCCAGG TGGCCAAGCCCACTAGGTGGCAGCAGAGAACCAGTCAGTGGCCAG CAAGGTCTAGCAGGTACAAATCCTAACCCTGGGGCTGGTGTGGGG GCTGAGCTGCCTCACAGTAACAGTTCACACTATTGTTGCTAGGCGT ATTCTGTGCCACGTGCTGTACCAAGCACTTCCAACAGGATCTCGTT TAACTCTCACCATGAACCTATGAAGCAGATGCTGCTATTATCCCTTT CATTTCAGATGCGTGCACTGGGGCTTTCAGAGGTTTGAGATTTGCC CAAGGTCACACACACTGAAGAGATGAAGTGCAAGTGGTTTGGACT

	CTAGAGCTCCTGCTGTTAGTTTGGGACTAGATTGCCAGGGGAGAAA<br>AGGAAGCCACTTGGAAGTGTTGAAAATGAAGGCAGGTAGGGCCTG<br>ACTTGGGCTGGCTTTGGTGTGGACTCTGAGGCCCAAGGCCGGCTC<br>AAAAACAGAGACTAAGAGCCTAGTTAGAGGGAACTTCTAGGGGGC<br>TTCCGGGAAACAGGATGTGGTAGGTTTTTCAAGTTGAGGTGCCCA<br>GGGAACCCCTATTTGAAGAAGCGATCGCCAGCTTATCCTGTTCACA<br>TAGCCCTGAAAGGGCCAGGCACCTCAGAAGCTGCAAACCACTCAA<br>AGACCCAAAGGGATGCCTGTGAAAGAGCTGGTCAGGTCTCCCTCG<br>TTGAGCTCAAAGAACAAACAAGGGCCAGGTGCAGTGGGTCGCGTC<br>TGTAATCCCAGCACTTTGGGAGGCAGAGGTGGGCGGACCACTAGA<br>GGTCAGGAGTTCGAGACCAGCCTGGCCAAAATAGCAAAATCCTGT<br>CTCTACTAAAAATACAAAAACTAGCTGGGCATGGTGGTGCATACCT<br>GTGGTCCCAGCTACTCAGGAGGCTGAGGCAGGAGAATCGCTTGAA<br>CCCAGGAGGTGGAGGTTGCAGTGACCCGAGATCACACCACTGCAT<br>TCCTGCCTGGGCAACAGAACAAGACTGTCCCCCAACCCACCCACC<br>AAAAAATATCTCAAACAAGCTGGAGAGCATGTTCCCAGACTGGCC<br>ATTGTGTGCCCAGCATGCTGAGTCTGGTCCTGTAGCATCCTCTGGG<br>GGAGGGGATGATGCCAGCTGCCTGTTGTCTCCACCCTGGAAGAATG<br>CCAGGGCACACTCAATCCCCAAGTCAGCTCACATCACCAGAGAAC<br>TTGAATGAGACCTGGGAACTTCACCTTGCCCTGAGATCCAGGTCAG<br>TACTCCCTCCTCTGTGGTCTTGCCACCAGTCATATATGGGCCAGGCT<br>ATTATAGAACCCAGCTCAGTGCAATGGACTTTTCAGCTCAGAACTG<br>CATCCCTCTTTGTAGACTAGTACTCACCACCAGGACTTTCTGGGATT<br>TTCAGGCATAACCCTGAAATTCAGGTTGGAGTTGCTCTCTCCAAAT<br>CTATTGACACAAGGGGCTTATATCCTTCCCAATTTTTGCACAAAATT<br>CCCTGAGATAGCCTCCACTCTGGGAACTCAGTCTAAGTCATGGTCT<br>ATTGTTCTGCCTCAAGGTCTCCAACTGGTTGTTCTTTCTGAAATGCT<br>CTCTCTCCATCTTTTCATGGCTGACTGCTTCTTACCATTTAGGTCTCA<br>GTTTAAATTTTCCCTCCTTTACAGATAGGCTCTCCCTGGCTTGCCTA<br>ATAAAGATCAACCCTTCTTTGTTTTCTTTCTCCCTTACCATTTTCAGT<br>GGTGACTATCCAGTATCTGGTGGGTTGGTACTGCCCTTACACAGGA<br>CAGCCTTTGTCCATAGCTCACGATGGAAACCAGAAAAATCACAGGA<br>TCTGAAAGGAATGGGTCCTCAGGTATTATGGGAAAGGGGACCTTAG<br>AGGTCATTTAATCTAAGTCTTGAGCTGCTAGGACTGTCATATTCCGG<br>TGGGGTTTTTTTTCTTTGTTTTGTTTGTTCACAATGGCTATGGAGAG<br>CTGGTGAGCAAAGGTGACTGTTGTGTCCCACTGAAATGCTTCTGGG<br>CTGTCCAAGATAGATCATGATGTTAAGTAGAATCAGCTCTGGGGTC<br>AATTATTAGGTGAGTGCAAAAGTAACTGTGATTTTTGCACTGTCGG<br>ACTTTGCTGTTTGATATTAAATACATTCTTAAATAAATGTGGTTATGT<br>TATACATCATTTTAATGGGCATTTCTCACTTTATGTATTTTTGCTAAAG<br>ACATCACTTGTTCTTTGTTGTATGTTTATTTTAGACTATGGAAATGTT<br>GGACAAAAAGCAAATTCAAGTGATTTTCTTATTCGAGTTCAAAATG<br>AGTTATAAAGCAGCAGAGACAATTTGCAACATCGACAATGCATTTG<br>GCCCTGGAACTGCTAACGAATGTACAGGGCAGTGGTGGTTCAAGA<br>AGTTTTGCAAAGGAGATGAGAGCCTGGAAGATGAGGAGCTGGCCA<br>CTGGAAGTTGACAACGACCAGTTGAGAACAATCATCGAAGCTGAT

	CCTCTTTCAACTACACTAGAAATTGCCGAAGAACTCAACGTCGATC<br>ATTCTACAGTTGTTCAGCATTTGAAGCAAATTGGAAATGTGAAAAA<br>GCTCATTTAGTGGTTGCTTCATGAGCTGAGTGAAAATTTAAAAAC<br>GGTCTTTTTGAAATGTTGTCTTATCTTATTCTACACAACAACAAGGA<br>AGAATTTCTCAATTGAGTTGTGATGTACAATGAAAAGTGGATTTTAT<br>ATGATAACTGGCAATGACCAGCTCAGTGGCTGGTCTGAGAAGAAG<br>CTCCAAAGCACTTCCCAAAGCCAAACTTGCACCGAAAAAAGGTCA<br>TGGTCACTGTTTGGTGGTCTCTGCTGGTCTGATCCACTACATCTTTC<br>TGAATCCCGGTGAAACCATTATATCTGAGAAGTATGCTCAGCAAATC<br>AATGAGTTGCACCGAATAGTACAACACCTGCAGCTGGCATTAATCA<br>ACAGAAAAAGCCCAATTGTCCATGACAGCACCTAACCACATGTCA<br>CACAACCAATGCTTCAAAAGTTGGAATAAATTGGGCTACGAACTTT<br>TGCCTCATCTGCCATATTCACCTGACCTCTCATCAACCGACTACCAC<br>TTCTTCAATCATCTTAACAACTTTTTGCAGGGAAAATCCTTCCACAA<br>CCAGCAGGATGCAGAAAATGCTTTCCAAGAGTTCATCGAATCTTGA<br>AGCATGAATGTGTATGCTACAGGAATAAACAAACTTATTTTTCATTG<br>GCAAAAATGTCTTCATTGCAATGGTTCCTATTTTGATTAATAAAGAT<br>GTGTTTGAGGCTGGGCGCGGTGGCTCATGCCTGTAATCCCAGCACT<br>TTGGGAGGCTGAGGCAGGTGGATCATGAGGTCAGGAGATCGAGAC<br>CATCCTGGCCAACATGGTGAAACCCTGTCTCTACTAAAAATACAAA<br>AATTTGCCAGGTGTGGTGGCGTGCACCTGTAGTCCCAGCTACTCAG<br>GAGGCTGAGGCAGGAGAATTGCTTGAACCTGGGAGGCAGAGGTTG<br>TAGTGAGCTGAGATCCTGCCACTGCACTCCAGCCTGGGCGACAGA<br>GTGAGTCTCCATCTCAGAAAAAAAAAATGTGTTTGAGCCTAGTTAT<br>AATGATTTAAAATTCATGGTCCGACACCGCAATTACTTTTGCACCAA<br>CCTAATTGATGTCTAAGTAGGTCATATTCTACCTGCAAAAAGAAAAT<br>TTCATCTATCCCTTTCACATAGATGGAAACCCACTATCTCCAGTGGA<br>CAGTTAACACCAAAGGCATCACAGAGAACTCATGGAGCTCAGCTG<br>AGGAGGTTTCAGGGATTTTTCTATTTCCTTTTCTTGATTATGAGAGT<br>CTGGGACTAGATGCTCTCCAGACCTGTGCCTAAAGACTCTTCAACC<br>CTTTGAGATGGAGATGAGGGAGGGAATAGGGAACCCAGTTTAGTTT<br>GGATTTCAGATCCTTTTGTGGGTCATAAGCGTGATGATTGGGTTTCC<br>ATGTTCACGTGTGAGATATGCCTCCCTCAAACCTTGTTACAATGACA<br>TGGGCACCTTACCTATCTGACATGAGAAAAACAAATGTGGATTTCA<br>GATAAACAAAAAATAACTCTTTTAGTGTATATGTCCCATAGAATATG<br>TGGACATATTTATCCTAAAAATATTGTATGGGACATAGTTGTATTAAG<br>AAACTGTTCATTGTTTATCTGAAGTTCAAATTTAACTGGGCATCCTC<br>CTCAGCTGAGCTCCATGAGTTCTCTGTGATGCCTTTGGTGTTAACTG<br>TCCACTGGAGATAGTGGGTTTCCATCTATGTGAAAGGGATAGATGA<br>AATTTTCTTTTTGCAGGTAGAATATGACCTACTTAGACATCAATTAG<br>GTTGGTGCAAAAGTAATTGTGGTGTCGGACCATGAATTTTAAATCAT<br>TATAACTAGGCTCATGTCATATTTTATGTGACATGGCAATCCTATGGA<br>GGAGGGACCAACATTTAAAATAAATGGCTTCCCTAGGATAGAGCAC<br>TGGGACTGGGGAAAACAGAGGCCACAGTCAGCTGTGACTTTTTGA<br>AGGAAGGAATAAAGTTGGTTTCTTTCATGCCAATTTAGCAATTACA<br>GACGACCCCGTCAGAAATCTAAACCCGTGACTATCATGGGACTCAA

	AACCAGGAAAAAAAATAAGTCAAAACGATTAAGAGCCAGAGAAG<br>CAGTCTTCATACACGCGGCCAGCCAGCAGACAGAGGACTCTCATTA<br>AGG
MMP1	CTCTGTCTCAATAGAAAAAAAAAAAAGAAAAAGAATTGACTGGTTC<br>AAGGAAAACCAGCTGACCAGCCCAATGGTCATCATAAATGATTATA<br>ACTTTAGGAGGCTGATGAGGGAGGATCACTTGAGGCTAGGAGTTT<br>GAGACCAGCCGGAACAGCATAGCAAGACCCTGTCTCTACAAAAAT<br>AATAATAATAACAATTAGCTGGGTGTGGTGGCATACACCTGTAGTTC<br>CCCAGTTGCCGTACGAGAGCTGTAGTCCCAGCTACTTGAGAGGCTG<br>ATGTGGGAGGATCACTTGAGCCCAGGAGGCGGAGGCTGCAATGAG<br>CCATGGTTATGCCACTGCATTCCAGCCTGGGCAACAGAGCAAGACT<br>CTGCCTCAAAAAGTATAATAATAAAAAAATAAAGAAAGAATTATGG<br>CTGAGGCATAAGTAGTTAAGAGTAATTAAAAAGAACTGGGTAGCAG<br>GCTTAGAGAACATTTCACTTTGTGAAAAACAGAAAAATTCTGAAA<br>CAATGGAATATAGTTTGAATTCTGAATCATGGACTAAAAGCTCTGCA<br>GGCCACACACGTGTGTATGTCACAGACTCCAAACAAGCAAGTCAT<br>AGTCTGGGCACATGATCAAAATCTCACAGGAAGCTTCCTCACATAT<br>TTCAAATCCATCTCAAATTCACATTCACAGATGTAAGAGCTGGGAA<br>AGGACGGTTTTGACAGGGCTGAACTGAGCTATGGTATGAGTAGCAC<br>TCATCCCCAGAAAGTCTCTTGGTTTGAATTTCCTTTAAAAGGAGCTA<br>TAGCTGCAAAAATCTGTTTCACAAATGTGCTAACTATAAGCATTTTC<br>CACAGTGTTTAATAAACCATGCAGATAAGAAAATATTATTGACAAAC<br>AAATTAATAAAATGCTCAAAATAATCTGATACTAAATGCTTGTAGCA<br>TGGCATGCAAATCACCAAAAATAAATGTGCTATGCTTCATATAAAAT<br>CTCCAGTAAGGCTGAGTGTGGTGGCTCACACCTATAATCCCAACAC<br>TCTGGGAGGCCGAGGTGAGAGAACTGCTTGAGGCCAGGAGTTTGA<br>GACTAGCCTGGCCAACATAGTGAGACCTCATCTCTACAAAAAATCT<br>TAAAAATCAGTGGGACATGGTGGTGCACATCTGTAGTTCTAGCTAC<br>TTGGGAGTCTGAGGCAGGAAGATTGCTTAAGCCCAAGAGTTTGAG<br>GTCCCTACACTCCAGCCTAAGCGACAGAGGGAGACCTTGTCTCTAA<br>ATAAATAAATTAGTTAATTGAATGTCCAGTCAGTTGATATATCCAAAT<br>TCTTCCCATGGTAATTTTAAAAACTTTAGTCTTAGGAGAGTAAAAGT<br>CATGGGCATAAGACTTCTTATAAACAACTCAGCCTAATGAGAAATA<br>GACCCTTTATTTAAGTGTCATTTAAGTATCTATTTCTTCATTGATCTAT<br>TCATTTATTAACTCATGTAACAATCATTTGCAGACACCTACTATGTTG<br>AGGTAGTATAAACTATAAATTCAACAAGTTTGATAAGGGAAATAAG<br>AGAGATTGAGTGACAGCTTGAAGGGGAGGATTCTTTCAGGCCTGT<br>GGGACCGGGTGGTGGCATGGAGACATTATTGTGGACTTGAGGGAG<br>TTAATGTGACAGTCCTCGTGTCTCCAGACACTTTCTCTCTGTTAGGG<br>AAGCAAGATTTCTATCCCCAGAGTATGTATGTGTTATGTCTGGACTG<br>CAGTGGCACAGAACTGTGTTCAACGAGTGACTACCGCTCTGCTGT<br>GTGCCCTGGGACTTGGGGTTAATTGATCAATCATTTCTACCCAGAA<br>GGTAACCATGAGGACTGACGGAACCAGTGTGTACCAAGTGTCTGTT<br>AAGTGTCTGGTCAATGGTTATCCATAAAGCTACTGCATGGCCATATG<br>TAGGAAGAATACAGACCGTGGGGCAAATTTTTCCCACGTGTAACTC<br>TCACAACAAAATAGCATTAAATACTTAATGTTTCTGGCTAAAGACCA

	TTTCAAGACTTGCAGGACAAAAAAATAGAAAAAATATCTGACACTC<br>AAATGGAGTTACAAAATTAAAACGGCTGAATTCCCCAGCATAAAAA<br>AATATGAAGCAAGATTGAAATTTCAAGACTAAGTTTAATATGGAAA<br>AATACAAATATGTTTGAGGCCTTTCACAGAGCAGCCAGCATGAAGC<br>AACCAAGAAAACCACGGAAATAATCTGGCTGCCTGGAAATAGTCC<br>GGAGTCAGCTGACACAGCCACACGAGAGCCCTCTTATGCTTGTCAT<br>AAGGGGTAAAGGAATAATTTCAGAAAATTACATTTAAAAGAGAATT<br>ATGGGGGAAGAAGATGCTCCCAGAGGAAACAAATAGTATGGATGT<br>GAAGAGCAAATACAACTTTAACATGTTCTGAACTTCTTGGAAACTA<br>TGCTAAGTTTAGGCATTGCTAGGATTTGGTATGATTTAATCCCCAGC<br>TTTCTGTTCTAAATTTTTGTTTTCTTTTTTACTCTCAAATAAATCATAT<br>GCTAGCACCAGCTGCAAAGTTACATATGTTGTATTAGACGATCTTCC<br>ATGAATACCTAACTGGAAATTCCAAGATTCAGGGCCATGTGAATCT<br>AGGCTGGCTGCTTAACCAAAACTTAATTTAATTTTTTTCGTTTATTTT<br>AGGAAAAAAAATTAACGAAAAGATGTTTCAAGCAACCAGTTTCCA<br>ATCCACGTCAGCAACTATGACATTTAATGAAACACTATGAGCATTTA<br>GCATGAGAGCTCTGGACTCAGATGCAGGGAGCTTTGCTAGAGAAG<br>GGAGGAAAAAGCAGGCATGATGTGGCGGGTTGTGGGGGACTCCAA<br>GGCTCTATTTCCAACTTCCATCAGAGAACTTCTGTTTTCACCTGGTT<br>TTCAAATTTGCTTTCCAAAAGGGATTTTGTTTAAGTAAAGGATACA<br>GAGGTTTATAAAAGTTTGAAAACTTCTACATTGCAGGATGTGCAGG<br>CTCTTGCCAGATGGGACAGTGTATGAGACTCTTCCAGGGTGACGTC<br>TTAGGCAATTTCCTGTCCAATCACAGATGGTCACATGCTGCTTTCCT<br>GAGTTAACCTATTAACTCACCCTTGTTTCCCAGGCCTCAGTGGAGCT<br>AGGCTTGTCACGTCTTCACAGTGACTAGATTCCCTCACAGTCGAGT<br>ATATCTGCCACTCCTTGACTTTTAAAACATAGTCTATGTTCACCCTCT<br>AATATGAAGAGCCCCTTTCACTATTTTCTTTGTCTGTGCTGGAGTCA<br>CTTCAGTGGCAAGTGTTCTTTGGTCTCTGCCGCACCCTCCCTCTGAT<br>GCCTCTGAGAAGAGGATTTCCTTTTTGTGAGAATGTCTTCCCATTCT<br>TCTTACCCTCTTGAACTCACATGTTATGCCACTTAGATGAGGAAATT<br>GTAGTTAAATAATTAGAAAGGATATGACTTATCTCAAATCAATCCAA<br>GATATACTGAAGTATTGTTTATGAGTAAGATATCAGTCTTGACGCAG<br>AAAGAAAACAGGAATCCATAAGGGGAGGAAAGTGTTGAAAAGCA<br>AACCTGATACAGTGGGAAAGGTGGGAGACACCATAAGGTGCTGAA<br>GTGATAAAACAGGTCAGTGTTTCTCCACTGTATGTTTTCAATAAATG<br>CTTCCAAGGAAGGAGAGTGGGGCATGAGTAGGGGAGCTACAGAGA<br>TAAACCAACTTTTCTTACCAGGAATGCTACAGATAGCACTGGTGAC<br>ACCGGTCACCAGTACCCAAGACAATTTAATGTGGAACATAAGTACA<br>GGAATACACATCTTTCATTACAGAGCCATGTATTTATTTTAATGGGCA<br>GGAGATGCTAAATAAGATCTTTTGAATGGAGGAATGCATAAATATAT<br>GAATGAATGCATACATGAAAGAATAAATAAATGCTGCCTAGCACCA<br>AGGAGCGAAGATAGACTCATATCAAGGGAAACAAGTATGATTAAAA<br>ATAAGACCCCAGAGTCACGCTCAGTCTCTTTCCAGCCTTTTCATCAT<br>CCGGTACATTCAGACAAGTTTCAGGGAAGGATCCTATTTGTCCCAT<br>GATAATGATGGGCAAGGGGTGGGGAGTTATCTCATACTCCGCCTGT<br>GGATGAGGGGTCTTCTCAGGTAAGGCTCTTAAATCCTAGGCCTGAG

	TAAATTTTTTCAAATTTTATTTTAGACAGGGTCCCTCTCTGTTGCCTAGGCTGGAGTGCAGCGGCACAATCACAGCTCAATGCAGCCTCAACCTCCCAGGCCCAAGTGATCCTCCCACCTCAGCCTCTTCAGTGACTAGGACTACAGGTGCATGACTCCATGCTTGGCTAACTTTAAAAAATGTTTGTTTGTTTGTTTGTTTTTTACAGAGATGGGGTCTCACCATGTTGCCCAGGCTGATCTTGAACTCCTGGGCTCAAGTGATTCCCCTGCCTCGGCCTCCTGAAATTCTGGGATTATAGGCTTGAGCCACCATGCCTGGCTCTGAGTAAAGATTAAGGGAAGCCATGGTGCTATCGCAATAGGGTACCAGGCAGCTTAACAAAGGCAGAAGGGAACCTCAGAGAACCCCGAAGAGCCACCGTAAAGTGAGTGCTGGGGGAGCTGAACTTCAGTCAGTACAGGTGCCGAACAGCCATCAGGTGCGCAGTGTTAGTAATTCCACCCTCTGCCCTGGGAGCAAGGTGTGTGGAGAAACCTGTAGCACTTTATGACCATCAGAACCAGTCTTTTTCAAAAAGACCATGGAGTACTCTTTGACCTGTGTATATAACAAGAACCTTTCTCAAATAGGAAAGAAATGAATTGGAGAAAACCACTGTTTACATGGCAGAGTGTGTCTCCTTCGCACACATCTTGTTTGAAGTTAATCATGACATTGCAACACCAAGTGATTCCAAATAATCTGCTAGGAGTCACCATTTCTAATGATTGCCTAGTCTATTCATAGCTAATCAAGAGGATGTTATAAAGCATGAGTCAGACAGCCTCTGGCTTTCTGGAAGGGCAAGGACTCTATATATACAGAGGGAGCTTCCTAGCTGGGATATTGGAGCAGCAAGAGGCTG
MMP3	AGTATTTGATAGCACAACAGGGTGACTACAGTCAACAATTTATTGTACATTTTAAAATAACTAAACAATAAGTTGGAATGTTTGTAACACAAGGAAGTGATCAATGCTTGAGGTGATGAATACCCCCATTTACCCCAATGTGATTGCTACATATTGCATACCAGTATCAAAATATTTTATTGTTAGGCATAGTGGCTCATGTCTGTAATCTTAGCACTTTGCGGGGCCAAGGTGGGCAAATCACTCAGGCCTAGGAATTTGAAACCAGTCTGAGCAACATGGCCCTATAATTCAGATGGAATATGTTGCCTTGTTAGTACTTCTGTCCAGCTAGTGATATTCAAAATTGTGGAAAATATAGATGAAATTTTATAAACTCCCATGATCAGCATGATGTGAAAAAGAATACACAGGTATAAAGAAATTAGAGGAAATACATAGTTAATACTGTTAGTAGTGTTTTTCTTTTTGGGTCATGACTATAAAAGATTTATTTTCTTCTTTGTACTTTTTTATATATTCTAAATTTTCTATAATAAACATTTAACGTTTTATTTAAGTATGTAAAATTTATTTAAAATGTTTTGTAGAAACTAGTGTATTAGTTTGTTTTCACACTGCTGATAAAGACATACCAGAGACTGGAAAGAAAAAGAGGTTTAATATGGACTTACAGTTTCACGTGGCTGGGAAAGCCTCACAATCATGGCAGAAGGTAAGGAGGAGCAAGTCATGTCTTACGTGGAAGGCAGCAGGCAAAAAAAGTGCTTGTGCAGGGAAACTACCATTTTTAAAACCATCAGATCTCATGAGACTCATTCACTATCATGAGAACAGCGCAGGGAAGACCCACCCCCATAATTCAATCACCTCCCACTGGGTTCTTCCCACGACATGTGGGAATTGTTGGAGTTACAATTCAAGATGAGATTTGGTTGGGGACACAGCCAGACCATATCACATGGCTTTCCACCAACAATGGAATAAGCTCTAAGCACCTTAGCTACAGACTAATGGTCTTCTATGACTGGACTCCAACCTATCTTTTCACAACTTTATTTTTTATTTTCCATTATTACCCTTCACATACCTTTTATTTCAATAAAAAGTAACTCTTTGACGTTTTCCATATAGAGTCTTTCTTGACTCTGCATACTTGTATACTCTGTTACTGAATTTTCCTCCATTTCCACCCTTGTTCTTAGCCAAAAGGCCA

	AGAAGCAATTTTCCTCCATTTCCAGATCATTCCAGGCTCACTTTATC<br>TGCTACTTTATCTATGAGTTTTTCTAAAGGAAACACCTAAAGTAAG<br>CAAGCACCTTCTGCTTAATTCGTGTACAACTTTTACTTTTTTTTTT<br>TTGAGACAGGGTCTTGCTCTCTTGCCCAGGCTGGAGTGCAGTGGC<br>ACCATCACAGCTCACTTCCAGGGCTCAAGTGATCCTCCTGCCTCAG<br>CTTCCCAAATAGCTGGGACTACAGGTGTGCACTGCCACAAGTGGCT<br>AATTATTTTTATTTTTTCGTGGAGCTGAGGTCTCCCTATGTTGCCTAG<br>GTGGGTCTTCAACTTCTGGGCTCAAGCAGTCCTCCTATCTCAGTCT<br>CCCAAAGTGCTGAGGTTATAGGCAGGAGCCACCATACCAGGCTTAT<br>TTCTTATAGAATTAATTATATAGGGGATTCTTTCCTTGGCTAGGTTATA<br>TTCTCTCCTTCCCTCCTTCCCTCCCTCCCTCCCTCCTTCCCTCCCTCC<br>CTCCCTCTTTTCCTTCCTTCCTTCCTTTCTTCCTTCCTTCCTTTCTTC<br>CTTCCTCTACCTTCTTCCCTTCCTTCCTTCCTATACCTCCTTCCCTTC<br>CTTCCTTTCTTCTTTCTTTCCTGCTTTTAAACAGCTAGTTTGTTCACT<br>TTTTTTTTTTTACTAAAATTTTCTAAACTTTATCTCCAAATCTATACTC<br>TTAGTTGGGGTATTCTGAAGCCCTTGAGACTCAGATACTTGATAAAT<br>GAATGTATAGCTGAAATAATGATTATATCAGTTATCAGCTAGAGGTA<br>GCAAGGATCCTGCACAATCATGTCTGATTAGTCATGGAGAAGTGTG<br>ACAAATAGGAAGACAAATGTGAAAAGTTACGAGATAGAATGTTACC<br>TGGGATCACTGCCAGTTTTTAAATCCAAGGATTAGATTGAATGTAAA<br>TTTAATTCTAAGCCGCTGATCCCTGGGAGGTCAGATTTATAAAACTA<br>GCCATTATCTGACCCTTGTATTTATTTAACCATATAACAAATGATTGT<br>TCTGTGGAACAGTCATTTTGATTTCTTGCTGGAAATGCCCACTTTTT<br>TTCCGCTAGTATTCAGTTCCTCCCTCTACACCTGGAGTGTTTGCTCT<br>CCTCCTCCCCTCCCCTCATCTTCCTCCCCCTTTCTCTTCTCCCTCCTT<br>CTCCCTCCGTTCCTTCTTACACTCAGTTGCTCTTTAATTTTACCTATT<br>CCTTCTCATTACTGCATTGATTTTTAAAACATACTCTACAAAATGCAA<br>TTTGACTGTCTCATGCACGGTTAGAAAACTTCTGGTTCTACAAGATT<br>AGTTTGAGTGTTCTGCAGACATATGAATGAGGACTTCAATTATATAT<br>TAGAGAGATTTTAGATGATAGCTTGATCTTTTCTTGTGGAAATACAA<br>CTTCTAAAAAATTTAGTTTTTAGAGTATTTAGTATATATGACATGTAG<br>AATCTGCAAATCTAGAAATGTCTGCATGATTTTTGGATTTTTTGACT<br>TTTAATTTACCTGTTTGACATTTGCTATGAGCCTTTCACTCATAACTA<br>ATATATTATTTAGTTCTCTAAGTAATTTTTGGTTACCTACTATATATCA<br>GATACCATGCTAAGTACTAGGAATACAGAATCAAATGAGGCATGGT<br>CCATACCCTCAAGTAGCTTACATTAGAATGAGAGAGACAGATAAAC<br>CATTTCACTACAGTTCAGTGTGGAAAATAGAGTAGCAGAGGCAGGT<br>ACAAGGTACCATTGAACAATGATTAATGACTCTTCCTGGGACTTGG<br>GAAACATCTTCCAGGGAAGTCGTCGAAGCTGTTTTAAAATATAGCA<br>AACTTTTGTATTTAGTTCAGGAACAGCATGGCCCATTTTGCCAATCA<br>CATCTTAACAGTTGGAAAAGCAAACATATTATCTATCAGGCTTTCCT<br>CTAAACTTTAAATATGTTTTATAAGTTATAACTCCAGAGAAAATTTAC<br>AAAGGATAAACCTTAATATAGAAGGAATTAGAGCTGCCACAGCTTC<br>TACACTTTTAACCTCTCAATATTTTATCTGTTGGGCTCCACTGTTTCT<br>TCCTGGAATTCACATCACTGCCACCACTCTGTTCTCCTTGTCCTCAT<br>ATCAATGTGGCCAAATATTTTCCCTGTATTTCAATCAGGACAAGACA

	TGGTTTTTTCCCCCCATCAAAGGAATGGAGAACCATAGAATACTAG TTTTAAAATGTCTTTAGGCCAGGTGCCGTGACCCATGTCTGTAATCC TAGCACTTTGAGAGGTTGAGGCAGGAGAATCACTTGATCCCAGAG CTCGAAACCAGCCTGGGCAACATAGTGAAACCTCTGTCTCTATTTT TTAAATAAAATTTGAAAAAGTCTTTAGACATAATCTAGTCTAAAAAT GAAGGCTTAAATGTGATGTATAGCCCCCTGCCAAGTGGCTATCACCT GTGTGGGCATCTTCAGTCATAGGGATCTTATTGCCACAGAGAAATC CCTTTAAACTTATTGGGTAAAATCTCTCCAATGTTTATTAAGAAACA CACAAAAAATAAAGCAAAGAAGAAAATGCAAAAGAGTTATAAATG AGAGGAAGCAAAATGGGCACTTATTAAAGGTCTAATAAATGCACAT TTGTATCCATCATTCTACTGAGTTCTTACTCCCAAGATGTTCTTCCCT TTAGCAAACAAATAAGCAAGTCAGCAAAGAAAGAAAGAACAAAC AAAATGTGGTGATCAGGGAAGCATTGAGGAGATGGATGGTGGCAG GTGGCAAGAGGACTATAAAAGTTTTACAAAATGTCTTCCTCTGAAT ATGTTTAGAGTCTTGCATTCAAGCATTTATTATACACCAATAATGTGA GCAACACTTTACTTGACAAAGAAACAGAAAAGAAAGGAAAGGAA GAAAACAGAAGAGCATGAAGAGAAAATTTAGGATGGATTCTGTTC TTCAACTTCAAAGCATCTGCTAATTTGAATTTAGGGAGGAGGGGAA AAGGTTGAAAGAGAATAAGACATGTGTAGAAGACAAGGACAGAG AGAATTTCAGTCCGGTAAGCAATGTAATTCATTTCAATTCTACAACT ATTTATGGAGCAGCTACGTGGGCCCATCACCCATTAATAAATTGGTT ACAGAATTAAAACCAACCCAAAGGGAATATACTTCCTTCTTTTTCA CAGACCCTCTTTGTTCTATTCTGCCCATGAGGTTTTCCTCCTCAAGA ACCAGCAAATCCAACGACAGTCAATAGCAGGCATTACAAATCAGAT TCAGAAAAATAAATCACCCCTTCTAAATTTCTTCTAGATATTATCTTT TATGTTTTGAGTATAATTGTATATAGTATAGACTATAGCTATGTATGTA CACTTTCCACTTACATCTTTTATTTGCTTTTATAATGTCTTTCTTAAAA TAAAACTGCTTTTAGAAGTTCTGCACAATTCTGATTTTTACCAAGTC AACCTACTTCTTCTCTCAAAAGGACAAACATAAATTGTCTAGTGAA TTCCAGTCAATTTTTCCAGAAGAAAAAAAATGCTCCAGTTTTCTCC TCTACCAAGACAGGAAGCACTTCCTGGAGATTAATCACTGTGTTGC CTTGCAAAATTGGGAAGGTTGAGAGAAATTAGTAAAGTAGGTTGTA TCATCCTACTTTGAATTTGGAATGTTTGGAAATGGTCCTGCTGCCAT TTGGATGAAAGCAAGGATGAGTCAAGCTGCGGGTGATCCAAACAA ACACTGTCACTCTTTAAAAGCTGCGCTCCCGAGGTTGGACCTACAA GGAGGCAGGCAAGACAGCAAG
MSH2	ACCAGTCTTAACATGGTGAAACCCTGTCTCTTCTAAAAATATAAAAA TTAGTGGGGCGTGGTGGCCTGCACCTGTAGATGCAATTACTTGGGA GGCTGAGGTAAAAGAGTCACCTGAGCCTGGGGAAGTGGAGGCTTC AGTGAGCTCTGATCATACCACTGCAATCCAGCCTGGGCGACAGTTA GACCCTGTCTCAAAAAAAAAAAAAAAAAAAATTAAAAAAAGAATTT ATCATGAACAGTTAATACACGCACATGGTTGGAAACAAAGGATACT GTTAAAACAGTTTCAGCTGGGCGCAGTGGCTCACACCTGTAATCCC AGCACTTTGGGAGGCCGAGTTGGGTGGATCATGAGGTCAGGAGAT CGAGACCATCCTGGCTAACACGGTGAAACCCCGTCTCTACTAAAAA TACAGAAAAATTAGCCGGGAGTGGTGGCAGGCGCCTGGGAAGGCT

	GAGGCAGTAGAATGGAGTGAACCCAGGAGGCGGAGCTTGCAGTG<br>AGCCGAGATCACACCACTGGAGTCCAGCCTGGGCGACCGAGCGAG<br>ACTCCGTCTCAAAAAACAAACAAACAAACAAACAAAAAAAGTTT<br>CTTAGACAAATTGATTACAATTGCATATCAAATTTTAATTTCAAGCTT<br>ATTTCCTTTCCAAGCAGGCTAAATATTCCTCTTAGACATTTTGTAAA<br>AAAGAAGCATATTAGAATCATCAGTATTAGCCTGGAGAAAGCAAAC<br>AATGTCTGGAATTATTAGCTATCTTCTAAGTCTAGTTAAAGGTAACT<br>GTCTTGTGCCAAGACTGACAGTGAGCTAAATGAACTGTTTTTTTTT<br>GAGACAGGATCTTGCTCTGCTGCCCAGGCTGGAGTGCAGTGGTGC<br>TATCATAGCTCAATGCAGCCTTAAACACCTGGACTTAAGGAATTTTT<br>CCACCTCGGCCTCCCAAGGATTACAGGTACATGCCTGGCTGGTTTT<br>TTAAACTTTTTTTTTTTTTTTGAGACAGAATCTTGCTCTGTTGCCC<br>CCACTGGAGTGAAGTAGTGTGATCTTGGCTCACTGCAACCCCCACC<br>TCCCGGGCTGAAGCTATCCTCCTGCCTCAGCCTCCCAAGCAAATAG<br>AACTACAGGCATGTGCCACCAAGCCTGACTAATTTTTGCATTTTTAG<br>TAGAGACAAGGTTTCACCATGTTGCTCAGGCTGGTCTTGAACTCCT<br>GAGCTTAAGCGATCCTCCCACCTCAGCCTACCAAAGTGCTGGTATT<br>ACAGGCATGAGTCGCTGTGCCTGCCTTACAGTTTTTTGTAGAGATG<br>AGATCTTGCTATGTTGCCAGGCTGGTCTCCAACTCCTGGGCTCAAG<br>TGCTCCTCTGGCTTCAAGTCTTCTAAAATGTTGGGACTACAGGCAT<br>GAGGTACCATGCCTGGCCAGCTCATTAAAGTGTATCAGAAATGGTA<br>GTAGCTTCTCTAAAGGGATAGCACATGACATCAGACTATAATACTCA<br>GGTTTGAGGCTGGGTGTGGTGGCTCACACCTGTAATCCCAGCACTT<br>TGGGAGGCCGAGGTGGGCGGATCACTTGAGATCAGGAGTTCCAGA<br>CCAGCCTGGCCAACGTTGTAAAACTCATCTCCACAAAAATTACAAA<br>AATTAGCCAGGTGTGATGGTGTGCACTTGTAGTCCCAGCTATTTGG<br>GAGGCTGAGGCAGGAGAATCACTTGAAACCAGGGCGGGTGGAGG<br>TTGCAGTCAGCCGAGATTGTGCCACTGTACTCCAGCCTGGGAGAC<br>AGAGCGAGACTCTGAAAAAAATAAAAATATAAATATAAAATAAATA<br>AAACTCAGGTCAGAAACACATCTTCTTTCTCAGTATACAGTCTTGC<br>TGCAGATTCTGTAAGCACACAGACGGTTAAGCATACAGAATCTGGA<br>AGAAGACTGGATTCTAGATCTATCACTTACTAGCTCTGTGATCTGGC<br>GCAAGGCCAGGCAATTGAATTTTAATCTGTAGGCCCGAGATTTCTTT<br>CTTTTTTTTTTTCTATGGAGACGGAGTCTCGCTCTGTCGCCCAGGCT<br>GGAGTGTAATGGCGTGGTCTCGGCTCACTGCAACCTCTGCCTCCCG<br>GATTCAAGCGATTCTCCTGTCTCAGCCTCCTGAGTAGCTGGGATTAC<br>AGGTGCCTGCCACCATGCCAGGGTAATTTTTGTATTTTTAGTAGAGA<br>CGGGGTTTCACCATGTTGGCCAGGCTGATCTTGACCCGCTGACCTC<br>GGCCTCCCAAAGTGCTGGGATTACAGGCGTGAGCCACTGTGCCCA<br>CCAGAGATTTCTAAATTGAGCTGATTGTCAGGAACATCCAGGGAGC<br>TTTCAAAAATGATTCCAGGCTGAGGCTGGGCGGGGTGGCTCATGCC<br>TGTAATCTCAGCACTTGGGGAGGCCGAGGTGGGTGGATCACCGGA<br>GGTTGGGAGTTTGAGACCAGCCTGACCAACTTGGAGAGACCCTAT<br>CTCTACTAAAAATACAAAAATTAGCTGGGCGTGGTGGCGCATGCCT<br>GTAATCCCAGCTACTCAGAAGGCTGAGGCAGGAGAATTGCTTGAA<br>CCCAGGAAGTGGAGGTTGCAGTGAGCTGAGATTGTGCCATTGCAC

	TCCAGCCTGGGCAACAAGAGCGAAACTCCATCTCAAAAAACAAAC<br>AAACAAACAAACAAAAAACAAAACCAAAAAAAAAAAAAATGATT<br>CCAAGCTGGGCATGGTGGCTCACACCTGTAATCCCAGCACTTTGGG<br>AGGCCGAGGCGGGCAGAACAGGAGGTCAAGAGATCGAGACCAGA<br>CCATCCTGGCCAACACAGTGAAACCCCGTCTCTCCTAAAAATACCA<br>AAATTAGCTGGGGGTGGTGGTGCACACCTGTAGTCATAGCTACTTG<br>GGAAGCTGAGGCAGGAGGATCGCCTGAACCCGGGAGGCAGTGAG<br>CCGAGATTGCGCCACTGCACTCCAGTGTGGTGACAGAGCCAGACT<br>TCCTCTCAAAAAAACAAACAAAGGTTCCAAGGCTCTACTCCAGAA<br>ATTAAGACCCATTATGTGTGGGCTGGGCACAGGAATATGTTTAAATC<br>CCCCCCCACCTCCCCAGGGTGATTCTGCTGCTGAATTAGGTTTTGG<br>AACCACTTCCATGGGGAAAGGGTAAACTAAACTGGAGAATGCAAA<br>AACCTTTTTTTTTTTTTTTTTTTTTTTTGAGACAGAGTCTCACCCTGT<br>TGCCCAGGCTGGAGTGCAATGGCGCGACCTCGGCTCACTGCAACC<br>TCCACCTCCCAGGTTCAAGGGATTCTCCTGCCTCAGCCTCCCAAGT<br>AGCTGGGATTACAGGTGCCCACCACTACACCCGGCTAATTTTGTGT<br>GTGTTTTTAGTAGAGACGGAGGTTCACCATGTTAGTCAGGCTGGTC<br>TCGAACTCCTGACCTCAGGTGATCTGCCCGCCTCGGCCTCCCAAAG<br>TGCTGGGATTACAGGCGTGAGCCACCGCGCCCGGCCAGCAAAAAC<br>CTTTTCTAAGAGTTAATTTTGCAGGATGGATCCTGAGCTCCTTCAGG<br>TGTTTGATAACATTTTATTTATTTTTTGAGACAGAGTCTCGCTCTGTC<br>ACCGAGGCTGGAGTGTAGTGGCGCGATCTTCGCTCACTGCCCCCTC<br>CACCTCCCAGGTTCAAGAGATTCTCCTGCCTCAGCCTCTCGAGTAG<br>CTGTGATTACAGGCGCCCACCACTATGCCCGGTAATTTTTGTATTTTT<br>AGTAGAGACGGGGTTTCGCCATGTTGGCCAGGCTGGTCTCAAACT<br>CCGGACCTCAGGTGATCCACCCGCGTTGGCCTCCCAATACGCTGGG<br>ATTACAGGCGTGAGCCACTGCGCCTGGCTGTTTGCTAACATTTTAA<br>AATTTCAACCTTCCTCTTCCTTAACTGGTCTTCCCCTACCCCCCCTC<br>AAGACGGGGTCTCTCGCTCTGTCGCCCCAGCTGCGGTGCAGTGGC<br>GTGAACATAGCTGACGGCAGAAGACCTCCTGGGCTCAAACGATCC<br>TCCTGGCTCGTGCTCCCAAAGTACTGGGATTATGGCGTGTGACACC<br>ACGCCTGGCGTCAAACGTTTGTCTTTTTATTTATTTTATTTTGTATTT<br>TTTGAGACAGGGTTTTCAATCTGTCGCCCACGCTGGAGTGCAGTGG<br>CACAATTTACGGCTCACCGCAGCCTCGACCTCCCGGGCTCAGGTGA<br>TCCTTTCGCCTCAGCCCTGCTAATATCTGGGATCACAGACGTGGGTT<br>TTACCATGTTGCCCAGGATGGTGTCAATCTCCTGGGCTCAAGTGAT<br>CCGCCCACCTCGGCCTCCCAAATTGCTGGGATTACAGGCGTGAGCT<br>ACCGCGCCCTGCCACAAACGCATATCTTCTAACGTACCATTTCATTT<br>ACTTGCTATATTCATTATCTGAATTTTCTCATATTAGAATGTAAGCAG<br>AATAAAGGCAGTGATTTTTCTTTTTACTGGCGATCCTCAGAGCCAA<br>GAAGAGTCTGGGACATAGCAGGCCATATAAATGTTTTCGAATGAGT<br>GAATCATCAACGAGTGGATGAAACGATAATGTGGCTAACAGGCAGC<br>AGTAAGGAGGCTGTGTAGAATAAACCCGTAATCCCGATGTTGGCAG<br>TTTGCTTAGAAAGAAAAAGGGAGGCAGTCGGAGAGGGGCACACG<br>TTTTAACAAAATACTGGGAGGAGGAGGAAGGCTAGTTTTTTTTTG<br>TTTTCAAGTTTCCTTCTGATGTTACTCCCATGCTTCCGGGCACATTA

	CGAGCTCAGTGCCTGCCGGAAATCTCCCACCTGGTGGCAACCTACCCTTGCATACACCCCACCCAGGGGCTTCAAGCCTTGCAGCTGAGTAAACACAGAAAGGAGCTCTACTAAGGATGCGCGTCTGCGGGTTTCCGCGCGACCTAGGCGCAGGCATGCGCAGTAGCTAAAGTCACCAGCGTGCGCGGGAAGCTGGGCCGCGTCTGCTTATGATTGGTTGCCGCGGCAGACTCCCACCCACCGAAACGCAGCCCTGGAAGCTGATTGGGTGTGGTCGCCGTGGCCGGACGCCGCTCGGGGGACGTGGGAGGGGAGGCGGGAAACAGCTTAGTGGGTGTG
MYH7	CTGCTTGTGCAATCTCTAATGGCCTGAGTAGCCCTGGAAACAAAATGTGGCTATCCCCATGTTACAGATGACAAAACTGGCTCAAAGCAATTATATGACTGACGTAGACCACACAGCTAGAAAATGGAATCACTGGGTTTCAAAGTGGATTTTTGTGACTGACTCCAAAGCTGACCAGACTGCTCATCCTGTATGCTTCTGAGGCCTCTGGCCCTCCAGACCGGTCACTCCCCACAAAGTCTCCCTTTTGGTGAGTTGTGTCCCTCCAAACACTCCCTGAAGGCAAGATCAACCTGCCCTAACTACCCCAAAGTGAACTAGGCCAGCCCCAAGCCTGTCTCCGGCCCTCCATCTCTCTCTTTGTTCACATCCATCAGAAAGCAGAGACCTAGAGTAGGGAATGAGAGGGGGTGCACAAGTACAAAAGATTTTGAAGCCAGCAGGAAGACAAAAGAATGACAAGAAGGAGCAGCTGTTTGTAAAGGGAAATCTGAGACAGGCAGAAAGCTCAGCTCCCTAATCCTAGGCTCGTGTGGAGCCAGGGCAGGGTCCCCCCAAGGTCAGGGATTCTGGGGGCAGGCGGATGGGAGAGTCAGCCTCCACTCCTGGGTGGCTCCCCTCTGCCCTGTGCACAAACAACGAGTGCCTCATTCCTCCCCTGCACCAAGTGCCCTGCCTCTTCCTGCCCCGTTTCTTCCTTCCCTTTGGGTACCGGAGCTTTTTCTGATTCCCCTCTCTCCATGCTGACATCGCTTCTAATTCGCCCTGGTGTCTCCCTCCCTCACAATTACTGGGAGTAATTTTAGCAACTTTTCCTCTGAACTACCGCTGGCGTGTTCACAGCAAATGGCCCCTGTTTTGGCTTGACACTTGATCAGGGTGGTGACAGAGTGCAGACAAAGCCGGCACAATGCGGGGAGGGGAGGACGGGGGACAGATCACCTTCCTTTCACTTCAGGCCTTATTTGGTCTACCTTGCCGAAGCGGCCCTCCTTCCAGCCTCTCTTTATTTATAGCATTTTGCTTTGCTCTCCTTTATCTTGCTATCACTTTGTAGCACACTCTGCACAATCCCCACCCACCCCAGCATGGTGGGGCCCCTGGCCCCTCACTCTCCCCACAAGGAATGTGCATGTGTGCAAAGGGTAGGCCTGGATGCCATTTCTACAAACAGTTGACTAAAGCAGTCCAGAGCAGGGATCTTTCTTTGGGTCATTGTTATGTCCTTATTGACCAGAATAATGCCTGGCACATAGTAGGCACTCAATAAATACTTGTTGAATGGAGGAATCTGCAAAGCTGCTCAGTCTTCAGACAGCAGCAAGCAGCAGGAAGAGAATCATGATGTAACCAAGAGCAGGTGCTGGAGGAGCTAGGGAGTGAGTGGGGATCTTTAAGGACAGCAGGCCCTCTGATCTGTCTGAGCTGTGAGGCAAAGCCTAGTTAGGGAGGTGAGTTCTATCTGCAATGAGAGGGGGCATGGGTGGAGAACAGTAACACCCAAACACCAACAGACCAGAGCCTCCCAAACCCGAAAGGAGAGAAACAAAGTGTGATTGATTTCCCAAATGCAGAAAAGACTACCCAAGTCCCCAGCCAAGGAAGGACGCTGGCCTGTGGTGAAGACACTGGCTTTGAAATGGCTCAACTACGTCCCAAGCACCCTTTGTGCATCTTAATTGCCCTGGGAAATCAGGGAGGGGTGGCAGTTTGGAGCTTGGAAAGCTGGAAGGTGGGG

	ACGAAAGGAGTTTTTGCCCCTCATCGCCAATCCTTGAGGTTCCTAC<br>AGGGAACCTCAGACAACAGCTGTCTTAGGGGTTTCCTTCCAGCTCC<br>AAGCAGCTGTATTATCTATAGCTTTGAGAGGGTCTGTCTCTGTCAAT<br>GGGGCTGATTCCTCAGCACCTGGCAGGGCCTGGGGGGGACTGCCA<br>GAGTCTGTTCTGCCGGGCATGTTAGTAGCACAGACTACTGGGATAA<br>CCTCAGCAACTGTATACCTTAGCACTGCAGAGCTGCGGTGTTGTCA<br>GACACAGCCCTTGCAGTTCGTGGTCTACCCCCACTTGCCTGCACCC<br>CCCTTATAGCTGCTCAATGTCCTCAGACTGTACCTTAGTGCCTTGGT<br>TACCAAGCAACCCCCACAGAGGCCTCCTCCTGGATTTTCCCTTCCC<br>TGCTATGCCCACCCCACCTTCAAAATGTACCTGCTGCACCCATCCCC<br>CAGGCCTGCCTCACTCCTTCCAAGCTGACAGCTAATCAGATGGACT<br>AATCCGCATGCGCGGAAGAGCTGTGCTTGGGAAGAAGGGGGAGAT<br>TTCACACCAAATCGTGCAGTGGTTGCCTAGACACGGAGCCAGTCA<br>AGCTTGCATGCCCCGTTGCTAGAAGAGCATGGCATCGGTTGTGTTC<br>TGTGCTCGTAGGCTGGGCCCCAGCTCCCTTTCCTCCCCTCCTTTGTG<br>GCTGTTCCTCAGGCCTGCAGAGGGGAAGATTTTTCCTGTCCTGTTA<br>CTCTGCCTCACTTGCCAGAACACTAGTCGCCTCCAGCTCTGGATAA<br>AGCTGAGGCTGGGTGGGCCAGACAACAGCTGTCTTAGGGGTCTCC<br>TTCCAGCTCCAAGTAGTTGTATTATCTATAGCTCTGAGAGGGTCTGT<br>CTCTGGGCTCAGGCCTTTAGTACCTGTGGGGCAGAGCCAGGAACG<br>TGAGTCAGGCAAACCGCTCCAAACTAGGACCAATGCAGCTGGGTC<br>CTGCTCTCTGCGCATAGGGGGTCCCCACATCTTTGGAATCCCAGCC<br>CACCTTTCCAGGCTACCCTCCACAGGCCGGGCTCCACTCCCATCTG<br>CTGAGGTTTCCCACCTTGAATCCTGCTGCCCCGATTAGCTGTATAAC<br>CTTAAAGAATGCACTGTCCCTCTCCATTAAATGAAGTGCTTGGATG<br>GATTGCTAAAGGCCTGTCTGGCTCGGAGGCTTGGTGACCTCAACAC<br>ATTGCCTGCTGGTCCAAGGAAATCAGTGCCTGAGCCAGAGTCCCCA<br>TCTCTAAGCTCCATGGTTATTGTTCTTGCCACCTGGCTAGGAAATGT<br>CCTTCCAGCTGCCCCAGTCTAGCTGCCTCACCCTGGGGCCATGCCC<br>CCAACTCTGTCCTACCCTTCTCTGCTGCTGACACTCAGCCCCTTCCC<br>AGCTTCCAGTTGGATACAGGACCTGGGCCAGGAGAGCAGGGAGGA<br>CACTGTGGAAATGCGGCCAGGCCATCAGGGGCCTCGCAGCAGGGG<br>ACTGGAGGGGGAGCAGTGTCCAGGGCCAGAAGTGCCCTGCGGGA<br>GAGCCAGGACATTGGCTGCCTGTGGTCTTGGTGGTCGTGGTCAGTT<br>CCCTCTCCTGCCAGCTGTGGAATGTGAGGCCTGGCCTGGGAGATAT<br>TTTTGCTGCACTTTGAGCCACCCCGCCCCCTGGAACTCAGACCCTG<br>CACAGTCCATGCCATAACAATGACGACCACTTCCAATTGTTTCCTAG<br>CTGGAGAGGCGGGGAGGGGAGCACTGTTTGGGAAGGGGGGGAGC<br>CTGGGGGAAATGCTTCTAGTGACAACAGCCCTTTCTAAATCCGGCT<br>AGGGACTGGGTGCCGTTGGGGGTGGGGGTGCCCTGCTGCCCCATAT<br>ATACAGCCCCTGAGACCAGGTCTGGCTCCACAGCTCTGTCCTGCTC<br>TGTGTCTTTCCCTGCTGCTCTCAGGTAGGAGCGGGAGCTGGAGGCT<br>TTACTCTGGGATAAGGGGGCTCCAGGCTTAGGAAGGGATTCCTCTT<br>GGAATAGCAAGCTTCATGCAGGACTTCATGCAGAGTACCAGGTCCA<br>GTCACTGGGCACACATGTGCAGGTCTAAACATGGGCGTATGTGCCA<br>CAGGAGTTCCTAGGGGAAGATATCTGCATCTGAGCATATGGGACCA

	ATATGCATTACAGGGTGTGTGTGTGTGTGTGTGTGTGTGTGTGTGTGTG<br>TGGACAAGCCTGCATAGTCTGAATGTGAGAAAGGGTATGTACCTCA<br>CTGACTGATACAGAAATATCAAGTGGGAGATGTGGGTGTATGATCA<br>CGTCTGTGAACGAGGAGGTTCCTTTGCAGGGAGGGAGAAAGAATG<br>GTGGAGAAGGATTCTAGGAAGTGTGTATGCCTTCATGTGATTCTGT<br>GAGGCCTGGAATGTGTAGCTACCCCTGTCCATGCATGCAGAGGTTC<br>TTGTCTATGCCTCCACGAGTCATGACTGTTATAGTATCTGTACCCTCT<br>GCAGAAGGAACTCACAGGCACGGGGGTGTAGGATGTTCCAGGTCT<br>TTGTGTACTGAAGAACACGCAGTGATGTGATCTACTTACGTGGCAC<br>ACATCCTGAAATGTGCACATTTGCTGTTGAGGCATATATGGCACCCT<br>GGGGTTGGGGTGCGGATTGAGGTATGGGAAGGAAGCCTGTCTTCA<br>TGTGAGAGGCATGCCCAAGCAGCAGGCATGAGTGTTCATCAGAGG<br>GCTTGTGGAGGGTGTTTGGCTATGTGATGGGTGTGTCTGTGAGAGG<br>AAGCCCACAGTGTCTTCTCTCTGAGAATGTCCACAAAGTCTGACAA<br>CAAAATGGGGACAGGATGGAAAAGAAGTCTCTTTAGAGTCATCAG<br>ACTTGCATTCAAGTCTTGGCTCTCCCACTTGTTGTCATGGGCTCTTG<br>GCCAAGCAAGCCATTTAACCTCGATTTCCTCATCTGTCAAATGGGG<br>TGAAGACTGAGTAGGAGAAAGTGCATTGAAGTGCTTTTAGACTGG<br>AAAGTGCCGAACAAATGTGACCATTAGTTGTCTCTCAGATGGCTCA<br>AAGAAAGGTACCACTAGATGGGTACAGGCAGGGGTGGGACCATCT<br>CGCAGGAGTGAAGAGGATTAGGGTTCAGGTTGAGTTGACACCAGG<br>AAGCTGTTAGAACTAGTTCTTGAGGAAAAAGGGAATAGGGTTTGA<br>AAAACAAGAAGGATGGGACCAGAGAAGCTGACCACCCTTTCCACC<br>TTGTTTTGGAAGAGGGTGGATGCTGAAGAACAGAGCCAGGGAGAA<br>GCAGGAAGGTGGGACTGGTCAGGTTGGGCACAGCCTGCCCTGACA<br>CAGCCTCTTCCCTCTCTCCAGGTCCCCTGC
NAT1	TTGTAGATTGGGTGCTGTGAGTCTGGGGAATTAGTGAGAATTTCCT<br>TTATTTGTTATATAACTGGTTTTCCCTCTGGAGTCCCCATGATGAGAT<br>CAGGTTGTTCATTTTTTAGGTATGCATATGGAGGGTGAGCCACAAA<br>GGAGAAATTTGGTATCCAGTTTCTACAACAGCCTGCCAGTCCCCGA<br>AATCCTCTAAGTTGTTTCTTAGTGATGGGCATTGGGAAAGCTAAAAT<br>TTCTCTCACATTATCAGGGTTAATTTAATACTCAGTCCTTTGACCAA<br>GATAATATGCCCCAAATACTTAACTTCCGGTAAGCACAGCTGAAGTT<br>TGTCTTCAGACGCTTTGCGTCCCTTGCTGGCTGAGTAAATGTAGAC<br>TATCTTCCTGGGAGGAAGATGCTGTGCCTGGACAAAGGAGAAGGT<br>CATCCACATACTGAACGAGTGTTGAGCCCTGGGGAAAAATTAAATC<br>CACTAAATCAGCTTTTAGTATTTGGGAAAAGTAAGTGGGACTTTCT<br>GTATACCCTTGGGGCATGACAGCCCACATATATTGCCATTCTTTCCA<br>AGTAAAAGCAAACAAATATGGCTGTCTGGATCTACAGGAATACTGA<br>AAAAGGCACCGCAGAGATTCACAACTGAGAAATACTGGCTGGTAG<br>TGAGTATAGCTGATAAAACGGCATGTGGGTTGGGGACTACTGGGTG<br>CCTGGGTATTACAATATTGTTTATTGCCCTCAAGTCCTGCACAAATC<br>TCCATTTTCTCCTGCTTTGTTTCTTTACAGGGAACATAGGGCTGTTG<br>CAGGGGCTGGTGCAGGGAATAATGAGCCCCTTTTTAGATAGTCTTG<br>TATAATAGGGGCAATTCCATTTGCGGCTTCCTGCTATAGAGTGTATTG<br>TTTAAGGCTAGGCAGGGGTTTCTTTGGATTTATCTCTACCTTTATTG

	GAGTGGCTGAGAATATTTTCCCTATATCTGTATTGACTGAGACCGTA<br>AGTGGGAGGAGACATCCTTGAGCAAGCGTTCCTCTTCTGGTGTTAA<br>CAGGAAGACTGGGGAAGCTAAAAGGAGTTTTACCGTTTCCTGCTC<br>TTTATTCCAGTGTTTTCTCTTCTCCTCTATTATTTCCTTCTGTGTCTCT<br>ATTTCATATTCCCGGCCACAAGACGATGTTTCAACACTACTAGTACT<br>AAATTGTGGTACGTTGTCAGGACATTTTGTAATTCGGTATTCTTGGT<br>CTCCTGGCCCCAATTCTAAAAAAAGTTCACGTTTTGATGAAAAAAA<br>GATATGGGCATTATGGATGTTGAGGAGATCACAGCTCAAAAGATTG<br>ACAGGGGCCCCAGGGCATATAAGAAACATATGGTACCTACGGAGCA<br>TGTCACACACAATTAGAGCTTGCGGCGGGGGAGCTGAACCCGATG<br>GAATTGTAAGGTATAGGTTTGGACTTAAAACGTGAGATCATTTTATT<br>AGACGCAGCCACCATGTTAATTCTTTCATTACTCTGAGGAATGGGCT<br>TTCTAAATCAGGTGGGATTTGTCAGAGATATAGTTGTGTCTGTGACA<br>ATTAAGGCAGTTGTAAGCTCATGATTTATTATTATATTAATGTCTCCC<br>AATTTGTTTGTCATGGTGAAGTTTTAACAGCTCACTAGGAGTTAACT<br>TGTGGGCAAAATATGGGGGAGGCATGTGGCTTTTCATCTTGTAGCC<br>ATCTTATTCAGGAACCAAAAAGGGGGAGGCAGGTTTGCATGACCC<br>ACTTACTAGCTTGACTTTTCCCTTTGGCTAAATGAGTTTCGGGGGTC<br>CCACAATTTAATTTCCTTTCACAGACTCCTCAGTTTTCTTTAGTGGA<br>GGTGAGGGGAGGTGGCTATGACAAGAGATGCTCTGATAGCGGATCT<br>TCTGTATATAGATGGTCCTTGTTTCTTTTGAGTAGCATCATACATTAA<br>GGGCCTTACCTTGTTTATCTGCCTCAACATGCCGAATTATAGCTGTA<br>CCTAGAGGAAGACCAGGTGCTAGCACTACATCCTGGTCTTTGATGT<br>GTTTGGTATATGTCATGGTAGTGACTGGGGGCATGGAACTTGGTTC<br>ACCCTTCCTTGCTGATCTCATGACAGTTCATCAGCTGGTGGGTTGCC<br>TCGGGAGGCTGTGGAAGCCCCGCTGCTGGTGGCTTCCACAGCTTC<br>CAGGTTTTTAAGCCAGAAGGCTGTACCCCATCAGCGTTGCACCCAT<br>CTCCATAGTTGTAGTTTCCACACACTTATACAACTCAAATGTTCACT<br>TTACTGTGCAGTCCATTCCCTCTGTTTTGTGATTTTTAGGAAATCTTC<br>ATAATTTACGGTCTACAAAACTATTATTTTGTTTTCAAGGAAGTCAT<br>GTTATATATTTCTGACTGTCTTTTCTCTTATTTCTAGAATTCAAGCCA<br>GGAAGAAGCAGCAATCTGTCTTCTGGATTAAAACTGAAGATCAAC<br>CTACTTTCAACTTACTAAGAAAGGTATTAAGCGCCTTTCTGAGAGC<br>TCTCAGTGGGCTTCCTAAGCACGTTCACTCTGCCTCCTTTAAGTCTT<br>ATATTTATGATCAAGATGAAAGGGAGGGATGTATCACTGCACAGAG<br>ATTCTACAAGTGGATATATAAAGCTAATAATGTTTTCAGTGAGCTCTT<br>CTCTGACAACTACTTTCCAAGTGCTTTCAGCATTCCACCTTGAAGC<br>ACAACGTTAATAATGCACAATTTCTTATGTTCAGCCACAGAATGTTC<br>ACGTGTGATTTGGTTACAACATATTGCTCTATCCAGGCCTCTAACAA<br>AACTGACTTCTTTCAAATGGACTTGCAGTTGCTGAATTCCAAATCA<br>ACACTTTATCTTAAAAACAGAAAAGGAGGGGAAGGGTGGAGGGA<br>GGGGAACAGAGAGGGTACAAGAGGCAAAAAAAGAAAAAGAGGA<br>AAACAAATGCTTGTAAACAAGTTAAACCAAATAAAAGAGATATAAA<br>GTAATTTCTTAAAAGAAGGAATGATATTCTAAGCAAGAATCAAATAT<br>CCAGGTATGTATAAACCGCCTTCGTCCTTTTAGATCGGCTTCTCTCT<br>CTTCTTTACATACTCAGTACAAATTCTAGGTGAGGGGGCAGAGAGG

	GGTTGGCATTGTCTGACTAAATACATCTTGTTCTCTCTCTGAGTGGT<br>AGAACTTTAGAGCTTGACCAAATGTTACGAAAGGGTGCTGGTGGA<br>AGTTAGGATGAGAAGAAATGCAATCTAATAGATTTGGAGAAATGAT<br>GTTGTAGACACCAAGAACAGAGAAAGTTGGAAACTGGCAATATGG<br>GGAGGATAGCTGGGTCAGAAAGGAAAAATAGGAGAATATAGAAGA<br>AATGTTATGAGAAGGGAAAATAAGAGATGCTCCACTTATCACCATT<br>CATCTCGGGGCAGGTCCCTTTGTACAAGGTCATCCTCAGGCCATAC<br>CACATACAAAAGAGTTATACCTGAGGTCTGTACAGCACTGTTACAA<br>TCATAGTCTCAAAAGGTTAAGGCTCTATGATGGCAGGTTTAAAAAA<br>CCTTTTCTAAAATATTTAACAAAATAAGGGGCATTTATTTTTTTTGG<br>TTCACATTACCTGAAAGTTAAAAGATAGCTTTCGGCTTCATTCAGGT<br>CTTTCCTCATTCTACCTTCTGTTGTTTTTCATCTCTTGGTAAACTTGG<br>TGAACTCTTTGTTAGTGTTGTTCTTCAGTAGGTTCTCACCAATAGCT<br>TTGAATGAATACCCCACCAGCTTCTACTCACCAATAGCTTTGAATGA<br>ATACCCCACCAGCTTCTACCTTGTAGCAGAATGTACACGCTTAGGTT<br>TCAGCAAAAGTTCCTCAGTAAAGTCTCAGATCTACTTAGATCATATG<br>TTATCCATGGGCCAAGCACTGGTCCTAGAGCATATGTGTTCAAATTG<br>GTCCCACCTAGTCCATGTGACTATCCATAAGGCGCAATATGGGCAGC<br>CTGTTCTCTACACAGTATCCAGAAACATCACTTTGATTGTGGCAAA<br>CATTGCTCAATTACAACCCTTCTCTCAGGCCTCTCTCATTTACATTTC<br>TCATTTCACTTACAGTAAAAACCAAAATGCCAGTCTTAGCTCCAAG<br>GCTGCAGTTGCCTGTCAGATCTCATCTCAATCCACTCTCCTTCTTAC<br>TCATGTGCTCCAATTACCCTTGCTTCCTGAATGTTTTTTGAACGCTC<br>AAGGCAGTTTCCAGCTTAAGGCCTTGTTATTTTCTGTTTTCCTACTT<br>GGAACAATCCTCCTCACGCATATCCTTAGGCCTTATTCTCTGGCATC<br>CTTCAAATCTTAGCGAGTGTTTTTTTTTTGGTGTGTTTTTTTTTTTTT<br>TAAACCAACTAATTTAAAATTGGAGCTCTCCCTCTGTACTTACTGGG<br>TCCTTTCTTTACTTTAGTTTTCCCAGGGCACTTACTACAATCTAACA<br>GATTGCATGTTTTATCCATGTATTCAATTTCCTATATATGTCACTCCAA<br>CTCCCACTAAAATATAAGATTTTTGAGAGTAAGCAAGCACTAGAAC<br>AGTAGGAAACACATTATAGCTATTCAGTGAACTATTTCTTGAGTTAA<br>TCAATCAATGGATGAGTGAATCAATACAGTTCTTTGAGCGGAAAC<br>TTTGTATAAGGCTCAGCTAAAAGGGAAATTGAGTGGGTCAGGTACC<br>ACGGATACTATACACTCTATTGCATGATTCTCCTGCCTACATCAGAA<br>GACGTTTATAAGCCTATTTTAAAGGATACCAGTTGGAATCTCTCTTTT<br>ATTAATCACCAAGAGAACCATGAACAAGCTGTTTATCATTTGACTCA<br>TCATTTAATCTTGATTTCCAGCTTCTCACACTTGAAAGAAGACATAA<br>TACATTTCTCACAGGATTCTGGGACTATTAACTGAACTTATGTGTGT<br>AAAAGGAATTCATACAATGAAAGCACTAGAAATAATTATTATACTTA<br>TAACCATTGTATTTTTACATGTTTAAAATATAGCCATAATTAGCCTAC<br>TCAAATCCAAGTGTAAAAGTAAAATGATTTGCTTTCGTTTTGTTTTC<br>CTTGCTTAGGGGATCATGGACATTGAAGCATATCTTGAAA
NCF4	TGATGTGAGGTCCAAATCTTACTCTGCCGGTCATTAAGCAAGCCGC<br>TTTGGTTTATTTTCCTAATCCCGTAAAGCCTGGGTTTCCTTATCTCCC<br>GGTGGGGATAAGAATACCACTATTTTATGGGGGTTGGTGTGAGGATT<br>AGGTAACAACAATCAGAGCCAACATGGACCAAGCCAGGCTGTGGG

	CCAGGCACTGTACTAAGGGCTTTGCAAATGCTAGCTCTCGTGCTCC<br>TCCTGGCAATTTCACGGTGTAGGGGTGGTTACACAGTCAACTCTCA<br>GAGTGACTTGGCTGTTACCGTATTTATGATGGTGGAGAGGAGATTC<br>AGGATTCTTAGCCCCTGGGGGAAGCTTCCCCTGAGTGCAGCTCAGA<br>TTCTGGCTGATAGGGCTGTCTGGAGAGGTTGTCTGAGCCAGCAGG<br>CTGCGCGCTCTGCCTGGCCAGCTGCAACTTGACTCTCGACTTGGCC<br>GGGTCCTTCTAGTGCGGCTTATAGCATTCAACCTCCTCTAGGTGGCG<br>CTGCGGAGCGGGGACTCGGCTGCTGATCGCGGCGCCCCCTGGCGG<br>TCTGTTCCCGTCAACATTTAGATTAAGCGCAGAGCCGGTTCCGCCA<br>GGGAAGTGCGCGTCCCGGTGCCCCCGCGTGATGAGAGCGGATGAC<br>AGATTGCGGGAAAAAGTTAGAATGTAACGTTAGTTGTATGACAGAG<br>AGGACCAAGCTGTGCAGCAGAGGGAGACAGAAAAGGGAGGGACC<br>AAGCCACAGCAAGGAATGATCGTCAATAACATTAACGTCTAGAATT<br>TATTGACCACCTGCTATGCGCGAGCCACTGTCCTAAACATCTGGTAT<br>TCATTCCCTTAGTTAACACACCTCTAGTAAGTGCGTGTCTCAAGAA<br>CAGTGTCTGTGTTTCATTTAGGGCCTAGCACACAGTGGGTGTCTGG<br>CACATAGTAGGTTCTCAACACATTTGGAAATGAATAATACAATAATC<br>CTGCAATTTTTCTCTCCAATTAATAATGTAGAGGCAAATATTATCTCC<br>ATTTAGAAAACTGAACAATGGTCGGAGAGTTTGCACAACTAATGCG<br>TGACACAGCGAGGGCACACGAGGTTTAGACCCAGCTCAGCCGTGT<br>GCCAGGGCTTCTCAGAGAAGAGAAGACCCTCTGCCTTCAGGGAGT<br>TTGCTGTCTAATTGGCCACGTCTACACCCTCCCTGCCTGGGTGGCAT<br>GCCAGGGATGGCTATTTATGGCTCAGGGAGGAGGTCCAGTGAAGTT<br>CCCCTCATAAATTATAAAGGGAGACACATGTGGGTCAAGATTGGGG<br>AATTCAGTGTGTGAGGGATCCCCAGGAATCTTACACTGTCCAATCA<br>GGGATTTTCCTTCTCTTACCCTGGAGAGTGGCTGATGTAGGCAGTG<br>GGGATTTGCTGAGTACCTATAATGAGCTCTCAAGGGTATCTCCTGGT<br>CCTGTAGAAGAGGCTCACCTCCTCCCTATCACAGAGTTCCAGTTCC<br>TATGGCTGGAGCTCTCAGCCCCAGCCGCTGGCTGCTCAAAGGAGA<br>GTTTCCTCATGTCCAAAATGGGGGCTATGGTTTATCTTGTATACCCC<br>AGCACCTGCATGAGGAAGGACTGAAAAGTGCTGTGCACTTTTGTG<br>ATTCGCACATCCAGTCCCCATTCAGGCTCTGGGGATCCCCCAGGCA<br>TGGTCTTGGTGTATTAGACTTTTTCCTGGAGATGTGATTATGAATGG<br>GCATTAGTAAGTGGTGTTATGCAGAGATGCCCTTCTCTGAATGGCCT<br>CTGGTATTTGTTTTTTGAGTTCTTGAGAACATATATATTTTTTGGTATT<br>CCATGGTCTTTATCATTGTCAGGCTGTCACTGAACTGTCGCTTGCAT<br>TTGGCTGCCCCTGGTAATTTTCTTCCTTCCCCTAACTCACTGCTTCA<br>AGTTCTTGAGTTGGCAGGAAGAGAGGACCAGGCTCCTTTTATATTT<br>CTTCTGTGAATAAAATATGCCAGTGTCAATGCATAGACTTTGAGCTA<br>TCAATTCTAACCATTCAATGGCTTTTCTCTTTTTTTGGTTTATTCACA<br>GGCTGTGGGATGTGGAGGTTATCAAAAATCAGGTCTCCATAAGTGA<br>GAGGTCACTTCAGGCATTTCTTTGCACTTATCCAAAAAAGATTCCAT<br>CCATACTTGTTTAATGGAACGTGTGAAGAGCTTGGCTCCTCTTCCA<br>GGTCATCCCTCTGCCTCATTCTCCCCCTGCAGCAGGAGCAGCAAGA<br>GCCCGGTTCTGGAGTGGGAGTAGAAGGTACAGGCATTGCTGCAGC<br>GGCAGGACAGGAGCCCATTGCTGACAGAGTGCCTGGCCTCAAATT

	ACACTGTATCAGAGCTCGGGCATAAAATTGGCAGCAGCGTTCTGTG<br>TATGAGGAGACAGAGAAATACCTGTATTCAGCAGAACCACGGGCA<br>GGGACAGAAGAAAAGGAGAGAAAGAGGGGAGGAGGGGAAGAGC<br>TGGCCCGTCGCGGGCTCTTCCCTGGACATCTTTGTCCCCCAGGTGC<br>AGAGGTGGCTTCCCAGTGGCTGAGTCAGGGCCACAGGAGGTTTCT<br>TGGGGGGTTGGGGGGGCCGGGGTTTGGAGGGCAAGCCATGAAAAT<br>ACCTGGGAAGGTAAACTTGAGGGCTTAACCCACCTGAAACACACA<br>CATACAGATATACACACTGAAAACGATTAAAAAAGGAATAAGAAAT<br>GTCCATGTGTCTAGGCACTTTTATACAGTTTTCTATTAATTCCTACTA<br>CAACTCCATTAGGCAAGACTGTTTCACAGATTGGTGAAGAAGAAA<br>GGTTCAAAAAGGACATGTTATCTTGCCTGAGTTCACCTATTATAAAT<br>GTACTTGTGTTTATACTCATTTGTCACCGACTGCAACCTAAACATCT<br>AAACACATTCTCTCACAGCCACAATGCCACCACTATACACATTATAT<br>GGTCTTTTATAGACACAACACAAATGCACTTACACTTGCCTATGCGA<br>GCTGTATGTGTTTATACACAAATATGTGTTTCCAACTATAGGCATATA<br>CTACACACACAGTTTACACAATCACCTACAAATTCTGTATCATGCTT<br>GCTTGCTAAAATTGTTTTGCCACTTCCTGTCCTTCCTGACCAACTGT<br>AGGCCAATGTCCTCCTCTTCCCTCAGTTATGGCTGTGTGCTTGCACC<br>TGTTGGTACTTACAGACACCTGCACTAATAACCCTGCTCTGTGTTTC<br>ATTTCATCCTCTAGCCTGGGAGCCCCGAGACACCAGCAACTCATCA<br>GACTTATTGTTTGTATTATTTCTAGTTCTTTGGGTGTGAATTCTTCAT<br>GATGTGGAGGCTCTGAGCTGTCTTTCACAGCTCACGATTTGTAGTT<br>TTGGTCTGTGAGTTGACAGAGTAGGCCCTGCAGAGTCACTGACTC<br>CAAGCTGTTTTTATATAATGCCTCAGCTTAGGATTTCTGCACCACGT<br>GGGTAGCTTATATCGGGGCCTTACACCAGTGCAGAGGGCAGGCTTG<br>AGGCTGATCTGGTACACACGTCCCTTGTTCCACCCATGGACTGTGA<br>GAGGACCTCAGCTCCTGCCGTGACTCTAGGCTGATGGCTGAGAAA<br>CTCTGTTCCCATAATCAGTTCTGACACCCACTTTACACTGAGAACTC<br>AGATTTGACTTTCTGCTAACAGCAGGACCTGTGGCCTGTACCTAGT<br>CCCAGATTTGGCATTAAAATCTCAGCCCAACAATGCATATCCAATCT<br>TGGTTTTCCCATGGGCTGTTGGCCACGTTCCTCTCCCCCTGGTATAG<br>ATTCCTGACCCCTCTCTTTGATCCTACCTAAAAATTATGCTTTCTTGT<br>TTTTTTTAGCTGGGACTTGTATGTTTAAAGTAGTTTAAAACAACTTT<br>CTGCTCTGCATGTATGAATACGAGTGTGTGAGCGTGTGTAGCTGGC<br>AGGGAGTTACTGGGTGTGGCTGTGCCAATGACTCCTGTGGACCTCT<br>TCTCAGAGAAGTCCCTTCAGAATCAAATACCCATACCTGTTAGGAG<br>CTGCAGTCACTGACTGGCAGCGTTTCATTCATTCACTGAATATTGGT<br>TGAGCACTTGCAATGTGCCAGGCACGCTGCTAGCCAAGGAGGCAG<br>TCATTAAACATACAGATAAACAAACAAATGTGAGATGACAATAGTT<br>ATGAGTGCTCTGCAGTAGACGTGCAGGGACCTGTGAGAGAATGTA<br>GTAGGGAGGGCCTTAATTTAGGGTGTGTGGTCAGGGAAGGCCTCG<br>CTAAAGATGTAGCTTCTAAGCTGAGATGCAAAGGAGGCAGTGAAG<br>AGCTTTTTCCAGCAGAGGGAACACCATGAGCATAGGCCTTGGGGC<br>GGACGTCTTAGAACATTCTAGAAACGGAAAGGAGGCCAGTGAGAA<br>AAAGGGAGCTCAGGAGCAGAGGCTGGGGAAGGCTGCAGGGCCTC<br>AGGGGCATTGGAGGCAGGCAGGGCAAGGACTCTGGACTTTATCCT

	GAGCACAGGGTGATGGGGCAGGAGAGTTTAAGCAGGGAGGGTGA<br>AACTCACTCTGATTGGCAGCCCTGTGAAAGAGGATTGGCTGGGCAT<br>GGAGATCTAAGGATGAGGAAAGGATATCCCAGAGTGCCAGGGCGC<br>GCAAACCAAACTTCACACTCTAGGAAGACAGAGGCCAGAGGAAC<br>CAGGAGGGCTTCCTGGAGGAGGTGGCATTTACTCTGGGCCTAAGA<br>GATCACAGGAAAATGGAGGCCAGCATTCTAGGCCTGGGGAAGAGT<br>TTGGGCAAAGGCTTGGCAGTAAGAGAATAAAGGTGGGGCTTGATG<br>GGCCAGAGGATAAGTGACTCCTCAAGGTCACAAGACACCCTGATG<br>GCTGGGACCCCATCTCTGATGGAGCAAGGCTTCTGATACCCAACTG<br>AGGCTTGGCGCCAAGGACTGACATCAAGGAAGTGGATTCCTGCAA<br>ACCTGGCACCCGAGGGTCCACTTCCTCAATTTCTGTGCAGCTGGGA<br>GGTGAGGTAAGAGGAAGTGTGGGCCACAGGCTAGGCCCAGAGAG<br>CCTGGAGGAGGAGCCTCTGCCAGACTGGAGAGAAGCAGGCCTGA<br>GCCTCCCCAAAG
NDRG1	ACATTGCACAACTCTTCATTTATGTTCAAAGTAAAGGATTTATTACA<br>CATCCTGAACATAGCCTACCCTTCACCCTCATTTGGAATTGTTTTCC<br>TTTCATGGTTTTTTTTTTTTTTAATTTTCATTGCTCACAACATGATATT<br>ACAGGCCAGGCATGCTTGCAGATAACTGGGATAATTTACTTTTACTT<br>AAGTAAAAATCCCAGATGGCAGTCCCTCCACCTGGGATCATGAAGC<br>AGGTAGGATCCCCTTTTCCCTCCTAACAGAGTCCAAGCAGTAAGAC<br>CGAATAAGATGGGATGAATTCAAAGAGAAACATCTGCATTTGCTCA<br>GTTCTTCAACCAATATTTACTGAACTTCTCTACGGTCAGGTGTGCAA<br>GATGCCAGGAGGGATGAGACAAGATGATGATGATGATGATGATGAT<br>GATGATGATGGTGATGATGACAGTGAGGATGAGGGTGAACACTTTA<br>ATGAGAGCCTTCTATATGCCAGGGACTGTTCTAGGCACTTTACATAT<br>ATTAACACATATATTTATCACAATGACCCTGTATTGTTATGTGTATCTT<br>ATACATGAGGAGGCAGAGGAAGGAAATGAAATAACTTGCCCAAGA<br>GCATAGAGTCTGCAAGTGGCAAAGATAAGATAGAAATAATTCTCCA<br>AGAGCAGAGAGTGGTGACTTCTGGGGATAGGGAAGTGGAAAAGT<br>GGGGAGACAAAGGCTGTTGTTTTTCCTAACAAGTTTTGAAGACTTA<br>TTTGACCAGGTTCAAGAAAAACTTGATAATCACTACAAAGTAAAAG<br>GAAGAGGAGATGAGGTGGGCCTTTGGGTACAGATGCTTGTTTTGG<br>AAAGTTTAGGGAGGAAGGGGTGAGAGGAACTGGGCTGCAGTACA<br>GGAATGGGGGACTTGGGAATCAGAGGGGATTGGTTAGGGTGGGTC<br>ATTGATTGCAGTGAATGTTTACTTGTTGATTGGGCTGCAGTACAGGA<br>GTGGGGGGCTTGGAATTAAGAGGGGATTGGTTTAGGGTGGGTCATT<br>GATTGCAGTGAATATTTACTTGTTGATTAGTTTGATCCAGAAGAAAG<br>GGAGAGATGGATGCTGGAGGAGAGAGGGGGTCCCGAGGGAGCCC<br>AGGACTGGAGGTGGGAAGACACTTTTACGGACACTTTTTCATTTTG<br>ACAGACAGAAAAGATGATGGTCTGCAGCAGTAGACCCATAAATTGT<br>GATTGAAAGGTCTGATGGTTTCTCTCACTCGTTTCCTCCATGCACAG<br>AGAGGTGGAGTAATTTGCCCCAGGTACTACAGCTCATAAGTGGTGA<br>AGTTGGAGCTGGGGGCCACAGACCACCGGGTTCTTCTCCATGAATC<br>CCTGCTGCATCTTGGTCTCTCCCAACCCCGAGTACCCATGAGTTTTA<br>AGAAAGACAGTTGGGAACAGGTGATTCCTATGGTCATTTTTGGAAC<br>ATTTGGTTAGAATATTCTAGTATAAAATCTAAGTTGTTGTTAAGTAGA

	AATTTCCAGTGGACATTAGTCCAGAAAATCCAATCTCATCCTGTGA CGGATTTGCAGCGCGTGTGCATTTACTAAGCAAATCTGATTCTACTG CTGACCAGCGAGAAAGAGGAATAATGTAAACAGTCAGTGCAATTG CACCCACATTCAGCTCCGTGAGCTTCTGCCACCTCAGCTGGCTGCG GTGGGGGATGTGGAGGTGCATGCAGCTCCCAGGTCCCAGTGAGGG GCCTCTGTATAGGGTGGGCGTCCTCTATGCTTTATGTGCATCCAGGC CTGTACTAAAGACAGTCTTTCTCCTGCAGCGTGACACTTAGCTAAG AAAACTACTTCATCTGGTGCTCAGCGGAAGAAAACATCCTTTTCAA CAGGGAAGGAAAATTGGCTGTGTTGGGCTGAGGGAGGGGAGTGT GGAATCCAAATGGTGTCTTCCTCAGGCCTTCTCCAAGGTGATGTGG ACTCTGATTTTAGAATGCCATCACTTGGAAAATTGTGTTCCATTGCT CAAATTCTTGCCTCCCTTCTCTGCTCTTTTCTCTTCACTCCACTCTCC TTCCTCTGTCTTCTGACCTATTCAATGCTAATGGTCTTCCAGAAGGT TAGGCTTCCCACACAAGCCCAGCCTCCTGCCAGGCAACCTCCTCG GGGTTTCAAGCTGCAAATCTGGGCTGCTCTTCGCATTCTGTCCTGA GGACCTCCTCAAAGAGACAGGCAAGGGCCTCCCCTGGCAAAATGC CCTTCTAATTCCATGCACTAGTTCCATTTGTTACTGAAGAAGTGGGC CGCCTTCCCTGCGCCTTTGAACTTGACATTCCTTATCTTGACACTCC GCACTTTTGAACTTGACACTCTTGTATCTTTAGCAGGTAAAAAACC AAAGGTGCTCACTCAATAAATCGCCGCAATGGGGAGCCTATGGGAA AATGGGTATGTGTAAGCTGGCTAAAGGATAACAGGCACACAGGCTG CTTGATCTGGGAGGGTCTTTAGACAGCAGGATGGCTCCCAGAGATG CACTTTTGTTGTTGCTGTTGTTCATGATCATGTCCCCAGGGTCTAGA ATGGTGCCTGGGCATAGTAGGTCCTTAATATAGTTAGGGAATGAATG AGTTCATCTCAACCACGCTGTATGGATGGAGAAACTGCGTCTTGGG GCTGTACCATGCTATGCACCTGTAGAAAATGTTTTTGCCTTTTCAGT CCTTTCCCAGTGGCGGAGTACAGGACCCAGCTCATAAGAGCAGCT CAATTCTGTCATTTGTGGAATGACAGAATAAATAAGTTTCAGGGCCT AGGGAGGCAAGGCTCCTGCCTTAGCCTCCCAAGTAGCTGGAACTA CAGCTGCATGCCACTAAGCCCACCTGATTTTTATTTTCTTGTAGAG ATGATGTCTCATTATGTTGCTCAGACTTTTCTGGAACTCCTGGGCTC AAGCAATCCTCCCACCTCTGCCTCTGAAGTAGCTGGGACTACAGGT ACTTGCCACCAGGCCTGGCTAGCTTTCATCTTTTTTGTAGAGATGGG ACATCACTATGTTGTGAAGGCTGGTCTCAAACTCCTGGACTCAAGA GAGGCTTCTGCATTGGCCTCCCAAAGTGCTGGGATTACAGGTGTAA GCCACTCTACCTGGACACAACATTTTATTTGAAATAAGAAAAATTTT CCCTCACTGGGAATGCTGCAGTTGAACTAGTTCAAATCCATAAAGA ATGGAAGACTGCCCAGTGGTCATTCATTCACTTATTAATTCTAGATG TTAAATTTTCTAGGGTGGGGGAATGTGCTCCTACATTCAAGGCATTC TTGAATGTAGCCAAACTTTGTAGTTTAGCCAAACTTCATAGCTAATA GCCAAACTTTGTAGTTTAGCCAAACTCTTCTAGCAGCAAAACCCAT GACACTGGAACTCTGTTCTTCACACTGGTCATTTGTAATAGAATAAC AGAGCTTTAGCACTAGATAGGTTCTGAAAGATCATTGAATCTAATCC TCTCCCTTTGCCAGTGAGAATTCTGAAGCCCAGATTTGTTGATTTGT GATCGATAGTGTCAAAGACAGGCCTGAAACACAGATGTCCTGGGT CCTAGAGGTGCTGTTTGCCCCTCTCCATATTTCTTTTGTTCCAGAAA

	ACCCTTCTCCAAAACTGGCCCTAATAATCAGAGGGGAAAGCCATGG<br>CCCCTGCCTTGGGGACAGCATGGGTTGGCACAGAAAAGAGGTTTA<br>CAATTCAGCAGGAAGTGTTGTGCGTGCGCGCGTGTGTGTCTGTGG<br>AGGCGCAGGGAGGGTCACCTGAGCTTGCCCTGGGTCTGGTCCTGG<br>GCTCAGTGGCAAATTCAACGCTGGGCAGGTGGCCTGAGGACAATG<br>ACGGTGCCAGCTGTGAGTCAGACTTCCTTTATTCATAAAATCATGTT<br>CCTCCATGCAGTGCTTCTCAACCTGGCTGCACGTCAGGAGCCACCT<br>GGGTAGCTTTGTAAAAACCCCAATGCCCAGTTGGTTTGCACCCCCT<br>GAGATTCTGATTAAACTGTTCTGGAGTCCATCTCTCTCTTTTTATTAT<br>CTATCTATTTATTTATTTTTTAATTTGGCGGCTCCTCTGGGACTTCTCA<br>TATGCAGTAGGGGTTGAGAATTGCTGGTTTCTTTGGCCAGATGGGA<br>ACATGCAGGCCAATAGTTACGCACAAACAGAGCAGCAGAGCAGGA<br>CGGTGCTAAGGTTGGAAAGGGTGAGTCATGAAAGTTCAGGAAATC<br>AGAATAGGAAGGGGTGAGAATCCTCTCATCTGGGCTTGTGGGGCA<br>GGAGAGAAGAGCCCAGTGGGCAAGGTCCAGTGAGTGTGCTTAGA<br>ACTGGGAAGATGAGGGGAAGGGTAAGCGCTGGGCGGCGGTGGGC<br>AAGATGGACGAGATTAAACGGGAGCGAGGATTTCAGCGAGAGCTT<br>TGGCGACACGGAAGGCTGAGAATCGGAGGGAGCCTGTCGGGCGA<br>AAGCGAAGGCGGCTGCTGGGCAGGCAGCCGAATCCGGCTGGAGA<br>GCCGAGCTGGTGAGACCTACAGGAAGTGAAGGGAGTCGCTCAGG<br>GCGTGGCGCAACGAGACTCTTAGAAGAAACTCTGAGGCAGAGATG<br>GGGGGCCTCCGCCCATACGGAGACACAAGGAAGTCCACATGCACA<br>CGCACGAGCGCGCACATGAACACGCACAAGCACACAAACGCCTCC<br>TCCGGGCAGGGCACACGCGCCCGCTGCACAGGCCGAGGCCCTGGA<br>CTCGGAGGGGACTGCAGAGCCGACCCACAACCCGGGCCCCGATGC<br>CCCTCCCGGCCGCGCCCCTACGACTGCTTGCGCAACAGGCGGCGG<br>CTCCAGTGGGCGCCCGCCGCGCGCTGCCGGAGCCCAGCCCAGCCC<br>GGCGCGCCCGGGAGGAGGGAGCAGGGAGCGGGGAAGGGGTGTGT<br>CCCGGCTGCGTGCTGGGACTGCGAGGGTCTGGGAGGGGCGAGGC<br>GCGGGGGCGGGGCCGCGGCGCCTATAAAGTCGCCCTCCGCCCGGA<br>CGTAAACAAACCTCGCCTGGCTCCCAGCTGGTGCTGAAGCTCGTC<br>AGTTCACCATCCGC
NEUROG3	GGGGCGGTAGGTGGGGGTGGAAGACGGGGGGTGGGGGGTGGAGG<br>AGAGCTAAACTCTCAAAAGGCGAGGCCAAAGCGCCTGGCCCAGGT<br>ATTATGTTGGGAGAAGGTGAGGAGAGCAGCTGCTCCTTCTCGCGG<br>GGTGTGTATGGGAGCGAGGGTGCATGTGTCCCTGTGAAGAGTACA<br>CAACGATCTGCTCAACATATGTCGCCACAAATCACAGTGTCTCTCC<br>CTCCTCCCACCCCCCGCTCCCTGCGCGGCTCCTGACCGAGCGCAGA<br>GCAGAGAGGGGCGCTGAGAAGAGGAGCCGCTTCAACAGGTGTCT<br>TGGTGCGCAATGCACCTTTTCACGCATTGACGGTCTCAGCCGCCGG<br>CGAGGATAATGTGGACACTTTGTTTCCAGGCGCCGCGCGCGAGCTG<br>CGGACGCCTAGGGGAGGCGGCCCTGAGCAGGGAGGGGGGATCTG<br>GAAATAAAGACCTCCGGCTCTCCTGAGCGCCCGGGCCCTCTCTTCC<br>CTTTGTTCGAAGACTCCCTGGGCCGGAGCTCCCGTGTGCGCCCTGG<br>GGGGTGGCATATGGCGCCCTGTCACCTACCCAAGAAAAAGAACCG<br>GCCACCCTTTCAAGAGCGCACGAGGTCCCTGAGCCTGGGGAGGGG

	GAGTCTCTGAGGGAACGGCTTCTCTCCGAAAGTGGGTGAGGAGCC<br>CGTCTTATCTCTCCCTACCCTGCCTCCACCATCCCCTCAGCTGGCGC<br>AGCAGCCGTTGGGTGGGGAGCTGCGCGCTGGGCAGCACCAGCCTG<br>CGGAAGCGCTCCAGCTCCTGGCAGATGAAATATGGGTCTGGTGAG<br>CCCCTGGAGACCATATGCTTTCAATAAAAGAAGACAAATAGGACCG<br>AGATAGGCCTTGCAGCCAACCTGTTATAAATGAGTGGCAATTGGGC<br>CAATGTACACGGGGGCAGGGCCCCCGCGGGGAGGCCTGCTTGGTG<br>CCAAATCCATGTGTCAGCTTCTGGGACAGGTGTGTCCAGGGGCCA<br>GGGGCCAGGTCTCCCCCCTCCGGGTTTATCACGGCAAAGGTAATAT<br>TGTGCTAACTATGAGTAAACAGTCATTGTGAAAATGAGGGAGAGTT<br>TATCAGGGCGAACTAGTTGGGGGGTGGCTTAACAATAAAGTTGTTC<br>GCCTTAGGAGCAGGTGACGGCTGGCTGCACGGACTCCCGCGGTAG<br>ATGTTTTCCAAAGCACTCCACTTTACAATCTCTTGTAATTATTTATTA<br>AACGAAATCTATTTATTATTATTTTAGCAAACACTGGAGACAGGTGG<br>GGCTTTCTTTTCGTCGTCTCCTTTTGTTTGAAGGGCTGTTTGAAGTG<br>GCCAGTCTCGGCCCGGGCAGCTCGCTCGCCAGATTTTTGTCTTCCC<br>CTCTTTCACTGCTGAGCTGAAGGCGAGAGAAAGAATCCCCCTCCTC<br>TGGCAAGCCATTAGCTATTCAGCTTCCCATGGCCCCCCTCCCTCCCT<br>GCACAATTCTCAAAGAGCCCCCAGGCAATGCTAAAACTACCCCCGC<br>CGACAAGAAGGCAGGAAGGCGGGCCGCTGCCTTCCTTCTTTTCCA<br>ATGTGCCCGCGGTCTCCAGGCGTCTGGCTACCGCCTCTCTCCGGGA<br>CCCCTCAGCCTCCAGGCAAACTTAGGTCTCAGGAGGTGTAGGGGT<br>GCGGGAGTCCTGGAGGTCTTAGGGGCGGTGGATGACCAGGAGCTG<br>CAGGAACAGACTCTGCTGTCTGCGGGAAGAGCCATTTCACAAACA<br>CTGCCGGTGCGCACGGAGTCATTACCGCCTGCGCCTCCGCAGCCA<br>AAGGCACAGGAATTAACTTTGGAGAGGGGTCTATGTGTCCCCTGCA<br>GCTGAGACAGAGGGCATGGATCCTCAATCTCTAATCGCCTAAGACT<br>CCTTGACCTCCGGCCCTGGCCTCCTGGCTGGAAGAGGACTGCGGG<br>AAATCCTTGCATCCCTCAAGAAGGCGGCTGCCCCCAGCCACCGCG<br>CGCTCCTCGCCCTGGCTGCTTTTTGCGCAAAGCAGGAGTGATGGAG<br>AACTGCAAGCCCTGGAGGGCCCTGAGCCGGAGGGGTTTGGGGGA<br>GGCTGGATAAGAATTCCAAGCAGGTCCCAGAATTCTCTACCTATCA<br>AGAAAGCTTGAAAGAATCCCCAGAGTCTTTCCTGGCATGGGAAGC<br>TCACCCTGCGAACCTTGTCAGTATCCATTAGCCGCTCCAGGCAGGC<br>CCCAGCCAGAGTGAGACCTACCCCTATCTGAGGTCCACTCACCCCC<br>ATCTGAGGTCCACCGCCAGATGAGAGTCATCCACAGGCTTCTGGGA<br>AACCAGAGCGGTGCTTGGATCCCTCGTGGCCAGGGTTCCCTCCAA<br>GGTGCTTAGCCAGGTCAGGAGGCCCTAGAGAAGCATGGTTTGGATT<br>TTCTTTCCCAGACCAAAAAAGCTCCAAGTTGGTTCTCTCCCAGTTT<br>CTAACTTGCAGTTAAATAAATCAGGCAAGGCTGGCCTATGAGGCAG<br>ACAAGTGTGAAGAAGGAGAAGGAGGAGGAGAAGGAGAAGGAGA<br>AAGAAGAAGAAGGAGGAGAAGAAGAAGAAGAAGAAGAAGAGGA<br>GGAGGAGGAGGAGGAGGAGGAGGAGGAGGAAGCAGCAGCAGCA<br>GCAGCAGCTTGAATGGACAGTGGTTCCCCTTGCCTAGAAAATGGG<br>ACCATTATTTCTTTTCTAATCTGACCCCCAGACTCAGGACTTCCTCT<br>ATTTTCTGCATTTTGGGGTCTCTTGTTTTGCCTTGAAAAAAAATGTT

	TTCTCCCAAATCAAGGAGCAGTAGCTGGTGCAAGGGAAAATCTAG
	GGCTAGGAGTCTTAAGATATGACTTCTATGTGGTTCTGATAGAACTT
	GCTGTGTGACCTTGAGAGAGTCACTCCCCCTCTCTGGGCCTTGATT
	TTTTCATCTTTAAAGAAGGCCTCAAATTCCCATTCTTATGAGAAGAA
	GACAAGCTCCTAGTGAGTGGTGACCTAAGGGAGCAGCTGCAGCAA
	AATGCTAACCTGACAGTCCCAGATGGTCCCTTTATTGGTTCTGACCC
	TGGTCTCAGGCTTCATTTCCCCACAGCAAGGGAAGGAGCCTGCTC
	ACAGAGCACCAGCTAAGATCAGCAGGACCGCGCCACACCCCCGCC
	CAGTCCTAGAGCCCCCCTCTCGCTGGTTCCTGAGCATACCACCCTC
	TTCCTTGGAGGAAAATTTGCCCCCAAGCAGCCTAGGCGGTAAGAG
	GCTATCACTAGGGCAGACTCACAGACCTACCTCATCCCCTCACCCC
	ACCCTACAGTCTCGAAGTCGGGTCCTGTCCCCTCCTGCAGTTTCCG
	GGAGACTCAGGATATCTGGACCTGCTAGAAAGAGAAGCCTTCCTC
	GCCTAAGGAGACTTAAACCGGGATACTTAAACCTCCCGCCTCGGCG
	TCTTCCTCCAGGCACGACCGGGTCAAGAGAGAGAAGCGGAAGCTG
	CAACCCCTCACTCTGAGTGACCGGAAGCAGAAGACCACGGGATGT
	CCCAGGCGGGGACAAGAGGAGGGGCTGGGGAAGAAAGGAGGGAT
	GATGAGTTCAGAGTCCCTTTGGAAAGGTTTCCAGAGAGCGCTACC
	AGGGACAACCCAAGGGGCTGGGGAAGTCCCTGCCTTGTGCTCTCT
	GTGCGATGCCCGAGTGATGCAGAGGCAGGGGGCTGGAGCAGGTGA
	CTGCTGGCAGCTGCTGTCTGTCTGTGATTGGACCGGAGGACTAAGG
	GGAGAAAAAGTTTATCAGCTTCTCCCAGTGCCTGCACGCTGTGGTA
	GTTCAAAAGACACGAGGGGGAGGGGCACAGCAGCTCTGCTTCCCA
	GCGCCTTGGGAGACTGAAGTGAAAGGAACGCTTGAGCCCAGGAG
	TTCGAGACCATCCTGGGCAACAAAGCAAGACCGCCCCTCACCCCA
	TACAAAATAAAAATACAAATAAATTAGCCGGGCACAGTGGCGCATG
	CCTGTAGTCTCAGCTACTGGGAAGGCTGAAGTGGGAGGATAGCTT
	GAGCCCAGGAGATCAAGGCTGCAGTGAGCTGTGATTGCACCACTG
	CAGTCCAGCCTGGGCGACAGAAGGAGACCGTTTTTTGGTTTTGTTT
	GTTCGTTTAAAAAAAAAAAGAAGCAAGAGCTCACTGTGAACTCCT
	GGTTCCTTCCTCCCCTCCTCACACTTCCCAGAACTCTTCCTGTCACG
	GTTCCTGGCCAGAACGCTGGGATACTATCTACAAGCTGTAGTAGGC
	TTGTAGTAATGGAATGTCCGCTTGAGGGGTCCCCGCACAGCCAACC
	CCTGCCTCTGGAGTGGGATCTATGGGGGTGGGGTTCTAAGCGCCTC
	TGGGGAGTGTGAGGTAGCATCTCAGGGTGTGGCAGAGGCTCGGAC
	ACCCCCAAAAGGTCTGCGAATGGAAGGGACATAGGCAGGATCTCT
	CTCAGTGATGTCCCCTGTCTTCCAGGATGAAGAGAGGCAGTGAAA
	CACCAGGAGAGCAGGGCGTCCTTTAGAATTCCTGGACCCTTCTCCA
	GGCTGCTAGTCAGGACAATGAGCTCGTGGTTGTCTTTGCCACTATC
	TTCCTGTGCGATTTCAGACAAGCCACCTCCCTCACTAAGCCTAAAT
	TTCCCCATGTGTAACGTGCAGGCATTGTACCCTAGAGGCATCAAAG
	TCCCCTCCAGGACAGATGCTAAGGAAAGATAGGCTAGGAGCAAAG
	CCGTCTGAGGTGGCCTGACCAGAGCCACACGAGGCTCTTCTCACT
	GGGCGAGGCTCTTTGAGGAACCGAGAGTTGCTGGGACCCAGCCCG
	CCCTCGAGAGAGCAAACAGAGCGGCGCTCCCCTCCCCCGACCCCG
	GCCCTTTGTCCGGAATCCAGCTGTGCCCTGCGGGGGAGGAGCGGG

	CTCGCGTGGCGCGGCCCCAGGGCCCCGGCGCTGATTGGCCGGTGG<br>CGCGGGCAGCAGCCGGGCAGGCACGCTCCTGGCCCGGGCGAAGC<br>AGATAAAGCGTGCCAAGGGGCACACGACTTGCTGCTCAGGAAATC<br>CCTGCGGTCTCACCGCCGCGCCTCGAGAGAGAGCGTGACAGAGGC<br>CTCGGACCCCATTCTCTCTTCTTT
NFKB1	ATGTCCTTATAAGACGAAATGATTTGGACGTGGACACAGAGGGACA<br>ACCACATGAGGACAGGGAGAAGATGGCCATCTGCAAGCCAAGGAG<br>AGAAGCCCTCAAGTAAACCAATGCTGCCAACACATTGCTGTTGAA<br>CTTCCAGTCTCTAGAACGATAAAAAATATTTCTGTTGTTTAAGCCAC<br>CCAGTCTGAGATATTTTGTTTTGGCAGCCCTAACAAACACATTCTCT<br>AAGGGTTTATTCTCATTTTTGGTTTAGTGTATTTCAGCTGATGATTAT<br>AGCCTAATCCTCCCCCTCTTCTTGGGGCCTCTCTTCTCTGTACTCTT<br>CCTCCTCATATCTCACACAGGCAGATTTGTTCCTGCCTCCTCACCTA<br>CATTACGAGCTCCGCACAGTCTCCTTTCTTCTGGCTCCGGCTCCCTC<br>ATTAATCTTTCTGTTATTAACGCTATTAACTCGTTAAACTATCTCTCC<br>ACCTTTCCAAACTTCCATTAACTCTGGCACTCCATAAGTGTTAGTCA<br>AATAAATGAAGAATCACACTAACTCCTCTTTCCTTCCCTCTCACTCC<br>AAACTCTTGGTGAGATTCTTTTGCACTTATGCCCTCGTTTCCCCATT<br>TCCCACTCACTTGTCAGTCTCCTCATTCTTGGTTTTCCTCATTATATT<br>GAAACACATCTGAAAAAGTCATGTAAGACCACAGCCACTGCGTATC<br>AGTGCTATTTGAGAGTTAATAAAGTTCCCTTTTCCTCCAAGACAGC<br>GAGCAGAGTGAACAGAAGAAAGACTGGTTTCTGCCCTTGGCTGCA<br>CAGCCATGCCCCGAGGCCGGGCTTAGGATTTTATAATTCACAAAGT<br>CAACTGTCTCTTCCCCTCTCCATCCTTTAGGAACTGTCTGCAGTATC<br>TGACACTGTTGAGAGCTCTCATACCCTTTAAACTCTCTCTTCTCTGG<br>CTTTCTGAGCTTTCCTCCCACTTTTGATGGCACTGCAGGAGTTTTTA<br>AGCATCTCTCTCTCATACCAACCCCAGAAATAAAGTCATTCCTCGAA<br>TTCCTGTACTTAGCCCCTTGTCTCCTAACTCTTTGACCACTAATGTTT<br>TAGAAACACTCTCCTGACAACTATCGCATCTTTGCAGTTGTTTCCAT<br>TTCCAACGCCATGTCTCTCTGAGCAGCAGCCTTGAGTTTTCAATTG<br>CATATTTATTAGTCATTTCCATATGGACATCTCACTAAATCTGTCATAC<br>TAAACATGTCTAAAATATGTATGTTTACATGTTACATGTATGTTTGGT<br>ATGTGGGAATGCATTACATTCCCACAGATTTTTTCCCAGTTACAGTG<br>TTACTATTCTAATCACTCAGACTAGAACACTCAGAGCTATCTATGAG<br>TCTTCCTCTCACTCAAACTGCATATATAATCTATAAACAGGTCCTGTT<br>GACCTCATCTTCTAAAAATCTCTTACATGCTCCTTTTTATATCATTGG<br>ATAATAGTTTAGTGTGGTGGTTAATATCATGCACACTGGAGCCAAAT<br>CTGTTAGGTTCAAATCCTGACTCTCCTTCTATTTACTAATTATGAAAC<br>CTTAGGAAAATTATTCAACTTTTCATCTGTAAATATGGGGATTATAAT<br>GGTGATCCACTTTATATGACTGACAACCAAATAAGTGAATACGTGTA<br>TAGCAGGTATTAACCATATGCTTAGCCGACCTATTTCAACCTCCTAA<br>CCAACCAGAAGAACTGTAGTTCCCTGCTCTCTTGCATCTAAGTGGG<br>GCCATGGAATGTGGGCAGAAGTGTTGTGTCACCTTTACTCAGAGGC<br>AATTAAAAAGAAGACATACTTTTCCTATGTTCTCATTGCCAATGAAC<br>ACACTACACAGATCTCAACCTCAGAATTCTCTCCACACTGGTATTTC<br>CAAAATAGCTTCATGAGAAGGGAAATTTGTGTAGTTTCAATGATTTA

	GTGATGCTATGGTTTGGGTATTTGTCTCCTCTAAACCTCATGTTGAA<br>ATGTGACCCCAATTTTGGAGGTGGGGCCTAATGGGAGATGTTTCGG<br>TCATGGAGGTGGATCCCTCACAAATAGATTGATGCTATTCCCTGCAA<br>GGAGGAGAGGATGAGTGAGTTCTCACTGCATTAGTTCCTATGAGAG<br>CTGGTTGCTAAAAAGTTTGGCATCTCTCCCCTCTCTCTCGCCATACG<br>ATCTCTCTGCACACACCACCCGCCTTTGCCTTCCATTGAGTGGAAG<br>CAGCCTGAGGCCCTCACCAGACGCATATGCTGGTGCCATGCTTCTT<br>GTACAGTCTGAAGACTGTGAGCCTAATAAACCTCTTTTCATTATAAG<br>CTACCTAGTCTTGTGTATTCTGCCATAGCAACACAAATGAACTAAGA<br>CAAGTGACAAACACAAAATAACTTGTCTTTTAGACAATATTGATGT<br>CTGAAATTCCACATTGAGGCATTTTTTTCCTCTTCGTAGCCTGATAA<br>TGGCAAATACCTTCTGACACCTTAGGATGGATGAATTTGTAAGAGAT<br>ATTCCTGGAAGCAGGTTTGTACAGTGTAGAGAAAATGTTGGGTAAC<br>AAAGCAGGAATGGAGGAGGGTTAATCAGCAAGCCAAAAAGCTTTT<br>ATAGTTTTGCCTGCTGTGCTGCATGGAATTACATCCCCCCAAAACAT<br>ATGTTCAAGTCCTAACCACAGGTACCTGTGAACATGAGTTTATTTG<br>GAACTAGGATCTTTGCAAATGTTATCAAGTTAAGATGAGGTAATACT<br>GGATTGGGGTGGGCTCTAAATCCAATGACTGGTGTCTTCATAAAAG<br>AAAGGTGAGGGAGAGTTGAATATAGCGACACAAAGGAAACACGG<br>GGAAGGAGACCATGTGACAATGTAGGCACAGAATGGAGTGTTGCA<br>GCCAAGCCAAGAATGGCAAGGATTGCTGGAGCTACCAGAAGCTAG<br>GAGGAGGCAAAGGATTCTTCCCTAGAGCCTTCAGAGGGATCATGG<br>CCTTAATGGCACCTTGAGTTCAGAATTTTAGGCTCCAGGACTGTGA<br>CAGAATAAATTCCCGTTGTAAGCTGTTCAGTCGGTGGTAAATTTTA<br>CAGCAGCCCCAGGAAAATATGCCTGCTCAACACCCTACCCTGAATT<br>TTAAGTTCATTTTATGGAATCATTTTGAGTTTGGAGTTTGATTTAATC<br>CTTTTCAGTAATTAACGCTCTTCATTTTTTACTCCCAAATATGTCACA<br>CTGGCTCTACTCTTATAACAATTGTATTTATCTATCTTTTGGTAATGG<br>GGCTGCCACCCCTTGCAGAATGAAAAGTAGAGTGTGTATGCTCACA<br>CCTCCCTCCCTCCCTCCCTCTCTCCCTCCCTCTCTCTTTCTTTCTTTC<br>TCAGAGTATCGCTCTGTCTCGCCCAGGCTGGAGTGCAGTGGCGCAA<br>TCTTGGCTCACTGCAACCTCTGCCTCCAGGGTTCAAGCGATTCTCC<br>TGCCTCAGCCTCTGGAGTAGCTGGGATTACAGGCGCCCGCCACCAC<br>GCCCGGCTAATTTTTTTGTATTGTTAGTAGAGATGGGGTTTCACCAT<br>ATTGGTCAGGCTGGTCTCAAACTCCTGACCTCAGGGGATCCACCCG<br>CCTTGGCCTCCCAAAGTGCTGGGATTACAGGTGTGAGCCACCGCA<br>CCCGGCTCACAGAATTTCTTAAAAAGTCATTGTGTTCAGTCTTTATG<br>GATCCTCTAAATTCCAGCTGCTCAGCCTCCCGGAGCTAAATGAGAT<br>CACTTAAAGGTCGACTGTTGGGGCCTCCAAGGTCGCCTCGGAGAC<br>TCACCTCATAGGCAATCAAATGGATCGCTGGGTTTTTGTGTGTGTG<br>GGTTTTTTTTGTTTATTTTTTTTTTCCAGAAAAACACTCCACCAAGA<br>AGGTTTTTATAACCTACTGGAGGAGGAGGATGGAGAGTGGGCACG<br>AAAGGACTAGGATGAACATGCCATAAGCAATCTAAATGCCACTGCA<br>ATATTGCCTGGTGAGGACCTGATTACTGATGTTTTAAAGCTAAGCTT<br>TCAGTTGTCACTCCACCCAGTAGTGAAACAATGAGCTCTAAAATAT<br>ATATTTCGGCTCAAGCTTTCTTATGTGGGGAGGTAATCCACCCGAAG

	GTATCCCCAGCCTGTACCTAATACAGTGCCCAGCACTAAAGCAGCTCAGATGCCAGTGAATGGTGGCCACTGGGAGGCCTGTCAGTGGGTGCCAGTAGCGGTCTCTTCAGAGAAAAAGAAAACTCCCCTCTGCCAGATCAGTATTTTATGAGCTGTGAACCAAAACCATTGCTACCACCATCACTATAATTCTATCCACAGTAATTATCATAAAGGCCTAACAATGCCTTGTAGATGAACATTCTGAGTAACTGCTCTATAACCAGGAGATTTAAGACCGCACCAAAAACCAGTAGAGGGTTATACTTTACTGGGCACAAGTCGTTTATGATAACGAAATTGTAGTTTAATCTGTGAAGAGATGTGAATGTAACTGAGACACGCTTAAATGGAATATACAGATGAGCTTTATTTTTATATCTGGCATGCTTGGATCCATGCCGACCCTCCAGCTGCTCGGGCCTGCCCTTAGGGGCTATGGACCGCATGACTCTATCAGCGGCACTGCCACCGCCGCCGCCTCCGTGCTGCCTGCGTTCCCCGACCATTGATTGGGCCCGGCAGGCGCTTCCTGGGGGCTTCCCTACCGGCTCCAGCCCTTGGGATTCGGGAGCGCCCTGCTAGGAAGCCAGAGCCCCGCAGGGGCCGCGGCGTCCAGGCCGCCTAACGCGCGCCCCTCGCCCGGCGCCCCGAAGCGGCCCCGAGGGGCGGGAGCCGAGGCGAGCGGCAAGGCCGGGCCGGGGGCGCACAGCGCCCCTAGAAGTGCGGGCTTCCCCCACCCCCGGCAGCGACCCTACCTCCCGCCCCCGCTGCGTGCGCGCGTGTGTCCGTCTGTCTGTATGCTCTCTCGACGTCAGTGGGAATTTCCAGCCAGGAAGTGAGAGAGTGAGCGAGACAGAAAGAGAGAGAAGTGCACCAGCGAGCCGGGGCAGGAAGAGGAGGTTTCGCCACCGGAGCGGCCCGGCGACGC
NOD1	TGGCCATGAGGGACAGAAGTTGGAACTCTAGCTGCTTCTTTAGCTACCTTATCAGCATAAGCATTGCCCTGAGTGATGGGATCTGATGCCTTTTGATGGCCCTTGCAGTGAATGACTCCAGCTTCCTTTGGAAGTAAAGTGGTCTTGAGAAGAGTTTTTATTAAAGAGGCATTAATGATGGAGGACCTTTGCGTAGTGAGGGAACCTTTTTCTGCCTATATAACAGCATGGTGGTGCAGGATATGGGAGGCATATTTAGAGACAGTATAAATATTGACGTGTAGTCCTTTTGCAAGAGTGAGGGCTCAAGTTAAGGCAATGAGTTTGGCTTGCTGAGAGGTAGGGGAGCGGGGCAGAGTGGTAGCCTCAAGGATAGATGTGGAAGATACTACTATAGCATAGCCTGCCTTTGCTGGTGAATGGCAATTAGGCCTGGTGGAACTGCCAACAATAAACCAAGTGCGATCAAGGTGAGGAACAGGAAAGAAAGAAATATGGGGAAATGGAGTGAATGCCAGGTGGATTTAGAGAGATACAGTCATGGGGGTCAGGTGTGGTATCAGGAATAATGGGGGCTAGCTAAAACAGTAGGGTTAAGTTATTTGGACAGAAAGGCTACAGGGCACAATCCCAGCTCTTGTGTAAGAATTCTGACTGCACAGCCCTGCACTTCGGCTGTGTGTAATGAAAAAAGGGTTGGGATGAGTTACGGAGAGCTAGTGTGGGAGCAGCTTTTAGGGCTGTTTTTTAAGGAACGGAAAGAGGAGTGGGGAAAGGATTTAGGATTAATGGGGTCAGCTAGGTTTGCTTTTGTGAGTTTATATAATGGTTTAGTCAGGATGGTAAAACTAGGTATCCAAAGGCGGAAGTACCTACCCATGCCTAGGAAGGAAAGGAGTTGTTGTTTTGTAGAAGTGGCTGGGGTTTGGGAGATTAGCCAGTCAGGATCAGCAGAGAGAGCATGTGTTTTTTCATGAAGAATTATGCCAAGACAGGTAATGGATGAGGAAGAAATTTGGGCTTGACTGAAGCAATGGGGGCTGTCCATGAAGCCTTGTGGCAGTACAGCCCAAGTAAGTTGCTGAGGCTGATGGATGTCAG

	GATCAGTCTAAGTGAGAGCAAAGAGAGGCTGGGATGAAGGGTGCA AAGGAATAGTAAAGAAAGCATGTTTGAGATCCAGAACAGAATAAT GGGCTATGGAGGGGTTGTGGAGGGAGGTATTGAGGATAGGAGAGT ATATGGGTTTGGCACCACAGGGTGGATAGGCAAGACAATTTGGTTG ATAAGGCGCAGATCCTGAACTAACCTGTAAGGCTTGTCCGGTTTTT GGACAGGTAAAATGGGGGAATTGTAAGGAGAGTTTATAGGATTTAA AAGGCCATGCTGTTAACAGGTGAGTGATAACAGGCTTTAATCCTTTT AAAGCGTGCTGTGGGATGGGATATTGGCGTTGAGCGGGGTAAGGG TGATTAGGTTTTAATGGGATGGTACGGGTTGCGTGATCGGTTGCCA AGGAGGGAGTAGAGGTGTCCTACACTTGTGGATTAAGGTGGGGGG ATACAAAAGGAGGATGCAAAGGAGGCTTTGAACTGGGAAAAAGG GCAGCATTGAGGTGTGGCTGTAGCCTAGGAATAGTCAGGGAAGCA GATAATTTAGTTAAAATTTCTCGACCTAATAAGGGAACTGGGCAGG TGGGGATAACTAAAAAGGAGTGCATAAAAGAATGTTGTCCAAGTT GGCACCAGAATTGGGAAGTTTTAAGAGGTTTAGAAGCCTGGCCATC AATACCCACAACATTTATGGAGGCAAAGGAAACAGGCCTTTGTAAA GAAGGTAATGTGGAGTGGGTAGCCTCCGTATTAATTAAGAAGGGGA TGGACTTACCCTCCACTGTAAGAGTTACCCAAAGCATCTGTGATGG TCCAGGAGGCTTCTGAGGCGATCGGGCAGCGTCAGTCCGAGAAGA TCTGTAAAGGAGTCAGTCAGAGAGCCTTGGGCCAGACAGAGTTCC AGGGGCTCTAGCAGTGGCTGCTGGGCGAGTTGGACAGTCTGATTT CCAGTGGGGTCCTGCACAGATGGGACATGGCTTGGGAGGAATCCC GGGCTGCACACATTCCTTGGCCCAGTGGCCAGATTTCTGGCACTTG AAGCAAGATCCTGATGGAGGAGGTCCTGTAGGAATGCTTGACCAC TGCGGCTTAGGCATTCTGATGTTCTTGTGTGCTGGAGGTGCGGCTG GGTTTTGTCTTACAGCAGAGGCAAGTAATTGTAACTCAGAAATGCA TTGCTGTCTGGCTGCCTCCTTTCTATTATTGTACACCTTGAAGGTGA GGTTGATTAATTCCTTTTGTGGGGTTTGAGGGCCGAATTCTAATTTT TGGAGCTTTATGTAACGTCGGGAGTGGATTGGGTACTAAAATGCAT ACTAAGACAGCCTTCTGACCTTTCAGAGTCTAGGGCTGTAAAGTGT CTAAGGGTTGTTGCCAAAAGAGCCATGGACTGGGCTATGTTTTCAT ATTTGATGAAAAAAAGCCTAAATGCTAATTGATTTGGGAGAGGTTG GATAAAGAAAAAGGAGCATTAACCTTGAGGAAATTGTTGGGTAGGT AGGGGAGGGCTAGTTGCGGAACGAAACTGTAAGCTGGACCGGGTG TGAGGAGGTGAGGTGATAGAAGGATTATAGGGTGGGGGAGTGGAG GCAGAGGAAGAATTGGGACCTGGCTCAGCCTGGCGAGGAGCAGC CTGGGGAGGAGGGGAGAGGTTAGATGGGTCCATAGAAAAGGAGGA TTCACAGGACTCAGAGCTTGGGGTGGAGACTGAAGGAACAGACA GGAGAGAAAGAAGATTTGGGATGAGTCACATTGGGAGCAGAGATT AGGAAGGGACCAATGTGTAAAAGAATGCCTGGACATCAGGCACCT CAGACTATTTGCCCATTTTACGACAATAATTATCTAGCTCATGTAGGA TGGAGAAATTGAAAGTGCCGTTTTCTGGCTATTTGGAACCATTGTT GAGTCTGTATTGGGGTCAAACGGTATTGCAGAAGAAAATAAGGCGT TTAGGTTTTAGGTCAGGTGTGAGTTGAAGAGGTTTTAAGTTCTTGA GAACACAGGCTAAGGGAGAAGAAGGGGGAATGGAGGGTGGAAGG TTGCCCATAGTGAAGGAGGCAAGCCCAGAGAAAAGAAGGTAGAG

	ACCCGGAGAAGGGGGGTGGTGAGCAGCCCTGGGCTGCAATGTGGC<br>TGAGCAGCCAAAGCAGGCGACCCACAATTGACTTGCCACCAAGGG<br>AATGTGGGTGAATGACCAAGGCAGGCATCCCCACGGTGATCAGAC<br>ACCAATGAAATGTACGTGAATAATCAGGCAGGTGTCCCTGCAGTGA<br>TTAAACACCAAGGGACGACTGTTTTCCTGAGTCCGTGACCGGAGC<br>TGGAGTTTTGGGTCCATGGATAAAATATGTCTCCTTTGTCTCTACTA<br>GAGAGGAGAAAGAACTGGAATTGGAAATAGAGGGAGATTTAAGGG<br>TAGTGAGAGAGGCTGGAGAAGACAGTGAAAAGACCGCTTACCCGA<br>TTTGAAATTGGTGAGATGTTCCTTGGGCTGTTTGGTCTGAGGACCC<br>GAGGTCATAGGTGGATCTCCTCACAGAGTGAGGGTGAGGACAGGG<br>GACTGGTCTCCTGAAGGAGTCCTCCTGTCCCGGGTCTTCGGCACCA<br>AATGTCACACGTGTCCGTGTGAAGAGACCACCAAAAGGCTTTGCG<br>TGAGCAATAAAGCTGTTTATTTCACCTGGGTGCAGGTGGGCCGAGT<br>ACGAAAAAAAGAGTCAGCAAAGGGAGTTAGGGGTGTGGTAGCTTT<br>ATAGGATTTGTGTGGGTAGTGGAAAATTACAGTCAAAGGGGGTTGT<br>TCTCTGGTGGGCAAGGCGGGGGCCGGGGGGGTGGAAGGTCTACA<br>AAGTGCTCAGTGGGGGAGCTTCTGAGCCAGGAGAAGGAATTTCAC<br>AAGGTAATGTCATCAGTTAAGGCAGGAACCAGCCATTTTCACTACC<br>TTTGTGATTCCTCAGTTGCTTCAGGCCATCTGGATGTATACATGCAG<br>GCTTGGGCTCAGAGGCCTGACACAAGTTGGCACATTTGATCCTATC<br>AACTTTAGTTTGCACTTTCACTTTTGATTTAAGAACATACTTTCTGT<br>TTCTCAGAGGGCAGCCTCTGATGGGAATTTTAATACTCACAAGCAT<br>CAGTGGCTATATGATTGAATGTACTTCTTCAGTGTGAGTAAGTGACA<br>ACTTTCTGTAGCATGATATCCCTTATGGACACGATCCCCATTCAAAA<br>CGTTTTAAAACTATTTTATTGGGGACATTTATTAGCATGTTAAAACTC<br>TTTTACAACCTATTAGAGCATATGCATAGAACATAAAATGTACACTG<br>CCAAGAACAAATTTAAGGATAGGAAGAAAAGCTTACATTTTTTAAA<br>AAGTATAAATTGAAATGTATGCAGTGTAATTTAAATTTGATTACGTTA<br>ACCTCCATTTCATCAGGACCACGTGTCGCGTTTTAACAAGAGTTTC<br>CCTATCATGTCCAGGCTTTGGCTCTGGTTGCAGGCGCTGGGTAGGC<br>ACTTTTACGAGGAGGGACCTGGCACGATGGCACCACAAATTCCCA<br>AGTGTGTCACCTCTGGGCGAACTTGGGCCTGTCCACTCGGCCCTGC<br>GCGGTGGCCAGGGGCTCACCCTCTCGCACCGGGCGTCCCTCTGCG<br>CGCAGCTTCTCTCGCCCCCCCGCGCCAGACCCGGGCGAATGGCAG<br>CACCGTGGGACCCTGCCTTGACCGCCCCCGCCCTTCGGCGGCCTCT<br>CCCAGCAGCCGGCAGGCTCTTGGGCGCGCCAACAGAGGGGCGCG<br>GCTGCGGCTGTAGTCGCAGCCAGTTCCCGTTCCGGGCCCGCGAGG<br>CAGCCGCCCCGGTCCTGCCCCTCCCTCGCGCTACTGCGGGAGCAG<br>CGTCCTCCCGGGCCACGGCGCTTCCCGGCCCCGGCGTCCCCGGAC<br>CATGGCGCTCTCCGGGCTCTTCTCTAGCTCTCAGCGGCTGCGAAGT
NOS1	CTATAATGCTCCTACCTCTCCTGGGGGTTCTGGGCTCTCAGACAACC<br>CTGTTCGCTGTTCATTTTTCCTGGGTCCTACAGAAGCATGCCCCCGG<br>CCACTTTAGTGACACCCACTTTGCTCTTAGGAGGCTCAGACCCTGC<br>ATCCTGAACTGGGAGTTAGGACAGGAAACTGATCTGTCACTGATAG<br>GCTGCTGCCCCTGGATGAATTGTCAAACCTTCCTGGATCTTGGCCT<br>CCCCAGTGTAAAACGCTAGGTTGGAGGCAGCCATCTCCAAGGGCC

	CTTCCTTACTCTAACTGCAGAACTGACCAATGGGTGCCATCCTTCA<br>CAACAAGCAGGTCCAAATCACTTTCCCAGCCAAATACCCAGAGAC<br>AGGGTCAAAAGCATAATTCCTTTCCAGAATCAGAAGCCCTGGGTGG<br>ACGGGGCTGCTTAAGGGAGAGAGGGGACCTACGCACCATGGGAAC<br>AGGAAGGACACAGAGGAGGCACCCCATGGGTCTTCCAACACCTCC<br>AACGGGCCAGCAAGGCAGGACAATGAGGCAGACCAAGGGGCATT<br>TCTTGGGGATGAAGTAGTGTGGGAAATGGTAAGTGATGGATAAAGG<br>GATGGCTTGGTAGGTTCAAGGGCATAATGCAGAGGACTAGGTATAT<br>AGTTGGTGTCTAATAAGGGAGACATTGTCATCACATAGCTGAACCC<br>CAGCTTAATAGTAAACTCTTCTTCTACTTATTTAAACAAACATTTGTT<br>CGGTTTTTGGTGGGAACAGAGGTTCTCAAACTTGAGCATGCATCAG<br>AATTACCTGGCTGGCTTGTTTAAAATGCAGATTGCAGGGCCCCACC<br>CTGAGGGTCTAATTCAGGTGGTCTGAGGTGGGGACTTGGAATCAG<br>CATCACTAGCAAGTTCTCAGGTGATGCTGACCTCATCATTCAGGGA<br>ACACGCTTTGAGAACCACTGCCTTGGAGTGACGGATGAGACAGAC<br>CACCCTGCCCTCATGAGGGCATTGAACAAGTAAATATCACAGAACT<br>TGTATGTTCTGAAGAGGGACATGCAGAGTGCTTGGGGCTGCTCCCA<br>AGTGGGGGAGTTCGCGCTAAGACTTGAAGGCCGAGTAGAGCCAGC<br>CCAGGAAGAGGGATGAAAATTTTGTGAAACACTTACTTAGAATGTG<br>CCAGGCTCTCTCTCATATCGTTCATCTGCTCCTCCCAGTCAATGTCA<br>GGAAGGTAGGATTAACGCCCATTTTACAGATGAGGAAGGTGAGGC<br>TGAGAGAGAGAAATAACTGGCCCCAGAACTCATGGCAAGAAAGA<br>AGCCCAGGTCTGTTTGTGCACAGGACACCTACCAGCAGCCACATC<br>CCCTCTGCTGCACCCCCAGGAACCCCCTTCTTCCCAGGGGAGCTGC<br>CACCTGGACACAGCTGCCGGACCCGGCCAGCCACACTCTGTCCAT<br>TTCCGCCCCCTGAAGCGCTTCAAGTGTTTTCACTGCTGGGTGAATT<br>CAGCAGATGCCTTTCGATCACAGCCTATTAAATTAGCCCCTCTCTGC<br>AGCCAAAAGAAGATAATTATGTTTTCATTAGCCCTGGATAATGTATT<br>ACCCCCTCAGTGACAGCCACGTCAGTGAGGCCAGCCTCCTCCCTTC<br>CTCCTCCTGCCCAGGGCTCCGCCAGAGCACTCCACTCCCCTGGGGT<br>CCACCTCTGCACTGAGGGGTGGGCACCAGTGAGGGTCCAGAGTGG<br>CTGGGAGGCTGGGGGGGAAGGGACACCCTTTACACCCATCCACCA<br>TCCATGCAGGGGTTGAGGGGTGTGGTCCTGAGAGAAGGACCAAGA<br>ATAATTGTTATCACGGTGACCATTTATGAAGTGCCTGCCCTACGCTG<br>ATGCCTGGATGGGTCTCAGCTGGTTTGATCTTCCAATAGCTCGAGA<br>GAAGGGTGGCACAACCCTCATCTCCCCCTGAACCCCAGAGAGGTC<br>CTGGTGGAATCAGGTTATTGTGACAGCCCAGAGCAGCTTCCAAGG<br>CACTACACCCAAGGGGCATTTTGGGGACTGTCCCAAGGCCACCTG<br>GTTGATGGTAAAAGTCTCTTCCCTCCACTTGCTGGGGATCTCTGGA<br>GACACTGTCCGTACTCATGTGGTAGGGGTCCCCAAACACCAGGAG<br>CCACTCTCCCCAAACCCTAGACACTCCCCAGATGGCTCTGACATCC<br>TCCCTCTCCCTCTTTCTCCACAAGACAGCGGCGAGGAGGCAGGAA<br>AAGCTCCACTTTGGGGTCCCGAGTTCTAATATCTGCTTTACCACAGA<br>TTACCTGGGTGACCTGGAGCAAGTCACTTTGCCTCCAGAGCCTCTA<br>TTTTCTCCCTCTGTAAAATGGGAATAAGAAAGGGAGTCATTTCTTAC<br>CATTTTTATGATGGTTCAGTGAGTTATACCTATTAAATGCGTGGCTCA

	AAACCAGCAAAATGGGAAGTGCTCAACAGAAGATGGCCATTGTTC<br>TCTTTACTACCGCAACTAGTGTTGTCCATAGTTCCCGTTAATCCTGG<br>GGAAACCACCAGAGGGAGGGAAGGGAAGTATCTGGCTCTGTCTGT<br>CACTGTCCGTCTTTCTCTGTATCTCTGTCTCTCTTTCCTGTCTCTGCC<br>ATTCTCTCTCTGCCAATCTCTCTCTCTCTCTGCCAATCTCTCTGCCAA<br>TCTACCAATCTCTCTCTGCCTCTCTCTCTTCTCTTACTCTGTCTCTCT<br>TTTTCTCTCTGCCAATATGTCTCTCTGTGTATTTTTTCTTTCTGTCTCT<br>GCCAATCTGTCTCTCTCTCTCTGCAAATCCCTCTGCCTCTCTCTTCT<br>CTTACTCTCTTTCTGCCAATCTGTCTCCCTCTGTTTTCTCTGTCTTTC<br>TCTCTGTCTCTGCCAATAAGTCTCTGTCTTCCTGTCAATCTCTCCGT<br>CTCTCTCTTTTTCTGTGTGTTTTTTCTATCTCCGTCTCTGCCAATCTC<br>TCTCTCTCTGCCAGTCTTTCTGTGTGTTTTTCTATCTCTGTCTCTCAC<br>TGTCTCTGCCAGTCTCTGCTGTCTCTGTCTCTCTCTTTGTCTATAAAT<br>CTCTCTGCCTCTCTCTCTTCTCTGACTCTCTCTGTTTGTGTGTGTGT<br>GTGTGTGTCTGTCTGTCTGTCTTCGTCTCTCATCTACACACACACAC<br>AGAGGGCACCTCATGGTCCCTGGCTCCACCCGTGGGCTCTGAGGC<br>TCTGTCTTCCCAGATCTCTGTCTCAAGAGCTACATTCTAAGGCTTTG<br>CTTTTAATCGCACCAGGGCACCTGGAAGTGAGGAGAGAACTGAGG<br>GGAGTGGGGTTTCTGTCCCCCTCTCCCCAAACCCAAGCCAACTCTG<br>CCTTCTGCAGGCACAGCCAGCCTTGGCCCGACCCCACTATCTCCAC<br>CATCTCGTGGCCGTGCATCCCCTCCCAACCACCCTTCTCCCCAATTT<br>CAAAAAAGCTTGTGCTCCCAGAGAGGAAGTGCCCCTACTCTGAGC<br>TTCTTTTGGCCTTGACCCACACATAGGCAGACGTGGACTCTGTTTT<br>GAGCAGGTGAACCGCCAGCCCAGTGGAAGAGCAGCTTCCCTTCCC<br>CTAGCTTCCTGATTTTCCCCAGTTGCCATGGTAATTGAGGGAGCAGT<br>GTTCTGAGAATGACACCACTGAAACCCAGCGCTCAACTTTGAAAT<br>GGTTCGGGCAAGGGACGCTGGACCCAAAACAGGAGGGGACCAGA<br>CTGCAAGGACTCAGCTCCAGCCTCAATTTTCTGGGACCCACCCCCC<br>ATCCAGCCTGTATGCTGGATTATTCTCATCATCATCAAAATCACCATT<br>ATTGTTGGCAACTGTTTCCTGAGCATTTACTATATGTCTAGCACCAT<br>GTCAAGGGCTTCACCAGCATCATCTCATTGTGTATATCCTCACGACA<br>TCCCCAGGCCCATCCATGGCTCAGGAAACAGGCTCTGAAACTTGCC<br>CTTGTGGCAGCCAGGAAGTGGCAGAGCCCTGGGGCTGGTGGCATT<br>TCTCCAGGCCTCCTTCCAGCATGTGGAAGACAGCATAGACCTCCAG<br>GCACCCTCGTCAGCTTATTCTTTATTCCTTCCTCCCCTCCTCATCCCC<br>CACACCCTTCTTCCTGCCCCCAAAGTCACACACCCCCCTCCCCTGCT<br>GGATGTGCAAGACGATCTGAAAAGCATTTATTAGTGACAAAGGGG<br>GTGGTGGTTTTCCTCTTTGCCTCTTCTCTGCGCAACTCGAAGCCTG<br>AGTGGGTTTGCTCCGCTTGGCAAGGCTGGGGAGGAGGAAAAGATC<br>AAATCACCCAGAGCCAGGTGCCAAGTGTGTGTGTGTGTGTGTTTGT<br>GTGTGTGTATGTGCGCGTGTGCGCGTGCGCGTGTGTGTGTCCTCTC<br>TTTCCTTGATTTTCCCCCGTTTTCCCAAGACTGTGTGAGCAGCAGC<br>AACAGCAACGGGGAGATGCAAAATCCTTTGCAATCTGCCCAGGAA<br>AAGACAGATTTTGAGTCGGGTTTATATGGTTACCCATATGTTAGGGC<br>CCCTAAGAAAGGAAGGGAAGCTGCGTTCTCTGTGTCTTCCCTCCTC<br>CAGCCCAGACCTTGGCCTCTCCACACCTGTGGGGCTCCCAGGCCA

	CAGATGAACATGTGCCCTCCATCCTTCACTCTCCCCAGATGCCAGG<br>CCTGAGTGTGGCTCCAGAAAAGACCAGAACCAGGCAACATTTCCT<br>GAACAGGCTGCTTTTCTCCGTCTGTCCACCCGAGAGCCAAGGCCA<br>GAACCCAGGCTGCAACCTCTGCGGGCTCCTGGGAGGGGCTCCCAG<br>CACTGCCCAGCCTGGGAGGCTGTGTGTTCCACTGTCCCATTAAGGA<br>ATGCTATCCCAACACTGATGTAGAGGCAGAAATAACCATCCCTGCC<br>TCCCATGGGGCCTTCTCTGCCCAAATAGACCTGCCCTGCCCTGCGT<br>GGCTACTACATTCAGCCCTATCCAGGCTCCCAGAAATTGTCATTGTG<br>TGTGTGTGTGTGTGTGTGTGTGTGTGTGTATGTGTGTGTGTGTGTGT<br>GTGTGTGTGTTTCCTGATAGAAAAAAAAAAAATGGAAATTAGGTTAT<br>ATAAAAGATGTATGCTTTGGAGCCCAGAGCGGCTCTTTTAATGAGG<br>GTTG
NOS3	CCCAGAAGCCGCCCCAACGCCCTCACCAAAGGCTCAGCACCAATT<br>CTGCAATCTGGAAGCTGAGCAAATCTCTCCGCCCCCCAGCACTCAG<br>CATGAGTTACATCCTCGGGAGGGGGAGGCCTGGCGGCAGCTGCTC<br>CCCGCCCCTCTCCCGCTGCTCCCGCCTCCCTTCCCTCCTTTCCCCGC<br>TCCCCACCCGTCCTTGCCAGCAGGCAGGAAGGAAGCTGCTCATAG<br>TGCAAGGGTGGGGTACTTCCAGCTCACACTGAACAGCCCCGGGCT<br>GGGCTATAAAGAGTCAGGGACTTCCGCTCAGTCACCCCTGCCCTCA<br>CCACCCAGGCCCCCATGCACACACCCCAGGCCCAAGGCCTTCCCTT<br>CCCAGCACAGCCCTCCAAGGTCAAGGCGTCCCCCGCCCAGCCCTG<br>CAGAGCACTCTGGTCTGAGAGGGAAGGCAGGGGCAAGATTTGCCG<br>CCTCTTTGGCATTATGGAGGGGGCAGGGGGCACAGGGCACAGCCC<br>TCCCCAGGGAGGGATCCCTACTCACCCACAGAAGGCTGTCACCTTA<br>CCCCTGGGGTCTGGGCCAGCCCTCCCCGAGGCGGAAACACAGCCA<br>CTGTCAGGGACAAGGGGGCTCAGTTCCCAACCAGATCATCTCCAA<br>AGCCCATTCAGCTTCAGATCTCATGATTGAAGATGGAAATTCTTTCA<br>GCCGAACACCAAATCTCCAACCTTGAAAACTGTTTTTCGGAAAGT<br>CCTCTTTAAAACATGGTTACCAATATGAGCAGTCAGCCACTAAGAT<br>GCATGGGCATCATTCTTGGCAGGGCTGCAGGCATAGAGCAGGCCA<br>GCACCAGGCAGAGCCCTGGGGACGGCAGGCCTCCTCCGACAGGC<br>GGGGGAATCAACTGTTCTCATCAGAGCACCCTGATGGAAGGCAGG<br>ACACTCCGGACAACACTGGCCACGGCCCAAGAAAGGGGAAGCAG<br>GGAATCCCCTGTCCAGAGAGCATTCATCTCTGACTTTTTCTGTCTGT<br>TTAACTTTTTTTTTTCCTCCTTTTCTTAAGACCCTGCCATGGCATCCT<br>CTGCCCCAATTCAAACCCAATCCAGGCCTACTCTGACCCTCAGCTT<br>TCAGCGCTGACTGTACTGAACTGGACCCCATCCTCCCTGTGACCCT<br>CAGCCTTCAGTGCTGGCTGTACTGAACTGTAACCCATCCTCCCTGT<br>GGCCACAGAGTGCTCCTTCTTGCACTCCTCCCCTTCACCTAGACTG<br>ACTTCTTTCTGCCCACACAGACCCATCTAGCCTCAGCCAATTCCCTT<br>CTCAGTGGACCCTCTCCATTGCCTCCAGAGCACAGCTTCCAGTCCC<br>CAGGCGGCTGCACACATTTGCAACAAAACAAGCCAATGTATGAAG<br>CAATCCCACTCAGTGCAGGAGCTCAACCCAGCAGAGGATCCCCAC<br>AGAGAGGAACTGACCTGTGTCTTCCAGAGCACACAGGTTCAGAGC<br>ACACAGGTTCATTTAAGTGAGAACCCACACTTACAGAGCAAGCTG<br>CCCAAAAACCACGACCAGGAGGGCCCAGAGAAAGAGCTGTCCCC

	GGGGCCTTGGGGACAGGGTGACAGCCACCCAGAGATCATGGAGAA
	GGGGACGTAAGGAAGACCTCACAGAGGAGTCATCCTGCGACTGTG
	TTGGTTGGGTCCTTCAGGAAGCAGAGTCCCAGGAGTTGGAAGCAT
	AAGAGGAATACTGCGGGCAATGCCTGAGAAAGATAACAGGGACCG
	GGAGCAGGAGTGAGTTGGGCAGGGGAAGGATCAGGCCCACAATG
	CCAGGCTCACACCTGCAGAGGAGGGAAGAAGAAGGGCCTCA
	CATCAGCCCAGCGGGGGATGTTACGCCCACAGACGCCCCGGGGCT
	CAGTTACTGTCTAAGTGTTAGAAATAAATTTTCGGTGCCACAAAAG
	AAATAGCACTCAGATATAAATGTTCCCAGCAAGGCAATTTTACTTCT
	ATAGAAGGGTGCATCTCACAGATGGAGCAATGGCAAGAGCACACC
	TGAACAAGGGAAGGGAAGGGGTTTTTATCCCTAAGGCAGGTAGCC
	CCTACAGCTGTGTTGTTCCCCTATTGGCTAGGGTTGGACCACACCG
	TCTGAGCTAATTGTTACTGGCTATTTTAAAGAGAGCAGGGGTAAGA
	GCCGGATTGGCAGGGTAAGTAGTTTGGCAGGAAGGACGGTCACAG
	AACAGGTGACTCAGGATGACTCAGGTCAGAGCAGGTGACCAGTGG
	TGACTCAGTTCGGAGCAGGTGATAGAAGCTAGGAGGGGGTTGTTT
	ACTGAAACTAGGGGCAAGGAGACGAAGAGAACATGAAAGTTAAA
	CTTTAAGATGAAGAACAAAGCTGAACATACTGATGCATTGGATCTT
	TGGAGAGGATCTCAGAACTCATTGTACTTAATTTACAGGCTAAAAC
	CTTAGAAGAGGAATTTATTATATCCTACACAAGACTCCAGGGAAGC
	ACATGGCCTTGGACTGAAGGCTGGCATCTGGAAGCTGTCAGCCAC
	CAGCACCTTCTGCAGCAGGTACCTGCTCTCTAAGAGGGAGGCCTG
	GGTGGTGCACCTCCAGAGCTGCCCAGGCTGGGCCTCAAGGAAGAA
	AAAGATTTTCATTTGTCAGAGGCGGAAGGGAGAGGTGGAGGGAAC
	AGCACAGCAGCGGCCCAGGGGCAGGGAAGCACAGGACCATTAGG
	GAGACACGAGAAAGCCCATTTGTCTAGAACAGAGGATTCAAGCAG
	TGCACCAAGGAAAATGAGGGCCAGGCCAATGTGCTGGAGTGGCTT
	TGTTCTTGGCTGAGGGTTTTGGGTAGTGCCAAAGCGTAAGGTAAGC
	CCTGCTTTCCAGAAGAATCTAGCAGAGTGTGGAGCCCAGATGGGA
	CTGGAAGGCCTGGGAGGGGTCAGGTGGCCACAGGGACGGGCCAC
	AGCCAGTGGTGCAGGCAAGAAGACAATGGCCATCCATGGTGGCTC
	ACACCTGGAATCCCAGCCCATTGGGAGGTCGAGGCAGGTGGATCA
	CCTGAGGTCAGGAGTTCGAGACCAGCCTGGTCAACATGGTGAAAC
	CCTGTCTCTAATAAAATTATAAAAATTAGCCGGGCGTGGTGGTGGGT
	ACCTGTAATCTCAGCTACTCAGGAGGCTGGGTCAGGAGAATCGCTT
	GAACCCAGGAGGCGGAGGTTACAGTGAGCTGAGATAGCACCATTG
	CATTCCAGCCTGGACAACAAAAGCGAGACTCTGTCTCAAAAAAAA
	AAAAAAATTAGCCAGGCGTGGTGGTGGGTGCCTGTCGTCCTCGGG
	AGGCTGAGGCATGAGAATCACTCCGGGAGGCAGAGGTTGCAATGA
	ACCAAGATCACACCACTGCACTCCAGCCTGGGTGACAGAGCAAGA
	CTCTGTCTAAAAAAAAAAAAAAGACAGAAGGATGTCAGCATCTGA
	TGCTGCCTGTCACCTTGACCCTGAGGATGCCAGTCACAGCTCCATT
	AACTGGGACCTAGGAAAATGAGTCATCCTTGGTCATGCACATTTCA
	AATGGTGGCTTAATATGGAAGCCAGACTTGGGATCTGTTGTCTCCTC
	CAGCATGGTAGAAGATGCCTGAAAAGTAGGGGCTGGATCCCATCCC
	CTGCCTCACTGGGAAGGCGAGGTGGTGGGGTGTGGTGGGGCCTCA

	GGCTTGGGGTCATGGGACAAAGCCCAGGCTGAATGCCGCCCTTCCATCTCCCTCCTCCTGAGACAGGGGCAGCAGGGCACACTAGTGTCCAGGAGCAGCTTATGAGGCCCCTTCACCCTCCATCCTCCAAAACTGGCAGACCCCACCTTCTTGGTGTGACCCCAGAGCTCTGAGCACAGCCCGTTCCTTCCGCCTGCCGGCCCCCCACCCAGGCCCACCCCAACCTTATCCTCCACTGCTTTTCAGAGGAGTCTGGCCAACACAAATCCTCTTGTTTGTTTGTCTGTCTGTCTGCTGCTCCTAGTCTCTGCCTCTCCCAGTCTCTCAGCTTCCGTTTCTTTCTTAAACTTTCTCTCAGTCTCTGAGGTCTCGAAATCACGAGGCTTCGACCCCTGTGGACCAGATGCCCAGCTAGTGGCCTTTCTCCAGCCCCTCAGATGGCACAGAACTACAAACCCCAGCATGCACTCTGGCCTGAAGTGCCTGGAGAGTGCTGGTGTACCCCACCTGCATTCTGGGAACTGTAGTTTCCCTAGTCCCCCATGCTCCCACCAGGGCATCAAGCTCTTCCCTGGCCGGCTGACCCTGCCTCAGCCCTAGTCTCTCTGCTGACCTGCGGCCCCGGGAAGCGTGCGTCACTGAATGACAGGGTGGGGGTGGAGGCACTGGAAGGCAGCTTCCTGCTCTTTTGTGTCCCCCACTTGAGTCATGGGGGTGTGGGGGTTCCAGGAAATTGGGGCTGGGAGGGGAAGGGATACCCTAATGTCAGACTCAAGGACAAAAAGTCACTACATCCTTGCTGGGCCTCTATCCCCAAGAACCCAAAAGGACTCAAGGGTGGGGATCCAGGAGTTCTTGTATGTATGGGGGGAGGTGAAGGAGAGAACCTGCATGACCCTAGAGGTCCCTGTGGTCACTGAGAGTGTGGGCTGCCATCCCCTGCTACAGAAACGGTGCTCACCTTCTGCCCAACCCTCCAGGGAAAGGCACACAGGGGTGAGGCCGAAGGCCCTTCCGTCTGGTGCCACATCACAGAAGGACCTTTATGACCCCCTGGTGGCTCTACCCTGCCACTCCCCAATGCCCCAGCCCCCATGCTGCAGCCCCAGGGCTCTGCTGGACACCTGGGCTCCCACTTATCAGCCTCAGTCCTCACAGCGGAACCCAGGCGTCCGGCCCCCCACCCTTCAGGCCAGCGGGCGTGGAGCTGAGGCTTTAGAGCCTCCCAGCCGGGCTTGTTCCTGTCCCATTGTGTATGGGATAGGGGCGGGGCGAGGGCCAGCACTGGAGAGCCCCCTCCCACTGCCCCCTCCTCTCGGTCCCCTCCCTCTTCCTAAGGAAAAGGCCAGGGCTCTGCTGGAGCAGGCAGCAGAGTGGACGCACAGTAACATGGG
NPY	CTGATGGTTTTATAAAAGGTTTTTTCCCCTTTGCTCGCCACTTCTCTCTCCAGCTGCAATATGAAGAAGGACGTGTTTGCTTCCCCTTCCACCATAATTGTAAGTTTCCTGAGGGCTCTCCAGCCATACAGAACTGTGAGTCAATTAAACCTCTTTCCTTTATAAATTACCCAGTCTTGGATATTTCTTCATAGCAGCATGAGAATACACTGCCTGAAGGAGTCATCCAGGCTACAGTGCAGAAGGAAGGAATTGAATCATCCTGGCCAAGGAGCTGGGGCTGGAAGTGTAGGGAGCCAGGGCAGCTAGTGTCCACAGGAAGATTATCAGGTAAAAGAAAGAATACTCTGAAAGTCTGAAGGGTGTCCCCCTTGAGGATTTAGTTGATTATTGATCAGATTATACACAAGAACAAACCACCTGAGACCAGGAAAAGAACTACACAAAAAGATAAGGGTAAACTGTGTCTGATGCTGACCAAGGGCTGGGAATAGTGTCTATCTGAACAGCCAGAGGGGAGAGCTGTGCAATTTGAGGTGTGCGTGTTGGGGGGAAGTCAGTTACCAGTTAAAGTAAAAAGAAGGAATTTTGCCTCAAAGTGTGGAAAGAGTAAACCTAGTCTAAAACCAGCTCTGGTCCCACCTAACAAAACTCACATCCAAGACCTGAATGGATAAAACTGTCTTT

	AAATTTTTAATTGTCTCAGAACAAGCTCAAGAACAGTCATAGAAAG<br>ACAAAAAAACAAATAACAAGGTAAAATCCACAATATCTGACATCCT<br>ATCAAAAATTATCAGGCACACAAAGAAGAAGAAAAATATAAGCCAT<br>AATGTGGAGAAAAATCAATCAACCCAAAGTATACAAAAATCACAC<br>AGATGTTAGTAGTACAAAAGTTATTATTAATGTATATTCAAGAAGCT<br>AGAGAGAAGAGTGGACACGTTTAGATACGGAAGATATAAAAAAGA<br>TTCATTGCTTTATATTGTCCTGTAACTTAAAAAAAAGATTCAAGTTG<br>AAACTCTAGAGATGAAAGCTACCAGGTATAAGGTGCAAAATACACT<br>GGATAGGATTGGCAGTAATCAGATATTACAGAAGAAAACATTAGTG<br>AACTCACAGACATAGCAATAGAGACTATCCAAAATAAAATATGAAG<br>GAAAAAATGAGCAAAACAGAAGCAAAAACAGATCACCAACAAAC<br>TGTGGGCCAATATACACTCACCGTCTGACTCAGCCATTCTGGTTTTA<br>AGTATTTTCCAAGAGAATTAAAGGCTTTATGTCCATACAAAGACTTG<br>CATATGAATGTCAAGGCAGCTTTATTCGTAAAAGTTTCAAGTGAAA<br>ATAACCAAAAATTCATGAGCAGTAGAATTGATTAAAAATTTTGAAGT<br>ATATCATATAATGAAATACTGCCCAGCAATAAAAAAGAATAAACTAT<br>TGCTACATTTAACAATGTGAGTGGATTCAAAAATAATGATGCTAAGT<br>GAAAGAAGTCAGACAAAGAGTACCTACCGTGGAACTATATTTATGT<br>GCAATTCTAGAAAACGCAAACAAATCTCTAGTGATAGCAGATCAAT<br>GGAGGAGGGGGTGAGGAAGAGGCAAAAGGAAGGAATTACAAAGG<br>TACATGTGGAAATTTGGGGAAGTGATGTATAATCTTCATTTTCTTGG<br>TTTCAGTGATGGCTTCCTGAGCATATAACACACATGCATATGTGTAA<br>GAATATATCCAATTGAATATTTTACACATTTGTAGCTTTGTATATATTC<br>AGCAGTATCTATTCATTCCTTCTGCTTCTTTACCTTAGGTTTAGTCAT<br>GAAACCACTGGGTTTCATGTGGTCCAGGTTCTATCTGAATCAAGCT<br>ATGAACCTGCTATCAGTAAGACCTCCTAATTGCAAACCACATGGAC<br>CTTTACCGGTTATTTGACTTCTCTCGGTCATTACCTTCTCTAGTCTCC<br>ACTGATTCACCTCCTTTTGAATCTCAACAAGCTCAACAGTATCTCAA<br>TGTTATCTTAACAAAGCTGTTAGAAATAAACAACTTGACTTTGAAA<br>CAAAGGAGAGAAATGGCTCAGCAGCTAGGAGAAGATGCAGGGTT<br>GAGTTGCCAACAGTTGGTTTTGTTTGTATTTTGGCCAGGGGATGTG<br>GCTTGGACTGGAGAGAAAGGAGATAAGGATGTAAGCACATGTAGG<br>GCATATCACCCCCTATTTTTTATTCTCTGAATCCTTAACCCTCAGAAT<br>AAGTTCTTATTCTTGAGAATCAATGACATTATCTTAAGCTAAATTAAT<br>CAAGCCTCCACAGTGTTCTTCTCTCAATAGTGGTGTGGGCCTTCCT<br>AGAAGTAATTTTTCCCAAATTCAGTGATACATTTTAAGTTCAGATTT<br>TAATTGATATGAATCTGTGATACACTCTAAAATAAGATTATTTTATTG<br>AAAAGTGGACTGTAACTTTCCCTTTATCTAGGAAGAGCTCTAAGTT<br>AGAAGATGTTTTGCACTTTTACCGAAGGCTGTGTCTTGTAAGCACC<br>CCCGAGCAACTCTGAGAGCCTTGATTTTTGTGTCCTCAGCATATGTT<br>TGTGTAATACAGAAAGAGAAGCAGTTGCCAAGTGAAAGGGATGTT<br>GGTCTCCAAAATTATAGTTTGATCCCACAAACACACAAACACATAC<br>ATGCAAAGGATTGTTTGCTTCACGGTTTTTGATATTTAATTCAATGCT<br>GTTGGAACAGCACAAAAACTAAGTGTCAGTTTAACAGAATCACTT<br>GTCCTTTTAGCATTAAAATAACATGGAACTTAATGCTTTAATTTCCC<br>AACATGCCTTTTTATTTAGAAAGATTCAGACTTATATTTCATTTAGAA

	ATAAAATGCCATTTTATTTAGAAAGATACAGGAGCATTCATTCACGG<br>AACTTTCAGATCTCAGTCCACTGCATAAAATCTTGATCCTGTAATAA<br>TAGTTTCTGTATCTTGCATATTCATTCAACAGGTTTAACGCGATGAG<br>CAAATTAATGTTCATCGTTTTTAACATGTTTCATCTTAATCAGAACCC<br>ACATTCTCAACGTTAATTGAACGTACATAGGACTATACAAGGGTTAG<br>TAAATAAGACAGAAACTGTTGTTCATTTAACCACCGTCACTTTGGA<br>CCAAAAAAGAAAAAATATATATTTTTAAAATTGAGCTTAAAAGAGT<br>CTCTAGAAGCTGGAAGCGTGGCTCTTTTTCAGCAAACTGGGGGAAT<br>AGGTTTACCGTGTTCCCCCTCTGGGGAATTTTGAGTCGCCACACTC<br>ATGTCTCGACCGAGCCTGGCTCGCTGCGTCTGAGCGAGTACTTGAG<br>GAAGGCTGATCTAGAAAAACCAGCTGAGAGAAGGGGCAGAAGCC<br>CCTGAAACCACGGGCGGGGGTGGGGTGGGGAGCGCAGCTTTGGG<br>ACCCTCTAGCCGGAGACTTCCGGCAGCTGCCTCCGACTTGTTCTAA<br>GTACAGGAAAAATCTGTGCGCCCAGTTGCCTCACTCCAACAGCGC<br>GCAGTTGTGCCCGGCGAGGATGCCGCGCTAGTCGTGGAGATGCCC<br>CACCACAAAGAGGATTCAGGTGCTTCCTACTCCGGCACCCAGTGG<br>GTTGGTAGTCCTGTTGGCAGGAGACAAGAATCGTCTGGGCTGCTCC<br>TATCTCTGGCAGGACTAGACGGGGCGTGAAGGAAAGAAGGAAAG<br>AAGGAAAGCAGGGATCGGGCACTGCCCGAGGGCAGATACTTGGGC<br>TTTGGTGTTGTCCAGCGCGCTCGGAGTGCGCTGCCTCGCTCACGCG<br>GTCCCAGGCCCCGCTTCTTCAGGCAGTGCCTGGGGCGGGAGGGTT<br>GGGGTGTGGGTGGCTCCCTAAGTCGACACTCGTGCGGCTGCGGTT<br>CCAGCCCCCTCCCCCCGCCACTCAGGGGCGGGAAGTGGCGGGTGG<br>GAGTCACCCAAGCGTGACTGCCCGAGGCCCCTCCTGCCGCGGCGA<br>GGAAGCTCCATAAAAGCCCTGTCGCGACCCGCTCTCTGCACCCCAT<br>CCGCTGGCTCTCACCCCTCGGAGACGCTCGCCCGACAGCATAGTAC<br>TTGCCGCCCAGCCACGCCCGCGCGCCAGCCACCGTGAGTGCTACG<br>ACCCGTCTGTCTAGGGGTGGGAGCGAACGGGGCGCCCGCGAACTT<br>GCTAGAGACGCAGCCTCCCGCTCTGTGGAGCCCTGGGGCCCTGGG<br>ATGATCGCGCTCCACTCCCCAGCGGACTATGCCGGCTCCGCGCCCC<br>GACGCGGACCAGCCCTCTTGGCGGCTAAATTCCACTTGTTCCTCTG<br>CTCCCCTCTGATTGTCCACGGCCCTTCTCCCGGGCCCTTCCCGCTG<br>GGCGGTTCTTCTGAGTTACCTTTTAGCAGATATGGAGGGAGAACCC<br>GGGACCGCTATCCCAAGGCAGCTGGCGGTCTCCCTGCGGGTCGCC<br>GCCTTGAGGCCCAGGAAGCGGTGCGCGGTAGGAAGGTTTCCCCGG<br>CAGCGCCATCGAGTGAGGAATCCCTGGAGCTCTAGAGCCCCGCGC<br>CCTGCCACCTCCCTGGATTCTTGGGCTCCAAATCTCTTTGGAGCAAT<br>TCTGGCCCAGGGAGCAATTCTCTTTCCCCTTCCCCACCGCAGTCGT<br>CACCCCGAGGTGATCTCTGCTGTCAGCGTTGATCCCCTGAAGCTAG<br>GCAGACCAGAAGTAACAGAGAAGAAACTTTTCTTCCCAGACAAGA<br>GTTTGGGCAAGAAGGGAGAAAAGTGACCCAGCAGGAAGAACTTC<br>CAATTCGGTTTTGAATGCTAAACTGGCGGGGCCCCCACCTTGCACT<br>CTCGCCGCGCGCTTCTTGGTCCCTGAGACTTCGAACGAAGTTGCGC<br>GAAGTTTTCAGGTGGAGCAGAGGGGCAGGTCCCGACCGGACGGC<br>GCCCGGAGCCCGCAAGGTGGTGCTAGCCACTCCTGGGTTCTCTCTG<br>CGGGACTGGGACGAGAGCGGATTGGGGGTCGCGTGTGGTAGCAGG

	AGGAGGAGCGCGGGGGGCAGAGGAGGGAGGTGCTGCGCGTGGGT GCTCTGAATCCCCAAGCCCGTCCGTTGAGCCTTCTGTGCCTGCAGA TGCTAGGTAACAAGCG
NR1D1	CCCCAGCATTGCCATTTTCATTGTGACGCTGACAGTGGTAGTTCAA GAGATAGCTAGAGGGGAGATTTAGCCCCTCCAATAATCTCCATCCG CGCTATCATTCTTTGATGGACATCTTCTCAGTCTTAAGTGCTATGCA AATTACAGCTTCAAGACTAATCACCTAGACCCTCTGGCACCCTGGG CAAGTTAATAAAGTTCTCTAAGTCTCAGTTTCCCCATCTCTAAAATA AGAATGAGACCTACAGCATAGGGTTGTTGTGAATATTAAATGAGAT AATGTGTACAAAACACTTTGCACAGTGCCTGGCATACGGTTGGTAC TTAATACATATTTATGGTTTCCTTTCCTTGGAAAAAGCAGTAGTGAG GTAAAGATAGAATCTGAGGCAAAACACATCCATGGAACCCAGCCCT GGCTCATGCCTCGCCCTTGCTTTCTNCCATCCCCCTCCCCCCACCAC ACTGAGAAACAAGTTAGCTCAGACTCTGTCCTTCCTGTGGCATGTT CTTAAAGTTCTCCCCAAAACATCCAGGCTTTGGGTGGGGTTGGGAA AGATAGCTGGCCTTTTAGAGACAAAGAAAAGGATTTAGGAGGGCA GGGAGTGAGTCTCTCCCAGGCACATGGCTGGTTCCCTGATTCAGGG GCATCCCCAGCATTTCTATACCAGGTTTGCATCACCTGGGCAGACA GGTTTCTACTCTGGGCAATGGTTTCTTGGTGAGAAAGGAGGGGGAT GGGAAGGCATCTACAGAAGCCTGACCTGTTTCCAGCAAGGACCCA CCCTCAGTCAGTCAGTCAGTCAGTCAGTCAGTCAGTCAAGCAGTT GGCCCAAGATTTAGGCCACCCAGAATTTGCACAGGGTGTTGCCCTG TTACCCAGAACAACCTACTGACAGGGAGAACAGATCTCAGATCAA ACATGCACACCCACACACACAGCATTCACACTCACACACAAACAT GCAGTCTCACTCATAGTCATAGAATCACACACACACACACACACAC GTGCACACATACATAACTGACCAGAGCTTAGTGTTCTCAATACCTTT CCTTCTTCTCCTCTGTGTCTGCTGTCCTCTGCAGTCCAAATAAATCA AGGAAAATTAAAGACATATTTGGAAACACACAGGCAGGATCCAAC ACTGCCCCTAATGGGAAGGTTCGTCCCCAGAATCCCAGACTTGGGA ATCCAAGTGGAAGTGTATGCAGAACCAGGGCAGTGCCTTGCTGTAC CAGGTTGTGAATATGCATCTTTTCTACCTGGATCCAATCTGAGTACT ATTTATGAAGAGTTAAGTGCTTTCTTGAGTTGAACTGGGCTATGGG AGAAATGGGGTGTGTGTGTGTGTGTGTAGAAGTGAACAGCAGGCC GGGCATGGTGGCTCACGCCTGCTATCCCAGCACTTTGGGAGGCTGA GGTGGGTGGATCACCTTAGGTCAGGAGTTCAAGACCAGCCTGACT AATATGGTGAAACCCCATCTCTACTAAAAATATAAAAAAAATTAGCC AGGCATTGTGGCGTGCTCCTGCAGTCCCAGCAACTTGGGACGCTG AGACAGGAGAATGGCTTAAACCCGGAAGGTGGAGGTTGCAGTGA GCCACTGCACTCCAGCCTGGGCGATAGAGTGGGACTCTGTCTCAA AAAAAAAAAGAAGTGAACAGCAATGTCCAGCTGGAGGGATAGGT GAGGAAGGTTGTGTTGAGATACACCTGTTGTCTTAAGGTCTTACAG CCAAGGTTGAGGTCAAAGAGAAACCAAGATTCTACTCCGAATCAC CCAGAGCCAGACAACCCAGCCTTTCCCCCCTCCCTTTGGTGGCTTT GGGTCTCAGAGTTTCTGCCCTTCTCCCAGCAGCTGCTGACAGAGA AAGGAGCAGGGTACACAGACTGACTAGAAGCTTCTGGGGCCAAGA GAGAACCCTGCCTTTTCATTCTGTACTCCTCTCACCAGCTGTAGAA

	GAAAGACACCCGAATTACTTGTGAGGGAGAAAAGGAAGGGCCCTA<br>TAAGAAATGGAAAGGAAGGAAGGAAGGAGCAAGGGGGTAACCCC<br>TCTCCTTTGCCCCCTCCCTCCCAGCCTCTGCCTTCACACCACACAC<br>AGACTGCAGAACCACCCAGGTTTTGCCAAGAGGAGAGTCAGGCTG<br>ACTGAATTTCAGCTCTCTCTACCTCTGCTCCCTCCTCCTTCTCACTG<br>ATTGCAGCAGGCGAGGAGTCTTGACCCACCCCAGCTCCCACTCCTA<br>GAAGAACAAACATCCAGCAAGTATACAGGGGGTGGGTAGGACCTC<br>CACAGCAGCAAACAGAGCTGTGGGCCTTGGGGAGGACCCCGGCC<br>AAGTAAAGGGAGGGTGCTAGTGGTGGTGCTGTGCTGTGGGGAGCT<br>AGATGCACGCCAGGGGTCCTGATTTTGAGTCCAGCCCTGACAGAC<br>ACCTGGGCTCTGGCCCTGGCTCTGACTTGGAGTCAAGGTCACAGA<br>GAGCAGGCAAGGGCAGGACAGGGCAATTCACCTCCTAGCTTTCCC<br>TCCAGAGCCCAGAGGGTGGAGGGAGAGGGGGTGGAGGCTGAGAA<br>CCAAGTTTTCCTGAAAAGCCTCTTTCCTTGTTTGTTTTAGCCCCTCC<br>CAGTTGCTCCCACACTCTGCTGCAGCTTCCCAACTCTGGGCCAAAG<br>GGCCAGAGAAGAGCCCACTGCCCTCTCTCTCCCACTTTTCTTGGGA<br>GCAGGAGAACAAAGCTAAAGCCTAGCCCATTCCAGGAAGCTCATC<br>TTCTACAAGGAGTCAGAGGGTGAGTGGTCAAGCTCCTGCAGTCAA<br>CCTCACTGTCCTCACTACCCAGTTTACTCCTGTGCTCATATTATTATT<br>CTTTTTTTTTTTTTCTTTTTTTGAGATGGAGTTTCGGTCTTGTTGCCC<br>ATGCTGGAGTGCACTGGCGCGATCTCAGGTCACTGCAACCTCTGCC<br>TCCCGGGTTCAAGCAATTCTCCTGCCTCAGCCTCCCGAGTAGAGTA<br>GCTGGGACTACAGGCGTGCGCCACCACGCCCAGCTAATTTTGTAT<br>TTTTAGTAGAGACGAGGTTTCTCCATGTTGGCCAGGCTGGTCTCAA<br>ACTCCTGACCTCGGGTGATCCACCTGCCTCGGCCTCCCCAAGTGCT<br>GGGATTACAGGTGTGAGCCACCACCACACTTGGCCTTTTTTTTTTTT<br>CTTTGAGATGGACTCTCACCCTGTTGCCCAGGCTGTTGTGCAGTGG<br>CATGATCTCGGCTCACTGCAATCTCCATCTCCTGGGTTCAAGCAATT<br>CTCCTGCCTCAGCCTCCCGAGTGGCTGGGACTACAGGCGTGCGCC<br>ACCACGCCCAGCTAATTTTTGTATTTTTAGTAGAGATGGGGTTTCAC<br>CATGTTGGCCAGGCTGGTCTCGAACTCCTGACCTCAGGTGATCTGC<br>CCACTTCAGCCTCCCAAAATGCTGGGATTACAGGTGTCAGCCACCA<br>CCCCCGGACTCCCATATTCTTTTTCTCTACCTTTTCGGTGGCTTTCAT<br>GTTTCCCAGGCATCCATCAATACCATCCCAGGAGCTGCCTGTGGAG<br>AAGGGCTTCCTATGTGAAGAAAACCCTCTCTAGAAGCACTGGGAC<br>TGGGGAGGAATTAGCGGGAGCAGCAGGTGGCTCAGGCTCCCTCTC<br>CCTTCGCTGCCTAAGAAGCTTCCATCCCCTCCATGACCCAAGCCCT<br>CTAACATGATAGATCTCCTCTACTTGAGATCTGTTATTACTCATGGGA<br>CAGTTGCTGCTCTGAAGCGAAATACTGGCTGTTTTTTGTTTGTTTGT<br>TTTGGAGACAGAGTCTCACTCTATCCCCCAGGCCGGAGTGCAATGG<br>CGATCTCGGCTCACTGCAACCTCCACCTCCCGGGTTCTAGCGATTC<br>TCCTGCCTCAGCCTCCTGACTAGCTGGGATTACAGGCACCCACCAC<br>CACATCCGGCTAATTTTTGTATTTTTAGTAGAGACGTGGTTTCACCG<br>TGTTGGTCAGGCTGGTCTCAAACTCCTGACCTCAGGTGATCAACCC<br>ACCTCAGCCTCACAAAGTGCTGGGATTACAGGCATGAGCCAAAGC<br>ACCCGGCAATGCTGGCTGTTTCTAACCCCTGTTCAGTATTTCACTTG

	TACATCTACCCACCTTCCCATTCGGGGTGGGCAGATGAAACTAGCA<br>ATGGACGTCTGACCTTGGGTCGGTCACTTCTCCTAAGCTTCCTGTT<br>CCCCACTAGTAAAAAGAGGGAGGCTTAAGATGATCTACATGTTCCC<br>CTCTGAGTAGTAATCTTCTGTGGAATTCATATTTTATCCTCCAGCACC<br>GAGGGGCAGGGGTGTCACTCTGCCCCCACCCCCTGCCTCACCTCTT<br>CCCCATTACTTTAGGACCTCAAAGCACTTTCACTATTAGTTCCCCTC<br>TGTTGTCCTTTTTATTTCCCAGACAAAGGGAAATGACTCACCCCAA<br>AGTCAACTGGAGTGGGTGGAATGGTGTCATACAAGCAAACAGGGA<br>GTCCCTACAGACATCCCTACCTCTGTGGGAACTCCTTCCCCTGGAG<br>GTGTTCTCCCTAAGGCGAGTAGAAGGGAAAGGGGGTCACATTTCC<br>TTTCCTTCTCTGGACTTTGCCCTGAAGCAGAGGGCAGCCTAAGCTC<br>CTGACTCCAGGGAAATCTCCCTCCCCGGCTTCTCTCTCTCCCGGTC<br>ACCAGTAACCTCAGGACGAGGTCAGTCCTGCAATCACGTGAAGCC<br>CTCACGTTTGCAAGGTTTGCAGAAAGGGCCTCTTAGCTTTGATCTC<br>CCAGACAGCAAACAAGCTTGCCAGTCCCTCCCCAGAAATTCACAT<br>GCCCCTGCCATACAGGCTTTCTAAACACGCCACCCTGACTCTTCAG<br>CGCACCCCACCCCACCCCACTCTCAGCTCCTCCCAGGTCCCGGCAA<br>GCGCTTTGCCAGGCAGAAAGGGGAAAGGCACGCAGTCCGCCCACT<br>TTGTCGGTGGACTACAAATCCCGACAGTCTTGTCGTTGCGCAGGCG<br>CGCAAGAGCTCAACGTGCCGGCTGTTGGAAAAGTGTGTCACTGGG<br>GCACGAGGCGCTCCCTGGGATCACAT
NR3C1	TCTTTATAAATCAGTACTATTATTATCCCCATTTTACAGATGATGGAA<br>AAAGGCACAGAGAGTTAGCCTGCCCAAGGTTACATTCCTTTTAGTG<br>GTGGAGACTGTATTTGGCCTTTATAGTCTGGCTTTTAACTGCTACAC<br>TATATCAATGCATTACAGGTATAGTTTATACACACACTAAAGAAGAC<br>ATGGAACTCCAGTCAGTGGGCTCATAAAAGCTTTAGACCTTCATCA<br>AAAGATTAGAAAATAAATGTTCAATCACAGGTTTTAGGTTTCAAAA<br>GTTTCAAAAGTTTAAAGTTTGCAAGTTATCTTTCTTAAAATTATTTC<br>CTTCTTTTAACTAATTTTTAAATTAAACTCCAGTTCAATCACATCCAC<br>TAGAAGGCTTCACTTTCTACAAAGGGGTAGGTAAAGGTGATGATCT<br>TAAATACCATACTGGGAGTTGGGATCTGTGTAGCACTAGAATCAAG<br>AGTTATTGTATATACTTGAGAAGGAGCACAGCCTGATTTAGTTGATG<br>TTTTGGAGGGGTAATCTGGCAGTACAGCTTGGATTATTTTAGTGGGA<br>ATGGAGATGACAAAACATGAAGAGAATGGGAGAACTCACTTGGGT<br>AATATATCAGTTTCTGAAATCAATGTATCAAGTGGTTTGGTTATTAGT<br>CAGGCTTAAGGTTTTATTGGCCTTAGGACATAGTCTCTAAGACCATA<br>TATTATCTTCATTCATTCATTTAACCAACACCCACTGAGCACCAACTA<br>TGCACTAGTGATACAGGGGTGAACAAACAGACACAGTCCCTGCCT<br>TTGCAGAACTTATGTTCTGGTGGGTGATACAGAGAAATAACAAATG<br>AGGGCACAAATAATGAACTATCATTGTAATGTGCTAACAAAGTATGC<br>AGAATACTATAAGAACATACAATCAAGAATCTAATCTAGTTTATTTTA<br>AAAAAAAGTGGGGGGAGCACTCAGATGTTTAAACACAAAACAATA<br>AAAATGAGGATCTTGAGGGTTATCTGTACACTTTTTATTAACCCATT<br>TGGCCACAACAATAACCCAGTAAAAGTGATTTTACATCTAGTTAACT<br>AGTTGACTCAATGTTATTATAACCCTGTAATGTATTTGTTGGGTGCCT<br>GCTATGTAGTGGGCTGTGTTCCAGGAGTGGGACATAAAGCTATGAC

	AAGATAGATCAGGTCCTTGCCTTGACTGAGTCTGAATACATCACCTT<br>CATTTCCACCTCTGCTTCTAAGACAATCAAATACCTTCCCACTCAGA<br>TAGGGATTCATCCTATTTCTTGTAGTAATTATAAGTTCCCAGGGTTAC<br>AGATTAGTAAATGCCATACCTAAAGACTTAGGATACTGTGCATAAAA<br>CACTTTCAATAGTGTCTGTCAGACAGCAGATGCGCAATAAATGTTG<br>ACTTTCGTTAGAATTGTGTTATTATTATAATGCTAAATCTCACGTCCT<br>TTCTGGCACACTGGAGTTCATCTCTTCAATTAAATTCCGTAAGCACA<br>TATTGTAAGTAGTCTATCAGAATTGATATACTACTTCATGTTAATGAT<br>TGAGATCAGTGAGGAAAACCACTTTAATATTGTCTAATTCCTCTCTT<br>AATGCATAGGAGCCATGTGAACTTAGACAAGCATCAACCTCTGTAA<br>GCCTGTTGTCCTCATATTGAAAAATCGAATAACAATACCTATCTCATT<br>TAATTGTTGTCAGCAAAAGTGGGCAGCAGAAGAGTGGGGATCAAT<br>AAATGTTAGCCACAAATCATAACTATTTGTATTACTCTGAAAAAGAG<br>GGGTTAACTTATAGTTCATGCATGCTTCCAAATGAATGTGAAAGACT<br>AAAGAATGAGAATTTTTTTGCTATCAAATTAAAAAATTAACAGGCA<br>CATTTAGATTGCTGAAGAGAGAAATTAGGATAAGTTTTTTTTTTTT<br>TTGCTCAATTAAAAACGTTTTCAATGGCATATTTAAAACTACGTATT<br>CTTCCCCATTAAGATCTGCAGAGAAAAAAAAATGAAGGGAGAATA<br>ATTGGGAACTCTTGTGAAATCATAAATTATTTAAGCAGAGTACCTAC<br>CACTAAGGATTTGAGCATGTATGAGCGATGAGGTGGATTCTTAGAG<br>CAGAGATTCAGCAGTGAGACAGGCTAATGGCATGGGGAGATGTGT<br>TCTATTAGACAACTGTATTGTCCCCTTAATTATATACATATATATGTAT<br>ATAATATATATGTGTATACATATGTATACATACATGTATATATAAATAGA<br>ATTCTTAATCATTTTAAGCAGTGTACAAGGATAATTATTAGCATGGGC<br>TATTTAACTCACTTTTAAAACGTGTAAAACATTATTGTAGGGTTGGG<br>TCTTGATTTGTTTCCCATGAAACTGTTGGTAGTTTAGGGGCCAAATT<br>AACGAAAGACATCTCATTCTAGTGCTTGAGGCTCAGAAATTGGAAA<br>AACGGGCAACTAGGTCAAGCAGTGTTCTCATAGCCTCACAGATGA<br>GCATCCAAAGCAAGGGCCTCCTTCTAGTTGACTTGTGCCAAGGGA<br>CAGGAGAAGCGGAGTCTGCCTTCTGGGTCCAGAAGGGGGTTTTGT<br>TAACATGGAGTTGCTCAGCGCCCTTCATAAAAATTCTTCTGGCTGA<br>GGGTTCTACGTTGGCATACGGTTGGTTCCCTCTTCTTTTCCGAGGTG<br>GCGAGTATCTCTTCCTTTGCCAAGATGGCGGCTCCAGAATCCTCTG<br>GAGGCGGCCCCCGTAGATCGTCTCCGGACAAGAGGCTTGCTGAAA<br>GCCTACTTCTTTCCTTTCACATCAGACAATGCACAGGGAACCGTTT<br>ACCCTTGAGAACCAAGGAAGGACGGCTTAGGCTACCCGCGATCGC<br>GAACCTTTGCCAAGATGGTGGCCGCGGGGACGGGCTGGCGACACT<br>GTACCCTACCAAGATGGCGGCGGGCGGCTTCCGGGACGCGCTTCC<br>CCAATCGTCTTCAAGATGTCAGAGCAGGGGGAGCCGCCGTCAGTC<br>TGAGCGCGGCGGGAGGTGAGAGAGTGGCTGTGGCCGAGCGCCCG<br>AGCAGGATTAGGTGGAGCTGCGGCAGCCCCCGCCCGTGTCAGGAG<br>CTGGCAAGCGATGTCACCTGTGGGGGCGCAAAAGTTACCTCCCCA<br>AACCCTAAACCCACACAGCACAACCTTTCCCAGAGTCACAAAAAT<br>CATAATCTGTGCCGCACAAGGTAGGAGGCTCGGTCCCGGCATCGTC<br>CAAGCCTTCCCGACGCGGCGAGCTGGGGAAGGGAGCTGGGGCGG<br>GGGCTTCCCGCACGGGCACCCCTCGCCCCACGGCCCTCTCCTTTCT

	CAGGACGGACCACGAGTTCCCTTCCCCTTGGACTGAGGGGGAAGC<br>TCCTAACAGGAACATCTGTAGGGAGTTGAACGCTGGCATTTTAAAG<br>CTGCCTGTATTTTGTTTTATTTGTAGGGGCAGGGGTCCTATGAACGT<br>GATAGGGTGAGCAACGCACAGAGTCGAGGGCAGCAAATGTCAAGA<br>TTCGGGGGTGGGGCCTGCACCGGGAACTTGGACGCGGGCCCTGGC<br>CGGGGTGGAAGAAGAGGTCAGGAGTTTCGGAAGGGGGGCTATATT<br>TCGCCAGCAACTTACTATTTCGCCTGCAACTTGCTTTTAAGCCTGCC<br>GCCCCCTGCTTTCCTTAATCATAATAATAAAAAAAAAGTGCAAAGA<br>AATCCAGCTCGCTGGAGGTTTTGCATTTGGCGTGCAACTTCCTTCG<br>AGTGTGAGCACATTGGGCGGGAGGGGTGGGGGTTGAACTTGGCAG<br>GCGGCGCCTCCTTCTGCCGCCGCCGCCGCCTCGCAGACTCGGGGA<br>AGAGGGTGGGGGACGGTCGGGGCGCGGGGGAGGGTGGGTTCTGC<br>TTTGCAACTTCTCTCCCAGTGCGAGAGCGCGGCGGCGGCAGCTGA<br>AGACCCGGCCGCCCAGATGATGCGGTGGTGGGGGACCTGCCGGCA<br>CGCGACTCCCCCCGGGCCCAAAGTACGTATGCGCCGACCCCCGCTA<br>TCCCGTCCCTTCCCTGAAGCCTCCCCAGAGGGCGTGTCAGGCCGCC<br>CGGCCCCGAGCGCGGCCGAGACGCTGCGGCACCGTTTCCGTGCAA<br>CCCCGTAGCCCCTTTCGAAGTGACACACTTCACGCAACTCGGCCCG<br>GCGGCGGCGGCGCGGGCCACTCACGCAGCTCAGCCGCGGGAGGC<br>GCCCCGGCTCTTGTGGCCCGCCCGCTGTCACCCGCAGGGGCACTG<br>GCGGCGCTTGCCGCCAAGGGGCAGAGCGAGCTCCCGAGTGGGTCT<br>GGAGCCGCGGAGCTGGGCGGGGGCGGGAAGGAGGTAGCGAGAAA<br>AGAAACTGGAGAAACTCGGTGGCCCTCTTAACGCCGCCCCAGAGA<br>GACCAGGTCGGCCCCCGCCGCTGCCGCCGCCACCCTTTTTCCTGGG<br>GAGTTGGGGGCGGGGGGCGAAGCGCGGCGCACCGGGCGGGGCGG<br>CCACGCCAGGGGACGCGGGCGTGCAGGCGCCGTCGGGGCCGGGG<br>TGGCGGGGCCCGCGCGGAGGGCGTGGGGGCAGGGACCGCGGGCG<br>CCCCTGCAGTTGCCAAGCGTCACCAACAGGTTGCATCGTTCCCCGC<br>GGCCGCCGCGCGGCCCCTCGGGCGGGGAGCGGCCGGGGGTGGAG<br>TGGGAGCGCGTGTGTGCGAGTGTGTGCGCGCCGTGGCGCCGCCTC<br>CACCCGCTCCCCGCTCGGTCCCGCTCGCTCGCCCAGGCCGGGCTGC<br>CCTTTCGCGTGTCCGCGCTCTCTTCCCTCCGCCGCCGCCTCCTCCAT<br>TTTGCGAGCTCGTGTCTGTGACGGGAGCCCGAGTCACCGCCTGCC<br>CGTCGGGGACGGATTCTGTGGGTGGAAGGAGACGCCGCAGCCGGA<br>GCGGCCGAAGCAGCTGGGACCGGGACGGGGCACGCGCGCCCGGA<br>ACCTCGACCCGCGGAGCCCGGCGCGGGGCGGAGGGCTGGCTTGTC<br>AGCTGGGCAATGGGAGACTTTCTTAAATAGGGGCTCTCCCCCCACC<br>CATGGAGAAAGGGGCGGCTGTTTACTTCCTTTTTTTAGAAAAAAAA<br>AATATATTTC
OPRM1	GAGACCATCCTGGCTAACACGGTGAAACCGCGTCTCTACTAAAAAT<br>ACAAAAACAAAATTAGCTGAGTTTGGTGGCGGGCGCCTGTAGTCC<br>CAGCTACTCAGGAGTCTGAGGCGGGAGAAAGGCGTGAACTCGGG<br>AGGTGGAGCTTGCAGCGAGCTGAGATCGCGCCACTGCACTCCAGC<br>CTGGGCGACAGAGTGAGACTCTGTTTTAAAATAAATAAATAAATAA<br>AATAAAATATAATGATAAAGAAATGTTTTTATAGAGCTCTCAGTTTTA<br>ATTTCTGAAGTGATAGACTGTGATAAAGATAACCTAAATAAGAAAA

	GCTTTTTGGATCCTCAATAATTTAAAAATGTGTAAAGAGTCCTGAGA<br>CTGAAACATCTGACAACTGTTTATCTATTAGAAAGTGATAAGGGGC<br>TGTACTAGGGTAATGGCAAACCTCTGTAACTGGAAATTGAAGAGCA<br>AGACTTGAATAGAAATGAGGTTCCAGATTTTAGAAAGACATTTAGG<br>CTTATGTATCATTTAAACTAGTTGTTTTTGGATAAATTTTCAAGAGTT<br>GATTTAGTTGGACCCTAGATACTACCTTCAGGCCCTTAAAAGAATCA<br>GGTCCTTTAAGGACGATTCTGTACAGTTAAATATAGTGTATTATTTAT<br>AATAATTCTTCTTGCTAATTTCTAGGCCACATACAACAGGATATAAA<br>AAGCCAAACAACAAAGGATAAATTCTTTCATATGTGTGTAATCCTAT<br>AAACCCTCTTTGAAGTTGATTCTCATATTTTTATTTTACAGAAAAGG<br>AAACCAAATATCAGGAAGTATAAGTAATTTAGTAAGTCACAAGGAA<br>GTAAATAGTTGAGACTGGATTTTGAACTGCTTTTTTTTTAATCAAAA<br>GTCCGGGGCTTTCATCCACTTTATTCTAGATGGCTTTGTTTTAGGTTT<br>ATTGGGTAGAAGTTGGTGTGAGGAGTTTTTTGCTTTCATTTACTTAT<br>TTATTTTTTTACCATGCAAATAAAGAGTGCCTATTCCATTTTACTTGA<br>TATGTATGCAACAATCCAGTGATCAAGGATAATATTGTATCACAATG<br>CATTTGCATTCTCATTAGCTATGCCCATTTAAAAGTTAGATAACATAG<br>AAAATATCTATAAACAAATACATAAAATGTTCCTCCAATGAAACCTT<br>GGATGTCCCATTTTTCTGAGCTTCAAACCACTGTAATTGTTTCCTGC<br>ATAGTATTTATGTTTGTGTTCCTCATTAATAGCCATTAATCATGTGTTC<br>CATTAACAGCCACTCTCAACAGAATCAGGACTAAGCTATTAAAGTA<br>AGTGTAGGATGAAGGCAATTCTATGAATAACTAAGCCAATAGATGTT<br>GAAATTCAAAGAAGAGAAGTGACTTTTAGCACTTTCCTGGGATTCA<br>TCCGTGCTATGAATAAATACGTTGCTTTGAAACAAATAATTTTTGTG<br>AAAAATGTTGCATGATATGGGTTTGATAGCAATGTTCAATCTAAATG<br>TATAGATAATAAATTATTCTTGCTTCGAATAGAAAGCCTGGGCTAAG<br>CAAGAAGCATCAACAAAAAGACAGATATTTCTATTGTACAAATTAC<br>ATATTAAGGCTCAATGAAATAAAAGATATATTTGGGATCAATACTGTA<br>TGAAATAATTATGTTTGTAATAAATTGACTCATTTATTTGCAGTCCAT<br>CTCCTTTTATGAGAGACTTGAACTTTCACAACAGACTTCAGACCAA<br>TTGAGTAGGAAATGAACAATTATGGAGGAAACAGGGAATATACCAT<br>AGGGAAACTCTGAAATAACAAAAACTTGAAATTGAGGTGCTTTTAT<br>ACTACATATTAAGTGGTATTAATTTATTATTTCTGGAACAGTTTTTCT<br>CTAGGTTTCTCAAGACTGTCAATATATTTGAAAGAAAATTAAATTTG<br>TTCTGCAGCTTCCTGAATTGTATATTAAATGGGTGGAAGATACCAAG<br>ATATAAATGTTAAACAACTTAAGGAAGGACATGCAATGATAACCAG<br>AATATTCTATATTCAAAATAGAAGGATAATAACTATCCTCACTGAAA<br>GATGATAAAGACCAATAGAAAACAAAAAAATCAATCTCAGCTGAG<br>AATATGAAGCACAAAGTTCAAGTCCTGCTCTAGAGAGTAATTGCAT<br>TTTTCTATAAAACAGAATAAAAAAACAACTGTTTCTAGAAAAGGGC<br>AATGGGAATGGGGTTGCTCTTTGCCTGTAATTCTAGAAGAAAAGGC<br>ATATGGCTAGTCCATAAGTCTATACATTTAAGTCCTGGTTGGTTGAC<br>AGACTATATTGAGTCAAAAAGGACAAAACGTCTTCATTAAGGTTTT<br>CCCATAAAGATGTTTTATTTTCAAACTGTGTCATTAAAAAAATCACC<br>CAGATTGTTTTATTTTTATTTTATATTATTTATCACCCAGATTGCATATA<br>TTTTATGCCATTATCTGGGTATAATGGCATAAAATATATGCTAATCATT

	TTTTCAACTGAATTCAAATATTATGCACATTAATATTCATATATGTTTA<br>ATATAGAAAGAAACACAGAGAGTGAGGGAGGGAGTCCACTATGTA<br>TTAAGTACTGTGTTAGTGAGCAGACCTCCCTTAGGAACCTTATTACG<br>GAGTACAAAGCTAGGAGAGTAAATAAAGTATATTAAAAAATGCATA<br>CAAAAGATGACAGAATCACCATTCCAAAAGATCTTGGTGGATAAGA<br>ATCATGAATTGGATCTAACAAGATGTAACTTAAAAGTAAAAAAATCT<br>ATAGTGTTGTACTGAGCTCCCTCCAAAGCAACTATAAATTTATAGGA<br>GATGAAACATATGATTCACCAGGCATAAGAAGAAAGTTTCCGTAAT<br>CAAACACTATTGTATCCATCTTTTTAAACTCCAGCTCCTATCACAGC<br>ACCTGGTCCAAAGCAGATCTTTAGTATTTGTGGAACTGGCTTGGAT<br>TGTGTTTAGGAAATTTTGTCATTGGTAAACCTAAGGAGAGTCAAGA<br>GAACAACGTGACCAAAAAATAAAACTAAAAAAAAAAAAAAAGGGA<br>CTTTCATTGTACTGGTAGAAAGACAAAGTTTATAATCTGGCTTAGTT<br>TCTTTTTTTGTTGTTGTTTGTTTTTTGGTCAGGGCAAATTTAGGTCAT<br>TATTTTTAACACTGGAACTGTAGTTTCAGAGCAGATAGACAAACTAT<br>AATGAGAATAGATGAACAGCAAGGCCACTGAAAGGACTCAGAACT<br>ACATCTTATAAGAAACAACTGAATGATGCTAATGTTTAACTTGCAAA<br>AGAGAAAACTCAGTTGATTTCAAATATATGAAATATAGTGGTAAGG<br>AGTTATCACTTATTAAGCAATTACTATTGCAATGTATACTCATTTAAT<br>CCTGCTAACAGACATATGAGGTGAATATTATTAGCCTACCCTCGCCT<br>TTTTTAAGTAATGAGAAGACTGTCATCCTGTAGGGTAAAGTAACAT<br>GTCCAAACTCATACAGCTACAAAGTTACAAAGCTGATTTATAAAAT<br>GATTGACTCCAAGGTCAGGAATTATTATACTGTGTCTTGTCTTCCAC<br>ATGAACTAAGCACAAAGGAACTGAATGCAGGCAGACAGATTTCAG<br>CTCAATATAAGAGAATTGTTACATTAGTTCATGGAAGAATATGTTTTA<br>AGGTATTTTTGTTAGTCTCTAGGAAATCTCTGTAACATTTTATTGTGT<br>AAATTATATGCTTTAATGTAAGAGGATAAAAATAATAGTGAACATTG<br>GCAAAATAGCCTATGATTAATAGAGTTTACCTATGAGTTATCTGTTTC<br>TAAGATAAATGCCAAAAAATAATATTGGAATTAAATGTTCCTTTCAA<br>GATCTTCCCTCCCTGCTCCCTGAAATTGCAGTGAATTTTTCAAGACC<br>AACTGAGGACATGTATTTTCAATGTTTATGGTTAAAAGATATGTACA<br>TGCACAGATATATACATGTACAGAAATGAGAATTACTTCAGAATTGG<br>TGTTAACTTTAGAAAAAAAAAGACCAAGAACTTACTCTTGGTATTT<br>ACAAATTTATTTCTAAAATAGAAGCACTCATGGACTTAGAAGTAAG<br>GTATAAAATTCAAAAACGTATCCATGTTTCTCAAGGATCTTGTTGTA<br>GGCCACTCTAATTCCATATATTATGTGGCTTTTCCTAGAATTTTTACA<br>CTAGAAAACAGACTGAATGCAAATTTTGTTTTGTTTTAACAACCTT<br>CTTCTCAGAAGCATATGTCTATCGAGGAAGTCTTCAGATAAAAAAG<br>ATAAACAATTCCAAACAGGTCTATGAGATTTAAGATGTGAAAGATC<br>AACATTATCTTTAGTTGACTTTACTGGATGCCACAACCTTCTGATTT<br>CTGTAACCACTTCTTATGCCTCCTACCCACTGAAACAAAATCAGAG<br>GCAAACAGAGCTTCACCCTAGAAATTGGGGAAAATGAGGAACAGG<br>TTTTCTGCACAAAAGTTTATTTGTTTCTCATTTCTTTTTCAGAAAATA<br>AAGGATCGCTGTTGTTCCCAACAGGTTTGTAGGGAAGAAAATTGG<br>AGAAACATTATTACCTTTTCTTAGATGTTGGCAACGGAGGCAACAA<br>GGACTGCAAAAGAAAATTGTGTGTCCCCCATTCCTAAATAATCAAA

	ATTGGCAGTAGGGATGGAAGAGCATTGGGGTTTTAGGGCTGTTAGG<br>GTTTCATCAAGCCAATGTATTCCCTGCCAGATTTTAAGGAGAAAAA<br>GGCGCTGGAAAATTGAGTGATGTTAGCCCCCTTTCTTATTTTTCACT<br>GCTACCAAAGACTAACTCTATCTCTCTCCCCAACCCTTCTCTCCATC<br>TCCCTCCTTTAGATGTGTTTGCACAGAAGAGTGCCCAGTGAAGAGA<br>CCTACTCCTTGGATCGCTTTGCGCAAAATCCACCCCTTTTCCCTCCT<br>CCCTCCCTTCCAGCCTCCGAATCCCGCATGGCCCACGCTCCCCTCC<br>TGCAGCGGTGCGGGGCAGGTGATGAGCCTCTGTGAACTACTAAGG<br>TGGGAGGGGGCTATACGCAGAGGAGAATGTCAG
P53AIP1	TATAATCTAGCTAAAAGCAGACACGTGAAACCATCTAAGGTGATAA<br>ATATGCCTAAGATGGTGTAGTGAAATGTACATATTTTATAGCATCCAA<br>GTCTTCTAATAGCAAGAAAGAACACTTATGAATTATTCTGCTTCACT<br>GATTGCTTATTTATCATAAATGCTACAGTCAGTGCATTATTGGAGTTG<br>TTGACTACCTTTGGCTTCCTCTGAGAAATTTTAATGTACATGTACAA<br>TCCAAAGAGTTGTATTTATTAATAACCACTAGGACCTATAAATGTGG<br>TATAAAATAACAGGCAAGAGAGTAGGCCCAGGAGCCAGACTGGCT<br>GGGTGTGAATCCAGGGCTGGCTACTTACTAGTGTGGTGAGCTTGGT<br>TAAGTCATTTGACCTCACTAGCCCTCTGTTTGCTGTTCTGTAAAGTA<br>AGGATAAAAACACCTATCCCGTGACATGTTTTGCAGAACAGCTGGG<br>TTCGTGTACATTATGCATTGAGAATAAGGTCTGGCACATGGCGGAG<br>CACTCCATATAAATTCTTATTCTTCTTAGGCAGGCATCCCCAACCCCT<br>GGGCCACAGACCGGTATGGGTCTGTGGGCTGTTAGGAACTGGGCT<br>GCAGGAAGTGAGTGGTGGGTGAGCCAGTGAAGCTTCATCTGTATTT<br>ACAGCCACTCCCCGTCGCTCACCTTACTGCCTGAGCACCACCTCCT<br>GTCAGATCAGGGGCGGCATTCGATTCTCATAAGAGCGCGCATGAAT<br>TCTATCGTGAACTCCACATGCGAGGGATCTAGGCTGCATGCTTTTTA<br>TGAGAATCTAATGCCTGATGATCTGTCACTGTCTCCCATCACCCCCA<br>GATGGAACTGTCTAGTTGCAGAAAAACAAGCTCAGGGCTCCCACT<br>GATTCTACATTAGTGAGTTGTGTAATTATTTCATGATATATTAGAATGT<br>AACAATAATAGAAATAAAGTACACAATAAATGTAATGCACTTGAGT<br>CATCCTGAAACCATCCCCCTCCTCAGTCCATGGAAAAATTGTCTTCC<br>ATGAAACCGGTCCCTGCTGCCAAAAAGGTTGAGGACCGCTGTTCTT<br>AAGGTGCAATTATGGTTAAACATTTACCAGGGGGAATTGAAGCATA<br>AACAGAAGTATACTTTGTGAATTATTACCAACAACATCCACATAAAG<br>TGGGAAAGAGGAAAATCCATTTTATTCCCAGCCAAGAGACATTTAA<br>AGCACATACCCTCCCTGCCCCTACTCAAATGGGAGGTGTAGAAAGC<br>ACCCTCCTCTTTGTCCTTCCAGAATATCTTTTTGATGTGTCTTATACT<br>CACATCTGAAAAGGTCAGCTGGTGACATTTCTTTCTGGAGGCAATT<br>TTACTGAAAGCATTTTCACTTTCTCAGTGTGCAGGGACTGGAGGCT<br>CAATGAAGAGGATACAGCTATGTCCACATCATGTCAACTCTCACTG<br>ATTGTAGAGCTGTTCTCCCACCCAACTCAGTCCTCTCTCCCTCTACC<br>CCTAACAGGAACATTTAACTCTGGTTATTGACTACATCTCATTGTGA<br>TTACTGAGCAAATTTATTAAATAGCCTGGGATAATACTTTTTTTTTAG<br>ATGGATTATTGCTCTGTCGCCCAGGCTGGAGTGCAGTGGTACAATC<br>TCGGCTCACTGCAACCTTCACCTCCTGGGTTCAAGTGATTCTCCGG<br>ACTCAGCCTTCTGAGTAACTGGGAATAAAGGTGTGCACCACCATGC

	CTGGCTATTTTTTTTTTTGTATTTTTAGTAGACACACAGTTTCGCCAG<br>GTTGGCCAGGCTGATCTTGAACTCCTGACCTCAAGTGATCCACCTA<br>CCTTGGCCTCCCAAAGTGCTGGGATTACAGGTGTGAGCCACCGTGC<br>CCGGCCCTGGGACAATACTTGAATGGGGCTCCAGGGACTGATTACT<br>ATCCATCAAAACATTCTGTCCTATTGAAATATAAATTTATCTGAAGCA<br>GACACATGAAAACATGCTTTCCCATATAAGCCCCCATGCACTGGGC<br>AACTTCCCTTCTGCCTAGCCCTTTAATGTTGGTGTTCTTGAAGCTTT<br>GGCCTCAGTGCCTTTTCTTTGCTCTCACATTCTTCCTGAGAAATTGC<br>ATCTACTTTAAAGATTTCCTTTCCCACCTATTTGGATCTTTTGCTTGG<br>AACTTTCTCCTAAGCTCCAGGTCCATGGATCCTACTTCCTGCTGGAC<br>TGCTCCCATCTGGAGATCCCACAGGCCCCTCAACTCAGCCCCTGTT<br>TCTGAATCCCCTAATTATCCTCTATTTACTTGGTCACTCAAGCCAGA<br>GACTTGCCCTGGCCTCCTTGTCTCGCTGTACCCCGCCTGATGATTGA<br>GCTCAGCTCATCAAGGTCTGTCACCTGCAGCACTACAGCAATCGCT<br>TGTCTGGCCTCCAGTTTTTAAAATCCGTCCCACAAAACATGACCAA<br>AGTGGCCAATCTGATGACACCATCCCTTGGCTTAGATTATTCAGTTA<br>ACATCCTGATCAGTGACGTAGGCACAAGAACCTCCTAGAATGGAA<br>GTTCCTGGGCCCCACCCACAAAGATTCTGATCCAGCAGACCCGGAA<br>TGCGGTTCAAGAACCAACACATTTAACACTCAGACTGCTCTGGGG<br>CAGGTGGGCTGTGGGCATGTTCTAAGACGCACAGCACTGCAGGAT<br>GTTCCAGCGCCTCGATGTGGCCCGGACACTCACCGTCTTCTGGCAT<br>GCAAACACCTCTCTAACCTCATCCATGTCTAACCCCTTCATGTCCTA<br>AAGTCCAGCAGGCAGGGTCAAGGACTAGGGTGACATTAGGCCAGG<br>CCTGGGAATGCCTGGATCTTGGCCTGCTGTTCACTCATTGTTGCTTT<br>GAGGGTCTAATTGGAGGAGCTCTGTCCCCAAGCTCTAATAGGCAAA<br>AGTAAGAAAAGTAACCCATTGTTAGTAGCTCAGACTAAGCCCAATA<br>CCCTTGAATTCATGGTTCTCTTATAAAAACTGAAATGCTGAATATGG<br>CTTAGCAGAGGATAATTGGCAGGAAGCAAGACTTTCCCGCTGTGTC<br>AAGAGATGGGAATAGGCCAAGTCTACAAAGCTGACCTGAATCCATT<br>TGTCCAATTGGTGAGCCTGTCTGGCCTATCCCCAGGGGCCAGACCC<br>TGCCCTCGCCCCAAGCAGTAGATGCAAGAGCAGAGAAGGATGGTC<br>AAGTCTATCTAAGGAGACAGGCTGTATAGAAGTGTTCTGATTGCAT<br>GGGAGCAAATTTTAAGAGAAGTACCATGCATGGATAACAATGTTCA<br>AGCCACAGTAACCGCACACTGGTAAACTGTGGTTAAACTTGGTGC<br>AAAAGTAATTGTGGTTTTTGTCATTATTTTCAAATGGGTCTTAAAAA<br>GGAGTGTTGGGGGCCAGGTGCGGTAGCTCATGCCTGTAATCCCAGC<br>TCTTTGGGATGCTGAGGTGGGCAGATCACTTGAGGTCAGGAGTTCG<br>AGACCAGCCTGGCCAACATGGTAAAACCCCATCTCTACTAAAAAAT<br>ACAAAAGTTAGCTGGGTGTAGTGGTGGGTGCCTATAATCCCAGCTA<br>CTCCGGAGGCTGAGGCAGGAGAATTGTTTGAACCCAGGAGGTGGA<br>GGTTGCAGTGTGCTGGAGGTTGCGGTGTGCTGAGATGGCACCACT<br>GCACTCCAGCCGGGGTGACAGAGTGAGACTCTGTCTCAAAAAAAA<br>AAAAAAAAAAAAGATTGCTAGGGGAAGGCTGGAAGCCCAGGGAG<br>GAAGCAGGTGTTCCCTGCCCAGCAGTGATTGCTCCCTGGTTGCTGG<br>AGGAACGATGGAATCAGAGTCACCCGAGGACCAAAGCTCCACACC<br>TCTTGCTATCCCAGACTCTCTGGAACACCAGGAAGAAAACATATTT

	GGACAAAAATTATTCTTTGCAACATTAGAACTTTTGATTCTGGAATG<br>AGACAAGGAAATATGTATGTATCTCAGAGAGACAATGAATTTTTAA<br>AAGGAAATTGGGACCGTTTCTCAGGGTGAGATGTCTTATCCGGTTA<br>ACTGCGAGCCCCCAGGGATTTCTACTCAGAGTGTTCATCACATCAG<br>GTTTCCATTCTCTACAATAAAAACATGGCTAGGGAGAAATTACCCA<br>GCACTGGCAAGAAAGAGCTCCAAAATAGAAGCAGCTTTTGAGGAA<br>TTTGGAGGGATAAAACGGAATAGTATGTCTGAAAGTTGGCAACATG<br>TAAAAAGGCTAAAAATGACAATTCTGCCTGTGATACTATGTCTAGTG<br>TGCAACGCTTCATTCAAAAGGCAGGCACGTCTCCCCTCCAGGCGG<br>GAGTGGGAATCTCAGTGGGGAACCGGGAACTGCGTTTGAGCTGAT<br>GCAGACTCAGGGTCAACTCGACAAGGGAGCCCGGCACCCAGACA<br>CAGCGGCGTTCACCTCACCTTGCACCGTCTTTGTCGGTCTTACAGG<br>CATCAGCTTTCCCAGCCTCATTTGAGCCGGGATCTGAGTTGCATTTC<br>TTTCTGGGTCAGGCCTTGCCATTAGGGAGAGATGAAGTCAACTGAT<br>GAGCGTGCCAGCCTTGAGGAAGGACAGAGTGCGGGAAGCAGAGT<br>GTGGGAAGTTGGAAAAAATAATCTGCAAGGTAAACACCTTCAAGT<br>CAATTTCAAGAGGCTGGCTAGAAACCGCCAGGACTTCAGGGGGCC<br>CTGTCTCGCGGGTCTCCCTGCGCCCTCAGCACTTTGGCTTGGTATA<br>AGGCCACCTTGTCCCTGAAGAGAGGCCTATTTTAAGCTGAGCAAG<br>AAAAGGTGAGAAAGACTAAAATCAGCTTTGTGCAGAGACGCCAAT<br>TTCTGCTGCACACATGGAAACGATGTGCACATACAGCGAGCTGGCC<br>CAGCTCCCGGCCACGTGGCTCACACTGTCCGTGCTCACCCACCCGT<br>TGCCTTCACCCCCAGCAGCACTGGTTGTGAAAAGGGTAACTGCCT<br>GAGCCACCCCACCACTGCGGGCCTGCCAAGGACAGGCAGGAACTC<br>AGCCATGCCCCGCAAGGAGCCCAGGGGCATCTCCAGGGACGGCTC<br>CCCAGCCTATGGGCTCGCTCCCTGAGAGGCCGTGCTCGGGCTCG
PAX5	TCAGCAGCAAACCTGAAAGATTTTTAGAGCACAGTTTTATTTCCGG<br>CACGTTCTCAAATCCAGACTTCATATCCTGTAGCCCCCTGACCTTCC<br>CAAAAGACTTCCCTCCCAAAAGAAAAAATAAAAATCAGCCCACAA<br>CCTGCCCTGAGGCTGAGGTATTCACTTGGCGATTTGCAGGTTCACA<br>AGGCAGGCTCCAGGGAAAGCACCCCAATCCTGGGGTGATCCTCTG<br>CCACGGCTCTGAAGTTATTACAGAAAAATGGAGAATGAAGAAACG<br>AGAATATTAAAGGGCTTACTTAAAGGCGCGCTTCACTTCCGTTTAA<br>CACTCTGATCTGCGTCCAAAACTAAAAGAATCGTTTAGCTTGTGCA<br>CTCACCCTCACTCAAAATCGCTTGTGCAGTAGGCTTTTTTATGTTCC<br>AGTACTTCATGGAATCAAATTATATCACATTAAAATGTTATTAGTTTA<br>AAACACATTAAATAAATGTAAGTCTGGGCCGACGCATGAATATTTAA<br>AGCTCACATTACACGGTGAACTAGAAGAAAAGGGGGTGAGGAGA<br>GCCAACAGAACATCGTATAAATACTGATAGAAATAAAGCATCCTATG<br>TGCAAACTTTCTTTAGCAAAATATGGTTTTATTACTTACACTTATTAT<br>GGTATTGTTAAATTTGAAAAGAAATATAAAGAGAAATGAACTGATTT<br>ACGCTTGTTTTCAATATTAAACAACCAGAATGCCAGTAAATCTTTTC<br>AATGCCCCATTTTGGATTCTTAGGTGAGAGTGGATTGCTGGGACGT<br>GTTGGCAAATTTTAAATACAACTGATTGCCTGTTTTCCATATTCATAT<br>ATTGCGAATGTAACTCCAGGAGAGTGAATTTTATCTCAAAATTCTCC<br>AAGAGTTGCCTTCAAATAGCGTCTCTGTGCATATGCATACGGCATAC

	ACTTGACGTCTACCATGTGTACCTGTGCGCTGGAAACTAGTCGTGG<br>AACTGAATTGAATTAGTGGATTTGAGTTGAAATGAACCCATCCAAA<br>CGACCCCAGGTCTGCGCTCTTTGAGGCTTCTCTCAGTAACATTTCTA<br>ATGAAAGCAAGAAAAGCAGTAAAGTAAAACAACAATAGTAATAAC<br>CCACCATGAGCACCAGGTTACCCGAACACAGACCCATTGCGTATTC<br>TAGCGCAACCCAAGTATTTCCGGGTGGCGAGTCGCTGGCACATGGT<br>GCCCCAGTGAGCCCGCACCCGGAGCTCGAAGCCCTGTAGGCGCAG<br>AGGACCCCACTGCCAACTGAAATGGGTGTTTTGACCCAAACCAAT<br>CCTCAGCTCCTTTTTGCCCCGGGCGCCGCCTGAAACCCCGAGGCG<br>ACGGCTGGCCCGCGATGTGCACTTTTTGCGCTCTGCGCTGGGGGAG<br>TTGCAGGTGGAGAGGCGCCCCCATCCATCCCGGCACCGCCCCTAG<br>GGAAGCCCCAAGTTTATACCGCAAGGGACCAACTCGCCGCGACTC<br>GGCGTCCACCCTCAATGCTGACACAGTTCAGGCCCGGCAAACGAA<br>GCAGGGCGGGGCTGGGCATTAGGGGCGCCGCCTGCCGAAGATGCG<br>GTACCACGTGCGCGCTGTACGGACCCCGAATCCTGTGAGTCCAGCT<br>CCGGCCACAGGCTTTCGAAGAACTGGGCCCTCGGCTTTAGGGCGG<br>GGCCTCTTCTTTGCCTCCTGCCTGGATGTCGACTCCCACCCACGCT<br>CTGCGCTAGCTCCGGGGCCGAGCCCCAGTGCCAGAGTCGTTCCCT<br>GAGGCTTCTCCCAGGGCTCGCACAGAGGGAGGCCCAGTGGCGGAC<br>GGCGATGGCCCAGCCTTTGCTGGATTCCGGGCTAGGGGTCTGGGG<br>AAGAGGGGCATCGGGCCGGAAATGAGCCCCAGTTCGGGCTCCACG<br>AGCCCTGGAGCCCCCAACGATGTTTCCTGGCTGAGTTAAAGAGCC<br>CGACCTTGGCCCCTCGCCTGGCGATGTCGGCCTCCCCCTCCCTTGC<br>CCGCAGCGTCTTAGCTGGAGGGCAGCTCGCAGCGTGCCTAGGCCA<br>ACCCCTTCTGCGTCTCTTTTCTTTTCCTGTCACTCTCTGTTCTCGGC<br>TTCTGCTTTCTCCTTCTCACAGAATGCCAGAGCGCCCAGGGGCCTG<br>GAGGAGCTTCCGCACCAGCCCCTTTTCTGGGCCTCCAATTTGCCTT<br>CCCCACAGTCTAGTCTCCCCCCGTTCCATATTTCTTTCTGTTTAGAC<br>TCTGCTCGTATTTCCTCGCCGATTGAGCCAGCATGTGTCTCTGCCTC<br>TAAATCTTCTCCCTCGTGTAACATTAAACAAACCAAGGTGTGTTGA<br>GCTGGAGAAGTCCTGGCAACGTGGACAGAAGGAATGGCTTATTCT<br>GTCCTGGGGACATTTCCGGTCACTACAGTTCGGTTTCCCCTTGAGG<br>CGGCGCCAGCTCCGGTTCCGCCCCGCAGGCCGCTGCTACGGAGGC<br>GCCCGCTCGCTGTCTCGCTCTCTGGCGGTGTCTGCGTGTGTTCGGG<br>TGTGGGCACATGTATCTGTGCTTGCAAACCAGGGCGGGCTCTCCAA<br>ACCGAAAGCTGCGCGGAGTTGGGTTTCTGGGATTCTAGGGCTGCG<br>GGCAAGGGAGATGGGGGATTTTCCCGGGATCCCGACTCCGATCTGA<br>GCCTAGACTCCCCGCGTTCTGGCCCCTTGTCCCGTATCTCTAGGGCC<br>GCAGTGTCAATTCCGTGTCCTCCGAAGTGGTTCGTCACCTGCTCCG<br>CCGAGGGGAGCCGCCCCTTGGGCCAACGCTGCGGGAGTCCGGACG<br>GTCCGAAGCATGCTGGCCAGGCGCAAGGGGAGACCGACTCACAG<br>GGGAGAGGGTGGGCAAGGGGACGGCGGCTGGGCCTCGGTACCCC<br>GCGAGGGGGGCTCTGCGATTCTGACCCAGCAGCGGCGCCTCCGGG<br>CCCGCGAGGTTTTGACGACCAGCGGGCGCCCCCTCGGGAACCACA<br>TTTGTTTCCCGCTGGTCTCGAGCGTCCAGAGCGCGAAGCGGCGGA<br>GCACGGCGAACCTACGATTCGTGATGCTTGGCCTAATCCTCTGCCC

	TTCATGGGTGCGGGCAGGAACGGAGTAGCGGCTGATGTCCCCTCC<br>CTAATCCCAAAGCCCCACAAAAGCCAAAATACACACGCAAACTCT<br>CACCAGCGCATCTCACAAACACAAAAACTAAAGACAACACATGAA<br>AATATAACCCGATCCCCCACCAAAACGTACAGTTCAAAAAAATAAA<br>ATTCAAATCGAAGGAAACAAAACACACACAACACGAAATTCCCTA<br>AGGCAACACACACACAGACGACACCCACTCACACCAAATCAAAAC<br>ACATCTAAACACCGCGGAGAAGGAAAAGCCTTACAAACGACACAC<br>AAAAAGACAGCCTACAAGGACAAAGTACAAAATTACACAAAATCA<br>AAACTCACCCCAAAGACCAGGACTTACAGAGCAAAACCCGCTAAA<br>ATCAATCCCACGAAAAAACAACACGCAAAACACCCGAAACAAAA<br>GAATATCCCATGGAAAAATGAAAACTCACAGAAAACAAACAAAAA<br>CAAAACAAGCCCTCTAAATGGAAACAACAACAACAACAAAACAC<br>CAACACCCCCCGCCCCCAGCCCGGGGTGAAATGTCAATTCCGGGA<br>GGGCTTTTGATCTCCAGGCGCGGACAGGCCGCTCCTCCCAGGCGCT<br>GCCTCCCGGCGCGGGCGAGGCGGGAGGGCGGGAAGCCTGGGCTG<br>GGGCTGGGCTGCGGAGGGAAAGAGTCGAGCACTCGGAGATGGCC<br>AAAAATCAAATCTCGGGGTAGGGACACGGGCCCGCAGGCTGCAGG<br>GAGGAGGCGGCGCTGGGGAACTGCGCAGACCACTTGGTGGGGGG<br>GCCGCCCGGGAATCCACAGCCACATTTCCGATTGTGGCGAAATCTG<br>CTCAGTGGATATGTGATGTCCAGTTGCCGCCGGGGGCCCCCCTTTC<br>CTCGAGCTCTAGGCTCTTCCCCCTAGGTTGCGACCCAGCCTCGTGA<br>CCACCCCCTCCAAAAAACAAACAACACTCTTGCTGAGGACGATTC<br>ACTCTCCAAAACTGCCATTGTCCGGCGGGCCAGGAGTCCTCCTACG<br>GAGCGCTCCCCCGCCCTGGAGAACCTTTCCCCTGCGACCACCCCG<br>CTGCAGGGCCCCACAGGCTTTCAGTGGCTAGGCCCAGCAGCTGAA<br>CTAACTCAAGGCTGGGGAGGGGAACAGGGAGGGGAGGCTTGAGT<br>CTGGCCCGAAGAGAGAGGGCTGGACATGCCACACCTCTGCTCGGT<br>CTCTGTGGATCTGATTTCCTCTCTGGAATCAAGTCCTGGGGCTCTGG<br>GACTCCACAACGTCTCAGGGCTCGAGGGCAATGCGATTCCACTTAT<br>GGGCCGGGGTAAGGTGTCTGGAACTCCCGCCAATCTCCAGAAACT<br>ACTGAGATGTTCTGCTCTGCCCGGGTGCTCCAAAGCGGAAGCCCG<br>GATTTCATCTCCAGTATTTCACAGCTTAACCTCCATGCTCTGGGCCA<br>GAATGGAACTTGATGTGGGGAGGGGTTCTGGGACTAGAAGACTGG<br>ATGGGACCTGGGGGCTTTAGGGATCTGGCGCTGCCCTAGGGTCAGG<br>GGCCCAGCAATAGTCAGGACCCCAACAAATTGGGGTGGGGGTTGG<br>ATGGCTGGGAACCCTGGGGTCGGGCGCCGCCCTAGGGGTGAATCG<br>GAGTAAACCGGAACGTCGCCTCGGTGCCTGGCAACACTGCCAGCC<br>TTTGCAACCCCTTTCAAAAGCACCTGCTTGGCCGAGCAGTTAATTT<br>CTTCAAAAACAAAAACCCGGCCTGCGCTCGTCTAAGCAGCGGGGT<br>TTGCACATGGAGATGTCACAGGCCCCGCGCACAGCGCAGAGGGCC<br>GCGACCCCCAAGCGCATGTCTTAATAGAAGGTGCGGCTGGAAGAC<br>CCGGGCTCCCGGGCTCCGCTTCGGTCTGCCCCTTCCCGTAGGTGCG<br>CTGGCTAGCGCCCGGCGCAGGCTGAAGCCTTCCTTCCCTCCCCCCA<br>ACCCCTATAAAAGTCTGGGGCGGCGCGGCAGCAGCACTGCTGCTC<br>TCCCGGCTTCCC
PCNA	AATAAAAGGAGGCTTGGAGAATCAATGCACATAAGTCAGAATTATA

	GAATGCACAGCATGATTACTAGGATGGATAACATGTGGTCTGAACA<br>AACTTCTCATCCATTACACTCAGCCCTCTCAGGTTAATGGAACTCAG<br>ATCAAGTATATTCCTGAAGTCAGTACTGTGTGACGAACAGGGGTCT<br>GGTGGAAGTGATGCTAATAGGGCTTTAGGCCAGCTTTAGAGCCCTT<br>CTCTCTCTCTCTTTTCGTTTTTTGTTTTCTTTTTGTTTGTTTTTTGTTT<br>TTTTTTTTTAGACAGTTTCCCTCTTGTTGCTCAGGCTGGAGTGCAAT<br>GGTGTGATCTCGGCTCACTGCAACCTCCGCCTCCCAGGTTCAAGCA<br>ATTCTCCTGTCTCAGCCTCCCGAGTAGCTGGGACTACAGGCGCCCG<br>CCACCACAGCTGGCTAATTTTTGTATTTTTAGTAGAGACAGGGGTTT<br>TAGGTCAGGCTGGTTTTGAATTCTTGACCTCAGGTGATCCGCCCAC<br>CTGGGCCTCCCAAAGTGCTGGGATTACAGGCATCAGCCACTGCACC<br>TGGCTCAAGAAACATTCTTAAGCATGCTTGATCTTGGTAAATGCCCC<br>TGATTTCTTCTGTAGGAAAAAACCCTAGAGATCGAATTTCTGTGTC<br>AAAGAATAAGTAAAATTCTAAGGCTTTTGATACACATTTCTAAGTTT<br>CCACCAGAAAAATTATACCAATGTATACTCCCACTTGCAATGTATTT<br>AAGTGCCTACTTCCTTGAATCCTTCCTAACACACCAGGTGTTAACAT<br>GATTTTTATTAATTTGGTAGGTTAAAATTCTGTCTCCATTGAATTTAC<br>ATTTCTTTGCTTATTAATGTGGCTAACAAACTTCACGTTTATGAAGA<br>CTAGGACTGGACAGTGGAGGCCAGATACCTCTGGCTTTTTGTTTAG<br>ATGAATAAATTTTCTATTTCTCTTAATCCATTCAAGTTGAGTTCTCTG<br>TCACTGAAACCTAAACTATACTAACCAATATAATCCTTTGAGGCATA<br>CTGAAGTCTTAAAACTTGTATGGTTATTCATTATGTTTTATGGGTTTT<br>TTTCTTATGTTTGGAAGCCCATAGGAAGATCTCATAAATATTTACCA<br>AAATATTTTTCCATTTCTTATTGGGTCAAGATTTGAATTTGAATTCTT<br>TATCTAGAATTTAGTGAATAATTATTAGGAAAAGTCTTTTTTTTTTT<br>TTTGAGATGGAGGTTTGCTCTGTCACCCAGGCTGGAGTACAGTGGC<br>GCAATCTTGGTTCACTGCAACCTCTGCCTCCCGGGTTCAAGCAATT<br>CTCCTGCGTCAGCCTCCCTAGTATCTGGAATTACAGGTGTGCACCA<br>CCACGCCCAGCTAATTTTTGTATTTTTAGTAGAGACGGGGTTTCACC<br>ATGTTGGCCTGGCTGGTCTCGAACTCCTGACCTCAGATGATCCACC<br>TACCTCAGCCTCCCAAAGTTCTGGGATTACAGGCATGAGCCACTGC<br>GCCCATCCAGAAAAGTCTACAGCTTTTATTCTTTTCTAATAGTTTAA<br>CCAATTATCTGATCAAAATTTACTTCATTCTTTCTCCCACTGATCCAA<br>TAGTACCTTTACTAGATGTTAGAGTAGCCACTTAAAATGTAAATTGG<br>ATCACATCAAAGATCTGCTGTTGACCACATTCCCTTGCACTTTTAGA<br>GAGCCTACAAAGTCCTGCAAGAGCTCACCCCAGCCTCTCTGCAAC<br>CTCACCTTGCACCACTCTTGCTTCAGACACATCAGTCTGAGTTCTA<br>CATCAAACTTGTCTGAATATATTCCAAGTATGCTGACCTTTATAATCA<br>TGCTATTCTCTCAGCCTAGAATGCTTTTTTCCAACTCTCCAAGCAGC<br>TGTCTTCTCTGATAAGATGCTACCTCCTCAGAGAGGCCTTCTCTGAC<br>CACAGTCATAGGCATGTTTATGTCCTCATTTCAGTTTAACATAAAATT<br>TACGTAACTGTACACCTCTTTATCATTTGACACTTTTAGGAGGACAT<br>CACTTCTGTAATAGAGCAGAAATAAACAGATTGATGTACATGGTGG<br>ATCCCCCATGGATAGAGTTTGTCATTTAAAATGTCTTCAAATCTATTA<br>CATGTAATTTGGCATTAAACCAAAATGTTATTATGTAAGCAGACAGA<br>ACTGAAACAGAGTAGTGGAGCATATGCATTTGTTTTTAGTGAAGCA

	AATGTTCCTCACTGGAGGAAGGAACACATTCCACATTTTGCAAAGG<br>AACTGAGATTTTAACGGACCCAAGAAAGGAAAAGAACCACAAAC<br>AGATGATGCTGTATTATGTTTTGATATTGAGATGTATGCAAATGGGTA<br>GTCCATCACACATAAAGCAAGAAAGTGCCAACATCAAAACTTACA<br>AATGAGTAGCAGAGGCTTGCAAGAAATTCCAGAGACAATAGAATA<br>GCACTATTGAAAAATACTGCATCATCAATACCCTTGACAACACAGA<br>GTAGGATATTATACAGAAAAACAGCAGAATCAGCAACTAACTGAGT<br>GGAGAAACAGTAGGAATGCCTTAACCAATGTAGCTTAAATTTTTTC<br>ATGTTTGCACAAGAGTAATATATAATAAGAATGTTTTTAAAGTCTAA<br>AAGTTCTTTTAAAACAAAATAGACCCGGGCGCGGTGGCTCATGCCT<br>GTAATCCCAGCACTTTGGGAGGCCAAGGCAGGCAGATCACATGAG<br>GTCAGGAGTTCAAGACCAACCTGGCCAACATGGCAAAACCCCGTC<br>TCTACTAAAAATACAAAAATTAGCCAGGTGTGATGGCGCATGCCTG<br>TAATCCTAGCTATTCAGGAGGCTGAGGCATGAGAATTGCTTGAACC<br>CAGGAGGCGGAGATTGCGGTTAGCCAAGATCACACCACTGCACTC<br>CAGCCTGGGTGATAGAGTGAGACTCTGTCTCAGAAAAAAAAAAAAA<br>GAAAAAGAAAAAGAAAAAAAAAAAAGAAATTCTAAGTGATAAAGC<br>ATTGTGTAACAGTTTGAGGCTGGGCACAGTGGCTCATGCCTGTAAT<br>CTCACACTTTGGGAGACCGATTGGGAAGATTGCTTGAGCCCAGGA<br>GTTGGAGACCAGCCAGAGCGACATAGTGGGACCTTCTCTCTACAA<br>AAACATTTAAAAATTAGCCAGGTACAGTGGTGCACCCCTGTAGTCC<br>CAGCTATTTGGGAGGTGGCAAGAGGATCTCTCGAGCCTAGAAGTTG<br>GAGGTGGCAGTGAGTAGTCATCTTGCCACTGCACTCCAGCCTGGGT<br>GTCAAAGGGAGACTCCCCTCTCTAGAAAATAATAAAAAAAAAAAGT<br>TTGACAGTTTTCTTGCTTGTGCTACATAAAATAATGGTACATATTACA<br>ATTGAAGGCATCTTATAATAGAAATGCCCTTTCCTGTTCCCTTTTAA<br>GGCTGACCCAGTGCTTTAAGAGGCTGATACAGAAGGGTAAAGTTA<br>AGTCTCCACAAAATCCAGGGAAGAGACTGTGAAACTCATCTTAGA<br>ACCTTGTATGGAGTCACAGCTGAACTAGGAAAAAAAAAAAAAAAA<br>AAAAAAGGAAAGAAAGGCATTCCAGAGATAAAGAATAGACTAAGG<br>AGAGGAGATATTTGAAGCCATAATGATTGAGAATATGTCAGACTTG<br>AAATGAATCTTCAGTTCCCTCAACAAGACAAATAAAACTAACTCCA<br>TACCTAGATACATGGTAGAAAAACTAAAGAATTCTGCTGACCAAGG<br>TATTAAAAGTAACTAAAGAGAAGTGGTGTGAAGAAAGCAAGAGAG<br>AAACAACAAATCCTGTCCATCCTGTAACAATTGAAAATTTCTGGCT<br>GGGCGTGGTGGCTCACGCCTGTAATCCCAGCACTTTGAGAGGCCG<br>AGGCAGGTGGATCACCTCAGGTCAGGTGTTCAAGACCAGCCTGGC<br>CAACATGGTGAAACCCCGTCTCTACTAAAAAAAAATAATAATAATAA<br>TACAAAAATTAGCCGGGGGTGGTGGTAGGCACCTGTAATCCCAGAT<br>ACTCGGGAGGCTGAGGCAGGAGACTCACTTGAACCTGGGAGGCG<br>GAGGTTGCAATGAGCTGAGATCGCGCCACTGTACTCCAGCCTGGAT<br>GACAGAGCAGGACTCCATCTCAAAAAGAAAGGAAAAGAAAAGAA<br>AAAATATTAAATGTGTACGCTCTTTGACTCAGCTGTATTACTTCAAG<br>GAGTTGATATCACCAAAATTGCCTAAGTGCTCAAAGGTGTTTGTAG<br>TTAAACAACAGGAGATTGATAAATTATGTTATATACATGTGATGCTAT<br>GTTTTAAAGAGGTACTGATATGATAAAAGATGTACGTGGCATAAAAT

	TAAATGTACTTTATTAAGTACTTTTCCAAGTGTTTACGGAATGAGTG<br>CATTTTTGAAAAAAAAAAAAGTGTATTCGAACTTTTAAAAAAGCTTT<br>AAAAGCTTTATACAATGAACGATTGAGTGATTATAAGAGCTGGCGG<br>GGGAATGTTAAGAGGATGATAGGGAGCTAAGTTTAACAGAACAATT<br>CACCTCTTTATCTTGTGACACCTACGAGCGCATCAATTCTGTAATTG<br>AAAAATAAAGTGCATATTTGCAGCAGCTGTACTCTCTTCAGGCTGC<br>AAGGAGGCTTTTCCTCCCGGTAGGCTTGATTTGCATTTCACTTTCAC<br>TTTCGTGGCTGGAAACTTTCTACCCACGTAGTGGGAGGCTGAGGA<br>GCCACCATAAAGCTGGGGCTTGACGAGCCGGGACCGGGACCCGAT<br>CTCCACATATGCCCGGACTTGTTCTGCGGCCGGGTTCAGGAGTCAA<br>AGAGGCGGGGAGACCTGCGCGACGCTGCCCCGCCCTGCGCCCGCT<br>TCCTCCAATGTATGCTCTAGGGGGCGGGCCTCGCGGGGAGCATGGA<br>CACGATTGGCCCTAAAGTCTTCCCCGCAAGGCCGTGGGCTGGACA<br>GCGTGGTGACGTCGCAACGCGGCGCAGGGTGAGAGCGCGCGCTTG<br>CGGACGCGGCGGCATTAAACGGTTGCAGGCGTAGCAGAGTGGTCG<br>TTGTCTTTCTAGGTCTCAGCCGGTCGTCGCGACGT
PDGFB	TGGCACTCACAGGCCAGTGGCCAGAGCCTCATTAGGGTGAGAAGG<br>TCTGAGAGAAGTGGCGGGAGTTCTATGTAAGAACAACCTCCACCC<br>TCTCCAGGTGAAGACCTGTGGCCCCTCTCCTAGCGCTGGCTCTGGA<br>GCTCAGAGGTGAATTTGCAAGTGAGGTAGGGGTGTGTGTGTGTGT<br>GTGTGTGTGTGTGTGTGTGTGTGTGCGCGCGCGCGCAGAGCCTGC<br>AGTAATAACCATCACTGTGATGACCAACATCTGTGGGGTGCTCACT<br>CCAGGCTTTATCACTTCAGGTGAATTAACTCATTTAACCCAGTGAG<br>GTAGGGACTATTGTCCCTACGACTTACAGAGGAGGAACGTGAGGCT<br>CAGGAAGGTTAAGGAATTTGCTTAAGGTCACAGAGTTAGTAGATGG<br>TAGGGCAGGGCTTTGAACCCAGGTGGTCTTTGACGGGAAGCCTAG<br>TCCCTTGCCCTCTCTCAGCCTCACTTTCCTCATCTGTACAATGGGCA<br>TATACGATGGCAATGCCTTCTCCCTGGGCGACTGTGAGGGTGAGGC<br>AGGCAGAGGGGAAGGAAGTGCTTTGTGGACAGCCAAAGGCCCTG<br>TAGACAGGGAGGCAGGGGATGGGGCAGCAAGGGGCATGGGGGAG<br>CTGATGAGGGCCACGCGGAGTGGGGTAGGGCTTGGAGTTTCAGAC<br>CATTTGTGGCATGGTCTTGGACGGTCTCCAGCTTAGAGCCTCACTT<br>CCTCTCCATCAAGTATGGAGAAGCAGAGCTGCCTGGCCCAGGTGTT<br>GCTGGCCCCTCTCAGTGGCGGGGAGGCTCCCAGTGATCATCGCTCT<br>CACCTTTCTGGACTTGAGGGCGTGAGTTTGCCCCCGGGATGGAACC<br>CAACTCCTTCCTTGGTATTTCTTCACCCCAGCCAGTGTCCTGGACCC<br>CCACGCCCAGGGTGTAAGATGCTCTTCATGGATGAGGGCTTGGATA<br>GCTCTGAGCACACAGTCTAAAAAGCCCCCAACCTTGGGCAGCACC<br>CCCATCCCCCACGGCTGTGGCCACTCTTTTTCCCTTTGGCTCAGATC<br>TGTCCTGGAGTGGAGGGTGGAGAGTTACGGGGGTGTGGGCGCCCC<br>CATCCCTAGCTCTGCTGGAGAACCTTATTTCCCAGTGGCCTGAACG<br>CGAAGATGGGCGGGGGCCTTCCCAGTTCTCTCATTCAAGTGCTCCT<br>CAGACCCAGCCCTGATGAAGTTCCGAGGTCAGATGAGACCACGTG<br>CATTCAGGGTGGTGTGGCTTCAGACCCTTCCCAGTTCTGATACCTC<br>CAGAAGGGGTGCCCTTCAGGGGCCCTGTGCTTCCAAGCTTAGAGG<br>CTCATGCTACAAGCCCCTCCCCAACTCTTTCCACCCTTTAGGAAGG

	GCCTGGCATCTCTGCAATAACTGGTCAGGACTCAGAAAGAGAAATT<br>GGCAGGATTTTAATCATTTATTCATTCCTTCAGCGAGCGGCACTTCC<br>TGAGCAGCCACTGTATGCTAGGAGCTGGCAGGAGGCCCTTGAACA<br>CTCTGGACTCAGTCTAGTGCTAGATTGATGATCAGGCCATGACGAT<br>GCCAGGTGTGCATGCTGTGGAGCCTGGAACAAGTGCCAAGCCTGG<br>TGGGCCTTCTGGAGGAGGTGCCTGCTACACCCAGCCTGGCAGGAG<br>GCAGACGATCAGGTGTTATTAAAAAAAAATTTTTAGGCTGGGTGTG<br>GTGGTTCATGCCTGTAATCCCAGCACTTCGGGAGGCTGAGTGGGGC<br>AGATCACTTGAGGTCAGGAGATCGAGAGCACCCTGGTCAACATGG<br>TGAAACCGTCTCTACTAAAAATACAAAAATTAGCTGGGCATGGTGG<br>CAGGCGCCTGTGGTCTCAGCTACTCGGGAGGCTGAGGCAGGAGAA<br>TCGCTTGAACCTGGGAGGCAGAGGTTGCAGTGAGAAGAGATCGCA<br>CCACGGCACTCCAGCCTCGGCGACAGACCGAGACCCCGTCTCTGA<br>GATGGCTCACTGCAGTCTTGAACTCCTGGGCTCAGGGGATTCTCCC<br>TCCTGACTCAGCCTCCTGAGTAGCTGGGGCCACACTCAGATATATAT<br>ATATATATATATATATATATATATATATATATATATATATATATTTTTTTTTTT<br>TTTTTTTTTTTTTTTGGTAATTTTTTTTAAGAGATGGATCTCGCTAT<br>GTTGCTGAGGCTGGTCTCGAACTCCTGGCTGCACATGATCCTCTGG<br>CCTCAGCCTCCCAAATTGCTGGGAATACAGGGGTGAGCCACCGTG<br>CCTGGGGAGGTGTTCTTTACAGACTGTTCCTCTGCACAGAGAAGGT<br>ACAAGAAAGGAGCCCTTAGCACAACATTCAGGCCCAGGTGTCACG<br>CAGGTGTCACGTAGCATTCAGGCCCAGGTGTCTGCTGGGCCCTCTT<br>CTGCCACTTCTAACCCATGTACTGGCACCTGCACTGCAATGATTTGG<br>GTCTATCTCACTGAGAGCATGGGAAGTCCCTGAGTGCCGAGGCTG<br>GGACTCCTTCCTCATTCCATTTCTCAGCGCTTCACACAGGGTCTGC<br>AAACAGTAGGCGTTCTACGATGCTGCTCTGAGTAGCCTTCCCTCTC<br>CCTCAACTTGTACCTAAGTCAGAGTTCATATGGGTCCATCACTTTTT<br>AGCTGGCAGAGCAGGTTCCCACATATTAATGCATGTGGCGCTCACC<br>AAAGCGCTGTGTCAAAAGTGTCATCATTGTCATCAGCATCACCTGC<br>CAGAAAATTGCACAGCACGGTGGTCTCAGCATGTGGGCTCTGTAG<br>CTGGACTGCTCAGATTCAAGTCCTGGCCTTACCACTTACGGGCTGT<br>GTGACCTTGAACAAGTGTATTGGCCTCTCTGTGCTTCCATTTCCCCA<br>TCTGTAAGATAGGAATAACACATCTCACAGGAGCATGGAGAGGCTA<br>CAGGTAGAGTAAGAAAAACACTAGCTACCACATAGTCAGCTCACTA<br>ATCATGGTAGTTGTTACCATTCTATTTATAGAAGACACAGAAGGTCA<br>AGGTCAAATGACCCAGCTGTCCGGTGGAAGAGGGTTTTTATCTCTG<br>AGTCGTTAGACTTCAAATCTAGTGTTTGGGGGTTATCTCCTCGTCCC<br>GCTGCCCCATCTCCCTTGTCACCCTGTCTCCTAATTTATCTGTTTATA<br>CATCTGGTTGTCCTTCTCAATGGGGCTCTTCAAAGGCAGGGGCCTG<br>CATTAGGCTGGTTCACACCTGTCTCCCCAGTGCCTGGAGCAGTGCG<br>GGGCCACCAGTGAACTTGAAGGCTGCATGGAAGGTTCCAGGCGCT<br>TGCTTCCTGCTAAGGTGTTTTGCTGAGCGCCTCCACATGGAGGACC<br>TCATGGGAGCCTCCAGACCACTCCAGGAGCAGGGTAAAAATTCTCT<br>ACAACTGCACTGAGGTACAACAAACTGCACACATTTTGTTGTGATG<br>TACGTATACACCCATAAGCCCATCACCACAATCAAAAATGACAAAC<br>ATTTCCATCACCACTAAAATTTTGCCATGCCTCTCTGTTCTCTCTCA

	CTCCTTCCCATTATAGATTTATTTTTATTTCTTTAGAGATAGGGTCTTG CTCTGTTGCCCAGGCTGGAGTGCAGTGGCACAGTCACAACTCACT GCAGCCTCAAACTCCTGGGCTCAAAACGATCCACAGTCTCCTGAGT AGCTGGGACTACAGGAGCTTGTTACCACACCCAGCTCCAGTTTATA AATTCATCTCCAGTTTATAAAGGAGGAAACCGAGGTACTGAGAGGT TAAAAAACCTTCCTGCAGACACTTGTCCAGCAAGTGGCCACTCCA GGATTTGGACCAAGGTGATGTGTCTTCAGGCTGTGTCTCTGCCACT GTGCCACGCTGCTGGGTGGTAGGCAGCAGTGGGTGGGTGCCTGCA GTGGTCTGTAAAGACCACCTGAGATGTCCTTCCTCCTCTGTTCCAC CCTGTCCAGGTCCAAGAAGACAGTCTATGAAGAGAGAGCAGGTGT GACTCTCTCAGTGTGCTCCTCTGTGAGAAGCAGGCTGACATCCCAA AGGGAAGGGCGGATAACAGAGACAGTGCAAGCGGAGGAGATGAG GGTGCCTCAAAGCCGGGAGGCTGGGTGATGCAGGAGCCTGCGTGT CCCGAGGGGGGTGCTGGGCCCAGTGTGAGTACGTGTGACTGTGAC TGAGACAGTGTGACTGCTGAAGGCAGGGACACAGCAGCTCCCTGA CTGGGGGCAGAAGGCGTTAACTGTGTGAAGGCTGGTTGTGGGTGG GTGGGCTCTGGGCCTCGAACCCGGGGGCTGAGGGAGATAGTAAAC AGCAGGGTGACTGACGGGAAGATCATGTTGGTAGCCCTGCGAAGA TGCTGCAGGGCTGTGGGGGTTTGTGTGACTTTGCAGTTCAACAAAT TCAAATTCAGCCAACGCTGGCAGGGCCTGTTGTGCCAGGCAACCA GCTAGGAGGAGGAGACTCGGACCCAGCTTGCAGCTGAAGGGCGCT GGCTGCCGGGTTCTGTGGGTTCACCTTGCGGTGTCTTCCCTTGCTA ACACTGAGTCCTTACAATAGCCCCATCTCCAGGTTGAGGCTAGATG GAGGGGACAGAGGGAAGTGACTTGCCCAAGGTGACCCAAGCTCC CGAGTGCCAGGGCAGGATCTGAATTCAGGCTCTCAGACTGCAGAG CCTGAGTCCCTCCCTGCCATGCCTGTGCCAGGGTGGAAATGTCTGG TCCTGGAGGGGAGCGTGGACTCCTGGCCTTGGCTCTGGAGACATC CCCCTAGACCACGTGGGCTCCTAACCTGTCCATGGTCACTGTGCTG AGGGGCGGGACGGTGGGTCACCCCTAGTTCTTTTTTCCCCAGGGCC AGATTCATGGACTGAAGGGTTGCTCGGCTCTCAGAGACCCCCTAAG CGCCCCGCCCTGGCCCCAAGCCCTCCCCCAGCTCCCGCGTCCCCCC CCTCCTGGCGCTGACTCCGGGCCAGAAGAGGAAAGGCTGTCTCCA CCCACCTCTCGCACTCTCCCTTCTCCTTTATAAAGGCCGGAACAGC TGAAAGGGTGGCAACTTCTCCTCCTGCAGCC
PDK4	GACAAACCCGTCACTTGCACTACTGGTCTTTCCTTAGTCAACCTATC CTTTTCTCCTGACGCTTTTGACTTCCCTGCTTCTATCAATAAAATCA CCTCCTCCCAGCAGCACAGGTTTAGAATATTAGAGAACTTTAAAAA ATTATTCATTAACAGATTTGACAAAGCCTAGAGATACTACCAACAGA TGGCTCTTTTCGTTCCCTCTCCTCACTGCTCCAATGCCTTTCTTCTTT GTCTGCCTATGAATGTCTGCTCCACCTTTATAAGTCAGATGACCTAT GACTTCCTCCAAGGAAGCCTCCCCTGGCTGCTGCTTGTCCCCCCAG TACACATACCATATATGCCACATTCTATTTTTTAGATTATTTTCATGTC TGTCTCCCGTCTTTGAAGACAAACTATGCCTTCTTCATCTTTATGTC CTCAGCATTAATCACAGTGCCTAGTACATAGTAGGTGTTCAATAAAG TTTAGTAGAATTATTTAACTGCTAACAAATTTAAACAGAAAACACAT TGCTCCCTGATTTAAAGGCTTCAACAGTTCCCTGCTGCTTACTCTAG

	AAATCTCATAGACCTCATACTAGCATTTCAGGCTTTCATGAGATAAC<br>TGAATCTTCCTTTCCTTCTGCCCTTAGGGTACTCTAGACAGCAGCCA<br>GGTGAAACTAGTCATTATTTCTCCAATGTATCTCTAGCTCCTACATCT<br>GTGCTTTTACTCAGTCTTGGACATTTTTCCCCACTGTCTACCAATTAT<br>TTGCCAAAATCTTGCTCATCTTTCTACAGCCATCCCAAATGCCAATT<br>CTAGTAGCATTTCCTGATCCCCAACATAGCTCTTCCTCCAAACACCC<br>CAGATCACCCTTAACATATCCACTGTCTGGCACCATCACTACTACCA<br>CCAATACCACCACAACCAGCATTGCCCATGGCGTGAACCTTTCTTAT<br>GGGCTGCTTTTTAAATGCATTTAATTATAGCATTTTGCCCTGAGGATT<br>AAATGAAAATCTAATAAATTAAAAATAATTAGTTATTGTTTTCCATCA<br>TCAGCTAATAATCTGAGGTGCATGAGAGCAGACGCTTTTGCACCCA<br>TCAGGGCACCCAGTCCAGCTCCTGGCACCTAGTATCCTCTAGGTTA<br>ACAGTAACTATAAGAAACATCAGAGACCAAATTGTGGGTTAGATCC<br>TGCTTTTCCCAGTAGCTTCACCAATATGCTTTCACCAGTATTCCCTC<br>TCATCCCCAATAGAAGCTCATTCCATTTCTTTTAATTGTCGTTTACTC<br>AAGAAGACCTTCCTTGACCACCAAATCTAAGGTAGTTCCAGCCTAT<br>TACACTCTTCACAATCATTTTCTTCTTAGCCCTTGTCCTATCTTAATG<br>TATAGGCTTATTGGCCATATGCTTTTGTAATGCCAATTTCCCCCACAA<br>GTCTATCATCTCCATGAGGGAATAGATCATGACCGAATGGTTCCCTA<br>CTGAAACAGCCATCTCTTAAATCATCATTTGTCTATTACGTATTCTGT<br>AGATATGTTCTCCAAGTCTGCTGTTCATATTTTAACTTTAATATTCTAT<br>GGTCAAGTCTGTTAATCTTTCCATCATGTCTTCAGAGTTCCTGTTAC<br>GGGCATTTTAAATTGCTTCCTTGGAATACGATCCTGAGGTGGAAAA<br>ATTGTATAGACTTCCCTTTAAGGGATCTGATGGATTTTATCAAATTGG<br>CCATCCCCCACCCTCTGGAAAAAGTGTGACTTGCATTCTCAAAGTT<br>CTACCCGTGGCCTTTTGTTATTAGATTTTTTCCAGGCAAGCTGGACA<br>GAACTTATTTAATGCTAGGTAAGGTGCCACTGAAAATTTTAGGAAC<br>CAGGTATCTCAAGCAGGGATGTTCAGCATTGTATGCAAGGGAACAG<br>CTTTGATTACTTCTTCTATTCCTTAGCATTAGTCATTTAATAGTCTTCT<br>CATGGTTTACTTTTACTCAAATGAATGCATGGATTTGTCATATCCATT<br>GCCCCCAAATAATATTTTTGCTTCACCTCTGTCCCAACTGGATCACC<br>TCCAAGATTTGTGTTCAACCTGAGAAGAGAAGTGCCACTCTTTTCC<br>CAGGTCACTCTCTAATCATGGTAATACATAGCATCTTAGCCAACTGG<br>GTAAACAACTTGACCTGACCTTTCTAGGCCAGCTATCATGAATGCT<br>GCCAATTATAATAACAGTTCCCATCATACAGAAATGACACAGAAGA<br>ATGCAATGCACTGTGAGAGTAACTTCATAAATATTATTAGAAGGGAT<br>GACATAGAGACAAAATTTTGGCAACCTCAGTTCACATTTAGCCCTA<br>GGTCATCTCTGTGAACATCATATGATTTCACCTGATCTTTAATCAGCC<br>CACAGCACATAGAATAGTAGTGCTGGGTAAGCTAGGCTGGGCAATC<br>AATGTACTTAGGGACATGGGACTTTCCTAAATTAGACTTGCCTGTAA<br>TGCATAATTTCCATTGCCTTTCCTCTGAAAATCAGAGTATAATTCTTT<br>GTAATATATATTTTTGCTTATTCCAGGTTCCATATACAGTGAGAAAAG<br>CAAATGCATGCTTTACTTTTACTTATGTTCTTAAGACCCAATCTAAG<br>AAAAAGAAGGGATTTTTACCTTAAATTTACTTGCTTCATCAAAGAG<br>AGTCTTATTCAGTTTTTTGGCTACTTGAACACATTTCGTTGCTGCAT<br>CAAGCTATGCAAATATTTAATAAAGGATTCCAAGTACAAAAATATAA

	ATAATAGTGGCATCAAAACCTGTCTACAAGTGGAGATGCAATTTGG<br>AACTTGCCCATATTCCCACAGCTGTTTCAGCTTAAGATAAGTGCTTG<br>AGCTGTTGTGTTTTTGGTTTTTTTGTTTTTCTGTCCCACTCAAGTGC<br>AACTGCTAGAGTCTATACTTGGATCATCCTTGAATATTCTCTAAGAA<br>CCCAGGCCCCATCCTCTTCATTGGAAGATCATAAATATTGCATGTCT<br>TCACATAGCTGTTTCTCAAAGGTCTGAAAAAAATATTTGGTCATAAC<br>AGGAAGTCTCAAATTCTCGACCATCTATAGGTAATGTATAGACAATG<br>GGAGAAATAGTAGGAGAGCTAATATGGTGCCTCCAAACAGGCCACA<br>TAACTGACACAGGGTTTGCCAGGACTGTTTCAGATCCCTCATAATG<br>GTGTTGATTTTAGAGAAGCAAAATTGATACCTATTAGACATCCAAAT<br>GGAGAAACCAAATAGGCAATTGAATAGGAGAATCTGGCTCTTGGA<br>AGAAAGTTTGTGCTAGAGATAGAATTGGAAATCTGTCCTGTGATTT<br>AATGCAGTCTCCTAGACGAAGAGGGCAAAGGATGACAGCAGAGCC<br>TAGGATCACTCATGACTTTCAGAGTTGCCCGTTCTGCTTATAAATGC<br>AGGGCAGGCTGGGCGCAATGGCTCACTCCTGTAATCCCAGCACTAT<br>GGGAGACCGAGGTGGGTGGATCACTTGAGGTCAGGAGTTTGAGAC<br>CAGCCTGGCCAACATGGAGAAACCCCATCTTCACCAAAAATAGAA<br>AAAATTAGCTGAGTGTGGTGGCGTGTGCCTGTGATCCCAGCTACTC<br>AGAAGACTGAGGCAGCAGAATCGCTTGAACCTGGGAGGCCGAGG<br>TTGCAGTGAGCCGAGATGGCACCACTGCACTCCAGCCTGGGAGAC<br>AGTGTGACTCTGTCTCAATAAATAAAGTAATTAATTAATTAATTAAA<br>ATAAAATAAACGCAGGGGGAGGAGGCCAAAAAGGTTTTTTATAAC<br>AAACTATCATTTTTAACACCTCATAAATTCCCATTCTATTACATTTGT<br>AACCAACTCTCTTACTCCCAACTTTCCCAAACACGGTGGCTTCATT<br>CCCTAGTCCTTTTAGAGTGTCGGGAGAGGAAAAGGAGGTTAGTATG<br>ACAGGGTAATGTGTCTCAACCACTGTCATTAATTTAATAAATATTGA<br>GTAGTGAAAAAGTAGGAATCACAAAGCTGCATCAGACAGGGTCCC<br>TACCCTGATCTCTTTCAGGAAAGGGGGGAGTCAGAGAGGGATAATA<br>TAAAAACATAAATAGCTATAGAATAGTACCATGAAAGACCAAGGCC<br>TTATTTGGAAGAAGTGAGGAGAAGTAATCAAGGAAGACCTTTTGC<br>AGCAGACAGCTTGTGCCAGACATTTGAGGGTAGAATGGAATGTCTT<br>AGGTAAAGATTTGGAGTAAGGACATTTCGTACAGGGCAGAGTGAA<br>CAAATTCTCTGATCACCGCAAAAGGTAAGGCAAACTGACAAATTGA<br>TCCCTTTTTAATTTTGTTTTTAATTAACAGAACTAAACCTAACGGAA<br>AGAATGTGTTGCCGGAGAAGAACGAATTGATGGCCTTTTATTACCA<br>CAGGAAAAAGTAAATGTATCTGGGCAGCCCCTCAAGTCTCAGCAA<br>AAGGAACGTACAGTTCCTGCCATGTGCCGGTCTCTTCTGAAGAAA<br>AGGGGGCGGTGGGGGTGGGGGCGCAGGTCTTTAGGTTGTTTATTCC<br>TTTCTCTCTGTCCAGTGGCAGAGAAGCCCATACTTTTGAACGCAGC<br>TTGTTGGCTACTGTAAAAGCCCTGCTCTGAGCAAGGACCAATGAGC<br>ACGCGGAGTCCAAACTCTTGGAAACAGTTTGTGGCCAGGAGTACT<br>TGACATTGAGACAGCCTCCGAGTTGTAAACAAGGGCGAGCCTGGG<br>CGGGACCCCAGCCCACGCGACTCGGAGCCCCGTCCCAAGAGGCTA<br>ATCTTAAGCCCACGTTGCCCCAGATACCTGTTTCTGCTTCCTCTTCC<br>CTGTTCTTCCCACCCTTTTTCCGTCACAGCCGCGGGCACCGGTGCC<br>AGGGCACTCCGTGGTCACCGTGCCAGGCCATTCTGTGATGACCGCG

	GCTGGAGGGGCGGAGCCCATAGTTCTTTCTCTGATCTGATTGGCGC<br>GACCTGGAGTTCAGGACGCGTTTCCAAGTTCCAGTGACTCCTCCTG<br>TTTGGGACTCGGGGGGAGAGTGCGGGGAGACAAATAAAACCTCGG<br>GCGGCGGCGGCTGGTGGGAAGACTTGAACTTGAATCTCGAACCA
PGR	TGCTCCATGACCTAATCACTCCCCAACACCTCCTCCTACTAAGACTA<br>TCACATTGGTGATTGGGTTTTGACACACAGATTTGGGGAGAACACA<br>AACACTCATATCACAGCACCTACTAATTTATCCAGCCATTTCACTGC<br>TAGGTATTTACCCAGAAAAAATGAAAACACATGTTCATACAAAGAC<br>TTGTACAACATTTTCATAGTAGCTCTATTTATAATACCAAAAAAATTG<br>GAAACAACTCAGATATTTATCCACAGTTAAATGGATAAACAAACTAT<br>AGTATATAAATAAAAAGAATAAAAAGAATTAACTATTGATACATACT<br>ATAATTTAGCTGAATCTCAAAATAATTATGCTGAGTGAGAGAAACCA<br>AATGAAAAAAAGATACACTATATTATATACAATTCTAAGAAATAAAA<br>CCTAATTTATTTGGCACCCAGATCTTCATTTCTATACCAGTTTCAAAT<br>AAAAAGTCTCAAACTTCCTTGGATAAATGGCTGATTCTACGACTGG<br>GGCAGGAAATATACAAGATGAGTCTGATGAGTCTGTAGCATCTTGT<br>AATACCAGAAAGTAGGAAAGTCCACACATACACACACATACACAC<br>AATACAGAGAGTGGGTCAAGGAGCACAAGAGTCAACTGAAAAAG<br>CCCCCAGTGCCAAAAACTGGAAAAATTTGAGCAACAAAGTATTCA<br>TGAATCCATACAAAGAAAAGACTGAATAAAGAAGTAAATGGGGGA<br>GAAGAAGCAAATCTTCCTCACAGAATTCCAAATAAATTATGTAGATA<br>CTCCACTCCAAAGGAGGGCTAGCATAACTCCCTACTCCCCCACACT<br>TCCTTAAGTGACTCATAATGATTTCCTTCCAAAGAGTACAGAGTATG<br>CAAAGAGGGAAAAAAGAGTAACTATGCAATGGAGAAACCTGACA<br>AACACTACTTCAGCCAGGTGATGGAGAACAACATTAACAGTCATAA<br>GTCATGTTGACAATATGTACCCTTCATATGATGTAATAAATACAGTAC<br>ATTACCTCTTTGATTTCCACCCCGCCAAAACCCAAAACCCTAGTCTA<br>CTCATGAGGATACATCAAATCCCAATAGAAGGGCATCTTGCAATATA<br>CCTCACCAGTACTCCTAAGGACTGTCAAGGTCATCAAATACAAGGA<br>AAATCTGAGAAACTCTCACAGCCAAGAGGAGCACAAGACATCATA<br>ACAATTAAATGTGGTGTCCTGGATGGAATCCTGGAAAGGAAAAAG<br>GACATTAAGTGAAAATGAAGAAAATCTAAATAAACTATGAATATTAG<br>TTAATAACAATTTATCAGTATTAGTTCATTAATTGTAACAAATGTACC<br>CTATTAATTTAAAATGCTAACAGAGAAAATTGTACAAATGATATATG<br>AGCATTCTCTATATTATCTGCTCAATTTTTCTGTAAATCCAAAATCGT<br>TTCCCAAAAGTAAAATCTATTAATAAAACATCTAAAGCAAACAGAC<br>CTAAAAGGCATATGCAAATGTATAATTTAATGTTTAATAAATCAGATA<br>TAAATATTTTTAAAAACCTAATATTTGGTGACAGAAAGCAGATTAGT<br>GGCTTGCCAGAAAGTGGTGATTAGTACTAGGGGAAGGGGATGGGA<br>CAGAACAGTATGAAGGGATTTAAAAAATGGCATGAGGAAACTTTTG<br>GAGGCAAAGGGTATGTTTATCATTTTAGTTTCACAGGTCTAACAAC<br>AAATTATACAATTTAAATATATGCAGCTTATTATATGACAACTATATCT<br>TGATAAAGTGGTTTTTTTCCCCAAACTATAGAAAAAATACTTCCACA<br>TTTAAATCCTCTTCTCCAAAAACAGTCTTCAGGAATATTTGAAGTTA<br>GCCCCATCACACCTGAATTTAATCAGTGAATTTTGTTCATTCAAAGA<br>TTCATACATTTGGGAAGTTCCTGTGCCAAAAATGCTAATCACCCAG

	CATCAAAATTTATGGAATAATGGTGATTTAGGACATGCTGGACATCT<br>TTATTGTTGTTTTCCCACTTCAAGATTTTCAATCTTTTGGAAACAAC<br>CTCCCCAGACATTCTGACAAGGGAATTATTATAACCAACCAAAATC<br>AAGCCAAATATAGTTTGTCAGATTTAAAATGAGGCCAAGTGCCAAT<br>TTTACAGCAGCGACCGGAAAATCAAATTATATGGCATCTGACATTTA<br>AAATCACACTCCTAAGAAGGCTGTCATTTGATGTCTTGGGTTTGGG<br>TTACAGTACTTAACTTTATAATACTGGGTAACCTTGTGCCTCAGTTTT<br>CCCAACTATGAAATTGGGATAACAGTACCTATTTTGTGAGACTGTTG<br>AAGGATTACAGTTAACACAGGTAAAAGGCTTACAAGACTACCTGG<br>CAATGCTGTCCTGATTTTACAAATTCTATACACTGTGTGGCATTAGC<br>CATCAGGAAAATATAAATAAATCAGTGAGATACCACTTCACACACTT<br>GGGGGGCTATAACAAAAAAGACAGATAAAAAATGTTGTCAAAGAT<br>GTGAAGAAACTAGGACCCTTATTCACTGCTGGTGGAAATGTAAGAA<br>GTACAATCATTTGAACCATAGTCTGGCAGTTACTCCAAAGACCACA<br>CCTAGAGTTGCCATATGAACCAGCAATTCCACTCCTATGCATATATC<br>CAGGAGAAAGGAAAATATACGCTGCACAAAAACTTGTACATGAAT<br>GTTCATTAGCAGGATTTTTCATGGTAGCCAAAAGGTGTAAACAACC<br>CAAATGTCCAACAACTGATAGAGATGGATAAATAAAATGTGGTTTAT<br>CCATACAATGAAATATTATTTGTCAATAAAAAGGAGTGAAGGAGTG<br>AAGTACTGATTCATGTGGAACATGGATGAACCTTGATCCAAAATAG<br>CCAAATCACAGGTACAAAAAGCATATTAGTGGCTGCTGGGAGATTT<br>AGGCGGAAATGTGGAATAACTGCTAGTGGGTATTGAGATTTTAGAG<br>TCATACTCATGTTACAAAATTAATAGTGCTGATGGTTGCACAACTCT<br>GAGTACATGAAAAATCAATGAACTGATACTTTGAGTGAGCTGTATG<br>ATACTGGAATTACACCTCAATAAAGCATGGTAACTGTTTTAAGATAG<br>GCTGGAAAGAGAAAGCCTGAAAACAACAATAATGATATTAATAAAT<br>TAGTTTACTTCTCTAGTCTCATATACTTCTGTGCCCACACTTGCTCCT<br>GTTCTATTCATAATGGTCCCCTTGCAGTTGCCATATTATATCCTGCCA<br>TTTGATGCCCGGTGAACATTCTATACCTGCTTCCCAGAATTCTCTTT<br>ACCTTTCCTCTATCTGCCTAACTTCCACATATCTAAAATTAATCAGAG<br>TAAACTATTTACTAGAACAACCAACTCCAAATCCTAGTAACCTAACA<br>TGATAAAGGTTTGTTTCTCACTCATATAGCCCCTCCCCAGATGATCG<br>AGGGGTCCAGGCTCCTTACCTCTAGTGGCTCCCCCACCTTCTGGAG<br>TCTTCTGCATTCTTTATACATGGTTGAGATAAACTATGAGTCATTAGC<br>ACAGCTAGACCTTGAGGTCCTACAAGAAAATTTGCAAATCATTCAC<br>TCTGTTTTGAACAAGGTATATTTAAGATGATGTTAAAATACCCAATG<br>GTCTTGGGTCAAATACAGTTTATGACTGTGTATCTAAAATATATATTG<br>CAATATTCTTCCCTTTTTCTACTGACTTCATGAATTTAGCGGGGATCC<br>ATTTTATAAGCTCAAAGATAATTACTTTTCAGACTAAGAATATTTAGG<br>GTAAAAAGTACTGTTCAACATCTCTACTGAGGATGTTATGATGTAGC<br>ACACTGTATAAGCTGGAGCTAAAGGAAACTTTCCTTAAAGTGCTAT<br>TTACTAAAAATTGGAACACATTCCTTAAGACAAATCGAAGTGTGGC<br>ACACAACATCCAAACTTCCATCATAGATACAGAGGTGTTACCATCTC<br>CCACTCCCAAATTTCTTTGTCACGCTGAGGATACTCAAGAGGAGCA<br>GGACATGTTGGTCGCAGCAGGAGAAACTTGAAAGCATTCACTTTTA<br>TGGAACTCATAAGGGAGAGAATCTCTTATTTAGTATCGTCCTTGATA

	CATTTATTATTTTAAAAGATAATGTAGCCAAATGTCTTCCTCTGTGTT<br>AAATCTTTACAAAACTGAAATCTTAAAATGGTGACAAAAATTCTAC<br>TTCTGATAGAATCTATTCATTTTTCCAATTAGATAGGGCATAATTCTT<br>AATTTGCAAAACAAAACGTAATATGCTTATGAGGTTCCATCCCAAA<br>GAACCTGCTATTGAGAGTAGCATTCAGAATAACGGGTGGAAATGCC<br>AACTCCAGAGTTTCAGATCCTACCGGTAATTGGGGTAGGGAGGGGC<br>TTTGGGCGGGGCCTCCCTAGAGGAGGAGGCGTTGTTAGAAAGCTG<br>TCTGGCCAGTCCACAGCTGTCACTAATCGGGGTAAGCCTTGTTGTA<br>TTTGTGCGTGTGGGTGGCATTCTCAATGAGAACTAGCTTCACTTGT<br>CATTTGAGTGAAATCTACAACCCGAGGCGGCTAGTGCTCCCGCACT<br>ACTGGGATCTGAGATCTTCGGAGATGACTGTCGCCCGCAGTACGGA<br>GCCAGCAGAAGTCCGACCCTTCCTGGGAATGGGCTGTACCGAGAG<br>GTCCGACTAGCCCCAGGGTTTTAGTGAGGGGGCAGTGGAACTCAG<br>CGAGGGACTGAGAGCTTCACAGCATGCACGAGTTTGATGCCAGAG<br>AAAAAGTCGGGAGATAAAGGAGCCGCGTGTCACTAAATTGCCGTC<br>GCAGCCGCAGCCACTCAAGTGCCGGACTTGTGAGTACTCTGCGTC<br>TCCAGTCCTCGGACAGAAGTTGGAGAACTCTCTTGGAGAACTCCC<br>CGAGTTAGGAGACGAGATCTCCTAACAATTACTACTTTTTCTTGCGC<br>TCCCCACTTGCCGCTCGCTGGGACAAACGACAGCCACAGTTCCCC<br>TGACGACAGGATGGAGGCCAAGGGCAGGAGCTGACCAGCGCCGC<br>CCTCCCCCGCC
PHOX2A	GTGATCGTGCCACTGCACTCCAGCCTGGGCAACAGAGAGACATCC<br>TGTCTCTAAAAAACAAACAAAAACTAGTTTGCAGATGAGAAAACT<br>GAGGCACAGAGAGGTTGAATAACTTACCCTAAGTCACACAGCTCAT<br>CAATGGTGGAACCAGAGTTGAAACCCAGCAACCAGGCTCCAGAAT<br>TTGTCATCATTCTTGGAAGTGAAGCTGTCCTGCTCTGCAAGGAGGC<br>TTCACAGCAAGATTGCAGTGACAGTGTTTCATGCTAGAGCATTTTT<br>AGCTCCCCCAGGAGTACACACATTTTGGGGGTGGAGGCCTTAGTCA<br>GGATGAGCTCCCCATATCAGACTCTCAACCACCAGCCGTTCTGGTA<br>TGAAGCCCTTGCAGAGTCCTTCCCACTCTGCCTGTCTCCTTCTTCTT<br>TGATGGTGAGTTCTCAGGAAGCCTCTTTGCCTACCCCTATCACACAT<br>GCACACACACACGTGCACACATGTGCAGGCTGGCTTCCAAAGTTC<br>CCTCACCTCTGCCATCTGAGAGACCTGTCTATATAGGTCTGAACCCA<br>GTAGTGGACATGGTTTAAAACACTTCCTTGGAATGCTTCTCAGCTC<br>CTATGCTGATCAGCAGATGCAAATGGATGCCTGCAGAGCAGAAACC<br>CCAACTCCACATCCTGCGGCCTGGAAAGGCCAGTTCCTGCCCAGCT<br>AACCCTGTAGCATCTTACAATAAAGCTTCTAATCACCACTACCATTT<br>GTTTGATTACCTATAAATGTGTTAAGTCTTTAAAGATTGTATTGACCA<br>GAACTCTTGGTTGCAAGTGTCAGAAGCCCAACCAAAAGTAGTTTA<br>GACAGAAGGGAATTTATTGATTCAAGGACCAAACTGTAGAAATGGC<br>AGGGCTTAAGCCAGGCATGGTGGCTCATGCCTGTAATCCCAGCACT<br>TTGGGAGGCTGATGTGGGTGGACCACTTAAGGCCAGGAGTTCAAG<br>ACCAGCCTGGCCAACATGGTGAAACCCCGTCTGTACTAAAAACAC<br>AAAAATTAGTTGGTCATGGTGGCAGCGCCTGTAATCCCAGCTACTC<br>GGGAGACTGAGGCAGGAGAATCACTTGAACCCAGGAGGCAGGGG<br>TTGCAGTGAGCCGAGATTGCGCCACTGCTCTCCAGCCTAGGCAAC

	AGTGGGAGACTCTGTCTCAAAAAGAAAAAAAAAAAAAAAGAAAAAA<br>AGAAAAAAAAATGACAGGGCTAGATCATGGTCTCTGAGACAGCTG<br>GAGCCAGGGCCTTAGGCAGCACTGGGACTCTGCCTCCCCTTATATC<br>TCTGCTTGTCCCTGCATGCAAGCTTCAGTTGCTCCTAGTGCTGGTC<br>AGCTTCACTGGGGAAGGGGTATGGCCACCTGCAAATTCCTCAGATG<br>AGGAAGGACTTTCTTCCAGGCAGAGGTTGACTCGTGAGGGAAGAA<br>GTTTCCTGTCCCTGGAGAGAGAGGAAATGAAGAGGGCAGGATGAT<br>CCCTCTTCAGAGAGTGCAGAGTGGAGTTAGGGGATGAGAATTTCTT<br>CCCTGGAAGCAAGGGCAGGATTCTCCAACACGAAGATTTCAGATT<br>AGCCTAATCCATTCATTTGACAAACAGATAAGAGAGGACCTATGAC<br>ATGCAGAGTGATGGGCTAGGCACCACGGTAGGGCTGGGGAGTGAG<br>TGAGAAACAAGATGACATAGTTCTGCCCTAAGACCTTGCACTGTAT<br>TGCAAACACTTCAGCAAGGTCATTTGAGTGACCAAAAGAGAGGAA<br>GTACTACTATGGGACATGGCAGGGATGCCCAACCTAGTCTAGCAGC<br>ATCAGGGAAGGCTTCCAGGAAGCAATGATATCCAAATTGAGACCCA<br>ATGGATAAACAGGAGTCAACCAGGCTTGGGTTTGTTCGGTTTTGTT<br>TGTTTTGTGTGGGAGGGCAGGGAATGAAATGGGCATTCCAGCCAG<br>AGAGAACAGCTCTGAACCAAAAGATTAGTGTGTGATGGCTGCAAA<br>GAAAGCCAAGAACAGGGGGAGGCAGGCTGGAGCGCTAGCACTGA<br>GGACGTGGCGTGCCAGCAAGAGCTTGGACTTGATCATGAGGGCAA<br>TGGAGAGCCATGGGAGGGTCTGAGAGCAGTGATCCCATCTGATTTC<br>CCAATTCTGAGATCCCTTGGACTCCCTGTAGAGTATATATTGAGGGG<br>GAGAGGTGCACCTCACACACCAGGAGGGATAACTTTTTGAAGCAG<br>TTGAGCTGTCAGGTCTCAATCTCTACAAGTTTAGACCTAAAAAGTC<br>ATTTGAGAAAAACAGAAAATAGCAAGTGTTGGCAAGGATGTGTCA<br>AAATTGGAATACGGATGCATTGCTGGTGAGAATGTAAAATGGAAAA<br>GCCTCTGTGGAAAACATTGTGGCAGTTCCTTGAAAAACAAAAAGA<br>GAATTACTGTATGATCCAGGAATTCCACTTCTGGGTATATATTGAAA<br>AGAATTGAAAGCAGATACAAATATGTTAGGCAGATATGTGTACACC<br>CATGTTCATAGCAGCATTATTCACAACAGCTAAAATGTGGAAGCAA<br>CCCGAATGTCCACGGATGGATGGATGGATAAGGAAATGGGATATATA<br>AAAGGAGTTATGCCAAAAAGACAAATACTGGGCTGAGTGTGGAGG<br>CTCACACCTGTAATTCCAATACTCTGGGAGGCTGAGGTGGGAGGAT<br>CGCTTGAGCCCAGAAGGTTGAGGCAGCAGTGAGCTATGATCCCAC<br>CTCTGCACTGCAGCCTGGGTGACAGAGTAAGAGTATCTCAAAAATA<br>AGTAAGTAAACAGACAAATACTGTATGATTCCACTTATACGAGGTCC<br>CTGGTCAAAATAACAGAGACCAGCCTGGGCAACATAGTGAGACCC<br>CCATCTCTACAGAAGATTTAAAAATTAGCTAGGCATGGTGGTGTGC<br>ACGTGTAGTCCCAGCTACTCAGGACGCTGAGGCGGGAGGATCGCC<br>TGAGCCAAGGAATGCAAGACTGCACCATGATCTCGTTGCTGTACTC<br>CAGCCTGAGTGACAGAGTGAAAGACTGTCTGAAAAAAAAAAAACA<br>CAAAATCATAGGGACAGAAAGAGGAATGGCGGTTTGCAGGTAGGA<br>TGGGGAAATGGAAGGTGTTGTTTAACGGGTAGAGTTTTGATTTTAC<br>AGGATTCAAAGAGTTATGGAGATGGATGTATTAATACATAAATATGA<br>ATGTATTTACTACCACAGAACTATAGCTCAAAGTGGCTAAGATGGTA<br>AATTTTATGTTATGTGTATTTTGCCACAATAAAAACATTTGACAAGA

	GGTAATTTGAGAAACCTCAAAGAAATCCCCTGGCATGGAGTGGTTCCGGAGATTGGGGATAGAGAGTCCTCCTGGGAAGCCCAGAACGGATGATGGGGGAAGGATTACCTGAGGAGAGGCGCACTCCCCTCTGTCCTGACAAACTTGGGGAGAATTCCAGGAGTCCTTCTATCAACCCCCAAGGACTTCAGCTGGAAACAAAGACACGCCCTTCCCGCGGCTTCTCCATGGGTCTAGGACTCCCCATCTACAAGACCTGCCAAGATCGGTGGAAGAGGCTGGGGGTTGAGTGGACGAGCTGCCCAACCCAGCTCAGGAGCGACGGAAAAGTTATCTGTTCCCTTCTCTTGGTAAGGATTCTGAATGTGTCCCGCTGTCCCACAGCACGAGGGAGCTGGTGACTGAATCTGTGCAGGGATGTCTGGGCCAGGTGCAGACTGAGTCTAGGTGGTTTCCCTGGAGAAAAAAAGCGGGGGGCCCTGGAGAACAGGGGTAAGAGACTGAAGAACAATAAAGAGGTCACGGGGTACGTTCCCTTTCTGACATCCATCGCATCCTGAGCCCAAGGTCACAGGAGAGGGTCGCATACTGCAGATGGAGCTGAGGGAACGGGGGGAGAGGCAGGCGGGCCCTGAAGGTGCAAGGTCGTGTGGGCCTCGGGACAAGTCAGGGTAGAGGGACCTGGCTCCCACGACCCTCCCTCTCCGCAGCCCCGTGGAGGCCGCACCCCAGAGCCTCCAGCATCATCCCTGGGCCCCTCTGCGCTCCAGCCCTCGTGTCTAAGCTCCCCGGAGGCGTTTTCTCTCCGCTCTCAGCCTCTGCTCCCGCGCTTGTGGCTGTCCCTGGCCCTGTCTCTCTCAGACTGTCCCTGGGCATATGTGTCTGTCTCACGATTCCTGCCTCTGTTTTGTCTCATCCCTCCCTTGCTCTCCACCGCAGTCCTGGTTGCCGTCCATGTCTCATCCCGACCCCTTCTGTCTCCATGCCTCCCTCACTCTCTTTCTCGTCCCCCTGTCTCCCTGTCTCCTCTTGTCTCTCTCTTAACCTCATCTCCTGTCTCTGCCCTGTCCCCGCTCCATCCTGTCTCTCTCTTACCCTCATCTCCTGTCTCTGCCCTGTCCCCGCTCCATCCTGTCTTTCGCTGTGTATGTTCCGTCCTTGTCTCCCTCTGTGTACCTGTATCTCTTGTCCCTGTCTCCATCACTGTTTCTGCCCCTTTCTCTCCCGGTCTCTGTTTCCTCTGTCTATCCACGTCCCTTCCCTGTCCCCATCCCAGGCTCCGTCTCTTCCCGGTCTCCATCCCCCTTCCTGGGCCGCCTCCGCAGTCTCCGCCGGGATGGGACCTCGCTGGGGGCGGGGCTGAGGGGCCAGATCGGAATAACTCGGTTATCAGAGCGAATTGAATTAACTTCTTCACACGAGCGCCGCCAGGCCTGCCTCCCCTCCCTTCCGCGGGGCCGCAGGTGGGGAGGCCCGGGAAAAGGGTCGGGACCCTCTGGCCTCGGACGCTGTGTAGAGACCGAAGGTCTGGTCCGGATTGGGGCAGGATAGGGACTCCGGGGATAGGGTCCCGCGCATCCCAGCTCCACACCTCTGAGCCCTAAGACGGCGGCGGGGACCCTTCGGGGTGGGGGTCGGGGCGGCTTTGGGCGGTGTCTGGGCCTCCTAGGCCCCGCCCCTTCGGAGCTCGCCCCGCCCCGCCCCCCTCCGCGGGGTCCTGCGCGTTAAAAGGCGCGCGGGCCGGGCTGGCTGCACTTGCGTTGCACCCGGGCTGAGTGC
PIGR	TGAGCAGTGGAGAGTGAAGAAGGGCTATCTAGCTTCCCTTTCTCTGTTGGTGATTGTGTCAACCCATGGTTGTGGAGCATCAAGCTAAAGACAGCCTGAAGGGTTGGATTTGTGTGAGCCTGTGGGCTTGGTCATCTGCAATATTCTCTGGCTAAACCAAGGGTGTAAACGGACGTCCCTTGCTGTCTGTAGAAATATCATCCAAAAAATTCATCCTGACAGCAAAAGTTTTGGGAAGTGGAGGAAAATGCTTTCTAGAAAACCTTGGTTCTAGACCCATCTGTGTGATGACCTCCTGTGCAAACTGGAGCAAGTCATTTCA

	CTTTTCTGTTCTTGGTCCTTCATCATTCAGTGGAGAATCACTCTAGT
	TACGCCATCATGTTCTGGTACATGCTCCTAGTAGTCTCCAAAACTGT
	CTTAGCAGACAGTGCCAGACACGTATGCATGTACACACACACACTT
	GCCTTTTGCTGTCCCGTTTATTTGCTGCTGAATCATAAGCGCCTATTA
	GATGCTAGTCACTGAACATATTATAGCTAATCTTTTTTTAGATTTTTAT
	TTTGGGGAGATAGGGTCTTGTTCTGTCACCCAGGCCAGAGTGCAAT
	GGTGCAATCATAGCTCATTTCAGACTGCAACTCCTGGATTCAAGCA
	ATCCTCCCACCTCAGCCTCCCAAGTAGCTGGGACTACAGGGGTGTG
	CCACCACCCTGGCTAATTTTTTTGTTTTTGTAGAGACTGGGTCTTGC
	TATGTGGCCCAGGCTAGTCTCCAACTCCTGACCCCAAGTGATCCTC
	CCACCTTGGCCTCCCAAAGTGCTGGGACTAGAGGTGTGAACCACT
	GAACCTGGCCTATACTTAATCTTTATAACATACCTATACAGTAAGTAT
	AATTTTCCCCAGAGACATTAAATAATTTGTTCAAGGTTCCCTGGCTG
	GCAGGTGGTAAGGCTTGGATGTGAACCCAGGGCGTCGAGGTCCAG
	AGCCCCTGCTCTTTTCCCAAGGCTACACAGGCTCTCTATGCTTGCTA
	CCTCCTGGCTTGTGTTGTTCCTTGACAGTATTGCATGTGAGTGATGA
	CTTCTCAATTGGACTACTTGCCCTTGTGGAACAGGGGTGCACTCTC
	CTGCATGTCTCAAATCTCCCTGGACCTTGAGCTTGTCATGGTTGGTA
	GGCCGAGAAATAACCACCATCCCTCTTCCCCAAGGTTTGTCTATGT
	CTCAATCTCTGGAGCCCAGAAGTGTGACCTTATTTGGAAAAGAGTC
	TTTGTAGATGTGATGAAGTTCAGGATCTTGAGATGAGGACATCATCT
	TGGATTAGCTGGGTGGGCCCTAAATACCATCCATATAACAGAGAAA
	CAGAGAGTTATTTGAGACATACACAGAAGCCAATGTGACTATGAAG
	GCAGAGGTTGGAGTGACACAGCTACGTGCCAAAGAACACCAAAG
	AATGTCAGCAGCAACCAGAAGCTAGAAGAGGCAAGCAACGGATTA
	TCGTCTAGAGCCTGGAGGGTGGCCCTGCTGGCACCTTGATTTCAGG
	CTTCTGGTCTCCAGAACAGAAAGAGCATACATTTCTGTTTTCTTAA
	GCCACTAAAGTTGTGTTAATTTGTTATAGCAGCTGTCAGAAACTAAT
	ACGGTGCTCAACCAAAACACAGAATCCTAGAACTGAAAGAGCCTT
	GGGAACAATTTGGCACTGTCCTCTGATGGAACAAACAATCCTGCAG
	ACCTACAGGATGAAGTGATCTGGCAAAGGTCACTCGGCAATTAGA
	GGCAGAATCAGGAACAAAACAAAGGTGTTCCACAGTGACTTATGC
	TGAGTCCCAGAGTCAGGAAAGTCCAGTGAGAAGCTGTTCTTCTGC
	TGGTATATTTTCTTGTATGATTACTTTAAAAGAAGCAGGGAGGGTTG
	AGCAGATGAAGTGTTGTAAGTTTGGAAAGTATAATCACTGCTGTTAT
	CATCATTATTATTGATTTGGGCAATGGACTCTCTTGGCCTGTCCCTCC
	TCCCCCAGGGCTGAGAAATAGTACTATTTTAGGGCCACTGGGTTCC
	AGATGGGGAAACCACAGTGCGAGAGGGGAAGCAAGTGAGTCCAA
	TCACACCACCGGCGTGTGGCTGAGCCCAGAATAGGACGTAGGATTT
	TTGACTCCCTGCCTCTTCCCAGGGCTCTACTTCTCATTTTCCCCTTT
	CTGTAAACTGGATCAGTAGGATCAGTGGGCTCCAGGTATGTGGGGT
	GTGTGTGTGTGTATGTGTATGTGTGTGTGTCTGACATGGGTCAGCTA
	AGACAGTTTTGGAGACTATTGAAAACATGCAGCAGGACATGATCTA
	GTAAATTACCCACCATCATGAAATAGCAAAGCAGGGAAGGAAATCT
	GCAACCTGTCCTCCACGCCCACCAAGGCAGCATGAGAAGAAATGA
	TTTAATGAGTGTTCTCCCCTTCCTCCCTGCAAGCCTTTGCTCGTCAA

	ATTTCTGCACTAGTGTTCTGTGAACCTGCCTTGACTTCTACTCCAGC<br>CTGGACAACTGGACACAACTTATTTCTTGCTTTGGGAAGGAAGAA<br>ACAAACCCTACAAATATTTGACTATTGAGTTCAAATGGAGAGAGG<br>TATAAATCGGGAATCCTTTGAAAGGAGCTATAATCTGACAGAACCA<br>GGCTTGTCTCATCTCTGCACCTGCATATCTTGTCTCCCCTCGCATCC<br>TCCTGCTAGACATTAACCCCAGAACTGGGGAGGAGAAAGACTGCT<br>GAAGAGTAAAAAGGAGAGCTTAGTTACTGATGTGTCTTGGTGCCCT<br>CCCACCCACCACCATGGGGCAGAGTCCAGGCTACAGTAGGGGAGA<br>GGGGATTAACAATGATAGAGATATCCAGGTGGGCTTGAAAACTCCA<br>GATTGAGGGGCCCTCACCTTCGCCCATCCTGTAGAGAACTTTGGTG<br>GTCCACAGTCTTGCTGATATCCATTGTACCCCCAATTATATGGGAAC<br>ACTGGCACACAGGAGGTTACAAGAGTGTTGGGGGAAAATGTGACT<br>CTGCTGCACAGGATCTCCCAAAGGCCTCAGGAGTCTGTGATCAGCC<br>AAACTCACCCTGCACCCACAAGGTGGGCAAGGAGAGGCTGAGGG<br>CCTGGAAGACCCACCCCAGGGAGCTCAGTGTATGGACTAGGAGGG<br>CACAGTGTGGACCACAGAACCCTCAGGATGAGCAGGGACAAAGC<br>CCACTGACCAAGCAGGGGAAGTCACATCTCAGTAGACAAAACTAT<br>TTCCTTCTGGAGCCCAAGGCCAGCACAGCATCGTGACAAAGCAAG<br>ACCAAAATAATAATGGGCCACCCCTTTTCACTGCCTCAGCAGTAAC<br>ATTGACCACCCCCTCACCCAAGACCGCTCTCTCCAGACTGGACCCC<br>AGGGGAGAGAAGGGAGACAAAAAGGAAGGGAACTCCATAATGAG<br>GCTAAATTTTAAATCTGGAGTGAAGTGACTGAGAGATCACTGAATT<br>GGGCTGAGCTTGCCTAGAAGTGACTAGGTAACTTGTTTTCTTTTATC<br>AGTCAGATTATGGGTTTAGGGTTTGAAATTAAGCTACATCTGGAAG<br>CCATCATCCTCATCAAACTAACACAGGAACAGAAAACCAAACACC<br>ACATGTTCTCACTCATAAATGGGAGCTGAACAATGAGAACACACGG<br>ACACAGGGAGGGGAACATTACACATCCGGGCCTGTTGGGGGGGT<br>GGGGGGAGGGAGAGCATTAGAACTAATAGCTAATGCATGTGGGGCT<br>TAATACCTAGGTGGTGGGTTGACGGGTGCAGGAAACCATCATGGCA<br>CACGTTTACCTGTGTCACAAACCTGCACGTTCTGCACATGTAATCCT<br>GGAACCTAAAACAAAATAAAAAAAGAAAATCTAAGTTTGCAAACC<br>ACAAAAAAAGAGAGAAATTAAGCTACATAAAGAAATTATGGGGTTT<br>TTTTGCTTGCCATTGAAATCTGTGATTGTGTGTTAACACCCACAAGT<br>GAGCCAGTGGTTAGCTAAAATAAGACCCAGGGCTCTGGCTGCCTTG<br>TGTTTCTCCAGGGCTCTATTTCTCTATTTTTCCTTCCCCTTCTGGTGT<br>TTGGCCTATAGGACTCTGTAGGGAGAGCCTGGGTCAGCCCTGCACA<br>GGTAGGATGGGCAGCATTTCTGTGGGGGTTTGCAGTGAGGGGTCG<br>GGGGGCCATACACGTGTGTGTTGGGGAGTGGGCTTCACTTTTGTCA<br>CAGGACTGCCACGATCATTTGGGGAACACCTGGGAGTCCCAACCA<br>GGTCATGTTTTGGCATGGACTAACAGATAGGGAAGGAGGTGCTACT<br>TCAGCTCAGCAGGTCAGCACCGTCCTTGCTATGGAGCCGAGCCCG<br>GGTCCTAACAAGACTCTGAGGTCAGCCATGTTCAGGGGAGCCCAG<br>AAGCAGCGAGTCTTTCATGACTCTGGGGAGTGAAACAGGAAGGGC<br>CACAGTCCTGCCAGTTCCTGGAGCTCTGGGACTGGTAAAAGGGGC<br>TTGGCAGGAGGTGTATAAGGGGCATTTTTGTCCAGGCTAAGTAACC<br>TAGGGAGTCGGAGGGGATTCCAGAGCAACTGGGGATATGAGACCA

	AGGACTACGACAGCCACTCCTGCCACCTGTGCCCCATCAGATGATGTCAACTTCAAATCAAGCATTGGGCCAGGTATTTTAGAGCTAATACCGGGCTATATCCTCTACCTGTAGATTTGGTATTACCATCCCTCTTTTCCAGATGAAAAGAAATAGGAAGGTGACTTGCCAAAGGTCTTGCAGCTAGAAAGCGACAGAACAGCATCTTCACGCTTGACATTCTGTCCCTCATCCTGAAGCTGCAACGATGGAGGATTCCCAAGTAACAGAGTCTCCCCAAGGTCAAAGGAAACCAAATGGAGCCAGCCAGGAAGGCCAAAATGAAAGGAAAGCAAGGGATCTGTGAGAGTCACATGACCCTGGCTGGCCACGGTGCCTGTGGGAGAGTGGCCCTTTAAGAGCCCAGGTGTGGGTCAAACACTGAGCAGAGTTTCAGTTTTGGCAGCAGCGTCCAGTGCCCTGCCAGTAGCTCCTA
PLOD1	ACTATTATTATTATTAGGGAGGCTTAAGTGCTAGTGAGAGGTGATCATCCATCTAGCAGCACAGAATATGGCCATCCTACCGAAGCCCTTGCTCTTCCTGGGGCCAAAAAAGGAAATGAATAAAAGAAAAGGATGAAAATGAGAAAAGCCCCTCATGTCACCGGGCAGCTGCCCTCCAGGCCAGCTATCCTTTCTACACCTTCCCTAGAAATCTGAGTCCTGCGCCTTTAACTGTCCACAGCACGTGCAGCACGTACCAGAAGTAACATCTGGACGGCAGCCACCACTGGTTAAAGAGACAGGCCCCCTTTCTCCATGAAGCTTGGGCAGGGTTCAAGTTTCAGCAATTTGGTGAAGGGAACAGGAAACAGATACCAAAGGAGGAGAGGGCAGGCTTTGCCAGAGTGAATCCTTTCTCCCAGAGAGGCCAGCAGCCTCCAAGATTGATTCCCCCTCCTGACTTAAAATGACTTTTGGGCATGGGACTCCCACTGCCCAAGGGGTTTTTTCTTTTCCTTTTTTTTGAGACAGAGTCTTACTCTGTAACTCAGGCAGAAGTACAGTGGTACAATTACGGCTCACTGCAGCCTCAACCTCTTGGGCTCAAGTGATCCTCCCACTTCAGCCTCCTGAGAAGCTGAGATTACAGGCGCACACCACCATGCCCTCCTAATTTTTTTTGTGGAGACGGGAGGTGGGTGGGGGCGGAGGGACGGGTCTATGTTGCCCAGGCTGGTCTTGAACTCCTGGTCTCAAAGGATCCTCCCACCTCAGCCTCCCAAAGTGTTGGGATTACAGGCGTGAGTCATGGCACCTGGCCTCAAGGTTTTGTTTTTTTTTTCTATTCATAGTACATCACCATTGAGGTTCTCCTTGGACTACAGCAAATGGAGGGTTAGAGCTGGGTTCCAATGCTGCCTCCCTTACTGTAGTTCTGCATCAGTTGTGTGGCGTCGAGCAGATTGCTTCACTTCTCTGGGTCTGTTTTCTCATTTTGCAAATGGGGCAATACTGTTTAATTAGCAAGGCTGTTGCGAGCAGGGAATGAGGGAGAGTTTTGAAAACTGAACCAGGGGCCGGGAGCATTGGCTCACACGTGTAATCCCAGCACTTTGGGAGGCTGAGGAAGGCAGATGTCTTGAGCTCAGGAGTTTTAGACCAGCCTGGCCGACATTGTGAAACCCTGTCTCTACAAAAAAAAAAAAAAAAAAAAAAAATTAGCCAGGCAAGGTGCATGCCTGTAGTCCCAGCTACTCAGGAGGCTGAGGTGGGAGAATCACCTGAGCCCAGGAAGTTGAGGCTGCAGTGGGCTGTAATCACACCACTGCACTCCAGCCTGGGCAGCAAAGTGAGACCCTGTCTCAAAAAAAAAGAGAACATGGAACCAGCCAACAAATGCTCATTCTCCTCCCTCTGCTCCTCCTGGCATTTCCTCTTCCTCCAGGGGTCTGGAGTCTGAGTCTGCATCCTCCTGAATCCAAAACATCCGGTGGATTCTGGCACCAAGTCCTGTCTCCAGATGCTGAAACTCCCAGCCCTGGATCCCAGCTGGGAGGTAGGGGCCCTGGATTCTGCTCAGGCCTCTTCCTTTTACTTGCCTTGG

	GACCTTGAGCTTGCTGCCCCATGCCTATGGATATCACTTGTGCTATC<br>TGTGCAATGAGCACATCTCCATACAATGAGATGATTTCCACACAATG<br>AGATGATTTCCAAGGTTCCCAGTGACTGACTCTATCTTTGTTTTTTT<br>AATAGAGATGGGGGAAGGGTTTTGCTATAGTGTCCAGGCTGGTCTG<br>GAACTCCTGGGCCCAAGCAGTCCTCCTGCCTTGGCCTCCCGAAGT<br>GCTGGGATTACAGGCATGAGTCTCTGTGCCGGCCTGGACCTATATTC<br>TAAAAATAGCATGTATTGATTTTGTTTGAATATATATATATATTTTTTC<br>AAGACGGAGTTTTGCTCTTGTTGCCCAGGCTAGAGTGCAATGGTGC<br>GATCTCGGCTCACTGCAATCTCCGCCTTCCAGGCTCAAGAAATTCT<br>CCTGCCTCAGCCTCCCAAGTAGCTGGGATTACAGGAATGCGCCACC<br>ATGCCCAGATAATTTTGTATTTTTGGTAGAGACAGGGTTTCTCCATG<br>TTGGTCAGGCTGGTCTTGAAATCCCGACGTCTGGTGATCTGCCTGC<br>CTCCACCTCCCAAAGGGCTGGTATTACAGGCATAAGCCACCATGCC<br>CCGCCGATTTTGTTTGAATTTTACAAATAGTATATATTTATTGAAAAA<br>AAAAGAAAAATATAAATAACAAAATAAATACTTGGAAATAATATAA<br>AACATCAAAAATTGTCTATATAAATATGAAATAATCCCACAACCTGG<br>AAATAATCACAGTAAATATTTGGCCATAATTTTCTCCTAGCATTGTCT<br>CTATGCATGTATACATCGATAAGAATATATTTTTGGCTGGGCGCGATG<br>GCTCACGCCTATAATCCCAACACTTTGGGAGGCCGAGGCTGGTGGA<br>TCACGAGGTCAGGAGTTCAAGACCAGCCTGGCCAAGATGGTGAAA<br>CCTCATCTCTACTAAAAATACAAAAATGAGCCAGGCATGGTGGCAG<br>GCACCTGTAATCCCAGCTACTCGGAAGGCTGAGGCAAAGAATTGCT<br>TGAACCAGGGAGGCGGAGGTTGCAGTGAGCTGAGATTGTGCCACT<br>GCACTCCAACCTGGGCTATACAGTGAGACTCCATCTCAAAAAAAA<br>AAAAAAAGAAGGTATTTTTACTGAGTGGAACCATACTATATATTCTG<br>CTTTTCCACTTAACATCATATGGGTAGTGTATTTCTCTGGCTGCAGCA<br>TCTGAATGCTATTTTGTTTTTATTTTTATTTTTTGGTTTGTTTGTTTTT<br>TTTGAGGCAGGTTCTGACTCTCTTGCCCAGGCTGGAGTGCAGCGGT<br>GTGATCATGGCTCACTGCAACCTCCACCTCCCGGGCTCAAGTGATC<br>CTCCCCAGTCAGCCTCCTGAATAGCTGGGACTACAGGCACATGCCA<br>CCATGCCCAGCTAATTTTTGTTTTTTTAGTAAAGAAGGGGTTTCACC<br>ATGTTGCCCAGGCTGGTCTTGAATTCCTGGGCTCAAGCAATCTGGC<br>TGTCTTGGCCTCCCAAAGTACTGGGATTACAGGCATGAACCACCGC<br>ACCCAGCTCATCTGAATGCTGTTGATTTATTAATATCTTTTCCCAGCA<br>CAGACGCATAGCCTGCTCTTTGTCGTCCATTGACATTCGTGACATTC<br>CTTCTCTCCCACCCCTTTGCCCCCTGCAGGGAACCCCCTGCCAGCC<br>TAGGGTTACACCTGCCACCCACTCCTCTGGAGCCTAATCAGCTGGG<br>AGAAGGCTCCTGGGTCCCTGTCTAAGGTCTTTTTTTTCTTTTTTTTT<br>TTTTTTGAGACAGAGTTTCGCTCTGTCGCCTAGGCTGGAGTGCAGT<br>GGTTCAATCTTGGCTCACTGCAAACTCAGTCTCCTGGGTTCAAGTG<br>ATTCTACTGCCTCAGCCTCCTGAGTAGCTGGGATTACAGGCGCGCG<br>CCACCACGCCTGGCTAATTTTTGTATTTTAGTAGAGACAGAGTTTCA<br>CCATGTTGGTCAAGCTGGTCTTGAACTCCTGACCTTGTGATCCGCT<br>CGCCTCAGCCTCCCAAAGTGCTGGGATTACAGGTGTGAGCCACCG<br>TGCCCGGCCTCTGTCTGAGGTTTCTTCCAAGGAAGGAAGGATTTTT<br>GTTCAGAGGTGGCTGCCCCTGTCTGACATCTGGTCCAGGAGCCCTT

	ACTCTAGAGACCGTTGAGTTGTTTGTTGGGCTTTGGGTATCTGTGA<br>AGAGGCCCTAAAAACTTTCATCGATTCGTTTACTCATTTAACAATTA<br>ATCTCTCTAGGGTGTGGTGGCTCAGCCTGTAATCCCAGCACTTTGG<br>GAGGCCAAGGCGGGTGGATCATCTGAGGGTGGGAGTTCAAGACCA<br>GCCTGACCAACATGGAGAAACCCCACCTCTACTAAAAATACAAAAT<br>TAGCTGGGCATGGTGGCACATGCCTGTAATCCCAGCTACTCGGGAG<br>GCTGAGGCAGGAGAATTGCTTGAACCCGGGAGGCGGAGGTTGCCG<br>TGACCCCAGATCTCGCCGTTGCACTCCAGCCTGGGCAACAAGAGT<br>GAAACTCTGTCTCAAAAAAAAGGTAGAAAAATTAGCCAGGGATGG<br>TGGCTCAGGCCTGTGATCCCACCTACTTGGGAGGCTGAGATGGGA<br>GGATCGCTTGAGCCCGGGAGGTCGAGGCTGCAGCAAGCGGTGATC<br>ACGCCACTGCACTCCAGCCTGGGCAACTGAAAGCAACCTTGTCTC<br>AAAAAAATGAAAAAAAAAAAAACAAAAAACAAAAAAACCTCAGAA<br>GCAAGACTTTCCTTGGCTCAGAGGTTTGCCTCCCAGGGACACAAA<br>GGGAGATCAAGTGATTGCCTTTTGGGGAGAGTGGAGACGGGCTTC<br>CTGCATTTCCAGTGCCTGGGTGGGCCACGTAGGGGTGTCCGTGTCT<br>CTGAACGCATCCGTGCCCACCCTCACCCCTTGGGTTTTGGGAAGGA<br>CTAATGAAATCATGAAAACTGACTTCCTTGGGGCTTGACCTGGGGC<br>TGCTCTAAGTGCGCTACAGGTAACGCCATTAACCCGTGCAAGACCC<br>AATGAACAGACGCTATCATAAGCCCCACTTTTCAGGCGAGCAAACA<br>GGGGCTCGTGGCTATCGATGCCATGGAGCCTTGGCCCAGAACCTGG<br>GGTCTAGAAATCGTGGCCTCTCTCTCCTCTCACCGCCCTCTTTCTGG<br>TCGGAGTGAACCCCCCAGAGAAGGGAGGGAGACGGCAGGGCCTG<br>ACGGAAGCCCCGGGAGCTGCACAGAGAGGCGGAGGGAGCTGCCC<br>TAATAAGGAGAGGCCCGGACTTGGGCATATCTGCAGAAAAACCCTT<br>CCCCACTAGGCAGGCGCGGGGGAGGGCGTGGAGGGGCGGGGTGG<br>TGCCGCCCCCGGGGCGGGCCCAGTGCGTGGCAGCGGGACCTGCGG<br>CCCCGTCGCGAAGTTTCCAGCCCTGCGAGCGCCGCCGGGTCGGCC<br>GATCGTCCCCCATA
PLTP	TGATATGATTGAGAGCGATTAATAGATAGGCAGGCAGGGAAGGCCT<br>CTCTGAAAAAACGCCATTTAAACTGAGACAAAATGCTGGGCGTGG<br>TGGCTCGAGCCTGTAATCCCAGCACTTTGGGAGGCTGAGGCGGAC<br>AGATCACTTGAGGTCAGGAGTTCGAGACCAGCCTGGCCAACATGT<br>GAAACCCCATCACTACTAAAAATACAAAATTAGCTGGGCGTGGTGG<br>TGCACGCCTGTAATCCCAGCTACTCAGAAGACTGAAGTAGGAGAAT<br>TGCTTGTACTCTGGAGGTAGAGTTTACAGTGAGCCGAGATCATACC<br>ACTGCACTCCAGCCTGGGTGACAGAGCGAAGCTCCGTCTCAAAAA<br>ATAAATAAATAAAATAAGCTGAGACAAAATGACAAGGAAGATCAG<br>GTGGAAAAGCATTCCAGGTAGAGGGAACAGCAAATGCAAAGGCTC<br>TGAGCAAGAAGAACTTGCACATTCAAGGAACAGAAAAACCAGTTT<br>GGCTGGAGCCGAATGCAAAGGGCAAGAGAGGTGAGAGGTGAGAC<br>AGAGAGAGTAGGCCCTATCACATGCCAGGGAATCAGGGTACTGAG<br>GCAGGAGTCTATTCCAGTATGGTGAAAAGCCACTGATGGCTGATCA<br>TGCTAATGATTGTAGGTAACATTTTTTGAGCCCTTAGGAAATGCCAA<br>GTACTTATTAAACCCTTTCAATGCATTAACTCATTTAATCCTCACAAG<br>CAATCCTAAGAGGGAGGTACCTGGATCATCCTCATCTTACAGATGA

	GGAAACTGAGATCCAGAGGGGTTAGGCCACTGGCCAGTGAATTGC<br>ATGGGACAAGACCATTTAAGAGATAAAAATGATACAAAGATGAAGC<br>CTGGATTCATCTGGGTACACAACTGAGGCTAGAAAAGCATTTCTTT<br>CCCATCCCTTTGATTGCTGGGAAGCCTCTCCTTCAGTCTTCAAAGC<br>CAGGAAGAGTGAGGCCAGGCACAGTGGCTTACGCCTGTAATACCA<br>GTATTTTGGGAAGCCAAGATCGGAGGATCTTTTGAGGCCAAGAGAT<br>TGAGACCAGCAAGGGCAATATAGCGAGAAATATTTCACCTAAGAAA<br>ATGAGAGCAAGGCTGGGCACACTGGCTCACGCCTGTAATCCCAGC<br>ACTTTGGTAGCTCAGGAGTTCGAGACCAGCCTGGCCAACGTGGTG<br>AAACCCCATCTCTACTAAAAATACAAAATTAGTCAGCTGTGGTGGT<br>GTGTGCCTGTAATCCTAGCTACTCTGGAGGCTGAGGCGGGAGAATC<br>GCTTAGAACCCGGGAGGTGGAGGTTGCAGTGAGCCGAGACAGCA<br>CCACTGCACTCCAGCCTGGGTGACAGAGTAAGACTCTGTCTCAAA<br>AAAAAAAAAAAAAAAGAAAAAAAGAAAAAGAAAACGAGAGGAA<br>AGGCCAGATGTGGTGTAACACCAGCACTTCGGGAGGCTGAGGTGG<br>GTAGATCTCTTGAGCTCAGGAGTTCAAGACCAGCCTGGGCAACAT<br>GGTGAAACCTCATCTCTGTGAAAAATACAAAAATTAGCTGGGTATG<br>GTGGCACATACCTGTAGTCTCAGCTACTCAGGAGGCTGAGGTGGG<br>AGGATCACCTGAGCCTGGGGAGGTTGAGGCTGCAGTGAGCCGTAA<br>CTGCACCACTGCACAGCAGTCTGGGCAACAGAATGAGACCCTCTC<br>TCAAAAAAGAGGGAAAAAAAGAAAGAAAAGAAGAGAAAACAGC<br>CAGGTATGGTGGTGCACACCTGTAGTCCCAGCTACTTGGGGGGCTG<br>AGGTGGGAGGATTGCTTGAGCCCGAGAGTTGGAGATCAGCCTGAG<br>CACATGGTGAGACCCTATCTTAAATAAATAAAATGTATTTTGTTGTT<br>GTTGTTAAAAAAAAATAAAAAAGGGCCAGGTGCACTGGCTCATGC<br>CTGTAATCCCAGCACTTCGGGAGGCCAAGGCGGGCGGATCACCTG<br>AGGTCAGAAGTTCGAGACCAACCTGGCCAACACGGTGAAACCCTG<br>TCTCTAAAAATACAAAAATTAGCTGGGCATGGTGGCAGGCGCCTCT<br>AGTCCCAGCTACTCGGGAGGCTGAGGCAAGAGAATCACTTGAACC<br>TGGGAGGTGGAGGTTGCAGTGAGCCGAGATCGTGCCACTGCACTC<br>CAGCCTGGGTGACAGAGCGAGACTCCACCCCACAAAAAAAAAAA<br>AAGAAAAGAAAGGAAATAAGAGGAAAGAAAACTAACATCCACCA<br>GGTTTTCTGCTTGAGCCAGGCTCTAGGAAGGTTACTAGAGTTCAAA<br>GGAACACAGATACTTCCTCCTCTGGATACTCTTAACACTTTCTCTGT<br>TCTCCCTCTACACCTTTTCTGCTTTGTTTGCTCGTGGTATGTGCCCCT<br>AATTAAGATAGCTAGCATTTATTTGGCACTAATGATGTGTCATGTACC<br>AGGCCCATTTATTCTTGCAATAACCTTAAATTATTGTTTCCATTTTAG<br>GATGAGGAAATTGAAGATCTGAGTGCTTAAGCAAGGGATCAATGTC<br>ATACACCTAAGAACTGACAACCTGGGGTGTAAACTCTTGTCTGTCA<br>GACACAAGAGTCTAAGTACATAACTACCGGGCTATAAAGCCCACCT<br>TCCACTCTTCCATGAACAGTCTACGGATTCCCTAATAGCAGCCACA<br>GAATTGCTTCAACCTCTACTTCCTGTTAAAGAGGGTTCATAATAGTG<br>CCAGTCTGACAGGTTGTTAAGGTTAAATAAATTACCTAGAATTCGGA<br>TGACCAGTTGTAAGGTTTCCCAGGGACTGAGTGGGTTCCCAGGAA<br>TGGTTTCAAAATGGGGATGGCTGGATGTGTGGCTCACGCCTGTAAT<br>CCCAGCACTTTGGGAGCCCAAGGCAAGAGGATCACTTGGGTCCAG

	GAGTTCAAGATCAGCCTGGGCAACATGGTGAGACCTGTCTCTACA<br>AAAAATACAAAACTTAGCTGGACATGACATGGTACCACCTGCCTGT<br>AGTCCTAGCTACTCAGGAGGCTGAGGTGGGAAGATCGCTTGAGCC<br>CAAGAGGTGGAGGCTTCAGTGAGCCGTGATTGTACCACTGCACTC<br>CAGCCTGGGCAACAGAGCAAGACCCTGTCTCTAAAAATATAAATAG<br>GCGGCCGGGCGCGGTGGCTCACGCCTGTAATCCCAGCACTTTGGG<br>AGGCCAAGGCAGGTGGATTGCCTGAGGTCAGGCGTTCGAAACCAG<br>CCTGGCCAACATAGCGAAACCATATCTCTACTAAAAATACAAAAAA<br>TTAGCTGAGCGTAGTGGTGGGTGCCTGTAATCCCAGCTACTTGGGA<br>GGCTGAGGTAGGAGAATTGCTTGAACCTGGTAGGCGGAGGTTGCA<br>GTGAGCCGAGATTGCGCCATTGCACTCCAGCCTGGGCAACAAGAG<br>AGAAACTCCATCTTAAATAAATAAATAAAAATAAATAAATAGGCTGG<br>GCATGGTGGGTCACACCTGTAATCCCAGCACTTTGGGAGGCTAAGG<br>CAGGCGGATCACTTGAGGGCAGGAGTTCAAGACCAGCCTGGCCAA<br>AATGGCAAAACCCTGTCTCCACTAAAAATACAAAAATTAGCCAGGT<br>GTGGTGGCACATGGCTGTAGTCCCAGCTACTTGGGAGGCTGAGGC<br>AGGAGAATCACTTGAACCCAGGAGGCAGAGGTTGCAGTGAGCCA<br>AGATCACACCACCGCACTGCAGCCTGGTGACAGAGCACGACTGTG<br>TCTCAAAAAAATTAATTAATTAATTAAATAAAAAAGGAAATGGAACT<br>ATTTTTGTGAATCTGTGGATTATATCAGAAAAAAAAGACACAATGG<br>GGAAAGTCCTAGGCAAATCAGGATGAGTTAGTCATCCTTCCTAGAT<br>GAGTGTTTGGTGCTAAATACATGCTCAGCAGACATGATTATTGCTTC<br>CCCTTTCTTTCGTCCATTTGGCAACAAAAAGGTGGCAAGCACCCAC<br>TCTGTGCCCTGTCCTAGGGTCCGGGAACCCTGTAAGCAGTAGATGG<br>AGGTGGGGGTGGGGGTGGGGGCGGGGATGCTGTTCAGAGCACCTT<br>GCTCCAAGGGTTCATTAAAAAATCCACCAGTGGACCGGGCGCGGT<br>GGCTCATGCCTTTAATCCCAGCACTTTGGGAGGCCGAGGCGGGCG<br>GATCACAAGGTCAGGAGATCGAGACCATCCTGGCTAACACGGTGA<br>AACCCCGTCTCTACTAAAAATACAAAAAAAATTAGCCGGGCGTGGC<br>AGCGTGCGCCTGTAGCCCCAGCTGCTGGGGAGGCTGAGGCAGGAG<br>AATGGCGTGAACCCGGGAGGCGGGGCTTGCAGGGAGCCGAGATCG<br>CGCCACTGCACTCCAGCCTGGGGGACAGAGCGAGACTCCGTCTCA<br>AAAAATAAAAATAAAAAAAATAGAAAAAACAATCCACCAGCCACG<br>ATAAATGGCAGACCTCCTTCTGATTTCAGCCGGTGTGGTATGTTCCT<br>GGGCTGACAGCACTTGTCTAGTCTTGCTTTCCCAAGTGGGAAAGGT<br>CTCTGGGACCTTAAGGTCCCCAGGTGGTGACACAGAGACAGGTAG<br>GGGGGCCCATAGCAAAGCCAGGCAAGGAGGTCCCGAGATGATTGT<br>GGGTGGCAGGGAAAGAAAAAATATTCCTTGACTTTGTGCCTGGAC<br>CTGGTTGTAATAAAGGCCCAAGAGGTAGTTCCTATCATCGTGCACAT<br>TTCGCTGAAGGAAGAAACTGAGGGTCAGTGACCCAAGTGAAGTG<br>ACTTGCCCAAGATCATGCAGGAAGACATGGATAATTGTAATTTGAA<br>CCAAGGTCCCAGCAAAGTGGGATTGTTGGGGCTGAGTGGGCCGGC<br>TCCTGCATTTCCTTCCCTCTCCCTGGGCTTGGGTCTCCCACTTGTCC<br>AGACAGCGGCCGGGCTTGTCACGGGGCTCTGTGCAGCCTTTTCCA<br>CTCTCCCGGCTGCCAGCGTCCCGCCCCGTCCCCTCCCAGCCCCCAA<br>GGGAGGAGGGGAGAGCTGCAGAGAGGAGGAGGGGTCGGGGAGG

	CCGGCTTTATAAAGGCGGCTGGAACAACCCTGCCCGCCAGACCCC<br>GTCGCCCGGATCCCCTG
POLB	AGTGATGTCTAACAATTATTGAAGGCTTACAACTACATGTATGCATT<br>ATCTTATTTAATCCTTGGAACACTTAATTCCGATGATGAGGAGGAAG<br>GTAGCATTGTTCCCAGAAGCCTGTCCCCACCTGCACCATTGGTACT<br>ACTGCTGAGTATTTGTGTGTTGAGTGCTGGGCTGGGAGCAGGAAC<br>CAGCCATGGCCTTGTCCTCAGGGCCTTGGAGCTTAGTGGTAGGTAG<br>TACTAGTTATGAGAAAACTAGCCAAGTTACAGGTACATTAAGACAA<br>AGCTTCCATTGTTGAGAGAATCCTGCATCATTTGCTTAGATCTTTAC<br>ATGGCCTTTACCTGCTAATCACTTGCTAACCTCCCCTAACTTTAAGC<br>TTTTGGAAGAATTACAAGGGATACCACTGTGAATTGCAGCCTTTGC<br>TGATGTAGTCGCATGTCCTCCTCCAGTCTGTGGACATTCTATATCTCT<br>TGCTGCTCTCCTCCTCCTGGCATGATAATGTTTTGATCAGTTCATTG<br>CTTACTTGCATGTCTGTGGGAGGAAGAGGAAGTCTCATCAGGCCA<br>AGTCATGTGGTGAGGCTGGAAATCCATAACTGAGATTAGCTACCCA<br>CAGTGAGCATGGGAGTGAGTGCTGTGAGCCACAGTGGTACTGACA<br>TCGACTTTCTAATTCCCACAGTCACAGAATCTCAAGGACTCTGAGC<br>CAATTCAAAGAAAAGATCCTGTACAGTTTGAATATGTAACTTAGCTT<br>AAGAGTACCCTGCAGTCACAAGATTTATTAATAAAAGGGTTTGGCT<br>TTTTAATTTGTATTTGTTTAATTTATTTGTTTTATGCAGCCTGGGTCTT<br>GCTATGTTGCCCAGGCTGGTCTTGAACTCCTGGCCTCAAGCGATCC<br>GCTCGCCTCTGCCTCCCAAAGTGCTGGGACTATAGGCATGAGCCAC<br>CATACCCAGCCAATAAAGGGGTTTCAATATATTTCTTTTTATTTTTA<br>TTTTTTGAGATGGAGTCTTGCTCTTTTGCCCAGGGTGGAGTGCAAT<br>GGCACGATCTCAGCTCACTGCAACCCCTACCTCCCAGGTTCAAGTG<br>ATTCTCCTGCCTCAGCCTCCTGAGTAGCTGGGACTATAGGAGCTTG<br>CCACCACGCCTGGCTAATTTTGTATTTTTAGTAGAAATGGGGTTTCA<br>CCATTTTGGCCAGGCTGGTCTCGAACTCCTGACCTCATGATCTGCC<br>CACCTTGGCCTCCCAAAGTGTTGGGATTATAGGCATGAGCCACTGT<br>GCCTGGCTGGGTTTTAGTATATTTCTCAATTGTCCTGATTTCCTCCTG<br>AAGAGGAAAAATAACCTGAAATGTGTTGGTGGCTGTTGTTTTTTTA<br>ACATGTCTGTTGACTTTCAGGCCCTAGACTGGAGCTGGTTACAGAC<br>GGAAGAAGAAGAGCACAGCTGCCTGGAGCAGGCCTCATGATGTGG<br>GGGGACTCGACCCCCTGACATGGGGCAGCCCATAGCAGGCCTTGT<br>GCGGTGGGGGGACTCGACCCCCTGACATGGGGCTGCCTGGAGCAG<br>GCCGCGTGACGTGGGGCTGCCTGGCCGCGGCTCTCACATGGTGGT<br>TCCTGCTGCACTGATGGCCCAGGGGTCTCTGGTATCCAGATGGAGC<br>TCTCGCTTCCTCAGCAGCTGTGACTTTCACCCAGGACCCAGGACGC<br>AGCCCTCCGTGGGCACTGCCGGCGCCTTGTCTGCACACTGGAGGT<br>CCTCCATTACAGAGGCCCAGCGCACATCGCTGGCCCCACAAACGTT<br>CAGGGGTACAGCCATGGCAGCTCCTTCCTCTGCCGTGAGAAAAGT<br>GCTTGGAGTACGGTTTGCCACACACGTGACTGGACAGTGTCCAATT<br>CAAATCTTTCAGGGCAGAGTCCGAGCAGCGCTTGGTGACAGCCTG<br>TCCTCTCCTGCTCTCCAAAGGCCCTGCTCCCTGTCCTCTCTCACTTT<br>ACAGCTTGTGTTTCTTCTGGATTCAGCTTCTCCTAAACAGACAGTTT<br>AATTATAGTTGCGGCCTGGCCCCATCCTCACTTCCTCTTTTTATTTCA

	CTGCTGCTAAAATTGTGTTTTTACCTACTACTTTGGTGGTTGTCCTC<br>TTTTCGGCAAAGTTGGAGCGAGTGCCAAGCTCTCCATCTGTGGTCC<br>TTTCTGCCAAGAGCGACTCATAGTAACCAGGATGGGAGAGCAGCT<br>GCCTTATTCTGAATCCCAAAAATTACTTGGGGGTGATTGTCACAGA<br>GGAGGGACAGAAAGGGTATCTGCTGACCACCAGCCTGCCTACCCA<br>TGCCCATGTCTCCATTCCTGCTCAAGCGTGTGTGCTGGGCCGGGGA<br>GTCCCTGTCTCTCACAGCATCTAGCAGTATTATTAAATGGATTCATTT<br>TAAAAATAGCTCCTATATTTTGTAACATGTCTCAAACACTCATACTG<br>GGTTCCACAATCCACTGTTAGAATACCTATGGTTAGGGCTTCTGAAC<br>TAAAATAATGGAAAATTTTAACAATTTGTATAGTGCCTGGATCATTA<br>CTAGTGCCATAACCCTGCTTCTTCAACATTTCACAGAACTTCTCTTT<br>TATATAAAGGCAAGAGCACAAAATGAGTTCAGATGATCACAAACAG<br>GTGAGTTTTGTTGGAGAAGAAAGTTGGAGTAGGAGACTTTCACAA<br>GTGGTTTCCATGGAGATAGAATGAAGCATTCTGTGGTCAAGTAAGT<br>TTAGGGAGCTATTCATGTTTCACTTGCTTTGTGGAGATTCACACTAT<br>GCACTGGGAAAGTATCTGAAAAGTCTTATAATAAAGAAACAGGCTT<br>AACTTTGTGTAAGAACACTGTTTATCAATGTCATTTGGCTATAGAAA<br>CATTTTCTCCTGCTGATTGTGTGTGTGAAACATGTATTAACATTCCA<br>ATGAACTAGCATTTAATAAAGCACAATTTTGGAAACCCTGGTAAAT<br>GACAGTGGGAAATAACACCCGAAAGGCAAGGACGGGCAGATTGG<br>GGAGGGAAAGGATGTTGGGCTAAGGGCTGTGAGCTTATGTTACAG<br>GCAACTGAGCCACTGAAGAATTTTGACGAAGAAAATGCCAACCAA<br>AGCAGTCATTTTAAAAGTTTATGGCTGTTCAGTTACAGGACAAGTT<br>GTGAAAAGAAAGAAAAAAAAAATGGAAAAAAAAAAAGTTTATGGC<br>TGAAACAGTGTAATTGATTAAAAAGTGAAAATCCAGGCCGGGCGC<br>GGTGGCTCACGCCTGTAATTCCAACACTTTGGGAGGCTGAGGCAG<br>GCAGATCACCTGAGGTCGGGAGTTCGAGACCAGCCTGACCAATAT<br>GGAGAAACCCCGTCTCTACTAAAAATACAAAAGCCAGGCGTGGTG<br>GGACATGCCTGTAATCCCAGCTACTCGGGAGGCTGAGGCAGGAGA<br>ATCGCTTGAACCCGGGAGGCAGAGGTTGCAGTGAGCCGAGATCGT<br>GCCATTGCACTCCGGCCTGCGGGACAAGAGTGAAACTCTGTCTCA<br>AAACAAAAAAATTTGTAAAACTGATCCCACAATTCCCTACATTTGT<br>AACTTTTAAAATTCAGAGGTAAATACATTTTTATTTAAGGAGGAATA<br>ATTCTTCAGAGCAAAATAATCTGAATATCACCTGGAATAGAGATTGG<br>ACAGAAAAAAGTGTGAACAGGGAAAACTAGGACAAGTCATATATG<br>TAAATGAGAGTATAAAGAGTAAATGAGAGTGGTCACTGCTCACTGC<br>AGCTTAACTAATCAGATAGTTAAGTTGCAGGCATAAAGTTTTTTTAA<br>CTTTTATTCCTCAAGTAATAAATTGAGGAAAATACAAATATGTACAA<br>AATTTTTACAAAAGCTATCCATAAAAGGGCAAGTAGTAATATATAGA<br>AAAGATCTACCAGATTAGTAACAAACTACCCCCATTAGGAAAATGG<br>TAAATGATATGAATAGGAAATTCAAAAAAAGAATTACAGATTTCCAA<br>TAAGCATAACTTAAGATATACACTGATATTGTAAGGAAACACAATCA<br>CCACAACCTTGCTAATCAAAGAAACTAATGATATTTATTGGAAAATA<br>ATGGGAATTTTTAATAGACTGGGTTAGAAGACTAGAAGTGTACGTC<br>CCAAAATATTAATAGTTCAGCTGTAGGGTGAGATCACTGATTTTAAT<br>TTTCTCCTTTTATACATTTTTCCAGATTTCTCTACAGCAACTACGTAT

	CATTTTGTAATAAATTTTAATTTTTCGATGCACAAAGCAATTGCCTG<br>GAATTGCAGTTCGATATGAACCCAGGAGTTACGCTTGTAAAATAAA<br>CTATTGCTGATGTGACATTTCCATTTCTCAAGCAGACTGTAAATTTC<br>TCAGATGTAGGTCATCATGTTATAATTAGGGTTCCATTGCTCATGATC<br>TAGATGAAACACTTCTCCCTTTCTTGTGGTCTCTTCATTAATTTGGT<br>CCCTACCGCAAAGGCAGGGGGATTAAAAATAAAATATCTTTTGACA<br>CTGGTTGGCAGGTGTACTAACACGTGTGCGGGAGGATTCCTGTTCT<br>CGGCATGGTTCACGCCGGGCTCCGGCAGCGCCCTAGTACGTAAGG<br>GGCTGCACTGGAGTGTGGACTGCCCGGTATCCTTAGAGAAAGTTTT<br>GACAGTGTGACGCCAGTGTCGCCTCTAACCACGTACAAAACGCGC<br>CATGCATTGGGTAGCTATTACAGTGCACATTTTTGTGCAGGAAGCTA<br>CCCCACCAGCGCAAGTATTTCAGAATCAAGATCGCACTCCCGTTTC<br>CCCTTCTAGGGAAAGGATTCCAGATAAACACTGAGAAAACAGATTT<br>AATATCTTAGGTCAAAACTCCAGGTCTTCCCATAGGAAGGCCCCTG<br>GGCTGTCATTCTGAGGTGCCTATTTCCCCCGCGCCGTGTCCACGTC<br>CACGAGTCCACGAACCCTCCGAGTTCTCTCTCCTCACCTGCCGAGG<br>GCTCGCCCCTCTAGCCCCGCCCCGCCCCGCCCAGGACGCGTGACG<br>TCACAACAAGCGCCGTGCGCCCCGCCCCCCATCGGGGGCAACCAT<br>TGTTCCGCCGGTCGCGCCGGAGCTGGGTTGCTCCTGCTCCC
PPARG	AGCAAGTTATTTTGTGAAGGGCAACATCGGAGGAGGCAGAATAAA<br>CAGAGGAGGAGAAAAGGACCTTAATGAAATTCAAAGTAAAATTGG<br>GAGGTTGTGGGTAAAATTTCATAACTAGACATAAAAATCACAAGTG<br>GGAACATGTCAGAGTCATTCAAAAAGTAGATACATTTGTGGAAGTG<br>TGTGTGTGTGCATATGTGTATGTTTAACCTGCCTGGCATGTTCAACT<br>GGTTATGATTTTTGAAATTTTAAATTAAGATCTTCTAAACCTCCTGG<br>GAATTTATAAGGCTTAGAGATTGATAAATGGTGTCTCAGATAAAGAG<br>AAGGAGCAAATGTCAGCGCTATGATGATACACAGCTGCTATAAATG<br>AGTGGCTTGATTGCTTGACTTCAAATACAGCACACTAACTTTCCTA<br>AACTCAGTCTGCAAAATGGTTTACATGTCACTATTCTCAATGCCCAG<br>TTTTGCCGAACACTCGGATGATAATGAAAAAGTTTTGCTACAATTTT<br>AAATTTTTCCTTTAAGAAGTAGCATAACACTACTATAAAATATCTGA<br>GGTCTGTCCAGTGAGACCAGTTTAAATATAGCTGGAGTAAAATGGA<br>AAACAATTCAGTTATGGTAAACTTCTATTTCTAGACTTGCACTCTCT<br>TCTTAATATAATAAACATAGTATGTAATTATAAACCTGGGAACAAAG<br>TCCTCTTTATTGAAAGAAAAGCAAATATGTATGCAAATCTCAAATGA<br>GATAATCAACTAAGCATTAATTGCAAAAAAAAAAAAAAAAAAAAGA<br>AGTCCCATGGGACCATGGGACCATGGGACCAAACACCAATATTTGG<br>AAGGAGTCCTTGTGGTTTGGAAAAATTGAAACGGGCATAGTCTTTC<br>GTCTCTAGGAACCCCATATGGATGGCACTGCCTATGTGCAAAGTTG<br>CAGGGTGGGGACCATGTGCTGTTTATCATTTTATTCCCCGCACTTAG<br>CAGAGGGCCTGGCACAAAATAAGCACTCAATAAAATGTTTGTGGA<br>AAGAAAGAATTCTACAGCTATAGTTTATACAGTGTTTTTCCAACTTT<br>AAAAGATATGGCCCACAGTAAAAACCACATTTTATACCAACTCCTG<br>GTACACTCAGATACACACACACATGCATTACACAAAAGTTACGAAG<br>CAGCACTTTCTTTAATACAGTAGGCATGTTCTGATTATTTCTGTCCTT<br>TTTGCTTGACTAAAAAATACTGATTATGACTGAATAAATTGATTTCAT

	AACCTACTAAATCAAGCCTTGCAATTTGAAAAACAGACATATTATTT CAGTTTACATTACTTAACCTCTTACATTTATACACTTCACTACCCATA AGTCTCCTTAAAAAAAAAAATCACAAAATTTTTGATCCAGAGGGAA TTTAGAAAATATTCAGCTCAACTCTAAAGTTTTGCACCACTTACGAA TTCACTGTTGTGGGGTTCCCCTTAGTCACTGTTGTTGAATTATGGGA ATTTTCCGACCAAAAAAACAGATTGGCCCCCATCTCACATCATATAC AAAATTTAACTCAACATGAATTTAAAACCTAAAGGTAAGAACTAAA CCTATAAAACTCTTAAAATAAACATTAGGAGCAAATTTCATGACTTT GGGTTAGTCACTGACACGACACCAAAAGAAAAAACAGGTAAACTG GACTTCATCAAAATTAAAAACTCAAATGACAACATCAAGAAAGTAA AAAGAACCAAGCATGGTGGCATCTGCCTGTAATTCCAGCTATTCAG AAGGCTGAGGCAGGAGGATCACTTGAGCCCTAAAGTTCAAGACCA GCCTAGGCAACATAGCAAGACCTCGTCTTTAAATAAATAAATAAATA ATAAATAAATAAATAAATAAATAATTATTTTAAAATGTAAAAAGACA ACTTACAGCCTGGAATAATATATTGGCAAATCATGTATCTGATAAGA GACTCGGATATAGACTATATAAATAAATAACTCTCACAACTCAACAA TAGAAAGAAAAATAATCCAATTTAAAAACAGGGAAAGGATTTAAG CAGACTTTCTTCAAAAAAGATAAATGGCCAATGAACACATGAAAA GATGCTCAACGGGATAGTCACCAAGGCAATGCAAATCAAAACCAT GAGACACCGGCTCGGGCAACATAGTGAGATCCCATCTCTACAAAA GAATACAAAAATTAGCTGGTGTGGTGGCATGCACCTGTTGTCCCAG CCACTTGGGAGGCTGAGGTGGGAGGATTGCTTTAGCCCAGGAATT CGAGGCTGCAGTGAACTATGATTGCACCACTGCACTCCAGCCTGGG TGAGAGAGCAATACCTTGTCTCAAAACAAACAAACAAACAAAACC CCATGAGATATCACTTCATACCCTTTAGGTTGGCTAAAATAAAAAAG ACTATAACAAGTGTTGACAAGGATGTGGAAAAACTGGAACCCTGA CACATTGCTGGTGGGATTGTAAAATGGTGTGCCCACTTTGGAAAAC AGACTGGCAGTTCCTCAAAAACACCGAGTTACCTTATGATCCTGCA GTTCTGTCCCTAGGTATATACTCAAGAGAAATAAAAATATATGTCCA CAAGTAACCTTGTACATGAATGCTCACAGCAGCATTATTCATAATAG CCCATAAAAGTAGAAACAACCTAAATATTCATCAATTCATGGGATGA ATAAACAAAATGTGGTATATGTGTATAATGGAATATTGACCATAAAA AGGAATGAAATATTAATATAAGCTATAACATGGATGAGCCTCACAAA TACTATGCTAAGTGAAAGAAGAAAGTCACAAAGGACTTCATATTCT ATGATTCTATTTATATGAATTGTCCAGAATAGGTAAATCTATAGAGAA AGAATATCTCTATCTAGAGTTGGTGGAATGACTGTTAATGGAGAGG GGGTTCCTTTTTGGAGTGATGAAAATGTTCTAAGGGTAGATTTGGT GATGATGGCACAACTCTGTCAATAAACTAAAACTCATTGAACTGTA CATTTTATTTATTTATTTTTGAGATGGAGTCTTGCTCTGGGGCTGAAG TGCAGTGGCGCAATCTCGGCTTGTAACCTCTGCCTCCCAGGGTCAA GCGATTCTACTGCCTCAGCCCCCCGAGTAGCTGAGATTACAGGCAC GTGCCACCACGCCCAGCTAATTTTTGTATTTCTTAGTAGAGATGGAG TTTCACCATGTTGGCCAGGCTGGTCTTGAACTCCCGGCCTCAAGTG ATCCACCTGCCTCGGCCTCCCAAAGTGCTGGGATTACAGGCGTGAG CTGCCATACCCGGCCTGAATTGTACATTTTACTTCTATGGTATTTACA TTTTAGATTATATTAATTATTCCTCAATAAAGCTGTGATTTTAAAAAG

	CAGGCTAGGCGCAGTGGCTGGTGCCTATAATCCCAGCACTTTGGAA<br>AGCTGAGGCAGGAGGATCACTTGAGCCCAGGAGTTTCAGACTAGT<br>CTAGGCAACATGTCAAGACACAGTCTCTACTAAACAATTAAAATTA<br>AAAAAAAAAATTAGCCAGGCATGGTGGTGTGCACCTGTAGTCCCA<br>GCTACTTGGGAGCCTGGGGTGGGAGGATTCCTTGAGCCCGGGAAG<br>TCGAGGCTGAAGTGAGCCGTGATTGCGCCACAGCACTCCAGCCTG<br>GGCGACACAGCAACACCCTGTCTCATGGAAGAAAGAAAGAAAAG<br>AAAGGAAGAAAGAAAAAAAAAAAGCAGATTGGAACTCTGGAATT<br>AACAAGAAGTAGGACGCACGGAGCACTTCCGCCTGAGTGGAGACT<br>GTGGATCCGGGTCAACCTGACTACCTAAATCACAGGCCAATAAATG<br>GTCTTTCAGTGGTCAGTCCCTGTAAGATCCGTGGCTCTCAGCTTCTT<br>ATCTTAGGGGCTGTGGAGGAAGGACATGATTATGTTGATTTAAGCG<br>CTGAATATTTTCCCTTGTGATACCCATCCTCGCAAAACTTTGCTTCA<br>ACCACAAACGAGGACCTTCTGTACCAGAGGGGCAATAACCACAAT<br>GAAGCTAGGAAGAAATGCAGAGCACCCCAGCATACAGTCCATAAG<br>CTTCCTGAAGTGGGGGGCCTCAGGCATCGCTGCCTCCCCAAAGAG<br>GATCAGGCCCAGAACAGTATGCTCCAGAAATAAGACTGGAAAAAG<br>GGAAAGAGGGGCCTCAAGTCCAGGAGACCAGCGGCTTTCTGAAC<br>GCGCACCTGCCAACCCACTTTGGACAGGTCACGATGGACAGCGTG<br>GCAGGAAAAGAAAAGGTCACTGTCTACCCAACACATGAGAAACTG<br>TTTCTCGTGCCTCACGTCCCCACTCCGTCCCCACCCATGTTGTCTGA<br>GTCCCTCGGTGTCAGAAACACTGCTAAGAAATTTAAGAAATTCTGT<br>TAATGAGTTTAAGAAATGTTTTTAATGATTAAAAGTCAGTGACTTGT<br>GAATAACCATGTAACTTACAAACGCAAGGAACTCTGAAAGTGTGC<br>AGCACCACCGATCAGAAGAGAAAACCAAGGGACCCGAAATATGCT<br>TTAATTAAATTTTCTTTTAAAATGTCACTGGAAAGAACATCTTGGGA<br>AGACGGCCTGGCCGATCGCCGTGTGAAGGGCAAGCCACTCTGGCC<br>GAGAGGGAGCCCCACACCTCGGTCTCCCCAGACCGGCCCTGGCCG<br>GGGGCATCCCCCTAAACTTCGGATCCCTCCTCGGAAATGGGACCCT<br>CTCTGGGCCGCCTCCCAGCGGTGGTGGCGAGGAGCAAACGACACC<br>AGGTAGCCTGCCGCGGGGCAGAGAGTGGACGCGGGAAAGCCGGT<br>GGCTCCCGCCGTGGGCCCTACTGTGCGCGGGCGGCGGCCGAGCCC<br>GGGCCGCTCCCTCCCAGTCGCGCGCCGCCGCCCCCGCCCCCGCCC<br>CCGCCCCCGCCCCCACCCCCACCCCCACCCCCACCCCCAGCCGGC<br>GCCCGCGCCCGCCCCCGCGCCGGGCCCGGCTCGGCCCGACCCGGC<br>TCCGCCGCGGGCAGGCGGGGCCCAGCGCACTCGGAGCCCGAGCCC<br>GAGCCGC
PRKCA	CAGAGGTACAATCTTGGCTCCTGTAGCCTCAATCTCCTAGGCTCAA<br>GGAATTCTCCTGCCTCAGCCCTCCTGAGTAGCTGAGACTACAGGCA<br>CATGCTACCATGCCTGGCTAATTTTTTATGTATTTTTTGTAGACACGG<br>ATCTCATTATATTGTCCAGCCTAGTCTCAAACTCCTGGGCTCAAATG<br>ATCCTCTCACCTTGGCCTCCCAAAATGCTGGGATTATAGGCATGAGC<br>CACTGCATCCAGCCCAGATATTTTAAATGTATATTTATATATAATCTTT<br>TTAAAAACCCATACATGCTAACATTCTATGCACATTTCTGCATTTTGC<br>ATTTTCACTCATACATCTGGAAGATATTTCTATATCAGTACATATAATT<br>AAAAAAAAAAAAAATCTCATTTTTTAAACAGCTGCAAATAATTCTA

	GCAAATGAATGCATAATTGATCTAACTAATCTCCTGTGGATAGACAT<br>TTAATAATTGTTTGCTACTGGAGTCAGTGCTGCCTTTATACATATGCC<br>TTCATGTACACGTGCGTTTATATATGCAAGATACATTTATAGAAGTGG<br>AAGTGCTAGGATTACATTTATAAAATGTATCAACCTTTTAAAAGTAC<br>ATGCATCAAAATGTTTGATACATTTTTAAATGACAATTATTGCCAAAT<br>TGCTCTGTATCATGTCTGTGTGCGACAATTTACAATCCCGCTAATGA<br>TGTATGAAACCATTTGATTCTGTACACCCTTAATGAAACAGTGTGTT<br>CCTTACACTTTTTATGTGTGCCCAACTCTTAGGTCACAGTGGGGTTT<br>CAATATGATCTTAATTTACATTTATCTTTTTATGAGACATGTTGAGAA<br>TTTTATCATAGGAACTATTTGTATTTCCTTTTCTGAGAACTATCTGTT<br>CACGACATTTGCCATTATTTTTTCTTGGGCTTGTTATTCTTTTTGTTC<br>TTGATTAATATCTGGTGGGATATTTATTCCTACTTTTTTTTTCATTTGG<br>ACTGTTGAATTAGCTTGTTTACTTCTAAAAAAATTCTACTTTCATTG<br>GGCTAAGTTACATTTATGGTTTAATTAAGAAAAACTGACATTTCAAT<br>GTCTCTTCCTGTATGAGAACAGGGCATATCTTTCCATTTATTCAAGA<br>CATAAAGTGGGTTTAAACAGGTACATTTTTAAAAGATGGCTACTTGT<br>GGAGTCCAAATTGAGAAGGGCAAGAACGAAAGCAGGAAGACCAA<br>TTAAGTGGTCCTGCAGTGGCCCAGGAAAGAGATGATAGTGCTTAGG<br>CTAGGGTTGGGGGTGGATAGATTTGAGATCTGTCCTGGAGGAAGCA<br>CCAATTAAACTTTCTCATGGATTGGATATGGGAGTAAGACCAAGGG<br>AAGAATAAGGATGATGCCTAGGTTTTCAACTTGAGCCATTGAGGGA<br>CTAGTGATGCTGTGAGATACAGGAGACAGAAAGAGATCCTAGAAG<br>AAGGCTTGCTTAGAAGCAAAGTCCCCAAGCCTCAAACTCCAGGGA<br>GGCTGCCCACAGAAGCTCTTTCAGTGGAGACACAACCAACTGGAA<br>TCAATCAACTGTAATCACAACCAACCAGGGCCTTCATTAACCCATG<br>TGGTACCTTGGTGTGAATTTAAAAATGGTGGCTTTTCTGGGAAGAC<br>AGAGGCCCAAAGGTACACTTGGCAAGGAGTATGCGAGTTGAGTTT<br>TAGCCCCTACTTAGTCTCTTGACTGGGAGCTCCTGTGCTATGAGCA<br>ACCTGCAGGACCAGACCTGGAAGCCTTGGAACTAACCTCTGACTG<br>GGGCATCTGACTCATCCACTCCTATACAGTACTACCCTGCTCCTTAA<br>ACTTACCCCAAAATGGCCCCTGACTCTAGTGTTCTGCTACTCAGGT<br>AGATCCTCACCTCTACCTGTTTAGAGATCTGCCAGTTACAGTGAATC<br>TGACTGCTGCCTGCTTACGCATTTCGGCAGTTGCCTTGCCTCTCCTA<br>AACCTTGAATATGGTGCATAGAATAAAATATAAGCCTTAAGTCATCT<br>TGGTTACCATAAATCTCAGAATGATGCTTTTTCGATACTTATGCAATT<br>CCTATTGCCCAATTTGAAAATGTCCTGGTTCATTTGAAAACAGTCCA<br>GTTAGCTGCATAGAGCAGCAAGCTGTAAATTGTATCTGAAAGACCG<br>CTGGCTTCAAACCATTGGGATTCTGAGTCATCTGTTTGGTGGTCTG<br>ACTTTGATCCCAGCCTCCAGCCTCTGGGTCCCCCTGGTCTAGGTGA<br>CACCTCTCCAAACAGTGTTGAGCTTACTCCTCAAAGGCAGAGGAC<br>AGAAGCGGATTCTGAAAAACTCTGCGTTCAGTCTGACATTCCCTGT<br>TCTTTTCCCGCACCTTCCCCATGAAATAGCAGACTCGAAAATAAAA<br>TTTACTCATGCCCTCATGACTGACTGACTACCCCCAACCCAGGGGG<br>TTCTCCCGTCCTTAATCTCCTCCATTTTAGTTATACCATGGGGACTTC<br>AGGTAGGCTGCAGGAGTAGATGCTGGCCCACATTTCCATCAGTCCT<br>TGCTAGGGACAGTGTTACACTGCTTGGTAATTGATAAATCCCCACTC

	TGGGTGCACAGTCCCCTCTCCCCAACTGAGCTAAGATGTGGGCTAT<br>TGATGCTTTCATGCCCAGCCCAACCATGAATCAGTTGACTTCCAGT<br>CAATCATTCCTCCTCGTACCCACCAGGAACAACACTAACAACTGAG<br>GCAAGAATGCAAAGCCACAACCTAAAAGAAAACAGACACATTGTT<br>CCATCTGATCTACTGACAGCTAAGACAGTCACTCCATGGAGTACAC<br>AGGAGATTCGCAGGCTGCTGCCAGGGCTCATTCTGTTCTTGTCTTT<br>CCTATGACTTTACTCAGGATTCCATTATCAGCAACTTCACCCTCCAG<br>CTCTCTCCATCCTCCTATGAAAGCCTCAGGGACATAGAAATAAGCTT<br>GTCTTTGTAAAATGTGCCAGGATCTCCGTCTTAGATAAAAGACGAC<br>TATGAGAAAAGAAATTATTTCATTCCACTATGTTCCATTTGATTTCTG<br>CTTTCAAGCAATAAACCACCATGGCTTGAGATGCTTCTAGTGTTTCA<br>TGGAGAATTGGGACTATTTCCCCAACATGCCCTGCTCTTCATTCCTC<br>TTTGCTTCTTCCCTTTTTGACTCCTTGGACCCGAGGGGCGACCATG<br>AGTGGCGAAATTTCTGACCAGCTAGAATACCAGGCAGGTGCAGGC<br>TCTTGAAGCACCAAGACTGGCTCAAGAGATTGTAAATGGATCAGTC<br>AAGTGCATGCACCACTGACGGCCAGTCAGGAGAGCTTCCATTTGTT<br>AAAAATATAAAGGACATTCCTATCATCAAGCATCACAATTAGGCAAT<br>CTCAAAGAAAACAATATTAGCTCTACTTGGCCTATGTTACATGCTCT<br>AAGAAAGTATTTTAGAAAAGTTTTTTTTTCAACATAAATTTATTGA<br>AGGATCAAACGTGTTCTCTTTCTTGGTCTCTTTAGCTCGTAAAGTCG<br>TTTTGGGGATTTCGTGAATCTGGCAAATAAAACAAAACACCAATGA<br>AACGCTGAGAAATGATTATTTCCCTTATGTATTTTAGCGGCCTCCAG<br>GCAGACCTGATAAATATTCAGCGAGTGATTAGCTGGTGCATCCCCA<br>CCTGCGTCCTTTGCATAGCCATGCCCGCTACTCTTTCACAGGCAGTT<br>TTACTTGGTAAGTTACAAACGTACTGGCTGCACTCTGCACCTGACA<br>GTATTGCAGTAATTAGCTTGGGATGCAAAATGATTTCTAAATTAAGG<br>TCCTGGACTTGTAAAATCAAGAAAGCCTGATAATCAACATCGTCGC<br>TCTGGTGGCCATTGAATAACGGGATAAATACCTAAGACAAGTCCTTT<br>AACCATCCCACGTGGTGATAAATGCCCGACGTACAAATTAAAAGTC<br>TCTACCACACGCAGGTGAAATAAGGTATAGGTCAAGAAGAGAAGG<br>TAGGGGAAAGGGTCAATGCTTAATTATTTAAAAATCGCTCTCTTCCT<br>CCTGTAGGAAGGTCTAGGGGCTAGGTCTTTTAGTGCACACGTGATG<br>AAAGTGACATTTTGGCCAGGTGAAACCTCAAGCTGGTACAGGCAG<br>CTAAAACACGGGCGTCGTGCCCTGGAGACACCGGGGGTCCTGAGG<br>ATGGGGAAGGGGCTCCCTGCTGCGGGTGCTGAGGGGTTAGGAGGT<br>GAGCACCGGGCGGGAGGCTCCGGGGCAGGGGCTGCGCGTGGAGC<br>CCACTCCCGGACGCGGGCTCCTCTCGGCCCGGCACGGGTGGCGCA<br>GGACTCCGAGCCCGTCGACAAGAGAGAGAGCGGGAGTGGGAGAA<br>GGAAGACCGGGAGAGGAGGAGGCCGGGCGGCAAGAGCGGAAGA<br>AGAGGAGGAGAACTGAGGCGGAGGAGGGCGAGAGGGAGGAGAG<br>CCGGGCGCAGGAAGGGGAGGAAAGAAAAAGGCGGCGGAGGAGC<br>GCGGACCCGCCACTCCCGGCCTCCTCCCCCGCATCCCCACTCGGGC<br>CGGCCCTCCCCCGGCCGCCGCCGCCGCCGCCGCCGCCGCCGCCGC<br>CGCCGCCGCCGCCTCCCCTTGCCCAGTGTTCCCAGCACCGCAAGG<br>CACTCGCTGCCTCCTCCGCACTCGGTCCGGTCCGCTCAGCGCCGCC<br>GCCGCCGCACCCTCGCCGCACCCCCGCGCGCGCCCGGCCCGCGCG

	CCCCCGCCGCTCGTCCTCGCGGCCTCGCGCTCGGGGCATGCGCGGT<br>GGGCGGTGCCGCTCCGGGCCGCCTCTGCCGCCGCGAGTAGGAAGC<br>GCGAGCGCCGGGCACCGGGCTGTCAGTGAGCCTGGGGCCAGCGG<br>GCGAGCCAGCGGCTCCGGCTCCCGCTCCCGCTCCGCGCAGCACCA<br>GCCCGACTCTCCCCGGCCCCCGCCGCGCCCCCTCGCCGCGACCTCG<br>GCCACCGGCCCGCGCCCCGCGCCCGGGGTCGCCCCGAGCCCGCAC<br>CTCTCCCCCGCCGCCCCCGCCCACCCGGCCCTCCGCGGCCGCAGCT<br>CCCCGGCGGAGGCAAGAGGTGGTTGGGGGGGA
PSEN1	GGTAGCCTCCGTATTGATGAAGAAGGGGATGGACTTACCCTCCACT<br>GTAAGAGTTACCTAAAGCATCTGTGATGGTCCAGGAGGCTTCTGAG<br>GCGATCGGGCAGCGTCAGTCTTCAGCCACTAAGCTGAGTGGGTCT<br>GGGAAGGAGTCGGCCAAGGAACATTGGGTTTGAGCTCCAGGACCT<br>TTAGGAGCAGTGGCGATGTGAGTCGGACAGTCTGACCTCCAGTGG<br>GGGCCCGCACAGACAGGGCATGGCTTAGGAGGAATCTCGGGCTGT<br>GGGCATTCCAAGGCCCAGTGGCCAGGCTTTTGGCATTTGAAGCAA<br>GGTCCACGAGGAGGTTTTGAAGGAGCCCCTGGGAGCTGTGGCTTG<br>GATGTTATGAAGTTTTTGTATGCTGGAGACGTGGTTGTGGGTTGTCT<br>TACAGCAGAGGCAAGTAGCTGTAACTCAGAGATGCGTTGTCACTTG<br>GCTGCCTCTTCTCTATTATTGTACACCTTGAAGGCGAGGTTAATTAA<br>GTCCTGTTGTGAGGTTGAGGGCTGGAATCCAATTTTTTGGAGTTTT<br>TTTTTTAATGTTGGGAGCAGATAAAATGCATATTGAGAATAAGATGG<br>CCTTAGGGCTGGGCGCGGTGGCTTACACCTGTAATCCCAGCACTTT<br>GGGAGGCCAAGGCGGGCAGATCACCTGAGGTCAGGAGTTCGAGA<br>CCAGCCTGACCAACATGGAGAAACCCCGTCTCTACTAAAAATACAA<br>AACTAGCCTGGCGTGGTGGTGCATGGCTGTAACCCCAGCTTCTCGG<br>GAGGCTGAGGCAGGAGAATCGCTTGAACCCGGGAGGCGGAGGTT<br>GCAGTGAGCTGAGATCGGGCCATTGCACCCCAATCTGGGCAAAAA<br>GAGTGAAACTCCATCTCAAAAAAAAAAAAAAAAAAAAAAGAATAA<br>GATGGCCTTCTGGCCCTTCTGGGTCTAGGGCAGTAAAGCATCTAAG<br>AGTTGTTGCCAAATGGGCCATGAACTGGGCTGGGTTTTTATATTTGA<br>TGAAAAGGAGCCTAAGCACTAACTGATTTGGGAGAGGTCAGATAA<br>AGAAAAAGGAGCATTAACCTTGACTGTGCCTTCAGCTCCAGCCAC<br>CTCTTTAGGAGGAAATTGTTGGGCAGGTGGGGGAGGGCTAGTCAC<br>GAAACAAAACTGTAAGCCAGACCAGGTATGAGGAGGGGAGGTGAT<br>AGAAGGATTATAGGGTGGGGGAGTGGAGGCCGAGGAAGAATTGGG<br>ACCTGGCTCGGCCTGGTGAGGAGCAGCCTGGGGAGAAGGGGAGA<br>GGTCAGATGGGTCCGTAGAAAAGGAGGATTTAAAGGACTCAGAGC<br>TTGGGGTGGAGACTGAAGGAACACACAGGAGAGAAAGAAGAAAG<br>ATTTGGAACGAGTTGCATTGGGAGCAGAGACTAGGGAGGGACCAA<br>TGTGTGAAAGAATGCCTGGATGTCAGGCACCTCAGACCATTTGCCC<br>ATTTTTCGACAAAAATTATCTAGATCTTGTAGGATGGAGAAATCAAA<br>AGTGCCGTTTTCTGGCTATTTGGGACCATTGTCGAATTTGTACTGGG<br>GCCAAGCAGGGTTGTAGAAGAAAATAAGGTGTTTTGGTCTTAGGTC<br>AAGTGTAAGTTGAAGAGGTTTTAAGTTCTTGAGAACACAGGCTAA<br>GGGAGAAAGGGGAATGGAGGGTGGAAGTTTGCCCATAGTGAAGG<br>AGGCAAGCCCAGAGAAAAGAGAGGGTAGAGACTTGGAGTGGGTG

	GTGGTACTTGCCACCCAGGGGAGGAGGTACTTGCCACCAAGGGGA<br>GGTGGTACTTGCCACCAAGGTGAAGGATCAAGGCAGGCATCCCCG<br>TGGTGATCAGACACCTCTGAAATGTGAGTGAATAATCAAGCAGGCG<br>TTCCTGCAGTGATTGGACACCAAGGGAAAACTGTCTTCCCGAGTCT<br>GTGACCAATGCCAGAGTTTTGGGTGCACAGATAAAATGTGTCTCCT<br>TTGTCTCTACTAGAGAGGAAAAAACTGGAATTGGAAGGACAGGGA<br>GATTAAAGGGTAGTGAGAAGGCTGGAGAAGAGAGTGAAAAGGCC<br>GCTTACCCAATTTTAAATTGGTGAGATGTTCCTTGGGCTGATTGATG<br>GGAGGACGCGAGGTCATAGGTGGATCTCCTCATGCAGTGAGGGTG<br>AGGACAGGGGACCAGTTTCCCGAAGGAGTCCTCCTGTCCTGGGTC<br>TTCAGCACCAAATGACACACACGTCCGTGTGAAGAGAGTCCACCA<br>ACAGGCTTTGTGTGAGCAACAAGGCTGTTTATTTCACTTGGGTGCA<br>AGTGGGTTGAGTCCGAAAAGAGAGTCAGCAAAGGGAAATGGGGG<br>TGGGGCAGTTTTATAGGATTTGGGTAGGTAGTGGAAAATTACAGTT<br>AAAGGGGGTTGTGCTCTTGTGGGCAGGGGCGGGGGTCACAAGGTG<br>TTTGGTGGGGAGCTCCAGAGACATTATCCAGGAGAAGGAATGTCA<br>CAAGGTAATGTCATCAGTTAAGGCAGGAACTGGCCATTTTCACTTC<br>TTTTGTGGTCCTTCAGTTGCCTCAGGCCATCCGGATGTATACGTGAA<br>GGCTTGGGCTCAGAGGCCTAACAGTAGGACATAAAGAAAATGAGA<br>AGAAAATTATCTGAAGGCTGGTTATTCAATGTTAGGGTTTGCTATTT<br>TTTTAAAATCTTACTGCCTTTTTGTTGCAGGACTTTTTTTTTTTTTTG<br>GGAAAAACAGAGTCTCACTGTGTCTCCCAGGCTGGAGTGCAGTGG<br>TGCAAACTCGGCTCACTGCAAACTTTCCTTCTCAGATTCAAGCCTC<br>AGCCACCTGAGTAGCTGAGATTACAGGCCCACACCACCATGCCGG<br>GCTAATTTTTGTATTTTTAATAGAGACGGGGTTTCACCATGTTGTCC<br>AGGCTGGTCTTGTACTCCCAACCTCAAGTGATCCTCCTGCCTTGGC<br>CTCCCAAAGTGCTGGGATTACAGGCATGAGCCACCGTGACCAGGC<br>GTTCATGAAAAATTTTCATGACATTCTGTGAGGTGGGTGGACCATA<br>ATTTATTGTACCACATTTATTTATTTATTTATTTATTTATTTATTTA<br>TTTATTTATTTTTATTTTTTGAGACATAGTTTCGCTCTTGTTGCCCAG<br>GCTGGAATGCAATGGCGCAATCTCGGTTCACTGCAACCTCTGCCTT<br>CCAGGCTCAAGCAATTCTGCCTCAGCCTCCCAAGTAGCTGGGATTA<br>CAGGCATGCGCCACCACGCCTGGCTAACTTTTTTTGTATTTTTAGTA<br>GAGACGGGGTTTCTCCATGTTGATCAGGCTGGTCTCGAACTCCTGA<br>CCTCAGATGAGCCGCCCACCTTGGCCTCCCAAAGTGCTGGAATTAC<br>AGGCGTGAGCCACCGCACCCTGCCTATTGTACCATATTTAGTTTCCC<br>AATTTTTTGCTATATAATATTCCATGAATATCTTTGCCCAAAATTTTTG<br>TAGATTGTTTCTAAGAATAGTTCATCAGCATTTTAAGACATCCTATTA<br>ATATTAGCGGACTGCAATCAGATTGAAGTAGTTCACAAATAGATGG<br>GTTTTAGCCTGTATTTGTTCAATACTGAACTATTGTATGTATCAGGCA<br>CTGTTATGTATCAGGCACTGTTCTCAGCACTTGTAAAATGTATATGT<br>GCTGCTGACAAGCATGAGCACTTGTAAATGAACAAACTAAAATCCC<br>TACTCTTGTTAAAGTGGGGGAGGTGAAGAATTTTAAAAACATCTTT<br>TTTCGTTCAACCTTCTCCCACAACTTATCTTTCATTCTGCTAATGGA<br>AAATATTACTTTTGTTCTTAAATATGAAGCTTTTGTGTGTAAAAAGTA<br>TTAGAATCTCATGTTTTTGAACAAGGTTGGCAGTGGGTTGGGAGGA

	GGGATTGGAGATTGATGCGATAGGAATGTGAAGGGATAGCTTGGGG TGGATTTTATTTTTTAATTTTAATTTTTATTTTTTGAGATGGAGTCTTG CTCTGTCTCCCAGGCTGGAGTGCAGTGGTGTGATCTCAGCTCACGG GTTCAAGCGATTCTCCTGCTGCAGCCTCCCGAGTAGCTGGGATTAC AGGAGCGCGCCACCACACCCGGCTAATTTTTTTGTATTTTTAGTAGA GACGGGGTTTCACCATGTTGGTTAGGCTGGTCTAGAACTCCCAACC TCATGATCCGCCTGCTTCGGCCTCCCAAAGTGCCGGAATTACAGGC GTGAGCGACTGCACCCGGCCGCTTGGGGGTGGATTTTTAAAGAAA CTTTAGAAGAATGTAACTTGCCCAGATACCATGTACCGTTAATTTCA TTTTCGGTTTTTTGAATACCCATGTTTGACATTTCTCCGTTCACCTTG ATTAAATAAGGTAGTATTCATTTTTTAGTTTTAGCTTTTGGATATATGT GTAAGTGTGGTATGCTGTCTAATGAATTAGACATTGGTACTGTCTTT ACCAAAACTGGACAAAGAGCAGGCAGATGCAAAAATCAAGTGAC CCAGCAAACCAGACACATTTTCTGCTCTCAGCTAGCTTGCCACCTA GAAAGACTGGTTGTCAAAGTTGGAGTCCAAGAATCGCGGAGGATG TTTAAAATGCAGTTTCTCAGGTTCTCGCCACCCACCAGAAGTTTTG ATTCATTGAGTGGTGGGAGAGGGCAGAGATATTTGCGATTTTAACA GCATTCTCTTGATTGTGATGCAGCTGGTTCGCAAATAGGTACCCTAA AGAAATGACAGGTGTTAAATTTAGGATGGCCATCGCTTGTATGCCG GGAGAAGCACACGCTGGGCCCAATTTATATAGGGGCTTTCGTCCTC AGCTCGAGCAGCCTCAGAACCCCGACAACCCACGCCAGCGCTCTG GGCGGATTCCGTCAGGTGGGGAAGGCCAGGTGGAGCTCTGGGTTC TCCCCGCAATCGTTTCTCCAGGCCGGAGGCCCCGCCCCCTTCCTCC TGGCTCCTCCCCTCCTCCGTGGGCCGGCCGCCAACGACGCCAGAG CCGGAAATGACGACAACGGTGAGGGTTCTCGGGCGGGGCCTGGGA CAGGCAGCTCCGGGGTCCGC
PTH	GTATCACAGGGATAACATGTATGTCCAAACTCATCACATTGTACACA TTAAATATGAACAGTTCTTTGTATATCAATTGAACCTCAGTAAATTTG TTTTTTTTTTTTAAAAAGGGAGTAGAAATTATCCAATCTGTTCACAA AACATGGAATTAGAGAGATGTAGCTGGATTTTTCAGCCCTGGAAGC TTGTTGATGTCAAATTCCCAAAGCTGTACTAATTCCTATTGAGCAAA CAGAAGGAAAAAGTATCCGTCTTAACTTAAACCTTCCATAAGGGAA CAGGGCACAACACATGGTATTTTGTCCTTCCCAAATGTGAGGAATA CATTAGATTGTTTGACCCTAGCTTCAGTACTTTAGAATGCTAAGAAA AGCTGACGTTTTCAAAGCAAAAAGCTGTGCTCAGCTCTTCTCTGTC ACGGTGTCATCAGTCTCTTGTGCCCACATATCCCCTAACCCTGTCCT CCTAAGTGATTTCCTTCTATCTAATACCTGCAGGTCCAGAACGTGAT GGTTCAGATAAACTATATATCAAGTCTTGGCAGCTATATTACTGCTTT GTGGGAAGGTCAAGTAGTCCTTTGAAAAATTTGAAGGACACAACA ATATTACTCATGCACACATTTAGAGTTTAGAGAGGACTGCAAGACA AAACTCCTTGGCATTCACACAATAATCTTCCAAAATTGTACCCAAAT CTTACTTCTTTGGAGTCATCATCCATTTCCCCATTTTCCCTCTCCATC CACAAGCATATACTCAATATTCAGTCTTTTTTTTTTTTTTTTTGAGAC AGGGTCTCACTCTGTCGCCCAGGCTGGAGTACAGTGGCATGATCAG AGTTCACTGCAGCCTCCATCTTCCAGGTTTGGGTGATCCTCCCATCT CAGCCTCCTGAGTATCTGAGACTACAGGTGTGCACCACCACATCCT

	GCTGATTTTTTTTTTAACTTTTAGGAGAGATGAGGTCTTGCTATGTT<br>GCCCAGGCTGGTCTCAAACTCCTAAACTCAAGCAATCCCCTTGCCT<br>TGGCCTCCCAAAGTGCTGAGATTACAAGTGTGAGCCACTGCGTCTA<br>TTCAGTCTTCATTGCCTGCATGCCTGTGCGCATGCAGTTCCCTCTGT<br>CCTTTCTTACCTTCTCTGATGGAAAACACAATCACCTGTCAGGACA<br>GTTCAGCTTTTCAGTAAAACAGCTTCCAACAACTTTAGGGAAATTG<br>TCACTTCCTCTTCTGTGCTTTTATAAGTCTTTGTTCATAACCCTATTAT<br>AATACCTATCACATTATATTATAATTATGTGTTAAATTTCAAGGCATTG<br>ATTATGTCTCAATTATCCAGTGATTAATACAGCCCCTGGCAGATAGTA<br>TTCAACGCATTTTTTTGAGTGAACAAATGAGTGACCACTGTCTGAA<br>CTATTTGCTTTATTTTTCTGTAAAACTAGTTTAATAATGTGGTTGCCT<br>TGGTATGACAATTTATTCTATAAATAAGTGTAAGAGAAAGAAGAAC<br>AAGAAAGTGATAATTGCATCACTCCTAGAAGGCTGTCAATTACATTA<br>AACTTACCCAAAGCGGGAAAAATCTCCAAATCAGAATATTAAGCAT<br>GGCTTTATAATAACAATGCAAAAAATGTGTTTATAAAACCATTTGTG<br>TATGCAGAAGATTGAAACTGGACCTGTTCCTTACACTATATACAAAA<br>ATTAACTCAAGATGGATTAAAGATCCGAAACTATAAAAACCCTGGA<br>AGACAACATTGGCAATACTGTTATAGACATAGGAATGAGCAAAGAT<br>TTCATGAAGAAGATGCCAAAAGCAATGGCAATGAAAGCAAAAATT<br>AACAAATGAGATATAATTAAACTAAAGAGCTTCTGCACAGCAAAAG<br>AAACTATCAACAGAGTAAAGAGACAACCTACAGAATGGGAGAAAA<br>TTTTTGCAAACTATGCATCTGACAAAGGCCTAATATCCAACATCTAT<br>AAATTAAACAAATTTACAAGAAACAAACAACCCCATTAAAAAGTG<br>GGTAAAGGACATGAACAGACATTTTTCAAAATAAGACATACATGTG<br>GCCAACAAGCGTATGGAAAAAAGCTCAACATCACTGATCATCAGA<br>GAAATGCAAATCAAAACCATAACGAGCCACCATCTCACACCAGTC<br>AGAATGGCTGTTATCAAAAAGCCAAAAAATAACAGGTATGGGTGA<br>GGTTGTGGAGAAAAAGGAACATTTATACACTGTTAGTGGGAGTGTA<br>AATTAGTTCAACAATTGTGGAAAACAGTGTGGCGATCCCTCAAAGA<br>CTAAAAGCAAAACTACAATTTGATCCAGCAATCCCATTACTGGGTAT<br>ATACTCAAAGGAATATAAATCATTCTATAATAAAGACATATGCATGCG<br>TATGTTCATTATAGCACTATTCACAATAGCAAAGACATGCAATCAAC<br>CTAAATGCCCAACAGTGGTAGACTGGATAAAGAATACGTGGTGCCT<br>ACATACCATGGAATACTAGGCAGCCATAAAAAAGAACAAGATGGA<br>GCTAGAGGCCACTATCCTTAGCAAACTAACATAGGAACAGAAAACC<br>AAATATCTCATGGTCTCACTTATAATTGGGAGCTAAAAGATGAGAAC<br>ACATGGACACAAAGAAGGGAACCACACACACTGGGGCTTACTGAA<br>GGATGGAGAGTGGAAGGAGGCGGAGGATCAGGAAAAATAACTAAT<br>GGGCACTAGGCTTAATACCTGGGTCATGATATAATTAGTACAATAAA<br>CCTCCACGACATGAGTTTACCTATATAACAGGCCTGTACATGTACCC<br>CTGAACTTAAAAGTTAAAAAATAAAAATCCATTAGTGGCTGGGTGT<br>GGTGGCTCATGCCTGTAATCCCAGCACTTTGGGAGCGAGAGGTGG<br>GAGGACTGCTTGAGCCAAGGACAGCCTGGGCAATTTAGCTACACT<br>CTGCCTCCACAAAAATTTAAAAATTAGCTGGTTGTGGTGCTGTGTG<br>CCTATAGCCCCCACTACTCAGAAGGCTGAGGTGGAAGGACTGCTTG<br>AGCCCCGAAAGTTGAGGCTGCCATGTCCATACCACTGCACCACTCC

	AGTCTGAGTGACAGAGTGAGATCCTATCTCAAAGAAAAAAGAAAA<br>ACCATCTGTATTTCCAATTCTTCTTCTCAACAGAGTATAGCAGAAAA<br>ATGACACACAAACATCACATGTAGAGTTTGCATCAGAAGAGGTCTT<br>TAAAAAGATCAAGACAACAGATTTTTACAGTTAAAAGAGACCTCTT<br>GGTTATCTAGTTCAACTTTCTTCTATGTATGAATCTCTATAGTATGCC<br>TGATAAATGCTCATCTACCTTCTACTTGAATATTCTCAGTGATGGAG<br>AAATCACTATTTGAGAAGGTACTTGATTCCTTATTTTGACAATTATAA<br>TGTCCAAGTTAAACCCAATGAACCCAGCCCTTCCTTCTTAAACCAC<br>AGAGAATACTCCTTCTTCCTTATGGTAACCCTATAAATAATTAAACA<br>CAGCTATAAAGTCATCCCGTCTTTTTTTCCTCAGACTTTTCTAGTTTC<br>ATTCAACTACTTCTCATGTTATAGTTTTCAACATTTTCACCAAACTGA<br>TTGATATCCTCAGTGTATGTTCCCATTTGTTTCTACTGCTTCTAAAAT<br>GGAGCCTGGAGCAACACTCTAAGTGAGGTTAACCCAGAGTGGAGA<br>ATCTCCTAGGACAGGACTAGTATATATCTCTCATTATAAACTCCATAC<br>GGTTACTATTCAAATTTGTACTGGCTCTTACCTAAGGTAGAGTCAAA<br>CCTTGACAATGGCTAGTAGGAAAAAGGATGACATGAAGCCAGGAT<br>GGGACCAGAGCTGAGAGAACTGATCCCACTCTGGCACCGGATCTT<br>TGGTGGGAAGGCAAAGCTGTCTAAAGGAGTCCTAGATATATCAAAG<br>ACATTCAGGGTGTTTTGACAGGTGGACCAGGCAAATATTGCTGGAG<br>GAATATTTGAAGTCAGACATGTGGCAGCATCATGCCTCCAATAGGTT<br>TTGGAGCAGGCTCTCATCATCCCTTAGGGAACATGAGAATAAAGAC<br>CTAACCCCTAGGGGAAAATGTTGCAAAGAGCTTTTGCTGTGTTTTC<br>AGATGACTTTGCAAATTAAAGTCAAGAAGACTGGAGTTCAAAGAA<br>GGGCTGTATTTCTAGGCAGAAACTGAGGTAGTAAGAATCTGGTGTC<br>ATAAAGACCTTTGTTGAATTCCATCTTTTCTGCTTTCCAAGCCTAAG<br>TTTCCTCATGTAAAAATGGGGATAAGCACTTAACTTTAGTACTTTAA<br>GTACTAACTTTAAAGGACTGCTGTGAAGATCTAGTGGGATAATATAT<br>GTAGTTAGGCATGCAGTTAGTGCTTATCAAATGTTATTATTATAGATT<br>AAGATGCACAAATAAAATATAAGTTAAATAAATTTTAAAATAATTTC<br>ACTTTTGAAGCTTTTAAAGTAATTATGTACTAAGAGCATTTTCTCAA<br>ATTATTCTTAACACTTCCTTTAAGAAAAGGGCTATATCTGTTTTTTGA<br>AAGATGACCAGAAGTGACATGGATTGGTTGAAAATGGCTTGTAAA<br>GTAAGCCTAACATTTATGATTTATTACCATAAAAACTGTACCAACAG<br>TACGGTTATAACAAATACACTTATTTTTGGATTTTATTTTCAAGTAAG<br>ATAATGACTTTATCATAAACCTTTGAAATCAGTCTTTTTACAGTATAA<br>ATTCAGATTCATTAATCCACATAGAATTTTTCTCGATGGTATAATTCT<br>GTATTTGTTAAAAGTCTTTGCATAAGCCCCTTGTCAAGCCAAATGCT<br>GTTTTCCTTTTAGTATCCAATTATCTGAAACTTAAGAAGAGTGTGCA<br>CCGCCCAATGGGTGTGTGTATGTGCTGCTTTGAACCTATAGTTGAGA<br>TCCAGAGAATTGGGAGTGACATCATCTGTAACAATAAAAGAGCCTC<br>TCTTGGTAAGCAGAAGACCTATATATAAAAGTCACCATTTAAGGGGT<br>CTGCAGTCCAATTCATCAGTTGTCTTTAGTTTACTCAGCATCAGC
RAGE	CCTCCCAGGCTTAAATGGTCCTCCCACCTCAGCCTCCTGAGCAGCT<br>GGGACTACAGGCATGCACCACCATGCCCAGTTAAGTTTTGCATTTT<br>TTTTTTTTTTTTTGAGACGGTGTCTCCCTCTGTTGCCCAGGCTGGAG<br>TGCAGTGGTGCCATCTTGGCTCACTGCAACCTCCGCCTCCCGGGTT

	CAAGCAATTCTCTGCCTCAGAGTAGCCAGGATTACAGGTGCCCACC<br>ACCACGCCTGGCTAATTTTTTATAGTTTAGTAGAGACAGGGTTTC<br>ACCATCTTGGCCAGGCTGGTCTTGAACTCCTGACCTTGTGATCTGC<br>CGGCCTCAGCCTCCCAAAGTGCTGGGATTACAGGCATGAGCCACC<br>GCGCCTGGCCAATTTTTGTATTTTTTGTATTATTATTGTTATTTTTTGA<br>GACAACGGCTCTCTCCTGTTGCCCAGGCTGGAGGTCAGTGGTGCA<br>AACATGGCTTATTGCAGCCTCGATTTCCCAGGCTCAGGTGATTCTCC<br>CACTTTAGGCTTCTGAGTAGCTGGGACTGCAGGCACCTGCCACCAA<br>GCCCAGCTAATTTTTTGTATTTTTAGTAGATAAGGGGTTTCACCATG<br>TTGCCTCGGCTGGTCTCGAACCCCTGAGCTCAAGCCATCCGCTTGC<br>CTCGGCCTCCTAAAGTGCTGGGATTACAGGTGTGAGCCACTATTCC<br>TGGCCAGGAAACCTTTTCTTACACTTAAAAAATTCTAAATGTATTTC<br>TTTGAGTTCCTTTTTCTCAGAAACCTACACAGACTTCTTCGTAGAC<br>AGAATTTTACCTATTTTTGCATCCACACTAGACCAGGATCTAATTTCT<br>AGAGTGCTTGGTTAACTACCATCCTTCATGTTAATTTCAGTGGCTAA<br>TGTTTTTGCTCCTCCTTATTTAATATCCTTGTTCAAGCTTAGTTTTCC<br>CTGAGATGTGTTTATTATTTTACAAAATCAATCTAATTTCTAATATCTT<br>TAGGTAGATTTGCGTTTTACCCACTTTAACAGTGTTAACACGTTTGA<br>AATGTCTGTCATCCCTAGCTTTTCTGACTTGTTTCCTGGTTTGAACC<br>TTAATTCAGATGCTCAGCAGAAGGAAATAGGTTGTCTGCTAAACAT<br>AGCCACATGGCTGAATCCCTTGACATTGGCAAATAGGGAAAAACTA<br>ATGCTAGAATATGGGATGAACAGTCTGAGGGAGTTTTAGAACGATT<br>CAAAAAGCTCAAGTGGTTATAATTAGGTAGTGTTCATACAGTTTTAA<br>AAGCGGAGCTCTGGAACTGGGCGCGGTGGCTAACGCCTGTAATCC<br>CAGCACTTTGGGAGGCTGAGGCAGGTGAATCACCTGAGGTCAGGA<br>GTTCGAGACCAGCCTGACCAATATGGTGAAACCCCATCTCTACTAA<br>AAATACAAAAATTAGCCAGGTGTGGTGGCACGTGCCTGTAGTCCCA<br>GCTACTTGGGAGGCTGAGACAGGAGAATTGCTTGAACCTGGGAGG<br>CAGAGGTTGTAGTGAGCCGAGATCAAGCCACAGTACTCCAGCCTG<br>CGTGACAGAGCAAGACTCTGTCTCAAAAAAAAAAAATTTAAAAATA<br>ATAACTTAATAAAAAAAAAAAGCAGAGCTCTGAGATGCGTGGTGG<br>TTCCATTTCACAAGCGTTTCCTGAGCATGTAATATGTGCTGAGCAGG<br>CTCCTGGCTCTGGGTGAGCTGGGCAGGTGAGAGGCTTGGGGTGAA<br>GACCCTCTCTGGAGGAGTTCACAGCCTGGTACTTCTTACTTATCTGT<br>CCATTCAGGGCTGGAGTGGCCCAGCTAGGCAGGTGCTGAGGCCAC<br>CCAGCTGAAGCCTGAACAGGTTGACCCCTCCGCTGATGTTTGAGC<br>GTGTTTACCACAGGAAGAGAAGCTCCTCAGCAATTGTTCCTTCTTT<br>TCAAAGCAGCATTTCCTATTTCATTTAGAATTAAAGAAAGAATAAGG<br>AATTCTCTAGGCCAGTGGTTTTCAAAATATGGCCACTGGTGCAGCA<br>TCACCTGGAATTTTGTGAGAGCTGCAAACTATCCCCCACTCCAGAC<br>TTACAGATTTAGAAATAATGGGGCGGAAATCCCTGATCGAATGGAA<br>CATTCTGGAGTTCGTCCCCTCACCCAACCTTAAGATGAACTTGGAA<br>CAAGGGTGCTCAGCCAGAGGGGCAGGTGAGGCCGCAAGTCCAGC<br>TGCCCCTGTTCACCTAGGTAAGCACATAGACTGTGTCTACCCCCTG<br>GGGGCACCTTGCTCTAAATCATCTGCCCAATCAGGACCAACATGGT<br>CTCTACAACGTCACTTCTGAGAACCACTTGTCCACAATGAAGGGA

	AGAAGAAAATCCATCAGCACCTGGTGAATTGGGAAACAATGGGGC<br>TCTCACTTGCTAAATGCATTCACCTGTTCACTTATTTAATACACTCTG<br>CCCTGCAGGGTAGAGAAAAGACACATGAGGGGCCCAAGGAACTTA<br>GAACCCACCTGGTTAGAAGAAACTAACAAGCAAGAGACAGGGTTA<br>GAAATATAAATGTGACATAAGGGCAAGCCCCCAAATAAGAAAGAA<br>CACAAGAAGAGAAGAAGGGATAGATCAAATCCTAGTGGTCTGGAG<br>AGATTAGATGTGGCTTCTCAAAGCCAACAAAGATAGCACCACCAAC<br>AATAATACACCAGCAATAATCGCTATTTATTTTTCAACTTTTTTATT<br>GAGAGAAAATTTACATAAAATTCACCATTGTAACCATTTATTTATTTA<br>TTATTTTTTGAGATGGAGTCTTGCTCTGTCGCCCAGACTGGAGTGTA<br>GTGGCAGGATCTCGACTCACTGTAGCCTCCACCTCCTGGGTTCAAG<br>TGATTCTCCCGCCTCAGCCTCCTGAGTAGCTGGGATTACAGGAGCG<br>CATCACCACACCTGGCTAATTTTTGTATTTTTAGTAGTGACAGGGTT<br>TCACCATGTTGGTTGGCCAGCTGGTCTCGAATTCCTGACCTCAGTT<br>GGTCCATCCTCCTCGGCCTCCCAAAGTGCTGGGATTATAGGTGTGA<br>GCCACCACGCCTGGCTTGTTTTGTATTTTATTTATGTATTTGCTTTTTT<br>AGACGGAGTCTCACTCTGTCGCCCAGGCTGGAGTGCAGTGGCGCA<br>ATCTCGGCTCACTGCAACCTCCGCCTCCCGGGTTCAAGCAATTCTC<br>TGCCTCAGCCTCCCGAGTAGCTGGGATTACAGGTGCCTGCCACCAC<br>ACCCGGCTCATTTTTCTATTTTCAGTAGAGATGGGGTTTCACCATCT<br>TGGCCAGGCTAGTCTTGAACTCCTGACCTGGGGATCCACCCGCCTT<br>GGCCTCCCAAAGTGCTGGGATTACAGGCATCAGCCACTGTGCCCA<br>GCCGTTTTGTATTTTTTTAGTAGAGACGGGGTTTCCCCCATGTTGGC<br>CAGGCTGGTCTCGAACTCCTGGACTCAAGTGATCTGCCTGCCTCGG<br>CCGCCCAACGTGCTGGGATTGCAGGTGTGAGCCACCGCGACCGGC<br>CGCTTTCTCTTTCTTTCGAGACAAGGTCTTGCTTTGTCACCCAGGCT<br>AGAGTGCAGTGGTGCTATCATAGCTCACTGAGCCTAGAACTCCTGG<br>TCTCAAGATACCTCCGCCTTATTCTCCTGAATAGCTGGGTTTACAGG<br>CATGTGCCACCGTGCCCGGCTTTTTTTTTTTTTTTTTTTTTTTTTGA<br>GTCTCTTGCTCTGTCGCCCAGGCTGCAGTGCAGGTGGTGCAATCTC<br>GGCTCACTGCAAGCTCCGCCTCCAGGGTTCACGCCATTCTCCTGCC<br>TCAGCCCCCGGAGTAGCTGGAACTATAGCGCCCGCCACTACGCCCG<br>GCTAATTTTTTTGTATTTTTAGTAGAGATGGGGTTTCACCGTGTTAG<br>CCAGGATGGTCTCGATTTCCTGACCTCGTGATCTGCCCGCCTTGGC<br>CTCCCAAAGTGCTGGGATTACAGGCGTGAGCCACCACGCCCGGAG<br>TTTTTTTTTTTTTTTTTTTAAGAGGGAAGTGGGGTTTCTCTATGTTGC<br>CCAGGCTGGTCTTGAATTTCTGGCCTCAAGCGATCCTCTTGCCTCG<br>GCCTTCCAAAGTGCTGGGATTACAGGTGTGAGCCACCACACCTGG<br>CCAATTCAGTCTTAAACCAATTTTCTGAGGCTTCGTTTTGGCAAGG<br>CTACTTATCTGTTTTAAAAATAAACGATGTATTTTAGTAAAAGAAAA<br>AAAATGGAGGAAAAAACAGGGTTTTGATCTCATGGAATAATTATGA<br>GGCTAATCTAATGAGCTTCATAAAATACTATACCCATACATAGTATGC<br>TTGTTATTTTATTGTTTTGTCAGGGTCCAGAGAACTAGGCCACTGTG<br>TTGCTTAGGCACCGTGTCTGCACATAGTTCATTCCGGTGTACATTCA<br>TTCATCTTTACACTAGAATGTTAGTTTCAAGAACTCAGTCACCTCGT<br>CTACACTGTCCTTTAATCCCCAATACCTGCGACAGTTCCTGGGGCA

	AAAACAAATGCTTGTTGAATTAAAAAGAAAAACTGGCCGGGCGT GGTGGCTCACGCCTGTAATCCCAGCACTTCGGGAGGCCGAGGCAG GCGGATCACCTGAGGTCAGGAGTTCGAGACCAGCCTAACATGGTG AAACCCCGTCTCTACTAAAAATACAAAAACATTAGCCGGGTGTAGT GGTGGGTGCCTGTAATCCCAGCTACTTGGGAGGCTGAGGCAGGAG AATCGCTTGAAGCCGGGAGGCGGAGGTTGCAGTGAGCCGAGATCA CGCCATTGCACCCCAACCTGGGTAACAGAGCGACACTCTGTCTCA AAAAAAAAAAAAAATTCCTGTAGAAGCTCCCAAGCCCAATTAAAG CTCAAGGCTATCGTCCACGTAGTGGGTTCCAAATCGCTAAGTTCCC AGGCCAGCAGGGAGAGCGAGCCGAGCGAAGGTCGCCTGTGTCAA AGGCGGGGATTTGCACAGTATTTACATGGAAGCGGAGGAAAGGCT GCTTAGCAACCGGGCTGAGGGGACGGGCTGCACTGCGCACGCGTA CATGGGCGGGGCATCTTGCGCATGCGCAGGCGACAGCTCATGG
RARA	TGGCCCCGTCAACAGATGTTGCCCTGGGTGGGGTGAGGCAAGGGA AAGCAGGCCGACTGCTGAGGGGCACCAAGGTGGTGGCAAGAGGC ATGGGGATGGAGGGCTCCAGTTGCCCGGGCCTCCCCTGTGCCAAG CTCTGGTCTGGGTGCTTTCCAGGACTTCCTGTCTGAAATCGCACAG CTCGTTGTTGAGGTAGGCAGTTCACAGGCGAGTAAACAGATGCTTA GAGAGGTTAAGTGACTTGCGTAAGGTCACCCAGAGTGGCAGAGCT GAGATTTGACCCTTCCGACTTCTGGTTCTTTCTCCCATCTTCGTGGC TCAGAAAGCTCTCACCTTTTTCTCTAGGTGTGTGACTCTGGGGTGT AACGCGTGGGCAGCTAGGGATGCTGATGCTGGAGAAGGGATCCTG GCACCTTCTGAGGGTGAGAGCGGCCCCAGTGGGCAGGAGTGTGGG GCTGGTTGCAGGTGCTCTAGCCCCCTACCCCTTTTTCTGCTGTGCTT AGGCTAGGCTCCATATATTGGAGGTGTGGGCCTGTGGTTTTCTCTCT CTCAGGGGAGGGTGCCCGTGGGCAGTGGAGGTGGCTGGCATAGGG TGGAGGTTGGATGTGGGTAGTTTGTTTTCCCTCTCTTTGGTACTGGT TTCGATTCTCTGCCGAGGCCTTCTCCACCCCTGGGGTGTAGGGGGC TGGGGCCCAGGTGTCTCTCTGAGGGTCATAGCTTCTGATGGACCTT CTGAGGCAGAGTCTGTGCCCTTTTGGGCCCACCCTGGTCATCCCTA CCCTTGCCCATCCCTGTTCTCTGGTCTTTCTGGCCCTTGGCCTATTT CTGTGACCTTTAGCCCTCCTGCGGCTCAAGGCCCTCCCCAGGGAGC TGAGTAAATTTGCACTGAAATTTGCAGGCAGAGCTGGGTTAGGCTT TTTGGAGGCTCTGATAGGGGTCTGGGGAGGCCTTCCAGCCTTGCCC TGTAGTGTGTGTGGGTGGTGGGCTGGGGGTGAGGTGGGGGTAGTG GGGATTGAAGTTTGATTCAATTTCAAACTCCACTGGGGCAGAGTTG AGGTGGCTGTGGTTTTCTCTTCTCTGGGAGCAAGGGTGGGTTCCAG AGGGTCCAGGCTGGACTGACTAAACTGGCCTCCCGACCCCCAAAG GCTGGGTCAGGATGCCTGAGATCTGGAGTGATGGCTTTCTGGGCCT CAGCGTGGGTTACAGAGGCCTCACTTTGGGTTAGCTGCCCCTTGGG CCTCACCTTGAGAGAGGCAGGCCTCTGTGGATGTGAATTCACCTGA GTGTTTGTGGACCTGGTGCATGGGCTGGGCCCACAGGGAGGACCT CAGAGCATCTTCTGGCAGAAGCAGCAGGTACAAGGGCCACCCTGG CTGTGTGAGGTTCGGAGAATGTACAGTTTGGGAGACTGTGGGGCT GTGGTTGTGTCTTTGGGGAGCTGTGTACTGGGGCCCATGCATGCAG TTTCTGAGCTGTGTTCATTGTGTGGTGGAGCCCAGGTGTGGAGAGA

	TGGTATCTCCAGGTTCCTTCTCCTCACATGCATGCCTGCACACACAC AGGCTGGGGCTGCAGGCGTGCTCTTGCCCACCCAGATGTTTTCTGT AGACCTGCATGCATGAGTTTGTATGTGTATTTGTGTGTGAGGGTCCA ACTGTAGGAACTGATGAGGTTCTGCCAGCCTCTTCTTCCCTTGCAT GGCTCCCCCAGCTCCAGTTCCCGTTGTGTGGGGCCCCACAGGAGTT GGGAGGGGGCCAGGGCGGCAACTGGTCCAGTGGGTGGGGCCCCT GTTCCTCTGACATCAGCCCTCTCAGCCTAGGAGGGAATTGGCAGGG GCCTGGGGTAGGAAGGGAGCTCTTTATTCAGGAGTGAGGAGAGAG GCCCTATGATTGTCATAATGAGTAACAGTAAAAATCGTGGTTATTAG CAAGGTCCAGATTTTACTCCTGGGGCAGCCTCCCCTCCCTAGCCCA GTACCTGTCCCCNTCCCACCCTCTCTCCCTCAGTTCCACACAGGCT GGCTGGCTCAGCTGGGCCAGAATTCCTGGCCTCTTCCACAGTGGCC TTGGCAGGAACTCCCCCTTCCCAGGCCTGGTCTTCAACCAAATGCT GGCCATGTGCTTGTCCCCTGGACTCCAGATTTGAGGCTAGAGTCTG GAGTTCAGAGCTTGGAACCTAGTGTAGCCTGAGCTTGGGGTGGAG TGGCAAGGTATAGGGGGTGTTGGAAATGGACAGTCCTTATTTTGAG TCTCTGTGGCCCTGTTTCTTTTCTTTCTTTCCTCTTCTTTTTAAAACA GCTTTATTGAGATATAATTCACATACCATAAAATTTACCCATTAAAGT GTACAATTCAGTGGTTTTAGTATATTCAGAGTTGTGCAACTACCACC ACAATCAATTTTAGAACACTTTCATCACCCCAGAAAGAGCCCCCAT ACCCAGCAACAGTCACTTCCCATATTCCTCAAATCCACGCCCCAGC TCCTGGTAACCACAAGTCTGCTTTCTGTCTCTATAAATGTGCCTATC CTGGATATTTCATATAAATGGAATCCTACAATACATGGTCTTTGTAAC CGGTATAATGTTTTCAAGGAATATCCATATCATAGTATCAGTACTTCA TTCTTTTTTTGGCAGAATAATGTTCCATTGTAGGGACATACAACATT TTATTTATTCGTCAGTTGATGGACTTTTGGGTTGATTCCACTTTTTGA CTATTAGGAATAACGCTGCTGTGAATGTTCATGTATAAGTCTTTCTG TGGACTTACGTTGTCATATCTCTTGGGTAGATACCTAGGAGTAGAAT TGCTGGGTCATATGCCATCGCTGTGTTTAACCTTTTGAGGAACTGCC AAACTGTTTTCCAAAACAGCTATTCTATTTACATTCCCACCAGCAA TATATGAGGATTCCAATTTCTCCACATCCTCGCCAGTACTTGTTATTA TCTGTCTTTCTTATTAGAGCTAGCCTAGTGGGTATGAAGTGGTATCT CATTGTGCTTTTTTTTTTTTTGAGATGGAGTCTCACTCTGTTGCCCA GGCTGAAGTGCAGTGACACAATCTCGGCTCACTGCCTCCCAGGTTC AAGCAATTCTCGTGCATCAGCCTCCTGAGTAGCTGGGATTACAGGC ATGTGCCACCACGCCTGGATAACTTTTGTATTTGTGTGTGTGTTTTT TTTTTTTTGGAGACGGAGTCTTGCTCTGTTACCCATGCTGGAGTGC AATGGTGTGATCTCAGCTCACTGCAACCTCCACCTCCCTGGTTCAA GCGATTCTCCTGCCTCAGCCTCCCAAGTAGCTGGGATTACAGGCAC CTGCCACCATGCGCAGCTAATTTTTTAGTAGATTTTTGTATTTTTTAG TAGAGATGGGGTTTCGGCATGTTGGTCAGGCTGGTCTCAAACTCCT GACCTCAGGTGATCCACCTGCCTCAGCCTCCCAAAGTGCTGGGATT ATAGGTGTGAGCCACCGCACCCAACTGACTTTCGTATTTTTAGTAG AGACAGGATTTTGCCATGTTGGCCAGGCTGGTCTTGAACTCCTGAC TTAGGTGATCTACCTGCCTTAGCCTCCCAAAGTGGTGGGATTACAG GCGTGAGCCACCGCCCCCAGCCTCATTGTGCTTTTGATTGGCCATG

	TTTCTTTTTGTCTGTCTCTTTTCTTTTGTCTTTCATATATGTATTTTTC ATATATAAATACATGCATATGTATTTATATATAACCTGTACCCAAAATG GATTTGAAACATCCTCTTGTCCTCGTATGGATATGTTCTTCGTGTTCT CTCTCTTTGCTCTTGTTGCTGCCTATCTGTCTCACTGTGTTATGGAAT CTCTGGGTTCATCTCTGTCTCTGTCTCTACCCCATGGTATTATCTGTG CATATCCTCTGGTGCCAGCTGTTCAAACAGGGTGTGGATCTGTATTC ACACTGGTGAAGGGGGCATCTGGCCTTTCATCTTCTGCCCACCTCC CACCCCAGTTCATGTGTTTTGCTTCTGCAGACAGAAGTATGTGGGT GAACCTTGAGATCAATCTCTCCTTCATTGCCTGGGGGTAGGAGGCT TCCTGGGCGGTCCTTGCCCTCCTACACATCGCCTACCCCTGGACCAT CCATGCTGTTAAGCATTGGAGGTGATACCTAGGAGTCCTTGGTTCC CATTTTCCTATTAGTGTGTACGTAAATGGGCCTCCTCCCAGGGCCTG TGGGACAAACCCGTCTGGGGTGCTGGGGCTTGGATCTGGGGGGGA AGTGGTGTAAGAGGGGCCCCCTGGCTTAGGAGTCATTTTTCTGAAA AGTGCCAATGCTTTAGGGAAGCGGCTTTTCCTCCCCCTCCCTCTTC CCCCCCAGCACACGTTCTCTGCATCTTGACCTCAGGTAACCCTCAC TGGGGGAAGGAGGGCCAGCTGCCCGTTTCTGCTGCAGTGATCCCT TGAGGTGGCACTGTGGACCCTTTCCCCTCAGGACTGTGGGTGAGA GGCCCTNCCAAGGTTTTGGGTTNCTGTCCCTGTTTCCCCNAGCCTN GGCTGAGCTATCTGNNCTACCCCCAGCAGCGTTTNGGGCCCTGCNT TCCAGGNTCATCTCTGCCAGCCAGGCATGGTGCCAGCTGTCTGCTC ANNGCTGGGTGCCACGGGAAAATCTCCGGCAGCCCANGGTTGGGG AGCTCCACGGGGAATGCCTGTGTGCCTGTTCTTCAGTGCCCACTCA CCTGTCTTTCTTTTTTCTGCAGCATCACAGGACATGGCCCCCTCAGC CACCTAGCTGGGGCCCATCTAGGAGTGGCATCTTTTTTGGTGCCCT GAAGGCCAGCTCTGGACCTTCCCAGGAAAAGTGCCAGCTCACAGA ACTGCTTGACCAAAGGACCGGCTCTTGAGACATCCCCCAACCCAC CTGGCCCCCAGCTAGGGTGGGGGCTCCAGGAGACTGAGATTAGCC TGCCCTCTTTGGACAGCAGCTCCAGGACAGGGCGGGTGGGCTGAC CACCCAAACCCCATCTGGGCCCAGGCCCCATGCC
RBL2	ACAGATTTCAGTTGGGCAGGGCTCAGTGGGAAGGTTCATCTCTGTT GTATACAACATCAGCTAGGACAATTTGACTGAAGGCTGGAGGATCT GCTTCGGCTTCCAAACTGGCTTATTCAACACAGCTGAGGGCCAGA GGCCTTGGGTCCTATCCACATGGTGATAACTGCAGAATTCTTGGCA CATAAAGGCTCCTTCCAAACCTCTGCACTTAAGAGATGACCATATCT CATGAGCAAAAGAAAAGAGAGAGAGAGAGAGGATCAAATTTTTGC ATTGCTTCTGGGAGCTTAGGGTCAATTCTCTGGAATGATCCTGGGCT CCCTGCCCCCACCCTCCCATTGAAATCATCAGGAAGAATAGGGGTT CTGTCTTCCAGTCTGTGTAGGGGACAGTAGCCTGACTTTGATAGTT GCTGGCTAGTAGACACAACTAGCCTAATGACATTAACATTGACCCA CCTTTTGTAATTTTTTACTCAAGCTCTGCTCTCTCCCCCTCCTCCTTT ACTCTTTCTTTAAAAGGCCAAGTCACATCTAAAAAAGTTGGAATGA AGCTCAGCTCTTTCCCTGCTGTCTGTCAGTAGTTACTCAGTAAAATC CGTTTTTCACACTTTAACTAATGTTCGGCTTTGTTTATCTTTGACAAT AGGTGACCTGGTCTTTCCCACAGTATGTTGGCTGGGTACAAAGAGT GAATGAAGAATGACTGAGAGAATGAGAGACAGAGAGAGCACATGT

	GCCAGTCAGGTGAAAGACCTCTTGCATTTTATGACTAAGCTTCATA<br>AGTCAAATAATAACCTCAAGGAAATTTAACTTCTCCATATCAGAGGC<br>ATCCAAACCAGAGTGACTCCATCTTGAGTGATGGCTAGGAAAAATG<br>GGGCTGGGACTGGCTGGGCTGCATTCCTAGAAAGTTAGGTATTCCT<br>AGCCCCTAGATGTTTATAGTTAAGGGAACACATTGATAACATTTACT<br>AAACAGACCCAGACTTAGGAGTGTCCTGATATCCCGATATCTTGAG<br>AACAGAAGCATTCCTAATTTTGCTTTAAAGATAATATCAATTCTTGC<br>AAAATATAGTAATTAGGAAGATTAATCCTTTATCACAAACCCTTGTG<br>GCAGAGCACATCTCCTCATGATCTTTCTAAATCCTATTTATAAACAA<br>GTATTGTACCTAGGGTGGATGCATTCCTCCTCCTACTTTCGGGAACG<br>TCCTACTCTCTATGGAGTAACTGTTCTCTCATCACTTTACTTTCTTAA<br>TAAACTTTCTTTTGCTTTGCACTGTGGACTCGCCCTGAATTCTTTCT<br>TACATGAGATCCAAGAACCCTCTCTTGGGGTCTGGATCGGGACTCC<br>TTTCAGGTAACACTCACACCCCCATTCCACCCCACCCCCATCCCAA<br>AAGGCTTTGAAAACATTGGTATTAACAAGCAAAGGATATTTTAATA<br>AATACATATTTGATGGTTTATTGCCACCAATTTTGCTTACATTGCAAT<br>AATTATTTAAATTACATTAACTTTTACTAAAAATAGTAAAGATAATTT<br>CACATCGTTTGTTTAAGTTAAACAGTTTGAAGTTAAACTTTAATTGT<br>TAAAACAATTTAAGTTTTTCTCTTAGTTGCTAAAAGAAAAACTTTG<br>GACAAGTTAACAGTTTAAGCAAACAACAATTCATGAATTGGGCACC<br>ACTTAAAACAAGAAGATATTCAGAGCGCTTCTGCAGGTGAGCAGT<br>AAGCTTTAATAGAAAAGTAAAACAGTTACTTGATTGGCTACAGCTG<br>GGTGTTTGCCTTATTTGGGTGTGAGGGCTGGGCCCGGTGGCTCATG<br>CCTGTAATCCCAGCACTTTGGAAGGCCGAGGTGGTTGGATCATTTG<br>AGGTCAGGAGTTCAAGACCAGCCTGGCCAATGTGCTTAAACCCCG<br>TCTCTACTAAAAATACAAAAATTAGCCGGTGTGGTGGTTGGCTCCT<br>GTAATCCCAGCCACTCTGGAGGCTGAGGCAGGAGAATCGCTTGAA<br>CCTGGGAGGCAGAGGTTGCAGTAAGCCGAGATTGCATCATTGCACT<br>CCAGCCTGCGTGACAAGGCAAGACTCTGTCACCAAAAAAAAAAA<br>AAAAAAAAAAAAAGGAAAATCCCTAATTAGAGGCTAGTTGGCAGT<br>TTCTGATTGGTTAAGCTTATGTTTTGTTTTACTGTTGTCTTACTGTTT<br>ACACTGAATTCTGGTTTGCTTACATATAAGCTCCAGCTACAGAGAC<br>AGATGATATCAGGCTCATGGCCTCTTTATTTGCTTTGACATAGTAAA<br>GTGTTGATAATGATTTCCAAAGGAAGAAATATCATTATTTTAAAATC<br>AATATTCCCAGTGTATATACAGGTAAAATACTAAGATTTTAATACACT<br>TATTATTTATTAATTTATTTATTTATTGAGATGGAGTTGCTCTGTCGCC<br>CAGGCTGGAGTGCAGTGGCATGATCCCAGCTAACTGCAGTCTTTGC<br>CTCCTGGGTTCCAGCAATTCTCCTGCCTCAGCCTCCTGAGTAGCTG<br>GGATTACTGGCACCAGCCACCACTCCTGGCTAATTTTTTGTATTTTT<br>AGTAGAAATGGGGTTTCACAATGTTTGCCAGGCTGGTCTTAAACTC<br>CTAACCTTAAGTGATCTGCCCGCCTTGGCCTCCCAAAGTGTAGGGA<br>TTACAGGCGTGAGCCACCGCGCCTTGCCAAATTATTTATTATTATTTT<br>TTTGAGACAGGGTCTCTGTTGCCCAAGCTGTAGTGGCATGGTCACA<br>GTTCACTGCAGACTCCCCAGGCTTAGGCGTTCCTCCCACCTCAGTC<br>TCCCAAGTAGCTAGGATTACAGGCGTGTACCACCACTCTGGGTTAA<br>TTTTTCTATTTTTTGTAGAGACAGGGTTTCACTATGTCGCCCAGGCT

	GGTCTCGAACTCCTGTACTCAAGCAGTCCTCCCACCTTGACCTCCC<br>AAAGTGCTGGATTTACAGGTGTGATCCACAACGTCCAGCCTATATA<br>CTTAAGATACTTCTAAACCATTTGTGTTCAACTTCTGTTCTTGCCCC<br>ATAGTCACCTTGAGACTCATCACTTAGCCAACTCCAAAAGCATTGC<br>TGATTACTGTGAATTTTACTAAGGTTTTCTTAAGAGGGTTCCATTGT<br>CTCAAAATTGTTCCTGAAATATCCTGTTACCTGTCTACCTGATTTTCT<br>CCTATCTTCAGAGTTCCATTTCCTGTCCTCCCGCCTGTCATTATACCT<br>TCCATAAGCCCCTACTTTTGTCCCAGCACTTTTCCCTCTGTCAGTTT<br>ACATATCCCACCAAGCAAAACAAAAATAGCAAAACAGTAATGCCTT<br>CTGAATCCTCAAATTGCTCAATCCTCAGATTGCTCCTCAATCTGGAA<br>AATGTTTTATATCAAGCCCATTTATAAATCAAGGATTGGCAATTTAAA<br>AAATTAAAATAAAGAAAGGAGAATTGGAAATAAAATGAATTGGCTG<br>GGCACGGTGGCTCACGCCTGTAATCCCAGAACTTTGGGAGGCCGA<br>GGTGGGTGGATCACTTGAGGTCAGGAGTTCGAGACCAGCCTGGCC<br>AACATGGTGAAACCCTGCCTGTACTGAAAATACAAAAATTAGCTGG<br>GTGCGGTGGCGCACACCTGTAATCCCAGATACTCAGGAGGCTGAG<br>GCAGGAGAATCGCTTGAACCCAGGAGGCGGAGGTTGCAGTGAGCC<br>GAGATCGTGCCACTACACTCCAGCCTGGGCAACAGAGCCAGACTC<br>TGTCTCAAAAAAAAAAAAAAAAGTTTAATTCACGCAGAGCCAGCTG<br>AACGGCAGACAGGAGTTTGGTTATTCAAATCAGCCTACCAGAAAAT<br>TCGGAGACTGGGGTTTTTAAAGAATGACTTGGCGGGTAGGGGGCC<br>AGGGATTGGCGAATGCTAATTTGTCAGGTGGGAGGTGAAATCACA<br>GGGGGTTGAAGTGGGCTCTTGCTGTCTTCTGTTACTGAGTGGAATT<br>GCAGAACTTGTTGAGCCAGATTATGGTCTGAGTGGCGCCAGCTAGT<br>GCATCGGAATGCGCGGTCTGAAAAGTATCTCCAGCACCAATCTTAG<br>GTTTTACAATAGTGATGTTATCCCTGAGAGCAATTGGGGAGGTCAG<br>GAATCTTATAGCCTCTGGCTGCAAGCCTCCTAAATCATAATTTCTAAT<br>CTTGTGGCTAATTTGTTAGTTCTACAAAGGCAGACTGATCCCCAGG<br>CAAGAATGGGGTTTGTTTTTGGAAAGGACTGTTACAATCTTTGTTT<br>CAAAGTGAAATTAGAAATTAAATTCCTCCTGTAGTTAGTTAGGTCTT<br>CGCCCAGGAATGAACAAGGGCAGCTCGGAAGTGAGAAGCGTGGA<br>GTCATTTAGGTCAGATCCCTTGCACTGTCATAACTTTCTCACTGTTA<br>GGATTTTTGCAAAGGCAGTTTCGTGAACGTACAGAGACAGGCCCT<br>TGCTATTATCCCTATTTTTTAGATAAGGATATCCAGGCGATGAGGAA<br>GTTTTACTTCTGGGAACAGCCTGGATACGAAACCTTCACACGTCAG<br>TGTCTTTTGGACATTTTCTCGTCAGTACAGCCCTGTTGAATGTTCTC<br>ACGGTGGGGAGGTACGTGTTTAAAATGCGGGGAAGGTGCTTTTATT<br>TCACCCCTGGTGAAACTAGGGGAGCTAATTTTTTTAAACATGATTTT<br>TGGCCCCCTTGAACCGCCGGCCTGGACTACGTTTCCCAGCAGCCCG<br>TGCTCAAGACTACGGGTGCCTGCAGGCGGTCAGCGTCGTTTGCGG<br>CGGCGCAGGCGCGGTGCGGGCGGCGGACGGGCGGGCGCTTCGCC<br>GTTTGAATGGCTGCGGGCCCGGGCCCTCACCTCACCTGAGGTCCG<br>GCCGCCCAGGGGTGCGCTATGCCGTCGGGAGGTGACCAGTCGCCA<br>CCGCCCCCGCCTCCCCCTCCGGCGGCGGCAGCCTCGGATGAGGAG<br>GAGGAGGACGACGGCGAGGCGGAAGACGCCGCGCCGCCTGCCGA<br>GTCGCCCACCCCTCAGATCCAGCAGCGGTTCGACGAGCTGTGCAG

	CCGCCTCAACATGGACGAGGCGGCGCGGGCCGAGG
REL	AGTTACTTGGGAGGCTGAGGCAGAAGAATCGCTTGAACCTGAGAG<br>GTGAAGGTTCCAGTGAGCCGAGATTGCGCCACTGTACTCCAGCCT<br>GGGCGATAAAGCAAGACTCCGTCTCAAAAAAAAAAAAAAAAAGAA<br>AGAAAGGAAAGGAGTTCAGCATCTCTAGTTATCAGGGAAATGTATA<br>TAAAAACCACAAGAAGGTACTGCTACACCTCCATCAGACTGGCTAA<br>AATTAAAAGGACAATACCAAGTGTTGGTGAAACAAATGGAATTATC<br>TTAAGTTTTGTTGTTGTTGTTGTTGTTGTTTTTAGAAGGAGTTTCA<br>CTCTTGTTGCCCAGGCTGGAGTGGAGTGCAGTGGTGCAATCTCAGC<br>TCACTGGAACCTCCGCCTCCCGGGTTCAAGCGATTCTCCTGCCTCA<br>CCCTCCCAAGTAGCTGGGATTACAGGCATGCGCCACCATGCCGGGC<br>TAATTTTGTATCTTTAGTAGAGACAGGATTTCTCCATGTTGGTCAGG<br>CTGGTCTCGAACTCCCGACCTCCGGTGATCCACCTGCCTCGGCCTC<br>CCAAAGTGCTGGGATTACAGGCGTGAGCCACCGTGTCCGTCCGGC<br>CTCTTAAGTTTTTTTGTGGGAGTATAATTTGGTACAACAACCCTGGA<br>AAACTGGCAGTGTCTACTAAAACTAACATGTATTTACCATATGATCC<br>AGCAGTACTACTCTTGGGTGTATATCAACAGAAATAAGTGCTTATAA<br>CCACCAAAAGATGGACAACTATGTGGACACAACTCAAATATTTATC<br>AACAGTAGACTGGATAAATAAAATGTGCTATATTCATACAATGGATT<br>TCTCCCCAATAATGAAAAAGAATGTACTACTGTCACACAGTACGGA<br>CATGACACATACAATTGTTAAGTGAGAAAGTCATACTTAAAAGAGT<br>ATACACTGTATGAATCCATTCATATCAAGTTCAAAAACAGATACAAC<br>TAATTATTGTCAAGGAGGTCAGAATATCGGGTGCCTTTTGGGGGTG<br>GATATTGACTAGAAGCAGCTCCGAGGCAGCCTTCTGAGTGGCTGGC<br>TAGAAGCGTTCTGTATCTTAATCCGGGTAGTGGGTTACAAAGACTC<br>TCCTTGATGACACTCTGAAGAGGCTTCTCTGAGCCCTGTTCTCCAC<br>TAGGCTTCAGCCTTGGCCTGTAACTACTGCAGATACTGAGCACACA<br>TTATTTTATCCATGCCCCACACTAACAGACCTAAACAAATACTAGCA<br>TGCCCCTAGGATGATGACTTCGGTCACCTTTAGTTACTGCCTGGGA<br>AAGCTCAAGAATGCCAAAATAATATATGTTTGTTCCAGTTCTATGAA<br>CCTATGGTGACCACAACGTTTGATTTTATTCTGAAACTGTTGTTAAC<br>TGTATCCATTCAATTTAATGATGGGTGTCTTGTTCTTTCAACAACAA<br>AAAAATATCTTATGGAGCAATTTCACTTTTGACCCCTCACTCTTACT<br>TTTTCATATTCTGCGATCACAGACTCAGAACACATCCATTTGCTAAT<br>CAGATTTGTTTCTTTCCTGGAGGATTGTGACTTTTTAACTGCTGCAT<br>CATTAAAAAATTTTTTTTCATATGTTTAAGGTAAGTTCTTTAGCATCA<br>ACAGTTCTAAAGGTTCTAAAGGTTCTCTGCCCTTTCAGATCAGTGC<br>CCACTGTTTTATAACATATATTTGTAACATCCCCTTTAGTAGGTTGAA<br>GTGAAATTCATAATCTACCTTACATGAATATTTTCAAATAAATAAAAA<br>TTATGTTCTAACAGCAATATCAAGAATTAAAAAGAAAGCAATTTCTA<br>GGAAAATAATATCGACTACAGTATGTAAATGCTTGGGCATAACTACA<br>TCAAAAGACATAATGGGGCCAGATGCAGTGGCTCATGCCTATAATC<br>CCGGCACTTTGGGAGGCTGAGGCGGGAAGATCATTTGAGCCCAGG<br>AGTTCGAGACCAGCCTGGGCAACATAGCAAGACCCCGTCTCTTAA<br>AAAAAAAAGACATAATGGGTTGGGTGTGGTAGCACGGGCATGTAG<br>TCCCAGCTGTTTGGGAGGATGAGGTAGGAGGATAGCTTGAGCCCA

	GGAGGTGGAGGCTGCAGTGAGCTGTGTTCATGCCAGTGCATTCCA<br>GCCTTGGTGACAGAGCAAGACCCTGTCTCCAAAAAAAAAAAAAA<br>GACATAATGAAGTTGCCAGATGTTTGTACTTGTACCTAGAATCAGTG<br>TGAATGCAAGTGCTGCAAATGCAGCCTGATACTACTGTGGTTTTTT<br>GTTTGTTTGTTTTCTGAGACACAGTCTTGCTCTGTCATCCAGGCTAG<br>AATGCAATGGTGTGATCTCGGCTCACTGCAACCTCCACCTCCCCAG<br>TTCTAGCGATTCTCGTGCCTCAGCCTCTCAAGTAGCTGGGATTACA<br>GGAGCACACCACTACGCCCAGCTAATTTTTGTATTTTTAGTAGCGAC<br>AGGGTTTCACCATGTTGCCCAGGCTGGTGTTGAACTCCTGACCTCA<br>GGTAATCCACCCGCCTCGGCCTCCCAAAGTTCTGGGATTACAGGTG<br>TGAGCCACCACAGCTGGCATACTGTGTTGTATTGATGAGCTGTCAG<br>AGTTACAATTTTCCAACTTTTAAAACATTTTTTAATAAAAGTCTGCC<br>CTCAATTTATACAATATTTGCATTCCTGGGAAATTCAGTATATATTAA<br>AAACCATACAAAAATATTTTATATCTATATATGAAAGGGAGTTGTGTT<br>CTAACCATATATTATTAAAATAGGCATTTCAACTACATGAAAGTGAAT<br>GTTTGATGAGACGTTTGAAGGTCATGAGGATGAGGGACAATTCTTG<br>ACTGTGCTCTTTCCTATCTCAAGGACCTGCTGCCTGCTGTTCCCCCA<br>GCTGGAATGTTCTTTCCCTGAATCTCCCTGTGAATGGCCTTTTTTTT<br>TTTTTTTTTTTTTTTCTGAGACGGAGTCTCGCTCTGTCGCCCAGGCT<br>GGAGTGCAAGTGGCATGATCTCGGCTCACCGCAACCTCCGCCTCCC<br>AGGTTCAAGCAATTCTCTACCTCAGCCTCCTGAGTAGCTGGGGTTA<br>CAGGCGCCCGCCACCACGCCTGGCTAATTTTTGTATTTTTAGTAGAG<br>ACGGGGTTTCACCATCTTGGCCAGACTGGTCTTGAACTCCCAACCT<br>CGTGATCCACTTGCCTCAGCCTCCCAAAGTGCTGGGATTACAGGCA<br>TGAGCCACTGCGCCTGGCCGGCTCTTTCTTAAACTGAGGACTCAGC<br>TCAAATGTCACCTCCTCAGAACTTTCCTCTCCCCCAGTCACTTTTCA<br>TTTTGAAATTCCTTCAGAGAACTGAAAATGTTTTCCGTCTCTTTGAA<br>ATGTATGTACATATTTTAAAAAGCTAAATAAGCCTCTTGCTAGTTTTG<br>CGTCCTCAGAAGGTCTTTCTTGCGGGCCTCGGAGCCATCTCTTGGA<br>AATGTGAACATCAAGGATGGTGCTCCATGTTTCTGTCTCTAGGAGG<br>GTTTAAGCCTTGGCTCTTGGTTCCAAGATAACTTCCCATTATTTTCC<br>TTTGGATAAAGACAATTAATAGGTATGTAATGGATTGTACGTGCCTG<br>ACCATATGAAAAGGTGAGATTTCCTTTTTTTTGGTCTTTGCACTCTC<br>TTCAGTGGATTGCCTGTGATGTGCATCATGGTACGTTTTAGTACATAT<br>CCAATAATAAAACTGTTTCATTCTTTTGTTGTACATTGCGCATTTCGT<br>GGAAAGAACAGTTTGGGGTTGGCAGGATATTTTAAATTATTTGCCC<br>ATATGGGTCCCCCAGTTTTGGGCACTTGTATGGCAGGCTGATATTCC<br>CCATTACCTCTCCGTACTAATTTTAACTAGTAAGGGACAGGGGCAA<br>GGGCATGGAGCTTTGGAGTCAGACAACCCTGGCTTTAAACTGTGC<br>CTTTTATCAGCATTCTGAAGTACTGTATGTTGAAGTAACTTCTCTGA<br>ACCCCGAATACTTCATCTATAAAGTTAGGAATAATAAATACGTCATA<br>GATTAAGTATGGACATTATCTGATGCATAGCAGGTACTATTCAAATG<br>AAGGAAACGAAGTTATCCTAGGTCCTTTAATGCACTAAATGTGTGC<br>TCCATTTCCGCCTTTCTTGGCATCTGTGTGAAATCTCAGGATATAGT<br>GTCCACTAAACTTCAGTGCACTGCCTAATGAGACAACGAACTCCAA<br>GTGCGGACCTTTTGAGTTTAAACGGGTTTCAGTAGGTAAAATACGT

	AAAGTTCACAGTACCCTCGCAATTTAGATGGAGGAAAAAAAATCA<br>GTCGGGGGGGGGTCCCCGTATGCAAATACAGGAATTACAGAAGA<br>AGACAGGACCCCCGCCGCAGCACCACTTCCCCCTACAGGGCGCAA<br>CGTCCCGCCGCTGGCGCGGGGGTGGAGATTTGCATGTGCGAAGTC<br>GGGAAGAGGGGGAACCACCTCTCGAAAACCCCGGCCGTCGCCCTC<br>CCAGGCCGGGAGAGTCGGCCCCGCGCGCCCCCAAAAGCTCCCCAG<br>CGGCCGGCCCTCTCTACGTCATCCGCACCCTCCCGCCCGCTCCTGA<br>ACTTTAAACCACACTCGGAAGAACAACCTGGAGGAGGGGGATTTC<br>TCAGGAGCCAGCGGCCTTCTCACGCTGCTAGCCGTCACCTCCCGGC<br>TCCCGCCGTTCCCTGCGCTCCCGAAGCCCACCCCAAGCTCCCGCTC<br>CCTCGACCCCCTCCCAAGCCAGCCGCCGCAGCAGCGCCGGGGGCG<br>TATGCGTGGGGGCCGGCGGGGAGCGTGCGCGGGGACAGGCAGGG<br>GCGGGGCGCTGGGGGCGGTCGCGCGCGCGGCGGCCGCGAGGTCG<br>CCCCGCCCACGCCTCCGGGTGAGGGTTGCTGCGTCGGGCCTACGT<br>CAGCCGCGGGAAATTCCCCTCCCGCCAGGCTGCTCGCCTCTCGGCT<br>GGGCCAGCACTCGGCTCTCCCCGCTCCGCCCCCTGCCCCTGGCTCC<br>CGTACGGTGGACGGCGACGCTGGGTGACCCGGGGTGCAAGAATTC<br>AGGGGTTGGGAAGGTGTGAGCCGCAAACCCAG
RELA	GAGGTTGCAGTGAGCCGAGATTGCACCATTGCACTCCAGCCTGGG<br>CAGTAAGAGCGAAACTCCATCTCAAAAAAAAAAAAAGGAAAAAGA<br>AAAGAAATATCTTTTCATACCAAACAGCAAAGAAGCTATCACAGGC<br>TACTGTAATTGCTTTTTTAAAATTATTTTTTTAATAGACATAGGGATC<br>TTGCCATGTTGACCAGGCTGGTCTTAAACTCCTGGCCTCAATCAGT<br>CTTCCCCATTCAGCCTTCCAAAGTGCTGGGATTACAGGTATGAGCC<br>ACCAAGACTCATATATTGGAATTGTGTCAAAAGGACTGAAGAACAA<br>CCAACTTGAAGAAGTTCTCCCTAACCAAAGACGAATAATTTGAATT<br>TCAATAACTATACTTGATTAAAACACAGCTAATATATTTAAATCCATG<br>CATTCATTAAGATTTTAAAAAACTCAGCCGGGTTTGGTAGTTCATGC<br>CTGTAATCCTAACACTTTGGGAGGCTGAGACAGAAGGATCGCCTGA<br>GCTCAGAGGTTCGAGACCAGCCTTGGCAACATAGCAAGATCTTGT<br>GTCTACTAAAAATTAAAAAAAAGAAATTAGCTGAGTGTGGTGGTGC<br>ACGCTGTTAGTCCTAGCTACTTGGGAGGCTAAGGTGGGAGGATTGC<br>TTGAGCCCAGGAGTTTGAGGCTGCAGTGAGCAACGAGTCATGCCA<br>CAGCATTCCAGCATGGCGACAGAGTGAGACCCTGCCTCAAAAAAT<br>TGTTTTCTTTTTAATTAGCTGTGTGTGGTGGTGAGTGCTTATAGTCC<br>CAGCTACCTGGGAGGCCGAGGTAAGAGGATCCCAACTGATAGGGG<br>CGTTGGTGGGAGAAAAAGTAGGTTTACTGATTTATTCTGAAAGCCA<br>GCAAGACTGAGAGGACGGTGTACTAGCGTTCTAAAGTACCATTTTA<br>AGTCAGTACAATCTTAGGCTCTTTCTATGTTAAGGGCAGGGAAGAG<br>AAGGAGGGTGTGGTCAAGAAGTACCCCATGGCAGACATCTGGGCA<br>GCAGCGAGGGTCTGAGGAGGTTGGGAACTTCTTTGTCCTTGGTCC<br>AGTCACAATGCTCCTATAAATCTTTAAGAAAACATAGTTGTTTACAT<br>ACTTCTTTAACCCCAGACTAAGTTTTAAAAACTACATGATTGCTGTT<br>TTTATATATTACTCAGTGCTCTAAAGCTTATGGCAGCAAAAAATTAG<br>AAGTCCAAATGCCCATGGCTGGGCACGGTAGCTCACGCCTGTAATA<br>GCACTTTAGGAGGCCGAGACGGGCAGATCACCTGAGGTCAGGAGT

	TAGAGACCACCCTGGCCAACATGGTGAAACCCTGTCTCTACTAAAA<br>GTACAAAATTAGTCAGATGCAGTGGTTCATGCCTGTAATCCCAACAT<br>TTTCAGAGGCTGAGGCAGGTGGATCACCTAAGGTCAGGAGTTTGA<br>GACCAGCCTGGCCAACATGGTGAAACCCCGTCTCTACTAAAAATAC<br>AAAAAGTTAGCCAGGCATGGTGGCAGGCACCTGTAATCCCAGCTA<br>CTCAGGTGGCTGAGGCAGGAGAATCACTTGAACCTGGGAGGCAGA<br>GGTTGCTGTGAGCCGAGATTGTGCCATTGCACACAACAAGAGCGA<br>AACTCTGTCTCAAAAGAAAAAAAAAAATACAAAATTAGCCGGGCAT<br>GGTGATGCATACCTGTAATCCTAGCTACTCAGGAGGCTAAGGCAGG<br>AGAATCACTTGAACCTGGGAGGCAGAGGTTGCAGTGAGCCGAGAT<br>GGTGCTATTGCACTCCAGCCTGGGCCACAAGAGAGGAACTCCATCT<br>CAAAAAAATAAAAATAAAAATGCCTATCTTCAGGATGAATAAACAA<br>ACTGTGGTATATTTACACAACAAAATAGTATACAGCAGTGAAAAAG<br>TGAATTAACTGTGAAACATGAATCATAGTAACAGGTTGAGTGAAAA<br>AAGCAAGTAACAAAGGACAACAAATCATATGATGCCCTCTTCATAA<br>AAGTTCAAAACTGGCTGGGTGCGGTGGCTCACACCTGTAATCTCA<br>GCACTTTGGGAGGCAGAGGCTGGCGAATCACCTGAGGTCAGGAGT<br>TCGAGACCAGCCTGGCCAACATGGTGAAACCCCGTCTCTACTAAA<br>AAAATACAAAAATTAGCTGGGCGTGGTGGTTGGCCCCTTTAATCCC<br>AGCTACTCAGGAGGCTGAGGCAGGAGAATCGCTTGAACCTAGGAG<br>GCAGAGGTTGCAGTAAGCAGAATGCCACTGCATTCCAGCCTGCGC<br>GACAGAGTGAGACTTTGTCTCAAAAAAAAAAAAAAAAAAAGTTCA<br>AAACTAAGGAAAACCAAAAGATATATTGTGTAGGCATGCAGATATA<br>ACTTTTTTTTTCTTTAAAATACAGAATTCTGGGCCAGGTGTGGTGGC<br>TCATGCCTGTAATCCCACTTTGGGAGGCTGAGGTGGGAGGATCGCT<br>TGAGCCCAGGAGTTTGAGACCAGCCTGGGCAACACAGTGAGACCC<br>ATCTCTATTTACAATAAATTAATTAATTAATTAATTTAAAAAATACAG<br>AATTCTGGCCCAGCGTGGTGGCTCACGCCTGTAATCCCAGCACTTT<br>GTGAGGCCAAGCGGGGAGGAAAGTTGAGCTCAGGAGTTCAAGAC<br>TAGCCTGGACAACAAGGTTAAACCACTTCTTTACAAAAAATACAAA<br>AACTTAGCCGGGCATGGTGGTGTGCTCCCGTAGTCCCAGCTATGCA<br>AGAGGCTGAAGTGGGAGGATTGCTTGAGCCCAGGAGGTAGAGGCT<br>GCAGTGAGCAGAGATTGTGCCACTGCACTCCAGCCTGGGCGACAG<br>AGACAGACCGTGTCTCAAAAAATAAATAAATAAATAAATACAATAC<br>AATACAATACAATACAATACAATACAATACAATACAATACAGAATTC<br>TGGATGGTGGTGGCCCCTTGAGTTTTGCAATGGGTACATGGGTGTT<br>TATCGTATTAATTTAAAAGTTATAAATATACATGAATTTTAAGAAGAG<br>GATCCATGCAAGGTTCAATGATAATTGTATATCATTAACCAAAGATTT<br>TAATTAATCCATTCTGTCACCTGAAGTCGGCCTCCCCCGCCCCCTGC<br>CAAAAGTAAACACTTTTCTAATCAGGGCCAGCCACTGGAGAAACT<br>GAAATGAGTGGGCGGGGTGCTAACTCTATTTTCACTTTTCTCTTCCT<br>GTTTCTTCAGAGCCTGGGAAACAAGTCAGAATTTCCAATCTTTTCC<br>AATTCCCACATGATTTCCTAATTTAAGACGAATTCAGAGAAAATAAA<br>AAAGTCTTTCTCCATCTGGAGAAGACTCCGCCCCCTGCAGTGGAGC<br>ATCCTCCTTTGGGGATGAGGCCTGGGGTGGGGGTGCGTCAGCCCC<br>AGCCTCCACACGGACGCGCCTTGGGCTTGGTCTTGGGGGAGCTAC

	CTGCACTGTGGGGTCACATGACAGAATTAGTGGTCAGCCCTAGCTG<br>GAGGTCCAGGGCAAACACAGGCGGGGGCAGGGGGGTGGGGGGTC<br>GTGAGCCTGCTGATTCAAGTGTCCCTGTGGGTCCCGCACCTTCTGA<br>GACACAAGCGCCAGGCTTCAGAGACGAGGCTCCGGGATTTACCCC<br>TGGGGGCCTCCTGCTCCCACAGCCGATGAGAGCCGGCAGTTTGGC<br>TGTATCGTAAGCTGGCGCTTTAGCTGGAGAGGGACGAGCCCTCGGC<br>CAGCTCGCCAGCGGGAGGGCCTCAGTCGTCCCATCCGGGCTGTAG<br>GCTCCGCAAAGCTCTTGATGCACTGTCAGGCTGGGAGGTCCGGCC<br>GCTCCCATTCCCCCACAGCCGCATCTAGATTGGGGTGGGTAGGACA<br>TTTTAACGTGCTTCTGAGACAGCCACCACCGAAAGGCACCTTTAGC<br>GGTTAGGAAACCCGCGCGGAGTTCAACCACCCGGCCTCTGCGGAG<br>CAGAAGGCCCGGTCGGGCTTTTCCCTGCGCGGGGCGGGCGGCCGC<br>AGCCCAGTAGCCCTGGCTTTGCTCCAGACCCGGCCCAGAGTGACA<br>TCACCAAACTCCGCCGATCGGCGGGAGGGGGCCCTGAAATCCCCT<br>AAAAACAAAGTGAGTAATCGGCGGACCCACCCTCCAGGCGGGGCC<br>GGGACCCGGGAGCTAGCCGAGCCACCGGGCACGCCAGCCTCTTCG<br>TCCTCGGCGAGGCGCGCACTTGGCCCCGACCCCCGGCAGCGGCTG<br>TGCGTGCAGCCTCTTCGTCCTCCGCGCGGCGTGCACTTGCTCCCGG<br>CCCCTGCGCCGGGCGGCGGCGGGGCAGCGCGCAGGCGCGGCCGG<br>ATTCCGGGCAGTGACGCGACGGCGGGCCGCGCGGCGCATTTCCGC<br>CTCTGGCGAATGGCTCGTCTGTAGTGCACGCCGCGGGCCCAGCTGC<br>GACCCCGGCCCCGCCCCCGGGACCCCGGCCATGGACGGTGAGGTC<br>GCCCTCTGACCCCGCGGGGTGGCATCGCCGGGGCCGCCGCGGCTG<br>TGGCGGGAGCCCCCGCGCCGCTTTCCGCCCCTTCCTGCGCCGACG<br>GGCGCTGCGCAGGGAGCGGTCGGCCTGCCGGATGGTGGCGCGGGG<br>CGGGGCAGGCGGGGAGGGGTCTGACTCAGTTTCCCCTCTGGGTGG<br>AGGGGGTGGCCCTTGACTCAGCATCCTCCCTGGGTGGGGGAGCAG<br>AGGGAACCTTGACTCAGTTTCCCTCCACACCGTCCATGGGGGCAG<br>CTCTGGCTTCGTTTCCCTATTAGGGGAGAACGCATCTGATTCAGTTT<br>CCTCTCTGGAGGGAGGTGGGGCGTGCCCTCCCTCAATTTTCCTTAA<br>GAAAGTACAACAGGCCCTGATTCAGCTTCCCTCTGGAGAAGAAGG<br>ATTCCTTACCCCGTTTTCCCTCAAGAAGCAGGTGTTTCTGGTTCCTT<br>TCCTCCACCCCTTAGTTTCACCGCAGGTTCTAGGGAGCAGGTCCTG<br>ACTCAGTTCCCCTGGGAGGGAGCCTGGCTTCTCCTCTCCGTGGAGA<br>GCAGATTCCTCCTTGTGAGAATGGGCATTGGGTGTGTGCTGACCCT<br>GATCTCCCTCCCCTTTCAGAACTGTTCCCCCTCATCTTCCCGG
RET	GTCCACAGACCAGCCGGCCTCTGAGCCCCTCGGGCTGGGGCTGCC<br>TTATGTGAGCCAATCCCCCAGGAGCCTGGGTGGCCGCAGACATTAA<br>TCAACCCACCACTCCCGAATGTGTAATTCAAGGTCAGATAAGTGGT<br>CCTCTGCGGCAAATAGGACTCTGGGGACCCAGGTCCCTCCAGCAC<br>TGAGAAGGCTTCTGGTTGCATGGGGAGGGCTCAAAGCCTGTGGCC<br>AAGCCAGGATGCAGAAGACAGGTGTGTGCTCTAGCAAGCCAGTCT<br>CGTTGACCACCAGCCCCACACAGCACACAGGTGCGTGCTGTCACC<br>TGTGCAGGTGGGAAGCCCGGACTGTTTGGGTCCAGGGCCCCCTGG<br>CTTTCTATGCCACCCTAGCACCATCCAGTGACTGCTTGGGCCTCATG<br>GCCTCAGCTCCTACAGCTGACAGGACAGAGGCTGATGACAGCGGC

	AGCTCGTGCTGATGGCCAGCGTCCCTGGGCTAAGGACTCTGTGCA<br>GACTGACTGTCTGCATCCCCGGGGAACTTGGGGAGAAGGAGCTCT<br>GCCTTCCCACTGAGGCACGGAGACTCTGGGTAACCCGCCAAGGAC<br>ATGGGGACAAGAGACAGAGCGCAGACACACTCAGAGCCCCCTGC<br>AGCCTTCCCACCATGCTACTGCCCCAGGCCACACCTAGACAGCCCC<br>CTGTAGGAGAGAAAACCCTGGCAGAGGGGCAGGAAACACTGCCA<br>CTGTCTGCTGAGCCCACTGATGCATCATTGGGCTTTACCCTCAAGG<br>CAGTGCCCATGTTATTGCTCTCATGTCACAGAGGATGACACCGAGG<br>CCCAGAGAGGACCACAGAAGCTGCTGAGTGGTGTGGCTAGCCTGG<br>CTCTGGTGCTGAGCGAAAAGGATGGAGATTCCTCCAAGGGAAGGG<br>GAGGAGAAGACGGGCAGAGGGGAAAGAAGAAAGGAGGGCGAGG<br>GAAGGAAATTCACAAGAGAACAAAGTGTGGGGACATGGAAAACT<br>GAAGTGGGCAAAGTGATAATGATAGGTTAGTGGGAGTAGGTGGGC<br>AGGCTGGTGGGAGGAGGTGGGCTTTCTCTGAACCCCCCAGGGTCT<br>CTGATGGATATGTAGGCCCCTGTTGCTTTTGTTCGGGCACCCGGCTC<br>CTAATCAGGCCCCAGGGAGGATGCAAAAGGGGTCTTCTCCCAGGC<br>CAGGTTGAGGGCTCTTCTGCAAACACCCCTGAGGGCACCTGAGGC<br>CCACAGGCAGGCAGGACCTGCACAGTTAGGTAAAGCCTGTCCTGC<br>ACAGCCACACCCTGCAGGCCCAGGCCAGGTGTTCTGTGAGCATGC<br>TCATGGAAGCAGCATATTTGGCATAGGGTGGGCTGGCTGAGCAGCC<br>CCTCTGAGTGCCCTGAGAGCTAGCTGGTGGTCACTGGCTTCCTCCT<br>CGCAGCTGCCCTGGAGGGCACCAACAGCAAGGCCAGGGAGGCAA<br>ACCGTGGGGAGCAGTAGACCTGCCCCTCACCTCAATAGCCCGATGG<br>GGGAGCAGAGGGCAGTGGCAGACAGATGGGAAACTGAGGCACCG<br>ACCACTTTGCTAACAGTGCACACAGCCAGCCTCAGAGTCTGTGCTT<br>CCAACCACCATGTCACACTGCCCATGGGAGGGACCTGGACCCCTG<br>CCCACGCCCACTCTGAGAGGCCTCGGTCAGCAGTTCCAGGAATGC<br>CACCCAGCCTTCCAGTATCACCAGGACCGAGCTGCGCTCTTGAATG<br>TGTTTCCCCCTACCTGCAAGTCACGAGGCACCCCCAGAGCCAGAG<br>TTTCCTGTCACTGCACTCAGCCTCACTCCAACCATGCTTCTCAGTG<br>CAGGCAGAGACCCCCATGTCACCTGGGTCAGAGGACACAGAGCAT<br>CCCGCCCGCCTCCCGCCAGGCAAACCTGCACAGGCATGGCCCAGA<br>TCTCTGTCCATGATGGGTTTCCCTCCTCCCTCCCTTCCCTGTGCTGC<br>GTTTTGGTGCTTTGGGGTCAGACCTGAGGTGCTCCAGGTAATCTTC<br>ACCTGCTCAGAAGCAATGAATGACCTCCAAGTTCTCACATGCTACA<br>GGAAGATGGTGTGAGCCGCCTGCCCACTGAGCATCTTACCCTGCAC<br>TGGGGCAGAGCCTTGAAAATACCATCTCCTCACAGTCACCAGGGA<br>AGGACACTGTTTCAGTCCCCATTTGAGAGATGACAAAACTGAAGC<br>TGAGGAGTAATGTATGCAATGTCACACAACTATGAATGGCAAAATT<br>CAAACTCAGGTCTAATCACTCCAAGCAGATGCCTGCACCAGAACCT<br>CTTGGGGAATTTTTTTTTTTTTCTTAGAGATAGCGTCTCACTGTGCT<br>GCCCAGGCTGGTCTCGAACTCCTGGCCTCAAGTGATCTTCTTGCCT<br>TGGCCTCCCAAAGTACTGGGATTACAGGTGTGAGCCCTTAGCCTGG<br>CCCCTGAACTTTTTTTTTAACATGGATTTTTAGGGAGTGGGGGACCC<br>AGGATGTGTGTCTTCATAAAAGCTGCCCTGAAGATTCTCCAAGGGG<br>ACCTGCGGGCAGGGACAGGAAGCACTGACCACACTGTGATGTGCC

	CTCCTCCAGCTCCCAGAGAGCCCGCGACCCTCAGCAGGGCAGTGT<br>GGCCAGGGTGCCCGAGCCCAGGCCCAGGAAGGCCCAATGGCTACA<br>ACTTATTAAGGGCTTCCTGTTTGTCGGGCACTGGGCTCTACCCTCAC<br>ATGCACCCCTCTTTCTAGTTTCACCCCTTCTTCTGAAAGGGAGATGT<br>TTATAGCTGGGGAAACTGAGGCTCAGAGAGATGACTAAGGGGAGT<br>CAAGAAAGAGAGGCAGAGCCCCCTGCAGGCCCTCCCCACCCCATG<br>GACTGAGAGAAGCCAGGGCCAGGACTCCTGGGCAGCCAAAGACC<br>GGCACGTCTGCTTTGCACTTCACCTGGCCCCCACCTCCCCTTGTGA<br>GACCTCAGGGGGTGGCCTCGCTTCCCCTCTGCTGGGCAAGGAGCT<br>CCTTCCTGCTGCCTGATGTTCTGGATACTGAGCCTACACCCAGGCA<br>GGGCTGAATCTCCTTTCCCCGCAGTCCCTGAGGCCAGCAGGCTCTG<br>GGCCTCACAACTGTCCTCTGCTGAGCGACCCTTTGGTCAGATCCCT<br>GGGCGTGCACGTAGCAGGGAGGTATGCATCTGTGTGCGTGTGTGTC<br>CACGTATGGGAACACAGGTGTATGCATGTATGCATGTGTACAGGGT<br>ACACCCATGTGAGGGCCCCCCAGACCTTTTTCCAAGGAGTAAATGA<br>CACCCTCTCATAGTGTCAGGTGTTCACTCAGGACCCCCAAGGGTGA<br>GGGTGGAGCGAGAGCGTGGAAAGGCCTAAGGTAGGCTAGGCAGG<br>AGCACAGCCCCAGATGGCCCCTTAAGATGGCCACACAGAGCCCAG<br>CCAGACATCAATGTCAGCCAAGGGCTGGCCAGGGTGGTCCCCGGG<br>AGGTGCAGGGGTGGGGCTATTTAAGCCTACCCACTTCTCTCTTCATT<br>CACCTAGGAGGCAATCACAGGAGCTTGTCTGTAGCCAAAAGATGC<br>CCCCAGCCCCTCCCTACCTATCAGGTCCCTTCCCTGGGCACAGGCC<br>TGAGTGTGTAGCCTAGGTAGCCACTGGCACCAGAGATGGAACCTG<br>TCTGAACCCAAGGGAGCCCCTTGTGGCCAACTCTAGCTCCATAAAC<br>TGGAAGGGTGTGGCAGGCAGCCGGAGTCCAGTGGCCCACAGTGTC<br>TCTCCTGGGCTGGCAGGCCTGTGCCCCGGCCACCTGCCCCTCAGCA<br>AGCCAGGTCTGGACATCACGGTTGGCTGAGTGCAAGCCAGCCTCT<br>GTCTTGGCAGCTCCCCCTCCTGCCTGTGAGGGTGACTTCACTTTGA<br>CTTCATATCTGCAGTGATGTGCAGATCACTTATTCAGCCCTGAGCTC<br>AGCCAGGCAAGAGGGCGGGGTTCTGCACACAGCAAGCAAGGGAG<br>CGGCTGGCTCAAGGGTGCCCTGGGCCCAGGTTCCCTGGCTTTTCCT<br>CCTCAAGGGCCCATGGGCTTCCCTTCTTCCATCCCTCTGCGCCTAA<br>GTGCAGGCCAGACCTCAAAAAGCAGCCGGGGATGGGGCGAGAAC<br>AGAGGACTTCCCCACTGAGCCCACATCCAACCTCACCCAGAACCC<br>CTTCTCAGGTCCAGTTGCTGCTCCCTATTAGGGGTAAATGTGTTTAT<br>GCGCATCATTTCCATCATTTCTTTGGGGGAAAATTGGATTCTTTCCT<br>CCTCCTCGCTACTCGCTCTCCGACTCTGCCAGCCCATCTCCTGAGA<br>GTTCTCCTGTGTAAGGGATGCAAGGGCCACCAGCACCAGGTCTCT<br>GAAAGGACGCCGGAGCCAGTTTCCGGTTTCCAAGGGTTGGACCAG<br>TGCTGGAGGGGGGAATCACAAACTGCCCTTCCAGGACCCTACCCT<br>CTCTACTACCCCAGGATGCTGAAAAAGGCTATTCTGCCTCTCTTCCC<br>CTGCTCAGCTCTTCCCCGCCTCCTGCCTCCGGCCCCAGCAGAGGCC<br>GCGCTCCCACTGCGCCTCTCGCCTCTGAGAAATGGCGCAGCAAGC<br>GCTCCGCGGAGAGCCTGGAGGCGGGGCGCCCTTCTCTGAGTCCGC<br>GGGGTCGCACCCCGAGCCAGTCGGCCAGACCTGCATCCCGCGTAG<br>CATCCCTGCCCTCTCTGTGCAGCGGAAAGGGCAAAAGGCAGGGAC

	TGCAAGCGGGCGCGCACCGGGTAGGAAGAGCGGCTCTGCGTAGGT<br>GCGCGGACCCGGGCTCCTGGGTTCCATCCCCGCCGCGCACCTCGG<br>GGTCCGCACCCGGCTCCTGCCGGGCCCTTTTCGGCCGCACCCCGCT<br>CCCGCACCCCGCTCCTCCCCAAGCCCCACCCGGCCCAAGCCGCGC<br>GTCCCGCACTGAGCTCCTACACGCGCCGCGCCCCGGCCGCACCCC<br>GCGCAGCCAGAGCAAGCACTGGAGCCCCGCCCCTTCCCGCACCCC<br>ACCCGCCTCCGGCCCCGCCTGGCCCACCCCTGGACCGCCCCCGCC<br>CCGCCCCGCCCCTACCCGCTCCTCCGGCGCAGCCGGCGCTTACCTC<br>GCTTCAGTCCCGCGACCGAAGCAGG
RHO	GTGGGGGACCAGGAGAAAGAAAGCCAAGGAAGAGGAGGAGGAG<br>GAGGAGAAGGAGGAGAAGGATGCTGACCTAGCAGCTCCTCTCACA<br>GCAGCTCCTCTCTTGCAGAGGCTGAAGAGCGATTTGTGCCCTGCAA<br>GCTAAGCCCCTAATCCACCGAGGCAAAGGCAAAGCCCCTAGCCGG<br>GCTCCCGAGGGCTGGGACTCGGGTGCCCCAAGATGGCTGCATCCA<br>GCCATCTTGGCTTAGAAAGCCCCCCACATGCCAGCTTGGCCAACAC<br>CCACACCATGGGTTTCTCTGGACTGCCCGACACAAGGTGTGGGTG<br>CTGGCCAGGCCTGTGTTCAAATCCCAGCTCTGCAGAGGAACTTTGA<br>CCCTGCATACCCCAGATTCCTCAGTGGTCAGTGGGGAGTTAGACCC<br>TCTTCATAGGGGGCAGGAGGAGTTGTTCATTCATTCAACAAATGTTT<br>ATTGAACACCTCCTATGGGTTGTGAGCTCAGAGGCAGCGATGAACA<br>GGCCAGGCTGGTCCTGCATTCTAGAAATAGATGGGAAGTCAGTCAA<br>TAAGTAGACAAATGAGGCCAGGTGTGGTGGCATGCCTGTAGACCC<br>AGTTACTCGGGATGCTGAGGTAGGAGGATCACTTGAGCCTAGGAC<br>AGGAATTCAAGGCTGCAGTAAGCTATGATTGCGCCACTGCACTCCA<br>GCCTGGGCAACAGAGCAAGACTCATCTCTAAAAAACATTTAAAAAT<br>TGTTTTAAGAAGACAAATGAGATAGTCGCTGATGGTAATGACTGTG<br>TAAAAACTGAACATGGCTGGGTGTGGTTGCTCACACCTATAATCTC<br>AGCACTTTGGGAGGCTGAGATTACAGCCTCCCAAGTGGCTCCCAA<br>GGCAGGAGGATCACTTGAGCCTGGGAGTTAGAGAACAGCTTGGAC<br>AATATAGGGAGAGCCCAACTCTACAAAAATGAAAATAAATTAGCCA<br>GGCATGGTGGCACACACCAATGGTCCCAGCTACTCAGGGGTTGAG<br>GTGGTGGACCGCTTGAGCCCAGGAGGTTGAGGCTGCAGTGAGCCA<br>TGATCATGCCGCTGCACTCCAACCTGAGTCACAGAGTGATACCCTG<br>TCTCAAAAAACAATAGGCCAGGTGTGGTGGCTCACGCCTGTAACC<br>CCAGCACTTTGGGAGGCCGAGGCGGATGGATCACTTGAGATCAGG<br>AGTTAGAGACCAGCCTGGCTAACATGGCAAAATCCTGTCTGTACTA<br>AAAATATAAAAATTAGCCAGGCATGGCAGTACATGCCTGTAGTCCT<br>GGTTACTTGGGAGGCTAAGGCAGGAGAATCGCTTGAACCCAGGAA<br>GAGGAGGTTGCAGTGAGCCAAGATCACACCACTGCACTCCAGCTT<br>GGGTGACAGAGTGAGACCCTGTCTCAAAACAGCTAAACCTGGTGG<br>GGGTGCCTGGTGTGTAGGATGGTCAGGGGTGGTCTCTCCAAGGAC<br>ATGAGTGTGAGCGGAGACCTGAAGGAGACTCAGGAAGAGATTAAT<br>ACTGTCAGCAACAAATATATTGATCACTTACAAGCACTCCCAATAAT<br>CCTATTAGGTAGGCACTATTATCATTCCCATTTTACAGAGTGGAGAA<br>CCGAAGCACACTCTCGGGAGGGCGGGGTAGCTGGCTGCACCCAGG<br>CTGTGTAGCCTCAGTCCAGATGTAAGGGTGGGTGGAAAAGAGCCT

	TGCCCAATGAGGGAGAACAGTGAAACCAAGGCCATAGGGTCTAAA<br>GATTCACGAACCAGGCTCTCATGGAGAAAGCAGGTGAGGTTTACT<br>GTATAGATGGGTGTGCCCCTACCCCACACTGAGGCTTCCTCGTCTG<br>AGCAAACTGAGGCCCAGAGAGGGGAAGGAAGCAGGACTACCATG<br>GTGACTCAAAGACCAGCTAGAATCCAGCCTCCTCTCCTCGAGGCTT<br>CCACTGCCCCACGCCAGGCCTGTGTGACTCAGTCTAGGGCCTTTCC<br>ATTACCCCAGCTAAACCTTTCTTTAGTCATTTATACCATGGTGTGAAT<br>GGCTGGCTGGTCTTTCCTGAGAGCTATCTTTGATGAGGGGAGGGAG<br>GCATAGCCAGGTTTGGGAAGCTGATACCCCAGGAAGCCCAGTTGA<br>CTGTGTGGGTTATAGCCCAGGCTGTCACTGATTTGTAACGGGACCT<br>GAGCAACTCTGCAGAGCTAGGCCTCAGTCTTTTCATCTGCAAAATG<br>GATATAGCAGAGATGGTCAGAGTAGGTGACTTCGAATGACCCTTCC<br>AGCTCACTATGAGTCTGTTTTCCTGAACAAAGAGCATTTTTTGTTTA<br>AAAAAAAATTTCTTGGGCCGGACACGGTGGTTCACTCCTATAATCC<br>TGGCACTTTGGGAGGCCGAGGAGGGTGGATCGCTTGAGCCAGGAG<br>TTCAGGACCAGCCTGGGCAACATAGCGAGACTCCACCCCTACAAA<br>AAATACAAAAACTAGTGGTGTGCACTTGTGGTCCCAGCTACTCAGG<br>AGGCTGAGGTGAGAGGATCGCTTGAGCCCAGGAGGCAGAGGCTAC<br>AGTGAGCTATGATTGTGGCACTGCACTCCAGCCTGGGCGACAGAG<br>ACCTTGTCTCAAAACTTTTTTTTTCTTCGTCAAGCTTTACAGAATAA<br>AGAGCACTGTCACCTCAGTGATGGCTGTTAGTTCCCCATCACCAGG<br>GCTCCATGAGGTTGCAATTGTGAAACTCACAAAGGAGGAACCTGA<br>GACAGAGAGGGGAAGTACTGAGATCATCTAGGTCCATTCCCCCACT<br>CACTCGTTCATTCAACAAATATTCAGGAGCACCTTCTAGGTGCCAG<br>GCCCTGGAGACACATCAGTGAACAAAACAGACATCATCCCACCTC<br>TTTCCACTACAGGCCAAGCACCATGCTGGTCTCTGGGAACCCTGTT<br>GTGAGCAAGACAGACCCAGGCTTACCCTTGTGGACTCATGTTACAG<br>GCAGGGAGACGGGCACAAAACACAAATAAAAAGCTTCCATGCTGT<br>CAGAAGCACTATGCAAAAAGCAAGATGCTGAGGTACTGCTAAGCT<br>GTGTGGGATGGGGGCTCAGCCCGGCCAGGGAGGGGCCAGTTGTGG<br>GTCAGTCTTGACCCAAGGCATCCAGGACACCCTCCTTCTGGCCATG<br>AGGGTCCACGTCAGAATCAAACCCTCACCTTAACCTCATTAGCGTT<br>GGGCATAATCACCAGGCCAAGCGCCTTAAACTACGAGAGGCCCCAT<br>CCCACCCGCCCTGCCTTAGCCCTGCCACGTGTGCCAAACGCTGTTA<br>GACCCAACACCACCCAGGCCAGGTAGGGGGCTGGAGCCCAGGTG<br>GGCTGCAGGGAAGGGGGCACTCTTCTGAGCAGACAGATCTGGGAA<br>TCCTGGGTGGGAAGAGAGACAGTGAGAGAGAGATTAAGGGATATT<br>TCCCAGGCATCAGGGCTTTGCACTCTCAGGGGTCCTTCCGCCTGGA<br>TGTCCTTCCCCTGAAGCTTCCTCCTGTTGTTCCGTTCTCAGCTCAAG<br>CTCCAGCTTCTCAGAGAAGCCTCCTGTGTTGGGAGTGGCTGCGACT<br>GAACTGTCCCTACTGTTATTCGCTCTTCTATTTGTTTGTGGTCCCTGT<br>GCCCCCTCACCCCACAAAAACACTGGCTTCTTGTGAGCAGGAGCT<br>TGCTCTTTCGTGTACCCTGTGTGTCCCCAAGGACCAAGCACCTTGT<br>CTGGGCCACAGTAGGTGCTCAATACACATGTTGGCTGGACAGTGGT<br>CACTGAGCGGCCGCACGTCGGGCACTCTCAGCACTTGCACAGGCC<br>GCCCCAGACACCCCACTTCATTCCTGGGAGGTGTCATCATGTTGCT

	TGGACGACGGGGAGAGGGGGACCTGCCAGTGTTGGCCTCCATTTT CCCCCAGTCATCTGCCCCCAAGGCTCTGACTACTTTCTTTCTCACG GTACATCCTGCTATTCTGGAATCGGCCCTCGTGGGGCCACCTGGTA CATGGCATTTGAGGCCCTCGTGGCTGATTAGGCCTCCCCCAACAGT GCCCTGTCTGCTGCCTCCAGGGCCAGCCTCCCCTTCAGACTGGAGT CCCCTGAAGGGTTCTGCCCCTCCCCTGCTCTGGTAGCCCCCTCCAT CCTCCCTCCCTCCACTCCATCTTTGGGGGCATTTGAGTCACCTTTCT ACACCAGTGATCTGCCCAAGCCACTGCTCACTTTCCTCTGGATAAA GCCAGGTTCCCCGGCCTAGCGTTCAAGACCCATTACAACTGCCCCC AGCCCAGATCTTCCCCACCTAGCCACCTGGCAAACTGCTCCTTCTC TCAAAGGCCCAAACATGGCCTCCCAGACTGCAACCCCCAGGCAGT CAGGCCCTGTCTCCACAACCTCACAGCCACCCTGGACGGAATCTG CTTCTTCCCACATTTGAGTCCTCCTCAGCCCCTGAGCTCCTCTGGGC AGGGCTGTTTCTTTCCATCTTTGTATTCCCAGGGGCCTGCAAATAAA TGTTTAATGAACGAACAAGAGAGTGAATTCCAATTCCATGCAACAA GGATTGGGCTCCTGGGCCCTAGGCTATGTGTCTGGCACCAGAAACG GAAGCTGCAGGTTGCAGCCCCTGCCCTCATGGAGCTCCTCCTGTCA GAGGAGTGTGGGGACTGGATGACTCCAGAGGTAACTTGTGGGGGA ACGAACAGGTAAGGGGCTGTGTGACGAGATGAGAGACTGGGAGA ATAAACCAGAAAGTCTCTAGCTGTCCAGAGGACATAGCACAGAGG CCCATGGTCCCTATTTCAAACCCAGGCCACCAGACTGAGCTGGGAC CTTGGGACAGACAAGTCATGCAGAAGTTAGGGGACCTTCTCCTCC CTTTTCCTGGATCCTGAGTACCTCTCCTCCCTGACCTCAGGCTTCCT CCTAGTGTCACCTTGGCCCCTCTTAGAAGCCAATTAGGCCCTCAGT TTCTGCAGCGGGGATTAATATGATTATGAACACCCCCAATCTCCCAG ATGCTGATTCAGCCAGGAGCTTAGGAGGGGGAGGTCACTTTATAAG GGTCTGGGGGGGTCAGAACCCAGAGTCATCCAGCTGGAGCCCTGA G
S100A2	AGTGGCATGATCTCGGGTCACTGCAACCTCTGCCTCCCAGGTTCAA GCGATTTTCCTGGCTCAGCCTCCCTGAGTAGCTGGGACTACAGGCT TGTGCCACCACGCCTGGCTAATTTTTGTATTTTTAGTAGAGATGGGG TTTTGCCATGTTAGGCAGGCTGGTCTTGAACTCTTGGCTTCAGGTG ATCTGCCTGCCTCAGCCTCCCAAAATGCTGGGATTACAGGTGTGAG CCACCATGCCCAGTCTCTTTTATATTTATATTTAATCCGGGCTAGGCT GGGCATGGTGGCTCACGTTGGTATCCCAGCACTTTGGGAGGCCGA GGCAGGAGGATTGCTTGAGCTCAAGAGTTCAAGACCCGTCTGGGC AAGATGGCAAAACTCCATCACCACAAAAGATGCAAAAAGATGCGC ACAGTGGCGCACACCTATAGCCCCAGTTACTGAGGAGGTTAATGTG GGAGGATCACATGAGGCTGCAGTGAGCTGTGATGGTGCCACTGTA CTCCAGCCTTGGCGACAGTGAGTCTATGTCTCAAATAAGTAAGTAA ACAAAAATTAAAAAGAATCCAGTCCACAGGGCATTTGAAGGCAAG AGGAAAAGATGCCAGAATCAGAGATGGGGAGAAGATGGGCTTCTG GCGCCTGCTGAGGTTGAGAAATGAGACAGATAGGCTGAGTGTGGG GTGGAGAGAGGATGGGCAGAGAGACTGAGGCTGGTCTGAATGGA AATGAAATGTTAGGGCTCTCAGGGTTATCGGGGAATAATTGGAGCT TCTAGGAAAGGTTTAACGTTGTGACCACCTGTGTTCGTCATGCCTC

	CCCACCCCTTACTAATTGTGTGAATTTGGCAGACTTTGAGTCTCAGT GTTCTCCTCTGTGAAGTGGGGTCATCTTATTCCAACTCCTGGGATTG TTGTGTGAATTAAATGGGGTAATGTACGGAGAGCACCTGACGCACA GCGAGTGCTTCAAAATTTCAGTCTGCACCCCCCAGCAAAGGATATG CACACGCCCATTGTGAGTGACAAATCCAGGATGACCTGAACCCAAT GTGATAACGTGGGTCCTCGCATGCTGGTCATGCTGCCGGGAGACAC TTATGGATCCAATTAGTACAACAGGGGAAATAAATTATTTAATGCAT TTTGCTAAGACAGAATACCTCAGAACTTATTTTGTGGGGCGGGGCA TAATAAAGGGGGTCCTTCTGCTGAAAACGTTTAAGCTCAGGTTCGT GGCACCACTCAACCAAGGTCGACAGTCACACAGTAAGCCAGAGGC AATGTCAGGACTTAAACTAAACCTGTGGCCCCCACAATGAGGCCAT TTCTCTTTCCCCTGAACGGCCTGGGGAAAGGGGGTGGGTGGGCAG AACTTGGCAGTGGCCAATCCCTCACTTCTGTCCCCTGGTTTTCTCCT GCCCTTATCTCTAGGCTTGCATTGATTGATTGATTGAGACAGGGTCT TGCTCTGTCGTCCAGGCTGGAGTGCAGTGGCACGATCATGGCTCAC TGCAGCCTCAAACTCCTAGGCTCAAGTGGTCTTTCCGCCTCCTATCT CCCGAGTACCCATATCCCTAGGCTTTTAAAATGGCTTCCAGGTATCT GGCTGCCGTCTCAGACATCCACCTGGGCTTCTGGGCAGGGACTGTC CGGGAAACCTCATCTATGTGAAGCAGGTGTGGGTGTAGGAAGGCC GCTTGGAAATGAATCAGCACTGTCTCCTGTTTGAGTCGTAAGCAGG GCGCCAGAGGGTCTGGCGGACAAGAAAGGGAGGATGACAGGAGG CCGGCACTGCAATGACACGCCTTAGCCACCAGAGGGCACGAAGCA GCTGGGCAAAATCCCGCGGGGCCGCTGGTGGAAAATTTCTGGCAC CTGGAGCCCGGAGATGGGGTGGACGGAATGTGAGGACCCAGCTTC CTGAGGCTGGGCCGGGGCAGAGTCACTGCTTTGGATGTCCGCAGG GCCTGCTTGTGTCTTGACTACTCTGCCTTTGTAGACAGCTGGAGAA TGTGAGAGTGGGATTGGGATCGGACTCTAGGGGCCATTCCGTACAA CTCTCCTGCCCTGCCGTGGGGGAGGGAGTTGCCCAAGGTTACGCA GCAAGTTAGTGGCAAATGAATACGATTATCACCAGTCTCAGGTATAT GGCCATTTGATGGGCGCAGTCGCAGCCTCAGTTCCTGAGACAGAG ACACCTGATTAAGGACAGGCCTTCAGGAGCTGACCCTAGTGACCC GCGGCTCTGCTGCTGTCTCTGTTTTTCTCCCTGGCTTTTCCATCTGA CTGACTCTTTGTCTTCTTCGTCTGCCTGCCTGTCTCCGTCTCTGCCC GCTGGGGGGTTTGCTCAACTCCCTCACTGGGTCCTGGGAGCCGCA GTTTCCTGCTGTCACTCCTCAGGGATTTGTAGCTCTCTGAAGCTCTT TTCCGACCCGTTGTCTCGGTTCCACTCTTGGGATCCAGAGGAGAGG TGATTATTTCGTAGCATAGTCAGTGGTGTGATTTCACGGGGTGAGA AGGACTCCCTTGCTCCTAAGCACTCCTCCAGTGACCCCTGTTGCCA TGTGGTAGCCGTAAGCACTGGTTGGCACCTGGTGTGGGCGAGACC CTTACCTCATGCAGAAATGAGTAAGACCGGTGAGCTCACCATGTGG GGGTGAGGCTGAGAGAAAACAAGTACACAGGTGATTCAGTCAAAA TCAGAATTCTCTAAGTACACACGAAAAGGGCAAAAGGGGCGCTTT GTACAGGACAGAACAGGTAGACACTGAATCCGGTTGGGCCCTGGG AAGGCTCCCTGCAGTGGCCTTTGAAGGGGGGGTTGGATTTCAGCA GGATAGAGGGCATGGGCATGTGTGGGCACGTTCTGAACAGAGGGG TCAGCGCAAGCCGAGGGTCTTGGCCACACTAGTTGCATGTGCCGG

	TGTGTTTAAGGGACACGCAGCAGCAGGCCGAGTCTGGAGCGCCTC<br>ACTGCCAGGCTTTTTAAAAATTTTTAATTTTAATTTAATTTTATTTTAT<br>TTTTACTTTAAGTTCTGGCATACATGTGCAGAATGTGGTTTGTTACAT<br>AGGTATACATGTGCCATGGTGGTTTGCTGCACCTATCAACCCATCAT<br>CTAGGTTTTAAGCCCCGCATGCATCAGGTATTAGTCCTAATGCTCTC<br>CCTCCCCTTGCCCCCATCCTTCTCCCCGCAACTGCCCACAGGCCCT<br>GGTATGTGGTGTTCCCTTCCCTGTGTCCATATGTTCTCATTGTTCAAC<br>TCCCACTTATGAGTGAGAACATACCGCCTGGCTTTAAGGGACAGCC<br>ATGGGGATGCACTGCAGTTTCTGAGCAGGGAAGGCCCTGTGGAGG<br>CCCTTAGTTAAAAGGAAAGAATGGCTGTGAAAATCGATGCATTGCG<br>CTCCCTTGTCCCTCACCCTCAGTGTGAAGGGTTTTTATTCCGAGTTC<br>TACTTGAAGTAGGCCTCGATGGGAAGACAAGTAGCATGAGGGGTT<br>CAAGTACTGAGGGGAGCAAGGGACACTCGGTGGCTGTGCCAAGGT<br>GTAGAAGAGGACACTGGGGGCCCCAAGGCCTGACTTCATGTACAC<br>TGCTCAGGCTGGCCCCCAAGTCACACGGTGACCGCTAGGAAGGGA<br>CCAGCCTGTTCTCAGTCTGATCCTCCTGCCATGTCATTATCCAAAGC<br>TCCTCCTGGCAGGGCCTGTTTGGGGTCTCTGTGCCAGTGCTTTCCC<br>TGCCAGGCTGGGCTGGGGCTTCCACCTACTGCTCTGGGACTGCTGC<br>TGCCCTGGCCCTGGGGGAGGAGGGTGTGCCGCTGAGTCACTGCCT<br>GGGCATCTGGGCCTGGAACCTCGGGTGAGTCACTTAGGGCTGAGG<br>TAGAGGGGCTGGGGGAGGGGAAGAAGCTACTCGACAGCTGGAGC<br>AGGGAGGGGAGCTGGGGCCACAGGAAGGGCGGTGCCCTGATGCC<br>CAGACGGGCCGGGATAGACAAAGGGCCAAGGAGGAAGGGGCCCT<br>GGGAGGGGGCAGCCCTCCCTTGGGCTGGGCTCTGAATGGCCACAG<br>TGTTTGCCTTTCTCCGGGTCTGGGGAGGACATGTGTGTGGGGGGCA<br>GTGAGAGAGGGCTGTGGCTGAGGGCTGTGCTTCAGGCCTGGATTC<br>TGGCTTGGGAAGCTGTCCAGCTGGTGTTTTCAGCCTTGGGTAGGGA<br>TGTACCCCTACCCACCCACCCAGCCCTCAAGCTGGAGAAGAGGAG<br>GCCAAAGTTTTCCTGTTCAGCCTTTAACTACTTGGGACTTCCTTATG<br>CTCCCCACAGACTGTGGCCCAGCCCAACTGCGGCTGTGTGTAGAG<br>CAACCCCATTTCTCACTGCTTCCCCATCCTTCCAGACACCTTCCTAC<br>ACAGAGGGACCTTCCCAGGTATTTCTAAGCACACTTAGTTACCTCA<br>TTACCTCATTAAGAGGTATTCTGGTGCTGGCCATTAAAAGTCACTCC<br>ACTTCATCCATGCCCTGAAGTCAGTCCTGTCCTTCTCCTCCTGATGT<br>CCCCCAGCTGCCTCCTCTGGCCCCCAGCTTCCTAAGGGTGGCCCCA<br>GGTTGCTTCTCTCTCACACACACAGGCGCATGTATGTACACGAGCA<br>CTGGACCATGAAGTCTCAGCGTGTGCTCACAGCCTCTCACACAGG<br>AGTGGGCTGTGACTCACAGGCATGTCATGAGAATGAGGCCTGGCA<br>CCAGTCTCCAGGCCCCAGAGCAGGGGTTGCCTCCCCTCACCCCGG<br>TCCAGGATGCCCAGTCCCCACGACACCTCCCACTTCCCACTGTGGC<br>CTGGGTGGGCTCAGGGGCTGCCCTTGACCTGGCCTAGAGCCCTCC<br>CCCAGCTGGTGGTGGAGCTGGCACTCTCTGGGAGGGAGGGGGCTG<br>GGAGGGAATGAGTGGGAATGGCAAGAGGCCAGGGTTTGGTGGGAT<br>CAGGTTGAGGCAGGTTTGGTTTCCTTAAAATGCCAAGTTGGGGGCC<br>AGTGGGGCCCACATATAAATCCTCACCCTGGGAGCCTGGCTGCCTT<br>GCTCTCCTTCCTGGGTCTGTCTCTGCCACCTGGTC

S100A4	TTCAAGGAAAGTTGTTTTGGTGCTGAGCAGAGCAGGGGAAGAAGG<br>AAAACAGCCCTTAAGGAGCTCCAGCCACTGGCCAGCCTTCATGTG<br>ACTCTAGCCCAAATTCATTCCCATCACCTGGGGTGGAAGGGCCAGA<br>AATCTCAAGCTTAGACTCTGGAAGTGCCCCTGGGAGGAACACACC<br>TCCTTCCTGTGCCTCAAAGACATCCCACCTGATGATTTCCCCCCTGG<br>GAAGCAAGCAGTTCACAGCCAAAAGCTGGGATCTGGTCAGGCAAG<br>TTCAGACTTCTCGTCTGTAAAGGGGAGGCTTAGCCACGTGCTCTCC<br>AAGTTCTACCCGGAATAGAGATTTGTAGGGGTGCAACCTGAGCGA<br>GGCAGAAGTGTGTCCCCACTTGGTATTCTGGGGGCCTCTGCAGAGT<br>GAAGAGATGGGGCTGTGATGTGATGGATAAGCCAGACTGTCTCCTC<br>CAACTGAGCTGGAAGGACTTGGCAGGAGACTGTCCCCTGTTCCCA<br>ACTCTGGGGGAGGGCAGATGGGGAATGAAAGGGGAGTGGTGTCTG<br>AGCCCAGACTGGCCTTTGATGTGTGCGTGGATAGGTGGGCAGGTG<br>GGGCCTGTAATGGGTCTGCTGAGGGATCCCTGAGGGCATCCTCAGT<br>GTTGACCTACTTGTGTCCCCCATAATTGCTTCCTTCCTTTCTTGGGA<br>GGCTACAAATAGTGACGCCCTGTCTCTAAGCTCCTACAGCTGAAAC<br>GCCAGCACCAGTGGTGAGTGCCAGCTGCTTGGGTCCTGCCCTGGG<br>TTGAGTGTCAGGGCAGTTTGGGGACCCCCAGTAGCCAGCCTGACT<br>CCTGACCCAGGGTCTGGCATTGGAAGCAGAGAGATGACCACAGGC<br>GGGCCTGACACGGGGAGGGAAGTCAGATATAGTGGACGCAGAGGT<br>TTCCTTACACAGTGAGGGGCTGTTGGGGAAGTTTGGGCAGGGAGG<br>TGTGTAGAGCTAAGGTCTGCCTTGGGGTGGGAGCACTGGGCTCAA<br>GGCTCTGGTCTTCCCCTTGATCAGGCCCCTCACCTGCTCCTTGCCTC<br>CTTGCTTCACCTCTGAGTCTCCTCATTTCTGAGGATCATGGCCCTTT<br>CCTCTCCTGGGAACCTGGACACCTTGCTCTAATCCTGGGGTGTTTA<br>CAATGAATTAAATCCTGGCAGTGCTTGGCACTCCCCACTCCCCTTC<br>CCCTACTTTCCTGCCAGCCACAACCATGCCAGCCATTTGCTCCCAG<br>CAGGGGGCCCAGCAGTGGGCAAATCTTCAGGGAGAGTCTTGGCCC<br>TGGCCGCCCTGTGCTGCAGGACTCTTGTCCCGTGGGGGAATCAGG<br>AGTGAGTCACTGGGCTAGCATGGAAAAAACTCTTGCCTTCCTGGCT<br>GCCTTCGGGTTTTCCCAGGCTGGAGGCATCATCCCAGAAGGGCGGT<br>GGAAGGGGACAGTGGAAGTGGGGGGAGGTGAGCAATTCCAGGAA<br>GGGCTTCCCCTCCAATAGAACAGAGTATCCCTATACAGAACCTCCC<br>TGCTCAAAACCCCCAGGGGTCGGGAGGGTAACCTCAGGGGAGGCC<br>AGTCTCAGATTGGTAAACACCCGAACTGGTCAACTCTCAAGAGAC<br>CATCTGGTTCAGGTTCCTGACTGGGCCAGCGAGGTGAGATGAACC<br>CCAACTTACAGATCAGGCCCCGCCCCTGAGACCCAGAGAGACAAA<br>TGGCTCACCAAGGTTGCACAGCTGGCGACAGGCAGAGAGGATCCC<br>ACAAGTTTTTCTTCTGGGTGCAGCTTCCTCGAGTTCAGGGCAGGAG<br>GCAGGGTGAGGGCAGTGGTGGTGAGGCTGTGGGGGCGCCTCTCAG<br>ACCTGCCCCTCCCTGCTAAGGCCTAGCTTCTCCAAGGGCTGAGGGC<br>AGAGAAGCACAGGGCCAGGCTCTCCTTGTGCAAGTTCCTGCAGGG<br>AGGGAGGCTTCTAGGGCATCTCATGGGCTGGGGCAGACTGGGACC<br>GAGCCATCCCTGGGTGTTGGGGATAAGAAGAGGTGGCAGATAATCT<br>GAGAGTGCTTAAGGAGCCCCACGGTGGGGTGTGTGGGTGGTGGGG<br>TAGGATGCTGTCTCTCTGAGGAGTTTACAGCGATGTTGGGGCAATG

	AGACCTCTACATGGGGAAATAACAACAGGACAGCAGGGGGATAAA<br>TTAGGAGGGGCTGGTGTGGTCTGGGAGGGCTACTCAGAGGAGGGG<br>GAGACTGCGGGGCCTGAAGCCTGATGGAGGTTAAGATGGGTCTGG<br>GAACAGTCTCTGAGAAACACAGAGGGCAGAGGTGAGCTGGGGGG<br>CTGGCCTGGAACTCTGCTGTGAGCCACACCCTTGTTCTGTGACAGT<br>GAGGATGGCCAGGCCTCTGGAGCAGGCGGTAGCTGCCATCGTGTG<br>CACCTTCCAGGAATACGCAGGGCGCTGTGGGGACAAATACAAGCT<br>CTGCCAGGCGGAGCTCAAGGAGCTGCTGCAGAAGGAGCTGGCCA<br>CCTGGACCCCGGTGAGCAGTCAGAGTGTCCGTCCCCTCCTCCTCCG<br>GGGGGAGGCACTGCCTAGACTCTGCAAAGAGAGACCTGGGGACA<br>ACAAGCATGAGCACAGGACAGCAAGGGAGGGGGCTGGATTGTGA<br>GGAGGGAGTGGGAGGGGGAGAACTGACACAGCAGAAACCTCAGC<br>TTCCTCATCTGTAAAATGGGATGACAATCATCCCTGCTGCCTTGCAG<br>AGCTGTTGAGAGGACTGAGTGAGAGCACCATGAAGAATGCCTAAC<br>ACTTGGTAGGCACTCAGTAGCAAATAGTAGGTGCTCGATTAATAATC<br>TGAGAGGACAGATTTGGGAGGGGATTGCAACGAGTGCATTATCAG<br>AGCAGGGAGGTTGAAGTCGGCCCTCAGAAGGGTGTAGTTTGGGGT<br>GGGGTGGGTCCACTTGGGAGAGGAAGGAGGGAGGTGGCGTGTGA<br>GGCTGTTCTGCCCTCCTCATCCTACTCCCTTACCTCTCCCAACCCTT<br>CCCTTGCACAGACTGAGTTTCGGGAATGTGACTACAACAAATTCAT<br>GAGTGTTCTGGACACCAACAAGGACTGCGAGTGGACTTTGTGGAG<br>TATGTGCGCTCACTTGCCTGCCTCTGTCTCTACTGCCACGAGTACTT<br>CAAGGACTGCCCCTCAGAGCCCCCCTGCTCCCAGTAGCCTCTGCTC<br>CAGGGGGTGCGCTGGCTGTCGGGGGCTGGGCATGTCTCCCACACC<br>CCCTCCTACCCTCTCTCCTGTACCCCTTTCAATCTGGACTTGCCCAG<br>GTCTTCTGCGATCAGTTAACCCATTTTACCTAGGAGGCCCAGAGAT<br>GTGAGGGCTCCTTCCTCAGGATGCCCAGCGAATGAGGGGTAGAGC<br>CACTCTGGGGCCCAGCCTGCCTGCCGCACCCCTGTGGCCTCCCTTG<br>TGGATGGGAGGAGGCGGGATCTGCTCTGAGGCCCTCGAGGCTCAG<br>CAGAGCGTGCACCAATGAGACCACGATGGGAAAGGGCCTATTTAA<br>CTCCTAATAAAAAACTGGCATCAGCTTGGTTTTGTTTGGTTCTTCTC<br>TGAGGACGCGTCTCCTGAGCTCTTTGCTCCTCTCTGGCCTTTTCCA<br>CTGCGGGAAGGCAGCTGAGGGCAGGGGCAGGGTGCTCCCTGAGG<br>GAAGTTGCAGACCCTGCAGAAATGCAGTGGCCTCCAGGGTCCAGC<br>AGGAGGGCGTGGGGACAAAACAAGGACCCCTGTCCTCCCTTCCTC<br>TCTCTCTTTCTTTGGGATCTTTGAGAGAGGACTGCCTGAGTTTCCCC<br>TGGGACTCAGTGCTCATGGTTGGAGGAGGGTGGGGCCGTGGGTGC<br>AGTGGGGGTGTTTGTTCCCTGCCTCGGGCTGTGGGAGCCCAGAGA<br>GCAGCACTAATGGGATTAGGGTGTCTGAGGTGTTTCTCCCAGCCAG<br>GAAGGGGTGCTCTGTATGGAGGGGTAAGGGAATAACTTGAGCTAG<br>CCCCTATCCCTGAAGCCGCAGCCCCCTGAGGGCCTTAACCCCCTAC<br>TTCTAACCCTCACTGGGTTTGTAGCCCACCCTGAGAGGTTGACCCG<br>AATTATAACTCCCCTATTTCATGCCATTTCACCTCTAACTCTCCACCC<br>CAACCTGGATTCTTCATTCCTGACACTCATCCCAACTTTAAATGGCC<br>CCTCCTGATACCCTCTCCGAACCTGAGATCTATCCGTGAGCCCCCAC<br>GCCTCACTGCCACTCCACTCCATCACTACCTCACCCAGGACCTTTC

	CCACTGACGTTCCTGAGGTGGTCCCAGAGCCTCCTTTGGGTGTGAG<br>CCTGTTCCCCTCCAGATCCCCCCGCCCCGACCCTGAGCCTTACTTG<br>GCATGGCAGACAGTACCGGGCATGGGGATCCCCACCCCAGTTTTTG<br>TTTCTGAATCTTTATTTTTTTAAGAGACAAGGTCCTCTGTGTTGCTC<br>AGGCTGGAGAGCAGTGGCTTGAGCATAGCCAACTGCAGTCTCGAA<br>CTCCTGGGCTCAAATGATCCTCCTGTCTCAGCTTCCTGACTAGCTG<br>GGACTACAGGCTACAGCCATGCTGCCCAGCTAATTAAAAAAAAAAA<br>TTGTTTTTCCTTTTTATAGAGACAGAAGTCTCTCTATGTTGCCTAGG<br>CTGGTCTTGAACTCCTGGCCTCAGGCGATCCTCCCATCTCCCCCCTA<br>GCTTTTGTGTCACCACATTTCCAGGGCAATCTCCCACCTGTCACCC<br>ACCACCCCCTGCATCTCCTTTCCTAGGTCCCCATGGGACTACTCCCT<br>GTCCCCCATGCTCCAGGCACAGGCTGCCCCTTCCTCCACCTCTCTA<br>AAACTCAGGCTGAGCTATGTACACTGGGTGGTGCCCATCTCATCCA<br>GTCCCCTGCTAGTAACCGCTAGGGCTTACCCGTTACCCACGGGTGC<br>CCACCTGGGAACAGGAGGCTTGGTTCCACGGCTGGGCTGGTGGAG<br>GGTGCTGTGGCACTTACCGCATCAGCCCACAGCAGGAAGGCAGTA<br>TCCGCTCTCCCCTGTCCCCTGCTATGGGCAGGGCCTGGCTGGGGTA<br>TAAATAGGTCAGACCTCTGGGCCGTCCCCATTCTTCCCCTCTCTACA<br>ACCCTCT
SELE	CAGAGTGCCTCAGGAAGGCAGGAGGACACCACTGCCCCTGCTTCC<br>CGGTCACATTCACCAAATATAACTTCACTCCTCGATAGATGCTTTTG<br>CCACGTGTGGTTGTGGCCAGTGCTGCTTTCCACTAAACCCAATAAT<br>CTCCTGTCCACAGGGTGGCCTTGGTTAAAATTCTAACATTTGTCTTG<br>GGGTCCCCAATCTCTTCTTTCTGGCTATCTCTCTGTCTCCCACTCCC<br>TTTGCCATAAAAGTGAGAATGAGTGGGCCTGGGCACACAGCAAAA<br>CTGGTTAAAGTCTTTGATGCCTGATTAAAGGGTGTGGAGGAGAGGA<br>CAAGAGACTTCCAGGAAGAGACAATAAAGCCAGGAAGGGTGACA<br>CCTCAGAGATTAAATAGCACTGGTGGCAGACACCGGGGTGTGCCT<br>GAGTTTGCACCGTCTTCTTTCCTCTTTTCATTCCCATCTATGTAGCGG<br>ATGCTTCCCCCATGCGAGAAACTGCCAAGAGGGTCCATGTGAGGC<br>GAGGCTTGAAAACCGGCAATCCGGTAACAGCTAGCTACATCTTCAG<br>AGCTTGGGAGATTCAGTGGAGCCTTGAAGCACCTCTAATCAGCCTG<br>TTTCTTGGGGGTATATTTTGGATTATTTGAAGAGGGAGATGATATTCA<br>TAATATTCAACATGCCCAACTATTCAAATCTTACCCAATTTCCTCTTC<br>AACTCCCCCTGATTTGCCCCCACCCTCAAAAAACAAGCAATTGCCT<br>CTTATGAAATCCTGATAGGAAAAAGCAGGTGAAGAAGCTATAGTCT<br>GGTTGTAATCCCACTCCCACGCAGCGCTGGCCCAGGGCCTCTCCAC<br>GAGAGGAAAGCTGGGTCTGTCTATGGCCCCTTCAGCAGCCCAGGT<br>AGCCAGAGGGGAGCCCATCAGCTCCCAGTATCCATGTACCTGATTT<br>GCAGTGACAACCTCGGGGGCAGGGACACAGAAAATACTGTGGGA<br>GAATGCAGGTAATTGTAAGGATGAATGGATTTTGAATAACATACCTC<br>TGGTTAAAACAAGTACATATATTATATACATATAAATTATATACATACA<br>TGTACATATTGTAAACCAAAAAGTAACTGAAGCATATCTCAACAGA<br>TTAGAGGTTTATTTTGTCCCAGGTAAAAGAAACACAAATCACTGTA<br>GGATCTGTGACCTGCGCTTTTTCCAAAGAGAGTTTTGGGAACTTCC<br>ATCTTTAGAGGGGAAAGAGCAAGCAGGAGGGGAAGAAAATAAAC

	GAAAGATGGGTTGGCAATGAGACAAGTGTTTACATTGTGGCAAGG<br>CTCTGTAACACTCAGTGAGTCTACACTTTACATGTAAAAAGAAGGG<br>ACTAGAGGGAAATTATGCATGGATCTCATGCTCAGTACATCTACATT<br>TTATATAAGATAAAGTAAGCATGTGAAATTACAGGTATTTGGGAACA<br>AAAGGAAGGCAGTTTTTGCATGACTCATTTCCCAAGCTTAACTTTC<br>CCTTTGGCATAGTGAATTTGAGGTCTCAAGATTCTATTCTTCTTTCA<br>CAGTATTTTACATATATTTATATATAATATATATAAAAATATCTATATTTA<br>TATATAAAATATAAAAATATCTATATTTATATATAAAATATAAAAATATC<br>TATATATATAAAATATATAAATATATATAAATAATATATATATTCCACATA<br>TATAATATATTGTAATTATATATATATACTCTACATATATTATATATTATAT<br>ATAGTCTACATTTAAGTTACTGTTACTCACCTATCTCTTCCACTCCTC<br>ATTTCTCTTATCCTTCGAATATTGGGATCTCTCCCTGAAGCTTTCTCA<br>ATTTAGAAATAATCAGTTTAGAATTTAGAATAATCAGTTTGCCCTTT<br>GACCTTAGAGAAAATTTGCTTTTGAATCAGGAACTTCTGGGAACTG<br>TGGCTTAGGGATCAACTTTACCTAGGCATGGCTCCACCTGCCTTGCT<br>TGTTTTTTCCTTTGTCCACTTTCTCCCTGCCTATGCACCGGGGATCC<br>AGGGTCTGTTCTCACTGCATCCTCTCCCTACTGCTCACTGTCCCTGG<br>GGTCCCTTCCAGCCCCCATGTCTCAGAACCCAGGACACAGTTATCT<br>CAGTGGAGAGCTGTGATGGTCAAGGATGTCTCAGGAAGAAGAGGG<br>CAAGAAGAGTACAGGAAACTTGGGTAGAACAAGGAATCTCGGGGC<br>ATAACAACAAAAGAAATCATGGTTTGGAGTAGCAGGTAAGGGAGT<br>GGGCTCTGGATATCAACTGCCTGGTTTCAACCCTTAGCTCTGTGCTT<br>TTGAATGAGATAAAATCCCCAGATTCTCAATTTCCTCATCTGTAAAA<br>TGGGGATAATAGGAGTACCTATTTCATAGGCTGTTGTACGGATTGAA<br>TAATCAATAGGTGAACATACAAAATGCTCAGAATATGGCCTGGCAC<br>ATAGTAAGCACTCAATAAATGTTAGAGATTATAATTGGGGGTCCAGG<br>ACATATATATGTAAGTAAAATAGAAACTTCATCACCACTGGGGCTAT<br>TTTTAGATATCTTTGAACTAATTTTAAAAAGGGGAAGAGATAAACTA<br>TAAACTTAGTTATGAAAAATTCGCAATAATAAAGTTAGTTATCTAGG<br>GATGTTAACGACTAATTTCTATTAGCAATATAATTATTAGGTACTTTAT<br>TGATATCGAAGATAAATTGTTTTTTAAGATTTGGCTACTCTATACATT<br>TGACCATGCTTTTTAAAAATAGCAGATTATGTATTCATACTTTATTAA<br>AAATAAAATACATACAGATAATGTATTAACCCAACTTGACATTCTGC<br>CCTAAAATCTAAATAAACACTATATGAGTGATTACTCTTCTGAATTTA<br>TTGAAGCAACAACCACATTGAGCTAAGTAAAGTGGGATAAAGAAT<br>TGGAATAATGTCAGTATCTGGATCATGTAAGAGAGTGGTAAGGTGG<br>AGGGAATAAATTCTATGCTTTTTGATAGGGGTTGAATTAAATTCGTT<br>AATTAAGTTATTTTTTTCTAATCAGTTGTCTTTTTTGGAATTTTTCTT<br>CTCTTTTCTTGTTTTTTTTTTGGGTCTTGACATCTTTATGAACACAGA<br>AAGACCAGTGCTAACAAGTAAAGAGAAAAACGAGGCTTTTACCAC<br>TACTCGTTCAGGTCTGCTGACAGTACATTTTTAGATAGACCTCCTAT<br>TTTGAAAGGCTGTAGATATAGAATATTAGCTTGACTTGAGCTCTGAA<br>AACCTACAGAATATGGAAATTAAAATGATCCCCATTTACTGTTACCA<br>TCATTTAGATAGGGTAAGATGCAGAGATGGGGAATGTATACAATCAC<br>CCAGAAGAAATGTCAAAATGAAGAGCAGGAATATAGGATACCACAT<br>ACATCTCCTTCTGTTGCATTTGGAAACAAATTTCTTTTCTTTTCTTTT

	TCTTTTTTTCTTTTTTTTTGAGATGGAGTCTTGCTCTGTCGCCCAGG CTGGAGTGCAATGGTGTGATTTCAGCTCACTGCAACCTCTGCCTCC TGGGTTCAAGCGATTCTCTTGCATCAGGCTCCCCAGTAGCTGGGAT TACAGGCATGCGCCACTACACCCCGGCTAATTTTTGTATTTTTAGTA GAGACAGGGTTTCACCATATTGGCCACGCTGGTCTGGAACTCCTGA CCTCAGGTGATCCACCCACCTCAGCCTCCCAAAGTGCTGGGATTAC AGGCATGAGCCATCATGCCTGGCCTAGAGACAAGTTTCTTTAAACT GTATTCTTGTTACTATAAAGGTGAAAAAAAGGGAAATTGAAAAAAA CTTTAAAATCCTGAAAATAATAATGAAGGATATAAAAATTATCCCAA ATCCTGCCATCCAGAGTTAGTCACTGTTAACATTGTGGAAGAATTTT TATTCTTTGCACATTAACATTTCAAAAATTTTAAACAAAATTTTGAC CAATTAAATATATATATATAGTTTTCCGTTTTATTTTTTTTCTTTTCTTT TCTTTTTTTTTTTTTTTTTTTAAGACAGAGTTTCACTCGTTTCCCAGG CTGGAGTGCACTGGCGTGATCTTGGCTCACTGCAACCTCCACTTCC CGGGTTCAAGCGATTCTCCTGCCTCAGCCTCCCGAGTAGCTGGGAT TACAGGCATGCGCCACCATGCCCAGCTAATTTTGTATTTTTAGTAGA GATGGCGTTTCTCCATGTTGGTCAGGCTGGTCTTGAACTCCCGGCC TCAGGTGATCCGCCTGCCTCGGCCTCCCAAAGTGGTGGGATTACAG GCGTGAGCCACTGTGCCTGGCCTCCTTTTTATTTTTTTCACTGAACA AACCATGAAACTTTCCCAGATGTAAATATCTATTTCCCATTTTTCTTT TTTTAAAATAAGGCATTATTTTAACCATTTGAGTGTTAGATATTATTT TTAGATAATATTTTAATTTAGGCATAACTGCCGTGCAAAATCTGAAG ATTAATATCTACCTTGTGAGTCATTCCTCTGTGAGACAGTGCATGTT AAATATGTTGAATTGGCAGGTGAAAAAGGAAGAAAAAATGAGTAG TGATTGGTTATCCACAGCTATGAATGAGAAATTGAAGGTAGTAGACT ATGGATGACAAACCTATTCTTGGTTTCCTTCTGTTTCTGAAATTCTA ATTACTACCACAACTACATGAGAGACACTACTAACAAGCAAAGTTT TACAACTTTTTAAAGACATAGACTTTATGTTATTATAATTAAAAATCA TGCATTTTTGTCATATTAATAAAATTGCATATACGATATAAAGGCATG GACAAAGGTGAAGTAGCTTCAAGAGACAGAGTTTCTGACATCATT GTAATTTTAAGCATCGTGGATATTCCCGGGAAAGTTTTTGGATGCCA TTGGGGATTTCCTCTTTACTGGATGTGGACAATATCCTCCTATTATTC ACAGGAAGCAATCCCTCCTATAAAGGGCCTCAGCCGAAGTAGTG TTCAGCTGTTCTTGGCTGACTTCACATCAAAACTCCTATACTGACCT GAGACAGAGGCAGCAGTGATAC
SELP	TTGGTTGGCAGTTTTTTCTCTTTCAACACTTTGAATATATCATCTCAC TCTCTCTGGGCCTAGAAGCATTCTGCTGAGAAATCTTGTGATAGCCT TATTGCAATTCTTGTCTGTGATATGCTTCTTTTCTCTTGCTGATTTTA GGATCCTGTCTTTGTCTTTGATTTTTGATAGTTTGATTATTATGTCTT GGTGTAGTCTTTTTTGGATTGAACCTGACTGGAGATTTTTAAGCTTC CTGTACCTGAATATTAATATTTTATTTCAGATTTGGGAAATTTTCAGC TAGCAATCTTTAAATATGCTTTCTGACCCCCTTTTCCTCTATTTTCTC CTTCTTAAACTACTGTAATGTGAACATTAGCTCTCTTTTTTAATTTTT AATTTAATTTTTGTTTTTATTTTTTGAGATGCAGTCTCACTCTGTCAC CCAGGCTGGAGTGCAGTGGCATGATCTCAGCTCACTGCAACCTTTG CCTTCTAGGTTGAAGAGATTCTGCTGCCTCAGTCTCCCCAGTAGCT

	GGGATTACAGGCATGTGCCACAATCCCTGGCTAATTTTTTTGTATTT<br>TTAGTAGAGACTGGGTTTCACCATGTTGGTCAGGCTGGTCTTGACT<br>CCTGACCTCAGGTGATCCACTCACCTTGGCCTCCCAAAGCGCTGGG<br>ATTATGGCATGAGCCACTGAGTCTGGCTGAATGTTAGCTCTCTTGAT<br>GCTGTCCCATAAATCTTGTAGGCTTTCATCATTTCTTTTCATTCTTTT<br>TTCTCCTCTCACTGTATATTTTCAAAAACCTGTCTTCAGTTCACAGA<br>TTCTTTCTTCTGCTTGATCAAGTCTGCTACTGGTGATTTCTACTGCAT<br>TTCTCACTTCATTCATTATATTTTTCAGCTCCAATTTCTTTTATGATTT<br>CAATCTTTCTGTTACATTTCTTATGTTGTGCATTTATTGTTTCTCTGAT<br>TTCACCAAATTGTTTCTCTGTGTTTGCTTCAAAGTAACTGAGCTTCT<br>TTAAAAACAATTATCTTGAATCCATTGTCAGGCCATTTGTAGTACTC<br>CATTTCTTTTGGGTCAGCTACTGGGAAATTATTGTGTTTCTTAGGTG<br>GTGATATTTTAATTTGGGTTTTCATGTTTCTTGCTGCCTTACACTGCT<br>GTCTGAGCATCTGGTGGATCTGCCCCAATTTCAGGCTGTATGGGCT<br>GACTTTGGTGGAGAAATACCTTCTTATGTGGAATAATGCGAGGATG<br>CTGGCTGGGTGGGATGCAAAAGTTCTGACTTCAGTAGGGGCAAAG<br>CTGTGTGGTCTCCATGCAGATCTGTCAGCTGAGGTTGGTGTTAGTG<br>AATACTACAGGGATCCTTAGAGGCCAACACTGTGGGTATCTACAGT<br>GGCAATGAGGCTGTTGAGGTTTTCAATTGTGACAAGTCCTCCATAT<br>CTCTTTTTTTCCCCACCTGGGAAGTCATGACTGAGGACATCCCTCTT<br>GGAATTAGGTCTAACTTGCAGGCCTGCTCCTGGTGGTGGTGACACT<br>GGTGTCTGATGAACAGTGCCCATGGAGTGGCCAAGAGCCAAGGCC<br>TGAAGCATGGGCATGCATGGAGGGACCACAGCACCAGATTCAATT<br>GTAGCAATGGTACCAGTGCCCAAGGCACAGGCATACTTACTATCAC<br>ATTGATAATGGTGTGTAAAATGCAGGTACTTATAAAGCAGCTAAGG<br>AGCCAGGGACTTTACTGCATGCATACGCAGAGCTACAGTGGCTCCA<br>GGATCCAGGGTGTGGGCTAGCTCTCCTTGGTGGCTGAGCTGGTGAC<br>TAGAGCATGGACAGGCACAGAGAAACCTTGACTCTAGGACCCAGG<br>GTGTTCACTAGCTCACTATAGTGGTGGCTCTGGTGTTGGAGGTGTG<br>GGTGTGTGTAGTACAGCCTCAGAGACAGGGTCTGGAGCGCAGGTG<br>TGCACATTACTACAGCAGCTCTGGAGTTGAGAATATGGGTTACCTTT<br>CTACAGTGGCTGAACTAGTGTCTGGAGCAAAGACTTTCACAGAGA<br>GAACTTGGCTTGGGGTCCCAGGGTGAGATCTAGTTCACAACAGCA<br>GTGACTCCAGTGTCTGAGACATGAGGAGGTGCACTGCAGCCACAG<br>AGCCACAGTCCAGAGTGTGAATATCTGTAGAGCAGCCACAACTTTT<br>GGGGATCAGGAACACACATAGACTTGTGAGAGGTGGTAACCCTGG<br>CCCCAGTCCTGGGGCAGTGCAACAATAGCTGCTTCTTGGTGAGGG<br>GGTGTGAGGGGTAGTGCAACTGTGTTTCCCTTTTTAGCATCCTGCTA<br>TGGGAATGGCTGTTGGATAAAAGATGCCAGTGTCCTCTGTGGAGCA<br>GGACACTGGGGGCCTCAGTGGCTCTGTGTCACATGACTGACACAG<br>ATAGCCTACAAATTTCTTTATTCGTAGCTATCTCCTGGTGTCTCATAT<br>ATGCCAGTCTCACCGGTGATTCTTCTACATGGATATTCTTTCTTTTCT<br>CCATTGTGTTGTTCCAAATTCTTTAACAGGCTCTTGAGCCCCATCCC<br>CCAACTCCCCACCCTTGTGAGGGCTATTTTGGTTTGTGTATAACTGT<br>CTATGTTTGTTTTTTTGTTGGGGCATAAGGCTGACATCTCCTACTCC<br>ACCATCTTGCTAATGTCACCTGCATAGGAATCTTTTTATGCTTTCCTT

	ATATTCACTAAAATTTAACAATATCAAACTTAAAAACATATGATCAAT TGAACTTATTAATATCAAACTTATTATAAATAAGAAACTACCAGGCT GGGCATGGTGGCTCATGCCTGTAATCCCAACATTTTGGGAGGCTGA GGTGAAAGGATCACTTGAGCCCAGGAATTCAAGACCAGCCTGGGA AATATAGAGAGACCCTATCTCTAGAGATTTTTTTTTTTAATTAGCCAG TAGTGATGGCACACATCTATAGTCCCAGCTACTCAGGAGGCTGAGG TGGGAGAATTGCTTGAGCCCAGGAGGTCAAGGCTGGAGCAAGCAG TAATCATGCCACTGCACTCCAGCCTGGGCCGCAGAGTGAGACCCTG TCTCAAAAAAAGAACCTACTAGTCTACATACCACACTTCCTCATCC CCATCTGAGACTATATATATTTTTTCTAACATGAGGCAATGCCAAAA AGAGGGGCTGGTGAGTGAAAGTAAGAACAGAAAGACATGGAGGC AAGTCTTATAGAATAATAGCCAACACTTAAACTTACACTTAACAGCG TGATAGGTATTGTTCCAAACACATTAAATTCATTTAATGGTCCTTACA TGTCTATGTATTTGGTGATTATTATCCTTATTATTCACATTGCTGAGTG TATTATTCTGTTCTCATGATGCTGATAGAGACATACCCGAGACTGGA TAACTTATTAAAAAAAAAAAGGTTTAATGGACTCACAGTTCCACGT GGATGGGGAGTCCTCACAATCATGGTAGAAAGCAAAAGACACGTC TTACATGGCAGCAGGGAAGAGAGAGAAATGAGAACCAAACAAAA GGGGTTTCCCCTTATAAAACCATCAGCTCTCATGCGACTTATTCACT ACCATGAGAACAGTATGGGGGAAACCACCCCCATGATTCAATGATC TACCAGGTGCCTCCCACAACCTGTGGGAATTATGGGAGCTACAATT CCAGATGAGATTTGGGTGGGGACACAGCCAAACCACATCACTGAG GAAACTGAGTTATAGGGAGATTAGTAACGCCCAACACAGCTGGTAG GTGGTGGAGCCAGGCAGTCTGACTCTAGGGTCTGGACTCTGAACT GCATCATGCTGCCAAGAAGTTCCTCATTTTTTCCTCTCTCTAAGTTT CCCTTATTCCCCTACAGTCATTCCTTCAACAGCATTTCCTTCACCATC TTTTCTACTTCTACTATATAATTAATTTTTTCTTCTTGGTCCCAAATTC CAACGTGCAAATGCAGCCTTATATACCCTAATTCATCTTTACCTTTAG ACTTTCTTCCAATGTTTCTACTTCATTCCATTTTAAATTTATCCATGA GATGCCTATTTACAAGCTGTAACCATCATGAAGTGAATGAAGAATAA TACCTACTACTGTACAATAGAATTCCAAGAGTATAAATAGGAGTTAT GGCTTTCTGACTTGAAACTAAATACTTGATACTTGATTTTGCTGTCT GAGATCAATCTGAAAAGTAATAATAATCACTAACATTTGTTGAGCAT CAATTGTGGGCCAAGTGTCATTTCAATCACTCTGTACATATTAACTC ATTTCATCCTACAACAACCCGGTGAGGCAAGTTCTGTTATTCTGTTT TACAGTTGAGGAAACAGAGGCATAGAGAGCTTAAGTAGTTTGCCC AGTAGATAGCCAGAAGAGGAGCCAGGATGGGTCTCGGGCAGTTTA ACAGCACAGCTGAAGTCTTAACCACTATGCCAACAGCTTTTTGGTC CTACACATCCCATGGGAAGAGGAAAATAAAAAGGTATCTATTTGTAT ACCTTTTTATTTCTGATATAAGAAGCAGAATTCCTTTCACATGACCTA TGTCTATTTAATACGTCATTTTGAAACTTACCAATAAAATTTCCCAA GCGCCAGAAAACTGTTAGTGGCTTTTTCCATTTCTCTCTATTTTTTTT TGTGCTACTAATTTTGCTTCTTTCCCTCAGAAGGCTGCCGGAATAGT AAACATTCACTGACATGTCATAATTACTGGAAAATGGGCACTGGAA AATCACATTGTAATTAATTCAAAGCATGTTTTCCAAATGTACTACTTT AAATTGGAGCTTATATCATAATCCAAGGAAACCTTTGTGTGTGTACT

	GTTCCCACATTGCTCAGCCTGGGATATCCAGGAGTAATTCACCTTGC<br>GCCTGCCTCCAGACCATCTTCCATGGAAGGGGGTGACCCCTTGCCT<br>CTTGGCAACCACTATTTCTAAGCTGCCAACATTACTCTTGCATTATC<br>AACATTCTAACTTCATGGGAAGGGCTGTGGTGAGTTTCTGGAATGT<br>GAATAGGAAGTTGTTTTTCTAAACAGCCTGACACTGA
SERPINB5	CTGTTTAACTCTATTTTTTTTCACTGTGGTCTTTTTTTTTTTCTCTTAA<br>GTTTGCTTCTTAGAAAATTTCACACAGCCTGTTTTCTGTAATATAGT<br>ACAGTTTATCATTTAATATAAAATTTCTTCTAGACAAACACCAGAAG<br>TTGAAAAGACAAAAAGTATGAAATAAATCCTCAAAATAGAGTAGTT<br>AGGAAAAAGAATTATAAATCTGGGGCTTTTAAAATAGTTTTTTTTCC<br>ATCTCAGTCAGAAACTTTAAAGCAGTCACTTTATCTTCCCTGACTCA<br>GTTTCTCCACAAGTGAAAAGACATAATAGTCCACAGTGACTAGTTA<br>GGATCCTTTATAGTCAACTGTGAATGGCTGGAAACGTGGACAGCTA<br>ATATGATTCCACTATTGTATGGGAGAGGCATCATGTGAATTAGTGGG<br>AATCACCTTTATTAGTGAAGAAAAAAATCACTTGATGATTTGTAGG<br>AATCCGGGGAGAATGCTGAGAATTTGTGGCTGAAACTTCTCTTAGG<br>AAAAGAGAGAGATTGAGTGAGAGCGAGAGCGAGACAGAGAGAGA<br>GAGAGAGAGAGAAAGCGAGAGGCACATGCTAGAACAAAGCTCCT<br>TTCATTTGTTCCTGGGAGTGGCTGGGTGTGTCAAATCATCCCTCAG<br>CCTCGATGCTGAGTAATCAAGTATAATATTTGCAAAGCTTCAACCTA<br>TAGGTAAGAAAAAGCACCAACAAAACAGTCTCCTAGGTTATTAAAT<br>ATTTAGTCCAATGATTACTTGTGTTGAACTAGAGGAAGTTATTGCTT<br>CATACAATTGTATAATGAATGAATGAATAATTCATTCTTTGAGCTATG<br>TATCTCCCCTCCAAAAAATCTACTCTTTTAAATATATAGTCAATAAAA<br>TGGTTTTTGTGGCAAATAAAGAGAGGGGAAAACATTATGGATTATT<br>TTTGAAATTATAGAGCAAATGCTTTTTCCTACAAACAGGTGTATTCT<br>TTGTCACTAAATTTTTTTAAATAAATCATTTATTTGGTTGGGTGCGGT<br>GGCTTACGCCTGTAATCCCAGCACTTTGGGAGTCTGAGGCAGGTGG<br>GTCACAAGGTCAGGAGATCAAGATCATCCTGGCTAACAAGGTGAA<br>ACCCCGTCTCTACTAAAAAATAAAATAAAAAAAAAAATTAGCTGGGC<br>ATGGTGGCGGGCGCCTGTAGTCCCAGCTACTTGGGAGGCTGAGGC<br>AGGAGAATGGCATGAACCCAGGAGGTGGAGCTTGCAGTGAGCCGA<br>GATCGTGCCACTGCACTCCAGCCTGGGCGACAGAGCGAGACTCCG<br>TCTCAAAAAAAAAAAAAAAAAAAAAAAAAAAAAAAAAAAAAAA<br>AGAATCATTTATTTTACAAATGTAAAGTGTTTGGGATGAATCAGATG<br>TACTGGTCTTCATATACAAAGAGAACTGGAGGAAAGGTAGCTGGTA<br>TTTAAGGAAATGTCAAACTGACTTCAAATTTTGGGTTGAATTCATCC<br>TTAGTTGAATTCCTGTCGGGTAGGGTTATTTCCCACTCATTCATTCTC<br>TACCCACTATGTGCTAAGTACCCACTATGTGCCAGATACTATTCTAA<br>GTTCTTGGACTACATCAGTGAAAAAAACAGACCAAAAAAATCTGC<br>TTAGATTTCAGCTGAAGGAGGCCATCAACAGTAAACATAATAAGTG<br>AATTGTATAGTATATCATATAGAAAACTATAAAATCTGGCCTTATATCT<br>GCTTCTTATAGGTTCAAAGTTCATCAAGAAGAAGGGAGATCAAAAA<br>TACTCTGGAGTTGACTATTCATGGGCCCACATAAAAAGCTTTTCATG<br>AGGCAAGAGACTGAAGATTATAACAGGATAATAACACCATATTGTTA<br>TTTTTATGTTGTTGCTCTCTGAGTTATTAGTATGAGTAGAACCTTTTT

	TCTTATAAGTCTGATGCTTCTAAGTCCACTTGTTAACATAATTGACAT<br>GACAACTTTGTTCTTTCCAGGCATTTCCTTCAGGATGTATGTTTTTT<br>TTTTTTTTTTTTTTTTTTTTTTACAGTATAACAATTAGTGTAGGTATCT<br>GACTTATAGAAATGCAAAGCTAAACTGTAGTCGGCTTGAGATCCAT<br>AGTTAGGTCTTATTCTGGGTCTTGCCCAGGGTCTGACACACTCAAA<br>AAATGCTTATTCAGTCAATCACAGACTCCAATCAAAATGTCCAGTTA<br>AAACAGGGAAGCAGAGTAGAAACCTATTGAATGCAGAAGACTTTA<br>TTGAGGCCACTCTTTCCTAAAATTAATCAGCAGGCATGTTCTCCAG<br>AAACTAGTTTGAAATTCTGTAATTTAAAAAATGTGAACTGCTTGTAT<br>AACTCCATCCTCTCCCACTTTTCTGTTTTCTAGTGTCTTTTTAGGTAG<br>AATTCTAAAGAACTTAAAATTTTGGCTGCCGTGGAAAAGAATATTA<br>CTGGGAAATGGGTCTTCTCAGTCAAATAGAATCACAGTTTTAATACT<br>CTTTCCACTGAATGGGAACCACTTTTTTTTTTCCTGCAATTTTGCCT<br>AAATCAACAGTGTGTTTTGTCAATGATGTTACACCTGAGACTTGTG<br>AGTCCTTGCCTTACTTGGGTTCAATGAGAGGGTGAGAACATTCACG<br>TACAGGGTCTGACCTCCAGAGATACTTCAACAAGTGAAAGCTTTTG<br>ACAGGAAATGTTCTGTTTAATCTTTTGTTAACATATCTGTCCAATATA<br>CCTTGTATCATCCCAGCTCTGGACCTCCGTTCTCAGGCTTAAATGGT<br>CTCCTGATGTCCAATGATGCTGCCTCCATGATCCTTCCCCAGTCATA<br>GCCAGACTGAAAAGGTCACACCCATCTCTGAACTACTTTTGTCATT<br>TGCACCTCTCTTAAGTCCCTTATTACATGCCTCCAGATACTTTCTTTT<br>GTTTCTTGTCTTCCTACTGTACCCTTTGGGGAGAGGGAACATGTCTT<br>ATAGCCCCAGTCGAATGGTGCCTCTAATTTTCACCCAGTTAGAATCA<br>CCCAAAGGCATTTCACTTTGCTAGAACTTCCTTGGGTATTGTTGAAT<br>TCCTGTCATTGGAAATGTTTCTGCCCCGGTTGAGCAAGCACTGAAC<br>AGGAGTGTTTTAGAGGGAATTAAAACTTCAAAAAGGAGAATAGAG<br>AGGCTCTACCATTTAAATGCAAGTCGCTTGAAATCTGTAGACCACA<br>GTAGCAAGCACGAAGCAGGTATTCAATAAATGTGGTGAATTCAATG<br>TCAATAACATGCTCCAGTCTTTTGTCCACTATTCCCCGAGCCTACCT<br>AACCAATGGGCAAAGTGGTACTAATTGCCATCATTAGGAAGCACAT<br>GCTATGTGTCAGAAAGTATGTAAGAATTGTCTCTTCATCCATCTCTG<br>TCTATATCTCATTTTATCGATCTTATTGACAAATGAGAACTATATATAG<br>ATCTCATTTTACAGATGAGGAATCTGAAGATAAAGGGATTAAGCAA<br>TTTGCTCAGGATTTCTCAAGCAATGAGTGGTTAGACTTAGTTTGCA<br>AATTTATGACTCTGCTCCCAAATTCTCATTTTCCCTCCTACAGCTGG<br>ATAATAGAATTTTTAAGAAGCATCCTTCCACACCTCCTCACCTATGA<br>CCTTCCCTCCACTTGCTCCACTGATGCCTAAAATTCTGTTTTTGGAG<br>TTCACTTAGTTTATGACTTTGATTCTGGTTAACATTAAAATACCCAG<br>GACATGGGGGAGGGGAAGCTGTTATCAATCTTAGTTTAGTCAGTTA<br>ATATAGCGAGTTGCTCTTATTCATTACTCAACAAGCACTTATTGACT<br>GCATACTGTACATCAGGTCTGTACCAAGCTTTGGTGTTATAAAAATG<br>AATGAAGTTTGGCACTTACCTTTAACATTGTTGTAGTCACATATAGG<br>AGAATGTAAGAGATTATTATGAAACAATGTAGTAAATGTACAATAGG<br>GAATTCTAGATAAGCACAGCAGAGAAGCAACCAGCTCCGTTTCAG<br>GTCCTTTCCTGAGACTGATTCGGCTGGAAGGGAGTAGGTCCCGCCA<br>AATGAAGAAGCTGTGGGAAGACAGGAGGACAAGAACAGGCTCCA

	CGAAGAGATTTCAGAGCAGAGCTGCGTACTCCTTTTTCTTTTTGTTT<br>CTTTTGCTCTGTCACCCAGGCTGAAGTACAGTGGTTAGCTCACGGC<br>TCACTGCAGCTTTGACCTCCCAGGCTCAAGTGATCCTCTCGTCTCA<br>GCTTTCCAAGTAACTGGGACCACAGGCATGCATCACCACACTAGGC<br>TATTGTTTTACATTTTTTGTAGAGATGGGGTCTCACCATGTTGCCCA<br>GGTTGGTCTCAAACTCCTGGGCTCAAGCAATCCGCTCACGTCAACC<br>TCCCCAAATGCTGGGATTACAGGCGTGAGCCACCGCGCCAGGCCT<br>GAGTAATCCTAATCACAGGATTTTAAAAAGAAACTTCCTGCGCCAC<br>CCATTAAACAATATCTCCTACCAATTTGGTAGTAAATATTTTGCTAAT<br>AGTACCTAATTTTTAGGTAGGCACTGTGTTTATACATATATCCATTCC<br>TTCTTTTTTGATTGTCTTTCTGTTTAATGGGCAGCTACCTCTCTTGGC<br>ATCTAGCAGAATGAGCTGCTGCAGTTTACACAAAAAGAATGGAGAT<br>CAGAGTACTTTTTGTGCCACCAACGTGTCTGAGAAATTTGTAGTGT<br>TACTATCATCACACATTACTTTTATTTCATCGAATATTTCACCTTCCG<br>GTCCTGCGTGGGCCGAGAGGATTGCCGTACGCATGTCTGTACGTAT<br>GCATGTAACTCACAGCCCCTTCCTGCCCGAACATGTTGGAGGCCTT<br>TTGGAAGCTGTGCAGACAACAGTAACTTCAGCCTGAATCATTTCTT<br>TCAATTGTGGACAAGCTGCCAAGAGGCTTGAGTAGGAGAGGAGTG<br>CCGCCGAGGCGGGGCGGGGCGGGGCGTGGAGCTGGGCTGGCAGT<br>GGGCGTGGCGGTGCTGCCCAGGTGAGCCACCGCTGCTTCTGCCCA<br>GACACGGTCGCCTCCACATCCAGGTCTTTGTGCTCCTCGCTTGCCT<br>GTTCCT
SFN	GGTCACCCAGCTGGCAAAGCAGCAAATCCCAGATCGGAACCTGAT<br>CTCTGCCCCGAGCTCTGAGCCATCTGCACTACCCAAGGAATGAATA<br>CAGCGGTGGGAGGATGAGATCTTGGAGAAACCCTAAAATTAGAGA<br>ATGTCATAGCCAGTAGAGGGCTTAGAGTTGATCTGGGCCAGCCTCC<br>TTGTTTTACTGATGGAGAAATTGAAGCCCAGAGGCAGGAAGGGAC<br>CTGCCCAAGGCCTTATAACAGAGCTGGGATGCAGTCCCACACTCTG<br>ACCTCATTCCATTCTCTCTCCATAAATTCTGCACTGTCTCTAGACTG<br>GACTGGTTTAGATGTGGGATACTCTAAACAGCAGTGCCTTCAAGAG<br>AAAAAGAATCAGAACTACGAATCACTTAAAAGTAATGTAAGCTACT<br>CTGGGCACACTGCCTATGGGGTCGCCCTGCTCCACAAGGAGCCAC<br>AAAAATAATTAAAATAATTTAATATCCCTTCCCAAAGGTAACCAGTA<br>AAGTAAGCTCTTGGCTAGGTAACTGGACTCTTGTTCACAACTAGCC<br>AGTGGGAAAAGGTGCTAGAGCTTCCTCTGGCCACCTGTTTAATTTG<br>ATCATTCCAAGACAGAAACATTTCTTAGGAAGTTCTTTCTAGAATCT<br>ACCTGGTGTCCCTCCCACTGCTATCAGAGCCCTGTCCTCTGTCCTC<br>AGTGGAGGTAGAGAGCAAATGGTTGCTGCTTTCTTCATCACAACCC<br>TTCAAAGCCTATTATTACCAGCTAAGAAGGATTGGTTGACTATGGGG<br>CCAGAGCCCCTGAGCCTGCTGGTAGAATGGATGCTGTACAGGAGG<br>GTGGGGAGGTAGCAGGCAGAATGAGGAAAGCCCCTTTGAGCTGCA<br>ACCCCAGCTCCTGTCCTGCTGACTCAGACAGCTGACTGTGGAGCTC<br>CATGCCCTGCCAGGGCCTGCTGCCTCCTGCCCGTCTGAGCTCCTGA<br>ACTTGGGAAATGGAGGCCCAGAGGCAAAGGGAGGTACCTGAGAC<br>AGGAACTGAGTCAGGATCAACAGGCCAGAGCGGGCAGGAGGTATC<br>AGGCAGCCTGGCTCCCAGATGCACCCCTGAGCTCCAGCAGGGGAG

	GAGTAGGAATGAAGGGGCTTCCTTGCCCTTGCTCATGGCTATGCGG<br>AGGGCGTGAACCACCACCAGGTCCTCTGGCTTAAGTGGCGGGAAG<br>CAAATGGTCCCTCCCTGGACTCAGGCTCCAAAGTTCCTGGGCCTGC<br>CTTCCAGGTTCCCAGTGTCCTGGGATCTCCAGCTTTCCCCAGGACT<br>TGGGGAAGCCCCGGCTGGATGACTAGTACAAATGAAGGCCCCTGA<br>GGTTCCAGGACCTGCTGAGGTCACAGGAATATCCTAGATCAAGCTT<br>GTCCAACCCACGGCCCACAGGCTGCATGTGGCCCAGAATGGCTTT<br>GAATGCAGCCCAACACAAATTAGTAAACTTTCTTAAAACATTATGA<br>GATTTTTTTGCAAATTTTTTTTTTTTTTTAGCTCATCAGTTATTGGTA<br>GTGTTGGTATATTTTATGTGTGGCCCAAGACAATTCTTCCAATGTGG<br>CCCAGGGAAGCCAAAAGATTGGACACGCCTGTCCTAGATGGAGAG<br>GAAGGAGGCAGTGCTGAGCACATCTGGCCATTCATCCATCTGGAGA<br>GAGAAGGCTATGGGCAAACTGCTTCCTCTCCCCTGTAGACACCCAG<br>CTGGGAAGGTCTGGCCTTTGGCTAAGTCCTGGCTTGGGGTCCTTCC<br>TCATTTCACAGAACCTAACTCTATGTTAGTGCTTTGTGAGTATATGTT<br>GATCATAATAAAGTTGACGGGATTTTTTCACATGATAATAATAGTTGT<br>CATCTGGCCGGGCATGGTGGCTTATGCCTATAATTTCAGCACTTTGG<br>AAGGCTGAGGCAGGTGGATCACTTGAGGTCAGCTGTTCGAGACCA<br>GCCTGGCCAACATGGTGAAACCACATCTCTACTTAAAAAAAAAAAA<br>AAATACAAAAATTAGCTGGGTGTGGTGGTGCACCCTTGTAATCCCA<br>GCTACTCGGGAGGCTGAGGCAGGAGAATCACTTGAACCCAGGAGG<br>TGGAGGTTGCAGTGAGCTGAGATTGTGCCACTACACTCCAGCCTG<br>GGTGACAAGAGCGAAACTCCGTCTCAAAAAAAAAGAAAATAATAAT<br>AATAATAGTTGCCATCCATTCTACTGTGCTTTCCATTAACTCGTGTAA<br>TCCTCACAAGTCCCATTTTATAGTTACAGGAACTGAGGCTCACAGA<br>GCTTAAATCACTTGGCCAAGGCCACAAACAGCTATAAGAATTACAT<br>TTAGGCAGTCTGATTCCAAAGATACTAGTCTATTCTGTATCTCATAG<br>ACAAACAATACATATTCACTTTTTTTGTTGTTGTTTTGTTTTGAGACG<br>GAGTCTTGCTCTGTCACCCAGGCTGGAGTGCAGTGGCGCCATCTCG<br>GCTCACTGCAAGCTCCGCCTCCCGGGTTCAAGCGATTCTCCTGCCT<br>CAGCCTCCCGAGTAGCTGGGACTACAGGCATGTGCCACCATGCCCG<br>GCTAATTTTTTGTATTTTTAGTAGAGACAGGGTTTTCCTGGGTTAGC<br>CAGAATGGTCTCGATCTCCTGACCTTGTGATCCACCCACCTCAGCC<br>TCCCAAAGTGCTGAGATGACAGGCGTGAGCCACCGCGTCCGACCT<br>ATATTCACTATTTATAAATTGGAGAGAATAAGAAAATCAAAAGGGCC<br>AGGTGTAGTGACTCACACCTGTAATCCCAGCACTTTGGGAAGCCAA<br>GGCAGGAGGATTGCTTGAACCCAGAAGTTCGAGACCAGCCTGGGC<br>AACATGGTGAGACCCTGTCTCTACAAAAAAATACAAAAATTAGCTG<br>GGCGTTGTGGTGAGCACCTTATTCTTAGGAAGCTGAGGCAGGAGG<br>ATCACCTGAGGCCAAGGAGGTTGAGACTGCAGTGAGCTGTGATCA<br>TACCACTGTACTTCAGCCTGGACATCAGAGTAAGACCCTATCTCTA<br>AAAAGGAAATTGAGAAGAAAGAAAATCAAAGGGAAGCAAAATCA<br>CTCACTCTCACTACCTCAAGATACCCTCTAGAAGTTGGTATTTTAGT<br>GTGGTTCCTATTGTTTTCTGTGTCAGTTCTCTGATTTGAGCAAAATC<br>TTTGGGACGTCAAACTTAAAATCCCCTTTACTTCCTTGGAAACCCT<br>GTAGCATTAGCCCAGACATGTCCCTACTCCTCCTTGTGGCAAAGAG

	AAGGATCTCGTCTTTGGTCCCCAGAGTTCTGGCCTAAGCCTCCCTC<br>CAGGAGGGAAGATGAGTGTTCAGACACTCAGAGTAGCTGGGGGA<br>GACACAGGCCTGTGAAATTATCCTGGCTCAACTATTAGGTCGGCAG<br>AATCCCAGTGAAGGGAGCCCTACCTCTGAGCCCCATCTAAGCTTTG<br>GCTATGGGTGGGGCAGATAAGCAGGAATCCATCCCTATAGGCTCAA<br>TGCCAACACCCTTAGGTGAAACTCTTGATGAAACTTGAGGCCAGG<br>GCTCCGGCAAGCAGGGAAAGAACGTTGGCAACAGAGGTCTCCATC<br>TCTGAGGACTCTGCCAGGGGTCAGAGATGGGGCAATGGTCAAAAG<br>GAAGGAACAGGCCAGGCACAGTGGCTCATGCCCATAATCCCAGCA<br>CTTTGGGAGGCTGAGGCAGGAGGATCGCTTGAGCCCAGGAGTTTG<br>AGACCTGCCTGGGCAATGTAGTGAGATCTGCTCTCTATTTAAAAAA<br>AAAAAAAAGGAAAGAACAAGTAAACTTCTGAGAAACAGGCTGGG<br>GGAGGCATCACGTAGCTGGAATTGCTGCCCCATAAAACAGAATGGT<br>ATGTGTCACTGCCACCTCCCTTTCTCAGTCCTCTCTCTCCCCAGGTT<br>GCTAGCGTCCCCCCTGGGGGATCAAACTGGACTGCTTCCCAGCCTC<br>AGACAGAGAGCAGTCTGAGTCAGGCAGGAAAGTGGGACAGCCGG<br>GGAGCTGGACCCCACCCTCTGTGAGCCCCGCTGGTACCTGATGGCA<br>TGTGGCTTGGAGAGGGCAGGTGACCTGGCGTGGAGGGCCAGAGG<br>GTAAATCCTCAAACAAGTGGCAACAGGCCACCAACTTGAAAGGGA<br>AAATTGTGTAGTGATGGGAAATGTGTCCAACAAACCTACTGGGTGA<br>CTAATTACAAAGGCTGGGCTGGAGCTTCAGAGGCTGCTTGTTAAAC<br>ACTTCATTAAGCGGCACTCTGAAAGCTGCCACCTGCGCATTCTGGG<br>AGCTCAGAGGGGACCCTGAGGGGGAATGAGGCCTGGAGGATGGA<br>ACCATCTTCAGGTAGACTGAGAAGGAGCCTGGATCTCACTTCCAAA<br>CACAGTCTGGAGCTCATAGGTCAGAGGCCTCAATGGGAGAAAAGC<br>TAAAGGAAGAGGGTGCAGAAAGGAGTTTCAGGGAATTGGTGGCTA<br>TGTGACTTTGAGCAAATCTCACCCCTCTCTGAGACTTAGTGTTCCC<br>ATCTCTATGGTCCTGTGTGTGTCACAGAGACATGGTGGGGATTAAAT<br>TCGATCGTGAATATGAAAGTGCTTGGGAAACTCCATGGCCCTACCT<br>AAACATGAGTTATCCTCACCTGAACCAAGGGGGGAAGTTACCTGG<br>CAGGATTAGGAACCCCATCCTCCTGAACCTTTATGGGCTCTGTCGA<br>GGCTGAAGCAGCCAGGGGCTAAAGCCGTCCTTAGCCCCTGGAAGG<br>GCACTGTGAAAGTGGATCTGATTTGAGAAGCCGTTTCCTGATGTGG<br>GCAGCCATGTGATGCCAGCCCCGAACAAGAGGGGGCAGCCTGGAG<br>CCTGGAAAGGTGCCAGTGCAGGTGGGGCCCACGCCCAGATTTCTC<br>CTGCTGACTGTTCTGATGATTCACCCCCACATCCCAGCCTTTTTACC<br>TTTACTGCAGAGCCGGAAAGGGTGTGGGGAAGAGAGGAGAGGGA<br>GGCAGGTCTTGGGCCCTGGTCCCGCCCCCTGCTCCTCCCCACCCTT<br>CTCTGGGCCTGGCCACCCAGCCAAAAGGCAGGCCAAGAGCAGGA<br>GAGACACAGAGTCCGGCATTGGTCCCAGGCAGCAGTTAG
SHBG	TCCTGACCTCAGATGATCGACCCGCCTCGGCCTCCCAAACTGCTGG<br>GATCACAGGCATAAGCCACTGAGCCCGGCCAGGCATGAGCTTTTGT<br>AGGGCATACCAGATTGAAAATGAAGACTGTCAGGCCAGGCGTGGT<br>GGCTCACGCCTGTAATCTCAGCACTTTGGGAGGCAAGGTGGGTGG<br>ATCACGAGGTCAGGAGATCGAGACCATCCTGGCTAACACGGTGAA<br>ACCCCGTCTCTACTAAAAATACAAAAACTTAGCCGGGCGTGGTGGC

	GGGGGCCTGTAGTCCCAGCTGTTCAGGAGGCTGAGGCAGGAGAAT<br>GGTGAGAACCCAAGAGGCAAAGCTTGCAGTGAGCCAAGATCTCAC<br>CACTGCCCTCCAGCCTGGGTGACAGAGCGAGACTCTGTCTCAAAA<br>AAAATAAAAAGAAAAAGAAAAGACTGTGGGCTGGGCGCGGTGGC<br>TCACACCTGTAATCCCAGCACTTTGGGGGGCCGAGGCAGTCGGACT<br>ACCTGAGGTCAGGAATTTGAGACCAGCCTGGCCAACAAGGTGAAA<br>CCCCGTCTCTACTAAAAATACGAAAATTAGCCGCATGTGGTAGCAG<br>GCGCCTGTAATCCCAGCTACTCGGGAGTCTGAGGCAGGAGAATCA<br>CTTGAACCCGGGAGGCGGATGTTGCAGTGAGCCAAGATCAAACCT<br>TTGCACTCCTTGCACTCTGGCCTGAGGACAACAGCAAGACTTGATC<br>TCAAAAAAAAAAAAAAAGACTTTGGACTGGGTGCAGTGGCTCAC<br>ACCAGTAATCCCAGCACTTTTCGAGGCCGAGGCGGACGGATCACG<br>AGATCAGGAGTTGGAGACCAGCCTGGCCAGTATGGTGAAACACCG<br>TCTCTACTAAAAATTAGCCGGGCATGGTGGTGCGCACCCGTAGTCC<br>CAGCTACTCGGGAGGCTGAGGCAGGATAATTGCTTGAACCCGTGA<br>GGCAGAGGTTGCAGTGAGCGGAGTTCGCACCACTGCACTCCAGCG<br>TGCGCGACAGAGACTCTGTCTCAAAAAAAAAAAAAAAAAAGAAAT<br>AAAAGAAAGACCCAACACCACATCAGGACATGTAATTCTTATTTAT<br>TTTTCACCCTCAACAAGGAAGAAAGGTCTCTCCCTCAATTCTGCTC<br>TTCCAATACTTGAGGATAGGCACCCCTAACCCTCCTTCCTCCAGGG<br>AGGCCTCAGCATCAGTGTCTGTGGACGTAGTCTCTGAAGAGTGCTT<br>CAGCTGATGGGGAAGGAGAAACTCAAGACAGAGATCCTCCTAGGG<br>ATGGCGTCACTTTCCTGCCAACTTTCTCGTTGCCTCTCCTTGAAAGC<br>AGAAGAAGTGCCAGCCCTCAGCTTCCGTCAGATCTTGGGCTCCTAG<br>GGCCTTGTACAAGTCCATGGCCCTCTGGTTCCAGTCCAGGACGGCC<br>AGGCGGAATTGGGAGCAGCCCTTATCCAAGGCCACCTGTGGGAGA<br>AGACAACACTAACTTTTCTGGGGTCAAAAAAGAAAAAGGTTTTCT<br>CACTCCCTCTTTCAACCCGGGTCCCCCCTCCATATACCCTTGCTTCT<br>TCAGGTCCTCACTTGTCGCCCCACCCATCTCCTCACCTCAGCCACC<br>TTTTTGATTATTTTGGAACCAATCCCTTGACCTGTTGTGGAGAGAAA<br>GAGGCAAAAAATAGCTATTGTTTGAGCTGAAGGGGATCAGAAAAT<br>GACACCGGCTGGGCTCTGGGGACAGGGGATAACAGTGGGGTCTGT<br>GGGGTGCTTTGCTCCCACCCCAGCCTCAGCTTCTGCCCAGTACCCC<br>GATATTCCGGCATCACATAGATATCCTCCAGATAAATGGTGCGTCCC<br>TTCCATGTACTGTAGATGAAATAGTATATCCCATAGCCCACCACGCA<br>GGGCCCTGAGAGAGAGAAAAGGGGAGTAAGGCTTCTGGAAGCCT<br>GTGGGGAGACCTCTGAGGCCGGCTGGAGAGGTGGACTTCTAAGGG<br>CCAGGTGCTCTTACCCAGTAGCTTCCCGGGCGCTGGAAGAATCTCT<br>GCTACCAAACAGTGATAGAAAGGATTGTCTCCAAAGCCATCTGCTC<br>TCAGGGCTGCCGAGATTGGAGTTGTGACAAAGAGATAGAGAAAGA<br>GGACGTGGGTGTATGCCCAGCCTGGAGCTGTCGCCTGGGGAACCC<br>ATCCCTCATTTCCTCCCCAGCCTCCGCCAGAACCTGGGCGCTGAGC<br>CCCCACCTTCTTCACTGATCTTCACCTGATCCGAGAGTTTTTCGAAT<br>TCGGCTAGCTCCTAAGGCGTGGGTACGGAAGCTAGATTAGAGCAG<br>AAGGGCCCCGCTGCTCCCCGAGCAGGTTCCCAAGGCGAGCCCCTC<br>CCCCTGCCCCCGCCTCCTACGACCCCGCTCTGGCCGCGCCACTCTG

	ACCCCCGGGTTACCGGCCTGCAGTCTTCACCCGAATCAGCCTCAGG<br>ATATCTCCACAGTCTCCCTCCTTGGCCTCTCGGATCCGCACGGAAG<br>CCATCCGGATCCCCGCTGTCTGGGACCAAAGTCCCAGGGCCTCGCA<br>AACGGCAACTAGACCCCTTAAAGGGCCTACGGACTTGGATCCTGA<br>AGAGCCTGAGAGAGCGGGGTGGCGGGAGTCGGGGGGGACGGCGG<br>GGTAGCCGCGGCCTGGTAAGTGGAGCTGGGATTCCGGCGCCGTAC<br>GGGAGGAGAGAGTAGGCCAGCGAGGCGATCCTCTGTCCGGGCATA<br>GCCCCACCCCCTCGAATTCTGTCGCAGCAGGGGGCACAACTGTCA<br>GCCAATCAGCTTGGAGAACAGGCACGGCCGCGTCCCCCCCAAGCC<br>CCACCCCCGACAGCTGGATCTTGTGACTGGGCTCCTGGGTAGAGTT<br>CAAGGTTGGAGTGAAGCGGCTTCCTTGCGGTTGTGTGGGTGTCCC<br>AACCTGGGTCGAGATACCCCGCGGTTCAAAGGCTCCCCCGCAGTG<br>CTTTTTAAATTGACATATGCAGTGATAACCTGCTTTAGCCTCAGGCT<br>CACTCACCCGCCCAGACCCTGGGTAAGCCTTAAGACCCTCAGCTCT<br>GAAAGCTGTTTCCTGCAGCTCTTGAGTAGCATGAAGTGTTACCTCT<br>TGGGGGCATTTGCATTTTTTAAATGTTTTATTTTTATATTTATTTATTTA<br>TTTATTTTTGGAGATGGTGTATTGCTTTGTCGCCCAGGCTGGGGTGT<br>AGTGGCGCGATCTCTGCTCACTGCAGCCTCCACCTCCCGAGTTCAA<br>GCGATCCTCCTGTCTCAGCCTCCGGAGTAGCTGGAACTACAGTCGC<br>GCACCGGCACGCCCGGCTAATTTTTTTTTCTTTTTTCTTCTCTTTTTT<br>TTTGTTTTAACGGATTCTCACTCTGTCACTCAGGCTGGAGTGCAGT<br>GGCGCGATCTCGGCTCACTGCAACCTCTCCCTCCTGGGTTCAAGCG<br>ATTCTCCTGCCTCACCCTCGGAGTAGCTGGGATTACAGGCATGGGC<br>AACCATACCTGGCTAATTTTTGTATTTTTAGTAGAGACGGGGTTTCA<br>CTATGTTGGCCAGGCTGGTCTGGAACTCCTGACCTCAAGTGATCTG<br>CCCGCCTCAGCCTTCTAAAGTGCTGGGATTACAGATGTGACCCACC<br>AAGCCCGGTCTGTCATTTGCATTTTAAAATGGGTCATGGGGTGGGC<br>ACAGTGGCTCACACCTGTAATCCCAGCATTTTGGGAGGCAGAGGC<br>AGGCGGATCACCTGAGATCAGGAATTTGAGACCAGCCTGACCAAC<br>ATGGTGAAACCCCGTCTCTACTAAAAATACAAAAATTAGACAGGTG<br>TGGTGGCGCATGCCTTTACTCCCAGCTACTCGGGAGGCTGAGACAG<br>GAGAATCGCTTGAACCTGGGAGGTGGAGGTTACAGTGAGCCGAGA<br>TCGTGCCATTGCACTCCAGCCTGGGCAACAAAAGCGAAACTCCAT<br>CTCAAAATAAATAAATAAAATAAAATGCGTCAGGGAGGGTCGGGCC<br>TTGTGGCTAATGCCTGTAATCCAGGTACTTTGGGAGGCTGAGGTGG<br>GCGGATCATTTCAGGTCAGAGGTTCGAGATCAGCCTGGGCAACGT<br>GGTGAAATCCCCGTCTCTACTAAAAACACAAAAAAATTTGCTGGGC<br>GTGGTGGTGCGTGCACCTGTAGTCCCAACTACTAAGGAGGCTGAG<br>ACAGGAGGATCGCTTGAACTCGAGAGGCAGAGGCAGCAGTGAGC<br>CGAGATCACGCCACTGCACTCCAGCCTGGGTGATAGAGCAAGACT<br>CTGTCTAAAATAAAATAAAATAAAATAAAATAAAATAAAATAAAATG<br>GGTCAGGGAGTGGGTGATTTCTACTGCTAGACTGTTTAGGCCCTGT<br>AATAAATGGATAAGGGAAGATAACTGAGAGGCGGGGGGCAGGTCC<br>CTTCTTAATATTCACTGAATCATACACACAGACAATACCTTCTTGGG<br>AGACAGGCCTCAGAGGCCTGGGAAAAGACTGGGGGAGGAGTTCA<br>GACCAGATGCCAGGCACTGTGCCTGCATTTTCTCAATGAACCCTCT

	TTCACAGTCACCCCGTAAAGTATTATTTTCCTCATTTACAGACAAGGACACTGAAGCACAGAGGTGAAGTGACTTGCCCAAGGTCACTCAGCTAGAAATTTAGGATTCCATTATCTCATTTCTAGTCCTGATACAGGATGCTACTTGGGACGCAGGGGAGGACTGTTTCTAGACCTCAGGCCTGTGAATGCAGGCTCCCCGAGTGGACAGAAATCTTGGAGGACCTAGATCAGGCCCTAGAGGAGGAGAGGGGAGATGGAATATCCTCTCCCAGTTCAGAAACTTTCTCGGCAGTGGAGGATGATAGTGGAGGGGACTCTGTCCTTCACCCCATTGATCCCCAGAGGGGTGATAGCTGAGTCTTGTGACTGGGCCCCTGGGCAGGGGTCAAGGGTCAGTGCCCCTGTTTCCTTTACCCCCTCCTCCCCGGGCAACCTTTAACCCTCCACCGCCCACACGCAAGGCTGCCTGCCTCTACACATTCTCCCAAGAGTTGTCTGAGCCGCCGAGTGGACAGTGGCTGATTATGGAGAGCAGAGGCCCACTGGCTA
SLC25A3	TCTACTAAAAATACAAAAAAAATCAGCCGGGCATTGTGATGGGCGCCTGTAGTCCCAGCTACTCGGGAGGCCAAGGCAGGAGAATGGCGTGAACCTGTGAGGTGGAGCTTGCAGTGAGCCAAGATCGTGCTACTGCACTCCAGCCTGGGCGACAGTGCGAGACTCCATCTCAAAAAAGAAATAAAAATAAAAAAAAGAAAGCAAGGTGTTGGGTTGACCAAGCAGATGCTTTGGATTGGTTTGCTTCTTCTAATTATGTTGGATAACTGGGGGAAAGAAAATGATAACTTAGGGCTTTAACTTCTCAGTTCAACATCAGATAAAGGACCAGAAAGCCTTGACTGCTTTGTTTGTTTTTTGAGATGGAGTCTCGCTCTGTTGCCCAGGCTGGAGTGTGATAGTGTGATCTCGGCTCACTGCAACCTCTGCCAGCCTCCTGGCTTCAAGCGATTCTCCTGCCTCAGCCTCCTGAGTAGCTGGTACCACAGCCGCGCGCCACCACGCCCAGCTAATTTTTGTATTTTTAGTAGAGACAGGGTTTCACCGTCTTGGCCAGGCTGGTCTTGAACTCCTGACCTTGTGATCGTCCACCTTGGCCTCCCAAAGTGCTGAGATTACAGGCATGAGCCACCGCACCTGGTCGCCTTGACTGCTTTGCTGTATCTGCAAGACTGTACTTTCTAAAAATCAAACTCAAAATCTAATTCTGAATTACAATGCAAATTGAATTCCCAATCTCATAGTGTTTAAAGTTAGGGCAATGTTTTGAGACCCAAAATTTTGGAATAGTGACACCTGGGCAGATTATGATTCCACTAGGACCATGAACCCCCATATTCTTTTTTTTTTTCTTTTTTGAGACAAGATCTCTAACTGTTGCTTAGGCTGGAGTACAATGGCGCGATCTCGGCTCACTGCTACCTCTGCTTCCCAGGCTCAAGTGATCCTCCCTCCTCAGCCTCTTGCGTAGCTGGGACTGCAGGGGCATGCCACTATGCCTGGCTTTTTTTTTTTTTTTTTTTTAAGAGACAGGGTTTCACCATGTTGTCCAGGCTGGTTTTTAACTCCTGGACTCAAGAGATCCACCTACCTCGGCTTCCCAAAGTTCTGGGATTATAGGCGTGAGCCACCATGCCCAGCCAGAACCCCTAAATTCTGTTGAGCCTCCTTGTCAGTCAAAGAAGCCCTTCCTCTCCTGTCTGAGGAGGTTATGTTCCTCGTCCCTGTGAAATCTGTAATGGCCTCACCTGAGGTGGTTGCCTGGCGGGATCTGCTGATTCTTCTTAAAACCTACTGTCCACTTTGGGAGGCCGAGGCAGGTGGATCACCTGAGGTTGGGAGTTCGAGACCAGACTGACCAACATGGAGAAACCCCATCTCTACTAAAAATACAAAATTAGCCGGGCATGGTGGCGCGTGCCTGTAATCCCAGCTACTCCGGAGGCTGAGGCAGGAGAATTGCTTGAACCCGGGAGGCAGATTTGAATCTCCACAGTGGAGAGTTATGGTCCTTCAGGCAAT

	TTCCCTACTTGTGTCAGGTTGGAGGCCGAGAGCCTCTTGAATGTTA<br>AGGAGGCCAACCCCCTTGTCTTAGTCCATACCTGCTGCAATAACAT<br>CACCTAAAGACGGATAAAAGGATTTTGTATCCTCTTTTGGGTAGAAT<br>TAGCTTGTTCACAATTGTCCAAGGGACAGCTGCTTCATGTTAGGTA<br>GAAGTCTGATTTTGCTCTTTAGTTTTTGTCTGGAAGTTAAATATGATT<br>ACAAGAGGTGTTCATTGATGCCATGTTGACAACCGATAGACTCTGG<br>TACTTTTTTTGTTGCTGTTGTTAATTAGTTTTTATTTCATAATCATAAA<br>CTTAACTCTGCAATCTAGCTAGGCATGGAAAGGAACAAGGAAAAC<br>ATAGAACCCAAAGGGAACTGCAGTGAGAGCACAAAGATTCTAGGA<br>TACTGCGAGCAAATGGGGTGGAGGGTGCTCTCCTGAGCTACAGAA<br>GGAATGGTCTGGTGGTTAAGATAAAACACAAGTCAAACTTATTAGA<br>GTTGCCCACAGTCAGCAATGGTGATCTTCTTACTGGTCTTGCCATTC<br>CTGGACCCAAAGCACTCCATGGTCTCCACAATATTCATGCCTTCTTT<br>CACCTTGCTGAAGACCACATGCTTGCCATCCAACCACTCAGTCTTG<br>GCAGCACAGATGAAAAACTGGGAACTGTTTGCGTTGGGTCCAGCA<br>TTTGCCATGGACAAGATGCCAGAAACTGTATGCTTCCGGATGAAGT<br>TCTTGTCATCAAATTTCTCCCTGTAGATGGACTTGCCACCAGTGCCA<br>TCATGGCATGTGAAGTCTCCACCCTGACACATAAACCCTGGAACAA<br>TTCTGTGAAAGCAGGAACCCTTATAGCCAAATCCTTTCTCTCCAGT<br>GCTCAGAGCATGAAAGTTTCCTGCTGTCTTTGGAAACTTGTCTGCA<br>AACAGCTCAAAGGAGATGCGGCCCAAGGGCTCCGTGTCAAAGAAC<br>ACGGTGGGGTTGACCATGGCTGATAGCACAGGGCTCCTGGCAGCA<br>GCGGTGCCTGCAAAGCCCAGTGGTGTTTTTTTTACCATGTCAACTT<br>AGCTAGGCTAGAACCACATTTCCTAGATTCCTTTCCCTGCATAGTTT<br>TGGGTTAGGGCTAGCTATAAGAGAAATGTGTGCAGTTTGGAAGGCA<br>GAAGTGGAGCAACAGTCATTACATTCTGAAGGTCACCAGCTCCCA<br>CATGGGGTAGGAGCTGGGCCTGCAGCTCCTTCAGTTACCACTGTAT<br>CTTCTAGATCAACTTCTGTGAGTTCTGGGCCACGAGCATGTGCAGC<br>CCCATGGTGAAGGGTACCAGCTGTTCCAGCTGAAAAAATGTGCAG<br>CAAAGGGTCACGGTATTACCCGGTGCTGGGGGAAGGGAATAGGAA<br>GATTCACTACAGTCATATATTTTTACATAATATTTGACTTTTTCATGAC<br>AAAGGATTTTTAAATTAGAAAAGCACCATAAAGTTGAATTAACCAA<br>AAAAGAGAGAGAGAAAGTCCAGACCCTTCAGTGACTACCTCTGTC<br>TACATAAGATCAGACTCCTCAGTCTGGTATGTGAGGCCCTGGACAA<br>TCAGGTGCTAGTGTACTTTTTTAACTTTATCTGTCACTACTTCCCTTC<br>CTGTGCCCTCCACTCTGGAATTCTTCCTGGGACTGTAGGACTTTAGT<br>TGGGCCAGAAGGGCTGGGTCTGGGATAGGGTTGCTTAGAGAGTGC<br>CGTGATGCCGCAGCCTCAATGCTAACTTTTGTTTTTTTGTTTTTTTTT<br>TTTTTTGAGACGGAGTCTTGCTCTGTCGCCCAGGCTGGAGTGCAGT<br>GGCATGATCTCGGCTCACTGCAAGCTCTGCCTCCTGGGTTCACGCC<br>ATTCTCCTGCCTCAGTCTCCTCAGTAGCTGGGACTACAGGCGCCCG<br>CCACCTCGCCCGGCTAATTTTTTGTATTTTTAGTAGAGACGGGGTTT<br>CACCGTGTTAAACAGGATGGTCTCGATCTCCTGACCCCGTGATCTG<br>CCCGCCTCGGCCTCCCAAAGTGCTGGGATTACAGGCGTGAGCCAC<br>CTCGCCCGGCCAATGCTAACTTTTTTTAGGNTAACATCTTCTGTAGA<br>CTTTTAGACATTATCTCATTGAGTTCTCATAGACTCTGCAAGGTAAG

	TATTACTCCCATCCAGAACCAGACACCTGATTTGCAGGGTCCAGTG CAAACTAAAAAAAGCGTGGCCCCTTGTTCATACATGTATTAAGAAT TTCAAGCCGGGCATGGTGGCTCAGGCCTGTAATCCCAGCACATTGG GAGGCCGAGGTGGGCGGATCACCTGAGCTCAGGAGTTCGAGACCA GACTGGCCAACGTGACGAAACTCTGTCTCTACTAAAAATAGAAAA CTTAGCCGGGCGTGGTTGTGCACGCCTGTAGTCCCAGCTACTCCGG GGGCTGAGACAGGAGAATCGCTTGAACCCGGGAGGCGGAGGTTG CGGTGAGCTGAGATCGCCCCACTGCACTCCAGCCTGGGCGACAGA GCAAGACTCCATCTCAAAAGAAAAAAAAAAAAAATCAAAATGACA GCAGAGCATTAAATGAAGTGTAAGGCCATGTGTGACTGTACCAGTG TGAACCTATGAGGCCAGTCCCACCCCTATCTTACATCTAGGGAACA GGTACACAGAGATGAAGTAAATTCCCAAGGTTACTTTGTCAAGAGC TTGGAAAATATAAGAGTCAGAAGTCTAACAGGTTGAATCCCTATTA AACTCTCCCCAACTAAACTACGCCCCTCCCTGTTGAGCTGAATCTA ATGAAAGCTTCCTAAATGTATTAACCACACACACGCACAATCTGCTA TCAGTCACTTTCAGTAGTTCATCGCTGTAACATCTCAGGAGTTCAAT GTTTCAACGTGTTCTGTAGATTACTGTGATAACGCGGCAAGCTGTC GTAAGACTTCTTGCTGCACATTTCTTTTTAAAATATTCTCCAGGAGT CTGATGAAAAATACAGACATACCTATGTTTAATGTCAATGGATGGAA CTTATTTTAAAAAGTACTGTAATGGCAGAAGTTGTGACACAAAGTA CTGCAGTGATGTTGTAACTCCAACACCTAAATATATTATCAAGCATT CGCGTTTTGTTTTGTTTTTTAAGTGACTACTAGATACTGTGATTCACT TAAGTGAAGCGAACTGAAGCCCGTGGTCAAGGAGAGACACCTGA AGGTGAAGGGACAAAGCGAGCAGGGTAAGAGCTCGAGCCCGACC CCTCTCAAAAAATATAGACCCGGAACGCGCGCCTGCGCAGAACGA ATCCTCCCGCGCACAAGCCGGGCGGAAGAGCTGGGGCGGGCAGG GGCGGGAATAAGGTTGCTAGGAGAACGCGATGACGCTACGGGCTC GGCGCTCTTCTCCACCAATGGGCGACGCGTACTTGCAGTGGCGCGC TGTGTGCGTCACGCCGACGACGCGCGAAGGGCACACATCTTAGGA CCCGGAGGACGTCCGGCCTCTGTGAGCCGCAACCTTTCCAAGGGA GTGGTTGT
SLC6A2	ATGCCTATTGTCCCCTGATCAAGACAGAAAGACAGAATGAAAAGG AAGAAGGAAGGAAGGAAGGGAGGGAGGGAGGGAAAAAAGAGGA AGGAAAGAAATCAGGGAGAGAAAGAAGGAAAGGAAGGAAGGAA GGTGGGAAAGGAGAGAGAAAGAAAGGGACTAAGGGAGAAAGAA AAAAGGAAGGAAGGAAAGAAAAGAGAAAGAAAAAGAAAGAAAG AAAAAAGAAAAGAAAAGAAAGAAGGAAGGGAGGGAGGGAGGGA GGAAGAGGAAGGAAAGAAGTGAGGGAGGGAAAGAAGCAAAGGA AGGAAGGTAGGAAAGGAGAGAGAAAGAAAGGGAGGAAAGGAGA AAGAAAAAAGGAAGAAAGAAAAGAGAAAGAGAAAGAAAAGAAA GAAGGAAGGAAGGAAGGAAAGAAAAGAGGGATGGAGGAAACAA ATATGTAGGCAAACCTCTCCTCCTTTTTTTCCTTAGTCTCCTCATTGG TGCCATGGAGGTGTAGGTTCTGATAGCGTCCTCAGCGGACACAGGC CCTTGGATTCTAAATGTGTCCCAGCCCAGCTGTTGTGTGTCAGGGC CCCAGTGTCTGTGGGGAGATGGCCAGAGATGGACTCACAGCATCA GCCATTGCCTTTTACCCCATGGCCTGTGTCACCAAGTGCCATGGTAA

	GTGGAAGTGATGGCTCCCCAGAGATCACATTAGCTCTGATAATGCT<br>CCAGCCTCCCATGCACAACTTGCCCTCAGGCCACCTGGCTGGGCA<br>GGAAGAAGGGCTCCCAGAGAAGCCACATGGCCCCATGGCGGTGAG<br>TCTGGGCGAGAGATGGAGAGAGACGCCTTCCCTTTTAGCCCAGTC<br>CCAGCCTAGTGTCCTCACTGCTGACCCCCGGTAGTCTCTGAAACCA<br>CAGATGGAGCTCCCAGACTTGCTGATTGGCCCCCGTGATGGCGTGC<br>GCATTAGGAGGAAATGCCTCCCTCCACCCTTGTAGCAAACACTTCC<br>AGCTCCATGCCCACACCCCTTATCATCCACTGTTCCTGCCAGTGCA<br>GACCCAACCCAATGGCTTCCTGCTGCCAGTACCTGGGGCTCTGCTG<br>TTAGCCTTTCTCTGGCAGCAGGACAGGCTCATCCCTCTTATCAGAC<br>AGGCTGGACTTGCTGGGAAGTTGACACTCTGGGGGCGGCCTTCAT<br>GGATAAATACTCTAGTTTTCTTGCCCCTCAAGTGAGACAACCCCAA<br>GGCGTGCTCTGTGCAGTCTCCCGAGGCCCCCACCAGGGCTGAGCA<br>CCAGTTTCCCCAGCAGCAAGTGCTCCTTAATCTACTTTCTCCTGGTT<br>TCCTTCCCTTTGCTGTCTCACTTCTCCTCTCCCCAACGATTGCTTCC<br>AGGATTATCTCCTAAATAAATTACTTGTATTCTAATCTTTGTTTCCAG<br>TTGTTTCTTGAGGAATCCAATACAAAACGAAACCAATCAAAGACAC<br>CAAACCCAACAAAAGAAAGAGAACTCTTCTGGTTCTGTATTGTACA<br>TGTTTCTGGAGCACCGTTTAAGTAACAGAATACATCACGCACTGTC<br>TTTGACGCTGGTAGGGAGGTCTCACAAATAACTGTACAGGCAAATA<br>AACAGCAGTATAAAAGAGGAGTTTGACTCTGTTGTAGGAGGTGGTT<br>ATCAGGGAAGGCTTCACAGTGAGGGTGACTGGAGGTTTTGGAGGA<br>TGGGTAGGAGTTCTCCAGGTAGAAAAAAGAATGATGGAAACAGCA<br>TATTAAAAAACCCAGAAGTGTAAAAAAGCACAAGGTACTCAGGGA<br>ACCACTAGTAGTTTAGTATCTCTCATACTACTGGAGAATAAAGCATA<br>TTTTGGAAGTGATAAAGGATTTGTAGGAAAAATGAGGAGAGAATTA<br>GAATAGCAGGAGGTGTGGTTAAATAGCCAGAGTCTTGTCTATACTC<br>CTCAAAGTGTTTAGGCATTATCTTGGAAAGGTTGGAGGATTTTTAA<br>AATTGTGGGTTATCTTTTTCTGATTGCATAAATATCAAATGCTCATTG<br>TGGAAAAGTTGGCATATACAGAGAAGTATAGAGAAGAAAATAAAAT<br>TACCTCAAATCCAGAGTTAATTAAATCACATTTATGTGTTGGTGTGT<br>TTCAGAGGAGTTTCAGGCAGAGGAGTGAGGTGGTCGCACTTGAGT<br>TCTAGGTAGAGCTCTGGCAGCTGGTGACGATCATGCAGCTGGAAA<br>GATACTGAACGTGGGGTGACCAGCTAGGAGGCTGCTGCAGTGAGG<br>GGTGATGAGGGTCCTCTGTTGTGGCTTTGCAGGGTGTTTCAGAGGT<br>GGGATTCTCAGCACTTGAAAACTAGTTGGATGGGGTGTGGGGTAG<br>AGGAGAGAAGGGGACCATGACTACTAGAGAGATTCTGAGTGTCTC<br>CACCATGACTTGGTGGAGGGAGCAGAGGCTGGAGGCCAGGAGAC<br>TTGGGTTCTCATTTCCACTCTGTGACTTCCAATCTGTGGGACCTTGG<br>ACAAGTCCCTTTAGTCCACAGAGCCACAGTTTCCTGACCTGATACG<br>TGGAAAGATGATTCTCTTCCTGCTTTCCTTACAGGATTGTCATGATC<br>GTCCAATAGATCAAAGGAGATAAACACCTTTCATGTGTGGAAACCT<br>ACAGTTCTGAGCTCTTGTCCTCAAACCTCTGCTCAATCTTTTCCCTC<br>ACCTGCCTTCTGTCATCTTCCCCAGAAGCCAGGGGAACCCATACCC<br>CTCTAGGTTTCCCTTCCTCTCCCCTCTGCATCCTGCCCAGAGGACAT<br>TTGTCATGATTGTCCCTCTGGTACAGATGCTCTAAACACCGCCACAC

	ACCACCATTCTTTCATTCATTAACTCATTCACTTCACAGGCTGGTTG<br>ATGCTCTAGTACGTTCCAGGTGCCAGAACACGTGCTGTAAAAGACA<br>GACAAGGTCCCTGTGCTCATGGAGCTCACATACCACCCAAGGGGG<br>GACATAAAATTAAACAACTAGGACAAATTCGGATAGCGATGTAACA<br>AATGAATAAATAGGGTGATAGGATAGAGTGAGCGTGTATTTTCGATT<br>TGAGTCGTCGGGAAGGGACCTCTGGACTGAGACCTGAACAACTCA<br>GGCAGAGAAAACAGCAAGTGTAAAGGTCTTGGGGTGGGTTTGAGC<br>TTAGGATGTTTACAGAATGGAAAGGTCAGTGTGGCTGGGCTGATCC<br>AAGCCCCACCCAACCCACTTAGCACGGCCACCTCTCTGAAGCCTC<br>CTTGGATCAACAGGATGCTTCCCTTGCTGATTCAGCTCCACCCTACT<br>TCCACACATCTGTGGTCACAACACATCCTGACCACACTGGGTTGTG<br>ATAGACTCTATACACTGGTCTGGGAGCTCCTTGAAGGCAGGGGCTA<br>TCTCCTCTCTAGCCAGCACTGTTCACGCACACAGCAGATGATCAGC<br>ATCTGCTAAAGGGATTAATACATTAATCAATAGATGAGTGAATGAAT<br>GAGAAAACGACTCTCAGGTCCAAATGGCTCACTCCTTTACTTGTCT<br>AATTGTTACCTTCTCAAATTCCCTGACCCCGTATGCAAACTGCAGCC<br>CCACTGTCCCCCGATTCCCTCACCCATCATATAACGTGTGTATTTATT<br>ATGTTTCCCGTTTCCTCTGTCTCCGCCAGCAGAATGTAAACTCCATG<br>AGGTCAGGAATCTCCGAGTTATGTTGCGCCAGTGTAATCCAAGAGC<br>CCGGAACAGTGCCTGGCACACAGCGGGCATATGGAAGAACAAATG<br>TGTGAAGGTGTGAATGAATGAATAATTGAAAGAATAAATAGTAGTT<br>CTCAGCCTCACAGAACACGGGTCACAACCTCAAATGACCTGCTAC<br>CCTGCCCATAAATAACAGAGATGCAGGAGTAAGTGCTGGGCTGTGA<br>CCTGTCAACATGCTAAGCCGCTCAAACAAAACTGCCCAACAGCCC<br>GCTGGCCGCCTATTTGCAGCACTGGGCCCTGAGCCGCACATTCCCA<br>TTTCGTTGATAAAGAAACTGACCAGATAGTTTAAGTGGCCTGCTGC<br>GGAAGACAGGGCTGGTGCTGCACCGGTCGCTGCTTCCCCAGTCCT<br>TTTTTGGCCTCCTTTCTGACGCGACGCAGACCCCAGTTCTGGAGAG<br>TCTGTCACTCGCTCCCCGTGGTGGGAGATCAGAGGCCTGGTGTCCT<br>TGGGAGCGGCGAGCGGTGCTCGGCGCAGGATAGAAAGGGAGTGC<br>GCGCCCGAGTCCCCCAGATCCCTGGGAACCCGCGCCACCCTCCCG<br>CCCCTGCCCATCCCCGGCCGCGCTGTCAGTCTCCATTAGCGCTAAC<br>AGGCTCCAGACGGAGCGGGCCGGGCGCTGGGTTAATGCAATCGGC<br>GCGTTACCTGGGGCGCAGGCTACATTACCAGCCCGGCCCCCGCCAG<br>GCACGGCCAGAACCAGTCAGCCCGCGCCCTGCCGGCCGCCCCGCG<br>CCTCCAGCTCTTCCCCGGGCCCCGCCCGAACGCCACACGGCGGAG<br>CCCAGCCCCAGCCCGCGCCCTAGAGCCTGCCAAGGCGCCGCCGGT<br>CGGGGGCCGGCAGGGCGCAAGGCACCAGGGATCCCCTCGCCGCCG<br>GACACGTGAGTGCGCCCTGAGCGCGGGACAGGGCTAGGTCTGCCT<br>GGGAGGCCCGGGCCGAGACGCGCCAGCAGAGGGCTAGCGAGTTT<br>GTAGTGCAGTGACGTTAAGTGTCCGAGAAGGCTCCTGTGGCTGTTG<br>AAGTGTCGCGGACCTGAGCTGGGGAGGGGGTCGGCACGCTGCCCT<br>CAGCCTCGGTGAGTTCAATCCCAGCCATTTGGGGCAGGCGAGAGT<br>GGGTGAACGAGGAAAAGTGCTGCAGGGTCTTCAGCCGCCCCCAGA<br>GGGCTGTCAGAAGTCTCCAACTCTTGAGTTCCGGCGTGCCCCAAC<br>CTCTGTTTCCAAATTTTTCCAGCGGACGCGCGCTCTTTTCTGGGAA

	CCCTGCGTCCGCTCAGCGCGCGCTCATCCCAGTGTCTAAGGCGCTCCCGGGTGGTCTTGGGAGTTGCAAGTAGGGAGGAACGGCCGGGTAACCACCTCTTTTCCCTTTATCCAAGCAGAGCCTCGGCGTGCCCCCAGGACCGGTA
SLC9A3R1	TTATCCACAGAGACCAAGGGAAAAACACAACCAGAAAAGTCCACACATACATCCAGGGCAAGTTGCAAGAAAGTGACTCAGTCAGACAGAGTGAGTGGTTGTATCCTCACAACCAAACTATTATAGAGACAAAAATTTGATAAATTCAAGCACCAATTTTGTTCACGACATTGTATAGGTTTCATGAATCCCCTGACCTCAAGGACAGTTTGCTGATAAGCAAACTAGGAGAATAAAACGTTTATATAGAAAGAGGAAAATCCATGGCACTCATACTCCTACCTCCAACCCCATGCTCATGGCAGACATCACTAATCAATCACAGTACTTTTGATCACTGAAACCCTTATGTGGTCTTAGAATCTTTAACAGGACACTCCAAGAAATCACTGCTGACAGCCAACTGATTTGTGAGATAAGGTCTCCATGCATCTGGATCTTCCATAGAACTGATAGTTGCACAGCATAAAATGGTGAGGGTGGGGCCATTGTGGGTTGAGCCACCAAGGAAGGCCATCCAGGCCTGGATGGGCCAGAACAAAGGTACAGATGAGAGAACGCACAGGGTATCGTGTTCAAGGTAGTGAGTAACTGAGGATAGTCAAACGGAGCAGAAGAAGAAAGGGGCAGCAGGAGGAAGAGAATGCCAGTCTCGCACGCCCTCTCCCACAGGCCTGGCTCACTGAGATTCATGAGTATGCCCAGAGGGACGTGGTGATCATGCTGCTAGGCAACAAGGTGAGTGGCTCCGGGGCAGGGTCAGCCCAGCCCTGCACTTCCTCAGCCCTAGCCGGCCCCATAACCACCCAAGAACAGTTATCTAGGCATCCTTCCTGAAAAGGACTCTGCAGCCTCCAGCTCAGGGGTCAGACATATCTGGAGGCTTCTGCCCATCCCATCTGCCCCTTCCAGGGAAAGTCCAAGTTGTTGCCTGAGAAATCAAGGGGTGCCCAGTTCTCAGCCCCCATTAGAGCAGAGTGAACAGGGTCCCAGGTCAGGGGCTAAGAGTGCAAAGGGTTAGCCCCAACTGCTGTCCTATTCCAAGACCCTTTACCAAAGGTGAGATCCCAGAGCTGGGAGCTACACTGGGCAGAAACCCTGGCCCCAGGCCAATCACACCTGCCTGCAGTCCCTTGGGCCACCAGCAGAGGGCAGGCAACGCCTGCTTCTGGGGCAAAATATGGGCCCGCTGGGGCGGAGGCCTCCTTCCCCAGAGTGACCCATTTGGGCTTGACAGGCGGATATGAGCAGCGAAAGAGTGATCCGTTCCGAAGACGGAGAGACCTTGGCCAGGGTAAGTGATTGTCTGTGGGACAGGGTGAAGGGTGGGGGCAACCCGACGCTGGCCCTGAGGACACTCTCTCCCGGGCAGGAGTACGGTGTTCCCTTCCTGGAGACCAGCGCCAAGACTGGCATGAATGTGGAGTTAGCCTTTCTGGCCATCGCCAAGTGAGAGCTGGGCAGGGAAGGGAAGTGTGCGGGGCAGGGCGGCACACTCCAGGAATCCAGTAGGGCCCGGCCCCTGGCCCAGCCCCTGGACACACCTGCATTCTGCAGGCTGAGGTCCATTTGCTCTGGGAGCACTGGGCCACTGGGAGAGGGGAGGGGGCGGCTCAGCTCCTCACCCCAGCCCAGCCCAGCCCAGCCCAGCCCATTGTCTCTTCTTCAAGGGAACTGAAATACCGGGCCGGGCATCAGGCGGATGAGCCCAGCTTCCAGATCCGAGACTATGTAGAGTCCCAGAAGAAGCGCTCCAGCTGCTGCTCCTTCATGTGAATCCCAGGGGGCAGAGAGGAGGCTCTGGAGGCACACAGGATGCAGCCTTCCCCCTCCCAGGCCTGGCTTATTCCAAGAGGCTGAGCCAATGGGGAGAAAGATGGAGGACTCACTGCACAGCCGCTTCCTAGCA

	GGGAGCTATACTCCAACTCCTACTTGAGTTCCTGCGGTCTCCCCGC<br>ATCCACAGGGAGGGTAAAACACTTAGCTTTTATTTTAATAGTACATA<br>ATTTAATACCAAAAAAGGCGCCTGGATCCCCAAAAAACCGAGGCT<br>GGGAGCTAGTGGCCCTTTTGCTTTCTAGGACTTGGGGGGCCGGCCC<br>TCCCTCCTAAGCATAACAAAGGTGGTGTTGCTCCAGCTCAGCCCCA<br>GGGGACACAGATGCACTTTGGGGGTGAGGGCAGGTAATGACTCCA<br>TCGCACCCTCAGTTCAGCTGGACAGAGGCTCAGGTGACCCCAGCC<br>TTCACTGTCTCCCGCTCTCCAGGAGCTTATCTTCGCCCCATCTCCCA<br>AATAAGTGGGCCCTTGTGCTGTGAGGAAGACCAAAGCCTCAGGGA<br>AGATAAGAGATATGGAGATGGGAGGGGGAGGACAAGGGGCAGAG<br>AGTAGGGTCTAGCTGGCTATCTCTGGCCTTACTAACACCCCCCTGG<br>AGGCATGCCCCTTTTCTCCAGCACACAAGCACATTGGGGCACCTGG<br>AAATATTGGTTCCAGGCTCCTGTTCTCTGGACTTCAGATCCTGGGG<br>GAGCCCCTCCCCCCCCTGAATCCCTGGCTTAGCTACCTTCCTGCCTG<br>TGCACCTAAAAACCTCAGGTCAGAACTAGGAAAAGAGTTTTGTTTT<br>TATTTTTTTGAAATGGAGTCTCGTTCTGTCGCCCAGGCTGAGGTGC<br>AGTAGTGCAATCTCCGCTCACTACAACCTCCACTCCCTGGGGCTCA<br>AGCGATCCTCCCACCTCAGCCGCCGAAGTAGCTGGGACTATAGGTG<br>TGTACCATCACACCTGGCTAATTTTTGTATTTTTTGTAGACACAGGG<br>TTTCGCCATGTTGCCCAGGCTGGTCTTGAATTCCTGAGCTCAAGCA<br>ACCTGCCGGCCTCGGCCTCCCAAAGTACTGGGATTACACGCAGAA<br>GGCACCATGCCCAGGCTAGATGTGTCTTATCCCAATCCTTTGGCAG<br>GCATGCAGCTCCACAGGCGATTTCTTCAAGCAGCTGAAGTGTTTAG<br>CCCTCCTGGGTTAAGAGCCAGATAAGGAGAAATCCCTTTCCTAGGT<br>TTGGAATGTGTTGTGAAAAAAAAGAGAAATCCCTGGCTCCTGGAG<br>CTGGTGGGAGACAAGATTAAGCAAACCTCCCCTGACATGTATCCCT<br>TTGACCCCAAGCTCTGCCTCCTCCCTGACCACCCATGCCCTTTCCTT<br>TAACTTCTCAAACAGATACCAGGGCCTAAACTGCTTTACCTCCCCT<br>CCTACTGAGTCAGGTTAGGTGGTGGGAGGTCACCCATTTCCGAGTT<br>AAACCAATGCAATATGAGTAAAACAAAGTCATGTGGGTATGTCTGG<br>GGTAGAGAGAGGGGTAGCAAGTTCATGTGTCCTCCTTGGTCACATA<br>TCTCCCAAAGCTCCGATCCCTGCCATGGGAAGTGGACAGGAAACA<br>TGAGGTCATGACCTGCAGGCATCTTTACTGCAGCTCTGCCGGCCTG<br>GAGGGGGAGAGGGGGAGGAAGAAGTATGCGCTGCACATTTCTGAG<br>GCTACTGCATTTGCTTTCAAGGCAGAAATCTTGCTCTGAGCAGTCA<br>GCGGCTCCAGTTTGGGCCCGATAAGGAAGTTCTCCGTGGCCTCCCT<br>CAGGCAGAGCAGGGAGGAGGCTGACATTGCCAGTCTCTTCTGGGG<br>CCCAAGGCAGGTTGCAGGAGATCCAATCCCATAGACAGCTCTGGG<br>CCTCTTGCATTTGAGTTTTTCAGAATTAAACTGCAGTATTTTGGAAA<br>GCACATCCTGTCCACTGTTTCTTTGAAGTGAGTGGGGGGGGGGGG<br>TCTTGTTGAAGGAATTGTCATTCACTGCCAAAATCATTCCATCCTCC<br>TTCCTCAGTGTCTGTCCTCAGATGGTCAGCTCCCCGCTCAACAGAC<br>TGTCTCCCGCCTCTGTGACCAGCCTCTCTTTGGCAAGAGGGAGCTA<br>GAAGGCTTTACAGTCCTAATCATTTTTCTGTTGGAAAAAAAAAAAA<br>AAAACCAAGGCTCCTTTCCCTGTGGCGTGTACCCAGAGGTTGATTA<br>CCTGAGTCTGTCCTGCCTCTCCCCACCCCACCTCCCTAGCCAAACG

	CTGCTGCCAAAGCCCACGCTATTGCCCTAGATGGCCTGTCTTCAGC<br>GGGCTGCCCCTCGAGGTCCCAGGCTCTCCGCGGAGCCCTCACCTTC<br>CCAGCAGGGATCAGAACCTGCACTCCTCTATGCGAGTCCTGGGAC<br>AGCACAAAGTGGATTAGGGTTAGGGTTCCCACAAACGGAAAAATG<br>TTATTCAAACAACTCTGTAGGGTCCGAGGAGGCCCTCCGTCTTAAT<br>TCTCGAGACTGACCGGCCCTCGCTGCCCCGAGCGGGAGCAGTTGC<br>CCCGGCAACAGCCGCTCCCTCTCAACTGGAGCTGCACCCAGGCTT<br>TGGCTAAAGGCTGTTAAAACGTTGGCCAGGTGCGGTGGCTCACGT<br>CTGTAATCCCAGGGCGGATCACCTGAGGTCAGGAGTTTGAAACCAT<br>CCTGGCCAACATGGCGAAATTTCGTCTCTACTAAAAATACAAAAAT<br>TAGCGGGGCGTGGTGGTGCGCGCCTGTAACCCCAGCTGCTCGGGA<br>GGCTGAGGCAGGGGAATCGCTTGAACCCGGGAGGCGGAGGTTGC<br>AGTGATCCGAGATCGCGCCACGGCAGTCCAGCCTGGGCGACAGAG<br>CGAGACTCCGTCTCAAAAAAAAAAAAAAAAGTTAGGGTCCTTTAC<br>CCGAGGGCCGGCTTTCCTCACTCCCCGCCACAGGTAGGGGAAACC<br>AGGCCGGAGCCGGCGGGCCCACCCGCCCAGAACCGGGAATTCGGC<br>GAGCCCCGCCCCTGCCACCCCAGCGCCGGCCGCTCGGTAACAAAC<br>ACTTCCACTTCCTGAGCGCTAGTCTTCGCCCGCCGCGGGGCGCCGC<br>GCCGAGCGCAGGCCCCGCCCCGCGCGTTCCCAATGGCCGGCGCCG<br>TTCACCCGGCCGGAGCGCCCAGGCCTGCAGCCCCCTATTGGCCCGC<br>GGAGGTCCCCACCCTCAGCGCGGCCCCGCCCCCGGGGTAAGGAGC<br>CGGGGCGGACTCTGGGACGCTCAGACGCCGCGCGGGGCGGGGATT<br>GGTCTGTGGT
SLCO1B1	CTCTGCAGTGTAACTTCATGGTTTTTGAGGCAAAGGAAGGCTGGCA<br>GTAAACAAAATATGTGAGTAAAATTTATAGTATGATGGTGAGAGAAT<br>AAAAATTGAGTGTCAAAGGGAGTAATATTAAAAAATTGTTAGAGAA<br>GACTTCGCTGGGAAAGTGTCATTTGTTTGGGTACTAGAATATGGTG<br>AGGGAGTAAGTTATGCAGCTATAGGGAAAGAACATTCCAGACAGA<br>AAGAAAAATAGGAACAAAGATTCTCAGTAGAGGATCTATAACTTAT<br>AAGGCCTTAAAGCTGTTCTTTGGATTTTTGAGTGATATGTGAAGCC<br>AATGGGGGATTTGAGCAGAGCTATGACATGGTATGACCCTCACTTAT<br>ATGTTAAGATTTACACAGGGGAAGGAGGTTGGGGAAGAGGGGCTG<br>TGAGTGGTCAGGAGGAAAGCTGAGATACAAGTATGAGGGTATTGC<br>AATAATTCAGGACCAGCCTGAAAGTTGTGGAGGAGGTAAGAAATC<br>ATCAGATTCTGGGTATATTTGGAAATTAGAGCCTAATGTGAGTGTGA<br>GAAATAGAGAAGAATGATTCTAATATTGTATGTGGCTGTGCATGTGC<br>ACGCATGTGTGCAACTGGAATGACAGAATTACAATGTATGGGGATT<br>ATTGTCTTCCCTGGACAATAATCCCTGGAAATGGGGATTATTACAAG<br>AGGAGAGGTTGGGGAAGGAAAGACAAAGATACCATGTTAGAAAAT<br>ATTAAGTGTAAGATGCCTATTAGAGATATATAGGAAGATGGTGAGAA<br>GTCAATGGATAAATCTAGAGTTCATGAGTAACTTGGCTAGAGAAAA<br>AATTTTGGAATTATCAACGTATGGATAAGATTTAAAGACATGAGAAT<br>AGATGAGGTCACAAACACACACAGTAAGGTTATTGTCTGACATGCT<br>TCAATTTAGAAGACAAAGATAAAGAATTTAGAGTAAATGAAGTGTA<br>AATAAGTAAGTCAGTGAGTTAGAAGGCAAACCACAAAGGGTAGTG<br>TCCTGCAAAGTTTTGCTTAGGGAGGGAATTCTCAGTTCTGAAAAT

	GCTGCTAATAGTTCAAGTCAGATGAGGACTATTAGATGTAGCATTTA<br>GGTAATTTATGACTTAGGAAAAGTAGTTTCAGTGGAAATTTGGGGG<br>CAAAAGCCTGATAAGAGTGTGCTCAAGAGAGAATGTAGAGAGAGA<br>GATTGAAAGCAATAATTAGACAAGCTATTTCTCTGTAAAAATAGCAC<br>AGAAATAGGGCAATATTAGGAAAAAATGGGATCAAAAGAGAGATT<br>TTTGGAAGATGAAAAAGTGACAGCATTCTTACATGCTGATGGAAAC<br>ATTTAAAAAGAGGATAGTGCTATCCTTAAAAACACAAGGGGAGGA<br>GTCAATGTCATATGCAAGTGGAGATTGGCCTTAGCTTAAACTTGGGT<br>AGTTCACTAAGGCAGACCATGTGGTGGTACTGGGGCTTTACATAGA<br>TAAATGTGGTCAAGGGAGCTTTTGAAGTTTCTTCTTTATTTATTTATT<br>TTTTCAGTAAAATAATTCATCAGCTGAAGGTGGGGAAGGGATGTATT<br>AGAATTTCAAAAAAAAAAAAAAAAAGATGAAGGCACAAAATGCTC<br>AGAGTGCAAAATGGCGTGAATGATTTCTTGGCAAAATGAAAGGTC<br>CATTTGGGCTTCCTAATCATACGTTTAAAGTCAGCATGGTTGAGAGA<br>TTTTCCTCAGTTACATTTAGCTTCATAAGGGAAATGTGCAGAGTTAT<br>TTGAGAGTTAAATTTAATCAAGATTGTGGCTTTGACATGCAATTAAA<br>ATAATGCAAAACAGTCAAAATTATATGAAGTTATATAGAAGAGAGTG<br>ATTACCGTGACCATTAAATTAACCCAGATGTATAGATAAGGAACACC<br>GAAATCTCTGGCAAAATCAAATTGTAGGTTATACATCCTGGTCAGTG<br>GAAGCATGTGGATCTAGGGTGTTACTGAGTGTGAGCTAGGAGTGAT<br>AAAGAGTGGTGCTCCAATCTTTGATATTATGGAGAGATTACAGTGG<br>TGTGAAAAGCATAGGATATGCCCATAGCAGTAGGTAGAAGCCAAGA<br>TCATTGAGGAGAAGGCTAAAAAATAAAAAGAAAATAAAAAGAAAA<br>CAGAGAATTAAAAAGATTCTCTTCATGGTCATTACCATCATGAATAA<br>TTAAAATAGTAGTAGCATTTTGAGAGAATGGCTTTGGATCGAGAGC<br>TAAAATTGTCATGAAATGAGTGGGAGTGACTGAGCGTTGATAGATG<br>ATTTCAACAAGAGGAATAAGTGATAGAGTCTTTCAATATGAGATCC<br>AAAACTATGGATTATGAGTGTAAAGGGTGGGGAAATGTAAAGAAA<br>CTAGCAAAGTGAGGCACATTGGAAACCAGCCCATCTGGTTTGCAG<br>CAATATGGGGGATAAGGCAACCCCTATGTATCACTGCTGCAGGAGA<br>GGGAGTGTCTCAGGGAAGACCAATTTTTAAGTTCAAGGCAGAAGT<br>GAAGCAGAATAACTCAGAGAAGAGGTAAAGGTAAGGGGAGTTTCA<br>GTCATGACTAGTGATGGGTTCCAGAAAACTCAAGAGAAGAATTTC<br>AGTCCAGGATTGGGAGAAAGAGAGAAGATGGGGGCAAACATAGG<br>AATGTGCAGAGCCCTGTGGGGATTAGAGAAGAGGTGATGAGTCAT<br>CAGGGAATCCCTACCTTCTTATGGTGGTCACTACCATAAGAGCGATA<br>ATTGCCATGGTAATATTAGTCTTGATGATGCCAGAGCTTTGATCATTG<br>GTGGGTATAGAAACATGTTTGGAGAATAATATGTAGAATAGGAGTTC<br>TTTCCAGGAGAGTGTAGCTTTCTGGAGCTGTCTCTTAATCAGCACC<br>AACAGAAGTGAAATGTTCGGGTAAGGGCGGATCTGCTTGGAGCCT<br>GACGGTTCCCTCTTGAGGTGTGCCTGTGGTACACACCTGAGAACTC<br>TGGGGCTAAAACCTATTGGACATAGGTTTTACAGATCTACAGGATAC<br>AGATCTCAGAGATTTTATTTGTATTCATTTAATATAAATTAACTGCTCT<br>AAAATTTATAATATGCAAATATCATACAATTAATCTAATTAGGTGTTG<br>AATCTATAATGTGCCAGGCATTATGTAAGGCACTTTACATACACTAA<br>ATCTTTATTCCAAATATAGACTTCTTACTTTATAGATGAGTGCACTGA

	TGCTCAGAAATGGTAAATAACCTACTGATGTTTATACTGCTGGCAGGTAGCAGAGACATATCGGCATTTAAGTCTTTCAGACTTCAAAGGCCATGATATTTCATCAGAGCTGTGATAGCCGTTCCTGAAAAAAATATCAGCTGATTCTTTAAATCAATTTTTGTCATCTAACTGATGCGTGGCTGTTAGCATAATATTGATCTTGAAAGATGTTTTGCAACATCTTTCCCCTGGTGTACTCTTGTTTTTCCATGATCCCACAAAATGAGCAGTCTAATTATTTACACAATTAGGAAGAGAAAAGGGGCACAGAGAATGCTCTTTGACCTCTGAAAATATTGGAGAATTTTACAACTGGCACCTTTAGCTCAGGATTATAAAGGTTGTTAGTTAGTTTGTACTGTTTTATCTTCATTGTATATAATATATATATTAGTCTCCAAACATGTTGATGTGTTTTCAATGAAATGGATGTCTGAGGAGAAAACCATTAGCCTGAGAAAACCCAAACTGTATTCCCATTGTGAATAAAAGGAAGTCCATAAAAATGATGGAAAATGTTCTGCATTCCTGTTATGATATCAAAATCTGGCAGTACATGAAAATTTTCAAAGTGCTTATTTAACAGGCATAATCTTTGGTCTCCTGAGCCAGAATCTGCTGGGTATGGGACTGGATTGCTATTTTGACAACTCGCCAGTAGATTCTTACTCAGCAGAGTATTTGGAAGCCTTACTCTAATATTTTGGCCTTGGGTCTACATTTCTCAGTTCTGCACAGTCATTCTTCCCCTCTACACTACTCTTTAGTTTGTCTCATGATTCCAATACTCTCAATAATTAACCAAGAATAGAACTAATCAATCAGATAACTGTGGCACAGACATCAAATACATTTTGCTGCAACCATATCAACAAATGTCCCATGAATGATAAGGGGTAACCATATTCTCATATATGCATCCTCACATTACCACATATATATATGTGCATATGTGTATACAGGTAAAAGTGTGTATATATGTATACATGTATGTTTGTGTGTATATACATACATATATCTTCACACTTTTCTGAAATATATATATTTATGTGAGAGAAGGGTCTGTACTTTATTTCAGAAGAGAGCTTAATGTCCAAGGTATAATTGAGAGTCTAAAATGTTTGAGTTATTGAATTAATTAAACTTCATCTCTACTCAAGAAAACTTTTAACTGAGTTAAGCTCTTCCTTTCTCCACAAGTCAAGTCAATAAAAGGAAACTGTGATATTAATAATTCTTTCCTGTTTTGATGTAAAGAATCTATCGCATAAAGCAGTCTTAATTTTCATCATTCAGAAAAATGGTCTTGCAGTTAATTGGGACTCTCTTATTCCAGGTGGTATCTCCAGTCTCCATACATACCACGTTAGAACCATACTTATGTACCAAGCAAAGAGGGTATATTTTAATTTTTAAATGCCAATGTAACCTGTAGGCATATTTTTTATTTGTCTTAAATTATTTCCTATTTGGAAGTTTTAAATACCTGGAATAATTTATTGTACTCATATTTTTAAAGAAAAAAATCTTATGCCACCAACTTAATTGAATAAACAAGTAAAAGCCATTCCCAAAAGTAAGGTTTACTTGTTAAGATTAACAAAAAATAATGTGAGAATTCTGAGAAATATAATCTTTAAATATTGGCAACTGGAGTGAACTCTTAAAACTAACTAGGTTTTATATGTTTGACTAGAGCAATGACATAATAAGGTGGTTAATCATCACTGGACTTGTTTTCAAAAAGCCAACTACTTTAAGAGGAATAAAGGGTGGACTTGTTGCAGTTGCTGTAGG
SLCO1B3	CATTTAAAGTGAGACTTTAACATCAGAAAAAGGATGGACTTGTTGCAGTTGCTGTAGCATTCAAAGTCAAGGTAAGAACCGTCGAGGTTGTCTAATTAAAACATTTCTTTTATGTAAGAAATTTTAACAAATTTCAGTGAAAATGTTTTTGGTAGTTATGATAAATATTACAGATTTTATGCTCTGTGTCTTCTCCACACTTTTGTTCTGAGCCCTTAAAGCCTTGTAAATATCCCTTCTAGAAATAGAAGAGCCTCAGATTTATATACTCAAAAATAAGA

	GTAAATCATACCTTTATAAAGTGAAAAATAAAGTTTGGAGTCCTATT<br>TCTATTGCTTTCTAAACAAATGAAACATGAAGTCTGAAAGGATGGA<br>CTTCTAGTTACTCTCTTAAAATATACTATAAGATAATTATTTGTATAAA<br>CTGTATTGAATTACTATACCAGGCTCCCTATTTCTTTGATTTCACTTT<br>AACTGGATAAGTAGGAGTGGGCGGGTGGAGGGGTGGAAAATATGT<br>TTCAAAGTCACTTAATCACCTCACTTTCCTCCATTCAACTCGTTTGA<br>TTTAGGGTGAGGGGAAAGAGATTTTCTAGATAACTTTTTTATCTCTG<br>TTCAAATAAGAGAAAAAAATCTTCTGGGCACAGCTCTTGGAAAGA<br>ATTAACAGCTCAATAAATAATGGTCCCCTTCACCCATTCAGGAAGGT<br>GGCTTCCCTGATAAGACAGTTATTTTTATTGTGAAAGTAAGGTGGG<br>GATGCAGAAATTCTCTCCTCTGAACAATCTAAGAAGTTAGGAAATG<br>CTGTTTTCCCTTTTATCCTCCTCAGTTCCATAGGTACATGCTAGAAA<br>CTGGGTCACTTGGCCTGCACTGGTTTTCTATAACCCCTTAAATATGA<br>AAAGCTAAACAGAAATAAGCAGAGGCTGGTAAGATTCTGCCCTCTA<br>CTTCAGCCCAATCAAACTGTGAAGAACTGACCTGTTCTGTTCAGGG<br>AAGAGTTGCAATGCTGCATACAAATGCCTGTGGCTCTGTCTATAAA<br>CATATTGTGTCCTCAACATTTATTTATGTCAATAATTTGGAACTCAGA<br>AATAATATGTTCAGAGCAAAATATATTAAACGCTTCTAAATAAGGCT<br>TAAAATGCCACAATGAAGTAAAAAGTGTGGTATTTATTCAAGGCTA<br>AATTGATATTCAAGTTTTGTAGGTACTTTGTACTTACTTGATACATTT<br>TTTTTTTTTTTTTTTTGAGAAGGAGTTTCGCTCTTGTTGCCCAGGCT<br>GGAGTGCAATGGCGTGATTTCAGCTCACCACAACCTCAGCCTCCTG<br>GGTTCAAGTGATTCTCCTGCCTGAGCTTCCAGAGTAGCTGAGATTA<br>CAGGCATGTACCACCACACCCAGCTAATTTTGTATTTTTGGCAGAAA<br>TGGGGTTTCTCTGTGTTGGTCAGGCTGGTCTCGAACTCCCAACCTC<br>AGGTGATCTGCCCGCCTCGGCCTCCCAAAGTGCTGGGATTACAGGC<br>ATGAGTCACTGCTCCCGGCCTACTTGATACAATTTAACCTTTCTGAG<br>AGATGAGAAACCAAGTGCCCACATTTCTTATTTTTCTGGATGGGTAT<br>TTTATCTGAACATAAAAGAGAGAGGTGTGGAATAGTTGTCTTGGAA<br>AGTACGGGAAATAACAGAAAAAAGTAATGATTATTCGGGTATGTTC<br>TCCAGTGGTACAGAAGGATGAGAGGATGAGAGAGTTTATTTCAACT<br>AGAGGTAAATAGGGAAACATTAAAGTGGTGTGATTTGGAGCCGCAT<br>TACAGCCAAGAGATAGGGAAGGACATTCCAGGAATGGAAGACATAT<br>ACATGAAGGTAGAGAGCTTTTCTGTTGGGACAGTGGTTCCATACAT<br>CTGCAAGTGCTTATGGTTGTATTGGGAAATGGGACTAAAAACTTGG<br>ATTCATTTAGGCTGTGAAGACCCTCAAATAGGAAGTGTAAGTTTTAT<br>TTTTGTTGTAAGAGGGATCTTTGAATGATTCCAAGAATGGACATGAT<br>ATGCATAGGAAAGAAAGCAAGCTGGAAGACAGGACAAGTAGAAAT<br>TAATCAATTGAAAGAAACAAGGCTTGAGTGGTGAGATAATAGTCTT<br>CAAGCCTTCAAGAATATTTGTTTAGTATAAAGAAAAAGAAAACTCT<br>AAAGCTGAAGGAAGACTATCTGCCTTGCAAGTCGTTCAAAAAGAT<br>TTGCAATTATAGAATTAATTGGTAAAAAATAAATATTTTCCAACTTTT<br>AAATTATGAAGAAACAGAGATATTCGTGGAAAAAGTGGTGTAAATT<br>TATTAAGGGGCTGCTTATTACCTTGAGACCACCAAATTATTGATTTTT<br>TTCCTCACTTTACCTACAAAACACATACAAAAAGTGGAATAATTGA<br>ACTTCTCTTAGAGATACATAGGTATGGGTTTCTCCATTCATGAGTGTA

	TTTATATACACTCGCATATACATAAGTGATAATATCTTCAGATATTGAT
	TTTTCCATATTCATGCATTATCCACTAAATATGCATGATATATATTTATG
	GTTTTTATGCATTTATAATATAGAGAATCAATAAATCCTTGTTGAGTA
	AATTAATGCATAAAATATGCAGTAGATCTTCATTATTCACAGATTCTA
	TATTAGAAAATTTGTCTAAACACTAAAATTTGTTTGTGACACCAAAA
	TTAATACTGCTGGGCTTTTGTGATCATTGGCATGCATGAGCAGAGTG
	GTGAAAAATTTGAGTTGCTCTGTTTCCAGTTGAAGTGGAACAAAGT
	GAAGCTCTGCCTTCCTCTCTCAGCTCCCATACTATAAACTTCACAGT
	GCATTTATCGCCACATTGTTTCCATAGTTGTGCTTTGCGATGCTGAG
	TTTACTGTTTTAAATGGACTTCAAGCATAGCCCTGAAGTGTTGTCCC
	GTTGTTCCCACCTCCTAGAGGGCCTGACAGAAGAGGTACATATGCT
	ATGTAAGTTTCCCTCAGGCATGAACTACAGTGCTATTGGCCACTAAT
	TCAATGTTAATGAATCAACAGTATATACTAAGGTGTCTTTAAACAAA
	AACATACATAAAACATTATGTATTAAGTTATGTATTGATCCGTTGATT
	AAAATATTGTGACCAAAGCCACACAGTCACCTAACACTGTATCTCT
	TCTAGGAATGATGGATTAATATTTGCAAATTCAGTGTTCCCAGCAAC
	TTTATGGAACATAATTATTGCAAAACGTGAGAATCAATTGTACATAT
	AACCTATATCATACATATTATATAATCAGTATCCTATTACAAATCGGAT
	GCTTATATAGATAACATTGATATTTACATACTTAGATATTTACATATCT
	AAGGTATCTATATGAGCAGCTACATAGCAAGTATCTACATAGCATGTA
	TCCACATATATATGCTATATACATAACATATACCTATATCTATGTTATGT
	GTCTGCTTATATACTTTATATACATAAATATGTGTTATGCATATATGTAT
	GAACAAGAATACATCTCTAAACCTTATAAATATTCCACTGTTCTATAT
	AAAAGAGTCAACTGACGTAAAAAGTTGACTCATTTTACTGGTCTTT
	AAAATAGGGGCTTAATAACTCTGAAACTTATGATTTCTCATATACTTT
	GACTTCAAAACTTCACTTGTAGGAATTTATCCTACAGATATGACCAC
	ACAGAAAATGACATATAACATATTAAGAACATTTTCTAATATGAAAT
	AATTAGATATAAGCTAAATAGTTAACGATATAGAACTAGTTACATATA
	TTATGAACAACAGAATACCCTGCTACCATAAGCAAAATGAGGACTT
	TTATTAATATAAAGTATATACAGAAGATAAAAAGGTAGAGTGCAGTA
	ATACTGGTATGCTTACATTTATATTAAAAGAGAAAATATTATGTATTT
	AATTAAAAATGCATATTAAGGCTGGGCGCGGTGGTTCACGCCTGTA
	ATCCCAGCACTTTGGGAGGCCGAGGCGGGCGGATCAGGAGGTCAG
	GAGATCGAGACCATGCTGGCTAACACGGTGAAACCCTGTCTCTACT
	AAAAATACAAAACAATTAGCCGGGCGTTGTGGCACGCACCTGCGC
	CTGTGGTCCCAGCTACTCAGGAGGCTGAGGCAGGAGAATGGCGTG
	AACCTGGGAGGCAGAGCTTACAGTGAGCCGAGATCACGCTACTGC
	CCTCCAGCCCGGGAGACAGAGCAAGACTCCGTCTCAAAAAAAAA
	AAATAAAAAAGCATACCAGATTTCTGGATAAGGGGACAAAGGTTAT
	AAGCAGAATCTTGCCTTTTCTTTTCTTTTCTTTTTTTTTTTTTTTGCA
	TTTTTCAACTACATGCATGTATTTTCTCTTGAAAAATAAGTGGATAA
	ACCCTAAATACAGTAAGATCATGATGATGGTAAGGTCAAGCATGGC
	CAGAGTCTCTCTGAGAGATACTTGGAATATATTTTGCTCTTTTAAAC
	AAATCCAAACTTTATCTGCTTTTTGAGCCTTGTTCCATAGGCATCTC
	CTCTATGAAACTGACTGAGGCTATCCTAATCATTATTATTTACATTAT
	TTTTCAACCCATTTTGAGTATCCACTATGTGCAAGTCATCATTTGGC

	ACTTTATAAACTTATAAGGATAACGTAAACATAAATTTCCTTTCATAA ATCTTGTAATCTGGATATGTAGAAAATATAAAAATTTAATTTCTATA ATTTAAAATTGTTGTTCATACAATCTAGTGTGTGGTTTTATATTATTTA CTTGTTTCAAATTTCTCTCTATGAAAATTATTTTTCTAAGCAAATTAT AATCTCTTTAGGCTAGGAGTTTGTCTCTGTCTTTCCTCCTCTGTGTC CAGCATTGACCTAGTCCTGTGGTCAGGAAATAGCAGGCCCTGAATG AATATTAGAGAATGATTGATTGATTGATATTGAGCTTGTGGCTTTTCC TATTTTTAAATTGTATATTGTTAAAGTAAAATAAATTATACTTTTTCTT TTTTAACAGGTGATCATTTCAAACCAAGCATCAGCAACAATTAAAA ATATTCAC
SLPI	GAGGAAGCAGGATGAAGCTGTTTCTTAGAGTCACAGTTACCGTCTT GGGACAGGTAGAGGAACTGAGGCCCAGGGAAAGGTAGTTGCTGC CTTATTCACACAGGAATGAGCTGTGAAAACCTGGTTGTCTGCCAGA TTCTGAAAGAGTTTTTTGTTCTGTGTTGTTTTTTGGTTTTGAGACTT GGAAACATAGTGAATTTTAGGTAAGAACACAGTGACTTTTAGTTAA GGGTGAAGCTGGCATCTGCTATCAGAATGGGGCGACCCGAAACAC ATTCTTCCTTAGACCTGAGCAACGGGGCTCCTTCCCTGCACCTCAC ACTTCAGGTCGCTCTGTGCCTTGGGGCGTCTTCCTCAAAATTGTCTA TGTTCCCCTGGAGTGCCTGAAGCCAGCCAGGATCAATGGTGCCATG GGGCAGAGAATCCCAGCTTGGACTGAGAACTGCATGGGTTCTAGT CCAAGGGGGTGCTTCTCACTCTGTACTCCTTCCCCCATTGCTAGAAT CTAGGGCAGTCATCCTACCACCTTGGATGGCTCCTGGCAGGAGCCT GTGGCTGCTCCTGTCTCTTCCTCAGGGCTTTCCAGGCCATCTCTGTG CAACTCCAAAGGGGCTTTTCAGTTGATGTCTTTTTTTTTTTCCTCTG ATTGACCTATGCTCTCTCGGGTCAGTGTTGAGCCATAATGGATTAGG CAGAGACATTTCTTGTGCAAATGAGATGCTCCTCTCACTTCAACCC TGAGCCCCTCCAGGGCTGGCTACATAATTTACAAGACTCAATGCAA AGTGAAAATGTGGAGCTGCTTGTTCAAAAGCAGGAAAAAAAGTGT TAACGGTACTAAAATTAATTTTTTAACTTTCTTTGGCAGTTTCTCTCT AGATCTGCCATGGTGTTAAAAAAATTATTTACTGTTGCTCCTCCACA AGTATGTGGATACTCACAGGGAGAGTGCAGACTCCCAGAGGTGCG CAGGGTCCCTGTCCCGCTACTTGGTGTGTGCAGGACCCACCAGCTG CCAGGGTCCCCCTGTCACCAGCACCCTGGTAGTCAGGGACAAGCG GCCACACTAACAACATTTGAGACAGTAATTGGAAGCCAGTATTCAG AGAAGGCATAAAGGATGATGCAGCCTCACAAGTAATGATGTAACCA ACTTTAATAAATGGCATTGGGAAGTGCAGAGGCAAGCAGGTTCCAT CAGCCAGGGTTTTCCAGAGAAACAGAACCAATAGTTGATTATCTGG CTATCTATCTATCTATCTATCTATCTATCTATCTATATCTATCTATCTATC TATATCTATCTGTCAGTTAAGATCTGTCTATCTGTCTGTCTACCTACC TACCAATAGGCCCAATCAGGTCTATTTTGTCAAGAAATAAGACCATG TTTTGTCAAACATTTAATGGGACAGAGCCAGATGGGTCCAGACCCA AGGACTAAGCTATTTAGTTAAAGACTGACAGCTCCCTGTGCGCTTA ATGTGTGCAGGGACACGCCGGTAATAAAAACAATGTGTAATGTACT GAGGGGACCAGACAGTTTGCTGAGTGCTTTCCAAATACTCCTCACA ACAACTGTACGACTCCAGAGGCAGCAACACTCTCATTTTTCAGTGG AGGTAGCTGAAGTTCAAAGAGGTTCAGTGATTTGCTTCCAACCTGT

	AAGTCTGACTTTGAAGTTCAGGTTCTTAACAACCATGTGGTGTCGA<br>CTCCGTGGGCGTGCTCTTTTCTGTAGCCCTAGGCCTAGTGCAATACC<br>TGACATGTGGTAAGAAATGTTAAACAACTTTTGTGTGAATGGTTTG<br>TATAAATGAATGAAGGAATCTATAGGATTTAGGTAGAAGGCAATAAA<br>GGGTGTGTGAAATAGATTTTTTTTTTGAGACAGAGTGTCACTCTT<br>GTAGCCCAGTGGCATGACCTCAGCTAACTGCAACTCTGCCTCCCAG<br>GTTGAAGTGATTCTCCTGCTTCAGCCTCCTGAGTATTACAGGCACA<br>CACCACCATGCCCGGCTGATTTTTGTATTTTTAGTAGAGATGGGATT<br>TCACCATGTTGGCCAGGCTGGTCTCAAACTCCTGACCTCAAGTGAT<br>CCACCCACCTCAGTCTCCCAAAGTTCTGGGATCACAGGAGTGAGC<br>CACCACTCCCAGCCTTGAGATAGATTGAATGAGTCAGTCTCAGGTT<br>AAGATTTGGTTAGGAGTGGAAGTGTCCTCCACCAGACAGGTTCTG<br>GGGATCAGCATGTGAGTAACCTGTTTTAGAAAAGCTCAATCTTGTG<br>TGTGTCGTTAATCCGCTATGCAGCTTTGGGCCTGGATCTTCTTCTAA<br>GGGATTCAGTCTCTTTTATGCAAGCTGTGGGTTTGGTCTAGGGGTTA<br>GCATTTTCTGTAAAGGACCAGACGATAAGTATTTTAGGCTGTGCAGT<br>AAATATTTTAGGTCCCTGTTGCCATTGTAGTGGAAAGATAGCCTTAG<br>ACAATACATAAATTAATGGATGTGTGGCTATGTGCCAATAAAACTTT<br>ATGAAAACAGGTAACTAGGTGGATTTGGTCCAAGGACTGTAGTTTG<br>CTGATCTAGAAAACCCTTGAAGTCCTCTTTTTAGTCTTAACATTCTA<br>TGATGTAAACATTTAGAACCAATTATGTCAAAGATAATAATGATTATA<br>TACTATTATCTTCTTATGACACCCATGGAAGACATTGCTAATTGGGC<br>ACAGGGCTCTTTTCTGATGAGAAAGGAGCTGGCTCTAATCAGTCAA<br>TCACAGATTGAGCAATCAGTCAATCACAGATTTAAAAAAAACTTAA<br>GCTGTAGCATTTTCCTTTTAATTTTTTTTTTTTTTGAGACGGAGTCTC<br>AGTTTGTTGCCTAGGCTAGAATGCAATGGTACAATCTCAGCTCACT<br>GCAACCTCTACCTCCAGGGTTCAAGTGATTCTCCTGCCTCAGCCTC<br>CTGAGTAGCTGGGATTACAGGTGTGTGCCACACTGCCTGGCTAATT<br>TTTGTATTTTTAGTGGAGATAGGGTTTCACCATGTTGGCCAGGCTGC<br>TCTTGAACTCCTGACCTCAAGTGATCCGCCCACCTTGGTTTCCCAA<br>AGTGCTAGGATTACAGGCATAAGCCACCGTGCCCAGCCCCTTTTCA<br>TTCTTTAAGAAATAAATATTTAGAAAACTCCAGATAATTTCTTGAGT<br>GTGGCACTGAGGAAAATAGGAACAAACAGAGAGAGAGAGAGAGA<br>GAGAGAACAGGAACTCTGTGAGAGAAATCAAATTATCCCCTGGGT<br>GGTGGAAGTGAGAACAAAAATCTTGACCTGAGAGTGTCATTACCC<br>ATTGGTGGAAATCATAGCCTCCATTTCTCTCTTACCTACCCTACTAA<br>GTACTCCAACTAAGAGTGAATGCACCTGGCTCATAGTAGTTACCTT<br>GTGCATATTAATTTTCTTCCTTTTCTTTTTCCACCAAAATTTGACTGA<br>TGATTGATTATGTCCAAGTCTTCTGATCCACCAAAATTCAAACCATG<br>GAGTGACTTTTGTATAAAGGGATCTGCTGCCAGTTGCCTGTTTGGTT<br>GTGGAATACTTAAATACTTCATGTGGGGCCATCGCTACTTGATAATG<br>AGTGGGTCTTTCATGTCTGTGGAAAGTTCCACAAACCTTCACCTTA<br>GGAATGAATGGGAGAATTCCAAGCATGAAGATAATGAGTCAAGAG<br>CTTGGAGTTTGTAGCTAGATGAGCTTTGGTTGAATTTTATTTTATTTT<br>ATTTTTTTAAGACAGGGTATCGCTCTGTCCCCCAAGCTGGAATGCA<br>GTGGCACAATCATGGCTCACTGCAGCCTCAAACTCCTGGGCTAAAG

	CGATCCTCCTGGCTCAGCCTCCCAAGTAGCTGGGACTACAGGCATA<br>CGTACGTCATCATGCCTGGCTAATTTTTACATTTTTTTGTAGAGATG<br>GGGTCTCAATATGTGGCCAGGGCTGGTCTCAAACTCCTACTCTCAA<br>GGAATCCATACACCTCAGCCTCCTGGGCAGCTGAGACAGCAAGTG<br>TGCGACCCTACACTCAGCTATGGGCTGAATTTTAGAGATAATGGTCG<br>CTCTCTTTATAATTAGAAGCAACCTATGCAGACTGGGTAGCAAATAG<br>AATGGGTTTAATTTTTTGCTGTCATGTGAGATCTGTAAGGGATTTTG<br>GGGAATTTTAGGAAGCAATCCTCTAAGATCTCAAATTATCTCACAGC<br>TAAATGTAGATTACAGTGACTGATGAGCTGCTTTCCCCCTTTATCTC<br>AGATTCATTTCAATTCTCTTTAGTGGGAAGGGATACTATTCATTTGTT<br>CTTTTCATTCAGAGTCCCTTCATGCCCTTAATTTCATAACCCTCTGA<br>GAAGGGCTGACTTGTTAGTATCATTTCATTTCACAGCTGAGACAAC<br>TGAGCTCCAGAGAGATTTGTGGAGAGCGGAGCTCTTCTTCAGCTTT<br>CATTTGTGAGTGCTTTTCCTGTGTCAGGCACAGAACAGGCACTGGG<br>GATATAACGGTGTAAATATTTCAGGGAACTAAGTATCAGTTGGTTGA<br>ACGAGCTGAACTTTTGAGAAAGAAACTGCATTGAGTAATCAGCAG<br>AGTTTCACAATGCCTGAGAGTCCAGTAATGTGAGAATCAGAATTAG<br>CAATGTGAGAATAGAATGTATTGCACAAAGTCTCAGCAGGGAGTCT<br>GTGTCTGGTTTTAGTTCCAGGTCCGGGTAGCACCTTTGCAATTGAC<br>CACTTCTTCCCTCTCTCCACCTATAAGGCTAATGGCCTGGGATCTTG<br>TGATGTTTAGGGCTCAGATGGACACTGAGATGGCCTCTTTAATCAA<br>CCAACTTCCCAGGCCAATCTCTTCCCTTTCTTTTCTGATAGTTGCTG<br>TGTTGGCCTCATAGCCTTACCTGGCATAGGAAAGATAAACAATCTCC<br>TTGGTGTCAGGATTTCTGGTCTCTGGCTACGTTTCCTGCTTATGCAA<br>TAGTAGCTGGGAGAGGCCGAAAGAATTCTGGTGGGGCCACACCCA<br>CTGGTGAAAGAATAAATAGTGAGGTTTGGCATTGGCCATCAGAGTC<br>ACTCCTGCCTTCACCATG
SOAT1	GATCATGCCACTGCACTTCAACCTGGGCGACATGGCATGACTCCGT<br>CTCAAAAAAAAAAAAAAAATGCCGGGCGCGGTGGCTCACACCTGT<br>AATCCCAGCACTTTGGGAGGCCGAGGCAGGCGGATCACCTGAGGT<br>TGGGAGTTCGACACCCCATCTCTACTAAAAATACAAAATTAGCTGG<br>GTGTGGTGGCCTGTAACCCCAGCTGCTCCAGAGGCTGAGGCAGGA<br>GAATCGCTTGAATCCAGGAGGCGGAGGTTGCGGTGAGCTGAGATC<br>GTGCCATTGCACTCCAGTCTGACCAACAAGAGTGAAACTCTGTCTC<br>AAAAAAAAAAAAAGATTGATGGTGTTTAAGGGGGTGGTGGTGAGT<br>GGCAGGCAATGCAGAAGTTACTAGATCATGGTAAGGACTTAGGATG<br>TTTGAAGGGGTTTAAGTTGTGAAGTGACTTGATCTAACTTGAGTGC<br>AATGGTGTGATTGAGGTTCACTGCAACCTCCACCTCCGGGTTCAAG<br>CGATTCTTCTGCCTCAGCCTCCTGAGTAGCTGGGACTACAGGTGCC<br>TGCCACCACACCTGGCTAATTTTTTGTATTTTTAGTAGAGACGAGGT<br>TTCACCGTGCTAGGCTAGCCAGGATGGTCTCGATCTCCTGACCTGG<br>TGATCTGCCCACCTCGGCCTCCCAAAGTGCTGGGATTACAGATGTA<br>TTCCACCATGCCCGGCCCTGTTTTAGCTTTTGATGTAAGGTTTCCTC<br>TAGAGTAGCTTGATGGTTGAGGAACCATCTTATTACCAAGGACTCC<br>CTTTTTATATTTCTTCTTTTGGAGACAGGGTTTCACTCTGTTGTCTGG<br>GCTGGAGTGCAGAGGTGCGACCTGTGCTCACTGCAACCTTCGCCT

	CCTGGGTTCAAGTGATTTTCCTGCCTCAGCCTCCTGAGTAGATGGG
	ATTACAGGTGCTTGCCACCACGCCGGGCTAAATGTTTTTGGGTAG
	TTTTAGCCTAGTGTACCTTAAATATGCTCCAAACACTTAATATTAGCC
	TACAGTTGGGCAAAATTATTGAACACAGACTATTTTACAATAAAGT
	GTTCATTATCTCATGTAATTTATTGAATACTATACTGAAAGTGAAAAA
	CAGAATAGTTGTATGAGTACTCAAAATATGATTTCCACAGAATGCAT
	GTTGCTTTTGCACCATCATAAAGTTGAAAAATTTTAAATTGAACCAT
	CATAAGTTGGGGACTGTCTTGTGCTTAGAATAATTGGTTACATATTA
	ACTGATACATTTGCTTTCTTTCTTAATGCCTGCCTGTTGTACTCCAG
	GTACTCCACACTAGATTTGGGGGAGTCTTACAGAAAATACTATGTAA
	TCCCTATCTTTGAGAACTTAAGCCTAATGTAAGAGACAGAGAGTAT
	TGCTCTCTGATTATAGGACAATGTAACATGTGACAGGATGCTCAGTG
	TAGTGGGACCGCAGAGAGGATGAGATGAGAGGTGGTGGTAAAGGA
	AACTTCAGAGGGTGAGTTGGAGGCGGTTTAGCGATTAGAGGGCAG
	GTTGTAGAATCTGATAGTCTTTGTTTTGAATTCTAGTTCTGCTGTTG
	GAGAATTATTTGATTTGGGACAAGTTAAATCACTTCCCTAAACTCAT
	CTGTAAAATGAGGATAATACTTATCTAAGGTTGTAAAGATTAAATGT
	AATGTAAAAAGTTTAGCAGCACAGTGCCTAGAGGTACATAATAGGT
	GCTCATTAAATCAGAACTGTTGGTATTAATTCTATTACTGCTGTAGCT
	GTGATTCTTTGAAGGCTTGAGTAGGAGTTAGCTACATAAAAAAAGG
	GCAGAGGAGAAATAGCTTTCTATAGACAGGGAAGAGTATATGGGAC
	ACTATGGTGGCCAAAGAATATTTGGAAAATAACAATAGTATCTGTCT
	TGATTCTGTTGAGATGAAAATAATACATGTAAAACACATAAGCACTA
	AAAAAAATCAGCTGTTATTATTCTACTTTGAGAAATATTGCATTGTG
	CTATACTGTTTCATATGGCTTGGGTAATAAACTTTAATCTGGCATTTA
	TTTTTTAATCATTTTGCTATCTTTTTTTTTTTTTTTTTTTTTTTTTTGAG
	ACGGAGTCTTGCTCTGTTGCCCAGGCTGGAGTACAGTGGTGTGATC
	TCAGCTCACTGCAAGCTCCGCCTCCTGGGTTCATGCCATTCTCCTG
	CCTCAGCCTCCCGAGTAGCTGGGACTACAGGCGCCTGCCCCCATGC
	CCAGCTAATTTTTTTTTTTTTTTGTATTTTTCGTAGAGATGGGGTTTCA
	CTGTATTAGCCAGGATGGTCTTGATCTCCTGACTTCATGATCTGCCC
	ACCTCAGCCTCCCAAAGTGCTGGGATTACAGGCATGAGCCACCGC
	ACCCGGCTTCATTTTGCTGTCTTTTATGCTTTAAACTTTTGCTATATG
	CTCTTTAAAAGAGTATCTACTTTAAATTCCCCATCATCTGGCCAGAA
	AAAAAAAGTCTCTGTTCATCTGGCCAGAAAAAAAAAAATTCCCATT
	ATTCCTTGTAAAATTCCTCTCTTACTGAAACATCTTGGTTCCTTTTGT
	GTCTTTCTTTTTCTTCCCTGATTTCCTCTACTTTGAGGCTGTCCCTCA
	AAGTTTAGTTTTCAGTTCTTACTTCTCTCATCATTTCTGATGAAGAG
	ATAGATGCTCTTGTGGCTTCCTCTGTCACTTTTACACAGAATTGGAC
	CATTGTAAGTTGGGCATCGTCTTGTGATTTATTAGCCCTGACTTTTC
	CCTTAAGTTCCTCTTAATTAGATCTTATGATATCATAACATTTCTACTT
	GATATATTCATCTGCTGTTCCACAATTGTTTCATTTGTTTGATTTCAG
	AAATAATTAGAAGAAAGCCGATCTAGATTTGTCAGATTTTTACTTTG
	CCAAGCTGTGATAGTTGAAAAACAACCAAACCCACCATGTACTATA
	TTTCATCCTTTTATACAGAAAAGGATATTTTAGTACAAAAATTAAGA
	AAAACTGTAGTCTCAGAATTTAAATATATATAGCTTTAAAATAATGTA

	ATGAGTATTTCACAGTTGTTTTCTTCTTGCTTTAGACTCTTGAAAAC<br>TTTATCACATAGTGAAACACTGCTTTACTTTTTATTTAAAATTGTTTT<br>TTTGGCCGGGTGCGGTGGCTCATGCCTGTAATCCCAGCACTTTGGG<br>AGGCTGAGGCGGGCGGATAACTTGAGGTCAGAAGTTCGAGACCAG<br>CGTGACCAACATGGTGAAACCCTGTCTCTACTAAAATACAAAAATT<br>AGCCACGTGTGGTGGCAGTCACCTGTAATCTCAGCCACTCGGGAG<br>GCTGAGGCAGGAGAATTGCCTGAACCCAGGAGGCGGAGGTTGCA<br>GTGAGCCGAGATTGCACCATTGCACTCCCACCTGGGCAAAGCGATA<br>CTCCTTCTCAATAAATAAATAAATAAATAATTTTGAGATGTGGTCTTG<br>CTATGTTTCCCATGCCAGACTCAAACTCCTGGGTTCAAGTCATCCTC<br>CCGCCTGAGCCTCCCAAGTAGCTGGGACTACAGCTGTGTGCCACCA<br>TGCCCAACTAGGGAAACACATGGCTTTAGAGTAAACCTTGTACTAT<br>CTTGATATTGATCTTTGATCATGTTTTTTAGTGCTTAAAGTGTCTCAC<br>CACTGCCTTCCTGAAAAACTTCTAATTTCTTAGGCCTTTATAGTTTG<br>ACTCCTGTCCTCCTTTCTAGTCTTTTCCCTCATCACATTTCTCCATTA<br>CCAACTATTTTCCCCCAGTAGTTAGTGGTCTTTGTAGTTTGGATCCA<br>ACTAATGCCAGTTCCAATCGGGCTCTGTCGTTAGCTGTGTGGTCTTG<br>GGCAAGTTACTGTAATGAGAAGAAAAGCAGACTCTAGTACCTGGG<br>GCTGTGGTGTGCATTCCTATAAATTCAGCTTGTTCAACTGCAGGTGT<br>ATTCCTGGTACATGGGTGGAGGACACTGATAGTAACTACTTTAAATC<br>ATTGTTTTCTCATGTGAAAACTGAGGGTAATTTTTTTCAGAATTGTT<br>ATGAAGATTAAATGAGAAAACTTAAAGCATCTGGCAGGGTCCTTGC<br>ACTTAGTAGATACTTAACAAAAACTTGTTTTTGTCGTAGTTCAATTC<br>CTTCATGGTTCCCTGAACGGGGCTTATAAGGTCCTTGTTTTAATCCT<br>TTCTTGCACATGGAATGCACAGTTCTCATCCTTCAAGACTGTGTCTA<br>AAATACCTTCTTTTCTGTGAAACCTTTACATCTTCTGCTAATCTCTGC<br>TGTCTCTGAACTTAAGGTATTGTTTTTACCATTGACTTTGTTATTAAA<br>TCATACTCCACCCTGTGGCATCTTTGTAGTTACCTGTTACTATTTAAT<br>CCTTTTTTTTTTTTTTTACATACTTAATGCTTTGTTCAACTAAATTGT<br>TCTTTAAGAGTGGGGTCTATGTTTAAAAAGGGGCTTCAAAACCATT<br>CCAATTATTTTATAGGACCTGATTTCTTCAGATTTGTTATCTTGAGTA<br>TCTTGTACCCAGAAAATGCTTTGAAATGATTAATGCAAAAGGAAGA<br>GAATGTATCATCACATTTCACCTCTCCTAAATATAGTGGTATTGTGAA<br>ATTCACGTAAGCCAGATGCTTAGTTCAAAGCTGATATATTGGTTTGC<br>TTCTGTGTCCCCACACAAATCTCATCTCTAACTGTAATCACCACGTG<br>TCTAGGGAGGGAACTGGTGGGAGGTGATTGGATCATGGGGGCAGT<br>TTCCCCCATGCTGTTCTCGTGATAGTGAGGGAGTTCTCATGAGAGC<br>TGATGGTTTTAAATGTGGCACTTCCTTGCTCTGTCTCTCCTGCCGCC<br>ACGTAAGACATGCCTTGCCTCCCCTTGGTCTTCTGCCATGATTGTAA<br>GTTTCCTGAGGCCTTTCCAGCCATGCGGAACTGTGAGACAGTTAAA<br>CCTCTTTCCTTTATAAATTACCCAGTCTCAGGTAGTGTATAGCAGTG<br>TGAAAACGGACTAATACGAAAGCTTTTTACACCTTCTTTCCTAGAC<br>AATACAATGGTGGGTGAAGAGAAGATGTCTC
SOD2	CCTCCCAAGTAGCTGGGACTACAGGTGCGTGCCACCACGCCTAGCT<br>AATTTTTGTATTTTTAGTAAAGACAGAGTTTCACCATATTGGCCAGG<br>CTGGTCTCCAATTCCTGACCTCATGATCCACCTGCCTCAGCCTCCCA

	AAGTCCTGGGATTATAGGCGTGAGCCACTGGGCCCTGCCAATTTTT<br>GTATTTTTAGTAGAGATGGGGTTTCACCATGTTGGCCAGGCTGATCT<br>CGATTTCCCGACCTCAGATGATCCGCCCACCTCGGCCTCCCAAAGT<br>GTTGGGATTACAGGCGTGAGCCATGGTGCTGGGCATATACGCAGAT<br>TTCTGACTGTGAAGGGACCAGCACCCCTGACACTGCATCATTCAGG<br>GGTCAACTGTACATTCATATCCCCCTTACTACCTACTAGCTCTATGTG<br>TGACCTTGGTCAAGTCACTGTCTTTGCTTACAGCAGGAATAAAGCA<br>TTTGACTTAGTAGCTAGCAACATGGAGGATCTGCTCAATGATGCCG<br>ACTCCCTTTGCCCTTTATGGACGTAATCAACTCATGATTACCTGCGA<br>CACATGCCCACTTTATCCCTTTGTTTAACAGAACACTTTTGTTCTGA<br>ACCAAGCTCCTCCTTTCCTTTCCAGTAGTTAATGTGTTCTTTGAGGA<br>TGCAATCTAATCGTAATTTTGCCCGAAGTGGGGAAAAAAATTAAGC<br>TCACATTGAAACCTTAATATAGCTCTATAATCATTTTCTCTTTTAGCC<br>TAGATCACTTCATTTTGGCAATTTAAGTACTACTTTTGGTCCTATTAA<br>CAATACCACTCTTGCAAAGGACAGGTTACAACCAATTCTCAGATTC<br>CTGCCTGGATAAGATTTGTGACCCCAAATTTTAAAAAAGCAGAAAA<br>AAACCCCACAAACCACAGGCAGGAAGAAAGAAAAACAAGAGAAT<br>GTAGCTAAGGTAATTCCATTAATGATCAGTTTATGTCAAAGCATGTG<br>GAAATAATGCCTTATAGGAGGATATTTAGCAACAACGATTTTTTTAA<br>ACTCATTTTCTCCTGTAGGTACAGAATTGCAAGTTTTTTCTGACTTC<br>AAATGTGTATAGCTGACTGCGTGTGTGTGTGTGTGTGTGTGTGT<br>GTGTGTGTGTGTGTGTGTGTCAGGGTCTCCTTCTCTTGCCCAGACT<br>GGAGTAAGGTGACATAATCATAGCTCATTGCAGCCATGAACTCTTG<br>GGCTCAAGTGATCCTCCTGCCTTCAGCCTCCTGGCGTAGCTAGGAC<br>TGCAGGCACATGCTACCACACCCAGCTACTGTTTTTTGGTAGAGAC<br>AGAGTCTTGCTATGTTGCCTAAGCTGGTCTCAAACTCCTGGCCTCA<br>AGCAATCTTCCCACCTCAGCCTCTCAAAGAGCTGGGATTACAAGCA<br>TGAACCACTGCACCTGGCCCCATACCTGACTACATTTCTCAGCTTCC<br>TTTGTGGCTAGGCTGTGCTAGGTAGCCTTGTACCTAAATTTTGACCA<br>GTGAGCAGCAAGTGGAATTTGTTGGATGAATCTACCACAAAGGATC<br>CTTACAATGGAGATAGTGGGGCCAGGCATGGTGGCTCATGCCTGTA<br>ATCCCAGCACTTTGGGAGGCTGAGGCAGGCAGATCACTTCAGTCTA<br>GGAGTTCGAGACCAGCCTGGCCAACATGGTGAAACCCCATCTCCA<br>CTAAAAATACAAAAATTAGCCAGGCATGGTGGCACGCACCTGTAAT<br>CCCAGCTACTCAGGAGGCTAAGGCAGGAGAATCACTTGAACTCAG<br>GAGGTGGAGGTTGCAGTGAGCCGAGATCGCACCACTGCACTCCAG<br>CCCAGCAACAAAGCAAGACTCTTGATTCGGAAAGAAAAAATAAAA<br>AAAGGTTGGGGGAGACAGTGGGAGCCCAGACTTTTGTCCTTCCCC<br>TTGCCTAGAAGGGAGATGAGGTTGCTGGTGCTGTGGAAGCTATTAT<br>GGACCATGAGGCAGCTTTGAAGACAGAAAGCCTGCATCCTTGATG<br>ACTCAATGGAATTTCATCCCAACCCAGGACTGACTGCCCCAGATCT<br>TTGTTACATGAGGGAATACACTCTTCTGTGTTTAAGAAACTGTGGTT<br>GGATCTGTTACTGGAATATGAATGCAGTTAATAACTGGCTCAAAATG<br>ACTTCATCTCAACCCACATTGGTTGCTTCTTCCAAACAATATATAGG<br>TCTGTACTGTGGTGGGTCTCAGGATGGCTGTGATGTAGCCTTAGGA<br>AGTTTATCTATGGGAAATCCATATTCATGGTGTCCTGATGTTGCAGA

	GGACATCCTGAGCTGGCTGGAGTAACTTGGGACACAGGTCAATCG<br>ACTGTAATCTAACTTCTGAGGCCATTCAGTACCCTCTACAGTGGCCA<br>CCTAAAAAAAAGGCAGCCAGGTGTGGTGGCTCAAGCCTATATAGAT<br>CCCAGAACTTTGGAAGGCTGAGGTGGGAGGATCACTTGAGCCAAG<br>GAGTTTGAGGCCAGCCTGGGCAACATGAAGAAACTCTGTTTCTAC<br>AAAAAATAAAAAAAATTAGCCAGGCATGGTGGTATGCACCTGTAGT<br>TCCAGCTTCTTGGGAGGCTGAGGTGGAAGAATGACATGAGCCCAG<br>CAAGTCGCTGCAATCAGCCGTGATCACGCCGCTGCACTCCAGCCTG<br>GGCGACAAAAAGAAAAAGAAAACGGAGCCTGTTCACTGGGTGTG<br>GTAGACAAGGTAAACTTTTCTTTACCTCCCATATCCCACAACCTTGG<br>ATGTGCTCACAGTCATGGTAGTGTTTTGTAATGATGTAGCTGATGAC<br>AGGTGTGATGTTGGAGATTCTTCTACCTGACTGCTGCTATCAGTCCT<br>ACCAGCCCCCAACGTTTGGTGCTTGTTCTAAAGGGCATGTCCTAGG<br>AGTCGCTTTAAACTCTCAAAGTATCACTCTCTATACAAACAAGAAG<br>TGCAAGTAAGTAGCCTGAGCTCAGCCTCCCAATAGGAATATTTCATT<br>ATCACTAGATCAAGTCTTTCCATTACAATGACTGATCTGTCTCTGAA<br>TCCTGTGGATTCATCCTTCAAAATGCCCTTTTCTTCCAGTTCTCATA<br>GCTAGTGCCCTAAAAGTGACCTGCAGTACCTCCTGCTGAGACGAAT<br>GTACCAGCTTCCTAACTAGCCTGCACTCCCTTCATCCCCCCAAGTC<br>AGTGCCAGACCACCTTGCCTGAAAAACCACTTTCAGTGTGTCTCAC<br>CTCAGCAGAAATGTTTCTCAGCTTCCAATTAACAATCACATCAAAC<br>CCCTGCTCTTGTCTGCGTTTTAAGGGTATCTATAGGCCGGGCGCCGT<br>GGCTCCTACCTGTAATCCCAGCACTTTGGAAGGCCGAGGCGGGCA<br>GATCACTTGAGGTCAGGCGTTCGAGACCATCCTGACCAACATAGTG<br>AAACCCCGTCTCTACCAAAAATACAAAAAAAAAAAAAAATAGTGGG<br>GCGTGGAGGTGCACGCCTGTAATTCCAGCTACTCGGGAGGCTGAG<br>GCAGGAGAATCGCTTGAACCCGGGAGGCAGAGGTTCCAGTGAGCC<br>GACATCGCGACACAGTACTCCAGCCTGAGCGACAGAGCGAGGCTG<br>TGTCTCAAAAATAAATAAATAATAAATTAAAAAAATAAGAGTATCTA<br>TAACCTGGTCCCAGCCTGAATTTCCTTTTTCACCCCAACACGTAGC<br>CCTAGTTACATTCTTCTGACGTCTGTAAACAAGCCCAGCCCTTCCTG<br>TTGTGAAGCCAAGTTCAGGTGGTTCCTCTTCGCCTGACTGTTTTCC<br>CATTCCACTTACCGGAAGCCTAGTCATCCTTCGGAGGGCTGTACGG<br>GGGTTGCAAGAAGCAACGGAAACGGTTCAGCACCTGCTACCTTCC<br>ATCATATTCTTTTCAATAAAGGGGCAACTCCCGCCAATGGCAGTGTA<br>GATTTCCTAACCTCTACACATGGAAGATTCACACCATTCAGGATTGT<br>TGTTTAACTGTTGAGAGAGCACTTGATACTTAACAGCTTACTAGGCT<br>ACAAGACAGCGCAGGAAAGAATCCTCTGTTGTCCTTTTATGTTATC<br>CTGAACAGTTGGTTCACAGAGTTACTGTAAACACACAAAACATGA<br>CTGCCAGGGCTTAGTAGTGAGGAAGGTGGGAACTAGTCCTGACTC<br>AGTTAACTGTGCCCAGGAGAAGCTGCTTAACCTCAAAGGATTTCAC<br>TATTACTAGAATCAATAATACCAACCCTAGGGGTAAAAATAAAGATA<br>AATGTGTGCAAATCCTGCCTGCAGTCTCGGGCACGTCGTGGGTGTC<br>CAAGAACTGTTCTTAGGCAGCCGGTGGGGACAAAGTCTGTGTGCC<br>TCCTGTCCTGGAATAGGTCCCAAGGTCGGCTTACTTGCAAAGCAAG<br>GGTACGGCGCAAGAGTACTGAATACGGGTTGGAAGGGCGCTGGCT

	CTACCCTCAGCTCATAGGCCGGCTGGGCGGCGCTGACCAGCAGCTA<br>GGCCCCGTCTTCCCTAGGAACGGCCACGGGGGCCCTGGGAGGGTA<br>TGAATGTCTTTTTGCAGTGAGGCCTCTGGACCCCGCGGCCCCCCGG<br>CAGCGCAACCAAAACTCAGGGGCAGGCGCCGCAGCCGCCTAGTGC<br>AGCCAGATCCCCGCCGGCACCCTCAGGGGCGGAGCCGGAGGCAG<br>GGCCTTCGGGCCGTACCAACTCCACGGGGGCAGGGGCCGCCTCCC<br>TTCGGCCGCGCGCCACTCAAGTACGGCAGACAGGCAGCGAGGTTG<br>CCGAGGCCGAGGCTAGCCTGCAGCCTCCTTTCTCCCGTGCCCTGGG<br>CGCGGGGTGTACGGCAAGCGCGGGCGGGCGGGACAGGCACGCAG<br>GGCACCCCCGGGGTTGGGCGCGGCGGGCGCGGGGCGGGGCCCGC<br>GGGGGGGGGGGGCGGGGCGGCGGTGCCCTTGCGGCGCAGCTGGG<br>GTCGCGGCCCTGCTCCCCGCGCTTTCTTAAGGCCCGCGGGCGGCGC<br>AGGAGCGGCACTCGTGGCTGTGGTGGCTTCGGCAGCGGCTTCAGC<br>AGATCGGCGGCATCAGCGGTAGCACCAGCACTAGCAGCATGTTGA<br>GCCGGGCAGTGTGC
SOX3	AGAGCCTTCCTTCCTCCGGGAAAATGGCAACTTTCGGGTGGTGAG<br>GTCAGCGAGTAAAAGCAGAAGGCAAAGGTTGTCAAATTCCTTGGA<br>GTTGCTGCGCACATAGGGAAAAGAGAGCTGGTGATTAACACCTGA<br>ATACTCTGTGGGTGGAAGGAAACTGAGCGCCAACGTGTGGGCCGA<br>GGGGACTCCCAGTGTATTTTCCAAGTACAGCTCAGCCAACCAAGTG<br>AATTGAATGCGGAAACTTTTTTAAAAGTACCCCCGCACCCTCCCAA<br>AAAAGAGCAACACCAAACTACGATAGAATACAGTTGTACAGCCCG<br>CAGGAGACTTTATGGTACCTCGAGGACCTTGTTTTCCCATACCTCGC<br>AGCTCCTAGCAACCTTTCTTTAGAGACCATCGACTAAGTTATTACAA<br>TCTACACCCACCAGCCTAGGGTCTTCAGCTGCTTCTGTGACCTGTA<br>AGAAAGAGTACTTCCGCTGGAGACGGTGCTACGCGTTGGCGGAGT<br>GGGGCTGGGGTTCCTACTGAATGCTCCAGGAACTGGAAGGTGCTG<br>TCTCTACCACCCCGTTGAAATGATGGTGACCTTTCTGACCTTTTTTT<br>TTTTTTTTTTTTACAAGGGATGGTTCTACAATAGACCTAAGCCTATG<br>ATTTTTTGATCAAGGCTCTCGATCAGCGGCCTAAATACACAATCTTG<br>GCTAGAAGCCAGAGTAAACAGACAAGGGGTGTTCTCAAGGGAAA<br>AGATAGAAATGACTGCTTTAAGAAACGCAAACTGTTGCGGGTTTGT<br>TTTTGGTTTTGTTTGTCTGTTGAAAATCAAGGCGTCTCCGAGAGTC<br>CCCAGGATAAGTTGTTCTCACTTCCTAAAAGCCAGACCCCGAGCTG<br>GGCTTGGCTACACTTGAGAAACCTGAAAGGCGAACGCTAGGCCAC<br>AGCCGGAGGGCAACCTTTGCTGACCCTATTGTCAGGCTTCAGCGG<br>GCCCAGGCGAGCCCAAATACCGCCCACCCCACCCCACTCTCCGAC<br>TCTCCGTCCCCCAAGCGACACAGACACACATCGGCCCCAAACCAC<br>CCAGCACGCAGAGGACACCCTTCTTCTTCTTCTCCTTCTTTTCTTTT<br>CTTTTTTTTTTTTTTTTTTGAGACGGAGTCTCTCGCTCTTGTCGCCCT<br>GGCTGGAGTGCAGTGGCGCAATCTCGGCTCACTGCAACCTCCGCC<br>TCCCGGGTTCAAGGGATTCTCCTGCCTCAGCCTCCTGAGTAGCTGG<br>GATTACAGGCGCACGCCACCAAGCCAGGCTAATTTTTGTACTTTTA<br>GTAGAGACGGGGTTTCGCCATGTAGGCCAGTCTGGTCTCGAGCTCC<br>TGACCTTAGGTAATCCACCCGCCTCGGCCTCCCAAAGTGCTGGTATT<br>ACAGGCTTGAGCCACCGCGCCCGGCTGAGGGCACCTCTTCTAATAA

	GTTGGGTCCTAATCATGCCAGGGACCTTGAAAAAGGTCCCCTATGA<br>GCAGTTACTGAAGAGGAATCAATGCAGATACCTCCGCGCGAGTCGT<br>GGGACAGCTCCTTTCCTGCTCTTTCCACCAGTAGCTCTAAGGCACA<br>ATTGCTGAGTGTTCTTTGCAGATATAAGGCACTTGGGACACCTGAT<br>GTCGGCCAAGCCTAGAAATAGGAAGAAGGCAGAGCAGGGGTGCC<br>CTATGTACCCAAAGCCCATTGGGCCAGTCACGCTAGAGTCTTGGTC<br>CCGGAGTTTTGCTATAACCCGCCCTCTAAGGATAGGCATCACATCTG<br>CTCCGCCGCAAATCCGCTTGGTCTCTTTCGAAGGTTGGGGCGACCT<br>CCAAGCCCCGCAGGGCGAAGCGGCAAGAGGCTACGCTGGGTGAA<br>GTTTCCAACCGAAGCCAACAAACGGCGCTATTGTCGCGCCCTGGG<br>TTCTGGGAGCCCCAGGGCTGGCTCCAGTCGGAGGGAGCGGGAGCT<br>CTCTAGGAAGGGCGGGGGAATGGGGAGGAGGCCTTGGTCGGGTTC<br>CCAGCGCCAGATCCGGAGCTCTGTGAACCGCCCAAGGGCACTTGG<br>AGGCTGGAGAGGGCGAGAGAGCTGCTGAGCGCGCTCTCTTCTCCT<br>GAAGAGGTTGAAGGATCGGTCCTGGTTCCCACTGCGTTCCTCTGCT<br>TTCCTCGCTGGGTGCAGGGTGCGCGTCGGAGACACTGGAGTTGGG<br>GCTCCGCCAGCTGCCCTGCCTTCCCGCAGCCCCAGCCGGAACCGA<br>GGCCTCCTGCGCTGTGGTACCTGTCGCGACACCTCTCCCGACCCTT<br>AGCGAAGGTTGCGCCGCTGGGCTGGTGGCTAAGCCTGCGCTGCAG<br>GTGGGCTAAGAACTGATTTATTTCCAGCCTTCCCCCATCTCCCCACG<br>CCCGCAATGGTAACCTTGCTTGTCTATTGAAATGATAGCTTGTATTT<br>AGAAAGTGTCCTGGCAAGAGTTTCCAAGGAAATCTGGCGTGGCTC<br>TTACTTGAGAATGTGCAGATCACCCCAGAACCACCAGATACGGAGA<br>TTTTATGTATCTACCCCCCGAGAGGGAGCTGGCCTCAGCGAGAAGC<br>CGGGAGAATTGGGAGAGTGTGCCTGGTCCTGGCAAAAGACTGCTG<br>TAAATCTGGCAGGAGGATGTAGGGCAAAAATGAGCTCACCTAAGC<br>CTCGGGCTAAGTGTGATCAAATGTCAGACAGCTGGGAGCAGTGAC<br>TAGTTTCCCCTGGTAATCTGCTGCGGGGAGGGTGTCGGCCCAGCAC<br>CGTATCCCCTGCCTTGGGAAATATGCAGCCCAGGCGCTGTCACTGC<br>AACGTTTACTTGGGTCCTAAAACTTGGAAAGAAACCTGACATCTGT<br>GAAGCTACTGGACGCCAGGCCGCGCGTCGGTTGGGTAGTTTCCTA<br>AAACGCGCTTCACTTAGCAACAGCCTCCAGGGCCCAGAAAAGCCC<br>AGAGAGGGCATGCGGGAAAGACGGCAGGAGGGCAGAGATATTGC<br>ACATTGGCTTCTGTAGGAGAGGATGGATTTGCCTAAGAAAGTTTGA<br>CCTTGTCGGCCACTTCGCCAAGAAAACAGGTGCAGGTAGGGATTG<br>CCTAATCATTATGTTTTCTGAGACGTACTTATCAGCTCACGTTCGCTA<br>CGCGCGCGCGCGCGCGCGCGCACACACACACACACACACACACA<br>CACACACACACTGTATCTACACACAGAAGCCTTCCAGAGAGTTTAC<br>TTACATTTAAAACCCTAAATCCGTATACCCATCCAGTTGACTGCAGC<br>CCTTGGCTGCAGAATACTCAGATCAAGTGGAACACAAACTTAAGG<br>GGCAAGTTTCCAGACCCCAAACTTCCGAGAGATGATGCCGACTGG<br>GAGCCGGGATTCTTTTGCCAGAGGTCCGCCTGGCCGAGAGGGGGC<br>TGGCCTGCCCTGAGCCTCCACTCCCCCCCTGACCCCACTAACAAGC<br>CGAAGAGGCGTACTTTTTAATTCGTGGAGAGAAAGGGGGAAGAGC<br>CCAGCATGCAGAAGTCCCGGGATCGCCTGGTCTTGCATTAACGCTA<br>AGACGCTAGAAACCTGGGATTCCCCCATGCGCCCCCCACCCACTTA

	AGATTATGCTTTCCGTTTAGTTCCCCACCTTTCCACGGGGCTAGGGA<br>TGATGGGGGGGCGAGACCCCACACCGTATGGACACCTTATTTTAGT<br>TTCTGCCAAATCTGCCGGGATGGCTTCTCATTCCCACTCGCTACCTC<br>GGGCTGCTCCGAGAGTTTCAAACGCTGTGCCACTCGCTTCAGCTG<br>GCAGTGGCGATTATCGGGGTTTCCTCTCCCCGCAGCCAGAGTTCCC<br>GCGCCGCTAGTCTTTGGCAGATAACCCAACAAGCAAGTGAATGTCC<br>CGCTCCGAATCTCAGGAAGTCTCTAAAACAATAAAAGAGACGGAC<br>GGAATCCGTTAGCAGAGCCTGCTCCCGGGGGAGGCGGCGAGGCTG<br>CGCGCCCAGAGTAGCGCTCAGCGGTCCCGGCAGGTGCGGGGGCAG<br>TGGAGGGGGCCCGGCGGTTCGCCCGGAGGACGGCCTCGGCTCCGC<br>TCCGGCCAACGTTCCCCCCTCCAAGAAACTTTTCTTTCACTCTGAG<br>AGGCTCCGTGTTGCCATGAAAACGGATTTTCCAAGGGGCCCGACC<br>CTCCCCCTCAGGGGCATCGGACTGGAAAAACCGAAATGGCCACAG<br>AGCAGAGACAAAAAGGGGCCGCTAAGCGGGGGCCAGGATCGAGA<br>GAGCGGAGGCCAGTTCACCCCCTACGTCCCACACATACTTTCCACA<br>CCACCTTGTGCGAGCGCCTCCGACAGGCTCAGGAGACGGGCGACT<br>GACTGCTTGCCCGCCCTCCTGCAAACCCCCAAGAGCCTCACAGCC<br>GAATGCACCCCGCAGTCCTGAAGCCTGTCTCCCATGCCCGGGCCCC<br>TGACCCACGCTGCGTCCTGGACGCACTGGCCCTCCTAGAGAAAGA<br>TCTGGACAACCAGCGGCCAATTCCAGGGAGAGCTTCTTGGAGACG<br>CAGCCAGGAGCACTTCCAATGAGCCTGGAGTCCCTAAGCCGGCCT<br>GGGTAGCTGGTGGTGCCCTGCGTTCCTAGCGCTGGCAGCAGAAGC<br>GGCCAGAAAACCTCCGTGCAGACAACGGGAAGCTTTGCGGGCTGG<br>CCGGGGGATGGGGCGCCGGTCTGCCTTGACAGGGTTGCAAAGTTG<br>TTTTCTAAATTCCGAAGGCGCCCCTCTGCCCCCTCCCCCCAATCTGC<br>TTGCGTGCCCCCTCCCCCCTCCCCCCGTCACCTCCTCAGGTTTCGTT<br>CTTTCAAACTTTTTGAAACCCTAATTGGTGGCCTCTGAGTGGGCCT<br>CGTGGACTCCCGCCTCCTAAGTAACTCTTACCACGTCACTAGGCCA<br>AAGAGGGGCGTGGGGTGAACGAAAGGGCTCCCCGAACTTTTTTTT<br>TTCCAGCCAGGCCGAACGGGGGCTCGGTAATGATTGGCCAGGGCG<br>CATCACTGCGAACCTGTCAATCACGGGTCCTCCGGGTTGCGAGGG<br>GCGGACCAAGCCCCAACCCCGGGGAATCCGAGCAGGTATATAAGG<br>GGCCCAGCTAGAGCCCAGGCAGACTGTGAATGCGACCTGTTCGAG<br>AGAACTCAT
SP1	CCCAGCTACTGGGGAGGCTGAGGCAGGAAGATCACTTGAGTCTGG<br>GAGGTTGAGGCTGCAGTGAATCATGATTGCACCACTGCACTCCAGC<br>CTGGGCGACAGAGCAAGACCGTCTCAAAAAAAGAAAGAAACATTT<br>AACAGGGACTTACAAACAGAAGCCCTGTTTGTATCTCTGGCAGTGG<br>TGAGACAAGACAGTGTATCCCTGTGCCATTAATCTCCAGATCCAGA<br>ATTTATATACCATAGGGAAGGAATGTGTAGGACAATTGAAGTCGAC<br>CTCTATGGGAAAGGCAAGAATGTTCTGTGAATCTGCCTAAGGGCAA<br>GATTCATGGTCAAGGTGGTCTTGACCTAAGGGCAGGATTTATGATA<br>AGTATATGCTCTTACCCAAAGATCAGTAGATAAAATAAAAATTTTAG<br>AGGCATTCCTGGAGCTGGGGTTATTCAGAAGTCAACATAGCAGATT<br>AACATCCAAGATGGAGTTGCTTCAGCCTCCATTCTGTAAAACTCTA<br>GAAAAAAATAAATCTAGGGGCCAGGCACAGTGGCTCATGCCTATAA

	TCCCAGCACTTTGGTAGGCCGAGGCGGGTGGATCATGATGTAAAGA<br>GATTGAGACCATCCTGGACTCCATCTCAATTAAAAAAAAAAAAAAA<br>TTAGCTGAGCGTGGTGGCAGGCAACAAGAACAAAACTCCTGTCAA<br>AAAACAAAACAAAACAACAACAACACCTGCAGCGTGTGTAGAATT<br>TAAAAAAAGACAATCCATAGGGAGAAAATATTTGTCATATTTTAAAC<br>AAATAATTAATATCTAATACACATAACAAGCTTCTTTAAGTCAAACA<br>ATGCAATACAAAAATATAGCCTGAGCCAGGCGCGGTGGCTCACGCC<br>TGTAATTCCAGCATTTTGGGAGGCCGAGGTCGGTTGATCACTTGAG<br>GTTGGTAGTTCAAGACCAGCCTGACCAACATGGAGAAACCCTGTC<br>TCTACTAAAAAATATAAAATTAGACGGGCATGGTGGCACATGCCTCT<br>AATCCCAGATGCTCGGGAGGCTGAGGCAGGAGAATCACTTGAACC<br>TGTGAGGCAGAGGTTGCTGAGCCGAGATTGCACCATTGCACTCCA<br>GCCTGGGCAACAAGAGTGAAACTCCATCTCAAAAAAAAAAAATGTA<br>GGCTGAGCACAGACCCTGTCTCTACAAAAAATAAAAACAGTTGGC<br>TGGGCACTCGCAGTGGCTCATGTCTGTAATCTCAGCACTTTGGGAG<br>GCCAAGGCAGAAATATCATGAGTCCAGGAGTTTGAGTCCAGCTTG<br>GGCAACGTATTGGGACTCCATCTCTAGAAAAAATTTTAAAAATTAG<br>CTGGGTGTGGTAGTCTGCGCCTGTAGTCCCAGCTACTTAGGAAGTT<br>GAGAGAGAAAGATCACTTGAGCCCAGGAGGTGGAGGCTGCAGTG<br>AACCATGACTGTACCACTGCATTCCAGCCTGGATGACACCTAAGAC<br>CCTGTCTCAAAAATTTATTTTAAGGTTGGGCAAGGTGGCTTATGCCT<br>ATAATCCCAGCACGTTGGGAGGCCAAGGCAGGAGGATCCCTTCAG<br>CCCAGGAGTTCAAAACCAGCCTGGAAAACATAGTGGGACCTTTTC<br>TCTACAAAAAAAAAAAAATTAGCCTGGTATGGTGACAGGCACCCAT<br>GGTCCCAGCTACTTGGGAGGCTGAGGCAAGAGGATTGCTTGAGCC<br>CAGGAGGTGGAGGCTGCAATGAGCTGAGATCCCGTGACTGCACTC<br>CAGGCTGGGTGACAGTGAGACCGTATCTCAAAAACAAAAACAAAC<br>AAATTATTTATTTTTGTTTGTTTTTGTTTTTATTTATTTTTTGTTTTGTT<br>TCTGTTTTAAGACAAAAGCCCTGTCGCCCATGCTAAAGTACAGTGG<br>TACAATCTGGGCTCACTGCAACCTCTGTCTCCCAAATTCAAGCGAC<br>CCTCCCACATCAGCCTCTGGAGTGGCTTGGGACTTCAGGTGTGCAG<br>CACCATGCCCAGTTAAAAAAAAAAATTTCTTTTTAATAAAAATAAA<br>AATATGGCCGGGCTCAGTGGCTCATGTTTGTAAGCATAGCACTTTG<br>GAAGGCCAAGGCAGGTGGAATGATTGAGCCCAGGAGTTCAAGACC<br>AGCCTGGTAACATGGCAAAACCTCTTCTCTACAAAAAATACCGATG<br>ATTGAGCCACTGCACTCCAGCCTGGGCGACAGAGTGAGACCCTGT<br>CACAGAAATAACTGAAATTCACGCCTGTAATCCCAGCACTTTGGGA<br>GGTCGAGGCGGGCGGATCATGAGGTCAAGAGATCAAGATCATCCT<br>GGCCAACATGGTGAAACCCCGTCTCTCCTAAAAATACAAAAATTAG<br>CTGGGCATGGTGGTGCACGCCTGTAATCCCAGCTACTTGGGAGGCT<br>GAGGCAGGAGAATTGCTTGAACCCGGGAGGTGGAGATTGCAGTGA<br>GCCAAGATCATGCCACTACATTCCAACCTGGTGACAGAGCAAGACT<br>CTGTTTCAAAAAGAAAAAAAAAAAAGAAAAGAATTTGTTGGGCACG<br>GGGGCTCATGCCTATAATCCCAGCACTTTGGGAGGCCGTCCAGGCA<br>GATCACAAGGTCAGGAGTTTGAGACCAGCCTGGCCAATATGGTAA<br>AACCCCGTCTCTACTAAAAATACAAAAATTAGCCAGGTGTGGTGGT

	GAGCGCCTGTAGTCCCAGCTACTCGGGAGTCTGAGGCAGAAGAAT<br>CACTTGAACCCGGGACGGGGAGGTTGCAGTGAGGCGAGATCATGC<br>CACTGCACTCCAGCCTGGGCAACAGAGCTAGACATTGTCTTAAAA<br>AAATAAATAAATAGGCTGGGTGTGGTGGCTCATGCCTGTAATCCCA<br>GCACTTTGGGAGGCCAAGGCGGGCGGATCACAAGGTCAAGAGATT<br>GAGACCATCCTGGCCAATGTGGTGAAACCCCGTCTCTACTAAAAAC<br>ACAAAAATTAGCTGGGCATGGTGGCACACGCCTATAGTCCCAGCTA<br>CTCAGGAGGCTGAAGCAGGAGAATCACTTGAACCCAGGAGGCGG<br>AGATTGCAGTGAGTCAAGGTCGTGCCACTGCACTCCAGCCTGGGC<br>GACAGAGCGAGACACTGTCTCAAAAAAAAAGGAAGGAGCCAGGT<br>GTCGTGGCTCATGCCTGTAATCNCAGCCCTTTGGGAGGCCGAGGTG<br>GGTGGATTTCTTGAGGTCAGGAGTTCAAGACCAGCCTGGCCAACA<br>TGGGGAAACCCTGTCTCTACTAAAATTACAAAATAGTCAAGCGTGC<br>TGGTGCACACCTGTAATCCCAGCTACTCAGGAGGCTGAGGCAGGA<br>GAATTGCTTGAACCTTGGAGGCGGAGGTTACAGTGAGCTGAGATT<br>GTGCCACTGCACTACAGAATAGGCGACAGAGCAAGACTCTGTCTC<br>AAAAAAATAAAATAAAATAAAATAAGGAGCTAGATTTTGAGTGGGA<br>TTTTTGAAGGTGAAGCATTGAAACCATTACTCTTAGTCTCTGCCCTG<br>CCTTACTTTTAAAACTGCTAAGTTCTACCTTCCTTACCGAGTACTCC<br>CCAACCTTTATCTAGTTTTCTTCCTCACGCCCCCGCCAAAAGGAAA<br>ATATACTTGAAGTTTTTTGTTTTTTGTTTTTTTTTTGAGACAGAGTCT<br>CTGTCGCCCAGGCTGGAGTGCAGTGGCGCGATCTCGGCTCATTGCA<br>AGCTCCGCCTCCCGGGTCCACGCCATTCTCCTGCCTCAGCCTCTCC<br>GAGTAGCTGGGACTACAGGCGCCCGCCACCACGCCCGGCTAATTTT<br>TTGTATTTTTAGTAGAGACAGGGTTTCACTGTGGTCTTGATCTCCTG<br>ACCTCGTGATCTGCCCGCCTTGGCCTCCCAAAGTGCTGGGATTACA<br>AGCGTGAGCCACCACGCCCAGCCACTTGAAGATTTTTCTTGTTTTT<br>GATCACCTTCTCATACTGGCCTTACTCAGCTTAGTAGAGTAAGCACT<br>GAATTGGGTAAGGTAACCTCACTTCCTAACATGTCAGCTTCTTATCT<br>TTTCTTCCTTTACCTAGGTGAGAAGAAATTTGCCTGCCCTGAGTGTC<br>CTAAGCGCTTCATGAGGAGTGACCACCTGTCAAAACATATCAAGAC<br>CCACCAGAATAAGAAGGGAGGCCCAGGTGTAGCTCTGAGTGTGGG<br>CACTTTGCCCCTGGACAGTGGGGCAGGTTCAGAAGGCAGTGGCAC<br>TGCCACTCCTTCAGCCCTTATTACCACCAATATGGTAGCCATGGAGG<br>CCATCTGTCCAGAGGGCATTGCCCGTCTTGCCAACAGTGGCATCAA<br>CGTCATGCAGGTGGCAGATCTGCAGTCCATTAATATCAGTGGCAAT<br>GGCTTCTGAGATCAGGCACCCGGGGCCAGAGACATATGGGCCATAC<br>CCCTTAACCCCGGGATGCAAGGTAGCATGGGTCCAAGAGACATGG<br>AAGAGAGAGCCATGAAGCATTAAAATGCATGGTGTTGAGAAGAAT<br>CAGGAGAGGGATACAAGAGAGGAGATGGGGTCCCGGCACCCATCT<br>GTATCATCAGTGCCTCTTTGAAGGTGGGAAACATTAGTGAAAATTC<br>TGTTGGTGCCACGCTTTGATGAGCATTTGTTTGACCCCAGTTTCTTC<br>TTACACTTCTTACCCCAGCCTACCCTTCCTGCATTTCTCTTCTCAGC<br>TCTTCCATGATGGATTCCCCCCCCTTTCCTAAAGCCATCATGCCTTG<br>ATAAATATATATGATCATTGAAATACTTTTTAACAAAAAACAGATTCT<br>ATATTATTATATATATATATATATATATATAAAGATATATAGAGATGCATT

	CACAGGGGTTGGCTGGGAGGAGGAAGACCATTCTGTGACCAAAAT<br>ACCTTGGTCATTTTTTTATATTGCCTTATTTCCCTATGGCTGAGCCT<br>TGTTGTGACACATCAAGCTTTTCTGTAGATGTTGTCTTGGCTTCCCA<br>CCAGCTTAAGCGTTCATATGCTCTGCTTTTAGTTCATATATACATACA<br>TAATGTTTTTCCTTTCTTAATTTTGTCTTTTTGTTTGGGATCAGCTTC<br>TTGCACTCCTTCCCTAAC
SPIB	AAAGCCACAGTGTGAAGGGATGTTGCTGCTTAGATTCTCCTGAGGC<br>TGGGGCCTTGGTTTGGGAAGCCTCCAAGGCCTTGGGCTTTGTTACC<br>CTGGATGGCATCTGTTCTTTGAAAACCCTGTCGCTGTCCCTGGTTTT<br>GAGGTGTCACAGTGTCCCTGGTTTTGAGGTTTCCCAGTACCTGTCA<br>GATGTGGAGCTGCCCTCCACGCAGGGACCCTGATGGGGGTGCACC<br>TTAGATCTGTGCACCATTGCCCCTGGCCCTCTGAGTGCGGCACACT<br>TGGTCCCAGGAAACCTTGCACCATATTTTCTTTTGAAGAGTCTGGG<br>TCTCGCTCTGTCGCCCAGGCTGGAGTGCAGTGGCGAGTTCACAGC<br>TCACTGCAGCCTCCAACTCCTGGGCTCCGGCGATCCTCCCGCCTTG<br>GCCTCCTGAGCCGCCACGCCCAGTTCTCACACTGTACTGTTTCCCC<br>GTGGCTTAACGCTCTCTCGTGCCAGGCCTTGGACTTGGAGCCTCGC<br>AGAGTGGGGAGGTCTCTGCCTCAGGGCTCACCCAGCCGTAGTTTC<br>TCCTCTCTTTCATGGGATGCTTCATCGACTCGCCTGTCTGGATCTCA<br>TGTTTTAGGGACTATCTGGATTTATCCCAGTCTCTGTTTCAGATTGG<br>GGTCTCTGGTGTGGGGGCTCTGGCATTGCCGAGGGGCCTCAGGAG<br>CCACTTCCAGCCATACAGTGTCTCGGTCCATCCCACCAGGCTCAGG<br>GCACGGCTCCCATGTCCTCAAACTGCGTCTGGGACCCTGTCTACCT<br>TCAGTTTCTGGGGGGCGTCTCCAGATTGGGGCTTGGCCTCCTCAGG<br>CTCAGGGTCTTGGCCATGGCTCCCTCCCAGGTCTACTTCCCATACCT<br>GCTTATCAGCAAGAAGCGCTACGCGGGCCTGCTCTTCTCCTCCCGG<br>CCCGACGCCCACGACCGCATGGACTGCAAGGGCCTGGAGGCCGTG<br>CGCAGGGACAACTGCCCCCTCGTGGCCAACCTGGTCACTGCCTCA<br>CTGCGCCGCCTGCTCATCGACCGGTGTGTGGGGCCTCCTCCCTCAG<br>ACTCAGGGGGCTGGGCCCCAAACCCCTCCTCCCTCAGACCCAGGA<br>GTCTAGGCCCCAGCCCCTCCTCCCTCAGACCCACGGGTCCAGGCCC<br>CCAGCCCCCTCCTCCTCAGATCCCGGGGTCAGGGCCCCCAGCCCCT<br>TCCTCTCTTAGATACAAGAGTCCAGACCCCCAGCCCCTCCTCCTCT<br>GGGAACACAGGTGTCAGGCCCAGCTCTTCCTCGGTGGTGAAGCAG<br>TGGAAAGAGTCGGCTTGGGCAGCTGTGGGTTCAGGCCCTGCCTCT<br>GCCACTCTGCAGCCTATGGTAGGAAGGGCCCCTCTCTGCCCTGAGC<br>CTCAGCCCTAAGAGCTCATCCTGGTCTCCAGCCCTGAGACCCGTGG<br>AGGCACCAGCCTGGCCCTCAGTGCCTTTTGGTGACGCTGTGCGGC<br>CCGCTCTCCTACAGAGACCCTGAGGGCGCGGTGGCTCACGCACAG<br>GACGTCATCTCGGACCTGCTGTGCAACCGCATCGATATCTCCCAGC<br>TGGTCATCACCAAGGAGCTGACCCGCGCGGCCTCCGACTATGCCG<br>GCAAGCAGGCCCACGTGGAGCTGGCCGAGAGGTCCTGCGCGGGG<br>CGGGTGGCCTGGCCAGAAATAACCCCCTCCTTCCTGCCAGCTGGGC<br>CCACTTCCTACACCCTCGCCCCCACCCCCGCCACCCACCTGCCCTC<br>ACCCACCCGCCACCCCATCTCCACGCAGGATGAGGAAGCGGGACC<br>CCGGGAGTGCGCCCAGCCTGGGCGACCGCGTCCCCTACGTGATCAT

	CAGTGCCGCCAAGGGTGTGGCCGCCTACATGAAGTCGGAGGTCAG<br>GCCCACCTGGCTGCCTGCTCCCGCCCAGCCCCCTCGCTCTCACTTC<br>TGCTTTCCGAGATGGGCGGGCCTGCGGGAAGGGTGGGGCCTCCCG<br>TGCCCTGTGGGGCCCTGAGAACCGCCCCCCATGGCAGCCTGGGTG<br>TGGCCTCGGCCCCCTCTGGAGGTCCCCCCTCTATTCTGACCCCTCC<br>CTTGCTTCCCTATGGAAGGGGCCCCAGTCCCTCCCCTGTGCATACA<br>GCTCCCCAGCCGGGGGTTCCCTTGGATTCATAATCCTCCAGCTCTAC<br>CCCCACCCCCAGTGACCCACATCTTAGCCCCATGACCTCTGACCTC<br>CTGACCCCTTCCTCATCCTTGCTTCCCCTTGTGAACTCTGACCCTTC<br>CGTGGTGACCTGAGAGCCCTACAGACTCTGTGGCCCAGAGACTCC<br>GTGACCTCCGACCCCATCCCAGACCCAGGCCCCCCCCATGTCACAG<br>CCCGCAGGCAGGCCTAGGCCCTAAGCCCCAGGCCCCATGACCACC<br>CCGTGTCCACCCCGGTGCCCTTTCCCTGGCTGCCCGGGTGTGACTG<br>CCATGTGGCCGCAGGACCCGCTGTTCGTGCTGGAGCACAGCCTGC<br>CCATTGACACGCAGTACTACCTGGAGCAGCAGCTGGCCAAGCCCC<br>TCCTGCGCATCTTCGAGCCCATCCTGGGCGAGGGCCGTGCCGAGGC<br>TGTGCTACTGCGTACGGGGGCACCAGGGGACTGGGGGCACCCTGG<br>GGGGGCAGAGGAGATCACCGGCCCACCACCTGCCTCCTCTCCTGC<br>AGGGGGGGACCACACGCGCTGCAAGACGGTGCTCACGGGCAAGG<br>TGGGCGGCCTCCTGGCCTTCGCCAAACGCCGCAACTGCTGCATTGG<br>CTGCCGCACAGTGCTCAGCCACCAGGGTGAGCGGCCCTGGCCACT<br>GGGCCCCCACTGGCCCTCAACCTGCTCTGCCGTCACCCCAGATCCT<br>AGACACCCACCCCATCGGCTGGCACTGCCACCCAGTGGGCCCAGG<br>GCCCCTGGGTGGGTGGCCCCTGTCTCCACCCCCCACAGAGGGTCC<br>TCGGCCCCCACCCCGGGAGTTCCCCAGGGGAGTTTTCCCAGCACA<br>CTTGCTCCGTTGTTCTCCAGCCTCTGGGGACTTTCAGAAGCTGGGA<br>TTGGCAGTGGGCAGGGATGGGGTGGCCCAGTTCCTGGCTGGGCCC<br>CAGCACTTGGGCTGACCCGCCTCCCCACAGGAGCCGTGTGTGAGT<br>TCTGCCAGCCCCGGGAGTCTGAGCTGTATCAGAAGGAGGTGAGAG<br>GGCCGGGAGGTGAGGAGGGGCCAGGTGGGGAGGCGGGGGCGCCC<br>TGCTCAGCCGCTGCCGTCCCCAGGTATCCCATCTGAATGCCCTGGA<br>GGAGCGCTTCTCGCGCCTCTGGACGCAGTGCCAGCGCTGCCAGGG<br>CAGCCTGCACGAGGACGTCATCTGCACCAGGTGTGTGCCATGTCCC<br>GACCCTGGGCTGCCCCGCCCCTTCCCAGCTCCCAGGCCTGTGGGTT<br>GTGGACCCAACCTCTGACTCCAAGGCCTCTCCTGAGCATCCTCCCC<br>GCCCATCCCCTTCCAGCTGTGAGCTGTCCTGAGCCTCAGTGTCCTT<br>GTCTGAAAAATGGGCCCATCCCAGCCCCCCAGAGGGGGTGGGTGC<br>TTAGGGAGGCAGCGCCCGGCACCCAGTGTTGCCCGGGAAACGGCT<br>GTCCTCCCGACACACCCAGCCAGCCTGGCCCCCTCCCCAGGGCGG<br>GGCCGGGCTTTCCCCAGGGAACGGGTAGGCGGGGTGGGTTCCCAC<br>GCCAGGTGACAGGTGATATACGGCCAGCCATGGCCCTGCACCTCGC<br>CAGGCACCCGCTGTTATCGGCTCCCCCCCCCCACCCCCCCCGTGCC<br>TGCTGAGCAAACAGCCCGCTGCGGGAGGGGGCGGGTGGGCTGGG<br>CAGCAGGCGGGGACCAAAGTCCTGGGAACAGCCCCCACCCCTCTC<br>CCAGGCTGGGCACTGGGCCTTGGCTGGTCCTGACCCTGCCCCTGCC<br>CCCACCCGCAGCCGGGACTGCCCCATCTTCTACATGCGCAAGAAG

	GTGCGGAAGGACCTGGAAGACCAGGAGCAGCTCCTGCGGCGCTTCGGACCCCCTGGACCTGAGGCCTGGTGACCTTGCAAGCATCCCATGGGGCGGGGGCGGGACCAGGGAGAATTAATAAAGTTCTGGACTTTTGCTATATGGTGCTTTGTGGTCTCTGGGGGACACTGTCTGGTTTCATAAGCCCTGGCCTTGGCTGGGACCCTCCTGAGGCCCGCACCGCCCCTCATCCTCAGCAAAAGCCCCCGGACTCCCACCTGTGCTGCCTGGTTCATGTGGGCCAAGCCTGCCTCAGTTTCCTCTCTGGCCCTGAAGGAACCCTTATACCAGCGCTTCTCTGCCCTGCTTAGGGCCCCCAGGTGTCTGGCCCCGGCCCAGCCCTCCCTGGTGATGACCCTGGGGACCCTGGTGGCTGCCCCGCCCTCCAGGTGCTGACCTGTTTCCTCCAGGCCCTGGGGCAGCCCCAGGCGCACCGGGGCTTCCACTCCTCAGCCCGGCCAAGGGGACCCCCCCATCCCCCGCCGGACCACACACCCACGATGTTTCCACGTCGGGGTCCTGGCAATGGGCTTTGGGTCTGGGTTTCCCCCCAGCGACTCCCATCTCTATCCCCGCAGCCTCTAACTGCCGTCAGATGCCCTCGGCCCTCCCGTCACCTCACACATCCCCCGGGGAGAGTCTTGTGCTTGCCCCTCGGCCCCCAGCCCGCCCCTGCTGGAATTCGGCTCCTGGGTCCAGATCCTCCGTTTCCTGATTCTCCTCTTCAGTGAACCCCAGCCCTTCCTCGATGTTTTCAGGGTTCCCCCAAATCCAGCTCCTGTCCCATCTCCAGCCCTCAAGGTCAGCCCCCCAAACCCAGCCCCACGGGGATCCCCAAATCCAGACAGCCCCTGGCCCGCCATCCCTGCCATGTGACAAAGGGGTTGTGCAATCCAGCCCCCTCCCCTGACCCCCTCCCTGAGGGGATTTTTGAGGAGGGCCGAGCTTGTGGCCAGCGGGCACCCTCCTCCCCCCGGCAGGGGCTGGGCCAAGATATAAATAGGGTTGGCGGCTGCAGCGGGCGGCAAACAGCCCGCCCGGCACCACCATGCTCGCCCTGGAGGCTGC
SPP1	GGCTGGAGAGACAGAAGAATGCCAGACCCCAGAGGACCTCATGTTATGTAAGAAATAGCAATCACCTGGGCAGTCCTTTTTAGGCACAGGAAGCATGCCTGAAGTTCTCAGGTGTAATTCACTCAAGCGGAACACTATCTATATTGGAATCTGAAGACGTTTAATTCAGGAGGCCGGGAGCGGTGGTTGATGCCTGTAATCCCAGCACTTTGGGAGGCTGAGGTGGGCCAATCACTCAAGGCCAGGAGTTTGAGACCACCCTGGCCAACATGATAAAATTTCATCTCTACTAAAAAAAAAAAAAAAAAAAAAATTAGCTGGGCATGGTGGTGCATGCCTGTAATCCCAGCTACTTGGAAGGCTGAGGCACAAGAATCGCTTGAACCCAGGAGGCAGAGGTTGCAGTGAGCCGAGATCGTGCCACTGCACTTCAGCCTGGGTGACAGAGTGAGACACTGTCTCAAAAATAAATAAATATGTTTAATCTGGGGCTTTATGAAGGCAACAGAAAATAATCAGCAAGAATAACTCAATTTATGTAAGAACTATATACATGTCATAATTGAGCTAAATTTCTTCACTGAATAAATATTTTTGGCACATAATCTGCAGAGGGATTAGCATATCATGGAACTTACACTCTATGAGGAATTCAAACATTAAAGAAGAAATCAAATTTTAATATAGAATATTTTAAATTACAGGTTTAAGAAACATAAATGTTATGCTATGGGATAGGGGCATTAATGTAAACTATGCCTTGACAAGAATTGTCGAAACAAAAAACCTTAGCCAAGTGTAACCAGAAAAATATCTGACTGAGCTGCTTTTGTTGTCTAGTTTTACTCCCCTTTCTTTTGATTTTGTTTCTCTTTTGCACTGAAATTGTCACCTTTTCATATCAACCTTATGATAGATTCTCTACCAAAAATTACTCAAACATCAGATTTTAAGCAAGAATTCTCATAAACC

	AATTTCGGAGAATGATTTTCTCCCCTCAAATATATCTGCAAAATGAC<br>TGTCACTGACCGCTTATTGCCACAAAATGTCTTTTTCTGAACAAAC<br>AAGGTCCTCCTGCAGCTGCTAGACTTTCTATTTGTGCTTTTCCACCT<br>TGTTCTATCTCCCTCTTTGGATCCTCTCTGATTACAGCCTCCTCTATT<br>GTTCCCCAGGTTGTTATAGGTCTCGAGTTTATGCTGCTGTGGTTGAT<br>GTTGAGTGGGAAGGGGGACTTTCTGTTTGGATCCAATCCCAGAATA<br>CCCATCCCTTCAACTCCAATGTGGACACAGGGTCTCTACCATATACC<br>TTCTCTACCCAGGGTGCTGCCAAGTTTACTGTTTAGCCACACCCCC<br>TTAATCTCATTGGTTACTCAAGTTTCAAATTTCCTAGTTGATTTTCAT<br>GAACAAATGGGCTCCATGAGGGTAAGAATGGTGTCATTTAAGAAA<br>AAAAAGTTAGTAATTTAAGTCTACCTTAAAAAAGTCTATCATTTAAA<br>GAGCATTAGAATGTCCTCAGAGTTACTAATACTACTTTAATGAAGAA<br>ATAAATGCTAGATTCTTGAGTACAGGCAGATATTAGATATCAATATAT<br>GAGATATACTCTGAATTCAAGCATAGTGCCTATGCATTGTATTCATAA<br>GCAAAAACTATAAGGTCAGAATTCGTGTCATAGATGCAGGGCTGAG<br>TGGGCAATCACCAACAATTTATTGAGCACTTACTCCATAACAGTTCT<br>ATGTGTCTAAAGGAAAACTTTTTTTTATTTGTTTTAAATTGAAACTA<br>ATGTTTACCAATGGTACGACCACTTTGTCAGGAGCTGACTTTTGTTG<br>TCTAAGCTATTGAGCAATGGAATAAAAGAGTTTATGGTGTTTTTGTT<br>TTTTTTTTTTTGTATTTCTGAAATGCTCTAATTTCATGTCTGAACTTG<br>AAAATAGCACCCTCTTATCATCCACCTTTGCTTGCTACCCTGTGATA<br>AATAATGCCCCTTTTCCCAGTCTATATATGATGAATGGTGCTAGGCTT<br>CCGATTAGCTTTAATTCCAAACTTCTTTATGACAGATTAGTGGTGTT<br>ACCAAAATATCAGAGTCATGTCTGAACCTTGACTCAAGGGAAAAA<br>AATAATTACCTGTGGAGCTGAAAACACTGAAATAGTTGGTTACACT<br>TTACTAAGTAAAATATAATTAGAAATGTTCAAATAAACTGGAAGTGA<br>GTTTAATAAAGTTGTCCACCAAAGTAGTGACATTGAACACTTACTTT<br>TTATTATATGGGAATTTTCATGTAATTCAGGAAAAAGACCTTAAGGC<br>TCTTTATTTGTTTCCATGTGTCCTCTTAGTGTGTATAATTTAAGATCA<br>CAAATTACTCTTTGCATTCTAGTTTGACAACTAAACTTTTTTTACTTT<br>CCACTCACAAGATTTATAGGGAATTTGTGCATTTTGACCTTTTGATT<br>CTAACAACCAATCATTAGCCAATCAAGAATGGAAACTTTGGAAATA<br>TTACAGATAGAGCCACTGTGATTTAAATCATTTAACATGTTAATTCT<br>GACAGAAAATCCTACTCAGAAAAAACAACCAGCAGCAGCATGTTT<br>GAGTGCAAACAGCAAGAAACATCCCAAAACATGACCTCCCCGATA<br>GTCAGAATGACGCAGCATCCATTTCCTACTTTACAGAATTGCCTCCC<br>ATTATAAATCACTTTATCTTCTCAACTCCAAAGCATCCCAGACACAT<br>TCTTTACCCGTGAATTCACAAGCCTTTTCTCTGAGAGAGGCCTTGG<br>GACTAGGAACTTTTTGAATGAGTGTAGAAGTCGGGAAGGAGACAA<br>TAGTGTCAACTTGGGATTGCCTAAGGCAACAACAGAGCAAAACAA<br>GAACGCTTTGGTTCTCTGGGTCTCTGTCCCTGATTGCATAGCGGGT<br>CATTGTTGGGAAATATTTCCTCACCTGGCATTCCAAGAAATGGAGA<br>GCTCCACAGCTGTATATAGTCCTGTCATTAAATACAGGAGTGTTCTA<br>TCCCGCTGGAATTAAGAAAATTGGTAGAACCAGATTGTGGTCTGAA<br>ATCTTTTTTCAGAAATGCTGCCATCGTGTGGCACTGCGGAGCTATG<br>ACCAGAAGAGTCCTGTAAAGGGTCGTATGGTTCATCTCAAGATGGC

	TGGGCTCCAGCATAATCTATTCCTATAATTAATTCTAGCTTCATATTG<br>AATCATTCCCGTGGGCACAGAGTAAACTACAGTAAATCCTGTGGAA<br>ATTTTGTTGTTTTTAGAATTTTCAGACTTCCCTCCACTAAATTGACA<br>ACATGACACGCTTATGCGGGTATGTTTAAAGGAAAAAAAATAGTTTTT<br>AGAAGCAGAAAAAAGAAGTCTATTTTGCAACTTTATAATCTGTGTG<br>CTTTCTATTTTATAGAGATAGTCGTCATCTTACTTATTAAAATGGGTG<br>CTTATTACCTACAAACCAATCATATCAATTCATCTGGAATACATCCAA<br>TTTAAGGGAGACATATTTCCCCCTACCAAATGTTCATGAAACCTATG<br>AATTAGCTATACACTATCACTGCAAGACATTATTTAATCTATATTTATA<br>TTAAAAGTAATATTTGGCAAAAGGAAGCTGACACTTTAGGACTAAT<br>AAAAACCACAATTACTTTTGCAGCAACCTAATAATAAATAGGACCA<br>TTTATTTTTCATCTCAATTACACACAAGTCTTAACAATAAAGGTGTA<br>AGGTAAATAAATAGTGCAATCTGCATTTCACAACTGAGAAAGCAAA<br>TGAAGATAAGTAATCTCAAGGCAATATTAAATATTTTAAAAGGACCC<br>AGAGCTCTGCTATCCCTGAATTCTGCTCTAATATTCGGACTTTCCCT<br>GTAATTTTCTTTCATTCAGACACCTTTTAAATACCTAGTAAAGTGTTT<br>TTTAATACAGAAATTTTTAAAAATGTTTTTCTTTTTAAGTGGCCTACT<br>TTACATACCTTGGGAGAAAAACTAGAAAAAAAGATGATTCCAAAAT<br>CGAATCTGTTCCTTTAGAAATGTGCAAAATTTCCTTATTGATGCATA<br>CAATTTAAAGATCTTACGTCTACTCTCATTTTAATAACCTGTTCTTTT<br>AAAGGACATTACAATTCGTGACTGCCTGCCCCTCTTAAAAATTTCAT<br>AATAGTTAACACACATATAGTCCTTAAGATACGCAGAGCATTTGCAT<br>CTAATATGTGCTAAGCATTGCTAGTTTAACATACTAATTCATTTAAAC<br>CCCTCAAAAACCCCATGACCTAGGTAATAGTATTGCATTTCATGGAT<br>GAGGGAACAAGGATAGGTAGGCTGGGCGATTTGCCCAAGGTTGCA<br>CAGGTCAGCAGTGACACAGCGGAATTCAGAACCACGGTCTGGCTC<br>CTGAAGCAGCCCTCTCAAGCAGTCATCCTGCTCTCAGTCAGAAACT<br>GCTTTACTTCTGCAACATCTAGAATAAATTACCATTCTTCTATTTCAT<br>ATAGAATTTTATATTTTAATGTCACTAGTGCCATTTGTCTAAGTAACA<br>AGCTACTGCATACTCGAAATCACAAAGCTAAGCTTGAGTAGTAAAG<br>GACAGAGGCAAGTTTTCTGAACTCCTTGCAGGCTTGAACAATAGC<br>CTTCTGGCTCTTCAATAAGTACAATCATACAGGCAAGAGTGGTTGC<br>AGATATTACCTTTATGTTACTTAAACCGAAAGAAACAAAAATCCATT<br>CTATTTAATTTTACATTAATGTTTTTCCCTACTTTCTCCCTTTTTCATG<br>GGATCCCTAAGTGCTCTTCCTGGATGCTGAATGCCCATCCCGTAAAT<br>GAAAAAGCTAGTTAATGATATTGTACATAAGTAATGTTTTAACTGTA<br>GATTGTGTGTGTGCGTTTTTGTTTTTTTTTGTTTTAACCACAAAACC<br>AGAGGGGGAAGTGTGGGAGCAGGTGGGCTGGGCAGTGGCAGAAA<br>ACCTCATGACACAATCTCTCCGCCTCCCTGTGTTGGTGGAGGATGT<br>CTGCAGCAGCATTTAAATTCTGGGAGGGCTTGGTTGTCAGCAGCAG<br>CAGGAGGAGGCAGAGCACAGCATCGTCGGGACCA
SPRR1B	TGTGGGCTTTTTTGGCATTCATTCCATTTACGATGTTGTATGTCTTTG<br>TTTATATATATATATATGTGTGTGTATGTGTATGCATGTGGGTTGGTGG<br>AAGATTAAAAAAATCCCGATACTTACTATAGCAAAATATAAACAGTG<br>GTTATTTCTGGCTGTTTAATGTTAGTGTTCATTTAATAGCTATCTGTTT<br>TATACATTATTAATTATATAATAATTTTTTTTTCTTTTTTTTATTTTTTG

	AGATGGAGTCTCGCTCTATCACCCAGTCTGGAGTGCAGGGGCTCAA<br>TCTTGGCTCGCTGCAACCTCTGCCTCCCTTTCTTTCAGATATTGCTA<br>AGTAACAAGAATAACGTTCTTCTAGCAGTAGCCTTGCCAGCCAGCT<br>CCTGTAATCTTACCTGCATATCCTGTTACATATTCTTCCTGGGGTTGA<br>GAGACATAGCCTTCAAGTGTCCAGAACTTTTCACGTTTCCACAAAG<br>CCCGCCTCACCACTGTCCACCACCACATTGTCTCTCCTCAGTGTCT<br>ACCTCTTCAGTAACCACCTAGACAGTGTCTGCCCAAGTCTACTTTG<br>AAACCTTCTTCATCTGGCCAGCAGCAACACATTCCCCAAAGCAGTC<br>TCAGAAGCTGGCAGGGGGAAAACATGACAAAGACAAATGGATCCT<br>CCTTCTCATGACTCTCCATCAAATGTTCTTCAAAGCCTGAAATGGTG<br>AAGAAGGCCTCTTCACCAAGCTTCAGTCTCTGATCTCCCTGGGATG<br>AGGCTAAACTAAGAGAAGGATTCTTTCCTGAATTTATTGTCATGCA<br>GCATTCCAGGGTAAGCGAGGAAAGGACAAGCCCCAGCCACATCAA<br>CTTTTCTGGAGTCTCCAAAGAAAGAACTTAAATCAAGTTCAACTTT<br>CTTACAGTGATCTGTCCAACTTTCTGTGCTCTGGGCATTGATTTCCA<br>TCCTTATAATGATTGTGTTTATATATCCACTTCCTGAATTATGTGCCTC<br>TTTCATGATTGTCTTAACCTGATTCCTACAGTAAGTTGTGCCTGACA<br>CTAATGCCTCATAGGCAGGAAGTTTATTTGGGAATGCAATCCCAAG<br>GAAGAGGAATAATGGACAAAGGGAAATACAAGAGGGAAGGAATG<br>TCCATTCAAGAAGGCATTACCATTTTGCACATCACCCAGAACAGCT<br>GACAGTCAAGTCCATGGGACTTTTTTTAATAGCAACTCAGTAAATTT<br>ATTGACTTATTTTAAGACTGTAACAATAGAATTGTTTTCATTTTAGCA<br>CTTAGTCATTTATAATTGCAAAGACATTGCCTTATCGGTCCTTGGATA<br>ATGGGAAAAGGGAGAGAAAGTGACTATGTAACTTAATTAGCATATA<br>TGATGTCAGTTAATGATTATTGAACAATCCCTGTACCAGTTACTGTT<br>GTAAACACTCTACTGTTATTAACCCATTTATTCCTCCCAGAAACACT<br>GTGAGATACCTACTACTATCTTCATTTTATGTACAAAGAAACTGAAA<br>CACAGGTTAGGGTAATAACTTGCCCAGAGTCACCAATAGTTCAGCT<br>GGGACCAGAACTCAGTAAGTCTGGCTGCAGAGTTCACACTTGTAA<br>CCATTATTTTACACTCACTTCTCAAACATAAAGTACCTAATATGGGT<br>GCTGTACTGATAGCTCAACAATGGTTAGTTCCTCCCAACCATGTGCT<br>TTCTTCCTGAAATATTTATTATAATTACTACAAAGCCATTCAAAATT<br>GAATGCTGAAACTTGTGTCTTACCTCTTTTTTTATTGGGTCATATAGT<br>AGTCCCCTTCCCCCCTCCCCCCTCCCTCCTTCTCCTTCTCCTTCCTC<br>GTCTTGTTTGTTCCAGAAGCACACATGCAGGCTTGTACATGAGTAA<br>ATAACGTCCTGCTCAGGGTTGGTGTACAAATGATTCCATCACCCAA<br>GTTGTGAGCACAGTACCTGACAGGTAGCCTTCCAACCCATTCCCAT<br>CTTCCACCTTCCCCCCAAGCACTCCTGAGTGTCTATTGTTCCCACCT<br>TTGTGTCCACGTGCATTCAATGCTTCGCTCCCACTTATAAGTAAGAA<br>CATGCAGTAGTTGGTTTATTCTTGCATTAGTTCCCTTAGGATAATGGC<br>CTTCAGCTTCAGCTATATTGTACAAAGAACATATTTTAGCAAAGACA<br>TGAAATCAATGAAGATGCCCAGCAACAGTGAACTGAATAAAGAAA<br>ATGTGGTACATATACATACATCATAGAATACTATACAACCATAAAAAA<br>GGTCCATGGATGGGACTTTCTGATGATCTTTAAGAAGTAGTCTCAG<br>AACTCTCTGTCCTGGAGATGAAGGCAGGAAGTATTTATCCATTGTCT<br>TATTTCACTTCCCCTCACTATCATCATTTGTCAAATCTGGGCCCATAT

	GTATTGACTCCCCCACACTTCCCAGTTGTACACACAAAAGCATTCC<br>ATGAGTGCCCTGCACTGCAGTATCATAGGAGCCCCAGATCAAGAAG<br>AAAGAGGTATGTAGTATGGTCTGCAGAGCAACTTTATCAGGTATCTC<br>CTGCACAAAGCCCATCCAAGCCTATATGAAAAGAGCCACATGACTG<br>TGTATGGATAAAAGGCAGGGCAGAGAGAATTTGTAGGGATATCCAA<br>GAGGTGTCAAGTACAGTTAATCAGAAAGGTCAGGCTTTGATGTTAA<br>TAAAAACAATCAGCAGCATTATTTCATCCTGAGAAGGACAGACATA<br>AAAGTCAGAGGCATATGGGGAGATGTTAGTTGCAATCTGCAGCCAT<br>ATGGCTTCCCAGTAAATCCGTGGCTGATCCAGCACTACTGATGAGG<br>AAATTGAGCTTTCTTGTACAGTGAAAAAAAAAAGCAGCAGGGGGA<br>TCTTTACACCTAGCTATTCAATGTAATTTAGAAAAAAACAGCAGAA<br>GAGTAAAATGAGAAAGTCTAGAAGCTAGAATGAGAACAGTGCTGA<br>CTGAGGGAGAGGAGATATGTCAAGTGAGTGCATCAGGTCAACACC<br>TAAACAGCATATTGAATGAAAGCTCCCCCAAGACATGTGGGTCTAG<br>GAAAGACATGGGAGGCAGGGGCCCAGCCTCAGAGCTGAGGTTCA<br>GGTCAGCCAGGTCCTGGGCCTGGCGGCATGGAGGACCGAGAAGCA<br>GGAAGCAGGTTGCCTTGGACCTGATAATCCCACCATATTGCAAACA<br>AAGTGTGCTTATAATTTCCTTCGTAGGGTCTACAGGGAGCAGGTTT<br>GAGGAAAGGCTTGTGTCCAAGTCTAGAAAAGAGCTCAACTGATGC<br>AAGAGGCATGGTGATGACAGTGGCCACAGACCAAAGTGAGGTGAC<br>TACTTAAACAAGGAGAGAGAGAAATAGATAAGTGGATGTATTCATC<br>CATTTTCATGCTGCTGACAAAGACATAACAGGGACTGGGCAATTTA<br>CAACAGAAGAGGTTTAATGGACTTACAGTTCCACATGGCTGGAGA<br>GGCCTCACAATCATGGCAGAAGGCAAGGAGAAGCACATCACATCT<br>TATATGGATGGCAGTAGGCAAACAGAGAGCTTGTACAGGAGAACTC<br>CTCCTTTTCCAAAACCATCAGATACCTTGAGACGTATTCACTATCAT<br>GAGAACAGCACTGGAAAGACCTTCCCCCATGATTCAATTACTTCCC<br>ACCGGGTCCCTCCCACAACACGTGGGAATTCAAACTGAGAGTTGG<br>GTGGGGCAACAGCCAAATCATAATGGATAAAATGACAGAAATATCT<br>GTCTCTTGCTAAGCTCTTACAGCTTTTCTCAGCTTATACCATGAGGC<br>CTGCCACAAGGAAGGATTTCCCCTATAAAAAAATTTCCTGATAATTC<br>TCAAAACAGCCTCTGGCGTCTCTTCACGAATAACATCATCCACCCA<br>TCTCTCCATCCTTCCATTCATTCAGTTATTCTATAATTGTCAATTCTCT<br>GAGCAGCTACTACACACCAGGCATCACGTGAAGATGGAAGGTAGA<br>ACAATGGCTAAGCAGAGGAACTTCTGGTGGTAGCTGGCTGGTCCT<br>GGGCTCAAGTTCATGAGAGGTGACACAGCTGTGCAGCCAGCAGTT<br>GCTCTACAAACATTTAGATGAGTGAAAATAATGAGGGAGGGACCAT<br>CCGACACCTGGATTTGAAGGATAGTTTGGATTTTCCAAAAACCACA<br>CTTGCAACAGGTTGTGTACTGCAGGTAGACCTGGATTAGTGACCTA<br>AGTTTCATCTTATCTTGAGGAGAGTGAACTTTGATATAAGGGCTCAG<br>GGAATCCCAGCTCCTTTGTTCCTTCCCATGTTGGCTGCAGAGAAGG<br>GATTCCCCATGATAGCCAGGGTCTTCAGGGCCTACTGTCACAATGT<br>ACAGAATAAATGGATGGACTAAAAGGGCCAGGTTGCTCCAGAGCT<br>TCCGAACGTGACCATTCTTCCCAGAGTTTCACTGCTCCGAACTCTC<br>CTTCACAGACTGAGGTCAGCCTGCCCCTATGTTGTTTACCCTGAGT<br>TCCTCCTTGGAGAAAAGGCTTTTTAGAACCGAAATAATCATTTTAGT

	TTTTCCTTTCCCCTGCCAGGCACGAGTATACTGAGTGATGGATTAAC<br>AGAATATTACTATTTTTCACCTAGCCACCATGCCCTTGGCCTCCCAG<br>AATAACCAGAAATACCTATTGTTTATCTCTACATCCACTTCATATATT<br>AAACAGCCCTACAAGTGTCACCTGTGCTGAGGATTAGACTCTTCCA<br>GAAGATAGGACAGTTTCTGGTTCCAGCAGCCCCAGAATGTCTTCCT<br>TCTCTCTCTTTCAGCCCACACCACCCTTCCTGTAAACACTACACCTG<br>GGAGCAAAGGGTGTTCAGGGGGATAAAGCCCAGGTGACATCCTTG<br>TCAGACAGCAAGTGCCACAAGTTTCATCACAAAAGTTGAGTCAAC<br>AGGTGGGTGAGGGAAGAGGGGTGAATCACATCTGACAGGTAAGG<br>AATGTAGGCACAGCAGCCCAGATGGATCCTGTTTCCTTGAGGCAGG<br>GCTTGTTCCATGCATAAAAAGCCAGTTGGCTGGGAACACTACACCA<br>GTTCTAAGGGACCATACAGAG
SPRR2A	TCTTAATCTCATTAACATTAAGCAACAGGAGTACTTTACATTACTAAT<br>AGCTCTTGAAATTATGCTCTTAAATATTCTGATCTAACATATAGAGAA<br>AATAACAGTAGAATCTATAAGGTCTATTCACTTTGGGAAGGTTAGTG<br>CTAATGAGCTATTCAGTTTCTTATAAGGATTTAGCCTAAAATTTAGGT<br>AATTTGCCACCAGAAGAAATGTGATATTTGAATGAGTGTTTTTTATT<br>AATGTCCTAAACAGCTAACCACATAACAGTAAACTAAATTGGAGGT<br>AACATTATATATATTTTCACATGGAATGCTAAATATCTATGTATGTTAA<br>TATTTTACAAATGAACACATCCACATATTGCTATTTACTGTATTTAGA<br>TTTATTTTCTTGTATTAACTGAATTCAGAGGAAAGTCTTAGAGAGTA<br>CGAACAACTAGGAACTGATCTAAATTTCTGCTTAGATTAACTCTCAT<br>TTTTATCACATAAGAATAAACGTCAGGATCAGCCCATCTTATTATTTC<br>TGCCCACTTCATTCAAATAATATATTTGCATTTGCTTTGTCCTAAAAA<br>CTTCTGGCACACAGTTAAAATAAGGATAATATAAAGATCTTAAAG<br>AGAAAGCAAGTATCTTTCTTAACCATAAACAACTTGAAATTGATATT<br>TTGTCTCTATTTCTTTCATTAACAATGTGTTACTTTAGCATTTTGAGC<br>ACTATCTGTAGCCTGTGCTAAGTATTTATATGCAGTCTCTGATTTAAT<br>TGTTTTCTCATGACAATTTAGTGGTAGTTACTATTATTATCACCACGT<br>TCTACTATCCTTACTACCTGTCCTGCTAATCATCCATCTCTTCCAATT<br>TCATCATATTATGGCCAATTTATGCTTTCTATCCACCTGGCTCTTGGA<br>TAGTAGACAAACTCATTCCTGATTTTGTTATCCTAGAATGATCTCCC<br>AATTTTGGTGAATGATTTCACAGACACATACAGCTATTGGGTGTCTG<br>TGGTTACTACGATTCTCACATTTGGAGAGAACACCATTTGTAAACAT<br>CTGTGTGTATATGTCAATACTCTGAGGAAGGGGGATTATTATTGGAG<br>TTTTCTTCGAGAGAGATTTAGAAGGGATGTGATCAAATGTAGAGC<br>TATTACAATAAAACTACTGCTACTTACATCCTGCCCCACCTTTAGTG<br>TCTAGCCAATAATGCTTAAAAAAAACCCAAAAAACCCACTATCTCA<br>CTAACTGAGCAATGGACAGGCTTGTCTCTTGTTAGCTTTTCAAAGT<br>AGAATTTTAAGATTCCTTACTCAATAATAGGTAACATTTGAAGCATA<br>CTTTGAAAAGTTAAATAAGAACATAAACATCATCTGTAATTATACCA<br>GCCATATATAATCACATTTAGTATGTTGTTTTATGTTTCCCATCTTTTA<br>ATGAAAATGTTATCATGCTTTACATACATTTTTTAGTATAATTTGAATA<br>TTTTAAATAATATTACATAATCTCCCAAAACATTCTTCTTAATTACTTT<br>CAGAATTCTACCATATGTTTATCTAATTCATCGAATTATGGAAGGACA<br>ATGGAGACTAAGCCTGCTATTCAGAAATAAAAGCAGAAGGCAGATA

	TGCATGGGGACTGGTAGTTACCATTTCTCTTAACATATCAAGAATAT<br>TTTGCAACAATTCCTGTTTCTTTGTTTTTCATATTTTATTTTAAATCT<br>TCACTTGCCAGCACTATTTTGTATAGCAGAATGTATCATAGTAAAAT<br>GTGAGATAAAATGTTGAATCAAATATTACACCAAATAAAAGTTTTTG<br>TTCTTTCCTGTCATTTTTATGAACTAATAATTCGTACTTAATGATCCC<br>TTACAAAATTAGGAAGTAGAAATAATAGGAATGAATCTAAGGCTATC<br>AGACTGTTAAGAAACATAAAGCTAAGCTCTTTAGACATGAAGATAG<br>ATGATAGATAAATAAAGACCCATGGAGTGCGTGTGTATGTGTGTGTG<br>TGTGTGCATGTGTGAAGTATGCCTCGCTCCATGTATCAAGGCCTGGA<br>TGTCCTAACTTGGGGAAGCTGTGACTGTTAGCTGCCCCTCTTCATAT<br>TGTGGCCGTTCCTCATGTGGCTGCTCTGTGTGGAACTCTGAGTTTC<br>AAGTTCACCACCTCAACTTGTAAAGTGAGCCTGTCTGAGATGCGG<br>GGGGCACTAGGGCTGCTGCCATTCTAGTGCCTGCACTGGTGTGATA<br>AGCCTCCTGTTGGAATGCGGTGTTCTCAGAAGGGGTGTCGCAGGG<br>TGTGACTCAGACTGTGTTATCTCACACAGTGATCTCATCTGGCAGC<br>AAATTCTTCAATTTTGCATGGCACAAATCAGATATTTCTTTAAGGAT<br>TTGTGTTTTAGATGTGATGTGAGGCTGTTGGGGAGTAGTAGGGGAC<br>CTATTGAGTAAAGGCCCTTTACTTTGCCCAGTGAAGTGCTTGACAT<br>GTGTAATAGTCTATAGGAAACACTTGGTGAGAACACCATTTGTAAA<br>CACCTGGCCTCAAGGTTCTGATATGTTGGCTCTATTTCTGTAGTGTC<br>CTGGCCAGTGATGATCTTAGCCAAAAGCCCTTCCTTACTGACAGGA<br>GAAATAGCAGTTCTGGGTACCTAACCTGCTTAACTGAGGGATGTGA<br>TGCTGGGTTCATTCAACACATGTTCTTCCTGGAGCATAGCTGGTGA<br>CTGCTCTGATGATTTTGTCTTTCCTCTTCCTCATTCCCCTTTCTTCTT<br>ATCTACTTACAAAACCACATTCTTGGAAAGCTTCCTGATTAATGGTC<br>CAGATAAGGAAGCTCTAGCAATATCACTTTAAATGCTTAATATACAA<br>TATTTAGAAAACCTTATGATTGTAAAAGAGCTTAAAAAAGATGTGA<br>AAGAACAATCACTAAATGCATTGACAACATATGTGTTAAGTGAACA<br>ATATGTACCAAAATGGACAGATGAGAGGTGTACATTGGGGTGTGAG<br>TTGATAATCCAGCAGACTGTGGGACTAGAGGGTCTACGGAAAAAA<br>CAAAGGAAGAACATACAAACAAATTTTAAAACACTGTCTTTCAAA<br>CACCTAAAACCTGTTTGGAAATAGAGAGCAAGAATACATATTGGAT<br>TACACTTAGCTTTTATTTTCCTCTAGATTTCAGACTTTAAGTGCTCTG<br>AACTTCTGCACTCTGCAAACCTACTTCTAATTATTTACATATGATAAC<br>ATGAGTCTATGCAGCTTGTTCTCTATAGGATATTCACAGGACACTAC<br>AGAATGTTTTGGGAGATTATGTAATATTTAAAATATTCAAATTATACT<br>AAAAATGTATGTAAAATGTATTGAACATAGGCAAGTTTCAATACATA<br>GATTTTGAGTGAATGCTTGCAACTTTGGTTCCATTCTCTCTACTTCC<br>TTAAGGTGGTGTCCAAGAGTACATTTTTATAAATAAAAAGTTATAGT<br>ACACATCCCTAAGGGCAGCAAGTAGAAAACGTGCTAGGGAGACTC<br>GATCTCACTTTGGAATCTATCCTGGGAGACAAATGCCTCTACAAATG<br>GATTAGAGAAGACAGTTTTAAAGAGGAAGATAATACAGGTAAAATC<br>TGGGGTTTTATGAGAGAAAGAAAGAGGTAGAAGAAAAAATTTCAA<br>GCTCGAACAGGGTCATCAGGTGGCACAATGCGGTCAATGCCTGCA<br>AACTCAGGGGTAAGTATTATTCTCCCTGTTTACAGTTCCGTGGAGG<br>AGAAGTGACTTGCCTGTGGTCATACAACAGAGCAAAGAAAAGGCT

	TGAGCTAGAACTCAGGCCTTTGTTAGGTCTCCCCTTCCTCCTAGCA<br>CATTGGCAAATTGCATGAGGAAAGTAGAGGTACAGTTGAGTTCATG<br>TACAACAATAAGGCATTCAGGTAAAGTGAATGAGGGCAGAAGTTTT<br>ATGATTTAGGGAAGGTGTAAGACAGGAAAATATCTTTGTTCCCAATT<br>AAGAAAGAGATCCCTTGACCATCAGTTAGAGATTCCCCCAAGTCCC<br>TCTTTGCCATAAGTCACTGAAACTGAGATCCAAGGCATGGCTTCTG<br>TGAGTCAGGAGAGCTTAACCCAGAGGAGAGATTTCAGAACAGGAT<br>ATTTCCTATTTTGAGTATCCTGCTCATGCCAGTCATGGATAAATTTGC<br>ATCTGGCTTAAGAAATTACTGGATCAGCATTGTTTTGGGTAGTTTCA<br>CTTCCTGCTGGGTGGGGTAGCAGGCTCTATAAAGAGATCCTCTGCT<br>GCACGACTCTTAAACCCCTGGTACCTGAGCACTGATCTGCCTTGGA<br>GAACCTGGTGAGTCGGCTTCCTTGAGTTCCTCTGTTCTTTGTGCCC<br>TGAAATGTTGAGTTTAATCTGAATATGGCAAGTTTGGTGGATCCAAT<br>CCTATGAAAATTGACTTGATGCTACTTAGTGGATGAAAATTTAAGAT<br>TAGAGCACAATTATATGCTATTTTAGCTTTCTTTTGTTATACAGGTAG<br>GTATCCATATGGACAGAGAAGTTAAGGGGTAACCTTTGATATGAAG<br>AAGAAAAAAGAACAAAGTATTTTTCTTTATTCTCTGTCTTTCTAGTG<br>TCCTTTACAAAGGTTTGTGTCTTAGCAGGTGTGAAAGACTACAATT<br>CTCCCTGAGCAGCCCTTTGCTCTATGCCCAAGTCAGCCCACTTGGA<br>CTTTATAACAGATAATGATGATAGGAATAGCATATTAGATTGCCCAG<br>GGTGTCTGAACTTGTGACTGCCTTTCTTGAATTGGTTATTTTCAGGG<br>AAATAAGATGCTTGATTCTTTATAACAGAGATAATTTATTTGGAAAA<br>ATTGTATGAGAAAACACAGGATTTCCTAGGGACAATGAAGCAATTT<br>GTTAAAGTGGAAGGGAGAAACCAGAAAGTCTTGAAAAGGTAATTA<br>AGAATTTAAATAATTTCTTGGAGATTGGAGAAATAATATGCCATGGT<br>ATTACACAAGCTTTGGCTTCTCTCTCTGGAGGATTCCCTTCCCACGA<br>ACACTGTTGTATCATTTCTTTCAGATCCTGAGACTCCAGCAGGATGT<br>CTTATCAACAGCAGCAGTGCA
SRC	GCCCCCTGTCTGCTGAGGAGTCCAGAGCCCACGGTGAGGAGTGGC<br>TTCCCGAAGGAAAGGGGCCACCTTTCCCTGGGAACCTCAGGCAGC<br>CCCATTGTGGGGCCCCCACTGCTTCCCCTTGACCCCCGCAAGGGTC<br>TATCTCTACCTTGGAGGCTGCAAGTGGCGGAAGAGATGAGGGGGG<br>TTGGCCCCTCTCCAAGGAGCAACTCATGGTGACTCGGCAGTTGGG<br>CCCCAGAACCAGGAAGTGCAAGGATAGAGCTCTGTGCCAGGGAGG<br>AGGGTGGTCTCTCATGAGGTCCCACGGCATGGAAGCAGCCCTCCG<br>AGACTAACTCCCACCCTCACTGAGTCATCGAGTGAAGAGCCGTGTA<br>GCTTTCGAGACCAGCCCAGCCGACATGGTGGAACCCCGTGTCTACT<br>AAAAATACCAGAATTAGCCGGGAGTGGTGGCACTCACCTGTAATCC<br>CAGCTACTCAGGAGGCTGAGGCAGTAGAATTGCTTGAACCTGGGA<br>GGCGAAGGGTGCAGTGAGCCGAGATCACACCATTGCACTCCAGCC<br>TGGGCGACAGAGGAGACTCTGTCTCAAAAAAAAAAAAATAATAATA<br>ATAATTAGCCAGGCGTGGTATTCGGTGACTGTAATCCCAGCTACTTG<br>GGAGGCTGAGGCAGGAGAACCGCTGGAACCCGGGAGGCGGAGGT<br>TGCAGTGAGCCGAGATCGCGCCACTGCACTCCAGCCTGGGCAATA<br>GAGTGAGACTCCATTTCAAAAAAAAAAAAAAAAGGAAAAGAAAA<br>AGAACGAGAGAGCTTGGAGTAAAAAAAAAAAAAAAAAAAAAAA

	AAGGAAAAGAAAAAAGAAAAAGACCGAGAGAGCTTGGAGTGGCC<br>TGAGAAGATGCTCTGAACACACTAACACATAAAGTGAAAAGAGGG<br>AGGCGGCAGTGTAGAATCTCCTCTGGGTATCTCAGGGCCTCAGTTT<br>CCTCATCTGGAAAGTGGGGGCCACAGTGCTACCCGCATGACCCTCA<br>GTGGGCCATTCTTGCTCAAGGACGTTCTGCTCGGAGCTTTCTCAGG<br>CTTTCTCTGGGAGTGGGGTGGGCTGCTTTATCCTCTCACCCCTCTTC<br>CAAGGCCATCTGGCTGCTTTGTGAGAGAGATCCCTGGGGGTAGGA<br>GGTGGGGCCTGTGAGTTTCAGCCCCTGATTCCCACCTGCCCAGTGT<br>GGTGGCCCCTTTTCACTCTCACCCATGCTGTGTGACTTCAGGCAAG<br>TTCTTCAACCTCTCTGTGTCTTAATGTCCTCAACAGTAAGATAAAGA<br>CATGATTAGTATTTACCTCCTAGATTATCATGAGGAGCCGATGAGTC<br>AATGTTTGCAAAGTACTTAGAGCAAGGTCCAATTCAAGGTCTGAGC<br>TCTGGGAGTATTTGAATACCACGGAATGATATTTAGTCTTTTCAGTG<br>TGGCATTGCAGGAACAAAGTAGGATGCAAATTATACATCCATGCCC<br>ATTTAGTCTTTTGTCTATTCTGCAAATAGTTCCAGAGCACCTAGTGG<br>GTGTCAGGCCCGTCCTGGTCTCTGGGGGCATGGAGATGGATCCTGC<br>CTGGCCCTGACAGCCGCGAGTGCACAGTGGAGGGCACTGGGTGGA<br>CCCCAGTTTGTTGACCGTCTTGCGTTGCTGTGACGCTGGAAGGAAA<br>CGCACCACCACACCCAGGTGTTTGTCTTCTAGGGGCAGAATTATGG<br>GGCATTTTCTTTTATTTTTTCTTTTCTTTTTTTTTAATCTTCTTGTAGT<br>TCTCAAAGAGTCCACTCTGAATGATAAAAGTTACCATTTACGGAGG<br>CCTGGTAACGTTCCGTGCCATCCTGAGCATGTTCCGTGTACTTTTCT<br>CACGGATTCTCCCAGCAGTCCTGACACGGGTGTTATTCCCGATCGAT<br>AGAGAGGAAAACTGAGGCGAAGGGCGGCCGGCCAGGATTCAAAC<br>CAGGGAGTCTGCTCCAGCACTCCGGCTCTTAACCTCAACCGTCTGC<br>CTCTCCACAAACACCAGGATCAACCACCAAGACCAAAAAAACAGT<br>CTCACAAACCATCAAACATTGCACTTGGTGGCTCAGGACCTTAGCT<br>TCGTCTTAAAGGTCCCTGTTATGCTTTTTCTGAGTGCCCCAGTGTGG<br>AGTGGTCTTCGTGTTTGTGAGTGCAGGGGTCAGGGGTTGTGTCTTT<br>TCTTCTTGTCCCCTTCCAAGAGGTGACATGTATCCTTGATACTGGAA<br>GGGCCCTTAAGGTCTAGCCCTGGCTCTCTGTCTCCCTTCATGGCACA<br>TCAGCCAAGTACCCATTCTCCCGTCTTTGATTACCTCCAGTGACAG<br>GGAGCTCACTCCTCAACATTTATTTTATTCTAAAGAAAATTCAGGCT<br>CTTAGATAGTTCTTCCTCCATAAAAATTTTTTAAAAAAATTTTTACTT<br>CCTCTTAAAGTCTCATAAACAAGTCCCCTGCTTTGGGGTCCTGAGG<br>CAGGACCTGGCTAATTCGTCTCTATGTCCCCCTATAGTGAGGAGCAC<br>AGGGCCTGCTAGTAGGTACTATAAATGCTGCACCAGGAAGCAAGTG<br>GCTAGGGGGCGGGCTGGTTGGGCCCCCATCAATCCTTGGGCTGCA<br>GGTAACCAGGTCAGCAGTTCTTGTGGGACACCAGCTGGGTGAGTG<br>CTGATGGGTATCGTGACCTCAATGGAATAGGTTGCCATAGAAACTG<br>ACTTTGGACCTGTCCCCAGGTCCATTGACTTGTCCCTCAGGAGACC<br>ATGGTAATCCCTTGAGCTCTTCAGCAGGTCTCCCCAGGGCCTCCCC<br>AACCCCACATTCTCTGAGCTGGGTGGTCCCCAGGGAGCATGCAAA<br>CCATACCCCCATTGTGCAGCTGGGTAGCCTGAGGCCCCAGGGGCAC<br>AGGGGACAGGCTGAAATTCACACAGGCAGGGGGCGTCTCATAGCT<br>GGGCTGGGCGCTCAGGGCAAACAGCCTTGCCCGAGGACCCAGCCA

	GTCAGGGGGCCTTTTCCCCACTCATGGGTTCTCAGAGGAAATTCTG<br>GACCGATTCCCAGGCCTTCTGCCTCCCCAAAGAATAATCGGAACCT<br>GCCTCCCTGGTGTGTCCAGAATTACACAATATCAATAACGGCTGAG<br>GTGACCTGTGATGCCCCTAGCACCCTACAGTGTCTTGTGCATTTATC<br>ACAATGACCCCATCGTTACAGATGAGGAAACGGCACAGAGAGAAG<br>TTAAGTCACATGCCTAAGGTTACACAGCTATTTAGGGGTAGAGCCA<br>GGATCTGGATGTGGAGAGCCCAGCTCCAGAGCCCACTCTCACCAC<br>CATGCCATACCCTGTCTCGCTCTTGCTTTGAGAGGGTTGAAAAGAC<br>TTTGGGAGGGTTGAAGGATATTTCAGGTCTAGGAGGCTCTTAGCAC<br>GGGCCTGCTAGAGCCATAGCCTGATGAACAGTGATCAGAGAGTGG<br>GCGCCATTATGATTTCTCTTTCTAATGACATCCTCAAAACAGTGCTG<br>AGATGTCTGCTTTATCCCTGCTTTGCAGATGAGGACGCTGAGGCCC<br>AGAGAGGGAAAGCCACTTGCCTAGGGACACACAGCGGGGAGAGG<br>TGGAGCAGGGCCTCTATTTCGAGACCCCTGACTCCACACCTGGTGT<br>TTGTGCCAAGACCCCAGGCTGCCTCCCAGGTCCTCTGGGACAGCC<br>CCTGCCTTCTACCAGGTGAGATGATGGCCCTATCAGGGCTCAAGAT<br>GGGTGGGGAGATCCCAGCGAATCTGGGTGGGAGCTGGGACCAGCC<br>AGGGCCTGCGGATGGGGAAATGGGAGGCAGAGGGTGGTGGCAGG<br>ACTGTGGGCGTCAGGGGGAGTTTTCAACTTGGTTTACTCTGTGTTT<br>CCGTCTCTCTGGACCTCCCAGGTTTCTGGCTCTGAAGTCCCTTAGC<br>TCTTAAATAGTCACTCATTCGTTCAGCTGTTTACTGAGCACCTACTG<br>TGTGCCACACCCCATGCTAGGCACTGGGAGCACAGCAGTGAACAA<br>GATGAACAAAAGCCCACGACAGTAGTCACATAACCACATGAATTCA<br>AACAACAAAAACAAAACAGGACCACATGTTATCAAAGACAGACAC<br>AAGTCAGGGGGACCCAGTGAAAGGATTTGGGGAACACTTCCCAAG<br>GAGGGGACTTTTGAGTTGAGAACGGAAGGAGGCACCAGTCACCCT<br>GGTGAAGTGGCCAGGGAACAGCATTTGGGCAGTGGGAATGGTATG<br>TGTGAAGGCCCTGTGGCTGGAGTGAGCTCCCAGCCATCTCTGCTTC<br>AAACCTCCTGCTGTATGGCCTTGGCTGCCACTTTTAGCACCTTTCCC<br>CAGCGTTAGGCAATTCCCGTCAGCCACGTATTCATTCAACAAACCC<br>TCAATGAGCCCCCTCCATGTGCTAGGTGCCCGGGACATAGGCGTGG<br>CTCAGATACTGTCCCTGCCAGGAGGGAGGGAAACAGTGACTCAGG<br>GGGAGGTGGGTGGGTAAGGTCAGGGCTTCTGATTATGAGGCATTTC<br>TTCCGTTCCTGGCGCTGGACCGAGGAAGCCCTTTACGGATGGATGC<br>CATCCTTACGCTCATTTTACAGATGAAGACATCGAGGTTTTGAGAG<br>GCTAACTCTCCCAAAAAGGCTGGTGCCCCTGTCCCCCATTTTTTTCT<br>TTTCTTTCTTTTTTTTTTGAGACAGAGTATTCCTCTGACGCCCAGGC<br>TGGAGTGCAGTGGCAGGATCTGAGCTCACTGCAGCCTCTGCCTCCC<br>CTGTTCAAGCGATTCTCCTGCCTCACCCTCCGGAGTAGCTGGGACT<br>ACAGGCGCGTGCCACCATGCCCGGCTAATTTTTTTTTTTTTTTCAAG<br>TAGAGCCCGGGTTTCCGCGTGTTGGCCCGGCAGGTCTCAAACTCCT<br>GATCTCAAGTGATCCGCCCGCTTCGGCCTCCCAAAGTGCTGGGATT<br>ACAGGCGTGAGCCACCGCGCCCGGCCCTGCTGCCTCTCCTTGGGC<br>CTCGTTTTCCCTATCTGTGGAATGGGTGGGAGGGAGGCCGGCCAAG<br>GGGCCCCGGCAGCCCTGCCTGTTCCAGTGTCTTCTCTCTCTCCTGC<br>CAGGACCATGGGTAGCAACAAGAGCAAGCCCA

SRY	TGACCTTCATTTTATGGAGAGAAACAAGCTATAACATGTAGTATCTA<br>AGCTGATTAGAAGAACTAAAAAGAGAAGCTCATACTTGTGCATCAG<br>AAGGTAAATGAAAGAGTGAAGTTACCTCTTTGTTTTAAGGAAGAA<br>AGGAAAATTGTGGATGTCATCTGTTTTCTGTTTACATATTTCAGGCA<br>TGGATAGCCACAATGTGATTTTAAGACGGTTAGTTACAACTGATTTG<br>AAAAAAAAAAAAAAATGCTTCACTCTATGAGAAATTTCTTCCCAAGT<br>ATGAAACCTTGTTTTTACAGGCAATTTCCTATACTTTGAAAAAATCA<br>AAATAATAAAGTAAAAGAAAAATAATTCAGGTGAAGTTAGAGAAA<br>AAAACAGGCAGCATTATTTTAAAGTTGTAAACTATTTTGTTTACTTAT<br>AGTTTAATTTACATGTAGTAGATATGCATTTGTAAGGTTCTTCGGCTC<br>AGGTAGGAGATCATTCTATTTCCCACTGCACCCTACTTCATCCTCCC<br>ACTGGCAAATAATTAGATTATCCCTGGGAAAAAAAGATGCCAGTAA<br>AATTGATCATGTTTAAATGCATCAGTTGCTAGGTGATTTATCTGATTA<br>AGTCTTGAAACAGTAGAACCTAGCAATTAAAGTGAGCATTAACTTC<br>TACCTACCAAATCAGAAGACTATTCTAACTTTTTGAGAATTAGATGT<br>TGAAAATATGGCCCATGAATTTAGCATGGTTAAAATAAAAAACATGC<br>AAACAAAACAAACCCAACATCTTGAAAGGACATTTGACTCTAAAG<br>TCCCAAAAATAATCACAAGTCTAAAAATCCTAAGTTTAGTGTTACTC<br>TATTACACCTTTTTATTTGTAAGTGTCCTTTCACAAAAGTTTTAAATT<br>TTGCTCTTGTGCATTTTATTTACCTTTTCTTTTGTTGTTTGTGTCTTTG<br>GTGACCTGCCAACCATTAGACTTCAAAAAACAGCCTATAGCCAAGC<br>TGCAGGATAAATGAACACATAAGTTGACTTAGAATAGTCAACTCTG<br>TCTAGTATACAATTTATGGGGGATGGTTTATGACCACATATATTTCTA<br>CTTTGATGGGAATATCTTGAGATAAAATTAGAGAGAATGAGTGGAG<br>TAATATTCACAACATTTTTGCTGCATTCATCCCTGAATTTGAAGAAAT<br>ACCAAAGTACATCTTGTGAGGAGAAAAAATAAATAAATTCATATAA<br>AATGTTGTGGGTTTTATTCTTTATGCAGTGGTAAACTGTGTTTGCAT<br>ACACCATAGCAATTAAATTAGGGCTACAAAGGGTATTTAACTAATGA<br>GCATAAAATACCTTAATGTACCTCAAATGCAATTAATTGCATTGGAC<br>CAATCTAAGTTACTATTCTTCAGTTTTCATTTTTATTTCATTATTCATT<br>TCATTTTTATTCTGATATAAAAATGAACCAGGATCTGTGTGAAATTAT<br>TTGAATCTAATGTCTTTGAACATTTTTCTTACCATACCTTAAGATTAA<br>AAAAACAAAAAAAAATCCCTTAGTTTGGCAACTTTTGCTGTTGGTT<br>AAGCCCGTTTGGATTTAACATTGACAGGACCAGCTAACTTCCTACC<br>AGTTAACATTGCTTGTGGGCCTGACTGATGTAAAAATATTTAACAAC<br>TGTTGAGAAATAGTCATCAGTGAATCTTAAAGGTCGTACTCATATAA<br>AACAATATAGCGCTTTAACTTTTTATTTACTGAATTTGAGAGAAAGG<br>AATAGTGTTCCAGAAACTGGGATCACTCAGGCGCTCAGGGAAGCAT<br>ATGGTTAGCTGATGATATAAAGGCCCAATATTGGAAGAACTGTTAAT<br>GTAGTCCCCTGGAGACAGTGAGTGAATTATTAGCCCTGGTTGAGAG<br>ATTAAAATGTGTTGATATGATATTATAGAGGCAAATTTAATGCTCTCG<br>GCCTCCCCCTGCATCCTTATTTTTGCATGTAGGTTTATTACTTTACAG<br>GAGAACCAACACTTTTTTCCTTTTTTTTTTTTTAAGTGGTTGAGTTT<br>GTTTTTCTGAACCCAGAGGCATGTTTACACATTTCATTAATTTAAGT<br>ACCAGAGAAGTATACTGAGAACCTACTACATGTCACACACTATCCT<br>AGGAGCTGTAGACAGAAAAATAATAAAATCTCGTGTCTTTATTTAAA

	TTTTGTTGACTGCCACCCCCAAGTGAGTCTCCTCACACACCCCGTC<br>CTGTAATATATGCATCTGGGAGGTCTTTTTTGCCTTCTTAAAAACATA<br>TAGATGGTTGGACATATGTATATAAGAATATAAAATTCACCACTTTAT<br>CTTTTGTGAATGTGTGCTGTGAAGAACTCCTTTACTGGGGTGATGG<br>AACCAGTGGCTACAAAGTAAGGAGCTGGTTTACTGCTGTAAAGGG<br>TTCGCGGCTTTGAATTTCAAGCTCTGGTTCTGTGTCCTTGGGCACCT<br>GCGCGTGAATCGTTGCCGCGAGGCTGGGCCAAGTTAAGGCCCCAC<br>GCAGTTTGGCTTCCGGGCCAAGGAAGCCCCACAGGGTGCCCCACA<br>GGGTGAAGCCCCATGCCCTACAGGGTGAAGCGGCTGAAGCTGGTA<br>GTGGTTCCGAGGAAGCGGTCAAAGTCCCGCTCCAGAGGTTCCTCT<br>TCTTGGTTGTCACTCCCGGAACCCCGCCAGGGGTCTGGCTTCCCCA<br>TCGACACCTCCTCCTGTTCAGTCACCATCACGTATCCCACGAGGCC<br>GGACGGCACTGCCACCTCGTCGCCCCGTAGACTGCGGCCCCGAAA<br>CTACACTTAAGAGTCCCTCGGGGCCCTGGCGGATGGCAGGCGTGAT<br>GAAGCGCCACACCGTGGTGGGCCGCAGGGTATCAGGTGTAGTGTG<br>GCGGGGGCGGCGTCCTGCAGTGTGGCTGGGCGCAACTGGACGCTG<br>TACCTCTCCATAGCCGCCTAGTCGCCGCTCTCCATCCTCGCTCCTGT<br>GCGACGCCACTTTATCCCTCTTAAACTGGACGATTCAGTAGTACCG<br>GGGAACGAAGGCAACAAGACCTAACCATAGCAACAACATTTTGTT<br>TTTGAAACCCATCTTGCAACTGCCTGACTGCAAAATGTACTTGCAG<br>ATTCTGCAGACAAAAATTGCTGCCACTTATTAAAAGACTTCATAGT<br>GAAAACTGACTTTTTACCCAAACCACTGATTCAAGGACTTTTGCGT<br>AGAAACTCATGAACTGTTAAGATAACCCTTCATTACCAATTAAGATA<br>ATAGGTTGTAAGTAACCTAATGAGTACCAACTTTCTCTTATTTCCTTT<br>AAAAACTGTCTCTCAAAACAGCCGGTCACGACACAATGTCAGATG<br>CTTCTGGAATCTGTTTCTCTAATTACAGTTCTAAAACTGCCAACAAA<br>CTCCTTATTTGGACTTCAGTTTCTCTGACTCTTTGGTTCACCATGTT<br>GTGCAGCCATCACCTCTCTCTAGTTCCAGAGCATATTTTATCAATCC<br>TCAAAAGAAACCGTGCATCCACCAGCAGTAACTCCCCACAACCTC<br>TTTCATCCAGTCCTTAGCAACCATTAATCTGGTTTTAGTCTCTATTCA<br>TTTGCCTTTCCTGGATATTTCATATACATGGGATCATTCAGTATCTGG<br>CCTCTTGTATCTGACTTTTTCACACAGTGTAACATTTTCAAGGTTCA<br>CCTATGTGGTGCCTTGTGTCATTTTTTGCTATTTTCATGTCAGGTTTT<br>GAAATGGTATGTTTTTTCTCATCTTGTAAGGGATTTCCTGTTGTAGA<br>AAATCTGTTTCTGTGTATGACTGCAATTTAACCATTTTATTTGCTATC<br>CAGATACATGTTTTAACTTGAGCCAACTATATTTCCACTTTGTATAAT<br>GAATACTAAATTTTGTATCTTAGATACTAAACTTTAGTAGATAACTAG<br>GATACAAATTACAGTTTTCATATTTTTCTTTTCATGGAATATAATTTTT<br>TAAAGTTTCTGATATTAATTCATTATAACATTTACTGGACAAACCAAC<br>CACATTTGGTTCTATGAACTCTTTTCATATTTTACGGGATTTTTATTTT<br>GTTGTCTTAGGAATTGTGGCATCATTCAGTTTTGCTGAGAGTTTATT<br>TTTATTAATAGGTTCTGTGTACTATGCAACCTGTAGTTGTTACTCCTG<br>TTTTGCAAATATGGCATTAAAACATTGGTGAGTGGGGGGACTTTCC<br>TCCCATTTTATTTCAGCAAAGAAATCTTGTCATTTCTCTTCAGACTTT<br>AAGATTTATTGAGGAGAGTTATTCACTTGTTTAGTCTGGTAAACTGT<br>GACCTTTAAATTTTGAAGGAGGTTGAACTAAAAGGTGAAATATGAT

	AAATTGATGGCTCTATTTGTTGTGAATGGAAATTTAATTTAGACTTGT AAAAGATTGTTTTTTAAATTGCTAATTGTGTGACAGTGAAGAGAAT GACTTCAAAATATCCCACTATACAATCTGCAAAGAAAAGAATTGTG TCCCCTTTTTAGTGTAGCTTAACACTTCACTGAAACTGTTTTGAGTT CTTAGGTCATATTTTTTTTTCTCTAAACGAAACAATTACTTTTCTAAA AGTCAAATGTTAGCCATCCTAGAAGTTGGGCATAAAATACTTGTAA GTATATGCTAATATTCTGATACTTAATGCCTGTGAAAAATGTGTATAG AATTTTCAATTTTTAAATAGAAGTGAAGAAAAAGCGATAATAATTAC TATAAATTCAATATGCAGTTATGTATGTATGTGTGTGGTTAAGACAAT TAGGTTCTCATTAAGCTTTGTTTTTTTAAAGATAACATACACATATAT TGATAATGATAAACAATTCATATAGCTTTTTGTGTCCTCTCGTTTTGT GACATAAAAGGTCAATGAAAAAATTGGCGATTAAGTCAAATTCGCA TTTTTCAGGACAGCAGTAGAGCAGTCAGGGAGGCAGATCAGCAGG GCAAGTAGTCAACGTTACTGAATTACCATGTTTTGCTTGAGAATGA ATACATTGTCAGGGTACTAGGGGGTAGGCTGGTTGGGCGGGGTTGA GGGGGTGTTGAGGGCGGAGA
STAT1	AAATGTCACAATGTCACATCTGCTGCAAGGTGGGGAAATAACAGA GCAGGGAATAATCTGGCCATTATTGAAAATCATTGATCAGGAGAGC CTTCTTAGTTTCAGTTTCATATAATGAAAACTACGTAACTGATATCTT TTTGCCCTGGTTTGCAGGCTGTACTGTATTCTTTATCTAGTTATTGTA GATATTTCTGCTATAAGTTGCTTTAAAATGCTTTGTGCAACAAGGCA GATAAACTAAGTAAATAGAACTGGATGATTTGGGTATAAAAGAAAC AGTGAGTGAAGAATAGGAATGATACTGTCTTTTCTCCAAGCAAAAT ACAGTTCCTCCAAATGGGAAGTGTGGAGTAGTTCTTAAGTCTTTCT ACTGTAACATCATGTGAACTTATGAGTTACTCCGGTGACTTCACATC AGTTACTCATCATATAATAGTATTTTCTGGATTCCTCACTGTCCTTGA CTCTCCTTTGTCTGTACACCAGTTCAGGGGTGGCAAATAGGAAATT TCACCTTACCTGTTGCTTCTGATTGATTGCTGGTGACAGTGTACCCT GTCATTTGTTGCTCTTTTAAATCCAATACAAGTAGTTGCATTTATTGT TTTAAATGTTGTCCAGTTGATGGGAGGCTGAGGGAGGAGAATCGTT TGAACCTAGGAGGGGGAGGTTGCAGTGAGCCAAGATCGCGCCACT GCACTCCAGCCTGGGCGACAGAGTGACACTTGGTCTCAAAAAAAA AAAAAGTTATCCAGTTGAGGCCAGGCACAGTGGTTGACACCTGTA ATCCCAGCACTTTGGGAGGTCAAGGCAGACAGATCACCTGAGGCC GGGAGTGTGAGACCAGTCTGGCCAACATGGCAAAACCCTGACTCT ACTAAAAATACAAAAAATTTGACGGGTTTGGTGATGTAGGCCTATA ATCCCAGCTACTTGGGAGGCTAAAGAATGAGAATCGCTTGAACTCA GGAGGCGGTGGTTGCAGTGAGCCGAGATTGCACCAATGCACTCCA GCCTGGGCGACAGAGCGAGACTCTGTCATCAATACATACATACATA CATATATACATACATACATAGTATCCAGTTGGTTTCAGACCAGCAGC AGTCCGTTAGAATATTTAGATTATTTAGAATTTTGATTTAGCGTGGGA ACCTGAGGCATGCTGTCTCCTAGCAGAGGGAAGGAGCACACAAGA ACAAGTTGATTACAAAGAACACGCAGTTTCAGGACAGATGGTATAT ACATCACAACTGTTCATATTCCTTTATCCAGAACAAGTCACATAGCC AAGGTCAGAGTTACAGCACACAGAGAGGTATACTCCACCCTTAGTG AACCATGGCAAGCAGTGGGAAGGGACAGAAGAGATTTGAAAATAG

	TACAATCTACAGACATAGTGTTAGCTGTTTTTTCTAGTTTTGTGTCTT<br>TTGCAAATCTGACATTGTTGATATATAGATATAGCTTTCCAAGCTGAC<br>TGTTATAAGGCAACCGTCACACATTTGTATCTGTCAAATTCTGGATT<br>TCTAGGTTTTTGTTTGGCTTTGGTTTGATTGCTTTTTGAAGCAGTCA<br>ATATGTTATACAGTAAGAAAATATAAATAGTTTATCCTTCAGGAATAT<br>TGAATCTTGGAGGCATAAGGATTGGACACTTCAAAAACCATAATATT<br>AGAGAGTGTATAGTTATTGTCCAAATAAATAGAGACACATATATTATT<br>TGGTCATTCAGAGACCCCAGATTAATTGCTACAGGTTGAGGTGGTT<br>CTAGAAAGAGAATATAGTATTTGGGCTGGGCTTTAACAGGTGAATA<br>AGAATTTGGTAAGTGAAGAGAGTGAGGAGCAAGACTTGCCATTCA<br>GTATGTACAAAGGGGCAGAAATCAGGAAATTAAAAGCATGGGGGG<br>AGTGCTGGAGCTAGACGAGGTTAGTTGGGAAATGCGACAATGATA<br>GAAAAGGCTGGAAAGAAGGAAAGAAACATTTTGTGGAGGATCCA<br>GACTAGCAGACAACATTTGTGTTTATCATATGTGCAAGGAGAGTTG<br>TTAAGGCTTTTGAGCAGGGAATGACTATATCAATGCAGTATTTTAAG<br>GAATTGAATTTGGCAAATATAGTGAAGGAGAAAGAGACAAGAAAA<br>AGGTAGTATACCTAGAGATTAGCATAGCATTCCAATTCTCAAGTGAT<br>GAAGGCTTATGGTAGGTATTGCTGTGGATGCTAAAATAAAGGGGGG<br>TAGGGAAGGAAGCATCCACAGATCTTGATGATCCAAATTGGGATTT<br>GGAGTGCCAAATAATCCTATTTGCAGATGTTAGGATTATTCCATTCCT<br>TCATCAATATTGAAGCTAATATCTGTGCAAGTTTTATAGTACCAGAA<br>AAAAAATTTTTAACATGTAATCTACCCTTCCTAGAGTCAACTTTTGA<br>TATAGTTTGGCTGTGTCCCCACCCAAATCTCATCTTGAATGATAACT<br>CCCACAATTCCCACCTGTCATGGGAGGAAACTGGTGGGAGGTGATT<br>AAATTATGGGGGCGAGACTTTCCTGCGCTGTTTTTGTGATAGTGAAT<br>GAGTCTCGAGGATCCGATCATTTTAAAAATGGGAGTTTCCCTGCAC<br>AAGCTCTTTGCCTGCCGCCATCAATGAAAGATGTGACTTGTTCCTC<br>CTTGCCTCCCGCCATGATTGTGAGGCCTCCCCAGCCATGTGGAACT<br>GTAAGTCCATTAAACCTCTTTATTTTGTAAATTGCCCAGTCTCAGGT<br>CTTTATTAGCAGCGTTAAAAATGGATTAATACAGCTTTTTTCAGAGA<br>ACAGTAAAACATTGTCTTGGTTCTTGGGTGAGTAAGAGCAGAAGG<br>GACACTTTTGGTGCCGTGGCATCTCTTGGCAGAGAAGTCTAATTTC<br>ACTGCTAACTTGCTTTGTGAGGGTGCATTTGTGACAAGAAGAACA<br>AATAGATGTGCAAAGAAAAAAAACTCATACACACACATATAGGCAT<br>ATCCATGGGAAATTATTCAGATCCTTCTCCTACCCCAAAGTGCCTAA<br>TTATTCAAGGCTTGACTCAAATCCCGTCTACTTGAAGGAACTAATTG<br>ATTTTATGAGCATATAGTCCAAAAAGATTTAGTTTGTCTGGCTTCAG<br>CAATGCCTACCTGGAGAAGTGACATTTAAGCTGAGATTTTTAAACT<br>AAGATCTAAAAGATGAGTTAGCCAGCGAAGAGTTGGGTGAAGCAG<br>ATGTTGGCTCAAGGGGTTTTCCAAACTCAGGGTGAGAAGTCCCTG<br>AGTTTGGAGAGTGTGGTTTATTTGAAGAACTAAATGCTGAAGTAAG<br>CAAAGCAGAGTGGCACATTATGGGGTTGGACAAAGAGATCGGGCA<br>ACCAGGGCTTTATAGTATGTATTAAGAAACTGAATTTTATAAGAGCA<br>AAGAAAAGCCATGGAAGGATTTTCAGGTTGGCAAATGACATGATCT<br>GCTTTTCAATTAAAAAAAAAAACACTCAAACTCTTGTATGGAGAAT<br>GGATTAAAAGGTAGATAAGGGCTGAAGTGAGGCAGCCATTCGGGA

	ATCTACTGCAGAAGGAGGCAGCAGAAGAGATGGAGCAGAGTAAAT<br>GAGACTTGGGAAATATTTAGGAGGAAGAATCAATTAAGATTTGAGG<br>ATCAGGTGTTGCGAGGTGAGGGAGAAGGCAATGTCAACTATGAAT<br>CCTAGGTATCTGTCATGTGCCATTTCTAGAAAAAGAGCAGGTTTGA<br>GGAGAAAGATGTTTGGTTCTGAACATAGTTTGTAGAGCTCACTGCA<br>CATACAAGTGGAGAGGCAAGTGGGAGTTGTAGGTGTGAAGCCCAG<br>AGGAGAGGTGTGGACGGGATAAGCATTTAAGACTCCTCCATCTAGA<br>AGGAAACTGAAGCTGTGGGTGAGGTCATCACAGCACAGCGTTTAG<br>GAGAAGCCCAGGTAAAGAAGCTGACGAATGTCTGGACCCTGACAA<br>CCTTAACATATAATGGTTTGATAGTGGAGGTGGAGGCAATGTAGAA<br>AGAATGCCAGAGGCAGGAAAAAGCAAGAAGGATGTGTTATCATCA<br>TGACCAAGGAAGAAACGTGTTTCAAGAACAAAGGCGTCAACTCTG<br>CCCCATGCTTCCGAGCTGTCAAGTAAAGTGAGAAAAACAGAAAAG<br>CGTTCCCTGGGTTTAGCAACACGGAGGTCAGTTGCTAAAGGGAGC<br>TTCTAGAATGACGACGTCGCCAAATCTGTCCTCTGCCTGGATTCTC<br>GGCGATGAAACTACTACAGAGACCTCCAAGTTTGGGCTTCTGCAA<br>ACACAGCACGTCCTTCTGATCGTTCTCTAAGATATGTAAACAGAAC<br>GCCAGTTCCCAGCGTGGCAACACGGGACTGGGCTGCAGCTCACCC<br>AGCCGGCGGCCCCCGCCGGAAGCCGGCGGAAATACCCCAGCGCGT<br>GGGCGGAGCAGCGGCCCGCAGAGGGAGGCGGTGGCGCCCACGGA<br>ACAGCCGCGTCTAATTGGCTGAGCGCGGAGCCGCCCGGTGATTGG<br>TGGGGGCGGAAGGGGGCCGGGCGCCAGCGCTGCCTTTTCTCCTGC<br>CGGGTAGTTTCGCTTTCCTGCGCAGAGTCTGCGGAGGGGCTCGGCT<br>GCACCGGGGGGATCGCGCCTGGCAGACCCCAGACCGAGCAGAGG<br>CGACCCAGCGCGCTCGGGAGAGGCTGCACCGCCGCGCCCCCGCCT<br>AGCCCTTCCGGATCCTGCGCGCAGAGTGAGTGGCCGTGAGGTTCC<br>GGGTGCCGGGGGTGGGACGCGCAGGGACAGAGTCCTCGGGCCGT<br>GCAGTTGGACGCCGGGCGAGGACGGGCCGTCTGTGCTGACCCGCG<br>AATAGTGATCCCAGAGGAAGAACGCGTCCTGAGTATTCCAAGTGCG<br>AGCAGTGCCACCTGTTCTCGGAGATGGCACTGCTCAGCACGATTGT<br>CCTTGCCAGTCGTGCTCTGGCAGTGAAGAGGACTTGTGAAATATAA<br>TTTTCCTCTAGAAGGCACTGCACCTTCCCATTTACCGTCACTTTCTC<br>CCGTTTCCACCCCTTTCCATCAGTCACGTTTTCTTCTTTTCGCAGAA<br>AGTTTCATTTGCT
STAT4	AATCCCAAATCTGTCCTTACCTCTGATAAGCCTCACTAAAGCTCACT<br>AAACCTCTGTCCCCTTATCTGCCAATTGGAGATAATCATGATATTTAA<br>TCCTATGGAGTGCTGTGAGGATTAACTAAAACAATGCATTTGAAGT<br>TCTTGGCACTATGTCTGGCATGGAGGTATATGACTATGATTTGTAAAT<br>TGCAATGCTTCTGAGATGGCAGTAGAGGGAGCAGAGTCAATGGGA<br>ACTGGACTTCAGAAAATTAGCAAGATTGATATTAAACCCTTTTACAA<br>GACAATGTTCCTGTTCTGATGGAGCTAGAGCAACCTGCACACAGAT<br>GAGTCGATGAGATTCACACCAGGCCATCTTATCCCAATAATCCTTGC<br>TGTCCCACTCATGTGGTACTAGCATATGCAACCAGGGAACTCAACG<br>CATCTTTCTGCGTGTACATATTGTCTGTCCAACCTCCAACTCCAGCT<br>CACCCCAGTTTCCTCAGAACACTTAAGAGGGGAGAGGAGAAGTAT<br>GGTGATATCACAAAACTGAAACTAGCAAATGTTTATATTCTTTGTAC

	AAAAATTGGGAGAAGGTGGAGCTGGTTTACCTCCCTTACAGCGGC<br>AAGAGTGATATCCTCTCTCTTCCTCTCTCTGTTTCCCACAAAAGCAG<br>CTCCACTGGGCAGAGTGGGAAAAGCACAATCAGAACCGATTTTGA<br>ATCCACCCTATCTTCTATTTACAACTGTGAACCTGCCTATCCCACCC<br>AGACTCTCTTCATCTCCATTTCCGCTCTGTACAGCAAGGTGGCAGG<br>GAAGCTGGAGGGTTAACCTGGTGAGTGCCAAAGTTACCCATAACTT<br>GTTTATTATTCTATTATTAACAGCTATTGATTCAGCATTTAACCTGATC<br>CCATTTCTTACATTATATCTTTGACTTTTCCCAGCAATTTCAGGTGGG<br>ACATAATATCCCCACTTTTATAGACAGGGAGTTTTGGGGTTAGAGAA<br>GTTAGGTAACTTATATAACTTTGTGCCACTAGAAATTGACACACCAG<br>AACTAGAAGCTACTTTTCTGAAAGTCTTTAATCTGGGATTTTCTTCT<br>TAGCATGATGCAGCAAAAAATTAGATATAGGGCTTATCACCAGGGC<br>AACTGGGTGGCTGTTTGAGAAAGGAGACAGGAATCTGTGGGGGCC<br>TTTGAATTTGGAAAACCTTCCAGGGGTTTCATTTTAGCATTTGGTTT<br>GTTCTTCATTTTACTTCGAATTTTTTTATGTCTTTGTCTCTGAGGGAC<br>TGACAAGGCCAGGGCCAGTGTAGGGTCAAGAAACTGCAGACCTGC<br>TTATTTAGTTTTGATTCTAAAAAAGAGAAACAAATTGGACATAGAA<br>GCGCCCTTTCTTAGATGTCTTCCTCCACTCATGAAATACAGAAGGA<br>AGACAGAGGTTCAAGGCAGAAGATTTAACCAAGAAAAAAGTGCGT<br>ATTTGGGCAGAGAGTCCCTCATGATAGACATCTCTGGATAAACGAG<br>TTACAGTTGGCAGCATATGGCAAATAGAGCAGAGGTTCTCTAAGTG<br>TTACCTGCAGATCCCTGGGAGTGGTCCCCGAGATCTTTTTAGAGCA<br>TCTGCAAGGTTAAACTATTTTTATAATAATACTAATACAGTTTTGTCT<br>TTTCACCCTGCTGACATTTGCACCAATGGTACCAAAGCAAAGGTGG<br>GTGAAACTGTTGGGGCCTTAGCACAAAGCAAGGAAGTGGTGCCAA<br>ATTATACTAGCAGTTGTATATTTCACCACCATGCACTTACAATAAAAT<br>GCCAGTTTGACTTAAGAATGTTTTTGATGAAGCAGTAAAAATTATTA<br>ATTTTATTAAATCTCAGACCTTAAGTACACGTCTTTTTAATATTCTTT<br>GTGATGAAATGGAAAAGGCACATATTAAAGGATGATGGTCATCTCG<br>AGGAAAAGCACTTGCGTGATTGTTTGAGTTGCCAAGACGAGCCTG<br>TCACTTTAAGGAAAACAACTGGTAGTATCTGCTGCCAATAATAAAA<br>CTGAGCTTTCAAGCAAAAATTAGAATTTGAGCTTGACTGCTTCCCA<br>GTATTTAAAATACTTACATGATGAGATCAACATTGATATTACCTGACA<br>TGAATTTCTGAACACTGTATGATGAAATGTTGTATTCATTGTATGATG<br>AAGTAGGCAGCATTTAGAAAATCTGCATACTTCAGTGGACCAATGT<br>TTTTTAAATGACCAGTGTCTGACATTGCAAAATAATTTGTGGGTAAT<br>AGTTTCATTCAAAATATATACAACAAAAGATTTGAATGTAACAGTAT<br>ACATAAAGTATATGTTTACATAAAGTATACATAAAGTATATGTTGATA<br>AGGTTTCAGATTTCACAATGCAACTAGCCCTTCAGAAACTACCACT<br>CATTGAGTTTTGATGTGGTATCAAAGAAGAATAATTATTTGAAAAGG<br>TCATTATAATACTCCCCTTTTCCAACTACATAAATGTGTGAGTCCATA<br>CTTTCTTCAAATATGTCAACCAAAATAATACTTTGCAGCAGATTAAA<br>TGCAGAAACAGATATGAGAATCCAGCTGTCTTCTCTCAAACCAGAC<br>ATTAAAGAGATTTTTAAAATGCCACTCTTCTCATTACGTTTTTCATGT<br>TGGAAGATATATTTGTTTTTCTTAAAACATGTTTAACATGGAGTGGG<br>TTTATTATTGTCATTTTGAAGGAATAAATACATATTTTAATTTTTTCTC

	AGATTTAAGTTTTAACACAGTAAATAAAAATTTCATCCAGAACAGT<br>AATACACAAACATTCCTTGGGTTTGTCAACAATTTTTAAGGGAATAA<br>TGGTATCTTGAGAACAAAAGGTTTAAAAACTGCTGTGGAAGCCGC<br>AAGGTAAGGCCCTGAAAAGGAGGAGGAGAACAAACTCTGTTTTGT<br>ACAGCAGGCACTCAACAATTGAATATGATAATGATAAGGACATGTA<br>AAACCAGTGTGACTAACACTATGAGGCAGGAAGTGAAAAGTGCTA<br>AATAAGTGGTCTAGATGTGCACTGTCCAACACGGCAGCCACTAGCC<br>ATATGTGGCTTGGAATGTGGCTAGTCCTAACTGACATGCCCTGTAAG<br>TGCTAAATATACACCAGACTTCAGAGATTGAGTATGAAATAAGGAA<br>TGCAAAGTATCTTATTGATAATTTTTTATATTGATTATATGCCAAAATG<br>TTAAGATTCTTGTATATATTGGGTTATTTACAATATTAAAATTAATTTC<br>ACCTGTGCTTTTTTCCTTTTTAATATGGCTATGAGAAAATTTACAATT<br>ACATATGTGGCTTACATTTTATTTCTCTTGGAGAGCGCTGGTTTGGA<br>CAGGAAGTATGAAGAATTTGGAGCAGGGAAACATTGCTGTGGGTT<br>CAGGTCATCGGGAAGTCTTCACTGAGAACTATAGGATTTCACTAAG<br>TAGGTAGGCTCAGGGAGGAATATCCAGTTGAAGAAAGCCTCGGTA<br>GTGAAATATGAGGTTAATCTGGGCAGCTGAGGTGACTGCTTGGGTC<br>ACATCATGGGCTCTTATAGGGGAGAAAAGTTTCCTGGATAGGTTGG<br>GACCAAACTGTACAGAACTTAAACTGCCAAGAATTTGGATTTTTTT<br>CCTATAGGCAGTGACAGCTTCTCCAGAAGAAAATCAGACTTTGCTT<br>AGTAGCTTATGGAATAGCCTGAAATTGGCACTTTCTTATGTAGGGTC<br>AGAGCAGCATAAGCTCCTGGAAACTGCCTGCTCAACAAGCAGTGG<br>GGACCAGGCCTACCTAACCAGGAAATTTACAGTGATATGGAAAGTG<br>TGACTTTGGATTGATTGACCTATTTGGGGGTGGGTGGTAAGTTTAA<br>GCACATGATAATGTCACCTGGGAGAGAAGAATCCAGAGGGTGTAC<br>CTGTGTGAAACCCGTCAGGGAAGAGGGAGGCAGGCCTTTCACCTT<br>GGTATATTGCTCTAAGCATGTGTATAACCAAATTGAGTGACACTTTC<br>TACCTTTCTTTCAGTCTGTCTGTCTGTCTTTATTTTTGTCTTTGTTTT<br>ACTATTTTTACTTTCTTAAACATTTTTATTGATTTTTTAATATACGGTG<br>GGATTTTACTGTGTTGCCTGGGCTGGTCTCAAACTCCTGGGCTCAA<br>GGAGTCCTTCCACTTCAGCCTCCCAAAGTGCTAGGATTACAGGTGT<br>GAGCCACACGCCTGGACTTCTCTTTATTTTATTGAACCCCTACTGTG<br>AATCAAGGGGTTAAGAGCTAACTACAAGGAGATGAAATGAAAAGA<br>CCACTGAAGTGGGAAGGGAAACACCTGGGCTATTATCGGGCTCCG<br>TGGCTAACTTATTGAGTCCTCTTGGTCACCTCACTGGGTCTCTTCAG<br>ATATGAAAGAGGATTTAGCATTAGATGGGACCAAGACTTCCTCCAA<br>CTCTGTGATTCTAGAACAGGCTGATGTCACAAAGGGAGAAGAACG<br>CACCGTAGCAACCAGGGGCAAGGCTACTCAAGGAGAGTAGGAGG<br>AGGCTAGGTCAGGAAGGGCAAGCACTGGTGGCCTACCCAGGACTT<br>GGCCCAACCCTCTGGGTCAGGTCACCCTCGTCGGAGTGAGCGGAC<br>CCCGCTGGAATTGGAGCCCAGTAAGGTCTATGAGTTTGGTGTTTTG<br>ATCCTGGCTACCCATCCCTTCCATCCCAGGGGTGGTTCACCAGGCT<br>GAGTGGAGCCTTATACTAGGGAGAGAGGAAGCTGAAGAACTGGGC<br>TCCAGGTAAGTAACTCTGCAGAGGATCTCAGATTGTCTAAGGAAAC<br>CACTGAAGAAAATGTATGCCCCGCCCCTCCCCTTTTTCCTGGCACT<br>GCTGAGCCACCTGCAGTTGCGAGAGCCGCTGGGAGGGATAAGAGG

	GAAGAGGACGCCCGGTGAAGGGGCTCCAGCCTGGCAGTTTCTGCG<br>TGTTAGCATTTCTAGAATAGAGTGGGTGGGAACTGACCCAAGTAAA<br>GTCCCAGAGACTCGAACACTGACGCACAGGAAAGCCTCAAGTGG<br>GAGGAGAAATGCAAATCCCCTACTGATGATGGCGTCAGCGGCTTTC<br>TCCTAGGGACTGTGAGGGG
SYN1	TATCCTCATTCAACCCCGTGAGGTGAGTATTAATATTACTATTCCTAC<br>CATTCTCATTTTGCAGATGAGGGAAACCGAGGCACAGAGAAGCTA<br>AAGAAATTGCCCAATATCCTATAGCTAAAAAGTAGTGTTGGGAGGC<br>CAGGCACAGTGGCTCACGCCTGTAATCCCAGCAACACTTTGGGAG<br>GCTGAGGCAGGAGCATCACTTGAGCCCAGGAGATTGAGGCTGCAG<br>TGAGCTATGATTGTGCCACTGCACTGCAGCCTGGGCAACAGAGTG<br>AGACCCTGCCTCAAAAAAAAAAAAAGGAAAAAAAAAAAAAAAAA<br>AAGTAGCCCTCGGCATATAGCAGGCACTCAATAAGAATTGAATGCA<br>TTCTTGCCTTCCCTGAGATTCTCCCTTCCGTTCCTCCCCACCCCTAA<br>TGCCTCAGGCCCACCGTTTCCATGGTCGAAGGTCAGGGCGAGAAG<br>AACGTGACCTTCTGGGGGAGACCGCTGCCACGGTGTGAGGAGCTA<br>CAAGGGCAGAAGCTGGTGGTGGAGGAGAAACGACCATGTCTACAC<br>GTGCCTGCTTGCAAAGACCCTGAGGAAGAGGAACTCTAACACTTC<br>TCTCCTCCACTCTGAGCCCCCTGACCTTCCAAACCTCAATAAACTA<br>GCCTCTTCGAGTTCGTCTACGATTCCTTAAAGGAGGAAAAACAACC<br>TATCCCCTTCCCCAAAAGTAGGGTGATAGCATCTCATAGGGCAAAC<br>AGCACAGCACGGGCCAAGACACAGCAGCCTGTTGCGGAAAACAG<br>TATTTTCCACTGTGGTCGCAGGTTGGCAGGAGTGAAAGCGAGTGT<br>GCAGTCCCAGCCTCTCCCCGCCGCTGCCATAACCCCAATGTCTGGG<br>CCCTGCGCAGCCCCCTTAGAGCCTCAGGGCCGCCCGGCACGTTCCT<br>CTGCAGAGCTCCGCGTTACGGGTTTCCTGATTGGCTACTTGTCCTG<br>GCAGGCACTTCTTGATTGGCCGGCGTGCTTGTTCGTTGTCTCCCCG<br>TCTCCTGGACCTCCCTAGATTCCCACCAGCCGGAACCCTGGAGGAG<br>CCTCTGCCGGCCGACTCCGACTCCGCGTCAGTCGAGGATGAACGG<br>CAGAGGCGTGGCCGAGGCTGGCACCCTGCCGCCCGAGCACCTGCA<br>CCCACCCACTCCCGACAGCCCAGCTCCAATGCCACAATTACCAGCG<br>ACAAATAGCTCGGCGAGGCGGGGGGCGGGCCGAGGAAGTGCCTG<br>TGTTGCGCTCTGAGCTGAGGAGGGGCAAAGAAGCTAGAGTCAGTA<br>TCCGCCCGGAGGAGCCCCCAAGACAAATCCAGACACTTTCCTCTG<br>GTAATTTGTTCATTCATTCAACACTTATTAGCGCCTGCTGTATGCTAA<br>AGCTGAGTCTGAGCATCTAGGGTGGAGGAGGGCTGGTAGAAAGAG<br>GACATCTGAGTGAGGGAACAGACTCAGAAGGTATTCCTTTCGTGTG<br>AACATCTATTCAACACTTAACGCCTACTGTATACTAACAATAGTGCT<br>GGACATCACGGAGAATAGAGAAGAAGAGGAATCAGAGCGTTACGT<br>GTCTCCGCAGTAAAGGGAACAAAGCCAGACACCAGTGCTTTTAGC<br>ATTTGCTCATTCATTTACCACTTACTGAATGCCGGGCCCCTTGTGAA<br>GAGACGCATCATAAGAATCATAATAATCATGGTAATAGCTACTACTT<br>AGAAAGTACTTACTGTGTGCTTAGTAGTGTCTTAAGGCTTTCCATAG<br>CATGAACTCATTTCGTCCACAGCAAACCCATGTGGGTAAGTATTATT<br>ATCCCCATTTTACAAAGGAAACACGCACAGAAAGGTAGTAGGCTG<br>GACATTTTTGAAGCATCATGGATTGTGATGAGGTCTTAAATGTCAAG

	AGTGCTGTGGGTGGAGGGGACCCGTCTGTGTGGGAAGGTTCGGGT<br>AGGAGGTTTGTGACATGCACACCCAGAGTATATTGGTACACTTACT<br>TGACTTCTCTGGTTCGTTTTCCCCTTATTCAAGTGGACCAGTCAATG<br>AGTTGAAGTGAAGATGAAGTTGATGAGTACTTACTTGGTCATCTTG<br>GGAAGACTGAGCCAAAGTGAGGGGTTCTGGTTGCTACTACAGGGT<br>ATGAGAGAGGTATACGTTTACAAGAAAGTGGGAATTCAGCTGTGTG<br>TGTGTGAGAGAGAGATGCAGACCTTTGGGCCTGTGCAAGAATTAA<br>GGATGTATTGATATGTGGCATGTGTGTGAGGTTACAAATGTATGTAT<br>GCCATAACAATGCAGGAGTCCCGTGTGTGCACATACACATTAAGGT<br>GCCATGGACATGTATGTGTGAGTCACAAACATGGGTACAATACTGA<br>ATGTGAACATATGTCAGGGGTGTGTGTTTGAGAGTCGTTGCTCACC<br>AATGTACAGCAAAATCATTATTGGGGCCCCTTTTGTACGCCTATTCC<br>TGCTGAACAAGATGGAGACAGTTTGGGGGAGCCTAACCTTTGCCTT<br>AGTGAATTGAGGCACTCCTGAGGTGAATCCTCATTCCTCTACAAAA<br>CAATCTACAAAAAATTAATTTCTGGAAATTGAGCCACGTGGAAAAA<br>TGAAATGGTGCTGGGGCCACAAGTACTAGGAACCTAGGAATGGGG<br>ACCCCAGGAAGAGGCGCAGCCTGGAGATAGATTCCCGAGGAACCT<br>GAATTCAGACCTAAAGGAGTCAGAAAGATCTGTAGACCTGCAGCT<br>GTTCCTGTCCCCAAAGAGGGCACCGCGCCCTGCACAGCTTGTTTGC<br>TTCCGTTACTGGGCAAAAAACCAGGGAGCACATACTTCAAGACATT<br>GGAAAATATCTGATTTGCGGGGAGATCCCCATCTGGGTAACACTAG<br>GATTTGGAATGCCAGAGAAGGAATCCTGAAGGGAGGAGAAAAGA<br>GTGGCCAAATCAAAGGCCTGGAGGTGGGACTGAAGCTGGCTGGGT<br>TGGAGGAGGGAGTAGAGGATGGTTTGGGAAGTGTTTGACTTGGGT<br>ACTGTGGAAGCCTAGGGGAGGAGGGGATAGAATCTGAGACCTGGG<br>GGTGAGGGGTTCCTGCAGACAGTGGCCAAAGGCAGCCCTCGAGA<br>GAGAAGGAGAGGACATTCCTGGCAGAAGTTACAACACATGCAAAG<br>GTACAGAGGTTGCCCCCTTCCTACCCCTCTCCTTAGAGGTGGGTTA<br>GAGATGTATCCTTTTTACAGATGAGGAAACCAAATCTCAGAAAGAT<br>TAAGTCACTTTCCCAAGTGTATGGTGGAGGCCCCACTTGAACCCAG<br>GCACTGTGTCTCCAGACCCCACACTATTACTGCCTTGTTTAAACCA<br>GCCAATGATTTAATGAATAAAGGATGAACAAATGAATAAGTGGATG<br>AGTCACCTGAAAATTCTGCAGGCAAAAGAGACTCCATATCTACTTA<br>CTTCTTGCCTATCTTCTGCCACCTCTCCTAGTCCACCATCACTGCTC<br>ACTATGGTCAAGGTCCTACCCAATCTGGCCCCTGCTACCACAACCC<br>CCTTCAGCTTGTTCCAGCCACATTGGCACTGGATGTTTCCTCTTCCT<br>GGCACATTCTTAAAAAAATGTGTTGATCATAAAGTGAACATGACCC<br>TTTGGGAATTAACTGGAGTTCTTGTATTCCCTCATCTGTAAAATAGA<br>CATTATATTATCCACCCCACTGGATTGTTGTGAGGGTTGGATGAAAT<br>GATGCATGTAAACAGCTTAGTTAAGAGTTGGGTACAATCAGTGAAC<br>AAATGATTATGAATTAGTGCTTTTATTGTAGTCAGAATCATAAAGATT<br>TGACAGGTTCCCATATCCCACCTCTGCTTGGACTACCTCATTTGCTC<br>ATATGCAAACATTATTTGGTACCTACTGTGTGTGCACCATGGGATGG<br>GCCTGCCTCTGTGGAAAGTTCTTGGGTGCAGGGGGAGACAGCCAT<br>GGGCACTGATGACATCAGGTAGTTATCGTGAGTTTTGGCGGTGTCC<br>AGAGCAAAGGGATGGTGGCGTATATACCAAGTGTGTTCTGGTGTGG

	GGGTGGACACGCACCAGGGCTAGGGCTGCAGAGAATGTCTGTGTT GCAGATCTAGGTTTCTCCATGATCATCGGTGGGAATGTGTTTGTCT GCAAGTGTATGCTCATATGAGTTTCCCTGGGTCTCTGTGTGTCAGTG TGTTACCTGTGTGTGTGGGGGTATGGGTGTATGCATGCATGTATGTA ACATGCCCATGTGTGTTACTCTGGACTTGTATGTCTGTATGTATACCT AGATTGGCGTGTGTTCTGTCTGTACATGCCCTCGTATGTTTCCTCAC TTTTGTGTGTGTTTATATGTGTGTCATTTCTTGTGTGCCTCCAGGCCC CCCTTGCCACCTTGGGCAAGGGTGTGTACACCACCCAAGTGTCCA CCTCCGCTTGTCTGATGCTGTCTGTGACGCCCCCGCTCTCTGCCTAG CTGAGCCTGTGTGGATGTGGGAGACTAATCTCCCCGCGGGCACTGC GTGTGACCTCACCCCCCTCTGTGAGGGGGTTATTTCTCTACTTTCGT GTCTCTGAGTGTGCTTCCAGTGCCCCCCTCCCCCCAAAAAATGCCT TCTGAGTTGAATATCAACACTACAAACCGAGTATCTGCAGAGGGCC CTGCGTATGAGTGCAAGTGGGTTTTAGGACCAGGATGAGGCGGGG TGGGGGTGCCTACCTGACGACCGACCCCGACCCACTGGACAAGCA CCCAACCCCCATTCCCCAAATTGCGCATCCCCTATCAGAGAGGGGG AGGGGAAACAGGATGCGGCGAGGCGCGTGCGCACTGCCAGCTTCA GCACCGCGGACAGTGCCTTCGCCCCCGCCTGGCGGCGCGCGCCAC CGCCGCCTCAGCACTGAAGGCGCGCTGACGTCACTCGCCGGTCCC CCGCAAACTCCCCTTCCCGGCCACCTTGGTCGCGTCCGCGCCGCCG CCGGCCCAGCCGGACCGCACCACGCGAGGCGCGAGATAGGGGGG CACGGGCGCGACCATCTGCGCTGCGGCGCCGGCGACTCAGCGCTG CCTCAGTCTGCGGTGGGCAGCGGAGGAG
TCF7	GCCGAGAGCCAGCAGCCCAGCCTCCCGGCGGCTTTGGGCAAGCCC CAACAGGATGCCAGGCGGGCTGGTGAGCAGGAAGAGACGCCGCT CACAACCTCTCCTTGCTGTAATTTACTAAATCCATCTGGGGCTCCTT TGAGACGTTTTCTGGAAATGCCACATTTCAAGGGTCTCCCTGTCAG AAAAGGTCAGAGTTCACGTGGGCCTTTCATGTTAAAGGAGCTTGA AAGAAAAGGAAAGGAAATGTATATTTCAGCTCTAGCCATGATTAA GTGGGCTCAGGCCATGAGAACATTCAGATGATTTACATGACGTTAC ATGAAGAGCCAATTAGCCTAACAACTTCCGAAAAATATCTCGTTCA AGAGCACCCATTAGACACTTGGAATGCACCAGGCTGCTGTTCTAAG CTCTTCACTAGGGTACAGTGGTTCATCTCGACTGCACTCCAACAGA GCAGGGCAGGCCTGCGTATGACATCCATTTAACCTATGGGGAGACT GAGGCTCAGAGAGGGTCAGCAATCTGCCTGGGGTCCCACAGCTAG TAAGTGGCACGGCAGAGATGGAAACTTAAGACTGCCCAAATCTAA AGCTGGAGCTCATCTGCTCCAACAACCCTCAAGCCTTGGGAGTCCT GACTTAGTCCTGTTTCCAGCATCTGACTAGTGCAAGCTCATTTGCGT GAAGATGAAGCCTTATTAAATCACCAGCCCTGGCCTGGTCTGGATG AATCCAGAAGACTCTGGGGCCTCCTTGCAGCTGGTCCTGGTCCTGA AACTCCCCGGTCACCTCCAGGGGGGCAACCACAGCAGATTGCACT CACTACCCTGTGGAAGAACCCCAGTGTCTATCATCTGTGTATGTGAT CTGAGCCAATTCCCAGAACAGGTGGCCTCGGGTGGGCTCAGCAGA GAGATGGTACACACTGAGATCTGTCTGGTTCAAGGGAGAGATTCTA GGCCCTCTCTGCCAGCAAAGGTCTTGGGCCCCCTATACAGACCACC AAGATGATTCCCTAATCCTGTTGATGGCCCCCTTCATGCAACATGAG

	CCACCCAGAAAGTCAGGGCTCCATATTAGTGACAGCTTATTTGTCA<br>GCATTTGTCTGCCAAGACCACAAGGCAAATAAGGACTCCCTCTGG<br>AATTTTATAATGGAACACTAGAGTGTGATAGTTAGGATGTGGGCTCA<br>GGAGTCAGACTGCCTGGGTTTCAAATTCTGACACCTTCATTTTCAA<br>GCTGTGAGAACTTAGGTGACTTCATCTCTCTGAGCTTCAGTTTCCT<br>CATTAGTCAAATGGGGATACGAGTCCCTATGTCACAGGGTTGGTGT<br>GATGATTAAATGAGATAATGTGTGTGAAGCTCTTGACAGGATGCCT<br>GGCACATAGTAATCACTCAGTATATGAGGTTGGTCGTTGTGTAGACG<br>ATGGTGAAGGAATCAGTGCTGACTAGACCAGGGACTCCTGTGGGG<br>CCGCACCAACTTCTGGGGAACAAATCAGAGCTCTTGTTCCTTTCCA<br>GCTTTCCAAACCCTCCTAAGCCATCCAGGCCCACATGAAATCTACC<br>TCCTTCAGGAAGCCACCCTGACTACTCTAGTCCACATTGATCTCAC<br>CATATCTGAGCTCTTTCTGTCCATTACCTGTATAGACTACTCTAGCAC<br>CTAAATCGGCTCTAATAACTCTTCAGCTTCTCAAAGCAGCCAATGG<br>CTCCTGGAAGGCAGACACCTCTAGCCCTTCCCTCTCCACAGCCCAA<br>GCCAGGGCTAGGTACACAGTAGATGATCCCTTAGCAAGCCTTCAGA<br>CTGATTTCAGTGTGGCTGTGGATGCCTGTGACTCAGCCCTTCTCTCC<br>CTCACGCCTCCTCACAGTCCCACTCTCCCTACTCACCCACCCTCTC<br>CTCTGGTTCCAGCCAGCAGGGTGGGGAGTTGGAGCTATGACCCCAT<br>AGGACAGTTGTTCCCAGACTATAAAATGTCCAAAGAAACTTGAGG<br>GTGGGGGAGTTAACAGATGGGGAAAAAAATGTTCCTTATGTTCTTT<br>CACCAAATAAAGACATGAAAAAAAACACTTGTCCCACCACCATTTT<br>AGTAAAGAAAGGATGTTTTCACACCAATAAAGCAGTAAGGGCATAA<br>TCCTTGATTAAAGGCTAATACGAAGCTGGAAAGTTTTAACCTTATTG<br>AGAAAACAAAACAAAACAAAAAGAACCTCACTGTTGTTGTGACTT<br>TTCTCCTTTCTCCAAGGACTGGAGAACACTTCAGCCTCCACTGATC<br>TGTGTCGGTGGAACTCATGGGTCTGGGGACAGCTATTCTGGAGGAC<br>GTTCCTGCTGAACCAGGGTTCCCTACAAACACATGAGGACCCCTAA<br>AGCCATTGGGTTGGGCAGATCCGGATTCCCTTTCAGCTGTCCCAGA<br>GGAGGCTCACCCTATGCCAGACCCTACCTATAACCTCAGGAGTTGA<br>GGTGGCGGAGGCTCTGAACTCCCGTCCCTGTACCTCTCCCCTCCGG<br>GCAGGGCCGAGCCCCCCCTTCCCCGCCAATCCGGAGCAGATGAGG<br>CGAGACTAAGCGGCAAGCCCACCCTGGGAAGAGGCTGGGGAGAC<br>CTAATGGGCAGGGTCCCCCTCATCTCCATCACCAAGCTGAGCACTC<br>GTGCCCATGCCACCCCCCAGCCCCAGCCTGGGGAAAGGAGGAAAA<br>GTGGGCACCCTAGCCCGGAGGTGGCGGGGGAGGGGGGTGTGGGG<br>GCTGGTGTCCGAGACACCCGGCTGGGCGGGCACAGATAGGATCTG<br>AACACAGGGAAGCATGGCCTAGCTCTTTGATCCACAGCCAGCCAC<br>GCTGGGCCTGGACCCGGAGGGAGGCACGGCTGTCTTCCGGCCCGG<br>CTTTGCACCGCTGCCAGGGGTCCTGTGGGGCCCTCAGAGGGAGAG<br>GCGTCCAATACCCGGCCCTCCCCCAAATACCCTCTATCCCAACTGCT<br>CTCAGAAGAGGCGTGAGCGTCTACAGTGAACCCAGCACAGAAACC<br>TGCTAGGGGAGCTGCTGTTGACTGCATCGCGATCCAAAGGACCGG<br>CGTCTTTTGTAGATGCAGGGGCTGAGCCAGGCCAAGCGCGCCGTG<br>GAGGCCCGCGTGGACGCAGACCCGGGTGCCATACAGATGCGCTGG<br>AATCCAGGCACTTTCCCGCGCTCCGCAAATCTAGATGTTTCAGCCT

	GGATCGAGCGAGGGTTAGAGGGTTAGTCAGGCCAGGGTCAACTTC<br>AGAGTCATGGGCTCCCTAAATGCGACTTCTAGGGTTGAGTTGCTGT<br>GGACGAGCGACCCATGTCGGAATCCCGCGCCCACGTGGCTGCCCA<br>AAGTTCCGAGTCTCCGGGCTGCAGGTTCTAGTCACGGAACCGAGT<br>TGGGAGAGTCATAGGGGCTGGGACTTGGAGGATCGGCTGAGGTCC<br>GGTGCTCTTGGCTGTGTTCGCGGCTCGGAGCCGTCGCCTGACTGAG<br>GGGCCCGTCACAGATGTGTGATGTATAAGCTCTGCACGCAACAGGA<br>GCTCAATAAATGTGCGAAGGGGGGTATACTTATGTTCGCACTGTATG<br>CAGGCGGCCTAGAAGGAAGTCCCTGATTGGCACAGGGATGGAGGA<br>TGGGGCAAGAGCCGCAACAGCGCCGCGGAGTTCCAACGCTGCCG<br>GTTCCCTGGGGTACGAGCACAGCCTCAAGCAGCCTCAAGCCCTAG<br>GAAGCCCCCAGTTCAAAGCACAGGGCGCATTGGAGCCTGGGCACG<br>ATACAGTTCACACCACGGCTGCGATGGTAAGCCACGCCCAAGTCCC<br>AAGGGCCTAGGGGACCCCCGCCCTCCACAGCCGGAGGAGAAACCT<br>GGGCGCAGAAAGCAGGGGGAATATCTGGTTGTAGGTGAGTAAGCG<br>GGGTCAGGAGTTCCCGTTAGAGTCTCTGCGTTTCGGGAGAAGGGT<br>GATCATTCCCAGGCTTGTCCGACGTCTCTCTCAGGGTGCGCTCCGG<br>AAGAGCGAGCCCTTTAAGGCTATGCCGAGTGGGCGCGTCCCGGCC<br>TCTCCCGGGAGAGGAGAGGCGGGGCGGACCTGTGTCCCGCCCCCG<br>GCCCGGCCCGCCCCCAGTGCCCGCCCCGCCCCCGGCACTCGGCCG<br>GCGGCGCCTTTGATGTTCCGACCCGCCAGCTCGCGGAGCCGCTCTG<br>CCCCGCGCCCTAGCCCGCGCCTGCAGCCCGCCCAGGCGGAGTCAG<br>CCCGCGCTCCGCCCGCCGCGATCCGAGCTCGGAGGTTCGGACTCC<br>GGGCTCGCCGCCCCCCGGGCCGGCTCCGCGCCCCGCACTCCCGGC<br>GCCCAGCGCCCCGCGCCCCGGCGGGCGGAGCGCACCATGCCGCAG<br>CTGGACTCCGGCGGGGGCGGCGCGGGCGGCGGCGACGACCTCGG<br>CGCGCCGGACGAGCTGCTGGCCTTCCAGGATGAAGGCGAGGAGCA<br>GGACGACAAGAGCCGCGACAGCGCCGCCGGTCCCGAGCGCGACC<br>TGGCCGAGCTCAAGTCGTCGCTCGTGAACGAGTCCGAGGGCGCGG<br>CCGGCGGCGCAGGGATCCCGGGGGTCCCGGGGGCCGGCGCCGGG<br>GCCCGCGGCGAGGCCGAGGTGAGCCCCCGCCGGCGCCGGCTCCTC<br>CCCCGCGGTCGCCGCGCCGCGCCGCCCCAGTTGCGCGCGGCCCTC<br>GGGGTCTCCAGCGCGCAGAGCGTCCCTGCCCCGGCGTCGGCCCCG<br>ACCCCCGCGGTCCCACCGCCCCTCACTCCCCTCCGGTTCTCCCTCC<br>AGGCTCTCGGGCGGGAACACGCTGCGCAGAGACTCTTCCCGGACA<br>AACTTCCAGAGCCCCTGGAGGACGGTGAGTTTCTGCCCGGCCCGG<br>CTTCCCTTCGTCGCGCTCAGGCCCTGGCCTCGGTGGGACGGGGAC<br>GCCAAGGACCGCGGGGAGCCGGGTGCCTCCCCCACCGCAGCTCAG<br>GAGGCGGCAGAACCCAGGGGTGGAGAGTGGGGGGCGGGCTTCCC<br>GGGCGCCGCCGGGTCGAGTCACTTCCGGTGCCCTGACCTTTATAGG<br>AGTAAACAGACCCCCGCCATCCCCGCCTCCCCTCCTGCCCAGGTGA<br>CTGACTAATCCGCCG
TF	TTTCAATCCTTTTACTTACTATTTCTTTTTCTTGCCTGACTAAACTAG<br>CAAGGGCCTCCCGTAAAAAGGATGACCAGAAATGATAATAGTAAAC<br>ATTCTGGTCTTATTACTGATCTCAACAGAAAAGCTTTATTCAGCATG<br>CCACATTAAGTATGATTTCTGTAGATTTTTATGGTACACTAATACTTG

	ATATCAGACTGAGAAAGGTTTGTCGAATAAATTTGCTAAGAGGTTT<br>TGTTATGAATGGATATTGAATTTTATGAAATGATGTTCCCATACTGAG<br>TGATAGGGTCATACCGTTTTCCTTTATTATTCCACTAATATGATATATT<br>ACAGTGATTGATACTTGAAAAGATAGCTTACATTCCTGGAATAAACC<br>CAATTTGGTAATTATTTATTAATCTTTTTTACATTTTGCTAGATTCTAT<br>TTGCTAATCTTTTGTGTAGAAATTTTGCATCTGAGTTCATAGAAGTTA<br>TTTTTTTATCTTTGTCATGATGTCTTTGTTAGTTTTTCCTTTCTAAGTA<br>TACATACTGTCCTCAAAACATTAGTTAAGGAGTGTTCCTACTTTTCT<br>GTTCCTGGAGGAATATATTGGTTTAATCCTTTTTCTTTATTTAGAGAC<br>AGGGTCTCACTTTGTTGCCCAGGCTGGAATGCAGTGTTGTGATCTT<br>GGTTCACTGCAGCCTTGAACTCCTGGGCTCAAGCAATCCTCTTACC<br>TTAGCCTCTTGGGTAGCTGGCACTACAGGTGCATGCCACTGTGTCC<br>AGCTGATTTAAATTTTTTGTAGAGACGAGTTCTCACTATATTGCCCA<br>GGCTGGTCTTGAACTCGTGAGCTCAAGCAATCCTCCTGCCTCAGGC<br>TTCCAAAGCCCTAGGATTACAGGCATGAGCCACCACCCCCACGCTG<br>TAATTCTTAAATGTTTGATGGAATTCATTCCACCTGGCCCTGGACTA<br>TTCTTCATGAGACAGGTATTTTTGCAGACTCCCCTGTGAAAAGAAT<br>GGAAACCTTGAGAATCTTCATTGTCCAAGGACTTCTGAGAGGTAGA<br>ACAGAGATAGAGAAAGGGAAAGAGAAGGAGAGAGAGAGAGAGA<br>AAGAGAGAGAGAGTGAGAGCGAGTGAGCAGTGGTGTCATGTGGC<br>ATTTCAAAATGCCTGGCATTTTCCTAAAAGTACCCTGCCCAGTCCCC<br>AACCCCCTAGCCATACAGCCCGACCCAGCGGGCTTGCAGGGTGAC<br>TGATTTCGTGAATGTCGGTTCTCAGGCTGCCAGGCTGCTGCTTCTC<br>AAGGATGACCCAGACTTAGAGAAGTCTCTGGTGTCAGGCAGAGGA<br>CACTGGGTGGGGGACAGGAGGAACTGCTCAAAACAGACAGAGGC<br>TCTTTGTTTGCTTTGCTTCTGTGTCAACTGGGCAACATTTGGAAAC<br>AACAAATATTGGTTCAGAGGCCCACTGCTTTCTTACCCACCTCCTG<br>CTGGTCAGCTTTTCCAGCTTTCCTGCACGTACACACAAGCGCAGCT<br>ATTTCTTTAAGGGCCAACTCGTGGTTGAAATTGAGAGTTTTGCCC<br>TGTTTGCGGTGAAAGATTTGGCTCATGCTTGGGTTGGTCTAGAACT<br>GGAAGCTTCCTTTATGTCCTCTATCAATCTCTTTCTCTTTCTCTTTCT<br>CTCTCACCTCCCTCCCTCTCTTTCCTCCCCCACTAAATAAATAAAGA<br>GAAGGTCAATATCAAGAAGGCAGCTGTGGTCTGGAGAAGGGCAAG<br>ATTTAGGAAGTTTGAGTTCAGGTCTCAGAGCTCCATGAATAAGGAC<br>CCATCATTTGACTGTAATGACCACATGTGATAAATGGAGGTACTGAT<br>CTTATCTTGCATGGCTGCCCCAATGATCTTGCCCCAGGACAGTGCCT<br>GACACATACAAGGGGATAAAAACATGATATTTTCTTCCAGCTGAGC<br>ATAATCAAGCCAGTCCATGTCTAAGGATCTGCAGTTGGTACTCAGA<br>CCTCTCCCAGACTGACCAGCCCCTGTGCTGGCAAAGCCCTGGGGG<br>CTGTGTGCTTTTGGAGAAATGGCAAGGCTGGGAGTCTCATGTCTGT<br>CCCTCTCTCGGTGTGACCCCAGATGAGCTCCTTGGCCCCTGCATGT<br>CCTGGTTTCCTGGGAGAGGCAGTGTCCCTGGTGGATGTGTACACTC<br>TGGAACCAGACTGCCTTCTTTTGTATTCTGACTTTGCAGCTGACCTT<br>GAACAAGATATCATCCTCTCTTGTCCTCAGTTTCCTTGTCTCATCTGT<br>AAGATACAGATGCTAAAGCACCAAAGTCAGAGGGGTATGGTAAGT<br>GCTCAGTGAAGCTCCCAGTAAATCTCAGCCATCATTAAGCTATTAAA

	AGGGACAGTGATTCTCCAACTCCAGAACATCTCTGGTATAGAATTA<br>AAGAATATAAATGAGAGCACAGGTACAAGTGTCATGGACATGGAG<br>CGCTGTAATGTTAACCTCTTACCTTCACATGCTCCTATAGACATATAC<br>AGGCATAGGCACCCCATACAGCTAGGGCCATGCACACATGTACACC<br>CCAAAGCTGGCCTCACCCCTGCCTACCCCATCCAAGGAATGGCAA<br>GCACGAAGCTCTGCTGTTGTCCTCTTGAGCAAAAGCCAAGGCCCT<br>GGCCTCTCCATCTAGGTGTGTGTAGATGACGTGGGAGCACCAGTTC<br>TCTATGTCTTGTCTGAGCTTCCTTCATATCCCATGAGGCTGTATATTT<br>CCTTCCCAGCACCTGTCTTGGGCTTGGACTCAAAGGACCCTTCTGA<br>GGACAGGGCATAGAGCTGGCTGCTCTGGAGAGGACTCAGCCTGGC<br>CAGTAGGTAGTTGCTCCCTGCTGAGGGGCTCTGGCTAGGCTATGGG<br>CCTGTGGCTGGAGGACTGGCCTCAGCGGGGCTTAGTCTACGAATGT<br>AGATGGGCTCCAGAAAAGGAGAATCACTGACCATCTTCTTTCCATG<br>CCCTGCGTTGAGCTTCCCTGAGTTTGTGGCATGGGCTCAACCTTTC<br>GTAACTGCACTTCCTGCTCTCTGGCAGACCCTGCAAGCTTACTACA<br>GGGCCTGTCCATGCATCAGCTGTATGTGTGCATGCTGCTCTGCTGG<br>GTGCCTTCCAGTTAGCCTTGCCCCTGACTTTTCCTGCTCATCTCACA<br>TCCTTACCTCAAGGTCTCCAAATGAATCCAGACCACTGCCTAGAGC<br>CTGCTGCAGGGGCAGTAGGAATATGGCCCTCATGCTAACAGTCTTC<br>TTTCCCTATGGCTCAAGCACAAGGGGCCCGTGGACGGGCAAGGGG<br>GGAGTAAGACAGGGCAGCAGTGGTCACTCTCCCTCGCTGGTGAGG<br>CTGATGTGTTGGGAAAGCCAGACTTGACTTCTGTCCTCACTGTAAA<br>CAGAAAGCTTCTGTAGTGGACTCTAAAGTGGGAGGTATGTAACCCC<br>TTTGCATATGAGATAATCTTTTGGAGTGTGGGAGAACATAATCTAAC<br>TTCCAGTTATATTTATTGTTATTGCAAAAGATTAACAAATTATCATTC<br>ATTCATATTGAATGTGTGAATTGGCCCTATCTCCCACCTGAGTCTGC<br>AGTCATGACAGATGGAGCCTTTCAGGTGTCCTAAGGGAAGATGGG<br>GTTCACCTCACAATGCTAAAGGTGAAAAGGGGAGTGCCGACCCTG<br>CCCTTTGTTTTGGCATTTTTATTTCCATACTTTGTGGTTTGTATGTGC<br>CCAGTTAAACGGATGTACAGATTAGATAATCTGGCTTTAACTCCAAC<br>CTTCACGAGATGCACACATGCCTTAAAAAGATTCTTACAAAGAAAC<br>TAGAATTAGGAGACAATTCTGACAACACAAAAACAAGTGAACAAA<br>AATCAAATGACTGACCTGACGTGTCCACTAGGCTTGATATGAGCTT<br>GGTCAACTAAAACATGAGACAGAATGAATGACCATCTGGTCAGGTT<br>GGATAAGAAGTAACTTTTGGCAAAATCTCTGAAGTCTGGATTTACA<br>CTTCGTGGAAGGAATCTTGACCTTTCCCCAAGTATATTATGCCTTGA<br>GATAATAATCCTGTATAAGGCCGCATAAGCAAAACGACATTTGAAA<br>ATGAGTTTCATCTTTAGCTCATCCTTTTACAAATTTCTATTCCTTGTG<br>CATGATTGAAGTGTACACATTTTTTAGTTAAAATATAAGCATATAGA<br>AGGCACAAGTTCAATATTTTTCATGATAAGGATGTGTAATTAAACTG<br>GTTAGGAGACTCATCGACTAGAGGGGGACATGGTGGCCCCAGGCT<br>GTAAGAACAGGCCACACCGTCCACTGGGCCGCTTGCTTTGTGCTAA<br>GATGACACTTTGTTCTGAGCCTCACAGTGTCTTGACCATGTTCCTG<br>GAACCTTCTTGTTGGAGGGAGTTCATCTTCCCCTATGACTCTGTCCC<br>TAGTCTAAGGTGTCCCACAGGAAGCTTGAGGGCGGGAAGTTTTCC<br>AGCCCAGGAGCCTGAGCTCAGCGGGGCAGGAAGAGGGAGCAGCT

	CCTCCGTGGGGGACCTTTGAGAGCCCAGGAGCAGGATTTCGAGGG ACACCTGGTGGGGAGCAAAAGGTGCTGAGTCTGTCTTTGACCTTG AGCCCAGCTTGTTTCTCCTGCATCCTCCCCCAAAAGGGGCTTTGCC TGTCATTCTGCAGTTCTAGTGTGGGGTCTGGGCGCAGTTCTTTTCCC TCTCCAGCCTCGGAGTCTTCCTCTGTGGACTGCGCAGATAGGACTG GTGGCACGGACCAGCTCTGCAGCCCTGGAGTCAGGAGCAGAGCCC CCCGGCTCCCAGCCCGCCGTAGCCGCTCCTGGCACCGAGCGAGCC GCGATGACAATGGCTGCATTGTGCTTCATGTCCCTTCCCATCAACAT TTCTGTGCTGGACTCCTTCCACTCGCGGGTCGTCTCCAGAGCTCAG AAAATGAGGTGATCAGTGGGACGAGTAAGGAAGGGGGGTTGGGA GAGGGGCGATTGGGCAACCCGGCTGCACAAACACGGGAGGTCAA AGATTGCGCCCAGCCCGCCCAGGCCGGGAATGGAATAAAGGGACG CGGGGCGCCGGAGGCTGCACAGAAGCGAGTCCGACTG
TFAM	CTTAGCACAGTGTCTGGCACAGTATTGGCCTCTCCTAGGAACTGAG TTTTTAAAAAATTATCACTAAAAGATGAATATTACTATTGTGTTTTAC AAGAAGACTAAGATAATATATAAATTAAAAAATGAGTACCACCCATG CACTCCTGCAATACTCAGATACAATCACTATTAAACCAAATTTTGTG TACTATTCTGCACTCTTACTTTTGCACGCACACTCATGCATATGTGTA TGTGTAGTTCTTTAAATTCAAACCTATGTTTTTGGGTGACTGAATAC TTTTTAAGAGAAAGGGTCTTGCTATGTTGCCCAAGCTAGATTCAAA CTTCTGGGCTCAAGCAATCCTCCTGCTTCAGCCTCCCAAGTAACTG GGACTACAGGCATGTGCCATTGCCCTGGCTTAAGATATTATTTTAGT AAGCCACTCAGATAAATAAGAAAGCATTTATGGGGACTGGAAATAA TAGAATGTGAGCAGGTGTTTAAGAAAACTACCGCTTCATGACTGAT TTACCTATCCTGTATCTACGTAGAGTCATATATAACTTCAAAGCTGCA AACTGTGGATTCAGAGGTGGCAATTTTTGTTATTAACATGCTAACAA GGAAGATATCCTTCCTATTATTTATACAGTTTTCATAGCTGTACAGTA GAAATCATAGCACCTTAGAGGATTTATGAAAGGATTAGTGATAATGT ATGTAAAATACTTGGCATGTAGTTTAGCAATAAATTATAATTATTACC ATTTGTATTGATCAGATACTTTACCGTGGTATGAGGGCAGACACATT ATATTGTCCTATTAACAGCATTATTCCTTCAATACAATCATTCTGGAT TTCTTCCGATCTTTAAGTTAGCTTTCAGTAATATGAAGTCAGCTTTC AGTAACATCCAGTAATATGAATTGGGGTATTATACATTTGCATAAAAT TATAGCTGTTCTTTTTGCTTGTCCTACCTCCTGCTGACTCATAGAATC AGCTTTAAAATAGACAAGCTACATTTATTTTTTGTAATGCTGCTACA GCAATGGCTTAAAATAATTCTGATATGCCCTTAATAATTAAGGCTGCA TGCAGACTTTGTTCTAATTGAGAAATATTTCACAGATAATAATAATTT CGGGTAGCTGGGAACTATTAACATGCAACAGGTCTTTCTTCTAATTT CTCTATTTCTCTTTCCCTTCCTTCTTTTTGTATATTGATTTTAAAGGCA AACTCCAGTGGCATGAAAACAGACTCTATGTCAATAAAAATGGTTT GAAAGAAAAAATTATACAAGTAGTTTTCCATACATAATTTATTAAAA TCTTGACTCACAGAAAAAATAAGCTAGGAGATATTCAAGGTGTAAA ATTTTTTCTCATTGCTGCAATGCACTGTTATACTTTTATTCCTTTTGT GTCTTTATGCTTTGTTCCCCCAATCACAGTGTCAGCTCTTTGGGGCA GGATCAATATCTTATGCTTTTTGTGCCCCTTTCAAGTGCTTATCACAG TGCTTTGTAATTGCTTATGAGGAAATTGTCTTTAAATGTGGTGTCCAT

	AGTATGATCTTATTTCCAGAGATATTTTACTTACAAGTTCAGAATCAC<br>ATCTATTTATCCTGCTAAAACCTATTAGGTAATTGCTCCTGTTGTAGC<br>TAAATTATTTATCTATTATAATTACAAAAGCCAAGACTGCTTTCTCTT<br>AGCAACATTTTTTCTTCATTTGAGTGATATCTGTGCATCTAACTCCAT<br>TATTCAGCAGGGAATTATCCAGGTTTCTGGCTGCTCCTGCTGTTTAT<br>TGGCACTACCTTCTGCTTTACCAATGTGCAAGTATCAATGAACCATA<br>ATCATTACTTTCATGTGTATATATATTTCAGAATTTTACATGGTTTCCA<br>TCTCCACACACTGGCAAATGTGTCCTTCTATTGGCAAATGACACTT<br>CTAACAGGTAGTTGTTCTCTATGGGGCTAAATTTATATATAAATAATT<br>TGTGCAGATGCTAATCTTTTCACACTATTCATCTCTCTCTGTGTATCT<br>ATATGTCTGTCTGTCATCTATTATCTATCTATCTATATATCTGTCAGTTC<br>ACCTATCCATCCGTTTGTCCCTTCATCCATCTATCATGCTCAGTGTAC<br>TGTGCTAGGTACAATAGGGGATAAAAGAAGGCATAAGATTTAGTCT<br>TTTCCTAATATAACTTACCATCTATTGTGGAAAAAAATATGTATACAT<br>AGAGAAAAAGATCAGAAACCAGAGTGGTACATGATGTCACAAGTA<br>AGTGATGTGAGCTCACACACTATAGATGATCCACTAAAGATATTTTG<br>GGCTGGAATGATGTGGCATGTTCACTGAGTCTGAGTGATCTGATAC<br>AAACGAAAAGCACAGGACAATGAATTGCAATTTTTACCTACACAAC<br>TTACTGTTGGAAGCTTATGAGTTGGGCTTTCTGAACTTCATTTTTCT<br>CATCTGGAAATTGGGAATAATGATCATGACAATACCTTCTCTACCTT<br>TTCCTTTGATATTTGAATCATACAGTCAAATAAAGTTATTTAAAAATT<br>TCTGAAAATATTAAAGTGCAATCCAACTTCAGGGTGTGTTGTTTTAA<br>TTTTTCATCAACTACCACAATGTGTCTGCTCAAATATTACTAATTTAA<br>GAAATATTAAAAGTTCAACAGGAAGAAAGGAGCAATGCTGGTTAT<br>GTAATATGTATGTTTGCATGTTAACAGGAAAAGAAAAATTTGTCAGT<br>AGCATGAAATCTGCCAATGGTGGGAGTATTTACACCATAGAACTCA<br>GCAAACACCACAAATTGGGGCTTTCTTTTTTGGAGAGCTATTTACC<br>AGCATATTACTGGCCTGAACAAAAGCAACAGCAATGGGACTAGAG<br>AGAAGGAAATACATACTAGAGGGATGAATGAGGCAGAATCAGCAAT<br>ACTCATCATCAGTTGGTTAATGGCAGGTGGAGTGTCAATGGGGAGA<br>TTCTAAAATGATTTATCAAGTTATTGGCTCAGATGATTAAAGAGGAA<br>AACATGTGCATCTTACCAAGAGAAGGACTTCAAGATGGGTGAAAG<br>ATAGTGGATTTTGTTCAGGGAATGTTGAAATTGAAGTACTTGTGGG<br>ACATGATGGTGAAAATGTACAGAAGACAGTGGTTATGTGGATACGG<br>AACTCAAAGGAGAGGACTGGGTTCAATATGGCAGTCATGAGGCTAT<br>AACTAATAGCTGAAGTCATGGGACTAGAGAACTTTACAACAAAGG<br>GACCCTGGGAAAGGCTGCAGTAAGTCTTCATGTTTTATAAAGTAATT<br>ATATACCATGTCTTTTTGTTAAAAGACAGTTTTAGTGGATAAAGTTAT<br>GTTTCTTTGTAAATTCTGCTTTTAGAAGTAAAATTATCACTGCAATGT<br>AAATGAGTTTTAAGGAAGATTGGTATCTGATGAAAAATGTTATGGAT<br>TTGATTTTTAAGGTCTTTACAGCAAGATTTATACAAATGAAAGTCGA<br>AAACAACAAAAAGTTTAATATCCAAAAGAAGTTTATGTCAAACATT<br>TGAAAGAACAAAAACATTCTAGAACAAAAAGGGAAACAAATTATT<br>TTAGAAATCAACTTAAATGTAACTTCTGTCTGAGCCCCATGTTGTAA<br>TTATTTGCTACTTTGCCTCACCTTTGGGGCTGGGGGCGGGGGGTGG<br>GCTGGGGCACAACACTGTGTCTTTTATCTATGTTTAGGGCGTTGTAC

	ATAGTATATACTCAGGAAAAAAACCTGTTTATTCCATGCCAGAATGG<br>GAGGTTTGAACAGATGACTTGGAAGGTCCCTTTCAACCCTGGGAG<br>TCTATAAAAGGGAAGTGGTTATTACCGCTTAAAAGAAGAAGTAGGC<br>TTAGTCCTACTTCCAGAGAGCCAATGTTTTCATAGTTACAGAAAGGT<br>AAATAGACTGTCAGCTCAATATGACACTGGTAAGAATTTTCTAACAA<br>TTACGGCTATCCAACAGAAGAAAGGCTGTCTCAGAAGGTGGTTAG<br>CTCTGAAACAACTGGTATTTAAGGGCTGGAAGGACCCTTAGGTATC<br>TTTAGGTCTTCCTAATCTTTTTCATGCCATTGCCTATACAGAAAATGG<br>TATTTCTATAACACAAATAAAAAAATTATACAGCAATTTTTGACACA<br>TCAATGTCTCAAGGCATACTTGTCACACGTTCTCAGGGCTTTGGCT<br>GGGAAAACTGATTTAGTCTAACATCCTCGTTTGCAGAGGGGGAAAC<br>TGAATCTTAACGAGGGAATGCAATATTCCCAAGTCAAGACGTTAAT<br>GTTAATACTGGAACTAGAAGAATTGAGACTAGCGACTGTGGACAAC<br>TAGCCAGTTTCCTCTGCTCAAATTAAGCAAGCTGGAGGCCAGACTG<br>AAAGACTGCAGTAGATGGTAGAATAAACAGTCTAAAGCTATATTGC<br>TTTAAATGTGGTCTCAAGACCACCGACATTAGAACTTGTTAAATTCT<br>GGGGAACTCGCTAAAATGAAGATTAATGGGCCCCACATAGACACAC<br>GGAATTAAGCTCTGCGGTAAGGCCTTGGAATCTGCATTTTTAGGTTT<br>GCGAATCCCCGCCTCTCCCGTTACTATTTCTGAACTCCGAGCTCCAG<br>CCCTGGCTTGAACTGAGACGCTCCGCTGGGCGCGCAGCAGCCGCC<br>GATCGGACCTCGGGGTCCTGGATGCAGGACTGTCTGTTACGTACAG<br>CCCTTGTGACCGTCACGGGCGGACACCGGCCAACGCCGGGTTGGG<br>GTGAGGCCGCCGCCGCGGTCCCTCCATCACCCTCCTGGCCCGGCA<br>GAGGAACCCACTGCTCCGGGCGGCCGGGGACAGAGGTGGCTCAA<br>CAGCGCCGCCTCGAAGCCAGAGCCCTCCGCAGGCTAGAGGATTGC<br>GGTTTCCCTTCATCTCCGCGGCTCTTATTCCTCCCCCGCAAGGCCGC<br>CCACCGGGGTACGCTCTCCCGCGCCTGCGCCAATTCCGCCCCGCCC<br>CGCCCCCATCTACCGACCGGATGTTAGCAGATTTCCCATAGTGCCTC<br>GCTAGTGGCGGGCATGATAAC
TFAP2C	AGCAGCTTCTGTACAAGTCTGATTTCTCCTGCCTGGCCTGGGTGAT<br>CACAGTGGGTCCCGGGATGTTTAAACTGATGGTAGAGTCAACCATC<br>AGCATTTATTTCTGTGTTTACAGTCTTCTTATTCTATTTTACTGGGCT<br>CTTGGTTTTTGCTTGCCTGCCTACCTTCCTTTCTTCCTTCCTTCCTTC<br>CATGTACTTTGCCCCTTTCTTTAAGGCAAATTAGCATGTAATGTGCA<br>AACCAATCTCTCTGGCACTTAAGGAAGACCCCAAACCCCACTCCCT<br>TTCTTCCCCTACCCCAAGAATTCACTACCCCTGGTTCAATTTCAGCT<br>GAACCCGGAAACCTGTGATGTGACATGAAGGCTGAGAATTCCCAG<br>AAATGTTAATACTTTTGGTCTTAGAAACTAACCAACTAAAAAAAAA<br>AATCCCTTGCCAGTAAGAGGTAGGCTACATTTAAAATAATTTCTGAC<br>TGAAGAAGTCCTACGTACCGCTTGTCATATACATATGTAAAGATAAT<br>TTAAAACCCTAGGGTATACCCTTTTCAAATGCAGAGTTTATAGTTTC<br>CAAGGGGAGGAGGGCAGGGGAGGAGTAACCAGGACCTTTCCTGC<br>CAGTCTTCCAATCGCGATAGCTCGTTTTGGCTGTGAATTTCCCCCCT<br>TCCCCTCCCCCTCCCGTTAATTAAATAAACGCTGGAAGCGTGTGTTT<br>ACCGCACACGACTCGGCCCTCGGGGAACTCTTTTTCTACGTCTCTC<br>CTACTCTTAAACTCCTCAACTCACAGAGGGTTGGAGAGCTGGCACC

	ATGAGCTTCCAGTTCCTACGGGTGAGAAAAGAGGTGTCTGTGTGG<br>AGGGCAGGTGCGACCCCGGGGGCTAGCAGAGAGGAGAGGCTGCG<br>GGAGGACTCGCTGCGAAGGGCGAGGGGTGGCGCTGGGTGGGACG<br>GGCGCCTGGGGCCGTGCAGGGTGGCTCCTGGGTGCTGCCCGGGCT<br>GCCTGTCGCCCAGTACTGGCGCAGGAAGACGGGTCCGCGCAGCGT<br>CTGGCAACAGTGGCCTGCTCCACGCCTGGAGGCGACCAAGTTCTG<br>AGTTAAAGGCGCCGGGCTTAGCCGGGAAGTGTATGGCGACCCAGA<br>ACACGGAGCGGAGAAGGCCTCAGGGGACACGAGGCTACCGCCTTA<br>GTCCTCGGGCCTGCGCTCCGAGCGGTTAGGGTGCGTACGGATGGG<br>CTCCGGGATGTTAGTGGAGAGGTGACAGGAGTCTACCATCCCACG<br>GCCAAGCGTGTGAGGCCTCCGGCGGGCGGGGGTCCCCCCCGGGGC<br>AGGGCAGGGGGAGAGTCAGGCATGTCTCTAACGGGCTCCCTAGTA<br>GAACCGCTAGGAAAGTGGCCTTCAACCCTAGGCACCTTTGTGGCTT<br>TCAGGTAGTTGGGCGACTAAAAATTCACTTTTGCCTGCTGTGAAAT<br>GGGACAGGTGGTTGTCAGGTTAGAGACTGTGCCCACGAAGTCTCG<br>TTTGGCGGGCCGGGACTGACGTGGACCCGGGCCCTCCTCGGAAGC<br>TTGGGAACCTGTGAGCCCCTCAGCTCCTCGCCTCACTTTGCCTGTT<br>TGAAAAAAGGAGTTGTTTGAGAGGAAGAGTTAAGGCTCAAAAGG<br>TTCTAAACGAAGAGCATGCGGCCTGACGCGGAGCGCAAAGAAGCG<br>AGCAGTGAACTGAATCTGGTGGGACTGAGACCAGAGGCGTCTGTA<br>TGCGGCGGCCGCTTCGCGCCCTATTAAAGCAATCTGTTATTTACGAT<br>AATCATTTTTGTAATTATTTTGGAGTGGCTGTGACTTGATGTCTGTG<br>GTGCCCCAGGGCATGCCCTTACTGGACATAGATACTTCAGGGTGCC<br>CTTTGACGCCAGTGTCGGTCCAGTGGGTGCCAGTGCCGCGAAGGG<br>AGGGTGGCGGGCGTGAATGCCCCCTCCTCCTGGGTTTGCCGCAGG<br>CCCCGGCCTTTCCACCTTGCTTGATCCTGGCGGGTCTGCCACTTACT<br>GAGGTCCCTACCGGCGCTCACGTTGTCCCGAGAGGCGGGACAAAC<br>GTCCGGCGCGTGGCGCGGAGTCTGCGAAAGCGCTCGGGGGCCTCC<br>ATCTTCTGCTCGGGAGTCCCCTCCCTGGGGCGGGAAGAGGAGTCG<br>CAGATCTGCCCGAATCTTGCTCTACCCGCCTGGCCGCCTTCTTCCGT<br>CTGGGGTGGGGGCAGGGGTGGCGGATGACTCAGGCCTGGGGCCG<br>GGAGCCCTGTTTCCAAAGGCACCACCTCGCGCCTCTTTGGAAGTG<br>GCGCGCTGTTGGGGCGCTCCCGCTGTTGCAGAGGCCTGGTTTGCG<br>GTTTCATAGGGGCGTGGGTCCAGGGCTTCCAGGGAGTAAGTGAGT<br>TTAGGCTCCCTAGGGGGCGGTAGAGGGGAACCCCCGACAACCTTC<br>CATCCTCTTCTCCAGGCGGCTGCGAGGCCTCCGGGGTTCCAGAGG<br>GGTCCCCTTCTGCCGGTGCCGGCTGTCTTGAGCGCGGCCACGCAGT<br>CCGCGTCTTCACGTGGGCAAGGGGCGGGGACGTTGGAGGGAGGCT<br>GTCTTGGATGCTGGAGCCTTCGGTTCCCGGCTCTGCTGAGCAGAAA<br>AGTTGAAGTCAAGGAAGGGTTGAGGCTTCCTCACAAGGGCGCGCG<br>CGTGCCTGGGTGCCGGCCCAGAAGCCGAGAATGTGCGTATTGGGG<br>AAGGGTCTCCCGTGCCGAGAAGACAGAGGATTTGTTCTAGCTGCC<br>TTCCGTACAGAGGGCGCGGAGGTTGCGCTCCAGTTCGAACGCTTA<br>CCCATTGGAAAGAGGGCAGCGCCGGGGTCCAGGGAAGCTCCTTGG<br>GAATGAATGGCCTTTGCCAAGCGGTTCCGGATCCTCTGGGTCCTTT<br>GGGCCCACGGCACGGTGCTGCGCGAGCCCTCAGTGCCCATCGGCT

	CCCTTCGCCTCCTGCGTAGACGCTCCCAGGCGGGGAGGCATATCGG<br>TTCCTCCGGGCAGCTTTGGCTAGTGTTGCTGTGGGAAAGGAGAGC<br>CAGGGCCTGGGATGGGGGATGAGCACCTTCTTGCCCATTCCGGGCC<br>CCAGCGTGCAGGAGGTAAACTTGCCAGCACAGACAAGACAGCTTG<br>TTCAAGCTGCACCTCAGGCCGGGTCAGAGAATAAAACCGAGGGCT<br>AGAAGGCCCAGAATGTCGGACAGCCCAGCGGCACCCGTCAGGGA<br>GTCCCAGGCGCCCGAAAGAGGCGCCGCACCTCTGGCGAGTCTAGG<br>ACCCATCTTCCTGGACCTGTGCTCTGGAGTGCCTGCGGGCCTGGGT<br>CTAATTTCTGCTTCTGGCAGGTGTCCCCCTCCCCCGCCGCTAATACC<br>AGGAGCGCTGCTTCTGCGGTAACTTATTTACCCCCAGAAGCCTGT<br>TTTGGGACCGAAGTGTCAGGGTCCTGTGTGTCTTTTTATGCACTGT<br>TCTCCTTAGTGCAAAGCCCTGCAGATATGCTTGGCCGAAAAGTCTC<br>AGTGGTTTCAACTTCCAGATTTTGTTCCCGCGTCCGGTCGGAAAAT<br>CACCTGGGATTTGGCTGCTGTCTAAGGCCGGGGGAAAGTTTCCCTT<br>GGAGGACGCCTATTATTATTATTTCTTTTAGGCCCACCTGGGTCTAA<br>ATAAATGCTAAAGTTCAAACGAACCCGAGAAGGAACAGCGAGGCA<br>GTGGATGCCGCCATTCCGTGGATGGGAGAGTCATGTCTACTGCAGG<br>TGCGGACACCGTACTGATAGGCATTGATTATTGCAAAATCGTATTCA<br>GTATCAAATAGAAATAGTCTTGTCCTCTAATCCCCACAGAATGTTTA<br>AATCCAGCCCAGGGAGCCAGTTGGGGCGTCCCAACAAATACGCCC<br>TCGAGAATTAGCGATGTTCCCCTTACAATGAAAGTCAATTGCTGCAT<br>AATAACTGGGCCAAAGAATTTCGTTAGTTTAAATTTTAAAAATTAGT<br>ATTGCACTTTGCTTTCAAGTTTGGGTGGCAAGTACCGAAGGGGAG<br>AGGGAAGCAGCGTTTGTGTGGAAGGAAGTTTCGTTTTCTGTCTAA<br>ACATGTGGGACCTGGGTGTCTACCTCTCCAGTCGAGAGCTCTCTGC<br>AGGCTCTCTCCCAACTTCCTGCACCTCCTCACCCTCACCTTACCCG<br>TGGCGCTCGGGTTTTATTCCTGCCCGGCGCTTCTTTCCCAGGGCGA<br>GTAGAGGCTTCTGAGTGACCGGCCCGCCAGAGGCAGCTGCAGAGC<br>CGGCGTTCCGCAGGGCAGGGCAGGGCCGGGCCGCTGGGCTGGTCC<br>AGTGCGCGCGGGTTCCCCCTGGCCTCAGCCAGCCGCGGGAGATGC<br>CACTTCGGGCGACAGCGGCGAGACGCCGCTGTCCGCAACTCCTGG<br>GCGGGTAGGACCGTCTGCTGCCTCTGCGGCCCTTGGGGCAGACTC<br>CCCAGGAGTCCCTTCCTCTCTCCTCCCGCCCGGGGGCCGGGACCGC<br>GCTGTGCCCACGGAGGGAACTGGGCGTCTCTGGCGCACTGGGGCT<br>CCAGCTGCATGATCCCAGCTCAGACCTTAAGCCTCAGTGGGGTGTA<br>GGTGGGATGGGATGTGTTTTTAGGAGATGAGGAGACCCCGGTTGTT<br>GAGTCGCTCCCCAAATGCGCAGTTTCTTAACTCCAGGTGACATCCG<br>TCTTTTGGAGGAACGAGTGAAGGCCGTTTAGGCGAAAGAGGGGTG<br>GGTTTCAGTATTTGGATCCCTTCCCCCAGCTCTGCCAATCTCAGGTG<br>TGTTTACGGTGGAGGTGACAGAAGGAAGGCGCGTCGGGGGACCC<br>AGGCCTCGCAGGTCTTCGGCTGTCAACGAGGCACCGCCCACTGAC<br>TCCGCGCTTCGTCCCTCACAGCGTGGGGCTCGGCGCTGCGCCAGG<br>CCCGGGTGCGGGGCGGAGCTAGGCTGGGACTGGCTGGGGGCCGCC<br>CCGCCCGGCGCCTGGGCCTCCGCGCCGGCCCCCGGGGAGGAGTTA<br>TGATAATTTCCTTCTCATTAAGGCGCTCGGGTCCCCCGGCTATCGCC<br>AGGACACACTGTTCGGGCGCGGCTTTCCCCGTCCGCGGAGCGGTC

	TTGACACTCGCGGCGGCAGCATCTACGCTCGCAGAGCCGCCGATG<br>CGTGTCCA
TFF2	TTGAGTAAATAATACTGTGTTATCAGTCATTATTGTTATTATGTTCTCC<br>TCCATGCTGTATGTCTTTGTCTTAGCCTATTTTCTGTTGCTAAGTTGA<br>AACTGGTCATTTATAAGAAATGAACTTCATCTCTTACAGTTCTGGAG<br>ACTGGGATGTCCAAGGTTGAGGGGTTGTATCTCTTGAGCGCCTTCT<br>TGCTGGTGGGGACTCTCAGATGTGTCCCAAGGCAGCCCAGGGTATT<br>CCATGGTGAGGGCGCTGAGTGTGTTCATGTCCTAGCACAGGTCTCT<br>CTTCTTATAAAGCCACCAGGTCCCCTTCCACAATAACCCATGAATCC<br>ATCATCCCATGAATCCATTAATTCATGAGTGGATTAATTCATTCATGA<br>GAGCAGAGCTCTCACGATCCAATCACCTGTTAAAGGCCCCACCTCT<br>CAATACGGCCACATTGGGGATTAAGTTTCGACATGAGTTTGGAGGG<br>ACATTCAAATTATAGAAGTCTTTTCCTCCAGCTGCTTTAAGAGTTTT<br>TTAAATCACCGGATTTTCAACAGACGAATCATGCTGTGCCTTGATGT<br>GATATTTTTGGTATTTATCTTCCTTGAAGTTCTTTGAGCCTCTAAGAT<br>TTGTAGGTTTATACTTTTCACCAACTTCAGAAAAATCTAATGAATAT<br>TTACTCAATACTCATTCTTGATCAAGTATTTCTTTAAAAGATTTTTTT<br>CTCTCCTCTCCTACTGGTACATCAATTACATGTATGTTAGATTGCTTG<br>ACATTGTCTCACCGGTCATTGGAATCTTGTTTATTTGTTTAGCCCTTT<br>TTCACTTTATGCTCTAGTTCGCATAGTTTATCTTGCACTGGCTTCACT<br>TCAAGTAAACTGACCATTCCTCTGCAGCATCTAATAGTATCTAATCA<br>AAAACCCATACAGTGTATTTTTCATTTCAGATGTTGTATTTTTTATTT<br>CTAAAATTTGTACCTGGTTAATTTTTAGTTTCCATTTATCTACTGAGA<br>TTCCTTTTTGTTCCTTTATTATATTATGTTTTTCTTTCAATCTTTGAAC<br>ATTTTATGCCATGTCTAAGTCTGTTTTTGTTCACTGATTTTTCTAATG<br>ATTATGGGCCATATTTCCTAGTTCTCTCCACACATCTAGTAACTTCTG<br>ATTTTCTGTTAGACGTTAGGGATGTTATGTTGTGTATTTAGGTTTTGC<br>TGTCTTCATTAGTAAAGTGTTGAATGCTTTGCTCTCTCAGGTAGTGT<br>TATCGGTTGAATTATTTCCCCCAAATTCATATATGAAGCCTTAAATTC<br>CATTACCTAAGAATGTGACTACATTTGGAGACAGTGTCTTAAAAGG<br>GGTAATCAGGTTAAAATGAGGCTCTTAGGGTGGGCCCTGGTTCAAT<br>TTGACTGGTGTCCTTATAAGAAAAGAGTCGCCAGAGATGTGCGTGC<br>ACAGAGGAAAGACCATGTGAGATATGGGGAAAAAAAGCCATCTGC<br>AAGCAGAAAGAGGCCTCAGTAGAAACCAGATGTGCTGACACCCTA<br>TCTTGGACTTCTGGGCACCAGAATTGTGAGAAAGTACACTTCTGTT<br>GGTTAAGCTACACAATGTGTAGTATTTTGTTACAGCAACCTGAGTG<br>AACTAATACAAGCAGTTAATTTACTTGCAGGTTAACTTGATTCCTGC<br>AAGACTTGTTTTAAAGTTTTGTTCGAATGGATTTTGAGTAGCTTTCC<br>TCTAGAAAGATCAGCCTTCTTTTTACGGCATGGCCTTTATTGAATGC<br>TTGAAGTGGTCAACACCTCTGGCTGCTCTGAGCTCAAATGCTTTCC<br>AGCCCCACGAACCCTACACTAGTTGTTCAGTTTAGCCTCCCCAGTG<br>ATTGTTCTGTCCCCAGGGGCTACTTAGCCTGGCCTTGTGGGCACGA<br>ACAGCATAGTATTTGATGAAAAGCTCAAAGGACACACTTTTATATTT<br>CTGGAGCTTCCTTCTCTTACCTTGATAACCCGCCTGCCTCAGAAGCT<br>TCCAATTCTGACCTCTGTCTCCTCAGCCCGATGGGACTTCAGTGCTA<br>TTTGGGTTCCCCTCCCTGTGCTGTAGTCTGGAGAGCGTCTCCAGGA

	AACCAAGGCAGCCATGGGACTCCCCCTATTGTTTCCCTTCTCTCAG<br>GGATCGCTGTCTGGCACTGCCTGCTGCTCAGCATCTGCAGACTGTT<br>ACTTATGTAGTTTTAGAGTTACTCATGGTGGGAGGCTAGCCTGGTAC<br>CAGTGACTCCATAGTCAAAAGCGGAAGTCCACATGGGTGATAGCTG<br>AGGTGGTTCCCATTTGTTGACTTTCTATGTGAACTGATGGAAAGTAT<br>CATCCCCAAAGTGTGCAAAATGGAAGTGAGCAGGGGTGGAGAAG<br>GATAAACAGAAAGAGGAGGGCGCCAGCCCTGAGTGCCTGGATGGC<br>GCTGAGATCATGCGTTGAGAGTGGCAACAATTCAAGGTAGGTGTC<br>GGCCATGCTAGGCAGATGGCATAGGCACAGAGACAGTGGGAAACA<br>GCAGTGAGGAAGACGGTGGGTGAAGTGAGCACTGTGGAAATCAG<br>GCTGGAGAAGCAGGAAGGAAGGATAGACAGGAGGGGGCTCACAG<br>GGGAATAGGGGATTGGAGGGCTTTAGGATTCAGACAAAGGGAGAA<br>TGTTAGGAGCTTAGGACATCCTGGGCAGAAAGGACATTTGCATACC<br>CTCTTTCAGAGGTGGAGCTCAAGTTCTGAGTGTGGTCATGGGAGG<br>GTGGCAGAAGTGGCAGAAGTGGAGTGGAGATGGAAGTTCCTAGA<br>GATGAGGAAAAATAAAATCTGAGCGACTGTGAATTTTGATGAAACA<br>TCGACCGCCATGTTGAAGATGGCTGGGATGTGGCCAAGTCTTAGCA<br>TGACAAGGAACACAGTGAACTCAGTGCTGAAATTCTTAACGAAAG<br>GGACAGGCTGCACAAAGGTCGGCAGATGACAGCAGGGCAGGGAC<br>TCCGGACATGGCGCCCTTCCCAGCCTGCATCTCCAAGGAGGATGGT<br>TTTTGCATGAGGGTGAAGACTTGGGGCTCAAAGTCAATGTCCAGCC<br>CCCAGTCTGAGCCCATGTGATGGGGACGTGGCCACCAAGTACAGT<br>TGTGGAGGTTGTGAACAGCTCCACTTTCAACTAGCGATGCCACTCT<br>ATTGCAGACGTCCCATCTCGTGTAATGGTTTCCCAACTCTGTGGGC<br>ACTCAGTGTCCCTGCGTGGGGGCCTGAGCAGGATTCCAGGGAAGC<br>ATGCCTATGACAACACAGGCTTCCGCCAGGGCCAGGAGAGTGAGA<br>GAGGCTGAGAAGTGGTCTCTTTTGCATGGGTCAGGTGGTCGCAGC<br>AGGAATCCTGAAGGGGCTGCAGTGAGCGGTGGGAAGTCGAGTTGA<br>TGGGGAGGAAGCAGAGAGGAGAGGTGGGTGGGAATATGAGAGAA<br>AACAATGACTCAGTCACTTATATCATGATGGTGACTAAAGTGACAG<br>GGATGTAGAACAAGGAAACTGACCTTGCCAGAGTTTTCATGGATCG<br>AGTCCCTGTGGTTGCCCAGTGGTGAACAGTCAGCAGAGAGCCTGG<br>AGGTACTTGACCCGGACCCAGAGGAGCCCTGGAGGATGGAGCCTG<br>GAGGACGGGGGCCTGGAGAATGAGGGTCTGGAGGACGAGGGTCT<br>GGAGGAACAGGGGCCTGGAGGACGAGGGTCTGGAGGAAGAGGGC<br>CTGGAGGACAGGGGCCTGAAAGATGGGGGCCTGGAGGATGAGGG<br>TCTGGAGGATGAGGGTCTGGAGGAGCAGGGGCCTGGAGGATGAG<br>GGCCTGGAGGACAAGAGCCTGGAGGACAGGGGCCTGAAAGATGG<br>GGGCCTGGAGGATGGGGCCTGGAGGATGGGGGCCTGGAGGACGG<br>GGGCCTGGAGGATGATGGCCTGAAGGATGAAACCTGGAGGATGGA<br>GCCTGGAGGATGGAGCCTGGAGGATGAGGGCCTAGAGGATGGGGG<br>CCTGGAGGACAGGGGCCTAGAGGATGGAGCCCTGGAGAATGGGG<br>GCCTGGAGGATGGAGCCTGGGGGACGAGGGCCTGGAGGATGGGG<br>GCCTGGAGTATGGGGCCTGCTCTCCAGACCCTGAGGACAGCCCCG<br>TGGTGGACCAAGGATTTTGGGTTTCCTGCGTCTCTGGGCCCCTGAC<br>TGCTCAACCATCAGAAACAGACTGGCAACCCCCTGTCATTTCCCTG

	GCGTGGGGAACTTCGGGTCCCCTCTGTCCTTCCCACCACACTTTTC<br>CCTCTTTCTTTCCGGGTGTCTACTCTCTGGCTTCTGTCTTCTCTGTC<br>AGGTCCACAGAATCCTTCTCCAGCACATCCTACCCCAGGAAGGCCA<br>TGGGCTGGGTCCCAGGTGCCATCTTTCAGAAGATGTAGAGCATTCC<br>CATGGAACAAAAATAACCCATTTCAGGGGTTGGCTGAAAATGAACT<br>TATTAAAACCTGCCTGTCACAGGCTACTCCGCTGACCCTGTCAGCC<br>TCATCTCCATGGAGAGCAGCCCCTCCTGCTGAAGATGGGACAAAG<br>GGCATCGTGCTGCGGTTGGGGAGGCTCTAACCACAGCCCTGGGAG<br>CAGTCTCTTACCTCCTCTGAGATGCTTCCCTTCCTCAGGGAGGGGA<br>CTTTTCCATGCTATCTGCTGGCCTGTACATTTTCCCCAGTAAACTTG<br>GCCCTAATATTTTCTAAATTCCTGTGGTCCCTGCCCACTCTATCAATA<br>GAAATGCATAGCTTATCCCTTCCTGGGTGTGACCCTGTGTGTGCCCA<br>GCCCCAGACCTGCACGTGGCCGGTTTTCCACGCTGGCAGCCTGGC<br>ATGACCCAACTCTCTGTCCAGGGCAGGAAGAGGTATCACCGAGCA<br>GGGAGAGAGTCACCCTGGCCCGGAAGCCTCGCCTGCACAGGGCAC<br>AGCTGCCTCTTGCCTCCTCTTCGCCTCCACGGTGGAAGGGCTGGGG<br>CCACGGGGCAGAGAAGAAAGGTTATCTCTGCTTGTTGGACAAACA<br>GAGGGGAGATTATAAAACATACCCGGCAGTGGACACCATGCATTCT<br>GCAAGCCACCCTGGGGTGCAGCTGAGCTAGACATGGGACGGCGAG<br>ACGCCCAGCTCC
TGFB2	CTGGAGAGCTAAAATTGTAGAGTTATGAGTGGCCCAAAAGCCACA<br>AGCAGTAATCAATCAACTAATCATAATTCCAAAGGGTGAGATAATGT<br>TCCCACAGTGTATCATTAGTGTGATGAGTTTATCTCTTTAAAGCCTC<br>ACAACCTGCTGATATGCTGTGGTTTTTCTTGGTCCTATCTCTTTATCT<br>TTTACCACCTCTGATCTCTTTAAACCCACCCTGTTTCCATTTTCCTCC<br>CCGCCCATTGTTTCCTCATTTTTCCTTAACACTTCTTCCTTAGATTAA<br>CCTTTAAATACAAATGGAAATAATAATCGGATTATTTTTTGTAAGGA<br>AACCACCTTTCTGTTTCATTATTCATAACTAGAATAATGAAATAAAG<br>CTGTTTATAGAAATGCATCTAGAACATGAGACAAAATTGATATAGAC<br>CAAACATTGTTCTTGAATAGTGGTATCAAATATAAAATTATAAATAA<br>AATCTTAAAAGACTTTTGGTGTATTTTCATATTAAAGGGTTATTTTGC<br>ATTTATCAAGTATCACTATTTGGGGGGAAATGTAATCAATTACCGTC<br>GTTCCATGTATTTCTTTTTTTCACCTCCTGATTTTTCATGCTTGAAAC<br>CGTTCTGGAAAGCTTCCAGAATTGTTACAAATGAAGTTATGCTTTG<br>GTCAATGTTAGTAAGGACCTATGATCCATAGGCGTAAAACTCCAGG<br>ACAGGGATGAGTAGTTTTGTTTTCTTCAGCCTCTTGTAGAAATCCCA<br>CATTACTTAAGTGAATAGACCCATTGATGAGTGGAGAATGGCTAAG<br>AGTGGAAACAGCTCCACTCTCTACCTCTGTAATTTCAAAACAAAAG<br>ATTCCATTTGAGGGCTTTAATAAGAGACTTTAATCGTGAATGCTTAA<br>CTGAACTTTGATAGTCATTTAGCATTTTATTCACCAAAAGTGTATTTT<br>CTTGTCCTTCCCTCCTATAATTACTGGGGGCAAAATACCATAATATTT<br>AAAATGTGCCTACAGAAACTACTAGGAGGCAGAACTAGAAGCAAA<br>TTCTTCTTGGGTTCTTTCCTGGCTTTTCAGAGAGAGCGAGAGAGAG<br>AGAGAGAGAAGGCCAGATATTTTTATTATAAATTCCTTGACAGAAAT<br>AAAGACATGATAGGATAAGAAACTCACAGAGTCAGTGAGTAACTT<br>GTTCAGGATCACAAAGCCAGAAAGTGACAGAAATCTGAACCCAAA

	AAACCATGACTGGAGCCTGCCCTTTAAACCACTGTGCTCTCCTGCC<br>TCCTTGAAGTAATCAAGATGGGTGTATATCATTTAAATACCCTTTTAC<br>CATGAAGACTGTAGAGACCCCGGCACATAAGACCTCCATTAAATAT<br>AGAGATGAAAAGGCATTATAAGAGCCATTTAATAGAAGAAATGATT<br>CATTTACGGTTACCAGGGAGACAGCTCTAGCAATCTTGCCGGTTAG<br>GTCTTCAAAGAACTGATCATTTGAGAGGTGGAAAGGGGCTCCTCCT<br>CTCCTGCTATACTCACTTACTGCACTTACCCATTCCAGAAGCTTTTA<br>CCAATACCTCCCGTCTTACCCTCCTGGGCTTTGGGGAAATTAAAGTA<br>GCCTCTTATGAGTAAGTCAGGGGTCTCAGGTCTAAGGAGTTGTTAA<br>GTGAGCAATAGGTACTCAGTAAACTAAGTATTATGAACAAAAGTGT<br>GTATGTCTATGTCAGGAAGAGGGGTGGCCATCAGAATTTATGGCTT<br>GCGCTGTTTTCCTAGAAGTGATGTAAATGAACTTTTGCTACTCTATC<br>CACACTCTAATCTGAATCTACTTAAGGTGCATCAGTGTCTGTACCAA<br>GAAGGTTGTCTATAAACATGAAAGATGCTCACTGGCTTGTGGAAGC<br>TGAACCTGTATCCTCAGCAAAATACAGTGATAGCTAATTCAGGTAA<br>CCAGCCATATTCCACAGCAGCATCTTCTCTCAGTAGCTCTGGTTTGG<br>AGCTCCTGCTCTGTGTCTATAATGGCCACAGGTGTAAGAATATTCAC<br>TTTTTGTCCAATCTGTAGAGCTAGCCTACTGCAGTTCTCAAACTGA<br>ACTCAGAGGGAGGACCTAACTGGATGAAACTACTAGTCTGACAGT<br>AGCGCCTCTTGATTATCTTTTTCTTGGGCTACTGGGATGGTAGCTTT<br>GCTTCAACTCAAAACTGGTATCAAGGAAAGGAACCTGCTGGTGCT<br>GATTTATACATAATTTTTAGAATTATTCAGAAGTGGGTTGGAACAATT<br>ATTTTATTCCAGAGTTTTTCAATGTGTGATAATGGAAAAAATTCTGT<br>ATTCAAGGGAGTTTGGAAAATGCTGGGTTAAAAGAGTGAAAAGGT<br>TTTCTCTTCTACAGGAGTTTCAGAGCCTTTAACATGATAATGTTCCA<br>GAATGAGGAATCTAAGAGGACAGGAGAGTACCCAGTATCTCCCAA<br>ACTTGTTTGACTCCAGAATTCCTGTTTGTCAGAACATATTCTGGGAC<br>CATTGTTTCTCAGAAGTACATAGTAGGTAAGAACATAGTGGATCCTG<br>ACTGCAAAAATCCAGCTCTACCACTTACTGTGGTCTCGAACAAAGT<br>ACTTAACTTCTTTGTACCTCAGTCTCCTCATCTGCCAGATATGGATAA<br>TAAGACCCACTTTATAGGTTCATAGTGAAGATTAAATGACCATACAC<br>AACACACATCAAATTACTAAGTGTAGTATATGTTAGCTATTATTATTT<br>TATTTATTCAGTGCTCTACTAATAACCTAGGCCCCATACACAACTGA<br>AGTATAAATTCCAAAAGTGATAGAAAGTTCTTTGTGACTTTTCTGA<br>ACTCAGGAACATCTGAAGTAGAGAACAGTATAGAGATCTTGGGTTT<br>GGGAGTACATTCAACAGAGTTTCCAGTTTAAATCATCTGTCTGGTC<br>AGTATGGCTGCAGAGTCATGCCGAAATGAAAATGTTGACTTTGAGT<br>AACTAAAGGTAAAATAAAAGAAAAAGGGAAGGTGGAACAGTGGT<br>AAGAGTTATTCTTGTATTCATCTAATTTAGACTTAGTTGAAATTGAA<br>AATGTCAAGTTATGAGTAGTGTAGAACAGTAGACATCAAACACTTA<br>AAATTCCAGCTTCCTGGATTAATGCTATGGAAAGAATGAAGTTGGT<br>TGATAATGTTTAGCCTAGCAAGAAGGTGAAGAAGAAAGCCATACA<br>AGAAGTGGCTTAGGCAGCAAATTATAAAGGTGACCATTCATTCAAA<br>TCAGTAAAACAAACAAGTATACCTTATTCTTTAGGTAAAATTGATGG<br>ATCTCTGTTTTCCAGCAGTTCACAAACAGAGGGGTACATTGTAAAC<br>AACAAACTAACAAAATAAATTCTGGGATGGCAACCTGCTAAGGTAT

	CCCAGAAAATAAGAGGTAGGACATGAATTTAAAAGATTGGAAGGTA<br>TGTCTTCAGTACTGGCCTGGCCCTGAGTAGACTAGTGCTCCCTCCC<br>ATAGGGGTGCGTGTGCACACATAATACAGGAGGGAAGCCTTCCCTT<br>CTAGAGCAAGTGATTCAGCTTGGGAGGCTGTGACTGAGCTACACTA<br>AGTAAAAACGGGAGACTTGATTGTCCTTCCTTCAACAGACCTGTCC<br>AAAATGACTGGAAAGTAAATACCGTAAATCACTGTTGTCAGGGCGC<br>ACATTCCACCTCCTTCCTCCCTTACCCACAGCGGTCCTCATTTCCAC<br>ACTCCCTCAACGGTTCGGGGAGAGCTCGTGGTCTAAGTAACGAGA<br>GGACTTCTGACTGTAATCCTAGCACGTCACTTTGTTGAAGGCAGAC<br>ACGTGGTTCAGAGAGAACTTATAAATCTCCCCTCCCCGGCAAGATC<br>GTGATGTTATCTGCTGGCAGCAGAAGGTTCGCTCCGAGCGGAGCTC<br>CAGAAGCTCCTGACAAGAGAAAGACAGATTGAGATAGAGATAGAA<br>AGAGAAAGAGAGAAAGAGACAGCAGAGCGAGAGCGCAAGTGAA<br>AGAGGCAGGGGAGGGGGATGGAGAATATTAGCCTGACGGTCTAGG<br>GAGTCATCCAGGAACAAACTGAGGGGCTGCCCGGCTGCAGACAGG<br>AGGAGACAGAGAGGATCTATTTTAGGGTGGCAAGTGCCTACCTACC<br>CTAAGCGAGCAATTCCACGTTGGGGAGAAGCCAGCAGAGGTTGGG<br>AAAGGGTGGGAGTCCAAGGGAGCCCCTGCGCAACCCCCTCAGGAA<br>TAAAACTCCCCAGCCAGGGTGTCGCAAGGGCTGCCGTTGTGATCC<br>GCAGGGGGTGAACGCAACCGCGACGGCTGATCGTCTGTGGCTGGG<br>TTGGCGTTTGGAGCAAGAGAAGGAGGAGCAGGAGAAGGAGGGAG<br>CTGGAGGCTGGAAGCGTTTGCAAGCGGCGGCGGCAGCAACGTGG<br>AGTAACCAAGCGGGTCAGCGCGCGCCCGCCAGGGTGTAGGCCACG<br>GAGCGCAGCTCCCAGAGCAGGATCCGCGCCGCCTCAGCAGCCTCT<br>GCGGCCCCTGCGGCACCCGACCGAGTACCGAGCGCCCTGCGAAGC<br>GCACCCTCCTCCCCGCGGTGCGCTGGGCTCGCCCCCAGCGCGCGC<br>ACACGCACACACACACACACACACACACACACGCACGCACACACGT<br>GTGCGCTTCTCTGCTCCGGAGCTGCTGCTGCTCCTGCTCTCAGCGC<br>CGCAGTGGAAGGCAGGACCGAACCGCTCCTTCTTTAAATATATAAA<br>TTTCAGCCCAGGTCAGCCTCGGCGGCCCCCCTCACCGCGCTCCCGG<br>CGCCCCTCCCGTCAGTTCGCCAGCTGCCAGCCCCGGGACCTTTTCA<br>TCTCTTCCCTTTTGGCCGGAGGAGCCGAGTTCAGATCCGCCACTCC<br>GCACCCGAGACTGACACACTGAACTCCACTTCCTCCTCTTAAATTT<br>ATTTCTACTTAATAGCCACTCGTCTCTTTTTTTCCCCATCTCATTGCT<br>CCAAGAATTTTTTTCTTCTTACTCGCCAAAGTCAGGGTTCCCTCTGC<br>CCGTCCCGTATTAATATTTCCACTTTTGGAACTACTGGCCTTTTCTTT<br>TTAAAGGAATTCAAGCAGGATACGTTTTTCTGTTGG
TGFBR1	TTTACACTGTTGGTGGGAGTGTAAATTAGTTCAACCATTGTGGAAG<br>ACACAATGTGTGTGTGGCGATTCCTCAAGGATCTAGAATTAGAAAT<br>ACCATTTGACCCAGCAATCCCATTATAGGGTATATACCCAAAGGATT<br>ATAAATCATTCTACTATAAAGACACATGCACCTGTATGTTTATTGTGG<br>CACTGCTCACAATAGCAAAGACTTGGAACCAACCCAAATGCCCAC<br>CAATGATAGATTGGATAAAGAAAATGTGGCACATATACACCATGGAA<br>TACTATGCAGCCATAAAAAAGGATGAGTTCATGTCCTTTGCAGGGA<br>CATGGATGACACTGAAAACCATCATTCTCAGCAACTAACACAAGAA<br>CAGAAAACCAAACATCATATGTTCTCACTCATAAATGGGACCTGAA

	CAATGAGAACACAGGGACACAGGGAGGGGAACATCACACACAGG<br>GGCCTGTCGGGGGTGGGGAGCTAGGGGAGGGATAGCATTAGGAGA<br>AATACCTAATGTAGATGATGGGTTGATGGATGCAGCAAACCACCATA<br>GCACGTGTATACCTATGTAACAAACCTGCATGTTCTGCCCATGTACT<br>CCAGAACTTAAAGTATAATTTAAAAAGAAAGAAAGAAAGAAAGAA<br>ATGTGACCTCCAATGTTGGAGGTGGGCCTAGCAGAAGGTGTATGAG<br>TCATGGGGGTGGAAAAAAAAGTGTTCGTAAACATGTACACACTTAT<br>TTTCTCTTGATATCCTTGCCTCTTTGCAATGTGATTTTGCAGTTCCTT<br>TTTATCAAAAAAAATGGAGTCTTTTTCCCCACCCTTTGAATTTGGGC<br>TCAGCCCCAGTTCATGCCCAAACCAATGGGATATGGTAGAAGTGAT<br>GTTGTGCCAGTGGATGTTTTTTTGTTTTGTTTTGTTTTTGAGACAGG<br>GTTTCACTCTTGTCACCCAGGCTGGAGTGCAGTGGTGTAATCATGG<br>CTCACTGCAGCGTTGACTTTCTGGGCTCAAGCGATCCTCCAACCTC<br>TCAGCTTCCTGAGTAGCTGAGACTACAGGTGTGAACCACTATGCCT<br>GGCTAATTTTTGTATTTTTTGTAGATAAGTGGTTTTGCCATGTTAACC<br>AGGCTGGCCTCAAACTCCTGGGCTCAAGCCATCGCCCCATCTTGGC<br>CTCCCAAAGCGCTGGGATTACAGGCATGGGCCACCATGCCTGGCCG<br>GTGCCAGTTCTAAACCTAGACTCAAGGCTCTTGCACGCTTTCATTC<br>ATTATCTTGGATAATGAATGTCATGTTATCAAGCCTAGGCTAGCCTG<br>CTGGAGGATGAAAATCCACATGGAGGAGCTGATCTAGCTCATTACC<br>CTCTTCCACCCCCTTCCACCTGACAACCGACTCATAAGTGAGCTCA<br>GTGGAAATCAGCATAAATTGGCCTAAACCTGAAAAACTGTCCTACA<br>GTCTCATGAAAGATGATATATGGTTATTATTCTAAGCCACTAAATTTT<br>GAGGTGGTTTGTTTTTCAGTTAGGAGCACCAAGAATTAGCAATAGC<br>TAATAATGGCAGAAGTCAATAATGTCTTGTAGGTTAGAAAATAAGAT<br>AGAAATAAAATATGCAACAATAAATAGGATGGACAAATGAGCAGAA<br>TAGAAGCATTCTAAGGTGAATAAAAATAGATTAATTTTTCTCATATAA<br>TAAGAAATAAGCATTATGGAGCTGGTGCAGTTATTCAAAGTGAATA<br>AAAAATAGATTAATTTTTCTCATATAATAAGAAATAGGCATTATAGAG<br>CTGGTGCAACTGTTCAGGAAATTTATCAAAAACCTAGACTCTCTCC<br>ATTTTTTCTCTCTACAGTCCTCAGTTTTGGCTATCGCCTTTATGTTTA<br>CAAAATGGCTGCTGTACCTCCAGGCATTGCAACTGTGTTCCAGGGA<br>GAAAGAGAGAATATGATGAGTCTCTTCTTTTTTTAACTAGGAAGTAT<br>CTTTTGTACAGGCTCCGCCTTGTGGAAAGTAGATTTCTGTTTACATG<br>TCACTGGCCAGAACTCTTGTCACATGGCCATACCTAGGTACAAGGA<br>GCCTGGGAAATTGACATATATATTTTTTCTAGGTGATCTGCTTTCCCA<br>AAGAAAAACAGGACTCCATTATAAAGCAAATGTAAGAATGGGTGTA<br>GTTTAGATGACAAGCAGTCTCTGCTCTCTATTTTGGAGAAATCCTCT<br>TACTTAAGGAACTAATCCAAAAGGAAAAGCAAACAATGTCCTTGG<br>CATAAAGATTAAGAGACAACATTACACAGGGTACAAGGCACTGGA<br>GTGAGACTCCTAGTCCTAGCTCTGCAGCTGACTTGCTGGGGAACTG<br>ATCCTCAGTTTCCTTCCCTTTAAATTAAGACATTGGAGTAAATAACA<br>TGTAAGCATTTTAAACAACAAGTAAGTCTGTAAAGACTGAAGGGAA<br>TTAACATTTATGACAGATTCCTTAATGTGCAATAGCTCATTTAGACCT<br>CACAACAGTATCTCCCTTTACCCTGGGGGATGCTAAATCTCAAAGC<br>AGTCAAATAGTGTTCCCAGACCCACATCTAGTGAGTGGCAGCCAGA

	ATTTCAAGTCGGGTCTGTAGGCCTCCAAAGCCAGAGGCTTCTTTCT<br>ACTGCCCATCCAACCTTTCTCAAAAGGCAAATAAGGTCATGCATTT<br>TTATTTACTGAATTTTAAGTAAAGCAGTGTCTTTTGAAGCGGGCCCA<br>GTGCCACTTGCAGCAGAATCACCTGGGATGTCTATTACAAATGCAG<br>ATTCCTGAACCTTCTCCACATGAACTGAAGCAGACTTGCTAGGGCC<br>GAGGGAGACCCAGGAATCTGTATTATTACAAGCTCTCCAGGTAATT<br>GGAGAGACTTTACAATTTGGGAACCATGATTTTAAACTAATAATTTA<br>GTGTTCCATTCTAGTGTAGTTTATTTATGGATCTGTGGCTCATGGAAA<br>TTTCCTCCTAGTATCAGGAAGATACAACTTAAAAATAGTCTGTTTTT<br>CCTTCCTATTGTATCCACTGAATTCCACGGAAAATGTTAAGGAACTG<br>GAGGTGTGATTAACACTGATTAATCTAGGCTCCGTACGTCAAAGGC<br>ACAGACTGACATGAACATACATGCTGATTAGAATCATACAGAGCTG<br>GAAGGGGATGGGCAAAGAAATGGCATTAGCTAAATATTTACTATGT<br>TCCAGACACTGTACCAGACACATTACGTCATTTAAGGGGCCCAAGA<br>CAAGTGATTCAATCCCTTTTATTTATAAACGGGGTAGGTACTATTATT<br>ATTTACATTTTAGAGATGAAGAAGCTAATGGCCAGAGAAAAACAGT<br>CACTTTCTGAGATCATTCATTCATTCATACAATAAAAATCTCATTAGC<br>TCTTATCTTATGAAAGACATTGCAAATAGAGCAGCAAAAAGGCAGT<br>CTAGGGACCTCAGGTGGCACAAAGTCCACCAAATCTCCATTGAAG<br>AAACTGGAACTGAGAGGGCAAATTGGGACTGGAGCCTGCCAGAA<br>GCCATCTCTCTGGAATCCCTCAGCCTGAGCAAGAGGGGAGTTTGCT<br>GCTGAGGCTGCTGGAGCTGCGGTGTAGAGCAAGTTGCAGGCCTCT<br>GAGCCTGTTGGAGGTTCAGAAAGGTTAGCTGACTTGCTTAAGTAA<br>GTGTCCTAGACAGGAGGGAGAAAGCGCGCGGGTCTCAGATCCCAG<br>CTCCAATATCCACGCCTTTTCCCTTTTCATCTCCCGGAAGGAAGCCT<br>TCACAGGTGGGGAAAATGCTCACCCAGTGACTCTAAAGTAGTGCA<br>CAAATTGGCATTAATCAGAACCATTCTGATTCCTGCTATCAAGGTTT<br>ATTTAATTCTAAGCTCAAATGACACAGAAAATAAAATTTCCTCCTTA<br>AAAGGTTCTGCGGAAAAATATTCTCTGTTCTACGTAACAGCCTCCT<br>TGGCTGTGTTTGTGTCTGAAGAAAGGACTAGTCTGACAGGAAGAA<br>TTACAAATCTGGAGCTCATTTTGGCGTCGCAGAGGGAAGGTGGGT<br>GGAGCGTCTCGCAGTAAATTAGGAATTCAGAATGAAAACGTAGAG<br>TTTATTTGGGTTTTTAGTGACACCTCAGGATTATTATACAGCAGTGT<br>CAGACACAGCTCAAAGTAATGATGACTGCTCCCCCTCCGCCCCGCG<br>GGAGCTGGGTGCTAAGAAGGGGGAACCCCTCCTTTGAGGGGTTGG<br>GGTGGGGATCCTCCCTTTCCAACCTGGATCGGGAAGGGGTTTGAG<br>AGGAGTGCGGCTTGGATCCCCATCCTGGATCTGTGCTGGAGGGAG<br>GCGGGAGGCTATTTGGGGGTGTGTTTGGGCTCAGGATCTGGGTCTG<br>ACGCTGGGATCTGAGGACTTTCTGGGTTTGGAACTGGGTGGGGGA<br>GCTGGGTTTGGGGTGACCGCTGGGAGCAGGAGGAAATAGGAGAGT<br>GAGTCGCATGTGAAGGGCACTGAGGAGGGATCTTCGGGGGAATGC<br>TGAAGTTGATGCGTGGACTTGGAGAGGGGAAGTTGAAGTGGATGC<br>AGGGGCCTGGCGCTGCAGCGGATCTGGGTGGCATGTGCTGGGATT<br>GCCAATGCGGAGCTGTGAATGGGGTCAGGGTGGGGCTGAGGCAGT<br>ACCAGATCTTCCGCGCCTAGAGGAGGTTAGAAGAAAAGAGCGTCG<br>AACGGCCACAGCCTTTTTCCGAAAGAGACTCACACAGACACACCC

	ATCGCTCTACCCGGCCCTCCGGGGCTAGCGGCTGAGCGGTGCTGGGGCTGGCAGACCCCGCCCCCACGCGGCCTCTCGGAGCCGGGAGCCGATCGGGCCGGGGCTGGGGTCTGGCGGCCCAGCCCCCGGGGAGCGTGGGGCGTGGCCAGAAACCGGCGCTCGCGGCTGCGGATTGGCTGCCTGGCGGGCCCGCAGGCGGGGCTCCCGGCTGGGTCCCGCTTGGCAGCTCGCGGCGGCGGGGAGGCGGGGCCGGCGGGAGCCCGGCAGCCAATGGACGCGCGTCCTCCGAGCAGTTACAAAGGGCCGGAGCGAGGCCGCCGCGGCGGCTAGGGAGGTGGGGCGAGGCGAGGTTTGCTGGGGTGA
TK1	ACTCCAGCCTGGGCAACAAGAGCGAAACTCTGTCTCAAAAACAAAAAAAAGAAAGAAAGAAAAGGCGGCCAGGCACGATGGCTCATGCCTGTAATCCCAGCACTTTGGGAGGCCGAGGCAGCCAGATCACGAGGTCAAGAGATGGAGACCATCCTGGCCAACATGGTGGCCCCATCTCTACTAAAAATACAAAAATCAGCTGGGTGTGGAGAGGTGCGCCTATAGTCCCAGCTACTCAGGAAGCTGAGTCAGGAGAATCGCTTGAACCCGGGAAGCAGAGGTTGCAGTGAGCTGAGATCGCGCCACTGCACTCCAGCCTGGGCAACAGAGCAAGACTCAGTCTCAAAAAAAAAAAGAGGCTTCATTGCTTTTTTTTTTTTTTTGAGACAGGGTCTCACTGTTACCCAGGCTGGCATGCAATGATGTAATCTCAGCTCACTGCAACCTCTGCCTCCCAGGTTGAAGCAATTCTCATGCCTCAGGTTCCCGAGTAGCTGGAATTACAGGGGTCCAGCTAATTTTTGTATTTTTTTGGAGAGATAGGGTTTTGCCATGTTGGCCAGGCTGGTCTTGAACTCCTGGCCTCAAGTGATATACCTGCCTCGGCCTCCCAAAAATTGCNTTTTTAGGAATAGTGACCGCATTCCACATTCAGCAGCGGATGGGGAACGTTCCTCTGTTCACTTCACCTCCCTATGCCAACCACAGCCCGCAGCCTTACGGGCCTGGGTATGGATGGCTGAGCAAGAAAGGCCAGGCATGGTCGACTGAGCCGTGGACCTCCCCCGGACCTCTCCTGCCTCCAGTAGTCCCTGCAGCAAGGGGTCCTGGGCACCTGCCCCTGAGCAGGGAGGTTTATAAAGTGCCTGGTGTTTGGGAAATGCCCCTCGAGGCCCAGGTGGTGCAGCACAGCCCCCCCAACCCCCAGCATTCTGAATCACTGCCACATGCTTAAGGACACCCAATGCGGCCACGGAGAGGTCACACTAGTACCTTCAATTCCTATCTGTGAGTAGGTCCTCAAGGCTTCCTCTGCTCCCAGTCGGACAACCCATCGGCTGGGACAGTACTGATTCTCCAGCTCCTGCAGGGCACAAACATGAGGCTCCGTAAGACTTTCCTTCTCAACAGAGTGTGCCCCTCCCCCTCGCCCATGCCCGGCTGCTCCTCTCTGCCTTAGAAGCCCTCGCCCCAGTATCCCAGGGTCTCCAAGATGCCCTCAGATCCATGGTCTAGAGGTATACCAGCGACCGCTGTGCCTTTAGCCAGCTGGCAGCCTTAAGGGGAGATGAGGTCCCCCAAACGAATTCAGTTAATGCCATCATGGGCACCACTCCCACAGCAGTTACGACCAGGGGAGGCCAGGTGGCCCGGTGGCTCACGCCTGTAATCCTAGCACTTTGGGAGCCTGAGGCAGGTGGATCACTTGAAGTCAGAAGTTTGAGACCAGCCTGGCCAACATGGCAAAACCCCATCTCTACTGAAAACAAAAACTAGCCGAGCGTGGTGGTGTGCACCTGTAATCCCAGCTACTTGGGAGGCTGAGGCAGGAGAATTGCTTAAGCCTGGGAGGTGGAGGTTGCAGTGAGCCGAGATCGCACCACTGCACTCCAGCCTAGCGACAGAGCAAGACTCTACCTCAAAAAAATTAAAAAAGGAGGAGCTGATCTGCGCTGAGAGGGTGCGATTCT

	GACCACAGGAGGTCACTGCACAGTCAAAGGCACAGAGGTGAGGA<br>GGAAGGAATGAAAGGTCAGTTTGCAAATGCTGGCAGGCCCTATGG<br>CTGACACACAGAGGTGAGGCTGGGAGGCTGGGCTGGAGGAGGAG<br>GGGCGGTGAATGTCGGGCTAACACCTTCACACTTTATGCAGAAAGT<br>AACAAGGAACCATTCTGAGGCAGTTTGGAGAATTAAAAAAAGAAA<br>AAAAAAAACAGAGGCTGGGCGCGGTGGCTCACGCCTGTAATCCCA<br>GCACTTTGGGAGGCGGAGGCGGGATCACGAGGTCAGGAGTTCAAG<br>ATCAGCCTGACCAACATGATGAAACCCCATTTCTACTAAAAATACA<br>AAAATTAACCAGGCATGGTGACGCGCGCCTGTAATCCCAGCTACTC<br>AGGAGGCTGAAGCAGGAGAATAGCTTGAACCAGGGAGGCAGAGG<br>TTGCAGTGAGCCGGGATCATGCTACTGCACTCCAGCCTGGGCGACA<br>GAGTGAGACCCCATCTCAAAAAAAAAAAAAAAAAAAAACCAACAGA<br>AAATGCTGGACACGGTGGCTCTCGCCTGTAATCCCAGCACTTTGGG<br>AGGCCAAGATAGGCAGATCACTTGAGTCCAGGAGTTCCAGACCAG<br>TCTGGGCAATATGGCGAAACACTATCTCTACGAAAAATATGAAAAA<br>TCAACCGGGCATGGTGGTGGACACCTGTAGTCCTAGCTATTCGGGA<br>GGCTAAGGCAGGAGAATCGCTTGAGCCCAGGAAGCAGAGATTGTA<br>GTGAGCCAAGATTGCGCCATTGCACTCCAGCCTGAGAGAGCAAAA<br>CCCTGTTTCAAAAAAAAAAAAAACAAAACACTGGGCGTGGTGGCTC<br>ACACCTGTAATCCCAGCACTTTGGGAGGCTGAGGCGGGTGAATCA<br>CCTGAGGTCAGGAGTTCGAGACCATCCTGGCCAACATGGTGAAAC<br>TCCGTCTCTACTAAAAATACAAAAATTAGCTGGGCATGGTGGCGGG<br>CGCCCGTAATCTCAGCTACTCGGGAGGCTGAGGCAGGAGAATCGC<br>TGGAACTCGGGAGGTGGAGGTTGCAGCGAGCTGAGATCCCACCAT<br>TGCACTCTAGCCTGGGTGACAACAGCGAGACTCCATCTCAAAAAA<br>AAAAAAAAAAAAAAGTAACAAGGAACCAGATTTTGAAGAAAGGG<br>CTCAAAACCATCATGAGAAATGGGAAGTCAGGGCAGCCTCATGGC<br>AAGGCCTGAGGTCAAATGCCATCTGACCTCCCTCACTTACTTACTG<br>TTTTATCCTCAACAAGTTACTTAGTCTCTGAGATTCAGTTTCCTGAC<br>TGGTAGAAGGTATCTATCCAGGCCCGGCACAGTGGCTCACACCTGT<br>AATCTCAGCACTTTGGGAGGCCGAGGCGGGTGGATCACTTGAGGC<br>CAGGAGTTCAAGACCAGTCTGTCCAACATGGCGAAACCCCATCTCT<br>ATTAAAAATACAAAAATTGGCCAGGCGCAGTGGCTCATGCCTGTAA<br>ACCCAGCACTTTGGGAGGCCAAGGGGGGCGGATCATGAGGTCAGG<br>AGTTGGAGACCATCCTGGCTAACACAGTGAAACCCCATCTCTACTA<br>AAAATACAAAAAAATTAGCCGGGCATGGTTGCGGGCGCATGTAGTC<br>CCAGCTACTCAGGAGGCTGAGGCCGGAGAATGGCGTGAACCCTGG<br>AAGAAGAGGTTGCAGTGAGCCCAGATCGCGCCACTGCACTCCAGC<br>CTGGGCGATAGAGCGAGACTCTGTCTCAAAAGAAAAAAAAAAAA<br>AATTAGCCAGGCATGTAGTACCAGCTACAGTGTGCCTGTAATCCCA<br>GCCACTCGGGAGGCTGAGGCAGGAGAATCACTTGAACCCGGGAG<br>GTAGAGGTTGCAGTGAGCCGAGATCATGCCACTGCACTCCAGCCT<br>GGGTGACTGAGCGAGACTGTGTCTCAAAAAAAGAAGGCATGTATC<br>CAAATCACAAGGTTAAAAGAGATAAAGCATGCGAGTAAAATAAAG<br>CAAGCCAGTCAGTGTGGGTTGCTTCTTCCTCCCAGTGAAGGAGCTC<br>TTTGTCAGAGGTCCTTGGATCTGTCCAATCTGTACCTGGAAAGGTTA

	TTACCTGTAGGATCCTTACAGCCACACCTGGCACACTCTGTGATCA<br>CTACCACCATCTTTGTTGCTATTATTTATGATCATGATTATACAATGGG<br>TTTCTTTTCTTTCTTTCTTTTTTTTTTTGAGCCACCGCACCCGGCCTG<br>TATGTTTCTAACCTTCACAACCCCAATGTAAATACCAGCTGTTCTGA<br>ATCCTTCAGCCTGTGCCACATAAGCACAGACCCAAAAACATTGTGT<br>AACCTCAGATCAAGGTCCAGAACTTTGTCCCCTAACCTCCTGCGGG<br>TCTATGGATTTTTTTTTTTTTTTTTTTTTTTTTTTTTTGAGACTAAGTCT<br>TGGTCTATCGCCCAGCCTTCCAGGCTGGAGTACAAATGGCATGATC<br>TCGGCTCACTGCAACCTCCACCTCCTGGGTTCAAGCGATTCTCCTG<br>TCTCAGCCTCCCCAGTAGCTGGGATTACAGAGGTACATCACCACGC<br>CCGGCTAATTTTTGTATTTTTAGTAGAAACGGGGTTTCACCATGTTG<br>GCCAGGCTGGTCTCGAACTCCTGACCTCGTGATCCGCCAAAAGTG<br>CTAGGATTACAGGTGTGAGCCGCCACGCCTGGCAGGGTCTACGGAT<br>ATTATTAGCATAGTCAGGACTTCAATTTTCTTTATCCTGAAAACCAG<br>GTTAACAAAAATCCTTAGGAGAAGGTAAAATACACCATGGTGCTCA<br>AGCACTGGGGACCTGGCACGCACTAGGCGCTCTGCATGCCCACAG<br>GAGTGCTCTAGACGGCTTCTAAGCTTCCTTCTTGGAATTCCAATCTA<br>ATAAATGAGCTAACTCCGCCCCAGCCCCTTAGTCCCTCCCTGCAATC<br>CACCTACCTCTGCAGACATCTTCTTCCAAGGAACCTTGCTTGGGAA<br>ACCCACACCAGACACATCCATCATGGCGTCTACAGCCGCATGGGCG<br>TGCGTCCCTCTGTTTATATGGCCAGAGCCCCGCCTCGCTCCGCCCCT<br>TTAAACTTGGTGGGCGGACCGAGGCGGGGCTCAGACCAGGCCCCA<br>CCCCGATCAGCCACGTCCATCGCCCTGATTTCCAGGCCCTCCCAGT<br>CCCTGGGCGCACGTCCCGGATTCCTCCCACGAGGGGGCGGGCTGC<br>GGCCAAATCTCCCGCCAGGTCAGCGGCCGGGCGCTGATTGGCCCC<br>ATGGCGGCGGGGCCGGCTCGTGATTGGCCAGCACGCCGTGGTTTA<br>AAGCGGTCGGCGCGGGAACCAGGGGCTTACTGCGGGACGGCCTTG<br>GAGA
TNFRSF1<br>0B	AATTCTGTGGTGACTCATTCATTAACTTTTCTCTCCCTTCCCAGGAT<br>CTCATAGATCAGAATATACTGGAGCCTGTAACCCGTGCACAGAGGG<br>TGTGGATTACACCATTGCTTCCAACAATTTGCCTTCTTGCCTGCTAT<br>GTACAGTTTGTAAATCAGGTACAGAATGTGTGGACCCCTTGCCCAG<br>AGGTGGAGTGCATAGTGATGAGGTGGGAGCTGTAGCCGATGTGAG<br>TCGGGGAACCAAGTTCTAGCCCAACTTGGTAGTCTTCATCATGCCC<br>TGTTCCATGATTATGACTAGTTGATTAAAAAAAAAAAATAGAAATCT<br>TTTTTTTCTTTTCTTTTATTTATTTATTTTTTTGAAGACAGTTTCACTC<br>TTGTTGCTGAGGCTTGAGGGCAATGGCATGATCTTGGCTCACTGCA<br>ACCTCTGCCTCCCAGGTTTAGTGATTCTTCTACCTCAGCCTCCTAAG<br>TAGCTGGGATCACAGGCATGCACCACCACACCCAGCTAATTTTTTT<br>TTTTTTTTTTTTTTTTTGTGAGACGGAGTCTCGTTCTGTTGTCCAGG<br>CTGGAGTGCAGTGGCGCGATCTCGGCTCACTGCAAGCTCCGCCTCC<br>TGAGTTCACGCCATTCTCCTGCCTCAGCCTCCTGAGTAGCTATGACT<br>ACAGGTGCCCGCCACCACACCCGGCTAATTTTTTTTTGTATTTTTAG<br>TAGAGACGGGGTTTCACCGTGTTAGCCAGGATGGTCTCGATCTCCT<br>GACCTCGTGATCCACCCACCTCGGCCTCCCAAAGTACTGGGCTTAC<br>AGGCATGAGCCACCGTGCCCAGCCAGTTTTTTGTGTTTTTAGTAGA

	GACAGGGTTTCTCCATGTTGGCCAGGCTGGTCTCGAACTCCCGACC<br>TCAGGTGATCCGCTTGCCTCAGCCTCCCAGAGTGCTGTGATTACAG<br>GTGTGAGCCACTGCGCCTGGCCAAAATCTTTTCTGTAATGCATGCC<br>ACCAGGCTGGGAAATCTTCTAAACTATTGCCTGCAGCCGGGTGGGG<br>GCATCATTACAGCATAGAGGAGGCCTGGCCCAGTGGACAGGGCTG<br>GGTTCCAGCAGTGGCACAGGTCTGCTCCGTGGCCAGGCTCTCTCCT<br>CCCGGGCTCTGTGCTCCTGAAAGGTGCTTGGCCTCACTTAGCCTTC<br>AGGTTCTCAGGCCCCTTCCTCTTCCCCAGCAAGGCCGGGGCTTTTG<br>GGAAGTCCCCCAAGGGCCCCTCTCCTTGCAGAGTCTCTCAGGTGAT<br>GCCCTTGCGCCTCCCTTGCTACTGACCATGCCATCTAAACAATGCTT<br>TTCCCCTAACCTGATATAATAAATAATGAATCACGGTCCTTTTGTATT<br>GGTCCTTACATACAGGGTGGGTGTCCTGTATGAGTCCCGAGTGTGC<br>TGGTGCAGAAACCAGCCCTCAGAACTCCTTGGTGAACTGAGTTGA<br>GCTGAGTGGGAGGGGATGGGGTGGTGAGGAAGGGTGGACAGACA<br>TGTCAGGGAAACACATTCTCAAAACCTTATGCTCTGTTGTCAGGTC<br>AAACAAATAAAAGTTCCTGTACCACGACCAGAGACACCGTGTGTC<br>AGTGTGAAAAAGGAAGCTTCCAGGATAAAAACTCCCCTGAGATGT<br>GCCGGACGTGTAGAACAGGGTAAGACAGCAGCCAGGGGCTCCCA<br>ACAGCTTTCAGGAACCTGAAAAGACCTGATTCACCTGGTACCTCTA<br>TCTCTCCGCTGACCCCATGGATCCTCCAGGCCCGAGGCCTCCCTGA<br>GCCTGTGGGGTTTCCTGAGCCTGCGTTGGCCCCTCCTGGTCCATCT<br>GCCTGTCCCCACCCCTACCCCCTGCAGCAGCCCCTTCCCATACCCT<br>CCCCAGCAGGAGTCCCTTTGTCCAGGCTGAATTGTTCACTGGGCAC<br>AGGAGGTGACTTTCTAGGTCCCTTGAACCTTTTCAGGCAACCTAGG<br>AAATGTAATTTCCTTTTGAAATCAGAAGAAAACACTTGAATCCAGC<br>CTGGCTTGTATTTTTCCTTACTCTAAGTTGGTTTAAAAAATGTAATTT<br>TTAATATTTTTTATGTGGGCAGGGACCCATAAAGACAAAAGTGTCC<br>GTAGCCCACAAAAGTCATAATAAGGCCATGGGTGGAGCTCCCTTAA<br>GTTCCCATGAGGACCTGAGCCCACCCAGCCGGCCTCACCCCTGGC<br>CCCCGATTTCTCACTAAGTCCTTCTTTATCCTATTTGGGTTTTAGGGG<br>CAAAGAGTAACTTGCTCTTCTCTGGCCCTCCAGTGTTTGCTGTGGT<br>TGGGAGGAACCAGCGGCAGACAGGGTGGGGCGAGGACTCACTTG<br>TACTGTGTGTTAACTGGAGGAAGGGGCTCAGCTTGTGGCCACAGG<br>GGCTTGTGATCTTCCCCTGGAGTGCGGCTCCTCCTAACGCAGGCCT<br>CCCCTGCAGCCCAGGGAAGCTCAGTGTGTGCCCAGCACAGAAGGC<br>TCCTGGATCTACAGCTCTGACCCCAGAGCAGGGAGGCCGAGGGGC<br>TGAAGTGTGCATGCTCAGACCCTTCCCCAGCCTCAAGAAGGGAGC<br>ACAGGGGGACCCACTGCTGACACGAGGAGCCTGTCCTCCTGACGC<br>CTTCTCAGGGACTTTGGGCAGGGAGGGTGGCTCCTCTTTCATCCCA<br>CCTGGCCAGCTTTCCATCAAGAGTCCCTGTCTCCCTCTCTGTGTGTA<br>CCCAGGTGTCCCAGAGGGAT
TNFRSF1<br>0C	CCGTTCCCTCTGTCAGGTCCTAAAAGCAGGACAGGTCTCAGCAAA<br>CATGGAGCTTCCCCCTCCCAGATATCTGGAATAATTGAGCTAAGAG<br>ACAATGTCATCTCCTTCTCAGTATTACTGTAATATAAGATTTCCCTCA<br>TTACATAAACCATGTATTCATTCCTTTACTCTCAGCAACTCCTCCTCC<br>TCGTTTTCATTTCTACTCAAACTTTTCTACTTTGGAAATAACTGTGAT

	GGGCTGGCAGCAATACTAGTGTAACAAGTGTGTCCCTTCAGTGCAC<br>ATCCATTCATACAGGGCAGGGTTACACAGGTGTAGAACTAGTGGGC<br>TATTTTACCAATAGGCAACAAATTAGTCCATTGTGTGTTGCTATAAA<br>GGAATATCTGAGGCTGGGTCATTTATAAAGAAAAGAAGTTTATTTG<br>GCTTACGGTCCTGCAGGCTGCATGAGCATGGCATCTGCATCTGCTC<br>AGCTTCTGGTGAGGGCCTCAGGGAGCTTCCAATCATGGAAGAAGG<br>CAAAGGAGGAGCCAGCACATCACATGGTGAGAGGGAGCAAGAGA<br>TAGAGGTGAGAGGTCCCAGACTCCTTTAAACAACCAGCTCTCTAGT<br>GAACTAACAGAGTGAGAACTCACTCATTACTGTGGAAAGGGCAGT<br>GAGCCATTCATGAGGGTTCTTCCCCCATGACGCTAACACCTTCACC<br>AGGCCCATACCAACCTCAGGGATCACATTTCAATATGACATTTGGA<br>GGGGACACACATGCAAACTGTATCAGGCTATGTAGCTGCATTTACT<br>GTTAGCCCCAGTTTTGCAAGACTGGGGTCCCATCAGGTCCATAAGA<br>AAATTGACAGAAAAGTCTAAACACGTAGTCTGTTCCTGGCTTAGGG<br>GTCGTCGCTGCTTCCAGCGTTTATAGTTGTAGCCCTGGCCCTGTGGC<br>CAAGGTACCAGGTGGTGGGTGGGGAGAAGGGGAGAGGTGAGGTA<br>ATACAGGGAAGACAGACAAAAATGACGGTCACTGGCCGGGCGCTG<br>TGGCTCATGCCTATAATCTCAGCACTTATGGGATGCCAAGGCAGGTA<br>GATTACCCGAGGTCAGGAGTTTGAGACCTGCCTGACCAACACAGT<br>GAAACCCCGTCTCTGCTAAAAATATAAAAATTAGCTGGGCGTGGTG<br>GCGGGAGGCTAAGGCAGGGGAATCACTTGAACCCAGGAGGTGGA<br>GTTTGTAGTGAGCCAAGATCATGCCATTGCACTCCAGCCTGGGCAA<br>CAGAGCGAGACTGTGTCTCAAAAAAAAAAAAAAAAACCACTACCA<br>CCACCACCACCACCACCAACAAAATGACAGTCATCACACTAGCATC<br>CTCCCTACCTCCTCATCCCCAGATCCACTGGAAAAATGGAGGAAAG<br>TCTGGTGGGGACACTCCTTTAGCCCACTCGTGTGGAGTAGGGGCAC<br>ACACCAGTGAAGGTGTGGAAGCCAGCCCTTCATGCCTGTGTCTCCC<br>CCCATTTTAGACAATCAATGTTTCAGTTGACTGTTCTGCTTCCCTGT<br>CAAATTATTACTCTAAGGAGGGTATCTCTCTGCCCATTGCTAAACAT<br>TATGCACTGAACGGCATGTTTCTTGGTCCAAGGAAGTGCGACTTAG<br>TGGTCCAAACTAGAATAATACCTTATTTCAGTTCTTTTATATTAATAG<br>CACTCTGGGCATTTGCAGCCTCCACCAGGTAAGCAAAAGACAATC<br>CACAGTCAGTTTCTGTTCCTGTCAAGACTCACCTGTACCCCCCAGG<br>GCACTGCCCTCAGTCTCACTTTCCAAGTATGTTGAGGGCCTCCCCA<br>TCAGGGAATCTGCCTCATAGCTATAGGCAGTCTGTGTCTCTCCCGCT<br>GCTAGACAGAACAGCTCTTATTGGCATTATGTGCCTGAGAGAGAGC<br>AAGAGGAATGTGTTTCGATTCAGCCCATCTCTGCATCACTGTGGTT<br>CCCCCATAACCACTCCTTTTATTTAATGGCCTCTGGTGACCACCTCA<br>AGGGAGTACATGGTGGGTGGTATCTCTTGCTGATGTCAATCACTTTC<br>CAAACCTGAGGCAGGTTCTACTGAGCAGTATGGATGTGTCCTACTT<br>TAATGCGCTCCTCAAGTCCCCCTAGAGATTGCCATGGGGTCAGGCC<br>TCATATGGACATCCCTTCAATAGGCCAGTTTTCCATTATTCTTGGAA<br>ACAATGGGGAATGTAGAGACGAATATCCAAATTTAATACACTTTATT<br>TGAAAAGCAAGAATTGCAATGCTTGGCATATACAAAGACCAACTTG<br>TCTTTGATATGTCCAAAGACCAAAAAGAGAAGTTTAGAGGCTTTTT<br>TAAAAGGAGAAATTCTGGCCAGGTGCAGTGGCTCATGCCTGTAATC

	CTGGCACTTTGGGAGGCCAAGGCGGGCGGATCACGAGGTCACGAG<br>ATCGTGACCATCCTGGCTAACACGATGAAACCCCATCTGTACTAAA<br>AACACAAAAAATTAGCTGGGCGTGGTGGCAGGCACCTGTAGTCC<br>CAGCTACTTGGGGGGCTGAGGCAGGAGAATGGCGTGAACCCAGGA<br>GGCAGAGCTTGCAGTGAGCCGATATCGCACCACTGCACTCCAGTCT<br>GGGTGACACAGCGAGACTCCATCTCAAAAAAAAAAAAAAAAAA<br>AGGAGAAATACTAATTATTTTTTTTCTAGGAAGCTCATTGGCACTG<br>TCAAATTTGAAGAAGAGGCGTGCTCTGACTGGTGAGTGACTGCAG<br>TGGGTCAGGCTGGTCTTAGAGCAGTAGCAGGTTATGTCCATAGATAT<br>TAGATAGGACTATTAATAGTTTCAGGTTACAGCTGCCAGGCTTACAG<br>AGAATTGCAGTTTGGGGGTAATACAGTGATTTTTCTTCCCCATGGCC<br>TCTTGACTCTGTTTTAGTTGGGTGTGACAAGAATGACCCAATTTGT<br>GCAATCAACTTTCACATTCTGCTTCCCAACTCTATGACCAAGACGTT<br>GAGGACTGTTCATGAGCCAGAAAACAACTCAAGTACAGGGGCTTT<br>TGCTGCTGTCAAATCTTCCATCACGCTAGGAAAACAGCATGCAATC<br>CAACCCACCTAGCCAATTTGCCTTTGCCTTCTTCCATCAGAGGGGT<br>GGCCTTCCAAATAGGATGTTGACCATTCACCTAGGAATGACTGTCT<br>ACAAACTAAGTAGCTCTTGGTTGGTCAGTAAAGAGCTGCGTATAGG<br>GCAGTGTCCAAGTGACCACAGAATCCAGCAGCTCCTCACTCAGTT<br>CCAGACTCAGTCTGTGGGGAACAGAGGCTCCCTGCTCATGAGTGT<br>CTCCTCTTTGCCTTCCTCAGGCACCAGGATCCCGTATCAACCATTTC<br>CATTTTGTAATAGAACTCTTCGGGGACTGCCCTCCCCGTTAGAGCTT<br>TTCCAAGATCTCTGAAGACATCATGAGTTTTTCAGGTTTCAAGATTA<br>TTTTATGTCCTTCAGTCACAGGGGGAGCTTCAGCTAGTGTTCCATCG<br>TAAGCTAGTAAGTGCCTCTCAAATGGAAACTTTATAGTCTAAAGGC<br>CCAGTTCCCTGGAGGTGCTCACAGCCTTTTGCCATTAGCCCTAGGC<br>AGTGCTCCACACAGGGCACAGAGAATGTGTGTTACCTGCAGTCTG<br>GTTTCTAGTCTACACTGTGTAGTCCACCCTCCAGGGCCAAGGGGCC<br>TGGAGTGCTCTAAAGACTTTCCTGCAGGCCCTCGTGGCTCTGTGTT<br>AATGCTGTCAGTCTTCCTGAAGTCCTGTCCTCTCACCTCCTGCCTAG<br>ATCTGCCCCCAGCACACATTCCTGACTCCACACATTTCTCAGTGCCT<br>GCGACCTGGCACACATTTCTGATCCGAACTCAGTGCCTGGGGCCTG<br>GCAGGTGCCCAGGGCACTCCATTGCTCAAATCCAAGCCTGTGGAG<br>AGGATGACATATCATTCCCAGGTTTCTGATGAGCAGAAAAAAAAAT<br>CTTTGTTTCTATAGTGCTATCCTCTTTTATCCACCATGTGAGAGGTCA<br>GGAGCCGACTCACCCCAAGGGTCTAGGAGTTGTGCCATTGACCTTA<br>CTGCCTAGGGTCACAGGCTGGCACCCTTCAGTACCACACATTGGAT<br>CTCTCCACACAGCAGCAGTGTGCTTAACTCAATGGGGAGGTGAGT<br>AGCCCAAGGTCAGATAGTTGGACCTGGATAGACACTTGACTGGGG<br>GACAGGCTTTGGAAGCCACTCCCCTGGGCCTCCTCTGTCCATGGGG<br>CCTCAATACTAGAGCCCACAAGGTCGGCCAGAGAGTGTCTGTGCCT<br>CGACCATGCAAAGGGTGCCAGGTTGTGGCTAGGGATGCCCCCGGG<br>GTTCCTCTCCCTCCTACCTACTCCTCAGCCTCTGCATGTGCCCGTCA<br>TGGCCCCTGTGTCCTTCATTCTGTCCACTCATGTGTTCACTGGGCCC<br>CTCCTTGGTGCCAGGCACCCGCTACTGAGGGATACCAAGCCCCTGC<br>CAGAGAAGCTTGGAGCTGGGTCACAGCAGCAAGGAGGCTGGAGA

	CATCAGTGGCATCAGTGCACTGGTCAGTGAGGCCGCTGTGGGAGG AGGGAGTGGGAGCAGAAGCTAAGAGTGGACTGAAGTCCTTAGGCC AGGAGGTAGTTCAGGGTTTAAGAAGAGGAGAGACAGGCGTCCCG GGGAGGGAGCAGGCTCAGGATGGGCCTCCAGTGCCTCCTCTGGAG CTCACTACCCAGACTACAGCAGCGCAGTGGGCAGCGCCTCAGCTC CTTCCCCTTCTGCTGCCTACAGCCTGGACAGGACCCGGAATCAAAC CGCAGGCCCTGGGTCACCGCTGCCGGAAAGAGCCAGTTCCTGTCC GTCCATGCACCCACCACCAAAACCCAGGCCTTCCTGGAGGTGCTA GGGGAGGCCATGCCCCTTTTCTGAGTGCTTGGAAGTGACTGCTGCA AGTGACAAGTGACCACGCCTTTTCCCCCGCGGGTATAAATTCAGAG GCGCTGCGCTCCGATTCTGGCAGTGCAGCTGTGGGAACCTCTCCAC GCGCACGAACTCAGCCAACGATTTCTGATAGATTTTTGGGAGTTTG ACCAGAGATGCAAGGG
TOP1	CATACTACAGATCTATCCACAAGAAGACAGATGGAACCACTCTTTT CTTCTGAATTCTAGAACCTTAAGGAAGTTAACCTGAGTGGCTCAGC TTGGATCAAGTGCCCATCCTGGTCCAAACAACTGTAGCAGCACATC TGTGTATTTGTGTATCTTGTTCCTCCATAATCTTGCAGTCCGGGGAA AGTGTGCAGATGGATGGGTCCCTGAAAAGCAAGAGTTGTTTAGAC AACGCTAGAGGTGTTCAGTTCTGGGAGACAAGAGTTGGGCCAGGT TGATAACTAGCCTAAGAGAGTCGGAATCAGATGAATAAGAACTAGA TCTTGGGTGACAACCTAGATCAAGAGTCTTGAGGTTCAGTCATTTC AAAAAGCTCAAGTCTCATTGCTTCTTGGCCTTCTGGCTAAGATCAT GTGCAGTATCTGTTCTTACCAGTTTAATATCTGATACATCCTCTATCT GAGGATAATATAATAAATAGATTTTTGGAGCAAGGAGATGGAATAGG AGCTTGCTCTGTCCACTCTACACATTGGCCTGGATTGCAGTACCTCC AGGAATGGTGCACCCTCTCTACCACCCCCAAAAAGCCCAAGTCTC AAGAGGTAGTCTGGAAAAAAGTGTTTTTGGATTTACCTACATTTTTT TTTCCAAGATGGAGTCTCGCTCTGTCACCCAGGCTGGAGTGCAGTG GCGCCATCTTGGCTCACTGTAACCTCTGCCTCCAGGATTCAAGCAG TTCTCCTGCCTCAGTCTCCCAAGTAGCTGGGATTACAGGTGTGTGC CAACCATGCTCAGCTAATTTTTCTATTTTTAGTAGAGACAGGGTTTC ACCATGTTGGCCAGGCAGGTCTCAAAGTCCTGACCTCAGGTGATCT GCCTGCCTTGGCCTCCCGAAGTGCTAAGATTACAGGCGTGAGCCAC TGCGCCCGGCCTAATTTTTGTAATTTTAGTAGAGACGAGTTTTCACC ATGTTGACCAGGCTGGTCTCGAACTCCTGACCTCAGGTGATCCACC CCCGCCCTCGGCCTCCCAAAGTGCTGGCACTACAGGCATGAGCCA CCGCTCCTGGCCGGGTTTGCCTACATTTTGGAGATGCTGCACACCA TGTCCCTCTTTTCAAGAGTCACGTAAGTATTTACATATGGAAGACCC TGAGAAGGCACCTGGGAAAAAACCTGTTTAACTGTAAATATAATAA TGTATGTATTTGTATGTGGAGCTCTTCTTTTAGTGGATTATCTATAAA CCGTCCACTGTTCCCAATGCATACTTTGGAGACAGACCTACACTAC CAGTTTTCACCTGCTAGTTATTTTTATAAGGGTTGGGATGGTCCATG AGTGAACAGTCTTGATTTAAAGCAGTGTTTCCCAAACCTCATTTATT TGCCTATAGCCTTCCTGGAGTTTGTCTTATATGTCCGCTCTGTGCACT ATTCCTATATGTTTTTATGTAAATCAATCACTTTTTTAAAAAGCCTCA TCCCAGTTATATCCTTAAAACAATGGTTATTTTTTCTCATACACATCG

	AAATACATATCTATTAGAATTAAAATGCTTGTCCATGCACTATCAATA<br>ATCTCCTCAAGTACCATATGGTGGGGACATGTGTTTGAAGTGTTAG<br>GAGTCAGGAGTGGTCTCTGTAAGACAGAGTAAGTCAGTCAACCTC<br>TGAGCACTGCCAGCCACACATGCTGGCAGAGAAGTTCAGCCTGTG<br>ACAAGACTGCCCGGATATTTCCATGTCTGTGCACGGGGATGTCCTC<br>GGTCTGCCATTGTGAAGTCTGTGCTGTTTTCATGCCTACCTAGTCTT<br>CTTTGGAGGGGCCTGGAAAGCAAATGCAGGCTGGGTACAGTGGCT<br>CATGCCTGTAATCCCAGCACTTTGGGAGGCAAAGGTGGGCAGATC<br>ACGAGGTCAGGAGTTTGAGACCAGCCTGACCAACATAGTGAAACC<br>TTGTCTCTACTAAAATTGCAAAAATTAGCTGGGTGTGGTGGTACGT<br>GGCTGTAATCCCAGCTACCCGGGAGGCTGAGGCAGGATAATTGCTT<br>GAACCCAGGAGGCGGAGGTTGCAGTAAGCCCAGATGGCACCACTG<br>CACTCCAGCCTGGGTGACAGAGACTCCATCTCAAAAAAAAAAAAA<br>AAAAGAAAAAAAAGCAAATGCAGATTCTCCCTGGGAAACGGGGG<br>GTTGGGAGAGTGGAGTAGTGTGGATGCATTGTTATTCCTGAAGGCC<br>GTCCTCAAATTATCTTGGACTCACTGCAGCAAGCAGATGCCTCATC<br>TTCTGGCCAGGGATTGATCCTGAGATGGGAAGAGTATGAGAAGAG<br>ACACCAACAGCAAGGCAAAACTGGCTCTGGCAGCTTGCTTCAGAG<br>AAAGAGTATAGCTTTGGAGTCAGGCCGACCTGAGTATGAATCCAAG<br>ACTCAGCACTTACTAGGTGCCTGCCCTCAAACCATTATTTTTGATA<br>GTCATTACATACCCACAATGATCACCATGGAGTGGATGAGGAAACA<br>GGGACTCAGAGAAGTTAAACAACTTGCCTGATATCACACAGGTAA<br>CAAACAGCAGAGCAGAATTCAAACCTAAGTTGATTTGACTTCCAAA<br>TGTGTGCTCCTAACCAAAGCAGGGTAACCTGCTTTGATTTGCCCAT<br>TTCTTGAGCACCTCTTCTGTAGGAGGGCCTGTTGCTGGGTGCTGGG<br>AATTCAGTGGTAGGCAAGATGGACAAGATTCCCCCCTCTCACAGAC<br>CTTGCTTGCTATAGGGGAAATAGACAGGAAACCAATTACTACATTT<br>GTAACTTAATAACAAGTGTCATAAGTGTTACAAAGGAGATAGATACT<br>ATAAGGCACCTTTTATTTTTATTTGTTTATTTATTTTTTGAGACTCACT<br>CGCTCTGTTGCCCAGGCTGGGGTACAGTGGTGCGGTCTCGGCTCAC<br>TGCAACCTCTGCCTCCCAGGTTCAAGCGATTCTCCTGCCTCAGCCT<br>CCCGAGTAGCTGGGATTACAGGTGTGAACCACCACACTCTGCTAAT<br>TTTTGTATTTTTAGTAGAGACAGGGTTTCACCATGTTGGCCAGGCTG<br>TTGTTGAACTCCTGACCTCAAGTGATTGATCCACATTGACCTCCCA<br>AAGTGCTGTGATTACAGGCGTTAGCCACCGCGCCTGGCCTATAAGG<br>CACCCTTGGTCAGCCAGTGGAAGCAGGGTCAGTAGTGCGCCGGAG<br>AGTCACTTTGAAAAAGTGATATTTAGGTTGATCTTGAAGCATTCAC<br>AAGATGTGGTAAACACATCTAATGGTAAACATGTGATGGTAAACATT<br>AGATGTGATAAAGATACTGATGGTCCACTGGATGAACAGTTTTCATA<br>TTTACTTCCAAATGATTTCATAGATATTATTTCACAGATAACACTTAG<br>GATGAAGGTAAATAAAAAGGAGATCAATTAAAATATGAAATAATAAT<br>AGTGAAAGTGGTAATAGATGTGGCAATATCCACAAAGGAGTAATGG<br>TTAGATTAGCATATTGAGAAGATCACTTTGGCAATTGTTGGAATGCA<br>GTTTACGAGAGGGCCAAGAATGGAAGCAGCAATCCCTGTTAGAAG<br>TGTAGTGTTTTGGAGTGCGGTGTTTTTTGTTGGTTTGTTTTTTGTTT<br>TTTTTAAACCAGGTCTCACTCTGTTGCCCAGGCTGGAGTGCAGTGC

	AGCCTCGACCTCCTCCTCGGGCTCAGGTGATCCTCCCACCTCAGCCTCCCGAGTAGCCGGGACTACAGGCACATGCCACCACACCCCGCCAGTTTTTGTATTTTTTTGTAGAGTCAGGGTTCTGCCATGTTGCCCAGGCTGGTCTTGAACTCCTGGGTTCAAGTGATCTGCCAGCCTCGGCCTCCCAAAGTGCTAAGATTACAAGTGTGAGACACCACCCCCAGCCAGCACATTGTATTAATCCAAGCAGGAGAACGCAGACTTCAAAGGCTTATGGGCAGCAGAGAGAAGTAGATGAAGCAAAGAGATGCAATCAGCGGGACTAGTGGGAAATGAGGGAAAGGAAGGTGTCAGGATGGGGCTTTTTCTAGCTGAGTGGAGTTCAGTTCATTCCCAGAGAAGCTTGAGAGGTATCCAACTATAGATGCAAGGTAGCTGGCTGTTGGTCCCTTGTCTTTAGGACAGTTTGACTTTTGTAATACAGGCGACCTCACAGAGCTATTGTGAGAATTCAGAGCTGGGCACAGAATAGATGCTCTATAAACGGTAGTTCTATTCCCCATTTGCCCCGTCCTCCCAGCATGCCAGGGATACAGGGGCGGATAGAGACAGGTTCAGTTCCCTTTGGTCTCTTGCATTCCCCCTTCCCTCAGCACAATTCCTGGGCTCATAGCGGATGCTCAATAAGTATTTGTAGAATGAACCAAAGAAATCAGACCCTTGGTGACCCAGGGCTACATGTACTCGGCGGAAAACTGGGGTTAAACCGGACCCTTGGTTGGTTGAAAAATTAAAATAATTTTTAAAAAGTGGGCGTGAAATAATCCAGAAGTTGGAGGGCCTGGACAAAGGCTGGGGAGGGGACTCCTCCCACCAGGCGGCCTCAGGGTCGGCTTAGCAAGAAAGGAGGCGCTGGGGAGACCTCTGGGGTCCCAGCTGCCAAGGTACTCGCCGCTCCAAGGAGATGCGGACGCCGGATACCAGCTCTGCCCAAGATTGCTGGGTCCCGCCGCGGCCTTTAAAGGCCCACCACGTGGCTGCGCGGCCAGCCCTGGTCCGGAGGCGGTGCCCGGGTGGCGCCTGCGCACTGCCTAGCAGAGGGGCTGGGGTTCCGAGAAAAAGCGTCTGGAGAGGAAGGGGAGGCTGCTTGGGGTGGGACGCCGTGGGAGGAGTCGGCTCCTTATGCAAATCACAGCGGAGCGCGCACGGTCCGGAGGCGGGGCTTGCGATGCAAAGACAGGTCCGTCTGGCGAACAGCGAGGGGGCGGGCCGCAACCCTCTGCCTCTTTCCGCGAGCGCTGACGTCGCCGACGTGTTGTTTAAAAGCGGCCGCGCAGGCGCAGTGAGCCCAAATGCGAACTTAGGCTGTTACAC
TP53INP1	AATAGGTCAGGTGTGGTGGTTCATGCCTGTAATCCCAGCACTTTGGGAGGCTGAGGCGGGCGGATCATGAGGTCAAGAGATCAAGACCATCCTGGCCAACATGGTGAAACCCTGTCTCTACTAAAAACACGAAAATTAGCTGGGCTTGGTGGCACACGCCTGTAGTCCCAGCTACTTGGGAGGCTGAGGCAGGAGAATCTCTTGAACCCGGGGGGCGGAGGTTGTAATGAGTGGAGATCACGCCACTGCACTCCAGCCTGGCGACGAAGCGAGATTCCATCTCAAACAAAAAACAAAACAAAAAAACTCATCTTGCAAAATGTAATGGATATTTAAGAGAAATAAATGCCTACTCCTATCAAAGAGGAATAAAGCAGGAAAAGAGGCCCCGAAAGGATACTATAGAAATAAAAAACCTTGCCTTGGATGATCAAAACGTTTAGGCCTGGCTTTGCCAAAGCCATGAGAAACATGGACATTTAAAAGCATAAATTCATCTTGCAAAATGAAATGGATATTTAAGGTAGTTCATTCATGCAAAAAGAAAAAAAAAGAATCCAATAAACGAGTTTTCCTTAAATAGGTAGATTATTTAGGCAGTGTGTGGCTTATGTAAGTAGTAACCATATCATCTGCATCCTTTTGAGAAGAGCAGAAAGGCACTTGCACTCATGCTGTCACCCTAA

	CAAAGCAATTCATGATTACAGACCCACCTCAGGTTGCCTCTGGCAT<br>ATGCGGACTGTTGGGCAGTGCCCAGAAGTATCAGTGTGAGTGAAG<br>AAGTCGCTTTGGCAGCGGGAGTTACAAAAGCCCTTTGCTCACACA<br>CTTCAACTAATCTCTGACAACCGCCCTAAGAACGAGTCACATCTGC<br>CCCACACAGTGCGCCTCTTCAAGCTTCATGGCTCCTTGGAGCTAGG<br>GTGGTGTATGAAGGGCCATTCTGTATCATGTCTCTAGACCATATCCA<br>GTAAGGGATGACCTTGCTTTCTGCTCAAAGGTTTTTTTTTGTTTTT<br>TTTTTTCTTGAGATGGAGTTTCCGTCTTGTTGCCCAGGCTGGAGTG<br>CAATGGTGCGATCTCGGCTCATGCAACCTCCGCCTCCCCGGTTCAA<br>GCGATTCTCCTGCCTCAGCTTCCCGAGTAGCTGGGATTACAGGAAT<br>GCACCACCATGACCAGCTAATTTTGTATTTTTAGTAGAGACGGGGTT<br>TCTCCATGTTGGTCAGGCTGGTCTTGAACTCCCGACCTCAGGTGAT<br>CCACCTGCCTCGGCCTCCCAAAGTGCTGGGATTACAGGCGTAAGCC<br>ACCGTGCCCAGCCTCTGCTCTAAGTTTTAACATAAATAATTGGGTTA<br>CCTGTACATTCAGCCAGAAACTAAGAAGCCTTGAAAGTACTTTCCA<br>GAAACTAGAGAAAATACTCTAGGCCTGAAGCGATCATTAGTTACAA<br>TCCAGCGCAGGCTATCCCACCTCTTGGGGACTACCCCCTAGCACTG<br>GTTACTGAATGAAGGGGGCCCGCCTCCTTTTTCCTTTCTACTGCCTC<br>AGCCAGCACACAGACCCTTTTTATTTTAAGGATTTTATTTCTAATCA<br>CCACGTGGAAATAGGGTTAAACAAAAAGACAAACATACAAAAGTC<br>ACAAAGGTTGCAGGGTCTTGAAAAGCTATTACACTTATGGCCCTGG<br>AATAATTCTTGACTCCTTTCTGAGATGGTAACTGGCAGCCCAGTTAA<br>ACGCCTGTTTTCTGTTTTTATTTCAGAAAGCAGAAATCACAGAATCT<br>CTTGCTTCTTTGTTCCAGGCTTTCTGGAGACCCAGTCAGATCCTTTC<br>TTCCCTTAAGACAATAGATGTCACTGGAATGCTAAGTGATTGTTTAT<br>CTAAAATGCAATTTAAAAAAATTCTTGTCTTTCCCAGACACTTGGC<br>AACATGCTGTGTCCATGGTAGAAGCCCAGCAGATCCTAACTGTTGA<br>TGGGTAGATAGAAAACGACCTAAGTGGACTTCACTTTGCCATTCTC<br>CTCAGTACAGACAGAACAACTTGTTGCTTTGTGTTCTGAGGCTGCC<br>CCAAGTAGCCATCCTATGGGTTGGTAGGAAACAACATTCTTTAGCA<br>GCCTCGATGCAACTGGCAGGGTTATGGCCACAGCCTGCTTTCATAA<br>GCAGGAGGCAGAGGCATTAGTTGCATTGTATCTGTTTGTTGGTGTG<br>AATGTGGTCCTGCACTGTGAGTCTTCCAAACAAAACTTGAGGTTGA<br>ATCCCTTTTGTTAGGGTAGATAAGGGAGCGTATTGAGCTCCATGGA<br>AAACAGACAGGAGAACTCTGACAGATGGGGAAAGGGTCACCCCC<br>ACAGCAGGGGGTCAGCCTGGAAGTGGGTACTATAGCAACACGCTA<br>AGAATTATTCTGTGAATTAAAGGATGGTTGGATTCACTGGATCTGCC<br>CAGGCAGGGAACCTGGCTGGGTCTTTGTCAGTGAACAGATGAAAA<br>GCTTGCTTGCATTTGTGTAATTTGACAGTTTGGTTGTGTTCAGACAG<br>CTCTACGCTATGGAGATGACATTACTCCTGAGTTTCACTGCATGCAT<br>TTATGTAAACCAGTGGCATACCAGGAAGGGAGGCAGTGGGAGTGA<br>TCCACCCCAGGTACAGGCAGTACACAGTAAATTTGTGTGTCTGCAA<br>AGAATTTTAAAATAAAATTGACTAAAAGTCAGTTTGCTTTTTATTATT<br>ATGCTCCAACAGTTCCAAACAATGTCGTGGTAAAGTACTCCTCCCT<br>CCTAGGGACAGACTTCACCTATGCTGCCCTGAGGCACGCCCTCCTT<br>GGTGCACCACTGGGAATAAACAAGCACCAGCTGGGAAGATTATAA

	ATCAGCAAGAGAGTAAACAGAAAGCAAGTGACTCCTGAGACAAG<br>ATGTTTTCATCACAAACACTATTTGGTAGAGTTTAGAGTCACTGATC<br>TGTGATGGGCATTTCTGTTAGCTTTGTGTTTTGGTTATCGCTGCTTTA<br>AAAACTACCCCAACATTTAGTGCCTTAAAACAATGATCATTTATTTG<br>TTCACAATGCCGTCATTTTGGCAGTGCTCCGTGGGGATGGCTTATCT<br>CTCCTCTGTTCAGTATGGTGTCTGCTGGAGCTGGAACTTCCTAAGG<br>GCTTTTCCACTCATGTGTCTGGTAGCTCAGCTGGGATGAGCTGAAG<br>AGCTAGAGCTGGCCAGGCATCTCTTTCTTTTCACATAGCCTCTCCCT<br>GTGGCTAGCAGGGTGGTCTCTCCTCATGGGTGGCTGGCATTTTCCA<br>GAGTACAAAGCAGAAGCTGTGGGGTTTGCTTAATGCTTTTGCTTAA<br>TGACGAGGCTTGGAAGTCCTAGAACATTCTTTCTGCCACATTCTATT<br>GGTCAGAGCAATCACAGGGTCAGCCCAGATTCAAAGGGAAGAAA<br>AATACACCCACTTCATGATGGAGATACTGTTGTGGTCATCTTTGGAA<br>AAATGATCTACTTCATATTGAATTTTTCTATCATAACAAGATGATGAT<br>TTCACATGTTGCTTTAACCATGATAAACCTTATCAAACTGGAAAAA<br>GGTAGGCCTTTTTCTTTTTAAAAATTAGCCCCTTGTAATTGTCATCAT<br>ACCTTGGTTCTGAATGTGGAGATGGCCTCTTCAATGGATTCCTAGG<br>GAGAAACAAGAGATCATGGGGCTCCTACCATAGCATGTGGGAGAA<br>AATATTAGAACAAGCCTGATTTGCAAAAACCTAAAATCCAAAGCAT<br>GTTCAAGTCTGTGAGACTATCTAGAACACAGTTTTAGCTCTTTCTCC<br>CAAGATAAGGTCCAATGACCATCATATTTTGATCATTACTCCTTGGT<br>GTCTGACAACTCCCTGCACTGCAATTTACATCCTTTAAAACCAGCAT<br>TGTTGGAAATGGACTGACTACATTGTGAGGGTCAGAATGTGAGTGT<br>CTTTCAGATACTGTAATGTGTGATAGGCATCCCTCCAGGGTTGCTAT<br>ACAGCTCACCCCAGGTCAAAGAACTACATCCCAGTCCAAACAGTAT<br>CCACATGAGAGCTGATAGGCATCAACGTTACTCACCTGCCTTTGCC<br>AGACAGTTAATCCACCTATATGTGGGACAGAGCCTTGGTCTGGAAA<br>ACACTTTCAAGCCCACTGTCTCACTCTCTTGTCACTATAAATAACCT<br>GGTGAGTTGGCAGTCCCGGCGTGAATGCCCACAATTTGCAGGTATG<br>CAAACAGAACAGAGGGGCTAAGTTCCGTGGGTCACATGAGAGCTA<br>GGCTGCAGCGCCTGGGGAGGCGGCCGTTCCCGCCCGGTGCGCTTG<br>TTGTCCGCTGGAGCAGAGGCCCCCGAGCCTGCTGACCTCTCTGCCT<br>TCAGGCCTGGTCTCGCAGTCCCTTGTGCCTCGCCTATAGGCAGGCG<br>GAATCCAGAGCGTGCAGCAAGCCCGGCCGGCTCTCGGTGGCGGTT<br>TCTACAGCAGGTGGGGCCTGGCTCCGTGATTGAGCGGCCTCTACCC<br>CTCTATCTGGGGCCCAGCAGCGGCCCCCTCCCAGCACCCTGGCTAC<br>ACGTCTAACCCTAGGCTGACCAGGTGGGGCTCTCGGAGGCGTTAG<br>CCCCAGCCCTCCCAGGAGTCTTAATGTTCCTCTCACAGGAAAAAAC<br>GTTCTGCGCCCTTTGTGCCCCAAACCCTCGACCCTTCACTCGGCGA<br>GAGGGGTCGCTGAGGGAGAGATCCACCTCTGCCCTTCCCGTTGCC<br>CGCCGGTTCTTCCTCGCCCGCCTCTTACGCACCCCGCTCCCGCCGG<br>CTCCTGGAAAGCCGGCCCCGGCCCCAGCGCTTCCCTCCCCGGCCTA<br>GCCCCTCTCTCCCACCCTTCCGCTCGCCCAGGCCTTCACGCTCAGC<br>TCCCGGTTTTTGTTGCCCGGGCTTGTTTCGGAGCTGAGCGCGCCGC<br>CGGCCGGGCGCCAGGTCACGTGCGTTGGTGACAACCTCTCGCAGG<br>GCGGCCCCGGGGCCCCCGCACCGCCGATTGGTGGGGCCCGGCCTG

	GCGCGGCCCGGGGCGGGGCAGCGGGGCCCGACGGCGAGCGGAGC<br>GGCCGATCAGCTGTTGCGAGCGCTGCACAACAACAAAAGGACTTG<br>GACTGGC
TRH	CTGGGTGAACTGTACCCATGATCTCTTGGTGTTATTTCTTACCACTG<br>CATGCAAATCTACAATGGTCTCAAAATTAAAAGTTTAAATTAGGCCA<br>GGCGCAGTGGCTCACGCCTATAATCCCAGCACTTCGGGAGGCTAAG<br>GCCGGCGGGTCACCTGAGGTCAGGAGTTTAAGCCCAGCCTGGCCA<br>ATATAGTGAAACCCTGTCTTTACTAAAAATACAAAAAAAAATTAGCT<br>GCGCATGGTGGTAAAATTACACGCCTGTAATTCTAGCTAGTTGGGA<br>GGCTGAGGCAGGATAATCGCTTGAACCCAGGAGGCGGAGGTTGCA<br>ATGAGCCGAGATTGCGCCACTGCACACCAGCCTGGGTGGCAGAGC<br>GAGGCTGTCTCTTAAAAAAAAAAAAAAGTTTAAATTAAAAAAACCC<br>ACCATAAACAAAATTCAAAAGTAAACTCAGTGAAACTGCTTTAGGC<br>CTGTCTGCAGCTTAGGGTTGTTAAAGACTCTTAACCATCTGCAGGA<br>CATCTATGGATAGGAATAGGGAGCAGAGAGGCTTTGAGAGGGAAA<br>AAGAATTCACGGAGGCAGGAATTAGGAAATCAGAGCCTACCTACC<br>CTGAGACTCAATACACCTCCTACAAGGAGTGTCGTGTGTCAGAGTG<br>AGTGTGTGAGTGTGTGTGTCAGTGTGTGAGTTCGTGAGACTGTGTG<br>AGTGTGTGTGTGTGAGACAGTGAATGTATGTATATGAGTGTGTGTG<br>AGTATGTGTGAGTGTATGAGTGTGTGTGTGTGTATGTGTGTATTGTG<br>GGTGGCACTGGGAGTTAGAAATAGTGTACATACTCACCATTAAATTA<br>TCCCATTATTCATTTTCAAAAGCATAGCTGTATTAATCTGATAAGCAA<br>CTACAGTCTTGAGATTCAAGCCCATTTGCCTTATCAGAGAAAAAAA<br>TTGCACGCTAGAAAAATATATGCAACACACTGACTAGATTCTAAGC<br>TCTGTGAGAGAGGGAACAGGGTCCTGACTTCTTGCCCTGCTGGGC<br>CCCTGCTTTAAAGTGTAGGTGTCATTATTATTCAGATATGGCGAGGC<br>CAACAGATCTTCGTTACTTACAGTTCCCAAGAGGAGTGGGCACACC<br>AAGATCTGCAGAGCCATGGGGGAAGCACCGGGGTCAGTCAGGAG<br>GCACAGGGAGTGGGTGAAAACAGGAGAGAAATCCTTTCTTCTGGC<br>TTCCTCAGGGAGGAACGGACGAGACAGTGTGAGCAGGTTTACGAT<br>TGGCTAGTTTGGATAATTTCAATGGGCTCTGGGTCATCGGGGCTGTC<br>CCCTAGTTGTCTGGTACGTGGCCCTGAGGTGACTAGGGAGGGGGAT<br>AGTGGCCCTGAGTGTGAGAACTCCATAGAGAAGGTGGTGGGGAGA<br>GAAAGCTCTGGATTGGTTGGTTTGCACATGAAAGGGGCGCCCCTA<br>GGAGAGTTGTTTACTTTCTGGGAGGTGGCTAACCCTAGAAGTCTCT<br>CCAAGGTCAGCAAGACCTCAGATGTCAAAGCATCAGAAAAAACAC<br>ATGGTAAATACAATCCCCAAGGTCCAGAACAGGTCCTGGTACTTAG<br>GAAGGGCTCAGTAAATATTTGTTTAGTGAGGGAGAGAAGAAAGGA<br>TGGAAGGGAGGAAGGGAGGGAGGAAGGGAGGGAGGGAGGAAGG<br>GAGGGATGTCTCCCCCCCCCTCTCCTTTCTTTCTTCCCCCGTGCCTC<br>TTCGCTTCCCCGTGCTCGTTCCTTCCCCCTTTCGCTTCCTCCTTCCC<br>CCTCCCTGCCCTCCCTCCTTTCCTCTTCGTTTTGTCTTCGTTTTCCTT<br>CCTTCCCCTCTTCCTCCCTCCTCTTCCTTCCCTCGCCCTTCGCTCCC<br>CTCTCTTTCGTGTGTCTCGTCCGCCTTGTTCCGGCTCCCCTGTCGCT<br>TTCCGTTTGTTCCTCCCCGTTTCCTTTCCTTCCCCCCCCTCCTCCAA<br>ACTCAAATCNNNNNNNNNNNNNNNNNNNNNNNNNNNNNNNNNN

	NNNNNNNNNNNNNNNNNNNNNNNNNNNNNNNNNNNNNNNNNNN<br>NNNNNNNNNNNNNNNNNNNNNNNAGGGAGGGAGGAGGGAAGGA<br>AGGAAGGGAGGGAGGGAGGGAGGGAGTTGGCTTGCTAAGGACAC<br>CGCAGAGGCCTCTACCATCCTCTCTGGTTCCATGGGCTGCAGGGGA<br>GCTCTCCTGACTCCTCCCGATCCAACCAGGACCTATCCTCCAAACA<br>GGCCACTCAAATCCCTGATGATAGAAAGGCACCTAATGGGCTCACC<br>AGGGAGCAGTGTTCCAGCCATCTTACCCTTCAGGAGGCAGTTTTGC<br>CATGCATCCATGCTCACCAAGCCTTTGGTGCCTTAGGTGGGATTTCT<br>GTTGTTCTTATTGTTGGGAGTTTTGGTGCTGTTTGTTTGTTTGTGGA<br>CAGTAATCCTGCAGAGAGAAAATTCAAACAGGGCTAAGAGCTGAC<br>AGTGAAAAGCAAGCCTCTCCACAACCCTCTCTGTCCTCTCTTTCCC<br>TGGCTTCTAGGTTTCCTTCTTCATGGGTCTTGTCTAAGAACATAAGA<br>AGGATATGTGTGGTGGCTCAGGTCTGTAATCCCAGCACTTGGGAGG<br>CCAAGGCTGGAGGATTGCTTGAGGCCAGGAGTCCGAGACTAGCCT<br>GGGCAATATAGTGAAACCCTGTTTTTACAAAACAAAAACAAAAAC<br>AAAAATCTAGCCTGGTGTGGTACTGGTGCGTGCCTGTAATCCCAGT<br>GCTTTAGGAGGCTGAGGCAAGAGGATTGCTTGAGCCCAGGAGTTT<br>GAGGCTGCAGTGAGCTAGTGATTGTGCTGCTGCACTCCAACCTGG<br>GCAACAGTGCAAGACCCCATCTCCAAACAATAACAAAACAAAACA<br>AAAAGAACATAAGAGCCTGGCAGCCCATCTTCCTCACACTCCACA<br>AATGGCAGCATCATATACACATAGTTCTCTACTCTGCTTCTGGGAGC<br>TTATTCCACATGAGTGCATATGGAACTGCCTTATTCTTTTAAAGGCT<br>GCATAGTACCCCACAGTTTTAGATGGGTCTTAGTTGACTTAAGTGTC<br>CCCCTCCTGATGGACAGACATCCAGGCTGGTTCCAATTCTGGCTTG<br>TATAAACCATGCTGCACTGAATACCCTGAGGTATACATTCTCATGTAT<br>ATAGGTGGGAGTATCTGTATTATGAATTCTGAAAAACAGAATTGTCT<br>CATGGAAGGTACATATGTGTTAAATTTTCAGATTGCCACCCAAAAGA<br>TTGGATCAATTTATACCCTCGTCATTGTGTACAAAAGACAACAGGTC<br>TATCTGATTTCATCTTAGCTAGGGTGGCATTTCTTCAAATCCTGGAG<br>TCTAGTTGGGAGACCAAGCCTGAGGTTTCATGTTTGCAGTGTCTCC<br>CTCACTGCCTCTGAACTTTTTGAGGGCTGGGGCTGAGCCTCATTCA<br>CTTCTAGGCCTTCAGGACCTACCAAGGAGTCTGACACCATAGTGGG<br>TGCTTGTTAAATGTTTAGAGATGGGCCGGGTGTGGCGGCTCATGCC<br>TGTAATCCCAGGACTTTGGGAGGCCAAGGAGGAGGATTGCTAGAG<br>TCTAGGAGTTCGAGACCAGCCTGGGCAGCATAAGAAGACTCCATCT<br>CTACAAGAAATAATAATAAAAAATAGCCATAAAAATTATGGTGCCTG<br>GCTGTAGTCCCAGCTACTTCGGAGGCTGAGGTGGGAGGATCCCTTG<br>AACTGGGGAGGTCAAGGCTACAGTGAGGTCAGATTGCACCACTGC<br>ACTCCAGCCTGGGTGACAGAATGAGACCCTGTTTTAAAAAAAAAG<br>TTTCAAGATGGAGGCAGTCCCCAGGGCTAGATGTGGCAGTCCCACA<br>TCTGGGACTTTGGGAACTGACTCGGGGGGCAAGCTCCTTGGCACC<br>CATGTGTTTCTCCTCCCTGCTTCCCCTCCCCTCCTTTGCTAGAGTTG<br>GGTGAACTCCAATAGATTCCTGTGCAGGGGCTGCAGACTCGAATGT<br>CCCTCCTCAGTGACAGGGGCAACACATTGGAAGACATTAGGGAGT<br>GGTGGAGACTGTGGTGGCCGAGAGCAGCCAGGCTCAGCTCCACCT<br>GGCTGTTGACAGGAGGAAATGGGGGCCCATTCTTTTTCAAGAGAA

	GCCATAAATCCAAAATTTTCTAAGTTATCTTGGTTTTAAAATGTTGG<br>TTATTCAGATTTTTTTCAAAACACTGTGGAAGTCAATCAAAATTGTC<br>CATGAACTGGTTGTAGCCTACAGGCCGCCACTTTTGGAATTCTGAA<br>AGCTTTCCTCTGGCTCCTATTACACAGAGGAGAAAACTGAGGCCCA<br>GAGAAACGGAGGCACTAGCCAGGAATGACACAGTTAAGATTAGCA<br>CCCTGGAGCCTGCGGGGTGGCTCACGCCTGTAATCCCAGAGACGC<br>AGGAGTCTGAGGCAGGAGGATGACTTGAGCCCAGGAGTTCAAGGC<br>TGCAGTGAGCCGTGATCGCGCCACCGCACTCCAGTCTGGGGGACA<br>AAGCGAGATCCCATCTCTTTAAAAAAAAAAAAAAAAAAAGTAGAG<br>CTCCGCTCCCACTTGCCCCCCATTCTCTCTCTCTCTCTCTTTCTCTCT<br>CTCTCTCTCTTGCTCTACTCTCGCCCGTCTGCGTTCTACATTGGTTCT<br>GCGGCCGTGTCCCTTTCGTGTGTGTCTGCCCTGTGTTTCTCCGTCGT<br>CTCCGTGTGGATCTCGGTCTTTGTCTCGCCCTCTAGCTCTCTCGTGC<br>GCCTGTGTTTAATCGTGCGCTTCTCTCAATCTCTCTGTCCCTCCCTT<br>TGCCCCCTACACGCGTGTTCTCGTCAACAGATCTCCAGAGGTGGTG<br>CAGAAACGACCCCGCGCCGGCGCCCCATCCTGCGGCCAGTGCCTC<br>CGCGCCCCGGCTCCGGTCCCCACCGTCCCCGCCCCAGATTTCCGGA<br>GGAGCAGGCGGGCGGGGTCCCGCGGGGCCGGCTGCCGTCAGCGC<br>CCCTTCCCGGCGGCCGCGACCCCTCCCCGCTGACCTCACTCGAGCC<br>GCCGCCTGGCGCAGATATAAGCGGCGGCCCATCTGAAGAGGGCTC<br>GGCAGGCGCCCGGGGTCCTCAGCG
TSHB	AGCTGATCAGACTTTGTCCAAGTAAGGCAAGTGCTGATGTATAATG<br>AAATCAAGCTGTTTCGATATCTCATTTCTACTTTCTGTCCATAAATGC<br>TGTCTGCCCACATTGAGGAGCAGAGCTCTGTGAATCTCTTCTGATT<br>CTGAGGGCTGCCTGATTTGACAATCATTCTTTTTTCAATTAAACTGA<br>CAAAAACAAGCAATGGGGAAAAGATTCCCTGTACAATAAATGGTG<br>CTGAGATAACTGGCTAGCCATATGCAGAAGACTGAAACTGGATCCC<br>TTCCCTACACCATATAGAAAAATCAACTCAACGTGGATTAAAGACTT<br>ACATGTAAAACCTAAAACTGTAAAAACCCTCGAGGATAACCTAGGA<br>AATATCATTCCAGATGTAGGACCGGGCAGAGATTTCATGACGAAGA<br>CGACAAATGGAGTTTCAACAAGAACAAAAGTTGACAAATGGGACC<br>TTTAACTAAAGGGCTTCTGCATTATGGCCCAGAAACTTTCTCCAGG<br>CAGTTTCTCCCTGTTTGTTTCCCATCTCTCTAGGATCGTGTACTTTTG<br>TTACCTGATGTAACAGGAAAAGAAACTATCAACATAATAAACACAC<br>AACCTACAGAATGGGAGAAAATATTTGCAAACTATGCATCTGACAA<br>AGGTCTAATATCCAGAATCTATAAGGAGCTTAAACAATTTATAAGCA<br>AAAACTGAAGAACCCCGTTAAAAAGTGGGCAAAAGACATAAACA<br>GACACTTTTCAAAAGAAGATATATACATGGCCAATAAGCATATGAAA<br>AAATGCTCACACCACAAATCATTAGAGAAGTGATATTACCTTTTAAT<br>AGAAACTGAAGAGGTCAAGCATGCCAATGTCATATGGCAAGTAAAT<br>GGCAGGGCCAAAATTTTATCTCAGGTCAGCTTGACATCCTGTCAGA<br>TGTGGCCATGTTCCTCAGAGTCATTCTTTCACATGTGTAAATTTTAC<br>TTAATATCTTAAAATCCCCCAAAAATATTTTGAACTACACATTTTGTT<br>CTAAACAAAATTTTTAGATGAGGAATAATTTTATATATCTTTACATGA<br>ACATTTAGGTAAAACTTGAAGCCCTCAAGCCCCAAATAAATTTTTAT<br>TCCCAAGACTTATTTAGATGATGTTAGAGTGTTTTTTGAATTTGGTT

	TTTCCCATTCTGTATCTATCTTTTATTAATATTAATAAATAAATATTAAT<br>AAAGAATATATTTAAAAGGTAATTTTATTTTGAAACAGAAGAGAGG<br>AAAATGCATGCTTTAATAATGTCAGTTTCCAGGTAAAGATATTGTGA<br>GCTTGTTTGTCTAAATACATTTTATTTGGAATTCAGTATGAATTTTCA<br>ATAGATGCTTTTCAGATAAGAAAGCAGTAAATCAAATGCAATTGTAT<br>AAACAAGAAGATCAGAGGGATTATCCTGAAGGGTATAAAATGAAC<br>CAAGAGCTTTTAGTTTGGGTCACCACAGCATCTGCTCACCAATGCA<br>AAGTAAGGTAGGTGTCTATAGTGAGAAGGCTCTGTGAAATGATATG<br>TTCTTAAGCTATTTTTTTTCACTTTCACCATTTTATCATTTTTTAGAAA<br>GTGGCAAATGCTGAAAGATAATTTGCTTCAACAGGCTGTAATATCTT<br>AAATTACTGCTCCAAATTGTTTAGTAGTAGATGGAGGTGAGAGAAC<br>TACCTACATAAGGATAAGGAAAAAATTCATTGACTTAAAGAAAGAG<br>AAACAACTGATTAGTTCCCTAATGAAGGATATTTGATATTCATGGCT<br>TTCTTCTGTTTTCATTTTGATTTAAATATTGTTGTCCATTTAATGATGT<br>GTGTATTAAATCCTCTCAATTACTGTACTAATGGCCATCTTTTATGAA<br>AATGTACTTCCGGGAATGTTAGAGCAAATTCATTTTTGAGTACCTGC<br>TGGGGTAATGTACTCTGTATTATAAGTTCTTTTTAGTTATTTAATAGCT<br>AAACCTCCTTTGATAGTGTTCAACACACACACAGCTGCTGTATAGT<br>ACATTTTGCTTTCTAGAAAAATATACTGCTTTAGTGAAGTTCCAGGA<br>TTATTCATAGGATGGGCAATTAGTTCTTTTCATAGTAAGGGATTTGG<br>AGAGTAAAAATTAATGAAGGAAATTAGCAGAGCAGAGAGGAAAGT<br>GTTTATTAAGAATGAAGCATTGGGAGCAAAAATAAGAGAAAAAAA<br>GAGAGGAAAGAGGCAAAGGAAAGAACATATCTTGGAATAAGAAAA<br>TACGTTCAAAGTAGGGACATATATGTCCATGCATCTGAAGAAGGCAT<br>CTCATTATTTAGGCCCTGCCTGAAACCATGCCTTCTATGTTTAGGTA<br>CCCAACAAATTATTCATGGTGATTTCAGAATAAGCAATTAAGTGGCA<br>AAGATGAGAACCCAGATGACAATGAGGAAGAAATTGTGGCATATC<br>ACAGAGGAAGGGATTAACTAACGCGGATTTGTACAGTGTTATACAG<br>CTATGTGATCTTTCTTATATAGAGAGCCAAGTGTTTCACATGTATTGT<br>TTTATTCTTTTTATGATTTTAGGAAGGGACATATGGAAAAATGAAGG<br>CACAAAGATATTAAATTGCTCAGTTGCACACAATTACAATTAAATCC<br>TTAAGTAGAATTTAGATCACTAACTCTTAATCAATCAATCAGATCTG<br>ACACTAATTTATCATATAGAATTACAGACTATTTGTGCTGCGAAGATT<br>TTCATGATCATCTTCTGGCTCTAAGCCCTGCTTTTATAGGTGAGAAA<br>GTTGAGGCCCAGAAAGATTCTGTGGGTGAAGTTTTACTATTCGCAA<br>TAAAGAAGTAAAGGAAAAGAAAAGTTCTTGCTTTCTTTATGCCCAC<br>AAAGTTGTACAGATACTGTGGCTGGTAATTCAGCACTTCCATTTTCT<br>ACTTTTGTTAGTTTAGAGTTCTACATTTATAGGAGAAACTTCAAAAA<br>AATATAGAGTATCCATAGTTAGTATGACAATAGGAAAACCAAAACA<br>AATTTTCATCCTACATTTTAAAAAGGGTCAGGTGTACTTGTCACTTT<br>CTATTTTGCAGATGGGGAAATAACACTCAAACAGATTATATATTTTA<br>GTCAAGTTCTCATAGATTTATGGAAGAATTCTGTTTCTTAATTTTTAG<br>ACCAATATTTTCATCACCAGGTCACCACATTGATTTTATACAGAATTA<br>TAGAGCTAAAAGTTAAAATATGTGAAGTTAGTTGTTTCCTATAGAAA<br>AAACTCAGTAGCTGGCTGTCTAAACAAGCAAGAACTATAATGGCCA<br>TGATGGGTAAGCATTTTACATAATTTACCAGTTAGTAATTTTAAGATC

	CTACAGAGGATCATCAAGTTCTGTATCTCTAAAATACTGGAAGCAACTCTGTACCATAGAGGGAGCTCACATTTGAGAAGCGCTTGAGAAAGCCAGATTCCATGAGTAAGGAATTTGTATGTTTTTGCTCCTTAACTCAAATACCAAACAAACCTTCCGTGGAATGTTTGAATATGCAACATTTCATTTAAAAGAATTTGTGAGAAAAACCCCTGTTATTTTAACCTTTAAAAGTTAAGTCTTCCTAAAATGGGTTAGTTTACTTTCTTGTGTAATAAGAAGTATAGTAAACATAAATCTTTGATAATTGTAGGTCTTAAAGTCTTAATTTGGACTCTTTCAGAGCACTAGAACAATCTGCGTTTTTATTATTTGTGTTCATATTAGCTCCTCCACTGGACAGTCAGCCCTTTAGGTAAAGGACCGTAGCTTTTCCGTTTGTGTTTGTCAGTGCCTGGTTCAGATGCTCAATATATGTTTATCTAATTTATCTACAATTCTCATTCTAGGAATAGAAGGTTGATTGAAATGACCACTGATCTCTGGAATTTGACATTTAAAATAAATCCCTGGTAACCTGTGTAATGTGGTTTTCATGGTTTCTTATAAGTTGATTGAAAAAAAAATCCTGGTTAACCAAGGCCGAACCTATGAGGTTTCAGTTTACAGAGGTTTTAGTGTATATAGCTTAAAGATGCTGTTGGGATATAACTGACAGATTTGGGGAAATCTTGGTACTTCTGATTATTGAACAAAATATTTAGTAAATTATATTTTATTTAATAGAACAAGCAAACAAGTTTATGTTCATAGACCACAATACTACTCCATTAAGGTATATATTTTCTTTACTGACAATGATACTTGAGAAGGAAAAAAATCCAAACACATCTAGCTAGCTGTACAATATTTGTAAGATTAAAAAAATTCTTCTTAATCTTCAAGGTGATCAACTTACATTCAAAAGTCTGAAATTTAATTACCCTTCCTCAGTAGTAGACCTAAAATAATTATGTCATAACATTATCCAGAGGGATAATGATATCAATATCAATATCATATCAATATCATATTGATATGATATATCCAATATGATATCATATCCAATATAACTATATCCAATCAATACATATTGATAATGATATCCAATTGATATTGAACAAGTTCTTCATCACGCAGGCTCTATTGAGATGCATGTTCTGTTTCTGCCTGAGGCACTGTGGATCCTCTATTTTCTTCCATGCATCCAAAAATTATTTAATATTCCTGGCTAACCCATAATGGAGCTGTCATCCTTGATTGTATTCTTCTAATTGTGTCAACCTTTTTTTTTTTTTTGCCTGTTAATCTTTTTGAGAACAAATTACATACCTGTTCTGTGTAAAATAATCTTTAATTTGTCCCAGCTGTACATATTTCCACCTTAAAGGGATATCCTAAGGGTTTGGAAGTGGGATCAGGGGGTTCCTAGATTTCTGAGTTAGCCCCTTAACACCAGTTGTAATTTCAGTTGACCTTTTTTGGACTTTATCTTTCTGGTGTCTTCCTTGACCAAATGGTAGAATTATAAGCATGATCATATGCATTGGGATGGTACTGAAGTTTGGTTATACTTTTTCTTGGTTTCTTTGCCCTTTCTGATTTTAACAAATAGGTTCTTTAATTTTATCTTTGATTTAGCATGACTGCTCTCTTTCTGATGTCCA
TTR	GGCTCTCCTGCAGTCCGGATGAAATACTCTCAAAAAATTAGCCCGGGCCCTTTGCTCCAATTTTTCGCTTACCTAGCAACCATCTAACTATTAATTAAATTGGTATTATGGTTTTAACATGAATCTTTTATGATTTGCTTACCATTAATCAAACCCCCGAGGCTTATTCACCTCAAGGGGAGCTGACAAAGTTGAATTATTCAACCTGCAAAGATCCAGGGCCCCCAAATACTGTCATTTCCACTCTCCCCTAACCCCCACCATGAGGCCCAGTCTCAGCACTCGGCCAGCCTATGCCCAACTCGGGGTAATCAGCTTAGACATATTAATATTAGTGGGCATTTCAGTATCAACAGATCACTGTCTAGCAGCTGACAGGCACCCTCAGAAAATAAACCAAGAAGAAAGGGTTTATCTATAA

	TATCAAAATTTTTCATAGATAAACCTGCCCATTATAAGGAAGAGGGC<br>AGAAGAACCCTAAACTAAGAGCCAGGCAACTTGTTCATTAATCACA<br>GCATATTCCATAGAAGGAGGAGAAATGTTGTCATCAAATTCATCTTT<br>TTCACCTCAATTAAACATCTATGCTACAGCTCCACAGTCAGATTGAG<br>AGGAAAAACAGTACGTAGCTAAGAAAAGACATAGACTTGTAACTG<br>AAATGCTTCACTGGTGCTCCTTTTGTTTTAAGGCATTGGATCTTCAT<br>AGCTACTGATCGTGCCCAAGCACACAGTATCTGCAGCAACCACTTA<br>GGCCTCCAGGAATGTGGTGACCATTGACCCTAATTCATTCCCCTTCA<br>TGGATCCTATGTAACCATCCTCCAAAAAGAGCTTTCGCAAACTCAA<br>ATAAACACAGGAAAGGAAGACCTTCTTATCTTTGAGAGTATATGTTT<br>AGCCCTATAACCCTCTCTTATCATAAATTGCTTCTTAGGCAAGAAAC<br>ACTGGATTTTTCTTGTATTTGTCATTGCCATTGGTTCCATACAAGCAT<br>TCATTTAACAAATAATACATTCCATCTCCGTTTTTGCTTTTTCCTTC<br>ATGCTTCGGCCTTGGTTTTCTCTCACCTAAAACACTACAAGCTTCTT<br>CTCCCAGAGCTCTCACTTTGACTCCAGACTACCTACATTTAATCTTT<br>AATTCTCTACCAAAATTTTCTCTTATCTTTATATCTTTTTAATTTTAAT<br>TTTATTTTTTGTGGATACATAGTAGGTGTATATATTTATGGGGTACATG<br>CAATGTTTTAATACAGGCATGCAATGTGAAATAAGGACATCATGGA<br>GAATGGGGTATCCATCTCCTCAAGCATTTATCTTTTCAGTTACAAAC<br>AATCCAATTACACTCTTTATTTTAAAATATACAATTATTAATTATAGTC<br>ACTCTGTTGTGCTATCAAATAGTAGATCTTATTCTTTCTATTTTTTGTA<br>CCCTCTCGTCTTTTTATATTAAAAAATAATCTTTATCTCTGTAAGCTT<br>CATCAGTGATTTTCCAATGAAATTTAGGATCTTCTCTATACCCTGAAT<br>TGCCTTACTTTCTCCCCACTTCCTTGTCTTATTCAAATGCAGATTTCA<br>TTAATGATTTCCAGATCAATGATAGTTCAGAAAGCAAGCAAGTCAA<br>AGTGACCAAGGGCATGGCCTGAAAACTGTTCTAAGAGAGGAATTT<br>ACAGAACAACTATTAAATGATGTCAATAGGATTGTATTAGTCCGTTT<br>TCATACGGCTATAAAGAACTGCCTGAGACTGGGTAATTTATAAAGG<br>AAAGAGGTTTAATTGACTCACAGTTCAGCACAACTGGGCAGGCCT<br>CAGGAAACTTACAATCATGGTAGAAGGTGAAGGGGAAGCAAAGCA<br>CCTTCCTCACAAGGCGTCAGGAAGAAGTGCCAAGCAAAGGGGGA<br>AAAGCCCCTTGTAAAACTACCAGAACCTGTGAGAACTCAATCACTA<br>TCACAAGAACAGCATGAGGGAACCGCCCCTCGTGATTCAATTACCT<br>CCACCTGGTCTCTCCCTTGACACATGGGGATTATGGGTGTTACAATT<br>CAAGATGAGATTTGGGTGGGGACACAAAGCCTAACCATATCAAGGA<br>TCAAGTGGTGGGTTGAAACTAACAGGATGAGATATATCAGATACAA<br>ACACAGGGTCCCATATTTGGGTTAAAATTCATAAATGATCAAAGCA<br>CAGGATGACAGATAATATAGGTCATTTTAGATTATTGTGGCCAACAG<br>ATCACAGTGGGTAGTGTTATGACGAAGGGAGGGTCACAGTTACTAC<br>AGTTACAGATGGATTCTGGGTACAACATTTGCACTAAAGTGCCTTT<br>GCCAAGGGAGGCAACAGTCTCGACATCCTGTGGCCTGATCTACTTC<br>AGGGACTGTGTCTTGTTCAGAGCATCACATTTGAAGAGAACTTTGA<br>CCAAGGGGAATATGCCAGAAAAGGAAGTTCGGGATGCTGAGGATC<br>TTAGGAACTATGTCTAAACAAGATTCATTCACAGAAGTGGGAATGT<br>CTATTTGGCAAAAAGAAAATACTACTTACATGGCTGTTGGAAGACC<br>AGCAATCACAAACTCAGTTTTTCAAAAGGCTGGGCAGAAACACAG

	ATGAAAGAAACAGGCCATGTTTAAGAAAAGATAAAAGCTCACGCA<br>TGATATGCCACTAGAGAATCACCTAGCCTCAGTGTTGGCGGGGAGG<br>CCTGGGGAGTCTTGATGTCTGAGAGTGACATTCTGATGATCACTGT<br>CATGTGTAAATGTTGGCCTAAAGCTGCCAATATTTTTGATTTAAGAG<br>AAGCAAGAAATGCAAATTTTTATGCAGCATGTCTCAATTTTTAATTT<br>TGGCAACTATTACAAAATGTTTAAAGAGACTCTGTGCAGCCCAAAT<br>ATAACATATCTATGGGCTGATGGCAGCCCAGCGTTGCCAGTTCACA<br>GGGTCTACAAGAGATGATTCTTAGTTTCAACAGGGTGCAGTGCTGA<br>AACGCGTGCACAGTAGATTTTGCTTCGGTTATGAAAGAACTTCCAA<br>ATATTTATGATTCATAGCCAGAGAAAAGGCTCTCTATCCAGGTTCTG<br>AACAATAGGAAATCATCAAGAGGATATTGGATGACAATATATGAAA<br>GATGTTATTTGAGAAAGGATTCTCTCCTGAGGCATAGATGTTGAACC<br>AAATTCTATTAGTTATGCTTTTACAGCAAGATAGTGGTTTACAGCTT<br>ACAAAAGGCTTGTACATCCTCTCATATTAAAAGTTATTAGAACAGTC<br>CTTTGAAGTAGAAAAGTAGGCATTTCTATTTTACAAACGAGTTGGC<br>CGAGTATCTGAGATAGTAGATAACTCATAGAAGGTCATCCGGGAAA<br>CGGGGCAGCAGAACTGGGATCGAATGACTCTGGTCATCCAACTCC<br>AAATGCAAAAGTCTTTCTGCTGCTGCTTCCTAGTTAAACTCTAAGG<br>GTCTAAGACTCCATTCCTAGTTATGGTCTCAACTACATTTGCTCATT<br>GCTGTGAGGGGTCAACCCACCTCCCGGAGTCCTCTCCTGCACATTC<br>TCATGTTCCTGAAAGGCTTTTCTGTCCCTTCCACTACTCCCTGTAAG<br>CTCCTGTGCTTCACAATTTCTTGTTGAATTTTTTCTAATCTGACTCTA<br>TCAGTTATGGGAATGTTCCCTCAATTCTTAGTGCTCCAAACCGGACT<br>TGCTCTTGGCTTGTATTTGTCCAAAATATTTGTCTTCTCTATGTTTTC<br>TACATGTTTGTCTTATAAGGACAAAAACCTGCCTTAGTTTATCCATG<br>AACAAAGCCACGCATGCTAGTGGACACACACACACATGCGCGTGC<br>GCGCGCACACACACACACACACACACATACACACAGAGACTTTGTAT<br>GTGAGTAATGAATCATCAAATCATCATAATTTCTGGACTTGTATTAAT<br>AAGTCGGCCAGGAGGAAAAGAATCTGCTGTCAATCATGGCTTCTG<br>GTTCTCACAGTCATCTCTACTTTCTTCCAGCAAGTTTGGTTCTGTCA<br>AAAACCAGCTGTCAGCCTTGTTCCTGCATGCCCAATGCAGAAGAGT<br>CAGTAAAGAAGATTTGGTTCTCTGTATTTCAGGGGCATCAATGCCA<br>GGTTGAAATATGCCATTCTGGCCCAGCTCAGTGGCTCACACGTGTA<br>ATCCCAGCACTTTGGAAGGCCAAAGCGGGTGGATTGCTTGAGCTC<br>AGGAGTTCGAGACCAGCCTGGGCAAGAGGCTGAGGTGGGAGGAT<br>GACCTGAGCCCGGGAGGTCAAGGCTGCAGCGAGCTGTGATCGTGC<br>CACTGCACTCGAGCCAGGGCGTTGGAGTGAGACCCTGTCAAAAAA<br>AAAAAAAAAAAGGAAGGAAAAAAGGAAGGAAGGAAGGGAGGGA<br>GGGAAGATGCCATTCTTAGATTGAAGTGGACTTTATCTGGGCAGAA<br>CACACACACACATACACACATGCACACACACATTGTGGAGAAATTG<br>CTGACTAAGCAAAGCTTCCAAATGACTTAGTTTGGCTAAAATGTAG<br>GCTTTTAAAAATGTGAGCACTGCCAAGGGTTTTTCCTTGTTGACCC<br>ATGGATCCATCAAGTGCAAACATTTTCTAATGCACTATATTTAAGCC<br>TGTGCAGCTAGATGTCATTCAACATGAAATACATTATTACAACTTGC<br>ATCTGTCTAAAATCTTGCATCTAAAATGAGAGACAAAAAATCTATAA<br>AAATGGAAAACATGCATAGAAATATGTGAGGGAGGAAAAAATTAC

	CCCCAAGAATGTTAGTGCACGCAGTCACACAGGGAGAAGACTATT<br>TTTGTTTTGTTTTGATTGTTTTGTTTTGTTTTGGTTGTTTTGTTTTGG<br>TGACCTAACTGGTCAAATGACCTATTAAGAATATTTCATAGAACGAA<br>TGTTCCGATGCTCTAATCTCTCTAGACAAGGTTCATATTTGTATGGGT<br>TACTTATTCTCTCTTTGTTGACTAAGTCAATAATCAGAATCAGCAGG<br>TTTGCAGTCAGATTGGCAGGGATAAGCAGCCTAGCTCAGGAGAAG<br>TGAGTATAAAAGCCCCAGGCTGGGAGCAGCCATCACAGAAGTCCA<br>CTCATTCTTGGCAG
TYR	CACTAAATACACAAAACTTTAGAAGTCTTCACTACAGTTAAAAATAT<br>ATCATGTTTAGAGGTTGTTAGGTAGCCACATTATTATTCTGAAAATT<br>GAAAAATGAAGGACAAGAATCAAACATTTACCCCGCTTTTCTTGTA<br>CAGGGTAACTAAATAGTTCATGAAGAAAGTTATTCTTTACAAAAAA<br>AAGGGGATAACCATTCTTGCAACCTTTAATAAAATACCAAATGTAG<br>GATATACTTATCAGTGAAAATAAAACCATATAAAAGTTTGCTAGAGA<br>ATTATGTAATTGAAAGGACAGTCTGACATCACTTCAAGCTGCTGATA<br>AATTTTAAAAACAAATACTGAGAAAACCAGATAATATGGTCCTCTA<br>GTATATCAAAATAAGTATTACTTAACAATGCCTGTGAAGTAATTGTT<br>AAGAAAAGCAATAACAAAATTCAACTTGTATATAATAAAATCTCTAG<br>ATCTAACTCCCTGATTGTAGAAAATGCAAGATATTATTATTGAAAAT<br>GTATAAAATGATAGAAAATACAGGATAATGAGCATGTTAAATTCATA<br>GAGATAAAAGTTGTTAACCCCGAAATGTGAGAAATCCTTAGGGCAA<br>AAACTCAGTTTCCTCTAAAAATAAATTGCATTTTTTTTTTTTTTTTT<br>TTTTTTTTGAGACGGAGTCTCGCTCTGTCGTCCAGGCTGGAGTGCA<br>GTGGCGGGATCTCGGCTCACTGCAAGCTCCGCCTCCCGGGTTCACG<br>CCATTCTCCTGCCTCAGCCTCCCAAGTAGCTGGGACTACAGGCGCC<br>CGCCACTACGCCCGGCTAATTTTTTGTATTTTTAGTAGAGACGGGGT<br>TTCACCGTTTTAGCCGGGATGGTCTCGATCTCCTGACCTCGTGATCC<br>GCCCGCCTCGGCCTCCCAAAGTGCTGGGATTACAGGCGTGAGCCA<br>CCGCGCCCGGCCGCATTTTTTTTTAAAAAAGGAAACTTATAACTTAA<br>TAGAAATTGATAAAATAGATCAACCAAAATGCAGTGTATATACCTGT<br>TTGAATCTTGACAAAAATACTTTTGAGATAATTAAAGTAAATTAACC<br>ATAAATTTGATACCTGATATTAAAGAATTATTGTTCTTTTTGTTTGAG<br>ATGTGTTTGTGTAAGATACGTGGTTTTGTTTTTATGTTCTTAATTGTT<br>AGCAATATATACCGAAATTAGAGGTAGAATATGCTATCTGAGTTTTG<br>TTTTAAAACATTCCAGAATTTTTAAACTAGTAGAAACAAAACAGAA<br>AAATGCTGGATGTTGAAACTTGGAGGTAAGTAAATGCTAGTTTATTA<br>TATTATTTTTTACTGATTTGAGCATATTTCTTTCCACAGTAAATTTTTT<br>TCTTTAAATGGTAGCTACTGATACTGAGATAATTATATTAGTTGTATTT<br>AATAAATGGTAGCTACCATTATAATTGAAAGGAGGGCTGAGAAACA<br>AAGACAGAAAATGGATTCAAACTACTACCAAATAAAAGAGCAAAT<br>ACCAGTGATCTGAGTGAGGAAAATGAGGAAAATTGACCAGGTTAA<br>CACAAGTGAGTGGCTGAATAATCTATACAAATACCAGAAGTAGAGG<br>ACCAGTGATTGTGGCTAGTTGATACTCAAACTCAACTTATCTAAAAT<br>TGAGCCTATATTCAATTCCCATAAACATTTATCCTCTATTGTTTCTTAT<br>TTTAGTTAATGGCACAAGCCAAAAACCTAGCACATAGCCCTAAATC<br>TTCTTTCCCTTCACTTCTTACTGCTCTTGAATTTATCACCTATGTATCT

	CTCAAATTCATTAACTTATGCCCATCTCAGAGATCATTACTCTAGTCT<br>AAGCAATGTCATGTTTGCCTGGTTTACATCAATAATATCCTAACAGT<br>AATCTCCACATTTAATCTAGCTCCCACCCCACATCTGTTCTCACCTC<br>AGTAGCTTGAGTGAAAAGTTTTAAATTCATGTATCGTTACCCAAAAT<br>TTTGCTACAAAGCCTTTATGGACTATGAGCTGGTCCATACACACACC<br>TTCATTCTTATATCACGCCTCTACCATATTGGGGGTTGCAGCCACCTT<br>AACTGTCCTTAAATCCCTTGAATATACCAACCTACTTCCACTTTGGA<br>GACTTTGCACATGCTATTCTCTCTGCCTGGAAAGCTCACACTCCTAC<br>TGTTCTCTCTGTCATCAAACAGACTTCTAGTGATCCCTCAAGTGTCA<br>CTTGAAATGTCACTTCTCCCATAAAATTATCCCTGACTATCCCTTCTC<br>TTGTGAGACTAGAAAGGCCCTTCTGTCATGTTCCCTAATTTCTCTCT<br>TTAATTTTCTCTTATAGCAACCTAAAAAGTAATTTAATCCAGAAAAA<br>AAGTTATTTAAAGTAATGTAGTTAAAATTATTATTTAAAATCACTAAA<br>ATTAAAATAGTATTTTAAGAGGCAATATCCTTCCTGATGATAGAGATT<br>TTTGTTGTGCTTACCATCACATTCCCATCTCTCCCCATGTCTGCAAGT<br>AAATACTTGCAGAGTATTTACTGAACCCCTAATTCTACTTTATAGAG<br>CATTGCAGTAATTTCATGTCAGGTCATACAATAATCTCCCAAAATAG<br>GATAGGACCCTTTCAAATCCCACAACTGAAGATACCTAAAGAATGT<br>ACATCTAGAGTCAGGAAGAAAGCACTCTAAATGGGATTTAATATTT<br>AGGAACCCAAGACTGGCAATAAGACTGAAGACTACAACACGTGTA<br>GGCCAGAGGAGACAGTGGCCTATACTGGGGACAAATAAAGAGGTC<br>TGTCCTATTTAAGAAAATCAACCCTGTAAAGGAAATTAATAGGACTA<br>AGTACATTTTAGTAATTCCTCTAAGCAGGCTCTAAAGATTATGAAAA<br>ATAGACGGGACAGCAGACACAAAAGCCCTTAAAGAGCATGATGAA<br>GACTTTCTAAGTTATTTCACTGGAAGCCTGATAGTGGGGCAAGTGT<br>AAGGCAAAATTCTTAATTAAATTGAACATGATAAGTTGAATTCTGTC<br>TTCGAGAACATAGAAAAGAATTATGAAATGCCACATGTGGTTACAA<br>GTAATGCAGACCCAAGGCTCCCCAGGGACAAGAAGTCTTGTGTTA<br>ATCTCTTTGTGGCTCTGAAAGAAAGAGAGAGAGAAAAGATTAAGC<br>CTCCTTGTGGAGATCATGTGATGACTTCCTGATTCCAGCCAGAGGC<br>AGCATTTCCATGGAAACTTCTCTTCCTCTTCACCCACACACTGCTCC<br>ATGTACCTGCAAAGCCTGTTCTGTCTCAAAAAAGTTGTTTGGATGA<br>GCCGTGACTTTTTTTTTCTTAAATAATGAGACAAACTCCAGAAAA<br>AGAGAAAAAAGCAGAGCAGTCTGACATTCCGGCATCATCGAAATA<br>GTGATGGCTTTTCCTAGAATGCTTCAGCTAAGGACCCAAATACTAAT<br>GATCTCCTCAAAGCTTTCACTTTCTTTTACTTTTTCATTAATTTCAGT<br>GGACCCCCAAACTTTAAGTATGGAAGAGGACAAAGAAGGAAGCTT<br>CAGAGGGGCAACTTTGATTTGACTACTCTTTTTGTCACTCTTCAGCT<br>CACAAAAGAGCTCACTTTAGTTCAAAACACAAAGTCTTTAAGCCC<br>CTCCATAGATTGGTCCCAGGTTTAATTTTCTATGATGTGTGGAGGCC<br>TCAGTTTAATGCTCCAACTTGATAGATGAAACACAGTTCCCACCTCT<br>ACACATTTCCCCTGTCTCAGGAGTTGTATATATTCTCAGTTGTCTGT<br>CCAACTTATGCCCACTCTTTGAGATATTAATCAAGGCACTCCCTTGA<br>TAACACTTGCATATTATTATCAAAATTATGCAATTCTTTCTAATATCAG<br>CCCACAAATACATCTCTTCCATTAAAAGTTTGACTTAATTATCTATAC<br>TACTCATTTGAAAACTAACATAGTTAAGTTGTATTTTTAGCCATGAAT

	TTCAGTTTCCCTAGCTCACTATACACAGAGAAGGAACTTTTGAAAT<br>AATTGAGATGATCAAAAATATTTGCTGAAGAAATATATTTCTCCTTTT<br>TCATTCACTCACTAATTGAGAATGTCTTTGCACAAAACACATTGCA<br>AAAACATTTTCAAAAAAATTCCTAATTTCTAGAATTGATAGGAAAA<br>ACAATATGGCTACAGCATTGGAGAGAGAGAGAAAGGAGAGAGGA<br>GAAAGGAGAGAGAGAGAAAGGAGAGAGGAGAGAGACAGAGGAG<br>AGAGAGAGAGGATAGAGGGGAGAGAGAGAGAGGAGAGAGACAG<br>AGGAGAGAGAGAGAGGATAGAGGGGAGAGAGAGGGAGAGGGAG<br>AGAGAGGGAGAGAGAGGGAGAGAGAGAGAGAGAGGGAGAGA<br>GAGAGAGAAAGAGAGAGAGAGGGAGAGAGAGAGAGAGAGCTCT<br>TTAACGTGAGATATCCCCACAATGAAGCAAATCGCCCAGTTATCAA<br>AGTGAGCTATCCTTAGGAGTTGTCAGAAAATGCATCAGGATTATCA<br>GAGAAAAGTATCAGAAAGATTTTTTTTTCTGATACGTTGTATAAAAT<br>AAACAAACTGAAATTCAATAACATATAAGGAATTCTGTCTGGGCTC<br>TGAAGACAATCTCTCTCTGCATATTGAGTTCTTCAAACATTGTAGCC<br>TCTTTATGGTCTCTGAGAAATAACTACCTTAAACCCATAATCTTTAAT<br>ACTTCCTAAACTTTCTTAATAAGAGAAGCTCTATTCCTGACACTACC<br>TCTCATTTGCAAGGTCAAATCATCATTAGTTTTGTAGTCTATTAACTG<br>GGTTTGCTTAGGTCAGGCATTATTATTACTAACCTTATTGTTAATATT<br>CTAACCATAAGAATTAAACTATTAATGGTGAATAGAGTTTTTCACTT<br>TAACATAGGCCTATCCCACTGGTGGGATACGAGCCAATTCGAAAGA<br>AAAGTCAGTCATGTGCTTTTCAGAGGATGAAAGCTTAAGATAAAGA<br>CTAAAAGTGTTTGATGCTGGAGGTGGGAGTGGTATTATATAGGTCTC<br>AGCCAAGACATGTGATAATCACTGTAGTAGTAGCTGGAAAGA
TYRP1	TATTAGAGTGCGTAAGGAGGAAACAGAGGTAAATTTTGGTTCTGAT<br>GAAGAGGTAGAAGGGGTCAGGAGGTTTCCATCATTGTCTGTCTTCT<br>GCAAGGTTATCTAAAAAGTGAAGAGACATCCTTAAAGTGAGTATTG<br>ACCAAATGTCAGTTGTCTCTTCAGACTTCCTATGGTTTAAAAATAAA<br>TGAAAAGAAAAAAGCTCTGTTTTCTTACCTAAATTTGAGTTATATTA<br>CCAAAGGCCCCTTAGGTAATGGGGCCTTTATACGTTCCAGAGTAAG<br>TAGCATATGGTGTGTGCATGCATGTTTCTCTCTCTCTCTCTCTCTCTC<br>ATTCTGTGTGTGTGTGTGTGTGTGTGTGTGTGTGTGTGTGTGTGTCA<br>GTGGAGAGAGAGAGAGACGAAGAAAAGGGAGGAGTGATAATCCT<br>TCTAAACGTATACAAGTTGCTTGTGAGAAAAATAGCAGTAATCATA<br>ACACACCAAGTCCCTTACTGCTGGTAATGCTTTTCATGTGTCTCTCC<br>AGATTGCCTAATTTTAACTTCTTAAATGTCTCATCTCCTCCTCTATAC<br>CCTGGAAAATTATAATATGCTGATTCAATTTAAAAAAGTTATTTGAA<br>ATGTAGCCAAACTCATTTGATCTTCCTCAATTCATCACTGCATATGG<br>GTTTCATCCCATTTTATTAGTAAATTTGACACTGATTATGATAAATCC<br>ATCCAGTGCCACACATCTCCATCTGCAAATTTCAAGGTTTAATTTTA<br>AACTTTTTAGGGTAAGAGGTTTTTAAAATATTTTCAACCTGTAGGAT<br>TTCCAAGACCTTATTTCTAAATGTTTCAATATGACCAATCTTATACAT<br>GTGATTTTATATTTCTATATCAGATATAAAAAAGATTTAGATTATTTAG<br>GCTTTCAGTTAGTTCTTTTTATACATGAAGCTACTTTTACGAGCAAG<br>ATGAAGGGGAAGTGAGATAGTGGCAGTTGCTGTCTTTTGGATGACA<br>GGCCAATTTACTTCTTGCTTCTAAAGGAAGTAGGCTTTACCCTTTAA

	ACCTCCCCCATCATATGTTCTTGCATGTACTGTTTCCTTTCATCAGTA<br>AGTTCTCCTGATCTTCTCTGTCTCTCCCTCTCTCTCTATCTCTCTCTC<br>TGTTTGACTGTTTCTCTGACATTACCCAAAGATAACTGTTTTTTTTT<br>CTGGTCTCAGAATTATTCGATGTCCTTTAGTAGTGAGGTCAGCTTCC<br>ACAAATGCCTAATTAAAAAAAATTTTCCTTAAAATAGGCAAGAGGA<br>GGAGGAGTTGAATTCCAAACACCTGGAGTCTCCTTTGATTAGCAGT<br>ATTTAATTTCTGATAGTACCCAATTTATGGCATTTTAGTGAAGACAAT<br>TCTAAAAGTTAACAGAGAAGTCAGGCCAATCTTTCCGATAGGATGA<br>CACACTCCTTTGTAAATCTTTAAATGCTTTTCATTGCATTATTTGTGA<br>GTAGGGACCAGCAGTGTTCCTAAATAGGAAGTGTGTTTTTTGCCAA<br>CAGTAAGTCCCATAGATCTCTGGTTTTCCTGTTTAGTGGAGGGTTAC<br>TAAGAGAGAGTACTTAATTTCTCACAGTTGGAAATTTTCCTGGTTTC<br>TCTAGACCATGTAATTCTTCAAAATGTTATAGGGTAGACCAAGTTCT<br>GAATTTTACCTGTGCTTTTGATTAGGCTTCAGTTTGTTTTACAGATTA<br>TTGTATTTCTTGTCTCCTTCTGTGCTTTTTCCACAGTGCAGGCAGTA<br>ATCACAATATCCTTCATAGAGTGTACTACCTTGCCCTTAAGGATGTT<br>AGAAACGCATGGTATCGATTCCCATTGTGCATTTAGATAATAGCAAT<br>ACAAGCACAGAATATTATCTTGGCACCATAGAAGCTCTACAATCCCC<br>AAGTGTCAGTAGTACATTTTCTTCAAACGTTAGCAACCTCAATCCCT<br>GGCAAGAAAAAAAACAGATTATGCAATCTATAATGATCTTACTCTTC<br>AGCAAAATTCTATTTTTTTGTGTTCGTTATTAGAAATTTCCACAGAC<br>ATATTTTAGCAGTCATATTTCTGCCTGCCCAGCTAGAGTAAGCTCAG<br>TGAGGAAGGGGGTCTGTTGCTACTCTTCTTTAATGATGGAAACACT<br>GAGGAGTATATGAGGAATTTTACCCATTAGATGTGTGAGGCTAAGA<br>GATCTTTGATCATGCCACTGCTCTCCAGCCTGGGCGACAGAGTGAG<br>ACCCTGTCCCAAAAAAAAAAAAAAAATGATATGTCCACTCATTAAAA<br>ACTTCTGTCAGACCACTCTTTTTTATAAATTTTTAAATTTTTTAATGA<br>TCTAACAAGCTTTATTTTTATCATTGTTAAAAAAATCCTTCTTTTGAG<br>TAACACAATCACAAAGTAAAGAATGTTATCATTGATTTAACCAGTAA<br>AAAAACAATACTTAACAATAAAATTTTTCAAACATGGCTCCAGGCC<br>ATCCAGATGACTTAGTTATCCTCCTTTGATGCCCTTCTCTTCTTCGAA<br>TGAATCACAATGAGGCAGAAAAATTTTCATGGGTGCAGAGTAGGT<br>GTATATATTTATGGAGTATGTGAGATATTTTGATACAGGCATACAACA<br>TGAAATAAGCACATCATGGAGAATGGGGTATTCATACCCTCAAGCA<br>TTTATCCTTTGAGTTACAAACAATCCAATAACACTTTTTAAGTTATTT<br>AAAAATATACAGTTATGATTGACTATAGTCACCCTATTGTGCTATCAA<br>ACAGTAGGTCTTATTCATTCTTGCTAATTTTTTTGTACCTATTGACTA<br>TCCCTACTTCCCTACTCCCCTCAGACACCCACTACCTTTCCCAGCCT<br>CCATTCTTCTACTCTTTATGTCCATAAGTTCAATTGTTTTGAGGTTTA<br>GATTCCACAAATAAGTGAGAATAAGTGATGTTTGTCTTTCTGTGCCT<br>GGCTTATTTCACTTAACAGAATGTCTAGTTCCATCCATGTTGGTGCA<br>AATGACTGGATCTCATTGTTTTACTGCATTTTGCATATGTACCATATT<br>TTCTTAATCCATTCATCTATTGATGAACACTTAGGTTGCTTCCAAATT<br>TTAGCTTTGTAAACAGTGCTGCAATAAACATAGGAGTGCAGATATCT<br>CTTCAATATATTGATTTCCTTTCTTTTGGGTATATATCTAGCAGTGGGA<br>TTGCTGGATCATATAGTAGCTCAATTTTTAGTTTTTTGAGGAAACTC

	CAAACTTTGCTCCATAGTGGTTGTACTAATTTACATTACTACCAACG CTGTATGAGGGTTTCCTTTTCTCCACATCCTTGTCAACATTTCTTATT GCCTGAATTTTGGACATAAGCCATTTAATTGGGGTAAGAGGGTATC TCACAATAGTTTTGATTTGTACTTCTCTAATGATCAATAATGTTGAGT ATCTTTTCATAAGTCTGTTTGCCATTTGTATGTCTTCTTTTGATAAAT GTCTGCTCAAATATTTTGCCCATTTTTTTATTGGATTATTAATTTTTTT CTATAGAATTTTTTGAGCTTCTCATATATTCTGGATATTAATCCCTTGT CAAATGGGTAGTTTACAAATATCTTATCCCATTCTGTGGGTTGTCTCT TCACTTCATTGATTGTACTCTTTGCTGTGCAGAAGATTTTTAACTAG ATGTGATCCCATTTGTTCATTTTTGCTTTGGCTACCTGTGCTTGTGTG GTATTGCTCAAGAAATCTTTGTCAAGACCAATGTCCTCAAGATTTTC CTCAATGTTTCTTCATTGTAGTTTCATAGTTTGAGGTCTCAGATTTAA ATATTTAATCAAATCATTTTTTATTTGATTTTTGTATATGGAACGAGAT AGGGACCTAGTTTCATTCTTTTGCATATGGGTATGTAGTTTGCCAGC ACCATTTATTAAAAAGACTGTCTTTTCCCCAGTGTATGTTCTTGGTAT CTTGGTGAAAAATGTCTTTACTGTAGGTGTGTGATTTTGTTTTTGGG TTCTCCTGTTCCATTGCTCTATGTGTCTGTTTTTAGGCCAGTACCATG CTGTTTTTGCTACTATCACTCCGTAGTATAATTTGAAGTAAAGTAATA TGATTCCTCCAGTTTTGTTATTTTTGCTTAGGATAACTTTGCCTCTTC AAGGTTTGGTGTTCCATATAAATTTTAGGATTTTCTTTCTGTTTCTGT GAAGAATGTCTTTGGTATTTTGATAGGAATTGCATTGAATCTGTAGA TTGCTTTAGGTAATACGGACTTTTTAACAATCTTGATTCTCCCAATCC ATGAACATATAATATTTTTCCATTATTTGGTGTCTTCTTCAATTTCTTT CATCAATGTTTTGTAGTTATCATTATAGAGACCTTTCATTCATTGGTT AATTCCTAGGTATTTAATTTTATGTGTGGCTATTGTAAATGGGATTAC TTTTTTATTTCTTTTTCACATTTTTCACTGTTGGTATATGGAAACTTT GCTGATTTTGGTATGCTGATTTTGTATCCTGCAACTTTACTGAATTTG TTTGTCAGCTCTAATAGTTTTTTAGAGGAGTCTTTAGGTTTTATTCA AATATAAGATCTTATCATCAGCAAACAAGGATAATTTGACTTCTTCA TTTCCAATTTGGATGCTCTTTATACCTTCTCTTGCCTGGTTGCTCTAG CAAGGTAATATGTTGAATAACAGTTGAATAACAGAATAAAAAAAAA TCTCTGCAAAGTAAACAAATCTCACTAGTTTATCTGACTTGTATTCC AAATTAGTGCTTCTGGCCTTTTCTTAAAACTTTAAGCATCACAAGG AAATCAGTTGGAAGGGAATCATGTGCTGATCAAGTCCTTAAAGGGC AGAAATATTCACTGAAGTGAAAAGGATTAGTAAAGGGTGGAAAAA AAGACCAGCCCCCCGCCTAGTTTGGGTGAGCAGATTTGGGATTAAT TATCAGGCAGCAATCCACATGCACTTAACAGTTCTGACGTGAGAGG ACAAGAAACACAAGCAAATATAAAACATTCAATTCTAAGAGAAGTT CATCAGAGACATCCTTCAGGATT
UCP2	GCCTGGGCAACAAGAGTGAAACTCGGTCTCAAAAAAAAAAAAAA AGAGAAGAAGAAGAAAGAAAACTAGGTGGAGTGTGGTGGCTTGC ACCTATAATCCCAGCACTTTGGGAGGCCGAGGTGGGTGGATCTATT GAGGCTAGGAGTTCGAGATCAACCTGCCAACATGACGAAACCCCA CCTCTACTAAAAATACAAAAAATTAGCACGGCGTGGTGTGTGTGCC TGTAATCCTAGCTACTTGGAAGGCTGAGGCAGGAATCGCTTGAACC TGGGGGGCAGAGGTTGCAGTGAGCCAAGATCTTGCCACTGCACTC

	CAGGCTGGGCGACACAGCACAACTCTATCTCAAAAAAAAAAAAAAGA
	AAAAACAAAAGAAAACTAATATATCAAAATAATTTCTAGTTAGTTG
	GATTCACTCACTTATTCATTCAATGACTTATTGAATTATCATATATTAC
	TAGTGCTTTTTAATACATACCTTCTACAATTTTTCAACTGAAAATTAC
	TTCATTGATCAGGGCTCTTTAAACTGATCTCCATTTGCATTGTTTTAC
	TAACTATAGTTATTATTCATGTATTAGCACTCTGAGCCTACTGTAATG
	ATGTGTACCTTAATAAAGAACTGAATATTTGTAATGGCTGGCAGTGA
	ATTTAGTAGTTCTTGAATTTAGAGCTCAAAATATGGGAGTAATTTGC
	TGCTTTATTTCCTTTGAGAGGTAATAGAGGAAAAACAGAATCTAATA
	ACAATCACAGATTTTCGGGAAAGCACTGTAAAACCATATGATCAAT
	TCTAGCTTCTTATGTAAACATGGAAAGATTGCCAGCTGAAGACCTG
	TCATGCTCTAAGAAGTTGGGGAGAATTTGCATTTTTAGAACTGTGA
	GCAAAATGAGAACGACTGCTATGTTCATGCTTTGTGAATTTAGCTTT
	ATTTCATTCACACAATTCATGGGAAAAAATGCATCTTTTAACTCGGT
	GTTTTTCAATTCAACTTTTAAAATACAGGAGTGGGCCAGACCCGGT
	GGCTCACACCTGTAATCTCATCACTTTAGGAGGCCGAGGCAGGTGG
	ACCACAAGGTCAAGAGATAGACACCATCCTGGCCAACATGGTGAA
	ACCCCATCTCCACTAAAAATACAAAAATTAGCTGGGCATGTTGGCA
	CGTACCTGTAATCCCAGCTACTCGGGAGACTGAGGCAGGAGAATC
	GCTTGAACCTGGGAGATGGAGGTTACAGTAAGCCGAGATCGCGCC
	ACTGCACTCCAGCCTGGCGACAGAGCAAGACTCCATCTCAAAAAA
	AAAAAAACAAAAAAAAAAAAAAACAAAAAAAAAAACCAGGATGTGT
	TACCAAGGAAAATTTATTTACAATGGTTAATTACGTGACAAACATGT
	CAAGTAATTCCATCTGGCTTTGTGTCACCATTTCCCCACCCTTTTTT
	CAGAAACCAAAACCAAGAAGAAGAACAAACATCAAAATGGACAT
	GGAAATTAACAAATATATGATTCAATTTAATCTCCTAAGAGGTTTTTT
	AAAATTATTTTATTTTGAGACGGAGTCTTGCTCTGTCGCCAGGCTGG
	AGTGCAGTGGCAGGATCTCAGCTCACTGCAACCTCCATCTCCCAGG
	TTCAAGCGATTCTCCTGCTTCAGCCTCCCAAGTAGCTGGAACTACA
	GGCAAGCACCACCACACCCAGCTAATGTTTGTATTTTTGGTAGAGA
	TGGGGTTTCACCATGTTGGCCAGGATGGTCTCGATCTCTTGACCTCA
	TGATCCACCCGCCTTGGCCTCCCAAAGTGCTGGGATTACAGGTATT
	TTTTATTTTTTTTGAGACAGGGTCACCCTGTCACCCAGGCTGGAGT
	GTAGTGGCACAATCATGGCTCACTGCAGCCTCAACCTCCCAGGCTC
	AGGTGATCCTCCATGTCAGCCTCCCAAGTAGCTGGAACTATAGGCG
	TGCAACACCATGCCCAGCTAATTTTTGTATTTTTTGTAGAGACAGGG
	ATTTGCCATGTTGGCCAGGCTGGTCTTCAACTCCTGGCCTCAAGTG
	ATCCACCCGTCTCAACCTCCCAAACTGCTAGGATTACAGGTGTGAG
	CCACCGTGCCCCATCTCATCTGCTAAGTGGGTTTAAAGAAATTCAG
	TTTCATGTCAATTTTTAAAATGTATGGTTATCAAATTCGACTTCTTTT
	TAAAAATGCAATCAGATAACTGTATGCTTGTTTGATGAGGGGAGGA
	AAGTTAATATAGCCAATCTACTCAATATTTTTAGCAGAAATTATCAGA
	GACTAAGGAAATGATTAAGTTTTTCTCATGTTGGTTTTAATTACCTA
	ATGTTTTCAGTTTTCTCTTTCATTCTTGTGTCTTTTTTTCATTTTCAGT
	GTTTCAAATACAGTTTGTATTTAAAGATTTAGAAGTTCCAAAACTGT
	AAGCACAGTGGATTGTTTCCTGGGATGATGTTAAAATTATACAACA

	AAATATATGAAACTTTGTCAATTTGGTTATTGGCACATACAAAATATT<br>TACAAATAAACGTGTGTGTGTGTGCGTGTACACACAATTCAATGAA<br>ATAGATGTGAAACAAGTTTTCTTTTTTTTTTTTTTTGAGACAGAGTC<br>TTGCTCTGTCGCCCAGGCTGGAGTGCAATGTCGCAGTCTCAGCTCA<br>CTGCAACCTCTGCCTCCCGGGTTCAAGCGATTCTCCTGCCTCAGCC<br>TCCCGAGTAGCTGGGACTACAGGCACCTACCACCACTCCCAGCTAA<br>TTTTTGTGTTTTTAGTAGAGACAGGGTTTCACCATGTTAGCCAGGCT<br>AGTCTCCAACTCCTGACCTCAGGTGATCTGCCCGCCTCAGCCTCCC<br>AAAGTGCTGGGATTGCAGGCGTGAGCCACCTCACCTGGCTACAAG<br>TTTTCAAAATACATTTATCTGTACCCATACATTCTCCAGTTTGTCCAC<br>AGGACATCTTATGACTTGAGCAAGCTGCTAAAAATCCAAGGGTGCA<br>GCGTTTGTATGTCTATAGGATTGCTCAGATCTGCCCCCACCCTGAAA<br>GAATTTAAGAGAATTTCTTGAGGCCAGGCACAATGGCTCACACCTG<br>TAATTCCAGTACTGTGAGAGTCCGAGGTCAGAGGACTGCTTGAGG<br>CCAGGAGTTCAAGAGCAGCCTGGACAACATAGGGAGACCTGTCAC<br>TACAAAGAATAAATAAATTAGCCAGGCTTAGTGGCTCATCCCTGTG<br>GTCCCAGCTACTAGGGAGGCAGAAGTAGGACTGCTTGTCCCAGGA<br>GGTCAAGACTGCAGTGAGCTGAGACCCAGCCACCTGCATTCCAGC<br>CTGGGCAACAAAAAGAGACCCTGTCTCAAAAAATAAGTTAAATAA<br>ATAAATAATAAAAATAGTTTAAACCCTAAACACATCTTCTTTTTCAA<br>AGAGGACTTCTTAAGGACTTCATGCTGCGTCCTGTTGATCTCCACT<br>TCCCTTTTTCAGCGTCCACACTTTTAACAGTCTCTTTTGCCAAGGAT<br>AATAAGTATATAGTTTCTGGAATCCAGATTCTTCCCTGTTTGGACAG<br>CCAGGGGGACAATTTTTGGTCTGCAGGCCTTTGCATCTGTTCTGCT<br>GTTGCTCAGCAATCTCACAGCAAATTTGCCGAGCCTCTCCGGAATG<br>CACAGCCAGACAGAGCTCAGCGCAAAAGCTAGAGAACCTGGCGG<br>AGGGAGACTCACAGTGCCACAAAAAAACTTTATCTTTTCTTTTTTT<br>TTTTCTTTTCTTTCTTTCTCTTTCTTTCTTGTCTTTCTGTCTTTCCTCT<br>CTCTCTCTCTGTCTTTCTTTCCTCTCTTTCTTTCTTTTTTCCTACATG<br>GCAAGATCTCCTCATGGCAGAAATAATCTGCCTTGACTTCTGTTTCC<br>ACGCTGCTTCTGCCAGGACCATGCGCTCGGCGTGTTTTTCTTTCCG<br>CTATAATTATCCAGGCCCATCCCAGCTCTGGTCCCCTCAGCTGTTCC<br>CTGGCAGTCCCTTCTGCTGGTGAAAACACATATGGCGCCGGCCTGA<br>CCAGGGTGTAAGTGTGTGAATATCAGGAAGATGACTGAACGTCTTT<br>GGGACTCCGTTTCCTCATTGTAAAATGGAGGTTAATACCAGCCTTCT<br>TCTACTCCCCAAACGCACGTGTTTGTCCCGGCCAGAGGGCCCAATT<br>GTTGGCTGTTCACGCATCAGTTACCCCCACAGGACGGGTCAGCCAA<br>TTAAAGGCGAACCAGGCCCGGTCCATCTCCTGACGCCTTTTCTCAT<br>CCCAGGGCTGGACAGGCAGCTGGCCTGGGCCCGGCTCTGCCTTGT<br>CACGTGCGGGGGCCGGCCCGTTTGCTTGTCTGTGTGTAGGAGCGT<br>GAGGTCACGCTGGGTGCTCCCGCCCCGCCGGGGCCTTTAGTGTCCC<br>TGGTCCCTAAACGCCAGGCCGCTCCACCGGGGGAGAAGGCGCGAA<br>CCCCAGCCGAGCCCAACGGCTGTTGTCGGTTGCCGGGCCACCTGT<br>TGCTGCAGTTCTGATTGGTTCCTTCCCCCGACAACGCGGCGGCTGT<br>AACCAATCGACAGCGAGGCCGGTCGCGAGGCCCCAGTCCCGCCCT<br>GCAGGAGCCAGCCGCGCGCTCGCTCGCAGGAGGGTGGGTAGTTTG

	CCCAGCGTAGGGGGGCTGGGCCCATAAAAGAGGAAGTGCACTTAA<br>GACACGGCCCCGCTGGACGCTGTTAGAAACCGTCCTGGCTGGGAA<br>GGCAAGAGGTGTGTGACTGGACAAGACTTGTTTCTGGCGGTCAGT<br>CTTGCCATCCTCACAGAGGTTGGCGGCCCGAGAGAGTGTGAGGCA<br>GAGGCGGGGAGTGGCAAGGGAGTGACCATCTCGGGGAACGAAGG<br>AGTAAACGCGGTGATGGGACGCACGGAAACGGGAGTGGAGAAAG<br>TCATGGAGAGAACCCTAGGCGGGGCGGTCCCCGCGGAAAGGCGGC<br>TGCTCCAGGGTCTCCGCACCCAAGTAGGAGCTGGCAGGCCCGGCC<br>CCGCCCCGCAGGCCCCACCCCGGGCCCCGCCCCCGAGGCTTAAGC<br>CGCGCCGCCGCCTGCGCGGAGCCCCACTGCGAAGCCCAGCTGCGC<br>GCGC
UGT1A1	GGACTGCCAAAGAGCCTGCGGGGCACTGGCACAGCGAGCGGCAA<br>GGCTGCCAGCACCCGCGCACAGGTCAGAGGCTTGGCGACCTGGGC<br>CGCCTGGAGGGCCGCCCCTTATGACGCAGCCACATCTCATTGGCCG<br>AGGCCTGTGAGCGCCTCGCATCCCAAGATGCAGTGCTCCTGGGACT<br>GGCCCTGCTCTCTGTGAGGCTCTGTGAGGCCCTGTGATGCTCCAAG<br>ACCAGGCCCCGCCCACTCCGGCCTCCAACCAGCCATGGTCTCCAA<br>AAAGGATGGGAAAAAGAGGTTGGGGAAAAGAGAGGGCCTTGACT<br>TTGGCTGCCTGAAGAACTGTTTTTCTTAAAGTAGGCTTTATATCAGT<br>CTTTTTCCTCGGCCACAGGAGGGAAGAGGGTGGTGGGAGTGAGTT<br>TAGTCTGACCGGGGCTGAAGACATCCTGTTGTTTAGGACTGCGGTT<br>CTCCAACGTTCCAGCCCCGGTGCCCATTTGCTTTTGTTCATCTGGAT<br>TATGCCTATCATATGTACTGCATTAGAGATTAAAACAGAATTAAAAA<br>GACATATTCATTGGGCAATTTAAGAAGAATAAACCCATGACACACT<br>AACAAACCTTTTTATGTAACTTTTTTTGAGACAAAATGTAGTGAGA<br>AGAGTGGCATCGTTTTACAGTTTTTGCATCTCTCTCTTTAGTACTTG<br>GCTCTATAGAGAGGTGGATTCTCATGTCAGCTTCTGCATTCTATCTAT<br>TGTGATATTACACATCCCCCATGTAGCTTCTGGAAAACTCCACTGTA<br>CACTTGTGGGAGAATGACAATGAGAAAATCAAGTAACATTATTACG<br>GAAATAGTTTTGACTTTGTAAAATTCTCCTGAAAAATTACTGGGGAT<br>CCCTAGGATTTCCTGGCTCATACTTTGAGAATCGCTAGTCTAGCAGA<br>GTAGTCCCTGGTATTCTGAAGGGATTAGTTTAGGACAACCCTCCTTC<br>CCCATACCAAAATCTAGATGCTCAAGCTCCTTTTATAAAATGACACA<br>GTATTTGTATATAACCTACCCATATCCTCCTTTAAACCTCTAGTCATC<br>TCTTGATTACTTTTACCTAATAAATGTAAATGCTATGTAAATAGTTGT<br>TTTACAGTATTGGTTTTTTATTTGTATTATTTGTACTGTTTTTTTTTCA<br>TTGTTGTTCCCCCCAAATATTTTCAATCTGCTGTTGGCTGAATCTGC<br>AGATGTGAAGCCCAAGTATATGGAGGGTCAAATGTGCATGTTATTC<br>ACTTTTCTTGACTGCTAAAACAACCAGGGAGATCCTCTCAGACAAA<br>AGGAAATACAGCACTATTTACTGTATCGAAACCATTAAGACTTGCA<br>GGCCGTGTGTATAGCACTGGGGATAAACATGGGATGCAGTGATTATT<br>TCCGCTAGAACTGCTATATAATGACGATGAATTTTGGGGGGACTTTT<br>TTTGAGATCTGAGTTCTCTTCACCTCCTCCTTATTCTCTTTTTGACAC<br>TGGATTCTTTGCTTTGATAAATTGTGGGGCAATACACTAGTAAAGGT<br>CACTCAATTCCAAGGGGAAAATGATTAACCAAAGAACATTCTAACG<br>GTTCATAAAGGGTATTAGGTGTAATGAGGATGTGTTATCTCACCAGA

	ACAAACTTCTGAGTTTATATAACCTCTAGTTACATAACCTGAAACCC GGACTTGGCACTTGGTAAGCACGCAATGAACAGTCATAGTAAGCTG GCCAAGGGTAGAGTTCAGTTTGAACAAAGCAATTTGAGAACATCA AAGGAAGTTTGGGGAACAGCAAGGGATCCAGAATGGCTAGAGGGT AAGAGGCAGAGGGAGGGGGCAAGCAGAAGGGCTAGAGAGGAGG AATGAGCTTGGACAGGTGGGCTGGGGTCTATCCCAGAGTTTTGAG AGCAAGGCAGAGGACTCTGAATTTTCTGTGCCCAGGAAGCTGCTG ACCAAGGTTCCAGAAGTGGTGGTGAGGTGGGGTTATTCAGGTGGC AGCCGATGCAATGATTCAGAAGGGACAGCTGGGGGTTGGGGGACA GGGGGGCTGGGGCCCTGAAATGGGACCATGACAGCTGGGTCTGAG AGACAGTGGTAGAAACATCCAGATTCAGCACTTACTTGCTGGCTTG GATGCAGGGTCTAGAACGAAAAGAGAAGAAAAGTCACTTCTATAC AGAAACATGTCCAGAGCGCTTACTGTCTCCAAAACCATGGACTGG CACCTGAGTGATAGCATGATTCCAAAGCCAAAATCTTGCCTGTAAG GAATATATATATATATATATATATATATGTATATATGATATAGCTATAGTCT AATAGCAAGGACAGATATGCAAACTGCTAAAAGATACAAGGCAGA ACAGAACAAAATGCTGTTTTTCTGGGATTTTGGAAATTCAAGGAAT TCAAGGGATTCAAGGAAGGTGGCTTTGCTTCCCGGGAGGGTCCTG TAGATGATCTACAGGGCACTGGACATGTTTATGTTGCTCCTTTAGTA ATAAGCCTGTCATTCTGATTTGATGAAAGGAGATGAAAGGAGCTGG TAGTGTGTCTGATGGTGGCCTACTAACTTATGTCTTCAGCTTAAAAA GAAAGTAGCTTCAAAAGGGTTCCAGAAACACTTTCCATGGACGTG TCACTCTTTAGCAGCCCCCAAAGCAAGACCATCATATTGCTGCCCT GCTGTGTGATTTCTCAGCCCCTAGAGCACCATCCCCTGTAATTGCCT GGTCATGAGTTTGTCTCTGTCTACCTGACCCCTCCTTTCAGGCAAG GACCATTTCTAACTTGACTTTCTGGGCCTAGTTCCTAGCATAGTGAC TGCCATCCAGTAGGGCTCACACGTTCCATAAATATTTGGCAGATGA GGGAATTAGCAATGGGTTCTGCTTTGGTTTCAGAGCAGATATTAATT GGATTGCTTAGTAGTGGTTCTCTGTTGTAATTCATGAGCATGAATGT GGATTGCCCACTATTCAGATTAGTAAGTATTTCTTGGTCAAGGGCAG AGCTGTGGCCACAAACCATCCAGGTACACAGCAGAAGCAGCCTCA AAAAGCTTGGAAGCTCTGCATGATGCAGGAAAGTCATAAAATCATT ACAGTGGTGACTTATGTGTTTATAGCCCCTTTACTGTCTATAATCTGC AAATGAACTCACACAGCATTGGGACTTTGGAAGAATTATCACCCTT AAGGTTTAAATTAAACTGTGAATTTCAGAATTTCTAATAAGGACAC AACAAAGAGTGAAAGCATTGCTATGTCTATTCTGCTTGCCCAGAAT CTTGGTCCTAAAAAATGAAGAGTGTTTGGGTGTGGGGAGGAGCTT CAGTGTGCATGTGCATGCAAAGTACCTACTCTAAGGAGAAGAATGA GAGGGTACCCTAATTACCTGTTAATATGTCCCATAGGACACCAAAAC TCTAGTTAGCTGTTTCTCTATGATCCTCTAAGCACATCCCCAAGTAT GGCTGGCCAGTGATGTGTATGGTTCAAATGTTGGGATCTGTGCAGT TATCTTGGAATTGTATAGTACAGCAGTATATCCCCCCCAAAAAGAGT GTAATACTTCCAATTCTGGCTGCACAATACTTGCCCCATAGTCCATG GTCAATAAATACAAATTTGAGTTGTTTTTGCTCATCTTTCCCTTTTGA CTTCAAATCAGTCATCAGAATTTCCCCAAATGCCTTTCCCCTGGATC TTGGGCCAGTGGAATGAGTACAATTTAACTTAATTGAATTTGCTTAT

	CTATTTGGTTTCCTGTTGTGAACAAAAGTTCTCTGAAAAGGAATTT<br>GGAAGAAAGAGACTTTGTTCTAGTGAACAGTTTGCAAACCAGGGA<br>GTTACAGCCTCTGGTACGCAATGAAGGTGAGTTCCACAGAACACA<br>AGGCAGGCAGGTTTCACGGCAAAAAGTTCCTTCCCAGGTTCCCAA<br>TCAGGTCCATTTATGCAAATGAAGGATGGAAACTTGCTTAGTTCTTA<br>TTGGTCACTGCAGCTGCATTCTGATTGGTTGATGAAGCTGAGCCCT<br>GAGTGGCTGAGGTGGGTGAGCTTTAATTGGTTGGTTCAGGTGAGC<br>GCTGAAAATCTCAACTATAAAAAGGTACAGGTTTTCAGGATACTCA<br>GAGTAACCGTGTGACCTGTAGTAAGCAAAGGGCCAGTTGGCTCTAT<br>TTTAAATCCAGGCCCAGTTAGCCACTCAAGATCTATCTTACAGGACT<br>GGCTCTTTCAGGTTCACACTAATAAAGGCCTGTCCTTGGGGAAGAC<br>TTCTGTTCACATGCGCTCCAGTGAATTTCCCTTTCTGGTCATTCTCT<br>ACCCCAGCACGCCCCCCACCCCCGACCCGCCCCACCCACCCACCT<br>GTTCATTTCCTTCTTAGCATGCTTCACGATTTCTAAGTTCCTGCTCAT<br>GTGTTTAAATTGTGAGTCTGGCTCACCTCATGGCGCGTGCTCGTGT<br>GGTGGGCTCTGCTGCAGCCTCAAGACCCCACACTGTGCTGGACTC<br>AATAAATATTGTTGGACGAAGGAATGAAACACATGATACAAGTGAG<br>CAGGCAGTACCGGGGGAGCTGTGGAGTGGGCACTCTTACAGGTTT<br>CCATGGCGAAAGCGGGGGTACAGTTGTGTTCTTTTCTTTCTAAAAG<br>GCTTTCTAAAAAGCCTTCTGTTTAATTTCTGGAAAAGAAGCCTAAC<br>TTGTTCACTACATAGTCGTCCTTCTTCCTCTCTGGTAACACTTGTTG<br>GTCTGTGGAAATACTAATTTAATGGATCCTGAGGTTCTGGAAGTACT<br>TTGCTGTGTTCACTCAAGAATGTGATTTGAGTATGAAATTCCAGCC<br>AGTTCAACTGTTGTTGCCTATTAAGAAACCTAATAAAGCTCCACCTT<br>CTTTATCTCTGAAAGTGAACTCCCTGCTACCTTTGTGGACTGACAG<br>CTTTTTATAGTCACGTGACACAGTCAAACATTAACTTGGTGTATCGA<br>TTGGTTTTTGCCATATATATATATATAAGTAGGAGAGGGCGAACCTCT<br>GGCAGGAGCAAAGGCGCCATGGCTGTGG
UGT1A8	AGCATCTGTTCTTTCCTGAGTTTTAATGATCGCCATTCTAACTGGCA<br>TGAGATGGTATCTCATTGTGGATTTGATTTGCATTTCTCTAATGACCA<br>GTGATGATGAGCTGTTTTTGATATGTTTGCTGGTCATATAAATGTCTT<br>CTTTTGTGAAGTGTCTGTTCATATCCTTTGCGCACTTTTTGATGGGG<br>TTGTTTTTTTCTTGTAAATTGGTTTAAGTTCCTTTTAGATTCTGGATA<br>TTAGCCCTTTGTCAGATGGATAGGTTGCAAAACTTTTCTCCAATTCT<br>TTAAGTTGCCTGTTCCATCTGATGATAGTTTCTTTTGCTGTGCAGAA<br>GCTCTTTGGTTTAATTAGATCCCATTTGTCAATTTTGGCTTTTGGTTG<br>CCATTGCTTTTGGTGTTTTAGTTATGAAGTCTTTGCCCATGCCTATGT<br>CCTGAATGGTTATTGCCTAGGTTTTCTTCTAGGGTTTTCATGGTTTTA<br>GGTCTTATGTTTAAGGCTTTAATCCATCTTGAGTTAATTTTTGTATAA<br>GGTGTAAGGAAGGGGTCCAGATTCAGTTTTCTACAAATGGCTAGCC<br>AGTTTTCCCAACACCATTTATTAAATAGGGAATCGTTTCCCCATTTC<br>TTGTTTTTGTCAGGTTTGTCAAAGGTCAGATGGTTGTAGATGTGTG<br>GCATAATTTCTGAGGCCTCTGTTCTGTTCCATTGGTCTATATATCTGT<br>TTGGGTACAAGTACCATGCTGTTTTGGTTACTGTAGTCTTGTAGTAT<br>AGTTTGAAGTCAAGTAGCGTGATGCCTCCAGCTTTGTTCTTTTTGCT<br>TAGGATTGTCTTGGCTATACTGGCTCTTTTTTAGTTCCATATGAACTT

	TAAAGTAGTTTTTTTCCAATTCTGTGAAGAAATTCAATGGTAGCTTG<br>ATGGGGATAGCATTGAATGTATAAATTACCTTGGACAGTATGACCAT<br>TTTCACGATATTGATTCTTCCTATCCATGAGCGTGGAATGTTTTTCCA<br>TTTGTTTGTGTCCTCCCTTATTTACTTGAGCAGTGGTTTGTAGTTTTC<br>CTTGAAGAGGTCCTTCCCATCCCTTGTAAGTTGTATTCCTAGGTATT<br>TTATTCTCTTTGTAGCAATTGTGAATGGGAGTTTACTCATGATTTGG<br>CTCTGTTTTTCTGTTATTGGTGTATAGGAATGCCTGTGATTTTTGCAC<br>ATTGATTTTTGTATCTTGAGACTTTTCTGAAGTTGCTTATCAGCTTAA<br>GGAGATTTTGGGCTGAGATGATGGGGTTTTCTAAATATACAATCATG<br>TCATCTGCAAACAGAGACAATTTGACTTCCTCTCTTCCTATTTGAAT<br>CCCTATATTGCTTTCTCTTGCCTGATTACCTTGGCCAGAACTTCCAAT<br>ACTATGTTGAATAGGAGTGGTGAGAGAGGGCATCCTTATCTTGTGC<br>CAGTTTTCAAAGGGAATGCTTCCAGCTTTTGCCCATTCAGTATGATA<br>TTGGCTGTGTGTTTGTCATAAAATACTCCTATTATTTTGAGATATGTT<br>CAATCAATACCTGGTTTATTGAGAGGTTTTAGCATGAAGGGCTGTTG<br>AATTTGTTGAAGGTCTTTTCTGCATCTATGAGGATAATCATGTGTTT<br>TTTGTCATTGGTTCTGTTTATGTAATGGATTACGTTTATTGATTTGCAT<br>ATGTTGGACTAGCCTTGCATTCCAGAGATGAAGCCAACTTCATCAT<br>GGTGGATAAGCTTGTTGATGTGTTGCTGGATTTGGTTTGGCAGTATT<br>TTATTGAGGATTTTCACCATTGATGTTCATCAGGGATAGTGGTCTGA<br>ACTTTTTTGTTGTTGTTGTTGTGTCTCTGCCAGGTTTTGGTATCAAG<br>ATGATGCTGGCCTCATAAAATGAGTTATGGAGGAGTCTCTGTTTTTC<br>TATTGTTGGGAATAGTTTCAGAAGGAATGGAACCAGCTCCTTTTTGT<br>ACCTCTGGTAGAATTAGGCTGTGAATCCATCTGGTCCTGGGCTTTTT<br>TTGGTTGGTAGGCTATTAATTACTGCCTCAATTTCAGAACCTGTTATT<br>GGTTTAGTCAGGGATTCGACTTCTTCCTGGTTTAGCCTTGGGAGGG<br>TGTATGAGTCCGGGAATTTATCCATTTCTCCAAGATTTTTTAGTTTAT<br>TTGCATAGAGGTGTTTATAGTATTCTCTGATGGTAGTTTGTATTTCTC<br>TCGGATCAGTGGTGATCTCCCTTTTATCATTTTTTATTGTGTCTATTT<br>GATTCTTCTCTCTTTTCTTCTGTATTAGTCTGGCTAGTGGTCTATCTA<br>TTTTGTTAATCTTTTCAAAAAACCAGCTCCTGGATTCATTGATTTTTT<br>GAAAATATTTTGTGTCTCTATCTTCTTCAGTTCTGCTCTGATCTTAGT<br>TTTTTCTTGTCTTCTGCTAGCTTTTGAATTTGTTTGCTCTTGCTTCTC<br>TAGTTCTTTTAATTGTGATGTTAGGGTTTTGATTTTAGATCTTTCCTG<br>CTTTCTCCTGTGGGCATTTAGTGCTATAAATTTCCCTCTAAACACTG<br>CTTTAGCCATGTCCCAGAGATTCTGGTACGTTGTGTCTTTGTTCTCA<br>TTGGTTTCAAAGAGCTTATTTATTTCTGCCTTAATTTCATTATTTACC<br>CAGTAGTTATTCAGGAGCAAGTTGCTCAGTTTCCATGCAGTTGTGT<br>GGTTATGAGTGAGTTTCTTAATCCTGAGTTCTAATTTGATTGCTCTG<br>TGGTCTGAGAGACTGTTTTACTTCCAATTATGTGGTCAATTTTAGAA<br>TAAGTGCAGTGTGGTGATGAGAAGAATGTATATTCTGTTGATTTGGG<br>GTGGAGAATTCTGTAGATGTCTAGTAGGTCTGCTTGATCCAGAGCT<br>GAGTTCAGGTCCTCAATATCCTTGCTACTTTTCTGTCTCATTGATCTA<br>TCTAATATTGACAGTGGGGTGTTAAAGTCTTCCACTGTTATTGTATG<br>GGAGTCTAAGTCTCTTTGTAGGTCTGTAAGAACTTGCTTTATGAATC<br>TGGGTGCTCCTGTATTGGGTGCATATGTACTTAGGATGGGTAGCACT

	TCTTGTTGCATTGATCTCTTTACCATTATGTAATGCCCTTCTTTGTCT<br>TTTTTGATCTTTGTTCATTTAAAGTCTATTTTATCAGAGACTAGGATT<br>GCAACCCCTGCTTTTTTTTTCTTTCCATTTGCTTGGTAAATATTCCTC<br>CCTCCCTTTATTTTGAGCCTATGTGTGTCTTGCACGTGAGATGGGTC<br>TCCTGAGTGCAGCACACCGATGGGTCTTGACTGTTTATCCAATTTG<br>CCAGTCTGTGTCTTTTAATTGGGGCATTTAGCCCATTTACATTTAGG<br>GTTAATATTGTTATGTGTGAATTTGATCCTGTCATTATGATGCTAGCT<br>GGTTATATTGCCCACTAGTTGATGCAGTTTCTTTATAGTGTCAATTGT<br>CCTTACAACTGGGTATGTTTTTGCAGTGGCTGGTACTCATTTTTCCT<br>TTCCATATTTAGTGCTTCCTTCAGGAGCTCTTGTTTAAGGCAGGCCT<br>GGTGGTGACAAAATCTCTCAGCATTTGCTGGTCTGTAAAGGATTTTA<br>TTTCTCCTTCACTTATGAAGCTTAGTTTGGCTGGATATGAAATTCTG<br>GGTTGAAAATTCTTTTCTTTAAGAATGTTGAATATTGGCCCCCACTG<br>TCTTCTGGCTTGTGGGGTTTCTGCAGAGATATCTGCTTTTAGTCTGA<br>TGGGTTACCTTTGTGGGTAACCAGACCTTTCTCTCTGGTTGCCCTTA<br>ACATTTTTTCCTTCATTTTAACCTTAATGAATCTGATGATTCTGTGTC<br>TTGGGGTTGCTTTTCTCGAGGAGTGTCTTTGTGGTGTTCTCTGTTTT<br>TCCTGAATTTGAATGTTGGCCTGTCTTGCTAGCCTGGAGAAGTTCT<br>CCTGGATAATATCCTAAAGAGTGTTTTTGGTACATACGTTTAAACAA<br>CCAGATCTCCTGAGAAATCACTCACTATTGGGAGGACAGTGCCAAG<br>CCATTCATGAGGGAACCATGCCCATGATCCAAACATCTCCCACCAT<br>GCTTCACCTTCAACTCTGGGGATTATATTTGACCATGATATATGGAG<br>GGGACAAACATACAAATTATATCAAAGGCATAGCATGGCTACTGTG<br>AAAGGAGGGTAAAAACACAAAGATGACATCACCACTGACCTCAGG<br>GAGTGCCCAGTAGACTGAGGGAGACAAGTACATATTTTCCTGAAG<br>GAGGGCACTGGATTGATGGCATGTTTAGAATGTGGAAGTAGAGCGG<br>CCACAGAAAGGCAGCCCGGCAGAGTGCTCGCACAAAGACTGGGC<br>TAGAGACTCCCCACTGCATGTGATGTATCTGAGGAAAGTCATTAAA<br>ATAGGTGATGGTCACTTTCCATCAAGTCCCTGGTATGGTCCATGGAG<br>GCAGGGTTGTCAATGTCATTTCGGCATTTCAGAGGCCTCTCAGGGT<br>TTGGATATGGCAGAAGACAACCAGCAATGATTATGCATTGCAGAGA<br>TGCAGGTGAGCCCCAATTTCTTGCCAGCTGGGAAGTCGGTGCTAA<br>GGGGCCTTGCTGTCTTTCCTTGGAACATAGGATACCAATTTCTTTCT<br>GGACAGAGAGTATTTGGTTGGCTAAAGTTAAAATCTAAATTTTGCTT<br>TGGGATGAATTCCAAAAATATTAGCTTTATTCAAATTTACTTTCACTT<br>TATCTTTCTGAATCTTCAAGGTCCTAAAGCATTGCTTAGTAATTTTGT<br>TTCTAAACTCACGTTACAGCACTGGGCATGATCTGTCCAAGGCAGA<br>GACTATAAGCTACTCTTATAGTACTCTTATGAGATACATACAAGTAG<br>GTATCTCAAAAAATGATACTCATGTATTCCTGTTCTTATGAGTAAATC<br>ATTGGCAGTGAGTGTGATTTTTTTTTTTTTTATGACAGGATAAATAC<br>ACGCCCTCTATTGGGGTCAGGTTTTGTGCCTGTAGTTCTTCCGCCTA<br>CTGTATCATAGCAGCTTAGAATCCCAGCTGCTGGCTCGGGCTGCAG<br>TTCTCTCATGGCTCGCACAGGGTGGACCAGCC
UGT1A9	GTGGTAGTTTGCTGAGAATGATGGTTTCCAGCTTCATCAATGTCCCT<br>ACAATGGACATGAACTCATCATTTTTTATGGCTGCATAGTATTCCATG<br>GTGTGTATGTGCCACATTTTCTTAATCCAGTCTATCATTGTTGGACAT

	TTGGGTTGGTTCCAAGTCTTTGCTATTGTGAATAGTGCTGCAATAAA<br>CATACGTGTGCATGTGTCTTTATAGCAGCATGTTTTACAATCCTTTGG<br>GTATATACCCAGTAATGGGATGGCTGGGTCAAATAGTATTTCTAGTT<br>CTAGATCCCTGAGGAATAGCCACACTGACTTCCACAATGGTTGAAC<br>TAGTTTACAGTCCCACAAAAACAAGAAATGGGGAAAGGATTCCCTA<br>TTTAATAAATGGTGCTGGGAAAACTGGCTAGCCATTTGTAGAAAGC<br>TGAAACTGGATCCCTTCCTTACACCTTATACAAAAATTAATTCAAGA<br>TGGATTAAAGACTTAAATGTTAGACCTTAAACCATAAAAACCCTAG<br>AAGAAAACCTAGGCATTACCATTCAGGACATAGGCATGGGCAAGG<br>ACTTCATGTCTAAAACACCAAAAGCAGTGGCAACAAAAGCCAAAA<br>TTGACAAATGGGATCTCATTAAACTAAAGAGCTTCTGCACAGCAAA<br>AGAAACTACCATCAGAGTGAACAGGCAACTTACAGAATGGGAGAA<br>AATTTTGGAATCTACTCATCTGACAAAGGGCTAATATCCAGAATCTA<br>CAATGAACTCAGACAAATTTACAAGAAAAAATCAAACAGCCCCAT<br>CAAAAAGTGGGTGAAGGATATGAACAGACCCTTCTCAAAAGAAGA<br>CATTTATGCAGCCAAAAGACACATGAAAAAATGCTCATCATCACTG<br>GCCATCTGAGAAATGCAAATCAAAACCACAATGAGATACCATCTCA<br>CACAAGTTAGAATGGCAATCATTAAAAAGTCAGGAAACAACAGGT<br>GCTGTAGAGGATGTGGAGAAATAGGAACACTTTTACACTGTTGGTG<br>GGACTGTAAACTTTTTATTTTACAATAAATTTAGATTTAAAGCAAGG<br>GTGCAAAAAATTGCACTAAGAATTCCCATATATCTTTTGCCAGTTTC<br>CCCTATTGCTAAATGTTAGTTTACTGTGCACATTTTTTTTATTATACT<br>TTAAGTTCTAGGGTACATGTGCACAACATGCAGGTTTGTTACATATG<br>TATACATGTGCCGTGTTGGTTTGCTGCACCCATTAACTCGTCATTTA<br>CATTAGGTATTTTTCCTAATGCTATCCCTCCCCCATACCCCCACCTCT<br>CGACAGGCCCCGGTGTGCGATATTCCCCGCCCTGTGTCCAAGTGTT<br>CTCATTGTTTAAATCCCACCTATGAGTGAGAACATGCAATGTTTGGT<br>TTTCCGTCCTTGCGATAGTTTGCTCAGAATGATGGTTTCCAGCTTCA<br>TCCATGTCGCTACAAAGGACATGAACTCATCCTTTTTTATGGCTGCA<br>TAGTATTCCATGGTGTATATGTGCCACATTTTCTTAATCCAGTCTACC<br>ACTGATGGACATTTGGGTTGCTTCCAAGTCTTTGCTTTTGTGAATAG<br>TGCCCCAATAAACATACGTGTGCATGTGTCTTTATAGTACCATGATTT<br>ATAATCCTTTGGATACATACCCAGTAATGGGATGGCTGGGTCAAATG<br>GTATTTCTAGTTCTAGATCCTTGAGGAATTGCCACACTGTCTTCCAC<br>AATGGTTGAACTAGTTTACACTCCCACCAACAGGGTAAAAGCGTTC<br>CTATTTCTCCACATCTTCTCTAGCACCTATGTACATTTATCACAACTC<br>ATTAATCAACACTGACACATTATGTTGAACCAAAGTCTATGTTTTCT<br>TTAGATGTCATTAATTTTTACCTAATGTCCTTGTTCTTTTCTGGGATC<br>CCATCCAGGATACCGCATGACATTTACTCATCACATTTGTTTCATCTC<br>CTCTAGCCTGTGACAGTTTTTCAGAGTTTCCTTGGTTTTGATTACTT<br>TGTCAGTTTTGAGCAATTCTGGTGAGGTATTCTGTAGAATGTTCCTC<br>AGTTTGAGTTTGTCTGATGTTTTGATTATGGTAACACAGGGGTTATA<br>GGTTTTTAGGAGGAAGACCACAAGGTAATGTGTTTTCCCGCTTTCT<br>TGCGAATGGATACCCAACTGTTGAAGAGTCATCTTTTCCCCACAGA<br>ATTGCCTGTGCACCTTTGAAAAAACTTGGTTGCTCATACTTCTATTT<br>CTGGGTATTCTATTCTGTTCCAATGATCTTTTTGTGAAATATTTGATT

	ACTATAGATTTGTAATAAGTCTTGAAATTAGGTAATAATTGCTCTCCA<br>TTTTTTTTTCTATTTTTTAAAAGTTATTTTTGCTATTGTAGGTCTTTTA<br>CATTTCCCTATGAATTTAGAATCAGTTTGCCAATTTGTACAAAAAG<br>ATAGCCTGCTGGAATTTAGATTGGGGTTGTATTAAATTTATAGATCAA<br>CTAAGGAAAAATAACATTTTAACAATTTTGAGTTTTCTGATTCATGA<br>ACATATCTATTTATTCAGGTCTCTCACCAATGTTTTGTTGTTTTCAGT<br>GTACAATCTTTCACATCTTTTGTCAGATTTATCTGTTAGTATTTTTTAT<br>TTTTGATGCTATTTAGGTGCTGTTTTCATTCCAATTTTCAATTGTTCA<br>TTGCTAGTACATTTGACCCTTGAAGAATGTGAGGATTGAGGTACCA<br>ACCCCCGAGCAGTAAAAAATTAGCATAGAAAGGTTTTGTGGGTTTC<br>TTTTTGTTTTTTATTTTTGAGACAGAGTCGTGCTGTTTTGCCCGGGC<br>TGGAGTATAATGGCGTGATCTCAGCTCAATGCAACCTCCGCTTCCC<br>GGGTTCAAGTGATTCTCCTGCCTCAGCCTCCAGAGTAGCTGGGATT<br>ACAGGCATGCACCACCACCTGCAGCTAATTTTTTGCATTTTTAGTAG<br>AGATAGGGTTTCACCATGTTGGCCAGGCTGGTCTCCAACTCCTGGC<br>CTCCCGTGATACGCCCACCTTGACCTCCCAAAGTGCTGGGACTACA<br>GGTGTGAGCCACCACGCCCAGCACACATAGAATTTTTGACTCCCTA<br>AAAATTTAACTATTAATAGCCTACTGTGCACTAGAAGCCTTACCAAT<br>AACAGAAACAGTTGCTTAACACATATTTGGCATGTTATATGTGTTATA<br>TACTGTATTATCATAATGAAGTCAGCTAGAGAAAAGAAAATGTTATT<br>AAGAAAATCTTAAGGAAGAGAAAATTAAGTATTCATTAAGTGGAAG<br>TGGATCATGATAAAGGTCTTCCTCTTGATTGTCCTCCATTGAGTAGG<br>CTGAGAAGGAGGAAGAGGTGGGTTGGTTTTGCTGTTTCAGGGGTG<br>GCAGAGGGGGAAGAAGTGGAGGAAGAAGGAGGAGAGACAGGTA<br>CACTTGGTGTAACTTTACAGAATTACATCATAATTATTATTTGACTTT<br>TTTGCCTTTGCAATTCTTTGAAAATGCTTTTTTACAGTACTAGTCCTT<br>CTTCCCCATTTGCTTTAGTTTCAGTGCCCATTCATGGAAGGGTTTGT<br>GTTGTAAAATAAGTCAAAAGTAGTCTTAATAATTGGAAGCCTTTGC<br>CAAACTGTTTAATAGGAATTTGTTTTCTGGCATGGCTTCTTCTATGT<br>CTTCTTTAGTATCTGGTACTGATTCAAAAGCACTCATCTCCATCAAG<br>TCATCTTCTGTTGATTCCTCTGGTGTGGTGTCTATTCATTCTTGAATT<br>TCTCACAGATTCATATCTTGAAAGACCATATCCCCCACCTTTTGTTG<br>CTGAATTAGAGATGTTGGGTTTGCAGGCAAGTAGACCACTTTGACA<br>CCTTCAGTGTTGAACTCATGGGTTCTGGGTGGCTAGGGGCATTGTC<br>CAAAAATCAAAAGAACTTTGAAAGACCGTCTCTTACTGGCAAGATA<br>TTACCTGACTTCAGGGACAAAGTAATGATAGAACCAATCCAGAAAA<br>AGTGTTCTTGCCGAGGCCTTCTTGTACAACAAAAAAACTGGCAGT<br>GGGTATTGATCTTTTCCCTTTAAGGCTTGGAGGCTAGCAGGCTTATG<br>GATGGGGGCAGTCCTATTTGTAAACCCAAACATACAAACATACAAA<br>CTATGTCAAAGGCATAGCATGGGTACTGTGAAAGGAGGGTGAAAA<br>CACAAAGTTGACATCACCTCTGACCTCAAGGAGTGCTCAGCAGAC<br>TGAGAGAGACAAGTACATATTTTCCTGAAGGAGGGCACTGGAGTG<br>ATGGCGTGTTTAGAATGTGCAAGTTGAGCGGTCACTGAGAGGCAG<br>CTCAGCAGAGTGCTCTCGCAAGGATTGGGCGGGCAACTTCCCACT<br>GCGTGCGATGTATCTTAGGAAAGCCATTTAAAATAGGAGACGGTTA<br>CTTTCCATCAAGTCCCTGGTATGGTCCATGGAAGCAGGGTTGTCAG

	TCTCATTTCAGCATTTTAGAGGCTTCTCAGGGTTTGGAAATGGAAG<br>AAGAGAAGCAGCAATATGTATGCATTGCAGAGACACAGGCGAGCC<br>CCAATTTAGGAGGTTAGGAGGTCAGTGCTAAGGGCCTTGTTTTCTT<br>TGCTTAGAGCATGAGTTGCCATCTTCTCTGGACAGAGAGTATTTGG<br>TTGCCTAAAGGTAAAATCTAAATTTTGCTCTGGGACAAATTCCAAA<br>AAAAATTAGCTTTAATCAAATTTACTTTTACTTTATCTTTCTGAACCT<br>TCAAGGTCCAAAAGCATTGGTTAATAATTCTGCTTCTAAACTTAACA<br>TTGCAGCACAGGGCATGTTCTGCCCCCAAGGCAAAGACCATAAGCT<br>ACTGTTGTCTGGAAAACATACAAATAGATATCTCAGCAAAAGCTAC<br>TCATATATTCTTGTTCTTTTGGGTAAATCATTGTCAGTGACTGATTTT<br>TTTTTTATGAAAGGATAAAAACACGCCCTCTATTGGGGTCAGGTTTT<br>GTGCTGGTATTTCTCCCACCTACTGTATCATAGGAGCTTAGATTCCC<br>AGCTGCTTGCTCTCAGCTGCAGTTCTCTGATGGCTT
UGT2B17	ACGGTTTGGTAAGTAGAGCGAAGGAGGAAACCCAGAGCTTAAAAT<br>ATCCTGCTAATCCTGGAAAAGAGAGAATTTCTTGCTTAGTTTGTGG<br>AGGCGGGAGGGACAGAAGGAGGGAGTATGTGGTCTGTTGTGAGCC<br>CTTGCATTCAGGGGTAAGAGCTAGGCCTAGATAGGTGACTGGGGGG<br>TGCATATTTGGGCTTTCTTAGGGGAGACCTGATACCCCTGTTCTGCC<br>AAGAGGTTTAAAAGGGAGATAGTATGTATGGGTGTTGCAGTCTCTT<br>TGAGACGGGCTACACAGGAGCAGATTATTAACATATTAAAGAAGAG<br>AGGATGATTTAGGGATAAAGTACAGAAGTTACTAGCAAGGGCCTG<br>TCCAAAAAGGTGGGGGCTGTCTCTAAAACCTTGAGGTAGTACGCA<br>CCAGGTGAGCTGACCTGAAAGGTGGGTGTCGGGGTTTTCCCAGGT<br>AAAGGCAAAGAGGTTTTGGGAATCAGGGTGTAAAGGAATTGTGAA<br>AAAGCATCATTTAGGTTTAGAACAGAAAAATGGGTGGTATTCGAGG<br>GAAGTGAGGAAAGTAAAGTATATGGGTTAGAAACTACTGGACACA<br>CTGGGAGTACAGCTTGGTTAATGAGCCTGAGGTCCTGGACTAAGTG<br>ATAAGTTCCATCTGGCTTTTTAACAGGTAGAACTGATGTGTTAAAAG<br>GAGAGTTTGTTGGGCAGAGTAGGTGACTGGTGAGGAGGAGAGAA<br>ATGATAGGCTTTAGGCCTATGAGAGCTGCTTGGGGGATGAGATACT<br>GCTTCTGTGATAGGAACGGGGTGGGCTCTTTAAGGGTGATGTGGAT<br>GGGGGTGTGGTGCTCTGTGACCGAGGGTGTGGAAGTATCCCAAAC<br>AGTGGGGTTAACTACAGGTGCGGGATAAGGAAAAGTTGCATGTTTC<br>AGGGTGGGAGGTCGGAGGAGTAGAAGAAGGTTAGAAGCCTGTTA<br>GAAGCCTTGGAGGGGTCTGGGTTGATGTGTTGGGTACTATGGGGAA<br>TGTGGAAGTGGAGAGTAGTGTGGAGTTTTGAAAGAATATCTCTGCC<br>TAGGAGCGGAGCTGGGCATGAAGGCAGGACTAAGAAAGAGTGAC<br>TGAAGGAAAAGGTGTTCAGGGAGCAGAAAAGTGGAGGGGGGTCT<br>TGGGGTTTGGAGACTTGTCCATTGATTCCCACAACAGAGACTTGGG<br>AGGACTGGGTGGGTCCTGAAAATTTAGGTAAAGCAGAGTAGGTTG<br>CTCCAATATTAATTTTTAAAAAACATGCATGCCTACCTGCCACCATC<br>AGAGTTACCCTTGGCTCAGATGAAGCAATGGTAGTTGCTGGGACAT<br>CTGTTCCAGGGCACTGTCAGTCTTCAGTGGCAAGGCCGATGAGATC<br>CGAGTAGGAGGTTTTGGCCAGCTCGGAAAGGGATGGAGGCGATCC<br>TTGCTGGAGCCGCTCACAGCCTGACTTTCACTGAGTCCTCCACAGA<br>GGGGGCACAGCCTGGTGAGCACAGCTGGGTTTGGGCATGGCCTGG

	ACCAGTAGCCTTCATTGCCACACTTGAAACAGGCGCCAGGTGGAG<br>GTGTTTTGCTAGGAGGCTTCTGTATGGAGCTGTGGCCCTGTGGGCC<br>TGCAGGGTAAAGGCAAGCATTGGAAACTGTTTTTTTCTTTTACTTTC<br>CTCATCACAACTGTTAAAGACTTTGAAGGCTAAATTAAGAAGGTCC<br>CACTGTGGGGTTTGAGGGCCGTCATCAACCTTCTGAAGCTTGCGCC<br>AAATATCATGGTAGATTGGGTGAGGAACTGAAGGTTTGAAATAGTG<br>GTTCCTTCTGGGCTGGCTGGGTCCAGGTTGGTAATTTTCTCATGGCT<br>TCAGTTAAACAAGAGAGAAAAAGGGCTGGGTTTTTGTCAGGACCT<br>TCGGTGATTTCTGAAAGTTTTTCATAGTTTACCACTTTATGGGCACC<br>CTTTTTGAGTCCTGCAAGGAGACACATAATCATGAGGTCTTGATGG<br>CTGTGTCCAGAGGCCCTGTCTTGATAATCCCAGTGGGGGTCCTGGT<br>TGGGAACTGCCTCTGTGCCAGTAGGCTGGGCAGGAGCTTGATGAT<br>GAATTGTATTAGCATGCACCTGAGCTAGGGTCCAAGTACGGTGCTG<br>GTCTTCTGGGGTGAGGGTGGAAGAGAAGATAAGTAGCTGTCATGC<br>CAGGTTAGTTCATAAGACTGAGTAAGGTACTGAATCTCCCTAATATA<br>AGAGGTAGGATCTTCTGGAAATGAACTGAGTCTTTTGTTAGTTTGA<br>GAGAGATCAGTGAGGGAGAAGGGAACATGAACTCTAACAATACCT<br>TCAGTTCCTGCTACTTCCCGAAGGGGGCATTCTAGCACTGGCGCTA<br>AAGTACGGGTGGGGCATGGGGGCCAAAGATGGTGCCGGAGCTAGT<br>ATGGGCAGGAGAGAAGGAAGAAGCCAGAAGGGGTTCCTGCTGAG<br>GGTTTGAAGAGGGAAGGGGGGTTGAGTTGATAGGCATTGGAGGAT<br>AGATACGGGTGTAAGGTGGCGGGATGGGTTTACAGGCTCCGTGAG<br>AGGGCTGTAAGGAAGGAGAATGAGTACAGGCAATGCTAGAGTTTT<br>CCTGAGGAGAGGATGGTATAGGAAAAGAAGTGGGTGATGCTGGCT<br>GGGAAGATGGTGGCTGGGAAGATGGTGGCTGAGAAGACAATGAG<br>GAAGCTTGGGGAGTTAATGAAGTTGGTTGAGATGGAGGGGTAGGG<br>TCTGGGAGGGGTGGACAGCAGTCTGCTCCATCCAATGAGGAAAAA<br>GAGGTAGGGTCGGGAGGATAAAGGCAATGAGGGCGGCGAGAATG<br>AAGAAGGATTTGAACAGGTAAGCAAGAATTGCAGAGGTCGGGCTG<br>TGATCTGAGTGCAAAAAGGCCTGGACACAAGGAATTTCTCCCCATT<br>TTCCCAGTCATCTGCAATAACTGCTTAAGTCAGTTAAAACTGTAAA<br>GCTGAGTGTTCCATTTGCGGGCCATTTGGACCCGTTATCTAATTCAT<br>ACTGCGACCAGGCTTTATCGTATAAAAAAGACAAGGCACTTATGGC<br>AGATATCTTGCCTGAGGCCTAAGGTTTGCAGATTTTTAATGAGGCA<br>GGCTGGAGGGCTGTTTTTTGGAATTGAGGACCGGGAGTTTCCCATA<br>ATGAAGGGTAGCTCGAGAGAACAGGGAAAAAGGAGACCATCCTG<br>GACAGCTGGAGGGAGACGATAAAAGGAGTTATCGTCTCCGCTGCC<br>TTTTTGGTTCCTGGAATGGGATGAAATGGCTTAGAGGCATCCCCCTA<br>AGACCAGATGACCAGCGAGTGCCTGGCGCACGCTGGCGCCTTCTT<br>GGATCAGTGTTGGATTTTTGGACTGGAGAAACCAAGAGAGGCCAT<br>GTAGATTTTGCCCTGTTAACCGGGCTCCAAGGGAAACTTACCAGTA<br>GGCGAGATCAGTGGCTGATGCGCATGCACAGAGAGGCGACTGGAG<br>GCTGAGGAGCTTCCTCTGTCTAATTGCTGTGGCCTGCTCTCTGGGG<br>TGGAGGGGTAGGTCCACAAGCGACACAGACCTGAGCCCCTCCTAG<br>GATTTGGCACCAGATGTAAGGTTCTTGAATCAGTTTGAACCCCAAC<br>AGCGCGCCAACAGACAACAGGAGGCGGTGTGGAGCAACATGCTG

	TTTTAATGAACGCCTGGTTGCAGGTGGGCTGAGGCCTAAAATGGCA<br>TCAGCCCCCAGTGAGGATGGGACAGGGGTTTTGTAGTCCTCTGTAA<br>ACAGTAAGTGTCCCAGTCTGTTGTGACTGCTATGTAGTACCTGGAT<br>GGCCTATTTCTTGATCTTCAGGGGTACATCTCTTCCAGCCAGGGTAA<br>GTGTCTTCTGGCCGGCTCTCTTCCTGCTTCTGCTATCTTGCTGACAC<br>ACGCTGCTGATGCAAGTGGTCTTGCATCTTGGGGCTGGGCCTGAGA<br>AGGGAGGAGTTACTCATCCCTTCAAGCCTTCAGGCCCTGAGGAGA<br>ATCTTTGAACCCCCTGCATCACTGGCCACCCTCCTGATAGATCCCTG<br>TGATGCCAGGCAGGAATGAGCCGCTTGGGTATCCAGAGAGCTCCC<br>AGTGCCTTTCTGCTACTTCCTCTACCCCTGTATTTTGCTTGGCTTGG<br>TTCTCTAATTTGACTCAGCTTCACATAAAGTCAGGAACTTCTCCTGC<br>CAACAGAACTTCAGCTTCTCCAGTGGGGATGTGTATTCTGGAGAGG<br>GGGATCACCCTTTCCCACTTCCATTGTTGGGGCACTCACAGTGTTT<br>GGGATGTCTCCCAGGTCCTGCAGGAGCAGTACGCTTCCTGCAGAG<br>GGTGTGTGGATCCTCTCAGAATTGCTGGTCTGTTCTTGCAGTTGATC<br>TGCAGCTAAAATTCACAATGCAAGCCTCTGCATGCTGCTCTGTCTG<br>GAGCTGCAATCTAGTCCTGCATCCCATCTGCCATGATCACTGGAAA<br>ACCCTCATTTATTTTTTAAAGGGTCCAGAAAATGCTAATCTATAGAG<br>ATAGAAATTAGATTAGTGGTTGCCTAGGGTAGGATGGATGCAAAATT<br>TCAGAGTGGGGGGTTAGAGGCTATTGTATAGAATCTTTTGGAGATAA<br>TACTGATTATTGTAGTGAAAGTAAAATTCTGTGAATATACTAGGAAA<br>CATTGAACTGTACACACTAATTGGTGAGTCATATGGCATATGAATTA<br>TGTGTCAACAAAGTTTTAGAAGACATTACTTGCACCACGATATTAA<br>AAAATGCCGTTTGAGTTGTATAATTACTTCTTCTCTCTATGTCAAGG<br>GCACCGAACAGGCAGGAGCCTCTCACTTGCCACTGTTCTTAACAGT<br>ATTATAAAATAATTACATAAGACAGGTTACTTACATATTCTAGGTCAT<br>AAAAATTATTGCTTGACTAGAGTAATTGTAAACATAAAAGAACACC<br>AAACACACTAAAATAAATATGAGGTCATCAATCTTTTGTTGGTCTCC<br>TTGGCATGCACCTATTCAGACTGTTAGTATTATGTATTTACTTCAAAT<br>TTTAGCAGTTATATTTTAACTTGATTGATTTTTCCTCAGATATAAGTAT<br>GAGAAATGACAGAAAGAAACAACAACTGGAA
UGT2B7	TTCTCTGGAACTCTAAACACATCCTGTATTACTCATGTGATTCAGAT<br>CAAAATAGTCTTTATATTATAGCTATTCAATGTATTATCAGCTAATTAG<br>TCGGGGTCCACATAGGTGAAGCAATGTAAAATATTTGTTTTCCAAA<br>ACCACCTTGTACCTTTGGGTTTTGTGTGGAATGAAAGAAAGTGTGT<br>GTATGTTTGTGTTTTGTGTGAATTAGAAACTTTAAAGATTTTGTTTTT<br>TTTTTCTGTCATTGAAAATATAGATTAGTACAGTTATATTTCATGTATT<br>TTTATTTTTTAAAGGTTGGAACAAATTTTTCTGGATACATAATTTTTT<br>GATTCCCGTAATAAACATCAAGTTTCAAAATAAAGCGTGGCCATTCA<br>TATTTAATATTTAATTTTTAACATCTGTGTATATTTATTTCAATTCGGA<br>GCCCTGTAAACTCTTAATCTCTGTGAGCAGTGCGTCTGGGAGCTAC<br>CATCACAGTTTCTGAGAAGTGGGCTTCAAGTATTCTGCACCTGCAG<br>CTCTGCTGTGCTGGTTGTGCATTCTGTGGGAAAGTCCTGGTTGGCC<br>TTGTGACATGAGCCATTAGCTTAATGTCAAAAACATTCCAGATAAA<br>CTCATAGAAAGGGGCCACAAAGTAACAATGTTTACTTACTACAGGC<br>TTTTCTCATTGACTACAAGAAGCCTTCTTCATTGAACTTCAAGGAG

	GTCCATGTGGCACATGACAAAGATAATCTTGAGAATTCAAATGATG<br>AATTTTTTGACCTAGCTTATTATTTTCTTTTATTTTCTATTTGAGACAA<br>GGTCTTGCTCTGTCACCCAGGCTGAAGTGCAATCATGGCTTGCTTC<br>AGCCTCGAACTCTCAGGCTCAAGTGATCCTCCCCACCAAAGCCTCC<br>CAAGTAGCTAGGACTGTAGGCACACCCCAGCACACCTGGTCAATTT<br>TTTTGTAAAATGTTTGTACTGAGTAGGTCTTGTTATGTTACCCAGGC<br>TGGTCTCAAACTCTTGGCCTTAAATGATCCTCTCATCTTGCCTCCCA<br>AAGTTCTGGGATTACAGGTTGAGCCACTATATTTGGCTTGTAGACCC<br>AGTTCTCAGTATCTTTCCTGGCTTATCATCTGGTAATGGAAGGTAAA<br>ACTAAATATATTCTTTAATTGAATTGGGTGGAATTTTTAAACCACTAT<br>GTGAGTGTTGTCTACAGTCAGGCACTCATGAAGCAGCTGCAGGAA<br>ATGAATCATTACAGAGTAATGGTTTACTGACCCTATAATTTTCTCTGG<br>GGAGCTAGTGGCTGAGTTGCTTGCAGTCCCTTTTGTGCTCACACTT<br>AGGATGCAACAGGGAGTGATGCTTCAGGAAACTTCTAGCTCCACC<br>TTCCTATGTACCTGTGCACATCGTAGGACTAACAGACAGAGTGACC<br>TTTCTGGAAAAAGTAAAATATAAAACATAAAATGTGAAAAATATAA<br>GTTTTCTTTACCTTTTGTATCCAGGATTATGACTTTCATTTTTGGTAA<br>TCATTTTATAGTGAGGCATTAGATAAGTGTCTTTTATTTTAAATGAGT<br>TATCAAAATAAATAGAAGAGAGTTAAATGAAATCTATGTAAATATAA<br>ATAATACATTTTTATATAGCTATCCTACATGCAAAACTCTAACAGTGA<br>TACCAATGAAAGAATAATCAAAATCTAATTGGGAATAGAAGTAATAC<br>ATTTCTTAAAATAGTTACACTTTCTTTAGAGTCAGTTAACCATTTTTC<br>TTGATACTTGTTAAGAGTCATGCCAAAAATAATACCCCCACCCCAG<br>AAAAAAAAAAAAAAACAATATTAATACTTTGGAAGATTTGGAAGACA<br>TATGGTTTGTCATACATAATCTCCAAAGCCCACAGGCTGGAATTGCT<br>AAGTCACCCAAACAGCAAAGATGGCGGCCCATATCTCTGTGAGCTC<br>TCTCCCAGGGAGTTTTCAAATCTCTGCTGGCCTAAGAACAATGGTG<br>GAAATGACTGGAGTCCCCTGTTGGGAGGTCCCACCCAGTGAAGTG<br>GAACAGGATCTGCAACCTACTTGAAGAAGCAGTCTGGCCATGTTTT<br>GGTAGAGTAGCTGTGTTGTGCTGGGGAATTCCTTCCTTCTGTGGTC<br>GATTTGGATGCTCCAAAGCTCAAATGCCAGAACAGCTAAGTTGCAC<br>AAACAGCAAAGATGGTGGCCCACCCCACGCGGCTGAGAGTTCCAT<br>TCCAGGGAGGCCCAACACTGCTAGCACGGCTGGTTGAAATTCCAA<br>GCCAGTGGATCTTATCCTGTGAGGTGCTGTGAAAATGGGGCCTGCA<br>GACTGTCACTGCTCAGCCCCCTGAATTCAGCCTCCCACTTTGCAAT<br>TCTAACCTTCCACTTTGCAAGAGCTACAGCTACTTTTGCCAGGAAG<br>CCCTGAAAGCCAGAGTATCTAAAGGCATCACGTCTCTACACGTGCC<br>TGAGTGGCTGCTCTGCTGAGACTCCACATACCTTTGTGTGTCAGAC<br>TGAAGACCTGGTTAAGTGGGTTCATGAGGGGATCTTGTGACCTGAG<br>GGTTACAAAAATCTGTGAAAGAAGCATGTTATTTGGGGTAGCACAT<br>TCACTCACCACTTTCCTGGGTGAGGAAATTTTCCTTGGTTCTGTGTT<br>GATGCCAGGTTGGCAGTCATCCTGCCTTACTTTTCTTCATTCTCTGT<br>GGATCAAGTTGTTTCCTTAAATAGTCCCAATGCGAGTGCCCGCATGT<br>TTCAGTTGAAGGGGCTGTATTTACTCACCCCTTCTGTTCATCTTTGT<br>GAGAGCCATGCATATTACCTACTTCCGGTCAGTCATCCTGGCCACTC<br>CTCCAGCTTAAAATATTTTTAGAGTATATCTCATATTAAGTGTTTTCA

	CTAATACAAGGCATACTTCTGAGATGTTGCAGATTTGGTTCTAGAAT<br>ACTAAAATAAAGCAAATATTGCAATGAAGCAAATCACACAATTTGT<br>TTTGGTTTCTAAGGCATATAAAAAGTTATGTTTACACAATGCTATAGT<br>ATATTAAGTCTGCAATAATATTAAGTCTAAAAAAATACATGCCTTAAT<br>TTAAAAACACTTAATTGCTAAAAAGTGCTAATGATCATCTGAGTCTT<br>CAGTCAGTGCAACTTTTGTTGATGAAGATTCTCATATCAATGTTGAT<br>GGTTGCTAACTCATTTTGGTGGTGGCCGCTAGTAGTTAGCATGGCTG<br>TGGCAATTTCTTAAAAATAAGACAACAATGGCATTTGCCACATACAT<br>TAACCCACCCTTTCACAAAAGATATTTTTAGCAAGCAATGCTGTCA<br>GATAGCATTTTACCCATAGAAGATCTTCTTTCAAAATTGGAATTAAT<br>CCTCTCAAACTCTAATGCTGCTTTATAAAGTAAGTTTAAGTAATATTC<br>TAAATCTCCTGTTGTCATTCAACAATGTTCATAACATCTTCACCAGG<br>AGTAGATTCCATCTCAGGAAACAAGTTTCTTTGCTCTTCCAAAAGT<br>AGTAACTCCTCACTTATTTGTTTTATCATGAGATTTCATCAATTCTGT<br>TACATCATTAAGTTCCACTTCTAATTATAGTTATCTTTCTAGTTTTATT<br>ATATTTGCAGTTACTTATTCCATTAAAGTTTTGTTATAGGACCAACAG<br>TTTCATATGCTTACTGTCCAATAATAGTCCAATTACACTGAGACGTC<br>AGATTTGGCAGCAAAGAAGGAGTTTAATAATTGCAGGGCACTGAG<br>CAAGGAGATAAGAAGGGAACCCCAAATCCAGCTCCTCAAGGAGTT<br>CTGGGTTGGAGGTTTTAAGGAGATCATAGATGGTGATAGGCTGGAA<br>AATTTGGGTAATTGATTAGTCAGGGTAATAGGGTAAAGTCATCAGA<br>ACAAGAAACTACATTATTTGCTGAGTCAGTTCCTTGTGGGGTCCTCT<br>AGATCAGCTGGCATCAGTGAAGTCTCTCAGATCAGCTGACATCAGT<br>GAGATCCTTCAGACCAGCCGAGTCAGTAGTTTTAGCAGTATGCTGG<br>ACCTGAAGGAATACCTCAAAAGGAAAGTTGATCATTTCATAATGTT<br>TAAGTTAATATTTATAGAAGGATTAAGAGGAACTATAGTCTTGTAAC<br>AGGGTCTGCATAATTCTAGGACAACAGCCAAATAACTGTGAGGAA<br>GTGAGTCAGAGAACAAGCTAACCTAATGATTAATGCTGAATGTACT<br>ACAAGCTTGGTTTACTTTTCTCTTTTTCTTTCTTTATCTTTGATTACT<br>TTAACAAAGTTTAAAAAGATAGTGTCATCTTGAAACTCTCAATATCT<br>TACATGATGGTTGTAAGAATCAACATGTATACGCTATATCATAAATGA<br>ACCTTTAAAATATTATGTTACATAAAAGAAGCCAGTCACAAAATACC<br>ACATATTGTATGATTCTATGCATTAAAAATTGTCCAGAATAGGCAAAT<br>CTATAGAGATAGAAATTCAATTAGCAATTACCTAGGACAGGGGAAA<br>TGCAGAAACTAGGGGTGGAGAGAAAAAGGCTAAGGACTATAGGGC<br>TTATTTTGGGGAGATAAAAGGGCTCTCCAACTGATTGTTATGGTAGA<br>TGCACAATTCTGTGAATATACTATGAAACATTAAATTTTACACAATAA<br>TTGATAAATAGTATGATATTTAAATTACATGTCAACAAAGTTTACAA<br>AAATATGTGGACCATGTTTAGTCATTTAATCTTTAGTTTTGTGTCAAA<br>TGGACTGCAGAAACAGGATCTGTCACTGCTACTGTTCTGGACACTC<br>TTCTAAAATATATTGCATAAGACAGATGGCATGTCCATACAAGATCC<br>TTGATATTAGCTGAAGGATAGCACTCATAAACATAAAAGGGAAATTA<br>ATCACATCTGTGTGAACAGATCATTTACCTTCATTTGTCTCCTTGCC<br>ATCCACATGCTCAGACTGTTGATTTAATGATATTGTATGTACTTTGAC<br>TTATAAGGGTTACATTTTAACTTCTTGGCTAATTTATCTTTGGACATA<br>ACCATGAGAAATGACAGAAAGGAACAGCAACTGGAAAACAAGCA

	TTGCATTGCACCAGGATGTCTGTGAA
VIP	TATGTCAACCAGATGTGAAATGAAGCCAAGTTAGCCTCTATTGTTC<br>AGCCCCTACACTTGATAACACAGGGTTCTGTCATCCATCCCTTCCAC<br>TCTACCATAAACTCACACCAGCTTCCCCATCTCAGAGGCTCTTCCCT<br>GGCCTGTGGAGAATAGATGTAGTGGTAGAGGGGCCATGAGAACTC<br>ACTCAGAAAATATTCTTGCCAAATCTTCATTCTAACATCTAGACTGC<br>AGCTTTCTTAAGGTTAAAAGCCAGAAATTAGACATGGTTGTTTTTCA<br>TTTGATCCATATAGTAAACACAGAGGGTGATTGAGTTGAAGGCTGG<br>TTGCATGGAATGTGTACAGTCATCTCCCAACACCCCGGCAGACGCC<br>TGAAACCACTGACAGCACCTAACCCTACATAAACCATGTATTTTCCA<br>TCTGTCAACAAATTGGCTAAGTGACTACCTGGGAGGTAGCATAGAC<br>AGCACAGATATGCTGGACAAAGGGATGATTCACATCCCAGGCTGGA<br>CTGGGCAGGATGGCAGAAGATTTCATCATGCTATTTGGAATGGCAC<br>ACAGTTTAAAGCAAATAAATGGTTTATTTCTGGAATTTTCCATTAAT<br>GTTTTCAGACTGCTGTTGACCACAGGTAACTGAAATCATGGAAAGA<br>GAAACTGCAGATAAGGGAGGACTACTCTATGTGGTGGGGGAACATA<br>TACATAAGAGATGACATTGTGAAGTAGGTAAAATTGGTTTTAAAATC<br>TTTAATACTCTATTTTTCAACTTCCACATCTTAGTATTGTGTGTTCCT<br>GTGATTTCTCTATACCCTTCTTTTTGAAATCTATTTTCAGGAAATCGT<br>CTGATACATACAACCCTAATAAAGTTCGAATGACTTGCAGCACTTGC<br>ATTGTTCTTTCTACATGTAATTTGATTTGAAGCAAAAAAATTCTGAG<br>ACAAAGGAATAATTTTCTCTAAACTCAGCATCAGTAAGTGGGACAG<br>ATAACGAGGTTTTGGAGTTAAGGGAGAAGTTGTCCTCGATCATATC<br>ATTTAGATCATTATTTGTTTTGCTTTATATTTGCTGATATATTTTTAGAT<br>TCCGAAATGAATTCTAATAGGTAACTTTAAAGGGAAAATTTACCTTT<br>ATTGTAATATTATTTTCATCAAATCACTAATATGACAAGAATACAAAA<br>TCCTGAAAATAAGTTCAAATTTTCTAATTTAAAAGAATTGAAAGTGT<br>TTATATATACTCTAATATAGATGCTTTAAATATATAAACTTTTAATATGC<br>TATACAAATATACGTATGTATATACATCTTATGCAAATGTACACTTTTA<br>GTAAAAGTATACATGTATATTAAATGTATGTATATATTCTGTATGTTAT<br>ACATATATACATGTATAATATATTGTATAGAATATATGTATTTATGTATAT<br>ATAACATATACAGTACATAATACATTATACATTAGGTATATAAAATATA<br>TATACGCAAATATATATTTCCTAATACAAGTTATTGAAAGATATTGGA<br>TTTAAGACTATTAAAGCAAACATATCATCTTAAATATCTGCCAAACA<br>GGAGGATCTAGGGGATTGAAAAACTTTTTTTGTGAAAGATCAAATA<br>GTAATTATTTTAACCTTTGAGAGCTGTGCAGTTTGTTGCAACTATCC<br>AACTTTGCCGTTGTAGCACAAAAGCAGCTATAGAGAATACATATATG<br>ATGAAATGCTTTTATGTTCCAACAAAACTTTATTTATGGACACTGAC<br>GTTTAAATTATATATAAGTTTTGGCCAGGGAGGTGGCTCACACCTGT<br>AATCACAGCACTTTGGGAGTCCGAGGCAGGCGGATCACTTGAGCT<br>CAGGAGTTCCAGACCAAAGCCCGACCAACATGGTGGGCACCAGTG<br>GTCCCAGCTACTCTGGGGGCTGAGGCACGAGAATCGCTTGAATCTG<br>GGAAGCAGAGGTTGCAGTGCACCAAGATTGCACCACTGTACTCCA<br>GCCTGGATGATAGAGTGCATGAGACTCCGTCTCAAAATAAAATAAA<br>ATAATAACGTATAATTGTCATGTGTCACAAAAAGTTCATTTAATTTTT<br>TTAACCATTTAAAAATATAAACATCATTCTTAGCTTGAGAGATGTAA

	ACAAACAAAAACAAATGAATGAAGAAACAAACAAACAAAATCCA GAGGGGTCAGTCTATTTAGCTCACAGCCTGTAGTTTCCAGACCTCT GATCTAGACTTACCCACTCAGGATTACCAACGCAGACATTGGCTAA GTCTCAGGACTGGAGGTGAAATAGGGTGAATTAGGCAGGTAAAAC CACCATAAAAAGAGGCAGAAGAGCACAAGCTTAGGCATTTAATCC ATAATAAGAATATGGACCCTCTATTAGTATTTTGCACTAAAGAAAAA AAGGCAAATTTGGATTTCTGCTAATATCCTGAGCTTGAAAATCCCTT CCATCTAATTTCCTTGCATCCAGTATGTTTGAATTTAGAATGCCAGTT TTATTTCCCTGCTCTTGGTTCTGTTGGTGAAAAAAGGGGAGAGGAG ATGCCAATATTTTTATGAGCTGTCAATTTCCATATCTGACCTGCTAGC ATCAAAAAGGTGATAGCAACAATTACAGAATATTACCAATTCTAAAT TGAAAGAATGCTGACTCAAAATCAGACGGTCTATCTTCCAATCTCG TATTTGTTATTAACCACCTGAGTGGCAAAAATTACTGAAACTTTCTG GAGATCAGTTTACTTATTTGTAAAAGAAGGATCTTGAAGATTTTTCT ATCCATGTGAATGAATTATAGTTCCCCTTATTACAAACATCACTTTAC TGAGAAGGCCTAAGGCAAAAATAAACTTTGCCAGAAATGTAGGCA AATTATGGAAGAATATGTCAAAGTACGGAAAAGACACATTTAAACA GTAAATTCAAGTTTGTATCTTTGTACTTATTTACTAAGTTTTATGGTA ACCGAGAATTCAGGATAAATTTGACTGCAGTACTTTTTCTTATGTTT ATTTTTTCTGGAAGCATCCAAAAGTTAAGCTGGCTACAGGACCTCT GGTTTACAATTCATTTCCTTGAAAGAAAAGAAGAAATAAACACAAT TCTATAAAGTCTGTGAATTAAAACTTTGAACCTGCAGATGGTGCTAT GATTCCATTTTCAAATTGTTTTGCTTTCTAGTTTAAATTCATAAGAGA AAATGCAGTTGTTTGGTTGAGTTTACTTTTATTCAGAGGTAATAAAA TTGAATTATTTCTTTTTATTTTATCTTATTATTATTATAATAGAAGCAAT TGTCTTTTGTATTGGGAAAAGCTACTATAGTTACTCTTTGTAAATGCT AAGTGCTTGCCTAGGCCAAATAATGATCAGCTTTACATCTTTTATGT TTAAAATTTTTCTTCTTATCATTCAGCAGTCATTTGTCTGTGACCCTC AATTAAGTTGCTTTGCTGTTTCTGCTCTTAAATAAAATTGGTTCACT AAGAATTGGGTGATGAAGAGCTAAGGATAAAAAAATTCCATATGAG AACTGTTTCAACCTCTAGTTTGTAATAATCAGGTAAAAAAGATTTCC TGGAATTAAGCCACAGGAACTCTGGCTTAAGTCAGAGCTGTCAACT GGGAAACAAATTTCCAGACATTTTGAAACTTAATTCATTTAATTTTT CTGGTAACTGGATTAGAAAATATGATTAAGCATAGCAGGATATTCTT TTACTGGATCAGTCTGACTTTGAACGAAGGAAATAGATACTACAAT CTTAAAGTAGAAGAAAGAGGACTGTAAAGCCATTCTGTCTTATGAA ACTTAGAAACAAAATGTAAAAATAATACACATGGCAGAAAGCCTTT AAAATAAAAGGAAAAATGGCATTTCCTCTGTTGTTTAGGAAATTT TTCATGTGGCTGCATTTTTACCTTAAACTCTGATTGAATGTGTGCTA GACTACCTTTAGTTACAAATCCACAAATAGAAGTGAAGTATTTATCA AAGTACTATATAAATGCTTTTCAACTATTTGAAGCCACCTGTCAAGT GATTGAATCAGAAATGTTCAAAACTGGTTTGAGTATGTGTGTGTGT TGGGGATGAGGGTAGAAAACTAATTCTACATTATGCAAAGACTTATA AAAGTGATGAGGAAAATCTTTAAAAAGAAAAGACAATTAAATTCCT TGAGGTATATAGCCTGACAACAATTTATTTTTTGGATCATTTGACAC AGTGTATGTAAAAGTGCATTGGGAATTGTGCCATTTTATACAAATAT

	GAAATAGCACTACTTGTACTGCTTTGTACAACAGCGTGCATATCTTA<br>CTATCTTAGAGACTTTCTGAAACACTTATCTTGGAATTAGTTTCAAC<br>AAAGACGAAATGTAATAATAGGAAGGTGAAAATTATCTCTAGAGCT<br>TTTAAAGTTAGGATAGATTTCAAGAAAGATTAAATCTGGAACTATAT<br>GAATAGCATAAAAATTAAAAATATTAAGAGAATTGATACTTTGTAAT<br>AAAGTATAATTATACTTTTATAAGCGTAATGAACATAAGTGTTTGTTG<br>CTTTATGCATTTGTTTCTACACATGAAGTCTTTATTTTCTGAAGGAAT<br>CCCAATGGCCCTGGCTATAACACTACAATCATTTTCTTATTTTACAAT<br>TAATTAAAGGTTAAAACATTTCACATTGAGCTTGCTACTTTGTTTGT<br>TTAAAGAAAAGTAAATTTCCTCTTATCTTACATTTTGAAGTTGCTCA<br>CTATAAACTTATATAAATTAGGCATAATGACAATTAAGTAAGAACTTC<br>AAGCCCTATTCATCCCATGGCCGTCATACTGTGACGTCTTTCAGAGC<br>ACTTTGTGATTGCTCAGTCCTAAGTATAAGCCCTATAAAATGATGGG<br>CTTTGAAATGCTGGTCAGGGTAGAGTGAGAAGCACCAGCAGGCAG<br>TAACAGCCAACCCTTAGCCATTGCTAAGGGCAGAGAACTGGTGGA<br>GCCTTTCTCTTACTCCCAGGACTTCAGCACCTAAGACAGCTCCAAA<br>ACAAACCAGAAC
VPREB1	CATGCCGTTATACTATAGTGAACAATAAACTCTTGAGGAATGTCGGG<br>TTTAGGGGAGCCAGTCCCCTGTTCAGTTGAAAATCTGCCTGGCTAT<br>GTATAAATTTTGACTCCCCCAAAACTTAACTACTAATAGCCTGCTGT<br>TGACTCGAAGCCTTACTGATGATATCAACAGTCAATTAACACATACT<br>TTGAATTTTATACGTATTATGTACTGTATGCATTTTTTTTTCTTTGAGA<br>TGGAGTCTCACTTTGTGGCCCTGGTTGGAGTGCAGTGGCACCATCT<br>CTGCTCACTGCAACCTCCCAGATTTAAGTGATTCTCCTGCCTCAGC<br>CTCCCAAGTAGCTGGGATTACAGGCATGTGCCACCAAGCCTGGTTA<br>ATTTTTGTATTTTTAGTAGAGATGGGGTTTCACCATGTTGGCCAGGC<br>TAGTCTCAAACTCCTGACCTCAAGTGATCCACCTGCTTCGGCCTCC<br>CAAAGTGCTGGGATTACAGGCGTGAGCCACTGCACCCTTCCTGTAC<br>TGTATTCTTATAATAAAGTAAGCTAGAGAAAAGAAAACGTTATTAAG<br>ATAATCTTAAGGAAGATAAAATCTATTTACTATTCATTAGGTAGAAA<br>CAGATGGATTATCCTACAGGAGGAGGAGGAAGAGGAGGGGGTTGG<br>TTTTACAGTCTCAGGGGCGAAAGAGGCATAAGAAAATCTGCAAATA<br>AGTGGACCCACGAAGTTCAAATTCCTGTTGTTCACAGTTAGTGGTA<br>CACAGGAGACACTAGGTAAGCTAAAGGTTGGGCTGGGCATGGTGG<br>CTCACACCTGTAATCTCAGCACTGTGGGAGGTCAAGGCGGGAGGA<br>TCGCTTGAGGCCAGGAGTTTGAGACCAGCCTGGCCAACATGGTGA<br>AATGCTGTCTCTACTAAAAATACAAAAAGTAGCTGGGCATTGTGGC<br>ATGTGCCTGTAATCCCAGCTACTTGGGAGGCTGAGGCACAAGAATC<br>ACTTGAACCTGGAAGGCAGAGGTTGCAGTGAGCCAAAATAGTGCC<br>ACTTCACTCCAGCCTGGGTGACAGAGTGAGACTGTCTCAAAAAAA<br>AACAAACAAACCAAAAAGATTAGCTAAAGGTTGAACTTTCAGTGA<br>TGAACTAAACAGAAACAATCTCATAGAGATTGTGGACAGGAGAAG<br>AAACAAGCACAGGTGCAGTTAAAACCCGTGATAAATGCCCCGGCA<br>GAAACAAGTGTTAGGCCAGGCGTGATGGCTCATGTCTGTAATCCCA<br>ACACTGTGGGAGGCCAAGACAGGAGGACTGCTTGAGGCCGGGGG<br>TTTCAAGCCTGCAGTGAGCTATGATTACACCACTGCTCTCCTGCCTG

	GGCGGTAGAGACCCTGCTTAAGGAAAAAAAAAAAGAAAGGCTGGG<br>CGCCGTGGCTCACGCCTGTAATCCCAGCACTTTGGGAGGCCGAGG<br>TGGGCGGATTACGAGGTCAGGAGATCGAGACGATCCTGGCTAACA<br>CAGTGAAACCCCGTCTCTACTAAAAATACAAAAAAAATTAGCAGG<br>GCGCGGTGGCAGGCGCCTGTAGTCCCAGCTACTCGGGAGGCTGAA<br>GCAGGAGAATGGCGTGAACTTGGGAGGTGGAGCTTGCAGTGAGCT<br>GAGATTGCGCCACTGCACTCCAGCCTCGGCGACAGAGCAAGACTC<br>TGTCTCAAAAAACGAACAAACAAAAAAACAAAACAAAACAAAAC<br>AAAAACCTAAACCAAAACCAAAACGCAGCATAGTCCATGAGAGAG<br>GAAGAGCAGGGCAGGCCTACTCACCTGGGGCTGTCGGGGAGGCTG<br>CCCAGGAAGGGACTCTGAAGTGAGCACCTGGAGGAAGGAGGTTA<br>GTTCTGAGGGACTGGAATTCATCCTGGCATTGGGATCACGGGCAAA<br>GGTCCTGTGGTGACAAGGAGACTGGTGCGAGCCTGGCTTAGCACC<br>ATGTGAGAGCAGAGGGGTGGAAAGGCACTAGGTAAGCATGCCTTC<br>GGATGTGAAATGAGGGAGACGGGGACTGGGGGACCCTGACATGAG<br>GGAGGAGGAAAGGAGATGGGAAGAATGGGTGACCAGAGGGACCA<br>CTAGACCACCGAGGGTGAGAGCTTGCTAATATCAGAGCCATGTGTA<br>GGAGAATGAAGTGCAATCTTGGATAGACCTCCAGTGCAGGTGGTC<br>CAGCCATCAATGACCATAACCACATGCATGATTGTAGGTGGAACCA<br>GAAGAACCACCCAGGCAACTCACAGAACTGGGGAACTAATCAAGT<br>CTTGTTTTAAATGACTACATTACGGGTGGTTTGCCACGTAGCAGTGG<br>ATCGCTGAAACAGTTTAAAATATCTATTCAGCCGGGCGTGCGGTGG<br>GTAGCTCACACCTGTAATCCCAGCATTTTAGGAAGCTGAGGCAGGA<br>GGATCTCTCGAACGCAGGAATTTGAGACCAGCCTTGAGCAAAATG<br>GCAAGACCCTGTCTCCACAAAAAGTAAAAAAAATTAGCTGGGTGT<br>GGTAGCATGTGCTTGTAGTCCCAGCTACTTGGGAGGCTGGGACAGG<br>AGAATTGTTTGAGCCCAGGAGTTCGGAGGCTGCAGTGAGCTGTGA<br>CTGCACCGCTACACTCCAGCCTGGACAACAGAGTGAAACCTTGTC<br>TCTAAAAAAAAAAAAAAAATCTACAAACCAGGCAGGAGCACTGAA<br>TAGCTCCTGTCTCCCCTGAGACCCTTGTAGACCTTGAGGTCTGTTA<br>CAGAGCTCACCAACCTTCTCTTGGTCTAGAATTATCTAAAAAGAGT<br>TGAGTGCAGCCAGAAAGGACTGAATACATGGTGTTCTTCAATCCCA<br>TCAAACTGCTGTGCCCTGCAGATGATCCCTGAGTCCAGGTGTTGGT<br>GGGTTGAGGACAAGGCTGAGGGAACAGAGGTGGGAACAGTGGCC<br>CAGGACCAGGAGAATGCAGAGTTCAGAGTGCATGACCTGCATCTA<br>ACTCTAGATGCTCCAGGGGTCTGAGACCATCTCCAGCAGGGTCTGG<br>AAGGCCCAGTCATTCCCCAGCACGCACAGTCACCACTGGATACTAT<br>CTTGCTCTGTGAGTGATGCAAAGGTCCTGGGTCAGTGAAGCCTGTG<br>AAACAAAGTTGGGACCAGAGTGTCTCTACGGTTGAGGGCTGGGCT<br>TATTTGCTCTCTAAGTATAATTGTAAGAGCAAATAGAAACACCTATC<br>CTCTCTGAGCTTAAAAGGATCAAAAAGCTCTGACAGTGTCAGTGGT<br>AGGGCTAGTTCAGGGGCTGCAGTAGTGCTGGTGCCCACAGGTGGA<br>ACCTTCTCCTGCCCTGATTCCCAAGGACTGGTGCTCTCGGAGGGGT<br>CTGGGAGGTATATTTCTCTTTCACAAATACGTGCACAACACATGAAT<br>GAGCTACTGGATGAGGGAATAAATGAATGAATGAGTGAAAAAAGC<br>TTCCTTTCTCCTCTCCAGGAGTTAGCAATCACTGGTTAGTTCCCCAC

	TGCCCACCAGCCTGTGCAGAGGAGGGAGGAGCAACAGGTCATAGA TGTGGTTGCAGCTGTTACTGGACAGAGTGGTCAAGACATCCTGTGT CTGCAGAGGCCCTCCCCAGCCCTCACCTGGACAGTGGAGCTGTTG GCCAGAGAGGCAGTGGGAAGAAGGGTGGGGCTCCCAGAGTCGAC CCAGCGGGCTGAAACTGGTGCAGTCTGCGCCTCACCAGGCAGAGA ATGGATGTGGGGCAGCTTGACACCGAGCAGGGGCTCATCTGAGCC TCAGGGGTGGTTGCTCTATCAAAGTGTTTGCAAGTAAACAGTCCCA GTGCACACCCAGCTGCTCAGTGCCACCTCGTGCACCCAGGGCCAC TTCAGCCTTGATCACAGGCCTGCACAGGTTCCAGGGGAGGAGACA CTCCAGCTTTTTGAGGGAGGGTGAGCATGTGGGATCAGAGACTTTA AGTCCAAATTTCCCCTTAATTTTTTTTAAATCTTGCAGGTAGTATGC AAACATACATTTAATGTATTATTTTATTCTCATTTATTTTTTGAGGC AGGGTCTCCCTCTGTTGGCCAGGCTGGAGGGCAGTGGCGAGATCT TGGCTTGCTGCAGCCTCAACTCAACCTCCCAAGCTCAAGTGATTTT CCCACCTCAGCCTCCCAAGTAGTTGGGACTGCAGGTGCCTGTCCGC CATGACCAGCTAATTTTTAAATTTTTTTGTAGAGATGGGGTTTTGC CATGTTGCTCAGGCTAGACTCGAACTCCTGAGCTCAAGTGATCCTC CCACCTTGGCCTCCCAAAGTGCTGGGATTATAGGTGTGAGACACTG CATCCGGCTCCCAAATTTCCCCTTTTTATAAGGACACCAGTTGTACT GACCGGGGCCCCACCTCTATGACCTCATCTAACCTTCATTAACTCCT TAAAGGCCCTATCTCCAAATACAGGCACACTGGGGGTTAGGGCTTC AACCTATGGATTTGGGGGATGCAATTCAGTCTATCACAGTGCCAAG CTGCACCTGCTCGGTGAGCCAGAACAGGGGTGGGGAAGGCACCA GGCCCTGGTGCTTCTTTCCCATGCCTGAGTCACTCTACTTGGTTCTA GGAGGGAGAGGAGCATGGGCTGAAGGCAGTGCTGCAACATGGAA GGGGCAGAACATCCCAACCCGCTCTGGGCCCCCAGTGTCTTCTGCT GTACAAAGCAGGTGTTTTCACCCCCCGAGGATGTGTCCTCCCTTCC TGGCTCACCAAGCATTTAAATGTAGACAGAAAATTTATTTATCTGGG GTGCGGTTTCCTCAGGGGGAAGTTGAGGTCACGACCCCTGAGGTA CCTTAACCCAAAGGCCTCCAGGGCACTGGCCCCAGAGTCTCCAGC AGGTGCTTCCTCCCTGAATGCTTCCATATGGCAAGATCAGAGCCAC AAATGCTGCCCCGACACTGTTCCGGTAACCGACGTTGCAGACCCA AGAAAGTCTGGGATGGTAGTGGCCAGGAACGGGGTGTGCCACGGC CCCACCTCTCAGAGCAGGGAGGAGGATGCTGGGCCAGCCCTTGGG GACCCCAGGCACCGTGGCCACAGGAGTCAGAGCTCTGCATGTCTG CAC
WEE1	AATTCTTGTCCTCCAAGAGCTTAAAATTAAGTTTGCATGACCATATA AAAAACACATTTATACACCAGTTAGCAAATGTTAGGAGAAAAGACA GCTGTTGATCCTTCACTGTGTCTTCAGTTGTGTGATCTCAGGAAATT CCCGATTTGATGTGTTAAGTACTACCGTGTGTTTCAAGAAAAATTCA GCTTGAGTTTATTTAGCCTTTGAAAAGCATTAGAACAACATATATGA CAAAGTGTTAATATGCATAAAGTGCTAACAATAAGAAAAATAGGCA ATAGAGATAAATGTAAATTATCATTAAACCCATGACAACTGTTCAAC AATATGAATCAAAACAACAAAACCACATCAACGAACATCCACCGAT GTCCTGAATTCACAGAGTGTGCTCTAATCCACTGCTAGTGAGAATG TATTGTGTTTCTGGAGGGGGAGTTTGCCAGTTTATATCCTTTGACTG

	AGGAATTCTAACTCCAGGGAGGTATCCAAAAGAGTATTTTAAAAAG<br>GCCAGGCGCGGTGGCTCACGCCTGTAATCCTAGCACTTTGGGAGGC<br>CGAGGCGGGCAGATCACCTGAGGTCAGGAGTTTGAGACCAGCCTG<br>CCAACATGGTGAAACTCCGTATTTTGCATTTTGTAAAAATACAAAAA<br>TTGGCCAGGCCTGGTATGTGCTTGTAATACCAGCTACCCGGGAGAC<br>AGGAGAATCGCTGGAACCCGAGAACCCGGGAGGTGGGGGCTGCA<br>GTGAGCCGAGATCGCGCCACTGCACTCCAGCCTGGGGCGACAGAG<br>CGAGACTCCGTGTCGAAAAAAAACCAAACCAAACAAAAAACATG<br>GAGAAGGAAGCATATACACCACGGGGTTGTTTACAACAGAAAACT<br>GGAAGAGGCCGGGAGCGGTGGTTCACGTCTGTAATTCCAGCACTT<br>TGAGAGGCCGAGGCGGGCGGATCACTTGAGATCAGGAGTTGGAGA<br>CCAGCCTGGCCAACATTGGGAAGTTCGTCTCTACTAAAAATACAAA<br>AATTAGCCAGGTGTGGTGGCAGCCGCCTGTAATCCCAGCTACTCGG<br>GTGGCTGAGGCATGAGAATCGCTTGAACTCTAAAGGCGGAGGTTG<br>CAGGTTGTAGTGAGCCGAGATCGTGCCACTGCACTCCAGCCTGGG<br>TGACAGAGCAAGACCCCGTCTCAGAAAAAAAAAAAAAAAAAATA<br>AACAAAGAAAGAAAAAAGAAAACTGGAAGAAACGTGTAAGTGCA<br>TTAAGGGACAGATTAAATCAGTTTAACGAAGCACTAGGGAAATTTT<br>TTAAAATTGCGTTTGAGTTTGCCGCGA
WT1	TAAAGCAGGGTGTTACTAAAGAAAGAAGGAAGAAATTGGCTAAAC<br>GAATTTAGAACACAAATAAAATTACTGCACAAATAAAATCTCTGCTT<br>ACGATTAAGCTGGAATGGCGGTCAAAAAAGAAAAGGAAAAAAA<br>ATTCCTAGATGCAGGTAGATTTCTATAATTAAGCCATTTCTCAAAGG<br>GGAAGTAAATGTGACAGAATATTTAGCAAGCAAATTTACTGAGGAA<br>TATTTTGTTCCTATATAATTAAATTCAACACTGAGGTAAAACTGACA<br>ACTATTAAACTGAGGGCAGTTGGGAGAGGGAAAGGGGATGAGATA<br>AAATCCAGTCTATAAAACATTCCATTATCTTTTATAGGTTAAAAAAC<br>ACAAAAGAAATACTTGGAAAGAATTGTCTAAGACGGAAAGATTTT<br>AAGATTGTGACTTTTTTTTTTTTTTTTTTTTTTTTTTTTTTTTTTTACA<br>GGTGCAGAATAATTTTTTTTCCCTCTCTCGTGGTTTTTCTTTTCCCCA<br>TTTTTTTCCCCTGTTGTTTGCAAAGTTGAATCTGGGGTCAGGAGTTC<br>TAATTGTAACCTCAGACTGGAATTCCAGATGGTGCGCTCAGGACTG<br>GAAGCAACTCTCAGTTCATCAATTTCATGGGCCTACGTATCTGCTCT<br>GGCCACGGCCTGGCCTCCATCCCCACTGTGGCATTAATGCAGAACA<br>AATTGAGACATATCCTCTATATAATTAGTTCCAGAATTTAAAAAGATT<br>TACGGTATAATAATAGAGGCAGTGCCGCGGAATCCTTTCACTGAGG<br>ACTCTCGGGGAGCTCGGGGACCATCTCGCGGTGGCATCAGAACAG<br>ACGAGTAACCCCAGCGGCTCCAGGCACTGCGAACTGGGGGAGAG<br>GGGGAAATTTTGCACCGCATTCGGCAGTACCTAACGGTTCCGGGCT<br>CCCAGATAGGGTCAGCGAGGAGAGTTAGGACAGTAAGTGGGGACT<br>GGTTAAAAAAAATCGACTTAATTTTGAAATGATCAGAAGAAGCTGG<br>GAGTAGTTATCACAAAGCCCCTCCCTTGCCCCCCCCTTTTTTTAATT<br>GAAGGAATGACGTTGAAATTTTCACCGCCAGCGCGGGGCTGGGAA<br>CCATCCTAAACACTTGCCCTGCAGCCCCGAGTGGGCAGATTCCGAA<br>GAGTTGGAGTCGGCGGGGAGGGGAGGAACAGCTGTTGGGCTGTCT<br>TGCGCGTGGGGCTCGGGGCGCGGGCCCCGGGGAGAGGCGCTAGTC

	GAAAGCTTGTTCCTCTCCCGCGACAGGCAGCAGCGAGGTCGAGCC<br>ACTCTTTATTACGGCCTGCGGGCCCCGCGCGCAGTGTGGCTGTCCC<br>CGCCCTTGACCGACCGCAGCGCTTTGGGGTGTTTATTCAGTTGTCC<br>GCGGCCGCTGGGTGACTCCCGGAGGGCGGCCCAGGCGTGCGCACT<br>GGTCCCTGGGGTTGCGGCTGTAGCAGCCCAGCTCCGCGCTCTGTCA<br>GATGCAGTGGACAGCGCCGGGGTGAAAGTAAGGGTTGAGAAATCC<br>TCACTGCCCTGCTCCTACCCAGCCTCGATTTTTTCATATTGCAATAAT<br>TATGCACCTTCGAGGCCGGGATGCCTGCGCTTTGCCAGTGTATCAA<br>CGCCACATGGTTTTCCAGGGGCTGTCCTCGCCTCTGCATCCCATTAG<br>CTCTGAAGAGGTAGAGGGGTGGTGGGTAAAACCTCCAACTGGACG<br>TTGAGAGACCCGGGTTTCAGCTCGGCGTTGCCACTAACTTGTTTCC<br>TGTCCTTAAACAGTAGTAGTAACAAAAACTGTAAAGTTGATTGAGT<br>CAAAGGCCCACACTGCTCTCCTATCTAATCCTCAGAACAACTCTAT<br>GAGGTGGGCGCTAGTATCCCATTATCCCGTTTGACCAGTAAGGAAA<br>CTGAGGCTCAGAGAGACTGCGGAACTCGTCTAGGGTCACACAGCT<br>GTTGGTTACCTGGCAGAGCTGAGGTTCCATTCTGGGGCCACCAACC<br>CCTTGCTCTTTACCGCTGCGCTCCATAGACTGCCTCTCCTTCGGCTG<br>TCTGGGCCTCAGTTTCCTTCTCTGTAAAACGAGAAGTTTAATCTGC<br>AGCTTTCTTTGCCAATCGGTGGTTCAGACCAGGAACCTCTGCTACA<br>GATCTCGGCGCTGACAGGGGTAAGAAGGTGGGTGAAAGAAGGGG<br>GCGCCCAGTTTTCCACAGCCTGCTTTTCTCTGGGACCTCGCGAAGG<br>GCGGGCCTCGCGAGCTAAAGGAGGTACAGGAGAGCGCCTATCGTC<br>CGCGGCGGGTGAAGGTGCTACCTGCCTTCGTGCTAGGCTGTGAGTC<br>CTGGTGCTTAGCTCAGGGCGCCAAGGCCAGTGTAGCTGGCATGTCC<br>CCCTTGGAAAACCTCAGGTCTCCCGCAGAGAACGTTACCCACAAC<br>AAAGAAGAGGACAGAGAGGCATGGAGCGCCCTGCGACTGCAGGA<br>GTACGTCAGTTCCCCAGCGCTGGCTTAGTGTCGCCTGGGCTTCCGG<br>GCATGTGGATCCGTTGGGGTCGTACGGAGACTTCCTGTCGGGTCCC<br>TGGGGTCCTCCGACTGCGGCTCCTCAGCTTAGCACTTTCTTCTTGG<br>CCCCGCAGGCTGCAGGGAACTCCTCCCACCTCTTTAGTCGGAGAA<br>GTCCAAGTCGGGCGAGGGGGCACCCCGGGGTTCGCACCGGTGCTC<br>TTCCCCTCCCCGCCCCCACAAGGATTCTGAGAAAATAAATGGCAGA<br>GGAGAGAGGAGTTCTACATTTGCTTGGCTCTCCTTTCCTCCTATCCA<br>CCCCTACATCCCTCACCCCGGGGCAAAAACTTATTTTTGAAAAATG<br>TTGGCAGAGATTTACGTGTCTTTGCCTTACCTGGGTTTCACAAACA<br>CAACGACTCACATTCAAGCCAGCCTCCCTTCAGATAACCTCCTCTC<br>CCCCCGCTAAAAGTGCCAAGGATGGTAAAAGAAGAAACAATCTCA<br>ATCTTTTCGTTTGGAAATGAAAGTCCCCGGCTTTTCATAAAGGGCT<br>CCTCGCCCCTCACAGTTGAGTCCTAGTTAAGAAAAACGACTTCCAA<br>GTAGAAATAATAGGCGGGGAGAAGGAAGGGAGATACAGGGATCTG<br>GGGGGTTCTTAGGGCAACTGGCAGTGAATTTTGTCTCGAGAGTCCT<br>TTCTCCACTCAAAAAACCAAACGCGCGAGCCCCGCGAAAGGTTTA<br>GGGATAGATCGTGTGGGAGAGGACTGAGCAGAGAGCGTGGGGGC<br>AGTGTCTTGTAGAATCTTTCTTTTCTTAATAATAATTTTAAAAGCTTC<br>TGAGTGGAGACGACGCAAAGTCAAGCAGCAAAGGTGGCCTGGGA<br>GGCAAGCGGAGGGCTCAAGTGCCGCATCTTTACCCTCAGGTCTC

	CTGCGCCTACGGGATGCGCATTCCCAAGAAGTGCGCCCTTCGAGTA<br>AGTCCTGGGCCCGCACACACTTCGGGTCCGCAGCCAGAATTTAATG<br>GCGACAACGTTTATGCAATGCAAGCTAAAAACCAAAGCGTAAAAA<br>ATTACTATGTCATTTATTGAAACGCCATTCTTTGTCAAACTGCAACT<br>ACTTTGCTTCACATAAGTTTGGCTGGAAAGCTTGCAGCCCCAGCCC<br>GGGCCAGCCAGGTACAGGAGGCCGGACTGCAACCGGTTGCTTCCC<br>TCCCGTCGCGCCTGGCCGTCCCACGCTGCGCCGTCGCTGCTGCCTC<br>CTGGCGCCCCTGGGATTTTATACGCACCTCTGAAACACGCTCCGCT<br>CCGGCCCCCGGTTCTTCTCCTTGCCTAGGGGTTGTTTCCCAATAGAT<br>ACTGACTCCTTTAGAAGATCCAAAAACCAAACCAAAACACCCCCT<br>ACCCGCCCCAAACACCTGCTCTGGGGCGCGGGGGCTGCCAAACAG<br>AGACTAGACGAAGGGAGTCAGATTTAGCGAAGCTCTTCGAGCTCC<br>CAAAGATTCGAACACTAACTCGCGCCCGTGGGCCGATGGAGGTTC<br>TCCCTACTCCACTCCTTGGTCCCCTTAACTGGCTTCCGCCTCCTGGT<br>CAATCACTGAGCAACCAGAATGGTATCCTCGACCAGGGCCACAGG<br>CAGTGCTCGGCGGAGTGGCTCCAGGAGTTACCCGCTCCCTGCCGG<br>GCTTCGTATCCAAACCCTCCCCTTCACCCCTCCTCCCCAAACTGGG<br>CGCCAGGATGCTCCGGCCGGAATATACGCAGGCTTTGGGCGTTTGC<br>CCAAGGGTTTTCTTCCCTCCTAAACTAGCCGCTGTTTTCCCGGCTTA<br>ACCGTAGAAGAATTAGATATTCCTCACTGGAAAGGGAAACTAAGTG<br>CTGCTGACTCCAATTTTAGGTAGGCGGCAACCGCCTTCCGCCTGGC<br>GCAAACCTCACCAAGTAAACAACTACTAGCCGATCGAAATACGCCC<br>GGCTTATAACTGGTGCAACTCCCGGCCACCCAACTGAGGGACGTTC<br>GCTTTCAGTCCCGACCTCTGGAACCCACAAAGGGCCACCTCTTTCC<br>CCAGTGACCCCAAGATCATGGCCACTCCCCTACCCGACAGTTCTAG<br>AAGCAAGAGCCAGACTCAAGGGTGCAAAGCAAGGGTATACGCTTC<br>TTTGAAGCTTGACTGAGTTCTTTCTGCGCTTTCCTGAAGTTCCCGC<br>CCTCTTGGAGCCTACCTGCCCCTCCCTCCAAACCACTCTTTTAGATT<br>AACAACCCCATCTCTACTCCCACCGCATTCGACCCTGCCCGGACTC<br>ACTGCTTACCTGAACGGACTCTCCAGTGAGACGAGGCTCCCACAC<br>TGGCGAAGGCCAAGAAGGGGAGGTGGGGGGAGGGTTGTGCCACA<br>CCGGCCAGCTGAGAGCGCGTGTTGGGTTGAAGAGGAGGGTGTCTC<br>CGAGAGGGACGCTCCCTCGGACCCGCCCTCACCCCAGCTGCGAGG<br>GCGCCCCCAAGGAGCAGCGCGCGCTGCCTGGCCGGGCTTGGGCTG<br>CTGAGTGAATGGAGCGGCCGAGCCTCCTGGCTCCTCCTCTTCCCCG<br>CGCCGCCGGCCCCTCTTATTTGAGCTTTGGGAAGCTGAGGGCAGCC<br>AGGCAGCTGGGGTAAGGAGTTCAAGGCAGCGCC
YBX1	TCAAATTGATGTGCAAACTTAACACAATTTCTATCAAAATCCCAGCA<br>AGACTTTTTGTATGTATAGATAAAATTATTCTAAAATGTACATAGAAA<br>GGCAAAGGAACTCATACAGCTAAAATAATTTTATACAAGTAAAAAT<br>AAAGTGGAAGGAATCACTATTGCTGGCTTTAAGATTTTTTACATAGC<br>TACAATAATCTTGTGAAGGGTACACAAGACCATGTAGTATTGGTAG<br>AGGAACAGACACGTAGATCAATGAAACAGAATAGAGAGTCAGGAA<br>ATAACCCCACATAAGTATGTCCAATGATTTTTGACAAAAGTGCAAA<br>AGCTATTCAATGACATAAGAATAGCCTTTGCAATAAACGGTACTGG<br>AGCAATTGAATATCCATAGGAAAAAAACAAAAACAAAAACTTTAA

	CCAAAGTCTCATACATTGTGCAAAAATTAACTCACAAGAAAACCAG<br>ATCAAGATGGCTGACTAGGGATGCCAAACACTCATCTCCTGCACAA<br>AGAAGAAGAAAACAACAAATAACTGTACTGTGAGTAGAGCAACTA<br>AAGGAGAACACGGAAATACAGCAAGAAAGTGACAAAAACCCTCT<br>GAGGCATGGATGTGATGGCAGCATAGACAGGGAAGTGAAACATTC<br>AGCTGGGGTCAGCTCAGAGTCAAAAGGGACTTCTCATTGTGGGGA<br>AAAGGTAAGCAGGAGCTCCTCAGCAGTCCCCATTTCAACTGAGGA<br>CATCTGCAATCTTAGCTGCTGGCATGCCTCACAAGTCTCACAAGCC<br>TTGAATCCATAATAGGGAGCTGCCTGGAGTCTGCATAACCTCATTGC<br>TCTAAAGAAGGAACACACACCAAGTGCCCCCTTACTCCCAGGACC<br>AAAGCTGCTACAGCATGGTGGCCATTTGGGGAATGAGGTTGCTGTT<br>GGTGTGCATCTTGACCTGGGATCCTATAGCCACTACATGTCTACATT<br>TTTGGAGACCAGCCATCATCCCCTGATACCCATATGGAAAACTGCA<br>CCACCACCCCAGCTAGACCCAGCAGTACAGCCTTAACCCCAGCAC<br>CTGAGTCTATGCATTGCCCTACACTCCAGGGAGCAGGCAGTTCAGG<br>ACAGCATGGAGGCCACCCTCAGGACTGGAGGAACAGACATACACA<br>TTCCTTAGAGCCATAAAACAAGCTGCCTGATACCCACCACCACCAA<br>TGACTCTGTCCCCTCCAATGGCAGAACTGTCACGCATTGCAAACAT<br>ATTCCAGGAACCCAGAACTGGTCCAACTGGTACCCACCACTGCCA<br>GTCACCCTGCCCCCTCCAATCGTCTCATATTATATATGCCCCCTAGGG<br>ACGAAGAACCTGCCTGTGTGGTGCCCACTGCCACCAGCAACCCCA<br>TCTCCTTCAACTGCAAAGTAGCCATGCACCATGTATACCCCCCAGG<br>AACCAAGGACCAGCTCGTCCAGTGCCCACTGCAGCTGGCAACACC<br>ATCCTGACAACTGGTGGAGACACTATGCTCAGTGCACACCCCCACC<br>GCAAGAACTTAAGAACCAACCCATCTAGTAGCCCCTGCCTCTAGCA<br>AAGCCACACAACAGCCTCCACAAACACCTTCAGTCTAGGCCACTG<br>AGGCACTCACAGACACCACCACTGACATTGATTAGAGCTAAAGAA<br>ATCACATGGAGACTGCATTACTGCACTCACAGAACAAAATTCAAAG<br>CACCCTACCAAACCAACAATACAGGACACATCTACAGGAAAAAGG<br>CTTTCCCTATGGAAGCTACTCCATAAATTTGGAAGGGCTGACTGCTC<br>CATTAGATGCACAAATATCAGTGTAGGAACACAAGAAACATGAAAA<br>ACAAGGAAACATGACACCTTCAAAGGAACACAATAATTCTCTAATA<br>ACAAAACCAAAGGAAAGGAAATCTATGACGTGTCTGAAAAGGAAT<br>TCAAAATAATGATCCTAGGAATCTCAGTGAGATACAAAAGAATATC<br>GATAGATAATTCAATGAAATCAGAAAAACAATTCATGATCACAATG<br>AGAAATTCAACAAAGAGAAAGAGTTCATAAAAAAGAACTAAACAA<br>ACCTTGGAGCGGAATAACTCAAAGAATAAAAATACAATTGAGAGCT<br>TCAACAACAGAGAAATAATTTTTCAACTTAAAGACAGATCTTTTGA<br>AAAAATAACCCAGTGGAACCAAAAAAAAAAAAAGAAAAGACAAA<br>ATAATTTAAAAGAATGAAGAAAACCTACATGATTTATGGGATACCAT<br>TAAGCAAACAAATATTGCTATTATGGGAATTCCAGAAGGAATAGAG<br>ATTAGAAAGGCATAAAAAACCTACTTACTGAAATATTAACTGAAAA<br>CTTCCCAAGTCTTTGTAAAGATACAGACATCCAGATCCAGGAAGTT<br>CAAAGATCCCCACACAAAGTCAACTCAAAAAGGTCCTTTCCAAGG<br>CACATTATAGTTAAATTGTCAAAAGCCAAAAACAAAGAGAAAAATT<br>TAAAGACAGCAGGAGAAAAGAATCAAGGCATATACAAGGGAATAC

	CTATTAGACTAATAGTGGATTTCTCAGCAAAAACCTTATGGCCAGGG AGAACGATATATTCAAAGTACTGAAAGGAAAAACTGGCAGCCAAG ATTACTATACCTAGCAAAGCTATCCTTCAGAAATGAAGGGGAAATA GAGTCTTTTCAAGACAAGCAAAACAAGAAAATGCATGACTAGACC AGCCTTACAAGAAATGCTCAAGGGAGTCTTACATCTGAAGTGAAA GGATAATAACTACCATCATGAAAACATGTGAAAGTATAAAACTCTCT GCTAGAGCAGATAAACAAATGAGAAAAAGAAAAGGATCAAACCTT ATCAACTACACAGAATCACCAAACCACAAAGATAAGCAATAAAAG AAGAATAAAGAAACAAAGGATATATAAAACAACCAGAAAACAAAT AACAAAATGACAGGAGTAAGTATTCACCTATCAATAATAACCTTGA ACGTAAACAAATTAAATTATCCATTTAAAAGATATAGACTGGCTGAA TGAATAAAAAGAACAAGACTCAACTATATTCTGCCTACAAGAAACT AATTTTACCTGTAAAGACAAACACAGACAGAAAGTGAAGGGATAG AAAAAGATATTCTACACAAATGGAAACCAAAAATGAGCAGGAATA GCTATATTTATACTAGATAAAATAGACTTTAAATTAAAAATTGTAAAA AGAGACAAAAGGGTCACCCTCCAATGATAAAGGGATTAATTCCACA GGAGGATTATATGACAATTGTAAATATTTATAAACCTAACACTGGAG AACCCAGATATATAAAGCAATCGTTAGATCTAAAGAGGGAGAGAGA CCATAATACAATAATGGTTAAGGACTTTACCACCCCATTGTCAGGAT TGGGCAGATCATCTAGACAGAAAACCAAAAAAGAAACATTGGATT TAAACTGCGCTATGAACCAAACAAATCTTACAGACATTTGCAGAAT ATTTCATCCACCAGCTGCGGCATACACATTCTTCACCTCAGTATATG CAACATTCTCCAGGATAGACTGTATGTTAAGCCAAAAAATAAATCTC AACAAATTTTTAAAAGTTGAAATAATATCAAATATATTTTTTGACCA CAATGGAATAAAAATATAAATGAATAACAAGAGGAACTTTAGAAAT GTAATGAATAGATGGAAATTAGAAAACATGTTTCTGAATGACCAGT GAGTCAGTGAAGAAATTAACAAGGAAATTTAAAATCTTCCTGAAAT AAATGAAAATAGAAACACAACATGCCAAAATGGGACATAGCAAAA GCAGTATTATGAGGGAAGTTTACAGCAATAAACATCTACATTAAAA AAGTAAAAAGAATACAAATAAACAACCTATTGGGGTACCTCAAGTA AATAGAAAAGGAAGAACAAACCAAACCCCAAATCAGTAGGAGGA AAGAAATGATAAAGATTAGAGCATAAATAAATGAAAGAGTATTTAA AAAGTATATAAAAGATCAAGGAAACAAAAATTTGGTCTTTTGAAAA GATAAATAAAATTGGCAAACCATCAGATAGACGGAGAAAAAAAAG AAAAGGCCCAAGTAAATAAAATCATAAATAAAAAAAGGAGACATT ACAACCGATACAACAAAAATGCAAAGAATCATTAGAAACTATTATA AATAACTACACATCAACATACTGGAAAACCTAGATGAAATGGGTAA ATTCCTGGACACATACAACCTACTAAGGTAGAACCAGAAATAAACA GAAAACCTGCACTGACTAATAATGAGTAACAAGATTGAAGTAGTAA AAAAGTCTTCCAACAAAGAAAAGCTCAAGTCCAGTGGCTTTACTG TTGAATTCTACTTCAGATTTAAAGAAGAGTTAACACCAATTCTTCAC AAACCATTCCAAAAAATTGAAGGGGAGGAAATTTTTCCAAACTTAC TCTATGAGGTCAGCATTACCCTGATACCAGAAGCAGAAAGGGACAC AACAAAAAAAGAAATGCAGGCCAATATTCCTGATGAACATAGATGC AAAGTCTTCAACAAAATACCAGCAAATCAAACCTGACAGCACATC AAAAAATGTATACACCATGATCAAGTAGGATTTATGTCAGGGATGCT

	TGCAAGGATGGTTCAACATACGCAAATTAATAAATGTGATTATCACA<br>TCAACAGAATGAAGGAAAAATACCATAGGATCCTCTCAATAGATGC<br>AGAAAAATGATTTGATAAAATTTAACATTCTTTCATGATAAAAATTC<br>TCAACAAAACAGGTATAGAAGAAATGTACCTCAACACAATAAAGG<br>CCACATATGACAAACCCACAGCTAACATCATATTAAGTGAGAAAAA<br>GTTGAATGCTTTTTCTCAAAGAACTGGAACAAGACAAGGATGCCA<br>CTTAGAACCAAAAAATGAAATCAGTAAAGTAGCAGGACACAAAAA<br>TTAATACACAAAATAAGTAGTGTTTCTATAAACCAATAACAAACTAG<br>CTGAAAAAGAAATCAAGAATGCAATTTT
ZNF267	TGAGGAAGAAAGCAGAGGGGTGAGAAATGAAGTGACCACTTTGC<br>CTCTGGAGCCCAAATGAGGACTTAGCTCCCGGATGTGGGTGGGAAT<br>GTATTCTTTCACAGAATTCTCCCAAATTAGGGCCTAGCCCCCAACA<br>AAACCTTTGGCATCCTCCACGAAGTCCCCCACTCACGGCAATTGGT<br>CTGCTGTGTGGCTGCTCTCAAAGGAGCTGAGACTGAAACCTGAGG<br>TTGAAGAGCCACCTACTAGGTGGGAGGATTCTGGATTCCACAGGAC<br>TAAGCATTTCCTTTAGTTCAAGCAGGCAACGTTTCTCGTACTCAGT<br>GGCAAACACACCACAGTACACACACATGCACATACGCATGCACCA<br>CAAGCACCATTTTAATCTATTTGCATGTATTTCATCCCAGGAGTGATT<br>AGTCCTGTCCCCGATTCAATCTGGGTAATTTTTTTTTCTTCTCATGGT<br>AATGCATGGAAATAGAGTTTCTCAGGTGTCTTTAGTGAGAGTACAC<br>ACCCTGTTATTTTTTTTCTTATTTTTTTGGAATGCTGTTGCCAATTGG<br>ATTGATGTGCTGGTAGCAGGTTTCATCCAATCATTGCATGAAAGCAC<br>AGATACACCACCCACAGATGAATTAGGACTTTGTTATAGAATATATT<br>TTTTCTTTTTAAAATAAAGCCTGTAGTCAAGTTTCTTTCTTCTTCCTT<br>TTTTCTTCATTGTGAGGAAAGAATGTACTATGTTTCCATGAAGCGGA<br>ACTTCCATTTTGCTTCCCATAGTCTATTTGTCATCTTCCTCTCTCTCC<br>TGTCACACCCCCTCCAGCTTGTCATGTGGCACAGTCCTTTTCTGAG<br>CTCATGCACTTCTCAGCTTACCCTGACAATGGCAGTCATGAACACG<br>TGAGTCAACCACTTCTAGCCTGTTGTTTTTTCCTTCAAAGCATAAGG<br>AGGATGTCTCATTTCCTTCCTCTCAGGTTTTGTCAGCAGATAGAGCT<br>TTACTGTAGTTTGCAGTTGCATGAGATGTGTTGCCCTCTATCCAGGG<br>TGCAATGTGGCTTGGTATCACCCATGGACTGACCCCTTCTTGGGTTA<br>ATTTACCTATATAACTTTTCTGGAAGGGTGTGACCCTGGCAGCCTAC<br>GGAGACCCTGGGAATATCTTCCAGAGGGCCCTGGACCAGGGATGT<br>CTGCCTGGGTGTGAGACAGACAACCTCTCACCCTCCATGTACCTCC<br>TGGGTCAGGGCATGGAAATCTCACAGGCACCCTGCTCCAGGCAGA<br>AGAGGAGGGGGCAGCCTTAGAGGGTGTGGGCAGTTCCCTGGCTCC<br>TGGGGGGACTTCAAACTTATTGCAGACGGAAACCCCTTCTTCCTCA<br>TTTTCAGTCCAACCAACACACTTGGTGGCTAGAGAAACTTTACTTC<br>CTGTTTTTATTTATATATTAAATGTTTTCTTTTCATTTTACACAAGATG<br>TGTATTTAAACAACCAGATCCTGGATTTGAAGAAGAACATATCTGG<br>AATCCGTTCTGTTCTTTTAATTGTCACTTAGTCCTGTGTGAGGACAG<br>ATTTGTTCTATGTGTAACCGCTACATTTAAAAAGTTATAAAATTGTTC<br>TTAGCGTTACAATGATATATTTTTAAAAATAAAATTTTCTAATTGAAC<br>AATGTATGCAAAGGTTATTCCCCCTTTTTTTTTCCTGCCCCCAACAA<br>ACCTAAAGGATCACTATATTTTTTATACTGTTGTTGTTAAAGTCCTGG

	AATATAGAGATAAACAGTGAGTGAGCAGTCCCTGGTGGAGAAAGA<br>GAGTCTTTTTCACAGAAACAGTGTTTTCCCTCAGCTGTCTTCATTG<br>GAAATCACTCAACACCAATGACTCAATGGCCATTGGAAATCAATGA<br>TCGTTGTTCATTCAGTACAAACAAACAAGCAAACAAAAATTATCAA<br>TATGTTTGTACAGCTACGCCCGATAATTTATGTACAATAAAGGAATG<br>AGGAACAGGAAAGTGAAGAAGAGAGAAAGGGATGCTGGACCAGT<br>AGATGTGATGGAAACATATTGCAATTTTCTACGTGAGAATGTCTGCT<br>TGGTCTGTAGAAACAGAGTTCTGGAGTAAAGCAGTGAGTATGTTCT<br>CAGTAGACCCTCCTGTCTGCTTCTGGAACACATCAACTGAATCTTT<br>GTCCTCCATTTCCAGTTCTGCAGATGTGTCTGTTTCACTGATTGGCC<br>CCAGTCAAAATGGAATCTGATCTGTGTCATGTAGAAACTCTGCCTT<br>TCACCAAAGGCTTTCACTCATTCACTAAGTGGTGTGCACCTCTTCA<br>ACATCAACTGCATCCTGGGACCATCCTACCTGCCACCTTCAAATTCT<br>GCCATAGCGGAGCCTGAAGGCTCTTCAGCTGCAGCTTCACCAAAG<br>AAGTCCAGAGTGTGCACCAAACTAGTGAGCCAGCAGCTCCCGATG<br>CCGCAGGTGAAGGAGGCAGCAGAGCCTTACTCGCTGTTTTTATCAC<br>CATCCCTCCAGCAACAGCAGCAGCAGCAGCATCCACAACTACTATT<br>TCTAATTGTGGTAATAATGATGATCACACTTAGAATAATGATAAATAA<br>CAGTAGTTGTAATTGCTGACATGTTTAGTTTATATATAGATTTATTATT<br>TGTAATGTTCTATAATTTACTTTTAGTAATATACACATTAATTGTGTCA<br>TAAATATCAGGACCTCAAAACACCCCACCTAATGAGTGATTCTCAC<br>ATATGGACCCATGGCTGCCCACCTGCTAGCGTGGGTCCTCTCACCA<br>CTCCACCCCCAGAGCTTCGATCTGTCTGGTCAACATTCTGGGACAA<br>TGAAGTAGTTAAGTCATATGGTCTGACTGTGGTCTTGGCAAATAGCT<br>TGACTTGTTAGAAATCAAAGGCTGACTTTAAGACGTACTCAGCTGT<br>TTGGCTAAATTGTGGCAGAAAAAAATGTCTTCTCAGCAGATATACC<br>TTTGTATCTCTTGCTTTCAGAGTAAAAGATTGTTTCAGTCACAGTAG<br>TTTGAGTTAATCTGTATTGAAAATCATCAACAGCTTACAAATACTGA<br>AAGGGGACAAAAATAATTTGCAGACACATTGAGTCCATTATAAGTG<br>CTTTCAAATCTCTCCTATAAGAAATGAAAAATAGAATGTTAGGCCTT<br>GATTTCTTAAACTCAGCCTTCAGCCATTTTCCCAAATTATTCTACATG<br>TTTCCAAGACATTTCAGAAGCATTCTCTGGTGGGGATTATTTCCAAT<br>TGTGAAGAGTACTAGAAGGATGAGAATCAAAAGGAATCTTTCTTCT<br>ATCGCAAAAGAGTCAATCTCCAGGATTTCAAAGGCCTAGGGAAGC<br>TGCTTGAGTTGTGAGGTCCATGGAGGTGCATGGGCTTTGGAGTCAG<br>GCAGAGTTGATCCTGAGTTCCAGCCCAGCCCCTTAGTAGGCCCTAA<br>GGACTCCCGTGGTGCTGTCACAAGCAGTGCTGCCTTTGGGAGCCC<br>CTAACCTCCTGTCTTTTCTCCCTTCAGTGTGGGAAAGGGGTTTGGT<br>CAGCCAGTGGTGTCAGCAGCCCTGTCCCACACCCGTTGTCTACACC<br>TAATCTGGTGGCAGGGAGGCCACAGAGTGCAGCGACTAGAACTGG<br>GTGAGAGGGTGAGAGGCAGCCATGCCCCAGGCCAAGACAAGTGC<br>ATGGAATCCTGACTGTCAGCAATCAGGGATGTTTCTGCTTTGGGGA<br>GGGAAGCATTTGTTCCACACGTCTCACTGAATAAGGCACAGAGCCT<br>GATCTGTCCCAAGTGGGAGTTTAACCAAGCTAGATGGCGGCCGAG<br>GGCCCTGGCCCAGTTTGACACCCACCTCCTCTCCCACCTTCTACTC<br>AGCCAGAAGACTAGGAGAATCTACTCAGCCGGAAGACTACGAGGT

	GCTGCAGCGCCACTAGCTCCAAGACTCCAGAAAAACAGGAATAGG<br>GCATTCTGGCAGGTCAAAGCAGACAGAATACTTGAGCTCAGGAGT<br>TGGCGAACTGACTATTAACCTGTAGCCCCAGTTACTTGGGAGGCTA<br>AGGTGGGAGGATCGCTTGAGCTCAGGAGATGGAGACCAGCCTGGG<br>CAACATGGTGAAACCTCATCTCTACAAAAAATACAAAAATCAGCCG<br>GGCATGGTGGCATGTGCCTGTAGTCCCAGCTACTCAGGAGGCTGAG<br>GTGGGAAGATTGCTTGAGCCCCGGAGGCTGCAGTGAGCCATGACT<br>GCACCACTGCACCAGCCTGGGCAATAGAGTGAGACCCTGTCTCAA<br>AACAAAAGCAAAAACCAGAGGTAGGGCTGAGGAGTCCGTGGCCA<br>CAGAGCCTGAGCCAGCACGGCCCAGAGGAACTGAGCTCCCAGCCC<br>TGTCGCTGCCAAGGACCCTTCTTCCCTGAGCTTGAAGGGATTGAGA<br>AGGAGAGACAGACGGACAGTCAGCGGCCCCGTTACCAGTGATGCA<br>GACTCCAGGAGATGCGGGCAGAGTTGTAGATTAGAAGAATAGGCC<br>AGCCCCTCTTCCACGTTCCACCACGGCCCGCAACATCCTCCTGGGA<br>CCCCAGCCTGCATGCCCCTATAAGATGCTTTTGCACTGGTTCAGTCT<br>ATGTATCATGCGAGGGCCTAATTGAAAACACGTGAATGAAGTGTAC<br>GGCCTTCTGTTCCCAGCTGAGGGGCCCCCTCCCACGGTGGCGTTAG<br>CAGTGGGGTACAGTGCGGTAAGAGAGGTGCTAAGGGACGGGATGA<br>GGGTCTTTCTTGCTTTAAAGGTGTCTAAATATACTTCTTCTTAAACTT<br>AATTAGCTAATAAGATGTCTTATACAAGCAGAACCAGGTAAGGGAA<br>AGAGCAAGTGTATTTCAAGTCGCAGCTCACCCCTTAATTAGCTGTA<br>ATACCCGCAGCAGGCGGCCCCACCTCAATGAGCCAGTCTCCCTTTC<br>AAAAAAAAAAAAAAAAAGGCACTAATAATGAGGGGGCGGGGCA<br>TGGAGCTTTATCCAATCAGGGGCGCTGGAGTGGAAACCGTCCAATC<br>AGGAGTGAAGCCGGAGAGCAGGGGCGGCTTCCGGGATTTGGCGG<br>GGGCCTTCGTCGGCTCCAGTTAGAGCTCGGGTCTCCTCGC

TF of interest: ABF1
Target genes predicted by AdaFuzzy:

PUP1	MRPL36	UME6	SEC18
PMT2	RRP7	YHR080C	FIP1
RPB8	ZIP1	YPR045C	MRPL16
TIF1	RRP45	LTV1	RER1
APS2	CDC24	PET112	GBP2
RSM19	KAP95	AME1	APL2
DHR2	SCD6	THR4	SAP4
LYS4	HOS2	GCN1	ERV25
TFG1	ALG1	RET1	APE3
WSC4	YDR340W	RPN8	SPT8
SWI3	CKA1	YBR284W	SEC14
COP1	PAN6	MRPS18	SSF2
CST6	MDM20	CYB5	STP3
IME4	YDJ1	SPR3	SSH1
YLR400W	SUP35	BET3	SRB2
MNN11	IDP2	NUP133	ECM33
EGD1	NPR1	SSC1	GUK1
YDR327W	ERV46	RAD23	UBP6
NUP84	FIN1	LAC1	END3
CAT8	NEO1	UBP1	STE14
RPA12	TYS1	RPC82	PRP4
AIP1	RLF2	NRD1	GUA1
EBP2	RPO31	COX6	MRPL28
YER158C	YPR148C	BCP1	KIN2
MRPL8	SIP1	NMD2	CIK1
PUP2	TOM70	MKT1	NOP58
STI1	YDL032W	TOM6	ATP7
NCE103	RPN4	YNL213C	PRM2
SSM4	NTH1	PMA2	NTA1
KRE1	RIF2	FUI1	UBP14
YNL313C	NUP57	YDL012C	UFD2
NHP2	CCT8	MRP20	NGR1
MLS1	RPC25	SLA2	YKR015C
PUF3	CHS5	NPT1	HST1
NAS6	MSS116	BRX1	YTA7
YAP1801	SYF2	TPM2	RPP1A
FAS1	URA7	RNA14	SNF8
SOD1	ARC35	PHB2	RFA2
KRE9	ARO3	HCR1	REF2
YIP3	NFU1	YML081W	YER071C
MCK1	YOR309C	SUI1	NHX1
GIS2	SEC21	YJR116W	SCL1
CNB1	ARC19	LPD1	POM152
IQG1	GCN2	ADE3	NCB2

RSC1	PIK1	ULP2	YOR304C-A
AUR1	IPP1	DBP6	RPS8B
ARP5	PZF1	OPY1	SIP2
CCT7	ECM25	SEC72	AAC3
MKC7	MRP49	PTA1	YDR316W-B
SGT1	MRPL32	VID22	NUP85
YOL036W	SYS1	NOC2	GRH1
PMP2	IOC2	YIL032C	MTD1
YOR262W	SNF7	CMD1	HXT14
MSH3	ERG3	MSN1	ZPR1
SLC1	YNG2	SKY1	FYV6
TIF11	RNP1	RPN10	FAR3
LCB1	HNM1	DAL81	YPR202W
GZF3	TFA1	SWI4	HAP3
MNN10	FHL1	CLF1	KRE34
FBA1	CDS1	CTF18	YUH1
RPT5	VMA6	YHR078W	USV1
ROT1	CHO1	RPS28B	POB3
ARC18	STP1	MAS1	OM45
IDH1	MAE1	YDR266C	YFL052W
YNL115C	RSM27	MLC1	PCL5
NFS1	INO1	IML1	SLH1
ACS1	YPR063C	BSD2	YCR050C
GSP1	PRC1	SLA1	MND2
QCR9	CET1	KEM1	YNL091W
YJL202C	LAS21	NUP1	YIL169C
SRP1	ECM17	RPS28A	YGL066W
EDC1	DBP2	SPT23	GPI1
HSP60	TRM2	SOF1	YML133C
MHR1	VID27	VPS33	YOR255W
PEX3	YHR162W	RPO26	YMR291W
DPP1	RPB7	POP2	RPC53
DPH2	VTI1	MRP7	YDL037C
DHH1	RIB1	EFT2	CDC10
NAB2	CDC42	UBR2	UBC6
ARP9	YNL057W	BRE1	AAC3
BDF1	MCM16	SHE10	YDR084C
BGL2	NUP170	DCP2	CDC54
MRPS9	EMP47	RIM101	SAC3
YLR179C	DCC1	PHO2	LYS12
BET2	YGR053C	ADE8	YMR102C
CAP1	PGS1	PUT3	YJL068C
GPR1	IRR1	YCR001W	RAD27
RNA1	STE7	ARP8	YMR293C
YKT6	DSS4	YFL032W	YPR011C
SMX2	SSO1	MAF1	PFK1

PIG1	AAD6	YJL122W	GFA1
YBR096W	YKR083C	ATF1	CTK1
YOR227W	HMI1	YPL159C	YLL014W
YOR197W	PLP2	YGL217C	SDH3
YPR158C-D	RPC19	YJR157W	SEC11
XKS1	YDR401W	ZRT1	ARO8
YGL217C	YLR162W	TIF2	YNL310C
HXT2	LYS7	YCL034W	YNL227C
RPS0B	FUN26	PAK1	YER159C-A
ALR1	RNP1	YGR161W-B	ESC2
MKK2	ISU1	YNL143C	AFG1
DBF20	DCC1	PRR2	YPR1
YOR287C	YDR444W	YGR250C	YET1
MRP21	CHL1	DIG1	YJR054W
BUD3	DPL1	GDA1	YFR035C
MUP1	ECO1	YIM1	YOL032W
YCR095C	SIR3	YFR046C	MRPL37
MAL12	RGA2	YIL169C	YHL005C
YMR185W	RPN2	SPH1	YOR271C
RGS2	YJR001W	YML002W	RPO31
YGL109W	GRE3	DBP8	YDR442W
TIM18	YJL060W	PRE7	SEC20
YLR253W	ADR1	VPS29	ATP4
COX6	YCR062W	THP1	NEJ1
TOP1	NAN1	MRS6	SPT15
YPR143W	MSS2	MRPL13	PRM7
YGR164W	YGL161C	REX4	BUD22
YJL027C	ALP1	RPL26B	CBT1
FRS1	RPL41A	CDC33	YDL100C
TAF1	RPL14A	YOR215C	STN1
YLR108C	YOR082C	ACE2	YMR093W
YLL067C	YPL136W	YGR290W	UBP13
SEC2	THS1	POL5	THO1
YKR073C	SAP1	SEC23	YPL257W
RPS8B	YDR199W	EMG1	YBL048W
PRM5	YML084W	HSH155	YNL213C
YHR140W	VAM6	YOR146W	AAH1
UFD1	NTH1	SEC24	YCR013C
YOR342C	YML107C	BOI2	ERB1
YGL239C	SHU1	YIL176C	NRG2
MNS1	IES6	PET112	CDC28
PEP7	YPT1	MRPL28	APC1
YJL107C	YUH1	MGM101	YNL087W
SNF11	TPS2	MAL12	NAT3
RAV1	RRN6	YMR293C	PEP4
YMR051C	MDM10	TAT2	YMR157C

TFP3	OLE1	MSN5	YPL105C
YDL113C	YOL048C	YJL070C	RPC53
STP3	YNR020C	FYV11	LSM7
MSM1	HIS2	RPP0	YLR350W
YJR136C	FIG4	MATALPHA1	YMR148W
MSD1	SHP1	MSG5	KGD2
KIN4	YOL053W	YFL040W	YGR106C
LRS4	TDH1	MAK21	CLU1
YCL020W	YCR045C	SEC3	PPA1
YRA1	YKL223W	YPR027C	GEA2
LEU3	RAD57	IPK1	YRR1
CHS7	YOR390W	YOR304C-A	ORC1
RNR2	PKR1	MAK21	
YMR084W	FYV14	BNR1	

TF of interest: ACE2
Target genes predicted by AdaFuzzy:

YJR146W
MDJ2
MRPL4
SCW11
PMA1
MSR1
EGT2
TSL1
BMH1
MCR1
YKR040C
YMR262W
YGL007W
FRS2
YHL012W
VID30
ITC1
YCK2
AAD6
YJR149W
EGD2
YML003W
WSC4
SNQ2
GLO1
ZWF1
HMS2
SCP160
PET122
PCL2
YGR283C
HSP150
YNR018W
YDR010C
YAP1
RAS2
AFG2
SHE10
RPA14
MRP8
HOM2
MCM2
YHB1
BUD9
RPS30A
MUM2
ISR1
TPS3
YJL160C
RSP5
CTS1
TPM1
YFR017C
PCL9
NVJ1
LCP5
YER189W
YER078C
PRY3
YKL151C
YGR125W
YRF1-2
YMR135W-A
YDR154C
UTH1
YDR157W
YER152C
ROT2
BAT2
YJL075C
RPS4A
GDH3
OXA1
YPL025C
SAP4
SER3
ICS2
ERG6
SFL1
PSA1
YER079W
GAT1
YML076C
TTR1
RPC53
YFL043C
YDL094C
PCT1
SAM3
RPS18A
YER160C
SPC24
YDL209C
ITR2
FAP1
YPT11
PAC1
YOR186W
YAR066W
SMY1
INP52
YBR266C
WBP1
YIL060W
YIL121W
YOR203W
YER083C
RPS16B
ACN9
TIR1
YBR267W
YLR386W
UTR1
YML003W
MPM1
YER160C
YJR044C
ERG10
YMR278W
CHS6
YOR223W
NDD1
YIL058W
YGL111W
YGL177W

TF of interest: FKH1

Target genes predicted by AdaFuzzy:

PRY3	IFM1	DBF2	MDM1
ALG5	APM3	YHP1	CDS1
IME1	UME1	YDR370C	LSM6
MSH3	RPN11	TOM71	YJR111C
YBR071W	HOG1	FIR1	NUP145
GLO3	PBN1	YPR195C	YDR179W-A
TUB2	SEC28	YNL176C	YIL077C
YPT1	YNL174W	GND1	CDC20
CWP2	RPL39	YKL069W	AAD15
TEM1	DYN1	JSN1	STE12
GSH1	RPL37B	YAR070C	SEC14
PES4	YBR139W	YOL114C	CDC39
BUD9	APE2	RPL20B	SKM1
ESP1	YBL083C	RPN12	YFR018C
SSO2	YLR400W	PTC3	PDS5
FAB1	YPR013C	MKK2	NNF1
YBL081W	PNP1	CDC7	YNG2
YCK3	RPL31B	YKL097C	YNL213C
YMC2	PDS1	ADH4	DEM1
YPT31	MFA2	RNR1	BUB1
NEW1	SUR7	IDI1	HOS3
KIP2	YMR086W	ARP7	YCG1
BUD8	TIF1	ALG1	CLN1
YTA7	OPT1	YGR050C	YPT53
YDR115W	CTS1	YMR144W	CLB4
YLR407W	SUB2	CHA1	YOL106W
TSL1	CTF18	SNF5	YEL043W
BRN1	LEU4	AFR1	YNR029C
SET1	YHR095W	RET2	BIO4
VTI1	RPN10	FHL1	MNN1
UTR2	ACE2	RSP5	HLJ1
MSM1	EXG1	BUD4	KAP95
CLB2	ERS1	ERP3	YNL152W
BAR1	BDF1	GLN1	YKU70
DPP1	RPS22A	GSG1	RPO26
CIK1	HXT1	SCW11	YNL054W-B
SPC24	YPL141C	HAP5	YOR155C
MNT2	ECM33	DIN7	YGL261C
YIL158W	KAR4	PHO11	TRF5
RHO4	TEL2	CMD1	CCL1
ORC6	SVL3	OPY2	SLF1

TF of interest: FKH2
Target genes predicted by AdaFuzzy:

YER078C	CDC7	UBR1	IFM1
SPO12	WHI4	OPT2	SUR7
ALK1	VTI1	YPL025C	PMP1
NUM1	YGR050C	PTR2	YPL142C
ECM33	MND1	CIK1	RIM4
YGL007W	YNL046W	ZWF1	CDC20
KIN3	RRM3	SSF2	MTF2
RIB1	YBR139W	GAT1	YHR159W
HSL7	YAP6	DIN7	SCC4
JSN1	CPA2	YML050W	YDL114W
OSH7	CHS2	EXG1	RPL14A
PRY3	YNL176C	IDI1	HUL5
KGD2	CLN1	MNT2	SMY2
YMR144W	ACE2	KRR1	YCL019W
MYO4	RKI1	RPS1B	GPA1
YKL097C	SMI1	YNL170W	YIM2
MRPL4	ECM2	DBF2	YOL098C
YLR400W	YER189W	GLN1	YHL049C
TIF1	FKH1	YPL141C	UFO1
PDS5	TUB3	CWP2	RPL42B
BNS1	YKR040C	SCO1	SPT3
ADH4	FRS2	CIS3	YMR075C-A
GIC1	YHP1	RPL37B	DIA4
AMD1	SSO2	YCG1	YOR380W
BDF1	MDG1	UTH1	YLR231C
NCE102	YER079W	PGM2	HIP1
PRY1	PLM2	YNL040W	YGR027W-B
NDD1	SRL1	RAX2	GRX3
GDH2	PDS1	TEM1	CBT1
YDR115W	SWI5	ERP3	PRP39
YIL169C	YRF1-2	YNL174W	YLR004C
HOS3	CKS1	PUP3	YPL162C
SDC25	YIL158W	SUT1	YMR304C-A
RPL20B	RNR1	RAD51	YDR338C
ATR1	YGL114W	KIP2	DAN2
SCW11	COT1	BRN1	TUB3
YML053C	EKI1	CTS1	YOL154W
RPS30A	RSP5	PES4	MPM1
CWP1	YGL102C	LTE1	GLO4
HXT1	MRH1	BUD4	YRF1-4
SPC24	ACS1	PHO3	PEX19
YNL058C	YOR246C	YFR017C	CAR1
PMA1	HHF1	HHT1	PTA1
SIM1	APC1	CLB2	YDR067C

PCD1	SSC1	BUD23	EMG1
YDR324C	YDR411C	CST6	PRE3
YLR456W	CTK1	YFR018C	ECM2
PRI2	GCD2	ERG3	PRI2
FYV6	IKS1	RME1	YGL214W
MET6	YHR182W	AAT2	HAP3
GLY1	ELP3	DCP1	CDC42
NOG1	RAD53	YOL154W	NFU1
ADY3	HRP1	MAK11	PUT2
TIF35	BUD5	PWP1	YNL269W
PIR1	YNR053C	YGL047W	NRG1
SEC61	DEG1	POP4	RCE1
YER181C	CCT5	YAR009C	

TF of interest: GCN4
Target genes predicted by AdaFuzzy:

PTC2	ECM17	ILV6	YLL056C
SNZ1	YLR356W	CAF16	SDC1
ARO8	ARG1	HIS4	YML039W
GLT1	MET22	YOR203W	YJR001W
CIT3	YJR018W	MET13	YDR267C
ORT1	YOR302W	ARG8	YOL063C
HIS7	SPO75	YPR038W	YCR015C
ALD5	CYC8	HOM3	MET6
PYC2	STR3	ARG3	YHR219W
TRP4	RAD16	YEL076C	DPH2
YDR341C	ILS1	YHL026C	YCR082W
YCR023C	PCL5	IQG1	YCR101C
YGR269W	CAN1	STT4	YAR069C
STB4	MTO1	SFB2	SFB3
RIM9	ARG2	OSH2	GCN20
ATP7	ADH5	YER053C	YER097W
CIT2	YPR039W	MCK1	YLR297W
CAR1	TEA1	YMR310C	KTR5
PHO8	NPR2	YHR085W	KRE5
MRP7	POP4	YML036W	DCG1
LYS2	HIS1	YML023C	YOR112W
TRP3	ILV5	RVB1	SNF5
YAH1	ODC2	ADE1	YLR410W-A
RIB5	ARG4	YBL071C	VTI1
ARO3	STB1	BUD5	PCT1
ECM18	MCT1	HAL9	TAD3
YHR162W	LYS9	YCR051W	FUS3
CPA1	NNF1	RPL7A	PUF3
DED1	YJR111C	YMR140W	SUT1
ILV1	HIS3	YGR090W	YDL016C
RPC40	ILV3	YJL182C	VID22
TRP2	LEU3	SRB4	COS5
FOL2	SNO1	SAC1	HOM2
UGA3	LYS20	OM45	YTA7
ARO1	ECM40	YMR147W	YJL070C
ARO4	SAM4	YCR062W	YHR173C
PUT3	VAS1	YPR084W	IDP2
BNA1	YBR144C	VMA10	GAL3
HRB1	LEU4	YNL207W	YPL056C
YMC1	LYS14	ECM14	YOR088W
CPA2	MET4	YPT11	FCY22
ORC3	YMC2	PET130	YJL175W
YDL183C	YAP1801	YJR136C	YHC3
DBF20	YER085C	ERP1	CSG2

YHL026C	YLR072W	YKR029C	PET18
YGR161W-B	PLO1	HEM2	SPO73
YDR094W	YAR030C	MUM3	NUP159
RPL25	YIL082W	RPT4	YJL193W
YLR279W	YJR024C	ESC4	REM50
YJR011C	EFT1	NGR1	GUT2
YMR087W	YDL247W	YLR232W	YHR029C
DPB3	MSC2	GPD2	ECM25
ELC1	GRX5	RRS1	YDR034C-A
YBL104C	AAH1	GAL2	GIM4
RPS18A	YDR049W	HXT5	DIN7
YJL118W	ELP4	YAL037W	VPS9
RPL35A	FZO1	YME1	YKL027W
CIN8	CBP4	STE18	PHO87
PMT3	YML050W	MCM6	YGL242C
YJR129C	PRS4	YKR079C	LCB4
ERP4	YOR192C-A	RUD3	YDL026W
YJR039W	GPE1	NEO1	YOR086C
RPL25	YHR177W	MFT1	FAR3
AGE1	TFB3	PRT1	YDR098C-A
YDL027C	PPR1	AAD3	YOR165W
ERF2	PHO85	YDR431W	SRB6
TRX3	RFX1	YJL045W	TAF65
DMC1	RAD16	URE2	RHC18
PET18	YPR158C-D	EGD2	RPS18B
YER184C	YFL046W	YDR109C	RRP9
YOR345C	FIT1	RPS28A	ARA1
NCE101	ETF-BETA	YBL073W	XDJ1
RPL34A	KRE30	YGR046W	SNX4
VAM7	YDR438W	YOR051C	PHO4
MPM1	SRB5	YMR171C	PRR2
SRP1	RUB1	ECM38	
YER010C	BOP3	BCK1	

TF of interest: LEU3
Target genes predicted by AdaFuzzy:

YUR1	IST2	RPS23B	MSR1
RPS14B	ERR2	YOR263C	CHL4
YLR356W	MFA1	PTP1	YBR197C
POL1	RSM27	FUS1	FIG2
YJR018W	HO	YGR011W	YHR177W
PMP1	YPT52	YOL134C	AMA1
VTC1	CHS7	VAN1	PRB1
RPL36A	ARE1	YDR071C	CDC123
MAK32	FUN19	PMP3	YLR247C
GLG1	TNA1	SSE1	GIS3
RPS11B	YOR154W	ERG28	RHK1
YDR042C	YFL030W	RPL4A	YEL041W
RML2	YML023C	HAP1	YMR041C
YCR018C-A	YMR187C	YGR212W	YMR160W
YGR190C	RRS1	YML122C	BFR1
RPL7B	YMR087W	YBR203W	TIM8
DFR1	ADY4	VPS28	BUD26
BUD22	SEN1	YEL077C	FKH2
MRPL24	YIM1	YLR401C	YLR070C
RPA34	YOR263C	CAN1	YPL112C
YER137C	MOT1	YPL150W	YJL068C
YKL118W	EGD2	YGL111W	YGR022C
ERG26	YGR002C	ELP4	YNL326C
YJR100C	YPL064C	KRE29	YGL080W
SKS1	IPL1	PHM7	FCY1
RNR3	KTR4	YLR266C	YGR161W-A
YPR009W	SIP2	YLR169W	KAR4
YDR274C	FUN59	COS4	LSM2
ALF1	SLH1	YLR282C	CDC11
GPD2	YLL055W	YPR081C	ADP1
YNL063W	GDH2	NUP188	PRT1
ZPR1	MUP3	UGA1	YMR103C
NIP100	YBR242W	CLN1	YIL077C
MGM101	YLR054C	YFL043C	MET6
URA6	YGL149W	YIL152W	YOL017W
YFL054C	RER1	NIP1	SHE3
YMR254C	PPA2	HMS2	YKL076C
SIR2	CUS1	CAF40	PCK1
AFR1	DIG2	THI13	YFR048W
NCA2	OCH1	PLO2	YGR090W
RPL21A	YDR063W	YNL018C	RHO1
PSE1	FIG1	AAD6	YLR063W
RPL42B	YKL066W	MEF2	FUN34
YDL186W	VPS33	YLR143W	YLL029W

YSA1	NCL1	YER181C	YHM2
YBR144C	YBR147W	PCL10	YNR009W
YHR151C	AMD1	UBP7	YLL023C
UNG1	YER034W	YKL162C	YKL153W
YLR407W	RRN10	SDH2	BRE5
UFD1	YKR078W	SPS2	ERR2
YJL065C	SCO2	CBT1	YBR075W
YDR131C	IMG1	VMA21	YDR063W
MCD1	ERG25	RAD51	HTZ1
MST1	YAL048C	CLB5	YAL065C
YGR122W	RHO3	SSP120	IMH1
YGR038C-B	YDL033C	PEX21	DCP1
CDC10	YMR074C	MPS1	YGR110W
YPR116W	ROM2	YFR057W	AIP1
STE12	YKL099C	PDX3	RSM27
YNL026W	DBR1	YIP1	VPS28
PEP5	YIL130W	YPK2	NIT1
RPL3	RPS23A	RPS7B	ANB1
KRE25	YBL109W	BPH1	YBL113C
YJR029W	YOR055W	YOR392W	HKR1
YER007C-A	YPL251W	UNG1	CAF40
RPS28A	VMA4	OSH2	YCR102C
YOL117W	YMR291W	GLC8	

TF of interest: MBP1
Target genes predicted by AdaFuzzy:

TPM1	MNN9	ELO1	MOT3
OCH1	YRF1-2	SCW10	POT1
GIC1	TSL1	PUP3	YHL012W
VTC1	RNR4	RPL37B	CLB2
SCP160	CIN8	SEN34	RAD53
SMC3	NDD1	MCD1	YFR017C
RNR1	TSA1	MSH6	SPO12
MNN5	YJL045W	YCR064C	NUD1
PCF11	YDR115W	HLR1	RPL9A
MCR1	YOX1	YMR144W	FKS3
YMR304C-A	YDR526C	MRP8	YOR246C
RAD51	YJR030C	PDS5	GIN4
CRH1	AFG2	RPL7B	YPL056C
YER079W	HTA2	NCA3	NVJ1
PDR16	YLR104W	SWI4	NPL6
HXT10	YLR049C	VPS8	SWI6
GDH3	CLA4	MOT1	YPL158C
RFA2	RAD27	CLN1	RPL13B
YER071C	ASF1	CDC45	GEA1
NAM2	CDC21	RPS22B	ERF2
YNL313C	SHO1	EGD2	PAI3
SPO73	HCM1	RPS9A	ARF3
UFE1	YMR279C	TAL1	VPS5
RSP5	GIC2	CWP2	SPP41
TOS1	SIM1	RRD2	IOC3
YNL089C	IRR1	RPA34	ERG11
MRC1	DIN7	PLM2	TRS23
YER189W	PDS1	YML003W	SWM1
SCS2	LSM4	YHP1	YDL237W
OPY2	YDR509W	SRL1	YOL162W
REG2	CDC55	TOS2	YPL063W
ABF1	CLB5	RPL21A	DBP2
DUN1	YKL151C	VTC3	RSM10
ECM33	EXG1	YBR190W	YMR135W-A
YER078C	SWE1	BUD31	MET32
YNR018W	RFA1	HTB2	THS1
CLB6	YCK2	JEM1	YOL163W
SEN2	MRPL4	CDC7	CDC48
MCM2	YKL097C	AGA1	YLR042C
HO	ZWF1	BUD9	YLR193C
BUB1	CDC6	ERP3	ZDS1
PCL1	TOF1	SKI7	YER010C
YEL077C	SPT21	YBR071W	YKL034W
YGR125W	YJL185C	GLO1	YPL109C

YPL185W	GIP1	CIN1	YOR147W
YLL058W	TFA1	RAD53	YGR161W-B
YNL303W	FUN36	RPS24A	YNR062C
YGL041C	YMR111C	GCV1	GAL3
UBP9	YLR073C	YGR117C	HSP104
SET1	YGR127W	RPN9	GPD2
ZRC1	TUB2	YBL059C-A	MAD1
QDR1	YBR116C	SCC2	SBP1
YPR109W	YML100W-A	VAC8	YHR035W
RPS19A	ISU2	PZF1	SRY1
GUP1	YMR119W	SEC72	PHO2
YDR165W	YKR040C	NAT1	PWP2
YOL162W	RPL7B	POP2	YDL199C
STI1	YGR058W	SWP1	PFS1
BUD31	YCL044C	ERB1	YIL163C
YMR320W	YJL037W	CYT2	YML018C
GLT1	SSS1	ATF2	SCM4
YLR001C	HEM14	KAP114	PMC1
YNL194C	DEP1	PGS1	YBR033W
YDR360W	SPO1	TFC6	MEX67
YBL094C	PBI2	RNQ1	PRP5
ORC5	YAR068W	YMR315W	YPL183C
YBR022W	YDL076C	YBR206W	RIB3
YMR204C	YBR273C	YKR041W	PGS1
IME4	SHG1	RAD4	YMR132C
DOG2	YKR075C	YJR029W	NGL2
YDR255C	BCK1	YPR050C	MET7
YPL166W	YJL075C	YML005W	APG10
APG9	YLR252W	YHL008C	SYS1
SLN1	YOL075C	YCR062W	HNM1
YOL138C	YBL004W	PUT2	SEC24
BUD16	HMI1	TFA1	SPO22
YCK1	YDJ1	DIG2	YKR065C
NUT2	CDC4	CDC19	MLH1
VPS16	YML100W-A	YJR149W	

TF of interest: MCM1
Target genes predicted by AdaFuzzy:

YJL160C	PIR1	KGD2	SMD3
BUD4	DBF2	PLB3	GCN5
SFB3	SAC7	UTR2	MCR1
LSM2	ELO1	ACE2	CTP1
HSP150	CLN3	LSM4	GCD14
STE2	PCL1	YNL058C	STP22
SUR7	PDE2	CPR8	PRB1
PIS1	KIN3	FRQ1	YDR131C
YPL044C	YML053C	TCM62	YBL098W
UFD1	MFA1	YEL045C	ADE2
HLR1	YNR063W	HTZ1	HSL7
FDH1	MFA2	SCO1	OM45
BUD9	CDC6	GLY1	YLR003C
YJL049W	BET3	EKI1	YGR039W
ZDS2	SWI4	YFR041C	BET1
NFU1	YMR253C	YBT1	RAP1
MCM6	PIG1	TDH1	LEM3
YGR168C	CLA4	YEL016C	YFR024C-A
YBL059W	YDR179W-A	YOR102W	BUD16
TFC4	CDC19	GRR1	SMI1
MSG5	YDR042C	YIM2	YDR078C
MTF2	SEC28	RIM101	PPA2
STB6	IES6	CTF13	TBS1
YPT6	SEN2	YML117W-A	YCL045C
SPO12	COQ5	YFL057C	MEI5
MRD1	YOR389W	CHK1	YKR029C
SIM1	YHP1	RPL10	YOR012W
TRA1	PIR3	YIL141W	YIP1
PTM1	YLR302C	AFT2	MUP1
YKR040C	HIP1	PMR1	EPL1
CDC20	UTH1	HUG1	YCL005W
RPS24B	TOA2	RPI1	YLR236C
CIS3	JSN1	HXT13	RPL21B
YPL276W	YIL077C	MUC1	LAC1
MSC6	AGA2	YLR031W	YLR386W
CLB2	MET1	YIL019W	YPL030W
RPA34	CHS2	STP22	YKT6
BAR1	YDR278C	TIF5	PUT4
AGA1	SWI5	PHM6	VPS16
ARO8	RAX2	GRX1	SPB4
GPA1	NUM1	COS5	NAN1
ERG28	YNR062C	YDR066C	MDJ2
HCM1	YIL158W	PRP45	OSM1
YPL278C	YPL277C	HCS1	YNR020C

BET5	CET1	LST4	MDS3
TRS33	SCM4	TRM2	ELP3
GPM1	YCR006C	YBL073W	RET2
ECM30	WTM1	YCL044C	GAL7
YBL073W	PDX1	GCD14	GPM1
YMR315W	ENA1	FIT2	BOP2
SWI5	CDC123	HYP2	YMR191W
LAP4	YBR016W	YLR280C	MMT2
RPL43B	NTC20	YBR178W	YFR042W
MET10	YJR136C	IMD1	YMR313C
VPS9	FIT2	TES1	OXA1
YPL197C	YDR093W	YSH1	YOR283W
UFD4	RPP1A	YGR259C	STE23
YAT2	PRM9	ERG1	GAT1
LSM2	YNL008C	YGR111W	PAN2
YMR304C-A	YMR098C	YMR279C	RIM9
YAL048C	YOL157C	YLR031W	YDR199W
FCY21	PAU2	HLR1	GPI16
PHD1	YLR049C	PHO85	YGR081C
SFA1	RPG1	YGR182C	XRS2
YIL003W	APC11	CAF130	URA8
PPH22	RAI1	ITC1	YOL022C
PFY1	YIL163C	UAF30	YIL169C
PHD1	YBR267W	YIL127C	ASP3B
YKR035C	CDC91	ASG7	YDR271C
YJR087W	OSM1	YNL207W	SKI3
AHC1	YJL218W	YDL146W	FUN31
TPK1	NIP29	YDR319C	YLR051C
SIP18	HNT1	HEM1	LYS9
TIR4	BOP2	(FUN34)	ERC1
ERP3	MSB4	AUS1	TOS11
DCD1	YGL232W	FLO9	CDC6
TMT1	PGI1	PEX22	YPL257W
BUD21	RFA1	YOL073C	SSK1
RPS9B	ERV46	VAC7	YLR361C
ADE16	YFL027C	SLG1	TFC8
YGL224C	SXM1	RPS9A	RPL2B
YER085C	YFR018C	YAR061W	YLR161W
KKQ8	SIR1	YDR230W	GGA1
VCX1	PGM1	PIF1	AAT1
YDL146W	NCB2	SPE3	YCR051W
RPS6A	YGR237C	MNN4	STE2
SUA5	YGL185C	YKL118W	SFT1
YDR275W	RNH1	COX11	YDR179C
PYC2	CLB6	PPZ1	GLO3
YOR343C-A	YHR085W	HUL5	PAC11

UGP1	GAT4	YPT52	SOR1
YKL086W	HIR1	YDL246C	YIL092W
RAX2	IST3	YHR009C	YPD1
SSA1	ARO4	YOR227W	
SPR28	MAK10	YOL157C	

TF of interest: NDD1
Target genes predicted by AdaFuzzy:

CLB2
(MLP1)
CHC1
YHR054C
YDR154C
PRE6
URA4
UME6
SED5
ERG13
YBL070C
YJR024C
YAT2
ASG7
RPL43A
URK1
PET127
CLP1
YFR003C
SPP2
MNN1
MNN10
YDR210W-D
HMS1
YHR214C-C
YGR212W
DIS3
YDR327W
YOR258W
YIL086C
SNF11
YER079W
YJR023C
RPL9B
CCH1
YRO2
YPR128C
YJR037W
YOL092W
STE13
ERV25
HYS2
YHR199C
YIP1
RPL32
YDR384C
YCL002C
ADE12
YHL009W-A
YBR085C-A
YGL050W
RKI1
YPL068C
YHR100C
RPL3
YIR040C
YOR322C
AFG1
CBF1
YEL018W

TF of interest: RAP1
Target genes predicted by AdaFuzzy:

RPS18B	YCL065W	RPS6A	YDR186C
YRF1-7	RPS25A	ALD4	RPS17B
RPS4A	RPL13B	DPB11	YDR543C
FUN19	YML089C	YEL074W	YFL034W
RPS26B	YPL056C	YGL072C	RPL16B
YHL049C	YGR117C	RPS7B	RPL31A
HMLALPHA1	YNL010W	DUR3	SEC10
RPL17B	RPS23B	YOR387C	RPL20B
RPL1B	RPS10B	YGR283C	RPL41A
RPL20A	SIP4	YKL097C	PRO1
PTK2	PLM2	MATALPHA1	RAP1
APS2	HMS2	YAP6	ISR1
RPS2	YOR268C	TEM1	YOR338W
YPL080C	YDR544C	UBC11	IDP2
HMS1	SGT1	TYE7	FRS2
YDL133W	RPL23B	YOR302W	YBL111C
SNF4	RPL21A	RPL25	YGR149W
CPA1	YNL144C	PGI1	RPS27B
MRE11	YEL023C	LCB3	PRY1
RKI1	RPL39	SGV1	MRPL15
RPS15	HSP12	MATALPHA2	RPL16A
MON1	RPL34A	RPL36A	BDF1
YGR050C	TSR1	DDR2	CCA1
YGL188C	TEF1	RPS12	CRZ1
YMR144W	PFK2	CLN3	GPD2
YMR265C	GEA2	YNL338W	SEC59
RAD2	PRY3	EDC2	FBA1
FOB1	YJR146W	SFT1	CCW14
RPS0A	UFO1	RPL17A	RPL12A
GPM1	YML6	PMR1	YEL075C
TKL2	RPL26B	AAC3	RPL5
TDH3	YLR463C	LEE1	CFT1
GPD1	HXT5	MSR1	COT1
APL4	RPP1B	RPS24B	YFL015C
YKL151C	YER130C	YJL135W	YNL165W
HOM6	YOR342C	KTR5	CTS1
YBL029W	SWI1	YOR292C	STB1
KES1	RPS22A	HIS4	SRO9
RPL42B	CBP2	YLR312C	RPS1A
BCK2	YOR343C	GPR1	NTC20
PFK27	YRF1-5	RPL24A	PIM1
HMLALPHA2	RPS31	RPL35B	YNR018W
SST2	RPS6B	RPS20	RPL6B
RPS25B	ENB1	YIL057C	RPL14B

YLR326W	BNI5	RPS19B	RPS30B
RPL12B	TEF2	ACS1	TEF4
YIL177C	AST2	MLP2	RPS1B
UBP2	FYV12	RPS30A	RPL9B
RPL1A	RPL6A	SCP1	YRF1-1
AZF1	RPS8B	SLX8	NCE102
RPS16A	ERG5	RPL18A	RPL40A
RPS10A	YML133C	YLR407W	YFL064C
YDR109C	YPR148C	RPL35A	RNR2
MRS3	KCC4	RPL21B	RPS14B
FIR1	RGS2	RPP0	CAF40
RPL37B	YLR112W	ADH1	YHR033W
RPL24B	RPL28	RPL38	OPI3
RPS19A	FPS1	MPS2	ZEO1
YJL225C	YRF1-4	SSF2	RPL2B
RPL26A	PDR12	UGO1	SHE9
YIR016W	VMA6	RPS29B	MDJ1
CWP2	GCN4	YLR400W	RPL33B
CSR2	RPL8B	PPH22	YER158C
RPL9A	TIM8	RPS8A	DNM1
MCR1	FKH2	RPL13A	RPS7A
RPL43A	RPL23A	YHR122W	SMP1
SKN1	GIM3	YLL037W	NAT3
YGL074C	RPS13	AGP1	RTA1
RPL19A	RPC40	YDL186W	CCT6
STE23	RHR2	RNR4	PMP1
RPS21B	RPL37A	RPP2A	GAT1
HAP1	RRP5	EPL1	RPL33A
RPS18A	RPL14A	DBF20	SRP40
CHS7	RPS24A	ANP1	TOS4
RIA1	RPS26A	YEL008W	YLR184W
RPS23A	RPL32	YCG1	RPS9B
DOT6	YNL190W	RPL42A	YLR255C
YDL032W	YLR465C	YRF1-6	RPL27B
CRC1	RAS1	YJL027C	CUP2
YNL011C	YIL012W	YNL337W	ADA2
ASC1	RPS29A	GSC2	FAS1
RSN1	YOR365C	PDR11	RPL31B
PDC1	PEX6	RPS0B	RPS17A
RPS4B	RPL30	YLR402W	RPH1
YBR190W	YCR025C	ECM12	ECM19
RPL40B	RPS16B	RPL2A	PEP5
RPS27A	YBL081W	YPT31	NMD5
SGM1	MOG1	RPL11A	RPL22A
UBI4	POP5	FAA1	RPS11A
FYV1	SPO73	PRE2	PAC1

ROX3	ERD2	YOR170W	YBL029W
MSN4	KCS1	ORC2	AAD3
HSF1	MRPL23	YJL075C	YKL075C
TOS1	YDL046W	FOX2	YLL055W
MHP1	HST3	CBC2	YNL140C
PRE10	YOR059C	ACS2	THI6
ZAP1	YOR243C	SDL1	HSP78
BBC1	YAR009C	YDL062W	YIL130W
SWI4	IES2	UBP5	UBC11
PDI1	YDR186C	SFB2	DOG2
VPS38	YBL095W	YMR265C	YFL061W
LUC7	YDR071C	HIR1	HEM14
YCR090C	YKL031W	RPL18A	TAT2
HTM1	YIP3	YOR286W	TEM1
MSN4	DBP2	RPN13	CDC19
YPL261C	RTG2	YBR030W	RPL37A
LST4	CLU1	DPB11	AAD14
ISW1	YGL138C	PSP2	YLR331C
YGR068C	ERG3	HHF1	YGL165C
FUS3	YIL082W-A	MAK10	IQG1
YIL113W	RPB7	YGL050W	YJR114W
BOP1	SPS100	YDR094W	RPT1
MED1	YLR199C	YIL087C	MPS1
SEC31	YOL015W	YOR379C	YJL175W
RPS8B	ERG5	SPT7	AAD14
APL4	ARG82	YER158C	ASP3B
YDJ1	MRPL13	THI12	YDL115C
GTT2	POL1	YOR227W	RSM26
PHM7	ELP6	YNL179C	YPL191C
YLR437C	MAK10	YBL036C	SHG1
ULP1	YGL220W	TSC10	CDC23
YPR053C	NDH1	ULA1	ESBP6
CKS1	YER189W	YJL207C	TPS2
YBL059C-A	FLO5	FAT1	SPC2
MAC1	HST1	YNL181W	SPO16
MSS51	RPN7	YMR130W	YNR022C
TPM2	YLR046C	CDC10	YKL070W
CYT2	YDR153C	YNL174W	BNI5
HMLALPHA2	SEC66	MPT1	ALD5
YNR022C	YML013C-A	NAS6	CDC11
BTN2	RPL27B	SNU66	YLR257W
TYE7	YLR352W	SNU23	YDR179W-A
RIB2	COQ3	YIL091C	YDR278C
PRP16	ALD2	CCT5	LUV1
CIT2	YOR268C	TRX1	AXL1
SPS1	YEL015W	YBR095C	MAK10

SYF2	ECM22	YJL103C	YEN1
DAL3	PAU7	FUN50	HXT3
YFR032C	ERP1	LSC1	SIP18
YMR031W-A	DAP2	PCL10	YHL035C
YIL157C	PAN2	CTA1	YJL215C
YLR122C	GOG5	BUR6	PEX12
YDR338C	YEL015W	YPL185W	NCA2
YGR243W	ERG11	TUB4	YDR029W
APC4	ECM8	YER140W	SGN1
TOF2	MRPL40	YMR31	DAL3
LCP5	HHT1	HTM1	GAR1
YCL049C	ECM27	YOR218C	NUP192
ZRG8	NUP85	ARC1	NUT2
DDR2	YCL012W	YGR141W	PAF1
YMR278W	YDR482C	YNL087W	FUN59
YGR081C	TDH3	CDA1	YPR126C
HIF1	HXT1	YAL037W	YPL186C
YGL214W	IMP4	YCL019W	MEP2
DNA2	SPT20	FRDS1	ARC40
SBH2	YPL158C	HVG1	YHR052W
SPS100	SIR2	PTC1	UFD1
NTH2	RAD9	TRL1	YPR127W
YBR262C	MSH3	BNR1	BEM3
YGL193C	FYV2	YJR119C	YAR064W
YDR339C	RSC3	ELP3	HXT12
TRS130	YER166W	CDC28	GCD1
PRM10	YGL198W	MAL33	MGT1
KTR4	YLR021W	MRPL17	GEA2
YHB1	MET17	RPL17B	YDL124W
SRB4	YPR083W	NAS6	TAF65
YMR291W	RPF1	FAD1	RPL1B
YOR142W-B	NGL1	YLR294C	YCR050C
DIA2	PCF11	EDC2	YDR417C
CDC40	YAP1802	REC107	YOL131W
YHR218W	SBA1	YBL104C	YNL165W
POL12	ALG5	YPL261C	MSS4
YPT53	YBR052C	YTA6	YGR263C
MRP4	YBR174C	HUL4	GAL83
YLR101C	YOR322C	APC2	SDC25
YPR045C	OGG1	YOL103W-A	SUR4
YGR165W	YOL157C	YOR121C	HIS7
CPR3	RPL9B	ALG1	YGR250C
YIL041W	RVB2	YBR216C	PHO8
BAT1	HGH1	YDR261W-B	BEM4
UBC8	NOP10	DAL5	SCM4
YDR098C-B	YER034W	RAM2	GNA1

YCR006C	HTA1	ECM22	SMM1
YLR232W	YIL165C	YBR168W	AZF1
BCK1	RPL22A	POP7	YLR091W
ICL1	YPL035C	YBT1	SEO1
YMR157C	YDR090C	YHL049C	CAD1
SHD7	YOR051C	OYE2	YBR101C
YPL280W	LSM4	YBR267W	CBP1
COS10	ACP1	YHL045W	ISU2
COX20	RPL13A	YDL009C	ECM9
YNL143C	APL1	PZF1	ZRT2
YIL087C	RIF1	ERO1	YDL204W
YDL086W	YGR115C	SIM1	HOR7
YGR190C	YLR410W-A	PSA1	SPI1
RIM2	YBL059C-A	YMR265C	DIA2
KRE5	PIG1	YPL278C	YNL253W
BRR1	YOR225W	ALG8	RTT101
ALO1	YIL028W	YOR146W	YPR045C
RIP1	YOR068C	YCF1	RPA12
CDC73	SAP30	SPB4	RPT3
YCL036W	YIL015C-A	YOL138C	MSW1
YPR013C	GIN11	YIL105C	YBL005W-B
BUD8	TAF19	PZF1	YGL223C
YDL011C	YDR493W	PET122	NOP12
YBR287W	RPL34B	YGR042W	SPR6
RER2	RDH54	CIK1	CDA2
YKL179C	AAT2	MAK11	FOB1
RTG1	HTA2	LST4	YFL040W
YNL043C	REX3	YBR232C	YGR117C
YPR202W	HSP78	BSD2	YLR256W-A
YLL056C	FBP26	YDR274C	GPM1
ESP1	YIR035C	YOR251C	YJL195C
HTB2	PEX12	YLR408C	ARA1
PSP1	YML040W	YLR003C	YNL101W
SIR3	YMR119W	YBR095C	YBL081W
YHR121W	EGD2	SET2	RPL6B
RLI1	SCS3	YFR012W	YOR225W
SOR1	URA6	YDR537C	SEC35
TIF1	YOR015W	RHO2	CDC45
YNL310C	HAP5	RPB8	CYB2
YIL165C	YLR118C	YMC2	YNL146W
FIT3	MPP10	YKL084W	YLR157C-A
STD1	ZMS1	NTH1	YML040W
YJR030C	TGL2	YPR142C	FOL1
MOG1	YDL166C	CDC8	RPL24B
ECM7	SIR1	ATP2	YBR074W
NBP1	YLL067C	MSN2	BUD23

RPS4A	HIS2	YJL075C	TRS31
MDH2	YKR040C	YKL097C	APG2
YJR096W	YEL023C	YOL159C	CAT8
YOR324C	CIK1	PRM6	YPR100W
YCL034W	YDR428C	TSC3	YCR105W
YOR050C	YAL004W	HEM4	AEP1
TIR4	ATS1	YEL074W	YLR365W
YOR166C	YPL033C	DHR2	TCI1
YBR235W	SCO2	COX12	YGR024C
YPT32	ENT3	YNR024W	NAS6
TFB3	BUD26	YKL033W	SPC34
WRS1	TPS2	YBL059C-A	SWM1
RPO21	DUS1	YOL054W	YDR131C
NFI1	SEC61	YAH1	YOR380W
CHL4	MEP2	YOL015W	MTL1
ZEO1	GEA1	PRS3	PGI1
YDR336W	YNL213C	YUR1	YDR267C
STE23	SRY1	YLR279W	YAK1
YDR387C	HXT8	RPL6A	
SWD3	YGR226C	RPC19	
YHR122W	YKR007W	SFL1	

TF of interest: REB1
Target genes predicted by AdaFuzzy:

YOR093C	VPS13	UBR1	ARL1
BPH1	SMP3	GLN3	RGM1
SUI2	ADE2	MET1	YGL182C
YGR079W	YNL171C	SHP1	YAP1802
YIL108W	ASK1	SNT309	PPH22
YER156C	CLB2	YDR157W	PUS1
NUT1	YKR015C	YGL193C	TUF1
YMR122W-A	PIB1	NHP6A	EPL1
PRE4	FMT1	YBL059C-A	SIN3
GCV2	SEC61	RPB11	YGL088W
YLR224W	SPC24	EMP24	CCL1
GFA1	MRPL23	PAC10	POL2
STB6	MRP10	UFE1	MLS1
PEP7	RPA14	ATH1	PSD1
ARF3	YPR099C	ABD1	PRE3
ABC1	MAM1	YNR040W	CDC5
PRP22	CYB5	IFH1	CAT8
RRN6	ISA2	TEF4	PRP5
SCJ1	RAT1	TIF3	TIM11
TIM44	COX14	RMT2	YBR096W
NUP157	HVG1	YOR318C	CBK1
SMX3	PRP2	TOA2	YDL186W
UBC8	YEL023C	YML119W	HAP2
CIS3	MF(ALPHA)2	SHR3	YJR087W
COX13	OST1	TOP1	RHO2
GCS1	RPO31	EMP70	GPI12
YGR235C	HSH49	SKI7	TKL1
RPO21	NAS6	HRR25	MRPL10
PHO91	YPL182C	YIR003W	GRR1
YNL115C	YTM1	APS1	LIP5
NDI1	DCP2	SNC1	TOR2
YDR154C	CDC50	ELO1	YGR012W
RPN2	YGL152C	CSE1	URK1
ARF2	MPH1	ALA1	YPR076W
YJR085C	YPR053C	SEC24	SUB1
NUP116	SEC8	CDC21	HOM2
RTF1	KRE5	SHS1	SRP21
YUH1	SDH1	SPP2	GOS1
NAT2	ATP4	RPB9	CSM3
RPL15B	YIF1	BET3	SEC4
YJR098C	RPC19	VMA10	RPA34
SEC23	YPT7	MLP2	YJR100C
HSC82	RPT5	GEA2	ZRG17
PHB2	CSR1	GTS1	RPL40A

APL4	YCR050C	SSN3	YKL027W
STE18	LSM2	ZRG17	YDR306C
PFY1	PTP1	YDL038C	YOR055W
PRP43	NUT1	PDS1	CAD1
AKR2	RTG3	YNL205C	GPI13
YGR164W	TPP1	KRE11	CUP5
YGR149W	SKN7	YOR223W	SNF2
YLR053C	RIM4	CKI1	RPL37A
RPL26A	LRE1	MCM3	HEM3
TUB2	RPB11	YNR014W	PUF6

TF of interest: STB1
Target genes predicted by AdaFuzzy:

YKL097C	YHR133C	CLN3	YFL042C
ELC1	POL30	MUS81	THI20
FUN26	URA4	YJR160C	RTF1
YMR306C-A	YCR102C	HOC1	YME1
GIC2	YHR149C	NGL2	YHL013C
YGR277C	PRE6	CDC42	TAH11
SPO16	ADP1	PAU4	YJL184W
GIC1	MEP2	YMR034C	LOT5
NDD1	PRS3	YIR036C	WTM2
TOS2	YLR236C	ADK2	SKN1
RPL37B	YLR023C	SIR2	YPR011C
YFR011C	YAR047C	YJR116W	YGR015C
SNO3	GAP1	URA6	YDR198C
YOR246C	PPE1	ZRG17	APL2
CLN2	YBL112C	RSE1	YML058C-A
CLB6	EST2	HXT2	ILV3
CSI2	YAF9	REF2	YMR009W
YPL056C	YDR152W	YGL081W	YOR305W
HSL1	ERR1	YDR063W	ORT1
MDM1	TAF60	YMR209C	VTC1
YNR065C	BUR6	YKL146W	HAT1
FLX1	YML107C	YMR226C	SIR4
YDR179W-A	YPL114W	SPB4	SPS1
MCM16	MDM12	YCL065W	XPT1
SIT1	YIL058W	YKR018C	RGT2
YIL127C	GUT1	YCL002C	YDR455C
YBL112C	MEP3	END3	NTF2
ARF2	YER139C	YMR031W-A	SDS23
PPS1	YFR006W	LSM2	YDL133W
MFA2	IMP3	MRP1	RAD55
HMO1	PFS1	SEN1	YER093C
YGL109W	DAL3	MET30	SHE10
SEC53	YDR229W	ICS3	SRB7
YCR049C	YJL103C	YGR011W	APG12
YPL136W	VPS17	YLR031W	INP53
YDR327W	SIR4	RPS24B	

TF of interest: SWI4
Target genes predicted by AdaFuzzy:

YKL097C	YHR133C	CLN3	YFL042C
ELC1	POL30	MUS81	THI20
FUN26	URA4	YJR160C	RTF1
YMR306C-A	YCR102C	HOC1	YME1
GIC2	YHR149C	NGL2	YHL013C
YGR277C	PRE6	CDC42	TAH11
SPO16	ADP1	PAU4	YJL184W
GIC1	MEP2	YMR034C	LOT5
NDD1	PRS3	YIR036C	WTM2
TOS2	YLR236C	ADK2	SKN1
RPL37B	YLR023C	SIR2	YPR011C
YFR011C	YAR047C	YJR116W	YGR015C
SNO3	GAP1	URA6	YDR198C
YOR246C	PPE1	ZRG17	APL2
CLN2	YBL112C	RSE1	YML058C-A
CLB6	EST2	HXT2	ILV3
CSI2	YAF9	REF2	YMR009W
YPL056C	YDR152W	YGL081W	YOR305W
HSL1	ERR1	YDR063W	ORT1
MDM1	TAF60	YMR209C	VTC1
YNR065C	BUR6	YKL146W	HAT1
FLX1	YML107C	YMR226C	SIR4
YDR179W-A	YPL114W	SPB4	SPS1
MCM16	MDM12	YCL065W	XPT1
SIT1	YIL058W	YKR018C	RGT2
YIL127C	GUT1	YCL002C	YDR455C
YBL112C	MEP3	END3	NTF2
ARF2	YER139C	YMR031W-A	SDS23
PPS1	YFR006W	LSM2	YDL133W
MFA2	IMP3	MRP1	RAD55
HMO1	PFS1	SEN1	YER093C
YGL109W	DAL3	MET30	SHE10
SEC53	YDR229W	ICS3	SRB7
YCR049C	YJL103C	YGR011W	APG12
YPL136W	VPS17	YLR031W	INP53
YDR327W	SIR4	RPS24B	

TF of interest: SWI5
Target genes predicted by AdaFuzzy:

YDR112W	CTS1	BOS1	ECM4
DLD1	DBP9	YFL064C	YGR257C
YER079W	VID30	BUL2	PAU3
YNL337W	MSR1	YAL018C	SMX3
MFA2	PRY3	YDL119C	MTW1
CYK3	PIR1	HOR7	PCM1
PCL9	YPL158C	FRS2	RPS29A
YRF1-7	AYT1	PIR3	GAA1
PUT4	YHR138C	YDL173W	YHR078W
YDL114W	AST1	GAT1	ACF2
MDJ2	TSL1	TPM1	YBR022W
YRF1-2	SIC1	ICS2	INP51
SFL1	RPL31B	EXG1	RPS2
MRPL4	YER078C	YLR407W	YNL224C
ASH1	YLR194C	ERG25	AAD15
FAA3	CRH1	YLR049C	VTC3
BNI1	YNR018W	YMR173W-A	EMP47
RPL2A	YDL032W	YML131W	YOR287C
MTR2	YFL063W	YGL108C	YCR001W
YMR262W	DDR48	HRD3	MES1
CHS1	LOT6	YMR172C-A	YLR434C
ZWF1	HXT3	YLR346C	YDR014W
YLR012C	DFR1	YJL160C	YBR178W
YBR071W	PST1	GPR1	YCR072C
VTH1	YHB1	CDC34	RVS161
YFR017C	SCP160	YGL101W	YTA7
DPM1	SCW11	YER064C	YPR118W
SEN2	KEX2	GLC7	YKL160W
PCL2	DJP1	HSH155	YER159C-A
YGL140C	EGT2	MRL1	FBP1
GAT3	TAO3	YLR218C	YLR076C
YMR135W-A	MMF1	NOP8	YOR365C
COS1	SHS1	YOL162W	YDR333C
YER189W	YJL225C	YKL097C	MRS5
YRF1-3	MET18	YEL067C	TAF65
GCS1	BUD9	YGR154C	TPI1
YDR543C	ENO1	PRP40	YLR392C
MNN9	YNL324W	YBR056W	YBR197C
YBL108W	OST5	RAD54	NUP170
TEC1	FUN26	GIS2	REX4
YEL077C	NCB2	PRY1	YKU70
YFL065C	WHI4	SRB4	CWH43
PRP9	HSP150	GRX4	YDR339C
PCL7	TPS3	YNR004W	DCC1

CKA2	YBL049W	PWP2	ZRT2
YBL048W	YLR455W	YGR126W	YMR316C-B
SOM1	YMR172C-A	FTH1	YEL033W
YLR053C	PHO13	YOR343C	YDR415C
YIL141W	LEU1	YLL023C	BEM2
YMR188C	YGL146C	YPR094W	YNL099C
YDR261W-A	YDR210W-D	PUT4	CUP1-2
IXR1	YML023C	RPE1	YCR087W
YIL083C	YKL133C	YBR071W	YHR033W
SRO7	RPL14A	PIF1	TYE7
YGL098W	RPS9A	PBP2	PMU1
YDR306C	MRP21	YJR096W	YNL205C
DBR1	KRE21	PCD1	YFR056C
MAD3	MKT1	ZEO1	CIT2
STE2	SSP120	CDC45	YBR266C
YHL029C	YPR115W	GPX2	YKL099C
RPS21B	YPL216W	SAD1	UFD1
NOP13	YMR265C	GLN4	TOF1
FYV5	ADP1	YMR160W	NRG2
BFR2	PAN6	YKR071C	

TF of interest: SWI6
Target genes predicted by AdaFuzzy:

YOR246C	ELO1	MCR1	YHR049C-A
ERP3	RPL37B	SCW10	MOT3
FKS1	ECM33	YCR101C	IFM1
PDS1	PCL2	YMR172C-A	NAM2
YKL066W	PCL1	YJR100C	YKL086W
SWI4	TOS3	POR2	YPL246C
TOK1	CLB5	MLP1	YOR238W
UTR2	HCM1	YPR105C	YDL089W
GIN4	LAC1	RPL11B	SYG1
RPL18B	MNN1	YER119C-A	YFR026C
PUP3	RNR4	NAB2	YDL096C
YER078C	OCH1	TOA1	MPS2
MNN5	YJR054W	TOP2	YGR110W
RNR1	SWE1	YDR482C	SLG1
PDS5	IRR1	YLR266C	YDR179W-A
TOS6	RAD27	RPL32	YCR024C
YHP1	CLB6	SEC8	YBL005W-B
HO	YOX1	GPM2	YNL226W
SIM1	FKS3	ARF1	NTF2
PLM2	SVS1	VPS20	YGR052W
YOL114C	TOS2	YGL121C	MID1
OPY2	GAS1	CNA1	LTV1
YMR306C-A	BBP1	MATALPHA1	TEP1
CWP2	NDD1	LAP4	YJL043W
CDC21	DUN1	BDF2	YHR196W
SMC3	CLN1	POL5	STR3
MRPL4	GIC1	KKQ8	RAD16
YPT52	CDC6	GPE2	SUR7
YJL185C	YNL089C	CCT2	YBL005W-B
YNL313C	SNU71	RPO41	PPE1
SRL1	SOK2	GCD11	NFI1
YDL211C	MNN4	YJR015W	FUN36
PRY2	GRH1	NIT2	YJL123C
DIN7	YOR343C	YBR174C	HAS1
CCW12	YBR012W-B	APS3	CDC73
TYE7	YKL086W	ARE2	SCC2
SCW10	YLR412W	YLR320W	PLO1
BUB1	GCR1	RMS1	RFC1
HHO1	YPR137C-B	HSP82	RPL6A
CRH1	ARO1	IPL1	YCR041W
CLN2	CCR4	SPS4	NUP1
GIC2	YDL072C	ATF2	YOR322C
GAT2	YDR078C	FRE1	SNU66
CDC45	YKL177W	YNR025C	SDH1

YHL049C	YDL109C	TIR1	CCT6
YCR033W	YPL071C	YGL010W	YKL121W
YKL005C	UBP3	YDR455C	ECM12
IKI1	PPT2	YMR097C	SKO1
SPE4	GIS4	YLR257W	DUT1
BCS1	YLR410W-B	SUR1	YBL064C
YLR202C	YGR154C	SFT1	IRA2
YPR013C	SKI7	YHR198C	YDL057W
DUS1	CWP1	QRI5	YPL201C
MED4	YFR026C	STE14	RSC58
ISM1	YOL125W	RPL19A	RSM18
KIP2	YNL143C	YDR352W	YML076C
HYS2	YPR027C	YOR051C	PTK2
YDR163W	OPY2	YCR103C	
YNR034W-A	YLL044W	YPL276W	
SPT7	YAR060C	OPI1	

TF of interest: HSF1
Target genes predicted by AdaFuzzy:

CPR6	SUM1	FYV11	PPS1
SSH4	VMA21	YNR014W	ALR1
EDC2	DIA1	RIB5	YPL062W
YHR173C	BUD31	MYO2	YJR128W
ENO2	YLR198C	RPC82	YBR101C
SEC1	JNM1	FYV4	SPR1
STI1	VPS34	HOR2	YDR051C
HSC82	YLR376C	SMB1	YNL213C
YLL032C	NUP133	MDS3	PRE4
HSP78	ROT1	YNL168C	YLR278C
YDL176W	POR2	YLR181C	YOR114W
KRR1	YDL199C	ZRC1	UGA4
UTR5	YGL064C	YOR291W	YOR083W
GRC3	MSN1	YPR204W	CKB2
YDL177C	CDC25	HOS1	YMR244W
YKL123W	YGR226C	STS1	ARC18
ILS1	ALD4	SEC11	YPL070W
HSP10	TOR1	KTR4	GCN5
PGK1	RPC11	PHO4	YHR212C
YGR205W	IMD4	YPL017C	SHE9
YNL134C	YNL136W	YML014W	ADE12
YGL218W	INO4	YER185W	YOR088W
SDH3	HOP1	IMP3	YMR119W-A
DUO1	HIS4	TEL2	YDL109C
SGV1	RPC53	RPE1	YCL068C
YBL064C	ZRT3	KRS1	MRC1
UBC6	APC9	YIL012W	YDR261C-D
LCB3	DUS1	PDE2	YHR202W
YGR111W	SRB2	BIK1	PUS2
ARO80	UGX2	YGR126W	YLR227W-B
YGR210C	YGL010W	YBR100W	YMR206W
YNL266W	MSN2	RSM19	YGL152C
YNL144C	CDC40	BNI1	YNR005C
ATP14	YMR162C	HAL9	HPA3
YGR038C-B	SMI1	NUP2	YLR041W
CDC55	LSM2	VIK1	DNL4
YBR101C	BOP2	PSR1	CDC37
WHI2	(MSF1)	SFL1	YJL147C
YBR232C	YMR073C	YBR100W	YMR031W-A
ERG6	MPA43	URA7	SNF1
HYM1	LEU3	SWA2	SEC21
YHR132W-A	YER128W	URA1	YKL223W
YJL032W	GUF1	ARP5	YPR076W
YLR437C	HDA1	CRC1	BFR1

PBI2
GLN4
YGL110C
SRT1
SEC24
CIK1
YKL111C
YSC83
ARA1
RPE1
YJL217W
SAG1
CIS3
SAE3
SEC72
YMR087W
YLR361C
PRY1
YCR007C
TOS6
FOL2
YGR125W
HEM2
ERG11
YJL215C
RAD4
IST3
RPS8A
UGX2
MRP10
BUD19
BEM3
RPL26B
ISU1
QCR6
DOT1
YCR090C
FRE6
YKL077W
YLL053C
YNL194C
RRN10
MEP3
BUD3
MOD5
SPS2
YNL295W
YLR050C
COX15
SEC22
RIS1
YGR176W
ATX2
CIN1
UBC11
RPB2
YDR384C
SWH1
YCL049C
CTF18
YFR057W
YDR366C
YDL036C
SMD1
YGL152C
YLR012C
ERP1
FUS1
LHS1
YPR012W
YNL019C
ZIP2
YOR380W
YPL166W
YGL149W
YEL064C
RPL2B
CLN1
YCR013C
YNL127W
TRP2
PHO2
PPQ1
SMM1
PMT5
YMR147W
HLR1
YDL001W
YDR476C
AUT1
YDR271C
YOR139C
YMR318C
YOR121C
YBL062W
GRH1
SOD2
YSC83
FKB2
UBP2
RRP7
VPS38
HYP2
MED2
APG10
RLF2
RNH1
ATP16
EKI1
PAN2
BUD3
FAP1
HXT8
MSH6
TOF2
YDR370C
LUV1
KNS1
GAT1
PET130
DPL1
YCR023C
YFR044C
YDR306C
YOL111C
ALO1
DOC1
YLR326W
YGR198W
MRT4
PCL9
YLR343W
YIL152W
YHR177W
CYB5
MSH3
ORC1
TBS1
YKR017C
YGR138C
TFC4
YMR067C
TUB1
YNL208W
CAF4
YIL058W
HTB1
RRP1
BAG7
BZZ1
YHR020W
RPE1
STB6
SBE22
YNL200C
RSC30
MRP20
YLR269C
YPL064C

TF of interest: MSN4
Target genes predicted by AdaFuzzy:

YPL150W	COS1	TOS1	YLR266C
IRE1	SSA1	HSH49	POL4
YGR210C	YRF1-5	BUD21	BUB1
YBR074W	HSP104	NUP60	KRE35
MMF1	YLR465C	YDR540C	YCL034W
PRE2	IPT1	YPL060C-A	TRP2
YPR202W	EFB1	YIL090W	YOR258W
MSR1	AFR1	PDR11	YLR428C
HSP26	SNQ2	PYC2	PRM10
YEL077C	RAD53	GLG2	LYS12
YRF1-4	SNF11	ERG1	YKL047W
YLR462W	FHL1	HXK2	YGL138C
PCF11	YMR119W-A	CDC24	YDR210W-D
GDB1	YNL337W	COX5A	YDL023C
YJL225C	IST2	YIP2	UTH1
YHR035W	UPF3	CTT1	TWF1
YIL174W	YEL073C	ESC4	YBR241C
PCL7	BTN2	YOR093C	YGL138C
HOR7	PRP46	GLK1	PTR2
RPL11A	YPR203W	YAR1	URA2
YNL338W	YHR033W	POL32	YNL123W
YGR071C	YRF1-3	PRP43	MVD1
CMP2	RPL7B	MRPL8	SEC63
COS3	YLR464W	YMR018W	PIR1
YDR544C	YIL175W	MRPL7	(MRF1)
YRF1-7	BUD19	VPH2	FAS2
YML131W	VTH1	SPC3	YOR267C
DFR1	AYT1	MAF1	TRP1
YRF1-2	YPR092W	YPL201C	STE11
YER189W	RAD53	YPS7	AGP2
RDH54	YDR008C	RGA1	UFE1
YBL108W	YPR171W	RIB3	RSM22
SSE1	YLR124W	YIL082W	KAP122
SSA4	SSH1	RAX1	YCR050C
YIL176C	YIL001W	YKR046C	YMC2
CAF20	YFR042W	YBR025C	YME1
YTH1	RPL2B	RPS26B	HMLALPHA1
YLR463C	PRS2	PCM1	YHC1
MRPL4	YDR526C	ATP17	RPS23A
YDR010C	ALG5	YLR422W	YJU2
YRF1-1	YOL071W	YKL063C	MDH2
RPL2A	YOR161C	YDR307W	IES2
YIL177C	ISM1	YPR123C	SRT1
GSC2	YPL205C	YBL071C	ZRT3

YGR203W	SDC1	YOR165W	YLR157C-B
VPS17	RRP7	YBR277C	YJL217W
RNT1	YPR097W	YOL047C	YNL158W
MED8	CDD1	BET5	YJL135W
HMT1	ASC1	RPL8B	VPS41
ARG8	RPS0B	YJL204C	HAL5
YKR049C	FCY1	UTR5	ZRG17
REC102	MAK10	NDI1	MSH1
RHO4	PET122	CDC16	KRS1
FAA2	YKL044W	PET123	YLR414C
FPR4	RPL1A	CCT4	KRE32
SSP2	YEL059W	DIA2	BTN2
PBP1	NMD5	YPR091C	RTT102
PNC1	DBP7	DAM1	VCX1
KRS1	YJL016W	YAP6	CIT3
CCW14	OST1	YJL018W	HFI1
FIP1	GAT1	ENO2	YAT1
MRPS18	PLP1	YDR049W	RPL17B
OPT1	YDR489W	ULP1	YDR261C-D
YDR223W	YJL046W	SSF1	DCP1
INP52	YLR352W	EST2	SPT20
YOR302W	ATP3	PDR13	MST1
PGM1	POS5	NOG1	PRM8
HIF1	YIL001W	MSN2	RTA1
YLR080W	YHR130C	YPL201C	HDA1
YLR266C	YMR244W	YOR364W	FPR4
PCL2	MMS21	RVB1	NDH2
SOM1	YMR215W	FBP1	SIC1
YML045W	GRX4	RVS161	RPS17A
PBP1	CNS1	DBF20	YMR318C
YKL099C	YLR035C-A	YMR221C	GUK1
YDR273W	TIF4631	YPR1	PPZ2
MFA2	SMM1	YLR064W	
EMP70	YBR089W	SUA5	

TF of interest: SKN7
Target genes predicted by AdaFuzzy:

IES6	YDR157W	YKL097C	MAS6
RPA34	KRE6	YPR013C	YLR002C
GAT2	FKS3	RAS2	NDH1
SCW10	YMR173W-A	NRG1	YMR221C
YMR252C	DDR2	YIR020C	YLR076C
APC1	AHP1	MDG1	RAD23
KNS1	HAA1	YML089C	GLE1
YMR172C-A	GIC2	HRD1	YJL028W
HOR7	MSN4	APG12	ELP2
USV1	UTH1	NUP159	RDH54
ROX1	RPP2A	YDR339C	ECM21
OLE1	NCE102	YML090W	STM1
SOK1	MGA1	YMR153C-A	PMC1
SGV1	RPM2	YBR273C	RAD9
YLR108C	YDR442W	MAL13	YNL028W
RPA14	CIN1	YPL013C	UBR2
YML053C	SPO20	DNA2	YMR082C
SDS23	YDR154C	GRH1	PTR2
HOT1	YMR304C-A	SPP41	YMR099C
SUR7	HEF3	YER093C-A	YOR325W
SGA1	YEL045C	TOS6	YDL096C
MSN2	KNH1	FLO5	DIA1
PMP2	DDR48	YOR333C	RNQ1
FRS2	CWP2	IMD2	YLR463C
CBF2	ARF2	ERC1	SNC2
NCA3	YNL170W	YKR070W	YDR018C
GSY2	GAT1	FLO5	IDI1
RGS2	UFO1	MCX1	RPL5
PUT4	YKR040C	BAR1	DNA2
TIR1	YMR018W	YOR318C	YEL023C
STB4	SCW4	NIT2	APG12
YLR257W	ASF1	YDR090C	YOL153C
GPH1	CIS3	TRS23	PCK1
ERG6	CLN1	YJR071W	EAP1
GLY1	SOK2	GRD19	MUQ1
YLL020C	MET13	RER2	YOR054C
HAP4	GSC2	PPH3	YBR194W
CCW12	YDR526C	YDR029W	MHR1
YBL029W	TAL1	RTT101	YOR322C
YAP1	APT2	ATF2	YDR248C
ICS2	YPL025C	YNL058C	UBP1
YLR111W	HOM2	YDL183C	END3
RPS15	PCK1	RPS29A	YMR045C
SSK22	CTT1	YKT9	PAN6

YGR038C-A	XPT1	SER2	TEA1
YDL146W	NEO1	RPL30	YLR190W
ATP16	OSH1	YOL159C	VMA13
YJL010C	DUO1	WHI3	EMG1
YPL035C	SSA4	YLR104W	YNL245C
RPS17B	YHL009W-B	YKL056C	EFD1
YCL033C	RPL27B	ACN9	ALG5
YJL163C	YRF1-5	YDR516C	PDR1
YFR012W	YGR081C	SAP155	FRE6
YNL083W	YDR248C	NPL3	LSM4
YAL045C	MBP1	YDR057W	MCX1
HAT2	PIG1	MGM1	UGA4
YLR356W	PIB2	YNL091W	MLC1
PEX10	YBR210W	EFD1	YKL056C
KIM3	YPT6	SLA2	SAC3
PRC1	HRP1	CTP1	SMY1
MET13	PHO87	YDR070C	BUD8
DAN1	YGL211W	SHP1	YKL005C
YVH1	VID21	MMS2	YOR137C
SAC7	YCL044C	YDR317W	YBR277C
GCR2	YEL064C	ARP3	KIP1
HCR1	YDR330W	RTG3	UBC12
ARK1	ERG3	YDR036C	YDR116C
VTI1	STE13	YBT1	HCA4
RPN6	RNA14	VPS1	TRK2
GLO1	ASE1	MGM101	MIG1
ZRC1	GPD2	GRE1	YGR042W
BUD9	PEP1	PEP3	TIF5
PRP42	GPI10	SSA2	YDL026W
MST1	TFC1	YBR075W	YOR243C
ERG28	YLR402W	YDR444W	YOL098C
LSC2	SPS2	YGR042W	YDL062W
DBP3	IST3	YMR173W-A	SOK1
YER087C-A	YBR116C	RFC2	RCE1
FUN21	YOL073C	MAC1	YDR154C
CWH41	YLL023C	HPR5	DNM1
REC102	MAG1	SPS19	KTR7
YJL162C	YPL276W	PEX3	MLS1
YOR138C	YCL020W	YPL133C	YNL041C
YBR246W	BAP2	YLR301W	LAP4
YPL272C	SPC3	YEL010W	YOR086C
YDL133W	YPR014C	DDI1	SYF1
SPO75	SRP14	HMLALPHA2	MON2
TIF4631	YGR122C-A	RPL6B	YDL203C
ERV29	GLE2	YPL066W	PEX1
BNI5	YDL109C	ASH1	XKS1

YLR266C	YPR105C	YOR285W	YHR150W
PAN3	PRT1	YPL276W	YHL044W
CRH1	HOR2	HXT4	DBP1
GAL3	FTH1	GCV1	GND2
PHM5	BUD13	YPR142C	CSL4
YGL039W	YOR175C	YML089C	FUN30
YCR090C	OAF1	YML005W	YJR026W
ZRT1	YDL223C	YGR273C	YMR031W-A
CAF130	SEC4	YPR151C	CAD1
YFL002W-A	LYS21	VMA4	YFL006W
MSD1	YBR032W	GDH2	

TF of interest: YAP1
Target genes predicted by AdaFuzzy:

SSA2	OYE2	RPN4	YIL110W
RPL21A	YDR307W	YPR002C-A	RSM19
WHI2	CDC7	LYS20	TIM44
GLG1	SRB7	YDL183C	YPL233W
SCP160	MRPL4	RPL10	SNL1
YER079W	YNR018W	YBR190W	YBR203W
GSH1	SOD1	YFR017C	SEC26
IES6	HIP1	HXT9	CYT2
DDP1	MSN2	YGL114W	YPL166W
PLM2	YKL151C	YER130C	YLR049C
SNQ2	YLR460C	ROD1	LEM3
EXG2	AYT1	BUD20	RMA1
YKL071W	YAL037W	YGR210C	STR2
YFL034W	UGA3	DFR1	YAL058C-A
ADO1	SEN2	GLY1	YFL006W
MRS4	TAH18	YAP6	SEC72
YOL118C	YLR302C	NOP1	KTR1
ERG25	RPS5	YLL058W	YMR040W
GRE2	DYN1	RPS26B	MSS11
POP8	TSA1	YEL045C	YKU70
YDR010C	AHP1	PDR16	YSY6
VAN1	ATR1	SAP185	YNL105W
GTT2	YLL059C	YRR1	ECM15
YHB1	DOA4	QRI7	ZRT1
AAD6	YML131W	YMR313C	VID22
TIF4631	ERO1	YNL269W	YKR022C
ERG28	ROX1	YER053C	YNL200C
ARO8	CPA2	LAP3	YBR271W
VTC2	RPL1B	NDT80	ISW1
RHO4	TDH3	LIP5	ASF1
YDL180W	YLR108C	YNL105W	SAN1
FSP2	GLT1	HMF1	AGP1
RPA34	YMR144W	YDR344C	ESC2
YNL134C	CAR1	YGR150C	NUP120
YDR132C	SIR4	YNL235C	RNQ1
YLR346C	YGR011W	TOS9	YBR246W
SEC9	YER078C	YNL108C	MEI5
RIB1	ZPR1	YER138C	YJL169W
ADE2	RPL23A	HYS2	ISR1
MAK21	PRE1	YER053C	VPS20
RPS25A	ECM4	YLR073C	YMR041C
FEN2	RPS13	NCP1	RPO41
SSA1	RPS22B	PYC1	PEP4
RPL37B	EFB1	UBP6	RPL8A

PMP1	RLM1	CYC7	PTC4
YBR028C	YPL113C	YGR130C	YBR137W
YGR265W	YGL010W	ERG26	SPO20
PSR1	YEF3	YHR100C	YHR111W
NUP60	FDH1	RPN6	ALO1
GCN20	LIP2	PHO23	THP1
YGL242C	REF2	MRPS28	PTI1
YOX1	ALR1	NPR1	YJR098C
YGL177W	OPY2	PEX11	YHM2
PMT1	RPL28	FUN19	YGR293C
YJR100C	YEL075C	YNR042W	CRZ1
MUM3	APE2	BCK2	FLX1
RAD4	YMR158W	GIT1	YOR161C
TOR1	NHA1	MED6	RPL23B
YNL207W	YBL065W	CDC34	IOC4
QCR6	YFL047W	MUD1	RDI1
FLO1	USO1	SSN8	YGL217C
RRP5	MGT1	DFG16	YJR029W
TMT1	YMR157C	YNL190W	YGL139W
CAM1	YIL096C	AAD6	ORC1
FUN11	SOD1	RAT1	YOR019W
YIL163C	CDC4	NUP53	YOR353C
YDL163W	THP1	SPP381	PTC5
ISC10	YDR042C	SSQ1	YNL054W-B
NMD5	MRPL16	YBR224W	ZRC1
GTS1	YLL020C	PGK1	NDI1
LEM3	YLR254C	YJL220W	BPT1
YJR039W	MTR4	YIM1	RPS1A
YDR210W-A	ORC4	RAD3	RPN11
MRPL23	ECM25	YPR116W	RPS4A
MUD1	CRH1	OSH6	YPL039W
YJR128W	SWD2	YPL105C	MSC1
YLR152C	PBP2	YDR525W	ARP5
RPB2	YOL026C	PKH2	STE6
MRPL33	YEL072W	NOT3	KEL3
YGR137W	PDS1	TOS1	BOI1
SHR5	POT1	GPX2	YJR072C
PRY1	YDR482C	CBK1	ALD5
CDC48	PHO4	SRB8	YDR250C
PRK1	YJL105W	YPR143W	YDR136C
DIA3	SPO21	ASF1	YDR198C
SPB4	YNR046W	TOM40	EMP47
YHL029C	OPY1	YGR012W	NSR1
DUR1	MGT1	MCX1	RPS31
YGR237C	TIR1	AGA1	MSM1
YNL022C	YOR205C	YEL001C	YIL113W

YMR294W-A	KAR1	KRE24	NUP84
YER004W	LSM4	CAF130	RRP40
YLR456W	YOL114C	YDR061W	GUT2
YBR030W	YFR018C	YAR053W	FUN55
RPL34B	ARF1	YGR093W	BEM3
POP1	YML079W	HSN1	MTR3
TGL1	YDR332W	TRR2	GYP1
YLR003C	YDR215C	YBL004W	YOR105W
SCS7	YGR131W	RET2	PET9
CTR3	FAR3	YER087W	YOR112W
YIM2	AHT1	GYP1	VPS21
VHT1	YFL066C	YMR071C	SRB8
SSP2	YLR431C	RPL27B	IQG1
ARP7	CUP2	YGL034C	YHR036W
FUN16	MRPL40	MRL1	GDH1
ARC19	RPN4	RPL5	STE24
SOK2	MEK1	LAT1	UTR2
LEE1	RET3	YOL015W	YNL127W
YBL104C	FUN59	CBP6	YLL029W
PRE10	YBL101W-B	RPL35A	SSF2
SRY1	GRR1	IDS2	RSR1
YDL180W	YMR184W	YLR410W-B	FZO1
CDH1	SML1	KGD2	RPS23B
ANP1	SPT23	YHB1	FSP2
YBR071W	YGL104C	FIT3	YPL158C
PHM6	YKL223W	APM1	UBA2
COS4	YOR255W	YMR317W	YML018C
YPR143W	NPT1	MCX1	TAO3
YPR044C	PTR3	STE12	YND1
YJL120W	HIS1	TCI1	GAL11
YKR065C	ROM2	SMB1	YFL019C
RIM13	YFR054C	AAP1	ARP2
BET2	YLR169W	PAC11	TFG1
YPL197C	AAT1	APC4	YDR365W-A
FUN21	YCR026C	SPO16	CPS1
RPB10	RNR4	APT2	TES1
VAC8	YKL151C	YPL168W	YOR387C
BUD3	IST2	NSR1	KIP2
YOR256C	YIR036C	SCM4	YPL186C
RBL2	ERD2	YPR153W	YJL097W
YER030W	PFS1	YGL109W	CBT1
YMR233W	GIM4	ODC2	YPR146C
ZUO1	STB1	DAM1	YKL160W
RPS18B	YHR182W	FUN31	TRX1
FUN31	TOA1	YMR322C	ADY3
THP1	YIH1	TRR2	YFR048W

YMR119W-A	YDR116C	AMS1	HRB1
MYO3	YLR428C	SMD2	EBP2
FYV14	YEL008W	SPG1	RPS13
ASK1	YLR247C	YHR199C	MSW1
HOG1	YGL161C	GPI13	YBL083C
YPT7	YIL100W	YPR139C	YJL064W
YLR408C	CDC5	ABD1	GPR1
YFL034W	RPL26B	GSC2	YET1
UFD2	RPN12	YDR324C	YGR109W-A
DIM1	TOM40	YHL017W	SLT2
MRPL38	RLR1	ADE6	YFR038W
MCM21	YJR116W	YNL157W	YGR111W
HOM3	YDR371W	YKL014C	FRS2
YCL041C	TDH3	MAC1	TFC7
YLR437C	YLL032C	ISY1	CST29
RPL5	MID1	SPO14	PRP24
RPL4B	YCL074W	YDR411C	SMM1
SSQ1	TFC5	YLR462W	TOS6
ESBP6	INP53	YGL160W	NAS6
YOR279C	YOL014W	YML035C-A	YBR052C
SWI3	GDH1	SEC8	COR1
PDX1	YHR003C	COX14	KTR1
SCS7	YOR169C	YLL017W	SNU66
SYG1	SEC11	TRM1	PRM8
MRPL38	YBL089W	MPD2	YMR322C
SPC105	YPL233W	IKS1	HAP1
YER077C	FBA1	GAL83	COQ2
TIF34	ADK2	IPK1	CDC33
GCV3	YGR290W	YOR376W	YER130C
YLR253W	RCS1	JIP1	OSH2
YDR316W-B	YNL140C	YNL245C	BRE2
GUF1	FUN12	PHO2	YDR031W
KRE32	CEP3	CDC43	VID24
ERP4	MMP1	COX13	NAT2
YAP1802	VPS38	YDR444W	YPL095C
YMR114C	YOL048C	DPM1	SUM1
SPT2	MAL33	TOM1	YHR125W
LSC2	ERV41	UBP9	RPB4
UBP5	CLG1	PDE1	YDR291W
CTK1	YMR279C	TEL2	APC2
PTI1	YDL123W	HMS2	YBL012C
RRP42	YMR087W	UGP1	CSE2
YDR008C	PIN4	SSK22	MND1
YMR007W	YHL026C	YJL027C	RPA14
ALD5	RTS1	YGR145W	RPS8B
RPS15	PPG1	ABM1	CNA1

YIL012W	YNL058C	AFG3	YLR400W
SEC63	SEC61	PFY1	RPL13A
CCA1	AIP2	TAF17	ARC15
BUD2	ADY2	YER066C-A	TOS3
SNO3	CVT9	SMD1	BNI1
PEP4	SSB1	YNL081C	CDC8
PRC1	YGR031W	YOL131W	YCR016W
YKL206C	PDS1	YLR374C	YIL055C
YAP1801	MLF3	KRE32	YPL257W-A
TOS6	AEP2	ARH1	DOT5
SUL2	PLO2	ILM1	NRG1
YGR064W	MSB1	HCR1	GCN3
HDR1	IKI1	(MRF1)	LEA1
YMR252C	STB5	YPL105C	MSB4
SGE1	YML003W	COX5A	ILV6
YKR033C	YDL001W	COQ6	TIR4
YDR316W-B	YKL066W	UGT51	SPP1
SIW14	YME1	RMA1	PIN3
MRP2	YLL044W	PRP18	YNL303W
YGL101W	YLR287C	LSM6	ANP1
ERG2	FBP1	AOS1	ICL1
PRP24	YHR150W	YML122C	RAD18
YMR171C	YJL181W	OSM1	HXT10
YLR412W	RPL27B	RRM3	MAK21
YPR148C	YKL051W	YDR346C	CEP3
YHR186C	MET1	SEC2	RPS4B
YML119W	HXT14	OPI1	HFA1
YOR083W	YNL337W	BUD23	ARR1
SMY2	LSM6	YOR108W	BRE5
YDR210W-B	YML107C	SPT8	TRP3
FUN12	SMP3	YLR104W	JEM1
SGT1	PCL6	AGP3	YDR494W
YKL107W	PHM7	GLO1	YDR015C
YCL033C	BNI1	YHR095W	ABF2
YNG2	YGR089W	TIF34	ISA1
MRS5	YMR153C-A	YLR159W	YJL207C
ATP5	YKL160W	PRT1	YOL079W
YDL204W	PRM8	CEM1	CCA1
RSM25	YGL114W	OAF1	GCD14
YNL247W	YGR278W	ECM16	PAT1
NHP10	PHO87	CLA4	YGL121C
PDX1	YER093C	TOS10	MET17
HAL9	BOI1	RPB9	ORT1
MRPL6	YLR334C	WTM2	HRB1
PIN3	NOP58	YIL137C	YNL311C
SMD1	RPL24B	TAF47	YVH1

TMT1	YNL091W	CDC42	YNL022C
NEO1	FUN30	YCR023C	RNR2
YRF1-2	YLL066C	ARR2	TAF90
YGR038C-B	CAX4	CUP1-2	HYS2
DAN2	BUD32	ISW1	MUP3
YHR035W	TAF145	YMR206W	YKR017C
NCR1	PAN5	YPR202W	YPR002C-A
YGL079W	YNR066C	YKL036C	YPL261C
RSN1	YMR119W	YKR087C	OPT2
ASP1	YOR304C-A	YDR020C	PRP21
CDC14	TOM22	SWI6	CTF13
YER078C	SNF4	SWD2	FYV14
CMP2	PRO1	BRR2	TCP1
YDR094W	SEC72	IMH1	FKB2
BUD4	YCR090C	NUT2	YOR390W
ATP5	GSP2	YJL181W	YBR281C
YDR034C-D	YKR051W	SWA2	YML072C
ATM1	YNL123W	YLR157C-A	YDR223W
PDE2	SFI1	YLR465C	YNR005C
ADE8	FYV9	YIL059C	KRE35
SPO71	YBR273C	MAL31	APG2
YNL234W	PAU6	NCE102	YNL337W
ADE16	YDR014W	YBR022W	ERV14
MLF3	SLI15	YMR206W	SUM1
YNK1	GPT2	NAP1	YDR061W
MOB2	AYR1	NEW1	RPA190
YKR071C	YIL104C	YGR294W	PLB2
PPH3	PDR10	YJL204C	YDL057W
YGR122C-A	YDR048C	YNL198C	YLR205C
YOR284W	SPS19	FOL2	YDL233W
RPL27A	SFP1	YDR056C	YBR280C
MDM12	YOR387C	PIR1	MTF1
YPR045C	YKL115C	MMM1	YDR249C
KTI12	ATP5	CPR8	SSA1
YML003W	MNN1	TIF3	PET9
ATP7	GIS3	SKI8	PRS3
YMR254C	YGR150C	SSA1	YKR074W
YLR224W	TFC4	UBS1	SAT2
PRR2	RPS8A	STH1	FUN26
YOL057W	IDH1	YPR130C	CBT1
YDL115C	GRH1	CHS3	YGL261C
PSP2	VPS38	VAS1	YDR066C
RNY1	HOR7	YDR112W	YLR101C
YIL060W	YMR132C	ALG8	CSE4
AAD3	YNL326C	HCR1	MDH2
ICS2	CIK1	YOR192C	YHL008C

CMD1
AQY1
ZTA1
RAM1
YIL077C
YNL254C
YHL021C
HAS1
GCN1
NIF3
RAD5
KAP120
RPA14
YDL038C
YIL082W
YPR063C
GCR2
YDR034C-C
YNL177C
SOV1
YFR039C
YDL199C
GPH1
RNH35
ECM25
YGR161C-D
ICL1
ORC3
YCR085W
YRF1-5
YCG1
BFR1
YLR072W
NTH2
MDH2
YDL176W
YDR476C
NAT2

GIC2
SSQ1
NDH2
YOL053W
CTK3
TUB2
YMR279C
NTG2
RAD51
CYC1
FLO9
EFR3
SPO69
YPR061C
YLR345W
RRP3
ACS1
YGR071C
YML036W
YHL026C
YDR514C
ALG5
CUP5
YBR014C
YER049W
WSC2
YNL218W
PMT1
QRI7
YBR178W
YOL008W
YKR046C
IME4
SCW10
YLR434C
DOT6
YLR041W
GAT4

DPL1
GLG1
YOR024W
ICY1
RPL18A
PEX2
KEX1
SAS4
MRPL27
YIL108W
YCL045C
YKR065C
THP1
YBR030W
YGR201C
YJR080C
YLR253W
MSH3
SCW10
RAD2
YBL112C
YSY6
FLO9
LOS1
YMR324C
DCC1
ARL1
QRI5
YOR302W
SOD2
ESBP6
SSY5
ERG28
KRE11
SDL1
ISF1
OSH6
OM45

SRB4
YLR224W
YBR016W
APD1
YMR196W
SHE1
YLR257W
LIP5
VPH1
TUB2
PFS2
MCR1
ATP4
ELA1
KRE32
OPT2
YMR122C
YER186C
YKR077W
YMR254C
YIL028W
YBR033W
RPL1A
MAD3
BSD2
YMC2
PEP3
YFR035C
CLN1
SRP21
MSN2
YLR236C
YFR047C
MRP17
YNL224C
YLR434C
